# Transcriptomic analysis of female and male gonads in juvenile snakeskin gourami (*Trichopodus pectoralis*)

**DOI:** 10.1038/s41598-020-61738-0

**Published:** 2020-03-23

**Authors:** Surintorn Boonanuntanasarn, Araya Jangprai, Uthairat Na-Nakorn

**Affiliations:** 1 School of Animal Technology and Innovation, Institute of Agricultural Technology, Suranaree University of Technology, 111 University Avenue, Muang, Nakhon Ratchasima 30000 Thailand; 2 Department of Aquaculture, Faculty of Fisheries, Kasetsart University, 50 Ngamvongwan Road, Chatujak Bangkok, 10900 Thailand

**Keywords:** High-throughput screening, Transcriptomics

## Abstract

The snakeskin gourami (*Trichopodus pectoralis*) exhibits sexual dimorphism, particularly in body size. Since the snakeskin gourami is usually marketed during sexual maturation, the sexual size dimorphism has become an economically important trait. Sex-biased gene expression plays a key role in phenotypic sexual dimorphism. Therefore, using high-throughput RNA sequencing (RNA-seq) technology, we aimed to explore the differentially expressed genes (DEGs) in ovary and testis during sex differentiation in juvenile snakeskin gourami. Our results revealed a number of DEGs were demonstrated to be overexpressed in ovary (11,625 unigenes) and testis (16,120 unigenes), and the top 10 female-biased (*rdh7*, *dnajc25*, *ap1s3*, *zp4*, *polb*, *parp12*, *trim39*, *gucy2g*, *rtbs*, and *fdxr*) and male-biased (*vamp3*, *nbl1*, *dnah2*, *ccdc11*, *nr2e3*, *spats1*, *pih1d2*, *tekt3*, *fbxo36*, and *mybl2*) DEGs were suggested to be mainly associated with ovary and testis differentiation, respectively. Additionally, using real-time reverse transcription polymerase chain reaction (qRT-PCR), validation of the differential expression of 21 genes that were previously shown to be related to gonad development was performed (*ar*, *bHLH*, *cyp19a1*, *daz*, *dead-end*, *esrb*, *esrrg*, *gnrhr*, *gpa*, *gsg1l*, *hsd17B*, *mospd1*, *nanos-1*, *nanos-2*, *p53*, *piwi-1*, *piwi-2*, *rerg*, *rps6ka*, *tgf-beta*, and *VgR*). The results showed a significantly positive correlation (0.84; *P* < 0.001) between the results of RNA-seq and qRT-PCR. Therefore, RNA-seq analysis in our study identified global genes that were associated with ovary and testis differentiation in the juvenile phase of the snakeskin gourami. Our findings provide valuable transcriptomic bioinformation for further investigation of reproductive biology and applications of sex manipulation.

## Introduction

Sexual states in fish are diverse and have been widely classified as gonochorism or hermaphroditism. Thus, fish are a suitable model to study reproductive strategies among vertebrates, particularly for sex development. Additionally, sexual plasticity in fish diversifies sexual development systems, including genetic and environmental sex-determination factors^1^. Several mammals possess sex chromosomes that allow the identification of genetic markers for sex determination and gene expression associated with sex differentiation and maturation, providing various approaches for reproductive manipulation^2,3^. Although fish exhibit the largest biodiversity among vertebrates, bioinformation regarding their sex chromosomes is limited. For example, single-nucleotide polymorphisms, linkage genes, and quantitative trait loci in a few genes or loci have been demonstrated to be related to sex determination in several fish species^4–6^. Furthermore, different sex-determining genes have been shown to exhibit diversity across different fish lineages^7^, and it has been demonstrated and suggested that young Y chromosome drives the sex-biased expression of genes^8^. Currently, to provide a holistic view of the evolution of reproduction biology, genetic studies on sexual system in fish have focused on sex-specific genetic differences and/or sex-biased gene expression, particularly in hermaphroditic and gonochoristic fish, which exhibit strong sexual dimorphism^9–18^.

Occasionally, phenotypic sexual dimorphism in fish has been observed in aquaculture-related and ornamental fish^8^. The snakeskin gourami (*Trichopodus pectoralis*), which is native to Southeast Asia, is an economically important food source as well as an aquarium fish. It is a type of labyrinth fish that is able to breathe air directly, in addition to absorbing oxygen through its gills, enabling it to inhabit oxygen-poor water. Snakeskin gourami naturally occurs in shallow ponds, swamps, flooded forests, and rice paddies. Morphologically, the snakeskin gourami is elongated and compressed. Compared with female fish, male fish have a slimmer and more compressed body. Snakeskin gourami mature when they are 8 months old, having a body length>12 cm, and this is also the harvesting size of farmed fish. Therfore, the sexual size dimorphism has become an economically important trait, and female populations are more productive and preferable. Therefore, the establishment of all-female stock is attractive. Although the snakeskin gourami is one of the most important aquaculture-related species in Thailand, its genomic resources are limited. The identification of sex-biased gene expression would provide a genetic approach to understanding not only sexual dimorphism but also comparative bioinformatics in fish reproduction.

Recently, high-throughput RNA sequencing (RNA-seq) technology has been used as a novel tool for studying the global networks of gene expression. RNA-seq has enabled a rapid and cost-effective approach for preliminarily examining differences in gene expression without the requirement of prior sequence information. A genetic approach to understanding sexual dimorphism during sex differentiation would contribute to application of biotechnology for future monosex culture. In our study, therefore, comparative transcriptomic analysis during sex differentiation in juvenile snakeskin gourami was conducted for preliminarily screening sex-biased gene expression in gonads, which are the primary organs responsible for sexual development. The *de novo* transcriptome assembly served as a reference for read mapping and comparing gene expression. Functional annotation and enrichment analysis of the genes demonstrating sex-biased expression were performed. Additionally, the level of differential gene expression of 21 genes that had previously been associated with gonadal development were validated using real-time reverse transcription polymerase chain reaction (qRT-PCR).

## Results

### Histological study

**Figure 1:**
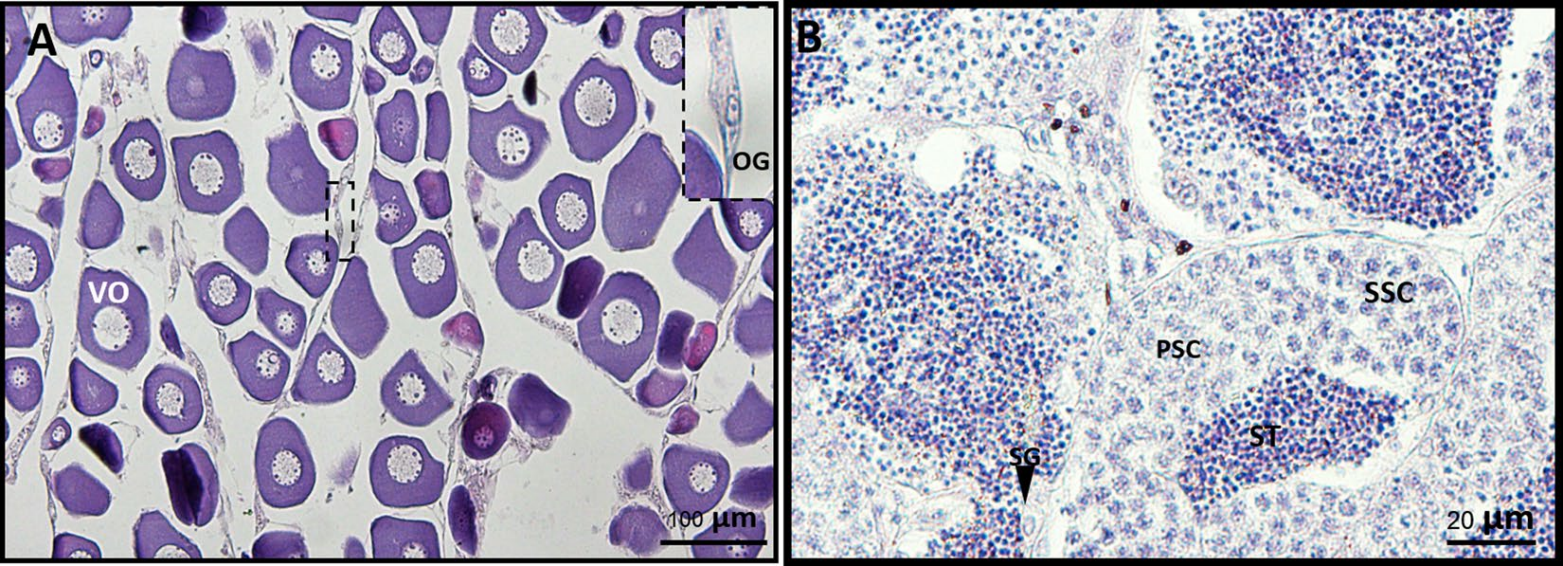
Histological characterization of ovary (**A**) and testis (**B**) of juvenile snakeskin gourami. Various stages of ovarian cells were found, including vitellogenic oocyte (VO) and oogonia (OG). Different stages of testicular cells were observed, including spermatogonia (SG), primary spermatocytes (PSC), secondary spermatocytes (SSC), and spermatid (ST). Scale bars represent 100 µm (**A**) and 20 µm (**B**).

Although gender dimorphism of external morphology was not observed in snakeskin gourami during the juvenile phases, ovary and testis were distinctly observed (Fig. 1). The ovaries of sexually immature juvenile female fish contained primary oocytes and oogonia. The testis of sexually immature juvenile male fish contained various stages of testicular germ cells, including spermatids, spermatocytes, and spermatogonia.

### Sequencing assembly and functional annotation of assembled unigenes

Approximately 56 Gb of raw reads were generated, including 388,645,886 raw reads with 370,116,966 cleans reads (Table 1). Moreover, Phred quality scores of the clean reads at Q20 and Q30 ranged 95.18%–96.32% and 88.54%–90.79%, respectively. The *de novo* assembly of each clean read resulted in>80% of each JO and 76% of each JT mapping to the reference transcriptome. Among the 54,353 assembled unigenes, 50,517 (92.9%) were able to be annotated in at least 1/7 databases, (nr, nt, Swiss-Prot, KO, GO, KOG, and Pfam) (Table 2). There were 5,300 unigenes (9.8%) annotated in all seven databases. Among teleost databases, the experimental transcripts had high level of sequence identity with *Larimichthys crocea* (31.5%), *Stegastes partitus* (29.1%), *Oreochromis niloticus* (9.1%), *Maylandia zebra* (5.2%), and *Haplochromis burtoni* (4.2%) (Fig. S1). A Venn diagram was constructed to indicate the distribution of the expressed genes between testis and ovary of the juvenile fish (Fig. S2). There were 47,626 and 30,805 unigenes expressed in testis and ovary of juvenile fish, respectively, and among them, 24,078 were co-expressed between testis and ovary.

**Table 1:** Summary of the sequencing results.

Sample	Number of reads	Number of clean reads	Number of clean bases	Total mapped (%)	Error (%)	Q20 (%)	Q30 (%)	GC content (%)
JO1	51,743,352	48,749,338	7.3 Gb	39,541,700 (81.11)	0.02	95.87	89.94	46.56
JO2	52,352,342	50,173,912	7.5 Gb	40,528,680 (80.78)	0.02	96.05	90.50	47.11
JO3	49,695,566	46,330,550	6.9 Gb	37,638,188 (81.24)	0.02	96.32	90.79	46.46
JO4	47,515,470	45,539,184	6.8 Gb	37,123,730 (81.52)	0.02	95.89	90.08	47.58
JT1	43,092,642	49,018,366	7.4 Gb	37,674,564 (76.86)	0.02	95.18	88.54	47.85
JT2	45,252,560	48,342,728	7.3 Gb	37,553,264 (77.68)	0.02	95.86	89.99	47.43
JT3	47,702,228	42,090,162	6.3 Gb	32,983,988 (78.37)	0.02	96.12	90.52	47.53
JT4	51,291,726	39,872,726	6.0 Gb	31,520,992 (79.05)	0.02	95.85	89.79	47.61

**Table 2:** Annotation of RNA-seq results.

Database	Number of unigenes (%)
Total unigenes	54,353 (100)
Annotated in nr	46,052 (84.7)
Annotated in nt	46,413 (85.4)
Annotated in KO	14,915 (27.4)
Annotated in Swiss-Prot	42,698 (78.6)
Annotated in Pfam	39,268 (72.2)
Annotated in GO	39,290 (72.3)
Annotated in KOG	27,647 (50.9)
Annotated in all databases	5,300 (9.8)
Annotated in at least one database	50,517 (92.9)

NCBI non-redundant protein database (nr), NCBI nucleotide sequences database (nt), KEGG ortholog (KO), a protein sequence database (Swiss-Prot), Protein Family (Pfam), Gene Ontology (GO), and Eukaryotic Ortholog Groups (KOG) databases were used for alignment of all the assembled unigenes.

### GO classification

**Figure 2:**
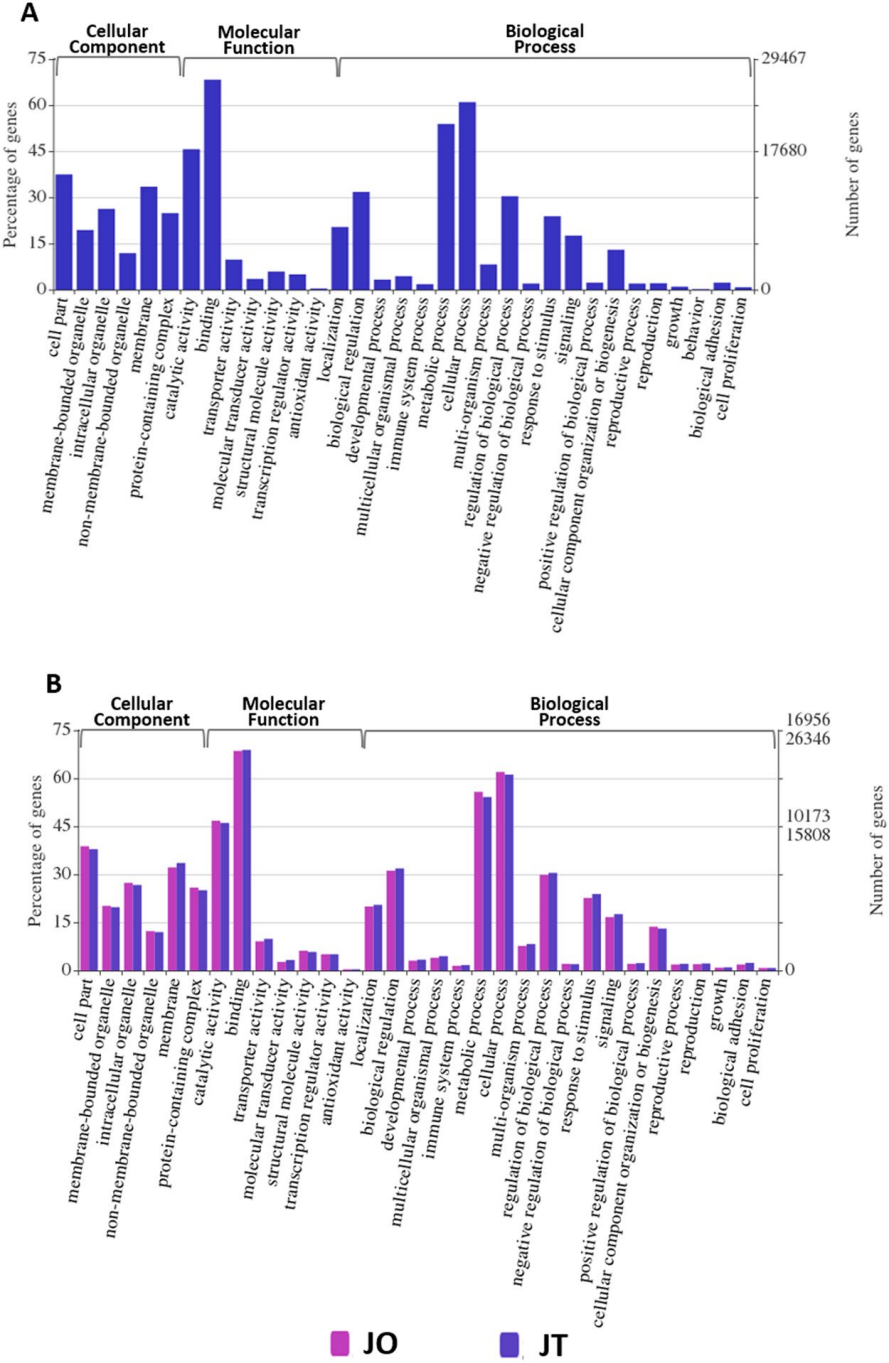
Histogram of GO classification (assigned by WEGO). (**A**) All genes that were expressed in ovary and testis. (**B**) Comparative view of GO annotations that were expressed in ovary (pink) and testis (blue). The results are presented in three main categories: biological process, cellular component, and molecular function. The left y-axis indicates the percentage of a specific category of genes in the main category. The right y-axis indicates the number of genes in each category.

**Figure 3:**
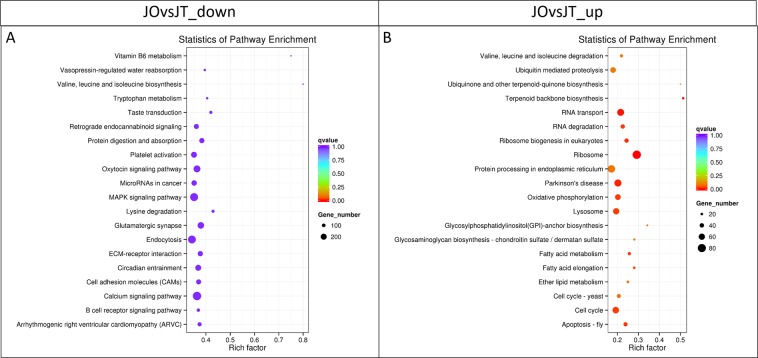
Scatter plot of the top 20 enriched KEGG pathways of DEGs. DEGs in JOvsJT_down (**A**) refer to male-biased expressed genes, and those in JOvsJT_up (**B**) refer to female-biased expressed genes. The x-axis represents the rich factor, which refers to the ratio of DEG numbers annotated in the pathway term to all gene numbers annotated in the pathway term. The greater the rich factor, the greater the degree of enrichment. The y-axis shows each KEGG pathway name. Each round point represents a specific KEGG pathway. The circle size indicates the number of DEGs that are associated with each significant pathway. The circle color indicates the significance level as a q-value. A q-value < 0.05 was considered significantly enriched. Light purple shows least significant, and orange represents most significant.

To interprete global gene function, GO classification revealed 39,290 unigenes annotated into 76 functional groups of three ontologies (Fig. 2A). GO enrichment of unigenes that were expressed in either ovary or testis were provided in Table S1. In addition, Tables S2 and S3 displayed GO enrichment of unigenes that were found in ovary and testis, respectively. Our results revealed that the genes most expressed during sex differentiation were those involved in biological processes (cellular process, metabolic process and regulation of biological process, etc.). Enriched GO terms identified in ovary and testis demonstrated similar patterns (Fig. 2B). The highest enriched GO terms were matched to “binding,” “cellular process,” and “metabolic process.” Fig. 3 presents a scatterplot of the top 20 enriched KEGG pathways of DEGs. KEGG analysis of over-expressed genes in testis (JOvsJT_down) showed a number of genes in various pathways such as amino acid metabolism, endocytosis, cell signaling and hormone metabolism (Fig. 3A, Table S4). KEGG analysis of over-expressed genes in ovary (JOvsJT_up) displayed a number of genes in various pathways, including amino metabolism and synthesis, RNA transport and degradation, ribosome and its biogenesis, fatty acid metabolism and elongation, glycomolecule synthesis, cell cycle and apoptosis (Fig. 3B, Table S5).

### DEG and real-time RT-PCR analysis

**Figure 4:**
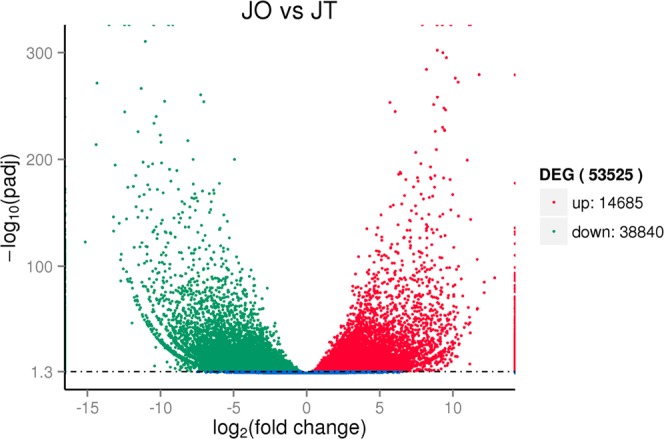
Volcano plot displaying DEGs identified between ovary (JO) and testis (JT). Significantly upregulated ((female-biased genes) and downregulated (male-biased genes) expressed genes (padj <0.05) are denoted as red and green dots, respectively.

**Figure 5:**
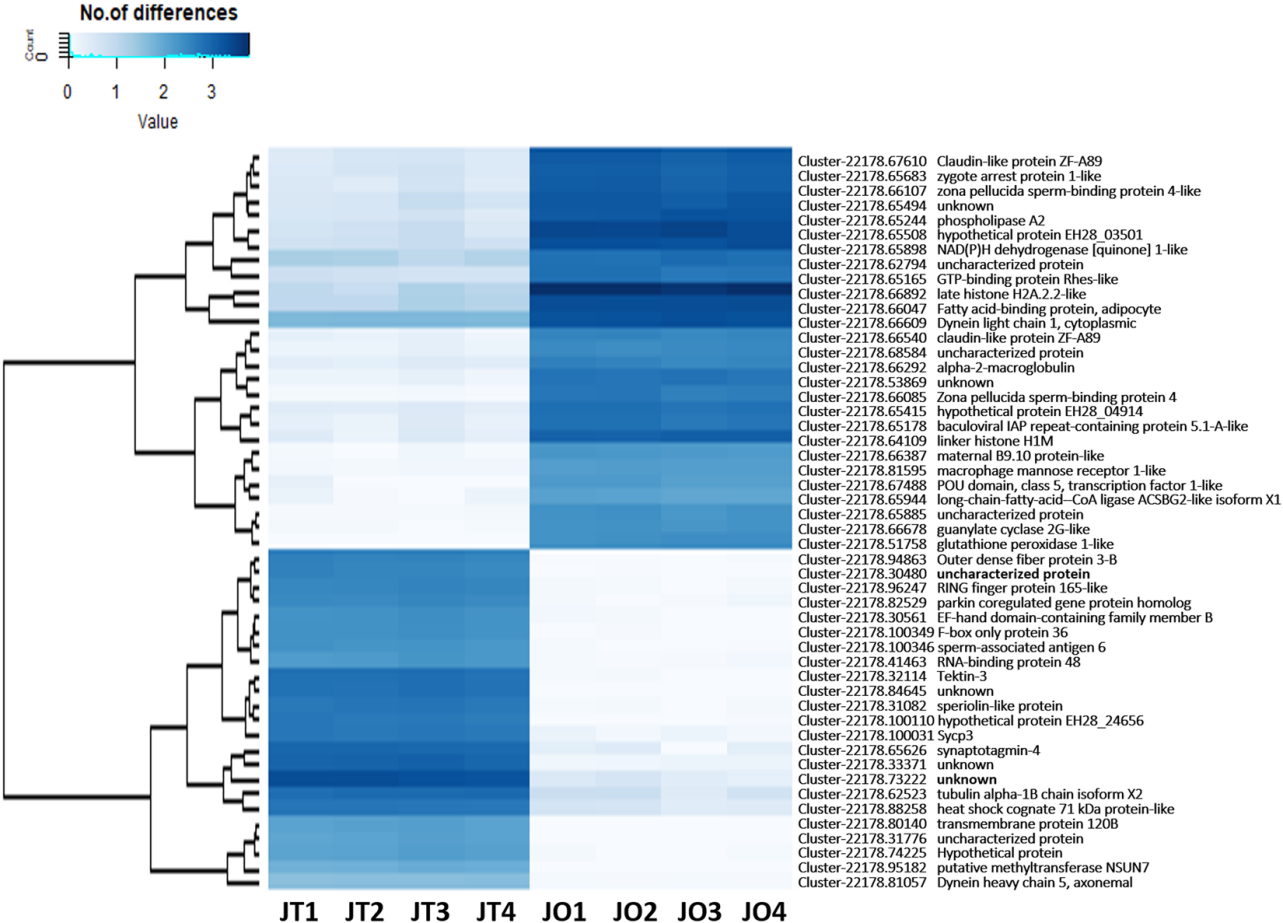
Heatmap plots for differentially expressed transcripts between male and female snakeskin gourami. The intensity of the blue color in the matrix indicates the log_2_ fold change. Dark blue, high log_2_ fold change; light blue, low log_2_ fold change. Only the top 50 DEGs were used to construct the heatmap.

**Figure 6:**
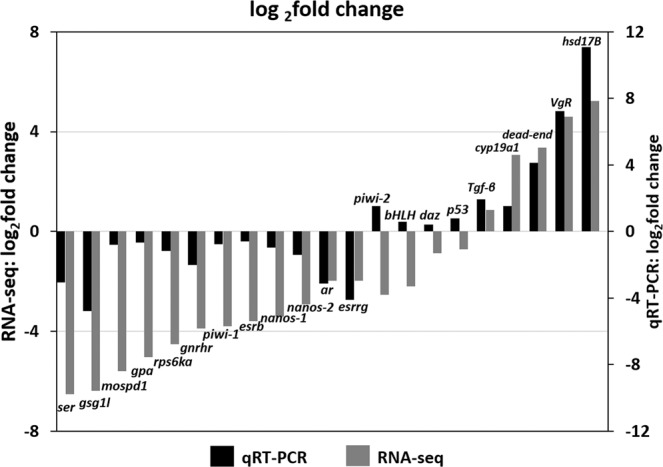
Validation of DEGs (JO vs JT) generated from RNA-seq by qRT-PCR (n = 4). By qRT-PCR, the expression levels of the 21 selected genes were each normalized to that of elongation factor 1 alpha (*ef–1α*). The functions of the selected genes were related to gonad development and included androgen receptor (*ar*), basic helix-loop-helix domain-containing protein (*bHLH*), cytochrome P450 aromatase (*cyp19a1*), deleted in azoospermia-like protein (*daz*), dead end (*dead-end*), estrogen receptor beta (*esrb*), estrogen-related receptor gamma (*esrrg*), gonadotropin-releasing hormone II receptor (*gnrhr*), gonadotropin common alpha subunit (*gpa*), germ cell-specific gene 1-like protein (*gsg1l*), estradiol 17-beta-dehydrogenase 12 (*hsd17B*), motile sperm domain-containing protein 1 (*mospd1*), nanos homolog 1 (*nanos–1*), nanos homolog 2 (*nanos-2*), cell death-inducing p53-target protein 1 (*p53*), piwi-like protein 1 (*piwi-1*), piwi-like protein 2 (*piwi-2*), ras-related and estrogen-regulated growth inhibitor-like protein (*rerg*), ribosomal protein S6 kinase alpha-3 (*rps6ka*), transforming growth factor-beta receptor-associated protein 1 (*tgf-beta*), and vitellogenin receptor isoform 2 (*VgR*).

The volcano plotting of differential expression analysis of the assembled transcripts between ovary and testis was depicted (Fig. 4). Our study focused on the DEGs that had a log_2_ fold change>1 and q-value <0.01. The results showed that 11,625 unigenes were overexpressed in ovary when compared with that in testis (Table S6), and 16,120 unigenes were overexpressed in testis when compared with that in ovary (Table S7). The top 50 log_2_ fold changes of DEGs were chosen to construct a hierarchical clustering, and two clusters of DEGs were delineated (Fig. 5). Table 3 presents the top 10 female-biased and male-biased genes, respectively. Additionally, the 21 expression genes that had been reported as involved in reproductive processes were validated using real-time RT-PCR analysis (Fig. 6). The log_2_ fold change (JO vs JT) results obtained from qRT-PCR demonstrated a significant positive correlation (0.84; *P* < 0.001) with that of the RNA-seq results. Furthermore, the coefficient of determination of a linear regression analysis (*y* = 0.796*x* − 2.066) of expression levels between RNA-seq (*x*) and real-time RT-PCR (*y*) was 0.706 (*P* < 0.001).

**Table 3:** Top 10 DEGs between female and male of juvenile snakeskin gourami.

Gene name	Gene description	Gene ID	Sex demonstrating upregulation	Log_2_ fold change
*rdh7*	retinol dehydrogenase 7	Cluster-22178.61468	F	12.86
*dnajc25*	DnaJ homolog, subfamily C, member 25	Cluster-22178.55749	F	12.14
*ap1s3*	AP-1 complex subunit sigma-3-like isoform X2	Cluster-22178.79126	F	12.14
*zp4*	Zona pellucida sperm-binding protein 4	Cluster-22178.66085	F	11.80
*polb*	DNA polymerase beta	Cluster-22178.45476	F	11.66
*parp12*	Poly (ADP-ribose) polymerase 12 isoform X2	Cluster-22178.72728	F	11.66
*trim39*	E3 ubiquitin-protein ligase TRIM39-like	Cluster-22178.64478	F	11.21
*gucy2g*	Guanylate cyclase 2G-like	Cluster-22178.66678	F	11.20
*rtbs*	RNA-directed DNA polymerase from transposon BS	Cluster-22178.58026	F	11.19
*fdxr*	NADPH:adrenodoxin oxidoreductase, mitochondrial-like isoform X1	Cluster-22178.65491	F	11.06
*vamp3*	Vesicle-associated membrane protein 3	Cluster-22178.99880	M	15.14
*nbl1*	Neuroblastoma suppressor of tumorigenicity 1	Cluster-22178.31453	M	13.21
*dnah2*	Dynein heavy chain 2, axonemal	Cluster-22178.100689	M	13.21
*ccdc11*	Coiled-coil domain-containing protein 11	Cluster-22178.100133	M	13.10
*nr2e3*	Photoreceptor-specific nuclear receptor-like	Cluster-22178.30265	M	12.72
*spats1*	Spermatogenesis-associated serine-rich protein 1 isoform X2	Cluster-22178.28827	M	12.68
*pih1d2*	PIH1 domain-containing protein 2	Cluster-22178.89036	M	12.54
*tekt3*	Tektin-3	Cluster-22178.32114	M	12.47
*fbxo36*	F-box only protein 36	Cluster-22178.100349	M	12.45
*mybl2*	Myb-related protein B-like	Cluster-22178.100072	M	12.33

F, female; M, male.

## Discussion

In our study, transcriptomic analysis between testis and ovary demonstrated global differential expression of transcripts during sex differentiation in juvenile snakeskin gourami. Our results showed that 9.8% (5,300) of the assembled unigenes had a significant annotation to all databases, which was considerably high when compared with previous reports of ovarian transcriptomic analysis of ricefield eel (*Monopterus albus*), showing only 3.36% of assembled matching to all databases^12^. Therefore, our transcriptome sequencing and annotating were considered acceptable for further analysis. The expressed transcripts were involved in various functional roles, indicating that a number of biological and physiological functions are involved in testis and ovary development. Similarly, high-throughput transcriptomic analysis has been applied to investigate global DEGs between sexes in gonads, brain, liver, muscle, and caudal fin in several teleosts, including gonochoristic and hermaphroditic fish^9–18^. These high-throughput transcriptome analyses could provide a holistic approach that will be a useful tool for further investigation of various aspects of reproductive biology.

The snakeskin gourami strongly exhibits sexual dimorphism in growth and external morphology. Our study focused on genes that were differentially expressed between sexes in juvenile fish. The histological study of ovary and testis revealed that they contained all the developmental stages of differentiated ovarian and testicular cells, respectively, demonstrating the sex differentiation phase of gonad development. By means of transcriptome analysis, global functional profiling of gene expression during sex differentiation in males and females revealed that a number of genes involved in biological processes, cellular components, and molecular functions. Our results implyed that these genes might be associated with biological and physiological processes during gonad differentiation. Similar findings were demonstrated in bluehead wrasses (*Thalassoma bifasciatum*)^9^, southern bluefin tuna (*Thunnus maccoyii*)^10^, red porgy (*Pagrus pagrus*)^13^, common pandora (*Pagellus erythrinus*)^13^, yellow perch (*Perca flavescens*)^15^, and ricefield eel (*Monopterus albus*)^12^. Our results identified similar enriched GO terms between ovary and testis, with the highest enriched GO terms being for binding, cellular processes, and metabolic processes. In contrast, enriched GO terms for metabolic processes were observed in ovary and for signal transduction and receptor activity in testis of bluehead wrasses^9^.

Our results demonstrated that the divergence of transcriptomic profiles was observed in both the number of expressed genes and their expression levels. Since the early stages of ovary development comprise vitellogenic oocytes that contain a high amount of maternal 5 S rRNA^19^, the high proportion of 5 S rRNA might be the reason for the relatively low number of expressed genes in ovary. Low RIN values have been reported in several transcriptomic analyses of ovary^15^. Therefore, during the preparation of RNA samples in our study, although low RIN values of were found in some ovary samples, all RIN values> 5.0 were used for further library preparation to ensure a similar amount of analyzed RNA between the testis and ovary samples. Our study found a higher number of transcripts detected in testis compared with ovary. Additionally, the number of male-biased overexpressed transcripts was higher than that of female-biased transcripts. These findings were similar to those of transcriptomic analysis of gonads of southern bluefin tuna^10^ and bluehead wrasses^9^.

DEGs that were overexpressed in ovary compared with testis were considered female-biased. From the 11,625 unigenes that were overexpressed in ovary, our study focused on the top 10 unigenes that were upregulated when compared with testis. These DEGs were identified as involved in several physiological processes in the reproductive system. For example, retinol dehydrogenase 7 (*rdh7*), also is known as cis-retinol/androgen dehydrogenase type 2 (*crad2*), was demonstrated as involved in the regulation of retinol and androgen metabolism in mice (*Mus musculus*)^20^. DnaJ homolog, subfamily C, member 25 (*dnajc25*) encodes a protein belong to heat shock protein (HSP) 40 subfamily C that functions as co-chaperone by binding to HSP 70 to stimulate adenosine triphosphate hydrolysis. In the Pacific white shrimp (*Litopenaeus vannamei*), high levels of HSP 40 transcripts were detected in ovary, and it was suggested to play important role in ovarian development^21,22^. Zona pellucida sperm-binding protein (*zp*) is one of the major proteins of egg envelopment and is essential for sperm penetration and fusion of the plasma membrane to prevent polyspermy^23^. The expression of DNA polymerase beta (*polb*), which performs base excision repair of DNA, was demonstrated in ovarian tissue, and its overexpression increased the potential for mutagenesis^24^. Poly (ADP-ribose) polymerases (*parp12*) and E3 ubiquitin-protein ligase TRIM39 (*trim39*) were reported as important proteins of DNA damage repair^25^. Additionally, inhibition of the expression of *polb*, *parp12*, and *trim39* has been investigated for the potential to manage ovarian cancer in mammals^25–27^. Guanylate cyclases (*gucy*) catalyze the conversion of guanosine triphosphate to pyrophosphate and cyclic guanosine monophosphate, which is a second messenger and involved in a number of physiological processes including oocyte maturation^28^. Transposons in germlines were recently demonstrated as a factor driving genome evolution^29^. Our study identified high expression levels of RNA-directed DNA polymerase from transposon BS (*rtbs*), which is involved in retrotransposon processes, in ovary. Adrenodoxin oxidoreductase type I (*fdxr*), which obtains electrons from nicotinamide-adenine dinucleotide phosphate (NADPH) via mediation by ferredoxin, was demonstrated to localize on mitochondria and was expressed in steroidogenic tissues including ovary^30^. Our study provided some indication of the top 10 female-biased genes expressed, which included several related to the reproductive development of the ovary in the juvenile stage. For a holistic view of ovary development in fish, a number DEGs that were upregulated in ovary still require elucidation.

The top DEGs that were upregulated in testis when compared with ovary were identified, and they were involved in several biological mechanisms related to testis development. This included vesicle-associated membrane protein 3 (*vamp3*), a component of ternary trans-soluble N-ethylmaleimide-sensitive factor attachment protein receptor complexes of membrane structure and whole capacitated sperm^31^. Using transcriptome analysis, upregulation of the expression of neuroblastoma suppressor of tumorigenicity 1 (*nbl1*) was detected when zebrafish (*Danio rerio*) were exposed to 17α-ethinylestradiol, and its fertility decreased^32^. Additionally, *nbl1* was proposed to be a candidate tumor marker for prostate cancer^33^. The axonemal dynein family is one of the cytoskeletal motor protein families, and its heavy chain (*dnah2*) is attributed to motor activity^34^. The coiled-coil domain-containing (*ccdc*) protein family was demonstrated as essential for sperm development and male fertility in mammals^35,36^. The PIH1 domain-containing proteins (*pih1*) have been described in the preassembly of axonemal dyneins and are required for cilia and flagella motion^37^. Additionally, tektin–3 (*tekt3*) is an axonemal structural protein essential to sperm motility^38^. It was described in mammals that disruption of the expression of *dnah*, *ccdc*, or *tekt3* led to asthenozoospermia^36,38–41^. Furthermore, in zebrafish, genome editing to generate mutants of genes encoding PIH proteins resulted in abnormal sperm motility^37^. The photoreceptor-specific nuclear receptor (*nr2e3*) is an orphan nuclear receptor essential for the development and function of photoreceptor cells^42^. The function of *nr2e3* was related to testis development^43^. The expression of spermatogenesis-associated serine-rich protein 1 (*spats1*) was demonstrated as testis-specific and expressed during spermatogenesis, and a particularly high expression level was observed during meiosis of the first spermatogenic wave, mainly in pachytene spermatocytes^44,45^. F-box proteins contain an F-box domain that mediates protein-protein interaction^46^. Although information regarding the function of the F-box only protein 36 (*fbxo36*) relating to the reproductive system is limited, a high level of *fbxo36* transcripts was observed in our study. Similarly, by means of proteomic analysis, upregulation of the FBXO36 protein was detectable during sperm maturation and capacitation in the bivalve *Pecten maximus*^47^. Myb-related protein B (*mybl2*) was reported to activate the transcription of mitotic genes^48^. Taken together, these testis-upregulated genes appear to be involved in spermatogenesis and sperm motility function. Further investigation of specific roles of these and the other testis-upregulated genes are necessary to determine the entire physiological process of testis development.

qRT-PCR is a highly sensitive and specific technique used to quantitatively analyze gene expression. qRT-PCR should rather be complementary to than replaced by RNA-seq.^49^ and used to validate and confirm DEGs. Our study validated DEGs between testis and ovary using qRT-PCR for 21 genes that had previously been demonstrated as involved in gonadal development in teleosts and were specifically expressed in germ cells in various animals^50–58^. Our findings showed a significantly positive correlation of DEG analysis results between RNA-seq and qRT-PCR, particularly for DEGs that had a high log_2_ fold change. Similarly, several investigations of transcriptomic analysis have described a positive correlation between RNA-seq and qRT-PCR data^11,59,60^. With both methods of gene expression analysis, the differential expression of several genes that play an important role in regulating reproductive functions were similar to previous transcriptomic analysis findings. For example, *hsd17B* and *cyp19a1* are involved in regulating the level of biologically active estrogens and androgens and in steroid hormone biosynthesis^61^. A similar overexpression of *hsd17B* and *cyp19a1* were observed in ovarian cells of southern bluefin tuna, bluehead wrasses, common pandora, and red porgy^9,10,13^. Additionally, the expression of *gnrhr* and *gpa*, which are related to endocrine regulation-related genes, were overexpressed in testis. Also, the testicular overexpression of *nanos–1* and *nanos–2*, which are classified as germ cell-specific genes, has been previously detected^10^. Our study results identified a high expression level of *piwi–1* in testis, which was similar to the findings demonstrated in bluehead wrasses^9^. Our study also revealed the high expression of *esrb* and *esrrg* in testis, correlating with those reported in testis of bluehead wrasses (*esr1*, *esr2a*, and *esr2b*), common pandora (*esrr1a*), and red porgy (*esr1* and *esrr1a*)^9,13^. Nevertheless, no significant differences in the expression levels of *esr1* and *esrr2b* were observed in common pandora and red porgy, respectively^13^. Moreover, several estrogen receptors were demonstrated to be female-biased genes in common pandora (*esrrb* and *esrr2b*) and red porgy (*esrrb*)^13^. Furthermore, contradictory results of sex-biased gene expression were observed in *dead-end* and *ar*. For instance, while overexpressed *dead-end* was observed in ovary in our study, no significant expression differences were detected for *dead-end* in southern bluefin tuna^10^. Additionally, our results showed that *ar* was overexpressed in testis. However, it exhibited no sex-biased expression in bluehead wrasses, and androgen receptor alpha and beta (*ara* and *arb*) were demonstrated as female-biased genes in common pandora and red porgy^9,13^.

## Conclusions

In conclusion, our study compared transcriptomic analysis between testis and ovary, demonstrating sex-biased gene expression in juvenile snakeskin gourami. Most expressed genes were annotated and categorized as involved biological processes. A number of DEGs in ovary and testis were demonstrated, and their expression was proposed as female- or male-biased, with their functions suggested to be related to ovary and testis differentiation, respectively. A positive correlation of DEGs obtained from RNA-seq and qRT-PCR were observed. Our findings demonstrated sex-associated gene expression database during sex differentiation in juvenile snakeskin gourami. This bioinformatics would offer various application for reproductive biotechnology such as sex-biased RNA-based molecular markers.

## Methods

### Ethics statement

The study protocols of fish care and experiments were reviewed and approved by the KASETSART UNIVERSITY Institutional Animal Care and Use Committee (ACKU 61-FIS-004) and conducted in accordance with relevant guidelines.

### Fish sampling and experimental design

 The snakeskin gourami (*T. pectoralis*) used in our study were obtained from four commercial farms in central and north-east Thailand. Fifty male (body weight, 19.22 ± 0.23 g; gonad weight, 0.01 ± 0.001 g; gonadosomatic index [GSI], 0.05 ± 0.003%) and 25 female (body weight, 18.68 ± 0.39 g; gonad weight, 0.14 ± 0.008 g; GSI, 0.73 ± 0.03%) snakeskin gourami were collected for gonad sampling from each farm. The fish were euthanatized using 150 mg/L MS222 before dissection. The sampled gonads were divided into two portions. One portion was stored at −80 °C, and the other portion was used for histological study to confirm the sex microscopically. For each replication (pond), pools of ten testis or five ovaries were collected in a sterile tube, and five tubes of each pond were kept at −80 °C for RNA extraction.

To perform transcriptomic analysis for comparison of differentially expressed genes (DEGs) between testis and ovary, the testis and ovary samples, each containing four replications, were used. Each replication of testicular RNA was obtained from pooled equal amounts of RNA from five tubes each containing 10 testes. Therefore, each replication of testicular RNA was obtained from 50 fish. Each replication of ovarian RNA was obtained from pooled equal amounts of RNA from five tubes each containing five ovaries. Therefore, each replication of ovarian RNA was obtained from 25 fish.

### Histological study of gonad samples

The presence of testes or ovaries in the juvenile fish were confirmed histologically. Briefly, a portion of either ovary or testis tissue was fixed in Bouin’s solution for 16 h at 4 °C. Then, the solution was replaced with 80% ethanol, and the tissues were stored at 4 °C until use. The fixed gonad tissues were dehydrated using a standard xylene-ethanol series and embedded in a paraffin block using a standard method. The paraffin tissue block was cut into 5-μm transverse sections. The sections were then dewaxed, rehydrated, and stained with hematoxylin and eosin.

### RNA extraction and sequencing

Total RNA was extracted from the sampled tissues (approximately 100 mg) using TRIzol reagent (Invitrogen, Carlsbad, CA, USA) and digested with RNase-free DNase I (Promega, Madison, WI, USA) according to the manufacturer’s instructions. The quantity of the isolated RNA was determined using a NanoDrop® ND-1000 UV-Vis Spectrophotomoter (Thermo Fisher Scientific, Waltham, MA, USA). Agarose gel electrophoresis was also performed to assess RNA degradation. Additionally, the quality and integrity of the isolated RNA was analyzed using an Agilent Technologies 2100 Bioanalyzer (Agilent Technologies, Santa Clara, CA, USA). The RNA obtained from the testes samples that had an RNA integrity number (RIN) value>7.0 were used for further analysis. Since the RNA isolated from ovary samples, particularly during the juvenile stage, contained a high amount of low molecular weight 5 S RNA, the RNA obtained from ovary samples with a RIN value>4.5 were used for RNA processing. Four replicates of testicular and ovarian RNA samples (2 µg) were used to perform mRNA paired-end library construction with a TruSeq RNA Sample Preparation Kit v2 (Illumina, San Diego, CA, USA) according to the manufacturer’s protocol. mRNA enrichment was carried out via poly-A mRNA isolation with oligo-dT beads. Subsequently, mRNA fragmentation was performed. First-strand cDNA synthesis was then carried out on all samples using random hexamer primers and reverse transcriptase. To generate the second strand by nick-translation, a custom second-strand synthesis buffer (Illumina) was added with dNTPs, RNase H, and *Escherichia coli* polymerase I. Agencourt AMPure XP beads were used to purify the cDNA (Beckman Coulter, Brea, CA, USA). Terminal repair, A-tailing, ligation of sequencing adapters, size selection, and PCR enrichment were applied to produce the cDNA libraries. Four librarites each were prepared for ovaries (JO1, JO2, JO3, and JO4) or testis (JT1, JT2, JT3, and JT4). Subsequently, the cDNA libraries were quantified using a Qubit 2.0 fluorometer (Life Technologies, Carlsbad, CA, USA) and then diluted to 1 ng/µL. The insert size (150–200 bp) was checked using an Agilent 2100 Bioanalyzer, and the concentration was determined by quantitative PCR (library activity>2 nM). Cluster generation was performed on a cBot cluster Generation System using a TruSeq PE Cluster Kit v3-cBot-HS (both Illumina). All libraries were loaded onto a HiSeq. 2500 Sequencing System (Illumina) as per the manufacturer’s protocol at Novogene Bioinformatics Institute, Beijing, China.

### Preprocessing, *de novo* assembly, and read preprocessing

**Figure 7:**
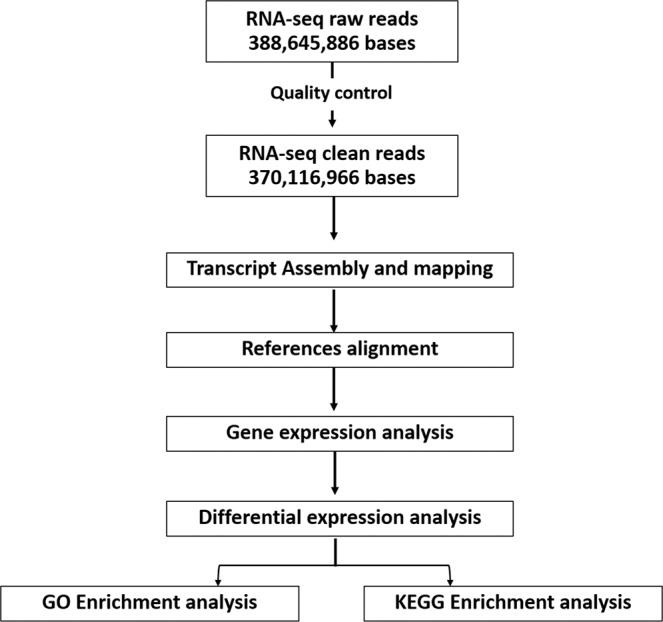
Workflow of RNA sequencing, data processing, and analysis.

Figure 7 displayed the workflow of RNA-seq and differential expression analysis. The original raw data from sequencing were converted to sequenced reads by base calling. In total, 388.65 million bases (Gb) of raw reads were generated by the Illumina HiSeq platform. (Table 1). The raw sequence files generated from 8 files (fastq) has been deposited to NCBI’s Sequence Read Archive (SRA) database with the accession number PRJNA597181 (https://www.ncbi.nlm.nih.gov/sra/PRJNA597181). Then, to obtain clean reads, the raw reads were filtered to remove those with adapter sequences, containing poly-N, or of low quality (low quality nucleotides constitute more than 50% of the read). The base calling and quality assignment were evaluated using the Phred score, calculated as Q_phred_ = −10 log_10_(*e*) (Table 1). Eight sets of clean reads were assembled *de novo* using Trinity 2.0.6 software with the default parameters (k-mer, 25; minimum length, 200 nucleotides)^62^, and then Corset software was used to perform hierarchical clustering to remove redundancy^63^. Finally, the longest transcript of each cluster was selected as the unigene.

### Transcriptome functional annotation

For transcriptome functional annotation, seven databases were searched. A similarity search of nr (National Center for Biotechnology Information [NCBI] non-redundant protein sequences), nt (NCBI BLAST 2.2.28 + ), and Swiss-Prot databases was performed with the E-value threshold at 10^−5^ to obtain the top 10 alignment results for annotation of the assembled transcripts. The unigenes were also searched against the Eukaryotic Orthologous Groups (KOG) database, with the significant E-value threshold set at 10^−3^. Additionally, the unigene annotations were searched against the Protein Family (Pfam) database using the HMMER 3.0 software package with the E-value threshold at 0.01. For gene ontology (GO) mapping, based on the protein annotation results of the BLAST search and Pfam, Blast2GO 2.5^64^ was used to assign the GO annotation associated with the hits obtained by the BLAST search and Pfam, with a significant E-value threshold of 10^−6^ for describing biological processes, molecular functions, and cellular components. The sequences were also annotated and characterized by Kyoto Encyclopedia of Genes and Genomes (KEGG), with a significant E-value threshold of 10^−10^. The species distribution of the top BLASTX results matched to the nt database was also analyzed.

### Analysis of gene expression levels and differential expression

The *de novo* transcriptome assembly served as a reference for read mapping. The clean reads were mapped back onto the assembled transcriptome with Bowtie 2 software, and the read count of each gene from each sample was estimated. The gene expression levels of each sample were quantified using RSEM software^65^. The read count for each gene of each sample was normalized to fragments per kilobase of transcript sequence per million base pairs sequenced (FPKM)^66^. Contigs with a low expression (FPKM < 0.3) were excluded. The read count and FPKM for each gene of each sample were demonstrated in Tables S8 and S9. A Venn diagram was constructed to present the number of unigenes that were uniquely expressed and co-expressed in ovary and testis. Differential expression analysis was estimated at unigene level by pairwise comparisons between males and females using the R package DEGseq.^67^. The false discovery rate (FDR) *P*-value was adjusted using the q-value. A q-value <0.01 and log_2_ fold change>1 were set as the thresholds for significant DEGs. GO enrichment analysis of the DEGs was carried out for biological processes, cellular components, and molecular function by WEGO 2.0 software (http://wego.genomics.org.cn/). Enrichment analysis of DEGs in KEGG pathways was carried out for KEGG enrichment scattered plots to determine the top 20 significant DEG enriched pathways. A volcano plot for inferring the overall distribution of DEGs was constructed using a padj (FDR) threshold of <0.05. Heatmaps of DEGs were constructed using the R package gplots to display the top 50 significant DEGs.

### qRT-PCR analysis

qRT-PCR analysis was used to quantitatively validate 21 genes that had been demonstrated as involved in the reproductive system, including androgen receptor (*ar*), basic helix-loop-helix domain-containing protein (*bHLH*), cytochrome P450 aromatase (*cyp19a1*), deleted in azoospermia-like protein (*daz*), dead end (*dead-end*), estrogen receptor beta (*esrb*), estrogen-related receptor gamma (*esrrg*), gonadotropin-releasing hormone II receptor (*gnrhr*), gonadotropin common alpha subunit (*gpa*), germ cell-specific gene 1-like protein (*gsg1l*), estradiol 17-beta-dehydrogenase 12 (*hsd17B*), motile sperm domain-containing protein 1 (*mospd*), nanos homolog 1 (*nanos-1*), nanos homolog 2 (*nanos-2*), cell death-inducing p53-target protein 1 (*p53*), piwi-like protein 1 (*piwi-1*), piwi-like protein 2 (*piwi-2*), ras-related and estrogen-regulated growth inhibitor-like protein (*rerg*), ribosomal protein S6 kinase alpha-3 (*rps6ka*), transforming growth factor-beta receptor-associated protein 1 (*tgf-beta*), and vitellogenin receptor isoform 2 (*VgR*). Breifly, using the same RNA samples (2 µg) as for the RNA-seq library preparation, the first-strand cDNA was synthesized using the ImProm-II^TM^ Reverse Transcription System kit (Promega). qRT-PCR amplification (in duplicate) was conducted using LightCycler^®^ 480 SYBR Green I Master (Roche Applied Science, Indianapolis, IN, USA). Elongation factor 1 alpha (*ef-1α*) was used as an internal reference for data normalization. Table S10 lists the primers and annealing temperatures used in our study and the expected size of the amplicons. PCR samples were prepared in a final volume of 15 µl consisting of 7.5 µl of LightCycler^®^ 480 SYBR Green I Master, 500 nM of each primer, and 1.5 µl of cDNA template. PCR was performed at 95 °C for 10 min followed by 50 reaction cycles consisting of 15 s at 95 °C, 15 s at the annealing temperature (Table S1), and 20 s at 72 °C. Upon completion of amplification, all samples were subjected to melting curve analysis to distinguish between the PCR products. Each PCR assay included triplicate PCR amplification and the negative controls (reverse-transcriptase- and cDNA-template-free samples). To analyze the mRNA levels, relative quantification of target gene expression was carried out using the Roche Applied Science E-method. The data were analyzed using the comparative cycle threshold method. PCR efficiency was measured by the slope of a standard curve constructed using serial dilutions of cDNA. In all cases, PCR efficiency values ranged between 1.8 and 2.2. The expression of *ef-1α* was used as an internal reference gene prior to calculating DEGs using log_2_ fold change. In addition, Pearson’s correlation coefficient was used to calculate the relationship of the results of RNA-seq and qRT-PCR. Moreover, regression analysis of the results of RNA-seq and qRT-PCR and goodness of fit (R^2^) were performed.

### Ethics approval

This study followed the Kasetsart University Institutional Animal Care and Use Committee. Fish care and experiments were approved by the Kasetsart University Institutional Animal Care and Use Committee (ACKU-61-FIS-004).

## Supplementary information

Table S1.

Table S2.

Table S3.

Table S4.

Table S5.

Table S6.

Table S7.

Table S8.

Table S9.

Table S10.

## Data Availability

All data from this work are public and included in this article in the main manuscript or as additional files. Additional datasets and information are available from the corresponding author under reasonable request.
