# Supplementary material for: Transcriptomic analysis of female and male gonads in juvenile snakeskin gourami (Trichopodus pectoralis)
Source: Sci Rep. 2020 Mar 23;10:5240. doi: 10.1038/s41598-020-61738-0 (PMC7090014; doi:10.1038/s41598-020-61738-0)
Supplement: Supplementary file 10 — Table S10. [file 41598_2020_61738_MOESM10_ESM.docx]

**Supplementary information**

**Transcriptomic analysis of female and male gonads in juvenile snakeskin gourami (*Trichopodus pectoralis*)**

Surintorn Boonanuntanasarn^1*^, Araya Jangprai^1^, Uthairat Na-Nakorn^2*^

^1^ School of Animal Technology and Innovation, Institute of Agricultural Technology, Suranaree University of Technology, 111 University Avenue, Muang, Nakhon Ratchasima, 30000, Thailand

^2^ Department of Aquaculture, Faculty of Fisheries, Kasetsart University, 50 Paholyothin Road, Chatujak, Bangkok 10900, Thailand

*Corresponding author:

S. Boonanuntanasarn; surinton@sut.ac.th, Tel: +6644224371, Fax: +6644224150

Uthairat Na-Nakorn; e-mail: [ffisurn@ku.ac.th](mailto:ffisurn@ku.ac.th)

Table S10 List of primers used for qRT-PCRs

| Genes | 5’/3′ Forward primer | 5′/3′ Reverse primer | Amplicon size (bp) |
| --- | --- | --- | --- |
| *ef* | GAAAGACAGCTGGTGACCGT | GGAGAGCAGCTTCATCCTCA | 249 |
| *ar* | GAAAGACAGCTGGTGACCGT | GGAGAGCAGCTTCATCCTCA | 249 |
| *bHLH* | AGCTGAGGAACCAACTACCA | CCGTTCCAGGGACTGATACT | 191 |
| *cyp19a1* | GCTGTCTGACGACTGCATAG | TTACCATGGCGATGTGCTTG | 206 |
| *daz* | TGACATCTTCGTGTTGTGGAC | GCTTGTACAGTGAGGTTGCT | 245 |
| *dead-end* | TGGCCAGCCACTCTATGAAA | GGCAAGCTGCTCGTCATAAA | 211 |
| *esrb* | AGAGGGAGGAGTACGTCTGT | TGGTCCATGCCCTTGTTACT | 250 |
| *esrrg* | CGGACATCTGTTTGAAACGC | GGTATCTCTGTACCGACGCA | 237 |
| *gnrhr* | CATCGTGCTGTCCTTTCTGG | GGTATCGGGAAGAGGCTTCA | 236 |
| *gpa* | CGTCTTCTCCAGGGATCGTC | AAATAGCAGGTGCTGCAGTG | 213 |
| *gsg1l* | GCTCCTCTCCTAAAGACGCT | ACTGTCATCGAGTTCAGGCA | 220 |
| *hsd17B* | AGGTGGAATGCAGGACCATT | ATCTGGCACACAGAGGTCAT | 208 |
| *mospd1* | GGCAGTGAGACAGCAGAGAG | AGAAGACCCAGTGTGTAGGC | 249 |
| *nanos-1* | ATCTTCTGAACGCACGAGAC | ACACACCCACACCACTACAA | 231 |
| *nanos-2* | CAGCAACGGGACTTCCAC | CAGATGACTTTCCCGTCGTT | 120 |
| *p53* | TGGTAAATGTTGGCCGTGTC | ACATATGGCCAGAGGTGTCA | 211 |
| *piwi-1* | GCAGCACAAAGGACAGGAAA | ACTGAGCTTGGGCATGTAGT | 217 |
| *piwi-2* | TAGCAGGTCACGTCAGTCAG | AGACACACCGTCACGATACA | 205 |
| *rerg* | AGGAGAACCGGTGTCACTTC | ACTGCACAAACCATCACACC | 207 |
| *rps6ka* | GACCGGGTCAGGACAAAGAT | ATCCCTAGGCTGTGAAGGTG | 236 |
| *tgf-beta* | CAGGATTCCACGAGGTCTGA | AGCACCAGGTGTTCCAAGTA | 242 |
| *VgR* | AACCTAAATGACCCGCAGGA | TGTGCTCACATTGGCTTCAG | 222 |


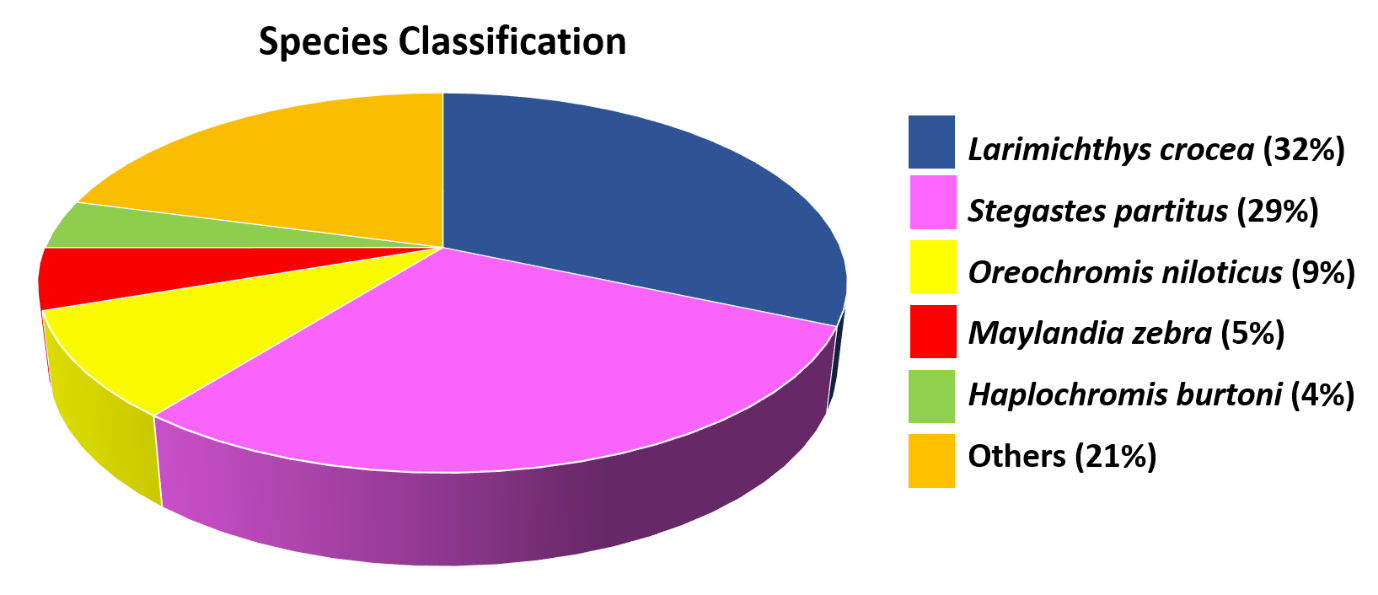


**Figure S1** Species distribution of the top BLAST hits obtained from the experimental transcripts.


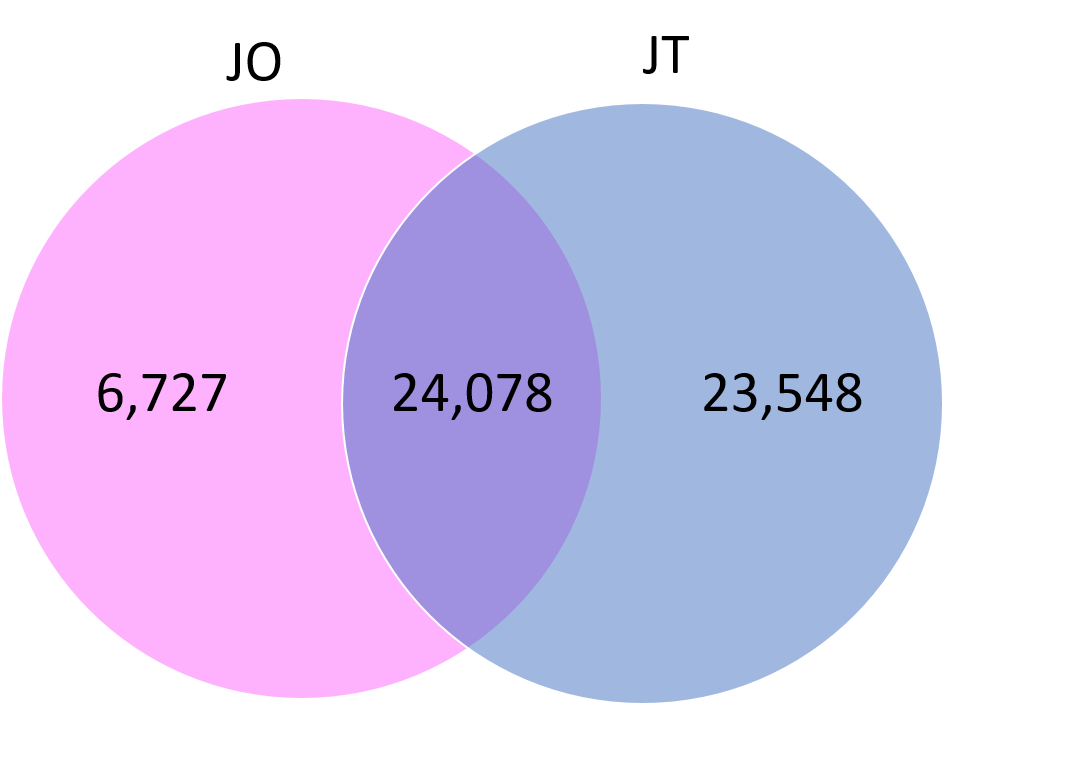


**Figure S2** Venn diagram of the number of genes differentially expressed between ovary and testis. Pink: genes observed in ovary. Light blue: genes observed in testis. Purple (intersection): genes co-expressed between ovary and testis.
