# Supplementary material for: Transcriptomic analysis of female and male gonads in juvenile snakeskin gourami (Trichopodus pectoralis)
Source: Sci Rep. 2020 Mar 23;10:5240. doi: 10.1038/s41598-020-61738-0 (PMC7090014; doi:10.1038/s41598-020-61738-0)
Supplement: Supplementary file 2 — Table S2. [file 41598_2020_61738_MOESM2_ESM.pdf]

Transcriptomic analysis of female and male gonads in juvenile snakeskin gourami (Trichopodus pectoralis)

Surintorn Boonanuntanasarn1*, Araya Jangprai1, Uthairat Na-Nakorn2*
1 School of Animal Technology and Innovation, Institute of Agricultural Technology, Suranaree University of Technology, 111 University Avenue, Muang, Nakhon Ratchasima, 30000, Thailand
2 Department of Aquaculture, Faculty of Fisheries, Kasetsart University, 50 Paholyothin Road, Chatujak, Bangkok 10900, Thailand

*Corresponding author:
S. Boonanuntanasarn; surinton@sut.ac.th, Tel: +6644224371, Fax: +6644224150
Uthairat Na-Nakorn; e-mail: ffisurn@ku.ac.th

Table S2 GO enrichment of unigenes that were expressed in ovary.

Cluster-21138.1 GO:0042254 GO:0006412 GO:0003735 GO:0005622 GO:0005840
Cluster-21823.0 GO:0006511 GO:0004175 GO:0019773
Cluster-22178.100024 GO:0006355 GO:0007155 GO:0005198 GO:0008270 GO:0046872 GO:0043565 GO:0003700 GO:0005667 GO:0031012
Cluster-22178.100025 GO:0006355 GO:0007155 GO:0005198 GO:0003700 GO:0008270 GO:0046872 GO:0043565 GO:0005667 GO:0031012
Cluster-22178.100030 GO:0003677
Cluster-22178.100033 GO:0015986 GO:0031175 GO:0015992 GO:0006826 GO:0005516 GO:0005506 GO:0030507 GO:0015078 GO:0008849 GO:0045263 GO:0005737 GO:0008091
Cluster-22178.100050 GO:0006952 GO:0004190 GO:0005576 GO:0016021
Cluster-22178.100060 GO:0046983
Cluster-22178.100096 GO:0007264 GO:0000287 GO:0003924 GO:0005525 GO:0030976
Cluster-22178.10012 GO:0045454 GO:0006118 GO:0016301 GO:0015035

GO:0009055
Cluster-22178.100127 GO:0007264 GO:0006886 GO:0005543 GO:0003924
GO:0005525
Cluster-22178.100197 GO:0006281 GO:0006139 GO:0006310 GO:0007264
GO:0006000 GO:0006206 GO:0006013 GO:0003723 GO:0005525
GO:0003873 GO:0098519 GO:0009378 GO:0003724 GO:0004127
GO:0016887 GO:0005524 GO:0009379 GO:0005657
Cluster-22178.100212 GO:0006520 GO:0042302 GO:0005856
Cluster-22178.10031 GO:0006468 GO:0004672 GO:0005524
Cluster-22178.100370 GO:0006366 GO:0006384 GO:0008017 GO:0003700
GO:0005515 GO:0045298 GO:0016021 GO:0005667 GO:0005634
Cluster-22178.10038 GO:0006955 GO:0043087 GO:0030153 GO:0005089
GO:0015643 GO:0019814 GO:0005737
Cluster-22178.100466 GO:0046872
Cluster-22178.10047 GO:0072519 GO:0006508 GO:0006118 GO:0016567
GO:0016491 GO:0008233 GO:0009055 GO:0004842 GO:0004222
GO:0031012 GO:0016020
Cluster-22178.100473 GO:0055114 GO:0005507 GO:0016491
Cluster-22178.100482 GO:0016567 GO:0008270 GO:0046872 GO:0005515
GO:0004842 GO:0005680
Cluster-22178.100525 GO:0019901
Cluster-22178.100528 GO:0009405 GO:0005576
Cluster-22178.100536 GO:0006468 GO:0007623
Cluster-22178.100582 GO:0006468 GO:0004672 GO:0005524 GO:0005515

Cluster-22178.100610 GO:0015886 GO:0017004 GO:0005515 GO:0016021
GO:0016020
Cluster-22178.100620 GO:0005515
Cluster-22178.100640 GO:0006811 GO:0055085 GO:0005216 GO:0016020
Cluster-22178.100679 GO:0015631 GO:0045298
Cluster-22178.100681 GO:0007156 GO:0005509 GO:0016020
Cluster-22178.100693 GO:0004867
Cluster-22178.100703 GO:0019497 GO:0006771 GO:0005515 GO:0003993

GO:0046872
Cluster-22178.100708 GO:0007264 GO:0005525 GO:0003924
Cluster-22178.100721 GO:0006355 GO:0005515 GO:0004222 GO:0003677
GO:0005634
Cluster-22178.100722 GO:0006355 GO:0006560 GO:0005985 GO:0006814
GO:0006090 GO:0006525 GO:0071436 GO:0005982 GO:0006012
GO:0004575 GO:0005515 GO:0008948 GO:0003677 GO:0004564
GO:0015081 GO:0004222 GO:0017177 GO:0005634 GO:0016020
Cluster-22178.100783 GO:0006508 GO:0004252 GO:0019031
Cluster-22178.100808 GO:0007165 GO:0008158 GO:0016020
Cluster-22178.100837 GO:0006479 GO:0006464 GO:0046500 GO:0008171
GO:0004719 GO:0008168
Cluster-22178.10086 GO:0046983
Cluster-22178.100867 GO:0006836 GO:0007304 GO:0006812 GO:0005328
GO:0005213 GO:0005576 GO:0042600 GO:0016021
Cluster-22178.100893 GO:0005524 GO:0005515 GO:0003824
Cluster-22178.10090 GO:0003723
Cluster-22178.100915 GO:0007264 GO:0006355 GO:0015684 GO:0005524
GO:0015093 GO:0016887 GO:0003924 GO:0005525 GO:0008134
GO:0005515 GO:0016021 GO:0005667
Cluster-22178.100964 GO:0006355 GO:0006914 GO:0042981 GO:0005515
GO:0008134 GO:0042803 GO:0045502 GO:0003700 GO:0043565
GO:0005667 GO:0030286
Cluster-22178.10098 GO:0042254 GO:0006412 GO:0003735 GO:0005622
GO:0005840
Cluster-22178.100991 GO:0006464
Cluster-22178.100994 GO:0006464 GO:0009247 GO:0016758
Cluster-22178.10101 GO:0008152 GO:0042254 GO:0006412 GO:0003735
GO:0016491 GO:0003723 GO:0005840
Cluster-22178.10102 GO:0006164 GO:0006531 GO:0042254 GO:0006412
GO:0006144 GO:0008152 GO:0006522 GO:0003723 GO:0050662
GO:0003735 GO:0016491 GO:0005525 GO:0003824 GO:0004019
GO:0005840

Cluster-22178.101061 GO:0006281 GO:0006310 GO:0008270

Cluster-22178.101091 GO:0030694

Cluster-22178.101116 GO:0006952 GO:0006310 GO:0046872 GO:0000150 GO:0003677 GO:0005515

Cluster-22178.101172 GO:0006570 GO:0006470 GO:0019988 GO:0043399 GO:0004725 GO:0008138

Cluster-22178.101217 GO:0006413 GO:0006813 GO:0006446 GO:0005516 GO:0031369 GO:0003743 GO:0015269 GO:0005852 GO:0005840 GO:0016021

Cluster-22178.101236 GO:0007049 GO:0006308 GO:0019901 GO:0008855 GO:0016021 GO:0009318 GO:0005815

Cluster-22178.101244 GO:0055114 GO:0008152 GO:0016491

Cluster-22178.10125 GO:0006810 GO:0016020

Cluster-22178.101307 GO:0006468 GO:0004672 GO:0005524 GO:0016020

Cluster-22178.101311 GO:0016021 GO:0005886

Cluster-22178.101325 GO:0006396 GO:0005515 GO:0000176

Cluster-22178.101373 GO:0007049 GO:0006308 GO:0019901 GO:0008855 GO:0009318 GO:0005815 GO:0016021

Cluster-22178.10142 GO:0009089 GO:0016310 GO:0004673 GO:0008270 GO:0008837 GO:0009365

Cluster-22178.101452 GO:0005515 GO:0008537

Cluster-22178.101456 GO:0050830 GO:0007165 GO:0006811 GO:0004864 GO:0046872 GO:0031083 GO:0016020

Cluster-22178.10146 GO:0008146 GO:0016021

Cluster-22178.101481 GO:0042546 GO:0005199 GO:0005515 GO:0009277 GO:0005618

Cluster-22178.101484 GO:0042546 GO:0005515 GO:0005199 GO:0005618 GO:0009277

Cluster-22178.101500 GO:0016301

Cluster-22178.101548 GO:0005515

Cluster-22178.101565 GO:0042981

Cluster-22178.101590 GO:0005515

Cluster-22178.10160 GO:0006506 GO:0010181 GO:0017176 GO:0016021

Cluster-22178.101663 GO:0055085 GO:0016021
Cluster-22178.101664 GO:0009890
Cluster-22178.101721 GO:0043066 GO:0003950 GO:0051015 GO:0016788

Cluster-22178.101724 GO:0007010 GO:0003779
Cluster-22178.101728 GO:0055114 GO:0016620
Cluster-22178.101731 GO:0009085 GO:0055114 GO:0009089 GO:0051287
GO:0008839 GO:0016620
Cluster-22178.101756 GO:0006812 GO:0008324 GO:0016021
Cluster-22178.101820 GO:0005524
Cluster-22178.101831 GO:0046677 GO:0016779
Cluster-22178.101855 GO:0006355 GO:0006351 GO:0005515
Cluster-22178.101912 GO:0005515
Cluster-22178.101978 GO:0005911 GO:0016020
Cluster-22178.101980 GO:0006355 GO:0003677
Cluster-22178.101984 GO:0048193 GO:0008053 GO:0000921 GO:0030168
GO:0006914 GO:0007165 GO:0007155 GO:0051258 GO:0009405
GO:0006397 GO:0005102 GO:0030674 GO:0046872 GO:0003924
GO:0005198 GO:0005577 GO:0005940 GO:0005741 GO:0016020
GO:0019031 GO:0000445 GO:0016021 GO:0019028
Cluster-22178.101996 GO:0030991
Cluster-22178.102002 GO:0007049 GO:0048309 GO:0006914 GO:0051028
GO:0005198 GO:0019031 GO:0019028
Cluster-22178.10201 GO:0005515
Cluster-22178.102065 GO:0006123 GO:0015992 GO:0016788 GO:0004129
GO:0045277 GO:0005743
Cluster-22178.102182 GO:0055114 GO:0042773 GO:0016651 GO:0016209
GO:0016491 GO:0008270 GO:0005622
Cluster-22178.102232 GO:0005516
Cluster-22178.10228 GO:0006879 GO:0008199
Cluster-22178.10229 GO:0005509
Cluster-22178.102302 GO:0015684 GO:0006468 GO:0007264 GO:0005525
GO:0004672 GO:0005524 GO:0015093 GO:0016887 GO:0003924

GO:0016021

Cluster-22178.102397 GO:0004222 GO:0016012

Cluster-22178.102483 GO:0006355 GO:0003677 GO:0016021

Cluster-22178.102494 GO:0007155 GO:0005515 GO:0016021

Cluster-22178.102561 GO:0006355 GO:0005634

Cluster-22178.10257 GO:0005515 GO:0016021

Cluster-22178.10259 GO:0005515

Cluster-22178.10262 GO:0005201 GO:0005581 GO:0005578

Cluster-22178.102678 GO:0055114 GO:0006606 GO:0005506 GO:0020037 GO:0016705 GO:0016021

Cluster-22178.102726 GO:0055114 GO:0008033 GO:0006351 GO:0055085 GO:0006260 GO:0006269 GO:0006810 GO:0007165 GO:0003896 GO:0005515 GO:0016491 GO:0008270 GO:0005524 GO:0042626 GO:0050660 GO:0005509 GO:0003677 GO:0016021 GO:0005578 GO:0005657 GO:0005730

Cluster-22178.102739 GO:0001558 GO:0015031 GO:0006464 GO:0007049 GO:0005515 GO:0005520 GO:0005576 GO:0016942 GO:0017053

Cluster-22178.102799 GO:0005515

Cluster-22178.102882 GO:0006508 GO:0039693 GO:0007165 GO:0019784 GO:0005509 GO:0005515 GO:0005578

Cluster-22178.102899 GO:0006886 GO:0043248

Cluster-22178.10291 GO:0005576

Cluster-22178.10292 GO:0055114 GO:0015979 GO:0015995 GO:0016491

Cluster-22178.102951 GO:0003677

Cluster-22178.103012 GO:0007155 GO:0003677 GO:0019028

Cluster-22178.103100 GO:0048870 GO:0006810 GO:0007165 GO:0008289 GO:0004871 GO:0008270 GO:0046872 GO:0005515 GO:0016021 GO:0031514 GO:0016020

Cluster-22178.10312 GO:0003723 GO:0019843 GO:0005622

Cluster-22178.103146 GO:0006351 GO:0006206 GO:0015074 GO:0006144 GO:0003677 GO:0003899 GO:0005730

Cluster-22178.103290 GO:0005515

Cluster-22178.10332 GO:0003824
Cluster-22178.103326 GO:0005515
Cluster-22178.103344 GO:0015631 GO:0045298
Cluster-22178.103440 GO:0005201 GO:0005578 GO:0005581
Cluster-22178.10347 GO:0055085 GO:0022857 GO:0016021
Cluster-22178.103485 GO:0007165 GO:0006355 GO:0030521 GO:0003677
GO:0003700 GO:0005496 GO:0008270 GO:0004882 GO:0043565
GO:0005634 GO:0005667
Cluster-22178.10351 GO:0009847 GO:0046983 GO:0008168 GO:0016021
Cluster-22178.103541 GO:0005515
Cluster-22178.103606 GO:0005515
Cluster-22178.10366 GO:0000902 GO:0005975 GO:0016773
Cluster-22178.103738 GO:0017111
Cluster-22178.10375 GO:0005515
Cluster-22178.103751 GO:0006811 GO:0009405 GO:0030170 GO:0005216
GO:0008483 GO:0005576
Cluster-22178.103829 GO:0005515 GO:0001772 GO:0097197 GO:0016021
Cluster-22178.103841 GO:0042921 GO:0006355 GO:0043402 GO:0043401
GO:0007165 GO:0008270 GO:0043565 GO:0004883 GO:0003700
GO:0005496 GO:0003677 GO:0005634 GO:0005667
Cluster-22178.10388 GO:0055114 GO:0006879 GO:0007010 GO:0016491
GO:0003779 GO:0046872 GO:0008199
Cluster-22178.103887 GO:0005615
Cluster-22178.103932 GO:0040007 GO:0008283 GO:0007165 GO:0008083

Cluster-22178.10397 GO:0008270 GO:0046872 GO:0003676 GO:0003677
GO:0005634
Cluster-22178.103984 GO:0008270
Cluster-22178.103994 GO:0006396 GO:0007165 GO:0006144 GO:0004114
GO:0005622
Cluster-22178.104037 GO:0030155 GO:0007165 GO:0007155 GO:0045995
GO:0008033 GO:0055114 GO:0030334 GO:0005102 GO:0017150
GO:0050660

Cluster-22178.104071 GO:0005515 GO:0061630
Cluster-22178.10416 GO:0006351 GO:0008270 GO:0003677 GO:0005634
Cluster-22178.104252 GO:0007165
Cluster-22178.10428 GO:0016068
Cluster-22178.104335 GO:0006355 GO:0006810 GO:0006351 GO:0007275
GO:0008270 GO:0005524 GO:0030250 GO:0046872 GO:0042626
GO:0005576 GO:0045202 GO:0016021
Cluster-22178.104345 GO:0006508 GO:0042254 GO:0006412 GO:0016787
GO:0003735 GO:0042802 GO:0008236 GO:0005515 GO:0005622
GO:0005840
Cluster-22178.104346 GO:0016021
Cluster-22178.104348 GO:0004222
Cluster-22178.104374 GO:0046872 GO:0019012
Cluster-22178.10438 GO:0006412 GO:0042254 GO:0003735 GO:0005840
Cluster-22178.104391 GO:0003676 GO:0003723
Cluster-22178.104397 GO:0005515 GO:0030234 GO:0016020
Cluster-22178.104545 GO:0030001 GO:0055085 GO:0046873 GO:0016020
Cluster-22178.10457 GO:0005524 GO:0003774 GO:0016459
Cluster-22178.104590 GO:0032801 GO:0006812 GO:0019076 GO:0042254
GO:0006412 GO:0003735 GO:0005261 GO:0005840 GO:0033644
GO:0005622
Cluster-22178.104592 GO:0005201 GO:0005581 GO:0005578
Cluster-22178.104734 GO:0048870 GO:0055114 GO:0006352 GO:0007049
GO:0015991 GO:0030001 GO:0006355 GO:0010508 GO:0007094
GO:0006914 GO:0015992 GO:0055085 GO:0007155 GO:0006810
GO:0007165 GO:0003700 GO:0004871 GO:0015078 GO:0016491
GO:0003774 GO:0016987 GO:0046873 GO:0005509 GO:0003677
GO:0033179 GO:0031514 GO:0005886 GO:0005667 GO:0016459
GO:0016020
Cluster-22178.104735 GO:0007049 GO:0006352 GO:0048870 GO:0055114
GO:0007094 GO:0010508 GO:0030001 GO:0006355 GO:0015991
GO:0055085 GO:0015992 GO:0006914 GO:0006810 GO:0007165
GO:0007155 GO:0015078 GO:0003700 GO:0004871 GO:0016987

GO:0003774 GO:0016491 GO:0003677 GO:0005509 GO:0046873
GO:0031514 GO:0033179 GO:0005886 GO:0016020 GO:0016459
GO:0005667
Cluster-22178.104746 GO:0005509 GO:0005515
Cluster-22178.104804 GO:0003723 GO:0003676
Cluster-22178.104814 GO:0006351 GO:0003714 GO:0005667
Cluster-22178.104819 GO:0007186 GO:0004930 GO:0016021
Cluster-22178.10488 GO:0006629 GO:0042254 GO:0006412 GO:0003735
GO:0005622 GO:0005840
Cluster-22178.104913 GO:0019028
Cluster-22178.104930 GO:0006144 GO:0006206 GO:0006351 GO:0003723
GO:0003899 GO:0003677 GO:0005730
Cluster-22178.104931 GO:0008643 GO:0006629 GO:0008982 GO:0009357
Cluster-22178.104951 GO:0006334 GO:0003677 GO:0005634 GO:0000786
Cluster-22178.10508 GO:0005515
Cluster-22178.105184 GO:0016055 GO:0007275 GO:0007165 GO:0005102
GO:0005576
Cluster-22178.105210 GO:0005515
Cluster-22178.10525 GO:0045766 GO:0043066 GO:0055085 GO:0043123
GO:0016021
Cluster-22178.105373 GO:0019083 GO:0005524 GO:0016887 GO:0003924
GO:0005525 GO:0016020
Cluster-22178.105386 GO:0005515
Cluster-22178.10543 GO:0005515
Cluster-22178.105519 GO:0007186 GO:0007165 GO:0003676 GO:0004871
GO:0003924 GO:0031683 GO:0008270 GO:0019001
Cluster-22178.105545 GO:0055114 GO:0016491
Cluster-22178.105594 GO:0031012
Cluster-22178.105601 GO:0048786
Cluster-22178.105724 GO:0007186 GO:0004930 GO:0016021
Cluster-22178.10574 GO:0006412 GO:0042254 GO:0003735 GO:0005840
Cluster-22178.105865 GO:0003333 GO:0006865 GO:0015171 GO:0016020
Cluster-22178.10591 GO:0007165 GO:0001664

Cluster-22178.105913 GO:0055085 GO:0030436 GO:0006508 GO:0006817
GO:0006810 GO:0005215 GO:0005315 GO:0005525 GO:0004190
GO:0016021 GO:0016020
Cluster-22178.10596 GO:0035556
Cluster-22178.106059 GO:0043113 GO:0006810 GO:0007213 GO:0005515
GO:0043236 GO:0005215 GO:0016020
Cluster-22178.106069 GO:0006468 GO:0016773 GO:0005524 GO:0004672
GO:0016020
Cluster-22178.106167 GO:0005515
Cluster-22178.10617 GO:0006813 GO:0009103 GO:0008918 GO:0005242
GO:0008076 GO:0016021
Cluster-22178.106208 GO:0005515 GO:0003824
Cluster-22178.10630 GO:0000413 GO:0006457 GO:0003755
Cluster-22178.106342 GO:0000087 GO:0006470 GO:0006570 GO:0004725
GO:0005622
Cluster-22178.106429 GO:0030036 GO:0003779 GO:0017048 GO:0005737
Cluster-22178.106437 GO:0006123 GO:0015992 GO:0004129 GO:0045277
GO:0033588
Cluster-22178.106578 GO:0007411 GO:0005515
Cluster-22178.106631 GO:0016192 GO:0005515 GO:0005198 GO:0016021
GO:0019031
Cluster-22178.106654 GO:0005975 GO:0006396 GO:0080009 GO:0009451
GO:0016758 GO:0008174 GO:0003723 GO:0016020
Cluster-22178.10666 GO:0006355 GO:0003677
Cluster-22178.106660 GO:0034755 GO:0006826 GO:0005381 GO:0016021
Cluster-22178.10677 GO:0046854
Cluster-22178.106771 GO:0006364 GO:0055114 GO:0016705 GO:0020037
GO:0008168 GO:0005096 GO:0005506
Cluster-22178.10678 GO:0046854
Cluster-22178.106801 GO:0046983
Cluster-22178.106818 GO:0005515
Cluster-22178.106846 GO:0030168 GO:0006914 GO:0043093 GO:0007165
GO:0006457 GO:0051382 GO:0051258 GO:0000917 GO:0000774

GO:0005102 GO:0030674 GO:0051087 GO:0042803 GO:0005577
GO:0005737 GO:0000776 GO:0019028
Cluster-22178.10688 GO:0005515
Cluster-22178.10689 GO:0005515
Cluster-22178.106971 GO:0015074 GO:0051726 GO:0005515 GO:0003677
GO:0005634 GO:0016020
Cluster-22178.106984 GO:0046872
Cluster-22178.10699 GO:0046039 GO:0009190 GO:0006182 GO:0006144
GO:0035556 GO:0004383 GO:0016849 GO:0020037
Cluster-22178.107076 GO:0006355 GO:0006525 GO:0003700 GO:0005667
Cluster-22178.107116 GO:0006771 GO:0002532 GO:0007165 GO:0019497
GO:0005515 GO:0046872 GO:0004896 GO:0003993 GO:0016021
Cluster-22178.107181 GO:0005515
Cluster-22178.107310 GO:0005515
Cluster-22178.107355 GO:0016021
Cluster-22178.107456 GO:0006310 GO:0006378 GO:0008152 GO:0055114
GO:0003677 GO:0003729 GO:0016491 GO:0005634 GO:0005849
Cluster-22178.10748 GO:0016021
Cluster-22178.10753 GO:0006144 GO:0007165 GO:0004114
Cluster-22178.10754 GO:0006144 GO:0007165 GO:0004114
Cluster-22178.107602 GO:0008152 GO:0008484 GO:0003824
Cluster-22178.10766 GO:0006412 GO:0042254 GO:0003735 GO:0005840
GO:0005622
Cluster-22178.107755 GO:0006270
Cluster-22178.107843 GO:0006468 GO:0016773 GO:0005524 GO:0004672
GO:0016020
Cluster-22178.107844 GO:0006468 GO:0006814 GO:0055114 GO:0015992
GO:0006744 GO:0006120 GO:0008137 GO:0016773 GO:0004672
GO:0005524 GO:0016020
Cluster-22178.108092 GO:0007268 GO:0009405 GO:0007165 GO:0046872
GO:0030550 GO:0005576
Cluster-22178.108245 GO:0019042 GO:0031224 GO:0033644
Cluster-22178.108332 GO:0005515

Cluster-22178.108334 GO:0006888 GO:0006886 GO:0008270 GO:0005515
GO:0030127
Cluster-22178.108360 GO:0003824 GO:0050660
Cluster-22178.108363 GO:0003824 GO:0050660
Cluster-22178.108474 GO:0006821 GO:0006508 GO:0004252
Cluster-22178.108753 GO:0005201 GO:0005581 GO:0005578
Cluster-22178.108819 GO:0055114 GO:0005975 GO:0004553 GO:0016616
GO:0016491
Cluster-22178.108825 GO:0006468 GO:0009069 GO:0007178 GO:0016310
GO:0004675 GO:0004672 GO:0005524 GO:0016020
Cluster-22178.108965 GO:0019082 GO:0016032
Cluster-22178.108966 GO:0016032 GO:0019082
Cluster-22178.109007 GO:0006364 GO:0032040
Cluster-22178.109013 GO:0019031
Cluster-22178.109143 GO:0001558 GO:0005520 GO:0005576 GO:0016942
Cluster-22178.109271 GO:0005515
Cluster-22178.109292 GO:0008283 GO:0040007 GO:0007165 GO:0006955
GO:0005975 GO:0008083 GO:0005509 GO:0005515 GO:0005135
GO:0004553 GO:0005578 GO:0005576 GO:0005894
Cluster-22178.109348 GO:0009877 GO:0009312 GO:0008757
Cluster-22178.109408 GO:0007165 GO:0055114 GO:0005515 GO:0005044
GO:0016020
Cluster-22178.10944 GO:0009271 GO:0050832 GO:0006355
Cluster-22178.109461 GO:0009306 GO:0046740 GO:0006915 GO:0003677
GO:0016021
Cluster-22178.109589 GO:0005615 GO:0005576
Cluster-22178.109592 GO:0046872 GO:0005615 GO:0005576
Cluster-22178.109594 GO:0046872 GO:0005615 GO:0005576
Cluster-22178.109595 GO:0046872 GO:0005615 GO:0005576
Cluster-22178.109627 GO:0006754 GO:0043248 GO:0005515 GO:0016887
GO:0005524 GO:0000275
Cluster-22178.109745 GO:0005515 GO:0005509
Cluster-22178.109808 GO:0005515 GO:0008440 GO:0016020 GO:0005737

Cluster-22178.109870 GO:0006955 GO:0019882
Cluster-22178.110000 GO:0030168 GO:0000921 GO:0030334 GO:0007165
GO:0030155 GO:0007155 GO:0045995 GO:0051258 GO:0048870
GO:0007094 GO:0005102 GO:0030674 GO:0008092 GO:0005515
GO:0042803 GO:0008134 GO:0045502 GO:0019898 GO:0005577
GO:0030286 GO:0005940 GO:0005667 GO:0016021 GO:0005737
GO:0031514
Cluster-22178.110132 GO:0007186 GO:0004930 GO:0016021
Cluster-22178.110218 GO:0015684 GO:0007264 GO:0005525 GO:0005524
GO:0015093 GO:0003924 GO:0016021
Cluster-22178.110284 GO:0005515
Cluster-22178.110316 GO:0005515
Cluster-22178.110472 GO:0015074
Cluster-22178.110484 GO:0007165 GO:0006955 GO:0005164 GO:0016020
Cluster-22178.110642 GO:0016042 GO:0035556 GO:0009395 GO:0046339
GO:0006289 GO:0007165 GO:0006629 GO:0004435 GO:0008270
GO:0005515 GO:0005509 GO:0004003 GO:0000439 GO:0005657
GO:0005634
Cluster-22178.110695 GO:0006468 GO:0004672 GO:0005524 GO:0008270
GO:0005515
Cluster-22178.110729 GO:0006810 GO:0006468 GO:0008200 GO:0004672
GO:0005524 GO:0005576
Cluster-22178.110799 GO:0006508 GO:0004222
Cluster-22178.110866 GO:0016055 GO:0007165 GO:0007275 GO:0005102
GO:0005576
Cluster-22178.110930 GO:0051056 GO:0005096
Cluster-22178.110963 GO:0007186 GO:0004930 GO:0016020
Cluster-22178.111011 GO:0006355 GO:0007186 GO:0015684 GO:0007165
GO:0007264 GO:0008134 GO:0000166 GO:0003723 GO:0001671
GO:0004871 GO:0005525 GO:0043531 GO:0003924 GO:0031683
GO:0019001 GO:0051087 GO:0003677 GO:0003724 GO:0016887
GO:0005524 GO:0015093 GO:0016021 GO:0016020 GO:0005667
Cluster-22178.111079 GO:0015074 GO:0003676 GO:0008270

Cluster-22178.111081 GO:0015074 GO:0008270 GO:0003676
Cluster-22178.111204 GO:0070140 GO:0032183
Cluster-22178.111298 GO:0006281 GO:0016032 GO:0035556 GO:0006464
GO:0055114 GO:0005506 GO:0016151 GO:0008270 GO:0046872
GO:0016491
Cluster-22178.111453 GO:0007186 GO:0004930 GO:0016020
Cluster-22178.111516 GO:0051225 GO:0008092 GO:0046983 GO:0030430
GO:0019898 GO:0070652 GO:0005737
Cluster-22178.111560 GO:0007275 GO:0030178 GO:0005515 GO:0005576
Cluster-22178.111652 GO:0007186 GO:0004930 GO:0016021
Cluster-22178.111714 GO:0022900 GO:0006355 GO:0003677 GO:0005747
GO:0005739
Cluster-22178.111820 GO:0045892 GO:0003677
Cluster-22178.11185 GO:0042742
Cluster-22178.112204 GO:0006144 GO:0015074 GO:0006206 GO:0006351
GO:0003899 GO:0003677 GO:0005730
Cluster-22178.112245 GO:0000724
Cluster-22178.112457 GO:0003676
Cluster-22178.11267 GO:0006811 GO:0007165 GO:0007268 GO:0004970
GO:0016020
Cluster-22178.112671 GO:0042254 GO:0006412 GO:0007186 GO:0001558
GO:0005520 GO:0004930 GO:0003735 GO:0005515 GO:0005622
GO:0016021 GO:0005840 GO:0016942 GO:0005576
Cluster-22178.112678 GO:0007264 GO:0007155 GO:0007165 GO:0016192
GO:0071973 GO:0030168 GO:0008053 GO:0000226 GO:0090063
GO:0006886 GO:0051258 GO:0006935 GO:0030674 GO:0003774
GO:0005096 GO:0005102 GO:0005515 GO:0003924 GO:0004871
GO:0005741 GO:0016020 GO:0005815 GO:0005577 GO:0019033
GO:0009288 GO:0005801 GO:0019867 GO:0016021 GO:0000922
Cluster-22178.11268 GO:0007268 GO:0007165 GO:0006811 GO:0005234
GO:0004970 GO:0004222 GO:0008270 GO:0005524 GO:0016020
GO:0016021
Cluster-22178.112809 GO:0006468 GO:0005524 GO:0004672 GO:0005515

Cluster-22178.112834 GO:0005515
Cluster-22178.112843 GO:0042254 GO:0006412 GO:0006355 GO:0003677
GO:0003735 GO:0003700 GO:0005667 GO:0005840 GO:0005622
Cluster-22178.112890 GO:0050832
Cluster-22178.112937 GO:0007165 GO:0005158 GO:0005899
Cluster-22178.112980 GO:0019087 GO:0015031 GO:0019068 GO:0005198
GO:0016021 GO:0016020
Cluster-22178.113060 GO:0006355 GO:0046983 GO:0003677
Cluster-22178.113520 GO:0006470 GO:0006570 GO:0008138 GO:0004725

Cluster-22178.113678 GO:0006836 GO:0006826 GO:0034755 GO:0006812
GO:0005328 GO:0005381 GO:0016021
Cluster-22178.113934 GO:0006468 GO:0030474 GO:0005524 GO:0004672
GO:0016773 GO:0005200 GO:0003676 GO:0005739 GO:0016020
GO:0005747 GO:0005856 GO:0005823
Cluster-22178.114072 GO:0007264 GO:0005525
Cluster-22178.114192 GO:0055085 GO:0006811 GO:0005216 GO:0016020
Cluster-22178.114258 GO:0009058 GO:0008152 GO:0016788 GO:0016787
GO:0005576
Cluster-22178.114273 GO:0004252 GO:0070008
Cluster-22178.114408 GO:0006032 GO:0008061
Cluster-22178.114435 GO:0005515 GO:0016020
Cluster-22178.114591 GO:0005515
Cluster-22178.114674 GO:0016020
Cluster-22178.114708 GO:0030001 GO:0055085 GO:0046873 GO:0016020
Cluster-22178.114793 GO:0005576
Cluster-22178.114955 GO:0006468 GO:0004867 GO:0004672 GO:0005524
GO:0005515
Cluster-22178.115199 GO:0006508 GO:0016805
Cluster-22178.115227 GO:0007156 GO:0005509 GO:0016020
Cluster-22178.115461 GO:0005515
Cluster-22178.115468 GO:0003676

Cluster-22178.115651 GO:0008134 GO:0003677 GO:0005667 GO:0005634
Cluster-22178.11573 GO:0016021
Cluster-22178.115845 GO:0005975 GO:0055114 GO:0016758 GO:0016491
GO:0016020
Cluster-22178.11591 GO:0006352 GO:0007186 GO:0004930 GO:0005669
GO:0016021
Cluster-22178.116090 GO:0009307 GO:0006308 GO:0009036 GO:0003677
GO:0009359
Cluster-22178.11622 GO:0005515 GO:0016020
Cluster-22178.11624 GO:0005515 GO:0016020
Cluster-22178.116251 GO:0005201 GO:0005581 GO:0005578
Cluster-22178.116252 GO:0005201 GO:0005578 GO:0005581
Cluster-22178.116283 GO:0006816 GO:0006810 GO:0009097 GO:0009099
GO:0055114 GO:0009098 GO:0006090 GO:0003852 GO:0005506
GO:0005246 GO:0020037 GO:0016705 GO:0042030 GO:0016020
Cluster-22178.116285 GO:0070072 GO:0006508 GO:0004197
Cluster-22178.116286 GO:0070072
Cluster-22178.116314 GO:0008152 GO:0006206 GO:0006013 GO:0006000
GO:0015976 GO:0006144 GO:0006096 GO:0006351 GO:0046486
GO:0006020 GO:0006094 GO:0004807 GO:0003899 GO:0005730
GO:0005634
Cluster-22178.116328 GO:0019058 GO:0006461 GO:0005739
Cluster-22178.116352 GO:0006355 GO:0043401
Cluster-22178.116398 GO:0005634
Cluster-22178.116401 GO:0009307 GO:0006308 GO:0009036 GO:0003677
GO:0009359
Cluster-22178.116402 GO:0046983
Cluster-22178.116412 GO:0005515
Cluster-22178.116423 GO:0007029 GO:0016021 GO:0005789
Cluster-22178.116469 GO:0006364 GO:0006412 GO:0042254 GO:0003735
GO:0005840 GO:0005622
Cluster-22178.116476 GO:0006457 GO:0006270 GO:0006351 GO:0006260
GO:0006950 GO:0051082 GO:0003887 GO:0003677 GO:0005524

GO:0042575
Cluster-22178.116520 GO:0007186 GO:0004930 GO:0016021
Cluster-22178.116531 GO:0004222 GO:0005524 GO:0008270 GO:0016021
Cluster-22178.116532 GO:0071555 GO:0030288
Cluster-22178.116548 GO:0006633 GO:0007186 GO:0006813 GO:0003824
GO:0004930 GO:0016747 GO:0005267 GO:0016021
Cluster-22178.116550 GO:0006633 GO:0007186 GO:0006813 GO:0003824
GO:0004930 GO:0016747 GO:0005267 GO:0016021
Cluster-22178.116551 GO:0006813 GO:0007186 GO:0006633 GO:0005267
GO:0016747 GO:0004930 GO:0003824 GO:0016021
Cluster-22178.116557 GO:0044030 GO:0005634
Cluster-22178.116589 GO:0015074 GO:0005515
Cluster-22178.116607 GO:0007165 GO:0005515 GO:0005096 GO:0003729

Cluster-22178.116634 GO:0019905
Cluster-22178.116635 GO:0006450 GO:0006119 GO:0006118 GO:0015992
GO:0008121 GO:0009055
Cluster-22178.116641 GO:0045766 GO:0043066 GO:0055085 GO:0043123
GO:0016021
Cluster-22178.116642 GO:0055085 GO:0043123 GO:0045766 GO:0043066
GO:0016021
Cluster-22178.116657 GO:0043087 GO:0006355 GO:0005089 GO:0019902
GO:0005737 GO:0005634
Cluster-22178.116679 GO:0007186 GO:0004930 GO:0016021
Cluster-22178.116706 GO:0005975 GO:0007186 GO:0008270 GO:0004930
GO:0008184 GO:0003677 GO:0019904 GO:0016021
Cluster-22178.116708 GO:0009059 GO:0030176
Cluster-22178.116719 GO:0055085 GO:0006260 GO:0022857 GO:0003677
GO:0016021 GO:0005664
Cluster-22178.116728 GO:0015995 GO:0055114 GO:0015979 GO:0016491

Cluster-22178.116746 GO:0034314 GO:0001558 GO:0030041 GO:0005520
GO:0005515 GO:0015629 GO:0005885 GO:0016942 GO:0005576

Cluster-22178.116751 GO:0035091 GO:0005515
Cluster-22178.116795 GO:0006814 GO:0071436 GO:0006560 GO:0006525
GO:0006352 GO:0006090 GO:0008152 GO:0008948 GO:0003677
GO:0016788 GO:0015081 GO:0016020
Cluster-22178.116817 GO:0016042 GO:0046339 GO:0009395 GO:0004435
GO:0005509 GO:0016021
Cluster-22178.116818 GO:0016042 GO:0009395 GO:0046339 GO:0005509
GO:0004435 GO:0016021
Cluster-22178.116833 GO:0030246
Cluster-22178.117041 GO:0006508 GO:0004197
Cluster-22178.117045 GO:0006486 GO:0008378 GO:0016020
Cluster-22178.117056 GO:0006355 GO:0003677
Cluster-22178.117078 GO:0006508 GO:0071973 GO:0005198 GO:0004198
GO:0009428 GO:0005622 GO:0030288
Cluster-22178.117095 GO:0016020
Cluster-22178.117108 GO:0055114 GO:0006397 GO:0051252 GO:0006120
GO:0004540
Cluster-22178.117166 GO:0042254 GO:0006412 GO:0003735 GO:0005622
GO:0005840
Cluster-22178.117205 GO:0007186 GO:0007010 GO:0004930 GO:0003779
GO:0016021
Cluster-22178.117213 GO:0006505 GO:0006886 GO:0098599 GO:0016788

Cluster-22178.117218 GO:0005515 GO:0008270
Cluster-22178.117245 GO:0046983
Cluster-22178.117322 GO:0043130
Cluster-22178.117445 GO:0006471 GO:0009405 GO:0006355 GO:0003700
GO:0003956 GO:0043565 GO:0005576 GO:0005667
Cluster-22178.117462 GO:0006351 GO:0006144 GO:0006206 GO:0003899
GO:0003677 GO:0003723 GO:0005730
Cluster-22178.117497 GO:0035556 GO:0009190 GO:0006468 GO:0016849
GO:0005524 GO:0004672
Cluster-22178.117498 GO:0009190 GO:0006468 GO:0035556 GO:0016849

GO:0004672 GO:0005524
Cluster-22178.117533 GO:0015074 GO:0006355 GO:0003700 GO:0005667
Cluster-22178.117540 GO:0042157 GO:0030001 GO:0006869 GO:0042262
GO:0055085 GO:0006950 GO:0003690 GO:0046873 GO:0005198
GO:0008289 GO:0019028 GO:0005576 GO:0016020 GO:0016021
GO:0019031
Cluster-22178.117598 GO:0005509 GO:0016020
Cluster-22178.117611 GO:0051056 GO:0005096
Cluster-22178.117613 GO:0051056 GO:0005096
Cluster-22178.117620 GO:0005975 GO:0055114 GO:0016491 GO:0016616
GO:0004553
Cluster-22178.117623 GO:0019068 GO:0019904 GO:0008134 GO:0005667
Cluster-22178.117690 GO:0055114 GO:0006826 GO:0046168 GO:0006827
GO:0006468 GO:0005524 GO:0004672 GO:0016616 GO:0005381
GO:0051287 GO:0033573 GO:0016020
Cluster-22178.117756 GO:0007165 GO:0006508 GO:0004252 GO:0005044
GO:0016020
Cluster-22178.117779 GO:0007165 GO:0040007 GO:0008283 GO:0008083

Cluster-22178.117793 GO:0005515
Cluster-22178.117890 GO:0009405 GO:0015684 GO:0042742 GO:0005525
GO:0005524 GO:0015093 GO:0016887 GO:0003924 GO:0016021
Cluster-22178.117898 GO:0006813 GO:0009103 GO:0005242 GO:0008918
GO:0008076 GO:0016021
Cluster-22178.117906 GO:0006468 GO:0006811 GO:0006810 GO:0005230
GO:0005524 GO:0004672 GO:0016020
Cluster-22178.117932 GO:0070836 GO:0006952 GO:0005576
Cluster-22178.117939 GO:0005515
Cluster-22178.117966 GO:0006310 GO:0007165 GO:0009190 GO:0006171
GO:0006281 GO:0000160 GO:0035556 GO:0006144 GO:0006308
GO:0004520 GO:0004016 GO:0016849 GO:0004871 GO:0005886
Cluster-22178.118114 GO:0006355 GO:0043401 GO:0008270 GO:0043565
GO:0003700 GO:0005634 GO:0005667

Cluster-22178.118160 GO:0016310 GO:0018149 GO:0009069 GO:0006468
GO:0004674 GO:0005515 GO:0005524
Cluster-22178.118166 GO:0004222 GO:0008233 GO:0031012
Cluster-22178.118252 GO:0046677
Cluster-22178.118321 GO:0006468 GO:0016773 GO:0005524 GO:0004672
GO:0016020
Cluster-22178.118328 GO:0006955 GO:0030153 GO:0043087 GO:0005089
GO:0015643 GO:0019814 GO:0005737
Cluster-22178.118349 GO:0003677
Cluster-22178.118354 GO:0045454
Cluster-22178.118433 GO:0043395 GO:0005578 GO:0016020
Cluster-22178.118828 GO:0008272 GO:0006355 GO:0015116 GO:0003677
GO:0016021
Cluster-22178.11916 GO:0008152 GO:0003824 GO:0016491 GO:0050662

Cluster-22178.120082 GO:0044419 GO:0006144 GO:0006508 GO:0003968
GO:0017111 GO:0004197 GO:0031379
Cluster-22178.120144 GO:0051258 GO:0007010 GO:0009405 GO:0030168
GO:0042157 GO:0007067 GO:0007165 GO:0006869 GO:0003779
GO:0008289 GO:0005198 GO:0005515 GO:0005102 GO:0030674
GO:0019031 GO:0005576 GO:0072686 GO:0019028 GO:0042729
GO:0005577 GO:0016020
Cluster-22178.120177 GO:0005515
Cluster-22178.120225 GO:0006355 GO:0003677
Cluster-22178.120804 GO:0006810 GO:0015858 GO:0005337 GO:0016021
Cluster-22178.12099 GO:0055114 GO:0006508 GO:0050792 GO:0004190
GO:0016491 GO:0050662 GO:0042025
Cluster-22178.12209 GO:0055114
Cluster-22178.122476 GO:0016020
Cluster-22178.12250 GO:0006694 GO:0046660 GO:0006397 GO:0006270
GO:0003676 GO:0008168 GO:0004190 GO:0016021 GO:0005634
Cluster-22178.12506 GO:0006464 GO:0046500 GO:0006479 GO:0008610
GO:0004719 GO:0008168 GO:0008171

Cluster-22178.12593 GO:0004045
Cluster-22178.12730 GO:0006629
Cluster-22178.12774 GO:0016567 GO:0004842 GO:0005515 GO:0005507

Cluster-22178.13162 GO:0005515
Cluster-22178.13221 GO:0035556 GO:0007165 GO:0005515
Cluster-22178.13265 GO:0006817 GO:0006813 GO:0006814 GO:0005515 GO:0005890
Cluster-22178.13342 GO:0007155 GO:0005198 GO:0019028 GO:0031012
Cluster-22178.13343 GO:0007155 GO:0005198 GO:0031012
Cluster-22178.13383 GO:0006811 GO:0055085 GO:0005515 GO:0005216 GO:0016020
Cluster-22178.13475 GO:0020037 GO:0005515
Cluster-22178.13567 GO:0009231 GO:0006508 GO:0006771 GO:0008152 GO:0016787 GO:0008236 GO:0003919
Cluster-22178.13598 GO:0006414 GO:0006448 GO:0003746 GO:0005853 GO:0005840
Cluster-22178.13628 GO:0045892 GO:0006260 GO:0032968 GO:0006351 GO:0006367 GO:0006270 GO:0008270 GO:0003887 GO:0003677 GO:0032040 GO:0005634 GO:0042575
Cluster-22178.13629 GO:0042967 GO:0008080
Cluster-22178.13741 GO:0005198 GO:0019028 GO:0019031 GO:0005882
Cluster-22178.13834 GO:0043395 GO:0005515 GO:0005578 GO:0016020
Cluster-22178.13838 GO:0043087 GO:0007264 GO:0005085
Cluster-22178.13859 GO:0006486 GO:0016757 GO:0008378 GO:0016020
Cluster-22178.13878 GO:0016020
Cluster-22178.13887 GO:0006355 GO:0043565 GO:0003677 GO:0003700 GO:0005667 GO:0005634
Cluster-22178.14108 GO:0005516
Cluster-22178.14126 GO:0042381 GO:0005524 GO:0005576
Cluster-22178.14160 GO:0006388 GO:0005524 GO:0003972
Cluster-22178.14183 GO:0006812 GO:0015074 GO:0008324 GO:0016021 GO:0005886

Cluster-22178.14273 GO:0016310 GO:0006629 GO:0009069 GO:0006468
GO:0004674 GO:0005524 GO:0004672
Cluster-22178.14347 GO:0005515
Cluster-22178.14351 GO:0005515
Cluster-22178.14441 GO:0006144 GO:0032465 GO:0006915 GO:0016567
GO:0006351 GO:0005524 GO:0003968 GO:0004842 GO:0003723
GO:0005509 GO:0005198 GO:0031379 GO:0019031
Cluster-22178.14453 GO:0005515
Cluster-22178.14586 GO:0008270 GO:0005515 GO:0009295
Cluster-22178.14691 GO:0000041 GO:0005509 GO:0016021 GO:0016020
Cluster-22178.14785 GO:0005975 GO:0000902 GO:0016773
Cluster-22178.15189 GO:0042254 GO:0006412 GO:0006355 GO:0003735
GO:0003700 GO:0043565 GO:0008270 GO:0005667 GO:0005840
GO:0005622
Cluster-22178.15191 GO:0007165 GO:0006144 GO:0005515 GO:0004114

Cluster-22178.15193 GO:0006886 GO:0016567 GO:0006888 GO:0005515
GO:0004842 GO:0046872 GO:0008270 GO:0030127 GO:0019031
GO:0000228
Cluster-22178.15412 GO:0016021
Cluster-22178.15522 GO:0035556 GO:0005515
Cluster-22178.15756 GO:0005515 GO:0043565 GO:0005634
Cluster-22178.16081 GO:0008283 GO:0040007 GO:0007165 GO:0000902
GO:0006955 GO:0008083 GO:0016020
Cluster-22178.1618 GO:0020037 GO:0005515
Cluster-22178.16187 GO:0006355 GO:0003676 GO:0003677
Cluster-22178.16190 GO:0019354 GO:0016117 GO:0006779 GO:0055114
GO:0008033 GO:0016705 GO:0043115 GO:0005515 GO:0071949
GO:0050661 GO:0050660 GO:0004499 GO:0016491 GO:0008270
Cluster-22178.16347 GO:0006448 GO:0006414 GO:0003746 GO:0005840
Cluster-22178.16363 GO:0006810 GO:0043565 GO:0005515 GO:0005634
GO:0016020
Cluster-22178.16372 GO:0006144 GO:0007165 GO:0004114 GO:0005515

Cluster-22178.16401 GO:0005515
Cluster-22178.16469 GO:0005515
Cluster-22178.16845 GO:0005515
Cluster-22178.16865 GO:0003676
Cluster-22178.16892 GO:0019856
Cluster-22178.17546 GO:0006468 GO:0016773 GO:0005524 GO:0008270 GO:0004672 GO:0005515 GO:0016020
Cluster-22178.17565 GO:0006333 GO:0005634
Cluster-22178.17632 GO:0006812 GO:0034755 GO:0006826 GO:0006836 GO:0005381 GO:0005328 GO:0016021
Cluster-22178.17682 GO:0006355 GO:0003677
Cluster-22178.17784 GO:0005515 GO:0004867
Cluster-22178.17931 GO:0009405
Cluster-22178.17994 GO:0007264 GO:0005524 GO:0003924 GO:0005525 GO:0019013
Cluster-22178.18091 GO:0007165 GO:0008283 GO:0040007 GO:0008083 GO:0016020
Cluster-22178.18115 GO:0006355 GO:0030474 GO:0070206 GO:0051607 GO:0007049 GO:0005515 GO:0043565 GO:0005200 GO:0003700 GO:0005667 GO:0005856 GO:0005823
Cluster-22178.18148 GO:0006468 GO:0006810 GO:0008200 GO:0005515 GO:0005524 GO:0004672 GO:0005576 GO:0016020
Cluster-22178.18314 GO:0009405 GO:0007165 GO:0005198 GO:0005515 GO:0016021
Cluster-22178.18322 GO:0070588 GO:0006816 GO:0015002 GO:0005515 GO:0005262 GO:0016020
Cluster-22178.18408 GO:0042254 GO:0006412 GO:0003735 GO:0005840
Cluster-22178.18452 GO:0006457 GO:0006520 GO:0016743
Cluster-22178.18480 GO:0035556 GO:0005515
Cluster-22178.185 GO:0042981 GO:0005515
Cluster-22178.18567 GO:0006265 GO:0007165 GO:0005057 GO:0003918 GO:0003677 GO:0005694

Cluster-22178.18781 GO:0030674 GO:0051015
Cluster-22178.19104 GO:0006508 GO:0007165 GO:0008270 GO:0005125
GO:0004222 GO:0031012
Cluster-22178.1930 GO:0016042 GO:0046486 GO:0030682 GO:0004806
Cluster-22178.19360 GO:0008152 GO:0016791 GO:0008641
Cluster-22178.19480 GO:0015074 GO:0003676 GO:0008270
Cluster-22178.19509 GO:0008289
Cluster-22178.19583 GO:0006570 GO:0006470 GO:0019988 GO:0008138
GO:0004725 GO:0043399
Cluster-22178.19658 GO:0006955 GO:0007165 GO:0030247 GO:0016787
GO:0005524 GO:0005044
Cluster-22178.19694 GO:0015992 GO:0006744 GO:0006120 GO:0006355
GO:0006814 GO:0006118 GO:0003954 GO:0008137 GO:0005515
GO:0005198 GO:0005739
Cluster-22178.19900 GO:0007605 GO:0005515
Cluster-22178.19935 GO:0008152 GO:0016746
Cluster-22178.19936 GO:0008152 GO:0016746
Cluster-22178.20181 GO:2000123
Cluster-22178.20226 GO:0006955 GO:0007165 GO:0040007 GO:0008283
GO:0009098 GO:0005515 GO:0005143 GO:0008083 GO:0042022
GO:0005576
Cluster-22178.20635 GO:0007015 GO:0003779 GO:0003785
Cluster-22178.2077 GO:0008146 GO:0016021
Cluster-22178.20821 GO:0019089 GO:0010508 GO:0006606 GO:0006914
GO:0070206 GO:0005198 GO:0008134 GO:0042803 GO:0045502
GO:0008017 GO:0019028 GO:0045298 GO:0019031 GO:0005882
GO:0005667 GO:0030286
Cluster-22178.20879 GO:0005198 GO:0005882
Cluster-22178.20881 GO:0006606 GO:0006633 GO:0070206 GO:0030168
GO:0000921 GO:0006914 GO:0007165 GO:0007155 GO:0006090
GO:0005975 GO:0051258 GO:0010508 GO:0006355 GO:0004553
GO:0005102 GO:0003989 GO:0008270 GO:0030674 GO:0003700
GO:0043565 GO:0005198 GO:0005577 GO:0009317 GO:0005940

GO:0005882 GO:0005667 GO:0019031 GO:0016021 GO:0019028
Cluster-22178.20882 GO:0000917 GO:0006355 GO:0007049 GO:0006810
GO:0007264 GO:0006260 GO:0070206 GO:0010508 GO:0006090
GO:0019058 GO:0007155 GO:0043093 GO:0006633 GO:0006914
GO:0008134 GO:0045502 GO:0003700 GO:0005096 GO:0003989
GO:0005198 GO:0005515 GO:0042803 GO:0043565 GO:0019028
GO:0016021 GO:0005882 GO:0009317 GO:0034464 GO:0030286
GO:0005886 GO:0005737 GO:0005667
Cluster-22178.21032 GO:0007156 GO:0005509 GO:0016020
Cluster-22178.21233 GO:0006308 GO:0007165 GO:0007186 GO:0009307
GO:0007264 GO:0005525 GO:0004871 GO:0031683 GO:0009036
GO:0019001 GO:0003924 GO:0003677 GO:0009359
Cluster-22178.21234 GO:0007264 GO:0007165 GO:0007186 GO:0006013
GO:0006000 GO:0003873 GO:0016301 GO:0019001 GO:0005524
GO:0031683 GO:0003924 GO:0005525 GO:0004871
Cluster-22178.21257 GO:0006281 GO:0004519
Cluster-22178.21265 GO:0006470 GO:0016032 GO:0006570 GO:0004725
GO:0005525 GO:0005515 GO:0008138 GO:0019013
Cluster-22178.21379 GO:0046872
Cluster-22178.21388 GO:0005198 GO:0003677 GO:0019028 GO:0005634
Cluster-22178.21443 GO:0006807 GO:0005515 GO:0016810
Cluster-22178.21450 GO:0005615
Cluster-22178.21614 GO:0042254 GO:0006412 GO:0007165 GO:0040007
GO:0008283 GO:0003735 GO:0008083 GO:0005840 GO:0005622
Cluster-22178.21736 GO:0007076 GO:0000796
Cluster-22178.21760 GO:0006468 GO:0016772 GO:0005524 GO:0008270
GO:0004672 GO:0016773 GO:0003677 GO:0016020
Cluster-22178.21796 GO:0009607 GO:0016021
Cluster-22178.21803 GO:0030089
Cluster-22178.21864 GO:0042998 GO:0071253 GO:0005802
Cluster-22178.21963 GO:0000041 GO:0008270 GO:0005509 GO:0016021
Cluster-22178.22163 GO:0006457 GO:0000413 GO:0003755
Cluster-22178.22181 GO:0006570 GO:0006810 GO:0009405 GO:0019988

GO:0006470 GO:0043399 GO:0019870 GO:0008138 GO:0004725
GO:0005576
Cluster-22178.22241 GO:0043401 GO:0006355 GO:0003700 GO:0043565
GO:0008270 GO:0005667 GO:0005634
Cluster-22178.22340 GO:0007165 GO:0007186 GO:0006771 GO:0019497
GO:0005515 GO:0003993 GO:0004871 GO:0019001 GO:0046872
GO:0031683 GO:0003924 GO:0016020
Cluster-22178.22471 GO:0007268 GO:0006811 GO:0007165 GO:0004970
GO:0005234 GO:0016020
Cluster-22178.22491 GO:0009405 GO:0045735
Cluster-22178.22511 GO:0006118 GO:0009055 GO:0016491 GO:0016020
Cluster-22178.22533 GO:0005515
Cluster-22178.22554 GO:0006508 GO:0008270 GO:0004222 GO:0031012
Cluster-22178.22644 GO:0006468 GO:0006979 GO:0004672 GO:0019031
Cluster-22178.22711 GO:0006508 GO:0008152 GO:0008270 GO:0016788
GO:0004181 GO:0005515
Cluster-22178.22748 GO:0005515 GO:0008270
Cluster-22178.22751 GO:0005515
Cluster-22178.22773 GO:0016021
Cluster-22178.22860 GO:0006810 GO:0005515 GO:0016021
Cluster-22178.22999 GO:0007010 GO:0018342 GO:0042393 GO:0020037
GO:0008318 GO:0003779
Cluster-22178.23050 GO:0035556 GO:0005515
Cluster-22178.23268 GO:0055114 GO:0007155 GO:0070072 GO:0005515
GO:0005509 GO:0005576 GO:0016020
Cluster-22178.23290 GO:0006355 GO:0007094 GO:0051258 GO:0007165
GO:0070206 GO:0030168 GO:0048193 GO:0045502 GO:0005198
GO:0042803 GO:0008134 GO:0003700 GO:0030674 GO:0005102
GO:0019028 GO:0019031 GO:0016021 GO:0005667 GO:0016020
GO:0030286 GO:0005577
Cluster-22178.23457 GO:0070403
Cluster-22178.23468 GO:0007165 GO:0006869 GO:0042157 GO:0007155
GO:0030168 GO:0051258 GO:0030674 GO:0005102 GO:0008289

GO:0005576 GO:0016021 GO:0005577
Cluster-22178.23492 GO:0006629
Cluster-22178.23625 GO:0007186 GO:0004930 GO:0016021
Cluster-22178.23663 GO:0005515
Cluster-22178.23712 GO:0017004 GO:0015886 GO:0016021
Cluster-22178.23780 GO:0035556
Cluster-22178.2379 GO:0042742 GO:0005576
Cluster-22178.23880 GO:0055114 GO:0016491 GO:0005507
Cluster-22178.23886 GO:0006508 GO:0004252
Cluster-22178.23887 GO:0006508 GO:0004252
Cluster-22178.23935 GO:0007186 GO:0004930 GO:0016021
Cluster-22178.23944 GO:0007156 GO:0005509 GO:0016020
Cluster-22178.23989 GO:0005515
Cluster-22178.24031 GO:0030178 GO:0007275 GO:0005576
Cluster-22178.24278 GO:0006955 GO:0000902 GO:0016020
Cluster-22178.24343 GO:0005515
Cluster-22178.24358 GO:0042254 GO:0006412 GO:0019076 GO:0006812
GO:0032801 GO:0005261 GO:0003735 GO:0005622 GO:0033644
GO:0005840
Cluster-22178.24429 GO:0019836 GO:0005515 GO:0005576
Cluster-22178.24434 GO:0000975
Cluster-22178.24448 GO:0005509 GO:0005515
Cluster-22178.24506 GO:0042254 GO:0006412 GO:0003735 GO:0005840
Cluster-22178.24594 GO:0006364 GO:0005515 GO:0008270 GO:0032040
GO:0005737
Cluster-22178.24613 GO:0006506 GO:0004584 GO:0000136
Cluster-22178.24685 GO:0006813 GO:0008556 GO:0016021
Cluster-22178.24690 GO:0006468 GO:0005524 GO:0004672
Cluster-22178.24747 GO:0005515
Cluster-22178.24814 GO:0003950
Cluster-22178.24823 GO:0005856
Cluster-22178.24840 GO:0042254 GO:0006412 GO:0008152 GO:0003735
GO:0003824 GO:0005840

Cluster-22178.249 GO:0030212 GO:0004867
Cluster-22178.24934 GO:0005975 GO:0005515 GO:0016846 GO:0004415

Cluster-22178.24972 GO:0006817 GO:0008270 GO:0046872 GO:0000166
GO:0005315 GO:0016020
Cluster-22178.24995 GO:0030001 GO:0055085 GO:0046873 GO:0016020
Cluster-22178.25034 GO:0003676
Cluster-22178.25086 GO:0005515
Cluster-22178.25126 GO:0005515
Cluster-22178.25329 GO:0019013
Cluster-22178.25342 GO:0051260
Cluster-22178.25356 GO:0030308 GO:0060047 GO:0055010 GO:0042694
GO:0055003 GO:0005509 GO:0003785 GO:0030016 GO:0015629
Cluster-22178.25405 GO:0032784 GO:0004222 GO:0003677
Cluster-22178.25521 GO:0005515 GO:0008270 GO:0046872
Cluster-22178.25544 GO:0008152 GO:0005515 GO:0016758 GO:0008270
GO:0005622
Cluster-22178.25545 GO:0006357 GO:0001104 GO:0005515 GO:0016592
Cluster-22178.25566 GO:0006508 GO:0004197
Cluster-22178.25569 GO:0004672
Cluster-22178.25589 GO:0006811 GO:0055085 GO:0006816 GO:0006874
GO:0006118 GO:0051536 GO:0009055 GO:0005219 GO:0005216
GO:0005509 GO:0005622 GO:0016021 GO:0016020
Cluster-22178.25860 GO:0006368 GO:0004867 GO:0005576 GO:0008023
Cluster-22178.25866 GO:0006935 GO:0006955 GO:0007165 GO:0008009
GO:0036128 GO:0005576
Cluster-22178.25871 GO:0015840 GO:0071918 GO:0007155 GO:0005509
GO:0015204 GO:0005515 GO:0016021 GO:0005576
Cluster-22178.25986 GO:0051726 GO:0005634
Cluster-22178.26223 GO:0046486 GO:0007165 GO:0040007 GO:0008283
GO:0008654 GO:0006464 GO:0008083 GO:0008715 GO:0003697
GO:0016020
Cluster-22178.26360 GO:0006807 GO:0005509 GO:0004668 GO:0005737

Cluster-22178.26405 GO:0071988 GO:0030474 GO:0005515
Cluster-22178.26435 GO:0008272 GO:0015116 GO:0016021
Cluster-22178.26457 GO:0006508 GO:0042254 GO:0006412 GO:0016787
GO:0005515 GO:0008236 GO:0042802 GO:0003735 GO:0005622
GO:0005840
Cluster-22178.26458 GO:0006508 GO:0042254 GO:0006412 GO:0016787
GO:0005515 GO:0042802 GO:0008236 GO:0003735 GO:0005622
GO:0005840
Cluster-22178.26561 GO:0005515
Cluster-22178.26578 GO:0006415 GO:0006449 GO:0003747 GO:0018444
GO:0005840
Cluster-22178.26592 GO:0055114 GO:0008270 GO:0047134 GO:0005622
Cluster-22178.26676 GO:0009405 GO:0016747 GO:0016020 GO:0016021
GO:0005576
Cluster-22178.26678 GO:0006468 GO:0005524 GO:0004672
Cluster-22178.26799 GO:0006725 GO:0055114 GO:0006265 GO:0019337
GO:0006807 GO:0009399 GO:0003690 GO:0016853 GO:0003677
GO:0016163 GO:0016610
Cluster-22178.26805 GO:0009231 GO:0007275 GO:0008686 GO:0005578
Cluster-22178.26857 GO:0015074 GO:0003676 GO:0008270
Cluster-22178.26951 GO:0006355 GO:0005515 GO:0005634 GO:0005668
Cluster-22178.26965 GO:0046740 GO:0005509 GO:0003677 GO:0033644
GO:0042765 GO:0016021
Cluster-22178.27145 GO:0051258 GO:0030168 GO:0007165 GO:0006457
GO:0005102 GO:0000774 GO:0051087 GO:0042803 GO:0030674
GO:0005577
Cluster-22178.27147 GO:0007165 GO:0006457 GO:0030168 GO:0051258
GO:0030674 GO:0051087 GO:0042803 GO:0000774 GO:0005102
GO:0005577
Cluster-22178.27372 GO:0019497 GO:0006771 GO:0046872 GO:0005515
GO:0016788 GO:0003993
Cluster-22178.27588 GO:0006412 GO:0042254 GO:0003735 GO:0005840
Cluster-22178.27608 GO:0007017 GO:0005875

Cluster-22178.27613 GO:0007304 GO:0043087 GO:0007165 GO:0007010 GO:0007264 GO:0004872 GO:0005213 GO:0003779 GO:0005085 GO:0016021 GO:0042600 GO:0005576

Cluster-22178.27627 GO:0004659 GO:0016020 GO:0016021

Cluster-22178.27637 GO:0007264 GO:0007186 GO:0007165 GO:0003924 GO:0031683 GO:0019001 GO:0004871 GO:0005525 GO:0032040

Cluster-22178.27647 GO:0042147 GO:0000079 GO:0019901 GO:0005856

Cluster-22178.27751 GO:0006468 GO:0016773 GO:0005524 GO:0004672 GO:0016020

Cluster-22178.27779 GO:0006508 GO:0004197 GO:0008234

Cluster-22178.27780 GO:0006508 GO:0008234

Cluster-22178.27781 GO:0006508 GO:0016226 GO:0008234 GO:0004197 GO:0051536 GO:0005506

Cluster-22178.27792 GO:0006355 GO:0003700 GO:0005667

Cluster-22178.27824 GO:0005515

Cluster-22178.27926 GO:0005515

Cluster-22178.28059 GO:0042816 GO:0008615 GO:0055114 GO:0005515 GO:0050570 GO:0051287

Cluster-22178.28066 GO:0009395 GO:0046486 GO:0035556 GO:0007205 GO:0016301 GO:0005509 GO:0004143

Cluster-22178.28076 GO:0055114 GO:0005507 GO:0016616 GO:0016491

Cluster-22178.28131 GO:0016746

Cluster-22178.28140 GO:0016301

Cluster-22178.28245 GO:0016021 GO:0016012

Cluster-22178.28249 GO:0005976 GO:0005515 GO:0016779

Cluster-22178.28305 GO:0007186 GO:0004930 GO:0016020

Cluster-22178.28399 GO:0016192 GO:0006629

Cluster-22178.28401 GO:0016192

Cluster-22178.28415 GO:0055085 GO:0016020 GO:0016021

Cluster-22178.28535 GO:0006259 GO:0005524 GO:0003824 GO:0003677 GO:0005694

Cluster-22178.28574 GO:0005975 GO:0019497 GO:0007155 GO:0006771

GO:0046872 GO:0004553 GO:0003993 GO:0005515 GO:0016021
Cluster-22178.28578 GO:0006479 GO:0006554 GO:0018024
Cluster-22178.28588 GO:0019684 GO:0006118 GO:0004859 GO:0005515
GO:0045156 GO:0016021 GO:0030077 GO:0005576
Cluster-22178.28589 GO:0006118 GO:0019684 GO:0004859 GO:0045156
GO:0016021 GO:0030077 GO:0005576
Cluster-22178.28597 GO:0006825 GO:0006606 GO:0006090 GO:0006560
GO:0071436 GO:0006814 GO:0006525 GO:0035434 GO:0015081
GO:0019904 GO:0008948 GO:0005375 GO:0016021 GO:0016020
Cluster-22178.28629 GO:0043066 GO:0003950 GO:0016788 GO:0051015

Cluster-22178.28711 GO:0016567 GO:0004842
Cluster-22178.28831 GO:0046487 GO:0007010 GO:0008152 GO:0055114
GO:0009396 GO:0004488 GO:0050662 GO:0003824 GO:0016491
GO:0003779
Cluster-22178.28856 GO:0006914 GO:0051607 GO:0043093 GO:0051301
GO:0007059 GO:0007049 GO:0006355 GO:0006118 GO:0007094
GO:0000917 GO:0008017 GO:0003954 GO:0017048 GO:0043565
GO:0046872 GO:0003700 GO:0045502 GO:0005515 GO:0008134
GO:0042803 GO:0000444 GO:0030286 GO:0005887 GO:0005667
GO:0005739 GO:0005737 GO:0045298
Cluster-22178.28871 GO:0009405 GO:0006355 GO:0051258 GO:0007165
GO:0048193 GO:0000921 GO:0030168 GO:0005198 GO:0016818
GO:0003677 GO:0030674 GO:0005102 GO:0008270 GO:0005524
GO:0005634 GO:0019028 GO:0016021 GO:0019031 GO:0016020
GO:0005577 GO:0005940
Cluster-22178.28895 GO:0046872
Cluster-22178.28940 GO:0006144 GO:0007165 GO:0004114 GO:0003677

Cluster-22178.28941 GO:0006355 GO:0003677 GO:0016787
Cluster-22178.28943 GO:0046034 GO:0006144 GO:0006464 GO:0004017
GO:0016151 GO:0046872 GO:0005761
Cluster-22178.29094 GO:0006508 GO:0042773 GO:0015979 GO:0055114

GO:0019684 GO:0004197 GO:0016651 GO:0009523 GO:0016021
Cluster-22178.29157 GO:0006357 GO:0005515 GO:0001104 GO:0016592
Cluster-22178.29204 GO:0007264 GO:0007010 GO:0003779 GO:0003924
GO:0005525
Cluster-22178.29207 GO:0043113 GO:0006810 GO:0007213 GO:0005515
GO:0043236 GO:0005215 GO:0016020
Cluster-22178.29215 GO:0050909 GO:0007186 GO:0003779 GO:0004930
GO:0016021
Cluster-22178.29243 GO:0050765 GO:0005515
Cluster-22178.29268 GO:0005515
Cluster-22178.29374 GO:0006355 GO:0007049 GO:0006260 GO:0006606
GO:0006914 GO:0008134 GO:0003677 GO:0003723 GO:0016787
GO:0003724 GO:0016887 GO:0005524 GO:0005667
Cluster-22178.29379 GO:0005615
Cluster-22178.29409 GO:0016021
Cluster-22178.29447 GO:0003677
Cluster-22178.29504 GO:0006355 GO:0007165 GO:0003700 GO:0004871
GO:0005634 GO:0005667
Cluster-22178.29559 GO:0007165 GO:0009401 GO:0009405 GO:0005615
Cluster-22178.29588 GO:0005975 GO:0008608 GO:0006118 GO:0008270
GO:0008440 GO:0004553 GO:0030246 GO:0003954 GO:0005576
GO:0005739 GO:0072686 GO:0042729
Cluster-22178.29592 GO:0007165 GO:0055114 GO:0043087 GO:0007264
GO:0016491 GO:0005085 GO:0046872
Cluster-22178.29610 GO:0042384 GO:0010826 GO:0036038
Cluster-22178.29627 GO:0006370 GO:0015991 GO:0016192 GO:0006886
GO:0009451 GO:0004482 GO:0016820 GO:0000221 GO:0030117
GO:0030131
Cluster-22178.29662 GO:0005515
Cluster-22178.29668 GO:0006865 GO:0055085 GO:0003333 GO:0006810
GO:0015171 GO:0016020
Cluster-22178.29713 GO:0016032 GO:0044780 GO:0019033
Cluster-22178.29743 GO:0007275 GO:0006468 GO:0045893 GO:0008270

GO:0004672 GO:0005524 GO:0046983

Cluster-22178.29854 GO:0051260 GO:0016020

Cluster-22178.29896 GO:0007166 GO:0045892 GO:0007165 GO:0050792 GO:0004871 GO:0046872 GO:0008270 GO:0004518 GO:0005515 GO:0005509 GO:0005634

Cluster-22178.29924 GO:0007165

Cluster-22178.29927 GO:0003824

Cluster-22178.29995 GO:0007186 GO:0004930 GO:0016021

Cluster-22178.30013 GO:0006810 GO:0055085 GO:0005215 GO:0016020

Cluster-22178.30128 GO:0005515

Cluster-22178.30131 GO:0006508 GO:0008152 GO:0008270 GO:0004181 GO:0016788

Cluster-22178.30132 GO:0042967 GO:0016747 GO:0008080

Cluster-22178.30142 GO:0098542 GO:0006118 GO:0009055 GO:0005509 GO:0005515 GO:0009512 GO:0005887 GO:0019031

Cluster-22178.30149 GO:0005515

Cluster-22178.30197 GO:0008152 GO:0016787

Cluster-22178.30214 GO:0008270

Cluster-22178.30312 GO:0006525 GO:0006560 GO:0071436 GO:0006814 GO:0007304 GO:0006090 GO:0005509 GO:0008948 GO:0015081 GO:0005213 GO:0005576 GO:0016020 GO:0042600

Cluster-22178.30323 GO:0042549 GO:0015979 GO:0006807 GO:0005515 GO:0008270 GO:0016814 GO:0009523 GO:0009539

Cluster-22178.30396 GO:0006206 GO:0006144 GO:0055114 GO:0006351 GO:0015991 GO:0033743 GO:0003899 GO:0005515 GO:0016820 GO:0000221 GO:0005730

Cluster-22178.30404 GO:0006364 GO:0032040

Cluster-22178.30459 GO:0006144 GO:0032509 GO:0006182 GO:0046039 GO:0043130 GO:0020037 GO:0004383 GO:0000813

Cluster-22178.30559 GO:0006468 GO:0005515 GO:0005524 GO:0004672

Cluster-22178.30830 GO:0005515 GO:0046983 GO:0008270

Cluster-22178.30924 GO:0008063 GO:0051252 GO:0006869 GO:0004540

GO:0005319 GO:0019031
Cluster-22178.30958 GO:0015031 GO:0030168 GO:0006810 GO:0007165
GO:0016567 GO:0051258 GO:0006308 GO:0007049 GO:0015628
GO:0016032 GO:0006351 GO:0008565 GO:0008855 GO:0004842
GO:0005102 GO:0030674 GO:0004866 GO:0005886 GO:0005577
GO:0017053 GO:0015627 GO:0019028 GO:0009318
Cluster-22178.30961 GO:0015628 GO:0006534 GO:0042967 GO:0015031
GO:0016032 GO:0006535 GO:0004866 GO:0008565 GO:0009001
GO:0015627 GO:0005737
Cluster-22178.31005 GO:0007186 GO:0004930 GO:0016021
Cluster-22178.31049 GO:0030170 GO:0003676
Cluster-22178.31065 GO:0006355 GO:0043565
Cluster-22178.31088 GO:0006695 GO:0006694 GO:0032297 GO:0003677
GO:0004631 GO:0005737
Cluster-22178.31136 GO:0009058 GO:0003824
Cluster-22178.31137 GO:0009058 GO:0003824
Cluster-22178.31199 GO:0006355 GO:0006260 GO:0003700 GO:0005667
Cluster-22178.31293 GO:0050790 GO:0005515 GO:0035091 GO:0030234
GO:0016773
Cluster-22178.31327 GO:0016772
Cluster-22178.31366 GO:0006771 GO:0019497 GO:0007016 GO:0003993
GO:0005515 GO:0046872 GO:0016010
Cluster-22178.31368 GO:0005515
Cluster-22178.31404 GO:0023052
Cluster-22178.31407 GO:0042254 GO:0006412 GO:0003723 GO:0003735
GO:0005840
Cluster-22178.31421 GO:0030246
Cluster-22178.31464 GO:0007165 GO:0009847 GO:0006869 GO:0042157
GO:0007067 GO:0009069 GO:0006260 GO:0006355 GO:0006468
GO:0016310 GO:0007049 GO:0004674 GO:0005096 GO:0005198
GO:0004871 GO:0003700 GO:0008289 GO:0043565 GO:0016020
GO:0005667 GO:0005815 GO:0042729 GO:0072686 GO:0019028
GO:0005576 GO:0019031

Cluster-22178.31511 GO:0009069 GO:0006468 GO:0009405 GO:0016310
GO:0005524 GO:0004672 GO:0004674
Cluster-22178.31525 GO:0003676
Cluster-22178.31560 GO:0006869 GO:0007186 GO:0004930 GO:0005319
GO:0005515 GO:0016021
Cluster-22178.31571 GO:0055114 GO:0015992 GO:0006744 GO:0006814
GO:0006120 GO:0042773 GO:0008137
Cluster-22178.31572 GO:0007275 GO:0005515 GO:0004867 GO:0005578
Cluster-22178.31635 GO:0031492 GO:0000785 GO:0005634
Cluster-22178.31639 GO:0005515
Cluster-22178.31715 GO:0006810 GO:0005215 GO:0016020
Cluster-22178.31716 GO:0003676 GO:0005515
Cluster-22178.31734 GO:0007165 GO:0040007 GO:0008283 GO:0008083

Cluster-22178.31745 GO:0019882 GO:0006955 GO:0042613 GO:0016020
Cluster-22178.3178 GO:0016021
Cluster-22178.31807 GO:0006508 GO:0006915 GO:0004222 GO:0008270

Cluster-22178.31821 GO:0007049
Cluster-22178.31823 GO:0070072
Cluster-22178.31837 GO:0010923 GO:0019902
Cluster-22178.31850 GO:0007275 GO:0009231 GO:0008686 GO:0005578
Cluster-22178.31859 GO:0007186 GO:0050909 GO:0004930 GO:0016021
Cluster-22178.31865 GO:0006470 GO:0008138
Cluster-22178.31902 GO:0006468 GO:0004672 GO:0005524
Cluster-22178.31924 GO:0006351 GO:0006270 GO:0006275 GO:0008152
GO:0006144 GO:0055114 GO:0006206 GO:0003899 GO:0050660
GO:0043565 GO:0016627 GO:0005524 GO:0005730
Cluster-22178.31929 GO:0007155 GO:0016755 GO:0016020
Cluster-22178.31987 GO:0005515
Cluster-22178.31990 GO:0051016 GO:0007264 GO:0005525 GO:0008290
Cluster-22178.32013 GO:0046983
Cluster-22178.32061 GO:0005509 GO:0005544

Cluster-22178.32070 GO:0007154 GO:0055085 GO:0016021
Cluster-22178.32078 GO:0007186 GO:0004930 GO:0005515 GO:0016021
Cluster-22178.32079 GO:0007411 GO:0007186 GO:0004930 GO:0016021
Cluster-22178.32087 GO:0007218 GO:0005198 GO:0005515 GO:0005184
GO:0046872 GO:0005576
Cluster-22178.32090 GO:0005515
Cluster-22178.32105 GO:0005515
Cluster-22178.32118 GO:0055114 GO:0016491
Cluster-22178.3212 GO:0006525 GO:0006560 GO:0006814 GO:0071436
GO:0006090 GO:0008948 GO:0015081 GO:0016020
Cluster-22178.32137 GO:0006811 GO:0006810 GO:0006508 GO:0016805
GO:0005215 GO:0016020
Cluster-22178.32172 GO:0016021
Cluster-22178.32207 GO:0008270
Cluster-22178.32235 GO:0006886 GO:0008536
Cluster-22178.32246 GO:0042254 GO:0006412 GO:0006886 GO:0006888
GO:0003735 GO:0008270 GO:0030127 GO:0005840
Cluster-22178.32254 GO:0006260 GO:0006281 GO:0003911 GO:0004222
GO:0008270 GO:0005524 GO:0016021
Cluster-22178.32268 GO:0007165 GO:0006955 GO:0006412 GO:0042254
GO:0030247 GO:0003735 GO:0005044 GO:0005840 GO:0005622
Cluster-22178.32280 GO:0008152 GO:0003824
Cluster-22178.32303 GO:0042981 GO:0005515
Cluster-22178.32318 GO:0016020
Cluster-22178.32339 GO:0006388 GO:0003950 GO:0016772
Cluster-22178.32362 GO:0015992 GO:0006123 GO:0004129 GO:0045277
Cluster-22178.32369 GO:0006508 GO:0016787 GO:0008236
Cluster-22178.32371 GO:0006206 GO:0006144 GO:0006352 GO:0006355
GO:0006351 GO:0016987 GO:0003700 GO:0003677 GO:0005515
GO:0003899 GO:0005730 GO:0005667
Cluster-22178.32391 GO:0000287
Cluster-22178.32413 GO:0004190 GO:0016021
Cluster-22178.32422 GO:2001256 GO:0030176

Cluster-22178.32464 GO:0046983

Cluster-22178.32502 GO:0005515

Cluster-22178.32511 GO:0042254 GO:0006412 GO:0030414 GO:0003735 GO:0005525 GO:0005840

Cluster-22178.32512 GO:0006412 GO:0042254 GO:0005525 GO:0003735 GO:0030414 GO:0005840

Cluster-22178.32514 GO:0003677

Cluster-22178.32520 GO:0015992 GO:0006120 GO:0006744 GO:0006355 GO:0006118 GO:0006814 GO:0003954 GO:0008137 GO:0005515 GO:0005739

Cluster-22178.32578 GO:0055114 GO:0016705 GO:0020037 GO:0005506

Cluster-22178.32580 GO:0001522 GO:0009451 GO:0003723 GO:0009982

Cluster-22178.32597 GO:0007049 GO:0006355 GO:0043093 GO:0000917 GO:0043565 GO:0003700 GO:0003677 GO:0005737 GO:0005634 GO:0005667

Cluster-22178.32605 GO:0030414 GO:0005509

Cluster-22178.32610 GO:0006144 GO:0006206 GO:0015074 GO:0006351 GO:0003899 GO:0003677 GO:0005730

Cluster-22178.32631 GO:0051382 GO:0048870 GO:0007049 GO:0051225 GO:0006886 GO:0051607 GO:0070206 GO:0006914 GO:0031023 GO:0006606 GO:0007165 GO:0006952 GO:0009264 GO:0008253 GO:0045502 GO:0042803 GO:0008134 GO:0005198 GO:0005515 GO:0003677 GO:0019031 GO:0016021 GO:0000776 GO:0005576 GO:0019028 GO:0031514 GO:0030286 GO:0005815 GO:0005667 GO:0005882 GO:0005783

Cluster-22178.32663 GO:0046983

Cluster-22178.32677 GO:0003676

Cluster-22178.32720 GO:0005515

Cluster-22178.32728 GO:0055114 GO:0016972

Cluster-22178.32729 GO:0042967 GO:0008080 GO:0016747

Cluster-22178.32732 GO:0006355 GO:0000975 GO:0042302 GO:0003700

GO:0005667
Cluster-22178.32740 GO:0006355 GO:0003700 GO:0043565 GO:0005634
GO:0005667
Cluster-22178.32744 GO:0007264 GO:0005525 GO:0003924 GO:0005524

Cluster-22178.32749 GO:0005515
Cluster-22178.32751 GO:0055085 GO:0022857 GO:0005887 GO:0016021
GO:0036128
Cluster-22178.32759 GO:0007186 GO:0009236 GO:0004930 GO:0048472
GO:0016021
Cluster-22178.32760 GO:0007186 GO:0004930 GO:0016021
Cluster-22178.32761 GO:0009236 GO:0007186 GO:0004930 GO:0048472
GO:0016021
Cluster-22178.32762 GO:0005515
Cluster-22178.32780 GO:0007217
Cluster-22178.32805 GO:0009966 GO:0007275 GO:0045444 GO:0016020
Cluster-22178.32823 GO:0009890
Cluster-22178.32824 GO:0006570 GO:0006470 GO:0004725 GO:0008138

Cluster-22178.32841 GO:0006118 GO:0008608 GO:0005975 GO:0030246
GO:0003954 GO:0004553 GO:0008270 GO:0008440 GO:0042729
GO:0005576 GO:0072686 GO:0005739
Cluster-22178.32843 GO:0005515
Cluster-22178.32895 GO:0006810 GO:0000917 GO:0006355 GO:0043093
GO:0003700 GO:0043565 GO:0005667 GO:0005886 GO:0005737
Cluster-22178.32935 GO:0007212 GO:0048268 GO:0032051 GO:0008270
GO:0016021
Cluster-22178.32945 GO:0006108 GO:0006810 GO:0006099 GO:0006090
GO:0015976 GO:0055114 GO:0004471 GO:0005471 GO:0051287
GO:0005524 GO:0016021
Cluster-22178.32946 GO:0006099 GO:0006108 GO:0055114 GO:0015976
GO:0006090 GO:0051287 GO:0004471 GO:0005515
Cluster-22178.32963 GO:0006468 GO:0004672 GO:0005524

Cluster-22178.32975 GO:0016021
Cluster-22178.32976 GO:0006836 GO:0006812 GO:0005328 GO:0016021
Cluster-22178.33026 GO:0006468 GO:0016773 GO:0004672 GO:0005524
GO:0016020
Cluster-22178.33067 GO:0006816 GO:0006810 GO:0006814 GO:0015992
GO:0006744 GO:0006120 GO:0005246 GO:0008137 GO:0046872
GO:0042030 GO:0016020 GO:0005743
Cluster-22178.33072 GO:0006629 GO:0016627 GO:0005737 GO:0016021
Cluster-22178.33086 GO:0018874 GO:0042207 GO:0006568 GO:0018822

Cluster-22178.33094 GO:0006950 GO:0006508 GO:0007186 GO:0008270
GO:0004930 GO:0004190 GO:0005198 GO:0005634 GO:0019028
Cluster-22178.33114 GO:0007606 GO:0016021
Cluster-22178.33118 GO:2001070
Cluster-22178.33140 GO:0005509
Cluster-22178.33149 GO:0031412 GO:0009405 GO:0005524 GO:0003774
GO:0005515 GO:0031411 GO:0016459 GO:0016020
Cluster-22178.33153 GO:0007165 GO:0005509 GO:0005578
Cluster-22178.33171 GO:0007049 GO:0017053
Cluster-22178.33174 GO:0006814 GO:0006813 GO:0005890
Cluster-22178.33183 GO:0006629 GO:0042967 GO:0008374
Cluster-22178.33188 GO:0006448 GO:0006414 GO:0003746 GO:0005515
GO:0005840
Cluster-22178.33211 GO:0009308 GO:0055114 GO:0008131 GO:0048038
GO:0005507
Cluster-22178.33222 GO:0006355 GO:0008270 GO:0003700 GO:0005515
GO:0005634 GO:0005667
Cluster-22178.33224 GO:0006355 GO:0005515 GO:0003700 GO:0008270
GO:0005634 GO:0005667
Cluster-22178.33228 GO:0006810 GO:0019083 GO:0055085 GO:0003924
GO:0016887 GO:0005524 GO:0042626 GO:0005525 GO:0016021
Cluster-22178.33235 GO:0006629 GO:0009058 GO:0005524 GO:0016874

Cluster-22178.33242 GO:0016310 GO:0009069 GO:0006468 GO:0004674
GO:0005524 GO:0000287
Cluster-22178.33258 GO:0006807 GO:0016310 GO:0009069 GO:0006468
GO:0005524 GO:0004672 GO:0016773 GO:0004668 GO:0016151
GO:0004674 GO:0005509 GO:0005737 GO:0016020
Cluster-22178.33293 GO:0006511 GO:0007186 GO:0007218 GO:0004930
GO:0016021 GO:0005576
Cluster-22178.33352 GO:0031505 GO:0006817 GO:0005315 GO:0016020
Cluster-22178.33361 GO:0009056 GO:0031047 GO:0006308 GO:0005975
GO:0004560 GO:0016888 GO:0008270
Cluster-22178.33368 GO:0006810 GO:0046034 GO:0006144 GO:0005515
GO:0004017 GO:0042626 GO:0046872 GO:0005524 GO:0016021
Cluster-22178.33387 GO:0006355 GO:0043401 GO:0043565 GO:0008270
GO:0003700 GO:0005667 GO:0005634
Cluster-22178.33406 GO:0007165 GO:0008283 GO:0040007 GO:0008083

Cluster-22178.33415 GO:0042742
Cluster-22178.33425 GO:0043087 GO:0035023 GO:0005089
Cluster-22178.33426 GO:0043087 GO:0035023 GO:0005089
Cluster-22178.33427 GO:0006355 GO:0007165 GO:0006810 GO:0055085
GO:0005524 GO:0042626 GO:0003712 GO:0005667 GO:0016021
Cluster-22178.33439 GO:0006468 GO:0000746 GO:0005524 GO:0004672
GO:0005515
Cluster-22178.33460 GO:0006281 GO:0006260 GO:0016787 GO:0046872
GO:0005515 GO:0003911
Cluster-22178.33525 GO:0005515
Cluster-22178.33530 GO:0006470 GO:0006570 GO:0004725 GO:0008138
GO:0005515
Cluster-22178.33537 GO:0008017 GO:0045298
Cluster-22178.33545 GO:0006825 GO:0006396 GO:0019087 GO:0035434
GO:0005375 GO:0019031 GO:0016021 GO:0005622
Cluster-22178.33574 GO:0043065 GO:0005740 GO:0016021
Cluster-22178.33617 GO:0007165 GO:0005515

Cluster-22178.33628 GO:0016567 GO:0004842
Cluster-22178.33634 GO:0055114 GO:0032922 GO:0016491 GO:0000976

Cluster-22178.33666 GO:0016567 GO:0008270 GO:0046872 GO:0005515
GO:0004842 GO:0005680
Cluster-22178.33685 GO:0051260 GO:0016020
Cluster-22178.33692 GO:0046813 GO:0061025 GO:0007165 GO:0006355
GO:0043565 GO:0005515 GO:0016021 GO:0019031 GO:0005634
Cluster-22178.33693 GO:0007165 GO:0005515
Cluster-22178.33698 GO:0042330
Cluster-22178.33708 GO:0009405 GO:0042254 GO:0006412 GO:0005198
GO:0003735 GO:0005840
Cluster-22178.33726 GO:0006412 GO:0042254 GO:0003735 GO:0005622
GO:0005840
Cluster-22178.33727 GO:0005515
Cluster-22178.33733 GO:0003677
Cluster-22178.33736 GO:0042254 GO:0006412 GO:0003735 GO:0005840
GO:0005622
Cluster-22178.33739 GO:0019911
Cluster-22178.33771 GO:0015074 GO:0006355 GO:0008270 GO:0003700
GO:0003676 GO:0005667
Cluster-22178.33776 GO:0042254 GO:0006412 GO:0003735 GO:0005840
GO:0005622
Cluster-22178.33781 GO:0006468 GO:0005524 GO:0004672 GO:0003677

Cluster-22178.33792 GO:0006886 GO:0006888 GO:0008270 GO:0030127
Cluster-22178.33798 GO:0006013 GO:0006000 GO:0009298 GO:0004615
GO:0005737
Cluster-22178.33840 GO:0001558 GO:0005520 GO:0005515 GO:0016942
GO:0005576
Cluster-22178.33855 GO:0005198 GO:0016787
Cluster-22178.33856 GO:0006508 GO:0016787 GO:0005198 GO:0008236

Cluster-22178.33861 GO:0003774 GO:0005506 GO:0005524 GO:0005515 GO:0016459

Cluster-22178.33865 GO:0006355 GO:0015684 GO:0015093 GO:0005622 GO:0016021

Cluster-22178.33891 GO:0006355 GO:0003677

Cluster-22178.33905 GO:0003676

Cluster-22178.33920 GO:0019058

Cluster-22178.33923 GO:0006270 GO:0016567 GO:0006260 GO:0006355 GO:0006351 GO:0003682 GO:0003677 GO:0008270 GO:0004842 GO:0003887 GO:0005515 GO:0005680 GO:0005634 GO:0042575 GO:0005737 GO:0000775 GO:0000785

Cluster-22178.33944 GO:0016021

Cluster-22178.33955 GO:0009847 GO:0008168 GO:0046983 GO:0016021

Cluster-22178.33962 GO:0016301

Cluster-22178.33963 GO:0006810 GO:0005215 GO:0046872 GO:0016020

Cluster-22178.33973 GO:0042254 GO:0005622

Cluster-22178.33984 GO:0009250 GO:0046527

Cluster-22178.33985 GO:0006810 GO:0055085 GO:0016020

Cluster-22178.33991 GO:0007264 GO:0005096 GO:0008270

Cluster-22178.34003 GO:0006508 GO:0007165 GO:0008158 GO:0004197 GO:0016020 GO:0031225

Cluster-22178.34016 GO:0055114 GO:0016491 GO:0016209

Cluster-22178.34030 GO:0030435 GO:0006351 GO:0005509 GO:0043565

Cluster-22178.34039 GO:0003677

Cluster-22178.34061 GO:0005515

Cluster-22178.34072 GO:0016301

Cluster-22178.34105 GO:0007187 GO:0006468 GO:0009069 GO:0007608 GO:0016310 GO:0007178 GO:0005549 GO:0004675 GO:0004984 GO:0004672 GO:0005524 GO:0016020

Cluster-22178.34107 GO:0003723

Cluster-22178.34121 GO:0008191

Cluster-22178.34125 GO:2001070 GO:0046983 GO:0008270 GO:0005515

GO:0003677 GO:0019867
Cluster-22178.34129 GO:0007165 GO:0006508 GO:0004197 GO:0008234
GO:0005179 GO:0005576
Cluster-22178.34182 GO:0009968 GO:0005737
Cluster-22178.34202 GO:0006308 GO:0009307 GO:0003677 GO:0009036
GO:0009359
Cluster-22178.34210 GO:0006814 GO:0042773 GO:0055114 GO:0015992
GO:0006120 GO:0006744 GO:0008137 GO:0016651
Cluster-22178.34215 GO:0005524 GO:0003774 GO:0016459
Cluster-22178.34228 GO:0006672 GO:0006807 GO:0016811 GO:0005524
GO:0016021
Cluster-22178.34232 GO:0008270 GO:0005634
Cluster-22178.34235 GO:0043087 GO:0035023 GO:0035556 GO:0019825
GO:0005089 GO:0005515
Cluster-22178.34238 GO:0005515
Cluster-22178.34272 GO:0005515 GO:0003677 GO:0008270
Cluster-22178.34276 GO:0042254 GO:0006412 GO:0003735 GO:0005622
GO:0005840
Cluster-22178.34278 GO:0006118 GO:0009055 GO:0020037 GO:0005515

Cluster-22178.34285 GO:0042981 GO:0043065 GO:0016192 GO:0006886
GO:0005198 GO:0005737 GO:0030130 GO:0030132
Cluster-22178.34291 GO:0006457 GO:0006366 GO:0003677 GO:0005737
GO:0005665
Cluster-22178.34292 GO:0006366 GO:0006457 GO:0003677 GO:0005665
GO:0005737
Cluster-22178.34299 GO:0046486 GO:0009058 GO:0006886 GO:0008218
GO:0006508 GO:0006629 GO:0016042 GO:0008152 GO:0006505
GO:0008236 GO:0016788 GO:0016787 GO:0003847 GO:0008247
Cluster-22178.34303 GO:0005515
Cluster-22178.34338 GO:0003676 GO:0005524
Cluster-22178.34344 GO:0006355 GO:0005524 GO:0005634
Cluster-22178.34346 GO:0006464

Cluster-22178.34372 GO:0006820 GO:0008509 GO:0016021
Cluster-22178.34379 GO:0019079 GO:0003723 GO:0005515
Cluster-22178.34392 GO:0006810 GO:0016887 GO:0003924 GO:0005524
GO:0003676 GO:0005525
Cluster-22178.34398 GO:0007034
Cluster-22178.34416 GO:0006570 GO:0006493 GO:0006470 GO:0008138
GO:0000030 GO:0004725 GO:0016020 GO:0000136
Cluster-22178.34420 GO:0006118 GO:0015992 GO:0055114 GO:0009060
GO:0006123 GO:0005506 GO:0020037 GO:0009055 GO:0004129
GO:0045277 GO:0016021
Cluster-22178.34432 GO:0005198 GO:0003677
Cluster-22178.34443 GO:0000917 GO:0006355 GO:0043093 GO:0007049
GO:0055085 GO:0003700 GO:0046983 GO:0043565 GO:0016020
GO:0005667 GO:0016021 GO:0005737
Cluster-22178.34448 GO:0005524 GO:0003774 GO:0005515 GO:0016459
Cluster-22178.34450 GO:0007165 GO:0016020
Cluster-22178.34454 GO:0046872
Cluster-22178.34459 GO:0030246
Cluster-22178.34463 GO:0005515
Cluster-22178.34481 GO:0005525
Cluster-22178.34496 GO:0006355 GO:0043401 GO:0007186 GO:0004930
GO:0016021
Cluster-22178.34502 GO:0005515
Cluster-22178.34503 GO:0042254 GO:0006412 GO:0009058 GO:0003824
GO:0003735 GO:0005622 GO:0005840
Cluster-22178.34506 GO:0006886 GO:0003677 GO:0030176
Cluster-22178.34523 GO:0005515
Cluster-22178.34527 GO:0005515
Cluster-22178.34542 GO:0046856 GO:0030246 GO:0034597
Cluster-22178.34557 GO:0008152 GO:0006508 GO:0004197 GO:0003824
GO:0003676
Cluster-22178.34573 GO:0005576
Cluster-22178.34574 GO:0005576

Cluster-22178.34575 GO:0005856
Cluster-22178.34583 GO:0009294 GO:0008152 GO:0016787
Cluster-22178.34600 GO:0015074 GO:0004222
Cluster-22178.34613 GO:0003950 GO:0005634 GO:0016020
Cluster-22178.34651 GO:0005783 GO:0016021
Cluster-22178.34656 GO:0007186 GO:0004930 GO:0016021
Cluster-22178.34670 GO:0006355 GO:0003682 GO:0003677 GO:0005634 GO:0000775 GO:0000785
Cluster-22178.34681 GO:0005509 GO:0005515 GO:0005576
Cluster-22178.34690 GO:0030036 GO:0003779 GO:0030054
Cluster-22178.34691 GO:0006508 GO:0004198 GO:0005515 GO:0005622
Cluster-22178.34697 GO:0006355 GO:0003682 GO:0043565 GO:0008270 GO:0003700 GO:0005667 GO:0000785
Cluster-22178.34700 GO:0008168 GO:0019028
Cluster-22178.34702 GO:0005515
Cluster-22178.34736 GO:0008253 GO:0000287 GO:0005737
Cluster-22178.34738 GO:0016773
Cluster-22178.34740 GO:0007186 GO:0004930 GO:0016020 GO:0016021
Cluster-22178.34745 GO:0006412 GO:0008168 GO:0051537 GO:0016021
Cluster-22178.34768 GO:0006468 GO:0005515 GO:0005524 GO:0004672

Cluster-22178.34779 GO:0006468 GO:0016773 GO:0008270 GO:0005524 GO:0004672 GO:0005515 GO:0016020
Cluster-22178.34812 GO:2001070 GO:0005515
Cluster-22178.34819 GO:0006118 GO:0009405 GO:0045735 GO:0015035

Cluster-22178.34844 GO:0006508 GO:0008270 GO:0004222 GO:0031012
Cluster-22178.34882 GO:0046836 GO:0046872 GO:0051861 GO:0017089 GO:0005737
Cluster-22178.34889 GO:0046486 GO:0005975 GO:0046168 GO:0055114 GO:0006072 GO:0051287 GO:0016616 GO:0004367 GO:0009331
Cluster-22178.34897 GO:0006810 GO:0006629 GO:0005215 GO:0016627 GO:0016021 GO:0005737

Cluster-22178.34911 GO:0007165 GO:0006807 GO:0016811 GO:0005515

Cluster-22178.34917 GO:0040007 GO:0008283 GO:0007165 GO:0008083

Cluster-22178.34924 GO:0000226 GO:0006355 GO:0005515 GO:0015630
GO:0016020

Cluster-22178.34926 GO:0006013 GO:0006000 GO:0005524 GO:0003873

Cluster-22178.34932 GO:0005869

Cluster-22178.34942 GO:0005515

Cluster-22178.34948 GO:0007275 GO:0005578

Cluster-22178.34950 GO:0016020

Cluster-22178.34951 GO:0016020

Cluster-22178.34956 GO:0016567 GO:0008270 GO:0046872 GO:0005515
GO:0004842 GO:0005680

Cluster-22178.34964 GO:0009898 GO:0016021

Cluster-22178.34967 GO:0006621 GO:0046923 GO:0016021 GO:0005783

Cluster-22178.34974 GO:0008219 GO:0009405 GO:0007339 GO:0005509
GO:0005515 GO:0005576

Cluster-22178.34980 GO:0007165 GO:0006955 GO:0005164 GO:0016020

Cluster-22178.34982 GO:0006807 GO:0016151

Cluster-22178.34984 GO:0005515

Cluster-22178.34995 GO:0006355 GO:0046872 GO:0005634

Cluster-22178.35003 GO:0006508 GO:0009405 GO:0004252 GO:0005576

Cluster-22178.35013 GO:0005515 GO:0016020

Cluster-22178.35015 GO:0008152 GO:0005515 GO:0016746 GO:0043565
GO:0005634

Cluster-22178.35018 GO:0019062 GO:0019058 GO:0007155 GO:0006355
GO:0005515 GO:0043565 GO:0005634

Cluster-22178.35047 GO:0006810 GO:0008200 GO:0005576

Cluster-22178.35063 GO:0019068

Cluster-22178.35071 GO:0030908 GO:0019867 GO:0016020

Cluster-22178.35096 GO:0006313 GO:0046872 GO:0003677 GO:0004803

Cluster-22178.35136 GO:0006508 GO:0004252
Cluster-22178.35146 GO:0007165 GO:0004252 GO:0019028 GO:0055036
Cluster-22178.35195 GO:0016020
Cluster-22178.35200 GO:0055085 GO:0006811 GO:0055114 GO:0015992
GO:0006118 GO:0006119 GO:0006810 GO:0042626 GO:0005524
GO:0008121 GO:0005509 GO:0005216 GO:0016021 GO:0016020
Cluster-22178.35214 GO:0004112 GO:0005515
Cluster-22178.35216 GO:0006468 GO:0008152 GO:0004672 GO:0005524
GO:0016773 GO:0016791 GO:0016020
Cluster-22178.35222 GO:0009089 GO:0016310 GO:0008270 GO:0004673
GO:0008837 GO:0009365
Cluster-22178.35240 GO:0005515
Cluster-22178.35241 GO:0046935 GO:0005944
Cluster-22178.35246 GO:0002949 GO:0015684 GO:0006576 GO:0007264
GO:0005525 GO:0016887 GO:0003924 GO:0005524 GO:0015093
GO:0016021
Cluster-22178.35256 GO:0006355 GO:0046872 GO:0003677
Cluster-22178.35260 GO:0007165 GO:0005179 GO:0005576
Cluster-22178.35264 GO:0050909 GO:0007156 GO:0007186 GO:0004930
GO:0005509 GO:0019031 GO:0016021 GO:0016020
Cluster-22178.35269 GO:0019028
Cluster-22178.35288 GO:0006935 GO:0007165 GO:0006955 GO:0008009
GO:0005576
Cluster-22178.35292 GO:0045859 GO:0006771 GO:0006000 GO:0006013
GO:0019497 GO:0007049 GO:0016538 GO:0003873 GO:0005524
GO:0003993
Cluster-22178.35307 GO:0008544 GO:0006468 GO:0005524 GO:0004672
GO:0005515
Cluster-22178.35334 GO:0005515
Cluster-22178.35352 GO:0005509
Cluster-22178.35354 GO:0005515 GO:0003676
Cluster-22178.35370 GO:0006206 GO:0006144 GO:0006470 GO:0006351

GO:0006570 GO:0004725 GO:0008138 GO:0003899 GO:0005730
Cluster-22178.35374 GO:0046983
Cluster-22178.35395 GO:0006468 GO:0005524 GO:0004672
Cluster-22178.35403 GO:0006508 GO:0004222
Cluster-22178.35416 GO:0007165 GO:0005509 GO:0005515 GO:0005578
Cluster-22178.35420 GO:0042981
Cluster-22178.35425 GO:0006355 GO:0009607 GO:0003677 GO:0004222
GO:0016021 GO:0005634
Cluster-22178.35426 GO:0006355 GO:0055085 GO:0006811 GO:0009607
GO:0003677 GO:0005216 GO:0016020 GO:0005634 GO:0016021
Cluster-22178.35434 GO:0005515 GO:0016757 GO:0016020
Cluster-22178.35471 GO:0005515
Cluster-22178.35472 GO:0003677
Cluster-22178.35473 GO:0003677
Cluster-22178.35475 GO:0006468 GO:0009069 GO:0006807 GO:0016310
GO:0005509 GO:0004674 GO:0016151 GO:0004672 GO:0005524
GO:0004668 GO:0016773 GO:0016020 GO:0005737
Cluster-22178.35482 GO:0005515
Cluster-22178.35487 GO:0055114 GO:0006120 GO:0046872
Cluster-22178.35516 GO:0042967 GO:0007015 GO:0047961 GO:0003785
GO:0005739
Cluster-22178.35532 GO:0005982 GO:0005985 GO:0015031 GO:0016157
GO:0008565 GO:0016021
Cluster-22178.35538 GO:0015986 GO:0015992 GO:0015078 GO:0000276
Cluster-22178.35549 GO:0046872
Cluster-22178.35558 GO:0007165 GO:0008289
Cluster-22178.35560 GO:0008270
Cluster-22178.35562 GO:0055114 GO:0006118 GO:0008270 GO:0016655

Cluster-22178.35564 GO:0035556 GO:0006511 GO:0004175 GO:0005515
GO:0019773
Cluster-22178.35578 GO:0043087 GO:0032012 GO:0005086 GO:0005515

Cluster-22178.35614 GO:0051262 GO:0044212 GO:0005515
Cluster-22178.35619 GO:0009069 GO:0006468 GO:0016310 GO:0004672
GO:0005524 GO:0000287 GO:0004674
Cluster-22178.35632 GO:0005509 GO:0005515 GO:0004252 GO:0005886
Cluster-22178.35643 GO:0006508 GO:0008237 GO:0008241 GO:0016020
Cluster-22178.35645 GO:0015074 GO:0016310 GO:0004673 GO:0003677
GO:0008907 GO:0009365
Cluster-22178.35648 GO:0055085 GO:0006811 GO:0005216 GO:0016020
Cluster-22178.35657 GO:0006810 GO:0015893 GO:0006855 GO:0055085
GO:0015297 GO:0015238 GO:0016020
Cluster-22178.35661 GO:0006355 GO:0043401
Cluster-22178.35663 GO:0005515
Cluster-22178.35664 GO:0005515
Cluster-22178.35690 GO:0003677
Cluster-22178.35694 GO:0006468 GO:0004672 GO:0005524
Cluster-22178.35699 GO:0042981
Cluster-22178.35714 GO:0055085 GO:0019120 GO:0016021
Cluster-22178.35720 GO:0006886 GO:0016192 GO:0005515 GO:0030117
Cluster-22178.35721 GO:0007264 GO:0003924 GO:0016887 GO:0005524
GO:0005525
Cluster-22178.35727 GO:0006629 GO:0007186 GO:0004930 GO:0016021
Cluster-22178.35743 GO:0016192 GO:0006886 GO:0005515 GO:0030117
Cluster-22178.35753 GO:0007165 GO:0043565 GO:0005515 GO:0016020
Cluster-22178.35760 GO:0003723 GO:0016021
Cluster-22178.35764 GO:0032509 GO:0043130 GO:0000813
Cluster-22178.35773 GO:0006355 GO:0043401 GO:0008270 GO:0043565
GO:0003700 GO:0005634 GO:0005667
Cluster-22178.35780 GO:0009890 GO:0005515
Cluster-22178.35781 GO:0009890 GO:0043401 GO:0006355 GO:0003700
GO:0043565 GO:0008270 GO:0005515 GO:0005634 GO:0005667
Cluster-22178.35796 GO:0046872 GO:0005515 GO:0045202
Cluster-22178.35797 GO:0005515 GO:0046872 GO:0045202
Cluster-22178.35801 GO:0007218 GO:2000123 GO:0030036 GO:0018990

GO:0003779 GO:0008255 GO:0030054
Cluster-22178.35802 GO:0006810 GO:0009405 GO:0019855 GO:0005576
Cluster-22178.35811 GO:0006457 GO:0010951 GO:0006355 GO:0015074
GO:0005515 GO:0005198 GO:0004866 GO:0003700 GO:0019028
GO:0005667
Cluster-22178.35833 GO:0006955 GO:0007165 GO:0005164 GO:0016020
Cluster-22178.35842 GO:0006269 GO:0019354 GO:0006260 GO:0006779
GO:0016117 GO:0006351 GO:0055114 GO:0008033 GO:0071949
GO:0008270 GO:0016491 GO:0050660 GO:0016705 GO:0005515
GO:0043115 GO:0003896 GO:0005730 GO:0005657
Cluster-22178.35850 GO:0006508 GO:0004222
Cluster-22178.35852 GO:0006508 GO:0004222
Cluster-22178.35862 GO:0006308 GO:0008855 GO:0051082 GO:0009318
Cluster-22178.35871 GO:0006355 GO:0003677 GO:0008270 GO:0046872
GO:0046983
Cluster-22178.35874 GO:0008152 GO:0016787
Cluster-22178.35885 GO:0006508 GO:0008033 GO:0000166 GO:0004252
GO:0005524 GO:0016879 GO:0005737
Cluster-22178.35887 GO:0005975 GO:0016773
Cluster-22178.35907 GO:0019031
Cluster-22178.35939 GO:0016021
Cluster-22178.35940 GO:0016021
Cluster-22178.35955 GO:0006506 GO:0017176 GO:0016021
Cluster-22178.35958 GO:0016310 GO:0009069 GO:0006468 GO:0004674
GO:0005524 GO:0004672
Cluster-22178.36001 GO:0009235
Cluster-22178.36004 GO:0007186 GO:0004930 GO:0016021
Cluster-22178.36017 GO:0005509 GO:0005544
Cluster-22178.36021 GO:0006935 GO:0006955 GO:0007165 GO:0008009
GO:0005576
Cluster-22178.36023 GO:0007017 GO:0007018 GO:0003777 GO:0030286
GO:0005874
Cluster-22178.36024 GO:0005515

Cluster-22178.36036 GO:0005515
Cluster-22178.36042 GO:0005515
Cluster-22178.36043 GO:0005634
Cluster-22178.36045 GO:0071630 GO:0031144 GO:0006508 GO:0006457
GO:0008270 GO:0046872 GO:0051082 GO:0003677 GO:0005515
GO:0004252 GO:0005622 GO:0009424 GO:0016272 GO:0005634
GO:0042025
Cluster-22178.36069 GO:0007165 GO:0005096
Cluster-22178.36075 GO:0006886 GO:0016192 GO:0005515 GO:0030117
Cluster-22178.36084 GO:0051252 GO:0006396 GO:0008608 GO:0003723
GO:0003676 GO:0000175 GO:0042729 GO:0072686
Cluster-22178.36093 GO:0008033 GO:0000166 GO:0005524 GO:0016879
GO:0005737
Cluster-22178.36113 GO:0007165
Cluster-22178.36118 GO:0006606 GO:0003774 GO:0005198 GO:0016459
GO:0005882
Cluster-22178.36124 GO:0005524 GO:0005515
Cluster-22178.36126 GO:0009405 GO:0051603 GO:0004298 GO:0005839
GO:0005576
Cluster-22178.36127 GO:0009405 GO:0051603 GO:0004298 GO:0005576
GO:0005839
Cluster-22178.36134 GO:0006364 GO:0051252 GO:0006955 GO:0000902
GO:0006379 GO:0006811 GO:0008033 GO:0004540 GO:0003723
GO:0004190 GO:0016020 GO:0030677 GO:0000172 GO:0016021
Cluster-22178.36139 GO:0005515
Cluster-22178.36141 GO:0005515
Cluster-22178.36149 GO:0005515
Cluster-22178.36156 GO:0009069 GO:0000746 GO:0007165 GO:0015031
GO:0006914 GO:0006606 GO:0008360 GO:0006355 GO:0006468
GO:0006886 GO:0016573 GO:0007049 GO:0016310 GO:0005524
GO:0008565 GO:0004674 GO:0051082 GO:0046872 GO:0043565
GO:0003700 GO:0005667 GO:0016020 GO:0005815 GO:0005737
GO:0016021

Cluster-22178.36157 GO:0008219 GO:0005976 GO:0006886 GO:0016779
GO:0017119 GO:0000775
Cluster-22178.36172 GO:0016032 GO:0005198 GO:0016020
Cluster-22178.36202 GO:0006468 GO:0005515 GO:0005524 GO:0008270
GO:0004672 GO:0016773 GO:0016020
Cluster-22178.36225 GO:0005509 GO:0016020
Cluster-22178.36234 GO:0046872
Cluster-22178.36238 GO:0000917 GO:0043093 GO:0006355 GO:0003677
GO:0030234 GO:0003700 GO:0043565 GO:0016020 GO:0005634
GO:0005667 GO:0005737
Cluster-22178.36240 GO:0010024 GO:0015886 GO:0055114 GO:0015232
GO:0050897 GO:0016636
Cluster-22178.36241 GO:0015994 GO:0006788 GO:0055114 GO:0004392
GO:0000975 GO:0008270
Cluster-22178.36247 GO:0071973 GO:0030288
Cluster-22178.36256 GO:0007186 GO:0004930 GO:0016021
Cluster-22178.36266 GO:0006355
Cluster-22178.36284 GO:0007186 GO:0004930 GO:0016020 GO:0016021
Cluster-22178.36286 GO:0051382 GO:0048870 GO:0015991 GO:0015031
GO:0000921 GO:0006260 GO:0006810 GO:0005975 GO:0048309
GO:0006397 GO:0006886 GO:0051028 GO:0015992 GO:0006914
GO:0006606 GO:0008092 GO:0045502 GO:0042802 GO:0008134
GO:0015078 GO:0051082 GO:0042803 GO:0016758 GO:0008565
GO:0003677 GO:0043015 GO:0016021 GO:0031514 GO:0033179
GO:0030286 GO:0005886 GO:0005940 GO:0019898 GO:0005737
GO:0000445 GO:0045298 GO:0000776 GO:0005667 GO:0016020
Cluster-22178.36292 GO:0006355 GO:0003677 GO:0003682 GO:0000775
GO:0000785 GO:0005634
Cluster-22178.36293 GO:0006355 GO:0003682 GO:0003677 GO:0005634
GO:0000775 GO:0000785
Cluster-22178.36297 GO:0006629
Cluster-22178.36298 GO:0006629
Cluster-22178.36299 GO:0006629

Cluster-22178.36301 GO:0006486 GO:0008378 GO:0016020
Cluster-22178.36310 GO:0006122 GO:0008146 GO:0005750
Cluster-22178.36363 GO:0006468 GO:0004672 GO:0005524
Cluster-22178.36372 GO:0007049 GO:0051258 GO:0030168 GO:0006357
GO:0031110 GO:0007165 GO:0006457 GO:0005102 GO:0001104
GO:0051082 GO:0030674 GO:0005577 GO:0016272 GO:0016592
Cluster-22178.36388 GO:0055085 GO:0030154 GO:0007219 GO:0030001
GO:0007275 GO:0046873 GO:0005515 GO:0016021 GO:0016020
GO:0045095
Cluster-22178.36403 GO:0042967 GO:0006355 GO:0003712 GO:0008270
GO:0004402 GO:0000123 GO:0005634 GO:0005667
Cluster-22178.36404 GO:0006355 GO:0042967 GO:0004402 GO:0008270
GO:0003712 GO:0005667 GO:0005634 GO:0000123
Cluster-22178.36405 GO:0005515
Cluster-22178.36406 GO:0005515
Cluster-22178.36412 GO:0006955 GO:0051249
Cluster-22178.36419 GO:0004252 GO:0016021
Cluster-22178.36422 GO:0044030 GO:0005634
Cluster-22178.36424 GO:0007016 GO:0019031 GO:0016010
Cluster-22178.36426 GO:0044030 GO:0005634
Cluster-22178.36429 GO:0003676 GO:0005739
Cluster-22178.36431 GO:0006402 GO:0051252 GO:0004535 GO:0046872
GO:0003676 GO:0003723 GO:0005737 GO:0005634
Cluster-22178.36436 GO:0008270
Cluster-22178.36450 GO:0004222
Cluster-22178.36457 GO:0015074 GO:0006355 GO:0003700 GO:0005667
Cluster-22178.36479 GO:0006470 GO:0006144 GO:0006206 GO:0006570
GO:0006351 GO:0004725 GO:0003899 GO:0008138 GO:0005730
Cluster-22178.36481 GO:0046339 GO:0016042 GO:0035556 GO:0042742
GO:0009069 GO:0007165 GO:0009395 GO:0055114 GO:0016310
GO:0006468 GO:0006629 GO:0006570 GO:0005096 GO:0046935
GO:0004674 GO:0004511 GO:0005509 GO:0004435 GO:0005515
GO:0005944 GO:0005576

Cluster-22178.36497 GO:0016310 GO:0031047 GO:0009069 GO:0006468
GO:0004674 GO:0004672 GO:0005524
Cluster-22178.36505 GO:0016788
Cluster-22178.36512 GO:0016310 GO:0006468 GO:0009069 GO:0009405
GO:0004672 GO:0005524 GO:0004674 GO:0045735
Cluster-22178.36513 GO:0046872 GO:0016820 GO:0000166
Cluster-22178.36522 GO:0005515 GO:0016020
Cluster-22178.36525 GO:0055114 GO:0005506 GO:0000287 GO:0030976
GO:0020037 GO:0016705
Cluster-22178.36534 GO:0003676 GO:0003723
Cluster-22178.36545 GO:0046034 GO:0006144 GO:0004017 GO:0003676
GO:0046872 GO:0016787
Cluster-22178.36572 GO:0032922 GO:0000976
Cluster-22178.36587 GO:0006355 GO:0045893 GO:0043565 GO:0003677
GO:0005634
Cluster-22178.36592 GO:0007165 GO:0005057
Cluster-22178.36601 GO:0003676
Cluster-22178.36613 GO:0007165 GO:0006566 GO:0006563 GO:0006783
GO:0042967 GO:0006544 GO:0006778 GO:0003870 GO:0005179
GO:0030170 GO:0005576 GO:0005759
Cluster-22178.36614 GO:0005515
Cluster-22178.36616 GO:0015991 GO:0015992 GO:0015078 GO:0033179
Cluster-22178.36626 GO:0006508 GO:0008152 GO:0016042 GO:0046486
GO:0016787 GO:0004806 GO:0008236
Cluster-22178.36638 GO:0009252 GO:0006807 GO:0004356
Cluster-22178.36640 GO:0016114 GO:0006694 GO:0055114 GO:0050660
GO:0004506 GO:0016021
Cluster-22178.36644 GO:0007010 GO:0003779 GO:0003676
Cluster-22178.36649 GO:0019049 GO:0042998 GO:0006355 GO:0043565
GO:0046872 GO:0008270 GO:0003700 GO:0071253 GO:0005515
GO:0005634 GO:0005667 GO:0005802
Cluster-22178.36654 GO:0005515
Cluster-22178.36659 GO:0006355 GO:0001104 GO:0005515 GO:0016592

Cluster-22178.36665 GO:0031110 GO:0006457 GO:0051082 GO:0016272
Cluster-22178.36678 GO:0035556 GO:0005515
Cluster-22178.36684 GO:0006508 GO:0004197 GO:0005515 GO:0005739
Cluster-22178.36686 GO:0006119 GO:0006118 GO:0015992 GO:0008121
GO:0009055
Cluster-22178.36700 GO:0046872 GO:0016787 GO:0005515 GO:0003676
GO:0031966
Cluster-22178.36711 GO:0031225
Cluster-22178.36718 GO:0006814 GO:0005272 GO:0016020
Cluster-22178.36729 GO:0005515
Cluster-22178.36748 GO:0008544
Cluster-22178.36749 GO:0008544
Cluster-22178.36754 GO:0000226 GO:0008608 GO:0000910 GO:0006886
GO:0008017 GO:0008536 GO:0005515 GO:0045298 GO:0042729
GO:0072686
Cluster-22178.36755 GO:0007275 GO:0006511 GO:0006886 GO:0000910
GO:0008608 GO:0000226 GO:0005515 GO:0008270 GO:0008536
GO:0008017 GO:0072686 GO:0005634 GO:0042729 GO:0045298
Cluster-22178.36777 GO:0006355 GO:0005975 GO:0016773 GO:0005524
GO:0003677
Cluster-22178.36786 GO:0005515
Cluster-22178.36790 GO:0006915 GO:0005622
Cluster-22178.36792 GO:0007219 GO:0045944 GO:0008440 GO:0003713
GO:0005667 GO:0016607
Cluster-22178.36797 GO:0019068 GO:0042438 GO:0005507
Cluster-22178.36798 GO:0003779
Cluster-22178.36814 GO:0006810 GO:0005471 GO:0005524 GO:0016021
GO:0005634
Cluster-22178.36823 GO:0006457 GO:0007165 GO:0005509 GO:0005578
Cluster-22178.36825 GO:0042254 GO:0006412 GO:0003735 GO:0005622
GO:0005840
Cluster-22178.36827 GO:0007165 GO:0004864
Cluster-22178.36828 GO:0007264 GO:0005525

Cluster-22178.36861 GO:0006355 GO:0006457 GO:0051082 GO:0005515 GO:0003677 GO:0043565 GO:0003700 GO:0005667 GO:0005634 GO:0016272

Cluster-22178.36870 GO:0005515

Cluster-22178.36873 GO:0006260 GO:0003677 GO:0008270 GO:0019033

Cluster-22178.36880 GO:0007165 GO:0043130 GO:0032266 GO:0005515

Cluster-22178.36887 GO:0008643 GO:0015693 GO:0055085 GO:0016310 GO:0009069 GO:0006810 GO:0005524 GO:0004674 GO:0005351 GO:0015095 GO:0016021 GO:0000139 GO:0016020

Cluster-22178.36897 GO:0004252 GO:0016021

Cluster-22178.36901 GO:0043565 GO:0005515 GO:0005634

Cluster-22178.36902 GO:0005515

Cluster-22178.36906 GO:0006955 GO:0007165 GO:0040007 GO:0008283 GO:0005515 GO:0005509 GO:0008083 GO:0005135 GO:0005894 GO:0005576 GO:0005578

Cluster-22178.36915 GO:0006807 GO:0005509 GO:0004668 GO:0005737

Cluster-22178.36916 GO:0005515

Cluster-22178.36922 GO:0007165 GO:0006810 GO:0007268 GO:0006811 GO:0015288 GO:0004970 GO:0005234 GO:0016020

Cluster-22178.36926 GO:0016567 GO:0004842 GO:0008270 GO:0046872 GO:0061630 GO:0005515 GO:0005680 GO:0005622

Cluster-22178.36934 GO:0009607 GO:0016021

Cluster-22178.36935 GO:0006397 GO:0003723 GO:0005515 GO:0005634 GO:0005737

Cluster-22178.36939 GO:0005524 GO:0003924 GO:0016887 GO:0005525

Cluster-22178.36944 GO:0055114 GO:0006355 GO:0006413 GO:0006351 GO:0045893 GO:0006269 GO:0006446 GO:0043565 GO:0003700 GO:0003896 GO:0008270 GO:0004386 GO:0047134 GO:0003743 GO:0003677 GO:0005840 GO:0005634 GO:0005657 GO:0005667 GO:0005730

Cluster-22178.36969 GO:0003676 GO:0008376 GO:0032580

Cluster-22178.36983 GO:0006511 GO:0006886 GO:0007275 GO:0000910
GO:0000226 GO:0008608 GO:0005515 GO:0008270 GO:0008017
GO:0008536 GO:0005634 GO:0072686 GO:0042729 GO:0045298
Cluster-22178.37005 GO:0016757 GO:0005515 GO:0016020
Cluster-22178.37009 GO:0051258 GO:0030168 GO:0007165 GO:0030674
GO:0005102 GO:0005577
Cluster-22178.37013 GO:0009405 GO:0005515 GO:0003682 GO:0000785
GO:0005576
Cluster-22178.37020 GO:0055085 GO:0046872 GO:0016020 GO:0016021
GO:0045202
Cluster-22178.37023 GO:0035556 GO:0009190 GO:0020037 GO:0016849
GO:0019825
Cluster-22178.37024 GO:0035556 GO:0009190 GO:0016849
Cluster-22178.37036 GO:0006182 GO:0046039 GO:0009190 GO:0006144
GO:0035556 GO:0016849 GO:0004383 GO:0020037
Cluster-22178.37043 GO:0005525 GO:0003924 GO:0016887 GO:0005524

Cluster-22178.37047 GO:0005515
Cluster-22178.37048 GO:0005515
Cluster-22178.37051 GO:0006508 GO:0045893 GO:1902208 GO:0016579
GO:0004843 GO:0003677 GO:0008270 GO:0051536 GO:0036459
Cluster-22178.37067 GO:0005515
Cluster-22178.37072 GO:0006605 GO:0007165 GO:0006811 GO:0004190
GO:0016021 GO:0016020
Cluster-22178.37074 GO:0006468 GO:0006355 GO:0003700 GO:0004672
GO:0005524 GO:0008270 GO:0043565 GO:0016773 GO:0016020
GO:0005667 GO:0005794
Cluster-22178.37110 GO:0007165 GO:0005515
Cluster-22178.37134 GO:0006310 GO:0019079 GO:0055085 GO:0006260
GO:0006206 GO:0006281 GO:0006355 GO:0006139 GO:0003677
GO:0009378 GO:0003724 GO:0004127 GO:0016887 GO:0005524
GO:0008134 GO:0016301 GO:0003723 GO:0022857 GO:0003678
GO:0009379 GO:0005667 GO:0005657 GO:0016021

Cluster-22178.37153 GO:0046872
Cluster-22178.37192 GO:0005515
Cluster-22178.37198 GO:0006284 GO:0016787 GO:0003677 GO:0003905
GO:0016021
Cluster-22178.37199 GO:0001503 GO:0005515 GO:0005509
Cluster-22178.37206 GO:0050909 GO:0007156 GO:0007186 GO:0005509
GO:0004930 GO:0016020 GO:0019031 GO:0016021
Cluster-22178.37210 GO:0005515
Cluster-22178.37218 GO:0007165
Cluster-22178.37219 GO:0005515
Cluster-22178.37222 GO:0001503 GO:0007155 GO:0005515 GO:0005634
Cluster-22178.37225 GO:0042802 GO:0005515
Cluster-22178.37232 GO:0007156 GO:0005509 GO:0016020
Cluster-22178.37233 GO:0003924
Cluster-22178.37241 GO:0007165 GO:0008283 GO:0040007 GO:0008083

Cluster-22178.37257 GO:0005515 GO:0016020
Cluster-22178.37259 GO:0007165 GO:0006629 GO:0005524 GO:0005179
GO:0016627 GO:0016021 GO:0005737
Cluster-22178.37260 GO:0006629 GO:0007165 GO:0005524 GO:0005179
GO:0016627 GO:0005737 GO:0016021
Cluster-22178.37261 GO:0006629 GO:0007165 GO:0005524 GO:0005179
GO:0016627 GO:0005737 GO:0016021
Cluster-22178.37263 GO:0005515
Cluster-22178.37273 GO:0005515
Cluster-22178.37282 GO:0008168
Cluster-22178.37303 GO:0006123 GO:0015992 GO:0004129 GO:0005515
GO:0045277
Cluster-22178.37314 GO:0005515
Cluster-22178.37319 GO:0006508 GO:0004222
Cluster-22178.37345 GO:0006811 GO:0006816 GO:0005244 GO:0005262
GO:0016021
Cluster-22178.37354 GO:0046872

Cluster-22178.37355 GO:0006879 GO:0008199
Cluster-22178.37356 GO:0006879 GO:0008199
Cluster-22178.37363 GO:0006935 GO:0007154 GO:0007165 GO:0009451
GO:0006955 GO:0001522 GO:0009982 GO:0003723 GO:0005509
GO:0008009 GO:0005515 GO:0005576 GO:0016020
Cluster-22178.37373 GO:0006352 GO:0006606 GO:0006355 GO:0003700
GO:0016987 GO:0019904 GO:0003677 GO:0005667
Cluster-22178.37386 GO:0006508 GO:0016787 GO:0008236 GO:0005576
Cluster-22178.37388 GO:0007186 GO:0004930 GO:0016021
Cluster-22178.37391 GO:0008270
Cluster-22178.37398 GO:0008270
Cluster-22178.37400 GO:0030163 GO:0008914
Cluster-22178.37404 GO:0005515
Cluster-22178.37407 GO:0005515
Cluster-22178.37408 GO:0006811 GO:0006270 GO:0004190 GO:0046983
GO:0016020 GO:0016021
Cluster-22178.37413 GO:0016032 GO:0019012
Cluster-22178.37414 GO:0006811 GO:0006810 GO:0005230 GO:0016020
Cluster-22178.37417 GO:0030153 GO:0006955 GO:0015643 GO:0019814
Cluster-22178.37430 GO:0008608 GO:0006950 GO:0005515 GO:0072686
GO:0042729
Cluster-22178.37481 GO:0006807 GO:0009399 GO:0019337 GO:0055114
GO:0007165 GO:0006355 GO:0016163 GO:0004872 GO:0003677
GO:0016020 GO:0016610
Cluster-22178.37486 GO:0005737
Cluster-22178.37500 GO:0006810 GO:0007165 GO:0042626 GO:0005524
GO:0005158 GO:0016021 GO:0005899
Cluster-22178.37504 GO:0009116
Cluster-22178.37511 GO:0009878 GO:0046872 GO:0005923
Cluster-22178.37518 GO:0034080 GO:0008270 GO:0000775
Cluster-22178.37519 GO:0055114 GO:0010024 GO:0009058 GO:0050897
GO:0005524 GO:0016779 GO:0016636
Cluster-22178.37533 GO:0005515

Cluster-22178.37535 GO:0005515
Cluster-22178.37539 GO:0006955 GO:0007165 GO:0005164 GO:0016020
Cluster-22178.37558 GO:0015979 GO:0016310 GO:0006468 GO:0009069
GO:0016773 GO:0004672 GO:0005524 GO:0004674 GO:0009523
GO:0009539 GO:0016020
Cluster-22178.37568 GO:0016567 GO:0004842 GO:0008270 GO:0046872

Cluster-22178.37577 GO:0016012 GO:0016021
Cluster-22178.37579 GO:0016012 GO:0016021
Cluster-22178.37592 GO:0051726
Cluster-22178.37597 GO:0006355 GO:0043565 GO:0003700 GO:0005667
Cluster-22178.37602 GO:0008152 GO:0019521 GO:0055114 GO:0006098
GO:0004616 GO:0016616 GO:0051287
Cluster-22178.37605 GO:0006470 GO:0006570 GO:0004725 GO:0008138

Cluster-22178.37615 GO:0055114 GO:0009308 GO:0007067 GO:0006355
GO:0043565 GO:0003700 GO:0048038 GO:0005507 GO:0008131
GO:0019901 GO:0072686 GO:0005667 GO:0042729
Cluster-22178.37622 GO:0006357 GO:0007165 GO:0005179 GO:0042393
GO:0001104 GO:0005576 GO:0016592
Cluster-22178.37627 GO:0055114 GO:0005515 GO:0016491
Cluster-22178.37662 GO:0007186 GO:0004930 GO:0005509 GO:0016021
Cluster-22178.37664 GO:0006885 GO:0006814 GO:0016021 GO:0019028
Cluster-22178.37671 GO:0042802 GO:0005515
Cluster-22178.37678 GO:0006355 GO:0005634
Cluster-22178.37683 GO:0003677 GO:0003824 GO:0030151 GO:0030170
GO:0005634
Cluster-22178.37686 GO:0006810 GO:0046849 GO:0006355 GO:0006352
GO:0030420 GO:0003677 GO:0008200 GO:0016987 GO:0003700
GO:0005576 GO:0005667
Cluster-22178.37688 GO:0005515
Cluster-22178.37690 GO:0009405 GO:0019867
Cluster-22178.37691 GO:0055085 GO:0006811 GO:0005975 GO:0016857

GO:0005216 GO:0016020
Cluster-22178.37695 GO:0005975 GO:0004553 GO:0030248 GO:0005576
Cluster-22178.37708 GO:0006355 GO:0005515 GO:0003700 GO:0043565
GO:0051082 GO:0005737 GO:0005667
Cluster-22178.37721 GO:0031556 GO:0031554
Cluster-22178.37729 GO:0006098 GO:0006144 GO:0009165 GO:0019825
GO:0000287 GO:0004749 GO:0020037
Cluster-22178.37731 GO:0006098 GO:0009165 GO:0006144 GO:0019825
GO:0000287 GO:0004749 GO:0020037
Cluster-22178.37739 GO:0015074
Cluster-22178.37745 GO:0006633 GO:0042967 GO:0055114 GO:0004318
GO:0005835
Cluster-22178.37747 GO:0006355 GO:0003677
Cluster-22178.37761 GO:0005509 GO:0005515
Cluster-22178.37776 GO:0004252 GO:0005886 GO:0016021
Cluster-22178.37790 GO:0016021
Cluster-22178.37808 GO:0051262 GO:0006357 GO:0001104 GO:0046983
GO:0016021 GO:0016592
Cluster-22178.37813 GO:0009877 GO:0009312 GO:0016758 GO:0005515
GO:0016021
Cluster-22178.37817 GO:0071203
Cluster-22178.37821 GO:0006144 GO:0003968 GO:0031379
Cluster-22178.37826 GO:0006265 GO:0003690
Cluster-22178.37827 GO:0055085 GO:0006811 GO:0006554 GO:0006367
GO:0006479 GO:0034968 GO:0016817 GO:0008270 GO:0005515
GO:0005216 GO:0018024 GO:0019015 GO:0016020 GO:0005672
GO:0005634
Cluster-22178.37867 GO:0005096 GO:0005515
Cluster-22178.37875 GO:0071816 GO:0016765 GO:0005515
Cluster-22178.37886 GO:0007165 GO:0006144 GO:0004114
Cluster-22178.37888 GO:0009253 GO:0006807 GO:0009252 GO:0008745

Cluster-22178.37893 GO:0019836 GO:0006744 GO:0005515 GO:0005576

Cluster-22178.37907 GO:0015876 GO:0008521 GO:0016021
Cluster-22178.37920 GO:0006566 GO:0006468 GO:0006563 GO:0006778
GO:0006783 GO:0042967 GO:0006544 GO:0003870 GO:0030170
GO:0005524 GO:0004672 GO:0016773 GO:0016020 GO:0005759
Cluster-22178.37926 GO:0016020
Cluster-22178.37935 GO:0004252 GO:0016021
Cluster-22178.37940 GO:0015979 GO:0005524 GO:0009522
Cluster-22178.37942 GO:0007275 GO:0005515 GO:0008440 GO:0042600
Cluster-22178.37950 GO:0008440
Cluster-22178.37953 GO:0006355 GO:0003677
Cluster-22178.37957 GO:0007155 GO:0005515 GO:0016020 GO:0016021
Cluster-22178.37958 GO:0055114 GO:0016730
Cluster-22178.37969 GO:0016301
Cluster-22178.37972 GO:0005515 GO:0046872 GO:0008270 GO:0003723

Cluster-22178.37975 GO:0055114 GO:0051082 GO:0016638 GO:0031072

Cluster-22178.37990 GO:0005515
Cluster-22178.37993 GO:0004659 GO:0016021
Cluster-22178.37998 GO:0006355 GO:0006144 GO:0006464 GO:0046034
GO:0004017 GO:0005515 GO:0003677 GO:0008270 GO:0046872
GO:0016151 GO:0019031
Cluster-22178.37999 GO:0046034 GO:0006144 GO:0006464 GO:0006355
GO:0016151 GO:0008270 GO:0046872 GO:0004017 GO:0003677
GO:0019031
Cluster-22178.38000 GO:0005576
Cluster-22178.38006 GO:0005515 GO:2001070
Cluster-22178.38011 GO:0016192 GO:0006886 GO:0006355 GO:0003676
GO:0003677 GO:0005198 GO:0005515 GO:0005622 GO:0030117
Cluster-22178.38019 GO:0006355 GO:0005622
Cluster-22178.38031 GO:0006915 GO:0006919 GO:0006974 GO:0001836
GO:0006412 GO:0008168
Cluster-22178.38033 GO:0006412 GO:0001836 GO:0006974 GO:0006919

GO:0006915 GO:0008168
Cluster-22178.38038 GO:0007264 GO:0007186 GO:0007165 GO:0003924
GO:0031683 GO:0019001 GO:0004871 GO:0005525
Cluster-22178.38067 GO:0006810 GO:0005515 GO:0016021
Cluster-22178.38090 GO:0007165 GO:0006955 GO:0005164 GO:0016020
Cluster-22178.38098 GO:0007165 GO:0005515
Cluster-22178.38100 GO:0006812 GO:0007165 GO:0006820 GO:0006835
GO:0005515 GO:0017153 GO:0016020
Cluster-22178.38102 GO:0006144 GO:0007165 GO:0004114
Cluster-22178.38109 GO:0055114 GO:0016209 GO:0051920 GO:0016491
GO:0005198 GO:0019028
Cluster-22178.38114 GO:0007607 GO:0050912 GO:0007187 GO:0010951
GO:0008527 GO:0004866 GO:0016021
Cluster-22178.38122 GO:0006626 GO:0005741
Cluster-22178.38123 GO:0006814 GO:0006183 GO:0006813 GO:0006228
GO:0006165 GO:0006241 GO:0006206 GO:0006144 GO:0004550
GO:0005890
Cluster-22178.38124 GO:0006183 GO:0006814 GO:0006813 GO:0006228
GO:0006165 GO:0006241 GO:0006206 GO:0006144 GO:0004550
GO:0005890
Cluster-22178.38140 GO:0007165 GO:0040007 GO:0008283 GO:0008083

Cluster-22178.38145 GO:0007049 GO:0005198 GO:0004045 GO:0005882
Cluster-22178.38148 GO:0006468 GO:0006810 GO:0006811 GO:0005230
GO:0004672 GO:0005524 GO:0016020
Cluster-22178.38150 GO:0005515 GO:0016755
Cluster-22178.38165 GO:0007156 GO:0005509 GO:0016020
Cluster-22178.38170 GO:0016757
Cluster-22178.38176 GO:0003950
Cluster-22178.38184 GO:0006355 GO:0003677
Cluster-22178.38187 GO:0007165 GO:0016020
Cluster-22178.38206 GO:0046872 GO:0008270 GO:0005634
Cluster-22178.38211 GO:0015074

Cluster-22178.38248 GO:0055085 GO:0007155 GO:0030001 GO:0008270
GO:0016853 GO:0051015 GO:0003682 GO:0046873 GO:0000785
GO:0016020
Cluster-22178.38249 GO:0006555 GO:0006556 GO:0004478
Cluster-22178.38257 GO:0006464 GO:0055114 GO:0008152 GO:0007165
GO:0006298 GO:0005524 GO:0016491 GO:0030983 GO:0019901
Cluster-22178.38261 GO:0005515
Cluster-22178.38276 GO:0005515
Cluster-22178.38286 GO:0006355 GO:0030001 GO:0055085 GO:0046873
GO:0003677 GO:0005634 GO:0016020
Cluster-22178.38290 GO:0005515
Cluster-22178.38296 GO:0055114 GO:0015992 GO:0006744 GO:0006814
GO:0006120 GO:0008137
Cluster-22178.38303 GO:0006355 GO:0003700 GO:0000975 GO:0005667
GO:0005622
Cluster-22178.38311 GO:0032968 GO:0007217 GO:0006367 GO:0006351
GO:0006270 GO:0045892 GO:0006260 GO:0003887 GO:0003677
GO:0008270 GO:0003676 GO:0005634 GO:0042575
Cluster-22178.38317 GO:0055114 GO:0016817 GO:0033905 GO:0016972

Cluster-22178.38318 GO:0071973 GO:0006935 GO:0048870 GO:0006308
GO:0006355 GO:0006412 GO:0006914 GO:0019069 GO:0042254
GO:0010508 GO:0008855 GO:0004663 GO:0045502 GO:0008134
GO:0005524 GO:0003774 GO:0008270 GO:0016755 GO:0016818
GO:0003677 GO:0003735 GO:0042803 GO:0005198 GO:0030286
GO:0005968 GO:0005882 GO:0005840 GO:0019031 GO:0019028
GO:0009318 GO:0031083 GO:0031514 GO:0005622 GO:0005667
GO:0030133 GO:0016020 GO:0009288 GO:0005634
Cluster-22178.38331 GO:0006886 GO:0046653 GO:0006355 GO:0006563
GO:0015991 GO:0006544 GO:0006528 GO:0016192 GO:0006566
GO:0016820 GO:0005515 GO:0008418 GO:0003677 GO:0008115
GO:0008270 GO:0030117 GO:0000221
Cluster-22178.38339 GO:0005887

Cluster-22178.38346 GO:0020037 GO:0016021
Cluster-22178.38347 GO:0005515
Cluster-22178.38348 GO:0006351 GO:0006206 GO:0006144 GO:0003677
GO:0003899 GO:0008242 GO:0005730
Cluster-22178.38349 GO:0005856
Cluster-22178.38359 GO:0045892 GO:0048870 GO:0006935 GO:0000160
GO:0016310 GO:0007049 GO:0015991 GO:0010508 GO:0000917
GO:0006914 GO:0015992 GO:0043093 GO:0007165 GO:0003924
GO:0015078 GO:0005525 GO:0005515 GO:0004673 GO:0000155
GO:0003677 GO:0005737 GO:0009365 GO:0031514 GO:0033179
GO:0005815
Cluster-22178.38360 GO:0000160 GO:0016310 GO:0007049 GO:0045892
GO:0048870 GO:0006935 GO:0010508 GO:0000917 GO:0015991
GO:0006355 GO:0006914 GO:0015992 GO:0007165 GO:0043093
GO:0003700 GO:0015078 GO:0005525 GO:0003924 GO:0005515
GO:0003677 GO:0000155 GO:0004673 GO:0009365 GO:0005737
GO:0031514 GO:0033179 GO:0005815 GO:0005667
Cluster-22178.38361 GO:0016310 GO:0007049 GO:0000160 GO:0006935
GO:0048870 GO:0045892 GO:0000917 GO:0010508 GO:0006355
GO:0015991 GO:0015992 GO:0006914 GO:0007165 GO:0043093
GO:0005525 GO:0015078 GO:0003700 GO:0003924 GO:0005515
GO:0003677 GO:0000155 GO:0004673 GO:0009365 GO:0005737
GO:0033179 GO:0031514 GO:0005815 GO:0005667
Cluster-22178.38369 GO:0055114 GO:0016491 GO:0005515
Cluster-22178.38371 GO:0015074
Cluster-22178.38373 GO:0007275 GO:0030178 GO:0005576
Cluster-22178.38383 GO:0006804 GO:0019079 GO:0006749 GO:0006979
GO:0055114 GO:0005515 GO:0003723 GO:0004602
Cluster-22178.38386 GO:0008053 GO:0009069 GO:0007165 GO:0016310
GO:0006468 GO:0005096 GO:0015631 GO:0004674 GO:0003924
GO:0045502 GO:0051082 GO:0042803 GO:0008134 GO:0030286
GO:0005667 GO:0005741 GO:0016021 GO:0045298
Cluster-22178.38392 GO:0055114 GO:0006364 GO:0005506 GO:0008168

GO:0005096 GO:0016705 GO:0020037
Cluster-22178.38395 GO:0007155 GO:0007165
Cluster-22178.38401 GO:0006812 GO:0032801 GO:0006090 GO:0019076
GO:0071436 GO:0006814 GO:0006560 GO:0006525 GO:0004222
GO:0015081 GO:0008948 GO:0005261 GO:0033644 GO:0016020
Cluster-22178.38418 GO:0003723 GO:0005515
Cluster-22178.38421 GO:0006355 GO:0003723 GO:0016887 GO:0005524
GO:0008134 GO:0003724 GO:0005667
Cluster-22178.38436 GO:0007178 GO:0016310 GO:0009069 GO:0006468
GO:0004672 GO:0005524 GO:0004675 GO:0016020
Cluster-22178.38455 GO:0006355 GO:0006810 GO:0007165 GO:0055085
GO:0005524 GO:0003712 GO:0042626 GO:0005667 GO:0016021
Cluster-22178.38457 GO:0006468 GO:0006270 GO:0005975 GO:0035556
GO:0004672 GO:0005524 GO:0016773 GO:0016020 GO:0032040
Cluster-22178.38463 GO:0006412 GO:0042254 GO:0003735 GO:0005622
GO:0005840
Cluster-22178.38470 GO:0005737
Cluster-22178.38479 GO:0006508 GO:0006468 GO:0003676 GO:0008270
GO:0004672 GO:0005524 GO:0005515 GO:0005634
Cluster-22178.38480 GO:0006468 GO:0006508 GO:0003676 GO:0004672
GO:0008270 GO:0005524 GO:0005515 GO:0005634
Cluster-22178.38483 GO:0006355 GO:0005515 GO:0005634 GO:0016021
Cluster-22178.38488 GO:0006955
Cluster-22178.38489 GO:0006955 GO:0016021
Cluster-22178.38493 GO:0016020
Cluster-22178.38495 GO:0006298 GO:0030983 GO:0005524
Cluster-22178.38503 GO:0006464 GO:0006355 GO:0046872 GO:0003677
GO:0016151
Cluster-22178.38514 GO:0007275 GO:0035091
Cluster-22178.38518 GO:0005507
Cluster-22178.38520 GO:0005975 GO:0006308 GO:0031047 GO:0016888
GO:0004560
Cluster-22178.38521 GO:0006308 GO:0031047 GO:0005975 GO:0004560

GO:0016888
Cluster-22178.38525 GO:0006355 GO:0005622
Cluster-22178.38529 GO:0006313 GO:0019901
Cluster-22178.38539 GO:0006811 GO:0055085 GO:0005515 GO:0005216
GO:0016020 GO:0005634
Cluster-22178.38540 GO:0003676
Cluster-22178.38545 GO:0016567 GO:0046872 GO:0008270 GO:0004842
GO:0005515 GO:0005680 GO:0042470
Cluster-22178.38552 GO:0003677 GO:0003676 GO:0008270 GO:0016021
Cluster-22178.38557 GO:0009405 GO:0005615
Cluster-22178.38559 GO:0035556 GO:0051726 GO:0005515 GO:0016021
Cluster-22178.38561 GO:0006355 GO:0003677
Cluster-22178.38569 GO:0005576
Cluster-22178.38573 GO:0055085 GO:0006810 GO:0006820 GO:0008509
GO:0005215 GO:0016021 GO:0016020
Cluster-22178.38574 GO:0006090 GO:0006811 GO:0071436 GO:0006814
GO:0006560 GO:0006525 GO:0015081 GO:0008948 GO:0005515
GO:0005198 GO:0016020 GO:0019028
Cluster-22178.38582 GO:0046500 GO:0006464 GO:0006479 GO:0008168
GO:0004719
Cluster-22178.38590 GO:0046872
Cluster-22178.38598 GO:0008270 GO:0005198
Cluster-22178.38609 GO:0046872 GO:0003677
Cluster-22178.38619 GO:0033014 GO:0055114 GO:0015994 GO:0004418
GO:0016491
Cluster-22178.38624 GO:0055114 GO:0007264 GO:0008272 GO:0016491
GO:0003924 GO:0005525 GO:0015116 GO:0016021
Cluster-22178.38626 GO:0005515
Cluster-22178.38628 GO:0043401 GO:0006355 GO:0003700 GO:0008270
GO:0043565 GO:0005667 GO:0005634
Cluster-22178.38640 GO:0009405 GO:0008219 GO:0005515 GO:0005198
GO:0005509 GO:0005576 GO:0019028
Cluster-22178.38650 GO:0016020

Cluster-22178.38653 GO:0019031
Cluster-22178.38655 GO:0016055 GO:0008013 GO:0016342
Cluster-22178.38656 GO:0006084 GO:0006508 GO:0004222 GO:0003824
GO:0031012
Cluster-22178.38660 GO:0016820 GO:0000166 GO:0046872
Cluster-22178.38692 GO:0006814 GO:0006813 GO:0005890
Cluster-22178.38704 GO:0016567 GO:0005515 GO:0004842 GO:0046872
GO:0008270 GO:0005680
Cluster-22178.38727 GO:0043401 GO:0006355 GO:0003700 GO:0043565
GO:0008270 GO:0005634 GO:0005667
Cluster-22178.38741 GO:0035556
Cluster-22178.38751 GO:0016567 GO:0004842 GO:0005515 GO:0008270
GO:0046872 GO:0005680
Cluster-22178.38779 GO:0005515
Cluster-22178.38787 GO:0016310 GO:0015074 GO:0003677 GO:0008907
GO:0004673 GO:0009365 GO:0005576
Cluster-22178.38791 GO:0004190 GO:0005515 GO:0016021
Cluster-22178.38794 GO:0006465 GO:0008233 GO:0016021 GO:0005787
Cluster-22178.38813 GO:0019064 GO:0007165 GO:0005515 GO:0046789
GO:0016788 GO:0009986 GO:0019031
Cluster-22178.38814 GO:0008152 GO:0046872 GO:0008484 GO:0003824

Cluster-22178.38826 GO:0006468 GO:0006355 GO:0016310 GO:0007049
GO:0009069 GO:0006869 GO:0009847 GO:0007165 GO:0006260
GO:0043565 GO:0003700 GO:0008289 GO:0005096 GO:0004674
GO:0005576 GO:0005667 GO:0016020 GO:0005815
Cluster-22178.38829 GO:0009372
Cluster-22178.38834 GO:0006810 GO:0015858 GO:0005337 GO:0016021
Cluster-22178.38840 GO:0007186 GO:0004930 GO:0016021
Cluster-22178.38846 GO:0005634
Cluster-22178.38847 GO:0005515
Cluster-22178.38863 GO:0016042 GO:0008203 GO:0016298
Cluster-22178.38867 GO:0008152 GO:0005515 GO:0016491

Cluster-22178.38872 GO:0006508 GO:0016032 GO:0006506 GO:0016758
GO:0008233 GO:0016021
Cluster-22178.38884 GO:0007166 GO:0005515 GO:0016021 GO:0016020
Cluster-22178.38889 GO:0046872 GO:0005515
Cluster-22178.38894 GO:0015886 GO:0015232
Cluster-22178.38906 GO:0016032 GO:0005515 GO:0042025
Cluster-22178.38907 GO:0055114 GO:0016671 GO:0003756 GO:0005783
Cluster-22178.38909 GO:0003677
Cluster-22178.38922 GO:0015684 GO:0006576 GO:0007264 GO:0005525
GO:0016887 GO:0003924 GO:0005524 GO:0015093 GO:0016021
Cluster-22178.38924 GO:0009405 GO:0003950 GO:0005576
Cluster-22178.38933 GO:0016020
Cluster-22178.38942 GO:0006508 GO:0008270 GO:0004222
Cluster-22178.38959 GO:0055114 GO:0050660 GO:0004499 GO:0016491
GO:0050661
Cluster-22178.38964 GO:0008376 GO:0016021 GO:0032580
Cluster-22178.38968 GO:0006508 GO:0008270 GO:0008233 GO:0004222
GO:0031012
Cluster-22178.38971 GO:0006887 GO:0001514 GO:0006448 GO:0020037
GO:0003723 GO:0005525 GO:0003746 GO:0000145 GO:0005840
GO:0005737
Cluster-22178.38972 GO:0008152 GO:0055114 GO:0003824 GO:0016836
GO:0016491
Cluster-22178.38973 GO:0008152 GO:0016787
Cluster-22178.38975 GO:0016055 GO:0006355 GO:0005515 GO:0043565
GO:0008013 GO:0003700 GO:0005667 GO:0016342
Cluster-22178.38976 GO:0003774 GO:0016459
Cluster-22178.38993 GO:0006260
Cluster-22178.38997 GO:0007165 GO:0006955 GO:0005164 GO:0016020
Cluster-22178.39004 GO:0003333 GO:0006865 GO:0015171 GO:0016020
Cluster-22178.39020 GO:0005515
Cluster-22178.39023 GO:0007154 GO:0016021
Cluster-22178.39025 GO:0006412 GO:0042254 GO:0006810 GO:0003735

GO:0016021 GO:0005840
Cluster-22178.39027 GO:0016020
Cluster-22178.39036 GO:0002949 GO:0015948 GO:0015684 GO:0006810
GO:0007264 GO:0055114 GO:0006576 GO:0006355 GO:0003724
GO:0016887 GO:0015093 GO:0005524 GO:0003677 GO:0005525
GO:0003924 GO:0008134 GO:0000166 GO:0003723 GO:0005667
GO:0016021
Cluster-22178.39044 GO:0006468 GO:0016773 GO:0004672 GO:0005524
GO:0016020
Cluster-22178.39056 GO:0055114 GO:0016706 GO:0008168
Cluster-22178.39077 GO:0007165 GO:0009446 GO:0040007 GO:0006508
GO:0008283 GO:0016579 GO:0006807 GO:0005515 GO:0008083
GO:0004843 GO:0004668 GO:0036459 GO:0005125 GO:0016021
Cluster-22178.39113 GO:0051321 GO:0005515
Cluster-22178.39114 GO:0055085 GO:0016021
Cluster-22178.39127 GO:0006355 GO:0003700 GO:0043565 GO:0005634
GO:0005667
Cluster-22178.39130 GO:0055085 GO:0006811 GO:0005216 GO:0016020
Cluster-22178.39132 GO:0055085 GO:0006811 GO:0005216 GO:0016020
Cluster-22178.39135 GO:0005861
Cluster-22178.39143 GO:0006355 GO:0007165 GO:0042974 GO:0005667
Cluster-22178.39145 GO:0007165 GO:0006355 GO:0042974 GO:0005667
Cluster-22178.39149 GO:0006468 GO:0006355 GO:0003677 GO:0004672
GO:0005524 GO:0016773 GO:0016020
Cluster-22178.39155 GO:0005515
Cluster-22178.39168 GO:0007605 GO:0005515
Cluster-22178.39178 GO:0051260
Cluster-22178.39195 GO:0006298 GO:0009405 GO:0005524 GO:0030983
GO:0033644
Cluster-22178.39209 GO:0006402 GO:0051252 GO:0003676 GO:0046872
GO:0004535 GO:0003723 GO:0005737 GO:0005634
Cluster-22178.39211 GO:0007586 GO:0016042 GO:0006952 GO:0006486
GO:0008047 GO:0008373 GO:0005576

Cluster-22178.39228 GO:0006810 GO:0005215 GO:0016020
Cluster-22178.39232 GO:0016021
Cluster-22178.39236 GO:0006542 GO:0006807 GO:0009252 GO:0004356

Cluster-22178.39238 GO:0042254 GO:0006412 GO:0008270 GO:0046983
GO:0003735 GO:0005622 GO:0005840
Cluster-22178.39246 GO:0006520 GO:0055114 GO:0006779 GO:0044564
GO:0008152 GO:0019354 GO:0016491 GO:0051287 GO:0043115
Cluster-22178.39260 GO:0006355 GO:0030234 GO:0004222 GO:0003677
GO:0005634 GO:0016020
Cluster-22178.39272 GO:0019752 GO:0016831 GO:0030170
Cluster-22178.39276 GO:0007010 GO:0003779
Cluster-22178.39279 GO:0015946 GO:0055114 GO:0004022
Cluster-22178.39287 GO:0042330
Cluster-22178.39293 GO:0007276 GO:0043093 GO:0000917 GO:0007165
GO:0006310 GO:0051082 GO:0005179 GO:0003677 GO:0005515
GO:0005737 GO:0005576 GO:0005634
Cluster-22178.39307 GO:0016567 GO:0004842 GO:0019031
Cluster-22178.39308 GO:0006269 GO:0019354 GO:0016117 GO:0006260
GO:0006779 GO:0006351 GO:0008033 GO:0055114 GO:0071949
GO:0016491 GO:0008270 GO:0050660 GO:0016705 GO:0043115
GO:0005515 GO:0003896 GO:0005730 GO:0005657
Cluster-22178.39313 GO:0005515 GO:0004252 GO:0005509 GO:0005886
Cluster-22178.39324 GO:0005515
Cluster-22178.39337 GO:0009117 GO:0008270 GO:0000287 GO:0000166
GO:0008253 GO:0005737
Cluster-22178.39349 GO:0005515
Cluster-22178.39369 GO:0006629
Cluster-22178.39380 GO:0007165 GO:0019064 GO:0016788 GO:0005515
GO:0046789 GO:0019031 GO:0009986
Cluster-22178.39385 GO:0000266 GO:0008270 GO:0005739
Cluster-22178.39397 GO:0032780 GO:0042030 GO:0005739
Cluster-22178.39407 GO:0006281 GO:0006836 GO:0006310 GO:0006812

GO:0005328 GO:0016021
Cluster-22178.39409 GO:0006144 GO:0006206 GO:0006351 GO:0046872
GO:0003899 GO:0003677 GO:0005730
Cluster-22178.39410 GO:0006629 GO:0006505 GO:0006886 GO:0016787
GO:0016788
Cluster-22178.39419 GO:0016567 GO:0006355 GO:0004842 GO:0003700
GO:0046872 GO:0008270 GO:0005515 GO:0005622 GO:0005667
Cluster-22178.39422 GO:0006355 GO:0003677 GO:0016787
Cluster-22178.39433 GO:0051260 GO:0016020 GO:0019033
Cluster-22178.39435 GO:0005739
Cluster-22178.39436 GO:0007186 GO:0007165 GO:0007264 GO:0003677
GO:0000166 GO:0004871 GO:0005525 GO:0003924 GO:0019001
GO:0031683 GO:0005524
Cluster-22178.39440 GO:0006935 GO:0007165 GO:0004888 GO:0016020
Cluster-22178.39443 GO:0008033 GO:0006771 GO:0019497 GO:0046872
GO:0005515 GO:0003993 GO:0016021
Cluster-22178.39446 GO:0006281 GO:0009058 GO:0006310 GO:0016742

Cluster-22178.39447 GO:0006310 GO:0009058 GO:0006281 GO:0016742

Cluster-22178.39457 GO:0006120 GO:0055114
Cluster-22178.39461 GO:0003774 GO:0016459
Cluster-22178.39463 GO:0016579 GO:0006508 GO:0045893 GO:1902208
GO:0003677 GO:0004843 GO:0036459
Cluster-22178.39466 GO:0005515 GO:0016021
Cluster-22178.39468 GO:0042254 GO:0006412 GO:0003735 GO:0005840
Cluster-22178.39477 GO:0006486 GO:0008378 GO:0016020
Cluster-22178.39480 GO:0006508 GO:0015996 GO:0008152 GO:0015994
GO:0016787 GO:0008236 GO:0047746 GO:0005576
Cluster-22178.39484 GO:0008152 GO:0016042 GO:0007586 GO:0008047
GO:0016746 GO:0005509 GO:0008236 GO:0005515 GO:0005576
Cluster-22178.39493 GO:0055114 GO:0006694 GO:0008210 GO:0008209
GO:0008207 GO:0003824 GO:0050662 GO:0003854 GO:0016616

Cluster-22178.39515 GO:0003950
Cluster-22178.39522 GO:0007186 GO:0004930 GO:0016021
Cluster-22178.39542 GO:0006812 GO:0008270 GO:0016887 GO:0045735
GO:0003676 GO:0016021
Cluster-22178.39548 GO:0006355 GO:0006352 GO:0006606 GO:0019904
GO:0003677 GO:0003700 GO:0016987 GO:0005667
Cluster-22178.39557 GO:0006810 GO:0006811 GO:0005216 GO:0016021
Cluster-22178.39558 GO:0021549 GO:0030182
Cluster-22178.39562 GO:0008270
Cluster-22178.39563 GO:0005515 GO:0016020
Cluster-22178.39569 GO:0055085 GO:0035556 GO:0016021 GO:0019028
Cluster-22178.39575 GO:0005515
Cluster-22178.39579 GO:0055114 GO:0006396 GO:0003723 GO:0008168
GO:0005515 GO:0016706
Cluster-22178.39602 GO:0006811 GO:0055085 GO:0006813 GO:0050790
GO:0015269 GO:0016286 GO:0030234 GO:0005216 GO:0005516
GO:0016021 GO:0016020
Cluster-22178.39612 GO:0009069 GO:0006468 GO:0006355 GO:0015986
GO:0016310 GO:0007178 GO:0015992 GO:0003677 GO:0004675
GO:0015078 GO:0005524 GO:0004672 GO:0016020 GO:0005634
GO:0000276
Cluster-22178.39633 GO:0016757
Cluster-22178.39637 GO:0009236 GO:0006891 GO:0048472 GO:0030173
GO:0016021
Cluster-22178.39643 GO:0006865 GO:0003333 GO:0016747 GO:0015171
GO:0005634 GO:0016020
Cluster-22178.39658 GO:0006486 GO:0009405 GO:0008376 GO:0005198
GO:0008378 GO:0016757 GO:0032580 GO:0016020
Cluster-22178.39666 GO:0032065 GO:0006355 GO:0003700 GO:0005543
GO:0043565 GO:0003677 GO:0005515 GO:0005938 GO:0005667
GO:0005634
Cluster-22178.39678 GO:0008615 GO:0055114 GO:0010181 GO:0004733

Cluster-22178.39690 GO:0055114 GO:0006694 GO:0015936 GO:0050662 GO:0004420
Cluster-22178.39696 GO:0006810 GO:0007186 GO:0004930 GO:0005215 GO:0016020 GO:0016021
Cluster-22178.39697 GO:0007186 GO:0004930 GO:0016021
Cluster-22178.39698 GO:0015672 GO:0006812 GO:0005261 GO:0016020
Cluster-22178.39711 GO:0030154 GO:0016021
Cluster-22178.39718 GO:0005515 GO:0046872 GO:0008270
Cluster-22178.39728 GO:0046872 GO:0046935 GO:0005944 GO:0005576
Cluster-22178.39729 GO:0046872
Cluster-22178.39730 GO:0005515
Cluster-22178.39733 GO:0006470 GO:0006570 GO:0004725 GO:0005515

Cluster-22178.39734 GO:0006402 GO:0055114 GO:0051252 GO:0005507 GO:0004535 GO:0046872 GO:0016641 GO:0003723 GO:0005737 GO:0005634
Cluster-22178.39736 GO:0006886 GO:0055114 GO:0007049 GO:0006606 GO:0005506 GO:0020037 GO:0016705 GO:0005634 GO:0016020
Cluster-22178.39740 GO:0051726 GO:0007186 GO:0035023 GO:0035556 GO:0055114 GO:0043087 GO:0005515 GO:0005089 GO:0004930 GO:0047134 GO:0016020
Cluster-22178.39744 GO:0006313 GO:0015074 GO:0005515 GO:0003677

Cluster-22178.39746 GO:0006355 GO:0000917 GO:0009405 GO:0007049 GO:0043093 GO:0006914 GO:0006744 GO:0006260 GO:0043565 GO:0008813 GO:0003700 GO:0003677 GO:0005576 GO:0005737 GO:0005667 GO:0005815
Cluster-22178.39750 GO:0007283 GO:0005515
Cluster-22178.39751 GO:0006355 GO:0007267 GO:0046872 GO:0003677

Cluster-22178.39758 GO:0009405 GO:0034061 GO:0003677 GO:0005515 GO:0005576 GO:0005634
Cluster-22178.39761 GO:0006355 GO:0009607 GO:0003677 GO:0004222

GO:0016021 GO:0005634
Cluster-22178.39762 GO:0040007 GO:0008283 GO:0006355 GO:0007186
GO:0007165 GO:0043401 GO:0043565 GO:0008270 GO:0003700
GO:0004930 GO:0008083 GO:0005515 GO:0016021 GO:0005667
GO:0005634
Cluster-22178.39774 GO:0055114 GO:0008152 GO:0016491
Cluster-22178.39776 GO:0006811 GO:0006508 GO:0004197 GO:0016020
Cluster-22178.39783 GO:0006396 GO:0000154 GO:0016433
Cluster-22178.39795 GO:0007029 GO:0005789
Cluster-22178.39798 GO:0019752 GO:0030170 GO:0016831
Cluster-22178.39825 GO:0007067 GO:0005975 GO:0004553 GO:0072686
GO:0042729
Cluster-22178.39827 GO:0006810 GO:0009405 GO:0019870 GO:0005576
Cluster-22178.39829 GO:0003677
Cluster-22178.39838 GO:0007264 GO:0005525
Cluster-22178.39865 GO:0007165 GO:0007186 GO:0005525 GO:0004871
GO:0031683 GO:0019001 GO:0003924
Cluster-22178.39868 GO:0002949 GO:0009058 GO:0006614 GO:0007264
GO:0016114 GO:0006694 GO:0007018 GO:0006576 GO:0007017
GO:0006355 GO:0003724 GO:0016887 GO:0005524 GO:0003676
GO:0005525 GO:0003777 GO:0003924 GO:0016301 GO:0008134
GO:0004161 GO:0003723 GO:0030286 GO:0005667 GO:0005874
Cluster-22178.39877 GO:0016021
Cluster-22178.39878 GO:0008270
Cluster-22178.39881 GO:0005515
Cluster-22178.39884 GO:0046836 GO:0035556 GO:0051861 GO:0046872
GO:0017089 GO:0003677 GO:0005737
Cluster-22178.39885 GO:0006355 GO:0016032 GO:0009405 GO:0006810
GO:0016070 GO:0016779 GO:0003677 GO:0005515 GO:0019871
GO:0005576 GO:0019012
Cluster-22178.39899 GO:0005515
Cluster-22178.39906 GO:0006811 GO:0055085 GO:0005216 GO:0016020
Cluster-22178.39907 GO:0003676

Cluster-22178.39908 GO:0006570 GO:0006470 GO:0004725 GO:0008138

Cluster-22178.39913 GO:0046935 GO:0005944

Cluster-22178.39919 GO:0007018 GO:0007017 GO:0008017 GO:0003777 GO:0005524 GO:0045298 GO:0005874

Cluster-22178.39920 GO:0007165 GO:0040007 GO:0008283 GO:0008083 GO:0046872 GO:0016020

Cluster-22178.39926 GO:0007010 GO:0006468 GO:0006508 GO:0006357 GO:0016805 GO:0001104 GO:0004672 GO:0005524 GO:0046872 GO:0003779 GO:0016773 GO:0016592 GO:0016020

Cluster-22178.39932 GO:0006810 GO:0042302 GO:0016021 GO:0016020

Cluster-22178.39933 GO:0005515

Cluster-22178.39935 GO:0006508 GO:0004222 GO:0003779 GO:0005622

Cluster-22178.39938 GO:0016757

Cluster-22178.39946 GO:0006355 GO:0043087 GO:0035023 GO:0003677 GO:0005089

Cluster-22178.39949 GO:0006357 GO:0001104 GO:0016592

Cluster-22178.39952 GO:0006144 GO:0003968 GO:0005515 GO:0031379

Cluster-22178.39958 GO:0006468 GO:0042742 GO:0006508 GO:0006827 GO:0006826 GO:0005381 GO:0004672 GO:0005524 GO:0016773 GO:0004222 GO:0005576 GO:0016020 GO:0033573

Cluster-22178.39966 GO:0006470 GO:0006570 GO:0008138 GO:0004725 GO:0004721

Cluster-22178.39967 GO:0043395 GO:0005578 GO:0016020

Cluster-22178.39971 GO:0006265 GO:0003677 GO:0005524 GO:0003918 GO:0005634

Cluster-22178.39977 GO:0015979 GO:0005515 GO:0009507 GO:0009523

Cluster-22178.39979 GO:0015979 GO:0005515 GO:0009523 GO:0009507

Cluster-22178.39997 GO:0006468 GO:0009069 GO:0016310 GO:0005516 GO:0004683 GO:0016773 GO:0004672 GO:0005524 GO:0016020

Cluster-22178.39998 GO:0006468 GO:0005524 GO:0004672 GO:0016773 GO:0016020

Cluster-22178.40003 GO:0016567 GO:0008270 GO:0046872 GO:0005515

GO:0004842

Cluster-22178.40012 GO:0005737

Cluster-22178.40013 GO:0005737

Cluster-22178.40015 GO:0055114 GO:0000723 GO:0007017 GO:0006281 GO:0007018 GO:0005524 GO:0008270 GO:0016972 GO:0003678 GO:0008017 GO:0003777 GO:0005515 GO:0045298 GO:0005874 GO:0005657

Cluster-22178.40023 GO:0006508 GO:0004222

Cluster-22178.40024 GO:0006508 GO:0004222

Cluster-22178.40033 GO:0006812 GO:0055085 GO:0008324 GO:0016021

Cluster-22178.40034 GO:0043039 GO:0016876 GO:0005524 GO:0003676

Cluster-22178.40036 GO:0006813 GO:0005242 GO:0003677 GO:0016021 GO:0008076

Cluster-22178.40037 GO:0006813 GO:0005242 GO:0003677 GO:0008076 GO:0016021

Cluster-22178.40041 GO:0046872

Cluster-22178.40043 GO:0006355 GO:0006814 GO:0006118 GO:0015992 GO:0006744 GO:0006120 GO:0003954 GO:0005515 GO:0008137 GO:0005739

Cluster-22178.40049 GO:0006570 GO:0045892 GO:0006470 GO:0042754 GO:0008138 GO:0005515 GO:0004725 GO:0016020

Cluster-22178.40050 GO:0006570 GO:0006470 GO:0008138 GO:0004725

Cluster-22178.40065 GO:0005515

Cluster-22178.40077 GO:0006313 GO:0008063 GO:0015074 GO:0003677

Cluster-22178.40079 GO:0006313 GO:0015074 GO:0003677

Cluster-22178.40088 GO:0005515

Cluster-22178.40092 GO:0008152 GO:0006260 GO:0006123 GO:0015992 GO:0005975 GO:0016624 GO:0004129 GO:0004649 GO:0005524 GO:0003689 GO:0042575 GO:0005663 GO:0045277

Cluster-22178.40108 GO:0005515 GO:0003723

Cluster-22178.40109 GO:0006468 GO:0005524 GO:0004672
Cluster-22178.40110 GO:0006468 GO:0005524 GO:0004672
Cluster-22178.40114 GO:0035556
Cluster-22178.40131 GO:0006470 GO:0006570 GO:0055114 GO:0008138
GO:0004725 GO:0033743
Cluster-22178.40140 GO:0055114 GO:0016671 GO:0003756 GO:0005783
Cluster-22178.40150 GO:0005515 GO:0008270
Cluster-22178.40151 GO:0016255 GO:0042765
Cluster-22178.40152 GO:0008643 GO:0008982 GO:0009357
Cluster-22178.40157 GO:0005515
Cluster-22178.40161 GO:0007264 GO:0005525
Cluster-22178.40168 GO:0006814 GO:0006886 GO:0018149 GO:0006560
GO:0007411 GO:0006090 GO:0006525 GO:0071436 GO:0006810
GO:0006888 GO:0005515 GO:0008948 GO:0015081 GO:0003810
GO:0016020 GO:0030127
Cluster-22178.4017 GO:0006269 GO:0018142 GO:0006260 GO:0003723
Cluster-22178.40176 GO:0006887 GO:0000145
Cluster-22178.40182 GO:0005975 GO:0004553
Cluster-22178.40191 GO:0046486 GO:0009395 GO:0009231 GO:0055114
GO:0032065 GO:0035556 GO:0007205 GO:0016301 GO:0005515
GO:0005543 GO:0008686 GO:0004143 GO:0047134 GO:0005938
Cluster-22178.40193 GO:0006468 GO:0004672 GO:0005524 GO:0043565
GO:0005515 GO:0005634
Cluster-22178.40195 GO:0007165 GO:0005057
Cluster-22178.40200 GO:0046872 GO:0005887
Cluster-22178.40214 GO:0006505 GO:0006629 GO:0006886 GO:0042967
GO:0016788 GO:0008374 GO:0005739
Cluster-22178.40229 GO:0046872 GO:0008270 GO:0016021
Cluster-22178.40231 GO:0019069 GO:0006810 GO:0005886 GO:0016021
Cluster-22178.40240 GO:0042981 GO:0006955 GO:0005515
Cluster-22178.40244 GO:0007218 GO:0005515
Cluster-22178.40250 GO:0003676
Cluster-22178.40254 GO:0031554 GO:0031556

Cluster-22178.40261 GO:0006479 GO:0046500 GO:0008152 GO:0006464 GO:0003723 GO:0003724 GO:0004719 GO:0008168
Cluster-22178.40263 GO:0007156 GO:0005509 GO:0005515 GO:0016020
Cluster-22178.40268 GO:0016192 GO:0005515 GO:0016021
Cluster-22178.40274 GO:0016070 GO:0006355 GO:0016032 GO:0003677 GO:0019012
Cluster-22178.40276 GO:0022900 GO:0005747 GO:0005739
Cluster-22178.40278 GO:0005515
Cluster-22178.40281 GO:0005515
Cluster-22178.40294 GO:0000287 GO:0008253 GO:0005737
Cluster-22178.40299 GO:0050690 GO:0005537
Cluster-22178.40300 GO:0007010 GO:0003779 GO:0008168
Cluster-22178.40302 GO:0006813 GO:0005249 GO:0008076
Cluster-22178.40313 GO:0007165 GO:0005179 GO:0005576
Cluster-22178.40315 GO:0006355 GO:0043087 GO:0035023 GO:0003677 GO:0005089
Cluster-22178.40318 GO:0007010 GO:0006816 GO:0005388 GO:0003779 GO:0016529
Cluster-22178.40328 GO:0009069 GO:0042157 GO:0007165 GO:0006869 GO:0009847 GO:0006260 GO:0006355 GO:0006468 GO:0016310 GO:0007049 GO:0005096 GO:0004674 GO:0005198 GO:0043565 GO:0008289 GO:0003700 GO:0005667 GO:0016020 GO:0005815 GO:0005576 GO:0019028 GO:0019031
Cluster-22178.40329 GO:0009986 GO:0016020
Cluster-22178.40333 GO:0051537 GO:0043231
Cluster-22178.40346 GO:0006955
Cluster-22178.40349 GO:0007268 GO:0007165 GO:0006230 GO:0006206 GO:0005515 GO:0030550 GO:0004797 GO:0005524 GO:0005576
Cluster-22178.40357 GO:0006952 GO:0015940 GO:0005515 GO:0008897 GO:0003677 GO:0004190 GO:0000287 GO:0016021
Cluster-22178.40358 GO:0006376 GO:0005515 GO:0008270 GO:0003729 GO:0005622 GO:0005685
Cluster-22178.40360 GO:0016020

Cluster-22178.40364 GO:0007049 GO:0051301 GO:0006355 GO:0003700
GO:0016021 GO:0005667 GO:0005634
Cluster-22178.40370 GO:0008152 GO:0016829
Cluster-22178.40389 GO:0006465 GO:0008233 GO:0005515 GO:0016757
GO:0005787 GO:0016021
Cluster-22178.40397 GO:0009103 GO:0008918
Cluster-22178.40398 GO:0046872 GO:0008270
Cluster-22178.40402 GO:0055085 GO:0019953 GO:0000105 GO:0006308
GO:0055114 GO:0030001 GO:0006355 GO:0004399 GO:0008855
GO:0008270 GO:0051287 GO:0046873 GO:0016021 GO:0016020
GO:0009318
Cluster-22178.40409 GO:0042981
Cluster-22178.40411 GO:0009117 GO:0000166 GO:0008253 GO:0000287
GO:0005737
Cluster-22178.40412 GO:0016787
Cluster-22178.40413 GO:0030170 GO:0003676
Cluster-22178.40417 GO:0042742 GO:0007264 GO:0005525 GO:0003924

Cluster-22178.40423 GO:0006418 GO:0004812 GO:0005524
Cluster-22178.40432 GO:0005509 GO:0005544
Cluster-22178.40435 GO:0043565 GO:0005634
Cluster-22178.40438 GO:0006811 GO:0016020
Cluster-22178.40445 GO:0035064 GO:0005515
Cluster-22178.40456 GO:0016310 GO:0000160 GO:0007165 GO:0006281
GO:0008270 GO:0046872 GO:0000155 GO:0009365 GO:0016021
Cluster-22178.40459 GO:0006289 GO:0043087 GO:0007264 GO:0006355
GO:0005085 GO:0000439
Cluster-22178.40467 GO:0009451 GO:0008033 GO:0006879 GO:0070072
GO:0009020 GO:0008199
Cluster-22178.40472 GO:0019064 GO:0019867
Cluster-22178.40474 GO:0005975 GO:0004553
Cluster-22178.40483 GO:0016881 GO:0005524
Cluster-22178.40488 GO:0006629 GO:0006505 GO:0006886 GO:0016787

GO:0016788
Cluster-22178.40494 GO:0006950 GO:0009415 GO:0005515
Cluster-22178.40502 GO:0009103 GO:0055085 GO:0005515 GO:0016021
GO:0016020
Cluster-22178.40505 GO:0034968 GO:0006479 GO:0042254 GO:0006412
GO:0006554 GO:0018024 GO:0003735 GO:0005515 GO:0008270
GO:0005634 GO:0005840
Cluster-22178.40510 GO:0007009 GO:0051082 GO:0003779
Cluster-22178.40517 GO:0007186 GO:0016598 GO:0004930 GO:0004057
GO:0016021
Cluster-22178.40520 GO:0007165 GO:0005179 GO:0046872 GO:0005576
Cluster-22178.40529 GO:0006807 GO:0006355 GO:0003700 GO:0000982
GO:0003677 GO:0016812 GO:0005515 GO:0000978 GO:0005634
GO:0005667
Cluster-22178.40531 GO:0007067 GO:0005515 GO:0000775
Cluster-22178.40537 GO:0003824
Cluster-22178.40539 GO:0046872
Cluster-22178.40541 GO:0000245 GO:0001558 GO:0005520 GO:0016942
GO:0005634 GO:0005576
Cluster-22178.40545 GO:0007049
Cluster-22178.40549 GO:0005515
Cluster-22178.40551 GO:0048870 GO:0031505 GO:0007049 GO:0006355
GO:0043565 GO:0003700 GO:0019905 GO:0004252 GO:0003677
GO:0005667 GO:0005634 GO:0031514
Cluster-22178.40556 GO:0015992 GO:0006123 GO:0030436 GO:0006810
GO:0009297 GO:0006355 GO:0003774 GO:0005524 GO:0004129
GO:0005515 GO:0008134 GO:0005667 GO:0016459 GO:0019867
GO:0042601 GO:0045277
Cluster-22178.40573 GO:0008152 GO:0016746 GO:0005524
Cluster-22178.40580 GO:0046836 GO:0051861 GO:0017089 GO:0005737
GO:0019028
Cluster-22178.40582 GO:0007059 GO:0051301 GO:0000444
Cluster-22178.40585 GO:0005515

Cluster-22178.40590 GO:0055114 GO:0007165 GO:0008283 GO:0040007
GO:0006259 GO:0047134 GO:0003824 GO:0016627 GO:0005524
GO:0003677 GO:0008083 GO:0005694
Cluster-22178.40608 GO:0055114 GO:0016491
Cluster-22178.40618 GO:0016310 GO:0009069 GO:0006449 GO:0006468
GO:0006415 GO:0007165 GO:0005524 GO:0004672 GO:0016149
GO:0004674 GO:0005737 GO:0005840 GO:0018444
Cluster-22178.40620 GO:0016310 GO:0007165 GO:0006449 GO:0009069
GO:0006468 GO:0006415 GO:0004674 GO:0004672 GO:0005524
GO:0016149 GO:0005840 GO:0005737 GO:0018444
Cluster-22178.40623 GO:0055114 GO:0016055 GO:0016491 GO:0008013
GO:0016342
Cluster-22178.40627 GO:0006355 GO:0003677
Cluster-22178.40632 GO:0051252 GO:0055114 GO:0006402 GO:0016641
GO:0003723 GO:0046872 GO:0004535 GO:0005507 GO:0005634
GO:0005737
Cluster-22178.40636 GO:0005634
Cluster-22178.40638 GO:0006694 GO:0008299 GO:0005515 GO:0050518

Cluster-22178.40644 GO:0006468 GO:0005524 GO:0004672
Cluster-22178.40645 GO:0036065 GO:0009246 GO:0055114 GO:0020037
GO:0016705 GO:0008417 GO:0005506
Cluster-22178.40649 GO:0015979 GO:0000062 GO:0005515 GO:0009522
GO:0016021 GO:0009579
Cluster-22178.40652 GO:0015684 GO:0015093 GO:0016021
Cluster-22178.40654 GO:0006118 GO:0055114 GO:0009916
Cluster-22178.40656 GO:0000122 GO:0007050 GO:0009405 GO:0045859
GO:0043303 GO:0017069 GO:0004861 GO:0005737 GO:0005634
GO:0005576
Cluster-22178.40659 GO:0006508 GO:0045444 GO:0005515 GO:0004198
GO:0005622
Cluster-22178.40672 GO:0006888 GO:0071436 GO:0006810 GO:0006525
GO:0007411 GO:0006090 GO:0018149 GO:0006886 GO:0006814

GO:0006560 GO:0003810 GO:0015081 GO:0005515 GO:0008948
GO:0016020 GO:0030127
Cluster-22178.40673 GO:0016310 GO:0009069 GO:0008384 GO:0008385
Cluster-22178.40674 GO:0006858 GO:0006468 GO:0004672 GO:0005524
GO:0016773 GO:0016020
Cluster-22178.40684 GO:0006355 GO:0005992 GO:0006811 GO:0055085
GO:0005515 GO:0005216 GO:0003700 GO:0003824 GO:0016020
GO:0005667
Cluster-22178.40689 GO:0051016 GO:0006468 GO:0007165 GO:0007172
GO:0004713 GO:0004871 GO:0030479 GO:0005925
Cluster-22178.40690 GO:0048544 GO:0008270 GO:0046872 GO:0005515
GO:0061630 GO:0005622
Cluster-22178.40703 GO:0007067 GO:0000090 GO:0006468 GO:0007059
GO:0031110 GO:0051301 GO:0008017 GO:0005524 GO:0004672
GO:0045298 GO:0005876 GO:0000940
Cluster-22178.40708 GO:0016567 GO:0006355 GO:0007049 GO:0051382
GO:0003700 GO:0004842 GO:0043565 GO:0005667 GO:0000776
Cluster-22178.40718 GO:0035556 GO:0005515 GO:0019013 GO:0016021
Cluster-22178.40722 GO:0015992 GO:0015986 GO:0015078 GO:0000276
Cluster-22178.40729 GO:0035556 GO:0006144 GO:0006182 GO:0046039
GO:0009190 GO:0046872 GO:0020037 GO:0016849 GO:0004383
Cluster-22178.40732 GO:0055114 GO:0009395 GO:0016310 GO:0006629
GO:0006468 GO:0006570 GO:0046339 GO:0016042 GO:0035556
GO:0042742 GO:0009069 GO:0007165 GO:0004435 GO:0005515
GO:0005096 GO:0004674 GO:0004511 GO:0005509 GO:0005576
Cluster-22178.40739 GO:0055114 GO:0046872 GO:0016491
Cluster-22178.40740 GO:0015694 GO:0015097 GO:0016020
Cluster-22178.40743 GO:0006355 GO:0000917 GO:0043093 GO:0006457
GO:0006914 GO:0006260 GO:0010506 GO:0051082 GO:0005515
GO:0043565 GO:0003700 GO:0005507 GO:0005506 GO:0003677
GO:0008270 GO:0008017 GO:0005634 GO:0005737 GO:0045298
GO:0005615 GO:0005667 GO:0016272
Cluster-22178.40746 GO:0043401 GO:0007186 GO:0006355 GO:0004930

GO:0016021
Cluster-22178.40762 GO:0006508 GO:0004198 GO:0005515 GO:0005622
Cluster-22178.40770 GO:0008376 GO:0032580
Cluster-22178.40771 GO:0016021 GO:0019012
Cluster-22178.40775 GO:0006914 GO:0005515
Cluster-22178.40788 GO:0006412 GO:0042254 GO:0005524 GO:0008270
GO:0003735 GO:0005622 GO:0005840
Cluster-22178.40791 GO:0006281 GO:0006310 GO:0009378 GO:0005515
GO:0009379 GO:0005657
Cluster-22178.40800 GO:0006865 GO:0055085 GO:0006810 GO:0003333
GO:0015171 GO:0016020
Cluster-22178.40821 GO:0009405
Cluster-22178.40829 GO:0006915 GO:0005622
Cluster-22178.40845 GO:0035556
Cluster-22178.40853 GO:0005975 GO:0016901 GO:0048038
Cluster-22178.40859 GO:0006779 GO:0006470 GO:0055114 GO:0019988
GO:0006570 GO:0004725 GO:0008138 GO:0043399
Cluster-22178.40861 GO:0006807 GO:0016151
Cluster-22178.40864 GO:0098542
Cluster-22178.40872 GO:0006275 GO:0046872
Cluster-22178.40874 GO:0055114 GO:0016491 GO:0016021
Cluster-22178.40876 GO:0055114 GO:0016491 GO:0016021
Cluster-22178.40878 GO:0008152 GO:0008484 GO:0003824
Cluster-22178.40881 GO:0051726 GO:0005634
Cluster-22178.40883 GO:0015074 GO:0016310 GO:0004673 GO:0008907
GO:0003677 GO:0009365
Cluster-22178.40884 GO:0007275 GO:0042600
Cluster-22178.40897 GO:0032801 GO:0006812 GO:0019076 GO:0005515
GO:0005261 GO:0033644
Cluster-22178.40898 GO:0005515
Cluster-22178.40907 GO:0055085 GO:0006810 GO:0005215 GO:0043565
GO:0016020 GO:0016021 GO:0005634
Cluster-22178.40914 GO:0006486 GO:0008373 GO:0003677

Cluster-22178.40925 GO:0005515
Cluster-22178.40930 GO:0006355 GO:0046983 GO:0003677
Cluster-22178.40941 GO:0016021
Cluster-22178.40943 GO:0006355 GO:0042393 GO:0003677 GO:0005515
GO:0003723 GO:0003700 GO:0008270 GO:0046872 GO:0043565
GO:0005667 GO:0016021
Cluster-22178.40944 GO:0006355 GO:0042393 GO:0003677 GO:0005515
GO:0003700 GO:0008270 GO:0046872 GO:0043565 GO:0005667
GO:0016021
Cluster-22178.40950 GO:0035429 GO:0015725 GO:0015128 GO:0003677
GO:0016020
Cluster-22178.40957 GO:0005515 GO:0005737
Cluster-22178.40966 GO:0016021 GO:0005886
Cluster-22178.40968 GO:0006826 GO:0055114 GO:0006827 GO:0006468
GO:0006508 GO:0046168 GO:0042742 GO:0004672 GO:0004222
GO:0005524 GO:0016773 GO:0005381 GO:0016616 GO:0051287
GO:0033573 GO:0005576 GO:0016020
Cluster-22178.40970 GO:0009878 GO:0046872
Cluster-22178.40980 GO:0006468 GO:0005524 GO:0004672
Cluster-22178.40990 GO:0006810 GO:0006811 GO:0005230 GO:0016020
Cluster-22178.40997 GO:0009405 GO:0005515 GO:0003682 GO:0000785
GO:0005576
Cluster-22178.41000 GO:0006468 GO:0005515 GO:0004672 GO:0005524

Cluster-22178.41009 GO:0006811 GO:0006355 GO:0003677 GO:0005216
GO:0016020 GO:0005634
Cluster-22178.41010 GO:0006811 GO:0005216 GO:0016020
Cluster-22178.41017 GO:0019013
Cluster-22178.41019 GO:0006810 GO:0055085 GO:0003723 GO:0005524
GO:0016887 GO:0003724 GO:0042626 GO:0016021
Cluster-22178.41026 GO:0007186 GO:0007606 GO:0004930 GO:0005198
GO:0016021 GO:0019028
Cluster-22178.41030 GO:0006464 GO:0006412 GO:0042254 GO:0016151

GO:0046872 GO:0008270 GO:0003735 GO:0005840 GO:0005622
Cluster-22178.41049 GO:0005515
Cluster-22178.41053 GO:0008270 GO:0005515 GO:0005783
Cluster-22178.41054 GO:0008270 GO:0005515 GO:0005783
Cluster-22178.41055 GO:0015700 GO:0005198 GO:0005515 GO:0015105
GO:0019028 GO:0016021
Cluster-22178.41056 GO:0015700 GO:0005515 GO:0005198 GO:0015105
GO:0019028 GO:0016021
Cluster-22178.41058 GO:0016020
Cluster-22178.41060 GO:0005783
Cluster-22178.41074 GO:0009058 GO:0008483 GO:0030170
Cluster-22178.41087 GO:0019089 GO:0006886 GO:0006606 GO:0006807
GO:0051258 GO:0006118 GO:0051607 GO:0030168 GO:0006891
GO:0008053 GO:0019551 GO:0007165 GO:0004352 GO:0003924
GO:0042803 GO:0005198 GO:0005524 GO:0008092 GO:0045502
GO:0008134 GO:0019901 GO:0005102 GO:0030674 GO:0005737
GO:0030173 GO:0005577 GO:0005667 GO:0005741 GO:0016020
GO:0019031 GO:0016021 GO:0019028 GO:0030286 GO:0005815
GO:0019898 GO:0017119
Cluster-22178.41123 GO:0045454
Cluster-22178.41125 GO:0051304 GO:0005515
Cluster-22178.41132 GO:0007186 GO:0004930 GO:0016021
Cluster-22178.41141 GO:0006139 GO:0004003 GO:0016787 GO:0003677
GO:0016818 GO:0008026 GO:0005524 GO:0003676 GO:0005634
GO:0005657
Cluster-22178.41150 GO:0005515 GO:0005856
Cluster-22178.41163 GO:0005856
Cluster-22178.41178 GO:0004222
Cluster-22178.41179 GO:0004222
Cluster-22178.41185 GO:0055114 GO:0046872
Cluster-22178.41188 GO:0009405 GO:0019835 GO:0019867
Cluster-22178.41202 GO:0006418 GO:0005524 GO:0004812 GO:0003676
GO:0000166

Cluster-22178.41203 GO:0006418 GO:0019079 GO:0003676 GO:0005524
GO:0000166 GO:0003677 GO:0008026 GO:0016787 GO:0004812
Cluster-22178.41211 GO:0006468 GO:0005524 GO:0004672
Cluster-22178.41214 GO:0006486 GO:0016757 GO:0008378 GO:0016020
Cluster-22178.41226 GO:0007009 GO:0007165
Cluster-22178.41238 GO:0055114 GO:0016616
Cluster-22178.41245 GO:0055114 GO:0008152 GO:0016491 GO:0051287

Cluster-22178.41253 GO:0005515
Cluster-22178.41261 GO:0019229 GO:0003676 GO:0005576
Cluster-22178.41265 GO:0006811 GO:0009405 GO:0006810 GO:0055085
GO:0005215 GO:0045735 GO:0016020
Cluster-22178.41275 GO:0042967 GO:0008080
Cluster-22178.41283 GO:0009405 GO:0007623 GO:0019835 GO:0006351
GO:0006355 GO:0003700 GO:0043565 GO:0005667 GO:0005634
GO:0019867
Cluster-22178.41291 GO:0007264 GO:0015684 GO:0015093 GO:0005524
GO:0003924 GO:0005525 GO:0016021
Cluster-22178.41301 GO:0000266 GO:0018149 GO:0003810 GO:0005739
Cluster-22178.41303 GO:0005515 GO:0003682 GO:0000785
Cluster-22178.41305 GO:0006810 GO:0009405 GO:0005515 GO:0019870
GO:0046872 GO:0008270 GO:0005576
Cluster-22178.41306 GO:0055114 GO:0051287 GO:0016620
Cluster-22178.41307 GO:0055114 GO:0016620
Cluster-22178.41310 GO:0000398 GO:0034258 GO:0034257 GO:0019901
GO:0005634 GO:0016020
Cluster-22178.41312 GO:0046872 GO:0005515
Cluster-22178.41329 GO:0006810 GO:0005471 GO:0005524 GO:0005634
GO:0016021
Cluster-22178.41338 GO:0009405 GO:0006508 GO:0005515 GO:0004197
GO:2001070
Cluster-22178.41342 GO:0006457 GO:0051082 GO:0005509 GO:0005783
Cluster-22178.41352 GO:0008270 GO:0046872 GO:0061630 GO:0005515

GO:0005622 GO:0019867
Cluster-22178.41356 GO:0006397 GO:0016043 GO:0030036 GO:0017048
GO:0003723 GO:0051082 GO:0003779 GO:0005634 GO:0005737
Cluster-22178.41357 GO:0030212 GO:0004867
Cluster-22178.41368 GO:0005515
Cluster-22178.41374 GO:0007283 GO:0003677 GO:0000786 GO:0005634
Cluster-22178.41410 GO:0006508 GO:0016787 GO:0008236 GO:0003950
GO:0005634
Cluster-22178.41417 GO:0035556 GO:0005515
Cluster-22178.41428 GO:0006468 GO:0030150 GO:0005524 GO:0004672
GO:0005742
Cluster-22178.41429 GO:0048870 GO:0030246 GO:0003774 GO:0005524
GO:0005515 GO:0016459 GO:0031514
Cluster-22178.41432 GO:0006355 GO:0006281 GO:0042128 GO:0055114
GO:0006807 GO:0003677 GO:0008270 GO:0008942 GO:0016491
GO:0008168 GO:0051537
Cluster-22178.41436 GO:0003677
Cluster-22178.41438 GO:0042254
Cluster-22178.41441 GO:0019497 GO:0006771 GO:0003993 GO:0005615
Cluster-22178.41448 GO:0043087 GO:0007165 GO:0005057 GO:0030695
GO:0005089 GO:0005737
Cluster-22178.41449 GO:0043087 GO:0000723 GO:0007165 GO:0005057
GO:0030695 GO:0005089 GO:0005515 GO:0003677 GO:0005737
GO:0000784
Cluster-22178.41450 GO:0005515
Cluster-22178.41465 GO:0055114 GO:0047134 GO:0005515
Cluster-22178.41468 GO:0046872
Cluster-22178.41475 GO:0019031
Cluster-22178.41477 GO:0006355 GO:0055085 GO:0007049 GO:0043565
GO:0046983 GO:0003700 GO:0005667 GO:0016020 GO:0016021
Cluster-22178.41484 GO:0006446 GO:0006413 GO:0003743 GO:0005840
Cluster-22178.41485 GO:0017004 GO:0015886 GO:0015232 GO:0016020
Cluster-22178.41487 GO:0006259 GO:0006308 GO:0004531

Cluster-22178.41489 GO:0007165 GO:0046872 GO:0005179 GO:0005576
Cluster-22178.41497 GO:0005515
Cluster-22178.41498 GO:0003676
Cluster-22178.41499 GO:0003824
Cluster-22178.41500 GO:0009607 GO:0016021
Cluster-22178.41501 GO:0006118 GO:0003824 GO:0005515 GO:0009055

Cluster-22178.41505 GO:0006468 GO:0005524 GO:0004672
Cluster-22178.41514 GO:0051726 GO:0005634
Cluster-22178.41525 GO:0015992 GO:0015986 GO:0015078 GO:0000276
Cluster-22178.41526 GO:0015986 GO:0015992 GO:0015078 GO:0000276
Cluster-22178.41529 GO:0006402 GO:0051252 GO:0003676 GO:0046872
GO:0004535 GO:0003723 GO:0005737 GO:0005634
Cluster-22178.41538 GO:0050852 GO:0050853 GO:0016310 GO:0016772

Cluster-22178.41546 GO:0016747 GO:0016787 GO:0008270 GO:0005515

Cluster-22178.41547 GO:0043248 GO:0006892 GO:0006278 GO:0006605
GO:0003964 GO:0051018 GO:0019901 GO:0005886 GO:0012505
GO:0005829 GO:0005802 GO:0045211 GO:0030054
Cluster-22178.41570 GO:0006352 GO:0009236 GO:0015979 GO:0006270
GO:0006468 GO:0004672 GO:0005524 GO:0005669
Cluster-22178.41573 GO:0006378 GO:0003729 GO:0016787 GO:0005849
Cluster-22178.41578 GO:0006955 GO:0007165 GO:0006935 GO:0008009
GO:0005576
Cluster-22178.41594 GO:0008152 GO:0003824
Cluster-22178.41596 GO:0042967 GO:0008080 GO:0016747 GO:0016021
Cluster-22178.41601 GO:0015992 GO:0006744 GO:0006120 GO:0006468
GO:0006814 GO:0016773 GO:0004672 GO:0005524 GO:0016787
GO:0008137 GO:0005739 GO:0016020
Cluster-22178.41604 GO:0007264 GO:0005525 GO:0005524 GO:0016887

Cluster-22178.41610 GO:0032481 GO:0002218

Cluster-22178.41611 GO:0003824
Cluster-22178.41619 GO:0007268 GO:0033130 GO:0005515 GO:0042802
GO:0043495
Cluster-22178.41627 GO:0005515
Cluster-22178.41633 GO:0032775 GO:0031167 GO:0007165 GO:0007275
GO:0006306 GO:0006298 GO:0016055 GO:0030983 GO:0098519
GO:0030414 GO:0005102 GO:0009007 GO:0008168 GO:0005524
GO:0005576
Cluster-22178.41635 GO:0008152 GO:0055114 GO:0003824 GO:0050662
GO:0016491
Cluster-22178.41644 GO:0005615
Cluster-22178.41657 GO:0007128 GO:0007130 GO:0070193 GO:0005515
GO:0000795
Cluster-22178.41663 GO:0005515
Cluster-22178.41665 GO:0030001 GO:0055085 GO:0046873 GO:0016020
Cluster-22178.41667 GO:0030001 GO:0055085 GO:0046873 GO:0016020
Cluster-22178.41670 GO:0016192 GO:0006886 GO:0004789 GO:0005198
GO:0005515 GO:0030117
Cluster-22178.41671 GO:0005515
Cluster-22178.41672 GO:0046740 GO:0016021
Cluster-22178.41674 GO:0006338 GO:0006355 GO:0003676 GO:0016818
GO:0005524 GO:0005634
Cluster-22178.41680 GO:0055114 GO:0016226 GO:0006464 GO:0006479
GO:0046500 GO:0008168 GO:0051536 GO:0004719 GO:0005506
Cluster-22178.41686 GO:0003924 GO:0005615
Cluster-22178.41687 GO:0004672 GO:0005515
Cluster-22178.41691 GO:0019363 GO:0055114 GO:0008033 GO:0006742
GO:0008168 GO:0016491
Cluster-22178.41699 GO:0016021
Cluster-22178.41715 GO:0007010 GO:0051260 GO:0003779
Cluster-22178.41718 GO:0006418 GO:0019079 GO:0005524 GO:0003676
GO:0004812 GO:0016787 GO:0000166 GO:0008026 GO:0003677
Cluster-22178.41728 GO:0009306 GO:0016020

Cluster-22178.41730 GO:0006810 GO:0007165 GO:0005215 GO:0008158
GO:0016020
Cluster-22178.41733 GO:0007021 GO:0015631 GO:0051082 GO:0005783
GO:0045298
Cluster-22178.41734 GO:0007021 GO:0051082 GO:0015631 GO:0045298
GO:0005783
Cluster-22178.41740 GO:0007165 GO:0009446 GO:0040007 GO:0006508
GO:0008283 GO:0016579 GO:0006807 GO:0005515 GO:0008083
GO:0004843 GO:0004668 GO:0036459 GO:0005125 GO:0016021
Cluster-22178.41745 GO:0051258 GO:0048870 GO:0035307 GO:0007165
GO:0030168 GO:0006260 GO:0007218 GO:0003723 GO:0005184
GO:0016787 GO:0030674 GO:0003677 GO:0005524 GO:0016887
GO:0003724 GO:0005102 GO:0005576 GO:0031514 GO:0005577
GO:0071595
Cluster-22178.41748 GO:0030570 GO:0005576
Cluster-22178.41749 GO:0016032 GO:0019012
Cluster-22178.41750 GO:0016032 GO:0019012
Cluster-22178.41751 GO:0016032 GO:0019012
Cluster-22178.41759 GO:0006281 GO:0006310 GO:0005524 GO:0043565
GO:0009378 GO:0003677 GO:0005657 GO:0009379
Cluster-22178.41762 GO:0006825 GO:0035434 GO:0005375 GO:0016021
Cluster-22178.41779 GO:0006468 GO:0005524 GO:0004672
Cluster-22178.41796 GO:0007165 GO:0035023 GO:0043087 GO:0005089
GO:0005515
Cluster-22178.41805 GO:0006281
Cluster-22178.41823 GO:0035556 GO:0006468 GO:0006355 GO:0030255
GO:0005524 GO:0004672 GO:0016773 GO:0005634 GO:0005668
GO:0016020
Cluster-22178.41829 GO:0009069 GO:0006468 GO:0016310 GO:0005524
GO:0005515 GO:0004674
Cluster-22178.41830 GO:0000077 GO:0030896 GO:0019012
Cluster-22178.41831 GO:0006355 GO:0003677 GO:0005507 GO:0003700
GO:0005667 GO:0005634

Cluster-22178.41832 GO:0050830
Cluster-22178.41838 GO:0005975 GO:0051260 GO:0046872 GO:0003677
GO:0004553 GO:0016020 GO:0016021
Cluster-22178.41839 GO:0051260 GO:0003677 GO:0046872 GO:0016020
Cluster-22178.41849 GO:0050690 GO:0005537
Cluster-22178.41855 GO:0006891 GO:0009878 GO:0046872 GO:0030173
Cluster-22178.41861 GO:0042967 GO:0046660 GO:0006807 GO:0006397
GO:0006897 GO:0016814 GO:0008270 GO:0008080 GO:0001527
GO:0005634 GO:0016020 GO:0042470
Cluster-22178.41862 GO:0006897 GO:0006397 GO:0046660 GO:0042967
GO:0006807 GO:0008080 GO:0008270 GO:0016814 GO:0042470
GO:0016020 GO:0005634 GO:0001527
Cluster-22178.41878 GO:0016598 GO:0004057
Cluster-22178.41880 GO:0003676
Cluster-22178.41893 GO:0006629 GO:0006886 GO:0006605 GO:0005742
Cluster-22178.41894 GO:0000103 GO:0006144 GO:0016301 GO:0005515
GO:0004020 GO:0005524
Cluster-22178.41904 GO:0008270 GO:0003676
Cluster-22178.41906 GO:0002949 GO:0007264 GO:0006576 GO:0098519
GO:0005525 GO:0005524 GO:0003924
Cluster-22178.41924 GO:0035556 GO:0006508 GO:0005515 GO:0008234
GO:0004197
Cluster-22178.41931 GO:0008152 GO:0016836 GO:0003824
Cluster-22178.41936 GO:0007165
Cluster-22178.41937 GO:0006570 GO:0006470 GO:0055114 GO:0004725
GO:0016730 GO:0005680
Cluster-22178.41938 GO:0006570 GO:0006470 GO:0055114 GO:0004725
GO:0016730
Cluster-22178.41950 GO:0035556 GO:0016579 GO:0043087 GO:0007264
GO:0008270 GO:0036459 GO:0005085 GO:0032183 GO:0070140
Cluster-22178.41952 GO:0007264 GO:0005525
Cluster-22178.41956 GO:0006260 GO:0006810 GO:0003887 GO:0001882
GO:0042575 GO:0016020 GO:0016021

Cluster-22178.41965 GO:0008270
Cluster-22178.41967 GO:0008152 GO:0019497 GO:0015976 GO:0006094
GO:0006013 GO:0006000 GO:0006096 GO:0046486 GO:0006771
GO:0006020 GO:0004807 GO:0046872 GO:0005515 GO:0003993
GO:0003677 GO:0016020
Cluster-22178.41970 GO:0009058 GO:0019079 GO:0008152 GO:0003677
GO:0008026 GO:0016787 GO:0003824 GO:0003676 GO:0005524
Cluster-22178.41971 GO:0003676 GO:0008270 GO:0005622
Cluster-22178.41974 GO:0007165 GO:0005509 GO:0005515 GO:0005578
Cluster-22178.41982 GO:0008033 GO:0055114 GO:0016117 GO:0006779
GO:0019354 GO:0016491 GO:0008270 GO:0050660 GO:0071949
GO:0005515 GO:0043115 GO:0016705
Cluster-22178.41993 GO:0006355 GO:0003700 GO:0005667
Cluster-22178.41996 GO:0046983
Cluster-22178.42006 GO:0006144 GO:0006260 GO:0002949 GO:0007264
GO:0006355 GO:0005524 GO:0008270 GO:0046983 GO:0016887
GO:0003677 GO:0046914 GO:0003924 GO:0005525 GO:0004114
GO:0008134 GO:0005667
Cluster-22178.42008 GO:0006118 GO:0045454 GO:0005515 GO:0015035
GO:0009055
Cluster-22178.42019 GO:0046872
Cluster-22178.42026 GO:0007165 GO:0005057
Cluster-22178.42028 GO:0006355 GO:0007165 GO:0043248 GO:0035257
GO:0005515 GO:0003713 GO:0046983 GO:0005634 GO:0005667
Cluster-22178.42036 GO:0006486 GO:0008378 GO:0016021 GO:0016020
Cluster-22178.42037 GO:0006486 GO:0008378 GO:0016021 GO:0016020
Cluster-22178.42041 GO:0016021
Cluster-22178.42049 GO:0006811 GO:0055085 GO:0005216 GO:0005515
GO:0016020
Cluster-22178.42054 GO:0006118 GO:0006810 GO:0015979 GO:0005506
GO:0020037 GO:0005524 GO:0042626 GO:0009055 GO:0031361
GO:0016021
Cluster-22178.42070 GO:0016485 GO:0005975 GO:0043085 GO:0035091

GO:0031419 GO:0005515 GO:0016773 GO:0046872 GO:0016021
Cluster-22178.42072 GO:0008299
Cluster-22178.42079 GO:0005515 GO:0016020
Cluster-22178.42081 GO:0005515
Cluster-22178.42083 GO:0000160 GO:0016310 GO:0006935 GO:0005515
GO:0004673 GO:0000155 GO:0009365 GO:0005737
Cluster-22178.42104 GO:0006351 GO:0000917 GO:0035556 GO:0043093
GO:0005515 GO:0008270 GO:0005737
Cluster-22178.42108 GO:0001503 GO:0005509 GO:0005515
Cluster-22178.42112 GO:0006355 GO:0003677
Cluster-22178.42117 GO:0007165 GO:0007275 GO:0030168 GO:0006606
GO:0030178 GO:0001558 GO:0048870 GO:0005975 GO:0051258
GO:0030674 GO:0030246 GO:0005520 GO:0008270 GO:0004553
GO:0005102 GO:0051082 GO:0005515 GO:0016942 GO:0005577
GO:0005576 GO:0031514 GO:0016021
Cluster-22178.42127 GO:0008270
Cluster-22178.42129 GO:0003779
Cluster-22178.42131 GO:0007166 GO:0003677 GO:0005515 GO:0046983
GO:0016020
Cluster-22178.42134 GO:0046983
Cluster-22178.42135 GO:0008152 GO:0003824
Cluster-22178.42136 GO:0006096 GO:0006094 GO:0006144 GO:0015976
GO:0004743 GO:0030955 GO:0000287 GO:0046872
Cluster-22178.42137 GO:0015976 GO:0006144 GO:0006096 GO:0006094
GO:0046872 GO:0000287 GO:0030955 GO:0004743
Cluster-22178.42139 GO:0019058 GO:0019915 GO:0003676 GO:0005524
GO:0003774 GO:0019031 GO:0005789 GO:0016459
Cluster-22178.42162 GO:0008152 GO:0055114 GO:0003824 GO:0016491
GO:0016836
Cluster-22178.42168 GO:0016310 GO:0009089 GO:0008270 GO:0004673
GO:0005515 GO:0008837 GO:0009365
Cluster-22178.42173 GO:0055085 GO:0006359 GO:0006914 GO:0015743
GO:0030001 GO:1901031 GO:0046872 GO:0046873 GO:0016020

GO:0005634
Cluster-22178.42175 GO:0006357 GO:0006811 GO:0006813 GO:0006446
GO:0005249 GO:0001104 GO:0003743 GO:0005737 GO:0005840
GO:0016592 GO:0005852 GO:0008076 GO:0016020
Cluster-22178.42177 GO:0005515
Cluster-22178.42179 GO:0007165 GO:0009069 GO:0006914 GO:0006468
GO:0016310 GO:0048870 GO:0004674 GO:0005096 GO:0005198
GO:0042803 GO:0008134 GO:0045502 GO:0008092 GO:0005882
GO:0005667 GO:0019898 GO:0030286 GO:0031514 GO:0019028
GO:0019031 GO:0005737
Cluster-22178.42181 GO:0006406 GO:0007264 GO:0005524 GO:0016887
GO:0005525 GO:0003723 GO:0005737 GO:0005634
Cluster-22178.42182 GO:0006281 GO:0006816 GO:0006874 GO:0006730
GO:0006555 GO:0004013 GO:0008270 GO:0046872 GO:0005219
GO:0005622 GO:0016021
Cluster-22178.42188 GO:0071630 GO:0031144 GO:0030212 GO:0003677
GO:0004867 GO:0008270 GO:0005634
Cluster-22178.42194 GO:0015992 GO:0006123 GO:0004129 GO:0005507
GO:0045277 GO:0016020
Cluster-22178.42199 GO:0006355 GO:0005515 GO:0016021 GO:0005634
Cluster-22178.42201 GO:0000287 GO:0010333 GO:0016829
Cluster-22178.42210 GO:0035091
Cluster-22178.42216 GO:0006468 GO:0004672 GO:0005524 GO:0016773
GO:0016020
Cluster-22178.42217 GO:0006313 GO:0046872 GO:0008270 GO:0004867
GO:0003677 GO:0005515 GO:0004803
Cluster-22178.42218 GO:0018193
Cluster-22178.42228 GO:0008152 GO:0003824
Cluster-22178.42234 GO:0005515
Cluster-22178.42238 GO:0001522 GO:0009451 GO:0003723 GO:0009982

Cluster-22178.42251 GO:0008270 GO:0003676
Cluster-22178.42252 GO:0008270 GO:0003676

Cluster-22178.42273 GO:0007218 GO:0007165 GO:0006355 GO:0003700
GO:0005102 GO:0043565 GO:0005184 GO:0003677 GO:0016021
GO:0005667 GO:0005634
Cluster-22178.42274 GO:0046872
Cluster-22178.42278 GO:0005515
Cluster-22178.42281 GO:0015031 GO:0016021
Cluster-22178.42282 GO:0035556 GO:0005515
Cluster-22178.42285 GO:0048193 GO:0005515 GO:0005783
Cluster-22178.42286 GO:0009878 GO:0005515 GO:0008270 GO:0046872
GO:0046983
Cluster-22178.42288 GO:0040007 GO:0008283 GO:0007165 GO:0008083
GO:0003676
Cluster-22178.42289 GO:0006269 GO:0006351 GO:0005506 GO:0046872
GO:0008270 GO:0004386 GO:0003896 GO:0005730 GO:0005657
Cluster-22178.42291 GO:0006950 GO:0007186 GO:0004930
Cluster-22178.42294 GO:0055114 GO:0006464 GO:0008113
Cluster-22178.42306 GO:0006355 GO:0003700 GO:0043565 GO:0005667
GO:0005634
Cluster-22178.42308 GO:0035556
Cluster-22178.42311 GO:0030036 GO:0005975 GO:0005515 GO:0017048
GO:0003779 GO:0004649
Cluster-22178.42314 GO:0016787
Cluster-22178.42317 GO:0015031 GO:0016021
Cluster-22178.42323 GO:0006935 GO:0055114 GO:0006468 GO:0035556
GO:0030472 GO:0071973 GO:0004672 GO:0016779 GO:0005515
GO:0047134 GO:0005524 GO:0003774 GO:0016773 GO:0009288
GO:0042729 GO:0072686 GO:0016020
Cluster-22178.42324 GO:0005783
Cluster-22178.42327 GO:0055114 GO:0009405 GO:0006813 GO:0005267
GO:0005524 GO:0003677 GO:0020037 GO:0016705 GO:0005506
GO:0016787 GO:0016020
Cluster-22178.42329 GO:0006355 GO:0006352 GO:0003700 GO:0005667
GO:0005669

Cluster-22178.42336 GO:0006260 GO:0005975 GO:0006397 GO:0006810 GO:0016758 GO:0008092 GO:0019898 GO:0000445 GO:0005737 GO:0005886 GO:0016020

Cluster-22178.42339 GO:0006118 GO:0017004 GO:0019058 GO:0005515 GO:0045158 GO:0009512

Cluster-22178.42340 GO:0006801 GO:0004784 GO:0016151 GO:0016209

Cluster-22178.42343 GO:0046486 GO:0009058 GO:0008152 GO:0016042 GO:0016787 GO:0016788 GO:0003847 GO:0008247

Cluster-22178.42346 GO:0006109 GO:0006402 GO:0003723

Cluster-22178.42348 GO:0006109 GO:0006402 GO:0003723

Cluster-22178.42358 GO:0008284 GO:0001819 GO:0007165 GO:0010884 GO:0005515 GO:0005509 GO:0005578

Cluster-22178.42360 GO:0032979 GO:0042578 GO:0005743

Cluster-22178.42371 GO:0016788 GO:0016881

Cluster-22178.42374 GO:0009058 GO:0055114 GO:0006520 GO:0016491 GO:0030170 GO:0016829

Cluster-22178.42375 GO:0008270 GO:0005634

Cluster-22178.42378 GO:0016567 GO:0006810 GO:0004842 GO:0046872 GO:0008270 GO:0005515 GO:0030288 GO:0005680

Cluster-22178.42379 GO:0019239

Cluster-22178.42388 GO:0007165 GO:0005158 GO:0005899

Cluster-22178.42390 GO:0006576 GO:0015684 GO:0015889 GO:0031419 GO:0016887 GO:0003924 GO:0005524 GO:0015093 GO:0005525 GO:0016021

Cluster-22178.42395 GO:0007186 GO:0004930 GO:0046872 GO:0016021

Cluster-22178.42398 GO:0007224

Cluster-22178.42402 GO:0000917 GO:0043093 GO:0005737

Cluster-22178.42422 GO:0006281 GO:0006144 GO:0006464 GO:0046034 GO:0035556 GO:0004017 GO:0008270 GO:0046872 GO:0016151

Cluster-22178.42423 GO:0006355 GO:0042254 GO:0046872 GO:0043565 GO:0003700 GO:0003735 GO:0005840 GO:0005667 GO:0005763

Cluster-22178.42426 GO:0005515

Cluster-22178.42436 GO:0006810 GO:0006816 GO:0005246 GO:0042030
GO:0016020
Cluster-22178.42448 GO:0006313 GO:0003677 GO:0004803
Cluster-22178.42454 GO:0006835 GO:0006820 GO:0006812 GO:0017153
GO:0016020 GO:0016021
Cluster-22178.42458 GO:0016021
Cluster-22178.42460 GO:0007049
Cluster-22178.42467 GO:0016567 GO:0019058 GO:0006464 GO:0005198
GO:0005515 GO:0004842 GO:0016151 GO:0046872 GO:0008270
GO:0005680
Cluster-22178.42485 GO:0042742 GO:0007165 GO:0005515
Cluster-22178.42488 GO:0015031 GO:0006206 GO:0006139 GO:0006606
GO:0008565 GO:0016887 GO:0005524 GO:0004127 GO:0005737
GO:0005634
Cluster-22178.42493 GO:0055114 GO:0008615 GO:0010181 GO:0004733

Cluster-22178.42495 GO:0055114 GO:0008615 GO:0010181 GO:0004733

Cluster-22178.42496 GO:0007010 GO:0006479 GO:0006481 GO:0004671
GO:0003779 GO:0016021
Cluster-22178.42497 GO:0003676 GO:0003677 GO:0016787 GO:0005524

Cluster-22178.42500 GO:0007339 GO:0005515 GO:0005576 GO:0016020
Cluster-22178.42501 GO:0009976
Cluster-22178.42529 GO:0046872 GO:0046983 GO:0019012 GO:0016021
Cluster-22178.42543 GO:0046740 GO:0016021
Cluster-22178.42544 GO:0030414 GO:0005576
Cluster-22178.42549 GO:0006568 GO:0006813 GO:0006552 GO:0006550
GO:0055114 GO:0008033 GO:0006574 GO:0015940 GO:0006631
GO:0018874 GO:0019354 GO:0006554 GO:0016117 GO:0006633
GO:0006779 GO:0003857 GO:0016705 GO:0043115 GO:0008677
GO:0071949 GO:0051287 GO:0016616 GO:0016491 GO:0050660
GO:0050151

Cluster-22178.42551 GO:0008017 GO:0045298

Cluster-22178.42560 GO:0008209 GO:0008207 GO:0008210 GO:0006694 GO:0008152 GO:0055114 GO:0016616 GO:0003854 GO:0050662 GO:0003824 GO:0016491

Cluster-22178.42562 GO:0005515 GO:0003824 GO:0016020

Cluster-22178.42565 GO:0000372 GO:0007005 GO:0003677 GO:0016787 GO:0003676 GO:0005524 GO:0016887 GO:0032865 GO:0030529

Cluster-22178.42572 GO:0016192 GO:0005515 GO:0016021

Cluster-22178.42573 GO:0016021

Cluster-22178.42580 GO:0006355

Cluster-22178.42584 GO:0006355 GO:0043130 GO:0003700 GO:0005622 GO:0005667

Cluster-22178.42592 GO:0007155

Cluster-22178.42598 GO:0016310 GO:0009069 GO:0004674

Cluster-22178.42604 GO:0046872

Cluster-22178.42607 GO:0006464 GO:0006888 GO:0006886 GO:0008270 GO:0046872 GO:0016151 GO:0030127

Cluster-22178.42608 GO:0035556 GO:0006464 GO:0016151 GO:0046872

Cluster-22178.42613 GO:0031412 GO:0006606 GO:0006914 GO:0019064 GO:0051382 GO:0010508 GO:0003677 GO:0019901 GO:0008134 GO:0042803 GO:0045502 GO:0005815 GO:0030286 GO:0005667 GO:0031411 GO:0019031 GO:0000776

Cluster-22178.42621 GO:0043565 GO:0005515 GO:0005634

Cluster-22178.42622 GO:0005515

Cluster-22178.42623 GO:0005515

Cluster-22178.42639 GO:0006144 GO:0004114 GO:0016020

Cluster-22178.42640 GO:0005515

Cluster-22178.42642 GO:0055070 GO:0005507

Cluster-22178.42647 GO:0046034 GO:0035556 GO:0006144 GO:0006464 GO:0016151 GO:0046872 GO:0008270 GO:0004017

Cluster-22178.42651 GO:0015994 GO:0033014 GO:0005515 GO:0004418

Cluster-22178.42659 GO:0003677
Cluster-22178.42668 GO:0007156 GO:0006935 GO:0007165 GO:0004888
GO:0005509 GO:0016020
Cluster-22178.42674 GO:0004252 GO:0005515 GO:0070008
Cluster-22178.42675 GO:0006351 GO:0008270 GO:0003677 GO:0005634
Cluster-22178.42678 GO:0055036
Cluster-22178.42683 GO:0000723 GO:0006281 GO:0055085 GO:0007165
GO:0006810 GO:0003924 GO:0043531 GO:0003676 GO:0003678
GO:0005525 GO:0005515 GO:0016301 GO:0000166 GO:0016887
GO:0005524 GO:0008270 GO:0042626 GO:0003677 GO:0098519
GO:0016021 GO:0005657 GO:0016020
Cluster-22178.42687 GO:0006260 GO:0006308 GO:0016567 GO:0006281
GO:0005525 GO:0004842 GO:0017108 GO:0008270 GO:0005515
GO:0003723 GO:0033557 GO:0005634
Cluster-22178.42691 GO:0006260 GO:0003677 GO:0005634
Cluster-22178.42692 GO:0005515
Cluster-22178.42693 GO:0006418 GO:0043039 GO:0003676 GO:0005524
GO:0000166 GO:0000049 GO:0004812 GO:0005737
Cluster-22178.42695 GO:0005515
Cluster-22178.42705 GO:0009405 GO:0006468 GO:0005198 GO:0005515
GO:0004190 GO:0004672 GO:0005524 GO:0016773 GO:0016020
GO:0016021
Cluster-22178.42711 GO:0019497 GO:0006508 GO:0006771 GO:0008242
GO:0046872 GO:0003993 GO:0016740 GO:0005515 GO:0004197
Cluster-22178.42714 GO:0006613 GO:0005784 GO:0030176
Cluster-22178.42718 GO:0006811 GO:0007165 GO:0007268 GO:0006508
GO:0005234 GO:0008233 GO:0004970 GO:0005524 GO:0016021
GO:0016020
Cluster-22178.42727 GO:0006032 GO:0005515 GO:0008061
Cluster-22178.42731 GO:0006397 GO:0006355 GO:0006470 GO:0042981
GO:0003677 GO:0005507 GO:0003700 GO:0004721 GO:0005667
GO:0005634
Cluster-22178.42732 GO:0005515

Cluster-22178.42743 GO:0035556 GO:0009082 GO:0006468 GO:0005515
GO:0005524 GO:0004672
Cluster-22178.42750 GO:0043087 GO:0055114 GO:0035556 GO:0035023
GO:0007186 GO:0004930 GO:0047134 GO:0005089 GO:0005515
GO:0016020
Cluster-22178.42754 GO:0007186 GO:0035023 GO:0035556 GO:0055114
GO:0043087 GO:0005515 GO:0005089 GO:0047134 GO:0004930
GO:0016020
Cluster-22178.42758 GO:0006352 GO:0006470 GO:0008138 GO:0005861
GO:0005669
Cluster-22178.42759 GO:0023052
Cluster-22178.42763 GO:0055085 GO:0006810 GO:0005524 GO:0042626
GO:0016021
Cluster-22178.42764 GO:0006614 GO:0016301 GO:0003723 GO:0003724
GO:0005525 GO:0003924 GO:0016887 GO:0005524
Cluster-22178.42765 GO:0005515
Cluster-22178.42766 GO:0005515
Cluster-22178.42767 GO:0030420
Cluster-22178.42771 GO:0007165 GO:0007186 GO:0006468 GO:0007264
GO:0006576 GO:0006777 GO:0005515 GO:0005525 GO:0004871
GO:0004672 GO:0005524 GO:0031683 GO:0019001 GO:0003924
Cluster-22178.42773 GO:0006260 GO:0046872 GO:0008270 GO:0005524
GO:0003689 GO:0061630 GO:0005515 GO:0004867 GO:0005622
GO:0042575 GO:0005663
Cluster-22178.42782 GO:0006396 GO:0003723 GO:0016779
Cluster-22178.42783 GO:0008270
Cluster-22178.42790 GO:0006355 GO:0003677 GO:0005634
Cluster-22178.42794 GO:0007165 GO:0005179 GO:0005887 GO:0005576
Cluster-22178.42795 GO:0007010 GO:0003779 GO:0046872
Cluster-22178.42801 GO:0042254 GO:0006412 GO:0003735 GO:0046872
GO:0005840 GO:0005622
Cluster-22178.42803 GO:0005975
Cluster-22178.42810 GO:0055114 GO:0015992 GO:0006123 GO:0009060

GO:0006118 GO:0010508 GO:0004129 GO:0009055 GO:0005506
GO:0020037 GO:0016021 GO:0045277
Cluster-22178.42812 GO:0005509 GO:0005515
Cluster-22178.42815 GO:0006914 GO:0007155 GO:0010508
Cluster-22178.42818 GO:0008270
Cluster-22178.42832 GO:0008152 GO:0016746 GO:0019028
Cluster-22178.42834 GO:0006355 GO:0043401 GO:0008270 GO:0043565
GO:0003700 GO:0005667 GO:0005634
Cluster-22178.42835 GO:0006355 GO:0043401 GO:0043565 GO:0008270
GO:0003700 GO:0005667 GO:0005634
Cluster-22178.42836 GO:0006355 GO:0043401 GO:0043565 GO:0008270
GO:0003700 GO:0005634 GO:0005667
Cluster-22178.42838 GO:0009252 GO:0046436 GO:0006750 GO:0004363
GO:0008716 GO:0046872 GO:0005524
Cluster-22178.42841 GO:0006468 GO:0003677 GO:0008270 GO:0005524
GO:0004672 GO:0016773 GO:0016772 GO:0016020
Cluster-22178.42843 GO:0005515
Cluster-22178.42845 GO:0015986 GO:0042254 GO:0015992 GO:0035556
GO:0006355 GO:0006119 GO:0006412 GO:0003677 GO:0046961
GO:0003735 GO:0008270 GO:0046933 GO:0045259 GO:0045261
GO:0005622 GO:0005840
Cluster-22178.42857 GO:0006366 GO:0046872 GO:0003677 GO:0005665
Cluster-22178.42864 GO:0006779 GO:0015994 GO:0055114 GO:0004109
GO:0005856
Cluster-22178.42865 GO:0015994 GO:0006779 GO:0055114 GO:0004109
GO:0005856
Cluster-22178.42867 GO:0030071 GO:0051028 GO:0005515 GO:0005680
GO:0005634
Cluster-22178.42868 GO:0006412 GO:0042254 GO:0003735 GO:0005622
GO:0005840
Cluster-22178.42870 GO:0003677 GO:0005634
Cluster-22178.42876 GO:0006465 GO:0008233 GO:0005787 GO:0016021
Cluster-22178.42879 GO:0006810 GO:0006816 GO:0005246 GO:0042030

GO:0016020
Cluster-22178.42894 GO:0023052
Cluster-22178.42895 GO:0003723
Cluster-22178.42897 GO:0007156 GO:0005509 GO:0016020
Cluster-22178.42899 GO:0007186 GO:0004930 GO:0016021
Cluster-22178.42901 GO:0007165 GO:0008601 GO:0000159 GO:0019031
Cluster-22178.42904 GO:0006811 GO:0006810 GO:0003677 GO:0005230
GO:0016020
Cluster-22178.42906 GO:0006355 GO:0003677
Cluster-22178.42911 GO:0006836 GO:0006812 GO:0005328 GO:0016021
Cluster-22178.42915 GO:0006206 GO:0006144 GO:0006351 GO:0003677
GO:0003899 GO:0005730
Cluster-22178.42918 GO:0006047
Cluster-22178.42920 GO:0006954 GO:0031167 GO:0007165 GO:0008168
GO:0005125 GO:0005576
Cluster-22178.42947 GO:0015684 GO:0006576 GO:0007264 GO:0005525
GO:0003924 GO:0005524 GO:0015093 GO:0016021
Cluster-22178.42949 GO:0006576 GO:0007264 GO:0015684 GO:0003924
GO:0005524 GO:0015093 GO:0005525 GO:0016021
Cluster-22178.42958 GO:0055085 GO:0007606 GO:0006810 GO:0007165
GO:0005524 GO:0042626 GO:0004888 GO:0016021 GO:0016020
Cluster-22178.42960 GO:0043087 GO:0005089 GO:0005737
Cluster-22178.42964 GO:0006357 GO:0006811 GO:0006813 GO:0006446
GO:0005249 GO:0003743 GO:0001104 GO:0005737 GO:0005840
GO:0016020 GO:0005852 GO:0008076 GO:0016592
Cluster-22178.42967 GO:0005515
Cluster-22178.42984 GO:0015031 GO:0005515
Cluster-22178.42988 GO:0008270 GO:0003676
Cluster-22178.43002 GO:0015693 GO:0004222 GO:0015095 GO:0005515
GO:0008233 GO:0016021 GO:0031012
Cluster-22178.43003 GO:0005982 GO:0005985 GO:0006075 GO:0003843
GO:0005515 GO:0016020 GO:0000148
Cluster-22178.43004 GO:0005509

Cluster-22178.43009 GO:0006566 GO:0006563 GO:0046487 GO:0046486
GO:0055114 GO:0006544 GO:0031388 GO:0008887 GO:0016491
Cluster-22178.43010 GO:0006563 GO:0006566 GO:0046486 GO:0046487
GO:0031388 GO:0006544 GO:0035091 GO:0008887
Cluster-22178.43017 GO:0007275 GO:0005201 GO:0005578 GO:0005581
Cluster-22178.43031 GO:0006450 GO:0005515
Cluster-22178.43032 GO:0016021
Cluster-22178.43040 GO:0051382 GO:0006281 GO:0005515 GO:0003712
GO:0005667 GO:0000124
Cluster-22178.43049 GO:0003676 GO:0016020
Cluster-22178.43054 GO:0007165 GO:0006955 GO:0008283 GO:0040007
GO:0005137 GO:0008083 GO:0005895 GO:0005576
Cluster-22178.4306 GO:0006508 GO:0004198 GO:0005622
Cluster-22178.43070 GO:0006629 GO:0006812 GO:0008324 GO:0008081

Cluster-22178.43071 GO:0006629 GO:0009607 GO:0006812 GO:0008324
GO:0008081 GO:0016021
Cluster-22178.43074 GO:0016310 GO:0000160 GO:0009405 GO:0006886
GO:0016192 GO:0000155 GO:0005622 GO:0009365 GO:0016021
GO:0005576 GO:0030131
Cluster-22178.43083 GO:0003950
Cluster-22178.43105 GO:0007186 GO:0004930 GO:0019031 GO:0016020
Cluster-22178.43107 GO:0007304 GO:0005213 GO:0042600 GO:0005576
Cluster-22178.43117 GO:0006810 GO:0042626 GO:0005524 GO:0016021
Cluster-22178.43143 GO:0006213 GO:0016763
Cluster-22178.43160 GO:0006820 GO:0016021
Cluster-22178.43171 GO:0005515
Cluster-22178.43177 GO:0055114 GO:0016491 GO:0051537 GO:0003676

Cluster-22178.43180 GO:0005515
Cluster-22178.43184 GO:0051252 GO:0004523 GO:0003676 GO:0003824

Cluster-22178.43186 GO:0046872 GO:0003824

Cluster-22178.43190 GO:0015833 GO:0000166 GO:0005524 GO:0016021
Cluster-22178.43192 GO:0006807 GO:0006396 GO:0006144 GO:0004000
GO:0003723
Cluster-22178.43193 GO:0005515 GO:0005509
Cluster-22178.43198 GO:0009058 GO:0006952 GO:0043565 GO:0003824
GO:0008289 GO:0005634 GO:0009279
Cluster-22178.43207 GO:0007165 GO:0005515 GO:0005509 GO:0005578
Cluster-22178.43208 GO:0007165 GO:0005509 GO:0005515 GO:0005578
Cluster-22178.43219 GO:0005515 GO:0015631 GO:0045298
Cluster-22178.43220 GO:0007165 GO:0006468 GO:0006531 GO:0035556
GO:0006522 GO:0042967 GO:0004672 GO:0005524 GO:0005057
GO:0047200 GO:0016773 GO:0016020 GO:0016021 GO:0005856
Cluster-22178.43225 GO:0034755 GO:0055114 GO:0006826 GO:0005381
GO:0016706 GO:0008168 GO:0016021
Cluster-22178.43226 GO:0006281 GO:0006310 GO:0006206 GO:0006139
GO:0002949 GO:0009378 GO:0016301 GO:0016791 GO:0005524
GO:0016887 GO:0004127 GO:0009379 GO:0005657
Cluster-22178.43227 GO:0055114 GO:0016616 GO:0005515
Cluster-22178.43230 GO:0071203
Cluster-22178.43231 GO:0007009 GO:0006281 GO:0071203
Cluster-22178.43238 GO:0006367 GO:0046983
Cluster-22178.43240 GO:0007165 GO:0006355 GO:0048193 GO:0004871
GO:0003700 GO:0008270 GO:0016020 GO:0005667 GO:0005634
Cluster-22178.43241 GO:0006468 GO:0005524 GO:0004672 GO:0016773
GO:0016020
Cluster-22178.43249 GO:0015727 GO:0007156 GO:0006629 GO:0005509
GO:0004857 GO:0015129 GO:0016020 GO:0042627 GO:0005887
Cluster-22178.43257 GO:0006383 GO:0007155 GO:0042157 GO:0006869
GO:0006206 GO:0006606 GO:0010508 GO:0042262 GO:0006810
GO:0006144 GO:0008053 GO:0006355 GO:0006351 GO:0003690
GO:0003677 GO:0042803 GO:0005198 GO:0003899 GO:0003924
GO:0045502 GO:0008134 GO:0008289 GO:0005730 GO:0005666
GO:0005667 GO:0005741 GO:0005882 GO:0005886 GO:0030286

GO:0005576 GO:0019028 GO:0019031 GO:0016021
Cluster-22178.43260 GO:0005515
Cluster-22178.43261 GO:0055114 GO:0017148 GO:0006355 GO:0006413
GO:0006260 GO:0010506 GO:0006206 GO:0006914 GO:0008998
GO:0003700 GO:0051082 GO:0016491 GO:0005516 GO:0005615
GO:0005845 GO:0031514 GO:0005667
Cluster-22178.43265 GO:0006357 GO:0001104 GO:0016592
Cluster-22178.43266 GO:0004867 GO:0031224
Cluster-22178.43267 GO:0006629 GO:0008081
Cluster-22178.43281 GO:0046983 GO:0003677
Cluster-22178.43300 GO:0005515
Cluster-22178.43301 GO:0019836 GO:0022904 GO:0005576 GO:0016020
GO:0016021
Cluster-22178.43307 GO:0005515
Cluster-22178.43321 GO:0005524 GO:0016773 GO:0016020
Cluster-22178.43327 GO:0000186 GO:0001678 GO:0032099 GO:0007186
GO:0008343 GO:0009267 GO:0005615
Cluster-22178.43329 GO:0006468 GO:0005524 GO:0004672
Cluster-22178.43331 GO:0016192 GO:0006886 GO:0005515 GO:0030117
Cluster-22178.43334 GO:0006633 GO:0016790
Cluster-22178.43339 GO:0005515
Cluster-22178.43345 GO:0006952 GO:0005515
Cluster-22178.43347 GO:0045502 GO:0042803 GO:0008134 GO:0005667
GO:0030286 GO:0016020
Cluster-22178.43351 GO:0006338 GO:0005524 GO:0016818 GO:0003676
GO:0005634
Cluster-22178.43352 GO:0000774 GO:0005515 GO:0005783
Cluster-22178.43355 GO:0005515
Cluster-22178.43358 GO:0005515
Cluster-22178.43360 GO:0006355 GO:0009271
Cluster-22178.43374 GO:0019911
Cluster-22178.43375 GO:0035091
Cluster-22178.43381 GO:0006468 GO:0016773 GO:0005524 GO:0004672

GO:0016020
Cluster-22178.43395 GO:0045859 GO:0016534 GO:0016533
Cluster-22178.43396 GO:0008033 GO:0006355 GO:0009451 GO:0006479
GO:0006554 GO:0006464 GO:0030488 GO:0035556 GO:0046500
GO:0006364 GO:0008168 GO:0004719 GO:0003700 GO:0003723
GO:0018024 GO:0016429 GO:0042025 GO:0005667 GO:0031515
Cluster-22178.43404 GO:0015992 GO:0015991 GO:0015078 GO:0033177
Cluster-22178.43411 GO:0007067 GO:0072686 GO:0042729
Cluster-22178.43412 GO:0006811 GO:0007016 GO:0005216 GO:0016010
GO:0016020
Cluster-22178.43415 GO:0006508 GO:0008236
Cluster-22178.43418 GO:0007165 GO:0019073 GO:0005515 GO:0016021
Cluster-22178.43430 GO:0005515 GO:0003677 GO:0046872
Cluster-22178.43431 GO:0003677 GO:0005515 GO:0046872
Cluster-22178.43435 GO:0042254 GO:0006412 GO:0003735 GO:0019843
GO:0005840 GO:0005622
Cluster-22178.43438 GO:0006366
Cluster-22178.43439 GO:0055085 GO:0022857 GO:0016021
Cluster-22178.43449 GO:0015948 GO:0008168 GO:0005515
Cluster-22178.43456 GO:0008152 GO:0016791 GO:0016021
Cluster-22178.43457 GO:0008152 GO:0016491 GO:0005515
Cluster-22178.43458 GO:0055085 GO:0022900 GO:0006810 GO:0016021
GO:0016020 GO:0005747 GO:0005739 GO:0071203
Cluster-22178.43460 GO:0007224 GO:0046872 GO:0008270 GO:0061630
GO:0005515 GO:0005622
Cluster-22178.43462 GO:0007165 GO:0005509 GO:0005515 GO:0005578
Cluster-22178.43471 GO:0003729 GO:0016787 GO:0005524 GO:0003676
GO:0003677
Cluster-22178.43475 GO:0006281 GO:0042254 GO:0006412 GO:0006310
GO:0009378 GO:0098519 GO:0003735 GO:0005524 GO:0016887
GO:0003678 GO:0009379 GO:0005657 GO:0005840
Cluster-22178.43487 GO:0006953 GO:0034364 GO:0005576
Cluster-22178.43489 GO:0006334 GO:0003677 GO:0005634 GO:0000786

Cluster-22178.43496 GO:0042967 GO:0008080

Cluster-22178.43500 GO:0008152 GO:0016746

Cluster-22178.43501 GO:0005515 GO:0005509 GO:0005576

Cluster-22178.43506 GO:0006812 GO:0006836 GO:0005328 GO:0016021

Cluster-22178.43510 GO:0007186 GO:0001503 GO:0004930 GO:0016021

Cluster-22178.43522 GO:0040001 GO:0000226 GO:0000281 GO:0005874 GO:0005819

Cluster-22178.43528 GO:0006807 GO:0006672 GO:0016811 GO:0016021

Cluster-22178.43533 GO:0005096 GO:0005515

Cluster-22178.43546 GO:0006836 GO:0055114 GO:0006812 GO:0007165 GO:0006118 GO:0006954 GO:0005125 GO:0009916 GO:0005328 GO:0016021 GO:0005576

Cluster-22178.43547 GO:0009607 GO:0019901 GO:0016020 GO:0016021

Cluster-22178.43561 GO:0005982 GO:0005985 GO:0006118 GO:0030683 GO:0030245 GO:0055114 GO:0015031 GO:0005515 GO:0008810 GO:0046983 GO:0016655 GO:0016020 GO:0019012 GO:0016021 GO:0019031

Cluster-22178.43571 GO:0006629 GO:0009607 GO:0006812 GO:0008324 GO:0008081 GO:0016021

Cluster-22178.43572 GO:0016579 GO:0036459

Cluster-22178.43577 GO:0046836 GO:0006508 GO:0017089 GO:0008236 GO:0051861 GO:0005737

Cluster-22178.43588 GO:0007165 GO:0005509 GO:0005578

Cluster-22178.43595 GO:0006810 GO:0006811 GO:0005230 GO:0016020

Cluster-22178.43596 GO:0008283 GO:0040007 GO:0007165 GO:0008083

Cluster-22178.43600 GO:0006094 GO:0006096 GO:0006144 GO:0055114 GO:0015976 GO:0004743 GO:0030955 GO:0000287 GO:0003824

Cluster-22178.43605 GO:0008237 GO:0008270

Cluster-22178.43613 GO:0007264 GO:0043087 GO:0016485 GO:0007165 GO:0005085 GO:0005515 GO:0016021

Cluster-22178.43614 GO:0050909 GO:0010038 GO:0007186 GO:0009058 GO:0004930 GO:0016021

Cluster-22178.43616 GO:0016021
Cluster-22178.43628 GO:0006351 GO:0003714 GO:0005667
Cluster-22178.43630 GO:0006281 GO:0000012 GO:0005515 GO:0003684
GO:0005634
Cluster-22178.43656 GO:0042967 GO:0008080 GO:0016747
Cluster-22178.43658 GO:0016021 GO:0005789
Cluster-22178.43659 GO:0007186 GO:0009405 GO:0004930 GO:0016021
Cluster-22178.43660 GO:0016853
Cluster-22178.43665 GO:0009069 GO:0016310 GO:0070009 GO:0008239
GO:0004674
Cluster-22178.43666 GO:0007165 GO:0016043 GO:0000921 GO:0016301
GO:0003779 GO:0016021 GO:0005940
Cluster-22178.43673 GO:0008641
Cluster-22178.43675 GO:0007264 GO:0005525 GO:0005524 GO:0003924
GO:0016887
Cluster-22178.43676 GO:0042254 GO:0006412 GO:0003735 GO:0005622
GO:0005840
Cluster-22178.43686 GO:0045892 GO:0042919 GO:0006486 GO:0006810
GO:0042925 GO:0008373 GO:0016021 GO:0005634
Cluster-22178.43690 GO:0006486 GO:0070072 GO:0008373
Cluster-22178.43691 GO:0030234 GO:0016020
Cluster-22178.43695 GO:0007059 GO:0008218 GO:0006260 GO:0008152
GO:0008168 GO:0005509 GO:0005737
Cluster-22178.43698 GO:0006891 GO:0030173
Cluster-22178.43700 GO:0006468 GO:0016773 GO:0005524 GO:0004672
GO:0016020
Cluster-22178.43710 GO:0006118 GO:0008152 GO:0055114 GO:0050660
GO:0003995 GO:0016627
Cluster-22178.43713 GO:0042981
Cluster-22178.43717 GO:0019069 GO:0006355 GO:0003677 GO:0005524

Cluster-22178.43720 GO:0035091
Cluster-22178.43723 GO:0006508 GO:0004197 GO:0008233

Cluster-22178.43736 GO:0009245 GO:0008915
Cluster-22178.43740 GO:0019087 GO:0000917 GO:0016021
Cluster-22178.43741 GO:0000917 GO:0019087 GO:0016021
Cluster-22178.43743 GO:0008033 GO:0042254 GO:0006412 GO:0009451 GO:0004809 GO:0003723 GO:0005515 GO:0003735 GO:0005622 GO:0005840
Cluster-22178.43744 GO:0009451 GO:0006412 GO:0042254 GO:0008033 GO:0003735 GO:0003723 GO:0004809 GO:0005840 GO:0005622
Cluster-22178.43745 GO:0019872 GO:0055085 GO:0008654 GO:0006021 GO:0030001 GO:0016817 GO:0004512 GO:0046873 GO:0005515 GO:0016020
Cluster-22178.43748 GO:0055085 GO:0006811 GO:0007165 GO:0005216 GO:0016020
Cluster-22178.43750 GO:0016020
Cluster-22178.43751 GO:0006811 GO:0007165 GO:0055085 GO:0005216 GO:0016020
Cluster-22178.43754 GO:0009267 GO:0032099 GO:0000186 GO:0007186 GO:0008343 GO:0001678 GO:0045502 GO:0005198 GO:0008134 GO:0042803 GO:0030286 GO:0005615 GO:0005667 GO:0005882
Cluster-22178.43760 GO:0006508 GO:0016032 GO:0007186 GO:0008241 GO:0003676 GO:0004930 GO:0008237 GO:0016021 GO:0016020
Cluster-22178.43770 GO:0016021
Cluster-22178.43772 GO:0055085 GO:0005741
Cluster-22178.43777 GO:0055085 GO:0016021 GO:0055036
Cluster-22178.43792 GO:0043087 GO:0006265 GO:0035023 GO:0003677 GO:0005524 GO:0003918 GO:0019905 GO:0005089
Cluster-22178.43798 GO:0043087 GO:0006265 GO:0035023 GO:0005524 GO:0003918 GO:0005089 GO:0003677 GO:0019028
Cluster-22178.43802 GO:0017025 GO:0005096 GO:0005634
Cluster-22178.43805 GO:0006915
Cluster-22178.43812 GO:0005515
Cluster-22178.43813 GO:0006289 GO:0006284 GO:0006810 GO:0042254 GO:0006281 GO:0006412 GO:0008270 GO:0003906 GO:0003684

GO:0003735 GO:0003924 GO:0005525 GO:0016799 GO:0005840
GO:0016021 GO:0005861
Cluster-22178.43821 GO:0018193 GO:0016192 GO:0006886 GO:0008061
GO:0005198 GO:0005515 GO:0016020 GO:0030126
Cluster-22178.43822 GO:0016020
Cluster-22178.43825 GO:0042981
Cluster-22178.43828 GO:0050776 GO:0014068 GO:0006118 GO:0007275
GO:0007165 GO:0006259 GO:0003824 GO:0009055 GO:0020037
GO:0005102 GO:0005524 GO:0043548 GO:0003677 GO:0016021
GO:0016020 GO:0005694
Cluster-22178.43829 GO:0016055 GO:0031167 GO:0032775 GO:0006298
GO:0006306 GO:0007165 GO:0007275 GO:0030414 GO:0030983
GO:0098519 GO:0008168 GO:0009007 GO:0005524 GO:0005102
GO:0005576
Cluster-22178.43832 GO:0006771 GO:0009103 GO:0019497 GO:0005515
GO:0046872 GO:0008918 GO:0008168 GO:0016817 GO:0003993
Cluster-22178.43833 GO:0007049
Cluster-22178.43835 GO:0009405 GO:0006810 GO:0008200 GO:0005576
Cluster-22178.43838 GO:0006355 GO:0008270 GO:0043565 GO:0003700
GO:0005667 GO:0005634
Cluster-22178.43842 GO:0000154 GO:0006396 GO:0004252 GO:0016433
GO:0055036 GO:0019028
Cluster-22178.43844 GO:0008152 GO:0008033 GO:0055114 GO:0005975
GO:0009451 GO:0005985 GO:0006479 GO:0006396 GO:0006464
GO:0005982 GO:0006400 GO:0046500 GO:0008757 GO:0008168
GO:0004719 GO:0008173 GO:0008810 GO:0008176
Cluster-22178.43845 GO:0008152 GO:0005985 GO:0005982 GO:0005975
GO:0008810 GO:0008168
Cluster-22178.43847 GO:0007165 GO:0005057
Cluster-22178.43852 GO:0042254 GO:0006412 GO:0006464 GO:0003735
GO:0008270 GO:0046872 GO:0016151 GO:0005622 GO:0005840
Cluster-22178.43855 GO:0006269 GO:0018142 GO:0009058 GO:0006260
GO:0003723

Cluster-22178.43862 GO:0006506 GO:0017176 GO:0016021
Cluster-22178.43863 GO:0006506 GO:0042254 GO:0006412 GO:0003735
GO:0017176 GO:0005622 GO:0005840 GO:0016021
Cluster-22178.43866 GO:0006633 GO:0008152 GO:0006574 GO:0006554
GO:0055114 GO:0018874 GO:0006550 GO:0006552 GO:0006631
GO:0006568 GO:0003824 GO:0016491 GO:0050662 GO:0051287
GO:0003857
Cluster-22178.43868 GO:0007186 GO:0007165 GO:0007264 GO:0004871
GO:0005525 GO:0003924 GO:0019001 GO:0031683
Cluster-22178.43874 GO:0003824
Cluster-22178.43883 GO:0005515
Cluster-22178.43884 GO:0006570 GO:0006355 GO:0051607 GO:0055114
GO:0005515 GO:0004511 GO:0003700 GO:0005667
Cluster-22178.43893 GO:0016020
Cluster-22178.43894 GO:0016020
Cluster-22178.43901 GO:0006303 GO:0009069 GO:0016310 GO:0005515
GO:0003677 GO:0004677 GO:0016773 GO:0005524 GO:0005634
GO:0005958
Cluster-22178.43921 GO:0005509
Cluster-22178.43922 GO:0005509
Cluster-22178.43938 GO:0005515
Cluster-22178.43942 GO:0006457
Cluster-22178.43949 GO:0009058 GO:0008152 GO:0006508 GO:0006629
GO:0008236 GO:0016788 GO:0016787
Cluster-22178.43953 GO:0005515 GO:0019901
Cluster-22178.43957 GO:0006355 GO:0003700 GO:0005634 GO:0005667
Cluster-22178.43968 GO:0006355
Cluster-22178.43975 GO:0035556
Cluster-22178.43978 GO:0008608 GO:0007155 GO:0048870 GO:0031514
GO:0042729 GO:0072686
Cluster-22178.43979 GO:0007155 GO:0048870 GO:0008608 GO:0072686
GO:0042729 GO:0031514
Cluster-22178.43981 GO:0006118 GO:0045454 GO:0009055 GO:0015035

Cluster-22178.43984 GO:0007517 GO:0006355 GO:0003677 GO:0005634
GO:0032040
Cluster-22178.43985 GO:0006094 GO:0006096 GO:0055114 GO:0006144
GO:0015976 GO:0004743 GO:0030955 GO:0000287 GO:0003824
Cluster-22178.43987 GO:0005198 GO:0046872
Cluster-22178.43993 GO:0019538 GO:0003677
Cluster-22178.44001 GO:0009058 GO:0006144 GO:0006508 GO:0004252
GO:0003877 GO:0030170
Cluster-22178.44003 GO:0046500 GO:0006400 GO:0006396 GO:0006480
GO:0006554 GO:0030488 GO:0006464 GO:0009451 GO:0006479
GO:0008152 GO:0008033 GO:0008176 GO:0016429 GO:0008173
GO:0018024 GO:0008168 GO:0004719 GO:0031515 GO:0005856
Cluster-22178.44006 GO:0015979 GO:0016020 GO:0009539 GO:0009523
Cluster-22178.44011 GO:0003677
Cluster-22178.44012 GO:0008270
Cluster-22178.44019 GO:0006355 GO:0008270 GO:0043565 GO:0005515
GO:0003700 GO:0005634 GO:0005667
Cluster-22178.44026 GO:0030153 GO:0006955 GO:0015643 GO:0019814
Cluster-22178.44028 GO:0008270
Cluster-22178.44030 GO:0072519 GO:0016567 GO:0004842 GO:0008270

Cluster-22178.44032 GO:0006355 GO:0016787 GO:0005507 GO:0003700
GO:0003677 GO:0005667 GO:0005634
Cluster-22178.44042 GO:0006771 GO:0019497 GO:0016192 GO:0003993
GO:0019031 GO:0016021
Cluster-22178.44046 GO:0006508 GO:0016787 GO:0008236
Cluster-22178.44048 GO:0005198 GO:0016021
Cluster-22178.44050 GO:0007165 GO:0005179 GO:0005576
Cluster-22178.44058 GO:0005198 GO:0003676 GO:0046872 GO:0019028
GO:0005622
Cluster-22178.44062 GO:0017004 GO:0006118 GO:0042254 GO:0003735
GO:0045158 GO:0009512 GO:0005840

Cluster-22178.44070 GO:0003676 GO:0016787 GO:0046872

Cluster-22178.44075 GO:0006874 GO:0006816 GO:0005219 GO:0046872 GO:0004252 GO:0016021 GO:0005622 GO:0005886

Cluster-22178.44086 GO:0006810 GO:0009405 GO:0008200 GO:0042151

Cluster-22178.44097 GO:0006406 GO:0007186 GO:0007165 GO:0007264 GO:0004871 GO:0005525 GO:0003924 GO:0031683 GO:0005524 GO:0019001 GO:0000166 GO:0003677 GO:0003723 GO:0005737 GO:0005634

Cluster-22178.44102 GO:0007165 GO:0006355 GO:0030521 GO:0003677 GO:0005496 GO:0004882 GO:0005634

Cluster-22178.44107 GO:0006812 GO:0015672 GO:0055114 GO:0016638 GO:0005261 GO:0016020

Cluster-22178.44123 GO:0005515

Cluster-22178.44126 GO:0007049 GO:0006355 GO:0006914 GO:0003700 GO:0005198 GO:0043565 GO:0005667

Cluster-22178.44127 GO:0007165 GO:0006355 GO:0006914 GO:0005198 GO:0016301 GO:0003700 GO:0043565 GO:0005667

Cluster-22178.44128 GO:0006355 GO:0007165 GO:0006914 GO:0005198 GO:0016301 GO:0043565 GO:0003700 GO:0005667

Cluster-22178.44135 GO:0005515

Cluster-22178.44136 GO:0007165 GO:0001819 GO:0008284 GO:0010884 GO:0005509 GO:0005515 GO:0005578

Cluster-22178.44150 GO:0055114 GO:0006940 GO:0006952 GO:0016620 GO:0005515

Cluster-22178.44153 GO:0005515

Cluster-22178.44159 GO:0030071 GO:0046983 GO:0042802 GO:0005515 GO:0005680

Cluster-22178.44160 GO:0006810 GO:0006771 GO:0015986 GO:0019497 GO:0003677 GO:0030674 GO:0042626 GO:0003993 GO:0043130 GO:0016887 GO:0005524 GO:0042802 GO:0005515 GO:0000166 GO:0005525 GO:0008092 GO:0003924 GO:0046872 GO:0033178

Cluster-22178.44164 GO:0006351 GO:0007275 GO:0006206 GO:0006144 GO:0003677 GO:0003899 GO:0005515 GO:0003774 GO:0005524

GO:0005730 GO:0016459

Cluster-22178.44166 GO:0005198 GO:0003682 GO:0000785

Cluster-22178.44167 GO:0042254 GO:0006412 GO:0003735 GO:0005840

GO:0005622

Cluster-22178.44170 GO:0016055 GO:0005515

Cluster-22178.44172 GO:0006355 GO:0003677 GO:0046872 GO:0005634

Cluster-22178.44173 GO:0007010 GO:0003779 GO:0008168

Cluster-22178.44174 GO:0007010 GO:0003779 GO:0008168

Cluster-22178.44175 GO:0007049

Cluster-22178.44181 GO:0005515

Cluster-22178.44185 GO:0006355

Cluster-22178.44186 GO:0006754 GO:0017004 GO:0055114 GO:0006811

GO:0005216 GO:0005509 GO:0005515 GO:0005524 GO:0016887

GO:0000275 GO:0016020

Cluster-22178.44191 GO:0005815

Cluster-22178.44195 GO:0007016 GO:0005515 GO:0016010

Cluster-22178.44196 GO:0006355 GO:0061024 GO:0007264 GO:0007186

GO:0007165 GO:0010033 GO:0043531 GO:0003924 GO:0031683

GO:0019001 GO:0004871 GO:0005525 GO:0008134 GO:0000166

GO:0016887 GO:0005524 GO:0003677 GO:0019867 GO:0005667

GO:0016020

Cluster-22178.44199 GO:0000045 GO:0005737

Cluster-22178.44207 GO:0006396 GO:0006807 GO:0006144 GO:0004000

GO:0003723

Cluster-22178.44210 GO:0006144 GO:0006396 GO:0006807 GO:0003723

GO:0004000

Cluster-22178.44218 GO:0006123 GO:0006807 GO:0015992 GO:0007067

GO:0004129 GO:0005507 GO:0016810 GO:0045277 GO:0016020

GO:0042729 GO:0072686

Cluster-22178.44219 GO:0015992 GO:0015031 GO:0006260 GO:0006891

GO:0015991 GO:0006355 GO:0046983 GO:0008565 GO:0045502

GO:0042803 GO:0008134 GO:0003676 GO:0015078 GO:0017119

GO:0005667 GO:0030286 GO:0033179

Cluster-22178.44226 GO:0055114 GO:0006801 GO:0046872 GO:0004784

Cluster-22178.44233 GO:0008152 GO:0008134 GO:0003824 GO:0005634
GO:0005667
Cluster-22178.44238 GO:0006468 GO:0016773 GO:0004672 GO:0005524
GO:0016020
Cluster-22178.44246 GO:0006090 GO:0006725 GO:0006814 GO:0071436
GO:0006560 GO:0006525 GO:0015081 GO:0008948 GO:0016853
GO:0016020
Cluster-22178.44250 GO:0051260
Cluster-22178.44251 GO:0006412 GO:0042254 GO:0003735 GO:0005622
GO:0005840
Cluster-22178.44258 GO:0030472 GO:0008608 GO:0005509 GO:0072686
GO:0042729
Cluster-22178.44259 GO:0030472 GO:0008608 GO:0005509 GO:0072686
GO:0042729
Cluster-22178.44260 GO:0003723 GO:0003676 GO:0016021
Cluster-22178.44261 GO:0005198 GO:0019028
Cluster-22178.44263 GO:0006468 GO:0006413 GO:0007165 GO:0006446
GO:0003743 GO:0005515 GO:0005524 GO:0004672 GO:0043565
GO:0016020 GO:0005840
Cluster-22178.44264 GO:0007165 GO:0042981 GO:0005515 GO:0003677

Cluster-22178.44268 GO:0009069 GO:0007021 GO:0016310 GO:0051082
GO:0015631 GO:0004674 GO:0005783 GO:0045298
Cluster-22178.44274 GO:0005515
Cluster-22178.44276 GO:0000746 GO:0019867
Cluster-22178.44280 GO:0006270 GO:0006446 GO:0006886 GO:0004190
GO:0003743 GO:0005852 GO:0005737 GO:0030176 GO:0005840
GO:0016021
Cluster-22178.44281 GO:0005515
Cluster-22178.44282 GO:0019033
Cluster-22178.44284 GO:0016021

Cluster-22178.44285 GO:0015979 GO:0009523 GO:0009539
Cluster-22178.44286 GO:0015979 GO:0009523 GO:0009539
Cluster-22178.44287 GO:0006810 GO:0042626 GO:0005524 GO:0016021
Cluster-22178.44290 GO:0008152 GO:0006570 GO:0042207 GO:0009072
GO:0003824 GO:0004334
Cluster-22178.44291 GO:0008152 GO:0042207 GO:0006570 GO:0009072
GO:0003824 GO:0004334
Cluster-22178.44292 GO:0006313 GO:0055085 GO:0003677 GO:0022857
GO:0016020 GO:0016021
Cluster-22178.44299 GO:0016021
Cluster-22178.44311 GO:0009607 GO:0016021
Cluster-22178.44314 GO:0005515
Cluster-22178.44318 GO:0046872
Cluster-22178.44325 GO:0005515
Cluster-22178.44333 GO:0016310 GO:0009069 GO:0006468 GO:0004674
GO:0000287 GO:0005524 GO:0004672
Cluster-22178.44334 GO:0006468 GO:0016773 GO:0004672 GO:0005524
GO:0016020
Cluster-22178.44340 GO:0035556
Cluster-22178.44342 GO:0006355 GO:0003700 GO:0008270 GO:0043565
GO:0005667
Cluster-22178.44349 GO:0007156 GO:0005509 GO:0003676 GO:0008270
GO:0016020
Cluster-22178.44352 GO:0005515
Cluster-22178.44360 GO:0015992 GO:0006820 GO:0006744 GO:0006120
GO:0006814 GO:0008137 GO:0005743 GO:0016021
Cluster-22178.44362 GO:0006413 GO:0003723 GO:0019012
Cluster-22178.44363 GO:0005515
Cluster-22178.44364 GO:0005515
Cluster-22178.44365 GO:0005515
Cluster-22178.44372 GO:0005515 GO:0008270
Cluster-22178.44375 GO:0016773
Cluster-22178.44378 GO:0007411

Cluster-22178.44383 GO:0004867
Cluster-22178.44385 GO:0006355 GO:0043565 GO:0008270 GO:0005515
GO:0003700 GO:0005667 GO:0005634
Cluster-22178.44388 GO:0016021
Cluster-22178.44392 GO:0003723
Cluster-22178.44393 GO:0003723
Cluster-22178.44395 GO:0003723
Cluster-22178.44398 GO:0006915 GO:0008285 GO:0007050 GO:0005515

Cluster-22178.44401 GO:0008270 GO:0046872 GO:0003676
Cluster-22178.44408 GO:0015992 GO:0006123 GO:0004129 GO:0005739
GO:0005576 GO:0045277
Cluster-22178.44420 GO:0008270 GO:0046872 GO:0005515
Cluster-22178.44422 GO:0005509
Cluster-22178.44427 GO:0007156 GO:0005509 GO:0016020
Cluster-22178.44428 GO:0016021 GO:0016020
Cluster-22178.44454 GO:0007156 GO:0005509 GO:0016020
Cluster-22178.44460 GO:0016192 GO:0006886 GO:0005515 GO:0008270

Cluster-22178.44464 GO:0009246 GO:0055114 GO:0036065 GO:0020037
GO:0016705 GO:0008417 GO:0005506
Cluster-22178.44466 GO:0006334 GO:0000398 GO:0005515 GO:0005681
GO:0005634
Cluster-22178.44472 GO:0006812 GO:0006836 GO:0016032 GO:0005328
GO:0016021
Cluster-22178.44473 GO:0006812 GO:0006836 GO:0016032 GO:0005328
GO:0016021
Cluster-22178.44481 GO:0046488 GO:0016307 GO:0005515
Cluster-22178.44484 GO:0008270 GO:0005515 GO:0005576
Cluster-22178.44488 GO:0009307 GO:0006308 GO:0009036 GO:0003677
GO:0005515 GO:0009359
Cluster-22178.44492 GO:0005515
Cluster-22178.44496 GO:0035556 GO:0045333 GO:0005739 GO:0005634

Cluster-22178.44515 GO:0005515
Cluster-22178.44521 GO:0005509
Cluster-22178.44531 GO:0002949 GO:0032065 GO:0007165 GO:0000062 GO:0016887 GO:0005543 GO:0003774 GO:0005524 GO:0005515 GO:0005938 GO:0005856 GO:0016459
Cluster-22178.44533 GO:0005524
Cluster-22178.44537 GO:0006470 GO:0006570 GO:0019988 GO:0008138 GO:0004725 GO:0043399
Cluster-22178.44542 GO:0005515
Cluster-22178.44544 GO:0006355 GO:0006260 GO:0003700 GO:0005667 GO:0005634
Cluster-22178.44547 GO:0005515
Cluster-22178.44548 GO:2001256 GO:0006355 GO:0005515 GO:0030176 GO:0005634
Cluster-22178.44549 GO:0008277 GO:0006886 GO:0015031 GO:0008565 GO:0016021
Cluster-22178.44552 GO:0007165 GO:0006289 GO:0006281 GO:0043161 GO:0003684 GO:0005179 GO:0005576
Cluster-22178.44554 GO:0006355 GO:0006357 GO:0003712 GO:0001104 GO:0005667 GO:0016592
Cluster-22178.44559 GO:0006511 GO:0007275 GO:0008270 GO:0005515 GO:0005634
Cluster-22178.44563 GO:0016192 GO:0006904
Cluster-22178.44565 GO:0009405 GO:0005576
Cluster-22178.44571 GO:0055114 GO:0004499 GO:0050661 GO:0016491 GO:0050660
Cluster-22178.44572 GO:0006811 GO:0006260 GO:0006351 GO:0006810 GO:0006269 GO:0006813 GO:0003896 GO:0005215 GO:0005267 GO:0008270 GO:0003677 GO:0005657 GO:0016020 GO:0005730
Cluster-22178.44574 GO:0006811 GO:0006351 GO:0006813 GO:0006260 GO:0006810 GO:0006269 GO:0005215 GO:0003896 GO:0005267 GO:0004190 GO:0008270 GO:0003677 GO:0016021 GO:0005657 GO:0016020 GO:0005730

Cluster-22178.44577 GO:0006508 GO:0005515 GO:0004252 GO:0005509 GO:0005576
Cluster-22178.44579 GO:0006260 GO:0000724 GO:0003677 GO:0003676 GO:0016021 GO:0005634
Cluster-22178.44581 GO:0006508 GO:0005515 GO:0004252 GO:0005509 GO:0005576
Cluster-22178.44592 GO:0008283 GO:0006259 GO:0040007 GO:0007165 GO:0055114 GO:0003677 GO:0008083 GO:0005524 GO:0016627 GO:0047134 GO:0003824 GO:0005694
Cluster-22178.44593 GO:0040007 GO:0006259 GO:0008283 GO:0007165 GO:0055114 GO:0008083 GO:0003677 GO:0016627 GO:0005524 GO:0003824 GO:0047134 GO:0005694
Cluster-22178.44598 GO:0007010 GO:0051260 GO:0003779
Cluster-22178.44599 GO:0005515
Cluster-22178.44600 GO:0006122 GO:0030866 GO:0005198 GO:0003779 GO:0008092 GO:0005856 GO:0005743 GO:0005750 GO:0016021
Cluster-22178.44604 GO:0006313
Cluster-22178.44610 GO:0046872
Cluster-22178.44611 GO:0005515
Cluster-22178.44612 GO:0009405 GO:0006810 GO:0019871 GO:0005515 GO:0005576
Cluster-22178.44617 GO:0007165 GO:0006355 GO:0005515 GO:0003713 GO:0004519 GO:0035257 GO:0046983 GO:0005634 GO:0005667 GO:0019031
Cluster-22178.44618 GO:0051258 GO:0006508 GO:0006355 GO:0030168 GO:0016567 GO:0007165 GO:0003700 GO:0043565 GO:0046872 GO:0008236 GO:0005515 GO:0005102 GO:0004842 GO:0008270 GO:0061630 GO:0030674 GO:0005577 GO:0005680 GO:0005622 GO:0005667
Cluster-22178.44619 GO:0055085 GO:0006811 GO:0006355 GO:0046325 GO:0043565 GO:0005216 GO:0016020
Cluster-22178.44620 GO:0005524 GO:0016887
Cluster-22178.44625 GO:0009059 GO:0030176

Cluster-22178.44626 GO:0048309 GO:0051028 GO:0006396 GO:0000176
Cluster-22178.44627 GO:0055114 GO:0047134
Cluster-22178.44634 GO:0004252 GO:0016021
Cluster-22178.44635 GO:0019229 GO:0003676 GO:0005576
Cluster-22178.44641 GO:0016787
Cluster-22178.44643 GO:0007050 GO:0045859 GO:0004861 GO:0005634
Cluster-22178.44644 GO:0045859 GO:0007050 GO:0004861 GO:0005634
Cluster-22178.44648 GO:0015031 GO:0006606 GO:0016787 GO:0003677
GO:0008565 GO:0005524 GO:0003676 GO:0005634 GO:0005737
Cluster-22178.44651 GO:0016787
Cluster-22178.44652 GO:0055085 GO:0030001 GO:0008272 GO:0015116
GO:0046873 GO:0016020 GO:0016021
Cluster-22178.44656 GO:0003676
Cluster-22178.44658 GO:0006355 GO:0016791 GO:0003677
Cluster-22178.44659 GO:0006260 GO:0006281 GO:0003676 GO:0043140
GO:0016874 GO:0005524 GO:0003677 GO:0016818 GO:0016787
GO:0010181 GO:0005657 GO:0005622 GO:0016020 GO:0005634
Cluster-22178.44664 GO:0006355 GO:0043093 GO:0007165 GO:0000917
GO:0030521 GO:0051056 GO:0005515 GO:0003677 GO:0004882
GO:0005096 GO:0005496 GO:0005634 GO:0005737
Cluster-22178.44665 GO:0030521 GO:0051056 GO:0006260 GO:0006355
GO:0043093 GO:0007165 GO:0000917 GO:0005096 GO:0004882
GO:0005496 GO:0005515 GO:0003677 GO:0005737 GO:0005634
Cluster-22178.44666 GO:0055114 GO:0006771 GO:0019497 GO:0003993
GO:0016671 GO:0003756 GO:0005783
Cluster-22178.44667 GO:0042742 GO:0003677 GO:0008270 GO:0005576
Cluster-22178.44668 GO:0005515
Cluster-22178.44670 GO:0009877 GO:0005515 GO:0016746 GO:0005829
Cluster-22178.44674 GO:0007172 GO:0000917 GO:0007165 GO:0006468
GO:0043093 GO:0004713 GO:0004871 GO:0005515 GO:0005737
GO:0005925
Cluster-22178.44676 GO:0030154 GO:0016021
Cluster-22178.44679 GO:0007010 GO:0005524 GO:0016787 GO:0003779

GO:0003677 GO:0003676
Cluster-22178.44688 GO:0046486 GO:0042967 GO:0004144
Cluster-22178.44689 GO:0006269 GO:0006351 GO:0006260 GO:0003677
GO:0008270 GO:0003896 GO:0005730 GO:0005657
Cluster-22178.44697 GO:0007264 GO:0000398 GO:0005524 GO:0003924
GO:0005525
Cluster-22178.44699 GO:0006914 GO:0007049 GO:0043093 GO:0006355
GO:0000917 GO:0043565 GO:0003700 GO:0003677 GO:0005515
GO:0005737 GO:0005667 GO:0005634
Cluster-22178.44700 GO:0003677
Cluster-22178.44703 GO:0055114 GO:0016616 GO:0016491
Cluster-22178.44712 GO:0006260 GO:0006276 GO:0005515 GO:0016020
Cluster-22178.44717 GO:0046856 GO:0035556 GO:0006464 GO:0003677
GO:0016151 GO:0034597
Cluster-22178.44719 GO:0008242
Cluster-22178.44722 GO:0006858
Cluster-22178.44726 GO:0043401 GO:0006355
Cluster-22178.44732 GO:0006886 GO:0006888 GO:0006464 GO:0016151
GO:0008270 GO:0046872 GO:0030127
Cluster-22178.44733 GO:0019028
Cluster-22178.44734 GO:0003723 GO:0004867 GO:0005524 GO:0003924
GO:0043531 GO:0005525 GO:0035064 GO:0003724 GO:0005576
Cluster-22178.44739 GO:0006260 GO:0005524 GO:0005634 GO:0000808
Cluster-22178.44744 GO:0003677
Cluster-22178.44752 GO:0022904 GO:0003676 GO:0008017 GO:0045298
GO:0016020
Cluster-22178.44757 GO:0015979 GO:0005515 GO:0005509 GO:0009654
GO:0019898 GO:0009523
Cluster-22178.44759 GO:0003676
Cluster-22178.44764 GO:0007275 GO:0009966 GO:0003723 GO:0032266
GO:0043130 GO:0034464 GO:0016020
Cluster-22178.44765 GO:0006468 GO:0005524 GO:0004672 GO:0016773
GO:0016020

Cluster-22178.44767 GO:0006144 GO:0035556 GO:0009190 GO:0046039
GO:0006182 GO:0006171 GO:0004383 GO:0016849 GO:0004252
GO:0004016 GO:0005886
Cluster-22178.44770 GO:0006260 GO:0006914 GO:0015992 GO:0000921
GO:0007049 GO:0006886 GO:0015991 GO:0003774 GO:0015078
GO:0005887 GO:0005940 GO:0005783 GO:0016459 GO:0019867
GO:0016021 GO:0033179
Cluster-22178.44781 GO:0007264 GO:0007010 GO:0009405 GO:0046718
GO:0003779 GO:0003924 GO:0005525 GO:0046812 GO:0039624
GO:0009986
Cluster-22178.44787 GO:0055114 GO:0016705 GO:0020037 GO:0005506

Cluster-22178.44788 GO:0042073 GO:0006355 GO:0008270 GO:0003682
GO:0003677 GO:0000775 GO:0032040 GO:0000785 GO:0005634
Cluster-22178.44789 GO:0006355 GO:0042073 GO:0003677 GO:0003682
GO:0008270 GO:0005634 GO:0000785 GO:0032040 GO:0000775
Cluster-22178.44801 GO:0008152 GO:0016747 GO:0016746
Cluster-22178.44806 GO:0003723 GO:0003676
Cluster-22178.44827 GO:0003824 GO:0005198 GO:0046872 GO:0008270
GO:0051536 GO:0019028
Cluster-22178.44828 GO:0006355 GO:0003700 GO:0043565 GO:0046872
GO:0008270 GO:0005667
Cluster-22178.44833 GO:0008152 GO:0006468 GO:0003824 GO:0005524
GO:0004672
Cluster-22178.44843 GO:0035556
Cluster-22178.44846 GO:0006811 GO:0006810 GO:0005230 GO:0016020
Cluster-22178.44854 GO:0035556 GO:0005515 GO:0016021
Cluster-22178.44855 GO:0006816 GO:0006874 GO:0006270 GO:0035556
GO:0004190 GO:0004518 GO:0003677 GO:0005515 GO:0005219
GO:0005622 GO:0016021
Cluster-22178.44864 GO:0006811 GO:0007052 GO:0035058 GO:0055085
GO:0031122 GO:0007165 GO:0005515 GO:0005216 GO:0004888
GO:0016021 GO:0005813 GO:0016020 GO:0097546

Cluster-22178.44865 GO:0009847 GO:0006508 GO:0003676 GO:0008233
GO:0000166 GO:0003723
Cluster-22178.44869 GO:0006886 GO:0006260 GO:0008134 GO:0042803
GO:0005198 GO:0045502 GO:0016020 GO:0005667 GO:0005801
GO:0030286
Cluster-22178.44878 GO:0005515
Cluster-22178.44885 GO:0016021
Cluster-22178.44886 GO:0046872 GO:0008270 GO:0003677
Cluster-22178.44889 GO:0007186 GO:0055114 GO:0004930 GO:0005509
GO:0016702 GO:0016021
Cluster-22178.44893 GO:0006355 GO:0048870 GO:0005515 GO:0003700
GO:0043565 GO:0031514 GO:0019028 GO:0005667
Cluster-22178.44898 GO:0016567 GO:0004842
Cluster-22178.44899 GO:0016021
Cluster-22178.44902 GO:0015940 GO:0019521 GO:0055114 GO:0006574
GO:0006552 GO:0006550 GO:0006568 GO:0006633 GO:0006554
GO:0006631 GO:0018874 GO:0006098 GO:0008677 GO:0003857
GO:0016491 GO:0004616 GO:0051287
Cluster-22178.44905 GO:0006811 GO:0006813 GO:0005249 GO:0008076
GO:0016020
Cluster-22178.44906 GO:0051205 GO:0016021
Cluster-22178.44910 GO:0006355 GO:0009271 GO:0016021
Cluster-22178.44911 GO:0046872 GO:0016021
Cluster-22178.44916 GO:0006874 GO:0030234 GO:0030176
Cluster-22178.44920 GO:0006355 GO:0003677
Cluster-22178.44925 GO:0022900 GO:0016021
Cluster-22178.44931 GO:0055085 GO:0016021
Cluster-22178.44936 GO:0007050
Cluster-22178.44938 GO:0003779
Cluster-22178.44944 GO:0016020
Cluster-22178.44950 GO:0051258 GO:0009405 GO:0006904 GO:0006886
GO:0030168 GO:0015031 GO:0006810 GO:0007165 GO:0019871
GO:0005198 GO:0005102 GO:0030674 GO:0005801 GO:0005576

GO:0005577 GO:0005882 GO:0016020 GO:0000145
Cluster-22178.44956 GO:0003677
Cluster-22178.44962 GO:0003677
Cluster-22178.44966 GO:0005975 GO:0006355 GO:0008270 GO:0003824
GO:0003682 GO:0043169 GO:0003677 GO:0000785 GO:0000775
GO:0005634
Cluster-22178.44973 GO:0051056 GO:0055085 GO:0006811 GO:0009968
GO:0007268 GO:0007165 GO:0005096 GO:0004970 GO:0005216
GO:0005737 GO:0016020
Cluster-22178.44976 GO:0006816 GO:0006874 GO:0005219 GO:0046872
GO:0004252 GO:0016021 GO:0005622 GO:0005886
Cluster-22178.44980 GO:0005515
Cluster-22178.44982 GO:0015940 GO:0015937 GO:0004140 GO:0005524
GO:0005921
Cluster-22178.44985 GO:0016567 GO:0006810 GO:0046872 GO:0008270
GO:0004842 GO:0005515 GO:0030288 GO:0005680
Cluster-22178.44989 GO:0007165 GO:0005515 GO:0005509 GO:0005578
Cluster-22178.44995 GO:0006139 GO:0031167 GO:0008168
Cluster-22178.44996 GO:0031167 GO:0006139 GO:0008168
Cluster-22178.44997 GO:0006139 GO:0031167 GO:0008168
Cluster-22178.44999 GO:0009405
Cluster-22178.45001 GO:0005515 GO:0008270
Cluster-22178.45005 GO:0042981
Cluster-22178.45006 GO:0030071 GO:0005515 GO:0046983 GO:0005680
Cluster-22178.45007 GO:0008152 GO:0008168
Cluster-22178.45011 GO:0005515
Cluster-22178.45012 GO:0008152 GO:0016491
Cluster-22178.45015 GO:0016021
Cluster-22178.45016 GO:0005515 GO:0016021
Cluster-22178.45027 GO:0005515
Cluster-22178.45031 GO:0006813 GO:0006811 GO:0008270 GO:0005249
GO:0016020 GO:0008076
Cluster-22178.45033 GO:0055085 GO:0022857 GO:0016021

Cluster-22178.45039 GO:0006886 GO:0006888 GO:0003677 GO:0008270
GO:0046872 GO:0045202 GO:0030127
Cluster-22178.45047 GO:0015979 GO:0009523 GO:0009539 GO:0016020
Cluster-22178.45051 GO:0035556 GO:0005515 GO:0003677
Cluster-22178.45053 GO:0031047 GO:0045454 GO:0006118 GO:0015035
GO:0009055
Cluster-22178.45054 GO:0006355 GO:0003700 GO:0043565 GO:0005667
Cluster-22178.45057 GO:0007059 GO:0051301 GO:0005515 GO:0008017
GO:0045298 GO:0000444
Cluster-22178.45061 GO:0035556 GO:0009279
Cluster-22178.45063 GO:0007268 GO:0006811 GO:0007165 GO:0005234
GO:0004970 GO:0016020
Cluster-22178.45067 GO:0009424
Cluster-22178.45071 GO:0043087 GO:0032012 GO:0005086
Cluster-22178.45076 GO:0005515
Cluster-22178.45079 GO:0006479 GO:0009451 GO:0046500 GO:0008152
GO:0006464 GO:0030488 GO:0008033 GO:0016429 GO:0004719
GO:0008168 GO:0031515
Cluster-22178.45080 GO:0007165 GO:0005515
Cluster-22178.45088 GO:0006355 GO:0008270 GO:0003677 GO:0005634
Cluster-22178.45096 GO:0022904 GO:0016651 GO:0005743
Cluster-22178.45101 GO:0006810 GO:0055085 GO:0005215 GO:0005515
GO:0016021 GO:0016020
Cluster-22178.45102 GO:0006810 GO:0055085 GO:0005215 GO:0005515
GO:0016021 GO:0016020
Cluster-22178.45103 GO:0016462 GO:0005509
Cluster-22178.45106 GO:0042967 GO:0016747 GO:0008080 GO:0016021
Cluster-22178.45118 GO:0006355 GO:0005515 GO:0046983
Cluster-22178.45125 GO:0002098 GO:0034227 GO:0000049 GO:0003677
GO:0046872 GO:0008270 GO:0003676 GO:0005634
Cluster-22178.45129 GO:0006230 GO:0006470 GO:0006888 GO:0006206
GO:0006886 GO:0006570 GO:0003824 GO:0004725 GO:0051536
GO:0008270 GO:0004797 GO:0005524 GO:0008138 GO:0030127

Cluster-22178.45130 GO:0035556 GO:0032065 GO:0055114 GO:0016301
GO:0005515 GO:0047134 GO:0005543 GO:0005938

Cluster-22178.45134 GO:0006355 GO:0008191 GO:0043565

Cluster-22178.45135 GO:0006355 GO:0005634

Cluster-22178.45145 GO:0009395 GO:0035556 GO:0008654 GO:0006506
GO:0046486 GO:0004143 GO:0016746 GO:0005515 GO:0005789
GO:0016021 GO:0016020

Cluster-22178.45148 GO:0046486 GO:0006506 GO:0008654 GO:0035556
GO:0009395 GO:0005515 GO:0016746 GO:0004143 GO:0016020
GO:0016021 GO:0005789

Cluster-22178.45152 GO:0005515

Cluster-22178.45155 GO:0042203 GO:0032402 GO:0019261 GO:0046232
GO:0018874 GO:0042184 GO:0055114 GO:0009712 GO:0005506
GO:0018576 GO:0042470

Cluster-22178.45156 GO:0006308 GO:0016888

Cluster-22178.45157 GO:0006308 GO:0016888

Cluster-22178.45159 GO:0005515

Cluster-22178.45165 GO:0006544 GO:0008654 GO:0046486 GO:0006563
GO:0006566 GO:0008270 GO:0046872 GO:0005515 GO:0004609

Cluster-22178.45167 GO:0006357 GO:0006470 GO:0006570 GO:0004725
GO:0008138 GO:0001104 GO:0016592

Cluster-22178.45175 GO:0005509 GO:0005515

Cluster-22178.45178 GO:0007165 GO:0006869 GO:0042157 GO:0019915
GO:0008289 GO:0005789 GO:0005576 GO:0008537

Cluster-22178.45183 GO:0042254 GO:0005975 GO:0003735 GO:0004553
GO:0005840

Cluster-22178.45185 GO:0006886 GO:0050909 GO:0016021 GO:0005783

Cluster-22178.45189 GO:0055114 GO:0051287

Cluster-22178.45197 GO:0006811 GO:0051056 GO:0055085 GO:0007165
GO:0007268 GO:0009968 GO:0005096 GO:0004970 GO:0005216
GO:0005737 GO:0016020

Cluster-22178.45200 GO:0003677

Cluster-22178.45201 GO:0008152 GO:0016746

Cluster-22178.45203 GO:0046983 GO:0036128
Cluster-22178.45207 GO:0042262 GO:0006090 GO:0006357 GO:0006914
GO:0006633 GO:0048870 GO:0051258 GO:0030168 GO:0070206
GO:0006260 GO:0007165 GO:0042803 GO:0005198 GO:0001104
GO:0008017 GO:0003690 GO:0045502 GO:0008134 GO:0003989
GO:0005102 GO:0030674 GO:0045298 GO:0005577 GO:0005667
GO:0016021 GO:0019031 GO:0019028 GO:0031514 GO:0030286
GO:0009317 GO:0005882 GO:0016592
Cluster-22178.45214 GO:0016021
Cluster-22178.45215 GO:0005975 GO:0048870 GO:0001558 GO:0030178
GO:0006914 GO:0006606 GO:0007275 GO:0007165 GO:0005515
GO:0008270 GO:0005520 GO:0004553 GO:0030246 GO:0005576
GO:0031514 GO:0016942
Cluster-22178.45216 GO:0030178 GO:0001558 GO:0005975 GO:0048870
GO:0007165 GO:0007275 GO:0006606 GO:0006914 GO:0005515
GO:0030246 GO:0004553 GO:0008270 GO:0005520 GO:0031514
GO:0005576 GO:0016942
Cluster-22178.45219 GO:0042254 GO:0006118 GO:0017004 GO:0045158
GO:0003735 GO:0005840 GO:0009512
Cluster-22178.45223 GO:0007165 GO:0009372 GO:0005515 GO:0005509
GO:0005578
Cluster-22178.45224 GO:0006355 GO:0009271
Cluster-22178.45225 GO:0006119 GO:0006118 GO:0006351 GO:0055114
GO:0006144 GO:0015992 GO:0008121 GO:0003723 GO:0003968
GO:0019033 GO:0031379
Cluster-22178.45240 GO:0003676 GO:0008270 GO:0046872
Cluster-22178.45248 GO:0006284 GO:0008725 GO:0008440
Cluster-22178.45253 GO:0006308 GO:0055114 GO:0016114 GO:0006694
GO:0006810 GO:0003924 GO:0005525 GO:0003723 GO:0004506
GO:0016887 GO:0008855 GO:0005524 GO:0003724 GO:0050660
GO:0098519 GO:0016021 GO:0009318 GO:0016020
Cluster-22178.45254 GO:0007049 GO:0006260 GO:0048870 GO:0006914
GO:0006810 GO:0005886 GO:0031514 GO:0005634

Cluster-22178.45257 GO:0035064
Cluster-22178.45259 GO:0046872
Cluster-22178.45272 GO:0032979 GO:0042578 GO:0005743
Cluster-22178.45274 GO:0019031
Cluster-22178.45276 GO:0006289 GO:0055085 GO:0000105 GO:0055114
GO:0006308 GO:0006355 GO:0030001 GO:0008855 GO:0008270
GO:0004399 GO:0051287 GO:0046873 GO:0008168 GO:0000439
GO:0016020 GO:0009318
Cluster-22178.45283 GO:0008643 GO:0055085 GO:0005351 GO:0000139
GO:0016020 GO:0016021
Cluster-22178.45287 GO:0016787
Cluster-22178.45291 GO:0030246
Cluster-22178.45296 GO:0055114 GO:0016310 GO:0005506 GO:0004673
GO:0016705 GO:0020037 GO:0005198 GO:0019028 GO:0009365
Cluster-22178.45301 GO:0007275 GO:0006468 GO:0045893 GO:0008270
GO:0005524 GO:0004672 GO:0046983
Cluster-22178.45305 GO:0015937 GO:0015940 GO:0005524 GO:0004594

Cluster-22178.45307 GO:0007165 GO:0005509 GO:0005515 GO:0005578
Cluster-22178.45314 GO:0016020 GO:0016021
Cluster-22178.45317 GO:0008270
Cluster-22178.45318 GO:0030153
Cluster-22178.45322 GO:0006470 GO:0006570 GO:0008138 GO:0004725
GO:0005515 GO:0004721 GO:0008270
Cluster-22178.45323 GO:0006486 GO:0008417 GO:0016020
Cluster-22178.45324 GO:0006355 GO:0006486 GO:0003682 GO:0003677
GO:0008270 GO:0008417 GO:0005634 GO:0016020 GO:0005789
GO:0000775 GO:0000785
Cluster-22178.45326 GO:0055085 GO:0006812 GO:0006835 GO:0006820
GO:0030001 GO:0006508 GO:0017153 GO:0046873 GO:0016020
Cluster-22178.45328 GO:0003677
Cluster-22178.45334 GO:0016757
Cluster-22178.45336 GO:0005515

Cluster-22178.45337 GO:0005515
Cluster-22178.45340 GO:0006508 GO:0008152 GO:0008270 GO:0005515
GO:0004181 GO:0016788 GO:0005576
Cluster-22178.45347 GO:0035556 GO:0005515 GO:0019013 GO:0016021
Cluster-22178.45351 GO:0001558 GO:0005520 GO:0016942 GO:0005576
Cluster-22178.45353 GO:0007050
Cluster-22178.45357 GO:0008152 GO:0016757
Cluster-22178.45364 GO:0055114 GO:0042773 GO:0016651 GO:0019031
GO:0016021 GO:0016020
Cluster-22178.45365 GO:0055114 GO:0042773 GO:0016651 GO:0016020
GO:0016021 GO:0019031
Cluster-22178.45366 GO:0042773 GO:0055114 GO:0016651 GO:0019031
GO:0016021 GO:0016020
Cluster-22178.45367 GO:0046740 GO:0016021
Cluster-22178.45371 GO:0006888 GO:0007010 GO:0006886 GO:0008270
GO:0003779 GO:0003677 GO:0030127
Cluster-22178.45374 GO:0055085 GO:0022857 GO:0016021
Cluster-22178.45375 GO:0006355 GO:0007165 GO:0006955 GO:0005179
GO:0003700 GO:0005576 GO:0005667
Cluster-22178.45377 GO:0006355 GO:0007165 GO:0006955 GO:0005179
GO:0003700 GO:0005576 GO:0005667
Cluster-22178.45380 GO:0055085 GO:0006810 GO:0006308 GO:0007010
GO:0030001 GO:0043395 GO:0008855 GO:0046873 GO:0005215
GO:0003779 GO:0005198 GO:0005515 GO:0016020 GO:0019031
GO:0009318 GO:0019028 GO:0005578
Cluster-22178.45383 GO:0006691 GO:0019530 GO:0006749 GO:0006693
GO:0006486 GO:0051252 GO:0003840 GO:0008373 GO:0004540
Cluster-22178.45390 GO:0008270 GO:0005515 GO:0005509
Cluster-22178.45391 GO:0009069 GO:0006468 GO:0016310 GO:0000287
GO:0004674 GO:0016773 GO:0004672 GO:0005524 GO:0016020
Cluster-22178.45392 GO:0005975 GO:0004571 GO:0005509 GO:0016020
Cluster-22178.45396 GO:0046983 GO:0005515
Cluster-22178.45397 GO:0006629

Cluster-22178.45399 GO:0036065 GO:0009246 GO:0055114 GO:0020037 GO:0016705 GO:0008417 GO:0005506

Cluster-22178.45400 GO:0055114 GO:0020037 GO:0016705 GO:0005506

Cluster-22178.45410 GO:0006811 GO:0006813 GO:0005249 GO:0016020 GO:0008076

Cluster-22178.45413 GO:0006570 GO:0006470 GO:0004725 GO:0008138

Cluster-22178.45417 GO:0006355 GO:0007155 GO:0046872 GO:0008270 GO:0043565 GO:0003700 GO:0005198 GO:0005515 GO:0009288 GO:0019031 GO:0031225 GO:0005667 GO:0019028

Cluster-22178.45418 GO:0000226 GO:0000910 GO:0008017 GO:0019031 GO:0045298

Cluster-22178.45419 GO:0005515

Cluster-22178.45420 GO:0003824 GO:0051536

Cluster-22178.45422 GO:0005515 GO:0005516

Cluster-22178.45423 GO:0006813 GO:0055114 GO:0016705 GO:0020037 GO:0005506 GO:0005267 GO:0016020

Cluster-22178.45433 GO:0006508 GO:0015031 GO:0008236 GO:0016787

Cluster-22178.45434 GO:0048013 GO:0019497 GO:0006771 GO:0006468 GO:0003993 GO:0004672 GO:0005524 GO:0046872 GO:0005515

Cluster-22178.45448 GO:0009405 GO:0006351 GO:0051716 GO:0006810 GO:0006364 GO:0006383 GO:0006260 GO:0006206 GO:0009236 GO:0006144 GO:0003887 GO:0003899 GO:0005515 GO:0008200 GO:0004190 GO:0016817 GO:0003677 GO:0042575 GO:0005576 GO:0005634 GO:0034457 GO:0032040 GO:0005789 GO:0016021 GO:0046658 GO:0016020 GO:0005730 GO:0005666 GO:0005732

Cluster-22178.45453 GO:0009082

Cluster-22178.45455 GO:0006468 GO:0005524 GO:0004672 GO:0016773 GO:0016020

Cluster-22178.45460 GO:0007165

Cluster-22178.45468 GO:0034080 GO:0008270 GO:0000775

Cluster-22178.45476 GO:0003677 GO:0016779 GO:0034061 GO:0005634

Cluster-22178.45485 GO:0043087 GO:0042742 GO:0035023 GO:0005089

Cluster-22178.45486 GO:0035023 GO:0042742 GO:0043087 GO:0005089

Cluster-22178.45489 GO:0016049 GO:0006468 GO:0010215 GO:0005524 GO:0004672 GO:0031225

Cluster-22178.45500 GO:0007264 GO:0005525

Cluster-22178.45506 GO:0005515

Cluster-22178.45508 GO:0008270 GO:0046872 GO:0005515

Cluster-22178.45513 GO:0003924

Cluster-22178.45517 GO:0005975 GO:0005509 GO:0004571 GO:0016020

Cluster-22178.45518 GO:0005975 GO:0004571 GO:0005509 GO:0016020

Cluster-22178.45529 GO:0005515

Cluster-22178.45532 GO:0006508 GO:0003676 GO:0008233

Cluster-22178.45536 GO:0003677

Cluster-22178.45538 GO:0006886 GO:0008536

Cluster-22178.45549 GO:0046656 GO:0006457 GO:0015948 GO:0030269 GO:0016021

Cluster-22178.45554 GO:0006281 GO:0003824 GO:0003908

Cluster-22178.45563 GO:0005515

Cluster-22178.45566 GO:0007186 GO:0004930 GO:0016021

Cluster-22178.45575 GO:0035556 GO:0046872

Cluster-22178.45576 GO:0016758 GO:0005789

Cluster-22178.45579 GO:0019988 GO:0006470 GO:0006570 GO:0004725 GO:0008138 GO:0043399

Cluster-22178.45580 GO:0007031 GO:0005524 GO:0019031 GO:0005777

Cluster-22178.45586 GO:0008152 GO:0016491

Cluster-22178.45589 GO:0005509

Cluster-22178.45591 GO:0006260 GO:0016192 GO:0019079 GO:0005524 GO:0003678 GO:0003676 GO:0016787 GO:0005515 GO:0003677 GO:0016021 GO:0005657

Cluster-22178.45597 GO:0005515

Cluster-22178.45600 GO:0005515
Cluster-22178.45601 GO:0003677 GO:0046872 GO:0008270
Cluster-22178.45604 GO:0006355 GO:0003700 GO:0043565 GO:0005667
Cluster-22178.45607 GO:0008152 GO:0008484 GO:0003824
Cluster-22178.45609 GO:0006310 GO:0003677 GO:0005515 GO:0005634
Cluster-22178.45613 GO:0048870 GO:0045502 GO:0008134 GO:0042803
GO:0008092 GO:0005667 GO:0031514 GO:0005737 GO:0030286
GO:0019898
Cluster-22178.45614 GO:0042254 GO:0006412 GO:0007165 GO:0006891
GO:0003735 GO:0005515 GO:0016817 GO:0017119 GO:0005840
GO:0005622
Cluster-22178.45616 GO:0042254 GO:0006412 GO:0007165 GO:0006891
GO:0005515 GO:0003735 GO:0016817 GO:0017119 GO:0005840
GO:0005622
Cluster-22178.45617 GO:0006886 GO:0007186 GO:0050909 GO:0007031
GO:0000921 GO:0051276 GO:0005198 GO:0005515 GO:0000166
GO:0003677 GO:0008773 GO:0004930 GO:0005524 GO:0019028
GO:0005801 GO:0016021 GO:0019031 GO:0016020 GO:0005694
GO:0005940 GO:0005777
Cluster-22178.45619 GO:0007165 GO:0043087 GO:0007264 GO:0016485
GO:0005515 GO:0005085 GO:0016021
Cluster-22178.45641 GO:0055114 GO:0008168 GO:0016706 GO:0005515

Cluster-22178.45649 GO:0003697
Cluster-22178.45650 GO:0000723 GO:0003697
Cluster-22178.45665 GO:0015684 GO:0007264 GO:0005525 GO:0015093
GO:0016021 GO:0019028
Cluster-22178.45670 GO:0031167 GO:0006139 GO:0008168
Cluster-22178.45671 GO:0007186 GO:0004930 GO:0016021
Cluster-22178.45672 GO:0008270 GO:0003676
Cluster-22178.45677 GO:0042254 GO:0006412 GO:0051252 GO:0008033
GO:0003735 GO:0004540 GO:0015934 GO:0005840
Cluster-22178.45678 GO:0008033 GO:0042254 GO:0006412 GO:0051252

GO:0003735 GO:0004540 GO:0005840 GO:0015934
Cluster-22178.45682 GO:0005515 GO:0061630 GO:0046872 GO:0008270

Cluster-22178.45686 GO:0003677 GO:0005634
Cluster-22178.45690 GO:0007268 GO:0009405 GO:0007165 GO:0006355
GO:0048193 GO:0030550 GO:0003700 GO:0008270 GO:0016020
GO:0005667 GO:0005634 GO:0005576
Cluster-22178.45692 GO:0008210 GO:0008207 GO:0046487 GO:0008209
GO:0009396 GO:0055114 GO:0008152 GO:0006694 GO:0003854
GO:0050662 GO:0004488 GO:0016616 GO:0016491 GO:0003824
Cluster-22178.45698 GO:0016579 GO:0006511 GO:0006508 GO:0004843
GO:0005622
Cluster-22178.45700 GO:0043565 GO:0005515 GO:0005634
Cluster-22178.45701 GO:0003677
Cluster-22178.45703 GO:0006810 GO:0006816 GO:0005515 GO:0042030
GO:0005246 GO:0016020
Cluster-22178.45706 GO:0006508 GO:0007155 GO:0042254 GO:0006412
GO:0003735 GO:0004222 GO:0016020 GO:0005622 GO:0005840
Cluster-22178.45708 GO:0005515
Cluster-22178.45711 GO:0006810 GO:0015893 GO:0006855 GO:0055085
GO:0015238 GO:0015297 GO:0016020
Cluster-22178.45714 GO:0005515
Cluster-22178.45716 GO:0009405 GO:0003333
Cluster-22178.45725 GO:0044237 GO:0006904 GO:0006355 GO:0003700
GO:0003824 GO:0043565 GO:0003677 GO:0000145 GO:0005667
GO:0005634
Cluster-22178.45734 GO:0008270
Cluster-22178.45735 GO:1901031 GO:0055114 GO:0004190 GO:0005634
GO:0016021
Cluster-22178.45736 GO:0042254 GO:0006412 GO:0003723 GO:0003735
GO:0005622 GO:0005840
Cluster-22178.45739 GO:0006470 GO:0006570 GO:0004725 GO:0008138

Cluster-22178.45741 GO:0007165 GO:0005515
Cluster-22178.45744 GO:0006506 GO:0007009 GO:0005515 GO:0005509
GO:0003676 GO:0017176 GO:0016021 GO:0005737
Cluster-22178.45746 GO:0016310 GO:0007178 GO:0009069 GO:0006468
GO:0005524 GO:0004672 GO:0004675 GO:0016020
Cluster-22178.45753 GO:0015979 GO:0009523 GO:0009539 GO:0016020
Cluster-22178.45754 GO:0007186 GO:0004930 GO:0019031 GO:0016020
Cluster-22178.45756 GO:0006605 GO:0006886 GO:0005515 GO:0005742
Cluster-22178.45758 GO:0006493 GO:0000030 GO:0016020 GO:0000136
Cluster-22178.45761 GO:0006508 GO:0008236
Cluster-22178.45763 GO:0006481 GO:0006479 GO:0004671 GO:0016020
GO:0016021
Cluster-22178.45764 GO:0007224 GO:0015074
Cluster-22178.45767 GO:0006885 GO:0006812 GO:0055085 GO:0015299
GO:0016021
Cluster-22178.45769 GO:0055085 GO:0016020 GO:0016021
Cluster-22178.45770 GO:0055114 GO:0016491
Cluster-22178.45772 GO:0006013 GO:0015074 GO:0019497 GO:0006000
GO:0006771 GO:0008270 GO:0005524 GO:0003993 GO:0003873
Cluster-22178.45773 GO:0006013 GO:0006000 GO:0006096 GO:0046486
GO:0006771 GO:0006020 GO:0008152 GO:0019497 GO:0015976
GO:0006094 GO:0003993 GO:0003677 GO:0004807 GO:0046872
GO:0005515 GO:0016020
Cluster-22178.45774 GO:0006096 GO:0046486 GO:0006771 GO:0006020
GO:0006013 GO:0006000 GO:0006094 GO:0008152 GO:0019497
GO:0015976 GO:0003677 GO:0003993 GO:0005515 GO:0004807
GO:0046872 GO:0016020
Cluster-22178.45777 GO:0030182 GO:0006281 GO:0021549 GO:0006310
GO:0005524 GO:0009378 GO:0005657 GO:0009379
Cluster-22178.45790 GO:0005515 GO:0046872 GO:0031225
Cluster-22178.45792 GO:0015886 GO:0017004 GO:0005509 GO:0005515
GO:0016021
Cluster-22178.45800 GO:0005515

Cluster-22178.45806 GO:0005515 GO:0016021

Cluster-22178.45811 GO:0006858

Cluster-22178.45815 GO:0015992 GO:0015986 GO:0015078 GO:0000276

Cluster-22178.45816 GO:0009401 GO:0006812 GO:0055085 GO:0008324 GO:0016020 GO:0016021

Cluster-22178.45820 GO:0003677

Cluster-22178.45821 GO:0005975 GO:0006013 GO:0006886 GO:0008536 GO:0004553 GO:0003824 GO:0004559 GO:0008270 GO:0015923

Cluster-22178.45822 GO:0005515 GO:0035091

Cluster-22178.45823 GO:0035091 GO:0005515

Cluster-22178.45824 GO:0035091

Cluster-22178.45828 GO:0009271 GO:0006355

Cluster-22178.45834 GO:0055085 GO:0030001 GO:0045040 GO:0007268 GO:0033130 GO:0046873 GO:0043495 GO:0005198 GO:0005515 GO:0042802 GO:0005742 GO:0019028 GO:0016020

Cluster-22178.45838 GO:0006471 GO:0003956

Cluster-22178.45839 GO:0006471 GO:0003956

Cluster-22178.45843 GO:0006940

Cluster-22178.45845 GO:0046488 GO:0005515 GO:0016307

Cluster-22178.45846 GO:0005515

Cluster-22178.45849 GO:0005515

Cluster-22178.45851 GO:0006816 GO:0006874 GO:0006397 GO:0046660 GO:0006811 GO:0004190 GO:0005219 GO:0005634 GO:0016020 GO:0005622 GO:0016021

Cluster-22178.45864 GO:0000184 GO:0008270 GO:0005524 GO:0003677 GO:0004386 GO:0005634 GO:0005737

Cluster-22178.45873 GO:0005515

Cluster-22178.45874 GO:0005515

Cluster-22178.45877 GO:0015074 GO:0006364 GO:0008270 GO:0003676

Cluster-22178.45883 GO:0006432 GO:0000162 GO:0055085 GO:0006571 GO:0008033 GO:0009094 GO:0000049 GO:0000287 GO:0004826 GO:0008168 GO:0005524 GO:0005741 GO:0009328

Cluster-22178.45885 GO:0006810 GO:0009405 GO:0008200 GO:0051537
GO:0005515 GO:0042151 GO:0005643
Cluster-22178.45893 GO:0015992 GO:0015991 GO:0015078 GO:0005856
GO:0033179
Cluster-22178.45902 GO:0015979 GO:0009522 GO:0009538
Cluster-22178.45904 GO:0008152 GO:0007160 GO:0008168 GO:0005509
GO:0005576
Cluster-22178.45909 GO:0006771 GO:0019497 GO:0046872 GO:0005515
GO:0003993
Cluster-22178.45914 GO:0005515
Cluster-22178.45920 GO:0006359
Cluster-22178.45924 GO:0006313 GO:0007165 GO:0009069 GO:0000184
GO:0006470 GO:0016310 GO:0000087 GO:0006570 GO:0004674
GO:0016773 GO:0004803 GO:0008144 GO:0004725 GO:0008168
GO:0005198 GO:0005515 GO:0005622 GO:0019031 GO:0019028
Cluster-22178.45933 GO:0015992 GO:0006119 GO:0006118 GO:0008121
GO:0009055 GO:0004222
Cluster-22178.45940 GO:0009405
Cluster-22178.45943 GO:0005515
Cluster-22178.45944 GO:0006771 GO:0019497 GO:0046872 GO:0005515
GO:0003993
Cluster-22178.45948 GO:0005515
Cluster-22178.45951 GO:0048013 GO:0005515
Cluster-22178.45965 GO:0000160 GO:0006935 GO:0000156 GO:0005515
GO:0008984 GO:0005737
Cluster-22178.45969 GO:0008152 GO:0003824
Cluster-22178.45974 GO:0035556 GO:0007218 GO:0055085 GO:0005185
GO:0016747 GO:0016021 GO:0005576
Cluster-22178.45977 GO:0007186 GO:0051260 GO:0004930 GO:0016020
GO:0016021
Cluster-22178.45984 GO:0006323 GO:0003677 GO:0019012
Cluster-22178.45986 GO:0006468 GO:0006351 GO:0006144 GO:0003723
GO:0005524 GO:0004672 GO:0003968 GO:0031379

Cluster-22178.45990 GO:0003676
Cluster-22178.45991 GO:0003676
Cluster-22178.45993 GO:0005634
Cluster-22178.45994 GO:0007165 GO:0005179 GO:0005634 GO:0005576
Cluster-22178.45996 GO:0006355 GO:0003677
Cluster-22178.45998 GO:0003723 GO:0003676
Cluster-22178.46007 GO:0006096 GO:0006094 GO:0015976 GO:0055114
GO:0006144 GO:0000287 GO:0030955 GO:0004743 GO:0003824
Cluster-22178.46008 GO:0006144 GO:0003968 GO:0031379
Cluster-22178.46012 GO:0006468 GO:0004672 GO:0005524
Cluster-22178.46013 GO:0007264 GO:0007186 GO:0007165 GO:0003924
GO:0019001 GO:0031683 GO:0004871 GO:0005525
Cluster-22178.46022 GO:0006771 GO:0006468 GO:0048013 GO:0019497
GO:0005515 GO:0003993 GO:0004672 GO:0005524 GO:0046872
GO:0019028
Cluster-22178.46027 GO:0005982 GO:0005985 GO:0005986 GO:0003677
GO:0050307
Cluster-22178.46028 GO:0005985 GO:0005982 GO:0005986 GO:0050307
GO:0003677
Cluster-22178.46030 GO:0005982 GO:0005985 GO:0005986 GO:0003677
GO:0050307
Cluster-22178.46031 GO:0009306 GO:0016020
Cluster-22178.46037 GO:0006914
Cluster-22178.46038 GO:0006260 GO:0006323 GO:0019012 GO:0005634
Cluster-22178.46039 GO:0009966 GO:0007275 GO:0016020
Cluster-22178.46040 GO:0007155 GO:0030155 GO:0006810 GO:0007165
GO:0030334 GO:0000921 GO:0048193 GO:0055085 GO:0006260
GO:0030001 GO:0045995 GO:0046873 GO:0005102 GO:0004871
GO:0016020 GO:0005940 GO:0005886 GO:0019028 GO:0005634
GO:0016021
Cluster-22178.46041 GO:0009451 GO:0007155 GO:0006260 GO:0030488
GO:0008033 GO:0016429 GO:0031515 GO:0005634
Cluster-22178.46050 GO:0007130 GO:0007283 GO:0007131

Cluster-22178.46053 GO:0006355 GO:0003677 GO:0004252 GO:0005515
GO:0005886 GO:0005634
Cluster-22178.46060 GO:0015074 GO:0006313 GO:0003677
Cluster-22178.46066 GO:0008616 GO:0006281 GO:0046034 GO:0006144
GO:0006366 GO:0016853 GO:0004017 GO:0003677 GO:0004867
GO:0016740 GO:0046872 GO:0008270 GO:0005576 GO:0005665
Cluster-22178.46067 GO:0005515
Cluster-22178.46074 GO:0007264 GO:0005525 GO:0005524 GO:0016887
GO:0003924
Cluster-22178.46076 GO:0007264 GO:0005525 GO:0003924 GO:0016887
GO:0005524
Cluster-22178.46080 GO:0009405 GO:0006813 GO:0055114 GO:0003677
GO:0020037 GO:0016705 GO:0005506 GO:0016787 GO:0005267
GO:0005524 GO:0016020
Cluster-22178.46082 GO:0042254 GO:0006412 GO:0007264 GO:0003735
GO:0005525 GO:0003924 GO:0005840 GO:0005622
Cluster-22178.46091 GO:0006313 GO:0015074 GO:0003677
Cluster-22178.46092 GO:0015979 GO:0005198 GO:0016020 GO:0009522
Cluster-22178.46097 GO:0055114 GO:0030976 GO:0050660 GO:0003824
GO:0016491 GO:0000287
Cluster-22178.46101 GO:0035556 GO:0005515 GO:0016021
Cluster-22178.46102 GO:0010024 GO:0055114 GO:0008152 GO:0016491
GO:0050897 GO:0016636
Cluster-22178.46103 GO:0008641
Cluster-22178.46107 GO:0005515 GO:0046872 GO:0008270
Cluster-22178.46113 GO:0006206 GO:0006144 GO:0016310 GO:0009069
GO:0006468 GO:0006351 GO:0005524 GO:0004672 GO:0004674
GO:0003677 GO:0003899 GO:0005730
Cluster-22178.46115 GO:0006464 GO:0051537 GO:0016151 GO:0008270
GO:0046872 GO:0070403 GO:0003677 GO:0005634
Cluster-22178.46129 GO:0055114 GO:0016491 GO:0016616
Cluster-22178.46130 GO:0055114 GO:0006468 GO:0051287 GO:0005524
GO:0004672 GO:0016616

Cluster-22178.46133 GO:0005515
Cluster-22178.46141 GO:0032065 GO:0005515 GO:0005543 GO:0005938
Cluster-22178.46144 GO:0016020
Cluster-22178.46145 GO:0006807 GO:0005515 GO:0016812
Cluster-22178.46149 GO:0006144 GO:0006807 GO:0006396 GO:0003723 GO:0004000
Cluster-22178.46157 GO:0005515
Cluster-22178.46159 GO:0005515
Cluster-22178.46160 GO:0005515 GO:0035091 GO:0005737
Cluster-22178.46161 GO:0007283 GO:0007130 GO:0006468 GO:0044780 GO:0007131 GO:0006308 GO:0006914 GO:0004672 GO:0005524 GO:0008855 GO:0009318
Cluster-22178.46165 GO:0006468 GO:0009069 GO:0006807 GO:0016310 GO:0005509 GO:0016151 GO:0004674 GO:0004668 GO:0016773 GO:0005524 GO:0004672 GO:0016020 GO:0005737
Cluster-22178.46166 GO:0016567 GO:0005515 GO:0061630 GO:0004842 GO:0046872 GO:0008270
Cluster-22178.46168 GO:0005198 GO:0016021
Cluster-22178.46174 GO:0006470 GO:0019988 GO:0006570 GO:0007165 GO:0003676 GO:0004725 GO:0016829 GO:0005515 GO:0008138 GO:0043399 GO:0005179 GO:0009424 GO:0005576
Cluster-22178.46178 GO:0008152 GO:0006508 GO:0004190 GO:0016491

Cluster-22178.46181 GO:0008152 GO:0055114 GO:0006508 GO:0016491 GO:0004190 GO:0051287
Cluster-22178.46183 GO:0046983
Cluster-22178.46184 GO:0005509 GO:0005576
Cluster-22178.46186 GO:0005975 GO:0008063 GO:0016773
Cluster-22178.46187 GO:0046872 GO:0008270 GO:0005515
Cluster-22178.46189 GO:0003723
Cluster-22178.46201 GO:0045454 GO:0042254 GO:0006412 GO:0006810 GO:0003735 GO:0005515 GO:0003924 GO:0005525 GO:0005861 GO:0016021 GO:0005840

Cluster-22178.46203 GO:0043087 GO:0007264 GO:0007165 GO:0005085

Cluster-22178.46204 GO:0006531 GO:0006522 GO:0006529 GO:0004066

Cluster-22178.46210 GO:0016567 GO:0008270 GO:0046872 GO:0004842 GO:0005515 GO:0005680
Cluster-22178.46211 GO:0035556 GO:0006464 GO:0006355 GO:0016151 GO:0005507 GO:0003700 GO:0008270 GO:0046872 GO:0003677 GO:0005667 GO:0005634
Cluster-22178.46212 GO:0006468 GO:0007165 GO:0016773 GO:0004672 GO:0005524 GO:0005179 GO:0005840 GO:0005576 GO:0016020
Cluster-22178.46216 GO:0006351 GO:0006357 GO:0016055 GO:0005515 GO:0001104 GO:0016592
Cluster-22178.46220 GO:0034314 GO:0030833 GO:0005885 GO:0005856
Cluster-22178.46223 GO:0005515
Cluster-22178.46226 GO:0055085 GO:0006470 GO:0006811 GO:0006570 GO:0004725 GO:0008138 GO:0003677 GO:0005216 GO:0019031 GO:0016020
Cluster-22178.46231 GO:0042254 GO:0006412 GO:0006810 GO:0006858 GO:0009306 GO:0003735 GO:0003723 GO:0042626 GO:0005840 GO:0005622
Cluster-22178.46238 GO:0009607 GO:0016021
Cluster-22178.46240 GO:0019076 GO:0006812 GO:0032801 GO:0006508 GO:0016032 GO:0008233 GO:0005261 GO:0033644
Cluster-22178.46244 GO:0006281 GO:0006310
Cluster-22178.46246 GO:0032955 GO:0051301 GO:0009405 GO:0005515

Cluster-22178.46251 GO:0055085 GO:0006810 GO:0006355 GO:0003700 GO:0005215 GO:0008270 GO:0016020 GO:0005667 GO:0005634
Cluster-22178.46262 GO:0055114 GO:0016491 GO:0005525 GO:0005515

Cluster-22178.46264 GO:0009607 GO:0006355 GO:0003677 GO:0016021
Cluster-22178.46267 GO:0015992 GO:0015991 GO:0015078 GO:0005198

GO:0019028 GO:0016021 GO:0033179
Cluster-22178.46269 GO:0015992 GO:0015991 GO:0015078 GO:0005198
GO:0033179
Cluster-22178.46276 GO:0000226 GO:0043085 GO:0008033 GO:0016485
GO:0000910 GO:0016226 GO:0008017 GO:0005524 GO:0000166
GO:0016879 GO:0045298 GO:0016021 GO:0005737
Cluster-22178.46279 GO:0005515
Cluster-22178.46281 GO:0003779 GO:0005622
Cluster-22178.46290 GO:0007165 GO:0019012
Cluster-22178.46294 GO:0007218 GO:0007165 GO:0005057 GO:0005184
GO:0005576
Cluster-22178.46298 GO:0010508 GO:0009405 GO:0007049 GO:0016020
Cluster-22178.46305 GO:0018342 GO:0008318 GO:0008270 GO:0005515
GO:0004663 GO:0005968
Cluster-22178.46306 GO:0055085 GO:0030001 GO:0006813 GO:0006904
GO:0016192 GO:0005249 GO:0046873 GO:0005515 GO:0008076
GO:0016020
Cluster-22178.46307 GO:0008033 GO:0006418 GO:0009094 GO:0000162
GO:0006571 GO:0006432 GO:0043039 GO:0005524 GO:0004826
GO:0000287 GO:0004812 GO:0000049 GO:0000166 GO:0009328
GO:0005737
Cluster-22178.46313 GO:0006468 GO:0004672 GO:0005524
Cluster-22178.46317 GO:0055114 GO:0016616
Cluster-22178.46323 GO:0006397 GO:0000445
Cluster-22178.46325 GO:0046872
Cluster-22178.46331 GO:0005515
Cluster-22178.46332 GO:0005515
Cluster-22178.46333 GO:0006886 GO:0005515 GO:0008536
Cluster-22178.46335 GO:0006886 GO:0008536 GO:0005515
Cluster-22178.46339 GO:0030150 GO:0006118 GO:0009055 GO:0046872
GO:0008270 GO:0051536 GO:0061630 GO:0005515 GO:0005742
Cluster-22178.46341 GO:0015031 GO:0051607 GO:0055085 GO:0006260
GO:0006457 GO:0048870 GO:0015991 GO:0006355 GO:0042384

GO:0006904 GO:0000917 GO:0006914 GO:0015992 GO:0048193
GO:0008360 GO:0006606 GO:0007155 GO:0043093 GO:0030001
GO:0046873 GO:0003700 GO:0045502 GO:0008134 GO:0000975
GO:0003677 GO:0043565 GO:0015078 GO:0051082 GO:0005198
GO:0042803 GO:0030286 GO:0034464 GO:0000145 GO:0016021
GO:0019028 GO:0033179 GO:0031514 GO:0016272 GO:0005667
GO:0016020 GO:0005737
Cluster-22178.46348 GO:0003677
Cluster-22178.46353 GO:0005576
Cluster-22178.46358 GO:0048870 GO:0046872 GO:0031514
Cluster-22178.46359 GO:0008152 GO:0009452 GO:0006306 GO:0031167
GO:0001510 GO:0005201 GO:0008168 GO:0003677 GO:0005515
GO:0008170 GO:0016021 GO:0005581 GO:0019013 GO:0005578
Cluster-22178.46362 GO:0006310 GO:0006508 GO:0006281 GO:0006260
GO:0008152 GO:0003677 GO:0016788 GO:0004181 GO:0008270
GO:0005634
Cluster-22178.46370 GO:0055085 GO:0006811 GO:0003677 GO:0015075
GO:0016020
Cluster-22178.46372 GO:0006813
Cluster-22178.46374 GO:0006118 GO:0015986 GO:0045454 GO:0015992
GO:0015078 GO:0009055 GO:0015035 GO:0000276
Cluster-22178.46382 GO:0046872 GO:0008270 GO:0005515 GO:0005622
Cluster-22178.46386 GO:0006119 GO:0006355 GO:0015991 GO:0015992
GO:0046961 GO:0003700 GO:0033178 GO:0005667
Cluster-22178.46399 GO:0000226 GO:0016310 GO:0000910 GO:0009069
GO:0006468 GO:0008017 GO:0004674 GO:0005524 GO:0004672
GO:0045298
Cluster-22178.46402 GO:0005515
Cluster-22178.46403 GO:0006260 GO:0015979 GO:0006468 GO:0004715
GO:0005524 GO:0005515 GO:0005198 GO:0019031 GO:0009523
GO:0009539 GO:0016020 GO:0019028
Cluster-22178.46406 GO:0016032 GO:0046872
Cluster-22178.46408 GO:0008152 GO:0055114 GO:0016491

Cluster-22178.46412 GO:0005515
Cluster-22178.46413 GO:0005515
Cluster-22178.46424 GO:0006560 GO:0009405 GO:0006814 GO:0006570
GO:0006090 GO:0006470 GO:0006525 GO:0071436 GO:0015031
GO:0046856 GO:0005272 GO:0008948 GO:0004725 GO:0034597
GO:0015081 GO:0031224 GO:0016021 GO:0016020
Cluster-22178.46429 GO:0005524 GO:0016787 GO:0003677
Cluster-22178.46431 GO:0015743
Cluster-22178.46436 GO:0006260 GO:0003887 GO:0042575
Cluster-22178.46437 GO:0006694 GO:0009058 GO:0008299 GO:0016779
GO:0050518
Cluster-22178.46438 GO:0016192 GO:0006886 GO:0006284 GO:0016114
GO:0006694 GO:0005198 GO:0005515 GO:0003677 GO:0008661
GO:0005634 GO:0030117
Cluster-22178.46442 GO:0008270
Cluster-22178.46443 GO:0019048 GO:0019031
Cluster-22178.46445 GO:0006032 GO:0016998 GO:0004568
Cluster-22178.46451 GO:0007165
Cluster-22178.46453 GO:0006355 GO:0007165 GO:0003723 GO:0016763
GO:0005179 GO:0003677 GO:0046983 GO:0005634 GO:0019028
GO:0005576
Cluster-22178.46456 GO:0006281 GO:0006952 GO:0006464 GO:0035556
GO:0008270 GO:0046872 GO:0016151 GO:0005576
Cluster-22178.46462 GO:0042802 GO:0043015 GO:0008017 GO:0045298
Cluster-22178.46465 GO:0032065 GO:0005515 GO:0005543 GO:0005938
Cluster-22178.46466 GO:0043039 GO:0006418 GO:0000166 GO:0000049
GO:0004812 GO:0003676 GO:0005524 GO:0019031 GO:0005737
Cluster-22178.46468 GO:0005515 GO:0001533
Cluster-22178.46477 GO:0055085 GO:0016021
Cluster-22178.46482 GO:0010181 GO:0005509 GO:0005515
Cluster-22178.46484 GO:0015886 GO:0005515 GO:0008270 GO:0015232

Cluster-22178.46486 GO:0042262 GO:0009405 GO:0015979 GO:0006531

GO:0042157 GO:0042619 GO:0051726 GO:0006869 GO:0006308
GO:0006522 GO:0006164 GO:0006144 GO:0008053 GO:0006457
GO:0003924 GO:0042803 GO:0005198 GO:0004019 GO:0003690
GO:0005525 GO:0008289 GO:0016836 GO:0000774 GO:0008855
GO:0051087 GO:0005634 GO:0005741 GO:0009538 GO:0009522
GO:0016021 GO:0019031 GO:0019028 GO:0005576 GO:0009318
Cluster-22178.46487 GO:0019054 GO:0005509 GO:0004857
Cluster-22178.46489 GO:0006777 GO:0004222 GO:0051539 GO:0019008
Cluster-22178.46490 GO:0006886 GO:0006351 GO:0005515 GO:0008536

Cluster-22178.46491 GO:0006886 GO:0006351 GO:0008536 GO:0005515

Cluster-22178.46492 GO:0006468 GO:0055114 GO:0004497 GO:0016706
GO:0008168 GO:0005524 GO:0004672 GO:0016715 GO:0005507
Cluster-22178.46495 GO:0003676 GO:0005515
Cluster-22178.46496 GO:0003677 GO:0003676 GO:0005515
Cluster-22178.46497 GO:0003676 GO:0005515
Cluster-22178.46500 GO:0008610 GO:0042967 GO:0008152 GO:0006633
GO:0004315 GO:0016747 GO:0005835
Cluster-22178.46502 GO:0008152 GO:0008610 GO:0016747
Cluster-22178.46505 GO:0034477 GO:0004518 GO:0005515
Cluster-22178.46506 GO:0006098 GO:0046168 GO:0009094 GO:0055114
GO:0019521 GO:0006571 GO:0015940 GO:0000162 GO:0051287
GO:0004616 GO:0016616 GO:0008977 GO:0008677 GO:0004665
Cluster-22178.46508 GO:0051056 GO:0006284 GO:0006144 GO:0043547
GO:0006508 GO:0003676 GO:0003968 GO:0005096 GO:0016817
GO:0008270 GO:0005515 GO:0017111 GO:0004197 GO:0003677
GO:0031379
Cluster-22178.46509 GO:0008270 GO:0046872 GO:0061630 GO:0005515

Cluster-22178.46512 GO:0015979 GO:0022900 GO:0009539 GO:0005739
GO:0009523 GO:0016020 GO:0005747
Cluster-22178.46515 GO:0005515

Cluster-22178.46521 GO:0006396 GO:0006807 GO:0006144 GO:0004000 GO:0003723

Cluster-22178.46524 GO:0042619

Cluster-22178.46525 GO:0007165 GO:0005515

Cluster-22178.46526 GO:0002098 GO:0034227 GO:0000049

Cluster-22178.46529 GO:0006810 GO:0016021

Cluster-22178.46534 GO:0006355 GO:0055114 GO:0046872 GO:0047134 GO:0003677 GO:0008289

Cluster-22178.46536 GO:0016021

Cluster-22178.46538 GO:0008152 GO:0016829

Cluster-22178.46542 GO:0006310 GO:0006073 GO:0002949 GO:0006508 GO:0006281 GO:0009378 GO:0004222 GO:0016887 GO:0005524 GO:0016301 GO:0016762 GO:0003678 GO:0009379 GO:0048046 GO:0005618 GO:0005657

Cluster-22178.46544 GO:0006914 GO:0005515 GO:0008092 GO:0019898 GO:0005737

Cluster-22178.46549 GO:0003677 GO:0046872

Cluster-22178.46550 GO:0006313 GO:0006355 GO:0006352 GO:0015074 GO:0000049 GO:0003677 GO:0003700 GO:0016987 GO:0005667

Cluster-22178.46552 GO:0007165 GO:0019953 GO:0000723 GO:0043047 GO:0003677 GO:0003697 GO:0003676 GO:0000772 GO:0005576 GO:0000784

Cluster-22178.46555 GO:0003676 GO:0003723

Cluster-22178.46561 GO:0000723 GO:0019953 GO:0007165 GO:0000772 GO:0003676 GO:0003697 GO:0003677 GO:0043047 GO:0000784 GO:0005576

Cluster-22178.46562 GO:0046872 GO:0003677

Cluster-22178.46565 GO:0006914 GO:0051082

Cluster-22178.46570 GO:0019079 GO:0006810 GO:0004386 GO:0003676 GO:0008026 GO:0005524

Cluster-22178.46573 GO:0000413 GO:0006457 GO:0003755 GO:0003676

Cluster-22178.46576 GO:0055085 GO:0006810 GO:0016021 GO:0016020

Cluster-22178.46579 GO:0005515
Cluster-22178.46581 GO:0009405 GO:0005576
Cluster-22178.46583 GO:0005201 GO:0008168 GO:0005581 GO:0005578
Cluster-22178.46584 GO:0005201 GO:0008168 GO:0005578 GO:0005581
Cluster-22178.46587 GO:2001070 GO:0005515
Cluster-22178.46591 GO:0009228 GO:0004789
Cluster-22178.46592 GO:0006891 GO:0030173
Cluster-22178.46595 GO:0009451 GO:0046999 GO:0003824 GO:0051539
GO:0019867
Cluster-22178.46601 GO:0005515
Cluster-22178.46603 GO:0016573
Cluster-22178.46607 GO:0005515
Cluster-22178.46613 GO:0006531 GO:0006468 GO:0007165 GO:0006522
GO:0042967 GO:0035556 GO:0016773 GO:0047200 GO:0005057
GO:0005524 GO:0004672 GO:0016020 GO:0005856 GO:0016021
Cluster-22178.46615 GO:0006810 GO:0042626
Cluster-22178.46619 GO:0009058 GO:0015979 GO:0005515 GO:0016779
GO:0016772 GO:0016020 GO:0009523 GO:0009539
Cluster-22178.46625 GO:0005524 GO:0016773 GO:0016020
Cluster-22178.46626 GO:0019082 GO:0016032 GO:0006508 GO:0000723
GO:0006879 GO:0005515 GO:0004252 GO:0003677 GO:0070009
GO:0008239 GO:0005576 GO:0000784
Cluster-22178.46627 GO:0006355 GO:0046983 GO:0008270 GO:0005515
GO:0005634
Cluster-22178.46629 GO:0005515 GO:0046872 GO:0016021 GO:0045202
Cluster-22178.46630 GO:0006810 GO:0008152 GO:0009405 GO:0008200
GO:0004867 GO:0005576 GO:0042151
Cluster-22178.46633 GO:0045947 GO:0008608 GO:0008190 GO:0003677
GO:0072686 GO:0042729
Cluster-22178.46635 GO:0007156 GO:0006886 GO:0016192 GO:0005515
GO:0005509 GO:0030117 GO:0016020
Cluster-22178.46642 GO:0006355 GO:0003677
Cluster-22178.46645 GO:0005515

Cluster-22178.46646 GO:0031110 GO:0030168 GO:0015031 GO:0006891
GO:0007067 GO:0000090 GO:0042157 GO:0006869 GO:0007165
GO:0051301 GO:0051258 GO:0007059 GO:0006813 GO:0006904
GO:0015269 GO:0008017 GO:0005102 GO:0030674 GO:0005516
GO:0008289 GO:0000940 GO:0005577 GO:0005876 GO:0017119
GO:0000145 GO:0045298 GO:0016021 GO:0005576
Cluster-22178.46648 GO:0003676 GO:0003723
Cluster-22178.46653 GO:0008270
Cluster-22178.46654 GO:0008152 GO:0008134 GO:0003824 GO:0005667
GO:0005634
Cluster-22178.46657 GO:0019068 GO:0043565 GO:0005198 GO:0005515
GO:0005634
Cluster-22178.46663 GO:0090263 GO:0016567 GO:0005515 GO:0008270

Cluster-22178.46667 GO:0006694 GO:0008299 GO:0050518
Cluster-22178.46679 GO:0009405 GO:0005515 GO:0003682 GO:0000785
GO:0005576
Cluster-22178.46685 GO:0042254 GO:0006412 GO:0003735 GO:0005840
Cluster-22178.46686 GO:0051276 GO:0007031 GO:0051082 GO:0008773
GO:0005515 GO:0005524 GO:0005694 GO:0005777
Cluster-22178.46691 GO:0006412 GO:0042254 GO:0008270 GO:0046872
GO:0005515 GO:0003735 GO:0004867 GO:0005840 GO:0005622
Cluster-22178.46696 GO:0016021
Cluster-22178.46697 GO:0016021
Cluster-22178.46698 GO:0006308 GO:0010508 GO:0006397 GO:0008855
GO:0016021 GO:0009318 GO:0000445
Cluster-22178.46712 GO:0006351 GO:0003677 GO:0005634
Cluster-22178.46713 GO:0008217 GO:0042312 GO:0007165 GO:0006355
GO:0000245 GO:0006508 GO:0004222 GO:0008270 GO:0005515
GO:0005179 GO:0031012 GO:0005576 GO:0005634
Cluster-22178.46719 GO:0015031 GO:0015979 GO:0055114 GO:0030145
GO:0008270 GO:0008565 GO:0016817 GO:0016972 GO:0009523
GO:0016021

Cluster-22178.46726 GO:0042981 GO:0016788 GO:0051499 GO:0005634
Cluster-22178.46727 GO:0006012 GO:0009069 GO:0005982 GO:0007178
GO:0016310 GO:0006813 GO:0006468 GO:0005985 GO:0004564
GO:0005524 GO:0004675 GO:0005249 GO:0004672 GO:0030955
GO:0004575 GO:0008076 GO:0016020 GO:0016021 GO:0017177
Cluster-22178.46728 GO:0051276 GO:0005515 GO:0005524 GO:0005694
Cluster-22178.46729 GO:0019083 GO:0019229 GO:0006144 GO:0003968
GO:0005576 GO:0031379
Cluster-22178.46732 GO:2001256 GO:0030176
Cluster-22178.46734 GO:0035023 GO:0051321 GO:0043087 GO:0005089
GO:0043531 GO:0005737
Cluster-22178.46735 GO:0006355 GO:0006771 GO:0019497 GO:0008152
GO:0000287 GO:0016791 GO:0008134 GO:0005524 GO:0003993
GO:0008253 GO:0005667 GO:0005737
Cluster-22178.46737 GO:0005515
Cluster-22178.46742 GO:0000012 GO:0006281 GO:0003684 GO:0005515
GO:0005634
Cluster-22178.46746 GO:0007264 GO:0007165 GO:0007186 GO:0031683
GO:0005524 GO:0019001 GO:0003924 GO:0005525 GO:0004871
GO:0000166 GO:0003677
Cluster-22178.46754 GO:0006281 GO:0016567 GO:0006308 GO:0006260
GO:0003723 GO:0005515 GO:0008270 GO:0017108 GO:0004842
GO:0005525 GO:0005634 GO:0033557
Cluster-22178.46759 GO:0005975 GO:0006367 GO:0008184 GO:0005634
Cluster-22178.46760 GO:0005515 GO:0061630 GO:0008270 GO:0046872

Cluster-22178.46761 GO:0046872 GO:0008270 GO:0005515 GO:0061630

Cluster-22178.46762 GO:0005515 GO:0061630 GO:0046872 GO:0008270

Cluster-22178.46768 GO:0006914
Cluster-22178.46777 GO:0016021
Cluster-22178.46779 GO:0005975 GO:0004553 GO:0005515

Cluster-22178.46785 GO:0007155 GO:0005515 GO:0016021
Cluster-22178.46787 GO:0016491
Cluster-22178.46789 GO:0000160 GO:0016310 GO:0006928 GO:0000155
GO:0004673 GO:0009365
Cluster-22178.46791 GO:0006139 GO:0005515 GO:0003676 GO:0008408
GO:0043395 GO:0016020 GO:0005578
Cluster-22178.46792 GO:0045040 GO:0005515 GO:0005742
Cluster-22178.46798 GO:0035556 GO:0007264 GO:0005525
Cluster-22178.46800 GO:0005515
Cluster-22178.46802 GO:0046872
Cluster-22178.46806 GO:0030071 GO:0005515 GO:0005680
Cluster-22178.46808 GO:0042967 GO:0016747 GO:0008080
Cluster-22178.46811 GO:0006520 GO:0005515 GO:0008483 GO:0016829
GO:0030170
Cluster-22178.46817 GO:0009069 GO:0006468 GO:0016310 GO:0005516
GO:0004683
Cluster-22178.46823 GO:0006725 GO:0016853 GO:0055036
Cluster-22178.46824 GO:0005515 GO:0035091
Cluster-22178.46827 GO:0003677
Cluster-22178.46828 GO:0016579 GO:0006511 GO:0006508 GO:0004843
GO:0005622
Cluster-22178.46833 GO:0007186 GO:0004930 GO:0016020 GO:0016021
Cluster-22178.46835 GO:0006420 GO:0006357 GO:0006560 GO:0019089
GO:0006525 GO:0005524 GO:0004814 GO:0001104 GO:0016592
Cluster-22178.46844 GO:0006464 GO:0035556 GO:0006355 GO:0046872
GO:0008270 GO:0016151 GO:0005507 GO:0003700 GO:0003677
GO:0005634 GO:0005667
Cluster-22178.46846 GO:0006464 GO:0006281 GO:0006355 GO:0006351
GO:0008270 GO:0046872 GO:0016151 GO:0003700 GO:0005507
GO:0003676 GO:0003677 GO:0005515 GO:0005667 GO:0005634
Cluster-22178.46851 GO:0003677
Cluster-22178.46855 GO:0007275 GO:0009966 GO:0016020
Cluster-22178.46862 GO:0008270 GO:0046872

Cluster-22178.46868 GO:0045454 GO:0006118 GO:0005515 GO:0009055
GO:0015035
Cluster-22178.46870 GO:0005515
Cluster-22178.46871 GO:0055114 GO:0016705 GO:0020037 GO:0005506

Cluster-22178.46878 GO:0021987 GO:0005515
Cluster-22178.46879 GO:0055114 GO:0006813 GO:0005524 GO:0005267
GO:0016787 GO:0005506 GO:0020037 GO:0016705 GO:0003677
GO:0016020
Cluster-22178.46882 GO:0006606 GO:0007017 GO:0007018 GO:0005524
GO:0008017 GO:0003777 GO:0045298 GO:0005874
Cluster-22178.46883 GO:0005515
Cluster-22178.46887 GO:0015994 GO:0033014 GO:0005515 GO:0004418

Cluster-22178.46894 GO:0015979 GO:0046872 GO:0009523 GO:0016021
Cluster-22178.46896 GO:0045454 GO:0055114 GO:0016972
Cluster-22178.46899 GO:0006631 GO:0018874 GO:0006298 GO:0009396
GO:0006554 GO:0006633 GO:0006568 GO:0006552 GO:0046487
GO:0006550 GO:0055114 GO:0006574 GO:0008152 GO:0050662
GO:0004488 GO:0016491 GO:0005524 GO:0003857 GO:0003824
Cluster-22178.46901 GO:0016773
Cluster-22178.46903 GO:0009405 GO:0000184 GO:0004386 GO:0005524
GO:0008270 GO:0003677 GO:0019867 GO:0005737
Cluster-22178.46908 GO:0006355 GO:0003700 GO:0043565 GO:0005667
GO:0005634
Cluster-22178.46909 GO:0006886 GO:0008536
Cluster-22178.46910 GO:0055114 GO:0016491
Cluster-22178.46911 GO:0055114 GO:0051287 GO:0016491 GO:0016616

Cluster-22178.46914 GO:0008544
Cluster-22178.46922 GO:0006955 GO:0007165 GO:0003723 GO:0005125
GO:0003676 GO:0005576
Cluster-22178.46923 GO:0005887

Cluster-22178.46926 GO:0007411
Cluster-22178.46932 GO:0006284 GO:0042254 GO:0006412 GO:0016799
GO:0003735 GO:0003906 GO:0005840 GO:0005622
Cluster-22178.46946 GO:0008146 GO:0016021
Cluster-22178.46958 GO:0005515
Cluster-22178.46959 GO:0051205 GO:0016021
Cluster-22178.46961 GO:0055114 GO:0047134
Cluster-22178.46965 GO:0003677 GO:0003676 GO:0005524 GO:0016787
GO:0019031
Cluster-22178.46977 GO:0007276 GO:0009405 GO:0006412 GO:0042254
GO:0005515 GO:0005198 GO:0003735 GO:0005509 GO:0005840
GO:0005622
Cluster-22178.46979 GO:0003676 GO:0016020
Cluster-22178.46980 GO:0006486 GO:0008373
Cluster-22178.46981 GO:0006486 GO:0008373
Cluster-22178.46982 GO:0006486 GO:0008373
Cluster-22178.46984 GO:0007165 GO:0005515
Cluster-22178.46985 GO:0016567 GO:0005515 GO:0004842 GO:0008270
GO:0046872 GO:0005680
Cluster-22178.46986 GO:0019988 GO:0006470 GO:0008152 GO:0006570
GO:0008168 GO:0004725 GO:0043399 GO:0008138
Cluster-22178.46988 GO:0006886 GO:0005737
Cluster-22178.46989 GO:0006810 GO:0016567 GO:0006606 GO:0006914
GO:0010508 GO:0016032 GO:0007049 GO:0048870 GO:0004842
GO:0042803 GO:0008134 GO:0045502 GO:0005667 GO:0005886
GO:0030286 GO:0031514 GO:0019867
Cluster-22178.46990 GO:0000902 GO:0042254 GO:0006412 GO:0003735
GO:0005840 GO:0005622
Cluster-22178.46993 GO:0007165 GO:0005179 GO:0005576
Cluster-22178.47001 GO:0042254 GO:0006412 GO:0005515 GO:0003735
GO:0016817 GO:0005840 GO:0005622
Cluster-22178.47003 GO:0006810 GO:0006260 GO:0003677 GO:0016787
GO:0004386 GO:0003676 GO:0003688 GO:0005524 GO:0046809

Cluster-22178.47005 GO:0043395 GO:0005515 GO:0005578 GO:0016020
Cluster-22178.47008 GO:0008641
Cluster-22178.47025 GO:0098542 GO:0006470 GO:0008138
Cluster-22178.47030 GO:0005515
Cluster-22178.47031 GO:0006554 GO:0006479 GO:0007165 GO:0006355
GO:0034968 GO:0005515 GO:0005509 GO:0018024 GO:0005578
GO:0005694
Cluster-22178.47034 GO:0000226 GO:0016226 GO:0016485 GO:0000910
GO:0008033 GO:0043085 GO:0008017 GO:0005524 GO:0000166
GO:0016879 GO:0045298 GO:0016021 GO:0005737
Cluster-22178.47038 GO:0030036 GO:0015684 GO:0007264 GO:0030976
GO:0005525 GO:0003924 GO:0003779 GO:0015093 GO:0000287
GO:0016021 GO:0030054
Cluster-22178.47039 GO:0007094 GO:0055114 GO:0016620 GO:0051287

Cluster-22178.47041 GO:0009405 GO:0000184 GO:0003677 GO:0004386
GO:0008270 GO:0005524 GO:0005576 GO:0005737
Cluster-22178.47044 GO:0019836 GO:0005525 GO:0005524 GO:0016887
GO:0005576
Cluster-22178.47045 GO:0055085 GO:0006812 GO:0009607 GO:0007165
GO:0006890 GO:0008324 GO:0016021 GO:0016020
Cluster-22178.47052 GO:0006351 GO:0006355 GO:0003677 GO:0005634
Cluster-22178.47053 GO:0006694 GO:0009058 GO:0008299 GO:0007165
GO:0050518 GO:0005044 GO:0005515 GO:0016779 GO:0019031
GO:0016020
Cluster-22178.47054 GO:0007165 GO:0040007 GO:0008283 GO:0008083
GO:0016020
Cluster-22178.47055 GO:0007165 GO:0008283 GO:0040007 GO:0008083
GO:0016020
Cluster-22178.47063 GO:0046660 GO:0006812 GO:0006397 GO:0003676
GO:0016887 GO:0016021 GO:0005634
Cluster-22178.47069 GO:0016055 GO:0007275 GO:0007165 GO:0005102
GO:0005515 GO:0005509 GO:0005576 GO:0045095

Cluster-22178.47071 GO:0006955
Cluster-22178.47076 GO:0055114 GO:0006355 GO:0005515 GO:0016491

Cluster-22178.47079 GO:0055114 GO:0044237 GO:0006412 GO:0008168
GO:0016491 GO:0003824
Cluster-22178.47085 GO:0006418 GO:0000166 GO:0004812 GO:0005524

Cluster-22178.47086 GO:0008152 GO:0006694 GO:0016114 GO:0008661
GO:0030976 GO:0003824 GO:0016624
Cluster-22178.47090 GO:0007156 GO:0005509 GO:0016020
Cluster-22178.47093 GO:0031505 GO:0016020
Cluster-22178.47096 GO:0006468 GO:0005524 GO:0004672 GO:0016020
Cluster-22178.47098 GO:0016192 GO:0006886 GO:0005515 GO:0030117
Cluster-22178.47099 GO:0006355 GO:0003700 GO:0043565 GO:0005667
Cluster-22178.47103 GO:0046872
Cluster-22178.47104 GO:0008270
Cluster-22178.47106 GO:0006955 GO:0009058 GO:0007165 GO:0006479
GO:0006554 GO:0030170 GO:0018024 GO:0008270 GO:0005125
GO:0005576
Cluster-22178.47118 GO:0006468 GO:0016773 GO:0005524 GO:0004672
GO:0019028 GO:0016020
Cluster-22178.47119 GO:0006468 GO:0016773 GO:0004672 GO:0005524
GO:0016020
Cluster-22178.47124 GO:0008270 GO:0005515 GO:0005783
Cluster-22178.47125 GO:0006265 GO:0003677 GO:0003918 GO:0005524
GO:0005815 GO:0005634
Cluster-22178.47127 GO:0007186 GO:0004930 GO:0016021
Cluster-22178.47129 GO:0006825 GO:0003676 GO:0016531 GO:0005507
GO:0005758
Cluster-22178.47134 GO:0000154 GO:0006396 GO:0016433 GO:0004252
GO:0055036 GO:0019028
Cluster-22178.47142 GO:0006298 GO:0007165 GO:0005515 GO:0030983
GO:0005524 GO:0005096

Cluster-22178.47146 GO:0016021
Cluster-22178.47147 GO:0016021
Cluster-22178.47152 GO:0015979 GO:0009539 GO:0009523
Cluster-22178.47160 GO:0005515
Cluster-22178.47171 GO:0006814 GO:0007275 GO:0015846 GO:0015813
GO:0007219 GO:0007154 GO:0007016 GO:0005509 GO:0005515
GO:0003677 GO:0015501 GO:0016020 GO:0016010 GO:0005576
GO:0016021
Cluster-22178.47172 GO:0006357 GO:0006508 GO:0004252 GO:0001104
GO:0016592
Cluster-22178.47174 GO:0004252 GO:0070008
Cluster-22178.47182 GO:0006886 GO:0006955 GO:0006468 GO:0008152
GO:0007165 GO:0042254 GO:0016192 GO:0005515 GO:0005198
GO:0003824 GO:0046872 GO:0005125 GO:0004672 GO:0008484
GO:0030117 GO:0005576 GO:0005730
Cluster-22178.47184 GO:0055114 GO:0006355 GO:0016491 GO:0003824
GO:0042393
Cluster-22178.47188 GO:0006351 GO:0009451 GO:0006269 GO:0008033
GO:0009020 GO:0003896 GO:0005730 GO:0005657
Cluster-22178.47189 GO:0006269 GO:0006351 GO:0003896 GO:0005730
GO:0005657
Cluster-22178.47192 GO:0006465 GO:0008233 GO:0016021 GO:0005787
Cluster-22178.47198 GO:0007010 GO:0004222 GO:0003779 GO:0031012
Cluster-22178.47199 GO:0005515
Cluster-22178.47203 GO:0018874 GO:0019354 GO:0006631 GO:0006554
GO:0006633 GO:0006779 GO:0016117 GO:0006813 GO:0006568
GO:0006550 GO:0006552 GO:0006574 GO:0055114 GO:0008033
GO:0015940 GO:0071949 GO:0051287 GO:0016616 GO:0016491
GO:0050660 GO:0050151 GO:0003857 GO:0016705 GO:0043115
GO:0008677
Cluster-22178.47204 GO:0006807 GO:0009252 GO:0003676 GO:0004356
GO:0008270 GO:0005923 GO:0005634
Cluster-22178.47206 GO:0008270 GO:0005515

Cluster-22178.47209 GO:0042254 GO:0006412 GO:0003735 GO:0005840

Cluster-22178.47211 GO:0055114 GO:0016616 GO:0016491

Cluster-22178.47214 GO:0009401 GO:0006812 GO:0055085 GO:0008324
GO:0016021 GO:0016020

Cluster-22178.47218 GO:0005515

Cluster-22178.47219 GO:0006308 GO:0009307 GO:0005515 GO:0003677
GO:0009036 GO:0009359

Cluster-22178.47230 GO:0006810 GO:0016114 GO:0006694 GO:0006308
GO:0055114 GO:0003724 GO:0050660 GO:0016887 GO:0008855
GO:0005524 GO:0098519 GO:0005525 GO:0003924 GO:0003723
GO:0004506 GO:0016020 GO:0016021 GO:0009318

Cluster-22178.47231 GO:0006308 GO:0007264 GO:0015684 GO:0015093
GO:0005524 GO:0003924 GO:0008855 GO:0016887 GO:0005525
GO:0016021 GO:0009318

Cluster-22178.47236 GO:0006914 GO:0019028 GO:0005737

Cluster-22178.47240 GO:0015074 GO:0016310 GO:0006139 GO:0006281
GO:0006412 GO:0006206 GO:0002949 GO:0009069 GO:0006313
GO:0006810 GO:0042254 GO:0006310 GO:0003924 GO:0005525
GO:0003723 GO:0016301 GO:0005524 GO:0016887 GO:0004674
GO:0004127 GO:0003724 GO:0009378 GO:0098519 GO:0003677
GO:0003735 GO:0005840 GO:0005622 GO:0005657 GO:0009379

Cluster-22178.47241 GO:0019835 GO:0050829 GO:0016021

Cluster-22178.47247 GO:0016310 GO:0009069 GO:0006468 GO:0004674
GO:0005524 GO:0004672

Cluster-22178.47252 GO:0005525 GO:0005515 GO:0008270

Cluster-22178.47254 GO:0030001 GO:0046872 GO:0008270 GO:0005515
GO:0000166 GO:0019956

Cluster-22178.47255 GO:0016114 GO:0055114 GO:0046429

Cluster-22178.47257 GO:0006457 GO:0005515

Cluster-22178.47260 GO:0046872 GO:0005634

Cluster-22178.47262 GO:0006355 GO:0008270 GO:0003677 GO:0003682
GO:0000775 GO:0000785 GO:0005783 GO:0005634

Cluster-22178.47266 GO:0055085 GO:0016021 GO:0055036

Cluster-22178.47271 GO:0006629 GO:0046486 GO:0008152 GO:0016042
GO:0016787 GO:0003847 GO:0019028 GO:0008247
Cluster-22178.47280 GO:0015031 GO:0006606 GO:0019684 GO:0009772
GO:0006355 GO:0006118 GO:0009405 GO:0003700 GO:0045502
GO:0045156 GO:0005198 GO:0008134 GO:0042803 GO:0030286
GO:0005667 GO:0005882 GO:0019031 GO:0019028
Cluster-22178.47281 GO:0007275 GO:0042600
Cluster-22178.47283 GO:0008270
Cluster-22178.47286 GO:0009435 GO:0051539 GO:0008987 GO:0016020
GO:0019804
Cluster-22178.47287 GO:0009069 GO:0006468 GO:0016032 GO:0007165
GO:0010508 GO:0016310 GO:0007131 GO:0005515 GO:0005096
GO:0004674 GO:0003676 GO:0000794
Cluster-22178.47288 GO:0006355 GO:0042981 GO:0008152 GO:0005515
GO:0016757 GO:0003677
Cluster-22178.47294 GO:0035556 GO:0009190 GO:0016849
Cluster-22178.47314 GO:0016757
Cluster-22178.47317 GO:0032968 GO:0007165 GO:0006367 GO:0050750
GO:0008201 GO:0003677 GO:0005634 GO:0005783
Cluster-22178.47321 GO:0006367 GO:0005672
Cluster-22178.47324 GO:0006367 GO:0005672
Cluster-22178.47325 GO:0016226 GO:0006464 GO:0046872 GO:0008199
GO:0016151
Cluster-22178.47326 GO:0032012 GO:0043087 GO:0005086
Cluster-22178.47329 GO:0003924 GO:0046872 GO:0005525
Cluster-22178.47342 GO:0006779 GO:0016117 GO:0055114 GO:0008033
GO:0019354 GO:0050660 GO:0008270 GO:0016491 GO:0016705
GO:0043115 GO:0005515 GO:0071949
Cluster-22178.47347 GO:0016021
Cluster-22178.47348 GO:0043087 GO:0035023 GO:0005089
Cluster-22178.47349 GO:0006278 GO:0017038 GO:0005524 GO:0003964
GO:0003676 GO:0016787 GO:0003677 GO:0016020
Cluster-22178.47354 GO:0008152 GO:0003824 GO:0008484

Cluster-22178.47355 GO:0022904 GO:0003676 GO:0008017 GO:0045298
GO:0016020
Cluster-22178.47358 GO:0016747
Cluster-22178.47359 GO:0016747
Cluster-22178.47363 GO:0008152 GO:0006614 GO:0005524 GO:0005525
GO:0016301
Cluster-22178.47364 GO:0008152 GO:0006614 GO:0005525 GO:0016301
GO:0005524
Cluster-22178.47368 GO:0006351 GO:0007034 GO:0006269 GO:0003896
GO:0005657 GO:0005730
Cluster-22178.47369 GO:0043401 GO:0006355 GO:0003700 GO:0008270
GO:0043565 GO:0005667 GO:0005634
Cluster-22178.47371 GO:0009308 GO:0055114 GO:0003676 GO:0016638
GO:0003723 GO:0042597
Cluster-22178.47374 GO:0016567 GO:0023052 GO:0005515 GO:0004842

Cluster-22178.47383 GO:0006355 GO:0005634
Cluster-22178.47384 GO:0006118 GO:0030001 GO:0015991 GO:0051258
GO:0006308 GO:0007165 GO:0019069 GO:0055085 GO:0030168
GO:0015992 GO:0020037 GO:0015078 GO:0009055 GO:0046873
GO:0005506 GO:0030674 GO:0005102 GO:0008855 GO:0009318
GO:0033179 GO:0016020 GO:0005577
Cluster-22178.47386 GO:0006886 GO:0006468 GO:0007264 GO:0006606
GO:0006914 GO:0004672 GO:0005524 GO:0005096 GO:0016773
GO:0005783 GO:0016020 GO:0016021
Cluster-22178.47388 GO:0042254 GO:0006412 GO:0003735 GO:0046872
GO:0005840 GO:0005622
Cluster-22178.47405 GO:0046872
Cluster-22178.47407 GO:0009058 GO:0030170
Cluster-22178.47409 GO:0035091
Cluster-22178.47410 GO:0015689 GO:0055114 GO:0009405 GO:0007165
GO:0006816 GO:0006874 GO:0005219 GO:0016491 GO:0015098
GO:0016021 GO:0005622 GO:0005576

Cluster-22178.47420 GO:0007165 GO:0000917 GO:0007172 GO:0043093
GO:0006468 GO:0005515 GO:0004871 GO:0004713 GO:0005096
GO:0005925 GO:0005737
Cluster-22178.47425 GO:0003676
Cluster-22178.47428 GO:0006687 GO:0009058 GO:0001733 GO:0008146
GO:0005794 GO:0016021
Cluster-22178.47439 GO:0006165 GO:0006144 GO:0006228 GO:0006206
GO:0006183 GO:0006241 GO:0004550
Cluster-22178.47441 GO:0005634
Cluster-22178.47442 GO:0008270
Cluster-22178.47443 GO:0008270
Cluster-22178.47449 GO:0055085 GO:0006811 GO:0046983 GO:0005216
GO:0005515 GO:0016020
Cluster-22178.47452 GO:0006468 GO:0016773 GO:0005524 GO:0004672
GO:0016020
Cluster-22178.47453 GO:0006360 GO:0001168
Cluster-22178.47456 GO:0035556 GO:0005515
Cluster-22178.47460 GO:0007165 GO:0005096
Cluster-22178.47465 GO:0006206 GO:0006144 GO:0006351 GO:0003677
GO:0003899 GO:0005730
Cluster-22178.47466 GO:0016020 GO:0016021
Cluster-22178.47477 GO:0008483 GO:0030170
Cluster-22178.47478 GO:0006260 GO:0006351 GO:0007165 GO:0006269
GO:0003896 GO:0005657 GO:0005730
Cluster-22178.47481 GO:0006869 GO:0006468 GO:0042157 GO:0009399
GO:0007131 GO:0008219 GO:0005515 GO:0008289 GO:0004672
GO:0005524 GO:0005576
Cluster-22178.47483 GO:0016310 GO:0009069 GO:0006303 GO:0005524
GO:0004677 GO:0003677 GO:0005958 GO:0005634
Cluster-22178.47487 GO:0016021 GO:0016020
Cluster-22178.47488 GO:0042998 GO:0005198 GO:0071253 GO:0016021
GO:0019031 GO:0005802
Cluster-22178.47494 GO:0005525 GO:0016887 GO:0005524

Cluster-22178.47495 GO:0006351 GO:0003677 GO:0043565
Cluster-22178.47496 GO:0009607 GO:0019069 GO:0016021
Cluster-22178.47497 GO:0043087 GO:0051321 GO:0061136 GO:0035023
GO:0043531 GO:0005089 GO:0005737
Cluster-22178.47498 GO:0043087 GO:0051321 GO:0035023 GO:0043531
GO:0005089 GO:0005737
Cluster-22178.47500 GO:0006338 GO:0005524 GO:0046872 GO:0016818
GO:0003676 GO:0005634
Cluster-22178.47501 GO:0006355 GO:0005515 GO:0061630 GO:0042393
GO:0003677 GO:0043565 GO:0008270 GO:0046872 GO:0003700
GO:0005667 GO:0016021
Cluster-22178.47504 GO:0019058
Cluster-22178.47506 GO:0016021
Cluster-22178.47508 GO:0008152 GO:0006206 GO:0055114 GO:0006144
GO:0006351 GO:0006270 GO:0006118 GO:0006275 GO:0050660
GO:0016627 GO:0005524 GO:0043565 GO:0003995 GO:0003899
GO:0005730
Cluster-22178.47511 GO:0006468 GO:0004672 GO:0005524 GO:0016773
GO:0005515 GO:0016020
Cluster-22178.47515 GO:0071973 GO:0006606 GO:0006935 GO:0042803
GO:0008134 GO:0005515 GO:0045502 GO:0003774 GO:0016020
GO:0005667 GO:0030286 GO:0009288
Cluster-22178.47516 GO:0016055 GO:0005515
Cluster-22178.47517 GO:0016055 GO:0005515
Cluster-22178.47518 GO:0042254 GO:0006412 GO:0003735 GO:0005840
Cluster-22178.47526 GO:0019012 GO:0016021
Cluster-22178.47529 GO:0035556 GO:0005515
Cluster-22178.47530 GO:0007018 GO:0000077 GO:0005515 GO:0005868
Cluster-22178.47534 GO:0043087 GO:0006051 GO:0006040 GO:0032012
GO:0005086 GO:0047465 GO:0016772
Cluster-22178.47535 GO:0045116 GO:0016881 GO:0005524 GO:0008641

Cluster-22178.47536 GO:0042325 GO:0005737

Cluster-22178.47549 GO:0030155 GO:0006886 GO:0007165 GO:0007155
GO:0045995 GO:0006914 GO:0055114 GO:0019049 GO:0030334
GO:0005102 GO:0016702 GO:0031224 GO:0016020 GO:0005801
Cluster-22178.47554 GO:0003774 GO:0005524 GO:0005515 GO:0016459
Cluster-22178.47564 GO:0030168 GO:0051258 GO:0006308 GO:0055085
GO:0030001 GO:0007165 GO:0008855 GO:0005102 GO:0046873
GO:0030674 GO:0005515 GO:0005577 GO:0009318 GO:0016020
Cluster-22178.47565 GO:0006468 GO:0009069 GO:0016310 GO:0005524
GO:0004674
Cluster-22178.47566 GO:0006357 GO:0001104 GO:0016592
Cluster-22178.47572 GO:0015992 GO:0006123 GO:0004129 GO:0045277
GO:0005751 GO:0005743
Cluster-22178.47575 GO:0005515
Cluster-22178.47576 GO:0005515
Cluster-22178.47577 GO:0033617 GO:0005739
Cluster-22178.47578 GO:0033617 GO:0005739
Cluster-22178.47579 GO:0003774 GO:0005524 GO:0016459
Cluster-22178.47584 GO:0006047
Cluster-22178.47586 GO:0016579 GO:0032324 GO:0008270 GO:0036459

Cluster-22178.47587 GO:0006351 GO:0006412 GO:0042254 GO:0046983
GO:0003735 GO:0005622 GO:0005840
Cluster-22178.47589 GO:0015074 GO:0016042 GO:0006629 GO:0006468
GO:0006313 GO:0046486 GO:0005524 GO:0004672 GO:0004806
GO:0003677 GO:0005515
Cluster-22178.47590 GO:0008270 GO:0005634
Cluster-22178.47593 GO:0051259 GO:0005975 GO:0016773 GO:0005515

Cluster-22178.47594 GO:0051259
Cluster-22178.47596 GO:0051259 GO:0005975 GO:0005515 GO:0016773

Cluster-22178.47599 GO:0008152 GO:0008168
Cluster-22178.47609 GO:0008152 GO:0005515 GO:0016791 GO:0008270

GO:0043395 GO:0016020 GO:0005578 GO:0019028

Cluster-22178.47618 GO:0006144 GO:0008283 GO:0006508 GO:0040007 GO:0007165 GO:0008270 GO:0016817 GO:0070008 GO:0003968 GO:0004197 GO:0017111 GO:0008083 GO:0004252 GO:0031379 GO:0016020

Cluster-22178.47620 GO:0006468 GO:0005524 GO:0004672

Cluster-22178.47622 GO:0042254 GO:0006412 GO:0006915 GO:0005515 GO:0003735 GO:0005509 GO:0005840 GO:0005622

Cluster-22178.47623 GO:0008152 GO:0003824

Cluster-22178.47626 GO:0016021

Cluster-22178.47631 GO:0016787

Cluster-22178.47633 GO:0006260 GO:0006351 GO:0007165 GO:0006269 GO:0003896 GO:0005657 GO:0005730

Cluster-22178.47634 GO:0046983

Cluster-22178.47643 GO:0006814 GO:0006813 GO:0016787 GO:0005890

Cluster-22178.47644 GO:0006144 GO:0006468 GO:0006171 GO:0000287 GO:0005524 GO:0004672 GO:0004016

Cluster-22178.47645 GO:0008270 GO:0046872 GO:0003677

Cluster-22178.47646 GO:0016567 GO:0004842 GO:0016020

Cluster-22178.47651 GO:0055085 GO:0035556 GO:0006811 GO:0006816 GO:0006810 GO:0042030 GO:0005246 GO:0005216 GO:0005515 GO:0016020

Cluster-22178.47652 GO:0006284 GO:0016787 GO:0003677 GO:0003905 GO:0016021

Cluster-22178.47655 GO:0035091 GO:0005515

Cluster-22178.47658 GO:0005198 GO:0005882

Cluster-22178.47663 GO:0006470 GO:0008138 GO:0070403

Cluster-22178.47669 GO:0031554 GO:0009086 GO:0019069 GO:0042558 GO:0031556 GO:0005524 GO:0046872 GO:0008705 GO:0031419 GO:0005622 GO:0005887

Cluster-22178.47672 GO:0046836 GO:0017089 GO:0051861 GO:0005737

Cluster-22178.47673 GO:0046872 GO:0003950

Cluster-22178.47677 GO:0006313 GO:0046872 GO:0004803 GO:0003677

Cluster-22178.47678 GO:0006355 GO:0006816 GO:0070588 GO:0006352
GO:0003677 GO:0005515 GO:0016987 GO:0003700 GO:0005262
GO:0005667 GO:0016020
Cluster-22178.47680 GO:0006355 GO:0032065 GO:0016579 GO:0005515
GO:0003677 GO:0043565 GO:0036459 GO:0005543 GO:0003700
GO:0005667 GO:0005634 GO:0005938
Cluster-22178.47681 GO:0006355 GO:0003690 GO:0005739
Cluster-22178.47691 GO:0006810 GO:0009405 GO:0006310 GO:0006051
GO:0006040 GO:0003677 GO:0005515 GO:0047465 GO:0008200
GO:0019905 GO:0005576 GO:0005886
Cluster-22178.47692 GO:0017025
Cluster-22178.47694 GO:0003677
Cluster-22178.47695 GO:0046872 GO:0043130
Cluster-22178.47705 GO:0051259 GO:0007034
Cluster-22178.47707 GO:0005515
Cluster-22178.47708 GO:0007155 GO:0042246 GO:0046872 GO:0008270
GO:0005515 GO:0016021
Cluster-22178.47711 GO:0006508 GO:0004222 GO:0019902 GO:0008270
GO:0005576
Cluster-22178.47713 GO:0006508 GO:0008270 GO:0004222 GO:0019902
GO:0005576
Cluster-22178.47717 GO:0006355 GO:0003700 GO:0003677 GO:0005667
Cluster-22178.47718 GO:0055114 GO:0016705 GO:0020037 GO:0005506

Cluster-22178.47725 GO:0000746 GO:0004252 GO:0055036 GO:0019028
GO:0019867
Cluster-22178.47732 GO:0007186 GO:0007165 GO:0015684 GO:0007264
GO:0004871 GO:0005525 GO:0003924 GO:0016887 GO:0015093
GO:0019001 GO:0031683 GO:0005524 GO:0016021
Cluster-22178.47734 GO:0015991 GO:0015992 GO:0015078 GO:0033177
Cluster-22178.47735 GO:0018874 GO:0008209 GO:0008207 GO:0006631
GO:0006554 GO:0006633 GO:0008210 GO:0006568 GO:0006550

GO:0006552 GO:0006574 GO:0055114 GO:0006694 GO:0008152
GO:0003854 GO:0016616 GO:0016491 GO:0003857
Cluster-22178.47737 GO:0055114 GO:0006260 GO:0032266 GO:0043130

Cluster-22178.47741 GO:0030036 GO:0003779 GO:0005515 GO:0030054
Cluster-22178.47749 GO:0042254 GO:0006412 GO:0005515 GO:0003735
GO:0005622 GO:0005840
Cluster-22178.47750 GO:0005515
Cluster-22178.47751 GO:0042254 GO:0006412 GO:0005515 GO:0003735
GO:0005622 GO:0005840
Cluster-22178.47752 GO:0005515
Cluster-22178.47756 GO:0006351 GO:0003677 GO:0003676 GO:0008270
GO:0005634
Cluster-22178.47757 GO:0015979 GO:0008146 GO:0030145 GO:0009523
GO:0016021
Cluster-22178.47763 GO:0043087 GO:0007264 GO:0035023 GO:0005096
GO:0005089 GO:0016021
Cluster-22178.47777 GO:0007165 GO:0007186 GO:0043934 GO:0015937
GO:0008152 GO:0015940 GO:0016301 GO:0004871 GO:0019001
GO:0031683 GO:0005524 GO:0004140 GO:0003924 GO:0016887
GO:0016020
Cluster-22178.47781 GO:0006355 GO:0016021 GO:0005634
Cluster-22178.47784 GO:0045947 GO:0008190
Cluster-22178.47786 GO:0006323 GO:0003677 GO:0005515 GO:0019012
Cluster-22178.47787 GO:0005975 GO:0008270 GO:0008184 GO:0003723
GO:0003676
Cluster-22178.47793 GO:0006260 GO:0008270 GO:0003677
Cluster-22178.47794 GO:0019497 GO:0006771 GO:0055114 GO:0003756
GO:0016671 GO:0003993 GO:0005783
Cluster-22178.47796 GO:0016787
Cluster-22178.47805 GO:0005515
Cluster-22178.47808 GO:0045892 GO:0042754 GO:0006281 GO:0006310
GO:0005524 GO:0009378 GO:0005657 GO:0009379

Cluster-22178.47809 GO:0005515
Cluster-22178.47811 GO:0009405 GO:0006811 GO:0008375 GO:0005216
GO:0016020 GO:0005576
Cluster-22178.47813 GO:0007005 GO:0006265 GO:0003917 GO:0003677
GO:0003918 GO:0005694 GO:0031305
Cluster-22178.47817 GO:0007165 GO:0005515 GO:0004871 GO:0016021
Cluster-22178.47821 GO:0006355 GO:0016791 GO:0043565
Cluster-22178.47823 GO:0055085 GO:0022857 GO:0016021
Cluster-22178.47825 GO:0030334 GO:0015031 GO:0030155 GO:0007165
GO:0045995 GO:0008033 GO:0006904 GO:0051252 GO:0006355
GO:0005102 GO:0003677 GO:0005507 GO:0003700 GO:0004540
GO:0000145 GO:0005667 GO:0016021 GO:0005634
Cluster-22178.47828 GO:0016021
Cluster-22178.47831 GO:0006351 GO:0006260 GO:0006269 GO:0003896
GO:0005657 GO:0005730
Cluster-22178.47833 GO:0016747
Cluster-22178.47839 GO:0006464 GO:0046872 GO:0016151 GO:0003677
GO:0005737
Cluster-22178.47841 GO:0003682 GO:0000785
Cluster-22178.47843 GO:0055085 GO:0006810 GO:0016887 GO:0003924
GO:0005524 GO:0042626 GO:0005525 GO:0016301 GO:0016021
Cluster-22178.47849 GO:0007049 GO:0006260 GO:0006355 GO:0043093
GO:0006457 GO:0006810 GO:0000917 GO:0043565 GO:0003700
GO:0051082 GO:0005509 GO:0005737 GO:0005667 GO:0016020
GO:0005783
Cluster-22178.47855 GO:0009235
Cluster-22178.47856 GO:0007165 GO:0042254 GO:0006412 GO:0007016
GO:0003735 GO:0005200 GO:0000062 GO:0005158 GO:0003779
GO:0005925 GO:0001726 GO:0005840 GO:0005622 GO:0005856
GO:0005899
Cluster-22178.47859 GO:0043087 GO:0035023 GO:0007165 GO:0005057
GO:0005089
Cluster-22178.47861 GO:0005198 GO:0019028

Cluster-22178.47863 GO:0005515 GO:0016021
Cluster-22178.47868 GO:0008608 GO:0006396 GO:0051252 GO:0003676 GO:0000175 GO:0003723 GO:0042729 GO:0072686
Cluster-22178.47869 GO:0006396 GO:0008608 GO:0051252 GO:0000175 GO:0003676 GO:0003723 GO:0072686 GO:0042729
Cluster-22178.47870 GO:0040007 GO:0008283 GO:0008406 GO:0007165 GO:0008083
Cluster-22178.47872 GO:0016114 GO:0019385 GO:0006694 GO:0003824 GO:0030976 GO:0008661 GO:0000287
Cluster-22178.47874 GO:0006468 GO:0004672 GO:0005524 GO:0016773 GO:0016020
Cluster-22178.47875 GO:0006468 GO:0016773 GO:0004672 GO:0005524 GO:0016020
Cluster-22178.47876 GO:0046872 GO:0020037 GO:0016020
Cluster-22178.47883 GO:0006470 GO:0006570 GO:0004725 GO:0008138 GO:0016787
Cluster-22178.47885 GO:0046872
Cluster-22178.47887 GO:0008168 GO:0005515
Cluster-22178.47889 GO:0006351 GO:0006468 GO:0006144 GO:0006206 GO:0003899 GO:0005524 GO:0004672 GO:0005634 GO:0005730
Cluster-22178.47895 GO:0016052 GO:0005985 GO:0005982 GO:0004556 GO:0005509
Cluster-22178.47898 GO:0055114 GO:0005506 GO:0020037 GO:0016705

Cluster-22178.47903 GO:0007166 GO:0016020
Cluster-22178.47904 GO:0006355 GO:0043402 GO:0043401 GO:0007165 GO:0042921 GO:0003677 GO:0043565 GO:0008270 GO:0004883 GO:0005496 GO:0003700 GO:0005667 GO:0005634
Cluster-22178.47905 GO:0043401 GO:0006355
Cluster-22178.47908 GO:0005615
Cluster-22178.47909 GO:0006144 GO:0055114 GO:0018144 GO:0016818 GO:0005198 GO:0016491 GO:0051537 GO:0003968 GO:0030430 GO:0031379

Cluster-22178.47910 GO:0016032 GO:0016020 GO:0030430 GO:0019031
Cluster-22178.47921 GO:0019497 GO:0015979 GO:0006771 GO:0046872
GO:0003993 GO:0005515 GO:0009523 GO:0016020
Cluster-22178.47926 GO:0030001 GO:0006813 GO:0006355 GO:0015940
GO:0008608 GO:0055114 GO:0007155 GO:0016117 GO:0055085
GO:0015979 GO:0016705 GO:0008677 GO:0016614 GO:0005540
GO:0003677 GO:0016787 GO:0046873 GO:0050660 GO:0004222
GO:0016491 GO:0005524 GO:0042729 GO:0072686 GO:0016020
Cluster-22178.47928 GO:0035556 GO:0007165 GO:0003677 GO:0005515

Cluster-22178.47929 GO:0019028
Cluster-22178.47936 GO:0035023 GO:0043087 GO:0006355 GO:0005089
GO:0005515 GO:0042393
Cluster-22178.47938 GO:0005985 GO:0005982 GO:0005975 GO:0004045
GO:0004650
Cluster-22178.47948 GO:0016462 GO:0005509
Cluster-22178.47949 GO:0043087 GO:0032012 GO:0005086 GO:0046872
GO:0008289
Cluster-22178.47953 GO:0030246
Cluster-22178.47956 GO:0006779 GO:0016117 GO:0055114 GO:0008033
GO:0019354 GO:0050660 GO:0008270 GO:0016491 GO:0043115
GO:0005515 GO:0016705 GO:0071949
Cluster-22178.47961 GO:0006470 GO:0006570 GO:0004725 GO:0008138

Cluster-22178.47964 GO:0009405 GO:0007155 GO:0005515 GO:0005509
GO:0003824 GO:0051536 GO:0016020 GO:0005576
Cluster-22178.47967 GO:0022904 GO:0016651 GO:0005743
Cluster-22178.47971 GO:0007264 GO:0006338 GO:0005525 GO:0003676
GO:0005524 GO:0003924 GO:0016818 GO:0005634
Cluster-22178.47972 GO:0006338 GO:0007264 GO:0003676 GO:0005525
GO:0003924 GO:0005524 GO:0016818 GO:0005634
Cluster-22178.47980 GO:0006206 GO:0006464 GO:0055114 GO:0006230
GO:0007010 GO:0005524 GO:0008270 GO:0004797 GO:0046872

GO:0003779 GO:0050660 GO:0016151 GO:0016614
Cluster-22178.47983 GO:0006810 GO:0006144 GO:0015992 GO:0006396
GO:0006807 GO:0051252 GO:0009405 GO:0016891 GO:0004000
GO:0003723 GO:0003726 GO:0019855 GO:0015078 GO:0055036
GO:0033644 GO:0005576
Cluster-22178.47989 GO:0006355 GO:0005524 GO:0003677 GO:0005634
Cluster-22178.47990 GO:0042254 GO:0006412 GO:0003735 GO:0005622
GO:0005840
Cluster-22178.47999 GO:0006468 GO:0005524 GO:0004672
Cluster-22178.48001 GO:0008284 GO:0001819 GO:0010884 GO:0005515
GO:0042802 GO:0016020
Cluster-22178.48004 GO:0007264 GO:0005525
Cluster-22178.48007 GO:0006508 GO:0009002
Cluster-22178.48008 GO:0007186 GO:0006526 GO:0042967 GO:0015979
GO:0004358 GO:0004930 GO:0016020 GO:0016021 GO:0009523
GO:0009539
Cluster-22178.48017 GO:0006468 GO:0005524 GO:0004672 GO:0016773
GO:0016020
Cluster-22178.48020 GO:0006468 GO:0004672 GO:0005524
Cluster-22178.48021 GO:0006355
Cluster-22178.48023 GO:0006836 GO:0005515 GO:0051539 GO:0019905
GO:0016020 GO:0005887
Cluster-22178.48026 GO:0042325 GO:0005737
Cluster-22178.48030 GO:0005515
Cluster-22178.48037 GO:0005515
Cluster-22178.48038 GO:0007617 GO:0006355 GO:0003700 GO:0008270
GO:0009289 GO:0005667 GO:0005634 GO:0005576
Cluster-22178.48040 GO:0042967 GO:0008080 GO:0016747
Cluster-22178.48041 GO:0006270 GO:0046983
Cluster-22178.48042 GO:0042981 GO:0006355 GO:0003700 GO:0043565
GO:0005667 GO:0005634
Cluster-22178.48045 GO:0006310 GO:0006812 GO:0006281 GO:0006836
GO:0005328 GO:0016021

Cluster-22178.48046 GO:0016021
Cluster-22178.48048 GO:0005515 GO:0005576
Cluster-22178.48054 GO:0000105 GO:0055114 GO:0006537 GO:0009607
GO:0006355 GO:0003824 GO:0016491 GO:0016627 GO:0015930
GO:0003723 GO:0016638 GO:0005737
Cluster-22178.48061 GO:0016032 GO:0005096 GO:0019031
Cluster-22178.48062 GO:0006144 GO:0055114 GO:0046034 GO:0005506
GO:0005515 GO:0004017 GO:0020037 GO:0016705
Cluster-22178.48063 GO:0055114 GO:0016705 GO:0020037 GO:0005506

Cluster-22178.48065 GO:0051028 GO:0048309 GO:0030430
Cluster-22178.48066 GO:0006355 GO:0003700 GO:0005667 GO:0005622
Cluster-22178.48067 GO:0034219 GO:0008643 GO:0006810 GO:0005524
GO:0042626 GO:0015144 GO:0016021
Cluster-22178.48072 GO:0007165 GO:0007186 GO:0000166 GO:0003677
GO:0031683 GO:0005524 GO:0019001 GO:0003924 GO:0005525
GO:0004871 GO:0016020
Cluster-22178.48075 GO:0016310 GO:0004673 GO:0043015 GO:0042802
GO:0008017 GO:0005634 GO:0045298 GO:0016021 GO:0009365
Cluster-22178.48081 GO:0006570 GO:0006470 GO:0008138 GO:0004725

Cluster-22178.48083 GO:0016310 GO:0000160 GO:0000155 GO:0046983
GO:0009365 GO:0016021
Cluster-22178.48085 GO:0016021
Cluster-22178.48086 GO:0016567 GO:0008270 GO:0046872 GO:0003677
GO:0004842 GO:0005515 GO:0005680
Cluster-22178.48087 GO:0006464 GO:0007186 GO:0004930 GO:0016020
Cluster-22178.48099 GO:0005515
Cluster-22178.48101 GO:0006457 GO:0009116 GO:0005515 GO:0051082
GO:0016020
Cluster-22178.48102 GO:0046983
Cluster-22178.48106 GO:0005515
Cluster-22178.48120 GO:0050790 GO:0006355 GO:0055114 GO:0016491

GO:0030234 GO:0003677
Cluster-22178.48127 GO:0006508 GO:0009002
Cluster-22178.48128 GO:0006508 GO:0009002
Cluster-22178.48135 GO:0006935 GO:0051258 GO:0048024 GO:0010508
GO:0006886 GO:0030168 GO:0048193 GO:0007165 GO:0071973
GO:0003774 GO:0019905 GO:0005102 GO:0008017 GO:0030674
GO:0009288 GO:0045298 GO:0005634 GO:0005577 GO:0016020
Cluster-22178.48138 GO:0046983
Cluster-22178.48146 GO:0007130 GO:0000795
Cluster-22178.48147 GO:0007165 GO:0005179 GO:0005576
Cluster-22178.48155 GO:0048013 GO:0005515
Cluster-22178.48162 GO:0006352 GO:0006355 GO:0008270 GO:0003700
GO:0016987 GO:0005515 GO:0003677 GO:0005667
Cluster-22178.48164 GO:0043093 GO:0006260 GO:0000917 GO:0005737
GO:0045095
Cluster-22178.48166 GO:0006260 GO:0015979 GO:0016020 GO:0009523
GO:0009539
Cluster-22178.48174 GO:0003824
Cluster-22178.48184 GO:0003824
Cluster-22178.48186 GO:0005515 GO:0019028
Cluster-22178.48188 GO:0007304 GO:0007165 GO:0006355 GO:0003700
GO:0005213 GO:0043565 GO:0008270 GO:0005515 GO:0005179
GO:0042600 GO:0005667 GO:0005576
Cluster-22178.48193 GO:0005198 GO:0019028
Cluster-22178.48194 GO:0006810 GO:0055085 GO:0005215 GO:0016021
GO:0016020
Cluster-22178.48195 GO:0055085 GO:0006810 GO:0005215 GO:0016020
GO:0016021
Cluster-22178.48196 GO:0006260 GO:0009405 GO:0003677 GO:0008270
GO:0005664
Cluster-22178.48197 GO:0000917 GO:0010508 GO:0043093 GO:0005737
Cluster-22178.48199 GO:0007268 GO:0005515 GO:0043495 GO:0033130

Cluster-22178.48201 GO:0006412 GO:0008168
Cluster-22178.48211 GO:0006355 GO:0008270 GO:0046872 GO:0003700
GO:0003677 GO:0005667 GO:0005634
Cluster-22178.48218 GO:0001836 GO:0006974 GO:0006919 GO:0006915
GO:0003677
Cluster-22178.48220 GO:0006308 GO:0048870 GO:0030001 GO:0070193
GO:0006886 GO:0007130 GO:0000921 GO:0055085 GO:0031412
GO:0007128 GO:0006457 GO:0042803 GO:0035299 GO:0000774
GO:0008855 GO:0005524 GO:0046873 GO:0051087 GO:0000795
GO:0005801 GO:0016021 GO:0031514 GO:0009318 GO:0005940
GO:0031411 GO:0016020
Cluster-22178.48225 GO:0006355 GO:0043565 GO:0003700 GO:0005667
Cluster-22178.48227 GO:0008283 GO:0040007 GO:0007165 GO:0006886
GO:0016192 GO:0030674 GO:0051015 GO:0008083 GO:0008092
GO:0030131 GO:0005737 GO:0019898
Cluster-22178.48229 GO:0006468 GO:0005515 GO:0005524 GO:0004672
GO:0016773 GO:0016020
Cluster-22178.48232 GO:0005515
Cluster-22178.48235 GO:0015012 GO:0006810 GO:0006024 GO:0042626
GO:0005524 GO:0005515 GO:0003723 GO:0016021
Cluster-22178.48238 GO:0020037 GO:0019825
Cluster-22178.48240 GO:0007165 GO:0006811 GO:0007268 GO:0055085
GO:0005216 GO:0004970 GO:0016020
Cluster-22178.48242 GO:0005515 GO:0016020
Cluster-22178.48243 GO:0005515 GO:0016020
Cluster-22178.48244 GO:0008152 GO:0006260 GO:0016787 GO:0003677
GO:0003887 GO:0042575
Cluster-22178.48245 GO:0016255 GO:0003723 GO:0003676 GO:0042765
Cluster-22178.48246 GO:0001558 GO:0005515 GO:0005520 GO:0016942
GO:0005576
Cluster-22178.48248 GO:0007165 GO:0006955 GO:0006935 GO:0008009
GO:0005576
Cluster-22178.48252 GO:0016757

Cluster-22178.48253 GO:0006351 GO:0017111 GO:0005515 GO:0005524

Cluster-22178.48255 GO:0007015 GO:0003779 GO:0003785
Cluster-22178.48256 GO:0006468 GO:0004672 GO:0005524 GO:0016773 GO:0005515 GO:0016020
Cluster-22178.48259 GO:0042254 GO:0006412 GO:0003735 GO:0005622 GO:0005840
Cluster-22178.48260 GO:0016021
Cluster-22178.48261 GO:0006508 GO:0005515 GO:0004252
Cluster-22178.48265 GO:0006355 GO:0010024 GO:0006270 GO:0055114 GO:0003677 GO:0004252 GO:0050897 GO:0016636 GO:0003700 GO:0005634 GO:0005667 GO:0005886
Cluster-22178.48267 GO:0006355 GO:0005515 GO:0016021 GO:0005634
Cluster-22178.48271 GO:0006355 GO:0003700 GO:0003677 GO:0043565 GO:0005634 GO:0005667
Cluster-22178.48273 GO:0055085 GO:0022857 GO:0016021
Cluster-22178.48277 GO:0007275 GO:0006511 GO:0008053 GO:0003677 GO:0005515 GO:0016787 GO:0005524 GO:0008270 GO:0046872 GO:0003924 GO:0005741 GO:0005634 GO:0016021
Cluster-22178.48279 GO:0051252 GO:0006351 GO:0006396 GO:0007049 GO:0005515 GO:0003723 GO:0004525 GO:0017053 GO:0005887
Cluster-22178.48297 GO:0003676 GO:0003723 GO:0016021
Cluster-22178.48300 GO:0010033 GO:0007264 GO:0006355 GO:0061024 GO:0000166 GO:0003677 GO:0008134 GO:0005525 GO:0005524 GO:0016887 GO:0043531 GO:0003924 GO:0016020 GO:0005667 GO:0019867
Cluster-22178.48301 GO:0007165 GO:0007264 GO:0006629 GO:0035556 GO:0043087 GO:0046339 GO:0009395 GO:0005515 GO:0005085 GO:0008081 GO:0004435
Cluster-22178.48308 GO:0019836 GO:0005576
Cluster-22178.48313 GO:0045859 GO:0004860
Cluster-22178.48316 GO:0007264 GO:0007186 GO:0007165 GO:0003924 GO:0031683 GO:0019001 GO:0004871 GO:0005525

Cluster-22178.48324 GO:0007219 GO:0007165 GO:0007275 GO:0005179 GO:0016021 GO:0005576
Cluster-22178.48325 GO:0006468 GO:0005524 GO:0004672 GO:0016773 GO:0016020
Cluster-22178.48327 GO:0009103 GO:0008918
Cluster-22178.48333 GO:0030154 GO:0018189 GO:0016021
Cluster-22178.48334 GO:0055114 GO:0005515 GO:0010309
Cluster-22178.48353 GO:0005515
Cluster-22178.48360 GO:0000902 GO:0006955 GO:0016020
Cluster-22178.48366 GO:0006355 GO:0003677 GO:0003700 GO:0043565 GO:0016021 GO:0005634 GO:0005667
Cluster-22178.48369 GO:0015684 GO:0007264 GO:0000287 GO:0030976 GO:0005525 GO:0015093 GO:0003924 GO:0016021
Cluster-22178.48370 GO:0016310 GO:0009069 GO:0006468 GO:0004674 GO:0004672 GO:0005524 GO:0000287
Cluster-22178.48371 GO:0009069 GO:0006468 GO:0016310 GO:0004672 GO:0005524 GO:0000287 GO:0004674
Cluster-22178.48372 GO:0046034 GO:0006139 GO:0006461 GO:0019627 GO:0006144 GO:0006206 GO:0004127 GO:0016151 GO:0005524 GO:0004017
Cluster-22178.48374 GO:0006334 GO:0007165 GO:0006955 GO:0005044 GO:0030247 GO:0005634
Cluster-22178.48375 GO:0006468 GO:0006813 GO:0006814 GO:0004672 GO:0005524 GO:0016773 GO:0005890 GO:0016020
Cluster-22178.48379 GO:0006355 GO:0043565
Cluster-22178.48380 GO:0055114 GO:0016310 GO:0005506 GO:0004673 GO:0020037 GO:0016705 GO:0005198 GO:0009365 GO:0019028
Cluster-22178.48381 GO:0055114 GO:0008270 GO:0047134
Cluster-22178.48388 GO:0007165 GO:0005179 GO:0005576
Cluster-22178.48389 GO:0006064 GO:0006118 GO:0016491 GO:0008927 GO:0009055 GO:0016020 GO:0016021
Cluster-22178.48390 GO:0007165 GO:0051028 GO:0008283 GO:0040007 GO:0008083 GO:0005634

Cluster-22178.48397 GO:0007165 GO:0006950 GO:0005179 GO:0005576
Cluster-22178.48403 GO:0045046 GO:0055085 GO:0006886 GO:0006810
GO:0005524 GO:0032403 GO:0042626 GO:0016021 GO:0030176
GO:0005779 GO:0016020
Cluster-22178.48406 GO:0009165 GO:0006144 GO:0006098 GO:0020037
GO:0004749 GO:0000287 GO:0019825
Cluster-22178.48407 GO:0005515
Cluster-22178.48408 GO:0008152 GO:0055114 GO:0006574 GO:0006552
GO:0006550 GO:0046487 GO:0006568 GO:0006633 GO:0009396
GO:0006554 GO:0006631 GO:0006298 GO:0018874 GO:0003824
GO:0003857 GO:0016491 GO:0005524 GO:0004488 GO:0050662
Cluster-22178.48413 GO:0042967 GO:0006633 GO:0006574 GO:0006552
GO:0008299 GO:0046950 GO:0006550 GO:0006084 GO:0004421
GO:0004315 GO:0005835
Cluster-22178.48415 GO:0005515
Cluster-22178.48417 GO:0043087 GO:0007264 GO:0031412 GO:0035556
GO:0005085 GO:0005509 GO:0005815
Cluster-22178.48422 GO:0009877 GO:0009312 GO:0016758 GO:0016020
Cluster-22178.48430 GO:0006139 GO:0003676 GO:0016818 GO:0008026
GO:0005524
Cluster-22178.48432 GO:0008289
Cluster-22178.48438 GO:0006468 GO:0005524 GO:0004672 GO:0016773
GO:0016020
Cluster-22178.48439 GO:0006570 GO:0006470 GO:0019988 GO:0043399
GO:0004725 GO:0008138 GO:0008270
Cluster-22178.48441 GO:0007155 GO:0005515 GO:0009289
Cluster-22178.48444 GO:0046872 GO:0003950 GO:0005634
Cluster-22178.48454 GO:0055085 GO:0022857 GO:0016021
Cluster-22178.48460 GO:0007010 GO:0008270 GO:0003779 GO:0005515

Cluster-22178.48462 GO:0046677 GO:0008152 GO:0070569
Cluster-22178.48464 GO:0005515 GO:0008270 GO:0046872
Cluster-22178.48471 GO:0051191

Cluster-22178.48476 GO:0005515
Cluster-22178.48482 GO:0006468 GO:0005524 GO:0004672
Cluster-22178.48483 GO:0008152 GO:0050662 GO:0016491 GO:0003824 GO:0005515
Cluster-22178.48484 GO:0006886 GO:0006810 GO:0009405 GO:0006888 GO:0006887 GO:0005515 GO:0003677 GO:0000166 GO:0005524 GO:0008270 GO:0019855 GO:0005576 GO:0000145 GO:0030127
Cluster-22178.48490 GO:0032266 GO:0043130 GO:0034464
Cluster-22178.48492 GO:0007021 GO:0015631 GO:0051082 GO:0046872 GO:0045298
Cluster-22178.48501 GO:0006281 GO:0045893 GO:0008081 GO:0005634
Cluster-22178.48505 GO:0003723
Cluster-22178.48507 GO:0006552 GO:0006550 GO:0046487 GO:0006568 GO:0008152 GO:0055114 GO:0006574 GO:0006631 GO:0009058 GO:0018874 GO:0006633 GO:0009396 GO:0006554 GO:0003857 GO:0003824 GO:0004488 GO:0050662 GO:0016491
Cluster-22178.48508 GO:0008146 GO:0016021
Cluster-22178.48511 GO:0006388 GO:0051252 GO:0000213 GO:0000214
Cluster-22178.48514 GO:0006729 GO:0035556 GO:0016567 GO:0042254 GO:0006412 GO:0008270 GO:0004842 GO:0008124 GO:0003735 GO:0005515 GO:0005622 GO:0016021 GO:0032040 GO:0005840 GO:0005680
Cluster-22178.48515 GO:0045859 GO:0005515 GO:0004860 GO:0005576
Cluster-22178.48519 GO:0003676 GO:0005524
Cluster-22178.48520 GO:0006355 GO:0005198 GO:0003700 GO:0005667 GO:0019028 GO:0019031
Cluster-22178.48524 GO:0005515 GO:0046872
Cluster-22178.48530 GO:0006814 GO:0016829
Cluster-22178.48536 GO:0005515
Cluster-22178.48537 GO:0007021 GO:0017000 GO:0015631 GO:0042302 GO:0005515 GO:0051082 GO:0016787 GO:0045298
Cluster-22178.48541 GO:0046849 GO:0008152 GO:0008484 GO:0003824 GO:0005576

Cluster-22178.48550 GO:0008270
Cluster-22178.48552 GO:0042802
Cluster-22178.48555 GO:0008152 GO:0006355 GO:0006506 GO:0000917
GO:0006260 GO:0043093 GO:0006457 GO:0008484 GO:0043565
GO:0003824 GO:0003700 GO:0051082 GO:0045502 GO:0016740
GO:0005515 GO:0008134 GO:0042803 GO:0005737 GO:0005789
GO:0005861 GO:0030286 GO:0016272 GO:0005667
Cluster-22178.48556 GO:0006508 GO:0008233
Cluster-22178.48564 GO:0006468 GO:0006816 GO:0006810 GO:0009607
GO:0034508 GO:0005246 GO:0003677 GO:0005524 GO:0004672
GO:0042030 GO:0000776 GO:0016020 GO:0016021
Cluster-22178.48567 GO:0016567 GO:0005515 GO:0004842 GO:0008270
GO:0046872 GO:0005680
Cluster-22178.48568 GO:0016567 GO:0046872 GO:0008270 GO:0005515
GO:0004842 GO:0005680
Cluster-22178.48584 GO:0055114 GO:0006508 GO:0004197 GO:0020037
GO:0019825 GO:0016702
Cluster-22178.48585 GO:0006486 GO:0008373
Cluster-22178.48595 GO:0005509 GO:0005544
Cluster-22178.48600 GO:0006260 GO:0006629 GO:0016627 GO:0003677
GO:0003887 GO:0016021 GO:0005737 GO:0042575
Cluster-22178.48601 GO:0006260 GO:0006629 GO:0016627 GO:0003677
GO:0003887 GO:0005737 GO:0016021 GO:0042575
Cluster-22178.48606 GO:0006265 GO:0003677 GO:0003917 GO:0005694
Cluster-22178.48607 GO:0006265 GO:0006260 GO:0007059 GO:0006338
GO:0007005 GO:0003918 GO:0003917 GO:0003677 GO:0031298
GO:0031305 GO:0005694 GO:0005730
Cluster-22178.48610 GO:0006606 GO:0006508 GO:0005515 GO:0061630
GO:0008233 GO:0046872 GO:0008270
Cluster-22178.48613 GO:0042254 GO:0006412 GO:0003677 GO:0003735
GO:0005840
Cluster-22178.48620 GO:0007218
Cluster-22178.48626 GO:0007165 GO:0042254 GO:0016192 GO:1902209

GO:0006955 GO:0006886 GO:0006468 GO:0008152 GO:0006402
GO:0048027 GO:0005515 GO:0042802 GO:0005198 GO:0003824
GO:0004672 GO:0005125 GO:0046872 GO:0008484 GO:0005730
GO:0030117 GO:0005576
Cluster-22178.48629 GO:0042254 GO:0016192 GO:0007165 GO:0006468
GO:0006955 GO:0006886 GO:0008152 GO:0005515 GO:0005198
GO:0008484 GO:0005125 GO:0004672 GO:0046872 GO:0003824
GO:0005730 GO:0005576 GO:0030117
Cluster-22178.48633 GO:0008152 GO:0006508 GO:0006629 GO:0008236
GO:0016787
Cluster-22178.48636 GO:0007623 GO:0006351 GO:0006355 GO:0003677
GO:0003700 GO:0043565 GO:0005634 GO:0005667
Cluster-22178.48647 GO:0016887 GO:0005524
Cluster-22178.48651 GO:0006355 GO:0005515 GO:0005634 GO:0016021
Cluster-22178.48656 GO:0006506 GO:0017176 GO:0016021
Cluster-22178.48660 GO:0006520 GO:0016829 GO:0046872
Cluster-22178.48661 GO:0009451 GO:0001522 GO:0003723 GO:0009982

Cluster-22178.48665 GO:0007224 GO:0000077 GO:0003824 GO:0030896
Cluster-22178.48666 GO:0005515
Cluster-22178.48667 GO:0016310 GO:0000160 GO:0051252 GO:0008171
GO:0004673 GO:0000155 GO:0004540 GO:0009365 GO:0005737
GO:0016020
Cluster-22178.48668 GO:0051726 GO:0006281 GO:0004518 GO:0003677
GO:0017025 GO:0003824 GO:0005096 GO:0005634
Cluster-22178.48670 GO:0006355 GO:0008270 GO:0046872 GO:0005634
Cluster-22178.48673 GO:0016787 GO:0046872
Cluster-22178.48674 GO:0055085 GO:0016021
Cluster-22178.48677 GO:0005515
Cluster-22178.48678 GO:0005515
Cluster-22178.48685 GO:0008146 GO:0016021
Cluster-22178.48694 GO:0006265 GO:0007165 GO:0003918 GO:0004864
GO:0005524 GO:0003677

Cluster-22178.48700 GO:0006120 GO:0055114 GO:0042546 GO:0005199
GO:0005618 GO:0016021
Cluster-22178.48703 GO:0055114 GO:0006520 GO:0009058 GO:0016491
GO:0030170 GO:0016829
Cluster-22178.48710 GO:0042475 GO:0070175 GO:0003677 GO:0005198

Cluster-22178.48711 GO:0007165 GO:0005096 GO:0005515
Cluster-22178.48715 GO:0006672 GO:0015931 GO:0006807 GO:0033227
GO:0051033 GO:0016811 GO:0016021
Cluster-22178.48717 GO:0016755 GO:0008270 GO:0005515
Cluster-22178.48721 GO:0005975 GO:0004553 GO:0033925 GO:0005737
Cluster-22178.48724 GO:0005515
Cluster-22178.48727 GO:0005515 GO:0008270
Cluster-22178.48729 GO:0006355 GO:0006886 GO:0006955 GO:0019882
GO:0030521 GO:0007165 GO:0003700 GO:0004882 GO:0005496
GO:0042289 GO:0003677 GO:0042613 GO:0005634 GO:0042025
GO:0005667 GO:0016020
Cluster-22178.48731 GO:0008152 GO:0007050 GO:0045859 GO:0004861
GO:0016491 GO:0005634
Cluster-22178.48732 GO:0006771 GO:0019497 GO:0003993 GO:0016021
Cluster-22178.48736 GO:0007010 GO:0006508 GO:0006468 GO:0006357
GO:0001104 GO:0016805 GO:0003779 GO:0016773 GO:0005524
GO:0004672 GO:0016020 GO:0016592
Cluster-22178.48741 GO:0016021
Cluster-22178.48745 GO:0015937 GO:0015940 GO:0005524 GO:0004140
GO:0005921
Cluster-22178.48746 GO:0007264 GO:0005525
Cluster-22178.48747 GO:0006078 GO:0000910 GO:0000226 GO:0042546
GO:0008017 GO:0016021 GO:0045298 GO:0009276
Cluster-22178.48748 GO:0006468 GO:0004672 GO:0005524
Cluster-22178.48750 GO:0006309 GO:0006915 GO:0016787 GO:0005634
GO:0005737 GO:0005622
Cluster-22178.48754 GO:0055114 GO:0042981 GO:0016714

Cluster-22178.48757 GO:0009069 GO:0006468 GO:0016310 GO:0006812
GO:0000287 GO:0005515 GO:0005524 GO:0004672 GO:0008324
GO:0004674 GO:0016021
Cluster-22178.48763 GO:0051262 GO:0006357 GO:0007165 GO:0006355
GO:0003700 GO:0004871 GO:0045182 GO:0005515 GO:0001104
GO:0003723 GO:0016592 GO:0019028 GO:0005634 GO:0005667
Cluster-22178.48768 GO:0006355 GO:0043401 GO:0008270 GO:0043565
GO:0003700 GO:0005634 GO:0005667
Cluster-22178.48770 GO:0005515
Cluster-22178.48772 GO:0008233 GO:0008270
Cluster-22178.48777 GO:0016567 GO:0004842 GO:0005515 GO:0004867

Cluster-22178.48778 GO:0007218 GO:0016192 GO:0016579 GO:0005185
GO:0036459 GO:0005576 GO:0016021
Cluster-22178.48781 GO:0005509 GO:0005515
Cluster-22178.48785 GO:0009405 GO:0006810 GO:0008200 GO:0042151
Cluster-22178.48786 GO:0007052 GO:0035058 GO:0031122 GO:0006355
GO:0003700 GO:0043565 GO:0008270 GO:0005813 GO:0005634
GO:0005667 GO:0097546
Cluster-22178.48787 GO:0007186 GO:0004930 GO:0003729 GO:0046872
GO:0016021
Cluster-22178.48788 GO:0009116
Cluster-22178.48789 GO:0005515
Cluster-22178.48790 GO:0016021
Cluster-22178.48791 GO:0005515
Cluster-22178.48792 GO:0005515
Cluster-22178.48795 GO:0048193 GO:0005783
Cluster-22178.48805 GO:0007186 GO:0007165 GO:0007155 GO:0007264
GO:0004871 GO:0005525 GO:0003924 GO:0031683 GO:0019001
GO:0005540
Cluster-22178.48809 GO:0016998 GO:0007017 GO:0001510 GO:0006118
GO:0007018 GO:0033539 GO:0055114 GO:0008152 GO:0009452
GO:0005524 GO:0052862 GO:0008017 GO:0042162 GO:0042803

GO:0052861 GO:0003995 GO:0008168 GO:0003777 GO:0045298
GO:0005874
Cluster-22178.48810 GO:0009452 GO:0008152 GO:0033539 GO:0055114
GO:0007018 GO:0006118 GO:0007017 GO:0001510 GO:0008017
GO:0003777 GO:0008168 GO:0005524 GO:0003995 GO:0045298
GO:0005874
Cluster-22178.48812 GO:0006464 GO:0008152 GO:0046500 GO:0006479
GO:0009058 GO:0004719 GO:0008168 GO:0016742 GO:0019867
Cluster-22178.48826 GO:0006090 GO:0008152 GO:0005975 GO:0030259
GO:0006560 GO:0006814 GO:0071436 GO:0006525 GO:0003824
GO:0015081 GO:0016758 GO:0051536 GO:0008948 GO:0010181
GO:0016020
Cluster-22178.48832 GO:0003824
Cluster-22178.48840 GO:0005515
Cluster-22178.48841 GO:0046500 GO:0006400 GO:0006479 GO:0009451
GO:0030488 GO:0006464 GO:0008033 GO:0008152 GO:0008176
GO:0004719 GO:0008168 GO:0008171 GO:0016429 GO:0031515
Cluster-22178.48845 GO:0007186 GO:0004930 GO:0016021
Cluster-22178.48854 GO:0006281 GO:0006468 GO:0006351 GO:0007178
GO:0016310 GO:0009069 GO:0006206 GO:0006289 GO:0043161
GO:0006144 GO:0003899 GO:0004675 GO:0003684 GO:0005524
GO:0005634 GO:0005730 GO:0016020
Cluster-22178.48864 GO:0046332 GO:0016817
Cluster-22178.48865 GO:0046332
Cluster-22178.48871 GO:0019051 GO:0042025
Cluster-22178.48872 GO:0006525 GO:0007165 GO:0006560 GO:0006814
GO:0071436 GO:0014068 GO:0006090 GO:0050776 GO:0008948
GO:0005515 GO:0043548 GO:0015081 GO:0005102 GO:0016020
Cluster-22178.48874 GO:0019497 GO:0006979 GO:0006771 GO:0007155
GO:0006804 GO:0046872 GO:0004601 GO:0003993 GO:0005515
GO:0016021
Cluster-22178.48876 GO:0005515
Cluster-22178.48879 GO:0098542

Cluster-22178.48880 GO:0055085 GO:0006811 GO:0005216 GO:0016020
Cluster-22178.48883 GO:0046856 GO:0015992 GO:0043401 GO:0006355
GO:0015986 GO:0015078 GO:0003700 GO:0034597 GO:0008270
GO:0043565 GO:0000276 GO:0005667 GO:0005634
Cluster-22178.48901 GO:0009306 GO:0005886 GO:0016021 GO:0016020
Cluster-22178.48919 GO:0019497 GO:0006771 GO:0048013 GO:0046872
GO:0005515 GO:0003993
Cluster-22178.48927 GO:0006275 GO:0005515 GO:0016020 GO:0016021
Cluster-22178.48934 GO:0015031 GO:0009306 GO:0008270 GO:0015450
GO:0016021 GO:0009941
Cluster-22178.48935 GO:0007049 GO:0006032 GO:0006118 GO:0006119
GO:0006355 GO:0015991 GO:0006260 GO:0015992 GO:0003700
GO:0016765 GO:0043565 GO:0008061 GO:0005515 GO:0046961
GO:0003954 GO:0033178 GO:0005739 GO:0005667
Cluster-22178.48936 GO:0006281 GO:0003824 GO:0003677 GO:0004518

Cluster-22178.48940 GO:0006468 GO:0043093 GO:0000917 GO:0007172
GO:0007165 GO:0004713 GO:0004871 GO:0005515 GO:0005737
GO:0005925
Cluster-22178.48943 GO:0046436 GO:0009252 GO:0006090 GO:0006633
GO:0008716 GO:0003989 GO:0046872 GO:0005524 GO:0009317
Cluster-22178.48950 GO:0031110 GO:2001256 GO:0042470 GO:0030176
Cluster-22178.48953 GO:0016021
Cluster-22178.48955 GO:0046872
Cluster-22178.48956 GO:0007186 GO:0004930 GO:0016021
Cluster-22178.48962 GO:0007076 GO:0000796
Cluster-22178.48967 GO:0005515 GO:0003677
Cluster-22178.48970 GO:0007606 GO:0007186 GO:0004930 GO:0016021
Cluster-22178.48974 GO:0006508 GO:0008270 GO:0004222 GO:0031012
Cluster-22178.48975 GO:0006355 GO:0006508 GO:0006144 GO:0003677
GO:0004197 GO:0017111 GO:0043565 GO:0003968 GO:0003700
GO:0005667 GO:0005634 GO:0031379
Cluster-22178.48979 GO:0003676

Cluster-22178.48983 GO:0005985 GO:0005982 GO:0006769 GO:0046854
GO:0006012 GO:0009267 GO:0046497 GO:0003953 GO:0004575
GO:0004564 GO:0017177 GO:0016012 GO:0019033 GO:0016020
GO:0016021
Cluster-22178.48984 GO:0055085 GO:0016021
Cluster-22178.48987 GO:0048309 GO:0051028 GO:0030430
Cluster-22178.48988 GO:0051028 GO:0048309 GO:0030430
Cluster-22178.48992 GO:0006771 GO:0019497 GO:0003993
Cluster-22178.48997 GO:0008270
Cluster-22178.48999 GO:0008233
Cluster-22178.49002 GO:0006886 GO:0017119
Cluster-22178.49003 GO:0005515
Cluster-22178.49008 GO:0007010 GO:0051260 GO:0003779
Cluster-22178.49016 GO:0005515 GO:0016021
Cluster-22178.49017 GO:0006694 GO:0006284 GO:0016114 GO:0016192
GO:0006886 GO:0003677 GO:0008661 GO:0005198 GO:0005515
GO:0030117 GO:0005634
Cluster-22178.49018 GO:0005515
Cluster-22178.49019 GO:0006355 GO:0003677
Cluster-22178.49021 GO:0005789
Cluster-22178.49022 GO:0015979 GO:0016020 GO:0009539 GO:0009523
Cluster-22178.49023 GO:0016020
Cluster-22178.49031 GO:0005524 GO:0003677
Cluster-22178.49033 GO:0051382 GO:0007049 GO:0006355 GO:0042073
GO:0000917 GO:0000921 GO:0043093 GO:0006810 GO:0043565
GO:0003700 GO:0005737 GO:0016021 GO:0000776 GO:0005940
GO:0005886 GO:0005815 GO:0005667
Cluster-22178.49041 GO:0006144 GO:0002098 GO:0034227 GO:0046034
GO:0006468 GO:0005524 GO:0008270 GO:0004017 GO:0003677
GO:0016787 GO:0003676 GO:0046872 GO:0004672 GO:0020037
GO:0000049 GO:0005634
Cluster-22178.49042 GO:0006810 GO:0009405 GO:0008200 GO:0005576
Cluster-22178.49043 GO:0005515

Cluster-22178.49045 GO:0005634 GO:0005576
Cluster-22178.49047 GO:0006771 GO:0019497 GO:0046872 GO:0003993
GO:0005515
Cluster-22178.49051 GO:0018279 GO:0005789
Cluster-22178.49060 GO:0006810 GO:0007165 GO:0055114 GO:0016705
GO:0020037 GO:0005506 GO:0005215 GO:0005158 GO:0016020
GO:0005899
Cluster-22178.49063 GO:0006886 GO:0015031 GO:0008565 GO:0016020
GO:0005737
Cluster-22178.49065 GO:0009271 GO:0055114 GO:0006355 GO:0005975
GO:0016491 GO:0016209 GO:0030246
Cluster-22178.49072 GO:0015992 GO:0015991 GO:0006355 GO:0015078
GO:0003677 GO:0019031 GO:0005886 GO:0033179
Cluster-22178.49076 GO:0006950 GO:0016021
Cluster-22178.49077 GO:0003676
Cluster-22178.49081 GO:0009059 GO:0019073 GO:0008654 GO:0043772
GO:0005886 GO:0030176
Cluster-22178.49083 GO:0006810 GO:0005515 GO:0005509 GO:0016021
Cluster-22178.49084 GO:0005515
Cluster-22178.49087 GO:0016740 GO:0016021
Cluster-22178.49089 GO:0070072 GO:0007186 GO:0007165 GO:0007264
GO:0004871 GO:0005525 GO:0003924 GO:0019001 GO:0031683
Cluster-22178.49090 GO:0055085
Cluster-22178.49091 GO:0055085
Cluster-22178.49092 GO:0016846 GO:0003723
Cluster-22178.49095 GO:0006464 GO:0008152 GO:0046500 GO:0006479
GO:0008168 GO:0004719 GO:0003724 GO:0003723
Cluster-22178.49099 GO:0045892 GO:0019856 GO:0005515 GO:0003700
GO:0005667 GO:0016021 GO:0019012
Cluster-22178.49114 GO:0042967 GO:0006355 GO:0008270 GO:0003712
GO:0004402 GO:0000123 GO:0005634 GO:0005667
Cluster-22178.49117 GO:0006355 GO:0006914 GO:0043565 GO:0003700
GO:0005667

Cluster-22178.49142 GO:0008270
Cluster-22178.49147 GO:0006355 GO:0008270 GO:0003700 GO:0003676
GO:0005634 GO:0005667
Cluster-22178.49154 GO:0019497 GO:0006771 GO:0055114 GO:0016706
GO:0003993 GO:0008168 GO:0016021
Cluster-22178.49156 GO:0007186 GO:0055114 GO:0004930 GO:0016491
GO:0016020
Cluster-22178.49157 GO:0055114 GO:0033743 GO:0005515 GO:0016021
Cluster-22178.49160 GO:0019031
Cluster-22178.49161 GO:0035023 GO:0043087 GO:0009405 GO:0044179
GO:0005089 GO:0005515 GO:0005737 GO:0016020
Cluster-22178.49166 GO:0070206
Cluster-22178.49169 GO:0006820 GO:0008509 GO:0016021
Cluster-22178.49171 GO:0042254 GO:0006412 GO:0003735 GO:0005739
GO:0005840
Cluster-22178.49176 GO:0006563 GO:0006566 GO:0046653 GO:0006544
GO:0008115 GO:0005515 GO:0016020
Cluster-22178.49177 GO:0006563 GO:0006566 GO:0046653 GO:0006544
GO:0008115 GO:0005515
Cluster-22178.49179 GO:0055114 GO:0042128 GO:0030151 GO:0016491

Cluster-22178.49181 GO:0042254 GO:0006412 GO:0003735 GO:0005840
GO:0005622
Cluster-22178.49182 GO:0006275 GO:0005515 GO:0003677
Cluster-22178.49183 GO:0008270 GO:0003677 GO:0005515
Cluster-22178.49189 GO:0016032
Cluster-22178.49192 GO:0055114 GO:0016491 GO:0005201 GO:0005581
GO:0005578
Cluster-22178.49194 GO:0000921 GO:0006812 GO:0006355 GO:0015991
GO:0030474 GO:0006308 GO:0015992 GO:0051028 GO:0006914
GO:0006606 GO:0006807 GO:0010508 GO:0006886 GO:0048309
GO:0008855 GO:0045502 GO:0008134 GO:0016814 GO:0008092
GO:0005200 GO:0003700 GO:0008270 GO:0016887 GO:0008017

GO:0051082 GO:0042803 GO:0005515 GO:0043565 GO:0015078
GO:0030286 GO:0005940 GO:0005815 GO:0005823 GO:0019898
GO:0009318 GO:0033179 GO:0019031 GO:0005801 GO:0016021
GO:0005667 GO:0016020 GO:0005737 GO:0005856 GO:0045298
Cluster-22178.49195 GO:0048309 GO:0010508 GO:0006886 GO:0006914
GO:0015992 GO:0051028 GO:0006606 GO:0006807 GO:0006308
GO:0015991 GO:0006355 GO:0030474 GO:0000921 GO:0006812
GO:0043565 GO:0015078 GO:0051082 GO:0042803 GO:0005515
GO:0016887 GO:0008270 GO:0008017 GO:0008092 GO:0016814
GO:0003700 GO:0005200 GO:0045502 GO:0008134 GO:0008855
GO:0005737 GO:0005856 GO:0045298 GO:0005667 GO:0016020
GO:0005801 GO:0016021 GO:0019031 GO:0033179 GO:0009318
GO:0005940 GO:0030286 GO:0019898 GO:0005815 GO:0005823
Cluster-22178.49197 GO:0006810 GO:0043085 GO:0016485 GO:0009252
GO:0006914 GO:0006811 GO:0016755 GO:0004190 GO:0008092
GO:0016020 GO:0005737 GO:0019898 GO:0016021
Cluster-22178.49203 GO:0007218 GO:0046677 GO:0009116 GO:0007631
GO:0008270
Cluster-22178.49208 GO:0006351 GO:0006144 GO:0006206 GO:0003899
GO:0005634 GO:0005730
Cluster-22178.49209 GO:0016772
Cluster-22178.49210 GO:0055114 GO:0016491
Cluster-22178.49211 GO:0055114 GO:0016491
Cluster-22178.49217 GO:0005515
Cluster-22178.49219 GO:0046872
Cluster-22178.49220 GO:0046872
Cluster-22178.49223 GO:0006412 GO:0042254 GO:0008289 GO:0003735
GO:0005739 GO:0005840
Cluster-22178.49229 GO:0017004 GO:0020037
Cluster-22178.49230 GO:0005515
Cluster-22178.49233 GO:0007264 GO:0005524 GO:0016887 GO:0003924
GO:0005525
Cluster-22178.49235 GO:0006468 GO:0009103 GO:0009245 GO:0015940

GO:0009029 GO:0005524 GO:0004672 GO:0016773 GO:0016020
Cluster-22178.49236 GO:0006694 GO:0008152 GO:0016114 GO:0016624
GO:0030976 GO:0008661 GO:0003824
Cluster-22178.49250 GO:0035556
Cluster-22178.49252 GO:0045893 GO:0007275 GO:0008270
Cluster-22178.49254 GO:0006816 GO:0005388 GO:0016529
Cluster-22178.49257 GO:0006355 GO:0006606 GO:0007094 GO:0003700
GO:0005515 GO:0005667 GO:0042025
Cluster-22178.49262 GO:0006355 GO:0005515 GO:0003700 GO:0008270
GO:0043565 GO:0005634 GO:0005667
Cluster-22178.49264 GO:0006355 GO:0005506 GO:0003700 GO:0005515
GO:0005667
Cluster-22178.49265 GO:0007186 GO:0004930 GO:0016021
Cluster-22178.49266 GO:0019064 GO:0007165 GO:0046789 GO:0009986
GO:0019031
Cluster-22178.49272 GO:0016787
Cluster-22178.49275 GO:0008033 GO:0006351 GO:0006479 GO:0009451
GO:0035556 GO:0006260 GO:0030488 GO:0006464 GO:0006554
GO:0046500 GO:0004719 GO:0003887 GO:0018024 GO:0016429
GO:0003677 GO:0042575 GO:0031515
Cluster-22178.49278 GO:0005515
Cluster-22178.49286 GO:0006355 GO:0006260 GO:0046034 GO:0051028
GO:0006144 GO:0048309 GO:0005515 GO:0004017 GO:0003676
GO:0003700 GO:0046872 GO:0008270 GO:0043565 GO:0005667
Cluster-22178.49288 GO:0046872
Cluster-22178.49300 GO:0006952
Cluster-22178.49306 GO:0015979 GO:0003677 GO:0005515 GO:0008092
GO:0016020 GO:0005634 GO:0019898 GO:0009539 GO:0005737
GO:0009523
Cluster-22178.49307 GO:0035556 GO:0006812 GO:0055085 GO:0005515
GO:0008324
Cluster-22178.49308 GO:0042981 GO:0000902
Cluster-22178.49310 GO:0042981 GO:0000902

Cluster-22178.49314 GO:0005515
Cluster-22178.49318 GO:0000902
Cluster-22178.49319 GO:0035556 GO:0016787 GO:0005515 GO:0003677 GO:0005524 GO:0003676 GO:0005507 GO:0016020 GO:0016021
Cluster-22178.49323 GO:0006355 GO:0006281 GO:0055114 GO:0006725 GO:0019079 GO:0006310 GO:0006260 GO:0003723 GO:0005515 GO:0008134 GO:0003678 GO:0003824 GO:0009378 GO:0003677 GO:0005524 GO:0016887 GO:0003724 GO:0005667 GO:0009379 GO:0005657
Cluster-22178.49327 GO:0006886
Cluster-22178.49335 GO:0019370 GO:0008270 GO:0008237 GO:0005515

Cluster-22178.49337 GO:0016310 GO:0007178 GO:0006468 GO:0009069 GO:0005524 GO:0004672 GO:0016773 GO:0004675 GO:0016020
Cluster-22178.49342 GO:0008152 GO:0016787 GO:0005515
Cluster-22178.49354 GO:0006811 GO:0055085 GO:0006812 GO:0006816 GO:0070588 GO:0005262 GO:0008324 GO:0005216 GO:0016021 GO:0016020
Cluster-22178.49355 GO:0006281 GO:0009432 GO:0005524 GO:0003697

Cluster-22178.49356 GO:0046872 GO:0003677 GO:0005515
Cluster-22178.49357 GO:0007186 GO:0004930 GO:0016021
Cluster-22178.49361 GO:0007130 GO:0007283 GO:0007131 GO:0006520 GO:0008851 GO:0009350
Cluster-22178.49371 GO:0009405
Cluster-22178.49372 GO:0015996 GO:0015994 GO:0008270 GO:0005515 GO:0047746
Cluster-22178.49373 GO:0019076 GO:0006812 GO:0032801 GO:0005261 GO:0005515 GO:0033644
Cluster-22178.49376 GO:0006357 GO:0001104 GO:0016592
Cluster-22178.49381 GO:0000917 GO:0006457 GO:0006886 GO:0043093 GO:0006355 GO:0005515 GO:0005198 GO:0051082 GO:0003700 GO:0043565 GO:0005783 GO:0005667 GO:0016021 GO:0016272

GO:0005737
Cluster-22178.49384 GO:0005515
Cluster-22178.49385 GO:0005515
Cluster-22178.49387 GO:0008360 GO:0033588
Cluster-22178.49388 GO:0005515 GO:0046872 GO:0031225
Cluster-22178.49390 GO:0016021
Cluster-22178.49395 GO:0007186 GO:0004930 GO:0046872 GO:0008270
GO:0016021
Cluster-22178.49398 GO:0009405 GO:0006811 GO:0008375 GO:0005216
GO:0016020 GO:0005576
Cluster-22178.49409 GO:0006355 GO:0008270 GO:0043565 GO:0005515
GO:0003700 GO:0005634 GO:0005667
Cluster-22178.49413 GO:0008152 GO:0008610 GO:0016747
Cluster-22178.49415 GO:0003723 GO:0008017 GO:0005515 GO:0045298
Cluster-22178.49418 GO:0006629
Cluster-22178.49422 GO:0007165 GO:0003677 GO:0005515 GO:0005509
GO:0016021 GO:0005578 GO:0005634
Cluster-22178.49426 GO:0005515
Cluster-22178.49429 GO:0005515
Cluster-22178.49430 GO:0006144 GO:0000398 GO:0045454 GO:0006118
GO:0003968 GO:0015035 GO:0009055 GO:0031379 GO:0005681
Cluster-22178.49434 GO:0007010 GO:0003779
Cluster-22178.49436 GO:0003677 GO:0003676 GO:0005524 GO:0016787

Cluster-22178.49438 GO:0007165 GO:0006804 GO:0006355 GO:0006749
GO:0006979 GO:0006352 GO:0055114 GO:0003677 GO:0005515
GO:0005509 GO:0016987 GO:0003700 GO:0004602 GO:0005578
GO:0005667
Cluster-22178.49440 GO:0015992 GO:0005975 GO:0006260 GO:0008152
GO:0006123 GO:0016624 GO:0005524 GO:0004649 GO:0003689
GO:0004129 GO:0005663 GO:0042575 GO:0045277
Cluster-22178.49444 GO:0055114 GO:0016972 GO:0005515
Cluster-22178.49445 GO:0055114 GO:0016972 GO:0005515

Cluster-22178.49446 GO:0055114 GO:0005515 GO:0016972
Cluster-22178.49448 GO:0040007 GO:0015991 GO:0015992 GO:0055085
GO:0008283 GO:0007165 GO:0015858 GO:0006810 GO:0015078
GO:0008083 GO:0003774 GO:0005524 GO:0005337 GO:0016021
GO:0033179 GO:0016459
Cluster-22178.49450 GO:0005975 GO:0005515 GO:0098599 GO:0016832

Cluster-22178.49451 GO:0005975 GO:0098599 GO:0016832 GO:0005515

Cluster-22178.49452 GO:0006355 GO:0007267 GO:0046872 GO:0003677

Cluster-22178.49457 GO:0006974 GO:0051726 GO:0003677
Cluster-22178.49460 GO:0005524 GO:0003677 GO:0000166 GO:0005525

Cluster-22178.49465 GO:0046872 GO:0003677
Cluster-22178.49466 GO:0006568 GO:0008210 GO:0006552 GO:0006550
GO:0055114 GO:0006574 GO:0008152 GO:0006694 GO:0006631
GO:0008207 GO:0008209 GO:0018874 GO:0006554 GO:0006633
GO:0003857 GO:0003854 GO:0016616 GO:0016491
Cluster-22178.49470 GO:0016787
Cluster-22178.49477 GO:0008033 GO:0015992 GO:0015991 GO:0015078
GO:0008134 GO:0042803 GO:0045502 GO:0030286 GO:0033179
GO:0005667
Cluster-22178.49478 GO:0006446 GO:0006413 GO:0003743 GO:0003723
GO:0005840 GO:0005737
Cluster-22178.49479 GO:0006446 GO:0006413 GO:0003743 GO:0003723
GO:0005840 GO:0005737
Cluster-22178.49491 GO:0006508 GO:0008270 GO:0004222 GO:0016788

Cluster-22178.49493 GO:0006508 GO:0016788 GO:0004222 GO:0008270

Cluster-22178.49494 GO:0006355 GO:0006351 GO:0003677 GO:0005634
Cluster-22178.49496 GO:0005515

Cluster-22178.49500 GO:0005515
Cluster-22178.49503 GO:0006468 GO:0055114 GO:0005524 GO:0004672
GO:0051287 GO:0016616
Cluster-22178.49504 GO:0016021
Cluster-22178.49508 GO:0006270 GO:0005515
Cluster-22178.49510 GO:0007264 GO:0005525
Cluster-22178.49512 GO:0005515
Cluster-22178.49514 GO:0043087 GO:0035023 GO:0008017 GO:0005089
GO:0005515 GO:0045298
Cluster-22178.49521 GO:0006935 GO:0006308 GO:0006869 GO:0071973
GO:0042157 GO:0008289 GO:0003774 GO:0008855 GO:0009288
GO:0009318 GO:0016020 GO:0005576
Cluster-22178.49525 GO:0046872
Cluster-22178.49531 GO:0007165 GO:0005515 GO:0005509 GO:0005578
Cluster-22178.49541 GO:0071479 GO:0000077 GO:0005515 GO:0005634
Cluster-22178.49544 GO:0006352 GO:0003723 GO:0003676 GO:0003677

Cluster-22178.49546 GO:0005515 GO:0008270
Cluster-22178.49547 GO:0008270 GO:0005515
Cluster-22178.49549 GO:0006511 GO:0031625
Cluster-22178.49553 GO:0000921 GO:0006355 GO:0042254 GO:0006412
GO:0043565 GO:0003700 GO:0003735 GO:0003677 GO:0005940
GO:0005622 GO:0016021 GO:0005840 GO:0005667 GO:0005634
Cluster-22178.49564 GO:0006269 GO:0006351 GO:0003896 GO:0005730
GO:0005657
Cluster-22178.49566 GO:0004518 GO:0005515 GO:0019867
Cluster-22178.49572 GO:0046718 GO:0005515
Cluster-22178.49579 GO:0006144 GO:0046034 GO:0046872 GO:0008270
GO:0005515 GO:0004017
Cluster-22178.49581 GO:0015684 GO:0006576 GO:0007264 GO:0005525
GO:0015093 GO:0005524 GO:0016021
Cluster-22178.49583 GO:0016567 GO:0008270 GO:0046872 GO:0005515
GO:0004842 GO:0042470 GO:0005680

Cluster-22178.49585 GO:0012511 GO:0016021

Cluster-22178.49586 GO:0007165 GO:0015684 GO:0007186 GO:0007264 GO:0005515 GO:0005525 GO:0004871 GO:0015093 GO:0019001 GO:0031683 GO:0003924 GO:0016021

Cluster-22178.49597 GO:0046983 GO:0003677 GO:0005634

Cluster-22178.49599 GO:0005515

Cluster-22178.49602 GO:0008270 GO:0005622

Cluster-22178.49605 GO:0042254 GO:0006412 GO:0003735 GO:0005515 GO:0005840

Cluster-22178.49607 GO:0008152 GO:0006412 GO:0042254 GO:0016846 GO:0003735 GO:0005622 GO:0005840

Cluster-22178.49609 GO:0035064

Cluster-22178.49612 GO:0035023 GO:0043087 GO:0005515 GO:0005089

Cluster-22178.49616 GO:0007165 GO:0007009 GO:0006471 GO:0005515 GO:0019904 GO:0003950 GO:0016021 GO:0005737

Cluster-22178.49619 GO:0019076 GO:0022900 GO:0032801 GO:0006812 GO:0005261 GO:0033644 GO:0016021

Cluster-22178.49622 GO:0006468 GO:0005524 GO:0004672

Cluster-22178.49624 GO:0005515 GO:0003824

Cluster-22178.49626 GO:0006464

Cluster-22178.49627 GO:0006914 GO:0000921 GO:0006606 GO:0009069 GO:0007165 GO:0048870 GO:0006308 GO:0016310 GO:0006468 GO:0008855 GO:0005096 GO:0004674 GO:0045502 GO:0008134 GO:0042803 GO:0019901 GO:0005940 GO:0030286 GO:0005667 GO:0016021 GO:0031514 GO:0009318

Cluster-22178.49628 GO:0055114 GO:0015946 GO:0004022

Cluster-22178.49633 GO:0048870 GO:0031514

Cluster-22178.49635 GO:0015684 GO:0007264 GO:0005525 GO:0008289 GO:0015093 GO:0016021

Cluster-22178.49636 GO:0007165 GO:0006508 GO:0005515 GO:0016820 GO:0016021 GO:0016020 GO:0005739

Cluster-22178.49637 GO:0003682 GO:0000785

Cluster-22178.49638 GO:0003676

Cluster-22178.49640 GO:0006468 GO:0004672 GO:0005515

Cluster-22178.49645 GO:0006886 GO:0035091

Cluster-22178.49650 GO:0048203 GO:0034315 GO:1900025 GO:2000146
GO:0035650

Cluster-22178.49651 GO:0007212 GO:0006281 GO:0048268 GO:0044237
GO:0003824 GO:0032051 GO:0016021

Cluster-22178.49652 GO:0005515 GO:0042802

Cluster-22178.49656 GO:0007034 GO:0030001 GO:0007411

Cluster-22178.49663 GO:0046034 GO:0006144 GO:0006464 GO:0016151
GO:0046872 GO:0008270 GO:0004017 GO:0005634

Cluster-22178.49667 GO:0006468 GO:0006355 GO:0006754 GO:0008152
GO:0005515 GO:0016887 GO:0005524 GO:0004672 GO:0003824
GO:0000275 GO:0005634

Cluster-22178.49669 GO:0019882 GO:0006886 GO:0006955 GO:0007165
GO:0042289 GO:0042613 GO:0016020

Cluster-22178.49674 GO:0007130 GO:0000795

Cluster-22178.49675 GO:0035556 GO:0005515

Cluster-22178.49676 GO:0035556 GO:0005515

Cluster-22178.49677 GO:0016020

Cluster-22178.49679 GO:0055085 GO:0016021

Cluster-22178.49683 GO:0019068 GO:0009253 GO:0016567 GO:0009252
GO:0006807 GO:0008745 GO:0003677 GO:0000166 GO:0005515
GO:0046872 GO:0008270 GO:0005524 GO:0004842 GO:0019028
GO:0005680

Cluster-22178.49684 GO:0015684 GO:0055085 GO:0006812 GO:0006885
GO:0015093 GO:0015299 GO:0016021

Cluster-22178.49687 GO:0005515

Cluster-22178.49688 GO:0055114 GO:0005515 GO:0016491

Cluster-22178.49690 GO:0006955

Cluster-22178.49692 GO:0055114 GO:0043087 GO:0035023 GO:0006779
GO:0019354 GO:0043115 GO:0005089

Cluster-22178.49693 GO:0006298 GO:0009405 GO:0005524 GO:0030983

GO:0033644
Cluster-22178.49694 GO:0042254 GO:0006412 GO:0004222 GO:0005524
GO:0008270 GO:0046872 GO:0005096 GO:0003677 GO:0003735
GO:0005840 GO:0045202 GO:0016021 GO:0005622
Cluster-22178.49700 GO:0006355 GO:0003677
Cluster-22178.49703 GO:0016462 GO:0005509
Cluster-22178.49705 GO:0005096
Cluster-22178.49706 GO:0008152 GO:0003677 GO:0070569
Cluster-22178.49714 GO:0006281 GO:0004518 GO:0003677 GO:0003824

Cluster-22178.49716 GO:0006605 GO:0006886 GO:0045040 GO:0005515
GO:0046983 GO:0005742
Cluster-22178.49717 GO:0045893 GO:1902208 GO:0016579 GO:0006508
GO:0051536 GO:0036459 GO:0008270 GO:0004843 GO:0003677
Cluster-22178.49718 GO:0005515
Cluster-22178.49719 GO:0005515
Cluster-22178.49722 GO:0009403 GO:0016021 GO:0016020
Cluster-22178.49723 GO:0022904 GO:0016020 GO:0016021
Cluster-22178.49724 GO:0022904 GO:0016021 GO:0016020
Cluster-22178.49730 GO:0016310 GO:0007275 GO:0009069 GO:0006468
GO:0004674 GO:0004672 GO:0005524 GO:0046872 GO:0016773
GO:0045202 GO:0042600 GO:0016020
Cluster-22178.49737 GO:0006355 GO:0005515 GO:0003676 GO:0043565
GO:0005634
Cluster-22178.49740 GO:0000398 GO:0007165 GO:0005681
Cluster-22178.49744 GO:0006811 GO:0006810 GO:0005216 GO:0016021
Cluster-22178.49746 GO:0006614 GO:0006118 GO:0006508 GO:0020037
GO:0004252 GO:0005525 GO:0009055 GO:0005524 GO:0016887
GO:0003924
Cluster-22178.49751 GO:0006308 GO:0006281 GO:0008852 GO:0005515
GO:0019901
Cluster-22178.49754 GO:0016021
Cluster-22178.49757 GO:0051382 GO:0006308 GO:0048870 GO:0055114

GO:0006352 GO:0006355 GO:0009690 GO:0010508 GO:0035092
GO:0000921 GO:0070206 GO:0006914 GO:0006260 GO:0010506
GO:0019139 GO:0005524 GO:0008855 GO:0050660 GO:0016787
GO:0003677 GO:0016021 GO:0005615 GO:0000776 GO:0009318
GO:0031514 GO:0000228 GO:0005940 GO:0005815

Cluster-22178.49770 GO:0007264 GO:0007186 GO:0015684 GO:0007165
GO:0006810 GO:0055085 GO:0003924 GO:0015093 GO:0019001
GO:0031683 GO:0004871 GO:0005525 GO:0005215 GO:0016020
GO:0016021

Cluster-22178.49773 GO:0016192 GO:0016021

Cluster-22178.49775 GO:0006935 GO:0006955 GO:0007165 GO:0008009
GO:0005576

Cluster-22178.49788 GO:0006810 GO:0006508 GO:0046034 GO:0006144
GO:0004017 GO:0004190 GO:0042626 GO:0005524 GO:0046872
GO:0016021

Cluster-22178.49789 GO:0003677

Cluster-22178.49791 GO:0006355 GO:0055085 GO:0003700 GO:0005667
GO:0016021

Cluster-22178.49794 GO:0005515

Cluster-22178.49796 GO:0051252 GO:0006468 GO:0003676 GO:0016773
GO:0005524 GO:0004672 GO:0004523 GO:0016020

Cluster-22178.49797 GO:0042254 GO:0006412 GO:0003735 GO:0005622
GO:0005840 GO:0005763

Cluster-22178.49803 GO:0016032 GO:0007304 GO:0005213 GO:0042600
GO:0005576

Cluster-22178.49804 GO:0006355 GO:0003700 GO:0005515 GO:0008270
GO:0005634 GO:0005667

Cluster-22178.49809 GO:0035064

Cluster-22178.49813 GO:0006412 GO:0042254 GO:0003735 GO:0005840
GO:0005622

Cluster-22178.49816 GO:0005515 GO:0019031

Cluster-22178.49818 GO:0005515 GO:0019031

Cluster-22178.49823 GO:0006144 GO:0009165 GO:0006098 GO:0009116

GO:0000287 GO:0004749
Cluster-22178.49826 GO:0006486 GO:0008378 GO:0016020
Cluster-22178.49832 GO:0006355 GO:0003700 GO:0005667
Cluster-22178.49833 GO:0009279
Cluster-22178.49840 GO:0055114 GO:0016903
Cluster-22178.49846 GO:0015986 GO:0015992 GO:0015078 GO:0000276
Cluster-22178.49847 GO:0006355 GO:0003676 GO:0003677 GO:0005634
Cluster-22178.49848 GO:0006355 GO:0003676 GO:0003677 GO:0005634
Cluster-22178.49849 GO:0007049 GO:0006351 GO:0070176
Cluster-22178.49850 GO:0055085 GO:0006030 GO:0006821 GO:0005247
GO:0008061 GO:0016020 GO:0005576
Cluster-22178.49852 GO:0006355 GO:0005515 GO:0003700 GO:0043565
GO:0005524 GO:0005667
Cluster-22178.49856 GO:0046872
Cluster-22178.49859 GO:0006813 GO:0000184 GO:0046872 GO:0005524
GO:0008270 GO:0015269 GO:0003924 GO:0016887 GO:0005525
GO:0005242 GO:0005516 GO:0016286 GO:0016021 GO:0008076
Cluster-22178.49863 GO:0016310 GO:0009069 GO:0000184 GO:0008380
GO:0004674 GO:0005681
Cluster-22178.49872 GO:0055114 GO:0019497 GO:0006771 GO:0016671
GO:0003756 GO:0003993 GO:0005783
Cluster-22178.49878 GO:0006355 GO:0007165 GO:0006367 GO:0046983
GO:0005509 GO:0005515 GO:0005578
Cluster-22178.49879 GO:0015991 GO:0009069 GO:0046325 GO:0016310
GO:0016820 GO:0005515 GO:0016773 GO:0004674 GO:0005737
GO:0000221
Cluster-22178.49887 GO:0006355 GO:0005634
Cluster-22178.49890 GO:0006355 GO:0006508 GO:0006510 GO:0003682
GO:0003677 GO:0004176 GO:0005634 GO:0000775 GO:0000785
Cluster-22178.49894 GO:0015991 GO:0015992 GO:0015078 GO:0033177
Cluster-22178.49900 GO:0006486 GO:0008378 GO:0016757 GO:0016020
Cluster-22178.49903 GO:0007264 GO:0005525
Cluster-22178.49905 GO:0055085 GO:0051607 GO:0030168 GO:0006810

GO:0007165 GO:0006308 GO:0051258 GO:0030001 GO:0005102
GO:0008855 GO:0030674 GO:0046873 GO:0005577 GO:0016020
GO:0016021 GO:0009318
Cluster-22178.49906 GO:0019087 GO:0016021
Cluster-22178.49908 GO:0042254 GO:0006412 GO:0006355 GO:0006729
GO:0008124 GO:0003735 GO:0005515 GO:0003677 GO:0003682
GO:0005634 GO:0016021 GO:0000785 GO:0000775 GO:0005840
GO:0005622
Cluster-22178.49915 GO:0006810 GO:0009451 GO:0001522 GO:0006355
GO:0005515 GO:0003723 GO:0005215 GO:0009982 GO:0046983
GO:0016021
Cluster-22178.49916 GO:0006810 GO:0001522 GO:0009451 GO:0006355
GO:0005515 GO:0003723 GO:0005215 GO:0009982 GO:0046983
GO:0016021
Cluster-22178.49920 GO:0005975 GO:0016773
Cluster-22178.49922 GO:0006355 GO:0008270 GO:0043565 GO:0003700
GO:0005634 GO:0005667
Cluster-22178.49923 GO:0006355 GO:0003677
Cluster-22178.49924 GO:0042254 GO:0001522
Cluster-22178.49926 GO:0006355 GO:0043093 GO:0000917 GO:0006260
GO:0043565 GO:0003700 GO:0005667 GO:0005737
Cluster-22178.49927 GO:0006260 GO:0006355 GO:0043093 GO:0000917
GO:0043565 GO:0003700 GO:0005737 GO:0005667
Cluster-22178.49928 GO:0006260 GO:0015992 GO:0000917 GO:0015991
GO:0043093 GO:0006355 GO:0003700 GO:0015078 GO:0005737
GO:0033179 GO:0005667
Cluster-22178.49931 GO:0007186 GO:0006629 GO:0004930 GO:0000166
GO:0046872 GO:0016021
Cluster-22178.49934 GO:0006464 GO:0008608 GO:0051082 GO:0016151
GO:0031072 GO:0072686 GO:0005634 GO:0042729
Cluster-22178.49935 GO:0009086 GO:0031554 GO:0019069 GO:0042558
GO:0031556 GO:0046872 GO:0005524 GO:0008705 GO:0031419
GO:0005622 GO:0005887

Cluster-22178.49940 GO:0004252 GO:0016021
Cluster-22178.49941 GO:0006508 GO:0004252
Cluster-22178.49946 GO:0006554 GO:0008152 GO:0006479 GO:0008168
GO:0018024 GO:0042802 GO:0005515
Cluster-22178.49947 GO:0051260
Cluster-22178.49948 GO:0051260
Cluster-22178.49950 GO:0008270
Cluster-22178.49955 GO:0006355 GO:0005634
Cluster-22178.49958 GO:0003676
Cluster-22178.49962 GO:0003924 GO:0005525
Cluster-22178.49970 GO:0005515
Cluster-22178.49974 GO:0003723
Cluster-22178.49978 GO:0006886 GO:0008536
Cluster-22178.49982 GO:0005515 GO:0005198 GO:0019028
Cluster-22178.49985 GO:0015979 GO:0009538 GO:0009522
Cluster-22178.49986 GO:0006096 GO:0000162 GO:0006357 GO:0006144
GO:0009405 GO:0006397 GO:0006094 GO:0006571 GO:0009094
GO:0005524 GO:0004634 GO:0005198 GO:0001104 GO:0000287
GO:0003723 GO:0003968 GO:0016592 GO:0000015 GO:0005576
GO:0019028 GO:0005634 GO:0019031 GO:0031379 GO:0005737
Cluster-22178.49987 GO:0016021
Cluster-22178.49988 GO:0006605 GO:0006886 GO:0005515 GO:0005742
Cluster-22178.49989 GO:0006950 GO:0051087 GO:0001671
Cluster-22178.49991 GO:0006308 GO:0031047 GO:0005975 GO:0016888
GO:0004560
Cluster-22178.49993 GO:0015074 GO:0051537
Cluster-22178.49994 GO:0008299 GO:0008270
Cluster-22178.50001 GO:0016772
Cluster-22178.50002 GO:0055114 GO:0009405 GO:0051920 GO:0003677

Cluster-22178.50006 GO:0006355 GO:0003677
Cluster-22178.50009 GO:0007186 GO:0004930 GO:0016021
Cluster-22178.50010 GO:0051258 GO:0030168 GO:0000226 GO:0090063

GO:0007165 GO:0005102 GO:0030674 GO:0003677 GO:0005815
GO:0005577 GO:0000922
Cluster-22178.50011 GO:0016310 GO:0009069 GO:0007186 GO:0007165
GO:0003924 GO:0019001 GO:0031683 GO:0004871 GO:0016772
GO:0004674 GO:0005525 GO:0005515 GO:0016021
Cluster-22178.50016 GO:0008152 GO:0016192 GO:0006886 GO:0005515
GO:0003824 GO:0030117
Cluster-22178.50024 GO:0019497 GO:0006771 GO:0055114 GO:0003993
GO:0003756 GO:0016671 GO:0005783
Cluster-22178.50025 GO:0008270
Cluster-22178.50026 GO:0005634
Cluster-22178.50029 GO:0009405 GO:0009098 GO:0005198 GO:0005882
Cluster-22178.50032 GO:0008616 GO:0016192 GO:0006886 GO:0043248
GO:0016740 GO:0005515 GO:0016853 GO:0030117
Cluster-22178.50035 GO:0055114 GO:0009308 GO:0048038 GO:0005507
GO:0008131
Cluster-22178.50041 GO:0003676
Cluster-22178.50042 GO:0017004 GO:0006118 GO:0008610 GO:0008152
GO:0045158 GO:0016747 GO:0009512
Cluster-22178.50044 GO:0008207 GO:0006631 GO:0008209 GO:0018874
GO:0006633 GO:0006554 GO:0006552 GO:0006550 GO:0006568
GO:0008210 GO:0006813 GO:0006694 GO:0008152 GO:0055114
GO:0006574 GO:0016616 GO:0003854 GO:0050662 GO:0016491
GO:0003857 GO:0003824
Cluster-22178.50047 GO:0006812 GO:0051191 GO:0070836 GO:0006836
GO:0005328 GO:0016021 GO:0005634
Cluster-22178.50048 GO:0002062 GO:0035988 GO:0042246 GO:0005634
Cluster-22178.50049 GO:0006904 GO:0016192 GO:0046914 GO:0046983

Cluster-22178.50050 GO:0003676 GO:0005515 GO:0003723 GO:0008270

Cluster-22178.50056 GO:0006813 GO:0015269 GO:0016020
Cluster-22178.50059 GO:0046872 GO:0008270 GO:0003723 GO:0005515

Cluster-22178.50074 GO:0009399 GO:0005515 GO:0008270 GO:0016021
Cluster-22178.50077 GO:0005515
Cluster-22178.50081 GO:0009245 GO:0008270 GO:0008915 GO:0046872
GO:0061630 GO:0005515
Cluster-22178.50085 GO:0007005 GO:0006508 GO:0004222 GO:0031305
Cluster-22178.50086 GO:0006355 GO:0005515 GO:0005634
Cluster-22178.50091 GO:0046872
Cluster-22178.50093 GO:0007010 GO:0015031 GO:0006464 GO:0003779
GO:0005615
Cluster-22178.50096 GO:0006468 GO:0005975 GO:0035556 GO:0003779
GO:0033691 GO:0016773 GO:0005524 GO:0004672
Cluster-22178.50097 GO:0005524 GO:0035091 GO:0020037 GO:0016020
Cluster-22178.50098 GO:0005634
Cluster-22178.50099 GO:0005634
Cluster-22178.50104 GO:0005515
Cluster-22178.50107 GO:0055085 GO:0016567 GO:0006810 GO:0046872
GO:0005524 GO:0008270 GO:0042626 GO:0004842 GO:0005515
GO:0016021 GO:0005680
Cluster-22178.50110 GO:0019467 GO:0008073 GO:0004857
Cluster-22178.50111 GO:0009396 GO:0015992 GO:0006771 GO:0042254
GO:0055114 GO:0019497 GO:0008152 GO:0015991 GO:0006412
GO:0046487 GO:0016491 GO:0003993 GO:0050662 GO:0004488
GO:0003735 GO:0046872 GO:0003824 GO:0015078 GO:0005515
GO:0005840 GO:0033179
Cluster-22178.50116 GO:0070588 GO:0006816 GO:0006812 GO:0055085
GO:0006811 GO:0005216 GO:0005262 GO:0008324 GO:0016020
GO:0016021
Cluster-22178.50117 GO:0051258 GO:0042157 GO:0007165 GO:0006869
GO:0019064 GO:0051262 GO:0030168 GO:0005515 GO:0004871
GO:0008289 GO:0030674 GO:0005102 GO:0005861 GO:0019028
GO:0005576 GO:0019031 GO:0016020 GO:0005577
Cluster-22178.50121 GO:0000105 GO:0055085 GO:0006289 GO:0055114

GO:0006308 GO:0006355 GO:0030001 GO:0004399 GO:0008270
GO:0008855 GO:0046873 GO:0051287 GO:0008168 GO:0000439
GO:0016020 GO:0009318
Cluster-22178.50122 GO:0006030 GO:0006355 GO:0008092 GO:0008061
GO:0019898 GO:0005737 GO:0005634 GO:0005576
Cluster-22178.50126 GO:0008152 GO:0008168
Cluster-22178.50137 GO:0042254 GO:0003735 GO:0005739 GO:0005840
Cluster-22178.50139 GO:0035091 GO:0005515
Cluster-22178.50140 GO:0009968 GO:0007268 GO:0007165 GO:0051056
GO:0055085 GO:0006811 GO:0005216 GO:0004970 GO:0005096
GO:0016020 GO:0005737
Cluster-22178.50149 GO:0008270
Cluster-22178.50152 GO:0006825 GO:0035434 GO:0005375 GO:0016021
Cluster-22178.50156 GO:0006468 GO:0005524 GO:0004672
Cluster-22178.50157 GO:0006468 GO:0006355 GO:0005524 GO:0004672
GO:0043565
Cluster-22178.50159 GO:0006355 GO:0005622
Cluster-22178.50165 GO:0046872 GO:0005515 GO:0005737 GO:0045202
Cluster-22178.50166 GO:0000045 GO:0005737
Cluster-22178.50167 GO:0006355 GO:0003700 GO:0005198 GO:0019028
GO:0005667
Cluster-22178.50169 GO:0006355 GO:0003700 GO:0005667
Cluster-22178.50171 GO:0007264 GO:0010506 GO:0006260 GO:0009966
GO:0005096 GO:0005576 GO:0005615
Cluster-22178.50175 GO:0003676
Cluster-22178.50178 GO:0007165 GO:0042803 GO:0008134 GO:0045502
GO:0030286 GO:0005667
Cluster-22178.50182 GO:0005515
Cluster-22178.50187 GO:0018193 GO:0005515 GO:0046872
Cluster-22178.50188 GO:0015992 GO:0035556 GO:0015986 GO:0042254
GO:0006355 GO:0006412 GO:0006119 GO:0046933 GO:0008270
GO:0003677 GO:0046961 GO:0003735 GO:0005622 GO:0045259
GO:0045261 GO:0005840

Cluster-22178.50191 GO:0005515

Cluster-22178.50200 GO:0055114 GO:0010309 GO:0005515

Cluster-22178.50202 GO:0005515

Cluster-22178.50207 GO:0006310 GO:0000917 GO:0006302 GO:0006355 GO:0043093 GO:0003677 GO:0003700 GO:0043565 GO:0005634 GO:0005667 GO:0005737

Cluster-22178.50208 GO:0006464 GO:0046872 GO:0016151

Cluster-22178.50210 GO:0007017 GO:0005875

Cluster-22178.50211 GO:0003677

Cluster-22178.50219 GO:0016567 GO:0006810 GO:0005515 GO:0004842 GO:0008270 GO:0046872 GO:0016020 GO:0005680

Cluster-22178.50225 GO:0016192 GO:0016021

Cluster-22178.50228 GO:0008152 GO:0055114 GO:0003824 GO:0005515 GO:0016491 GO:0050662

Cluster-22178.50229 GO:0055114 GO:0042773 GO:0016651

Cluster-22178.50230 GO:0008380 GO:0003824 GO:0005515 GO:0005681

Cluster-22178.50236 GO:0005515

Cluster-22178.50240 GO:0071816 GO:0005975 GO:0016740 GO:0004649

Cluster-22178.50245 GO:0055114 GO:0006570 GO:0004511 GO:0005515

Cluster-22178.50250 GO:0003676

Cluster-22178.50252 GO:0016462 GO:0005737

Cluster-22178.50255 GO:0010468 GO:0015992 GO:0015991 GO:0022900 GO:0015078 GO:0033179 GO:0016021

Cluster-22178.50256 GO:0006355 GO:0046983

Cluster-22178.50266 GO:0006816 GO:0006811 GO:0055085 GO:0005244 GO:0005515 GO:0005262 GO:0016020 GO:0016021

Cluster-22178.50268 GO:0000398 GO:0019064 GO:0019031 GO:0005681

Cluster-22178.50273 GO:0015946 GO:0006481 GO:0055114 GO:0006479 GO:0004671 GO:0004022 GO:0016021

Cluster-22178.50275 GO:0005515 GO:0046872 GO:0045202 GO:0005737

Cluster-22178.50279 GO:0005515

Cluster-22178.50280 GO:0043087 GO:0005085
Cluster-22178.50287 GO:0006886 GO:0016192 GO:0019013
Cluster-22178.50291 GO:0007165 GO:0006355 GO:0005515 GO:0003677
GO:0005179 GO:0005576
Cluster-22178.50293 GO:0004252 GO:0016021
Cluster-22178.50294 GO:0055114 GO:0047134
Cluster-22178.50302 GO:0042254 GO:0006412 GO:0003735 GO:0005840
GO:0005622
Cluster-22178.50303 GO:0015940 GO:0015937 GO:0004252 GO:0004594
GO:0005524 GO:0055036 GO:0019028
Cluster-22178.50308 GO:0015991 GO:0005515 GO:0016820 GO:0000221
Cluster-22178.50316 GO:0000062 GO:0005515 GO:0016021
Cluster-22178.50318 GO:0007155 GO:0030246
Cluster-22178.50322 GO:0003678 GO:0005524 GO:0046983 GO:0005657
Cluster-22178.50324 GO:0005515
Cluster-22178.50330 GO:0006313 GO:0003677 GO:0004803 GO:0008537
Cluster-22178.50331 GO:0009069 GO:0006468 GO:0016310 GO:0005524
GO:0004672 GO:0004674
Cluster-22178.50332 GO:0006355 GO:0006810 GO:0002949 GO:0008134
GO:0003774 GO:0005524 GO:0016459 GO:0005667
Cluster-22178.50334 GO:0009069 GO:0006468 GO:0006810 GO:0007178
GO:0016310 GO:0016301 GO:0046872 GO:0005524 GO:0003774
GO:0005026 GO:0016459 GO:0016020
Cluster-22178.50335 GO:0007021 GO:0015631 GO:0046872 GO:0051082
GO:0045298
Cluster-22178.50338 GO:0005515 GO:0005887
Cluster-22178.50340 GO:0016567 GO:0004842 GO:0005515 GO:0046872
GO:0008270 GO:0005680
Cluster-22178.50341 GO:0006355 GO:0008270 GO:0043565 GO:0003700
GO:0005667 GO:0005634
Cluster-22178.50345 GO:0005515
Cluster-22178.50347 GO:0006418 GO:0005524 GO:0004812 GO:0000166

Cluster-22178.50354 GO:0007165 GO:0007264 GO:0005525 GO:0005509
GO:0005524 GO:0016887 GO:0005578
Cluster-22178.50356 GO:0008608 GO:0030472 GO:0005509 GO:0042729
GO:0072686
Cluster-22178.50357 GO:0030472 GO:0008608 GO:0005509 GO:0072686
GO:0042729
Cluster-22178.50362 GO:0006351 GO:0006281 GO:0006144 GO:0006206
GO:0006464 GO:0003899 GO:0005515 GO:0003677 GO:0016151
GO:0008270 GO:0046872 GO:0005730
Cluster-22178.50363 GO:0003677 GO:0046872
Cluster-22178.50368 GO:0003723 GO:0016021
Cluster-22178.50370 GO:0042802 GO:0005515 GO:0030695
Cluster-22178.50371 GO:0006810 GO:0009405 GO:0019870 GO:0005576
Cluster-22178.50372 GO:0071704 GO:0005515 GO:0016868
Cluster-22178.50374 GO:0008146
Cluster-22178.50375 GO:0055114 GO:0006576 GO:0007264 GO:0005525
GO:0003924 GO:0005524 GO:0005509 GO:0016616 GO:0031225
Cluster-22178.50378 GO:0006241 GO:0006206 GO:0006144 GO:0006183
GO:0006228 GO:0006165 GO:0003676 GO:0004550 GO:0005515
GO:0003723
Cluster-22178.5038 GO:0006693 GO:0006691 GO:0019530 GO:0006749
GO:0005515 GO:0003840
Cluster-22178.50382 GO:0007165 GO:0005179 GO:0005576
Cluster-22178.50384 GO:0006811 GO:0055085 GO:0006813 GO:0008270
GO:0003677 GO:0005216 GO:0005242 GO:0016021 GO:0008076
GO:0016020
Cluster-22178.50385 GO:0019867
Cluster-22178.50388 GO:0008191 GO:0004190 GO:0019031 GO:0016021
Cluster-22178.50390 GO:0006384 GO:0006366 GO:0003700 GO:0005667
GO:0005634
Cluster-22178.50395 GO:0015031 GO:0005886 GO:0016021
Cluster-22178.50396 GO:0007155 GO:0035556 GO:0005515
Cluster-22178.5040 GO:0006691 GO:0006749 GO:0019530 GO:0006693

GO:0003840
Cluster-22178.50400 GO:0016567 GO:0005515 GO:0004842 GO:0008270
GO:0046872 GO:0005680
Cluster-22178.50403 GO:0006470 GO:0006570 GO:0008138 GO:0004725
GO:0003677 GO:0004527
Cluster-22178.50405 GO:0019634 GO:0015716 GO:0019058 GO:0046872
GO:0005887
Cluster-22178.50415 GO:0043154 GO:0043066 GO:0019079 GO:0005515
GO:0071986 GO:0005737
Cluster-22178.50416 GO:0015693 GO:0015095 GO:0016020 GO:0016021
Cluster-22178.50419 GO:0008270
Cluster-22178.50425 GO:0046872 GO:0016020
Cluster-22178.50426 GO:0003677 GO:0046872
Cluster-22178.50430 GO:0006313 GO:0015074 GO:0003677 GO:0019028
Cluster-22178.50433 GO:0005975 GO:0004571 GO:0005509 GO:0016020
Cluster-22178.50447 GO:0015684 GO:0046487 GO:0009396 GO:0055114
GO:0004488 GO:0016491 GO:0005524 GO:0015093 GO:0003824
GO:0004329 GO:0016021
Cluster-22178.50452 GO:0005515
Cluster-22178.50455 GO:0006281 GO:0051726 GO:0017025 GO:0003677
GO:0004518 GO:0005096 GO:0003824 GO:0005634
Cluster-22178.50457 GO:0009847 GO:0016021
Cluster-22178.50462 GO:0006355 GO:0003677 GO:0005507 GO:0003700
GO:0046872 GO:0005667 GO:0005634
Cluster-22178.50463 GO:0008152 GO:0055114 GO:0003824 GO:0005515
GO:0050662 GO:0016491
Cluster-22178.50465 GO:0006412 GO:0042254 GO:0005515 GO:0003735
GO:0005622 GO:0005840
Cluster-22178.50467 GO:0006308 GO:0008239 GO:0008855 GO:0070009
GO:0009318
Cluster-22178.50469 GO:0006355 GO:0003676 GO:0005515 GO:0005634
GO:0019028
Cluster-22178.50472 GO:0042981 GO:0005524

Cluster-22178.50473 GO:0004190 GO:0016021
Cluster-22178.50475 GO:0005515
Cluster-22178.50476 GO:0005515
Cluster-22178.50480 GO:0006464
Cluster-22178.50481 GO:0006810 GO:0015986 GO:0042626 GO:0033178
Cluster-22178.50482 GO:0042254 GO:0006412 GO:0006355 GO:0045454
GO:0003700 GO:0003735 GO:0005840 GO:0005622 GO:0005667
Cluster-22178.50485 GO:0051782 GO:0009432 GO:0003824 GO:0009276
Cluster-22178.50487 GO:0007165 GO:0007186 GO:0007264 GO:0005525
GO:0004871 GO:0008270 GO:0031683 GO:0046872 GO:0019001
GO:0003924 GO:0005515 GO:0005622
Cluster-22178.50490 GO:0006144 GO:0006206 GO:0016570 GO:0006816
GO:0006874 GO:0006368 GO:0006351 GO:0005219 GO:0003899
GO:0005622 GO:0016021 GO:0005634 GO:0005730 GO:0016593
Cluster-22178.50491 GO:0019031
Cluster-22178.50495 GO:0006820 GO:0015992 GO:0006120 GO:0006744
GO:0006814 GO:0043565 GO:0008509 GO:0008137 GO:0016021
GO:0005743 GO:0005634
Cluster-22178.50497 GO:0046486 GO:0042967 GO:0045017 GO:0004144

Cluster-22178.50499 GO:0005975 GO:0004553
Cluster-22178.50502 GO:0006629 GO:0016627 GO:0016021 GO:0005737
Cluster-22178.50503 GO:0005524
Cluster-22178.50505 GO:0048280 GO:0006886 GO:0003676 GO:0005737
GO:0000139
Cluster-22178.50507 GO:0009159 GO:0006206 GO:0050144 GO:0070694

Cluster-22178.50508 GO:0006259 GO:0005515 GO:0003824 GO:0003677
GO:0005524 GO:0005694
Cluster-22178.50511 GO:0009306 GO:0005515 GO:0016020
Cluster-22178.50519 GO:0002949 GO:0019079 GO:0006310 GO:0006771
GO:0019497 GO:0006281 GO:0003993 GO:0003724 GO:0005524
GO:0016887 GO:0009378 GO:0003678 GO:0005515 GO:0003723

GO:0005657 GO:0009379
Cluster-22178.50522 GO:0003924 GO:0005525 GO:0005861
Cluster-22178.50525 GO:0043093 GO:0000917 GO:0007165 GO:0007275
GO:0005044 GO:0005737 GO:0005578 GO:0016020
Cluster-22178.50526 GO:0007165 GO:0007275 GO:0000917 GO:0043093
GO:0005044 GO:0016020 GO:0005578 GO:0005737
Cluster-22178.50532 GO:0006397 GO:0006370 GO:0005515 GO:0004484
GO:0016020
Cluster-22178.50536 GO:0006355 GO:0035091 GO:0003700 GO:0005515
GO:0016773 GO:0042025 GO:0005667
Cluster-22178.50539 GO:0005515
Cluster-22178.50543 GO:0006744 GO:0055114 GO:0005515
Cluster-22178.50545 GO:0006302 GO:0006265 GO:0003677 GO:0003917
GO:0005694 GO:0005634
Cluster-22178.50550 GO:0006366 GO:0006384 GO:0016226 GO:0001503
GO:0051536 GO:0003700 GO:0005506 GO:0005634 GO:0005667
Cluster-22178.50551 GO:0018149 GO:0009069 GO:0006468 GO:0016310
GO:0007178 GO:0004675 GO:0003810 GO:0005524 GO:0016020
Cluster-22178.50554 GO:0006468 GO:0003677 GO:0005515 GO:0005524
GO:0004672
Cluster-22178.50557 GO:0042254 GO:0006412 GO:0003735 GO:0005840
GO:0015934
Cluster-22178.50559 GO:0005515
Cluster-22178.50560 GO:0015992 GO:0015991 GO:0015078 GO:0033177
Cluster-22178.50561 GO:0015991 GO:0007165 GO:0007186 GO:0015992
GO:0019001 GO:0031683 GO:0003924 GO:0015078 GO:0004871
GO:0033177
Cluster-22178.50563 GO:0009966 GO:0007275 GO:0016020
Cluster-22178.50564 GO:0005198 GO:0045095
Cluster-22178.50565 GO:0005975 GO:0005982 GO:0005985 GO:0004650
GO:0004045
Cluster-22178.50566 GO:0005515
Cluster-22178.50571 GO:0008544 GO:0000166 GO:0046872 GO:0008168

Cluster-22178.50573 GO:0051015
Cluster-22178.50575 GO:0007018 GO:0000398 GO:0005515 GO:0005868
GO:0005681
Cluster-22178.50577 GO:0006508 GO:0055085 GO:0004197 GO:0016021
Cluster-22178.50579 GO:0051537 GO:0043231
Cluster-22178.50584 GO:0007264 GO:0007165 GO:0007186 GO:0031683
GO:0005524 GO:0019001 GO:0003924 GO:0005525 GO:0004871
GO:0016021
Cluster-22178.50586 GO:0006260 GO:0000723 GO:0003677 GO:0003676
GO:0000784 GO:0005634
Cluster-22178.50589 GO:0006355 GO:0003700 GO:0005667
Cluster-22178.50593 GO:0032040
Cluster-22178.50597 GO:0006810 GO:0003333 GO:0006865 GO:0055085
GO:0015171 GO:0016020
Cluster-22178.50600 GO:0005515
Cluster-22178.50602 GO:0048193 GO:0055114 GO:0007165 GO:0007186
GO:0005525 GO:0004871 GO:0031683 GO:0019001 GO:0016491
GO:0003924 GO:0050661 GO:0005783
Cluster-22178.50603 GO:0006886 GO:0008536
Cluster-22178.50605 GO:0006413 GO:0006446 GO:0003743 GO:0005840
Cluster-22178.50607 GO:0006308 GO:0009307 GO:0005515 GO:0003677
GO:0009036 GO:0009359
Cluster-22178.50618 GO:0007165 GO:0018106 GO:0000160 GO:0016310
GO:0035556 GO:0004673 GO:0046872 GO:0005524 GO:0016020
GO:0009365
Cluster-22178.50622 GO:0006313 GO:0004803 GO:0003677
Cluster-22178.50624 GO:0008152 GO:0003824
Cluster-22178.50626 GO:0007067 GO:0006807 GO:0006123 GO:0015992
GO:0005507 GO:0004129 GO:0016810 GO:0042729 GO:0016020
GO:0072686 GO:0045277
Cluster-22178.50627 GO:0007186 GO:0007165 GO:0007264 GO:0004871
GO:0005525 GO:0003924 GO:0031683 GO:0019001

Cluster-22178.50629 GO:0006807 GO:0042967 GO:0006537 GO:0055114
GO:0016407 GO:0003676 GO:0015930 GO:0005515
Cluster-22178.50632 GO:0006486 GO:0008373
Cluster-22178.50633 GO:0006486 GO:0008373
Cluster-22178.50638 GO:0050821 GO:0020027 GO:0006468 GO:0030097
GO:0006457 GO:0005524 GO:0004672 GO:0030492 GO:0005833
Cluster-22178.50639 GO:0009607 GO:0055085 GO:0030001 GO:0009405
GO:0046873 GO:0005515 GO:0005198 GO:0016021 GO:0016020
Cluster-22178.50640 GO:0006355 GO:0016791 GO:0004252 GO:0003723
GO:0008233 GO:0003700 GO:0016020 GO:0005667 GO:0005634
GO:0005618
Cluster-22178.50646 GO:0005515
Cluster-22178.50647 GO:0005515
Cluster-22178.50654 GO:0030001 GO:0000166 GO:0019956 GO:0005515
GO:0003682 GO:0046872 GO:0008270 GO:0000785
Cluster-22178.50656 GO:0006468 GO:0004672 GO:0005524
Cluster-22178.50659 GO:0003824
Cluster-22178.50660 GO:0007155 GO:0006886 GO:0030155 GO:0007165
GO:0055114 GO:0006914 GO:0030334 GO:0019049 GO:0045995
GO:0016702 GO:0005102 GO:0016020 GO:0031224 GO:0005801
Cluster-22178.50662 GO:0035556
Cluster-22178.50664 GO:0006367 GO:0032968 GO:0003677 GO:0005634
Cluster-22178.50666 GO:0006418 GO:0004812 GO:0005524 GO:0008270
GO:0004518 GO:0003677 GO:0000166
Cluster-22178.50668 GO:0003676 GO:0008270
Cluster-22178.50669 GO:0007186 GO:0004930 GO:0016021
Cluster-22178.50670 GO:0017009 GO:0007186 GO:0004930 GO:0016021
Cluster-22178.50674 GO:0005525 GO:0016887 GO:0005524
Cluster-22178.50677 GO:0033014 GO:0055114 GO:0015994 GO:0004418
GO:0016491
Cluster-22178.50678 GO:0005509
Cluster-22178.50679 GO:0046983
Cluster-22178.50680 GO:0007034 GO:0006308 GO:0008855 GO:0009318

Cluster-22178.50687 GO:0042254 GO:0006412 GO:0019843 GO:0003735 GO:0005622 GO:0005840

Cluster-22178.50688 GO:0008033 GO:0009452 GO:0006396 GO:0001510 GO:0009451 GO:0031167 GO:0008270 GO:0004809 GO:0008168 GO:0003723 GO:0008173 GO:0016021

Cluster-22178.50693 GO:0006118 GO:0051258 GO:0019551 GO:0007165 GO:0051607 GO:0030168 GO:0006891 GO:0008053 GO:0019089 GO:0006886 GO:0006606 GO:0006807 GO:0045502 GO:0008134 GO:0019901 GO:0008092 GO:0030674 GO:0005102 GO:0042803 GO:0005198 GO:0004352 GO:0003924 GO:0005524 GO:0019028 GO:0019031 GO:0016021 GO:0017119 GO:0030286 GO:0005815 GO:0019898 GO:0005737 GO:0005667 GO:0005741 GO:0016020 GO:0030173 GO:0005577

Cluster-22178.50698 GO:0008152

Cluster-22178.50700 GO:0006298 GO:0005524 GO:0030983

Cluster-22178.50705 GO:0009235 GO:0006576 GO:0019079 GO:0005524 GO:0005515 GO:0043657

Cluster-22178.50706 GO:0006281 GO:0004518

Cluster-22178.50709 GO:0055114 GO:0008152 GO:0016491

Cluster-22178.50710 GO:0042967 GO:0008080 GO:0005198 GO:0019028

Cluster-22178.50711 GO:0046740 GO:0007155 GO:0005515 GO:0016021 GO:0016020

Cluster-22178.50712 GO:0005515 GO:0016020

Cluster-22178.50713 GO:0055114 GO:0006464 GO:0008113

Cluster-22178.50714 GO:0006464 GO:0055114 GO:0008113

Cluster-22178.50718 GO:0008270 GO:0005515 GO:0042765 GO:0016021

Cluster-22178.50720 GO:0009405 GO:0043303 GO:0046872 GO:0005576

Cluster-22178.50721 GO:0043303 GO:0009405 GO:0046872 GO:0005198 GO:0005576

Cluster-22178.50723 GO:0006508 GO:0004252

Cluster-22178.50730 GO:0005515 GO:0016021

Cluster-22178.50731 GO:0016021

Cluster-22178.50733 GO:0022900 GO:0005515 GO:0016021

Cluster-22178.50737 GO:0016480
Cluster-22178.50738 GO:0006047
Cluster-22178.50740 GO:0051028 GO:0006464 GO:0015031 GO:0008643
GO:0048309 GO:0008982 GO:0005515 GO:0009357
Cluster-22178.50741 GO:0006355 GO:0005622
Cluster-22178.50744 GO:0008152 GO:0003824 GO:0016836
Cluster-22178.50750 GO:0055085 GO:0006821 GO:0008270 GO:0005247
GO:0016020
Cluster-22178.50771 GO:0055114 GO:0008641 GO:0051287
Cluster-22178.50772 GO:0055114 GO:0008641 GO:0051287
Cluster-22178.50775 GO:0007010 GO:0008270 GO:0003779 GO:0005515

Cluster-22178.50776 GO:0003676
Cluster-22178.50778 GO:0071705 GO:0004190 GO:0003676 GO:0045095
GO:0016021
Cluster-22178.50789 GO:0006468 GO:0005515 GO:0005524 GO:0004672

Cluster-22178.50793 GO:0006308 GO:0009307 GO:0006355 GO:0009036
GO:0003700 GO:0005515 GO:0003677 GO:0005667 GO:0009359
Cluster-22178.50797 GO:0046872
Cluster-22178.50798 GO:0006508 GO:0004197 GO:0008234
Cluster-22178.50800 GO:0006629 GO:0009058 GO:0016788
Cluster-22178.50801 GO:0009058 GO:0006629 GO:0016788
Cluster-22178.50802 GO:0016567 GO:0007165 GO:0007166 GO:0005515
GO:0005509 GO:0004871 GO:0004842 GO:0046872 GO:0008270
GO:0005634 GO:0005680
Cluster-22178.50804 GO:0009058 GO:0008152 GO:0016747 GO:0005524
GO:0016874
Cluster-22178.50805 GO:0006090 GO:0006525 GO:0006814 GO:0071436
GO:0006560 GO:0004672 GO:0015081 GO:0008948 GO:0016020
Cluster-22178.50807 GO:0009097 GO:0009099 GO:0009098 GO:0006438
GO:0000166 GO:0005524 GO:0004832 GO:0005737
Cluster-22178.50808 GO:0016337 GO:0045132 GO:0006813 GO:0007049

GO:0016286 GO:0016020 GO:0005634 GO:0016021 GO:0000775
Cluster-22178.50810 GO:0006813 GO:0016286 GO:0016021
Cluster-22178.50817 GO:0006468 GO:0016773 GO:0004672 GO:0005524
GO:0016020
Cluster-22178.50826 GO:0006629 GO:0006810 GO:0016787 GO:0005524
GO:0042626 GO:0016021
Cluster-22178.50829 GO:0005515
Cluster-22178.50830 GO:0006355 GO:0003676 GO:0005515 GO:0043565
GO:0005634
Cluster-22178.50833 GO:0006810 GO:0009405 GO:0019871 GO:0005576
Cluster-22178.50835 GO:0055085 GO:0006821 GO:0005247 GO:0016020
Cluster-22178.50837 GO:0003677 GO:0005515 GO:0046872
Cluster-22178.50842 GO:0006270 GO:0006323 GO:0016020
Cluster-22178.50843 GO:0006355 GO:0006260 GO:0003700 GO:0005634
GO:0005667
Cluster-22178.50844 GO:0006355 GO:0006260 GO:0003700 GO:0005667
GO:0005634
Cluster-22178.50846 GO:0007049
Cluster-22178.50851 GO:0006470 GO:0006570 GO:0004725 GO:0008138

Cluster-22178.50852 GO:0005515
Cluster-22178.50853 GO:0003677 GO:0008270 GO:0046872
Cluster-22178.50854 GO:0007264 GO:0005096 GO:0005525
Cluster-22178.50856 GO:0006206 GO:0006144 GO:0006352 GO:0006355
GO:0006351 GO:0016987 GO:0003700 GO:0003677 GO:0003899
GO:0005730 GO:0005667
Cluster-22178.50859 GO:0006396 GO:0009451 GO:0001522 GO:0009982
GO:0003723
Cluster-22178.50861 GO:0007156 GO:0005509 GO:0016020
Cluster-22178.50862 GO:0005975
Cluster-22178.50864 GO:0006468 GO:0008152 GO:0006606 GO:0008733
GO:0004672 GO:0005524 GO:0016773 GO:0016020
Cluster-22178.50865 GO:0006468 GO:0006270 GO:0016773 GO:0005524

GO:0004672 GO:0016020
Cluster-22178.50866 GO:0009396 GO:0055114 GO:0015684 GO:0046487
GO:0016491 GO:0005524 GO:0015093 GO:0003824 GO:0004329
GO:0004488 GO:0016021
Cluster-22178.50867 GO:0007218
Cluster-22178.50873 GO:0007264 GO:0006119 GO:0006118 GO:0015992
GO:0055114 GO:0035556 GO:0008121 GO:0005525 GO:0005507
Cluster-22178.50875 GO:0006310 GO:0055085 GO:0009396 GO:0015031
GO:0046487 GO:0006355 GO:0006281 GO:0008152 GO:0055114
GO:0004488 GO:0009378 GO:0003724 GO:0008565 GO:0016491
GO:0016887 GO:0005524 GO:0008134 GO:0003723 GO:0003824
GO:0003678 GO:0022857 GO:0005667 GO:0009379 GO:0005657
GO:0016021
Cluster-22178.50880 GO:0006355 GO:0043565 GO:0003700 GO:0005634
GO:0005667
Cluster-22178.50887 GO:0000062
Cluster-22178.50889 GO:0006506 GO:0016746 GO:0016021 GO:0005789
Cluster-22178.50891 GO:0006367 GO:0006355 GO:0008152 GO:0048870
GO:0017048 GO:0003677 GO:0003682 GO:0016491 GO:0008440
GO:0031514 GO:0005634 GO:0005672 GO:0000785 GO:0000775
Cluster-22178.50895 GO:0008643 GO:0055085 GO:0005351 GO:0000139
GO:0016020 GO:0016021
Cluster-22178.50900 GO:0007010 GO:0003779
Cluster-22178.50902 GO:0055114 GO:0016972 GO:0005515
Cluster-22178.50907 GO:0008146
Cluster-22178.50910 GO:0006468 GO:0007169 GO:0004672 GO:0005524
GO:0005515 GO:0004714 GO:0016020
Cluster-22178.50922 GO:0016787 GO:0016020
Cluster-22178.50926 GO:0008616 GO:0006886 GO:0016192 GO:0043248
GO:0016740 GO:0005515 GO:0016853 GO:0030117
Cluster-22178.50930 GO:0006355 GO:0003677 GO:0003682 GO:0000775
GO:0000785 GO:0005634
Cluster-22178.50932 GO:0007165 GO:0005057

Cluster-22178.50936 GO:0006118 GO:0006468 GO:0009055 GO:0003676
GO:0004672 GO:0005524 GO:0046983 GO:0016773 GO:0016020
Cluster-22178.50938 GO:0005737
Cluster-22178.50941 GO:0005515 GO:0016020
Cluster-22178.50944 GO:0016787
Cluster-22178.50946 GO:0022904 GO:0016020
Cluster-22178.50947 GO:0006470 GO:0006570 GO:0004725
Cluster-22178.50949 GO:0046872
Cluster-22178.50951 GO:0009878 GO:0046872
Cluster-22178.50952 GO:0007165 GO:0006811 GO:0007268 GO:0004970
GO:0005234 GO:0016020
Cluster-22178.50957 GO:0006357 GO:0006468 GO:0001104 GO:0005515
GO:0005524 GO:0004672 GO:0016592
Cluster-22178.50961 GO:0017176
Cluster-22178.50962 GO:0017176
Cluster-22178.50968 GO:0006814 GO:0006118 GO:0006744 GO:0006120
GO:0015992 GO:0008137 GO:0009055 GO:0016020
Cluster-22178.50972 GO:0006260 GO:0009966 GO:0007264 GO:0005096
GO:0005576
Cluster-22178.50974 GO:0019836 GO:0005525 GO:0016887 GO:0005524
GO:0005576
Cluster-22178.50979 GO:0006629 GO:0008081
Cluster-22178.50986 GO:0055085 GO:0006811 GO:0005216 GO:0016020
Cluster-22178.50990 GO:0005515
Cluster-22178.50991 GO:0009058 GO:0008201 GO:0030170 GO:0009512
Cluster-22178.50992 GO:0009058 GO:0030170 GO:0008201 GO:0009512
Cluster-22178.51000 GO:0055114 GO:0006355 GO:0016491 GO:0005515

Cluster-22178.51006 GO:0019068 GO:0003676 GO:0008233 GO:0008168
GO:0008047
Cluster-22178.51008 GO:0009058 GO:0008483 GO:0030170
Cluster-22178.51013 GO:0006629 GO:0016627 GO:0005737 GO:0016021
Cluster-22178.51014 GO:0007264 GO:0005096 GO:0005525

Cluster-22178.51018 GO:0007264 GO:0005096 GO:0005525

Cluster-22178.51021 GO:0019497 GO:1901031 GO:0006771 GO:0003993 GO:0008270 GO:0046872 GO:0005515 GO:0005634

Cluster-22178.51022 GO:0016020

Cluster-22178.51026 GO:0006955 GO:0005201 GO:0005578 GO:0005581

Cluster-22178.51033 GO:0006468 GO:0005515 GO:0005524 GO:0004672 GO:0008168

Cluster-22178.51036 GO:0051016 GO:0006468 GO:0016773 GO:0004672 GO:0005524 GO:0008290 GO:0016020

Cluster-22178.51038 GO:0007165 GO:0005509 GO:0005515 GO:0005578

Cluster-22178.51047 GO:0009245 GO:0016567 GO:0008915 GO:0008270 GO:0046872 GO:0008233 GO:0004842 GO:0003723 GO:0005515 GO:0005680 GO:0016020

Cluster-22178.51048 GO:0009245 GO:0005515 GO:0003723 GO:0008915 GO:0016020

Cluster-22178.51049 GO:0043087 GO:0005085 GO:0005515

Cluster-22178.51053 GO:0046872

Cluster-22178.51056 GO:0042254 GO:0006412 GO:0003735 GO:0005515 GO:0046872 GO:0005840 GO:0005622

Cluster-22178.51057 GO:0016192 GO:0006886 GO:0005515 GO:0042802

Cluster-22178.51059 GO:0000160 GO:0016310 GO:0006576 GO:0007264 GO:0015684 GO:0006109 GO:0003924 GO:0015093 GO:0004672 GO:0005524 GO:0005525 GO:0000155 GO:0016021 GO:0009365

Cluster-22178.51062 GO:0006313 GO:0004803 GO:0003677

Cluster-22178.51063 GO:0048870 GO:0006935 GO:0006357 GO:0000921 GO:0006606 GO:0071973 GO:0008092 GO:0016817 GO:0005198 GO:0001104 GO:0003774 GO:0005737 GO:0009288 GO:0016021 GO:0031514 GO:0005940 GO:0019898 GO:0016020 GO:0016592 GO:0005882

Cluster-22178.51064 GO:0006355 GO:0003677 GO:0003700 GO:0005667

Cluster-22178.51065 GO:0035023 GO:0035556 GO:0055114 GO:0043087 GO:0006955 GO:0007165 GO:0040007 GO:0008283 GO:0005089

GO:0047134 GO:0005137 GO:0005515 GO:0008083 GO:0005895
GO:0005576
Cluster-22178.51067 GO:0006118 GO:0022900 GO:0055114 GO:0008827
GO:0016021 GO:0009319
Cluster-22178.51069 GO:0006820 GO:0006835 GO:0006812 GO:0005515
GO:0017153 GO:0016020
Cluster-22178.51073 GO:0007304 GO:0005213 GO:0005576 GO:0042600
Cluster-22178.51074 GO:0015693 GO:0015095 GO:0016020 GO:0016021
Cluster-22178.51081 GO:0055114 GO:0010309 GO:0005515
Cluster-22178.51082 GO:0019087 GO:0016021
Cluster-22178.51083 GO:0006468 GO:0005524 GO:0004672
Cluster-22178.51085 GO:0006355 GO:0009772 GO:0006118 GO:0019684
GO:0045156 GO:0003676 GO:0003700 GO:0005667
Cluster-22178.51086 GO:0055114 GO:0051920
Cluster-22178.51095 GO:0042254 GO:0006412 GO:0003735 GO:0000774
GO:0005783 GO:0005840 GO:0005622
Cluster-22178.51097 GO:0042254 GO:0006412 GO:0003924 GO:0003735
GO:0005525 GO:0005622 GO:0005840
Cluster-22178.51103 GO:0005515
Cluster-22178.51107 GO:0007010 GO:0005975 GO:0007165 GO:0006810
GO:0016192 GO:0006357 GO:0005198 GO:0001104 GO:0005515
GO:0003779 GO:0005215 GO:0016853 GO:0043395 GO:0004872
GO:0005578 GO:0019028 GO:0019031 GO:0016021 GO:0016592
GO:0016020
Cluster-22178.51113 GO:0006468 GO:0005515 GO:0005524 GO:0004672
GO:0016773 GO:0016020
Cluster-22178.51117 GO:0008152 GO:0007165 GO:0006955 GO:0030247
GO:0003676 GO:0003824 GO:0046872 GO:0008484 GO:0005044
GO:0016787
Cluster-22178.51120 GO:0050909 GO:0006506 GO:0016021 GO:0005789
Cluster-22178.51124 GO:0006355 GO:0003690 GO:0005739
Cluster-22178.51126 GO:0006355 GO:0003700 GO:0005622 GO:0005667
Cluster-22178.51133 GO:0055114 GO:0006479 GO:0006486 GO:0006355

GO:0006554 GO:0007165 GO:0034968 GO:0005507 GO:0008375
GO:0018024 GO:0016641 GO:0003677 GO:0005044 GO:0051920
GO:0005622 GO:0005763 GO:0016020 GO:0005694
Cluster-22178.51139 GO:0016032 GO:0019031
Cluster-22178.51140 GO:0015684 GO:0007264 GO:0005525 GO:0005509
GO:0005515 GO:0015093 GO:0016021
Cluster-22178.51141 GO:0006508 GO:0004198 GO:0005509 GO:0005622
Cluster-22178.51143 GO:0006047
Cluster-22178.51144 GO:0046872 GO:0008270 GO:0003676
Cluster-22178.51145 GO:0009058 GO:0016772 GO:0016779 GO:0031224
Cluster-22178.51150 GO:0007165 GO:0006313 GO:0005515 GO:0005509
GO:0005578
Cluster-22178.51152 GO:0007049 GO:0005515
Cluster-22178.51153 GO:0006955 GO:0030153 GO:0015643 GO:0019814
Cluster-22178.51158 GO:0019076 GO:0032801 GO:0006812 GO:0006468
GO:0007155 GO:0001503 GO:0004672 GO:0005524 GO:0016773
GO:0005261 GO:0033644 GO:0005576 GO:0016020
Cluster-22178.51162 GO:0016567 GO:0004842 GO:0005515 GO:0046872
GO:0008270 GO:0005680
Cluster-22178.51165 GO:0045859 GO:0046677 GO:0004860
Cluster-22178.51169 GO:0009405 GO:0008270 GO:0003723 GO:0004222
GO:0005576
Cluster-22178.51170 GO:0007205 GO:0035556 GO:0007165 GO:0046486
GO:0009395 GO:0004143 GO:0016301
Cluster-22178.51171 GO:0008152 GO:0051252 GO:0008033 GO:0004540
GO:0016491
Cluster-22178.51174 GO:0051225 GO:0071973 GO:0006935 GO:0003774
GO:0016020 GO:0009288 GO:0070652
Cluster-22178.51175 GO:0006351 GO:0008270 GO:0003677 GO:0005634
Cluster-22178.51177 GO:0006355 GO:0000975 GO:0003700 GO:0005840
GO:0005667
Cluster-22178.51180 GO:0006310 GO:0006281
Cluster-22178.51183 GO:0005515

Cluster-22178.51185 GO:0007186 GO:0004930 GO:0000975 GO:0046872
GO:0016021
Cluster-22178.51187 GO:0016021
Cluster-22178.51189 GO:0016579 GO:0006511 GO:0006508 GO:0004843
GO:0005622
Cluster-22178.51190 GO:0005515
Cluster-22178.51194 GO:0042254 GO:0003735 GO:0005739 GO:0005840
Cluster-22178.51195 GO:0042254 GO:0003735 GO:0005840 GO:0005739
Cluster-22178.51197 GO:0050776 GO:0035556 GO:0014068 GO:0006464
GO:0007165 GO:0005102 GO:0016151 GO:0046872 GO:0043548
GO:0016020
Cluster-22178.51199 GO:0046872
Cluster-22178.51200 GO:0008033 GO:0006379 GO:0006364 GO:0051252
GO:0003723 GO:0004540 GO:0000172 GO:0030677
Cluster-22178.51203 GO:0016197 GO:0000724 GO:0006270 GO:0030119
Cluster-22178.51206 GO:0008152 GO:0005975 GO:0005509 GO:0004571
GO:0016829 GO:0016020
Cluster-22178.51207 GO:0019478 GO:0016788 GO:0005737
Cluster-22178.51208 GO:0019478 GO:0016788 GO:0005737
Cluster-22178.51210 GO:0055085 GO:0006355 GO:0006270 GO:0019058
GO:0022857 GO:0003700 GO:0003677 GO:0004252 GO:0005886
GO:0019031 GO:0016021 GO:0042765 GO:0005667 GO:0016020
Cluster-22178.51212 GO:0004222
Cluster-22178.51213 GO:0004222
Cluster-22178.51214 GO:0005525
Cluster-22178.51222 GO:0006260 GO:0003677 GO:0005664
Cluster-22178.51223 GO:0006486 GO:0008378 GO:0016757 GO:0016020
Cluster-22178.51224 GO:0042967 GO:0019497 GO:0006771 GO:0007155
GO:0003993 GO:0008080 GO:0046872 GO:0016747 GO:0005515
GO:0016021 GO:0016020
Cluster-22178.51225 GO:0030168 GO:0051258 GO:0016192 GO:0006886
GO:0007165 GO:0005102 GO:0005198 GO:0005515 GO:0030674
GO:0005577 GO:0030117

Cluster-22178.51229 GO:0055114 GO:0006694 GO:0008152 GO:0008210 GO:0008207 GO:0008209 GO:0016491 GO:0003824 GO:0003854 GO:0050662 GO:0016616

Cluster-22178.51236 GO:0003723 GO:0000178

Cluster-22178.51238 GO:0009451 GO:0046999 GO:0051539 GO:0003824 GO:0019867

Cluster-22178.51241 GO:0006118 GO:0006820 GO:0008509 GO:0009055 GO:0016491 GO:0016020 GO:0016021

Cluster-22178.51247 GO:0005515 GO:0008270

Cluster-22178.51248 GO:0008270 GO:0005515

Cluster-22178.51252 GO:0071203

Cluster-22178.51253 GO:0016567 GO:0005515 GO:0004842 GO:0008270 GO:0046872 GO:0005680

Cluster-22178.51255 GO:0006355 GO:0009271

Cluster-22178.51258 GO:0005515

Cluster-22178.51260 GO:0005739

Cluster-22178.51265 GO:0016579 GO:0036459 GO:0032183 GO:0070140

Cluster-22178.51269 GO:0007264 GO:0006576 GO:0005524 GO:0003924 GO:0005525

Cluster-22178.51270 GO:1901031 GO:0000128 GO:0009847 GO:0006357 GO:0006914 GO:0001104 GO:0005634 GO:0016592

Cluster-22178.51273 GO:0006629 GO:0016627 GO:0005737 GO:0016021

Cluster-22178.51285 GO:0005515

Cluster-22178.51288 GO:0007165 GO:0005515 GO:0005179 GO:0008270 GO:0005576

Cluster-22178.51290 GO:0006139 GO:0043395 GO:0003676 GO:0008408 GO:0005515 GO:0005578 GO:0016020

Cluster-22178.51291 GO:0009069 GO:0006468 GO:0016310 GO:0005524 GO:0004674

Cluster-22178.51293 GO:0005543

Cluster-22178.51296 GO:0006260 GO:0055085 GO:0015074 GO:0006810 GO:0005525 GO:0003676 GO:0003678 GO:0042626 GO:0005524

GO:0003924 GO:0016887 GO:0005657 GO:0016021

Cluster-22178.51298 GO:0006468 GO:0004672 GO:0005524 GO:0008134

GO:0042803 GO:0045502 GO:0030286 GO:0005856 GO:0005667

Cluster-22178.51299 GO:0055085 GO:0005741

Cluster-22178.51301 GO:0005515

Cluster-22178.51304 GO:0016310 GO:0000160 GO:0000155 GO:0046983

GO:0009365 GO:0016021

Cluster-22178.51307 GO:0000002 GO:0003723 GO:0005524 GO:0016887

GO:0003924 GO:0005525 GO:0003724 GO:0045095 GO:0000262

Cluster-22178.51308 GO:0006534 GO:0046439 GO:0055114 GO:0019530

GO:0016702 GO:0005506 GO:0017172

Cluster-22178.51312 GO:0043093 GO:0006468 GO:0007165 GO:0007172

GO:0000917 GO:0005515 GO:0005096 GO:0004871 GO:0004713

GO:0005925 GO:0005737

Cluster-22178.51313 GO:0005515

Cluster-22178.51318 GO:0005975 GO:0008152 GO:0006508 GO:0006631

GO:0009058 GO:0016787 GO:0016746 GO:0008184 GO:0008236

GO:0016788

Cluster-22178.51319 GO:0045893 GO:0046872 GO:0005634

Cluster-22178.51322 GO:0006510 GO:0006310 GO:0006508 GO:0006281

GO:0016887 GO:0005524 GO:0003724 GO:0009378 GO:0003924

GO:0004176 GO:0003678 GO:0005525 GO:0003723 GO:0004252

GO:0005657 GO:0009379

Cluster-22178.51324 GO:0016021

Cluster-22178.51325 GO:0016021

Cluster-22178.51328 GO:0006013 GO:0006000 GO:0006012 GO:0006094

GO:0006098 GO:0006096 GO:0003872 GO:0005945

Cluster-22178.51329 GO:0006310 GO:0006281 GO:0003724 GO:0016887

GO:0005524 GO:0016829 GO:0005515 GO:0003723 GO:0009378

GO:0005657 GO:0042597 GO:0009379

Cluster-22178.51332 GO:0007165 GO:0006541 GO:0006457 GO:0004359

GO:0042302 GO:0005509 GO:0016021 GO:0005578

Cluster-22178.51333 GO:0007212 GO:0048268 GO:0044237 GO:0032051

GO:0003824 GO:0016021
Cluster-22178.51339 GO:0009069 GO:0016192 GO:0006886 GO:0016310
GO:0005515 GO:0004674 GO:0030117
Cluster-22178.51341 GO:0015986 GO:0017038 GO:0006511 GO:0031625
GO:0005198 GO:0046933 GO:0045259 GO:0016020 GO:0019031
Cluster-22178.51349 GO:0055114 GO:0016817 GO:0033743 GO:0042302

Cluster-22178.51357 GO:0006974 GO:0006260 GO:0006281 GO:0007067
GO:0051726 GO:0003911 GO:0050840 GO:0005576 GO:0072686
GO:0042729
Cluster-22178.51359 GO:0006810 GO:0055085 GO:0005215 GO:0004177
GO:0022857 GO:0016021 GO:0016020
Cluster-22178.51362 GO:0005515
Cluster-22178.51364 GO:0015979 GO:0008270 GO:0016020 GO:0009523
GO:0009539
Cluster-22178.51367 GO:0042476 GO:0042475 GO:0030345 GO:0005578
GO:0019867
Cluster-22178.51368 GO:0055114 GO:0009396 GO:0007608 GO:0007187
GO:0046487 GO:0004984 GO:0005524 GO:0003824 GO:0004329
GO:0004488 GO:0005549 GO:0016020
Cluster-22178.51371 GO:0006310 GO:0003677 GO:0005515 GO:0005634
Cluster-22178.51374 GO:0007155 GO:0006310 GO:0010181 GO:0051015
GO:0003677 GO:0005727
Cluster-22178.51376 GO:0004252 GO:0016021
Cluster-22178.51377 GO:0007165 GO:0045502 GO:0008134 GO:0042803
GO:0005667 GO:0030286
Cluster-22178.51378 GO:0043401 GO:0006355 GO:0003700 GO:0008270
GO:0043565 GO:0005667 GO:0005634 GO:0016021
Cluster-22178.51384 GO:0007165 GO:0005158 GO:0005899
Cluster-22178.51385 GO:0007165 GO:0005509 GO:0005578
Cluster-22178.51388 GO:0006355 GO:0005198 GO:0003700 GO:0043565
GO:0019031 GO:0005667 GO:0019028
Cluster-22178.51395 GO:0006464 GO:0055114 GO:0008113

Cluster-22178.51398 GO:0016021 GO:0019012
Cluster-22178.51401 GO:0046907 GO:0006367 GO:0005672
Cluster-22178.51402 GO:0006367 GO:0046907 GO:0005672
Cluster-22178.51408 GO:0007165 GO:0005515 GO:0005509 GO:0005578
Cluster-22178.51412 GO:0016021
Cluster-22178.51414 GO:0007165 GO:0005509 GO:0005515 GO:0005578
Cluster-22178.51416 GO:0006310 GO:0006260 GO:0006281 GO:0003677
GO:0005634
Cluster-22178.51419 GO:0055114 GO:0005506 GO:0020037 GO:0016705

Cluster-22178.51420 GO:0006000 GO:0006013 GO:0007186 GO:0007165
GO:0007264 GO:0004871 GO:0005525 GO:0003924 GO:0005524
GO:0031683 GO:0019001 GO:0003873
Cluster-22178.51422 GO:0006457 GO:0007165 GO:0005509
Cluster-22178.51423 GO:0005515
Cluster-22178.51424 GO:0007264 GO:0006576 GO:0005525 GO:0005524

Cluster-22178.51429 GO:0006357 GO:0006810 GO:0007018 GO:0007017
GO:0003777 GO:0019871 GO:0001104 GO:0005874 GO:0030286
GO:0016592 GO:0005576 GO:0019028
Cluster-22178.51430 GO:0042981 GO:0006606 GO:0005515
Cluster-22178.51431 GO:0042254 GO:0006412 GO:0003735 GO:0005622
GO:0005840
Cluster-22178.51435 GO:0050832 GO:0008376 GO:0016757 GO:0016020
GO:0032580 GO:0005576
Cluster-22178.51441 GO:0005515
Cluster-22178.51443 GO:0006468 GO:0004672 GO:0005524
Cluster-22178.51444 GO:0005515
Cluster-22178.51449 GO:0005515
Cluster-22178.51451 GO:0005515
Cluster-22178.51454 GO:0006355 GO:0007155 GO:0043565 GO:0005515
GO:0003700 GO:0005667
Cluster-22178.51455 GO:0006825 GO:0007186 GO:0004930 GO:0016531

GO:0005507 GO:0005758 GO:0016021
Cluster-22178.51457 GO:0055114 GO:0005975 GO:0016491
Cluster-22178.51458 GO:0019069 GO:0015743 GO:0006570 GO:0006810
GO:0009072 GO:0042207 GO:0004334 GO:0005886
Cluster-22178.51465 GO:0006811 GO:0042254 GO:0006412 GO:0006816
GO:0005262 GO:0003735 GO:0005244 GO:0016021 GO:0005840
GO:0005622
Cluster-22178.51469 GO:0007165 GO:0006308 GO:0008855 GO:0009318
Cluster-22178.51470 GO:0006308 GO:0007165 GO:0008855 GO:0009318
Cluster-22178.51472 GO:0007165 GO:0019905 GO:0016021
Cluster-22178.51473 GO:0007165 GO:0006955 GO:0006281 GO:0006355
GO:0003677 GO:0016818 GO:0042803 GO:0005044 GO:0042162
GO:0030247 GO:0008270 GO:0005524 GO:0005634
Cluster-22178.51475 GO:0006486 GO:0006811 GO:0008378 GO:0032797
GO:0016020 GO:0016021
Cluster-22178.51476 GO:0006303 GO:0030328 GO:0006631 GO:0009069
GO:0016310 GO:0055114 GO:0016670 GO:0003677 GO:0071949
GO:0050151 GO:0004677 GO:0016491 GO:0005524 GO:0005634
GO:0005958
Cluster-22178.51477 GO:0009405 GO:0007186 GO:0004930 GO:0005515
GO:0016021 GO:0016020 GO:0005576
Cluster-22178.51479 GO:0006508 GO:0006412 GO:0042254 GO:0003735
GO:0004198 GO:0005509 GO:0005622 GO:0005840
Cluster-22178.51480 GO:0042254 GO:0006412 GO:0006508 GO:0004198
GO:0005509 GO:0003735 GO:0005840 GO:0005622
Cluster-22178.51491 GO:0006355 GO:0003677 GO:0003700 GO:0005507
GO:0005634 GO:0005667
Cluster-22178.51493 GO:0055085 GO:0009405 GO:0006811 GO:0005216
GO:0005576 GO:0016020 GO:0016021
Cluster-22178.51495 GO:0006811 GO:0055085 GO:0005216 GO:0016021
GO:0016020
Cluster-22178.51501 GO:0008134 GO:0042803 GO:0045502 GO:0005667
GO:0030286

Cluster-22178.51506 GO:0009058 GO:0000103 GO:0006144 GO:0006468
GO:0015940 GO:0015937 GO:0008152 GO:0016773 GO:0005524
GO:0005515 GO:0016301 GO:0003824 GO:0004020 GO:0004140
GO:0004672 GO:0016020
Cluster-22178.51508 GO:0006470 GO:0042981 GO:0006397 GO:0006355
GO:0004721 GO:0003700 GO:0005507 GO:0003677 GO:0005667
GO:0005634
Cluster-22178.51510 GO:0008152 GO:0008168 GO:0003723
Cluster-22178.51511 GO:0005515 GO:0005509 GO:0019028
Cluster-22178.51513 GO:0005198 GO:0051082 GO:0005882 GO:0005815
GO:0019031 GO:0019028 GO:0048786
Cluster-22178.51517 GO:0046872
Cluster-22178.51518 GO:0008270 GO:0005515
Cluster-22178.51521 GO:0061024 GO:0016020 GO:0030176
Cluster-22178.51522 GO:0061024 GO:0016020 GO:0030176
Cluster-22178.51523 GO:0030001 GO:0046872
Cluster-22178.51529 GO:0005515
Cluster-22178.51531 GO:0005975 GO:0006013 GO:0004553 GO:0004559
GO:0015923 GO:0008270
Cluster-22178.51532 GO:0016310 GO:0007165 GO:0009069 GO:0006468
GO:0004674 GO:0016773 GO:0004672 GO:0005524 GO:0008601
GO:0005515 GO:0003677 GO:0016787 GO:0000159 GO:0016020
Cluster-22178.51539 GO:0003824 GO:0005615
Cluster-22178.51543 GO:0006508 GO:0006511 GO:0016579 GO:0004843
GO:0005622
Cluster-22178.51544 GO:0015074 GO:0006313 GO:0003677
Cluster-22178.51548 GO:0006355 GO:0043565 GO:0003700 GO:0005667
Cluster-22178.51549 GO:0006508
Cluster-22178.51558 GO:0016192 GO:0006904 GO:0046983 GO:0008270

Cluster-22178.51561 GO:0006904 GO:0016192 GO:0008270 GO:0046983

Cluster-22178.51568 GO:0019904 GO:0005515 GO:0005737

Cluster-22178.51571 GO:0055114 GO:0007165 GO:0016817 GO:0033743 GO:0005179 GO:0005576

Cluster-22178.51572 GO:0006810 GO:0006479 GO:0046500 GO:0008152 GO:0006464 GO:0008757 GO:0042626 GO:0008168 GO:0005524 GO:0004719 GO:0016021

Cluster-22178.51575 GO:0055114 GO:0016616

Cluster-22178.51577 GO:0016021

Cluster-22178.51578 GO:0043087 GO:0035556 GO:0007186 GO:0007165 GO:0004871 GO:0005089 GO:0005737 GO:0005834

Cluster-22178.51580 GO:0007264 GO:0003924 GO:0005525 GO:0016021

Cluster-22178.51584 GO:0005515 GO:0016021

Cluster-22178.51586 GO:0006298 GO:0006887 GO:0030983 GO:0005198 GO:0005524 GO:0000145 GO:0019028 GO:0019031

Cluster-22178.51590 GO:0006811 GO:0055085 GO:0015031 GO:0006810 GO:0005215 GO:0008565 GO:0016021 GO:0016020

Cluster-22178.51591 GO:0015833 GO:0046872 GO:0005524 GO:0000166

Cluster-22178.51592 GO:0005515 GO:0016020 GO:0016021

Cluster-22178.51594 GO:0019058

Cluster-22178.51595 GO:0007218

Cluster-22178.51597 GO:0006281 GO:0006310 GO:0005737

Cluster-22178.51601 GO:0007264 GO:0007186 GO:0015684 GO:0007165 GO:0003924 GO:0031683 GO:0019001 GO:0015093 GO:0005102 GO:0004871 GO:0005525 GO:0016021

Cluster-22178.51612 GO:0003676 GO:0008168

Cluster-22178.51613 GO:0005515 GO:0016020

Cluster-22178.51619 GO:0003824

Cluster-22178.51623 GO:0007275

Cluster-22178.51624 GO:0006352 GO:0006355 GO:0006334 GO:0016987 GO:0003700 GO:0003677 GO:0005667 GO:0005634

Cluster-22178.51633 GO:0005515

Cluster-22178.51638 GO:0005515

Cluster-22178.51640 GO:0005515

Cluster-22178.51645 GO:0006260 GO:0004190 GO:0003677 GO:0016021
GO:0005664
Cluster-22178.51652 GO:0030001 GO:0006801 GO:0055114 GO:0046872

Cluster-22178.51654 GO:0003824
Cluster-22178.51656 GO:0007186 GO:0007166 GO:0005515 GO:0004930
GO:0016020 GO:0016021
Cluster-22178.51657 GO:0046938 GO:0006355 GO:0010038 GO:0003676
GO:0046872 GO:0016756 GO:0003677 GO:0003723
Cluster-22178.51658 GO:0005515 GO:0008270
Cluster-22178.51662 GO:0015986 GO:0006355 GO:0006367 GO:0007165
GO:0015992 GO:0005515 GO:0005509 GO:0046983 GO:0015078
GO:0005578 GO:0000276
Cluster-22178.51666 GO:0000077 GO:0006281 GO:0030896
Cluster-22178.51672 GO:0006810 GO:0032259 GO:0055114 GO:0005215
GO:0016972 GO:0008168 GO:0016020
Cluster-22178.51676 GO:0005975 GO:0043169 GO:0004553 GO:0003824

Cluster-22178.51678 GO:0009372 GO:0009405 GO:0008233 GO:0016021
Cluster-22178.51679 GO:0003677
Cluster-22178.51688 GO:0007165 GO:0005515
Cluster-22178.51691 GO:0007165 GO:0005515
Cluster-22178.51692 GO:0006355 GO:0003700 GO:0003676 GO:0008270
GO:0043565 GO:0005622 GO:0005667
Cluster-22178.51693 GO:0006308 GO:0008855 GO:0009318
Cluster-22178.51694 GO:0015743 GO:0045859 GO:0006308 GO:0004860
GO:0008855 GO:0009318 GO:0005576
Cluster-22178.51699 GO:0071704 GO:0005975 GO:0016868
Cluster-22178.51702 GO:0015979 GO:0009579 GO:0016021 GO:0009522
Cluster-22178.51705 GO:0006367 GO:0005672
Cluster-22178.51709 GO:0005515
Cluster-22178.51711 GO:0006144 GO:0006464 GO:0046034 GO:0004017
GO:0003677 GO:0046872 GO:0008270 GO:0016151

Cluster-22178.51723 GO:0007017 GO:0007018 GO:0030150 GO:0005524
GO:0008017 GO:0003777 GO:0005742 GO:0045298 GO:0005874
Cluster-22178.51724 GO:0051015
Cluster-22178.51726 GO:0042742 GO:0003779 GO:0005543 GO:0005576
Cluster-22178.51727 GO:0006508 GO:0007264 GO:0007186 GO:0007165
GO:0003924 GO:0005524 GO:0031683 GO:0019001 GO:0004871
GO:0005525 GO:0000166 GO:0003677 GO:0004197
Cluster-22178.51728 GO:0046872
Cluster-22178.51732 GO:0007264 GO:0005524 GO:0016887 GO:0003924
GO:0005525 GO:0016020
Cluster-22178.51735 GO:0006886 GO:0015031 GO:0008565 GO:0016740
GO:0016021 GO:0016020 GO:0005737
Cluster-22178.51737 GO:0007165 GO:0006468 GO:0004872 GO:0004672
GO:0005524 GO:0016021
Cluster-22178.51742 GO:0008608 GO:0072686 GO:0042729
Cluster-22178.51746 GO:0009052 GO:0006098 GO:0015976 GO:0004751
GO:0030246
Cluster-22178.51747 GO:0015940 GO:0015937 GO:0005524 GO:0004594

Cluster-22178.51753 GO:0007275 GO:0007165 GO:0016055 GO:0005102
GO:0005576
Cluster-22178.51754 GO:0006633 GO:0055114 GO:0016491 GO:0005506

Cluster-22178.51756 GO:0008152 GO:0006400 GO:0008616 GO:0003824
GO:0008479
Cluster-22178.51758 GO:0006118 GO:0006804 GO:0006979 GO:0055114
GO:0006749 GO:0004602 GO:0016655
Cluster-22178.51761 GO:0045444 GO:0006418 GO:0000166 GO:0004812
GO:0005524
Cluster-22178.51762 GO:0016559 GO:0005779
Cluster-22178.51763 GO:0015684 GO:0015093 GO:0005525 GO:0016021
Cluster-22178.51764 GO:0015684 GO:0015093 GO:0005525 GO:0016021
Cluster-22178.51768 GO:0006355 GO:0009607 GO:0003677 GO:0016021

Cluster-22178.51769 GO:0006355 GO:0009607 GO:0003677 GO:0005739
GO:0005747 GO:0016021
Cluster-22178.51770 GO:0006744 GO:0008270 GO:0046872 GO:0005515

Cluster-22178.51771 GO:0006744 GO:0005515 GO:0008270 GO:0046872

Cluster-22178.51772 GO:0016020 GO:0016021
Cluster-22178.51773 GO:0005515
Cluster-22178.51774 GO:0006289 GO:0043130 GO:0004003 GO:0000439
GO:0005657 GO:0005634
Cluster-22178.51777 GO:0016021
Cluster-22178.51778 GO:0016021
Cluster-22178.51790 GO:0048870 GO:0007049 GO:0006355 GO:0070206
GO:0006914 GO:0006357 GO:0006260 GO:0043565 GO:0003700
GO:0045502 GO:0042803 GO:0008134 GO:0001104 GO:0005198
GO:0019031 GO:0016021 GO:0019028 GO:0031514 GO:0030286
GO:0005667 GO:0005882 GO:0016592
Cluster-22178.51794 GO:0005509
Cluster-22178.51796 GO:0009236 GO:0015992 GO:0055114 GO:0006119
GO:0006118 GO:0016627 GO:0008121 GO:0051073 GO:0008818
GO:0003697 GO:0070188 GO:0005856
Cluster-22178.51798 GO:0055114 GO:0016972 GO:0016627 GO:0003697
GO:0070188 GO:0005856
Cluster-22178.51800 GO:0006635 GO:0006637 GO:0055114 GO:0003997
GO:0005777
Cluster-22178.51801 GO:0046740 GO:0016021
Cluster-22178.51803 GO:0055085 GO:0005741
Cluster-22178.51804 GO:0006629
Cluster-22178.51806 GO:0006886 GO:0008536 GO:0005515
Cluster-22178.51810 GO:0030145 GO:0004177 GO:0016021
Cluster-22178.51812 GO:0006367 GO:0005672
Cluster-22178.51815 GO:0007017 GO:0007018 GO:0005524 GO:0008017
GO:0003777 GO:0045298 GO:0005874

Cluster-22178.51821 GO:0007165 GO:0005856

Cluster-22178.51826 GO:0005515

Cluster-22178.51828 GO:0005515 GO:0016021

Cluster-22178.51829 GO:0005509

Cluster-22178.51830 GO:0005509

Cluster-22178.51831 GO:0005515

Cluster-22178.51832 GO:0006810 GO:0006816 GO:0042998 GO:0071253 GO:0005246 GO:0042030 GO:0016020 GO:0005802 GO:0019028 GO:0016021

Cluster-22178.51833 GO:0046872

Cluster-22178.51834 GO:0006629 GO:0005515 GO:0004222 GO:0008270 GO:0005524 GO:0016021

Cluster-22178.51843 GO:0006355 GO:0005634

Cluster-22178.51845 GO:0010508 GO:0008284 GO:0015991 GO:0007009 GO:0001819 GO:0006308 GO:0007264 GO:0008053 GO:0015992 GO:0010884 GO:0000166 GO:0005525 GO:0015078 GO:0003924 GO:0003677 GO:0005524 GO:0016887 GO:0008855 GO:0009318 GO:0033179 GO:0016021 GO:0005741

Cluster-22178.51846 GO:0007165 GO:0005179 GO:0005576

Cluster-22178.51847 GO:0006468 GO:0055085 GO:0004672 GO:0005524 GO:0016773 GO:0022857 GO:0016020 GO:0016021

Cluster-22178.51852 GO:0007017 GO:0006281 GO:0007018 GO:0006260 GO:0006310 GO:0003777 GO:0005515 GO:0003887 GO:0016887 GO:0005524 GO:0008017 GO:0016787 GO:0009378 GO:0016818 GO:0003677 GO:0045298 GO:0005874 GO:0005634 GO:0042575 GO:0009360 GO:0005657 GO:0009379

Cluster-22178.51853 GO:0006352 GO:0006355 GO:0007165 GO:0003700 GO:0016987 GO:0005179 GO:0003677 GO:0019031 GO:0005667 GO:0005576

Cluster-22178.51855 GO:0005985 GO:0005982 GO:0016157

Cluster-22178.51856 GO:0006397 GO:0008152 GO:0003723 GO:0016788 GO:0005515 GO:0005737 GO:0005634

Cluster-22178.51862 GO:0006275 GO:0045947 GO:0008283 GO:0040007

GO:0007165 GO:0045893 GO:0006260 GO:0003677 GO:0008190
GO:0008083 GO:0003688 GO:0046809 GO:0042025
Cluster-22178.51863 GO:0007264 GO:0003924 GO:0005525
Cluster-22178.51864 GO:0042967 GO:0008080 GO:0016747
Cluster-22178.51865 GO:0008152 GO:0055114 GO:0016491
Cluster-22178.51868 GO:0030683 GO:0055085 GO:0006355 GO:0022857
GO:0043565 GO:0016021 GO:0019012
Cluster-22178.51872 GO:0015979 GO:0005515 GO:0009522 GO:0009538
Cluster-22178.51873 GO:0016310 GO:0009069 GO:0006468 GO:0004674
GO:0004672 GO:0005524
Cluster-22178.51874 GO:0001510 GO:0006306 GO:0031167 GO:0008152
GO:0009452 GO:0008170 GO:0003677 GO:0005515 GO:0008168
GO:0005201 GO:0005578 GO:0019013 GO:0005581 GO:0016021
Cluster-22178.51875 GO:0006886 GO:0006888 GO:0008270 GO:0030127
Cluster-22178.51880 GO:0006144 GO:0003968 GO:0031379
Cluster-22178.51884 GO:0007165 GO:0008601 GO:0000159
Cluster-22178.51886 GO:0007165 GO:0008270 GO:0008601 GO:0000159
Cluster-22178.51887 GO:0007165 GO:0008270 GO:0008601 GO:0000159
Cluster-22178.51889 GO:0005515
Cluster-22178.51892 GO:0006570 GO:0030639 GO:0006470 GO:0009117
GO:0019872 GO:0008138 GO:0004725 GO:0008830 GO:0016021
Cluster-22178.51893 GO:0006508 GO:0008233
Cluster-22178.51894 GO:0001558 GO:0005520 GO:0005515 GO:0005576
GO:0016942
Cluster-22178.51895 GO:0055114 GO:0042157 GO:0006869 GO:0015694
GO:0008289 GO:0050660 GO:0015097 GO:0003677 GO:0016614
GO:0005576 GO:0016020
Cluster-22178.51899 GO:0006508 GO:0005524 GO:0008233 GO:0016021
Cluster-22178.51903 GO:0005515
Cluster-22178.51917 GO:0019439 GO:0016833
Cluster-22178.51920 GO:0046872
Cluster-22178.51925 GO:0006613 GO:0005784 GO:0030176
Cluster-22178.51926 GO:0046034 GO:0006144 GO:0006464 GO:0004017

GO:0016151 GO:0008270 GO:0046872
Cluster-22178.51929 GO:0007165 GO:0005515 GO:0008270
Cluster-22178.51937 GO:0000921 GO:0006355 GO:0008270 GO:0005524
GO:0045502 GO:0008134 GO:0042803 GO:0003677 GO:0016818
GO:0005940 GO:0030286 GO:0016021 GO:0005667 GO:0005634
Cluster-22178.51938 GO:0006810 GO:0016021
Cluster-22178.51940 GO:0042470
Cluster-22178.51950 GO:0006486 GO:0009097 GO:0009098 GO:0006418
GO:0009099 GO:0007155 GO:0009312 GO:0006438 GO:0000166
GO:0008375 GO:0008455 GO:0098599 GO:0003676 GO:0002161
GO:0003677 GO:0004812 GO:0016787 GO:0005524 GO:0004832
GO:0005795 GO:0016021 GO:0005737
Cluster-22178.51956 GO:0003677 GO:0008270
Cluster-22178.51957 GO:0003676
Cluster-22178.51961 GO:0007018 GO:0005515 GO:0005868
Cluster-22178.51963 GO:0006355 GO:0000245 GO:0006508 GO:0007165
GO:0008217 GO:0042312 GO:0005179 GO:0005515 GO:0008270
GO:0004222 GO:0005576 GO:0005634 GO:0031012
Cluster-22178.51965 GO:0005515 GO:0008270
Cluster-22178.51967 GO:0016567 GO:0005515 GO:0004842
Cluster-22178.51968 GO:0015991 GO:0015992 GO:0015078 GO:0033180
Cluster-22178.51969 GO:0055085 GO:0006821 GO:0006952 GO:0006810
GO:0009405 GO:0008270 GO:0019855 GO:0005247 GO:0005576
GO:0016020
Cluster-22178.51974 GO:0006310 GO:0006281 GO:0005515
Cluster-22178.51977 GO:0046872 GO:0035091 GO:0005515
Cluster-22178.51978 GO:0006810 GO:0042626
Cluster-22178.51980 GO:0006119 GO:0006118 GO:0000128 GO:0015992
GO:0008121 GO:0009055 GO:0008023
Cluster-22178.51981 GO:0016032 GO:0005096 GO:0019031
Cluster-22178.51983 GO:0006470 GO:0008138
Cluster-22178.51986 GO:0006810 GO:0019871 GO:0003676 GO:0005576
Cluster-22178.51987 GO:0006468 GO:0016773 GO:0005524 GO:0004672

GO:0016020
Cluster-22178.51990 GO:0005975 GO:0004553
Cluster-22178.51991 GO:0006812 GO:0006506 GO:0005515 GO:0008324
GO:0016746 GO:0016021 GO:0005789
Cluster-22178.51994 GO:0005543
Cluster-22178.51998 GO:0008643 GO:0018401 GO:0006525 GO:0015948
GO:0055114 GO:0046656 GO:0006355 GO:0006560 GO:0030269
GO:0005351 GO:0004656 GO:0043565 GO:0016702 GO:0003700
GO:0000139 GO:0005667 GO:0016020 GO:0005783 GO:0016021
Cluster-22178.51999 GO:0016310 GO:0009069 GO:0006468 GO:0004674
GO:0005524 GO:0004672
Cluster-22178.52006 GO:0006468 GO:0019911 GO:0004672 GO:0005524

Cluster-22178.52009 GO:0005515
Cluster-22178.52011 GO:0006276 GO:0006355 GO:0043565 GO:0003700
GO:0005515 GO:0005667 GO:0016020
Cluster-22178.52012 GO:0015992 GO:0006811 GO:0009405 GO:0006118
GO:0006119 GO:0009055 GO:0005216 GO:0008121 GO:0019904
GO:0005515 GO:0005737 GO:0005576
Cluster-22178.52015 GO:0006813 GO:0006687 GO:0009058 GO:0005242
GO:0008146 GO:0046872 GO:0001733 GO:0008076 GO:0005840
GO:0005794 GO:0016021
Cluster-22178.52016 GO:0006687 GO:0009058 GO:0008146 GO:0001733
GO:0016021 GO:0005794
Cluster-22178.52020 GO:0004386 GO:0003676 GO:0005524
Cluster-22178.52021 GO:0006810 GO:0005524 GO:0016887 GO:0003676
GO:0004386
Cluster-22178.52024 GO:0000902 GO:0030683 GO:0006955 GO:0007010
GO:0006811 GO:0005216 GO:0005515 GO:0003779 GO:0019012
GO:0016020
Cluster-22178.52026 GO:0046872
Cluster-22178.52027 GO:0019012 GO:0016021
Cluster-22178.52029 GO:0006810 GO:0016021

Cluster-22178.52031 GO:0006821 GO:0055085 GO:0005247 GO:0020037
GO:0016020
Cluster-22178.52032 GO:0009069 GO:0007186 GO:0007165 GO:0016310
GO:0005515 GO:0003924 GO:0031683 GO:0019001 GO:0016772
GO:0004871 GO:0005525 GO:0004674 GO:0016021
Cluster-22178.52033 GO:0016068 GO:0015031 GO:0006606 GO:0008565
GO:0005737 GO:0005634 GO:0016021
Cluster-22178.52034 GO:0051607 GO:0051258 GO:0030168 GO:0007165
GO:0005102 GO:0005525 GO:0030674 GO:0051082 GO:0005577
Cluster-22178.52036 GO:0005975 GO:0004560 GO:0003676 GO:0008270

Cluster-22178.52039 GO:0005515 GO:0016020
Cluster-22178.52040 GO:0022904 GO:0016651 GO:0005743
Cluster-22178.52042 GO:0000077
Cluster-22178.52044 GO:0007010 GO:0003779
Cluster-22178.52045 GO:0006213 GO:0006090 GO:0007155 GO:0006525
GO:0071436 GO:0007186 GO:0006814 GO:0006560 GO:0016817
GO:0015081 GO:0004930 GO:0016763 GO:0008948 GO:0016021
GO:0016020
Cluster-22178.52047 GO:0018149 GO:0042710 GO:0008270
Cluster-22178.52048 GO:0042710 GO:0018149 GO:0008270 GO:0019031
Cluster-22178.52049 GO:0042710 GO:0018149 GO:0008270 GO:0019031
Cluster-22178.52051 GO:0019058 GO:0007283 GO:0007165 GO:0008601
GO:0000159
Cluster-22178.52054 GO:0006812 GO:0007165 GO:0033198 GO:0098655
GO:0001614 GO:0004931 GO:0005887
Cluster-22178.52056 GO:0007165 GO:0006812 GO:0033198 GO:0098655
GO:0004931 GO:0001614 GO:0005887
Cluster-22178.52058 GO:0006355 GO:0003700 GO:0043565 GO:0005667
Cluster-22178.52060 GO:0022904 GO:0046983 GO:0016651 GO:0005743
Cluster-22178.52065 GO:0016567 GO:0008270 GO:0046872 GO:0004842
GO:0003723 GO:0005515 GO:0005887 GO:0005680
Cluster-22178.52067 GO:0007186 GO:0006260 GO:0005515 GO:0003887

GO:0004930 GO:0016021 GO:0042575

Cluster-22178.52069 GO:0007264 GO:0015684 GO:0015093 GO:0003924

GO:0005525 GO:0016021

Cluster-22178.52070 GO:0008152 GO:0006338 GO:0003824 GO:0005515

GO:0016836 GO:0090544

Cluster-22178.52073 GO:0000105 GO:0005201 GO:0005581 GO:0005578

Cluster-22178.52074 GO:0010024 GO:0055114 GO:0017022 GO:0003677

GO:0016636 GO:0046983 GO:0050897 GO:0016459

Cluster-22178.52078 GO:0008270 GO:0005515

Cluster-22178.52082 GO:0006810 GO:0007268 GO:0015893 GO:0007165

GO:0006811 GO:0006855 GO:0055085 GO:0015238 GO:0015297

GO:0004970 GO:0005234 GO:0016020

Cluster-22178.52089 GO:0016310 GO:0006468 GO:0009069 GO:0004674

GO:0000287 GO:0005524

Cluster-22178.52093 GO:0008615 GO:0055114 GO:0010181 GO:0004733

Cluster-22178.52094 GO:0015876 GO:0006810 GO:0055085 GO:0008521

GO:0005215 GO:0016021 GO:0016020

Cluster-22178.52095 GO:0015876 GO:0055085 GO:0006810 GO:0008521

GO:0005215 GO:0016021 GO:0016020

Cluster-22178.52097 GO:0005975 GO:0005515 GO:0004553

Cluster-22178.52104 GO:0001503 GO:0007155 GO:0005515 GO:0005524

GO:0005576

Cluster-22178.52105 GO:0005524 GO:0005515

Cluster-22178.52106 GO:0006355 GO:0007165 GO:0006605 GO:0007268

GO:0043495 GO:0005515 GO:0033130 GO:0043565 GO:0030695

Cluster-22178.52114 GO:0005515

Cluster-22178.52119 GO:0006438 GO:0009098 GO:0009097 GO:0009099

GO:0004832 GO:0005524 GO:0000166 GO:0005737

Cluster-22178.52128 GO:0007617 GO:0006355 GO:0008270 GO:0003700

GO:0005667 GO:0005634 GO:0005576

Cluster-22178.52129 GO:0006355 GO:0007617 GO:0003700 GO:0008270

GO:0005576 GO:0005667 GO:0005634

Cluster-22178.52137 GO:0006457 GO:0000413 GO:0003755
Cluster-22178.52142 GO:0019872 GO:0006021 GO:0008654 GO:0004512

Cluster-22178.52143 GO:0016055 GO:0007275 GO:0007165 GO:0005102
GO:0005515 GO:0005509 GO:0005576 GO:0045095
Cluster-22178.52144 GO:0048013 GO:0005515
Cluster-22178.52146 GO:0055085 GO:0006865 GO:0003333 GO:0006810
GO:0015171 GO:0016021 GO:0016020
Cluster-22178.52148 GO:0005515
Cluster-22178.52149 GO:0005544 GO:0005509
Cluster-22178.52158 GO:0031047 GO:0003723 GO:0019012 GO:0016021
Cluster-22178.52160 GO:0006310 GO:0000150 GO:0003677
Cluster-22178.52164 GO:0000077 GO:0071479 GO:0005515 GO:0005634
Cluster-22178.52168 GO:0000723 GO:0042254 GO:0006412 GO:0003677
GO:0003735 GO:0005622 GO:0000784 GO:0005840
Cluster-22178.52169 GO:0006355 GO:0007165 GO:0042974 GO:0005667
Cluster-22178.52170 GO:0007165 GO:0006281 GO:0006464 GO:0005179
GO:0016151 GO:0046872 GO:0008270 GO:0005576 GO:0005742
Cluster-22178.52172 GO:0015991 GO:0006351 GO:0055114 GO:0006090
GO:0006206 GO:0015992 GO:0015031 GO:0006144 GO:0006633
GO:0003899 GO:0015078 GO:0003677 GO:0003989 GO:0047134
GO:0033179 GO:0016021 GO:0005730 GO:0016020 GO:0009317
Cluster-22178.52173 GO:0006090 GO:0055114 GO:0006351 GO:0015991
GO:0006633 GO:0006144 GO:0006206 GO:0015992 GO:0015031
GO:0015078 GO:0003899 GO:0047134 GO:0003989 GO:0003677
GO:0016021 GO:0033179 GO:0009317 GO:0016020 GO:0005730
Cluster-22178.52177 GO:0055114 GO:0020037 GO:0016705 GO:0005506

Cluster-22178.52180 GO:0006810 GO:0042626 GO:0005524 GO:0016021
Cluster-22178.52181 GO:0016310 GO:0006468 GO:0009069 GO:0001558
GO:0005520 GO:0005524 GO:0004672 GO:0008270 GO:0004674
GO:0016942 GO:0005576 GO:0016020
Cluster-22178.52185 GO:0043565 GO:0005515 GO:0005634

Cluster-22178.52186 GO:0006955 GO:0007165 GO:0006355 GO:0035023
GO:0035556 GO:0055114 GO:0043087 GO:0005515 GO:0003677
GO:0003700 GO:0005089 GO:0047134 GO:0005125 GO:0005667
GO:0005576
Cluster-22178.52187 GO:0006333 GO:0005634
Cluster-22178.52190 GO:0055114 GO:0006479 GO:0006486 GO:0006629
GO:0006355 GO:0006554 GO:0007165 GO:0034968 GO:0005507
GO:0008375 GO:0018024 GO:0016641 GO:0003677 GO:0051920
GO:0005044 GO:0005622 GO:0016020 GO:0005763 GO:0005694
Cluster-22178.52193 GO:0055085 GO:0034314 GO:0030833 GO:0006821
GO:0006364 GO:0005247 GO:0015629 GO:0005730 GO:0016020
GO:0005885
Cluster-22178.52196 GO:0005975 GO:0004415 GO:0005515
Cluster-22178.52200 GO:0042742 GO:0006629 GO:0006505 GO:0006886
GO:0009058 GO:0016788 GO:0005576
Cluster-22178.52203 GO:0006505 GO:0042742 GO:0006508 GO:0006629
GO:0006886 GO:0009058 GO:0008236 GO:0016788 GO:0005576
Cluster-22178.52207 GO:0009069 GO:0006468 GO:0006479 GO:0006554
GO:0016310 GO:0035556 GO:0018024 GO:0017048 GO:0005524
GO:0004672 GO:0004674 GO:0005615
Cluster-22178.52209 GO:0006554 GO:0016310 GO:0035556 GO:0006468
GO:0009069 GO:0006479 GO:0005524 GO:0004672 GO:0004674
GO:0018024 GO:0017048 GO:0005615
Cluster-22178.52215 GO:0003676
Cluster-22178.52220 GO:0006259 GO:0000166 GO:0003677 GO:0003824
GO:0005524 GO:0005694
Cluster-22178.52221 GO:0008063
Cluster-22178.52223 GO:0015992 GO:0043066 GO:0015986 GO:0045766
GO:0016567 GO:0043123 GO:0043130 GO:0004842 GO:0015078
GO:0003723 GO:0000276
Cluster-22178.52225 GO:0002062 GO:0042246 GO:0035988 GO:0003676
GO:0005634
Cluster-22178.52232 GO:0007217

Cluster-22178.52233 GO:0006355 GO:0006810 GO:0055085 GO:0008270
GO:0003700 GO:0005215 GO:0005634 GO:0005667 GO:0016020
Cluster-22178.52234 GO:0003677
Cluster-22178.52238 GO:0006771 GO:0006000 GO:0015074 GO:0019497
GO:0006013 GO:0003873 GO:0005524 GO:0003993
Cluster-22178.52239 GO:0008152 GO:0016788
Cluster-22178.52240 GO:0007218 GO:0005576
Cluster-22178.52242 GO:0005515
Cluster-22178.52246 GO:0007186 GO:0005975 GO:0003723 GO:0016853
GO:0046983 GO:0004930 GO:0003676 GO:0016021
Cluster-22178.52248 GO:0016032 GO:0016887 GO:0005524 GO:0005525
GO:0019013
Cluster-22178.52251 GO:0005515
Cluster-22178.52255 GO:0043087 GO:0007264 GO:0007165 GO:0005085

Cluster-22178.52257 GO:0019076 GO:0006352 GO:0006355 GO:0006886
GO:0006505 GO:0046718 GO:0075521 GO:0007165 GO:0003700
GO:0098599 GO:0016788 GO:0016987 GO:0005179 GO:0016787
GO:0003677 GO:0019031 GO:0005576 GO:0019028 GO:0042025
GO:0005667 GO:0044163 GO:0030430
Cluster-22178.52258 GO:0019031
Cluster-22178.52259 GO:0005975 GO:0004571 GO:0005509 GO:0016020
Cluster-22178.52260 GO:0005524 GO:0016787 GO:0003677 GO:0008017
GO:0003676 GO:0045298
Cluster-22178.52261 GO:0051260 GO:0005515
Cluster-22178.52262 GO:0007186 GO:0007165 GO:0007264 GO:0004871
GO:0005525 GO:0003924 GO:0019001 GO:0031683
Cluster-22178.52263 GO:0009228 GO:0033617 GO:0004789 GO:0005739
Cluster-22178.52264 GO:0008152 GO:0055114 GO:0016616 GO:0005515
GO:0051287
Cluster-22178.52265 GO:0055114 GO:0008152 GO:0051287 GO:0016616

Cluster-22178.52266 GO:0005975 GO:0004553

Cluster-22178.52278 GO:0007411 GO:0000045 GO:0005737
Cluster-22178.52279 GO:0007264 GO:0005515 GO:0005525 GO:0005096

Cluster-22178.52284 GO:0055114 GO:0006006 GO:0006749 GO:0006098
GO:0004345 GO:0050661
Cluster-22178.52286 GO:0002682 GO:0030246
Cluster-22178.52288 GO:0016772 GO:0005524
Cluster-22178.52293 GO:0008152 GO:0055114 GO:0016491
Cluster-22178.52296 GO:0003723 GO:0005515
Cluster-22178.52297 GO:0019872 GO:0008654 GO:0006355 GO:0006021
GO:0043565 GO:0004512 GO:0003700 GO:0005667
Cluster-22178.52307 GO:0007264 GO:0005525
Cluster-22178.52309 GO:0000902
Cluster-22178.52316 GO:0007165 GO:0030155 GO:0055085 GO:0008053
GO:0030334 GO:0019064 GO:0030001 GO:0045995 GO:0006265
GO:0003677 GO:0046873 GO:0005102 GO:0003918 GO:0003917
GO:0046789 GO:0005198 GO:0003924 GO:0005741 GO:0016020
GO:0005694 GO:0009986 GO:0019028 GO:0019031 GO:0016021
Cluster-22178.52321 GO:0005515 GO:0003677
Cluster-22178.52322 GO:0005515 GO:0003677
Cluster-22178.52328 GO:0007155 GO:0042157 GO:0006869 GO:0006367
GO:0015684 GO:0051726 GO:0006281 GO:0009432 GO:0005524
GO:0015093 GO:0016887 GO:0003697 GO:0017025 GO:0005525
GO:0008289 GO:0009289 GO:0005576 GO:0005672 GO:0005634
GO:0016021
Cluster-22178.52329 GO:0006281 GO:0008270 GO:0046872 GO:0005815
Cluster-22178.52330 GO:0016310 GO:0009401 GO:0030474 GO:0071988
GO:0007165 GO:0009069 GO:0006468 GO:0004674 GO:0005096
GO:0008376 GO:0051082 GO:0005815 GO:0032580
Cluster-22178.52331 GO:0055085 GO:0006468 GO:0006810 GO:0006351
GO:0016773 GO:0005524 GO:0004672 GO:0005215 GO:0005515
GO:0016021 GO:0016020
Cluster-22178.52334 GO:0006888 GO:0006810 GO:0009405 GO:0006886

GO:0008270 GO:0005524 GO:0019855 GO:0003677 GO:0000166
GO:0005576 GO:0030127
Cluster-22178.52335 GO:0006508 GO:0008270 GO:0004222 GO:0031012
GO:0016020
Cluster-22178.52336 GO:0009069 GO:0016310 GO:0005515 GO:0004674

Cluster-22178.52339 GO:0005515
Cluster-22178.52342 GO:0055114 GO:0016491
Cluster-22178.52348 GO:0006260 GO:0006914 GO:0003887 GO:0001882
GO:0008092 GO:0042575 GO:0019898 GO:0005737
Cluster-22178.52349 GO:0055085 GO:0016021
Cluster-22178.52354 GO:0006281 GO:0006310 GO:0005515
Cluster-22178.52356 GO:0005515
Cluster-22178.52358 GO:0006508 GO:0046872 GO:0004222
Cluster-22178.52360 GO:0008283 GO:0040007 GO:0007165 GO:0008083
GO:0003676
Cluster-22178.52362 GO:0045227 GO:0004252 GO:0016020 GO:0016021
Cluster-22178.52364 GO:0035556 GO:0016055 GO:0008013 GO:0016342
Cluster-22178.52367 GO:0035091
Cluster-22178.52368 GO:0006355 GO:0008134 GO:0005524 GO:0044599
GO:0005667
Cluster-22178.52372 GO:0003676
Cluster-22178.52374 GO:0006355 GO:0005515
Cluster-22178.52375 GO:0043087 GO:0007264 GO:0005085
Cluster-22178.52376 GO:0006123 GO:0015992 GO:0005515 GO:0004129
GO:0045277
Cluster-22178.52385 GO:0055085 GO:0046872 GO:0008270 GO:0005515
GO:0016021
Cluster-22178.52387 GO:0006144 GO:0002098 GO:0034227 GO:0046034
GO:0006468 GO:0005524 GO:0008270 GO:0004017 GO:0003677
GO:0016787 GO:0003676 GO:0046872 GO:0004672 GO:0020037
GO:0000049 GO:0005634
Cluster-22178.52388 GO:0005634

Cluster-22178.52390 GO:0006825 GO:0035434 GO:0005375 GO:0016021
Cluster-22178.52392 GO:0008152 GO:0003824
Cluster-22178.52393 GO:0006123 GO:0000256 GO:0006807 GO:0006144
GO:0015992 GO:0004129 GO:0004037 GO:0019901 GO:0045277
Cluster-22178.52395 GO:0016020
Cluster-22178.52398 GO:0005975 GO:0071704 GO:0016868 GO:0005515

Cluster-22178.52404 GO:0006260 GO:0005198 GO:0003887 GO:0042575
GO:0019028
Cluster-22178.52405 GO:0016192 GO:0016021
Cluster-22178.52411 GO:0008033 GO:0007165 GO:0016879 GO:0005524
GO:0000166 GO:0005737
Cluster-22178.52431 GO:0008283 GO:0040007 GO:0007165 GO:0006955
GO:0005138 GO:0005125 GO:0005576 GO:0005896
Cluster-22178.52433 GO:0055114 GO:0008033 GO:0004499 GO:0016491
GO:0050661 GO:0050660
Cluster-22178.52434 GO:0006810 GO:0055085 GO:0016887 GO:0005524
GO:0042626 GO:0003676 GO:0005525 GO:0016021
Cluster-22178.52438 GO:0008270
Cluster-22178.52441 GO:0009252 GO:0046436 GO:0006541 GO:0016787
GO:0008716 GO:0046872 GO:0005524
Cluster-22178.52443 GO:0007155 GO:0042967 GO:0016747 GO:0005515
GO:0008080 GO:0016021
Cluster-22178.52445 GO:0006508 GO:0004197
Cluster-22178.52456 GO:0000917 GO:0015991 GO:0006308 GO:0007165
GO:0006810 GO:0043093 GO:0006606 GO:0008053 GO:0015992
GO:0042802 GO:0015078 GO:0003924 GO:0043015 GO:0008855
GO:0009318 GO:0033179 GO:0019028 GO:0016021 GO:0045298
GO:0005737 GO:0005741 GO:0005886
Cluster-22178.52458 GO:0006952 GO:0009058 GO:0043565 GO:0003824
GO:0009279 GO:0005634
Cluster-22178.52460 GO:0016579 GO:0043248 GO:0036459
Cluster-22178.52463 GO:0006355 GO:0003700 GO:0043565 GO:0005667

GO:0005634
Cluster-22178.52464 GO:0016747
Cluster-22178.52465 GO:0003682 GO:0005515 GO:0000785
Cluster-22178.52466 GO:0006769 GO:0006741 GO:0008152 GO:0046497
GO:0003951 GO:0016301
Cluster-22178.52471 GO:0005975 GO:0055114 GO:0016491
Cluster-22178.52472 GO:0055114 GO:0005975 GO:0016491
Cluster-22178.52476 GO:0004867
Cluster-22178.52482 GO:0006269 GO:0006351 GO:0006260 GO:0003896
GO:0005730 GO:0005657
Cluster-22178.52483 GO:0007166 GO:0016020
Cluster-22178.52485 GO:0015743
Cluster-22178.52486 GO:0006206 GO:0015743 GO:0006144 GO:0006351
GO:0003677 GO:0003899 GO:0005730
Cluster-22178.52495 GO:0005515
Cluster-22178.52499 GO:0008270 GO:0046872 GO:0003677 GO:0003676
GO:0005634
Cluster-22178.52500 GO:0051259 GO:0007034 GO:0006457 GO:0047869
GO:0000774 GO:0051087 GO:0042803
Cluster-22178.52502 GO:0006260 GO:0009231 GO:0003678 GO:0008686
GO:0005524 GO:0016887 GO:0005657 GO:0033588
Cluster-22178.52508 GO:0046999 GO:0009451 GO:0051536 GO:0051539
GO:0003824 GO:0019867
Cluster-22178.52510 GO:0007275 GO:0007219 GO:0016021
Cluster-22178.52511 GO:0006464
Cluster-22178.52513 GO:0007165
Cluster-22178.52514 GO:0046740 GO:0016021
Cluster-22178.52516 GO:0042967 GO:0009401 GO:0008080
Cluster-22178.52519 GO:0043066 GO:0035444 GO:0015675 GO:0045766
GO:0043123 GO:0004222 GO:0015099 GO:0046872 GO:0016021
Cluster-22178.52524 GO:0040007 GO:0008283 GO:0007165 GO:0008083
GO:0003676
Cluster-22178.52526 GO:0007154 GO:0006812 GO:0016887 GO:0016021

Cluster-22178.52534 GO:0015979 GO:0031175 GO:0008017 GO:0030507
GO:0005516 GO:0045298 GO:0008091 GO:0009523 GO:0009539
GO:0016020
Cluster-22178.52537 GO:0006144 GO:0009190 GO:0006171 GO:0035556
GO:0016849 GO:0004016 GO:0005886
Cluster-22178.52542 GO:0007264 GO:0005525
Cluster-22178.52544 GO:0016192 GO:0009306 GO:0016020
Cluster-22178.52545 GO:0006355 GO:0043565 GO:0046872
Cluster-22178.52546 GO:0006355 GO:0043565 GO:0046872
Cluster-22178.52547 GO:0046339 GO:0009395 GO:0016042 GO:0007021
GO:0010124 GO:0007275 GO:0046872 GO:0004435 GO:0015631
GO:0051082 GO:0005509 GO:0045298
Cluster-22178.52548 GO:0007275 GO:0007165 GO:0006886 GO:0016192
GO:0016055 GO:0015991 GO:0015992 GO:0005515 GO:0015078
GO:0005102 GO:0033179 GO:0005576 GO:0016021
Cluster-22178.52549 GO:0016032 GO:0006508 GO:0005975 GO:0042967
GO:0006555 GO:0019281 GO:0008899 GO:0004649 GO:0008270
GO:0008233 GO:0004222 GO:0005737 GO:0031012
Cluster-22178.52553 GO:0006979 GO:0055114 GO:0006804 GO:0006118
GO:0006749 GO:0016655 GO:0004602 GO:0071203
Cluster-22178.52558 GO:0007155 GO:0006368 GO:0007165 GO:0006406
GO:0006914 GO:0048193 GO:0006891 GO:0006606 GO:0042246
GO:0006308 GO:0008855 GO:0005515 GO:0017119 GO:0016020
GO:0000446 GO:0009318 GO:0016021
Cluster-22178.52559 GO:0016055
Cluster-22178.52560 GO:0042742 GO:0005515
Cluster-22178.52562 GO:0006508 GO:0006144 GO:0004197 GO:0017111
GO:0004252 GO:0003968 GO:0031379
Cluster-22178.52568 GO:0005975 GO:0005515 GO:0004415
Cluster-22178.52576 GO:0051028 GO:0006406 GO:0050765 GO:0006810
GO:0005515 GO:0003723 GO:0005622 GO:0005737 GO:0005634
Cluster-22178.52577 GO:0007165 GO:0006479 GO:0034968 GO:0019953
GO:0006554 GO:0005515 GO:0018024 GO:0008270 GO:0000772

GO:0005634 GO:0005576
Cluster-22178.52578 GO:0007009 GO:0005515 GO:0005737
Cluster-22178.52579 GO:0005515
Cluster-22178.52583 GO:0042254 GO:0006412 GO:0003735 GO:0005840
GO:0005622
Cluster-22178.52587 GO:0006094 GO:0006096 GO:0009094 GO:0006571
GO:0000162 GO:0000287 GO:0004634 GO:0000015
Cluster-22178.52588 GO:0009094 GO:0006571 GO:0000162 GO:0006094
GO:0006096 GO:0004634 GO:0000287 GO:0000015
Cluster-22178.52590 GO:0000162 GO:0006571 GO:0009094 GO:0006096
GO:0006094 GO:0004634 GO:0000287 GO:0000015
Cluster-22178.52592 GO:0006886 GO:0008536
Cluster-22178.52594 GO:0000398 GO:0005681
Cluster-22178.52597 GO:0031556 GO:0031554
Cluster-22178.52598 GO:0007165 GO:0019856 GO:0006955 GO:0034229
GO:0005164 GO:0034228 GO:0016020 GO:0016021
Cluster-22178.52601 GO:0000723 GO:0003697 GO:0003676
Cluster-22178.52604 GO:0007186 GO:0004930 GO:0003677 GO:0016021
Cluster-22178.52605 GO:0007186 GO:0004930 GO:0016021
Cluster-22178.52609 GO:0055036
Cluster-22178.52610 GO:0006725 GO:0016853 GO:0055036
Cluster-22178.52611 GO:0006725 GO:0016853 GO:0055036
Cluster-22178.52622 GO:0042802 GO:0043015 GO:0008017 GO:0045298
Cluster-22178.52624 GO:0019079 GO:0016192 GO:0006260 GO:0016787
GO:0003677 GO:0005515 GO:0005524 GO:0003678 GO:0003676
GO:0005657 GO:0016021
Cluster-22178.52625 GO:0005739
Cluster-22178.52631 GO:0006364
Cluster-22178.52636 GO:0016310 GO:0009069 GO:0006468 GO:0004674
GO:0005524 GO:0004672
Cluster-22178.52641 GO:0055085 GO:0016021
Cluster-22178.52644 GO:0006810 GO:0042626 GO:0005524 GO:0016021
Cluster-22178.52646 GO:0006869 GO:0005319 GO:0016831

Cluster-22178.52649 GO:0007264 GO:0005525
Cluster-22178.52651 GO:0006355 GO:0005622
Cluster-22178.52652 GO:0006886 GO:0016192 GO:0005515 GO:0008270
GO:0030117
Cluster-22178.52656 GO:0009405
Cluster-22178.52660 GO:0007165 GO:0008289
Cluster-22178.52670 GO:0051056 GO:0019048 GO:0006355 GO:0005515
GO:0003677 GO:0005096
Cluster-22178.52679 GO:0045454 GO:0005515
Cluster-22178.52681 GO:0006355 GO:0003700 GO:0005622 GO:0005667
Cluster-22178.52685 GO:0007339 GO:0006771 GO:0019497 GO:0005515
GO:0003993 GO:0046872 GO:0016020 GO:0005576
Cluster-22178.52686 GO:0019049 GO:0005515
Cluster-22178.52695 GO:0045454 GO:0006118 GO:0015035 GO:0009055

Cluster-22178.52708 GO:0030500 GO:0042742 GO:0046848 GO:0005576
Cluster-22178.52709 GO:0008171 GO:0005634
Cluster-22178.52711 GO:0008152 GO:0008168
Cluster-22178.52712 GO:0006355 GO:0007049 GO:0003700 GO:0005667
Cluster-22178.52715 GO:0006810 GO:0016567 GO:0008270 GO:0046872
GO:0004842 GO:0005515 GO:0005680 GO:0030288
Cluster-22178.52717 GO:0006810 GO:0016567 GO:0005515 GO:0046872
GO:0008270 GO:0004842 GO:0005680 GO:0030288
Cluster-22178.52718 GO:0006364 GO:0051252 GO:0006379 GO:0008033
GO:0003723 GO:0004540 GO:0030677 GO:0000172
Cluster-22178.52719 GO:0055114 GO:0016491 GO:0016209
Cluster-22178.52722 GO:0042254 GO:0006412 GO:0035556 GO:0005515
GO:0003735 GO:0015934 GO:0005840
Cluster-22178.52723 GO:0046872
Cluster-22178.52724 GO:0071705 GO:0003676 GO:0045095
Cluster-22178.52725 GO:0071705 GO:0003676 GO:0004190 GO:0016021
GO:0045095
Cluster-22178.52726 GO:0051016 GO:0008290

Cluster-22178.52728 GO:0009405 GO:0016567 GO:0004842 GO:0005515

Cluster-22178.52729 GO:0006468 GO:0004672 GO:0005524
Cluster-22178.52731 GO:0007165 GO:0006351 GO:0006260 GO:0006269
GO:0003896 GO:0005657 GO:0005730
Cluster-22178.52733 GO:0006576 GO:0005525 GO:0005524 GO:0003924

Cluster-22178.52736 GO:0032183 GO:0070140
Cluster-22178.52737 GO:0016579 GO:0032183 GO:0070140 GO:0036459

Cluster-22178.52738 GO:0042254 GO:0005515 GO:0005730 GO:0016020
GO:0016021
Cluster-22178.52740 GO:0055114 GO:0006357 GO:0008013 GO:0016702
GO:0001104 GO:0016342 GO:0016592
Cluster-22178.52742 GO:0035023 GO:0043087 GO:0005089 GO:0003677
GO:0034061 GO:0005634
Cluster-22178.52746 GO:0051301 GO:0005515 GO:0003677
Cluster-22178.52748 GO:0006090 GO:0008152 GO:0006099 GO:0005515
GO:0008924 GO:0016829 GO:0016021
Cluster-22178.52752 GO:0032402 GO:0042470
Cluster-22178.52755 GO:0003723 GO:0003676
Cluster-22178.52759 GO:0005515
Cluster-22178.52761 GO:0030168 GO:0006606 GO:0055085 GO:0006810
GO:0007165 GO:0006457 GO:0051258 GO:0007049 GO:0009405
GO:0010508 GO:0005102 GO:0030674 GO:0005215 GO:0051082
GO:0045502 GO:0042803 GO:0008134 GO:0005198 GO:0030286
GO:0016272 GO:0005577 GO:0005667 GO:0005882 GO:0016020
GO:0019031 GO:0005578 GO:0019028
Cluster-22178.52762 GO:0055085 GO:0006810 GO:0022857 GO:0005215
GO:0005525 GO:0003924 GO:0046872 GO:0005515 GO:0004045
GO:0016021 GO:0016020 GO:0016012
Cluster-22178.52763 GO:0005515
Cluster-22178.52764 GO:0015979 GO:0005515 GO:0009522

Cluster-22178.52766 GO:0000079 GO:0019901
Cluster-22178.52767 GO:0051382 GO:0006914 GO:0006606 GO:0000776
GO:0031083 GO:0016021
Cluster-22178.52770 GO:0005515
Cluster-22178.52776 GO:0019497 GO:0006771 GO:0016192 GO:0003993
GO:0016021 GO:0019031
Cluster-22178.52779 GO:0005576
Cluster-22178.52780 GO:0006480 GO:0006118 GO:0045454 GO:0009055
GO:0015035 GO:0005515 GO:0008168
Cluster-22178.52781 GO:0006468 GO:0004672 GO:0005524 GO:0008168
GO:0005515
Cluster-22178.52783 GO:0006468 GO:0005524 GO:0004672 GO:0016020
Cluster-22178.52784 GO:0003824
Cluster-22178.52785 GO:0003824 GO:0005515
Cluster-22178.52794 GO:0006508 GO:0004222 GO:0008270
Cluster-22178.52795 GO:0005515
Cluster-22178.52796 GO:0006353 GO:0016758
Cluster-22178.52798 GO:0006508 GO:0004222 GO:0004659 GO:0005515
GO:0008270 GO:0016021 GO:0031012
Cluster-22178.52801 GO:0006144 GO:0035556 GO:0008152 GO:0006171
GO:0009190 GO:0016849 GO:0004016 GO:0005886
Cluster-22178.52802 GO:0009190 GO:0035556 GO:0016849 GO:0042802

Cluster-22178.52807 GO:0006388 GO:0006351 GO:0051252 GO:0003714
GO:0000213 GO:0005667 GO:0000214
Cluster-22178.52813 GO:0046486 GO:0016042 GO:0003924 GO:0004806

Cluster-22178.52814 GO:0006355 GO:0003700 GO:0003924 GO:0042025
GO:0005667
Cluster-22178.52815 GO:0016579 GO:0036459
Cluster-22178.52817 GO:0008033 GO:0009451 GO:0046872 GO:0009020

Cluster-22178.52818 GO:0042254 GO:0006413 GO:0005515 GO:0005509

GO:0005622
Cluster-22178.52822 GO:0006367 GO:0000398 GO:0005681 GO:0005672
Cluster-22178.52823 GO:0006118 GO:0019836 GO:0009055 GO:0009512
GO:0005576
Cluster-22178.52829 GO:0008152 GO:0003824
Cluster-22178.52833 GO:0006400 GO:0046500 GO:0000154 GO:0006364
GO:0006479 GO:0009451 GO:0006464 GO:0008033 GO:0006396
GO:0008176 GO:0003723 GO:0004719 GO:0008168 GO:0008649
GO:0005737
Cluster-22178.52834 GO:0006464 GO:0008033 GO:0006396 GO:0006400
GO:0046500 GO:0000154 GO:0006479 GO:0006364 GO:0009451
GO:0004719 GO:0008168 GO:0008649 GO:0008176 GO:0003723
GO:0005737
Cluster-22178.52835 GO:0016310 GO:0007178 GO:0006468 GO:0009069
GO:0005524 GO:0004672 GO:0016773 GO:0004675 GO:0016020
Cluster-22178.52838 GO:0022904 GO:0003676 GO:0008017 GO:0045298
GO:0016020
Cluster-22178.52840 GO:0005515
Cluster-22178.52843 GO:0016757
Cluster-22178.52846 GO:0006355 GO:0005524 GO:0003774 GO:0008134
GO:0016301 GO:0005515 GO:0016459 GO:0005667
Cluster-22178.52848 GO:0055114 GO:0006118 GO:0016627 GO:0003995

Cluster-22178.52850 GO:0006355 GO:0005524 GO:0003774 GO:0005515
GO:0005856 GO:0016459 GO:0009289
Cluster-22178.52858 GO:0046008 GO:0007165 GO:0005179 GO:0005576
Cluster-22178.52859 GO:0005515
Cluster-22178.52862 GO:0005515 GO:0005509
Cluster-22178.52863 GO:0042254 GO:0006412 GO:0046872 GO:0003735
GO:0005622 GO:0005840
Cluster-22178.52864 GO:0042549 GO:0015979 GO:0051205 GO:0016772
GO:0016301 GO:0009523 GO:0009539 GO:0016021
Cluster-22178.52865 GO:0006352 GO:0006355 GO:0006270 GO:0003700

GO:0016987 GO:0003677 GO:0019031 GO:0005667

Cluster-22178.52871 GO:0007165 GO:0005125 GO:0016020

Cluster-22178.52875 GO:0006784 GO:0055114 GO:0016627 GO:0016021

Cluster-22178.52877 GO:0006303 GO:0005515

Cluster-22178.52880 GO:0046332 GO:0016817

Cluster-22178.52883 GO:0007156 GO:0005509 GO:0005515 GO:0016020

Cluster-22178.52884 GO:0016788

Cluster-22178.52886 GO:0007224 GO:0016310 GO:0042967 GO:0006468
GO:0009069 GO:0005524 GO:0008270 GO:0004674 GO:0008080

Cluster-22178.52887 GO:0009069 GO:0006468 GO:0007224 GO:0016310
GO:0005524 GO:0008270 GO:0004674

Cluster-22178.52890 GO:0006355 GO:0003700 GO:0003677 GO:0043565
GO:0005634 GO:0005667

Cluster-22178.52893 GO:0055085 GO:0006508 GO:0022857 GO:0016805
GO:0016021

Cluster-22178.52896 GO:0006355 GO:0003700 GO:0005667 GO:0042025

Cluster-22178.52901 GO:0006259 GO:0005524 GO:0005515 GO:0003824
GO:0003677 GO:0005694

Cluster-22178.52902 GO:0006813 GO:0005242 GO:0003677 GO:0008076
GO:0016021

Cluster-22178.52903 GO:0042246 GO:0007155 GO:0005515 GO:0016021

Cluster-22178.52905 GO:0006468 GO:0004672 GO:0005524

Cluster-22178.52910 GO:0006812 GO:0032801 GO:0019076 GO:0005515
GO:0005261 GO:0008270 GO:0033644

Cluster-22178.52913 GO:0019076 GO:0006812 GO:0032801 GO:0008270
GO:0005261 GO:0005515 GO:0033644

Cluster-22178.52915 GO:0032801 GO:0006812 GO:0019076 GO:0005515
GO:0008270 GO:0005261 GO:0033644

Cluster-22178.52922 GO:0006771 GO:0019497 GO:0046872 GO:0003993
GO:0005515 GO:0016021

Cluster-22178.52926 GO:0015992 GO:0006886 GO:0051301 GO:0007067
GO:0000090 GO:0007165 GO:0006810 GO:0030168 GO:0031110
GO:0006891 GO:0015991 GO:0006351 GO:0051382 GO:0051258

GO:0007059 GO:0007049 GO:0008017 GO:0005198 GO:0015078
GO:0030674 GO:0005102 GO:0005783 GO:0005876 GO:0005577
GO:0000776 GO:0005634 GO:0045298 GO:0017119 GO:0017053
GO:0005886 GO:0000940 GO:0033179 GO:0016021
Cluster-22178.52929 GO:0031966
Cluster-22178.52931 GO:0046872
Cluster-22178.52936 GO:0006955
Cluster-22178.52939 GO:0006891 GO:0006355 GO:0006118 GO:0043565
GO:0015035 GO:0003700 GO:0019901 GO:0005515 GO:0017119
GO:0005667
Cluster-22178.52941 GO:0042981 GO:0008270
Cluster-22178.52943 GO:0006418 GO:0005524 GO:0004812 GO:0005515
GO:0000166
Cluster-22178.52944 GO:0006355 GO:0003700 GO:0005667
Cluster-22178.52946 GO:0006355 GO:0003700 GO:0005667
Cluster-22178.52947 GO:0046903 GO:0006807 GO:0009405 GO:0016151
GO:0019867
Cluster-22178.52948 GO:0005515 GO:0005509
Cluster-22178.52951 GO:0048193 GO:0006914 GO:0010508 GO:0016192
GO:0006886 GO:0007155 GO:0005515 GO:0005801 GO:0016021
GO:0009288 GO:0016020
Cluster-22178.52952 GO:0007264 GO:0005525
Cluster-22178.52958 GO:0042381 GO:0042742
Cluster-22178.52959 GO:0016310 GO:0000160 GO:0005509 GO:0000155
GO:0046983 GO:0009365 GO:0016021
Cluster-22178.52969 GO:0001510 GO:0006479 GO:0006306 GO:0031167
GO:0009452 GO:0046500 GO:0006810 GO:0006464 GO:0032259
GO:0005515 GO:0019871 GO:0046872 GO:0004719 GO:0008168
GO:0008170 GO:0003677 GO:0008270 GO:0005576
Cluster-22178.52971 GO:0006812 GO:0006885 GO:0055085 GO:0007165
GO:0015299 GO:0005179 GO:0016021 GO:0005576
Cluster-22178.52974 GO:0046872
Cluster-22178.52976 GO:0042254 GO:0006412 GO:0003735 GO:0005622

GO:0005840
Cluster-22178.52986 GO:0005515 GO:0000228
Cluster-22178.52989 GO:0051016 GO:0042254 GO:0006412 GO:0030414
GO:0003735 GO:0005515 GO:0005509 GO:0030479 GO:0005840
Cluster-22178.52991 GO:0005515
Cluster-22178.52992 GO:0017025
Cluster-22178.52996 GO:0007165 GO:0006811 GO:0007268 GO:0004970
GO:0016020
Cluster-22178.52998 GO:0015991 GO:0006810 GO:0009405 GO:0008200
GO:0016820 GO:0005515 GO:0005576 GO:0000221
Cluster-22178.52999 GO:0006886 GO:0042720 GO:0005737
Cluster-22178.53000 GO:0005515 GO:0016021
Cluster-22178.53003 GO:0006508 GO:0016787 GO:0008236
Cluster-22178.53006 GO:0015992 GO:0005975 GO:0006260 GO:0008152
GO:0006123 GO:0016624 GO:0005524 GO:0004649 GO:0003689
GO:0004129 GO:0005663 GO:0042575 GO:0045277
Cluster-22178.53007 GO:0006470 GO:0006754 GO:0006570 GO:0005524
GO:0016887 GO:0004725 GO:0008138 GO:0005515 GO:0000275
Cluster-22178.53009 GO:0006470 GO:0006570 GO:0006754 GO:0004725
GO:0016887 GO:0005524 GO:0005515 GO:0008138 GO:0000275
Cluster-22178.53016 GO:0005515
Cluster-22178.53020 GO:0006470 GO:0006570 GO:0004725 GO:0008138
GO:0005515 GO:0016021
Cluster-22178.53021 GO:0006084 GO:0006550 GO:0046950 GO:0008299
GO:0006552 GO:0006574 GO:0006633 GO:0042967 GO:0004315
GO:0004421 GO:0005835
Cluster-22178.53028 GO:0007275 GO:0003700 GO:0005667 GO:0005634
Cluster-22178.5303 GO:0030170 GO:0008483
Cluster-22178.53035 GO:0005634
Cluster-22178.53039 GO:0016757
Cluster-22178.53044 GO:0006508 GO:0004197 GO:0008234
Cluster-22178.53046 GO:0046872
Cluster-22178.53048 GO:0046872

Cluster-22178.53049 GO:0005634
Cluster-22178.53052 GO:0042720
Cluster-22178.53055 GO:0048870 GO:0009058 GO:0016787 GO:0046872
GO:0016788 GO:0031514
Cluster-22178.53056 GO:0016787
Cluster-22178.53059 GO:0016021
Cluster-22178.53064 GO:0030259 GO:0005975 GO:0005515 GO:0016758

Cluster-22178.53065 GO:0006468 GO:0004672 GO:0005524
Cluster-22178.53068 GO:0005515
Cluster-22178.53076 GO:0009395 GO:0046486 GO:0008654 GO:0005524
GO:0003676 GO:0004143 GO:0016020
Cluster-22178.53077 GO:0000226 GO:0090063 GO:0016192 GO:0051090
GO:0006886 GO:0005515 GO:0005815 GO:0000922 GO:0030117
GO:0005634
Cluster-22178.53083 GO:0051726 GO:0000736 GO:0030414 GO:0046872
GO:0070336 GO:0003677 GO:0005634
Cluster-22178.53086 GO:0006468 GO:0006479 GO:0006554 GO:0008152
GO:0018024 GO:0005515 GO:0004672 GO:0005524 GO:0008168
Cluster-22178.53088 GO:0051603 GO:0006511 GO:0004175 GO:0004298
GO:0005839 GO:0019773
Cluster-22178.53095 GO:0006508 GO:0008236
Cluster-22178.53097 GO:0015996 GO:0015994 GO:0047746
Cluster-22178.53102 GO:0055114 GO:0004800 GO:0016021
Cluster-22178.53103 GO:0004252 GO:0016021
Cluster-22178.53109 GO:0050909 GO:0006566 GO:0006563 GO:0006778
GO:0006783 GO:0006812 GO:0006544 GO:0042967 GO:0003870
GO:0030170 GO:0005515 GO:0008324 GO:0005759 GO:0016021
Cluster-22178.53110 GO:0005515
Cluster-22178.53111 GO:0016020
Cluster-22178.53117 GO:0009069 GO:0006468 GO:0006886 GO:0007165
GO:0006888 GO:0007178 GO:0035556 GO:0016310 GO:0003723
GO:0005515 GO:0004675 GO:0005524 GO:0008270 GO:0016020

GO:0030127
Cluster-22178.53118 GO:0007275 GO:0006040 GO:0009435 GO:0009607
GO:0006807 GO:0000105 GO:0006355 GO:0006051 GO:0008033
GO:0055114 GO:0006537 GO:0046497 GO:0007219 GO:0008152
GO:0016638 GO:0018580 GO:0015930 GO:0016491 GO:0050660
GO:0004514 GO:0047465 GO:0016627 GO:0016829 GO:0003824
GO:0017150 GO:0005737 GO:0016021
Cluster-22178.53120 GO:0006355 GO:0006817 GO:0006810 GO:0019087
GO:0055085 GO:0000978 GO:0000982 GO:0005315 GO:0005634
GO:0016020 GO:0016021
Cluster-22178.53124 GO:0006260 GO:0005515 GO:0003677 GO:0003887
GO:0016021 GO:0042575 GO:0009360
Cluster-22178.53128 GO:0055085 GO:0006355 GO:0055114 GO:0003700
GO:0016491 GO:0005741 GO:0005667
Cluster-22178.53129 GO:0007156 GO:0005509 GO:0016020
Cluster-22178.53130 GO:0055114 GO:0042128 GO:0016491 GO:0030151

Cluster-22178.53131 GO:0005515
Cluster-22178.53133 GO:0046872
Cluster-22178.53138 GO:0006355 GO:0005634
Cluster-22178.53140 GO:0006508 GO:0019079 GO:0004190 GO:0003678
GO:0005657 GO:0016020
Cluster-22178.53147 GO:0016310 GO:0009069 GO:0006468 GO:0005524
GO:0004672 GO:0016773 GO:0004674 GO:0016020
Cluster-22178.53150 GO:0006355 GO:0016787 GO:0003677 GO:0003700
GO:0005507 GO:0005634 GO:0005667
Cluster-22178.53152 GO:0016787 GO:0003725 GO:0008270 GO:0003676

Cluster-22178.53154 GO:0007264 GO:0007186 GO:0015684 GO:0007165
GO:0006888 GO:0003924 GO:0031683 GO:0019001 GO:0015093
GO:0004871 GO:0005525 GO:0019033 GO:0005622 GO:0005801
GO:0016021
Cluster-22178.53155 GO:0006888 GO:0007264 GO:0015684 GO:0007165

GO:0007186 GO:0015093 GO:0019001 GO:0031683 GO:0003924
GO:0005525 GO:0004871 GO:0005622 GO:0016021 GO:0005801
GO:0019033
Cluster-22178.53162 GO:0006355 GO:0032324
Cluster-22178.53164 GO:0005515
Cluster-22178.53165 GO:0008270
Cluster-22178.53166 GO:0006886 GO:0016192 GO:0005515 GO:0030117
Cluster-22178.53168 GO:0006886 GO:0016192 GO:0005515 GO:0030117
Cluster-22178.53169 GO:0019478 GO:0016788 GO:0005737
Cluster-22178.53170 GO:0010468 GO:0005515 GO:0016021 GO:0005634
Cluster-22178.53173 GO:0003677 GO:0005515 GO:0003676 GO:0005507
GO:0005524 GO:0016787
Cluster-22178.53174 GO:0005515 GO:0004222
Cluster-22178.53175 GO:0042254 GO:0006412 GO:0007264 GO:0043248
GO:0005509 GO:0003735 GO:0005515 GO:0005525 GO:0005840
GO:0005622
Cluster-22178.53176 GO:0005515 GO:0008270 GO:0005921
Cluster-22178.53181 GO:0005525 GO:0003723
Cluster-22178.53182 GO:0005525 GO:0003723
Cluster-22178.53184 GO:0006468 GO:0009069 GO:0007178 GO:0016310
GO:0004675 GO:0016773 GO:0005524 GO:0004672 GO:0016020
Cluster-22178.53185 GO:0006886 GO:0042720 GO:0005737
Cluster-22178.53193 GO:0016032 GO:0005524
Cluster-22178.53194 GO:0009069 GO:0006260 GO:0015031 GO:0015992
GO:0006914 GO:0006886 GO:0006468 GO:0015991 GO:0016310
GO:0004674 GO:0005524 GO:0008565 GO:0005515 GO:0003723
GO:0015078 GO:0016020 GO:0033179 GO:0005737
Cluster-22178.53195 GO:0016310 GO:0015991 GO:0006468 GO:0006886
GO:0006914 GO:0015031 GO:0015992 GO:0006260 GO:0009069
GO:0015078 GO:0005515 GO:0008565 GO:0005524 GO:0004674
GO:0005737 GO:0033179 GO:0016020
Cluster-22178.53196 GO:0009069 GO:0006914 GO:0015031 GO:0015992
GO:0006260 GO:0015991 GO:0006468 GO:0006886 GO:0016310

GO:0008565 GO:0005524 GO:0004674 GO:0005515 GO:0015078
GO:0016020 GO:0033179 GO:0005737
Cluster-22178.53198 GO:0005515
Cluster-22178.53199 GO:0000917 GO:0006355 GO:0043093 GO:0003677
GO:0003700 GO:0043565 GO:0005634 GO:0005667 GO:0005737
Cluster-22178.53211 GO:0006378 GO:0016192 GO:0016787 GO:0003729
GO:0005849 GO:0016021
Cluster-22178.53213 GO:0006313 GO:0006378 GO:0003677 GO:0004803
GO:0016787 GO:0003729 GO:0005849
Cluster-22178.53217 GO:0007186 GO:0009405 GO:0005515 GO:0004930
GO:0005576 GO:0016021
Cluster-22178.53219 GO:0006508 GO:0004185
Cluster-22178.53223 GO:0015979 GO:0015693 GO:0030436 GO:0006508
GO:0015684 GO:0006952 GO:0015093 GO:0004190 GO:0015095
GO:0009523 GO:0009539 GO:0016021 GO:0005576 GO:0016020
Cluster-22178.53228 GO:0006396 GO:0003723 GO:0003676
Cluster-22178.53236 GO:0015031 GO:0042147 GO:0008565 GO:0030904
Cluster-22178.53238 GO:0016192 GO:0006886 GO:0015031 GO:0008565
GO:0030117
Cluster-22178.53239 GO:0015743 GO:0003774 GO:0005524 GO:0005515
GO:0016459
Cluster-22178.53242 GO:0006144 GO:0015992 GO:0006206 GO:0006260
GO:0006270 GO:0055114 GO:0016032 GO:0006119 GO:0006118
GO:0006351 GO:0003677 GO:0003899 GO:0008121 GO:0003887
GO:0005730 GO:0032040 GO:0042575
Cluster-22178.53243 GO:0001514 GO:0006448 GO:0005525 GO:0003723
GO:0003746 GO:0005840 GO:0005737
Cluster-22178.53245 GO:0006281 GO:0006310
Cluster-22178.53248 GO:0006468 GO:0006412 GO:0055114 GO:0042254
GO:0007165 GO:0035556 GO:0005515 GO:0016788 GO:0005057
GO:0004672 GO:0003735 GO:0005524 GO:0008270 GO:0047134
GO:0005840 GO:0005622
Cluster-22178.53251 GO:0006144 GO:0046034 GO:0003723 GO:0046872

GO:0004017 GO:0003676
Cluster-22178.53255 GO:0006869 GO:0042157 GO:0055114 GO:0050662
GO:0008289 GO:0016491 GO:0031083 GO:0005576
Cluster-22178.53258 GO:0006355 GO:0043401 GO:0043565 GO:0046872
GO:0008270 GO:0003700 GO:0005667 GO:0005634
Cluster-22178.53259 GO:0046486 GO:0006313 GO:0006468 GO:0006629
GO:0016042 GO:0006260 GO:0015074 GO:0005515 GO:0003677
GO:0003887 GO:0004806 GO:0005524 GO:0004672 GO:0042575
Cluster-22178.53267 GO:0016787 GO:0046872 GO:0005198 GO:0003676
GO:0019028 GO:0005622
Cluster-22178.53271 GO:0009405 GO:0003676 GO:0005576
Cluster-22178.53274 GO:0007264 GO:0005096 GO:0005525
Cluster-22178.53277 GO:0006886 GO:0006605 GO:0005742
Cluster-22178.53279 GO:0007607 GO:0007154 GO:0050912 GO:0007187
GO:0005515 GO:0008527 GO:0005509 GO:0016021 GO:0016020
Cluster-22178.53282 GO:0010508
Cluster-22178.53284 GO:0006355 GO:0016817
Cluster-22178.53288 GO:0006486 GO:0008378 GO:0016020
Cluster-22178.53289 GO:0030414 GO:0005515
Cluster-22178.53290 GO:0006486 GO:0016757 GO:0008378 GO:0016020
Cluster-22178.53294 GO:0003677
Cluster-22178.53295 GO:0008270 GO:0046872
Cluster-22178.53296 GO:0006811 GO:0055085 GO:0015075 GO:0016020
Cluster-22178.53297 GO:0000271 GO:0007186 GO:0005515 GO:0004930
GO:0016021 GO:0016020
Cluster-22178.53301 GO:0015992 GO:0006120 GO:0006744 GO:2001256
GO:0006814 GO:0042254 GO:0006412 GO:0042773 GO:0003735
GO:0008137 GO:0005622 GO:0005840 GO:0005743 GO:0030176
Cluster-22178.53304 GO:0003677
Cluster-22178.53309 GO:0006260 GO:0006351 GO:0055085 GO:0003677
GO:0003887 GO:0042575 GO:0016021
Cluster-22178.53310 GO:0019028
Cluster-22178.53314 GO:0003723 GO:0005524 GO:0003724 GO:0003677

GO:0000166 GO:0005525
Cluster-22178.53316 GO:0006471 GO:0019087 GO:0003956 GO:0016021
Cluster-22178.53326 GO:0055114 GO:0004800
Cluster-22178.53327 GO:0019058 GO:0015992 GO:0006914 GO:0015991
GO:0006139 GO:0006308 GO:0008270 GO:0008855 GO:0005515
GO:0051082 GO:0015078 GO:0008168 GO:0055036 GO:0033644
GO:0009318 GO:0033179 GO:0019031
Cluster-22178.53328 GO:0033925 GO:0005737
Cluster-22178.53335 GO:0016787
Cluster-22178.53338 GO:0046983
Cluster-22178.53339 GO:0019079 GO:0003678 GO:0005657
Cluster-22178.53343 GO:0006468 GO:0004672 GO:0005524 GO:0016020
Cluster-22178.53344 GO:0043087 GO:0035023 GO:0035556 GO:0005089
GO:0005515 GO:0005576
Cluster-22178.53348 GO:0016740
Cluster-22178.53350 GO:0005515
Cluster-22178.53351 GO:0007264 GO:0005525
Cluster-22178.53352 GO:0009401 GO:0055085 GO:0006812 GO:0008324
GO:0016020 GO:0016021
Cluster-22178.53354 GO:0009401 GO:0055085 GO:0006812 GO:0008324
GO:0016021 GO:0016020
Cluster-22178.53355 GO:0051302
Cluster-22178.53358 GO:0006206 GO:0008152 GO:0006230 GO:0006629
GO:0006508 GO:0042254 GO:0006412 GO:0005524 GO:0004797
GO:0016787 GO:0003735 GO:0008236 GO:0005840 GO:0016020
Cluster-22178.53360 GO:0043565 GO:0005515 GO:0005634 GO:0005615
Cluster-22178.53363 GO:0006465 GO:0019087 GO:0008233 GO:0008270
GO:0016021 GO:0005787
Cluster-22178.53367 GO:0046983 GO:0005515
Cluster-22178.53368 GO:0006836 GO:0055114 GO:0008152 GO:0006118
GO:0008218 GO:0016491 GO:0019905 GO:0003995 GO:0016021
Cluster-22178.53371 GO:0006508 GO:0045947 GO:0045765 GO:0006144
GO:0008190 GO:0003968 GO:0008234 GO:0005737 GO:0031379

Cluster-22178.53372 GO:0016020
Cluster-22178.53373 GO:0005739
Cluster-22178.53374 GO:0008270
Cluster-22178.53375 GO:0008270
Cluster-22178.53378 GO:0022904 GO:0003676 GO:0008017 GO:0045298 GO:0016020
Cluster-22178.53379 GO:0006323 GO:0003677 GO:0005515 GO:0019012
Cluster-22178.53383 GO:0017004 GO:0015886 GO:0006914 GO:0007411 GO:0006260 GO:0015232 GO:0005198 GO:0008092 GO:0019028 GO:0016020 GO:0005737 GO:0019898 GO:0019031
Cluster-22178.53384 GO:0006886 GO:0016020
Cluster-22178.53385 GO:0006308 GO:0006948 GO:0006886 GO:0008855 GO:0005615 GO:0005634 GO:0009318 GO:0016020
Cluster-22178.53386 GO:0016567 GO:0042167 GO:0072519 GO:0015994 GO:0055114 GO:0003677 GO:0004842 GO:0004074 GO:0008270 GO:0005634
Cluster-22178.53389 GO:0006811 GO:0055085 GO:0051056 GO:0007268 GO:0007165 GO:0009968 GO:0009089 GO:0005096 GO:0004970 GO:0008837 GO:0005216 GO:0005737 GO:0016020
Cluster-22178.53395 GO:0003676
Cluster-22178.53397 GO:0051252 GO:0006402 GO:0004535 GO:0046872 GO:0003723 GO:0005634 GO:0005737
Cluster-22178.53399 GO:0005515
Cluster-22178.53401 GO:0019634 GO:0016567 GO:0016579 GO:0036459 GO:0004842 GO:0005680
Cluster-22178.53408 GO:0003723 GO:0003676 GO:0046935 GO:0005944 GO:0016021
Cluster-22178.53410 GO:0005887
Cluster-22178.53416 GO:0006886 GO:0016192 GO:0005515 GO:0030117
Cluster-22178.53417 GO:0005515
Cluster-22178.53419 GO:0007010 GO:0006813 GO:0030955 GO:0005249 GO:0003779 GO:0008076 GO:0016020 GO:0019033 GO:0016021
Cluster-22178.53423 GO:0043065 GO:0005740 GO:0016021

Cluster-22178.53425 GO:0019064 GO:0006914 GO:0007155 GO:0006457
GO:0006810 GO:0071988 GO:0006308 GO:0006886 GO:0030474
GO:0008855 GO:0000774 GO:0051087 GO:0003676 GO:0042803
GO:0005783 GO:0009288 GO:0016021 GO:0019031 GO:0009318
Cluster-22178.53429 GO:0006807 GO:0003824 GO:0003723 GO:0046914

Cluster-22178.53430 GO:0006468 GO:0016773 GO:0005524 GO:0004672
GO:0016020
Cluster-22178.53431 GO:0006468 GO:0004672 GO:0005524 GO:0016773
GO:0016020
Cluster-22178.53436 GO:0005515
Cluster-22178.53438 GO:0017038 GO:0003676 GO:0008270 GO:0005524
GO:0005515 GO:0003677 GO:0016787 GO:0003723 GO:0016020
Cluster-22178.53439 GO:0070588 GO:0006816 GO:0005515 GO:0005245
GO:0005891
Cluster-22178.53442 GO:0006032 GO:0008061 GO:0005869
Cluster-22178.53444 GO:0006952 GO:0006810 GO:0009405 GO:0019855
GO:0005576
Cluster-22178.53448 GO:0006396 GO:0006364 GO:0000154 GO:0008649
GO:0005737
Cluster-22178.53449 GO:0035556
Cluster-22178.53450 GO:0006886 GO:0003924 GO:0005525 GO:0005047
GO:0005786 GO:0005785
Cluster-22178.53452 GO:0007165 GO:0040007 GO:0008283 GO:0008083
GO:0005615
Cluster-22178.53454 GO:0005515
Cluster-22178.53455 GO:0005515
Cluster-22178.53458 GO:0008152
Cluster-22178.53459 GO:0055114 GO:0006694 GO:0006813 GO:0008210
GO:0008207 GO:0008209 GO:0003824 GO:0050662 GO:0003854
GO:0016616
Cluster-22178.53461 GO:0008152
Cluster-22178.53462 GO:0009405 GO:0006810 GO:0019871 GO:0005576

Cluster-22178.53463 GO:0008210 GO:0006813 GO:0008209 GO:0008207
GO:0055114 GO:0006694 GO:0050662 GO:0003854 GO:0016616
GO:0003824
Cluster-22178.53465 GO:0009405 GO:0009372 GO:0008233 GO:0016021
Cluster-22178.53467 GO:0016021
Cluster-22178.53468 GO:0006810 GO:0003333 GO:0006865 GO:0055085
GO:0015171 GO:0016020 GO:0016021
Cluster-22178.53469 GO:0006865 GO:0055085 GO:0006810 GO:0003333
GO:0015171 GO:0016020
Cluster-22178.53470 GO:0032324 GO:0006468 GO:0005524 GO:0004672

Cluster-22178.53471 GO:0005515
Cluster-22178.53473 GO:0043039 GO:0006531 GO:0016055 GO:0006522
GO:0006419 GO:0000166 GO:0003676 GO:0005524 GO:0016876
GO:0004813
Cluster-22178.53475 GO:0055085 GO:0005515 GO:0016021
Cluster-22178.53476 GO:0046872 GO:0005515 GO:0016020
Cluster-22178.53478 GO:0042254 GO:0006310 GO:0006810 GO:0009069
GO:0002949 GO:0006206 GO:0006412 GO:0006281 GO:0006139
GO:0016310 GO:0003735 GO:0098519 GO:0009378 GO:0003724
GO:0004127 GO:0004674 GO:0016887 GO:0005524 GO:0016301
GO:0003723 GO:0005525 GO:0003924 GO:0009379 GO:0005657
GO:0005622 GO:0005840
Cluster-22178.53482 GO:0016310 GO:0006139 GO:0006281 GO:0006412
GO:0006206 GO:0002949 GO:0009069 GO:0006810 GO:0042254
GO:0006310 GO:0003924 GO:0005525 GO:0003723 GO:0016301
GO:0005524 GO:0016887 GO:0004674 GO:0003724 GO:0004127
GO:0009378 GO:0098519 GO:0003735 GO:0005840 GO:0005622
GO:0005657 GO:0009379
Cluster-22178.53483 GO:0006281 GO:0006412 GO:0016310 GO:0006139
GO:0009069 GO:0006810 GO:0042254 GO:0006310 GO:0006206
GO:0002949 GO:0003723 GO:0016301 GO:0003924 GO:0005525
GO:0009378 GO:0098519 GO:0003735 GO:0005524 GO:0016887

GO:0004674 GO:0003724 GO:0004127 GO:0005840 GO:0009379
GO:0005622 GO:0005657
Cluster-22178.53484 GO:0007155 GO:0042157 GO:0009405 GO:0006869
GO:0008289 GO:0005576 GO:0016020
Cluster-22178.53487 GO:0046497 GO:0006012 GO:0009267 GO:0046854
GO:0006769 GO:0005982 GO:0005985 GO:0004564 GO:0004575
GO:0003953 GO:0016021 GO:0016020 GO:0019033 GO:0016012
GO:0017177
Cluster-22178.53495 GO:0006777 GO:0007165 GO:0005515 GO:0003779

Cluster-22178.53501 GO:0051607 GO:0016560 GO:0009405 GO:0005096
GO:0005515 GO:0005777 GO:0016021
Cluster-22178.53503 GO:0005515
Cluster-22178.53507 GO:0005515 GO:0046872 GO:0008270
Cluster-22178.53508 GO:0005515 GO:0046872 GO:0008270
Cluster-22178.53509 GO:0006281 GO:0006310 GO:0009378 GO:0003677
GO:0005524 GO:0043565 GO:0009379 GO:0005657
Cluster-22178.53510 GO:0042157 GO:0006869 GO:0005515 GO:0008289
GO:0005576
Cluster-22178.53511 GO:0016763 GO:0005515
Cluster-22178.53513 GO:0046872 GO:0008144 GO:0003676
Cluster-22178.53514 GO:0009507
Cluster-22178.53517 GO:0009968 GO:0007165 GO:0007268 GO:0051056
GO:0055085 GO:0006811 GO:0005216 GO:0004970 GO:0005096
GO:0016020 GO:0005737
Cluster-22178.53518 GO:0007268 GO:0007165 GO:0009968 GO:0006811
GO:0055085 GO:0051056 GO:0005216 GO:0005096 GO:0004970
GO:0016020 GO:0005737
Cluster-22178.53519 GO:0006811 GO:0051056 GO:0055085 GO:0007268
GO:0007165 GO:0009968 GO:0004970 GO:0005096 GO:0005216
GO:0005737 GO:0016020
Cluster-22178.53521 GO:0046872 GO:0005515
Cluster-22178.53525 GO:0008408 GO:0000166 GO:0003677

Cluster-22178.53528 GO:0008033 GO:0055114 GO:0033151 GO:0046872 GO:0043565 GO:0050660 GO:0017150
Cluster-22178.53533 GO:0008152 GO:0016787 GO:0005198 GO:0019028 GO:0016021
Cluster-22178.53534 GO:0006355 GO:0043565 GO:0003677 GO:0003700 GO:0005667 GO:0005634
Cluster-22178.53535 GO:0008152 GO:0003824 GO:0003677
Cluster-22178.53536 GO:0046872
Cluster-22178.53537 GO:0046872
Cluster-22178.53540 GO:0006935 GO:0000723 GO:0019048 GO:0050920 GO:0000160 GO:0006281 GO:0007017 GO:0009405 GO:0007018 GO:0008270 GO:0005524 GO:0008017 GO:0000156 GO:0003777 GO:0003824 GO:0003678 GO:0008984 GO:0005515 GO:0005657 GO:0016020 GO:0009288 GO:0005737 GO:0019031 GO:0005874 GO:0045298
Cluster-22178.53543 GO:0019497 GO:0006771 GO:0046872 GO:0008270 GO:0003993 GO:0051082 GO:0005515 GO:0005622
Cluster-22178.53546 GO:0005198 GO:0019028
Cluster-22178.53548 GO:0006355 GO:0005515 GO:0046872
Cluster-22178.53549 GO:0006109 GO:0009058 GO:0008033 GO:0006694 GO:0016310 GO:0016114 GO:0000160 GO:0000155 GO:0003723 GO:0004161 GO:0005524 GO:0004672 GO:0016887 GO:0003724 GO:0009365
Cluster-22178.53555 GO:0055114 GO:0006308 GO:0006574 GO:0006568 GO:0006355 GO:0006552 GO:0006550 GO:0006554 GO:0006633 GO:0006631 GO:0018874 GO:0003857 GO:0016491 GO:0008855 GO:0003677 GO:0009318
Cluster-22178.53559 GO:0007076 GO:0000796
Cluster-22178.53560 GO:0007275 GO:0006570 GO:0006355 GO:0006470 GO:0007219 GO:0030154 GO:0008138 GO:0005515 GO:0004725 GO:0003700 GO:0005667 GO:0016021
Cluster-22178.53561 GO:0032012 GO:0006952 GO:0043087 GO:0005086 GO:0005576

Cluster-22178.53563 GO:0007165 GO:0030414 GO:0005509 GO:0005578
Cluster-22178.53564 GO:0051321 GO:0055114 GO:0009401 GO:0018401
GO:0006525 GO:0006560 GO:0016491 GO:0016702 GO:0003677
GO:0004656 GO:0005634 GO:0005783
Cluster-22178.53565 GO:0007283
Cluster-22178.53567 GO:0008152 GO:0003824 GO:0016817
Cluster-22178.53568 GO:0031554 GO:0006355 GO:0031556 GO:0003677

Cluster-22178.53572 GO:0006281 GO:0006260 GO:0006310 GO:0003677
GO:0003910
Cluster-22178.53573 GO:0006310 GO:0006260 GO:0006281 GO:0003910
GO:0003677
Cluster-22178.53574 GO:0006313 GO:0035438 GO:0003677 GO:0046872

Cluster-22178.53575 GO:0016020
Cluster-22178.53578 GO:0005515 GO:0043565 GO:0005634
Cluster-22178.53582 GO:0007165 GO:0016055 GO:0006508 GO:0070008
GO:0004252 GO:0005515 GO:0005044 GO:0005179 GO:0016020
GO:0005576
Cluster-22178.53583 GO:0007165 GO:0016055 GO:0006508 GO:0004252
GO:0005515 GO:0005179 GO:0005044 GO:0070008 GO:0016020
GO:0005576
Cluster-22178.53584 GO:0009405 GO:0015631 GO:0005576 GO:0045298
Cluster-22178.53595 GO:0008152 GO:0003824
Cluster-22178.53596 GO:0008270 GO:0046872 GO:0003676
Cluster-22178.53597 GO:0044237 GO:0006810 GO:0010038 GO:0007049
GO:0006357 GO:0005515 GO:0001104 GO:0005215 GO:0016592
GO:0016021
Cluster-22178.53599 GO:0042967 GO:0008080 GO:0004252 GO:0016592
GO:0005886
Cluster-22178.53600 GO:0006508 GO:0005509 GO:0005515 GO:0004252
GO:0005576
Cluster-22178.53601 GO:0006508 GO:0005515 GO:0004252 GO:0005509

GO:0005576 GO:0019013

Cluster-22178.53603 GO:0035444 GO:0015675 GO:0015099 GO:0046872 GO:0016021

Cluster-22178.53604 GO:0006144 GO:0009116 GO:0030154 GO:0000105 GO:0006547 GO:0003824 GO:0004114 GO:0004635 GO:0016021

Cluster-22178.53609 GO:0006886 GO:0006281 GO:0006888 GO:0055114 GO:0006464 GO:0003677 GO:0016972 GO:0016151 GO:0008270 GO:0046872 GO:0030127

Cluster-22178.53611 GO:0016021

Cluster-22178.53614 GO:0009451 GO:0008033 GO:0009020 GO:0046872

Cluster-22178.53619 GO:0016787

Cluster-22178.53621 GO:0043087 GO:0035556 GO:0007264 GO:0009966 GO:0007165 GO:0007275 GO:0005085 GO:0005509 GO:0005578 GO:0016020

Cluster-22178.53626 GO:0006270 GO:0016055 GO:0006811 GO:0007411 GO:0006534 GO:0006423 GO:0000166 GO:0004817 GO:0005515 GO:0005524 GO:0016020 GO:0005576 GO:0005737

Cluster-22178.53628 GO:0006355 GO:0003700 GO:0043565 GO:0005667

Cluster-22178.53631 GO:0042254 GO:0006412 GO:0003735 GO:0005840 GO:0005622

Cluster-22178.53635 GO:0006355 GO:0006886 GO:0005515 GO:0005622

Cluster-22178.53636 GO:0055114 GO:0016714

Cluster-22178.53639 GO:0007165 GO:0043401 GO:0006355 GO:0003677 GO:0030284 GO:0005496 GO:0005634

Cluster-22178.53642 GO:0015684 GO:0006576 GO:0007264 GO:0005525 GO:0003924 GO:0005524 GO:0015093 GO:0016021

Cluster-22178.53644 GO:0016032 GO:0016973 GO:0006508 GO:0004197 GO:0005643 GO:0016021

Cluster-22178.53646 GO:0030178 GO:0007165 GO:0007275 GO:0005515 GO:0035091 GO:0005576

Cluster-22178.53647 GO:0006886 GO:0016192 GO:0030131

Cluster-22178.53650 GO:0006355 GO:0006099 GO:0006479 GO:0006108

GO:0055114 GO:0015976 GO:0006090 GO:0034968 GO:0022904
GO:0006554 GO:0018024 GO:0004471 GO:0046872 GO:0051287
GO:0016651 GO:0005743 GO:0005694
Cluster-22178.53651 GO:0006525 GO:0006560 GO:0006511 GO:0006433
GO:0006777 GO:0000166 GO:0051539 GO:0005524 GO:0051536
GO:0004175 GO:0004827 GO:0003824 GO:0019773 GO:0019008
GO:0005737
Cluster-22178.53654 GO:0006260 GO:0005515
Cluster-22178.53655 GO:0007165
Cluster-22178.53659 GO:0005515
Cluster-22178.53660 GO:0016579 GO:0055114 GO:0036459 GO:0016491

Cluster-22178.53661 GO:0016579 GO:0008270 GO:0036459
Cluster-22178.53663 GO:0016579 GO:0036459
Cluster-22178.53672 GO:0006139 GO:0015940 GO:0008152 GO:0015937
GO:0007186 GO:0006614 GO:0006810 GO:0007165 GO:0006206
GO:0016301 GO:0004140 GO:0003924 GO:0031683 GO:0019001
GO:0004871 GO:0005525 GO:0005524 GO:0004127 GO:0042597
Cluster-22178.53677 GO:0016757
Cluster-22178.53678 GO:0006260 GO:0006486 GO:0006270 GO:0016757
GO:0046983 GO:0003980 GO:0042802 GO:0005515 GO:0003887
GO:0042575 GO:0005727
Cluster-22178.53681 GO:0030639 GO:0019872 GO:0009117 GO:0008830

Cluster-22178.53685 GO:0035556
Cluster-22178.53693 GO:0007304 GO:0008063 GO:0005213 GO:0005515
GO:0008270 GO:0005576 GO:0042600
Cluster-22178.53695 GO:0006820 GO:0006355 GO:0008509 GO:0003676
GO:0005622 GO:0016021
Cluster-22178.53698 GO:0006270 GO:0046935 GO:0032040 GO:0005944
Cluster-22178.53701 GO:0019354 GO:0055114 GO:0006779 GO:0071949
GO:0005515 GO:0043115 GO:0016491 GO:0005576 GO:0016020
Cluster-22178.53702 GO:0055114 GO:0006779 GO:0019354 GO:0016491

GO:0071949 GO:0043115 GO:0005515 GO:0005576 GO:0016020
Cluster-22178.53706 GO:0006355
Cluster-22178.53707 GO:0015031 GO:0006904 GO:0007165 GO:0005515
GO:0000145
Cluster-22178.53711 GO:0009245 GO:0016747
Cluster-22178.53712 GO:0046872
Cluster-22178.53713 GO:0050853 GO:0050852 GO:0007165 GO:0007264
GO:0006813 GO:0005525 GO:0003924 GO:0015269 GO:0016020
Cluster-22178.53714 GO:0016114 GO:0006355 GO:0006468 GO:0055085
GO:0009058 GO:0042432 GO:0003700 GO:0004672 GO:0005515
GO:0016773 GO:0016844 GO:0005524 GO:0004064 GO:0016021
GO:0016020 GO:0005667
Cluster-22178.53715 GO:0006355 GO:0003700 GO:0005667
Cluster-22178.53718 GO:0016787 GO:0016020
Cluster-22178.53719 GO:0033925 GO:0005737
Cluster-22178.53723 GO:0006508 GO:0042254 GO:0006412 GO:0005509
GO:0004198 GO:0003735 GO:0005622 GO:0005840
Cluster-22178.53724 GO:0046983
Cluster-22178.53725 GO:0008270
Cluster-22178.53726 GO:0006096 GO:0006144 GO:0006357 GO:0000162
GO:0006094 GO:0006397 GO:0009405 GO:0009094 GO:0006571
GO:0004634 GO:0005524 GO:0003723 GO:0000287 GO:0001104
GO:0005198 GO:0003968 GO:0000015 GO:0016592 GO:0005634
GO:0019028 GO:0005576 GO:0005737 GO:0031379 GO:0019031
Cluster-22178.53727 GO:0015977 GO:0006351 GO:0006270 GO:0006099
GO:0006355 GO:0019643 GO:0006094 GO:0006260 GO:0008964
GO:0003677 GO:0003887 GO:0042575
Cluster-22178.53731 GO:0006123 GO:0030436 GO:0015992 GO:0009297
GO:0005515 GO:0004129 GO:0005524 GO:0003774 GO:0016459
GO:0019867 GO:0042601 GO:0045277
Cluster-22178.53738 GO:0006470 GO:0006402 GO:0051252 GO:0006468
GO:0003676 GO:0004672 GO:0005524 GO:0004535 GO:0046872
GO:0008138 GO:0003723 GO:0005737 GO:0005634

Cluster-22178.53740 GO:0008616 GO:0016740 GO:0016853
Cluster-22178.53744 GO:0005515
Cluster-22178.53749 GO:0007283 GO:0005515 GO:0003677 GO:0005634
GO:0000786
Cluster-22178.53751 GO:0055085 GO:0006885 GO:0006812 GO:0007165
GO:0015299 GO:0005179 GO:0016021 GO:0005576
Cluster-22178.53753 GO:0006904 GO:0006891 GO:0006887 GO:0015031
GO:0000145 GO:0017119
Cluster-22178.53761 GO:0007165 GO:0070008 GO:0005509 GO:0004252
GO:0005578
Cluster-22178.53763 GO:0015031 GO:0008277 GO:0006886 GO:0006313
GO:0008565 GO:0003676 GO:0004803 GO:0003677 GO:0016021
Cluster-22178.53765 GO:0006270 GO:0006275 GO:0005515 GO:0043565
GO:0005524
Cluster-22178.53766 GO:0007029 GO:0016021 GO:0005789
Cluster-22178.53768 GO:0006367 GO:0019069 GO:0004190 GO:0016021
GO:0005672
Cluster-22178.53769 GO:0046660 GO:0006397 GO:0005634
Cluster-22178.53770 GO:0016567 GO:0006457 GO:0055085 GO:0007034
GO:0030001 GO:0006308 GO:0046873 GO:0008855 GO:0031386
GO:0051082 GO:0003824 GO:0016881 GO:0016020 GO:0016272
GO:0009318 GO:0019028
Cluster-22178.53776 GO:0009405 GO:0006886 GO:0051258 GO:0006308
GO:0007155 GO:0007165 GO:0006869 GO:0006457 GO:0030168
GO:0006357 GO:0006606 GO:0051082 GO:0005198 GO:0001104
GO:0008289 GO:0004871 GO:0030674 GO:0008855 GO:0005102
GO:0005576 GO:0019028 GO:0009318 GO:0019031 GO:0016021
GO:0005801 GO:0016592 GO:0016020 GO:0016272 GO:0005577
Cluster-22178.53779 GO:0016567 GO:0005515 GO:0004842
Cluster-22178.53782 GO:0008152 GO:0016491
Cluster-22178.53787 GO:0009966 GO:0005515 GO:0005576 GO:0016021
Cluster-22178.53788 GO:0006470 GO:0050852 GO:0009231 GO:0009058
GO:0006570 GO:0006771 GO:0004725 GO:0003824 GO:0003919

Cluster-22178.53789 GO:0055114 GO:0020037 GO:0016705 GO:0005506

Cluster-22178.53790 GO:0016020
Cluster-22178.53792 GO:0010038 GO:0046938 GO:0046872 GO:0016756

Cluster-22178.53793 GO:0006355 GO:0006468 GO:0016567 GO:0007165
GO:0005515 GO:0004672 GO:0003682 GO:0003677 GO:0005524
GO:0008270 GO:0004842 GO:0005634 GO:0019028 GO:0005737
GO:0000785 GO:0000775 GO:0016020 GO:0005680
Cluster-22178.53794 GO:0006355 GO:0005622
Cluster-22178.53798 GO:0008152 GO:0008610 GO:0016747
Cluster-22178.53804 GO:0055114 GO:0006952 GO:0008017 GO:0047134
GO:0046872 GO:0005515 GO:0045298 GO:0005576
Cluster-22178.53805 GO:0007010 GO:0007186 GO:0004930 GO:0003779
GO:0016021
Cluster-22178.53806 GO:0046872
Cluster-22178.53809 GO:0006555 GO:0030494 GO:0006556 GO:0019685
GO:0004478 GO:0016836
Cluster-22178.53814 GO:0055085 GO:0030001 GO:0005975 GO:0016758
GO:0046873 GO:0016020
Cluster-22178.53819 GO:0006198 GO:0004115
Cluster-22178.53821 GO:0007165 GO:0006955 GO:0006457 GO:0005164
GO:0016020
Cluster-22178.53822 GO:0006457 GO:0006955 GO:0007165 GO:0005164
GO:0016020
Cluster-22178.53823 GO:0006576 GO:0006614 GO:0002949 GO:0016787
GO:0003677 GO:0016887 GO:0003924 GO:0005524 GO:0003676
GO:0005525
Cluster-22178.53826 GO:0008270
Cluster-22178.53829 GO:0006119 GO:0006118 GO:0015992 GO:0055114
GO:0008121 GO:0051537 GO:0016491 GO:0051536
Cluster-22178.53835 GO:0007165 GO:0035023 GO:0043087 GO:0005515
GO:0005089

Cluster-22178.53838 GO:0006355 GO:0006730 GO:0006032 GO:0008061
GO:0005515 GO:0008134 GO:0005524 GO:0008168 GO:0005576
GO:0005667 GO:0009279
Cluster-22178.53839 GO:0006355 GO:0009405 GO:0006914 GO:0006308
GO:0007049 GO:0003677 GO:0008855 GO:0019865 GO:0043565
GO:0003700 GO:0005634 GO:0005667 GO:0009318 GO:0019814
GO:0016021
Cluster-22178.53840 GO:0016567 GO:0008270 GO:0046872 GO:0004842
GO:0005515 GO:0005680
Cluster-22178.53841 GO:0005639
Cluster-22178.53842 GO:0007217
Cluster-22178.53845 GO:0005515
Cluster-22178.53848 GO:0006367 GO:0005515 GO:0005672
Cluster-22178.53852 GO:0005515
Cluster-22178.53853 GO:0043248 GO:0004252 GO:0016021
Cluster-22178.53859 GO:0006355 GO:0007165 GO:0043248 GO:0035257
GO:0003713 GO:0005515 GO:0046983 GO:0005667 GO:0005634
Cluster-22178.53860 GO:0045454 GO:0006729 GO:0008124
Cluster-22178.53861 GO:0005515 GO:0005516
Cluster-22178.53865 GO:0006355 GO:0009271
Cluster-22178.53871 GO:0008643 GO:0005351 GO:0000139 GO:0016021
Cluster-22178.53872 GO:0046872 GO:0008270
Cluster-22178.53873 GO:0008061 GO:0005515
Cluster-22178.53874 GO:0008061 GO:0005515
Cluster-22178.53875 GO:0005515
Cluster-22178.53879 GO:0055114 GO:0006779 GO:0019354 GO:0051287
GO:0008641 GO:0043115
Cluster-22178.53883 GO:0005515
Cluster-22178.53885 GO:0008270 GO:0005515 GO:0005921
Cluster-22178.53887 GO:0051262 GO:0015991 GO:0015992 GO:0015078
GO:0033179
Cluster-22178.53888 GO:0005515
Cluster-22178.53890 GO:0006352 GO:0051382 GO:0006366 GO:0006281

GO:0003677 GO:0005669
Cluster-22178.53898 GO:0060478 GO:0007342 GO:0006897 GO:0005886
Cluster-22178.53902 GO:0006355 GO:0003700 GO:0043565 GO:0008270
GO:0005667 GO:0005634
Cluster-22178.53904 GO:0006955 GO:0016021
Cluster-22178.53905 GO:0019882 GO:0006955 GO:0042613 GO:0016020
Cluster-22178.53906 GO:0006955 GO:0005201 GO:0005581 GO:0005578
Cluster-22178.53910 GO:0016579 GO:0036459
Cluster-22178.53911 GO:0046740 GO:0046872 GO:0008270 GO:0005515
GO:0016021
Cluster-22178.53915 GO:0016021
Cluster-22178.53916 GO:0016021
Cluster-22178.53920 GO:0005515
Cluster-22178.53931 GO:0009069 GO:0016310 GO:0016192 GO:0006886
GO:0004674 GO:0005515 GO:0030117
Cluster-22178.53932 GO:0045947 GO:0008190
Cluster-22178.53934 GO:0006812 GO:0008324
Cluster-22178.53943 GO:0006260 GO:0006351 GO:0006886 GO:0016192
GO:0008536 GO:0003887 GO:0003677 GO:0005515 GO:0042575
GO:0005576 GO:0030117
Cluster-22178.53950 GO:0042254 GO:0006412 GO:0006508 GO:0004197
GO:0003735 GO:0005840 GO:0005622
Cluster-22178.53954 GO:0016310 GO:0006139 GO:0006281 GO:0006412
GO:0006206 GO:0002949 GO:0009069 GO:0006810 GO:0006310
GO:0042254 GO:0003924 GO:0005525 GO:0003723 GO:0016301
GO:0005524 GO:0016887 GO:0004674 GO:0004127 GO:0003724
GO:0009378 GO:0098519 GO:0003735 GO:0005840 GO:0005622
GO:0005657 GO:0009379
Cluster-22178.53957 GO:0055114 GO:0019530 GO:0006534 GO:0046439
GO:0016702 GO:0017172 GO:0005506
Cluster-22178.53958 GO:0042254 GO:0006412 GO:0003735 GO:0005840
GO:0005622
Cluster-22178.53960 GO:0006771 GO:0006260 GO:0019497 GO:0008408

GO:0000166 GO:0003887 GO:0003677 GO:0003993 GO:0042575

Cluster-22178.53962 GO:0006691 GO:0006749 GO:0019530 GO:0006281

GO:0006693 GO:0006289 GO:0006284 GO:0016787 GO:0003906

GO:0005516 GO:0003684 GO:0016799 GO:0008270 GO:0003840

Cluster-22178.53963 GO:0019530 GO:0006749 GO:0006281 GO:0006691

GO:0006693 GO:0006289 GO:0006284 GO:0005516 GO:0016787

GO:0003906 GO:0003684 GO:0016799 GO:0008270 GO:0003840

Cluster-22178.53964 GO:0006812 GO:0006836 GO:0005515 GO:0005328

GO:0016021

Cluster-22178.53966 GO:0007165 GO:0004872 GO:0005515 GO:0016021

Cluster-22178.53969 GO:0006118 GO:0055114 GO:0045454 GO:0015035

GO:0009055 GO:0016209 GO:0016491

Cluster-22178.53971 GO:0000390 GO:0071008

Cluster-22178.53972 GO:0006468 GO:0005524 GO:0004672

Cluster-22178.53973 GO:0006468 GO:0004672 GO:0005524

Cluster-22178.53975 GO:0051260 GO:0005515 GO:0016021

Cluster-22178.53981 GO:0006241 GO:0006206 GO:0006144 GO:0006183

GO:0006886 GO:0006228 GO:0006355 GO:0006165 GO:0006508

GO:0004222 GO:0008536 GO:0004550 GO:0005634

Cluster-22178.53983 GO:0006998 GO:0006406 GO:0006611 GO:0031965

GO:0005576

Cluster-22178.53988 GO:0006887 GO:0000145

Cluster-22178.53989 GO:0007186 GO:0004930 GO:0016021

Cluster-22178.53992 GO:0006810 GO:0006886 GO:0016192 GO:0055085

GO:0005215 GO:0022857 GO:0016020 GO:0030117 GO:0016021

Cluster-22178.53997 GO:0051252 GO:0006402 GO:0046872 GO:0004535

GO:0003723 GO:0005634 GO:0005737

Cluster-22178.53999 GO:0005515

Cluster-22178.54001 GO:0006144 GO:0019079 GO:0003968 GO:0031379

Cluster-22178.54003 GO:0006570 GO:0007165 GO:0019988 GO:0006470

GO:0005179 GO:0005515 GO:0043399 GO:0008138 GO:0016829

GO:0003676 GO:0004725 GO:0005576 GO:0009424

Cluster-22178.54006 GO:0006687 GO:0006953 GO:0050290 GO:0005576

Cluster-22178.54007 GO:0006355 GO:0006352 GO:0003700 GO:0003677
GO:0016987 GO:0005667
Cluster-22178.54014 GO:0007165 GO:0042710 GO:0040007 GO:0008283
GO:0005975 GO:0008083 GO:0051015 GO:0043169 GO:0030674
GO:0003824 GO:0003676 GO:0008270
Cluster-22178.54015 GO:0006481 GO:0006383 GO:0006468 GO:0006351
GO:0006479 GO:0006206 GO:0006144 GO:0004671 GO:0003677
GO:0003899 GO:0005524 GO:0004672 GO:0005730 GO:0005666
GO:0016021
Cluster-22178.54020 GO:0055114 GO:0006813 GO:0005524 GO:0005267
GO:0003677 GO:0020037 GO:0016705 GO:0005506 GO:0016787
GO:0016020
Cluster-22178.54021 GO:0055114 GO:0016620 GO:0016491 GO:0051287

Cluster-22178.54024 GO:0005515
Cluster-22178.54031 GO:0043039 GO:0003676 GO:0016876 GO:0005524
GO:0016021
Cluster-22178.54033 GO:0008270 GO:0016020
Cluster-22178.54034 GO:0007186 GO:0004930 GO:0005634 GO:0016021
Cluster-22178.54036 GO:0006355 GO:0043401 GO:0046872 GO:0008270
GO:0043565 GO:0003700 GO:0005634 GO:0005667
Cluster-22178.54037 GO:0009058 GO:0016114 GO:0042432 GO:0005515
GO:0005509 GO:0016844
Cluster-22178.54038 GO:0016114 GO:0009058 GO:0042432 GO:0005509
GO:0005515 GO:0016844 GO:0004064
Cluster-22178.54039 GO:0016114 GO:0009058 GO:0042432 GO:0005509
GO:0005515 GO:0016844
Cluster-22178.54041 GO:0006508 GO:0008152 GO:0005524 GO:0016787
GO:0003676 GO:0008236
Cluster-22178.54043 GO:0005515
Cluster-22178.54045 GO:0005515
Cluster-22178.54046 GO:0007034
Cluster-22178.54050 GO:0005515

Cluster-22178.54052 GO:0005515
Cluster-22178.54054 GO:0009103 GO:0042967 GO:0055085 GO:0008811
GO:0005515 GO:0016020 GO:0016021
Cluster-22178.54055 GO:0055085 GO:0016021 GO:0005576
Cluster-22178.54057 GO:0016567 GO:0046872 GO:0008270 GO:0004842
GO:0005515 GO:0061630 GO:0005622
Cluster-22178.54061 GO:0006260 GO:0042254 GO:0006412 GO:0005524
GO:0003735 GO:0005840 GO:0005622 GO:0000808 GO:0005634
Cluster-22178.54068 GO:0030001 GO:0055085 GO:0007186 GO:0046873
GO:0004930 GO:0016020 GO:0016021
Cluster-22178.54069 GO:0007165 GO:0019882 GO:0055085 GO:0007017
GO:0030001 GO:0006955 GO:0006886 GO:0007018 GO:0016787
GO:0042289 GO:0046873 GO:0004190 GO:0046872 GO:0003777
GO:0016020 GO:0030286 GO:0042613 GO:0005874
Cluster-22178.54071 GO:0016021 GO:0005887
Cluster-22178.54074 GO:0005515
Cluster-22178.54075 GO:0043531 GO:0016887 GO:0003924 GO:0005524
GO:0003724 GO:0035064 GO:0005525 GO:0004867 GO:0003723
GO:0005576
Cluster-22178.54076 GO:0031224
Cluster-22178.54077 GO:0007631 GO:0007218 GO:0005094 GO:0005737
Cluster-22178.54079 GO:0048870 GO:0031514
Cluster-22178.54081 GO:0051539
Cluster-22178.54092 GO:0007186 GO:0004930 GO:0016021
Cluster-22178.54095 GO:0045040 GO:0005515 GO:0005742
Cluster-22178.54099 GO:0005515
Cluster-22178.54100 GO:0006508 GO:0008234 GO:0004197
Cluster-22178.54101 GO:0006508 GO:0008234
Cluster-22178.54105 GO:0046872 GO:0004222
Cluster-22178.54106 GO:0007218 GO:0009733 GO:0007010 GO:0007186
GO:0003779 GO:0004930 GO:0005185 GO:0005544 GO:0005509
GO:0016021 GO:0005576
Cluster-22178.54109 GO:0022904 GO:0016651 GO:0046983 GO:0005743

Cluster-22178.54112 GO:0006260 GO:0003677 GO:0005634
Cluster-22178.54118 GO:0006355 GO:0005515 GO:0005634
Cluster-22178.54122 GO:0051297 GO:0005813
Cluster-22178.54123 GO:0007186 GO:0004930 GO:0016021 GO:0016020
Cluster-22178.54124 GO:0008283 GO:0040007 GO:0007165 GO:0008270
GO:0003676 GO:0008083
Cluster-22178.54127 GO:0071816 GO:0031492 GO:0000785 GO:0005634
Cluster-22178.54130 GO:0016021
Cluster-22178.54131 GO:0016598 GO:0004057
Cluster-22178.54132 GO:0046080 GO:0016787
Cluster-22178.54134 GO:0017025
Cluster-22178.54143 GO:0009451 GO:0001522 GO:0003723 GO:0009982
GO:0008017 GO:0045298
Cluster-22178.54144 GO:0035556
Cluster-22178.54146 GO:0006886 GO:0006887 GO:0006891 GO:0048278
GO:0005737 GO:0017119
Cluster-22178.54148 GO:0006355 GO:0005634
Cluster-22178.5415 GO:0009405 GO:0006471 GO:0000398 GO:0003956
GO:0005576 GO:0005634
Cluster-22178.54150 GO:0005515
Cluster-22178.54155 GO:0051603 GO:0006511 GO:0004175 GO:0004298
GO:0005839 GO:0019773
Cluster-22178.54157 GO:0005515
Cluster-22178.54158 GO:0007186 GO:0004930 GO:0008270 GO:0016021
GO:0005622
Cluster-22178.54159 GO:0055085 GO:0048024 GO:0030001 GO:0008017
GO:0019901 GO:0046873 GO:0045298 GO:0016020 GO:0005634
Cluster-22178.54162 GO:0008152 GO:0046034 GO:0006464 GO:0008033
GO:0006144 GO:0051252 GO:0016151 GO:0046872 GO:0016846
GO:0004017 GO:0004540 GO:0016787
Cluster-22178.54166 GO:0006400 GO:0008616 GO:0008479
Cluster-22178.54169 GO:0006139 GO:0004003 GO:0016787 GO:0008026
GO:0016818 GO:0003677 GO:0005524 GO:0003676 GO:0005634

GO:0005657
Cluster-22178.54170 GO:0006850 GO:0005743
Cluster-22178.54171 GO:0006744 GO:0006120 GO:0006825 GO:0015886
GO:0015992 GO:0006814 GO:0006118 GO:0017004 GO:0005507
GO:0016531 GO:0008137 GO:0003954 GO:0016021 GO:0005758
GO:0005739
Cluster-22178.54174 GO:0006570 GO:0006470 GO:0004725
Cluster-22178.54177 GO:0006629 GO:0008081
Cluster-22178.54178 GO:0006629 GO:0008081
Cluster-22178.54179 GO:2001070
Cluster-22178.54183 GO:0006355 GO:0046983 GO:0016020
Cluster-22178.54185 GO:0006355 GO:0003824
Cluster-22178.54186 GO:0006355 GO:0003824
Cluster-22178.54188 GO:0006810 GO:0009405 GO:0019855 GO:0005576
Cluster-22178.54195 GO:0015991 GO:0042967 GO:0006555 GO:0019281
GO:0048309 GO:0051028 GO:0005975 GO:0042302 GO:0016820
GO:0004553 GO:0008899 GO:0005524 GO:0033178 GO:0005737
Cluster-22178.54199 GO:0005886
Cluster-22178.54202 GO:0005515
Cluster-22178.54203 GO:0006886 GO:0005543
Cluster-22178.54207 GO:0006468 GO:0016773 GO:0004672 GO:0005524
GO:0016020
Cluster-22178.54210 GO:0008270 GO:0046872
Cluster-22178.54211 GO:0008270 GO:0046872
Cluster-22178.54215 GO:0035556 GO:0005515
Cluster-22178.54218 GO:0006468 GO:0004672 GO:0005524 GO:0005515

Cluster-22178.54221 GO:0016818 GO:0008270 GO:0005524
Cluster-22178.54224 GO:0015992 GO:0015991 GO:0015078 GO:0016772
GO:0016301 GO:0033179
Cluster-22178.54225 GO:0016567 GO:0006308 GO:0006914 GO:0035091
GO:0005515 GO:0004842 GO:0008855 GO:0008270 GO:0046872
GO:0009318 GO:0005680 GO:0005622

Cluster-22178.54226 GO:0006779 GO:0055114 GO:0006464 GO:0019354
GO:0016151 GO:0016491 GO:0046872 GO:0016829 GO:0008270
GO:0043115 GO:0005515 GO:0003677 GO:0016616 GO:0051287
Cluster-22178.54230 GO:0016740 GO:0016021
Cluster-22178.54231 GO:0042578
Cluster-22178.54235 GO:0007165 GO:0019042 GO:0006955 GO:0005164
GO:0033644 GO:0016020
Cluster-22178.54237 GO:0030168 GO:0015992 GO:0008053 GO:0030472
GO:0015986 GO:0007165 GO:0048870 GO:0051258 GO:0006397
GO:0005102 GO:0030674 GO:0003924 GO:0015078 GO:0000276
GO:0005577 GO:0005741 GO:0016021 GO:0072686 GO:0031514
GO:0042729
Cluster-22178.54239 GO:0006810 GO:0006812 GO:0046931 GO:0052331
GO:0015267 GO:0035091 GO:0046930
Cluster-22178.54245 GO:0007275 GO:0006511 GO:0008270 GO:0005515
GO:0005634
Cluster-22178.54248 GO:0006144 GO:0006522 GO:0006531 GO:0006164
GO:0006955 GO:0006810 GO:0007165 GO:0015858 GO:0005125
GO:0005337 GO:0004019 GO:0005525 GO:0016021 GO:0005576
Cluster-22178.54249 GO:0005739
Cluster-22178.54256 GO:0032259 GO:0008168
Cluster-22178.54262 GO:0016310 GO:0035023 GO:0009069 GO:0043087
GO:0004674 GO:0005515 GO:0005089
Cluster-22178.54263 GO:0005515
Cluster-22178.54265 GO:0005515 GO:0043395 GO:0016020 GO:0005578
Cluster-22178.54266 GO:0005524 GO:0016773 GO:0016020
Cluster-22178.54268 GO:0046983
Cluster-22178.54270 GO:0006355 GO:0003723 GO:0003682 GO:0003677
GO:0005634 GO:0000785 GO:0000775
Cluster-22178.54271 GO:0005515 GO:0033588
Cluster-22178.54275 GO:0042742 GO:0042381 GO:0046872
Cluster-22178.54280 GO:0006355 GO:0008270 GO:0046872 GO:0003700
GO:0005667 GO:0042025

Cluster-22178.54281 GO:0022900 GO:0005747 GO:0019031 GO:0005739
Cluster-22178.54283 GO:0006357 GO:0001104 GO:0016592 GO:0071203
Cluster-22178.54285 GO:0006810 GO:0042254 GO:0006412 GO:0046872
GO:0005215 GO:0004867 GO:0003735 GO:0005622 GO:0005840
Cluster-22178.54290 GO:0005515
Cluster-22178.54292 GO:0006468 GO:0005515 GO:0003723 GO:0005524
GO:0004672 GO:0000178
Cluster-22178.54293 GO:0006508 GO:0008270 GO:0004222
Cluster-22178.54295 GO:0042157 GO:0006869 GO:0015694 GO:0055114
GO:0015097 GO:0003677 GO:0016614 GO:0008289 GO:0050660
GO:0005576 GO:0016020
Cluster-22178.54297 GO:0042254 GO:0006412 GO:0003735 GO:0005840
Cluster-22178.54299 GO:0007016 GO:0016010
Cluster-22178.54300 GO:0016021
Cluster-22178.54303 GO:0006955 GO:0009405 GO:0005515 GO:0043022
GO:0016788 GO:0003723 GO:0016020
Cluster-22178.54304 GO:0030150 GO:0005742
Cluster-22178.54305 GO:0015689 GO:0055085 GO:0015098 GO:0022857
GO:0016021
Cluster-22178.54309 GO:0006276
Cluster-22178.54310 GO:0007165 GO:0005975 GO:0005509 GO:0004571
GO:0008158 GO:0016020
Cluster-22178.54311 GO:0005515
Cluster-22178.54313 GO:0030001 GO:0007034 GO:0007411
Cluster-22178.54317 GO:0000042 GO:0007094 GO:0006606 GO:0005515
GO:0005789
Cluster-22178.54318 GO:0006468 GO:0005524 GO:0004672
Cluster-22178.54327 GO:0006468 GO:0055114 GO:0004497 GO:0016706
GO:0008168 GO:0004672 GO:0005524 GO:0016715 GO:0005507
Cluster-22178.54333 GO:0004045 GO:0003677 GO:0005198 GO:0019028
Cluster-22178.54335 GO:0046872
Cluster-22178.54336 GO:0009405 GO:0016021
Cluster-22178.54337 GO:0006897 GO:0007015 GO:0003677 GO:0008270

GO:0046872
Cluster-22178.54338 GO:0005975 GO:0051260 GO:0046872 GO:0003677
GO:0004553 GO:0016021 GO:0016020
Cluster-22178.54343 GO:0005525 GO:0003924
Cluster-22178.54349 GO:0006659
Cluster-22178.54352 GO:0007010 GO:0003779
Cluster-22178.54353 GO:0005515
Cluster-22178.54354 GO:0006313 GO:0004803 GO:0003677
Cluster-22178.54355 GO:0005515
Cluster-22178.54356 GO:0007186 GO:0004930 GO:0016021
Cluster-22178.54360 GO:0008284 GO:0007165 GO:0006952 GO:0009069
GO:0016310 GO:0005179 GO:0004674 GO:0005576 GO:0005887
Cluster-22178.54361 GO:0016579 GO:0036459
Cluster-22178.54362 GO:0000226 GO:0090063 GO:0005815 GO:0000922
Cluster-22178.54363 GO:0009415 GO:0006470 GO:0006950 GO:0006508
GO:0006570 GO:0000087 GO:0008270 GO:0004222 GO:0004725
GO:0031012 GO:0005622
Cluster-22178.54364 GO:0008270
Cluster-22178.54366 GO:0034314 GO:0030041 GO:0005885 GO:0015629
Cluster-22178.54367 GO:0003924
Cluster-22178.54372 GO:0046740 GO:0006810 GO:0042254 GO:0006412
GO:0006260 GO:0051082 GO:0003735 GO:0016798 GO:0016020
GO:0005886 GO:0005622 GO:0005840 GO:0016021
Cluster-22178.54379 GO:0010506 GO:0005615
Cluster-22178.54380 GO:0006979 GO:0055114 GO:0006804 GO:0006749
GO:0007264 GO:0005525 GO:0003924 GO:0004602 GO:0005524
Cluster-22178.54382 GO:0006468 GO:0005524 GO:0004672 GO:0016773
GO:0016020
Cluster-22178.54383 GO:0005634
Cluster-22178.54384 GO:0007165 GO:0050830 GO:0006032 GO:0008061
GO:0005515
Cluster-22178.54385 GO:0035556 GO:0005515
Cluster-22178.54389 GO:0006355 GO:0003700 GO:0005622 GO:0005667

Cluster-22178.54392 GO:0043087 GO:0003677 GO:0003676 GO:0005089
GO:0005524 GO:0016787 GO:0005737
Cluster-22178.54393 GO:0009395 GO:0016042 GO:0006355 GO:0043093
GO:0000917 GO:0006364 GO:0043565 GO:0003700 GO:0003677
GO:0005515 GO:0004629 GO:0005737 GO:0005667 GO:0005634
Cluster-22178.54399 GO:0005515
Cluster-22178.54407 GO:0019497 GO:0006771 GO:0046872 GO:0005515
GO:0003993 GO:0005576
Cluster-22178.54408 GO:0051028 GO:0009236 GO:0006144 GO:0006260
GO:0006355 GO:0048309 GO:0015994 GO:0046034 GO:0016993
GO:0004017 GO:0008270 GO:0005515 GO:0046872 GO:0043565
GO:0003676 GO:0003700 GO:0005667
Cluster-22178.54412 GO:0007165 GO:0006270 GO:0006886 GO:0008283
GO:0040007 GO:0005047 GO:0008083 GO:0005525 GO:0003924
GO:0005785 GO:0005786
Cluster-22178.54413 GO:0006886 GO:0007165 GO:0040007 GO:0008283
GO:0005525 GO:0003924 GO:0008083 GO:0005047 GO:0005785
GO:0005786
Cluster-22178.54415 GO:0006355 GO:0003700 GO:0003677 GO:0043565
GO:0005667 GO:0005634
Cluster-22178.54416 GO:0000160 GO:0016310 GO:0005975 GO:0046983
GO:0046872 GO:0016868 GO:0000155 GO:0016021 GO:0009365
Cluster-22178.54417 GO:0043093 GO:0006260 GO:0010506 GO:0008053
GO:0006355 GO:0000917 GO:0007049 GO:0043565 GO:0003924
GO:0003700 GO:0005667 GO:0005741 GO:0005634 GO:0005737
GO:0016021 GO:0005615
Cluster-22178.54420 GO:0006464 GO:0006144 GO:0046034 GO:0046872
GO:0016151 GO:0004017
Cluster-22178.54421 GO:0035556 GO:0007165 GO:0003774 GO:0005524
GO:0005515 GO:0016021 GO:0097197 GO:0001772 GO:0016459
Cluster-22178.54424 GO:0006801 GO:0055114 GO:0046872
Cluster-22178.54425 GO:0042967 GO:0006355 GO:0045132 GO:0003676
GO:0008080 GO:0005515 GO:0004402 GO:0000775 GO:0000123

GO:0005634
Cluster-22178.54426 GO:0030153 GO:0006955 GO:0015643 GO:0046872
GO:0000166 GO:0019814
Cluster-22178.54427 GO:0006468 GO:0005524 GO:0004672 GO:0005515

Cluster-22178.54428 GO:0006468 GO:0005524 GO:0004672 GO:0005515

Cluster-22178.54429 GO:0051056 GO:0005096
Cluster-22178.54430 GO:0042254 GO:0006412 GO:0008152 GO:0003735
GO:0003824 GO:0005840 GO:0005739
Cluster-22178.54431 GO:0006355 GO:0046034 GO:0042157 GO:0006869
GO:0006464 GO:0006144 GO:0051082 GO:0046872 GO:0043565
GO:0008289 GO:0016151 GO:0003700 GO:0004017 GO:0008270
GO:0005576 GO:0005667 GO:0005815
Cluster-22178.54433 GO:0005634
Cluster-22178.54434 GO:0046872
Cluster-22178.54436 GO:0015979 GO:0042549 GO:0005515 GO:0009523
GO:0009539
Cluster-22178.54439 GO:0007186 GO:0004930 GO:0016021
Cluster-22178.54440 GO:0007186 GO:0007010 GO:0004930 GO:0003779
GO:0016021
Cluster-22178.54445 GO:0008537
Cluster-22178.54447 GO:0009966 GO:0007275 GO:0016020
Cluster-22178.54449 GO:0016021
Cluster-22178.54450 GO:0003824
Cluster-22178.54451 GO:0045454
Cluster-22178.54455 GO:0042262 GO:0003690 GO:0008092 GO:0005737
GO:0019898
Cluster-22178.54457 GO:0006886 GO:0007264 GO:0006468 GO:0006606
GO:0006914 GO:0004672 GO:0005524 GO:0016773 GO:0005096
GO:0005783 GO:0016020 GO:0016021
Cluster-22178.54462 GO:0046813 GO:0061025 GO:0016021 GO:0019031
Cluster-22178.54463 GO:0009405 GO:0016560 GO:0005515 GO:0005096

GO:0016021 GO:0005777
Cluster-22178.54464 GO:0006355 GO:0003677 GO:0005515 GO:0003700
GO:0008270 GO:0043565 GO:0005634 GO:0005667 GO:0005815
Cluster-22178.54468 GO:0006013 GO:0006144 GO:0006000 GO:0000103
GO:0006614 GO:0015976 GO:0008152 GO:0006094 GO:0006099
GO:0005524 GO:0016887 GO:0098519 GO:0003873 GO:0003924
GO:0004020 GO:0005525 GO:0004612 GO:0016301
Cluster-22178.54470 GO:0000103 GO:0006144 GO:0004020 GO:0005524

Cluster-22178.54471 GO:0035091 GO:0016021
Cluster-22178.54472 GO:0005634
Cluster-22178.54478 GO:0009405
Cluster-22178.54482 GO:0015031 GO:0042147 GO:0007165 GO:0008565
GO:0005198 GO:0005515 GO:0019031 GO:0019028 GO:0030904
Cluster-22178.54483 GO:0006470 GO:0006570 GO:0008138 GO:0004725
GO:0005515 GO:0004721 GO:0008270
Cluster-22178.54484 GO:0006276 GO:0009236 GO:0055085 GO:0048472
GO:0016021
Cluster-22178.54490 GO:0000398 GO:0006355 GO:0008270 GO:0005515
GO:0003677 GO:0003682 GO:0000785 GO:0005681 GO:0000775
GO:0005634
Cluster-22178.54493 GO:0007275 GO:0034477 GO:0004518 GO:0005515
GO:0003700 GO:0046872 GO:0005634 GO:0005667
Cluster-22178.54494 GO:0016021
Cluster-22178.54496 GO:0005982 GO:0005985 GO:0005978 GO:0005515
GO:0004373
Cluster-22178.54500 GO:0008284 GO:0006952 GO:0003676 GO:0046872

Cluster-22178.54505 GO:0006510 GO:0006508 GO:0004176
Cluster-22178.54507 GO:0006144 GO:0006508 GO:0004197 GO:0003968
GO:0004252 GO:0017111 GO:0031379
Cluster-22178.54509 GO:0000921 GO:0006355 GO:0003700 GO:0043565
GO:0005198 GO:0016021 GO:0005940 GO:0005882 GO:0005667

Cluster-22178.54510 GO:0006811 GO:0019439 GO:0006813 GO:0005249
GO:0003677 GO:0016833 GO:0016020 GO:0008076
Cluster-22178.54514 GO:0006308 GO:0006260 GO:0006281 GO:0017108
GO:0047869 GO:0005515 GO:0005634 GO:0033557
Cluster-22178.54515 GO:0006811 GO:0055085 GO:0015075 GO:0016020
Cluster-22178.54516 GO:0006468 GO:0009190 GO:0046039 GO:0006182
GO:0006144 GO:0035556 GO:0004383 GO:0016849 GO:0005524
GO:0004672
Cluster-22178.54517 GO:0008654 GO:0055085 GO:0006812 GO:0016780
GO:0008324 GO:0016021 GO:0016020
Cluster-22178.54522 GO:0006418 GO:0005524 GO:0004812 GO:0000166
GO:0003676
Cluster-22178.54526 GO:0006468 GO:0004672 GO:0005524 GO:0016773
GO:0016020
Cluster-22178.54531 GO:0051252 GO:0006402 GO:0005975 GO:0003723
GO:0016832 GO:0003676 GO:0046872 GO:0004535 GO:0005634
GO:0005737
Cluster-22178.54533 GO:0006355 GO:0003723 GO:0005515 GO:0005634
Cluster-22178.54534 GO:0006470 GO:0015991 GO:0006351 GO:0006412
GO:0006570 GO:0015992 GO:0006260 GO:0042710 GO:0042254
GO:0004725 GO:0015078 GO:0003887 GO:0008138 GO:0003677
GO:0003735 GO:0005840 GO:0042575 GO:0033179 GO:0005622
Cluster-22178.54535 GO:0016021
Cluster-22178.54538 GO:0016021
Cluster-22178.54539 GO:0016197 GO:0000724 GO:0005515 GO:0030119
Cluster-22178.54541 GO:0006355 GO:0003700 GO:0008270 GO:0043565
GO:0005667
Cluster-22178.54542 GO:0008152 GO:0006614 GO:0003824 GO:0005525
GO:0016491 GO:0005198 GO:0050662 GO:0019028
Cluster-22178.54543 GO:0042254 GO:0006412 GO:0003735 GO:0005840
GO:0005622
Cluster-22178.54544 GO:0008270
Cluster-22178.54545 GO:0006633 GO:0006554 GO:0018874 GO:0006631

GO:0006098 GO:0015940 GO:0006574 GO:0019521 GO:0055114
GO:0006550 GO:0006552 GO:0006813 GO:0006568 GO:0016491
GO:0004616 GO:0051287 GO:0008677 GO:0003857
Cluster-22178.54546 GO:0051287
Cluster-22178.54547 GO:0006574 GO:0055114 GO:0008152 GO:0006020
GO:0006550 GO:0008218 GO:0006118 GO:0006552 GO:0016491
GO:0004491 GO:0003995
Cluster-22178.54548 GO:0055114 GO:0015992 GO:0006123 GO:0009060
GO:0006118 GO:0005524 GO:0004129 GO:0009055 GO:0005506
GO:0020037 GO:0035299 GO:0016021 GO:0045277
Cluster-22178.54549 GO:0070206 GO:0006406 GO:0006998 GO:0006611
GO:0051082 GO:0005198 GO:0019031 GO:0019028 GO:0031965
Cluster-22178.54551 GO:0006544 GO:0006546 GO:0006566 GO:0055114
GO:0006563 GO:0016616 GO:0004375
Cluster-22178.54553 GO:0006265 GO:0003677 GO:0003918 GO:0005524
GO:0016020
Cluster-22178.54554 GO:0008152 GO:0016491
Cluster-22178.54556 GO:0016310 GO:0015012 GO:0006024 GO:0009069
GO:0006468 GO:0004674 GO:0005524 GO:0016021 GO:0045095
Cluster-22178.54557 GO:0008152 GO:0016746
Cluster-22178.54559 GO:0006693 GO:0006691 GO:0006281 GO:0006749
GO:0019530 GO:0006284 GO:0006289 GO:0003684 GO:0016799
GO:0003906 GO:0016787 GO:0005516 GO:0008270 GO:0003840
Cluster-22178.54561 GO:0007050 GO:0006355 GO:0045859 GO:0000122
GO:0004861 GO:0003700 GO:0017069 GO:0005667 GO:0005634
GO:0005737
Cluster-22178.54563 GO:0007017 GO:0007018 GO:0005524 GO:0003777
GO:0008017 GO:0005874 GO:0045298
Cluster-22178.54565 GO:0007017 GO:0007018 GO:0005524 GO:0003777
GO:0008017 GO:0005874 GO:0045298
Cluster-22178.54567 GO:0005515 GO:0003950
Cluster-22178.54568 GO:0003950 GO:0005515
Cluster-22178.54569 GO:0035091 GO:0005515

Cluster-22178.54571 GO:0006364 GO:0004222
Cluster-22178.54572 GO:0006355 GO:0003677 GO:0005634
Cluster-22178.54573 GO:0007165 GO:0006606 GO:0006955 GO:0005044
GO:0030247
Cluster-22178.54574 GO:0006888 GO:0006886 GO:0007010 GO:0003779
GO:0008270 GO:0003677 GO:0030127
Cluster-22178.54575 GO:0003824 GO:0005515
Cluster-22178.54576 GO:0019354 GO:0055114 GO:0006464 GO:0006779
GO:0051287 GO:0043115 GO:0005515 GO:0016616 GO:0003677
GO:0016491 GO:0016829 GO:0008270 GO:0046872 GO:0016151
Cluster-22178.54579 GO:0007217 GO:0007275 GO:0007268 GO:0004867
GO:0005578
Cluster-22178.54580 GO:0035556 GO:0055114 GO:0007165 GO:0006468
GO:0047134 GO:0008270 GO:0004672 GO:0005524 GO:0005057
GO:0005515
Cluster-22178.54581 GO:0055114 GO:0035556 GO:0006468 GO:0007165
GO:0005057 GO:0005524 GO:0008270 GO:0004672 GO:0047134
GO:0005515
Cluster-22178.54582 GO:0007166 GO:0007165 GO:0004871 GO:0005634
Cluster-22178.54583 GO:0005515
Cluster-22178.54586 GO:0016787
Cluster-22178.54592 GO:0044419 GO:0006144 GO:0006508 GO:0003968
GO:0017111 GO:0004197 GO:0031379
Cluster-22178.54593 GO:0005515
Cluster-22178.54595 GO:0006555 GO:0006556 GO:0004478
Cluster-22178.54599 GO:0015991 GO:0015992 GO:0015078 GO:0033179
Cluster-22178.54603 GO:0006729 GO:0022900 GO:0045454 GO:0016651
GO:0008124
Cluster-22178.54605 GO:0008616 GO:0016853 GO:0016740
Cluster-22178.54606 GO:0019031
Cluster-22178.54609 GO:0006355 GO:0005622
Cluster-22178.54610 GO:0016788
Cluster-22178.54612 GO:0006355 GO:0005515 GO:0005634

Cluster-22178.54615 GO:0051260 GO:0007186 GO:0004930 GO:0016021 GO:0016020
Cluster-22178.54616 GO:0055114 GO:1901031 GO:0051537 GO:0051920 GO:0016491 GO:0005634
Cluster-22178.54617 GO:0006886 GO:0022900 GO:0006888 GO:0008270 GO:0005747 GO:0030127 GO:0005739
Cluster-22178.54621 GO:0043085 GO:0000290 GO:0003779 GO:0008047

Cluster-22178.54622 GO:0042254 GO:0006412 GO:0006506 GO:0016746 GO:0003735 GO:0016021 GO:0005789 GO:0005840 GO:0005739
Cluster-22178.54626 GO:0006357 GO:0006508 GO:0008237 GO:0001104 GO:0005515 GO:0016805 GO:0005618 GO:0005634 GO:0016592
Cluster-22178.54628 GO:0006468 GO:0007010 GO:0003779 GO:0004672 GO:0005524 GO:0019028 GO:0016020
Cluster-22178.54629 GO:0006468 GO:0005524 GO:0004672
Cluster-22178.54632 GO:0016192 GO:0006886 GO:0008616 GO:0005515 GO:0016853 GO:0016740 GO:0030117
Cluster-22178.54634 GO:0006810 GO:0009405 GO:0006952 GO:0019855 GO:0005576
Cluster-22178.54635 GO:0055085 GO:0006821 GO:0006952 GO:0009405 GO:0006810 GO:0019855 GO:0005247 GO:0005576 GO:0016020
Cluster-22178.54636 GO:0006952 GO:0009405 GO:0006810 GO:0019855 GO:0005576
Cluster-22178.54639 GO:0006313 GO:0055085 GO:0003677 GO:0022857 GO:0016020 GO:0016021
Cluster-22178.54641 GO:0007017 GO:0007018 GO:0005524 GO:0003777 GO:0008017 GO:0005874 GO:0045298
Cluster-22178.54647 GO:0055085 GO:0006811 GO:0005216 GO:0016020
Cluster-22178.54648 GO:0005861
Cluster-22178.54649 GO:0009264 GO:0008253
Cluster-22178.54652 GO:0006355 GO:0016032 GO:0007017 GO:0007018 GO:0003777 GO:0045502 GO:0042803 GO:0008134 GO:0003774 GO:0005524 GO:0008017 GO:0003677 GO:0005874 GO:0045298

GO:0005634 GO:0030286 GO:0016459 GO:0005667
Cluster-22178.54654 GO:0006352 GO:0006355 GO:0003700 GO:0016987
GO:0003677 GO:0005667
Cluster-22178.54655 GO:0055114 GO:0009395 GO:0035556 GO:0007205
GO:0042254 GO:0006412 GO:0046486 GO:0004143 GO:0047134
GO:0016301 GO:0003735 GO:0005515 GO:0005840
Cluster-22178.54658 GO:0043087 GO:0006914 GO:0032012 GO:0008053
GO:0003924 GO:0005086 GO:0016021 GO:0005741
Cluster-22178.54659 GO:0006355 GO:0006281 GO:0002949 GO:0006310
GO:0003678 GO:0043531 GO:0008134 GO:0016301 GO:0003723
GO:0003724 GO:0016887 GO:0005524 GO:0009378 GO:0005657
GO:0005667 GO:0009379
Cluster-22178.54661 GO:0007165 GO:0042254 GO:0015992 GO:0070206
GO:0006914 GO:0007034 GO:0006606 GO:0007218 GO:0015991
GO:0010508 GO:0030474 GO:0006412 GO:0006308 GO:0005179
GO:0003677 GO:0003735 GO:0008855 GO:0005198 GO:0005200
GO:0015078 GO:0005622 GO:0005823 GO:0005576 GO:0009318
GO:0033179 GO:0005856 GO:0005840
Cluster-22178.54669 GO:0016773
Cluster-22178.54670 GO:0015031 GO:0030176
Cluster-22178.54672 GO:0005515
Cluster-22178.54674 GO:0005198 GO:0019028
Cluster-22178.54675 GO:0005198 GO:0019028
Cluster-22178.54677 GO:0007186 GO:0004930 GO:0016021
Cluster-22178.54681 GO:0006468 GO:0006118 GO:0055114 GO:0009496
GO:0005524 GO:0004672 GO:0051537 GO:0042651 GO:0009512
Cluster-22178.54684 GO:0006687 GO:0009058 GO:0003677 GO:0008146
GO:0001733 GO:0016021 GO:0005794
Cluster-22178.54690 GO:0055114 GO:0016491
Cluster-22178.54691 GO:0051289 GO:0006914 GO:0070206 GO:0007165
GO:0006506 GO:0008140 GO:0005515 GO:0005509 GO:0016740
GO:0016021 GO:0005789 GO:0005886 GO:0005667 GO:0016012
GO:0005578

Cluster-22178.54695 GO:0006606 GO:0048870 GO:0031514

Cluster-22178.54697 GO:0006468 GO:0009405 GO:0051082 GO:0005515 GO:0004672 GO:0005524 GO:0019865 GO:0019814

Cluster-22178.54698 GO:0006355 GO:0016973 GO:0048870 GO:0003700 GO:0031514 GO:0005667 GO:0016021 GO:0005643

Cluster-22178.54701 GO:0030244 GO:0006011 GO:0005985 GO:0016567 GO:0005982 GO:0008270 GO:0046872 GO:0004842 GO:0005515 GO:0016760 GO:0005680 GO:0016020

Cluster-22178.54702 GO:0030244 GO:0061136 GO:0005982 GO:0005985 GO:0016567 GO:0019915 GO:0006011 GO:0004842 GO:0008270 GO:0046872 GO:0005515 GO:0016760 GO:0005789 GO:0005680 GO:0016020

Cluster-22178.54703 GO:0030244 GO:0006011 GO:0005985 GO:0016567 GO:0005982 GO:0008270 GO:0046872 GO:0004842 GO:0016760 GO:0005515 GO:0005680 GO:0016020

Cluster-22178.54706 GO:0006351 GO:0006206 GO:0006144 GO:0003677 GO:0003899 GO:0008270 GO:0046872 GO:0003676 GO:0005730

Cluster-22178.54708 GO:0007165 GO:0007155 GO:0005515 GO:0005198 GO:0005179 GO:0005576 GO:0031012

Cluster-22178.54710 GO:0006825 GO:0035434 GO:0005375 GO:0016021 GO:0019031 GO:0005887

Cluster-22178.54716 GO:0006352

Cluster-22178.54718 GO:0042981

Cluster-22178.54719 GO:0046872

Cluster-22178.54720 GO:0005576

Cluster-22178.54721 GO:0015031 GO:0006464 GO:0005515

Cluster-22178.54722 GO:0015031 GO:0006464 GO:0005515

Cluster-22178.54723 GO:0032040

Cluster-22178.54725 GO:0006729 GO:0008124 GO:0016021

Cluster-22178.54726 GO:0042981

Cluster-22178.54727 GO:0046872 GO:0003676

Cluster-22178.54736 GO:0080019

Cluster-22178.54738 GO:0007156 GO:0005509 GO:0005634 GO:0016020

Cluster-22178.54739 GO:0007156 GO:0005509 GO:0016020 GO:0005634
Cluster-22178.54740 GO:0007156 GO:0005509 GO:0005634 GO:0016020
Cluster-22178.54742 GO:0009405
Cluster-22178.54743 GO:0048203 GO:0034315 GO:1900025 GO:2000146
GO:0035650
Cluster-22178.54746 GO:0006629 GO:0035434 GO:0006825 GO:0009252
GO:0003677 GO:0005375 GO:0046983 GO:0008963 GO:0016021
Cluster-22178.54751 GO:0006468 GO:0009069 GO:0055114 GO:0016310
GO:0035556 GO:0008152 GO:0005515 GO:0005524 GO:0004672
GO:0016846 GO:0047134 GO:0004674
Cluster-22178.54754 GO:0006355 GO:0043565 GO:0003700 GO:0005667
Cluster-22178.54758 GO:0003677 GO:0005515
Cluster-22178.54759 GO:0055114 GO:0051920
Cluster-22178.54761 GO:0003824 GO:0051537 GO:0043231
Cluster-22178.54762 GO:0046872
Cluster-22178.54763 GO:0042157 GO:0006869 GO:0008289 GO:0005515
GO:0005576
Cluster-22178.54767 GO:0015074 GO:0055085 GO:0006260 GO:0006810
GO:0003924 GO:0016887 GO:0005524 GO:0042626 GO:0003678
GO:0003676 GO:0005525 GO:0016021 GO:0005657
Cluster-22178.54768 GO:0006308 GO:0006355 GO:0003677 GO:0008855
GO:0009318
Cluster-22178.54777 GO:0006659
Cluster-22178.54781 GO:0008270 GO:0005515
Cluster-22178.54789 GO:0006810 GO:0009405 GO:0035556 GO:0003677
GO:0005515 GO:0008200 GO:0005576 GO:0005634
Cluster-22178.54791 GO:0006488 GO:0004583 GO:0005789
Cluster-22178.54792 GO:0004867 GO:0005524 GO:0005576
Cluster-22178.54793 GO:0005515
Cluster-22178.54794 GO:0008270
Cluster-22178.54800 GO:0043087 GO:0035023 GO:0008152 GO:0003824
GO:0005089 GO:0017022 GO:0016459
Cluster-22178.54804 GO:0005515 GO:0046872 GO:0016021

Cluster-22178.54806 GO:0006508 GO:0006816 GO:0070588 GO:0006355
GO:0007165 GO:0005509 GO:0005515 GO:0043565 GO:0003700
GO:0005262 GO:0019033 GO:0005667 GO:0005578 GO:0016020
Cluster-22178.54807 GO:0006506 GO:0005789
Cluster-22178.54811 GO:0006355 GO:0005515 GO:0003723 GO:0005634
Cluster-22178.54812 GO:0032797
Cluster-22178.54817 GO:0006508 GO:0004197 GO:0008233
Cluster-22178.54825 GO:0007017 GO:0007018 GO:0005524 GO:0003777
GO:0008017 GO:0005874 GO:0045298 GO:0019028
Cluster-22178.54828 GO:0016021
Cluster-22178.54836 GO:0022900 GO:0006397 GO:0003723 GO:0005634
GO:0005739 GO:0005737 GO:0005747
Cluster-22178.54840 GO:0042254 GO:0006412 GO:0007411 GO:0003735
GO:0005515 GO:0044599 GO:0005622 GO:0005840
Cluster-22178.54841 GO:0051260 GO:0005515
Cluster-22178.54842 GO:0003677 GO:0003676 GO:0005524 GO:0016787

Cluster-22178.54847 GO:0007165 GO:0042254 GO:0006412 GO:0003735
GO:0005125 GO:0005840 GO:0005622
Cluster-22178.54848 GO:0008440 GO:0005515 GO:0005737 GO:0016020
Cluster-22178.54851 GO:0003723 GO:0003676 GO:0016021
Cluster-22178.54855 GO:0005576
Cluster-22178.54859 GO:0016192 GO:0006886 GO:0005515 GO:0005198
GO:0030130 GO:0030132 GO:0030117
Cluster-22178.54860 GO:0007165 GO:0008601 GO:0000159
Cluster-22178.54868 GO:0006886 GO:0005515 GO:0008536
Cluster-22178.54869 GO:0006811 GO:0006614 GO:0008312 GO:0003723
GO:0005216 GO:0005515 GO:0048500 GO:0016020
Cluster-22178.54871 GO:0046872
Cluster-22178.54875 GO:0016567 GO:0006915 GO:0032465 GO:0015031
GO:0006464 GO:0005515 GO:0004842 GO:0016020
Cluster-22178.54876 GO:0050832
Cluster-22178.54879 GO:0006355 GO:0003700 GO:0005515 GO:0005634

GO:0005667
Cluster-22178.54883 GO:0005525 GO:0005515
Cluster-22178.54886 GO:0042254 GO:0006412 GO:0003735 GO:0005840
Cluster-22178.54887 GO:0015948 GO:0008152 GO:0046656 GO:0030269
GO:0016746 GO:0016021
Cluster-22178.54888 GO:0016032 GO:0006541 GO:0042819 GO:0009236
GO:0042823 GO:0016070 GO:0016787 GO:0004359 GO:0003824
GO:0019012
Cluster-22178.54891 GO:0006396 GO:0051537 GO:0043231
Cluster-22178.54894 GO:0042981 GO:0005515
Cluster-22178.54895 GO:0042981 GO:0005515
Cluster-22178.54896 GO:0042981 GO:0005515
Cluster-22178.54903 GO:0006310 GO:0042138 GO:0003824
Cluster-22178.54906 GO:0007283 GO:0003677 GO:0005515 GO:0000786
GO:0005634
Cluster-22178.54907 GO:0006508 GO:0007031 GO:0005509 GO:0004252
GO:0005779
Cluster-22178.54910 GO:0006355 GO:0003677 GO:0008270 GO:0016021
Cluster-22178.54917 GO:0035556 GO:0042967 GO:0046339 GO:0009395
GO:0007165 GO:0006629 GO:0008080 GO:0004435 GO:0005515
Cluster-22178.54918 GO:0008152 GO:0003824
Cluster-22178.54919 GO:0006139 GO:0006206 GO:0006614 GO:0006810
GO:0005525 GO:0003724 GO:0004127 GO:0005524 GO:0016887
GO:0003924 GO:0000166 GO:0003677 GO:0098519 GO:0003723
Cluster-22178.54920 GO:0008152 GO:0003824
Cluster-22178.54921 GO:0042710 GO:0005524 GO:0003774 GO:0005198
GO:0005515 GO:0019031 GO:0019028 GO:0016459
Cluster-22178.54926 GO:0051537 GO:0043231
Cluster-22178.54929 GO:0006814 GO:0006886 GO:0006265 GO:0055114
GO:0006308 GO:0045995 GO:0006120 GO:0007165 GO:0006605
GO:0006313 GO:0030155 GO:0015992 GO:0030334 GO:0006744
GO:0008137 GO:0005515 GO:0003677 GO:0005524 GO:0008855
GO:0003918 GO:0005102 GO:0009318 GO:0016020

Cluster-22178.54930 GO:0006465 GO:0008233 GO:0016021 GO:0005887 GO:0005787
Cluster-22178.54931 GO:0015876 GO:0008521 GO:0016021
Cluster-22178.54932 GO:0015876 GO:0008521 GO:0016021
Cluster-22178.54934 GO:0007009
Cluster-22178.54937 GO:0007165 GO:0009405 GO:0008283 GO:0040007 GO:0051015 GO:0008083 GO:0030674
Cluster-22178.54940 GO:0006024 GO:0030206 GO:0006470 GO:0006570 GO:0004725 GO:0008138 GO:0030158
Cluster-22178.54941 GO:0005515 GO:0005198 GO:0005882
Cluster-22178.54942 GO:0007165 GO:0006468 GO:0016773 GO:0004672 GO:0005524 GO:0005179 GO:0005840 GO:0016020 GO:0005576
Cluster-22178.54946 GO:0006542 GO:0006807 GO:0006355 GO:0009252 GO:0003677 GO:0004356 GO:0005622
Cluster-22178.54947 GO:0008270 GO:0005515 GO:0042765 GO:0016021
Cluster-22178.54953 GO:0006183 GO:0006228 GO:0006165 GO:0006241 GO:0006206 GO:0006144 GO:0005515 GO:0003723 GO:0003676 GO:0004550
Cluster-22178.54956 GO:0031124 GO:0006468 GO:0030976 GO:0000287 GO:0005524 GO:0004672
Cluster-22178.54957 GO:0031124 GO:0006468 GO:0030976 GO:0005524 GO:0004672 GO:0000287
Cluster-22178.54958 GO:0009451 GO:0006879 GO:0008033 GO:0009020 GO:0008199
Cluster-22178.54960 GO:0006308 GO:0009307 GO:0009036 GO:0005515 GO:0003677 GO:0009359
Cluster-22178.54963 GO:0015994 GO:0015995 GO:0046406
Cluster-22178.54965 GO:0006468 GO:0004672 GO:0005524 GO:0016773 GO:0016020
Cluster-22178.54966 GO:0006891 GO:0007165 GO:0005515 GO:0017119
Cluster-22178.54970 GO:0005739
Cluster-22178.54973 GO:0005975 GO:0007165 GO:0004553 GO:0005509 GO:0005578

Cluster-22178.54974 GO:0009186 GO:0055114 GO:0006144 GO:0006206 GO:0006260 GO:0005524 GO:0004748 GO:0005971
Cluster-22178.54975 GO:0016125 GO:0006694 GO:0047750 GO:0016021
Cluster-22178.54977 GO:0016787 GO:0005524 GO:0003676 GO:0003677

Cluster-22178.54978 GO:0005524 GO:0003676
Cluster-22178.54980 GO:0005515
Cluster-22178.54981 GO:0005515
Cluster-22178.54984 GO:0042773 GO:0006814 GO:0015992 GO:0055114 GO:0008152 GO:0006744 GO:0055085 GO:0006120 GO:0006812 GO:0008137 GO:0008324 GO:0008168 GO:0016021
Cluster-22178.54987 GO:0055114 GO:0016491 GO:0010181
Cluster-22178.54990 GO:0006816 GO:0070588 GO:0015992 GO:0005262 GO:0016021 GO:0016020
Cluster-22178.54992 GO:0046872 GO:0005515 GO:0003677
Cluster-22178.54993 GO:0006084 GO:0015947 GO:0005515 GO:0018492

Cluster-22178.55000 GO:0007264 GO:0007186 GO:0007165 GO:0003924 GO:0019001 GO:0031683 GO:0004871 GO:0005525
Cluster-22178.55002 GO:0006508 GO:0042981 GO:0004197 GO:0005506

Cluster-22178.55004 GO:0042981 GO:0006508 GO:0004197 GO:0005506

Cluster-22178.55007 GO:0006281 GO:0003684 GO:0016020
Cluster-22178.55008 GO:0009405 GO:0007165 GO:0007186 GO:0006355 GO:0005509 GO:0005515 GO:0005198 GO:0004888 GO:0004930 GO:0003700 GO:0043565 GO:0005667 GO:0016021
Cluster-22178.55014 GO:0006270 GO:0005515
Cluster-22178.55015 GO:0006270 GO:0009306 GO:0005515
Cluster-22178.55017 GO:0017004 GO:0007165 GO:0015886 GO:0004871 GO:2001070 GO:0016021
Cluster-22178.55018 GO:0008270 GO:0008233 GO:0005634
Cluster-22178.55019 GO:0007275 GO:0046872 GO:0061630 GO:0042600

Cluster-22178.55028 GO:0046034 GO:0006144 GO:0006464 GO:0004017
GO:0016151 GO:0008270 GO:0046872
Cluster-22178.55029 GO:0006470 GO:0006570 GO:0042967 GO:0004725
GO:0008138 GO:0005515 GO:0008080
Cluster-22178.55030 GO:0005515 GO:0005509
Cluster-22178.55033 GO:0005509 GO:0005515
Cluster-22178.55036 GO:0005975 GO:0030682 GO:0008270 GO:0004553

Cluster-22178.55039 GO:0042256 GO:0043022
Cluster-22178.55041 GO:0005576 GO:0030133 GO:0031083
Cluster-22178.55042 GO:0035556 GO:0005524 GO:0046872
Cluster-22178.55043 GO:0003676
Cluster-22178.55044 GO:0015979 GO:0046872 GO:0016021 GO:0009523
Cluster-22178.55046 GO:0007165 GO:0010506 GO:0005515 GO:0005615
Cluster-22178.55052 GO:0007165 GO:0006954 GO:0006836 GO:0006812
GO:0005328 GO:0005125 GO:0005576 GO:0016021
Cluster-22178.55054 GO:0030695 GO:0005515 GO:0042802
Cluster-22178.55058 GO:0003676 GO:0031224
Cluster-22178.55066 GO:0007275 GO:0006511 GO:0006744 GO:0008270
GO:0046872 GO:0005515 GO:0005634
Cluster-22178.55067 GO:0006468 GO:0004672 GO:0005524
Cluster-22178.55070 GO:0006508 GO:0004252
Cluster-22178.55074 GO:0006351
Cluster-22178.55079 GO:0006508 GO:0016579 GO:0008270 GO:0004843
GO:0036459
Cluster-22178.55080 GO:0008270 GO:0046872 GO:0005634
Cluster-22178.55082 GO:0016021
Cluster-22178.55085 GO:0016021
Cluster-22178.55086 GO:0006904 GO:0003779 GO:0000145
Cluster-22178.55088 GO:0008537
Cluster-22178.55089 GO:0006556 GO:0019685 GO:0030494 GO:0006555
GO:0004478 GO:0016836
Cluster-22178.55090 GO:0006357 GO:0019051 GO:0001104 GO:0042025

GO:0016592

Cluster-22178.55091 GO:0070403 GO:0000287 GO:0030976 GO:0005515

Cluster-22178.55096 GO:0006260 GO:0009405 GO:0005515 GO:0005576

Cluster-22178.55097 GO:0007264 GO:0006355 GO:0008134 GO:0005524 GO:0003924 GO:0005525 GO:0005667 GO:0016020

Cluster-22178.55098 GO:0008152 GO:0006633 GO:0042967 GO:0004315 GO:0016747 GO:0005835

Cluster-22178.55099 GO:0006351 GO:0006468 GO:0006281 GO:0016310 GO:0007178 GO:0009069 GO:0006206 GO:0006289 GO:0043161 GO:0006144 GO:0003899 GO:0004675 GO:0003684 GO:0005524 GO:0005634 GO:0016020 GO:0005730

Cluster-22178.55104 GO:0051260 GO:0003677 GO:0046872

Cluster-22178.55105 GO:0051260 GO:0046872 GO:0003677

Cluster-22178.55110 GO:0005515

Cluster-22178.55113 GO:0005975 GO:0071704 GO:0016868

Cluster-22178.55116 GO:0007275 GO:0045893 GO:0008270

Cluster-22178.55122 GO:0007264 GO:0005525 GO:0003924

Cluster-22178.55130 GO:0006275 GO:0006270 GO:0005524 GO:0043565

Cluster-22178.55132 GO:0017004 GO:0006560 GO:0006525 GO:0015886 GO:0006418 GO:0006420 GO:0000166 GO:0004812 GO:0004814 GO:0005524 GO:0016021

Cluster-22178.55134 GO:0006355 GO:0003677

Cluster-22178.55135 GO:0006468 GO:0016773 GO:0005524 GO:0004672 GO:0016020

Cluster-22178.55136 GO:0042254 GO:0006412 GO:0015979 GO:0003735 GO:0005515 GO:0003677 GO:0016020 GO:0009523 GO:0005622 GO:0009539 GO:0005840

Cluster-22178.55140 GO:0046486 GO:0006071 GO:0005515 GO:0004371 GO:0008270 GO:0016829 GO:0005524 GO:0043565 GO:0005783 GO:0005634

Cluster-22178.55143 GO:0008270 GO:0005515

Cluster-22178.55145 GO:0006118 GO:0045454 GO:0006355 GO:0015035
GO:0009055 GO:0046872 GO:0005634
Cluster-22178.55147 GO:0006470 GO:2000123 GO:0006886 GO:0006570
GO:0004725 GO:0005525 GO:0005543 GO:0005515
Cluster-22178.55151 GO:0006047
Cluster-22178.55153 GO:0035556 GO:0046339 GO:0009395 GO:0007165
GO:0006629 GO:0004435 GO:0005509 GO:0005515 GO:0005578
Cluster-22178.55155 GO:0006118 GO:0051536 GO:0009055
Cluster-22178.55156 GO:0003676 GO:0016020
Cluster-22178.55158 GO:0006570 GO:0006470 GO:0019988 GO:0008138
GO:0043399 GO:0005515 GO:0004725 GO:0005634
Cluster-22178.55161 GO:0006817 GO:0008152 GO:0003824
Cluster-22178.55164 GO:0016973 GO:0006355 GO:0048870 GO:0003700
GO:0005667 GO:0031514 GO:0005643 GO:0016021
Cluster-22178.55165 GO:0016973 GO:0006355 GO:0048870 GO:0003700
GO:0005667 GO:0031514 GO:0005643 GO:0016021
Cluster-22178.55166 GO:0055085 GO:0016020 GO:0016021
Cluster-22178.55167 GO:0006351 GO:0006355 GO:0003712 GO:0003676
GO:0008270 GO:0003677 GO:0005634 GO:0005667
Cluster-22178.55170 GO:0044780 GO:0006468 GO:0007283 GO:0007130
GO:0006308 GO:0006914 GO:0007131 GO:0005524 GO:0004672
GO:0008855 GO:0009318
Cluster-22178.55171 GO:0016020
Cluster-22178.55172 GO:0007165 GO:0006355 GO:0005515 GO:0004871
GO:0003700 GO:0005667 GO:0071203
Cluster-22178.55174 GO:0005515
Cluster-22178.55176 GO:0030976 GO:0016787 GO:0000287 GO:0046872
GO:0070403
Cluster-22178.55177 GO:0007165 GO:0006886 GO:0005515 GO:0005783
GO:0016021
Cluster-22178.55179 GO:0003677
Cluster-22178.55181 GO:0055114 GO:0051321 GO:0018401 GO:0009401
GO:0006525 GO:0006560 GO:0016702 GO:0016491 GO:0004656

GO:0003677 GO:0005634 GO:0005783
Cluster-22178.55184 GO:0051252 GO:0006355 GO:0001682 GO:0008033
GO:0004526 GO:0003700 GO:0008270 GO:0016747 GO:0030677
GO:0005667 GO:0005634
Cluster-22178.55185 GO:0008152 GO:0003824
Cluster-22178.55186 GO:0035556 GO:0009395 GO:0046339 GO:0007165
GO:0006629 GO:0004435 GO:0005509 GO:0005515 GO:0005578
Cluster-22178.55189 GO:0006468 GO:0005524 GO:0004672 GO:0016020
Cluster-22178.55190 GO:0006468 GO:0005524 GO:0004672 GO:0016020
Cluster-22178.55193 GO:0006468 GO:0016773 GO:0005524 GO:0004672
GO:0016020
Cluster-22178.55196 GO:0015937 GO:0015940 GO:0006412 GO:0042254
GO:0005524 GO:0004594 GO:0003735 GO:0004252 GO:0005622
GO:0005840 GO:0019028 GO:0055036
Cluster-22178.55197 GO:0006120 GO:0006744 GO:0015992 GO:0006118
GO:0006814 GO:0008137 GO:0003954 GO:0005739
Cluster-22178.55198 GO:0015992 GO:0006744 GO:0006120 GO:0006310
GO:0006814 GO:0006118 GO:0046872 GO:0003954 GO:0003677
GO:0008137 GO:0000166 GO:0005739
Cluster-22178.55199 GO:0021549 GO:0006351 GO:0030182 GO:0006730
GO:0006144 GO:0006206 GO:0003899 GO:0003677 GO:0003676
GO:0008168 GO:0005730
Cluster-22178.55200 GO:0009058 GO:0006631 GO:0006508 GO:0008152
GO:0005975 GO:0008236 GO:0016788 GO:0016787 GO:0016746
GO:0008184
Cluster-22178.55203 GO:0005515
Cluster-22178.55205 GO:0003676 GO:0005515 GO:0046872 GO:0008270

Cluster-22178.55208 GO:0003824 GO:0052855
Cluster-22178.55209 GO:0055085 GO:0035556 GO:0032007 GO:0048011
GO:0008270 GO:0046872 GO:0005515 GO:0016021
Cluster-22178.55213 GO:0003824
Cluster-22178.55214 GO:0005515

Cluster-22178.55217 GO:0006810 GO:0005215
Cluster-22178.55218 GO:0019089
Cluster-22178.55219 GO:0019089
Cluster-22178.55222 GO:0005515 GO:0005737
Cluster-22178.55223 GO:0008063 GO:0008270 GO:0005515 GO:0019901
GO:0005634
Cluster-22178.55226 GO:0006206 GO:0006144 GO:0006351 GO:0046983
GO:0003899 GO:0005730
Cluster-22178.55230 GO:0006355 GO:0006260 GO:0003677 GO:0005664
Cluster-22178.55234 GO:0006821 GO:0055085 GO:0005247 GO:0016020
Cluster-22178.55235 GO:0006529 GO:0006522 GO:0006560 GO:0006526
GO:0006531 GO:0004055 GO:0005524 GO:0004066
Cluster-22178.55236 GO:0006468 GO:0006355 GO:0007264 GO:0006457
GO:0035556 GO:0007031 GO:0051082 GO:0042803 GO:0016820
GO:0005515 GO:0004672 GO:0046872 GO:0003924 GO:0005525
GO:0003700 GO:0051087 GO:0003677 GO:0005524 GO:0000774
GO:0004222 GO:0016021 GO:0005667 GO:0005779 GO:0005739
GO:0016272
Cluster-22178.55237 GO:0042967 GO:0008152 GO:0006633 GO:0016491
GO:0046983 GO:0004315 GO:0005835
Cluster-22178.55238 GO:0055114 GO:0032922 GO:0042967 GO:0006633
GO:0016567 GO:0000976 GO:0004842 GO:0004318 GO:0005515
GO:0005835
Cluster-22178.55241 GO:0016567 GO:0032922 GO:0000976 GO:0005515
GO:0004842
Cluster-22178.55243 GO:0016055 GO:0006468 GO:0006629 GO:0035091
GO:0005524 GO:0004672
Cluster-22178.55246 GO:0006171 GO:0006144 GO:0009190 GO:0035556
GO:0016849 GO:0004016 GO:0005886
Cluster-22178.55248 GO:0007275 GO:0016021
Cluster-22178.55250 GO:0007275 GO:0016021
Cluster-22178.55253 GO:0006378 GO:0051258 GO:0006369 GO:0006308
GO:0007165 GO:0006379 GO:0006260 GO:0030168 GO:0019064

GO:0051082 GO:0030674 GO:0005102 GO:0008855 GO:0016758
GO:0009318 GO:0005849 GO:0019031 GO:0005789 GO:0005815
GO:0005577
Cluster-22178.55256 GO:0005509 GO:0016758 GO:0005789
Cluster-22178.55259 GO:0019497 GO:0006771 GO:0046872 GO:0030414
GO:0003993 GO:0005515 GO:0005576
Cluster-22178.55261 GO:0006355 GO:0009252 GO:0006807 GO:0004356
GO:0005515 GO:0043565 GO:0003700 GO:0055036 GO:0005667
GO:0005634
Cluster-22178.55264 GO:0005515
Cluster-22178.55270 GO:0006355 GO:0003677 GO:0003676 GO:0003723

Cluster-22178.55273 GO:0003676
Cluster-22178.55276 GO:0008152 GO:0003824
Cluster-22178.55286 GO:0035556
Cluster-22178.55288 GO:0006952 GO:0055085 GO:0006812 GO:0008324
GO:0016817 GO:0016021
Cluster-22178.55289 GO:0008270 GO:0005622
Cluster-22178.55290 GO:0006812 GO:0008324 GO:0016747 GO:0005515

Cluster-22178.55291 GO:0006812 GO:0005515 GO:0008324 GO:0016747

Cluster-22178.55294 GO:0055114 GO:0016972 GO:0005515
Cluster-22178.55297 GO:0003676 GO:0008270
Cluster-22178.55299 GO:0015940 GO:0008897 GO:0016829 GO:0000287

Cluster-22178.55300 GO:0007034 GO:0008654 GO:0006544 GO:0006566
GO:0006563 GO:0046486 GO:0008270 GO:0004609 GO:0005515
GO:0019028
Cluster-22178.55303 GO:0006771 GO:0006000 GO:0006013 GO:0019497
GO:0016301 GO:0003873 GO:0003993 GO:0005524
Cluster-22178.55305 GO:0046872 GO:0005515 GO:0032040
Cluster-22178.55307 GO:0046872

Cluster-22178.55308 GO:0015979 GO:0008270 GO:0005515 GO:0009539 GO:0009523 GO:0016020
Cluster-22178.55309 GO:0007050 GO:0045859 GO:0004861 GO:0016020 GO:0005634
Cluster-22178.55310 GO:0007186 GO:0006890 GO:0004930 GO:0016021 GO:0019028
Cluster-22178.55317 GO:0006511 GO:0031625
Cluster-22178.55320 GO:0006457 GO:0006955 GO:0007094 GO:0007155 GO:0019882 GO:0005198 GO:0051082 GO:0019028 GO:0019031 GO:0016272 GO:0009288
Cluster-22178.55321 GO:0007094 GO:0006897 GO:0042254 GO:0006457 GO:0006955 GO:0007015 GO:0007155 GO:0019882 GO:0005198 GO:0051082 GO:0019028 GO:0019031 GO:0009288 GO:0005622 GO:0016272
Cluster-22178.55324 GO:0006955 GO:0042254 GO:0006457 GO:0006897 GO:0007094 GO:0007155 GO:0007015 GO:0019882 GO:0005198 GO:0051082 GO:0019028 GO:0019031 GO:0016272 GO:0005622 GO:0009288
Cluster-22178.55326 GO:0006807 GO:0008033 GO:0006725 GO:0055114 GO:0050660 GO:0017150 GO:0016491 GO:0016627 GO:0016853 GO:0018580 GO:0005737
Cluster-22178.55334 GO:0007156 GO:0005509 GO:0016020
Cluster-22178.55339 GO:0007165 GO:0006367 GO:0006886 GO:0016192 GO:0032968 GO:0007259 GO:0051607 GO:0050778 GO:0003677 GO:0005515 GO:0005125 GO:0030117 GO:0005634
Cluster-22178.55343 GO:0043087 GO:0007264 GO:0007165 GO:0005085

Cluster-22178.55344 GO:0006810 GO:0006816 GO:0015994 GO:0006779 GO:0004853 GO:0005246 GO:0042030 GO:0016020
Cluster-22178.55345 GO:0006090 GO:0006108 GO:0055114 GO:0006099 GO:0015976 GO:0004471 GO:0005515
Cluster-22178.55346 GO:0008270 GO:0003677
Cluster-22178.55349 GO:0009405 GO:0006810 GO:0019871 GO:0005576

Cluster-22178.55352 GO:0007186 GO:0019069 GO:0006836 GO:0004930 GO:0019905 GO:0004190 GO:0016021
Cluster-22178.55354 GO:0015992 GO:0015991 GO:0016567 GO:0046872 GO:0008270 GO:0015078 GO:0004842 GO:0005515 GO:0061630 GO:0005680 GO:0033179
Cluster-22178.55355 GO:0006355 GO:0046813 GO:0061025 GO:0003677 GO:0016787 GO:0005524 GO:0005634 GO:0016021 GO:0019031
Cluster-22178.55356 GO:0046813 GO:0061025 GO:0006355 GO:0005524 GO:0003677 GO:0016787 GO:0016021 GO:0019031 GO:0005634
Cluster-22178.55357 GO:0006464 GO:0015031 GO:0022900 GO:0005515 GO:0005743 GO:0070469
Cluster-22178.55359 GO:0005515
Cluster-22178.55361 GO:0032259 GO:0008168
Cluster-22178.55362 GO:0042742 GO:0035556 GO:0005515
Cluster-22178.55363 GO:0016021
Cluster-22178.55364 GO:0071973 GO:0030288
Cluster-22178.55365 GO:0015992 GO:0006123 GO:0004129 GO:0005515 GO:0005739 GO:0045277
Cluster-22178.55368 GO:0006351 GO:0017111 GO:0003700 GO:0005515 GO:0005524 GO:0005667
Cluster-22178.55369 GO:0005515
Cluster-22178.55373 GO:0005515
Cluster-22178.55377 GO:0005524 GO:0016772
Cluster-22178.55381 GO:0000398 GO:0007009 GO:0005634
Cluster-22178.55383 GO:0002949 GO:0006260 GO:0035988 GO:0006310 GO:0002062 GO:0006576 GO:0006281 GO:0006355 GO:0042246 GO:0016887 GO:0005524 GO:0016787 GO:0009378 GO:0003677 GO:0003924 GO:0003678 GO:0005525 GO:0008134 GO:0016301 GO:0005657 GO:0009379 GO:0005667 GO:0005634
Cluster-22178.55384 GO:0016765
Cluster-22178.55385 GO:0031505 GO:0009229 GO:0030975 GO:0019031
Cluster-22178.55389 GO:0006811 GO:0005216 GO:0016020
Cluster-22178.55391 GO:0005515

Cluster-22178.55394 GO:0006508 GO:0004222
Cluster-22178.55402 GO:0006260 GO:0015031 GO:0055114 GO:0016192
GO:0006886 GO:0007264 GO:0047134 GO:0070403 GO:0008270
GO:0008565 GO:0005096 GO:0016491 GO:0005515 GO:0005737
GO:0016020
Cluster-22178.55404 GO:0006412 GO:0042254 GO:0003735 GO:0005515
GO:0005622 GO:0005840
Cluster-22178.55405 GO:0006571 GO:0006694 GO:0015940 GO:0009094
GO:0019521 GO:0055114 GO:0008210 GO:0000162 GO:0046168
GO:0008209 GO:0008207 GO:0006098 GO:0004665 GO:0003824
GO:0008677 GO:0004616 GO:0016616 GO:0008977 GO:0003854
GO:0051287 GO:0050662 GO:0016021
Cluster-22178.55407 GO:0008152 GO:0003824
Cluster-22178.55409 GO:0006470 GO:0006402 GO:0006468 GO:0051252
GO:0004535 GO:0046872 GO:0005524 GO:0004672 GO:0003676
GO:0003723 GO:0008138 GO:0005737 GO:0005634
Cluster-22178.55410 GO:0005515
Cluster-22178.55411 GO:0005515
Cluster-22178.55414 GO:0015893 GO:0030436 GO:0006855 GO:0015238
GO:0005886 GO:0042601
Cluster-22178.55415 GO:0005515
Cluster-22178.55416 GO:0007219 GO:0045944 GO:0003713 GO:0005667
GO:0016607
Cluster-22178.55419 GO:0006468 GO:0006465 GO:0016773 GO:0005524
GO:0004672 GO:0008233 GO:0016757 GO:0005787 GO:0016020
GO:0016021
Cluster-22178.55421 GO:0007165 GO:0040007 GO:0008283 GO:0008083
GO:0005515 GO:0016787 GO:0004190 GO:0016021
Cluster-22178.55422 GO:0009405 GO:0046999 GO:0005198 GO:0019867
Cluster-22178.55423 GO:0007186 GO:0006270 GO:0007606 GO:0019236
GO:0006629 GO:0004932 GO:0016020
Cluster-22178.55424 GO:0042254 GO:0006412 GO:0003735 GO:0005622
GO:0005783 GO:0005840

Cluster-22178.55428 GO:0022900 GO:0006955 GO:0007165 GO:0016032
GO:0006355 GO:0005164 GO:0003677 GO:0003682 GO:0000785
GO:0000775 GO:0016020 GO:0019012 GO:0005747 GO:0005634
GO:0005739
Cluster-22178.55431 GO:0016787 GO:0019028
Cluster-22178.55436 GO:0005524 GO:0005515 GO:0003676 GO:0005634
Cluster-22178.55437 GO:0005515 GO:0003676 GO:0005524 GO:0005634
Cluster-22178.55440 GO:0005515 GO:0008146 GO:0015016 GO:0016787

Cluster-22178.55441 GO:0006952 GO:0005509
Cluster-22178.55443 GO:0055114
Cluster-22178.55445 GO:0005515
Cluster-22178.55450 GO:0017004 GO:0015886 GO:0004867 GO:0016021
Cluster-22178.55455 GO:0016887 GO:0003924 GO:0005524 GO:0005525
GO:0000166 GO:0003677
Cluster-22178.55458 GO:0040007 GO:0008283 GO:0006955 GO:0007165
GO:0006464 GO:0019752 GO:0030170 GO:0008083 GO:0016831
GO:0005134 GO:0016151 GO:0005576 GO:0005893
Cluster-22178.55460 GO:0042967 GO:0006355 GO:0003712 GO:0008270
GO:0004402 GO:0000123 GO:0005667 GO:0005634
Cluster-22178.55462 GO:0006479 GO:0006351 GO:0006468 GO:0006481
GO:0006383 GO:0006144 GO:0006206 GO:0003899 GO:0003677
GO:0004671 GO:0005524 GO:0004672 GO:0005730 GO:0005666
GO:0016021
Cluster-22178.55465 GO:0016043 GO:0000398 GO:0003779 GO:0005681
Cluster-22178.55466 GO:0016579 GO:0036459
Cluster-22178.55471 GO:0006629 GO:0008081
Cluster-22178.55472 GO:0016020
Cluster-22178.55474 GO:0045454
Cluster-22178.55475 GO:0048013 GO:0005515
Cluster-22178.55476 GO:0048870 GO:0006914 GO:0051607 GO:0006606
GO:0008092 GO:0045502 GO:0008134 GO:0042803 GO:0005737
GO:0009424 GO:0030286 GO:0019898 GO:0005667 GO:0031514

Cluster-22178.55477 GO:0006886 GO:0006468 GO:0005524 GO:0004672 GO:0030176
Cluster-22178.55479 GO:0005515
Cluster-22178.55484 GO:0007224
Cluster-22178.55490 GO:0003712 GO:0003724 GO:0016818 GO:0005667 GO:0005634
Cluster-22178.55495 GO:0007186 GO:0004930 GO:0016020 GO:0016021
Cluster-22178.55496 GO:0007186 GO:0004930 GO:0016021 GO:0016020
Cluster-22178.55497 GO:0005515
Cluster-22178.55498 GO:0006508 GO:0005515 GO:0004252
Cluster-22178.55499 GO:0005515
Cluster-22178.55502 GO:0007186 GO:0004930 GO:0016021 GO:0016020
Cluster-22178.55510 GO:0005515
Cluster-22178.55511 GO:0016579 GO:0036459
Cluster-22178.55512 GO:0046740 GO:0006771 GO:0006810 GO:0015992 GO:0006123 GO:0055085 GO:0006570 GO:0006814 GO:0019497 GO:0006470 GO:0008138 GO:0003677 GO:0008270 GO:0003993 GO:0003723 GO:0005515 GO:0046872 GO:0005215 GO:0004725 GO:0004129 GO:0005739 GO:0016020 GO:0033644 GO:0045277 GO:0016021
Cluster-22178.55514 GO:0006814 GO:0006810 GO:0006123 GO:0055085 GO:0015992 GO:0003723 GO:0004129 GO:0005215 GO:0008270 GO:0016020 GO:0005739 GO:0016021 GO:0045277
Cluster-22178.55516 GO:0006355 GO:0003700 GO:0005667
Cluster-22178.55519 GO:0007165 GO:0006811 GO:0007268 GO:0004970 GO:0016020
Cluster-22178.55521 GO:0006855 GO:0055085 GO:0009069 GO:0006810 GO:0007268 GO:0007165 GO:0015893 GO:0016310 GO:0006811 GO:0006468 GO:0005524 GO:0004674 GO:0004970 GO:0015297 GO:0005234 GO:0015238 GO:0016020
Cluster-22178.55523 GO:0007186 GO:0004190 GO:0005515 GO:0004930 GO:0016021
Cluster-22178.55526 GO:0005515

Cluster-22178.55528 GO:0032040
Cluster-22178.55529 GO:0008033 GO:0051252 GO:0007165 GO:0003676
GO:0005179 GO:0004526 GO:0030677 GO:0005576 GO:0005634
Cluster-22178.55536 GO:0005515
Cluster-22178.55543 GO:0018279 GO:0005789
Cluster-22178.55548 GO:0006376 GO:0003729 GO:0005685
Cluster-22178.55549 GO:0007186 GO:0004930 GO:0016020 GO:0016021
Cluster-22178.55550 GO:0007186 GO:0004930 GO:0016021
Cluster-22178.55551 GO:0006281 GO:0008270 GO:0046872
Cluster-22178.55555 GO:0055085 GO:0016021
Cluster-22178.55556 GO:0005515 GO:0005509 GO:0004064
Cluster-22178.55560 GO:0008152 GO:0016791 GO:0003824 GO:0046872
GO:0016021
Cluster-22178.55562 GO:0032183 GO:0070140
Cluster-22178.55563 GO:0003724 GO:0005525 GO:0016887 GO:0003924
GO:0005524 GO:0003774 GO:0005515 GO:0003723 GO:0016459
Cluster-22178.55565 GO:0015074 GO:0042967 GO:0050832 GO:0042742
GO:0003676 GO:0008080 GO:0019239 GO:0003677 GO:0031224
Cluster-22178.55567 GO:0050832 GO:0042967 GO:0015074 GO:0042742
GO:0008080 GO:0003676 GO:0003677 GO:0019239 GO:0031224
Cluster-22178.55568 GO:0006298 GO:0005524 GO:0030983
Cluster-22178.55573 GO:0007264 GO:0005525
Cluster-22178.55574 GO:0006397 GO:0005634
Cluster-22178.55575 GO:0005634
Cluster-22178.55577 GO:0015992 GO:0055114 GO:0006744 GO:0006814
GO:0006120 GO:0008137
Cluster-22178.55579 GO:0016787
Cluster-22178.55580 GO:0007264 GO:0007165 GO:0007186 GO:0006184
GO:0019001 GO:0031683 GO:0003924 GO:0005525 GO:0004871
GO:0016020
Cluster-22178.55582 GO:0006810 GO:0007155 GO:0005215 GO:0016021
GO:0005737
Cluster-22178.55583 GO:0003676

Cluster-22178.55586 GO:0005515
Cluster-22178.55587 GO:0019521 GO:0055114 GO:0008152 GO:0006098
GO:0007005 GO:0016491 GO:0004616 GO:0031305
Cluster-22178.55588 GO:0006879 GO:0046872 GO:0008199
Cluster-22178.55593 GO:0008063 GO:0008270 GO:0005515
Cluster-22178.55596 GO:0009236 GO:0006824 GO:0006355 GO:0015087
GO:0003676 GO:0003700 GO:0005667 GO:0016020
Cluster-22178.55597 GO:0006824 GO:0009236 GO:0006355 GO:0003700
GO:0003676 GO:0015087 GO:0005667 GO:0016020
Cluster-22178.55599 GO:0019064 GO:0007165 GO:0007172 GO:0006415
GO:0008283 GO:0000160 GO:0008608 GO:0006955 GO:0048193
GO:0016310 GO:0040007 GO:0006468 GO:0006449 GO:0004871
GO:0004713 GO:0046983 GO:0008270 GO:0005126 GO:0000155
GO:0016149 GO:0008083 GO:0018444 GO:0016021 GO:0019031
GO:0009365 GO:0005840 GO:0005576 GO:0005876 GO:0016020
GO:0005925 GO:0005737 GO:0042729
Cluster-22178.55606 GO:0006886 GO:0016192 GO:0005515 GO:0030117
Cluster-22178.55607 GO:0006886 GO:0016192 GO:0005515 GO:0030117
Cluster-22178.55608 GO:0009395 GO:0046339 GO:0035556 GO:0006355
GO:0006629 GO:0007165 GO:0003676 GO:0004435 GO:0005509
GO:0005515 GO:0005622
Cluster-22178.55612 GO:0047355 GO:0003677 GO:0016020
Cluster-22178.55618 GO:0042254 GO:0006412 GO:0019843 GO:0003735
GO:0005840
Cluster-22178.55619 GO:0009405 GO:0006811 GO:0008375 GO:0005216
GO:0016020 GO:0005576
Cluster-22178.55621 GO:0009405 GO:0006811 GO:0005216 GO:0008375
GO:0005576 GO:0016020
Cluster-22178.55622 GO:0009405 GO:0006811 GO:0008375 GO:0005216
GO:0016020 GO:0005576
Cluster-22178.55624 GO:0006260 GO:0003677 GO:0005664
Cluster-22178.55626 GO:0007016 GO:0007165 GO:0042254 GO:0006412
GO:0005158 GO:0003779 GO:0005200 GO:0000062 GO:0003735

GO:0005622 GO:0005856 GO:0005899 GO:0005840 GO:0001726
GO:0005925
Cluster-22178.55627 GO:0046931 GO:0006952 GO:0007165 GO:0006810
GO:0006812 GO:0052331 GO:0005126 GO:0015267 GO:0005576
GO:0046930
Cluster-22178.55629 GO:0006771 GO:0019497 GO:0006260 GO:0008408
GO:0000166 GO:0003887 GO:0003677 GO:0003993 GO:0042575
Cluster-22178.55632 GO:0005525 GO:0003924 GO:0005861
Cluster-22178.55637 GO:0006508 GO:0008234
Cluster-22178.55641 GO:0006334 GO:0007283 GO:0008270 GO:0005524
GO:0046872 GO:0003677 GO:0005515 GO:0000786 GO:0005634
Cluster-22178.55643 GO:0051258 GO:0070206 GO:0030168 GO:0016973
GO:0007165 GO:0005102 GO:0030674 GO:0003677 GO:0005643
GO:0005577
Cluster-22178.55644 GO:0007264 GO:0015684 GO:0015093 GO:0005525
GO:0019028 GO:0016021
Cluster-22178.55647 GO:0006308 GO:0006376 GO:0010508 GO:0006904
GO:0034508 GO:0015031 GO:0019069 GO:0042254 GO:0008855
GO:0008270 GO:0003729 GO:0000776 GO:0009318 GO:0005685
GO:0005622 GO:0000145
Cluster-22178.55648 GO:0042254 GO:0006904 GO:0015031 GO:0005515
GO:0000145
Cluster-22178.55649 GO:0007165 GO:0007186 GO:0008612 GO:0004871
GO:0005834
Cluster-22178.55651 GO:0055114 GO:0006813 GO:0006351 GO:0006144
GO:0006206 GO:0035556 GO:0016567 GO:0046872 GO:0005249
GO:0030955 GO:0003899 GO:0005515 GO:0008270 GO:0047134
GO:0004842 GO:0003677 GO:0005737 GO:0016021 GO:0005680
GO:0005730 GO:0008076
Cluster-22178.55652 GO:0006813 GO:0006351 GO:0006412 GO:0006886
GO:0042254 GO:0016567 GO:0006464 GO:0006206 GO:0006144
GO:0006888 GO:0030955 GO:0005515 GO:0003899 GO:0004519
GO:0046872 GO:0005249 GO:0016151 GO:0003676 GO:0003677

GO:0003735 GO:0008270 GO:0004842 GO:0005737 GO:0005840
GO:0016021 GO:0005730 GO:0030127 GO:0008076 GO:0005622
GO:0005680
Cluster-22178.55655 GO:0035556 GO:0006144 GO:0006206 GO:0016567
GO:0055114 GO:0006351 GO:0006813 GO:0004842 GO:0047134
GO:0008270 GO:0003677 GO:0046872 GO:0005249 GO:0005515
GO:0003899 GO:0030955 GO:0005680 GO:0008076 GO:0005730
GO:0016021 GO:0005737
Cluster-22178.55657 GO:0006144 GO:0006888 GO:0006464 GO:0006206
GO:0042254 GO:0016567 GO:0006886 GO:0006412 GO:0006351
GO:0008270 GO:0004842 GO:0003735 GO:0003677 GO:0046872
GO:0003676 GO:0016151 GO:0003899 GO:0005515 GO:0004519
GO:0005622 GO:0005680 GO:0005730 GO:0030127 GO:0005737
GO:0005840
Cluster-22178.55658 GO:0006206 GO:0006144 GO:0035556 GO:0016567
GO:0055114 GO:0006813 GO:0006351 GO:0008270 GO:0047134
GO:0004842 GO:0003677 GO:0046872 GO:0005249 GO:0030955
GO:0003899 GO:0005515 GO:0005680 GO:0005730 GO:0008076
GO:0005737 GO:0016021
Cluster-22178.55659 GO:0006351 GO:0006144 GO:0006206 GO:0005515
GO:0003899 GO:0003677 GO:0008270 GO:0046872 GO:0005730
Cluster-22178.55661 GO:0006464 GO:0006206 GO:0006144 GO:0042254
GO:0006281 GO:0006351 GO:0006412 GO:0008270 GO:0003677
GO:0003735 GO:0046872 GO:0016151 GO:0003676 GO:0003899
GO:0004519 GO:0005622 GO:0005730 GO:0005840
Cluster-22178.55662 GO:0042254 GO:0016567 GO:0006888 GO:0006144
GO:0006464 GO:0006206 GO:0006813 GO:0006886 GO:0006412
GO:0006351 GO:0003735 GO:0003677 GO:0008270 GO:0004842
GO:0030955 GO:0003899 GO:0005515 GO:0004519 GO:0046872
GO:0005249 GO:0003676 GO:0016151 GO:0005730 GO:0030127
GO:0008076 GO:0005622 GO:0005680 GO:0005737 GO:0016021
GO:0005840
Cluster-22178.55663 GO:0006351 GO:0006144 GO:0006206 GO:0003899

GO:0003677 GO:0046872 GO:0005730

Cluster-22178.55665 GO:0019370 GO:0008270 GO:0008237

Cluster-22178.55666 GO:0018342 GO:0008318 GO:0008270 GO:0005515 GO:0004663 GO:0005968

Cluster-22178.55668 GO:0004190 GO:0016021

Cluster-22178.55669 GO:0006810 GO:0009405 GO:0006281 GO:0000077 GO:0055085 GO:0003723 GO:0004222 GO:0008270 GO:0016020 GO:0005576 GO:0030896

Cluster-22178.55670 GO:0008376 GO:0016757 GO:0032580 GO:0016020

Cluster-22178.55677 GO:0006260 GO:0055114

Cluster-22178.55678 GO:0008033 GO:0006269 GO:0009451 GO:0006351 GO:0003896 GO:0009020 GO:0005657 GO:0005730

Cluster-22178.55681 GO:0003676

Cluster-22178.55685 GO:0006351 GO:0006412 GO:0006265 GO:0042254 GO:0006464 GO:0006206 GO:0006144 GO:0003899 GO:0016151 GO:0003676 GO:0003677 GO:0003735 GO:0003916 GO:0008270 GO:0005840 GO:0005694 GO:0005730 GO:0005622

Cluster-22178.55686 GO:0008033 GO:0009451 GO:0008175 GO:0005515

Cluster-22178.55688 GO:0006260 GO:0000166 GO:0003887 GO:0003677 GO:0008408 GO:0001882 GO:0008270 GO:0042575 GO:0005634

Cluster-22178.55689 GO:0045892 GO:0008270 GO:0005634

Cluster-22178.55690 GO:0030001 GO:0007034 GO:0005887

Cluster-22178.55692 GO:0006355 GO:0006955 GO:0007165 GO:0005515 GO:0003677 GO:0005125 GO:0005634 GO:0005576

Cluster-22178.55696 GO:0006820 GO:0008643 GO:0015725 GO:0006812 GO:0046411 GO:0015649 GO:0016021

Cluster-22178.55698 GO:0045893 GO:0034508 GO:0015672 GO:0006812 GO:0007275 GO:0010508 GO:0008270 GO:0005261 GO:0005515 GO:0000776 GO:0016020

Cluster-22178.55699 GO:0007165 GO:0006955 GO:0046872 GO:0005164 GO:0016020

Cluster-22178.55700 GO:0006508 GO:0008270 GO:0046872 GO:0004252

GO:0008236 GO:0005618
Cluster-22178.55701 GO:0007165 GO:0006955 GO:0005164 GO:0016020
Cluster-22178.55703 GO:0006355 GO:0005515 GO:0005634
Cluster-22178.55704 GO:0055085 GO:0006810 GO:0005524 GO:0016887
GO:0005215 GO:0042626 GO:0003676 GO:0000166 GO:0003677
GO:0016021
Cluster-22178.55705 GO:0016787
Cluster-22178.55707 GO:0007264 GO:0006811 GO:0055114 GO:0010181
GO:0005216 GO:0005525 GO:0016491 GO:0016020 GO:0005615
Cluster-22178.55709 GO:0006281 GO:0046872 GO:0008270
Cluster-22178.55710 GO:0003676
Cluster-22178.55714 GO:0006144 GO:0015992 GO:0006123 GO:0000256
GO:0006807 GO:0004037 GO:0004129 GO:0019901 GO:0045277
Cluster-22178.55716 GO:0009395 GO:0035556 GO:0007205 GO:0046486
GO:0004143 GO:0016301
Cluster-22178.55717 GO:0007205 GO:0035556 GO:0009395 GO:0046486
GO:0004143 GO:0008270 GO:0046872 GO:0016301 GO:0005515
GO:0005622
Cluster-22178.55719 GO:0006144 GO:0015992 GO:0006123 GO:0006807
GO:0000256 GO:0004037 GO:0004129 GO:0019901 GO:0045277
Cluster-22178.55720 GO:0046872
Cluster-22178.55721 GO:0000256 GO:0006807 GO:0006123 GO:0015992
GO:0006144 GO:0004129 GO:0004037 GO:0019901 GO:0045277
Cluster-22178.55726 GO:0046872
Cluster-22178.55731 GO:0051016 GO:0006508 GO:0008152 GO:0016787
GO:0008236 GO:0008290
Cluster-22178.55733 GO:0003950 GO:0046872
Cluster-22178.55734 GO:0006144 GO:0007165 GO:0004114 GO:0005515

Cluster-22178.55735 GO:0003950
Cluster-22178.55736 GO:0005975 GO:0004415 GO:0005515
Cluster-22178.55738 GO:0006355 GO:0010508 GO:0051028 GO:0048309
GO:0006606 GO:0008134 GO:0005524 GO:0003700 GO:0005667

Cluster-22178.55740 GO:0005515

Cluster-22178.55744 GO:0006576 GO:0007264 GO:0015684 GO:0003924 GO:0015093 GO:0005524 GO:0005525 GO:0016021

Cluster-22178.55745 GO:0007264 GO:0006576 GO:0015684 GO:0015093 GO:0005524 GO:0003924 GO:0005525 GO:0016021

Cluster-22178.55751 GO:0006367 GO:0007010 GO:0006270 GO:0032968 GO:0003677 GO:0003779 GO:0005634 GO:0032040

Cluster-22178.55752 GO:0015992 GO:0015986 GO:0015078 GO:0000276

Cluster-22178.55754 GO:0005515 GO:0046872 GO:0016021

Cluster-22178.55758 GO:0000002 GO:0016887 GO:0003924 GO:0005524 GO:0003724 GO:0005525 GO:0003723 GO:0016787 GO:0003677 GO:0000262 GO:0045095

Cluster-22178.55760 GO:0005515

Cluster-22178.55762 GO:0009877 GO:0009312 GO:0008757

Cluster-22178.55765 GO:0042254 GO:0006412 GO:0055114 GO:0015074 GO:0003735 GO:0003824 GO:0050660 GO:0016491 GO:0005840 GO:0005622

Cluster-22178.55766 GO:0055114 GO:0015074 GO:0042254 GO:0006412 GO:0003824 GO:0050660 GO:0016491 GO:0003735 GO:0005840 GO:0005622

Cluster-22178.55770 GO:0008152 GO:0003824 GO:0016836

Cluster-22178.55773 GO:0016579 GO:0016192 GO:0006886 GO:0006506 GO:0015991 GO:0036459 GO:0005515 GO:0016820 GO:0016746 GO:0016021 GO:0000221 GO:0005789 GO:0030117

Cluster-22178.55775 GO:0051252 GO:0004540 GO:0003677

Cluster-22178.55776 GO:0005515

Cluster-22178.55781 GO:0017038 GO:0007165 GO:0005158 GO:0008270 GO:0005524 GO:0003676 GO:0016787 GO:0003677 GO:0005899 GO:0005634 GO:0016020

Cluster-22178.55788 GO:0006470 GO:0006570 GO:0006355 GO:0004725 GO:0003700 GO:0043565 GO:0008138 GO:0016020 GO:0005667

Cluster-22178.55795 GO:0006468 GO:0016192 GO:0005524 GO:0004672 GO:0016021

Cluster-22178.55796 GO:0046872 GO:0008168 GO:0005515
Cluster-22178.55798 GO:0016746
Cluster-22178.55805 GO:0005737
Cluster-22178.55807 GO:0016020
Cluster-22178.55810 GO:0005515
Cluster-22178.55811 GO:0006144 GO:0006508 GO:0003968 GO:0017111 GO:0004197 GO:0016817 GO:0031379
Cluster-22178.55813 GO:0006886 GO:0006367 GO:0006888 GO:0008270 GO:0016021 GO:0030127 GO:0005672
Cluster-22178.55814 GO:0008376 GO:0032580
Cluster-22178.55816 GO:0006355 GO:0006468 GO:0006298 GO:0004672 GO:0005524 GO:0016773 GO:0043565 GO:0003700 GO:0030983 GO:0005667 GO:0016020
Cluster-22178.55817 GO:0055114 GO:0006631 GO:0071949 GO:0016491 GO:0050151
Cluster-22178.55819 GO:0006269 GO:0006351 GO:0003896 GO:0019825 GO:0005515 GO:0005198 GO:0020037 GO:0003677 GO:0005657 GO:0005634 GO:0005730
Cluster-22178.55821 GO:0016579 GO:0036459
Cluster-22178.55823 GO:0045766 GO:0043066 GO:0043123
Cluster-22178.55824 GO:0006308 GO:0000077 GO:0006376 GO:0006281 GO:0006355 GO:0006810 GO:0043565 GO:0000014 GO:0003700 GO:0003729 GO:0005685 GO:0016021 GO:0005739 GO:0005667
Cluster-22178.55826 GO:0046872 GO:0005515 GO:0032040
Cluster-22178.55828 GO:0006355 GO:0007165 GO:0035257 GO:0005515 GO:0003713 GO:0046983 GO:0005667 GO:0005634
Cluster-22178.55829 GO:0006355 GO:0005515 GO:0005634
Cluster-22178.55831 GO:0005515
Cluster-22178.55833 GO:0055085 GO:0032007 GO:0048011 GO:0005515 GO:0046872 GO:0008270 GO:0016021
Cluster-22178.55834 GO:0055114
Cluster-22178.55836 GO:0003824 GO:0030151 GO:0030170
Cluster-22178.55842 GO:0003676 GO:0005515 GO:0005509

Cluster-22178.55845 GO:0015992 GO:0007165 GO:0006952 GO:0006508
GO:0015986 GO:0015078 GO:0008270 GO:0004252 GO:0003677
GO:0004197 GO:0005044 GO:0016020
Cluster-22178.55846 GO:0006367 GO:0006123 GO:0015992 GO:0004129
GO:0046872 GO:0008270 GO:0005634 GO:0005672 GO:0045277
Cluster-22178.55848 GO:0019068 GO:0043565 GO:0005515 GO:0005198
GO:0005634
Cluster-22178.55853 GO:0005515 GO:0003677
Cluster-22178.55858 GO:0006744 GO:0006120 GO:0015992 GO:0006814
GO:0006118 GO:0005515 GO:0008137 GO:0003954 GO:0005739
Cluster-22178.55859 GO:0006468 GO:0006606 GO:0016773 GO:0004672
GO:0005524 GO:0016020
Cluster-22178.55860 GO:0006367 GO:0032968 GO:0003677 GO:0005634
Cluster-22178.55861 GO:0006359
Cluster-22178.55863 GO:0009772 GO:0019684 GO:0006118 GO:0045156

Cluster-22178.55866 GO:0022900 GO:0006904 GO:0006118 GO:0006260
GO:0006891 GO:0015031 GO:0006457 GO:0007067 GO:0009055
GO:0020037 GO:0051082 GO:0005506 GO:0000775 GO:0005634
GO:0042597 GO:0016272 GO:0000145 GO:0017119
Cluster-22178.55867 GO:0022900 GO:0006904 GO:0006118 GO:0006891
GO:0006260 GO:0015031 GO:0006457 GO:0007067 GO:0009055
GO:0020037 GO:0051082 GO:0005506 GO:0000775 GO:0005634
GO:0042597 GO:0016272 GO:0000145 GO:0017119
Cluster-22178.55869 GO:0006629 GO:0050909 GO:0007186 GO:0055085
GO:0004930 GO:0016021
Cluster-22178.55872 GO:0006355 GO:0005524 GO:0043565 GO:0005515
GO:0003700 GO:0005667
Cluster-22178.55873 GO:0019722 GO:0006352 GO:0006402 GO:0006730
GO:0007018 GO:0051252 GO:0007017 GO:0006355 GO:0008017
GO:0016987 GO:0005524 GO:0003677 GO:0003676 GO:0003700
GO:0003777 GO:0046872 GO:0004535 GO:0005515 GO:0003723
GO:0016740 GO:0005667 GO:0045298 GO:0005874 GO:0005737

GO:0005634
Cluster-22178.55874 GO:0006810 GO:0006811 GO:0005515 GO:0005230
GO:0016020
Cluster-22178.55875 GO:0043093 GO:0000917 GO:0008376 GO:0005737
GO:0032580
Cluster-22178.55876 GO:0006570 GO:0006470 GO:0004725 GO:0008138

Cluster-22178.55877 GO:0046500 GO:0006260 GO:0035556 GO:0030488
GO:0006464 GO:0006554 GO:0006479 GO:0006351 GO:0009451
GO:0008033 GO:0003677 GO:0016429 GO:0003887 GO:0018024
GO:0004719 GO:0031515 GO:0042575
Cluster-22178.55879 GO:0006355 GO:0046872 GO:0008270 GO:0005515
GO:0005634
Cluster-22178.55881 GO:0006952 GO:0008284 GO:0005515
Cluster-22178.55882 GO:0006260 GO:0006265 GO:0003677 GO:0005509
GO:0003916 GO:0005727
Cluster-22178.55885 GO:0030153 GO:0006367 GO:0006955 GO:0006357
GO:0015643 GO:0004252 GO:0001104 GO:0005672 GO:0016592
GO:0019814 GO:0005886
Cluster-22178.55886 GO:0006570 GO:0006470 GO:0004725 GO:0008138
GO:0046872
Cluster-22178.55891 GO:0007165 GO:0005509 GO:0005578
Cluster-22178.55894 GO:0006810 GO:0006811 GO:0005230 GO:0016020
Cluster-22178.55897 GO:0006355 GO:0003677 GO:0008270 GO:0005506

Cluster-22178.55898 GO:0007411 GO:0006355 GO:0003677 GO:0005506
GO:0008270
Cluster-22178.55899 GO:0014068 GO:0050776 GO:0006351 GO:0007165
GO:0008270 GO:0005102 GO:0003676 GO:0043548 GO:0016020
Cluster-22178.55901 GO:0016114 GO:0006694 GO:0055114 GO:0003676
GO:0050660 GO:0004506 GO:0016021
Cluster-22178.55905 GO:0019082 GO:0016032 GO:0006281 GO:0007049
GO:0030150 GO:0005515 GO:0003677 GO:0003824 GO:0005096

GO:0005742 GO:0034464
Cluster-22178.55906 GO:0016192 GO:0019217 GO:0006886 GO:0015992
GO:0006123 GO:0005515 GO:0000062 GO:0004129 GO:0030117
GO:0045277
Cluster-22178.55907 GO:0007049 GO:0030150 GO:0019082 GO:0006281
GO:0016032 GO:0003824 GO:0005096 GO:0005515 GO:0034464
GO:0005742
Cluster-22178.55909 GO:0045892 GO:0005634
Cluster-22178.55910 GO:0032065 GO:0005543 GO:0005515 GO:0005938
Cluster-22178.55914 GO:0032780 GO:0016820 GO:0042030 GO:0046872
GO:0005739
Cluster-22178.55915 GO:0006506 GO:0016758 GO:0016021
Cluster-22178.55916 GO:0005576
Cluster-22178.55917 GO:0016020 GO:0019033
Cluster-22178.55918 GO:0006418 GO:0005524 GO:0004812 GO:0000166
GO:0005515
Cluster-22178.55919 GO:0005576
Cluster-22178.55922 GO:0006741 GO:0046497 GO:0007218 GO:0008152
GO:0006769 GO:0016301 GO:0003951 GO:0005576
Cluster-22178.55923 GO:0008152 GO:0008134 GO:0003824 GO:0005667
GO:0005634
Cluster-22178.55924 GO:0007094 GO:0006355 GO:0005515 GO:0003700
GO:0042025 GO:0005667
Cluster-22178.55925 GO:0003677 GO:0008270
Cluster-22178.55927 GO:0007186 GO:0004930 GO:0000975 GO:0046872
GO:0016021
Cluster-22178.55930 GO:0009405 GO:0006810 GO:0007049 GO:0019855
GO:0017053 GO:0005576
Cluster-22178.55933 GO:0016021
Cluster-22178.55935 GO:0006355 GO:0043401 GO:0043565 GO:0008270
GO:0003700 GO:0016021 GO:0005667 GO:0005634
Cluster-22178.55937 GO:0016020
Cluster-22178.55940 GO:0008610 GO:0006396 GO:0016747 GO:0008452

Cluster-22178.55942 GO:0005978 GO:0005982 GO:0005985 GO:0004373

Cluster-22178.55943 GO:0006396 GO:0008610 GO:0008452 GO:0016747

Cluster-22178.55945 GO:0032259 GO:0055114 GO:0006810 GO:0008168 GO:0005215 GO:0016972 GO:0016020

Cluster-22178.55950 GO:0003676

Cluster-22178.55951 GO:0005634

Cluster-22178.55952 GO:0005634

Cluster-22178.55954 GO:0006555 GO:0006457 GO:0006355 GO:0003700 GO:0008270 GO:0005198 GO:0008134 GO:0042803 GO:0051082 GO:0045502 GO:0030286 GO:0016272 GO:0005882 GO:0005667 GO:0048786

Cluster-22178.55955 GO:0015992 GO:0006123 GO:0006508 GO:0005524 GO:0008242 GO:0016817 GO:0016887 GO:0004129 GO:0016740 GO:0016788 GO:0004197 GO:0045277 GO:0005680

Cluster-22178.55957 GO:0016788

Cluster-22178.55958 GO:0055114 GO:0006468 GO:0016616 GO:0051287 GO:0005524 GO:0004672

Cluster-22178.55959 GO:0006270 GO:0003723 GO:0003676

Cluster-22178.55960 GO:0055114 GO:0007186 GO:0046872 GO:0016972 GO:0004930 GO:0016021

Cluster-22178.55963 GO:0019497 GO:0006771 GO:0005975 GO:0046872 GO:0004553 GO:0005515 GO:0003993

Cluster-22178.55964 GO:0042262 GO:0006464 GO:0007165 GO:0006355 GO:0015986 GO:0046933 GO:0008270 GO:0005515 GO:0003677 GO:0001664 GO:0045259

Cluster-22178.55972 GO:0005634

Cluster-22178.55976 GO:0016042 GO:0019497 GO:0007586 GO:0006771 GO:0003993 GO:0008047 GO:0046872 GO:0005515 GO:0005576

Cluster-22178.55977 GO:0006351 GO:0006479 GO:0031167 GO:0006206 GO:0006144 GO:0008152 GO:0003677 GO:0003899 GO:0008168

GO:0005730
Cluster-22178.55981 GO:0006811 GO:0055085 GO:0006355 GO:0005515
GO:0005216 GO:0005761 GO:0016020
Cluster-22178.55982 GO:0003677
Cluster-22178.55985 GO:0005515
Cluster-22178.55986 GO:0046872
Cluster-22178.55988 GO:0009271 GO:0006355 GO:0003676 GO:0005524
GO:0003677 GO:0016787 GO:0004003 GO:0005657
Cluster-22178.55989 GO:0006508 GO:0004222
Cluster-22178.55991 GO:0003676
Cluster-22178.55992 GO:0003824
Cluster-22178.55994 GO:0005096
Cluster-22178.55995 GO:0016887 GO:0005524
Cluster-22178.55997 GO:0006334 GO:0006810 GO:0003676 GO:0005634
GO:0005643
Cluster-22178.55998 GO:0006468 GO:0005515 GO:0005524 GO:0004672
GO:0016021
Cluster-22178.55999 GO:0006810 GO:0055114 GO:0055085 GO:0016301
GO:0016627 GO:0005524 GO:0003924 GO:0016887 GO:0005525
GO:0042626 GO:0016021
Cluster-22178.56001 GO:0006457 GO:0000413 GO:0003676 GO:0003755
GO:0051082 GO:0005737
Cluster-22178.56004 GO:0007169 GO:0050830 GO:0006468 GO:0005524
GO:0016817 GO:0004714 GO:0005515 GO:0016020
Cluster-22178.56007 GO:0006508 GO:0006144 GO:0004197 GO:0017111
GO:0003968 GO:0004252 GO:0031379
Cluster-22178.56008 GO:0006998 GO:0006406 GO:0006611 GO:0031965
GO:0005576
Cluster-22178.56010 GO:0005515
Cluster-22178.56011 GO:0006355 GO:0001104 GO:0016592
Cluster-22178.56012 GO:0048193 GO:0005887 GO:0005783
Cluster-22178.56014 GO:0005515
Cluster-22178.56017 GO:0051694 GO:0005523

Cluster-22178.56022 GO:0055114 GO:0046872 GO:0016972 GO:0019028
Cluster-22178.56023 GO:0006511 GO:0031625
Cluster-22178.56024 GO:0046870
Cluster-22178.56027 GO:0006807 GO:0005524 GO:0070403 GO:0003933

Cluster-22178.56029 GO:0046872
Cluster-22178.56030 GO:0006144 GO:0046034 GO:0046872 GO:0004017

Cluster-22178.56031 GO:0046872
Cluster-22178.56033 GO:0015693 GO:0015979 GO:0015095 GO:0016020
GO:0016021 GO:0009523 GO:0009539
Cluster-22178.56035 GO:0006464 GO:0015031 GO:0032465 GO:0006915
GO:0016567 GO:0004842 GO:0005515 GO:0016020
Cluster-22178.56037 GO:0006206 GO:0006541 GO:0006221 GO:0016787
GO:0003883
Cluster-22178.56038 GO:0006206 GO:0006221 GO:0006541 GO:0016787
GO:0003883
Cluster-22178.56040 GO:0006355 GO:0007264 GO:0009271 GO:0005525

Cluster-22178.56041 GO:0009271 GO:0007264 GO:0006355 GO:0005525

Cluster-22178.56046 GO:0050840 GO:0005515 GO:0005576 GO:0019028
Cluster-22178.56047 GO:0055085 GO:0016021 GO:0016020
Cluster-22178.56051 GO:0090063 GO:0000226 GO:0005634 GO:0005815
GO:0000922
Cluster-22178.56056 GO:0006810 GO:0006468 GO:0015696 GO:0008519
GO:0016787 GO:0004672 GO:0005524 GO:0016020 GO:0031224
GO:0005794 GO:0016021
Cluster-22178.56058 GO:0019062 GO:0019058 GO:0007155 GO:0006355
GO:0003677
Cluster-22178.56059 GO:0016021
Cluster-22178.56062 GO:0005515
Cluster-22178.56063 GO:0005515

Cluster-22178.56065 GO:0006355 GO:0009271
Cluster-22178.56066 GO:0006412 GO:0006351 GO:0006144 GO:0006206
GO:0045116 GO:0007264 GO:0042254 GO:0003924 GO:0016881
GO:0005525 GO:0003899 GO:0005515 GO:0005524 GO:0003735
GO:0003677 GO:0005840 GO:0005622 GO:0005730
Cluster-22178.56067 GO:0030145 GO:0004177 GO:0019031
Cluster-22178.56068 GO:0006629 GO:0045892 GO:0003677
Cluster-22178.56076 GO:0006144 GO:0006464 GO:0006206 GO:0006281
GO:0006351 GO:0046872 GO:0008270 GO:0003676 GO:0016151
GO:0003899 GO:0003677 GO:0005730
Cluster-22178.56078 GO:0016192
Cluster-22178.56084 GO:0006468 GO:0016773 GO:0004672 GO:0005524
GO:0016020
Cluster-22178.56086 GO:0005515
Cluster-22178.56087 GO:0006302 GO:0006310 GO:0005515 GO:0003677
GO:0005634
Cluster-22178.56091 GO:0006122 GO:0005750 GO:0016021
Cluster-22178.56092 GO:0043085 GO:0000290 GO:0008047
Cluster-22178.56093 GO:0006122 GO:0005750
Cluster-22178.56094 GO:0015992 GO:0055114 GO:0006120 GO:0006744
GO:0006122 GO:0006810 GO:0042773 GO:0006814 GO:0005215
GO:0008137 GO:0005750
Cluster-22178.56105 GO:0006771 GO:0019497 GO:0005515 GO:0003993
GO:0046872
Cluster-22178.56108 GO:0007283 GO:0003677 GO:0000062 GO:0046872
GO:0000786 GO:0005634
Cluster-22178.56116 GO:0005515
Cluster-22178.56117 GO:0018189 GO:0030154 GO:0016021
Cluster-22178.56119 GO:0005975 GO:0004553
Cluster-22178.56121 GO:0000166 GO:0046872
Cluster-22178.56125 GO:0006810 GO:0005215 GO:0016021
Cluster-22178.56130 GO:0006468 GO:0016773 GO:0005524 GO:0004672
GO:0016020

Cluster-22178.56141 GO:0043065 GO:0046983 GO:0003677 GO:0016021 GO:0005740

Cluster-22178.56143 GO:0051252 GO:0016891

Cluster-22178.56147 GO:0006355 GO:0016032 GO:0043565 GO:0003700 GO:0005667 GO:0005634

Cluster-22178.56148 GO:0006397 GO:0006570 GO:0006310 GO:0006281 GO:0006807 GO:0006470 GO:0006260 GO:0006935 GO:0006370 GO:0050568 GO:0008138 GO:0004484 GO:0004725 GO:0003910 GO:0005524

Cluster-22178.56150 GO:0006468 GO:0004672 GO:0005524

Cluster-22178.56151 GO:0006355 GO:0006486 GO:0006811 GO:0004576 GO:0016020 GO:0008250

Cluster-22178.56153 GO:0003723

Cluster-22178.56155 GO:0008270 GO:0016021

Cluster-22178.56157 GO:0055085 GO:0006810 GO:0006118 GO:0009055 GO:0005525 GO:0042626 GO:0005524 GO:0003924 GO:0016491 GO:0016887 GO:0016301 GO:0016021 GO:0016020

Cluster-22178.56158 GO:0006464 GO:0016151 GO:0031072 GO:0051082

Cluster-22178.56160 GO:0043087 GO:0071929 GO:0042967 GO:0019799 GO:0005089 GO:0008080 GO:0005515 GO:0005737 GO:0005874 GO:0045298 GO:0016021

Cluster-22178.56161 GO:0035556 GO:0007165 GO:0003774 GO:0005524 GO:0005515 GO:0016021 GO:0001772 GO:0097197 GO:0016459

Cluster-22178.56166 GO:0005515

Cluster-22178.56167 GO:0003725

Cluster-22178.56168 GO:0006470 GO:0000287 GO:0003924 GO:0005525 GO:0030145 GO:0004721 GO:0003824

Cluster-22178.56171 GO:0006464 GO:0015031 GO:0005515 GO:0005634

Cluster-22178.56172 GO:0016787

Cluster-22178.56173 GO:0055085 GO:0008270 GO:0005515 GO:0016021

Cluster-22178.56174 GO:0019058 GO:0006886 GO:0005515 GO:0019031 GO:0005622

Cluster-22178.56179 GO:0006508 GO:0004252
Cluster-22178.56182 GO:0006260 GO:0003887 GO:0042575
Cluster-22178.56183 GO:0055114 GO:0010181 GO:0016491
Cluster-22178.56187 GO:0051260 GO:0005515 GO:0016021
Cluster-22178.56188 GO:0006355 GO:0003676 GO:0005515 GO:0043565 GO:0005634
Cluster-22178.56190 GO:0006457 GO:0005515
Cluster-22178.56191 GO:0008152 GO:0019087 GO:0006508 GO:0008236 GO:0008168
Cluster-22178.56195 GO:0006118 GO:0009055 GO:0005515
Cluster-22178.56196 GO:0006810 GO:0006260 GO:0003887 GO:0001882 GO:0016020 GO:0016021 GO:0042575
Cluster-22178.56197 GO:0009405 GO:0005576
Cluster-22178.56198 GO:0005515
Cluster-22178.56199 GO:0005515 GO:0008168
Cluster-22178.56200 GO:0006801 GO:0055114 GO:0006914 GO:0045502 GO:0042803 GO:0008134 GO:0046872 GO:0005667 GO:0030286
Cluster-22178.56202 GO:0006914 GO:0055114 GO:0006801 GO:0008134 GO:0042803 GO:0045502 GO:0046872 GO:0005667 GO:0030286
Cluster-22178.56207 GO:0006310 GO:0006281 GO:0009378 GO:0003723 GO:0003678 GO:0003724 GO:0005524 GO:0016887 GO:0009379 GO:0005657
Cluster-22178.56214 GO:0006144 GO:0006508 GO:0006355 GO:0003968 GO:0003700 GO:0043565 GO:0017111 GO:0004197 GO:0003677 GO:0031379 GO:0005667 GO:0005634
Cluster-22178.56215 GO:0006468 GO:0005524 GO:0004672
Cluster-22178.56216 GO:0003924 GO:0005198 GO:0019028
Cluster-22178.56218 GO:0007275 GO:0009966 GO:0016020
Cluster-22178.56221 GO:0051028 GO:0005634
Cluster-22178.56222 GO:0019089 GO:0006886 GO:0006355 GO:0008608 GO:0007049 GO:0048870 GO:0009271 GO:0051607 GO:0003700 GO:0043565 GO:0031514 GO:0042729 GO:0016021 GO:0005783 GO:0005667 GO:0005876

Cluster-22178.56224 GO:0055114 GO:0008152 GO:0016491 GO:0016620

Cluster-22178.56227 GO:0006508 GO:0055085 GO:0022857 GO:0004197
GO:0016021
Cluster-22178.56231 GO:0006614 GO:0046907 GO:0006355 GO:0006281
GO:0000723 GO:0003677 GO:0005515 GO:0016787 GO:0005525
GO:0003678 GO:0003676 GO:0005524 GO:0005657 GO:0009289
Cluster-22178.56233 GO:0015031 GO:0055114 GO:0016491 GO:0008565
GO:0004190 GO:0005515 GO:0016021
Cluster-22178.56244 GO:0006508 GO:0055085 GO:0046813 GO:0035556
GO:0061025 GO:0016805 GO:0016021 GO:0019031
Cluster-22178.56245 GO:0008270 GO:0046872 GO:0005515
Cluster-22178.56247 GO:0055085 GO:0015979 GO:0030150 GO:0006810
GO:0005215 GO:0005515 GO:0009539 GO:0009523 GO:0016020
GO:0005742
Cluster-22178.56248 GO:0007186 GO:0015684 GO:0007165 GO:0007264
GO:0004871 GO:0005525 GO:0003924 GO:0019001 GO:0015093
GO:0008270 GO:0031683 GO:0016021
Cluster-22178.56249 GO:0006269 GO:0006351 GO:0006260 GO:0046872
GO:0003896 GO:0005730 GO:0005657
Cluster-22178.56254 GO:0008152 GO:0006397 GO:0003723 GO:0004064
GO:0005515 GO:0016788 GO:0005737 GO:0005634
Cluster-22178.56259 GO:0006118 GO:0005515 GO:0015035
Cluster-22178.56261 GO:0006355 GO:0003700 GO:0043565 GO:0005667
Cluster-22178.56262 GO:0006355 GO:0003700 GO:0043565 GO:0005667
Cluster-22178.56264 GO:0019069 GO:0007165 GO:0006914 GO:0051258
GO:0030168 GO:0007049 GO:0030674 GO:0005102 GO:0005577
Cluster-22178.56266 GO:0048309 GO:0051028 GO:0030430
Cluster-22178.56267 GO:0051028 GO:0048309 GO:0030430
Cluster-22178.56272 GO:0000166 GO:0046872
Cluster-22178.56273 GO:0032259 GO:0046872 GO:0003723 GO:0005845
Cluster-22178.56274 GO:0006606 GO:0048870 GO:0007094 GO:0005198
GO:0008134 GO:0042803 GO:0045502 GO:0030286 GO:0005882

GO:0031514 GO:0005667
Cluster-22178.56279 GO:0006511 GO:0016747
Cluster-22178.56281 GO:0006885 GO:0006812 GO:0055085 GO:0015299
GO:0016021
Cluster-22178.56283 GO:0019645 GO:0016560 GO:0005515 GO:0016021
GO:0005777
Cluster-22178.56284 GO:0019521 GO:0055114 GO:0046168 GO:0009094
GO:0015940 GO:0000162 GO:0006571 GO:0006098 GO:0008677
GO:0004665 GO:0051287 GO:0008977 GO:0004616 GO:0016616
Cluster-22178.56286 GO:0008152 GO:0055085 GO:0015031 GO:0055114
GO:0009396 GO:0046487 GO:0003824 GO:0022857 GO:0008565
GO:0016491 GO:0004488 GO:0016021
Cluster-22178.56287 GO:0007219 GO:0055085 GO:0030154 GO:0006355
GO:0030001 GO:0007275 GO:0003676 GO:0046873 GO:0005622
GO:0016021 GO:0045095 GO:0016020
Cluster-22178.56291 GO:0042546 GO:0009664 GO:0007275 GO:0005199
GO:0005578 GO:0005618
Cluster-22178.56294 GO:0030246
Cluster-22178.56295 GO:0005509 GO:0005515
Cluster-22178.56296 GO:0005515
Cluster-22178.56297 GO:0005515
Cluster-22178.56299 GO:0008152 GO:0005198 GO:0016746 GO:0019028
Cluster-22178.56301 GO:0005198 GO:0019028
Cluster-22178.56303 GO:0042147 GO:0007165 GO:0006807 GO:0015031
GO:0005509 GO:0005179 GO:0008565 GO:0004668 GO:0030904
GO:0005576 GO:0016021 GO:0005737
Cluster-22178.56306 GO:0005509 GO:0005515
Cluster-22178.56308 GO:0016772 GO:0005739
Cluster-22178.56312 GO:0007155 GO:0001503 GO:0003676 GO:0046935
GO:0005576 GO:0005944
Cluster-22178.56313 GO:0006606 GO:0006914 GO:0006355 GO:0003700
GO:0043565 GO:0003677 GO:0016788 GO:0005634 GO:0005667
Cluster-22178.56318 GO:0006418 GO:0000166 GO:0003676 GO:0005524

GO:0004812
Cluster-22178.56320 GO:0046740 GO:0016021
Cluster-22178.56321 GO:0006355 GO:0008270 GO:0043565 GO:0003682
GO:0003700 GO:0005667 GO:0000785
Cluster-22178.56323 GO:0035307 GO:0071595
Cluster-22178.56326 GO:0006352 GO:0003677
Cluster-22178.56327 GO:0055114 GO:0009089 GO:0006508 GO:0009085
GO:0008839 GO:0004222 GO:0008270 GO:0005509 GO:0005515
GO:0031012
Cluster-22178.56328 GO:1900758
Cluster-22178.56329 GO:0005515
Cluster-22178.56334 GO:0030866 GO:0016192 GO:0006886 GO:0008092
GO:0005515 GO:0005856 GO:0030117
Cluster-22178.56336 GO:0006886 GO:0016192 GO:0030866 GO:0005515
GO:0008092 GO:0005856 GO:0030117
Cluster-22178.56337 GO:0005515 GO:0005739
Cluster-22178.56338 GO:0006355 GO:0006357 GO:0001104 GO:0016592
Cluster-22178.56342 GO:0006457 GO:0006355 GO:0006323 GO:0006891
GO:0000077 GO:0005515 GO:0051082 GO:0003700 GO:0046872
GO:0008270 GO:0043565 GO:0005667 GO:0017119 GO:0016272
Cluster-22178.56346 GO:0006308 GO:0007049 GO:0043093 GO:0006355
GO:0000917 GO:0008855 GO:0043565 GO:0003700 GO:0005737
GO:0005667 GO:0009318
Cluster-22178.56347 GO:0007049 GO:0006308 GO:0000917 GO:0006355
GO:0043093 GO:0003700 GO:0043565 GO:0008855 GO:0005737
GO:0009318 GO:0005667
Cluster-22178.56351 GO:0005975 GO:0070206 GO:0048870 GO:0016857
GO:0005198 GO:0005515 GO:0005882 GO:0031514
Cluster-22178.56352 GO:0009405 GO:0010508 GO:0051258 GO:0007165
GO:0019058 GO:0007067 GO:0007155 GO:0006260 GO:0006606
GO:0030168 GO:0006357 GO:0051262 GO:0005515 GO:0001104
GO:0005198 GO:0004871 GO:0046872 GO:0030674 GO:0005102
GO:0042729 GO:0019028 GO:0072686 GO:0000775 GO:0019031

GO:0019867 GO:0016021 GO:0005882 GO:0016592 GO:0016020
GO:0005577
Cluster-22178.56355 GO:0046034 GO:0006144 GO:0016567 GO:0004842
GO:0046872 GO:0008270 GO:0005515 GO:0061630 GO:0004017
GO:0005622
Cluster-22178.56358 GO:0005515
Cluster-22178.56359 GO:0055085 GO:0016021
Cluster-22178.56367 GO:0006813 GO:0055085 GO:0006811 GO:0005216
GO:0005242 GO:0016020 GO:0008076 GO:0016021
Cluster-22178.56368 GO:0009069 GO:0016310 GO:0004674 GO:0005515
GO:0019028
Cluster-22178.56370 GO:0006270 GO:0006367 GO:0015684 GO:0007155
GO:0005525 GO:0015093 GO:0016021 GO:0032040 GO:0009289
GO:0005672
Cluster-22178.56371 GO:0006820 GO:0008509 GO:0016021
Cluster-22178.56374 GO:0006810 GO:0005524 GO:0003676 GO:0004386

Cluster-22178.56375 GO:0006810 GO:0016787 GO:0005524 GO:0003676
GO:0004386 GO:0003677 GO:0000166
Cluster-22178.56376 GO:0006554 GO:0034968 GO:0006479 GO:0008270
GO:0018024 GO:0005515 GO:0016021 GO:0005634
Cluster-22178.56381 GO:0006810 GO:0007165 GO:0055085 GO:0006744
GO:0005179 GO:0003676 GO:0008813 GO:0016020 GO:0005576
GO:0016021 GO:0005737
Cluster-22178.56382 GO:0015675 GO:0035444 GO:0046872 GO:0015099
GO:0016021
Cluster-22178.56385 GO:0006260 GO:0008608 GO:0015992 GO:0015991
GO:0006355 GO:0003700 GO:0015078 GO:0005876 GO:0033179
GO:0042729 GO:0005667
Cluster-22178.56386 GO:0006506 GO:0016746 GO:0005789 GO:0016021
Cluster-22178.56387 GO:0042967 GO:0006355 GO:0008080 GO:0016747

Cluster-22178.56391 GO:0002949 GO:0006914 GO:0007018 GO:0007017

GO:0003724 GO:0016887 GO:0005524 GO:0005525 GO:0003777
GO:0003924 GO:0008092 GO:0016301 GO:0005515 GO:0003723
GO:0019898 GO:0030286 GO:0005874 GO:0005737
Cluster-22178.56394 GO:0005515
Cluster-22178.56396 GO:0005515
Cluster-22178.56400 GO:0009405
Cluster-22178.56403 GO:0006629 GO:0009405 GO:0005524 GO:0003677
GO:0000166 GO:0008081 GO:0005576
Cluster-22178.56405 GO:0003676
Cluster-22178.56409 GO:0007049 GO:0000917 GO:0006355 GO:0043093
GO:0003700 GO:0043565 GO:0003677 GO:0005815 GO:0005737
GO:0005634 GO:0005667
Cluster-22178.56410 GO:0048193 GO:0006357 GO:0016192 GO:0006869
GO:0006886 GO:0030001 GO:0006891 GO:0007165 GO:0030474
GO:0051225 GO:0005179 GO:0042803 GO:0005515 GO:0005198
GO:0001104 GO:0005200 GO:0046872 GO:0005319 GO:0008134
GO:0045502 GO:0016020 GO:0005667 GO:0019867 GO:0005856
GO:0070652 GO:0005823 GO:0030286 GO:0016592 GO:0017119
GO:0005801 GO:0016021 GO:0019031 GO:0019033 GO:0019028
GO:0005576
Cluster-22178.56415 GO:0070072
Cluster-22178.56416 GO:0006260 GO:0005515
Cluster-22178.56418 GO:0009405 GO:0097193 GO:0090200 GO:0051321
GO:0043065 GO:0005515 GO:0005576 GO:0005739
Cluster-22178.56419 GO:0097193 GO:0009405 GO:0090200 GO:0051321
GO:0043065 GO:0005515 GO:0005576 GO:0005739
Cluster-22178.56428 GO:1900758
Cluster-22178.56429 GO:0006289 GO:0004003 GO:0000439 GO:0005657
GO:0005634
Cluster-22178.56430 GO:0055114
Cluster-22178.56431 GO:0005515 GO:0005509
Cluster-22178.56439 GO:0006090 GO:0006525 GO:0006560 GO:0006814
GO:0007186 GO:0071436 GO:0015081 GO:0004930 GO:0008948

GO:0016020
Cluster-22178.56441 GO:0006952 GO:0019013 GO:0016021
Cluster-22178.56442 GO:0018279 GO:0006355 GO:0006810 GO:0008270
GO:0004579 GO:0005622 GO:0016021 GO:0005634 GO:0008250
Cluster-22178.56446 GO:0016255 GO:0042765
Cluster-22178.56449 GO:0006011 GO:0005982 GO:0005985 GO:0030244
GO:0016760 GO:0016020
Cluster-22178.56453 GO:0055114 GO:0033743
Cluster-22178.56454 GO:0055114 GO:0033743
Cluster-22178.56459 GO:0006446 GO:0006468 GO:0006413 GO:0003743
GO:0004672 GO:0005524 GO:0005840
Cluster-22178.56461 GO:0016021
Cluster-22178.56462 GO:0008270
Cluster-22178.56463 GO:0008270
Cluster-22178.56466 GO:0000917 GO:0006355 GO:0043093 GO:0007049
GO:0006260 GO:0008053 GO:0051259 GO:0003700 GO:0034618
GO:0003924 GO:0005741 GO:0005667 GO:0005634 GO:0016021
GO:0005737
Cluster-22178.56467 GO:0055114 GO:0006807 GO:0006281 GO:0006355
GO:0042128 GO:0008270 GO:0008168 GO:0008942 GO:0016491
GO:0051537 GO:0003677
Cluster-22178.56468 GO:0051424 GO:0016021
Cluster-22178.56470 GO:0004672
Cluster-22178.56471 GO:0004672
Cluster-22178.56474 GO:0035434 GO:0006825 GO:0005375 GO:0016021
Cluster-22178.56475 GO:0006071 GO:0046486 GO:0043565 GO:0016829
GO:0005524 GO:0005515 GO:0003677 GO:0004371 GO:0005783
GO:0005634
Cluster-22178.56476 GO:0006397 GO:0051252 GO:0006468 GO:0004540
GO:0004672 GO:0005524 GO:0016773 GO:0016020
Cluster-22178.56477 GO:0006355 GO:0003677 GO:0016021
Cluster-22178.56478 GO:0005515 GO:0003676
Cluster-22178.56480 GO:0006334 GO:0003677 GO:0005634 GO:0000786

Cluster-22178.56485 GO:0006810 GO:0009405 GO:0005515 GO:0019871 GO:0005576
Cluster-22178.56486 GO:0006810 GO:0009405 GO:0005515 GO:0019871 GO:0005576
Cluster-22178.56487 GO:0015684 GO:0007264 GO:0005525 GO:0003924 GO:0015093 GO:0001527 GO:0016021
Cluster-22178.56489 GO:0009116
Cluster-22178.56490 GO:0003677 GO:0005524 GO:0016787
Cluster-22178.56492 GO:0006606 GO:0042981 GO:0005515
Cluster-22178.56493 GO:0006807 GO:0009252 GO:0006355 GO:0003700 GO:0043565 GO:0004356 GO:0005634 GO:0005667 GO:0055036
Cluster-22178.56495 GO:0006269 GO:0006351 GO:0035556 GO:0006260 GO:0003677 GO:0046872 GO:0003896 GO:0005730 GO:0005657
Cluster-22178.56496 GO:0098599
Cluster-22178.56498 GO:0006886 GO:0009847 GO:0006508 GO:0008233 GO:0005515
Cluster-22178.56499 GO:0006310 GO:0006355 GO:0003700 GO:0003677 GO:0000150 GO:0005667 GO:0005737
Cluster-22178.56500 GO:0000077 GO:0051082
Cluster-22178.56501 GO:0055085 GO:0008643 GO:0005351 GO:0000139 GO:0016020 GO:0016021
Cluster-22178.56502 GO:0016787
Cluster-22178.56504 GO:0000723 GO:0006260 GO:0008270 GO:0003676 GO:0003677 GO:0005634 GO:0000784
Cluster-22178.56505 GO:0005515
Cluster-22178.56510 GO:0000077 GO:0000413 GO:0006260 GO:0006457 GO:0006816 GO:0006874 GO:0071479 GO:0005219 GO:0003755 GO:0016021 GO:0005622 GO:0005634
Cluster-22178.56511 GO:0007096 GO:0007165 GO:0040007 GO:0008283 GO:0008083 GO:0016020 GO:0005634
Cluster-22178.56513 GO:0055114 GO:0008152 GO:0016491
Cluster-22178.56518 GO:0008152 GO:0016624
Cluster-22178.56520 GO:0005515

Cluster-22178.56521 GO:0006355 GO:0003700 GO:0008270 GO:0042025
GO:0016020 GO:0005667 GO:0005634
Cluster-22178.56523 GO:0006955 GO:0006635 GO:0055114 GO:0006637
GO:0019882 GO:0005515 GO:0030234 GO:0003997 GO:0016020
GO:0005777
Cluster-22178.56525 GO:0006570 GO:0006470 GO:0004725 GO:0008138
GO:0046872
Cluster-22178.56528 GO:0006470 GO:0006570 GO:0004725 GO:0008138
GO:0004721
Cluster-22178.56529 GO:0006468 GO:0004672 GO:0005524
Cluster-22178.56534 GO:0006355 GO:0043565 GO:0003677 GO:0003700
GO:0005667 GO:0005634 GO:0016021
Cluster-22178.56540 GO:0003677
Cluster-22178.56541 GO:0006355 GO:0003677
Cluster-22178.56544 GO:0006355 GO:0003677 GO:0005634
Cluster-22178.56545 GO:0006032 GO:0005524 GO:0016887 GO:0003723
GO:0008061 GO:0003724 GO:0005515
Cluster-22178.56548 GO:0016567 GO:0005515 GO:0061630 GO:0043565
GO:0046872 GO:0008270 GO:0004842 GO:0005634 GO:0005680
Cluster-22178.56551 GO:0016567 GO:0004842
Cluster-22178.56562 GO:0002949 GO:0006260 GO:0005978 GO:0015992
GO:0070206 GO:0016567 GO:0006310 GO:0006869 GO:0042157
GO:0007049 GO:0015991 GO:0006281 GO:0006355 GO:0004842
GO:0016887 GO:0008270 GO:0005524 GO:0009378 GO:0003678
GO:0015078 GO:0008289 GO:0046872 GO:0016301 GO:0008134
GO:0005515 GO:0005657 GO:0005622 GO:0009379 GO:0005667
GO:0033179 GO:0005576
Cluster-22178.56564 GO:0055114 GO:0006631 GO:0071949 GO:0016491
GO:0050151
Cluster-22178.56568 GO:0007186 GO:0055114 GO:0016972 GO:0004930
GO:0046872 GO:0016021
Cluster-22178.56569 GO:0008654 GO:0016780 GO:0016020
Cluster-22178.56570 GO:0016772 GO:0016301

Cluster-22178.56577 GO:0016567 GO:0007165 GO:0006260 GO:0005179
GO:0005515 GO:0016818 GO:0003677 GO:0046872 GO:0008270
GO:0005524 GO:0004842 GO:0005634 GO:0005576 GO:0005680
Cluster-22178.56579 GO:0006955 GO:0030153 GO:0000166 GO:0015643
GO:0046872 GO:0019814
Cluster-22178.56582 GO:0042742 GO:0008270 GO:0005634 GO:0005576
Cluster-22178.56583 GO:0055114 GO:0003756 GO:0016671 GO:0005783
Cluster-22178.56589 GO:0046080 GO:0016787
Cluster-22178.56590 GO:0003676 GO:0005515 GO:0005634
Cluster-22178.56592 GO:0055114 GO:0016491
Cluster-22178.56593 GO:0055085 GO:0006810 GO:0005515 GO:0005215
GO:0016021 GO:0016020
Cluster-22178.56596 GO:0045454 GO:0042254 GO:0006412 GO:0006810
GO:0003924 GO:0005525 GO:0003735 GO:0005515 GO:0016021
GO:0005840 GO:0005861
Cluster-22178.56600 GO:0016567 GO:0005515 GO:0004842 GO:0008270
GO:0046872 GO:0005680
Cluster-22178.56601 GO:0005544 GO:0005509
Cluster-22178.56602 GO:0008237 GO:0008270
Cluster-22178.56609 GO:0005515
Cluster-22178.56610 GO:0046872 GO:0035091
Cluster-22178.56612 GO:0055114 GO:0043401 GO:0006355 GO:0003700
GO:0008270 GO:0016491 GO:0043565 GO:0005634 GO:0005667
Cluster-22178.56614 GO:0046872
Cluster-22178.56616 GO:0040007 GO:0005737
Cluster-22178.56617 GO:0005515
Cluster-22178.56619 GO:0006119 GO:0006118 GO:0015992 GO:0008121

Cluster-22178.56620 GO:0009069 GO:0006825 GO:0006744 GO:0006123
GO:0015992 GO:0006814 GO:0006118 GO:0016310 GO:0006120
GO:0016531 GO:0003954 GO:0004674 GO:0008137 GO:0004129
GO:0005507 GO:0005739 GO:0005758 GO:0045277
Cluster-22178.56621 GO:0003676 GO:0019013

Cluster-22178.56624 GO:0005515
Cluster-22178.56628 GO:0006537 GO:0055114 GO:0006807 GO:0016773 GO:0016972 GO:0035091 GO:0015930 GO:0005515
Cluster-22178.56633 GO:0030001 GO:0005515 GO:0046872
Cluster-22178.56634 GO:0006810 GO:0055085 GO:0006260 GO:0015074 GO:0003678 GO:0003676 GO:0042626 GO:0005525 GO:0003924 GO:0016887 GO:0005524 GO:0016021 GO:0005657
Cluster-22178.56637 GO:0016301 GO:0016772
Cluster-22178.56638 GO:0005525
Cluster-22178.56640 GO:0003676
Cluster-22178.56641 GO:0006807 GO:0016810
Cluster-22178.56642 GO:0003676 GO:0016820 GO:0016757
Cluster-22178.56644 GO:0017134
Cluster-22178.56646 GO:0005515
Cluster-22178.56647 GO:0008643 GO:0005351 GO:0016021 GO:0016020 GO:0000139
Cluster-22178.56648 GO:0016310 GO:0000160 GO:0048870 GO:0007165 GO:0006810 GO:0000103 GO:0006144 GO:0005515 GO:0008134 GO:0042803 GO:0045502 GO:0004020 GO:0000155 GO:0005524 GO:0031514 GO:0009365 GO:0016021 GO:0005667 GO:0030286 GO:0005886
Cluster-22178.56649 GO:0055114 GO:0046422 GO:0009507
Cluster-22178.56650 GO:0019752 GO:0006729 GO:0006520 GO:0009058 GO:0006506 GO:0016831 GO:0016829 GO:0008124 GO:0016740 GO:0030170 GO:0016746 GO:0016021 GO:0005789
Cluster-22178.56651 GO:0006123 GO:0035023 GO:0015992 GO:0043087 GO:0005515 GO:0005089 GO:0004129 GO:0045277
Cluster-22178.56652 GO:0043087 GO:0015992 GO:0035023 GO:0006123 GO:0004129 GO:0005089 GO:0005515 GO:0045277
Cluster-22178.56654 GO:0019064 GO:0006260 GO:0010508 GO:0016758 GO:0019031 GO:0005815 GO:0005789
Cluster-22178.56655 GO:0016310 GO:0043248 GO:0006886 GO:0016192 GO:0009069 GO:0004674 GO:0005515 GO:0030117

Cluster-22178.56659 GO:0045040 GO:0006289 GO:0006281 GO:0043161
GO:0003684 GO:0005515 GO:0005742
Cluster-22178.56661 GO:0002949 GO:0006260 GO:0035988 GO:0006310
GO:0002062 GO:0042246 GO:0006576 GO:0006281 GO:0006355
GO:0016887 GO:0005524 GO:0003677 GO:0016787 GO:0009378
GO:0003678 GO:0005525 GO:0003924 GO:0008134 GO:0016301
GO:0005657 GO:0009379 GO:0005667 GO:0005634
Cluster-22178.56662 GO:0043093 GO:0007165 GO:0006914 GO:0015992
GO:0015991 GO:0006355 GO:0010508 GO:0000917 GO:0048870
GO:0045892 GO:0006935 GO:0000160 GO:0007049 GO:0016310
GO:0000155 GO:0004673 GO:0003677 GO:0005515 GO:0003924
GO:0003700 GO:0005525 GO:0015078 GO:0005667 GO:0005815
GO:0033179 GO:0031514 GO:0005737 GO:0009365
Cluster-22178.56663 GO:0006486 GO:0035429 GO:0015725 GO:0015128
GO:0016787 GO:0008373 GO:0046872 GO:0016020
Cluster-22178.56665 GO:0007264 GO:0006614 GO:0042254 GO:0006412
GO:0005524 GO:0016887 GO:0003924 GO:0005525 GO:0003724
GO:0003723 GO:0098519 GO:0003735 GO:0005622 GO:0005840
Cluster-22178.56666 GO:0005991 GO:0005985 GO:0005982 GO:0007186
GO:0004930 GO:0005509 GO:0004555
Cluster-22178.56670 GO:0007186 GO:0005985 GO:0005982 GO:0005991
GO:0004555 GO:0005509 GO:0004930
Cluster-22178.56676 GO:0003676
Cluster-22178.56677 GO:0003676
Cluster-22178.56681 GO:0006468 GO:0004672 GO:0005524
Cluster-22178.56685 GO:0000746 GO:0004252 GO:0019028 GO:0055036
GO:0019867
Cluster-22178.56689 GO:0005515
Cluster-22178.56690 GO:0035023 GO:0007165 GO:0043087 GO:0005515
GO:0005089
Cluster-22178.56692 GO:0007157 GO:0009405 GO:0008270 GO:0046872
GO:0016021
Cluster-22178.56695 GO:0007264 GO:0007165 GO:0007186 GO:0031683

GO:0019001 GO:0003924 GO:0005525 GO:0004871
Cluster-22178.56697 GO:0009214 GO:0006468 GO:0004113 GO:0005524
GO:0004672 GO:0016020 GO:0019031
Cluster-22178.56699 GO:0007165 GO:0005179 GO:0005576 GO:0031224
Cluster-22178.56700 GO:0006351 GO:0006144 GO:0006206 GO:0051603
GO:0003899 GO:0003677 GO:0004298 GO:0005730 GO:0005839
Cluster-22178.56701 GO:0005509 GO:0005515
Cluster-22178.56706 GO:0097193 GO:0007165 GO:0090200 GO:0043065
GO:0016020 GO:0005739
Cluster-22178.56712 GO:0016021
Cluster-22178.56714 GO:0005506 GO:0046872
Cluster-22178.56715 GO:0055114 GO:0006508 GO:0006886 GO:0006118
GO:0008270 GO:0016655 GO:0004181 GO:0008536 GO:0005886
Cluster-22178.56716 GO:0016020
Cluster-22178.56718 GO:0019497 GO:0006470 GO:0006771 GO:0006570
GO:0046872 GO:0003993 GO:0004725 GO:0004222 GO:0005515
Cluster-22178.56719 GO:0006464 GO:0032259 GO:0006554 GO:0008152
GO:0046500 GO:0006479 GO:0008168 GO:0004719 GO:0018024
Cluster-22178.56721 GO:0008270
Cluster-22178.56722 GO:0042438 GO:0007264 GO:0007165 GO:0015684
GO:0007186 GO:0030036 GO:0015093 GO:0098519 GO:0019001
GO:0031683 GO:0003924 GO:0003779 GO:0005525 GO:0004871
GO:0005507 GO:0009295 GO:0016021 GO:0030054
Cluster-22178.56725 GO:0016787
Cluster-22178.56727 GO:0006281 GO:0006974 GO:0005634 GO:0070876
Cluster-22178.56730 GO:0006270
Cluster-22178.56735 GO:0045893 GO:1902208 GO:0046856 GO:0003677
GO:0034597
Cluster-22178.56741 GO:0006355 GO:0006351 GO:0005634
Cluster-22178.56745 GO:0009058 GO:0006687 GO:0001733 GO:0008146
GO:0043130 GO:0005794 GO:0016021
Cluster-22178.56748 GO:0002949 GO:0007264 GO:0006355 GO:0016887
GO:0005524 GO:0003724 GO:0005525 GO:0003723 GO:0008134

GO:0005667

Cluster-22178.56749 GO:0007165 GO:0006355 GO:0005515 GO:0016787
GO:0003700 GO:0043565 GO:0016817 GO:0005524 GO:0008270
GO:0005667 GO:0005737

Cluster-22178.56751 GO:0006355 GO:0007165 GO:0005524 GO:0008270
GO:0043565 GO:0003700 GO:0016787 GO:0005515 GO:0005737
GO:0005667

Cluster-22178.56755 GO:0006397 GO:0006508 GO:0006606 GO:0039693
GO:0006914 GO:0005198 GO:0003682 GO:0003723 GO:0019784
GO:0019028 GO:0005634 GO:0019031 GO:0000785 GO:0005737

Cluster-22178.56756 GO:0030866 GO:0008092 GO:0005856

Cluster-22178.56757 GO:0042176 GO:0005515 GO:0030234 GO:0000502

Cluster-22178.56759 GO:0006886 GO:0008536

Cluster-22178.56764 GO:0006400 GO:0000287 GO:0008193

Cluster-22178.56766 GO:0000723 GO:0006281 GO:0005524 GO:0046872
GO:0003678 GO:0003824 GO:0016787 GO:0003677 GO:0005657
GO:0016020

Cluster-22178.56769 GO:0006359 GO:0008270 GO:0005634

Cluster-22178.56770 GO:0005515

Cluster-22178.56773 GO:0006396 GO:0003723

Cluster-22178.56777 GO:0006119 GO:0006796 GO:0004427 GO:0000287
GO:0005737

Cluster-22178.56779 GO:0042773 GO:0006351 GO:0006814 GO:0006744
GO:0006120 GO:0015992 GO:0006206 GO:0006144 GO:0003677
GO:0008137 GO:0003899 GO:0005730 GO:0005743

Cluster-22178.56782 GO:0006355 GO:0046872

Cluster-22178.56790 GO:0006123 GO:0015992 GO:0055114 GO:0004129
GO:0016645 GO:0045277

Cluster-22178.56794 GO:0003676 GO:0008408

Cluster-22178.56800 GO:0008152 GO:0055114 GO:0016620 GO:0016491

Cluster-22178.56802 GO:0007165 GO:0003824 GO:0031704 GO:0005179

Cluster-22178.56808 GO:0009878 GO:0022900 GO:0046872 GO:0016021
Cluster-22178.56809 GO:0009878 GO:0022900 GO:0046872 GO:0016021
Cluster-22178.56810 GO:0006355 GO:0003700 GO:0043565 GO:0005667
Cluster-22178.56811 GO:0006355 GO:0003700 GO:0043565 GO:0005667
Cluster-22178.56815 GO:0009607 GO:0019069 GO:0016021
Cluster-22178.56817 GO:0016310 GO:0006919 GO:0006915 GO:0006468
GO:0009069 GO:0004674 GO:0004672 GO:0005524 GO:0016020
GO:0005739
Cluster-22178.56822 GO:0007186 GO:0006355 GO:0003700 GO:0004930
GO:0043565 GO:0008270 GO:0003713 GO:0003677 GO:0016021
GO:0005667
Cluster-22178.56832 GO:0006281 GO:0008270 GO:0046872
Cluster-22178.56833 GO:0006468 GO:0005524 GO:0004672
Cluster-22178.56837 GO:0009267 GO:0005515 GO:0003723 GO:0016020
Cluster-22178.56839 GO:0006144 GO:0006206 GO:0006098 GO:0008152
GO:0019521 GO:0055114 GO:0006351 GO:0006355 GO:0008641
GO:0003677 GO:0016616 GO:0004616 GO:0051287 GO:0003700
GO:0046872 GO:0003899 GO:0005515 GO:0005667 GO:0005730
GO:0005634
Cluster-22178.56840 GO:0003677 GO:0003824
Cluster-22178.56841 GO:0005515
Cluster-22178.56842 GO:0005515
Cluster-22178.56843 GO:0016773 GO:0005524 GO:0016020
Cluster-22178.56845 GO:0045893 GO:0006260 GO:0008283 GO:0045947
GO:0006275 GO:0040007 GO:0007165 GO:0003688 GO:0003677
GO:0008083 GO:0008190 GO:0046809 GO:0042025
Cluster-22178.56846 GO:0005515 GO:0008270
Cluster-22178.56849 GO:0019087 GO:0016021
Cluster-22178.56852 GO:0006465 GO:0008233 GO:0016021 GO:0005787
Cluster-22178.56854 GO:0030163 GO:0008914
Cluster-22178.56858 GO:0005515
Cluster-22178.56860 GO:0008152 GO:0016787 GO:0005198 GO:0019028
GO:0016021

Cluster-22178.56861 GO:0016567 GO:0004842 GO:0005515 GO:0008270
GO:0046872 GO:0005680
Cluster-22178.56862 GO:0008608 GO:0016787 GO:0042729 GO:0072686
Cluster-22178.56864 GO:0000042 GO:0005515
Cluster-22178.56866 GO:0006144 GO:0008033 GO:0006464 GO:0046034
GO:0008152 GO:0051252 GO:0016846 GO:0046872 GO:0016151
GO:0004540 GO:0016787 GO:0004017
Cluster-22178.56869 GO:0006364 GO:0005730
Cluster-22178.56873 GO:0042967 GO:0006633 GO:0055114 GO:0004318
GO:0005835
Cluster-22178.56875 GO:0008270
Cluster-22178.56876 GO:0006351 GO:0006206 GO:0006144 GO:0003677
GO:0003899 GO:0005730
Cluster-22178.56878 GO:0015992 GO:0015991 GO:0015078 GO:0033177
Cluster-22178.56881 GO:0055114 GO:0046422 GO:0009507
Cluster-22178.56885 GO:0008152 GO:0008168
Cluster-22178.56886 GO:0006265 GO:0009755 GO:0006364 GO:0003918
GO:0005515 GO:0003677 GO:0032040 GO:0005576 GO:0005694
Cluster-22178.56889 GO:0016192 GO:0016021 GO:0019031
Cluster-22178.56894 GO:0005515
Cluster-22178.56898 GO:0055114 GO:0008152 GO:0016491 GO:0005515

Cluster-22178.56899 GO:0009401 GO:0006812 GO:0055085 GO:0008324
GO:0016020 GO:0016021
Cluster-22178.56900 GO:0015992 GO:0006120 GO:0006814 GO:0006744
GO:0008137 GO:0005743
Cluster-22178.56901 GO:0042254 GO:0006412 GO:0007342 GO:0003735
GO:0046872 GO:0019843 GO:0005840 GO:0005622
Cluster-22178.56902 GO:0006531 GO:0006526 GO:0006144 GO:0006177
GO:0006164 GO:0006560 GO:0006536 GO:0006541 GO:0015074
GO:0008033 GO:0008152 GO:0006529 GO:0006522 GO:0016787
GO:0004066 GO:0005524 GO:0003922 GO:0004810 GO:0004055
GO:0003824

Cluster-22178.56905 GO:0006886 GO:0007165 GO:0005515 GO:0016021
GO:0005783
Cluster-22178.56909 GO:0006613 GO:0055114 GO:0006355 GO:0043401
GO:0008270 GO:0016491 GO:0016757 GO:0043565 GO:0016209
GO:0003700 GO:0005784 GO:0030176 GO:0005667 GO:0016020
GO:0016021 GO:0005634
Cluster-22178.56910 GO:0005524
Cluster-22178.56912 GO:0007264 GO:0035556 GO:0006468 GO:0006355
GO:0003677 GO:0005524 GO:0004222 GO:0016820 GO:0005515
GO:0004672 GO:0046872 GO:0003700 GO:0005525 GO:0005667
GO:0005739 GO:0016021
Cluster-22178.56913 GO:0006355 GO:0006468 GO:0007031 GO:0035556
GO:0006457 GO:0007264 GO:0003700 GO:0005525 GO:0003924
GO:0046872 GO:0004672 GO:0005515 GO:0042803 GO:0016820
GO:0051082 GO:0004222 GO:0000774 GO:0005524 GO:0003677
GO:0051087 GO:0016021 GO:0016272 GO:0005739 GO:0005667
GO:0005779
Cluster-22178.56914 GO:0007031 GO:0035556 GO:0007264 GO:0006457
GO:0006355 GO:0006468 GO:0000774 GO:0005524 GO:0003677
GO:0051087 GO:0003924 GO:0046872 GO:0004672 GO:0003700
GO:0005525 GO:0051082 GO:0005515 GO:0042803 GO:0016820
GO:0016272 GO:0005779 GO:0005667 GO:0005739 GO:0016021
Cluster-22178.56915 GO:0007155 GO:0006310 GO:0019079 GO:0002949
GO:0006355 GO:0006281 GO:0007049 GO:0009378 GO:0016887
GO:0005524 GO:0003724 GO:0003723 GO:0008134 GO:0003678
GO:0005667 GO:0009379 GO:0005657
Cluster-22178.56918 GO:0016787
Cluster-22178.56920 GO:0005515
Cluster-22178.56922 GO:0005515
Cluster-22178.56923 GO:0003723 GO:0003774 GO:0016459
Cluster-22178.56926 GO:0006355 GO:0005515 GO:0004252 GO:0003677
GO:0005634 GO:0005886
Cluster-22178.56932 GO:0046436 GO:0009252 GO:0006090 GO:0006633

GO:0019370 GO:0046872 GO:0003989 GO:0008270 GO:0005524
GO:0008237 GO:0008716 GO:0009317
Cluster-22178.56933 GO:0006355 GO:0046487 GO:0055114 GO:0009396
GO:0008152 GO:0050662 GO:0004488 GO:0003677 GO:0016491
GO:0003824
Cluster-22178.56934 GO:0008152 GO:0006355 GO:0003824 GO:0003677
GO:0050662 GO:0016491
Cluster-22178.56939 GO:0007017 GO:0007018 GO:0005524 GO:0003777
GO:0008017 GO:0019028 GO:0005874 GO:0045298
Cluster-22178.56940 GO:0015979 GO:0009523 GO:0009539
Cluster-22178.56944 GO:0006260 GO:0003697 GO:0003924
Cluster-22178.56945 GO:0046836 GO:0017089 GO:0003677 GO:0046872
GO:0051861 GO:0005737
Cluster-22178.56946 GO:0046983
Cluster-22178.56947 GO:0006506 GO:0005515 GO:0016746 GO:0016021
GO:0005789
Cluster-22178.56948 GO:0019069 GO:0005524
Cluster-22178.56950 GO:0009733 GO:0005515
Cluster-22178.56951 GO:0005515
Cluster-22178.56952 GO:0007165 GO:0005515
Cluster-22178.56953 GO:0008152 GO:0005975 GO:0005509 GO:0004571
GO:0016829 GO:0016020
Cluster-22178.56954 GO:0008152 GO:0005975 GO:0005509 GO:0004571
GO:0016829 GO:0016020
Cluster-22178.56955 GO:0006449 GO:0006415 GO:0006013 GO:0016226
GO:0006000 GO:0003873 GO:0016301 GO:0005506 GO:0016740
GO:0005524 GO:0016149 GO:0051536 GO:0018444 GO:0005840
GO:0005737
Cluster-22178.56957 GO:0006887 GO:0046488 GO:0016307 GO:0008289
GO:0019031 GO:0000145
Cluster-22178.56960 GO:0016192 GO:0016567 GO:0090263 GO:0016021
GO:0005634
Cluster-22178.56961 GO:0008152 GO:0003824

Cluster-22178.56962 GO:0005515 GO:0043395 GO:0008191 GO:0016020 GO:0005578

Cluster-22178.56966 GO:0032324 GO:0019033

Cluster-22178.56971 GO:0042254 GO:0006412 GO:0003735 GO:0005515 GO:0005622 GO:0005840

Cluster-22178.56975 GO:0030244 GO:0006886 GO:0003924 GO:0005525 GO:0016866 GO:0005047 GO:0005515 GO:0005786 GO:0005785

Cluster-22178.56978 GO:0006355 GO:0043565 GO:0003700 GO:0005515 GO:0005667

Cluster-22178.56979 GO:0006955 GO:0000902 GO:0016020

Cluster-22178.56985 GO:0008219 GO:0051262 GO:0005515 GO:0044212

Cluster-22178.56987 GO:0006355 GO:0007275 GO:0000398 GO:0006357 GO:0003682 GO:0003677 GO:0001104 GO:0008270 GO:0005634 GO:0016592 GO:0042600 GO:0000775 GO:0005681 GO:0000785 GO:0016021

Cluster-22178.56988 GO:0005515 GO:0008270

Cluster-22178.56991 GO:0016021

Cluster-22178.56995 GO:0005515

Cluster-22178.56997 GO:0045947 GO:0008190

Cluster-22178.57001 GO:0008201

Cluster-22178.57002 GO:0006486 GO:0008373

Cluster-22178.57003 GO:0042254 GO:0006412 GO:0006810 GO:0005515 GO:0003735 GO:0003924 GO:0005525 GO:0005861 GO:0016021 GO:0005840

Cluster-22178.57004 GO:0033227 GO:0006412 GO:0015931 GO:0045454 GO:0006810 GO:0006270 GO:0042254 GO:0003924 GO:0005525 GO:0004190 GO:0051033 GO:0005515 GO:0003735 GO:0005840 GO:0016021 GO:0005861

Cluster-22178.57007 GO:0003723 GO:0003676

Cluster-22178.57008 GO:0003676 GO:0003723 GO:0046872 GO:0005576

Cluster-22178.57010 GO:0006810 GO:0006270 GO:0006874 GO:0006816 GO:0004190 GO:0005246 GO:0005219 GO:0042030 GO:0016020

GO:0016021 GO:0005622

Cluster-22178.57013 GO:0046740 GO:0016567 GO:0046872 GO:0008270

GO:0004842 GO:0005515 GO:0005680 GO:0044156

Cluster-22178.57014 GO:0006446 GO:0006413 GO:0003743 GO:0003723

GO:0005840 GO:0005737

Cluster-22178.57016 GO:0005515

Cluster-22178.57017 GO:0005515

Cluster-22178.57020 GO:0003677

Cluster-22178.57022 GO:0006886 GO:0005801 GO:0016020

Cluster-22178.57023 GO:0006310 GO:0006281

Cluster-22178.57024 GO:0051607

Cluster-22178.57025 GO:0043087 GO:0005085 GO:0005515

Cluster-22178.57027 GO:0000723 GO:0042254 GO:0006412 GO:0003735

GO:0003677 GO:0005840 GO:0005622 GO:0000784

Cluster-22178.57030 GO:0005975 GO:0030001 GO:0055085 GO:0016758

GO:0046873 GO:0016020

Cluster-22178.57031 GO:0009116 GO:0007631 GO:0046677 GO:0007218

GO:0008270

Cluster-22178.57035 GO:0019882 GO:0007165 GO:0006955 GO:0016567

GO:0004842 GO:0005515

Cluster-22178.57037 GO:0005524 GO:0016787 GO:0003677 GO:0003676

Cluster-22178.57039 GO:0001558 GO:0005515 GO:0005520 GO:0005576

GO:0016942

Cluster-22178.57041 GO:0016311 GO:0035023 GO:0043087 GO:0009252

GO:0005515 GO:0005089 GO:0050380 GO:0016020

Cluster-22178.57042 GO:0016311 GO:0009252 GO:0043087 GO:0035023

GO:0005515 GO:0050380 GO:0005089 GO:0016020

Cluster-22178.57043 GO:0051539

Cluster-22178.57044 GO:0046872 GO:0003723 GO:0005515 GO:0016021

Cluster-22178.57046 GO:0005975 GO:0000902 GO:0016773 GO:0016787

Cluster-22178.57048 GO:0006468 GO:0016773 GO:0005524 GO:0004672

GO:0016020
Cluster-22178.57049 GO:0006139 GO:0008408 GO:0003676
Cluster-22178.57050 GO:0006139 GO:0008408 GO:0003676
Cluster-22178.57051 GO:0015031 GO:0051262 GO:0051082
Cluster-22178.57056 GO:0006367 GO:0006260 GO:0003676 GO:0005634
GO:0005672
Cluster-22178.57058 GO:0006468 GO:0009069 GO:0016310 GO:0004672
GO:0005524 GO:0004674
Cluster-22178.57059 GO:0016310 GO:0009069 GO:0006468 GO:0004674
GO:0005524 GO:0004672
Cluster-22178.57060 GO:0055085 GO:0030001 GO:0046983 GO:0046873
GO:0016020
Cluster-22178.57061 GO:0055085 GO:0007165 GO:0007268 GO:0030639
GO:0030550 GO:0005576
Cluster-22178.57062 GO:0009058 GO:0030170
Cluster-22178.57064 GO:0005634
Cluster-22178.57065 GO:0008270 GO:0005515
Cluster-22178.57066 GO:0006468 GO:0004672 GO:0005524 GO:0016773
GO:0016020
Cluster-22178.57068 GO:0006414 GO:0006448 GO:0055114 GO:0003677
GO:0016706 GO:0005515 GO:0003746 GO:0005853 GO:0005840
Cluster-22178.57070 GO:0035023 GO:0007165 GO:0043087 GO:0005089
GO:0005515
Cluster-22178.57072 GO:0006687 GO:0006027 GO:0005975 GO:0006012
GO:0046486 GO:0004565 GO:0009341
Cluster-22178.57073 GO:0046688 GO:0006118 GO:0009055 GO:0005515
GO:0020037 GO:0005507 GO:0042597
Cluster-22178.57075 GO:0048511 GO:0048870 GO:0006810 GO:0006355
GO:0003700 GO:0043565 GO:0005515 GO:0005198 GO:0016021
GO:0031514 GO:0019028 GO:0005667
Cluster-22178.57076 GO:0046688 GO:0006118 GO:0009055 GO:0005507
GO:0020037 GO:0005515 GO:0042597
Cluster-22178.57077 GO:0006351 GO:0043565

Cluster-22178.57078 GO:0015979 GO:0005515 GO:0008270 GO:0016020 GO:0009539 GO:0009523
Cluster-22178.57079 GO:0006413 GO:0006446 GO:0003723 GO:0003743 GO:0005737 GO:0005840
Cluster-22178.57082 GO:0006479 GO:0006554 GO:0018024 GO:0046872

Cluster-22178.57083 GO:0003676
Cluster-22178.57084 GO:0006281
Cluster-22178.57089 GO:0035556 GO:0055114 GO:0047134
Cluster-22178.57091 GO:0046872 GO:0030991
Cluster-22178.57092 GO:0008152 GO:0003824
Cluster-22178.57099 GO:0046872
Cluster-22178.57100 GO:0016021
Cluster-22178.57101 GO:0006545 GO:0009165 GO:0055114 GO:0046656 GO:0006761 GO:0004146
Cluster-22178.57102 GO:0006334 GO:0003677 GO:0005634 GO:0000786
Cluster-22178.57103 GO:0046656 GO:0006761 GO:0006545 GO:0009165 GO:0055114 GO:0004146
Cluster-22178.57109 GO:0004866
Cluster-22178.57111 GO:0006508 GO:0007186 GO:0061025 GO:0055114 GO:0035556 GO:0046813 GO:0006120 GO:0016805 GO:0004930 GO:0019031 GO:0016021
Cluster-22178.57115 GO:0005515
Cluster-22178.57116 GO:0046912
Cluster-22178.57117 GO:0007411 GO:0006914 GO:0006308 GO:0006814 GO:0006813 GO:0019069 GO:0016055 GO:0008239 GO:0008855 GO:0004190 GO:0070009 GO:0016021 GO:0009318 GO:0005576 GO:0005890
Cluster-22178.57118 GO:0007009
Cluster-22178.57119 GO:0006355 GO:0003700 GO:0042025 GO:0005667
Cluster-22178.57120 GO:0055114 GO:0015940 GO:0008677
Cluster-22178.57122 GO:0051028 GO:0045893 GO:0007275 GO:0008270 GO:0031012 GO:0005634

Cluster-22178.57125 GO:0005515
Cluster-22178.57133 GO:0006511 GO:0007275 GO:0005515 GO:0008270
GO:0005634
Cluster-22178.57136 GO:0055114 GO:0030151 GO:0016491 GO:0005515
GO:0035091
Cluster-22178.57141 GO:0016887 GO:0005524
Cluster-22178.57143 GO:0007187 GO:0006812 GO:0007608 GO:0005549
GO:0008324 GO:0004984 GO:0016020
Cluster-22178.57145 GO:0006359 GO:0006260 GO:0015992 GO:0006351
GO:0015991 GO:0090266 GO:0015078 GO:0005524 GO:0003677
GO:0003887 GO:0032040 GO:0005680 GO:0019031 GO:0033179
GO:0042575
Cluster-22178.57146 GO:0006694 GO:0008152 GO:0016114 GO:0030976
GO:0008661 GO:0003824 GO:0016624
Cluster-22178.57148 GO:0008152 GO:0016746 GO:0046872
Cluster-22178.57150 GO:0006952
Cluster-22178.57152 GO:0006508 GO:0007155 GO:0009058 GO:0006952
GO:0007165 GO:0008013 GO:0003824 GO:0004222 GO:0005126
GO:0008134 GO:0016342 GO:0019028 GO:0005667 GO:0005634
GO:0005576
Cluster-22178.57153 GO:0016020
Cluster-22178.57157 GO:0032259 GO:0015946 GO:0055114 GO:0006364
GO:0008168 GO:0005515 GO:0004022 GO:0005634
Cluster-22178.57158 GO:0006508 GO:0009405 GO:0004252 GO:0005576
Cluster-22178.57159 GO:0005975 GO:0006890 GO:0015926 GO:0008270
GO:0003676 GO:0004252 GO:0005515 GO:0016021
Cluster-22178.57160 GO:0055114 GO:0042546 GO:0006120 GO:0005199
GO:0016021 GO:0005618
Cluster-22178.57161 GO:0006338 GO:0006355 GO:0003677 GO:0016818
GO:0003676 GO:0005524 GO:0008270 GO:0005634
Cluster-22178.57162 GO:0009405
Cluster-22178.57163 GO:0003677
Cluster-22178.57167 GO:0006468 GO:0005524 GO:0004672 GO:0003677

Cluster-22178.57168 GO:0008152 GO:0008168 GO:0016021
Cluster-22178.57169 GO:0000160 GO:0006935 GO:0006904 GO:0016559 GO:0006396 GO:0055085 GO:0015031 GO:0006614 GO:0016310 GO:0009405 GO:0051252 GO:0006468 GO:0030001 GO:0006869 GO:0042157 GO:0008289 GO:0004672 GO:0042802 GO:0000175 GO:0004673 GO:0046873 GO:0005515 GO:0003723 GO:0046983 GO:0008312 GO:0005524 GO:0000155 GO:0016021 GO:0009365 GO:0048500 GO:0005576 GO:0000145 GO:0005737 GO:0016020 GO:0005779
Cluster-22178.57171 GO:0006955 GO:0019882 GO:0016020 GO:0042613
Cluster-22178.57172 GO:0006955
Cluster-22178.57173 GO:0006955 GO:0009405 GO:0004222 GO:0016020
Cluster-22178.57182 GO:0005975 GO:0007165 GO:0004553 GO:0005509 GO:0005578
Cluster-22178.57184 GO:0016787 GO:0046872 GO:0003676
Cluster-22178.57185 GO:0035556 GO:0006508 GO:0008239 GO:0005515 GO:0004252 GO:0070009
Cluster-22178.57196 GO:0006457 GO:0000774 GO:0051087 GO:0042803

Cluster-22178.57200 GO:0006357 GO:0006367 GO:0006810 GO:0009405 GO:0006468 GO:0046872 GO:0005524 GO:0004672 GO:0019870 GO:0005515 GO:0001104 GO:0016592 GO:0005576 GO:0005672
Cluster-22178.57205 GO:0006886 GO:0016192 GO:0005515 GO:0030117
Cluster-22178.57208 GO:0007165 GO:0007186 GO:0007264 GO:0005525 GO:0004871 GO:0019001 GO:0031683 GO:0003924
Cluster-22178.57209 GO:0006886 GO:0016192
Cluster-22178.57210 GO:0006886 GO:0016192
Cluster-22178.57211 GO:0006470 GO:0006570 GO:0004725 GO:0008138

Cluster-22178.57212 GO:0006508 GO:0004222
Cluster-22178.57215 GO:0007186 GO:0035556 GO:0035023 GO:0043087 GO:0055114 GO:0005515 GO:0004930 GO:0047134 GO:0005089

GO:0016020
Cluster-22178.57216 GO:0009116 GO:0003824
Cluster-22178.57219 GO:0006869 GO:0042157 GO:0005515 GO:0008289
GO:0005576
Cluster-22178.57226 GO:0007165 GO:0005044 GO:0016020
Cluster-22178.57230 GO:0006955 GO:0007165 GO:0005164 GO:0016020
Cluster-22178.57231 GO:0006614 GO:0008312 GO:0005515 GO:0048500
Cluster-22178.57232 GO:0055085 GO:0006810 GO:0005215 GO:0016020
Cluster-22178.57239 GO:0006486 GO:0009312 GO:0008375 GO:0008455
GO:0005795 GO:0016021
Cluster-22178.57240 GO:0016310 GO:0000160 GO:0000155 GO:0046983
GO:0009365 GO:0016021
Cluster-22178.57241 GO:0006270 GO:0006412 GO:0042254 GO:0006198
GO:0030866 GO:0003735 GO:0005198 GO:0008092 GO:0003779
GO:0004115 GO:0005622 GO:0005856 GO:0005840
Cluster-22178.57243 GO:0005515 GO:0008270
Cluster-22178.57244 GO:0005515 GO:0046983
Cluster-22178.57245 GO:0005515
Cluster-22178.57246 GO:0006352 GO:0003677 GO:0005669
Cluster-22178.57249 GO:0006779 GO:0015994 GO:0004853
Cluster-22178.57251 GO:0006355 GO:0043565 GO:0003677 GO:0003700
GO:0005634 GO:0005667 GO:0005815
Cluster-22178.57253 GO:0019836 GO:0005576
Cluster-22178.57256 GO:0007186 GO:0019236 GO:0006355 GO:0003677

Cluster-22178.57257 GO:0007186 GO:0006355 GO:0019236 GO:0003677

Cluster-22178.57258 GO:0019236 GO:0042742 GO:0007186 GO:0003677

Cluster-22178.57259 GO:0006383 GO:0006629 GO:0006351 GO:0006144
GO:0006206 GO:0055085 GO:0003899 GO:0003677 GO:0005730
GO:0005666 GO:0016021
Cluster-22178.57262 GO:0042254 GO:0006412 GO:0006310 GO:0006281

GO:0003678 GO:0005524 GO:0016887 GO:0098519 GO:0003735
GO:0009378 GO:0005840 GO:0005657 GO:0009379
Cluster-22178.57263 GO:0005515
Cluster-22178.57265 GO:0005975 GO:0005509 GO:0004571 GO:0016020
Cluster-22178.57271 GO:0005515
Cluster-22178.57272 GO:0009245 GO:0005515 GO:0008915 GO:0003723
GO:0016020
Cluster-22178.57273 GO:0019836 GO:0005576
Cluster-22178.57276 GO:0006118 GO:0006119 GO:0007264 GO:0035556
GO:0055114 GO:0015992 GO:0008121 GO:0005507 GO:0005525
Cluster-22178.57279 GO:0055114
Cluster-22178.57281 GO:0016021
Cluster-22178.57283 GO:0032007 GO:0055085 GO:0035556 GO:0048011
GO:0046872 GO:0008270 GO:0005515 GO:0016021
Cluster-22178.57287 GO:0006694 GO:0055114 GO:0015936 GO:0007165
GO:0008158 GO:0004420 GO:0050662 GO:0016020
Cluster-22178.57288 GO:0006355 GO:0003677
Cluster-22178.57289 GO:0003677 GO:0005515
Cluster-22178.57293 GO:0015743 GO:0005515 GO:0003774 GO:0005524
GO:0016459
Cluster-22178.57295 GO:0015992 GO:0006935 GO:0006120 GO:0006744
GO:0007165 GO:0006955 GO:0006118 GO:0006814 GO:0003954
GO:0008137 GO:0008009 GO:0005576 GO:0005739
Cluster-22178.57299 GO:0016579 GO:0070140 GO:0032183 GO:0036459

Cluster-22178.57300 GO:0006810 GO:0006355 GO:0055085 GO:0000103
GO:0006144 GO:0008134 GO:0016301 GO:0042626 GO:0004020
GO:0016887 GO:0005524 GO:0008270 GO:0005667 GO:0016021
Cluster-22178.57301 GO:0006522 GO:0048193 GO:0006419 GO:0043039
GO:0006531 GO:0003676 GO:0016876 GO:0004813 GO:0005524
GO:0005198 GO:0000166 GO:0016020
Cluster-22178.57305 GO:0043087 GO:0035023 GO:0005089
Cluster-22178.57308 GO:0008033 GO:0009452 GO:0008152 GO:0001510

GO:0006400 GO:0009451 GO:0006306 GO:0008168 GO:0016429
GO:0051537 GO:0003723 GO:0008176 GO:0008170 GO:0003677
GO:0043231
Cluster-22178.57310 GO:0006355 GO:0008270 GO:0005634
Cluster-22178.57311 GO:0019058 GO:0007165 GO:0046789 GO:0005515
GO:0043565 GO:0005634 GO:0019031 GO:0009986
Cluster-22178.57312 GO:0046872
Cluster-22178.57314 GO:0006886 GO:0006888 GO:0008270 GO:0030127
Cluster-22178.57315 GO:0016192 GO:0006886 GO:0006486 GO:0015991
GO:0043248 GO:0016820 GO:0008378 GO:0016020 GO:0030117
GO:0000221
Cluster-22178.57317 GO:0055085
Cluster-22178.57320 GO:0006812 GO:0006885 GO:0055085 GO:0006308
GO:0015299 GO:0008855 GO:0009318 GO:0016021
Cluster-22178.57326 GO:0007264 GO:0003723 GO:0005525
Cluster-22178.57335 GO:0000725 GO:0097196 GO:0005634
Cluster-22178.57336 GO:0019521 GO:0055114 GO:0006574 GO:0015940
GO:0008152 GO:0006508 GO:0006568 GO:0006813 GO:0006552
GO:0006550 GO:0009403 GO:0006520 GO:0006554 GO:0006633
GO:0006098 GO:0006631 GO:0006810 GO:0018874 GO:0008677
GO:0003824 GO:0005215 GO:0003857 GO:0016743 GO:0016836
GO:0016491 GO:0008233 GO:0016597 GO:0051287 GO:0004616
GO:0016616 GO:0016020
Cluster-22178.57340 GO:0006605 GO:0007165 GO:0051539 GO:0005515
GO:0016020 GO:0005887
Cluster-22178.57341 GO:0005515
Cluster-22178.57345 GO:0007165 GO:0005515 GO:0000166 GO:0046872
GO:0045202
Cluster-22178.57348 GO:0006914 GO:0006525 GO:0071436 GO:0048870
GO:0006090 GO:0006355 GO:0006886 GO:0006814 GO:0006560
GO:0015081 GO:0043565 GO:0003700 GO:0008948 GO:0005667
GO:0016020 GO:0005783 GO:0005801 GO:0016021 GO:0031514
Cluster-22178.57350 GO:0016567 GO:0061630 GO:0005515 GO:0004842

GO:0008270 GO:0046872 GO:0043565 GO:0005634 GO:0005680
Cluster-22178.57352 GO:0032012 GO:0043087 GO:0006355 GO:0008289
GO:0003700 GO:0046872 GO:0043565 GO:0005086 GO:0005667
Cluster-22178.57353 GO:0032012 GO:0043087 GO:0008289 GO:0005086
GO:0046872
Cluster-22178.57355 GO:0005975 GO:0006687 GO:0004869 GO:0004308

Cluster-22178.57358 GO:0006270 GO:0046983
Cluster-22178.57361 GO:0003779
Cluster-22178.57362 GO:0004386 GO:0005515 GO:0005524 GO:0031083
Cluster-22178.57363 GO:0006355 GO:0043565 GO:0008270 GO:0003700
GO:0005515 GO:0005667 GO:0005634
Cluster-22178.57364 GO:0048278 GO:0006887 GO:0006633 GO:0006090
GO:0051082 GO:0005198 GO:0003989 GO:0005737 GO:0009317
Cluster-22178.57369 GO:0009966 GO:0043565 GO:0005515 GO:0005634
GO:0005737 GO:0016020
Cluster-22178.57374 GO:0009451 GO:0001522 GO:0006810 GO:0007165
GO:0006355 GO:0009982 GO:0005215 GO:0046983 GO:0003713
GO:0005515 GO:0003723 GO:0035257 GO:0016021 GO:0005667
GO:0005634
Cluster-22178.57380 GO:0006334 GO:0003677 GO:0000786 GO:0005634
Cluster-22178.57381 GO:0006334 GO:0003677 GO:0005515 GO:0005634
GO:0000786
Cluster-22178.57383 GO:0006784 GO:0055114 GO:0006508 GO:0004252
GO:0016627 GO:0016020 GO:0016021
Cluster-22178.57384 GO:0005515
Cluster-22178.57385 GO:0005515
Cluster-22178.57387 GO:0035556 GO:0009190 GO:0016849
Cluster-22178.57393 GO:0008152 GO:0005524 GO:0016301 GO:0005634
GO:0042597
Cluster-22178.57396 GO:0016567 GO:0005515 GO:0004842 GO:0046872
GO:0008270 GO:0005680
Cluster-22178.57397 GO:0005985 GO:0006012 GO:0005982 GO:0004564

GO:0004575 GO:0017177
Cluster-22178.57398 GO:0007186 GO:0006310 GO:0006367 GO:0006281
GO:0006106 GO:0007411 GO:0016788 GO:0000287 GO:0000400
GO:0004930 GO:0016020 GO:0005672 GO:0016021
Cluster-22178.57402 GO:0006281 GO:0003824 GO:0004518 GO:0003677

Cluster-22178.57403 GO:0006810 GO:0051252 GO:0004386 GO:0003676
GO:0016887 GO:0005524 GO:0000166 GO:0003677 GO:0016891
Cluster-22178.57404 GO:0051252 GO:0006810 GO:0003677 GO:0016891
GO:0000166 GO:0003676 GO:0004386 GO:0005524 GO:0016887
Cluster-22178.57405 GO:0016757
Cluster-22178.57406 GO:0046034 GO:0006144 GO:0004017 GO:0003677
GO:0046872
Cluster-22178.57407 GO:0042967 GO:0008080 GO:0016747
Cluster-22178.57415 GO:0006468 GO:0006182 GO:0009190 GO:0046039
GO:0035556 GO:0006144 GO:0016849 GO:0004383 GO:0005524
GO:0004672
Cluster-22178.57416 GO:0006144 GO:0035556 GO:0046039 GO:0009190
GO:0006182 GO:0006468 GO:0004672 GO:0005524 GO:0004383
GO:0016849
Cluster-22178.57418 GO:0007010 GO:0003779 GO:0033926
Cluster-22178.57422 GO:0000413 GO:0006457 GO:0003755 GO:0003676

Cluster-22178.57423 GO:0055085 GO:0006810 GO:0005215 GO:0016020
GO:0016021
Cluster-22178.57424 GO:0006886 GO:0007165 GO:0006355 GO:0004871
GO:0003700 GO:0016020 GO:0005634 GO:0005667
Cluster-22178.57429 GO:0007049 GO:0017053
Cluster-22178.57431 GO:0006576 GO:0006281 GO:0006777 GO:0006310
GO:0007264 GO:0000103 GO:0002949 GO:0006144 GO:0003723
GO:0004020 GO:0005525 GO:0003924 GO:0043531 GO:0098519
GO:0009378 GO:0003724 GO:0016887 GO:0005524 GO:0009379
GO:0005657

Cluster-22178.57440 GO:0006468 GO:0005524 GO:0004672 GO:0016773 GO:0016020
Cluster-22178.57442 GO:0007264 GO:0043087 GO:0005085
Cluster-22178.57443 GO:0006351 GO:0006479 GO:0006383 GO:0006481 GO:0006468 GO:0006144 GO:0006206 GO:0003899 GO:0003677 GO:0004671 GO:0004672 GO:0005524 GO:0005730 GO:0005666 GO:0016021
Cluster-22178.57447 GO:0042967 GO:0007018 GO:0007017 GO:0003824 GO:0008080 GO:0008017 GO:0003777 GO:0051536 GO:0005524 GO:0045298 GO:0005874
Cluster-22178.57450 GO:0005515 GO:0005524 GO:0003774 GO:0016459
Cluster-22178.57451 GO:0006355 GO:0003723 GO:0008134 GO:0005515 GO:0016887 GO:0003924 GO:0003774 GO:0005524 GO:0003724 GO:0005525 GO:0005667 GO:0016459
Cluster-22178.57455 GO:0019953 GO:0007165 GO:0045893 GO:0003676 GO:0008430 GO:0000772 GO:0005576
Cluster-22178.57456 GO:0006119 GO:0006412 GO:0006355 GO:0042254 GO:0015986 GO:0035556 GO:0015992 GO:0003735 GO:0046961 GO:0003677 GO:0008270 GO:0046933 GO:0005840 GO:0045261 GO:0045259 GO:0005622
Cluster-22178.57458 GO:0043565
Cluster-22178.57459 GO:0016020
Cluster-22178.57463 GO:0006260 GO:0003677 GO:0003924 GO:0005615 GO:0005634
Cluster-22178.57471 GO:0008270 GO:0005515
Cluster-22178.57473 GO:0009058 GO:0005515 GO:0004866
Cluster-22178.57475 GO:0003676 GO:0016021
Cluster-22178.57477 GO:0008152 GO:0003824 GO:0005515
Cluster-22178.57478 GO:0006812 GO:0055085 GO:0008324 GO:0016021
Cluster-22178.57482 GO:0003723
Cluster-22178.57484 GO:0005515
Cluster-22178.57486 GO:0016021
Cluster-22178.57487 GO:0008063

Cluster-22178.57488 GO:0015992 GO:0034508 GO:0015991 GO:0015078 GO:0008270 GO:0003723 GO:0033179 GO:0000776
Cluster-22178.57489 GO:0005975 GO:0046373 GO:0009117 GO:0046556

Cluster-22178.57490 GO:0006508 GO:0008236 GO:0016787
Cluster-22178.57492 GO:0006810 GO:0030288
Cluster-22178.57494 GO:0005975 GO:0006013 GO:0034639 GO:0004553 GO:0004559 GO:0008270 GO:0046872 GO:0015923 GO:0016021
Cluster-22178.57495 GO:0019064 GO:0000413 GO:0006457 GO:0007165 GO:0046789 GO:0003755 GO:0016788 GO:0009986 GO:0019031
Cluster-22178.57498 GO:0006541 GO:0005515 GO:0004359
Cluster-22178.57501 GO:0003676 GO:0003723
Cluster-22178.57503 GO:0006821 GO:0055085 GO:0005247 GO:0046983 GO:0016020
Cluster-22178.57505 GO:0006974 GO:0006915 GO:0051260 GO:0005515

Cluster-22178.57506 GO:0055114 GO:0006098 GO:0005975 GO:0006749 GO:0006006 GO:0050661 GO:0004345
Cluster-22178.57507 GO:0006811 GO:0019439 GO:0006813 GO:0005249 GO:0008270 GO:0005515 GO:0003677 GO:0016833 GO:0005622 GO:0016020 GO:0008076
Cluster-22178.57508 GO:0006886 GO:0008144 GO:0046872 GO:0003676

Cluster-22178.57512 GO:0007186 GO:0007165 GO:0007264 GO:0004871 GO:0005525 GO:0003924 GO:0019001 GO:0031683
Cluster-22178.57513 GO:0016747
Cluster-22178.57516 GO:0006465 GO:0016032 GO:0008233 GO:0016021 GO:0005787
Cluster-22178.57517 GO:0007005 GO:0006508 GO:0004222 GO:0008270 GO:0031012 GO:0031305
Cluster-22178.57518 GO:0007005 GO:0006508 GO:0004222 GO:0008270 GO:0031305 GO:0031012
Cluster-22178.57523 GO:0019073 GO:0006139 GO:0006260 GO:0003676

GO:0003723 GO:0008408 GO:0005515 GO:0003887 GO:0003677
GO:0042575
Cluster-22178.57524 GO:0006355 GO:0005634
Cluster-22178.57525 GO:0005515 GO:0005509
Cluster-22178.57529 GO:0009069 GO:0006468 GO:0016310 GO:0004672
GO:0005524 GO:0004674
Cluster-22178.57532 GO:0006570 GO:0042207 GO:0006810 GO:0009072
GO:0015743 GO:0004334 GO:0005886
Cluster-22178.57533 GO:0003677
Cluster-22178.57540 GO:0016787 GO:0004222
Cluster-22178.57541 GO:0005515
Cluster-22178.57543 GO:0006886 GO:0005515 GO:0005763 GO:0016020
GO:0005801
Cluster-22178.57547 GO:0010038 GO:0045892 GO:0046938 GO:0042754
GO:0046872 GO:0016756 GO:0005515
Cluster-22178.57548 GO:0006468 GO:0035556 GO:0005524 GO:0004672
GO:0016773 GO:0005515 GO:0016020
Cluster-22178.57550 GO:0005515 GO:0046872
Cluster-22178.57551 GO:0005515
Cluster-22178.57553 GO:0015031 GO:0055114 GO:0016491 GO:0008565
GO:0005515 GO:0016021
Cluster-22178.57554 GO:0008376 GO:0005515 GO:0046872 GO:0032580
Cluster-22178.57555 GO:0006260 GO:0032259 GO:0006370 GO:0006397
GO:0006310 GO:0006281 GO:0003676 GO:0003910 GO:0005524
GO:0008168 GO:0005515 GO:0004484
Cluster-22178.57557 GO:0008283 GO:0040007 GO:0007165 GO:0005125
GO:0008083 GO:0005515 GO:0016021
Cluster-22178.57558 GO:0006464
Cluster-22178.57559 GO:0035556 GO:0031167 GO:0009451 GO:0006400
GO:0008033 GO:0008168 GO:0008176
Cluster-22178.57561 GO:0008146 GO:0016021
Cluster-22178.57562 GO:0051016 GO:0050840 GO:0016021 GO:0005576
GO:0030479

Cluster-22178.57565 GO:0015940 GO:0055114 GO:0003677 GO:0008677

Cluster-22178.57567 GO:0005515 GO:0019904 GO:0030246 GO:0005737
Cluster-22178.57568 GO:0030246
Cluster-22178.57569 GO:0030246
Cluster-22178.57573 GO:0005515
Cluster-22178.57574 GO:0003723 GO:0005515
Cluster-22178.57576 GO:0010207 GO:0007017 GO:0007018 GO:0003777
GO:0005515 GO:0005737 GO:0030286 GO:0005874
Cluster-22178.57577 GO:0015684 GO:0006576 GO:0007264 GO:0005525
GO:0003924 GO:0015093 GO:0005524 GO:0016021
Cluster-22178.57581 GO:0003676
Cluster-22178.57583 GO:0009405 GO:0003723 GO:0046872 GO:0003676
GO:0016788 GO:0043022 GO:0005634
Cluster-22178.57586 GO:0006555 GO:0009405 GO:0006810 GO:0006952
GO:0006629 GO:0009755 GO:0006556 GO:0019855 GO:0046872
GO:0004478 GO:0045202 GO:0005576
Cluster-22178.57587 GO:0009306 GO:0016021
Cluster-22178.57589 GO:0007186 GO:0004930 GO:0016021
Cluster-22178.57590 GO:0046872 GO:0045202
Cluster-22178.57594 GO:0045892 GO:0003676 GO:0005634
Cluster-22178.57598 GO:0050708 GO:0005515 GO:0051537 GO:0005737
Cluster-22178.57599 GO:0016567 GO:0001510 GO:0009452 GO:0032922
GO:0055114 GO:0005515 GO:0004842 GO:0051537 GO:0046872
GO:0000976 GO:0008270 GO:0008168 GO:0016491
Cluster-22178.57601 GO:0005515
Cluster-22178.57602 GO:0005515 GO:0070403 GO:0008168
Cluster-22178.57606 GO:0006281 GO:0003824 GO:0004518 GO:0003677

Cluster-22178.57607 GO:0006570 GO:0006470 GO:0004725 GO:0008138

Cluster-22178.57609 GO:0006468 GO:0005198 GO:0005524 GO:0004672
GO:0016773 GO:0016020

Cluster-22178.57610 GO:0042254 GO:0006412 GO:0003735 GO:0005856
GO:0005622 GO:0005840
Cluster-22178.57613 GO:0055085 GO:0030206 GO:0030001 GO:0006024
GO:0030158 GO:0046873 GO:0016020
Cluster-22178.57616 GO:0015992 GO:0006119 GO:0015991 GO:0006355
GO:0003700 GO:0046961 GO:0005667 GO:0033178
Cluster-22178.57618 GO:0044780 GO:0006886 GO:0005801 GO:0016020
Cluster-22178.57621 GO:0005515
Cluster-22178.57622 GO:0016117 GO:0009405 GO:0046872 GO:0003924
GO:0005525 GO:0003677 GO:0016705 GO:0005515
Cluster-22178.57628 GO:0015992 GO:0006118 GO:0006119 GO:0009055
GO:0008121
Cluster-22178.57629 GO:0009395 GO:0006955 GO:0030153 GO:0006629
GO:0006606 GO:0035556 GO:0006914 GO:0046339 GO:0007165
GO:0004435 GO:0008081 GO:0005515 GO:0042803 GO:0008134
GO:0045502 GO:0015643 GO:0005509 GO:0019814 GO:0019028
GO:0030286 GO:0005667
Cluster-22178.57634 GO:0008440 GO:0004519 GO:0003676
Cluster-22178.57635 GO:0006810 GO:0018279 GO:0006355 GO:0004579
GO:0008270 GO:0008250 GO:0005634 GO:0016021 GO:0005622
Cluster-22178.57639 GO:0006811 GO:0005509 GO:0005216 GO:0016020
GO:0005576
Cluster-22178.57645 GO:0005515
Cluster-22178.57646 GO:0006355 GO:0006313 GO:0004803 GO:0003677

Cluster-22178.57652 GO:0046856 GO:0034597 GO:0004190 GO:0016021
Cluster-22178.57656 GO:0007165 GO:0007186 GO:0007264 GO:0006508
GO:0000166 GO:0004197 GO:0003677 GO:0005525 GO:0004871
GO:0031683 GO:0005524 GO:0019001 GO:0003924
Cluster-22178.57657 GO:0006812 GO:0000166 GO:0046872 GO:0016887
GO:0016021
Cluster-22178.57661 GO:0032259 GO:0008168
Cluster-22178.57662 GO:0032259 GO:0008168

Cluster-22178.57663 GO:0046872
Cluster-22178.57664 GO:0006123 GO:0015992 GO:0004129 GO:0045277
GO:0005739
Cluster-22178.57668 GO:0009113 GO:0006144 GO:0009252 GO:0046436
GO:0008716 GO:0070140 GO:0032183 GO:0004637 GO:0005524
GO:0046872
Cluster-22178.57671 GO:0016787
Cluster-22178.57674 GO:0007264 GO:0005525 GO:0003924
Cluster-22178.57675 GO:0006629 GO:0000413 GO:0006457 GO:0003755

Cluster-22178.57677 GO:0006352
Cluster-22178.57678 GO:0006355 GO:0006281 GO:0006952 GO:0046872
GO:0008270 GO:0043565 GO:0003700 GO:0005667
Cluster-22178.57682 GO:0003677 GO:0005515 GO:0008017 GO:0034061
GO:0045298 GO:0005634
Cluster-22178.57683 GO:0016055 GO:0006270 GO:0006418 GO:0006423
GO:0006534 GO:0007411 GO:0006811 GO:0004812 GO:0005515
GO:0004817 GO:0000166 GO:0005524 GO:0005576 GO:0016020
GO:0005737
Cluster-22178.57684 GO:0006260 GO:0006281 GO:0003911
Cluster-22178.57685 GO:0006106 GO:0007411 GO:0006367 GO:0006310
GO:0007186 GO:0006281 GO:0004930 GO:0003677 GO:0016788
GO:0000400 GO:0000287 GO:0016021 GO:0016020 GO:0005672
Cluster-22178.57686 GO:0019439 GO:0055114 GO:0016708 GO:0005515
GO:0051015 GO:0051537 GO:0005506
Cluster-22178.57692 GO:0007165 GO:0007186 GO:0009069 GO:0016310
GO:0005515 GO:0005525 GO:0004674 GO:0016772 GO:0004871
GO:0031683 GO:0019001 GO:0003924 GO:0016021
Cluster-22178.57696 GO:0006470 GO:0006570 GO:0008138 GO:0004725

Cluster-22178.57698 GO:0000079 GO:0019901
Cluster-22178.57699 GO:0006914 GO:0048870 GO:0051607 GO:0006606
GO:0008092 GO:0045502 GO:0042803 GO:0008134 GO:0005737

GO:0030286 GO:0009424 GO:0019898 GO:0005667 GO:0031514
Cluster-22178.57700 GO:0006897 GO:0003676 GO:0016020
Cluster-22178.57702 GO:0005515
Cluster-22178.57704 GO:0005515
Cluster-22178.57705 GO:0006310 GO:0015743 GO:0006302 GO:0003677
GO:0005515 GO:0005789 GO:0005634
Cluster-22178.57707 GO:0006468 GO:0016773 GO:0046872 GO:0008270
GO:0004672 GO:0005524 GO:0005515 GO:0016020
Cluster-22178.57708 GO:0006308 GO:0008380 GO:0007155 GO:0008855
GO:0005200 GO:0005856 GO:0005681 GO:0005882 GO:0009318
Cluster-22178.57718 GO:0006355 GO:0010508 GO:0006914 GO:0007049
GO:0003677 GO:0008270 GO:0043565 GO:0003700 GO:0005667
GO:0005634
Cluster-22178.57719 GO:0006810 GO:0019079 GO:0008026 GO:0004386
GO:0003676 GO:0005524
Cluster-22178.57725 GO:0008152 GO:0016836 GO:0003824
Cluster-22178.57729 GO:0009405 GO:0045747 GO:0006221 GO:0007049

Cluster-22178.57732 GO:0006367 GO:0006355 GO:0008270 GO:0046872
GO:0003677 GO:0005515 GO:0003713 GO:0005672 GO:0005667
Cluster-22178.57733 GO:0055114 GO:0016310 GO:0005506 GO:0004673
GO:0016705 GO:0020037 GO:0005198 GO:0009365 GO:0019028
Cluster-22178.57734 GO:0007165 GO:0006355 GO:0005515 GO:0004871
GO:0003700 GO:0005667 GO:0071203
Cluster-22178.57736 GO:0046677 GO:0006810 GO:0006816 GO:0042030
GO:0005246 GO:0046872 GO:0016020
Cluster-22178.57738 GO:0016020
Cluster-22178.57739 GO:0007186 GO:0007165 GO:0005515 GO:0004930
GO:0008601 GO:0016021 GO:0000159
Cluster-22178.57744 GO:0007264 GO:0035556 GO:0006506 GO:0017176
GO:0005525 GO:0016021
Cluster-22178.57746 GO:0006687 GO:0009058 GO:0008146 GO:0001733
GO:0005794 GO:0016021

Cluster-22178.57750 GO:0005515 GO:0061630 GO:0008270 GO:0046872 GO:0016817

Cluster-22178.57751 GO:0007186 GO:0006825 GO:0005507 GO:0016531 GO:0004930 GO:0016021 GO:0005758

Cluster-22178.57761 GO:0015979 GO:0030071 GO:0005515 GO:0005680 GO:0009579 GO:0016021 GO:0009522

Cluster-22178.57764 GO:0015992 GO:0006123 GO:0007165 GO:0007005 GO:0016567 GO:0008270 GO:0004842 GO:0004129 GO:0016788 GO:0005515 GO:0005887 GO:0045277

Cluster-22178.57772 GO:0045892 GO:0005634

Cluster-22178.57773 GO:0008152 GO:0055114 GO:0016491

Cluster-22178.57774 GO:0055114 GO:0015031 GO:0005515 GO:0016706 GO:0008168 GO:0008565

Cluster-22178.57776 GO:0007165 GO:0016973 GO:0030168 GO:0051258 GO:0030674 GO:0005102 GO:0005577 GO:0005643

Cluster-22178.57777 GO:0006260 GO:0055114 GO:0005198 GO:0008270 GO:0016020 GO:0019028 GO:0019031 GO:0005622

Cluster-22178.57778 GO:0004867 GO:0005515 GO:0016020 GO:0019013

Cluster-22178.57779 GO:0006869 GO:0042157 GO:0006265 GO:0003917 GO:0003677 GO:0008289 GO:0005694 GO:0005576

Cluster-22178.57780 GO:0006396 GO:0006260 GO:0008270 GO:0005198 GO:0019031 GO:0005622 GO:0016020 GO:0019028

Cluster-22178.57783 GO:0007165 GO:0005509 GO:0005578

Cluster-22178.57784 GO:0007165

Cluster-22178.57785 GO:0030001 GO:0015992 GO:0006123 GO:0000166 GO:0046872 GO:0004129 GO:0045277 GO:0005743 GO:0005751

Cluster-22178.57787 GO:0032801 GO:0006812 GO:0019076 GO:0005261 GO:0005887 GO:0033644

Cluster-22178.57788 GO:0031554 GO:0006468 GO:0031556 GO:0016773 GO:0005524 GO:0004672 GO:0016020

Cluster-22178.57790 GO:0000723 GO:0003697 GO:0003676

Cluster-22178.57791 GO:0000723 GO:0003676 GO:0003697

Cluster-22178.57792 GO:0006468 GO:0007165 GO:0004672 GO:0005524

GO:0005515

Cluster-22178.57794 GO:0007264 GO:0005525 GO:0016020 GO:0005622

Cluster-22178.57796 GO:0006118 GO:0008270 GO:0046872 GO:0009055

GO:0020037 GO:0005198 GO:0005622 GO:0019031

Cluster-22178.57797 GO:0006118 GO:0005198 GO:0020037 GO:0046872

GO:0008270 GO:0009055 GO:0005622 GO:0019031

Cluster-22178.57798 GO:0006621 GO:0046923 GO:0005783 GO:0016021

Cluster-22178.57801 GO:0008152 GO:0016042 GO:0005975 GO:0009058

GO:0046486 GO:0006508 GO:0004806 GO:0016788 GO:0008236

GO:0008184 GO:0016787

Cluster-22178.57802 GO:0006813 GO:0009405 GO:0016567 GO:0005249

GO:0004842 GO:0030955 GO:0042802 GO:0005515 GO:0016021

GO:0008076

Cluster-22178.57804 GO:0055114 GO:0008033 GO:0046872 GO:0016491

GO:0071949 GO:0050660

Cluster-22178.57805 GO:0045892 GO:0005634

Cluster-22178.57809 GO:0008641

Cluster-22178.57811 GO:0006468 GO:0019079 GO:0004672 GO:0005524

GO:0008026

Cluster-22178.57812 GO:0003676

Cluster-22178.57815 GO:0006269 GO:0006260 GO:0006351 GO:0046872

GO:0003896 GO:0005730 GO:0005657

Cluster-22178.57817 GO:0005515

Cluster-22178.57818 GO:0005515

Cluster-22178.57820 GO:0004672

Cluster-22178.57823 GO:0006606

Cluster-22178.57827 GO:0016310 GO:0009069 GO:0006468 GO:0004674

GO:0005524 GO:0004672

Cluster-22178.57833 GO:0017004 GO:0006810 GO:0055114 GO:0016020

GO:0005886

Cluster-22178.57834 GO:0045893 GO:0006351 GO:0008270 GO:0005515

GO:0003700 GO:0005634 GO:0005667

Cluster-22178.57835 GO:0055085 GO:0006118 GO:0016758 GO:0005515

GO:0009055 GO:0016021
Cluster-22178.57836 GO:0003676
Cluster-22178.57839 GO:0006506 GO:0017176 GO:0008270 GO:0016021
Cluster-22178.57840 GO:0007049 GO:0006351 GO:0017053
Cluster-22178.57844 GO:0055114 GO:0000921 GO:0033743 GO:0016021
GO:0005887 GO:0005940
Cluster-22178.57845 GO:0030036 GO:0015743 GO:0016043 GO:0007067
GO:0003779 GO:0048037 GO:0017048 GO:0016740 GO:0042729
GO:0072686
Cluster-22178.57846 GO:0055085 GO:0006812 GO:0035556 GO:0005515
GO:0008324
Cluster-22178.57850 GO:0055114 GO:0010181 GO:0016491
Cluster-22178.57851 GO:0003677
Cluster-22178.57854 GO:0006952 GO:0006810 GO:0009405 GO:0006556
GO:0009755 GO:0006629 GO:0006555 GO:0006730 GO:0004478
GO:0016740 GO:0019855 GO:0046872 GO:0005576 GO:0045202
Cluster-22178.57859 GO:0005515
Cluster-22178.57861 GO:0006468 GO:0016773 GO:0005524 GO:0004672
GO:0016020
Cluster-22178.57864 GO:0009094 GO:0006103 GO:0009821 GO:0009074
GO:0006571 GO:0000162 GO:0006536 GO:0007275 GO:0006511
GO:0046872 GO:0030170 GO:0004838 GO:0005634
Cluster-22178.57866 GO:0008017 GO:0045298 GO:0016591
Cluster-22178.57868 GO:0042254 GO:0006412 GO:0003735 GO:0005840
Cluster-22178.57872 GO:0003677
Cluster-22178.57873 GO:0005515 GO:0043565 GO:0005634
Cluster-22178.57877 GO:0046500 GO:0006400 GO:0044419 GO:0006810
GO:0006952 GO:0030488 GO:0006464 GO:0009451 GO:0009405
GO:0006479 GO:0008033 GO:0008152 GO:0008176 GO:0016429
GO:0008168 GO:0004719 GO:0019855 GO:0031515 GO:0005576
Cluster-22178.57878 GO:0004867 GO:0005515
Cluster-22178.57879 GO:0020037 GO:0005515
Cluster-22178.57881 GO:0006508 GO:0005975 GO:0034599 GO:0016787

GO:0008184 GO:0008236 GO:0005789
Cluster-22178.57882 GO:0006886 GO:0005622
Cluster-22178.57887 GO:0030414 GO:0005515
Cluster-22178.57889 GO:0006446 GO:0006413 GO:0003743 GO:0003677
GO:0003723 GO:0005840 GO:0019031 GO:0005737
Cluster-22178.57892 GO:0051694 GO:0005523
Cluster-22178.57894 GO:0051028 GO:0005634
Cluster-22178.57895 GO:0006886 GO:0008092 GO:0019898 GO:0017119
GO:0005737
Cluster-22178.57896 GO:0051260 GO:0006915 GO:0006974 GO:0005515

Cluster-22178.57897 GO:0006508 GO:0060478 GO:0006281 GO:0007342
GO:0006310 GO:0006897 GO:0009378 GO:0004252 GO:0003677
GO:0046872 GO:0009379 GO:0005886 GO:0005657
Cluster-22178.57904 GO:0000917 GO:0043093 GO:0048870 GO:0031514
GO:0005737
Cluster-22178.57905 GO:0051028 GO:0005515 GO:0005634
Cluster-22178.57906 GO:0009052 GO:0006098 GO:0015976 GO:0004751
GO:0030246
Cluster-22178.57907 GO:0006313 GO:0004803 GO:0003677
Cluster-22178.57911 GO:0016310 GO:0044237 GO:0016772 GO:0005515

Cluster-22178.57912 GO:0008270 GO:0046872 GO:0005515 GO:0005634
GO:0005622
Cluster-22178.57916 GO:0005525 GO:0008270 GO:0046872 GO:0005634
Cluster-22178.57920 GO:0015979 GO:0009654 GO:0016020 GO:0009523
Cluster-22178.57922 GO:0006810 GO:0055114 GO:0016787 GO:0004497
GO:0003677 GO:0005524 GO:0003676 GO:0005507 GO:0016715
Cluster-22178.57927 GO:0009069 GO:0006468 GO:0006508 GO:0016310
GO:0007178 GO:0004675 GO:0004252 GO:0005524 GO:0016020
Cluster-22178.57931 GO:0046413 GO:0008152 GO:0016491 GO:0005515
GO:0018836
Cluster-22178.57934 GO:0006508 GO:0004190 GO:0005515

Cluster-22178.57937 GO:0004190 GO:0003677 GO:0016021
Cluster-22178.57938 GO:0006811 GO:0005216 GO:0016020
Cluster-22178.57940 GO:0006811 GO:0005216 GO:0016020
Cluster-22178.57942 GO:0016788
Cluster-22178.57943 GO:0016788
Cluster-22178.57944 GO:0006810 GO:0005515 GO:0005215 GO:0016020
Cluster-22178.57946 GO:0055085 GO:0006810 GO:0022857 GO:0005515
GO:0005215 GO:0016021 GO:0016020
Cluster-22178.57948 GO:0055085 GO:0006810 GO:0022857 GO:0005515
GO:0005215 GO:0016021 GO:0016020
Cluster-22178.57949 GO:0016021
Cluster-22178.57951 GO:0055114 GO:0006633 GO:0009058 GO:0005506
GO:0016833 GO:0016491
Cluster-22178.57954 GO:0016021
Cluster-22178.57955 GO:0005515
Cluster-22178.57958 GO:0006606 GO:0043248 GO:0015031 GO:0006886
GO:0016192 GO:0015991 GO:0004222 GO:0008565 GO:0005515
GO:0016820 GO:0016021 GO:0000221 GO:0005737 GO:0005634
GO:0030117
Cluster-22178.57959 GO:0006886 GO:0005515
Cluster-22178.57963 GO:0005515
Cluster-22178.57964 GO:0006470 GO:0005515 GO:0004721 GO:0008270
GO:0005634
Cluster-22178.57965 GO:0008270
Cluster-22178.57976 GO:0017013 GO:0006313 GO:0016567 GO:0000398
GO:0003677 GO:0005515 GO:0004803 GO:0046872 GO:0008270
GO:0004842 GO:0005634 GO:0005622
Cluster-22178.57977 GO:0046872
Cluster-22178.57978 GO:0015979 GO:0009523 GO:0009539 GO:0016020
Cluster-22178.57979 GO:0006355 GO:0042254 GO:0006412 GO:0006367
GO:0043565 GO:0003700 GO:0005515 GO:0003735 GO:0005622
GO:0005840 GO:0005672 GO:0005667
Cluster-22178.57982 GO:0005515 GO:0042802

Cluster-22178.57987 GO:0006629 GO:0008081
Cluster-22178.57991 GO:0007015 GO:0006265 GO:0006897 GO:0003918 GO:0005524 GO:0003677
Cluster-22178.57992 GO:0006541 GO:0009252 GO:0046436 GO:0005524 GO:0046872 GO:0008716 GO:0016787
Cluster-22178.57994 GO:0006468 GO:0019079 GO:0006260 GO:0030337 GO:0016773 GO:0005524 GO:0004672 GO:0016020 GO:0042575
Cluster-22178.57995 GO:0005515 GO:0046872
Cluster-22178.57996 GO:0006955 GO:0007165 GO:0005164 GO:0016020
Cluster-22178.57997 GO:0007165 GO:0006955 GO:0005164 GO:0016020
Cluster-22178.58000 GO:0005515
Cluster-22178.58001 GO:0032297 GO:0003677
Cluster-22178.58002 GO:0016055 GO:0006508 GO:0007165 GO:0070008 GO:0005179 GO:0005044 GO:0005515 GO:0004252 GO:0005576 GO:0016020
Cluster-22178.58003 GO:0006887 GO:0007165 GO:0048278 GO:0006810 GO:0016887 GO:0005524 GO:0003774 GO:0005737 GO:0016459
Cluster-22178.58005 GO:0006511 GO:0031625
Cluster-22178.58006 GO:0007018 GO:0007017 GO:0003777 GO:0008017 GO:0005524 GO:0016787 GO:0005874 GO:0045298
Cluster-22178.58007 GO:0015684 GO:0005525 GO:0015093 GO:0003924 GO:0016021
Cluster-22178.58008 GO:0000103 GO:0055085 GO:0006144 GO:0006810 GO:0006468 GO:0006355 GO:0042626 GO:0004020 GO:0016887 GO:0004672 GO:0005524 GO:0016301 GO:0008134 GO:0016021 GO:0005667
Cluster-22178.58012 GO:0016310 GO:0009069 GO:0004674
Cluster-22178.58013 GO:0006508 GO:0008234
Cluster-22178.58014 GO:0006468 GO:0007165 GO:0055114 GO:0035556 GO:0004672 GO:0005524 GO:0005057 GO:0047134
Cluster-22178.58015 GO:0006525 GO:0046740 GO:0006560 GO:0071436 GO:0006814 GO:0006090 GO:0008948 GO:0015081 GO:0016020 GO:0016021

Cluster-22178.58018 GO:0042254 GO:0006412 GO:0007165 GO:0007016
GO:0003735 GO:0003779 GO:0005158 GO:0000062 GO:0005200
GO:0005925 GO:0001726 GO:0005899 GO:0005856 GO:0005622
GO:0005840
Cluster-22178.58020 GO:0043123 GO:0045766 GO:0043066
Cluster-22178.58023 GO:0016579 GO:0006508 GO:0009405 GO:0007268
GO:0006952 GO:0007165 GO:0036459 GO:0008270 GO:0004843
GO:0030550 GO:0005576
Cluster-22178.58031 GO:0005515
Cluster-22178.58033 GO:0006570 GO:0019988 GO:0006470 GO:0008152
GO:0043399 GO:0008138 GO:0008168 GO:0004725
Cluster-22178.58034 GO:0034314 GO:0006355 GO:0030833 GO:0007165
GO:0007186 GO:0043565 GO:0004871 GO:0003700 GO:0005198
GO:0005856 GO:0005834 GO:0019867 GO:0005667 GO:0005885
GO:0005882
Cluster-22178.58035 GO:0007165 GO:0055085 GO:0000772 GO:0016020
GO:0016021
Cluster-22178.58036 GO:0006629 GO:0016787
Cluster-22178.58038 GO:0016746 GO:0005634
Cluster-22178.58039 GO:0006357 GO:0043565 GO:0005515 GO:0001104
GO:0005634 GO:0016592
Cluster-22178.58040 GO:0015992 GO:0006123 GO:0004129 GO:0045277
Cluster-22178.58041 GO:0007165 GO:0046008 GO:0005179 GO:0005576
Cluster-22178.58042 GO:0007165 GO:0046008 GO:0005179 GO:0005576
Cluster-22178.58043 GO:0006468 GO:0051016 GO:0016773 GO:0004672
GO:0005524 GO:0008290 GO:0016020
Cluster-22178.58044 GO:0048544 GO:0005515
Cluster-22178.58049 GO:0007165 GO:0006457 GO:0006955 GO:0005164
GO:0016020
Cluster-22178.58053 GO:0055114 GO:0042967 GO:0006633 GO:0046872
GO:0004318 GO:0005515 GO:0005835
Cluster-22178.58054 GO:0016226 GO:0051536 GO:0005737
Cluster-22178.58057 GO:0008168

Cluster-22178.58060 GO:0006506 GO:0016740 GO:0005789
Cluster-22178.58061 GO:0005975 GO:0005515 GO:0004553 GO:2001070

Cluster-22178.58063 GO:0006355 GO:0003723 GO:0005515
Cluster-22178.58064 GO:0006468 GO:0006355 GO:0007165 GO:0030521
GO:0003677 GO:0005524 GO:0004672 GO:0004882 GO:0016773
GO:0005496 GO:0005634 GO:0016020
Cluster-22178.58069 GO:0009404 GO:0016746 GO:0005737
Cluster-22178.58074 GO:0005739
Cluster-22178.58075 GO:0008654 GO:0019872 GO:0006355 GO:0006021
GO:0004512 GO:0003700 GO:0043565 GO:0005667
Cluster-22178.58076 GO:0006355 GO:0006281 GO:0006310 GO:0005978
GO:0006260 GO:0002949 GO:0009378 GO:0008134 GO:0016301
GO:0005524 GO:0016887 GO:0003678 GO:0005667 GO:0009379
GO:0005657
Cluster-22178.58078 GO:0009451 GO:0030488 GO:0008033 GO:0016429
GO:0008168 GO:0031515
Cluster-22178.58079 GO:0006099 GO:0015991 GO:0019643 GO:0046487
GO:0003994 GO:0005515 GO:0016820 GO:0000221
Cluster-22178.58080 GO:0006525 GO:0071436 GO:0006814 GO:0006560
GO:0006090 GO:0008948 GO:0015081 GO:0016020 GO:0016021
Cluster-22178.58081 GO:0060478 GO:0006897 GO:0023052 GO:0007342
GO:0003950 GO:0005886 GO:0005634
Cluster-22178.58082 GO:0016070 GO:0043087 GO:0019013 GO:0016021
Cluster-22178.58083 GO:0006260 GO:0004867 GO:0005515 GO:0061630
GO:0003689 GO:0046872 GO:0005524 GO:0008270 GO:0005663
GO:0042575 GO:0005622
Cluster-22178.58084 GO:0007264 GO:0015684 GO:0015093 GO:0005525
GO:0016021
Cluster-22178.58085 GO:0000398 GO:0046983 GO:0019031 GO:0005681
Cluster-22178.58086 GO:0046983
Cluster-22178.58090 GO:0005515
Cluster-22178.58091 GO:0006260 GO:0006810 GO:0006310 GO:0006309

GO:0006281 GO:0003724 GO:0005524 GO:0016887 GO:0003677
GO:0098519 GO:0009378 GO:0016787 GO:0003676 GO:0003887
GO:0000166 GO:0003723 GO:0005657 GO:0009360 GO:0009379
GO:0005737 GO:0042575 GO:0005634
Cluster-22178.58094 GO:0006397 GO:0006810 GO:0015986 GO:0046660
GO:0015992 GO:0003676 GO:0015078 GO:0008270 GO:0005634
GO:0000276 GO:0005643
Cluster-22178.58096 GO:0000281 GO:0000226 GO:0040001 GO:0030245
GO:0006353 GO:0016192 GO:0005985 GO:0005982 GO:0008810
GO:0005874 GO:0016021 GO:0005819
Cluster-22178.58102 GO:0010508
Cluster-22178.58103 GO:0007010 GO:0003779
Cluster-22178.58104 GO:0042254 GO:0006412 GO:0006827 GO:0006812
GO:0006836 GO:0006826 GO:0005328 GO:0003735 GO:0005381
GO:0016020 GO:0016021 GO:0005840 GO:0033573 GO:0005622
Cluster-22178.58108 GO:0006771 GO:0019497 GO:0051082 GO:0005515
GO:0046872 GO:0008270 GO:0003993 GO:0005622
Cluster-22178.58109 GO:0006355 GO:0043565 GO:0008270 GO:0003700
GO:0005667 GO:0005634
Cluster-22178.58111 GO:0007049 GO:0006357 GO:0044237 GO:0006810
GO:0010038 GO:0005215 GO:0005515 GO:0001104 GO:0016021
GO:0016592
Cluster-22178.58112 GO:0006351 GO:0006144 GO:0006206 GO:0003899
GO:0005730
Cluster-22178.58118 GO:0006769 GO:0055114 GO:0046497 GO:0070403
GO:0008750 GO:0030976 GO:0050661 GO:0000287 GO:0016021
Cluster-22178.58120 GO:0006886 GO:0016192 GO:0030866 GO:0005515
GO:0008092 GO:0030117 GO:0005856
Cluster-22178.58123 GO:0016226 GO:0051536 GO:0005737
Cluster-22178.58124 GO:0008152 GO:0016226 GO:0008168 GO:0051536
GO:0005737
Cluster-22178.58125 GO:0006400 GO:0016226 GO:0008033 GO:0009451
GO:0051536 GO:0008176 GO:0005737

Cluster-22178.58127 GO:0016226 GO:0008152 GO:0008168 GO:0051536 GO:0005737
Cluster-22178.58129 GO:0006313 GO:0003677 GO:0004803
Cluster-22178.58130 GO:0016020
Cluster-22178.58133 GO:0055085 GO:0016021
Cluster-22178.58134 GO:0009451 GO:0008610 GO:0006479 GO:0008033 GO:0046486 GO:0046500 GO:0042967 GO:0006396 GO:0006464 GO:0030488 GO:0008168 GO:0004719 GO:0016627 GO:0004144 GO:0016429 GO:0031515 GO:0005622
Cluster-22178.58135 GO:0006402 GO:0051252 GO:0003723 GO:0004535 GO:0046872 GO:0005634 GO:0005737
Cluster-22178.58137 GO:0055085 GO:0005741
Cluster-22178.58145 GO:0042254 GO:0006412 GO:0006355 GO:0019069 GO:0003700 GO:0003735 GO:0003677 GO:0005840 GO:0042025 GO:0005634 GO:0005667
Cluster-22178.58146 GO:0005515
Cluster-22178.58148 GO:0005515
Cluster-22178.58149 GO:0019290 GO:0006351 GO:0006144 GO:0006206 GO:0006826 GO:0003899 GO:0005515 GO:0003677 GO:0015343 GO:0005730
Cluster-22178.58153 GO:0000079 GO:0019901
Cluster-22178.58155 GO:0006886 GO:0006888 GO:0008270 GO:0030127
Cluster-22178.58157 GO:0005515
Cluster-22178.58158 GO:0016192
Cluster-22178.58159 GO:0006357 GO:0001104 GO:0016592
Cluster-22178.58160 GO:0005515
Cluster-22178.58163 GO:0015979 GO:0006621 GO:0046923 GO:0009523 GO:0009539 GO:0016021 GO:0016020 GO:0005783
Cluster-22178.58164 GO:0007165 GO:0007275 GO:0016055 GO:0005102 GO:0005576
Cluster-22178.58165 GO:0040001 GO:0000226 GO:0006606 GO:0071705 GO:0000281 GO:0015031 GO:0015991 GO:0008565 GO:0003677 GO:0016820 GO:0005515 GO:0000221 GO:0005874 GO:0005737

GO:0005634 GO:0005819
Cluster-22178.58167 GO:0051260 GO:0005515
Cluster-22178.58169 GO:0043401 GO:0006355 GO:0003700 GO:0043565
GO:0008270 GO:0005634 GO:0005667
Cluster-22178.58175 GO:0006270 GO:0046983
Cluster-22178.58176 GO:0005515
Cluster-22178.58181 GO:0009058 GO:0006546 GO:0006566 GO:0006563
GO:0006544 GO:0055114 GO:0004375 GO:0030170
Cluster-22178.58186 GO:0007050 GO:0006468 GO:0045859 GO:0004861
GO:0005524 GO:0004672 GO:0005634
Cluster-22178.58192 GO:0006629 GO:0006481 GO:0007165 GO:0006479
GO:0035556 GO:0004671 GO:0016627 GO:0016020 GO:0005737
GO:0016021
Cluster-22178.58194 GO:0006813 GO:0055114 GO:0003677 GO:0020037
GO:0016705 GO:0005506 GO:0016787 GO:0005524 GO:0005267
GO:0016020
Cluster-22178.58197 GO:0016255 GO:0042765
Cluster-22178.58198 GO:0006468 GO:0005524 GO:0004672 GO:0016773
GO:0016020
Cluster-22178.58199 GO:0006468 GO:0016773 GO:0005524 GO:0004672
GO:0035091 GO:0016020
Cluster-22178.58200 GO:0006554 GO:0006355 GO:0034968 GO:0006479
GO:0007165 GO:0018024 GO:0005515 GO:0005509 GO:0005578
GO:0005694
Cluster-22178.58201 GO:0005515
Cluster-22178.58202 GO:0005515
Cluster-22178.58204 GO:0001522 GO:0009451 GO:0009982 GO:0003676
GO:0046872 GO:0016787 GO:0003723 GO:0019031
Cluster-22178.58205 GO:0006355 GO:0009306 GO:0003682 GO:0043565
GO:0008270 GO:0003700 GO:0005667 GO:0016020 GO:0000785
Cluster-22178.58206 GO:0050832 GO:0046872 GO:0005576
Cluster-22178.58208 GO:0006479 GO:0032259 GO:0006464 GO:0046500
GO:0016787 GO:0008168 GO:0004719

Cluster-22178.58209 GO:0008643 GO:0005351 GO:0005507 GO:0000139
GO:0016021 GO:0016020
Cluster-22178.58210 GO:0006189 GO:0006355 GO:0003700 GO:0003824
GO:0043565 GO:0008270 GO:0005667
Cluster-22178.58214 GO:0006376 GO:0003729 GO:0016021 GO:0005685
Cluster-22178.58219 GO:0035556 GO:0009306 GO:0006464 GO:0051090
GO:0016151 GO:0046872 GO:0005515 GO:0003677 GO:0005634
Cluster-22178.58220 GO:0006310 GO:0006024 GO:0002949 GO:0006570
GO:0006281 GO:0006470 GO:0030206 GO:0008138 GO:0009378
GO:0003724 GO:0030158 GO:0016887 GO:0005524 GO:0016301
GO:0003723 GO:0003678 GO:0004725 GO:0009379 GO:0005657
Cluster-22178.58222 GO:0055085 GO:0006812 GO:0006885 GO:0015299
GO:0016021
Cluster-22178.58223 GO:0005515
Cluster-22178.58224 GO:0005509
Cluster-22178.58226 GO:0045947 GO:0008608 GO:0008190 GO:0072686
GO:0042729
Cluster-22178.58227 GO:0008608 GO:0045947 GO:0008190 GO:0003677
GO:0042729 GO:0072686
Cluster-22178.58233 GO:0007275 GO:0016021
Cluster-22178.58234 GO:0045454 GO:0055114 GO:0016972
Cluster-22178.58239 GO:0007165 GO:0046008 GO:0030474 GO:0005200
GO:0005057 GO:0008270 GO:0005179 GO:0005823 GO:0005856
GO:0005576 GO:0005634
Cluster-22178.58240 GO:0006621 GO:0046923 GO:0005783 GO:0016021
Cluster-22178.58243 GO:0016020
Cluster-22178.58245 GO:0003676
Cluster-22178.58246 GO:0015986 GO:0015992 GO:0007049 GO:0015078
GO:0000276
Cluster-22178.58247 GO:0005515
Cluster-22178.58248 GO:0003824
Cluster-22178.58251 GO:0006260 GO:0000723 GO:0003676 GO:0003677
GO:0005634 GO:0000784

Cluster-22178.58253 GO:0006281
Cluster-22178.58256 GO:0006355 GO:0005515 GO:0003676 GO:0043565
GO:0005634
Cluster-22178.58262 GO:0006597 GO:0008295 GO:0006560 GO:0006525
GO:0004014
Cluster-22178.58263 GO:0006118 GO:0006119 GO:0006950 GO:0015992
GO:0008121 GO:0016021
Cluster-22178.58264 GO:0006914 GO:0006891 GO:0006355 GO:0006397
GO:0043565 GO:0003700 GO:0003723 GO:0005737 GO:0017119
GO:0005667 GO:0005634
Cluster-22178.58265 GO:0006355 GO:0006811 GO:0055085 GO:0005216
GO:0005515 GO:0016020 GO:0005761
Cluster-22178.58266 GO:0006508 GO:0004177 GO:0008270
Cluster-22178.58267 GO:0000160 GO:0015031 GO:0005515 GO:0008270
GO:0008565
Cluster-22178.58268 GO:0043565
Cluster-22178.58269 GO:0015992 GO:0015986 GO:0015078 GO:0000276
Cluster-22178.58270 GO:0006351 GO:0006144 GO:0006206 GO:0003899
GO:0003677 GO:0005730
Cluster-22178.58271 GO:0009405 GO:0045735
Cluster-22178.58274 GO:0008270
Cluster-22178.58276 GO:0009069 GO:0006468 GO:0006357 GO:0016310
GO:0006260 GO:0001104 GO:0005524 GO:0004674 GO:0016592
Cluster-22178.58277 GO:0007017 GO:0004869 GO:0005875
Cluster-22178.58281 GO:0015031 GO:0016020
Cluster-22178.58284 GO:0006357 GO:0001104 GO:0016020 GO:0016592
Cluster-22178.58286 GO:0003677 GO:0016787
Cluster-22178.58287 GO:0005198 GO:0019028 GO:0019031
Cluster-22178.58288 GO:0035023 GO:0006260 GO:0043087 GO:0005515
GO:0005089
Cluster-22178.58289 GO:0006468 GO:0042742 GO:0004672 GO:0005524
GO:0016773 GO:0016020
Cluster-22178.58292 GO:0009847 GO:0003333 GO:0006508 GO:0004185

GO:0016021

Cluster-22178.58293 GO:0016021

Cluster-22178.58294 GO:0006508 GO:0005515 GO:0004222

Cluster-22178.58295 GO:0007034

Cluster-22178.58297 GO:0000774 GO:0005515 GO:0005783

Cluster-22178.58300 GO:0005515

Cluster-22178.58302 GO:0006098 GO:0055114 GO:0019521 GO:0016491
GO:0051287 GO:0004616

Cluster-22178.58306 GO:0043123 GO:0044564 GO:0007165 GO:0004871
GO:0005515

Cluster-22178.58309 GO:0006468 GO:0016773 GO:0004672 GO:0005524
GO:0005515 GO:0016020

Cluster-22178.58315 GO:0006468 GO:0009058 GO:0015937 GO:0008152
GO:0015940 GO:0005515 GO:0016301 GO:0005524 GO:0004672
GO:0016773 GO:0004140 GO:0003824 GO:0016020

Cluster-22178.58317 GO:0016567 GO:0008270 GO:0046872 GO:0005515
GO:0004842 GO:0005680

Cluster-22178.58318 GO:0035556 GO:0046872 GO:0005524

Cluster-22178.58319 GO:0035556 GO:0046872 GO:0005524

Cluster-22178.58320 GO:0016820

Cluster-22178.58323 GO:0032065 GO:0007165 GO:0005158 GO:0005543
GO:0005515 GO:0005899 GO:0005938

Cluster-22178.58324 GO:0005887

Cluster-22178.58325 GO:0006571 GO:0000162 GO:0009094 GO:0008033
GO:0006432 GO:0004826 GO:0005524 GO:0000049 GO:0000287
GO:0009328

Cluster-22178.58328 GO:0005634

Cluster-22178.58330 GO:0006952 GO:0006810 GO:0009405 GO:0019855
GO:0005576

Cluster-22178.58331 GO:0005524 GO:0016887

Cluster-22178.58332 GO:0007165 GO:0050830 GO:0005509 GO:0005578

Cluster-22178.58333 GO:0050830 GO:0051016 GO:0005509 GO:0030479

Cluster-22178.58334 GO:0045116 GO:0016881 GO:0008641 GO:0005524

Cluster-22178.58335 GO:0006486 GO:0008378 GO:0016757 GO:0016020
Cluster-22178.58336 GO:0042254 GO:0006412 GO:0003735 GO:0003824
GO:0005622 GO:0005840
Cluster-22178.58337 GO:0006531 GO:0009058 GO:0006520 GO:0006144
GO:0055114 GO:0006188 GO:0009152 GO:0006522 GO:0030170
GO:0004018 GO:0016829 GO:0010277
Cluster-22178.58338 GO:0008270 GO:0003676 GO:0005622
Cluster-22178.58339 GO:0055114 GO:0051536 GO:0016491 GO:0020037
GO:0003676
Cluster-22178.58341 GO:0005996 GO:0016853 GO:0048029
Cluster-22178.58344 GO:0008270 GO:0046872 GO:0003677
Cluster-22178.58346 GO:0006807 GO:0008033 GO:0006725 GO:0055114
GO:0050660 GO:0017150 GO:0016627 GO:0016491 GO:0016853
GO:0018580 GO:0005737
Cluster-22178.58349 GO:0005515
Cluster-22178.58350 GO:0007155 GO:0015031 GO:0000921 GO:0006606
GO:0009405 GO:0006904 GO:0051382 GO:0007049 GO:0045502
GO:0008134 GO:0042803 GO:0005198 GO:0008289 GO:0005667
GO:0000145 GO:0016020 GO:0005882 GO:0005940 GO:0030286
GO:0000776 GO:0016021
Cluster-22178.58358 GO:0008033 GO:0031167 GO:0009451 GO:0004809
GO:0046872 GO:0003723 GO:0008168
Cluster-22178.58360 GO:0005515
Cluster-22178.58361 GO:0055085 GO:0006812 GO:0008324 GO:0016021
Cluster-22178.58362 GO:0008641
Cluster-22178.58364 GO:0006810 GO:0005515 GO:0042626 GO:0005524
GO:0016021
Cluster-22178.58365 GO:0042254 GO:0006412 GO:0007264 GO:0045040
GO:0005525 GO:0046983 GO:0003924 GO:0003735 GO:0042802
GO:0005515 GO:0005840 GO:0005622 GO:0005742
Cluster-22178.58366 GO:0007156 GO:0005509 GO:0016020
Cluster-22178.58367 GO:0007165 GO:0006508 GO:0006355 GO:0008234

GO:0003677
Cluster-22178.58369 GO:0030150 GO:0005742
Cluster-22178.58371 GO:0008152 GO:0016787
Cluster-22178.58372 GO:0043631 GO:0004652 GO:0003676
Cluster-22178.58373 GO:0008152 GO:0016787
Cluster-22178.58375 GO:0006281 GO:0007264 GO:0006338 GO:0005525 GO:0005515 GO:0090544
Cluster-22178.58376 GO:0006355 GO:0001104 GO:0016592
Cluster-22178.58377 GO:0055114 GO:0006118 GO:0016655
Cluster-22178.58378 GO:0016114 GO:0008152 GO:0006694 GO:0003824 GO:0008661 GO:0030976 GO:0016624
Cluster-22178.58380 GO:0006694 GO:0008152 GO:0016114 GO:0016624 GO:0030976 GO:0008661 GO:0003824
Cluster-22178.58382 GO:0007165 GO:0051225 GO:0006355 GO:0007155 GO:0004871 GO:0003677 GO:0003723 GO:0009288 GO:0070652 GO:0016020 GO:0005634
Cluster-22178.58384 GO:0046872
Cluster-22178.58385 GO:0043087 GO:0007264 GO:0005085
Cluster-22178.58386 GO:0007165 GO:0005179 GO:0005576
Cluster-22178.58387 GO:0007165 GO:0006810 GO:0006310 GO:0014068 GO:0050776 GO:0006281 GO:0009405 GO:0005524 GO:0005102 GO:0005179 GO:0009378 GO:0043548 GO:0019871 GO:0005657 GO:0009379 GO:0016020 GO:0005576
Cluster-22178.58390 GO:0003677
Cluster-22178.58391 GO:0007186 GO:0005515 GO:0004930 GO:0042470 GO:0016021
Cluster-22178.58392 GO:0006810 GO:0055085 GO:0042626 GO:0005515 GO:0005524 GO:0016021
Cluster-22178.58394 GO:0015031 GO:0016021
Cluster-22178.58395 GO:0005509
Cluster-22178.58396 GO:0006525 GO:0006433 GO:0006560 GO:0007165 GO:0005179 GO:0000166 GO:0005524 GO:0004827 GO:0005576 GO:0005737

Cluster-22178.58400 GO:0007165 GO:0005515 GO:0008270
Cluster-22178.58401 GO:0006366 GO:0006281 GO:0051382 GO:0006352 GO:0003677 GO:0005669
Cluster-22178.58405 GO:0006355 GO:0006807 GO:0000105 GO:0009607 GO:0006537 GO:0055114 GO:0015930 GO:0018580 GO:0016638 GO:0003824 GO:0016627 GO:0016491 GO:0005737
Cluster-22178.58406 GO:0003676
Cluster-22178.58408 GO:0005985 GO:0005982 GO:0006096 GO:0006094 GO:0006098 GO:0005515 GO:0005198 GO:0004347 GO:0005737
Cluster-22178.58410 GO:0055114 GO:0005506 GO:0016705 GO:0020037

Cluster-22178.58412 GO:0071585 GO:0010273 GO:0005507 GO:0046870

Cluster-22178.58415 GO:0000077 GO:0051082
Cluster-22178.58418 GO:0009451 GO:0006396 GO:0008173 GO:0003723 GO:0008168
Cluster-22178.58420 GO:0008171
Cluster-22178.58422 GO:0006355 GO:0005634
Cluster-22178.58428 GO:0006465 GO:0008233 GO:0016021 GO:0005787
Cluster-22178.58429 GO:0005515
Cluster-22178.58431 GO:0016787
Cluster-22178.58432 GO:0016787
Cluster-22178.58433 GO:0006914 GO:0006355 GO:0019082 GO:0043565 GO:0003700 GO:0003677 GO:0005667
Cluster-22178.58434 GO:0008376 GO:0032580
Cluster-22178.58435 GO:0006144 GO:0006508 GO:0070008 GO:0003968 GO:0016817 GO:0004197 GO:0017111 GO:0005515 GO:0004252 GO:0031379
Cluster-22178.58437 GO:0006810 GO:0006355 GO:0016301 GO:0008134 GO:0005524 GO:0005667
Cluster-22178.58440 GO:0045947 GO:0008190
Cluster-22178.58441 GO:0015986 GO:0015992 GO:0006144 GO:0015078 GO:0003877 GO:0000276

Cluster-22178.58447 GO:0008053 GO:0006869 GO:0016192 GO:0042157 GO:0009405 GO:0006886 GO:0004185 GO:0005524 GO:0016887 GO:0008289 GO:0005525 GO:0003924 GO:0005198 GO:0005773 GO:0005741 GO:0016020 GO:0019031 GO:0016021 GO:0005576 GO:0019028

Cluster-22178.58450 GO:0006281 GO:0007631 GO:0007218 GO:0016817 GO:0046872 GO:0008270

Cluster-22178.58456 GO:0006355 GO:0008270 GO:0046872 GO:0003723

Cluster-22178.58457 GO:0035023 GO:0035556 GO:0055114 GO:0043087 GO:0007186 GO:0005089 GO:0004930 GO:0047134 GO:0005515 GO:0016020

Cluster-22178.58459 GO:0006810 GO:0009405 GO:0015858 GO:0055085 GO:0005337 GO:0005576 GO:0016021

Cluster-22178.58461 GO:0006633 GO:0008152 GO:0042967 GO:0008610 GO:0005524 GO:0016747 GO:0004315 GO:0005835

Cluster-22178.58464 GO:0006355 GO:0009451 GO:0001522 GO:0007165 GO:0006810 GO:0003723 GO:0035257 GO:0003713 GO:0005515 GO:0046983 GO:0009982 GO:0005215 GO:0005667 GO:0005634 GO:0016021

Cluster-22178.58466 GO:0007304 GO:0007275 GO:0006313 GO:0005515 GO:0005213 GO:0042600

Cluster-22178.58470 GO:0006811 GO:0005216 GO:0016020

Cluster-22178.58474 GO:0005515

Cluster-22178.58475 GO:0005515

Cluster-22178.58477 GO:0006813 GO:0030435 GO:0005249 GO:0030955 GO:0008076 GO:0016021

Cluster-22178.58479 GO:0003824

Cluster-22178.58484 GO:0055114 GO:0006355 GO:0016887 GO:0016491 GO:0005524 GO:0003677

Cluster-22178.58485 GO:0005515

Cluster-22178.58486 GO:0055114 GO:0006355 GO:0003677 GO:0016491

Cluster-22178.58487 GO:0003677
Cluster-22178.58488 GO:0009308 GO:0055114 GO:0003677 GO:0016787
GO:0008131 GO:0003676 GO:0005507 GO:0048038 GO:0005524
Cluster-22178.58489 GO:0005975 GO:0042967 GO:0046486 GO:0004571
GO:0005509 GO:0004144 GO:0016020
Cluster-22178.58491 GO:0006446 GO:0006886 GO:0003743 GO:0030176
GO:0005852 GO:0005840 GO:0005737
Cluster-22178.58493 GO:0007165 GO:0008152 GO:0055114 GO:0016706
GO:0005515 GO:0005509 GO:0016758 GO:0005578
Cluster-22178.58494 GO:0016192 GO:0006886 GO:0042981 GO:0005515
GO:0030117
Cluster-22178.58495 GO:0042981 GO:0006886 GO:0016192 GO:0005515
GO:0030117
Cluster-22178.58497 GO:0046740 GO:0005515 GO:0016021
Cluster-22178.58501 GO:0006952 GO:0008284 GO:0003676 GO:0046872

Cluster-22178.58502 GO:0003677
Cluster-22178.58505 GO:0009069 GO:0006468 GO:0016310 GO:0019087
GO:0007178 GO:0003677 GO:0005026 GO:0005524 GO:0046872
GO:0016020 GO:0016021
Cluster-22178.58508 GO:0005615 GO:0005576
Cluster-22178.58509 GO:0005515
Cluster-22178.58513 GO:0006313 GO:0015074 GO:0003677 GO:0019028
Cluster-22178.58516 GO:0046872
Cluster-22178.58524 GO:0007264 GO:0006886 GO:0007186 GO:0007165
GO:0008152 GO:0016836 GO:0003924 GO:0031683 GO:0019001
GO:0003824 GO:0004871 GO:0005525 GO:0005783 GO:0016021
Cluster-22178.58527 GO:0007267 GO:0046872
Cluster-22178.58531 GO:0007165 GO:0006508 GO:0004252 GO:0004197
GO:0005044 GO:0070008 GO:0008270 GO:0016020
Cluster-22178.58533 GO:0035091 GO:0005515
Cluster-22178.58535 GO:0042312 GO:0008217 GO:0007165 GO:0006508
GO:0000245 GO:0006355 GO:0004222 GO:0008270 GO:0005515

GO:0005179 GO:0031012 GO:0005634 GO:0005576
Cluster-22178.58539 GO:0006310 GO:0006302 GO:0003677 GO:0005634
Cluster-22178.58541 GO:0051056 GO:0005096
Cluster-22178.58542 GO:0051056 GO:0005096
Cluster-22178.58543 GO:0051056 GO:0005096
Cluster-22178.58544 GO:0006915
Cluster-22178.58545 GO:0030163 GO:0008270
Cluster-22178.58547 GO:0016567 GO:0005515 GO:0004842 GO:0008270
GO:0046872 GO:0005680
Cluster-22178.58550 GO:0006355 GO:0006289 GO:0000439
Cluster-22178.58552 GO:0008152 GO:0016491
Cluster-22178.58555 GO:0008152 GO:0016491
Cluster-22178.58556 GO:0007275 GO:0007219 GO:0016021
Cluster-22178.58557 GO:0006260 GO:0015940 GO:0042262 GO:0055114
GO:0015986 GO:0006355 GO:0008270 GO:0046933 GO:0046983
GO:0008677 GO:0003677 GO:0005515 GO:0045259
Cluster-22178.58558 GO:0006886 GO:0005622
Cluster-22178.58560 GO:0001514 GO:0006448 GO:0005515 GO:0003723
GO:0005525 GO:0003746 GO:0005840 GO:0005737
Cluster-22178.58561 GO:0008152 GO:0009878 GO:0005525 GO:0046872
GO:0016787 GO:0008168
Cluster-22178.58563 GO:0006265 GO:0016579 GO:0006355 GO:0003916
GO:0036459 GO:0003700 GO:0003677 GO:0005515 GO:0005694
GO:0005667
Cluster-22178.58565 GO:0016020
Cluster-22178.58566 GO:0045116 GO:0006468 GO:0016773 GO:0016881
GO:0005524 GO:0004672 GO:0016020
Cluster-22178.58567 GO:0016255 GO:0016627 GO:0042765
Cluster-22178.58571 GO:0007264 GO:0005525
Cluster-22178.58572 GO:0006508 GO:0055085 GO:0022857 GO:0016805
GO:0016021
Cluster-22178.58575 GO:0007165 GO:0007186 GO:0007264 GO:0005525
GO:0004871 GO:0031683 GO:0019001 GO:0003924

Cluster-22178.58577 GO:0006144 GO:0003968 GO:0005524 GO:0031379
Cluster-22178.58578 GO:0006323 GO:0006355 GO:0003700 GO:0005667
GO:0005622
Cluster-22178.58582 GO:0006118 GO:0019684 GO:0045156 GO:0016021
GO:0030077
Cluster-22178.58584 GO:0005975 GO:0004553
Cluster-22178.58585 GO:0042330 GO:0006508 GO:0016579 GO:0006511
GO:0004843 GO:0005622
Cluster-22178.58587 GO:0007018 GO:0007017 GO:0008017 GO:0003777
GO:0005524 GO:0045298 GO:0005874 GO:0019028
Cluster-22178.58588 GO:0006888 GO:0007165 GO:0006886 GO:0007606
GO:0008270 GO:0005515 GO:0004888 GO:0003723 GO:0030127
GO:0016020
Cluster-22178.58589 GO:0042710 GO:0007186 GO:0004930 GO:0016021
Cluster-22178.58592 GO:0019497 GO:0007154 GO:0006771 GO:0046872
GO:0003993 GO:0016820 GO:0051539 GO:0005515 GO:0016021
Cluster-22178.58597 GO:0006633 GO:0009396 GO:0006554 GO:0006631
GO:0006298 GO:0018874 GO:0008152 GO:0055114 GO:0006574
GO:0006552 GO:0006550 GO:0046487 GO:0006568 GO:0016491
GO:0005524 GO:0004488 GO:0050662 GO:0003824 GO:0003857
Cluster-22178.58598 GO:0006144 GO:0004114 GO:0005515 GO:0016020
Cluster-22178.58602 GO:0035556
Cluster-22178.58603 GO:0006464 GO:0006144 GO:0042157 GO:0006869
GO:0046034 GO:0006355 GO:0008270 GO:0004017 GO:0046872
GO:0043565 GO:0016151 GO:0008289 GO:0003700 GO:0051082
GO:0005815 GO:0005667 GO:0005576
Cluster-22178.58604 GO:0008270 GO:0003676
Cluster-22178.58605 GO:0006351 GO:0043565
Cluster-22178.58606 GO:0006412 GO:0042254 GO:0003735 GO:0005840
Cluster-22178.58608 GO:0005515
Cluster-22178.58609 GO:0006355 GO:0043161 GO:0006281 GO:0006289
GO:0043401 GO:0003684
Cluster-22178.58610 GO:0006810 GO:0034227 GO:0002098 GO:0042626

GO:0000049 GO:0005524 GO:0016021
Cluster-22178.58611 GO:0005975 GO:0004415 GO:0005515
Cluster-22178.58612 GO:0008270
Cluster-22178.58613 GO:0008270
Cluster-22178.58614 GO:0006874 GO:0006816 GO:0005219 GO:0016021
GO:0005622
Cluster-22178.58615 GO:0015992 GO:0043066 GO:0015986 GO:0045766
GO:0016567 GO:0043123 GO:0043130 GO:0004842 GO:0015078
GO:0003723 GO:0000276
Cluster-22178.58616 GO:0048870 GO:0051258 GO:0007165 GO:0030168
GO:0008053 GO:0006606 GO:0006260 GO:0045502 GO:0019901
GO:0008134 GO:0042803 GO:0003924 GO:0030674 GO:0005102
GO:0031514 GO:0016021 GO:0005667 GO:0005741 GO:0030286
GO:0005577
Cluster-22178.58617 GO:0006260 GO:0006950 GO:0007154 GO:0006313
GO:0007165 GO:0006281 GO:0003911 GO:0005179 GO:0016021
GO:0005576
Cluster-22178.58623 GO:0006457 GO:0005515
Cluster-22178.58627 GO:0006468 GO:0016055 GO:0007275 GO:0007165
GO:0004672 GO:0005524 GO:0005102 GO:0005576
Cluster-22178.58629 GO:0007165 GO:0055114 GO:0020037 GO:0016705
GO:0005158 GO:0005506 GO:0005899
Cluster-22178.58631 GO:0055114 GO:0016972
Cluster-22178.58634 GO:0007166 GO:0016567 GO:0007165 GO:0004871
GO:0004842 GO:0008270 GO:0046872 GO:0005509 GO:0005515
GO:0005680 GO:0005634
Cluster-22178.58638 GO:0045454 GO:0006118 GO:0009055 GO:0015035

Cluster-22178.58639 GO:0015996 GO:0015994 GO:0006811 GO:0006508
GO:0015991 GO:0006629 GO:0006355 GO:0006118 GO:0015992
GO:0016042 GO:0045454 GO:0046486 GO:0046872 GO:0015078
GO:0009055 GO:0015035 GO:0008236 GO:0047746 GO:0003847
GO:0016787 GO:0003677 GO:0019031 GO:0033179 GO:0005886

GO:0016020 GO:0008247
Cluster-22178.58640 GO:0006355 GO:0003700 GO:0005515 GO:0005667
GO:0042025
Cluster-22178.58642 GO:0042254 GO:0006412 GO:0003735 GO:0005840
Cluster-22178.58643 GO:0006891 GO:0000184 GO:0006914 GO:0005815
GO:0017119
Cluster-22178.58646 GO:0046872
Cluster-22178.58647 GO:0007275 GO:0005634
Cluster-22178.58648 GO:0006355 GO:0045893 GO:0003677 GO:0043565
GO:0005634
Cluster-22178.58650 GO:0009253 GO:0006807 GO:0009252 GO:0005198
GO:0008745 GO:0005882
Cluster-22178.58651 GO:0006308 GO:0008855 GO:0009318
Cluster-22178.58655 GO:0005515
Cluster-22178.58656 GO:0015684 GO:0007264 GO:0006576 GO:0005525
GO:0015093 GO:0005524 GO:0003924 GO:0016021
Cluster-22178.58660 GO:0007076 GO:0000796
Cluster-22178.58661 GO:0007049 GO:0005524 GO:0005515 GO:0009507
GO:0031011 GO:0017053
Cluster-22178.58663 GO:0006144 GO:0015074 GO:0003968 GO:0003677
GO:0005524 GO:0031379
Cluster-22178.58667 GO:0006302 GO:0006310 GO:0005515 GO:0003677
GO:0005634
Cluster-22178.58668 GO:0008152 GO:0003824 GO:0048037
Cluster-22178.58671 GO:0055085 GO:0006952 GO:0009405 GO:0006810
GO:0006821 GO:0019855 GO:0005247 GO:0008270 GO:0016020
GO:0005576
Cluster-22178.58673 GO:0055114 GO:0016491 GO:0046872 GO:0005515

Cluster-22178.58674 GO:0006355 GO:0005524 GO:0005634
Cluster-22178.58675 GO:0006811 GO:0006355 GO:0009405 GO:0043565
GO:0003700 GO:0005216 GO:0005515 GO:0005576 GO:0005667
Cluster-22178.58676 GO:0046740 GO:0006457 GO:0016021

Cluster-22178.58677 GO:0007165 GO:0098655 GO:0006812 GO:0033198
GO:0001614 GO:0004931 GO:0008270 GO:0005887
Cluster-22178.58678 GO:0005975 GO:0042325 GO:0004553 GO:0005737
Cluster-22178.58679 GO:0016021 GO:0012511
Cluster-22178.58680 GO:0016567 GO:0004842 GO:0005680
Cluster-22178.58684 GO:0003723
Cluster-22178.58686 GO:0009234 GO:0006810 GO:0019079 GO:0005524
GO:0016887 GO:0004386 GO:0003676 GO:0016787 GO:0000166
GO:0008026 GO:0003677
Cluster-22178.58687 GO:0006096 GO:0005982 GO:0051156 GO:0006012
GO:0042207 GO:0032259 GO:0006289 GO:0019872 GO:0043087
GO:0043161 GO:0009072 GO:0018149 GO:0005985 GO:0006570
GO:0006281 GO:0006094 GO:0045040 GO:0003684 GO:0004334
GO:0003810 GO:0005089 GO:0005524 GO:0004340 GO:0005515
GO:0008168 GO:0005742
Cluster-22178.58693 GO:0016114 GO:0006694 GO:0006886 GO:0006351
GO:0006284 GO:0006144 GO:0006206 GO:0016192 GO:0006383
GO:0003899 GO:0005198 GO:0005515 GO:0008661 GO:0003677
GO:0005634 GO:0030117 GO:0005730 GO:0005666
Cluster-22178.58697 GO:0006811 GO:0055085 GO:0055114 GO:0006810
GO:0007264 GO:0005215 GO:0005525 GO:0016491 GO:0005216
GO:0010181 GO:0005615 GO:0016021 GO:0016020
Cluster-22178.58699 GO:0005525 GO:0003924
Cluster-22178.58700 GO:0005524 GO:0003676 GO:0005634
Cluster-22178.58703 GO:0006355 GO:0006486 GO:0008270 GO:0008417
GO:0003682 GO:0003677 GO:0005789 GO:0000775 GO:0000785
GO:0005634 GO:0016020
Cluster-22178.58706 GO:0006024 GO:0015012 GO:0016021
Cluster-22178.58707 GO:0009405 GO:0003676
Cluster-22178.58710 GO:0007156 GO:0005509 GO:0016020 GO:0005634
Cluster-22178.58712 GO:0070206 GO:0048870 GO:0005515 GO:0031514
GO:0005639
Cluster-22178.58716 GO:0003677

Cluster-22178.58718 GO:0006955
Cluster-22178.58720 GO:0034227 GO:0005737
Cluster-22178.58721 GO:0009405 GO:0005515 GO:0003676 GO:0015485
GO:0008270 GO:0003723
Cluster-22178.58722 GO:0031175 GO:0030474 GO:0015031 GO:0042254
GO:0071988 GO:0007268 GO:0008017 GO:0033130 GO:0003735
GO:0030507 GO:0043495 GO:0005516 GO:0045298 GO:0005840
GO:0008091 GO:0005739
Cluster-22178.58725 GO:0006367 GO:0005672
Cluster-22178.58729 GO:0005515
Cluster-22178.58730 GO:0006554 GO:0006479 GO:0006886 GO:0018024
GO:0030176
Cluster-22178.58731 GO:0007017 GO:0007018 GO:0005524 GO:0008017
GO:0003777 GO:0045298 GO:0005874
Cluster-22178.58734 GO:0008152 GO:0007186 GO:0004930 GO:0008168
GO:0016021
Cluster-22178.58737 GO:0006144 GO:0006531 GO:0006526 GO:0015074
GO:0008033 GO:0008152 GO:0006529 GO:0006522 GO:0006164
GO:0006177 GO:0006536 GO:0006560 GO:0006541 GO:0005524
GO:0016787 GO:0004066 GO:0004055 GO:0003824 GO:0003922
GO:0004810
Cluster-22178.58739 GO:0030683 GO:0005524 GO:0008092 GO:0003676
GO:0005737 GO:0019898 GO:0016021 GO:0019012
Cluster-22178.58740 GO:0006090 GO:0015976 GO:0055114 GO:0006108
GO:0006099 GO:0004471 GO:0008201 GO:0051287
Cluster-22178.58741 GO:0017004 GO:0015886 GO:0015232 GO:0016020
Cluster-22178.58744 GO:0006367 GO:0008270 GO:0005672
Cluster-22178.58746 GO:0006464 GO:0015031 GO:0006508 GO:0006468
GO:0016773 GO:0005524 GO:0004672 GO:0004252 GO:0016021
GO:0016020
Cluster-22178.58748 GO:0007186 GO:0007165 GO:0006810 GO:0055085
GO:0003924 GO:0016887 GO:0019001 GO:0005524 GO:0031683
GO:0042626 GO:0004871 GO:0016021

Cluster-22178.58750 GO:0016310 GO:0007178 GO:0009069 GO:0006468
GO:0005524 GO:0004672 GO:0004675 GO:0016020
Cluster-22178.58752 GO:0007155
Cluster-22178.58753 GO:0006508 GO:0006629 GO:0008152 GO:0016787
GO:0008236
Cluster-22178.58754 GO:0019058
Cluster-22178.58756 GO:0005515
Cluster-22178.58757 GO:0005509
Cluster-22178.58758 GO:0035556 GO:0035023 GO:0043087 GO:0005515
GO:0005089
Cluster-22178.58761 GO:0042967 GO:0008080 GO:0016747
Cluster-22178.58763 GO:0055085 GO:0016021
Cluster-22178.58766 GO:0007186 GO:0007165 GO:0004871 GO:0005834
Cluster-22178.58768 GO:0048870 GO:0008152 GO:0006355 GO:0006367
GO:0016491 GO:0008440 GO:0003682 GO:0003677 GO:0017048
GO:0000775 GO:0000785 GO:0005634 GO:0005672 GO:0031514
Cluster-22178.58770 GO:0048870 GO:0008152 GO:0006355 GO:0006367
GO:0016491 GO:0008440 GO:0003682 GO:0017048 GO:0003677
GO:0000785 GO:0000775 GO:0005634 GO:0005672 GO:0031514
Cluster-22178.58773 GO:0006508 GO:0004190 GO:0005515
Cluster-22178.58774 GO:0006508 GO:0008234 GO:0004197
Cluster-22178.58779 GO:0016032 GO:0015991 GO:0006118 GO:0055114
GO:0015992 GO:0008152 GO:0016740 GO:0003995 GO:0016627
GO:0046872 GO:0005524 GO:0015078 GO:0050660 GO:0005525
GO:0033179
Cluster-22178.58780 GO:0006281 GO:0007186 GO:0006310 GO:0006367
GO:0000287 GO:0000400 GO:0003677 GO:0016788 GO:0004930
GO:0005672 GO:0016021
Cluster-22178.58781 GO:0006355 GO:0005515 GO:0004252 GO:0003677
GO:0005634 GO:0005886
Cluster-22178.58782 GO:0043666 GO:0009878 GO:0009966 GO:0046872
GO:0004864
Cluster-22178.58787 GO:0008608 GO:0003723 GO:0005876 GO:0042729

Cluster-22178.58789 GO:0007264 GO:0006576 GO:0007165 GO:0007186
GO:0098519 GO:0031683 GO:0005524 GO:0019001 GO:0016887
GO:0003924 GO:0005525 GO:0004871
Cluster-22178.58791 GO:0006355 GO:0003700 GO:0005667
Cluster-22178.58792 GO:0016746
Cluster-22178.58793 GO:0009405 GO:0006810 GO:0045444 GO:0008200
GO:0005576
Cluster-22178.58794 GO:0008270
Cluster-22178.58795 GO:0006396
Cluster-22178.58796 GO:0006886 GO:0035091 GO:0016020
Cluster-22178.58797 GO:0004222
Cluster-22178.58799 GO:0006364 GO:0019089 GO:0007034 GO:0008168
GO:0005737
Cluster-22178.58800 GO:0009512
Cluster-22178.58805 GO:0005515
Cluster-22178.58806 GO:0009405 GO:0005515 GO:0045735
Cluster-22178.58807 GO:0007165 GO:0006614 GO:0007067 GO:0000090
GO:0030168 GO:0031110 GO:0006355 GO:0007059 GO:0051258
GO:0048870 GO:0009069 GO:0006606 GO:0048193 GO:0006914
GO:0006468 GO:0016310 GO:0051301 GO:0030674 GO:0004674
GO:0016872 GO:0005102 GO:0005096 GO:0008134 GO:0045502
GO:0005525 GO:0003700 GO:0003677 GO:0008017 GO:0005524
GO:0005198 GO:0042803 GO:0005882 GO:0030286 GO:0000940
GO:0031514 GO:0005840 GO:0016021 GO:0016020 GO:0005667
GO:0005876 GO:0005577 GO:0045298
Cluster-22178.58809 GO:0005515
Cluster-22178.58813 GO:0005515
Cluster-22178.58814 GO:0055114 GO:0006520 GO:0008152 GO:0009245
GO:0044564 GO:0016747 GO:0016491
Cluster-22178.58816 GO:0051260
Cluster-22178.58818 GO:0006355 GO:0019048
Cluster-22178.58821 GO:0006468 GO:0005524 GO:0004672 GO:0008168
GO:0005515

Cluster-22178.58822 GO:0006744 GO:0006120 GO:0015992 GO:0055114 GO:0006814 GO:0006468 GO:0005524 GO:0004672 GO:0016491 GO:0008137 GO:0005515 GO:0005743

Cluster-22178.58826 GO:0006281 GO:0008270

Cluster-22178.58830 GO:0007275 GO:0016021

Cluster-22178.58832 GO:0006629 GO:0006355 GO:0015991 GO:0006886 GO:0016192 GO:0051276 GO:0015031 GO:0043248 GO:0006606 GO:0005515 GO:0016820 GO:0046872 GO:0003677 GO:0008270 GO:0005524 GO:0008565 GO:0016887 GO:0004222 GO:0030117 GO:0005634 GO:0005737 GO:0005694 GO:0005622 GO:0000221

Cluster-22178.58834 GO:0006281 GO:0000723 GO:0044564 GO:0003678 GO:0005657

Cluster-22178.58835 GO:0016567 GO:0009069 GO:0016310 GO:0005515 GO:0004842 GO:0004674 GO:0003676 GO:0046872 GO:0008270 GO:0005680

Cluster-22178.58838 GO:0007094 GO:0042742

Cluster-22178.58839 GO:0007224 GO:0006813 GO:0008063 GO:0006814 GO:0008270 GO:0005515 GO:0016021 GO:0005890

Cluster-22178.58840 GO:0006357 GO:0001104 GO:0016592

Cluster-22178.58849 GO:0007165 GO:0005515

Cluster-22178.58852 GO:0007165 GO:0040007 GO:0008283 GO:0008083

Cluster-22178.58859 GO:0022900 GO:0006118 GO:0006511 GO:0005506 GO:0020037 GO:0009055 GO:0042597

Cluster-22178.58860 GO:0055085 GO:0006508 GO:0022857 GO:0016805 GO:0046983 GO:0016021

Cluster-22178.58862 GO:0016021

Cluster-22178.58863 GO:0006355 GO:0009966 GO:0003677 GO:0005576

Cluster-22178.58865 GO:0006144 GO:0055114 GO:0006206 GO:0006383 GO:0006351 GO:0016491 GO:0003899 GO:0003677 GO:0005730 GO:0005666

Cluster-22178.58866 GO:0055114 GO:0016491

Cluster-22178.58868 GO:0005524

Cluster-22178.58871 GO:0007186 GO:0006464 GO:0015031 GO:0004930
GO:0005515 GO:0016021
Cluster-22178.58873 GO:0006935 GO:0000160 GO:0006355 GO:0000156
GO:0008984 GO:0005515 GO:0003677 GO:0005737
Cluster-22178.58879 GO:0009405 GO:0005576
Cluster-22178.58880 GO:0007264 GO:0015684 GO:0015093 GO:0005525
GO:0016021
Cluster-22178.58881 GO:0005515
Cluster-22178.58882 GO:0007154 GO:0051301 GO:0016021 GO:0016020
Cluster-22178.58883 GO:0006388 GO:0051252 GO:0006351 GO:0003714
GO:0000213 GO:0005667 GO:0000214
Cluster-22178.58885 GO:0006355 GO:0003677
Cluster-22178.58886 GO:0006576 GO:0003924 GO:0005524 GO:0005525
GO:0003677
Cluster-22178.58887 GO:0006355 GO:0005524 GO:0005634
Cluster-22178.58888 GO:0035556 GO:0030163 GO:0005515 GO:0008914

Cluster-22178.58892 GO:0006508 GO:0004222 GO:0016020
Cluster-22178.58896 GO:0055114 GO:0010309 GO:0005515
Cluster-22178.58898 GO:0005515 GO:0016773 GO:0046872 GO:0030414
GO:0005576
Cluster-22178.58906 GO:0019068 GO:0006310 GO:0005515 GO:0005198
GO:0003677 GO:0005634
Cluster-22178.58907 GO:0006355 GO:0006531 GO:0006419 GO:0006522
GO:0000166 GO:0005524 GO:0004813 GO:0032039 GO:0005622
GO:0016021
Cluster-22178.58908 GO:0046872
Cluster-22178.58909 GO:0004190 GO:0003676 GO:0016021
Cluster-22178.58910 GO:0006531 GO:0006355 GO:0006419 GO:0006522
GO:0000166 GO:0004813 GO:0005524 GO:0032039 GO:0005622
Cluster-22178.58911 GO:0007165 GO:0008270
Cluster-22178.58914 GO:0015991 GO:0006351 GO:0007094 GO:0055114
GO:0006144 GO:0015031 GO:0015992 GO:0006206 GO:0003899

GO:0015078 GO:0003677 GO:0047134 GO:0033179 GO:0016021
GO:0005730 GO:0016020
Cluster-22178.58915 GO:0051205 GO:0016021
Cluster-22178.58916 GO:0019856 GO:0045892 GO:0003700 GO:0005515
GO:0019012 GO:0005667
Cluster-22178.58917 GO:0006470 GO:0006570 GO:0004725 GO:0008138

Cluster-22178.58918 GO:0003676
Cluster-22178.58920 GO:0016021
Cluster-22178.58921 GO:0046813 GO:0061025 GO:0006955 GO:0030153
GO:0015643 GO:0019031 GO:0016021 GO:0019814
Cluster-22178.58923 GO:0006508 GO:0004252 GO:0004197
Cluster-22178.58924 GO:0042246 GO:0001558 GO:0007155 GO:0006508
GO:0008239 GO:0005520 GO:0005515 GO:0004252 GO:0070009
GO:0016021 GO:0016942 GO:0005576
Cluster-22178.58925 GO:0019497 GO:0006355 GO:0007264 GO:0006771
GO:0007165 GO:0003924 GO:0046872 GO:0004871 GO:0003700
GO:0005525 GO:0005515 GO:0016887 GO:0005524 GO:0003993
GO:0005634 GO:0005667
Cluster-22178.58926 GO:0006508 GO:0008236
Cluster-22178.58927 GO:0006468 GO:0005524 GO:0004672
Cluster-22178.58928 GO:0006935 GO:0006955 GO:0006303 GO:0007165
GO:0008009 GO:0005576
Cluster-22178.58929 GO:0006508 GO:0016579 GO:0004843 GO:0005515

Cluster-22178.58930 GO:0006486 GO:0004576 GO:0016021 GO:0008250
GO:0016020
Cluster-22178.58933 GO:0005739
Cluster-22178.58934 GO:0055114 GO:0003697 GO:0016638
Cluster-22178.58936 GO:0045859 GO:0019887 GO:0005956
Cluster-22178.58940 GO:0005515
Cluster-22178.58943 GO:0040007 GO:0008283 GO:0007165 GO:0006364
GO:0005515 GO:0008083 GO:0016020 GO:0016021 GO:0032040

Cluster-22178.58944 GO:0006351 GO:0009405 GO:0006144 GO:0006464
GO:0006206 GO:0016567 GO:0006511 GO:0007275 GO:0046872
GO:0016151 GO:0003899 GO:0005515 GO:0005198 GO:0008270
GO:0004842 GO:0003677 GO:0005634 GO:0005680 GO:0005730
Cluster-22178.58949 GO:0006479 GO:0034968 GO:0006508 GO:0006355
GO:0006554 GO:0004252 GO:0005515 GO:0018024 GO:0005694
Cluster-22178.58952 GO:0007067 GO:0016043 GO:0015743 GO:0048037
GO:0016740 GO:0003779 GO:0042729 GO:0072686
Cluster-22178.58953 GO:0016567 GO:0005515 GO:0004842 GO:0008270

Cluster-22178.58961 GO:0010508 GO:0006810 GO:0007264 GO:0008053
GO:0003723 GO:0003724 GO:0005525 GO:0016887 GO:0003924
GO:0005524 GO:0005741 GO:0016021
Cluster-22178.58965 GO:0005985 GO:0005982 GO:0009117 GO:0048040

Cluster-22178.58968 GO:0016032 GO:0005515 GO:0019031
Cluster-22178.58971 GO:0042476 GO:0007049 GO:0006351 GO:0042475
GO:0030345 GO:0005515 GO:0019867 GO:0070176 GO:0005578
Cluster-22178.58973 GO:0006464 GO:0015031 GO:0005515
Cluster-22178.58974 GO:0005515
Cluster-22178.58975 GO:0007155 GO:0016020
Cluster-22178.58976 GO:0006368 GO:0007155 GO:0042246 GO:0007165
GO:0006914 GO:0006308 GO:0006406 GO:0006606 GO:0006891
GO:0005515 GO:0008855 GO:0017119 GO:0009318 GO:0000446
GO:0016021
Cluster-22178.58977 GO:0016567 GO:0008270 GO:0046872 GO:0004842
GO:0005515 GO:0005680
Cluster-22178.58978 GO:0007631 GO:0007218 GO:0055114 GO:0016730

Cluster-22178.58980 GO:0005515
Cluster-22178.58982 GO:0006914 GO:0007155 GO:0007165 GO:0019058
GO:0004871 GO:0003723 GO:0005198 GO:0019031 GO:0019867
GO:0019028 GO:0016020

Cluster-22178.58984 GO:0001503 GO:0006760 GO:0046656 GO:0004150
GO:0008270
Cluster-22178.58985 GO:0001503 GO:0006760 GO:0046656 GO:0004150
GO:0008270
Cluster-22178.58989 GO:0055114 GO:0006468 GO:0005524 GO:0004672
GO:0016614
Cluster-22178.58990 GO:0007186 GO:0006952 GO:0007165 GO:0007264
GO:0004871 GO:0005525 GO:0003924 GO:0019001 GO:0031683
GO:0005576
Cluster-22178.58994 GO:0005515
Cluster-22178.58996 GO:0015948 GO:0051028 GO:0006914 GO:0046656
GO:0006886 GO:0048309 GO:0016310 GO:0015031 GO:0006260
GO:0048870 GO:0008608 GO:0007049 GO:0000160 GO:0000155
GO:0003774 GO:0005524 GO:0030269 GO:0008565 GO:0046983
GO:0016757 GO:0005198 GO:0005515 GO:0042803 GO:0045502
GO:0008134 GO:0008092 GO:0016459 GO:0005667 GO:0016020
GO:0042729 GO:0005737 GO:0005882 GO:0030286 GO:0019898
GO:0072686 GO:0031514 GO:0009365 GO:0016021 GO:0005801
Cluster-22178.58997 GO:0006118 GO:0051603 GO:0020037 GO:0004298
GO:0009055 GO:0005839
Cluster-22178.58999 GO:0003676
Cluster-22178.59000 GO:0003950 GO:0003723
Cluster-22178.59001 GO:0010112 GO:0015684 GO:0007264 GO:0005525
GO:0003924 GO:0015093 GO:0016021
Cluster-22178.59004 GO:0006144 GO:0002098 GO:0006468 GO:0046034
GO:0034227 GO:0016787 GO:0003677 GO:0004017 GO:0008270
GO:0005524 GO:0000049 GO:0020037 GO:0004672 GO:0046872
GO:0003676 GO:0005634
Cluster-22178.59005 GO:0003774 GO:0005524 GO:0016459
Cluster-22178.59006 GO:0003774 GO:0005524 GO:0016459
Cluster-22178.59008 GO:0042254 GO:0006412 GO:0003735 GO:0005622
GO:0005840
Cluster-22178.59010 GO:0019497 GO:0006771 GO:0003993 GO:0005515

GO:0046872 GO:0016020

Cluster-22178.59013 GO:0007130 GO:0000795

Cluster-22178.59019 GO:0006355 GO:0006270 GO:0019058 GO:0055085 GO:0003677 GO:0004252 GO:0003700 GO:0022857 GO:0005667 GO:0016020 GO:0005886 GO:0019031 GO:0016021

Cluster-22178.59021 GO:0006955 GO:0019882 GO:0016020 GO:0042613

Cluster-22178.59022 GO:0006952 GO:0009405 GO:0006810 GO:0019855 GO:0005576

Cluster-22178.59024 GO:0071705 GO:0003676 GO:0045095

Cluster-22178.59025 GO:0006807 GO:0007031 GO:0016814 GO:0008270 GO:0005779

Cluster-22178.59026 GO:0008152 GO:0016746 GO:0046872

Cluster-22178.59027 GO:0050830 GO:0050829 GO:0007009 GO:0003779 GO:0051082

Cluster-22178.59028 GO:0006355 GO:0003700 GO:0030695 GO:0003677 GO:0043565 GO:0005634 GO:0005667

Cluster-22178.59029 GO:0006468 GO:0005524 GO:0004672 GO:0005515

Cluster-22178.59035 GO:0035556 GO:0008152 GO:0016746 GO:0005515

Cluster-22178.59036 GO:0046856 GO:0034597

Cluster-22178.59040 GO:0006366 GO:0006281 GO:0051382 GO:0006352 GO:0003677 GO:0005669

Cluster-22178.59041 GO:0006275 GO:0006270 GO:0005524 GO:0043565 GO:0005515

Cluster-22178.59043 GO:0006355 GO:0003700 GO:0043565 GO:0005667

Cluster-22178.59044 GO:0005515

Cluster-22178.59048 GO:0017004 GO:0015886 GO:0015232 GO:0016020

Cluster-22178.59049 GO:0006355 GO:0003723 GO:0046872 GO:0008270

Cluster-22178.59050 GO:0042254 GO:0006412 GO:0003735 GO:0005840

Cluster-22178.59052 GO:0006525 GO:0006560 GO:0043039 GO:0006433 GO:0006418 GO:0004812 GO:0000166 GO:0016876 GO:0005524

GO:0004827 GO:0005737

Cluster-22178.59053 GO:0030420 GO:0008152 GO:0019497 GO:0006355 GO:0006771 GO:0008253 GO:0003993 GO:0005524 GO:0016791 GO:0008134 GO:0000287 GO:0005737 GO:0005667

Cluster-22178.59056 GO:0008270

Cluster-22178.59057 GO:0008270

Cluster-22178.59058 GO:0005515

Cluster-22178.59060 GO:0008270 GO:0016787

Cluster-22178.59061 GO:0055114 GO:0051536

Cluster-22178.59065 GO:0008270

Cluster-22178.59067 GO:0055114 GO:0016020

Cluster-22178.59070 GO:0006351 GO:0008270 GO:0003676 GO:0005515 GO:0005639

Cluster-22178.59071 GO:0006351 GO:0008270 GO:0003676 GO:0005515 GO:0005639

Cluster-22178.59073 GO:0005515

Cluster-22178.59078 GO:0055114 GO:0046872 GO:0016491 GO:0005576

Cluster-22178.59080 GO:0055114 GO:0016491 GO:0005507

Cluster-22178.59081 GO:0006281 GO:0006915 GO:0032465 GO:0016567 GO:0006310 GO:0009378 GO:0003723 GO:0005515 GO:0005524 GO:0016887 GO:0004842 GO:0003724 GO:0003678 GO:0009379 GO:0005657

Cluster-22178.59082 GO:0005978

Cluster-22178.59084 GO:0006355 GO:0043401

Cluster-22178.59090 GO:0006270 GO:0006397 GO:0006886 GO:0018342 GO:0006396 GO:0005515 GO:0008318 GO:0005634 GO:0005789 GO:0005622

Cluster-22178.59092 GO:0030154 GO:0016021

Cluster-22178.59093 GO:0006508 GO:0003676 GO:0008233

Cluster-22178.59094 GO:0006357 GO:0007165 GO:0005057 GO:0001104 GO:0016592

Cluster-22178.59095 GO:0003676 GO:0035091 GO:0005887

Cluster-22178.59099 GO:0003676 GO:0035091 GO:0005887

Cluster-22178.59100 GO:0006461 GO:0010508 GO:0019901 GO:0005739
Cluster-22178.59102 GO:0006355 GO:0006281 GO:0019497 GO:0019079
GO:0006310 GO:0006771 GO:0002949 GO:0005515 GO:0008134
GO:0003723 GO:0003678 GO:0009378 GO:0003993 GO:0003724
GO:0005524 GO:0016887 GO:0005667 GO:0009379 GO:0005657
Cluster-22178.59103 GO:0003677
Cluster-22178.59107 GO:0006355 GO:0008152 GO:0015937 GO:0015940
GO:0006139 GO:0006465 GO:0006206 GO:0016301 GO:0016740
GO:0003676 GO:0004140 GO:0004127 GO:0008233 GO:0005524
GO:0005787 GO:0016021 GO:0005622
Cluster-22178.59111 GO:0005975 GO:0006352 GO:0004553 GO:0003677

Cluster-22178.59112 GO:0007165 GO:0006355 GO:0042974 GO:0003677
GO:0005198 GO:0019013 GO:0005667
Cluster-22178.59113 GO:0006351 GO:0003677 GO:0005634
Cluster-22178.59115 GO:0006412 GO:0042254 GO:0034508 GO:0005975
GO:0003735 GO:0004553 GO:0030248 GO:0000776 GO:0005576
GO:0015934 GO:0005840
Cluster-22178.59116 GO:0006464 GO:0008152 GO:0009452 GO:0001510
GO:0046500 GO:0006479 GO:0008168 GO:0004719 GO:0008171
GO:0031224
Cluster-22178.59118 GO:0033588
Cluster-22178.59119 GO:0035023 GO:0035556 GO:0043087 GO:0009405
GO:0019835 GO:0005089 GO:0005515 GO:0019867
Cluster-22178.59121 GO:0035023 GO:0043087 GO:0005089
Cluster-22178.59124 GO:0007165 GO:0005515
Cluster-22178.59126 GO:0046872 GO:0003677
Cluster-22178.59127 GO:0006468 GO:0071973 GO:0006457 GO:0006914
GO:0051082 GO:0045502 GO:0005198 GO:0042803 GO:0008134
GO:0005524 GO:0004672 GO:0005667 GO:0030286 GO:0016272
Cluster-22178.59128 GO:0006468 GO:0042803 GO:0008134 GO:0045502
GO:0005524 GO:0004672 GO:0005667 GO:0019028 GO:0030286
GO:0005856

Cluster-22178.59129 GO:0071107 GO:0042254 GO:0006206 GO:0006144
GO:0006355 GO:0006351 GO:0006412 GO:0006352 GO:0003677
GO:0003735 GO:0016987 GO:0003899 GO:0003700 GO:0003676
GO:0005730 GO:0005667 GO:0016020 GO:0005622 GO:0005840
Cluster-22178.59133 GO:0007264 GO:0007186 GO:0007165 GO:0003924
GO:0019001 GO:0031683 GO:0004871 GO:0030976 GO:0005525
Cluster-22178.59140 GO:0016021
Cluster-22178.59141 GO:0030246
Cluster-22178.59142 GO:0007154 GO:0005515 GO:0004252 GO:0005509
GO:0005886 GO:0016020
Cluster-22178.59143 GO:0006570 GO:0006470 GO:0008138 GO:0004725

Cluster-22178.59146 GO:0006265 GO:0006302 GO:0003917 GO:0003677
GO:0005634 GO:0005694
Cluster-22178.59149 GO:0007165 GO:0005515 GO:0016020 GO:0016012
Cluster-22178.59150 GO:0007165 GO:0006468 GO:0009069 GO:0007217
GO:0016310 GO:0004674 GO:0005524 GO:0004672 GO:0005576
Cluster-22178.59151 GO:0055114 GO:0016787
Cluster-22178.59152 GO:0000413 GO:0006457 GO:0007010 GO:0008017
GO:0003755 GO:0045298 GO:0005887
Cluster-22178.59154 GO:0006278 GO:0004190 GO:0003964 GO:0016021
Cluster-22178.59155 GO:0009966 GO:0005515 GO:0005576
Cluster-22178.59156 GO:0031412 GO:0006260 GO:0006144 GO:0006281
GO:0003968 GO:0003911 GO:0046872 GO:0008270 GO:0005515
GO:0031379 GO:0031411
Cluster-22178.59159 GO:0015991 GO:0009069 GO:0016192 GO:0006886
GO:0051252 GO:0043248 GO:0006850 GO:0016310 GO:0005515
GO:0016820 GO:0004521 GO:0004674 GO:0030117 GO:0005743
GO:0000221
Cluster-22178.59161 GO:0007165 GO:0006891 GO:0008053 GO:0030168
GO:0051258 GO:0006308 GO:0030674 GO:0005102 GO:0003924
GO:0008855 GO:0009318 GO:0005741 GO:0017119 GO:0016021
GO:0005577

Cluster-22178.59170 GO:0007411 GO:0016055 GO:0006508 GO:0008270
GO:0005576
Cluster-22178.59172 GO:0055114 GO:0008152 GO:0051287 GO:0016616

Cluster-22178.59173 GO:0055114 GO:0019521 GO:0006098 GO:0008152
GO:0051287 GO:0016616 GO:0004616
Cluster-22178.59176 GO:0035556 GO:0046907 GO:0006469 GO:0045859
GO:0004862 GO:0005952
Cluster-22178.59180 GO:0000266 GO:0005739
Cluster-22178.59183 GO:0016567 GO:0008152 GO:0008168
Cluster-22178.59186 GO:0006886 GO:0005622
Cluster-22178.59190 GO:0042254 GO:0006412 GO:0051694 GO:0003723
GO:0003735 GO:0005523 GO:0016020 GO:0005622 GO:0005840
Cluster-22178.59191 GO:0006468 GO:0007165 GO:0004672 GO:0005524
GO:0005515
Cluster-22178.59192 GO:0006468 GO:0003677 GO:0004672 GO:0005524
GO:0005634
Cluster-22178.59193 GO:0006310 GO:0006511 GO:0007010 GO:0032259
GO:0005515 GO:0003677 GO:0008168 GO:0003779 GO:0004175
GO:0019773 GO:0005634
Cluster-22178.59195 GO:0007264 GO:0006576 GO:0015684 GO:0005509
GO:0005524 GO:0015093 GO:0016887 GO:0003924 GO:0005525
GO:0016021
Cluster-22178.59196 GO:0035556 GO:0005515
Cluster-22178.59197 GO:0005515 GO:0008270
Cluster-22178.59198 GO:0008033 GO:0015743 GO:0051252 GO:0006412
GO:0042254 GO:0003676 GO:0003723 GO:0004526 GO:0003735
GO:0005622 GO:0005840 GO:0005634 GO:0030677
Cluster-22178.59200 GO:0008152 GO:0016787 GO:0005198 GO:0019028
GO:0016021
Cluster-22178.59203 GO:0031412 GO:0007034 GO:0006457 GO:0051297
GO:0004672 GO:0003677 GO:0051082 GO:0005813 GO:0016272
GO:0019028

Cluster-22178.59207 GO:0055114 GO:0008168 GO:0016706
Cluster-22178.59208 GO:0008146
Cluster-22178.59210 GO:0005515
Cluster-22178.59211 GO:0006629 GO:0005515
Cluster-22178.59212 GO:0005739
Cluster-22178.59214 GO:0071973 GO:0015979 GO:0003774 GO:0016020 GO:0009425 GO:0009523 GO:0009539
Cluster-22178.59216 GO:0008152 GO:0019049 GO:0005975 GO:0003824 GO:0030246 GO:0016836
Cluster-22178.59217 GO:0006120 GO:0006744 GO:0008152 GO:0055114 GO:0015992 GO:0019049 GO:0005975 GO:0006814 GO:0003824 GO:0008137 GO:0016836 GO:0030246
Cluster-22178.59218 GO:0006367 GO:0032968 GO:0003677 GO:0005634
Cluster-22178.59219 GO:0015979 GO:0005515 GO:0016021
Cluster-22178.59220 GO:0046872
Cluster-22178.59221 GO:0006810 GO:0055085 GO:0005515 GO:0005525 GO:0005215 GO:0022857 GO:0046872 GO:0003924 GO:0016020 GO:0016012 GO:0016021
Cluster-22178.59224 GO:0016021
Cluster-22178.59225 GO:0004518 GO:0003677
Cluster-22178.59227 GO:0006869 GO:0007165 GO:0006955 GO:0008283 GO:0040007 GO:0042157 GO:0005138 GO:0008289 GO:0005576 GO:0005896
Cluster-22178.59229 GO:0055114 GO:0009058 GO:0003676 GO:0005524 GO:0016491 GO:0003677 GO:0010181 GO:0016787
Cluster-22178.59232 GO:0003677
Cluster-22178.59233 GO:0016021
Cluster-22178.59237 GO:0005198 GO:0019013
Cluster-22178.59238 GO:0019953 GO:0006554 GO:0007165 GO:0006479 GO:0034968 GO:0000772 GO:0008270 GO:0005515 GO:0018024 GO:0005634 GO:0005576
Cluster-22178.59241 GO:0006554 GO:0019953 GO:0034968 GO:0006479 GO:0007165 GO:0000772 GO:0008270 GO:0018024 GO:0005515

GO:0005634 GO:0005576
Cluster-22178.59243 GO:0007304 GO:0005213 GO:0046872 GO:0005515
GO:0045202 GO:0016021 GO:0042600 GO:0005576
Cluster-22178.59245 GO:0006810 GO:0005215 GO:0019028 GO:0016020
Cluster-22178.59247 GO:0007017 GO:0005875
Cluster-22178.59249 GO:0007018 GO:0007017 GO:0008017 GO:0003777
GO:0005524 GO:0005515 GO:0003723 GO:0045298 GO:0005874
Cluster-22178.59250 GO:0005515 GO:0016020
Cluster-22178.59251 GO:0006508 GO:0061025 GO:0055085 GO:0046813
GO:0035556 GO:0016805 GO:0016021 GO:0019031
Cluster-22178.59259 GO:0043087 GO:0005085
Cluster-22178.59261 GO:0007186 GO:0004930 GO:0016021
Cluster-22178.59263 GO:0009058 GO:0016788 GO:0019015
Cluster-22178.59264 GO:0016021
Cluster-22178.59270 GO:0006810 GO:0005622
Cluster-22178.59271 GO:0006457 GO:0000413 GO:0003755
Cluster-22178.59272 GO:0006457 GO:0000413 GO:0003755
Cluster-22178.59278 GO:0043087 GO:0005089 GO:0005515 GO:0016021
GO:0005737
Cluster-22178.59284 GO:0016021
Cluster-22178.59286 GO:0007165 GO:0005125 GO:0016020
Cluster-22178.59290 GO:0006413 GO:0006446 GO:0003723 GO:0003743
GO:0005737 GO:0005840
Cluster-22178.59292 GO:0043087 GO:0035023 GO:0035556 GO:0009405
GO:0019835 GO:0005089 GO:0005515 GO:0019867
Cluster-22178.59295 GO:0030570 GO:0005576
Cluster-22178.59299 GO:0046856 GO:0034597
Cluster-22178.59302 GO:0030246
Cluster-22178.59306 GO:0005515
Cluster-22178.59307 GO:0006281 GO:0006355 GO:0019079 GO:0006310
GO:0002949 GO:0016301 GO:0008134 GO:0003723 GO:0003678
GO:0043531 GO:0098519 GO:0009378 GO:0003724 GO:0005524
GO:0016887 GO:0009379 GO:0005667 GO:0005657

Cluster-22178.59309 GO:0019722 GO:0006402 GO:0051252 GO:0046872 GO:0004535 GO:0003676 GO:0003723 GO:0005515 GO:0005737 GO:0005634

Cluster-22178.59310 GO:0003676

Cluster-22178.59312 GO:0035023 GO:0005976 GO:0043087 GO:0016779 GO:0005089 GO:0043565

Cluster-22178.59316 GO:0000105 GO:0055114 GO:0008033 GO:0050660 GO:0017150

Cluster-22178.59318 GO:0007212 GO:0048268 GO:0019236 GO:0007186 GO:0032051 GO:0016021

Cluster-22178.59321 GO:0016772 GO:0005739

Cluster-22178.59325 GO:0009405 GO:0050765 GO:0016567 GO:0015979 GO:0019538 GO:0005515 GO:0008270 GO:0046872 GO:0016012 GO:0005576 GO:0016020 GO:0009539 GO:0009523

Cluster-22178.59327 GO:0040007 GO:0008283 GO:0006631 GO:0007165 GO:0055114 GO:0045300 GO:0008083 GO:0045735 GO:0005576

Cluster-22178.59329 GO:0043087 GO:0005089 GO:0005515 GO:0016021 GO:0005737

Cluster-22178.59331 GO:0016021

Cluster-22178.59335 GO:0008152 GO:0006479 GO:0006554 GO:0018024 GO:0008168

Cluster-22178.59337 GO:0005515

Cluster-22178.59338 GO:0055085 GO:0006397 GO:0006810 GO:0003924 GO:0016887 GO:0005524 GO:0042626 GO:0005525 GO:0016301 GO:0016021

Cluster-22178.59340 GO:0016567 GO:0061630 GO:0005515 GO:0004842 GO:0043565 GO:0046872 GO:0008270 GO:0005634 GO:0005680

Cluster-22178.59342 GO:0006950 GO:0005515

Cluster-22178.59346 GO:0006810 GO:0006614 GO:0006144 GO:0000103 GO:0002949 GO:0006260 GO:0016887 GO:0005524 GO:0003724 GO:0003723 GO:0016301 GO:0003924 GO:0043531 GO:0016765 GO:0003678 GO:0004020 GO:0005525 GO:0005657

Cluster-22178.59353 GO:0006810 GO:0016021

Cluster-22178.59356 GO:0006353 GO:0005739
Cluster-22178.59360 GO:0006468 GO:0016773 GO:0005524 GO:0004672
GO:0016020
Cluster-22178.59363 GO:0015992 GO:0006118 GO:0006119 GO:0008121

Cluster-22178.59366 GO:0005515
Cluster-22178.59368 GO:0006260 GO:0055085 GO:0000226 GO:0006810
GO:0006308 GO:0030001 GO:0007010 GO:0005524 GO:0008855
GO:0042626 GO:0003677 GO:0046872 GO:0003779 GO:0005515
GO:0005664 GO:0016021 GO:0009318 GO:0015630
Cluster-22178.59369 GO:0055085 GO:0000226 GO:0006260 GO:0006810
GO:0006308 GO:0030001 GO:0007010 GO:0008855 GO:0005524
GO:0042626 GO:0003677 GO:0003779 GO:0046872 GO:0005515
GO:0016021 GO:0005664 GO:0015630 GO:0009318
Cluster-22178.59370 GO:0006448 GO:0006414 GO:0055114 GO:0016706
GO:0003677 GO:0005515 GO:0003746 GO:0005853 GO:0005840
Cluster-22178.59377 GO:0035556 GO:0005515
Cluster-22178.59378 GO:0003677
Cluster-22178.59379 GO:0016021
Cluster-22178.59380 GO:0006464 GO:0046500 GO:0006479 GO:0008152
GO:0031167 GO:0004719 GO:0008168
Cluster-22178.59382 GO:0006468 GO:0004672 GO:0005524 GO:0016773
GO:0016020
Cluster-22178.59383 GO:0006468 GO:0004672 GO:0005524 GO:0016773
GO:0016020
Cluster-22178.59384 GO:0006351 GO:0006206 GO:0006144 GO:0003677
GO:0003899 GO:0005730
Cluster-22178.59385 GO:0006812 GO:0015672 GO:0005261 GO:0016020
Cluster-22178.59386 GO:0009405 GO:0003676 GO:0043022 GO:0016788
GO:0003723 GO:0046872 GO:0005634
Cluster-22178.59387 GO:0009451 GO:0001522 GO:0009982 GO:0003723

Cluster-22178.59388 GO:0009451 GO:0001522 GO:0009982 GO:0003723

Cluster-22178.59390 GO:0046872
Cluster-22178.59392 GO:0009512
Cluster-22178.59394 GO:0006508 GO:0004198 GO:0005509 GO:0005622
Cluster-22178.59396 GO:0005515
Cluster-22178.59397 GO:0005201 GO:0005515 GO:0005578 GO:0005581
Cluster-22178.59399 GO:0006355 GO:0006568 GO:0006357 GO:0019441
GO:0003677 GO:0020037 GO:0001104 GO:0004833 GO:0016592
Cluster-22178.59400 GO:0006094 GO:0006096 GO:0055114 GO:0006144
GO:0015976 GO:0004743 GO:0000287 GO:0030955 GO:0003824
Cluster-22178.59402 GO:0005515
Cluster-22178.59403 GO:0006915 GO:0019028 GO:0005622
Cluster-22178.59407 GO:0006355 GO:0042967 GO:0004402 GO:0008080
GO:0005515 GO:0005634 GO:0000123
Cluster-22178.59408 GO:0006508 GO:0004252
Cluster-22178.59411 GO:0003723
Cluster-22178.59413 GO:0006412 GO:0008168
Cluster-22178.59414 GO:0006364 GO:0015694 GO:0046872 GO:0008270
GO:0015097 GO:0005730 GO:0016020
Cluster-22178.59418 GO:0019251 GO:0009877 GO:0008152 GO:0006783
GO:0009312 GO:0016852 GO:0008168 GO:0008757
Cluster-22178.59420 GO:0006308 GO:0016888
Cluster-22178.59422 GO:0005515
Cluster-22178.59424 GO:0000160 GO:0006032 GO:0016310 GO:0051258
GO:0019497 GO:0030168 GO:0007165 GO:0006771 GO:0046872
GO:0005198 GO:0005515 GO:0008061 GO:0005102 GO:0003993
GO:0046983 GO:0030674 GO:0000155 GO:0016021 GO:0009365
GO:0005577 GO:0005882
Cluster-22178.59426 GO:0005515
Cluster-22178.59427 GO:0055085 GO:0006260 GO:0015074 GO:0006810
GO:0042626 GO:0003678 GO:0003676 GO:0005525 GO:0016887
GO:0003924 GO:0005524 GO:0016021 GO:0005657
Cluster-22178.59428 GO:0008295 GO:0010508 GO:0006560 GO:0006525

GO:0006357 GO:0001104 GO:0004014 GO:0008092 GO:0016592
GO:0019898 GO:0016021 GO:0005737
Cluster-22178.59430 GO:0042742 GO:0006281 GO:0006974 GO:0005975
GO:0004553 GO:0030248 GO:0005634 GO:0005576 GO:0070876
Cluster-22178.59431 GO:0006554 GO:0034968 GO:0006479 GO:0008270
GO:0018024 GO:0005515 GO:0016021 GO:0005634
Cluster-22178.59432 GO:0006486 GO:0016192 GO:0006886 GO:0005198
GO:0005515 GO:0016757 GO:0008378 GO:0030117 GO:0016020
Cluster-22178.59433 GO:0007264 GO:0016787 GO:0005525 GO:0005515

Cluster-22178.59434 GO:0006471 GO:0003950
Cluster-22178.59438 GO:0007218 GO:0005576
Cluster-22178.59439 GO:0031167 GO:0006351 GO:0006479 GO:0008152
GO:0006144 GO:0006206 GO:0003899 GO:0003677 GO:0008168
GO:0005730
Cluster-22178.59440 GO:0055114 GO:0008152 GO:0006694 GO:0008210
GO:0008209 GO:0008207 GO:0016491 GO:0003824 GO:0050662
GO:0003854 GO:0016616
Cluster-22178.59444 GO:0046872
Cluster-22178.59445 GO:0042254 GO:0006412 GO:0006810 GO:0003735
GO:0005622 GO:0016021 GO:0005840
Cluster-22178.59446 GO:0006508 GO:0009446 GO:0016579 GO:0006807
GO:0005515 GO:0004843 GO:0036459 GO:0004668
Cluster-22178.59449 GO:0006506 GO:0006468 GO:0005515 GO:0004386
GO:0005524 GO:0004672 GO:0016773 GO:0016020 GO:0031083
GO:0005789
Cluster-22178.59450 GO:0030001 GO:0055085 GO:0046873 GO:0016020
Cluster-22178.59451 GO:0055085 GO:0006506 GO:0006468 GO:0004386
GO:0016773 GO:0004672 GO:0005524 GO:0005515 GO:0005789
GO:0016020 GO:0031083
Cluster-22178.59453 GO:0051287 GO:0008191
Cluster-22178.59454 GO:0006355 GO:0006281 GO:0006310 GO:0019079
GO:0002949 GO:0016301 GO:0008134 GO:0003723 GO:0003678

GO:0009378 GO:0003724 GO:0016887 GO:0005524 GO:0005667
GO:0009379 GO:0005657
Cluster-22178.59456 GO:0016746
Cluster-22178.59459 GO:0016192 GO:0006886 GO:0016567 GO:0005515
GO:0004842 GO:0046872 GO:0008270 GO:0005680
Cluster-22178.59460 GO:0006812 GO:0008324
Cluster-22178.59464 GO:0008285 GO:0006396 GO:0045859 GO:0006915
GO:0007050 GO:0004860 GO:0005515 GO:0016021 GO:0000176
GO:0005576
Cluster-22178.59467 GO:0000166 GO:0046872
Cluster-22178.59468 GO:0006144 GO:0006508 GO:0006614 GO:0016817
GO:0008312 GO:0003968 GO:0004197 GO:0017111 GO:0048500
GO:0031379
Cluster-22178.59469 GO:0006508 GO:0004198 GO:0005515 GO:0005622
Cluster-22178.59471 GO:0007049 GO:0051382 GO:0044780 GO:0008239
GO:0070009 GO:0016021 GO:0000776 GO:0005634
Cluster-22178.59474 GO:0003677 GO:0046983
Cluster-22178.59476 GO:0016567 GO:0006118 GO:0005515 GO:0020037
GO:0003723 GO:0009055 GO:0004842 GO:0046872 GO:0008270
GO:0005680
Cluster-22178.59477 GO:0010972 GO:0030336 GO:0016579 GO:0036459

Cluster-22178.59478 GO:0016773
Cluster-22178.59480 GO:0016567 GO:0004842 GO:0005515 GO:0004867

Cluster-22178.59481 GO:0006270 GO:0009432 GO:0051782 GO:0005515
GO:0009276 GO:0019028
Cluster-22178.59484 GO:0005515
Cluster-22178.59488 GO:0005975 GO:0040007 GO:0008283 GO:0042710
GO:0007165 GO:0008270 GO:0003676 GO:0003824 GO:0030674
GO:0043169 GO:0008083 GO:0051015
Cluster-22178.59489 GO:0007186 GO:0007165 GO:0015684 GO:0007264
GO:0004871 GO:0005525 GO:0003924 GO:0031683 GO:0008270

GO:0015093 GO:0019001 GO:0016021
Cluster-22178.59490 GO:0005975 GO:0003824 GO:0043169
Cluster-22178.59492 GO:0006122 GO:0008242 GO:0005750
Cluster-22178.59493 GO:0006397 GO:0008270
Cluster-22178.59494 GO:0007076 GO:0000796
Cluster-22178.59496 GO:0006508 GO:0008152 GO:0016787 GO:0008236

Cluster-22178.59501 GO:0019904 GO:0005515 GO:0030246 GO:0005737
Cluster-22178.59502 GO:0007349 GO:0048598 GO:0072669 GO:0016020
GO:0005737
Cluster-22178.59504 GO:0006464 GO:0006554 GO:0008033 GO:0008152
GO:0046500 GO:0006400 GO:0006479 GO:0006886 GO:0016192
GO:0009451 GO:0004719 GO:0008168 GO:0018024 GO:0008176
GO:0030117
Cluster-22178.59505 GO:0032259 GO:0006310 GO:0008168 GO:0003677
GO:0005515 GO:0005634
Cluster-22178.59508 GO:0007165 GO:0009069 GO:0006468 GO:0035556
GO:0016310 GO:0005198 GO:0017048 GO:0004674 GO:0005524
GO:0004672 GO:0005882
Cluster-22178.59509 GO:0003779 GO:0005515 GO:0005622
Cluster-22178.5951 GO:0006096 GO:0006094 GO:0000162 GO:0006571
GO:0009094 GO:0000287 GO:0004634 GO:0000015
Cluster-22178.59510 GO:0034453 GO:0016021 GO:0005815
Cluster-22178.59511 GO:0006629 GO:0055085 GO:0006891 GO:0022857
GO:0005515 GO:0017119 GO:0016021
Cluster-22178.59514 GO:0020037
Cluster-22178.59515 GO:0009401 GO:0007186 GO:0006629 GO:0004930
GO:0005515 GO:0016021
Cluster-22178.59516 GO:0030250 GO:0005576
Cluster-22178.59518 GO:0030866 GO:0008092 GO:0005856
Cluster-22178.59519 GO:0006520 GO:0005515
Cluster-22178.59520 GO:0016567 GO:0061630 GO:0005515 GO:0043565
GO:0046872 GO:0008270 GO:0004842 GO:0005634 GO:0005680

Cluster-22178.59521 GO:0006694 GO:0008152 GO:0016114 GO:0030976
GO:0008661 GO:0003824 GO:0016624
Cluster-22178.59525 GO:0055114 GO:0006633 GO:0005506 GO:0016491

Cluster-22178.59526 GO:0006139 GO:0006465 GO:0006206 GO:0008233
GO:0004127 GO:0005525 GO:0016887 GO:0003924 GO:0005524
GO:0016020 GO:0005911 GO:0005787 GO:0016021
Cluster-22178.59527 GO:0006465 GO:0008233 GO:0016021 GO:0005787
Cluster-22178.59528 GO:0043087
Cluster-22178.59531 GO:0006570 GO:0006470 GO:0046872 GO:0004725
GO:0008138
Cluster-22178.59532 GO:0008643 GO:0055085 GO:0005351 GO:0000139
GO:0016020 GO:0016021
Cluster-22178.59533 GO:0006355 GO:0003700 GO:0005515 GO:0043565
GO:0005667
Cluster-22178.59534 GO:0006355 GO:0007275 GO:0043565 GO:0003700
GO:0005667
Cluster-22178.59535 GO:0043934 GO:0007264 GO:0005525 GO:0003924
GO:0016887 GO:0005524
Cluster-22178.59538 GO:0055036
Cluster-22178.59539 GO:0006468 GO:0005524 GO:0004672
Cluster-22178.59543 GO:0042330 GO:0006508 GO:0006511 GO:0016579
GO:0004843 GO:0005622
Cluster-22178.59545 GO:0006886 GO:0006888 GO:0030127
Cluster-22178.59547 GO:0006355 GO:0006098 GO:0055114 GO:0019521
GO:0004616 GO:0050661 GO:0051287 GO:0003676 GO:0005739
GO:0005622
Cluster-22178.5955 GO:0005515
Cluster-22178.59550 GO:0005515
Cluster-22178.59552 GO:0042254 GO:0006412 GO:0008152 GO:0003735
GO:0005515 GO:0005509 GO:0016746 GO:0016747 GO:0005840
GO:0005622
Cluster-22178.59553 GO:0007018 GO:0007017 GO:0003777 GO:0005874

GO:0030286
Cluster-22178.59554 GO:0008270 GO:0005096
Cluster-22178.59555 GO:0048309 GO:0009405 GO:0006886 GO:0030001
GO:0006606 GO:0030334 GO:0051028 GO:0006914 GO:0007155
GO:0045995 GO:0051258 GO:0006308 GO:0048870 GO:0000390
GO:0006355 GO:0055085 GO:0015031 GO:0000921 GO:0030168
GO:0006810 GO:0007165 GO:0019835 GO:0030155 GO:0042803
GO:0005198 GO:0015631 GO:0008565 GO:0003677 GO:0004871
GO:0008134 GO:0045502 GO:0005201 GO:0005102 GO:0008855
GO:0046873 GO:0030674 GO:0019867 GO:0045298 GO:0005737
GO:0005577 GO:0016020 GO:0005667 GO:0019031 GO:0016021
GO:0009318 GO:0031514 GO:0005578 GO:0019028 GO:0030286
GO:0005886 GO:0005581 GO:0005940 GO:0005882 GO:0071008
Cluster-22178.59557 GO:0015684 GO:0007264 GO:0005525 GO:0015093
GO:0016021 GO:0019028
Cluster-22178.59560 GO:0009712 GO:0042981 GO:0055114 GO:0042184
GO:0018874 GO:0046232 GO:0019261 GO:0006508 GO:0042203
GO:0018576 GO:0004197 GO:0005515 GO:0005506
Cluster-22178.59563 GO:0016021 GO:0005886
Cluster-22178.59569 GO:0006508 GO:0015992 GO:0006123 GO:0016740
GO:0004197 GO:0016788 GO:0005524 GO:0008242 GO:0016817
GO:0016887 GO:0004129 GO:0005680 GO:0045277
Cluster-22178.59570 GO:0006355 GO:0003677
Cluster-22178.59572 GO:0006355 GO:0006281 GO:0008168 GO:0008270
GO:0005515 GO:0003677 GO:0016020
Cluster-22178.59574 GO:0019217 GO:0006508 GO:0000062
Cluster-22178.59577 GO:0006508 GO:0019217 GO:0000062
Cluster-22178.59578 GO:0006508 GO:0019217 GO:0000062
Cluster-22178.59579 GO:0005515
Cluster-22178.59589 GO:0006357 GO:0006308 GO:0046983 GO:0008855
GO:0001104 GO:0009318 GO:0016592 GO:0016020
Cluster-22178.59590 GO:0048786
Cluster-22178.59591 GO:0048786

Cluster-22178.59592 GO:0048786
Cluster-22178.59594 GO:0045893 GO:0005515 GO:0001000 GO:0003677
GO:0008270
Cluster-22178.59595 GO:0030414
Cluster-22178.59596 GO:0035444 GO:0015675 GO:0015099 GO:0046872
GO:0016021
Cluster-22178.59597 GO:0016020
Cluster-22178.59599 GO:0016020
Cluster-22178.59600 GO:0007010 GO:0008017 GO:0045298
Cluster-22178.59601 GO:0005515
Cluster-22178.59606 GO:0043085 GO:0006897 GO:0000290 GO:0008047
GO:0016020
Cluster-22178.59608 GO:0006260
Cluster-22178.59610 GO:0007275 GO:0006810 GO:0006508 GO:0005215
GO:0005515 GO:0004198 GO:0005622 GO:0005576
Cluster-22178.59611 GO:0005515
Cluster-22178.59618 GO:0003333 GO:0009405
Cluster-22178.59619 GO:0055085 GO:0006270 GO:0030001 GO:0003676
GO:0005524 GO:0046873 GO:0004190 GO:0019031 GO:0016021
GO:0016020
Cluster-22178.59622 GO:0008061 GO:0005643
Cluster-22178.59626 GO:0019836 GO:0005576
Cluster-22178.59628 GO:0019836 GO:0005576
Cluster-22178.59630 GO:0051191 GO:0016042 GO:0006260 GO:0007586
GO:0006355 GO:0003700 GO:0043565 GO:0008047 GO:0003677
GO:0003887 GO:0009507 GO:0042575 GO:0005667 GO:0005576
Cluster-22178.59631 GO:0046872 GO:0000166 GO:0016820
Cluster-22178.59636 GO:0050909 GO:0005515 GO:0016021
Cluster-22178.59637 GO:0006810 GO:0005794
Cluster-22178.59638 GO:0016020
Cluster-22178.59639 GO:0005921
Cluster-22178.59641 GO:0005643
Cluster-22178.59642 GO:0006470 GO:0042967 GO:0019988 GO:0006570

GO:0006355 GO:0004725 GO:0008080 GO:0016747 GO:0043399
GO:0008138
Cluster-22178.59643 GO:0006355 GO:0009405 GO:0005515 GO:0005634
Cluster-22178.59644 GO:0007275 GO:0006446 GO:0006118 GO:0006413
GO:0045893 GO:0005515 GO:0003743 GO:0020037 GO:0009055
GO:0008270 GO:0005840 GO:0016021
Cluster-22178.59645 GO:0006413 GO:0007275 GO:0006446 GO:0006118
GO:0055114 GO:0045893 GO:0016706 GO:0005515 GO:0003743
GO:0020037 GO:0008270 GO:0008168 GO:0009055 GO:0005840
GO:0016021
Cluster-22178.59646 GO:0006413 GO:0007275 GO:0006118 GO:0006446
GO:0045893 GO:0003743 GO:0020037 GO:0005515 GO:0008270
GO:0009055 GO:0005840 GO:0016021
Cluster-22178.59648 GO:0046872
Cluster-22178.59649 GO:0006457 GO:0006555 GO:0006606 GO:0006886
GO:0006355 GO:0008270 GO:0008134 GO:0042803 GO:0005198
GO:0045502 GO:0051082 GO:0003700 GO:0016020 GO:0005882
GO:0005667 GO:0016272 GO:0030286 GO:0048786 GO:0005801
Cluster-22178.59650 GO:0010167 GO:0015706 GO:0005886 GO:0016021
Cluster-22178.59656 GO:0005515
Cluster-22178.59657 GO:0016579 GO:0008270 GO:0036459
Cluster-22178.59659 GO:0016579 GO:0045046 GO:0005515 GO:0032183
GO:0070140 GO:0032403 GO:0036459 GO:0008270 GO:0005779
Cluster-22178.59660 GO:0007155 GO:0010508 GO:0051015
Cluster-22178.59661 GO:0071766 GO:0016192 GO:0006446 GO:0008608
GO:0007009 GO:0071816 GO:0006355 GO:0003743 GO:0043565
GO:0019843 GO:0052636 GO:0003700 GO:0003723 GO:0005622
GO:0005667 GO:0008537 GO:0016021 GO:0005840 GO:0072686
GO:0042729
Cluster-22178.59662 GO:0008608 GO:0006355 GO:0071816 GO:0007009
GO:0006446 GO:0016192 GO:0071766 GO:0003700 GO:0019843
GO:0052636 GO:0043565 GO:0003723 GO:0003743 GO:0005840
GO:0016021 GO:0042729 GO:0072686 GO:0005622 GO:0008537

GO:0005667

Cluster-22178.59664 GO:0006355 GO:0004518 GO:0003677

Cluster-22178.59666 GO:0007165 GO:0006355 GO:0035091 GO:0000981 GO:0008270 GO:0005634

Cluster-22178.59667 GO:0006355 GO:0000981 GO:0008270 GO:0005634

Cluster-22178.59668 GO:0055114 GO:0046872 GO:0005515 GO:0008017 GO:0047134 GO:0045298

Cluster-22178.59669 GO:0006744 GO:0055114 GO:0046872 GO:0016491

Cluster-22178.59670 GO:0015031 GO:0021986 GO:0005789 GO:0016021 GO:0016020

Cluster-22178.59672 GO:0006508 GO:0000910 GO:0000226 GO:0008017 GO:0004252 GO:0045298 GO:0016021

Cluster-22178.59673 GO:0042546 GO:0000226 GO:0006078 GO:0000910 GO:0008017 GO:0009276 GO:0016021 GO:0045298

Cluster-22178.59675 GO:0007186 GO:0006614 GO:0006304 GO:0007165 GO:0007264 GO:0006000 GO:0006013 GO:0004871 GO:0005525 GO:0003924 GO:0031683 GO:0019001 GO:0003873 GO:0033821 GO:0005524 GO:0019012

Cluster-22178.59681 GO:0042742 GO:0005515 GO:0005576

Cluster-22178.59682 GO:0009405 GO:0006206 GO:0006231 GO:0004799 GO:0005576

Cluster-22178.59686 GO:0043571 GO:0006397 GO:0005524 GO:0003774 GO:0005515 GO:0004519 GO:0016459

Cluster-22178.59688 GO:0008152 GO:0016829

Cluster-22178.59690 GO:0009847 GO:0016021

Cluster-22178.59695 GO:0006479 GO:0034968 GO:0006554 GO:0005515 GO:0003677 GO:0018024 GO:0008270 GO:0005634

Cluster-22178.59696 GO:0042254 GO:0043093 GO:0006260 GO:0006357 GO:0006412 GO:0051252 GO:0000917 GO:0006355 GO:0007049 GO:0003735 GO:0001104 GO:0003887 GO:0004523 GO:0003700 GO:0043565 GO:0016592 GO:0005667 GO:0005815 GO:0005622 GO:0042575 GO:0005840 GO:0005737

Cluster-22178.59699 GO:0006367 GO:0006366 GO:0005674
Cluster-22178.59700 GO:0007169 GO:0006468 GO:0004714 GO:0005524
GO:0016020
Cluster-22178.59705 GO:0051028 GO:0008152 GO:0008272 GO:0008484
GO:0003824 GO:0015116 GO:0016021 GO:0005634
Cluster-22178.59706 GO:0046813 GO:0061025 GO:0005515 GO:0019031
GO:0016021
Cluster-22178.59708 GO:0005515
Cluster-22178.59710 GO:0005515
Cluster-22178.59711 GO:0009607 GO:0007154 GO:0005515 GO:0005922
GO:0016021
Cluster-22178.59712 GO:0055085 GO:0022857 GO:0016021
Cluster-22178.59714 GO:0006817 GO:0006270 GO:0005315 GO:0016020
GO:0005887
Cluster-22178.59715 GO:0007186 GO:0006890 GO:0004930 GO:0019028
GO:0016021
Cluster-22178.59717 GO:0005515
Cluster-22178.59719 GO:0006144 GO:0007165 GO:0009405 GO:0004114
GO:0005515 GO:0019028
Cluster-22178.59720 GO:0007157 GO:0009405 GO:0016021
Cluster-22178.59721 GO:0006406 GO:0006998 GO:0006470 GO:0006611
GO:0006570 GO:0004725 GO:0008138 GO:0031965
Cluster-22178.59722 GO:0034220
Cluster-22178.59725 GO:0051260 GO:0004190 GO:0016021
Cluster-22178.59726 GO:0000079 GO:0019901
Cluster-22178.59727 GO:0006629
Cluster-22178.59728 GO:0006886 GO:0008092 GO:0019898 GO:0017119
GO:0005737
Cluster-22178.59731 GO:0030474 GO:0071988 GO:0051082
Cluster-22178.59737 GO:0030163 GO:0006118 GO:0017004 GO:0008616
GO:0045158 GO:0008914 GO:0033739 GO:0005515 GO:0009512
Cluster-22178.59739 GO:0006886 GO:0007010 GO:0006506 GO:0003779
GO:0005515

Cluster-22178.59745 GO:0006621 GO:0005525 GO:0003924 GO:0046923 GO:0016021 GO:0005783
Cluster-22178.59746 GO:0043044 GO:0003676 GO:0046872 GO:0016787 GO:0016586
Cluster-22178.59748 GO:0015992 GO:0055114 GO:0006120 GO:0006744 GO:0030154 GO:0006814 GO:0008137 GO:0016021
Cluster-22178.59750 GO:0030001 GO:0008146 GO:0046872
Cluster-22178.59752 GO:0055114 GO:0016491
Cluster-22178.59753 GO:0006412 GO:0042254 GO:0006810 GO:0009405 GO:0003735 GO:0019871 GO:0005840 GO:0005576
Cluster-22178.59755 GO:0004869
Cluster-22178.59756 GO:0006865 GO:0008168 GO:0016021 GO:0016020
Cluster-22178.59759 GO:0040007 GO:0008283 GO:0006955 GO:0007165 GO:0006396 GO:0008083 GO:0005143 GO:0005576 GO:0042022
Cluster-22178.59760 GO:0016192 GO:0006886 GO:0015031 GO:0008565 GO:0030117
Cluster-22178.59763 GO:0006952 GO:0019069 GO:0042025
Cluster-22178.59765 GO:0050912 GO:0007187 GO:0007607 GO:0008527 GO:0016020 GO:0016021
Cluster-22178.59767 GO:0006468 GO:0006771 GO:0019497 GO:0048013 GO:0005515 GO:0046872 GO:0004672 GO:0005524 GO:0016773 GO:0003993 GO:0016020
Cluster-22178.59774 GO:0005515
Cluster-22178.59775 GO:0006468 GO:0005515 GO:0005524 GO:0004672 GO:0016020
Cluster-22178.59777 GO:0006486 GO:0016567 GO:0046872 GO:0005524 GO:0008270 GO:0004842 GO:0016757 GO:0003676 GO:0016787 GO:0016818 GO:0003677 GO:0005515 GO:0005680
Cluster-22178.59778 GO:0006402 GO:0009405 GO:0051252 GO:0004535 GO:0046872 GO:0003723 GO:0005737 GO:0005634
Cluster-22178.59779 GO:0006368 GO:0006406 GO:0000446
Cluster-22178.59781 GO:0000723 GO:0006281 GO:0006614 GO:0019079 GO:0006810 GO:0016887 GO:0008270 GO:0005524 GO:0003678

GO:0003676 GO:0005525 GO:0016787 GO:0008026 GO:0003677
GO:0005657
Cluster-22178.59783 GO:0006471 GO:0007049 GO:0055114 GO:0006351
GO:0050660 GO:0003950 GO:0008270 GO:0016491 GO:0003677
GO:0017053 GO:0005634
Cluster-22178.59785 GO:0006313 GO:0046872 GO:0003824
Cluster-22178.59788 GO:0009116
Cluster-22178.59789 GO:0008270 GO:0046872 GO:0005515 GO:0003677

Cluster-22178.59790 GO:0016567 GO:0004842 GO:0008270 GO:0005680
Cluster-22178.59791 GO:0006508 GO:0017148 GO:0007165 GO:0055085
GO:0005515 GO:0043565 GO:0008234 GO:0030598 GO:0005634
GO:0016021
Cluster-22178.59792 GO:0055114 GO:0015994 GO:0007165 GO:0006779
GO:0004109 GO:0005509 GO:0005578
Cluster-22178.59794 GO:0006367 GO:0006352 GO:0003677 GO:0005634
Cluster-22178.59796 GO:0005515
Cluster-22178.59798 GO:0015031 GO:0048193 GO:0006886 GO:0008565
GO:0005198 GO:0005737 GO:0016020 GO:0005882
Cluster-22178.59800 GO:0003677 GO:0005634
Cluster-22178.59806 GO:0008152 GO:0051028 GO:0006897 GO:0008272
GO:0015116 GO:0003824 GO:0008484 GO:0016021 GO:0016020
GO:0005634
Cluster-22178.59807 GO:0051028 GO:0008152 GO:0008272 GO:0006897
GO:0008484 GO:0003824 GO:0015116 GO:0016021 GO:0005634
GO:0016020
Cluster-22178.59808 GO:0019087 GO:0005515 GO:0016021
Cluster-22178.59809 GO:0007160 GO:0005509 GO:0005576
Cluster-22178.59815 GO:0007155 GO:0006457 GO:0006858 GO:0055114
GO:0006260 GO:0051082 GO:0016972 GO:0005634 GO:0016272
Cluster-22178.59816 GO:0007155 GO:0006457 GO:0006858 GO:0055114
GO:0006260 GO:0051082 GO:0016972 GO:0005634 GO:0016272
Cluster-22178.59818 GO:0015947 GO:0055114 GO:0030058 GO:0042597

Cluster-22178.59819 GO:0016567 GO:0005515 GO:0004842 GO:0008270
GO:0046872
Cluster-22178.59821 GO:0008152 GO:0016746
Cluster-22178.59824 GO:0006119 GO:0006118 GO:0055114 GO:0015992
GO:0008121 GO:0005515
Cluster-22178.59825 GO:0006508 GO:0008234
Cluster-22178.59826 GO:0000902 GO:0006308 GO:0009307 GO:0003677
GO:0009036 GO:0009359
Cluster-22178.59828 GO:0007186 GO:0015684 GO:0007165 GO:0006576
GO:0007264 GO:0004871 GO:0005525 GO:0003924 GO:0031683
GO:0005524 GO:0019001 GO:0015093 GO:0003723 GO:0016021
Cluster-22178.59830 GO:0009405
Cluster-22178.59837 GO:0006351 GO:0006269 GO:0003677 GO:0020037
GO:0005515 GO:0005198 GO:0019825 GO:0003896 GO:0005730
GO:0005634 GO:0005657
Cluster-22178.59839 GO:0006355 GO:0046872 GO:0003677 GO:0005634
Cluster-22178.59844 GO:0042254 GO:0006412 GO:0003735 GO:0005761
GO:0005840
Cluster-22178.59845 GO:0004867
Cluster-22178.59846 GO:0042384 GO:0010826 GO:0050821 GO:0015979
GO:0042301 GO:0036038 GO:0016020 GO:0005737 GO:0009523
Cluster-22178.59847 GO:0007017 GO:0007018 GO:0005515 GO:0005524
GO:0003777 GO:0008017 GO:0005874 GO:0045298
Cluster-22178.59849 GO:0007218 GO:0009733 GO:0007186 GO:0007010
GO:0004930 GO:0003779 GO:0005509 GO:0005544 GO:0005185
GO:0016021 GO:0005576
Cluster-22178.59851 GO:0016012 GO:0016021
Cluster-22178.59854 GO:0003723 GO:0046935 GO:0003676 GO:0005944
GO:0016021
Cluster-22178.59855 GO:0016021
Cluster-22178.59860 GO:0006464 GO:0051082 GO:0005515 GO:0016151
GO:0031072
Cluster-22178.59861 GO:0006397 GO:0030163 GO:0035556 GO:0003723

GO:0005515 GO:0008914 GO:0005634 GO:0005737
Cluster-22178.59862 GO:0006486 GO:0004576 GO:0016020 GO:0008250
GO:0016021 GO:0036128
Cluster-22178.59870 GO:0015012 GO:0006024 GO:0016021
Cluster-22178.59873 GO:0006570 GO:0006470 GO:0055114 GO:0004725
GO:0008138 GO:0033743
Cluster-22178.59874 GO:0006355 GO:0003723 GO:0003677 GO:0003676

Cluster-22178.59875 GO:0006355 GO:0003723 GO:0003676 GO:0003677

Cluster-22178.59877 GO:0006260 GO:0055085 GO:0006281 GO:0003911
GO:0046872 GO:0016787 GO:0016021
Cluster-22178.59879 GO:0019079 GO:0000045 GO:0043066 GO:0015979
GO:0043154 GO:0046872 GO:0016021 GO:0005737 GO:0071986
GO:0009523
Cluster-22178.59881 GO:0006260 GO:0006270 GO:0006355 GO:0006816
GO:0006874 GO:0003700 GO:0005219 GO:0000062 GO:0016884
GO:0016021 GO:0005622 GO:0005667
Cluster-22178.59886 GO:0007186 GO:0006536 GO:0009821 GO:0006103
GO:0009094 GO:0000162 GO:0006571 GO:0009074 GO:0004838
GO:0030170 GO:0004930 GO:0016021
Cluster-22178.59887 GO:0016226 GO:0005506 GO:0051536
Cluster-22178.59888 GO:0006355 GO:0008270 GO:0005634
Cluster-22178.59891 GO:0043401 GO:0006355 GO:0003700 GO:0008270
GO:0043565 GO:0005667 GO:0005634
Cluster-22178.59892 GO:0019058
Cluster-22178.59896 GO:0000917 GO:0006310 GO:0006302 GO:0043093
GO:0006355 GO:0003677 GO:0003700 GO:0043565 GO:0005634
GO:0005667 GO:0005737
Cluster-22178.59897 GO:0006355 GO:0043093 GO:0006302 GO:0006310
GO:0016573 GO:0006303 GO:0000917 GO:0003677 GO:0043565
GO:0003700 GO:0005634 GO:0005667 GO:0005737
Cluster-22178.59899 GO:0000413 GO:0006457 GO:0005515 GO:0003755

Cluster-22178.59900 GO:0006468 GO:0005524 GO:0004672 GO:0016773 GO:0016020

Cluster-22178.59902 GO:0010506 GO:0055085 GO:0016070 GO:0006415 GO:0010508 GO:0006886 GO:0030001 GO:0006449 GO:0006376 GO:0003729 GO:0046873 GO:0016149 GO:0005515 GO:0019901 GO:0003723 GO:0051082 GO:0005685 GO:0018444 GO:0016020 GO:0005783 GO:0005615 GO:0016021 GO:0005840 GO:0005737

Cluster-22178.59904 GO:0006281 GO:0006308 GO:0008855 GO:0003677 GO:0004518 GO:0005515 GO:0009318

Cluster-22178.59905 GO:0006355 GO:0003700 GO:0005667

Cluster-22178.59906 GO:0006355 GO:0005198 GO:0003700 GO:0005667 GO:0019028

Cluster-22178.59909 GO:0046740 GO:0042254 GO:0006412 GO:0006810 GO:0006260 GO:0051082 GO:0003735 GO:0005515 GO:0016798 GO:0016020 GO:0005622 GO:0005886 GO:0016021 GO:0005840

Cluster-22178.59910 GO:0046740 GO:0006810 GO:0042254 GO:0006412 GO:0006260 GO:0051082 GO:0005515 GO:0003735 GO:0016798 GO:0016020 GO:0005622 GO:0005886 GO:0005840 GO:0016021

Cluster-22178.59912 GO:0070836 GO:0007155 GO:0005515 GO:0016021

Cluster-22178.59913 GO:0005975 GO:0004553

Cluster-22178.59917 GO:0006952 GO:0005576

Cluster-22178.59922 GO:0042710 GO:0016787

Cluster-22178.59923 GO:0005515

Cluster-22178.59924 GO:0003779 GO:0005198 GO:0005856

Cluster-22178.59925 GO:0003779 GO:0005198 GO:0005856

Cluster-22178.59926 GO:0006396 GO:0019058 GO:0003676 GO:0003723

Cluster-22178.59928 GO:0019521 GO:0055114 GO:0009094 GO:0046168 GO:0015940 GO:0000162 GO:0006571 GO:0006098 GO:0008677 GO:0004665 GO:0051287 GO:0008977 GO:0016616 GO:0004616

Cluster-22178.59931 GO:0016570 GO:0006368 GO:0003779 GO:0016593 GO:0005622

Cluster-22178.59932 GO:0007264 GO:0005525 GO:0016021 GO:0042765
Cluster-22178.59934 GO:0006412 GO:0006814 GO:0006120 GO:0042254
GO:0006744 GO:0006820 GO:0015992 GO:0008137 GO:0008509
GO:0043565 GO:0003735 GO:0005634 GO:0016021 GO:0005743
GO:0005840 GO:0005622
Cluster-22178.59936 GO:0007264 GO:0006576 GO:0015684 GO:0005524
GO:0015093 GO:0016887 GO:0003924 GO:0005525 GO:0016021
Cluster-22178.59937 GO:0006468 GO:0016773 GO:0005524 GO:0004672
GO:0016020
Cluster-22178.59939 GO:0006614 GO:0008312 GO:0019825 GO:0020037
GO:0030942 GO:0005786
Cluster-22178.59940 GO:0007275 GO:0009405 GO:0006511 GO:0000921
GO:0005515 GO:0061630 GO:0046872 GO:0008270 GO:0005634
GO:0016020 GO:0005940 GO:0016021
Cluster-22178.59944 GO:0006323 GO:0005515 GO:0008270 GO:0019012
Cluster-22178.59945 GO:0016310 GO:0015979 GO:0016567 GO:0009069
GO:0006468 GO:0004674 GO:0004842 GO:0005524 GO:0008270
GO:0004672 GO:0046872 GO:0005515 GO:0005840 GO:0005680
GO:0030089
Cluster-22178.59947 GO:0008152 GO:0016491
Cluster-22178.59948 GO:0007172 GO:0007165 GO:0006468 GO:0004713
GO:0004871 GO:0004672 GO:0005524 GO:0005925
Cluster-22178.59952 GO:0007010 GO:0003779
Cluster-22178.59956 GO:0055085 GO:0006811 GO:0003677 GO:0005216
GO:0016020
Cluster-22178.59957 GO:0006355 GO:0003676
Cluster-22178.59958 GO:0006396 GO:0007186 GO:0009451 GO:0003723
GO:0004930 GO:0008173 GO:0016021
Cluster-22178.59959 GO:0007275 GO:0006614 GO:0000184 GO:0006400
GO:0048280 GO:0007218 GO:0045893 GO:0006886 GO:0008616
GO:0006281 GO:0000723 GO:0003677 GO:0016787 GO:0004386
GO:0005524 GO:0008270 GO:0005184 GO:0008479 GO:0003676
GO:0003678 GO:0005507 GO:0005525 GO:0000139 GO:0005657

GO:0005576 GO:0005737
Cluster-22178.59960 GO:0000166 GO:0046872
Cluster-22178.59961 GO:0005524
Cluster-22178.59963 GO:0016757
Cluster-22178.59965 GO:0051028 GO:0042254 GO:0005634
Cluster-22178.59966 GO:0009306 GO:0035091
Cluster-22178.59968 GO:0030001 GO:0007034
Cluster-22178.59969 GO:0016567 GO:0005515 GO:0061630 GO:0004842
GO:0008270 GO:0046872 GO:0005680
Cluster-22178.59970 GO:0016567 GO:0004842 GO:0061630 GO:0005515
GO:0008270 GO:0046872 GO:0005680
Cluster-22178.59971 GO:0016567 GO:0061630 GO:0005515 GO:0004842
GO:0046872 GO:0008270 GO:0005680
Cluster-22178.59973 GO:0046488 GO:0016307
Cluster-22178.59975 GO:0007029 GO:0016021 GO:0005789
Cluster-22178.59977 GO:0044030 GO:0005634
Cluster-22178.59978 GO:0007423 GO:0005212
Cluster-22178.59980 GO:0003950 GO:0004190 GO:0016020 GO:0016021
GO:0005634
Cluster-22178.59983 GO:0034314 GO:0030833 GO:0030041 GO:0005885
GO:0015629
Cluster-22178.59984 GO:0015979
Cluster-22178.59985 GO:0006468 GO:0016773 GO:0005524 GO:0004672
GO:0016020
Cluster-22178.59990 GO:0005515
Cluster-22178.59992 GO:0006396 GO:0031412 GO:0006139 GO:0003676
GO:0008408 GO:0000176 GO:0005622 GO:0031411
Cluster-22178.59993 GO:0042254 GO:0006412 GO:0015937 GO:0015940
GO:0003735 GO:0004252 GO:0004594 GO:0005524 GO:0055036
GO:0019028 GO:0005622 GO:0005840
Cluster-22178.59996 GO:0005515
Cluster-22178.59998 GO:0035556 GO:0006144 GO:0006357 GO:0006182
GO:0008152 GO:0006468 GO:0009190 GO:0046039 GO:0008270

GO:0005524 GO:0003824 GO:0004672 GO:0001104 GO:0003723
GO:0004383 GO:0016849 GO:0016592
Cluster-22178.59999 GO:0019867
Cluster-22178.60001 GO:0006241 GO:0006206 GO:0006144 GO:0006183
GO:0006228 GO:0007155 GO:0006165 GO:0003676 GO:0004550
GO:0005515 GO:0003723
Cluster-22178.60002 GO:0006810 GO:0015858 GO:0005337 GO:0005789
GO:0016021
Cluster-22178.60004 GO:0006260 GO:0019953 GO:0006351 GO:0006364
GO:0003887 GO:0003677 GO:0005198 GO:0005664 GO:0042575
GO:0030141 GO:0005576 GO:0030688
Cluster-22178.60005 GO:0016579 GO:0036459 GO:0005515
Cluster-22178.60007 GO:0006144 GO:0007165 GO:0016567 GO:0004114
GO:0004857 GO:0004842 GO:0032040
Cluster-22178.60012 GO:0009116 GO:0006506 GO:0016746 GO:0042393
GO:0005634 GO:0016021 GO:0005789
Cluster-22178.60014 GO:0007165 GO:0006914 GO:0046339 GO:0035556
GO:0006606 GO:0006629 GO:0009395 GO:0005509 GO:0045502
GO:0042803 GO:0008134 GO:0005515 GO:0004435 GO:0008081
GO:0005667 GO:0030286 GO:0019028
Cluster-22178.60015 GO:0006355 GO:0016787 GO:0003677 GO:0016818
GO:0008270 GO:0005524 GO:0003676 GO:0005634
Cluster-22178.60017 GO:0006750 GO:0005524 GO:0016787 GO:0004363

Cluster-22178.60018 GO:0015991 GO:0006508 GO:0006355 GO:0006629
GO:0006118 GO:0015996 GO:0015994 GO:0006811 GO:0045454
GO:0046486 GO:0015992 GO:0016042 GO:0008236 GO:0047746
GO:0046872 GO:0009055 GO:0015078 GO:0015035 GO:0016787
GO:0003677 GO:0003847 GO:0033179 GO:0019031 GO:0016020
GO:0008247 GO:0005886
Cluster-22178.60020 GO:0009966 GO:0007275 GO:0016020
Cluster-22178.60021 GO:0015991 GO:0030474 GO:0006547 GO:0006810
GO:0071988 GO:0016567 GO:0015992 GO:0045502 GO:0005515

GO:0008134 GO:0042803 GO:0046872 GO:0015078 GO:0003676
GO:0008270 GO:0004398 GO:0004842 GO:0033179 GO:0005667
GO:0030286 GO:0005886 GO:0005680
Cluster-22178.60022 GO:0006886 GO:0042967 GO:0006505 GO:0006629
GO:0008374 GO:0016788 GO:0005739
Cluster-22178.60023 GO:0000746 GO:0019867
Cluster-22178.60025 GO:0006355 GO:0007275 GO:0003700 GO:0000975
GO:0005667 GO:0042600
Cluster-22178.60027 GO:0009117 GO:0006888 GO:0019872 GO:0006886
GO:0006486 GO:0030639 GO:0016032 GO:0016757 GO:0008378
GO:0008830 GO:0008270 GO:0016020 GO:0030127
Cluster-22178.60029 GO:0006606 GO:0019904 GO:0005515 GO:0005737
Cluster-22178.60031 GO:0045454 GO:0006118 GO:0005515 GO:0009055
GO:0015035
Cluster-22178.60034 GO:0006396 GO:0003723
Cluster-22178.60037 GO:0006269 GO:0006952 GO:0006351 GO:0055114
GO:0008152 GO:0016846 GO:0008270 GO:0033743 GO:0004386
GO:0003896 GO:0005730 GO:0005657
Cluster-22178.60038 GO:0035556 GO:0009405 GO:0046488 GO:0046872
GO:0005524 GO:0016307 GO:0005198
Cluster-22178.60040 GO:0006534 GO:0042967 GO:0006535 GO:0005515
GO:0009001 GO:0016757 GO:0005737
Cluster-22178.60044 GO:0009405 GO:0006355 GO:0005515 GO:0005634
Cluster-22178.60045 GO:0004045
Cluster-22178.60048 GO:0016021
Cluster-22178.60049 GO:0009276 GO:0016021
Cluster-22178.60051 GO:0003723 GO:0055036
Cluster-22178.60053 GO:0042147 GO:0015031 GO:0003723 GO:0008565
GO:0030904
Cluster-22178.60056 GO:0003676 GO:0005515
Cluster-22178.60057 GO:0006468 GO:0005524 GO:0004672 GO:0005789
Cluster-22178.60058 GO:0009451 GO:0031167 GO:0001510 GO:0009452
GO:0006396 GO:0008033 GO:0008173 GO:0003723 GO:0008270

GO:0004809 GO:0008168 GO:0016021
Cluster-22178.60059 GO:0003723
Cluster-22178.60061 GO:0004672
Cluster-22178.60062 GO:0051302 GO:0006448 GO:0006414 GO:0016567
GO:0004842 GO:0003746 GO:0046872 GO:0008270 GO:0005515
GO:0005840 GO:0005622 GO:0005853
Cluster-22178.60063 GO:0006096 GO:0046486 GO:0006094 GO:0006020
GO:0008152 GO:0006013 GO:0006000 GO:0015976 GO:0004807
Cluster-22178.60065 GO:0006511 GO:0016747
Cluster-22178.60066 GO:0006357 GO:0001104 GO:0016592
Cluster-22178.60071 GO:0016853
Cluster-22178.60073 GO:0005515
Cluster-22178.60079 GO:0005515
Cluster-22178.60080 GO:0006357 GO:0006468 GO:0007172 GO:0007165
GO:0004713 GO:0004871 GO:0005198 GO:0001104 GO:0019031
GO:0019028 GO:0005925 GO:0016592 GO:0031083
Cluster-22178.60081 GO:0006511 GO:0051603 GO:0004298 GO:0004175
GO:0019773 GO:0005839
Cluster-22178.60082 GO:0006812 GO:0015948 GO:0046656 GO:0005515
GO:0008324 GO:0030269 GO:0016021
Cluster-22178.60083 GO:0003779
Cluster-22178.60084 GO:0016817 GO:0008168
Cluster-22178.60085 GO:0055114 GO:0003677 GO:0016706 GO:0005515

Cluster-22178.60086 GO:0009058 GO:0008152 GO:0006629 GO:0016788
GO:0016787
Cluster-22178.60087 GO:0007264 GO:0055114 GO:0006811 GO:0005216
GO:0010181 GO:0016491 GO:0005525 GO:0016020 GO:0005615
Cluster-22178.60088 GO:0055114 GO:0045454
Cluster-22178.60090 GO:0006359 GO:0046872 GO:0008270
Cluster-22178.60094 GO:0016787
Cluster-22178.60097 GO:0007155 GO:0042157 GO:0006869 GO:0008270
GO:0005515 GO:0008289 GO:0005576

Cluster-22178.60100 GO:0050790 GO:0006508 GO:0007165 GO:0006955
GO:0030247 GO:0016772 GO:0008234 GO:0030414 GO:0005044
GO:0004197 GO:0016301
Cluster-22178.60102 GO:0016567 GO:0004842 GO:0005515 GO:0046872
GO:0008270 GO:0003723 GO:0005680
Cluster-22178.60103 GO:0035556 GO:0005515
Cluster-22178.60107 GO:0005515
Cluster-22178.60108 GO:0006396 GO:0003723 GO:0016779
Cluster-22178.60109 GO:0006886 GO:0016192 GO:0010207 GO:0015979
GO:0004045
Cluster-22178.60110 GO:0016192 GO:0006886 GO:0015979 GO:0010207
GO:0004045
Cluster-22178.60111 GO:0016192 GO:0006886 GO:0005198 GO:0005515
GO:0030117
Cluster-22178.60112 GO:0006629
Cluster-22178.60114 GO:0000921 GO:0030168 GO:0051262 GO:0006817
GO:0043093 GO:0007165 GO:0051258 GO:0006355 GO:0000917
GO:0005102 GO:0030674 GO:0004871 GO:0005198 GO:0005940
GO:0005577 GO:0016020 GO:0005737 GO:0019031 GO:0016021
GO:0019028 GO:0005911
Cluster-22178.60118 GO:0006355 GO:0043401
Cluster-22178.60119 GO:1900758 GO:0003779
Cluster-22178.60121 GO:0006817 GO:0008654 GO:0055085 GO:0006812
GO:0008324 GO:0005315 GO:0016780 GO:0016020 GO:0016021
Cluster-22178.60123 GO:0046488 GO:0016307 GO:0019867
Cluster-22178.60124 GO:0035556 GO:0016567 GO:0046872 GO:0008270
GO:0004842 GO:0005515 GO:0005680
Cluster-22178.60128 GO:0045881 GO:0032183 GO:0070140 GO:0005515

Cluster-22178.60129 GO:0045881 GO:0070140 GO:0032183 GO:0005515

Cluster-22178.60132 GO:0006260 GO:0007264 GO:0007165 GO:0007186
GO:0006310 GO:0006355 GO:0006281 GO:0006576 GO:0005524

GO:0016887 GO:0003724 GO:0009378 GO:0003677 GO:0098519
GO:0019001 GO:0031683 GO:0003924 GO:0043531 GO:0005525
GO:0004871 GO:0003723 GO:0008134 GO:0005657 GO:0005667
GO:0009379
Cluster-22178.60133 GO:0016070 GO:0009271 GO:0006355 GO:0003677
GO:0005515 GO:0003723
Cluster-22178.60135 GO:0006308 GO:0048870 GO:0007049 GO:0006355
GO:0030474 GO:0070206 GO:0008053 GO:0006511 GO:0007275
GO:0006606 GO:0071988 GO:0046872 GO:0003700 GO:0045502
GO:0008134 GO:0008855 GO:0003924 GO:0003723 GO:0005198
GO:0005515 GO:0042803 GO:0005524 GO:0008270 GO:0004386
GO:0016021 GO:0019031 GO:0019028 GO:0071203 GO:0031514
GO:0009318 GO:0030286 GO:0005634 GO:0005667 GO:0005741
Cluster-22178.60137 GO:0071203
Cluster-22178.60138 GO:0007283 GO:0005515
Cluster-22178.60140 GO:0009405 GO:0033644
Cluster-22178.60141 GO:0006468 GO:0004672 GO:0005524
Cluster-22178.60144 GO:0005515 GO:0005737
Cluster-22178.60145 GO:0009058 GO:0042432 GO:0006355 GO:0016114
GO:0005515 GO:0004064 GO:0003676 GO:0016844
Cluster-22178.60146 GO:0009405 GO:0006886 GO:0009069 GO:0016310
GO:0005515 GO:0004674 GO:0008536 GO:0005576
Cluster-22178.60148 GO:0043087 GO:0016070 GO:0019013 GO:0016021
Cluster-22178.60150 GO:0008610 GO:0006633 GO:0008152 GO:0042967
GO:0004315 GO:0005524 GO:0016747 GO:0005835
Cluster-22178.60151 GO:0005856
Cluster-22178.60152 GO:0016021
Cluster-22178.60153 GO:0006357 GO:0001104 GO:0016592
Cluster-22178.60155 GO:0008643 GO:0042597
Cluster-22178.60158 GO:0006118 GO:0009055 GO:0020037
Cluster-22178.60161 GO:0006952 GO:0016772 GO:0005576
Cluster-22178.60162 GO:0009306 GO:0035091
Cluster-22178.60163 GO:0008270

Cluster-22178.60164 GO:0006810 GO:0042254 GO:0006310 GO:0009069
GO:0002949 GO:0006206 GO:0006412 GO:0006281 GO:0016310
GO:0006139 GO:0098519 GO:0003735 GO:0009378 GO:0004674
GO:0004127 GO:0003724 GO:0005524 GO:0016887 GO:0016301
GO:0003723 GO:0005525 GO:0003924 GO:0009379 GO:0005657
GO:0005622 GO:0005840
Cluster-22178.60165 GO:0008092 GO:0019898 GO:0005737
Cluster-22178.60169 GO:0045859 GO:0006468 GO:0005524 GO:0004672
GO:0004860 GO:0005515 GO:0005576
Cluster-22178.60172 GO:0006468 GO:0005524 GO:0004672
Cluster-22178.60173 GO:0008270 GO:0003676
Cluster-22178.60175 GO:0000226 GO:0000910 GO:0008017 GO:0045298
GO:0019028
Cluster-22178.60176 GO:0005515
Cluster-22178.60177 GO:0010508 GO:0015991 GO:0007264 GO:0008053
GO:0015992 GO:0005525 GO:0015078 GO:0016887 GO:0003924
GO:0005524 GO:0033179 GO:0005741 GO:0019013 GO:0016021
Cluster-22178.60178 GO:0006313 GO:0015031 GO:0006464 GO:0015074
GO:0005515 GO:0003677 GO:0005634
Cluster-22178.60182 GO:0016192 GO:0007165 GO:0030168 GO:0000921
GO:0048193 GO:0006891 GO:0006886 GO:0051258 GO:0030674
GO:0005102 GO:0008375 GO:0005515 GO:0005198 GO:0005507
GO:0017119 GO:0016020 GO:0005940 GO:0005577 GO:0019028
GO:0005801 GO:0016021 GO:0019031
Cluster-22178.60188 GO:2001070
Cluster-22178.60189 GO:0007275 GO:0005515 GO:0061630 GO:0008270
GO:0046872 GO:0042600
Cluster-22178.60190 GO:0006355 GO:0003700 GO:0043565 GO:0005667
Cluster-22178.60192 GO:0006355 GO:0042742 GO:0043565 GO:0003700
GO:0005667
Cluster-22178.60193 GO:0019031
Cluster-22178.60195 GO:0019031
Cluster-22178.60199 GO:0006897 GO:0003676 GO:0016020

Cluster-22178.60202 GO:0016757
Cluster-22178.60203 GO:0016757 GO:0046872
Cluster-22178.60204 GO:0016757 GO:0046872
Cluster-22178.60205 GO:0005975 GO:0042325 GO:0004553 GO:0005737
Cluster-22178.60206 GO:0045859 GO:0006355 GO:0007264 GO:0006468
GO:0007050 GO:0005096 GO:0043565 GO:0005524 GO:0004672
GO:0003700 GO:0005515 GO:0004861 GO:0005667 GO:0005634
Cluster-22178.60212 GO:0006810 GO:0005215 GO:0016020
Cluster-22178.60213 GO:0005737 GO:0005634
Cluster-22178.60214 GO:0006446 GO:0006413 GO:0003743 GO:0003723
GO:0005840 GO:0005737
Cluster-22178.60215 GO:0015979 GO:0005515 GO:0016773
Cluster-22178.60216 GO:0000226 GO:0006914 GO:0007165 GO:0008283
GO:0048870 GO:0000910 GO:0009405 GO:0006355 GO:0040007
GO:0008017 GO:0015631 GO:0005096 GO:0003700 GO:0043565
GO:0008083 GO:0042803 GO:0008134 GO:0005198 GO:0051082
GO:0045502 GO:0030286 GO:0005882 GO:0016020 GO:0005667
GO:0045298 GO:0031514
Cluster-22178.60217 GO:0008270
Cluster-22178.60218 GO:0006367 GO:0015979 GO:0032968 GO:0003677
GO:0005634
Cluster-22178.60222 GO:0006629 GO:0046339 GO:0009395 GO:0007165
GO:0035556 GO:0004435
Cluster-22178.60224 GO:0005515
Cluster-22178.60225 GO:0055114 GO:0006231 GO:0006206 GO:0006118
GO:0016491 GO:0051536 GO:0016625 GO:0009055 GO:0004799
GO:0005634
Cluster-22178.60226 GO:0007186 GO:0004930 GO:0016021
Cluster-22178.60227 GO:0005975 GO:0003676 GO:0016773 GO:0008184

Cluster-22178.60230 GO:0007166 GO:0007165 GO:0008270 GO:0046872
GO:0004871 GO:0016787 GO:0005509 GO:0005515 GO:0005634
Cluster-22178.60232 GO:0005515

Cluster-22178.60235 GO:0005515

Cluster-22178.60236 GO:0006118 GO:0006814 GO:0015992 GO:0006120 GO:0006744 GO:0003954 GO:0008137 GO:0005515 GO:0005739

Cluster-22178.60237 GO:0005198 GO:0019012

Cluster-22178.60238 GO:0006811 GO:0006355 GO:0009405 GO:0043565 GO:0003700 GO:0005216 GO:0005515 GO:0005667 GO:0005576

Cluster-22178.60240 GO:0015992 GO:0007264 GO:0015684 GO:0007165 GO:0007186 GO:0015991 GO:0006119 GO:0015093 GO:0046961 GO:0019001 GO:0031683 GO:0003924 GO:0005525 GO:0004871 GO:0005515 GO:0033178 GO:0016021 GO:0005634

Cluster-22178.60241 GO:0006814 GO:0006118 GO:0015992 GO:0006744 GO:0006120 GO:0003954 GO:0008137 GO:0005739

Cluster-22178.60243 GO:0006123 GO:0009060 GO:0055114 GO:0015992 GO:0007186 GO:0006118 GO:0004129 GO:0009055 GO:0004930 GO:0020037 GO:0005506 GO:0016021 GO:0045277

Cluster-22178.60245 GO:0007165 GO:0005509 GO:0005578

Cluster-22178.60246 GO:0006122 GO:0006508 GO:0004197 GO:0005515 GO:0005743 GO:0005750

Cluster-22178.60249 GO:0006468 GO:0005515 GO:0005524 GO:0004672

Cluster-22178.60250 GO:0000917 GO:0016192 GO:0015986 GO:0043093 GO:0005975 GO:0016052 GO:0015992 GO:0055114 GO:0030246 GO:0015078 GO:0004553 GO:0016491 GO:0005737 GO:0000276

Cluster-22178.60252 GO:0006351 GO:0042128 GO:0006206 GO:0006144 GO:0055114 GO:0006807 GO:0003677 GO:0003899 GO:0005515 GO:0008942 GO:0005730

Cluster-22178.60253 GO:0006397 GO:0006470 GO:0051260 GO:0032006 GO:0004721 GO:0005515 GO:0005634

Cluster-22178.60254 GO:0006807 GO:0016814 GO:0008270

Cluster-22178.60255 GO:0006367 GO:0005672

Cluster-22178.60256 GO:0005515

Cluster-22178.60257 GO:0055114 GO:0006351 GO:0042254 GO:0006412 GO:0008270 GO:0033743 GO:0046872 GO:0003676 GO:0003735

GO:0005840
Cluster-22178.60265 GO:0071805 GO:0006813 GO:0015079 GO:0016020
Cluster-22178.60267 GO:0033014 GO:0015994 GO:0046872 GO:0004655

Cluster-22178.60269 GO:0046660 GO:0006397 GO:0003723 GO:0003676
GO:0005634
Cluster-22178.60270 GO:0005515
Cluster-22178.60272 GO:0008152 GO:0016042 GO:0008203 GO:0006810
GO:0006816 GO:0042030 GO:0016298 GO:0005246 GO:0016787
GO:0016020
Cluster-22178.60276 GO:0008017 GO:0005515 GO:0003677 GO:0034061
GO:0045298 GO:0005634
Cluster-22178.60277 GO:0005515 GO:0042470
Cluster-22178.60279 GO:0009058 GO:0009878 GO:0030170 GO:0046872

Cluster-22178.60280 GO:0006355 GO:0009725 GO:0006412 GO:0042254
GO:0003677 GO:0003735 GO:0005515 GO:0005634 GO:0015934
GO:0005840
Cluster-22178.60282 GO:0006355 GO:0003700 GO:0005667 GO:0042025
Cluster-22178.60288 GO:0015696 GO:0006468 GO:0005524 GO:0004672
GO:0008519 GO:0016021 GO:0031224 GO:0016020
Cluster-22178.60289 GO:0006281 GO:0003677 GO:0003684 GO:0034061
GO:0005634
Cluster-22178.60291 GO:0007049 GO:0051301 GO:0005509 GO:0016021
Cluster-22178.60292 GO:0006614 GO:0005525 GO:0003724 GO:0005524
GO:0003723 GO:0016887
Cluster-22178.60293 GO:0006614 GO:0005525 GO:0003724 GO:0005524
GO:0003723 GO:0016887
Cluster-22178.60296 GO:0006915 GO:0006919 GO:0006974 GO:0001836
GO:0003677
Cluster-22178.60298 GO:0006468 GO:0006886 GO:0016192 GO:0004672
GO:0005524 GO:0005515 GO:0030117
Cluster-22178.60300 GO:0035429 GO:0015725 GO:0006486 GO:0046872

GO:0008373 GO:0016787 GO:0015128 GO:0016020
Cluster-22178.60302 GO:0009306 GO:0015031 GO:0009403 GO:0015450
GO:0008092 GO:0016020 GO:0009941 GO:0016021 GO:0019898
GO:0005737
Cluster-22178.60303 GO:0006397 GO:0008152 GO:0016788 GO:0005515
GO:0004064 GO:0003723 GO:0005634 GO:0005737
Cluster-22178.60308 GO:0005198 GO:0019028
Cluster-22178.60312 GO:0006355 GO:0009271 GO:0016070 GO:0003723
GO:0005515 GO:0003677
Cluster-22178.60316 GO:0006355 GO:0043093 GO:0000917 GO:0003677
GO:0043565 GO:0003700 GO:0005667 GO:0005634 GO:0005737
Cluster-22178.60317 GO:0016021
Cluster-22178.60318 GO:0030145 GO:0004177 GO:0005515
Cluster-22178.60320 GO:0005515 GO:0016021
Cluster-22178.60323 GO:0042254 GO:0006412 GO:0003735 GO:0005840
Cluster-22178.60326 GO:0006810 GO:0009733 GO:0005515 GO:0005622
Cluster-22178.60328 GO:0046486 GO:0006027 GO:0006687 GO:0006012
GO:0006812 GO:0006820 GO:0005975 GO:0006835 GO:0017153
GO:0004565 GO:0016020 GO:0005856 GO:0009341
Cluster-22178.60330 GO:0008146 GO:0046983 GO:0016021 GO:0016020
Cluster-22178.60331 GO:0046983 GO:0008146 GO:0016021
Cluster-22178.60332 GO:0006468 GO:0051016 GO:0005524 GO:0004672
GO:0016773 GO:0016020 GO:0008290
Cluster-22178.60333 GO:0007165 GO:0005515
Cluster-22178.60334 GO:0007275
Cluster-22178.60335 GO:0009102 GO:0051090 GO:0006768 GO:0042410
GO:0005524 GO:0005515 GO:0005634
Cluster-22178.60337 GO:0006886 GO:0007165 GO:0007606 GO:0006888
GO:0005515 GO:0003723 GO:0004888 GO:0008270 GO:0016020
GO:0030127
Cluster-22178.60342 GO:0004651
Cluster-22178.60344 GO:0006281 GO:0006260 GO:0016787 GO:0010181
GO:0003677 GO:0016818 GO:0005524 GO:0043140 GO:0003676

GO:0016874 GO:0005634 GO:0016020 GO:0005622 GO:0005657
Cluster-22178.60349 GO:0006470 GO:0006810 GO:0009405 GO:0006952
GO:0006570 GO:0004725 GO:0019855 GO:0046983 GO:0008138
GO:0005576
Cluster-22178.60351 GO:0045017 GO:0000160 GO:0042967 GO:0046486
GO:0005524 GO:0000156 GO:0003677 GO:0004144
Cluster-22178.60353 GO:0006486 GO:0009312 GO:0008455 GO:0008375
GO:0005795 GO:0016021
Cluster-22178.60354 GO:0006355 GO:0003700 GO:0005667
Cluster-22178.60355 GO:0006952 GO:0006313 GO:0006412 GO:0006310
GO:0015074 GO:0006471 GO:0003677 GO:0005515 GO:0008168
GO:0000150 GO:0003950
Cluster-22178.60357 GO:0016598 GO:0004057
Cluster-22178.60359 GO:0051262
Cluster-22178.60362 GO:0005509
Cluster-22178.60365 GO:0007155 GO:0006869 GO:0015992 GO:0006606
GO:0010508 GO:0007165 GO:0030150 GO:0030168 GO:0070206
GO:0006891 GO:0015991 GO:0044238 GO:0051258 GO:0042803
GO:0005515 GO:0005198 GO:0015078 GO:0030674 GO:0005102
GO:0045502 GO:0008134 GO:0008289 GO:0005667 GO:0005577
GO:0017119 GO:0005742 GO:0005882 GO:0030286 GO:0019028
GO:0005576 GO:0033179 GO:0016021 GO:0019031
Cluster-22178.60366 GO:0005739
Cluster-22178.60368 GO:0006508 GO:0019087 GO:0008152 GO:0008168
GO:0008236
Cluster-22178.60373 GO:0006355 GO:0006351 GO:0005634
Cluster-22178.60376 GO:0016310 GO:0006468 GO:0009069 GO:0004674
GO:0005524 GO:0004672 GO:0003774 GO:0042803 GO:0008134
GO:0005198 GO:0045502 GO:0030286 GO:0005882 GO:0005667
GO:0016459
Cluster-22178.60377 GO:0031175 GO:0030507 GO:0016818 GO:0005516
GO:0008270 GO:0005524 GO:0008091
Cluster-22178.60378 GO:0016020

Cluster-22178.60382 GO:0015031 GO:0006464 GO:0005515
Cluster-22178.60384 GO:0000160 GO:0008608 GO:0006955 GO:0019064
GO:0007165 GO:0007172 GO:0006415 GO:0008283 GO:0016310
GO:0040007 GO:0006468 GO:0006449 GO:0048193 GO:0004871
GO:0016149 GO:0008083 GO:0004713 GO:0046983 GO:0008270
GO:0005126 GO:0000155 GO:0016021 GO:0019031 GO:0009365
GO:0005840 GO:0005576 GO:0018444 GO:0005737 GO:0042729
GO:0005876 GO:0016020 GO:0005925
Cluster-22178.60386 GO:0006396 GO:0003723
Cluster-22178.60387 GO:0007275 GO:0006355 GO:0003700 GO:0000975
GO:0005667 GO:0042600
Cluster-22178.60389 GO:0016021
Cluster-22178.60399 GO:0006446 GO:0005515 GO:0003743 GO:0005737
GO:0005852 GO:0005840
Cluster-22178.60400 GO:1901031 GO:0055114 GO:0051920 GO:0005634
Cluster-22178.60402 GO:0007165 GO:0005509 GO:0005578
Cluster-22178.60405 GO:0009451 GO:0003824 GO:0051539 GO:0051536

Cluster-22178.60407 GO:0007275 GO:0016021
Cluster-22178.60408 GO:0004252 GO:0016021 GO:0005886
Cluster-22178.60409 GO:0055085 GO:0015743 GO:0030001 GO:0046873
GO:0004190 GO:0016020 GO:0016021
Cluster-22178.60410 GO:0008152 GO:0070569
Cluster-22178.60411 GO:0055114 GO:0009117 GO:0000287 GO:0016491
GO:0000166 GO:0008253 GO:0005737
Cluster-22178.60413 GO:0006355 GO:0003677
Cluster-22178.60414 GO:0000103 GO:0006144 GO:0006000 GO:0006013
GO:0006614 GO:0006777 GO:0006576 GO:0008312 GO:0005524
GO:0003677 GO:0003873 GO:0016787 GO:0004020 GO:0005525
GO:0016301 GO:0048500
Cluster-22178.60416 GO:0006570 GO:0006470 GO:0005515 GO:0008138
GO:0004725
Cluster-22178.60417 GO:0006570 GO:0006470 GO:0005515 GO:0008138

GO:0004725
Cluster-22178.60419 GO:0001522 GO:0043044 GO:0009451 GO:0009982 GO:0003723 GO:0016586
Cluster-22178.60420 GO:0009451 GO:0001522 GO:0043044 GO:0003723 GO:0009982 GO:0016586
Cluster-22178.60421 GO:0006355 GO:0003700 GO:0005515 GO:0005634 GO:0005667
Cluster-22178.60423 GO:0007165 GO:0004871 GO:0016021
Cluster-22178.60429 GO:0006383 GO:0006351 GO:0006206 GO:0006144 GO:0004190 GO:0003677 GO:0003899 GO:0046872 GO:0005730 GO:0005666 GO:0016021
Cluster-22178.60430 GO:0006260 GO:0005515
Cluster-22178.60431 GO:0006351 GO:0055114 GO:0015774 GO:0007165 GO:0016192 GO:0006206 GO:0006144 GO:0005515 GO:0003899 GO:0051920 GO:0005509 GO:0015159 GO:0005578 GO:0016021 GO:0005730 GO:0016020
Cluster-22178.60433 GO:0030246
Cluster-22178.60435 GO:0015074 GO:0006260 GO:0055085 GO:0006810 GO:0005524 GO:0016887 GO:0003924 GO:0005525 GO:0003676 GO:0042626 GO:0003678 GO:0005657 GO:0016021
Cluster-22178.60440 GO:0015992 GO:0015986 GO:0003723 GO:0051082 GO:0015078 GO:0016020
Cluster-22178.60441 GO:0006810 GO:0004386 GO:0003676 GO:0005524

Cluster-22178.60443 GO:0045893 GO:0016567 GO:0004842 GO:0003676 GO:0005515 GO:0042025
Cluster-22178.60444 GO:0005515
Cluster-22178.60445 GO:0042254 GO:0006412 GO:0003735 GO:0005840
Cluster-22178.60446 GO:0007034 GO:0006952 GO:0071973 GO:0006355 GO:0000982 GO:0005198 GO:0000978 GO:0030288 GO:0005576 GO:0005634 GO:0009428
Cluster-22178.60449 GO:0007165
Cluster-22178.60450 GO:0055114 GO:0016491

Cluster-22178.60451 GO:0046872
Cluster-22178.60452 GO:0006144 GO:0000103 GO:0008270 GO:0005524
GO:0046872 GO:0004020 GO:0016301 GO:0005515
Cluster-22178.60453 GO:0006270 GO:0046872
Cluster-22178.60454 GO:0050765 GO:0005515
Cluster-22178.60457 GO:0034061 GO:0003677 GO:0016779 GO:0005634
Cluster-22178.60459 GO:0051056 GO:0006355 GO:0019048 GO:0003677
GO:0005515 GO:0005096
Cluster-22178.60461 GO:0009058 GO:0008152 GO:0003824 GO:0048037

Cluster-22178.60465 GO:0006816 GO:0016042 GO:0006811 GO:0005262
GO:0005244 GO:0016021 GO:0019031
Cluster-22178.60467 GO:0006397 GO:0003676 GO:0016021
Cluster-22178.60469 GO:0035091
Cluster-22178.60470 GO:0006621 GO:0046923 GO:0005783 GO:0016021
Cluster-22178.60474 GO:0007155 GO:0006904 GO:0006914 GO:0015031
GO:0051082 GO:0019901 GO:0016020 GO:0000145 GO:0005886
Cluster-22178.60476 GO:0005975 GO:0004553
Cluster-22178.60478 GO:0006457 GO:0000413 GO:0006629 GO:0003755

Cluster-22178.60480 GO:0015992 GO:0015986 GO:0015078 GO:0000276
Cluster-22178.60481 GO:0043087 GO:0006308 GO:0006302 GO:0007155
GO:0008855 GO:0005515 GO:0009288 GO:0005634 GO:0009318
Cluster-22178.60482 GO:0003677 GO:0005524 GO:0016787
Cluster-22178.60484 GO:0006402 GO:0051252 GO:0003676 GO:0046872
GO:0004535 GO:0003723 GO:0005737 GO:0016020 GO:0005634
Cluster-22178.60485 GO:0009058 GO:0003824
Cluster-22178.60487 GO:0006810 GO:0009069 GO:0006468 GO:0035556
GO:0016310 GO:0005198 GO:0017048 GO:0005215 GO:0004674
GO:0003774 GO:0005524 GO:0004672 GO:0016020 GO:0005882
GO:0016459
Cluster-22178.60489 GO:0006098 GO:0006144 GO:0009165 GO:0004749
GO:0000287

Cluster-22178.60491 GO:0051287
Cluster-22178.60497 GO:0016567 GO:0006511 GO:0006915 GO:0001836
GO:0008608 GO:0006974 GO:0006919 GO:0034450 GO:0004842
GO:0042729 GO:0000151 GO:0005876
Cluster-22178.60502 GO:0000398 GO:0005681
Cluster-22178.60503 GO:0016043 GO:0000398 GO:0003779 GO:0005681
Cluster-22178.60504 GO:0007010 GO:0006508 GO:0055114 GO:0016972
GO:0008233 GO:0003779 GO:0046872 GO:0005634
Cluster-22178.60505 GO:0035556 GO:0005515
Cluster-22178.60508 GO:0042254 GO:0006412 GO:0006508 GO:0004198
GO:0005509 GO:0003735 GO:0005840 GO:0005622
Cluster-22178.60512 GO:0005515 GO:0046872
Cluster-22178.60513 GO:0006813 GO:0006814 GO:0005890
Cluster-22178.60514 GO:0046983 GO:0005515
Cluster-22178.60515 GO:0007186 GO:0004930 GO:0016021
Cluster-22178.60516 GO:0030168 GO:0008360 GO:0043093 GO:0007165
GO:0051258 GO:0007049 GO:0006355 GO:0000917 GO:0005102
GO:0030674 GO:0003677 GO:0043565 GO:0003700 GO:0005577
GO:0005667 GO:0016020 GO:0005737 GO:0005634
Cluster-22178.60519 GO:0006298 GO:0006355 GO:0030983 GO:0005515
GO:0003700 GO:0043565 GO:0005524 GO:0005667
Cluster-22178.60521 GO:0016310 GO:0009069 GO:0006468 GO:0004674
GO:0004672 GO:0005524 GO:0016773 GO:0016020
Cluster-22178.60522 GO:0006810 GO:0006024 GO:0015012 GO:0005515
GO:0003723 GO:0042626 GO:0005524 GO:0016021
Cluster-22178.60524 GO:0055085 GO:0030234 GO:0016020 GO:0016021
Cluster-22178.60525 GO:0008270 GO:0005515
Cluster-22178.60526 GO:0003676 GO:0003723
Cluster-22178.60527 GO:0003723 GO:0003676
Cluster-22178.60528 GO:0055085 GO:0007165 GO:0007186 GO:0007264
GO:0006576 GO:0005525 GO:0004871 GO:0005524 GO:0031683
GO:0019001 GO:0003924 GO:0030234 GO:0016021 GO:0016020
Cluster-22178.60531 GO:0035556 GO:0005515

Cluster-22178.60532 GO:0006508 GO:0004197
Cluster-22178.60533 GO:0006508 GO:0004197
Cluster-22178.60536 GO:0006144 GO:0006807 GO:0006396 GO:0003723 GO:0004000
Cluster-22178.60537 GO:0006144 GO:0006396 GO:0006807 GO:0003723 GO:0004000
Cluster-22178.60538 GO:0006687 GO:0050290
Cluster-22178.60539 GO:0007186 GO:0006144 GO:0006807 GO:0006396 GO:0003723 GO:0004000 GO:0004930 GO:0016020
Cluster-22178.60542 GO:0005515
Cluster-22178.60544 GO:0006570 GO:0042967 GO:0006470 GO:0005515 GO:0008080 GO:0004725 GO:0008138
Cluster-22178.60545 GO:0006914 GO:0006260 GO:0001882 GO:0003887 GO:0008092 GO:0042575 GO:0005737 GO:0019898
Cluster-22178.60547 GO:0055114 GO:0016616 GO:0016491
Cluster-22178.60549 GO:0007264 GO:0007186 GO:0006614 GO:0007165 GO:0006576 GO:0006355 GO:0003677 GO:0016887 GO:0005524 GO:0008270 GO:0005515 GO:0003924 GO:0031683 GO:0019001 GO:0004871 GO:0005525
Cluster-22178.60550 GO:0043547 GO:0046983 GO:0005096
Cluster-22178.60551 GO:0000746 GO:0006118 GO:0005515 GO:0003677 GO:0016491 GO:0009055 GO:0005634 GO:0016020
Cluster-22178.60552 GO:0043547 GO:0005096 GO:0046983
Cluster-22178.60553 GO:0005524 GO:0003774 GO:0016301 GO:0005515 GO:0016459
Cluster-22178.60554 GO:0055114 GO:0008152 GO:0016491 GO:0005515

Cluster-22178.60555 GO:0055114 GO:0004659 GO:0016021 GO:0016020
Cluster-22178.60559 GO:0006890 GO:0007186 GO:0004930 GO:0016021 GO:0019028
Cluster-22178.60561 GO:0003677
Cluster-22178.60562 GO:0006369 GO:0006378 GO:0016310 GO:0006379 GO:0009069 GO:0016773 GO:0016765 GO:0004674 GO:0008144

GO:0005515 GO:0003677 GO:0005849
Cluster-22178.60563 GO:0006914 GO:0006355 GO:0003700 GO:0043565
GO:0005667
Cluster-22178.60567 GO:0006974 GO:0006915 GO:0006919 GO:0001836

Cluster-22178.60569 GO:0019082 GO:0008270 GO:0003676 GO:0003677

Cluster-22178.60572 GO:0016567 GO:0004842
Cluster-22178.60574 GO:0055114 GO:0016114 GO:0006694 GO:0004506
GO:0050660 GO:0016021
Cluster-22178.60577 GO:0042475 GO:0006351 GO:0007049 GO:0042476
GO:0005515 GO:0030345 GO:0005578 GO:0019867 GO:0070176
Cluster-22178.60578 GO:0035556 GO:0046872 GO:0005515 GO:0045202
Cluster-22178.60583 GO:0055114 GO:0006357 GO:0005506 GO:0001104
GO:0020037 GO:0016705 GO:0016592
Cluster-22178.60587 GO:0016746
Cluster-22178.60588 GO:0042967 GO:0004379 GO:0016765
Cluster-22178.60589 GO:0008152 GO:0015066 GO:0008168
Cluster-22178.60590 GO:0055114 GO:0008168 GO:0016491 GO:0016706
GO:0003676
Cluster-22178.60591 GO:0008152 GO:0008168 GO:0015066
Cluster-22178.60592 GO:0006144 GO:0006526 GO:0006531 GO:0006529
GO:0006522 GO:0008152 GO:0008033 GO:0006541 GO:0006536
GO:0006560 GO:0006164 GO:0006177 GO:0005524 GO:0004066
GO:0016787 GO:0004055 GO:0003824 GO:0003922 GO:0004810
Cluster-22178.60597 GO:0003677 GO:0005634
Cluster-22178.60598 GO:0006511 GO:0007275 GO:0046872 GO:0005634
Cluster-22178.60601 GO:0005515
Cluster-22178.60602 GO:0015833 GO:0035092 GO:0005524 GO:0046872
GO:0000166 GO:0003677 GO:0000228
Cluster-22178.60606 GO:0007186 GO:0055114 GO:0004930 GO:0016972
GO:0046872 GO:0016021
Cluster-22178.60613 GO:0007165 GO:0006468 GO:0006355 GO:0008152

GO:0005515 GO:0005179 GO:0003824 GO:0004672 GO:0005524
GO:0005576 GO:0005634
Cluster-22178.60615 GO:0006308 GO:0008855 GO:0009318
Cluster-22178.60617 GO:0006468 GO:0006118 GO:0005524 GO:0004672
GO:0046872 GO:0016773 GO:0009055 GO:0020037 GO:0005515
GO:0016020
Cluster-22178.60620 GO:0005515
Cluster-22178.60621 GO:0005515 GO:0005509
Cluster-22178.60622 GO:0051205 GO:0016021
Cluster-22178.60624 GO:0035556 GO:0046872
Cluster-22178.60625 GO:0007049 GO:0006355 GO:0030474 GO:0046983
GO:0043565 GO:0005200 GO:0003700 GO:0005198 GO:0005856
GO:0005823 GO:0005667 GO:0005882
Cluster-22178.60627 GO:0006355 GO:0003700 GO:0000062 GO:0016884
GO:0005667
Cluster-22178.60629 GO:0015979 GO:0042549 GO:0051205 GO:0016021
GO:0009539 GO:0009523
Cluster-22178.60630 GO:0006260 GO:0003677 GO:0005664
Cluster-22178.60631 GO:0032780 GO:0006355 GO:0003700 GO:0042030
GO:0008270 GO:0005739 GO:0005667 GO:0005634
Cluster-22178.60633 GO:0003676
Cluster-22178.60634 GO:0000902 GO:0006270 GO:0003676 GO:0008270
GO:0016021 GO:0032040 GO:0005634
Cluster-22178.60635 GO:0016021
Cluster-22178.60636 GO:0006886 GO:0000921 GO:0003774 GO:0016459
GO:0016020 GO:0005940 GO:0016021 GO:0005801
Cluster-22178.60637 GO:0005515 GO:0005201 GO:0003723 GO:0008168
GO:0008270 GO:0005581 GO:0005578
Cluster-22178.60638 GO:0043571 GO:0006397 GO:0003774 GO:0005524
GO:0004519 GO:0005515 GO:0016459
Cluster-22178.60639 GO:0005515 GO:0008270 GO:0016021 GO:0042765
Cluster-22178.60640 GO:0007165 GO:0040007 GO:0008283 GO:0051028
GO:0008083 GO:0005634

Cluster-22178.60641 GO:0007010 GO:0007165 GO:0006629 GO:0035556
GO:0046339 GO:0032065 GO:0009395 GO:0003677 GO:0005515
GO:0004435 GO:0005543 GO:0034061 GO:0003779 GO:0005634
GO:0005938
Cluster-22178.60643 GO:0045859 GO:0019887 GO:0005956
Cluster-22178.60645 GO:0006260 GO:0019079 GO:0005524 GO:0003676
GO:0003723 GO:0005198 GO:0016818 GO:0003887 GO:0003677
GO:0008026 GO:0019028 GO:0005634 GO:0042575
Cluster-22178.60647 GO:0006139 GO:0008408 GO:0003676
Cluster-22178.60649 GO:0008270 GO:0016021
Cluster-22178.60650 GO:0016021 GO:0042765
Cluster-22178.60654 GO:0003677 GO:0005634
Cluster-22178.60656 GO:0050776 GO:0014068 GO:0007165 GO:0006446
GO:0006413 GO:0005102 GO:0003743 GO:0043548 GO:0003723
GO:0005840 GO:0005737 GO:0016020
Cluster-22178.60657 GO:0006904 GO:0016192 GO:0008270 GO:0046983

Cluster-22178.60659 GO:0003676 GO:0005515 GO:0003677 GO:0016787
GO:0005524
Cluster-22178.60660 GO:0005515
Cluster-22178.60662 GO:0007411 GO:0042742 GO:0005515 GO:0005576
Cluster-22178.60663 GO:0006493 GO:0006338 GO:0005524 GO:0003676
GO:0000030 GO:0016818 GO:0000136 GO:0005634 GO:0016020
Cluster-22178.60664 GO:0006338 GO:0006493 GO:0005524 GO:0003676
GO:0016818 GO:0000030 GO:0000136 GO:0005634 GO:0016020
Cluster-22178.60667 GO:0006355 GO:0005198 GO:0003700 GO:0005667
GO:0019028
Cluster-22178.60669 GO:0007165 GO:0008270
Cluster-22178.60673 GO:0003677 GO:0046872 GO:0008270
Cluster-22178.60674 GO:0048193 GO:0006457 GO:0051082 GO:0016020
GO:0016021 GO:0016272
Cluster-22178.60675 GO:0006465 GO:0008233 GO:0016021 GO:0005787
Cluster-22178.60678 GO:0030246

Cluster-22178.60679 GO:0015979 GO:0046872
Cluster-22178.60680 GO:0005515
Cluster-22178.60681 GO:0009306 GO:0055114 GO:0006118 GO:0045454 GO:0009055 GO:0015035 GO:0016491 GO:0016209 GO:0005509 GO:0005788
Cluster-22178.60682 GO:0006355 GO:0015994 GO:0006783 GO:0019079 GO:0033014 GO:0004852 GO:0003700 GO:0043565 GO:0003677 GO:0003682 GO:0004386 GO:0008270 GO:0005634 GO:0000785 GO:0000775 GO:0005667
Cluster-22178.60684 GO:0016757
Cluster-22178.60686 GO:0005515
Cluster-22178.60691 GO:0006024 GO:0007283 GO:0009264 GO:0015012 GO:0003677 GO:0004252 GO:0016757 GO:0008253 GO:0005634 GO:0016020 GO:0000786 GO:0016021
Cluster-22178.60693 GO:0007049 GO:0006897 GO:0007342 GO:0060478 GO:0051301 GO:0016021 GO:0005886
Cluster-22178.60698 GO:0015986 GO:0006355 GO:0015992 GO:0046872 GO:0008270 GO:0043565 GO:0015078 GO:0003700 GO:0005667 GO:0000276
Cluster-22178.60699 GO:0042254 GO:0006412 GO:0005515 GO:0003735 GO:0005622 GO:0005840
Cluster-22178.60700 GO:0006468 GO:0005524 GO:0004672
Cluster-22178.60701 GO:0006836 GO:0070836 GO:0006812 GO:0051191 GO:0005328 GO:0005634 GO:0016021
Cluster-22178.60703 GO:0001522 GO:0042254 GO:0006508 GO:0008239

Cluster-22178.60705 GO:0046872 GO:0008376 GO:0005515 GO:0032580
Cluster-22178.60706 GO:0005515 GO:0005737
Cluster-22178.60708 GO:0008270
Cluster-22178.60712 GO:0007275 GO:0007304 GO:0006313 GO:0005515 GO:0005213 GO:0042600
Cluster-22178.60713 GO:0019073 GO:0007165 GO:0005515 GO:0003677 GO:0016021

Cluster-22178.60715 GO:0006260 GO:0015992 GO:0042254 GO:0006470
GO:0006351 GO:0009405 GO:0006412 GO:0006570 GO:0015991
GO:0003677 GO:0008138 GO:0003735 GO:0015078 GO:0004725
GO:0003887 GO:0005622 GO:0005840 GO:0016021 GO:0042575
GO:0033179
Cluster-22178.60717 GO:0042254 GO:0001522 GO:0005515 GO:0030515
GO:0072588
Cluster-22178.60720 GO:0006508 GO:0042742 GO:0008152 GO:0016836
GO:0003824 GO:0008233 GO:0005576
Cluster-22178.60721 GO:0016049 GO:0010215 GO:0031225
Cluster-22178.60722 GO:0007165 GO:0005515
Cluster-22178.60725 GO:0010508 GO:0042254 GO:0006412 GO:0003723
GO:0003735 GO:0005622 GO:0005840
Cluster-22178.60727 GO:0006281 GO:0006886 GO:0016192 GO:0043161
GO:0006289 GO:0003684 GO:0003676 GO:0030117
Cluster-22178.60729 GO:0055114 GO:0008152 GO:0006480 GO:0010181
GO:0008168 GO:0016491 GO:0008171
Cluster-22178.60731 GO:0003677 GO:0008270
Cluster-22178.60732 GO:0007165 GO:0006810 GO:0044237 GO:0010038
GO:0007049 GO:0006357 GO:0006308 GO:0001104 GO:0005515
GO:0005215 GO:0004871 GO:0008855 GO:0009318 GO:0016592
GO:0016020
Cluster-22178.60733 GO:0006508 GO:0008234
Cluster-22178.60735 GO:0006281 GO:0009432 GO:0006367 GO:0015684
GO:0051726 GO:0007155 GO:0005525 GO:0003697 GO:0017025
GO:0005524 GO:0015093 GO:0016887 GO:0016021 GO:0005672
GO:0005634 GO:0009289
Cluster-22178.60736 GO:0006118 GO:0015991 GO:0016032 GO:0008152
GO:0055114 GO:0015992 GO:0003995 GO:0016740 GO:0050660
GO:0005525 GO:0015078 GO:0005524 GO:0046872 GO:0016627
GO:0033179
Cluster-22178.60738 GO:0009878 GO:0046872
Cluster-22178.60739 GO:0000398 GO:0008270 GO:0005681

Cluster-22178.60740 GO:0046488 GO:0016307
Cluster-22178.60743 GO:0006310 GO:0006355 GO:0035556 GO:0003677
GO:0050662 GO:0003824 GO:0003676 GO:0003700 GO:0046872
GO:0008270 GO:0043565 GO:0005634 GO:0005667
Cluster-22178.60744 GO:0005886 GO:0016021
Cluster-22178.60748 GO:0007165 GO:0030155 GO:0030334 GO:0006308
GO:0045995 GO:0005515 GO:0008855 GO:0005102 GO:0009318
Cluster-22178.60750 GO:0051260
Cluster-22178.60754 GO:0000413 GO:0006457 GO:0003755
Cluster-22178.60755 GO:0006457 GO:0000413 GO:0003755
Cluster-22178.60756 GO:0008270
Cluster-22178.60757 GO:0015992 GO:0006123 GO:0008270 GO:0046872
GO:0004129 GO:0005634 GO:0045277
Cluster-22178.60762 GO:0006313 GO:0006355 GO:0004803 GO:0003700
GO:0003677 GO:0005667 GO:0005622
Cluster-22178.60764 GO:0006505 GO:0008152 GO:0006508 GO:0006886
GO:0016787 GO:0016788 GO:0008236 GO:0005576
Cluster-22178.60765 GO:0030206 GO:0006024 GO:0030158 GO:0008233
GO:0008375 GO:0016020
Cluster-22178.60766 GO:0008610 GO:0006118 GO:0017004 GO:0008152
GO:0016747 GO:0045158 GO:0009512
Cluster-22178.60769 GO:0031175 GO:0005516 GO:0030507 GO:0008091
Cluster-22178.60771 GO:0006520 GO:0055114 GO:0006144 GO:0009152
GO:0006188 GO:0006522 GO:0006531 GO:0009058 GO:0004018
GO:0016829 GO:0010277 GO:0030170
Cluster-22178.60772 GO:0006412 GO:0042254 GO:0003735 GO:0005840
Cluster-22178.60774 GO:0042254 GO:0006412 GO:0006810 GO:0003735
GO:0016020 GO:0005840
Cluster-22178.60776 GO:0035556 GO:0016310 GO:0006468 GO:0009069
GO:0004674 GO:0004672 GO:0005524 GO:0016773 GO:0016020
Cluster-22178.60778 GO:0016032 GO:0006508 GO:0007155 GO:0019082
GO:0001558 GO:0042246 GO:0005520 GO:0008239 GO:0070009
GO:0005515 GO:0004252 GO:0016021 GO:0016942 GO:0005576

Cluster-22178.60779 GO:0006508 GO:0007606 GO:0007165 GO:0016787
GO:0005509 GO:0008236 GO:0005578 GO:0016021
Cluster-22178.60780 GO:0006816 GO:0006874 GO:0005515 GO:0005219
GO:0016021 GO:0005622
Cluster-22178.60781 GO:0016779
Cluster-22178.60782 GO:0006888 GO:0006886 GO:0008270 GO:0030127
Cluster-22178.60787 GO:0016567 GO:0008270 GO:0005515 GO:0003677
GO:0004842 GO:0009279 GO:0005680
Cluster-22178.60788 GO:0030168 GO:0055085 GO:0007165 GO:0051258
GO:0048870 GO:0030001 GO:0008270 GO:0005102 GO:0046873
GO:0030674 GO:0046872 GO:0005515 GO:0005622 GO:0005577
GO:0016020 GO:0016021 GO:0031514
Cluster-22178.60791 GO:0016567 GO:0008270 GO:0005515 GO:0004842
GO:0003677 GO:0009279 GO:0005680
Cluster-22178.60792 GO:0006576 GO:0006508 GO:0007264 GO:0007186
GO:0007165 GO:0015684 GO:0003924 GO:0031683 GO:0019001
GO:0004871 GO:0005525 GO:0016887 GO:0005524 GO:0015093
GO:0004222 GO:0005509 GO:0016021
Cluster-22178.60793 GO:0003723
Cluster-22178.60796 GO:0005515
Cluster-22178.60797 GO:0006090 GO:0009401 GO:0006412 GO:0006814
GO:0006560 GO:0006525 GO:0042254 GO:0071436 GO:0008948
GO:0005515 GO:0015081 GO:0016787 GO:0003735 GO:0016021
GO:0005840 GO:0015934 GO:0016020
Cluster-22178.60798 GO:0009401 GO:0006090 GO:0006814 GO:0006412
GO:0006560 GO:0006525 GO:0071436 GO:0042254 GO:0005515
GO:0008948 GO:0015081 GO:0016787 GO:0003735 GO:0016021
GO:0005840 GO:0015934 GO:0016020
Cluster-22178.60799 GO:0046935 GO:0005515 GO:0005944
Cluster-22178.60803 GO:0007342 GO:0007340 GO:0003676 GO:0001669
Cluster-22178.60805 GO:0006260 GO:0003887 GO:0005515 GO:0042575
Cluster-22178.60808 GO:0006914 GO:0051382 GO:0006308 GO:0048870
GO:0008608 GO:0006355 GO:0010508 GO:0008855 GO:0008092

GO:0043565 GO:0003700 GO:0051082 GO:0045502 GO:0008134
GO:0042803 GO:0030286 GO:0019898 GO:0005876 GO:0005667
GO:0005737 GO:0000776 GO:0005634 GO:0009318 GO:0042729
GO:0031514
Cluster-22178.60812 GO:0000902 GO:0006955 GO:0006351 GO:0009405
GO:0006355 GO:0006811 GO:0006464 GO:0005515 GO:0003676
GO:0003700 GO:0016790 GO:0016151 GO:0043565 GO:0046872
GO:0003677 GO:0008270 GO:0045202 GO:0016020 GO:0005667
GO:0005887
Cluster-22178.60813 GO:0051726 GO:0005634
Cluster-22178.60814 GO:0009089 GO:0006508 GO:0009085 GO:0055114
GO:0005509 GO:0005515 GO:0008839 GO:0004222 GO:0008270
GO:0031012
Cluster-22178.60815 GO:0016020
Cluster-22178.60816 GO:0016560 GO:0006468 GO:0005524 GO:0004672
GO:0016021 GO:0005777
Cluster-22178.60817 GO:0006468 GO:0005524 GO:0004672
Cluster-22178.60818 GO:0015031 GO:0016021
Cluster-22178.60823 GO:0055114 GO:0016887 GO:0005524 GO:0016616

Cluster-22178.60824 GO:0042254 GO:0006412 GO:0003735 GO:0005840
GO:0005622
Cluster-22178.60827 GO:0005515 GO:0016020
Cluster-22178.60830 GO:0006206 GO:0006144 GO:0007165 GO:0006351
GO:0015774 GO:0015159 GO:0005515 GO:0003899 GO:0016791
GO:0005509 GO:0016020 GO:0005730 GO:0005578
Cluster-22178.60833 GO:0019217 GO:0016567 GO:0005515 GO:0046872
GO:0008270 GO:0004842 GO:0000062 GO:0005680
Cluster-22178.60835 GO:0042803 GO:0003723 GO:0008270 GO:0042162

Cluster-22178.60837 GO:0030328 GO:0055114 GO:0016670 GO:0016491

Cluster-22178.60839 GO:0006298 GO:0007010 GO:0007264 GO:0000398

GO:0004519 GO:0005525 GO:0008270 GO:0005524 GO:0016887
GO:0003779 GO:0003924 GO:0019028
Cluster-22178.60843 GO:0043461 GO:0055085 GO:0016020
Cluster-22178.60844 GO:0006470 GO:0070403 GO:0008138 GO:0005515

Cluster-22178.60847 GO:0004867
Cluster-22178.60848 GO:0016021
Cluster-22178.60850 GO:0006508 GO:0019825 GO:0020037 GO:0004197

Cluster-22178.60851 GO:0006123 GO:0015992 GO:0004129 GO:0045277
Cluster-22178.60854 GO:0006887 GO:0008289 GO:0000145
Cluster-22178.60858 GO:0006355 GO:0046854 GO:0042967 GO:0004402
GO:0016787 GO:0008270 GO:0003712 GO:0019033 GO:0005667
GO:0005634 GO:0000123
Cluster-22178.60860 GO:0030071 GO:0005680
Cluster-22178.60861 GO:0006508 GO:0008233 GO:0003677
Cluster-22178.60864 GO:1903818 GO:0090063 GO:0030431 GO:0032222
GO:0000226 GO:0034235 GO:0005634 GO:0005815 GO:0000922
Cluster-22178.60865 GO:0006206 GO:0006144 GO:0006351 GO:0003677
GO:0003899 GO:0005730
Cluster-22178.60867 GO:0006355 GO:0032039 GO:0005622
Cluster-22178.60868 GO:0006419 GO:0006522 GO:0006531 GO:0006355
GO:0004813 GO:0005524 GO:0000166 GO:0005622 GO:0016021
GO:0032039
Cluster-22178.60869 GO:0018990 GO:0007218 GO:0008255
Cluster-22178.60870 GO:0006464 GO:0015031 GO:0005515
Cluster-22178.60875 GO:0007264 GO:0005525
Cluster-22178.60876 GO:0006396 GO:0006807 GO:0006144 GO:0004000
GO:0003723
Cluster-22178.60879 GO:0000390 GO:0071008
Cluster-22178.60881 GO:0007607 GO:0006402 GO:0009405 GO:0050912
GO:0051252 GO:0007187 GO:0046872 GO:0004535 GO:0003723
GO:0008527 GO:0005737 GO:0016021 GO:0005634

Cluster-22178.60882 GO:0045766 GO:0016567 GO:0043123 GO:0015986
GO:0043066 GO:0015992 GO:0003723 GO:0043130 GO:0004842
GO:0015078 GO:0000276
Cluster-22178.60883 GO:0006886 GO:0006570 GO:0006470 GO:0050852
GO:0005047 GO:0005515 GO:0044212 GO:0004725 GO:0005525
GO:0046872 GO:0003924 GO:0005785 GO:0016021 GO:0005786
Cluster-22178.60884 GO:0046872
Cluster-22178.60887 GO:0008152 GO:0055114 GO:0016491
Cluster-22178.60888 GO:0007275 GO:0009103 GO:0008152 GO:0055114
GO:0005201 GO:0016491 GO:0008918 GO:0005578 GO:0005581
Cluster-22178.60889 GO:0006357 GO:0001104 GO:0016592
Cluster-22178.60891 GO:0048870 GO:0019905 GO:0005515 GO:0031514
GO:0019028
Cluster-22178.60893 GO:0005515
Cluster-22178.60894 GO:0007155 GO:0001503 GO:0019028 GO:0005576
Cluster-22178.60895 GO:0016020
Cluster-22178.60896 GO:0003924
Cluster-22178.60900 GO:0015986 GO:0016567 GO:0006119 GO:0007005
GO:0015992 GO:0046961 GO:0046933 GO:0008289 GO:0004842
GO:0000275 GO:0045259
Cluster-22178.60904 GO:0046487 GO:0042128 GO:0016117 GO:0006807
GO:0009396 GO:0008033 GO:0055114 GO:0016705 GO:0004488
GO:0003824 GO:0050660 GO:0051537 GO:0008942 GO:0016491
Cluster-22178.60905 GO:0016567 GO:0006355 GO:0061630 GO:0005515
GO:0003677 GO:0016787 GO:0003676 GO:0003700 GO:0004842
GO:0046872 GO:0008270 GO:0005524 GO:0005667 GO:0005680
Cluster-22178.60906 GO:0015979 GO:0030494
Cluster-22178.60907 GO:0006355 GO:0006812 GO:0008324 GO:0003677
GO:0016021
Cluster-22178.60908 GO:0009405 GO:0005198 GO:0005882
Cluster-22178.60909 GO:0046872
Cluster-22178.60911 GO:0008168 GO:0070403
Cluster-22178.60913 GO:0009089 GO:0006355 GO:0008837 GO:0005634

GO:0005737
Cluster-22178.60915 GO:0016032 GO:0006874 GO:0006508 GO:0004197
GO:0030234 GO:0030176
Cluster-22178.60917 GO:0006874 GO:0030234 GO:0030176
Cluster-22178.60919 GO:0035556 GO:0006260 GO:0006269 GO:0006351
GO:0046872 GO:0003896 GO:0003677 GO:0005657 GO:0005730
Cluster-22178.60920 GO:0006464
Cluster-22178.60921 GO:0005975 GO:0033925 GO:0004553 GO:0005737
Cluster-22178.60923 GO:0009298 GO:0007219 GO:0042351 GO:0006012
GO:0019673 GO:0006000 GO:0008446 GO:0050662 GO:0070401
GO:0003824 GO:0005622
Cluster-22178.60925 GO:0035444 GO:0007264 GO:0015675 GO:0015099
GO:0005525 GO:0046872 GO:0016021
Cluster-22178.60926 GO:0007165 GO:0006614 GO:0007186 GO:0007264
GO:0006355 GO:0006576 GO:0008270 GO:0005524 GO:0016887
GO:0003677 GO:0005525 GO:0004871 GO:0019001 GO:0031683
GO:0003924 GO:0005515
Cluster-22178.60929 GO:0005975 GO:0030246
Cluster-22178.60930 GO:0046486 GO:0001575 GO:0006012 GO:0005975
GO:0004553 GO:0004557
Cluster-22178.60937 GO:0016579 GO:0036459
Cluster-22178.60940 GO:0006364 GO:0019068 GO:0005198 GO:0008168
GO:0005634
Cluster-22178.60944 GO:0006571 GO:0000162 GO:0009094 GO:0006096
GO:0006094 GO:0004634 GO:0000287 GO:0000015
Cluster-22178.60946 GO:0006571 GO:0000162 GO:0000413 GO:0009094
GO:0006096 GO:0006457 GO:0006094 GO:0004634 GO:0003755
GO:0000287 GO:0000015
Cluster-22178.60947 GO:0043087 GO:0007264 GO:0005085
Cluster-22178.60953 GO:0016021
Cluster-22178.60954 GO:0009966 GO:0007275 GO:0016020
Cluster-22178.60955 GO:0008608 GO:0003723 GO:0005876 GO:0042729
Cluster-22178.60956 GO:0010506 GO:0005515 GO:0005615

Cluster-22178.60960 GO:0005515

Cluster-22178.60961 GO:0006468 GO:0006886 GO:0016192 GO:0005524 GO:0004672 GO:0005515 GO:0030117

Cluster-22178.60963 GO:0043087 GO:0006260 GO:0006269 GO:0006351 GO:0007096 GO:0003896 GO:0005515 GO:0005657 GO:0005730 GO:0005634

Cluster-22178.60965 GO:0055114 GO:0016706 GO:0005506 GO:0031418

Cluster-22178.60966 GO:0006355 GO:0043565 GO:0003700 GO:0005667

Cluster-22178.60968 GO:0051694 GO:0005523

Cluster-22178.60969 GO:0055085 GO:0016567 GO:0006810 GO:0046872 GO:0008270 GO:0005524 GO:0042626 GO:0004842 GO:0005515 GO:0016021 GO:0005680

Cluster-22178.60972 GO:0016853

Cluster-22178.60973 GO:0016567 GO:0008270 GO:0046872 GO:0005515 GO:0004842 GO:0005680

Cluster-22178.60975 GO:0006119 GO:0006118 GO:0015992 GO:0055114 GO:0008121 GO:0005515

Cluster-22178.60976 GO:0007275 GO:0042600

Cluster-22178.60977 GO:0007165 GO:0008152 GO:0005179 GO:0003676 GO:0003824 GO:0005576

Cluster-22178.60979 GO:0055085 GO:0046872 GO:0016021

Cluster-22178.60981 GO:0006508 GO:0004198 GO:0005509 GO:0005622

Cluster-22178.60988 GO:0032006 GO:0019031

Cluster-22178.60991 GO:0008616 GO:0006400 GO:0008479

Cluster-22178.60993 GO:0009435 GO:0042742 GO:0046497 GO:0016763 GO:0004514 GO:0019901 GO:0005515

Cluster-22178.60995 GO:0007155 GO:0015992 GO:0006206 GO:0006144 GO:0006888 GO:0006351 GO:0006886 GO:0015991 GO:0003677 GO:0032183 GO:0070140 GO:0008270 GO:0003899 GO:0005515 GO:0015078 GO:0030127 GO:0016020 GO:0005730 GO:0033179

Cluster-22178.60996 GO:0006206 GO:0006144 GO:0006888 GO:0007155 GO:0006508 GO:0006351 GO:0006886 GO:0008270 GO:0003677

GO:0004197 GO:0017111 GO:0003968 GO:0003899 GO:0005515
GO:0005730 GO:0030127 GO:0016020 GO:0031379
Cluster-22178.60997 GO:0007155 GO:0006206 GO:0015992 GO:0006144
GO:0006888 GO:0006351 GO:0006886 GO:0015991 GO:0003677
GO:0008270 GO:0003899 GO:0005515 GO:0015078 GO:0030127
GO:0016020 GO:0005730 GO:0033179
Cluster-22178.60998 GO:0040001 GO:0000226 GO:0042254 GO:0000281
GO:0005874 GO:0005819 GO:0005634
Cluster-22178.61000 GO:0003676
Cluster-22178.61001 GO:0015979 GO:0009507 GO:0009523
Cluster-22178.61011 GO:0006508 GO:0008152 GO:0016042 GO:0046486
GO:0016787 GO:0004806 GO:0008236
Cluster-22178.61014 GO:0005543
Cluster-22178.61015 GO:0006355 GO:0003676 GO:0005622
Cluster-22178.61016 GO:0055114 GO:0050662 GO:0016491 GO:0003824

Cluster-22178.61017 GO:0006412 GO:0042254 GO:0003735 GO:0005622
GO:0005840
Cluster-22178.61023 GO:0055114 GO:0052855 GO:0016491
Cluster-22178.61024 GO:0008033 GO:0006888 GO:0006554 GO:0008152
GO:0006400 GO:0006886 GO:0009451 GO:0006479 GO:0008168
GO:0008270 GO:0008176 GO:0018024 GO:0019955 GO:0016020
GO:0030127
Cluster-22178.61025 GO:0009401 GO:0006355 GO:0006955 GO:0000902
GO:0043565 GO:0003700 GO:0051087 GO:0016021 GO:0005667
GO:0016020
Cluster-22178.61026 GO:0007049 GO:0051301 GO:0005515 GO:0005887
GO:0016021
Cluster-22178.61029 GO:0003676
Cluster-22178.61032 GO:0006355 GO:0005515 GO:0003677 GO:0005634
Cluster-22178.61034 GO:0006730 GO:0008270 GO:0016740
Cluster-22178.61035 GO:0006730 GO:0016740 GO:0008270
Cluster-22178.61037 GO:0016197 GO:0006308 GO:0030474 GO:0006886

GO:0000724 GO:0006914 GO:0019064 GO:0006457 GO:0071988
GO:0006810 GO:0007155 GO:0003676 GO:0042803 GO:0008855
GO:0000774 GO:0051087 GO:0016021 GO:0019031 GO:0009288
GO:0009318 GO:0030119 GO:0005783
Cluster-22178.61038 GO:0006364 GO:0003676
Cluster-22178.61040 GO:0070072
Cluster-22178.61041 GO:0006144 GO:0006206 GO:0016567 GO:0005975
GO:0006687 GO:0006351 GO:0008270 GO:0004842 GO:0003677
GO:0046872 GO:0004308 GO:0005515 GO:0003899 GO:0033644
GO:0055036 GO:0005730 GO:0016020 GO:0005634
Cluster-22178.61044 GO:0009405 GO:0006811 GO:0005216 GO:0005576
Cluster-22178.61045 GO:0006206 GO:0002949 GO:0006139 GO:0005524
GO:0004127
Cluster-22178.61047 GO:0008152 GO:0005542 GO:0016740
Cluster-22178.61048 GO:0003779
Cluster-22178.61055 GO:0016567 GO:0046872 GO:0004842 GO:0005515

Cluster-22178.61056 GO:0007017 GO:0010207 GO:0007018 GO:0005515
GO:0003777 GO:0030286 GO:0005737 GO:0005874
Cluster-22178.61058 GO:0034508 GO:0007059
Cluster-22178.61060 GO:0006576 GO:0007264 GO:0003723 GO:0016301
GO:0098519 GO:0003924 GO:0016887 GO:0005524 GO:0003724
GO:0005525
Cluster-22178.61063 GO:0005515 GO:0005737
Cluster-22178.61064 GO:0043401 GO:0006355 GO:0003700 GO:0008270
GO:0043565 GO:0005634 GO:0005667
Cluster-22178.61066 GO:0006508 GO:0004252 GO:0003676 GO:0016020
Cluster-22178.61067 GO:0006886 GO:0006313 GO:0035307 GO:0006888
GO:0003677 GO:0003676 GO:0008270 GO:0016020 GO:0030127
GO:0071595
Cluster-22178.61068 GO:0006888 GO:0006886 GO:0035307 GO:0006313
GO:0008270 GO:0003676 GO:0003677 GO:0071595 GO:0030127
Cluster-22178.61069 GO:0006778 GO:0042967 GO:0006544 GO:0006783

GO:0006563 GO:0006566 GO:0009058 GO:0016740 GO:0030170
GO:0003870 GO:0005759 GO:0005576
Cluster-22178.61071 GO:0016310 GO:0009069 GO:0009405 GO:0006886
GO:0004674 GO:0008536 GO:0005515 GO:0005576
Cluster-22178.61072 GO:0007264 GO:0006465 GO:0005525 GO:0008233
GO:0005787 GO:0016021
Cluster-22178.61075 GO:0030001 GO:0055085 GO:0046873 GO:0016020
GO:0019867
Cluster-22178.61079 GO:0007264 GO:0003924 GO:0005525 GO:0005634
Cluster-22178.61080 GO:0048870 GO:0007017 GO:0006560 GO:0007018
GO:0006420 GO:0006260 GO:0006525 GO:0004814 GO:0003777
GO:0045502 GO:0000166 GO:0042803 GO:0008134 GO:0005198
GO:0005524 GO:0008017 GO:0005737 GO:0005874 GO:0019031
GO:0045298 GO:0019028 GO:0031514 GO:0030286 GO:0005815
GO:0005667 GO:0005882
Cluster-22178.61089 GO:0006955 GO:0030153 GO:0015643 GO:0019814
Cluster-22178.61095 GO:0005515
Cluster-22178.61097 GO:0000166 GO:0003677 GO:0008408
Cluster-22178.61104 GO:0042254 GO:0006412 GO:0008270 GO:0003735
GO:0003677 GO:0005840 GO:0005622 GO:0005634
Cluster-22178.61105 GO:0042254 GO:0006412 GO:0008270 GO:0003677
GO:0003735 GO:0005840 GO:0005622 GO:0005634
Cluster-22178.61107 GO:0006457
Cluster-22178.61109 GO:0009401 GO:0008643 GO:0005515 GO:0005509
GO:0008982 GO:0005737 GO:0009357
Cluster-22178.61110 GO:0005515 GO:0016020
Cluster-22178.61111 GO:0008152 GO:0016491
Cluster-22178.61112 GO:0007067 GO:0000090 GO:0031110 GO:0051301
GO:0007059 GO:0008017 GO:0000940 GO:0045298 GO:0005876
Cluster-22178.61113 GO:0007067 GO:0000090 GO:0051301 GO:0031110
GO:0007059 GO:0008017 GO:0000940 GO:0005876 GO:0045298
Cluster-22178.61114 GO:0019058 GO:0007165 GO:0043565 GO:0005515
GO:0046789 GO:0019031 GO:0009986 GO:0005634

Cluster-22178.61116 GO:0006144 GO:0007156 GO:0009058 GO:0009113
GO:0005524 GO:0046872 GO:0016742 GO:0005509 GO:0051539
GO:0004637 GO:0016020
Cluster-22178.61117 GO:0006915 GO:0005622
Cluster-22178.61118 GO:0016192 GO:0006904 GO:0008144
Cluster-22178.61119 GO:0000902 GO:0005975 GO:0003676 GO:0016773
GO:0005524 GO:0032040
Cluster-22178.61125 GO:0016567 GO:0006886 GO:0016192 GO:0008270
GO:0046872 GO:0004842 GO:0005515 GO:0000775 GO:0005680
Cluster-22178.61126 GO:0015992 GO:0006123 GO:0003774 GO:0004129
GO:0005198 GO:0045277 GO:0016459 GO:0005739 GO:0005882
GO:0005740 GO:0005747
Cluster-22178.61127 GO:0042546 GO:0001575 GO:0009405 GO:0009247
GO:0005975 GO:0008107 GO:0005576 GO:0016020
Cluster-22178.61129 GO:0015992 GO:0019049 GO:0005975 GO:0055114
GO:0008152 GO:0006120 GO:0006744 GO:0006814 GO:0003824
GO:0030246 GO:0016836 GO:0008137
Cluster-22178.61134 GO:0015992 GO:0006123 GO:0004129 GO:0036128
GO:0005743 GO:0005751 GO:0045277
Cluster-22178.61135 GO:0006364 GO:0005515 GO:0032040
Cluster-22178.61137 GO:0006308 GO:0008855 GO:0005524 GO:0035299
GO:0009318
Cluster-22178.61141 GO:0006357 GO:0001104 GO:0016592
Cluster-22178.61142 GO:0005515
Cluster-22178.61143 GO:0005515
Cluster-22178.61144 GO:0005515
Cluster-22178.61147 GO:0035556 GO:0005515
Cluster-22178.61148 GO:0046034 GO:0035556 GO:0006144 GO:0006281
GO:0046872 GO:0008270 GO:0004017 GO:0003677
Cluster-22178.61149 GO:0055114 GO:0008152 GO:0050832 GO:0006694
GO:0008210 GO:0008207 GO:0008209 GO:0016491 GO:0003824
GO:0003854 GO:0050662 GO:0016616
Cluster-22178.61151 GO:0019722

Cluster-22178.61152 GO:0015631 GO:0045298
Cluster-22178.61153 GO:0055114 GO:0016491
Cluster-22178.61154 GO:0055114 GO:0016491
Cluster-22178.61155 GO:0006468 GO:0045859 GO:0007050 GO:0005524
GO:0004672 GO:0004861 GO:0005515 GO:0005634
Cluster-22178.61156 GO:0016055 GO:0008013 GO:0046872 GO:0016342
Cluster-22178.61157 GO:0042302
Cluster-22178.61158 GO:0006281 GO:0006310 GO:0016887 GO:0005524
GO:0003678 GO:0009378 GO:0016021 GO:0005657 GO:0009379
Cluster-22178.61162 GO:0055114 GO:0008168 GO:0016706 GO:0019028
Cluster-22178.61164 GO:0003676 GO:0003723 GO:0019013
Cluster-22178.61166 GO:0006470 GO:0006570 GO:0004725 GO:0008138

Cluster-22178.61169 GO:0007155 GO:0006144 GO:0005094 GO:0003877
GO:0005737
Cluster-22178.61172 GO:0005515
Cluster-22178.61176 GO:0006260 GO:0006351 GO:0006269 GO:0003896
GO:0005657 GO:0005730
Cluster-22178.61177 GO:0008270
Cluster-22178.61178 GO:0006468 GO:0005524 GO:0004672
Cluster-22178.61184 GO:0006281 GO:0003684
Cluster-22178.61185 GO:0007165 GO:0007186 GO:0031683 GO:0019001
GO:0003924 GO:0005525 GO:0004871
Cluster-22178.61188 GO:0005887
Cluster-22178.61191 GO:0005515
Cluster-22178.61193 GO:0005515 GO:0008270
Cluster-22178.61195 GO:0005515
Cluster-22178.61197 GO:0005515
Cluster-22178.61198 GO:0005515
Cluster-22178.61199 GO:0000390 GO:0071008
Cluster-22178.61200 GO:0006355 GO:0005515 GO:0005634
Cluster-22178.61202 GO:0006281 GO:0006094 GO:0045040 GO:0018149
GO:0005985 GO:0006570 GO:0051156 GO:0006096 GO:0005982

GO:0006289 GO:0032259 GO:0019872 GO:0043161 GO:0043087
GO:0009072 GO:0006012 GO:0042207 GO:0005515 GO:0008168
GO:0004334 GO:0003684 GO:0005524 GO:0004340 GO:0003810
GO:0005089 GO:0005742
Cluster-22178.61203 GO:0006486 GO:0006886 GO:0016192 GO:0005515
GO:0005198 GO:0008378 GO:0016757 GO:0016020 GO:0030117
Cluster-22178.61209 GO:0016192 GO:0006886 GO:0005198 GO:0030130
GO:0030132
Cluster-22178.61211 GO:0006098 GO:0055114 GO:0019521 GO:0008152
GO:0051287 GO:0016616 GO:0004616
Cluster-22178.61212 GO:0006098 GO:0055114 GO:0019521 GO:0008152
GO:0051287 GO:0016616 GO:0004616
Cluster-22178.61213 GO:0006098 GO:0019521 GO:0055114 GO:0008152
GO:0051287 GO:0016616 GO:0004616
Cluster-22178.61214 GO:0006468 GO:0005515 GO:0004672
Cluster-22178.61215 GO:0006508 GO:0070008 GO:0008236 GO:0004252

Cluster-22178.61216 GO:0007264 GO:0016887 GO:0003924 GO:0005524
GO:0005525 GO:0016020
Cluster-22178.61218 GO:0006388 GO:0051252 GO:0006351 GO:0003714
GO:0000213 GO:0005667 GO:0000214
Cluster-22178.61219 GO:0006144 GO:0006206 GO:0015992 GO:0015979
GO:0006123 GO:0050790 GO:0006351 GO:0003677 GO:0004129
GO:0005507 GO:0003899 GO:0030234 GO:0005730 GO:0016020
GO:0009523 GO:0009539 GO:0045277
Cluster-22178.61220 GO:0016052 GO:0005975 GO:0015992 GO:0055114
GO:0015986 GO:0043093 GO:0000917 GO:0016192 GO:0016491
GO:0015078 GO:0004553 GO:0030246 GO:0005737 GO:0000276
Cluster-22178.61221 GO:0019497 GO:0048013 GO:0006468 GO:0006771
GO:0016773 GO:0046872 GO:0005524 GO:0004672 GO:0003993
GO:0005515 GO:0016020
Cluster-22178.61224 GO:0006914 GO:0007165 GO:0004871 GO:0016020
Cluster-22178.61225 GO:0006260 GO:0005515

Cluster-22178.61226 GO:0042254 GO:0006412 GO:0006364 GO:0003735
GO:0005622 GO:0005840
Cluster-22178.61227 GO:0009405 GO:0045735 GO:0005515 GO:0030430
Cluster-22178.61229 GO:0007186 GO:0007275 GO:0007165 GO:0070206
GO:0006260 GO:0005179 GO:0004930 GO:0005576 GO:0016021
Cluster-22178.61230 GO:0006123 GO:0015992 GO:0004129 GO:0045277
GO:0005743
Cluster-22178.61232 GO:0006338 GO:0003677 GO:0016818 GO:0000049
GO:0003676 GO:0005524 GO:0046983 GO:0005634
Cluster-22178.61234 GO:0050708 GO:0005515 GO:0051537 GO:0005737
Cluster-22178.61235 GO:0006139 GO:0008168
Cluster-22178.61240 GO:0006118 GO:0007165 GO:0035556 GO:0004871
GO:0020037 GO:0009055 GO:0008270
Cluster-22178.61241 GO:0006355 GO:0005515 GO:0003677 GO:0008270
GO:0046872
Cluster-22178.61245 GO:0008440
Cluster-22178.61246 GO:0007411 GO:0016055 GO:0008092 GO:0019898
GO:0005576 GO:0005737
Cluster-22178.61247 GO:0006120 GO:0006744 GO:0015992 GO:0006118
GO:0006814 GO:0006629 GO:0016627 GO:0008137 GO:0003954
GO:0016021 GO:0005737 GO:0005739
Cluster-22178.61248 GO:0061024 GO:0046935 GO:0005944 GO:0030176
Cluster-22178.61249 GO:0000917 GO:0043093 GO:0006355 GO:0007049
GO:0006260 GO:0010506 GO:0051016 GO:0003700 GO:0043565
GO:0008290 GO:0005667 GO:0005634 GO:0005615 GO:0005737
Cluster-22178.61250 GO:0043065 GO:0016020 GO:0005737
Cluster-22178.61251 GO:0005975 GO:0006886 GO:0016832 GO:0005741
GO:0016020 GO:0016021
Cluster-22178.61252 GO:0006470 GO:0006570 GO:0008138 GO:0004725

Cluster-22178.61254 GO:0006464 GO:0006898 GO:0007165 GO:0005044
GO:0016020
Cluster-22178.61256 GO:0043093 GO:0006171 GO:0006810 GO:0002949

GO:0000917 GO:0009405 GO:0048870 GO:0016787 GO:0008294
GO:0003677 GO:0005524 GO:0016887 GO:0046983 GO:0003678
GO:0005657 GO:0005576 GO:0031514 GO:0005737
Cluster-22178.61257 GO:0015992 GO:0055114 GO:0055085 GO:0015986
GO:0006119 GO:0046933 GO:0016638 GO:0046961 GO:0005515
GO:0016021 GO:0045259 GO:0045261
Cluster-22178.61258 GO:0006468 GO:0005515 GO:0016773 GO:0004672
GO:0005524 GO:0016020
Cluster-22178.61263 GO:0006355 GO:0009405 GO:0005515 GO:0005634
Cluster-22178.61265 GO:0006119 GO:0015991 GO:0015992 GO:0046961
GO:0033178
Cluster-22178.61268 GO:0006508 GO:0004252
Cluster-22178.61270 GO:0055114 GO:0016491 GO:0071949 GO:0003950
GO:0005634
Cluster-22178.61273 GO:0006446 GO:0006413 GO:0003743 GO:0003723
GO:0005840 GO:0005737
Cluster-22178.61274 GO:0055085 GO:0006468 GO:0022857 GO:0005524
GO:0004672 GO:0016021
Cluster-22178.61275 GO:0006355 GO:0008270 GO:0016820 GO:0005634
GO:0005739 GO:0016021
Cluster-22178.61280 GO:0034453 GO:0000042 GO:0019370 GO:0005515
GO:0008237 GO:0008270 GO:0005815 GO:0016021
Cluster-22178.61283 GO:0005515
Cluster-22178.61284 GO:0055085 GO:0030001 GO:0046873 GO:0016020
Cluster-22178.61285 GO:0055085 GO:0030001 GO:0046873 GO:0016020
Cluster-22178.61288 GO:0005515
Cluster-22178.61289 GO:0005515
Cluster-22178.61293 GO:0005975
Cluster-22178.61294 GO:0006351 GO:0008152 GO:0055114 GO:0006144
GO:0006206 GO:0003899 GO:0003677 GO:0016491 GO:0005730
Cluster-22178.61295 GO:0055085 GO:0006888 GO:0006810 GO:0022857
GO:0005215 GO:0005515 GO:0005801 GO:0019867 GO:0016021
GO:0005622 GO:0016020

Cluster-22178.61298 GO:0030244 GO:0005985 GO:0005982 GO:0006011
GO:0016760 GO:0016020
Cluster-22178.61299 GO:0046656 GO:0055114 GO:0015948 GO:0006511
GO:0031625 GO:0047068 GO:0018537
Cluster-22178.61301 GO:0007617 GO:0006355 GO:0003700 GO:0008270
GO:0005667 GO:0005634 GO:0005576
Cluster-22178.61303 GO:0042254 GO:0006412 GO:0003735 GO:0005622
GO:0005840
Cluster-22178.61309 GO:0016310 GO:0006352 GO:0005975 GO:0009069
GO:0006468 GO:0003824 GO:0005524 GO:0004672 GO:0016773
GO:0005515 GO:0008384 GO:0005669 GO:0016020 GO:0008385
Cluster-22178.61310 GO:0005515
Cluster-22178.61311 GO:0006952 GO:0016787
Cluster-22178.61314 GO:0015886 GO:0055085 GO:0015232 GO:0022857
GO:0016021
Cluster-22178.61315 GO:0008152 GO:0005515 GO:0003824
Cluster-22178.61316 GO:0008152 GO:0005515 GO:0003824
Cluster-22178.61317 GO:0016020
Cluster-22178.61318 GO:0016567 GO:0004842 GO:0016787 GO:0005680
Cluster-22178.61320 GO:0016567 GO:0004842 GO:0016787 GO:0005680
Cluster-22178.61321 GO:0006879 GO:0008199
Cluster-22178.61323 GO:0009116 GO:0006144 GO:0009165 GO:0006098
GO:0004749 GO:0000287
Cluster-22178.61324 GO:0006406 GO:0055085 GO:0006998 GO:0035556
GO:0009584 GO:0006611 GO:0006355 GO:0018298 GO:0006810
GO:0005215 GO:0005515 GO:0016020 GO:0031965
Cluster-22178.61326 GO:0006468 GO:0016560 GO:0005524 GO:0004672
GO:0016021 GO:0005777
Cluster-22178.61327 GO:0006470 GO:0006402 GO:0051252 GO:0006468
GO:0003676 GO:0004535 GO:0046872 GO:0005524 GO:0004672
GO:0008138 GO:0003723 GO:0005737 GO:0005634
Cluster-22178.61329 GO:0003676
Cluster-22178.61330 GO:0017025 GO:0005634

Cluster-22178.61332 GO:0006446 GO:0003743 GO:0005852 GO:0005840
GO:0005737
Cluster-22178.61333 GO:0042981 GO:0006468 GO:0043565 GO:0004672
GO:0005524
Cluster-22178.61335 GO:0007010 GO:0055085 GO:0046872 GO:0003779
GO:0016021
Cluster-22178.61337 GO:0006413 GO:0006270 GO:0006886 GO:0006446
GO:0003723 GO:0004190 GO:0003743 GO:0005741 GO:0005840
GO:0016021
Cluster-22178.61341 GO:0005515
Cluster-22178.61342 GO:0055085 GO:0006811 GO:0000917 GO:0043093
GO:0006355 GO:0005515 GO:0005216 GO:0005737 GO:0016020
Cluster-22178.61343 GO:0005515
Cluster-22178.61345 GO:0009432 GO:0015991 GO:0051258 GO:0051782
GO:0007165 GO:0030168 GO:0051607 GO:0015992 GO:0005515
GO:0015078 GO:0030674 GO:0005096 GO:0005102 GO:0033179
GO:0005737 GO:0009276 GO:0005577
Cluster-22178.61346 GO:0003677
Cluster-22178.61347 GO:0005515
Cluster-22178.61348 GO:0007016 GO:0016010
Cluster-22178.61349 GO:0007016 GO:0016010
Cluster-22178.61350 GO:0003676
Cluster-22178.61351 GO:0055114 GO:0042254 GO:0006412 GO:0006457
GO:0003735 GO:0005515 GO:0005840 GO:0005622
Cluster-22178.61352 GO:0006355 GO:0006950 GO:0009415 GO:0043565
GO:0003700 GO:0005667
Cluster-22178.61353 GO:0006338 GO:0016818 GO:0005515 GO:0003676
GO:0005524 GO:0005634
Cluster-22178.61355 GO:0006486 GO:0008378 GO:0016020
Cluster-22178.61356 GO:0016055 GO:0003676 GO:0008270 GO:0008013
GO:0016342
Cluster-22178.61357 GO:0071918 GO:0006468 GO:0009069 GO:0016310
GO:0015840 GO:0007178 GO:0015204 GO:0004675 GO:0004672

GO:0005524 GO:0016020 GO:0016021
Cluster-22178.61358 GO:0006508 GO:0005515 GO:0004252
Cluster-22178.61360 GO:0006508 GO:0030436 GO:0035307 GO:0004190
GO:0071595
Cluster-22178.61364 GO:0003676
Cluster-22178.61368 GO:0006118 GO:0006119 GO:0016192 GO:0015992
GO:0008121 GO:0016021
Cluster-22178.61370 GO:0046034 GO:0006144 GO:0004017
Cluster-22178.61371 GO:0051252 GO:0006396 GO:0003723 GO:0004525

Cluster-22178.61373 GO:0016757
Cluster-22178.61374 GO:0007165 GO:0006886 GO:0005515 GO:0005783
GO:0016021
Cluster-22178.61375 GO:0007165 GO:0005515
Cluster-22178.61376 GO:0006464 GO:0046872
Cluster-22178.61377 GO:0005524 GO:0003676
Cluster-22178.61378 GO:0006468 GO:0005524 GO:0004672 GO:0005515

Cluster-22178.61380 GO:0007156 GO:0006396 GO:0051252 GO:0003723
GO:0004525 GO:0005509 GO:0016020
Cluster-22178.61382 GO:0006891 GO:0031110 GO:0015031 GO:0030168
GO:0007165 GO:0006869 GO:0042157 GO:0000090 GO:0007067
GO:0007059 GO:0051301 GO:0051258 GO:0006904 GO:0006813
GO:0008017 GO:0005102 GO:0015269 GO:0030674 GO:0005516
GO:0008289 GO:0005876 GO:0005577 GO:0000940 GO:0000145
GO:0017119 GO:0045298 GO:0016021 GO:0005576
Cluster-22178.61384 GO:0006355 GO:0046983
Cluster-22178.61385 GO:0006810 GO:0009405 GO:0019871 GO:0005576
Cluster-22178.61387 GO:0005518 GO:0005581
Cluster-22178.61388 GO:0009405 GO:0006810 GO:0019871 GO:0005576
Cluster-22178.61392 GO:0009235 GO:0006355 GO:0005515 GO:0046872
GO:0005634
Cluster-22178.61395 GO:0006508 GO:0004222

Cluster-22178.61399 GO:0016787
Cluster-22178.61403 GO:0006812 GO:0006885 GO:0055085 GO:0015299
GO:0016021
Cluster-22178.61404 GO:0008643 GO:0055085 GO:0006885 GO:0006812
GO:0009401 GO:0008982 GO:0005198 GO:0015299 GO:0009357
GO:0005737 GO:0016021
Cluster-22178.61406 GO:0042254 GO:0006412 GO:0005515 GO:0003735
GO:0015934 GO:0005840
Cluster-22178.61409 GO:0003676
Cluster-22178.61410 GO:0008270 GO:0046872 GO:0005515 GO:0005622
GO:0031224
Cluster-22178.61411 GO:0005515
Cluster-22178.61412 GO:0006351 GO:0006144 GO:0006206 GO:0004519
GO:0003899 GO:0003677 GO:0005730 GO:0030880
Cluster-22178.61413 GO:0006260 GO:0006352 GO:0051479 GO:0006355
GO:0016987 GO:0003724 GO:0008270 GO:0005524 GO:0016887
GO:0003677 GO:0005525 GO:0003700 GO:0046872 GO:0003924
GO:0050504 GO:0005515 GO:0003723 GO:0005667 GO:0005737
Cluster-22178.61416 GO:0015675 GO:0035444 GO:0030474 GO:0071988
GO:0007165 GO:0046872 GO:0015099 GO:0051082 GO:0005179
GO:0016021 GO:0005887 GO:0005576
Cluster-22178.61418 GO:0003676 GO:0016021
Cluster-22178.61420 GO:0008152 GO:0005515 GO:0016829 GO:0010333

Cluster-22178.61421 GO:0005515
Cluster-22178.61423 GO:0005515 GO:0016020
Cluster-22178.61426 GO:0006355 GO:0003677
Cluster-22178.61429 GO:0006308 GO:0009307 GO:0006281 GO:0009036
GO:0003677 GO:0016020 GO:0009359 GO:0005634
Cluster-22178.61430 GO:0009307 GO:0006281 GO:0006308 GO:0003677
GO:0009036 GO:0016020 GO:0009359 GO:0005634
Cluster-22178.61431 GO:0003333 GO:0051205 GO:0042967 GO:0006865
GO:0015171 GO:0008080 GO:0016020 GO:0019033 GO:0016021

Cluster-22178.61433 GO:0016021
Cluster-22178.61434 GO:0006355 GO:0003700 GO:0005667
Cluster-22178.61435 GO:0006260 GO:0043248 GO:0006270 GO:0006351
GO:0016032 GO:0006355 GO:0008270 GO:0003677 GO:0003682
GO:0003700 GO:0003887 GO:0005783 GO:0005667 GO:0000785
GO:0000775 GO:0005789 GO:0032040 GO:0042575 GO:0005634
Cluster-22178.61436 GO:0006629 GO:0005515 GO:0005509 GO:0008081

Cluster-22178.61438 GO:0018401 GO:0055114 GO:0015992 GO:0016567
GO:0006560 GO:0006525 GO:0015986 GO:0015078 GO:0004842
GO:0046983 GO:0016702 GO:0008270 GO:0004656 GO:0000276
GO:0005783
Cluster-22178.61439 GO:0016020 GO:0016021
Cluster-22178.61440 GO:0005634
Cluster-22178.61442 GO:0055114 GO:0016972
Cluster-22178.61444 GO:0008210 GO:0006568 GO:0006550 GO:0006552
GO:0006574 GO:0055114 GO:0008152 GO:0006694 GO:0018874
GO:0008209 GO:0006631 GO:0008207 GO:0006554 GO:0006633
GO:0003857 GO:0003854 GO:0016616 GO:0016491
Cluster-22178.61447 GO:0006364 GO:0006270 GO:0006355 GO:0003676
GO:0008270 GO:0005515 GO:0005732 GO:0005634 GO:0034457
Cluster-22178.61449 GO:0034220
Cluster-22178.61450 GO:0007165
Cluster-22178.61451 GO:0006886 GO:0008152 GO:0015074 GO:0015979
GO:0055114 GO:0035091 GO:0016491 GO:0016020 GO:0019028
GO:0019031 GO:0009539 GO:0009523
Cluster-22178.61453 GO:0008152 GO:0015979 GO:0006886 GO:0035091
GO:0009523 GO:0009539 GO:0016020 GO:0019028
Cluster-22178.61454 GO:0003676 GO:0008270 GO:0005622
Cluster-22178.61455 GO:0008270 GO:0003676 GO:0015066 GO:0005622
Cluster-22178.61456 GO:0006810 GO:0005622
Cluster-22178.61458 GO:0008152 GO:0006118 GO:0055114 GO:0050660
GO:0003995 GO:0016627

Cluster-22178.61465 GO:0008152 GO:0016491
Cluster-22178.61468 GO:0008152 GO:0016491
Cluster-22178.61470 GO:0055085 GO:0006810 GO:0006820 GO:0005215 GO:0016020 GO:0016021
Cluster-22178.61471 GO:0051537 GO:0043231
Cluster-22178.61474 GO:0007268 GO:0006508 GO:0030695 GO:0033130 GO:0004197 GO:0043495 GO:0042802 GO:0005515
Cluster-22178.61475 GO:0007165 GO:0014068 GO:0016226 GO:0035556 GO:0050776 GO:0043548 GO:0051536 GO:0005102 GO:0016020 GO:0005737
Cluster-22178.61478 GO:0055114 GO:0006725 GO:0006810 GO:0008199 GO:0003824 GO:0016021
Cluster-22178.61481 GO:0005515 GO:0005509 GO:0005576
Cluster-22178.61482 GO:0005509 GO:0005515 GO:0005576
Cluster-22178.61483 GO:0006144 GO:0046034 GO:0006355 GO:0043565 GO:0046872 GO:0003700 GO:0004017 GO:0005667
Cluster-22178.61484 GO:0006364 GO:0008033 GO:0005515 GO:0008168 GO:0003723
Cluster-22178.61485 GO:0006810 GO:0006629 GO:0042626 GO:0016787 GO:0005524 GO:0016021
Cluster-22178.61486 GO:0051301 GO:0007049 GO:0005509 GO:0016021
Cluster-22178.61488 GO:0006270 GO:0006816 GO:0006874 GO:0006383 GO:0006396 GO:0006144 GO:0006206 GO:0006351 GO:0030036 GO:0003677 GO:0003899 GO:0003723 GO:0005219 GO:0003676 GO:0003779 GO:0005730 GO:0005666 GO:0005622 GO:0030054 GO:0016021
Cluster-22178.61489 GO:0006468 GO:0006813 GO:0016032 GO:0006508 GO:0006118 GO:0055114 GO:0007219 GO:0008152 GO:0007275 GO:0006511 GO:0009236 GO:0035556 GO:0003995 GO:0020037 GO:0046872 GO:0016627 GO:0004672 GO:0048472 GO:0051287 GO:0071949 GO:0016616 GO:0008641 GO:0005524 GO:0008270 GO:0016491 GO:0016773 GO:0050660 GO:0008233 GO:0005634 GO:0016021 GO:0016020

Cluster-22178.61493 GO:0035091
Cluster-22178.61495 GO:0006260 GO:0006914 GO:0006370 GO:0006310
GO:0006397 GO:0006281 GO:0003910 GO:0005524 GO:0003677
GO:0004484
Cluster-22178.61498 GO:0055114 GO:0008168 GO:0016706 GO:0019028
Cluster-22178.61499 GO:0035556 GO:0007165 GO:0005515
Cluster-22178.61500 GO:0006810 GO:0019079 GO:0003676 GO:0004386
GO:0008026 GO:0043531 GO:0005524
Cluster-22178.61502 GO:0005515
Cluster-22178.61503 GO:0015031 GO:0006606 GO:0015991 GO:0006886
GO:0016192 GO:0008565 GO:0016820 GO:0005515 GO:0005737
GO:0000221 GO:0005634 GO:0030117
Cluster-22178.61504 GO:0006508 GO:0006810 GO:0005524 GO:0004386
GO:0003676 GO:0016787 GO:0003677 GO:0004197
Cluster-22178.61505 GO:0015684 GO:0007264 GO:0005525 GO:0015093
GO:0005524 GO:0003924 GO:0016021
Cluster-22178.61507 GO:0043039 GO:0016055 GO:0006531 GO:0006522
GO:0006419 GO:0000166 GO:0003676 GO:0016876 GO:0004813
GO:0005524
Cluster-22178.61508 GO:0006413 GO:0006446 GO:0031369 GO:0003743
GO:0005515 GO:0005852 GO:0005840
Cluster-22178.61511 GO:0006886 GO:0042254 GO:0006412 GO:0006888
GO:0003735 GO:0003824 GO:0008270 GO:0030127 GO:0005840
GO:0005622
Cluster-22178.61516 GO:0006508 GO:0005506 GO:0004197
Cluster-22178.61517 GO:0006810 GO:0016567 GO:0008270 GO:0046872
GO:0004842 GO:0005515 GO:0005680 GO:0016020
Cluster-22178.61519 GO:0032065 GO:0042998 GO:0007186 GO:0005543
GO:0004930 GO:0071253 GO:0005515 GO:0005938 GO:0005802
Cluster-22178.61521 GO:0015694 GO:0006367 GO:0015097 GO:0046872
GO:0016020 GO:0005634
Cluster-22178.61525 GO:0003723
Cluster-22178.61526 GO:0006522 GO:0006188 GO:0009152 GO:0006144

GO:0055114 GO:0006520 GO:0009058 GO:0006531 GO:0010277
GO:0004018 GO:0016829 GO:0030170
Cluster-22178.61530 GO:0016567 GO:0006486 GO:0005515 GO:0003677
GO:0016818 GO:0016787 GO:0003676 GO:0016757 GO:0004842
GO:0008270 GO:0005524 GO:0046872 GO:0005680
Cluster-22178.61531 GO:0006950 GO:0009415 GO:0061630
Cluster-22178.61532 GO:0016226 GO:0005515 GO:0051536 GO:0005506

Cluster-22178.61533 GO:0048870 GO:0031514
Cluster-22178.61534 GO:0006357 GO:0006308 GO:0008855 GO:0046983
GO:0001104 GO:0009318 GO:0016592 GO:0016020
Cluster-22178.61535 GO:0005515
Cluster-22178.61536 GO:0000917 GO:0043093 GO:0015876 GO:0008521
GO:0016021 GO:0005737
Cluster-22178.61538 GO:0006118 GO:0007586 GO:0008152 GO:0019684
GO:0016042 GO:0045156 GO:0008047 GO:0016758 GO:0030077
GO:0005576 GO:0016021
Cluster-22178.61539 GO:0009451 GO:0006886 GO:0006479 GO:0006400
GO:0008152 GO:0008033 GO:0006888 GO:0006554 GO:0008176
GO:0019955 GO:0018024 GO:0008168 GO:0008270 GO:0016020
GO:0030127
Cluster-22178.61542 GO:0015886 GO:0017004 GO:0015232 GO:0016020
Cluster-22178.61543 GO:0055085 GO:0019953 GO:0043248 GO:0022857
GO:0005198 GO:0016021 GO:0030141 GO:0005576
Cluster-22178.61545 GO:0006351 GO:0006355 GO:0007049 GO:0006206
GO:0006144 GO:0003677 GO:0003899 GO:0003700 GO:0043565
GO:0005730 GO:0005667 GO:0005634
Cluster-22178.61546 GO:0016301
Cluster-22178.61549 GO:0005515
Cluster-22178.61550 GO:0006811 GO:0055085 GO:0006816 GO:0006874
GO:0005219 GO:0005509 GO:0005515 GO:0005216 GO:0004064
GO:0016021 GO:0005622 GO:0016020
Cluster-22178.61552 GO:0008270

Cluster-22178.61554 GO:0008152 GO:0055114 GO:0003824 GO:0050662
GO:0016491
Cluster-22178.61555 GO:0005515
Cluster-22178.61556 GO:0006206 GO:0006144 GO:0008152 GO:0006351
GO:0006479 GO:0031167 GO:0008168 GO:0003677 GO:0003899
GO:0005730
Cluster-22178.61558 GO:0005634
Cluster-22178.61559 GO:0033617
Cluster-22178.61561 GO:0006744 GO:0055114 GO:0009271 GO:0016567
GO:0006355 GO:0004842 GO:0046872 GO:0008270 GO:0005515
Cluster-22178.61565 GO:0016021
Cluster-22178.61567 GO:0008152 GO:0003824
Cluster-22178.61568 GO:0006457 GO:0005509 GO:0051082 GO:0032040
GO:0005783 GO:0019031
Cluster-22178.61573 GO:0003676
Cluster-22178.61574 GO:0018279 GO:0006486 GO:0004579 GO:0008250
GO:0016021 GO:0005783
Cluster-22178.61575 GO:0016788
Cluster-22178.61580 GO:0006633 GO:0055114 GO:0016491 GO:0005506

Cluster-22178.61581 GO:0016055 GO:0008013 GO:0008270 GO:0003676
GO:0016342
Cluster-22178.61583 GO:0031124 GO:0005515
Cluster-22178.61584 GO:0019048 GO:0006355 GO:0007342 GO:0007340
GO:0003677 GO:0016020 GO:0001669
Cluster-22178.61585 GO:0050909 GO:0007186 GO:0004930 GO:0016021
Cluster-22178.61586 GO:0055114 GO:0015940 GO:0008677 GO:0016491
GO:0071949
Cluster-22178.61587 GO:0004672
Cluster-22178.61588 GO:0006355 GO:0003700 GO:0004190 GO:0016021
GO:0005667
Cluster-22178.61589 GO:0035556 GO:0019087 GO:0055114 GO:0006468
GO:0005524 GO:0016773 GO:0047134 GO:0003724 GO:0003712

GO:0016818 GO:0004672 GO:0005515 GO:0005667 GO:0016020
GO:0016021 GO:0005634
Cluster-22178.61590 GO:0006457 GO:0005515
Cluster-22178.61592 GO:0008643 GO:0008982 GO:0016787 GO:0015016
GO:0008146 GO:0009357
Cluster-22178.61596 GO:0005515
Cluster-22178.61597 GO:0006278 GO:0004672 GO:0003964 GO:0005515

Cluster-22178.61598 GO:0006355 GO:0006281 GO:0006525 GO:0006310
GO:0003677 GO:0000166 GO:0009015 GO:0008168 GO:0046872
GO:0008270 GO:0005634
Cluster-22178.61600 GO:0018342 GO:0008318
Cluster-22178.61602 GO:0009069 GO:0006468 GO:0006915 GO:0016310
GO:0006919 GO:0004674 GO:0005524 GO:0004672 GO:0016020
GO:0005739
Cluster-22178.61604 GO:0006378 GO:0006313 GO:0003729 GO:0016787
GO:0004803 GO:0003677 GO:0005849
Cluster-22178.61608 GO:0035091
Cluster-22178.61609 GO:0042254 GO:0006412 GO:0003735 GO:0005622
GO:0005840
Cluster-22178.61611 GO:0005515
Cluster-22178.61613 GO:0051205 GO:0055114 GO:0015948 GO:0016021
GO:0016020
Cluster-22178.61614 GO:0009116
Cluster-22178.61615 GO:0007165 GO:0005515 GO:0005096
Cluster-22178.61616 GO:0015979 GO:2001070 GO:0030145 GO:0005515
GO:0008146 GO:0009523 GO:0016021
Cluster-22178.61617 GO:0051260 GO:0005515
Cluster-22178.61618 GO:0006355 GO:0006468 GO:0007165 GO:0030521
GO:0003677 GO:0005524 GO:0004672 GO:0016773 GO:0004882
GO:0005496 GO:0005634 GO:0016020
Cluster-22178.61619 GO:0016973 GO:0006355 GO:0048870 GO:0003700
GO:0031514 GO:0005667 GO:0016021 GO:0005643

Cluster-22178.61621 GO:0042254 GO:0003735 GO:0005840

Cluster-22178.61622 GO:0051252 GO:0006388 GO:0000213 GO:0000214

Cluster-22178.61626 GO:0005544 GO:0005509

Cluster-22178.61627 GO:0006952 GO:0006313 GO:0006412 GO:0006310

GO:0006471 GO:0015074 GO:0003677 GO:0005515 GO:0003950

GO:0000150 GO:0008168

Cluster-22178.61630 GO:0005576

Cluster-22178.61631 GO:0016567 GO:0004842 GO:0005515

Cluster-22178.61632 GO:0007165 GO:0007186 GO:0006886 GO:0005525

GO:0004871 GO:0003924 GO:0005047 GO:0005834 GO:0005785

GO:0005786

Cluster-22178.61634 GO:0006508 GO:0004222 GO:0005515

Cluster-22178.61638 GO:0030474 GO:0019069 GO:0005200 GO:0005515

GO:0005823 GO:0005856

Cluster-22178.61641 GO:0016021

Cluster-22178.61643 GO:0016567 GO:0004842 GO:0061630 GO:0005515

GO:0046872 GO:0008270 GO:0005680

Cluster-22178.61644 GO:0007165 GO:0005179 GO:0005576

Cluster-22178.61646 GO:0005975 GO:0006013 GO:0008270 GO:0046872

GO:0034639 GO:0004553 GO:0004559 GO:0015923 GO:0016021

Cluster-22178.61647 GO:0003676 GO:0004386 GO:0003677 GO:0003723

GO:0016787 GO:0005524 GO:0005634

Cluster-22178.61650 GO:0071555 GO:0030288

Cluster-22178.61652 GO:0000160 GO:0016310 GO:0046983 GO:0000155

GO:0016021 GO:0009365

Cluster-22178.61653 GO:0007186 GO:0000271 GO:0004930 GO:0005515

GO:0016021 GO:0016020

Cluster-22178.61654 GO:0000271 GO:0007186 GO:0005515 GO:0004930

GO:0016020 GO:0016021

Cluster-22178.61656 GO:0006576 GO:0006355 GO:0030001 GO:0006281

GO:0006352 GO:0006139 GO:0006310 GO:0006206 GO:0055085

GO:0002949 GO:0003723 GO:0005515 GO:0008134 GO:0000166

GO:0003678 GO:0046873 GO:0009378 GO:0098519 GO:0003677

GO:0016887 GO:0005524 GO:0004127 GO:0003724 GO:0051537
GO:0005669 GO:0005667 GO:0009379 GO:0043231 GO:0016020
GO:0005657
Cluster-22178.61657 GO:0005515
Cluster-22178.61659 GO:0003676
Cluster-22178.61661 GO:0055114 GO:0006749 GO:0006804 GO:0006979
GO:0016209 GO:0004602 GO:0016491
Cluster-22178.61662 GO:0003723
Cluster-22178.61670 GO:0009405 GO:0006810 GO:0046486 GO:0006071
GO:0004371 GO:0008200 GO:0042151
Cluster-22178.61672 GO:0003676 GO:0016021
Cluster-22178.61673 GO:0008033 GO:0006400 GO:0006886 GO:0016192
GO:0009451 GO:0007165 GO:0046872 GO:0008176 GO:0005515
GO:0016301 GO:0016021 GO:0030117
Cluster-22178.61677 GO:0005515
Cluster-22178.61678 GO:0007165 GO:0005179 GO:0005576
Cluster-22178.61679 GO:0042476 GO:0046740 GO:0006355 GO:0042475
GO:0030345 GO:0046914 GO:0003700 GO:0008201 GO:0003677
GO:0016021 GO:0005667 GO:0005578
Cluster-22178.61682 GO:0007186 GO:0004930 GO:0016021
Cluster-22178.61683 GO:0006270 GO:0005198 GO:0019031 GO:0019028
Cluster-22178.61686 GO:0006457 GO:0008013 GO:0051082 GO:0016342
GO:0016272
Cluster-22178.61687 GO:0006457 GO:0051082 GO:0008013 GO:0016342
GO:0016272
Cluster-22178.61689 GO:0016021
Cluster-22178.61691 GO:0005515
Cluster-22178.61693 GO:0048193 GO:0070273
Cluster-22178.61695 GO:0055085 GO:0005741
Cluster-22178.61696 GO:0042254 GO:0006412 GO:0006506 GO:0016746
GO:0003735 GO:0016021 GO:0005840 GO:0005789 GO:0005739
Cluster-22178.61698 GO:0000281 GO:0000226 GO:0040001 GO:0005819
GO:0005874

Cluster-22178.61701 GO:0006508 GO:0008234
Cluster-22178.61702 GO:0055114 GO:0040007 GO:0006259 GO:0008283 GO:0007165 GO:0016627 GO:0005524 GO:0003824 GO:0047134 GO:0008083 GO:0003677 GO:0005694
Cluster-22178.61705 GO:0006541 GO:0006221 GO:0006206 GO:0003883 GO:0016787
Cluster-22178.61707 GO:0043039 GO:0016876 GO:0005524 GO:0003676 GO:0016021
Cluster-22178.61708 GO:0006123 GO:0006812 GO:0006820 GO:0055114 GO:0015992 GO:0006835 GO:0017153 GO:0016638 GO:0004129 GO:0016020 GO:0045277
Cluster-22178.61709 GO:0006364
Cluster-22178.61714 GO:0000398 GO:0019439 GO:0016833 GO:0019031 GO:0005681
Cluster-22178.61716 GO:0048870 GO:0006308 GO:0007130 GO:0006886 GO:0070193 GO:0030001 GO:0007128 GO:0055085 GO:0031412 GO:0000921 GO:0006457 GO:0035299 GO:0042803 GO:0005524 GO:0008855 GO:0000774 GO:0051087 GO:0046873 GO:0016021 GO:0005801 GO:0000795 GO:0009318 GO:0031514 GO:0005940 GO:0016020 GO:0031411
Cluster-22178.61717 GO:0050912 GO:0007187 GO:0007607 GO:0008527 GO:0016020 GO:0016021
Cluster-22178.61720 GO:0055114 GO:0015946 GO:0004022
Cluster-22178.61721 GO:0009405 GO:0006629 GO:0006508 GO:0008236 GO:0005515 GO:0005198
Cluster-22178.61722 GO:0006241 GO:0006183 GO:0006144 GO:0006165 GO:0006228 GO:0006206 GO:0004550
Cluster-22178.61726 GO:0006355 GO:0006614 GO:0006810 GO:0019079 GO:0016787 GO:0003677 GO:0008026 GO:0005524 GO:0016887 GO:0005525 GO:0003676 GO:0004386
Cluster-22178.61727 GO:0005515
Cluster-22178.61729 GO:0005515
Cluster-22178.61730 GO:0003676

Cluster-22178.61731 GO:0003676
Cluster-22178.61733 GO:0006355 GO:0005634
Cluster-22178.61734 GO:0006885 GO:0006812 GO:0055085 GO:0009401
GO:0008643 GO:0005198 GO:0008982 GO:0015299 GO:0009357
GO:0016021 GO:0005737
Cluster-22178.61735 GO:0016567 GO:0046983 GO:0004842
Cluster-22178.61737 GO:0006464 GO:0016151 GO:0031072 GO:0051082

Cluster-22178.61738 GO:0000162 GO:0006571 GO:0055114 GO:0009094
GO:0004665 GO:0005515 GO:0008977 GO:0005509 GO:0019028
Cluster-22178.61740 GO:0051258 GO:0016310 GO:0006468 GO:0007094
GO:0006397 GO:0030168 GO:0048193 GO:0009069 GO:0006457
GO:0007165 GO:0008092 GO:0051082 GO:0005515 GO:0005524
GO:0005102 GO:0004674 GO:0030674 GO:0005737 GO:0000445
GO:0016272 GO:0005577 GO:0019898 GO:0016020
Cluster-22178.61741 GO:0007165 GO:0035556
Cluster-22178.61744 GO:0006206 GO:0006013 GO:0045893 GO:0009451
GO:0001510 GO:0009452 GO:0006230 GO:0005975 GO:0008033
GO:0003677 GO:0015923 GO:0004553 GO:0008270 GO:0005524
GO:0003723 GO:0004559 GO:0004809 GO:0004797 GO:0046872
GO:0008168 GO:0005634
Cluster-22178.61747 GO:0006468 GO:0009069 GO:0016310 GO:0004683
GO:0005516 GO:0005524 GO:0004672 GO:0016773 GO:0016020
Cluster-22178.61749 GO:0016568 GO:0042967 GO:0004402 GO:0008080
GO:0000123
Cluster-22178.61751 GO:0006950 GO:0001671 GO:0051087
Cluster-22178.61752 GO:0008270 GO:0030246 GO:0005515
Cluster-22178.61753 GO:0030246 GO:0008270 GO:0005515
Cluster-22178.61755 GO:0009069 GO:0016310 GO:0004674 GO:0005515

Cluster-22178.61756 GO:0007178 GO:0016310 GO:0015840 GO:0009069
GO:0006468 GO:0071918 GO:0004672 GO:0005524 GO:0015204
GO:0004675 GO:0016021 GO:0016020

Cluster-22178.61757 GO:0006508 GO:0005515 GO:0004252

Cluster-22178.61758 GO:0005515 GO:0046872 GO:0045202 GO:0016021

Cluster-22178.61763 GO:0006629

Cluster-22178.61764 GO:0006952 GO:0006810 GO:0009405 GO:0019855 GO:0005576 GO:0019012

Cluster-22178.61765 GO:0035556 GO:0016567 GO:0004842 GO:0005515 GO:0046872 GO:0008270 GO:0005680

Cluster-22178.61768 GO:0046656 GO:0015948 GO:0030269 GO:0016020

Cluster-22178.61770 GO:0006777 GO:0006810 GO:0008152 GO:0005524 GO:0016301 GO:0005525

Cluster-22178.61771 GO:0008270

Cluster-22178.61772 GO:0055114 GO:0009395 GO:0006260 GO:0016042 GO:0051536 GO:0004623 GO:0005509 GO:0005576

Cluster-22178.61773 GO:0005509 GO:0005515

Cluster-22178.61774 GO:0032324 GO:0009245 GO:0007165 GO:0008270 GO:0008915 GO:0005179 GO:0005515 GO:0005737 GO:0005615 GO:0016021

Cluster-22178.61776 GO:0046938 GO:0042754 GO:0010038 GO:0045892 GO:0005515 GO:0016756 GO:0046872

Cluster-22178.61777 GO:0016567 GO:0006414 GO:0006448 GO:0051302 GO:0005515 GO:0008270 GO:0046872 GO:0003746 GO:0004842 GO:0005853 GO:0005622 GO:0005840

Cluster-22178.61779 GO:0008033 GO:0006396 GO:0009452 GO:0008152 GO:0001510 GO:0006629 GO:0006400 GO:0031167 GO:0009451 GO:0008168 GO:0008176 GO:0008173 GO:0005515

Cluster-22178.61780 GO:0007165 GO:0005515

Cluster-22178.61781 GO:0006367 GO:0006270 GO:0005672

Cluster-22178.61783 GO:0030430

Cluster-22178.61787 GO:0016021

Cluster-22178.61789 GO:0008033 GO:0032259 GO:0006364 GO:0008168 GO:0003723

Cluster-22178.61790 GO:0005515

Cluster-22178.61791 GO:0046872 GO:0008270 GO:0005515

Cluster-22178.61793 GO:0006650 GO:0047396
Cluster-22178.61795 GO:0006281 GO:0004518 GO:0005515
Cluster-22178.61798 GO:0043395 GO:0005515 GO:0005578 GO:0016020
Cluster-22178.61799 GO:0006298 GO:0005524 GO:0030983
Cluster-22178.61802 GO:0042254 GO:0006412 GO:0015979 GO:0005515
GO:0003735 GO:0003677 GO:0016020 GO:0005840 GO:0009523
GO:0009539 GO:0005622
Cluster-22178.61803 GO:0006777
Cluster-22178.61805 GO:0005515 GO:0016788
Cluster-22178.61807 GO:0005515
Cluster-22178.61809 GO:0006278 GO:0005515 GO:0004672 GO:0003964

Cluster-22178.61810 GO:0055114 GO:0016491 GO:0050661 GO:0016020
Cluster-22178.61812 GO:0008270
Cluster-22178.61813 GO:0006468 GO:0004672 GO:0005524
Cluster-22178.61814 GO:0007029 GO:0022900 GO:0005515 GO:0016651
GO:0005789
Cluster-22178.61815 GO:0008270
Cluster-22178.61816 GO:0015991 GO:0015992 GO:0006119 GO:0046961
GO:0035091 GO:0033178
Cluster-22178.61817 GO:0008270 GO:0005515
Cluster-22178.61819 GO:0006355 GO:0009755 GO:0043401 GO:0019281
GO:0006555 GO:0042967 GO:0008899 GO:0005576 GO:0005737
Cluster-22178.61820 GO:0000062
Cluster-22178.61823 GO:0006468 GO:0005524 GO:0004672
Cluster-22178.61825 GO:0006749 GO:0007264 GO:0015684 GO:0006804
GO:0055114 GO:0006979 GO:0015093 GO:0005524 GO:0003924
GO:0004602 GO:0005525 GO:0016021
Cluster-22178.61826 GO:0043087 GO:0005515 GO:0016020
Cluster-22178.61828 GO:0008152 GO:0009452 GO:0008033 GO:0006306
GO:0006479 GO:0009451 GO:0031167 GO:0001510 GO:0006396
GO:0042549 GO:0015979 GO:0006464 GO:0006400 GO:0046500
GO:0003676 GO:0004719 GO:0008168 GO:0008173 GO:0005515

GO:0003677 GO:0008170 GO:0008176 GO:0009539 GO:0009523
Cluster-22178.61829 GO:0009452 GO:0008152 GO:0008033 GO:0006479
GO:0006306 GO:0009451 GO:0031167 GO:0001510 GO:0006396
GO:0006464 GO:0042549 GO:0015979 GO:0046500 GO:0006400
GO:0003676 GO:0004719 GO:0008168 GO:0005515 GO:0008173
GO:0003677 GO:0008170 GO:0008176 GO:0009539 GO:0009523
Cluster-22178.61831 GO:0035556 GO:0006508 GO:0005515 GO:0004252
GO:0004672
Cluster-22178.61832 GO:0005515 GO:0003677
Cluster-22178.61833 GO:0015986 GO:0015992 GO:0015078 GO:0000276
Cluster-22178.61836 GO:0006281 GO:0003684
Cluster-22178.61838 GO:0005515
Cluster-22178.61839 GO:0046907 GO:0006635 GO:0006260 GO:0055114
GO:0006637 GO:0008408 GO:0003887 GO:0003677 GO:0003997
GO:0042575 GO:0005634 GO:0005664 GO:0005777
Cluster-22178.61842 GO:0006810 GO:0042626 GO:0005515 GO:0005524
GO:0016021
Cluster-22178.61845 GO:0005515
Cluster-22178.61847 GO:0008219
Cluster-22178.61848 GO:0006355 GO:0008270 GO:0005096 GO:0005544
GO:0005509 GO:0005515 GO:0008023
Cluster-22178.61852 GO:0007264 GO:0016817 GO:0003924 GO:0046872
GO:0005515 GO:0005525
Cluster-22178.61855 GO:0044564 GO:0006281 GO:0000723 GO:0003678
GO:0005657
Cluster-22178.61856 GO:0006449 GO:0006415 GO:0003677 GO:0005515
GO:0008270 GO:0016149 GO:0003747 GO:0042025 GO:0018444
GO:0005840 GO:0005737
Cluster-22178.61857 GO:0006629 GO:0006468 GO:0004672 GO:0005524
GO:0016787
Cluster-22178.61859 GO:0007165 GO:0008601 GO:0000159
Cluster-22178.61860 GO:0019058 GO:0007165 GO:0007283 GO:0008601
GO:0000159

Cluster-22178.61862 GO:0006508 GO:0007031 GO:0004252 GO:0005509
GO:0005779
Cluster-22178.61863 GO:0006144 GO:0006206 GO:0006351 GO:0003899
GO:0005515 GO:0003677 GO:0005737 GO:0005730
Cluster-22178.61865 GO:0005975 GO:0000902 GO:0016787 GO:0016773

Cluster-22178.61870 GO:0015074 GO:0007224
Cluster-22178.61872 GO:0030833 GO:0034314 GO:0005856 GO:0005885
Cluster-22178.61873 GO:0016746
Cluster-22178.61874 GO:0005506 GO:0016791
Cluster-22178.61876 GO:0004190 GO:0016021
Cluster-22178.61877 GO:0035556 GO:0003723 GO:0005515 GO:0000166
GO:0003677 GO:0005524 GO:0046872 GO:0003724 GO:0005525
Cluster-22178.61879 GO:0005515
Cluster-22178.61882 GO:0005869
Cluster-22178.61885 GO:0006284 GO:0015833 GO:0003677 GO:0000166
GO:0003905 GO:0005524 GO:0016020
Cluster-22178.61886 GO:0016021 GO:0016020
Cluster-22178.61887 GO:0006626 GO:0007264 GO:0015684 GO:0016829
GO:0015093 GO:0003924 GO:0005525 GO:0030976 GO:0000287
GO:0016021 GO:0005741
Cluster-22178.61888 GO:0003677 GO:0005634
Cluster-22178.61892 GO:0015947 GO:0006568 GO:0055114 GO:0006979
GO:0006804 GO:0004096 GO:0020037
Cluster-22178.61893 GO:0006367 GO:0005672
Cluster-22178.61894 GO:0003697 GO:0046872
Cluster-22178.61895 GO:0046983
Cluster-22178.61896 GO:0042981 GO:0007165 GO:0005515
Cluster-22178.61897 GO:0007165 GO:0015684 GO:0007186 GO:0007264
GO:0005525 GO:0004871 GO:0031683 GO:0019001 GO:0015093
GO:0003924 GO:0016021
Cluster-22178.61898 GO:0006811 GO:0055085 GO:0045735 GO:0005216
GO:0016020

Cluster-22178.61901 GO:0006457 GO:0051082 GO:0016272
Cluster-22178.61902 GO:0005524
Cluster-22178.61905 GO:0006631 GO:0055114 GO:0008033 GO:0016117
GO:0071949 GO:0051287 GO:0016705 GO:0005515 GO:0016491
GO:0050660 GO:0050151
Cluster-22178.61906 GO:0016032 GO:0005509 GO:0005515
Cluster-22178.61907 GO:0006355 GO:0043565 GO:0003676 GO:0005515
GO:0005634
Cluster-22178.61910 GO:0030261 GO:0009058 GO:0006614 GO:0007059
GO:0006777 GO:0000723 GO:0006281 GO:0005524 GO:0003677
GO:0016787 GO:0003678 GO:0003824 GO:0005525 GO:0005657
GO:0019031 GO:0009295
Cluster-22178.61911 GO:0046034 GO:0006144 GO:0008033 GO:0006464
GO:0051252 GO:0016151 GO:0046872 GO:0004017 GO:0004540
GO:0016787
Cluster-22178.61912 GO:0005509
Cluster-22178.61916 GO:0055085 GO:0006811 GO:0005525 GO:0005216
GO:0016020
Cluster-22178.61918 GO:0006554 GO:0016117 GO:0006633 GO:0006779
GO:0006631 GO:0019354 GO:0018874 GO:0008033 GO:0055114
GO:0006574 GO:0015940 GO:0006568 GO:0006813 GO:0006552
GO:0006550 GO:0016491 GO:0050660 GO:0050151 GO:0071949
GO:0051287 GO:0016616 GO:0008677 GO:0003857 GO:0016705
GO:0043115
Cluster-22178.61919 GO:0051603 GO:0004298 GO:0005839
Cluster-22178.61923 GO:0006281 GO:0006810 GO:0006310 GO:0010506
GO:0000400 GO:0000287 GO:0000166 GO:0016788 GO:0003677
GO:0005524 GO:0016887 GO:0005615
Cluster-22178.61925 GO:0006629 GO:0042254 GO:0006412 GO:0006007
GO:0046537 GO:0005515 GO:0003735 GO:0003824 GO:0005622
GO:0005840
Cluster-22178.61926 GO:0035556 GO:0007165
Cluster-22178.61928 GO:0006118 GO:0006814 GO:0006120 GO:0006744

GO:0015992 GO:0008137 GO:0003954 GO:0005739
Cluster-22178.61932 GO:0005789
Cluster-22178.61936 GO:0006629 GO:0005515
Cluster-22178.61940 GO:0046500 GO:0006400 GO:0006367 GO:0006464
GO:0006260 GO:0006355 GO:0006351 GO:0006479 GO:0031167
GO:0009451 GO:0008033 GO:0008152 GO:0003682 GO:0008176
GO:0003677 GO:0008270 GO:0008171 GO:0003887 GO:0004719
GO:0008168 GO:0005634 GO:0005672 GO:0042575 GO:0032040
GO:0000775 GO:0000785
Cluster-22178.61944 GO:0006811 GO:0006118 GO:0055085 GO:0015035
GO:0015075 GO:0016020
Cluster-22178.61946 GO:0009058 GO:0055114 GO:0003824 GO:0016209
GO:0051920 GO:0016491
Cluster-22178.61947 GO:0055114 GO:0009058 GO:0016209 GO:0016491
GO:0051920 GO:0003824
Cluster-22178.61948 GO:0006810 GO:0055085 GO:0006511 GO:0005215
GO:0031625 GO:0016020
Cluster-22178.61949 GO:0006560 GO:0007165 GO:0071436 GO:0006814
GO:0006525 GO:0006090 GO:0015979 GO:0050821 GO:0042301
GO:0008948 GO:0005515 GO:0015081 GO:0016020 GO:0009523
Cluster-22178.61950 GO:0006355 GO:0003700 GO:0043565 GO:0008270
GO:0005667
Cluster-22178.61952 GO:0006425 GO:0055085 GO:0004819 GO:0000166
GO:0005524 GO:0022857 GO:0005737 GO:0016021
Cluster-22178.61957 GO:0005515
Cluster-22178.61959 GO:0003676 GO:0005515 GO:0046872 GO:0008270

Cluster-22178.61961 GO:0046872
Cluster-22178.61967 GO:0006144 GO:0006614 GO:0006508 GO:0005525
GO:0003968 GO:0005524 GO:0004197 GO:0003677 GO:0017111
GO:0016787 GO:0031379
Cluster-22178.61971 GO:0006605 GO:0006457 GO:0006886 GO:0000413
GO:0003755 GO:0005515 GO:0005742

Cluster-22178.61974 GO:0006364 GO:0032259 GO:0008033 GO:0008168
GO:0003723

Cluster-22178.61975 GO:0006468 GO:0006816 GO:0006874 GO:0006397
GO:0051252 GO:0005524 GO:0004672 GO:0046872 GO:0005219
GO:0004540 GO:0005622 GO:0016021

Cluster-22178.61977 GO:0001558 GO:0005520 GO:0005515 GO:0005576
GO:0016942

Cluster-22178.61978 GO:0030436

Cluster-22178.61979 GO:0001558 GO:0005515 GO:0005520 GO:0016942
GO:0005576

Cluster-22178.61984 GO:0007156 GO:0005509 GO:0016020

Cluster-22178.61985 GO:0015991 GO:0006265 GO:0016567 GO:0015992
GO:0005515 GO:0003723 GO:0015078 GO:0046872 GO:0003677
GO:0061630 GO:0004842 GO:0005524 GO:0008270 GO:0003918
GO:0033179 GO:0019028 GO:0005680

Cluster-22178.61986 GO:0003677

Cluster-22178.61991 GO:0006357 GO:0001104 GO:0016592

Cluster-22178.61992 GO:0006508 GO:0008236

Cluster-22178.61994 GO:0006355 GO:0003677 GO:0046983

Cluster-22178.61997 GO:0006810 GO:0009405 GO:0006355 GO:0005515
GO:0019871 GO:0000166 GO:0043565 GO:0005576

Cluster-22178.61998 GO:0005515

Cluster-22178.61999 GO:0015979 GO:0016020 GO:0016021 GO:0009523

Cluster-22178.62000 GO:0007165 GO:0055114 GO:0051258 GO:0030168
GO:0006779 GO:0030674 GO:0005515 GO:0005102 GO:0005737
GO:0005577

Cluster-22178.62001 GO:0016192 GO:0006904 GO:0043395 GO:0005578
GO:0016020

Cluster-22178.62002 GO:0006047 GO:0005975 GO:0016773 GO:0005524

Cluster-22178.62003 GO:0006265 GO:0003677 GO:0003918 GO:0005634
GO:0005694

Cluster-22178.62004 GO:0006629 GO:0005515

Cluster-22178.62006 GO:0008152 GO:0006118 GO:0055114 GO:0003995 GO:0050660 GO:0016627
Cluster-22178.62007 GO:0010508 GO:0030001 GO:0006323 GO:0055085 GO:0006914 GO:0048870 GO:0005198 GO:0005516 GO:0051082 GO:0046873 GO:0031514 GO:0016020 GO:0019028 GO:0019031 GO:0016021
Cluster-22178.62009 GO:0008152 GO:0016747 GO:0016746
Cluster-22178.62010 GO:0008152 GO:0016747 GO:0016746
Cluster-22178.62011 GO:0007155 GO:0042398 GO:0006750 GO:0006749 GO:0004357 GO:0017109 GO:0016021
Cluster-22178.62013 GO:0006750 GO:0006749 GO:0007155 GO:0042398 GO:0004357 GO:0017109 GO:0016021
Cluster-22178.62014 GO:0009432 GO:0051782 GO:0009276
Cluster-22178.62015 GO:0005509
Cluster-22178.62018 GO:0005515 GO:0008270
Cluster-22178.62022 GO:0007631 GO:0007218 GO:0016817 GO:0046872

Cluster-22178.62025 GO:0007264 GO:0006576 GO:0007165 GO:0015684 GO:0019064 GO:0046789 GO:0005524 GO:0015093 GO:0003924 GO:0005525 GO:0042765 GO:0009986 GO:0019031 GO:0016021
Cluster-22178.62026 GO:0016020
Cluster-22178.62028 GO:0000723 GO:0006879 GO:0006508 GO:0016032 GO:0019082 GO:0008239 GO:0070009 GO:0004252 GO:0005515 GO:0003677 GO:0000784 GO:0005576
Cluster-22178.6203 GO:0009069 GO:0006468 GO:0016310 GO:0004672 GO:0005524 GO:0004674
Cluster-22178.62032 GO:0016021
Cluster-22178.62033 GO:0016021
Cluster-22178.62034 GO:0005515
Cluster-22178.62036 GO:0042254 GO:0008615 GO:0055114 GO:0016638 GO:0010181 GO:0004733 GO:0016021 GO:0005622
Cluster-22178.62037 GO:0005515
Cluster-22178.62039 GO:0006355 GO:0042967 GO:0004402 GO:0005515

GO:0008080 GO:0005634 GO:0000123
Cluster-22178.62042 GO:0016579 GO:0009228 GO:0004789 GO:0036459

Cluster-22178.62044 GO:0007165 GO:0019079 GO:0006260 GO:0005179
GO:0030337 GO:0005576 GO:0042575
Cluster-22178.62046 GO:0019062 GO:0006120 GO:0006355 GO:0006814
GO:0030521 GO:0015992 GO:0006744 GO:0042773 GO:0007165
GO:0008137 GO:0004882 GO:0005496 GO:0003677 GO:0005743
GO:0005634 GO:0019028
Cluster-22178.62047 GO:0008063 GO:0006355 GO:0043565 GO:0008270
GO:0003700 GO:0005515 GO:0005667
Cluster-22178.62048 GO:0016055 GO:0008013 GO:0016020 GO:0016342
Cluster-22178.62049 GO:0016020
Cluster-22178.62051 GO:0015031 GO:0006464 GO:0006355 GO:0043565
GO:0003700 GO:0003676 GO:0005515 GO:0005667
Cluster-22178.62052 GO:0005515
Cluster-22178.62053 GO:0042254 GO:0006412 GO:0006629 GO:0006007
GO:0005515 GO:0003735 GO:0046537 GO:0003824 GO:0005840
GO:0005622
Cluster-22178.62054 GO:0006007 GO:0042254 GO:0006412 GO:0006629
GO:0003824 GO:0003735 GO:0005515 GO:0046537 GO:0005840
GO:0005622
Cluster-22178.62055 GO:0006812 GO:0032801 GO:0019076 GO:0016567
GO:0004842 GO:0046872 GO:0008270 GO:0005515 GO:0005261
GO:0005680 GO:0033644
Cluster-22178.62060 GO:0043087 GO:0015031 GO:0007186 GO:0019058
GO:0007165 GO:0006364 GO:0007264 GO:0008277 GO:0006886
GO:0007606 GO:0004930 GO:0043130 GO:0005096 GO:0008565
GO:0030674 GO:0008092 GO:0005515 GO:0042802 GO:0004888
GO:0016020 GO:0016021 GO:0019031
Cluster-22178.62061 GO:0003676
Cluster-22178.62062 GO:0000290 GO:0043085 GO:0008047
Cluster-22178.62063 GO:0003779

Cluster-22178.62064 GO:0046872
Cluster-22178.62065 GO:0005634 GO:0005737
Cluster-22178.62068 GO:0006260 GO:0006355 GO:0006351 GO:0008270
GO:0003682 GO:0003677 GO:0003887 GO:0000785 GO:0000775
GO:0005634 GO:0042575
Cluster-22178.62072 GO:0015684 GO:0007264 GO:0005525 GO:0015093
GO:0003924 GO:0016021
Cluster-22178.62073 GO:0005525
Cluster-22178.62076 GO:0016021
Cluster-22178.62078 GO:0003677 GO:0005515
Cluster-22178.62079 GO:0009405 GO:0003723 GO:0008270 GO:0015485
GO:0003676 GO:0005515
Cluster-22178.62080 GO:0007165 GO:0035556
Cluster-22178.62085 GO:0003676 GO:0005515
Cluster-22178.62086 GO:0006886 GO:0055114 GO:0016740 GO:0005047
GO:0016301 GO:0005515 GO:0005524 GO:0051536 GO:0003924
GO:0016491 GO:0005525 GO:0016625 GO:0005786 GO:0005785
Cluster-22178.62088 GO:0005975 GO:0008270 GO:0008184 GO:0003676

Cluster-22178.62090 GO:0090263 GO:0046856 GO:0016567 GO:0034597

Cluster-22178.62091 GO:0042742
Cluster-22178.62094 GO:0042981
Cluster-22178.62099 GO:0033151 GO:0008033 GO:0055114 GO:0017150
GO:0050660 GO:0043565 GO:0046872
Cluster-22178.62100 GO:0006468 GO:0016773 GO:0005524 GO:0004672
GO:0016020
Cluster-22178.62101 GO:0043085 GO:0000290 GO:0008047
Cluster-22178.62102 GO:0006468 GO:0016773 GO:0005524 GO:0004672
GO:0016020
Cluster-22178.62103 GO:0006537 GO:0055114 GO:0006807 GO:0016773
GO:0016972 GO:0035091 GO:0015930 GO:0005515
Cluster-22178.62104 GO:0022900 GO:0005739 GO:0005747 GO:0019031

Cluster-22178.62106 GO:0005515
Cluster-22178.62113 GO:0042157 GO:0009405 GO:0006810 GO:0006869
GO:0015031 GO:0008565 GO:0008289 GO:0005576 GO:0016020
Cluster-22178.62114 GO:0008270 GO:0003729
Cluster-22178.62117 GO:0015991 GO:0015992 GO:0015078 GO:0033179
Cluster-22178.62118 GO:0006824 GO:0009236 GO:0003676 GO:0015087
GO:0016020
Cluster-22178.62119 GO:0006886 GO:0016192 GO:0005515 GO:0016021
GO:0030117
Cluster-22178.62120 GO:0016829 GO:0005576
Cluster-22178.62121 GO:0006094 GO:0006020 GO:0046486 GO:0006096
GO:0006000 GO:0015976 GO:0006013 GO:0008152 GO:0004807
Cluster-22178.62122 GO:0005515 GO:0008270 GO:0030246
Cluster-22178.62123 GO:0046872 GO:0046983
Cluster-22178.62124 GO:0003676
Cluster-22178.62125 GO:0006355 GO:0043565 GO:0003700 GO:0042765
GO:0005667 GO:0005634 GO:0016021
Cluster-22178.62126 GO:0055114 GO:0005509 GO:0016616
Cluster-22178.62128 GO:0007264 GO:0008270 GO:0003676 GO:0005515
GO:0005525
Cluster-22178.62133 GO:0006281 GO:0006310
Cluster-22178.62136 GO:0006396 GO:0003723
Cluster-22178.62141 GO:0016829
Cluster-22178.62145 GO:0008152 GO:0003824
Cluster-22178.62146 GO:0006260 GO:0006914 GO:0015031 GO:0015992
GO:0009069 GO:0016310 GO:0006886 GO:0015991 GO:0006468
GO:0004674 GO:0008565 GO:0005524 GO:0015078 GO:0005515
GO:0003723 GO:0016020 GO:0005737 GO:0033179
Cluster-22178.62149 GO:0008152 GO:0055114 GO:0003824 GO:0008484
GO:0016020 GO:0016021
Cluster-22178.62150 GO:0008152 GO:0055114 GO:0003824 GO:0008484
GO:0016020 GO:0016021
Cluster-22178.62152 GO:0007264 GO:0015684 GO:0006260 GO:0003677

GO:0005524 GO:0015093 GO:0016887 GO:0003924 GO:0005525
GO:0016021
Cluster-22178.62153 GO:0046872
Cluster-22178.62154 GO:0008270
Cluster-22178.62156 GO:0003723
Cluster-22178.62157 GO:0005515
Cluster-22178.62159 GO:0007596
Cluster-22178.62160 GO:0006118 GO:0017004 GO:0008616 GO:0030163
GO:0033739 GO:0005515 GO:0045158 GO:0008914 GO:0009512
Cluster-22178.62161 GO:0015031 GO:0005975 GO:0006886 GO:0008565
GO:0016773 GO:0005509 GO:0005737 GO:0016020
Cluster-22178.62162 GO:0016773
Cluster-22178.62163 GO:0006522 GO:0006188 GO:0009152 GO:0006144
GO:0055114 GO:0006520 GO:0009058 GO:0006531 GO:0010277
GO:0016829 GO:0004018 GO:0030170
Cluster-22178.62167 GO:0007283 GO:0009264 GO:0006024 GO:0015012
GO:0003677 GO:0004252 GO:0016757 GO:0008253 GO:0016020
GO:0005634 GO:0016021 GO:0000786
Cluster-22178.62171 GO:0043087 GO:0006508 GO:0007264 GO:0009847
GO:0005096 GO:0030250 GO:0008233 GO:0005515 GO:0005576
Cluster-22178.62172 GO:0006508 GO:0006323 GO:0006355 GO:0006771
GO:0019497 GO:0005515 GO:0004252 GO:0003993 GO:0008270
GO:0005634
Cluster-22178.62179 GO:0031556 GO:0031554 GO:0016021
Cluster-22178.62180 GO:0016598 GO:0004057 GO:0046983
Cluster-22178.62181 GO:0005515
Cluster-22178.62182 GO:0006260 GO:0006357 GO:0031110 GO:0070206
GO:0007186 GO:0007165 GO:0007275 GO:0000090 GO:0007067
GO:0007059 GO:0051301 GO:0008017 GO:0004930 GO:0005179
GO:0001104 GO:0005876 GO:0000940 GO:0016592 GO:0016021
GO:0045298 GO:0005576
Cluster-22178.62183 GO:0015979 GO:0042967 GO:0006629 GO:0009058
GO:0016788 GO:0008374 GO:0009523 GO:0009539

Cluster-22178.62185 GO:0042254 GO:0006412 GO:0009968 GO:0003735 GO:0005622 GO:0005737 GO:0005840

Cluster-22178.62189 GO:0005515 GO:0016757 GO:0016020

Cluster-22178.62190 GO:0016192 GO:0006886 GO:0005515 GO:0008536 GO:0030117

Cluster-22178.62192 GO:0042549 GO:0015979 GO:0009539 GO:0009523

Cluster-22178.62193 GO:0031167 GO:0008152 GO:0008168

Cluster-22178.62194 GO:0006511 GO:0005515

Cluster-22178.62200 GO:0015991 GO:0016192 GO:0006886 GO:0015031 GO:0006606 GO:0016820 GO:0005515 GO:0008565 GO:0008270 GO:0046872 GO:0005634 GO:0030117 GO:0005737 GO:0005622 GO:0000221

Cluster-22178.62201 GO:0015991 GO:0016192 GO:0006886 GO:0015031 GO:0006606 GO:0005515 GO:0016820 GO:0046872 GO:0008270 GO:0008565 GO:0030117 GO:0005634 GO:0005622 GO:0005737 GO:0000221

Cluster-22178.62202 GO:0016192 GO:0006886 GO:0015991 GO:0006606 GO:0015031 GO:0005515 GO:0016820 GO:0008565 GO:0046872 GO:0008270 GO:0005634 GO:0030117 GO:0000221 GO:0005737 GO:0005622

Cluster-22178.62205 GO:0007186 GO:0016787 GO:0005524 GO:0003676 GO:0004930 GO:0003677 GO:0016021

Cluster-22178.62209 GO:0022900 GO:0005515 GO:0005747 GO:0005739

Cluster-22178.62210 GO:0007165 GO:0005509 GO:0005515 GO:0005578

Cluster-22178.62212 GO:0006771 GO:0019497 GO:0003993 GO:0005515 GO:0046872

Cluster-22178.62213 GO:0006771 GO:0019497 GO:0005515 GO:0005509 GO:0003993 GO:0046872

Cluster-22178.62214 GO:0019497 GO:0006771 GO:0007165 GO:0046872 GO:0003993 GO:0005509 GO:0005515 GO:0005578

Cluster-22178.62215 GO:0019497 GO:0007165 GO:0006508 GO:0006771 GO:0008233 GO:0003993 GO:0046872 GO:0005515 GO:0005509 GO:0005578

Cluster-22178.62222 GO:0006334 GO:0000398 GO:0005515 GO:0005681
GO:0005634
Cluster-22178.62224 GO:0006807 GO:0005515 GO:0003824 GO:0046914

Cluster-22178.62226 GO:0008270
Cluster-22178.62227 GO:0006810 GO:0006814 GO:0055085 GO:0005215
GO:0016020
Cluster-22178.62228 GO:0043248
Cluster-22178.62229 GO:0009069 GO:0006468 GO:0016310 GO:0004674
GO:0004672 GO:0005524 GO:0016773 GO:0016020
Cluster-22178.62231 GO:0016310 GO:0006468 GO:0009069 GO:0004674
GO:0005524 GO:0004672
Cluster-22178.62232 GO:0009069 GO:0006468 GO:0016310 GO:0005524
GO:0004672 GO:0004674
Cluster-22178.62234 GO:0006355 GO:0043565 GO:0003700 GO:0005667
Cluster-22178.62239 GO:0035556 GO:0007155 GO:0005515
Cluster-22178.62240 GO:0051258 GO:0048870 GO:0006811 GO:0015991
GO:0015992 GO:0030168 GO:0006914 GO:0006606 GO:0006810
GO:0007165 GO:0071973 GO:0006457 GO:0008092 GO:0015078
GO:0042803 GO:0005198 GO:0003774 GO:0000774 GO:0005102
GO:0030674 GO:0051087 GO:0009288 GO:0005737 GO:0031514
GO:0033179 GO:0005886 GO:0005577 GO:0019898 GO:0005882
GO:0016020
Cluster-22178.62242 GO:0006812 GO:0008324
Cluster-22178.62245 GO:0007186 GO:0008360 GO:0015979 GO:0003677
GO:0042802 GO:0005515 GO:0004930 GO:0016020 GO:0016021
GO:0009539 GO:0009523
Cluster-22178.62246 GO:0051260 GO:0006468 GO:0004672 GO:0005524
GO:0008270 GO:0016773 GO:0005515 GO:0016020 GO:0005634
Cluster-22178.62247 GO:0051260 GO:0006468 GO:0016773 GO:0005524
GO:0008270 GO:0004672 GO:0005515 GO:0016020 GO:0005634
Cluster-22178.62248 GO:0006468 GO:0004672 GO:0005524
Cluster-22178.62250 GO:0055036

Cluster-22178.62252 GO:0006144 GO:0004114
Cluster-22178.62256 GO:0006629 GO:0004252
Cluster-22178.62257 GO:0016757
Cluster-22178.62258 GO:0005634
Cluster-22178.62259 GO:0021549 GO:0006351 GO:0006144 GO:0006730 GO:0006206 GO:0030182 GO:0003899 GO:0003677 GO:0008168 GO:0003676 GO:0005730
Cluster-22178.62260 GO:0006468 GO:0006886 GO:0004672 GO:0005524 GO:0030176
Cluster-22178.62262 GO:0007165 GO:0015684 GO:0006355 GO:0007264 GO:0006576 GO:0005509 GO:0005515 GO:0005525 GO:0005524 GO:0015093 GO:0016887 GO:0005578 GO:0005634 GO:0016021
Cluster-22178.62264 GO:0006974 GO:0051726 GO:0003677
Cluster-22178.62265 GO:0008654 GO:0016780 GO:0016020
Cluster-22178.62266 GO:0055114 GO:0031145 GO:0030071 GO:0016567 GO:0046872 GO:0008270 GO:0047134 GO:0004842 GO:0061630 GO:0005515 GO:0005680
Cluster-22178.62271 GO:0043087 GO:0035023 GO:0005089
Cluster-22178.62272 GO:0007165 GO:0007218 GO:0030141
Cluster-22178.62276 GO:0006144 GO:0006464 GO:0006206 GO:0006281 GO:0006351 GO:0046872 GO:0008270 GO:0016151 GO:0003899 GO:0005515 GO:0003677 GO:0005730
Cluster-22178.62277 GO:0006470 GO:0006570 GO:0008138 GO:0004725 GO:0004721
Cluster-22178.62278 GO:0003676 GO:0005515 GO:0005634
Cluster-22178.62281 GO:0009291 GO:0006811 GO:0006865 GO:0055085 GO:0003333 GO:0006810 GO:0005215 GO:0015171 GO:0016020
Cluster-22178.62283 GO:0005515
Cluster-22178.62287 GO:0007264 GO:0043087 GO:0005085
Cluster-22178.62289 GO:0006047
Cluster-22178.62290 GO:0051260 GO:0004190 GO:0016021
Cluster-22178.62292 GO:0006693 GO:0006691 GO:0019530 GO:0006749 GO:0003840

Cluster-22178.62294 GO:0006606 GO:0006260 GO:0015031 GO:0006370
GO:0006397 GO:0006310 GO:0006281 GO:0003910 GO:0005524
GO:0008565 GO:0005515 GO:0004484 GO:0005737 GO:0005634
Cluster-22178.62296 GO:0003677 GO:0003676 GO:0005515 GO:0005524
GO:0016787
Cluster-22178.62297 GO:0006886 GO:0003677 GO:0004003 GO:0005524
GO:0016020 GO:0005657
Cluster-22178.62299 GO:0055114 GO:0003723 GO:0016491 GO:0050661

Cluster-22178.62303 GO:0006511 GO:0031625
Cluster-22178.62304 GO:0007264 GO:0005525 GO:0003924
Cluster-22178.62305 GO:0006511 GO:0006418 GO:0000166 GO:0005524
GO:0031625 GO:0004812
Cluster-22178.62306 GO:0005634
Cluster-22178.62308 GO:0005515 GO:0008270 GO:0005634
Cluster-22178.62312 GO:0055085 GO:0008643 GO:0005351 GO:0000139
GO:0016020 GO:0016021
Cluster-22178.62313 GO:0008152 GO:0055114 GO:0016491
Cluster-22178.62316 GO:0009405 GO:0006886 GO:0008053 GO:0006869
GO:0016192 GO:0042157 GO:0005525 GO:0008289 GO:0003924
GO:0005198 GO:0004185 GO:0005524 GO:0016887 GO:0019031
GO:0016021 GO:0005576 GO:0019028 GO:0005741 GO:0005773
GO:0016020
Cluster-22178.62317 GO:0016192 GO:0006886 GO:0007264 GO:0006260
GO:0055114 GO:0015031 GO:0005515 GO:0047134 GO:0005096
GO:0016491 GO:0008565 GO:0070403 GO:0008270 GO:0016020
GO:0005737
Cluster-22178.62319 GO:0009405 GO:0007049 GO:0031505 GO:0006355
GO:0003700 GO:0043565 GO:0005667
Cluster-22178.62320 GO:0008168
Cluster-22178.62321 GO:0010508 GO:0015979 GO:0005794 GO:0016020
GO:0009539 GO:0009523
Cluster-22178.62323 GO:0006810 GO:0004659 GO:0016021

Cluster-22178.62324 GO:0006508 GO:0005515 GO:0004190 GO:0005861
Cluster-22178.62330 GO:0007169 GO:0035023 GO:0043087 GO:0006468
GO:0006508 GO:0005089 GO:0004185 GO:0004672 GO:0005524
GO:0004714 GO:0005515 GO:0016020
Cluster-22178.62332 GO:0007165 GO:0006302 GO:0004519 GO:0030145
GO:0016787 GO:0005125 GO:0005634
Cluster-22178.62335 GO:0000226 GO:0000910 GO:0007186 GO:0004930
GO:0008017 GO:0046983 GO:0003677 GO:0045298 GO:0016021
Cluster-22178.62337 GO:0016055 GO:0006468 GO:0007165 GO:0007275
GO:0005524 GO:0004672 GO:0005102 GO:0005576
Cluster-22178.62338 GO:0016567 GO:0004842
Cluster-22178.62339 GO:0006281 GO:0010447 GO:0003677 GO:0004518
GO:0003824 GO:0042597
Cluster-22178.62340 GO:0006281 GO:0010447 GO:0003677 GO:0004518
GO:0003824 GO:0042597
Cluster-22178.62343 GO:0005737
Cluster-22178.62345 GO:0007264 GO:0006119 GO:0006118 GO:0055114
GO:0015992 GO:0035556 GO:0008121 GO:0005507 GO:0005525
Cluster-22178.62347 GO:0007275 GO:0009966 GO:0016020
Cluster-22178.62351 GO:0007264 GO:0007165 GO:0007186 GO:0006013
GO:0006000 GO:0003873 GO:0005515 GO:0005524 GO:0031683
GO:0019001 GO:0003924 GO:0005525 GO:0004871
Cluster-22178.62352 GO:0006631 GO:0007304 GO:0005515 GO:0071949
GO:0005213 GO:0050151 GO:0046872 GO:0005576 GO:0045202
GO:0016021 GO:0042600
Cluster-22178.62353 GO:0007304 GO:0005515 GO:0046872 GO:0005213
GO:0005576 GO:0042600 GO:0045202 GO:0016021
Cluster-22178.62354 GO:0003723
Cluster-22178.62355 GO:0005515
Cluster-22178.62356 GO:0006464 GO:0015031 GO:0005515
Cluster-22178.62357 GO:0000902
Cluster-22178.62358 GO:0006810 GO:0009405 GO:0008200 GO:0051537
GO:0005515 GO:0042151

Cluster-22178.62359 GO:0006351 GO:0006144 GO:0006265 GO:0006206
GO:0005515 GO:0003899 GO:0003677 GO:0003676 GO:0008270
GO:0003916 GO:0005730 GO:0005694
Cluster-22178.62360 GO:0006355 GO:0008270 GO:0046872 GO:0003677

Cluster-22178.62361 GO:0016310 GO:0000160 GO:0000155 GO:0046983
GO:0009365 GO:0016021
Cluster-22178.62366 GO:0006357 GO:0009847 GO:0048193 GO:0001104
GO:0016592 GO:0005783 GO:0016020
Cluster-22178.62369 GO:0006298 GO:0009405 GO:0030983 GO:0005524
GO:0033644
Cluster-22178.62372 GO:0016779
Cluster-22178.62373 GO:0007049 GO:0006281 GO:0006355 GO:0002949
GO:0007155 GO:0006874 GO:0006816 GO:0019079 GO:0006270
GO:0006310 GO:0003678 GO:0005219 GO:0003723 GO:0004190
GO:0008134 GO:0005515 GO:0005524 GO:0016887 GO:0003724
GO:0009378 GO:0016021 GO:0005622 GO:0005657 GO:0009379
GO:0005667
Cluster-22178.62374 GO:0015992 GO:0006308 GO:0006914 GO:0006810
GO:0006886 GO:0015991 GO:0015078 GO:0008855 GO:0016021
GO:0009318 GO:0005783 GO:0033179
Cluster-22178.62377 GO:0015991 GO:0006508 GO:0004222 GO:0003712
GO:0003724 GO:0016818 GO:0016820 GO:0033178 GO:0005634
GO:0005667
Cluster-22178.62378 GO:0003723
Cluster-22178.62379 GO:0006303 GO:0006508 GO:0004222 GO:0003677
GO:0004003 GO:0005815 GO:0005657
Cluster-22178.62380 GO:0006355 GO:0006275 GO:0016032 GO:0006952
GO:0005515
Cluster-22178.62381 GO:0021549 GO:0030182 GO:0008270
Cluster-22178.62382 GO:0043087 GO:0035023 GO:0005089
Cluster-22178.62383 GO:0035023 GO:0016310 GO:0048870 GO:0019048
GO:0006468 GO:0043087 GO:0006952 GO:0007165 GO:0009069

GO:0003779 GO:0046914 GO:0005515 GO:0005089 GO:0004674
GO:0046983 GO:0005096 GO:0031514 GO:0005576
Cluster-22178.62384 GO:0006298 GO:0007010 GO:0006364 GO:0007017
GO:0006813 GO:0003779 GO:0005524 GO:0005267 GO:0030983
GO:0005875 GO:0034457 GO:0005634 GO:0005732
Cluster-22178.62386 GO:0055114 GO:0008152 GO:0006118 GO:0046872
GO:0016627 GO:0050660 GO:0016787 GO:0003995
Cluster-22178.62388 GO:0016567 GO:0004842 GO:0005515
Cluster-22178.62392 GO:0055085 GO:0022857 GO:0016021
Cluster-22178.62393 GO:0005680
Cluster-22178.62394 GO:0006555 GO:0030154 GO:0006556 GO:0019722
GO:0004478 GO:0016021
Cluster-22178.62395 GO:0016567 GO:0090263 GO:0043565
Cluster-22178.62398 GO:0006260 GO:0005524 GO:0005634 GO:0000808
Cluster-22178.62400 GO:0005515 GO:0003677
Cluster-22178.62402 GO:0005515
Cluster-22178.62405 GO:0006570 GO:0006470 GO:0004527 GO:0003677
GO:0008138 GO:0004725
Cluster-22178.62407 GO:0048870 GO:0008608 GO:0007155 GO:0016567
GO:0006511 GO:0007275 GO:0005515 GO:0016881 GO:0046872
GO:0061630 GO:0008270 GO:0031386 GO:0004842 GO:0005634
GO:0031514 GO:0042729 GO:0005876 GO:0005680
Cluster-22178.62409 GO:0004222 GO:0005524 GO:0008270 GO:0016021
Cluster-22178.62411 GO:0006531 GO:0006529 GO:0006522 GO:0008270
GO:0046872 GO:0004066 GO:0005515
Cluster-22178.62416 GO:0006260 GO:0006281 GO:0006308 GO:0017108
GO:0033557 GO:0005634
Cluster-22178.62417 GO:0016773
Cluster-22178.62419 GO:0007049 GO:0017053
Cluster-22178.62421 GO:0008233 GO:0008270 GO:0005634
Cluster-22178.62422 GO:0016020
Cluster-22178.62423 GO:0006811 GO:0055085 GO:0006812 GO:0046677
GO:0008324 GO:0005216 GO:0016021 GO:0016020

Cluster-22178.62424 GO:0050832 GO:0042967 GO:0015074 GO:0042742
GO:0008080 GO:0003676 GO:0003677 GO:0019239 GO:0031224
Cluster-22178.62429 GO:0006355 GO:0003700 GO:0005667
Cluster-22178.62433 GO:0006412 GO:0042254 GO:0003735 GO:0005622
GO:0005840
Cluster-22178.62434 GO:0006468 GO:0005524 GO:0004672 GO:0016020
Cluster-22178.62436 GO:0006260 GO:0006637 GO:0055114 GO:0046907
GO:0006635 GO:0003997 GO:0003677 GO:0005664 GO:0005777
Cluster-22178.62437 GO:0016787
Cluster-22178.62438 GO:0046497 GO:0008152 GO:0006769 GO:0006741
GO:0016301 GO:0003677 GO:0003951
Cluster-22178.62441 GO:0006468 GO:0009069 GO:0000910 GO:0000226
GO:0016310 GO:0005524 GO:0004672 GO:0008017 GO:0004674
GO:0045298
Cluster-22178.62444 GO:0019087 GO:0016021
Cluster-22178.62445 GO:0055114 GO:0019521 GO:0009094 GO:0006574
GO:0015940 GO:0006571 GO:0006568 GO:0006813 GO:0006552
GO:0006550 GO:0006554 GO:0000162 GO:0006633 GO:0006098
GO:0006631 GO:0018874 GO:0008677 GO:0004665 GO:0003857
GO:0016491 GO:0051287 GO:0008977 GO:0016616 GO:0004616
Cluster-22178.62446 GO:0006810 GO:0016021 GO:0005886
Cluster-22178.62447 GO:0042254 GO:0006412 GO:0003735 GO:0005840
Cluster-22178.62449 GO:0030866 GO:0008092 GO:0005856
Cluster-22178.62450 GO:0030866 GO:0008092 GO:0005856
Cluster-22178.62457 GO:0007165 GO:0007186 GO:0007264 GO:0005525
GO:0004871 GO:0019001 GO:0031683 GO:0003924
Cluster-22178.62459 GO:0007165 GO:0007186 GO:0006508 GO:0008152
GO:0016746 GO:0003723 GO:0004190 GO:0004871 GO:0003676
GO:0019001 GO:0005524 GO:0031683 GO:0008270 GO:0004672
GO:0003924
Cluster-22178.62466 GO:0019031
Cluster-22178.62468 GO:0005515
Cluster-22178.62469 GO:0006281 GO:0007165 GO:0034061 GO:0008601

GO:0003684 GO:0003677 GO:0000159 GO:0005634
Cluster-22178.62470 GO:0006260 GO:0005509
Cluster-22178.62471 GO:0005515
Cluster-22178.62472 GO:0043087 GO:0050790 GO:0035023 GO:0030234
GO:0005089
Cluster-22178.62476 GO:0000226 GO:0000910 GO:0008017 GO:0004190
GO:0045298 GO:0016021
Cluster-22178.62478 GO:0016021
Cluster-22178.62481 GO:0006814 GO:0055114 GO:0015992 GO:0006120
GO:0006744 GO:0008137 GO:0003677 GO:0008270
Cluster-22178.62482 GO:0006260 GO:0000917 GO:0006355 GO:0043093
GO:0003700 GO:0005667 GO:0005737
Cluster-22178.62483 GO:0006397 GO:0046983 GO:0005515
Cluster-22178.62484 GO:0006355 GO:0008270 GO:0005634
Cluster-22178.62485 GO:0035091
Cluster-22178.62487 GO:0006468 GO:0005524 GO:0004672
Cluster-22178.62489 GO:0007165 GO:0005509 GO:0005578
Cluster-22178.62490 GO:0005515
Cluster-22178.62493 GO:0016021
Cluster-22178.62494 GO:0006468 GO:0004672 GO:0005524
Cluster-22178.62497 GO:0043154 GO:0043066 GO:0019079 GO:0005515
GO:0005737 GO:0071986
Cluster-22178.62498 GO:0007275 GO:0009966 GO:0006464 GO:0016151
GO:0046872 GO:0008270 GO:0016020
Cluster-22178.62499 GO:0008270
Cluster-22178.62500 GO:0003676
Cluster-22178.62502 GO:0003723 GO:0003676
Cluster-22178.62503 GO:0006464 GO:0007275 GO:0009966 GO:0046872
GO:0016151 GO:0016020
Cluster-22178.62504 GO:0006118 GO:0009055 GO:0051536
Cluster-22178.62505 GO:0006464 GO:0007275 GO:0009966 GO:0046872
GO:0008270 GO:0016151 GO:0016020
Cluster-22178.62507 GO:0042254 GO:0006412 GO:0003735 GO:0005840

GO:0005622
Cluster-22178.62510 GO:0008270
Cluster-22178.62511 GO:0005509
Cluster-22178.62512 GO:0005515
Cluster-22178.62514 GO:0006468 GO:0005524 GO:0004672 GO:0016773
GO:0016020
Cluster-22178.62517 GO:0005509
Cluster-22178.62519 GO:0006270 GO:0015684 GO:0008270 GO:0015093
GO:0003924 GO:0005525 GO:0005634 GO:0032040 GO:0016021
Cluster-22178.62520 GO:0006098 GO:0006631 GO:0018874 GO:0006554
GO:0046168 GO:0006633 GO:0006099 GO:0006568 GO:0006552
GO:0006550 GO:0055114 GO:0019521 GO:0051321 GO:0006574
GO:0008152 GO:0051287 GO:0004616 GO:0016616 GO:0016491
GO:0003857 GO:0005515 GO:0016829
Cluster-22178.62521 GO:0007050 GO:0006508 GO:0006915 GO:0007264
GO:0008285 GO:0006144 GO:0043087 GO:0017111 GO:0004252
GO:0004197 GO:0005085 GO:0003968 GO:0031379
Cluster-22178.62523 GO:0003924
Cluster-22178.62525 GO:0005524 GO:0016787 GO:0003677 GO:0003676
GO:0005515
Cluster-22178.62526 GO:0003676 GO:0005515 GO:0003677 GO:0016787
GO:0005524
Cluster-22178.62527 GO:0010508
Cluster-22178.62528 GO:0016787 GO:0005524 GO:0003676 GO:0005515
GO:0003677
Cluster-22178.62529 GO:0003676
Cluster-22178.62533 GO:0003723 GO:0008270
Cluster-22178.62534 GO:0006144 GO:0006206 GO:0006351 GO:0007618
GO:0006468 GO:0016773 GO:0005524 GO:0004672 GO:0046872
GO:0003899 GO:0003677 GO:0016787 GO:0016020 GO:0005730
GO:0005576
Cluster-22178.62535 GO:0043087 GO:0005515 GO:0016020
Cluster-22178.62536 GO:0007009 GO:0007165 GO:0006471 GO:0005515

GO:0019904 GO:0003950 GO:0005737 GO:0016021

Cluster-22178.62537 GO:0006468 GO:0004672 GO:0005524

Cluster-22178.62538 GO:0055085 GO:0007156 GO:0030001 GO:0005509

GO:0046983 GO:0046873 GO:0016020

Cluster-22178.62539 GO:0045454

Cluster-22178.62542 GO:0016773

Cluster-22178.62544 GO:0006629 GO:0005515

Cluster-22178.62547 GO:0055114 GO:0008152 GO:0006118 GO:0016627

GO:0008440 GO:0050660 GO:0003995

Cluster-22178.62548 GO:0006886 GO:0016192 GO:0046872 GO:0030131

Cluster-22178.62549 GO:0006351 GO:0006355 GO:0006352 GO:0006144

GO:0006206 GO:0003899 GO:0003677 GO:0003700 GO:0016987

GO:0046872 GO:0005667 GO:0005730

Cluster-22178.62551 GO:0007165 GO:0015684 GO:0006355 GO:0007264

GO:0006576 GO:0005525 GO:0015093 GO:0005524 GO:0016887

GO:0005515 GO:0005509 GO:0016021 GO:0005578 GO:0005634

Cluster-22178.62553 GO:0006614 GO:0005524 GO:0043531 GO:0005525

GO:0016301

Cluster-22178.62554 GO:0006457 GO:0007165 GO:0006807 GO:0006914

GO:0016301 GO:0051082 GO:0016810 GO:0016272

Cluster-22178.62556 GO:0006446 GO:0006413 GO:0003743 GO:0005840

Cluster-22178.62557 GO:0016311 GO:0030001 GO:0055085 GO:0035023

GO:0009252 GO:0043087 GO:0005515 GO:0046873 GO:0005089

GO:0050380 GO:0016020 GO:0005737

Cluster-22178.62558 GO:0016192 GO:0006886 GO:0030117 GO:0030131

Cluster-22178.62559 GO:0006281 GO:0008270

Cluster-22178.62560 GO:0046983 GO:0003677

Cluster-22178.62561 GO:0055114 GO:0003676 GO:0003677 GO:0016787

GO:0016491 GO:0046872 GO:0005524

Cluster-22178.62562 GO:0016032 GO:0006118 GO:0006119 GO:0006270

GO:0006351 GO:0006144 GO:0055114 GO:0015992 GO:0006206

GO:0008121 GO:0003899 GO:0003677 GO:0005730 GO:0032040

Cluster-22178.62564 GO:0003676

Cluster-22178.62565 GO:0042254 GO:0006412 GO:0003735 GO:0005622 GO:0005840

Cluster-22178.62566 GO:0018149 GO:0003723 GO:0003810 GO:0005515

Cluster-22178.62567 GO:0009228 GO:0006464 GO:0006772 GO:0015031 GO:0006886 GO:0005525 GO:0052855 GO:0008270 GO:0003924 GO:0005047 GO:0004417 GO:0005515 GO:0005785 GO:0005786

Cluster-22178.62568 GO:0005515

Cluster-22178.62570 GO:0016020

Cluster-22178.62572 GO:0008299

Cluster-22178.62574 GO:0007165 GO:0007186 GO:0007264 GO:0006508 GO:0000166 GO:0004197 GO:0003677 GO:0005525 GO:0004871 GO:0031683 GO:0005524 GO:0019001 GO:0003924

Cluster-22178.62576 GO:0006118 GO:0009055 GO:0020037

Cluster-22178.62577 GO:0016021

Cluster-22178.62578 GO:0008152 GO:0055114 GO:0006520 GO:0016491

Cluster-22178.62579 GO:0016787 GO:0046872 GO:0005515 GO:0016012 GO:0016021

Cluster-22178.62580 GO:0008152 GO:0046872 GO:0016866 GO:0031419

Cluster-22178.62582 GO:0007165 GO:0005515 GO:0004872 GO:0016020 GO:0016021

Cluster-22178.62584 GO:0042254 GO:0007009 GO:0005515 GO:0003735 GO:0051082 GO:0043565 GO:0005634 GO:0005840

Cluster-22178.62586 GO:0006355 GO:0003700 GO:0043565 GO:0008537 GO:0005667

Cluster-22178.62590 GO:0048511

Cluster-22178.62591 GO:0007165 GO:0006955 GO:0006118 GO:0006814 GO:0015992 GO:0006120 GO:0006744 GO:0005179 GO:0003954 GO:0008137 GO:0005164 GO:0005576 GO:0005739 GO:0016020

Cluster-22178.62593 GO:0005515

Cluster-22178.62594 GO:0006355 GO:0009252 GO:0006807 GO:0006542

GO:0003677 GO:0004356 GO:0005622
Cluster-22178.62596 GO:0006508 GO:0004190
Cluster-22178.62598 GO:0031554 GO:0031556
Cluster-22178.62599 GO:0005975 GO:0042254 GO:0006412 GO:0009405
GO:0004553 GO:0019843 GO:0003735 GO:0005515 GO:0005509
GO:0005840 GO:0005576
Cluster-22178.62600 GO:0016579 GO:0005515 GO:0036459 GO:0032183
GO:0070140
Cluster-22178.62602 GO:0007010 GO:0003677 GO:0005524 GO:0003779
GO:0004003 GO:0005657
Cluster-22178.62603 GO:0004003 GO:0005524 GO:0003677 GO:0005657
Cluster-22178.62604 GO:0006270 GO:0006874 GO:0006816 GO:0006355
GO:0006260 GO:0016884 GO:0003700 GO:0005219 GO:0000062
GO:0005667 GO:0016021 GO:0005622
Cluster-22178.62607 GO:0006479 GO:0008168
Cluster-22178.62609 GO:0006302 GO:0006310 GO:0003677 GO:0005634
Cluster-22178.62610 GO:0015031 GO:0006464 GO:0005515
Cluster-22178.62611 GO:0016740 GO:0016887 GO:0005524 GO:0005515
GO:0016301
Cluster-22178.62613 GO:0051225 GO:0007165 GO:0009058 GO:0005179
GO:0016740 GO:0070652 GO:0005576
Cluster-22178.62614 GO:0003676
Cluster-22178.62615 GO:0007155 GO:0030155 GO:0007165 GO:0006308
GO:0030334 GO:0045995 GO:0005515 GO:0008855 GO:0046872
GO:0005102 GO:0009318
Cluster-22178.62616 GO:0055114 GO:0008152 GO:0006313 GO:0016491
GO:0003677 GO:0004803
Cluster-22178.62617 GO:0006308 GO:0007049 GO:0008270 GO:0046872
GO:0008855 GO:0003723 GO:0005515 GO:0005634 GO:0009318
Cluster-22178.62620 GO:0006139 GO:0016310 GO:0008152 GO:0006281
GO:0002949 GO:0006206 GO:0006310 GO:0006810 GO:0009069
GO:0005525 GO:0003924 GO:0016301 GO:0003723 GO:0004127
GO:0003724 GO:0004674 GO:0016887 GO:0005524 GO:0098519

GO:0009378 GO:0005657 GO:0009379
Cluster-22178.62623 GO:0006955 GO:0031342 GO:0015675 GO:0009636
GO:0035444 GO:0015643 GO:0046872 GO:0015099 GO:0019814
GO:0016021
Cluster-22178.62625 GO:0046872 GO:0008270 GO:0005515
Cluster-22178.62626 GO:0006355 GO:0043565 GO:0008270 GO:0003700
GO:0005667
Cluster-22178.62627 GO:0006955 GO:0009405 GO:0031342 GO:0006629
GO:0050920 GO:0009636 GO:0015643 GO:0003824 GO:0019814
GO:0009288
Cluster-22178.62628 GO:0051260
Cluster-22178.62629 GO:0005515
Cluster-22178.62631 GO:0003676
Cluster-22178.62633 GO:0006397 GO:0003676 GO:0016021
Cluster-22178.62635 GO:0005515
Cluster-22178.62637 GO:0008152 GO:0016747
Cluster-22178.62638 GO:0046872 GO:0000166
Cluster-22178.62639 GO:0006811 GO:0055085 GO:0005216 GO:0016020
Cluster-22178.62643 GO:0016310 GO:0000160 GO:0006109 GO:0006355
GO:0005524 GO:0003774 GO:0004672 GO:0008134 GO:0005515
GO:0000155 GO:0009365 GO:0016020 GO:0016459 GO:0005667
Cluster-22178.62646 GO:0008152 GO:0055114 GO:0003824 GO:0050662
GO:0016491
Cluster-22178.62647 GO:0005515
Cluster-22178.62648 GO:0006468 GO:0004672 GO:0005524
Cluster-22178.62652 GO:0051726 GO:0006974 GO:0003677
Cluster-22178.62654 GO:0046907 GO:0005515 GO:0008270
Cluster-22178.62655 GO:0051287
Cluster-22178.62656 GO:0051287
Cluster-22178.62657 GO:0070403 GO:0000287 GO:0030976 GO:0005515

Cluster-22178.62658 GO:0005515
Cluster-22178.62660 GO:0055114

Cluster-22178.62663 GO:0006355 GO:0006626 GO:0004518 GO:0003700
GO:0005622 GO:0005667 GO:0005741
Cluster-22178.62664 GO:0000184 GO:0006281 GO:0006355 GO:0055114
GO:0003677 GO:0004386 GO:0016972 GO:0047134 GO:0005524
GO:0008270 GO:0046872 GO:0005737
Cluster-22178.62665 GO:0006270 GO:0005515 GO:0008242
Cluster-22178.62667 GO:0045454 GO:0009306 GO:0019013 GO:0005788
Cluster-22178.62669 GO:0006570 GO:0006952 GO:0006810 GO:0009405
GO:0006470 GO:0008138 GO:0046983 GO:0019855 GO:0004725
GO:0005576
Cluster-22178.62670 GO:0006413 GO:0006265 GO:0006446 GO:0007186
GO:0015684 GO:0007165 GO:0007264 GO:0004871 GO:0005525
GO:0003924 GO:0031683 GO:0019001 GO:0003743 GO:0003690
GO:0015093 GO:0016021 GO:0005840
Cluster-22178.62671 GO:0035091
Cluster-22178.62674 GO:0005515
Cluster-22178.62676 GO:0016567 GO:0006260 GO:0005524 GO:0003678
GO:0004842 GO:0005657
Cluster-22178.62677 GO:0007050 GO:0005515 GO:0004672 GO:0005887
GO:0016020
Cluster-22178.62678 GO:0007050 GO:0005515 GO:0004672 GO:0005887
GO:0016020
Cluster-22178.62680 GO:0007050 GO:0004672 GO:0005515 GO:0005887
GO:0016020
Cluster-22178.62681 GO:0007165 GO:0006221 GO:0030155 GO:0007049
GO:0045747 GO:0045995 GO:0030334 GO:0005515 GO:0051082
GO:0005102 GO:0005777
Cluster-22178.62682 GO:0007050 GO:0005515 GO:0004672 GO:0016020
GO:0005887
Cluster-22178.62684 GO:0006351 GO:0042254 GO:0006412 GO:0003735
GO:0046983 GO:0005840 GO:0005622
Cluster-22178.62686 GO:0000398 GO:0005681
Cluster-22178.62687 GO:0000398 GO:0005681

Cluster-22178.62689 GO:0005634
Cluster-22178.62690 GO:0006260 GO:0010508 GO:0006355 GO:0035091
GO:0003700 GO:0043565 GO:0005667
Cluster-22178.62692 GO:0006313
Cluster-22178.62693 GO:0006570 GO:0006470 GO:0004725 GO:0008138

Cluster-22178.62695 GO:0006355 GO:0003677 GO:0034061 GO:0005634
Cluster-22178.62696 GO:0004867 GO:0005515
Cluster-22178.62698 GO:0006811 GO:0006470 GO:0055085 GO:0006570
GO:0004725 GO:0005216 GO:0003677 GO:0008138 GO:0019031
GO:0016020
Cluster-22178.62699 GO:0006886 GO:0015991 GO:0043248 GO:0016820
GO:0005515 GO:0008536 GO:0005783 GO:0032040 GO:0000221
GO:0019031
Cluster-22178.62700 GO:0051287
Cluster-22178.62701 GO:0007165 GO:0040007 GO:0008283 GO:0008083
GO:0005096
Cluster-22178.62702 GO:0006355 GO:0003700 GO:0003677 GO:0043565
GO:0016021 GO:0005667 GO:0005634
Cluster-22178.62703 GO:0005515 GO:0035091
Cluster-22178.62704 GO:0043161 GO:0006281 GO:0006289 GO:0003684

Cluster-22178.62705 GO:0005975 GO:0006807 GO:0006355 GO:0043093
GO:0000917 GO:0009058 GO:0008270 GO:0043565 GO:0003933
GO:0004553 GO:0005737
Cluster-22178.62709 GO:0010884 GO:0008284 GO:0001819
Cluster-22178.62715 GO:0007165 GO:0007606 GO:0003676 GO:0008270
GO:0004888 GO:0016020
Cluster-22178.62716 GO:0005975 GO:0055114 GO:0042254 GO:0006412
GO:0043631 GO:0004553 GO:0004652 GO:0016706 GO:0003677
GO:0003735 GO:0005515 GO:0005840 GO:0005622
Cluster-22178.62719 GO:0007049 GO:0000917 GO:0043093 GO:0006355
GO:0003700 GO:0005667 GO:0005737

Cluster-22178.62721 GO:0006810 GO:0016021

Cluster-22178.62723 GO:0006367 GO:0007165 GO:0005515 GO:0046983 GO:0019031 GO:0005669

Cluster-22178.62727 GO:0007165 GO:0006955 GO:0005125 GO:0003723 GO:0003676 GO:0005576

Cluster-22178.62728 GO:0003677 GO:0004527

Cluster-22178.62730 GO:0006506 GO:0017176 GO:0008270 GO:0016021

Cluster-22178.62733 GO:0006508 GO:0006351 GO:0055114 GO:0015979 GO:0009094 GO:0030494 GO:0000162 GO:0009073 GO:0006260 GO:0006571 GO:0003856 GO:0004252 GO:0003677 GO:0016491 GO:0042025

Cluster-22178.62734 GO:0055085 GO:0016021

Cluster-22178.62738 GO:0005515

Cluster-22178.62739 GO:0016310 GO:0004673 GO:0009365

Cluster-22178.62740 GO:0046487 GO:0006355 GO:0015940 GO:0008152 GO:0055114 GO:0006418 GO:0019354 GO:0006779 GO:0009396 GO:0043115 GO:0000166 GO:0003824 GO:0008677 GO:0004488 GO:0051287 GO:0050662 GO:0004812 GO:0016757 GO:0016491 GO:0005524 GO:0016020

Cluster-22178.62741 GO:0006810 GO:0005622

Cluster-22178.62744 GO:0005515 GO:0003824 GO:0046872 GO:0008270

Cluster-22178.62748 GO:0009234 GO:0008299

Cluster-22178.62749 GO:0055114 GO:0016491

Cluster-22178.62751 GO:0003723 GO:0005515 GO:0003676

Cluster-22178.62753 GO:0042393 GO:0005515

Cluster-22178.62759 GO:0006413 GO:0006446 GO:0003723 GO:0003743 GO:0003677 GO:0005737 GO:0005840 GO:0019031

Cluster-22178.62760 GO:0008289 GO:0020037 GO:0005783

Cluster-22178.62761 GO:0006360 GO:0001168

Cluster-22178.62764 GO:0019354 GO:0055114 GO:0016117 GO:0006779 GO:0071949 GO:0043115 GO:0016705 GO:0016491

Cluster-22178.62765 GO:0042254 GO:0006412 GO:0003735 GO:0005840

Cluster-22178.62766 GO:0005515 GO:0005509
Cluster-22178.62768 GO:0006351 GO:0007050 GO:0006915 GO:0016579
GO:0006206 GO:0019083 GO:0006144 GO:0008285 GO:0055114
GO:0003677 GO:0003899 GO:0008270 GO:0036459 GO:0016491
GO:0005730
Cluster-22178.62770 GO:0003677 GO:0003676 GO:0005524 GO:0016787

Cluster-22178.62771 GO:0006879 GO:0042742 GO:0008199 GO:0005576
Cluster-22178.62774 GO:0005515
Cluster-22178.62777 GO:0007160 GO:0043248 GO:0043087 GO:0032012
GO:0005086 GO:0005509 GO:0005634 GO:0005576 GO:0005783
Cluster-22178.62778 GO:0005515
Cluster-22178.62779 GO:0006355 GO:0005515 GO:0003677 GO:0008270

Cluster-22178.62785 GO:0042742 GO:0007094 GO:0016021
Cluster-22178.62786 GO:0042742 GO:0007094 GO:0016021
Cluster-22178.62788 GO:0006464 GO:0006665 GO:0006807 GO:0006396
GO:0019058 GO:0016567 GO:0005975 GO:0006687 GO:0006468
GO:0005524 GO:0008270 GO:0016773 GO:0004842 GO:0004348
GO:0046872 GO:0004672 GO:0016151 GO:0003676 GO:0003723
GO:0005515 GO:0005680 GO:0016020
Cluster-22178.62789 GO:0019058 GO:0007165 GO:0007283 GO:0008601
GO:0000159
Cluster-22178.62791 GO:0006886 GO:0015991 GO:0006629 GO:0016192
GO:0006606 GO:0043248 GO:0051276 GO:0015031 GO:0005515
GO:0016820 GO:0046872 GO:0004222 GO:0008565 GO:0016887
GO:0005524 GO:0008270 GO:0005634 GO:0030117 GO:0005737
GO:0005694 GO:0000221 GO:0005622
Cluster-22178.62793 GO:0046872 GO:0008270
Cluster-22178.62794 GO:0007049 GO:0006275 GO:0007155 GO:0020037

Cluster-22178.62796 GO:0005515
Cluster-22178.62797 GO:0006334 GO:0003677 GO:0016021 GO:0000786

GO:0005634
Cluster-22178.62798 GO:0006810 GO:0016021
Cluster-22178.62800 GO:0006465 GO:0007264 GO:0005525 GO:0008233
GO:0003924 GO:0016021 GO:0005787
Cluster-22178.62801 GO:0007165 GO:0006810 GO:0055085 GO:0006898
GO:0005044 GO:0042626 GO:0016887 GO:0005524 GO:0016020
GO:0016021
Cluster-22178.62807 GO:0006811 GO:0055085 GO:0005216 GO:0016020
Cluster-22178.62808 GO:0004859 GO:0005576
Cluster-22178.62811 GO:0005524 GO:0035299
Cluster-22178.62813 GO:0007411 GO:0050776 GO:0014068 GO:0055114
GO:0007165 GO:0006446 GO:0045454 GO:0006413 GO:0005102
GO:0003743 GO:0043548 GO:0003723 GO:0005840 GO:0005737
GO:0016020
Cluster-22178.62817 GO:0045227 GO:0004252 GO:0016021 GO:0016020
Cluster-22178.62820 GO:0007165 GO:0016032 GO:0004871 GO:0019867
GO:0016021
Cluster-22178.62821 GO:0007165 GO:0016032 GO:0004871 GO:0019867
GO:0016021
Cluster-22178.62822 GO:0006313 GO:0055114 GO:0004803 GO:0003677
GO:0016702
Cluster-22178.62824 GO:0006468 GO:0004672 GO:0005524
Cluster-22178.62825 GO:0007264 GO:0003924 GO:0005509 GO:0005515
GO:0005525
Cluster-22178.62828 GO:0006119 GO:0006412 GO:0006355 GO:0035556
GO:0015992 GO:0042254 GO:0015986 GO:0008270 GO:0046933
GO:0003677 GO:0003735 GO:0046961 GO:0005840 GO:0005622
GO:0045259 GO:0045261
Cluster-22178.62829 GO:0043066 GO:0045766 GO:0043123
Cluster-22178.62830 GO:0017000 GO:0043085 GO:0000290 GO:0003779
GO:0008047
Cluster-22178.62832 GO:0009069 GO:0006468 GO:0016310 GO:0035556
GO:0005515 GO:0005524 GO:0004672 GO:0004674

Cluster-22178.62836 GO:0005515
Cluster-22178.62839 GO:0003676 GO:0003723
Cluster-22178.62841 GO:0016021
Cluster-22178.62842 GO:0006355 GO:0006465 GO:0008233 GO:0003676 GO:0005787 GO:0005622 GO:0016021
Cluster-22178.62843 GO:0003723 GO:0003676
Cluster-22178.62845 GO:0016021
Cluster-22178.62847 GO:0005515
Cluster-22178.62850 GO:0006000 GO:0006013 GO:0019497 GO:0015074 GO:0006771 GO:0003993 GO:0005524 GO:0003873
Cluster-22178.62855 GO:0006886 GO:0006779 GO:0015994 GO:0055114 GO:0008536 GO:0004109
Cluster-22178.62857 GO:0005543
Cluster-22178.62862 GO:0007018 GO:0006355 GO:0007017 GO:0003777 GO:0008017 GO:0005524 GO:0008270 GO:0016818 GO:0003677 GO:0005874 GO:0045298 GO:0005634
Cluster-22178.62867 GO:0008440
Cluster-22178.62869 GO:0007264 GO:0005525 GO:0003924
Cluster-22178.62870 GO:0007264 GO:0005525
Cluster-22178.62872 GO:0046488 GO:1901031 GO:0016307 GO:0005634
Cluster-22178.62873 GO:0006370 GO:0006260 GO:0006281 GO:0006310 GO:0006397 GO:0005524 GO:0003910 GO:0004484 GO:0003677
Cluster-22178.62874 GO:0006298 GO:0005524
Cluster-22178.62875 GO:0043087 GO:0005089 GO:0005737
Cluster-22178.62876 GO:0043087 GO:0005089 GO:0005737
Cluster-22178.62878 GO:0016021
Cluster-22178.62880 GO:0009435 GO:0006040 GO:0009607 GO:0000105 GO:0006807 GO:0007275 GO:0006537 GO:0055114 GO:0008033 GO:0046497 GO:0008152 GO:0007219 GO:0006051 GO:0006355 GO:0016491 GO:0050660 GO:0016638 GO:0018580 GO:0015930 GO:0016829 GO:0016627 GO:0003824 GO:0017150 GO:0004514 GO:0047465 GO:0005737 GO:0016021
Cluster-22178.62882 GO:0007050 GO:0045859 GO:0004861 GO:0016462

GO:0005737 GO:0005634
Cluster-22178.62884 GO:0006355 GO:0005622
Cluster-22178.62885 GO:0007165 GO:0042981 GO:0005515
Cluster-22178.62886 GO:0007015 GO:0003785
Cluster-22178.62887 GO:0006438 GO:0009098 GO:0008152 GO:0009099
GO:0009097 GO:0009405 GO:0016758 GO:0004832 GO:0005524
GO:0000166 GO:0005737 GO:0016020 GO:0005783
Cluster-22178.62889 GO:0043039 GO:0005524 GO:0016876 GO:0016021
Cluster-22178.62890 GO:0015991 GO:0015992 GO:0015078 GO:0033179
Cluster-22178.62896 GO:0019087 GO:0006769 GO:0015031 GO:0055085
GO:0046497 GO:0008152 GO:0035556 GO:0006741 GO:0006810
GO:0008565 GO:0005525 GO:0003951 GO:0016021 GO:0016020
Cluster-22178.62897 GO:0016787
Cluster-22178.62899 GO:0006810 GO:0007218 GO:0008610 GO:0015947
GO:0006741 GO:0046497 GO:0008152 GO:0006308 GO:0055114
GO:0006769 GO:0016747 GO:0008855 GO:0019871 GO:0016301
GO:0003951 GO:0046332 GO:0030058 GO:0042597 GO:0005886
GO:0009318 GO:0005576
Cluster-22178.62900 GO:0006364 GO:0032259 GO:0008033 GO:0003723
GO:0008168
Cluster-22178.62901 GO:0006359 GO:0006260 GO:0015992 GO:0006351
GO:0090266 GO:0015991 GO:0015078 GO:0005524 GO:0003887
GO:0003677 GO:0005680 GO:0019031 GO:0032040 GO:0033179
GO:0042575
Cluster-22178.62902 GO:0009877 GO:0009312 GO:0008152 GO:0008757
GO:0008168 GO:0005634
Cluster-22178.62906 GO:0006606 GO:0006914 GO:0039693 GO:0006397
GO:0006508 GO:0019784 GO:0005198 GO:0003682 GO:0003723
GO:0000785 GO:0019031 GO:0005737 GO:0005634 GO:0019028
Cluster-22178.62907 GO:0000921 GO:0006397 GO:0003682 GO:0003723
GO:0000785 GO:0016021 GO:0005737 GO:0005940 GO:0005634
Cluster-22178.62909 GO:0004867
Cluster-22178.62912 GO:0006418 GO:0002161 GO:0005515

Cluster-22178.62917 GO:0003723
Cluster-22178.62918 GO:0006508 GO:0050790 GO:0008234 GO:0004197

Cluster-22178.62919 GO:0046677 GO:0009116 GO:0007631 GO:0007218
GO:0008270
Cluster-22178.62921 GO:0005634
Cluster-22178.62926 GO:0006637 GO:0055114 GO:0006260 GO:0006635
GO:0046907 GO:0003997 GO:0003677 GO:0003887 GO:0008408
GO:0005777 GO:0005664 GO:0005634 GO:0042575
Cluster-22178.62927 GO:0006508 GO:0006813 GO:0006355 GO:0001522
GO:0042254 GO:0055114 GO:0051287 GO:0016616 GO:0008239
GO:0003700 GO:0005667 GO:0005622
Cluster-22178.62928 GO:0006144 GO:0035556 GO:0009190 GO:0006171
GO:0051537 GO:0016849 GO:0005515 GO:0004016 GO:0005886
Cluster-22178.62932 GO:0030866 GO:0005198 GO:0003779 GO:0008092
GO:0005856
Cluster-22178.62936 GO:0003723
Cluster-22178.62937 GO:0006281 GO:0008270 GO:0046983
Cluster-22178.62938 GO:0061024 GO:0010033 GO:0019867
Cluster-22178.62939 GO:0005515
Cluster-22178.62940 GO:0005509 GO:0005515 GO:0019028
Cluster-22178.62943 GO:0043066 GO:0043027
Cluster-22178.62944 GO:0015937 GO:0015940 GO:0042254 GO:0006412
GO:0005524 GO:0004594 GO:0003735 GO:0004252 GO:0005622
GO:0005840 GO:0019028 GO:0055036
Cluster-22178.62946 GO:0005975 GO:0004553 GO:0016020
Cluster-22178.62947 GO:0007267 GO:0046872
Cluster-22178.62948 GO:0006914 GO:0006357 GO:0006206 GO:0006606
GO:0006886 GO:0006457 GO:0007165 GO:0006144 GO:0030168
GO:0015031 GO:0006260 GO:0006355 GO:0006351 GO:0051258
GO:0007049 GO:0003677 GO:0008565 GO:0051082 GO:0003899
GO:0001104 GO:0043565 GO:0030674 GO:0005102 GO:0003700
GO:0005667 GO:0005730 GO:0016020 GO:0016272 GO:0005577

GO:0005737 GO:0016592 GO:0019028

Cluster-22178.62949 GO:0042157 GO:0046486 GO:0016567 GO:0006869 GO:0009395 GO:0008654 GO:0061630 GO:0046983 GO:0008270 GO:0004842 GO:0004143 GO:0005198 GO:0005515 GO:0046872 GO:0008289 GO:0016020 GO:0005622 GO:0019028 GO:0005576 GO:0019031

Cluster-22178.62950 GO:0015940 GO:0007186 GO:0006952 GO:0015937 GO:0004930 GO:0005524 GO:0004594

Cluster-22178.62952 GO:0009058 GO:0019054 GO:0004857 GO:0016740 GO:0008191

Cluster-22178.62953 GO:0042742 GO:0009435 GO:0046497 GO:0004514 GO:0016763 GO:0005515 GO:0019901

Cluster-22178.62955 GO:0004867

Cluster-22178.62957 GO:0006352

Cluster-22178.62958 GO:0008270

Cluster-22178.62959 GO:0007165 GO:0005057

Cluster-22178.62961 GO:0030168 GO:0051258 GO:0007165 GO:0005102 GO:0005515 GO:0030674 GO:0042720 GO:0005577

Cluster-22178.62963 GO:0006357 GO:0001104 GO:0016592

Cluster-22178.62964 GO:0006281 GO:0006144 GO:0006366 GO:0046034 GO:0004867 GO:0004017 GO:0003677 GO:0046872 GO:0008270 GO:0005576 GO:0005665

Cluster-22178.62968 GO:0006508 GO:0004252

Cluster-22178.62969 GO:0006468 GO:0006560 GO:0006814 GO:0055114 GO:0006090 GO:0006525 GO:0019354 GO:0071436 GO:0006779 GO:0005515 GO:0043115 GO:0008948 GO:0004672 GO:0008641 GO:0015081 GO:0005524 GO:0016021 GO:0016020

Cluster-22178.62970 GO:0005515

Cluster-22178.62971 GO:0006508 GO:0006886 GO:0016192 GO:0006506 GO:0005515 GO:0005509 GO:0004198 GO:0030131 GO:0005622

Cluster-22178.62972 GO:0006570 GO:0006470 GO:0008138 GO:0004725

Cluster-22178.62976 GO:0042254 GO:0006412 GO:0003735 GO:0005840

Cluster-22178.62977 GO:0006355 GO:0009966 GO:0003677 GO:0005576
Cluster-22178.62978 GO:0006355 GO:0043565
Cluster-22178.62981 GO:0055114 GO:0006278 GO:0016579 GO:0003964
GO:0036459 GO:0004800
Cluster-22178.62984 GO:0031124 GO:0046872
Cluster-22178.62986 GO:0006824 GO:0009236 GO:0003676 GO:0015087
GO:0016020
Cluster-22178.62987 GO:0005515 GO:0005201 GO:0005581 GO:0005578
Cluster-22178.62988 GO:0006811 GO:0005216 GO:0016020
Cluster-22178.62989 GO:0009235 GO:0006355 GO:0005515 GO:0046872
GO:0005634
Cluster-22178.62992 GO:0005515
Cluster-22178.62993 GO:0008092 GO:0005886 GO:0005737 GO:0019898
Cluster-22178.62994 GO:0003676
Cluster-22178.62995 GO:0006464 GO:0015031 GO:0015992 GO:0015986
GO:0015689 GO:0006886 GO:0052855 GO:0008270 GO:0005047
GO:0015078 GO:0005525 GO:0003924 GO:0005515 GO:0015098
GO:0005785 GO:0000276 GO:0016021 GO:0005786
Cluster-22178.62997 GO:0016042 GO:0008152 GO:0008203 GO:0016298
GO:0016787
Cluster-22178.62998 GO:0005515
Cluster-22178.62999 GO:0005515 GO:0008270 GO:0046872
Cluster-22178.63001 GO:0007340 GO:0007165 GO:0007342 GO:0046872
GO:0003676 GO:0005179 GO:0003677 GO:0005887 GO:0005576
GO:0045095 GO:0001669
Cluster-22178.63003 GO:0006810 GO:0005215 GO:0016020
Cluster-22178.63005 GO:0005516 GO:0005515
Cluster-22178.63008 GO:0006412 GO:0042254 GO:0009372 GO:0017148
GO:0042710 GO:0016032 GO:0004672 GO:0005524 GO:0003735
GO:0005840 GO:0005763
Cluster-22178.63009 GO:0016567 GO:0004842
Cluster-22178.63010 GO:0006412 GO:0042254 GO:0003735 GO:0005840
GO:0005622

Cluster-22178.63011 GO:0006397 GO:0035023 GO:0043087 GO:0003723
GO:0005089 GO:0046872 GO:0005634 GO:0005737

Cluster-22178.63013 GO:0005515

Cluster-22178.63015 GO:0070206 GO:0007049 GO:0006355 GO:0043565
GO:0003700 GO:0003677 GO:0005667 GO:0005634

Cluster-22178.63017 GO:0004518 GO:0003677

Cluster-22178.63018 GO:0006825 GO:0016531 GO:0005507 GO:0005758

Cluster-22178.63020 GO:0007067 GO:0007049 GO:0005634 GO:0000775

Cluster-22178.63021 GO:0007049 GO:0006450 GO:0007067 GO:0051082
GO:0000775 GO:0005634

Cluster-22178.63022 GO:0042967 GO:0006479 GO:0007165 GO:0006481
GO:0008080 GO:0016747 GO:0005198 GO:0004671 GO:0019031
GO:0016021 GO:0019028

Cluster-22178.63025 GO:0016020

Cluster-22178.63027 GO:0008152 GO:0006355 GO:0003824 GO:0005634

Cluster-22178.63028 GO:0021987 GO:0016021

Cluster-22178.63029 GO:0042254 GO:0006412 GO:0003735 GO:0005840

Cluster-22178.63032 GO:0005515

Cluster-22178.63034 GO:0007264 GO:0006355 GO:0006576 GO:0015684
GO:0015093 GO:0005524 GO:0003924 GO:0016887 GO:0005525
GO:0003677 GO:0016021

Cluster-22178.63035 GO:0006418 GO:0000166 GO:0003676 GO:0005524
GO:0004812

Cluster-22178.63036 GO:0005515

Cluster-22178.63037 GO:0006810 GO:0006811 GO:0055085 GO:0005975
GO:0016868 GO:0005216 GO:0005215 GO:0016020

Cluster-22178.63039 GO:0006367 GO:0015684 GO:0007155 GO:0017025
GO:0005525 GO:0015093 GO:0005672 GO:0016021 GO:0009289

Cluster-22178.63040 GO:0045017 GO:0007155 GO:0006914 GO:0030334
GO:0042967 GO:0048870 GO:0051258 GO:0045995 GO:0071973
GO:0046486 GO:0030155 GO:0007165 GO:0030168 GO:0000921
GO:0051607 GO:0005515 GO:0005198 GO:0042803 GO:0045502
GO:0008134 GO:0008092 GO:0030674 GO:0004144 GO:0005102

GO:0005737 GO:0005667 GO:0005577 GO:0019028 GO:0031514
GO:0016021 GO:0005940 GO:0030286 GO:0019898
Cluster-22178.63042 GO:0003676
Cluster-22178.63044 GO:0016020
Cluster-22178.63048 GO:0006412 GO:0008168 GO:0003676
Cluster-22178.63049 GO:0016567 GO:0006457 GO:0007165 GO:0051607
GO:0030168 GO:0000921 GO:0015031 GO:0007017 GO:0006355
GO:0070193 GO:0007018 GO:0006904 GO:0048870 GO:0051258
GO:0006914 GO:0006357 GO:0051028 GO:0048193 GO:0006606
GO:0007128 GO:0010508 GO:0007130 GO:0007094 GO:0048309
GO:0030674 GO:0051087 GO:0000774 GO:0031386 GO:0005102
GO:0045502 GO:0008134 GO:0019901 GO:0003700 GO:0003777
GO:0005524 GO:0008017 GO:0051082 GO:0042803 GO:0001104
GO:0005198 GO:0005515 GO:0016881 GO:0000145 GO:0005882
GO:0016592 GO:0005940 GO:0030286 GO:0005815 GO:0019028
GO:0031514 GO:0000795 GO:0016021 GO:0005874 GO:0019031
GO:0005667 GO:0016020 GO:0016272 GO:0005577 GO:0005634
GO:0045298
Cluster-22178.63050 GO:0006771 GO:0009231 GO:0042128 GO:0009058
GO:0003919 GO:0035091 GO:0003824
Cluster-22178.63051 GO:0007276 GO:0055085 GO:0006355 GO:0007155
GO:0009725 GO:0003677 GO:0016021 GO:0005634
Cluster-22178.63054 GO:0006364 GO:0042254 GO:0006412 GO:0005515
GO:0003735 GO:0005622 GO:0005840
Cluster-22178.63057 GO:0006281 GO:0043093 GO:0000917 GO:0007165
GO:0005125 GO:0008270 GO:0043130 GO:0016020 GO:0005737
Cluster-22178.63060 GO:0005515
Cluster-22178.63061 GO:0007018 GO:0030071 GO:0007017 GO:0005515
GO:0008017 GO:0003777 GO:0005524 GO:0045298 GO:0005874
GO:0005680
Cluster-22178.63063 GO:0046413 GO:0008152 GO:0005515 GO:0018836
GO:0016491
Cluster-22178.63067 GO:0006468 GO:0005515 GO:0005524 GO:0004672

Cluster-22178.63068 GO:0006144 GO:0046034 GO:0035556 GO:0008270 GO:0046872 GO:0005506 GO:0004017 GO:0003677

Cluster-22178.63070 GO:0006118 GO:0006355 GO:0003677 GO:0003682 GO:0015035 GO:0046872 GO:0016020 GO:0005634 GO:0000785 GO:0000775

Cluster-22178.63073 GO:0006355 GO:0006260 GO:0016787 GO:0043565 GO:0003700 GO:0005667

Cluster-22178.63074 GO:0016021

Cluster-22178.63075 GO:0051726 GO:0006355 GO:0006352 GO:0003677 GO:0017025 GO:0016987 GO:0003700 GO:0005667 GO:0005634

Cluster-22178.63076 GO:0005975 GO:0042254 GO:0006412 GO:0006948 GO:0004553 GO:0003735 GO:0005840 GO:0005622 GO:0019028

Cluster-22178.63077 GO:0016021

Cluster-22178.63078 GO:0055085 GO:0006811 GO:0001522 GO:0042254 GO:0007165 GO:0004872 GO:0005216 GO:0030515 GO:0072588 GO:0016021 GO:0016020

Cluster-22178.63080 GO:0007005 GO:0016567 GO:0006313 GO:0004803 GO:0004842 GO:0003677

Cluster-22178.63084 GO:0006508 GO:0016757 GO:0004198 GO:0016020 GO:0005622

Cluster-22178.63089 GO:0042254 GO:0006412 GO:0042981 GO:0003735 GO:0005515 GO:0008270 GO:0019843 GO:0005634 GO:0005840

Cluster-22178.63090 GO:0005515 GO:0004519

Cluster-22178.63097 GO:0003723

Cluster-22178.63098 GO:0007186 GO:0035023 GO:0043087 GO:0005089 GO:0004930 GO:0016021

Cluster-22178.63100 GO:0042254 GO:0006412 GO:0003735 GO:0005840

Cluster-22178.63103 GO:0005515

Cluster-22178.63104 GO:0003676

Cluster-22178.63105 GO:0006448 GO:0001514 GO:0003723 GO:0005515 GO:0003746 GO:0005525 GO:0005737 GO:0005840

Cluster-22178.63106 GO:0035092 GO:0003677 GO:0000228

Cluster-22178.63110 GO:0006508 GO:0015031 GO:0016787 GO:0008236

Cluster-22178.63112 GO:0051252 GO:0006402 GO:0003723 GO:0003676
GO:0004535 GO:0046872 GO:0005634 GO:0005737
Cluster-22178.63113 GO:0006051 GO:0006355 GO:0006537 GO:0008033
GO:0055114 GO:0046497 GO:0008152 GO:0007219 GO:0007275
GO:0009435 GO:0006040 GO:0009607 GO:0000105 GO:0006807
GO:0004514 GO:0047465 GO:0016829 GO:0016627 GO:0003824
GO:0017150 GO:0016638 GO:0015930 GO:0018580 GO:0016491
GO:0050660 GO:0005737 GO:0016021
Cluster-22178.63115 GO:0015684 GO:0015093 GO:0003924 GO:0005525
GO:0016021
Cluster-22178.63118 GO:0007218 GO:0006629 GO:0016021 GO:0005576
GO:0019033
Cluster-22178.63120 GO:0008207 GO:0008209 GO:0008210 GO:0006694
GO:0055114 GO:0016616 GO:0003854 GO:0050662 GO:0003824
GO:0016491 GO:0005634
Cluster-22178.63121 GO:0007165 GO:0006468 GO:0009069 GO:0016310
GO:0035556 GO:0017048 GO:0005198 GO:0004674 GO:0005524
GO:0004672 GO:0005882
Cluster-22178.63122 GO:0046983
Cluster-22178.63128 GO:0006355 GO:0003700 GO:0005667
Cluster-22178.63129 GO:0006355 GO:0003700 GO:0043565 GO:0005634
GO:0005667
Cluster-22178.63130 GO:0006633 GO:0006554 GO:0006631 GO:0018874
GO:0045454 GO:0055114 GO:0008033 GO:0006574 GO:0006552
GO:0006550 GO:0006508 GO:0006568 GO:0050660 GO:0016491
GO:0016616 GO:0071949 GO:0051287 GO:0008236 GO:0003857
GO:0005576
Cluster-22178.63132 GO:0042254 GO:0006412 GO:0003735 GO:0005622
GO:0005840
Cluster-22178.63133 GO:0016192 GO:0006886 GO:0006810 GO:0016787
GO:0005515 GO:0005198 GO:0003677 GO:0005524 GO:0003676

GO:0005634 GO:0030117
Cluster-22178.63134 GO:0006357 GO:0001104 GO:0016592
Cluster-22178.63136 GO:0005975 GO:0006012 GO:0000917 GO:0046486
GO:0001575 GO:0004553 GO:0004557 GO:0016021
Cluster-22178.63137 GO:0043093 GO:0006355 GO:0000917 GO:0006260
GO:0043565 GO:0003700 GO:0005667 GO:0005737
Cluster-22178.63138 GO:0061630
Cluster-22178.63139 GO:0008152 GO:0006554 GO:0006479 GO:0006468
GO:0008168 GO:0004672 GO:0005524 GO:0005515 GO:0018024
Cluster-22178.63140 GO:0005515
Cluster-22178.63143 GO:0006412 GO:0042254 GO:0003735 GO:0005840
GO:0005622
Cluster-22178.63145 GO:0006810 GO:0006418 GO:0000166 GO:0004812
GO:0005524 GO:0005886
Cluster-22178.63147 GO:0006418 GO:0006810 GO:0005524 GO:0004812
GO:0000166 GO:0005886
Cluster-22178.63149 GO:0006412 GO:0042254 GO:0003735 GO:0005761
GO:0005840
Cluster-22178.63150 GO:0006412 GO:0042254 GO:0003735 GO:0005840
Cluster-22178.63151 GO:0003924
Cluster-22178.63154 GO:0007165 GO:0008158 GO:0016020
Cluster-22178.63155 GO:0006355 GO:0003700 GO:0043565 GO:0005783
GO:0016021 GO:0005667
Cluster-22178.63157 GO:0007264 GO:0009082 GO:0005525
Cluster-22178.63162 GO:0005975 GO:0004553
Cluster-22178.63163 GO:0005515
Cluster-22178.63164 GO:0007186 GO:0006810 GO:0009405 GO:0008200
GO:0005515 GO:0008270 GO:0004930 GO:0005576 GO:0016021
Cluster-22178.63166 GO:0006413 GO:0006446 GO:0003743 GO:0005840
Cluster-22178.63167 GO:0003676
Cluster-22178.63168 GO:0006812 GO:0032801 GO:0019076 GO:0006355
GO:0003677 GO:0005261 GO:0033644 GO:0005634
Cluster-22178.63169 GO:0046872

Cluster-22178.63170 GO:0005515
Cluster-22178.63172 GO:0016037 GO:0031404 GO:0005515 GO:0030089
Cluster-22178.63173 GO:0006888 GO:0006886 GO:0008270 GO:0046872
GO:0005515 GO:0030127
Cluster-22178.63175 GO:0009403 GO:0006886 GO:0006605 GO:0005886
GO:0005742 GO:0019867 GO:0016020
Cluster-22178.63176 GO:0051252 GO:0016891
Cluster-22178.63178 GO:0006402 GO:0006810 GO:0051252 GO:0005215
GO:0003676 GO:0004535 GO:0046872 GO:0003723 GO:0005737
GO:0005576 GO:0005634
Cluster-22178.63186 GO:0000079 GO:0019901
Cluster-22178.63187 GO:0055114 GO:0008270 GO:0004190 GO:0016706
GO:0003677 GO:0005515 GO:0016021
Cluster-22178.63188 GO:0007018 GO:0007017 GO:0008017 GO:0003777
GO:0005524 GO:0045298 GO:0005874
Cluster-22178.63189 GO:0055114 GO:0006810 GO:0055085 GO:0006118
GO:0016655 GO:0005215 GO:0016020
Cluster-22178.63190 GO:0016773 GO:0016020
Cluster-22178.63192 GO:0005515 GO:0061630 GO:0008270 GO:0046872

Cluster-22178.63195 GO:0008053 GO:0006810 GO:0010508 GO:0007264
GO:0005525 GO:0003724 GO:0005524 GO:0003924 GO:0016887
GO:0003723 GO:0016021 GO:0005741
Cluster-22178.63196 GO:0003950
Cluster-22178.63198 GO:0006418 GO:0000166 GO:0005524 GO:0004812

Cluster-22178.63199 GO:0007264 GO:0005515 GO:0005525 GO:0016887
GO:0003924 GO:0005524
Cluster-22178.63200 GO:0055114 GO:0009058 GO:0005524 GO:0016491
GO:0003676 GO:0016787 GO:0010181 GO:0003677 GO:0005634
Cluster-22178.63201 GO:0006355 GO:0003723 GO:0003676 GO:0005634
Cluster-22178.63207 GO:0035556 GO:0005515
Cluster-22178.63212 GO:0006508 GO:0004222

Cluster-22178.63213 GO:0005515
Cluster-22178.63214 GO:0005096 GO:0005515
Cluster-22178.63216 GO:0005198 GO:0003779 GO:0005856
Cluster-22178.63220 GO:0006355 GO:0006338 GO:0005524 GO:0008270
GO:0003676 GO:0016787 GO:0003677 GO:0016818 GO:0005856
GO:0005739 GO:0005634
Cluster-22178.63224 GO:0003824
Cluster-22178.63225 GO:0003824
Cluster-22178.63227 GO:0006629 GO:0007186 GO:0009401 GO:0005515
GO:0004930 GO:0016021
Cluster-22178.63228 GO:0005515 GO:0016020
Cluster-22178.63229 GO:0007018 GO:0007017 GO:0008017 GO:0003777
GO:0005524 GO:0045298 GO:0005874
Cluster-22178.63230 GO:0055114 GO:0016627
Cluster-22178.63231 GO:0048511 GO:0048870 GO:0006810 GO:0006355
GO:0003700 GO:0043565 GO:0005198 GO:0005515 GO:0016021
GO:0031514 GO:0005667 GO:0019028
Cluster-22178.63234 GO:0006510 GO:0006508 GO:0016567 GO:0042710
GO:0008270 GO:0004176 GO:0046872 GO:0004842 GO:0061630
GO:0005515
Cluster-22178.63236 GO:0045454 GO:0005515
Cluster-22178.63240 GO:0006886 GO:0016192 GO:0030131 GO:0005622
Cluster-22178.63242 GO:0006144 GO:0030494 GO:0006206 GO:0061024
GO:0019685 GO:0007165 GO:0006351 GO:0016836 GO:0005179
GO:0003899 GO:0003677 GO:0030176 GO:0005576 GO:0005730
Cluster-22178.63244 GO:0006898 GO:0007165 GO:0005044 GO:0016020
Cluster-22178.63246 GO:0006468 GO:0004672 GO:0005524 GO:0016773
GO:0016020
Cluster-22178.63248 GO:0006950 GO:0051087 GO:0001671
Cluster-22178.63257 GO:0006888 GO:0016032 GO:0006508 GO:0003676
GO:0008233 GO:0005622 GO:0005801
Cluster-22178.63261 GO:0006355 GO:0004112
Cluster-22178.63262 GO:0005515

Cluster-22178.63263 GO:0007283
Cluster-22178.63264 GO:0019835 GO:0009405 GO:0006122 GO:0035556
GO:0055114 GO:0005515 GO:0016491 GO:0005750 GO:0019867
Cluster-22178.63269 GO:0005509
Cluster-22178.63271 GO:0006505 GO:0005975 GO:0045454 GO:0006886
GO:0005985 GO:0005982 GO:0008810 GO:0016788 GO:0005509
Cluster-22178.63272 GO:0016032
Cluster-22178.63273 GO:0006468 GO:0016773 GO:0005524 GO:0004672
GO:0016020
Cluster-22178.63274 GO:0005515 GO:0008270
Cluster-22178.63275 GO:0006886 GO:0016192 GO:0005198 GO:0005515
GO:0030117
Cluster-22178.63276 GO:0016021
Cluster-22178.63278 GO:0006281 GO:0006355 GO:0051276 GO:0005515
GO:0003677 GO:0008168 GO:0005524 GO:0008270 GO:0005694
Cluster-22178.63280 GO:0008270
Cluster-22178.63282 GO:0006098 GO:0006094 GO:0006096 GO:0006013
GO:0006000 GO:0006012 GO:0016301 GO:0003872 GO:0005945
Cluster-22178.63284 GO:0009186 GO:0055114 GO:0030246
Cluster-22178.63286 GO:0055114 GO:0009186 GO:0030246 GO:0016021
Cluster-22178.63287 GO:0006355 GO:0006508 GO:0043087 GO:0003677
GO:0008233 GO:0005524 GO:0005634 GO:0016021
Cluster-22178.63288 GO:0001522 GO:0051090 GO:0042254 GO:0030515
GO:0005515 GO:0005524 GO:0005634 GO:0072588
Cluster-22178.63291 GO:0055114 GO:0007165 GO:0008152 GO:0016758
GO:0005509 GO:0016706 GO:0005578
Cluster-22178.63293 GO:0006402 GO:0019722 GO:0051252 GO:0003676
GO:0004535 GO:0046872 GO:0005515 GO:0003723 GO:0005737
GO:0005634
Cluster-22178.63295 GO:0009228 GO:0033617 GO:0004789 GO:0005739
Cluster-22178.63296 GO:0006355 GO:0006810 GO:0022900 GO:0016301
GO:0008134 GO:0005524 GO:0005739 GO:0005667 GO:0005747
Cluster-22178.63297 GO:0042967 GO:0046486 GO:0004144

Cluster-22178.63298 GO:0006355 GO:0003700 GO:0042025 GO:0005667
Cluster-22178.63301 GO:0016021
Cluster-22178.63304 GO:0006260 GO:0006144 GO:0006206 GO:0015979
GO:0016567 GO:0009186 GO:0055114 GO:0006886 GO:0004748
GO:0004842 GO:0005524 GO:0046872 GO:0005198 GO:0000166
GO:0005680 GO:0016020 GO:0009538 GO:0005801 GO:0005971
GO:0009522 GO:0019031 GO:0019028
Cluster-22178.63305 GO:0035307 GO:0030436 GO:0006508 GO:0004190
GO:0071595
Cluster-22178.63306 GO:0006260 GO:0006914 GO:0003887 GO:0001882
GO:0008092 GO:0042575 GO:0019898 GO:0005737
Cluster-22178.63307 GO:0006468 GO:0051301 GO:0016310 GO:0009069
GO:0006914 GO:0006606 GO:0048193 GO:0006355 GO:0051258
GO:0048870 GO:0007059 GO:0007067 GO:0000090 GO:0007165
GO:0006614 GO:0030168 GO:0031110 GO:0005198 GO:0042803
GO:0003677 GO:0005524 GO:0008017 GO:0045502 GO:0008134
GO:0005525 GO:0003700 GO:0030674 GO:0005096 GO:0004674
GO:0016872 GO:0005102 GO:0045298 GO:0005667 GO:0016020
GO:0005876 GO:0005577 GO:0031514 GO:0005840 GO:0016021
GO:0005882 GO:0030286 GO:0000940
Cluster-22178.63310 GO:0009236 GO:0042819 GO:0016070 GO:0042823
GO:0016032 GO:0006541 GO:0004359 GO:0003824 GO:0016787
GO:0005515 GO:0019012
Cluster-22178.63311 GO:0007076 GO:0000796
Cluster-22178.63315 GO:0003676
Cluster-22178.63316 GO:0003723
Cluster-22178.63317 GO:0005201 GO:0005578 GO:0005581
Cluster-22178.63318 GO:0055114 GO:0030328 GO:0016491 GO:0016670

Cluster-22178.63319 GO:0007186 GO:0007165 GO:0004871 GO:0005834
Cluster-22178.63320 GO:0007165 GO:0007275 GO:0006468 GO:0016055
GO:0005102 GO:0004672 GO:0005524 GO:0005576
Cluster-22178.63321 GO:0006468 GO:0005524 GO:0004672 GO:0005515

Cluster-22178.63322 GO:0055114 GO:0008152 GO:0051287 GO:0050662
GO:0016491 GO:0003824
Cluster-22178.63323 GO:0008210 GO:0008207 GO:0008209 GO:0055114
GO:0006694 GO:0050662 GO:0003854 GO:0016616 GO:0003824
Cluster-22178.63326 GO:0045947 GO:0008190
Cluster-22178.63328 GO:0005515
Cluster-22178.63330 GO:0007264 GO:0043087 GO:0007165 GO:0005085

Cluster-22178.63332 GO:0051090 GO:0005634
Cluster-22178.63333 GO:0015675 GO:0035444 GO:0008152 GO:0046872
GO:0003824 GO:0015099 GO:0016021
Cluster-22178.63336 GO:0007018 GO:0007017 GO:0005515 GO:0008017
GO:0003777 GO:0005524 GO:0045298 GO:0005874
Cluster-22178.63337 GO:0006276 GO:0005515
Cluster-22178.63339 GO:0006508 GO:0004185
Cluster-22178.63341 GO:0009069 GO:0042742 GO:0006388 GO:0009401
GO:0016310 GO:0003972 GO:0005524 GO:0004674
Cluster-22178.63344 GO:0009405 GO:0006468 GO:0019865 GO:0003779
GO:0005524 GO:0004672 GO:0005515 GO:0051082 GO:0019814
Cluster-22178.63345 GO:0003676
Cluster-22178.63346 GO:0055085 GO:0006811 GO:0005216 GO:0016020
GO:0016021
Cluster-22178.63347 GO:0008152 GO:0016829 GO:0010333 GO:0005515

Cluster-22178.63348 GO:0046034 GO:0034227 GO:0006468 GO:0006144
GO:0002098 GO:0004672 GO:0046872 GO:0003676 GO:0000049
GO:0020037 GO:0005524 GO:0008270 GO:0016787 GO:0003677
GO:0004017 GO:0005634
Cluster-22178.63349 GO:0007264 GO:0051087 GO:0005096
Cluster-22178.63350 GO:0008270
Cluster-22178.63352 GO:0006351 GO:0003676 GO:0005515 GO:0008270
GO:0005639

Cluster-22178.63354 GO:0016579 GO:0034477 GO:0007276 GO:0006364
GO:0006508 GO:0003676 GO:0004222 GO:0036459 GO:0008270
GO:0004518 GO:0004857 GO:0032183 GO:0070140 GO:0030246
GO:0031012
Cluster-22178.63355 GO:0007186 GO:0007166 GO:0004930 GO:0008017
GO:0045298 GO:0016020 GO:0016021
Cluster-22178.63357 GO:0005515
Cluster-22178.63360 GO:0006810 GO:0005622
Cluster-22178.63361 GO:0006090 GO:0006633 GO:0005198 GO:0016874
GO:0003989 GO:0009317
Cluster-22178.63362 GO:0045859 GO:0006469 GO:0046907 GO:0035556
GO:0004862 GO:0005952
Cluster-22178.63365 GO:0006139 GO:0003676 GO:0008408
Cluster-22178.63366 GO:0006576 GO:0006413 GO:0007264 GO:0006446
GO:0015684 GO:0003743 GO:0005515 GO:0046983 GO:0003924
GO:0005524 GO:0015093 GO:0005525 GO:0005576 GO:0016021
GO:0005840
Cluster-22178.63368 GO:0005515 GO:0003677
Cluster-22178.63369 GO:0006260
Cluster-22178.63370 GO:0008270
Cluster-22178.63371 GO:0006367 GO:0005515 GO:0005672
Cluster-22178.63372 GO:0006352 GO:0005634
Cluster-22178.63374 GO:0055114 GO:0009058 GO:0003676 GO:0016491
GO:0005524 GO:0003677 GO:0010181 GO:0016787
Cluster-22178.63377 GO:0070403 GO:0046872 GO:0016787 GO:0000287
GO:0030976
Cluster-22178.63379 GO:0016772
Cluster-22178.63383 GO:0006481 GO:0006479 GO:0004671 GO:0016021
Cluster-22178.63384 GO:0006260 GO:0055085 GO:0006364 GO:0006270
GO:0030001 GO:0005515 GO:0046873 GO:0032040 GO:0016020
GO:0005732 GO:0034457 GO:0005634
Cluster-22178.63386 GO:0006355 GO:0003677
Cluster-22178.63388 GO:0035556 GO:0009190 GO:0016849

Cluster-22178.63389 GO:0009399 GO:0006040 GO:0032509 GO:0006051
GO:0043130 GO:0033926 GO:0047465 GO:0005515 GO:0030151
GO:0016021 GO:0000813
Cluster-22178.63390 GO:0009056 GO:0006308 GO:0016888 GO:0008270

Cluster-22178.63391 GO:0006468 GO:0005524 GO:0004672
Cluster-22178.63392 GO:0006351 GO:0006355 GO:0006260 GO:0003887
GO:0003677 GO:0003682 GO:0008270 GO:0042575 GO:0005634
GO:0000785 GO:0000775
Cluster-22178.63393 GO:0007155 GO:0005540 GO:0016021
Cluster-22178.63395 GO:0003723
Cluster-22178.63397 GO:0019079 GO:0003678 GO:0005657
Cluster-22178.63400 GO:0000045 GO:0005737
Cluster-22178.63401 GO:0008152 GO:0031419 GO:0016866 GO:0046872

Cluster-22178.63403 GO:0006811 GO:0006888 GO:0006810 GO:0007172
GO:0007165 GO:0006886 GO:0006468 GO:0004713 GO:0004871
GO:0005524 GO:0004672 GO:0005230 GO:0030127 GO:0016020
GO:0005925
Cluster-22178.63404 GO:0000041 GO:0016021
Cluster-22178.63415 GO:0019058 GO:0055114 GO:0000398 GO:0045454
GO:0016209 GO:0016491 GO:0005681
Cluster-22178.63416 GO:0035556 GO:0005525 GO:0003724 GO:0005524
GO:0046872 GO:0000166 GO:0003677 GO:0005515 GO:0003723
Cluster-22178.63417 GO:0006810 GO:0003333 GO:0006355 GO:0055085
GO:0006865 GO:0015171 GO:0003700 GO:0008270 GO:0016020
GO:0005634 GO:0005667
Cluster-22178.63419 GO:0019497 GO:0006144 GO:0006807 GO:0050830
GO:0006771 GO:0006164 GO:0031423 GO:0046872 GO:0004643
GO:0003937 GO:0003993 GO:0005515 GO:0042720 GO:0005576
GO:0019012
Cluster-22178.63421 GO:0005975 GO:0016773
Cluster-22178.63426 GO:0042578

Cluster-22178.63427 GO:0006886 GO:0048870 GO:0051258 GO:0045995
GO:0007155 GO:0030155 GO:0006810 GO:0007165 GO:0030334
GO:0030168 GO:0000921 GO:0006606 GO:0045502 GO:0042803
GO:0008134 GO:0008092 GO:0030674 GO:0005102 GO:0031514
GO:0005737 GO:0005801 GO:0005794 GO:0016021 GO:0005667
GO:0016020 GO:0005940 GO:0030286 GO:0005886 GO:0005577
GO:0019898
Cluster-22178.63428 GO:0006000 GO:0019497 GO:0006013 GO:0006771
GO:0005524 GO:0003993 GO:0016301 GO:0003873
Cluster-22178.63429 GO:0007067 GO:0007049 GO:0006367 GO:0005672
GO:0005634 GO:0000775
Cluster-22178.63430 GO:0003676
Cluster-22178.63431 GO:0005515
Cluster-22178.63433 GO:0006355 GO:0000917 GO:0006119 GO:0015986
GO:0043093 GO:0015992 GO:0016817 GO:0043565 GO:0003700
GO:0003677 GO:0046961 GO:0046933 GO:0005634 GO:0005737
GO:0005667 GO:0045259 GO:0045261
Cluster-22178.63434 GO:0006119 GO:0000917 GO:0006355 GO:0043093
GO:0015986 GO:0015992 GO:0003700 GO:0043565 GO:0046961
GO:0003677 GO:0046933 GO:0005634 GO:0005737 GO:0045261
GO:0045259 GO:0005667
Cluster-22178.63435 GO:0005515
Cluster-22178.63436 GO:0015992 GO:0006123 GO:0004129 GO:0005739
GO:0045277
Cluster-22178.63438 GO:0042254 GO:0006412 GO:0003735 GO:0005840
Cluster-22178.63440 GO:0042254 GO:0006412 GO:0003735 GO:0005840
GO:0005622
Cluster-22178.63441 GO:0006118 GO:0006119 GO:0055114 GO:0015992
GO:0008121
Cluster-22178.63447 GO:0015986 GO:0046933 GO:0045259
Cluster-22178.63452 GO:0006281 GO:0006260 GO:0016787 GO:0010181
GO:0003677 GO:0016818 GO:0005524 GO:0016874 GO:0003676
GO:0043140 GO:0005634 GO:0016020 GO:0005622 GO:0005657

Cluster-22178.63456 GO:0042254 GO:0006412 GO:0000723 GO:0003735
GO:0003677 GO:0005840 GO:0005622 GO:0000784
Cluster-22178.63457 GO:0042254 GO:0006412 GO:0000723 GO:0003735
GO:0003677 GO:0005840 GO:0005622 GO:0000784
Cluster-22178.63459 GO:0007017 GO:0007018 GO:0005515 GO:0035091
GO:0005524 GO:0003777 GO:0008017 GO:0005874 GO:0045298
Cluster-22178.63460 GO:0006355 GO:0005515 GO:0003677 GO:0003700
GO:0008168 GO:0043565 GO:0005667 GO:0005634
Cluster-22178.63461 GO:0008207 GO:0008209 GO:0006813 GO:0008210
GO:0008152 GO:0006694 GO:0055114 GO:0016616 GO:0050662
GO:0003854 GO:0003824 GO:0016491
Cluster-22178.63464 GO:0005515 GO:0005509
Cluster-22178.63466 GO:0005509 GO:0005515
Cluster-22178.63467 GO:0007264 GO:0005525 GO:0005524 GO:0003924
GO:0016887 GO:0016020
Cluster-22178.63468 GO:0008152 GO:0006886 GO:0046872 GO:0008484
GO:0003824 GO:0030176
Cluster-22178.63469 GO:0008612
Cluster-22178.63471 GO:0007186 GO:0004190 GO:0005515 GO:0004930
GO:0016021
Cluster-22178.63475 GO:0007165 GO:0006955 GO:0005125 GO:0016817
GO:0005576 GO:0005789 GO:0016021
Cluster-22178.63485 GO:0003676
Cluster-22178.63486 GO:0055114 GO:0030246 GO:0050485
Cluster-22178.63488 GO:0006810 GO:0005215 GO:0005515 GO:0008270

Cluster-22178.63489 GO:0008033 GO:0006364 GO:0003723 GO:0008168
GO:0005515
Cluster-22178.63492 GO:0007186 GO:0015684 GO:0007165 GO:0007264
GO:0046500 GO:0006554 GO:0006464 GO:0006479 GO:0006576
GO:0008152 GO:0005524 GO:0015093 GO:0016740 GO:0018024
GO:0004871 GO:0005525 GO:0003924 GO:0008168 GO:0004719
GO:0031683 GO:0019001 GO:0016021

Cluster-22178.63495 GO:0006886 GO:0048280 GO:0015031 GO:0008565 GO:0016020 GO:0000139 GO:0005737
Cluster-22178.63496 GO:0009186 GO:0055114 GO:0030246 GO:0016021
Cluster-22178.63500 GO:0055085 GO:0016021
Cluster-22178.63502 GO:0015992 GO:0046034 GO:0003723 GO:0003924 GO:0016887 GO:0043531 GO:0005524 GO:0003724 GO:0005525
Cluster-22178.63503 GO:0034508 GO:0004867 GO:0005515 GO:0000776
Cluster-22178.63505 GO:0008270 GO:0005524 GO:0004222 GO:0016021
Cluster-22178.63506 GO:0009058 GO:0055114 GO:0016787 GO:0010181 GO:0003677 GO:0016491 GO:0005524 GO:0003676
Cluster-22178.63508 GO:0004672 GO:0019028
Cluster-22178.63512 GO:0006265 GO:0003677 GO:0003917 GO:0005694
Cluster-22178.63515 GO:0008152 GO:0016846
Cluster-22178.63516 GO:0006508 GO:0006468 GO:0005515 GO:0008233 GO:0004672 GO:0005524 GO:0045182 GO:0005634
Cluster-22178.63517 GO:0007275 GO:0006355 GO:0003700 GO:0005667 GO:0042600
Cluster-22178.63518 GO:0008152 GO:0016846
Cluster-22178.63519 GO:0005515
Cluster-22178.63520 GO:0006468 GO:0006508 GO:0004672 GO:0005524 GO:0045182 GO:0008233 GO:0005515 GO:0005634
Cluster-22178.63521 GO:0006468 GO:0006508 GO:0009405 GO:0005515 GO:0004672 GO:0005524 GO:0045182 GO:0008233 GO:0005634
Cluster-22178.63522 GO:0006811 GO:0035556 GO:0055085 GO:0006810 GO:0006816 GO:0042030 GO:0005515 GO:0005216 GO:0005246 GO:0016020
Cluster-22178.63524 GO:0015992 GO:0006260 GO:0042254 GO:0006470 GO:0015991 GO:0006570 GO:0006412 GO:0009405 GO:0006351 GO:0003735 GO:0008138 GO:0003677 GO:0004725 GO:0015078 GO:0003887 GO:0005622 GO:0016021 GO:0005840 GO:0033179 GO:0042575
Cluster-22178.63526 GO:0006633 GO:0008152 GO:0042967 GO:0008610 GO:0005524 GO:0016747 GO:0004315 GO:0005835

Cluster-22178.63532 GO:0006464 GO:0006144 GO:0046034 GO:0006281
GO:0046872 GO:0005524 GO:0008270 GO:0016151 GO:0004017
Cluster-22178.63534 GO:0006281 GO:0006144 GO:0006464 GO:0046034
GO:0004017 GO:0046872 GO:0008270 GO:0016151
Cluster-22178.63535 GO:0016020
Cluster-22178.63537 GO:0016757
Cluster-22178.63538 GO:0006376 GO:0003729 GO:0005685
Cluster-22178.63539 GO:0006554 GO:0008152 GO:0006468 GO:0006479
GO:0008168 GO:0005524 GO:0004672 GO:0018024 GO:0005515
Cluster-22178.63540 GO:0005515
Cluster-22178.63541 GO:0007264 GO:0042254 GO:0006412 GO:0006614
GO:0003723 GO:0003735 GO:0098519 GO:0016887 GO:0003924
GO:0005524 GO:0003724 GO:0005525 GO:0005622 GO:0005840
Cluster-22178.63542 GO:0015991 GO:0006412 GO:0006119 GO:0015992
GO:0042254 GO:0003924 GO:0005525 GO:0051082 GO:0003723
GO:0005524 GO:0016887 GO:0003724 GO:0046961 GO:0003735
GO:0005840 GO:0005622 GO:0033178
Cluster-22178.63545 GO:0006284 GO:0006576 GO:0005525 GO:0003924
GO:0005524 GO:0003905 GO:0003677 GO:0030246
Cluster-22178.63546 GO:0008152 GO:0003677 GO:0008168 GO:0016021
Cluster-22178.63547 GO:0006816 GO:0006874 GO:0018279 GO:0006355
GO:0006506 GO:0006810 GO:0004579 GO:0003677 GO:0017176
GO:0000062 GO:0005219 GO:0005634 GO:0008250 GO:0005622
GO:0016021
Cluster-22178.63550 GO:0006817 GO:0008654 GO:0055085 GO:0006812
GO:0005315 GO:0016780 GO:0008324 GO:0016020 GO:0016021
Cluster-22178.63551 GO:0042254 GO:0006412 GO:0007218 GO:0003735
GO:0005185 GO:0005576 GO:0005840 GO:0005622
Cluster-22178.63553 GO:0008152 GO:0008168
Cluster-22178.63554 GO:0006270 GO:0006355 GO:0003700 GO:0043565
GO:0005667
Cluster-22178.63558 GO:0006000 GO:0015976 GO:0006013 GO:0005975
GO:0006096 GO:0006094 GO:0006098 GO:0042578 GO:0042132

Cluster-22178.63559 GO:0016567 GO:0004842 GO:0005515 GO:0008270 GO:0046872 GO:0005680

Cluster-22178.63562 GO:0004672

Cluster-22178.63563 GO:0006094 GO:0006098 GO:0006096 GO:0006000 GO:0006013 GO:0006012 GO:0003872 GO:0005945 GO:0019031

Cluster-22178.63564 GO:0006576 GO:0006351 GO:0055114 GO:0008152 GO:0007264 GO:0042254 GO:0006144 GO:0006206 GO:0003723 GO:0003899 GO:0005525 GO:0003677 GO:0016491 GO:0005524 GO:0003724 GO:0005634 GO:0005730

Cluster-22178.63565 GO:0006397 GO:0003723 GO:0005634 GO:0005737

Cluster-22178.63569 GO:0006940

Cluster-22178.63570 GO:0006366 GO:0006265 GO:0005524 GO:0003918 GO:0003677

Cluster-22178.63571 GO:0006418 GO:0000166 GO:0005524 GO:0004812 GO:0003723

Cluster-22178.63572 GO:0006118 GO:0055114 GO:0005515 GO:0003677 GO:0016655

Cluster-22178.63575 GO:0016773 GO:0046872 GO:0030414 GO:0008270 GO:0005515 GO:0005509 GO:0005576

Cluster-22178.63577 GO:0042254 GO:0006412 GO:0003735 GO:0005840 GO:0005622

Cluster-22178.63582 GO:0009058 GO:0003824

Cluster-22178.63583 GO:0046872

Cluster-22178.63584 GO:0042967 GO:0008080 GO:0016747

Cluster-22178.63585 GO:0007186 GO:0055085 GO:0006812 GO:0008324 GO:0004930 GO:0005886 GO:0016021

Cluster-22178.63586 GO:0006355 GO:0006281 GO:0003677 GO:0008270 GO:0008168 GO:0016020

Cluster-22178.63587 GO:0006270 GO:0009306 GO:0005515

Cluster-22178.63590 GO:0006144 GO:0006807 GO:0006396 GO:0003723 GO:0004000

Cluster-22178.63592 GO:0005524

Cluster-22178.63593 GO:0005524

Cluster-22178.63599 GO:0006446 GO:0006413 GO:0003743 GO:0005840
Cluster-22178.63600 GO:0032040
Cluster-22178.63601 GO:0009235
Cluster-22178.63603 GO:0008152 GO:0005524 GO:0003824
Cluster-22178.63605 GO:0055114 GO:0006554 GO:0006574 GO:0006633
GO:0008152 GO:0006568 GO:0006552 GO:0006631 GO:0018874
GO:0006550 GO:0016491 GO:0003824 GO:0016836 GO:0003857
Cluster-22178.63607 GO:0030244 GO:0005515 GO:0019867
Cluster-22178.63610 GO:0008210 GO:0008209 GO:0008207 GO:0055114
GO:0008152 GO:0006694 GO:0003854 GO:0050662 GO:0016616
GO:0005515 GO:0046872 GO:0016491 GO:0003824
Cluster-22178.63611 GO:0005515 GO:0005509
Cluster-22178.63613 GO:0090200 GO:0043065 GO:0097193 GO:0007165
GO:0005739 GO:0016020
Cluster-22178.63615 GO:0015628 GO:0040007 GO:0015031 GO:0008283
GO:0006614 GO:0006810 GO:0007165 GO:0005525 GO:0003676
GO:0008083 GO:0005515 GO:0005524 GO:0016887 GO:0008565
GO:0004386 GO:0015627
Cluster-22178.63616 GO:0003676 GO:0016020
Cluster-22178.63618 GO:0006468 GO:0005524 GO:0004672
Cluster-22178.63619 GO:0006396 GO:0016779 GO:0003723
Cluster-22178.63620 GO:0016020
Cluster-22178.63621 GO:0030261 GO:0003677 GO:0009507
Cluster-22178.63622 GO:0015074 GO:0006810 GO:0007186 GO:0008200
GO:0004930 GO:0005576 GO:0016020
Cluster-22178.63623 GO:0007165 GO:0006606 GO:0007218 GO:0007034
GO:0006914 GO:0070206 GO:0051028 GO:0015992 GO:0030474
GO:0010508 GO:0015991 GO:0031144 GO:0071630 GO:0006308
GO:0048309 GO:0003677 GO:0005179 GO:0008855 GO:0005198
GO:0005200 GO:0015078 GO:0005823 GO:0033179 GO:0009318
GO:0005634 GO:0005576 GO:0005856
Cluster-22178.63624 GO:0008283 GO:0040007 GO:0007165 GO:0008083

Cluster-22178.63625 GO:0006118 GO:0055114 GO:0003995 GO:0003697 GO:0070188 GO:0005856

Cluster-22178.63626 GO:0006355 GO:0003700 GO:0005515 GO:0042025 GO:0005667

Cluster-22178.63630 GO:0005515 GO:0051539 GO:0016020 GO:0005887

Cluster-22178.63632 GO:0006810 GO:0055085 GO:0005215 GO:0022857 GO:0016021 GO:0016020

Cluster-22178.63634 GO:0008270

Cluster-22178.63635 GO:0043087 GO:0035023 GO:0005089 GO:0005515

Cluster-22178.63636 GO:0006813 GO:0005249 GO:0008076

Cluster-22178.63637 GO:0006605 GO:0006886 GO:0048193 GO:0009297 GO:0005515 GO:0016020 GO:0005742 GO:0019867

Cluster-22178.63638 GO:0015991 GO:0006814 GO:0042773 GO:0055114 GO:0015992 GO:0006120 GO:0006744 GO:0008137 GO:0015078 GO:0033177

Cluster-22178.63641 GO:0008033 GO:0051252 GO:0006364 GO:0004540 GO:0008168

Cluster-22178.63642 GO:0006508 GO:0006771 GO:0006355 GO:0019497 GO:0004252 GO:0005515 GO:0008270 GO:0003993 GO:0005634

Cluster-22178.63643 GO:0006468 GO:0006817 GO:0005524 GO:0004672

Cluster-22178.63645 GO:0005515

Cluster-22178.63646 GO:0015992 GO:0055114 GO:0019069 GO:0015991 GO:0009405 GO:0008270 GO:0004800 GO:0015078 GO:0003723 GO:0005515 GO:0033179 GO:0016020

Cluster-22178.63647 GO:0006423 GO:0006534 GO:0005524 GO:0004817 GO:0000166 GO:0005737

Cluster-22178.63648 GO:0015031 GO:0006355 GO:0008270 GO:0008565 GO:0016021 GO:0005634

Cluster-22178.63649 GO:0006355 GO:0005198 GO:0003700 GO:0005667 GO:0019028 GO:0042025

Cluster-22178.63650 GO:0006468 GO:0016310 GO:0051301 GO:0009069

GO:0006606 GO:0048193 GO:0006914 GO:0006355 GO:0007059
GO:0051258 GO:0048870 GO:0007165 GO:0006614 GO:0007067
GO:0000090 GO:0030168 GO:0031110 GO:0005198 GO:0042803
GO:0003677 GO:0008017 GO:0005524 GO:0008134 GO:0045502
GO:0005525 GO:0003700 GO:0030674 GO:0004674 GO:0016872
GO:0005102 GO:0005096 GO:0045298 GO:0016020 GO:0005667
GO:0005876 GO:0005577 GO:0031514 GO:0005840 GO:0016021
GO:0005882 GO:0030286 GO:0000940
Cluster-22178.63652 GO:0006338 GO:0003677 GO:0090544
Cluster-22178.63653 GO:0042254 GO:0006412 GO:0016773 GO:0005515
GO:0003735 GO:0005622 GO:0005840
Cluster-22178.63657 GO:0006355 GO:0006468 GO:0030071 GO:0007010
GO:0031145 GO:0005515 GO:0005524 GO:0004672 GO:0003779
GO:0005634 GO:0019028 GO:0016020 GO:0005680
Cluster-22178.63658 GO:0006468 GO:0007010 GO:0005524 GO:0004672
GO:0003779 GO:0019028
Cluster-22178.63661 GO:0006123 GO:0015992 GO:0004129 GO:0045277
GO:0005739
Cluster-22178.63662 GO:0009401 GO:0006810 GO:0005886
Cluster-22178.63663 GO:0006270 GO:0000398 GO:0003677 GO:0005681
GO:0032040
Cluster-22178.63666 GO:0006508 GO:0008234 GO:0004197
Cluster-22178.63668 GO:0006508 GO:0004197
Cluster-22178.63672 GO:0005198 GO:0003779 GO:0005856
Cluster-22178.63673 GO:0006950 GO:0051087 GO:0003677 GO:0001671

Cluster-22178.63674 GO:0006351 GO:0045893 GO:0003700 GO:0005515
GO:0008270 GO:0005667 GO:0005634
Cluster-22178.63675 GO:0046872
Cluster-22178.63679 GO:0009214 GO:0004113 GO:0005515 GO:0016020
Cluster-22178.63680 GO:0005509
Cluster-22178.63681 GO:0006032 GO:0006270 GO:0006397 GO:0006367
GO:0003723 GO:0004190 GO:0008061 GO:0005737 GO:0016021

GO:0005634
Cluster-22178.63683 GO:0006470 GO:0006570 GO:0042967 GO:0004725
GO:0008138 GO:0005515 GO:0008080
Cluster-22178.63685 GO:0046660 GO:0006397 GO:0005634
Cluster-22178.63693 GO:0007165 GO:0009069 GO:0006468 GO:0016310
GO:0008152 GO:0005515 GO:0015016 GO:0016746 GO:0016787
GO:0004674 GO:0005096
Cluster-22178.63694 GO:0003723
Cluster-22178.63695 GO:0006807 GO:0050830 GO:0006144 GO:0019497
GO:0006771 GO:0006164 GO:0003937 GO:0003993 GO:0046872
GO:0031423 GO:0004643 GO:0005515 GO:0042720 GO:0019012
GO:0005576
Cluster-22178.63698 GO:0006511 GO:0031625 GO:0005515
Cluster-22178.63704 GO:0006468 GO:0004672 GO:0005524
Cluster-22178.63707 GO:0006355 GO:0016310 GO:0004673 GO:0003700
GO:0016791 GO:0005667 GO:0009365
Cluster-22178.63708 GO:0006355 GO:0046983 GO:0003700 GO:0005634
GO:0005667
Cluster-22178.63709 GO:0006308 GO:0006281 GO:0006260 GO:0017108
GO:0005634 GO:0033557
Cluster-22178.63711 GO:0019843 GO:0003723 GO:0005622
Cluster-22178.63713 GO:0006468 GO:0005524 GO:0004672
Cluster-22178.63714 GO:0008053 GO:0010508 GO:0003924 GO:0016021
GO:0005741
Cluster-22178.63716 GO:0016310 GO:0009069 GO:0006468 GO:0004674
GO:0005524 GO:0004672
Cluster-22178.63718 GO:0006629 GO:0006508 GO:0006144 GO:0004197
GO:0017111 GO:0004252 GO:0003968 GO:0031379
Cluster-22178.63719 GO:0008152 GO:0006508 GO:0008236 GO:0016787

Cluster-22178.63720 GO:0055114 GO:0000372 GO:0007165 GO:0005509
GO:0005515 GO:0016616 GO:0030529 GO:0005634 GO:0005578
Cluster-22178.63721 GO:0055085 GO:0046872 GO:0016021 GO:0016020

Cluster-22178.63722 GO:0005515
Cluster-22178.63725 GO:0007275 GO:0007186 GO:0004252 GO:0005515
GO:0004930 GO:0070008 GO:0003676 GO:0042600
Cluster-22178.63726 GO:0042967 GO:0008152 GO:0006633 GO:0016747
GO:0004315 GO:0005835
Cluster-22178.63728 GO:0009405 GO:0006221 GO:0005985 GO:0005982
GO:0007049 GO:0045747 GO:0006012 GO:0004575 GO:0004190
GO:0004222 GO:0004564 GO:0017177 GO:0016021
Cluster-22178.63729 GO:0005515
Cluster-22178.63734 GO:0008270 GO:0005515
Cluster-22178.63735 GO:0045893 GO:0016032 GO:0007165 GO:0005158
GO:0004866 GO:0005899
Cluster-22178.63736 GO:0043066 GO:0008207 GO:0008209 GO:0019079
GO:0055114 GO:0043154 GO:0006694 GO:0008210 GO:0016620
GO:0080019 GO:0043130 GO:0003854 GO:0050662 GO:0051287
GO:0016616 GO:0032266 GO:0003824 GO:0071986 GO:0005737
Cluster-22178.63737 GO:0006470 GO:0051260 GO:0006397 GO:0032006
GO:0004721 GO:0005515 GO:0005634
Cluster-22178.63740 GO:0055114 GO:0042967 GO:0008152 GO:0000902
GO:0006412 GO:0042254 GO:0006351 GO:0016491 GO:0008080
GO:0003714 GO:0003735 GO:0005622 GO:0005840 GO:0005667
GO:0005761
Cluster-22178.63742 GO:0030683
Cluster-22178.63744 GO:0051276 GO:0006281 GO:0006355 GO:0008168
GO:0008270 GO:0005524 GO:0005515 GO:0003677 GO:0005694
Cluster-22178.63745 GO:0006383 GO:0006351 GO:0006144 GO:0006206
GO:0003899 GO:0003677 GO:0005666 GO:0005730
Cluster-22178.63746 GO:0009069 GO:0006468 GO:0035556 GO:0016310
GO:0005515 GO:0004674 GO:0005524 GO:0004672
Cluster-22178.63751 GO:0043044 GO:0006338 GO:0055085 GO:0031491
GO:0016818 GO:0003677 GO:0016787 GO:0003676 GO:0005524
GO:0008270 GO:0005634 GO:0000785 GO:0016021
Cluster-22178.63754 GO:0006355 GO:0007165 GO:0046872 GO:0004871

GO:0003700 GO:0016787 GO:0005667 GO:0005634
Cluster-22178.63757 GO:0005783
Cluster-22178.63759 GO:0003677 GO:0005634
Cluster-22178.63765 GO:0005515
Cluster-22178.63766 GO:0006508 GO:0008234
Cluster-22178.63769 GO:0006355 GO:0006368 GO:0007155 GO:0055114
GO:0050660 GO:0003824 GO:0003700 GO:0008270 GO:0016491
GO:0043565 GO:0005667 GO:0008023
Cluster-22178.63770 GO:0006396 GO:0005789
Cluster-22178.63771 GO:0006396 GO:0005789
Cluster-22178.63772 GO:0007186 GO:0007165 GO:0004871 GO:0005834
Cluster-22178.63774 GO:0030168 GO:0000921 GO:0051607 GO:0007165
GO:0006457 GO:0008608 GO:0051258 GO:0048870 GO:0006412
GO:0006355 GO:0042254 GO:0007155 GO:0005102 GO:0030674
GO:0003700 GO:0008134 GO:0045502 GO:0003735 GO:0005515
GO:0042803 GO:0051082 GO:0030286 GO:0001772 GO:0005940
GO:0005840 GO:0016021 GO:0097197 GO:0031514 GO:0072686
GO:0005577 GO:0005622 GO:0016272 GO:0005667 GO:0042729
Cluster-22178.63775 GO:0005515 GO:0008270
Cluster-22178.63776 GO:0019058 GO:0006260 GO:0000166 GO:0003887
GO:0003677 GO:0008408 GO:0042575 GO:0019031
Cluster-22178.63779 GO:0006879 GO:0008199
Cluster-22178.63786 GO:0006355 GO:0006270 GO:0003682 GO:0003677
GO:0005515 GO:0008270 GO:0005634 GO:0000775 GO:0000785
Cluster-22178.63789 GO:0005634
Cluster-22178.63790 GO:0006355 GO:0043093 GO:0000917 GO:0006914
GO:0010506 GO:0003677 GO:0043565 GO:0003700 GO:0005634
GO:0005667 GO:0005737 GO:0005615
Cluster-22178.63792 GO:0006468 GO:0003676 GO:0005524 GO:0004672
GO:0005634
Cluster-22178.63794 GO:0006468 GO:0003676 GO:0004672 GO:0005524
GO:0005789 GO:0005634
Cluster-22178.63795 GO:0019051 GO:0005515 GO:0042025

Cluster-22178.63796 GO:0043565
Cluster-22178.63797 GO:0040007 GO:0008283 GO:0007165 GO:0008083
GO:0005615
Cluster-22178.63798 GO:0009847 GO:0016021
Cluster-22178.63799 GO:0005975 GO:0000902 GO:0016773 GO:0005524
GO:0003676 GO:0032040
Cluster-22178.63804 GO:0006308 GO:0055114 GO:0006260 GO:0006281
GO:0017108 GO:0016021 GO:0005634 GO:0016020 GO:0033557
Cluster-22178.63806 GO:0005515 GO:0005869
Cluster-22178.63809 GO:0006468 GO:0004672 GO:0005524 GO:0005515
GO:0016021
Cluster-22178.63811 GO:0000917 GO:0008299 GO:0043093 GO:0006694
GO:0005525 GO:0050518 GO:0005524 GO:0046983 GO:0016887
GO:0005737
Cluster-22178.63812 GO:0006270 GO:0021987 GO:0051028 GO:0005515
GO:0016021 GO:0016020 GO:0005634
Cluster-22178.63813 GO:0055114 GO:0008168 GO:0016706 GO:0005515

Cluster-22178.63814 GO:0007165 GO:0005515
Cluster-22178.63815 GO:0055114 GO:0004800
Cluster-22178.63817 GO:0006270 GO:0006275 GO:0043565 GO:0005524

Cluster-22178.63818 GO:0019722 GO:0003676 GO:0003723
Cluster-22178.63822 GO:0008152 GO:0055114 GO:0016491
Cluster-22178.63825 GO:0006309 GO:0007155 GO:0006810 GO:0007165
GO:0005173 GO:0016787 GO:0005737 GO:0005634 GO:0043190
GO:0016020
Cluster-22178.63827 GO:0006355 GO:0008270 GO:0046872 GO:0005515
GO:0005525 GO:0003677
Cluster-22178.63829 GO:0042254 GO:0006412 GO:0032957 GO:0047325
GO:0003735 GO:0005515 GO:0000287 GO:0052725 GO:0052726
GO:0046872 GO:0008270 GO:0005524 GO:0005840 GO:0005622
Cluster-22178.63830 GO:0043087 GO:0005085

Cluster-22178.63831 GO:0006886 GO:0016192 GO:0005515 GO:0051537 GO:0030131 GO:0043231
Cluster-22178.63833 GO:0006468 GO:0006817 GO:0005524 GO:0004672

Cluster-22178.63836 GO:0006891 GO:0006355 GO:0005524 GO:0003713 GO:0003677 GO:0017119 GO:0005634 GO:0005667
Cluster-22178.63837 GO:0051252 GO:0016891
Cluster-22178.63839 GO:0005515 GO:0008270
Cluster-22178.63842 GO:0006378 GO:0003729 GO:0003677 GO:0005849
Cluster-22178.63843 GO:0043401 GO:0006613 GO:0006355 GO:0003700 GO:0008270 GO:0043565 GO:0005667 GO:0005634 GO:0030176 GO:0016021 GO:0005784
Cluster-22178.63845 GO:0005198 GO:0005515 GO:0019028
Cluster-22178.63846 GO:0006576 GO:0008608 GO:0007264 GO:0007165 GO:0007186 GO:0006457 GO:0006950 GO:0051082 GO:0019001 GO:0031683 GO:0003924 GO:0005525 GO:0004871 GO:0005524 GO:0016887 GO:0072686 GO:0042729 GO:0016021 GO:0016272
Cluster-22178.63848 GO:0007049 GO:0006144 GO:0006206 GO:0006351 GO:0006355 GO:0003700 GO:0043565 GO:0003899 GO:0003677 GO:0005667 GO:0005634 GO:0005730
Cluster-22178.63849 GO:0006468 GO:0016773 GO:0004672 GO:0005524 GO:0016020
Cluster-22178.63850 GO:0007096 GO:0005634
Cluster-22178.63851 GO:0007165 GO:0034227 GO:0006308 GO:0005179 GO:0008855 GO:0005096 GO:0009318 GO:0005634 GO:0005576 GO:0005737
Cluster-22178.63854 GO:0006355 GO:0016051 GO:0005515 GO:0005634 GO:0016021
Cluster-22178.63857 GO:0019058
Cluster-22178.63859 GO:0055085 GO:0007186 GO:0006812 GO:0008324 GO:0004930 GO:0005886 GO:0016021
Cluster-22178.63860 GO:0009405
Cluster-22178.63861 GO:0015931 GO:0033227 GO:0016055 GO:0006457

GO:0008013 GO:0051082 GO:0051033 GO:0005509 GO:0016342
GO:0005789 GO:0016021 GO:0005783 GO:0016020
Cluster-22178.63862 GO:0006914 GO:0006606 GO:0009069 GO:0051301
GO:0016310 GO:0006468 GO:0030168 GO:0031110 GO:0007067
GO:0000090 GO:0006614 GO:0007165 GO:0051258 GO:0048870
GO:0007059 GO:0005524 GO:0008017 GO:0003677 GO:0042803
GO:0005198 GO:0005096 GO:0004674 GO:0005102 GO:0030674
GO:0005525 GO:0045502 GO:0008134 GO:0005577 GO:0005876
GO:0005667 GO:0045298 GO:0030286 GO:0000940 GO:0005882
GO:0005840 GO:0016021 GO:0031514
Cluster-22178.63863 GO:0006457 GO:0005515
Cluster-22178.63866 GO:0042967 GO:0008811 GO:0005515 GO:0005856
Cluster-22178.63867 GO:0007264 GO:0005525
Cluster-22178.63869 GO:0045454 GO:0019354 GO:0008033 GO:0055114
GO:0006779 GO:0071949 GO:0043115 GO:0016491 GO:0046872
GO:0050660
Cluster-22178.63870 GO:0042967 GO:0006629 GO:0008081 GO:0008080

Cluster-22178.63871 GO:0046872
Cluster-22178.63873 GO:0006338 GO:0006487 GO:0005524 GO:0003676
GO:0003677 GO:0016818 GO:0016021 GO:0005634 GO:0008250
Cluster-22178.63874 GO:0003676
Cluster-22178.63875 GO:0055085 GO:0006811 GO:0005216 GO:0016020
Cluster-22178.63876 GO:0007186 GO:0042998 GO:0004930 GO:0071253
GO:0005802
Cluster-22178.63878 GO:0042254 GO:0006412 GO:0009103 GO:0098519
GO:0003735 GO:0005524 GO:0008918 GO:0005783 GO:0005840
GO:0016021
Cluster-22178.63879 GO:0020037
Cluster-22178.63880 GO:0006376 GO:0003729 GO:0016021 GO:0005685
Cluster-22178.63881 GO:0006355 GO:0043565 GO:0003700 GO:0005515
GO:0005667 GO:0005634
Cluster-22178.63882 GO:0006464

Cluster-22178.63884 GO:0005525 GO:0003924
Cluster-22178.63885 GO:0008270 GO:0003676
Cluster-22178.63887 GO:0000902
Cluster-22178.63888 GO:0090263 GO:0016567 GO:0005515
Cluster-22178.63889 GO:0007155 GO:0005515 GO:0016021
Cluster-22178.63890 GO:0005634
Cluster-22178.63891 GO:0009186 GO:0006260 GO:0006206 GO:0055114 GO:0006144 GO:0004748 GO:0005524 GO:0005971
Cluster-22178.63892 GO:0042981
Cluster-22178.63893 GO:0019905
Cluster-22178.63894 GO:0006886 GO:0006605 GO:0008201 GO:0004867 GO:0046914 GO:0016021 GO:0005742
Cluster-22178.63895 GO:0019905
Cluster-22178.63898 GO:0042710 GO:0090063 GO:0035556 GO:0009890 GO:0000226 GO:0005515 GO:0003774 GO:0005524 GO:0016459 GO:0005815 GO:0000922
Cluster-22178.63899 GO:0006412 GO:0042254 GO:0003735 GO:0005840 GO:0005622
Cluster-22178.63901 GO:0009069 GO:0006468 GO:0006979 GO:0016310 GO:0004674 GO:0016773 GO:0005524 GO:0016020
Cluster-22178.63902 GO:0005515 GO:0003723
Cluster-22178.63905 GO:0015031 GO:0006464 GO:0005515
Cluster-22178.63906 GO:0003723
Cluster-22178.63907 GO:0042157 GO:0006869 GO:0008270 GO:0003677 GO:0008289 GO:0005576 GO:0019028
Cluster-22178.63908 GO:0042158 GO:0009249 GO:0016757 GO:0003677 GO:0016020
Cluster-22178.63909 GO:0055114 GO:0006629 GO:0016491 GO:0016021
Cluster-22178.63910 GO:0007155 GO:0008152 GO:0042246 GO:0016491 GO:0016021
Cluster-22178.63911 GO:0007186 GO:0006890 GO:0004930 GO:0019028 GO:0016021
Cluster-22178.63912 GO:0019722 GO:0006556 GO:0006555 GO:0030154

GO:0004478 GO:0016021
Cluster-22178.63915 GO:0007049 GO:0006355 GO:0003700 GO:0046872
GO:0043565 GO:0019901 GO:0042803 GO:0008134 GO:0045502
GO:0030286 GO:0005667
Cluster-22178.63920 GO:0042742
Cluster-22178.63922 GO:0006741 GO:0006769 GO:0008152 GO:0046497
GO:0003951 GO:0016301
Cluster-22178.63923 GO:0007165 GO:0008601 GO:0005515 GO:0000159
Cluster-22178.63926 GO:0015947 GO:0006084 GO:0018492 GO:0005515

Cluster-22178.63929 GO:0008201 GO:0009512
Cluster-22178.63932 GO:0045893 GO:0005515 GO:0005634
Cluster-22178.63933 GO:0005515
Cluster-22178.63934 GO:0005515
Cluster-22178.63935 GO:0055114 GO:0006323 GO:0016491 GO:0005198
GO:0005882
Cluster-22178.63936 GO:0006412 GO:0042254 GO:0003735 GO:0005622
GO:0005840
Cluster-22178.63937 GO:0000045 GO:0046486 GO:0006071 GO:0004371
GO:0005737
Cluster-22178.63939 GO:0008152 GO:0016491
Cluster-22178.63940 GO:0009405 GO:0006508 GO:0006629 GO:0005198
GO:0008236 GO:0005515
Cluster-22178.63943 GO:0046872 GO:0005515 GO:0003677
Cluster-22178.63946 GO:0006355 GO:0003677
Cluster-22178.63948 GO:0015992 GO:0015991 GO:0015078 GO:0033177
Cluster-22178.63950 GO:0006541 GO:0009252 GO:0046436 GO:0008716
GO:0016787 GO:0005524 GO:0046872
Cluster-22178.63952 GO:0006206 GO:0006144 GO:0006351 GO:0003677
GO:0003899 GO:0005730
Cluster-22178.63954 GO:0042254 GO:0006412 GO:0003735 GO:0005622
GO:0005840 GO:0005789
Cluster-22178.63955 GO:0006468 GO:0042254 GO:0006412 GO:0005524

GO:0004672 GO:0003735 GO:0005622 GO:0005840

Cluster-22178.63956 GO:0006338 GO:0009405 GO:0090544

Cluster-22178.63957 GO:0042254 GO:0006412 GO:0003735 GO:0046872 GO:0005840 GO:0005622

Cluster-22178.63959 GO:0042254 GO:0008615 GO:0055114 GO:0010181 GO:0016638 GO:0004733 GO:0016021 GO:0005622

Cluster-22178.63962 GO:0055114 GO:0005507 GO:0005515

Cluster-22178.63963 GO:0007165 GO:0035023 GO:0043087 GO:0005089 GO:0005515 GO:0005057 GO:0030170

Cluster-22178.63964 GO:0006122 GO:0006355 GO:0006508 GO:0008270 GO:0043565 GO:0004222 GO:0003700 GO:0005515 GO:0031012 GO:0005743 GO:0005750 GO:0005634 GO:0005667

Cluster-22178.63966 GO:0039694 GO:0006144 GO:0003723 GO:0003924 GO:0046872 GO:0003968 GO:0005525 GO:0031379

Cluster-22178.63968 GO:0006470 GO:0006570 GO:0004725 GO:0008138

Cluster-22178.63970 GO:0007264 GO:0005525

Cluster-22178.63971 GO:0006355 GO:0006813 GO:0009252 GO:0006807 GO:0006811 GO:0006542 GO:0004356 GO:0003677 GO:0005249 GO:0016020 GO:0008076

Cluster-22178.63974 GO:0003723 GO:0003676

Cluster-22178.63975 GO:0045454 GO:0006508 GO:0055114 GO:0008236 GO:0016491 GO:0005576

Cluster-22178.63976 GO:0008270

Cluster-22178.63977 GO:0006468 GO:0004672 GO:0005524 GO:0016773 GO:0016020

Cluster-22178.63978 GO:0006886 GO:0006397 GO:0015991 GO:0048193 GO:0006914 GO:0015992 GO:0007155 GO:0015078 GO:0005525 GO:0003924 GO:0042802 GO:0003774 GO:0043015 GO:0045298 GO:0016021 GO:0005801 GO:0033179 GO:0005861 GO:0016020 GO:0016459

Cluster-22178.63980 GO:0007165 GO:0007186 GO:0007264 GO:0005525 GO:0004871 GO:0031683 GO:0019001 GO:0003924

Cluster-22178.63981 GO:0016055 GO:0008013 GO:0016020 GO:0016342
Cluster-22178.63983 GO:0006919 GO:0006915 GO:0006974 GO:0001836

Cluster-22178.63984 GO:0006950 GO:0005515
Cluster-22178.63986 GO:0009451 GO:0001522 GO:0009982 GO:0003723

Cluster-22178.63987 GO:0005524 GO:0016787 GO:0003677 GO:0003676 GO:0005515
Cluster-22178.63990 GO:0006952 GO:0045454 GO:0055114 GO:0005509 GO:0016491 GO:0016209
Cluster-22178.63993 GO:0006672 GO:0006139 GO:0006807 GO:0003676 GO:0005524 GO:0008270 GO:0016818 GO:0008026 GO:0016811 GO:0016021
Cluster-22178.63995 GO:0055085 GO:0015992 GO:0055114 GO:0006119 GO:0015986 GO:0046933 GO:0046961 GO:0005515 GO:0016638 GO:0016021 GO:0045259 GO:0045261
Cluster-22178.63996 GO:0005515
Cluster-22178.63997 GO:0019491 GO:0005515 GO:0033990 GO:0016021
Cluster-22178.63998 GO:0008299
Cluster-22178.63999 GO:0046080 GO:0016787
Cluster-22178.64000 GO:0006388 GO:0002949 GO:0008152 GO:0006260 GO:0016301 GO:0003677 GO:0003972 GO:0003723 GO:0004003 GO:0003724 GO:0005525 GO:0003924 GO:0016887 GO:0005524 GO:0005657
Cluster-22178.64001 GO:0008253 GO:0000287 GO:0005737
Cluster-22178.64003 GO:0008270
Cluster-22178.64004 GO:0005515
Cluster-22178.64005 GO:0042254 GO:0001522 GO:0005515 GO:0030515 GO:0072588
Cluster-22178.64007 GO:0005515
Cluster-22178.64008 GO:0042742 GO:0005576
Cluster-22178.64010 GO:0006468 GO:0055114 GO:0016787 GO:0003723 GO:0003697 GO:0061630 GO:0005515 GO:0003677 GO:0008270

GO:0005524 GO:0004672 GO:0046872 GO:0003676 GO:0047134
Cluster-22178.64011 GO:0005789 GO:0032040
Cluster-22178.64012 GO:0006659 GO:0042967 GO:0016747 GO:0008080

Cluster-22178.64013 GO:0006468 GO:0005524 GO:0004672 GO:0016773
GO:0016020
Cluster-22178.64014 GO:0015031 GO:0008152 GO:0042254 GO:0006412
GO:0008565 GO:0016747 GO:0016746 GO:0003735 GO:0005509
GO:0005622 GO:0016021 GO:0005840
Cluster-22178.64015 GO:0008152 GO:0042254 GO:0006412 GO:0016747
GO:0003735 GO:0005509 GO:0016746 GO:0005840 GO:0005622
Cluster-22178.64016 GO:0006355 GO:0005634
Cluster-22178.64017 GO:0006123 GO:0015992 GO:0004129 GO:0045277
Cluster-22178.64020 GO:0006355 GO:0009405 GO:0008092 GO:0005198
GO:0005737 GO:0019898
Cluster-22178.64021 GO:0008270 GO:0003676
Cluster-22178.64022 GO:0051258 GO:0048870 GO:0045017 GO:0045995
GO:0007155 GO:0006810 GO:0007165 GO:0030155 GO:0046486
GO:0030334 GO:0051607 GO:0000921 GO:0030168 GO:0006914
GO:0042967 GO:0045502 GO:0008134 GO:0042803 GO:0005515
GO:0030674 GO:0004144 GO:0005102 GO:0031514 GO:0016021
GO:0005667 GO:0030286 GO:0005940 GO:0005577
Cluster-22178.64023 GO:0006355 GO:0051258 GO:0007165 GO:0007155
GO:0030168 GO:0000921 GO:0051607 GO:0005515 GO:0008134
GO:0042803 GO:0045502 GO:0003700 GO:0030674 GO:0005102
GO:0016021 GO:0097197 GO:0005667 GO:0005577 GO:0001772
GO:0005940 GO:0030286
Cluster-22178.64025 GO:0006952 GO:0007165 GO:0005576
Cluster-22178.64027 GO:0042254 GO:0006412 GO:0003735 GO:0005840
GO:0005622
Cluster-22178.64028 GO:0003676
Cluster-22178.64030 GO:0016567 GO:0008270 GO:0046872 GO:0004842
GO:0005515 GO:0061630 GO:0005680

Cluster-22178.64031 GO:0005515
Cluster-22178.64033 GO:0006338 GO:0000228
Cluster-22178.64035 GO:0030001 GO:0006355 GO:0006308 GO:0055114 GO:0055085 GO:0019953 GO:0000105 GO:0051287 GO:0046873 GO:0008855 GO:0008270 GO:0004399 GO:0016020 GO:0009318 GO:0016021
Cluster-22178.64037 GO:0003677
Cluster-22178.64039 GO:0016042 GO:0015992 GO:0046486 GO:0045454 GO:0015994 GO:0006811 GO:0015996 GO:0006118 GO:0006629 GO:0006355 GO:0015991 GO:0006508 GO:0003847 GO:0003677 GO:0016787 GO:0015035 GO:0015078 GO:0009055 GO:0046872 GO:0047746 GO:0008236 GO:0005886 GO:0008247 GO:0016020 GO:0019031 GO:0033179
Cluster-22178.64040 GO:0006378 GO:0003677 GO:0008270 GO:0046872 GO:0003729 GO:0005849
Cluster-22178.64041 GO:0003723 GO:0003676
Cluster-22178.64044 GO:0006468 GO:0004672 GO:0005524 GO:0016020
Cluster-22178.64045 GO:0008270 GO:0008168
Cluster-22178.64047 GO:0016032 GO:0005515 GO:0016021
Cluster-22178.64049 GO:0015940 GO:0019521 GO:0055114 GO:0006574 GO:0006552 GO:0006550 GO:0006568 GO:0006813 GO:0006633 GO:0006779 GO:0046168 GO:0006554 GO:0006631 GO:0019354 GO:0018874 GO:0006098 GO:0008677 GO:0043115 GO:0003857 GO:0016491 GO:0004616 GO:0016616 GO:0051287
Cluster-22178.64050 GO:0006355 GO:0007275 GO:0043565 GO:0003700 GO:0005667
Cluster-22178.64053 GO:0005515
Cluster-22178.64054 GO:0006206 GO:0009972 GO:0006807 GO:0046087 GO:0008270 GO:0004126
Cluster-22178.64055 GO:0051289 GO:0007165 GO:0006506 GO:0008140 GO:0005509 GO:0005515 GO:0016740 GO:0005789 GO:0016021 GO:0005886 GO:0005578 GO:0016012 GO:0005667
Cluster-22178.64056 GO:0006955 GO:0030153 GO:0005515 GO:0015643

GO:0019814
Cluster-22178.64062 GO:0016021 GO:0005886
Cluster-22178.64063 GO:0015992 GO:0009297 GO:0006123 GO:0030436
GO:0003774 GO:0005524 GO:0004129 GO:0005515 GO:0019867
GO:0042601 GO:0045277 GO:0016459
Cluster-22178.64067 GO:0055114 GO:0050661 GO:0016491 GO:0016020
Cluster-22178.64068 GO:0046872
Cluster-22178.64069 GO:0016192 GO:0006904 GO:0046983 GO:0046914

Cluster-22178.64074 GO:0006355 GO:0009098 GO:0003677 GO:0005515

Cluster-22178.64077 GO:0015992 GO:0006123 GO:0016310 GO:0009069
GO:0016192 GO:0006886 GO:0003676 GO:0004129 GO:0004674
GO:0005515 GO:0045277 GO:0030117
Cluster-22178.64080 GO:0005515
Cluster-22178.64081 GO:0055114 GO:0006355 GO:0010309 GO:0045735

Cluster-22178.64082 GO:0019497 GO:0006771 GO:0005515 GO:0003993
GO:0046872 GO:0016020
Cluster-22178.64083 GO:0003723
Cluster-22178.64084 GO:0006412 GO:0042254 GO:0007067 GO:0051087
GO:0003735 GO:0005515 GO:0005509 GO:0005840 GO:0005622
GO:0042729 GO:0072686
Cluster-22178.64085 GO:0006367 GO:0006364 GO:0006270 GO:0009236
GO:0006260 GO:0006633 GO:0006355 GO:0006351 GO:0006352
GO:0015940 GO:0003677 GO:0016987 GO:0005515 GO:0003887
GO:0008770 GO:0003700 GO:0005667 GO:0005732 GO:0034457
GO:0005634 GO:0005672 GO:0042575 GO:0032040
Cluster-22178.64086 GO:0006355 GO:0007165 GO:0042254 GO:0006412
GO:0005179 GO:0003677 GO:0003735 GO:0005840
Cluster-22178.64087 GO:0006508 GO:0030436 GO:0004190
Cluster-22178.64088 GO:0046034 GO:0015991 GO:0015992 GO:0016820
GO:0005524 GO:0033178

Cluster-22178.64090 GO:0007017 GO:0007018 GO:0006351 GO:0006144
GO:0006914 GO:0006206 GO:0003899 GO:0003677 GO:0005524
GO:0008017 GO:0003777 GO:0005730 GO:0045298 GO:0005874
Cluster-22178.64091 GO:0007218 GO:0030436 GO:0006206 GO:0006144
GO:0006383 GO:0001682 GO:0008033 GO:0051252 GO:0006351
GO:0003677 GO:0004526 GO:0003899 GO:0005515 GO:0030677
GO:0005730 GO:0005666 GO:0042601 GO:0005576
Cluster-22178.64092 GO:0000398
Cluster-22178.64093 GO:0006560 GO:0016567 GO:0015986 GO:0006525
GO:0018401 GO:0015992 GO:0055114 GO:0004656 GO:0015078
GO:0004842 GO:0008270 GO:0046983 GO:0016702 GO:0005783
GO:0000276
Cluster-22178.64094 GO:0003924
Cluster-22178.64095 GO:0006914 GO:0007155 GO:0007165 GO:0019058
GO:0004871 GO:0003723 GO:0005198 GO:0019031 GO:0019867
GO:0019028 GO:0016020
Cluster-22178.64096 GO:0055085 GO:0007165 GO:0000772 GO:0016021
GO:0016020
Cluster-22178.64099 GO:0006139 GO:0005524 GO:0003676 GO:0016787
GO:0004003 GO:0016818 GO:0008026 GO:0003677 GO:0005657
Cluster-22178.64103 GO:0055114 GO:0000160 GO:0008152 GO:0016310
GO:0006109 GO:0016491 GO:0004672 GO:0005524 GO:0003824
GO:0050662 GO:0051287 GO:0000155 GO:0005198 GO:0009365
GO:0019028
Cluster-22178.64104 GO:0042981 GO:0043531 GO:0005515
Cluster-22178.64109 GO:0006334 GO:0003677 GO:0005634 GO:0000786
Cluster-22178.64110 GO:0005515 GO:0008270
Cluster-22178.64111 GO:0006955 GO:0006457 GO:0007165 GO:0005164
GO:0016020
Cluster-22178.64114 GO:0055114 GO:0008152 GO:0006355 GO:0043401
GO:0006511 GO:0016491 GO:0003824 GO:0016872 GO:0031625
GO:0050662 GO:0016020
Cluster-22178.64115 GO:0055114 GO:0016638

Cluster-22178.64117 GO:0042254 GO:0006412 GO:0005515 GO:0003735 GO:0005840 GO:0005622
Cluster-22178.64124 GO:0007264 GO:0046872 GO:0005525 GO:0031419

Cluster-22178.64126 GO:0006468 GO:0004672 GO:0005524
Cluster-22178.64129 GO:0035556 GO:0006313
Cluster-22178.64130 GO:0006397 GO:0006367 GO:0006270 GO:0006032 GO:0008061 GO:0004190 GO:0003723 GO:0033691 GO:0005634 GO:0016021 GO:0005737
Cluster-22178.64131 GO:0015992 GO:0015986 GO:0000902 GO:0015078

Cluster-22178.64133 GO:0018101 GO:0006570 GO:0006807 GO:0006470 GO:0008138 GO:0005509 GO:0004668 GO:0004725 GO:0005737
Cluster-22178.64134 GO:0055085 GO:0022857 GO:0016021
Cluster-22178.64135 GO:0006810 GO:0009405 GO:0006952 GO:0019855 GO:0005576
Cluster-22178.64136 GO:0006468 GO:0005524 GO:0004672
Cluster-22178.64137 GO:0017004 GO:0017003 GO:0006468 GO:0005515 GO:0005524 GO:0004672 GO:0005886
Cluster-22178.64139 GO:0003779 GO:0005543
Cluster-22178.64140 GO:0006508 GO:0004185
Cluster-22178.64141 GO:0006457 GO:0006952 GO:0006351 GO:0006950 GO:0006260 GO:0009236 GO:0003677 GO:0003887 GO:0051082 GO:0005524 GO:0008270 GO:0042575 GO:0005634
Cluster-22178.64142 GO:0008270 GO:0016021
Cluster-22178.64144 GO:0006468 GO:0005515 GO:0005524 GO:0004672

Cluster-22178.64147 GO:0005515
Cluster-22178.64149 GO:0007018 GO:0007017 GO:0000921 GO:0003777 GO:0008017 GO:0005524 GO:0005874 GO:0045298 GO:0016021 GO:0005940
Cluster-22178.64151 GO:0006260 GO:0046983 GO:0003887 GO:0005515 GO:0042575

Cluster-22178.64153 GO:0006904 GO:0016874 GO:0000145
Cluster-22178.64154 GO:0006446 GO:0006413 GO:0003743 GO:0005515
GO:0031369 GO:0005852 GO:0005840
Cluster-22178.64158 GO:0005524 GO:0008270 GO:0003676 GO:0004222
GO:0016021
Cluster-22178.64159 GO:0051258 GO:0009432 GO:0006281 GO:0008053
GO:0030168 GO:0007165 GO:0006310 GO:0006457 GO:0006302
GO:0003824 GO:0003924 GO:0008092 GO:0003697 GO:0051082
GO:0005102 GO:0005524 GO:0003677 GO:0030674 GO:0016021
GO:0005737 GO:0005634 GO:0019898 GO:0005577 GO:0033588
GO:0016272 GO:0005741
Cluster-22178.64162 GO:0006464 GO:0006914 GO:0010506 GO:0006084
GO:0006367 GO:0055114 GO:0007049 GO:0030001 GO:0006355
GO:0010508 GO:0006886 GO:0046872 GO:0003824 GO:0008113
GO:0005783 GO:0005615 GO:0005669 GO:0016021
Cluster-22178.64164 GO:2001070 GO:0005515
Cluster-22178.64168 GO:0006886 GO:0006355 GO:0046034 GO:0006464
GO:0006888 GO:0006144 GO:0004017 GO:0016151 GO:0046872
GO:0008270 GO:0030127 GO:0005634
Cluster-22178.64169 GO:0006260 GO:0006468 GO:0003677 GO:0004672
GO:0005524 GO:0016773 GO:0016020
Cluster-22178.64170 GO:0006355 GO:0003824 GO:0003700 GO:0004252
GO:0016021 GO:0005886 GO:0005882 GO:0042025 GO:0005667
Cluster-22178.64171 GO:0010884 GO:0006810 GO:0006270 GO:0019079
GO:0001819 GO:0008284 GO:0006355 GO:0004386 GO:0005524
GO:0016887 GO:0008026 GO:0003676 GO:0008134 GO:0004190
GO:0005667 GO:0016021
Cluster-22178.64172 GO:0042254 GO:0005622
Cluster-22178.64173 GO:0006355 GO:0004252 GO:0003824 GO:0003700
GO:0042025 GO:0005882 GO:0005667 GO:0016021 GO:0005886
Cluster-22178.64176 GO:0001819 GO:0006355 GO:0008284 GO:0010884
GO:0006810 GO:0019079 GO:0006270 GO:0003676 GO:0004190
GO:0008134 GO:0016887 GO:0005524 GO:0004386 GO:0008026

GO:0016021 GO:0005667
Cluster-22178.64179 GO:0006508 GO:0008270 GO:0008233 GO:0004222
GO:0031012
Cluster-22178.64180 GO:0007059 GO:0031110 GO:0051301 GO:0000090
GO:0007067 GO:0008017 GO:0004867 GO:0045298 GO:0005876
GO:0000940
Cluster-22178.64183 GO:0015992 GO:0006308 GO:0006914 GO:0015991
GO:0006810 GO:0006886 GO:0008855 GO:0015078 GO:0016021
GO:0005783 GO:0009318 GO:0033179
Cluster-22178.64184 GO:0046872 GO:0005509 GO:0005515
Cluster-22178.64185 GO:0003924 GO:0005525 GO:0012511 GO:0016021
Cluster-22178.64186 GO:0007165 GO:0003774 GO:0005524 GO:0016459
Cluster-22178.64190 GO:0006508 GO:0005515 GO:0004222
Cluster-22178.64191 GO:0007165
Cluster-22178.64193 GO:0006355 GO:0009271 GO:0016567 GO:0005515
GO:0046872 GO:0008270 GO:0004842 GO:0005680
Cluster-22178.64194 GO:0006144 GO:0007156 GO:0009058 GO:0009113
GO:0005524 GO:0046872 GO:0016742 GO:0005509 GO:0051539
GO:0004637 GO:0016020
Cluster-22178.64195 GO:0006144 GO:0007156 GO:0009113 GO:0005524
GO:0046872 GO:0005509 GO:0004637 GO:0016020
Cluster-22178.64198 GO:0003676
Cluster-22178.64199 GO:0043401 GO:0006355 GO:0003700 GO:0008270
GO:0043565 GO:0005667 GO:0005634
Cluster-22178.64200 GO:0005198 GO:0005882
Cluster-22178.64201 GO:0006520 GO:0055114 GO:0009058 GO:0045454
GO:0016829 GO:0016209 GO:0016491 GO:0005509 GO:0030170
GO:0032040
Cluster-22178.64202 GO:0045893 GO:0016567 GO:0003676 GO:0005515
GO:0004842 GO:0042025
Cluster-22178.64203 GO:0043039 GO:0006531 GO:0006522 GO:0048193
GO:0006419 GO:0000166 GO:0003676 GO:0004813 GO:0016876
GO:0005524 GO:0016020

Cluster-22178.64205 GO:0006144 GO:0055114 GO:0006807 GO:0006260
GO:0006396 GO:0003723 GO:0004000
Cluster-22178.64206 GO:0016567 GO:0008270 GO:0046872 GO:0004842
GO:0005515 GO:0005680
Cluster-22178.64207 GO:0007264 GO:0005525
Cluster-22178.64209 GO:0009733 GO:0005515
Cluster-22178.64210 GO:0006631 GO:0055114 GO:0050151 GO:0016491
GO:0071949
Cluster-22178.64211 GO:0006810 GO:0005515 GO:0005622 GO:0016021
Cluster-22178.64214 GO:0016567 GO:0004842 GO:0046872 GO:0008270
GO:0005524 GO:0005515 GO:0061630 GO:0005680
Cluster-22178.64215 GO:0006606 GO:0006357 GO:0006914 GO:0015992
GO:0051028 GO:0048309 GO:0010508 GO:0006260 GO:0030168
GO:0006457 GO:0071973 GO:0006810 GO:0007165 GO:0006811
GO:0048870 GO:0051258 GO:0015991 GO:0003774 GO:0015078
GO:0042803 GO:0005198 GO:0001104 GO:0005102 GO:0000774
GO:0051087 GO:0030674 GO:0008092 GO:0005577 GO:0016020
GO:0005737 GO:0009288 GO:0019898 GO:0005886 GO:0005882
GO:0016592 GO:0016021 GO:0033179 GO:0031514
Cluster-22178.64217 GO:0042967 GO:0047961 GO:0005515 GO:0005739
Cluster-22178.64219 GO:0046872
Cluster-22178.64223 GO:0019069 GO:0006355 GO:0042254 GO:0006412
GO:0003677 GO:0003735 GO:0003700 GO:0005667 GO:0005634
GO:0042025 GO:0005840
Cluster-22178.64225 GO:0015977 GO:0043039 GO:0019643 GO:0006094
GO:0006099 GO:0006418 GO:0000166 GO:0008964 GO:0000049
GO:0004812 GO:0005524 GO:0031225 GO:0005737
Cluster-22178.64229 GO:0006355 GO:0006090 GO:0006633 GO:0003989
GO:0005524 GO:0005634 GO:0009317 GO:0005737
Cluster-22178.64231 GO:0043065 GO:0046983 GO:0005515 GO:0005740
GO:0016021
Cluster-22178.64232 GO:0006123 GO:0015992 GO:0004129 GO:0005743
GO:0005751 GO:0045277

Cluster-22178.64236 GO:0055114 GO:0016972
Cluster-22178.64237 GO:0006397 GO:0046872
Cluster-22178.64239 GO:0016772
Cluster-22178.64240 GO:0006935 GO:0007165 GO:0006270 GO:0006955
GO:0008009 GO:0005576
Cluster-22178.64241 GO:0007005 GO:0031305
Cluster-22178.64243 GO:0006470 GO:0006570 GO:0008138 GO:0004725

Cluster-22178.64245 GO:0009069 GO:0008608 GO:0007049 GO:0016310
GO:0048870 GO:0009405 GO:0006468 GO:0004674 GO:0005524
GO:0005198 GO:0005815 GO:0016020 GO:0019031 GO:0042729
GO:0031514 GO:0019028 GO:0072686
Cluster-22178.64246 GO:0003723 GO:0003676
Cluster-22178.64247 GO:0006810 GO:0000917 GO:0043093 GO:0006334
GO:0042262 GO:0003690 GO:0019012 GO:0005634 GO:0016021
GO:0005737
Cluster-22178.64248 GO:0000917 GO:0042262 GO:0016310 GO:0006810
GO:0006334 GO:0009069 GO:0043093 GO:0008053 GO:0051082
GO:0003924 GO:0003690 GO:0004674 GO:0005634 GO:0016021
GO:0005737 GO:0005741
Cluster-22178.64249 GO:0005515 GO:0008270
Cluster-22178.64250 GO:0006614 GO:0006520 GO:0000921 GO:0006396
GO:0046856 GO:0006606 GO:0006355 GO:0008312 GO:0003723
GO:0045502 GO:0008134 GO:0042803 GO:0005515 GO:0043565
GO:0003700 GO:0034597 GO:0005667 GO:0005940 GO:0030286
GO:0048500 GO:0016021
Cluster-22178.64253 GO:0004252 GO:0016021 GO:0012511
Cluster-22178.64254 GO:0043039 GO:0005524 GO:0016876
Cluster-22178.64255 GO:0005096
Cluster-22178.64256 GO:0030488 GO:0006464 GO:0032259 GO:0008033
GO:0046500 GO:0006364 GO:0006479 GO:0009451 GO:0004719
GO:0008168 GO:0016429 GO:0003723 GO:0031515
Cluster-22178.64257 GO:0006260 GO:0006269 GO:0006351 GO:0008270

GO:0003896 GO:0003677 GO:0005657 GO:0005730
Cluster-22178.64258 GO:0009094 GO:0006103 GO:0009821 GO:0009074
GO:0006571 GO:0000162 GO:0006536 GO:0007165 GO:0004872
GO:0030170 GO:0004838 GO:0005515 GO:0016021
Cluster-22178.64259 GO:0007186 GO:0004930 GO:0016021
Cluster-22178.64262 GO:0007165 GO:0005179 GO:0005576
Cluster-22178.64264 GO:0007264 GO:0007165 GO:0007186 GO:0002949
GO:0003723 GO:0019001 GO:0031683 GO:0003924 GO:0005525
GO:0004871 GO:0003724
Cluster-22178.64265 GO:0006281 GO:0006308 GO:0005515 GO:0003677
GO:0004518 GO:0003723 GO:0008855 GO:0009318
Cluster-22178.64266 GO:0006281 GO:0006308 GO:0003677 GO:0004518
GO:0005515 GO:0008855 GO:0009318
Cluster-22178.64268 GO:0005515
Cluster-22178.64271 GO:0006914 GO:0007165 GO:0031423 GO:0005198
GO:0019028 GO:0019012
Cluster-22178.64275 GO:0003723
Cluster-22178.64277 GO:0008053 GO:0006810 GO:0010508 GO:0007264
GO:0005525 GO:0003724 GO:0005524 GO:0016887 GO:0003924
GO:0003723 GO:0016021 GO:0005741
Cluster-22178.64279 GO:0006351 GO:0006468 GO:0007618 GO:0006206
GO:0006144 GO:0003677 GO:0003899 GO:0016787 GO:0046872
GO:0004672 GO:0005524 GO:0016773 GO:0016020 GO:0005576
GO:0005730
Cluster-22178.64280 GO:0007165 GO:0016255 GO:0005515 GO:0016021
GO:0042765
Cluster-22178.64284 GO:0042254 GO:0006412 GO:0006355 GO:0003700
GO:0043565 GO:0008270 GO:0003735 GO:0005840 GO:0005667
Cluster-22178.64285 GO:0042998 GO:0019049 GO:0071253 GO:0005802
Cluster-22178.64286 GO:0006811 GO:0004190 GO:0051424 GO:0016021
GO:0016020
Cluster-22178.64287 GO:0042967 GO:0006886 GO:0006629 GO:0006505
GO:0016788 GO:0008374 GO:0016787

Cluster-22178.64291 GO:0006355 GO:0045132 GO:0006576 GO:0007264
GO:0015684 GO:0043565 GO:0003924 GO:0005525 GO:0003700
GO:0003676 GO:0016787 GO:0003677 GO:0005524 GO:0015093
GO:0016887 GO:0005634 GO:0000775 GO:0016021 GO:0005667
Cluster-22178.64293 GO:0007276 GO:0006744
Cluster-22178.64295 GO:0007607 GO:0050912 GO:0007187 GO:0008527
GO:0016021
Cluster-22178.64296 GO:0046500 GO:0030488 GO:0006464 GO:0032259
GO:0006355 GO:0006479 GO:0006412 GO:0009451 GO:0008033
GO:0008152 GO:0016491 GO:0016429 GO:0004719 GO:0008168
GO:0043565 GO:0003700 GO:0005667 GO:0031515 GO:0005634
Cluster-22178.64297 GO:0043066 GO:0015992 GO:0016567 GO:0043123
GO:0045766 GO:0015986 GO:0043130 GO:0015078 GO:0004842
GO:0003723 GO:0000276
Cluster-22178.64298 GO:0003723
Cluster-22178.64299 GO:0006364 GO:0032040 GO:0005732 GO:0005634
GO:0034457
Cluster-22178.64301 GO:0006508 GO:0006468 GO:0006144 GO:0017111
GO:0004252 GO:0004197 GO:0016773 GO:0004672 GO:0005524
GO:0003968 GO:0016020 GO:0031379
Cluster-22178.64303 GO:0006570 GO:0006470 GO:0004527 GO:0008138
GO:0004725 GO:0003677
Cluster-22178.64304 GO:0006470 GO:0006570 GO:0004725 GO:0008138
GO:0003677 GO:0004527
Cluster-22178.64308 GO:0007165 GO:0005509 GO:0005578
Cluster-22178.64311 GO:0030259 GO:0055114 GO:0005975 GO:0042773
GO:0016651 GO:0016758 GO:0005515
Cluster-22178.64312 GO:0006817 GO:0006468 GO:0004672 GO:0005524

Cluster-22178.64313 GO:0006281 GO:0006144 GO:0006974 GO:0015074
GO:0005975 GO:0003677 GO:0030248 GO:0004553 GO:0003968
GO:0005524 GO:0005634 GO:0005576 GO:0070876 GO:0031379
Cluster-22178.64314 GO:0006468 GO:0005515 GO:0005524 GO:0004672

Cluster-22178.64318 GO:0006817 GO:0006468 GO:0005524 GO:0004672

Cluster-22178.64323 GO:0006457 GO:0016192 GO:0007165 GO:0030521 GO:0006355 GO:0006886 GO:0009372 GO:0009405 GO:0005509 GO:0003677 GO:0004882 GO:0008270 GO:0008233 GO:0005496 GO:0051082 GO:0005515 GO:0005783 GO:0005634 GO:0030117 GO:0016021

Cluster-22178.64325 GO:0005515

Cluster-22178.64326 GO:0006468 GO:0005524 GO:0004672 GO:0016773 GO:0016020

Cluster-22178.64327 GO:0005515

Cluster-22178.64328 GO:0006886 GO:0006888 GO:0008270 GO:0030127

Cluster-22178.64329 GO:0006510 GO:0006508 GO:0006355 GO:0004176 GO:0003677 GO:0003682 GO:0000785 GO:0000775 GO:0005634

Cluster-22178.64330 GO:0055114 GO:0005506 GO:0016705 GO:0020037

Cluster-22178.64332 GO:0015992 GO:0006123 GO:0004129 GO:0045277

Cluster-22178.64334 GO:0005515 GO:0016020

Cluster-22178.64335 GO:0016021

Cluster-22178.64336 GO:0006508 GO:0006355 GO:0006144 GO:0005198 GO:0004252 GO:0017111 GO:0004197 GO:0003968 GO:0003700 GO:0005667 GO:0019028 GO:0019031 GO:0031379

Cluster-22178.64337 GO:0006355 GO:0006260 GO:0003677 GO:0005664

Cluster-22178.64341 GO:0006260 GO:0005634

Cluster-22178.64343 GO:0046983

Cluster-22178.64344 GO:0046983

Cluster-22178.64345 GO:0055114 GO:0005515

Cluster-22178.64347 GO:0042967 GO:0016747 GO:0008080

Cluster-22178.64349 GO:0006486 GO:0008378 GO:0016757 GO:0016020

Cluster-22178.64351 GO:0051028 GO:0042254 GO:0005515 GO:0005634

Cluster-22178.64352 GO:0006570 GO:0018101 GO:0006807 GO:0006470 GO:0005509 GO:0008138 GO:0004668 GO:0004725 GO:0005737

Cluster-22178.64354 GO:0005515
Cluster-22178.64357 GO:0008152 GO:0055114 GO:0016491
Cluster-22178.64361 GO:0006355 GO:0005515 GO:0005634 GO:0016021
Cluster-22178.64367 GO:0006468 GO:0009190 GO:0046039 GO:0008152
GO:0006182 GO:0006144 GO:0006357 GO:0035556 GO:0004383
GO:0003723 GO:0016849 GO:0001104 GO:0004672 GO:0003824
GO:0005524 GO:0008270 GO:0016592
Cluster-22178.64368 GO:0008201 GO:0046914
Cluster-22178.64370 GO:0006935 GO:0000160 GO:0016310 GO:0004673
GO:0000155 GO:0005737 GO:0009365
Cluster-22178.64371 GO:0016567 GO:0046872 GO:0004842
Cluster-22178.64372 GO:0009228 GO:0046497 GO:0006206 GO:0005975
GO:0009435 GO:0006207 GO:0004789 GO:0004514 GO:0016857
GO:0004590
Cluster-22178.64376 GO:0007010 GO:0003779
Cluster-22178.64378 GO:0006817 GO:0006355 GO:0005315 GO:0003677
GO:0016020 GO:0005634
Cluster-22178.64379 GO:0006367 GO:0032968 GO:0003677 GO:0005634
Cluster-22178.64380 GO:0042254 GO:0006412 GO:0003676 GO:0003723
GO:0003735 GO:0005622 GO:0005840 GO:0019013
Cluster-22178.64385 GO:0008092 GO:0019898 GO:0005737
Cluster-22178.64390 GO:0006744 GO:0019836 GO:0005515 GO:0005576
Cluster-22178.64391 GO:0006099 GO:0016829
Cluster-22178.64392 GO:0042254 GO:0006412 GO:0003723 GO:0003735
GO:0003676 GO:0005622 GO:0005840
Cluster-22178.64394 GO:0016021
Cluster-22178.64395 GO:0006480 GO:0008152 GO:0055114 GO:0015979
GO:0006118 GO:0017004 GO:0006468 GO:0045158 GO:0047134
GO:0008168 GO:0004672 GO:0005524 GO:0009512 GO:0016021
GO:0009523
Cluster-22178.64397 GO:0007015 GO:0003785
Cluster-22178.64398 GO:0005507
Cluster-22178.64402 GO:0007165 GO:0005515

Cluster-22178.64403 GO:0006355 GO:0007165 GO:0016787 GO:0005515
GO:0005524 GO:0008270 GO:0043565 GO:0003700 GO:0005667
GO:0005737
Cluster-22178.64404 GO:0006355 GO:0003700 GO:0043565 GO:0005667
Cluster-22178.64406 GO:0045227 GO:0007165 GO:0006887 GO:0005179
GO:0004252 GO:0005576 GO:0000145 GO:0016020 GO:0016021
Cluster-22178.64407 GO:0006629 GO:0006355 GO:0008270 GO:0005634
Cluster-22178.64408 GO:0016021
Cluster-22178.64409 GO:0046872 GO:0008270
Cluster-22178.64410 GO:0090307 GO:0005515
Cluster-22178.64411 GO:0006397 GO:0006366 GO:0006914 GO:0005515
GO:0000445
Cluster-22178.64413 GO:0007165 GO:0006955 GO:0006886 GO:0006355
GO:0019882 GO:0030521 GO:0003677 GO:0042289 GO:0005496
GO:0004882 GO:0016020 GO:0005634 GO:0042613
Cluster-22178.64414 GO:0035556 GO:0006144 GO:0009190 GO:0046039
GO:0006182 GO:0020037 GO:0004383 GO:0016849
Cluster-22178.64416 GO:0007017 GO:0007018 GO:0005524 GO:0003777
GO:0008017 GO:0005576 GO:0005874 GO:0045298
Cluster-22178.64420 GO:0006468 GO:0005524 GO:0004672
Cluster-22178.64422 GO:0006260 GO:0006270 GO:0006355 GO:0003678
GO:0005524 GO:0016887 GO:0003677 GO:0008134 GO:0005657
GO:0042555 GO:0005634 GO:0005667
Cluster-22178.64423 GO:0006412 GO:0042254 GO:0003735 GO:0005622
GO:0005840
Cluster-22178.64424 GO:0006338 GO:0000228
Cluster-22178.64425 GO:0003676 GO:0003677 GO:0016787 GO:0005524

Cluster-22178.64427 GO:0016567 GO:0004842 GO:0005515 GO:0008270
GO:0046872 GO:0005680
Cluster-22178.64428 GO:0003676 GO:0003723
Cluster-22178.64430 GO:0006605 GO:0006886 GO:0005742
Cluster-22178.64431 GO:0009214 GO:0006605 GO:0006886 GO:0006013

GO:0006000 GO:0003873 GO:0005515 GO:0042802 GO:0008641
GO:0005524 GO:0004113 GO:0016887 GO:0005742 GO:0016020
Cluster-22178.64433 GO:0003723
Cluster-22178.64434 GO:0007155 GO:0055114 GO:0016491 GO:0016616

Cluster-22178.64436 GO:0015031 GO:0030176
Cluster-22178.64437 GO:0046872
Cluster-22178.64438 GO:0006367 GO:0003682 GO:0005524 GO:0005672
GO:0000785
Cluster-22178.64439 GO:0009405 GO:0005515
Cluster-22178.64440 GO:0035556 GO:0006281 GO:0006464 GO:0003677
GO:0016151 GO:0008270 GO:0046872
Cluster-22178.64441 GO:0015931 GO:0033227 GO:0051033 GO:0016021
Cluster-22178.64442 GO:0006303
Cluster-22178.64443 GO:0006303 GO:0005515
Cluster-22178.64445 GO:0006355 GO:0007165 GO:0005179 GO:0003677
GO:0016818 GO:0004252 GO:0005524 GO:0008270 GO:0005576
GO:0005634 GO:0005886
Cluster-22178.64446 GO:0005634
Cluster-22178.64447 GO:0005198 GO:0005515 GO:0019028
Cluster-22178.64448 GO:0019058 GO:0005515
Cluster-22178.64449 GO:0006629 GO:0046339 GO:0009395 GO:0007165
GO:0035556 GO:0004435 GO:0005515
Cluster-22178.64450 GO:0006270 GO:0000746 GO:0006816 GO:0006874
GO:0006383 GO:0055085 GO:0006144 GO:0006206 GO:0006351
GO:1901031 GO:0030001 GO:0006352 GO:0003677 GO:0046873
GO:0003899 GO:0005515 GO:0004190 GO:0005219 GO:0046872
GO:0016020 GO:0005666 GO:0005730 GO:0005622 GO:0005634
GO:0019867 GO:0005669 GO:0016021 GO:0019031
Cluster-22178.64451 GO:0015684 GO:0006446 GO:0007264 GO:0006413
GO:0006576 GO:0005525 GO:0015093 GO:0005524 GO:0003924
GO:0046983 GO:0005515 GO:0003743 GO:0005840 GO:0016021
GO:0005576

Cluster-22178.64452 GO:0055085 GO:0005741
Cluster-22178.64453 GO:0016567 GO:0046872 GO:0008270 GO:0004842
GO:0061630 GO:0005515 GO:0005622 GO:0005680
Cluster-22178.64454 GO:0046872
Cluster-22178.64455 GO:0006570 GO:0006470 GO:0005515 GO:0008138
GO:0004725
Cluster-22178.64458 GO:0050992 GO:0019288 GO:0046872 GO:0005515
GO:0051745 GO:0005634
Cluster-22178.64460 GO:0019079 GO:0015994 GO:0006783 GO:0033014
GO:0004386 GO:0004852 GO:0008270
Cluster-22178.64461 GO:0005515
Cluster-22178.64462 GO:0007010 GO:0003779
Cluster-22178.64463 GO:0019370 GO:0055114 GO:0008237 GO:0008270
GO:0016491
Cluster-22178.64465 GO:0006118 GO:0015035
Cluster-22178.64467 GO:0008242
Cluster-22178.64468 GO:0007165 GO:0004190 GO:0005515 GO:0004872
GO:0016021 GO:0016020
Cluster-22178.64469 GO:0006368 GO:0016570 GO:0016593
Cluster-22178.64473 GO:0003677 GO:0046872 GO:0046983
Cluster-22178.64474 GO:0006811 GO:0007186 GO:0006813 GO:0004930
GO:0005249 GO:0016021 GO:0008076 GO:0016020
Cluster-22178.64477 GO:0015992 GO:0015986 GO:0015078 GO:0005543

Cluster-22178.64478 GO:0042157 GO:0006869 GO:0046486 GO:0016567
GO:0009395 GO:0008654 GO:0061630 GO:0008270 GO:0046983
GO:0004842 GO:0004143 GO:0005198 GO:0005515 GO:0046872
GO:0008289 GO:0016020 GO:0005622 GO:0005576 GO:0019028
GO:0019031
Cluster-22178.64479 GO:0004190 GO:0016021
Cluster-22178.64481 GO:0006355 GO:0042254 GO:0006412 GO:0007165
GO:0005179 GO:0003735 GO:0003677 GO:0005840
Cluster-22178.64482 GO:0016567 GO:0006355 GO:0004842 GO:0046872

GO:0008270 GO:0003677 GO:0005515 GO:0003682 GO:0005680
GO:0000775 GO:0000785 GO:0005634
Cluster-22178.64483 GO:0003676
Cluster-22178.64484 GO:0005198
Cluster-22178.64485 GO:0006886 GO:0016192 GO:0005515
Cluster-22178.64486 GO:0006357 GO:0001104 GO:0008270 GO:0016592
Cluster-22178.64487 GO:0015986 GO:0009405 GO:0016226 GO:0006357
GO:0015992 GO:0001104 GO:0051536 GO:0015078 GO:0016020
GO:0016592 GO:0005737 GO:0000276
Cluster-22178.64488 GO:0042254 GO:0006412 GO:0003735 GO:0005840
GO:0005622
Cluster-22178.64491 GO:0043093 GO:0010508 GO:0006364 GO:0000917
GO:0048870 GO:0070206 GO:0031514 GO:0005737
Cluster-22178.64493 GO:0003676 GO:0016021
Cluster-22178.64495 GO:0007186 GO:0004930 GO:0016021
Cluster-22178.64496 GO:0006355 GO:0007186 GO:0005524 GO:0008134
GO:0004930 GO:0005667 GO:0016021
Cluster-22178.64497 GO:0022904 GO:0008270 GO:0016020
Cluster-22178.64499 GO:0006270 GO:0006629 GO:0003676 GO:0003723

Cluster-22178.64500 GO:0016192 GO:0019497 GO:0006771 GO:0003993
GO:0019031 GO:0016021
Cluster-22178.64501 GO:0016032
Cluster-22178.64502 GO:0006096 GO:0006098 GO:0006020 GO:0006094
GO:0006013 GO:0015976 GO:0006000 GO:0004332
Cluster-22178.64503 GO:0008270
Cluster-22178.64506 GO:0006531 GO:0016567 GO:0015992 GO:0006144
GO:0006188 GO:0009152 GO:0006522 GO:0006123 GO:0004018
GO:0004842 GO:0004129 GO:0005740 GO:0045277
Cluster-22178.64510 GO:0006816 GO:0006874 GO:0005219 GO:0005622
GO:0016021
Cluster-22178.64511 GO:0043248
Cluster-22178.64513 GO:0000245 GO:0005634

Cluster-22178.64516 GO:0006364 GO:0046500 GO:0030488 GO:0015031
GO:0006464 GO:0009451 GO:0007010 GO:0006479 GO:0006468
GO:0055114 GO:0008033 GO:0006725 GO:0016772 GO:0016429
GO:0016491 GO:0010277 GO:0005524 GO:0016301 GO:0008198
GO:0003723 GO:0003779 GO:0008168 GO:0004719 GO:0004672
GO:0031515
Cluster-22178.64517 GO:0015684 GO:0015093 GO:0003924 GO:0005525
GO:0016021
Cluster-22178.64518 GO:0015684 GO:0015093 GO:0003924 GO:0005525
GO:0016021
Cluster-22178.64521 GO:0006355 GO:0005975 GO:0003677 GO:0016773
GO:0005524
Cluster-22178.64522 GO:0006810 GO:0006281 GO:0000723 GO:0004386
GO:0003678 GO:0003676 GO:0005525 GO:0016887 GO:0003924
GO:0005524 GO:0005657
Cluster-22178.64524 GO:0007264 GO:0005524 GO:0005525
Cluster-22178.64525 GO:0008063 GO:0005515 GO:0008270
Cluster-22178.64527 GO:0006355 GO:0032968 GO:0006367 GO:0003682
GO:0003677 GO:0003700 GO:0005667 GO:0005634 GO:0000775
GO:0000785
Cluster-22178.64528 GO:0006351 GO:0006260 GO:0003887 GO:0003677
GO:0042575 GO:0016021 GO:0005634
Cluster-22178.64529 GO:0007165 GO:0040007 GO:0008033 GO:0008283
GO:0008083
Cluster-22178.64530 GO:0007264 GO:0006576 GO:0007165 GO:0007186
GO:0031683 GO:0005524 GO:0019001 GO:0016887 GO:0003924
GO:0005525 GO:0004871 GO:0098519
Cluster-22178.64531 GO:0006468 GO:0009405 GO:0006605 GO:0006886
GO:0005524 GO:0004672 GO:0016773 GO:0005742 GO:0016020
Cluster-22178.64532 GO:0006355 GO:0019058 GO:0043565 GO:0003700
GO:0005634 GO:0005667 GO:0019031
Cluster-22178.64533 GO:0006355 GO:0006265 GO:0006464 GO:0003677
GO:0008270 GO:0003916 GO:0016151 GO:0005694

Cluster-22178.64534 GO:0055114 GO:0009690 GO:0050660 GO:0019139

Cluster-22178.64535 GO:0006355 GO:0003677
Cluster-22178.64537 GO:0007059 GO:0051258 GO:0051301 GO:0007018
GO:0007017 GO:0030168 GO:0031110 GO:0007165 GO:0007067
GO:0000090 GO:0003777 GO:0008017 GO:0005102 GO:0005524
GO:0030674 GO:0005874 GO:0045298 GO:0005577 GO:0005876
GO:0000940
Cluster-22178.64538 GO:0006298 GO:0006810 GO:0019083 GO:0055085
GO:0007021 GO:0051082 GO:0030983 GO:0005524 GO:0016887
GO:0015631 GO:0042626 GO:0045298 GO:0016021
Cluster-22178.64540 GO:0006771 GO:0019497 GO:0046872 GO:0005515
GO:0003993
Cluster-22178.64542 GO:0006813 GO:0005242 GO:0008076 GO:0016021
Cluster-22178.64543 GO:0055114 GO:0016491 GO:0051920 GO:0016209

Cluster-22178.64546 GO:0046872 GO:0005515 GO:0008376 GO:0032580
Cluster-22178.64549 GO:0016117 GO:0000413 GO:0008033 GO:0055114
GO:0006457 GO:0050660 GO:0016817 GO:0016491 GO:0004174
GO:0016705 GO:0003755 GO:0005515 GO:0071949
Cluster-22178.64550 GO:0007186 GO:0004930 GO:0016020 GO:0016021
Cluster-22178.64552 GO:0006810 GO:0016021
Cluster-22178.64555 GO:0046872
Cluster-22178.64557 GO:0007187 GO:0050912 GO:0007607 GO:0008527
GO:0016021
Cluster-22178.64558 GO:0046872 GO:0005515 GO:0003677
Cluster-22178.64559 GO:0006508 GO:0008654 GO:0008270 GO:0016780
GO:0004181 GO:0016020
Cluster-22178.64561 GO:0009396 GO:0055114 GO:0046487 GO:0004488
GO:0003824
Cluster-22178.64563 GO:0016226 GO:0008199
Cluster-22178.64564 GO:0015940 GO:0019482 GO:0004592
Cluster-22178.64566 GO:0006418 GO:0043039 GO:0006425 GO:0005524

GO:0016876 GO:0000166 GO:0004819 GO:0004812 GO:0005737
Cluster-22178.64567 GO:0019722 GO:0006402 GO:0006817 GO:0044341
GO:0051252 GO:0008270 GO:0015321 GO:0046872 GO:0004535
GO:0003676 GO:0003723 GO:0005737 GO:0005634 GO:0016020
Cluster-22178.64569 GO:0006486 GO:0016757 GO:0008378 GO:0016020
Cluster-22178.64570 GO:2001256 GO:0042710 GO:0008270 GO:0030176
Cluster-22178.64571 GO:0008270 GO:0003677
Cluster-22178.64575 GO:0015979 GO:0005515 GO:0008270 GO:0016020
GO:0009539 GO:0009523
Cluster-22178.64578 GO:0006508 GO:0006950 GO:0004197 GO:0004222
GO:0003700 GO:0008270 GO:0005667 GO:0031012
Cluster-22178.64579 GO:0000012 GO:0006468 GO:0006281 GO:0009069
GO:0006397 GO:0016310 GO:0003723 GO:0003684 GO:0005524
GO:0004674 GO:0005634 GO:0005737
Cluster-22178.64580 GO:0051716 GO:0006284 GO:0003906 GO:0003676
GO:0016799 GO:0046658
Cluster-22178.64581 GO:0006468 GO:0005524 GO:0004672
Cluster-22178.64583 GO:0006556 GO:0006555 GO:0006144 GO:0006206
GO:0006414 GO:0006448 GO:0006351 GO:1901031 GO:0055114
GO:0051920 GO:0004478 GO:0003746 GO:0003899 GO:0005515
GO:0003676 GO:0005730 GO:0005634 GO:0005840
Cluster-22178.64585 GO:0043395 GO:0005515 GO:0005578 GO:0016020
Cluster-22178.64586 GO:0006464 GO:0015031 GO:0005515
Cluster-22178.64592 GO:0006338 GO:0007049 GO:0005524 GO:0016818
GO:0005515 GO:0003676 GO:0005634
Cluster-22178.64593 GO:0006412 GO:0042254 GO:0003735 GO:0005622
GO:0005840
Cluster-22178.64594 GO:0006355 GO:0006807 GO:0009252 GO:0004356
GO:0003700 GO:0043565 GO:0005634 GO:0005667 GO:0055036
Cluster-22178.64595 GO:0042967 GO:0018193 GO:0008080 GO:0016747

Cluster-22178.64597 GO:0007018 GO:0007017 GO:0003677 GO:0003777
GO:0008017 GO:0005524 GO:0005874 GO:0045298

Cluster-22178.64598 GO:0007018 GO:0007017 GO:0003777 GO:0008017
GO:0005524 GO:0003677 GO:0005874 GO:0045298
Cluster-22178.64602 GO:0040007 GO:0008283 GO:0007165 GO:0008083

Cluster-22178.64604 GO:0015689 GO:0009405 GO:0055085 GO:0015098
GO:0022857 GO:0016021
Cluster-22178.64606 GO:0046740 GO:0000398 GO:0030246 GO:0005634
GO:0016021
Cluster-22178.64608 GO:0006457
Cluster-22178.64609 GO:0016758
Cluster-22178.64610 GO:0000724 GO:0017004 GO:0016197 GO:0015886
GO:0030119 GO:0005783 GO:0016021
Cluster-22178.64611 GO:0006531 GO:0006144 GO:0006164 GO:0006522
GO:0004019 GO:0005525
Cluster-22178.64619 GO:0016042 GO:0045454 GO:0006468 GO:0007586
GO:0004672 GO:0005524 GO:0008047 GO:0005576 GO:0019028
Cluster-22178.64620 GO:0009058 GO:0016740 GO:0030170 GO:0019012
Cluster-22178.64627 GO:0006888 GO:0003676 GO:0019900 GO:0005801
GO:0005622
Cluster-22178.64628 GO:0055114 GO:0006118 GO:0009496 GO:0051537
GO:0009512 GO:0042651
Cluster-22178.64631 GO:0016772 GO:0008134 GO:0005509 GO:0008270
GO:0005667 GO:0005634
Cluster-22178.64634 GO:0030334 GO:0051607 GO:0030168 GO:0007165
GO:0030155 GO:0007155 GO:0045995 GO:0051258 GO:0006355
GO:0005102 GO:0030674 GO:0003700 GO:0008134 GO:0042803
GO:0045502 GO:0005577 GO:0030286 GO:0001772 GO:0005667
GO:0016021 GO:0097197
Cluster-22178.64637 GO:0046983
Cluster-22178.64640 GO:0000910 GO:0006118 GO:0006355 GO:0000226
GO:0006974 GO:0007186 GO:0051726 GO:0007275 GO:0003700
GO:0009055 GO:0043565 GO:0008017 GO:0004930 GO:0046983
GO:0051536 GO:0008270 GO:0003677 GO:0016021 GO:0045298

GO:0005634 GO:0005667
Cluster-22178.64643 GO:0006355 GO:0003677 GO:0005515 GO:0046872
GO:0008270
Cluster-22178.64644 GO:0006614 GO:0006520 GO:0000921 GO:0046856
GO:0006606 GO:0006396 GO:0006355 GO:0008312 GO:0003723
GO:0045502 GO:0042803 GO:0008134 GO:0005515 GO:0043565
GO:0003700 GO:0034597 GO:0005667 GO:0030286 GO:0005940
GO:0048500 GO:0016021
Cluster-22178.64645 GO:0006355 GO:0016055 GO:0006886 GO:0000975
GO:0008536 GO:0003700 GO:0005667
Cluster-22178.64646 GO:0006446 GO:0003743 GO:0005840 GO:0005852
GO:0008023 GO:0005737
Cluster-22178.64647 GO:0006886 GO:0006355 GO:0016055 GO:0000975
GO:0008536 GO:0003700 GO:0005667
Cluster-22178.64649 GO:0008063 GO:0005515 GO:0019901 GO:0008270
GO:0005634
Cluster-22178.64653 GO:0015948 GO:0008168 GO:0005515
Cluster-22178.64654 GO:0042254 GO:0006412 GO:0003735 GO:0005622
GO:0005840
Cluster-22178.64655 GO:0042254 GO:0006412 GO:0003735 GO:0005622
GO:0005840
Cluster-22178.64656 GO:0042254 GO:0006412 GO:0003735 GO:0005840
GO:0005622
Cluster-22178.64658 GO:0016773
Cluster-22178.64659 GO:0006566 GO:0006563 GO:0006546 GO:0006520
GO:0055114 GO:0006544 GO:0006352 GO:0003677 GO:0004375
GO:0016829
Cluster-22178.64660 GO:0005509 GO:0003676 GO:0005515
Cluster-22178.64663 GO:0005515
Cluster-22178.64664 GO:0006886 GO:0006888 GO:0055114 GO:0016706
GO:0003677 GO:0008168 GO:0046872 GO:0008270 GO:0030127
Cluster-22178.64665 GO:0051252 GO:0006402 GO:0003723 GO:0004535
GO:0046872 GO:0003676 GO:0005634 GO:0005737

Cluster-22178.64666 GO:0008152 GO:0016624
Cluster-22178.64668 GO:0055114 GO:0015994 GO:0006470 GO:0006355
GO:0000087 GO:0006570 GO:0006260 GO:0006788 GO:0004725
GO:0004392 GO:0008134 GO:0005524 GO:0016887 GO:0003677
GO:0005622 GO:0005667
Cluster-22178.64669 GO:0007155 GO:0006275 GO:0007049
Cluster-22178.64670 GO:0006260
Cluster-22178.64672 GO:0042254 GO:0015986 GO:0035556 GO:0015992
GO:0006119 GO:0006412 GO:0006355 GO:0003677 GO:0046961
GO:0003735 GO:0008270 GO:0046933 GO:0045259 GO:0045261
GO:0005622 GO:0005840
Cluster-22178.64675 GO:0006486 GO:0055114 GO:0006725 GO:0016757

Cluster-22178.64676 GO:0006355 GO:0009405 GO:0043565 GO:0003700
GO:0005515 GO:0005198 GO:0005509 GO:0005667
Cluster-22178.64677 GO:0042254 GO:0006412 GO:0003735 GO:0003723
GO:0005840
Cluster-22178.64680 GO:0015992 GO:0055085 GO:0006123 GO:0006811
GO:0007165 GO:0007268 GO:0004129 GO:0004970 GO:0005216
GO:0005515 GO:0045277 GO:0016020
Cluster-22178.64682 GO:0006879 GO:0055114 GO:0008199 GO:0046872
GO:0016491
Cluster-22178.64685 GO:0005524 GO:0003676 GO:0000228
Cluster-22178.64686 GO:0007165 GO:0006355 GO:0051262 GO:0006357
GO:0005515 GO:0001104 GO:0003723 GO:0004871 GO:0003700
GO:0045182 GO:0016592 GO:0005667 GO:0005634 GO:0019028
Cluster-22178.64687 GO:0008652 GO:0000398 GO:0009086 GO:0035556
GO:0003871 GO:0005515 GO:0008270 GO:0005681
Cluster-22178.64688 GO:0035556 GO:0005515
Cluster-22178.64690 GO:0042254 GO:0006412 GO:0003735 GO:0005840
Cluster-22178.64691 GO:0006096 GO:0006094 GO:0000162 GO:0006571
GO:0009094 GO:0000287 GO:0004634 GO:0000015
Cluster-22178.64693 GO:0000012 GO:0006281 GO:0003684 GO:0005634

Cluster-22178.64694 GO:0042254 GO:0006412 GO:0005515 GO:0003735 GO:0019843 GO:0005840 GO:0005622

Cluster-22178.64695 GO:0006352 GO:0006355 GO:0016987 GO:0003677 GO:0003700 GO:0005667

Cluster-22178.64698 GO:0006118 GO:0051536 GO:0009055

Cluster-22178.64702 GO:0030246

Cluster-22178.64703 GO:0016567 GO:0005524 GO:0004842

Cluster-22178.64705 GO:0043248 GO:0006418 GO:0019079 GO:0005524 GO:0003676 GO:0016787 GO:0004812 GO:0008026 GO:0003677 GO:0000166

Cluster-22178.64706 GO:0006886 GO:0015991 GO:0016192 GO:0006606 GO:0000226 GO:0040001 GO:0015031 GO:0043248 GO:0000281 GO:0005515 GO:0016820 GO:0008565 GO:0030117 GO:0005819 GO:0005634 GO:0005874 GO:0005737 GO:0000221

Cluster-22178.64709 GO:0016020

Cluster-22178.64711 GO:0015991 GO:0006886 GO:0048280 GO:0005515 GO:0016820 GO:0004869 GO:0005737 GO:0000139 GO:0000221

Cluster-22178.64714 GO:0006118 GO:0007186 GO:0055114 GO:0015992 GO:0006123 GO:0009060 GO:0005506 GO:0020037 GO:0004129 GO:0009055 GO:0004930 GO:0016021 GO:0045277

Cluster-22178.64715 GO:0042274 GO:0003677 GO:0030686

Cluster-22178.64716 GO:0003677

Cluster-22178.64717 GO:0008289

Cluster-22178.64718 GO:0043248 GO:0016310 GO:0009069 GO:0006886 GO:0016192 GO:0004674 GO:0005515 GO:0030117

Cluster-22178.64720 GO:0005509 GO:0005544

Cluster-22178.64724 GO:0006886 GO:0016192 GO:0005515 GO:0005198 GO:0030117

Cluster-22178.64726 GO:0006418 GO:0004812 GO:0005524 GO:0000166

Cluster-22178.64728 GO:0006396

Cluster-22178.64729 GO:0005515

Cluster-22178.64730 GO:0015979 GO:0042254 GO:0006412 GO:0003677

GO:0003735 GO:0005515 GO:0009539 GO:0005622 GO:0009523
GO:0005840 GO:0016020
Cluster-22178.64734 GO:0006813 GO:0006811 GO:0005249 GO:0016020
GO:0008076
Cluster-22178.64735 GO:0007268 GO:0003723 GO:0033130 GO:0005515
GO:0043495 GO:0019867
Cluster-22178.64736 GO:0006355 GO:0042254 GO:0006412 GO:0003735
GO:0003677 GO:0003700 GO:0005667 GO:0005622 GO:0005840
Cluster-22178.64737 GO:0048870 GO:0006914 GO:0034508 GO:0007093
GO:0000776 GO:0019028 GO:0031514
Cluster-22178.64741 GO:0006353 GO:0007275 GO:0006355 GO:0007219
GO:0008608 GO:0030154 GO:0003677 GO:0003723 GO:0042729
GO:0072686 GO:0016021
Cluster-22178.64742 GO:0006631 GO:0055114 GO:0050151 GO:0016491
GO:0071949
Cluster-22178.64743 GO:0015684 GO:0007264 GO:0005525 GO:0003924
GO:0015093 GO:0016021
Cluster-22178.64744 GO:0006811 GO:0055085 GO:0005216 GO:0016020
Cluster-22178.64745 GO:0016055 GO:0008013 GO:0016342
Cluster-22178.64746 GO:0055114 GO:0033014 GO:0008883 GO:0050661

Cluster-22178.64747 GO:0005515 GO:0005509
Cluster-22178.64748 GO:0035434 GO:0006825 GO:0005375 GO:0008270
GO:0005524 GO:0004222 GO:0003676 GO:0016021
Cluster-22178.64749 GO:0006508 GO:0004177 GO:0005622
Cluster-22178.64750 GO:0035556 GO:0009890 GO:0000226 GO:0042710
GO:0090063 GO:0005524 GO:0003774 GO:0005515 GO:0005815
GO:0000922 GO:0016459
Cluster-22178.64751 GO:0006481 GO:0006629 GO:0007165 GO:0006479
GO:0035556 GO:0004671 GO:0016627 GO:0016020 GO:0005737
GO:0016021
Cluster-22178.64752 GO:0006357 GO:0008270 GO:0001104 GO:0003676
GO:0016592

Cluster-22178.64753 GO:0006807 GO:0016811
Cluster-22178.64755 GO:0008152 GO:0003824 GO:0003677
Cluster-22178.64756 GO:0007264 GO:0006281 GO:0015684 GO:0055114
GO:0005515 GO:0015093 GO:0008270 GO:0003924 GO:0016491
GO:0005525 GO:0016021
Cluster-22178.64757 GO:0043065 GO:0046983 GO:0003677 GO:0005740
GO:0016021
Cluster-22178.64758 GO:0005975 GO:0042742 GO:0007094 GO:0016853

Cluster-22178.64759 GO:0031124 GO:0000398 GO:0006406 GO:0006369
GO:0000166 GO:0005515 GO:0003723 GO:0003714 GO:0003676
GO:0005667 GO:0016605 GO:0005681 GO:0016607
Cluster-22178.64760 GO:0046872
Cluster-22178.64761 GO:0031047
Cluster-22178.64762 GO:0003677 GO:0005634
Cluster-22178.64763 GO:0019217 GO:0016567 GO:0046872 GO:0008270
GO:0000062 GO:0004842 GO:0005515 GO:0005680
Cluster-22178.64764 GO:0006260 GO:0000921 GO:0006457 GO:0048870
GO:0000917 GO:0044780 GO:0006355 GO:0015991 GO:0006606
GO:0048193 GO:0015992 GO:0006914 GO:0043093 GO:0007155
GO:0007094 GO:0010508 GO:0017048 GO:0003700 GO:0008134
GO:0045502 GO:0005524 GO:0008270 GO:0003677 GO:0016818
GO:0015078 GO:0043565 GO:0042803 GO:0005198 GO:0051082
GO:0033180 GO:0030286 GO:0005940 GO:0005882 GO:0016021
GO:0031514 GO:0019028 GO:0016272 GO:0016020 GO:0005667
GO:0019867 GO:0005737 GO:0005634
Cluster-22178.64765 GO:0006281 GO:0006308 GO:0006260 GO:0017108
GO:0005634 GO:0033557
Cluster-22178.64766 GO:0006206 GO:0006144 GO:0006351 GO:0006355
GO:0003700 GO:0046983 GO:0003677 GO:0003899 GO:0005730
GO:0005667
Cluster-22178.64767 GO:0006206 GO:0006144 GO:0006351 GO:0046983
GO:0003899 GO:0005730

Cluster-22178.64769 GO:0006355 GO:0003700 GO:0005667
Cluster-22178.64771 GO:0035023 GO:0043087 GO:0005089 GO:0005515
GO:0005509
Cluster-22178.64772 GO:0006355 GO:0005515 GO:0005634
Cluster-22178.64773 GO:0043401 GO:0006355 GO:0003700 GO:0043565
GO:0008270 GO:0005667 GO:0005634
Cluster-22178.64774 GO:0046872
Cluster-22178.64777 GO:0016567 GO:0004842 GO:0005515 GO:0046872
GO:0008270 GO:0005680
Cluster-22178.64779 GO:0055114 GO:0016052 GO:0005975 GO:0015992
GO:0043093 GO:0015986 GO:0016192 GO:0000917 GO:0016491
GO:0004553 GO:0015078 GO:0030246 GO:0005737 GO:0000276
Cluster-22178.64780 GO:0006816 GO:0070588 GO:0005509 GO:0005515
GO:0005262 GO:0019033 GO:0016020
Cluster-22178.64782 GO:0005515
Cluster-22178.64783 GO:0007155 GO:0001503 GO:0046935 GO:0003676
GO:0005944 GO:0005576
Cluster-22178.64784 GO:0006355 GO:0003700 GO:0003677 GO:0005667
Cluster-22178.64786 GO:0006355 GO:0055114 GO:0055085 GO:0016491
GO:0003700 GO:0005667 GO:0005741
Cluster-22178.64788 GO:0000398 GO:0005681
Cluster-22178.64792 GO:0006810 GO:0005215 GO:0016020
Cluster-22178.64793 GO:0005515
Cluster-22178.64797 GO:0006886 GO:0007165 GO:0006614 GO:0006352
GO:0004888 GO:0005047 GO:0003677 GO:0098519 GO:0003924
GO:0005525 GO:0005786 GO:0016021 GO:0005785
Cluster-22178.64798 GO:0006508 GO:0006144 GO:0004197 GO:0005515
GO:0004252 GO:0017111 GO:0008289 GO:0003968 GO:0005887
GO:0031379
Cluster-22178.64799 GO:0007165 GO:0030695 GO:0005057
Cluster-22178.64805 GO:0035556 GO:0005515
Cluster-22178.64806 GO:0007165 GO:0007275 GO:0016055 GO:0006468
GO:0005102 GO:0004672 GO:0005524 GO:0005576

Cluster-22178.64810 GO:0009405 GO:0016055 GO:0006629 GO:0006468
GO:0044564 GO:0035091 GO:0016773 GO:0004672 GO:0005524
GO:0016020 GO:0005576
Cluster-22178.64811 GO:0015986 GO:0015992 GO:0006364 GO:0015078
GO:0000276 GO:0032040 GO:0016021
Cluster-22178.64812 GO:0006886 GO:0016192 GO:0005515 GO:0030131
GO:0030117
Cluster-22178.64813 GO:0006206 GO:0009405 GO:0006230 GO:0004797
GO:0005524 GO:0005198
Cluster-22178.64814 GO:0004867 GO:0005576
Cluster-22178.64815 GO:0055114 GO:0015684 GO:0007264 GO:0005525
GO:0016491 GO:0003924 GO:0005524 GO:0015093 GO:0000166
GO:0003677 GO:0016021
Cluster-22178.64816 GO:0006355 GO:0005515 GO:0003723
Cluster-22178.64818 GO:0006486 GO:0016567 GO:0016787 GO:0016818
GO:0003677 GO:0005515 GO:0005524 GO:0008270 GO:0046872
GO:0004842 GO:0016757 GO:0003676 GO:0005680
Cluster-22178.64819 GO:0043248 GO:0006850 GO:0016310 GO:0009069
GO:0015991 GO:0051252 GO:0016192 GO:0006886 GO:0004674
GO:0004521 GO:0016820 GO:0005515 GO:0000221 GO:0005743
GO:0030117
Cluster-22178.64821 GO:0001522 GO:0051090 GO:0042254 GO:0008270
GO:0005524 GO:0030515 GO:0005515 GO:0072588 GO:0005634
Cluster-22178.64822 GO:0006810 GO:0005643
Cluster-22178.64823 GO:0006123 GO:0015992 GO:0004129 GO:0045277
Cluster-22178.64824 GO:0030001 GO:0009405 GO:0010508 GO:0006886
GO:0043093 GO:0007155 GO:0006357 GO:0006914 GO:0006606
GO:0000917 GO:0000398 GO:0051258 GO:0006935 GO:0048870
GO:0006308 GO:0006810 GO:0007165 GO:0071973 GO:0030168
GO:0015031 GO:0000921 GO:0006260 GO:0055085 GO:0042803
GO:0001104 GO:0005198 GO:0016817 GO:0005524 GO:0003774
GO:0008565 GO:0042626 GO:0045502 GO:0008376 GO:0008134
GO:0008092 GO:0046873 GO:0030674 GO:0008855 GO:0005102

GO:0005634 GO:0009288 GO:0005737 GO:0032580 GO:0005667
GO:0016020 GO:0005577 GO:0019028 GO:0009318 GO:0031514
GO:0019031 GO:0016021 GO:0005882 GO:0016592 GO:0030286
GO:0005940 GO:0005815 GO:0019898
Cluster-22178.64827 GO:0015979 GO:0005515 GO:0016773
Cluster-22178.64829 GO:0031047 GO:0003723 GO:0019012 GO:0016021
Cluster-22178.64831 GO:0006351 GO:0007165 GO:0007186 GO:0004871
GO:0019001 GO:0031683 GO:0003924
Cluster-22178.64832 GO:0009607 GO:0016021
Cluster-22178.64833 GO:0055114 GO:0016972
Cluster-22178.64834 GO:0006275 GO:0007155 GO:0007049
Cluster-22178.64836 GO:0042254 GO:0006412 GO:0042981 GO:0003735
GO:0005515 GO:0008270 GO:0019843 GO:0005634 GO:0005840
Cluster-22178.64837 GO:0006468 GO:0046486 GO:0000160 GO:0045017
GO:0042967 GO:0004144 GO:0003677 GO:0005524 GO:0004672
GO:0000156
Cluster-22178.64839 GO:0005515
Cluster-22178.64841 GO:0006814 GO:0006464 GO:0043087 GO:0015992
GO:0046436 GO:0009252 GO:0035023 GO:0006885 GO:0008716
GO:0005515 GO:0015385 GO:0005089 GO:0016020
Cluster-22178.64843 GO:0006814 GO:0006885 GO:0035023 GO:0009252
GO:0046436 GO:0015992 GO:0043087 GO:0006464 GO:0015385
GO:0005515 GO:0008716 GO:0005089 GO:0016020
Cluster-22178.64844 GO:0009405 GO:0007165 GO:0035023 GO:0043087
GO:0005515 GO:0005089
Cluster-22178.64845 GO:0008270
Cluster-22178.64846 GO:0048544 GO:0005515
Cluster-22178.64849 GO:0022900 GO:0006397 GO:0006355 GO:0035438
GO:0003723 GO:0003682 GO:0000785 GO:0005737 GO:0005747
GO:0005634 GO:0005739
Cluster-22178.64852 GO:0042157 GO:0006952 GO:0006810 GO:0009405
GO:0019855 GO:0005576
Cluster-22178.64854 GO:0006355 GO:0006468 GO:0005524 GO:0004672

Cluster-22178.64855 GO:0007169 GO:0006468 GO:0006508 GO:0006397
GO:0005524 GO:0003723 GO:0004714 GO:0004252 GO:0005737
GO:0005634 GO:0030430 GO:0016020
Cluster-22178.64856 GO:0006568 GO:0006550 GO:0018874 GO:0006631
GO:0006552 GO:0006574 GO:0006554 GO:0055114 GO:0008152
GO:0006633 GO:0003857 GO:0016836 GO:0016491 GO:0003824
Cluster-22178.64858 GO:0006260 GO:0006313 GO:0007154 GO:0006281
GO:0003911 GO:0016021
Cluster-22178.64859 GO:0006355 GO:0007010 GO:0003779 GO:0043565
GO:0008270 GO:0003700 GO:0005667
Cluster-22178.64861 GO:0042254 GO:0006412 GO:0003735 GO:0005622
GO:0005840
Cluster-22178.64862 GO:0042254 GO:0006412 GO:0003735 GO:0005622
GO:0005840
Cluster-22178.64863 GO:0055114 GO:0006118 GO:0008152 GO:0016627
GO:0050660 GO:0003995
Cluster-22178.64865 GO:0016579 GO:0006265 GO:0006355 GO:0003700
GO:0003916 GO:0036459 GO:0003677 GO:0005515 GO:0005694
GO:0005667
Cluster-22178.64866 GO:0006352 GO:0006355 GO:0008270 GO:0003700
GO:0016987 GO:0005515 GO:0003677 GO:0005667
Cluster-22178.64871 GO:0008152 GO:0008168 GO:0019028
Cluster-22178.64873 GO:0007264 GO:0006576 GO:0008201 GO:0005524
GO:0003924 GO:0005525 GO:0005886 GO:0016021
Cluster-22178.64874 GO:0006206 GO:0043087 GO:0006144 GO:0006351
GO:0007264 GO:0003677 GO:0003899 GO:0005085 GO:0005730
Cluster-22178.64877 GO:0006955 GO:0007165 GO:0006606 GO:0030247
GO:0005044
Cluster-22178.64878 GO:0006693 GO:0006486 GO:0006298 GO:0006691
GO:0019530 GO:0006749 GO:0008373 GO:0003840 GO:0005524
GO:0030983
Cluster-22178.64880 GO:0006749 GO:0006098 GO:0006313 GO:0046486

GO:0046168 GO:0006355 GO:0005975 GO:0006006 GO:0055114
GO:0006072 GO:0051287 GO:0003677 GO:0016616 GO:0004803
GO:0004367 GO:0016645 GO:0050661 GO:0004345 GO:0003676
GO:0009331 GO:0005622
Cluster-22178.64881 GO:0006072 GO:0005975 GO:0006006 GO:0055114
GO:0006355 GO:0046168 GO:0006313 GO:0046486 GO:0006098
GO:0006749 GO:0003676 GO:0016645 GO:0050661 GO:0004345
GO:0004367 GO:0003677 GO:0016616 GO:0004803 GO:0051287
GO:0005622 GO:0009331
Cluster-22178.64882 GO:0015031 GO:0006464 GO:0005198 GO:0005515

Cluster-22178.64883 GO:0006813 GO:0005515 GO:0005242 GO:0016021
GO:0008076
Cluster-22178.64885 GO:0043565
Cluster-22178.64890 GO:0000162 GO:0006571 GO:0016052 GO:0005975
GO:0009094 GO:0006568 GO:0004553 GO:0004834 GO:0030246
Cluster-22178.64893 GO:0007165 GO:0007606 GO:0003676 GO:0005524
GO:0005515 GO:0042393 GO:0003677 GO:0016787 GO:0004888
GO:0016020
Cluster-22178.64895 GO:0008152 GO:0003824
Cluster-22178.64896 GO:0007268 GO:0008152 GO:0007217 GO:0003824

Cluster-22178.64899 GO:0005509 GO:0005544
Cluster-22178.64900 GO:0006189 GO:0006355 GO:0003700 GO:0003824
GO:0043565 GO:0008270 GO:0005667
Cluster-22178.64901 GO:0005198 GO:0070140 GO:0032183 GO:0019028
Cluster-22178.64902 GO:0009089 GO:0055114 GO:0009085 GO:0016620
GO:0008839 GO:0016817
Cluster-22178.64903 GO:0006486 GO:0016192 GO:0006886 GO:0005198
GO:0005515 GO:0008378 GO:0016757 GO:0030117 GO:0016020
Cluster-22178.64905 GO:0006355 GO:0019058 GO:0003723 GO:0005515
GO:0008270 GO:0046872 GO:0003700 GO:0005667 GO:0042025
Cluster-22178.64907 GO:0015992 GO:0006744 GO:0006120 GO:0007165

GO:0006814 GO:0006118 GO:0006955 GO:0005179 GO:0003954
GO:0008137 GO:0005164 GO:0005576 GO:0005739 GO:0016020
Cluster-22178.64908 GO:0016787
Cluster-22178.64909 GO:0006468 GO:0004672 GO:0005524 GO:0016773
GO:0016020
Cluster-22178.64911 GO:0006412 GO:0042254 GO:0003735 GO:0005622
GO:0005840
Cluster-22178.64915 GO:0006886 GO:0009405 GO:0009069 GO:0016310
GO:0005515 GO:0008536 GO:0004674 GO:0005576
Cluster-22178.64917 GO:0006801 GO:0055114 GO:0046872 GO:0004784

Cluster-22178.64920 GO:0007264 GO:0051607 GO:0006357 GO:0007049
GO:0051082 GO:0003677 GO:0001104 GO:0005524 GO:0005525
GO:0019028 GO:0016592 GO:0016021
Cluster-22178.64922 GO:0005515
Cluster-22178.64926 GO:0009395 GO:0046339 GO:0006629 GO:0035556
GO:0007165 GO:0005515 GO:0004435
Cluster-22178.64927 GO:0004222 GO:0003676 GO:0005524 GO:0008270
GO:0016021
Cluster-22178.64929 GO:0007264 GO:0007165 GO:0007186 GO:0019001
GO:0031683 GO:0003924 GO:0005525 GO:0004871
Cluster-22178.64930 GO:0048306
Cluster-22178.64931 GO:0046656 GO:0007586 GO:0007165 GO:0006457
GO:0006730 GO:0006807 GO:0000413 GO:0016042 GO:0004888
GO:0003755 GO:0018759 GO:0008047 GO:0005576 GO:0016021
Cluster-22178.64932 GO:0009234 GO:0008152 GO:0050662 GO:0005515
GO:0005198 GO:0008270 GO:0046872 GO:0016491 GO:0003824
GO:0019028 GO:0005622
Cluster-22178.64934 GO:0005515
Cluster-22178.64935 GO:0006265 GO:0015031 GO:0008277 GO:0006886
GO:0008565 GO:0003917 GO:0003677 GO:0016021 GO:0005694
Cluster-22178.64936 GO:0006886 GO:0006605 GO:0008270 GO:0005515
GO:0016020

Cluster-22178.64937 GO:0007264 GO:0007010 GO:0007165 GO:0007186
GO:0005515 GO:0031683 GO:0019001 GO:0031423 GO:0003779
GO:0003924 GO:0005525 GO:0004871 GO:0019012
Cluster-22178.64938 GO:0006629 GO:0006308 GO:0031110 GO:0043565
GO:0008855 GO:0008081 GO:0009318
Cluster-22178.64939 GO:0055114 GO:0015031 GO:0000902 GO:0016645
GO:0008270 GO:0016021 GO:0005789
Cluster-22178.64941 GO:0035556
Cluster-22178.64944 GO:0006457 GO:0006886 GO:0008219 GO:0007304
GO:0051087 GO:0000774 GO:0008270 GO:0005198 GO:0005515
GO:0042803 GO:0005213 GO:0016020 GO:0005783 GO:0019028
GO:0005576 GO:0042600 GO:0016021 GO:0005801
Cluster-22178.64947 GO:0005515
Cluster-22178.64948 GO:0006820 GO:0019033 GO:0016021
Cluster-22178.64949 GO:0046872
Cluster-22178.64950 GO:0005509 GO:0005515
Cluster-22178.64951 GO:0042254 GO:0006412 GO:0003735 GO:0005622
GO:0005840
Cluster-22178.64952 GO:0006396 GO:0003723
Cluster-22178.64953 GO:0005634
Cluster-22178.64957 GO:0015992 GO:0015986 GO:0015078
Cluster-22178.64959 GO:0008152 GO:0005515 GO:0016746 GO:0019031
Cluster-22178.64960 GO:0015986 GO:0006119 GO:0006810 GO:0015992
GO:0046961 GO:0045182 GO:0046933 GO:0015288 GO:0016020
GO:0045261 GO:0045259
Cluster-22178.64961 GO:0006979 GO:0016579 GO:0055114 GO:0006118
GO:0006804 GO:0006281 GO:0006749 GO:0006508 GO:0036459
GO:0004602 GO:0016655 GO:0004843 GO:0016020 GO:0071203
Cluster-22178.64967 GO:0006351 GO:0006269 GO:0003896 GO:0005657
GO:0005730
Cluster-22178.64968 GO:0006412 GO:0042254 GO:0003723 GO:0003735
GO:0005840
Cluster-22178.64969 GO:0006468 GO:0032259 GO:0005524 GO:0004672

GO:0008168
Cluster-22178.64972 GO:0006520 GO:0055114 GO:0016491
Cluster-22178.64973 GO:0006541 GO:0004359
Cluster-22178.64974 GO:0003676
Cluster-22178.64975 GO:0006378 GO:0003676 GO:0008143 GO:0005737
GO:0005634
Cluster-22178.64979 GO:0048870 GO:0007165 GO:0005515 GO:0031514
Cluster-22178.64982 GO:0009405 GO:0005198
Cluster-22178.64985 GO:0006779 GO:0055114 GO:0016226 GO:0051536
GO:0005737
Cluster-22178.64986 GO:0005515
Cluster-22178.64987 GO:0005515
Cluster-22178.64990 GO:0070836 GO:0006468 GO:0016773 GO:0004672
GO:0005524 GO:0016020
Cluster-22178.64991 GO:0018279 GO:0005524 GO:0004579 GO:0016021
GO:0008250
Cluster-22178.64992 GO:0055085 GO:0007186 GO:0006812 GO:0004930
GO:0008324 GO:0016021 GO:0005886
Cluster-22178.64993 GO:0009236 GO:0006811 GO:0004190 GO:0016021
GO:0016020
Cluster-22178.64995 GO:0003677
Cluster-22178.64996 GO:0006413 GO:0006446 GO:0046983 GO:0003743
GO:0005515 GO:0005840
Cluster-22178.64998 GO:0006940
Cluster-22178.64999 GO:0006886 GO:0016192 GO:0005198 GO:0005515
GO:0030126 GO:0030117
Cluster-22178.65001 GO:0070403 GO:0005515
Cluster-22178.65002 GO:0008270 GO:0003676
Cluster-22178.65003 GO:0008270 GO:0003676
Cluster-22178.65004 GO:0006810 GO:0008152 GO:0016866 GO:0005215
GO:0031419 GO:0005737 GO:0016021
Cluster-22178.65007 GO:0006446 GO:0007218 GO:0006464 GO:0006144
GO:0006413 GO:0006355 GO:0046034 GO:0005975 GO:0006808

GO:0003677 GO:0003743 GO:0004017 GO:0004553 GO:0008270
GO:0030234 GO:0004519 GO:0005515 GO:0003723 GO:0016151
GO:0003700 GO:0003676 GO:0004672 GO:0046872 GO:0043565
GO:0005667 GO:0005840
Cluster-22178.65011 GO:0005515
Cluster-22178.65012 GO:0055114 GO:0015992 GO:0008152 GO:0006355
GO:0006118 GO:0006119 GO:0016491 GO:0003824 GO:0050662
GO:0008121 GO:0003677
Cluster-22178.65013 GO:0019031
Cluster-22178.65014 GO:0055114 GO:0008152 GO:0051287 GO:0016491
GO:0050662 GO:0003824
Cluster-22178.65015 GO:0006308 GO:0015991 GO:0000917 GO:0015992
GO:0008053 GO:0006606 GO:0043093 GO:0007165 GO:0006810
GO:0003924 GO:0015078 GO:0042802 GO:0008855 GO:0043015
GO:0005737 GO:0016021 GO:0045298 GO:0019028 GO:0033179
GO:0009318 GO:0005886 GO:0005741
Cluster-22178.65021 GO:0007165 GO:0005515
Cluster-22178.65022 GO:0016787 GO:0005882
Cluster-22178.65024 GO:0042967 GO:0016573 GO:0006355 GO:0008270
GO:0008134 GO:0003713 GO:0003677 GO:0004402 GO:0003682
GO:0000785 GO:0000123 GO:0005667 GO:0005634
Cluster-22178.65025 GO:0016787
Cluster-22178.65026 GO:0005515
Cluster-22178.65027 GO:0006812 GO:0016887 GO:0032040 GO:0016021
Cluster-22178.65032 GO:0006470 GO:0006355 GO:0006570 GO:0043565
GO:0003700 GO:0004725 GO:0008138 GO:0005667 GO:0016020
Cluster-22178.65033 GO:0003676
Cluster-22178.65037 GO:0042254 GO:0005524
Cluster-22178.65039 GO:0051028 GO:0003677 GO:0008270 GO:0005634
Cluster-22178.65040 GO:0006570 GO:0042207 GO:0006810 GO:0009072
GO:0015743 GO:0004334 GO:0005886
Cluster-22178.65041 GO:0006511 GO:0031625
Cluster-22178.65042 GO:0005515 GO:0009512

Cluster-22178.65043 GO:0005515
Cluster-22178.65044 GO:0016020
Cluster-22178.65048 GO:0015991 GO:0016192 GO:0006886 GO:0005515
GO:0016820 GO:0030117 GO:0000221
Cluster-22178.65049 GO:0006260 GO:0006355 GO:0006270 GO:0005524
GO:0016887 GO:0003678 GO:0003677 GO:0008134 GO:0005657
GO:0005667 GO:0005634 GO:0042555
Cluster-22178.65050 GO:0006506 GO:0006412 GO:0042254 GO:0003735
GO:0016746 GO:0005739 GO:0005840 GO:0005789 GO:0016021
Cluster-22178.65051 GO:0006364 GO:0032259 GO:0006370 GO:0006144
GO:0006396 GO:0000154 GO:0006479 GO:0031167 GO:0009451
GO:0008152 GO:0017111 GO:0004482 GO:0070008 GO:0008649
GO:0004252 GO:0016817 GO:0008168 GO:0004483 GO:0003968
GO:0031379 GO:0005737
Cluster-22178.65052 GO:0009405 GO:0006338 GO:0090544
Cluster-22178.65054 GO:0055085 GO:0005741
Cluster-22178.65057 GO:0034968 GO:0006355 GO:0006554 GO:0006479
GO:0018024 GO:0005515 GO:0005694
Cluster-22178.65058 GO:0007275 GO:0006511 GO:0005634
Cluster-22178.65059 GO:0006810 GO:0007165 GO:0043093 GO:0008053
GO:0006606 GO:0015992 GO:0000917 GO:0015991 GO:0006308
GO:0043015 GO:0008855 GO:0042802 GO:0015078 GO:0003924
GO:0005741 GO:0005886 GO:0033179 GO:0009318 GO:0019028
GO:0045298 GO:0016021 GO:0005737
Cluster-22178.65061 GO:0006352 GO:0006355 GO:0016987 GO:0003677
GO:0003700 GO:0005667
Cluster-22178.65063 GO:0043248 GO:0006886 GO:0016192 GO:0015991
GO:0016820 GO:0005515 GO:0000221 GO:0030117
Cluster-22178.65065 GO:0006629 GO:0042254 GO:0006412 GO:0006007
GO:0046537 GO:0005515 GO:0003735 GO:0003824 GO:0005622
GO:0005840
Cluster-22178.65066 GO:0008380 GO:0006308 GO:0007155 GO:0008855
GO:0005200 GO:0005856 GO:0005681 GO:0009318 GO:0005882

Cluster-22178.65067 GO:0032922 GO:0009452 GO:0055114 GO:0016567
GO:0001510 GO:0004842 GO:0051537 GO:0016491 GO:0008168
GO:0000976 GO:0046872 GO:0008270 GO:0005515
Cluster-22178.65068 GO:0006013 GO:0005975 GO:0007165 GO:0004553
GO:0008158 GO:0004559 GO:0008270 GO:0015923 GO:0016020
Cluster-22178.65069 GO:0042254 GO:0006412 GO:0006629 GO:0006007
GO:0005515 GO:0003735 GO:0046537 GO:0003824 GO:0005840
GO:0005622
Cluster-22178.65071 GO:0008608 GO:0048870 GO:0007094 GO:0006355
GO:0003700 GO:0031514 GO:0042729 GO:0072686 GO:0005667
Cluster-22178.65072 GO:0008210 GO:0019836 GO:0008207 GO:0008209
GO:0055114 GO:0006694 GO:0008152 GO:0003854 GO:0050662
GO:0005515 GO:0016616 GO:0016491 GO:0003824 GO:0005576
Cluster-22178.65073 GO:0016117 GO:0055114 GO:0006355 GO:0016705
GO:0016491 GO:0071949 GO:0005634
Cluster-22178.65077 GO:0009878 GO:0046872
Cluster-22178.65078 GO:0006412 GO:0042254 GO:0006260 GO:0003677
GO:0003887 GO:0003735 GO:0001882 GO:0008270 GO:0046872
GO:0042575 GO:0042025 GO:0005840 GO:0005622
Cluster-22178.65079 GO:0007050 GO:0045859 GO:0000122 GO:0004861
GO:0017069 GO:0005737 GO:0005634
Cluster-22178.65081 GO:0005515
Cluster-22178.65082 GO:0000917 GO:0043093 GO:0006355 GO:0007049
GO:0005515 GO:0003723 GO:0003700 GO:0008092 GO:0043565
GO:0046872 GO:0008270 GO:0005667 GO:0019898 GO:0005815
GO:0005737
Cluster-22178.65085 GO:0055114 GO:0006730 GO:0006813 GO:0006555
GO:0051287 GO:0004013
Cluster-22178.65086 GO:0016787 GO:0016765
Cluster-22178.65088 GO:0055114 GO:0048038 GO:0016151 GO:0016651
GO:0051287
Cluster-22178.65089 GO:0046935 GO:0003676 GO:0003723 GO:0016021
GO:0005944

Cluster-22178.65090 GO:0006260 GO:0005509
Cluster-22178.65092 GO:0000413 GO:0006457 GO:0003755
Cluster-22178.65093 GO:0000917 GO:0043093 GO:0006355 GO:0007049
GO:0006260 GO:0051259 GO:0003700 GO:0034618 GO:0005667
GO:0005634 GO:0005737
Cluster-22178.65094 GO:0022900 GO:0016021
Cluster-22178.65095 GO:0006412 GO:0042254 GO:0003735 GO:0005622
GO:0005840
Cluster-22178.65098 GO:0009312 GO:0008455 GO:0016021 GO:0005795
Cluster-22178.65099 GO:0005982 GO:0005985 GO:0006096 GO:0006098
GO:0006094 GO:0004347
Cluster-22178.65100 GO:0006468 GO:0016773 GO:0005524 GO:0004672
GO:0016020
Cluster-22178.65101 GO:0003676
Cluster-22178.65102 GO:0007165 GO:0006118 GO:0005509 GO:0016787
GO:0009055 GO:0016491 GO:0016020 GO:0005578 GO:0016021
Cluster-22178.65103 GO:0007186 GO:0007165 GO:0004871 GO:0005834
Cluster-22178.65104 GO:0016559 GO:0005779
Cluster-22178.65108 GO:0048193 GO:0046872 GO:0008270 GO:0003676
GO:0016020
Cluster-22178.65110 GO:0055114 GO:0006511 GO:0007275 GO:0006468
GO:0016627 GO:0046872 GO:0004672 GO:0008270 GO:0005524
GO:0005634
Cluster-22178.65111 GO:0016192 GO:0006886 GO:0006468 GO:0005524
GO:0004672 GO:0016773 GO:0016020
Cluster-22178.65116 GO:0009405 GO:0016114 GO:0006694 GO:0008152
GO:0016624 GO:0008661 GO:0003824 GO:0030976
Cluster-22178.65118 GO:0015684 GO:0007264 GO:0015886 GO:0006281
GO:0046497 GO:0006769 GO:0006310 GO:0002949 GO:0007131
GO:0055114 GO:0008033 GO:0009378 GO:0051287 GO:0050660
GO:0003724 GO:0008750 GO:0016491 GO:0050661 GO:0005525
GO:0003678 GO:0098519 GO:0015232 GO:0005524 GO:0015093
GO:0016887 GO:0016301 GO:0003723 GO:0003924 GO:0009379

GO:0016021 GO:0005657 GO:0000794
Cluster-22178.65120 GO:0006633 GO:0046436 GO:0009252 GO:0006479
GO:0006481 GO:0006813 GO:0006090 GO:0004671 GO:0005524
GO:0015269 GO:0003989 GO:0008716 GO:0046872 GO:0016020
GO:0009317 GO:0016021
Cluster-22178.65123 GO:0006144 GO:0015992 GO:0006206 GO:0015991
GO:0007155 GO:0006351 GO:0015078 GO:0070140 GO:0032183
GO:0005515 GO:0003899 GO:0003677 GO:0005730 GO:0016020
GO:0033179
Cluster-22178.65124 GO:0003676 GO:0046872
Cluster-22178.65126 GO:0031145 GO:0030071 GO:0007010 GO:0006355
GO:0006468 GO:0004672 GO:0005524 GO:0003779 GO:0005515
GO:0005680 GO:0016020 GO:0005634 GO:0019028
Cluster-22178.65128 GO:0005515
Cluster-22178.65130 GO:0007178 GO:0016310 GO:0009069 GO:0006468
GO:0006413 GO:0006446 GO:0005524 GO:0004675 GO:0003743
GO:0005840 GO:0016021 GO:0005576 GO:0016020
Cluster-22178.65131 GO:0043087 GO:0035556 GO:0007264 GO:0008283
GO:0019068 GO:0007165 GO:0015684 GO:0006270 GO:0007186
GO:0035023 GO:0040007 GO:0006576 GO:0015093 GO:0005524
GO:0016887 GO:0016757 GO:0005089 GO:0005179 GO:0046872
GO:0019001 GO:0031683 GO:0003924 GO:0005525 GO:0004871
GO:0005198 GO:0005515 GO:0008083 GO:0016020 GO:0016021
GO:0005576
Cluster-22178.65132 GO:0006355 GO:0006810 GO:0051537 GO:0003677
GO:0003713 GO:0005886 GO:0043231 GO:0005667
Cluster-22178.65133 GO:0005975 GO:0006012 GO:0006027 GO:0006687
GO:0046486 GO:0046872 GO:0004565 GO:0005515 GO:0009341
Cluster-22178.65134 GO:0008283 GO:0040007 GO:0007155 GO:0001558
GO:0007165 GO:0005515 GO:0008083 GO:0005520 GO:0005576
GO:0016942
Cluster-22178.65135 GO:0006364 GO:0005515 GO:0016021 GO:0032040
Cluster-22178.65137 GO:0007165 GO:0030695 GO:0005057

Cluster-22178.65138 GO:0008616 GO:0016192 GO:0006886 GO:0043248 GO:0016740 GO:0005515 GO:0016853 GO:0003676 GO:0030117
Cluster-22178.65140 GO:0007165
Cluster-22178.65141 GO:0035091 GO:0031083
Cluster-22178.65144 GO:0019058 GO:0051536 GO:0008289 GO:0003824 GO:0019031
Cluster-22178.65146 GO:0055085 GO:0030001 GO:0048024 GO:0008017 GO:0046873 GO:0019901 GO:0045298 GO:0005634 GO:0016020
Cluster-22178.65147 GO:0007275 GO:0042600
Cluster-22178.65150 GO:0005509 GO:0005515
Cluster-22178.65151 GO:0016020
Cluster-22178.65153 GO:0046907 GO:0000413 GO:0006457 GO:0004519 GO:0003755
Cluster-22178.65155 GO:0003723
Cluster-22178.65157 GO:0006109 GO:0055114 GO:0008152 GO:0016310 GO:0000160 GO:0000155 GO:0050662 GO:0005524 GO:0004672 GO:0016491 GO:0003824 GO:0009365
Cluster-22178.65158 GO:0006508 GO:0006511 GO:0016579 GO:0004843 GO:0005622
Cluster-22178.65160 GO:0005515
Cluster-22178.65161 GO:0015889 GO:0031419
Cluster-22178.65162 GO:0009235 GO:0006355 GO:0046872 GO:0005515 GO:0005634
Cluster-22178.65163 GO:0003676
Cluster-22178.65165 GO:0007264 GO:0005524 GO:0003924 GO:0005525

Cluster-22178.65167 GO:0007165 GO:0006614 GO:0007186 GO:0007264 GO:0006013 GO:0043087 GO:0006000 GO:0006355 GO:0035023 GO:0003873 GO:0005089 GO:0005524 GO:0005515 GO:0005525 GO:0004871 GO:0019001 GO:0031683 GO:0003924
Cluster-22178.65168 GO:0055114 GO:0006508 GO:0006281 GO:0043039 GO:0051536 GO:0046872 GO:0016876 GO:0005524 GO:0008270 GO:0008234 GO:0005515 GO:0005509 GO:0016020

Cluster-22178.65173 GO:0005515

Cluster-22178.65178 GO:0006568 GO:0006355 GO:0015947 GO:0006804 GO:0055114 GO:0006979 GO:0004096 GO:0020037 GO:0003700 GO:0005667 GO:0005634

Cluster-22178.65182 GO:0006323 GO:0005198 GO:0005882

Cluster-22178.65184 GO:0031047 GO:0005515

Cluster-22178.65186 GO:0046500 GO:0001510 GO:0006479 GO:0006306 GO:0031167 GO:0006464 GO:0032259 GO:0009452 GO:0008170 GO:0003723 GO:0003677 GO:0004719 GO:0008270 GO:0008168 GO:0003676

Cluster-22178.65187 GO:0035556 GO:0005515

Cluster-22178.65189 GO:0006468 GO:0006508 GO:0043087 GO:0007169 GO:0035023 GO:0004714 GO:0005515 GO:0005524 GO:0004672 GO:0004185 GO:0005089 GO:0016020

Cluster-22178.65191 GO:0007186 GO:0004930 GO:0016021

Cluster-22178.65192 GO:0055114 GO:0007165 GO:0035556 GO:0016717

Cluster-22178.65193 GO:0003677

Cluster-22178.65195 GO:0003676

Cluster-22178.65196 GO:0006021 GO:0019872 GO:0008654 GO:0004512

Cluster-22178.65197 GO:0035556 GO:0007264 GO:0005525

Cluster-22178.65198 GO:0006282 GO:0006355 GO:0008270 GO:0005096 GO:0005544 GO:0005509 GO:0005515 GO:0008023

Cluster-22178.65200 GO:0019079 GO:0043248 GO:0003676 GO:0003677 GO:0008026 GO:0016787 GO:0005524

Cluster-22178.65201 GO:0006281 GO:0007165 GO:0006144 GO:0006464 GO:0046034 GO:0005179 GO:0004017 GO:0008270 GO:0046872 GO:0016151 GO:0005742 GO:0005576

Cluster-22178.65203 GO:0003677 GO:0003676 GO:0005524 GO:0016787

Cluster-22178.65204 GO:0055114 GO:0006281 GO:0006355 GO:0000184 GO:0047134 GO:0004386 GO:0016972 GO:0005524 GO:0008270

GO:0046872 GO:0003677 GO:0005737
Cluster-22178.65205 GO:0007275 GO:0007219 GO:0016021
Cluster-22178.65206 GO:0006810 GO:0005515 GO:0016021
Cluster-22178.65212 GO:0015992 GO:0015986 GO:0015078 GO:0000276
Cluster-22178.65213 GO:0055114 GO:0006323 GO:0005198 GO:0016491
GO:0005882
Cluster-22178.65215 GO:0006397 GO:0046872
Cluster-22178.65216 GO:0051694 GO:0046782 GO:0005523
Cluster-22178.65217 GO:0006807 GO:0055114 GO:0006520 GO:0006118
GO:0019551 GO:0016491 GO:0004352 GO:0051287
Cluster-22178.65218 GO:0042254 GO:0006412 GO:0055114 GO:0061630
GO:0003735 GO:0005515 GO:0042393 GO:0016972 GO:0008270
GO:0046872 GO:0005840 GO:0005622
Cluster-22178.65219 GO:0006633 GO:0008152 GO:0042967 GO:0016747
GO:0004315 GO:0005835
Cluster-22178.65222 GO:0005509 GO:0005544
Cluster-22178.65224 GO:0006950 GO:0015743 GO:0004190 GO:0016021
Cluster-22178.65226 GO:0008152 GO:0006554 GO:0006479 GO:0006468
GO:0008168 GO:0005524 GO:0004672 GO:0005515 GO:0018024
Cluster-22178.65234 GO:0006414 GO:0006206 GO:0006448 GO:0006144
GO:0006555 GO:0006556 GO:0055114 GO:1901031 GO:0006351
GO:0003746 GO:0004478 GO:0051920 GO:0003676 GO:0005515
GO:0003899 GO:0005730 GO:0005840 GO:0005634
Cluster-22178.65236 GO:0007218 GO:0007264 GO:0007165 GO:0009847
GO:0006468 GO:0006118 GO:0018990 GO:0005524 GO:0016491
GO:0016887 GO:0004672 GO:0003924 GO:0008255 GO:0009055
GO:0005525 GO:0005515 GO:0005198 GO:0005739 GO:0016020
GO:0016021
Cluster-22178.65237 GO:0001503 GO:0030683
Cluster-22178.65243 GO:0045766 GO:0043123 GO:0016567 GO:0015986
GO:0043066 GO:0015992 GO:0003723 GO:0043130 GO:0015078
GO:0004842 GO:0000276
Cluster-22178.65244 GO:0005198 GO:0019028

Cluster-22178.65247 GO:0006355 GO:0003677
Cluster-22178.65248 GO:0055114 GO:0000051 GO:0008652 GO:0005525
GO:0016620 GO:0046983 GO:0003942 GO:0005737
Cluster-22178.65249 GO:0008152 GO:0006468 GO:0046039 GO:0009190
GO:0006144 GO:0006357 GO:0035556 GO:0006182 GO:0004672
GO:0003824 GO:0004383 GO:0016849 GO:0003723 GO:0001104
GO:0008270 GO:0005524 GO:0016592
Cluster-22178.65250 GO:0000226 GO:0090063 GO:0000922 GO:0005815
Cluster-22178.65251 GO:0006270 GO:0006367 GO:0006605 GO:0006886
GO:0032968 GO:0003677 GO:0005515 GO:0003676 GO:0016020
GO:0005742 GO:0005634
Cluster-22178.65253 GO:0070836 GO:0007165 GO:0051252 GO:0042254
GO:0006412 GO:0006355 GO:0003676 GO:0005515 GO:0003735
GO:0004523 GO:0005840 GO:0005622
Cluster-22178.65255 GO:0006807 GO:0006811 GO:0006672 GO:0055085
GO:0007165 GO:0006810 GO:0007186 GO:0004930 GO:0004872
GO:0005216 GO:0016811 GO:0016021 GO:0016020
Cluster-22178.65256 GO:0055114 GO:0006480 GO:0008152 GO:0008168
GO:0016491 GO:0010181 GO:0008171
Cluster-22178.65257 GO:0016740
Cluster-22178.65258 GO:0042254 GO:0006412 GO:0051252 GO:0007165
GO:0070836 GO:0004523 GO:0003735 GO:0005515 GO:0003676
GO:0005622 GO:0005840
Cluster-22178.65259 GO:0009401 GO:0006810 GO:0005886
Cluster-22178.65260 GO:0006468 GO:0019076 GO:0006812 GO:0032801
GO:0005261 GO:0004672 GO:0005524 GO:0016773 GO:0016020
GO:0033644
Cluster-22178.65263 GO:0007275 GO:0009966 GO:0005515 GO:0016020
Cluster-22178.65264 GO:0046872
Cluster-22178.65265 GO:0090063 GO:0000226 GO:0004190 GO:0008146
GO:0005815 GO:0016020 GO:0000922
Cluster-22178.65266 GO:0008270 GO:0005634
Cluster-22178.65273 GO:0055114 GO:0034599 GO:0005524 GO:0016887

GO:0016491 GO:0005789
Cluster-22178.65275 GO:0010508 GO:0006369 GO:0019064 GO:0006379
GO:0006260 GO:0006378 GO:0016758 GO:0005849 GO:0019031
GO:0005789 GO:0005815
Cluster-22178.65281 GO:0007049 GO:0000917 GO:0006355 GO:0010506
GO:0008053 GO:0006260 GO:0043093 GO:0003700 GO:0043565
GO:0003924 GO:0016021 GO:0005615 GO:0005737 GO:0005634
GO:0005741 GO:0005667
Cluster-22178.65282 GO:0042254 GO:0006412 GO:0003735 GO:0005622
GO:0005840
Cluster-22178.65286 GO:0042254 GO:0006412 GO:0003735 GO:0005622
GO:0005840
Cluster-22178.65288 GO:0006352 GO:0003677 GO:0005669 GO:0005634
Cluster-22178.65289 GO:0006007 GO:0006629 GO:0042254 GO:0006412
GO:0003824 GO:0046537 GO:0003735 GO:0005515 GO:0005622
GO:0005840
Cluster-22178.65290 GO:0009231 GO:0005985 GO:0005982 GO:0005991
GO:0005993 GO:0008531 GO:0008375 GO:0005509 GO:0004555
GO:0005737 GO:0016020
Cluster-22178.65291 GO:0006886 GO:0005975 GO:0016832 GO:0005741
GO:0016020 GO:0016021
Cluster-22178.65295 GO:0070206 GO:0048870 GO:0005515 GO:0005639
GO:0031514
Cluster-22178.65300 GO:0042254 GO:0006412 GO:0003735 GO:0005840
GO:0005622
Cluster-22178.65302 GO:0005524
Cluster-22178.65303 GO:0016192 GO:0006886 GO:0055114 GO:0005515
GO:0047134 GO:0070403 GO:0008270 GO:0016491
Cluster-22178.65305 GO:0004527 GO:0003676
Cluster-22178.65307 GO:0006270 GO:0030001 GO:0055085 GO:0019064
GO:0046873 GO:0004190 GO:0003676 GO:0046872 GO:0016020
GO:0016021 GO:0019867
Cluster-22178.65308 GO:0006919 GO:0006974 GO:0008608 GO:0001836

GO:0006915 GO:0016567 GO:0006511 GO:0004842 GO:0034450
GO:0005876 GO:0000151 GO:0042729
Cluster-22178.65309 GO:0019354 GO:0006779 GO:0055114 GO:0043115
GO:0008641 GO:0016491
Cluster-22178.65310 GO:0030150 GO:0005742
Cluster-22178.65312 GO:0045227 GO:0004252 GO:0016020 GO:0016021
Cluster-22178.65313 GO:0042254 GO:0006412 GO:0003735 GO:0005840
Cluster-22178.65315 GO:0046872 GO:0008270
Cluster-22178.65316 GO:0005515 GO:0016740
Cluster-22178.65317 GO:0006522 GO:0006419 GO:0043039 GO:0006531
GO:0016055 GO:0003676 GO:0005524 GO:0016876 GO:0004813
GO:0000166
Cluster-22178.65318 GO:0043039 GO:0006531 GO:0016055 GO:0006522
GO:0006419 GO:0000166 GO:0003676 GO:0005524 GO:0004813
GO:0016876
Cluster-22178.65322 GO:0035556 GO:0005515
Cluster-22178.65323 GO:0006397 GO:0006355 GO:0022900 GO:0003723
GO:0003682 GO:0035438 GO:0005747 GO:0005739 GO:0005634
GO:0000785 GO:0005737
Cluster-22178.65324 GO:0046487 GO:0015977 GO:0003677 GO:0016984
GO:0009573
Cluster-22178.65325 GO:0006260 GO:0006281 GO:0003910 GO:0003676
GO:0003723
Cluster-22178.65327 GO:0006118 GO:0055114 GO:0016625 GO:0009055
GO:0051536 GO:0016491
Cluster-22178.65328 GO:0055114 GO:0005975 GO:0016491 GO:0004553
GO:0016616
Cluster-22178.65329 GO:0016042 GO:0008203 GO:0008152 GO:0016298
GO:0016787
Cluster-22178.65330 GO:0006633 GO:0055114 GO:0005506 GO:0016491

Cluster-22178.65333 GO:0016020
Cluster-22178.65334 GO:0007155 GO:0006355 GO:0042246 GO:0005622

GO:0016021
Cluster-22178.65337 GO:0004064 GO:0005515
Cluster-22178.65338 GO:0005524 GO:0016787 GO:0003677 GO:0003676
GO:0005515
Cluster-22178.65340 GO:0055114 GO:0007010 GO:0046872 GO:0003779
GO:0016972
Cluster-22178.65341 GO:0042254 GO:0055114 GO:0008615 GO:0010181
GO:0016638 GO:0004733 GO:0005622 GO:0016021
Cluster-22178.65342 GO:0042254 GO:0008615 GO:0055114 GO:0016638
GO:0010181 GO:0004733 GO:0016021 GO:0005622
Cluster-22178.65343 GO:0007165 GO:0040007 GO:0008283 GO:0008083
GO:0051015 GO:0030674
Cluster-22178.65344 GO:0006508 GO:0009085 GO:0009089 GO:0055114
GO:0005509 GO:0005515 GO:0008270 GO:0008839 GO:0004222
GO:0031012
Cluster-22178.65346 GO:0045893 GO:0006351 GO:0008270 GO:0005515
GO:0003700 GO:0005634 GO:0005667
Cluster-22178.65347 GO:0005515 GO:0008270
Cluster-22178.65348 GO:0006355 GO:0015986 GO:0006260 GO:0007049
GO:0015992 GO:0003677 GO:0051082 GO:0015078 GO:0003700
GO:0043565 GO:0045263 GO:0005667 GO:0005634
Cluster-22178.65349 GO:0008210 GO:0006694 GO:0055114 GO:0043154
GO:0008207 GO:0019079 GO:0008209 GO:0043066 GO:0003824
GO:0032266 GO:0016616 GO:0051287 GO:0003854 GO:0050662
GO:0016620 GO:0043130 GO:0080019 GO:0005737 GO:0071986
Cluster-22178.65350 GO:0007165 GO:0016567 GO:0006260 GO:0016818
GO:0003677 GO:0005515 GO:0005179 GO:0004842 GO:0005524
GO:0008270 GO:0046872 GO:0043565 GO:0005576 GO:0005634
GO:0005680
Cluster-22178.65351 GO:0007264 GO:0007186 GO:0007165 GO:0003924
GO:0031683 GO:0019001 GO:0004871 GO:0005525
Cluster-22178.65352 GO:0003676
Cluster-22178.65354 GO:0006606 GO:0015031 GO:0042742 GO:0015991

GO:0008565 GO:0016820 GO:0005515 GO:0000221 GO:0005737
GO:0005634
Cluster-22178.65355 GO:0003677 GO:0005576
Cluster-22178.65356 GO:0006355 GO:0042254 GO:0006412 GO:0008270
GO:0043565 GO:0003700 GO:0003735 GO:0005840 GO:0005667
Cluster-22178.65357 GO:0005515
Cluster-22178.65358 GO:0008270
Cluster-22178.65359 GO:0055085 GO:0005741
Cluster-22178.65360 GO:0006817 GO:0005315 GO:0016020
Cluster-22178.65361 GO:0006118 GO:0009451 GO:0006418 GO:0008033
GO:0006308 GO:0045454 GO:0009307 GO:0035556 GO:0006464
GO:0030488 GO:0000166 GO:0005515 GO:0016151 GO:0009055
GO:0015035 GO:0003677 GO:0004812 GO:0016429 GO:0005524
GO:0009036 GO:0009359 GO:0031515
Cluster-22178.65362 GO:0005515
Cluster-22178.65363 GO:0006352 GO:0008152 GO:0055114 GO:0016491
GO:0005634
Cluster-22178.65364 GO:0005515
Cluster-22178.65366 GO:0006886 GO:0009308 GO:0055114 GO:0007264
GO:0007165 GO:0007186 GO:0006888 GO:0019001 GO:0031683
GO:0003924 GO:0005525 GO:0004871 GO:0005507 GO:0008270
GO:0008131 GO:0048038 GO:0030127
Cluster-22178.65368 GO:0006355 GO:0005524 GO:0016887 GO:0005515
GO:0008134 GO:0005667
Cluster-22178.65369 GO:0006886 GO:0006118 GO:0030001 GO:0007338
GO:0022900 GO:0006869 GO:0055085 GO:0020037 GO:0009055
GO:0008289 GO:0046873 GO:0005506 GO:0031083 GO:0005576
GO:0016021 GO:0016020 GO:0042597
Cluster-22178.6537 GO:0007094 GO:0005198 GO:0005882
Cluster-22178.65372 GO:0016310 GO:0008152 GO:0007178 GO:0009069
GO:0006468 GO:0005524 GO:0004675 GO:0016020
Cluster-22178.65373 GO:0015992 GO:0006123 GO:0008270 GO:0004129
GO:0005515 GO:0005740 GO:0045277

Cluster-22178.65374 GO:0006886 GO:0016192 GO:0005198 GO:0030132 GO:0030130
Cluster-22178.65375 GO:0006364 GO:0003676 GO:0008168 GO:0003723

Cluster-22178.65376 GO:0006606 GO:0000921 GO:0006914 GO:0003774 GO:0005198 GO:0019867 GO:0016021 GO:0005940 GO:0005882 GO:0016459
Cluster-22178.65377 GO:0006364 GO:0008168 GO:0003723 GO:0003676

Cluster-22178.65382 GO:0008152 GO:0016747
Cluster-22178.65383 GO:0055085 GO:0006810 GO:0016020 GO:0016021
Cluster-22178.65384 GO:0005515
Cluster-22178.65385 GO:0006308 GO:0009307 GO:0016787 GO:0000287 GO:0009036 GO:0003677 GO:0009359
Cluster-22178.65388 GO:0016020
Cluster-22178.65391 GO:0003676 GO:0003723
Cluster-22178.65392 GO:0046938 GO:0010038 GO:0016756 GO:0046872

Cluster-22178.65393 GO:0035023 GO:0043087 GO:0006810 GO:0006355 GO:0005089 GO:0003676 GO:0004386 GO:0043531 GO:0008270 GO:0005524 GO:0005515
Cluster-22178.65396 GO:0042254 GO:0006412 GO:0003735 GO:0003677 GO:0005096 GO:0008270 GO:0005524 GO:0046872 GO:0004222 GO:0005622 GO:0045202 GO:0016021 GO:0005840
Cluster-22178.65398 GO:0006814 GO:0006118 GO:0006744 GO:0006120 GO:0015992 GO:0008137 GO:0009055 GO:0016020
Cluster-22178.65399 GO:0008289 GO:0005515 GO:0005869
Cluster-22178.65400 GO:0005515
Cluster-22178.65403 GO:0016787
Cluster-22178.65405 GO:0016579 GO:0070140 GO:0032183 GO:0036459

Cluster-22178.65406 GO:0015979 GO:0019538 GO:0016567 GO:0009405 GO:0050765 GO:0008270 GO:0046872 GO:0005515 GO:0009523

GO:0009539 GO:0016020 GO:0016012 GO:0005576
Cluster-22178.65408 GO:0016579 GO:0036459
Cluster-22178.65409 GO:0007264 GO:0015684 GO:0003924 GO:0015093
GO:0005525 GO:0001527 GO:0016021
Cluster-22178.65411 GO:0006438 GO:0006418 GO:0009098 GO:0009097
GO:0009099 GO:0002161 GO:0004832 GO:0005524 GO:0004812
GO:0000166 GO:0005737
Cluster-22178.65413 GO:0006813 GO:0009405 GO:0006812 GO:0016051
GO:0005516 GO:0000166 GO:0016887 GO:0015269 GO:0046872
GO:0016840 GO:0043230 GO:0016021 GO:0009289
Cluster-22178.65415 GO:0006446 GO:0006313 GO:0006413 GO:0004803
GO:0003743 GO:0003677 GO:0003723 GO:0005840
Cluster-22178.65417 GO:0009432 GO:0051782 GO:0005515 GO:0005509
GO:0046983 GO:0009276
Cluster-22178.65422 GO:0006241 GO:0006183 GO:0006228 GO:0006206
GO:0006144 GO:0006165 GO:0004550
Cluster-22178.65423 GO:0006355 GO:0006098 GO:0019521 GO:0055114
GO:0050661 GO:0051287 GO:0004616 GO:0003676 GO:0005739
GO:0005622
Cluster-22178.65425 GO:0055085 GO:0019087 GO:0016021
Cluster-22178.65426 GO:0015684 GO:0007264 GO:0005525 GO:0003924
GO:0005524 GO:0015093 GO:0016021
Cluster-22178.65427 GO:0051603 GO:0006511 GO:0004175 GO:0004298
GO:0008658 GO:0005839 GO:0019773
Cluster-22178.65428 GO:0030168 GO:0007165 GO:0007155 GO:0006415
GO:0008608 GO:0006352 GO:0051258 GO:0006449 GO:0005102
GO:0003677 GO:0030674 GO:0016149 GO:0005577 GO:0018444
GO:0005840 GO:0005669 GO:0005737 GO:0042729 GO:0019028
GO:0072686
Cluster-22178.65429 GO:0005515
Cluster-22178.65431 GO:0043093 GO:0006171 GO:0006810 GO:0002949
GO:0000917 GO:0009405 GO:0048870 GO:0016787 GO:0008294
GO:0003677 GO:0005524 GO:0016887 GO:0046983 GO:0003678

GO:0005657 GO:0005576 GO:0031514 GO:0005737

Cluster-22178.65432 GO:0001558 GO:0003677 GO:0005515 GO:0016787

GO:0003676 GO:0005524 GO:0005520 GO:0005576 GO:0016942

Cluster-22178.65434 GO:0006228 GO:0007155 GO:0006165 GO:0006183

GO:0006206 GO:0006144 GO:0006241 GO:0003723 GO:0005515

GO:0004550 GO:0003676

Cluster-22178.65436 GO:0042176 GO:0030234 GO:0000502

Cluster-22178.65437 GO:0009058 GO:0009113 GO:0007156 GO:0006144

GO:0051539 GO:0004637 GO:0005509 GO:0016742 GO:0046872

GO:0005524 GO:0016020

Cluster-22178.65438 GO:0007264 GO:0015684 GO:0015093 GO:0003924

GO:0005525 GO:0016021 GO:0001527

Cluster-22178.65440 GO:0006810 GO:0009401 GO:0005886

Cluster-22178.65441 GO:0009401 GO:0006810 GO:0005886

Cluster-22178.65442 GO:0046872

Cluster-22178.65445 GO:0006265 GO:0003677 GO:0003918 GO:0005694

Cluster-22178.65446 GO:0007156 GO:0005509 GO:0016020

Cluster-22178.65448 GO:0006281 GO:0016192 GO:0006886 GO:0043161

GO:0006289 GO:0003684 GO:0003676 GO:0030117

Cluster-22178.65450 GO:0006448 GO:0001514 GO:0003746 GO:0005525

GO:0003723 GO:0005515 GO:0005737 GO:0005840

Cluster-22178.65453 GO:0015991 GO:0016820 GO:0005524 GO:0033178

Cluster-22178.65454 GO:0006270 GO:0006886 GO:0008536

Cluster-22178.65455 GO:0006355 GO:0005515 GO:0005525 GO:0003677

GO:0008270 GO:0046872

Cluster-22178.65456 GO:0007010 GO:0006281 GO:0006289 GO:0043161

GO:0003684 GO:0003779

Cluster-22178.65457 GO:0006355 GO:0043565 GO:0003682 GO:0008270

GO:0003700 GO:0005667 GO:0000785

Cluster-22178.65459 GO:0005887

Cluster-22178.65461 GO:0008152 GO:0016746 GO:0005509

Cluster-22178.65462 GO:0048280 GO:0016192 GO:0006886 GO:0005515

GO:0000139 GO:0030117 GO:0005737

Cluster-22178.65464 GO:0000746 GO:0019058 GO:0005975 GO:0006260
GO:0003677 GO:0003887 GO:0004553 GO:0042575 GO:0019867
Cluster-22178.65468 GO:0031124 GO:0005525
Cluster-22178.65470 GO:0006418 GO:0000166 GO:0004812 GO:0005524

Cluster-22178.65471 GO:0006508 GO:0006468 GO:0006144 GO:0004252
GO:0017111 GO:0004197 GO:0016773 GO:0004672 GO:0005524
GO:0003968 GO:0016020 GO:0031379
Cluster-22178.65472 GO:0005515
Cluster-22178.65473 GO:0005509 GO:0008168
Cluster-22178.65474 GO:0046872 GO:0003677
Cluster-22178.65476 GO:0006468 GO:0016773 GO:0004672 GO:0005524
GO:0016020
Cluster-22178.65477 GO:0001510 GO:0031167 GO:0009451 GO:0032775
GO:0006306 GO:0008033 GO:0006396 GO:0009452 GO:0003723
GO:0008173 GO:0035299 GO:0008168 GO:0009007 GO:0005524
GO:0004809
Cluster-22178.65479 GO:0003676 GO:0005886
Cluster-22178.65480 GO:0005515 GO:0005739 GO:0005576
Cluster-22178.65481 GO:0006364 GO:0032040
Cluster-22178.65486 GO:0005515
Cluster-22178.65493 GO:0006355 GO:0005515 GO:0005634
Cluster-22178.65495 GO:0006402 GO:0019722 GO:0051252 GO:0006817
GO:0044341 GO:0003676 GO:0015321 GO:0008270 GO:0046872
GO:0004535 GO:0003723 GO:0005737 GO:0016020 GO:0005634
Cluster-22178.65496 GO:0006511 GO:0031625
Cluster-22178.65497 GO:0046938 GO:0007049 GO:0010038 GO:0016756
GO:0005515 GO:0046872 GO:0017053
Cluster-22178.65498 GO:0000184
Cluster-22178.65499 GO:0015031 GO:0043248 GO:0006606 GO:0015991
GO:0016192 GO:0006886 GO:0008565 GO:0000166 GO:0016820
GO:0005515 GO:0005737 GO:0000221 GO:0030117 GO:0005634
Cluster-22178.65500 GO:0008283 GO:0040007 GO:0007165 GO:0008083

Cluster-22178.65501 GO:0006396 GO:0003723
Cluster-22178.65502 GO:0042254 GO:0006412 GO:0003735 GO:0005840
Cluster-22178.65504 GO:0007165 GO:0005509 GO:0005578
Cluster-22178.65506 GO:0015991 GO:0042742 GO:0015031 GO:0006606
GO:0016820 GO:0005515 GO:0008565 GO:0005634 GO:0005737
GO:0000221
Cluster-22178.65507 GO:0006886 GO:0006270 GO:0008536
Cluster-22178.65509 GO:0055114 GO:0045454 GO:0016972
Cluster-22178.65510 GO:0035556
Cluster-22178.65511 GO:0009298 GO:0006013 GO:0006000 GO:0004615
GO:0005737
Cluster-22178.65513 GO:0000045 GO:0005737
Cluster-22178.65514 GO:0006812 GO:0006820 GO:0006835 GO:0017153
GO:0016020
Cluster-22178.65515 GO:0016192 GO:0051090 GO:0006886 GO:0042742
GO:0009069 GO:0016310 GO:0005515 GO:0004674 GO:0005634
GO:0030117
Cluster-22178.65518 GO:0006810 GO:0005215 GO:0016020
Cluster-22178.65519 GO:0006355 GO:0003677
Cluster-22178.65520 GO:0006554 GO:0006633 GO:0016117 GO:0018874
GO:0006631 GO:0006574 GO:0055114 GO:0006090 GO:0008152
GO:0006813 GO:0006099 GO:0006568 GO:0006550 GO:0006552
GO:0016491 GO:0016620 GO:0016746 GO:0071949 GO:0008924
GO:0003857 GO:0016705 GO:0016021
Cluster-22178.65521 GO:0003723 GO:0008270 GO:0016021
Cluster-22178.65524 GO:0006810 GO:0016021
Cluster-22178.65525 GO:0055114 GO:0006915 GO:0006629 GO:0032465
GO:0009252 GO:0033014 GO:0006511 GO:0016567 GO:0003924
GO:0003676 GO:0003723 GO:0050661 GO:0008883 GO:0008963
GO:0004842 GO:0016021
Cluster-22178.65528 GO:0055114 GO:0016616
Cluster-22178.65530 GO:0006508 GO:0004185

Cluster-22178.65531 GO:0006351 GO:0006508 GO:0019083 GO:0006206
GO:0006144 GO:0003677 GO:0005515 GO:0003899 GO:0003723
GO:0008234 GO:0005730
Cluster-22178.65532 GO:0009058 GO:0006520 GO:0019752 GO:0016740
GO:0030170 GO:0016829 GO:0016831 GO:0019012
Cluster-22178.65533 GO:0006955 GO:0006457 GO:0007165 GO:0005164
GO:0016020
Cluster-22178.65535 GO:0003676
Cluster-22178.65537 GO:0015992 GO:0006123 GO:0004129 GO:0045277
Cluster-22178.65540 GO:0002949 GO:0007049 GO:0006310 GO:0019079
GO:0006281 GO:0003724 GO:0003678 GO:0016887 GO:0005524
GO:0003723 GO:0009378 GO:0005657 GO:0009379
Cluster-22178.65542 GO:0007017 GO:0007018 GO:0005524 GO:0008017
GO:0003777 GO:0005515 GO:0045298 GO:0005874
Cluster-22178.65543 GO:0015992 GO:0007275 GO:0006123 GO:0030154
GO:0004129 GO:0045277
Cluster-22178.65544 GO:0000226 GO:0007264 GO:0006576 GO:0005525
GO:0005524 GO:0015630 GO:0016020
Cluster-22178.65545 GO:0006520 GO:0055114 GO:0008152 GO:0016491

Cluster-22178.65549 GO:0055114 GO:0051920 GO:0016491 GO:0016209

Cluster-22178.65550 GO:0006570 GO:0006810 GO:0009405 GO:0006952
GO:0006470 GO:0008138 GO:0046983 GO:0019855 GO:0004725
GO:0005576
Cluster-22178.65551 GO:0016310 GO:0044237 GO:0006886 GO:0006397
GO:0003924 GO:0016772 GO:0005525 GO:0005515 GO:0005047
GO:0005786 GO:0005785 GO:0005634
Cluster-22178.65552 GO:0005886 GO:0016021
Cluster-22178.65553 GO:0006352 GO:0003677 GO:0005669
Cluster-22178.65555 GO:0006574 GO:0042967 GO:0006633 GO:0006084
GO:0006552 GO:0008299 GO:0046950 GO:0006550 GO:0004421
GO:0004315 GO:0005835

Cluster-22178.65558 GO:0032297 GO:0003677
Cluster-22178.65559 GO:0005975 GO:0042254 GO:0006412 GO:0004553
GO:0003735 GO:0042597 GO:0005840
Cluster-22178.65560 GO:0009451 GO:0001522 GO:0009982 GO:0003723
GO:0016021 GO:0016012
Cluster-22178.65565 GO:0008283 GO:0040007 GO:0007165 GO:0016567
GO:0006464 GO:0015031 GO:0055114 GO:0009245 GO:0016616
GO:0008083 GO:0008915 GO:0016491 GO:0004842 GO:0005576
GO:0019028
Cluster-22178.65566 GO:0016567 GO:0007165 GO:0040007 GO:0008283
GO:0009245 GO:0055114 GO:0006464 GO:0015031 GO:0008083
GO:0016616 GO:0004842 GO:0016491 GO:0008915 GO:0019028
GO:0005576
Cluster-22178.65568 GO:0006694 GO:0008168
Cluster-22178.65571 GO:0007349 GO:0016020 GO:0005737
Cluster-22178.65572 GO:0006771 GO:0051301 GO:0019497 GO:0007049
GO:0005515 GO:0043565 GO:0003993 GO:0005634 GO:0016021
Cluster-22178.65573 GO:0007165 GO:0005158 GO:0004252 GO:0005886
GO:0005899
Cluster-22178.65575 GO:0006449 GO:0006415 GO:0003747 GO:0005840
GO:0018444
Cluster-22178.65577 GO:0019902 GO:0005515
Cluster-22178.65578 GO:0030154 GO:0006418 GO:0000166 GO:0004812
GO:0005524 GO:0016021
Cluster-22178.65580 GO:0006811 GO:0005515 GO:0016020
Cluster-22178.65581 GO:0045454 GO:0055114 GO:0009306 GO:0005509
GO:0008270 GO:0016209 GO:0016491 GO:0005634 GO:0005788
Cluster-22178.65582 GO:0005515 GO:0043565
Cluster-22178.65583 GO:0006508 GO:0004177 GO:0005622
Cluster-22178.65584 GO:0016021
Cluster-22178.65585 GO:0008152 GO:0016758
Cluster-22178.65586 GO:0031072 GO:0051082
Cluster-22178.65588 GO:0006144 GO:0006206 GO:0006351 GO:0006508

GO:0003899 GO:0008236 GO:0003677 GO:0016787 GO:0005730
Cluster-22178.65589 GO:0006396 GO:0005515 GO:0005622
Cluster-22178.65593 GO:0006811 GO:0006351 GO:0006260 GO:0006367
GO:0032968 GO:0006816 GO:0003676 GO:0005262 GO:0003887
GO:0008270 GO:0003677 GO:0005244 GO:0032040 GO:0016021
GO:0042575 GO:0005634
Cluster-22178.65596 GO:0005525 GO:0046914 GO:0046983 GO:0003924

Cluster-22178.65597 GO:0034061 GO:0003677 GO:0016779 GO:0005634
Cluster-22178.65598 GO:0055085 GO:0006260 GO:0006914 GO:0070206
GO:0015992 GO:0007165 GO:0007049 GO:0006308 GO:0051382
GO:0015991 GO:0030001 GO:0008855 GO:0046873 GO:0004871
GO:0015078 GO:0005198 GO:0016020 GO:0033179 GO:0009318
GO:0000776
Cluster-22178.65599 GO:0009165 GO:0006144 GO:0006098 GO:0009116
GO:0000287 GO:0004749
Cluster-22178.65600 GO:0015991 GO:0006886 GO:0016192 GO:0005515
GO:0016820 GO:0030117 GO:0000221
Cluster-22178.65603 GO:0007275 GO:0007165 GO:0008152 GO:0005179
GO:0016624 GO:0047355 GO:0008270 GO:0005576 GO:0016020
Cluster-22178.65604 GO:0006886 GO:0016192 GO:0008152 GO:0003824
GO:0005515 GO:0030117
Cluster-22178.65605 GO:0006629 GO:0008081
Cluster-22178.65606 GO:0004518
Cluster-22178.65608 GO:0000398 GO:0030623 GO:0005515 GO:0008270
GO:0017070 GO:0003723
Cluster-22178.65609 GO:0006471 GO:0008270 GO:0003950 GO:0003677
GO:0005634
Cluster-22178.65610 GO:0009405 GO:0006355 GO:0003723 GO:0003700
GO:0031423 GO:0008270 GO:0043565 GO:0019012 GO:0005667
GO:0005634
Cluster-22178.65611 GO:0005515 GO:0003924
Cluster-22178.65612 GO:0006468 GO:0005524 GO:0004672

Cluster-22178.65613 GO:0006357 GO:0006446 GO:0003743 GO:0005515 GO:0001104 GO:0016021 GO:0005840 GO:0005737 GO:0005852 GO:0016592

Cluster-22178.65616 GO:0006094 GO:0005525 GO:0004611

Cluster-22178.65622 GO:0003779 GO:0005622

Cluster-22178.65623 GO:0008270 GO:0046872 GO:0003676

Cluster-22178.65624 GO:0006351 GO:0006270 GO:0009236 GO:0006260 GO:0005515 GO:0003887 GO:0003677 GO:0008270 GO:0005634 GO:0042575 GO:0005789 GO:0032040

Cluster-22178.65626 GO:0006468 GO:0016773 GO:0004672 GO:0008270 GO:0005524 GO:0003676 GO:0005515 GO:0016020

Cluster-22178.65627 GO:0005515

Cluster-22178.65628 GO:0005515

Cluster-22178.65629 GO:0003723

Cluster-22178.65631 GO:0006355 GO:0051087

Cluster-22178.65633 GO:0007165 GO:0019058 GO:0042254 GO:0006412 GO:0008152 GO:0046789 GO:0003735 GO:0016758 GO:0005840 GO:0019031 GO:0009986

Cluster-22178.65635 GO:0007165 GO:0051258 GO:0030168 GO:0005975 GO:0006914 GO:0030674 GO:0005102 GO:0004553 GO:0030248 GO:0005576 GO:0005577

Cluster-22178.65637 GO:0006811 GO:0005216 GO:0046872 GO:0016020

Cluster-22178.65640 GO:0005515 GO:0003677 GO:0046872 GO:0008270

Cluster-22178.65642 GO:0040007 GO:0008283 GO:0007165 GO:0008083

Cluster-22178.65644 GO:0006012 GO:0005975 GO:0018149 GO:0046486 GO:0006687 GO:0006122 GO:0006027 GO:0004565 GO:0008270 GO:0046872 GO:0005743 GO:0005750 GO:0009341

Cluster-22178.65648 GO:0003824

Cluster-22178.65649 GO:0016773

Cluster-22178.65651 GO:0010508 GO:0015858 GO:0006810 GO:0019076 GO:0032801 GO:0006812 GO:0007049 GO:0016787 GO:0005261

GO:0005337 GO:0016020 GO:0033644 GO:0016021
Cluster-22178.65656 GO:0006810 GO:0006465 GO:0008233 GO:0005622
GO:0005787 GO:0016021
Cluster-22178.65657 GO:0042254 GO:0006412 GO:0003735 GO:0005840
GO:0005622
Cluster-22178.65658 GO:0055114 GO:0006508 GO:0015947 GO:0016192
GO:0006886 GO:0030058 GO:0070008 GO:0005515 GO:0004252
GO:0005198 GO:0042597 GO:0030117
Cluster-22178.65659 GO:0006352 GO:0003677 GO:0005669
Cluster-22178.65660 GO:0006106 GO:0016020
Cluster-22178.65661 GO:0006468 GO:0016773 GO:0005524 GO:0004672
GO:0016020
Cluster-22178.65663 GO:0006355 GO:0005982 GO:0005985 GO:0005978
GO:0004373 GO:0005634
Cluster-22178.65664 GO:0009058 GO:0006260 GO:0006269 GO:0018142
GO:0003723 GO:0004672
Cluster-22178.65665 GO:0006355 GO:0006139 GO:0006260 GO:0006914
GO:0005515 GO:0003700 GO:0003676 GO:0043565 GO:0008026
GO:0016818 GO:0003677 GO:0004003 GO:0016787 GO:0005524
GO:0005634 GO:0005667 GO:0005657
Cluster-22178.65666 GO:0008152 GO:0003824 GO:0005524
Cluster-22178.65668 GO:0006334 GO:0006810 GO:0003676 GO:0005634
GO:0005643
Cluster-22178.65669 GO:0006468 GO:0005524 GO:0004672
Cluster-22178.65673 GO:0042262 GO:0030001 GO:0008053 GO:0031412
GO:0055085 GO:0006073 GO:0003924 GO:0008289 GO:0005198
GO:0016762 GO:0003690 GO:0046873 GO:0016021 GO:0019031
GO:0019028 GO:0005618 GO:0048046 GO:0016020 GO:0005741
Cluster-22178.65674 GO:0009405 GO:0007187 GO:0007631 GO:0007186
GO:0022410 GO:0016499 GO:0005887 GO:0016021 GO:0016020
Cluster-22178.65675 GO:0042967 GO:0008270 GO:0047961 GO:0003677
GO:0005739
Cluster-22178.65679 GO:0005515

Cluster-22178.65680 GO:0016485 GO:0016021
Cluster-22178.65681 GO:0016020
Cluster-22178.65684 GO:0003723 GO:0003824
Cluster-22178.65685 GO:0046677 GO:0006811 GO:0016020
Cluster-22178.65688 GO:0006120 GO:0006744 GO:0055085 GO:0015992 GO:0055114 GO:0006814 GO:0016192 GO:0007186 GO:0004930 GO:0008137 GO:0016021
Cluster-22178.65692 GO:0008152 GO:0016791
Cluster-22178.65693 GO:0005515 GO:0016021
Cluster-22178.65696 GO:0006613 GO:0007165 GO:0006465 GO:0004872 GO:0008233 GO:0005787 GO:0016021 GO:0030176 GO:0005784
Cluster-22178.65697 GO:0003824
Cluster-22178.65698 GO:0030154 GO:0007219 GO:0006351 GO:0007275 GO:0005515 GO:0016788 GO:0003677 GO:0016021 GO:0005634
Cluster-22178.65699 GO:0007283 GO:0003677 GO:0000786 GO:0005634
Cluster-22178.65700 GO:0019521 GO:0055114 GO:0006098 GO:0006355 GO:0003676 GO:0050661 GO:0051287 GO:0004616 GO:0005622 GO:0005739
Cluster-22178.65702 GO:0000910 GO:0008033 GO:0000226 GO:0005524 GO:0008017 GO:0016879 GO:0000166 GO:0005737 GO:0045298
Cluster-22178.65705 GO:0006364 GO:0006206 GO:0006144 GO:0006351 GO:0006355 GO:0003677 GO:0005524 GO:0016773 GO:0003899 GO:0003700 GO:0046872 GO:0016020 GO:0005732 GO:0005730 GO:0005667 GO:0034457 GO:0005634 GO:0019028
Cluster-22178.65706 GO:0006355 GO:0003677 GO:0003682 GO:0000775 GO:0000785 GO:0005634
Cluster-22178.65707 GO:0007264 GO:0007165 GO:0005515 GO:0005525

Cluster-22178.65708 GO:0000917 GO:0043093 GO:0006355 GO:0003700 GO:0043565 GO:0003677 GO:0005737 GO:0005634 GO:0005667
Cluster-22178.65709 GO:0045454
Cluster-22178.65710 GO:0006418 GO:0006886 GO:0004812 GO:0005524 GO:0002161 GO:0000166 GO:0005741

Cluster-22178.65711 GO:0006810 GO:0009405 GO:0006418 GO:0019871 GO:0000166 GO:0004812 GO:0005524 GO:0005576
Cluster-22178.65712 GO:0005509 GO:0005515
Cluster-22178.65713 GO:0007165 GO:0006468 GO:0009069 GO:0035556 GO:0016310 GO:0007178 GO:0004675 GO:0005515 GO:0005524 GO:0016020 GO:0016012
Cluster-22178.65714 GO:0006464 GO:0015031 GO:0005515
Cluster-22178.65716 GO:0051258 GO:0030168 GO:0007165 GO:0030674 GO:0005102 GO:0005577
Cluster-22178.65717 GO:0007017 GO:0007018 GO:0006357 GO:0001104 GO:0005198 GO:0005524 GO:0008017 GO:0003777 GO:0019028 GO:0016592 GO:0045298 GO:0005874 GO:0019031
Cluster-22178.65720 GO:0007165 GO:0035556 GO:0003774 GO:0005524 GO:0005515 GO:0016459
Cluster-22178.65722 GO:0050790 GO:0030234
Cluster-22178.65723 GO:0005515 GO:0005509
Cluster-22178.65725 GO:0006446 GO:0045892 GO:0006914 GO:0006413 GO:0003743 GO:0005840 GO:0005634
Cluster-22178.65727 GO:0007165 GO:0006479 GO:0006481 GO:0042967 GO:0004671 GO:0008080 GO:0016747 GO:0016021
Cluster-22178.65728 GO:0006413 GO:0006446 GO:0019079 GO:0016787 GO:0003743 GO:0003677 GO:0008026 GO:0005524 GO:0003676 GO:0005840
Cluster-22178.65731 GO:0006281 GO:0051382
Cluster-22178.65732 GO:0016567 GO:0030071 GO:0055114 GO:0031145 GO:0061630 GO:0005515 GO:0008270 GO:0046872 GO:0004842 GO:0047134 GO:0005680
Cluster-22178.65736 GO:0055114 GO:0045454 GO:0016209 GO:0016491

Cluster-22178.65737 GO:0035556 GO:0009405 GO:0019835 GO:0055114 GO:0005515 GO:0016491 GO:0019867
Cluster-22178.65740 GO:0003723 GO:0005515 GO:0003676
Cluster-22178.65742 GO:0007015 GO:0003785

Cluster-22178.65744 GO:0005634
Cluster-22178.65748 GO:0004866 GO:0003824 GO:0031224 GO:0005615
GO:0005576
Cluster-22178.65749 GO:0006629 GO:0006508 GO:0016787 GO:0004252

Cluster-22178.65751 GO:0015031 GO:0008375 GO:0016021 GO:0016020
Cluster-22178.65754 GO:0006576 GO:0007264 GO:0008201 GO:0005525
GO:0003924 GO:0005524 GO:0016021
Cluster-22178.65756 GO:0006355 GO:0008270 GO:0003676 GO:0003700
GO:0005667 GO:0005634
Cluster-22178.65762 GO:0006355 GO:0006468 GO:0019497 GO:0006771
GO:0048013 GO:0005515 GO:0003700 GO:0046872 GO:0004672
GO:0043565 GO:0003993 GO:0005524 GO:0008270 GO:0016773
GO:0005634 GO:0016020 GO:0005667
Cluster-22178.65768 GO:0006955 GO:0007165 GO:0006606 GO:0030247
GO:0005044
Cluster-22178.65773 GO:0009405 GO:0006355 GO:0003700 GO:0043565
GO:0005515 GO:0051539 GO:0004867 GO:0005667
Cluster-22178.65775 GO:0008152 GO:0009190 GO:0046039 GO:0006468
GO:0006144 GO:0006357 GO:0035556 GO:0006182 GO:0004672
GO:0003824 GO:0003723 GO:0016849 GO:0004383 GO:0001104
GO:0008270 GO:0005524 GO:0016592
Cluster-22178.65776 GO:0006412 GO:0042254 GO:0003735 GO:0005840
Cluster-22178.65777 GO:0009252 GO:0055114 GO:0046436 GO:0006814
GO:0006118 GO:0003824 GO:0016655 GO:0046872 GO:0005524
GO:0008716
Cluster-22178.65778 GO:0006367 GO:0005524 GO:0003682 GO:0000785
GO:0005672
Cluster-22178.65779 GO:0007165 GO:0016567 GO:0006260 GO:0016818
GO:0003677 GO:0005515 GO:0005179 GO:0004842 GO:0008270
GO:0005524 GO:0046872 GO:0005576 GO:0005634 GO:0005680
Cluster-22178.65781 GO:0005515
Cluster-22178.65785 GO:0007154 GO:0007607 GO:0007187 GO:0050912

GO:0005515 GO:0005509 GO:0008527 GO:0016021 GO:0016020
Cluster-22178.65786 GO:0006508 GO:0006486 GO:0004576 GO:0003676
GO:0004222 GO:0003677 GO:0008250 GO:0016020
Cluster-22178.65789 GO:0006886 GO:0006888 GO:0008270 GO:0030127
Cluster-22178.65790 GO:0006270 GO:0033014 GO:0006629 GO:0055114
GO:0050661 GO:0003723 GO:0003676 GO:0008883
Cluster-22178.65791 GO:0048870 GO:0015031 GO:0010508 GO:0006904
GO:0005515 GO:0005509 GO:0000145 GO:0031514
Cluster-22178.65793 GO:0006367 GO:0005524 GO:0003682 GO:0005672
GO:0000785
Cluster-22178.65794 GO:0006555 GO:0006556 GO:0006508 GO:0008234
GO:0004478
Cluster-22178.65796 GO:0006810 GO:0016787 GO:0005524 GO:0042626
GO:0016021
Cluster-22178.65797 GO:0006813 GO:0008152 GO:0009306 GO:0055114
GO:0009089 GO:0009085 GO:0015031 GO:0003824 GO:0015450
GO:0008839 GO:0051287 GO:0050662 GO:0016620 GO:0016491
GO:0016021 GO:0009941
Cluster-22178.65798 GO:0007165 GO:0016567 GO:0008283 GO:0040007
GO:0004842 GO:0008083 GO:0005576
Cluster-22178.65799 GO:0008152 GO:0016491
Cluster-22178.65800 GO:0004866 GO:0005615 GO:0005576
Cluster-22178.65801 GO:0006554 GO:0006479 GO:0018024 GO:0046872

Cluster-22178.65802 GO:0000245 GO:0005515 GO:0005634
Cluster-22178.65803 GO:0006118 GO:0015992 GO:0055114 GO:0009060
GO:0006123 GO:0005506 GO:0020037 GO:0009055 GO:0004129
GO:0045277 GO:0016021
Cluster-22178.65804 GO:0006359
Cluster-22178.65805 GO:0006355 GO:0042254 GO:0006412 GO:0043565
GO:0008270 GO:0003700 GO:0003735 GO:0005840 GO:0005667
Cluster-22178.65806 GO:0005515
Cluster-22178.65807 GO:0055114

Cluster-22178.65810 GO:0007165 GO:0015991 GO:0007155 GO:0051607
GO:0015992 GO:0006914 GO:0015078 GO:0008092 GO:0033179
GO:0019898 GO:0005737
Cluster-22178.65811 GO:0035556 GO:0016310 GO:0007178 GO:0007165
GO:0009069 GO:0006468 GO:0005524 GO:0004675 GO:0005515
GO:0016020
Cluster-22178.65813 GO:0008270 GO:0046872 GO:0005515
Cluster-22178.65815 GO:0030001 GO:0055085 GO:0046873 GO:0016020
Cluster-22178.65817 GO:0006355 GO:0055085 GO:0003700 GO:0022857
GO:0005667 GO:0016021
Cluster-22178.65819 GO:0016567 GO:0003824
Cluster-22178.65820 GO:0006810 GO:0003676 GO:0005622
Cluster-22178.65829 GO:0016817 GO:0005789 GO:0016021
Cluster-22178.65830 GO:0000398
Cluster-22178.65832 GO:0003824 GO:0004866 GO:0005576 GO:0005615
Cluster-22178.65834 GO:0006355 GO:0042710 GO:0017148 GO:0009372
GO:0005975 GO:0042967 GO:0004402 GO:0016787 GO:0008270
GO:0003824 GO:0003712 GO:0005667 GO:0005634 GO:0000123
Cluster-22178.65835 GO:0016567 GO:0004842 GO:0046872 GO:0008270
GO:0005515 GO:0061630 GO:0005680 GO:0005622
Cluster-22178.65836 GO:0055114 GO:0016491
Cluster-22178.65837 GO:0016567 GO:0004842
Cluster-22178.65838 GO:0006206 GO:0006308 GO:0006144 GO:0009307
GO:0006351 GO:0009036 GO:0003899 GO:0005730 GO:0009359
Cluster-22178.65839 GO:0006412 GO:0042254 GO:0003735 GO:0005622
GO:0005840
Cluster-22178.65840 GO:0043066 GO:0008207 GO:0008209 GO:0019079
GO:0006694 GO:0055114 GO:0043154 GO:0008210 GO:0016620
GO:0080019 GO:0043130 GO:0016616 GO:0032266 GO:0050662
GO:0051287 GO:0003854 GO:0003824 GO:0071986 GO:0005737
Cluster-22178.65842 GO:0006308 GO:0005515 GO:0046872 GO:0008270
GO:0008855 GO:0009318 GO:0005622
Cluster-22178.65846 GO:0006281 GO:0006260 GO:0016787 GO:0003677

GO:0005524 GO:0043140 GO:0003676 GO:0005657
Cluster-22178.65848 GO:0070072 GO:0043087 GO:0032012 GO:0005086

Cluster-22178.65851 GO:0006850 GO:0005743
Cluster-22178.65852 GO:0006725 GO:0016853
Cluster-22178.65853 GO:0006725 GO:0016853
Cluster-22178.65854 GO:0006694 GO:0008152 GO:0055114 GO:0008207
GO:0008209 GO:0008210 GO:0003824 GO:0016491 GO:0016616
GO:0003854 GO:0050662
Cluster-22178.65855 GO:0001514 GO:0006355 GO:0006351 GO:0006464
GO:0006448 GO:0006260 GO:0006269 GO:0005525 GO:0003896
GO:0016151 GO:0003723 GO:0008134 GO:0016887 GO:0003746
GO:0005524 GO:0003677 GO:0005737 GO:0005840 GO:0005657
GO:0005667 GO:0005730
Cluster-22178.65856 GO:0035988 GO:0006310 GO:0006260 GO:0002949
GO:0006281 GO:0006629 GO:0006355 GO:0006576 GO:0042246
GO:0002062 GO:0009378 GO:0016787 GO:0003677 GO:0005524
GO:0016887 GO:0016301 GO:0008134 GO:0003924 GO:0005525
GO:0003678 GO:0009379 GO:0005667 GO:0005657 GO:0005634
Cluster-22178.65857 GO:0009069 GO:0006468 GO:0006413 GO:0006446
GO:0007178 GO:0016310 GO:0004675 GO:0003743 GO:0005524
GO:0005576 GO:0016020 GO:0005840 GO:0016021
Cluster-22178.65858 GO:0016567 GO:0006915 GO:0032465 GO:0004842
GO:0003676
Cluster-22178.65859 GO:0051258 GO:0006308 GO:0015991 GO:0009405
GO:0010508 GO:0015992 GO:0030168 GO:0006914 GO:0006357
GO:0006606 GO:0008053 GO:0007165 GO:0003924 GO:0015078
GO:0051082 GO:0001104 GO:0008855 GO:0005102 GO:0030674
GO:0016021 GO:0009318 GO:0033179 GO:0005577 GO:0005923
GO:0005741 GO:0016592 GO:0016020
Cluster-22178.65860 GO:0008643 GO:0055085 GO:0005351 GO:0000139
GO:0016021 GO:0016020
Cluster-22178.65861 GO:0055114 GO:0003676 GO:0005515 GO:0005634

Cluster-22178.65862 GO:0006914 GO:0019497 GO:0006810 GO:0006771 GO:0003993 GO:0046872 GO:0005515 GO:0005794

Cluster-22178.65864 GO:0006629

Cluster-22178.65866 GO:0006508 GO:0008152 GO:0016836 GO:0003824 GO:0008233

Cluster-22178.65869 GO:0051539

Cluster-22178.65870 GO:0005739

Cluster-22178.65871 GO:0015992 GO:0006811 GO:0006355 GO:0045454 GO:0015991 GO:0006118 GO:0046872 GO:0009055 GO:0015078 GO:0015035 GO:0003677 GO:0005886 GO:0019031 GO:0016020 GO:0033179

Cluster-22178.65872 GO:0005515

Cluster-22178.65873 GO:0030001 GO:0006355 GO:0009451 GO:0055114 GO:0006352 GO:0006270 GO:0006396 GO:0055085 GO:0003723 GO:0008173 GO:0046873 GO:0003677 GO:0016491 GO:0008270 GO:0005634 GO:0016020

Cluster-22178.65875 GO:0007165 GO:0000772 GO:0046872 GO:0016020

Cluster-22178.65876 GO:0005525 GO:0031012

Cluster-22178.65878 GO:0005525 GO:0031012

Cluster-22178.65879 GO:0005975 GO:0006030 GO:0008061 GO:0004553 GO:0005576

Cluster-22178.65880 GO:0046872 GO:0003950 GO:0005634

Cluster-22178.65881 GO:0007186 GO:0004930

Cluster-22178.65882 GO:0007131 GO:0051598 GO:0005198 GO:0005634

Cluster-22178.65884 GO:0003677 GO:0004386 GO:0005524

Cluster-22178.65885 GO:0006811 GO:0016020

Cluster-22178.65888 GO:0016032 GO:0006508 GO:0004197

Cluster-22178.65890 GO:0016032 GO:0006508 GO:0004197

Cluster-22178.65893 GO:0005975 GO:0003723 GO:0004553

Cluster-22178.65896 GO:1901031 GO:0005634

Cluster-22178.65897 GO:0006508 GO:0044419 GO:0006144 GO:0017111 GO:0004197 GO:0003968 GO:0019028 GO:0031379

Cluster-22178.65898 GO:0016491

Cluster-22178.65899 GO:0007186 GO:0007165 GO:0007264 GO:0004871
GO:0005525 GO:0003924 GO:0031683 GO:0019001
Cluster-22178.65900 GO:0006606 GO:0010508 GO:0051607 GO:0006308
GO:0008855 GO:0009318
Cluster-22178.65901 GO:0005515
Cluster-22178.65902 GO:0005201 GO:0005578 GO:0005581
Cluster-22178.65904 GO:0030153 GO:0006355 GO:0006955 GO:0015643
GO:0003677 GO:0016021 GO:0019814
Cluster-22178.65906 GO:0006810 GO:0034227 GO:0002098 GO:0042626
GO:0000049 GO:0005524 GO:0016021
Cluster-22178.65908 GO:0006886 GO:0030001 GO:0051258 GO:0007165
GO:0055085 GO:0006891 GO:0000921 GO:0030168 GO:0008375
GO:0005198 GO:0005515 GO:0005507 GO:0004871 GO:0030674
GO:0046873 GO:0005102 GO:0019028 GO:0005801 GO:0016021
GO:0019031 GO:0016020 GO:0017119 GO:0005577 GO:0005940
Cluster-22178.65909 GO:0006468 GO:0004672 GO:0005524
Cluster-22178.65911 GO:0016829 GO:0016020
Cluster-22178.65919 GO:0051252 GO:0016891
Cluster-22178.65920 GO:0006952 GO:0007165 GO:0005515
Cluster-22178.65921 GO:0016021 GO:0016020
Cluster-22178.65923 GO:0006974 GO:0051726 GO:0003677
Cluster-22178.65925 GO:0018342 GO:0006270 GO:0006397 GO:0006886
GO:0006811 GO:0006396 GO:0008318 GO:0005515 GO:0005634
GO:0016020 GO:0005622 GO:0005789
Cluster-22178.65928 GO:0006952 GO:0006810 GO:0009405 GO:0019855
GO:0005576 GO:0005634
Cluster-22178.65929 GO:0016757
Cluster-22178.65930 GO:0007010 GO:0005515 GO:0016817 GO:0003779

Cluster-22178.65931 GO:0019048 GO:0006355
Cluster-22178.65934 GO:0016817
Cluster-22178.65936 GO:0003677
Cluster-22178.65938 GO:0006355 GO:0005524 GO:0008134 GO:0005634

GO:0005667
Cluster-22178.65939 GO:0005515
Cluster-22178.65940 GO:0015991 GO:0015992 GO:0046034 GO:0016820
GO:0005524 GO:0003924 GO:0043531 GO:0016887 GO:0005525
GO:0033178
Cluster-22178.65944 GO:0009085 GO:0009089 GO:0015031 GO:0006813
GO:0055114 GO:0009306 GO:0008152 GO:0050662 GO:0051287
GO:0016491 GO:0016620 GO:0015450 GO:0008839 GO:0003824
GO:0009941 GO:0016021
Cluster-22178.65945 GO:0007264 GO:0035556 GO:0005525
Cluster-22178.65946 GO:0009405 GO:0006810 GO:0006952 GO:0006334
GO:0019855 GO:0005576 GO:0005634
Cluster-22178.65947 GO:0006118 GO:0017004 GO:0015684 GO:0016818
GO:0003924 GO:0015093 GO:0003724 GO:0045158 GO:0003712
GO:0005525 GO:0005634 GO:0005667 GO:0009512 GO:0016021
Cluster-22178.65950 GO:0009405 GO:0006810 GO:0006952 GO:0006334
GO:0019855 GO:0005634 GO:0005576
Cluster-22178.65951 GO:0005515 GO:0005509
Cluster-22178.65952 GO:0005515
Cluster-22178.65954 GO:0019645 GO:0016560 GO:0009405 GO:0006810
GO:0008200 GO:0005515 GO:0005777 GO:0016021 GO:0005576
Cluster-22178.65955 GO:0046008 GO:0007165 GO:0005179 GO:0005576
Cluster-22178.65956 GO:0015031 GO:0016020
Cluster-22178.65957 GO:0003824
Cluster-22178.65959 GO:0055114 GO:0009308 GO:0006886 GO:0006888
GO:0007186 GO:0007165 GO:0007264 GO:0004871 GO:0005507
GO:0005525 GO:0003924 GO:0019001 GO:0031683 GO:0048038
GO:0008131 GO:0008270 GO:0030127
Cluster-22178.65960 GO:0006446 GO:0003743 GO:0005840 GO:0005852
GO:0016020 GO:0005737
Cluster-22178.65961 GO:0006869 GO:0016192 GO:0042157 GO:0008053
GO:0009405 GO:0006886 GO:0005524 GO:0016887 GO:0005198
GO:0008289 GO:0005525 GO:0003924 GO:0005741 GO:0016020

GO:0005576 GO:0019028 GO:0019031 GO:0016021
Cluster-22178.65965 GO:0006486 GO:0004576 GO:0046983 GO:0016020
GO:0008250
Cluster-22178.65968 GO:0006886 GO:0008536
Cluster-22178.65969 GO:0000087 GO:0006470 GO:0006570 GO:0004725
GO:0005622
Cluster-22178.65972 GO:0055114 GO:0006574 GO:0008152 GO:0006568
GO:0006552 GO:0006550 GO:0046487 GO:0009396 GO:0006554
GO:0006633 GO:0006631 GO:0006298 GO:0018874 GO:0003824
GO:0003857 GO:0016491 GO:0005524 GO:0050662 GO:0004488
Cluster-22178.65974 GO:0045859 GO:0004860 GO:0005576
Cluster-22178.65975 GO:0007016 GO:0005200 GO:0003779 GO:0001726
GO:0005925 GO:0005856
Cluster-22178.65976 GO:0006486 GO:0004576 GO:0008250 GO:0016020
GO:0036128
Cluster-22178.65977 GO:0006355 GO:0006260 GO:0016887 GO:0005524
GO:0008134 GO:0003677 GO:0005667
Cluster-22178.65979 GO:0005515
Cluster-22178.65983 GO:0055114 GO:0035556 GO:0006122 GO:0009405
GO:0019835 GO:0016491 GO:0005515 GO:0005750 GO:0019867
Cluster-22178.65984 GO:0006260 GO:0006355 GO:0008270 GO:0003676
GO:0003682 GO:0008408 GO:0003887 GO:0003677 GO:0000166
GO:0008201 GO:0000785 GO:0000775 GO:0005634 GO:0042575
Cluster-22178.65986 GO:0006013 GO:0006000 GO:0015976 GO:0006020
GO:0006094 GO:0006098 GO:0006096 GO:0009405 GO:0004332
GO:0016020
Cluster-22178.65987 GO:0055114 GO:0006508 GO:0015631 GO:0030699
GO:0050485 GO:0016779 GO:0004197 GO:0030700 GO:0045298
Cluster-22178.65988 GO:0006260 GO:0006355 GO:0008134 GO:0003677
GO:0016887 GO:0005524 GO:0005667
Cluster-22178.65989 GO:0055085 GO:0006865 GO:0006810 GO:0003333
GO:0015171 GO:0016021 GO:0016020
Cluster-22178.65990 GO:0016567 GO:0008270 GO:0046872 GO:0003723

GO:0004842 GO:0005515 GO:0005680
Cluster-22178.65991 GO:0006457 GO:0006955 GO:0007094 GO:0007155
GO:0019882 GO:0005198 GO:0051082 GO:0019028 GO:0019031
GO:0016272 GO:0009288
Cluster-22178.65992 GO:0006308 GO:0008239 GO:0008855 GO:0004252
GO:0051082 GO:0070009 GO:0005886 GO:0016592 GO:0009318
Cluster-22178.65994 GO:0006281 GO:0003677 GO:0003676 GO:0008270
GO:0005524 GO:0016787 GO:0016817
Cluster-22178.65995 GO:0006886 GO:0016192 GO:0005198 GO:0005515
GO:0030117
Cluster-22178.65996 GO:0006355 GO:0043565 GO:0003700 GO:0005667
Cluster-22178.65999 GO:0006413 GO:0006355 GO:0005975 GO:0006808
GO:0046034 GO:0006446 GO:0006464 GO:0006144 GO:0007218
GO:0003723 GO:0030234 GO:0004519 GO:0005515 GO:0004672
GO:0046872 GO:0043565 GO:0016151 GO:0003700 GO:0003676
GO:0003677 GO:0003743 GO:0004017 GO:0008270 GO:0005524
GO:0004553 GO:0005840 GO:0005667
Cluster-22178.66001 GO:0009058 GO:0042967 GO:0015696 GO:0008152
GO:0006633 GO:0055114 GO:0017111 GO:0004315 GO:0016788
GO:0008519 GO:0016491 GO:0016747 GO:0016020 GO:0005835
Cluster-22178.66002 GO:0042254 GO:0006412 GO:0003735 GO:0005840
Cluster-22178.66004 GO:0003924
Cluster-22178.66006 GO:0007267 GO:0006508 GO:0006355 GO:0008270
GO:0008233 GO:0003700 GO:0005667 GO:0005634
Cluster-22178.66007 GO:0003676 GO:0003950
Cluster-22178.66008 GO:0003676 GO:0003950
Cluster-22178.66009 GO:0005515
Cluster-22178.66010 GO:0007049 GO:0005515
Cluster-22178.66011 GO:0030150 GO:0005515 GO:0005744
Cluster-22178.66013 GO:0006396 GO:0005515 GO:0003677 GO:0003723
GO:0019013
Cluster-22178.66014 GO:0006629
Cluster-22178.66015 GO:0043066 GO:0043027

Cluster-22178.66016 GO:0046034 GO:0006144 GO:0000042 GO:0006281
GO:0008270 GO:0046872 GO:0004017 GO:0005515 GO:0003677
Cluster-22178.66020 GO:0003924
Cluster-22178.66021 GO:0016192 GO:0006886 GO:0015986 GO:0015992
GO:0015078 GO:0005515 GO:0000276
Cluster-22178.66023 GO:0009058 GO:0030170
Cluster-22178.66024 GO:0006355 GO:0003700 GO:0003677 GO:0005667
Cluster-22178.66025 GO:0030182 GO:0021549 GO:0008270
Cluster-22178.66026 GO:0006744 GO:0015992 GO:0042773 GO:0000290
GO:0006120 GO:0043085 GO:0005975 GO:0055114 GO:0000902
GO:0006814 GO:0016773 GO:0016787 GO:0005525 GO:0008047
GO:0008137 GO:0005515 GO:0016020
Cluster-22178.66029 GO:0006367 GO:0006506 GO:0006355 GO:0016746
GO:0016021 GO:0005789 GO:0005739
Cluster-22178.66030 GO:0051205 GO:0016021
Cluster-22178.66031 GO:0005515
Cluster-22178.66032 GO:0005515 GO:0016020
Cluster-22178.66033 GO:0009245 GO:0008915 GO:0003824
Cluster-22178.66035 GO:0005515
Cluster-22178.66036 GO:0006508 GO:0008270 GO:0004197 GO:0030695

Cluster-22178.66037 GO:0016579 GO:0042254 GO:0006811 GO:0006352
GO:0009405 GO:0006412 GO:0006355 GO:0007017 GO:0016987
GO:0003677 GO:0003735 GO:0005216 GO:0003700 GO:0036459
GO:0005622 GO:0019013 GO:0005667 GO:0005840 GO:0005875
GO:0005576
Cluster-22178.66038 GO:0007165 GO:0050660 GO:0005515 GO:0003824

Cluster-22178.66039 GO:0006281
Cluster-22178.66040 GO:0015693 GO:0015979 GO:0015095 GO:0016020
GO:0016021 GO:0009523 GO:0009539
Cluster-22178.66041 GO:0015992 GO:0015991 GO:0046034 GO:0005524
GO:0016820 GO:0033178

Cluster-22178.66042 GO:0007155 GO:0006508 GO:0003677 GO:0004197
GO:0051015
Cluster-22178.66043 GO:0006357 GO:0001104 GO:0016592
Cluster-22178.66045 GO:0008270 GO:0016021
Cluster-22178.66048 GO:0055114 GO:0035556 GO:0006122 GO:0019835
GO:0009405 GO:0016491 GO:0005515 GO:0019867 GO:0005750
Cluster-22178.66051 GO:0016567 GO:0008270 GO:0046872 GO:0004842
GO:0005515 GO:0005680
Cluster-22178.66052 GO:0006355 GO:0008033 GO:0055114 GO:0071949
GO:0003677 GO:0016491 GO:0050660 GO:0003700 GO:0005507
GO:0005667 GO:0005634
Cluster-22178.66053 GO:0006355 GO:0003700 GO:0005667
Cluster-22178.66058 GO:0006468 GO:0016773 GO:0005524 GO:0004672
GO:0016020
Cluster-22178.66060 GO:0016579 GO:0036459
Cluster-22178.66061 GO:0003677
Cluster-22178.66064 GO:0030474 GO:0071988 GO:0007165 GO:0035444
GO:0015675 GO:0051082 GO:0005179 GO:0015099 GO:0046872
GO:0005576 GO:0016021 GO:0005887
Cluster-22178.66065 GO:0005634
Cluster-22178.66067 GO:0006402 GO:0051252 GO:0003676 GO:0046872
GO:0004535 GO:0003723 GO:0005737 GO:0005634
Cluster-22178.66069 GO:0035091 GO:0046872
Cluster-22178.66070 GO:0006888 GO:0006464 GO:0015031 GO:0006886
GO:0006351 GO:0046983 GO:0008270 GO:0046872 GO:0005515
GO:0030127 GO:0005921
Cluster-22178.66071 GO:0016567 GO:0019835 GO:0009405 GO:0055114
GO:0016114 GO:0016491 GO:0046872 GO:0008685 GO:0008270
GO:0004842 GO:0019867
Cluster-22178.66072 GO:0007186 GO:0016310 GO:0009411 GO:0006289
GO:0004519 GO:0004930 GO:0005524 GO:0046917 GO:0016021
Cluster-22178.66074 GO:0006352
Cluster-22178.66075 GO:0016032 GO:0019012

Cluster-22178.66078 GO:0006308 GO:0048870 GO:0008855 GO:0009318 GO:0031514
Cluster-22178.66081 GO:0055114 GO:0019497 GO:0007165 GO:0006771 GO:0016620 GO:0004872 GO:0005525 GO:0003993 GO:0046872 GO:0005515 GO:0003677 GO:0004190 GO:0016021 GO:0016020
Cluster-22178.66084 GO:0006886 GO:0016192 GO:0005507 GO:0005515 GO:0005198 GO:0030117 GO:0030126
Cluster-22178.66085 GO:0055114 GO:0006270 GO:0033014 GO:0006629 GO:0003676 GO:0008883 GO:0003723 GO:0050661
Cluster-22178.66087 GO:0006468 GO:0005524 GO:0004672
Cluster-22178.66088 GO:0015991 GO:0015992 GO:0015078 GO:0033177
Cluster-22178.66089 GO:0055114 GO:0016972 GO:0003676 GO:0005634
Cluster-22178.66090 GO:0005509 GO:0005515
Cluster-22178.66091 GO:0033014 GO:0055114 GO:0050661 GO:0008883 GO:0005515
Cluster-22178.66092 GO:0006355 GO:0000978 GO:0000982 GO:0005515 GO:0005634
Cluster-22178.66093 GO:0006629
Cluster-22178.66095 GO:0016021
Cluster-22178.66097 GO:0006334 GO:0003677 GO:0005634 GO:0000786
Cluster-22178.66098 GO:0042254 GO:0006412 GO:0006355 GO:0003700 GO:0003735 GO:0005840 GO:0005622 GO:0005667
Cluster-22178.66099 GO:0007264 GO:0016817 GO:0003924 GO:0046872 GO:0005515 GO:0005525
Cluster-22178.66101 GO:0055114 GO:0016192 GO:0006886 GO:0006817 GO:0044341 GO:0015321 GO:0010309 GO:0005515 GO:0005198 GO:0016020 GO:0030117
Cluster-22178.66105 GO:0051090 GO:0000390 GO:0006914 GO:0005515 GO:0042803 GO:0008134 GO:0045502 GO:0071008 GO:0005667 GO:0005634 GO:0030286
Cluster-22178.66107 GO:0006508 GO:0045859 GO:0016817 GO:0008242 GO:0004860 GO:0016740 GO:0016788 GO:0004197 GO:0005576
Cluster-22178.66109 GO:0008152 GO:0007275 GO:0007165 GO:0047355

GO:0008270 GO:0005179 GO:0016624 GO:0005576 GO:0016020
Cluster-22178.66111 GO:0008207 GO:0008209 GO:0008210 GO:0006694
GO:0055114 GO:0016616 GO:0050662 GO:0003854 GO:0003824
GO:0016491 GO:0016021
Cluster-22178.66113 GO:0006334 GO:0003677 GO:0000786 GO:0005634
Cluster-22178.66114 GO:0006468 GO:0005515 GO:0005524 GO:0004672
GO:0016773 GO:0016020
Cluster-22178.66118 GO:0032007 GO:0048011
Cluster-22178.66120 GO:0008047 GO:0008233
Cluster-22178.66121 GO:0019722 GO:0006730 GO:0006352 GO:0006402
GO:0007017 GO:0006355 GO:0007018 GO:0051252 GO:0005524
GO:0008017 GO:0016987 GO:0003677 GO:0004535 GO:0046872
GO:0003676 GO:0003700 GO:0003777 GO:0003723 GO:0016740
GO:0005515 GO:0005667 GO:0005737 GO:0045298 GO:0005874
GO:0005634
Cluster-22178.66122 GO:0006334 GO:0003677 GO:0005634 GO:0000786
Cluster-22178.66124 GO:0055114 GO:0016301 GO:0005515 GO:0016740
GO:0016625 GO:0051536 GO:0016491 GO:0005524
Cluster-22178.66125 GO:0006468 GO:0005524 GO:0004672 GO:0016773
GO:0016020
Cluster-22178.66127 GO:0034314 GO:0015992 GO:0055114 GO:0006119
GO:0006118 GO:0030833 GO:0008121 GO:0015629 GO:0005885
Cluster-22178.66129 GO:0008616 GO:0034639 GO:0005515 GO:0033739
GO:0016021
Cluster-22178.66131 GO:0042981 GO:0009069 GO:0016310 GO:0005509
GO:0004674 GO:0005544
Cluster-22178.66132 GO:0051252 GO:0006351 GO:0007049 GO:0006396
GO:0005544 GO:0004252 GO:0005515 GO:0005509 GO:0003723
GO:0004525 GO:0017053 GO:0005887 GO:0005886
Cluster-22178.66133 GO:0006810 GO:0005215
Cluster-22178.66134 GO:0006914 GO:0030168 GO:0015992 GO:0006606
GO:0071973 GO:0006457 GO:0007165 GO:0006810 GO:0048870
GO:0051258 GO:0006811 GO:0015991 GO:0000774 GO:0003774

GO:0005102 GO:0030674 GO:0051087 GO:0008092 GO:0015078
GO:0005198 GO:0042803 GO:0005886 GO:0019898 GO:0005577
GO:0016020 GO:0005882 GO:0005737 GO:0009288 GO:0033179
GO:0031514
Cluster-22178.66136 GO:0006418 GO:0004812 GO:0005524 GO:0000166
GO:0005737
Cluster-22178.66137 GO:0008270 GO:0046872
Cluster-22178.66140 GO:0055114 GO:0008152 GO:0006940 GO:0006118
GO:0016627 GO:0050660 GO:0003995
Cluster-22178.66142 GO:0005515
Cluster-22178.66143 GO:0005515
Cluster-22178.66144 GO:0005515 GO:0008270
Cluster-22178.66145 GO:0006099 GO:0031412 GO:0016829 GO:0031411
Cluster-22178.66146 GO:0016020
Cluster-22178.66147 GO:0015684 GO:0007264 GO:0005525 GO:0015093
GO:0003924 GO:0016021
Cluster-22178.66148 GO:0006355 GO:0043565 GO:0003677 GO:0008236
GO:0016021
Cluster-22178.66149 GO:0005515
Cluster-22178.66150 GO:0006810 GO:0004519 GO:0003677 GO:0005622
Cluster-22178.66151 GO:0007264 GO:0005525 GO:0003924
Cluster-22178.66152 GO:0004386 GO:0003676 GO:0003677 GO:0003723
GO:0016787 GO:0005524 GO:0005634
Cluster-22178.66153 GO:0006171 GO:0006816 GO:0006810 GO:0006144
GO:0046677 GO:0000287 GO:0005246 GO:0004016 GO:0046872
GO:0042030 GO:0016020
Cluster-22178.66155 GO:0005515 GO:0003924
Cluster-22178.66156 GO:0006869 GO:0005319 GO:0016831
Cluster-22178.66158 GO:0017187 GO:0008488
Cluster-22178.66159 GO:0006260 GO:0005524 GO:0003677
Cluster-22178.66160 GO:0006508 GO:0006886 GO:0006605 GO:0007268
GO:0005515 GO:0042802 GO:0004197 GO:0043495 GO:0033130
GO:0030695 GO:0005742

Cluster-22178.66161 GO:0006468 GO:0005524 GO:0004672 GO:0019012
Cluster-22178.66164 GO:0006810 GO:0016021
Cluster-22178.66165 GO:0008152 GO:0008757 GO:0008168
Cluster-22178.66167 GO:0046849 GO:0005515 GO:0005576
Cluster-22178.66168 GO:0046849 GO:0006511 GO:0005515 GO:0005576
Cluster-22178.66170 GO:0006950 GO:0018342 GO:0006270 GO:0006351
GO:0006457 GO:0005524 GO:0051082 GO:0008318 GO:0003677
GO:0005634
Cluster-22178.66171 GO:0006013 GO:0006355 GO:0005975 GO:0006000
GO:0008270 GO:0004476
Cluster-22178.66172 GO:0007186 GO:0007165 GO:0007010 GO:0004871
GO:0005525 GO:0003924 GO:0003779 GO:0019001 GO:0031683
GO:0008270
Cluster-22178.66173 GO:0006486 GO:0016757 GO:0008378 GO:0016020
Cluster-22178.66174 GO:0009059 GO:0031072 GO:0051082 GO:0030176
Cluster-22178.66175 GO:0015684 GO:0007165 GO:0007186 GO:0007264
GO:0006749 GO:0030150 GO:0006804 GO:0006979 GO:0055114
GO:0015093 GO:0004602 GO:0005525 GO:0004871 GO:0031683
GO:0019001 GO:0003924 GO:0005742 GO:0016021
Cluster-22178.66179 GO:0006508 GO:0004185
Cluster-22178.66180 GO:0042254 GO:0006412 GO:0003735 GO:0005840
GO:0005622
Cluster-22178.66182 GO:0006570 GO:0006118 GO:0006119 GO:0046740
GO:0006470 GO:0055114 GO:0015992 GO:0008121 GO:0008138
GO:0016787 GO:0004725 GO:0016021
Cluster-22178.66183 GO:0006351 GO:0006144 GO:0006206 GO:0003899
GO:0005634 GO:0005730
Cluster-22178.66184 GO:0006461 GO:0010508 GO:0019901 GO:0005739
Cluster-22178.66185 GO:0016032 GO:0005198 GO:0005882
Cluster-22178.66186 GO:0005975 GO:0043169 GO:0003824
Cluster-22178.66187 GO:0006355 GO:0005515 GO:0016818 GO:0003677
GO:0016817 GO:0008270 GO:0005524 GO:0005634
Cluster-22178.66190 GO:0006950 GO:0018342 GO:0006270 GO:0006351

GO:0006457 GO:0005524 GO:0051082 GO:0008318 GO:0003677
GO:0005634
Cluster-22178.66191 GO:0006123 GO:0015992 GO:0004129 GO:0045277
Cluster-22178.66195 GO:0005509 GO:0019012
Cluster-22178.66196 GO:0006508 GO:0006364 GO:0007276 GO:0016579
GO:0034477 GO:0004857 GO:0030246 GO:0032183 GO:0070140
GO:0004518 GO:0036459 GO:0008270 GO:0003676 GO:0004222
GO:0031012
Cluster-22178.66201 GO:0000105
Cluster-22178.66203 GO:0031047 GO:0005515
Cluster-22178.66205 GO:0006265 GO:0042742 GO:0003916 GO:0005524
GO:0003677 GO:0019901 GO:0005515 GO:0005694
Cluster-22178.66210 GO:0061630 GO:0005515 GO:0046872 GO:0008270

Cluster-22178.66211 GO:0005515 GO:0005201 GO:0005581 GO:0005578
Cluster-22178.66212 GO:0055114 GO:1901031 GO:0051920 GO:0005634
Cluster-22178.66213 GO:0007165 GO:0007186 GO:0004871 GO:0005834
Cluster-22178.66215 GO:0043093 GO:0000917 GO:0007049 GO:0008092
GO:0051082 GO:0005737 GO:0019898
Cluster-22178.66219 GO:0030488 GO:0006352 GO:0006355 GO:0003700
GO:0016987 GO:0003677 GO:0005667 GO:0031515
Cluster-22178.66220 GO:0046872
Cluster-22178.66221 GO:0000045 GO:0005737
Cluster-22178.66222 GO:0000045 GO:0005737
Cluster-22178.66224 GO:0006355 GO:0008270 GO:0043565 GO:0003700
GO:0005634 GO:0005667
Cluster-22178.66226 GO:0055114 GO:0016491 GO:0046983
Cluster-22178.66227 GO:0009395 GO:0016042 GO:0006355 GO:0043093
GO:0006364 GO:0000917 GO:0043565 GO:0003700 GO:0004629
GO:0005515 GO:0003677 GO:0005737 GO:0005634 GO:0005667
Cluster-22178.66230 GO:0006810 GO:0004519 GO:0003677 GO:0005622
Cluster-22178.66231 GO:0055114 GO:0016491 GO:0000287 GO:0003824
GO:0030976 GO:0050660

Cluster-22178.66233 GO:0006605 GO:0007165 GO:0005921
Cluster-22178.66234 GO:0006355 GO:0006359 GO:0043565 GO:0008270
GO:0003700 GO:0005667 GO:0005634
Cluster-22178.66235 GO:0007165 GO:0005509 GO:0005578
Cluster-22178.66236 GO:0042254 GO:0006412 GO:0003735 GO:0005840
Cluster-22178.66237 GO:0005975 GO:0000902 GO:0006811 GO:0016773
GO:0005216 GO:0016020
Cluster-22178.66239 GO:0016787
Cluster-22178.66241 GO:0006950
Cluster-22178.66242 GO:0005975 GO:0016773 GO:0005615
Cluster-22178.66243 GO:0046983
Cluster-22178.66246 GO:0005515
Cluster-22178.66247 GO:0006118 GO:0000902 GO:0055114 GO:0000287
GO:0016651 GO:0051536 GO:0016491 GO:0009055 GO:0030976
Cluster-22178.66248 GO:0007165 GO:0030695 GO:0005057
Cluster-22178.66249 GO:0016192 GO:0006886 GO:0015991 GO:0043248
GO:0016820 GO:0005515 GO:0030117 GO:0000221
Cluster-22178.66250 GO:0007017 GO:0007018 GO:0005524 GO:0003777
GO:0008017 GO:0005874 GO:0045298
Cluster-22178.66251 GO:0016117 GO:0006355 GO:0055114 GO:0016705
GO:0071949 GO:0016491 GO:0005634
Cluster-22178.66252 GO:0006352 GO:0006355 GO:0007186 GO:0007165
GO:0004871 GO:0003700 GO:0016987 GO:0003677 GO:0005834
GO:0005667
Cluster-22178.66254 GO:0042967 GO:0016573 GO:0007005 GO:0016567
GO:0006355 GO:0004842 GO:0003713 GO:0004402 GO:0000123
GO:0005634 GO:0005667
Cluster-22178.66256 GO:0055114 GO:0015992 GO:0018401 GO:0006525
GO:0015986 GO:0016567 GO:0006560 GO:0016702 GO:0046983
GO:0008270 GO:0015078 GO:0004842 GO:0004656 GO:0000276
GO:0005783
Cluster-22178.66258 GO:0005515
Cluster-22178.66261 GO:0046677 GO:0006396 GO:0000154 GO:0008649

Cluster-22178.66263 GO:0006118 GO:0006814 GO:0009252 GO:0055114 GO:0046436 GO:0008716 GO:0016655 GO:0005524 GO:0046872

Cluster-22178.66265 GO:0055114 GO:0006508 GO:0015631 GO:0050485 GO:0030699 GO:0016779 GO:0004197 GO:0030700 GO:0045298

Cluster-22178.66266 GO:0051603 GO:0030494 GO:0015979 GO:0004298 GO:0005839

Cluster-22178.66267 GO:0030071 GO:0007018 GO:0007017 GO:0005515 GO:0003777 GO:0008017 GO:0005524 GO:0005680 GO:0005874 GO:0045298

Cluster-22178.66269 GO:0016567 GO:0004842 GO:0005515

Cluster-22178.66270 GO:0009069 GO:0006886 GO:0016192 GO:0006351 GO:0006260 GO:0016310 GO:0005515 GO:0003677 GO:0003887 GO:0004674 GO:0030117 GO:0042575

Cluster-22178.66271 GO:0003677 GO:0016020

Cluster-22178.66272 GO:0003723 GO:0005515

Cluster-22178.66273 GO:0006446 GO:0007034 GO:0006413 GO:0003743 GO:0005840

Cluster-22178.66275 GO:0006576 GO:0007155 GO:0007264 GO:0007186 GO:0007165 GO:0002949 GO:0005198 GO:0003924 GO:0019001 GO:0005524 GO:0031683 GO:0004871 GO:0005525 GO:0031012

Cluster-22178.66276 GO:0007165 GO:0042254 GO:0007186 GO:0019684 GO:0015708 GO:0006412 GO:0006955 GO:0006118 GO:0009772 GO:0007606 GO:0019236 GO:0003677 GO:0003735 GO:0004932 GO:0008270 GO:0005515 GO:0045156 GO:0003723 GO:0046872 GO:0005125 GO:0005622 GO:0005576 GO:0005634 GO:0005840 GO:0016021

Cluster-22178.66277 GO:0022900 GO:0016651 GO:0005634

Cluster-22178.66278 GO:0006812 GO:0016051 GO:0009405 GO:0046872 GO:0016887 GO:0016840 GO:0000166 GO:0009289 GO:0016021 GO:0043230

Cluster-22178.66281 GO:0051301 GO:0006468 GO:0032955 GO:0016773 GO:0005524 GO:0004672 GO:0016020

Cluster-22178.66283 GO:0006184 GO:0007165 GO:0007186 GO:0007264
GO:0005525 GO:0004871 GO:0019001 GO:0031683 GO:0003924
GO:0016020
Cluster-22178.66284 GO:0016070 GO:0010506 GO:0055085 GO:0006415
GO:0010508 GO:0006886 GO:0006449 GO:0006376 GO:0006355
GO:0003729 GO:0003700 GO:0046872 GO:0016149 GO:0043565
GO:0019901 GO:0005515 GO:0051082 GO:0003723 GO:0005685
GO:0005783 GO:0018444 GO:0005667 GO:0005840 GO:0016021
GO:0005615 GO:0005737
Cluster-22178.66288 GO:0030414 GO:0004866
Cluster-22178.66290 GO:0005515 GO:0003677 GO:0005634
Cluster-22178.66292 GO:0004866 GO:0003824 GO:0005576 GO:0005615
Cluster-22178.66293 GO:0007264 GO:0006119 GO:0006118 GO:0055114
GO:0015992 GO:0008121 GO:0005507 GO:0005525
Cluster-22178.66294 GO:0006260 GO:0005634
Cluster-22178.66295 GO:0007186 GO:0005515 GO:0004252 GO:0070008
GO:0004930
Cluster-22178.66299 GO:0006413 GO:0006270 GO:0006446 GO:0019087
GO:0003743 GO:0008270 GO:0019028 GO:0005840 GO:0016021
Cluster-22178.6630 GO:0007165 GO:0006887 GO:0005179 GO:0005576
GO:0000145
Cluster-22178.66300 GO:0006468 GO:0005524 GO:0004672
Cluster-22178.66301 GO:0043039 GO:0006418 GO:0016787 GO:0004812
GO:0016746 GO:0000049 GO:0000166 GO:0005524 GO:0005737
GO:0005680
Cluster-22178.66303 GO:0015031 GO:0006464 GO:0005515
Cluster-22178.66304 GO:0030908
Cluster-22178.66305 GO:0044237 GO:0005524 GO:0003676
Cluster-22178.66310 GO:0016567 GO:0008270 GO:0008017 GO:0005515
GO:0004842 GO:0045298
Cluster-22178.66311 GO:0009405 GO:0006508 GO:0004197
Cluster-22178.66312 GO:0043093 GO:0000917 GO:0046983 GO:0019901
GO:0005737

Cluster-22178.66313 GO:0016032 GO:0005524 GO:0005515

Cluster-22178.66314 GO:0006525 GO:0043039 GO:0006433 GO:0006560 GO:0006418 GO:0004812 GO:0000166 GO:0005524 GO:0016876 GO:0004827 GO:0005737

Cluster-22178.66315 GO:0008270

Cluster-22178.66316 GO:0008152 GO:0008168 GO:0008270 GO:0008237

Cluster-22178.66320 GO:0006418 GO:0030154 GO:0005524 GO:0004812 GO:0000166 GO:0016021

Cluster-22178.66321 GO:0006470 GO:0003924 GO:0000287 GO:0030145 GO:0005525 GO:0003824 GO:0004721

Cluster-22178.66322 GO:0006260 GO:0005515

Cluster-22178.66323 GO:0016579 GO:0042254 GO:0006352 GO:0006811 GO:0006412 GO:0009405 GO:0007017 GO:0006355 GO:0016987 GO:0003735 GO:0003677 GO:0005216 GO:0003700 GO:0036459 GO:0005622 GO:0019013 GO:0005667 GO:0005840 GO:0005875 GO:0005576

Cluster-22178.66325 GO:0006874 GO:0006816 GO:0005219 GO:0016021 GO:0005622

Cluster-22178.66326 GO:0003723 GO:0019843 GO:0005622

Cluster-22178.66328 GO:0007218 GO:0016310 GO:0006468 GO:0009069 GO:0004674 GO:0005524 GO:0046872 GO:0003677 GO:0005515

Cluster-22178.66329 GO:0006260 GO:0000077 GO:0006275 GO:0006281 GO:0008270 GO:0003677 GO:0030896 GO:0005634

Cluster-22178.66330 GO:0005737 GO:0005634

Cluster-22178.66331 GO:0051259 GO:0006468 GO:0006886 GO:0000921 GO:0008053 GO:0019684 GO:0009767 GO:0003924 GO:0004672 GO:0051082 GO:0016168 GO:0005524 GO:0016021 GO:0005940 GO:0009521 GO:0016020 GO:0005741

Cluster-22178.66332 GO:0005975

Cluster-22178.66333 GO:0003723 GO:0005515

Cluster-22178.66335 GO:0005515

Cluster-22178.66336 GO:0008033 GO:0016879 GO:0005524 GO:0000166

GO:0005515 GO:0005737
Cluster-22178.66338 GO:0016310 GO:0007178 GO:0006468 GO:0009069
GO:0005026 GO:0005524 GO:0004672 GO:0046872 GO:0004675
GO:0003723 GO:0016021 GO:0016020
Cluster-22178.66339 GO:0006886 GO:0008536 GO:0005515
Cluster-22178.66340 GO:0015940 GO:0015937 GO:0005524 GO:0004594

Cluster-22178.66341 GO:0055114 GO:0006631 GO:0042254 GO:0006412
GO:0045300 GO:0003735 GO:0005840
Cluster-22178.66342 GO:0006364 GO:0008168 GO:0005515
Cluster-22178.66343 GO:0006886 GO:0016192 GO:0005198 GO:0005515
GO:0003824 GO:0016020 GO:0030126 GO:0016021
Cluster-22178.66349 GO:0019079
Cluster-22178.66351 GO:0006629 GO:0006814 GO:0006118 GO:0015992
GO:0006744 GO:0006120 GO:0003954 GO:0008137 GO:0016627
GO:0005739 GO:0005737 GO:0016021
Cluster-22178.66352 GO:0005524 GO:0008270 GO:0016787 GO:0003677

Cluster-22178.66358 GO:0006470 GO:0006260 GO:0006570 GO:0042254
GO:0006412 GO:0006351 GO:0004725 GO:0003735 GO:0003887
GO:0003677 GO:0008138 GO:0005840 GO:0005622 GO:0042575
Cluster-22178.66363 GO:0009059 GO:0031072 GO:0051082 GO:0030176
Cluster-22178.66365 GO:0006364 GO:0005730
Cluster-22178.66366 GO:0005975 GO:0004553
Cluster-22178.66367 GO:0007050 GO:0016192 GO:0006886 GO:0007218
GO:0005515 GO:0005198 GO:0005185 GO:0005576 GO:0030117
GO:0019028 GO:0019031
Cluster-22178.66369 GO:0008152 GO:0008168
Cluster-22178.66371 GO:0016567 GO:0004842 GO:0005515 GO:0008270
GO:0046872 GO:0005680
Cluster-22178.66372 GO:0006629 GO:0007264 GO:0007165 GO:0046339
GO:0043087 GO:0009395 GO:0035556 GO:0005085 GO:0005515
GO:0008081 GO:0004435

Cluster-22178.66374 GO:0006355 GO:0000975 GO:0003700 GO:0005667
GO:0005840
Cluster-22178.66375 GO:0003723
Cluster-22178.66379 GO:0005515
Cluster-22178.66381 GO:0006448 GO:0045901 GO:0006452 GO:0045905
GO:0003746 GO:0043022 GO:0003723 GO:0005198 GO:0005840
GO:0019031
Cluster-22178.66385 GO:0006508 GO:0055114 GO:0050485 GO:0030699
GO:0016779 GO:0004197 GO:0015631 GO:0030700 GO:0045298
Cluster-22178.66389 GO:0016773
Cluster-22178.66392 GO:0006406 GO:0007186 GO:0007165 GO:0004871
GO:0005525 GO:0003924 GO:0019001 GO:0031683 GO:0005524
GO:0003677 GO:0000166 GO:0003723 GO:0005737 GO:0005634
Cluster-22178.66393 GO:0003779 GO:0005622
Cluster-22178.66394 GO:0019354 GO:0006468 GO:0006779 GO:0055114
GO:0005515 GO:0043115 GO:0004672 GO:0005524 GO:0008641
GO:0016021
Cluster-22178.66395 GO:0006351 GO:0006206 GO:0006144 GO:0003677
GO:0003899 GO:0005730
Cluster-22178.66396 GO:0015876 GO:0051016 GO:0006281 GO:0006412
GO:0042254 GO:0008521 GO:0046983 GO:0003676 GO:0003735
GO:0005622 GO:0030479 GO:0005840 GO:0016021
Cluster-22178.66397 GO:0006820 GO:0016021
Cluster-22178.66398 GO:0006629 GO:0045454 GO:0005515
Cluster-22178.66400 GO:0015979 GO:0019538 GO:0050765 GO:0009405
GO:0016567 GO:0046872 GO:0008270 GO:0005515 GO:0009539
GO:0009523 GO:0016020 GO:0016012 GO:0005576
Cluster-22178.66401 GO:0019538 GO:0015979 GO:0050765 GO:0009405
GO:0016567 GO:0046872 GO:0008270 GO:0005515 GO:0009539
GO:0009523 GO:0016020 GO:0016012 GO:0005576
Cluster-22178.66402 GO:0016567 GO:0004842
Cluster-22178.66403 GO:0055114 GO:0019521 GO:0008152 GO:0009306
GO:0006813 GO:0015031 GO:0006098 GO:0009085 GO:0009089

GO:0015450 GO:0008839 GO:0003824 GO:0016491 GO:0016620
GO:0050662 GO:0051287 GO:0004616 GO:0009941 GO:0016021
Cluster-22178.66404 GO:0055114 GO:0020037 GO:0016705 GO:0005506

Cluster-22178.66405 GO:0045454 GO:0006118 GO:0009055 GO:0015035
GO:0005515
Cluster-22178.66406 GO:0007606 GO:0007165 GO:0008270 GO:0004888
GO:0003676 GO:0016020
Cluster-22178.66407 GO:0019033
Cluster-22178.66410 GO:0035556
Cluster-22178.66411 GO:0006357 GO:0016787 GO:0001104 GO:0016592
Cluster-22178.66412 GO:0003676
Cluster-22178.66413 GO:0051252 GO:0055114 GO:0006402 GO:0016114
GO:0006694 GO:0003723 GO:0004506 GO:0004535 GO:0046872
GO:0003676 GO:0050660 GO:0005634 GO:0005737 GO:0016021
Cluster-22178.66416 GO:0006355 GO:0006914 GO:0043565 GO:0003700
GO:0005667
Cluster-22178.66418 GO:0015992 GO:0015976 GO:0016310 GO:0006123
GO:0006094 GO:0006096 GO:0004129 GO:0004618 GO:0005746
GO:0045277
Cluster-22178.66420 GO:0003677
Cluster-22178.66421 GO:0005975 GO:0003824
Cluster-22178.66422 GO:0006810 GO:0006355 GO:0005215 GO:0003677
GO:0046872 GO:0016020
Cluster-22178.66423 GO:0006260 GO:0005634
Cluster-22178.66425 GO:0006270 GO:0006260 GO:0006351 GO:0003677
GO:0003887 GO:0005515 GO:0042575
Cluster-22178.66429 GO:0006265 GO:0003918 GO:0005524 GO:0003677
GO:0005634
Cluster-22178.66432 GO:0006511 GO:0006310 GO:0019079 GO:0002949
GO:0006576 GO:0006281 GO:0006355 GO:0016787 GO:0009378
GO:0003677 GO:0016887 GO:0005524 GO:0003724 GO:0003723
GO:0016301 GO:0008134 GO:0043531 GO:0003678 GO:0009379

GO:0005667 GO:0005657
Cluster-22178.66433 GO:0006397 GO:0046660 GO:0005634
Cluster-22178.66434 GO:0006355 GO:0006109 GO:0000160 GO:0016310
GO:0000155 GO:0005515 GO:0008134 GO:0003774 GO:0005524
GO:0004672 GO:0005667 GO:0016459 GO:0016020 GO:0009365
Cluster-22178.66435 GO:0055085 GO:0030001 GO:0046873 GO:0016020
Cluster-22178.66436 GO:0015992 GO:0006814 GO:0006885 GO:0015385
GO:0005634 GO:0016020
Cluster-22178.66437 GO:0001514 GO:0006351 GO:0006355 GO:0006260
GO:0006464 GO:0006448 GO:0006269 GO:0003896 GO:0016151
GO:0005525 GO:0008134 GO:0003723 GO:0005524 GO:0003746
GO:0016887 GO:0003677 GO:0005840 GO:0005737 GO:0005657
GO:0005730 GO:0005667
Cluster-22178.66438 GO:0008152 GO:0003824
Cluster-22178.66440 GO:0007165 GO:0006950 GO:0005179 GO:0005576
Cluster-22178.66444 GO:0007276 GO:0016579 GO:0034477 GO:0006508
GO:0006364 GO:0036459 GO:0008270 GO:0003676 GO:0004222
GO:0004857 GO:0030246 GO:0032183 GO:0070140 GO:0004518
GO:0031012
Cluster-22178.66446 GO:0016747
Cluster-22178.66447 GO:0006357 GO:0008270 GO:0003676 GO:0001104
GO:0016592
Cluster-22178.66450 GO:0015937 GO:0015940 GO:0005524 GO:0004140

Cluster-22178.66456 GO:0015986 GO:0006464 GO:0015031 GO:0015992
GO:0006886 GO:0015689 GO:0005047 GO:0008270 GO:0052855
GO:0015098 GO:0005515 GO:0003924 GO:0015078 GO:0005525
GO:0000276 GO:0005785 GO:0005786 GO:0016021
Cluster-22178.66457 GO:0006886 GO:0016192 GO:0005515 GO:0008536
GO:0030117
Cluster-22178.66458 GO:0006915 GO:0006118 GO:0020037 GO:0009055
GO:0019028 GO:0005622
Cluster-22178.66459 GO:0016579 GO:0036459

Cluster-22178.66460 GO:0055114 GO:0006629 GO:0030001 GO:0016491
GO:0046872
Cluster-22178.66461 GO:0030001 GO:0006629 GO:0055114 GO:0046872
GO:0016491
Cluster-22178.66462 GO:0003723
Cluster-22178.66465 GO:0006508 GO:0004197 GO:0008234
Cluster-22178.66467 GO:0000917 GO:0006457 GO:0043093 GO:0051082
GO:0016272 GO:0005737
Cluster-22178.66468 GO:0055114 GO:0043093 GO:0000917 GO:0046872
GO:0008289 GO:0047134 GO:0005737
Cluster-22178.66469 GO:0006464 GO:0016151 GO:0005198 GO:0046872
GO:0019028
Cluster-22178.66470 GO:0006468 GO:0005524 GO:0004672
Cluster-22178.66472 GO:0006164 GO:0006531 GO:0006144 GO:0006522
GO:0005525 GO:0004019
Cluster-22178.66473 GO:0006446 GO:0005515 GO:0003743 GO:0005737
GO:0005840 GO:0005852
Cluster-22178.66474 GO:0005515
Cluster-22178.66476 GO:0005515
Cluster-22178.66477 GO:0006468 GO:0005524 GO:0004672 GO:0046872
GO:0016787 GO:0016773 GO:0016020
Cluster-22178.66479 GO:0035023 GO:0009405 GO:0043087 GO:0005089
GO:0005576
Cluster-22178.66481 GO:0005515 GO:0043565 GO:0005634
Cluster-22178.66482 GO:0008152 GO:0055114 GO:0046487 GO:0000917
GO:0045132 GO:0000226 GO:0009396 GO:0043093 GO:0003824
GO:0008092 GO:0005515 GO:0004488 GO:0005509 GO:0016787
GO:0016021 GO:0000775 GO:0005737 GO:0015630 GO:0005634
GO:0019898
Cluster-22178.66483 GO:0006357 GO:0007165 GO:0005057 GO:0001104
GO:0016592
Cluster-22178.66489 GO:0003676
Cluster-22178.66490 GO:0006397 GO:0005515

Cluster-22178.66491 GO:0006397
Cluster-22178.66493 GO:0015992 GO:0006119 GO:0015986 GO:0008289
GO:0046933 GO:0046961 GO:0045259 GO:0000275
Cluster-22178.66494 GO:0006810 GO:0006355 GO:0005215 GO:0003677
GO:0046872 GO:0016020
Cluster-22178.66495 GO:0055085 GO:0006811 GO:0005216 GO:0016020
GO:0016021
Cluster-22178.66496 GO:0006659
Cluster-22178.66497 GO:0006270 GO:0003723 GO:0003676
Cluster-22178.66498 GO:0006355 GO:0005634
Cluster-22178.66499 GO:0006260 GO:0055114
Cluster-22178.66500 GO:0019354 GO:0006468 GO:0006779 GO:0055114
GO:0005515 GO:0043115 GO:0005524 GO:0004672 GO:0008641
GO:0016021
Cluster-22178.66503 GO:0018342 GO:0006886 GO:0006270 GO:0006397
GO:0006396 GO:0008318 GO:0005515 GO:0005634 GO:0005622
GO:0005789
Cluster-22178.66504 GO:0019867
Cluster-22178.66505 GO:0006811 GO:0006396 GO:0018342 GO:0006397
GO:0006270 GO:0006886 GO:0008318 GO:0005515 GO:0005622
GO:0005789 GO:0005634 GO:0016020
Cluster-22178.66508 GO:0002949 GO:0007059 GO:0030261 GO:0003724
GO:0005525 GO:0003924 GO:0016887 GO:0005524 GO:0005515
GO:0003677 GO:0003723 GO:0009295
Cluster-22178.66510 GO:0006461 GO:0005739
Cluster-22178.66511 GO:0006397 GO:0080019 GO:0000445
Cluster-22178.66514 GO:0006298 GO:0008270 GO:0005524 GO:0030983
GO:0005634
Cluster-22178.66515 GO:0009307 GO:0051276 GO:0006308 GO:0003677
GO:0009036 GO:0009359 GO:0005634 GO:0005737
Cluster-22178.66519 GO:0055085 GO:0016020 GO:0016021
Cluster-22178.66521 GO:0055085 GO:0006811 GO:0005216 GO:0016020
GO:0016021

Cluster-22178.66522 GO:0005524 GO:0005856
Cluster-22178.66523 GO:0045947 GO:0008190
Cluster-22178.66525 GO:0006334 GO:0006952 GO:0009405 GO:0006810 GO:0019855 GO:0005576 GO:0005634
Cluster-22178.66526 GO:0006355 GO:0000917 GO:0007049 GO:0043093 GO:0007186 GO:0006914 GO:0006464 GO:0006744 GO:0006260 GO:0043565 GO:0008813 GO:0003700 GO:0016151 GO:0004930 GO:0005737 GO:0016021 GO:0005667 GO:0005815
Cluster-22178.66528 GO:0006952 GO:0007165 GO:0055114 GO:0016491 GO:0046872 GO:0005576
Cluster-22178.66529 GO:0007010 GO:0003779
Cluster-22178.66531 GO:0006412 GO:0042254 GO:0003735 GO:0005840 GO:0005622
Cluster-22178.66532 GO:0006468 GO:0004672 GO:0005524
Cluster-22178.66533 GO:0055085 GO:0006811 GO:0005216 GO:0016020
Cluster-22178.66534 GO:0005525
Cluster-22178.66537 GO:0006525 GO:0042254 GO:0071436 GO:0006412 GO:0006814 GO:0006560 GO:0006090 GO:0009401 GO:0016787 GO:0003735 GO:0015081 GO:0008948 GO:0005515 GO:0016020 GO:0015934 GO:0016021 GO:0005840
Cluster-22178.66539 GO:0005783
Cluster-22178.66540 GO:0016021
Cluster-22178.66541 GO:0006886 GO:0006468 GO:0006355 GO:0009767 GO:0007165 GO:0019684 GO:0008053 GO:0000921 GO:0003700 GO:0004871 GO:0004672 GO:0003924 GO:0005524 GO:0016168 GO:0016021 GO:0005741 GO:0016020 GO:0005667 GO:0009521 GO:0005940
Cluster-22178.66542 GO:0006812 GO:0006820 GO:0006835 GO:0017153 GO:0016020
Cluster-22178.66543 GO:0006144 GO:0006464 GO:0007165 GO:0004114 GO:0016151
Cluster-22178.66546 GO:0003824 GO:0004866 GO:0005576 GO:0005615
Cluster-22178.66549 GO:0006388 GO:0002949 GO:0006260 GO:0008152

GO:0004003 GO:0003723 GO:0016301 GO:0003677 GO:0003972
GO:0016887 GO:0003924 GO:0005524 GO:0003724 GO:0005525
GO:0005657
Cluster-22178.66550 GO:0042254 GO:0006412 GO:0003735 GO:0005622
GO:0005840
Cluster-22178.66552 GO:0045893 GO:0006413 GO:0007275 GO:0006118
GO:0006446 GO:0008270 GO:0009055 GO:0003743 GO:0020037
GO:0005515 GO:0005840 GO:0016021
Cluster-22178.66553 GO:0006260 GO:0006468 GO:0008641 GO:0005524
GO:0008270 GO:0004672 GO:0003677 GO:0005515 GO:0016021
Cluster-22178.66556 GO:0042254 GO:0006412 GO:0006810 GO:0045454
GO:0005515 GO:0003735 GO:0005525 GO:0003924 GO:0005861
GO:0016021 GO:0005840
Cluster-22178.66559 GO:0042254 GO:0006412 GO:0003735 GO:0005622
GO:0005840
Cluster-22178.66560 GO:0015992 GO:0015991 GO:0015078 GO:0033177
Cluster-22178.66562 GO:0006508 GO:0006468 GO:0035023 GO:0007169
GO:0043087 GO:0005515 GO:0004714 GO:0004185 GO:0005089
GO:0004672 GO:0005524 GO:0016020
Cluster-22178.66566 GO:0042998 GO:0015074 GO:0003677 GO:0008270
GO:0071253 GO:0005802
Cluster-22178.66567 GO:0006457 GO:0005515
Cluster-22178.66571 GO:0006810 GO:0016021 GO:0005886
Cluster-22178.66572 GO:0006955 GO:0007165 GO:0005164 GO:0046872
GO:0016020
Cluster-22178.66574 GO:0046008 GO:0007165 GO:0005179 GO:0005576
Cluster-22178.66575 GO:0006412 GO:0042254 GO:0003735 GO:0005622
GO:0005840
Cluster-22178.66576 GO:0006886 GO:0006531 GO:0006522 GO:0009152
GO:0006188 GO:0006144 GO:0004018 GO:0005737
Cluster-22178.66578 GO:0007275 GO:0006810 GO:0055085 GO:0005524
GO:0016887 GO:0005525 GO:0042626 GO:0042600 GO:0016021
Cluster-22178.66579 GO:0007218 GO:0005185 GO:0005576

Cluster-22178.66580 GO:0035556 GO:0005524 GO:0005515

Cluster-22178.66582 GO:0003950

Cluster-22178.66585 GO:0042254 GO:0006412 GO:0003735 GO:0005840 GO:0015934

Cluster-22178.66587 GO:0006468 GO:0051301 GO:0032955 GO:0016773 GO:0005524 GO:0004672 GO:0003676 GO:0016020

Cluster-22178.66591 GO:0015979 GO:0009539 GO:0009523 GO:0016020

Cluster-22178.66596 GO:0008146 GO:0016021

Cluster-22178.66597 GO:0006355 GO:0007049 GO:0046983 GO:0043565 GO:0003700 GO:0005667

Cluster-22178.66599 GO:0006357 GO:0001104 GO:0016592

Cluster-22178.66600 GO:0006388 GO:0051252 GO:0006351 GO:0003714 GO:0000213 GO:0005667 GO:0000214

Cluster-22178.66601 GO:0005975

Cluster-22178.66602 GO:0005975

Cluster-22178.66604 GO:0005524 GO:0005856

Cluster-22178.66605 GO:0055114 GO:0019646 GO:0015886 GO:0006118 GO:0015232 GO:0016491 GO:0009055

Cluster-22178.66608 GO:0055114 GO:0016117 GO:0016491 GO:0016705

Cluster-22178.66609 GO:0007017 GO:0005875

Cluster-22178.66610 GO:0008270

Cluster-22178.66612 GO:0005515 GO:0008270

Cluster-22178.66615 GO:0030261 GO:0003677

Cluster-22178.66616 GO:0006355 GO:0008134 GO:0003677 GO:0016787 GO:0005524 GO:0005667

Cluster-22178.66617 GO:0005982 GO:0006012 GO:0005985 GO:0004564 GO:0005515 GO:0004575 GO:0017177

Cluster-22178.66619 GO:0004659 GO:0016021

Cluster-22178.66620 GO:0006352 GO:0003677 GO:0005669

Cluster-22178.66622 GO:0006810 GO:0003676 GO:0004386 GO:0003677 GO:0000166 GO:0016787 GO:0005524

Cluster-22178.66623 GO:0003676

Cluster-22178.66625 GO:0046688 GO:0006118 GO:0009055 GO:0005515
GO:0005507 GO:0020037 GO:0042597
Cluster-22178.66627 GO:0042254 GO:0006412 GO:0006886 GO:0006605
GO:0005515 GO:0003735 GO:0005742 GO:0005840
Cluster-22178.66628 GO:0005515
Cluster-22178.66630 GO:0006914 GO:0007165 GO:0051082 GO:0016301
GO:0005515 GO:0016021 GO:0032039
Cluster-22178.66631 GO:0008219
Cluster-22178.66632 GO:0006144 GO:0046034 GO:0046872 GO:0004017
GO:0003676
Cluster-22178.66633 GO:0005975 GO:0006260 GO:0043169 GO:0003887
GO:0003824 GO:0042575
Cluster-22178.66636 GO:0006886 GO:0007094 GO:0006420 GO:0006606
GO:0044780 GO:0006355 GO:0006560 GO:0006308 GO:0051258
GO:0006302 GO:0006525 GO:0006457 GO:0007165 GO:0030168
GO:0042803 GO:0005515 GO:0005198 GO:0043565 GO:0005524
GO:0004814 GO:0003700 GO:0030674 GO:0051087 GO:0000774
GO:0008855 GO:0005102 GO:0005634 GO:0005667 GO:0005783
GO:0005577 GO:0009318 GO:0016021
Cluster-22178.66637 GO:0090307 GO:0006508 GO:0006355 GO:0043565
GO:0003700 GO:0004222 GO:0051082 GO:0033588 GO:0005667
Cluster-22178.66639 GO:0055114 GO:0007165 GO:0005506 GO:0005158
GO:0016705 GO:0020037 GO:0005899
Cluster-22178.66641 GO:0046872 GO:0004867
Cluster-22178.66642 GO:0051607 GO:0050778 GO:0007259 GO:0007165
GO:0005125
Cluster-22178.66643 GO:0006468 GO:0016773 GO:0005524 GO:0004672
GO:0016020
Cluster-22178.66644 GO:0006144 GO:0006206 GO:0016042 GO:0007586
GO:0009069 GO:0016310 GO:0006468 GO:0006351 GO:0005524
GO:0004674 GO:0003677 GO:0008047 GO:0004672 GO:0003899
GO:0005730 GO:0005576
Cluster-22178.66646 GO:0019076 GO:0075521 GO:0046718 GO:0019028

GO:0044163 GO:0042025 GO:0030430
Cluster-22178.66647 GO:0019028
Cluster-22178.66648 GO:0005634
Cluster-22178.66649 GO:0030833 GO:0006119 GO:0006118 GO:0055114
GO:0015992 GO:0034314 GO:0008121 GO:0005885 GO:0015629
Cluster-22178.66652 GO:0006811 GO:0055085 GO:0015075 GO:0005515
GO:0005886 GO:0016020
Cluster-22178.66653 GO:0015992 GO:0055114 GO:0034314 GO:0030833
GO:0006119 GO:0006118 GO:0008121 GO:0015629 GO:0005885
Cluster-22178.66654 GO:0006098 GO:0018874 GO:0006631 GO:0006554
GO:0006633 GO:0006568 GO:0006550 GO:0006552 GO:0006574
GO:0019521 GO:0055114 GO:0015940 GO:0051287 GO:0004616
GO:0016491 GO:0003857 GO:0008677
Cluster-22178.66655 GO:0006468 GO:0045454 GO:0007586 GO:0016042
GO:0003697 GO:0004672 GO:0005524 GO:0008047 GO:0005576
GO:0019028
Cluster-22178.66657 GO:0006355 GO:0006284 GO:0003905 GO:0003677
GO:0046983 GO:0003700 GO:0005634 GO:0005667 GO:0016021
Cluster-22178.66658 GO:0007005 GO:0016567 GO:0005515 GO:0016758
GO:0008270 GO:0004842 GO:0005783 GO:0016021
Cluster-22178.66659 GO:0055085 GO:0006812 GO:0006885 GO:0015299
GO:0016021
Cluster-22178.66660 GO:0006355 GO:0005515 GO:0005634
Cluster-22178.66661 GO:0008270 GO:0005096
Cluster-22178.66662 GO:0008643 GO:0009401 GO:0006950 GO:0006457
GO:0005524 GO:0008982 GO:0051082 GO:0016021 GO:0009357
Cluster-22178.66664 GO:0033014 GO:0006783 GO:0015994 GO:0008270
GO:0004852 GO:0004386
Cluster-22178.66669 GO:0006813 GO:0009405 GO:0016567 GO:0030955
GO:0042802 GO:0005515 GO:0005249 GO:0004842 GO:0008076
GO:0016021
Cluster-22178.66670 GO:0006118 GO:0009451 GO:0045454 GO:0006396
GO:0055114 GO:0008173 GO:0003723 GO:0015035 GO:0009055

GO:0016209 GO:0016491 GO:0008168
Cluster-22178.66671 GO:0006123 GO:0006812 GO:0055114 GO:0006820
GO:0006835 GO:0015992 GO:0017153 GO:0016638 GO:0004129
GO:0016020 GO:0045277
Cluster-22178.66672 GO:0006275 GO:0005515 GO:0003677
Cluster-22178.66673 GO:0005515
Cluster-22178.66674 GO:0006352 GO:0004190 GO:0005634 GO:0005669
GO:0016021
Cluster-22178.66675 GO:0015991 GO:0015992 GO:0015078 GO:0033177
Cluster-22178.66676 GO:0009607 GO:0005515 GO:0016021
Cluster-22178.66677 GO:0006355 GO:0003677
Cluster-22178.66678 GO:0035556 GO:0006144 GO:0006357 GO:0006182
GO:0008152 GO:0046039 GO:0009190 GO:0006468 GO:0005524
GO:0008270 GO:0003824 GO:0004672 GO:0001104 GO:0003723
GO:0016849 GO:0004383 GO:0016592
Cluster-22178.66679 GO:0006468 GO:0005524 GO:0004672 GO:0016773
GO:0016020
Cluster-22178.66680 GO:0006935 GO:0007165 GO:0006955 GO:0008009
GO:0005576
Cluster-22178.66682 GO:0006352 GO:0005634
Cluster-22178.66683 GO:0016021
Cluster-22178.66685 GO:0032007 GO:0030682 GO:0048011 GO:0043176

Cluster-22178.66687 GO:0055085 GO:0022857 GO:0016021
Cluster-22178.66689 GO:0007276
Cluster-22178.66690 GO:0006820 GO:0016021
Cluster-22178.66691 GO:0006355 GO:0003677
Cluster-22178.66692 GO:0048598 GO:0005515 GO:0072669
Cluster-22178.66693 GO:0005515
Cluster-22178.66694 GO:0006144 GO:0006206 GO:0000162 GO:0006432
GO:0006351 GO:0009094 GO:0006571 GO:0003677 GO:0003723
GO:0004519 GO:0003899 GO:0046872 GO:0003676 GO:0004826
GO:0005730 GO:0009328

Cluster-22178.66697 GO:0008152 GO:0031110 GO:0006308 GO:0006629 GO:0003824 GO:0008081 GO:0008855 GO:0043565 GO:0009318
Cluster-22178.66698 GO:0005515 GO:0008270
Cluster-22178.66700 GO:0001514 GO:0006355 GO:0006351 GO:0006448 GO:0006464 GO:0006260 GO:0006269 GO:0003896 GO:0016151 GO:0005525 GO:0003723 GO:0008134 GO:0005524 GO:0003746 GO:0016887 GO:0003677 GO:0005737 GO:0005840 GO:0005657 GO:0005730 GO:0005667
Cluster-22178.66701 GO:0001514 GO:0006355 GO:0006351 GO:0006464 GO:0006448 GO:0006260 GO:0006269 GO:0005525 GO:0016151 GO:0003896 GO:0003723 GO:0008134 GO:0005524 GO:0003746 GO:0016887 GO:0003677 GO:0005737 GO:0005840 GO:0005657 GO:0005730 GO:0005667
Cluster-22178.66704 GO:0006771 GO:0019497 GO:0007339 GO:0046872 GO:0005515 GO:0003993 GO:0005576
Cluster-22178.66705 GO:0006811 GO:0055085 GO:0005216 GO:0005515 GO:0012511 GO:0016021 GO:0016020
Cluster-22178.66706 GO:0046487 GO:0055114 GO:0009396 GO:0004329 GO:0003824 GO:0004488 GO:0005524
Cluster-22178.66707 GO:0071630 GO:0031144 GO:0005634
Cluster-22178.66708 GO:0008152 GO:0055114 GO:0016491
Cluster-22178.66709 GO:0005515
Cluster-22178.66710 GO:0006418 GO:0008270 GO:0005524 GO:0004812 GO:0000166
Cluster-22178.66711 GO:0042384 GO:0034464
Cluster-22178.66712 GO:0009405 GO:0006352 GO:0005576 GO:0070461 GO:0005669
Cluster-22178.66713 GO:0007264 GO:0006576 GO:0015684 GO:0015093 GO:0005524 GO:0003924 GO:0016887 GO:0005525 GO:0016021
Cluster-22178.66714 GO:0006355 GO:0003712 GO:0005515 GO:0005667
Cluster-22178.66715 GO:0006298 GO:0007010 GO:0006364 GO:0007017 GO:0006813 GO:0030983 GO:0003779 GO:0005524 GO:0005267 GO:0005875 GO:0005634 GO:0034457 GO:0005732

Cluster-22178.66717 GO:0006355 GO:0003712 GO:0005515 GO:0005667
Cluster-22178.66718 GO:0008152 GO:0016491
Cluster-22178.66719 GO:0008152 GO:0016491
Cluster-22178.66720 GO:0010024 GO:0055114 GO:0016636 GO:0050897

Cluster-22178.66724 GO:0042254 GO:0006412 GO:0003735 GO:2001070
GO:0005840
Cluster-22178.66725 GO:0042254 GO:0006412 GO:2001070 GO:0003735
GO:0005840
Cluster-22178.66728 GO:0051028 GO:0006508 GO:0005634
Cluster-22178.66729 GO:0048870 GO:0000917 GO:0044780 GO:0006355
GO:0015991 GO:0006260 GO:0000921 GO:0006457 GO:0007094
GO:0010508 GO:0006606 GO:0048193 GO:0015992 GO:0006914
GO:0043093 GO:0007155 GO:0003700 GO:0008134 GO:0045502
GO:0017048 GO:0015078 GO:0043565 GO:0042803 GO:0005198
GO:0051082 GO:0005524 GO:0008270 GO:0003677 GO:0016818
GO:0016021 GO:0031514 GO:0019028 GO:0033180 GO:0030286
GO:0005940 GO:0005882 GO:0019867 GO:0005737 GO:0005634
GO:0016272 GO:0016020 GO:0005667
Cluster-22178.66730 GO:0055114 GO:0035556 GO:0006122 GO:0009405
GO:0019835 GO:0016491 GO:0005515 GO:0005750 GO:0019867
Cluster-22178.66731 GO:0015979 GO:0046872
Cluster-22178.66733 GO:0008299
Cluster-22178.66734 GO:0019069 GO:0006816 GO:0006874 GO:0006260
GO:0000049 GO:0005219 GO:0016020 GO:0071203 GO:0005815
GO:0016021 GO:0005622
Cluster-22178.66735 GO:0007264 GO:0051087 GO:0005096
Cluster-22178.66737 GO:0005515
Cluster-22178.66738 GO:0046914 GO:0003924 GO:0046983 GO:0005525
GO:0005509
Cluster-22178.66739 GO:0006777 GO:0008152 GO:0006810 GO:0005524
GO:0005525 GO:0016301
Cluster-22178.66741 GO:0005515

Cluster-22178.66742 GO:0003950 GO:0004190 GO:0016020 GO:0016021
GO:0005634
Cluster-22178.66743 GO:0006355 GO:0006508 GO:0043565 GO:0004197
GO:0003700 GO:0005667
Cluster-22178.66744 GO:0006355 GO:0003700 GO:0005515 GO:0008270
GO:0043565 GO:0005667
Cluster-22178.66748 GO:0003950 GO:0016020 GO:0005634
Cluster-22178.66751 GO:0006355 GO:0006352 GO:0003677 GO:0016987
GO:0003700 GO:0005667
Cluster-22178.66752 GO:0009405 GO:0016772 GO:0016301 GO:0016020
Cluster-22178.66756 GO:0016567 GO:0045893 GO:0004842 GO:0005515
GO:0003676 GO:0042025
Cluster-22178.66757 GO:0006810 GO:0055085 GO:0005215 GO:0016020
Cluster-22178.66760 GO:0006355 GO:0009607 GO:0015979 GO:0046872
GO:0003700 GO:0005667 GO:0042025 GO:0009523 GO:0016021
Cluster-22178.66761 GO:0006470 GO:0006570 GO:0004725 GO:0008138

Cluster-22178.66762 GO:0006284 GO:0006576 GO:0005525 GO:0003924
GO:0005524 GO:0003905 GO:0003677 GO:0030246
Cluster-22178.66763 GO:0016021
Cluster-22178.66764 GO:0006914
Cluster-22178.66767 GO:0006914 GO:0070206 GO:0051289 GO:0007165
GO:0006506 GO:0008140 GO:0016740 GO:0005515 GO:0005509
GO:0005886 GO:0016021 GO:0005789 GO:0005667 GO:0016012
GO:0005578
Cluster-22178.66768 GO:0015992 GO:0015991 GO:0015078 GO:0005198
GO:0033180 GO:0019031 GO:0009424 GO:0019028
Cluster-22178.66769 GO:0006364 GO:0008168 GO:0005634
Cluster-22178.66771 GO:0009190 GO:0035556 GO:0016849
Cluster-22178.66772 GO:0045892 GO:0003677 GO:0051082
Cluster-22178.66774 GO:0055114 GO:0006570 GO:0004511 GO:0019028
Cluster-22178.66775 GO:0006351 GO:0003676 GO:0005515 GO:0008270
GO:0005639

Cluster-22178.66777 GO:0043087 GO:0032012 GO:0005086
Cluster-22178.66778 GO:0006687 GO:0006027 GO:0046486 GO:0006835
GO:0005975 GO:0006820 GO:0006812 GO:0006012 GO:0017153
GO:0004565 GO:0016020 GO:0009341
Cluster-22178.66779 GO:0006810 GO:0003333 GO:0055085 GO:0006865
GO:0006811 GO:0007016 GO:0015171 GO:0005200 GO:0005215
GO:0016020 GO:0005925 GO:0001726 GO:0005856
Cluster-22178.66781 GO:0051382 GO:0005515 GO:0000776
Cluster-22178.66782 GO:0006364 GO:0032040 GO:0031966
Cluster-22178.66783 GO:0006260 GO:0005975 GO:0019058 GO:0000746
GO:0004553 GO:0003677 GO:0003887 GO:0019867 GO:0042575
Cluster-22178.66788 GO:0031556 GO:0031554
Cluster-22178.66790 GO:0005515
Cluster-22178.66792 GO:0046872
Cluster-22178.66794 GO:0006810 GO:0051205 GO:0004386 GO:0003676
GO:0005524 GO:0003677 GO:0016787 GO:0016021
Cluster-22178.66795 GO:0003723
Cluster-22178.66797 GO:0006810 GO:0009733 GO:0005515 GO:0005622
Cluster-22178.66798 GO:0005515
Cluster-22178.66799 GO:0010506 GO:0007049 GO:0006260 GO:0006355
GO:0043093 GO:0000917 GO:0043565 GO:0003700 GO:0005737
GO:0005615 GO:0005667 GO:0005634
Cluster-22178.66801 GO:0006355 GO:0006468 GO:0016114 GO:0042432
GO:0009058 GO:0055085 GO:0005515 GO:0004672 GO:0003700
GO:0004064 GO:0016844 GO:0005524 GO:0016773 GO:0016021
GO:0005667 GO:0016020
Cluster-22178.66803 GO:0005515
Cluster-22178.66806 GO:0003723
Cluster-22178.66808 GO:0006144 GO:0006470 GO:0006508 GO:0006570
GO:0004725 GO:0003968 GO:0008138 GO:0004197 GO:0017111
GO:0031379
Cluster-22178.66812 GO:0042254 GO:0006412 GO:0003735 GO:0005622
GO:0005840

Cluster-22178.66813 GO:0007218 GO:0007165 GO:0030141
Cluster-22178.66814 GO:0006118 GO:0003954 GO:0005739
Cluster-22178.66815 GO:0019836 GO:0005576
Cluster-22178.66816 GO:0015992 GO:0016021
Cluster-22178.66817 GO:0006364 GO:0003676 GO:0003723
Cluster-22178.66819 GO:0045454 GO:0006508 GO:0008234 GO:0016491

Cluster-22178.66820 GO:0006508 GO:0055114 GO:0050485 GO:0030699
GO:0004197 GO:0016779 GO:0015631 GO:0030700 GO:0045298
Cluster-22178.66825 GO:0042254 GO:0006412 GO:0006457 GO:0055114
GO:0003735 GO:0005515 GO:0005840 GO:0005622
Cluster-22178.66826 GO:0006974 GO:0051726 GO:0003677
Cluster-22178.66828 GO:0006355 GO:0000736 GO:0051726 GO:0046872
GO:0043565 GO:0003700 GO:0003677 GO:0070336 GO:0005667
GO:0005634
Cluster-22178.66830 GO:0003676
Cluster-22178.66831 GO:0010508 GO:0000917 GO:0043093 GO:0006355
GO:0006914 GO:0003677 GO:0003700 GO:0043565 GO:0005634
GO:0005667 GO:0005737
Cluster-22178.66833 GO:0005515
Cluster-22178.66834 GO:0006206 GO:0006826 GO:0006144 GO:0006351
GO:0019290 GO:0015991 GO:0015343 GO:0003677 GO:0005515
GO:0003899 GO:0016820 GO:0000221 GO:0005730
Cluster-22178.66838 GO:0006355 GO:0003700 GO:0043565 GO:0005667
GO:0005634
Cluster-22178.66839 GO:0006355 GO:0007275 GO:0006353 GO:0008608
GO:0007219 GO:0030154 GO:0003723 GO:0003677 GO:0072686
GO:0042729 GO:0016021
Cluster-22178.66840 GO:0006468 GO:0006413 GO:0006446 GO:0005524
GO:0004672 GO:0003743 GO:0005840
Cluster-22178.66841 GO:0007165
Cluster-22178.66842 GO:0006313 GO:0003677 GO:0004803
Cluster-22178.66845 GO:0030570 GO:0005576

Cluster-22178.66846 GO:0006750 GO:0006749 GO:0007155 GO:0042398
GO:0004357 GO:0017109 GO:0016021
Cluster-22178.66847 GO:0006206 GO:0006139 GO:0005524 GO:0004127

Cluster-22178.66848 GO:0008283 GO:0040007 GO:0007165 GO:0006955
GO:0043087 GO:0035023 GO:0005126 GO:0003723 GO:0008083
GO:0005089 GO:0005576
Cluster-22178.66849 GO:0007165 GO:0006955 GO:0008283 GO:0040007
GO:0035023 GO:0043087 GO:0008083 GO:0005126 GO:0003723
GO:0005089 GO:0005576
Cluster-22178.66850 GO:0006449 GO:0006415 GO:0003747 GO:0005840
GO:0018444
Cluster-22178.66853 GO:0051262 GO:0055114 GO:0016491
Cluster-22178.66857 GO:0016567 GO:0005515 GO:0004842 GO:0008270
GO:0051082 GO:0046872 GO:0005680
Cluster-22178.66858 GO:0003676
Cluster-22178.66861 GO:0015979 GO:0005515 GO:0016773
Cluster-22178.66862 GO:0006935 GO:0007165 GO:0006810 GO:0004888
GO:0005643 GO:0016020
Cluster-22178.66863 GO:0006508 GO:0005516 GO:0004197
Cluster-22178.66864 GO:0006886 GO:0009069 GO:0006468 GO:0007155
GO:0016310 GO:0048193 GO:0006914 GO:0005515 GO:0004674
GO:0016757 GO:0005524 GO:0003774 GO:0016020 GO:0016459
GO:0005801
Cluster-22178.66865 GO:0005515
Cluster-22178.66866 GO:0042967 GO:0006355 GO:0003712 GO:0008270
GO:0004402 GO:0016787 GO:0000123 GO:0005667 GO:0005634
Cluster-22178.66870 GO:0005840
Cluster-22178.66871 GO:0006412 GO:0042254 GO:0007156 GO:0003735
GO:0005509 GO:0005840 GO:0005622 GO:0016020
Cluster-22178.66872 GO:0006289 GO:0016310 GO:0009411 GO:0007186
GO:0005524 GO:0046917 GO:0004930 GO:0004519 GO:0016021
Cluster-22178.66875 GO:0003677

Cluster-22178.66876 GO:0000746 GO:0003676 GO:0003677

Cluster-22178.66877 GO:0000746 GO:0003676 GO:0003677

Cluster-22178.66881 GO:0006777

Cluster-22178.66882 GO:0006118 GO:0045454 GO:0015035 GO:0009055

Cluster-22178.66883 GO:0016787 GO:0005524

Cluster-22178.66884 GO:0006352 GO:0006355 GO:0003677 GO:0016987 GO:0003700 GO:0005667

Cluster-22178.66885 GO:0007275 GO:0007165 GO:0008270 GO:0004888 GO:0016021

Cluster-22178.66886 GO:0035556 GO:0007165

Cluster-22178.66887 GO:0006355

Cluster-22178.66888 GO:0006183 GO:0006228 GO:0006165 GO:0006241 GO:0006206 GO:0006144 GO:0005515 GO:0003723 GO:0003676 GO:0004550

Cluster-22178.66889 GO:0009395 GO:0009372 GO:0009405 GO:0004620 GO:0008233 GO:0016021

Cluster-22178.66890 GO:0006355 GO:0042254 GO:0006412 GO:0043565 GO:0008270 GO:0003700 GO:0003735 GO:0005840 GO:0005667

Cluster-22178.66891 GO:0007049 GO:0000917 GO:0043093 GO:0006355 GO:0003700 GO:0043565 GO:0003677 GO:0005198 GO:0019031 GO:0005737 GO:0019028 GO:0005634 GO:0005667

Cluster-22178.66892 GO:0003677

Cluster-22178.66893 GO:0006281 GO:0046872 GO:0008270

Cluster-22178.66894 GO:0046872 GO:0000166

Cluster-22178.66896 GO:0006508 GO:0005509 GO:0004198 GO:0005622

Cluster-22178.66897 GO:0042254 GO:0006412 GO:0003735 GO:0005622 GO:0005840

Cluster-22178.66899 GO:0015991 GO:0045893 GO:0015992 GO:0015078 GO:0009512 GO:0033179

Cluster-22178.66905 GO:0016020

Cluster-22178.66909 GO:0006355 GO:0008168 GO:0016817 GO:0003676 GO:0003700 GO:0005507 GO:0003677 GO:0005667 GO:0005634

Cluster-22178.66910 GO:0006020 GO:0006094 GO:0046486 GO:0006096
GO:0006000 GO:0015976 GO:0006013 GO:0008152 GO:0004807
Cluster-22178.66911 GO:0003676 GO:0003713 GO:0005667 GO:0005634
Cluster-22178.66914 GO:0006511 GO:0006313 GO:0015074 GO:0005975
GO:0004560 GO:0005515 GO:0003677 GO:0046872
Cluster-22178.66916 GO:0055114 GO:0006809 GO:0010181 GO:0016491
GO:0004517 GO:0005515 GO:0016717
Cluster-22178.66917 GO:0055114 GO:0006809 GO:0006355 GO:0016491
GO:0016717 GO:0004517 GO:0010181 GO:0003677 GO:0005515
Cluster-22178.66919 GO:0016567 GO:0019058 GO:0015986 GO:0006396
GO:0006807 GO:0006665 GO:0006464 GO:0015992 GO:0006687
GO:0006468 GO:0005975 GO:0004348 GO:0004842 GO:0016773
GO:0005524 GO:0008270 GO:0005515 GO:0003723 GO:0003676
GO:0015078 GO:0016151 GO:0046872 GO:0004672 GO:0016020
GO:0005680 GO:0000276
Cluster-22178.66920 GO:0000105 GO:0006547 GO:0004635
Cluster-22178.66922 GO:0006355 GO:0008233 GO:0003700 GO:0003723
GO:0004252 GO:0016791 GO:0005618 GO:0005634 GO:0005667
GO:0016020
Cluster-22178.66926 GO:0005975 GO:0003824 GO:0043169 GO:0030089
Cluster-22178.66927 GO:0006355 GO:0004252 GO:0003700 GO:0003824
GO:0042025 GO:0005882 GO:0005667 GO:0016021 GO:0005886
Cluster-22178.66928 GO:0005739
Cluster-22178.66929 GO:0006560 GO:0006814 GO:0071436 GO:0006525
GO:0006771 GO:0006090 GO:0019497 GO:0003677 GO:0008948
GO:0005515 GO:0003993 GO:0046872 GO:0015081 GO:0016020
Cluster-22178.66930 GO:0006464
Cluster-22178.66931 GO:0006998 GO:0006470 GO:0006406 GO:0006570
GO:0006611 GO:0004725 GO:0008138 GO:0031965
Cluster-22178.66932 GO:0006886 GO:0006605 GO:0005515 GO:0008270
GO:0016020
Cluster-22178.66933 GO:0051028 GO:0005634
Cluster-22178.66934 GO:0006820 GO:0009236 GO:0006355 GO:0006270

GO:0019051 GO:0008509 GO:0003723 GO:0005515 GO:0003677
GO:0016021 GO:0042025
Cluster-22178.66935 GO:0051259 GO:0005515 GO:0017119
Cluster-22178.66936 GO:0006626 GO:0003824 GO:0005741
Cluster-22178.66937 GO:0045122 GO:0006351 GO:0006355 GO:0006206
GO:0051262 GO:0006144 GO:0046872 GO:0008134 GO:0005515
GO:0003899 GO:0044212 GO:0003677 GO:0019904 GO:0005634
GO:0005730 GO:0005667
Cluster-22178.66939 GO:0006687 GO:0009058 GO:0008146 GO:0001733
GO:0016021 GO:0005794
Cluster-22178.66940 GO:0007018 GO:0007017 GO:0009069 GO:0016310
GO:0005515 GO:0008017 GO:0003777 GO:0004674 GO:0016773
GO:0005524 GO:0045298 GO:0005874
Cluster-22178.66944 GO:0006351 GO:0006825 GO:0006144 GO:0006206
GO:0016531 GO:0003899 GO:0003677 GO:0005507 GO:0005634
GO:0005730 GO:0005758
Cluster-22178.66945 GO:0006355 GO:0005515 GO:0005634
Cluster-22178.66946 GO:0006364 GO:0003723 GO:0008168 GO:0003676

Cluster-22178.66947 GO:0019058
Cluster-22178.66948 GO:0006468 GO:0004672 GO:0005524
Cluster-22178.66950 GO:0015694 GO:0046872 GO:0008270 GO:0015097
GO:0003677 GO:0005515 GO:0016020
Cluster-22178.66951 GO:0006813 GO:0055114 GO:0051287 GO:0016616

Cluster-22178.66952 GO:0016021
Cluster-22178.66954 GO:0006418 GO:0005524 GO:0004812 GO:0000166

Cluster-22178.66955 GO:0006418 GO:0005524 GO:0004812 GO:0000166

Cluster-22178.66957 GO:0006468 GO:0005524 GO:0004672 GO:0016773
GO:0016020
Cluster-22178.66959 GO:0042254 GO:0005634

Cluster-22178.66962 GO:0006468 GO:0004672 GO:0005524 GO:0016773
GO:0016020
Cluster-22178.66963 GO:0019087 GO:0032259 GO:0008168 GO:0016021
Cluster-22178.66964 GO:0046856 GO:0034597 GO:0003677
Cluster-22178.66965 GO:0042254 GO:0006412 GO:0003735 GO:0019843
GO:0005840
Cluster-22178.66966 GO:0006508 GO:0006629 GO:0006144 GO:0004252
GO:0017111 GO:0004197 GO:0003968 GO:0070008 GO:0016817
GO:0031379
Cluster-22178.66970 GO:0021549 GO:0030182 GO:0008270
Cluster-22178.66973 GO:0008270
Cluster-22178.66975 GO:0009405 GO:0019835 GO:0019867
Cluster-22178.66978 GO:0006139 GO:0003676 GO:0005524 GO:0016818
GO:0003677 GO:0008026 GO:0016787 GO:0004003 GO:0005657
GO:0005634
Cluster-22178.66981 GO:0006308 GO:0006281 GO:0008852 GO:0016021
Cluster-22178.66983 GO:0007264 GO:0005525
Cluster-22178.66985 GO:0006520 GO:0000921 GO:0046856 GO:0006606
GO:0006396 GO:0006614 GO:0006355 GO:0008312 GO:0043565
GO:0003700 GO:0034597 GO:0003723 GO:0045502 GO:0008134
GO:0042803 GO:0005515 GO:0030286 GO:0005940 GO:0005667
GO:0048500 GO:0016021
Cluster-22178.66987 GO:0051568 GO:0006479 GO:0000902 GO:0006508
GO:0003779 GO:0042800 GO:0005515 GO:0003682 GO:0072357
GO:0000785 GO:0048188
Cluster-22178.66989 GO:0055114 GO:0016705 GO:0020037 GO:0005506

Cluster-22178.66995 GO:0035556 GO:0006357 GO:0006144 GO:0006182
GO:0008152 GO:0006468 GO:0009190 GO:0046039 GO:0008270
GO:0005524 GO:0003824 GO:0004672 GO:0001104 GO:0004383
GO:0003723 GO:0016849 GO:0016592
Cluster-22178.66996 GO:0006182 GO:0035556 GO:0006357 GO:0006144
GO:0006468 GO:0009190 GO:0046039 GO:0008152 GO:0008270

GO:0005524 GO:0001104 GO:0004383 GO:0016849 GO:0003723
GO:0003824 GO:0004672 GO:0016592
Cluster-22178.66998 GO:0071436 GO:0006814 GO:0006560 GO:0006525
GO:0006090 GO:0005515 GO:0008948 GO:0015081 GO:0008270
GO:0046872 GO:0016020 GO:0019013
Cluster-22178.66999 GO:0055114 GO:0007165 GO:0006810 GO:0005215
GO:0005158 GO:0016705 GO:0020037 GO:0005506 GO:0005899
GO:0016020
Cluster-22178.67000 GO:0006364 GO:0019722 GO:0003676 GO:0003723
GO:0008168
Cluster-22178.67001 GO:0055114 GO:0007165 GO:0005506 GO:0005158
GO:0020037 GO:0016705 GO:0005899
Cluster-22178.67003 GO:0006470 GO:0006400 GO:0008616 GO:0000087
GO:0006570 GO:0015694 GO:0004725 GO:0015097 GO:0008479
GO:0005622 GO:0019031 GO:0016020
Cluster-22178.67004 GO:0006457 GO:0006886 GO:0008219 GO:0007304
GO:0051087 GO:0000774 GO:0008270 GO:0042803 GO:0005515
GO:0005198 GO:0005213 GO:0016020 GO:0005783 GO:0019028
GO:0005576 GO:0042600 GO:0005801 GO:0016021
Cluster-22178.67006 GO:0006412 GO:0042254 GO:0003735 GO:0005840
GO:0005622
Cluster-22178.67007 GO:0006468 GO:0006118 GO:0016773 GO:0004672
GO:0005524 GO:0003676 GO:0009055 GO:0016020
Cluster-22178.67008 GO:0045859 GO:0007049 GO:0016538
Cluster-22178.67009 GO:0055114 GO:0016491
Cluster-22178.67010 GO:0006260 GO:0018142 GO:0006269
Cluster-22178.67011 GO:0042254 GO:0006412 GO:0003735 GO:0005840
GO:0005622
Cluster-22178.67012 GO:0003723
Cluster-22178.67013 GO:0003723
Cluster-22178.67014 GO:0051082 GO:0031072
Cluster-22178.67016 GO:0003723
Cluster-22178.67017 GO:0006396 GO:0051252 GO:0008033 GO:0003676

GO:0004526 GO:0030677
Cluster-22178.67018 GO:0006886 GO:0008277 GO:0006355 GO:0007606
GO:0007165 GO:0007186 GO:0019058 GO:0007264 GO:0043087
GO:0015031 GO:0005515 GO:0042802 GO:0004888 GO:0003700
GO:0008092 GO:0043565 GO:0030674 GO:0004930 GO:0043130
GO:0008565 GO:0005096 GO:0019031 GO:0016021 GO:0016020
GO:0005667
Cluster-22178.67020 GO:0006123 GO:0015992 GO:0004129 GO:0045277
Cluster-22178.67022 GO:0046872
Cluster-22178.67023 GO:0006508 GO:0006355 GO:0004197
Cluster-22178.67025 GO:0005634
Cluster-22178.67027 GO:0009755 GO:0006355 GO:0009405 GO:0007268
GO:0007165 GO:0042967 GO:0030550 GO:0003700 GO:0008080
GO:0005667 GO:0005576
Cluster-22178.67029 GO:0006836 GO:0006812 GO:0005335 GO:0003676
GO:0005887
Cluster-22178.67030 GO:0009405
Cluster-22178.67032 GO:0006633 GO:0009403 GO:0006554 GO:0006520
GO:0006631 GO:0006810 GO:0018874 GO:0006098 GO:0015940
GO:0008152 GO:0019521 GO:0055114 GO:0006574 GO:0006552
GO:0006550 GO:0006508 GO:0006568 GO:0006813 GO:0008233
GO:0016597 GO:0016491 GO:0016616 GO:0004616 GO:0051287
GO:0003824 GO:0005215 GO:0008677 GO:0003857 GO:0016743
GO:0016836 GO:0016020
Cluster-22178.67035 GO:0061630 GO:0005515 GO:0008270 GO:0046872

Cluster-22178.67036 GO:0016055 GO:0006531 GO:0043039 GO:0006419
GO:0006522 GO:0000166 GO:0004813 GO:0016876 GO:0005524
GO:0003676
Cluster-22178.67037 GO:0030336 GO:0010972 GO:0016579 GO:0036459

Cluster-22178.67041 GO:0006351 GO:0006269 GO:0006260 GO:0035556
GO:0003677 GO:0003896 GO:0046872 GO:0005730 GO:0005657

Cluster-22178.67042 GO:0006118 GO:0045454 GO:0006144 GO:0000398
GO:0005515 GO:0003968 GO:0015035 GO:0009055 GO:0005681
GO:0016021 GO:0031379
Cluster-22178.67044 GO:0006260 GO:0006355 GO:0006400 GO:0008616
GO:0003700 GO:0008479 GO:0005667 GO:0005634
Cluster-22178.67046 GO:0008616 GO:0006355 GO:0006400 GO:0006260
GO:0008479 GO:0003700 GO:0005667 GO:0005634
Cluster-22178.67047 GO:0023052 GO:0007165 GO:0005125
Cluster-22178.67048 GO:0016973 GO:0006355 GO:0048870 GO:0070206
GO:0003700 GO:0031514 GO:0005667 GO:0016021 GO:0005643
Cluster-22178.67050 GO:0022008 GO:0005764
Cluster-22178.67053 GO:0016567 GO:0007005 GO:0005515 GO:0008270
GO:0016758 GO:0004842 GO:0005783 GO:0016021
Cluster-22178.67054 GO:0055114 GO:0008152 GO:0006118 GO:0016627
GO:0050660 GO:0003995
Cluster-22178.67055 GO:0005515 GO:0008270
Cluster-22178.67056 GO:0006355 GO:0000921 GO:0006520 GO:0006396
GO:0046856 GO:0006606 GO:0006614 GO:0043565 GO:0034597
GO:0003700 GO:0003723 GO:0045502 GO:0005515 GO:0042803
GO:0008134 GO:0008312 GO:0048500 GO:0016021 GO:0005940
GO:0030286 GO:0005667
Cluster-22178.67058 GO:0006355 GO:0007165 GO:0046983 GO:0035257
GO:0003713 GO:0005667 GO:0005634
Cluster-22178.67059 GO:0006355 GO:0043565 GO:0003700 GO:0005515
GO:0005667
Cluster-22178.67060 GO:0055085 GO:0006810 GO:0005215 GO:0016020
Cluster-22178.67061 GO:0045859 GO:0007050 GO:0004861 GO:0005634
Cluster-22178.67062 GO:0006355 GO:0046983
Cluster-22178.67063 GO:0045454 GO:0000398 GO:0007067 GO:0005681
Cluster-22178.67065 GO:0042254 GO:0006412 GO:0003735 GO:0005622
GO:0005840
Cluster-22178.67068 GO:0008616 GO:0006281 GO:0046034 GO:0006144
GO:0006366 GO:0016853 GO:0004017 GO:0003677 GO:0016740

GO:0004867 GO:0008270 GO:0046872 GO:0005665 GO:0005576
Cluster-22178.67071 GO:0055114 GO:0016209 GO:0016491 GO:0008270
GO:0016021
Cluster-22178.67072 GO:0005515
Cluster-22178.67073 GO:0010508 GO:0006606 GO:0006308 GO:0051607
GO:0008855 GO:0009318
Cluster-22178.67074 GO:0008152 GO:0003824 GO:0008484
Cluster-22178.67077 GO:0008152 GO:0016746
Cluster-22178.67080 GO:0007049 GO:0006355 GO:0003677 GO:0003700
GO:0043565 GO:0005667 GO:0005634
Cluster-22178.67081 GO:0006412 GO:0042254 GO:0003735 GO:0005840
GO:0005622
Cluster-22178.67083 GO:0005201 GO:0005581 GO:0005578
Cluster-22178.67084 GO:0055114 GO:0005506 GO:0020037 GO:0016705

Cluster-22178.67085 GO:0005840
Cluster-22178.67087 GO:0008152 GO:0016787
Cluster-22178.67088 GO:0045132 GO:0005515 GO:0005634 GO:0000775
Cluster-22178.67090 GO:0019058 GO:0007283 GO:0007165 GO:0008601
GO:0000159
Cluster-22178.67091 GO:0042742 GO:0007165 GO:0009395 GO:0008654
GO:0016310 GO:0016042 GO:0000160 GO:0000155 GO:0004623
GO:0005509 GO:0005515 GO:0043772 GO:0005576 GO:0005886
GO:0009365
Cluster-22178.67094 GO:0055114 GO:0006879 GO:0016491 GO:0046872
GO:0008199
Cluster-22178.67095 GO:0006508 GO:0004198 GO:0016757 GO:0005622
GO:0016020
Cluster-22178.67097 GO:0006412 GO:0042254 GO:0003735 GO:0005515
GO:0005840
Cluster-22178.67099 GO:0009252 GO:0006807 GO:0006811 GO:0006542
GO:0006355 GO:0006813 GO:0005249 GO:0004356 GO:0003677
GO:0016020 GO:0008076

Cluster-22178.67100 GO:0005975 GO:0016773 GO:0005615
Cluster-22178.67101 GO:0055114 GO:0051920 GO:0016491 GO:0016209

Cluster-22178.67105 GO:0006355 GO:0005515 GO:0003676 GO:0043565
GO:0005634
Cluster-22178.67107 GO:0020037
Cluster-22178.67108 GO:0005524 GO:0016787 GO:0003677 GO:0003676

Cluster-22178.67110 GO:0006165 GO:0007155 GO:0006228 GO:0006183
GO:0006144 GO:0006206 GO:0006241 GO:0003723 GO:0005515
GO:0004550 GO:0003676
Cluster-22178.67111 GO:0006820 GO:0016021
Cluster-22178.67114 GO:0043248 GO:0015991 GO:0006486 GO:0016192
GO:0006886 GO:0008378 GO:0016820 GO:0000221 GO:0030117
GO:0016020
Cluster-22178.67115 GO:0006189
Cluster-22178.67118 GO:0006457 GO:0008270 GO:0042803 GO:0008134
GO:0045502 GO:0051082 GO:0016272 GO:0030286 GO:0005667
Cluster-22178.67120 GO:0022900 GO:0003676 GO:0005524 GO:0046872
GO:0003677 GO:0005515 GO:0016787 GO:0005743 GO:0070469
Cluster-22178.67121 GO:0006998 GO:0035556 GO:0006611 GO:0006406
GO:0005515 GO:0031965
Cluster-22178.67122 GO:0035023 GO:0043087 GO:0005089
Cluster-22178.67123 GO:0015992 GO:0006120 GO:0006744 GO:0006814
GO:0008137 GO:0005743
Cluster-22178.67124 GO:0006144 GO:0006508 GO:0004197 GO:0003968
GO:0005515 GO:0017111 GO:0031379
Cluster-22178.67125 GO:0016567 GO:0005515 GO:0004842 GO:0008270
GO:0046872 GO:0005680 GO:0005622
Cluster-22178.67127 GO:0005515
Cluster-22178.67128 GO:0016070
Cluster-22178.67129 GO:0007156 GO:0005509 GO:0016020
Cluster-22178.67130 GO:0015991 GO:0005524 GO:0016820 GO:0033178

Cluster-22178.67133 GO:0042138 GO:0006310 GO:0003824
Cluster-22178.67134 GO:0005509 GO:0005515 GO:0016846
Cluster-22178.67135 GO:0042138 GO:0006310 GO:0003824
Cluster-22178.67137 GO:0042981
Cluster-22178.67140 GO:0006310 GO:0006281 GO:0002949 GO:0003723 GO:0009378 GO:0003724 GO:0016887 GO:0005524 GO:0009379 GO:0005657
Cluster-22178.67141 GO:0005515
Cluster-22178.67143 GO:0019049
Cluster-22178.67144 GO:0030151 GO:0030170 GO:0003824
Cluster-22178.67145 GO:0016192 GO:0006886 GO:0003824 GO:0005515 GO:0005198 GO:0016021 GO:0030126 GO:0016020
Cluster-22178.67147 GO:0006397 GO:0016567 GO:0006508 GO:0004252 GO:0003723 GO:0008233 GO:0030430 GO:0005634 GO:0005737
Cluster-22178.67149 GO:0006813 GO:0071805 GO:0007165 GO:0015079 GO:0016787 GO:0005509 GO:0016021 GO:0005887 GO:0005578
Cluster-22178.67151 GO:0016567 GO:0006397 GO:0006508 GO:0004252 GO:0003723 GO:0008233 GO:0030430 GO:0005634 GO:0005737
Cluster-22178.67154 GO:0006979 GO:0055114 GO:0006804 GO:0006749 GO:0006400 GO:0008616 GO:0016491 GO:0004602 GO:0016209 GO:0008479
Cluster-22178.67155 GO:0003677 GO:0005576
Cluster-22178.67158 GO:0007275 GO:0007165 GO:0006468 GO:0016055 GO:0005102 GO:0004672 GO:0005524 GO:0005576
Cluster-22178.67159 GO:0016567 GO:0046872 GO:0008270 GO:0005515 GO:0004842 GO:0005680
Cluster-22178.67162 GO:0006952
Cluster-22178.67164 GO:0006810 GO:0005622
Cluster-22178.67166 GO:0006468 GO:0005524 GO:0004672 GO:0016773 GO:0016020
Cluster-22178.67167 GO:0007165 GO:0006457 GO:0000774 GO:0051087 GO:0016853 GO:0042803 GO:0035091
Cluster-22178.67170 GO:0015684 GO:0007264 GO:0005525 GO:0003924

GO:0015093 GO:0016021
Cluster-22178.67173 GO:0005515
Cluster-22178.67174 GO:0006470 GO:0006570 GO:0042967 GO:0008138
GO:0004725 GO:0008080 GO:0005515
Cluster-22178.67175 GO:0006396 GO:0042967 GO:0030488 GO:0006464
GO:0046486 GO:0046500 GO:0008033 GO:0008610 GO:0009451
GO:0006479 GO:0016429 GO:0004144 GO:0008168 GO:0016627
GO:0004719 GO:0005622 GO:0031515
Cluster-22178.67179 GO:0007586 GO:0016042 GO:0008152 GO:0016758
GO:0008047 GO:0005576
Cluster-22178.67180 GO:0001558 GO:0006508 GO:0005515 GO:0004252
GO:0005520 GO:0005576 GO:0016942
Cluster-22178.67183 GO:0055085 GO:0006744 GO:0007411 GO:0006355
GO:0030001 GO:0016055 GO:0003677 GO:0005198 GO:0005515
GO:0046873 GO:0019013 GO:0016020 GO:0005576
Cluster-22178.67187 GO:0042967 GO:0016747 GO:0005515 GO:0008080

Cluster-22178.67189 GO:0006810 GO:0006310 GO:0006309 GO:0006260
GO:0006281 GO:0098519 GO:0003677 GO:0009378 GO:0016787
GO:0003724 GO:0005524 GO:0016887 GO:0003887 GO:0000166
GO:0003723 GO:0003676 GO:0009379 GO:0005657 GO:0009360
GO:0042575 GO:0005634 GO:0005737
Cluster-22178.67191 GO:0045947 GO:0008190
Cluster-22178.67194 GO:0006888 GO:0003676 GO:0005801 GO:0005622
Cluster-22178.67195 GO:0006886 GO:0008536
Cluster-22178.67196 GO:0042981
Cluster-22178.67199 GO:0006260 GO:0006281 GO:0070206 GO:0003824
GO:0005515 GO:0005096 GO:0034464
Cluster-22178.67200 GO:0006807 GO:0005975 GO:0009058 GO:0000917
GO:0006355 GO:0043093 GO:0004553 GO:0003933 GO:0043565
GO:0008270 GO:0005737
Cluster-22178.67201 GO:0006260 GO:0006914 GO:0045454 GO:0005509
GO:0016491 GO:0005815

Cluster-22178.67202 GO:0006281 GO:0007017 GO:0007018 GO:0006310
GO:0006260 GO:0003887 GO:0005515 GO:0003777 GO:0009378
GO:0016787 GO:0016818 GO:0003677 GO:0005524 GO:0016887
GO:0008017 GO:0005634 GO:0042575 GO:0005874 GO:0045298
GO:0009379 GO:0009360 GO:0005657
Cluster-22178.67204 GO:0015992 GO:0006308 GO:0015991 GO:0015078
GO:0003676 GO:0008855 GO:0009318 GO:0033179
Cluster-22178.67205 GO:0055114 GO:0005515
Cluster-22178.67206 GO:0055114 GO:0005515
Cluster-22178.67208 GO:0006376 GO:0015743 GO:0003729 GO:0005685
Cluster-22178.67209 GO:0006376 GO:0003729 GO:0005685
Cluster-22178.67210 GO:0043565 GO:0005634
Cluster-22178.67211 GO:0005515
Cluster-22178.67215 GO:0006898 GO:0007165 GO:0006464 GO:0005044
GO:0016020
Cluster-22178.67216 GO:0006118 GO:0009055 GO:0051536
Cluster-22178.67218 GO:0006928 GO:0008356 GO:0051321 GO:0051653
GO:0030036 GO:0033206 GO:0016482 GO:0007163 GO:0006952
GO:0003779 GO:0005524 GO:0030478 GO:0042995 GO:0005576
GO:0005885
Cluster-22178.67219 GO:0051603 GO:0003677 GO:0005515 GO:0004298
GO:0005839
Cluster-22178.67220 GO:0006412 GO:0042254 GO:0003735 GO:0005622
GO:0005840
Cluster-22178.67222 GO:0034968 GO:0006554 GO:0006355 GO:0006479
GO:0018024 GO:0019901 GO:0005694
Cluster-22178.67224 GO:0008152 GO:0055114 GO:0006355 GO:0003700
GO:0016491 GO:0005667
Cluster-22178.67226 GO:0005524
Cluster-22178.67228 GO:0009231 GO:0006771 GO:0003919 GO:0008270
GO:0016021
Cluster-22178.67232 GO:0006468 GO:0005524 GO:0004672 GO:0016773
GO:0016020

Cluster-22178.67233 GO:0006810 GO:0055085 GO:0005524 GO:0016887 GO:0042626 GO:0016021

Cluster-22178.67234 GO:0006508 GO:0006122 GO:0005515 GO:0004197 GO:0003677 GO:0008270 GO:0005743 GO:0005750

Cluster-22178.67235 GO:0006270 GO:0019079 GO:0006310 GO:0006816 GO:0006874 GO:0007155 GO:0002949 GO:0006355 GO:0006281 GO:0007049 GO:0009378 GO:0003724 GO:0005524 GO:0016887 GO:0005515 GO:0004252 GO:0008134 GO:0003723 GO:0004190 GO:0003678 GO:0005219 GO:0005667 GO:0009379 GO:0005657 GO:0005622 GO:0005886 GO:0016021

Cluster-22178.67238 GO:0035556 GO:0000226 GO:0046486 GO:0009395 GO:0008654 GO:0007021 GO:0090063 GO:0006506 GO:0000725 GO:0004143 GO:0015631 GO:0016746 GO:0051082 GO:0005515 GO:0005815 GO:0016020 GO:0097196 GO:0005789 GO:0016021 GO:0045298 GO:0000922 GO:0005634

Cluster-22178.67239 GO:0006355 GO:0006289 GO:0005515 GO:0016020 GO:0000439

Cluster-22178.67240 GO:0010972 GO:0030336 GO:0016579 GO:0036459

Cluster-22178.67243 GO:0019491 GO:0007034 GO:0033990

Cluster-22178.67245 GO:0006812 GO:0052331 GO:0046931 GO:0006952 GO:0007165 GO:0006810 GO:0015267 GO:0005126 GO:0046930 GO:0005576

Cluster-22178.67246 GO:0006481 GO:0006479 GO:0006412 GO:0042254 GO:0004671 GO:0003735 GO:0005622 GO:0005840 GO:0016021

Cluster-22178.67248 GO:0005515

Cluster-22178.67252 GO:0006281 GO:0016032 GO:0006508 GO:0006351 GO:0006206 GO:0006144 GO:0006310 GO:0003678 GO:0003899 GO:0005524 GO:0008270 GO:0016887 GO:0004222 GO:0009378 GO:0003677 GO:0016021 GO:0005657 GO:0005730 GO:0009379

Cluster-22178.67253 GO:0046872

Cluster-22178.67254 GO:0046740 GO:0005515 GO:0016021

Cluster-22178.67255 GO:0008152 GO:0046872 GO:0016746

Cluster-22178.67256 GO:0018101 GO:0006807 GO:0005509 GO:0004668
GO:0005737
Cluster-22178.67257 GO:0006468 GO:0006810 GO:0015696 GO:0008519
GO:0016787 GO:0005524 GO:0004672 GO:0031224 GO:0016020
GO:0016021 GO:0005794
Cluster-22178.67259 GO:0046983 GO:0003677
Cluster-22178.67260 GO:0046983 GO:0003677
Cluster-22178.67265 GO:0007165 GO:0035556 GO:0003774 GO:0005524
GO:0005515 GO:0016459
Cluster-22178.67266 GO:0006904 GO:0016874 GO:0000145
Cluster-22178.67267 GO:0007165 GO:0035556 GO:0005515 GO:0003774
GO:0005524 GO:0016459
Cluster-22178.67268 GO:0006355 GO:0016055 GO:0006886 GO:0000975
GO:0008536 GO:0003700 GO:0005667
Cluster-22178.67269 GO:0006508 GO:0006260 GO:0019073 GO:0006139
GO:0003677 GO:0003887 GO:0008408 GO:0008270 GO:0004222
GO:0003676 GO:0042575 GO:0031012
Cluster-22178.67271 GO:0005515
Cluster-22178.67274 GO:0006606 GO:0006914 GO:0031083 GO:0016021
Cluster-22178.67277 GO:0055085 GO:0003677 GO:0022857 GO:0005515
GO:0016021
Cluster-22178.67279 GO:0042981
Cluster-22178.67281 GO:0016579 GO:0005515 GO:0036459 GO:0005096

Cluster-22178.67282 GO:0016310 GO:0006511 GO:0009069 GO:0006468
GO:0004674 GO:0005524 GO:0031625
Cluster-22178.67285 GO:0006810 GO:0009264 GO:0005515 GO:0005524
GO:0046872 GO:0043531 GO:0008253 GO:0004386 GO:0003676
GO:0016020
Cluster-22178.67287 GO:0016021
Cluster-22178.67288 GO:0042254 GO:0006412 GO:0003735 GO:0005840
GO:0005622
Cluster-22178.67289 GO:0050992 GO:0019288 GO:0046872 GO:0051745

GO:0005515
Cluster-22178.67291 GO:0007018 GO:0005515 GO:0005868
Cluster-22178.67293 GO:0007165 GO:0043401 GO:0006355 GO:0003700
GO:0005186 GO:0043565 GO:0008270 GO:0005615 GO:0005634
GO:0005667
Cluster-22178.67294 GO:0055114 GO:0010277 GO:0005515
Cluster-22178.67295 GO:0055114 GO:0019478 GO:0006355 GO:0003676
GO:0003700 GO:0016491 GO:0043565 GO:0016788 GO:0005515
GO:0050662 GO:0005737 GO:0016020 GO:0005667
Cluster-22178.67296 GO:0006886 GO:0019904 GO:0035091 GO:0019028
Cluster-22178.67298 GO:0030193 GO:0007155 GO:0005515 GO:0005639
GO:0005576
Cluster-22178.67299 GO:0005515
Cluster-22178.67300 GO:0006570 GO:0006470 GO:0050852 GO:0004725

Cluster-22178.67301 GO:0006687 GO:0050290
Cluster-22178.67302 GO:0006260 GO:0016192 GO:0019079 GO:0005524
GO:0003678 GO:0003676 GO:0016787 GO:0005515 GO:0003677
GO:0016021 GO:0005657
Cluster-22178.67303 GO:0050830 GO:0006807 GO:0006144 GO:0019497
GO:0006771 GO:0006164 GO:0003993 GO:0003937 GO:0004643
GO:0031423 GO:0046872 GO:0005515 GO:0042720 GO:0019012
GO:0005576
Cluster-22178.67306 GO:0005515
Cluster-22178.67307 GO:0006486 GO:0004576 GO:0016020 GO:0008250
Cluster-22178.67308 GO:0006886 GO:0006446 GO:0006270 GO:0006413
GO:0003743 GO:0003723 GO:0004190 GO:0016021 GO:0005840
GO:0005741
Cluster-22178.67312 GO:0006606 GO:0071988 GO:0006308 GO:0048870
GO:0006355 GO:0030474 GO:0070206 GO:0008053 GO:0006511
GO:0007275 GO:0003924 GO:0003723 GO:0042803 GO:0005198
GO:0005515 GO:0008270 GO:0005524 GO:0004386 GO:0046872
GO:0003700 GO:0045502 GO:0008134 GO:0008855 GO:0005634

GO:0005667 GO:0005741 GO:0016021 GO:0019031 GO:0019028
GO:0071203 GO:0031514 GO:0009318 GO:0030286
Cluster-22178.67313 GO:0006355 GO:0003677 GO:0005634
Cluster-22178.67315 GO:0006468 GO:0005524 GO:0004672 GO:0016773
GO:0016020
Cluster-22178.67317 GO:0006260 GO:0003677 GO:0005664
Cluster-22178.67320 GO:0006508 GO:0008234 GO:0005742
Cluster-22178.67321 GO:0006468 GO:0005524 GO:0004672 GO:0016773
GO:0016020
Cluster-22178.67322 GO:0006813 GO:0009405 GO:0016567 GO:0005249
GO:0004842 GO:0030955 GO:0005515 GO:0042802 GO:0016021
GO:0008076
Cluster-22178.67323 GO:0045893 GO:1902208 GO:0005975 GO:0006810
GO:0008270 GO:0005215 GO:0003950 GO:0016832 GO:0005515
GO:0003677 GO:0005737 GO:0016021 GO:0005634
Cluster-22178.67325 GO:0007186 GO:0004930 GO:0005515 GO:0016021
Cluster-22178.67326 GO:0006432 GO:0000162 GO:0006571 GO:0009094
GO:0005515 GO:0003723 GO:0004826 GO:0009328
Cluster-22178.67327 GO:0051603 GO:0006511 GO:0004175 GO:0004298
GO:0005839 GO:0019773 GO:0016021
Cluster-22178.67328 GO:0005634
Cluster-22178.67329 GO:0006807 GO:0000256 GO:0006144 GO:0004037
GO:0008168 GO:0005634
Cluster-22178.67330 GO:0006413 GO:0045881 GO:0006446 GO:0032183
GO:0070140 GO:0003723 GO:0005515 GO:0003743 GO:0005737
GO:0005840
Cluster-22178.67331 GO:0016021
Cluster-22178.67332 GO:0002949 GO:0006614 GO:0016887 GO:0003924
GO:0005524 GO:0005525
Cluster-22178.67333 GO:0005515
Cluster-22178.67334 GO:0007229 GO:0007155 GO:0007275 GO:0007165
GO:0046982 GO:0001968 GO:0004872 GO:0016021
Cluster-22178.67336 GO:0006412 GO:0042254 GO:0003735 GO:0005840

Cluster-22178.67338 GO:0009966
Cluster-22178.67339 GO:0008152
Cluster-22178.67341 GO:0007264 GO:0005525 GO:0003924
Cluster-22178.67343 GO:0007165 GO:0004872 GO:0016020
Cluster-22178.67345 GO:0006810 GO:0003677 GO:0003676 GO:0004386 GO:0005524 GO:0016787
Cluster-22178.67348 GO:0005515 GO:0046872 GO:0008270
Cluster-22178.67349 GO:0055085 GO:0006810 GO:0005215 GO:0016021 GO:0016020
Cluster-22178.67350 GO:0005515 GO:0003677
Cluster-22178.67351 GO:0016579 GO:0006276 GO:0005975 GO:0008657 GO:0004553 GO:0008270 GO:0036459
Cluster-22178.67352 GO:0007049 GO:0006355 GO:0009245 GO:0006260 GO:0010506 GO:0032324 GO:0007264 GO:0003700 GO:0008915 GO:0043565 GO:0005515 GO:0008017 GO:0008270 GO:0005096 GO:0005615 GO:0045298 GO:0016021 GO:0005815 GO:0005667
Cluster-22178.67353 GO:0006418 GO:0043039 GO:0004866 GO:0003676 GO:0005524 GO:0000049 GO:0000166 GO:0004812 GO:0005737
Cluster-22178.67354 GO:0006418 GO:0043039 GO:0005524 GO:0003676 GO:0004866 GO:0004812 GO:0000049 GO:0000166 GO:0005737
Cluster-22178.67355 GO:0007165 GO:0008601 GO:0000159
Cluster-22178.67356 GO:0005509
Cluster-22178.67357 GO:0008270 GO:0042162 GO:0003723 GO:0042803

Cluster-22178.67358 GO:0000290 GO:0042773 GO:0006744 GO:0015992 GO:0006814 GO:0000902 GO:0006120 GO:0055114 GO:0043085 GO:0005975 GO:0016787 GO:0016773 GO:0005515 GO:0008137 GO:0005525 GO:0008047 GO:0016020
Cluster-22178.67362 GO:0046660 GO:0006397 GO:0005634
Cluster-22178.67364 GO:0015940 GO:0006571 GO:0006694 GO:0019521 GO:0055114 GO:0009094 GO:0008210 GO:0000162 GO:0046168 GO:0017038 GO:0008207 GO:0008209 GO:0006098 GO:0004665 GO:0003824 GO:0008677 GO:0016491 GO:0008977 GO:0004616

GO:0016616 GO:0003854 GO:0050662 GO:0051287 GO:0016020
Cluster-22178.67366 GO:0006396 GO:0003677 GO:0005515 GO:0003723
GO:0019013
Cluster-22178.67367 GO:0000045 GO:0005737
Cluster-22178.67370 GO:0051205 GO:0016021
Cluster-22178.67371 GO:0016020
Cluster-22178.67373 GO:0016020
Cluster-22178.67375 GO:0006813 GO:0005515 GO:0005242 GO:0016021
GO:0008076
Cluster-22178.67376 GO:0005515
Cluster-22178.67381 GO:0006355 GO:0009252 GO:0046436 GO:0003700
GO:0008716 GO:0005622 GO:0005667
Cluster-22178.67384 GO:0007264 GO:0006357 GO:0001104 GO:0005515
GO:0005525 GO:0003924 GO:0008641 GO:0016592
Cluster-22178.67385 GO:0007264 GO:0006357 GO:0005515 GO:0001104
GO:0005525 GO:0008641 GO:0003924 GO:0016592
Cluster-22178.67386 GO:0005975 GO:0004553
Cluster-22178.67390 GO:1901031 GO:0006364 GO:0030688 GO:0005634
Cluster-22178.67391 GO:0019028
Cluster-22178.67392 GO:0006508 GO:0004181 GO:0008270
Cluster-22178.67395 GO:0006810 GO:0006816 GO:0042967 GO:0016573
GO:0006355 GO:0003713 GO:0004402 GO:0003712 GO:0008270
GO:0005515 GO:0005246 GO:0042030 GO:0016020 GO:0005667
GO:0000123 GO:0005634
Cluster-22178.67396 GO:0006950 GO:0042967 GO:0006270 GO:0016573
GO:0006457 GO:0006355 GO:0003712 GO:0008270 GO:0005524
GO:0051082 GO:0004402 GO:0000123 GO:0005667 GO:0005634
Cluster-22178.67397 GO:0005975 GO:0015926
Cluster-22178.67398 GO:0015979 GO:0007067 GO:0003677 GO:0009522
GO:0016020 GO:0042729 GO:0072686 GO:0005634
Cluster-22178.67399 GO:0006865 GO:0008168 GO:0016021 GO:0016020
Cluster-22178.67400 GO:0006122 GO:0006508 GO:0003677 GO:0004197
GO:0005515 GO:0008270 GO:0005750 GO:0005743

Cluster-22178.67401 GO:0006260 GO:0003887 GO:0005515 GO:0042575
Cluster-22178.67403 GO:0006118 GO:0020037 GO:0009055 GO:0016021
Cluster-22178.67405 GO:0006281 GO:0006289 GO:0043161 GO:0015031
GO:0005515 GO:0003684 GO:0030176
Cluster-22178.67406 GO:0050792 GO:0042025
Cluster-22178.67408 GO:0046872
Cluster-22178.67411 GO:0003677 GO:0005576
Cluster-22178.67413 GO:0006355 GO:0042173 GO:0005509 GO:0003676
GO:0003700 GO:0008270 GO:0005667 GO:0005737
Cluster-22178.67414 GO:0042254 GO:0003735 GO:0005840 GO:0005739
Cluster-22178.67415 GO:0042254 GO:0003735 GO:0005739 GO:0005840
Cluster-22178.67416 GO:0006098 GO:0007264 GO:0050792 GO:0006810
GO:0009396 GO:0046487 GO:0009405 GO:0055114 GO:0019521
GO:0004488 GO:0004616 GO:0008200 GO:0004518 GO:0003824
GO:0005525 GO:0005576
Cluster-22178.67417 GO:0042967 GO:0016573 GO:0006355 GO:0003713
GO:0004402 GO:0000123 GO:0005634 GO:0005667
Cluster-22178.67419 GO:0006626 GO:0016746 GO:0005741
Cluster-22178.67421 GO:0008152 GO:0007165 GO:0003676 GO:0003824
GO:0005179 GO:0005576
Cluster-22178.67423 GO:0015031 GO:0030176
Cluster-22178.67424 GO:0006820 GO:0006835 GO:0006812 GO:0017153
GO:0016020
Cluster-22178.67428 GO:0005524 GO:0005515 GO:0003676 GO:0005634
Cluster-22178.67430 GO:0007186 GO:0022904 GO:0004930 GO:0016021
GO:0016020 GO:0005741
Cluster-22178.67431 GO:0046872 GO:0045202
Cluster-22178.67433 GO:0006468 GO:0005524 GO:0004672
Cluster-22178.67436 GO:0009405 GO:0006952 GO:0007275 GO:0016567
GO:0006511 GO:0008270 GO:0046872 GO:0004842 GO:0005515
GO:0005680 GO:0005576 GO:0005634
Cluster-22178.67437 GO:0006950 GO:0005515
Cluster-22178.67438 GO:0006457 GO:0006810 GO:0006904 GO:0007155

GO:0006582 GO:0015031 GO:0042803 GO:0051087 GO:0003677
GO:0030411 GO:0000774 GO:0015288 GO:0016020 GO:0000145
Cluster-22178.67440 GO:0007283 GO:0003676 GO:0003677 GO:0008270
GO:0019013 GO:0000786 GO:0005634
Cluster-22178.67441 GO:0003723
Cluster-22178.67442 GO:0016192 GO:0006886 GO:0003824 GO:0005515
GO:0005198 GO:0016021 GO:0030126 GO:0016020
Cluster-22178.67444 GO:0003723
Cluster-22178.67446 GO:0006506 GO:0006468 GO:0005515 GO:0004386
GO:0016773 GO:0004672 GO:0005524 GO:0016020 GO:0031083
GO:0005789
Cluster-22178.67447 GO:0016301
Cluster-22178.67448 GO:0007165 GO:0008270 GO:0004888 GO:0016021
Cluster-22178.67449 GO:0007264 GO:0015684 GO:0015093 GO:0003924
GO:0005525 GO:0016021
Cluster-22178.67451 GO:0016787
Cluster-22178.67452 GO:0032259 GO:0008168
Cluster-22178.67453 GO:0040007 GO:0006955 GO:0042981 GO:0007264
GO:0008283 GO:0007165 GO:0006869 GO:0043087 GO:0048193
GO:0005515 GO:0008083 GO:0005319 GO:0005085 GO:0005134
GO:0005576 GO:0005893 GO:0016020 GO:0005887
Cluster-22178.67455 GO:0006355 GO:0006352 GO:0003700 GO:0003677
GO:0016987 GO:0005667
Cluster-22178.67457 GO:0006974 GO:0006118 GO:0051726 GO:0007275
GO:0006355 GO:0003700 GO:0009055 GO:0051536 GO:0043565
GO:0008270 GO:0005634 GO:0005667
Cluster-22178.67458 GO:0004869
Cluster-22178.67459 GO:0006468 GO:0016773 GO:0004672 GO:0005524
GO:0016020
Cluster-22178.67460 GO:0015992 GO:0015986 GO:0015078 GO:0000276
Cluster-22178.67461 GO:0008033 GO:0009094 GO:0000162 GO:0006571
GO:0006432 GO:0008168 GO:0005524 GO:0004826 GO:0000287
GO:0000049 GO:0009328

Cluster-22178.67462 GO:0007016 GO:0016010
Cluster-22178.67464 GO:0015991 GO:0007165 GO:0015992 GO:0046034
GO:0004888 GO:0016820 GO:0005524 GO:0033178 GO:0016021
Cluster-22178.67467 GO:0005515 GO:0046872
Cluster-22178.67468 GO:0006457 GO:0005515 GO:0005615
Cluster-22178.67469 GO:0007178 GO:0016310 GO:0009069 GO:0006468
GO:0005524 GO:0004672 GO:0004675 GO:0003677 GO:0016020
Cluster-22178.67470 GO:0006448 GO:0006414 GO:0007005 GO:0003746
GO:0003950 GO:0032865 GO:0005840
Cluster-22178.67477 GO:0008168 GO:0005515
Cluster-22178.67480 GO:0005515
Cluster-22178.67481 GO:0006508 GO:0055114 GO:0008152 GO:0008270
GO:0016787 GO:0047134 GO:0008236
Cluster-22178.67482 GO:0055114 GO:0042254 GO:0006412 GO:0006118
GO:0051537 GO:0003735 GO:0009496 GO:0005840 GO:0009512
GO:0005622 GO:0042651 GO:0005739
Cluster-22178.67483 GO:0009968 GO:0005737
Cluster-22178.67485 GO:0006367 GO:0006355 GO:0032968 GO:0003700
GO:0003677 GO:0003682 GO:0000775 GO:0000785 GO:0005667
GO:0005634
Cluster-22178.67487 GO:0046872
Cluster-22178.67488 GO:0006486 GO:0006355 GO:0003677 GO:0008417
GO:0003700 GO:0016020 GO:0005667
Cluster-22178.67489 GO:0006355 GO:0006275 GO:0007224 GO:0016032

Cluster-22178.67491 GO:0006351 GO:0042742 GO:0016998 GO:0006032
GO:0006206 GO:0006144 GO:0003677 GO:0003899 GO:0004568
GO:0003723 GO:0005730 GO:0005576
Cluster-22178.67492 GO:0005524
Cluster-22178.67493 GO:0006313 GO:0045444 GO:0003677 GO:0004803

Cluster-22178.67495 GO:0006508 GO:0004190
Cluster-22178.67498 GO:0008152 GO:0009877 GO:0009312 GO:0008168

GO:0008757 GO:0005634
Cluster-22178.67499 GO:0042981 GO:0005515
Cluster-22178.67503 GO:0006139 GO:0003676 GO:0008408
Cluster-22178.67505 GO:0006355 GO:0003700 GO:0005667
Cluster-22178.67507 GO:0007165 GO:0008601 GO:0008270 GO:0000159
Cluster-22178.67514 GO:0007275 GO:0007165 GO:0008152 GO:0016624
GO:0005179 GO:0047355 GO:0008270 GO:0016020 GO:0005576
Cluster-22178.67515 GO:0006355 GO:0003677
Cluster-22178.67517 GO:0006914 GO:0055085 GO:0006821 GO:0007005
GO:0005247 GO:0031305 GO:0016020
Cluster-22178.67519 GO:0007267 GO:0006508 GO:0006355 GO:0008270
GO:0008233 GO:0003700 GO:0005634 GO:0005667
Cluster-22178.67523 GO:0007264 GO:0005525
Cluster-22178.67524 GO:0006270 GO:0016020
Cluster-22178.67525 GO:0006397 GO:0046872
Cluster-22178.67527 GO:0006810 GO:0009405 GO:0008200 GO:0005515
GO:0042151
Cluster-22178.67528 GO:0006468 GO:0004672 GO:0005524 GO:0016020
Cluster-22178.67529 GO:0006574 GO:0008033 GO:0055114 GO:0008152
GO:0006813 GO:0006568 GO:0006550 GO:0006552 GO:0006554
GO:0046168 GO:0006779 GO:0006633 GO:0016117 GO:0018874
GO:0019354 GO:0006631 GO:0003824 GO:0003857 GO:0043115
GO:0016705 GO:0016491 GO:0050660 GO:0051287 GO:0071949
GO:0016616
Cluster-22178.67530 GO:0005509 GO:0005515
Cluster-22178.67532 GO:0046740 GO:0016226 GO:0005506 GO:0005515
GO:0051536 GO:0003676 GO:0005634 GO:0016021
Cluster-22178.67534 GO:0003779
Cluster-22178.67535 GO:0006464 GO:0015031
Cluster-22178.67536 GO:0051603 GO:0006511 GO:0004175 GO:0004298
GO:0005839 GO:0019773
Cluster-22178.67537 GO:0008033 GO:0055114 GO:0006914 GO:0006308
GO:0019049 GO:0006886 GO:0006605 GO:0008855 GO:0016491

GO:0050660 GO:0071949 GO:0005742 GO:0009318
Cluster-22178.67538 GO:0005515
Cluster-22178.67539 GO:0006935 GO:0042407 GO:0006351 GO:0006886
GO:0006206 GO:0006144 GO:0090263 GO:0016567 GO:0071973
GO:0016192 GO:0046872 GO:0005515 GO:0003899 GO:0003774
GO:0003677 GO:0009288 GO:0044284 GO:0016021 GO:0030117
GO:0061617 GO:0005730 GO:0016020
Cluster-22178.67542 GO:0003676
Cluster-22178.67547 GO:0005515
Cluster-22178.67548 GO:0006123 GO:0015992 GO:0004129 GO:0045277
Cluster-22178.67549 GO:0006468 GO:0046039 GO:0009190 GO:0008152
GO:0006182 GO:0035556 GO:0006144 GO:0006357 GO:0001104
GO:0004383 GO:0016849 GO:0003723 GO:0003824 GO:0004672
GO:0008270 GO:0005524 GO:0016592
Cluster-22178.67552 GO:0006810 GO:0005622
Cluster-22178.67553 GO:0006308 GO:0006289 GO:0006285 GO:0042384
GO:0006281 GO:0019900 GO:0003684 GO:0008534 GO:0034464
Cluster-22178.67554 GO:0006289 GO:0006281 GO:0006284 GO:0008270
GO:0003906 GO:0016799 GO:0003684
Cluster-22178.67555 GO:0007165 GO:0005975 GO:0003676 GO:0008270
GO:0008184 GO:0003723
Cluster-22178.67556 GO:0008152 GO:0006468 GO:0046039 GO:0009190
GO:0035556 GO:0006357 GO:0006144 GO:0006182 GO:0003824
GO:0004672 GO:0001104 GO:0003723 GO:0016849 GO:0004383
GO:0008270 GO:0005524 GO:0016592
Cluster-22178.67559 GO:0043087 GO:0035023 GO:0043565 GO:0005089

Cluster-22178.67560 GO:0006397 GO:0003723 GO:0005634 GO:0005737
Cluster-22178.67561 GO:0007218 GO:0042254 GO:0006412 GO:0005185
GO:0003735 GO:0005622 GO:0005840 GO:0005576
Cluster-22178.67563 GO:0006811 GO:0005515 GO:0016020
Cluster-22178.67564 GO:0046872
Cluster-22178.67565 GO:0005198 GO:0005515

Cluster-22178.67567 GO:0018342 GO:0055114 GO:0016491 GO:0008318

Cluster-22178.67568 GO:0018342 GO:0008318
Cluster-22178.67569 GO:0009636 GO:0006955 GO:0031342 GO:0015643 GO:0019814
Cluster-22178.67571 GO:0006355 GO:0043565 GO:0046872
Cluster-22178.67573 GO:0015031 GO:0006464 GO:0030488 GO:0046500 GO:0006364 GO:0008033 GO:0055114 GO:0006725 GO:0007010 GO:0006479 GO:0009451 GO:0008270 GO:0010277 GO:0016491 GO:0016429 GO:0016772 GO:0004719 GO:0003779 GO:0008168 GO:0003723 GO:0016301 GO:0008198 GO:0031515 GO:0005615
Cluster-22178.67575 GO:0006771 GO:0042546 GO:0009247 GO:0001575 GO:0005975 GO:0019497 GO:0003723 GO:0008107 GO:0005515 GO:0046872 GO:0003993 GO:0016020
Cluster-22178.67576 GO:0005515
Cluster-22178.67577 GO:0006144 GO:0006526 GO:0006531 GO:0006522 GO:0006529 GO:0008152 GO:0008033 GO:0006541 GO:0006536 GO:0006560 GO:0006164 GO:0006177 GO:0005524 GO:0004066 GO:0016787 GO:0003824 GO:0004055 GO:0004810 GO:0003922
Cluster-22178.67578 GO:0005515
Cluster-22178.67579 GO:0016021
Cluster-22178.67581 GO:0006397 GO:0003723 GO:0008270 GO:0005634 GO:0005737
Cluster-22178.67585 GO:0006260 GO:0006144 GO:0019064 GO:0043093 GO:0000390 GO:0006351 GO:0000917 GO:0006886 GO:0008270 GO:0005096 GO:0003968 GO:0003723 GO:0016020 GO:0071008 GO:0019031 GO:0005801 GO:0005737 GO:0031379
Cluster-22178.67587 GO:0009190 GO:0035556 GO:0007283 GO:0016849 GO:0003677 GO:0005634 GO:0000786
Cluster-22178.67590 GO:0006355 GO:0003677
Cluster-22178.67591 GO:0042254 GO:0006412 GO:0003735 GO:0005622 GO:0005840
Cluster-22178.67592 GO:0055114 GO:0016567 GO:0016706 GO:0004842

GO:0005515
Cluster-22178.67594 GO:0048870 GO:0019904 GO:0031514
Cluster-22178.67595 GO:0006265 GO:0005524 GO:0003918 GO:0003677
GO:0005634
Cluster-22178.67598 GO:0071436 GO:0006525 GO:0006260 GO:0006914
GO:0006560 GO:0006814 GO:0006886 GO:0006355 GO:0006090
GO:0048870 GO:0015081 GO:0008948 GO:0003700 GO:0043565
GO:0005783 GO:0016020 GO:0005667 GO:0031514 GO:0005801
GO:0016021
Cluster-22178.67601 GO:0055114 GO:0008033 GO:0045454 GO:0006355
GO:0016491 GO:0050660 GO:0003677
Cluster-22178.67603 GO:0006812 GO:0032801 GO:0019076 GO:0005261
GO:0033644
Cluster-22178.67605 GO:0006122 GO:0003676 GO:0005750
Cluster-22178.67609 GO:0005515
Cluster-22178.67610 GO:0015979 GO:0016021
Cluster-22178.67614 GO:0016310 GO:0009069 GO:0006468 GO:0004674
GO:0004672 GO:0005524 GO:0016773 GO:0016020
Cluster-22178.67615 GO:0006725 GO:0008033 GO:0055114 GO:0009451
GO:0007010 GO:0006479 GO:0006468 GO:0006464 GO:0030488
GO:0015031 GO:0006364 GO:0046500 GO:0003779 GO:0008168
GO:0004719 GO:0004672 GO:0016301 GO:0008198 GO:0003723
GO:0016772 GO:0016429 GO:0016491 GO:0010277 GO:0005524
GO:0031515
Cluster-22178.67617 GO:0042254 GO:0006412 GO:0000723 GO:0003677
GO:0003735 GO:0005622 GO:0000784 GO:0005840
Cluster-22178.67618 GO:0006508 GO:0008236
Cluster-22178.67620 GO:0003676 GO:0003677 GO:0016787 GO:0005524

Cluster-22178.67622 GO:0000166 GO:0046872
Cluster-22178.67625 GO:0006810 GO:0005215
Cluster-22178.67626 GO:0016579 GO:0016567 GO:0044237 GO:0019634
GO:0004842 GO:0003824 GO:0036459 GO:0005680

Cluster-22178.67629 GO:0006000 GO:0043087 GO:0006013 GO:0006012
GO:0006098 GO:0006457 GO:0006096 GO:0006470 GO:0006094
GO:0006570 GO:0003872 GO:0004725 GO:0051082 GO:0005515
GO:0005945 GO:0016272 GO:0005639 GO:0016020
Cluster-22178.67630 GO:0006281 GO:0006355 GO:0051276 GO:0003677
GO:0005515 GO:0005524 GO:0008270 GO:0008168 GO:0005694
Cluster-22178.67632 GO:0006352 GO:0042254 GO:0006412 GO:0006468
GO:0005524 GO:0004672 GO:0003677 GO:0003735 GO:0005840
GO:0005669 GO:0005622
Cluster-22178.67633 GO:0006486 GO:0006508 GO:0003676 GO:0004222
GO:0004576 GO:0003677 GO:0008250 GO:0016020
Cluster-22178.67635 GO:0006468 GO:0006886 GO:0007094 GO:0016310
GO:0009069 GO:0006606 GO:0006355 GO:0048870 GO:0006457
GO:0006810 GO:0032259 GO:0051607 GO:0015031 GO:0010506
GO:0003723 GO:0051082 GO:0042803 GO:0043565 GO:0008565
GO:0005524 GO:0045502 GO:0008134 GO:0003700 GO:0004674
GO:0005737 GO:0005615 GO:0005667 GO:0016020 GO:0016272
GO:0031514 GO:0005845 GO:0005801 GO:0016021 GO:0030286
GO:0005886
Cluster-22178.67637 GO:0006694 GO:0043093 GO:0000917 GO:0006869
GO:0008299 GO:0005524 GO:0016887 GO:0046983 GO:0005525
GO:0050518 GO:0005319 GO:0005737
Cluster-22178.67638 GO:0048193 GO:0003676 GO:0008270 GO:0046872
GO:0016020
Cluster-22178.67639 GO:0046488 GO:0006887 GO:0016307 GO:0008289
GO:0000145 GO:0019031
Cluster-22178.67640 GO:0005515
Cluster-22178.67643 GO:0051015 GO:0030674
Cluster-22178.67647 GO:0030150 GO:0055114 GO:0008033 GO:0045454
GO:0006118 GO:0016491 GO:0009055 GO:0050660 GO:0015035
GO:0005515 GO:0005742
Cluster-22178.67650 GO:0006914
Cluster-22178.67652 GO:0045132 GO:0000917 GO:0046487 GO:0007094

GO:0055114 GO:0008152 GO:0007049 GO:0043093 GO:0009396
GO:0000226 GO:0005515 GO:0008092 GO:0003824 GO:0016787
GO:0004488 GO:0005634 GO:0015630 GO:0005737 GO:0016021
GO:0000775 GO:0019898
Cluster-22178.67653 GO:0016787
Cluster-22178.67654 GO:0006418 GO:0006139 GO:0004812 GO:0008026
GO:0016818 GO:0000166 GO:0005524 GO:0003676
Cluster-22178.67655 GO:0003676 GO:0008270
Cluster-22178.67656 GO:0006810 GO:0007165 GO:0006935 GO:0004888
GO:0016020 GO:0005643
Cluster-22178.67657 GO:0005515
Cluster-22178.67658 GO:0006357 GO:0001104 GO:0016592
Cluster-22178.67659 GO:0015979 GO:0016020 GO:0009523 GO:0009539
Cluster-22178.67662 GO:0043066 GO:0016310 GO:0043123 GO:0045766
GO:0007165 GO:0009069 GO:0070008 GO:0016772 GO:0004674
GO:0004252 GO:0005515 GO:0005044 GO:0016020
Cluster-22178.67664 GO:0055114 GO:0016972 GO:0005515
Cluster-22178.67668 GO:0006359 GO:0030036 GO:0007275 GO:0016043
GO:0017048 GO:0003779
Cluster-22178.67670 GO:0008152 GO:0006974 GO:0006915 GO:0016491
GO:0000228
Cluster-22178.67671 GO:0006396 GO:0051252 GO:0008033 GO:0003676
GO:0004526 GO:0030677
Cluster-22178.67675 GO:0006357 GO:0035091 GO:0001104 GO:0016592
Cluster-22178.67676 GO:0055114 GO:0006000 GO:0006013 GO:0005515
GO:0016301 GO:0003873 GO:0016740 GO:0016625 GO:0016491
GO:0051536 GO:0005524
Cluster-22178.67679 GO:0015031 GO:0006772 GO:0006464 GO:0009228
GO:0006886 GO:0003924 GO:0008270 GO:0052855 GO:0005525
GO:0005515 GO:0005047 GO:0004417 GO:0005786 GO:0005785
Cluster-22178.67680 GO:0005515
Cluster-22178.67685 GO:0016192 GO:0006886 GO:0015991 GO:0055114
GO:0016820 GO:0005515 GO:0016491 GO:0030117 GO:0000221

Cluster-22178.67687 GO:0055114 GO:0006537 GO:0042967 GO:0006807 GO:0005515 GO:0015930 GO:0003676 GO:0016407

Cluster-22178.67688 GO:0042967 GO:0006807 GO:0055114 GO:0006537 GO:0015930 GO:0005515 GO:0003676 GO:0016407

Cluster-22178.67691 GO:0050832 GO:0042742 GO:0019239

Cluster-22178.67692 GO:0007268 GO:0033130 GO:0043495

Cluster-22178.67694 GO:0003677 GO:0005634

Cluster-22178.67696 GO:0042254 GO:0006412 GO:0019843 GO:0003735 GO:0005622 GO:0005840

Cluster-22178.67697 GO:0006355 GO:0006464 GO:0006265 GO:0003677 GO:0016151 GO:0003916 GO:0008270 GO:0005694

Cluster-22178.67698 GO:0008270 GO:0003676

Cluster-22178.67699 GO:0004866

Cluster-22178.67701 GO:0006810 GO:0009401 GO:0005886

Cluster-22178.67702 GO:0006810 GO:0007165 GO:0007186 GO:0000166 GO:0003677 GO:0016787 GO:0005525 GO:0004871 GO:0031683 GO:0005524 GO:0019001 GO:0016887 GO:0003924 GO:0005747 GO:0005739

Cluster-22178.67704 GO:0006457 GO:0051082 GO:0016272

Cluster-22178.67705 GO:0006468 GO:0016773 GO:0005524 GO:0004672 GO:0016020

Cluster-22178.67710 GO:0006231 GO:0006206 GO:0009405 GO:0004799 GO:0005576

Cluster-22178.67711 GO:0006955 GO:0006412 GO:0006000 GO:0006013 GO:0006004 GO:0042254 GO:0007165 GO:0005125 GO:0003676 GO:0008736 GO:0003723 GO:0003735 GO:0005737 GO:0005840 GO:0005634 GO:0005576 GO:0005622

Cluster-22178.67714 GO:0006511 GO:0016567 GO:0007017 GO:0007018 GO:0034450 GO:0005524 GO:0008017 GO:0004842 GO:0005515 GO:0005198 GO:0003777 GO:0019028 GO:0000151 GO:0016021 GO:0045298 GO:0005874

Cluster-22178.67718 GO:0006811 GO:0007155 GO:0006355 GO:0007165 GO:0051225 GO:0004871 GO:0003723 GO:0003677 GO:0009288

GO:0070652 GO:0005634 GO:0016020
Cluster-22178.67719 GO:0009405 GO:0045735 GO:0046872
Cluster-22178.67721 GO:0006508 GO:0006303 GO:0004003 GO:0004222
GO:0003677 GO:0005815 GO:0005657
Cluster-22178.67725 GO:0009405 GO:0005515
Cluster-22178.67729 GO:0009236 GO:0019236 GO:0016310 GO:0007606
GO:0007186 GO:0004932 GO:0003824 GO:0016772 GO:0016020
Cluster-22178.67731 GO:0015940 GO:0006139 GO:0015937 GO:0008152
GO:0030474 GO:0009405 GO:0030254 GO:0006206 GO:0006810
GO:0005200 GO:0004140 GO:0005515 GO:0016301 GO:0004127
GO:0005524 GO:0005856 GO:0005823 GO:0042597 GO:0030257
Cluster-22178.67732 GO:0006810 GO:0006206 GO:0009405 GO:0030474
GO:0030254 GO:0008152 GO:0015937 GO:0015940 GO:0006139
GO:0004127 GO:0005524 GO:0016301 GO:0005515 GO:0005200
GO:0004140 GO:0030257 GO:0005823 GO:0042597 GO:0005856
Cluster-22178.67733 GO:0009405 GO:0030474 GO:0030254 GO:0008152
GO:0015937 GO:0015940 GO:0006139 GO:0006810 GO:0006206
GO:0016301 GO:0005515 GO:0005200 GO:0004140 GO:0004127
GO:0005524 GO:0005856 GO:0030257 GO:0005823 GO:0042597
Cluster-22178.67735 GO:0016032 GO:0055114 GO:0008152 GO:0016491
GO:0019033
Cluster-22178.67736 GO:0006631 GO:0016310 GO:0009069 GO:0005515
GO:0004674 GO:0016746 GO:0005524
Cluster-22178.67737 GO:0006810 GO:0006206 GO:0030254 GO:0009405
GO:0030474 GO:0008152 GO:0015937 GO:0006139 GO:0015940
GO:0005524 GO:0004127 GO:0016301 GO:0005515 GO:0004140
GO:0005200 GO:0030257 GO:0042597 GO:0005823 GO:0005856
Cluster-22178.67741 GO:0009058 GO:0030170
Cluster-22178.67743 GO:0007304 GO:0006470 GO:0006570 GO:0016192
GO:0005213 GO:0004725 GO:0008138 GO:0042600 GO:0005576
Cluster-22178.67745 GO:0003824
Cluster-22178.67749 GO:0001522 GO:0009451 GO:0003723 GO:0005515
GO:0009982

Cluster-22178.67750 GO:0016579 GO:0006508 GO:0008270 GO:0004843
GO:0036459
Cluster-22178.67751 GO:0019229 GO:0000725 GO:0046983 GO:0003677
GO:0005634 GO:0097196 GO:0005576
Cluster-22178.67752 GO:0006096 GO:0006094 GO:0016579 GO:0030150
GO:0015976 GO:0006144 GO:0030955 GO:0000287 GO:0004743
GO:0003824 GO:0008270 GO:0036459 GO:0005742
Cluster-22178.67754 GO:0006915 GO:0006974 GO:0008152 GO:0016491
GO:0000228
Cluster-22178.67755 GO:0015979 GO:0005515 GO:0009539 GO:0009523
GO:0016020
Cluster-22178.67756 GO:0003723 GO:0005515 GO:0005198
Cluster-22178.67757 GO:0005515
Cluster-22178.67760 GO:0016021
Cluster-22178.67761 GO:0007219 GO:0030154 GO:0007275 GO:0006351
GO:0016788 GO:0003677 GO:0005515 GO:0016021 GO:0005634
Cluster-22178.67764 GO:0006355 GO:0006457 GO:0046872 GO:0043565
GO:0003700 GO:0051082 GO:0016272 GO:0019867 GO:0005667
Cluster-22178.67767 GO:0006118 GO:0006570 GO:0006886 GO:0008033
GO:0055114 GO:0015031 GO:0050660 GO:0008565 GO:0004097
GO:0016020 GO:0005737
Cluster-22178.67769 GO:0055114 GO:0008270 GO:0016491
Cluster-22178.67772 GO:0070206 GO:0051607 GO:0006606 GO:0009405
GO:0005515 GO:0019028 GO:0016020
Cluster-22178.67776 GO:0046487 GO:0009405 GO:0055114 GO:0019521
GO:0006098 GO:0007264 GO:0050792 GO:0006810 GO:0009396
GO:0008200 GO:0004518 GO:0003824 GO:0005525 GO:0004488
GO:0004616 GO:0005576
Cluster-22178.67777 GO:0090063 GO:0000226 GO:0005815 GO:0000922
Cluster-22178.67778 GO:0007049 GO:0006904 GO:0006123 GO:0015992
GO:0015031 GO:0006914 GO:0004129 GO:0008092 GO:0005198
GO:0003774 GO:0045277 GO:0005737 GO:0005740 GO:0019898
GO:0005882 GO:0000145 GO:0005747 GO:0016459 GO:0005739

Cluster-22178.67779 GO:0006820 GO:0015031 GO:0015992 GO:0015991 GO:0008565 GO:0046872 GO:0008509 GO:0015078 GO:0004190 GO:0005515 GO:0016021 GO:0016020 GO:0033179

Cluster-22178.67781 GO:0046912 GO:0005515 GO:0008270 GO:0008144 GO:0005622

Cluster-22178.67782 GO:0042254 GO:0006412 GO:0043395 GO:0005515 GO:0003735 GO:0005840 GO:0016020 GO:0005578

Cluster-22178.67783 GO:0015031 GO:0006769 GO:0046497 GO:0016567 GO:0004842 GO:0003953 GO:0016021 GO:0005789

Cluster-22178.67784 GO:0046872

Cluster-22178.67785 GO:0006355 GO:0051260 GO:0008270 GO:0003700 GO:0003676 GO:0005634 GO:0005667

Cluster-22178.67786 GO:0044237 GO:0006810 GO:0006351 GO:0006355 GO:0006144 GO:0006206 GO:0003899 GO:0003677 GO:0005730 GO:0016021 GO:0005639

Cluster-22178.67787 GO:0042254 GO:0006412 GO:0005096 GO:0046872 GO:0003735 GO:0003677 GO:0005622 GO:0045202 GO:0005840

Cluster-22178.67788 GO:0009306 GO:0009403 GO:0015031 GO:0015450 GO:0008092 GO:0016020 GO:0016021 GO:0019898 GO:0009941 GO:0005737

Cluster-22178.67791 GO:0015031 GO:0006464 GO:0003723 GO:0005515

Cluster-22178.67793 GO:0006771 GO:0009231 GO:0003919 GO:0008270 GO:0016021

Cluster-22178.67796 GO:0006813 GO:0006098 GO:0019521 GO:0055114 GO:0051287 GO:0016616 GO:0004616

Cluster-22178.67797 GO:0006470 GO:0006570 GO:0008138 GO:0004725 GO:0016021

Cluster-22178.67803 GO:0043087 GO:0006308 GO:0006302 GO:0007155 GO:0008855 GO:0005515 GO:0009288 GO:0009318 GO:0005634

Cluster-22178.67807 GO:0006810 GO:0005515 GO:0019901 GO:0005886 GO:0016020

Cluster-22178.67810 GO:0031505 GO:0006886 GO:0008536 GO:0008771

GO:0005515

Cluster-22178.67811 GO:0006816 GO:0006810 GO:0005246 GO:0042030 GO:0016020 GO:0005887

Cluster-22178.67814 GO:0055085 GO:0005741

Cluster-22178.67816 GO:0016567 GO:0005515 GO:0004842 GO:0008270 GO:0046872 GO:0005680

Cluster-22178.67818 GO:0006468 GO:0046872 GO:0005524 GO:0004672 GO:0016773 GO:0016787 GO:0016020

Cluster-22178.67819 GO:0046872

Cluster-22178.67820 GO:0007165 GO:0008270

Cluster-22178.67822 GO:0043087 GO:0035023 GO:0055085 GO:0006812 GO:0006885 GO:0007165 GO:0015299 GO:0005057 GO:0005089 GO:0016021

Cluster-22178.67823 GO:0055085 GO:0006810 GO:0005515 GO:0005215 GO:0016021 GO:0016020

Cluster-22178.67824 GO:0055085 GO:0005741

Cluster-22178.67826 GO:0046872 GO:0045202

Cluster-22178.67828 GO:0008643 GO:0008982 GO:0016787 GO:0008146 GO:0015016 GO:0009357

Cluster-22178.67829 GO:0009165 GO:0006144 GO:0009116 GO:0006098 GO:0006397 GO:0004749 GO:0000287 GO:0003723 GO:0005737 GO:0005634

Cluster-22178.67832 GO:0033206 GO:0016482 GO:0007163 GO:0030036 GO:0051653 GO:0051321 GO:0008356 GO:0006928 GO:0005524 GO:0003779 GO:0005885 GO:0042995 GO:0030478

Cluster-22178.67833 GO:0050821 GO:0015979 GO:0006357 GO:0001104 GO:0042301 GO:0009523 GO:0016592 GO:0016020

Cluster-22178.67836 GO:0046872

Cluster-22178.67839 GO:0055114 GO:0008152 GO:0016491

Cluster-22178.67840 GO:0006260 GO:0005524 GO:0016887 GO:0003677

Cluster-22178.67842 GO:0046656 GO:0006367 GO:0006506 GO:0042493 GO:0006761 GO:0055114 GO:0004146 GO:0005515 GO:0046872

GO:0008270 GO:0017176 GO:0005672 GO:0016021
Cluster-22178.67843 GO:0042254 GO:0006412 GO:0003735 GO:0005840
Cluster-22178.67846 GO:0006813 GO:0009405 GO:0005242 GO:0008270
GO:0003677 GO:0008076 GO:0016021
Cluster-22178.67847 GO:0009405 GO:0006351 GO:0006206 GO:0006464
GO:0006144 GO:0007275 GO:0006511 GO:0016567 GO:0016151
GO:0046872 GO:0005198 GO:0005515 GO:0003899 GO:0004842
GO:0008270 GO:0003677 GO:0005634 GO:0005680 GO:0005730
Cluster-22178.67849 GO:0006355 GO:0016787 GO:0016818 GO:0003677
GO:0008270 GO:0005524 GO:0003676 GO:0005634
Cluster-22178.67850 GO:0005515
Cluster-22178.67852 GO:0007283 GO:0046872 GO:0000062 GO:0003677
GO:0005634 GO:0000786
Cluster-22178.67855 GO:0006468 GO:0005524 GO:0004672 GO:0016773
GO:0016020
Cluster-22178.67856 GO:0005515
Cluster-22178.67860 GO:0019028
Cluster-22178.67861 GO:0000902 GO:0006955 GO:0016020
Cluster-22178.67862 GO:0042256 GO:0043022
Cluster-22178.67865 GO:0003676 GO:0005515
Cluster-22178.67866 GO:0006508 GO:0016192 GO:0006886 GO:0009847
GO:0005515 GO:0008233 GO:0030117
Cluster-22178.67867 GO:0005975 GO:0004553
Cluster-22178.67868 GO:0042254 GO:0006412 GO:0003735 GO:0005840
GO:0005739 GO:0005622
Cluster-22178.67869 GO:0043087 GO:0007165 GO:0035023 GO:0005057
GO:0005089
Cluster-22178.67870 GO:0007268 GO:0033130 GO:0003723 GO:0043495
GO:0005515 GO:0019867
Cluster-22178.67873 GO:0006468 GO:0005524 GO:0004672 GO:0005515

Cluster-22178.67874 GO:0006457 GO:0005737
Cluster-22178.67875 GO:0006606 GO:0009069 GO:0016310 GO:0006468

GO:0006886 GO:0007094 GO:0032259 GO:0051607 GO:0015031
GO:0010506 GO:0006457 GO:0006810 GO:0048870 GO:0006355
GO:0008565 GO:0005524 GO:0043565 GO:0003723 GO:0051082
GO:0042803 GO:0004674 GO:0003700 GO:0045502 GO:0008134
GO:0016272 GO:0005667 GO:0016020 GO:0005737 GO:0005615
GO:0030286 GO:0005886 GO:0005801 GO:0016021 GO:0031514
GO:0005845
Cluster-22178.67876 GO:0032259 GO:0003723 GO:0005845
Cluster-22178.67877 GO:0006352 GO:0006355 GO:0006412 GO:0006351
GO:0006144 GO:0006206 GO:0071107 GO:0042254 GO:0003676
GO:0003700 GO:0003899 GO:0016987 GO:0003735 GO:0003677
GO:0005840 GO:0005622 GO:0005667 GO:0005730 GO:0016020
Cluster-22178.67878 GO:0016482 GO:0033206 GO:0007163 GO:0006928
GO:0008356 GO:0051321 GO:0051653 GO:0030036 GO:0003779
GO:0005524 GO:0042995 GO:0005885 GO:0030478
Cluster-22178.67879 GO:0003676
Cluster-22178.67884 GO:0055114 GO:0006486 GO:0016491 GO:0046872
GO:0008373
Cluster-22178.67885 GO:0006351 GO:0008270 GO:0003676
Cluster-22178.67889 GO:0006355 GO:0006281 GO:0006310 GO:0016887
GO:0005524 GO:0003678 GO:0003724 GO:0003723 GO:0009378
GO:0008134 GO:0005657 GO:0005667 GO:0009379
Cluster-22178.67892 GO:0006144 GO:0006206 GO:0006241 GO:0007155
GO:0006165 GO:0006228 GO:0006183 GO:0004550 GO:0003676
GO:0003723 GO:0005515
Cluster-22178.67893 GO:0016567 GO:0008270 GO:0046872 GO:0004842
GO:0005198 GO:0005515 GO:0005622 GO:0019031 GO:0005680
GO:0019028
Cluster-22178.67897 GO:0006351 GO:0007165 GO:0003676 GO:0016817
GO:0005515 GO:0005509 GO:0004190 GO:0003714 GO:0016021
GO:0005667 GO:0005578
Cluster-22178.67898 GO:0007264 GO:0005525
Cluster-22178.67899 GO:0016567 GO:0006508 GO:0006771 GO:0019497

GO:0005515 GO:0004197 GO:0016740 GO:0004842 GO:0003993
GO:0008242 GO:0046872 GO:0008270 GO:0005622
Cluster-22178.67901 GO:0006402 GO:0006109 GO:0003723
Cluster-22178.67906 GO:0006820 GO:0008509 GO:0016021
Cluster-22178.67907 GO:0006351 GO:0006260 GO:0003677 GO:0003887
GO:0042575
Cluster-22178.67908 GO:0071816 GO:0006308 GO:0008855 GO:0009318
Cluster-22178.67909 GO:0008270
Cluster-22178.67910 GO:0006886 GO:0006367 GO:0006605 GO:0006270
GO:0032968 GO:0005515 GO:0005509 GO:0003677 GO:0003676
GO:0016020 GO:0005634 GO:0005742
Cluster-22178.67911 GO:0051016 GO:0008290
Cluster-22178.67912 GO:0046740 GO:0051205 GO:0006810 GO:0042254
GO:0006412 GO:0006260 GO:0051082 GO:0003735 GO:0016798
GO:0016020 GO:0005886 GO:0005622 GO:0005840 GO:0016021
Cluster-22178.67913 GO:0005515 GO:0005516
Cluster-22178.67914 GO:0030474 GO:0006270 GO:0071988
Cluster-22178.67916 GO:0006206 GO:0019083 GO:0055114 GO:0006144
GO:0008285 GO:0016579 GO:0006915 GO:0006351 GO:0007050
GO:0008270 GO:0036459 GO:0016491 GO:0003677 GO:0003899
GO:0005730
Cluster-22178.67917 GO:0008270
Cluster-22178.67918 GO:0006508 GO:0019058 GO:0006397 GO:0016740
GO:0008242 GO:0004197 GO:0000445
Cluster-22178.67920 GO:0006351 GO:0006486 GO:0018279 GO:0006915
GO:0008285 GO:0055114 GO:0019083 GO:0007050 GO:0016579
GO:0006144 GO:0006206 GO:0003899 GO:0036459 GO:0003677
GO:0004579 GO:0016491 GO:0008270 GO:0016021 GO:0008250
GO:0005783 GO:0005739 GO:0005730
Cluster-22178.67922 GO:0001514 GO:0006355 GO:0006351 GO:0006464
GO:0006448 GO:0006260 GO:0006269 GO:0016151 GO:0003896
GO:0005525 GO:0003723 GO:0008134 GO:0016887 GO:0003746
GO:0005524 GO:0003677 GO:0005737 GO:0005840 GO:0005657

GO:0005667 GO:0005730
Cluster-22178.67924 GO:0009229 GO:0030975
Cluster-22178.67927 GO:0008270 GO:0003676 GO:0015066 GO:0005622
Cluster-22178.67928 GO:0006412 GO:0042254 GO:0003735 GO:0005840
GO:0005622
Cluster-22178.67930 GO:0006270 GO:0008033 GO:0006508 GO:0004177
GO:0008168 GO:0016021 GO:0005622
Cluster-22178.67932 GO:0007010 GO:0003779
Cluster-22178.67934 GO:0007010 GO:0016567 GO:0003779 GO:0004857
GO:0004842
Cluster-22178.67935 GO:0006275 GO:0019073 GO:0006260 GO:0045893
GO:0005515 GO:0003677 GO:0003688 GO:0042025 GO:0046809
Cluster-22178.67936 GO:2000123 GO:0006508 GO:0004190
Cluster-22178.67937 GO:0005198 GO:0005094 GO:0005737 GO:0019028
Cluster-22178.67938 GO:0006118 GO:0019646 GO:0006629 GO:0009055

Cluster-22178.67940 GO:0055114 GO:0016620
Cluster-22178.67942 GO:0032040
Cluster-22178.67943 GO:0032040
Cluster-22178.67944 GO:0007165 GO:0006810 GO:0016192 GO:0046486
GO:0006633 GO:0006071 GO:0016573 GO:0006090 GO:0004371
GO:0003989 GO:0005515 GO:0004871 GO:0016020 GO:0009317
GO:0005886 GO:0016021
Cluster-22178.67945 GO:0000902 GO:0005975 GO:0016773
Cluster-22178.67946 GO:0000902 GO:0005975 GO:0016773
Cluster-22178.67947 GO:0007155 GO:0005515 GO:0016021
Cluster-22178.67948 GO:0016192 GO:0006886 GO:0030414 GO:0005506
GO:0030131 GO:0005576
Cluster-22178.67949 GO:0006357 GO:0001104 GO:0016592
Cluster-22178.67950 GO:0006779 GO:0055114 GO:0016567 GO:0016559
GO:0006355 GO:0004842 GO:0043565 GO:0008270 GO:0046872
GO:0005515 GO:0016020 GO:0005779
Cluster-22178.67951 GO:0042254 GO:0006412 GO:0003735 GO:0005622

GO:0005840
Cluster-22178.67952 GO:0015725 GO:0035429 GO:0006486 GO:0008373
GO:0046872 GO:0016787 GO:0015128 GO:0016020
Cluster-22178.67955 GO:0007264 GO:0005525 GO:0003924
Cluster-22178.67961 GO:0005869
Cluster-22178.67962 GO:0042967 GO:0008152 GO:0008270 GO:0016407

Cluster-22178.67964 GO:0007010 GO:0007018 GO:0007017 GO:0008152
GO:0003777 GO:0008289 GO:0008017 GO:0005524 GO:0016829
GO:0003779 GO:0010333 GO:0005739 GO:0005874 GO:0045298
Cluster-22178.67965 GO:0006364 GO:0016192 GO:0019031 GO:0016021
GO:0005730
Cluster-22178.67967 GO:0009405 GO:0006810 GO:0007186 GO:0005515
GO:0008200 GO:0004930 GO:0005576 GO:0016021
Cluster-22178.67970 GO:0005515
Cluster-22178.67972 GO:0016310 GO:0009069 GO:0005515 GO:0004674

Cluster-22178.67973 GO:0034314 GO:0006355 GO:0030833 GO:0003700
GO:0043565 GO:0005198 GO:0019867 GO:0005856 GO:0005882
GO:0005885 GO:0005667
Cluster-22178.67980 GO:0007411 GO:0019031 GO:0016021
Cluster-22178.67982 GO:0006886 GO:0006570 GO:0006576 GO:0006470
GO:2000123 GO:0007165 GO:0007186 GO:0007264 GO:0005525
GO:0004725 GO:0004871 GO:0019001 GO:0031683 GO:0003924
GO:0005524 GO:0005543 GO:0016887
Cluster-22178.67983 GO:0046813 GO:0061025 GO:0016021 GO:0019031
Cluster-22178.67985 GO:0007005 GO:0006511 GO:0042803 GO:0008134
GO:0005509 GO:0045502 GO:0030286 GO:0005667 GO:0031305
Cluster-22178.67987 GO:0006412 GO:0042254 GO:0003735 GO:0015935
GO:0005840
Cluster-22178.67988 GO:0007217
Cluster-22178.67989 GO:0006468 GO:0006479 GO:0006554 GO:0008152
GO:0018024 GO:0005515 GO:0008168 GO:0004672 GO:0005524

Cluster-22178.67990 GO:0005515
Cluster-22178.67994 GO:0006886 GO:0005525 GO:0005047 GO:0003924
GO:0005785 GO:0005786
Cluster-22178.67995 GO:0006260 GO:0015684 GO:0007264 GO:0006576
GO:0005525 GO:0005524 GO:0015093 GO:0016887 GO:0003924
GO:0003677 GO:0016021
Cluster-22178.67997 GO:0046872 GO:0005515
Cluster-22178.67998 GO:0006281 GO:0006355 GO:0006310 GO:0009378
GO:0003723 GO:0016301 GO:0008134 GO:0005515 GO:0005524
GO:0016887 GO:0003724 GO:0009379 GO:0005667 GO:0005657
Cluster-22178.67999 GO:0008033 GO:0055114 GO:0006355 GO:0016491
GO:0050660 GO:0005507 GO:0003700 GO:0071949 GO:0003677
GO:0005634 GO:0005667
Cluster-22178.68000 GO:0007178 GO:0019064 GO:0016310 GO:0006468
GO:0009069 GO:0004672 GO:0005524 GO:0004675 GO:0016020
Cluster-22178.68004 GO:0006118 GO:0009055 GO:0005515 GO:0009512
Cluster-22178.68005 GO:0006118 GO:0009055 GO:0005515 GO:0009512
Cluster-22178.68006 GO:0006118 GO:0009055 GO:0005515 GO:0009512
Cluster-22178.68008 GO:0048193 GO:0005515 GO:0016020 GO:0016021
Cluster-22178.68009 GO:0005975 GO:0004553
Cluster-22178.68010 GO:0048193 GO:0005515 GO:0016021 GO:0016020
Cluster-22178.68011 GO:0042710 GO:0042254 GO:0015992 GO:0006260
GO:0015991 GO:0006351 GO:0006412 GO:0006570 GO:0006470
GO:0003677 GO:0008138 GO:0003735 GO:0003887 GO:0015078
GO:0004725 GO:0005622 GO:0042575 GO:0033179 GO:0005840
Cluster-22178.68012 GO:0006355 GO:0008654 GO:0006260 GO:0000166
GO:0003677 GO:0008134 GO:0005524 GO:0016887 GO:0016780
GO:0005667 GO:0016020
Cluster-22178.68014 GO:0002949 GO:0007264 GO:0006614 GO:0007165
GO:0015684 GO:0007186 GO:0008152 GO:0006777 GO:0006576
GO:0005524 GO:0015093 GO:0016887 GO:0003677 GO:0098519
GO:0031683 GO:0019001 GO:0043531 GO:0003924 GO:0005525
GO:0004871 GO:0000166 GO:0016301 GO:0016021

Cluster-22178.68017 GO:0009405 GO:0005515
Cluster-22178.68018 GO:0042325 GO:0005789 GO:0005737
Cluster-22178.68023 GO:0007186 GO:0005515 GO:0004930 GO:0016021
Cluster-22178.68024 GO:0004867 GO:0005515
Cluster-22178.68025 GO:0005515
Cluster-22178.68026 GO:0005515 GO:0004867
Cluster-22178.68027 GO:0006367 GO:0006506 GO:0006355 GO:0016746
GO:0005789 GO:0016021 GO:0005739
Cluster-22178.68030 GO:0006891 GO:0006352 GO:0006355 GO:0016987
GO:0003700 GO:0003677 GO:0030173 GO:0005667
Cluster-22178.68031 GO:0006468 GO:0003677 GO:0016773 GO:0005524
GO:0004672 GO:0016020
Cluster-22178.68032 GO:0005515
Cluster-22178.68034 GO:0004190 GO:0016021
Cluster-22178.68035 GO:0043044 GO:0003676 GO:0016787 GO:0046872
GO:0016586
Cluster-22178.68036 GO:0006950 GO:0006508 GO:0008270 GO:0004222
GO:0003700 GO:0004197 GO:0031012 GO:0005667
Cluster-22178.68037 GO:0042967 GO:0008152 GO:0006633 GO:0016747
GO:0004315 GO:0005835
Cluster-22178.68038 GO:0008210 GO:0006694 GO:0043154 GO:0055114
GO:0008209 GO:0019079 GO:0008207 GO:0043066 GO:0003824
GO:0016616 GO:0032266 GO:0050662 GO:0003854 GO:0051287
GO:0080019 GO:0043130 GO:0016620 GO:0005737 GO:0071986
Cluster-22178.68039 GO:0007155 GO:0030155 GO:0007165 GO:0006914
GO:0051607 GO:0030334 GO:0030168 GO:0000921 GO:0006606
GO:0006886 GO:0051258 GO:0045995 GO:0030674 GO:0005102
GO:0045502 GO:0008134 GO:0042803 GO:0005515 GO:0008092
GO:0004871 GO:0005667 GO:0016020 GO:0005940 GO:0030286
GO:0005577 GO:0019898 GO:0048786 GO:0005737 GO:0005801
GO:0016021
Cluster-22178.68042 GO:0015992 GO:0006744 GO:0006120 GO:2001256
GO:0006814 GO:0042254 GO:0006412 GO:0042773 GO:0003735

GO:0008137 GO:0005622 GO:0030176 GO:0005743 GO:0005840
Cluster-22178.68045 GO:0055085 GO:0006811 GO:0005216 GO:0016020
Cluster-22178.68048 GO:0007186 GO:0007165 GO:0007010 GO:0003924
GO:0003779 GO:0019001 GO:0008270 GO:0031683 GO:0004871
GO:0005525
Cluster-22178.68050 GO:0006310 GO:0019079 GO:0006760 GO:0002949
GO:0006206 GO:0051016 GO:0006955 GO:0000902 GO:0006355
GO:0046656 GO:0006281 GO:0006139 GO:0003677 GO:0009378
GO:0003724 GO:0004127 GO:0046983 GO:0016887 GO:0005524
GO:0008134 GO:0003723 GO:0003678 GO:0004150 GO:0000150
GO:0043531 GO:0016020 GO:0008290 GO:0005667 GO:0009379
GO:0005657
Cluster-22178.68054 GO:0005515
Cluster-22178.68056 GO:0003676 GO:0003723
Cluster-22178.68057 GO:0006730 GO:0030163 GO:0005515 GO:0016740
GO:0008270
Cluster-22178.68058 GO:0007224
Cluster-22178.68059 GO:0046836 GO:0017089 GO:0051861 GO:0005737
Cluster-22178.68060 GO:0007224
Cluster-22178.68063 GO:0042254 GO:0006412 GO:0003735 GO:0005840
Cluster-22178.68066 GO:0016787
Cluster-22178.68068 GO:0008033 GO:0006508 GO:0006468 GO:0004222
GO:0008270 GO:0004672 GO:0005524 GO:0016437 GO:0004810
GO:0005576
Cluster-22178.68070 GO:0042254 GO:0006412 GO:0003735 GO:0005840
GO:0005622
Cluster-22178.68072 GO:0006465 GO:0008233 GO:0005787 GO:0016021
Cluster-22178.68076 GO:0015991 GO:0007155 GO:0006351 GO:0015992
GO:0006206 GO:0006144 GO:0070140 GO:0032183 GO:0003677
GO:0003899 GO:0005515 GO:0015078 GO:0005730 GO:0033179
GO:0016020
Cluster-22178.68077 GO:0055085 GO:0006811 GO:0005216 GO:0016020
Cluster-22178.68079 GO:0008440

Cluster-22178.68080 GO:0006355 GO:0005515 GO:0005634

Cluster-22178.68082 GO:0005515

Cluster-22178.68083 GO:0005515

Cluster-22178.68084 GO:0007186 GO:0006030 GO:0006468 GO:0030416 GO:0008152 GO:0008270 GO:0005524 GO:0016491 GO:0016773 GO:0004930 GO:0008061 GO:0005515 GO:0004672 GO:0016020 GO:0005576 GO:0016021

Cluster-22178.68086 GO:0016567 GO:0006915 GO:0032465 GO:0033014 GO:0055114 GO:0050661 GO:0004842 GO:0008883 GO:0016021

Cluster-22178.68088 GO:0015074 GO:0050832 GO:0042967 GO:0042742 GO:0008080 GO:0003676 GO:0019239 GO:0003677 GO:0031224

Cluster-22178.68090 GO:0007264 GO:0031419 GO:0005525 GO:0003924 GO:0046872

Cluster-22178.68092 GO:0006013 GO:0005975 GO:0015923

Cluster-22178.68093 GO:0015684 GO:0003924 GO:0015093 GO:0005515 GO:0005525 GO:0019033 GO:0016021

Cluster-22178.68094 GO:0006120 GO:0006744 GO:0055085 GO:0015992 GO:0055114 GO:0042773 GO:0006814 GO:0015986 GO:0015078 GO:0016651 GO:0008137 GO:0015002 GO:0009512 GO:0016021 GO:0016020

Cluster-22178.68095 GO:0006412 GO:0042254 GO:0003735 GO:0008289 GO:0005840 GO:0005739

Cluster-22178.68096 GO:0005515

Cluster-22178.68097 GO:0006974 GO:0006914 GO:0042254 GO:0009271 GO:0007155 GO:0007094 GO:0006886 GO:0006915 GO:0030001 GO:0006260 GO:0055085 GO:0030168 GO:0015031 GO:0007165 GO:0006810 GO:0006302 GO:0051258 GO:0048870 GO:0006412 GO:0030474 GO:0006355 GO:0008017 GO:0008565 GO:0003735 GO:0043565 GO:0005515 GO:0005198 GO:0042803 GO:0005102 GO:0046873 GO:0030674 GO:0005200 GO:0003700 GO:0008134 GO:0045502 GO:0005577 GO:0016020 GO:0005667 GO:0045298 GO:0005737 GO:0005856 GO:0005634 GO:0048786 GO:0005823 GO:0030286 GO:0005886 GO:0005840 GO:0031514

Cluster-22178.68098 GO:0006974 GO:0042254 GO:0009271 GO:0006886
GO:0006915 GO:0030001 GO:0055085 GO:0030168 GO:0015031
GO:0007165 GO:0006302 GO:0051258 GO:0048870 GO:0030474
GO:0006412 GO:0006355 GO:0008565 GO:0003735 GO:0005515
GO:0005198 GO:0042803 GO:0005102 GO:0046873 GO:0030674
GO:0005200 GO:0003700 GO:0008134 GO:0045502 GO:0005577
GO:0016020 GO:0005667 GO:0005737 GO:0005856 GO:0005634
GO:0005823 GO:0030286 GO:0005840 GO:0031514
Cluster-22178.68100 GO:0051539
Cluster-22178.68103 GO:0003824
Cluster-22178.68104 GO:0005515 GO:0016021
Cluster-22178.68105 GO:0006886 GO:0015991 GO:0006468 GO:0016310
GO:0009069 GO:0006260 GO:0006914 GO:0015992 GO:0015031
GO:0005515 GO:0015078 GO:0004674 GO:0008565 GO:0005524
GO:0033179 GO:0005737 GO:0016020
Cluster-22178.68106 GO:0042254 GO:0051090 GO:0006352 GO:0003735
GO:0003677 GO:0005763 GO:0005634 GO:0005840
Cluster-22178.68108 GO:0055114 GO:0006118 GO:0008152 GO:0016746
GO:0009916
Cluster-22178.68109 GO:0045454 GO:0055114 GO:0016491 GO:0016209

Cluster-22178.68110 GO:0007275 GO:0042600
Cluster-22178.68113 GO:0006771 GO:0019497 GO:0005515 GO:0003993
GO:0046872
Cluster-22178.68114 GO:0005525
Cluster-22178.68117 GO:0055114 GO:0016114 GO:0006402 GO:0006694
GO:0006629 GO:0051252 GO:0046872 GO:0004535 GO:0003676
GO:0050660 GO:0003723 GO:0004506 GO:0005737 GO:0016021
GO:0005634
Cluster-22178.68119 GO:0051694 GO:0005523
Cluster-22178.68123 GO:0055085 GO:0007005 GO:0006821 GO:0006914
GO:0005247 GO:0016020 GO:0031305
Cluster-22178.68125 GO:0006144 GO:0006508 GO:0044419 GO:0004197

GO:0003968 GO:0017111 GO:0031379
Cluster-22178.68126 GO:0006355 GO:0006508 GO:0090307 GO:0051082
GO:0004222 GO:0003700 GO:0043565 GO:0005667 GO:0033588
Cluster-22178.68127 GO:0000105 GO:0006807 GO:0000162 GO:0006013
GO:0009607 GO:0006040 GO:0006000 GO:0006004 GO:0006571
GO:0008152 GO:0009094 GO:0055114 GO:0008033 GO:0006537
GO:0006355 GO:0006051 GO:0050660 GO:0004425 GO:0016491
GO:0018580 GO:0015930 GO:0016638 GO:0017150 GO:0003824
GO:0016627 GO:0016829 GO:0047465 GO:0008736 GO:0005737
Cluster-22178.68128 GO:0005515
Cluster-22178.68133 GO:0042254 GO:0006412 GO:0046740 GO:0003735
GO:0005840 GO:0044156 GO:0005622
Cluster-22178.68135 GO:0016021
Cluster-22178.68142 GO:0005515
Cluster-22178.68144 GO:0006364 GO:0009755 GO:0005515 GO:0032040
GO:0005576
Cluster-22178.68145 GO:0000723 GO:0006281 GO:0003678 GO:0005524
GO:0003677 GO:0016787 GO:0005657 GO:0016020
Cluster-22178.68146 GO:0006412 GO:0006355 GO:0006281 GO:0006576
GO:0002949 GO:0007165 GO:0006810 GO:0042254 GO:0006310
GO:0007186 GO:0004871 GO:0046914 GO:0019001 GO:0031683
GO:0003924 GO:0008134 GO:0005524 GO:0016887 GO:0046983
GO:0003735 GO:0009378 GO:0005840 GO:0016021 GO:0005657
GO:0005622 GO:0005667 GO:0009379
Cluster-22178.68148 GO:0051603 GO:0004298 GO:0005839
Cluster-22178.68151 GO:0006897 GO:0005096 GO:0005515 GO:0016020
GO:0016021
Cluster-22178.68153 GO:0008270
Cluster-22178.68154 GO:0009395 GO:0006629 GO:0004620 GO:0016020
Cluster-22178.68155 GO:0005515 GO:0031224
Cluster-22178.68156 GO:0003677 GO:0003676 GO:0005524 GO:0016787

Cluster-22178.68157 GO:0045892 GO:0006308 GO:0046685 GO:0071816

GO:0008855 GO:0003677 GO:0009318 GO:0005634
Cluster-22178.68158 GO:0006811 GO:0006810 GO:0005230 GO:0016020
Cluster-22178.68160 GO:0006914
Cluster-22178.68161 GO:0055114 GO:0010309 GO:0005515
Cluster-22178.68162 GO:0055114 GO:0010309 GO:0005515
Cluster-22178.68165 GO:0046872
Cluster-22178.68166 GO:0006614 GO:0005515 GO:0003723 GO:0003724
GO:0005525 GO:0003924 GO:0016887 GO:0005524
Cluster-22178.68168 GO:0045893 GO:0003677 GO:0005634
Cluster-22178.68169 GO:0017119
Cluster-22178.68170 GO:0007015 GO:0003785
Cluster-22178.68171 GO:0001575 GO:0006012 GO:0046486 GO:0005975
GO:0004553 GO:0004557
Cluster-22178.68172 GO:0009089 GO:0008207 GO:0008209 GO:0009085
GO:0008210 GO:0006694 GO:0055114 GO:0016616 GO:0003854
GO:0051287 GO:0050662 GO:0016620 GO:0003824 GO:0008839
GO:0016491
Cluster-22178.68174 GO:0009607 GO:0016021
Cluster-22178.68176 GO:0032259 GO:0046872 GO:0003723 GO:0005845
Cluster-22178.68177 GO:0007165 GO:0005158 GO:0003723 GO:0005515
GO:0005899
Cluster-22178.68178 GO:0045859 GO:0004860 GO:0005576
Cluster-22178.68179 GO:0042967 GO:0004379 GO:0008080 GO:0003676

Cluster-22178.68182 GO:0009405 GO:0004859 GO:0005576
Cluster-22178.68183 GO:0006468 GO:0016773 GO:0004672 GO:0005524
GO:0016020
Cluster-22178.68186 GO:0007224
Cluster-22178.68188 GO:0003676 GO:0005921
Cluster-22178.68191 GO:0007018 GO:0006511 GO:0016567 GO:0007017
GO:0005515 GO:0034450 GO:0008017 GO:0004842 GO:0003777
GO:0005524 GO:0000151 GO:0045298 GO:0005874
Cluster-22178.68192 GO:0031167 GO:0009451 GO:0008033 GO:0004809

GO:0008168 GO:0003723
Cluster-22178.68193 GO:0005515
Cluster-22178.68197 GO:0015031 GO:0006464 GO:0005515
Cluster-22178.68199 GO:0016055 GO:0006531 GO:0043039 GO:0006419 GO:0006522 GO:0000166 GO:0004813 GO:0016876 GO:0005524 GO:0003676
Cluster-22178.68200 GO:0030145 GO:0004177
Cluster-22178.68203 GO:0007165 GO:0009451 GO:0006886 GO:0016192 GO:0006400 GO:0008033 GO:0016301 GO:0005515 GO:0008176 GO:0046872 GO:0030117 GO:0016021
Cluster-22178.68206 GO:0007264 GO:0007186 GO:0007165 GO:0003924 GO:0031683 GO:0019001 GO:0004871 GO:0005525
Cluster-22178.68209 GO:0019087 GO:0003676 GO:0016021
Cluster-22178.68210 GO:0006007 GO:0006629 GO:0006412 GO:0042254 GO:0003824 GO:0046537 GO:0005515 GO:0003735 GO:0005622 GO:0005840
Cluster-22178.68211 GO:0007186 GO:0004930 GO:0019031 GO:0016021
Cluster-22178.68212 GO:0008152 GO:0003824 GO:0008484 GO:0046872

Cluster-22178.68213 GO:0008152 GO:0016787 GO:0005198 GO:0019028 GO:0016021
Cluster-22178.68214 GO:0051258 GO:0006308 GO:0007155 GO:0007165 GO:0000921 GO:0030168 GO:0051607 GO:0006914 GO:0006606 GO:0045502 GO:0005515 GO:0042803 GO:0008134 GO:0005179 GO:0030674 GO:0008855 GO:0005102 GO:0005576 GO:0009318 GO:0016021 GO:0005667 GO:0030286 GO:0005940 GO:0005577
Cluster-22178.68215 GO:0051205 GO:0000042 GO:0005515 GO:0016021 GO:0016020
Cluster-22178.68217 GO:0006886 GO:0007067 GO:0005515 GO:0016020 GO:0042729 GO:0072686 GO:0005801 GO:0005737
Cluster-22178.68220 GO:0016020
Cluster-22178.68222 GO:0008270 GO:0005921
Cluster-22178.68223 GO:0008270 GO:0005921

Cluster-22178.68225 GO:0042254 GO:0006412 GO:0003735 GO:0005840 GO:0000228 GO:0005622
Cluster-22178.68227 GO:0006302 GO:0007155 GO:0043087 GO:0006308 GO:0005515 GO:0008855 GO:0009318 GO:0005634 GO:0009288
Cluster-22178.68230 GO:0006355 GO:0006412 GO:0042254 GO:0008270 GO:0043565 GO:0003700 GO:0003735 GO:0005840 GO:0005667
Cluster-22178.68231 GO:0030150 GO:0016579 GO:0008270 GO:0036459 GO:0005742
Cluster-22178.68232 GO:0006096 GO:0006094 GO:0006144 GO:0015976 GO:0005515 GO:0004743 GO:0000287 GO:0030955 GO:0003824
Cluster-22178.68233 GO:0009405 GO:0005887 GO:0016020
Cluster-22178.68235 GO:0006144 GO:0035556 GO:0006171 GO:0009190 GO:0071816 GO:0016849 GO:0004016 GO:0005886
Cluster-22178.68236 GO:0005515
Cluster-22178.68237 GO:0005515
Cluster-22178.68239 GO:0042157 GO:0006869 GO:0008270 GO:0003677 GO:0008289 GO:0019028 GO:0005576
Cluster-22178.68241 GO:0016255 GO:0016627 GO:0030414 GO:0042765 GO:0005576
Cluster-22178.68242 GO:0015992 GO:0006123 GO:0004129 GO:0005739 GO:0045277
Cluster-22178.68245 GO:0055114 GO:0008152 GO:0016491 GO:0005515

Cluster-22178.68247 GO:0044237 GO:0003676 GO:0005524
Cluster-22178.68249 GO:0006606 GO:0043087 GO:0006357 GO:0006302 GO:0006308 GO:0009405 GO:0006397 GO:0006355 GO:0008855 GO:0003700 GO:0001104 GO:0016592 GO:0016020 GO:0005667 GO:0009318 GO:0005634
Cluster-22178.68250 GO:0006457 GO:0006405 GO:0000921 GO:0006260 GO:0006606 GO:0006355 GO:0019089 GO:0010508 GO:0051382 GO:0045892 GO:0003677 GO:0051082 GO:0043565 GO:0003700 GO:0005667 GO:0016272 GO:0005940 GO:0005815 GO:0000776 GO:0005634 GO:0019028 GO:0042025 GO:0016021

Cluster-22178.68251 GO:0006614 GO:0008312 GO:0048500

Cluster-22178.68253 GO:0055114 GO:0020037 GO:0016705 GO:0005506

Cluster-22178.68254 GO:0055114 GO:0005506 GO:0016705 GO:0020037

Cluster-22178.68255 GO:0016021

Cluster-22178.68257 GO:0006355 GO:0003677 GO:0003700 GO:0005667

Cluster-22178.68258 GO:0005515

Cluster-22178.68259 GO:0008270 GO:0046872 GO:0005515 GO:0005622 GO:0005634

Cluster-22178.68261 GO:0006629

Cluster-22178.68262 GO:0006511 GO:0031625

Cluster-22178.68263 GO:0007165 GO:0008053 GO:0015031 GO:0030168 GO:0006464 GO:0048024 GO:0000917 GO:0006355 GO:0015991 GO:0007049 GO:0051258 GO:0006308 GO:0043093 GO:0007155 GO:0015992 GO:0006914 GO:0030674 GO:0005102 GO:0008855 GO:0003700 GO:0004871 GO:0008092 GO:0005198 GO:0051082 GO:0015078 GO:0043565 GO:0003924 GO:0019898 GO:0009318 GO:0033179 GO:0019028 GO:0016021 GO:0005741 GO:0016020 GO:0005667 GO:0005577 GO:0005634 GO:0005737

Cluster-22178.68264 GO:0006629

Cluster-22178.68265 GO:0005515 GO:0046872 GO:0008270 GO:0005634 GO:0005622

Cluster-22178.68267 GO:0007165

Cluster-22178.68269 GO:0009405 GO:0006810 GO:0019871 GO:0005576

Cluster-22178.68271 GO:0003924

Cluster-22178.68272 GO:0005515 GO:2001070

Cluster-22178.68273 GO:0055114 GO:0007156 GO:0006412 GO:0042254 GO:0016491 GO:0003735 GO:0005509 GO:0005622 GO:0005840 GO:0016020

Cluster-22178.68277 GO:0043565 GO:0004252 GO:0005515 GO:0019028 GO:0055036 GO:0005634

Cluster-22178.68279 GO:0007009 GO:0030001 GO:0006355 GO:0046872

GO:0005515 GO:0005743 GO:0005737 GO:0005747 GO:0005634
Cluster-22178.68280 GO:0005634
Cluster-22178.68282 GO:0005634
Cluster-22178.68283 GO:0055114 GO:0016705 GO:0020037 GO:0005506

Cluster-22178.68285 GO:0006298 GO:0006412 GO:0042254 GO:0003735
GO:0004519 GO:0008146 GO:0005840 GO:0016021 GO:0005622
Cluster-22178.68288 GO:0007010 GO:0003779
Cluster-22178.68290 GO:0005515
Cluster-22178.68294 GO:0006531 GO:0006529 GO:0006522 GO:0008270
GO:0046872 GO:0004066 GO:0005515
Cluster-22178.68295 GO:0030494 GO:0015979 GO:0016032 GO:0006355
GO:0003700 GO:0043565 GO:0005515 GO:0005667
Cluster-22178.68300 GO:0006508 GO:0006281 GO:0006412 GO:0042254
GO:0015876 GO:0051016 GO:0003735 GO:0008521 GO:0046983
GO:0003676 GO:0008239 GO:0030479 GO:0005622 GO:0016021
GO:0005840
Cluster-22178.68301 GO:0007165 GO:0007186 GO:0048193 GO:0031683
GO:0019001 GO:0003924 GO:0005525 GO:0004871 GO:0005783
Cluster-22178.68302 GO:0006355 GO:0043039 GO:0006418 GO:0006820
GO:0004812 GO:0003677 GO:0000166 GO:0016876 GO:0005524
GO:0043565 GO:0008509 GO:0003700 GO:0005667 GO:0016021
Cluster-22178.68303 GO:0044419 GO:0006508 GO:0006144 GO:0004197
GO:0017111 GO:0003968 GO:0031379
Cluster-22178.68304 GO:0003677 GO:0004859 GO:0005576 GO:0005634
Cluster-22178.68306 GO:0006554 GO:0034968 GO:0006479 GO:0008270
GO:0018024 GO:0005515 GO:0003677 GO:0005634
Cluster-22178.68307 GO:0007264 GO:0005525
Cluster-22178.68309 GO:0006412 GO:0042254 GO:0006298 GO:0003735
GO:0004519 GO:0008146 GO:0005622 GO:0016021 GO:0005840
Cluster-22178.68311 GO:0016032 GO:0006355 GO:0007094 GO:0048870
GO:0008608 GO:0003700 GO:0072686 GO:0005667 GO:0031514
GO:0042729

Cluster-22178.68314 GO:0006694 GO:0008152 GO:0055114 GO:0008209
GO:0008207 GO:0008210 GO:0019836 GO:0003824 GO:0016491
GO:0016616 GO:0005515 GO:0050662 GO:0003854 GO:0005576
Cluster-22178.68315 GO:0016567 GO:0005515 GO:0004842
Cluster-22178.68316 GO:0016746 GO:0005634
Cluster-22178.68318 GO:0016567 GO:0008270 GO:0046872 GO:0004842
GO:0005515 GO:0005680
Cluster-22178.68319 GO:0006355 GO:0043565 GO:0005515 GO:0005634
Cluster-22178.68323 GO:0051568 GO:0000902 GO:0006479 GO:0006508
GO:0003779 GO:0005515 GO:0042800 GO:0003682 GO:0000785
GO:0072357 GO:0048188
Cluster-22178.68326 GO:0023052 GO:0007165 GO:0005125
Cluster-22178.68327 GO:0019079 GO:0006810 GO:0004386 GO:0003676
GO:0008026 GO:0043531 GO:0005524
Cluster-22178.68331 GO:0023052 GO:0005515
Cluster-22178.68332 GO:0005886
Cluster-22178.68333 GO:0006355 GO:0043565 GO:0003700 GO:0005667
Cluster-22178.68334 GO:0008152 GO:0046872 GO:0008270 GO:0003824
GO:0016836 GO:0005515 GO:0061630 GO:0032040
Cluster-22178.68335 GO:0006464 GO:0051260 GO:0007186 GO:0004930
GO:0016020 GO:0016021
Cluster-22178.68336 GO:0005515
Cluster-22178.68337 GO:0006511 GO:0016567 GO:0033014 GO:0009252
GO:0032465 GO:0006915 GO:0006629 GO:0055114 GO:0004842
GO:0008963 GO:0008883 GO:0050661 GO:0003723 GO:0003676
GO:0003924 GO:0016021
Cluster-22178.68338 GO:0006891 GO:0008144 GO:0017119
Cluster-22178.68340 GO:0007155
Cluster-22178.68343 GO:0006270 GO:0003676
Cluster-22178.68344 GO:0043395 GO:0016020 GO:0005578
Cluster-22178.68347 GO:0006508 GO:0004177 GO:0008270
Cluster-22178.68348 GO:0005515
Cluster-22178.68351 GO:0006396 GO:0006807 GO:0006144 GO:0004000

GO:0003723
Cluster-22178.68353 GO:0007264 GO:0006351 GO:0006144 GO:0006206
GO:0043087 GO:0005085 GO:0003899 GO:0003677 GO:0005730
Cluster-22178.68354 GO:0006355 GO:0016055 GO:0006886 GO:0000975
GO:0008536 GO:0003700 GO:0005667
Cluster-22178.68355 GO:0016020
Cluster-22178.68356 GO:0022008 GO:0005764
Cluster-22178.68357 GO:0016020
Cluster-22178.68363 GO:0007224
Cluster-22178.68364 GO:0016021
Cluster-22178.68366 GO:0007049 GO:0055114 GO:0008033 GO:0048870
GO:0006118 GO:0030150 GO:0045454 GO:0009055 GO:0015035
GO:0005515 GO:0050660 GO:0016491 GO:0019904 GO:0016021
GO:0005737 GO:0031514 GO:0005634 GO:0005742
Cluster-22178.68367 GO:0005524 GO:0003676
Cluster-22178.68368 GO:0051782 GO:0045444 GO:0006281 GO:0009432
GO:0006260 GO:0006310 GO:0003723 GO:0005524 GO:0003689
GO:0016887 GO:0003724 GO:0009378 GO:0098519 GO:0016021
GO:0005663 GO:0042575 GO:0005657 GO:0009379 GO:0009276
GO:0016020
Cluster-22178.68369 GO:0000917 GO:0043093 GO:0006355 GO:0010506
GO:0006260 GO:0007049 GO:0051016 GO:0003700 GO:0043565
GO:0008290 GO:0005634 GO:0005667 GO:0005615 GO:0005737
Cluster-22178.68370 GO:0006468 GO:0016773 GO:0005524 GO:0004672
GO:0016020
Cluster-22178.68371 GO:0016192 GO:0007009 GO:0071766 GO:0052636
GO:0019843 GO:0003723 GO:0016021 GO:0005622
Cluster-22178.68373 GO:0007156 GO:0005509 GO:0016020
Cluster-22178.68374 GO:0006260 GO:0016255 GO:0003677 GO:0005524
GO:0042765
Cluster-22178.68377 GO:0034227 GO:0002098 GO:0000049
Cluster-22178.68379 GO:0005515
Cluster-22178.68381 GO:0016020

Cluster-22178.68383 GO:0006355 GO:0003700 GO:0005667 GO:0005634
Cluster-22178.68385 GO:0051016 GO:0006508 GO:0008152 GO:0016787
GO:0008236 GO:0008290
Cluster-22178.68386 GO:0006368 GO:0008023
Cluster-22178.68387 GO:0006397 GO:0015992 GO:0000398 GO:0015986
GO:0005515 GO:0015078 GO:0005634
Cluster-22178.68392 GO:0007018 GO:0007017 GO:0006357 GO:0001104
GO:0005198 GO:0003777 GO:0008017 GO:0005524 GO:0016592
GO:0019028 GO:0005874 GO:0019031 GO:0045298
Cluster-22178.68395 GO:0006508 GO:0045859 GO:0016740 GO:0016788
GO:0004197 GO:0008242 GO:0016817 GO:0004860 GO:0005576
Cluster-22178.68398 GO:0042967 GO:0004379
Cluster-22178.68401 GO:0016117 GO:0006779 GO:0055114 GO:0008033
GO:0019354 GO:0050660 GO:0016491 GO:0008270 GO:0043115
GO:0005515 GO:0016705 GO:0071949
Cluster-22178.68402 GO:0005515
Cluster-22178.68403 GO:0009399 GO:0006355 GO:0006468 GO:0003700
GO:0005524 GO:0004672 GO:0005667
Cluster-22178.68404 GO:0043161 GO:0006281 GO:0006289 GO:0007010
GO:0003779 GO:0003684
Cluster-22178.68405 GO:0046907 GO:0006446 GO:0003743 GO:0005840
GO:0005852 GO:0005737
Cluster-22178.68408 GO:0008270
Cluster-22178.68409 GO:0008152 GO:0016491
Cluster-22178.68413 GO:0015979 GO:0046872 GO:0008270 GO:0009523
GO:0009539
Cluster-22178.68414 GO:0006098 GO:0006094 GO:0005985 GO:0005982
GO:0006096 GO:0006352 GO:0004347 GO:0005669
Cluster-22178.68415 GO:0006355 GO:0003700 GO:0005667
Cluster-22178.68418 GO:0016757 GO:0016020
Cluster-22178.68423 GO:0008092 GO:0019898 GO:0005737
Cluster-22178.68425 GO:0007156 GO:0007155 GO:0019082 GO:0003677
GO:0005198 GO:0005509 GO:0008270 GO:0016020 GO:0031012

Cluster-22178.68427 GO:0006412 GO:0042254 GO:0005515 GO:0003735
GO:0005840 GO:0016021 GO:0019867
Cluster-22178.68429 GO:0010181 GO:0005759
Cluster-22178.68431 GO:0055114 GO:0009058 GO:0016209 GO:0051920
GO:0016491 GO:0003824
Cluster-22178.68432 GO:0006351 GO:0003677 GO:0005634
Cluster-22178.68433 GO:0006886 GO:0016192 GO:0007218 GO:0005515
GO:0005198 GO:0005185 GO:0043565 GO:0016798 GO:0019028
GO:0030117 GO:0005576 GO:0019031
Cluster-22178.68434 GO:0007218 GO:0006886 GO:0016192 GO:0043565
GO:0016798 GO:0005198 GO:0005515 GO:0005185 GO:0019031
GO:0019028 GO:0005576 GO:0030117
Cluster-22178.68435 GO:0006886 GO:0016192 GO:0005198 GO:0019867
GO:0030126
Cluster-22178.68439 GO:0016192 GO:0006886 GO:0005198 GO:0030126
GO:0019867
Cluster-22178.68440 GO:0034080 GO:0008270 GO:0000775
Cluster-22178.68442 GO:0055114 GO:0055085 GO:0006811 GO:0016209
GO:0016491 GO:0015075 GO:0016020
Cluster-22178.68443 GO:0006890 GO:0005975 GO:0005515 GO:0004252
GO:0003676 GO:0015926 GO:0008270 GO:0016021
Cluster-22178.68444 GO:0016773 GO:0005515
Cluster-22178.68445 GO:0006397 GO:0005515 GO:0003774 GO:0005524
GO:0016459
Cluster-22178.68446 GO:0009755 GO:0006364 GO:0005515 GO:0005576
GO:0032040
Cluster-22178.68448 GO:0046500 GO:0006400 GO:0006464 GO:0030488
GO:0001522 GO:0009451 GO:0006479 GO:0008033 GO:0008152
GO:0008176 GO:0009982 GO:0016429 GO:0008171 GO:0003723
GO:0008168 GO:0004719 GO:0031515
Cluster-22178.68450 GO:0007264 GO:0005525
Cluster-22178.68451 GO:0007165 GO:0006869 GO:0006955 GO:0008283
GO:0042157 GO:0040007 GO:0008289 GO:0005138 GO:0005896

GO:0005576
Cluster-22178.68452 GO:0055114 GO:0016491 GO:0046872
Cluster-22178.68453 GO:0070072
Cluster-22178.68456 GO:0016021
Cluster-22178.68459 GO:0055114 GO:0006355 GO:0019478 GO:0043565
GO:0016491 GO:0003700 GO:0003676 GO:0050662 GO:0005515
GO:0016788 GO:0005737 GO:0005667 GO:0016020
Cluster-22178.68462 GO:0055114 GO:0016491
Cluster-22178.68463 GO:0046486 GO:0006468 GO:0045017 GO:0000160
GO:0042967 GO:0004144 GO:0003677 GO:0000156 GO:0005524
GO:0004672
Cluster-22178.68465 GO:0016310 GO:0019089 GO:0007155 GO:0004673
GO:0009365 GO:0009288
Cluster-22178.68467 GO:0043087 GO:0035023 GO:0005089
Cluster-22178.68468 GO:0006464 GO:0030488 GO:0046500 GO:0006400
GO:0008152 GO:0008033 GO:0006479 GO:0001522 GO:0009451
GO:0016429 GO:0008171 GO:0009982 GO:0008176 GO:0004719
GO:0008168 GO:0003723 GO:0031515
Cluster-22178.68469 GO:0007283 GO:0006024 GO:0015012 GO:0003677
GO:0004252 GO:0016757 GO:0016020 GO:0005634 GO:0016021
GO:0000786
Cluster-22178.68471 GO:0006915 GO:0006355 GO:0006919 GO:0008608
GO:0001836 GO:0006511 GO:0016567 GO:0006974 GO:0003700
GO:0034450 GO:0004842 GO:0000151 GO:0042729 GO:0005667
GO:0005876 GO:0005815
Cluster-22178.68473 GO:0006571 GO:0008033 GO:0009094 GO:0006796
GO:0006432 GO:0006119 GO:0006508 GO:0000162 GO:0004826
GO:0008234 GO:0000049 GO:0000287 GO:0005524 GO:0004427
GO:0005737 GO:0009328
Cluster-22178.68478 GO:0006355 GO:0042967 GO:0016747 GO:0008080

Cluster-22178.68479 GO:0006468 GO:0042254 GO:0006412 GO:0055114
GO:0051287 GO:0003735 GO:0016773 GO:0005524 GO:0004672

GO:0016020 GO:0005840
Cluster-22178.68480 GO:0005515 GO:0046935 GO:0046872 GO:0008270
GO:0005944
Cluster-22178.68482 GO:0006464 GO:0055114 GO:0016226 GO:0046500
GO:0006479 GO:0004719 GO:0008168 GO:0051536 GO:0005506
Cluster-22178.68483 GO:0007304 GO:0005515 GO:0005213 GO:0046872
GO:0005576 GO:0016021 GO:0045202 GO:0042600
Cluster-22178.68491 GO:0007031 GO:0035556 GO:0007264 GO:0006457
GO:0006468 GO:0006355 GO:0000774 GO:0005524 GO:0004222
GO:0051087 GO:0003677 GO:0003924 GO:0004672 GO:0046872
GO:0003700 GO:0005525 GO:0051082 GO:0042803 GO:0016820
GO:0005515 GO:0016272 GO:0005667 GO:0005779 GO:0005739
GO:0016021
Cluster-22178.68492 GO:0016310 GO:0009069 GO:0006355 GO:0004674
GO:0034061 GO:0016779 GO:0003677 GO:0005634
Cluster-22178.68494 GO:0003723 GO:0005856
Cluster-22178.68495 GO:0005515
Cluster-22178.68497 GO:0007264 GO:0005525 GO:0005524 GO:0003924
GO:0016887 GO:0016020
Cluster-22178.68500 GO:0033691
Cluster-22178.68501 GO:0009372 GO:0004867
Cluster-22178.68502 GO:0006468 GO:0055114 GO:0003677 GO:0061630
GO:0005515 GO:0003697 GO:0003723 GO:0016787 GO:0047134
GO:0003676 GO:0005524 GO:0004672 GO:0008270 GO:0046872
Cluster-22178.68504 GO:0006260 GO:0007049 GO:0000917 GO:0006355
GO:0043093 GO:0003700 GO:0043565 GO:0003677 GO:0005737
GO:0005667 GO:0005634
Cluster-22178.68505 GO:0008152 GO:0006554 GO:0006464 GO:0008033
GO:0006479 GO:0009451 GO:0016192 GO:0006886 GO:0046500
GO:0006400 GO:0004719 GO:0008168 GO:0018024 GO:0008176
GO:0030117
Cluster-22178.68506 GO:0006260 GO:0006284 GO:0003906 GO:0016799

Cluster-22178.68507 GO:0006397 GO:0003682 GO:0003723 GO:0005737
GO:0005634 GO:0000785
Cluster-22178.68509 GO:0016020
Cluster-22178.68510 GO:0046872 GO:0003677
Cluster-22178.68514 GO:0006525 GO:0006560 GO:0055114 GO:0051321
GO:0018401 GO:0005515 GO:0004656 GO:0016702 GO:0005783
Cluster-22178.68515 GO:0009451 GO:0006886 GO:0008152 GO:0008033
GO:0016192 GO:0007165 GO:0006400 GO:0016301 GO:0005515
GO:0003723 GO:0008168 GO:0004809 GO:0046872 GO:0008176
GO:0030117 GO:0016021
Cluster-22178.68517 GO:0009252 GO:0006542 GO:0006807 GO:0004356

Cluster-22178.68518 GO:0006355 GO:0007155 GO:0042246 GO:0003677
GO:0005634 GO:0016021
Cluster-22178.68523 GO:0006144 GO:0006206 GO:0006355 GO:0044237
GO:0006351 GO:0006810 GO:0003899 GO:0003677 GO:0005639
GO:0016021 GO:0005730
Cluster-22178.68525 GO:0007411
Cluster-22178.68526 GO:0000160 GO:0016310 GO:0071555 GO:0005515
GO:0004673 GO:0000155 GO:0016021 GO:0009365
Cluster-22178.68527 GO:0007165 GO:0019079 GO:0006260 GO:0005179
GO:0030337 GO:0005576 GO:0042575
Cluster-22178.68528 GO:0034477 GO:0016579 GO:0007276 GO:0006364
GO:0006508 GO:0004222 GO:0003676 GO:0008270 GO:0036459
GO:0004518 GO:0030246 GO:0032183 GO:0070140 GO:0004857
GO:0031012
Cluster-22178.68529 GO:0007160 GO:0043087 GO:0043248 GO:0032012
GO:0005086 GO:0005509 GO:0005576 GO:0005634 GO:0005783
Cluster-22178.68530 GO:0003677
Cluster-22178.68532 GO:0007186 GO:0004930 GO:0016020 GO:0016021
Cluster-22178.68533 GO:0007165 GO:0005515 GO:0005737
Cluster-22178.68534 GO:0030153 GO:0007165 GO:0006955 GO:0005524
GO:0015643 GO:0016787 GO:0005515 GO:0005737 GO:0016021

GO:0019814
Cluster-22178.68537 GO:0006355 GO:0016747
Cluster-22178.68539 GO:0007264 GO:0005525
Cluster-22178.68542 GO:0005515
Cluster-22178.68544 GO:0055114 GO:0016209 GO:0016491 GO:0051920

Cluster-22178.68548 GO:0006779 GO:0006633 GO:0016117 GO:0006554
GO:0046168 GO:0018874 GO:0019354 GO:0006631 GO:0008152
GO:0006574 GO:0008033 GO:0055114 GO:0006550 GO:0006552
GO:0006813 GO:0006568 GO:0050660 GO:0016491 GO:0016616
GO:0051287 GO:0071949 GO:0003824 GO:0043115 GO:0016705
GO:0003857
Cluster-22178.68550 GO:0032922 GO:0000976
Cluster-22178.68551 GO:0006508 GO:0004222 GO:0008270 GO:0031012
Cluster-22178.68553 GO:0006487 GO:0005975 GO:0004553 GO:0016021
GO:0008250 GO:0019013
Cluster-22178.68554 GO:0006886 GO:0015031 GO:0006464 GO:0005047
GO:0005515 GO:0008270 GO:0003924 GO:0005525 GO:0052855
GO:0005786 GO:0005785
Cluster-22178.68555 GO:0006355 GO:0005515 GO:0003700 GO:0003677
GO:0046872 GO:0005667
Cluster-22178.68559 GO:0006412 GO:0042254 GO:0003735 GO:0005622
GO:0005840
Cluster-22178.68560 GO:0016192 GO:0006904 GO:0042742 GO:0003677
GO:0046872
Cluster-22178.68561 GO:0006531 GO:0009058 GO:0006520 GO:0055114
GO:0006144 GO:0009152 GO:0006188 GO:0006522 GO:0030170
GO:0016829 GO:0004018 GO:0010277
Cluster-22178.68562 GO:0005515 GO:0016021
Cluster-22178.68564 GO:0046983
Cluster-22178.68565 GO:0007165 GO:0006397 GO:0015743 GO:0030436
GO:0003723 GO:0005179 GO:0005634 GO:0005576 GO:0042601
GO:0016021 GO:0005737

Cluster-22178.68572 GO:0005515
Cluster-22178.68573 GO:0006144 GO:0006470 GO:0006508 GO:0006570
GO:0003968 GO:0004725 GO:0017111 GO:0008138 GO:0004197
GO:0031379
Cluster-22178.68574 GO:0006468 GO:0004672 GO:0005524 GO:0004190
GO:0016021
Cluster-22178.68575 GO:0005515 GO:0030250 GO:0005576
Cluster-22178.68576 GO:0042967 GO:0016407
Cluster-22178.68579 GO:0005643
Cluster-22178.68581 GO:0005975 GO:0008152 GO:0016042 GO:0006508
GO:0046486 GO:0009058 GO:0004806 GO:0008184 GO:0016787
GO:0016788 GO:0008236
Cluster-22178.68585 GO:0016567 GO:0004842
Cluster-22178.68586 GO:0019530 GO:0006749 GO:0006691 GO:0051252
GO:0006486 GO:0006693 GO:0004540 GO:0003840 GO:0008373
Cluster-22178.68587 GO:0008152 GO:0005975 GO:0005509 GO:0004571
GO:0016829 GO:0016020
Cluster-22178.68588 GO:0007224
Cluster-22178.68589 GO:0008152 GO:0005975 GO:0005509 GO:0004571
GO:0016829 GO:0016020
Cluster-22178.68591 GO:0006308 GO:0009307 GO:0042254 GO:0006412
GO:0009036 GO:0003735 GO:0003677 GO:0005840 GO:0009359
Cluster-22178.68593 GO:0009312 GO:0008455 GO:0016021 GO:0005795
Cluster-22178.68594 GO:0051603 GO:0006511 GO:0004175 GO:0004298
GO:0003677 GO:0005515 GO:0005839 GO:0019773
Cluster-22178.68595 GO:0006468 GO:0006886 GO:0015979 GO:0042967
GO:0005524 GO:0004672 GO:0008080 GO:0016020 GO:0009523
GO:0009539 GO:0030176
Cluster-22178.68596 GO:0016579 GO:0005515 GO:0036459
Cluster-22178.68597 GO:0005509 GO:0005544
Cluster-22178.68598 GO:0015979 GO:0042967 GO:0006468 GO:0006886
GO:0004672 GO:0005524 GO:0008080 GO:0009523 GO:0009539
GO:0030176 GO:0016020

Cluster-22178.68599 GO:0019073
Cluster-22178.68606 GO:0006955 GO:0030153 GO:0008152 GO:0006516
GO:0015643 GO:0016747 GO:0019814 GO:0005737
Cluster-22178.68607 GO:0009405 GO:0042254 GO:0006570 GO:0006412
GO:0006470 GO:0008138 GO:0003735 GO:0008270 GO:0004725
GO:0005576 GO:0005622 GO:0005840
Cluster-22178.68612 GO:0006355 GO:0006012 GO:0009117 GO:0003700
GO:0008108 GO:0005667 GO:0005634
Cluster-22178.68614 GO:0007165 GO:0035556 GO:0005515 GO:0016012
GO:0016020
Cluster-22178.68616 GO:0006281 GO:0009307 GO:0006308 GO:0003677
GO:0009036 GO:0005634 GO:0009359 GO:0016020
Cluster-22178.68617 GO:0007165 GO:0035556 GO:0005515 GO:0016012
GO:0016020
Cluster-22178.68620 GO:0006351 GO:0090266 GO:0006270 GO:0006260
GO:0051016 GO:0003887 GO:0004190 GO:0003677 GO:0008270
GO:0042575 GO:0005634 GO:0019028 GO:0005789 GO:0016021
GO:0005680 GO:0030479
Cluster-22178.68621 GO:0035556 GO:0042254 GO:0006412 GO:0032183
GO:0070140 GO:0005515 GO:0003735 GO:0005840 GO:0015934
Cluster-22178.68626 GO:0007275 GO:0007219 GO:0030154 GO:0005509
GO:0005515 GO:0016021
Cluster-22178.68627 GO:0006400 GO:0007172 GO:0007165 GO:0006487
GO:0006468 GO:0008616 GO:0006397 GO:0004713 GO:0004871
GO:0008479 GO:0005515 GO:0005925 GO:0008250 GO:0016021
GO:0019028 GO:0005634
Cluster-22178.68628 GO:0055114 GO:0008152 GO:0016491
Cluster-22178.68630 GO:0005515
Cluster-22178.68632 GO:0006508 GO:0004185
Cluster-22178.68634 GO:0009405 GO:0006486 GO:0007049 GO:0006270
GO:0007067 GO:0006260 GO:0005515 GO:0042802 GO:0003887
GO:0003980 GO:0016757 GO:0046983 GO:0042575 GO:0005634
GO:0005727 GO:0016021 GO:0000775 GO:0016020

Cluster-22178.68636 GO:0042967 GO:0006355 GO:0008270 GO:0003712
GO:0004402 GO:0005515 GO:0000123 GO:0005634 GO:0005667
Cluster-22178.68637 GO:0006355 GO:0042967 GO:0004402 GO:0005515
GO:0008270 GO:0003712 GO:0005634 GO:0005667 GO:0000123
Cluster-22178.68638 GO:0006118 GO:0006119 GO:0055114 GO:0015992
GO:0008121
Cluster-22178.68640 GO:0051252 GO:0004523 GO:0003676
Cluster-22178.68642 GO:0006412 GO:0006270 GO:0006614 GO:0042254
GO:0007264 GO:0005525 GO:0043531 GO:0003924 GO:0003723
GO:0003724 GO:0005524 GO:0016887 GO:0098519 GO:0003735
GO:0005840 GO:0005622
Cluster-22178.68643 GO:0006810 GO:0005215 GO:0016020
Cluster-22178.68644 GO:2000123 GO:0005515 GO:0016021
Cluster-22178.68647 GO:0006779 GO:0008033 GO:0055114 GO:0019354
GO:0045454 GO:0050660 GO:0016491 GO:0043115 GO:0071949
Cluster-22178.68648 GO:0006182 GO:0006508 GO:0046039 GO:0009190
GO:0006468 GO:0008152 GO:0035556 GO:0006144 GO:0008236
GO:0004383 GO:0016849 GO:0016787 GO:0004672 GO:0005524
Cluster-22178.68655 GO:0051603 GO:0004298 GO:0005839
Cluster-22178.68657 GO:0006810 GO:0005622
Cluster-22178.68660 GO:0042254 GO:0006412 GO:0005515 GO:0003735
GO:0005840 GO:0005622
Cluster-22178.68661 GO:0046034 GO:0006355 GO:0006144 GO:0006464
GO:0006869 GO:0042157 GO:0003700 GO:0008289 GO:0016151
GO:0043565 GO:0046872 GO:0051082 GO:0008270 GO:0004017
GO:0005576 GO:0005815 GO:0005667
Cluster-22178.68663 GO:0009253 GO:0016567 GO:0019068 GO:0006807
GO:0009252 GO:0000166 GO:0005515 GO:0008745 GO:0003676
GO:0046872 GO:0003677 GO:0004842 GO:0008270 GO:0005524
GO:0019028 GO:0005680
Cluster-22178.68665 GO:0016787 GO:0005524 GO:0003676 GO:0003677
GO:0005615
Cluster-22178.68666 GO:0015992 GO:0006144 GO:0006188 GO:0009152

GO:0006522 GO:0006123 GO:0006531 GO:0016567 GO:0004018
GO:0004842 GO:0004129 GO:0045277 GO:0005740
Cluster-22178.68668 GO:0006508 GO:0009847 GO:0003333 GO:0004185
GO:0016021
Cluster-22178.68669 GO:0006508 GO:0003333 GO:0009847 GO:0004185
GO:0016021
Cluster-22178.68671 GO:0007264 GO:0005525 GO:0003924
Cluster-22178.68672 GO:0000045 GO:0005737
Cluster-22178.68673 GO:0006357 GO:0001104 GO:0008270 GO:0016592
Cluster-22178.68674 GO:0007155 GO:0042246 GO:0051252 GO:0008033
GO:0006396 GO:0004526 GO:0003676 GO:0030677 GO:0016021
Cluster-22178.68675 GO:0051252 GO:0006396 GO:0008033 GO:0003676
GO:0004526 GO:0030677
Cluster-22178.68677 GO:0019497 GO:0055114 GO:0007165 GO:0006771
GO:0003993 GO:0005525 GO:0016620 GO:0004872 GO:0046872
GO:0003677 GO:0005515 GO:0004190 GO:0016021 GO:0016020
Cluster-22178.68679 GO:0035091
Cluster-22178.68680 GO:0007049 GO:0006935 GO:0048870 GO:0006355
GO:0000921 GO:0071973 GO:0016567 GO:0051259 GO:0048309
GO:0007094 GO:0006886 GO:0051028 GO:0006914 GO:0007155
GO:0003700 GO:0046872 GO:0008092 GO:0008134 GO:0045502
GO:0004842 GO:0031386 GO:0043565 GO:0016881 GO:0005515
GO:0005198 GO:0042803 GO:0008270 GO:0003774 GO:0016021
GO:0005801 GO:0031514 GO:0019898 GO:0030286 GO:0005940
GO:0005882 GO:0009288 GO:0005737 GO:0005680 GO:0005783
GO:0016020 GO:0005667
Cluster-22178.68681 GO:0006950 GO:0006508 GO:0004222 GO:0003700
GO:0008270 GO:0004197 GO:0031012 GO:0005667
Cluster-22178.68684 GO:0055114 GO:0008152 GO:0016491
Cluster-22178.68686 GO:0030001 GO:0007010 GO:0006308 GO:0006810
GO:0055085 GO:0000226 GO:0006260 GO:0005515 GO:0003779
GO:0046872 GO:0003677 GO:0008855 GO:0005524 GO:0042626
GO:0015630 GO:0009318 GO:0016021 GO:0005664

Cluster-22178.68688 GO:0032259 GO:0006511 GO:0006310 GO:0007010
GO:0004175 GO:0003779 GO:0008168 GO:0005515 GO:0003677
GO:0019773 GO:0005634
Cluster-22178.68690 GO:0016757
Cluster-22178.68692 GO:0007165 GO:0005515 GO:0016820 GO:0005739
GO:0016020 GO:0016021
Cluster-22178.68693 GO:0007165 GO:0003723 GO:0005158 GO:0005515
GO:0005899
Cluster-22178.68694 GO:0005515
Cluster-22178.68696 GO:0016192 GO:0005985 GO:0005982 GO:0030245
GO:0006353 GO:0000281 GO:0000226 GO:0040001 GO:0008810
GO:0005819 GO:0016021 GO:0005874
Cluster-22178.68698 GO:0006355 GO:0005515
Cluster-22178.68699 GO:0005515 GO:0003723
Cluster-22178.68700 GO:0006886 GO:0051258 GO:0048309 GO:0006308
GO:0042157 GO:0006869 GO:0007165 GO:0016567 GO:0016192
GO:0030168 GO:0051028 GO:0006260 GO:0005515 GO:0046872
GO:0008289 GO:0030674 GO:0008270 GO:0008855 GO:0004842
GO:0005102 GO:0019905 GO:0005576 GO:0005634 GO:0009318
GO:0016021 GO:0005783 GO:0005680 GO:0005577
Cluster-22178.68702 GO:0006355 GO:0006468 GO:0004672 GO:0003700
GO:0005634 GO:0005667
Cluster-22178.68707 GO:0006355 GO:0006338 GO:0003677 GO:0016818
GO:0005524 GO:0008270 GO:0003676 GO:0005634 GO:0005739
Cluster-22178.68708 GO:0016567 GO:0004842
Cluster-22178.68709 GO:0042578
Cluster-22178.68710 GO:0007050 GO:0045859 GO:0000122 GO:0004861
GO:0017069 GO:0005737 GO:0005634
Cluster-22178.68711 GO:0016887 GO:0005524 GO:0005515 GO:0005634
Cluster-22178.68713 GO:0090063 GO:0000725 GO:0006351 GO:0007021
GO:0006144 GO:0006206 GO:0000226 GO:0051082 GO:0003899
GO:0003677 GO:0015631 GO:0005634 GO:0097196 GO:0045298
GO:0000922 GO:0005730 GO:0005815

Cluster-22178.68715 GO:0051537 GO:0005509 GO:0005515 GO:0043231
Cluster-22178.68717 GO:0046413 GO:0045444 GO:0018836 GO:0005515

Cluster-22178.68718 GO:0006260 GO:0019953 GO:0006351 GO:0006886
GO:0003887 GO:0003677 GO:0005515 GO:0005198 GO:0005664
GO:0005576 GO:0042575 GO:0030141
Cluster-22178.68719 GO:0007049 GO:0006355 GO:0046872 GO:0043565
GO:0003700 GO:0045502 GO:0019901 GO:0042803 GO:0008134
GO:0030286 GO:0005667
Cluster-22178.68721 GO:0000090 GO:0007067 GO:0007059 GO:0051301
GO:0031110 GO:0008017 GO:0005876 GO:0045298 GO:0000940
Cluster-22178.68722 GO:0051225 GO:0006281 GO:0006260 GO:0031023
GO:0003911 GO:0005524 GO:0046983 GO:0003689 GO:0042575
GO:0005663
Cluster-22178.68728 GO:0015976 GO:0006144 GO:0006096 GO:0006094
GO:0003824 GO:0005515 GO:0000287 GO:0030955 GO:0004743
Cluster-22178.68731 GO:0009405 GO:0006810 GO:0007186 GO:0008200
GO:0004930 GO:0005576 GO:0016021
Cluster-22178.68732 GO:0006164 GO:0006531 GO:0006810 GO:0007165
GO:0015858 GO:0006955 GO:0006144 GO:0006522 GO:0005125
GO:0005525 GO:0005337 GO:0004019 GO:0005576 GO:0016021
Cluster-22178.68733 GO:0005515
Cluster-22178.68735 GO:0006816 GO:0022900 GO:0005388 GO:0005747
GO:0016020 GO:0016529 GO:0005739
Cluster-22178.68737 GO:0005524 GO:0005856
Cluster-22178.68738 GO:0016310 GO:0006090 GO:0006525 GO:0006560
GO:0006814 GO:0007186 GO:0071436 GO:0015081 GO:0004930
GO:0004673 GO:0008948 GO:0009365 GO:0016021 GO:0016020
Cluster-22178.68745 GO:0051603 GO:0006511 GO:0004175 GO:0004298
GO:0005839 GO:0019773
Cluster-22178.68746 GO:0006511 GO:0051603 GO:0004298 GO:0004175
GO:0019773 GO:0005839
Cluster-22178.68747 GO:0008152 GO:0016491

Cluster-22178.68748 GO:0035556 GO:0006469 GO:0046907 GO:0045859
GO:0004862 GO:0005952
Cluster-22178.68750 GO:0006260 GO:0005515 GO:0003887 GO:0005615
GO:0042575
Cluster-22178.68751 GO:0035556 GO:0009395 GO:0046339 GO:0007165
GO:0006629 GO:0004435 GO:0046872 GO:0005515
Cluster-22178.68753 GO:0006950 GO:0006508 GO:0008270 GO:0004222
GO:0003700 GO:0004197 GO:0031012 GO:0005667
Cluster-22178.68754 GO:0045454 GO:0022900 GO:0006729 GO:0016651
GO:0008124
Cluster-22178.68757 GO:0006309 GO:0006915 GO:0016787 GO:0005634
GO:0005737 GO:0005622
Cluster-22178.68758 GO:0043065 GO:0042981 GO:0015992 GO:0006119
GO:0015991 GO:0015986 GO:0015078 GO:0046961 GO:0031225
GO:0005737 GO:0045263 GO:0033178
Cluster-22178.68761 GO:0003723 GO:0003676
Cluster-22178.68762 GO:0007264 GO:0043093 GO:0042254 GO:0006457
GO:0006310 GO:0010506 GO:0006260 GO:0006281 GO:0006355
GO:0006412 GO:0000917 GO:0003735 GO:0005096 GO:0000287
GO:0051082 GO:0005515 GO:0043565 GO:0003700 GO:0005667
GO:0016272 GO:0005737 GO:0005615 GO:0005840
Cluster-22178.68764 GO:0000921 GO:0007096 GO:0007165 GO:0008092
GO:0004871 GO:0005515 GO:0005737 GO:0005940 GO:0005886
GO:0016021 GO:0019898 GO:0005634 GO:0016020
Cluster-22178.68765 GO:0055114 GO:0006629 GO:0030001 GO:0016491
GO:0046872
Cluster-22178.68766 GO:0019370 GO:0034453 GO:0000042 GO:0008270
GO:0008237 GO:0005515 GO:0005815 GO:0016021
Cluster-22178.68769 GO:0051252 GO:0000160 GO:0016310 GO:0004540
GO:0004673 GO:0000155 GO:0008171 GO:0016020 GO:0005737
GO:0009365
Cluster-22178.68770 GO:0051252 GO:0000160 GO:0016310 GO:0004540
GO:0000155 GO:0004673 GO:0008171 GO:0016020 GO:0005737

GO:0009365
Cluster-22178.68771 GO:0006810 GO:0005215 GO:0016020
Cluster-22178.68774 GO:0006570 GO:0007165 GO:0006470 GO:0005515
GO:0004721 GO:0004725 GO:0008138
Cluster-22178.68775 GO:0007264 GO:0005525
Cluster-22178.68776 GO:0016055 GO:0007165 GO:0007275 GO:0006570
GO:0006470 GO:0008138 GO:0005515 GO:0004725 GO:0004721
GO:0005102 GO:0005576
Cluster-22178.68778 GO:0003924
Cluster-22178.68779 GO:0006418 GO:0004812 GO:0008270 GO:0005524
GO:0000166
Cluster-22178.68780 GO:0006464 GO:0035556 GO:0046836 GO:0046872
GO:0016151 GO:0017089 GO:0051861 GO:0003677 GO:0005737
Cluster-22178.68781 GO:0035556 GO:0046836 GO:0017089 GO:0003677
GO:0051861 GO:0046872 GO:0005737
Cluster-22178.68784 GO:0006915 GO:0019028 GO:0005622
Cluster-22178.68789 GO:0006396 GO:0005622
Cluster-22178.68790 GO:0006825 GO:0016531 GO:0005507 GO:0005758
Cluster-22178.68791 GO:0044564 GO:0006468 GO:0005515 GO:0005524
GO:0004672
Cluster-22178.68792 GO:0046872
Cluster-22178.68793 GO:0043093 GO:0006355 GO:0006364 GO:0000917
GO:0009395 GO:0016042 GO:0005515 GO:0004629 GO:0003677
GO:0043565 GO:0003700 GO:0005634 GO:0005667 GO:0005737
Cluster-22178.68794 GO:0005515
Cluster-22178.68795 GO:0005515
Cluster-22178.68796 GO:0005515
Cluster-22178.68798 GO:0032008 GO:0007040 GO:0043410 GO:0032439
GO:0071230 GO:0001919 GO:0042632 GO:0031902 GO:0071986
GO:0045121
Cluster-22178.68799 GO:0006810 GO:0006397 GO:0051252 GO:0006402
GO:0016788 GO:0003723 GO:0003676 GO:0005215 GO:0046872
GO:0004535 GO:0005634 GO:0005576 GO:0005737

Cluster-22178.68800 GO:0051252 GO:0006810 GO:0006402 GO:0003723
GO:0003676 GO:0005215 GO:0004535 GO:0046872 GO:0005634
GO:0005576 GO:0005737
Cluster-22178.68801 GO:0006801 GO:0016765 GO:0016209 GO:0004784
GO:0016151
Cluster-22178.68802 GO:0007160 GO:0008152 GO:0003824 GO:0009976

Cluster-22178.68805 GO:0006364 GO:0006468 GO:0005515 GO:0005524
GO:0004672
Cluster-22178.68807 GO:0046339 GO:0009395 GO:0035556 GO:0006629
GO:0007165 GO:0004435 GO:0005515 GO:0005509
Cluster-22178.68809 GO:0004867
Cluster-22178.68810 GO:0046339 GO:0009395 GO:0035556 GO:0006629
GO:0007165 GO:0004435 GO:0005509 GO:0005515 GO:0005578
Cluster-22178.68812 GO:0006281 GO:0007059 GO:0006777 GO:0000723
GO:0006614 GO:0009089 GO:0009058 GO:0030261 GO:0005525
GO:0003678 GO:0003824 GO:0008837 GO:0003677 GO:0016787
GO:0005524 GO:0019031 GO:0009295 GO:0005657
Cluster-22178.68816 GO:0008152 GO:0007160 GO:0005509 GO:0008168
GO:0005576
Cluster-22178.68817 GO:0008152 GO:0046912 GO:0003824
Cluster-22178.68818 GO:0022900 GO:0046872 GO:0003676 GO:0005515
GO:0070469 GO:0005743
Cluster-22178.68819 GO:0035023 GO:0006807 GO:0005975 GO:0043087
GO:0005515 GO:0005089 GO:0016810 GO:0005737
Cluster-22178.68820 GO:0005975 GO:0043087 GO:0006807 GO:0035023
GO:0016810 GO:0005089 GO:0005515 GO:0005737
Cluster-22178.68821 GO:0035023 GO:0006807 GO:0043087 GO:0005975
GO:0005515 GO:0005089 GO:0016810 GO:0005737
Cluster-22178.68825 GO:0006508 GO:0008236
Cluster-22178.68827 GO:0005783
Cluster-22178.68828 GO:0003723 GO:0005515
Cluster-22178.68829 GO:0007154 GO:0016020

Cluster-22178.68832 GO:0006508 GO:0005515 GO:0004190 GO:0008270 GO:0005861

Cluster-22178.68836 GO:0008283 GO:0040007 GO:0007165 GO:0008284 GO:0005975 GO:0001934 GO:0005157 GO:0005509 GO:0004571 GO:0016020 GO:0005615

Cluster-22178.68839 GO:0009405 GO:0007165 GO:0006629 GO:0035556 GO:0046339 GO:0009395 GO:0005515 GO:0004435 GO:0046872

Cluster-22178.68844 GO:0009401 GO:0006812 GO:0006885 GO:0055085 GO:0008643 GO:0005198 GO:0008982 GO:0015299 GO:0009357 GO:0016021 GO:0005737

Cluster-22178.68847 GO:0003677

Cluster-22178.68848 GO:0002949 GO:0006310 GO:0006073 GO:0006508 GO:0006281 GO:0004222 GO:0016887 GO:0005524 GO:0009378 GO:0003678 GO:0016762 GO:0016301 GO:0005618 GO:0005657 GO:0009379 GO:0048046

Cluster-22178.68851 GO:0019048 GO:0005634 GO:0019031

Cluster-22178.68852 GO:0007165

Cluster-22178.68853 GO:0005515

Cluster-22178.68854 GO:0006813 GO:0006814 GO:0005890

Cluster-22178.68857 GO:0006470 GO:0006570 GO:0019988 GO:0043399 GO:0004725 GO:0008138

Cluster-22178.68863 GO:0006118 GO:0045454 GO:0015035 GO:0009055

Cluster-22178.68864 GO:0006357 GO:0001104 GO:0005515 GO:0003723 GO:0016592

Cluster-22178.68867 GO:0006355 GO:0006810 GO:0003677 GO:0005215 GO:0005634 GO:0016020

Cluster-22178.68868 GO:0006144 GO:0046034 GO:0003677 GO:0004017 GO:0005515

Cluster-22178.68869 GO:0055114 GO:0005515 GO:0016706

Cluster-22178.68870 GO:0016567 GO:0004842

Cluster-22178.68873 GO:0042254 GO:0006412 GO:2001070 GO:0003735 GO:0005840

Cluster-22178.68874 GO:0009405 GO:0016117 GO:0005515 GO:0016705
GO:0003677 GO:0005525 GO:0003924 GO:0046872
Cluster-22178.68875 GO:0009405 GO:0016117 GO:0046872 GO:0003677
GO:0005515 GO:0016705
Cluster-22178.68877 GO:0005887
Cluster-22178.68878 GO:0005887
Cluster-22178.68881 GO:0006955 GO:0030153 GO:0006355 GO:0003677
GO:0015643 GO:0019814 GO:0016021
Cluster-22178.68882 GO:0006364 GO:0016579 GO:0036459 GO:0005730
Cluster-22178.68884 GO:0006810 GO:0016021
Cluster-22178.68885 GO:0006446 GO:0003743 GO:0003676 GO:0003723
GO:0005840 GO:0005850
Cluster-22178.68886 GO:0008152 GO:0003824
Cluster-22178.68887 GO:0005515 GO:0016853
Cluster-22178.68890 GO:0007067 GO:0006073 GO:0016762 GO:0005618
GO:0042729 GO:0072686 GO:0048046
Cluster-22178.68893 GO:0005975 GO:0071704 GO:0005515 GO:0016868

Cluster-22178.68894 GO:0006357 GO:0005515 GO:0001104 GO:0003723
GO:0016592
Cluster-22178.68896 GO:0016114 GO:0006694 GO:0008661 GO:0016020
Cluster-22178.68899 GO:0006606 GO:0006891 GO:0006914 GO:0006308
GO:0042246 GO:0007165 GO:0007155 GO:0008855 GO:0005515
GO:0016021 GO:0009318 GO:0017119
Cluster-22178.68905 GO:0006810 GO:0016567 GO:0006915 GO:0033014
GO:0006783 GO:0015994 GO:0004852 GO:0005515 GO:0005215
GO:0004842 GO:0008270 GO:0046872 GO:0016020 GO:0005680
Cluster-22178.68906 GO:0033691
Cluster-22178.68908 GO:0055085 GO:0006811 GO:0005216 GO:0016020
Cluster-22178.68909 GO:0055085 GO:0006811 GO:0005216 GO:0003677
GO:0016020
Cluster-22178.68911 GO:0006355 GO:0055114 GO:0008152 GO:0015696
GO:0006352 GO:0009058 GO:0006633 GO:0042967 GO:0004315

GO:0016788 GO:0003700 GO:0008519 GO:0003677 GO:0017111
GO:0016491 GO:0016747 GO:0016987 GO:0005835 GO:0005667
GO:0016020
Cluster-22178.68913 GO:0015031 GO:0008565 GO:0016021
Cluster-22178.68914 GO:0048598 GO:0005515 GO:0072669
Cluster-22178.68915 GO:0043065 GO:0016020 GO:0005737
Cluster-22178.68916 GO:0006355 GO:0043565 GO:0003700 GO:0005667
Cluster-22178.68918 GO:0005515
Cluster-22178.68919 GO:0006915
Cluster-22178.68923 GO:0006952 GO:0004869 GO:0005576
Cluster-22178.68924 GO:0006952 GO:0004869 GO:0005576
Cluster-22178.68926 GO:0006355 GO:0035556 GO:0008270 GO:0046872
GO:0005515 GO:0005634
Cluster-22178.68927 GO:0030163 GO:0000398 GO:0006144 GO:0045454
GO:0006118 GO:0015035 GO:0009055 GO:0003968 GO:0005515
GO:0031379 GO:0005681 GO:0016021
Cluster-22178.68928 GO:0005515 GO:0005525 GO:0003924 GO:0046872

Cluster-22178.68929 GO:0006051 GO:0006355 GO:0006537 GO:0008033
GO:0055114 GO:0008152 GO:0007219 GO:0046497 GO:0007275
GO:0009607 GO:0006040 GO:0009435 GO:0000105 GO:0006807
GO:0004514 GO:0047465 GO:0016829 GO:0016627 GO:0017150
GO:0003824 GO:0016638 GO:0018580 GO:0015930 GO:0016491
GO:0050660 GO:0005737 GO:0016021
Cluster-22178.68931 GO:0008033 GO:0016779 GO:0003676 GO:0016437
GO:0004810 GO:0008270
Cluster-22178.68934 GO:0016567 GO:0004842
Cluster-22178.68937 GO:0046740 GO:0006810 GO:0042254 GO:0006412
GO:0006260 GO:0051082 GO:0003735 GO:0016798 GO:0016020
GO:0005886 GO:0005622 GO:0005840 GO:0016021
Cluster-22178.68938 GO:0006260 GO:0042254 GO:0006412 GO:0006810
GO:0046740 GO:0016798 GO:0003735 GO:0051082 GO:0016021
GO:0005840 GO:0005622 GO:0005886 GO:0016020

Cluster-22178.68942 GO:0016567 GO:0015684 GO:0007264 GO:0006812 GO:0006284 GO:0032801 GO:0006289 GO:0006576 GO:0006355 GO:0006281 GO:0019076 GO:0003684 GO:0003677 GO:0003906 GO:0004842 GO:0016887 GO:0015093 GO:0005524 GO:0008270 GO:0016799 GO:0005261 GO:0005525 GO:0003924 GO:0005680 GO:0033644 GO:0016021

Cluster-22178.68944 GO:0006570 GO:0006470 GO:0004725 GO:0008138

Cluster-22178.68950 GO:0007010 GO:0003779

Cluster-22178.68951 GO:0019082 GO:0035556 GO:0007010 GO:0003779 GO:0008270 GO:0005515 GO:0003677

Cluster-22178.68952 GO:0015740 GO:0015556 GO:0005789 GO:0032040 GO:0016021

Cluster-22178.68953 GO:0008270 GO:0003723

Cluster-22178.68954 GO:0003824

Cluster-22178.68957 GO:0006270 GO:0007005 GO:0016884 GO:0000062 GO:0032865 GO:0016021

Cluster-22178.68959 GO:0008643 GO:0005351 GO:0016021 GO:0016020 GO:0000139

Cluster-22178.68960 GO:0006813 GO:0030001 GO:0055085 GO:0046873 GO:0005515 GO:0005249 GO:0016020 GO:0008076 GO:0016021

Cluster-22178.68961 GO:0016787

Cluster-22178.68964 GO:0030001 GO:0006355 GO:0007009 GO:0046872 GO:0005515 GO:0005743 GO:0005737 GO:0005747 GO:0005634

Cluster-22178.68965 GO:0030001 GO:0006355 GO:0007009 GO:0005515 GO:0046872 GO:0005747 GO:0005634 GO:0005743

Cluster-22178.68970 GO:0000166 GO:0046872

Cluster-22178.68971 GO:0005515 GO:0061630 GO:0046872 GO:0008270 GO:0003723

Cluster-22178.68972 GO:0007049 GO:0016021 GO:0005634

Cluster-22178.68973 GO:0019058 GO:0019031

Cluster-22178.68978 GO:0006207 GO:0006206 GO:0009116 GO:0016310 GO:0004590 GO:0004673 GO:0009365

Cluster-22178.68980 GO:0006388 GO:0051252 GO:0000213 GO:0000214

Cluster-22178.68981 GO:0019028

Cluster-22178.68982 GO:0042967 GO:0006396 GO:0006397 GO:0008080 GO:0016747 GO:0005515 GO:0005622 GO:0005634

Cluster-22178.68984 GO:0006355 GO:0003700 GO:0043565 GO:0005667

Cluster-22178.68985 GO:0008270

Cluster-22178.68987 GO:0015675 GO:0035444 GO:0006508 GO:0046872 GO:0015099 GO:0004252 GO:0030288 GO:0016021

Cluster-22178.68988 GO:0046872 GO:0000166

Cluster-22178.68989 GO:0006260 GO:0016021

Cluster-22178.68990 GO:0008152 GO:0016491

Cluster-22178.68992 GO:0006468 GO:0004672 GO:0005524

Cluster-22178.68995 GO:0018101 GO:0006807 GO:0004668 GO:0016787 GO:0005509 GO:0005737

Cluster-22178.68996 GO:0006807 GO:0004668 GO:0005509 GO:0005737

Cluster-22178.68997 GO:0018101 GO:0006807 GO:0005509 GO:0004668 GO:0016787 GO:0005737

Cluster-22178.68998 GO:0016820 GO:0005739 GO:0016021

Cluster-22178.69001 GO:0006446 GO:0006413 GO:0003743 GO:0003723 GO:0005840

Cluster-22178.69003 GO:0005515

Cluster-22178.69010 GO:0005515

Cluster-22178.69012 GO:0006260 GO:0006355 GO:0006351 GO:0008270 GO:0003682 GO:0003677 GO:0003887 GO:0000775 GO:0000785 GO:0005634 GO:0042575

Cluster-22178.69013 GO:0000245 GO:0055114 GO:0008033 GO:0016117 GO:0071949 GO:0051287 GO:0016705 GO:0005515 GO:0008270 GO:0016491 GO:0050660 GO:0005634

Cluster-22178.69014 GO:0000245 GO:0008033 GO:0055114 GO:0016117 GO:0071949 GO:0051287 GO:0016705 GO:0005515 GO:0008270 GO:0016491 GO:0050660 GO:0005634

Cluster-22178.69017 GO:0006355 GO:0003700 GO:0005667

Cluster-22178.69018 GO:0016021

Cluster-22178.69019 GO:0005515
Cluster-22178.69020 GO:0045444 GO:0016020
Cluster-22178.69021 GO:0016787
Cluster-22178.69022 GO:1900758
Cluster-22178.69024 GO:0000002 GO:0000262
Cluster-22178.69026 GO:0007165 GO:0030001 GO:0008146 GO:0005179 GO:0046872 GO:0005576
Cluster-22178.69029 GO:0006270 GO:0006260 GO:0009236 GO:0006351 GO:0006355 GO:0006352 GO:0000398 GO:0003677 GO:0016987 GO:0008270 GO:0005515 GO:0003887 GO:0003700 GO:0005667 GO:0042575 GO:0005634 GO:0005681 GO:0032040
Cluster-22178.69034 GO:0019051 GO:0005515 GO:0042025
Cluster-22178.69036 GO:0006448 GO:0008544 GO:0045901 GO:0006452 GO:0045905 GO:0003746 GO:0043022 GO:0003723 GO:0005840
Cluster-22178.69037 GO:0006820 GO:0055085 GO:0006508 GO:0006810 GO:0008509 GO:0005215 GO:0004190 GO:0016021 GO:0016020
Cluster-22178.69038 GO:0007218 GO:0045893 GO:0007275 GO:0003676 GO:0008270 GO:0005524 GO:0005184 GO:0005515 GO:0005576
Cluster-22178.69041 GO:0006355 GO:0006265 GO:0016579 GO:0003677 GO:0005515 GO:0003916 GO:0036459 GO:0003700 GO:0005694 GO:0005667
Cluster-22178.69042 GO:0006367 GO:0005524 GO:0003682 GO:0000785 GO:0005672
Cluster-22178.69046 GO:0006338 GO:0003677 GO:0090544
Cluster-22178.69047 GO:0016787 GO:0005524 GO:0003676 GO:0003677

Cluster-22178.69048 GO:0006355 GO:0003677 GO:0016020
Cluster-22178.69049 GO:0015991 GO:0006811 GO:0048870 GO:0051258 GO:0006457 GO:0071973 GO:0007165 GO:0006810 GO:0006606 GO:0006914 GO:0030168 GO:0015992 GO:0005198 GO:0042803 GO:0015078 GO:0008092 GO:0051087 GO:0030674 GO:0005102 GO:0000774 GO:0003774 GO:0033179 GO:0031514 GO:0005737 GO:0009288 GO:0016020 GO:0005882 GO:0019898 GO:0005577

GO:0005886

Cluster-22178.69050 GO:0019722 GO:0035091 GO:0005515 GO:0003676

GO:0005887 GO:0016021

Cluster-22178.69051 GO:0042967 GO:0005515 GO:0008080 GO:0071203

Cluster-22178.69053 GO:0005975 GO:1902208 GO:0045893 GO:0006810

GO:0008270 GO:0003950 GO:0005215 GO:0016832 GO:0003677

GO:0005515 GO:0005737 GO:0016021 GO:0005634

Cluster-22178.69054 GO:0006446 GO:0003743 GO:0005737 GO:0008023

GO:0005852 GO:0005840

Cluster-22178.69055 GO:0015992 GO:0015986 GO:0015078 GO:0000276

Cluster-22178.69056 GO:0016032 GO:0005515 GO:0005576

Cluster-22178.69059 GO:0016032 GO:0005515 GO:0005576

Cluster-22178.69061 GO:0015031 GO:0030176

Cluster-22178.69062 GO:0006568 GO:0055114 GO:0015947 GO:0006804

GO:0006979 GO:0004096 GO:0020037

Cluster-22178.69063 GO:0006879 GO:0008199

Cluster-22178.69064 GO:0009052 GO:0006098 GO:0015976 GO:0004751

Cluster-22178.69066 GO:0008152 GO:0055114 GO:0016491

Cluster-22178.69071 GO:0006051 GO:0009405 GO:0006810 GO:0006310

GO:0006040 GO:0008200 GO:0003677 GO:0047465 GO:0005515

GO:0019905 GO:0005576 GO:0005886

Cluster-22178.69072 GO:0006810 GO:0007264 GO:0006396 GO:0051252

GO:0006355 GO:0006281 GO:0000723 GO:0003677 GO:0016787

GO:0005524 GO:0008134 GO:0003723 GO:0005525 GO:0003678

GO:0004525 GO:0005667 GO:0005657

Cluster-22178.69073 GO:0006468 GO:0006118 GO:0018990 GO:0007218

GO:0007264 GO:0007165 GO:0009847 GO:0003924 GO:0008255

GO:0004672 GO:0009055 GO:0005525 GO:0005515 GO:0005198

GO:0016491 GO:0016887 GO:0005524 GO:0016021 GO:0005739

GO:0016020

Cluster-22178.69074 GO:0031110 GO:0051301 GO:0007059 GO:0000090

GO:0007155 GO:0007067 GO:0006310 GO:0003824 GO:0008017

GO:0003677 GO:0051015 GO:0000940 GO:0045298 GO:0005876
GO:0005727
Cluster-22178.69075 GO:0043565 GO:0005515 GO:0005634 GO:0016020
Cluster-22178.69076 GO:0006355 GO:0006614 GO:0000921 GO:0006520
GO:0006606 GO:0046856 GO:0006396 GO:0045502 GO:0003723
GO:0005515 GO:0042803 GO:0008134 GO:0043565 GO:0034597
GO:0003700 GO:0008312 GO:0048500 GO:0016021 GO:0005667
GO:0030286 GO:0005940
Cluster-22178.69078 GO:0006281 GO:0003824 GO:0000166 GO:0046872

Cluster-22178.69080 GO:0006886 GO:0016192 GO:0006810 GO:0055085
GO:0022857 GO:0005215 GO:0030117 GO:0016020 GO:0016021
Cluster-22178.69081 GO:0006367 GO:0005672
Cluster-22178.69082 GO:0006468 GO:0016773 GO:0005524 GO:0004672
GO:0016020
Cluster-22178.69084 GO:0006886 GO:0005975 GO:0016832 GO:0005741
GO:0016021 GO:0016020
Cluster-22178.69088 GO:0055114 GO:0006979 GO:0015947 GO:0006355
GO:0006568 GO:0006804 GO:0003700 GO:0004096 GO:0020037
GO:0005667 GO:0005634
Cluster-22178.69089 GO:0006355 GO:0003700 GO:0005634 GO:0005667
Cluster-22178.69091 GO:0008152 GO:0016491
Cluster-22178.69093 GO:0006420 GO:0055085 GO:0006260 GO:0006525
GO:0007017 GO:0007018 GO:0006560 GO:0005524 GO:0008017
GO:0004814 GO:0022857 GO:0003777 GO:0000166 GO:0005737
GO:0045298 GO:0016021 GO:0005874
Cluster-22178.69095 GO:0006260 GO:0000184 GO:0046935 GO:0003677
GO:0005664 GO:0005944
Cluster-22178.69096 GO:0009058 GO:0006520 GO:0019752 GO:0016740
GO:0030170 GO:0016831 GO:0016829 GO:0019012
Cluster-22178.69098 GO:0007165 GO:0032780 GO:0006807 GO:0030168
GO:0010508 GO:0006886 GO:0007049 GO:0051258 GO:0030674
GO:0003933 GO:0008017 GO:0005102 GO:0042030 GO:0005783

GO:0005739 GO:0005577 GO:0045298 GO:0016021
Cluster-22178.69102 GO:0007165 GO:0005515 GO:0008270 GO:0005179
GO:0005576
Cluster-22178.69103 GO:0007165 GO:0005515 GO:0005179 GO:0008270
GO:0005576
Cluster-22178.69104 GO:0009877 GO:0007005 GO:0006448 GO:0006414
GO:0016746 GO:0003950 GO:0003746 GO:0005840 GO:0005829
GO:0032865
Cluster-22178.69105 GO:0007186 GO:0004930 GO:0016021 GO:0016020
Cluster-22178.69108 GO:0009250 GO:0046527 GO:0003676
Cluster-22178.69110 GO:0035556
Cluster-22178.69112 GO:0006400 GO:0007275 GO:0006614 GO:0045893
GO:0048280 GO:0007218 GO:0006281 GO:0008616 GO:0006886
GO:0000723 GO:0016787 GO:0003677 GO:0005524 GO:0008270
GO:0008479 GO:0005184 GO:0005525 GO:0003678 GO:0003676
GO:0005507 GO:0005657 GO:0000139 GO:0005576 GO:0005737
Cluster-22178.69113 GO:0008270 GO:0005515
Cluster-22178.69114 GO:0009297 GO:0006376 GO:0043087 GO:0003729
GO:0019867 GO:0005685
Cluster-22178.69116 GO:0043631 GO:0006355 GO:0006568 GO:0055114
GO:0005506 GO:0003723 GO:0045735 GO:0016779 GO:0004652
GO:0000334
Cluster-22178.69119 GO:0006468 GO:0016773 GO:0004672 GO:0005524
GO:0016020
Cluster-22178.69120 GO:0042981 GO:0005515
Cluster-22178.69121 GO:0042254 GO:0006412 GO:0003735 GO:0005840
GO:0005622
Cluster-22178.69124 GO:0006508 GO:0043547 GO:0006144 GO:0006284
GO:0051056 GO:0017111 GO:0005515 GO:0003677 GO:0004197
GO:0016817 GO:0005096 GO:0003968 GO:0031379
Cluster-22178.69125 GO:0006355 GO:0043565 GO:0003700 GO:0005515
GO:0003677 GO:0005667 GO:0005634
Cluster-22178.69133 GO:0006511 GO:0043401 GO:0006355 GO:0008152

GO:0055114 GO:0050662 GO:0031625 GO:0016872 GO:0003824
GO:0016491 GO:0016020
Cluster-22178.69137 GO:0051028 GO:0071973 GO:0003774 GO:0005515
GO:0009425 GO:0005634
Cluster-22178.69138 GO:0030168 GO:0051258 GO:0006352 GO:0007155
GO:0007165 GO:0005102 GO:0030674 GO:0003677 GO:0005669
GO:0005577 GO:0019028
Cluster-22178.69139 GO:0015684 GO:0005525 GO:0005524 GO:0015093
GO:0046872 GO:0003924 GO:0016021
Cluster-22178.69141 GO:0003676
Cluster-22178.69145 GO:0042254 GO:0006412 GO:0003676 GO:0003735
GO:0008270 GO:0005840 GO:0005622
Cluster-22178.69147 GO:0005515
Cluster-22178.69150 GO:0042981
Cluster-22178.69152 GO:0022900 GO:0007010 GO:0035556 GO:0046872
GO:0003779 GO:0003723 GO:0016021
Cluster-22178.69153 GO:0000041 GO:0042802 GO:0005515 GO:0016021
Cluster-22178.69154 GO:0042981
Cluster-22178.69157 GO:0016337 GO:0005515 GO:0003724 GO:0003723
GO:0016887 GO:0005524 GO:0016020
Cluster-22178.69160 GO:0006468 GO:0006182 GO:0046039 GO:0009190
GO:0006144 GO:0035556 GO:0016849 GO:0004383 GO:0004672
GO:0005524
Cluster-22178.69161 GO:0016021
Cluster-22178.69162 GO:0007165 GO:0005179 GO:0005576
Cluster-22178.69163 GO:0055085 GO:0030001 GO:0008146 GO:0046873
GO:0016020
Cluster-22178.69165 GO:0006281 GO:0010447 GO:0003677 GO:0004518
GO:0003824 GO:0042597
Cluster-22178.69166 GO:0016482 GO:0033206 GO:0007163 GO:0030036
GO:0051653 GO:0008356 GO:0006928 GO:0051321 GO:0003779
GO:0005524 GO:0005885 GO:0042995 GO:0030478
Cluster-22178.69168 GO:0006281 GO:0006139 GO:0006310 GO:0006810

GO:0007165 GO:0002949 GO:0006206 GO:0016301 GO:0005215
GO:0008158 GO:0009378 GO:0004127 GO:0016887 GO:0005524
GO:0016020 GO:0009379 GO:0005657
Cluster-22178.69169 GO:0006206 GO:0002949 GO:0006139 GO:0006281
GO:0006310 GO:0005524 GO:0016887 GO:0004127 GO:0009378
GO:0016791 GO:0016301 GO:0005657 GO:0009379
Cluster-22178.69171 GO:0008152 GO:0015940 GO:0042967 GO:0007034
GO:0055114 GO:0016573 GO:0006355 GO:0003824 GO:0016491
GO:0008677 GO:0008484 GO:0071949 GO:0004402 GO:0000123
Cluster-22178.69172 GO:0006810 GO:0005515 GO:0042626 GO:0005524
GO:0016021
Cluster-22178.69173 GO:0007034 GO:0055114 GO:0015940 GO:0008152
GO:0016491 GO:0008484 GO:0008677 GO:0003824 GO:0071949
Cluster-22178.69174 GO:0006816 GO:0006810 GO:0008203 GO:0006804
GO:0006979 GO:0016042 GO:0005246 GO:0016298 GO:0042030
GO:0004601 GO:0016020
Cluster-22178.69175 GO:0043039 GO:0006771 GO:0019497 GO:0005515
GO:0003993 GO:0016876 GO:0046872 GO:0005524
Cluster-22178.69178 GO:0006468 GO:0016310 GO:0005515 GO:0004673
GO:0005524 GO:0004672 GO:0034464 GO:0009365
Cluster-22178.69179 GO:0006468 GO:0006886 GO:0045667 GO:0016773
GO:0005524 GO:0004672 GO:0016020 GO:0030176
Cluster-22178.69181 GO:0006355 GO:0016579 GO:0032065 GO:0003677
GO:0005515 GO:0003700 GO:0005543 GO:0043565 GO:0036459
GO:0005667 GO:0005634 GO:0005938
Cluster-22178.69183 GO:0015979 GO:0016757
Cluster-22178.69185 GO:0005515
Cluster-22178.69186 GO:0004190 GO:0016021
Cluster-22178.69189 GO:0006804 GO:0006979 GO:0055114 GO:0015684
GO:0007165 GO:0007186 GO:0007264 GO:0006749 GO:0030150
GO:0005525 GO:0004871 GO:0031683 GO:0019001 GO:0003924
GO:0015093 GO:0004602 GO:0016021 GO:0005742
Cluster-22178.69190 GO:0007050 GO:0045859 GO:0004861 GO:0016020

GO:0005634
Cluster-22178.69193 GO:0006771 GO:0019497 GO:0005515 GO:0003993
GO:0030976 GO:0046872
Cluster-22178.69194 GO:0016579 GO:0006278 GO:0055114 GO:0004800
GO:0036459 GO:0003964
Cluster-22178.69195 GO:0005515 GO:0016020
Cluster-22178.69196 GO:0005515
Cluster-22178.69198 GO:0006570 GO:0000087 GO:0006355 GO:0006260
GO:0006470 GO:0008134 GO:0003677 GO:0004725 GO:0016887
GO:0005524 GO:0005667 GO:0005622
Cluster-22178.69200 GO:0006370 GO:0006260 GO:0006281 GO:0006310
GO:0006397 GO:0005524 GO:0008270 GO:0003910 GO:0004484
GO:0003677
Cluster-22178.69202 GO:0015693 GO:0046872 GO:0015095 GO:0000166
GO:0016021 GO:0016020
Cluster-22178.69205 GO:0006508 GO:0006355 GO:0006122 GO:0005515
GO:0043565 GO:0008270 GO:0003700 GO:0004222 GO:0005634
GO:0005667 GO:0031012 GO:0005743 GO:0005750
Cluster-22178.69206 GO:0006298 GO:0005524 GO:0030983
Cluster-22178.69208 GO:0015991 GO:0006355 GO:0006886 GO:0016192
GO:0006528 GO:0008418 GO:0005515 GO:0016820 GO:0003677
GO:0008270 GO:0030117 GO:0000221
Cluster-22178.69210 GO:0005515
Cluster-22178.69213 GO:0019236 GO:0007606 GO:0009772 GO:0015708
GO:0006118 GO:0006955 GO:0006412 GO:0019684 GO:0007165
GO:0042254 GO:0007186 GO:0005125 GO:0046872 GO:0045156
GO:0003723 GO:0005515 GO:0004932 GO:0008270 GO:0003677
GO:0003735 GO:0005840 GO:0016021 GO:0005576 GO:0005634
GO:0005622
Cluster-22178.69214 GO:0006357 GO:0006308 GO:0001104 GO:0008855
GO:0016592 GO:0009318
Cluster-22178.69215 GO:0008152 GO:0055114 GO:0003824 GO:0008484
GO:0016021 GO:0016020

Cluster-22178.69216 GO:0005515
Cluster-22178.69219 GO:0005515
Cluster-22178.69221 GO:0008219
Cluster-22178.69222 GO:0006464 GO:0000902 GO:0006955 GO:0006351 GO:0009405 GO:0006355 GO:0006811 GO:0003677 GO:0008270 GO:0005515 GO:0003676 GO:0016790 GO:0003700 GO:0016151 GO:0043565 GO:0046872 GO:0016020 GO:0005667 GO:0005887 GO:0045202
Cluster-22178.69223 GO:0055114 GO:0016620
Cluster-22178.69227 GO:0006816 GO:0070588 GO:0005245 GO:0005891
Cluster-22178.69229 GO:0007010 GO:0003779
Cluster-22178.69232 GO:0009405 GO:0044179
Cluster-22178.69233 GO:0009245 GO:0016567 GO:0008233 GO:0004842 GO:0046872 GO:0008915 GO:0008270 GO:0005515 GO:0003723 GO:0005680 GO:0016020
Cluster-22178.69234 GO:0006888 GO:0006281 GO:0006886 GO:0008270 GO:0046872 GO:0005515 GO:0003677 GO:0045202 GO:0030127
Cluster-22178.69235 GO:0035556 GO:0005515
Cluster-22178.69236 GO:0005515 GO:0003723
Cluster-22178.69237 GO:0016567 GO:0008270 GO:0046872 GO:0004842 GO:0005515 GO:0061630 GO:0005622 GO:0005680
Cluster-22178.69238 GO:0016567 GO:0046872 GO:0008270 GO:0004842 GO:0061630 GO:0005515 GO:0005622 GO:0005680
Cluster-22178.69240 GO:0042254 GO:0006412 GO:0003735 GO:0005840
Cluster-22178.69241 GO:0010972 GO:0030336 GO:0016579 GO:0036459

Cluster-22178.69242 GO:0003676
Cluster-22178.69247 GO:0006338 GO:0043044 GO:0055085 GO:0016787 GO:0031491 GO:0003677 GO:0016818 GO:0005524 GO:0008270 GO:0003676 GO:0005634 GO:0000785 GO:0016021
Cluster-22178.69248 GO:0007155 GO:0005515 GO:0016021
Cluster-22178.69249 GO:0007165 GO:0006351 GO:0009405 GO:0040007 GO:0008283 GO:0006144 GO:0006206 GO:0003899 GO:0008083

GO:0003677 GO:0005730
Cluster-22178.69252 GO:0016337 GO:0016020
Cluster-22178.69253 GO:0007010 GO:0016337 GO:0003779 GO:0016020
Cluster-22178.69256 GO:0006522 GO:0009152 GO:0006188 GO:0006531
GO:0006144 GO:0004018 GO:0005742
Cluster-22178.69258 GO:0006810 GO:0005215 GO:0016020
Cluster-22178.69259 GO:0005515
Cluster-22178.69265 GO:0006364 GO:0006810 GO:0042626 GO:0005524
GO:0032040 GO:0016021
Cluster-22178.69267 GO:0046872 GO:0005515
Cluster-22178.69268 GO:0006810 GO:0002949 GO:0005525 GO:0016301
GO:0005524 GO:0016887 GO:0003924
Cluster-22178.69269 GO:0043565 GO:0005634
Cluster-22178.69270 GO:0046487 GO:0006550 GO:0015940 GO:0006694
GO:0008207 GO:0008209 GO:0018874 GO:0009396 GO:0006554
GO:0006552 GO:0006568 GO:0008210 GO:0006813 GO:0055114
GO:0005975 GO:0006574 GO:0006631 GO:0006633 GO:0003857
GO:0003824 GO:0030976 GO:0008677 GO:0004488 GO:0051287
GO:0003854 GO:0050662 GO:0016620 GO:0016491 GO:0000287
GO:0016616 GO:0070402 GO:0004553
Cluster-22178.69272 GO:0005515 GO:0016021
Cluster-22178.69274 GO:0016310 GO:0009069 GO:0006468 GO:0004674
GO:0005515 GO:0005524 GO:0004672
Cluster-22178.69275 GO:0016021
Cluster-22178.69277 GO:0055114 GO:0016972
Cluster-22178.69282 GO:0000398 GO:0005515 GO:0005681
Cluster-22178.69283 GO:0019079 GO:0019048 GO:0016787 GO:0008026
GO:0003677 GO:0004519 GO:0005509 GO:0005515 GO:0005524
GO:0008270 GO:0003676
Cluster-22178.69286 GO:0019521 GO:0055114 GO:0008152 GO:0006355
GO:0006351 GO:0006206 GO:0006144 GO:0006098 GO:0046872
GO:0003700 GO:0005515 GO:0003899 GO:0008641 GO:0051287
GO:0004616 GO:0003677 GO:0016616 GO:0005634 GO:0005730

GO:0005667
Cluster-22178.69287 GO:0006529 GO:0006522 GO:0007218 GO:0006955
GO:0007268 GO:0006531 GO:0007217 GO:0030153 GO:0004066
GO:0015643 GO:0019814
Cluster-22178.69288 GO:0042981
Cluster-22178.69289 GO:0042981 GO:0005515
Cluster-22178.69290 GO:0006508 GO:0055114 GO:0006510 GO:0004252
GO:0016491 GO:0016829 GO:0004176
Cluster-22178.69291 GO:0016567 GO:0004842 GO:0004857
Cluster-22178.69292 GO:0007178 GO:0016310 GO:0006468 GO:0009069
GO:0005524 GO:0004672 GO:0046872 GO:0005026 GO:0005198
GO:0016021 GO:0016020
Cluster-22178.69298 GO:0050852 GO:0000226 GO:0006470 GO:0006570
GO:0004725 GO:0005515 GO:0005509 GO:0015630
Cluster-22178.69301 GO:0006508 GO:0006886 GO:0007268 GO:0006605
GO:0033130 GO:0030695 GO:0005515 GO:0042802 GO:0043495
GO:0004197 GO:0005742
Cluster-22178.69302 GO:0015992 GO:0006744 GO:0006098 GO:0055114
GO:0019521 GO:0008152 GO:0006120 GO:0006814 GO:0008641
GO:0051287 GO:0004616 GO:0016616 GO:0046872 GO:0008137
GO:0005515 GO:0005742 GO:0005743
Cluster-22178.69304 GO:0006355 GO:0019048 GO:0003677 GO:0016020
Cluster-22178.69305 GO:0006355 GO:0003700 GO:0005667
Cluster-22178.69307 GO:0009094 GO:0000162 GO:0000413 GO:0006571
GO:0006094 GO:0006457 GO:0006096 GO:0004634 GO:0000287
GO:0003755 GO:0000015
Cluster-22178.69308 GO:0008033 GO:0009451 GO:0006412 GO:0042254
GO:0004809 GO:0003723 GO:0005515 GO:0003735 GO:0005622
GO:0005840
Cluster-22178.69313 GO:0055114 GO:0019521 GO:0008152 GO:0006098
GO:0016491 GO:0051287 GO:0004616 GO:0016616
Cluster-22178.69317 GO:0016746
Cluster-22178.69318 GO:0015979 GO:0007275 GO:0007219 GO:0009539

GO:0009523 GO:0016021 GO:0016020
Cluster-22178.69320 GO:0006355 GO:0003700 GO:0043565 GO:0005667
Cluster-22178.69321 GO:0008152 GO:0003723 GO:0008168
Cluster-22178.69322 GO:0016579 GO:0036459 GO:0005783
Cluster-22178.69323 GO:0016579 GO:0036459 GO:0005783
Cluster-22178.69324 GO:0017004 GO:0006118 GO:0006952 GO:0009405
GO:0006869 GO:0042157 GO:0045158 GO:0008289 GO:0009512
GO:0016020 GO:0005576
Cluster-22178.69325 GO:0006914 GO:0006308 GO:0008855 GO:0008092
GO:0046935 GO:0005737 GO:0019898 GO:0005944 GO:0009318
Cluster-22178.69326 GO:0006412 GO:0042254 GO:0003735 GO:0005840
GO:0005622
Cluster-22178.69329 GO:0005544 GO:0005509
Cluster-22178.69330 GO:0005544 GO:0005509
Cluster-22178.69334 GO:0006351 GO:0006269 GO:0034508 GO:0003896
GO:0004386 GO:0008270 GO:0000776 GO:0005730 GO:0005657
Cluster-22178.69335 GO:0007165 GO:0006950 GO:0005509 GO:0005515
GO:0005578
Cluster-22178.69337 GO:0006468 GO:0005524 GO:0004672
Cluster-22178.69338 GO:0006418 GO:0004812 GO:0005524 GO:0000166

Cluster-22178.69340 GO:0006397 GO:0016567 GO:0046872 GO:0003723
GO:0004842 GO:0003676
Cluster-22178.69342 GO:0006811 GO:0006418 GO:0015992 GO:0007016
GO:0006119 GO:0015986 GO:0046933 GO:0046961 GO:0000166
GO:0005216 GO:0016020 GO:0045259 GO:0016010 GO:0000275
Cluster-22178.69344 GO:0055085 GO:0006810 GO:0003924 GO:0046872
GO:0022857 GO:0005215 GO:0005525 GO:0005515 GO:0016021
GO:0016020
Cluster-22178.69345 GO:0006355 GO:0003677 GO:0005515 GO:0003700
GO:0005667
Cluster-22178.69346 GO:0016567 GO:0046872 GO:0008270 GO:0004842
GO:0005515 GO:0061630 GO:0005680

Cluster-22178.69349 GO:0015886 GO:0055114 GO:0019646 GO:0006118 GO:0015232 GO:0016491 GO:0009055
Cluster-22178.69350 GO:0019882 GO:0042254 GO:0006412 GO:0006886 GO:0006955 GO:0007165 GO:0005125 GO:0042289 GO:0003735 GO:0042613 GO:0005622 GO:0005840 GO:0016020
Cluster-22178.69351 GO:0007049 GO:0005515 GO:0008270
Cluster-22178.69353 GO:0006260 GO:0035556 GO:0006310 GO:0006281 GO:0016887 GO:0005524 GO:0016787 GO:0009378 GO:0003677 GO:0003678 GO:0003676 GO:0005515 GO:0003887 GO:0009360 GO:0005657 GO:0009379 GO:0042575
Cluster-22178.69358 GO:0006468 GO:0005524 GO:0004672
Cluster-22178.69361 GO:0006313 GO:0006464 GO:0015031 GO:0055114 GO:0003677 GO:0004803 GO:0005515
Cluster-22178.69364 GO:0009069 GO:0006468 GO:0007178 GO:0016310 GO:0004675 GO:0003677 GO:0004672 GO:0005524 GO:0016020
Cluster-22178.69368 GO:0034314 GO:0006355 GO:0030833 GO:0003700 GO:0043565 GO:0005856 GO:0005885 GO:0005667
Cluster-22178.69369 GO:0007049 GO:0055114 GO:0006006 GO:0018990 GO:0006355 GO:0006260 GO:0007218 GO:0006098 GO:0006749 GO:0003700 GO:0008255 GO:0003887 GO:0050661 GO:0004345 GO:0008270 GO:0003677 GO:0016021 GO:0042575 GO:0005634 GO:0005576 GO:0005667
Cluster-22178.69370 GO:0007186 GO:0007165 GO:0007264 GO:0006000 GO:0006013 GO:0003873 GO:0004871 GO:0005525 GO:0003924 GO:0019001 GO:0005524 GO:0031683
Cluster-22178.69371 GO:0046872 GO:0016021
Cluster-22178.69372 GO:0006687 GO:0005975 GO:0006665 GO:0006807 GO:0004348 GO:0016021
Cluster-22178.69374 GO:0055085 GO:0022857 GO:0016021
Cluster-22178.69375 GO:0006013 GO:0005975 GO:0015923 GO:0008270 GO:0004553 GO:0004559
Cluster-22178.69376 GO:0006013 GO:0005975 GO:0008270 GO:0015923 GO:0004559 GO:0004553

Cluster-22178.69377 GO:0006351 GO:0006206 GO:0006144 GO:0003677
GO:0003899 GO:0005730 GO:0005634
Cluster-22178.69378 GO:0006804 GO:0006979 GO:0055114 GO:0006749
GO:0016209 GO:0004602 GO:0016491
Cluster-22178.69381 GO:0030246
Cluster-22178.69384 GO:0003677
Cluster-22178.69386 GO:0006355 GO:0006334 GO:0006352 GO:0003677
GO:0016987 GO:0003700 GO:0005667 GO:0005634
Cluster-22178.69389 GO:0008242
Cluster-22178.69390 GO:0015774 GO:0008270 GO:0015159 GO:0003676
GO:0016020
Cluster-22178.69391 GO:0042254 GO:0006412 GO:0003735 GO:2001070
GO:0005840
Cluster-22178.69394 GO:0007264 GO:0043087 GO:0005085
Cluster-22178.69399 GO:0003723 GO:0042162 GO:0008270 GO:0042803

Cluster-22178.69400 GO:0006355 GO:0006810 GO:0006614 GO:0019079
GO:0016787 GO:0003677 GO:0008026 GO:0005524 GO:0016887
GO:0005525 GO:0003676 GO:0004386
Cluster-22178.69403 GO:0046907
Cluster-22178.69406 GO:0009231 GO:0006771 GO:0003919 GO:0008270
GO:0016021
Cluster-22178.69407 GO:0007017 GO:0007018 GO:0005515 GO:0005524
GO:0008017 GO:0003777 GO:0045298 GO:0005874
Cluster-22178.69410 GO:0055114 GO:0008270 GO:0016491 GO:0003729
GO:0016020 GO:0016021
Cluster-22178.69411 GO:0005975 GO:0004553 GO:0016021
Cluster-22178.69412 GO:0010112 GO:0015684 GO:0007264 GO:0005525
GO:0003924 GO:0015093 GO:0016021
Cluster-22178.69413 GO:0007165 GO:0007275 GO:0008152 GO:0016624
GO:0005179 GO:0047355 GO:0008270 GO:0016020 GO:0005576
Cluster-22178.69415 GO:0006886 GO:0005515 GO:0005525 GO:0005741
Cluster-22178.69416 GO:0005975 GO:0006890 GO:0008270 GO:0015926

GO:0003676 GO:0005515 GO:0004252 GO:0016021
Cluster-22178.69418 GO:0006890 GO:0005975 GO:0005515 GO:0004252
GO:0008270 GO:0015926 GO:0003676 GO:0016021
Cluster-22178.69421 GO:0006919 GO:0006915 GO:0005739
Cluster-22178.69427 GO:0046872 GO:0016787 GO:0045502 GO:0008134
GO:0042803 GO:0030286 GO:0005667
Cluster-22178.69433 GO:0006813 GO:0055114 GO:0051287 GO:0016616

Cluster-22178.69437 GO:0006412 GO:0046487 GO:0015991 GO:0008152
GO:0055114 GO:0042254 GO:0009396 GO:0015992 GO:0005515
GO:0003824 GO:0015078 GO:0004488 GO:0003735 GO:0050662
GO:0016491 GO:0033179 GO:0005840
Cluster-22178.69439 GO:0030212 GO:0019068 GO:0003676 GO:0008233
GO:0008168 GO:0008047 GO:0005515 GO:0004867
Cluster-22178.69440 GO:0016310 GO:0005975 GO:0007178 GO:0005982
GO:0005985 GO:0006468 GO:0009069 GO:0016773 GO:0004672
GO:0005524 GO:0004556 GO:0005509 GO:0004675 GO:0016020
Cluster-22178.69441 GO:0016310 GO:0055114 GO:0006631 GO:0006303
GO:0030328 GO:0009069 GO:0004677 GO:0050151 GO:0005524
GO:0016491 GO:0003677 GO:0016670 GO:0071949 GO:0005958
GO:0005634
Cluster-22178.69443 GO:0006807 GO:0016810
Cluster-22178.69444 GO:0015694 GO:0005975 GO:0030150 GO:0015097
GO:0004553 GO:0005742 GO:0016020 GO:0016021
Cluster-22178.69446 GO:0006457 GO:0000413 GO:0016117 GO:0008033
GO:0055114 GO:0003755 GO:0005515 GO:0016705 GO:0004174
GO:0071949 GO:0050660 GO:0016491
Cluster-22178.69448 GO:0034453 GO:0005515 GO:0005815
Cluster-22178.69449 GO:0007018 GO:0006355 GO:0007017 GO:0003777
GO:0008017 GO:0008270 GO:0005524 GO:0016818 GO:0003677
GO:0005874 GO:0045298 GO:0005634
Cluster-22178.69453 GO:0005976 GO:0035023 GO:0043087 GO:0005089
GO:0016779 GO:0043565

Cluster-22178.69454 GO:0016032 GO:0055085 GO:0005506 GO:0045735 GO:0016021
Cluster-22178.69455 GO:0003676 GO:0005524
Cluster-22178.69457 GO:0006118 GO:0000746 GO:0009055 GO:0016491 GO:0003677 GO:0005515 GO:0016020 GO:0005634
Cluster-22178.69458 GO:0042167 GO:0006397 GO:0055114 GO:0015994 GO:0003723 GO:0003677 GO:0005515 GO:0008270 GO:0004074 GO:0005634 GO:0005737
Cluster-22178.69461 GO:0045893 GO:0005634
Cluster-22178.69462 GO:0006313 GO:0030866 GO:0015979 GO:0006914 GO:0048870 GO:0003677 GO:0004803 GO:0005198 GO:0008134 GO:0042803 GO:0030414 GO:0045502 GO:0008092 GO:0005882 GO:0016020 GO:0005667 GO:0030286 GO:0031514 GO:0009539 GO:0005856 GO:0009523
Cluster-22178.69463 GO:0005509
Cluster-22178.69464 GO:0006508 GO:0008234
Cluster-22178.69466 GO:0003677
Cluster-22178.69469 GO:0016567
Cluster-22178.69471 GO:0006891 GO:0006355 GO:0005524 GO:0003713 GO:0003677 GO:0005634 GO:0005667 GO:0017119
Cluster-22178.69472 GO:0042254 GO:0006412 GO:0007264 GO:0045040 GO:0005515 GO:0003735 GO:0042802 GO:0005525 GO:0046983 GO:0003924 GO:0005742 GO:0005840 GO:0016021 GO:0005622
Cluster-22178.69475 GO:0006118 GO:0016567 GO:0019684 GO:0006448 GO:0006414 GO:0005515 GO:0045156 GO:0003676 GO:0046872 GO:0004842 GO:0003746 GO:0008270 GO:0030077 GO:0016021 GO:0005840 GO:0005853
Cluster-22178.69476 GO:0007165 GO:0016874 GO:0001664
Cluster-22178.69477 GO:0006118 GO:0016567 GO:0006448 GO:0006414 GO:0019684 GO:0045156 GO:0005515 GO:0046872 GO:0003676 GO:0008270 GO:0003746 GO:0004842 GO:0030077 GO:0005840 GO:0016021 GO:0005853
Cluster-22178.69478 GO:0005515

Cluster-22178.69479 GO:0016567 GO:0006414 GO:0006448 GO:0019684
GO:0006118 GO:0003746 GO:0008270 GO:0004842 GO:0045156
GO:0005515 GO:0046872 GO:0003676 GO:0005853 GO:0030077
GO:0016021 GO:0005840
Cluster-22178.69482 GO:0007050 GO:0045859 GO:0004861 GO:0005634
Cluster-22178.69483 GO:0006694 GO:0008152 GO:0055114 GO:0008209
GO:0008207 GO:0008210 GO:0019836 GO:0003824 GO:0016491
GO:0016616 GO:0005515 GO:0050662 GO:0003854 GO:0005576
Cluster-22178.69484 GO:0015074 GO:0007017 GO:0007018 GO:0005524
GO:0008017 GO:0003777 GO:0008907 GO:0003677 GO:0045298
GO:0005874
Cluster-22178.69486 GO:0004386
Cluster-22178.69487 GO:0005515
Cluster-22178.69490 GO:0009405 GO:0005515 GO:0003779 GO:0005622
GO:0005576
Cluster-22178.69492 GO:0051252 GO:0006396 GO:0001682 GO:0008033
GO:0004526 GO:0030677
Cluster-22178.69493 GO:0007186 GO:0004930 GO:0016021
Cluster-22178.69494 GO:0007165 GO:0005515 GO:0005179 GO:0008270
GO:0005576
Cluster-22178.69495 GO:0007165 GO:0007050 GO:0045859 GO:0005515
GO:0004861 GO:0005179 GO:0008270 GO:0005634 GO:0005576
Cluster-22178.69496 GO:0004651
Cluster-22178.69497 GO:0006468 GO:0006464 GO:0015031 GO:0005524
GO:0004672
Cluster-22178.69498 GO:0015992 GO:0015031 GO:0006464 GO:0015986
GO:0006772 GO:0015689 GO:0009228 GO:0006886 GO:0008270
GO:0052855 GO:0005047 GO:0003924 GO:0005525 GO:0015078
GO:0015098 GO:0004417 GO:0005515 GO:0000276 GO:0005785
GO:0005786 GO:0016021
Cluster-22178.69499 GO:0008092 GO:0019898 GO:0005737
Cluster-22178.69500 GO:0006541 GO:0016787
Cluster-22178.69501 GO:0006351 GO:0008270 GO:0003676 GO:0003677

GO:0005634
Cluster-22178.69505 GO:0006397 GO:0008152 GO:0005515 GO:0016788
GO:0003723 GO:0004064 GO:0005634 GO:0005737
Cluster-22178.69506 GO:0003924 GO:0005525
Cluster-22178.69507 GO:0055114 GO:0034599 GO:0005524 GO:0016887
GO:0016491 GO:0005789
Cluster-22178.69509 GO:0006355 GO:0006914 GO:0055114 GO:0005515
GO:0043565 GO:0016491 GO:0003700 GO:0005667
Cluster-22178.69511 GO:0007165 GO:0035556
Cluster-22178.69513 GO:0000090 GO:0007067 GO:0007165 GO:0030168
GO:0031110 GO:0007017 GO:0007018 GO:0051258 GO:0051301
GO:0007059 GO:0030674 GO:0005524 GO:0005102 GO:0008017
GO:0003777 GO:0000940 GO:0005577 GO:0005876 GO:0005874
GO:0045298
Cluster-22178.69514 GO:0005515 GO:0046872
Cluster-22178.69515 GO:0006367 GO:0003677 GO:0005515 GO:0048786
GO:0005672
Cluster-22178.69516 GO:0006464 GO:0042254 GO:0006412 GO:0051536
GO:0016151 GO:0003735 GO:0005622 GO:0005840
Cluster-22178.69519 GO:0006464 GO:0006144 GO:0046034 GO:0006281
GO:0008270 GO:0046872 GO:0016151 GO:0004017
Cluster-22178.69520 GO:0005985 GO:0005982 GO:0009405 GO:0006221
GO:0006012 GO:0007049 GO:0045747 GO:0004575 GO:0004190
GO:0004222 GO:0004564 GO:0017177 GO:0016021
Cluster-22178.69523 GO:0005524
Cluster-22178.69524 GO:0016887 GO:0005524
Cluster-22178.69526 GO:0035091
Cluster-22178.69527 GO:0007165 GO:0007186 GO:0007264 GO:0005525
GO:0004871 GO:0031683 GO:0019001 GO:0003924
Cluster-22178.69529 GO:0009056 GO:0006940 GO:0046436 GO:0009252
GO:0008716 GO:0003824 GO:0046872 GO:0008270 GO:0005524
Cluster-22178.69530 GO:0006281 GO:0006464 GO:0015992 GO:0055114
GO:0015672 GO:0046872 GO:0008270 GO:0047134 GO:0016151

GO:0005451
Cluster-22178.69531 GO:0055114 GO:0006281 GO:0006464 GO:0008270
GO:0046872 GO:0047134 GO:0016151
Cluster-22178.69532 GO:0006412 GO:0042254 GO:0003735 GO:0005840
Cluster-22178.69533 GO:0043087 GO:0035023 GO:0006412 GO:0042254
GO:0046872 GO:0005089 GO:0003735 GO:0005840 GO:0015934
Cluster-22178.69535 GO:0008152 GO:0008168
Cluster-22178.69536 GO:0006298 GO:0005524 GO:0030983
Cluster-22178.69537 GO:0009405 GO:0006231 GO:0006206 GO:0004799
GO:0005576
Cluster-22178.69542 GO:0006298 GO:0030983 GO:0005524 GO:0030430
Cluster-22178.69543 GO:0031412 GO:0006396 GO:0006139 GO:0008408
GO:0003676 GO:0031411 GO:0005622 GO:0000176
Cluster-22178.69544 GO:0046660 GO:0006811 GO:0006397 GO:0006816
GO:0006874 GO:0005219 GO:0004190 GO:0016021 GO:0005622
GO:0016020 GO:0005634
Cluster-22178.69545 GO:0016310 GO:0009069 GO:0042981 GO:0005509
GO:0005544 GO:0004674
Cluster-22178.69546 GO:0005515 GO:0003677 GO:0008270 GO:0046872
GO:0005622
Cluster-22178.69547 GO:0006351 GO:0005975 GO:0003676 GO:0046872
GO:0008270
Cluster-22178.69548 GO:0006355 GO:0006508 GO:0003682 GO:0003677
GO:0008270 GO:0046872 GO:0004222 GO:0003676 GO:0005634
GO:0000775 GO:0000785
Cluster-22178.69551 GO:0016021
Cluster-22178.69553 GO:0007268 GO:0006468 GO:0016773 GO:0033130
GO:0005524 GO:0004672 GO:0043495 GO:0016020
Cluster-22178.69554 GO:0006570 GO:0016192 GO:0007304 GO:0006470
GO:0008138 GO:0004725 GO:0005213 GO:0005576 GO:0042600
Cluster-22178.69555 GO:0005515
Cluster-22178.69556 GO:0030435 GO:0046872 GO:0031160
Cluster-22178.69558 GO:0006367 GO:0006270 GO:0005672

Cluster-22178.69559 GO:0016310 GO:0006468 GO:0009069 GO:0004674
GO:0004672 GO:0005524 GO:0016773 GO:0016020
Cluster-22178.69562 GO:0006355 GO:0007049 GO:0008134 GO:0042803
GO:0019901 GO:0045502 GO:0003700 GO:0043565 GO:0046872
GO:0005667 GO:0030286
Cluster-22178.69565 GO:0006260 GO:0006241 GO:0006206 GO:0006144
GO:0006886 GO:0006183 GO:0006355 GO:0006228 GO:0006281
GO:0006508 GO:0006165 GO:0004222 GO:0003911 GO:0008536
GO:0004550 GO:0005634
Cluster-22178.69566 GO:0006886 GO:0006183 GO:0006165 GO:0006508
GO:0006355 GO:0006228 GO:0006241 GO:0006144 GO:0006206
GO:0008536 GO:0004222 GO:0004550 GO:0005634
Cluster-22178.69567 GO:0016021
Cluster-22178.69568 GO:0005975 GO:0032065 GO:0001575 GO:0006412
GO:0006012 GO:0035556 GO:0046486 GO:0042254 GO:0005515
GO:0004557 GO:0004553 GO:0008242 GO:0005543 GO:0003735
GO:0005840 GO:0005938 GO:0005622
Cluster-22178.69572 GO:0006355 GO:0007155 GO:0006508 GO:0008270
GO:0016747 GO:0004222 GO:0003700 GO:0005667 GO:0005634
GO:0016020
Cluster-22178.69573 GO:0006468 GO:0007264 GO:0004715 GO:0003924
GO:0005524 GO:0005525
Cluster-22178.69575 GO:0006468 GO:0004672 GO:0005524 GO:0003723
GO:0005515 GO:0000178
Cluster-22178.69578 GO:0005515 GO:0008270 GO:0043565 GO:0005634
Cluster-22178.69579 GO:0030683 GO:0019012
Cluster-22178.69580 GO:0007010 GO:0016579 GO:0009878 GO:0046872
GO:0036459 GO:0003779
Cluster-22178.69581 GO:0006769 GO:0006741 GO:0008152 GO:0046497
GO:0003677 GO:0003951 GO:0016301
Cluster-22178.69584 GO:0010508 GO:0019064 GO:0006369 GO:0006379
GO:0006260 GO:0006378 GO:0016758 GO:0005849 GO:0005789
GO:0005815 GO:0019031

Cluster-22178.69587 GO:0003723
Cluster-22178.69588 GO:0006355 GO:0006412 GO:0009451 GO:0006479
GO:0008033 GO:0008152 GO:0046500 GO:0006464 GO:0030488
GO:0032259 GO:0008168 GO:0043565 GO:0004719 GO:0003700
GO:0016491 GO:0016429 GO:0005634 GO:0005667 GO:0031515
Cluster-22178.69589 GO:0006119 GO:0006810 GO:0015986 GO:0015992
GO:0046961 GO:0015288 GO:0045182 GO:0046933 GO:0045261
GO:0016020 GO:0045259
Cluster-22178.69590 GO:0016192 GO:0016021
Cluster-22178.69591 GO:0003779 GO:0005198 GO:0005856
Cluster-22178.69593 GO:0003677 GO:0005515
Cluster-22178.69594 GO:0015996 GO:0015994 GO:0006811 GO:0015991
GO:0006508 GO:0006355 GO:0006629 GO:0006118 GO:0015992
GO:0016042 GO:0045454 GO:0046486 GO:0046872 GO:0009055
GO:0015078 GO:0015035 GO:0008236 GO:0047746 GO:0003847
GO:0016787 GO:0003677 GO:0019031 GO:0033179 GO:0005886
GO:0016020 GO:0008247
Cluster-22178.69597 GO:0009098 GO:0006418 GO:0006438 GO:0009099
GO:0009097 GO:0005524 GO:0004832 GO:0002161 GO:0000166
GO:0004812 GO:0005737
Cluster-22178.69600 GO:0043087 GO:0007165 GO:0035023 GO:0005057
GO:0030170 GO:0005515 GO:0005089
Cluster-22178.69603 GO:0005515
Cluster-22178.69604 GO:0006505 GO:0006448 GO:0006508 GO:0006629
GO:0006886 GO:0001514 GO:0015996 GO:0015994 GO:0008152
GO:0016787 GO:0003746 GO:0003723 GO:0008236 GO:0047746
GO:0016788 GO:0005525 GO:0005576 GO:0005737 GO:0005840
Cluster-22178.69605 GO:0006281 GO:0043240
Cluster-22178.69606 GO:0006415 GO:0006449 GO:0016149 GO:0003747
GO:0008270 GO:0005515 GO:0003677 GO:0005737 GO:0005840
GO:0018444 GO:0042025
Cluster-22178.69607 GO:0006508 GO:0006468 GO:0015031 GO:0006464
GO:0004252 GO:0016773 GO:0005524 GO:0004672 GO:0016020

GO:0016021
Cluster-22178.69608 GO:0006464 GO:0015031 GO:0006468 GO:0006508
GO:0004672 GO:0005524 GO:0016773 GO:0004252 GO:0016021
GO:0016020
Cluster-22178.69609 GO:0015031 GO:0006464
Cluster-22178.69613 GO:0006629 GO:0006355 GO:0007165 GO:0046339
GO:0009395 GO:0035556 GO:0005509 GO:0005515 GO:0003676
GO:0004435 GO:0005622
Cluster-22178.69615 GO:0005515
Cluster-22178.69620 GO:0015992 GO:0015986 GO:0015078 GO:0000276
Cluster-22178.69621 GO:0015992 GO:0015986 GO:0015078 GO:0000276
Cluster-22178.69622 GO:0055114 GO:0020037 GO:0016705 GO:0005506

Cluster-22178.69623 GO:0016055 GO:0006468 GO:0007165 GO:0007275
GO:0005524 GO:0004672 GO:0005102 GO:0005576
Cluster-22178.69625 GO:0008152 GO:0008484 GO:0016788
Cluster-22178.69627 GO:0005515
Cluster-22178.69628 GO:0006744 GO:0015992 GO:0042254 GO:0008152
GO:0006120 GO:0005975 GO:0006412 GO:0006814 GO:0006118
GO:0004553 GO:0003735 GO:0009055 GO:0003824 GO:0008484
GO:0008137 GO:0005622 GO:0016020 GO:0005840
Cluster-22178.69629 GO:0006821 GO:0046653 GO:0015991 GO:0006555
GO:0055085 GO:0055114 GO:0016820 GO:0005247 GO:0004489
GO:0016020 GO:0000221
Cluster-22178.69630 GO:0006555 GO:0055085 GO:0055114 GO:0006821
GO:0015991 GO:0046653 GO:0005247 GO:0004489 GO:0016820
GO:0000221 GO:0016020
Cluster-22178.69632 GO:0042742 GO:0006468 GO:0016773 GO:0004672
GO:0005524 GO:0005515 GO:0016020
Cluster-22178.69636 GO:0030494 GO:0015979 GO:0006355 GO:0016032
GO:0003700 GO:0043565 GO:0005515 GO:0005667
Cluster-22178.69640 GO:0016192 GO:0006886 GO:0030117 GO:0030131
Cluster-22178.69643 GO:0042254 GO:0006412 GO:0003735 GO:0005622

GO:0005840
Cluster-22178.69644 GO:0016310 GO:0004673 GO:0009365
Cluster-22178.69646 GO:0009405 GO:0006659 GO:0005198 GO:0004222
GO:0008289
Cluster-22178.69647 GO:0043039 GO:0005524 GO:0016876
Cluster-22178.69649 GO:0009069 GO:0006814 GO:0005975 GO:0015992
GO:0019049 GO:0055114 GO:0016310 GO:0008152 GO:0006744
GO:0006120 GO:0030246 GO:0016836 GO:0008137 GO:0004674
GO:0003824
Cluster-22178.69650 GO:0003676
Cluster-22178.69651 GO:0006270 GO:0000736 GO:0006364 GO:0070336
GO:0008270 GO:0005732 GO:0005634 GO:0034457
Cluster-22178.69653 GO:0007264 GO:0005525
Cluster-22178.69654 GO:0006355 GO:0051260 GO:0008270 GO:0003700
GO:0003676 GO:0005634 GO:0005667
Cluster-22178.69657 GO:0008152 GO:0006508 GO:0006412 GO:0042254
GO:0016787 GO:0008236 GO:0003735 GO:0005198 GO:0005622
GO:0005840 GO:0019028
Cluster-22178.69659 GO:0046872
Cluster-22178.69660 GO:0006914 GO:0048193 GO:0043093 GO:0051301
GO:0030036 GO:0031110 GO:0006260 GO:0007067 GO:0000090
GO:0048870 GO:0051382 GO:0007049 GO:0007059 GO:0006306
GO:0000917 GO:0046983 GO:0008017 GO:0008170 GO:0003677
GO:0003779 GO:0042803 GO:0005515 GO:0017048 GO:0045502
GO:0008134 GO:0005876 GO:0005667 GO:0016020 GO:0030133
GO:0005737 GO:0045298 GO:0000776 GO:0000940 GO:0030286
GO:0005815 GO:0017119 GO:0016021 GO:0031514 GO:0031083
Cluster-22178.69662 GO:0006308 GO:0006355 GO:0007017 GO:0007018
GO:0006812 GO:0007165 GO:0003777 GO:0035257 GO:0005524
GO:0016887 GO:0008855 GO:0008017 GO:0003713 GO:0005874
GO:0016021 GO:0045298 GO:0005634 GO:0009318 GO:0005667
Cluster-22178.69663 GO:0006687 GO:0005975 GO:0004308 GO:0033644
GO:0055036 GO:0016020

Cluster-22178.69664 GO:0007186 GO:0004930 GO:0016021
Cluster-22178.69665 GO:0006355 GO:0006281 GO:0006397 GO:0000917
GO:0006412 GO:0007264 GO:0043093 GO:0006310 GO:0042254
GO:0006457 GO:0006260 GO:0010506 GO:0051082 GO:0000287
GO:0005515 GO:0043565 GO:0003700 GO:0003735 GO:0005096
GO:0005737 GO:0000445 GO:0005840 GO:0005615 GO:0005667
GO:0016272
Cluster-22178.69666 GO:0019475 GO:0048870 GO:0006412 GO:0006355
GO:0006281 GO:0008053 GO:0010506 GO:0006260 GO:0006310
GO:0042254 GO:0006457 GO:0007264 GO:0003700 GO:0003924
GO:0043565 GO:0005515 GO:0000287 GO:0016740 GO:0051082
GO:0005096 GO:0003735 GO:0005615 GO:0016021 GO:0005840
GO:0031514 GO:0016272 GO:0005741 GO:0005667
Cluster-22178.69667 GO:0003676
Cluster-22178.69670 GO:0006352 GO:0000398 GO:0006351 GO:0006355
GO:0006260 GO:0000736 GO:0003700 GO:0070336 GO:0003887
GO:0046935 GO:0016987 GO:0008270 GO:0003677 GO:0003682
GO:0005681 GO:0000785 GO:0005789 GO:0032040 GO:0000775
GO:0042575 GO:0005634 GO:0019028 GO:0016020 GO:0005667
GO:0005944
Cluster-22178.69674 GO:0006206 GO:0006144 GO:0046034 GO:0006139
GO:0005524 GO:0004017 GO:0004127
Cluster-22178.69675 GO:0006570 GO:0016192 GO:0007304 GO:0006470
GO:0008138 GO:0005213 GO:0004725 GO:0005576 GO:0042600
Cluster-22178.69678 GO:0005975 GO:0004553 GO:0019013 GO:0016021
Cluster-22178.69679 GO:0055114 GO:0006788 GO:0015994 GO:0004392

Cluster-22178.69680 GO:0030246
Cluster-22178.69681 GO:0009186 GO:0055114 GO:0030246 GO:0016021
Cluster-22178.69684 GO:0016579 GO:0036459 GO:0008270 GO:0032183
GO:0070140
Cluster-22178.69685 GO:0016579 GO:0036459 GO:0008270 GO:0070140
GO:0032183

Cluster-22178.69687 GO:0019062 GO:0019058 GO:0007155
Cluster-22178.69688 GO:0019058 GO:0019062 GO:0007155
Cluster-22178.69690 GO:0006468 GO:0004672 GO:0005524 GO:0016773
GO:0016020
Cluster-22178.69692 GO:0015948 GO:0046656 GO:0003677 GO:0030269
GO:0016020 GO:0005634 GO:0005576
Cluster-22178.69695 GO:0005515 GO:0032040
Cluster-22178.69696 GO:0061025 GO:0046813 GO:0005634 GO:0005737
GO:0016021 GO:0019031
Cluster-22178.69698 GO:0045502 GO:0051082 GO:0005198 GO:0008134
GO:0042803 GO:0005667 GO:0048786 GO:0019028 GO:0005882
GO:0030286 GO:0019031 GO:0005815
Cluster-22178.69699 GO:0002949 GO:0007264 GO:0007165 GO:0007186
GO:0031683 GO:0019001 GO:0003924 GO:0005525 GO:0003724
GO:0004871 GO:0003723
Cluster-22178.69700 GO:0007165 GO:0046740 GO:0035556 GO:0043087
GO:0006814 GO:0006955 GO:0007010 GO:0003333 GO:0006508
GO:0006741 GO:0046656 GO:0046497 GO:0035023 GO:0008152
GO:0006769 GO:0005089 GO:0030269 GO:0008236 GO:0016301
GO:0003951 GO:0005133 GO:0003779 GO:0046872 GO:0012506
GO:0019028 GO:0005576 GO:0005737 GO:0044219 GO:0005643
Cluster-22178.69702 GO:0005975 GO:0006351 GO:0004553 GO:0003676
GO:0033925 GO:0008270 GO:0005515 GO:0005737
Cluster-22178.69704 GO:0006813 GO:0007010 GO:0030955 GO:0003779
GO:0005249 GO:0019033 GO:0016020 GO:0008076 GO:0016021
Cluster-22178.69710 GO:0046034 GO:0006144 GO:0006366 GO:0008616
GO:0006281 GO:0046872 GO:0008270 GO:0016853 GO:0004017
GO:0003677 GO:0004867 GO:0016740 GO:0005576 GO:0005665
Cluster-22178.69713 GO:0005515
Cluster-22178.69715 GO:0006479 GO:0006481 GO:0004671 GO:0016021
Cluster-22178.69717 GO:0004222 GO:0016020 GO:0016021
Cluster-22178.69718 GO:0015684 GO:0007264 GO:0005525 GO:0005515
GO:0015093 GO:0016021

Cluster-22178.69719 GO:0007165 GO:0008152 GO:0003824 GO:0003676
GO:0005179 GO:0005576
Cluster-22178.69720 GO:0030436 GO:0042601
Cluster-22178.69721 GO:0006269 GO:0018142 GO:0006260 GO:0004672
GO:0003723 GO:0000166
Cluster-22178.69722 GO:0055114 GO:0006464 GO:0008113 GO:0009279
Cluster-22178.69724 GO:0006613 GO:0051276 GO:0007029 GO:0005515
GO:0005524 GO:0005694 GO:0005784 GO:0016021 GO:0030176
GO:0005789
Cluster-22178.69728 GO:0016021
Cluster-22178.69729 GO:0008152 GO:0007165 GO:0005179 GO:0003824
GO:0003676 GO:0005576
Cluster-22178.69736 GO:0006464 GO:0005975 GO:0016868
Cluster-22178.69741 GO:0006888 GO:0007264 GO:0007186 GO:0007165
GO:0055114 GO:0009308 GO:0006886 GO:0008270 GO:0048038
GO:0008131 GO:0003924 GO:0031683 GO:0019001 GO:0005507
GO:0004871 GO:0005525 GO:0030127
Cluster-22178.69742 GO:0006402 GO:0019722 GO:0051252 GO:0006810
GO:0003676 GO:0046872 GO:0004535 GO:0008270 GO:0003723
GO:0005737 GO:0005634
Cluster-22178.69744 GO:0007268 GO:0033130 GO:0042802 GO:0005515
GO:0030695 GO:0043495
Cluster-22178.69747 GO:0042254 GO:0006412 GO:0006298 GO:0003735
GO:0004519 GO:0008146 GO:0016021 GO:0005840 GO:0005622
Cluster-22178.69749 GO:0003676 GO:0042393 GO:0005634
Cluster-22178.69750 GO:0055114 GO:0016491
Cluster-22178.69751 GO:0055085 GO:0006810 GO:0016192 GO:0006886
GO:0005215 GO:0022857 GO:0016021 GO:0016020 GO:0030117
Cluster-22178.69753 GO:0051262
Cluster-22178.69759 GO:0006525 GO:0006420 GO:0006560 GO:0005524
GO:0004814
Cluster-22178.69762 GO:0007264 GO:0007186 GO:0007165 GO:0015684
GO:0003924 GO:0031683 GO:0008270 GO:0015093 GO:0019001

GO:0004871 GO:0005525 GO:0016021
Cluster-22178.69764 GO:0006468 GO:0004672 GO:0005524 GO:0005515

Cluster-22178.69765 GO:0006352 GO:0005669
Cluster-22178.69766 GO:0006376 GO:0003729 GO:0005685
Cluster-22178.69768 GO:0006260 GO:0016567 GO:0006869 GO:0042157
GO:0006352 GO:0006308 GO:0006351 GO:0006355 GO:0004842
GO:0016987 GO:0008855 GO:0008270 GO:0061630 GO:0003677
GO:0003700 GO:0008289 GO:0046872 GO:0005515 GO:0003887
GO:0004190 GO:0005680 GO:0005622 GO:0016020 GO:0005667
GO:0016021 GO:0009318 GO:0042575 GO:0019028 GO:0005576
Cluster-22178.69770 GO:0006886 GO:0015031 GO:0005515 GO:0008565
GO:0016021 GO:0016020 GO:0005737
Cluster-22178.69771 GO:0006914 GO:0015031 GO:0006886 GO:0006298
GO:0008565 GO:0005524 GO:0030983 GO:0005815 GO:0005737
GO:0016020
Cluster-22178.69773 GO:0006470 GO:0006570 GO:0004725 GO:0043130
GO:0032266 GO:0008138
Cluster-22178.69776 GO:0003723
Cluster-22178.69777 GO:0051260 GO:0006355 GO:0006352 GO:0015074
GO:0003677 GO:0003700 GO:0005667 GO:0005669
Cluster-22178.69780 GO:0006119 GO:0015992 GO:0015986 GO:0046961
GO:0046933 GO:0045259 GO:0045261
Cluster-22178.69784 GO:0030244 GO:0061136 GO:0019915 GO:0006011
GO:0005985 GO:0005982 GO:0016567 GO:0008270 GO:0046872
GO:0004842 GO:0005515 GO:0016760 GO:0005789 GO:0005680
GO:0016020
Cluster-22178.69787 GO:0006355 GO:0005515 GO:0005634
Cluster-22178.69788 GO:0006357 GO:0042254 GO:0006412 GO:0001104
GO:0003735 GO:0005840 GO:0005622 GO:0016592
Cluster-22178.69789 GO:0006886 GO:0016020
Cluster-22178.69795 GO:0019491 GO:0033990 GO:0005515 GO:0016021
Cluster-22178.69796 GO:0006355 GO:0006418 GO:0004812 GO:0000166

GO:0016818 GO:0003677 GO:0008270 GO:0005524 GO:0002161
GO:0005634
Cluster-22178.69798 GO:0009405 GO:0006468 GO:0019865 GO:0005524
GO:0004672 GO:0005515 GO:0051082 GO:0019814
Cluster-22178.69799 GO:0016020
Cluster-22178.69800 GO:0030212 GO:0005515 GO:0004867
Cluster-22178.69801 GO:0015986 GO:0006810 GO:0006119 GO:0015992
GO:0046961 GO:0015288 GO:0046933 GO:0045182 GO:0045259
GO:0045261 GO:0016020
Cluster-22178.69802 GO:0015986 GO:0006810 GO:0006119 GO:0015992
GO:0046961 GO:0046933 GO:0045182 GO:0015288 GO:0045259
GO:0016020 GO:0045261
Cluster-22178.69803 GO:0008654 GO:0006278 GO:0016780 GO:0003964
GO:0016020
Cluster-22178.69804 GO:0031110 GO:0006260 GO:0007067 GO:0000090
GO:0051382 GO:0048870 GO:0007049 GO:0007059 GO:0006306
GO:0000917 GO:0006914 GO:0048193 GO:0043093 GO:0051301
GO:0030036 GO:0017048 GO:0045502 GO:0008134 GO:0046983
GO:0008017 GO:0008170 GO:0003677 GO:0003779 GO:0005515
GO:0042803 GO:0030286 GO:0000940 GO:0005815 GO:0017119
GO:0016021 GO:0031083 GO:0031514 GO:0005876 GO:0005667
GO:0030133 GO:0016020 GO:0005737 GO:0045298 GO:0000776
Cluster-22178.69806 GO:0007165 GO:0004872 GO:0016021
Cluster-22178.69807 GO:0006355 GO:0005515 GO:0046983
Cluster-22178.69808 GO:0006355 GO:0003333 GO:0046983 GO:0005515
GO:0016020
Cluster-22178.69809 GO:0006606 GO:0005515 GO:0019904 GO:0005737
Cluster-22178.69810 GO:0000921 GO:0042254 GO:0006412 GO:0016055
GO:0006355 GO:0003700 GO:0043565 GO:0003735 GO:0003677
GO:0016021 GO:0005840 GO:0005940 GO:0005622 GO:0005667
GO:0005634
Cluster-22178.69812 GO:0006412 GO:0042254 GO:0000723 GO:0003735
GO:0003677 GO:0005840 GO:0005622 GO:0000784

Cluster-22178.69814 GO:0005515
Cluster-22178.69817 GO:0005198 GO:0019012
Cluster-22178.69818 GO:0007165 GO:0005515
Cluster-22178.69820 GO:0005515
Cluster-22178.69821 GO:0030976
Cluster-22178.69823 GO:0003677 GO:0016020
Cluster-22178.69825 GO:0005576
Cluster-22178.69833 GO:0055085 GO:0016020 GO:0016021
Cluster-22178.69834 GO:0006468 GO:0016192 GO:0006886 GO:0005524 GO:0004672 GO:0005515 GO:0030117
Cluster-22178.69835 GO:0030001 GO:0055085 GO:0009306 GO:0046873 GO:0016020
Cluster-22178.69837 GO:0006412 GO:0042254 GO:0003735 GO:0005622 GO:0005840
Cluster-22178.69838 GO:0006888 GO:0006508 GO:0007606 GO:0006886 GO:0007165 GO:0008270 GO:0003723 GO:0004888 GO:0005515 GO:0004197 GO:0016020 GO:0030127
Cluster-22178.69844 GO:0006935 GO:0007165 GO:0004888 GO:0016020
Cluster-22178.69847 GO:0008283 GO:0040007 GO:0007165 GO:0008083

Cluster-22178.69848 GO:0006516 GO:0030153 GO:0006955 GO:0015643 GO:0005737 GO:0019814
Cluster-22178.69850 GO:0006357 GO:0001104 GO:0016592
Cluster-22178.69851 GO:0046872 GO:0005524 GO:0016787 GO:0003677 GO:0003676
Cluster-22178.69855 GO:0006810 GO:0005215 GO:0016020
Cluster-22178.69856 GO:0008152 GO:0005515 GO:0003824
Cluster-22178.69857 GO:0046854 GO:0006000 GO:0015976 GO:0006013 GO:0005975 GO:0006096 GO:0006098 GO:0006094 GO:0042578 GO:0042132
Cluster-22178.69863 GO:0005198 GO:0005515 GO:0003723
Cluster-22178.69867 GO:0007165 GO:0007155 GO:0048193 GO:0006606 GO:0035556 GO:0006357 GO:0006914 GO:0046339 GO:0006629

GO:0009395 GO:0005509 GO:0042803 GO:0008134 GO:0005515
GO:0001104 GO:0045502 GO:0004435 GO:0008081 GO:0016020
GO:0016592 GO:0005667 GO:0030286 GO:0019028
Cluster-22178.69868 GO:0006118 GO:0000917 GO:0006629 GO:0048870
GO:0009395 GO:0007165 GO:0035556 GO:0022900 GO:0043093
GO:0006606 GO:0006914 GO:0006357 GO:0046339 GO:0008134
GO:0045502 GO:0005509 GO:0042803 GO:0020037 GO:0005515
GO:0001104 GO:0004435 GO:0008081 GO:0009055 GO:0005506
GO:0019905 GO:0031514 GO:0019028 GO:0016592 GO:0030286
GO:0005737 GO:0005667 GO:0042597
Cluster-22178.69869 GO:0043531 GO:0005524
Cluster-22178.69870 GO:0042254 GO:0006412 GO:0003735 GO:0005622
GO:0005840
Cluster-22178.69871 GO:0006368 GO:0055114 GO:0016570 GO:0030151
GO:0035091 GO:0005515 GO:0016491 GO:0016593
Cluster-22178.69872 GO:0016567 GO:0006531 GO:0006123 GO:0006522
GO:0009152 GO:0006188 GO:0006144 GO:0015992 GO:0004129
GO:0004842 GO:0004018 GO:0005740 GO:0045277
Cluster-22178.69873 GO:0005515
Cluster-22178.69875 GO:0007165 GO:0005179 GO:0005576
Cluster-22178.69876 GO:0055114 GO:0007264 GO:0015684 GO:0015093
GO:0005524 GO:0016491 GO:0003924 GO:0005525 GO:0003677
GO:0000166 GO:0016021
Cluster-22178.69877 GO:0017004 GO:0006118 GO:0006468 GO:0008152
GO:0006480 GO:0015979 GO:0055114 GO:0047134 GO:0045158
GO:0004672 GO:0005524 GO:0008168 GO:0016021 GO:0009512
GO:0009523
Cluster-22178.69878 GO:0016579 GO:0036459
Cluster-22178.69879 GO:0003676
Cluster-22178.69880 GO:0006118 GO:0019684 GO:0006274 GO:0045156
GO:0003677 GO:0003676 GO:0030077 GO:0005737 GO:0016021
Cluster-22178.69882 GO:0007034 GO:0030001 GO:0005887
Cluster-22178.69884 GO:0007067 GO:0015743 GO:0016043 GO:0030036

GO:0048037 GO:0017048 GO:0016740 GO:0003779 GO:0042729
GO:0072686
Cluster-22178.69887 GO:0042967 GO:0004379 GO:0008080
Cluster-22178.69888 GO:0008152 GO:0009058 GO:0006886 GO:0006629
GO:0006505 GO:0016788 GO:0016787
Cluster-22178.69889 GO:0006189
Cluster-22178.69890 GO:0006811 GO:0006813 GO:0005249 GO:0016020
GO:0008076
Cluster-22178.69891 GO:0006412 GO:0000921 GO:0051607 GO:0042254
GO:0007155 GO:0005515 GO:0042803 GO:0008134 GO:0045502
GO:0003735 GO:0005840 GO:0016021 GO:0097197 GO:0019028
GO:0005622 GO:0030286 GO:0001772 GO:0005940 GO:0005667
Cluster-22178.69892 GO:0006886 GO:0000921 GO:0051276 GO:0007031
GO:0008773 GO:0051082 GO:0005515 GO:0005524 GO:0005694
GO:0016020 GO:0005777 GO:0005940 GO:0016021 GO:0005801
Cluster-22178.69893 GO:0006412 GO:0042254 GO:0003735 GO:0005840
GO:0005622
Cluster-22178.69899 GO:0005975 GO:0016192 GO:0006886 GO:0004553
GO:0005198 GO:0005515 GO:0019028 GO:0030117
Cluster-22178.69901 GO:0043087 GO:0035556 GO:0035023 GO:0005515
GO:0005089
Cluster-22178.69902 GO:0035556 GO:0035023 GO:0043087 GO:0005515
GO:0005089
Cluster-22178.69903 GO:0004190 GO:0016021
Cluster-22178.69904 GO:0007264 GO:0019079 GO:0006614 GO:0007165
GO:0006304 GO:0007186 GO:0006013 GO:0006000 GO:0043066
GO:0043154 GO:0003873 GO:0033821 GO:0005524 GO:0031683
GO:0019001 GO:0003924 GO:0005525 GO:0004871 GO:0071986
GO:0019012 GO:0005737
Cluster-22178.69905 GO:0043087 GO:0003676 GO:0005089 GO:0003677
GO:0016787 GO:0005524 GO:0005737
Cluster-22178.69910 GO:0006144 GO:0006206 GO:0006351 GO:0003899
GO:0003677 GO:0005730

Cluster-22178.69911 GO:0055085 GO:0006811 GO:0005216 GO:0012511
GO:0016021 GO:0016020
Cluster-22178.69914 GO:0006355 GO:0005515 GO:0003723
Cluster-22178.69916 GO:0003676
Cluster-22178.69918 GO:0003676
Cluster-22178.69919 GO:0046660 GO:0006397 GO:0003676 GO:0005634
Cluster-22178.69922 GO:0042742 GO:0051191 GO:0006352 GO:0005515
GO:0005576 GO:0005669
Cluster-22178.69923 GO:0006352 GO:0051191 GO:0042742 GO:0005515
GO:0005669 GO:0005576
Cluster-22178.69924 GO:0000103 GO:0006144 GO:0009058 GO:0008152
GO:0015937 GO:0015940 GO:0006468 GO:0005524 GO:0016773
GO:0004020 GO:0003824 GO:0004672 GO:0004140 GO:0016301
GO:0005515 GO:0016020
Cluster-22178.69925 GO:0007186 GO:0004930 GO:0046872 GO:0016021
Cluster-22178.69926 GO:0032780 GO:0042030 GO:0046872 GO:0016820
GO:0005739
Cluster-22178.69930 GO:0055114 GO:0006508 GO:0006886 GO:0015031
GO:0006260 GO:0007264 GO:0016192 GO:0005515 GO:0070403
GO:0008270 GO:0016491 GO:0005096 GO:0008565 GO:0004222
GO:0047134 GO:0005737 GO:0031012 GO:0016020
Cluster-22178.69931 GO:0005515
Cluster-22178.69935 GO:0006468 GO:0031124 GO:0000287 GO:0004672
GO:0005524 GO:0030976
Cluster-22178.69937 GO:0042176 GO:0030234 GO:0005515 GO:0000502
Cluster-22178.69938 GO:0015979 GO:0008152 GO:0016829 GO:0009523
GO:0009539 GO:0016020
Cluster-22178.69939 GO:0003950 GO:0005515
Cluster-22178.69942 GO:0006355 GO:0045132 GO:0003676 GO:0003700
GO:0005524 GO:0043565 GO:0003677 GO:0016787 GO:0000775
GO:0005634 GO:0005667
Cluster-22178.69943 GO:0006886 GO:0016192 GO:0005198 GO:0005515
GO:0030117

Cluster-22178.69949 GO:0006804 GO:0006979 GO:0055114 GO:0006568 GO:0015947 GO:0020037 GO:0004096

Cluster-22178.69951 GO:0007264 GO:0006468 GO:0005525 GO:0005524 GO:0003924 GO:0004715

Cluster-22178.69953 GO:0030154 GO:0016021

Cluster-22178.69954 GO:0055085 GO:0008053 GO:0015992 GO:0019722 GO:0006308 GO:0006355 GO:0030001 GO:0015991 GO:0008855 GO:0046873 GO:0015078 GO:0003700 GO:0003924 GO:0043565 GO:0005198 GO:0005741 GO:0016020 GO:0005667 GO:0019031 GO:0016021 GO:0009318 GO:0033179 GO:0019028

Cluster-22178.69955 GO:0006672 GO:0055085 GO:0006807 GO:0006811 GO:0007186 GO:0007165 GO:0006810 GO:0004872 GO:0004930 GO:0005216 GO:0016811 GO:0016021 GO:0016020

Cluster-22178.69958 GO:0006744 GO:0055114 GO:0046872 GO:0016491

Cluster-22178.69961 GO:0005515

Cluster-22178.69964 GO:0003723

Cluster-22178.69965 GO:0008053 GO:0007264 GO:0010508 GO:0006810 GO:0003924 GO:0016887 GO:0005524 GO:0003724 GO:0005525 GO:0003723 GO:0016021 GO:0005741

Cluster-22178.69966 GO:0003677

Cluster-22178.69969 GO:0055114 GO:0005996 GO:0005525 GO:0016491 GO:0016853 GO:0048029 GO:0050661 GO:0019867

Cluster-22178.69970 GO:0005515

Cluster-22178.69971 GO:0005515

Cluster-22178.69972 GO:0006886 GO:0006888 GO:0003677 GO:0046872 GO:0008270 GO:0030127

Cluster-22178.69973 GO:0007017 GO:0030001 GO:0007018 GO:0055085 GO:0004190 GO:0016787 GO:0046873 GO:0046872 GO:0003777 GO:0016020 GO:0030286 GO:0005874

Cluster-22178.69979 GO:0015992 GO:0006123 GO:0004129 GO:0005739 GO:0045277

Cluster-22178.69980 GO:0006508 GO:0042981 GO:0004197

Cluster-22178.69981 GO:0006955 GO:0006954 GO:0007165 GO:0040007 GO:0008283 GO:0005509 GO:0005515 GO:0005149 GO:0045323
Cluster-22178.69984 GO:0006749 GO:0006804 GO:0006118 GO:0055114 GO:0006979 GO:0016655 GO:0004602 GO:0071203
Cluster-22178.69986 GO:0000413 GO:0006457 GO:0003755
Cluster-22178.69988 GO:0009395 GO:0008654 GO:0042157 GO:0006869 GO:0016567 GO:0046486 GO:0046872 GO:0008289 GO:0005198 GO:0005515 GO:0008270 GO:0046983 GO:0004143 GO:0004842 GO:0061630 GO:0019031 GO:0005576 GO:0019028 GO:0005622 GO:0016020
Cluster-22178.69990 GO:0051082 GO:0005515 GO:0031072
Cluster-22178.69991 GO:0006811 GO:0006486 GO:0051225 GO:0007165 GO:0007155 GO:0006355 GO:0004871 GO:0004576 GO:0003677 GO:0003723 GO:0009288 GO:0070652 GO:0008250 GO:0016020 GO:0005634
Cluster-22178.69993 GO:0006260 GO:0003677 GO:0016818 GO:0005524 GO:0016021 GO:0005634
Cluster-22178.69995 GO:0006812 GO:0007304 GO:0008324 GO:0005213 GO:0042600 GO:0005886 GO:0016021 GO:0005576
Cluster-22178.69997 GO:0006468 GO:0005198 GO:0005524 GO:0004672

Cluster-22178.69998 GO:0006357 GO:0009405 GO:0006171 GO:0046983 GO:0001104 GO:0008294 GO:0016592 GO:0005576
Cluster-22178.69999 GO:0045454
Cluster-22178.70002 GO:0005515 GO:0003677
Cluster-22178.70004 GO:0005515
Cluster-22178.70005 GO:0008152 GO:0006355 GO:0055114 GO:0003700 GO:0016491 GO:0005667
Cluster-22178.70008 GO:0007049 GO:0006355 GO:0003677 GO:0003682 GO:0000785 GO:0000775 GO:0005634
Cluster-22178.70011 GO:0030001 GO:0006355 GO:0046872 GO:0005634
Cluster-22178.70012 GO:0005509 GO:0005515
Cluster-22178.70015 GO:0006351 GO:0015031 GO:0006464 GO:0005515

GO:0046983
Cluster-22178.70018 GO:0009069 GO:0016310 GO:0008608 GO:0007049
GO:0048870 GO:0009405 GO:0006468 GO:0004674 GO:0005524
GO:0005198 GO:0005815 GO:0016020 GO:0019031 GO:0042729
GO:0031514 GO:0019028 GO:0072686
Cluster-22178.70019 GO:0006281 GO:0006260 GO:0006310 GO:0046907
GO:0003677 GO:0005634
Cluster-22178.70020 GO:0009069 GO:0009405 GO:0006468 GO:0007049
GO:0016310 GO:0008608 GO:0048870 GO:0004674 GO:0005524
GO:0005198 GO:0016020 GO:0005815 GO:0042729 GO:0031514
GO:0019028 GO:0072686 GO:0019031
Cluster-22178.70021 GO:0009058 GO:0008483 GO:0030170
Cluster-22178.70022 GO:0006510 GO:0006508 GO:0006355 GO:0016567
GO:0008270 GO:0004176 GO:0046872 GO:0004842 GO:0061630
GO:0005515 GO:0005634
Cluster-22178.70023 GO:0043565 GO:0005515 GO:0019028 GO:0005634
GO:0016020
Cluster-22178.70024 GO:0043565 GO:0005515 GO:0005634
Cluster-22178.70025 GO:0043565 GO:0005515 GO:0005634 GO:0016020
Cluster-22178.70026 GO:0035444 GO:0015675 GO:0006508 GO:0015099
GO:0046872 GO:0004252 GO:0030288 GO:0016021
Cluster-22178.70027 GO:0008270 GO:0005515
Cluster-22178.70028 GO:0006418 GO:0019643 GO:0006094 GO:0006099
GO:0015977 GO:0043039 GO:0005524 GO:0004222 GO:0004812
GO:0000166 GO:0008964 GO:0000049 GO:0005737 GO:0031225
Cluster-22178.70029 GO:0009395 GO:0046339 GO:0035556 GO:0006629
GO:0007165 GO:0046872 GO:0004435 GO:0005515
Cluster-22178.70031 GO:0016757
Cluster-22178.70033 GO:0006260 GO:0003887 GO:0005515 GO:0042575
Cluster-22178.70034 GO:0019079 GO:0006810 GO:0008026 GO:0003676
GO:0004386 GO:0005524
Cluster-22178.70035 GO:0019079 GO:0006810 GO:0004386 GO:0003676
GO:0008026 GO:0005524

Cluster-22178.70037 GO:0048511 GO:0006118 GO:0045454 GO:0006357
GO:0015035 GO:0001104 GO:0016592
Cluster-22178.70039 GO:0005515
Cluster-22178.70040 GO:0005515 GO:0003677 GO:0019028
Cluster-22178.70041 GO:0042254 GO:0001522 GO:0030515 GO:0072588
Cluster-22178.70045 GO:0006118 GO:0006886 GO:0006449 GO:0006813
GO:0007165 GO:0016192 GO:0006415 GO:0009055 GO:0004871
GO:0005249 GO:0016149 GO:0020037 GO:0030955 GO:0005506
GO:0005840 GO:0016021 GO:0005737 GO:0005622 GO:0008076
GO:0030131 GO:0018444 GO:0016020
Cluster-22178.70046 GO:0006810 GO:0005215 GO:0005737 GO:0016021
Cluster-22178.70047 GO:0055114 GO:0005509 GO:0016717 GO:0016020
GO:0005576
Cluster-22178.70048 GO:0016021
Cluster-22178.70051 GO:0016310 GO:0006468 GO:0015031 GO:0015992
GO:0009069 GO:0004672 GO:0015078 GO:0008384 GO:0005515
GO:0016773 GO:0005524 GO:0016021 GO:0005789 GO:0008385
GO:0033644 GO:0055036 GO:0016020
Cluster-22178.70053 GO:0006412 GO:0042254 GO:0003735 GO:0005622
GO:0005840
Cluster-22178.70054 GO:0006412 GO:0042254 GO:0003735 GO:0005622
GO:0005840
Cluster-22178.70056 GO:0045040 GO:0006281 GO:0006289 GO:0043161
GO:0003684 GO:0005515 GO:0005742
Cluster-22178.70060 GO:0015031 GO:0016020
Cluster-22178.70061 GO:0007165 GO:0006955 GO:0005164 GO:0016020
Cluster-22178.70062 GO:0006807 GO:0006811 GO:0055085 GO:0006672
GO:0007165 GO:0004872 GO:0016811 GO:0005216 GO:0016021
GO:0016020
Cluster-22178.70063 GO:0006629 GO:0015991 GO:0006886 GO:0051276
GO:0015031 GO:0043248 GO:0006606 GO:0016192 GO:0046872
GO:0016820 GO:0005515 GO:0008270 GO:0005524 GO:0016887
GO:0008565 GO:0004222 GO:0061630 GO:0005737 GO:0030117

GO:0005634 GO:0005622 GO:0000221 GO:0005694
Cluster-22178.70064 GO:0006355 GO:0043401 GO:0008270 GO:0003677

Cluster-22178.70066 GO:0006469 GO:0045859 GO:0006355 GO:0003700
GO:0043565 GO:0008270 GO:0004862 GO:0005667 GO:0005634
GO:0005952
Cluster-22178.70069 GO:0060478 GO:0006897 GO:0007342 GO:0023052
GO:0003950 GO:0005886 GO:0005634
Cluster-22178.70070 GO:0006606 GO:0015031 GO:0051276 GO:0043248
GO:0016192 GO:0055114 GO:0006397 GO:0006886 GO:0006629
GO:0015991 GO:0004222 GO:0008270 GO:0005524 GO:0008565
GO:0016491 GO:0016887 GO:0051087 GO:0046872 GO:0005515
GO:0016820 GO:0000221 GO:0005622 GO:0005694 GO:0000445
GO:0005737 GO:0030117 GO:0005634 GO:0019028
Cluster-22178.70072 GO:0006886 GO:0006629 GO:0015991 GO:0006606
GO:0015031 GO:0051276 GO:0043248 GO:0016192 GO:0046872
GO:0016820 GO:0005515 GO:0004222 GO:0008270 GO:0005524
GO:0016887 GO:0008565 GO:0016021 GO:0005737 GO:0030117
GO:0005634 GO:0000221 GO:0005622 GO:0005694
Cluster-22178.70073 GO:0003676
Cluster-22178.70077 GO:0007010 GO:0003779
Cluster-22178.70080 GO:0016055 GO:0007165 GO:0007411 GO:0008289
GO:0005515 GO:0005576 GO:0016021
Cluster-22178.70082 GO:0050790 GO:0006351 GO:0006144 GO:0006206
GO:0015992 GO:0015979 GO:0006123 GO:0004129 GO:0005507
GO:0003899 GO:0030234 GO:0003677 GO:0009523 GO:0009539
GO:0045277 GO:0005730 GO:0016020
Cluster-22178.70084 GO:0045995 GO:0006308 GO:0030334 GO:0030155
GO:0007165 GO:0007155 GO:0005102 GO:0008855 GO:0046872
GO:0005515 GO:0009318
Cluster-22178.70086 GO:0006352 GO:0015031 GO:0006886 GO:0008565
GO:0003677 GO:0005515 GO:0005737 GO:0016020
Cluster-22178.70088 GO:0008152 GO:0003824

Cluster-22178.70090 GO:0006355 GO:0006412 GO:0042254 GO:0003735 GO:0003677 GO:0003700 GO:0005667 GO:0005634 GO:0005840
Cluster-22178.70092 GO:0055114 GO:0045454 GO:0005509 GO:0016209 GO:0016491
Cluster-22178.70093 GO:0006464 GO:0016151
Cluster-22178.70094 GO:0042967 GO:0016568 GO:0008080 GO:0004402 GO:0000123
Cluster-22178.70095 GO:0005975 GO:0003697 GO:0042302 GO:0004553

Cluster-22178.70096 GO:0006097 GO:0006099 GO:0006090 GO:0006418 GO:0055114 GO:0000166 GO:0004812 GO:0004474 GO:0016627 GO:0005524
Cluster-22178.70097 GO:0007154 GO:0016020
Cluster-22178.70100 GO:0007275 GO:0007165 GO:0008152 GO:0016624 GO:0005179 GO:0047355 GO:0008270 GO:0016020 GO:0005576
Cluster-22178.70102 GO:0015986 GO:0006119 GO:0055114 GO:0015992 GO:0046961 GO:0016491 GO:0046933 GO:0000275 GO:0045259
Cluster-22178.70103 GO:0005515
Cluster-22178.70106 GO:0006468 GO:0005524 GO:0004672 GO:0016021
Cluster-22178.70107 GO:0006281 GO:0043087 GO:0035556 GO:0032012 GO:0005086 GO:0046872 GO:0008270 GO:0005201 GO:0005578
Cluster-22178.70108 GO:0006144 GO:0007156 GO:0009113 GO:0009058 GO:0005524 GO:0046872 GO:0016742 GO:0005509 GO:0051539 GO:0004637 GO:0016020
Cluster-22178.70109 GO:0006470 GO:0006570 GO:0006412 GO:0042254 GO:0009405 GO:0008270 GO:0004725 GO:0003735 GO:0008138 GO:0005622 GO:0005840 GO:0005576
Cluster-22178.70110 GO:0042254 GO:0006412 GO:0008033 GO:0055114 GO:0000105 GO:0003735 GO:0017150 GO:0050660 GO:0005622 GO:0005840
Cluster-22178.70111 GO:0006355 GO:0007264 GO:0051289 GO:0043565 GO:0005096 GO:0008140 GO:0003700 GO:0005667
Cluster-22178.70115 GO:0016310 GO:0009405 GO:0006886 GO:0009069

GO:0004674 GO:0008536 GO:0005515 GO:0005576
Cluster-22178.70116 GO:0009405 GO:0051252 GO:0006402 GO:0003723
GO:0046872 GO:0004535 GO:0005634 GO:0005737
Cluster-22178.70118 GO:0006355 GO:0030833 GO:0034314 GO:0005198
GO:0043565 GO:0003700 GO:0005667 GO:0005885 GO:0005882
GO:0005856 GO:0019867
Cluster-22178.70120 GO:0005515
Cluster-22178.70121 GO:0005515
Cluster-22178.70126 GO:0055114 GO:0016114 GO:0009405 GO:0019835
GO:0016567 GO:0008270 GO:0046872 GO:0008685 GO:0016491
GO:0004842 GO:0019867
Cluster-22178.70129 GO:0006457 GO:0007128 GO:0055085 GO:0000921
GO:0007130 GO:0006886 GO:0070193 GO:0030001 GO:0048870
GO:0006308 GO:0051087 GO:0046873 GO:0005524 GO:0008855
GO:0000774 GO:0035299 GO:0042803 GO:0016020 GO:0005940
GO:0009318 GO:0031514 GO:0016021 GO:0005801 GO:0000795
Cluster-22178.70131 GO:0051082 GO:0005639
Cluster-22178.70132 GO:0035556 GO:0046872
Cluster-22178.70133 GO:0016973 GO:0071973 GO:0016192 GO:0006886
GO:0006935 GO:0005515 GO:0005198 GO:0003774 GO:0030117
GO:0005882 GO:0016020 GO:0009288 GO:0005643 GO:0016021
Cluster-22178.70134 GO:0006090 GO:0006633 GO:0051607 GO:0016973
GO:0003989 GO:0016021 GO:0009317 GO:0005643 GO:0019028
Cluster-22178.70136 GO:0006468 GO:0016773 GO:0005524 GO:0004672
GO:0016020
Cluster-22178.70138 GO:0046983
Cluster-22178.70139 GO:0006886 GO:0051258 GO:0045995 GO:0007155
GO:0030155 GO:0007165 GO:0006914 GO:0030168 GO:0000921
GO:0030334 GO:0051607 GO:0006606 GO:0045502 GO:0005515
GO:0042803 GO:0008134 GO:0008092 GO:0003824 GO:0004871
GO:0030674 GO:0005102 GO:0048786 GO:0005737 GO:0016021
GO:0005801 GO:0005667 GO:0016020 GO:0005940 GO:0030286
GO:0019898 GO:0005577

Cluster-22178.70140 GO:0005515 GO:0035091
Cluster-22178.70143 GO:0007186 GO:0015684 GO:0007165 GO:0007264
GO:0006888 GO:0004871 GO:0005525 GO:0003924 GO:0031683
GO:0015093 GO:0019001 GO:0005801 GO:0016021 GO:0005622
Cluster-22178.70145 GO:0003950
Cluster-22178.70146 GO:0006412 GO:0042254 GO:0003735 GO:0005622
GO:0005840
Cluster-22178.70147 GO:0006468 GO:0004672 GO:0005524
Cluster-22178.70151 GO:0045116 GO:0016881 GO:0005524 GO:0008641

Cluster-22178.70152 GO:0008152 GO:0003824 GO:0051536 GO:0010181

Cluster-22178.70153 GO:0008152 GO:0051536 GO:0010181 GO:0003824

Cluster-22178.70154 GO:0006914 GO:0019905
Cluster-22178.70156 GO:0042981
Cluster-22178.70157 GO:0006811 GO:0045859 GO:0007268 GO:0007165
GO:0006397 GO:0004970 GO:0004860 GO:0005515 GO:0005576
GO:0005634 GO:0016020
Cluster-22178.70162 GO:0042254 GO:0001522
Cluster-22178.70163 GO:0005515 GO:0042470 GO:0016020
Cluster-22178.70164 GO:0006570 GO:0055114 GO:0006470 GO:0050661
GO:0008138 GO:0005515 GO:0004499 GO:0050660 GO:0004725
Cluster-22178.70165 GO:0009405 GO:0005515 GO:0005576
Cluster-22178.70166 GO:0006357 GO:0003723 GO:0001104 GO:0005515
GO:0016592
Cluster-22178.70167 GO:0016567 GO:0004842
Cluster-22178.70168 GO:0008152 GO:0016491
Cluster-22178.70171 GO:0006811 GO:0055085 GO:0005216 GO:0005515
GO:0012511 GO:0016021 GO:0016020
Cluster-22178.70174 GO:0007165 GO:0055114 GO:0008152 GO:0005515
GO:0005509 GO:0016706 GO:0016758 GO:0005578
Cluster-22178.70176 GO:0006464 GO:0055114 GO:0006779 GO:0019354

GO:0008270 GO:0046872 GO:0016491 GO:0016151 GO:0051287
GO:0016616 GO:0003677 GO:0043115 GO:0005515
Cluster-22178.70177 GO:0000917 GO:0007049 GO:0006355 GO:0043093
GO:0003700 GO:0005667 GO:0005737
Cluster-22178.70179 GO:0015992 GO:0006412 GO:0042254 GO:0006118
GO:0006119 GO:0009055 GO:0030246 GO:0003735 GO:0008121
GO:0005622 GO:0005840
Cluster-22178.70180 GO:0048478 GO:0006974 GO:0007049 GO:0005634
Cluster-22178.70183 GO:0008152 GO:0003677 GO:0003824 GO:0008134
GO:0005667 GO:0005634
Cluster-22178.70184 GO:0006355 GO:0006122 GO:0006508 GO:0005515
GO:0008270 GO:0043565 GO:0004222 GO:0003700 GO:0005634
GO:0005667 GO:0031012 GO:0005743 GO:0005750
Cluster-22178.70186 GO:0006888 GO:0006886 GO:0007165 GO:0007606
GO:0008270 GO:0005515 GO:0003723 GO:0004888 GO:0016020
GO:0030127
Cluster-22178.70188 GO:0006886 GO:0042720 GO:0005737
Cluster-22178.70190 GO:0006814 GO:0005248 GO:0005634 GO:0001518
Cluster-22178.70191 GO:0015684 GO:0006270 GO:0006810 GO:0019082
GO:0015721 GO:0070140 GO:0032183 GO:0046982 GO:0015093
GO:0004252 GO:0003924 GO:0005525 GO:0005215 GO:0005783
GO:0016020 GO:0005886 GO:0005634 GO:0016021
Cluster-22178.70193 GO:0016787
Cluster-22178.70194 GO:0042967 GO:0003824 GO:0008080 GO:0051536

Cluster-22178.70197 GO:0007016 GO:0003779 GO:0005200 GO:0005925
GO:0005856 GO:0001726
Cluster-22178.70198 GO:0016787 GO:0046872
Cluster-22178.70199 GO:0046486 GO:0007205 GO:0035556 GO:0006260
GO:0009395 GO:0005515 GO:0016301 GO:0004143 GO:0005634
Cluster-22178.70200 GO:0005524 GO:0016887
Cluster-22178.70201 GO:0003676
Cluster-22178.70202 GO:0009396 GO:0055114 GO:0046487 GO:0009755

GO:0003824 GO:0016491 GO:0004488 GO:0005576
Cluster-22178.70203 GO:0007049
Cluster-22178.70204 GO:0019076 GO:0046718 GO:0006885 GO:0006812
GO:0055085 GO:0075521 GO:0015299 GO:0016021 GO:0019028
GO:0044163 GO:0030430 GO:0042025
Cluster-22178.70205 GO:0046488 GO:0016307 GO:0019867
Cluster-22178.70208 GO:0006820 GO:0022611 GO:0006333 GO:0006468
GO:0009432 GO:0005524 GO:0004672 GO:0005253 GO:0005887
GO:0005634
Cluster-22178.70209 GO:0016021
Cluster-22178.70210 GO:0006446 GO:0006413 GO:0005515 GO:0003743
GO:0031369 GO:0005852 GO:0005840
Cluster-22178.70212 GO:0051726 GO:0006974
Cluster-22178.70213 GO:0046740 GO:0006810 GO:0042254 GO:0006412
GO:0006260 GO:0051082 GO:0003735 GO:0016798 GO:0016020
GO:0005886 GO:0005622 GO:0005840 GO:0016021
Cluster-22178.70214 GO:0006260 GO:0042254 GO:0006412 GO:0006810
GO:0046740 GO:0016798 GO:0005515 GO:0003735 GO:0051082
GO:0016021 GO:0005840 GO:0005886 GO:0005622 GO:0016020
Cluster-22178.70215 GO:0006810 GO:0042254 GO:0006412 GO:0046740
GO:0006260 GO:0005515 GO:0003735 GO:0051082 GO:0005840
GO:0016021 GO:0005622 GO:0005886
Cluster-22178.70216 GO:0004222
Cluster-22178.70217 GO:0019211 GO:0005515 GO:0016746
Cluster-22178.70218 GO:0006811 GO:0006206 GO:0006144 GO:0006351
GO:0006816 GO:0006874 GO:0003676 GO:0005219 GO:0008270
GO:0003899 GO:0016021 GO:0005622 GO:0016020 GO:0005730
GO:0005634
Cluster-22178.70219 GO:0006355 GO:0008270 GO:0043565 GO:0003700
GO:0005667 GO:0005634
Cluster-22178.70220 GO:0007049 GO:0006367 GO:0007067 GO:0000775
GO:0005672 GO:0005634
Cluster-22178.70222 GO:0042254 GO:0006412 GO:0003735 GO:0005840

GO:0005622
Cluster-22178.70223 GO:0042967 GO:0006886 GO:0006810 GO:0006355
GO:0008080 GO:0042626 GO:0005524 GO:0005515 GO:0003677
GO:0016021 GO:0005783
Cluster-22178.70224 GO:0006810 GO:0006886 GO:0006355 GO:0042967
GO:0003677 GO:0005515 GO:0042626 GO:0008080 GO:0005524
GO:0005783 GO:0016021
Cluster-22178.70227 GO:0006508 GO:0051260 GO:0008234
Cluster-22178.70228 GO:0005515
Cluster-22178.70229 GO:0043044 GO:0003676 GO:0046872 GO:0016787
GO:0016586
Cluster-22178.70231 GO:0018342 GO:0030001 GO:0055085 GO:0046873
GO:0008318 GO:0016020
Cluster-22178.70234 GO:0006412 GO:0042254 GO:0046872 GO:0008270
GO:0003735 GO:0005515 GO:0061630 GO:0005840
Cluster-22178.70236 GO:0051252 GO:0006810 GO:0007264 GO:0006281
GO:0006396 GO:0000723 GO:0003677 GO:0003723 GO:0016787
GO:0003678 GO:0005525 GO:0004525 GO:0005524 GO:0005657
Cluster-22178.70238 GO:0009636 GO:0015675 GO:0035444 GO:0006955
GO:0031342 GO:0046872 GO:0015099 GO:0015643 GO:0016021
GO:0019814
Cluster-22178.70240 GO:0006810 GO:0006418 GO:0000166 GO:0005524
GO:0004812 GO:0005886
Cluster-22178.70241 GO:0006468 GO:0005515 GO:0005524 GO:0004672
GO:0016020
Cluster-22178.70242 GO:0006355 GO:0008270 GO:0005096 GO:0005544
GO:0005515 GO:0005509 GO:0008023
Cluster-22178.70245 GO:0051603 GO:0006888 GO:0004298 GO:0005801
GO:0005839 GO:0005622
Cluster-22178.70246 GO:0007154 GO:0016020
Cluster-22178.70247 GO:0006888 GO:0006260 GO:0051603 GO:0003887
GO:0003677 GO:0004298 GO:0005839 GO:0042575 GO:0005622
GO:0005801

Cluster-22178.70249 GO:0016787
Cluster-22178.70253 GO:0006351 GO:0006260 GO:0003677 GO:0003887
GO:0042575
Cluster-22178.70254 GO:0043085 GO:0016485 GO:0016021
Cluster-22178.70255 GO:0006811 GO:0043085 GO:0006914 GO:0009252
GO:0016485 GO:0006810 GO:0008092 GO:0016755 GO:0019898
GO:0016021 GO:0005737 GO:0016020
Cluster-22178.70256 GO:0043085 GO:0016485 GO:0016021
Cluster-22178.70259 GO:0016192 GO:0016021
Cluster-22178.70262 GO:0006520 GO:0019752 GO:0016829 GO:0030170
GO:0016831
Cluster-22178.70264 GO:0030435 GO:0046872 GO:0031160
Cluster-22178.70266 GO:0006144 GO:0005515 GO:0003968 GO:0031379
Cluster-22178.70267 GO:0019079 GO:0006355 GO:0007264 GO:0035023
GO:0043087 GO:0003677 GO:0008026 GO:0005085 GO:0005089
GO:0005524
Cluster-22178.70269 GO:0006511
Cluster-22178.70270 GO:0010951 GO:0007283 GO:0004866 GO:0003677
GO:0000786 GO:0005634
Cluster-22178.70271 GO:0005515
Cluster-22178.70272 GO:0006355 GO:0005634
Cluster-22178.70274 GO:0007186 GO:0007165 GO:0004871 GO:0005834
Cluster-22178.70277 GO:0016192 GO:0006886 GO:0051252 GO:0015991
GO:0009069 GO:0016310 GO:0043248 GO:0005198 GO:0005515
GO:0016820 GO:0004521 GO:0004674 GO:0045095 GO:0030117
GO:0000221
Cluster-22178.70280 GO:0042254 GO:0009058 GO:0006412 GO:0003735
GO:0030170 GO:0015934 GO:0005840
Cluster-22178.70282 GO:0017004 GO:0016192 GO:0006810 GO:0007165
GO:0006357 GO:0015886 GO:0007010 GO:0005975 GO:0016853
GO:0043395 GO:0004872 GO:0005515 GO:0005198 GO:0001104
GO:0003779 GO:0005215 GO:0016020 GO:0016592 GO:0019028
GO:0005578 GO:0016021 GO:0019031

Cluster-22178.70283 GO:0003824 GO:0005515
Cluster-22178.70284 GO:0006355 GO:0046034 GO:0006869 GO:0042157
GO:0006464 GO:0006144 GO:0051082 GO:0008289 GO:0016151
GO:0003700 GO:0046872 GO:0043565 GO:0004017 GO:0008270
GO:0005576 GO:0005667 GO:0005815
Cluster-22178.70286 GO:0006468 GO:0005524 GO:0004672
Cluster-22178.70287 GO:0006355 GO:0006479 GO:0006351 GO:0009451
GO:0031167 GO:0008033 GO:0008152 GO:0006400 GO:0046500
GO:0006367 GO:0006554 GO:0006464 GO:0006260 GO:0018024
GO:0003887 GO:0004719 GO:0008168 GO:0008176 GO:0003682
GO:0003677 GO:0008270 GO:0008171 GO:0005672 GO:0005634
GO:0042575 GO:0000775 GO:0032040 GO:0000785
Cluster-22178.70289 GO:0030001 GO:0042254 GO:0055085 GO:0046873
GO:0005730 GO:0016020
Cluster-22178.70291 GO:0005515
Cluster-22178.70295 GO:0006367 GO:0006084 GO:0010506 GO:0006464
GO:0006914 GO:0006886 GO:0010508 GO:0006355 GO:0030001
GO:0007049 GO:0055114 GO:0008113 GO:0003824 GO:0046872
GO:0005783 GO:0005669 GO:0016021 GO:0005615
Cluster-22178.70296 GO:0015992 GO:0006123 GO:0004129 GO:0045277
Cluster-22178.70298 GO:0019058 GO:0046872 GO:0005887
Cluster-22178.70303 GO:0006397 GO:0046660 GO:0005634
Cluster-22178.70304 GO:0006323 GO:0043087 GO:0006397 GO:0046872
GO:0005089 GO:0003676 GO:0005737
Cluster-22178.70310 GO:0003723 GO:0000178
Cluster-22178.70311 GO:0008152
Cluster-22178.70312 GO:0008152
Cluster-22178.70313 GO:0000226 GO:0030168 GO:0051258 GO:0007165
GO:0090063 GO:0005102 GO:0030674 GO:0000922 GO:0005577
GO:0005815
Cluster-22178.70314 GO:0005515
Cluster-22178.70316 GO:0019955 GO:0016020
Cluster-22178.70317 GO:0006886 GO:0016032 GO:0000160 GO:0016310

GO:0006888 GO:0003677 GO:0000155 GO:0046983 GO:0008270
GO:0030127 GO:0019028 GO:0016021 GO:0009365
Cluster-22178.70320 GO:0005515 GO:0016740
Cluster-22178.70321 GO:0017025
Cluster-22178.70322 GO:0006355 GO:0006281 GO:0003677 GO:0046872
GO:0008270
Cluster-22178.70326 GO:0007165 GO:0042254 GO:0006357 GO:0007169
GO:0051056 GO:0006355 GO:0006468 GO:0006412 GO:0004714
GO:0008201 GO:0003735 GO:0005524 GO:0005096 GO:0004872
GO:0042393 GO:0001104 GO:0046872 GO:0016592 GO:0005622
GO:0005887 GO:0005840
Cluster-22178.70327 GO:0005524 GO:0005515 GO:0016301 GO:0008289

Cluster-22178.70330 GO:0006357 GO:0051056 GO:0007169 GO:0042254
GO:0007165 GO:0006468 GO:0006355 GO:0006412 GO:0005096
GO:0005524 GO:0004872 GO:0003735 GO:0008201 GO:0004714
GO:0046872 GO:0001104 GO:0042393 GO:0005622 GO:0005887
GO:0016592 GO:0005840
Cluster-22178.70331 GO:0006468 GO:0005524 GO:0004672
Cluster-22178.70336 GO:0006144 GO:0006820 GO:0006835 GO:0006812
GO:0007155 GO:0003877 GO:0005094 GO:0017153 GO:0005737
GO:0016020
Cluster-22178.70338 GO:0007155 GO:0006812 GO:0006835 GO:0006820
GO:0006144 GO:0017153 GO:0005094 GO:0003877 GO:0016020
GO:0005737
Cluster-22178.70342 GO:0042254 GO:0006412 GO:0003735 GO:0005622
GO:0005840
Cluster-22178.70343 GO:0031110 GO:0006308 GO:0006448 GO:0006414
GO:0008855 GO:0003746 GO:0009318 GO:0005840
Cluster-22178.70348 GO:0045333 GO:0008643 GO:0004659 GO:0008982
GO:0016021 GO:0009357 GO:0005634 GO:0005739
Cluster-22178.70349 GO:0006915 GO:0006118 GO:0009055 GO:0020037
GO:0005622 GO:0019028

Cluster-22178.70352 GO:0007010 GO:0055085 GO:0046872 GO:0003779
GO:0016021
Cluster-22178.70353 GO:0016255 GO:0042765
Cluster-22178.70354 GO:0015986 GO:0006118 GO:0006364 GO:0015992
GO:0051536 GO:0009055 GO:0015078 GO:0000276 GO:0016021
GO:0032040
Cluster-22178.70355 GO:0016567 GO:0006914 GO:0006308 GO:0035091
GO:0005515 GO:0008855 GO:0008270 GO:0046872 GO:0004842
GO:0009318 GO:0005622 GO:0005680
Cluster-22178.70356 GO:0006355
Cluster-22178.70357 GO:0055114 GO:0016491
Cluster-22178.70358 GO:0006850 GO:0005743
Cluster-22178.70359 GO:0016310 GO:0009069 GO:0004674 GO:0005515

Cluster-22178.70360 GO:0015031 GO:0008152 GO:0042254 GO:0006412
GO:0016747 GO:0008565 GO:0016746 GO:0005509 GO:0003735
GO:0005622 GO:0005840 GO:0016021
Cluster-22178.70361 GO:0007165 GO:0005179 GO:0005576
Cluster-22178.70368 GO:0005515
Cluster-22178.70369 GO:0016579 GO:0036459
Cluster-22178.70370 GO:0016579 GO:0036459
Cluster-22178.70376 GO:0045892 GO:0005515 GO:0005634
Cluster-22178.70377 GO:0045892 GO:0005515 GO:0005634
Cluster-22178.70378 GO:0045892 GO:0005515 GO:0005634
Cluster-22178.70379 GO:0003676 GO:0003677 GO:0016787 GO:0005524

Cluster-22178.70380 GO:0003677 GO:0003676 GO:0005524 GO:0016787

Cluster-22178.70381 GO:0005615
Cluster-22178.70383 GO:0003676 GO:0003677 GO:0016787 GO:0005524

Cluster-22178.70385 GO:0007165 GO:0006898 GO:0005044 GO:0016020
Cluster-22178.70386 GO:0000226 GO:0046872 GO:0000166 GO:0016020

GO:0015630
Cluster-22178.70387 GO:0000226 GO:0046872 GO:0000166 GO:0015630
GO:0016020
Cluster-22178.70389 GO:0009405 GO:0006351 GO:0006807 GO:0006206
GO:0015031 GO:0006144 GO:0009252 GO:0003677 GO:0003899
GO:0004356 GO:0005730
Cluster-22178.70391 GO:0006351 GO:0006144 GO:0006206 GO:0006825
GO:0003899 GO:0016531 GO:0003677 GO:0005507 GO:0005634
GO:0005730 GO:0005758
Cluster-22178.70392 GO:0006810 GO:0006811 GO:0055085 GO:0005216
GO:0005215 GO:0022857 GO:0016020 GO:0016021
Cluster-22178.70393 GO:0006357 GO:0001104 GO:0005515 GO:0016592
Cluster-22178.70398 GO:0015986 GO:0001522 GO:0009451 GO:0015992
GO:0000045 GO:0003723 GO:0005515 GO:0015078 GO:0009982
GO:0045263 GO:0005737
Cluster-22178.70399 GO:0007186 GO:0043395 GO:0005515 GO:0004930
GO:0005578 GO:0016021 GO:0016020
Cluster-22178.70400 GO:0000045 GO:0009451 GO:0001522 GO:0003723
GO:0005515 GO:0009982 GO:0005737
Cluster-22178.70401 GO:0006144 GO:0055114 GO:0006520 GO:0006522
GO:0009152 GO:0006188 GO:0006531 GO:0009058 GO:0010277
GO:0016829 GO:0004018 GO:0030170
Cluster-22178.70408 GO:0016020
Cluster-22178.70409 GO:0003924
Cluster-22178.70411 GO:0009069 GO:0006468 GO:0016310 GO:0004672
GO:0005524 GO:0004674
Cluster-22178.70412 GO:0006355 GO:0009405 GO:0006397 GO:0006308
GO:0006302 GO:0006357 GO:0043087 GO:0006606 GO:0005515
GO:0001104 GO:0003700 GO:0008855 GO:0005634 GO:0009318
GO:0005667 GO:0016020 GO:0016592
Cluster-22178.70413 GO:0006355 GO:0005634
Cluster-22178.70414 GO:0006468 GO:0005524 GO:0004672 GO:0005515

Cluster-22178.70415 GO:0015979 GO:0005515 GO:0009523 GO:0009539
GO:0016020
Cluster-22178.70416 GO:0006355 GO:0043565 GO:0003700 GO:0005667
GO:0005634
Cluster-22178.70417 GO:0007156 GO:0051252 GO:0006396 GO:0003723
GO:0004525 GO:0005509 GO:0016020
Cluster-22178.70419 GO:0006355 GO:0046983
Cluster-22178.70424 GO:0007165 GO:0008283 GO:0040007 GO:0008083

Cluster-22178.70425 GO:0008270
Cluster-22178.70426 GO:0007165 GO:0008283 GO:0040007 GO:0008083

Cluster-22178.70427 GO:0006511 GO:0031625 GO:0005515
Cluster-22178.70433 GO:0006351 GO:0006144 GO:0006206 GO:0003899
GO:0003677 GO:0005730
Cluster-22178.70435 GO:0003676 GO:0005921
Cluster-22178.70436 GO:0003676
Cluster-22178.70438 GO:0006468 GO:0004672 GO:0005524
Cluster-22178.70439 GO:0042157 GO:0007186 GO:0006457 GO:0006869
GO:0034080 GO:0006355 GO:0051301 GO:0007059 GO:0003677
GO:0005524 GO:0004930 GO:0051082 GO:0035299 GO:0003700
GO:0003676 GO:0008289 GO:0005667 GO:0016272 GO:0000444
GO:0005576 GO:0016021 GO:0000775
Cluster-22178.70440 GO:0006886 GO:0016192 GO:0005515 GO:0005198
GO:0030117 GO:0030126
Cluster-22178.70444 GO:0009190 GO:0006171 GO:0035556 GO:0006144
GO:0005515 GO:0004016 GO:0016849 GO:0051537 GO:0005886
Cluster-22178.70449 GO:0009058 GO:0030170
Cluster-22178.70452 GO:0046872 GO:0005515
Cluster-22178.70457 GO:0043039 GO:0016876 GO:0005524 GO:0008270

Cluster-22178.70458 GO:0043039 GO:0016876 GO:0005524 GO:0008270

Cluster-22178.70459 GO:0005515 GO:0005887
Cluster-22178.70461 GO:0022900 GO:0005739 GO:0005747
Cluster-22178.70462 GO:0006355 GO:0003700 GO:0003677 GO:0005667
Cluster-22178.70463 GO:0051028 GO:0019049 GO:0048309 GO:0005515
GO:0005096
Cluster-22178.70464 GO:0006281 GO:0006310 GO:0005524 GO:0016887
GO:0003678 GO:0009378 GO:0005657 GO:0016021 GO:0009379
Cluster-22178.70468 GO:0019911
Cluster-22178.70471 GO:0000746 GO:0003676 GO:0003677
Cluster-22178.70472 GO:0000042 GO:0006281 GO:0046034 GO:0006144
GO:0004017 GO:0005515 GO:0003677 GO:0008270 GO:0046872
Cluster-22178.70474 GO:0051653 GO:0030036 GO:0006928 GO:0008356
GO:0051321 GO:0033206 GO:0016482 GO:0007163 GO:0003779
GO:0005524 GO:0030478 GO:0005885 GO:0042995
Cluster-22178.70477 GO:0035556 GO:0008270 GO:0005506 GO:0005515
GO:0003677
Cluster-22178.70481 GO:0055085 GO:0006367 GO:0008063 GO:0030001
GO:0046872 GO:0046873 GO:0016020 GO:0005672
Cluster-22178.70485 GO:0007018 GO:0007017 GO:0008017 GO:0003777
GO:0005524 GO:0045298 GO:0005874 GO:0030430
Cluster-22178.70486 GO:0090200 GO:0043065 GO:0097193 GO:0009405
GO:0005515 GO:0005576 GO:0005739
Cluster-22178.70488 GO:0003774 GO:0005524 GO:0005506 GO:0005515
GO:0016459
Cluster-22178.70492 GO:0007155 GO:0005515 GO:0016021
Cluster-22178.70493 GO:0046034 GO:0006144 GO:0006888 GO:0006464
GO:0006886 GO:0006355 GO:0016151 GO:0046872 GO:0008270
GO:0004017 GO:0030127 GO:0005634
Cluster-22178.70494 GO:0006979 GO:0006804 GO:0015947 GO:0055114
GO:0006568 GO:0020037 GO:0004096
Cluster-22178.70495 GO:0006468 GO:0006355 GO:0030255 GO:0035556
GO:0004672 GO:0005524 GO:0016773 GO:0005634 GO:0005668
GO:0016020

Cluster-22178.70496 GO:0007264 GO:0015684 GO:0006626 GO:0000287 GO:0016829 GO:0015093 GO:0003924 GO:0005525 GO:0030976 GO:0005741 GO:0016021
Cluster-22178.70497 GO:0015684 GO:0007264 GO:0006626 GO:0000287 GO:0005525 GO:0030976 GO:0016829 GO:0015093 GO:0003924 GO:0005741 GO:0016021
Cluster-22178.70498 GO:0009058 GO:0030170
Cluster-22178.70501 GO:0006955 GO:0040007 GO:0042981 GO:0007165 GO:0006869 GO:0007264 GO:0008283 GO:0048193 GO:0043087 GO:0005515 GO:0005319 GO:0008083 GO:0005085 GO:0005134 GO:0005576 GO:0005893 GO:0016020 GO:0005887
Cluster-22178.70503 GO:0042981 GO:0040007 GO:0006955 GO:0043087 GO:0048193 GO:0008283 GO:0007264 GO:0006869 GO:0007165 GO:0005319 GO:0008083 GO:0005515 GO:0005134 GO:0005085 GO:0005893 GO:0005576 GO:0005887 GO:0016020
Cluster-22178.70505 GO:0042981 GO:0040007 GO:0006955 GO:0043087 GO:0048193 GO:0008283 GO:0007264 GO:0006869 GO:0007165 GO:0005319 GO:0008083 GO:0005515 GO:0005134 GO:0005085 GO:0005893 GO:0005576 GO:0005887 GO:0016020
Cluster-22178.70507 GO:0007131 GO:0005515 GO:0003677 GO:0004867

Cluster-22178.70508 GO:0000226 GO:0016114 GO:0006694 GO:0008152 GO:0016624 GO:0008661 GO:0003824 GO:0030976 GO:0015630
Cluster-22178.70510 GO:0055114 GO:0006950 GO:0008270 GO:0016638 GO:0003697
Cluster-22178.70512 GO:0006355 GO:0003700 GO:0005667
Cluster-22178.70514 GO:0007186 GO:0007165 GO:0015986 GO:0055085 GO:0015992 GO:0004871 GO:0015078 GO:0003924 GO:0031683 GO:0019001 GO:0000276
Cluster-22178.70516 GO:0030001 GO:0055085 GO:0046873 GO:0016021 GO:0016020
Cluster-22178.70518 GO:0003676 GO:0031224
Cluster-22178.70520 GO:0006144 GO:0055114 GO:0009308 GO:0046034

GO:0046872 GO:0016638 GO:0004017 GO:0003677 GO:0042597
Cluster-22178.70521 GO:0046034 GO:0055114 GO:0006144 GO:0009308
GO:0006351 GO:0003676 GO:0046872 GO:0008270 GO:0004017
GO:0003677 GO:0016638 GO:0042597
Cluster-22178.70522 GO:0006281 GO:0008270 GO:0046872
Cluster-22178.70523 GO:0016021
Cluster-22178.70527 GO:0006281 GO:0006308 GO:0008852 GO:0003677
GO:0005515
Cluster-22178.70530 GO:0051302 GO:0006448 GO:0006414 GO:0006355
GO:0003746 GO:0003677 GO:0005840 GO:0005853
Cluster-22178.70533 GO:0016773
Cluster-22178.70534 GO:0006260 GO:0006508 GO:0016032 GO:0000398
GO:0004197 GO:0000808 GO:0005634
Cluster-22178.70535 GO:0000398 GO:0016032 GO:0006508 GO:0006260
GO:0004197 GO:0005634 GO:0000808
Cluster-22178.70538 GO:0016310 GO:0006096 GO:0046856 GO:0015976
GO:0006094 GO:0034597 GO:0004618
Cluster-22178.70539 GO:0006260 GO:0006351 GO:0003677 GO:0003887
GO:0042575
Cluster-22178.70540 GO:0006810 GO:0006950 GO:0016021 GO:0005921
Cluster-22178.70541 GO:0006885 GO:0006812 GO:0055085 GO:0015299
GO:0016021
Cluster-22178.70543 GO:0005515 GO:0042302 GO:0005549
Cluster-22178.70545 GO:0043401 GO:0006355 GO:0003700 GO:0008270
GO:0043565 GO:0016021 GO:0005634 GO:0005667
Cluster-22178.70546 GO:0006633 GO:0031412 GO:0019482 GO:0050080
GO:0031411
Cluster-22178.70549 GO:0006355 GO:0006281 GO:0000184 GO:0055114
GO:0003677 GO:0046872 GO:0005524 GO:0008270 GO:0047134
GO:0016972 GO:0004386 GO:0005737
Cluster-22178.70551 GO:0003723 GO:0000178
Cluster-22178.70552 GO:0051603 GO:0006511 GO:0004175 GO:0004298
GO:0005839 GO:0019773

Cluster-22178.70553 GO:0006568 GO:0006552 GO:0006631 GO:0018874
GO:0006550 GO:0055114 GO:0006554 GO:0006574 GO:0015074
GO:0008152 GO:0006633 GO:0003857 GO:0003677 GO:0016491
Cluster-22178.70554 GO:0005975 GO:0004553 GO:0042597
Cluster-22178.70555 GO:0005975 GO:0004553 GO:0042597
Cluster-22178.70556 GO:0006355 GO:0006352 GO:0003677 GO:0005515
GO:0016987 GO:0003700 GO:0008270 GO:0005667
Cluster-22178.70557 GO:0006525 GO:0006560 GO:0055114 GO:0018401
GO:0004656 GO:0016491 GO:0016702 GO:0005783
Cluster-22178.70561 GO:0006570 GO:0006298 GO:0009405 GO:0006355
GO:0006470 GO:0005515 GO:0004725 GO:0005524 GO:0005634
Cluster-22178.70563 GO:0006468 GO:0006810 GO:0015696 GO:0016787
GO:0008519 GO:0005524 GO:0004672 GO:0016020 GO:0031224
GO:0016021 GO:0005794
Cluster-22178.70566 GO:0006355 GO:0008270 GO:0003677
Cluster-22178.70567 GO:0015986 GO:0045766 GO:0043123 GO:0016567
GO:0015992 GO:0043066 GO:0003723 GO:0043130 GO:0004842
GO:0015078 GO:0000276
Cluster-22178.70570 GO:0006357 GO:0001104 GO:0016592 GO:0016020
Cluster-22178.70572 GO:0042254 GO:0001522
Cluster-22178.70573 GO:0006260 GO:0006744 GO:0006914 GO:0007186
GO:0043093 GO:0007049 GO:0006351 GO:0000917 GO:0006355
GO:0004930 GO:0003677 GO:0003700 GO:0008813 GO:0043565
GO:0003887 GO:0005815 GO:0005667 GO:0016021 GO:0005737
GO:0042575
Cluster-22178.70575 GO:0042254 GO:0006412 GO:0003735 GO:0015934
GO:0005840
Cluster-22178.70576 GO:0006810 GO:0055085 GO:0015979 GO:0005215
GO:0009579 GO:0016020 GO:0009522 GO:0016021
Cluster-22178.70581 GO:0019530 GO:0055114 GO:0046439 GO:0006534
GO:0017172 GO:0005506
Cluster-22178.70584 GO:0006825 GO:0016531 GO:0005507 GO:0005758
Cluster-22178.70585 GO:0007165 GO:0009069 GO:0006468 GO:0008152

GO:0016310 GO:0015016 GO:0005515 GO:0016787 GO:0016746
GO:0004674 GO:0005096
Cluster-22178.70586 GO:0007067 GO:0051382 GO:0003677
Cluster-22178.70587 GO:0005515
Cluster-22178.70590 GO:0000746 GO:0009512
Cluster-22178.70591 GO:0000746
Cluster-22178.70592 GO:0005515
Cluster-22178.70593 GO:0046487 GO:0055114 GO:0009396 GO:0003824
GO:0004488 GO:0004672
Cluster-22178.70594 GO:0005198 GO:0016021 GO:0005882
Cluster-22178.70596 GO:0005515
Cluster-22178.70597 GO:0006468 GO:0006355 GO:0004672 GO:0003700
GO:0005634 GO:0005667
Cluster-22178.70598 GO:0006355 GO:0042393
Cluster-22178.70599 GO:0015675 GO:0035444 GO:0046872 GO:0015099
GO:0016021
Cluster-22178.70601 GO:0042254 GO:0006412 GO:0003735 GO:0005840
Cluster-22178.70602 GO:0045893 GO:0042025
Cluster-22178.70603 GO:0035556 GO:0008053 GO:0003924 GO:0005741
GO:0016021
Cluster-22178.70604 GO:0016021
Cluster-22178.70607 GO:0042254 GO:0006412 GO:0006814 GO:0042773
GO:0006744 GO:0006120 GO:0015992 GO:0003735 GO:0008137
GO:0005743 GO:0005840 GO:0005622
Cluster-22178.70610 GO:0006520 GO:0055114 GO:0009058 GO:0016829
GO:0030170 GO:0016491
Cluster-22178.70613 GO:0055085 GO:0006888 GO:0006810 GO:0022857
GO:0005215 GO:0005515 GO:0016021 GO:0019867 GO:0005801
GO:0005622 GO:0016020
Cluster-22178.70617 GO:0006270 GO:0019028
Cluster-22178.70619 GO:0055114 GO:0006801 GO:0046872 GO:0004784

Cluster-22178.70620 GO:0006836 GO:0006457 GO:0006812 GO:0005515

GO:0005328 GO:0016021
Cluster-22178.70623 GO:0005515
Cluster-22178.70624 GO:0016012 GO:0016021
Cluster-22178.70626 GO:0005515
Cluster-22178.70628 GO:0055114 GO:0008152 GO:0016491
Cluster-22178.70629 GO:0019752 GO:0006520 GO:0006729 GO:0009058
GO:0016829 GO:0016831 GO:0008124 GO:0016740 GO:0030170
Cluster-22178.70634 GO:0006418 GO:0043039 GO:0016876 GO:0004812
GO:0005524 GO:0000166
Cluster-22178.70635 GO:0003676
Cluster-22178.70637 GO:0005515
Cluster-22178.70638 GO:0015992 GO:0006777 GO:0006123 GO:0006810
GO:0005524 GO:0005525 GO:0004129 GO:0045277 GO:0005743
Cluster-22178.70640 GO:0016021
Cluster-22178.70641 GO:0006506 GO:0009058 GO:0019752 GO:0006520
GO:0006729 GO:0008124 GO:0016746 GO:0016740 GO:0030170
GO:0016829 GO:0016831 GO:0005789 GO:0016021
Cluster-22178.70645 GO:0008152 GO:0008168
Cluster-22178.70647 GO:0005509
Cluster-22178.70650 GO:0005515
Cluster-22178.70658 GO:0019354 GO:0006464 GO:0055114 GO:0006779
GO:0051287 GO:0003677 GO:0016616 GO:0043115 GO:0005515
GO:0046872 GO:0008270 GO:0016829 GO:0016491 GO:0016151
Cluster-22178.70660 GO:0000287 GO:0070403 GO:0005515 GO:0030976

Cluster-22178.70661 GO:0007165 GO:0006886 GO:0005515 GO:0005783
GO:0016021
Cluster-22178.70662 GO:0006352 GO:0006355 GO:0007165 GO:0003700
GO:0016987 GO:0005515 GO:0003677 GO:0005667
Cluster-22178.70664 GO:0045490 GO:0005515 GO:0047487
Cluster-22178.70669 GO:0006955 GO:0031342 GO:0015675 GO:0009636
GO:0035444 GO:0015643 GO:0046872 GO:0015099 GO:0019814
GO:0016021

Cluster-22178.70671 GO:0030976 GO:0070403 GO:0008168 GO:0000287

Cluster-22178.70672 GO:0006779 GO:0015940 GO:0055114 GO:0008033
GO:0019354 GO:0050660 GO:0008641 GO:0008677 GO:0016491
GO:0043115
Cluster-22178.70673 GO:0006468 GO:0045893 GO:0007275 GO:0008270
GO:0005524 GO:0004672 GO:0046983
Cluster-22178.70674 GO:0006306 GO:0031167 GO:0032775 GO:0003677
GO:0008170 GO:0008168 GO:0009007
Cluster-22178.70675 GO:0033588
Cluster-22178.70677 GO:0007264 GO:0006355 GO:0006576 GO:0007155
GO:0002949 GO:0005198 GO:0008134 GO:0005524 GO:0005525
GO:0005667 GO:0016020 GO:0031012
Cluster-22178.70678 GO:0002949 GO:0006576 GO:0007155 GO:0006355
GO:0007264 GO:0005525 GO:0005524 GO:0005198 GO:0008134
GO:0031012 GO:0005667
Cluster-22178.70680 GO:0006397 GO:0003676
Cluster-22178.70681 GO:0019031
Cluster-22178.70682 GO:0006626 GO:0003824 GO:0005741
Cluster-22178.70683 GO:0006024 GO:0015012 GO:0016021
Cluster-22178.70687 GO:0051260
Cluster-22178.70688 GO:0000045 GO:0005737
Cluster-22178.70689 GO:0006955 GO:0007165 GO:0005164 GO:0016020
Cluster-22178.70690 GO:0061630
Cluster-22178.70692 GO:0007264 GO:0030623 GO:0005525
Cluster-22178.70693 GO:0043547 GO:0005096
Cluster-22178.70696 GO:0015992 GO:0006914 GO:0071988 GO:0007155
GO:0008608 GO:0006308 GO:0007094 GO:0010508 GO:0030474
GO:0015991 GO:0008855 GO:0005509 GO:0015078 GO:0005515
GO:0051082 GO:0005876 GO:0016021 GO:0042729 GO:0009318
GO:0033179
Cluster-22178.70699 GO:0006812 GO:0055085 GO:0008324 GO:0005886
GO:0016021

Cluster-22178.70700 GO:0006468 GO:0005975 GO:0055114 GO:0034080
GO:0016638 GO:0016868 GO:0005198 GO:0046872 GO:0005524
GO:0004672 GO:0046983 GO:0000775
Cluster-22178.70703 GO:0006468 GO:0006816 GO:0006874 GO:0006397
GO:0051252 GO:0005524 GO:0004672 GO:0046872 GO:0005219
GO:0004540 GO:0005622 GO:0016021
Cluster-22178.70705 GO:0046486 GO:0009395 GO:0055114 GO:0007205
GO:0035556 GO:0016301 GO:0005515 GO:0004143 GO:0047134
Cluster-22178.70706 GO:0006541 GO:0005515 GO:0004359
Cluster-22178.70712 GO:0006355 GO:0043401 GO:0043565 GO:0008270
GO:0003700 GO:0005667 GO:0005634
Cluster-22178.70714 GO:0071805 GO:0019089 GO:0009405 GO:0006904
GO:0006508 GO:0006687 GO:0006813 GO:0009401 GO:0006754
GO:0007067 GO:0006891 GO:0015031 GO:0050290 GO:0015079
GO:0004197 GO:0004867 GO:0046912 GO:0016887 GO:0005524
GO:0042729 GO:0000275 GO:0072686 GO:0005576 GO:0016021
GO:0000145 GO:0017119 GO:0005887
Cluster-22178.70715 GO:0006891 GO:0015031 GO:0006754 GO:0007067
GO:0009401 GO:0009405 GO:0006904 GO:0071805 GO:0019089
GO:0006687 GO:0006813 GO:0006508 GO:0005524 GO:0016887
GO:0004197 GO:0004867 GO:0050290 GO:0015079 GO:0005887
GO:0000145 GO:0017119 GO:0042729 GO:0005576 GO:0000275
GO:0072686
Cluster-22178.70716 GO:0007165 GO:0019087 GO:0005102 GO:0016021
Cluster-22178.70717 GO:0006289 GO:0006865 GO:0055085 GO:0009411
GO:0006397 GO:0006810 GO:0003333 GO:0015171 GO:0004519
GO:0005634 GO:0016020
Cluster-22178.70718 GO:0007165
Cluster-22178.70720 GO:0008152 GO:0008484 GO:0003824
Cluster-22178.70722 GO:0006355 GO:0000975 GO:0003700 GO:0005667
GO:0005840
Cluster-22178.70725 GO:0000921 GO:0006355 GO:0003700 GO:0043565
GO:0016021 GO:0005940 GO:0005667

Cluster-22178.70730 GO:0000105 GO:0055114 GO:0008033 GO:0006412 GO:0042254 GO:0017150 GO:0050660 GO:0003735 GO:0005840 GO:0005622
Cluster-22178.70732 GO:0006396 GO:0006807 GO:0006144 GO:0004000 GO:0003723
Cluster-22178.70735 GO:0006144 GO:0006396 GO:0006807 GO:0003723 GO:0004000
Cluster-22178.70739 GO:0006506 GO:0006468 GO:0055085 GO:0005515 GO:0004386 GO:0004672 GO:0005524 GO:0016773 GO:0031083 GO:0016020 GO:0005789 GO:0016021
Cluster-22178.70740 GO:0046872
Cluster-22178.70741 GO:0005515
Cluster-22178.70743 GO:0006812 GO:0055085 GO:0008324 GO:0016021
Cluster-22178.70746 GO:0055114 GO:0016491 GO:0010181
Cluster-22178.70748 GO:0006606 GO:0007094 GO:0000042 GO:0005515 GO:0005789
Cluster-22178.70750 GO:0040007 GO:0008283 GO:0007165 GO:0008083

Cluster-22178.70755 GO:0006807 GO:0006298 GO:0005524 GO:0030983 GO:0016151
Cluster-22178.70756 GO:0006298 GO:0006807 GO:0005524 GO:0030983 GO:0016151
Cluster-22178.70757 GO:0006810 GO:0016021
Cluster-22178.70763 GO:0016787
Cluster-22178.70764 GO:0090063 GO:0000226 GO:0004190 GO:0008146 GO:0005815 GO:0016020 GO:0000922
Cluster-22178.70767 GO:0016020
Cluster-22178.70768 GO:0055114 GO:0006979 GO:0006355 GO:0015947 GO:0006568 GO:0006804 GO:0003700 GO:0004096 GO:0020037 GO:0005667 GO:0005634
Cluster-22178.70769 GO:0007165 GO:0000166 GO:0005515 GO:0046872 GO:0045202
Cluster-22178.70772 GO:0005515 GO:0016773

Cluster-22178.70774 GO:0005515
Cluster-22178.70775 GO:0008152 GO:0015979 GO:0016829 GO:0016020
GO:0009523 GO:0009539
Cluster-22178.70779 GO:0006508 GO:0016567 GO:0006510 GO:0003723
GO:0005515 GO:0061630 GO:0046872 GO:0004176 GO:0008270
GO:0004842
Cluster-22178.70780 GO:0043565 GO:0005515 GO:0005634
Cluster-22178.70782 GO:0050832 GO:0006621 GO:0042742 GO:0046923
GO:0005783 GO:0016021
Cluster-22178.70784 GO:0006886 GO:0006888 GO:0008270 GO:0030127
Cluster-22178.70785 GO:0006457 GO:0005509 GO:0051082 GO:0005783
Cluster-22178.70786 GO:0006576 GO:0006777 GO:0006614 GO:0000103
GO:0006000 GO:0006144 GO:0006013 GO:0016301 GO:0005525
GO:0004020 GO:0003873 GO:0003677 GO:0016787 GO:0008312
GO:0005524 GO:0048500
Cluster-22178.70788 GO:0006888 GO:0006886 GO:0008270 GO:0030127
Cluster-22178.70789 GO:0006468 GO:0006397 GO:0051252 GO:0004540
GO:0016773 GO:0004672 GO:0005524 GO:0016020
Cluster-22178.70790 GO:0042742 GO:0005515
Cluster-22178.70793 GO:0055114 GO:0004190 GO:0016491 GO:0016021
Cluster-22178.70794 GO:0007165 GO:0015893 GO:0006308 GO:0006855
GO:0015238 GO:0008855 GO:0009318 GO:0005886
Cluster-22178.70795 GO:0006260 GO:0045893 GO:0006308 GO:0006355
GO:0006281 GO:0017108 GO:0003700 GO:0043565 GO:0003677
GO:0005515 GO:0033557 GO:0005634 GO:0005667
Cluster-22178.70796 GO:0042254 GO:0006412 GO:0003735 GO:0003677
GO:0005096 GO:0046872 GO:0005622 GO:0045202 GO:0005840
Cluster-22178.70798 GO:0006506 GO:0007165 GO:0005179 GO:0005576
Cluster-22178.70799 GO:0015977 GO:0046487 GO:0016984 GO:0003677
GO:0009573
Cluster-22178.70800 GO:0030168 GO:0051262 GO:0006357 GO:0006606
GO:0006260 GO:0007067 GO:0007155 GO:0007165 GO:0019058
GO:0051258 GO:0009405 GO:0010508 GO:0005102 GO:0030674

GO:0004871 GO:0005198 GO:0001104 GO:0005515 GO:0005577
GO:0005882 GO:0016592 GO:0016020 GO:0000775 GO:0019031
GO:0016021 GO:0019867 GO:0019028 GO:0072686 GO:0042729
Cluster-22178.70801 GO:0015992 GO:0055114 GO:0019069 GO:0015991
GO:0009405 GO:0008270 GO:0004800 GO:0015078 GO:0003723
GO:0005515 GO:0033179 GO:0016020
Cluster-22178.70803 GO:0007156 GO:0005509 GO:0016020
Cluster-22178.70805 GO:0006468 GO:0016773 GO:0005524 GO:0004672
GO:0005515 GO:0016020
Cluster-22178.70811 GO:0031047
Cluster-22178.70817 GO:0009059 GO:0006468 GO:0004672 GO:0005524
GO:0004519 GO:0003677 GO:0030176
Cluster-22178.70818 GO:0006555 GO:0006730 GO:0006816 GO:0006874
GO:0006281 GO:0005219 GO:0008270 GO:0046872 GO:0004013
GO:0016021 GO:0005622
Cluster-22178.70822 GO:0016021
Cluster-22178.70825 GO:0007155 GO:0051726 GO:0006367 GO:0015684
GO:0006281 GO:0009432 GO:0016887 GO:0005524 GO:0015093
GO:0017025 GO:0003697 GO:0005525 GO:0009289 GO:0005672
GO:0005634 GO:0016021
Cluster-22178.70830 GO:0006355 GO:0007186 GO:0008270 GO:0004930
GO:0003700 GO:0005667 GO:0005634 GO:0016020
Cluster-22178.70832 GO:0006260 GO:0005515
Cluster-22178.70833 GO:0006470 GO:0000226 GO:0050852 GO:0006570
GO:0004725 GO:0005509 GO:0005515 GO:0015630
Cluster-22178.70834 GO:0055114 GO:0006351 GO:0006206 GO:0006144
GO:0007165 GO:0015774 GO:0005515 GO:0003899 GO:0004888
GO:0015159 GO:0005509 GO:0051920 GO:0016021 GO:0005578
GO:0009289 GO:0016020 GO:0005730
Cluster-22178.70836 GO:0016787
Cluster-22178.70838 GO:0000398 GO:0006813 GO:0005249 GO:0030955
GO:0016021 GO:0005681 GO:0005886 GO:0008076
Cluster-22178.70839 GO:0035556 GO:0009086 GO:0000398 GO:0008652

GO:0008270 GO:0005515 GO:0003871 GO:0005681

Cluster-22178.70842 GO:0009405 GO:0035556 GO:0019835 GO:0005515 GO:0019867

Cluster-22178.70844 GO:0009405 GO:0005515 GO:0005198

Cluster-22178.70845 GO:0035091

Cluster-22178.70847 GO:0008063 GO:0005515 GO:0008270

Cluster-22178.70848 GO:0019068 GO:0007165 GO:0015992 GO:0055085 GO:0030001 GO:0015991 GO:0046849 GO:0046873 GO:0045502 GO:0008134 GO:0042803 GO:0015078 GO:0005667 GO:0016020 GO:0030286 GO:0005576 GO:0033179 GO:0016021

Cluster-22178.70849 GO:0006351 GO:0006508 GO:0000162 GO:0009073 GO:0006260 GO:0006571 GO:0055114 GO:0015979 GO:0009094 GO:0030494 GO:0004252 GO:0003677 GO:0003856 GO:0016491 GO:0042025

Cluster-22178.70851 GO:0015994 GO:0006811 GO:0015996 GO:0006118 GO:0006629 GO:0006355 GO:0015991 GO:0006508 GO:0016042 GO:0015992 GO:0046486 GO:0045454 GO:0015035 GO:0009055 GO:0015078 GO:0046872 GO:0047746 GO:0008236 GO:0003847 GO:0003677 GO:0016787 GO:0019031 GO:0033179 GO:0005886 GO:0008247 GO:0016020

Cluster-22178.70852 GO:0015074 GO:0006310 GO:0006313 GO:0000150 GO:0003677

Cluster-22178.70855 GO:0006118 GO:0045454 GO:0015035 GO:0009055 GO:0005515 GO:0005634

Cluster-22178.70859 GO:0006144 GO:0006206 GO:0050852 GO:0006470 GO:0006570 GO:0006351 GO:0046872 GO:0004725 GO:0044212 GO:0005515 GO:0003899 GO:0003677 GO:0005730

Cluster-22178.70860 GO:0034314 GO:0007165 GO:0006955 GO:0030041 GO:0003676 GO:0005125 GO:0003723 GO:0015629 GO:0005885 GO:0005576

Cluster-22178.70862 GO:0008152 GO:0046872 GO:0008484 GO:0003824

Cluster-22178.70865 GO:0007264 GO:0003924 GO:0005525 GO:0005634

Cluster-22178.70866 GO:0006813 GO:0015886 GO:0015232 GO:0016286
GO:0016021
Cluster-22178.70867 GO:0007165 GO:0035556 GO:0005509 GO:0046872
GO:0016787 GO:0005578
Cluster-22178.70871 GO:0008063 GO:0030001 GO:0006367 GO:0055085
GO:0046873 GO:0005672 GO:0016020
Cluster-22178.70872 GO:0007165 GO:0007067 GO:0000090 GO:0009069
GO:0006606 GO:0031110 GO:0006914 GO:0030168 GO:0006468
GO:0016310 GO:0007059 GO:0051301 GO:0048870 GO:0051258
GO:0030674 GO:0005102 GO:0008017 GO:0004674 GO:0005096
GO:0005198 GO:0042803 GO:0008134 GO:0045502 GO:0005882
GO:0005667 GO:0005876 GO:0005577 GO:0000940 GO:0030286
GO:0031514 GO:0045298
Cluster-22178.70873 GO:0006470 GO:0006570 GO:0004725 GO:0008138

Cluster-22178.70876 GO:0007264 GO:0007165 GO:0007186 GO:0006013
GO:0043087 GO:0006000 GO:0006355 GO:0035023 GO:0003873
GO:0005524 GO:0005089 GO:0005515 GO:0019001 GO:0031683
GO:0003924 GO:0005525 GO:0004871
Cluster-22178.70879 GO:0005515
Cluster-22178.70882 GO:0005515
Cluster-22178.70883 GO:0015992 GO:0015986 GO:0015078 GO:0003676
GO:0003723 GO:0071203 GO:0000276
Cluster-22178.70886 GO:0006397 GO:0006812 GO:0046660 GO:0003676
GO:0016887 GO:0016021 GO:0005634
Cluster-22178.70893 GO:0046034 GO:0006139 GO:0006144 GO:0006206
GO:0007186 GO:0050909 GO:0004127 GO:0004930 GO:0005524
GO:0004017 GO:0016021
Cluster-22178.70895 GO:0007165 GO:0005515
Cluster-22178.70901 GO:0042157 GO:0007186 GO:0006457 GO:0006869
GO:0034080 GO:0006355 GO:0051301 GO:0007059 GO:0003677
GO:0005524 GO:0004930 GO:0051082 GO:0035299 GO:0003700
GO:0003676 GO:0008289 GO:0005667 GO:0016272 GO:0000444

GO:0005576 GO:0016021 GO:0000775
Cluster-22178.70902 GO:0044406 GO:0006694 GO:0016114 GO:0019385
GO:0000287 GO:0008661 GO:0030976 GO:0003824 GO:0009289
Cluster-22178.70904 GO:0016310 GO:0006123 GO:0015992 GO:0006886
GO:0016192 GO:0009069 GO:0004674 GO:0004129 GO:0003676
GO:0005515 GO:0045277 GO:0030117
Cluster-22178.70905 GO:0006412 GO:0042254 GO:0003735 GO:0005515
GO:0005840 GO:0005622
Cluster-22178.70906 GO:0006351 GO:0043565
Cluster-22178.70907 GO:0006351 GO:0006144 GO:0005198 GO:0003723
GO:0003676 GO:0003968 GO:0019028 GO:0031379
Cluster-22178.70908 GO:0008152 GO:0016836 GO:0061630 GO:0005515
GO:0008270 GO:0046872 GO:0003824 GO:0032040
Cluster-22178.70910 GO:0006231 GO:0006206 GO:0009405 GO:0004799
GO:0045735 GO:0005576
Cluster-22178.70911 GO:0006351 GO:0006355 GO:0003677 GO:0005515
GO:0016021 GO:0005634
Cluster-22178.70912 GO:0005515 GO:0016021
Cluster-22178.70914 GO:0005515 GO:0043395 GO:0016020 GO:0005578
Cluster-22178.70915 GO:0042254 GO:0006412 GO:0043395 GO:0003735
GO:0005515 GO:0005840 GO:0016020 GO:0005578
Cluster-22178.70917 GO:0006139 GO:0006206 GO:0007009 GO:0005515
GO:0004127 GO:0005524 GO:0005887
Cluster-22178.70924 GO:0010508 GO:0006886 GO:0030001 GO:0006355
GO:0007049 GO:0055114 GO:0006367 GO:0010506 GO:0006914
GO:0006464 GO:0008113 GO:0046872 GO:0005669 GO:0005615
GO:0016021 GO:0005783
Cluster-22178.70926 GO:0006468 GO:0005524 GO:0004672
Cluster-22178.70927 GO:0005515 GO:0016021
Cluster-22178.70928 GO:0019236 GO:0007186
Cluster-22178.70931 GO:0042981 GO:0005515
Cluster-22178.70935 GO:0009512
Cluster-22178.70938 GO:0030418 GO:0006355 GO:0045892 GO:0030410

GO:0003677

Cluster-22178.70939 GO:0008152 GO:0008168

Cluster-22178.70940 GO:0009099 GO:0009097 GO:0009405 GO:0006438 GO:0009098 GO:0008152 GO:0000166 GO:0016758 GO:0004832 GO:0005524 GO:0016020 GO:0005783 GO:0005737

Cluster-22178.70941 GO:0006367 GO:0006952 GO:0006352 GO:0003677 GO:0005634 GO:0009279 GO:0005669

Cluster-22178.70945 GO:0007015 GO:0007155 GO:0007094 GO:0006897 GO:0006457 GO:0006955 GO:0019882 GO:0051082 GO:0005198 GO:0019028 GO:0009288 GO:0016272 GO:0019031

Cluster-22178.70948 GO:0007186 GO:0005515 GO:0004930 GO:0004190 GO:0016021

Cluster-22178.70951 GO:0006508 GO:0004185

Cluster-22178.70953 GO:0055114 GO:0006260 GO:0051536 GO:0016651 GO:0008270 GO:0005634

Cluster-22178.70954 GO:0046740 GO:0005515 GO:0008270 GO:0016021

Cluster-22178.70955 GO:0005515

Cluster-22178.70956 GO:0006605 GO:0007268 GO:0006886 GO:0006508 GO:0004197 GO:0043495 GO:0042802 GO:0005515 GO:0030695 GO:0033130 GO:0005742

Cluster-22178.70959 GO:0007217 GO:0007268 GO:0007275 GO:0004867 GO:0005578

Cluster-22178.70961 GO:0006260 GO:0005524 GO:0016301 GO:0003887 GO:0003677 GO:0009360 GO:0042575

Cluster-22178.70965 GO:0042967 GO:0006886 GO:0006505 GO:0006629 GO:0008374 GO:0016788 GO:0005515

Cluster-22178.70967 GO:0006094 GO:0005525 GO:0004611

Cluster-22178.70968 GO:0006511 GO:0051603 GO:0004298 GO:0004175 GO:0019773 GO:0005839

Cluster-22178.70972 GO:0007186 GO:0007165 GO:0004871 GO:0016829 GO:0005834 GO:0005576

Cluster-22178.70973 GO:0045454 GO:0006118 GO:0000398 GO:0006144 GO:0005515 GO:0009055 GO:0015035 GO:0003968 GO:0031379

GO:0005681 GO:0016021
Cluster-22178.70975 GO:0006310 GO:0002949 GO:0006886 GO:0006281
GO:0048870 GO:0009378 GO:0003724 GO:0005524 GO:0016887
GO:0003723 GO:0003678 GO:0046872 GO:0005783 GO:0009379
GO:0005657 GO:0031514 GO:0016021
Cluster-22178.70976 GO:0006378 GO:0003729 GO:0016787 GO:0005849
Cluster-22178.70977 GO:0009451 GO:0001522 GO:0003723 GO:0009982
GO:0016012 GO:0016021
Cluster-22178.70979 GO:0045046 GO:0032403 GO:0005779 GO:0008537
Cluster-22178.70980 GO:0006541 GO:0004359
Cluster-22178.70981 GO:0005634
Cluster-22178.70982 GO:0003924 GO:0005525
Cluster-22178.70985 GO:0006470 GO:0006402 GO:0051252 GO:0006468
GO:0003676 GO:0004672 GO:0005524 GO:0046872 GO:0004535
GO:0008138 GO:0003723 GO:0005737 GO:0005634
Cluster-22178.70987 GO:0006355 GO:0006118 GO:0055114 GO:0004190
GO:0005515 GO:0004252 GO:0016655 GO:0005886 GO:0016021
Cluster-22178.70989 GO:0007186 GO:0006614 GO:0007165 GO:0006576
GO:0002949 GO:0003676 GO:0004871 GO:0005525 GO:0016887
GO:0003924 GO:0005524 GO:0031683 GO:0019001
Cluster-22178.70991 GO:0055114 GO:0008152 GO:0016491
Cluster-22178.70992 GO:0010333 GO:0000287 GO:0016829
Cluster-22178.70996 GO:0003676 GO:0046872
Cluster-22178.70997 GO:0006400 GO:0009451 GO:0031167 GO:0016226
GO:0008033 GO:0008176 GO:0051536 GO:0008168 GO:0005737
Cluster-22178.70998 GO:0005515 GO:0003824 GO:0008270 GO:0046872

Cluster-22178.71001 GO:0051015
Cluster-22178.71003 GO:0006260 GO:0003677 GO:0005664
Cluster-22178.71007 GO:0006355 GO:0046854 GO:0042967 GO:0016787
GO:0004402 GO:0008270 GO:0003712 GO:0005667 GO:0005634
GO:0019033 GO:0000123
Cluster-22178.71009 GO:0005515

Cluster-22178.71010 GO:0000917 GO:0006355 GO:0043093 GO:0007049
GO:0006260 GO:0003700 GO:0005667 GO:0005737
Cluster-22178.71016 GO:0006570 GO:0006470 GO:0004527 GO:0008138
GO:0004725 GO:0003677
Cluster-22178.71020 GO:0015996 GO:0015994 GO:0006811 GO:0006118
GO:0006508 GO:0015991 GO:0006629 GO:0006355 GO:0016042
GO:0015992 GO:0046486 GO:0045454 GO:0015078 GO:0009055
GO:0015035 GO:0046872 GO:0008236 GO:0047746 GO:0003847
GO:0003677 GO:0016787 GO:0019031 GO:0033179 GO:0005886
GO:0016020 GO:0008247
Cluster-22178.71021 GO:0016567 GO:0004842 GO:0019033
Cluster-22178.71022 GO:0006418 GO:0006139 GO:0005524 GO:0003676
GO:0004812 GO:0008026 GO:0016818 GO:0000166
Cluster-22178.71023 GO:0046872
Cluster-22178.71027 GO:0007049 GO:0000917 GO:0006355 GO:0043093
GO:0003700 GO:0005737 GO:0005667
Cluster-22178.71028 GO:0005515
Cluster-22178.71029 GO:0006537 GO:0055114 GO:0009607 GO:0000105
GO:0006355 GO:0016491 GO:0016627 GO:0003824 GO:0016638
GO:0015930 GO:0005737
Cluster-22178.71030 GO:0005515
Cluster-22178.71031 GO:0055114 GO:0016972
Cluster-22178.71032 GO:0006468 GO:0016627 GO:0005524 GO:0004672

Cluster-22178.71034 GO:0006468 GO:0016627 GO:0005524 GO:0004672

Cluster-22178.71035 GO:0006310 GO:0007131 GO:0002949 GO:0008033
GO:0055114 GO:0015684 GO:0007264 GO:0015886 GO:0006281
GO:0046497 GO:0006769 GO:0098519 GO:0015232 GO:0016887
GO:0015093 GO:0005524 GO:0016301 GO:0003723 GO:0003924
GO:0051287 GO:0009378 GO:0003724 GO:0050660 GO:0016491
GO:0008750 GO:0050661 GO:0003678 GO:0005525 GO:0005657
GO:0000794 GO:0009379 GO:0016021

Cluster-22178.71037 GO:0007264 GO:0015684 GO:0006310 GO:0002949
GO:0006281 GO:0009378 GO:0098519 GO:0005524 GO:0015093
GO:0016887 GO:0003724 GO:0003723 GO:0016301 GO:0003924
GO:0005525 GO:0003678 GO:0009379 GO:0005657 GO:0016021
Cluster-22178.71038 GO:0006119 GO:0015986 GO:0015992 GO:0046961
GO:0008289 GO:0046933 GO:0045259 GO:0000275
Cluster-22178.71043 GO:0007165 GO:0005515 GO:0003677 GO:0005179
GO:0005576
Cluster-22178.71048 GO:0006520 GO:0019752 GO:0016831 GO:0030170
GO:0016829
Cluster-22178.71049 GO:0007275 GO:0005515 GO:0042600
Cluster-22178.71050 GO:0007275 GO:0005515 GO:0042600
Cluster-22178.71053 GO:0007165 GO:0004888 GO:0010181 GO:0005515
GO:0005509 GO:0016021
Cluster-22178.71054 GO:0005509 GO:0005515 GO:0010181
Cluster-22178.71055 GO:0016192 GO:0006886 GO:0005975 GO:0005515
GO:0005198 GO:0004553 GO:0030117 GO:0019028
Cluster-22178.71057 GO:0046872
Cluster-22178.71059 GO:0006355 GO:0006281 GO:0003677 GO:0030915
Cluster-22178.71061 GO:0006357 GO:0035091 GO:0001104 GO:0016592
Cluster-22178.71062 GO:0055114 GO:0000166 GO:0016616 GO:0051287
GO:0016491 GO:0008270 GO:0046872 GO:0016020
Cluster-22178.71063 GO:0006812 GO:0032801 GO:0007049 GO:0019076
GO:0019058 GO:0006351 GO:0005198 GO:0005261 GO:0070176
GO:0000228 GO:0033644
Cluster-22178.71064 GO:0019497 GO:0006281 GO:0002949 GO:0006310
GO:0019079 GO:0006771 GO:0003678 GO:0005515 GO:0003723
GO:0003724 GO:0003993 GO:0016887 GO:0005524 GO:0009378
GO:0005657 GO:0009379
Cluster-22178.71065 GO:0006281 GO:0019497 GO:0006771 GO:0006310
GO:0019079 GO:0002949 GO:0003723 GO:0005515 GO:0003678
GO:0009378 GO:0016887 GO:0005524 GO:0003724 GO:0003993
GO:0009379 GO:0005657

Cluster-22178.71066 GO:0003779
Cluster-22178.71067 GO:0009069 GO:0006468 GO:0048870 GO:0016310
GO:0005198 GO:0005524 GO:0004674 GO:0031514
Cluster-22178.71068 GO:0007264 GO:0006810 GO:0055114 GO:0006811
GO:0055085 GO:0005216 GO:0010181 GO:0016491 GO:0005525
GO:0005215 GO:0016020 GO:0005615 GO:0016021
Cluster-22178.71071 GO:0006313 GO:0004803 GO:0003677
Cluster-22178.71076 GO:0045490 GO:0047487 GO:0005515
Cluster-22178.71079 GO:0006886 GO:0005741
Cluster-22178.71080 GO:0005524
Cluster-22178.71081 GO:0008270 GO:0005515
Cluster-22178.71083 GO:0007264 GO:0005525
Cluster-22178.71087 GO:0006808 GO:0005975 GO:0046034 GO:0006413
GO:0006355 GO:0006506 GO:0006144 GO:0006464 GO:0007218
GO:0006446 GO:0043565 GO:0004672 GO:0046872 GO:0003700
GO:0003676 GO:0016151 GO:0003723 GO:0004519 GO:0005515
GO:0030234 GO:0008270 GO:0005524 GO:0004553 GO:0004017
GO:0003743 GO:0003677 GO:0005789 GO:0005840 GO:0005667
Cluster-22178.71098 GO:0015940 GO:0042967 GO:0008152 GO:0055114
GO:0007034 GO:0016573 GO:0006355 GO:0003824 GO:0016491
GO:0008677 GO:0008484 GO:0071949 GO:0004402 GO:0000123
Cluster-22178.71099 GO:0045892 GO:0019856 GO:0003700 GO:0005515
GO:0005667 GO:0019012
Cluster-22178.71103 GO:0043087 GO:0007169 GO:0035023 GO:0006468
GO:0006508 GO:0005524 GO:0004672 GO:0004185 GO:0005089
GO:0004714 GO:0005515 GO:0016020
Cluster-22178.71104 GO:0006468 GO:0007169 GO:0004672 GO:0005524
GO:0004714 GO:0005515 GO:0016020
Cluster-22178.71106 GO:0008152 GO:0016226 GO:0051536 GO:0008168
GO:0005737
Cluster-22178.71108 GO:0042967 GO:0016747 GO:0008080
Cluster-22178.71111 GO:0051726 GO:0006974
Cluster-22178.71113 GO:0055114 GO:0015675 GO:0035444 GO:0016491

GO:0046872 GO:0015099 GO:0005506 GO:0031418 GO:0016706
GO:0016021
Cluster-22178.71116 GO:0055114 GO:0015992 GO:0015991 GO:0019069
GO:0009405 GO:0004800 GO:0008270 GO:0015078 GO:0003723
GO:0005515 GO:0033179 GO:0016020
Cluster-22178.71117 GO:0015992 GO:0055114 GO:0019069 GO:0015991
GO:0009405 GO:0008270 GO:0004800 GO:0015078 GO:0003723
GO:0005515 GO:0033179 GO:0016020
Cluster-22178.71120 GO:0042254 GO:0006412 GO:0003735 GO:0005840
GO:0005622
Cluster-22178.71121 GO:0019835 GO:0009405 GO:0019867
Cluster-22178.71123 GO:0006355 GO:0019478 GO:0055114 GO:0016788
GO:0005515 GO:0050662 GO:0003676 GO:0003700 GO:0016491
GO:0043565 GO:0016020 GO:0005667 GO:0005737
Cluster-22178.71124 GO:0006265 GO:0003916 GO:0003677
Cluster-22178.71125 GO:0003712 GO:0005667 GO:0000124
Cluster-22178.71126 GO:0006812 GO:0008324
Cluster-22178.71131 GO:0006605 GO:0006886 GO:0005515 GO:0005742
Cluster-22178.71133 GO:0016310 GO:0006139 GO:0006412 GO:0006281
GO:0002949 GO:0006206 GO:0006810 GO:0006310 GO:0042254
GO:0009069 GO:0005525 GO:0003924 GO:0016301 GO:0003723
GO:0004674 GO:0003724 GO:0004127 GO:0005524 GO:0016887
GO:0098519 GO:0003735 GO:0009378 GO:0005840 GO:0005657
GO:0005622 GO:0009379
Cluster-22178.71135 GO:0005515 GO:0004252 GO:0043565 GO:0005634
GO:0055036 GO:0019028
Cluster-22178.71137 GO:0009405 GO:0009094 GO:0009073 GO:0006571
GO:0000162 GO:0004107 GO:0005515 GO:0033644
Cluster-22178.71138 GO:0055085 GO:0006812 GO:0008324 GO:0005886
GO:0016021
Cluster-22178.71139 GO:0006576 GO:0005524 GO:0003924 GO:0005525

Cluster-22178.71141 GO:0006779 GO:0009396 GO:0019354 GO:0008152

GO:0015940 GO:0055114 GO:0046487 GO:0016757 GO:0016491
GO:0004488 GO:0050662 GO:0051287 GO:0003824 GO:0008677
GO:0043115 GO:0016020
Cluster-22178.71145 GO:0005515
Cluster-22178.71146 GO:0042967 GO:0006810 GO:0006816 GO:0016573
GO:0006355 GO:0003712 GO:0008270 GO:0003713 GO:0004402
GO:0042030 GO:0005515 GO:0005246 GO:0000123 GO:0016020
GO:0005667 GO:0005634
Cluster-22178.71147 GO:0005975 GO:0043169 GO:0003824
Cluster-22178.71148 GO:0016787
Cluster-22178.71149 GO:0005515
Cluster-22178.71150 GO:0044237
Cluster-22178.71151 GO:0005515
Cluster-22178.71153 GO:0006508 GO:0016853 GO:0008233 GO:0046872
GO:0009507
Cluster-22178.71154 GO:0018342 GO:0019079 GO:0016787 GO:0008318
GO:0003677 GO:0008026 GO:0005524 GO:0003676
Cluster-22178.71155 GO:0006099 GO:0019643 GO:0006355 GO:0006094
GO:0015977 GO:0006270 GO:0008964
Cluster-22178.71159 GO:0008152 GO:0005515 GO:0008168
Cluster-22178.71162 GO:0019882 GO:0007155 GO:0007015 GO:0006955
GO:0042254 GO:0006457 GO:0006897 GO:0007094 GO:0051082
GO:0005198 GO:0016272 GO:0005622 GO:0009288 GO:0019031
GO:0019028
Cluster-22178.71163 GO:0005525
Cluster-22178.71164 GO:0006418 GO:0016567 GO:0006886 GO:0002161
GO:0005524 GO:0004842 GO:0004812 GO:0000166 GO:0005741
Cluster-22178.71165 GO:0006810 GO:0009405 GO:0006418 GO:0019871
GO:0000166 GO:0004812 GO:0005524 GO:0005576
Cluster-22178.71166 GO:0030089
Cluster-22178.71170 GO:0007049 GO:0006275 GO:0007155
Cluster-22178.71172 GO:0009405 GO:0006810 GO:0008270 GO:0046872
GO:0019870 GO:0005515 GO:0005576

Cluster-22178.71174 GO:0008270 GO:0005515
Cluster-22178.71176 GO:0005524 GO:0005856
Cluster-22178.71177 GO:0006260
Cluster-22178.71178 GO:0015979 GO:0019684 GO:0003723 GO:0009523
GO:0016021 GO:0019012
Cluster-22178.71179 GO:0005634
Cluster-22178.71180 GO:0043158 GO:0007165 GO:0003677 GO:0004252
GO:0005044 GO:0016020 GO:0005634
Cluster-22178.71182 GO:0008033 GO:0006508 GO:0004810 GO:0016437
GO:0008270 GO:0004222 GO:0005576
Cluster-22178.71184 GO:0016559 GO:0006355 GO:0016567 GO:0055114
GO:0006779 GO:0005515 GO:0043565 GO:0008270 GO:0046872
GO:0004842 GO:0005779 GO:0016020
Cluster-22178.71187 GO:0042254 GO:0006412 GO:0003735 GO:0005840
GO:0005622
Cluster-22178.71189 GO:0016310 GO:0033617 GO:0003677 GO:0016787
GO:0004673 GO:0003676 GO:0005524 GO:0008270 GO:0009365
Cluster-22178.71192 GO:0006013 GO:0006000 GO:0007165 GO:0007186
GO:0007264 GO:0007155 GO:0005525 GO:0004871 GO:0019001
GO:0031683 GO:0005524 GO:0003924 GO:0003873
Cluster-22178.71193 GO:0006400 GO:0008193 GO:0000287
Cluster-22178.71194 GO:0006281 GO:0006260 GO:0002949 GO:0006031
GO:0006310 GO:0005525 GO:0003678 GO:0043531 GO:0003924
GO:0003887 GO:0016301 GO:0004100 GO:0005524 GO:0016887
GO:0003689 GO:0003677 GO:0098519 GO:0009378 GO:0042575
GO:0005663 GO:0005657 GO:0009360 GO:0009379
Cluster-22178.71197 GO:0042254 GO:0006412 GO:0000902 GO:0042967
GO:0008152 GO:0055114 GO:0003735 GO:0008080 GO:0016491
GO:0005761 GO:0005840 GO:0005622
Cluster-22178.71198 GO:0055114 GO:0015979 GO:0008152 GO:0006480
GO:0006468 GO:0017004 GO:0006118 GO:0008168 GO:0004672
GO:0005524 GO:0045158 GO:0047134 GO:0009523 GO:0009512
GO:0016021

Cluster-22178.71199 GO:0006308 GO:0006955 GO:0006886 GO:0007131
GO:0007155 GO:0007165 GO:0005125 GO:0005057 GO:0005515
GO:0008855 GO:0008270 GO:0005737 GO:0000794 GO:0005576
GO:0009318 GO:0016020
Cluster-22178.71202 GO:0005515
Cluster-22178.71203 GO:0006120 GO:0042207 GO:0006744 GO:0015992
GO:0055114 GO:0006814 GO:0006570 GO:0006559 GO:0016817
GO:0004411 GO:0008137 GO:0005515 GO:0005739
Cluster-22178.71204 GO:0005975 GO:0006886 GO:0016192 GO:0004553
GO:0005198 GO:0005515 GO:0019028 GO:0030117
Cluster-22178.71205 GO:0005975 GO:0006886 GO:0016192 GO:0004553
GO:0005198 GO:0005515 GO:0030117 GO:0019028
Cluster-22178.71206 GO:0008152 GO:0016310 GO:0006120 GO:0006744
GO:0015992 GO:0019049 GO:0005975 GO:0055114 GO:0006814
GO:0009069 GO:0004674 GO:0003824 GO:0008137 GO:0030246
GO:0016836
Cluster-22178.71207 GO:0009405 GO:0006355 GO:0005515 GO:0005634
Cluster-22178.71208 GO:0006355 GO:0009405 GO:0005515 GO:0005634
Cluster-22178.71211 GO:0015992 GO:0055114 GO:0006817 GO:0035435
GO:0006119 GO:0006118 GO:0008121 GO:0015114 GO:0016021
Cluster-22178.71215 GO:0071973 GO:0008270 GO:0005198
Cluster-22178.71216 GO:0005515
Cluster-22178.71218 GO:0006144 GO:0007156 GO:0009058 GO:0009113
GO:0046872 GO:0005524 GO:0016742 GO:0004637 GO:0051539
GO:0005509 GO:0016020
Cluster-22178.71219 GO:0006139 GO:0006672 GO:0006807 GO:0003676
GO:0008270 GO:0005524 GO:0016811 GO:0016818 GO:0008026
GO:0016021
Cluster-22178.71220 GO:0019856 GO:0009405 GO:0032481 GO:0002218
GO:0005198 GO:0005515 GO:0008270 GO:0016021
Cluster-22178.71221 GO:0005515 GO:0033588
Cluster-22178.71223 GO:0006811 GO:0006813 GO:0005249 GO:0008076
GO:0016020

Cluster-22178.71224 GO:0006888 GO:0006886 GO:0006270 GO:0008270
GO:0030127
Cluster-22178.71225 GO:0007017 GO:0006511 GO:0016567 GO:0007018
GO:0034450 GO:0005515 GO:0005524 GO:0004842 GO:0003777
GO:0008017 GO:0000151 GO:0005874 GO:0045298 GO:0016021
Cluster-22178.71227 GO:0016021
Cluster-22178.71228 GO:0042176 GO:0005515 GO:0030234 GO:0000502
Cluster-22178.71230 GO:0006351 GO:0006813 GO:0016567 GO:0006144
GO:0006206 GO:0005515 GO:0003899 GO:0030955 GO:0046872
GO:0005249 GO:0003677 GO:0004842 GO:0008270 GO:0016021
GO:0005737 GO:0008076 GO:0005730 GO:0005680
Cluster-22178.71236 GO:0006508 GO:0055085 GO:0005515 GO:0008236
GO:0016021
Cluster-22178.71238 GO:0007165 GO:0005515 GO:0016020
Cluster-22178.71239 GO:0055085 GO:0006811 GO:0007010 GO:0003779
GO:0005216 GO:0016020
Cluster-22178.71240 GO:0055085 GO:0006811 GO:0006898 GO:0019068
GO:0007165 GO:0007010 GO:0003779 GO:0005044 GO:0005216
GO:0005198 GO:0016020
Cluster-22178.71241 GO:0006955 GO:0007165 GO:0040007 GO:0008283
GO:0005134 GO:0008083 GO:0035091 GO:0005515 GO:0005893
GO:0005576
Cluster-22178.71242 GO:0003677 GO:0046872
Cluster-22178.71246 GO:0046872
Cluster-22178.71247 GO:0008654 GO:0006508 GO:0016780 GO:0004181
GO:0008270 GO:0016020
Cluster-22178.71249 GO:0016567 GO:0006486 GO:0005515 GO:0016818
GO:0003677 GO:0016787 GO:0003676 GO:0004842 GO:0016757
GO:0005524 GO:0008270 GO:0046872 GO:0005680
Cluster-22178.71253 GO:0007165 GO:0030695 GO:0005057
Cluster-22178.71255 GO:0008033
Cluster-22178.71256 GO:0016192 GO:0006570 GO:0007304 GO:0006470
GO:0008138 GO:0005213 GO:0004725 GO:0005576 GO:0042600

Cluster-22178.71258 GO:0001510 GO:0006306 GO:0031167 GO:0009452 GO:0008152 GO:0008170 GO:0003677 GO:0005515 GO:0008168 GO:0005201 GO:0005578 GO:0019013 GO:0005581 GO:0016021

Cluster-22178.71259 GO:0035556 GO:0005515

Cluster-22178.71262 GO:0000226 GO:0000910 GO:0006554 GO:0006289 GO:0050690 GO:0043161 GO:0006479 GO:0006281 GO:0008017 GO:0005537 GO:0046872 GO:0005515 GO:0003684 GO:0018024 GO:0045298

Cluster-22178.71263 GO:0006865 GO:0055085 GO:0006355 GO:0003333 GO:0006810 GO:0008270 GO:0003700 GO:0015171 GO:0005634 GO:0005667 GO:0016020

Cluster-22178.71265 GO:0045892 GO:0005515 GO:0003677

Cluster-22178.71266 GO:0005515 GO:0046872 GO:0008270

Cluster-22178.71268 GO:0003676

Cluster-22178.71269 GO:0006886 GO:0005515 GO:0005525 GO:0005047 GO:0003924 GO:0005785 GO:0005786

Cluster-22178.71274 GO:0006687 GO:0009058 GO:0005515 GO:0001733 GO:0005794 GO:0016021

Cluster-22178.71275 GO:0000160 GO:0016310 GO:0006886 GO:0016192 GO:0009405 GO:0000155 GO:0016021 GO:0009365 GO:0005622 GO:0030131 GO:0005576

Cluster-22178.71276 GO:0006508 GO:0004197

Cluster-22178.71279 GO:0006508 GO:0004197 GO:0008234

Cluster-22178.71280 GO:0006323

Cluster-22178.71282 GO:0006278 GO:0007165 GO:0003964 GO:0005179 GO:0005576

Cluster-22178.71287 GO:0042254 GO:0006144 GO:0006508 GO:0006412 GO:0055114 GO:0071949 GO:0004197 GO:0016614 GO:0017111 GO:0003735 GO:0016491 GO:0050660 GO:0004252 GO:0003968 GO:0016020 GO:0031379 GO:0005840

Cluster-22178.71293 GO:0006364 GO:0009058 GO:0042432 GO:0016114 GO:0005515 GO:0016844 GO:0032040 GO:0016021

Cluster-22178.71294 GO:0005515

Cluster-22178.71296 GO:0005515
Cluster-22178.71297 GO:0006260 GO:0046983 GO:0003887 GO:0003677
GO:0005515 GO:0042802 GO:0042575
Cluster-22178.71298 GO:0006364 GO:0005515 GO:0032040
Cluster-22178.71303 GO:0016310 GO:0009069 GO:0005515 GO:0004674

Cluster-22178.71306 GO:0006412 GO:0042254 GO:0006364 GO:0003735
GO:0005515 GO:0005840 GO:0005622
Cluster-22178.71308 GO:0007218 GO:0006468 GO:0042811 GO:0004672
GO:0005524 GO:0016773 GO:0016746 GO:0005184 GO:0016020
Cluster-22178.71309 GO:0042811 GO:0006468 GO:0007218 GO:0005184
GO:0016746 GO:0016773 GO:0004672 GO:0005524 GO:0016020
Cluster-22178.71312 GO:0006464 GO:0032259 GO:0009452 GO:0046500
GO:0001510 GO:0031167 GO:0006479 GO:0008168 GO:0004719
GO:0003723
Cluster-22178.71314 GO:0006470 GO:0006570 GO:0008138 GO:0004725

Cluster-22178.71315 GO:0005515
Cluster-22178.71316 GO:0005515
Cluster-22178.71318 GO:0005515
Cluster-22178.71319 GO:0000413 GO:0000162 GO:0006571 GO:0009094
GO:0006457 GO:0006096 GO:0006094 GO:0004634 GO:0003755
GO:0000287 GO:0000015
Cluster-22178.71320 GO:0005515 GO:0016021
Cluster-22178.71321 GO:0006351 GO:0051252 GO:0006388 GO:0000213
GO:0003714 GO:0000214 GO:0005667
Cluster-22178.71324 GO:0006508 GO:0008233 GO:0005634
Cluster-22178.71325 GO:0006886 GO:0006605 GO:0009214 GO:0006000
GO:0006013 GO:0005515 GO:0042802 GO:0003873 GO:0004113
GO:0016887 GO:0008641 GO:0005524 GO:0016020 GO:0005742
Cluster-22178.71327 GO:0006281 GO:0000077 GO:0030896
Cluster-22178.71332 GO:0042742 GO:0006811 GO:0005216 GO:0005576
GO:0016020

Cluster-22178.71336 GO:0016032 GO:0009405 GO:0044179 GO:0042254 GO:0006412 GO:0043087 GO:0035023 GO:0003735 GO:0005515 GO:0005089 GO:0019012 GO:0005737 GO:0005840

Cluster-22178.71338 GO:0016310 GO:0000160 GO:0007268 GO:0033130 GO:0043495 GO:0005515 GO:0000155 GO:0003723 GO:0009365 GO:0016021 GO:0019867

Cluster-22178.71339 GO:0009308 GO:0055114 GO:0003676 GO:0016638 GO:0003723 GO:0042597

Cluster-22178.71340 GO:0006468 GO:0051016 GO:0005524 GO:0004672 GO:0016773 GO:0016020 GO:0008290

Cluster-22178.71342 GO:0051016 GO:0006468 GO:0016773 GO:0005524 GO:0004672 GO:0008290 GO:0016020

Cluster-22178.71343 GO:0046813 GO:0061025 GO:0005515 GO:0016021 GO:0019031

Cluster-22178.71346 GO:0007291 GO:0006520 GO:0016597 GO:0016743

Cluster-22178.71354 GO:0006260 GO:0019073 GO:0006139 GO:0006508 GO:0004222 GO:0003676 GO:0008270 GO:0003887 GO:0003677 GO:0008408 GO:0031012 GO:0042575

Cluster-22178.71355 GO:0006260 GO:0006139 GO:0019073 GO:0006508 GO:0008270 GO:0004222 GO:0003676 GO:0003677 GO:0003887 GO:0008408 GO:0031012 GO:0042575

Cluster-22178.71358 GO:0035023 GO:0006308 GO:0006468 GO:0006508 GO:0007169 GO:0043087 GO:0007165 GO:0015743 GO:0004672 GO:0005515 GO:0005089 GO:0004872 GO:0004185 GO:0005524 GO:0008855 GO:0004714 GO:0009318 GO:0016020

Cluster-22178.71359 GO:0005515

Cluster-22178.71360 GO:0010468 GO:0016021

Cluster-22178.71363 GO:0009405 GO:0005198

Cluster-22178.71364 GO:0009405 GO:0005198

Cluster-22178.71365 GO:0006950 GO:0006457 GO:0005524 GO:0051082

Cluster-22178.71366 GO:0009405 GO:0005198

Cluster-22178.71367 GO:0006493 GO:0000030 GO:0000136 GO:0016020
Cluster-22178.71371 GO:0008270
Cluster-22178.71375 GO:0006396 GO:0001522 GO:0009451 GO:0003723
GO:0009982
Cluster-22178.71376 GO:0006281 GO:0051382 GO:0003677
Cluster-22178.71378 GO:0007021 GO:0000160 GO:0016310 GO:0051258
GO:0009405 GO:0007131 GO:0015031 GO:0030168 GO:0007165
GO:0006869 GO:0042157 GO:0003676 GO:0008289 GO:0005198
GO:0003723 GO:0051082 GO:0015631 GO:0005102 GO:0046983
GO:0030674 GO:0000155 GO:0045298 GO:0016021 GO:0009365
GO:0019031 GO:0019028 GO:0000794 GO:0005576 GO:0005577
Cluster-22178.71380 GO:0006355 GO:0006858 GO:0046983
Cluster-22178.71381 GO:0005515
Cluster-22178.71382 GO:0006468 GO:0004672 GO:0005524
Cluster-22178.71383 GO:0002682 GO:0030246
Cluster-22178.71387 GO:0006508 GO:0004222
Cluster-22178.71389 GO:0046436 GO:0009252 GO:0008716 GO:0005524
GO:0046872
Cluster-22178.71390 GO:0007017 GO:0007018 GO:0005524 GO:0008017
GO:0003777 GO:0003723 GO:0005515 GO:0045298 GO:0005874
Cluster-22178.71395 GO:0016032 GO:0016567 GO:0090263 GO:0005515
GO:0046872 GO:0008270 GO:0004842 GO:0005680
Cluster-22178.71397 GO:0016567 GO:0090263 GO:0005515
Cluster-22178.71398 GO:0008033
Cluster-22178.71400 GO:0006355 GO:0008270 GO:0043565 GO:0003700
GO:0005667 GO:0005634
Cluster-22178.71403 GO:0008270 GO:0046872 GO:0005515
Cluster-22178.71406 GO:0007165 GO:0007275 GO:0016055 GO:0006468
GO:0005102 GO:0004672 GO:0005524 GO:0005576
Cluster-22178.71413 GO:0042254 GO:0006412 GO:0006355 GO:0003735
GO:0003677 GO:0005840 GO:0005622
Cluster-22178.71414 GO:0003676
Cluster-22178.71415 GO:0003676

Cluster-22178.71421 GO:0055085 GO:0016032 GO:0005506 GO:0016021
Cluster-22178.71423 GO:0055085 GO:0016032 GO:0005506 GO:0016021
Cluster-22178.71424 GO:0006468 GO:0006471 GO:0005515 GO:0004715
GO:0004672 GO:0005524 GO:0017176 GO:0003950
Cluster-22178.71425 GO:0006355 GO:0005515 GO:0003700 GO:0043565
GO:0005667 GO:0005634
Cluster-22178.71426 GO:0030150 GO:0046872 GO:0005742
Cluster-22178.71427 GO:0003723 GO:0055036
Cluster-22178.71428 GO:0006468 GO:0005524 GO:0004672
Cluster-22178.71429 GO:0005515
Cluster-22178.71432 GO:0006396 GO:0006810 GO:0051252 GO:0007264
GO:0005525 GO:0005524 GO:0004525 GO:0003677 GO:0003723
GO:0016787
Cluster-22178.71433 GO:0009966 GO:0006260 GO:0005783 GO:0005634
Cluster-22178.71434 GO:0006486 GO:0008373
Cluster-22178.71435 GO:0009452 GO:0006396 GO:0008033 GO:0009451
GO:0031167 GO:0001510 GO:0004809 GO:0008270 GO:0008168
GO:0008173 GO:0003723 GO:0016021
Cluster-22178.71438 GO:0042254 GO:0006412 GO:0003735 GO:0005622
GO:0005840
Cluster-22178.71440 GO:0009432 GO:0006281 GO:0051782 GO:0006310
GO:0007165 GO:0006260 GO:0051016 GO:0003723 GO:0005509
GO:0098519 GO:0009378 GO:0003724 GO:0016887 GO:0003689
GO:0005524 GO:0042575 GO:0005663 GO:0005578 GO:0009276
GO:0009379 GO:0005657 GO:0030479
Cluster-22178.71441 GO:0006629 GO:0042967 GO:0008080 GO:0008081

Cluster-22178.71444 GO:0005515
Cluster-22178.71445 GO:0004190 GO:0003676 GO:0016021
Cluster-22178.71446 GO:0005515
Cluster-22178.71449 GO:0005515 GO:0003676 GO:0003723
Cluster-22178.71450 GO:0019497 GO:0006771 GO:0046872 GO:0005515
GO:0003993

Cluster-22178.71455 GO:0045454 GO:0000398 GO:0005681
Cluster-22178.71457 GO:0007264 GO:0007186 GO:0007165 GO:0003924 GO:0019001 GO:0031683 GO:0005524 GO:0004871 GO:0005525 GO:0003677 GO:0000166
Cluster-22178.71458 GO:0009399 GO:0008270 GO:0005515 GO:0016021
Cluster-22178.71459 GO:0006402 GO:0051252 GO:0003676 GO:0004535 GO:0046872 GO:0003723 GO:0005737 GO:0005634
Cluster-22178.71460 GO:0006525 GO:0006560 GO:0018112
Cluster-22178.71462 GO:0006397 GO:0007264 GO:0005525 GO:0003924 GO:0046872 GO:0031419 GO:0016788 GO:0016787
Cluster-22178.71463 GO:0006855 GO:0055085 GO:0006810 GO:0015893 GO:0015297 GO:0015238 GO:0016020
Cluster-22178.71464 GO:0046872
Cluster-22178.71466 GO:0046325 GO:0005515 GO:0016773 GO:0005737
Cluster-22178.71472 GO:0007165 GO:0051258 GO:0030168 GO:0005102 GO:0030674 GO:0005577
Cluster-22178.71473 GO:0005985 GO:0005982 GO:0005978 GO:0005515 GO:0004373
Cluster-22178.71474 GO:0006571 GO:0000162 GO:0009094 GO:0006096 GO:0006094 GO:0004634 GO:0000287 GO:0000015
Cluster-22178.71475 GO:0005524 GO:0003676
Cluster-22178.71478 GO:0000103 GO:0006144 GO:0003824 GO:0004020 GO:0005524
Cluster-22178.71481 GO:0046872 GO:0003676 GO:0005198 GO:0005622 GO:0019028
Cluster-22178.71484 GO:0006461 GO:0042254 GO:0019627 GO:0016151 GO:0003735 GO:0005515 GO:0005840
Cluster-22178.71485 GO:0042967 GO:0005515 GO:0008080 GO:0016747

Cluster-22178.71490 GO:0006412 GO:0051258 GO:0048870 GO:0007049 GO:0071704 GO:0007165 GO:0030168 GO:0006260 GO:0006468 GO:0005975 GO:0016310 GO:0009069 GO:0042254 GO:0006606 GO:0045502 GO:0008134 GO:0030674 GO:0016868 GO:0005096

GO:0004674 GO:0005102 GO:0005198 GO:0005515 GO:0042803
GO:0003735 GO:0019028 GO:0031514 GO:0019031 GO:0005840
GO:0005882 GO:0030286 GO:0005667 GO:0005622 GO:0005577
Cluster-22178.71491 GO:0008092 GO:0005737 GO:0019898
Cluster-22178.71494 GO:0042147
Cluster-22178.71495 GO:0006508 GO:0004181 GO:0003950 GO:0016787
GO:0008270 GO:0005634
Cluster-22178.71496 GO:0035556 GO:0009069 GO:0015994 GO:0016310
GO:0015996 GO:0055114 GO:0006468 GO:0004674 GO:0047134
GO:0005524 GO:0016491 GO:0016773 GO:0004672 GO:0047746
GO:0005515 GO:0016020
Cluster-22178.71497 GO:0006813 GO:0000184 GO:0005516 GO:0046872
GO:0005524 GO:0015269 GO:0008270 GO:0003924 GO:0016887
GO:0005525 GO:0016021
Cluster-22178.71500 GO:0008283 GO:0040007 GO:0007165 GO:0006954
GO:0006955 GO:0005149 GO:0005509 GO:0005515 GO:0045323
Cluster-22178.71503 GO:0016579 GO:0036459
Cluster-22178.71505 GO:0006814 GO:0008152 GO:0006744 GO:0006120
GO:0005975 GO:0019049 GO:0015992 GO:0055114 GO:0008137
GO:0030246 GO:0016836 GO:0003824
Cluster-22178.71506 GO:0055114 GO:0006355 GO:0019478 GO:0016491
GO:0043565 GO:0003676 GO:0003700 GO:0050662 GO:0016788
GO:0005515 GO:0005737 GO:0005667 GO:0016020
Cluster-22178.71507 GO:0006952 GO:0031492 GO:0005634 GO:0000785
Cluster-22178.71508 GO:0006554 GO:0006479 GO:0034968 GO:0008270
GO:0005515 GO:0003677 GO:0018024 GO:0005634
Cluster-22178.71510 GO:0007268 GO:0033130 GO:0043495
Cluster-22178.71511 GO:0005515
Cluster-22178.71514 GO:0043093 GO:0000917 GO:0006260 GO:0005737
GO:0045095
Cluster-22178.71515 GO:0006631 GO:0016746 GO:0005515
Cluster-22178.71517 GO:0006552 GO:0006550 GO:0006568 GO:0015940
GO:0055114 GO:0019521 GO:0006574 GO:0006631 GO:0018874

GO:0006098 GO:0006633 GO:0006554 GO:0003857 GO:0008677
GO:0004616 GO:0051287 GO:0016491
Cluster-22178.71518 GO:0007304 GO:0046872 GO:0005213 GO:0042600
GO:0005576
Cluster-22178.71521 GO:0016787 GO:0005515
Cluster-22178.71522 GO:0008146 GO:0016021
Cluster-22178.71523 GO:0042742 GO:0051191 GO:0006352 GO:0005515
GO:0005576 GO:0005669
Cluster-22178.71524 GO:0051191 GO:0006352 GO:0042742 GO:0005515
GO:0005669 GO:0005576
Cluster-22178.71525 GO:0006352 GO:0051191 GO:0042742 GO:0005515
GO:0005669 GO:0005576
Cluster-22178.71527 GO:0042742 GO:0051191 GO:0006352 GO:0005515
GO:0005576 GO:0005669
Cluster-22178.71528 GO:0055114 GO:0007186 GO:0046872 GO:0004930
GO:0016972 GO:0016021
Cluster-22178.71529 GO:0016310 GO:0006144 GO:0006206 GO:0006351
GO:0009069 GO:0006468 GO:0004674 GO:0016773 GO:0005524
GO:0004672 GO:0003899 GO:0003677 GO:0016020 GO:0005730
Cluster-22178.71530 GO:0008270
Cluster-22178.71532 GO:0008270
Cluster-22178.71533 GO:0007165 GO:0004872 GO:0016021
Cluster-22178.71534 GO:0003676
Cluster-22178.71536 GO:0005515
Cluster-22178.71537 GO:0046872
Cluster-22178.71538 GO:0035556 GO:0016787
Cluster-22178.71541 GO:0006450 GO:0005515
Cluster-22178.71542 GO:0007018 GO:0007017 GO:0003777 GO:0008017
GO:0005524 GO:0003677 GO:0005874 GO:0045298
Cluster-22178.71543 GO:0016012
Cluster-22178.71544 GO:0016012
Cluster-22178.71545 GO:0016012
Cluster-22178.71547 GO:0006144 GO:0006807 GO:0000256 GO:0004037

GO:0005634
Cluster-22178.71552 GO:0008063 GO:0030001 GO:0055085 GO:0006367
GO:0046873 GO:0005672 GO:0016020
Cluster-22178.71553 GO:0000398 GO:0008270 GO:0005515 GO:0005681
Cluster-22178.71557 GO:0042981 GO:0008270 GO:0005515
Cluster-22178.71558 GO:0006508 GO:0006355 GO:0005515 GO:0003676
GO:0004252
Cluster-22178.71559 GO:0005515
Cluster-22178.71560 GO:0016746
Cluster-22178.71561 GO:0007165 GO:0035091 GO:0005515
Cluster-22178.71564 GO:0017004 GO:0015886 GO:0016021
Cluster-22178.71565 GO:0006355 GO:0005515
Cluster-22178.71567 GO:0030036 GO:0003779 GO:0016021 GO:0030054
Cluster-22178.71571 GO:0007165 GO:0006468 GO:0005515 GO:0005524
GO:0004672
Cluster-22178.71576 GO:0048306
Cluster-22178.71577 GO:0005515 GO:0016787
Cluster-22178.71578 GO:0007186 GO:0007275 GO:0005515 GO:0004252
GO:0003676 GO:0070008 GO:0004930 GO:0042600
Cluster-22178.71579 GO:0006281 GO:0000077 GO:0030896 GO:0005634
Cluster-22178.71581 GO:0035091 GO:0020037 GO:0005524
Cluster-22178.71582 GO:0005515
Cluster-22178.71583 GO:0007017 GO:0005875
Cluster-22178.71584 GO:0006351 GO:0035556 GO:0005515
Cluster-22178.71586 GO:0006468 GO:0004672 GO:0005524
Cluster-22178.71587 GO:0006118 GO:0045454 GO:0015035 GO:0009055
GO:0005515 GO:0005634
Cluster-22178.71590 GO:0006811 GO:0006888 GO:0007165 GO:0006810
GO:0007172 GO:0006886 GO:0006468 GO:0004713 GO:0004871
GO:0005524 GO:0004672 GO:0005230 GO:0030127 GO:0016020
GO:0005925
Cluster-22178.71592 GO:0006886 GO:0016192 GO:0005515 GO:0005198
GO:0030117

Cluster-22178.71594 GO:0043087 GO:0044419 GO:0035023 GO:0005089
GO:0005515 GO:0005887
Cluster-22178.71598 GO:0006874 GO:0006816 GO:0005219 GO:0003676
GO:0008270 GO:0016021 GO:0005622
Cluster-22178.71600 GO:0006298 GO:0006308 GO:0010506 GO:0006260
GO:0030983 GO:0008855 GO:0005524 GO:0005634 GO:0009318
GO:0005615
Cluster-22178.71603 GO:0051289 GO:0007264 GO:0006355 GO:0008140
GO:0003700 GO:0043565 GO:0005096 GO:0005667
Cluster-22178.71607 GO:0035434 GO:0006825 GO:0046983 GO:0005375
GO:0016021
Cluster-22178.71609 GO:0006468 GO:0006118 GO:0051536 GO:0005524
GO:0004672 GO:0009055
Cluster-22178.71613 GO:0006355 GO:0042967 GO:0046854 GO:0016787
GO:0004402 GO:0003712 GO:0008270 GO:0005667 GO:0005634
GO:0019033 GO:0000123
Cluster-22178.71614 GO:0006351 GO:0009297 GO:0016567 GO:0006144
GO:0006464 GO:0006206 GO:0003899 GO:0005515 GO:0003723
GO:0016151 GO:0046872 GO:0061630 GO:0004842 GO:0008270
GO:0005634 GO:0019867 GO:0005730 GO:0005680
Cluster-22178.71616 GO:0031224
Cluster-22178.71617 GO:0031224
Cluster-22178.71620 GO:0010508 GO:0006351 GO:0006144 GO:0006914
GO:0006206 GO:0005515 GO:0003899 GO:0003677 GO:0008191
GO:0005730
Cluster-22178.71622 GO:0055085 GO:0034314 GO:0030833 GO:0006821
GO:0006364 GO:0005247 GO:0015629 GO:0005730 GO:0016020
GO:0005885
Cluster-22178.71624 GO:0006886 GO:0005543
Cluster-22178.71625 GO:0006810 GO:0055085 GO:0030150 GO:0015979
GO:0005515 GO:0005215 GO:0016020 GO:0005742 GO:0009523
GO:0009539
Cluster-22178.71626 GO:0016021

Cluster-22178.71628 GO:0005515

Cluster-22178.71632 GO:0006183 GO:0006241 GO:0006165 GO:0006144 GO:0006206 GO:0006228 GO:0004550

Cluster-22178.71633 GO:0006241 GO:0006183 GO:0006206 GO:0006228 GO:0006144 GO:0006165 GO:0004550

Cluster-22178.71634 GO:0006352 GO:0005634

Cluster-22178.71635 GO:0005515

Cluster-22178.71637 GO:0006886 GO:0016192 GO:0006355 GO:0003700 GO:0030131 GO:0030117 GO:0005667

Cluster-22178.71641 GO:0006850 GO:0005743

Cluster-22178.71644 GO:0006468 GO:0004672 GO:0005524 GO:0003677 GO:0005515

Cluster-22178.71645 GO:0006468 GO:0016817 GO:0004672 GO:0005524 GO:0046872

Cluster-22178.71646 GO:0006468 GO:0046872 GO:0004672 GO:0005524 GO:0016817

Cluster-22178.71647 GO:0005643

Cluster-22178.71650 GO:0019062 GO:0030521 GO:0007165 GO:0006355 GO:0005496 GO:0004882 GO:0003677 GO:0005634 GO:0019028

Cluster-22178.71655 GO:0006351 GO:0030036 GO:0008033 GO:0003677 GO:0003779 GO:0046872 GO:0030054 GO:0005634

Cluster-22178.71656 GO:0006468 GO:0005524 GO:0004672

Cluster-22178.71659 GO:0030036 GO:0008033 GO:0006351 GO:0003779 GO:0046872 GO:0003677 GO:0030054 GO:0005634

Cluster-22178.71661 GO:0000226 GO:0051537 GO:0015630 GO:0043231

Cluster-22178.71662 GO:0000226 GO:0051537 GO:0015630 GO:0043231

Cluster-22178.71663 GO:0016192 GO:0006886 GO:0005515 GO:0000775 GO:0030117

Cluster-22178.71665 GO:0006355 GO:0006281 GO:0019079 GO:0006310 GO:0002949 GO:0003723 GO:0016301 GO:0008134 GO:0043531 GO:0003678 GO:0009378 GO:0098519 GO:0005524 GO:0016887 GO:0003724 GO:0005667 GO:0009379 GO:0005657

Cluster-22178.71668 GO:0019722 GO:0035091 GO:0005515 GO:0003676

GO:0016021 GO:0005887
Cluster-22178.71669 GO:0005515 GO:0042302 GO:0005549
Cluster-22178.71670 GO:0042302 GO:0005515 GO:0005549
Cluster-22178.71671 GO:0042302 GO:0005515 GO:0005549
Cluster-22178.71673 GO:0009306 GO:0035556 GO:0051090 GO:0005515
GO:0046872 GO:0005634
Cluster-22178.71676 GO:0043039 GO:0006531 GO:0006522 GO:0006419
GO:0051301 GO:0005515 GO:0000166 GO:0003677 GO:0005524
GO:0016876 GO:0004813
Cluster-22178.71679 GO:0005975 GO:0004553
Cluster-22178.71680 GO:0006465 GO:0006355 GO:0003676 GO:0008233
GO:0016021 GO:0005622 GO:0005787
Cluster-22178.71681 GO:0006813 GO:0003676 GO:0003677 GO:0016787
GO:0005267 GO:0005524 GO:0016020
Cluster-22178.71688 GO:0034453 GO:0000042 GO:0005515 GO:0005815
GO:0016021
Cluster-22178.71689 GO:0006810 GO:0006334 GO:0003676 GO:0005634
GO:0005643
Cluster-22178.71690 GO:0035556 GO:0005515
Cluster-22178.71691 GO:0003677 GO:0046983
Cluster-22178.71692 GO:0005634
Cluster-22178.71695 GO:0006308 GO:0008855 GO:0009318
Cluster-22178.71697 GO:0000226 GO:0000166 GO:0046872 GO:0015630
GO:0016020
Cluster-22178.71701 GO:0005515
Cluster-22178.71702 GO:0042254 GO:0006412 GO:0003735 GO:0005840
Cluster-22178.71703 GO:0006886 GO:0017119
Cluster-22178.71704 GO:0035023 GO:0016310 GO:0043087 GO:0007165
GO:0006468 GO:0009069 GO:0005089 GO:0004674 GO:0005096
Cluster-22178.71705 GO:0000226 GO:0000166 GO:0046872 GO:0016020
GO:0015630
Cluster-22178.71706 GO:0000226 GO:0000166 GO:0046872 GO:0016020
GO:0015630

Cluster-22178.71707 GO:0007010 GO:0006810 GO:0030001 GO:0006260
GO:0000226 GO:0055085 GO:0003677 GO:0005515 GO:0042626
GO:0046872 GO:0005524 GO:0003779 GO:0015630 GO:0005664
GO:0016021
Cluster-22178.71708 GO:0006471 GO:0003950
Cluster-22178.71710 GO:0008152 GO:0006886 GO:0016192 GO:0003824
GO:0005515 GO:0030117
Cluster-22178.71713 GO:0006355 GO:0003676 GO:0005515 GO:0005634
GO:0019028
Cluster-22178.71714 GO:0007165 GO:0007186 GO:0007264 GO:0006013
GO:0006000 GO:0003873 GO:0005525 GO:0004871 GO:0005125
GO:0005524 GO:0031683 GO:0019001 GO:0003924
Cluster-22178.71717 GO:0007165 GO:0008283 GO:0040007 GO:0008083
GO:0005515 GO:0005125 GO:0016021
Cluster-22178.71720 GO:0006281 GO:0006144 GO:0006464 GO:0046034
GO:0004017 GO:0003677 GO:0046872 GO:0008270 GO:0016151
Cluster-22178.71721 GO:0006355 GO:0009271
Cluster-22178.71723 GO:0007339 GO:0005576
Cluster-22178.71724 GO:0042710 GO:0007186 GO:0004930 GO:0016021
Cluster-22178.71729 GO:0006281 GO:0046872 GO:0008270
Cluster-22178.71732 GO:0003824 GO:0005886
Cluster-22178.71733 GO:0008063 GO:0006813 GO:0006814 GO:0007224
GO:0005515 GO:0008270 GO:0005890 GO:0016021
Cluster-22178.71735 GO:0006914 GO:0039693 GO:0006606 GO:0006508
GO:0006397 GO:0019784 GO:0003723 GO:0003682 GO:0005198
GO:0005737 GO:0000785 GO:0019031 GO:0019028 GO:0005634
Cluster-22178.71738 GO:0055114 GO:0015992 GO:0006118 GO:0006119
GO:0016491 GO:0051536 GO:0051537 GO:0008121
Cluster-22178.71741 GO:0019082 GO:0003677 GO:0005515 GO:0008270

Cluster-22178.71744 GO:0006355 GO:0005634
Cluster-22178.71746 GO:0006915 GO:0006508 GO:0006260 GO:0006974
GO:0003677 GO:0003887 GO:0004181 GO:0008270 GO:0042575

Cluster-22178.71747 GO:0006260 GO:0006974 GO:0006508 GO:0006915
GO:0004181 GO:0008270 GO:0003677 GO:0003887 GO:0042575
Cluster-22178.71748 GO:0005515
Cluster-22178.71750 GO:0015031 GO:0016021
Cluster-22178.71751 GO:0048309 GO:0016032 GO:0006355 GO:0006914
GO:0051028 GO:0051607 GO:0015684 GO:0003924 GO:0043565
GO:0003700 GO:0005525 GO:0005515 GO:0015093 GO:0016021
GO:0019033 GO:0005667
Cluster-22178.71752 GO:0007423 GO:0005212
Cluster-22178.71753 GO:0015979 GO:0030145 GO:0009523 GO:0016021
Cluster-22178.71758 GO:0006468 GO:0005524 GO:0004672 GO:0016773
GO:0016020
Cluster-22178.71759 GO:0006508 GO:0006281 GO:0006351 GO:0006144
GO:0006206 GO:0003723 GO:0004252 GO:0003899 GO:0005524
GO:0003724 GO:0005730
Cluster-22178.71761 GO:0005515
Cluster-22178.71764 GO:0008270 GO:0005515
Cluster-22178.71765 GO:0035091 GO:0020037
Cluster-22178.71767 GO:0008152 GO:0016226 GO:0008168 GO:0051536
GO:0005737
Cluster-22178.71768 GO:0017004 GO:0015886 GO:0004867 GO:0016021
Cluster-22178.71769 GO:0015886 GO:0017004 GO:0004867 GO:0016021
Cluster-22178.71773 GO:0008033 GO:0055114 GO:0006769 GO:0046497
GO:0006813 GO:0016491 GO:0008556 GO:0008750 GO:0050660
GO:0051287 GO:0071949 GO:0030976 GO:0050661 GO:0000287
GO:0016779 GO:0005886 GO:0016021
Cluster-22178.71774 GO:0003924 GO:0005525
Cluster-22178.71775 GO:0019051 GO:0006357 GO:0001104 GO:0016592
GO:0042025
Cluster-22178.71779 GO:0042967 GO:0006950 GO:0006355 GO:0016573
GO:0006457 GO:0005524 GO:0004402 GO:0051082 GO:0000123
Cluster-22178.71780 GO:0008270 GO:0005525 GO:0005634
Cluster-22178.71785 GO:0006464 GO:0051082 GO:0031072 GO:0016151

GO:0005515
Cluster-22178.71786 GO:0006464 GO:0005515 GO:0031072 GO:0016151
GO:0051082
Cluster-22178.71790 GO:0006771 GO:0006164 GO:0006144 GO:0019497
GO:0006807 GO:0050830 GO:0005515 GO:0046872 GO:0031423
GO:0004643 GO:0003937 GO:0003993 GO:0005576 GO:0019012
GO:0042720
Cluster-22178.71791 GO:0005515 GO:0008270 GO:0005783
Cluster-22178.71792 GO:0008270 GO:0005515 GO:0005783
Cluster-22178.71793 GO:0009253 GO:0006807 GO:0009252 GO:0016783
GO:0008745
Cluster-22178.71795 GO:0006123 GO:0015992 GO:0004129 GO:0045277
Cluster-22178.71797 GO:0006123 GO:0015992 GO:0004129 GO:0045277
Cluster-22178.71799 GO:0008270 GO:0016020
Cluster-22178.71802 GO:0005515
Cluster-22178.71803 GO:0007186 GO:0007165 GO:0007264 GO:0004871
GO:0005525 GO:0003924 GO:0031683 GO:0019001
Cluster-22178.71804 GO:0007264 GO:0007186 GO:0007165 GO:0003924
GO:0031683 GO:0019001 GO:0004871 GO:0005525 GO:0030976
Cluster-22178.71807 GO:0006412 GO:0042254 GO:0003735 GO:0005622
GO:0005840
Cluster-22178.71808 GO:0018279 GO:0004579 GO:0016021 GO:0008250
Cluster-22178.71809 GO:0006520 GO:0007291 GO:0016743 GO:0016597

Cluster-22178.71814 GO:0007094 GO:0042742 GO:0016021
Cluster-22178.71820 GO:0007165 GO:0005515 GO:0005096
Cluster-22178.71822 GO:0006270 GO:0015684 GO:0007165 GO:0007186
GO:0008283 GO:0007264 GO:0019068 GO:0035556 GO:0043087
GO:0006576 GO:0040007 GO:0035023 GO:0016757 GO:0005089
GO:0005524 GO:0015093 GO:0016887 GO:0008083 GO:0005198
GO:0005515 GO:0005525 GO:0004871 GO:0031683 GO:0019001
GO:0046872 GO:0003924 GO:0016020 GO:0016021
Cluster-22178.71825 GO:0006874 GO:0006816 GO:0006270 GO:0006810

GO:0004190 GO:0005246 GO:0042030 GO:0005219 GO:0016020
GO:0005622 GO:0016021
Cluster-22178.71827 GO:0016021
Cluster-22178.71830 GO:0006470 GO:0006570 GO:0004725
Cluster-22178.71831 GO:0006342 GO:0006476 GO:0006351 GO:0006355
GO:0006807 GO:0006144 GO:0006206 GO:0030976 GO:0003899
GO:0000287 GO:0008270 GO:0070403 GO:0016811 GO:0003677
GO:0051287 GO:0017136 GO:0000118 GO:0005730
Cluster-22178.71834 GO:0055114 GO:0008152 GO:0016491
Cluster-22178.71836 GO:0055114 GO:0008152 GO:0016491
Cluster-22178.71838 GO:0005515
Cluster-22178.71844 GO:0030683 GO:0008092 GO:0005524 GO:0003676
GO:0005737 GO:0016021 GO:0019898 GO:0019012
Cluster-22178.71846 GO:0009405 GO:0005515 GO:0005576
Cluster-22178.71848 GO:0006355 GO:0003700 GO:0003677 GO:0043565
GO:0008270 GO:0005667
Cluster-22178.71851 GO:0007186 GO:0004930 GO:0016021
Cluster-22178.71854 GO:0016579 GO:0036459
Cluster-22178.71855 GO:0006281 GO:0031412 GO:0006260 GO:0006144
GO:0005515 GO:0003968 GO:0003911 GO:0046872 GO:0008270
GO:0031411 GO:0031379
Cluster-22178.71857 GO:0003333 GO:0009847 GO:0006508 GO:0004185
GO:0016021
Cluster-22178.71858 GO:0043531 GO:0016887 GO:0005524
Cluster-22178.71860 GO:0005515
Cluster-22178.71861 GO:0006281 GO:0009432 GO:0003677 GO:0003824
GO:0003697 GO:0005524 GO:0033588
Cluster-22178.71862 GO:0006888 GO:0042254 GO:0006886 GO:0006412
GO:0003824 GO:0008270 GO:0003735 GO:0005840 GO:0005622
GO:0030127
Cluster-22178.71863 GO:0055114 GO:0005975 GO:0043085 GO:0006120
GO:0006814 GO:0000902 GO:0015992 GO:0006744 GO:0000290
GO:0042773 GO:0008047 GO:0005525 GO:0005515 GO:0008137

GO:0016773 GO:0016787 GO:0016020
Cluster-22178.71864 GO:0031072 GO:0051082
Cluster-22178.71865 GO:0055114 GO:0009308 GO:0048038 GO:0005507
GO:0031072 GO:0008131 GO:0051082
Cluster-22178.71867 GO:0016567 GO:0008270 GO:0046872 GO:0005515
GO:0061630 GO:0004842 GO:0005622
Cluster-22178.71868 GO:0006144 GO:0046034 GO:0006508 GO:0006810
GO:0005524 GO:0046872 GO:0042626 GO:0004190 GO:0004017
GO:0016021
Cluster-22178.71869 GO:0003676
Cluster-22178.71870 GO:0016310 GO:0009069 GO:0006468 GO:0004674
GO:0004672 GO:0005524
Cluster-22178.71871 GO:0009069 GO:0006468 GO:0016310 GO:0004672
GO:0005524 GO:0004674
Cluster-22178.71874 GO:0042802 GO:0005515
Cluster-22178.71876 GO:0006355 GO:0006118 GO:0006119 GO:0055114
GO:0015992 GO:0008152 GO:0050662 GO:0008121 GO:0003677
GO:0016491 GO:0003824
Cluster-22178.71877 GO:0005515 GO:0005509
Cluster-22178.71878 GO:0006468 GO:0009069 GO:0006915 GO:0016310
GO:0006919 GO:0004674 GO:0005524 GO:0004672 GO:0016020
GO:0005739
Cluster-22178.71887 GO:0055085 GO:0016021
Cluster-22178.71900 GO:0051259 GO:0005515 GO:0034618
Cluster-22178.71901 GO:0005515 GO:0008270
Cluster-22178.71904 GO:0006468 GO:0006351 GO:0006206 GO:0006144
GO:0016787 GO:0003677 GO:0003899 GO:0020037 GO:0046872
GO:0004672 GO:0005524 GO:0003676 GO:0005730
Cluster-22178.71908 GO:0043087 GO:0007264 GO:0005085
Cluster-22178.71909 GO:0022900 GO:0005747 GO:0019031 GO:0005739
Cluster-22178.71910 GO:0007131 GO:0009405 GO:0007283 GO:0007130
GO:0008270 GO:0016020
Cluster-22178.71911 GO:0007165 GO:0030168 GO:0051258 GO:0005102

GO:0030674 GO:0005577
Cluster-22178.71914 GO:0008283 GO:0040007 GO:0007165 GO:0008083
GO:0005615
Cluster-22178.71916 GO:0046872
Cluster-22178.71917 GO:0051607 GO:0070206 GO:0006606 GO:0009405
GO:0005515 GO:0019028 GO:0016020
Cluster-22178.71918 GO:0006355 GO:0007186 GO:0007165 GO:0055114
GO:0019337 GO:0006807 GO:0009399 GO:0005515 GO:0003677
GO:0004872 GO:0016163 GO:0004930 GO:0016610 GO:0016020
GO:0016021
Cluster-22178.71919 GO:0006914 GO:0006886 GO:0010508 GO:0008092
GO:0050840 GO:0004190 GO:0004252 GO:0005737 GO:0005886
GO:0016021 GO:0019898 GO:0005576 GO:0016020 GO:0005783
Cluster-22178.71924 GO:0006265 GO:0005524 GO:0003916 GO:0003677
GO:0005694
Cluster-22178.71926 GO:0046034 GO:0006144 GO:0008033 GO:0006464
GO:0051252 GO:0016151 GO:0046872 GO:0004017 GO:0004540
GO:0016787
Cluster-22178.71929 GO:0005515
Cluster-22178.71930 GO:0006000 GO:0006013 GO:0016255 GO:0007264
GO:0007186 GO:0007165 GO:0003924 GO:0019001 GO:0031683
GO:0005524 GO:0004871 GO:0005525 GO:0003873 GO:0042765
Cluster-22178.71932 GO:0006355 GO:0016747
Cluster-22178.71933 GO:0000917 GO:0015876 GO:0043093 GO:0008521
GO:0016021 GO:0005737
Cluster-22178.71937 GO:0016747
Cluster-22178.71939 GO:0007264 GO:0005525
Cluster-22178.71941 GO:0006511 GO:0046872 GO:0005515
Cluster-22178.71942 GO:0006367 GO:0005672
Cluster-22178.71943 GO:0006265 GO:0003677 GO:0005515 GO:0046872
GO:0008270 GO:0003916 GO:0005694
Cluster-22178.71944 GO:0006952 GO:0004869 GO:0005576
Cluster-22178.71945 GO:0008270 GO:0005515

Cluster-22178.71950 GO:0006388 GO:0051252 GO:0000213 GO:0000214
Cluster-22178.71954 GO:0000902 GO:0006955 GO:0006355 GO:0051087
GO:0003700 GO:0043565 GO:0016020 GO:0005667
Cluster-22178.71955 GO:0006955 GO:0000902 GO:0006355 GO:0051087
GO:0003700 GO:0043565 GO:0016020 GO:0005667
Cluster-22178.71956 GO:0008270 GO:0003676
Cluster-22178.71958 GO:0007005 GO:0003676 GO:0008270 GO:0031305
Cluster-22178.71960 GO:0009405 GO:0005634 GO:0005576
Cluster-22178.71961 GO:0019051 GO:0042025
Cluster-22178.71962 GO:0042254 GO:0006412 GO:0046872 GO:0008270
GO:0005515 GO:0061630 GO:0003735 GO:0005840
Cluster-22178.71964 GO:0016579 GO:0007186 GO:0036459 GO:0004930
GO:0016020
Cluster-22178.71965 GO:0008270
Cluster-22178.71966 GO:0006355 GO:0006352 GO:0003700 GO:0016987
GO:0003677 GO:0005667
Cluster-22178.71968 GO:0016310 GO:0004673 GO:0009365
Cluster-22178.71970 GO:0006355 GO:0003700 GO:0005667
Cluster-22178.71972 GO:0019064 GO:0008053 GO:0055085 GO:0006415
GO:0007165 GO:0071973 GO:0016567 GO:0030155 GO:0006935
GO:0006308 GO:0045995 GO:0030334 GO:0006265 GO:0006449
GO:0030001 GO:0031386 GO:0003747 GO:0008855 GO:0003918
GO:0005102 GO:0046873 GO:0003774 GO:0003677 GO:0003924
GO:0016881 GO:0046789 GO:0005198 GO:0018444 GO:0019031
GO:0005840 GO:0016021 GO:0019028 GO:0009318 GO:0009986
GO:0005741 GO:0016020 GO:0009288
Cluster-22178.71974 GO:0007018 GO:0007017 GO:0008017 GO:0003777
GO:0005524 GO:0030430 GO:0045298 GO:0005874
Cluster-22178.71975 GO:0006810 GO:0009264 GO:0005515 GO:0008253
GO:0004386 GO:0003676 GO:0005524 GO:0046872 GO:0043531
GO:0016020
Cluster-22178.71980 GO:0055114 GO:0005515 GO:0005198 GO:0016616
GO:0051287

Cluster-22178.71983 GO:0006355 GO:0005515 GO:0005634

Cluster-22178.71984 GO:0015031 GO:0008565 GO:0005515 GO:0016020 GO:0016021

Cluster-22178.71989 GO:0006457 GO:0005509

Cluster-22178.71993 GO:0006810 GO:0005515 GO:0016021

Cluster-22178.71994 GO:0003677 GO:0005634

Cluster-22178.71995 GO:0045454

Cluster-22178.71996 GO:0019370 GO:0008270 GO:0008237

Cluster-22178.71997 GO:0009878 GO:0046872

Cluster-22178.72001 GO:0016462 GO:0005737

Cluster-22178.72002 GO:0006810 GO:0006508 GO:0006816 GO:0005246 GO:0004222 GO:0042030 GO:0008168 GO:0016020

Cluster-22178.72003 GO:0055114 GO:0008033 GO:0006355 GO:0050660 GO:0005507 GO:0003700 GO:0016491 GO:0003677 GO:0071949 GO:0005667 GO:0005634

Cluster-22178.72004 GO:0006355 GO:0006810 GO:0006614 GO:0019079 GO:0016787 GO:0008026 GO:0003677 GO:0005524 GO:0016887 GO:0005525 GO:0003676 GO:0004386

Cluster-22178.72006 GO:0006468 GO:0005524 GO:0004672 GO:0005515

Cluster-22178.72012 GO:0007155 GO:0006606 GO:0051276 GO:0000166 GO:0003677 GO:0005515 GO:0003774 GO:0005524 GO:0016459 GO:0005694

Cluster-22178.72013 GO:0006260 GO:0006284 GO:0003906 GO:0016799

Cluster-22178.72015 GO:0007155 GO:0006816 GO:0006810 GO:0001503 GO:0042998 GO:0005246 GO:0071253 GO:0042030 GO:0019028 GO:0005802 GO:0005576 GO:0016020 GO:0016021

Cluster-22178.72017 GO:0046034 GO:0006144 GO:0006355 GO:0008270 GO:0046872 GO:0003677 GO:0004017 GO:0003682 GO:0000775 GO:0000785 GO:0005634

Cluster-22178.72019 GO:0033588

Cluster-22178.72022 GO:0019491 GO:0033990 GO:0016021

Cluster-22178.72025 GO:0006886 GO:0016192 GO:0005515 GO:0005198
GO:0003824 GO:0030126 GO:0016020 GO:0016021
Cluster-22178.72028 GO:0007165 GO:0005509 GO:0005578
Cluster-22178.72029 GO:0003676
Cluster-22178.72030 GO:0006468 GO:0016773 GO:0005524 GO:0004672
GO:0016020
Cluster-22178.72034 GO:0005515 GO:0003676 GO:0030250 GO:0005576
Cluster-22178.72041 GO:0005783
Cluster-22178.72047 GO:0008152 GO:0008033 GO:0009451 GO:0006886
GO:0016192 GO:0007165 GO:0006400 GO:0008168 GO:0004809
GO:0046872 GO:0016301 GO:0005515 GO:0003723 GO:0008176
GO:0016021 GO:0030117
Cluster-22178.72050 GO:0007264 GO:0005525
Cluster-22178.72052 GO:0006470 GO:0008152 GO:0030145 GO:0000287
GO:0005525 GO:0003824 GO:0004721 GO:0003924 GO:0016491
Cluster-22178.72053 GO:0008152 GO:0016846 GO:0016491 GO:0016021
Cluster-22178.72054 GO:0007219 GO:0007423 GO:0005739
Cluster-22178.72055 GO:0007165 GO:0015948 GO:0040007 GO:0046656
GO:0008283 GO:0043087 GO:0008083 GO:0030269 GO:0005125
GO:0016020 GO:0005576 GO:0016021
Cluster-22178.72056 GO:0006470 GO:0006270 GO:0003924 GO:0005525
GO:0004721 GO:0003824 GO:0000287 GO:0004190 GO:0030145
GO:0016021
Cluster-22178.72059 GO:0006355 GO:0005515 GO:0005737 GO:0005634
Cluster-22178.72061 GO:0022904 GO:0016651 GO:0005743
Cluster-22178.72062 GO:0006351 GO:0005515
Cluster-22178.72065 GO:0006505 GO:0006448 GO:0006508 GO:0006629
GO:0006886 GO:0015996 GO:0001514 GO:0008152 GO:0015994
GO:0016787 GO:0003746 GO:0003723 GO:0008236 GO:0016788
GO:0047746 GO:0005525 GO:0005576 GO:0005737 GO:0005840
Cluster-22178.72073 GO:0009405 GO:0055085 GO:0015689 GO:0022857
GO:0015098 GO:0016021
Cluster-22178.72074 GO:0005515

Cluster-22178.72075 GO:0006355 GO:0007067 GO:0005515 GO:0005634 GO:0072686 GO:0005737 GO:0042729

Cluster-22178.72077 GO:0008270 GO:0046332 GO:0046872 GO:0005201 GO:0005578

Cluster-22178.72078 GO:0006506 GO:0016021

Cluster-22178.72080 GO:0015991 GO:0015992 GO:0015078 GO:0033177

Cluster-22178.72081 GO:0006448 GO:0006414 GO:0003746 GO:0005840 GO:0005622

Cluster-22178.72082 GO:0007166 GO:0007165 GO:0004871 GO:0005634

Cluster-22178.72083 GO:0015992 GO:0055114 GO:0006118 GO:0006119 GO:0008121

Cluster-22178.72084 GO:0051607 GO:0006891 GO:0007049 GO:0043093 GO:0000390 GO:0000917 GO:0006886 GO:0005737 GO:0030173 GO:0016021 GO:0005801 GO:0005783 GO:0016020 GO:0071008

Cluster-22178.72085 GO:0006810 GO:0005794

Cluster-22178.72087 GO:0003824

Cluster-22178.72088 GO:0003824

Cluster-22178.72089 GO:0005515 GO:0016021 GO:0019013

Cluster-22178.72092 GO:0006144 GO:0007165 GO:0004114 GO:0005515

Cluster-22178.72095 GO:0005515 GO:0043565

Cluster-22178.72096 GO:0005515

Cluster-22178.72097 GO:0006355 GO:0005515 GO:0019031

Cluster-22178.72099 GO:0006810 GO:0006415 GO:0055085 GO:0034508 GO:0006355 GO:0006449 GO:0055114 GO:0003700 GO:0005215 GO:0016149 GO:0016020 GO:0018444 GO:0005667 GO:0000776 GO:0005840 GO:0005737

Cluster-22178.72101 GO:0055114 GO:0050660 GO:0016491

Cluster-22178.72104 GO:0042438 GO:0007186 GO:0007165 GO:0015684 GO:0007264 GO:0030036 GO:0015093 GO:0098519 GO:0004871 GO:0005507 GO:0005525 GO:0003924 GO:0003779 GO:0019001 GO:0031683 GO:0016021 GO:0009295 GO:0030054

Cluster-22178.72108 GO:0006820 GO:0016021

Cluster-22178.72111 GO:0003723
Cluster-22178.72113 GO:0046872
Cluster-22178.72114 GO:0055114 GO:0006281 GO:0008270 GO:0046872
GO:0005515 GO:0047134 GO:0005634
Cluster-22178.72115 GO:0006269 GO:0006351 GO:0046872 GO:0003677
GO:0003896 GO:0005730 GO:0005657
Cluster-22178.72116 GO:0015992 GO:0006123 GO:0003774 GO:0004129
GO:0005198 GO:0045277 GO:0016459 GO:0005882 GO:0005740
Cluster-22178.72117 GO:0008152 GO:0019521 GO:0055114 GO:0007005
GO:0006098 GO:0016491 GO:0004616 GO:0031305
Cluster-22178.72121 GO:0006457 GO:0000413 GO:0007010 GO:0008017
GO:0003755 GO:0045298 GO:0005887
Cluster-22178.72122 GO:0006468 GO:0007165 GO:0005125 GO:0004672
GO:0005524 GO:0005515
Cluster-22178.72125 GO:0006118 GO:0009060 GO:0006123 GO:0015992
GO:0055114 GO:0020037 GO:0005506 GO:0009055 GO:0004129
GO:0045277 GO:0016021
Cluster-22178.72127 GO:0016032 GO:0006810 GO:0042167 GO:0055114
GO:0015994 GO:0004074 GO:0046872 GO:0008270 GO:0042597
GO:0016021
Cluster-22178.72128 GO:0055114 GO:0015994 GO:0006810 GO:0042167
GO:0004074 GO:0008270 GO:0042597 GO:0016021
Cluster-22178.72129 GO:0006355 GO:0043401 GO:0043565 GO:0008270
GO:0003700 GO:0005667 GO:0005634
Cluster-22178.72130 GO:0043401 GO:0009405 GO:0006355 GO:0005515
GO:0005198 GO:0003700 GO:0008270 GO:0043565 GO:0005634
GO:0005667 GO:0016021
Cluster-22178.72131 GO:0009405 GO:0007165 GO:0043401 GO:0006355
GO:0005515 GO:0005198 GO:0008201 GO:0003700 GO:0050750
GO:0043565 GO:0008270 GO:0005783 GO:0005634 GO:0005667
GO:0016021
Cluster-22178.72132 GO:0015031 GO:0015992 GO:0006820 GO:0006352
GO:0015991 GO:0046872 GO:0008565 GO:0015078 GO:0008509

GO:0005515 GO:0016021 GO:0005669 GO:0033179

Cluster-22178.72133 GO:0007049 GO:0006886 GO:0006605 GO:0046914

GO:0004867 GO:0008201 GO:0016021 GO:0005742 GO:0017053

Cluster-22178.72136 GO:0035091

Cluster-22178.72139 GO:0051301 GO:0007059 GO:0000444

Cluster-22178.72140 GO:0045895 GO:0007531 GO:0008301 GO:0005634

Cluster-22178.72141 GO:0016579 GO:0006276 GO:0005975 GO:0008657

GO:0004553 GO:0008270 GO:0036459 GO:0005096

Cluster-22178.72143 GO:0006508 GO:0007010 GO:0003779 GO:0008270

GO:0004222 GO:0031012

Cluster-22178.72156 GO:0006468 GO:0005515 GO:0004672 GO:0005524

GO:0016021 GO:0019012 GO:0042765

Cluster-22178.72165 GO:0016787

Cluster-22178.72169 GO:0005975 GO:0016758 GO:0016020

Cluster-22178.72170 GO:0051607

Cluster-22178.72176 GO:0042742 GO:0016301 GO:0005576

Cluster-22178.72181 GO:0007049

Cluster-22178.72182 GO:0051603 GO:0006511 GO:0004175 GO:0004298

GO:0005839 GO:0019773

Cluster-22178.72183 GO:0016573 GO:0007049

Cluster-22178.72184 GO:0007049 GO:0016573

Cluster-22178.72186 GO:0043087 GO:0035023 GO:0007165 GO:0030170

GO:0005057 GO:0005515 GO:0005089

Cluster-22178.72187 GO:0046413 GO:0046983 GO:0005515 GO:0018836

Cluster-22178.72188 GO:0006508 GO:0006144 GO:0017111 GO:0005515

GO:0004197 GO:0003968 GO:0016020 GO:0031379

Cluster-22178.72190 GO:0019079 GO:0006260 GO:0003677 GO:0016787

GO:0003676 GO:0003678 GO:0005524 GO:0005657

Cluster-22178.72192 GO:0005515 GO:0016021

Cluster-22178.72196 GO:0034220

Cluster-22178.72199 GO:0006813 GO:0000184 GO:0005242 GO:0005516

GO:0016286 GO:0046872 GO:0005524 GO:0015269 GO:0003924

GO:0016887	GO:0005525	GO:0008076	GO:0016021
Cluster-22178.72200	GO:0046486	GO:0006071	GO:0004371
Cluster-22178.72201	GO:0040007	GO:0015628	GO:0006810	GO:0006614
GO:0007165	GO:0008283	GO:0015031	GO:0008083	GO:0005515
GO:0003723	GO:0003676	GO:0005525	GO:0030145	GO:0016787
GO:0004386	GO:0016887	GO:0008565	GO:0005524	GO:0015627
Cluster-22178.72202	GO:0006313	GO:0004803	GO:0003677	GO:0046872

Cluster-22178.72203	GO:0006355	GO:0007059	GO:0051301	GO:0006457
GO:0007186	GO:0006869	GO:0042157	GO:0034080	GO:0035299
GO:0051082	GO:0003676	GO:0003700	GO:0008289	GO:0003677
GO:0004930	GO:0005524	GO:0005576	GO:0016021	GO:0000775
GO:0005667	GO:0000444	GO:0016272
Cluster-22178.72205	GO:0042254	GO:0006412	GO:0003676	GO:0003735
GO:0008270	GO:0005840	GO:0005622
Cluster-22178.72206	GO:0007186	GO:0005515	GO:0004930	GO:0016021
Cluster-22178.72207	GO:0048193	GO:0006914	GO:0006144	GO:0071988
GO:0006810	GO:0044419	GO:0007165	GO:0043093	GO:0000917
GO:0030474	GO:0006508	GO:0016747	GO:0004197	GO:0017111
GO:0003968	GO:0016301	GO:0005886	GO:0016020	GO:0031379
GO:0005737
Cluster-22178.72208	GO:0007264	GO:0005524	GO:0016787	GO:0005525
GO:0003677
Cluster-22178.72209	GO:0035556	GO:0046339	GO:0009395	GO:0007165
GO:0006629	GO:0004435	GO:0046935	GO:0005944
Cluster-22178.72211	GO:0046872	GO:0000166
Cluster-22178.72213	GO:0006355	GO:0009271
Cluster-22178.72215	GO:0006094	GO:0006098	GO:0006096	GO:0005982
GO:0005985	GO:0004347	GO:0005515
Cluster-22178.72217	GO:0005515
Cluster-22178.72223	GO:0016559	GO:0005515	GO:0005779
Cluster-22178.72224	GO:0045046	GO:0016579	GO:0032183	GO:0070140
GO:0036459	GO:0008270	GO:0032403	GO:0005779

Cluster-22178.72227 GO:0006090 GO:0045116 GO:0006525 GO:0071436 GO:0006814 GO:0006560 GO:0016881 GO:0005524 GO:0015081 GO:0008948 GO:0005634 GO:0016020
Cluster-22178.72229 GO:0042254 GO:0019058 GO:0006289 GO:0006284 GO:0006508 GO:0006281 GO:0006412 GO:0006397 GO:0003906 GO:0003684 GO:0003735 GO:0004197 GO:0008242 GO:0008270 GO:0003723 GO:0016740 GO:0016799 GO:0005622 GO:0000445 GO:0005840
Cluster-22178.72232 GO:0010506 GO:0005515 GO:0004222 GO:0005615
Cluster-22178.72235 GO:0006811 GO:0055085 GO:0006816 GO:0005262 GO:0005244 GO:0005515 GO:0016021 GO:0016020
Cluster-22178.72241 GO:0015031 GO:0016021
Cluster-22178.72243 GO:0006221 GO:0006541 GO:0006206 GO:0003883 GO:0016787
Cluster-22178.72246 GO:0006570 GO:0006470 GO:0004527 GO:0008138 GO:0004725 GO:0003677
Cluster-22178.72248 GO:0006334 GO:0030170 GO:0003723 GO:0005515 GO:0003677 GO:0000786 GO:0005634
Cluster-22178.72249 GO:0006364 GO:0005515 GO:0032040
Cluster-22178.72250 GO:0007165 GO:0005179 GO:0005576
Cluster-22178.72251 GO:0006260 GO:0046983 GO:0003887 GO:0003677 GO:0005515 GO:0042575
Cluster-22178.72252 GO:0006950 GO:0009415 GO:0006890 GO:0004252 GO:0016021 GO:0097197 GO:0001772
Cluster-22178.72253 GO:0006950 GO:0006890 GO:0009415 GO:0004252 GO:0001772 GO:0097197 GO:0016021
Cluster-22178.72254 GO:0045995 GO:0030334 GO:0046168 GO:0055114 GO:0007165 GO:0030155 GO:0005102 GO:0016616 GO:0051287
Cluster-22178.72255 GO:0044780 GO:0006816 GO:0007165 GO:0030155 GO:0030334 GO:0045995 GO:0005388 GO:0005102 GO:0016529 GO:0019028
Cluster-22178.72256 GO:0005515
Cluster-22178.72258 GO:0051603 GO:0006260 GO:0006888 GO:0003887

GO:0003677 GO:0004298 GO:0042575 GO:0005839 GO:0005801
GO:0005622
Cluster-22178.72262 GO:0006310 GO:0042138
Cluster-22178.72263 GO:0042138 GO:0006310
Cluster-22178.72264 GO:0042138 GO:0006310
Cluster-22178.72265 GO:0006310 GO:0042138
Cluster-22178.72267 GO:0006355 GO:0003700 GO:0003677 GO:0005667
Cluster-22178.72268 GO:0005515
Cluster-22178.72269 GO:0006606 GO:0006418 GO:0005524 GO:0043565
GO:0005515 GO:0004812 GO:0003723 GO:0005634
Cluster-22178.72270 GO:0006334 GO:0005515 GO:0003677 GO:0030170
GO:0003723 GO:0000786 GO:0005634
Cluster-22178.72271 GO:0006334 GO:0003677 GO:0005515 GO:0005634
GO:0000786
Cluster-22178.72274 GO:0030435 GO:0046872 GO:0031160
Cluster-22178.72277 GO:0006355 GO:0055114 GO:0008033 GO:0071949
GO:0003677 GO:0016491 GO:0050660 GO:0005507 GO:0003700
GO:0005667 GO:0005634
Cluster-22178.72279 GO:0048870 GO:0031514
Cluster-22178.72281 GO:0015992 GO:0006123 GO:0004129 GO:0005746
GO:0045277
Cluster-22178.72283 GO:0003676 GO:0005515
Cluster-22178.72290 GO:0006897 GO:0016020
Cluster-22178.72291 GO:0008589 GO:0007218 GO:0007517
Cluster-22178.72294 GO:0043565 GO:0005515 GO:0005634
Cluster-22178.72296 GO:0005515 GO:0043565 GO:0005634
Cluster-22178.72297 GO:0015031 GO:0016021
Cluster-22178.72300 GO:0044564 GO:0000723 GO:0006281 GO:0003678
GO:0005657
Cluster-22178.72301 GO:0005515
Cluster-22178.72302 GO:0045454 GO:0055114 GO:0016491 GO:0016209

Cluster-22178.72303 GO:0055114 GO:0045454 GO:0016209 GO:0016491

Cluster-22178.72309 GO:0055114 GO:0019530 GO:0046439 GO:0006886
GO:0006534 GO:0005506 GO:0017172
Cluster-22178.72310 GO:0003700 GO:0005515 GO:0005667
Cluster-22178.72311 GO:0005515
Cluster-22178.72313 GO:0003824
Cluster-22178.72315 GO:0042742
Cluster-22178.72319 GO:0006355 GO:0003676 GO:0008017 GO:0005524
GO:0008270 GO:0016818 GO:0003677 GO:0005515 GO:0016787
GO:0045298 GO:0005634
Cluster-22178.72320 GO:0016020
Cluster-22178.72323 GO:0015991 GO:0019290 GO:0006351 GO:0006144
GO:0006206 GO:0006826 GO:0016820 GO:0003899 GO:0005515
GO:0003677 GO:0015343 GO:0005730 GO:0000221
Cluster-22178.72324 GO:0006276 GO:0005515
Cluster-22178.72326 GO:0006355 GO:0005634
Cluster-22178.72327 GO:0006886 GO:0006605 GO:0005515 GO:0005742
GO:0016020
Cluster-22178.72330 GO:0007010 GO:0000413 GO:0006457 GO:0003755
GO:0008017 GO:0005887 GO:0045298
Cluster-22178.72334 GO:0042147 GO:0030682
Cluster-22178.72335 GO:0055114 GO:0006559 GO:0006570 GO:0042207
GO:0004411 GO:0005515
Cluster-22178.72336 GO:0016567 GO:0090263 GO:0043565
Cluster-22178.72338 GO:0090263 GO:0016567
Cluster-22178.72339 GO:0006144 GO:0006206 GO:0006289 GO:0006355
GO:0006351 GO:0003899 GO:0000439 GO:0005730
Cluster-22178.72341 GO:0006886 GO:0048280 GO:0048870 GO:0015031
GO:0008565 GO:0031514 GO:0016020 GO:0000139 GO:0005737
Cluster-22178.72343 GO:0010508 GO:0006355 GO:0006260 GO:0003677
GO:0003700 GO:0043565 GO:0005634 GO:0005667
Cluster-22178.72344 GO:0006260 GO:0006355 GO:0010508 GO:0043565
GO:0003700 GO:0003677 GO:0005634 GO:0005667

Cluster-22178.72345 GO:0007018 GO:0007017 GO:0008017 GO:0003777
GO:0005524 GO:0045298 GO:0005874 GO:0005576
Cluster-22178.72349 GO:0006118 GO:0050776 GO:0014068 GO:0007165
GO:0007275 GO:0006259 GO:0020037 GO:0003824 GO:0009055
GO:0043548 GO:0003677 GO:0005102 GO:0005524 GO:0016021
GO:0016020 GO:0005694
Cluster-22178.72350 GO:0006259 GO:0007165 GO:0007275 GO:0006118
GO:0014068 GO:0050776 GO:0043548 GO:0003677 GO:0005524
GO:0005102 GO:0020037 GO:0003824 GO:0009055 GO:0005694
GO:0016020 GO:0016021
Cluster-22178.72351 GO:0006810 GO:0005622
Cluster-22178.72352 GO:0003677 GO:0046872
Cluster-22178.72353 GO:0008643 GO:0055085 GO:0005351 GO:0000139
GO:0016021 GO:0016020
Cluster-22178.72354 GO:0003676 GO:0016021
Cluster-22178.72356 GO:0046912
Cluster-22178.72357 GO:0006171 GO:0006144 GO:0004016 GO:0000287

Cluster-22178.72358 GO:0009847 GO:0051252 GO:0016891 GO:0016021
Cluster-22178.72359 GO:0006508 GO:0003676 GO:0004190 GO:0003723

Cluster-22178.72362 GO:0019013
Cluster-22178.72363 GO:0006935 GO:0006355 GO:0071973 GO:0003774
GO:0046872 GO:0003677 GO:0009288 GO:0012511 GO:0016021
GO:0016020
Cluster-22178.72367 GO:0048870 GO:0070206 GO:0005639 GO:0031514
Cluster-22178.72369 GO:0016021
Cluster-22178.72371 GO:0007165 GO:0005515
Cluster-22178.72372 GO:0008152
Cluster-22178.72374 GO:0006357 GO:0001104 GO:0016592
Cluster-22178.72376 GO:0009116 GO:0006506 GO:0016746 GO:0042393
GO:0005634 GO:0016021 GO:0005789
Cluster-22178.72379 GO:0005509 GO:0005544

Cluster-22178.72380 GO:0005509 GO:0005544
Cluster-22178.72382 GO:0007275 GO:0034477 GO:0004518 GO:0005515 GO:0003700 GO:0046872 GO:0005667 GO:0005634
Cluster-22178.72383 GO:0003676
Cluster-22178.72384 GO:0043087
Cluster-22178.72386 GO:0005515
Cluster-22178.72389 GO:0042262 GO:0005509 GO:0003690
Cluster-22178.72390 GO:0016114 GO:0006694 GO:0055114 GO:0050660 GO:0004506 GO:0016021
Cluster-22178.72391 GO:0010506 GO:0005515 GO:0005615
Cluster-22178.72392 GO:0006468 GO:0046872 GO:0005524 GO:0004672

Cluster-22178.72394 GO:0006508 GO:0006364 GO:0008237 GO:0016805 GO:0005515 GO:0005730 GO:0005618
Cluster-22178.72395 GO:0006364 GO:0005515 GO:0005730
Cluster-22178.72396 GO:0006355 GO:0043565 GO:0003700 GO:0005667
Cluster-22178.72398 GO:0016021
Cluster-22178.72400 GO:0003676 GO:0005515
Cluster-22178.72401 GO:0006508 GO:0016032 GO:0000398 GO:0006260 GO:0004197 GO:0005634 GO:0000808
Cluster-22178.72402 GO:0042967 GO:0006537 GO:0008285 GO:0055114 GO:0007050 GO:0006355 GO:0006915 GO:0003712 GO:0008270 GO:0015930 GO:0004402 GO:0016638 GO:0000123 GO:0005667 GO:0005634
Cluster-22178.72403 GO:0042967 GO:0006355 GO:0016573 GO:0004402 GO:0003713 GO:0000123 GO:0005667 GO:0005634
Cluster-22178.72404 GO:0048193 GO:0009059 GO:0070273 GO:0030176
Cluster-22178.72407 GO:0046872 GO:0008270 GO:0005515
Cluster-22178.72408 GO:0005975 GO:0016832 GO:0005515 GO:0035091

Cluster-22178.72409 GO:0007186 GO:0007165 GO:0055114 GO:0048193 GO:0050661 GO:0003924 GO:0016491 GO:0019001 GO:0031683 GO:0004871 GO:0005783

Cluster-22178.72411 GO:0006413 GO:0006446 GO:0003723 GO:0003743 GO:0005737 GO:0005840
Cluster-22178.72412 GO:0009069 GO:0006468 GO:0016310 GO:0004672 GO:0005524 GO:0004674
Cluster-22178.72413 GO:0006468 GO:0005524 GO:0004672 GO:0016773 GO:0016020
Cluster-22178.72416 GO:0042254 GO:0007009 GO:0043565 GO:0005515 GO:0003735 GO:0051082 GO:0005840 GO:0005634
Cluster-22178.72419 GO:0008270
Cluster-22178.72420 GO:0006511 GO:0007005 GO:0045502 GO:0008134 GO:0042803 GO:0031305 GO:0005667 GO:0030286
Cluster-22178.72425 GO:0006769 GO:0055114 GO:0046497 GO:0070403 GO:0008750 GO:0030976 GO:0050661 GO:0000287 GO:0016021
Cluster-22178.72431 GO:0006355 GO:0009405 GO:0006397 GO:0006308 GO:0006302 GO:0006357 GO:0043087 GO:0006606 GO:0005515 GO:0001104 GO:0003700 GO:0008855 GO:0005634 GO:0009318 GO:0005667 GO:0016020 GO:0016592
Cluster-22178.72433 GO:0007049 GO:0009405 GO:0006486 GO:0006260 GO:0006270 GO:0007067 GO:0003980 GO:0042802 GO:0005515 GO:0003887 GO:0016757 GO:0046983 GO:0016021 GO:0000775 GO:0042575 GO:0005727 GO:0005634
Cluster-22178.72435 GO:0016579 GO:0008063 GO:0036459
Cluster-22178.72436 GO:0006446 GO:0006464 GO:0006144 GO:0007218 GO:0006413 GO:0006355 GO:0006506 GO:0005975 GO:0006808 GO:0046034 GO:0003677 GO:0004017 GO:0003743 GO:0008270 GO:0005524 GO:0004553 GO:0003723 GO:0030234 GO:0004519 GO:0005515 GO:0004672 GO:0046872 GO:0043565 GO:0016151 GO:0003700 GO:0003676 GO:0005667 GO:0005840 GO:0005789
Cluster-22178.72437 GO:0070140 GO:0032183
Cluster-22178.72438 GO:0008152 GO:0016491 GO:0046983
Cluster-22178.72439 GO:0008270
Cluster-22178.72442 GO:0006508 GO:0004198 GO:0005622
Cluster-22178.72445 GO:0007155 GO:0016021 GO:0016020

Cluster-22178.72446 GO:0006355 GO:0035023 GO:0007264 GO:0007186
GO:0007165 GO:0006614 GO:0006000 GO:0006013 GO:0043087
GO:0005515 GO:0003924 GO:0019001 GO:0031683 GO:0004871
GO:0005525 GO:0003873 GO:0005524 GO:0005089
Cluster-22178.72447 GO:0007186 GO:0006614 GO:0007165 GO:0007264
GO:0006000 GO:0006013 GO:0043087 GO:0006355 GO:0035023
GO:0003873 GO:0005089 GO:0005524 GO:0005515 GO:0004871
GO:0005525 GO:0003924 GO:0031683 GO:0019001
Cluster-22178.72449 GO:0071918 GO:0015840 GO:0015204 GO:0003676
GO:0016021
Cluster-22178.72451 GO:0006281 GO:0006355 GO:0090263 GO:0016567
GO:0006310 GO:0003678 GO:0043531 GO:0008134 GO:0003723
GO:0003724 GO:0016887 GO:0005524 GO:0003677 GO:0098519
GO:0016787 GO:0009378 GO:0005657 GO:0009379 GO:0005667
Cluster-22178.72452 GO:0015992 GO:0006281 GO:0003824 GO:0015078
GO:0008289 GO:0005515 GO:0033644 GO:0055036
Cluster-22178.72453 GO:0006355 GO:0009607 GO:0003677 GO:0005739
GO:0005747 GO:0016021
Cluster-22178.72455 GO:0006468 GO:0005515 GO:0004672 GO:0005524

Cluster-22178.72456 GO:0008270 GO:0046872
Cluster-22178.72457 GO:0006047
Cluster-22178.72459 GO:0006355 GO:0042157 GO:0007186 GO:0006869
GO:0034080 GO:0035299 GO:0003700 GO:0003676 GO:0008289
GO:0003677 GO:0005524 GO:0004930 GO:0005576 GO:0016021
GO:0000775 GO:0005667
Cluster-22178.72461 GO:0000917 GO:0006355 GO:0043093 GO:0003700
GO:0043565 GO:0005737 GO:0005667
Cluster-22178.72462 GO:0043093 GO:0006355 GO:0000917 GO:0043565
GO:0003700 GO:0005737 GO:0005794 GO:0005667
Cluster-22178.72463 GO:0000290 GO:0005975 GO:0003824 GO:0043169
GO:0016787
Cluster-22178.72466 GO:0007219 GO:0007275 GO:0030154 GO:0016021

Cluster-22178.72467 GO:0007264 GO:0007165 GO:0005515 GO:0005525

Cluster-22178.72470 GO:0033014 GO:0055114 GO:0008152 GO:0006783
GO:0015994 GO:0050662 GO:0004852 GO:0016491 GO:0003824
Cluster-22178.72473 GO:0000398 GO:0015979 GO:0003677 GO:0019013
GO:0009523 GO:0009507
Cluster-22178.72476 GO:0005198 GO:0005882
Cluster-22178.72477 GO:0042254 GO:0006412 GO:0005515 GO:0003735
GO:0005622 GO:0005840
Cluster-22178.72480 GO:0016021
Cluster-22178.72481 GO:0006508 GO:0004252
Cluster-22178.72482 GO:0007275 GO:0030001 GO:0006812 GO:0055085
GO:0046873 GO:0008324 GO:0016020 GO:0016021 GO:0042600
Cluster-22178.72485 GO:0006351 GO:0006886 GO:0006508 GO:0007155
GO:0006206 GO:0006888 GO:0006144 GO:0005515 GO:0003899
GO:0003968 GO:0004197 GO:0003677 GO:0017111 GO:0008270
GO:0031379 GO:0030127 GO:0016020 GO:0005730
Cluster-22178.72487 GO:0070588 GO:0006816 GO:0006508 GO:0005515
GO:0005509 GO:0005262 GO:0016020 GO:0019033
Cluster-22178.72488 GO:0007166 GO:0007165 GO:0004871 GO:0005634
Cluster-22178.72489 GO:0006606 GO:0006914 GO:0016021 GO:0031083
Cluster-22178.72491 GO:0035556 GO:0009405 GO:0046488 GO:0005198
GO:0005524 GO:0046872 GO:0016307
Cluster-22178.72496 GO:0051016 GO:0006260 GO:0007165 GO:0006310
GO:0051782 GO:0009432 GO:0006281 GO:0005524 GO:0003689
GO:0016887 GO:0003724 GO:0009378 GO:0098519 GO:0005509
GO:0003723 GO:0030479 GO:0005657 GO:0009379 GO:0009276
GO:0005578 GO:0005663 GO:0042575
Cluster-22178.72497 GO:0046486 GO:0006687 GO:0006027 GO:0006012
GO:0005975 GO:0005515 GO:0004565 GO:0046872 GO:0009341
Cluster-22178.72498 GO:0006744 GO:0055114 GO:0009271 GO:0016567
GO:0006355 GO:0004842 GO:0046872 GO:0008270 GO:0005515
Cluster-22178.72501 GO:0046872 GO:0000166

Cluster-22178.72503 GO:0006508 GO:0008236
Cluster-22178.72508 GO:0015979 GO:0005198 GO:0009522 GO:0016020
Cluster-22178.72509 GO:0055114 GO:0016117 GO:0006090 GO:0006099 GO:0006468 GO:0016491 GO:0004672 GO:0005524 GO:0008924 GO:0071949 GO:0016705
Cluster-22178.72510 GO:0055114 GO:0016117 GO:0006090 GO:0006099 GO:0016491 GO:0008924 GO:0071949 GO:0016705
Cluster-22178.72514 GO:0055085 GO:0006811 GO:0005216 GO:0045735 GO:0016020
Cluster-22178.72519 GO:0006412 GO:0042254 GO:0003735 GO:0005840 GO:0005622
Cluster-22178.72523 GO:0007165 GO:0035091 GO:0005515
Cluster-22178.72524 GO:0007031 GO:0000166 GO:0005779
Cluster-22178.72529 GO:0006464 GO:0051082 GO:0016151 GO:0031072

Cluster-22178.72530 GO:0008152 GO:0006614 GO:0005525 GO:0016301 GO:0005524 GO:0005737
Cluster-22178.72531 GO:0005515
Cluster-22178.72533 GO:0008146
Cluster-22178.72536 GO:0006276 GO:0015992 GO:0006123 GO:0004129 GO:0005743 GO:0045277
Cluster-22178.72541 GO:0004867 GO:0019031
Cluster-22178.72542 GO:0015016 GO:0008146 GO:0005515 GO:0016787

Cluster-22178.72543 GO:0006355 GO:0043565 GO:0008270 GO:0003700 GO:0005515 GO:0005667
Cluster-22178.72544 GO:0006412 GO:0042254 GO:0016491 GO:0003735 GO:0005622 GO:0005840
Cluster-22178.72546 GO:0007275 GO:0030154
Cluster-22178.72551 GO:0006012 GO:0005982 GO:0005985 GO:0004564 GO:0004575 GO:0017177
Cluster-22178.72554 GO:0008270
Cluster-22178.72555 GO:0006448 GO:0001514 GO:0003746 GO:0005525

GO:0003723 GO:0005515 GO:0005737 GO:0005840
Cluster-22178.72557 GO:0006869 GO:0051262 GO:0005975 GO:0015031
GO:0051082 GO:0005319 GO:0016758 GO:0016020
Cluster-22178.72558 GO:0006355 GO:0016579 GO:0036459 GO:0003700
GO:0005515 GO:0005667
Cluster-22178.72559 GO:0015991 GO:0006904 GO:0006397 GO:0000910
GO:0008608 GO:0007067 GO:0006914 GO:0015031 GO:0015992
GO:0000226 GO:0005198 GO:0015078 GO:0070009 GO:0008017
GO:0008239 GO:0005634 GO:0072686 GO:0019028 GO:0033179
GO:0042729 GO:0045298 GO:0019031 GO:0000445 GO:0000775
GO:0000145 GO:0005876
Cluster-22178.72561 GO:0005576
Cluster-22178.72565 GO:0005975 GO:0006811 GO:0007049 GO:0055085
GO:0007268 GO:0051260 GO:0007165 GO:0016773 GO:0004970
GO:0005216 GO:0005515 GO:0016020
Cluster-22178.72566 GO:0006269 GO:0006351 GO:0019825 GO:0005515
GO:0005198 GO:0020037 GO:0003677 GO:0003896 GO:0005634
GO:0005730 GO:0005657
Cluster-22178.72567 GO:0006468 GO:0005524 GO:0004672
Cluster-22178.72568 GO:0006468 GO:0005524 GO:0004672 GO:0016773
GO:0016020
Cluster-22178.72569 GO:0042981 GO:0005515
Cluster-22178.72570 GO:0006355 GO:0006629 GO:0043401 GO:0008270
GO:0043565 GO:0003700 GO:0005634 GO:0005667
Cluster-22178.72573 GO:0042578
Cluster-22178.72576 GO:0005515
Cluster-22178.72578 GO:0003779 GO:0005515 GO:0005622 GO:0016020
Cluster-22178.72579 GO:0008270
Cluster-22178.72582 GO:0005634
Cluster-22178.72586 GO:0055085 GO:0006811 GO:0005216 GO:0005515
GO:0012511 GO:0016021 GO:0016020
Cluster-22178.72587 GO:0003676 GO:0005515 GO:0003723 GO:0008270

Cluster-22178.72591 GO:0019087 GO:0005515 GO:0016021
Cluster-22178.72593 GO:0055114 GO:0006574 GO:0008152 GO:0006568
GO:0006552 GO:0046487 GO:0006550 GO:0009396 GO:0006554
GO:0006633 GO:0006631 GO:0018874 GO:0006298 GO:0003824
GO:0003857 GO:0016491 GO:0005524 GO:0050662 GO:0004488
Cluster-22178.72594 GO:0005525 GO:0003924
Cluster-22178.72597 GO:0003676
Cluster-22178.72601 GO:0051262 GO:0008219 GO:0005515 GO:0044212

Cluster-22178.72604 GO:0008152 GO:0046413 GO:0018836 GO:0005515
GO:0016491 GO:0016020
Cluster-22178.72608 GO:0016021
Cluster-22178.72609 GO:0006874 GO:0006816 GO:0019069 GO:0006260
GO:0006468 GO:0006811 GO:0005244 GO:0005524 GO:0000049
GO:0005219 GO:0005262 GO:0004672 GO:0016020 GO:0005815
GO:0005622 GO:0071203 GO:0016021
Cluster-22178.72614 GO:0006338 GO:0008152 GO:0016836 GO:0005515
GO:0003824 GO:0090544
Cluster-22178.72618 GO:0008608 GO:0050690 GO:0006418 GO:0043039
GO:0003676 GO:0005524 GO:0005537 GO:0000166 GO:0000049
GO:0004812 GO:0005737 GO:0042729 GO:0072686
Cluster-22178.72619 GO:0005515
Cluster-22178.72620 GO:0051252 GO:0007165 GO:0042254 GO:0006412
GO:0006355 GO:0003676 GO:0003735 GO:0004523 GO:0005840
GO:0005622
Cluster-22178.72623 GO:0071973 GO:0003774 GO:0009425
Cluster-22178.72624 GO:0008289
Cluster-22178.72627 GO:0007283 GO:0055114 GO:0016491
Cluster-22178.72628 GO:0051260 GO:0006468 GO:0005515 GO:0016773
GO:0005524 GO:0004672 GO:0016020 GO:0016021
Cluster-22178.72629 GO:0016884 GO:0016874
Cluster-22178.72631 GO:0006355 GO:0005515
Cluster-22178.72635 GO:0005515

Cluster-22178.72636 GO:0005515
Cluster-22178.72640 GO:0016567 GO:0006810 GO:0033014 GO:0006915
GO:0006783 GO:0015994 GO:0005515 GO:0004852 GO:0004842
GO:0005215 GO:0046872 GO:0008270 GO:0016020 GO:0005680
Cluster-22178.72642 GO:0018342 GO:0008318
Cluster-22178.72645 GO:0006571 GO:0000162 GO:0006414 GO:0009094
GO:0006448 GO:0006432 GO:0004826 GO:0005524 GO:0003746
GO:0000287 GO:0003723 GO:0005840 GO:0009328 GO:0005853
Cluster-22178.72646 GO:0009094 GO:0006414 GO:0006448 GO:0006571
GO:0000162 GO:0006432 GO:0005524 GO:0003746 GO:0004826
GO:0000287 GO:0003723 GO:0009328 GO:0005840 GO:0005853
Cluster-22178.72647 GO:0055114 GO:0006694 GO:0007165 GO:0015936
GO:0008158 GO:0050662 GO:0004420 GO:0016020
Cluster-22178.72648 GO:0006508 GO:0005515 GO:0004190
Cluster-22178.72650 GO:0006144 GO:0051262 GO:0006206 GO:0006351
GO:0046872 GO:0005515 GO:0003899 GO:0003677 GO:0044212
GO:0005634 GO:0005730
Cluster-22178.72651 GO:0006357 GO:0005515 GO:0001104 GO:0043565
GO:0016592 GO:0005634
Cluster-22178.72652 GO:0006576 GO:0006118 GO:0006811 GO:0007264
GO:0007186 GO:0007165 GO:0015684 GO:0055085 GO:0003924
GO:0031683 GO:0019001 GO:0004871 GO:0005525 GO:0009055
GO:0003682 GO:0005524 GO:0015093 GO:0015075 GO:0005634
GO:0000785 GO:0016021 GO:0016020
Cluster-22178.72653 GO:0008219 GO:0051082 GO:0005515 GO:0005198

Cluster-22178.72656 GO:0003723 GO:0003676
Cluster-22178.72657 GO:0006952 GO:0006810 GO:0009405 GO:0003723
GO:0033897 GO:0019855 GO:0005576
Cluster-22178.72660 GO:0006310 GO:0006281
Cluster-22178.72661 GO:0006281 GO:0008270 GO:0046872 GO:0005815
Cluster-22178.72663 GO:0055085 GO:0006012 GO:0009058 GO:0046486
GO:0042432 GO:0006027 GO:0016114 GO:0006355 GO:0006687

GO:0006468 GO:0004565 GO:0016773 GO:0016844 GO:0005524
GO:0004064 GO:0003700 GO:0004672 GO:0016020 GO:0005667
GO:0016021 GO:0009341
Cluster-22178.72665 GO:0006412 GO:0007264 GO:0042254 GO:0006614
GO:0006270 GO:0003723 GO:0043531 GO:0003924 GO:0005525
GO:0003735 GO:0098519 GO:0016887 GO:0005524 GO:0003724
GO:0005840 GO:0005622
Cluster-22178.72666 GO:0006351 GO:0016787 GO:0015016 GO:0003677
GO:0005634
Cluster-22178.72667 GO:0043087 GO:0035023 GO:0044419 GO:0005089
GO:0005515
Cluster-22178.72668 GO:0010215 GO:0016049 GO:0031225
Cluster-22178.72672 GO:0006355 GO:0006260 GO:0016787 GO:0043565
GO:0003700 GO:0005667
Cluster-22178.72673 GO:0006355 GO:0016787 GO:0043565 GO:0005515
GO:0003700 GO:0005667
Cluster-22178.72674 GO:0007165 GO:0035556
Cluster-22178.72676 GO:0006807 GO:0003933 GO:0070403 GO:0005524

Cluster-22178.72678 GO:0006401 GO:0008272 GO:0006310 GO:0006355
GO:0006281 GO:0016887 GO:0005524 GO:0015116 GO:0003724
GO:0009378 GO:0003678 GO:0003723 GO:0004519 GO:0008134
GO:0005657 GO:0005667 GO:0009379 GO:0016021
Cluster-22178.72679 GO:0006355 GO:0006281 GO:0006955 GO:0006310
GO:0007165 GO:0003723 GO:0008134 GO:0003678 GO:0005126
GO:0009378 GO:0016887 GO:0005524 GO:0003724 GO:0005576
GO:0005667 GO:0009379 GO:0005657
Cluster-22178.72680 GO:0035556 GO:0016020
Cluster-22178.72681 GO:0006281 GO:0006355 GO:0006401 GO:0006310
GO:0008272 GO:0003678 GO:0008134 GO:0004519 GO:0005198
GO:0003723 GO:0015116 GO:0003724 GO:0016887 GO:0005524
GO:0009378 GO:0016021 GO:0019031 GO:0005657 GO:0009379
GO:0005667

Cluster-22178.72682 GO:0006355 GO:0006281 GO:0006401 GO:0006310
GO:0008272 GO:0003678 GO:0008134 GO:0004519 GO:0003723
GO:0003724 GO:0015116 GO:0016887 GO:0005524 GO:0009378
GO:0016021 GO:0005657 GO:0005667 GO:0009379
Cluster-22178.72685 GO:0016117 GO:0055114 GO:0016705 GO:0016491

Cluster-22178.72687 GO:0008152 GO:0003824
Cluster-22178.72688 GO:0006769 GO:0035023 GO:0046497 GO:0008152
GO:0006508 GO:0006741 GO:0046656 GO:0006814 GO:0007010
GO:0003333 GO:0043087 GO:0035556 GO:0046740 GO:0003779
GO:0046872 GO:0008236 GO:0016301 GO:0003951 GO:0030269
GO:0005089 GO:0005737 GO:0044219 GO:0005643 GO:0019028
GO:0012506
Cluster-22178.72692 GO:0022900 GO:0055114 GO:0006118 GO:0008827
GO:0009319 GO:0016021
Cluster-22178.72697 GO:0016192 GO:0006886 GO:0042981 GO:0005515
GO:0030117
Cluster-22178.72699 GO:0005515
Cluster-22178.72703 GO:0005515 GO:0016788 GO:0016021
Cluster-22178.72706 GO:0008299 GO:0006570 GO:0009058 GO:0007165
GO:0035556 GO:0006694 GO:0006470 GO:0005044 GO:0005515
GO:0016779 GO:0050518 GO:0004725 GO:0016020 GO:0019031
Cluster-22178.72709 GO:0005887
Cluster-22178.72710 GO:0005887
Cluster-22178.72711 GO:0015031 GO:0016021
Cluster-22178.72714 GO:0015979 GO:0031175 GO:0008017 GO:0005515
GO:0030507 GO:0005516 GO:0045298 GO:0008091 GO:0009523
GO:0009539 GO:0016020
Cluster-22178.72715 GO:0016579 GO:0015940 GO:0008897 GO:0005515
GO:0000287 GO:0036459 GO:0005096
Cluster-22178.72716 GO:0016579 GO:0005096 GO:0036459 GO:0005515

Cluster-22178.72717 GO:0006355 GO:0006270 GO:0006506 GO:0017176

GO:0003700 GO:0046935 GO:0005667 GO:0005944 GO:0016021
GO:0019031
Cluster-22178.72718 GO:0007067 GO:0000090 GO:0007059 GO:0031110
GO:0051301 GO:0006914 GO:0008017 GO:0045298 GO:0005876
GO:0000940
Cluster-22178.72719 GO:0003677 GO:0005198 GO:0005515 GO:0008270
GO:0016021
Cluster-22178.72720 GO:0006508 GO:0008234
Cluster-22178.72721 GO:0006352 GO:0003676 GO:0003677 GO:0003723

Cluster-22178.72722 GO:0045892 GO:0003723 GO:0005634
Cluster-22178.72724 GO:0006811 GO:0055085 GO:0015075 GO:0005515
GO:0005886 GO:0016020
Cluster-22178.72725 GO:0035556 GO:0006464 GO:0006144 GO:0006811
GO:0046034 GO:0009405 GO:0006351 GO:0006955 GO:0000902
GO:0006355 GO:0008270 GO:0003677 GO:0004017 GO:0016151
GO:0003700 GO:0016790 GO:0003676 GO:0046872 GO:0043565
GO:0005515 GO:0005887 GO:0016020 GO:0005667 GO:0045202
Cluster-22178.72728 GO:0006544 GO:0006563 GO:0006566 GO:0046653
GO:0003950 GO:0008115
Cluster-22178.72730 GO:0006338 GO:0016818 GO:0003676 GO:0046872
GO:0005524 GO:0005634
Cluster-22178.72731 GO:0006139 GO:0046034 GO:0006206 GO:0006144
GO:0007186 GO:0050909 GO:0004930 GO:0004127 GO:0005524
GO:0004017 GO:0016021
Cluster-22178.72732 GO:0006486 GO:0008378 GO:0016757 GO:0016020
Cluster-22178.72734 GO:0009072 GO:0015743 GO:0042207 GO:0006570
GO:0004334
Cluster-22178.72736 GO:0008152 GO:0009452 GO:0001510 GO:0031167
GO:0006306 GO:0008168 GO:0005201 GO:0008170 GO:0005515
GO:0003677 GO:0005581 GO:0016021 GO:0005578 GO:0019013
Cluster-22178.72737 GO:0005543
Cluster-22178.72740 GO:0000226 GO:0042254 GO:0006412 GO:0003735

GO:0015934 GO:0005840
Cluster-22178.72741 GO:0035556 GO:0008152 GO:0006144 GO:0006468
GO:0006182 GO:0046039 GO:0009190 GO:0006508 GO:0004672
GO:0005524 GO:0008236 GO:0004383 GO:0016787 GO:0016849
Cluster-22178.72744 GO:0006771 GO:0006000 GO:0019497 GO:0006013
GO:0016301 GO:0003873 GO:0003993 GO:0005524
Cluster-22178.72748 GO:0016998 GO:0006032 GO:0004568 GO:0016021
Cluster-22178.72752 GO:0006355 GO:0043565 GO:0003700 GO:0019905
GO:0005515 GO:0005667
Cluster-22178.72755 GO:0005515
Cluster-22178.72758 GO:0008270
Cluster-22178.72762 GO:0050909 GO:0005515 GO:0016021
Cluster-22178.72764 GO:0050909 GO:0005515 GO:0016021
Cluster-22178.72765 GO:0007264 GO:0006355 GO:0006576 GO:0019068
GO:0005524 GO:0003924 GO:0005525 GO:0003700 GO:0005667
Cluster-22178.72769 GO:0051028 GO:0031012 GO:0005634
Cluster-22178.72770 GO:0003677 GO:0005634
Cluster-22178.72773 GO:0007264 GO:0003924 GO:0005525
Cluster-22178.72775 GO:0006040 GO:0005975 GO:0016977 GO:0005576
Cluster-22178.72783 GO:0045444 GO:0046872
Cluster-22178.72784 GO:0045444 GO:0046872
Cluster-22178.72787 GO:0005515
Cluster-22178.72788 GO:0005515
Cluster-22178.72789 GO:0006118 GO:0051536 GO:0009055
Cluster-22178.72792 GO:0006118 GO:0010508 GO:0007186 GO:0003954
GO:0004930 GO:0016020 GO:0005739 GO:0031966 GO:0016021
Cluster-22178.72793 GO:0006355 GO:0006118 GO:0006914 GO:0006260
GO:0007186 GO:0007165 GO:0042974 GO:0005198 GO:0004930
GO:0003954 GO:0016021 GO:0005667 GO:0031966 GO:0005739
GO:0016020 GO:0005882
Cluster-22178.72794 GO:0007186 GO:0010508 GO:0006118 GO:0004930
GO:0003954 GO:0016021 GO:0005739 GO:0031966 GO:0016020
Cluster-22178.72795 GO:0006355 GO:0042967 GO:0016747 GO:0008080

Cluster-22178.72797 GO:0003677 GO:0009512
Cluster-22178.72798 GO:0055114 GO:0030699 GO:0050485 GO:0030700
Cluster-22178.72802 GO:0005201 GO:0046872 GO:0046332 GO:0008270
GO:0005578
Cluster-22178.72804 GO:0042254 GO:0006412 GO:0003723 GO:0003735
GO:0005840
Cluster-22178.72806 GO:0007155 GO:0006606 GO:0015031 GO:0000921
GO:0006904 GO:0051382 GO:0005198 GO:0042803 GO:0008134
GO:0045502 GO:0008289 GO:0000145 GO:0005882 GO:0005667
GO:0005940 GO:0030286 GO:0000776 GO:0016021
Cluster-22178.72807 GO:0051382 GO:0007049 GO:0006904 GO:0009405
GO:0015031 GO:0000921 GO:0006606 GO:0007155 GO:0008289
GO:0045502 GO:0005198 GO:0008134 GO:0042803 GO:0016021
GO:0000776 GO:0030286 GO:0005940 GO:0005667 GO:0005882
GO:0016020 GO:0000145
Cluster-22178.72808 GO:0006464 GO:0016151 GO:0005515 GO:0061630
GO:0008270 GO:0046872
Cluster-22178.72809 GO:0042254 GO:0001522 GO:0030515 GO:0072588
Cluster-22178.72811 GO:0006629 GO:0006355 GO:0043401 GO:0043565
GO:0008270 GO:0003700 GO:0016021 GO:0005634 GO:0005667
Cluster-22178.72812 GO:0006629 GO:0006355 GO:0043401 GO:0008270
GO:0043565 GO:0003700 GO:0005667 GO:0005634
Cluster-22178.72813 GO:0043401 GO:0006355 GO:0006629 GO:0003700
GO:0008270 GO:0043565 GO:0005634 GO:0005667
Cluster-22178.72817 GO:0005515
Cluster-22178.72821 GO:0006338 GO:0006355 GO:0008270 GO:0005524
GO:0003676 GO:0016818 GO:0003677 GO:0005634 GO:0005739
Cluster-22178.72826 GO:0019058 GO:0006810 GO:0046872 GO:0016021
Cluster-22178.72831 GO:0006508 GO:0004252
Cluster-22178.72832 GO:0019042 GO:0005524 GO:0003677 GO:0000166
GO:0033644
Cluster-22178.72834 GO:0050830 GO:0033588

Cluster-22178.72835 GO:0046872 GO:0016021
Cluster-22178.72836 GO:0055114 GO:0016491
Cluster-22178.72837 GO:0006820 GO:0004190 GO:0016021
Cluster-22178.72838 GO:0016020
Cluster-22178.72839 GO:0009058 GO:0055114 GO:0030170 GO:0016491

Cluster-22178.72840 GO:0016021
Cluster-22178.72841 GO:0051289 GO:0008140 GO:0005667
Cluster-22178.72842 GO:0051289 GO:0008140 GO:0005667
Cluster-22178.72843 GO:0030001 GO:0055085 GO:0046873 GO:0016020
Cluster-22178.72844 GO:0030001 GO:0055085 GO:0046873 GO:0016020
Cluster-22178.72845 GO:0006418 GO:0006355 GO:0005524 GO:0008270
GO:0002161 GO:0004812 GO:0016818 GO:0003677 GO:0000166
GO:0005634
Cluster-22178.72846 GO:0003676
Cluster-22178.72847 GO:0003824 GO:0005515
Cluster-22178.72849 GO:0035556 GO:0007165 GO:0004871
Cluster-22178.72850 GO:0042967 GO:0006355 GO:0003712 GO:0008270
GO:0005515 GO:0004402 GO:0000123 GO:0005634 GO:0005667
Cluster-22178.72854 GO:0007017 GO:0007018 GO:0005524 GO:0003777
GO:0008017 GO:0003677 GO:0005198 GO:0005874 GO:0045298
Cluster-22178.72855 GO:0032259 GO:0008168
Cluster-22178.72856 GO:0006974 GO:0051726 GO:0003677
Cluster-22178.72858 GO:0030153
Cluster-22178.72861 GO:0030472 GO:0035556 GO:0071973 GO:0055114
GO:0006935 GO:0006468 GO:0016773 GO:0005524 GO:0003774
GO:0047134 GO:0004672 GO:0005515 GO:0016779 GO:0016020
GO:0009288 GO:0072686 GO:0042729
Cluster-22178.72863 GO:0005975 GO:0035556 GO:0006954 GO:0007165
GO:0006952 GO:0005125 GO:0046872 GO:0004553 GO:0005515
GO:0003677 GO:0005576
Cluster-22178.72865 GO:0071816 GO:0031492 GO:0005634 GO:0000785
Cluster-22178.72870 GO:0008168

Cluster-22178.72872 GO:0006123 GO:0006812 GO:0006820 GO:0055114
GO:0006835 GO:0015992 GO:0004129 GO:0017153 GO:0016638
GO:0045277 GO:0016020
Cluster-22178.72873 GO:0050790 GO:0006508 GO:0009405 GO:0004197
GO:0005576
Cluster-22178.72878 GO:0019069 GO:0006816 GO:0006874 GO:0006260
GO:0000049 GO:0005219 GO:0071203 GO:0016020 GO:0005622
GO:0005815 GO:0016021
Cluster-22178.72879 GO:0005515
Cluster-22178.72880 GO:0006446 GO:0045454 GO:0006413 GO:0007411
GO:0055114 GO:0003743 GO:0003723 GO:0005840 GO:0005737
Cluster-22178.72885 GO:0043087 GO:0015886 GO:0035556 GO:0005085
GO:0015232 GO:0008270 GO:0005515
Cluster-22178.72886 GO:0035556 GO:0015886 GO:0043087 GO:0005515
GO:0008270 GO:0015232 GO:0005085
Cluster-22178.72887 GO:0006308 GO:0006289 GO:0006281 GO:0006285
GO:0008534 GO:0003684
Cluster-22178.72889 GO:0031167 GO:0009451 GO:0006479 GO:0006306
GO:0001510 GO:0009452 GO:0008152 GO:0008033 GO:0046500
GO:0006400 GO:0006396 GO:0006464 GO:0015979 GO:0042549
GO:0008173 GO:0005515 GO:0003676 GO:0008168 GO:0004719
GO:0003677 GO:0008176 GO:0008170 GO:0009523 GO:0009539
Cluster-22178.72890 GO:0008033 GO:0008152 GO:0009452 GO:0001510
GO:0009451 GO:0031167 GO:0006306 GO:0006479 GO:0042549
GO:0015979 GO:0006464 GO:0006396 GO:0006400 GO:0046500
GO:0008168 GO:0004719 GO:0003676 GO:0005515 GO:0008173
GO:0008176 GO:0008170 GO:0003677 GO:0009523 GO:0009539
Cluster-22178.72891 GO:0006898 GO:0006508 GO:0007165 GO:0008235
GO:0005044 GO:0030145 GO:0004177 GO:0005737 GO:0016020
Cluster-22178.72895 GO:0046660 GO:0006812 GO:0006397 GO:0003676
GO:0016887 GO:0016021 GO:0005634
Cluster-22178.72896 GO:0009847 GO:0007165 GO:0005102
Cluster-22178.72900 GO:0006355 GO:0042254 GO:0006412 GO:0003735

GO:0005515 GO:0043565 GO:0003700 GO:0005634 GO:0005667
GO:0005622 GO:0005840
Cluster-22178.72902 GO:0019497 GO:0006355 GO:0006771 GO:0006508
GO:0008270 GO:0003993 GO:0005515 GO:0004252 GO:0005634
Cluster-22178.72903 GO:0019497 GO:0006355 GO:0006771 GO:0006323
GO:0006508 GO:0008270 GO:0003993 GO:0004252 GO:0005515
GO:0005634
Cluster-22178.72904 GO:0007018 GO:0007017 GO:0003777 GO:0008017
GO:0005524 GO:0005874 GO:0045298
Cluster-22178.72909 GO:0005634
Cluster-22178.72912 GO:0055114 GO:0016491 GO:0016779
Cluster-22178.72913 GO:0030153 GO:0007186 GO:0006955 GO:0007165
GO:0015643 GO:0003924 GO:0019001 GO:0031683 GO:0004871
GO:0019814
Cluster-22178.72915 GO:0005515 GO:0016020
Cluster-22178.72916 GO:0007165 GO:0004872 GO:0005515 GO:0016021
GO:0016020
Cluster-22178.72918 GO:0015684 GO:0007264 GO:0000287 GO:0030976
GO:0005525 GO:0003924 GO:0015093 GO:0016021
Cluster-22178.72920 GO:0045444 GO:0016020
Cluster-22178.72922 GO:0005515
Cluster-22178.72925 GO:0006367 GO:0008270 GO:0046872 GO:0005515
GO:0005672
Cluster-22178.72929 GO:0007606 GO:0006508 GO:0007165 GO:0016787
GO:0008236 GO:0005509 GO:0005578 GO:0016021
Cluster-22178.72932 GO:0006525 GO:0006560 GO:0006814 GO:0017004
GO:0071436 GO:0051301 GO:0055114 GO:0007049 GO:0006090
GO:0008948 GO:0015081 GO:0016020 GO:0005886 GO:0016021
Cluster-22178.72933 GO:0006352 GO:0015979 GO:0009297 GO:0006355
GO:0003700 GO:0003677 GO:0003723 GO:0009522 GO:0005669
GO:0019867 GO:0005667
Cluster-22178.72934 GO:0055114 GO:0007165 GO:0051920 GO:0005509
GO:0005515 GO:0005578

Cluster-22178.72935 GO:0015992 GO:0006576 GO:0015986 GO:0007264
GO:0005524 GO:0005525 GO:0015078 GO:0000276
Cluster-22178.72941 GO:0042254 GO:0006412 GO:0006355 GO:0003677
GO:0003735 GO:0005840 GO:0005622
Cluster-22178.72943 GO:0008152 GO:0016491
Cluster-22178.72946 GO:0006364 GO:0015694 GO:0008270 GO:0046872
GO:0015097 GO:0016020 GO:0005730
Cluster-22178.72947 GO:0007342 GO:0006310 GO:0006897 GO:0006508
GO:0006281 GO:0060478 GO:0046872 GO:0004252 GO:0003677
GO:0009378 GO:0005657 GO:0005886 GO:0009379
Cluster-22178.72949 GO:0003676 GO:0003723
Cluster-22178.72951 GO:0006605 GO:0006886 GO:0009297 GO:0007154
GO:0005515 GO:0005922 GO:0005742 GO:0019867
Cluster-22178.72954 GO:0006144 GO:0004114
Cluster-22178.72956 GO:0006275 GO:0008033 GO:0016779 GO:0004810
GO:0003676 GO:0016437 GO:0008270 GO:0005634
Cluster-22178.72960 GO:0006032 GO:0007165 GO:0005515 GO:0008061

Cluster-22178.72962 GO:0005515
Cluster-22178.72963 GO:0008152 GO:0007275 GO:0007165 GO:0008270
GO:0047355 GO:0016624 GO:0005179 GO:0016020 GO:0005576
Cluster-22178.72967 GO:0016567 GO:0003723 GO:0005515 GO:0046872
GO:0008270 GO:0004842 GO:0005680 GO:0005887
Cluster-22178.72968 GO:0055114 GO:0051016 GO:0006376 GO:0003729
GO:0016491 GO:0008290 GO:0005685
Cluster-22178.72970 GO:0019441 GO:0046487 GO:0015684 GO:0007264
GO:0006568 GO:0005525 GO:0015093 GO:0004061 GO:0016021
Cluster-22178.72971 GO:0007165 GO:0007264 GO:0043087 GO:0005085

Cluster-22178.72972 GO:0007165 GO:0043087 GO:0007264 GO:0005085

Cluster-22178.72973 GO:0005515
Cluster-22178.72974 GO:0005515

Cluster-22178.72975 GO:0055114 GO:0006032 GO:0016705 GO:0008061

Cluster-22178.72980 GO:0006397 GO:0042967 GO:0006396 GO:0005515
GO:0008080 GO:0016747 GO:0005634 GO:0005622
Cluster-22178.72982 GO:0006914 GO:0000921 GO:0005515 GO:0005509
GO:0005940 GO:0016021
Cluster-22178.72983 GO:0006812 GO:0007165 GO:0006308 GO:0007018
GO:0007017 GO:0006355 GO:0008017 GO:0008855 GO:0016887
GO:0005524 GO:0003713 GO:0003777 GO:0005515 GO:0035257
GO:0005667 GO:0016021 GO:0045298 GO:0005874 GO:0009318
GO:0005634
Cluster-22178.72984 GO:0055114 GO:0006520 GO:0006807 GO:0006118
GO:0019551 GO:0004352 GO:0016491 GO:0051287
Cluster-22178.72988 GO:0006914 GO:0048870 GO:0051321 GO:0006606
GO:0007264 GO:0006457 GO:0003924 GO:0005525 GO:0005509
GO:0005515 GO:0005198 GO:0019031 GO:0019028 GO:0031514
Cluster-22178.72991 GO:0006570 GO:0006470 GO:0004725 GO:0008138

Cluster-22178.72993 GO:0007156 GO:0007155 GO:0005198 GO:0005509
GO:0008270 GO:0016020 GO:0031012
Cluster-22178.72995 GO:0006506 GO:0006355 GO:0018279 GO:0006357
GO:0006810 GO:0006367 GO:0000062 GO:0017176 GO:0001104
GO:0003677 GO:0004579 GO:0016021 GO:0005672 GO:0005634
GO:0016592 GO:0008250
Cluster-22178.72997 GO:0006817 GO:0031505 GO:0007186 GO:0005315
GO:0004930 GO:0016021 GO:0016020
Cluster-22178.72998 GO:0016579 GO:0006508 GO:0004843 GO:0036459
GO:0016020
Cluster-22178.73000 GO:0006811 GO:0005216 GO:0004190 GO:0016021
GO:0016020
Cluster-22178.73002 GO:0007059 GO:0044780 GO:0006355 GO:0051607
GO:0031110 GO:0007067 GO:0006415 GO:0000090 GO:0051301
GO:0006886 GO:0006449 GO:0006357 GO:0016192 GO:0019069

GO:0003700 GO:0016149 GO:0001104 GO:0005515 GO:0051082
GO:0008017 GO:0008270 GO:0005840 GO:0005801 GO:0016021
GO:0019033 GO:0000940 GO:0016592 GO:0018444 GO:0045298
GO:0005737 GO:0005634 GO:0005876 GO:0016020 GO:0005667
Cluster-22178.73003 GO:0051225 GO:0030474 GO:0006891 GO:0007165
GO:0030001 GO:0006886 GO:0006357 GO:0048193 GO:0006869
GO:0016192 GO:0046872 GO:0005200 GO:0045502 GO:0008134
GO:0005319 GO:0042803 GO:0005515 GO:0001104 GO:0005198
GO:0008270 GO:0005179 GO:0019031 GO:0005801 GO:0016021
GO:0005576 GO:0019028 GO:0019033 GO:0030286 GO:0005823
GO:0017119 GO:0016592 GO:0070652 GO:0005856 GO:0019867
GO:0005667 GO:0016020
Cluster-22178.73004 GO:0008270 GO:0000228
Cluster-22178.73008 GO:0006298 GO:0005524 GO:0030983 GO:0030430
Cluster-22178.73011 GO:0006555 GO:0006556 GO:1901031 GO:0055114
GO:0051920 GO:0004478 GO:0005634
Cluster-22178.73012 GO:0009297 GO:0006351 GO:0006144 GO:0006206
GO:0006464 GO:0016567 GO:0016151 GO:0046872 GO:0003899
GO:0005515 GO:0003723 GO:0004842 GO:0008270 GO:0061630
GO:0019867 GO:0005634 GO:0005680 GO:0005730
Cluster-22178.73013 GO:0045859 GO:0007049 GO:0016538
Cluster-22178.73015 GO:0006810 GO:0005515 GO:0016021
Cluster-22178.73016 GO:0035556 GO:0007165 GO:0003774 GO:0005524
GO:0005515 GO:0016459
Cluster-22178.73017 GO:0006915 GO:0018279 GO:0006351 GO:0006486
GO:0019083 GO:0008285 GO:0007050 GO:0006206 GO:0006144
GO:0016579 GO:0003899 GO:0036459 GO:0004579 GO:0003677
GO:0008270 GO:0016021 GO:0005730 GO:0005739 GO:0005783
GO:0008250
Cluster-22178.73018 GO:0006468 GO:0009058 GO:0016773 GO:0005524
GO:0004672 GO:0016779 GO:0016020
Cluster-22178.73019 GO:0006541 GO:0042823 GO:0042819 GO:0007186
GO:0050909 GO:0007031 GO:0051276 GO:0000166 GO:0005515

GO:0004359 GO:0003677 GO:0008773 GO:0004930 GO:0005524
GO:0016887 GO:0016021 GO:0005694 GO:0005777
Cluster-22178.73020 GO:0019087 GO:0055085 GO:0006817 GO:0006355
GO:0006810 GO:0005315 GO:0000982 GO:0000978 GO:0016021
GO:0005634 GO:0016020
Cluster-22178.73021 GO:0008283 GO:0040007 GO:0009405 GO:0007165
GO:0006351 GO:0006206 GO:0006144 GO:0003677 GO:0008083
GO:0003899 GO:0005730
Cluster-22178.73022 GO:0009307 GO:0042254 GO:0006412 GO:0006308
GO:0003677 GO:0003735 GO:0009036 GO:0009359 GO:0019013
GO:0005622 GO:0005840 GO:0016021
Cluster-22178.73023 GO:0048255
Cluster-22178.73026 GO:0003676 GO:0005524
Cluster-22178.73031 GO:0042138 GO:0006310
Cluster-22178.73032 GO:0016765
Cluster-22178.73033 GO:0005515
Cluster-22178.73034 GO:0009847 GO:0006355 GO:0003677
Cluster-22178.73036 GO:0006534 GO:0046439 GO:0055114 GO:0019530
GO:0017172 GO:0005506
Cluster-22178.73037 GO:0005515
Cluster-22178.73039 GO:0019491 GO:0033990 GO:0005515 GO:0016021
Cluster-22178.73040 GO:0005515 GO:0032040 GO:0000228
Cluster-22178.73042 GO:0046872 GO:0045202
Cluster-22178.73043 GO:0005975 GO:0019058 GO:0006886 GO:0008536
GO:0004553 GO:0005515 GO:0042025
Cluster-22178.73044 GO:0046487 GO:0015977 GO:0006950 GO:0016984
GO:0009573 GO:0005680 GO:0016021
Cluster-22178.73047 GO:0007165 GO:0008158 GO:0016020
Cluster-22178.73048 GO:0031144 GO:0071630 GO:0006355 GO:0005201
GO:0003677 GO:0003682 GO:0000785 GO:0000775 GO:0005581
GO:0005634 GO:0005578
Cluster-22178.73049 GO:0006810 GO:0005215 GO:0016021 GO:0005737
Cluster-22178.73050 GO:0005515

Cluster-22178.73054 GO:0032780 GO:0006310 GO:0006457 GO:0006281
GO:0051087 GO:0042803 GO:0000287 GO:0000774 GO:0042030
GO:0005739
Cluster-22178.73055 GO:0007165 GO:0030155 GO:0045995 GO:0046168
GO:0030334 GO:0055114 GO:0016616 GO:0051287 GO:0005102
Cluster-22178.73057 GO:0007275 GO:0007219 GO:0030154 GO:0005515
GO:0005509 GO:0016021
Cluster-22178.73058 GO:0009405 GO:0005576
Cluster-22178.73059 GO:0006570 GO:0006470 GO:0004725 GO:0008138

Cluster-22178.73060 GO:0007275 GO:0003677 GO:0003700 GO:0019028
GO:0005667 GO:0005634
Cluster-22178.73064 GO:0006508 GO:0008152 GO:0008168 GO:0008236
GO:0004252 GO:0070008 GO:0016020
Cluster-22178.73067 GO:0007186 GO:0004930 GO:0016021
Cluster-22178.73068 GO:0016020
Cluster-22178.73069 GO:0006606 GO:0010506 GO:0006355 GO:0003700
GO:0005615 GO:0005667
Cluster-22178.73075 GO:0006890
Cluster-22178.73078 GO:0005991 GO:0005993 GO:0005985 GO:0005982
GO:0009231 GO:0005509 GO:0004555 GO:0008531 GO:0005737
Cluster-22178.73079 GO:0006355 GO:0008270 GO:0046872 GO:0005515
GO:0005525 GO:0003677
Cluster-22178.73080 GO:0006355 GO:0003700 GO:0005515 GO:0005524
GO:0043565 GO:0005667
Cluster-22178.73081 GO:0016020
Cluster-22178.73082 GO:0051252 GO:0006364 GO:0006379 GO:0008033
GO:0003723 GO:0004540 GO:0030677 GO:0000172
Cluster-22178.73083 GO:0006511 GO:0004175 GO:0005515 GO:0019773
Cluster-22178.73091 GO:0009396 GO:0006144 GO:0042254 GO:0006531
GO:0033014 GO:0006522 GO:0008152 GO:0006783 GO:0015994
GO:0055114 GO:0006412 GO:0046487 GO:0006164 GO:0004019
GO:0016491 GO:0004488 GO:0003735 GO:0050662 GO:0003824

GO:0005525 GO:0004852 GO:0005622 GO:0005840
Cluster-22178.73093 GO:0009245 GO:0016747
Cluster-22178.73095 GO:0005515
Cluster-22178.73097 GO:0006367 GO:0005672
Cluster-22178.73098 GO:0006123 GO:0015992 GO:0004129 GO:0008270
GO:0046872 GO:0045277 GO:0005634
Cluster-22178.73101 GO:0006355 GO:0005515 GO:0005634
Cluster-22178.73103 GO:0006807 GO:0006355 GO:0006508 GO:0004252
GO:0005515 GO:0016812 GO:0005634
Cluster-22178.73105 GO:0009058 GO:0016740 GO:0003677 GO:0003824

Cluster-22178.73106 GO:0007010 GO:0003779
Cluster-22178.73107 GO:0016021
Cluster-22178.73109 GO:0006886 GO:0019058 GO:0005515 GO:0008536
GO:0042025
Cluster-22178.73114 GO:0055114 GO:0000287 GO:0016491 GO:0003824
GO:0050660 GO:0030976
Cluster-22178.73115 GO:0003924 GO:0005525
Cluster-22178.73116 GO:0051028 GO:0008270 GO:0003677 GO:0005634
Cluster-22178.73117 GO:0006276
Cluster-22178.73118 GO:0016567 GO:0051082 GO:0008270 GO:0046872
GO:0005515 GO:0004842 GO:0005680
Cluster-22178.73123 GO:0005515
Cluster-22178.73124 GO:0016787
Cluster-22178.73126 GO:0046039 GO:0006182 GO:0007218 GO:0006144
GO:0005515 GO:0004383 GO:0005543 GO:0005576
Cluster-22178.73128 GO:0055085 GO:0007186 GO:0019856 GO:0008440
GO:0004930 GO:0016021
Cluster-22178.73129 GO:0007186 GO:0055085 GO:0019856 GO:0008440
GO:0004930 GO:0016021
Cluster-22178.73132 GO:0009405 GO:0006355 GO:0090527 GO:0003700
GO:0008270 GO:0031423 GO:0043565 GO:0019012 GO:0005667
GO:0005634

Cluster-22178.73134 GO:0007165 GO:0005179 GO:0005576

Cluster-22178.73137 GO:0006457 GO:0000413 GO:0003755

Cluster-22178.73138 GO:0006457 GO:0046872 GO:0008270 GO:0051082 GO:0003677 GO:0005737

Cluster-22178.73139 GO:0003723

Cluster-22178.73140 GO:0006457 GO:0003677 GO:0046872 GO:0051082 GO:0005737

Cluster-22178.73141 GO:0007165 GO:0006955 GO:0008283 GO:0040007 GO:0035023 GO:0043087 GO:0008083 GO:0005126 GO:0003723 GO:0005089 GO:0005576

Cluster-22178.73144 GO:0016310 GO:0055114 GO:0006303 GO:0030328 GO:0006631 GO:0009069 GO:0050151 GO:0004677 GO:0005524 GO:0016491 GO:0003677 GO:0016670 GO:0071949 GO:0005958 GO:0005634

Cluster-22178.73148 GO:0042254 GO:0006412 GO:0006118 GO:0006355 GO:0045454 GO:0003735 GO:0003700 GO:0009055 GO:0015035 GO:0005667 GO:0016021 GO:0005840 GO:0005622

Cluster-22178.73149 GO:0006355 GO:0045454 GO:0006412 GO:0042254 GO:0006118 GO:0003700 GO:0009055 GO:0015035 GO:0003735 GO:0005622 GO:0005840 GO:0005667

Cluster-22178.73151 GO:0045454 GO:0006118 GO:0009055 GO:0015035

Cluster-22178.73153 GO:0007165 GO:0006614 GO:0006886 GO:0006352 GO:0004888 GO:0003677 GO:0098519 GO:0005047 GO:0003924 GO:0005525 GO:0005786 GO:0016021 GO:0005785

Cluster-22178.73154 GO:0042254 GO:0015992 GO:0006123 GO:0005515 GO:0005507 GO:0004129 GO:0005730 GO:0016020 GO:0045277

Cluster-22178.73160 GO:0005515

Cluster-22178.73161 GO:0035556 GO:0055114 GO:0006144 GO:0007165 GO:0006508 GO:0003968 GO:0004871 GO:0004197 GO:0016616 GO:0017111 GO:0004252 GO:0031379

Cluster-22178.73165 GO:0006206 GO:0006144 GO:0006351 GO:0006383 GO:0003677 GO:0003899 GO:0005730 GO:0005666

Cluster-22178.73166 GO:0006355 GO:0043565 GO:0003700 GO:0005515
GO:0005667
Cluster-22178.73167 GO:0007165 GO:0045122 GO:0006355 GO:0003677
GO:0005515 GO:0005634
Cluster-22178.73168 GO:0005515
Cluster-22178.73170 GO:0006886 GO:0015991 GO:0016820 GO:0008536
GO:0005515 GO:0000221
Cluster-22178.73172 GO:0007186 GO:0007165 GO:0004871 GO:0016772
GO:0005525 GO:0003924 GO:0019001 GO:0031683 GO:0005515
Cluster-22178.73174 GO:0007186 GO:0007165 GO:0007264 GO:0004871
GO:0005525 GO:0003924 GO:0019001 GO:0031683
Cluster-22178.73179 GO:0050832
Cluster-22178.73182 GO:0006118 GO:0007186 GO:0007283 GO:0005515
GO:0003677 GO:0009055 GO:0004930 GO:0016491 GO:0008270
GO:0016020 GO:0005634 GO:0016021 GO:0000786
Cluster-22178.73184 GO:0016021
Cluster-22178.73185 GO:0045893 GO:0006281 GO:0008081 GO:0005634
Cluster-22178.73186 GO:0006260 GO:0006351 GO:0006355 GO:0008270
GO:0003887 GO:0003677 GO:0003682 GO:0000775 GO:0000785
GO:0042575 GO:0005634
Cluster-22178.73191 GO:0000917 GO:0043093 GO:0015940 GO:0006260
GO:0055114 GO:0071949 GO:0016491 GO:0008677 GO:0008641
GO:0005737
Cluster-22178.73193 GO:0007165 GO:0007155 GO:0005173 GO:0016020
Cluster-22178.73194 GO:0005515
Cluster-22178.73196 GO:0016787
Cluster-22178.73197 GO:0019042 GO:0019013 GO:0016021 GO:0016020
GO:0033644
Cluster-22178.73198 GO:0046983 GO:0003677
Cluster-22178.73199 GO:0005515 GO:0005747 GO:0005739
Cluster-22178.73200 GO:0016567 GO:0006464 GO:0003723 GO:0005515
GO:0061630 GO:0008270 GO:0046872 GO:0016151 GO:0004842
GO:0005680

Cluster-22178.73201 GO:0005515
Cluster-22178.73202 GO:0004177 GO:0030145 GO:0016021
Cluster-22178.73204 GO:0007165 GO:0016310 GO:0007010 GO:0000160
GO:0000155 GO:0003779 GO:0009365
Cluster-22178.73205 GO:0006364 GO:0005515 GO:0032040
Cluster-22178.73213 GO:0016032 GO:0006355
Cluster-22178.73214 GO:0006508 GO:0016817 GO:0008242 GO:0016740
GO:0004197 GO:0016788 GO:0005634
Cluster-22178.73216 GO:0006525 GO:0008295 GO:0010508 GO:0006560
GO:0006357 GO:0001104 GO:0008092 GO:0004014 GO:0016592
GO:0005737 GO:0016021 GO:0019898
Cluster-22178.73218 GO:0018342 GO:0008318
Cluster-22178.73221 GO:0006468 GO:0004672 GO:0005524
Cluster-22178.73223 GO:0005783 GO:0016021
Cluster-22178.73224 GO:0015031 GO:0006807 GO:0042147 GO:0004668
GO:0008565 GO:0005509 GO:0005737 GO:0016021 GO:0030904
Cluster-22178.73225 GO:0006468 GO:0005515 GO:0003676 GO:0016773
GO:0005524 GO:0008270 GO:0004672 GO:0016020
Cluster-22178.73226 GO:0007010 GO:0003779
Cluster-22178.73229 GO:0006310 GO:0006281 GO:0006099 GO:0006508
GO:0016301 GO:0009378 GO:0004222 GO:0003678 GO:0005524
GO:0016829 GO:0016887 GO:0009379 GO:0005657
Cluster-22178.73231 GO:0009252 GO:0046436 GO:0006633 GO:0006090
GO:0006481 GO:0006813 GO:0006479 GO:0003989 GO:0015269
GO:0005524 GO:0004671 GO:0046872 GO:0008716 GO:0009317
GO:0016020 GO:0016021
Cluster-22178.73232 GO:0034453 GO:0005515 GO:0005815
Cluster-22178.73234 GO:0006810 GO:0005215
Cluster-22178.73235 GO:0006508 GO:0008654 GO:0008270 GO:0016780
GO:0004181 GO:0016020
Cluster-22178.73236 GO:0007264 GO:0055085 GO:0006885 GO:0006812
GO:0008146 GO:0015299 GO:0046872 GO:0003924 GO:0005525
GO:0016021

Cluster-22178.73242 GO:0016021
Cluster-22178.73243 GO:0009405 GO:0006955 GO:0004222 GO:0016020
Cluster-22178.73244 GO:0006955 GO:0042981
Cluster-22178.73246 GO:0006955
Cluster-22178.73248 GO:0006955 GO:0009405 GO:0004222 GO:0016020
Cluster-22178.73249 GO:0007264 GO:0006412 GO:0042254 GO:0051016
GO:0003735 GO:0005525 GO:0008290 GO:0005840
Cluster-22178.73251 GO:0003723 GO:0003676
Cluster-22178.73254 GO:0015992 GO:0006744 GO:0006120 GO:0006814
GO:0006118 GO:0003954 GO:0008137 GO:0005739
Cluster-22178.73255 GO:0046488 GO:0006887 GO:0016307 GO:0008289
GO:0000145 GO:0019031
Cluster-22178.73257 GO:0032006 GO:0051260 GO:0006470 GO:0006397
GO:0005515 GO:0004721 GO:0005634
Cluster-22178.73259 GO:0006468 GO:0004672 GO:0005524
Cluster-22178.73262 GO:0005515 GO:0016020
Cluster-22178.73263 GO:0015940 GO:0006139 GO:0006465 GO:0008152
GO:0015937 GO:0006355 GO:0006206 GO:0004140 GO:0003676
GO:0016740 GO:0016301 GO:0005524 GO:0004127 GO:0008233
GO:0016021 GO:0005787 GO:0005622
Cluster-22178.73268 GO:0006560 GO:0006525 GO:0006260 GO:0006420
GO:0006418 GO:0003677 GO:0000166 GO:0004812 GO:0004814
GO:0005524 GO:0005794 GO:0005815 GO:0005737
Cluster-22178.73269 GO:0007283 GO:0005794
Cluster-22178.73271 GO:0003950
Cluster-22178.73273 GO:0043683 GO:0007275 GO:0009966 GO:0016020
Cluster-22178.73275 GO:0008270 GO:0046872 GO:0003676 GO:0005515

Cluster-22178.73276 GO:0005515 GO:0016020
Cluster-22178.73277 GO:0055114 GO:0006355 GO:0005515 GO:0016491

Cluster-22178.73278 GO:0007283 GO:0005515
Cluster-22178.73280 GO:0006355 GO:0006606 GO:0006914 GO:0003677

GO:0016788 GO:0003700 GO:0043565 GO:0005667 GO:0005634
Cluster-22178.73284 GO:0009405 GO:0035091 GO:0016021 GO:0035792
Cluster-22178.73285 GO:0055114 GO:0016491
Cluster-22178.73286 GO:0042967 GO:0016747 GO:0008080
Cluster-22178.73289 GO:0006123 GO:0015992 GO:0004129 GO:0045277
GO:0005743 GO:0005751 GO:0036128
Cluster-22178.73291 GO:0006355 GO:0034968 GO:0006479 GO:0006554
GO:0018024 GO:0005524 GO:0003676 GO:0003712 GO:0005694
GO:0005667
Cluster-22178.73295 GO:0046872
Cluster-22178.73302 GO:0005515
Cluster-22178.73303 GO:0006886 GO:0016192 GO:0015991 GO:0005515
GO:0016820 GO:0000221 GO:0030117
Cluster-22178.73304 GO:0006508 GO:0005515 GO:0004252
Cluster-22178.73305 GO:0006351 GO:0006144 GO:0006206 GO:0003899
GO:0004190 GO:0016021 GO:0005730
Cluster-22178.73308 GO:0042967 GO:0016568 GO:0008080 GO:0004402
GO:0000123
Cluster-22178.73315 GO:0042254 GO:0006412 GO:0003735 GO:0005840
GO:0005622
Cluster-22178.73317 GO:0015986 GO:0015991 GO:0006119 GO:0015992
GO:0042981 GO:0043065 GO:0046961 GO:0015078 GO:0033178
GO:0045263 GO:0005737 GO:0031225
Cluster-22178.73320 GO:0007165 GO:0005515 GO:0004872 GO:0016021
Cluster-22178.73321 GO:0046872
Cluster-22178.73323 GO:0006260 GO:0006206 GO:0006144 GO:0006351
GO:0006269 GO:0003896 GO:0008270 GO:0046872 GO:0003677
GO:0003899 GO:0005657 GO:0005730
Cluster-22178.73325 GO:0006629 GO:0007606 GO:0019236 GO:0007186
GO:0004932 GO:0016020
Cluster-22178.73328 GO:0005509
Cluster-22178.73331 GO:0006508 GO:0004177 GO:0005622 GO:0016021
Cluster-22178.73332 GO:0000398 GO:0005681

Cluster-22178.73333 GO:0000902
Cluster-22178.73334 GO:0000902
Cluster-22178.73336 GO:0006298 GO:0007165 GO:0005096 GO:0005524 GO:0005515 GO:0030983
Cluster-22178.73337 GO:0006119 GO:0006118 GO:0042254 GO:0006412 GO:0016310 GO:0015992 GO:0008121 GO:0003735 GO:0030246 GO:0016772 GO:0009055 GO:0005840 GO:0005622
Cluster-22178.73340 GO:0006260 GO:0007049 GO:0006355 GO:0043093 GO:0000917 GO:0003700 GO:0005737 GO:0005634 GO:0005667
Cluster-22178.73341 GO:0007165 GO:0005044 GO:0016020
Cluster-22178.73346 GO:0042254 GO:0006412 GO:0016491 GO:0003735 GO:0005622 GO:0005840
Cluster-22178.73347 GO:0005543
Cluster-22178.73348 GO:0007165 GO:0005057 GO:0005515
Cluster-22178.73349 GO:0009306 GO:0008270 GO:0016020
Cluster-22178.73350 GO:0008152 GO:0048870 GO:0006367 GO:0006355 GO:0016491 GO:0008440 GO:0003677 GO:0017048 GO:0003682 GO:0000775 GO:0000785 GO:0031514 GO:0005634 GO:0005672
Cluster-22178.73352 GO:0006367 GO:0032968 GO:0008152 GO:0005515 GO:0004252 GO:0003677 GO:0004190 GO:0003824 GO:0005634 GO:0016021 GO:0005856 GO:0005886
Cluster-22178.73353 GO:0006351 GO:0090266 GO:0006260 GO:0005515 GO:0003677 GO:0003887 GO:0008270 GO:0042575 GO:0005634 GO:0032040 GO:0005680 GO:0019031
Cluster-22178.73356 GO:0005515
Cluster-22178.73360 GO:0017004 GO:0015886 GO:0005515 GO:0016021
Cluster-22178.73361 GO:0048268 GO:0007212 GO:0032051 GO:0016021
Cluster-22178.73362 GO:0035023 GO:0043087 GO:0005089
Cluster-22178.73363 GO:0006508 GO:0008234
Cluster-22178.73364 GO:0005515
Cluster-22178.73365 GO:0055085 GO:0006811 GO:0045735 GO:0005216 GO:0016020
Cluster-22178.73369 GO:0055114 GO:0016491 GO:0051287 GO:0016620

Cluster-22178.73371 GO:0006308 GO:0008855 GO:0009318

Cluster-22178.73379 GO:0007009

Cluster-22178.73380 GO:0007009

Cluster-22178.73382 GO:0005509 GO:0016021

Cluster-22178.73384 GO:0005515

Cluster-22178.73388 GO:0043093 GO:0006355 GO:0006457 GO:0006810 GO:0000917 GO:0006260 GO:0007049 GO:0051082 GO:0005509 GO:0043565 GO:0003700 GO:0005667 GO:0016020 GO:0005783 GO:0005737

Cluster-22178.73389 GO:0005515 GO:0008270

Cluster-22178.73390 GO:0006183 GO:0007010 GO:0006165 GO:0006228 GO:0006241 GO:0035556 GO:0006144 GO:0006206 GO:0005515 GO:0003779 GO:0004550

Cluster-22178.73392 GO:0005515 GO:0005509 GO:0016020 GO:0009986

Cluster-22178.73393 GO:0007186 GO:0006396 GO:0009451 GO:0004930 GO:0008173 GO:0003723 GO:0016021

Cluster-22178.73394 GO:0061630 GO:0005515 GO:0008270

Cluster-22178.73395 GO:0055114 GO:0006396 GO:0045859 GO:0004860 GO:0047134 GO:0005515 GO:0016021 GO:0000176 GO:0005576

Cluster-22178.73396 GO:0055114 GO:0006396 GO:0045859 GO:0004860 GO:0047134 GO:0005515 GO:0016021 GO:0000176 GO:0005576

Cluster-22178.73397 GO:0051028 GO:0003677 GO:0008270 GO:0005634

Cluster-22178.73400 GO:0006303 GO:0005515

Cluster-22178.73401 GO:0009069 GO:0006468 GO:0016310 GO:0007178 GO:0005198 GO:0005026 GO:0005524 GO:0004672 GO:0046872 GO:0016020 GO:0016021

Cluster-22178.73402 GO:0016567 GO:0004842 GO:0005515 GO:0046872 GO:0008270 GO:0003723 GO:0005680

Cluster-22178.73404 GO:0005975 GO:0004415

Cluster-22178.73407 GO:0015991 GO:0000390 GO:0045944 GO:0015992 GO:0007219 GO:0003713 GO:0015078 GO:0005667 GO:0033179 GO:0071008 GO:0016607 GO:0005923

Cluster-22178.73408 GO:0016021
Cluster-22178.73410 GO:0006468 GO:0005524 GO:0004672
Cluster-22178.73412 GO:0003924 GO:0005515 GO:0005525
Cluster-22178.73414 GO:0008270 GO:0005515 GO:0005622
Cluster-22178.73415 GO:0006355 GO:0000975 GO:0003700 GO:0005840
GO:0005667
Cluster-22178.73419 GO:0006508 GO:0004222 GO:0008270
Cluster-22178.73426 GO:0006904 GO:0006508 GO:0007155 GO:0006914
GO:0015031 GO:0019901 GO:0051082 GO:0004222 GO:0016020
GO:0000145
Cluster-22178.73427 GO:0006270 GO:0003676
Cluster-22178.73429 GO:0055114 GO:0007050 GO:0051536 GO:0016491
GO:0016625 GO:0005515
Cluster-22178.73435 GO:0006811 GO:0055085 GO:0006355 GO:0005515
GO:0005216 GO:0016020 GO:0005761
Cluster-22178.73436 GO:0006098 GO:0009165 GO:0006144 GO:0009116
GO:0000287 GO:0004749
Cluster-22178.73440 GO:0030150 GO:0006814 GO:0005272 GO:0005742
GO:0016020
Cluster-22178.73441 GO:0003924 GO:0005515 GO:0005525
Cluster-22178.73443 GO:0055114 GO:0008152 GO:0016491
Cluster-22178.73455 GO:0015992 GO:0015991 GO:0015078 GO:0033177
Cluster-22178.73457 GO:0006446 GO:0001503 GO:0007155 GO:0006413
GO:0003743 GO:0005515 GO:0003723 GO:0005840 GO:0005576
Cluster-22178.73461 GO:0042147 GO:0006614 GO:0030942 GO:0005047
GO:0008312 GO:0005786
Cluster-22178.73462 GO:0055114 GO:0006694 GO:0008152 GO:0050832
GO:0008210 GO:0008209 GO:0008207 GO:0016491 GO:0003824
GO:0050662 GO:0003854 GO:0016616
Cluster-22178.73463 GO:0009396 GO:0055114 GO:0046487 GO:0005524
GO:0004329 GO:0004488 GO:0003824
Cluster-22178.73464 GO:0003677 GO:0005634
Cluster-22178.73467 GO:0007186 GO:0009405 GO:0018193 GO:0050909

GO:0016788 GO:0003723 GO:0043022 GO:0004930 GO:0016021
GO:0019031
Cluster-22178.73468 GO:0006506 GO:0006810 GO:0006468 GO:0004386
GO:0005215 GO:0016773 GO:0005524 GO:0004672 GO:0005515
GO:0005789 GO:0016020 GO:0031083
Cluster-22178.73469 GO:0006468 GO:0006506 GO:0005524 GO:0004672
GO:0016773 GO:0004386 GO:0005515 GO:0005789 GO:0031083
GO:0016020
Cluster-22178.73471 GO:0006355 GO:0006626 GO:0003700 GO:0004518
GO:0005667 GO:0005622 GO:0005741
Cluster-22178.73472 GO:0006281 GO:0055114 GO:0015774 GO:0007165
GO:0006310 GO:0004888 GO:0005515 GO:0009378 GO:0051920
GO:0005509 GO:0005524 GO:0015159 GO:0005578 GO:0016021
GO:0009379 GO:0016020 GO:0005657
Cluster-22178.73473 GO:0007165 GO:0006351 GO:0015774 GO:0006206
GO:0006144 GO:0055114 GO:0005515 GO:0003899 GO:0005509
GO:0051920 GO:0015159 GO:0016020 GO:0005730 GO:0005578
Cluster-22178.73474 GO:0042254 GO:0007186 GO:0007165 GO:0007606
GO:0006955 GO:0006412 GO:0015708 GO:0019236 GO:0003735
GO:0008270 GO:0004932 GO:0003723 GO:0005515 GO:0005125
GO:0046872 GO:0005622 GO:0005576 GO:0016021 GO:0005840
Cluster-22178.73475 GO:0006629
Cluster-22178.73477 GO:0019068 GO:0006281 GO:0003677 GO:0046872
GO:0008270
Cluster-22178.73480 GO:0007155 GO:0016567 GO:0061630 GO:0005515
GO:0008270 GO:0046872 GO:0004842 GO:0009288 GO:0005680
Cluster-22178.73481 GO:0006260 GO:0006810 GO:0003676 GO:0004386
GO:0003688 GO:0005524 GO:0003677 GO:0016787 GO:0046809
Cluster-22178.73482 GO:0006508 GO:0005515 GO:0016021
Cluster-22178.73485 GO:0007165 GO:0006508 GO:0006355 GO:0008234
GO:0003677
Cluster-22178.73494 GO:0009405 GO:0006412 GO:0006355 GO:0007017
GO:0006811 GO:0006352 GO:0042254 GO:0016579 GO:0003700

GO:0036459 GO:0003677 GO:0003735 GO:0005216 GO:0016987
GO:0005875 GO:0005576 GO:0005840 GO:0019013 GO:0005667
GO:0005622
Cluster-22178.73500 GO:0006144 GO:0019079 GO:0006351 GO:0006303
GO:0016817 GO:0003968 GO:0004003 GO:0003677 GO:0031379
GO:0005657
Cluster-22178.73502 GO:0016021
Cluster-22178.73503 GO:0006418 GO:0008270 GO:0005524 GO:0004812
GO:0000166
Cluster-22178.73507 GO:0005515 GO:0046872
Cluster-22178.73508 GO:0005515 GO:0046872
Cluster-22178.73509 GO:0043130 GO:0032266
Cluster-22178.73510 GO:0046856 GO:0005515 GO:0034597 GO:0003677

Cluster-22178.73512 GO:0003677 GO:0005515
Cluster-22178.73513 GO:0046856 GO:0005515 GO:0034597 GO:0003677

Cluster-22178.73514 GO:0006814 GO:0042773 GO:0007010 GO:0015992
GO:0006744 GO:0006120 GO:0008137 GO:0003779 GO:0005743
Cluster-22178.73521 GO:0006811 GO:0005216 GO:0046872 GO:0016020
Cluster-22178.73522 GO:0006950 GO:0009415 GO:0006890 GO:0016790
GO:0004252 GO:0001772 GO:0097197 GO:0016021
Cluster-22178.73524 GO:0007275 GO:0005578
Cluster-22178.73526 GO:0006355 GO:0006139 GO:0006914 GO:0006260
GO:0005515 GO:0043565 GO:0003700 GO:0003676 GO:0004003
GO:0016787 GO:0016818 GO:0003677 GO:0008026 GO:0005524
GO:0005634 GO:0005667 GO:0005657
Cluster-22178.73527 GO:0006260 GO:0006914 GO:0006355 GO:0006139
GO:0003677 GO:0016818 GO:0008026 GO:0016787 GO:0004003
GO:0005524 GO:0005515 GO:0003676 GO:0003700 GO:0043565
GO:0005667 GO:0005657 GO:0005634
Cluster-22178.73529 GO:0006260 GO:0006914 GO:0006355 GO:0006139
GO:0016818 GO:0003677 GO:0008026 GO:0004003 GO:0016787

GO:0005524 GO:0005515 GO:0003700 GO:0003676 GO:0043565
GO:0005667 GO:0005657 GO:0005634
Cluster-22178.73531 GO:0006914 GO:0006260 GO:0006139 GO:0006355
GO:0005524 GO:0004003 GO:0016787 GO:0016818 GO:0008026
GO:0003677 GO:0043565 GO:0003700 GO:0003676 GO:0005515
GO:0005657 GO:0005667 GO:0005634
Cluster-22178.73536 GO:0007155 GO:0048193 GO:0006914 GO:0009405
GO:0006355 GO:0048870 GO:0016818 GO:0003677 GO:0005524
GO:0008270 GO:0005198 GO:0005882 GO:0016020 GO:0031514
GO:0005634 GO:0019028 GO:0019031 GO:0019867 GO:0009288
Cluster-22178.73540 GO:0007165 GO:0008283 GO:0035556 GO:0043087
GO:0006955 GO:0040007 GO:0035023 GO:0055114 GO:0043065
GO:0005089 GO:0047134 GO:0005137 GO:0005515 GO:0008083
GO:0005740 GO:0005576 GO:0016021 GO:0005895
Cluster-22178.73541 GO:0042967 GO:0006397 GO:0007165 GO:0005125
GO:0008080 GO:0003723 GO:0005737 GO:0005634
Cluster-22178.73543 GO:0006397 GO:0007165 GO:0042967 GO:0003723
GO:0005125 GO:0008080 GO:0005634 GO:0005737
Cluster-22178.73544 GO:0007165 GO:0006397 GO:0042967 GO:0003723
GO:0008080 GO:0005125 GO:0005634 GO:0005737
Cluster-22178.73545 GO:0005515
Cluster-22178.73551 GO:0003723
Cluster-22178.73553 GO:0009451 GO:0001522 GO:0009982 GO:0003723

Cluster-22178.73558 GO:0006950 GO:0046487 GO:0015977 GO:0016984
GO:0005680 GO:0009573 GO:0016021
Cluster-22178.73559 GO:0055085 GO:0015689 GO:0022857 GO:0015098
GO:0016021
Cluster-22178.73563 GO:0007010 GO:0003779
Cluster-22178.73566 GO:0006886 GO:0006772 GO:0006464 GO:0015031
GO:0009228 GO:0005515 GO:0004417 GO:0005047 GO:0003924
GO:0008270 GO:0052855 GO:0005525 GO:0005786 GO:0005785
Cluster-22178.73569 GO:0008270 GO:0005515 GO:0005576

Cluster-22178.73573 GO:0007218 GO:0006144 GO:0046039 GO:0006182 GO:0005543 GO:0005515 GO:0004383 GO:0005576
Cluster-22178.73575 GO:0005975 GO:0005515 GO:0016832 GO:0098599

Cluster-22178.73577 GO:0051382 GO:0016032 GO:0006914 GO:0043565 GO:0005525 GO:0000776 GO:0019013
Cluster-22178.73578 GO:0007049 GO:0009405 GO:0006355 GO:0031505 GO:0003700 GO:0043565 GO:0005667
Cluster-22178.73580 GO:0016042 GO:0008152 GO:0007586 GO:0016758 GO:0008047 GO:0005576
Cluster-22178.73581 GO:0055114 GO:0015992 GO:0006120 GO:0006814 GO:0006744 GO:0008137
Cluster-22178.73585 GO:0009058 GO:0006633 GO:0042967 GO:0006355 GO:0010508 GO:0055114 GO:0008152 GO:0015696 GO:0006352 GO:0008519 GO:0003677 GO:0017111 GO:0016747 GO:0016491 GO:0016987 GO:0016788 GO:0004315 GO:0003700 GO:0005667 GO:0016020 GO:0005835
Cluster-22178.73588 GO:0007016 GO:0016010
Cluster-22178.73589 GO:0005515
Cluster-22178.73590 GO:0005515
Cluster-22178.73593 GO:0006367 GO:0005515 GO:0008270 GO:0046872 GO:0005672
Cluster-22178.73597 GO:0042274
Cluster-22178.73600 GO:0006260 GO:0007049 GO:0007166 GO:0000917 GO:0006355 GO:0043093 GO:0003700 GO:0043565 GO:0005515 GO:0005737 GO:0016020 GO:0005667
Cluster-22178.73602 GO:0006508 GO:0004222 GO:0003676 GO:0008270

Cluster-22178.73607 GO:0008152 GO:0006464 GO:0009058 GO:0006479 GO:0046500 GO:0008168 GO:0004719 GO:0016742 GO:0019867
Cluster-22178.73609 GO:0002949 GO:0006355 GO:0006281 GO:0006310 GO:0016887 GO:0005524 GO:0003678 GO:0003724 GO:0003723 GO:0009378 GO:0008134 GO:0005657 GO:0005667 GO:0009379

Cluster-22178.73613 GO:0005515
Cluster-22178.73614 GO:0061024 GO:0010033 GO:0046872 GO:0019867
Cluster-22178.73615 GO:0006260 GO:0006914 GO:0070206 GO:0000921
GO:0006352 GO:0006308 GO:0048870 GO:0035092 GO:0010508
GO:0007094 GO:0006355 GO:0008855 GO:0005524 GO:0003774
GO:0003677 GO:0016787 GO:0005815 GO:0005940 GO:0000228
GO:0016459 GO:0016021 GO:0031514 GO:0009318
Cluster-22178.73617 GO:0006955 GO:0007165 GO:0006355 GO:0030247
GO:0008270 GO:0005524 GO:0042803 GO:0003677 GO:0016818
GO:0042162 GO:0005044 GO:0005634
Cluster-22178.73618 GO:0016021
Cluster-22178.73619 GO:0016192 GO:0006886 GO:0015991 GO:0016820
GO:0005515 GO:0000221 GO:0030117
Cluster-22178.73621 GO:0007218 GO:0005184 GO:0030695 GO:0005576
Cluster-22178.73622 GO:0009245 GO:0003824 GO:0008915
Cluster-22178.73625 GO:0007165 GO:0005856
Cluster-22178.73632 GO:0005515 GO:0005509 GO:0005576
Cluster-22178.73633 GO:0051537 GO:0043231
Cluster-22178.73639 GO:0006270 GO:0006260 GO:0006206 GO:0015992
GO:0006144 GO:0006351 GO:0006119 GO:0006118 GO:0016032
GO:0055114 GO:0003677 GO:0003887 GO:0008121 GO:0003899
GO:0005730 GO:0042575 GO:0032040
Cluster-22178.73640 GO:0006570 GO:0019988 GO:0006470 GO:0008138
GO:0004518 GO:0043399 GO:0003677 GO:0004725
Cluster-22178.73642 GO:0035307 GO:0071595
Cluster-22178.73645 GO:0046983 GO:0046872
Cluster-22178.73646 GO:0007166 GO:0007186 GO:0004930 GO:0008017
GO:0016020 GO:0045298 GO:0016021
Cluster-22178.73647 GO:0006468 GO:0006629 GO:0005524 GO:0004672
GO:0016787
Cluster-22178.73648 GO:0048870 GO:0070206 GO:0031514 GO:0005639
Cluster-22178.73650 GO:0055114 GO:0051262 GO:0016491
Cluster-22178.73652 GO:0008270 GO:0005622

Cluster-22178.73654 GO:0046872 GO:0000166
Cluster-22178.73657 GO:0019988 GO:0006470 GO:0007165 GO:0006570
GO:0016829 GO:0004725 GO:0003676 GO:0005179 GO:0043399
GO:0008138 GO:0005515 GO:0009424 GO:0005576
Cluster-22178.73659 GO:0006729 GO:0022900 GO:0045454 GO:0016651
GO:0008124
Cluster-22178.73662 GO:0006552 GO:0006550 GO:0006568 GO:0015940
GO:0019521 GO:0055114 GO:0006574 GO:0006631 GO:0018874
GO:0006098 GO:0006633 GO:0006554 GO:0003857 GO:0008677
GO:0004616 GO:0051287 GO:0016491
Cluster-22178.73665 GO:0007411 GO:0008152 GO:0008484 GO:0003824

Cluster-22178.73666 GO:0008152 GO:0003824 GO:0008484
Cluster-22178.73667 GO:0030154 GO:0007275 GO:0007219 GO:0005509
GO:0005515 GO:0016021
Cluster-22178.73668 GO:0005515
Cluster-22178.73669 GO:0016021
Cluster-22178.73674 GO:0090063 GO:0007096 GO:0000226 GO:0004190
GO:0003723 GO:0005515 GO:0008146 GO:0005634 GO:0016020
GO:0000922 GO:0005815
Cluster-22178.73676 GO:0006012 GO:0006260 GO:0019872 GO:0005982
GO:0006096 GO:0051156 GO:0006308 GO:0005985 GO:0010508
GO:0071816 GO:0006094 GO:0006355 GO:0008855 GO:0004340
GO:0005524 GO:0003700 GO:0005198 GO:0005667 GO:0009318
Cluster-22178.73677 GO:0042254 GO:0006412 GO:0046872 GO:0005515
GO:0003735 GO:0005622 GO:0005840
Cluster-22178.73678 GO:0006955 GO:0030153 GO:0006516 GO:0015643
GO:0019814 GO:0005737
Cluster-22178.73680 GO:0046872 GO:0005515
Cluster-22178.73685 GO:0005515
Cluster-22178.73687 GO:0007165 GO:0009069 GO:0035556 GO:0006888
GO:0006886 GO:0006468 GO:0016310 GO:0055114 GO:0007178
GO:0004675 GO:0047134 GO:0005524 GO:0008270 GO:0005515

GO:0003723 GO:0016020 GO:0030127
Cluster-22178.73688 GO:0007178 GO:0016310 GO:0035556 GO:0006468
GO:0009069 GO:0007165 GO:0005524 GO:0004675 GO:0005515
GO:0016020
Cluster-22178.73689 GO:0016032 GO:0006886 GO:0006888 GO:0016310
GO:0000160 GO:0000155 GO:0003677 GO:0008270 GO:0046983
GO:0019028 GO:0030127 GO:0009365 GO:0016021
Cluster-22178.73693 GO:0042254 GO:0006412 GO:0003735 GO:0005840
GO:0005622
Cluster-22178.73694 GO:0042254 GO:0007186 GO:0006412 GO:0046872
GO:0004930 GO:0003735 GO:0005622 GO:0016021 GO:0005840
Cluster-22178.73697 GO:0016788 GO:0005515
Cluster-22178.73699 GO:0016747
Cluster-22178.73700 GO:0019058 GO:0055114 GO:0020037 GO:0016705
GO:0005506
Cluster-22178.73702 GO:0006027 GO:0006687 GO:0046486 GO:0005975
GO:0006012 GO:0005515 GO:0046872 GO:0004565 GO:0009341
Cluster-22178.73703 GO:0018279 GO:0005789
Cluster-22178.73705 GO:0007264 GO:0006355 GO:0002949 GO:0008134
GO:0003723 GO:0003724 GO:0005525 GO:0016887 GO:0005524
GO:0005667
Cluster-22178.73707 GO:0006955 GO:0019882 GO:0016020 GO:0042613
Cluster-22178.73714 GO:0006090 GO:0006525 GO:0006814 GO:0071436
GO:0006118 GO:0006955 GO:0006560 GO:0015081 GO:0009055
GO:0008948 GO:0005515 GO:0016021 GO:0016020
Cluster-22178.73715 GO:0006470 GO:0006570 GO:0008138 GO:0004725

Cluster-22178.73717 GO:0006464 GO:0006914 GO:0008608 GO:0006606
GO:0006260 GO:0007165 GO:0045502 GO:0005515 GO:0008134
GO:0042803 GO:0030286 GO:0016021 GO:0072686 GO:0005667
GO:0042729
Cluster-22178.73718 GO:0008152 GO:0016491
Cluster-22178.73720 GO:0006508 GO:0005515 GO:0004198 GO:0005622

Cluster-22178.73725 GO:0008033 GO:0006479 GO:0009451 GO:0008610
GO:0006464 GO:0030488 GO:0006396 GO:0042967 GO:0046500
GO:0046486 GO:0004719 GO:0016627 GO:0008168 GO:0016429
GO:0004144 GO:0005622 GO:0031515
Cluster-22178.73726 GO:0031175 GO:0006265 GO:0005516 GO:0003677
GO:0030507 GO:0005515 GO:0046872 GO:0003916 GO:0008270
GO:0004222 GO:0008233 GO:0005694 GO:0031012 GO:0008091
Cluster-22178.73727 GO:0048037
Cluster-22178.73729 GO:0006357 GO:0005515 GO:0001104 GO:0016592
Cluster-22178.73731 GO:0006355 GO:0015986 GO:0015992 GO:0003677
GO:0015078 GO:0000276
Cluster-22178.73732 GO:0006355 GO:0006289 GO:0000439
Cluster-22178.73733 GO:0005515
Cluster-22178.73735 GO:0007017 GO:0030001 GO:0006355 GO:0007018
GO:0006952 GO:0055085 GO:0006606 GO:0003700 GO:0003777
GO:0046873 GO:0005524 GO:0008017 GO:0045298 GO:0005874
GO:0005667 GO:0016020
Cluster-22178.73736 GO:0007010 GO:0007165 GO:0055085 GO:0006898
GO:0006811 GO:0005216 GO:0005044 GO:0003779 GO:0016020
Cluster-22178.73739 GO:0055085 GO:0006812 GO:0006885 GO:0015299
GO:0016021
Cluster-22178.73740 GO:0007165 GO:0035556
Cluster-22178.73744 GO:0016020
Cluster-22178.73745 GO:0019538 GO:0005515 GO:0019028
Cluster-22178.73746 GO:0055114 GO:0019310 GO:0006355 GO:0044406
GO:0050113 GO:0005506 GO:0005515 GO:0005737 GO:0016021
GO:0009289 GO:0005634
Cluster-22178.73747 GO:0051016 GO:0008290
Cluster-22178.73750 GO:0005525 GO:0003924
Cluster-22178.73751 GO:0006355 GO:0006051 GO:0008033 GO:0055114
GO:0006537 GO:0007219 GO:0008152 GO:0046497 GO:0007275
GO:0009607 GO:0006040 GO:0009435 GO:0006807 GO:0000105
GO:0004514 GO:0047465 GO:0016627 GO:0016829 GO:0017150

GO:0003824 GO:0016638 GO:0015930 GO:0018580 GO:0016491
GO:0050660 GO:0005737 GO:0016021
Cluster-22178.73752 GO:0007186 GO:0004190 GO:0004930 GO:0005515
GO:0016021
Cluster-22178.73754 GO:0006355 GO:0005515
Cluster-22178.73757 GO:0002098 GO:0006629 GO:0034227 GO:0000049

Cluster-22178.73758 GO:0006508 GO:0004197
Cluster-22178.73761 GO:0046500 GO:0046486 GO:0006464 GO:0030488
GO:0042967 GO:0006396 GO:0008610 GO:0009451 GO:0006479
GO:0008033 GO:0004144 GO:0016429 GO:0008168 GO:0016627
GO:0004719 GO:0031515 GO:0005622
Cluster-22178.73762 GO:0006418 GO:0043039 GO:0005524 GO:0000166
GO:0000049 GO:0004812 GO:0016746 GO:0016787 GO:0005680
GO:0005737
Cluster-22178.73763 GO:0006308 GO:0008855 GO:0009318
Cluster-22178.73765 GO:0009405 GO:0006468 GO:0019865 GO:0005524
GO:0004672 GO:0005515 GO:0051082 GO:0019814
Cluster-22178.73766 GO:0006355 GO:0003677 GO:0005515
Cluster-22178.73767 GO:0006687 GO:0050290
Cluster-22178.73771 GO:0016567 GO:0005515 GO:0004842 GO:0008270
GO:0046872 GO:0005680
Cluster-22178.73772 GO:0009405 GO:0007165 GO:0006355 GO:0000160
GO:0016310 GO:0003677 GO:0000155 GO:0005576 GO:0009365
Cluster-22178.73774 GO:0003676
Cluster-22178.73776 GO:0009252 GO:0006807 GO:0008270 GO:0003676
GO:0004356 GO:0005634 GO:0005923
Cluster-22178.73778 GO:0015031 GO:0006606 GO:0015991 GO:0006886
GO:0016192 GO:0008565 GO:0016820 GO:0005515 GO:0005737
GO:0000221 GO:0005634 GO:0030117
Cluster-22178.73779 GO:0015991 GO:0005515 GO:0016820 GO:0000221
Cluster-22178.73782 GO:0009307 GO:0006464 GO:0030488 GO:0035556
GO:0009451 GO:0006308 GO:0008033 GO:0006418 GO:0004812

GO:0003677 GO:0005524 GO:0009036 GO:0016429 GO:0000166
GO:0016151 GO:0031515 GO:0009359
Cluster-22178.73783 GO:0007155 GO:0046740 GO:0005515 GO:0016020
GO:0016021
Cluster-22178.73786 GO:0006508 GO:0004252
Cluster-22178.73787 GO:0006468 GO:0004672 GO:0005524
Cluster-22178.73788 GO:0016310 GO:0015886 GO:0009069 GO:0006468
GO:0004674 GO:0016773 GO:0004672 GO:0005524 GO:0015232
Cluster-22178.73790 GO:0006270 GO:0007165
Cluster-22178.73791 GO:0006508 GO:0004252
Cluster-22178.73794 GO:0045454
Cluster-22178.73797 GO:0055085 GO:0006810 GO:0005215 GO:0016020
Cluster-22178.73798 GO:0055085 GO:0006810 GO:0005215 GO:0016020
Cluster-22178.73802 GO:0030001 GO:0048870 GO:0051258 GO:0007165
GO:0055085 GO:0030168 GO:0005515 GO:0046872 GO:0030674
GO:0046873 GO:0005102 GO:0008270 GO:0031514 GO:0016021
GO:0016020 GO:0005577 GO:0005622
Cluster-22178.73805 GO:0007165 GO:0007186 GO:0008643 GO:0055114
GO:0016641 GO:0005351 GO:0005525 GO:0005507 GO:0004871
GO:0031683 GO:0019001 GO:0003924 GO:0000139 GO:0016021
Cluster-22178.73806 GO:0004867
Cluster-22178.73810 GO:0006355 GO:0043565 GO:0003700 GO:0003677
GO:0008289 GO:0005667 GO:0005634
Cluster-22178.73811 GO:0006144 GO:0007165 GO:0004114 GO:0005515

Cluster-22178.73812 GO:0006144 GO:0007165 GO:0004114 GO:0005515

Cluster-22178.73813 GO:0007264 GO:0007186 GO:0007165 GO:0003924
GO:0019001 GO:0031683 GO:0004871 GO:0005525
Cluster-22178.73815 GO:0006886 GO:0008152 GO:0016836 GO:0003824
GO:0016021 GO:0005783
Cluster-22178.73816 GO:0006090 GO:0045116 GO:0006525 GO:0071436
GO:0006814 GO:0006560 GO:0016881 GO:0005524 GO:0015081

GO:0008948 GO:0016020
Cluster-22178.73818 GO:0019079 GO:0006810 GO:0006614 GO:0006355
GO:0003676 GO:0004386 GO:0005525 GO:0016887 GO:0005524
GO:0003677 GO:0008026 GO:0016787
Cluster-22178.73819 GO:0016021
Cluster-22178.73822 GO:0008643 GO:0006355 GO:0008270 GO:0003677
GO:0008982 GO:0003682 GO:0000785 GO:0000775 GO:0009357
GO:0019028 GO:0005634
Cluster-22178.73823 GO:0006952 GO:0006810 GO:0009405 GO:0019855
GO:0005576
Cluster-22178.73824 GO:0005515
Cluster-22178.73826 GO:0006811 GO:0005216 GO:0016020
Cluster-22178.73829 GO:0006810 GO:0055085 GO:0005515 GO:0046872
GO:0003924 GO:0005525 GO:0005215 GO:0022857 GO:0016020
GO:0016021
Cluster-22178.73831 GO:0055114 GO:0043123 GO:0045766 GO:0009186
GO:0043066
Cluster-22178.73835 GO:0046339 GO:0009395 GO:0016042 GO:0004435
GO:0005509
Cluster-22178.73843 GO:0006779 GO:0015994 GO:0004853
Cluster-22178.73844 GO:0005515
Cluster-22178.73846 GO:0005515
Cluster-22178.73849 GO:0006807 GO:0009252 GO:0006355 GO:0003700
GO:0043565 GO:0005515 GO:0004356 GO:0005667 GO:0055036
GO:0005634
Cluster-22178.73851 GO:0006544 GO:0055114 GO:0006563 GO:0006566
GO:0046653 GO:0003950 GO:0046983 GO:0046872 GO:0016705
GO:0020037 GO:0005506 GO:0008115
Cluster-22178.73852 GO:0004190 GO:0016021
Cluster-22178.73853 GO:0006886 GO:0019079 GO:0007165 GO:0006888
GO:0005515 GO:0016021 GO:0030127
Cluster-22178.73854 GO:0007165 GO:0006508 GO:0007155 GO:0005173
GO:0008236 GO:0016020

Cluster-22178.73855 GO:0000245 GO:0005634
Cluster-22178.73857 GO:0007155 GO:0006468 GO:0004672 GO:0005524
GO:0016020
Cluster-22178.73864 GO:0006313 GO:0006886 GO:0016192 GO:0003677
GO:0005515 GO:0004803
Cluster-22178.73865 GO:0006886 GO:0016192 GO:0005198 GO:0005515
GO:0030130 GO:0030132 GO:0030117
Cluster-22178.73867 GO:0005515
Cluster-22178.73868 GO:0005515
Cluster-22178.73869 GO:0006468 GO:0004672 GO:0005524 GO:0016773
GO:0016020
Cluster-22178.73873 GO:0009308 GO:0055114 GO:0008131 GO:0005507
GO:0048038 GO:0003676 GO:0005524
Cluster-22178.73874 GO:0006284 GO:0006412 GO:0042254 GO:0003735
GO:0016799 GO:0003906 GO:0005840 GO:0005622
Cluster-22178.73877 GO:0005515
Cluster-22178.73878 GO:0042254 GO:0006412 GO:0003735 GO:0005840
GO:0005622
Cluster-22178.73881 GO:0006418 GO:0043039 GO:0003676 GO:0004866
GO:0005524 GO:0000166 GO:0000049 GO:0004812 GO:0005737
Cluster-22178.73884 GO:0006629
Cluster-22178.73889 GO:0006811 GO:0034508 GO:0006810 GO:0005215
GO:0004190 GO:0016021 GO:0016020 GO:0000776
Cluster-22178.73894 GO:0006508 GO:0007606 GO:0006886 GO:0007165
GO:0006888 GO:0003723 GO:0004888 GO:0005515 GO:0004197
GO:0008270 GO:0016020 GO:0030127
Cluster-22178.73895 GO:0042254 GO:0006412 GO:0007411 GO:0005515
GO:0003735 GO:0044599 GO:0005622 GO:0005840
Cluster-22178.73897 GO:0008152 GO:0016791 GO:0016021
Cluster-22178.73898 GO:0016020
Cluster-22178.73899 GO:0016020
Cluster-22178.73903 GO:0055085 GO:0006811 GO:0006810 GO:0005215
GO:0016020

Cluster-22178.73906 GO:0006825 GO:0035434 GO:0003676 GO:0005375
GO:0016020 GO:0016021
Cluster-22178.73907 GO:0006357 GO:0006935 GO:0000160 GO:0000077
GO:0006260 GO:0016310 GO:0016032 GO:0004673 GO:0000155
GO:0019901 GO:0001104 GO:0005737 GO:0009365 GO:0016592
Cluster-22178.73911 GO:0007165 GO:0035556
Cluster-22178.73913 GO:0006281 GO:0000077 GO:0030896
Cluster-22178.73916 GO:0005515
Cluster-22178.73917 GO:0007304 GO:0046872 GO:0005213 GO:0005515
GO:0042600 GO:0016021 GO:0045202 GO:0005576
Cluster-22178.73919 GO:0006098 GO:0006096 GO:0006457 GO:0006013
GO:0043087 GO:0006000 GO:0006012 GO:0006094 GO:0006570
GO:0006470 GO:0003872 GO:0051082 GO:0005515 GO:0004725
GO:0016020 GO:0005639 GO:0016272 GO:0005945
Cluster-22178.73923 GO:0006570 GO:0006470 GO:0008138 GO:0004725
GO:0016021
Cluster-22178.73927 GO:0003924 GO:0005525
Cluster-22178.73928 GO:0005515
Cluster-22178.73930 GO:0055085 GO:0005525 GO:0016021
Cluster-22178.73932 GO:0000902 GO:0006816 GO:0006874 GO:0005198
GO:0005219 GO:0019013 GO:0016021 GO:0005622 GO:0001772
GO:0097197
Cluster-22178.73935 GO:0006278 GO:0003964
Cluster-22178.73936 GO:0003723 GO:0003676
Cluster-22178.73937 GO:0003723 GO:0003676
Cluster-22178.73939 GO:0003723 GO:0003676
Cluster-22178.73943 GO:0051262
Cluster-22178.73946 GO:0015992 GO:0006119 GO:0015991 GO:0005524
GO:0005515 GO:0008900 GO:0000287 GO:0005889 GO:0016020
Cluster-22178.73949 GO:0007021 GO:0051082 GO:0015631 GO:0045298
Cluster-22178.73951 GO:0006506 GO:0016757
Cluster-22178.73952 GO:0006355 GO:0005515 GO:0003700 GO:0005667
GO:0005634

Cluster-22178.73954 GO:0046872
Cluster-22178.73956 GO:0005515
Cluster-22178.73961 GO:0048268 GO:0007212 GO:0019236 GO:0007186
GO:0032051 GO:0016021
Cluster-22178.73965 GO:0030001 GO:0005515 GO:0046872 GO:0019028
Cluster-22178.73967 GO:0016021
Cluster-22178.73970 GO:0003774 GO:0003723 GO:0016459
Cluster-22178.73971 GO:0003723
Cluster-22178.73972 GO:0055114 GO:0015031 GO:0006118 GO:0005982
GO:0005985 GO:0030683 GO:0030245 GO:0016655 GO:0046983
GO:0005515 GO:0008810 GO:0016021 GO:0019031 GO:0019012
GO:0016020
Cluster-22178.73974 GO:0004867
Cluster-22178.73978 GO:0008152 GO:0016746
Cluster-22178.73979 GO:0006360 GO:0001168
Cluster-22178.73980 GO:0006813 GO:0006508 GO:0019497 GO:0016567
GO:0006771 GO:0005515 GO:0016740 GO:0046872 GO:0005249
GO:0004197 GO:0004842 GO:0003993 GO:0008270 GO:0008242
GO:0008076 GO:0005680
Cluster-22178.73983 GO:0046872
Cluster-22178.73986 GO:0015774 GO:0015159 GO:0016020
Cluster-22178.73988 GO:0006470 GO:0042254 GO:0006570 GO:0006412
GO:0004725 GO:0008138 GO:0003735 GO:0005840
Cluster-22178.73994 GO:0016020
Cluster-22178.73996 GO:0007154 GO:0006812 GO:0016887 GO:0016021
Cluster-22178.73997 GO:0007165 GO:0006955 GO:0006935 GO:0008009
GO:0005576
Cluster-22178.74001 GO:0008033 GO:0006400 GO:0031167 GO:0009451
GO:0035556 GO:0008168 GO:0008176
Cluster-22178.74003 GO:0005975 GO:0003824 GO:0043169 GO:0030089
Cluster-22178.74005 GO:0008152 GO:0009405 GO:0055085 GO:0007186
GO:0007165 GO:0006614 GO:0006810 GO:0003924 GO:0019001
GO:0031683 GO:0004871 GO:0005525 GO:0003723 GO:0016301

GO:0016887 GO:0005524 GO:0042626 GO:0003724 GO:0016746
GO:0016021 GO:0005576
Cluster-22178.74007 GO:0006352 GO:0006139 GO:0006576 GO:0006355
GO:0006281 GO:0006206 GO:0002949 GO:0006310 GO:0003678
GO:0003723 GO:0005515 GO:0008134 GO:0000166 GO:0016887
GO:0005524 GO:0004127 GO:0003724 GO:0051537 GO:0009378
GO:0098519 GO:0003677 GO:0005669 GO:0005657 GO:0005667
GO:0009379 GO:0043231
Cluster-22178.74008 GO:0019079 GO:0009235 GO:0006576 GO:0005524

Cluster-22178.74010 GO:0006979 GO:0008203 GO:0006804 GO:0016042
GO:0004601 GO:0016298
Cluster-22178.74011 GO:0006749 GO:0006098 GO:0006006 GO:0055114
GO:0050661 GO:0004345
Cluster-22178.74018 GO:0007186 GO:0007165 GO:0007264 GO:0004871
GO:0005525 GO:0003924 GO:0019001 GO:0031683
Cluster-22178.74020 GO:0006508 GO:0016787 GO:0008236
Cluster-22178.74024 GO:0009405 GO:0005576
Cluster-22178.74025 GO:0006554 GO:0006479 GO:0006355 GO:0034968
GO:0006508 GO:0005515 GO:0004252 GO:0018024 GO:0005694
Cluster-22178.74026 GO:0006464
Cluster-22178.74028 GO:0005515
Cluster-22178.74036 GO:0006810 GO:0006811 GO:0005230 GO:0016020
Cluster-22178.74037 GO:0003676
Cluster-22178.74038 GO:0035556 GO:0005515 GO:0016020
Cluster-22178.74040 GO:0042254 GO:0006412 GO:0003735 GO:0005515
GO:0005840 GO:0005622
Cluster-22178.74042 GO:0005515
Cluster-22178.74043 GO:0016192
Cluster-22178.74044 GO:0000723 GO:0006281 GO:0005524 GO:0046872
GO:0003824 GO:0003678 GO:0016787 GO:0003677 GO:0005657
GO:0016020
Cluster-22178.74045 GO:0043039 GO:0006531 GO:0006522 GO:0051301

GO:0006419 GO:0005515 GO:0003677 GO:0000166 GO:0016876
GO:0004813 GO:0005524
Cluster-22178.74052 GO:0006468 GO:0005524 GO:0004672 GO:0016773
GO:0016020
Cluster-22178.74061 GO:0046872
Cluster-22178.74062 GO:0007268 GO:0007165 GO:0007224 GO:0030550
GO:0005576
Cluster-22178.74063 GO:0003676
Cluster-22178.74064 GO:0006355 GO:0009451 GO:0001522 GO:0006810
GO:0003723 GO:0005515 GO:0046983 GO:0009982 GO:0005215
GO:0016021
Cluster-22178.74067 GO:0045892 GO:0005634
Cluster-22178.74069 GO:0040007 GO:0006576 GO:0035023 GO:0007264
GO:0008283 GO:0019068 GO:0015684 GO:0007165 GO:0006270
GO:0007186 GO:0043087 GO:0035556 GO:0005515 GO:0005198
GO:0008083 GO:0046872 GO:0019001 GO:0031683 GO:0003924
GO:0005525 GO:0004871 GO:0015093 GO:0005524 GO:0016887
GO:0016757 GO:0005089 GO:0016021 GO:0016020
Cluster-22178.74074 GO:0006506 GO:0017176 GO:0016021 GO:0016020
Cluster-22178.74076 GO:0046854
Cluster-22178.74077 GO:0006270
Cluster-22178.74078 GO:0006118 GO:0020037 GO:0009055
Cluster-22178.74082 GO:0048870 GO:0005975 GO:0001558 GO:0030178
GO:0006914 GO:0006606 GO:0007275 GO:0007165 GO:0005515
GO:0005520 GO:0008270 GO:0004553 GO:0030246 GO:0005576
GO:0031514 GO:0016942
Cluster-22178.74083 GO:0042967 GO:0004379
Cluster-22178.74084 GO:0015992 GO:0006119 GO:0006450 GO:0006118
GO:0007160 GO:0008121 GO:0009055
Cluster-22178.74087 GO:0007165 GO:0040007 GO:0008283 GO:0008083

Cluster-22178.74088 GO:0006810 GO:0006508 GO:0055085 GO:0004222
GO:0008270 GO:0016020 GO:0016021 GO:0031012

Cluster-22178.74089 GO:0006508 GO:0006810 GO:0055085 GO:0008270
GO:0004222 GO:0016020 GO:0031012 GO:0016021
Cluster-22178.74090 GO:0015684 GO:0007264 GO:0005525 GO:0015093
GO:0005524 GO:0003924 GO:0016887 GO:0043531 GO:0016020
GO:0016021
Cluster-22178.74091 GO:0032065 GO:0042254 GO:0006412 GO:0009103
GO:0008918 GO:0005543 GO:0005524 GO:0005096 GO:0098519
GO:0005515 GO:0003735 GO:0005840 GO:0005938 GO:0016021
GO:0005783
Cluster-22178.74092 GO:0051424
Cluster-22178.74093 GO:0005515
Cluster-22178.74094 GO:0006952 GO:0006351 GO:0006269 GO:0008152
GO:0055114 GO:0004386 GO:0003896 GO:0016846 GO:0008270
GO:0033743 GO:0005730 GO:0005657
Cluster-22178.74096 GO:0008152 GO:0016042 GO:0005975 GO:0046486
GO:0009058 GO:0006508 GO:0004806 GO:0016788 GO:0008236
GO:0008184 GO:0016787
Cluster-22178.74097 GO:0007049 GO:0005634
Cluster-22178.74098 GO:0019491 GO:0005515 GO:0033990 GO:0016021
Cluster-22178.74099 GO:0006508 GO:0008152 GO:0008270 GO:0016788
GO:0004181 GO:0005739
Cluster-22178.74100 GO:0003677 GO:0005634
Cluster-22178.74101 GO:0007606 GO:0016021
Cluster-22178.74109 GO:0019497 GO:0006771 GO:0003677 GO:0003993

Cluster-22178.74111 GO:0016021
Cluster-22178.74112 GO:0008152 GO:0016491
Cluster-22178.74113 GO:0006570 GO:0006470 GO:0007165 GO:0005515
GO:0004721 GO:0004725 GO:0008138
Cluster-22178.74116 GO:0006464 GO:0015031
Cluster-22178.74117 GO:0010508 GO:0048309 GO:0051028 GO:0015992
GO:0006606 GO:0048193 GO:0006355 GO:0007017 GO:0015991
GO:0007018 GO:0051258 GO:0048870 GO:0007165 GO:0016567

GO:0000921 GO:0030168 GO:0005198 GO:0005515 GO:0042803
GO:0016881 GO:0015078 GO:0005524 GO:0008017 GO:0045502
GO:0019901 GO:0008134 GO:0003777 GO:0003700 GO:0030674
GO:0031386 GO:0005102 GO:0005634 GO:0045298 GO:0005667
GO:0016020 GO:0005577 GO:0019028 GO:0031514 GO:0033179
GO:0005874 GO:0016021 GO:0005882 GO:0030286 GO:0005940
GO:0005815
Cluster-22178.74125 GO:0048870 GO:0070206 GO:0005515 GO:0005639
GO:0031514
Cluster-22178.74126 GO:0003677
Cluster-22178.74127 GO:0003677
Cluster-22178.74128 GO:0006812 GO:0006885 GO:0055085 GO:0007165
GO:0015299 GO:0005179 GO:0016021 GO:0005576
Cluster-22178.74129 GO:0051297 GO:0005813
Cluster-22178.74130 GO:0007165 GO:0006885 GO:0006812 GO:0055085
GO:0005179 GO:0015299 GO:0005576 GO:0016021
Cluster-22178.74131 GO:0055085 GO:0006885 GO:0006812 GO:0007165
GO:0015299 GO:0005179 GO:0016021 GO:0005576
Cluster-22178.74132 GO:0042157 GO:0006355 GO:0006869 GO:0006810
GO:0008270 GO:0003700 GO:0008289 GO:0005515 GO:0005886
GO:0005622 GO:0005667 GO:0005576
Cluster-22178.74137 GO:0006810 GO:0007218 GO:0006741 GO:0008152
GO:0046497 GO:0006769 GO:0006308 GO:0008855 GO:0003951
GO:0019871 GO:0016301 GO:0005198 GO:0005882 GO:0005886
GO:0009318 GO:0005576
Cluster-22178.74142 GO:0006355 GO:0006118 GO:0022900 GO:0055114
GO:0016787 GO:0005515 GO:0003677 GO:0043565 GO:0046983
GO:0008827 GO:0003700 GO:0005667 GO:0009319 GO:0016021
Cluster-22178.74147 GO:0005509
Cluster-22178.74148 GO:0005509
Cluster-22178.74152 GO:0006355 GO:0006511 GO:0043401 GO:0055114
GO:0008152 GO:0050662 GO:0031625 GO:0016491 GO:0016872
GO:0003824 GO:0016020

Cluster-22178.74153 GO:0055114 GO:0008152 GO:0006355 GO:0043401 GO:0006511 GO:0016491 GO:0003824 GO:0031625 GO:0050662
Cluster-22178.74154 GO:0055085 GO:0006812 GO:0009401 GO:0008324 GO:0016021 GO:0016020
Cluster-22178.74158 GO:0042254 GO:0006412 GO:0003735 GO:0005622 GO:0005840
Cluster-22178.74164 GO:0006355 GO:0043565 GO:0008270 GO:0003700 GO:0005634 GO:0005667
Cluster-22178.74165 GO:0006412 GO:0042254 GO:0003735 GO:0005840 GO:0005622
Cluster-22178.74166 GO:0007049 GO:0051301 GO:0005509 GO:0016021
Cluster-22178.74169 GO:0035307 GO:0000042 GO:0005515 GO:0071595
Cluster-22178.74170 GO:0006464 GO:0005524 GO:0046872
Cluster-22178.74171 GO:0051258 GO:0030168 GO:0006811 GO:0007165 GO:0005102 GO:0030674 GO:0005216 GO:0005515 GO:0005577 GO:0016020
Cluster-22178.74172 GO:0006364 GO:0042254 GO:0006412 GO:0005515 GO:0003735 GO:0005840 GO:0005622
Cluster-22178.74176 GO:0051301 GO:0007059 GO:0006355 GO:0034080 GO:0042157 GO:0006457 GO:0007186 GO:0006869 GO:0003700 GO:0003676 GO:0008289 GO:0051082 GO:0035299 GO:0005524 GO:0004930 GO:0003677 GO:0016021 GO:0000775 GO:0005576 GO:0016272 GO:0000444 GO:0005667
Cluster-22178.74179 GO:0006397 GO:0046660 GO:0006812 GO:0003676 GO:0016887 GO:0016021 GO:0005634
Cluster-22178.74180 GO:0006525 GO:0006560 GO:0006814 GO:0071436 GO:0006090 GO:0003677 GO:0008948 GO:0015081 GO:0016020
Cluster-22178.74181 GO:0006468 GO:0051252 GO:0016773 GO:0004672 GO:0005524 GO:0003676 GO:0004523 GO:0016020
Cluster-22178.74182 GO:0006364 GO:0005515 GO:0032040
Cluster-22178.74183 GO:0046872
Cluster-22178.74184 GO:0006355 GO:0003700 GO:0043565 GO:0005667
Cluster-22178.74189 GO:0008033 GO:0009452 GO:0008152 GO:0001510

GO:0006351 GO:0031167 GO:0009451 GO:0006206 GO:0006144
GO:0006396 GO:0006400 GO:0008168 GO:0003899 GO:0005515
GO:0008173 GO:0008176 GO:0003677 GO:0005730
Cluster-22178.74191 GO:0005515 GO:0005201 GO:0005581 GO:0005578
Cluster-22178.74195 GO:0006334 GO:0006955 GO:0007165 GO:0005044
GO:0030247 GO:0005634
Cluster-22178.74199 GO:0006260 GO:0006457 GO:0043093 GO:0008152
GO:0006506 GO:0000917 GO:0006355 GO:0003824 GO:0003700
GO:0008484 GO:0043565 GO:0005515 GO:0042803 GO:0008134
GO:0045502 GO:0016740 GO:0051082 GO:0016272 GO:0030286
GO:0005667 GO:0005789 GO:0005737 GO:0005861
Cluster-22178.74201 GO:0005524
Cluster-22178.74204 GO:0006771 GO:0019497 GO:0006013 GO:0006000
GO:0003873 GO:0016301 GO:0003993 GO:0005524
Cluster-22178.74205 GO:0007217 GO:0006260 GO:0003887 GO:0042575
Cluster-22178.74211 GO:0016021
Cluster-22178.74217 GO:0035556 GO:0006012 GO:0042254 GO:0046486
GO:0005975 GO:0032065 GO:0006412 GO:0001575 GO:0004553
GO:0005543 GO:0008242 GO:0003735 GO:0005515 GO:0004557
GO:0005622 GO:0005938 GO:0005840
Cluster-22178.74219 GO:0016567 GO:0046872 GO:0008270 GO:0051082
GO:0004842 GO:0005515 GO:0005680
Cluster-22178.74221 GO:0003677 GO:0046872 GO:0005634
Cluster-22178.74226 GO:0008152 GO:0055114 GO:0006520 GO:0016491
GO:0016746
Cluster-22178.74227 GO:0006397 GO:0008270
Cluster-22178.74229 GO:0048309 GO:0051028 GO:0019049 GO:0005096
GO:0005515
Cluster-22178.74233 GO:0042720
Cluster-22178.74235 GO:0000077 GO:0006281 GO:0030896
Cluster-22178.74238 GO:0015694 GO:0005975 GO:0030150 GO:0015097
GO:0004553 GO:0005742 GO:0016020 GO:0016021
Cluster-22178.74239 GO:0005515

Cluster-22178.74241 GO:0006418 GO:0005524 GO:0004812 GO:0000166

Cluster-22178.74245 GO:0055114 GO:0022900 GO:0006118 GO:0008827
GO:0009319 GO:0016021
Cluster-22178.74246 GO:0007165 GO:0050776 GO:0014068 GO:0005102
GO:0043548 GO:0016020
Cluster-22178.74251 GO:0006464 GO:0046872
Cluster-22178.74253 GO:0006570 GO:0006470 GO:0050852 GO:0004725

Cluster-22178.74255 GO:0055085 GO:0006811 GO:0005216 GO:0003677
GO:0016020
Cluster-22178.74256 GO:0016192 GO:0017004 GO:0007165 GO:0006810
GO:0015886 GO:0006357 GO:0007010 GO:0005975 GO:0016853
GO:0004872 GO:0043395 GO:0005515 GO:0001104 GO:0005198
GO:0005215 GO:0003779 GO:0016020 GO:0016592 GO:0019028
GO:0005578 GO:0016021 GO:0019031
Cluster-22178.74258 GO:0006364 GO:0006813 GO:0005267 GO:0032040
GO:0034457 GO:0005634 GO:0005732
Cluster-22178.74259 GO:0055114 GO:0006464 GO:0008152 GO:0006298
GO:0007165 GO:0016491 GO:0005524 GO:0030983 GO:0019901
Cluster-22178.74265 GO:0006574 GO:0006554 GO:0055114 GO:0006633
GO:0008152 GO:0006568 GO:0018874 GO:0006550 GO:0006552
GO:0006631 GO:0016491 GO:0003824 GO:0051287 GO:0050662
GO:0003857
Cluster-22178.74269 GO:0006820 GO:0016021
Cluster-22178.74270 GO:0002143 GO:0030683 GO:0003676 GO:0005524
GO:0008092 GO:0019898 GO:0005737 GO:0019012
Cluster-22178.74272 GO:0042254 GO:0006412 GO:0003735 GO:0005840
GO:0005622
Cluster-22178.74273 GO:0006189 GO:0005515
Cluster-22178.74275 GO:0048870 GO:0007165 GO:0005515 GO:0031514
Cluster-22178.74280 GO:0006468 GO:0004672 GO:0005524 GO:0016773
GO:0016020

Cluster-22178.74281 GO:0006468 GO:0009069 GO:0055114 GO:0016310
GO:0035556 GO:0005515 GO:0004672 GO:0005524 GO:0047134
GO:0004674
Cluster-22178.74282 GO:0016567 GO:0015684 GO:0007264 GO:0006812
GO:0006284 GO:0032801 GO:0006289 GO:0006576 GO:0006355
GO:0006281 GO:0019076 GO:0003684 GO:0003677 GO:0003906
GO:0004842 GO:0016887 GO:0015093 GO:0005524 GO:0008270
GO:0016799 GO:0005261 GO:0005525 GO:0003924 GO:0005680
GO:0033644 GO:0016021
Cluster-22178.74285 GO:0006118 GO:0016192 GO:0019684 GO:0006904
GO:0045156 GO:0016021 GO:0030077
Cluster-22178.74286 GO:0006468 GO:0005524 GO:0004672
Cluster-22178.74287 GO:0005515 GO:0046872 GO:0016020
Cluster-22178.74288 GO:0006144 GO:0007050 GO:0006508 GO:0045859
GO:0003968 GO:0004861 GO:0004197 GO:0017111 GO:0031379
GO:0005634
Cluster-22178.74289 GO:0000398 GO:0046983 GO:0005681 GO:0019031
Cluster-22178.74290 GO:0055114 GO:0006449 GO:0006355 GO:0055085
GO:0034508 GO:0006810 GO:0006415 GO:0003700 GO:0005215
GO:0016149 GO:0005840 GO:0005737 GO:0000776 GO:0018444
GO:0016020 GO:0005667
Cluster-22178.74291 GO:0007165 GO:0005158 GO:0005899
Cluster-22178.74293 GO:0003723 GO:0005737
Cluster-22178.74294 GO:0005515
Cluster-22178.74296 GO:0000902 GO:0005975 GO:0016787 GO:0016773

Cluster-22178.74297 GO:0006119 GO:0015986 GO:0015992 GO:0046961
GO:0016787 GO:0003824 GO:0046933 GO:0045259 GO:0000275
Cluster-22178.74300 GO:0043087 GO:0035023 GO:0005089
Cluster-22178.74301 GO:0055085 GO:0006810 GO:0006508 GO:0004222
GO:0008270 GO:0016021 GO:0031012 GO:0016020
Cluster-22178.74307 GO:0006801 GO:0006914 GO:0055114 GO:0046872
GO:0008134 GO:0042803 GO:0045502 GO:0030286 GO:0005667

Cluster-22178.74308 GO:0006270 GO:0046983
Cluster-22178.74309 GO:0000062 GO:0005634
Cluster-22178.74310 GO:0006355 GO:0009271 GO:0005198 GO:0019028
GO:0005882
Cluster-22178.74311 GO:0005525 GO:0003924
Cluster-22178.74314 GO:0006508 GO:0004252
Cluster-22178.74320 GO:0042742 GO:0006508 GO:0006260 GO:0006606
GO:0005515 GO:0004222 GO:0005576 GO:0005815
Cluster-22178.74321 GO:0042742 GO:0006508 GO:0006260 GO:0006606
GO:0004222 GO:0005576 GO:0005815
Cluster-22178.74322 GO:0055085 GO:0006260 GO:0006606 GO:0006810
GO:0007165 GO:0042742 GO:0008608 GO:0006886 GO:0006508
GO:0030001 GO:0004222 GO:0046873 GO:0004871 GO:0005515
GO:0005815 GO:0005886 GO:0016020 GO:0005801 GO:0042729
GO:0072686 GO:0005576
Cluster-22178.74323 GO:0006508 GO:0042742 GO:0004222 GO:0005576
Cluster-22178.74324 GO:0048280 GO:0009116 GO:0006508 GO:0006886
GO:0004222 GO:0003824 GO:0071949 GO:0005515 GO:0005737
GO:0000139
Cluster-22178.74325 GO:0035556 GO:0005515
Cluster-22178.74328 GO:0015979 GO:0043248 GO:0016020 GO:0009539
GO:0009523
Cluster-22178.74329 GO:0006886 GO:0006506 GO:0005515
Cluster-22178.74330 GO:0009236 GO:0003676 GO:0005634
Cluster-22178.74331 GO:0006810 GO:0005524 GO:0004386 GO:0003676

Cluster-22178.74333 GO:0015979 GO:0019538 GO:0016567 GO:0050765
GO:0009405 GO:0046872 GO:0008270 GO:0005515 GO:0009523
GO:0009539 GO:0016020 GO:0016012 GO:0005576
Cluster-22178.74335 GO:0006457 GO:0051082 GO:0005515 GO:0005509
GO:0005783
Cluster-22178.74336 GO:0051090 GO:0016192 GO:0006886 GO:0005515
GO:0008536 GO:0030117 GO:0005634

Cluster-22178.74338 GO:0009396 GO:0006779 GO:0006555 GO:0006098
GO:0019354 GO:0055114 GO:0006730 GO:0019521 GO:0006813
GO:0046487 GO:0051287 GO:0004616 GO:0004488 GO:0003824
GO:0004013 GO:0043115
Cluster-22178.74339 GO:0046034 GO:0006464 GO:0006144 GO:0016151
GO:0046872 GO:0008270 GO:0004017 GO:0005634
Cluster-22178.74343 GO:0046854 GO:0042967 GO:0006355 GO:0008270
GO:0003712 GO:0016787 GO:0004402 GO:0000123 GO:0005634
GO:0005667 GO:0019033
Cluster-22178.74347 GO:0051028 GO:0006406 GO:0006810 GO:0005515
GO:0003723 GO:0005622 GO:0005737 GO:0005634
Cluster-22178.74348 GO:0008270
Cluster-22178.74350 GO:0006511 GO:0031625
Cluster-22178.74351 GO:0046740 GO:0016226 GO:0005506 GO:0005515
GO:0051536 GO:0003676 GO:0005634 GO:0016021
Cluster-22178.74353 GO:0046740 GO:0016226 GO:0000398 GO:0005506
GO:0005515 GO:0051536 GO:0005681 GO:0016021
Cluster-22178.74354 GO:0051726 GO:0008152 GO:0003824 GO:0005634
Cluster-22178.74356 GO:0042742 GO:0006508 GO:0008152 GO:0016836
GO:0003824 GO:0008233 GO:0005576
Cluster-22178.74359 GO:0016021
Cluster-22178.74361 GO:0006471 GO:0008643 GO:0008982 GO:0003956
GO:0009357
Cluster-22178.74362 GO:0006810 GO:0007165 GO:0005215 GO:0008158
GO:0016020
Cluster-22178.74364 GO:0000160 GO:0003677 GO:0000156 GO:0005524

Cluster-22178.74366 GO:0006281 GO:0019497 GO:0019079 GO:0006310
GO:0006771 GO:0002949 GO:0005515 GO:0003723 GO:0003678
GO:0009378 GO:0003993 GO:0003724 GO:0005524 GO:0016887
GO:0009379 GO:0005657
Cluster-22178.74368 GO:0006355
Cluster-22178.74369 GO:0006351 GO:0006144 GO:0006206 GO:0003899

GO:0003677 GO:0005524 GO:0003676 GO:0005634 GO:0005730
Cluster-22178.74371 GO:0006260 GO:0006281 GO:0003676 GO:0043140
GO:0005524 GO:0003677 GO:0016787 GO:0005657
Cluster-22178.74373 GO:0006011 GO:0007165 GO:0006954 GO:0031167
GO:0005125 GO:0008168 GO:0005576 GO:0016020
Cluster-22178.74374 GO:0006810 GO:0003713 GO:0003676 GO:0019871
GO:0005634 GO:0005667 GO:0005576
Cluster-22178.74375 GO:0005634
Cluster-22178.74377 GO:0015991 GO:0046983 GO:0016820 GO:0033178
Cluster-22178.74379 GO:0007264 GO:0005524 GO:0005525
Cluster-22178.74381 GO:0009094 GO:0055114 GO:0006571 GO:0000162
GO:0004665 GO:0005515 GO:0005509 GO:0008977 GO:0019028
Cluster-22178.74382 GO:0016310 GO:0006449 GO:0009069 GO:0006468
GO:0006415 GO:0004674 GO:0005524 GO:0016149 GO:0003747
GO:0005840 GO:0005737 GO:0018444
Cluster-22178.74383 GO:0042802 GO:0005515
Cluster-22178.74386 GO:0009271 GO:0055114 GO:0006355 GO:0005975
GO:0016491 GO:0016209 GO:0030246
Cluster-22178.74387 GO:0035556 GO:0008270 GO:0005515 GO:0003677
GO:0045735
Cluster-22178.74388 GO:0009116 GO:0048280 GO:0006508 GO:0006886
GO:0003824 GO:0004222 GO:0071949 GO:0005515 GO:0005737
GO:0000139
Cluster-22178.74389 GO:0008033 GO:0008168
Cluster-22178.74391 GO:0030154 GO:0007275 GO:0005515 GO:0005634
Cluster-22178.74393 GO:0005975 GO:0005524 GO:0016773
Cluster-22178.74394 GO:0055085 GO:0006810 GO:0042626 GO:0016887
GO:0005524 GO:0016021
Cluster-22178.74395 GO:0000921 GO:0006606 GO:0006355 GO:0043565
GO:0008270 GO:0003700 GO:0003677 GO:0005940 GO:0016021
GO:0005667
Cluster-22178.74402 GO:0003677 GO:0005576
Cluster-22178.74403 GO:0008608 GO:0048870 GO:0007155 GO:0042729

GO:0031514 GO:0072686
Cluster-22178.74404 GO:0046872
Cluster-22178.74406 GO:0007049
Cluster-22178.74407 GO:0006351 GO:0003677 GO:0005634
Cluster-22178.74408 GO:0006351 GO:0003677 GO:0005634
Cluster-22178.74409 GO:0008152 GO:0008270 GO:0046872 GO:0003824
GO:0016836 GO:0061630 GO:0005515 GO:0032040
Cluster-22178.74411 GO:0042254 GO:0006412 GO:0015948 GO:0009082
GO:0046656 GO:0003735 GO:0005515 GO:0003723 GO:0030269
GO:0016021 GO:0005840
Cluster-22178.74412 GO:0006355 GO:0005515
Cluster-22178.74413 GO:0016117 GO:0009405 GO:0005525 GO:0046872
GO:0003924 GO:0003677 GO:0016705 GO:0005515
Cluster-22178.74415 GO:0016117 GO:0009405 GO:0005525 GO:0003924
GO:0046872 GO:0005515 GO:0016705 GO:0003677
Cluster-22178.74417 GO:0016117 GO:0009405 GO:0005525 GO:0046872
GO:0003924 GO:0003677 GO:0016705 GO:0005515
Cluster-22178.74418 GO:0016117 GO:0009405 GO:0046872 GO:0003924
GO:0005525 GO:0003677 GO:0016705 GO:0005515
Cluster-22178.74419 GO:0015979 GO:0031175 GO:0008017 GO:0005516
GO:0005515 GO:0030507 GO:0009523 GO:0009539 GO:0045298
GO:0008091 GO:0016020
Cluster-22178.74420 GO:0006355 GO:0043401 GO:0043565 GO:0008270
GO:0003700 GO:0005634 GO:0005667
Cluster-22178.74421 GO:0006457 GO:0004064 GO:0005515 GO:0042803
GO:0051087 GO:0008092 GO:0000774 GO:0005737 GO:0019898
Cluster-22178.74423 GO:0007018 GO:0007017 GO:0003677 GO:0003777
GO:0008017 GO:0005524 GO:0005874 GO:0045298
Cluster-22178.74424 GO:0032065 GO:0005515 GO:0005543 GO:0005938
Cluster-22178.74426 GO:0008152 GO:0006508 GO:0008236 GO:0016787

Cluster-22178.74429 GO:0006260 GO:0007067 GO:0006270 GO:0007049
GO:0009405 GO:0006486 GO:0046983 GO:0016757 GO:0003980

GO:0042802 GO:0005515 GO:0003887 GO:0016021 GO:0000775
GO:0005727 GO:0005634 GO:0042575
Cluster-22178.74432 GO:0006464 GO:0046872 GO:0005515 GO:0016151

Cluster-22178.74433 GO:0042254 GO:0006412 GO:0003735 GO:0005840
Cluster-22178.74434 GO:0007217
Cluster-22178.74435 GO:0006413 GO:0006446 GO:0003723 GO:0003743
GO:0005737 GO:0005840
Cluster-22178.74437 GO:0006303 GO:0006353 GO:0004003 GO:0003677
GO:0005525 GO:0005657
Cluster-22178.74438 GO:0006814 GO:0007186 GO:0016192 GO:0006120
GO:0006744 GO:0055085 GO:0055114 GO:0015992 GO:0005515
GO:0008137 GO:0004930 GO:0016021
Cluster-22178.74439 GO:0006629 GO:0008081
Cluster-22178.74440 GO:0035556 GO:0006171 GO:0009190 GO:0006144
GO:0004016 GO:0016849 GO:0005886
Cluster-22178.74442 GO:0006355 GO:0006228 GO:0006508 GO:0006165
GO:0006886 GO:0006183 GO:0006206 GO:0006144 GO:0006241
GO:0004550 GO:0004222 GO:0008536 GO:0005634
Cluster-22178.74445 GO:0005515
Cluster-22178.74448 GO:0019069 GO:0006367 GO:0004190 GO:0005672
GO:0016021
Cluster-22178.74449 GO:0005515 GO:0016021
Cluster-22178.74451 GO:0019646 GO:0006118 GO:0016740 GO:0009055

Cluster-22178.74453 GO:0006886 GO:0005622
Cluster-22178.74454 GO:0005515
Cluster-22178.74455 GO:0005634
Cluster-22178.74456 GO:0006508 GO:0004177 GO:0005622 GO:0016021
Cluster-22178.74457 GO:0006508 GO:0004177 GO:0016021 GO:0005622
Cluster-22178.74459 GO:0006886 GO:0016192 GO:0006810 GO:0055085
GO:0022857 GO:0005215 GO:0016020 GO:0030117 GO:0016021
Cluster-22178.74460 GO:0005515

Cluster-22178.74466 GO:0006355 GO:0003677
Cluster-22178.74470 GO:0007264 GO:0007165 GO:0007186 GO:0006013
GO:0006000 GO:0003873 GO:0031683 GO:0005524 GO:0019001
GO:0003924 GO:0005525 GO:0004871
Cluster-22178.74471 GO:0008033 GO:0055114 GO:0009228 GO:0006807
GO:0000105 GO:0050660 GO:0017150 GO:0018580 GO:0004789
Cluster-22178.74472 GO:0007264 GO:0007186 GO:0019079 GO:0006304
GO:0007165 GO:0043066 GO:0043154 GO:0033821 GO:0003924
GO:0031683 GO:0019001 GO:0004871 GO:0005525 GO:0071986
GO:0019012 GO:0005737
Cluster-22178.74473 GO:0016192
Cluster-22178.74475 GO:0012511 GO:0016021 GO:0016020
Cluster-22178.74479 GO:0006810 GO:0006334 GO:0043093 GO:0009069
GO:0008053 GO:0000917 GO:0042262 GO:0016310 GO:0003690
GO:0004674 GO:0051082 GO:0003924 GO:0005741 GO:0005634
GO:0016021 GO:0005737
Cluster-22178.74481 GO:0006605 GO:0006886 GO:0005515 GO:0005742
Cluster-22178.74487 GO:0006047
Cluster-22178.74493 GO:0006367 GO:0008270 GO:0005515 GO:0005672
Cluster-22178.74494 GO:0007010 GO:0003779 GO:0046872
Cluster-22178.74496 GO:0005515
Cluster-22178.74497 GO:0009607 GO:0007154 GO:0005515 GO:0016021
GO:0005922
Cluster-22178.74498 GO:0003924 GO:0005525
Cluster-22178.74499 GO:0003779
Cluster-22178.74500 GO:0006355 GO:0005634
Cluster-22178.74504 GO:0045893 GO:0007165 GO:0019953 GO:0008430
GO:0000772 GO:0003676 GO:0005576
Cluster-22178.74505 GO:0045893 GO:0019953 GO:0007165 GO:0008430
GO:0000772 GO:0003676 GO:0005576
Cluster-22178.74506 GO:0006030 GO:0008061 GO:0005576
Cluster-22178.74507 GO:0006357 GO:0001104 GO:0016592
Cluster-22178.74510 GO:0055085 GO:0016192 GO:0006886 GO:0006810

GO:0022857 GO:0005215 GO:0016021 GO:0016020 GO:0030117
Cluster-22178.74511 GO:0055085 GO:0006810 GO:0016192 GO:0006886
GO:0005215 GO:0022857 GO:0016021 GO:0016020 GO:0030117
Cluster-22178.74512 GO:0005524 GO:0003676
Cluster-22178.74514 GO:0019082 GO:0016032 GO:0006281 GO:0007049
GO:0030150 GO:0005515 GO:0003824 GO:0005096 GO:0005742
GO:0034464
Cluster-22178.74515 GO:0007165 GO:0030261 GO:0007059 GO:0002949
GO:0003677 GO:0005524 GO:0016765 GO:0016887 GO:0003924
GO:0005525 GO:0008158 GO:0016020 GO:0009295
Cluster-22178.74517 GO:0016560 GO:0006468 GO:0004672 GO:0005524
GO:0005777 GO:0016021
Cluster-22178.74522 GO:0006412 GO:0042254 GO:0003676 GO:0003735
GO:0005622 GO:0005840
Cluster-22178.74523 GO:0007165 GO:0006810 GO:0005524 GO:0003774
GO:0016459
Cluster-22178.74525 GO:0016032 GO:0005975 GO:0005509 GO:0004571
GO:0016020 GO:0019012
Cluster-22178.74527 GO:0006119 GO:0006412 GO:0006355 GO:0035556
GO:0015992 GO:0042254 GO:0015986 GO:0008270 GO:0046933
GO:0003677 GO:0003735 GO:0046961 GO:0005840 GO:0005622
GO:0045259 GO:0045261
Cluster-22178.74529 GO:0051252 GO:0006614 GO:0019079 GO:0006355
GO:0008026 GO:0003677 GO:0016787 GO:0004523 GO:0003676
GO:0004386 GO:0005525 GO:0005524
Cluster-22178.74530 GO:0035299 GO:0005524
Cluster-22178.74531 GO:0050829 GO:0051782 GO:0009432 GO:0050830
GO:0005515 GO:0051015 GO:0009276
Cluster-22178.74533 GO:0009432 GO:0051782 GO:0050829 GO:0050830
GO:0051015 GO:0005515 GO:0009276
Cluster-22178.74536 GO:0006090 GO:0006525 GO:0071436 GO:0006814
GO:0006560 GO:0015081 GO:0008948 GO:0003677 GO:0016020
Cluster-22178.74537 GO:0006814 GO:0071436 GO:0006560 GO:0006525

GO:0006090 GO:0008948 GO:0003677 GO:0015081 GO:0016020
Cluster-22178.74538 GO:0006457 GO:0051082 GO:0016272
Cluster-22178.74539 GO:0051082 GO:0005639
Cluster-22178.74542 GO:0006355 GO:0016032 GO:0005634
Cluster-22178.74546 GO:0005975 GO:0043085 GO:0016485 GO:0006355
GO:0008270 GO:0046872 GO:0016773 GO:0035091 GO:0031419
GO:0016021 GO:0005634
Cluster-22178.74547 GO:0006355 GO:0016192 GO:0006886 GO:0003700
GO:0030117 GO:0005667 GO:0030131
Cluster-22178.74548 GO:0016192 GO:0006886 GO:0030131
Cluster-22178.74549 GO:0016567 GO:0006376 GO:0004842 GO:0046872
GO:0008270 GO:0061630 GO:0005515 GO:0003729 GO:0005685
GO:0005622
Cluster-22178.74552 GO:0008270 GO:0005515 GO:0003676
Cluster-22178.74554 GO:0055085 GO:0022857 GO:0016021
Cluster-22178.74555 GO:0055085 GO:0022857 GO:0016021
Cluster-22178.74558 GO:0006118 GO:0045454 GO:0055114 GO:0009055
GO:0015035 GO:0016491 GO:0016209
Cluster-22178.74562 GO:0007049 GO:0005634
Cluster-22178.74563 GO:0006276 GO:0009236 GO:0055085 GO:0048472
GO:0016021
Cluster-22178.74564 GO:0006479 GO:0006306 GO:0031167 GO:0001510
GO:0009452 GO:0006810 GO:0046500 GO:0006464 GO:0032259
GO:0005515 GO:0019871 GO:0003723 GO:0003676 GO:0046872
GO:0004719 GO:0008168 GO:0003677 GO:0008170 GO:0008270
GO:0005576
Cluster-22178.74566 GO:0000226 GO:0090063 GO:0006508 GO:0006810
GO:0004197 GO:0005515 GO:0005815 GO:0016021 GO:0000922
Cluster-22178.74567 GO:0001510 GO:0006306 GO:0031167 GO:0009452
GO:0008152 GO:0008170 GO:0003677 GO:0005515 GO:0008168
GO:0005201 GO:0005578 GO:0019013 GO:0005581 GO:0016021
Cluster-22178.74568 GO:0016567 GO:0004842
Cluster-22178.7457 GO:0006468 GO:0004672 GO:0005524

Cluster-22178.74570 GO:0006265 GO:0003677 GO:0003918 GO:0005524
GO:0005634
Cluster-22178.74573 GO:0009271 GO:0006508 GO:0006355 GO:0004198
GO:0008270 GO:0005622
Cluster-22178.74574 GO:0046656 GO:0006955 GO:0000902 GO:0006760
GO:0006310 GO:0051016 GO:0005996 GO:0000150 GO:0003678
GO:0004150 GO:0048029 GO:0003677 GO:0016853 GO:0005524
GO:0046983 GO:0016020 GO:0008290 GO:0005657
Cluster-22178.74579 GO:0005515
Cluster-22178.74581 GO:0005515
Cluster-22178.74586 GO:0007165 GO:0005515 GO:0005509 GO:0005578
Cluster-22178.74588 GO:0051607 GO:0006891 GO:0007049 GO:0043093
GO:0000390 GO:0000917 GO:0006886 GO:0005737 GO:0030173
GO:0016021 GO:0005801 GO:0005783 GO:0016020 GO:0071008
Cluster-22178.74590 GO:0009094 GO:0006571 GO:0000413 GO:0000162
GO:0006094 GO:0006096 GO:0006457 GO:0004634 GO:0000287
GO:0003755 GO:0000015
Cluster-22178.74591 GO:0006355 GO:0019069 GO:0042254 GO:0006412
GO:0003700 GO:0003677 GO:0003735 GO:0005840 GO:0005634
GO:0005667 GO:0042025
Cluster-22178.74592 GO:0006094 GO:0006096 GO:0006457 GO:0009094
GO:0006571 GO:0000162 GO:0000413 GO:0000287 GO:0003755
GO:0004634 GO:0000015
Cluster-22178.74593 GO:0006270 GO:0005515 GO:0016021
Cluster-22178.74594 GO:0006468 GO:0004672 GO:0005524 GO:0005515

Cluster-22178.74597 GO:0006281 GO:0006367 GO:0007186 GO:0006310
GO:0006106 GO:0007411 GO:0000400 GO:0000287 GO:0003677
GO:0016788 GO:0004930 GO:0005672 GO:0016020 GO:0016021
Cluster-22178.74599 GO:0007010 GO:0030001 GO:0006308 GO:0006810
GO:0006260 GO:0055085 GO:0000226 GO:0005515 GO:0046872
GO:0003779 GO:0003677 GO:0042626 GO:0005524 GO:0008855
GO:0009318 GO:0015630 GO:0005664 GO:0016021

Cluster-22178.74608 GO:0006270 GO:0004190 GO:0008270 GO:0016021
Cluster-22178.74609 GO:0006355 GO:0008152 GO:0005515 GO:0003677
GO:0008168 GO:0043565 GO:0003700 GO:0005634 GO:0005667
Cluster-22178.74611 GO:0006479 GO:0008152 GO:0006464 GO:0046500
GO:0004719 GO:0008168
Cluster-22178.74612 GO:0042742
Cluster-22178.74613 GO:0005198 GO:0005578
Cluster-22178.74615 GO:0007276 GO:0051252 GO:0016891
Cluster-22178.74616 GO:0006040 GO:0006051 GO:0006310 GO:0006810
GO:0009405 GO:0019905 GO:0008200 GO:0005515 GO:0047465
GO:0003677 GO:0005886 GO:0005576
Cluster-22178.74617 GO:0006308 GO:0008855 GO:0009318
Cluster-22178.74620 GO:0007186 GO:0017009 GO:0004930 GO:0016021
Cluster-22178.74621 GO:0009405 GO:0046999 GO:0046740 GO:0005198
GO:0019867 GO:0016021
Cluster-22178.74622 GO:0015031 GO:0006813 GO:0006508 GO:0006814
GO:0016787 GO:0008236 GO:0005890
Cluster-22178.74625 GO:0006886 GO:0005515
Cluster-22178.74626 GO:0006511 GO:0007275 GO:0005515 GO:0008270
GO:0005634
Cluster-22178.74627 GO:0032012 GO:0043087 GO:0005086 GO:0005634
Cluster-22178.74629 GO:0006298 GO:0005524 GO:0030983
Cluster-22178.74630 GO:0006749 GO:0006750 GO:0042398 GO:0007155
GO:0004357 GO:0016021 GO:0017109
Cluster-22178.74633 GO:0006511 GO:0004175 GO:0005515 GO:0019773
Cluster-22178.74634 GO:0007165 GO:0008209 GO:0005982 GO:0008207
GO:0007186 GO:0009117 GO:0008210 GO:0005985 GO:0055114
GO:0006694 GO:0003854 GO:0050662 GO:0016616 GO:0048040
GO:0003824 GO:0004871 GO:0005834
Cluster-22178.74635 GO:0006570 GO:0006886 GO:0006576 GO:2000123
GO:0006470 GO:0007186 GO:0007165 GO:0007264 GO:0004871
GO:0005525 GO:0004725 GO:0003924 GO:0031683 GO:0019001
GO:0016887 GO:0005524 GO:0005543

Cluster-22178.74636 GO:0007264 GO:0007186 GO:0007165 GO:0006576
GO:0006570 GO:0006886 GO:2000123 GO:0006470 GO:0016887
GO:0005524 GO:0005543 GO:0003924 GO:0031683 GO:0019001
GO:0004871 GO:0005525 GO:0004725
Cluster-22178.74638 GO:0015979 GO:0030071 GO:0005515 GO:0005680
GO:0009579 GO:0016021 GO:0009522
Cluster-22178.74639 GO:0006511
Cluster-22178.74640 GO:0055114 GO:0006769 GO:0046497 GO:0043066
GO:0050661 GO:0008750 GO:0005521 GO:0016021 GO:0019031
Cluster-22178.74645 GO:0010506 GO:0005515 GO:0004222 GO:0005615
Cluster-22178.74651 GO:0016192 GO:0006886 GO:0042981 GO:0005515
GO:0030117
Cluster-22178.74655 GO:0015992 GO:0006119 GO:0006118 GO:0008121
GO:0009055
Cluster-22178.74661 GO:0009395 GO:0008654 GO:0042157 GO:0006869
GO:0046486 GO:0016567 GO:0046872 GO:0008289 GO:0005515
GO:0005198 GO:0008270 GO:0046983 GO:0004842 GO:0004143
GO:0061630 GO:0019031 GO:0005576 GO:0019028 GO:0005622
GO:0016020
Cluster-22178.74665 GO:0055114 GO:0030328 GO:0016491 GO:0016670
GO:0003677
Cluster-22178.74666 GO:0016021
Cluster-22178.74667 GO:0006355 GO:0006281 GO:0016114 GO:0006694
GO:0009058 GO:0006310 GO:0019079 GO:0006810 GO:0000103
GO:0002949 GO:0006144 GO:0016301 GO:0008134 GO:0004161
GO:0000166 GO:0003723 GO:0003678 GO:0004020 GO:0005525
GO:0043531 GO:0098519 GO:0003677 GO:0009378 GO:0003724
GO:0016887 GO:0005524 GO:0005667 GO:0009379 GO:0005657
Cluster-22178.74668 GO:0009058 GO:0042981 GO:0005515 GO:0030170

Cluster-22178.74674 GO:0042254 GO:0006413 GO:0005515 GO:0005509
GO:0005622
Cluster-22178.74675 GO:0006308 GO:0008380 GO:0007155 GO:0008855

GO:0005200 GO:0005856 GO:0005681 GO:0005882 GO:0009318
Cluster-22178.74680 GO:0006952 GO:0006355 GO:0003700 GO:0046872
GO:0005667
Cluster-22178.74681 GO:0016485 GO:0005506 GO:0016021
Cluster-22178.74682 GO:0006355 GO:0006464 GO:0006952 GO:0046872
GO:0003700 GO:0016151 GO:0005667
Cluster-22178.74687 GO:0006468 GO:0004672 GO:0005524 GO:0016020
Cluster-22178.74688 GO:0003677
Cluster-22178.74690 GO:0009073 GO:0006260 GO:0006571 GO:0000162
GO:0015979 GO:0009094 GO:0030494 GO:0055114 GO:0006351
GO:0006508 GO:0016491 GO:0003677 GO:0004252 GO:0003856
GO:0042025
Cluster-22178.74696 GO:0050765 GO:0005515
Cluster-22178.74699 GO:0007275 GO:0007165 GO:0016055 GO:0005515
GO:0005509 GO:0005102 GO:0045095 GO:0005576
Cluster-22178.74700 GO:0051536 GO:0005515 GO:0003824
Cluster-22178.74703 GO:0006355 GO:0019058 GO:0030036 GO:0003779
GO:0003677 GO:0030054
Cluster-22178.74705 GO:0042262 GO:0051607 GO:0005509 GO:0003690

Cluster-22178.74706 GO:0007186 GO:0004930 GO:0016021 GO:0016020
Cluster-22178.74707 GO:0005515
Cluster-22178.74708 GO:0006260 GO:0007217 GO:0003887 GO:0042575
Cluster-22178.74709 GO:0006508 GO:0055085 GO:0008236 GO:0005515
GO:0016021
Cluster-22178.74711 GO:0055114 GO:0005515
Cluster-22178.74714 GO:0035556 GO:0005975 GO:0006270 GO:0006468
GO:0005524 GO:0004672 GO:0016773 GO:0032040 GO:0016020
Cluster-22178.74715 GO:0006270 GO:0006468 GO:0035556 GO:0005975
GO:0016773 GO:0005524 GO:0004672 GO:0016020 GO:0032040
Cluster-22178.74716 GO:0015992 GO:0015991 GO:0015078 GO:0033179
Cluster-22178.74717 GO:0015991 GO:0015992 GO:0015078 GO:0033179
Cluster-22178.74718 GO:0042802 GO:0005515

Cluster-22178.74719 GO:0003677 GO:0003676 GO:0005524 GO:0016787

Cluster-22178.74723 GO:0008152 GO:0055114 GO:0016616 GO:0051287

Cluster-22178.74724 GO:0007165 GO:0008283 GO:0040007 GO:0005515 GO:0008083
Cluster-22178.74728 GO:0055114 GO:0006810 GO:0016491 GO:0003824 GO:0005515 GO:0050660 GO:0016020
Cluster-22178.74733 GO:0055085 GO:0015684 GO:0007165 GO:0007186 GO:0007264 GO:0006811 GO:0009405 GO:0006118 GO:0030254 GO:0006576 GO:0015075 GO:0005524 GO:0015093 GO:0003682 GO:0005525 GO:0009055 GO:0004871 GO:0031683 GO:0019001 GO:0003924 GO:0030257 GO:0016020 GO:0016021 GO:0000785 GO:0005634
Cluster-22178.74735 GO:0003682 GO:0005515 GO:0000785
Cluster-22178.74737 GO:0048193 GO:0007155 GO:0048870 GO:0006355 GO:0009405 GO:0005524 GO:0008270 GO:0016818 GO:0003677 GO:0005198 GO:0005882 GO:0016020 GO:0009288 GO:0019031 GO:0019867 GO:0019028 GO:0005634 GO:0031514
Cluster-22178.74738 GO:0002949 GO:0007059 GO:0030261 GO:0007165 GO:0008158 GO:0005525 GO:0003924 GO:0016887 GO:0016765 GO:0005524 GO:0003677 GO:0009295 GO:0016020
Cluster-22178.74739 GO:0003677 GO:0005524
Cluster-22178.74740 GO:0042254 GO:0006412 GO:0003735 GO:0008270 GO:0005840 GO:0005622
Cluster-22178.74742 GO:0048870 GO:0000917 GO:0009405 GO:0002949 GO:0043093 GO:0006171 GO:0006810 GO:0003678 GO:0005524 GO:0016887 GO:0046983 GO:0016787 GO:0008294 GO:0003677 GO:0005737 GO:0005576 GO:0031514 GO:0005657
Cluster-22178.74747 GO:0016055 GO:0007411 GO:0005576
Cluster-22178.74754 GO:0006904 GO:0006123 GO:0015031 GO:0015992 GO:0005198 GO:0004129 GO:0003774 GO:0005882 GO:0005740 GO:0000145 GO:0005747 GO:0016459 GO:0005739 GO:0045277

Cluster-22178.74755 GO:0006468 GO:0016773 GO:0004672 GO:0005524 GO:0016020
Cluster-22178.74757 GO:0055114 GO:0006508 GO:0070072 GO:0006118 GO:0016655 GO:0004252
Cluster-22178.74758 GO:0055114 GO:0007131 GO:0006032 GO:0016705 GO:0008061 GO:0000794
Cluster-22178.74762 GO:0005515
Cluster-22178.74765 GO:0015992 GO:0006281 GO:0008289 GO:0015078 GO:0003824 GO:0005515 GO:0033644 GO:0055036
Cluster-22178.74776 GO:0006812 GO:0007165 GO:0006308 GO:0007017 GO:0006355 GO:0007018 GO:0016887 GO:0008855 GO:0005524 GO:0008017 GO:0003713 GO:0003777 GO:0035257 GO:0005515 GO:0005667 GO:0045298 GO:0016021 GO:0005874 GO:0005634 GO:0009318
Cluster-22178.74778 GO:0015814 GO:0006811 GO:1902604 GO:0015846 GO:0005515 GO:0003723 GO:0015558
Cluster-22178.74781 GO:0030163 GO:0008270
Cluster-22178.74782 GO:0006508 GO:0004222
Cluster-22178.74784 GO:0034227 GO:0002098 GO:0000049
Cluster-22178.74785 GO:0034227 GO:0002098 GO:0000049
Cluster-22178.74786 GO:0005198
Cluster-22178.74787 GO:0006351 GO:0005975 GO:0005515 GO:0008270 GO:0033925 GO:0004553 GO:0003676 GO:0005737
Cluster-22178.74788 GO:0055114 GO:0006694 GO:0008210 GO:0008209 GO:0008207 GO:0016491 GO:0003824 GO:0050662 GO:0003854 GO:0016616 GO:0016021
Cluster-22178.74789 GO:0046168 GO:0000162 GO:0006098 GO:0008209 GO:0008207 GO:0009094 GO:0055114 GO:0019521 GO:0006694 GO:0006571 GO:0015940 GO:0008210 GO:0051287 GO:0003854 GO:0050662 GO:0004616 GO:0016616 GO:0008977 GO:0008677 GO:0003824 GO:0004665 GO:0016021
Cluster-22178.74793 GO:0006570 GO:0005985 GO:0018149 GO:0045040 GO:0006281 GO:0006094 GO:0005982 GO:0006096 GO:0051156

GO:0042207 GO:0006012 GO:0009072 GO:0043087 GO:0043161
GO:0019872 GO:0032259 GO:0006289 GO:0005515 GO:0008168
GO:0003684 GO:0004334 GO:0005089 GO:0003810 GO:0004340
GO:0005524 GO:0005742
Cluster-22178.74797 GO:0006457 GO:0000413 GO:0003755
Cluster-22178.74799 GO:0055114 GO:0043087 GO:0035023 GO:0035556
GO:0006355 GO:0006955 GO:0007165 GO:0005125 GO:0003700
GO:0005089 GO:0047134 GO:0005515 GO:0003677 GO:0005667
GO:0005576
Cluster-22178.74801 GO:0008270 GO:0005515 GO:0005634
Cluster-22178.74802 GO:0007010 GO:0003779
Cluster-22178.74803 GO:0008270 GO:0005515 GO:0005634
Cluster-22178.74804 GO:0006464 GO:0046872
Cluster-22178.74806 GO:0009451 GO:0006479 GO:0006400 GO:0046500
GO:0008152 GO:0008033 GO:0006554 GO:0006464 GO:0008176
GO:0018024 GO:0008168 GO:0004719
Cluster-22178.74807 GO:0006570 GO:0006397 GO:0006470 GO:0008138
GO:0003723 GO:0004725 GO:0005634 GO:0005737
Cluster-22178.74814 GO:0009405 GO:0008152 GO:0007186 GO:0006614
GO:0006810 GO:0007165 GO:0055085 GO:0003723 GO:0016301
GO:0003924 GO:0031683 GO:0019001 GO:0004871 GO:0005525
GO:0016746 GO:0016887 GO:0005524 GO:0003724 GO:0042626
GO:0005576 GO:0016021
Cluster-22178.74816 GO:0005515
Cluster-22178.74817 GO:0003779 GO:0005515 GO:0005622
Cluster-22178.74818 GO:0009424
Cluster-22178.74820 GO:0006470 GO:0006397 GO:0051260 GO:0032006
GO:0004721 GO:0005515 GO:0005634
Cluster-22178.74826 GO:0005515
Cluster-22178.74829 GO:0006810 GO:0009401 GO:0005886
Cluster-22178.74841 GO:0006508 GO:0042381 GO:0003676 GO:0005515
GO:0008233 GO:0005576
Cluster-22178.74844 GO:0045490 GO:0047487

Cluster-22178.74847 GO:0006418 GO:0000166 GO:0004812 GO:0008270 GO:0005524

Cluster-22178.74849 GO:0006887 GO:0006891 GO:0006308 GO:0010508 GO:0006886 GO:0008855 GO:0005801 GO:0016020 GO:0000145 GO:0009318 GO:0017119

Cluster-22178.74850 GO:0015994 GO:0055114 GO:0005975 GO:0042167 GO:0006890 GO:0006355 GO:0004559 GO:0016491 GO:0008270 GO:0004074 GO:0003677

Cluster-22178.74851 GO:0043395 GO:0005515 GO:0005578 GO:0016020

Cluster-22178.74852 GO:0006508 GO:0019087 GO:0008236 GO:0043395 GO:0016020 GO:0005578

Cluster-22178.74855 GO:0042167 GO:0006890 GO:0006355 GO:0015994 GO:0005975 GO:0055114 GO:0003677 GO:0051287 GO:0004559 GO:0016620 GO:0008270 GO:0004074 GO:0016491

Cluster-22178.74857 GO:0035556 GO:0007010 GO:0008289 GO:0005515 GO:0003779

Cluster-22178.74859 GO:0007264 GO:0005096 GO:0005515 GO:0005525

Cluster-22178.74860 GO:0006284 GO:0006576 GO:0003677 GO:0005525 GO:0003905 GO:0005524 GO:0003924

Cluster-22178.74862 GO:0006355 GO:0010508 GO:0006914 GO:0007049 GO:0003677 GO:0043565 GO:0003700 GO:0005667 GO:0005634

Cluster-22178.74863 GO:0006355 GO:0010508 GO:0006914 GO:0003677 GO:0043565 GO:0003700 GO:0005634 GO:0005667

Cluster-22178.74866 GO:0006508 GO:0019073 GO:0006139 GO:0006260 GO:0008408 GO:0003887 GO:0003677 GO:0008270 GO:0003676 GO:0004222 GO:0042575 GO:0031012

Cluster-22178.74867 GO:0007049 GO:0005515 GO:0008270

Cluster-22178.74874 GO:0006508 GO:0016805

Cluster-22178.74875 GO:0006508 GO:0016805

Cluster-22178.74877 GO:0006811 GO:0006334 GO:0006810 GO:0005215 GO:0003677 GO:0000786 GO:0005634 GO:0016020

Cluster-22178.74882 GO:0006886 GO:0016192 GO:0006355 GO:0005198

GO:0003677 GO:0003723 GO:0030131 GO:0005634
Cluster-22178.74883 GO:0006886 GO:0016192 GO:0006355 GO:0003677
GO:0030131 GO:0005634
Cluster-22178.74884 GO:0006355 GO:0003677 GO:0005634
Cluster-22178.74886 GO:0055114 GO:0006508 GO:0008234 GO:0016491

Cluster-22178.74889 GO:0005515
Cluster-22178.74890 GO:0042967 GO:0006534 GO:0006468 GO:0006535
GO:0004672 GO:0005524 GO:0016773 GO:0009001 GO:0005737
GO:0016020
Cluster-22178.74892 GO:0005515 GO:0046872
Cluster-22178.74893 GO:0005515
Cluster-22178.74895 GO:0006397 GO:0005634
Cluster-22178.74897 GO:0009405 GO:0005576
Cluster-22178.74898 GO:0009405 GO:0005576
Cluster-22178.74900 GO:0006265 GO:0005524 GO:0046872 GO:0003918
GO:0003677 GO:0005634
Cluster-22178.74902 GO:0006810 GO:0016021
Cluster-22178.74903 GO:0006355 GO:0016192 GO:0006886 GO:0003712
GO:0005515 GO:0030117 GO:0005667
Cluster-22178.74904 GO:0042254 GO:0006412 GO:0003735 GO:0005840
GO:0005622
Cluster-22178.74911 GO:0005515 GO:0019901 GO:0005634
Cluster-22178.74914 GO:0005515 GO:0016740
Cluster-22178.74915 GO:0003924
Cluster-22178.74916 GO:0007411
Cluster-22178.74917 GO:0007165 GO:0006955 GO:0008283 GO:0006468
GO:0040007 GO:0005524 GO:0004672 GO:0005135 GO:0016773
GO:0008083 GO:0005515 GO:0005615 GO:0005894 GO:0016020
GO:0005576
Cluster-22178.74918 GO:0006554 GO:0006479 GO:0046872 GO:0018024

Cluster-22178.74920 GO:0006012 GO:0045747 GO:0007049 GO:0005982

GO:0005985 GO:0009405 GO:0006221 GO:0004222 GO:0004564
GO:0004575 GO:0004190 GO:0016021 GO:0017177
Cluster-22178.74925 GO:0042262 GO:0006334 GO:0043093 GO:0006810
GO:0000917 GO:0003690 GO:0005737 GO:0016021 GO:0005634
Cluster-22178.74926 GO:0006144 GO:0006508 GO:0016817 GO:0003968
GO:0070008 GO:0004197 GO:0005515 GO:0004252 GO:0017111
GO:0031379
Cluster-22178.74927 GO:0005524 GO:0003774 GO:0005515 GO:0016459
Cluster-22178.74932 GO:0007275 GO:0005515 GO:0016020
Cluster-22178.74933 GO:0006525 GO:0006560 GO:0006511 GO:0006433
GO:0006777 GO:0000166 GO:0051539 GO:0005524 GO:0051536
GO:0004827 GO:0004175 GO:0003824 GO:0019773 GO:0019008
GO:0005737
Cluster-22178.74938 GO:0006865 GO:0008168 GO:0016020 GO:0016021
Cluster-22178.74940 GO:0004672 GO:0019028
Cluster-22178.74944 GO:0016192 GO:0006886 GO:0006694 GO:0006284
GO:0016114 GO:0003677 GO:0008661 GO:0005515 GO:0005198
GO:0030117 GO:0005634
Cluster-22178.74945 GO:0007165 GO:0006914 GO:0005515 GO:0016301
GO:0016021
Cluster-22178.74951 GO:0042262 GO:0051607 GO:0005509 GO:0003690

Cluster-22178.74953 GO:0019646 GO:0045454 GO:0000902 GO:0006118
GO:0006955 GO:0009055 GO:0015035 GO:0016020
Cluster-22178.74955 GO:0008239 GO:0070009 GO:0043565
Cluster-22178.74957 GO:0030435 GO:0005515
Cluster-22178.74958 GO:0007155 GO:0005540 GO:0042302
Cluster-22178.74961 GO:0002949 GO:0006310 GO:0019079 GO:0006281
GO:0006355 GO:0016887 GO:0005524 GO:0003724 GO:0009378
GO:0098519 GO:0043531 GO:0003678 GO:0003723 GO:0008134
GO:0016301 GO:0005657 GO:0009379 GO:0005667
Cluster-22178.74962 GO:0032065 GO:0005543 GO:0005515 GO:0005938
Cluster-22178.74964 GO:0005515

Cluster-22178.74965 GO:0034314 GO:0006541 GO:0030833 GO:0006508
GO:0008236 GO:0016787 GO:0005856 GO:0005885
Cluster-22178.74966 GO:0006144 GO:0006206 GO:0006351 GO:0004190
GO:0003899 GO:0005730 GO:0016021
Cluster-22178.74968 GO:0048870 GO:0010508 GO:0000917 GO:0043093
GO:0005198 GO:0005737 GO:0031514 GO:0005882
Cluster-22178.74970 GO:0055114 GO:0005975 GO:0006355 GO:0009271
GO:0016491 GO:0016209 GO:0030246
Cluster-22178.74971 GO:0006355 GO:0003700 GO:0005634 GO:0005667
Cluster-22178.74975 GO:0051016 GO:0005515 GO:0008290
Cluster-22178.74977 GO:0009069 GO:0035023 GO:0016310 GO:0043087
GO:0005509 GO:0005515 GO:0005089 GO:0004674
Cluster-22178.74981 GO:0006531 GO:0016055 GO:0043039 GO:0006419
GO:0006522 GO:0000166 GO:0005524 GO:0004813 GO:0016876
GO:0003676
Cluster-22178.74982 GO:0007275 GO:0007165 GO:0030178 GO:0016579
GO:0005179 GO:0005515 GO:0036459 GO:0005576
Cluster-22178.74983 GO:0007165 GO:0007275 GO:0030178 GO:0016579
GO:0005179 GO:0005515 GO:0036459 GO:0005576
Cluster-22178.74984 GO:0006807 GO:0004668 GO:0005509 GO:0005737
Cluster-22178.74985 GO:0009254 GO:0009058 GO:0016567 GO:0009451
GO:0001522 GO:0006040 GO:0007276 GO:0019538 GO:0003723
GO:0005515 GO:0016773 GO:0005524 GO:0009982 GO:0004842
Cluster-22178.74987 GO:0030488 GO:0008033 GO:0055114 GO:0009082
GO:0009451 GO:0042138 GO:0006310 GO:0016429 GO:0046872
GO:0016491 GO:0016021 GO:0031515
Cluster-22178.74988 GO:0009082 GO:0009451 GO:0006310 GO:0042138
GO:0030488 GO:0055114 GO:0008033 GO:0016429 GO:0016491
GO:0031515 GO:0005887 GO:0016021
Cluster-22178.74989 GO:0034453 GO:0016192 GO:0005515 GO:0016021
GO:0005815
Cluster-22178.74991 GO:0005975 GO:0030259 GO:0005515 GO:0016758

Cluster-22178.74993 GO:0006418 GO:0003677 GO:0004518 GO:0000166
GO:0004812 GO:0008270 GO:0005524

Cluster-22178.74995 GO:0016021

Cluster-22178.74996 GO:0006955 GO:0019042 GO:0007165 GO:0005164
GO:0016020 GO:0033644

Cluster-22178.74999 GO:0004867 GO:0005524 GO:0005576

Cluster-22178.75000 GO:0006807 GO:0003824 GO:0003723 GO:0046914

Cluster-22178.75002 GO:0035556 GO:0005515 GO:0046872

Cluster-22178.75003 GO:0035556 GO:0005515

Cluster-22178.75005 GO:0003677 GO:0046983

Cluster-22178.75014 GO:0046168 GO:0005975 GO:0055114 GO:0006072
GO:0030153 GO:0006955 GO:0046486 GO:0004367 GO:0015643
GO:0051287 GO:0016616 GO:0009331 GO:0019814

Cluster-22178.75015 GO:0055114 GO:0006276 GO:0020037 GO:0016705
GO:0005506

Cluster-22178.75016 GO:0005515 GO:0001533

Cluster-22178.75017 GO:0006096 GO:0006457 GO:0006094 GO:0006571
GO:0000162 GO:0000413 GO:0009094 GO:0003755 GO:0000287
GO:0004634 GO:0000015

Cluster-22178.75021 GO:0006629

Cluster-22178.75023 GO:0006367 GO:0005672

Cluster-22178.75024 GO:0006810 GO:0006457 GO:0019042 GO:0006206
GO:0051607 GO:0006914 GO:0006144 GO:0006351 GO:0006355
GO:0007049 GO:0003677 GO:0003899 GO:0051082 GO:0003700
GO:0043565 GO:0016817 GO:0016020 GO:0005730 GO:0005667
GO:0033644 GO:0016272 GO:0005634 GO:0016021

Cluster-22178.75025 GO:0016020

Cluster-22178.75026 GO:0006810 GO:0009405 GO:0015858 GO:0055085
GO:0005337 GO:0005576 GO:0016021

Cluster-22178.75028 GO:0015979 GO:0009523 GO:0009539

Cluster-22178.75030 GO:0006355 GO:0005634

Cluster-22178.75031 GO:0006888 GO:0055114 GO:0006886 GO:0008168

GO:0046872 GO:0008270 GO:0003677 GO:0016706 GO:0030127
Cluster-22178.75032 GO:0006333 GO:0005634
Cluster-22178.75033 GO:0051252 GO:0006412 GO:0016310 GO:0006465
GO:0000160 GO:0008033 GO:0042254 GO:0015743 GO:0005515
GO:0003723 GO:0003676 GO:0003735 GO:0000155 GO:0004526
GO:0008233 GO:0046983 GO:0005634 GO:0005787 GO:0009365
GO:0005840 GO:0016021 GO:0030677 GO:0005622
Cluster-22178.75035 GO:0035556 GO:0015886 GO:0005515 GO:0015232
GO:0008270
Cluster-22178.75036 GO:0016746
Cluster-22178.75037 GO:0003779 GO:0005622
Cluster-22178.75038 GO:0006144 GO:0035556 GO:0046034 GO:0046872
GO:0004017
Cluster-22178.75039 GO:0006281 GO:0042254 GO:0006412 GO:0006144
GO:0006464 GO:0046034 GO:0035556 GO:0005515 GO:0003735
GO:0004017 GO:0046872 GO:0008270 GO:0016151 GO:0005840
Cluster-22178.75040 GO:0006281 GO:0042254 GO:0006412 GO:0006144
GO:0006464 GO:0046034 GO:0035556 GO:0003735 GO:0005515
GO:0004017 GO:0046872 GO:0008270 GO:0016151 GO:0005840
Cluster-22178.75041 GO:0009396 GO:0015992 GO:0042254 GO:0008152
GO:0055114 GO:0006412 GO:0046487 GO:0015991 GO:0016491
GO:0004488 GO:0003735 GO:0050662 GO:0003824 GO:0015078
GO:0005515 GO:0005840 GO:0033179
Cluster-22178.75042 GO:0045766 GO:0043066 GO:0043123 GO:0005515

Cluster-22178.75044 GO:0018279 GO:0006355 GO:0006506 GO:0004579
GO:0003677 GO:0017176 GO:0005634 GO:0008250 GO:0016021
Cluster-22178.75047 GO:0006090 GO:0071436 GO:0006814 GO:0006560
GO:0045116 GO:0006525 GO:0016881 GO:0005524 GO:0015081
GO:0008948 GO:0016020 GO:0005634
Cluster-22178.75048 GO:0005515
Cluster-22178.75049 GO:0006368 GO:0016790 GO:0008023
Cluster-22178.75051 GO:0005840

Cluster-22178.75052 GO:0006511 GO:0031625 GO:0019028

Cluster-22178.75054 GO:0016020

Cluster-22178.75055 GO:0016757

Cluster-22178.75065 GO:0007165 GO:0005515 GO:0005096

Cluster-22178.75066 GO:0006468 GO:0006355 GO:0006412 GO:0042254 GO:0007165 GO:0006357 GO:0051056 GO:0007169 GO:0001104 GO:0042393 GO:0046872 GO:0003735 GO:0008201 GO:0004714 GO:0005096 GO:0005524 GO:0004872 GO:0005840 GO:0016592 GO:0005622 GO:0005887

Cluster-22178.75069 GO:0055085 GO:0016021 GO:0016020

Cluster-22178.75074 GO:0006470 GO:0006570 GO:0098542 GO:0008138 GO:0004725

Cluster-22178.75078 GO:0006118 GO:0009234 GO:0008152 GO:0050662 GO:0003954 GO:0005515 GO:0005198 GO:0046872 GO:0016491 GO:0003824 GO:0005739 GO:0019028

Cluster-22178.75079 GO:0005515

Cluster-22178.75082 GO:0016567 GO:0006914 GO:0006308 GO:0005515 GO:0035091 GO:0046872 GO:0008270 GO:0008855 GO:0004842 GO:0009318 GO:0005622 GO:0005680

Cluster-22178.75083 GO:0055085 GO:0005515 GO:0016021

Cluster-22178.75085 GO:0006355 GO:0005515 GO:0043565 GO:0005634

Cluster-22178.75086 GO:0004867

Cluster-22178.75087 GO:0006260 GO:0006955 GO:0051249 GO:0005198

Cluster-22178.75088 GO:0007155 GO:0001503 GO:0005524 GO:0005515 GO:0005576

Cluster-22178.75089 GO:0007155 GO:0001503 GO:0005524 GO:0005515 GO:0005576

Cluster-22178.75090 GO:0006470 GO:0006570 GO:0004725 GO:0008138

Cluster-22178.75096 GO:0006396 GO:0006260 GO:0055114 GO:0005198 GO:0008270 GO:0016020 GO:0019028 GO:0019031 GO:0005622

Cluster-22178.75098 GO:0006412 GO:0042254 GO:0003735 GO:0005840

Cluster-22178.75102 GO:0000917 GO:0007186 GO:0006281 GO:0043093
GO:0006355 GO:0006950 GO:0000077 GO:0005509 GO:0004930
GO:0003700 GO:0043565 GO:0005667 GO:0005737 GO:0030896
Cluster-22178.75103 GO:0008033 GO:0003676 GO:0016779 GO:0008270
GO:0016437 GO:0004810 GO:0005634
Cluster-22178.75112 GO:0055114 GO:0016616 GO:0016491
Cluster-22178.75113 GO:0006468 GO:0004672 GO:0005524
Cluster-22178.75114 GO:0015994 GO:0006783 GO:0006915 GO:0007130
GO:0007131 GO:0033014 GO:0006810 GO:0007283 GO:0016567
GO:0046872 GO:0005215 GO:0004852 GO:0005515 GO:0008270
GO:0004842 GO:0061630 GO:0005680 GO:0016020
Cluster-22178.75116 GO:0005515
Cluster-22178.75117 GO:0006914 GO:0016485 GO:0009252 GO:0043085
GO:0006810 GO:0016755 GO:0016021
Cluster-22178.75119 GO:0051537 GO:0043231
Cluster-22178.75123 GO:0003677
Cluster-22178.75128 GO:0006810 GO:0042626 GO:0005515 GO:0005524
GO:0016021
Cluster-22178.75129 GO:0006508 GO:0055085 GO:0008236 GO:0005515
GO:0016021
Cluster-22178.75130 GO:0055085 GO:0016021
Cluster-22178.75131 GO:0055085 GO:0016021
Cluster-22178.75132 GO:0006606 GO:0006468 GO:0005524 GO:0004672
GO:0016773 GO:0016020
Cluster-22178.75135 GO:0006626 GO:0016746 GO:0005741
Cluster-22178.75139 GO:0016772 GO:0016301 GO:0004045 GO:0003677

Cluster-22178.75141 GO:0016301 GO:0016772 GO:0004045 GO:0003677

Cluster-22178.75143 GO:0005515 GO:0046872
Cluster-22178.75145 GO:0006310 GO:0006810 GO:0007165 GO:0014068
GO:0050776 GO:0006281 GO:0009405 GO:0005524 GO:0005102
GO:0009378 GO:0005179 GO:0043548 GO:0019871 GO:0005657

GO:0009379 GO:0016020 GO:0005576
Cluster-22178.75147 GO:0006260 GO:0042254 GO:0043093 GO:0007049
GO:0051252 GO:0000917 GO:0006412 GO:0006355 GO:0003677
GO:0003735 GO:0003700 GO:0043565 GO:0003887 GO:0004523
GO:0005815 GO:0005622 GO:0005667 GO:0005840 GO:0005737
GO:0042575
Cluster-22178.75149 GO:0006406 GO:0055114 GO:0006357 GO:0006368
GO:0001104 GO:0020037 GO:0016705 GO:0005506 GO:0000446
GO:0016592
Cluster-22178.75150 GO:0005515 GO:0008270
Cluster-22178.75153 GO:0043087 GO:0007264 GO:0051260 GO:0006605
GO:0009264 GO:0006886 GO:0008253 GO:0005085 GO:0005515
GO:0005742 GO:0016020
Cluster-22178.75154 GO:0007264 GO:0006605 GO:0051260 GO:0009264
GO:0006886 GO:0043087 GO:0005085 GO:0005515 GO:0008253
GO:0005742 GO:0016020
Cluster-22178.75158 GO:0007165 GO:0007606 GO:0004888 GO:0016021
Cluster-22178.75164 GO:0006810 GO:0007018 GO:0006281 GO:0007017
GO:0051607 GO:0000723 GO:0070206 GO:0019901 GO:0003777
GO:0008017 GO:0003678 GO:0005524 GO:0005657 GO:0005874
GO:0045298
Cluster-22178.75166 GO:0006281 GO:0006355 GO:0008168 GO:0008270
GO:0005515 GO:0003677 GO:0016020
Cluster-22178.75167 GO:0006355 GO:0006281 GO:0008270 GO:0008168
GO:0003677 GO:0005515 GO:0016020
Cluster-22178.75168 GO:0005525 GO:0003924
Cluster-22178.75169 GO:0006468 GO:0006355 GO:0006886 GO:0000917
GO:0048870 GO:0016310 GO:0009069 GO:0043093 GO:0007186
GO:0007165 GO:0006914 GO:0015031 GO:0006606 GO:0005198
GO:0043565 GO:0003700 GO:0004871 GO:0070009 GO:0008565
GO:0005524 GO:0008239 GO:0004674 GO:0031514 GO:0005737
GO:0016021 GO:0005667 GO:0016020 GO:0005882 GO:0005834
Cluster-22178.75173 GO:0051382 GO:0006281

Cluster-22178.75174 GO:0015992 GO:0015986 GO:0015078 GO:0016020
Cluster-22178.75176 GO:0016021
Cluster-22178.75178 GO:0055114 GO:0009236 GO:0006118 GO:0016627
GO:0008818 GO:0003697 GO:0051073 GO:0003995 GO:0005856
GO:0070188
Cluster-22178.75182 GO:0055085 GO:0005741
Cluster-22178.75184 GO:0006508 GO:0004222
Cluster-22178.75185 GO:0006508 GO:0004222
Cluster-22178.75186 GO:0051603 GO:0004298 GO:0005839
Cluster-22178.75190 GO:0007165 GO:0006470 GO:0006570 GO:0005158
GO:0008138 GO:0004725 GO:0005899
Cluster-22178.75192 GO:0006357 GO:0019441 GO:0006568 GO:0006355
GO:0004833 GO:0001104 GO:0020037 GO:0003677 GO:0016592
Cluster-22178.75193 GO:0006260 GO:0006355 GO:0006915 GO:0032465
GO:0019058 GO:0016567 GO:0004842 GO:0003677 GO:0005664
Cluster-22178.75195 GO:0019058 GO:0006260 GO:0006355 GO:0003677
GO:0005664
Cluster-22178.75197 GO:0005515 GO:0008270
Cluster-22178.75199 GO:0008168 GO:0070403
Cluster-22178.75202 GO:0030001 GO:0046872 GO:0005515 GO:0019028
Cluster-22178.75203 GO:0019073
Cluster-22178.75205 GO:0004672
Cluster-22178.75206 GO:0005515 GO:0008270
Cluster-22178.75208 GO:0008152 GO:0055114 GO:0016491
Cluster-22178.75212 GO:0030001 GO:0005975 GO:0006810 GO:0007165
GO:0017004 GO:0016192 GO:0006357 GO:0015886 GO:0055085
GO:0005198 GO:0001104 GO:0005515 GO:0005215 GO:0046873
GO:0016853 GO:0043395 GO:0004872 GO:0005578 GO:0019028
GO:0031083 GO:0019031 GO:0019867 GO:0016021 GO:0016592
GO:0030133 GO:0016020
Cluster-22178.75214 GO:0009069 GO:0016310 GO:0004674 GO:0005515

Cluster-22178.75215 GO:0006352 GO:0045892 GO:0007010 GO:0006270

GO:0006355 GO:0003700 GO:0016987 GO:0003779 GO:0003677
GO:0032040 GO:0005667 GO:0005634
Cluster-22178.75217 GO:0045892 GO:0005634
Cluster-22178.75219 GO:0006355 GO:0043401 GO:0007165 GO:0043565
GO:0008270 GO:0003700 GO:0005186 GO:0005634 GO:0005667
GO:0005615
Cluster-22178.75225 GO:0009405 GO:0006810 GO:0006310 GO:0006051
GO:0006040 GO:0003677 GO:0047465 GO:0005515 GO:0008200
GO:0019905 GO:0005576 GO:0005886
Cluster-22178.75228 GO:0033227 GO:0006807 GO:0006672 GO:0015931
GO:0016811 GO:0051033 GO:0016021
Cluster-22178.75231 GO:0007275 GO:0006810 GO:0006508 GO:0005515
GO:0004198 GO:0005215 GO:0005576 GO:0005622
Cluster-22178.75236 GO:0006412 GO:0042254 GO:0003735 GO:0005840
GO:0016020 GO:0005622
Cluster-22178.75237 GO:0000413 GO:0006457 GO:0003755 GO:0003676

Cluster-22178.75239 GO:0016579 GO:0036459
Cluster-22178.75241 GO:0006351 GO:0019290 GO:0015991 GO:0006826
GO:0006206 GO:0006144 GO:0003677 GO:0015343 GO:0016820
GO:0005515 GO:0003899 GO:0005730 GO:0000221
Cluster-22178.75243 GO:0006827 GO:0006446 GO:0006413 GO:0006826
GO:0005381 GO:0003743 GO:0016020 GO:0005840 GO:0033573
Cluster-22178.75246 GO:0005515
Cluster-22178.75248 GO:0005515
Cluster-22178.75250 GO:0051258 GO:0048870 GO:0007049 GO:0006412
GO:0030168 GO:0006260 GO:0071704 GO:0007165 GO:0005975
GO:0016310 GO:0006468 GO:0006606 GO:0009069 GO:0042254
GO:0045502 GO:0008134 GO:0005096 GO:0004674 GO:0005102
GO:0030674 GO:0016868 GO:0005198 GO:0005515 GO:0042803
GO:0003735 GO:0019031 GO:0005840 GO:0019028 GO:0031514
GO:0030286 GO:0005882 GO:0005622 GO:0005577 GO:0005667
Cluster-22178.75251 GO:0006281 GO:0008270 GO:0046872 GO:0005815

Cluster-22178.75252 GO:0048870 GO:0006914 GO:0000921 GO:0048193
GO:0006606 GO:0007155 GO:0045502 GO:0042803 GO:0008134
GO:0005940 GO:0030286 GO:0016021 GO:0005667 GO:0016020
GO:0031514
Cluster-22178.75253 GO:0006118 GO:0006814 GO:0015992 GO:0006123
GO:0006120 GO:0006744 GO:0006825 GO:0003954 GO:0016531
GO:0008137 GO:0005507 GO:0004129 GO:0005739 GO:0045277
GO:0005758
Cluster-22178.75255 GO:0000062 GO:0005634
Cluster-22178.75257 GO:0007264 GO:0005525
Cluster-22178.75260 GO:0007165
Cluster-22178.75261 GO:0006479 GO:0006355 GO:0006554 GO:0034968
GO:0005515 GO:0018024 GO:0005694
Cluster-22178.75264 GO:0006811 GO:0006810 GO:0055114 GO:0005230
GO:0016020
Cluster-22178.75265 GO:0006813 GO:0055114 GO:0016620 GO:0016491
GO:0071949
Cluster-22178.75269 GO:0045893 GO:0006355 GO:0043565 GO:0003677
GO:0005634 GO:0016021
Cluster-22178.75274 GO:0007186 GO:0006810 GO:0009405 GO:0008200
GO:0004930 GO:0005576 GO:0016021
Cluster-22178.75275 GO:0006471 GO:0003956
Cluster-22178.75278 GO:0046486 GO:0006468 GO:0042967 GO:0045017
GO:0000160 GO:0003677 GO:0004144 GO:0000156 GO:0005524
GO:0004672
Cluster-22178.75279 GO:0009405 GO:0005576
Cluster-22178.75282 GO:0055114 GO:0046168 GO:0015940 GO:0008677
GO:0051287 GO:0016757 GO:0016616
Cluster-22178.75285 GO:0031124
Cluster-22178.75287 GO:0007155 GO:0031505 GO:0005540 GO:0042302

Cluster-22178.75291 GO:0019722 GO:0003723 GO:0003676
Cluster-22178.75292 GO:0005544 GO:0005198 GO:0005509 GO:0005578

Cluster-22178.75293 GO:0006357 GO:0001104 GO:0016592
Cluster-22178.75295 GO:0006811 GO:0016579 GO:0055085 GO:0006508
GO:0006511 GO:0008017 GO:0005216 GO:0004843 GO:0005622
GO:0045298 GO:0016020
Cluster-22178.75297 GO:0042546 GO:0009664 GO:0007275 GO:0005199
GO:0005578 GO:0005618
Cluster-22178.75302 GO:0055114 GO:0007283 GO:0016491
Cluster-22178.75303 GO:0007264 GO:0003676 GO:0005515 GO:0005525
GO:0008270
Cluster-22178.75304 GO:0006886 GO:0016192 GO:0016021 GO:0030131
GO:0030117
Cluster-22178.75306 GO:0016579 GO:0036459
Cluster-22178.75307 GO:0016579 GO:0036459
Cluster-22178.75310 GO:0006432 GO:0007165 GO:0035023 GO:0043087
GO:0005509 GO:0005515 GO:0000287 GO:0003723 GO:0005089
GO:0005524 GO:0005578
Cluster-22178.75313 GO:0006544 GO:0051258 GO:0055114 GO:0006308
GO:0046653 GO:0006563 GO:0006606 GO:0030168 GO:0007165
GO:0006566 GO:0046872 GO:0019901 GO:0020037 GO:0016705
GO:0003950 GO:0005102 GO:0008855 GO:0046983 GO:0008115
GO:0005506 GO:0030674 GO:0009318 GO:0005577
Cluster-22178.75314 GO:0006570 GO:0009058 GO:0008299 GO:0007165
GO:0006694 GO:0006470 GO:0035556 GO:0005515 GO:0016779
GO:0005044 GO:0050518 GO:0004725 GO:0016020 GO:0019031
Cluster-22178.75315 GO:0055114 GO:0006769 GO:0046497 GO:0070403
GO:0008750 GO:0030976 GO:0050661 GO:0000287 GO:0016021
Cluster-22178.75316 GO:0016567 GO:0006508 GO:0004197 GO:0005515
GO:0004842 GO:0046872 GO:0008270 GO:0005680 GO:0005622
Cluster-22178.75317 GO:0006355 GO:0016310 GO:0016772 GO:0003700
GO:0008270 GO:0016817 GO:0005667 GO:0005634
Cluster-22178.75318 GO:0006614 GO:0008152 GO:0016301 GO:0005525
GO:0005524
Cluster-22178.75320 GO:0006814 GO:0055114 GO:0019049 GO:0015992

GO:0005975 GO:0006120 GO:0006744 GO:0008152 GO:0016836
GO:0030246 GO:0008137 GO:0003824
Cluster-22178.75321 GO:0005515
Cluster-22178.75323 GO:0016567 GO:0004842 GO:0005515
Cluster-22178.75326 GO:0006351 GO:0051252 GO:0006396 GO:0007049
GO:0005509 GO:0005515 GO:0004252 GO:0005544 GO:0003723
GO:0004525 GO:0017053 GO:0005886
Cluster-22178.75331 GO:0007165 GO:0007275 GO:0005179 GO:0005576
Cluster-22178.75335 GO:0006094 GO:0006570 GO:0006470 GO:0006098
GO:0006457 GO:0006096 GO:0006000 GO:0006013 GO:0043087
GO:0006012 GO:0051082 GO:0005515 GO:0004725 GO:0003872
GO:0016020 GO:0016272 GO:0005945 GO:0005639
Cluster-22178.75338 GO:0006281 GO:0006144 GO:0046034 GO:0004017
GO:0004519 GO:0003677 GO:0046872 GO:0008270 GO:0003676
Cluster-22178.75339 GO:0016579 GO:0036459 GO:0016021
Cluster-22178.75340 GO:0009405 GO:0006810 GO:0019871 GO:0005576
Cluster-22178.75341 GO:0006694 GO:0055114 GO:0005985 GO:0008210
GO:0009117 GO:0008207 GO:0007186 GO:0005982 GO:0008209
GO:0007165 GO:0004871 GO:0003824 GO:0048040 GO:0016616
GO:0050662 GO:0003854 GO:0005834
Cluster-22178.75346 GO:0071766 GO:0052636
Cluster-22178.75347 GO:0007186 GO:0004930 GO:0016021
Cluster-22178.75349 GO:0007018 GO:0007017 GO:0008017 GO:0003777
GO:0005524 GO:0005515 GO:0045298 GO:0005874
Cluster-22178.75350 GO:0008152 GO:0006468 GO:0007165 GO:0004672
GO:0005524 GO:0003824 GO:0005179 GO:0005515 GO:0005576
Cluster-22178.75353 GO:0007218
Cluster-22178.75355 GO:0006571 GO:0000162 GO:0009094 GO:0055114
GO:0004764
Cluster-22178.75360 GO:0007339 GO:0055114 GO:0005515 GO:0051920
GO:0005576
Cluster-22178.75361 GO:0006560 GO:0007339 GO:0071436 GO:0006814
GO:0006525 GO:0006090 GO:0055114 GO:0008948 GO:0005515

GO:0051920 GO:0015081 GO:0016020 GO:0005576
Cluster-22178.75362 GO:0007264 GO:0005525 GO:0016020
Cluster-22178.75363 GO:0016567 GO:0006468 GO:0009069 GO:0016310
GO:0015979 GO:0005515 GO:0004674 GO:0004842 GO:0005524
GO:0008270 GO:0004672 GO:0046872 GO:0030089 GO:0005840
GO:0005680
Cluster-22178.75365 GO:0006281 GO:0006310
Cluster-22178.75366 GO:0015979 GO:0009523 GO:0009539 GO:0016020
Cluster-22178.75368 GO:0019722 GO:0003676 GO:0003723
Cluster-22178.75370 GO:0005975 GO:0003824 GO:0043169
Cluster-22178.75371 GO:0008152 GO:0016791
Cluster-22178.75375 GO:0008152 GO:0055114 GO:0016491
Cluster-22178.75376 GO:0008152 GO:0055114 GO:0016491
Cluster-22178.75377 GO:0006525 GO:0006420 GO:0006560 GO:0007018
GO:0007017 GO:0008017 GO:0005524 GO:0000166 GO:0005198
GO:0003777 GO:0004814 GO:0005882 GO:0005815 GO:0005874
GO:0045298 GO:0005737
Cluster-22178.75380 GO:0006729 GO:0008124
Cluster-22178.75381 GO:0018279 GO:0005789
Cluster-22178.75383 GO:0000266 GO:0007018 GO:0007165 GO:0007017
GO:0007605 GO:0008017 GO:0003777 GO:0005524 GO:0005179
GO:0045298 GO:0005874 GO:0005739 GO:0005576
Cluster-22178.75384 GO:0006576 GO:0005525 GO:0003924 GO:0005524

Cluster-22178.75385 GO:0007165 GO:0006566 GO:0006606 GO:0030168
GO:0006563 GO:0046653 GO:0006544 GO:0055114 GO:0051258
GO:0030674 GO:0008115 GO:0005506 GO:0005102 GO:0003950
GO:0046983 GO:0016705 GO:0020037 GO:0046872 GO:0005577
Cluster-22178.75386 GO:0043248 GO:0005783
Cluster-22178.75391 GO:0006420 GO:0006418 GO:0051262 GO:0007165
GO:0006560 GO:0006525 GO:0008289 GO:0005524 GO:0004814
GO:0003677 GO:0000166 GO:0004812 GO:0005737
Cluster-22178.75393 GO:0043547 GO:0046983 GO:0005096

Cluster-22178.75394 GO:0016021
Cluster-22178.75395 GO:0007283 GO:0005515 GO:0003676 GO:0046872
GO:0005794
Cluster-22178.75397 GO:0046872
Cluster-22178.75400 GO:0006355 GO:0003676 GO:0005515 GO:0005634
GO:0019028
Cluster-22178.75401 GO:0007186 GO:0055085 GO:0019856 GO:0004930
GO:0008440 GO:0016021
Cluster-22178.75402 GO:0006355 GO:0005507 GO:0003700 GO:0003676
GO:0005515 GO:0003677 GO:0019028 GO:0005634 GO:0005667
Cluster-22178.75404 GO:0051252 GO:0016891
Cluster-22178.75407 GO:0006457 GO:0051087 GO:0005515 GO:0042803
GO:0004064 GO:0008092 GO:0000774 GO:0019898 GO:0005737
Cluster-22178.75410 GO:0006508 GO:0051028 GO:0005634
Cluster-22178.75411 GO:0006571 GO:0000162 GO:0009094 GO:0006432
GO:0003676 GO:0004826 GO:0046872 GO:0004519 GO:0003723
GO:0009328
Cluster-22178.75412 GO:0015948 GO:0046656 GO:0008152 GO:0005515
GO:0005509 GO:0016746 GO:0030269 GO:0019031 GO:0016021
Cluster-22178.75413 GO:0006351 GO:0007186 GO:0006206 GO:0006144
GO:0055085 GO:0003677 GO:0003899 GO:0004930 GO:0005730
GO:0019033 GO:0016021
Cluster-22178.75415 GO:0006888 GO:0035556 GO:0006886 GO:0007165
GO:0008270 GO:0003723 GO:0005515 GO:0030127
Cluster-22178.75421 GO:0007155 GO:0051056 GO:0005096 GO:0005515
GO:0051539
Cluster-22178.75422 GO:0006355 GO:0009405 GO:0043565 GO:0003700
GO:0005198 GO:0005515 GO:0005509 GO:0005667
Cluster-22178.75423 GO:0071229 GO:0042710 GO:0032012 GO:0043087
GO:0070072 GO:0005086
Cluster-22178.75424 GO:0019073 GO:0015991 GO:0016192 GO:0006886
GO:0006351 GO:0005515 GO:0016820 GO:0000221 GO:0030117
Cluster-22178.75427 GO:0006865 GO:0010468 GO:0015979 GO:0050821

GO:0003333 GO:0009405 GO:0007165 GO:0004872 GO:0015171
GO:0042301 GO:0005515 GO:0016021 GO:0009523 GO:0016020
Cluster-22178.75429 GO:0055085 GO:0016117 GO:0007155 GO:0055114
GO:0008608 GO:0015940 GO:0030001 GO:0005524 GO:0016491
GO:0004222 GO:0050660 GO:0046873 GO:0016787 GO:0005540
GO:0016614 GO:0008677 GO:0016705 GO:0016020 GO:0072686
GO:0042729
Cluster-22178.75430 GO:0005515 GO:0003676
Cluster-22178.75433 GO:0006397 GO:0006886 GO:0016192 GO:0006355
GO:0005515 GO:0005198 GO:0030117 GO:0005634 GO:0005737
Cluster-22178.75438 GO:0006270
Cluster-22178.75441 GO:0005515 GO:0046872
Cluster-22178.75442 GO:0005634
Cluster-22178.75453 GO:0005515
Cluster-22178.75456 GO:0005515
Cluster-22178.75457 GO:0003677
Cluster-22178.75461 GO:0055114 GO:0016491 GO:0004252 GO:0005886
Cluster-22178.75462 GO:0006468 GO:0006260 GO:0016773 GO:0005524
GO:0004672 GO:0003677 GO:0016020
Cluster-22178.75464 GO:1902208 GO:0045893 GO:0003677 GO:0008270
GO:0005634
Cluster-22178.75465 GO:1902208 GO:0045893 GO:0043087 GO:0035023
GO:0008270 GO:0005089 GO:0003677 GO:0005634
Cluster-22178.75466 GO:1902208 GO:0045893 GO:0008270 GO:0003677
GO:0005634
Cluster-22178.75467 GO:0006457 GO:0008270 GO:0051082 GO:0045502
GO:0008134 GO:0042803 GO:0030286 GO:0016272 GO:0005667
Cluster-22178.75473 GO:0006486 GO:0008378 GO:0016020
Cluster-22178.75477 GO:0016021
Cluster-22178.75478 GO:0008270
Cluster-22178.75480 GO:0001522 GO:0043044 GO:0009451 GO:0009982
GO:0003723 GO:0016586
Cluster-22178.75482 GO:0009405 GO:0016021 GO:0016020 GO:0005576

Cluster-22178.75484 GO:0019042 GO:0005515 GO:0033644
Cluster-22178.75487 GO:0016192 GO:0006886 GO:0005198 GO:0005515
GO:0030132 GO:0030130 GO:0030117
Cluster-22178.75488 GO:0008270 GO:0003677
Cluster-22178.75490 GO:0016192 GO:0006886 GO:0005515 GO:0005198
GO:0030117 GO:0030130 GO:0030132
Cluster-22178.75493 GO:0016192 GO:0008053 GO:0003924 GO:0016021
GO:0005741
Cluster-22178.75494 GO:0006468 GO:0006118 GO:0004672 GO:0005524
GO:0009055
Cluster-22178.75499 GO:0006810 GO:0016021
Cluster-22178.75500 GO:0007155 GO:0046872 GO:0003677 GO:0016020
Cluster-22178.75503 GO:0006811 GO:0006144 GO:0006206 GO:0006351
GO:0006874 GO:0006816 GO:0003676 GO:0005219 GO:0008270
GO:0003899 GO:0016021 GO:0005622 GO:0016020 GO:0005634
GO:0005730
Cluster-22178.75506 GO:0051287
Cluster-22178.75507 GO:0006355 GO:0045893 GO:0003677 GO:0003700
GO:0005667 GO:0005737 GO:0005634
Cluster-22178.75512 GO:0042742
Cluster-22178.75515 GO:0006281 GO:0000012 GO:0003684 GO:0005634
Cluster-22178.75516 GO:0016226 GO:0042254 GO:0006412 GO:0051536
GO:0005506 GO:0003735 GO:0005622 GO:0005840
Cluster-22178.75518 GO:0005515
Cluster-22178.75519 GO:0006511 GO:0051603 GO:0004298 GO:0004175
GO:0019773 GO:0005839
Cluster-22178.75520 GO:0015074 GO:0007224
Cluster-22178.75525 GO:0006352
Cluster-22178.75529 GO:0005975 GO:0016868
Cluster-22178.75530 GO:0007005 GO:0007606 GO:0005515 GO:0043565
GO:0016021 GO:0031305 GO:0005634
Cluster-22178.75534 GO:0008152 GO:0006694 GO:0055114 GO:0008209
GO:0008207 GO:0006813 GO:0008210 GO:0003824 GO:0016491

GO:0016616 GO:0050662 GO:0003854
Cluster-22178.75535 GO:0006313 GO:0016192 GO:0006886 GO:0003677
GO:0005515 GO:0004803
Cluster-22178.75539 GO:0016192 GO:0006886 GO:0006810 GO:0055085
GO:0022857 GO:0005215 GO:0016020 GO:0030117 GO:0016021
Cluster-22178.75540 GO:0006886 GO:0016192 GO:0006810 GO:0055085
GO:0022857 GO:0005215 GO:0016020 GO:0030117 GO:0016021
Cluster-22178.75541 GO:0000166 GO:0046872
Cluster-22178.75542 GO:0008233
Cluster-22178.75545 GO:0005921
Cluster-22178.75546 GO:0051258 GO:0006935 GO:0030168 GO:0009405
GO:0007165 GO:0071973 GO:0003774 GO:0005102 GO:0030674
GO:0009288 GO:0005577 GO:0016020
Cluster-22178.75547 GO:0055114 GO:0006570 GO:0004511
Cluster-22178.75548 GO:0006952
Cluster-22178.75551 GO:0008152 GO:0055114 GO:0003824 GO:0016491
GO:0050662
Cluster-22178.75552 GO:0006531 GO:0006886 GO:0006144 GO:0006522
GO:0009152 GO:0006188 GO:0004018 GO:0005737
Cluster-22178.75554 GO:0055114 GO:0047134 GO:0016021
Cluster-22178.75556 GO:0006807 GO:0008270 GO:0016814
Cluster-22178.75557 GO:0051726 GO:0006974
Cluster-22178.75558 GO:0046488 GO:0016307 GO:0005515
Cluster-22178.75560 GO:0046488 GO:0006887 GO:0008289 GO:0016307
GO:0019031 GO:0000145
Cluster-22178.75561 GO:0016020
Cluster-22178.75564 GO:0046488 GO:0016307 GO:0005515
Cluster-22178.75566 GO:0046488 GO:0005515 GO:0016307
Cluster-22178.75567 GO:0071479 GO:0006629 GO:0000077 GO:0042967
GO:0008081 GO:0008080 GO:0005634
Cluster-22178.75569 GO:0006629 GO:0042967 GO:0008080 GO:0008081

Cluster-22178.75572 GO:0043066 GO:0007186 GO:0004930 GO:0005521

GO:0016020 GO:0016021
Cluster-22178.75575 GO:0005515 GO:0005634
Cluster-22178.75589 GO:0044419 GO:0006144 GO:0006508 GO:0003968
GO:0017111 GO:0004197 GO:0031379
Cluster-22178.75591 GO:0045893 GO:0046872 GO:0005634
Cluster-22178.75593 GO:0006469 GO:0045859 GO:0004862 GO:0005952
Cluster-22178.75594 GO:0050909 GO:0016021 GO:0005639
Cluster-22178.75595 GO:0032040
Cluster-22178.75598 GO:0055114 GO:0042254 GO:0051287 GO:0030250
GO:0005576 GO:0016021
Cluster-22178.75603 GO:0051260
Cluster-22178.75604 GO:0006888 GO:0006886 GO:0043565 GO:0046872
GO:0008270 GO:0030127
Cluster-22178.75608 GO:0007186 GO:0004930
Cluster-22178.75611 GO:0045859 GO:0007049 GO:0016538
Cluster-22178.75620 GO:0008152 GO:0055114 GO:0016491
Cluster-22178.75621 GO:0006571 GO:0000162 GO:0015940 GO:0009094
GO:0046168 GO:0019521 GO:0055114 GO:0006098 GO:0004665
GO:0008677 GO:0004616 GO:0016616 GO:0008977 GO:0051287
Cluster-22178.75622 GO:0007165 GO:0006357 GO:0005509 GO:0005515
GO:0001104 GO:0008270 GO:0016592 GO:0005578
Cluster-22178.75626 GO:0007187 GO:0050912 GO:0007154 GO:0007607
GO:0008527 GO:0005509 GO:0005515 GO:0016020 GO:0016021
Cluster-22178.75627 GO:0006265 GO:0003677 GO:0003917 GO:0005694
Cluster-22178.75630 GO:0006281 GO:0006310 GO:0005524 GO:0009378
GO:0005515 GO:0009379 GO:0005657
Cluster-22178.75631 GO:0006355 GO:0043565 GO:0010181 GO:0003700
GO:0005667
Cluster-22178.75632 GO:0005515 GO:0004064
Cluster-22178.75633 GO:0046413 GO:0046983 GO:0005515 GO:0018836

Cluster-22178.75634 GO:0046413 GO:0046983 GO:0018836 GO:0005515

Cluster-22178.75635 GO:0016567 GO:0005515 GO:0061630 GO:0003677
GO:0016787 GO:0003676 GO:0004842 GO:0005524 GO:0034061
GO:0008270 GO:0046872 GO:0005634 GO:0005680
Cluster-22178.75637 GO:0046872
Cluster-22178.75638 GO:0006364 GO:0034314 GO:0030833 GO:0005885
GO:0005730 GO:0015629
Cluster-22178.75640 GO:0006814 GO:0006885 GO:0035023 GO:0015992
GO:0006464 GO:0043087 GO:0046436 GO:0009252 GO:0015385
GO:0005515 GO:0008716 GO:0005089 GO:0016020
Cluster-22178.75641 GO:0006355 GO:0003700 GO:0043565 GO:0005667
GO:0005634
Cluster-22178.75642 GO:0035023 GO:0043087 GO:0005089 GO:0005515

Cluster-22178.75644 GO:0008270
Cluster-22178.75645 GO:0019882 GO:0030521 GO:0007165 GO:0006955
GO:0006886 GO:0006355 GO:0005496 GO:0004882 GO:0003677
GO:0042289 GO:0003700 GO:0016020 GO:0005667 GO:0042613
GO:0042025 GO:0005634
Cluster-22178.75650 GO:0055085 GO:0006810 GO:0005215 GO:0016021
GO:0016020
Cluster-22178.75651 GO:0051082 GO:0031072
Cluster-22178.75652 GO:0008152 GO:0019605 GO:0047989 GO:0070569
GO:0005615
Cluster-22178.75655 GO:0006807 GO:0005515 GO:0005509 GO:0004668
GO:0005737
Cluster-22178.75656 GO:0016337 GO:0016020
Cluster-22178.75660 GO:0016567 GO:0016787 GO:0004842 GO:0005680
Cluster-22178.75666 GO:0008152 GO:0007160 GO:0005509 GO:0008168
GO:0005576
Cluster-22178.75667 GO:0003824
Cluster-22178.75669 GO:0016579 GO:0005515 GO:0036459 GO:0032183
GO:0070140
Cluster-22178.75671 GO:0005515 GO:0005509

Cluster-22178.75679 GO:0005975 GO:0004553
Cluster-22178.75689 GO:0005515
Cluster-22178.75695 GO:0006355 GO:0008270 GO:0003677 GO:0003676

Cluster-22178.75699 GO:0007165 GO:0006605 GO:0008289
Cluster-22178.75700 GO:0007166 GO:0005515 GO:0016020
Cluster-22178.75702 GO:0032402 GO:0008289 GO:0042470
Cluster-22178.75708 GO:0016573 GO:0006355 GO:0042967 GO:0015940
GO:0055114 GO:0007034 GO:0071949 GO:0004402 GO:0008677
GO:0016491 GO:0000123
Cluster-22178.75709 GO:0046983
Cluster-22178.75714 GO:0006468 GO:0004672 GO:0005524
Cluster-22178.75717 GO:0006810 GO:0005215 GO:0016021 GO:0016020
Cluster-22178.75720 GO:0033691
Cluster-22178.75721 GO:0006308 GO:0006769 GO:0046497 GO:0008152
GO:0006741 GO:0007218 GO:0006810 GO:0019871 GO:0016301
GO:0005198 GO:0003951 GO:0008855 GO:0005576 GO:0009318
GO:0005886 GO:0005882
Cluster-22178.75722 GO:0009236 GO:0006811 GO:0004190 GO:0016020
GO:0016021
Cluster-22178.75724 GO:0043039 GO:0005524 GO:0008270 GO:0016876

Cluster-22178.75725 GO:0003824
Cluster-22178.75726 GO:0015991 GO:0015031 GO:0071705 GO:0006606
GO:0016820 GO:0005515 GO:0003677 GO:0008565 GO:0005634
GO:0005737 GO:0000221
Cluster-22178.75729 GO:0009058 GO:0006520 GO:0030170 GO:0016829

Cluster-22178.75730 GO:0055085 GO:0016020 GO:0016021
Cluster-22178.75733 GO:0006310 GO:0007155 GO:0003677 GO:0051015
GO:0010181 GO:0005727
Cluster-22178.75737 GO:0006555 GO:0006730 GO:0004013
Cluster-22178.75739 GO:0007186 GO:0004930 GO:0016021

Cluster-22178.75745 GO:0009231 GO:0006260 GO:0005524 GO:0008686 GO:0016887 GO:0003678 GO:0033588 GO:0005657

Cluster-22178.75752 GO:0042981 GO:0005524

Cluster-22178.75754 GO:0022900 GO:0055114 GO:0006118 GO:0006355 GO:0003700 GO:0008827 GO:0046983 GO:0043565 GO:0003677 GO:0005515 GO:0016787 GO:0016021 GO:0009319 GO:0005667

Cluster-22178.75755 GO:0006508 GO:0006144 GO:0016567 GO:0003968 GO:0046872 GO:0016817 GO:0004252 GO:0005515 GO:0004842 GO:0070008 GO:0008270 GO:0004197 GO:0061630 GO:0017111 GO:0031379 GO:0005680

Cluster-22178.75760 GO:0007005 GO:0031305

Cluster-22178.75761 GO:0006955 GO:0007165 GO:0005164 GO:0016020

Cluster-22178.75766 GO:0019031

Cluster-22178.75768 GO:0006457 GO:0048193 GO:0051082 GO:0016020 GO:0016021 GO:0016272

Cluster-22178.75771 GO:0009405 GO:0045735

Cluster-22178.75773 GO:0006260 GO:0016567 GO:0007165 GO:0008270 GO:0005524 GO:0046872 GO:0004842 GO:0005179 GO:0005515 GO:0003677 GO:0016818 GO:0005680 GO:0005634 GO:0005576

Cluster-22178.75775 GO:0008152 GO:0055114 GO:0016491

Cluster-22178.75776 GO:0035556 GO:0035023 GO:0043087 GO:0009405 GO:0019835 GO:0005089 GO:0005515 GO:0019867

Cluster-22178.75779 GO:0003723 GO:0005198

Cluster-22178.75780 GO:0003723 GO:0005198

Cluster-22178.75784 GO:0006950 GO:0016021

Cluster-22178.75785 GO:0006508 GO:0008236

Cluster-22178.75787 GO:0016567 GO:0055114 GO:0004842 GO:0005515 GO:0016491

Cluster-22178.75788 GO:0006891 GO:0006810 GO:0006355 GO:0005215 GO:0005524 GO:0003677 GO:0003713 GO:0016020 GO:0017119 GO:0005667 GO:0005634

Cluster-22178.75789 GO:0016192 GO:0006886 GO:0005515

Cluster-22178.75792 GO:0016192 GO:0006886 GO:0005515 GO:0030117

Cluster-22178.75794 GO:0007165 GO:0005179 GO:0005576

Cluster-22178.75796 GO:0005515 GO:0020037

Cluster-22178.75801 GO:0006310 GO:0006355 GO:0006281 GO:0003678 GO:0003724 GO:0005524 GO:0016887 GO:0005515 GO:0008134 GO:0009378 GO:0003723 GO:0005657 GO:0005667 GO:0009379

Cluster-22178.75802 GO:0006139 GO:0008168

Cluster-22178.75804 GO:0006950 GO:0006508 GO:0004197

Cluster-22178.75808 GO:0006659 GO:0009405 GO:0008270 GO:0046872 GO:0008289 GO:0004222 GO:0005198 GO:0005515 GO:0019031 GO:0019028

Cluster-22178.75810 GO:0030071 GO:0006506 GO:0016746 GO:0005515 GO:0005680 GO:0005789 GO:0016021

Cluster-22178.75811 GO:0006144 GO:0006508 GO:0044419 GO:0004197 GO:0003968 GO:0017111 GO:0031379

Cluster-22178.75812 GO:0019058 GO:0006886 GO:0005975 GO:0008536 GO:0004553 GO:0005515 GO:0042025

Cluster-22178.75813 GO:0055114 GO:0008152 GO:0006508 GO:0006118 GO:0016627 GO:0050660 GO:0004197 GO:0003995

Cluster-22178.75814 GO:0008152 GO:0055114 GO:0006118 GO:0007339 GO:0006508 GO:0006355 GO:0003700 GO:0050660 GO:0016627 GO:0005515 GO:0003995 GO:0004197 GO:0005667 GO:0005576

Cluster-22178.75816 GO:0006275 GO:0008033 GO:0004810 GO:0016779 GO:0016437 GO:0008270 GO:0003676 GO:0005634

Cluster-22178.75817 GO:0007606 GO:0006886 GO:0007165 GO:0006888 GO:0003723 GO:0004888 GO:0005515 GO:0008270 GO:0016020 GO:0030127

Cluster-22178.75819 GO:0008152 GO:0003824 GO:0046912

Cluster-22178.75821 GO:0007166 GO:0006974 GO:0007218 GO:0007165 GO:0051726 GO:0046872 GO:0008270 GO:0004871 GO:0005185 GO:0005515 GO:0005509 GO:0005576 GO:0005634

Cluster-22178.75822 GO:0008608 GO:0006464 GO:0051082 GO:0031072 GO:0016151 GO:0042729 GO:0072686 GO:0005634

Cluster-22178.75824 GO:0006464 GO:0008608 GO:0031072 GO:0016151

GO:0051082 GO:0072686 GO:0005634 GO:0042729
Cluster-22178.75825 GO:0005515
Cluster-22178.75832 GO:0051260 GO:0004190 GO:0016021
Cluster-22178.75834 GO:0042254 GO:0006412 GO:0007218 GO:0003735
GO:0005185 GO:0005576 GO:0005840 GO:0005622
Cluster-22178.75835 GO:0005515
Cluster-22178.75837 GO:0007009 GO:0005515
Cluster-22178.75838 GO:0007009 GO:0005515
Cluster-22178.7584 GO:0035023 GO:0043087 GO:0005089
Cluster-22178.75840 GO:0007009 GO:0005515
Cluster-22178.75842 GO:0008608 GO:0003723 GO:0003676 GO:0072686
GO:0042729
Cluster-22178.75844 GO:0006468 GO:0003723 GO:0005524 GO:0004672
GO:0005515 GO:0000178
Cluster-22178.75847 GO:0005515
Cluster-22178.75848 GO:0005515
Cluster-22178.75853 GO:0051082 GO:0031072 GO:0005515
Cluster-22178.75856 GO:0019236 GO:0007212 GO:0048268 GO:0007186
GO:0032051 GO:0016021
Cluster-22178.75857 GO:0005515
Cluster-22178.75859 GO:0016579 GO:0032183 GO:0070140 GO:0036459

Cluster-22178.75860 GO:0000226 GO:0051537 GO:0015630 GO:0043231
Cluster-22178.75861 GO:0048870 GO:0005739 GO:0031514
Cluster-22178.75862 GO:0007165 GO:0016310 GO:0009069 GO:0006468
GO:0004674 GO:0005096
Cluster-22178.75867 GO:0005515 GO:0008270 GO:0005634
Cluster-22178.75871 GO:0005515 GO:0016020
Cluster-22178.75873 GO:0035091
Cluster-22178.75876 GO:0055114 GO:0016705 GO:0020037 GO:0005506

Cluster-22178.75879 GO:0006508 GO:0006355 GO:0007165 GO:0042312
GO:0008217 GO:0005179 GO:0005515 GO:0008270 GO:0004222

GO:0005576 GO:0031012
Cluster-22178.75884 GO:0016998 GO:0006032 GO:0006206 GO:0006144
GO:0006351 GO:0042742 GO:0003677 GO:0003899 GO:0004568
GO:0003723 GO:0005730 GO:0005576
Cluster-22178.75885 GO:0009451 GO:0031167 GO:0006479 GO:0006306
GO:0001510 GO:0009452 GO:0008152 GO:0008033 GO:0006400
GO:0046500 GO:0006396 GO:0006464 GO:0015979 GO:0042549
GO:0005515 GO:0008173 GO:0003676 GO:0008168 GO:0004719
GO:0003677 GO:0008176 GO:0008170 GO:0009523 GO:0009539
Cluster-22178.75886 GO:0006811 GO:0055085 GO:0030036 GO:0005216
GO:0003779 GO:0030054 GO:0016020
Cluster-22178.75887 GO:0006468 GO:0005524 GO:0004672
Cluster-22178.75888 GO:0005515 GO:0046872
Cluster-22178.75889 GO:0007264 GO:0006576 GO:0015684 GO:0005524
GO:0015093 GO:0003924 GO:0005525 GO:0016021 GO:0031225
Cluster-22178.75892 GO:0030001 GO:0006367 GO:0055085 GO:0046873
GO:0046872 GO:0005672 GO:0016020
Cluster-22178.75895 GO:0015886 GO:0015684 GO:0007264 GO:0046497
GO:0006769 GO:0006281 GO:0007131 GO:0002949 GO:0006310
GO:0055114 GO:0008033 GO:0050660 GO:0003724 GO:0008750
GO:0016491 GO:0009378 GO:0051287 GO:0005525 GO:0003678
GO:0050661 GO:0015093 GO:0005524 GO:0016887 GO:0098519
GO:0015232 GO:0003924 GO:0016301 GO:0003723 GO:0009379
GO:0016021 GO:0005657 GO:0000794
Cluster-22178.75903 GO:0046080 GO:0016787 GO:0005515
Cluster-22178.75904 GO:0016567 GO:0008270 GO:0004842 GO:0005515

Cluster-22178.75906 GO:0015031 GO:0006464 GO:0005515
Cluster-22178.75908 GO:0006914 GO:0006368 GO:0015743 GO:0006352
GO:0055114 GO:0035092 GO:0010508 GO:0007094 GO:0009690
GO:0006355 GO:0050660 GO:0005524 GO:0003677 GO:0016787
GO:0019139 GO:0005815 GO:0000228 GO:0008023
Cluster-22178.75909 GO:0090063 GO:0000226 GO:0005815 GO:0000922

Cluster-22178.75910 GO:0090063 GO:0000226 GO:0005815 GO:0000922
Cluster-22178.75912 GO:0006260 GO:0007165 GO:0005179 GO:0005634
GO:0005576 GO:0000808
Cluster-22178.75914 GO:0006260 GO:0007165 GO:0005179 GO:0005634
GO:0005576 GO:0000808
Cluster-22178.75915 GO:0007165 GO:0006260 GO:0005179 GO:0000808
GO:0005576 GO:0005634
Cluster-22178.75916 GO:0006260 GO:0007165 GO:0005179 GO:0005576
GO:0005634 GO:0000808
Cluster-22178.75917 GO:0005515 GO:0016020
Cluster-22178.75918 GO:0035023 GO:0002949 GO:0043087 GO:0006810
GO:0007264 GO:0003676 GO:0005089 GO:0004386 GO:0005525
GO:0016887 GO:0043531 GO:0005524 GO:0005515
Cluster-22178.75919 GO:0007186 GO:0019236 GO:0007606 GO:0006629
GO:0004932 GO:0016020
Cluster-22178.75920 GO:0005515
Cluster-22178.75922 GO:0008152 GO:0031167 GO:0007160 GO:0005509
GO:0008168 GO:0005576
Cluster-22178.75925 GO:0009405 GO:0035556 GO:0046488 GO:0005198
GO:0005524 GO:0046872 GO:0016307 GO:0019028
Cluster-22178.75926 GO:0006281 GO:0010447 GO:0003677 GO:0004518
GO:0003824 GO:0042597
Cluster-22178.75931 GO:0005515
Cluster-22178.75932 GO:0046872
Cluster-22178.75940 GO:0046872 GO:0016021
Cluster-22178.75943 GO:0005524 GO:0016787 GO:0003677
Cluster-22178.75947 GO:0006448 GO:0045901 GO:0045905 GO:0006452
GO:0003746 GO:0043022 GO:0003723 GO:0005840
Cluster-22178.75952 GO:0006457
Cluster-22178.75953 GO:0043547 GO:0005096 GO:0046983
Cluster-22178.75954 GO:0006541 GO:0030833 GO:0006508 GO:0034314
GO:0008236 GO:0016787 GO:0005885 GO:0005856
Cluster-22178.75956 GO:0015992 GO:0008608 GO:0006123 GO:0016032

GO:0000902 GO:0006955 GO:0004129 GO:0005515 GO:0009424
GO:0045277 GO:0005751 GO:0005743 GO:0072686 GO:0042729
GO:0016020
Cluster-22178.75960 GO:0006355 GO:0046872 GO:0005622
Cluster-22178.75961 GO:0046872 GO:0003677
Cluster-22178.75964 GO:0046488 GO:0005515 GO:0016307 GO:0008270

Cluster-22178.75965 GO:0046907 GO:0006355 GO:0009607 GO:0005515
GO:0008270
Cluster-22178.75972 GO:0055085 GO:0006811 GO:0005216 GO:0016020
Cluster-22178.75973 GO:0030145 GO:0004177 GO:0019031
Cluster-22178.75974 GO:0006355 GO:0003700 GO:0043565 GO:0005667
Cluster-22178.75976 GO:0003779 GO:0005198 GO:0005856
Cluster-22178.75979 GO:0006334 GO:0005515 GO:0003677 GO:0005634
GO:0000786
Cluster-22178.75980 GO:0042754 GO:0045892
Cluster-22178.75981 GO:0006412 GO:0042254 GO:0006506 GO:0016746
GO:0003735 GO:0016021 GO:0005840 GO:0005789 GO:0005739
Cluster-22178.75984 GO:0031175 GO:0015979 GO:0008017 GO:0005516
GO:0030507 GO:0005515 GO:0009539 GO:0009523 GO:0008091
GO:0045298 GO:0016020
Cluster-22178.75985 GO:0042254 GO:0006412 GO:0003924 GO:0003735
GO:0005525 GO:0005840
Cluster-22178.75986 GO:0035556
Cluster-22178.75988 GO:0005515
Cluster-22178.75991 GO:0055114 GO:0010309 GO:0005515
Cluster-22178.75994 GO:0008152 GO:0003824
Cluster-22178.75997 GO:0006464
Cluster-22178.76000 GO:0046872 GO:0005515
Cluster-22178.76001 GO:0015074 GO:0003676 GO:0008270
Cluster-22178.76002 GO:0015074 GO:0008270 GO:0003676
Cluster-22178.76003 GO:0046486 GO:0008654 GO:0008715 GO:0016020
Cluster-22178.76006 GO:0006355 GO:0003700 GO:0043565 GO:0005634

GO:0005667
Cluster-22178.76007 GO:0045017 GO:0042967 GO:0046486 GO:0004144

Cluster-22178.76008 GO:0006165 GO:0006228 GO:0006355 GO:0006183
GO:0006144 GO:0006206 GO:0006241 GO:0003723 GO:0005515
GO:0003677 GO:0004550 GO:0003676
Cluster-22178.76017 GO:0005515 GO:0046983
Cluster-22178.76022 GO:0007165 GO:0075521 GO:0046718 GO:0006505
GO:0006886 GO:0006355 GO:0006352 GO:0019076 GO:0003677
GO:0005179 GO:0016787 GO:0016987 GO:0016788 GO:0098599
GO:0003700 GO:0030430 GO:0005667 GO:0044163 GO:0042025
GO:0005576 GO:0019028 GO:0019031
Cluster-22178.76023 GO:0016192 GO:0016021
Cluster-22178.76024 GO:0006355 GO:0006270 GO:0006352 GO:0003677
GO:0016987 GO:0003700 GO:0005667 GO:0019031
Cluster-22178.76025 GO:0003677 GO:0008270
Cluster-22178.76026 GO:0016787
Cluster-22178.76027 GO:0055114 GO:0005506 GO:0020037 GO:0016705

Cluster-22178.76029 GO:0006367 GO:0006281 GO:0051382 GO:0005634
Cluster-22178.76031 GO:0015833 GO:0006284 GO:0005524 GO:0003677
GO:0000166 GO:0003905 GO:0016020
Cluster-22178.76032 GO:0003723
Cluster-22178.76036 GO:0006355 GO:0005515 GO:0046983
Cluster-22178.76037 GO:0008207 GO:0008209 GO:0008210 GO:0006694
GO:0008152 GO:0055114 GO:0005515 GO:0016616 GO:0003854
GO:0050662 GO:0003824 GO:0016491 GO:0046872
Cluster-22178.76038 GO:0006468 GO:0016773 GO:0004672 GO:0005524
GO:0016020
Cluster-22178.76039 GO:0009098 GO:0006308 GO:0016888
Cluster-22178.76041 GO:0046872 GO:0016787 GO:0003676 GO:0005198
GO:0005622 GO:0019028
Cluster-22178.76042 GO:0006811 GO:0005216 GO:0016020

Cluster-22178.76043 GO:0006897 GO:0007342 GO:0006310 GO:0006281
GO:0060478 GO:0006508 GO:0046872 GO:0003677 GO:0004252
GO:0009378 GO:0005657 GO:0005886 GO:0009379
Cluster-22178.76045 GO:0006260 GO:0007165 GO:0006810 GO:0009058
GO:0018142 GO:0006269 GO:0005215 GO:0003723 GO:0016020
Cluster-22178.76047 GO:0006260 GO:0006810 GO:0007165 GO:0009058
GO:0018142 GO:0006269 GO:0005215 GO:0003723 GO:0016020
Cluster-22178.76048 GO:0009058 GO:0007165 GO:0006810 GO:0006269
GO:0018142 GO:0006260 GO:0003723 GO:0005215 GO:0016020
Cluster-22178.76049 GO:0006355 GO:0043565 GO:0005515 GO:0005634
Cluster-22178.76050 GO:0006397 GO:0006396 GO:0042967 GO:0005515
GO:0016747 GO:0008080 GO:0005634 GO:0005622
Cluster-22178.76051 GO:0016567 GO:0004842 GO:0005515
Cluster-22178.76052 GO:0055114 GO:0006570 GO:0006118 GO:0006351
GO:0004097 GO:0003677 GO:0005634
Cluster-22178.76053 GO:0016068 GO:0006606 GO:0015031 GO:0005515
GO:0008565 GO:0005634 GO:0016021 GO:0005737
Cluster-22178.76056 GO:0016020
Cluster-22178.76058 GO:0005515 GO:0000228 GO:0032040
Cluster-22178.76059 GO:0006506 GO:0005789 GO:0016021
Cluster-22178.76060 GO:0006810 GO:0007172 GO:0007165 GO:0006397
GO:0006487 GO:0006468 GO:0005215 GO:0004713 GO:0004871
GO:0005515 GO:0016021 GO:0008250 GO:0019028 GO:0005925
GO:0005634
Cluster-22178.76063 GO:0061025 GO:0046813 GO:0005515 GO:0016021
GO:0019031
Cluster-22178.76064 GO:0061025 GO:0046813 GO:0005515 GO:0016021
GO:0019031
Cluster-22178.76065 GO:0007165 GO:0005179 GO:0005576 GO:0016021
Cluster-22178.76074 GO:0007186 GO:0004930 GO:0016021 GO:0019031
Cluster-22178.76076 GO:0006334 GO:0003677 GO:0003723 GO:0005634
GO:0000786
Cluster-22178.76077 GO:0006886 GO:0006355 GO:0006413 GO:0046034

GO:0006808 GO:0005975 GO:0006446 GO:0035556 GO:0007218
GO:0006144 GO:0006888 GO:0006464 GO:0005515 GO:0004519
GO:0030234 GO:0003723 GO:0003676 GO:0003700 GO:0016151
GO:0043565 GO:0046872 GO:0004672 GO:0004017 GO:0003743
GO:0003677 GO:0004553 GO:0043130 GO:0008270 GO:0005840
GO:0030127 GO:0005667
Cluster-22178.76081 GO:0016567 GO:0004842 GO:0005515 GO:0046872
GO:0008270 GO:0003723 GO:0005680
Cluster-22178.76082 GO:0009405 GO:0016020
Cluster-22178.76088 GO:0005515 GO:0016020
Cluster-22178.76092 GO:0046034 GO:0006144 GO:0046656 GO:0046872
GO:0008270 GO:0005515 GO:0004017 GO:0003677 GO:0050524
Cluster-22178.76093 GO:0016301 GO:0005515 GO:0005524
Cluster-22178.76096 GO:0006810 GO:0003333 GO:0006865 GO:0055085
GO:0006811 GO:0015171 GO:0005215 GO:0016020
Cluster-22178.76097 GO:0006810 GO:0055085 GO:0016020
Cluster-22178.76102 GO:0006771 GO:0035023 GO:0019497 GO:0043087
GO:0005515 GO:0005089 GO:0003993 GO:0008270 GO:0046872
GO:0005634
Cluster-22178.76103 GO:0006355 GO:0006352 GO:0003700 GO:0016987
GO:0003677 GO:0005667
Cluster-22178.76105 GO:0016973 GO:0003677 GO:0005643
Cluster-22178.76106 GO:0016973 GO:0043565 GO:0005515 GO:0003677
GO:0005634 GO:0005643
Cluster-22178.76111 GO:0016973 GO:0043565 GO:0005515 GO:0003677
GO:0005634 GO:0005643
Cluster-22178.76112 GO:0016973 GO:0005515 GO:0043565 GO:0005643
GO:0005634
Cluster-22178.76114 GO:0009607 GO:0006355 GO:0032968 GO:0006367
GO:0003677 GO:0005634 GO:0016021
Cluster-22178.76116 GO:0042981
Cluster-22178.76117 GO:0043087 GO:0035023 GO:0003677 GO:0005515
GO:0005089

Cluster-22178.76119 GO:0007049 GO:0008608 GO:0006260 GO:0000917
GO:0006355 GO:0043093 GO:0003700 GO:0019905 GO:0043565
GO:0005737 GO:0042729 GO:0072686 GO:0005667
Cluster-22178.76120 GO:0006265 GO:0031175 GO:0008270 GO:0003916
GO:0046872 GO:0008233 GO:0004222 GO:0005516 GO:0005515
GO:0030507 GO:0003677 GO:0031012 GO:0008091 GO:0005694
Cluster-22178.76121 GO:0031175 GO:0006265 GO:0030507 GO:0003677
GO:0005515 GO:0005516 GO:0004222 GO:0008233 GO:0003916
GO:0008270 GO:0046872 GO:0005694 GO:0008091 GO:0031012
Cluster-22178.76124 GO:0007016 GO:0016010
Cluster-22178.76126 GO:0019722 GO:0003676
Cluster-22178.76129 GO:0055114 GO:0000042 GO:0016491 GO:0005515

Cluster-22178.76130 GO:0006629 GO:0006508 GO:0016787 GO:0004252

Cluster-22178.76132 GO:0006606 GO:0007165 GO:0006955 GO:0030247
GO:0005044
Cluster-22178.76138 GO:0001522 GO:0009451 GO:0015940 GO:0015937
GO:0003723 GO:0004140 GO:0005524 GO:0008017 GO:0009982
GO:0045298
Cluster-22178.76140 GO:0006355 GO:0006282 GO:0005096 GO:0008270
GO:0005515 GO:0005509 GO:0005544 GO:0008023
Cluster-22178.76143 GO:0055114 GO:0006438 GO:0008152 GO:0009098
GO:0009405 GO:0009097 GO:0009099 GO:0005524 GO:0004832
GO:0016758 GO:0000166 GO:0016706 GO:0005737 GO:0016020
Cluster-22178.76145 GO:0008152 GO:0055114 GO:0016706 GO:0016758

Cluster-22178.76150 GO:0055114 GO:0006508 GO:0043039 GO:0051536
GO:0046872 GO:0016876 GO:0005524 GO:0008270 GO:0008234
GO:0005515 GO:0005509 GO:0016020
Cluster-22178.76154 GO:0006464 GO:0015031 GO:0005515
Cluster-22178.76155 GO:0016567 GO:0004842 GO:0005515 GO:0019033
Cluster-22178.76157 GO:0016567 GO:0005515 GO:0004842 GO:0019033

Cluster-22178.76161 GO:0006302 GO:0016787 GO:0004519 GO:0030145
GO:0005634
Cluster-22178.76163 GO:0006357 GO:0001104 GO:0016592
Cluster-22178.76164 GO:0016114 GO:0005515 GO:0008685
Cluster-22178.76170 GO:0015031 GO:0006914 GO:0016787 GO:0005515

Cluster-22178.76174 GO:0009405 GO:0016791 GO:0005515
Cluster-22178.76177 GO:0016337 GO:0005524 GO:0003723 GO:0016887
GO:0005515 GO:0003724 GO:0016020
Cluster-22178.76181 GO:0006826 GO:0006827 GO:0005381 GO:0033573
GO:0016020
Cluster-22178.76182 GO:0006729 GO:0008124
Cluster-22178.76184 GO:0006729 GO:0008124
Cluster-22178.76185 GO:0005515
Cluster-22178.76190 GO:0055114 GO:0046872 GO:0016491
Cluster-22178.76192 GO:0006032 GO:0005515 GO:0008061
Cluster-22178.76193 GO:0006810 GO:0009069 GO:0006468 GO:0016310
GO:0035556 GO:0017048 GO:0005198 GO:0005215 GO:0004674
GO:0005524 GO:0004672 GO:0003774 GO:0016020 GO:0005882
GO:0016459
Cluster-22178.76196 GO:0019089 GO:0006886 GO:0006606 GO:0006807
GO:0051258 GO:0006118 GO:0051607 GO:0030168 GO:0008053
GO:0006891 GO:0007165 GO:0019551 GO:0003924 GO:0004352
GO:0042803 GO:0005198 GO:0005524 GO:0008092 GO:0045502
GO:0008134 GO:0019901 GO:0005102 GO:0030674 GO:0005737
GO:0030173 GO:0005577 GO:0005667 GO:0016020 GO:0005741
GO:0016021 GO:0019031 GO:0019028 GO:0030286 GO:0019898
GO:0017119
Cluster-22178.76198 GO:0004867 GO:0005576
Cluster-22178.76203 GO:0042176 GO:0030234 GO:0005515 GO:0000502
Cluster-22178.76205 GO:0006468 GO:0005524 GO:0004672 GO:0016773
GO:0016020
Cluster-22178.76210 GO:0009405 GO:0005515 GO:0004222 GO:0003723

GO:0008270 GO:0005576
Cluster-22178.76214 GO:0042254 GO:0006412 GO:0007186 GO:0003735
GO:0046872 GO:0004930 GO:0005622 GO:0016021 GO:0005840
Cluster-22178.76215 GO:0006869 GO:0042157 GO:0008289 GO:0005576
Cluster-22178.76218 GO:0007165
Cluster-22178.76220 GO:0006814 GO:0016829
Cluster-22178.76223 GO:0007224 GO:0015074
Cluster-22178.76224 GO:0006810 GO:0005215
Cluster-22178.76226 GO:0006355 GO:0003700 GO:0000975 GO:0005667
GO:0005840
Cluster-22178.76228 GO:0006144 GO:0006351 GO:0005524 GO:0003723
GO:0003968 GO:0031379
Cluster-22178.76229 GO:0008233 GO:0008270 GO:0005634
Cluster-22178.76230 GO:0005515
Cluster-22178.76232 GO:0046497 GO:0008152 GO:0006769 GO:0006741
GO:0016301 GO:0003677 GO:0003951
Cluster-22178.76236 GO:0016567 GO:0004842 GO:0005515 GO:0008270
GO:0046872 GO:0003723 GO:0005680
Cluster-22178.76241 GO:0016567 GO:0008270 GO:0046872 GO:0004842
GO:0003677 GO:0005515 GO:0005680
Cluster-22178.76243 GO:0009405 GO:0005576
Cluster-22178.76245 GO:0000921 GO:0006355 GO:0043565 GO:0003700
GO:0005667 GO:0005940 GO:0016021
Cluster-22178.76246 GO:0009405 GO:0005576
Cluster-22178.76252 GO:0003677 GO:0004386 GO:0005524
Cluster-22178.76254 GO:0016740
Cluster-22178.76255 GO:0048870 GO:0019904 GO:0031514
Cluster-22178.76258 GO:0006118 GO:0006119 GO:0055114 GO:0015992
GO:0008121
Cluster-22178.76259 GO:0008283 GO:0040007 GO:0007165 GO:0006351
GO:0009405 GO:0006206 GO:0006144 GO:0003677 GO:0003899
GO:0008083 GO:0005730
Cluster-22178.76262 GO:0006418 GO:0003676 GO:0000166 GO:0005524

GO:0004812
Cluster-22178.76264 GO:0006260 GO:0006355 GO:0019079 GO:0046983
GO:0003700 GO:0030337 GO:0005667 GO:0005634 GO:0042575
Cluster-22178.76268 GO:0006570 GO:0006470 GO:0004725 GO:0008138

Cluster-22178.76270 GO:0008146 GO:0046983 GO:0016021 GO:0016020
Cluster-22178.76273 GO:0009432 GO:0006281 GO:0005509 GO:0005524
GO:0003697
Cluster-22178.76275 GO:0009432 GO:0006281 GO:0003697 GO:0005524

Cluster-22178.76279 GO:0019354 GO:0006206 GO:0006231 GO:0055114
GO:0008033 GO:0006779 GO:0015940 GO:0043115 GO:0008641
GO:0016491 GO:0008677 GO:0050660 GO:0050797
Cluster-22178.76280 GO:0006355 GO:0015979 GO:0042549 GO:0006260
GO:0006606 GO:0019058 GO:0045502 GO:0042803 GO:0008134
GO:0005198 GO:0003677 GO:0009539 GO:0009523 GO:0005664
GO:0030286 GO:0005667 GO:0005882
Cluster-22178.76286 GO:0007165 GO:0043087 GO:0007264 GO:0005085

Cluster-22178.76288 GO:0007165 GO:0043401 GO:0006355 GO:0005186
GO:0003700 GO:0008270 GO:0043565 GO:0005634 GO:0005667
GO:0005615
Cluster-22178.76292 GO:0043087 GO:0005089 GO:0005515 GO:0016021
GO:0005737
Cluster-22178.76296 GO:0006355 GO:0045132 GO:0042967 GO:0005515
GO:0004402 GO:0008080 GO:0003676 GO:0005634 GO:0000775
GO:0000123
Cluster-22178.76297 GO:0008380 GO:0005681
Cluster-22178.76299 GO:0006308 GO:0008855 GO:0009318
Cluster-22178.76301 GO:0005515 GO:0005201 GO:0005581 GO:0005578
Cluster-22178.76308 GO:0007264 GO:0005525 GO:0005515 GO:0005576
Cluster-22178.7631 GO:0042330
Cluster-22178.76310 GO:0006260 GO:0016042 GO:0009395 GO:0005509

GO:0004623 GO:0005576
Cluster-22178.76311 GO:0019530 GO:0055114 GO:0046439 GO:0006534
GO:0017172 GO:0005506
Cluster-22178.76312 GO:0006508 GO:0016787 GO:0008236
Cluster-22178.76313 GO:0055114 GO:0007156 GO:0016972 GO:0005509
GO:0016020
Cluster-22178.76314 GO:0000045 GO:0005737
Cluster-22178.76317 GO:0006269 GO:0006351 GO:0006260 GO:0005515
GO:0003896 GO:0005730 GO:0005657
Cluster-22178.76318 GO:0006366 GO:0006367 GO:0005674
Cluster-22178.76320 GO:0030866 GO:0006366 GO:0006367 GO:0008092
GO:0005856 GO:0005674
Cluster-22178.76323 GO:0006366 GO:0006367 GO:0005674
Cluster-22178.76325 GO:0007268 GO:0005515 GO:0043495 GO:0043395
GO:0033130 GO:0005578 GO:0016020 GO:0016021
Cluster-22178.76329 GO:0005515 GO:0016020
Cluster-22178.76332 GO:0008270 GO:0005515
Cluster-22178.76335 GO:0016020
Cluster-22178.7634 GO:0000162 GO:0006571 GO:0009094 GO:0003855
Cluster-22178.76344 GO:0003676
Cluster-22178.76346 GO:0051603 GO:0004298 GO:0005839
Cluster-22178.76348 GO:0008152 GO:0003824
Cluster-22178.76357 GO:0019028
Cluster-22178.76358 GO:0042254 GO:0006412 GO:0003735 GO:0005622
GO:0005840
Cluster-22178.76359 GO:0055114 GO:0033743
Cluster-22178.76360 GO:0006813 GO:0016787 GO:0005267 GO:0005524
GO:0003676 GO:0003677 GO:0016020
Cluster-22178.76361 GO:0007186 GO:0004930 GO:0016021
Cluster-22178.76364 GO:0042254 GO:0043093 GO:0006260 GO:0006412
GO:0051252 GO:0000917 GO:0006355 GO:0007049 GO:0003735
GO:0003887 GO:0004523 GO:0003700 GO:0043565 GO:0005667
GO:0005815 GO:0005622 GO:0042575 GO:0005840 GO:0005737

Cluster-22178.76365 GO:0006109 GO:0006355 GO:0006694 GO:0016310
GO:0016114 GO:0000160 GO:0008033 GO:0009058 GO:0006260
GO:0004161 GO:0008134 GO:0016301 GO:0003723 GO:0005525
GO:0004672 GO:0003924 GO:0003677 GO:0000155 GO:0004003
GO:0003724 GO:0005524 GO:0009365 GO:0005667 GO:0005657
Cluster-22178.76366 GO:0009058 GO:0009069 GO:0006694 GO:0016310
GO:0000160 GO:0016114 GO:0008033 GO:0006109 GO:0004674
GO:0003724 GO:0005524 GO:0016887 GO:0000155 GO:0004672
GO:0004161 GO:0016301 GO:0003723 GO:0009365
Cluster-22178.76367 GO:0000160 GO:0016114 GO:0016310 GO:0006694
GO:0008033 GO:0006109 GO:0006355 GO:0006260 GO:0009058
GO:0004672 GO:0008134 GO:0016301 GO:0004161 GO:0003723
GO:0003724 GO:0016887 GO:0005524 GO:0003677 GO:0004003
GO:0000155 GO:0009365 GO:0005657 GO:0005667
Cluster-22178.76372 GO:0019915 GO:0005789
Cluster-22178.76373 GO:0007187 GO:0007608 GO:0006812 GO:0005549
GO:0008146 GO:0004984 GO:0008324 GO:0016020 GO:0016021
Cluster-22178.76375 GO:0007608 GO:0006812 GO:0007187 GO:0004984
GO:0008324 GO:0005549 GO:0008146 GO:0016021 GO:0016020
Cluster-22178.76376 GO:0035434 GO:0006629 GO:0006825 GO:0009252
GO:0003677 GO:0005375 GO:0008963 GO:0046983 GO:0016021
Cluster-22178.76381 GO:0008152 GO:0003824
Cluster-22178.76382 GO:0007010 GO:0005515 GO:0008270 GO:0003779

Cluster-22178.76386 GO:0003824
Cluster-22178.76389 GO:0008152 GO:0035556 GO:0005515 GO:0016746

Cluster-22178.76393 GO:0042254 GO:0006412 GO:0003735 GO:0005622
GO:0005840
Cluster-22178.76394 GO:0042254 GO:0006412 GO:0003735 GO:0005840
GO:0005622
Cluster-22178.76395 GO:0006355 GO:0003677 GO:0003700 GO:0030695
GO:0043565 GO:0005634 GO:0005667

Cluster-22178.76402 GO:0051252 GO:0016891
Cluster-22178.76405 GO:0005975 GO:0007165 GO:0008270 GO:0016817 GO:0004553 GO:0005515 GO:0005509 GO:0005578
Cluster-22178.76407 GO:0000154 GO:0006479 GO:0050776 GO:0008152 GO:0014068 GO:0006364 GO:0007165 GO:0006396 GO:0035556 GO:0006464 GO:0005515 GO:0016151 GO:0008168 GO:0046872 GO:0043548 GO:0008649 GO:0005102 GO:0005737 GO:0016020
Cluster-22178.76408 GO:0006811 GO:0055085 GO:0015075 GO:0016020
Cluster-22178.76411 GO:0009966 GO:0005515 GO:0016020 GO:0005737
Cluster-22178.76415 GO:0008152 GO:0055114 GO:0016491
Cluster-22178.76416 GO:0007067 GO:0051082 GO:0042729 GO:0072686
Cluster-22178.76417 GO:0055085 GO:0016032 GO:0045735 GO:0005506 GO:0016021
Cluster-22178.76421 GO:0005515
Cluster-22178.76423 GO:0055114 GO:0005506 GO:0016491
Cluster-22178.76424 GO:0055114 GO:0005506 GO:0016491
Cluster-22178.76427 GO:0006355 GO:0006810 GO:0046872 GO:0003677 GO:0005215 GO:0016020
Cluster-22178.76431 GO:0042254 GO:0006123 GO:0015992 GO:0005515 GO:0004129 GO:0005507 GO:0016020 GO:0005730 GO:0045277
Cluster-22178.76432 GO:0005515
Cluster-22178.76438 GO:0016032 GO:0006508 GO:0006813 GO:0006468 GO:0006118 GO:0055114 GO:0007219 GO:0008152 GO:0006511 GO:0007275 GO:0009236 GO:0035556 GO:0020037 GO:0003995 GO:0016627 GO:0046872 GO:0004672 GO:0048472 GO:0051287 GO:0071949 GO:0016616 GO:0016491 GO:0016773 GO:0008641 GO:0008270 GO:0005524 GO:0008233 GO:0050660 GO:0005634 GO:0016021 GO:0016020
Cluster-22178.76444 GO:0006869 GO:0006118 GO:0006508 GO:0004222 GO:0008289 GO:0046872 GO:0008241 GO:0008237 GO:0003954 GO:0016020 GO:0005576 GO:0005739
Cluster-22178.76445 GO:0003824
Cluster-22178.76449 GO:0055114 GO:0007186 GO:0046872 GO:0016972

GO:0004930 GO:0016021
Cluster-22178.76451 GO:0055114 GO:0008152 GO:0006269 GO:0006952
GO:0006351 GO:0016846 GO:0008270 GO:0033743 GO:0004386
GO:0003896 GO:0005657 GO:0005730
Cluster-22178.76456 GO:0006278 GO:0003964
Cluster-22178.76457 GO:0006355 GO:0043401 GO:0043565 GO:0008270
GO:0003700 GO:0005667 GO:0005634
Cluster-22178.76458 GO:0043093 GO:0000917 GO:0005737
Cluster-22178.76459 GO:0009058 GO:0055114 GO:0010024 GO:0016636
GO:0016779 GO:0050897 GO:0005524
Cluster-22178.76462 GO:0030866 GO:0055085 GO:0042254 GO:0006412
GO:0006198 GO:0006270 GO:0008092 GO:0003779 GO:0004115
GO:0005198 GO:0003735 GO:0005856 GO:0005622 GO:0016021
GO:0005840
Cluster-22178.76464 GO:0030866 GO:0055085 GO:0006198 GO:0006270
GO:0008092 GO:0003779 GO:0004115 GO:0005198 GO:0005856
GO:0016021
Cluster-22178.76466 GO:0006352 GO:0003677 GO:0046872 GO:0005634
Cluster-22178.76471 GO:0006468 GO:0004672 GO:0005524 GO:0016773
GO:0016020
Cluster-22178.76472 GO:0007165 GO:0015684 GO:0007186 GO:0007264
GO:0006576 GO:0005525 GO:0004871 GO:0031683 GO:0005524
GO:0015093 GO:0019001 GO:0003924 GO:0016021
Cluster-22178.76474 GO:0007283 GO:0006024 GO:0015012 GO:0004252
GO:0003677 GO:0016757 GO:0016020 GO:0005634 GO:0016021
GO:0000786
Cluster-22178.76476 GO:0051382 GO:0007049 GO:0006355 GO:0000917
GO:0006914 GO:0006260 GO:0006606 GO:0007264 GO:0043093
GO:0006457 GO:0043565 GO:0003700 GO:0042803 GO:0019901
GO:0000774 GO:0005096 GO:0051087 GO:0005737 GO:0005794
GO:0000776 GO:0005815 GO:0005667
Cluster-22178.76477 GO:0006355 GO:0003682 GO:0003677 GO:0005576
GO:0005634 GO:0000775 GO:0000785

Cluster-22178.76481 GO:0007275 GO:0005201 GO:0005578 GO:0005581
Cluster-22178.76483 GO:0003676 GO:0005515 GO:0003723 GO:0008270

Cluster-22178.76485 GO:0035023 GO:0040007 GO:0006576 GO:0035556
GO:0043087 GO:0015684 GO:0007165 GO:0006270 GO:0007186
GO:0007264 GO:0008283 GO:0019068 GO:0005525 GO:0004871
GO:0019001 GO:0046872 GO:0031683 GO:0003924 GO:0005198
GO:0005515 GO:0008083 GO:0016757 GO:0005089 GO:0015093
GO:0005524 GO:0016887 GO:0016021 GO:0016020
Cluster-22178.76487 GO:0003725
Cluster-22178.76489 GO:0006355 GO:0003677 GO:0008270
Cluster-22178.76491 GO:0006571 GO:0000162 GO:0015940 GO:0046168
GO:0009094 GO:0019521 GO:0055114 GO:0006098 GO:0004665
GO:0008677 GO:0004616 GO:0016616 GO:0008977 GO:0051287
Cluster-22178.76495 GO:0006260 GO:0007128 GO:0070206 GO:0048870
GO:0000917 GO:0007130 GO:0070193 GO:0006355 GO:0043093
GO:0003700 GO:0043565 GO:0005737 GO:0000795 GO:0031514
GO:0005667
Cluster-22178.76499 GO:0007165 GO:0007275 GO:0016055 GO:0005509
GO:0005515 GO:0005102 GO:0045095 GO:0005576
Cluster-22178.76500 GO:0006465 GO:0008233 GO:0003677 GO:0016021
GO:0005787
Cluster-22178.76501 GO:0008152 GO:0016829
Cluster-22178.76503 GO:0006629
Cluster-22178.76506 GO:0006334 GO:0003677 GO:0005515 GO:0046872
GO:0008270 GO:0005524 GO:0005634 GO:0000786
Cluster-22178.76509 GO:0019058 GO:0051536 GO:0008289 GO:0003824
GO:0019031
Cluster-22178.76511 GO:0042967 GO:0000077 GO:0006629 GO:0071479
GO:0008080 GO:0008081 GO:0005634
Cluster-22178.76514 GO:0006777 GO:0008152 GO:0003824
Cluster-22178.76515 GO:0005515
Cluster-22178.76516 GO:0006355 GO:0009405 GO:0006352 GO:0003677

GO:0003700 GO:0016987 GO:0005667 GO:0005576
Cluster-22178.76517 GO:0006355 GO:0043401 GO:0043565 GO:0008270
GO:0003700 GO:0005634 GO:0005667 GO:0016021
Cluster-22178.76518 GO:0006355 GO:0043401 GO:0043565 GO:0008270
GO:0003700 GO:0016021 GO:0005634 GO:0005667
Cluster-22178.76519 GO:0007264 GO:0015684 GO:0003924 GO:0015093
GO:0005524 GO:0005525 GO:0016021
Cluster-22178.76520 GO:0006352 GO:0006355 GO:0003700 GO:0003677
GO:0016987 GO:0005667
Cluster-22178.76521 GO:0046677 GO:0006270 GO:0003677
Cluster-22178.76522 GO:0008440
Cluster-22178.76523 GO:0006270 GO:0046677 GO:0003677
Cluster-22178.76526 GO:0016829
Cluster-22178.76527 GO:0006413 GO:0006914 GO:0045892 GO:0006446
GO:0003743 GO:0005634 GO:0005840
Cluster-22178.76531 GO:0006139 GO:0016787 GO:0004003 GO:0016818
GO:0008026 GO:0003677 GO:0005524 GO:0003676 GO:0005657
Cluster-22178.76532 GO:0006139 GO:0003676 GO:0005524 GO:0003677
GO:0008026 GO:0016818 GO:0004003 GO:0016787 GO:0005657
Cluster-22178.76533 GO:0006139 GO:0004003 GO:0016787 GO:0003677
GO:0008026 GO:0016818 GO:0005524 GO:0003676 GO:0005657
Cluster-22178.76534 GO:0006810 GO:0000724 GO:0005886
Cluster-22178.76536 GO:0043401 GO:0009405 GO:0006355 GO:0003700
GO:0008270 GO:0043565 GO:0005198 GO:0005515 GO:0016021
GO:0005634 GO:0005667
Cluster-22178.76537 GO:0000724 GO:0006810 GO:0005886
Cluster-22178.76538 GO:0006119 GO:0015991 GO:0015992 GO:0046961
GO:0033178
Cluster-22178.76539 GO:0015940 GO:0015937 GO:0005524 GO:0004594

Cluster-22178.76540 GO:0046872
Cluster-22178.76541 GO:0008152 GO:0016846
Cluster-22178.76542 GO:0007165 GO:0030155 GO:0007155 GO:0045995

GO:0030334 GO:0006308 GO:0005102 GO:0008855 GO:0009318
Cluster-22178.76549 GO:0006144 GO:0006470 GO:0006508 GO:0006570
GO:0003968 GO:0004725 GO:0017111 GO:0008138 GO:0004197
GO:0031379
Cluster-22178.76550 GO:0006570 GO:0006470 GO:0004725 GO:0008138

Cluster-22178.76551 GO:0006144 GO:0006470 GO:0006508 GO:0006570
GO:0004725 GO:0003968 GO:0004197 GO:0008138 GO:0017111
GO:0031379
Cluster-22178.76552 GO:0006144 GO:0006470 GO:0006508 GO:0006570
GO:0003968 GO:0004725 GO:0017111 GO:0008138 GO:0004197
GO:0031379
Cluster-22178.76555 GO:0051726 GO:0017025 GO:0005634
Cluster-22178.76556 GO:0000398 GO:0046740 GO:0030246 GO:0016021
GO:0005634
Cluster-22178.76559 GO:0000226 GO:0006355 GO:0090063 GO:0019087
GO:0055114 GO:0006308 GO:0003677 GO:0005201 GO:0008855
GO:0016491 GO:0005515 GO:0005815 GO:0009318 GO:0005578
GO:0016021 GO:0000922
Cluster-22178.76561 GO:0006355 GO:0043565 GO:0003700 GO:0005667
Cluster-22178.76567 GO:0055114 GO:0007165 GO:0046872 GO:0016491

Cluster-22178.76568 GO:0015031 GO:0006807 GO:0006810 GO:0007165
GO:0042147 GO:0005524 GO:0008565 GO:0004668 GO:0005179
GO:0005509 GO:0005737 GO:0016021 GO:0005576 GO:0030904
Cluster-22178.76570 GO:0055085 GO:0006338 GO:0043044 GO:0008270
GO:0005524 GO:0003676 GO:0016787 GO:0016818 GO:0003677
GO:0031491 GO:0000785 GO:0016021 GO:0005634 GO:0016020
Cluster-22178.76574 GO:0005515
Cluster-22178.76576 GO:0019482 GO:0007155 GO:0006886 GO:0015031
GO:0006633 GO:0050080 GO:0005515 GO:0008565 GO:0005524
GO:0016020 GO:0005737 GO:0009288
Cluster-22178.76577 GO:0006886 GO:0019482 GO:0007155 GO:0006633

GO:0015031 GO:0005515 GO:0050080 GO:0005524 GO:0008565
GO:0016020 GO:0009288 GO:0005737
Cluster-22178.76578 GO:0034508 GO:0006351 GO:0006269 GO:0003896
GO:0004386 GO:0008270 GO:0005657 GO:0000776 GO:0005730
Cluster-22178.76579 GO:0006351 GO:0006269 GO:0034508 GO:0003896
GO:0004386 GO:0008270 GO:0005730 GO:0000776 GO:0005657
Cluster-22178.76580 GO:0007218 GO:0006974 GO:0007166 GO:0051726
GO:0007165 GO:0004871 GO:0046872 GO:0008270 GO:0005515
GO:0005509 GO:0005185 GO:0005634 GO:0005576
Cluster-22178.76581 GO:0055114 GO:0008641 GO:0051287
Cluster-22178.76583 GO:0008270 GO:0003677
Cluster-22178.76584 GO:0006606 GO:0006508 GO:0005515 GO:0061630
GO:0008233 GO:0046872 GO:0008270
Cluster-22178.76586 GO:0042176 GO:0030234 GO:0008270 GO:0000502
Cluster-22178.76588 GO:0005525
Cluster-22178.76589 GO:0005515
Cluster-22178.76590 GO:0006309 GO:0006915 GO:0016787 GO:0005634
GO:0005737 GO:0005622
Cluster-22178.76591 GO:0055114 GO:0006801 GO:0046872
Cluster-22178.76593 GO:0006886 GO:0006355 GO:0046034 GO:0006464
GO:0006144 GO:0006888 GO:0003677 GO:0004017 GO:0016151
GO:0008270 GO:0046872 GO:0030127 GO:0005634
Cluster-22178.76596 GO:0008152 GO:0008168
Cluster-22178.76597 GO:0008152 GO:0008168
Cluster-22178.76606 GO:0042312 GO:0008217 GO:0006508 GO:0006355
GO:0007165 GO:0008270 GO:0004222 GO:0005179 GO:0005515
GO:0031012 GO:0005576
Cluster-22178.76607 GO:0030071 GO:0008270 GO:0005515 GO:0005680
Cluster-22178.76609 GO:0007067 GO:0005515 GO:0042729 GO:0072686
Cluster-22178.76611 GO:0016310 GO:0004673 GO:0003677 GO:0005524
GO:0008270 GO:0003676 GO:0005921 GO:0009365
Cluster-22178.76612 GO:0043093 GO:0006260 GO:0000917 GO:0005737
GO:0045095

Cluster-22178.76615 GO:0005576
Cluster-22178.76619 GO:0005515 GO:0016020
Cluster-22178.76620 GO:0007165 GO:0007096 GO:0008283 GO:0040007
GO:0008083 GO:0016020 GO:0005634
Cluster-22178.76623 GO:0006355 GO:0003677
Cluster-22178.76626 GO:0006355 GO:0006352 GO:0009297 GO:0015979
GO:0003677 GO:0003723 GO:0003700 GO:0005667 GO:0019867
GO:0005669 GO:0009522
Cluster-22178.76627 GO:0006457 GO:0000413 GO:0046907 GO:0003755
GO:0004519
Cluster-22178.76629 GO:0046872 GO:0008270 GO:0003676
Cluster-22178.76631 GO:0006323 GO:0043087 GO:0006397 GO:0046872
GO:0005089 GO:0003676 GO:0005737
Cluster-22178.76632 GO:0046872 GO:0008270 GO:0003676
Cluster-22178.76633 GO:0055114 GO:0016032 GO:0051537 GO:0016491
GO:0030430 GO:0019031
Cluster-22178.76638 GO:0016310 GO:0043248 GO:0006886 GO:0016192
GO:0051252 GO:0015991 GO:0009069 GO:0004674 GO:0004521
GO:0016820 GO:0005515 GO:0000221 GO:0030117
Cluster-22178.76642 GO:0006270 GO:0006355 GO:0006352 GO:0003677
GO:0005515 GO:0016987 GO:0003700 GO:0005667 GO:0016021
Cluster-22178.76643 GO:0006886 GO:0006397 GO:0015991 GO:0000160
GO:0016310 GO:0007049 GO:0006260 GO:0006914 GO:0015992
GO:0042802 GO:0005525 GO:0015078 GO:0003924 GO:0043015
GO:0000155 GO:0046983 GO:0003774 GO:0033179 GO:0005861
GO:0005801 GO:0045298 GO:0016021 GO:0009365 GO:0016020
GO:0016459
Cluster-22178.76644 GO:0003677
Cluster-22178.76645 GO:0003677
Cluster-22178.76646 GO:0005515
Cluster-22178.76650 GO:0005515 GO:0005634
Cluster-22178.76651 GO:0006278 GO:0030623 GO:0003964
Cluster-22178.76652 GO:0006355 GO:0008152 GO:0003824 GO:0005737

GO:0005634
Cluster-22178.76656 GO:0009432 GO:0006281 GO:0006260 GO:0005524
GO:0003697 GO:0003678 GO:0005657
Cluster-22178.76657 GO:0005515
Cluster-22178.76660 GO:0007165 GO:0030155 GO:0045995 GO:0030334
GO:0046168 GO:0055114 GO:0016616 GO:0051287 GO:0005102
Cluster-22178.76664 GO:0046999 GO:0009405 GO:0005198 GO:0019867
Cluster-22178.76665 GO:0006260 GO:0006308 GO:0006281 GO:0006355
GO:0006508 GO:0003700 GO:0017108 GO:0043565 GO:0003677
GO:0008236 GO:0005515 GO:0033557 GO:0005576 GO:0005634
GO:0005667
Cluster-22178.76668 GO:0006118 GO:0016567 GO:0019684 GO:0005515
GO:0045156 GO:0004842 GO:0046872 GO:0008270 GO:0030077
GO:0016021 GO:0005680
Cluster-22178.76669 GO:0046677 GO:0005515 GO:0016779
Cluster-22178.76672 GO:0000413 GO:0006457 GO:0003755 GO:0004190
GO:0016021 GO:0030430
Cluster-22178.76677 GO:0006807 GO:0006665 GO:0005975 GO:0006687
GO:0004348 GO:0016021
Cluster-22178.76678 GO:0048280 GO:0006886 GO:0015991 GO:0004869
GO:0016820 GO:0005515 GO:0000221 GO:0000139 GO:0005737
Cluster-22178.76679 GO:0003677 GO:0003676 GO:0005524 GO:0016787

Cluster-22178.76685 GO:0006468 GO:0005524 GO:0004672 GO:0016020
Cluster-22178.76688 GO:0007049
Cluster-22178.76691 GO:0005509
Cluster-22178.76692 GO:0006119 GO:0015991 GO:0015986 GO:0043065
GO:0015992 GO:0042981 GO:0046961 GO:0015078 GO:0045263
GO:0033178 GO:0031225 GO:0005737
Cluster-22178.76693 GO:0007165
Cluster-22178.76695 GO:0048280 GO:0009116 GO:0006886 GO:0006508
GO:0004222 GO:0003824 GO:0005515 GO:0071949 GO:0000139
GO:0005737

Cluster-22178.76697 GO:0009116 GO:0048280 GO:0006886 GO:0006508
GO:0003824 GO:0004222 GO:0005515 GO:0071949 GO:0000139
GO:0005737
Cluster-22178.76698 GO:0009069 GO:0006468 GO:0007178 GO:0016310
GO:0004675 GO:0016773 GO:0004672 GO:0005524 GO:0016020
Cluster-22178.76706 GO:0006468 GO:0005524 GO:0004672
Cluster-22178.76709 GO:0005515
Cluster-22178.76714 GO:0003677 GO:0046983
Cluster-22178.76716 GO:0055114 GO:0055085 GO:0006885 GO:0006812
GO:0016671 GO:0003756 GO:0015299 GO:0016021 GO:0005783
Cluster-22178.76717 GO:0006955 GO:0007165 GO:0005125 GO:0005576
Cluster-22178.76719 GO:0055085 GO:0016032 GO:0005506 GO:0016021
Cluster-22178.76722 GO:0008061 GO:0005643
Cluster-22178.76723 GO:0006486 GO:0008378 GO:0016020
Cluster-22178.76724 GO:0006836 GO:0006865 GO:0055085 GO:0006812
GO:0003333 GO:0005515 GO:0005328 GO:0015171 GO:0016021
GO:0016020
Cluster-22178.76726 GO:0009306 GO:0045454 GO:0055114 GO:0005509
GO:0016209 GO:0016491 GO:0005788
Cluster-22178.76729 GO:0008053 GO:0016192 GO:0003924 GO:0005198
GO:0019031 GO:0005741 GO:0016021
Cluster-22178.76730 GO:0008615 GO:0055114 GO:0005515 GO:0004733
GO:0046872 GO:0010181 GO:0016020
Cluster-22178.76731 GO:0006285 GO:0006281 GO:0006308 GO:0006289
GO:0006284 GO:0003684 GO:0008534 GO:0003677
Cluster-22178.76732 GO:0009069 GO:0006468 GO:0016310 GO:0004674
GO:0005524 GO:0004672 GO:0016773 GO:0016020
Cluster-22178.76734 GO:0016310 GO:0042742 GO:0009069 GO:0006886
GO:0051090 GO:0016192 GO:0004674 GO:0005515 GO:0005634
GO:0030117
Cluster-22178.76735 GO:0006885 GO:0006812 GO:0055085 GO:0015299
GO:0016021
Cluster-22178.76738 GO:0009968 GO:0005737

Cluster-22178.76741 GO:0007049 GO:0051301 GO:0005509 GO:0016021
Cluster-22178.76743 GO:0008152 GO:0055114 GO:0006813 GO:0016620
GO:0016491 GO:0016746 GO:0071949 GO:0016021
Cluster-22178.76745 GO:0006355 GO:0009607 GO:0003677 GO:0016021
Cluster-22178.76746 GO:0006355 GO:0009607 GO:0003677 GO:0016021
Cluster-22178.76748 GO:0055085 GO:0006810 GO:0016887 GO:0005524
GO:0042626 GO:0003676 GO:0005525 GO:0016021
Cluster-22178.76749 GO:0055085 GO:0006810 GO:0042626 GO:0005524
GO:0016021
Cluster-22178.76751 GO:0051016 GO:0043087 GO:0035023 GO:0005089
GO:0030479
Cluster-22178.76753 GO:0007018 GO:0007017 GO:0003777 GO:0005874
GO:0030286
Cluster-22178.76754 GO:0008608 GO:0005975 GO:0006914 GO:0006355
GO:0005524 GO:0016773 GO:0016887 GO:0005515 GO:0008134
GO:0042729 GO:0005667 GO:0072686
Cluster-22178.76757 GO:0006464 GO:0005515 GO:0016021
Cluster-22178.76765 GO:0042157 GO:0006869 GO:0003723 GO:0008289
GO:0005576
Cluster-22178.76767 GO:0035091 GO:0016021
Cluster-22178.76769 GO:0005515
Cluster-22178.76771 GO:0006754 GO:0005524 GO:0016887 GO:0000275
Cluster-22178.76776 GO:0009395 GO:0016042 GO:0006355 GO:0043093
GO:0000917 GO:0006364 GO:0043565 GO:0003700 GO:0003677
GO:0004629 GO:0005515 GO:0005737 GO:0005634 GO:0005667
Cluster-22178.76777 GO:0007165 GO:0008158 GO:0016020
Cluster-22178.76780 GO:0006118 GO:0019551 GO:0055114 GO:0006520
GO:0006807 GO:0051287 GO:0016491 GO:0004352
Cluster-22178.76788 GO:0007155 GO:0005509 GO:0003824 GO:0051536
GO:0016020 GO:0005576
Cluster-22178.76790 GO:0005978 GO:0005982 GO:0005985 GO:0004373

Cluster-22178.76791 GO:0016567 GO:0004867 GO:0004842 GO:0005515

Cluster-22178.76792 GO:0006468 GO:0005515 GO:0016773 GO:0005524
GO:0004672 GO:0016020
Cluster-22178.76795 GO:0006810 GO:0005524 GO:0042626 GO:0016021
Cluster-22178.76797 GO:0006886 GO:0006888 GO:0055114 GO:0003677
GO:0016706 GO:0008270 GO:0046872 GO:0008168 GO:0030127
Cluster-22178.76798 GO:0035091 GO:0005515
Cluster-22178.76803 GO:0007186 GO:0042254 GO:0006412 GO:0003735
GO:0004930 GO:0046872 GO:0005840 GO:0016021 GO:0005622
Cluster-22178.76804 GO:0006355 GO:0003700 GO:0005667 GO:0005634
Cluster-22178.76807 GO:0015992 GO:0046660 GO:0015986 GO:0006118
GO:0006810 GO:0006397 GO:0008270 GO:0003676 GO:0015078
GO:0009055 GO:0020037 GO:0000276 GO:0005643 GO:0005634
Cluster-22178.76808 GO:0007264 GO:0006810 GO:0043087 GO:0002949
GO:0035023 GO:0005515 GO:0016887 GO:0043531 GO:0005524
GO:0004386 GO:0003676 GO:0005089 GO:0005525
Cluster-22178.76810 GO:0006355 GO:0003700 GO:0043565 GO:0005667
Cluster-22178.76812 GO:0005515
Cluster-22178.76813 GO:0005515
Cluster-22178.76814 GO:0005515
Cluster-22178.76817 GO:0003924 GO:0005515 GO:0005525
Cluster-22178.76821 GO:0008608 GO:0006886 GO:0016192 GO:0005515
GO:0030117 GO:0072686 GO:0005634 GO:0042729
Cluster-22178.76822 GO:0008608 GO:0006886 GO:0016192 GO:0005515
GO:0005634 GO:0072686 GO:0030117 GO:0042729
Cluster-22178.76824 GO:0009069 GO:0006468 GO:0016310 GO:0004674
GO:0016773 GO:0005524 GO:0004672 GO:0016020
Cluster-22178.76826 GO:0006952 GO:0008284 GO:0005515
Cluster-22178.76829 GO:0007186 GO:0004930 GO:0016021
Cluster-22178.76830 GO:0055114 GO:0031124 GO:0006813 GO:0016491
GO:0005249 GO:0003676 GO:0030955 GO:0016021 GO:0008076
Cluster-22178.76833 GO:0005509 GO:0003676 GO:0005544 GO:0008270

Cluster-22178.76834 GO:0005096 GO:0005515
Cluster-22178.76835 GO:0055114 GO:0003677 GO:0016491 GO:0016021
Cluster-22178.76836 GO:0006820 GO:0016021
Cluster-22178.76837 GO:0006355 GO:0008270 GO:0005524 GO:0003677
GO:0016818 GO:0005634
Cluster-22178.76838 GO:0016192 GO:0006886 GO:0019013
Cluster-22178.76839 GO:0035023 GO:0051321 GO:0043087 GO:0005089
GO:0043531 GO:0005737
Cluster-22178.76842 GO:0006144 GO:0046034 GO:0035556 GO:0006355
GO:0007165 GO:0008270 GO:0004871 GO:0003700 GO:0005515
GO:0004017 GO:0003677 GO:0005667
Cluster-22178.76844 GO:0006810 GO:0007291 GO:0007049 GO:0007304
GO:0051082 GO:0005213 GO:0005576 GO:0016020 GO:0042600
Cluster-22178.76846 GO:0006886 GO:0006629 GO:0006508 GO:0008152
GO:0015994 GO:0015996 GO:0001514 GO:0006505 GO:0006448
GO:0016788 GO:0047746 GO:0008236 GO:0003723 GO:0005525
GO:0016787 GO:0003746 GO:0005576 GO:0005840 GO:0005737
Cluster-22178.76851 GO:0016021 GO:0005886
Cluster-22178.76852 GO:0006955 GO:0007165 GO:0006869 GO:0040007
GO:0042157 GO:0008283 GO:0008289 GO:0005138 GO:0005896
GO:0005576
Cluster-22178.76854 GO:0007264 GO:0006357 GO:0001104 GO:0005515
GO:0005525 GO:0008641 GO:0003924 GO:0016592
Cluster-22178.76855 GO:0045893 GO:0007018 GO:0006275 GO:0044780
GO:0007017 GO:0003777 GO:0003676 GO:0003723 GO:0005874
GO:0030286 GO:0031224 GO:0019028
Cluster-22178.76856 GO:0016567 GO:0035556 GO:0046872 GO:0008270
GO:0005515 GO:0004842 GO:0005680
Cluster-22178.76865 GO:0006231 GO:0055114 GO:0008033 GO:0006206
GO:0015940 GO:0006779 GO:0019354 GO:0016491 GO:0008677
GO:0008641 GO:0050797 GO:0050660 GO:0043115
Cluster-22178.76869 GO:0006260 GO:0046034 GO:0051028 GO:0048309
GO:0006144 GO:0006355 GO:0003700 GO:0003676 GO:0008270

GO:0046872 GO:0043565 GO:0004017 GO:0005515 GO:0005667
Cluster-22178.76872 GO:0006298 GO:0005515 GO:0030983 GO:0005524

Cluster-22178.76876 GO:0007186 GO:0005515 GO:0004930 GO:0003723
GO:0016021 GO:0019028
Cluster-22178.76878 GO:0045490 GO:0047487 GO:0005515
Cluster-22178.76879 GO:0007218
Cluster-22178.76882 GO:0005515 GO:0000228
Cluster-22178.76889 GO:0016310 GO:0009069 GO:0006468 GO:0005524
GO:0004672 GO:0016773 GO:0004674 GO:0016020
Cluster-22178.76890 GO:0007154 GO:0019497 GO:0006771 GO:0046872
GO:0003993 GO:0016820 GO:0051539 GO:0005515 GO:0016021
Cluster-22178.76893 GO:0009190 GO:0006171 GO:0006144 GO:0035556
GO:0016849 GO:0005515 GO:0004016 GO:0051537 GO:0005886
Cluster-22178.76894 GO:0043565 GO:0005515 GO:0005634 GO:0005615
Cluster-22178.76897 GO:0009069 GO:0006468 GO:0016310 GO:0005524
GO:0004672 GO:0004674
Cluster-22178.76900 GO:0005515
Cluster-22178.76901 GO:0006281 GO:0008270
Cluster-22178.76902 GO:0016567 GO:0006955 GO:0007165 GO:0019882
GO:0005515 GO:0004842
Cluster-22178.76904 GO:0006810 GO:0009405 GO:0008200 GO:0005576
Cluster-22178.76905 GO:0000226 GO:0051537 GO:0043231 GO:0015630
Cluster-22178.76907 GO:0015833 GO:0000166 GO:0005524 GO:0046872

Cluster-22178.76908 GO:0001503 GO:0006886 GO:0007155 GO:0006352
GO:0005047 GO:0005515 GO:0005525 GO:0003924 GO:0005576
GO:0005669 GO:0005785 GO:0005786
Cluster-22178.76925 GO:0007165
Cluster-22178.76930 GO:0006468 GO:0009069 GO:0035556 GO:0016310
GO:0005515 GO:0005524 GO:0004672 GO:0016773 GO:0004674
GO:0016020
Cluster-22178.76931 GO:0006468 GO:0005524 GO:0004672

Cluster-22178.76932 GO:0006468 GO:0004672 GO:0005524
Cluster-22178.76934 GO:0031224
Cluster-22178.76937 GO:0018342 GO:0008318 GO:0030288
Cluster-22178.76938 GO:0005515 GO:0046872
Cluster-22178.76940 GO:0006950 GO:0016021
Cluster-22178.76944 GO:0016817
Cluster-22178.76945 GO:0016567 GO:0006508 GO:0004842 GO:0008270
GO:0046872 GO:0004197 GO:0005515 GO:0005680 GO:0005622
Cluster-22178.76946 GO:0007165 GO:0006355 GO:0004871 GO:0003700
GO:0016817 GO:0005667
Cluster-22178.76947 GO:0005515
Cluster-22178.76950 GO:0015992 GO:0006914 GO:0008608 GO:0006120
GO:0006744 GO:0006118 GO:0006814 GO:0003954 GO:0008137
GO:0005739 GO:0072686 GO:0042729
Cluster-22178.76951 GO:0006919 GO:0006974 GO:0008608 GO:0001836
GO:0006915 GO:0006511 GO:0016567 GO:0004842 GO:0034450
GO:0005876 GO:0000151 GO:0042729
Cluster-22178.76956 GO:0005515
Cluster-22178.76959 GO:0016740
Cluster-22178.76960 GO:0018149 GO:0005515 GO:0003676 GO:0003810

Cluster-22178.76963 GO:0006812 GO:0006835 GO:0006820 GO:0017153
GO:0016020
Cluster-22178.76964 GO:0006950 GO:0009415
Cluster-22178.76968 GO:0007165 GO:0032065 GO:0005515 GO:0000062
GO:0005543 GO:0003774 GO:0005524 GO:0008242 GO:0016459
GO:0005938 GO:0005856
Cluster-22178.76972 GO:0007049 GO:0032012 GO:0043087 GO:0006355
GO:0003700 GO:0008289 GO:0043565 GO:0046872 GO:0005086
GO:0005667
Cluster-22178.76973 GO:0009405 GO:0006813 GO:0007157 GO:0008152
GO:0045444 GO:0003677 GO:0016301 GO:0016787 GO:0003676
GO:0005524 GO:0005267 GO:0016020 GO:0016021

Cluster-22178.76975 GO:0016021
Cluster-22178.76977 GO:0016021
Cluster-22178.76979 GO:0032797
Cluster-22178.76983 GO:0015992 GO:0007165 GO:0007275 GO:0006886
GO:0016192 GO:0015991 GO:0016055 GO:0015078 GO:0005102
GO:0005515 GO:0016021 GO:0033179 GO:0005576
Cluster-22178.76984 GO:0015992 GO:0016055 GO:0015991 GO:0006886
GO:0016192 GO:0007275 GO:0007165 GO:0005102 GO:0015078
GO:0005515 GO:0016021 GO:0005576 GO:0033179
Cluster-22178.76987 GO:0007165 GO:0007186 GO:0007264 GO:0005525
GO:0004871 GO:0019001 GO:0031683 GO:0003924
Cluster-22178.76989 GO:0006260 GO:0005524 GO:0016818 GO:0003677
GO:0005634
Cluster-22178.76991 GO:0055085 GO:0006810 GO:0016192 GO:0006886
GO:0005215 GO:0022857 GO:0016021 GO:0016020 GO:0030117
Cluster-22178.76992 GO:0000077 GO:0006351 GO:0007165 GO:0003676
GO:0003714 GO:0005509 GO:0005515 GO:0016021 GO:0005578
GO:0005667 GO:0005634
Cluster-22178.77001 GO:0004045 GO:0003677 GO:0016301 GO:0016772

Cluster-22178.77010 GO:0000921 GO:0003677 GO:0016021 GO:0005940
Cluster-22178.77011 GO:0006355 GO:0003700 GO:0043565 GO:0005667
Cluster-22178.77012 GO:0006470 GO:0006570 GO:0004725
Cluster-22178.77013 GO:0015991 GO:0006308 GO:0006139 GO:0019058
GO:0015992 GO:0006914 GO:0051082 GO:0005515 GO:0008168
GO:0015078 GO:0008270 GO:0008855 GO:0009318 GO:0033179
GO:0019031 GO:0055036 GO:0033644
Cluster-22178.77014 GO:0016310 GO:0007178 GO:0009069 GO:0006468
GO:0005524 GO:0004675 GO:0016021 GO:0016020 GO:0005576
Cluster-22178.77021 GO:0016301
Cluster-22178.77022 GO:0007067 GO:0000090 GO:0007186 GO:0007165
GO:0007275 GO:0031110 GO:0006357 GO:0070206 GO:0006260
GO:0051301 GO:0007059 GO:0005179 GO:0008017 GO:0004930

GO:0001104 GO:0016592 GO:0000940 GO:0005876 GO:0005576
GO:0045298 GO:0016021
Cluster-22178.77023 GO:0006412 GO:0042254 GO:0003735 GO:0005840
Cluster-22178.77029 GO:0006486 GO:0016757 GO:0008378 GO:0016020
Cluster-22178.77030 GO:0019882 GO:0006955 GO:0042613 GO:0016020
Cluster-22178.77032 GO:0007165 GO:0005509 GO:0051087 GO:0005578
Cluster-22178.77036 GO:0007009 GO:0042254 GO:0043565 GO:0051082
GO:0005515 GO:0003735 GO:0005840 GO:0005634
Cluster-22178.77039 GO:0016310 GO:0007178 GO:0009069 GO:0006468
GO:0005524 GO:0004672 GO:0004675 GO:0016020
Cluster-22178.77040 GO:0005515
Cluster-22178.77041 GO:0009264 GO:0007283 GO:0006024 GO:0015012
GO:0004252 GO:0003677 GO:0008253 GO:0016757 GO:0016020
GO:0005634 GO:0016021 GO:0000786
Cluster-22178.77042 GO:0042578
Cluster-22178.77044 GO:0005524
Cluster-22178.77045 GO:0040007 GO:0051252 GO:0009405 GO:0008033
GO:0006274 GO:0008283 GO:0007165 GO:0006364 GO:0006379
GO:0003723 GO:0004540 GO:0005198 GO:0005515 GO:0008083
GO:0016787 GO:0030674 GO:0003677 GO:0051015 GO:0005509
GO:0005737 GO:0005615 GO:0030677 GO:0000172
Cluster-22178.77046 GO:0015994 GO:0033014 GO:0004655 GO:0046872

Cluster-22178.77049 GO:0006479 GO:0008610 GO:0009451 GO:0008033
GO:0046500 GO:0046486 GO:0030488 GO:0006464 GO:0006396
GO:0042967 GO:0016627 GO:0004719 GO:0008168 GO:0004144
GO:0016429 GO:0031515 GO:0005622
Cluster-22178.77050 GO:0006313 GO:0009069 GO:0000184 GO:0016310
GO:0006470 GO:0000087 GO:0006570 GO:0004674 GO:0016773
GO:0004803 GO:0008144 GO:0004725 GO:0008168 GO:0005515
GO:0005198 GO:0005622 GO:0019031 GO:0019028
Cluster-22178.77052 GO:0006308 GO:0006281 GO:0008855 GO:0005515
GO:0003677 GO:0004518 GO:0009318

Cluster-22178.77053 GO:0009252 GO:0006825 GO:0035434 GO:0006629 GO:0008963 GO:0046983 GO:0005375 GO:0003677 GO:0016021
Cluster-22178.77055 GO:0070206 GO:0007165 GO:0008608 GO:0005515 GO:0072686 GO:0042729
Cluster-22178.77057 GO:0055085 GO:0016021
Cluster-22178.77059 GO:0070193 GO:0016567 GO:0007130 GO:0007128 GO:0016881 GO:0008092 GO:0031386 GO:0000795 GO:0005737 GO:0019898
Cluster-22178.77062 GO:0007186 GO:0007165 GO:0009069 GO:0006468 GO:0016310 GO:0000166 GO:0003677 GO:0004871 GO:0004674 GO:0003924 GO:0005524 GO:0031683 GO:0004672 GO:0019001
Cluster-22178.77063 GO:0044780
Cluster-22178.77064 GO:0042254 GO:0006412 GO:0045454 GO:0006355 GO:0003700 GO:0003735 GO:0005840 GO:0005622 GO:0005667
Cluster-22178.77068 GO:0007049 GO:0000917 GO:0043093 GO:0006355 GO:0003700 GO:0043565 GO:0003677 GO:0005737 GO:0005667 GO:0005634
Cluster-22178.77070 GO:0000917 GO:0006355 GO:0043093 GO:0007049 GO:0003677 GO:0003700 GO:0043565 GO:0005634 GO:0005667 GO:0005737
Cluster-22178.77076 GO:0042384 GO:0034464
Cluster-22178.77082 GO:0055085 GO:0016021 GO:0055036
Cluster-22178.77083 GO:0008152 GO:0003824
Cluster-22178.77087 GO:0009294 GO:0034477 GO:0007276 GO:0006508 GO:0016579 GO:0006364 GO:0003676 GO:0036459 GO:0004518 GO:0004222 GO:0008270 GO:0032183 GO:0070140 GO:0030246 GO:0004857 GO:0031012
Cluster-22178.77088 GO:0004252 GO:0005515 GO:0043565 GO:0055036 GO:0005634 GO:0019028
Cluster-22178.77089 GO:0004252 GO:0005515 GO:0043565 GO:0019028 GO:0005634 GO:0055036
Cluster-22178.7709 GO:0008146
Cluster-22178.77092 GO:0006355 GO:0003677

Cluster-22178.77094 GO:0006777 GO:0002949 GO:0007264 GO:0005525

Cluster-22178.77095 GO:0006811 GO:0006260 GO:0006281 GO:0019079
GO:0016567 GO:0046872 GO:0008270 GO:0003911 GO:0004842
GO:0030337 GO:0005515 GO:0005680 GO:0042575 GO:0016020
Cluster-22178.77096 GO:0006355 GO:0006730 GO:0006032 GO:0008134
GO:0005515 GO:0008061 GO:0008168 GO:0005524 GO:0005667
GO:0005576 GO:0009279
Cluster-22178.77099 GO:0005515 GO:0019904 GO:0005737
Cluster-22178.77104 GO:0005515
Cluster-22178.77106 GO:0016310 GO:0006468 GO:0009069 GO:0004674
GO:0004672 GO:0005524
Cluster-22178.77109 GO:0030261 GO:0006614 GO:0009089 GO:0009058
GO:0000723 GO:0006777 GO:0007059 GO:0006281 GO:0005524
GO:0016787 GO:0003677 GO:0005525 GO:0008837 GO:0003824
GO:0003678 GO:0005657 GO:0009295 GO:0019031
Cluster-22178.77110 GO:0046677 GO:0016779 GO:0005515
Cluster-22178.77111 GO:0007606 GO:0006886 GO:0007165 GO:0006888
GO:0003723 GO:0004888 GO:0005515 GO:0008270 GO:0016020
GO:0030127
Cluster-22178.77114 GO:0006355 GO:0006032 GO:0008134 GO:0005515
GO:0008061 GO:0005524 GO:0005667 GO:0009279
Cluster-22178.77116 GO:0006206 GO:0006144 GO:0007165 GO:0006351
GO:0008270 GO:0046872 GO:0003676 GO:0005179 GO:0003899
GO:0005730 GO:0005576
Cluster-22178.77117 GO:0009058 GO:0016742
Cluster-22178.77122 GO:0006457 GO:0005515 GO:0005615
Cluster-22178.77124 GO:0005515
Cluster-22178.77126 GO:0006106 GO:0016020
Cluster-22178.77129 GO:0042967 GO:0005515 GO:0008080 GO:0016747

Cluster-22178.77130 GO:0042967 GO:0008080 GO:0005515 GO:0016747

Cluster-22178.77132 GO:0006355 GO:0030153 GO:0007165 GO:0016192
GO:0006886 GO:0006955 GO:0015643 GO:0005198 GO:0005515
GO:0004871 GO:0003700 GO:0030117 GO:0005667 GO:0019814
Cluster-22178.77134 GO:0005515
Cluster-22178.77135 GO:0005515
Cluster-22178.77137 GO:0006030 GO:0055114 GO:0006468 GO:0016773
GO:0016491 GO:0005524 GO:0004672 GO:0005509 GO:0008061
GO:0019031 GO:0016020 GO:0005576
Cluster-22178.77139 GO:0008033 GO:0008168
Cluster-22178.77140 GO:0006355 GO:0008654 GO:0006260 GO:0003677
GO:0000166 GO:0008134 GO:0005524 GO:0016887 GO:0016780
GO:0005667 GO:0016020
Cluster-22178.77144 GO:0006816 GO:0006874 GO:0005219 GO:0005622
GO:0016021
Cluster-22178.77145 GO:0042254 GO:0006412 GO:0003735 GO:0005622
GO:0005840
Cluster-22178.77148 GO:0005509 GO:0016020
Cluster-22178.77150 GO:0006810 GO:0007186 GO:0004930 GO:0008200
GO:0016020 GO:0005576
Cluster-22178.77152 GO:0046486 GO:0006027 GO:0006687 GO:0006012
GO:0005975 GO:0004565 GO:0046872 GO:0008270 GO:0009341
Cluster-22178.77154 GO:0006820 GO:0006810 GO:0055085 GO:0005215
GO:0008509 GO:0016021 GO:0016020
Cluster-22178.77155 GO:0006935 GO:0000160 GO:0016559 GO:0006904
GO:0015031 GO:0006396 GO:0055085 GO:0006614 GO:0016310
GO:0006468 GO:0030001 GO:0051252 GO:0009405 GO:0042157
GO:0006869 GO:0004672 GO:0008289 GO:0042802 GO:0000175
GO:0046873 GO:0004673 GO:0003723 GO:0005515 GO:0008312
GO:0046983 GO:0005524 GO:0000155 GO:0048500 GO:0016021
GO:0009365 GO:0005576 GO:0000145 GO:0005737 GO:0005779
GO:0016020
Cluster-22178.77156 GO:0006935 GO:0000160 GO:0016559 GO:0006904
GO:0015031 GO:0055085 GO:0006396 GO:0006614 GO:0016310

GO:0006468 GO:0030001 GO:0009405 GO:0051252 GO:0042157
GO:0006869 GO:0004672 GO:0008289 GO:0042802 GO:0000175
GO:0004673 GO:0046873 GO:0003723 GO:0005515 GO:0005524
GO:0046983 GO:0008312 GO:0000155 GO:0048500 GO:0009365
GO:0016021 GO:0005576 GO:0000145 GO:0005737 GO:0005779
GO:0016020
Cluster-22178.77157 GO:0008270 GO:0003723 GO:0016021
Cluster-22178.77158 GO:0006614 GO:0015031 GO:0055085 GO:0006396
GO:0016559 GO:0006904 GO:0006935 GO:0000160 GO:0042157
GO:0006869 GO:0006468 GO:0030001 GO:0009405 GO:0051252
GO:0016310 GO:0004673 GO:0046873 GO:0000175 GO:0042802
GO:0004672 GO:0008289 GO:0000155 GO:0046983 GO:0008312
GO:0005524 GO:0003723 GO:0005515 GO:0000145 GO:0005576
GO:0048500 GO:0016021 GO:0009365 GO:0005779 GO:0016020
GO:0005737
Cluster-22178.77161 GO:0007010 GO:0006298 GO:0006364 GO:0006813
GO:0007017 GO:0005524 GO:0005267 GO:0003779 GO:0030983
GO:0005875 GO:0005732 GO:0034457 GO:0005634
Cluster-22178.77163 GO:0015631 GO:0005515 GO:0045298 GO:0005815
Cluster-22178.77164 GO:0045454 GO:0006813 GO:0008556 GO:0005509
GO:0005515 GO:0005886
Cluster-22178.77165 GO:0006771 GO:0019497 GO:0030908 GO:0003677
GO:0004519 GO:0005515 GO:0046872 GO:0003993
Cluster-22178.77174 GO:0003676 GO:0008270
Cluster-22178.77178 GO:0006289 GO:0006355 GO:0007034 GO:0005515
GO:0000439
Cluster-22178.77179 GO:0007165 GO:0006955 GO:0006886 GO:0007131
GO:0006308 GO:0005515 GO:0008270 GO:0005125 GO:0005057
GO:0008855 GO:0009318 GO:0016020 GO:0005576 GO:0000794
GO:0005737
Cluster-22178.77182 GO:0006269 GO:0006351 GO:0019825 GO:0003677
GO:0005515 GO:0020037 GO:0003896 GO:0005730 GO:0005634
GO:0005657

Cluster-22178.77188 GO:0015937 GO:0015940 GO:0009451 GO:0001522
GO:0005524 GO:0004140 GO:0009982 GO:0008017 GO:0003723
GO:0045298
Cluster-22178.77189 GO:0003723
Cluster-22178.77190 GO:0042128 GO:0003677 GO:0008168 GO:0008270
GO:0005634
Cluster-22178.77194 GO:0019068 GO:0006364 GO:0008168 GO:0005198
GO:0005634
Cluster-22178.77196 GO:0006355 GO:0005524 GO:0005634
Cluster-22178.77197 GO:0042254 GO:0006412 GO:0046983 GO:0046872
GO:0008270 GO:0003735 GO:0005515 GO:0005622 GO:0005840
Cluster-22178.77201 GO:0003676 GO:0003723 GO:0019013
Cluster-22178.77203 GO:0034453 GO:0005515 GO:0005509 GO:0005815
Cluster-22178.77205 GO:0016787
Cluster-22178.77207 GO:0005515
Cluster-22178.77213 GO:0051598 GO:0007131 GO:0000398 GO:0005198
GO:0008270 GO:0005681 GO:0005634
Cluster-22178.77215 GO:0005515
Cluster-22178.77216 GO:0006368 GO:0008023
Cluster-22178.7722 GO:0016021
Cluster-22178.77221 GO:0019079
Cluster-22178.77225 GO:0007049 GO:0006355 GO:0003677 GO:0003682
GO:0000785 GO:0000775 GO:0005634
Cluster-22178.77226 GO:0003676 GO:0005634
Cluster-22178.77227 GO:0000160 GO:0006935 GO:0015979 GO:0008984
GO:0000156 GO:0008270 GO:0016020 GO:0009523 GO:0005737
GO:0009539
Cluster-22178.77229 GO:0002949 GO:0016301 GO:0005524 GO:0003774
GO:0016459
Cluster-22178.77233 GO:0007155 GO:0001503 GO:0016758 GO:0008270
GO:0005515 GO:0005576 GO:0005789
Cluster-22178.77234 GO:0006479 GO:0008168
Cluster-22178.77236 GO:0006355 GO:0009097 GO:0006486 GO:0006351

GO:0006418 GO:0009098 GO:0007155 GO:0009099 GO:0006144
GO:0006438 GO:0006206 GO:0009312 GO:0098599 GO:0008455
GO:0008375 GO:0003899 GO:0000166 GO:0002161 GO:0003676
GO:0016787 GO:0004812 GO:0003677 GO:0004832 GO:0005524
GO:0008270 GO:0071203 GO:0005795 GO:0005737 GO:0016021
GO:0005730
Cluster-22178.77240 GO:0009099 GO:0007155 GO:0009312 GO:0006144
GO:0006438 GO:0006206 GO:0009097 GO:0006486 GO:0006351
GO:0006355 GO:0009098 GO:0006418 GO:0003677 GO:0016787
GO:0004812 GO:0004832 GO:0008270 GO:0005524 GO:0008375
GO:0003899 GO:0000166 GO:0098599 GO:0008455 GO:0003676
GO:0002161 GO:0005730 GO:0005795 GO:0071203 GO:0016021
GO:0005737
Cluster-22178.77241 GO:0006355 GO:0009097 GO:0006486 GO:0006351
GO:0006418 GO:0009098 GO:0007155 GO:0009099 GO:0006144
GO:0006438 GO:0006206 GO:0009312 GO:0008455 GO:0008375
GO:0003899 GO:0000166 GO:0002161 GO:0003676 GO:0016787
GO:0004812 GO:0003677 GO:0004832 GO:0008270 GO:0005524
GO:0071203 GO:0005795 GO:0005737 GO:0016021 GO:0005730
Cluster-22178.77243 GO:0006281 GO:0019497 GO:0006771 GO:0006310
GO:0019079 GO:0002949 GO:0003723 GO:0005515 GO:0003678
GO:0009378 GO:0016887 GO:0005524 GO:0003724 GO:0003993
GO:0009379 GO:0005657
Cluster-22178.77244 GO:0005198 GO:0005882
Cluster-22178.77248 GO:0006508 GO:0007186 GO:0004930 GO:0004252
GO:0016020
Cluster-22178.77251 GO:0006914 GO:0006308 GO:0051258 GO:0030168
GO:0007049 GO:0019069 GO:0007165 GO:0008855 GO:0008270
GO:0005102 GO:0030674 GO:0005577 GO:0009318
Cluster-22178.77253 GO:0004866 GO:0005515 GO:0008270
Cluster-22178.77259 GO:0007049 GO:0006355 GO:0003677 GO:0003700
GO:0043565 GO:0005667 GO:0005634
Cluster-22178.77260 GO:0005515

Cluster-22178.77261 GO:0007165 GO:0009069 GO:0006468 GO:0016310
GO:0004674 GO:0004672 GO:0005524 GO:0005576
Cluster-22178.77263 GO:0070072 GO:0006508 GO:0004252
Cluster-22178.77266 GO:0006355 GO:0010508 GO:0030474 GO:0006886
GO:0048870 GO:0050909 GO:0071988 GO:0006457 GO:0015031
GO:0006914 GO:0006606 GO:0042803 GO:0043565 GO:0003700
GO:0051087 GO:0000774 GO:0008565 GO:0031514 GO:0005737
GO:0005801 GO:0016021 GO:0005667 GO:0005783 GO:0016020
Cluster-22178.77267 GO:0005515 GO:0008270 GO:0005921
Cluster-22178.77268 GO:0050790 GO:0006508 GO:0004197 GO:0008234

Cluster-22178.77271 GO:0008270 GO:0005515 GO:0005576
Cluster-22178.77276 GO:0055085 GO:0005741
Cluster-22178.77280 GO:0051260 GO:0005515
Cluster-22178.77283 GO:0006355 GO:0046872 GO:0005524 GO:0003676
GO:0008134 GO:0005667
Cluster-22178.77290 GO:0016598 GO:0005515 GO:0004057
Cluster-22178.77291 GO:0019497 GO:0006771 GO:0046872 GO:0003993
GO:0005515 GO:0016021
Cluster-22178.77292 GO:0043039 GO:0016876 GO:0008270 GO:0005524

Cluster-22178.77296 GO:0005515
Cluster-22178.77298 GO:0016747
Cluster-22178.77299 GO:0046872 GO:0008270 GO:0005515 GO:0061630

Cluster-22178.77300 GO:0042819 GO:0055114 GO:0008033 GO:0000105
GO:0042823 GO:0017150 GO:0050660
Cluster-22178.77301 GO:0042254 GO:0006412 GO:0003735 GO:0016491
GO:0005840 GO:0005622
Cluster-22178.77302 GO:0006139 GO:0016818 GO:0008026 GO:0003677
GO:0004003 GO:0016787 GO:0003676 GO:0005524 GO:0005634
GO:0005657
Cluster-22178.77303 GO:0006139 GO:0005524 GO:0003676 GO:0004003

GO:0008026 GO:0016818 GO:0003677 GO:0005657 GO:0005634
Cluster-22178.77307 GO:0015940 GO:0015937 GO:0004140 GO:0005524

Cluster-22178.77308 GO:0006308 GO:0055114 GO:0016614 GO:0008855
GO:0005869 GO:0009318
Cluster-22178.77309 GO:0006858 GO:0006355 GO:0005515 GO:0016818
GO:0003677 GO:0016817 GO:0005524 GO:0008270 GO:0005634
Cluster-22178.77311 GO:0006355 GO:0005634
Cluster-22178.77312 GO:0006351 GO:0006260 GO:0006869 GO:0016567
GO:0042157 GO:0008289 GO:0046872 GO:0003887 GO:0005515
GO:0005198 GO:0004842 GO:0008270 GO:0003677 GO:0061630
GO:0019031 GO:0016021 GO:0042575 GO:0005576 GO:0019028
GO:0005680 GO:0005622 GO:0016020
Cluster-22178.77313 GO:0046872
Cluster-22178.77316 GO:0008033 GO:0008168
Cluster-22178.77317 GO:0006355 GO:0008270 GO:0005634
Cluster-22178.77318 GO:0006396
Cluster-22178.77319 GO:0046487 GO:0009396 GO:0055114 GO:0004488
GO:0003824 GO:0004672
Cluster-22178.77323 GO:0008152 GO:0050830 GO:0008168
Cluster-22178.77326 GO:0006355 GO:0016973 GO:0048870 GO:0003700
GO:0031514 GO:0005667 GO:0016021 GO:0005643
Cluster-22178.77330 GO:0007264 GO:0005525
Cluster-22178.77332 GO:0055085 GO:0016021
Cluster-22178.77334 GO:0015693 GO:0015095 GO:0016021 GO:0016020
Cluster-22178.77335 GO:0006355 GO:0003677 GO:0003700 GO:0005515
GO:0005667
Cluster-22178.77336 GO:0051082 GO:0005198 GO:0019028 GO:0048786
GO:0005815 GO:0005882 GO:0019031
Cluster-22178.77337 GO:0006779 GO:0015994 GO:0007165 GO:0055114
GO:0005509 GO:0004109 GO:0005578
Cluster-22178.77338 GO:0007009 GO:0007165 GO:0005515
Cluster-22178.77341 GO:0006576 GO:0007264 GO:0006355 GO:0006614

GO:0002949 GO:0005515 GO:0008134 GO:0003924 GO:0005524
GO:0005525 GO:0005667
Cluster-22178.77343 GO:0006614 GO:0006355 GO:0007264 GO:0006576
GO:0002949 GO:0005515 GO:0008134 GO:0005525 GO:0005524
GO:0003924 GO:0005667
Cluster-22178.77344 GO:0005515 GO:0005737
Cluster-22178.77345 GO:0003676
Cluster-22178.77347 GO:0005515
Cluster-22178.77349 GO:0005515
Cluster-22178.7735 GO:0055114 GO:0008152 GO:0016491 GO:0019031
Cluster-22178.77350 GO:0006525 GO:0042254 GO:0071436 GO:0006560
GO:0006814 GO:0006412 GO:0009401 GO:0006090 GO:0003735
GO:0015081 GO:0005515 GO:0008948 GO:0016020 GO:0015934
GO:0005840 GO:0016021
Cluster-22178.77352 GO:0008152 GO:0016746
Cluster-22178.77354 GO:0046983 GO:0005634
Cluster-22178.77356 GO:0019051 GO:0042025
Cluster-22178.77357 GO:0007186 GO:0004930 GO:0016021
Cluster-22178.77361 GO:0016567 GO:0006351 GO:0006144 GO:0006206
GO:0005515 GO:0003899 GO:0003677 GO:0046872 GO:0008270
GO:0004842 GO:0005730 GO:0005680
Cluster-22178.77368 GO:0006464 GO:0015031 GO:0005515
Cluster-22178.77369 GO:0006886 GO:0007268 GO:0006605 GO:0006508
GO:0005515 GO:0042802 GO:0043495 GO:0004197 GO:0030695
GO:0033130 GO:0005742
Cluster-22178.77370 GO:0006886 GO:0007268 GO:0006605 GO:0006508
GO:0030695 GO:0033130 GO:0005515 GO:0042802 GO:0043495
GO:0004197 GO:0005742
Cluster-22178.77371 GO:0016042 GO:0015074 GO:0046486 GO:0006313
GO:0006629 GO:0006468 GO:0004806 GO:0005524 GO:0004672
GO:0005515 GO:0003677
Cluster-22178.77377 GO:0009372 GO:0016310 GO:0009069 GO:0004674

Cluster-22178.77378 GO:0008537
Cluster-22178.77381 GO:0006355 GO:0006260 GO:0046034 GO:0051028
GO:0006144 GO:0048309 GO:0005515 GO:0004017 GO:0003676
GO:0003700 GO:0046872 GO:0008270 GO:0043565 GO:0005667
Cluster-22178.77382 GO:0005515
Cluster-22178.77385 GO:0006811 GO:0006810 GO:0051726 GO:0017025
GO:0005230 GO:0016020 GO:0005634
Cluster-22178.77387 GO:0015979 GO:0005515 GO:0009539 GO:0009523
GO:0016020
Cluster-22178.77388 GO:0006118 GO:0008152 GO:0009234 GO:0005515
GO:0005198 GO:0050662 GO:0003954 GO:0003824 GO:0016491
GO:0005739 GO:0019028
Cluster-22178.77390 GO:0006506 GO:0017176 GO:0016021
Cluster-22178.77392 GO:0051424 GO:0005515
Cluster-22178.77394 GO:0006355 GO:0003690 GO:0005739
Cluster-22178.77396 GO:0006468 GO:0046039 GO:0009190 GO:0006182
GO:0035556 GO:0006144 GO:0016849 GO:0004383 GO:0004672
Cluster-22178.77397 GO:0004045 GO:0003677 GO:0016301 GO:0016772

Cluster-22178.77403 GO:0043093 GO:0006355 GO:0006281 GO:0000917
GO:0007186 GO:0006950 GO:0000077 GO:0005509 GO:0043565
GO:0004930 GO:0003700 GO:0005667 GO:0030896 GO:0005737
Cluster-22178.77405 GO:0007165
Cluster-22178.77406 GO:0006281 GO:0006355 GO:0006310 GO:0005524
GO:0016887 GO:0003724 GO:0003678 GO:0009378 GO:0003723
GO:0008134 GO:0005657 GO:0009379 GO:0005667
Cluster-22178.77409 GO:0006508 GO:0055114 GO:0006510 GO:0004252
GO:0016491 GO:0016829 GO:0004176
Cluster-22178.77411 GO:0008152 GO:0015031 GO:0006508 GO:0008236
GO:0016787
Cluster-22178.77412 GO:0048280 GO:0016042 GO:0006508 GO:0006886
GO:0046486 GO:0004806 GO:0016787 GO:0008236 GO:0005737
GO:0000139

Cluster-22178.77416 GO:0005515
Cluster-22178.77417 GO:0003723
Cluster-22178.77418 GO:0006807 GO:0003824 GO:0046914 GO:0003723

Cluster-22178.77422 GO:0019079 GO:0006260 GO:0016787 GO:0003677
GO:0005524 GO:0003678 GO:0003676 GO:0005657
Cluster-22178.77423 GO:0006511 GO:0005515
Cluster-22178.77424 GO:0006511 GO:0005515
Cluster-22178.77425 GO:0016579 GO:0036459
Cluster-22178.77426 GO:0048870 GO:0019904 GO:0031514
Cluster-22178.77428 GO:0005515
Cluster-22178.77431 GO:0006464 GO:0008270 GO:0046872 GO:0003677
GO:0016151 GO:0005515
Cluster-22178.77432 GO:0006814 GO:0007186 GO:0046849 GO:0015992
GO:0055114 GO:0006744 GO:0055085 GO:0006120 GO:0008137
GO:0005515 GO:0004930 GO:0005576 GO:0016021
Cluster-22178.77433 GO:0005515
Cluster-22178.77434 GO:0007275 GO:0005506 GO:0005578
Cluster-22178.77435 GO:0006606 GO:0006508 GO:0061630 GO:0005515
GO:0008233 GO:0046872 GO:0008270
Cluster-22178.77436 GO:0005515 GO:0008270
Cluster-22178.77437 GO:0005524
Cluster-22178.77442 GO:0006457 GO:0051259 GO:0007034 GO:0051087
GO:0042803 GO:0000774 GO:0047869
Cluster-22178.77445 GO:0007186 GO:0042710 GO:0004930 GO:0016021
Cluster-22178.77447 GO:0006090 GO:0006418 GO:0006097 GO:0006099
GO:0005524 GO:0000166 GO:0004474 GO:0004812
Cluster-22178.77449 GO:0005515
Cluster-22178.77453 GO:0055114 GO:0004174 GO:0046332
Cluster-22178.77455 GO:0005515
Cluster-22178.77456 GO:0006281 GO:0006464 GO:0016151 GO:0003677
GO:0008270 GO:0046872
Cluster-22178.77457 GO:0006281 GO:0006464 GO:0008270 GO:0046872

GO:0016151 GO:0003677
Cluster-22178.77462 GO:0005515
Cluster-22178.77465 GO:0008152 GO:0016624
Cluster-22178.77469 GO:0004190 GO:0016021
Cluster-22178.77470 GO:0006278 GO:0003964 GO:0004190 GO:0016021
Cluster-22178.77471 GO:0007186 GO:0007165 GO:0004871 GO:0005834
Cluster-22178.77473 GO:0006486 GO:0008378 GO:0016757 GO:0016020
Cluster-22178.77474 GO:0006486 GO:0016757 GO:0008378 GO:0016020
Cluster-22178.77480 GO:0006554 GO:0007224 GO:0006479 GO:0018024
GO:0005515 GO:0016021
Cluster-22178.77481 GO:0016020
Cluster-22178.77483 GO:0055114 GO:0006810 GO:0016491 GO:0050660
GO:0005515 GO:0003824 GO:0016020
Cluster-22178.77484 GO:0006810 GO:0055114 GO:0005515 GO:0003824
GO:0050660 GO:0016491 GO:0016020
Cluster-22178.77486 GO:0061630 GO:0005515 GO:0008270
Cluster-22178.77487 GO:0006470 GO:0004721 GO:0003824 GO:0030145
GO:0000287
Cluster-22178.77489 GO:0016579 GO:0008270 GO:0036459
Cluster-22178.77491 GO:0035556 GO:0007165 GO:0005524 GO:0003774
GO:0005515 GO:0016459
Cluster-22178.77492 GO:0006508 GO:0055085 GO:0022857 GO:0016805
GO:0016021
Cluster-22178.77493 GO:0016567 GO:0008270 GO:0046872 GO:0061630
GO:0005515 GO:0004842 GO:0005622
Cluster-22178.77498 GO:0006123 GO:0015992 GO:0016567 GO:0007186
GO:0004842 GO:0004930 GO:0004129 GO:0008270 GO:0046872
GO:0005515 GO:0045277 GO:0005680 GO:0016021
Cluster-22178.77500 GO:0005509 GO:0019012
Cluster-22178.77501 GO:0006400 GO:0008193 GO:0000287
Cluster-22178.77502 GO:0006400 GO:0000287 GO:0008193
Cluster-22178.77503 GO:0006400 GO:0000287 GO:0008193
Cluster-22178.77506 GO:0007165 GO:0005179 GO:0005576

Cluster-22178.77513 GO:0007186 GO:0007165 GO:0006355 GO:0006807
GO:0009399 GO:0055114 GO:0019337 GO:0005515 GO:0003677
GO:0016163 GO:0004872 GO:0004930 GO:0016610 GO:0016020
GO:0016021
Cluster-22178.77515 GO:0006412 GO:0006118 GO:0006694 GO:0006522
GO:0006529 GO:0006531 GO:0042254 GO:0006457 GO:0009058
GO:0017004 GO:0008299 GO:0051082 GO:0016779 GO:0045158
GO:0050518 GO:0004066 GO:0003735 GO:0005840 GO:0016021
GO:0005622 GO:0016272 GO:0009512
Cluster-22178.77516 GO:0046872 GO:0008270 GO:0046983
Cluster-22178.77517 GO:0016462 GO:0005737
Cluster-22178.77518 GO:0016462 GO:0005737
Cluster-22178.77523 GO:0006073 GO:0015979 GO:0042549 GO:0016762
GO:0005515 GO:0048046 GO:0005618 GO:0009523 GO:0009539
Cluster-22178.77526 GO:0006919 GO:0006915 GO:0006508 GO:0004190
GO:0005739
Cluster-22178.77527 GO:0006508 GO:0006919 GO:0006915 GO:0004190
GO:0005739
Cluster-22178.77528 GO:0006915 GO:0006919 GO:0005739
Cluster-22178.77533 GO:0007165 GO:0005515 GO:0004872 GO:0016021
Cluster-22178.77539 GO:0006810 GO:0009405 GO:0006310 GO:0006051
GO:0006040 GO:0003677 GO:0005515 GO:0047465 GO:0008200
GO:0019905 GO:0005576 GO:0005886
Cluster-22178.77540 GO:0045454
Cluster-22178.77541 GO:0007165 GO:0006270
Cluster-22178.77544 GO:0048870 GO:0051382 GO:0006355 GO:0010508
GO:0000917 GO:0009405 GO:0055085 GO:0043093 GO:0006810
GO:0003700 GO:0045502 GO:0019871 GO:0008134 GO:0042803
GO:0005737 GO:0016021 GO:0005576 GO:0000776 GO:0031514
GO:0030286 GO:0005667
Cluster-22178.7755 GO:0007186 GO:0004930 GO:0016021
Cluster-22178.77555 GO:0016579 GO:0036459
Cluster-22178.77556 GO:0005524 GO:0004867 GO:0005576

Cluster-22178.77560 GO:0009405 GO:0005576
Cluster-22178.77561 GO:0009405 GO:0005576
Cluster-22178.77562 GO:0005515
Cluster-22178.77564 GO:0015031 GO:0016021
Cluster-22178.77565 GO:0007165 GO:0006812 GO:0000166 GO:0019829
GO:0046872 GO:0005158 GO:0016021 GO:0005899
Cluster-22178.77566 GO:0015986 GO:0006355 GO:0006810 GO:0015992
GO:0007049 GO:0006260 GO:0003677 GO:0043565 GO:0003700
GO:0015078 GO:0005667 GO:0005634 GO:0045263
Cluster-22178.77571 GO:0006811 GO:0006813 GO:0005515 GO:0005249
GO:0016020 GO:0008076
Cluster-22178.77572 GO:0005515
Cluster-22178.77573 GO:0016747
Cluster-22178.77574 GO:0006355 GO:0003700 GO:0005667
Cluster-22178.77576 GO:0006118 GO:0009055 GO:0008270 GO:0008237
GO:0051536 GO:0001533
Cluster-22178.77578 GO:0009271 GO:0006355 GO:0008270 GO:0000228
Cluster-22178.77579 GO:0016032 GO:0006508 GO:0042967 GO:0006555
GO:0019281 GO:0005975 GO:0008233 GO:0004222 GO:0008899
GO:0004649 GO:0008270 GO:0005737 GO:0031012
Cluster-22178.77583 GO:0003676
Cluster-22178.77585 GO:0003676 GO:0003723
Cluster-22178.77589 GO:0016310 GO:0009069 GO:0006468 GO:0005524
GO:0004672 GO:0016773 GO:0004674 GO:0016020
Cluster-22178.77590 GO:0006897 GO:0007342 GO:0060478 GO:0005886
Cluster-22178.77591 GO:0003677
Cluster-22178.77593 GO:0003676
Cluster-22178.77595 GO:0032007 GO:0030682 GO:0048011 GO:0043176

Cluster-22178.77597 GO:0045905 GO:0006452 GO:0045901 GO:0006448
GO:0003723 GO:0043022 GO:0003746 GO:0005840
Cluster-22178.77598 GO:0042981 GO:0007165 GO:0005515 GO:0003677

Cluster-22178.77599 GO:0006468 GO:0005524 GO:0004672 GO:0016773
GO:0016020
Cluster-22178.77608 GO:0005975 GO:0006351 GO:0003676 GO:0004553
GO:0033925 GO:0008270 GO:0005515 GO:0005737
Cluster-22178.77611 GO:0006898 GO:0007165 GO:0005044 GO:0016020
Cluster-22178.77612 GO:0016740 GO:0008270
Cluster-22178.77613 GO:0006118 GO:0006119 GO:0042254 GO:0006412
GO:0015992 GO:0008121 GO:0003735 GO:0030246 GO:0009055
GO:0005840 GO:0005622
Cluster-22178.77614 GO:0006118 GO:0006119 GO:0042254 GO:0006412
GO:0015992 GO:0003735 GO:0008121 GO:0009055 GO:0005622
GO:0005840
Cluster-22178.77617 GO:0007218
Cluster-22178.77618 GO:0005525 GO:0005515 GO:0003924
Cluster-22178.77619 GO:0006468 GO:0009069 GO:0016310 GO:0046872
GO:0005524 GO:0004674 GO:0005515
Cluster-22178.77621 GO:0055114 GO:0015994 GO:0006788 GO:0004392

Cluster-22178.77623 GO:0007165 GO:0005044 GO:0005515 GO:0016020
Cluster-22178.77624 GO:0019068 GO:0030212 GO:0005515 GO:0004867
GO:0003676 GO:0008233 GO:0008168 GO:0008047
Cluster-22178.77632 GO:0006471 GO:0006468 GO:0003950 GO:0005515
GO:0005524 GO:0004672 GO:0004715
Cluster-22178.77633 GO:0006813 GO:0019354 GO:0046487 GO:0055114
GO:0009396 GO:0006730 GO:0006520 GO:0006555 GO:0006779
GO:0051287 GO:0004013 GO:0043115 GO:0004488 GO:0016491
GO:0003824
Cluster-22178.77634 GO:0000910 GO:0000226 GO:0008017 GO:0045298
Cluster-22178.77636 GO:0043401 GO:0009405 GO:0006355 GO:0005198
GO:0005515 GO:0016021
Cluster-22178.77637 GO:0007165 GO:0043401 GO:0006355 GO:0003700
GO:0005496 GO:0043565 GO:0008270 GO:0030284 GO:0003677
GO:0005667 GO:0005634

Cluster-22178.77638 GO:0043401 GO:0006355 GO:0003700 GO:0043565
GO:0008270 GO:0005543 GO:0005667 GO:0005634
Cluster-22178.77643 GO:0005515
Cluster-22178.77644 GO:0006468 GO:0004672 GO:0005524 GO:0016773
GO:0016020
Cluster-22178.77646 GO:0005515
Cluster-22178.7765 GO:0005515 GO:0005509
Cluster-22178.77651 GO:0006355 GO:0005634
Cluster-22178.77652 GO:0007155 GO:0051536 GO:0005509 GO:0005515
GO:0003824 GO:0005576 GO:0016020
Cluster-22178.77653 GO:0007339 GO:0055114 GO:0005515 GO:0051920
GO:0005576
Cluster-22178.77654 GO:0032065 GO:0005515 GO:0005543 GO:0016817
GO:0005938
Cluster-22178.77655 GO:0006814 GO:0042773 GO:0055114 GO:0015992
GO:0055085 GO:0006744 GO:0006812 GO:0006120 GO:0008152
GO:0008137 GO:0008324 GO:0008168 GO:0016021
Cluster-22178.77657 GO:0019064 GO:0000413 GO:0007165 GO:0006457
GO:0016788 GO:0003755 GO:0046789 GO:0009986 GO:0019031
Cluster-22178.77659 GO:0012511 GO:0016021 GO:0016020
Cluster-22178.77661 GO:0006886 GO:0016192 GO:0005515 GO:0005198
GO:0004867 GO:0030117 GO:0005576
Cluster-22178.77663 GO:0005515
Cluster-22178.77667 GO:0006935 GO:0006308 GO:0042157 GO:0006869
GO:0071973 GO:0003774 GO:0008855 GO:0008289 GO:0009288
GO:0005576 GO:0009318 GO:0016020
Cluster-22178.77670 GO:0016310 GO:0007178 GO:0019087 GO:0006468
GO:0009069 GO:0005026 GO:0005524 GO:0046872 GO:0003677
GO:0016021 GO:0016020
Cluster-22178.77677 GO:0006396 GO:0006457 GO:0007034 GO:0051087
GO:0042803 GO:0000774 GO:0047869
Cluster-22178.77678 GO:0016020
Cluster-22178.77682 GO:0007165 GO:0023052 GO:0005125

Cluster-22178.77684 GO:0006313 GO:0006508 GO:0004252

Cluster-22178.77685 GO:0043087 GO:0035023 GO:0005089 GO:0005887

Cluster-22178.77687 GO:0006355 GO:0008643 GO:0003677 GO:0003682
GO:0008982 GO:0008270 GO:0005634 GO:0009357 GO:0019028
GO:0000775 GO:0000785

Cluster-22178.77688 GO:0006508 GO:0016579 GO:0006511 GO:0004843
GO:0070008 GO:0004252 GO:0005622

Cluster-22178.77689 GO:0016579 GO:0006511 GO:0006508 GO:0070008
GO:0004252 GO:0004843 GO:0005622

Cluster-22178.77692 GO:0003676 GO:0003677 GO:0016787 GO:0005524

Cluster-22178.77693 GO:0050830 GO:0051016 GO:0005509 GO:0030479

Cluster-22178.77697 GO:0005509

Cluster-22178.77698 GO:0005515

Cluster-22178.77700 GO:0005515

Cluster-22178.77702 GO:0006144 GO:0006807 GO:0006396 GO:0007186
GO:0004930 GO:0003723 GO:0004000 GO:0016020

Cluster-22178.77706 GO:0016020

Cluster-22178.77707 GO:0016787

Cluster-22178.77708 GO:0055114 GO:0008152 GO:0016491

Cluster-22178.77710 GO:0051260

Cluster-22178.77711 GO:0051260

Cluster-22178.77712 GO:0006950 GO:0009415 GO:0006890 GO:0016790
GO:0004252 GO:0001772 GO:0097197 GO:0016021

Cluster-22178.77714 GO:0016567 GO:0005515 GO:0003723 GO:0004842
GO:0046872 GO:0008270 GO:0005680 GO:0005887

Cluster-22178.77719 GO:0055114 GO:0050660 GO:0004499 GO:0016491
GO:0050661

Cluster-22178.77723 GO:0055114 GO:0016209 GO:0016491

Cluster-22178.77724 GO:0015031 GO:0000902 GO:0005789 GO:0016021

Cluster-22178.77725 GO:0016032 GO:0007304 GO:0005213 GO:0042600
GO:0005576

Cluster-22178.77727 GO:0006144 GO:0019064 GO:0006260 GO:0043093

GO:0019835 GO:0006355 GO:0006351 GO:0000390 GO:0009405
GO:0000917 GO:0006886 GO:0003677 GO:0003968 GO:0003723
GO:0071008 GO:0016020 GO:0005737 GO:0031379 GO:0019031
GO:0019867 GO:0005801
Cluster-22178.77732 GO:0006298 GO:0009405 GO:0006570 GO:0006355
GO:0006470 GO:0005515 GO:0004725 GO:0005524 GO:0005634
Cluster-22178.77733 GO:0006298 GO:0009405 GO:0006470 GO:0006570
GO:0004725 GO:0005515 GO:0005524
Cluster-22178.77737 GO:0006952 GO:0007165 GO:0005576
Cluster-22178.77739 GO:0003677
Cluster-22178.77742 GO:0046983
Cluster-22178.77743 GO:0006508 GO:0004222
Cluster-22178.77744 GO:0006508 GO:0004222
Cluster-22178.77745 GO:0051539
Cluster-22178.77749 GO:0042254 GO:0035556 GO:0003677 GO:0005515
GO:0003735 GO:0008270 GO:0046872 GO:0005840
Cluster-22178.77751 GO:0055114 GO:0008152 GO:0016491
Cluster-22178.77752 GO:0055114 GO:0008152 GO:0016491
Cluster-22178.77753 GO:0005516 GO:0030430
Cluster-22178.77754 GO:0007160 GO:0005509 GO:0005576
Cluster-22178.77756 GO:0005515
Cluster-22178.77757 GO:0006694 GO:0043093 GO:0000917 GO:0008299
GO:0005524 GO:0046983 GO:0016887 GO:0005525 GO:0050518
GO:0005737
Cluster-22178.77767 GO:0005515
Cluster-22178.77768 GO:0030001 GO:0005515 GO:0000166 GO:0019956
GO:0008270 GO:0046872
Cluster-22178.77769 GO:0034477 GO:0004518
Cluster-22178.77770 GO:0000746 GO:0019867
Cluster-22178.77773 GO:0007165 GO:0007264 GO:0006355 GO:0006771
GO:0019497 GO:0005515 GO:0004871 GO:0003700 GO:0003993
GO:0005525 GO:0003924 GO:0046872 GO:0005667 GO:0005634
Cluster-22178.77774 GO:0005515

Cluster-22178.77776 GO:0000062
Cluster-22178.77777 GO:0042967 GO:0016747 GO:0008080 GO:0005515

Cluster-22178.77778 GO:0030168 GO:0006606 GO:0006566 GO:0007165
GO:0055114 GO:0051258 GO:0006544 GO:0007049 GO:0006563
GO:0046653 GO:0046983 GO:0005102 GO:0003950 GO:0030674
GO:0005506 GO:0008115 GO:0046872 GO:0016705 GO:0020037
GO:0019901 GO:0005577
Cluster-22178.77779 GO:0006144 GO:0004114
Cluster-22178.77781 GO:0006886 GO:0006614 GO:0007165 GO:0006352
GO:0009399 GO:0003677 GO:0005047 GO:0098519 GO:0004888
GO:0005525 GO:0003924 GO:0005785 GO:0016021 GO:0005786
Cluster-22178.77784 GO:0005524
Cluster-22178.77785 GO:0016567 GO:0009271 GO:0006355 GO:0006744
GO:0055114 GO:0005515 GO:0004842 GO:0008270 GO:0046872
Cluster-22178.77787 GO:0006303 GO:0006353 GO:0005525 GO:0003677
GO:0004003 GO:0005657
Cluster-22178.77791 GO:0003676 GO:0008270
Cluster-22178.77804 GO:0055114 GO:0015992 GO:0015986 GO:0016972
GO:0015078 GO:0000276
Cluster-22178.77807 GO:0000166 GO:0046872
Cluster-22178.77811 GO:0005515
Cluster-22178.77812 GO:0007218 GO:0006260 GO:0006749 GO:0006098
GO:0007049 GO:0055114 GO:0006006 GO:0018990 GO:0006355
GO:0008270 GO:0003677 GO:0003700 GO:0008255 GO:0003887
GO:0004345 GO:0050661 GO:0005667 GO:0016021 GO:0042575
GO:0005634 GO:0005576
Cluster-22178.77814 GO:0006260 GO:0007218 GO:0006749 GO:0006098
GO:0055114 GO:0006006 GO:0007049 GO:0006355 GO:0018990
GO:0008270 GO:0003677 GO:0008255 GO:0003700 GO:0004345
GO:0050661 GO:0003887 GO:0005667 GO:0016021 GO:0005634
GO:0005576 GO:0042575
Cluster-22178.77815 GO:0006355 GO:0003677

Cluster-22178.77816 GO:0006260 GO:0005634
Cluster-22178.77819 GO:0009271 GO:0016567 GO:0006355 GO:0004842
GO:0046872 GO:0017176 GO:0008270 GO:0005515 GO:0005680
Cluster-22178.77822 GO:0006468 GO:0005515 GO:0005524 GO:0004672
GO:0016020
Cluster-22178.77827 GO:0055085 GO:0015979 GO:0043565 GO:0016021
GO:0009539 GO:0009523 GO:0016020 GO:0005634
Cluster-22178.77829 GO:0005515
Cluster-22178.77832 GO:0005515 GO:0016788
Cluster-22178.77833 GO:0007010 GO:0003779
Cluster-22178.77835 GO:0007165 GO:0017038 GO:0008270 GO:0005524
GO:0005158 GO:0003676 GO:0016787 GO:0003677 GO:0005899
GO:0005634 GO:0016020
Cluster-22178.77837 GO:0006887 GO:0006891 GO:0006308 GO:0006886
GO:0008855 GO:0005801 GO:0016020 GO:0000145 GO:0009318
GO:0017119
Cluster-22178.77838 GO:0005515 GO:0051424
Cluster-22178.77840 GO:0005975 GO:0006687 GO:0004308
Cluster-22178.77841 GO:0008152 GO:0006479 GO:0006554 GO:0042802
GO:0005515 GO:0018024 GO:0008168
Cluster-22178.77842 GO:0006554 GO:0006355 GO:0008152 GO:0006479
GO:0018024 GO:0008168 GO:0003677
Cluster-22178.77845 GO:0016567 GO:0006265 GO:0003677 GO:0005515
GO:0003916 GO:0008270 GO:0046872 GO:0004842 GO:0005694
GO:0005680
Cluster-22178.77846 GO:0016055 GO:0008013 GO:0046872 GO:0016342
Cluster-22178.77847 GO:0006814 GO:0006508 GO:0045444 GO:0006120
GO:0018144 GO:0006744 GO:0006144 GO:0015992 GO:0005198
GO:0016779 GO:0008137 GO:0000166 GO:0016740 GO:0003968
GO:0008234 GO:0016787 GO:0008233 GO:0019012 GO:0031379
GO:0005739
Cluster-22178.77849 GO:0007017 GO:0006355 GO:0007018 GO:0006412
GO:0006308 GO:0042254 GO:0007165 GO:0006812 GO:0035257

GO:0005515 GO:0043565 GO:0003700 GO:0003777 GO:0003713
GO:0003735 GO:0008855 GO:0016887 GO:0005524 GO:0008017
GO:0005634 GO:0009318 GO:0045298 GO:0016021 GO:0005874
GO:0005840 GO:0005667 GO:0005622
Cluster-22178.77853 GO:0006810 GO:0006811 GO:0005230 GO:0005515
GO:0016020
Cluster-22178.77856 GO:0051205 GO:0007155 GO:0007165 GO:0005173
GO:0016021 GO:0016020
Cluster-22178.77860 GO:0006928 GO:0015992 GO:0007165 GO:0000160
GO:0016310 GO:0055114 GO:0015991 GO:0006355 GO:0016491
GO:0005509 GO:0004673 GO:0000155 GO:0003700 GO:0015078
GO:0005515 GO:0009279 GO:0005667 GO:0016021 GO:0009365
GO:0033179 GO:0005578
Cluster-22178.77862 GO:0015631 GO:0008092 GO:0045298 GO:0019898
GO:0005737
Cluster-22178.77865 GO:0006355 GO:0042967 GO:0035556 GO:0004402
GO:0003712 GO:0008270 GO:0005634 GO:0005667 GO:0000123
Cluster-22178.77868 GO:0006465 GO:0016032 GO:0008233 GO:0016021
GO:0005787
Cluster-22178.77869 GO:0048870 GO:0009405 GO:0000917 GO:0002949
GO:0006810 GO:0006171 GO:0043093 GO:0003678 GO:0046983
GO:0016887 GO:0005524 GO:0003677 GO:0008294 GO:0016787
GO:0005737 GO:0031514 GO:0005576 GO:0005657
Cluster-22178.77870 GO:0004672
Cluster-22178.77871 GO:0042981
Cluster-22178.77872 GO:0006355 GO:0042981 GO:0043565 GO:0003700
GO:0005667 GO:0005634
Cluster-22178.77873 GO:0030639 GO:0007165 GO:0007268 GO:0030550
GO:0005576
Cluster-22178.77874 GO:0007165 GO:0007186 GO:0055114 GO:0048193
GO:0050661 GO:0031683 GO:0019001 GO:0016491 GO:0003924
GO:0005525 GO:0004871 GO:0005783
Cluster-22178.77875 GO:0006886 GO:0006355 GO:0003712 GO:0030176

GO:0005667
Cluster-22178.77876 GO:0006576 GO:0015889 GO:0006650 GO:0015684
GO:0031419 GO:0015093 GO:0005524 GO:0003924 GO:0016887
GO:0047396 GO:0005525 GO:0016021
Cluster-22178.77880 GO:0015992 GO:0006123 GO:0016032 GO:0000902
GO:0006955 GO:0004129 GO:0005515 GO:0009424 GO:0005751
GO:0005743 GO:0045277 GO:0016020
Cluster-22178.77881 GO:0000902 GO:0006955 GO:0016032 GO:0006123
GO:0015992 GO:0005515 GO:0004129 GO:0016020 GO:0005751
GO:0005743 GO:0045277 GO:0009424
Cluster-22178.77886 GO:0006614 GO:0008152 GO:0005525 GO:0016301
GO:0005524
Cluster-22178.77891 GO:0005515
Cluster-22178.77892 GO:0005515
Cluster-22178.77893 GO:0009403 GO:0016021 GO:0016020
Cluster-22178.77897 GO:0019217 GO:0006508 GO:0000062
Cluster-22178.77899 GO:0006144 GO:0006206 GO:0006383 GO:0006468
GO:0006481 GO:0006351 GO:0006479 GO:0004672 GO:0005524
GO:0004671 GO:0003899 GO:0003677 GO:0016021 GO:0005730
GO:0005666
Cluster-22178.77904 GO:0006144 GO:0045765 GO:0006508 GO:0045947
GO:0003968 GO:0008234 GO:0008190 GO:0005737 GO:0031379
Cluster-22178.77906 GO:0043087 GO:0005085 GO:0005515
Cluster-22178.77907 GO:0006464 GO:0015031 GO:0007186 GO:0005515
GO:0004930 GO:0016021
Cluster-22178.77909 GO:0006777 GO:0008152 GO:0003824
Cluster-22178.77911 GO:0019478 GO:0016788 GO:0005737
Cluster-22178.77912 GO:0010508 GO:0006606
Cluster-22178.77914 GO:0006457 GO:0000413 GO:0003755
Cluster-22178.77915 GO:0007165 GO:0006955 GO:0008283 GO:0040007
GO:0005134 GO:0008083 GO:0005893 GO:0005576
Cluster-22178.77919 GO:0006355 GO:0006270 GO:0008270 GO:0003682
GO:0003677 GO:0000785 GO:0000775 GO:0005634

Cluster-22178.77924 GO:0008152 GO:0034599 GO:0016042 GO:0005975 GO:0046486 GO:0009058 GO:0006508 GO:0004806 GO:0016788 GO:0008236 GO:0008184 GO:0016787 GO:0005789
Cluster-22178.77925 GO:0042773 GO:0055114 GO:0005515 GO:0016651

Cluster-22178.77928 GO:0006606 GO:0015012 GO:0030418 GO:0006024 GO:0030410 GO:0016021
Cluster-22178.77929 GO:0005515
Cluster-22178.77930 GO:0006468 GO:0005524 GO:0004672
Cluster-22178.77931 GO:0007165 GO:0007186 GO:0007264 GO:0006013 GO:0006000 GO:0003873 GO:0005525 GO:0004871 GO:0019001 GO:0031683 GO:0005524 GO:0005125 GO:0003924
Cluster-22178.77933 GO:0003676 GO:0003723
Cluster-22178.77934 GO:0003723 GO:0003676
Cluster-22178.77935 GO:0006310 GO:0009116 GO:0000150 GO:0003677

Cluster-22178.77941 GO:0009234
Cluster-22178.77942 GO:0043087 GO:0008283 GO:0040007 GO:0007165 GO:0005085 GO:0030674 GO:0051015 GO:0008083 GO:0005515
Cluster-22178.77946 GO:0031175 GO:0006265 GO:0008270 GO:0003916 GO:0046872 GO:0004222 GO:0008233 GO:0005516 GO:0030507 GO:0003677 GO:0005515 GO:0031012 GO:0008091 GO:0005694
Cluster-22178.77947 GO:0006355 GO:0035023 GO:0035556 GO:0055114 GO:0043087 GO:0005515 GO:0003677 GO:0005089 GO:0003700 GO:0047134 GO:0005667
Cluster-22178.77948 GO:0006810 GO:0019079 GO:0003676 GO:0004386 GO:0008026 GO:0016887 GO:0005524
Cluster-22178.77949 GO:0035091
Cluster-22178.77951 GO:0016853 GO:0005515
Cluster-22178.77955 GO:0016042 GO:0008152 GO:0007586 GO:0008047 GO:0016746 GO:0005515 GO:0008236 GO:0005509 GO:0005576
Cluster-22178.77956 GO:0007165 GO:0007218 GO:0030141
Cluster-22178.77958 GO:0035023 GO:0007165 GO:0009405 GO:0043087

GO:0005089 GO:0005515

Cluster-22178.77959 GO:0055114 GO:0009395 GO:0007205 GO:0035556 GO:0046486 GO:0006412 GO:0042254 GO:0047134 GO:0004143 GO:0005515 GO:0003735 GO:0016301 GO:0005840

Cluster-22178.77971 GO:0016579 GO:0006355 GO:0003700 GO:0036459 GO:0042025 GO:0005667

Cluster-22178.77973 GO:0042312 GO:0008217 GO:0006397 GO:0007165 GO:0005524 GO:0003774 GO:0005179 GO:0005515 GO:0016459 GO:0005576

Cluster-22178.77974 GO:0007264 GO:0005525

Cluster-22178.77975 GO:0000160 GO:0007165 GO:0046872 GO:0004871

Cluster-22178.77976 GO:0007165 GO:0000160 GO:0004871 GO:0046872

Cluster-22178.77978 GO:0042254 GO:0009069 GO:0016310 GO:0006412 GO:0006468 GO:0004674 GO:0005524 GO:0016773 GO:0035091 GO:0003735 GO:0004672 GO:0005198 GO:0005622 GO:0016020 GO:0005840 GO:0019028

Cluster-22178.77981 GO:0005634

Cluster-22178.77982 GO:0019482 GO:0006270 GO:0006633 GO:0050080 GO:0016021

Cluster-22178.77984 GO:0042773 GO:0006744 GO:0006814 GO:0006120 GO:0015992 GO:0008137 GO:0005743

Cluster-22178.77985 GO:0006412 GO:0042254 GO:0003735 GO:0005622 GO:0005840

Cluster-22178.77986 GO:0055114 GO:0005515 GO:0016491

Cluster-22178.77987 GO:0006355 GO:0003677

Cluster-22178.77990 GO:0009231 GO:0006260 GO:0005524 GO:0008686 GO:0016887 GO:0003678 GO:0033588 GO:0005657

Cluster-22178.77991 GO:0006355 GO:0005634

Cluster-22178.77994 GO:0003676

Cluster-22178.77998 GO:0030833 GO:0006355 GO:0034314 GO:0043565 GO:0003700 GO:0005667 GO:0005885 GO:0005856

Cluster-22178.78003 GO:0005515
Cluster-22178.78009 GO:0006508 GO:0003723 GO:0008234 GO:0005515

Cluster-22178.78010 GO:0006206 GO:0019083 GO:0006144 GO:0006508
GO:0006351 GO:0008234 GO:0003723 GO:0003677 GO:0003899
GO:0005730
Cluster-22178.78012 GO:0032040
Cluster-22178.78014 GO:0005975 GO:0004252 GO:0016773 GO:0016021
Cluster-22178.78016 GO:0019079 GO:0043066 GO:0055085 GO:0043154
GO:0005840 GO:0016021 GO:0005737 GO:0071986
Cluster-22178.78018 GO:0042981 GO:0008092 GO:0019898 GO:0005737
Cluster-22178.78019 GO:0008152 GO:0005198 GO:0016787 GO:0019028
Cluster-22178.78020 GO:0035556 GO:0003676 GO:0005507 GO:0005524
GO:0003677 GO:0005515 GO:0016787 GO:0016021 GO:0016020
Cluster-22178.78021 GO:0035556 GO:0005515
Cluster-22178.78023 GO:0016787
Cluster-22178.78025 GO:0007165 GO:0006144 GO:0005515 GO:0004114
GO:0043395 GO:0016020 GO:0005578
Cluster-22178.78027 GO:0042967 GO:0008080
Cluster-22178.78029 GO:0030168 GO:0031110 GO:0007165 GO:0007067
GO:0000090 GO:0007059 GO:0051258 GO:0048870 GO:0006606
GO:0006914 GO:0006310 GO:0009069 GO:0016310 GO:0051301
GO:0006468 GO:0004674 GO:0005102 GO:0005096 GO:0030674
GO:0008134 GO:0045502 GO:0008017 GO:0003677 GO:0042803
GO:0005198 GO:0030286 GO:0000940 GO:0005882 GO:0031514
GO:0005577 GO:0005876 GO:0005667 GO:0045298
Cluster-22178.78031 GO:0006784 GO:0055114 GO:0005515 GO:0005096
GO:0016627 GO:0016021 GO:0005737
Cluster-22178.78032 GO:0005515
Cluster-22178.78035 GO:0006511 GO:0031625
Cluster-22178.78039 GO:0055085 GO:0016021
Cluster-22178.78045 GO:0006355 GO:0006606 GO:0010506 GO:0019901
GO:0043565 GO:0003700 GO:0005667 GO:0005815 GO:0005615

Cluster-22178.78046 GO:0006508 GO:0008152 GO:0005975 GO:0004252 GO:0008236 GO:0043169 GO:0003824 GO:0070008 GO:0008168 GO:0016020

Cluster-22178.78047 GO:0005515

Cluster-22178.78048 GO:0035556

Cluster-22178.78049 GO:0006352 GO:0006355 GO:0003700 GO:0005669 GO:0005634 GO:0005667

Cluster-22178.78051 GO:0007018 GO:0005515 GO:0005868

Cluster-22178.78052 GO:0005515 GO:0016020

Cluster-22178.78053 GO:0046872

Cluster-22178.78054 GO:0009058 GO:0006520 GO:0016829 GO:0030170

Cluster-22178.78057 GO:0055085 GO:0006118 GO:0006810 GO:0042626 GO:0005525 GO:0009055 GO:0016887 GO:0003924 GO:0016491 GO:0005524 GO:0016301 GO:0005515 GO:0016021 GO:0016020

Cluster-22178.78060 GO:0016021

Cluster-22178.78062 GO:0009116 GO:0003824

Cluster-22178.78065 GO:0042254 GO:0006412 GO:0008289 GO:0003735 GO:0005739 GO:0005840

Cluster-22178.78066 GO:0009405 GO:0006810 GO:0003677 GO:0016779 GO:0019871 GO:0034061 GO:0005576 GO:0005634

Cluster-22178.78067 GO:0006744 GO:0008813 GO:0005737

Cluster-22178.78069 GO:0006744 GO:0008813 GO:0005737

Cluster-22178.78070 GO:0000160 GO:0016310 GO:0046983 GO:0000155 GO:0005509 GO:0016021 GO:0009365

Cluster-22178.78075 GO:0009058 GO:0006629 GO:0016788

Cluster-22178.78079 GO:0008152 GO:0003824

Cluster-22178.78080 GO:0006289 GO:0006355 GO:0000439

Cluster-22178.78082 GO:0007264 GO:0005096

Cluster-22178.78083 GO:0006047

Cluster-22178.78086 GO:0016032 GO:0006891 GO:0046872 GO:0005739 GO:0017119

Cluster-22178.78087 GO:0006891 GO:0016032 GO:0046872 GO:0017119

GO:0005739
Cluster-22178.78089 GO:0005975 GO:0004553
Cluster-22178.78090 GO:0005739 GO:0005747
Cluster-22178.78091 GO:0005739 GO:0005747
Cluster-22178.78092 GO:0005739 GO:0005747
Cluster-22178.78095 GO:0006508 GO:0005634
Cluster-22178.78097 GO:0035556 GO:0005515
Cluster-22178.78099 GO:0005515
Cluster-22178.78100 GO:0071816 GO:0031492 GO:0005634 GO:0000785
Cluster-22178.78101 GO:0009307 GO:0006308 GO:0009036 GO:0000287
GO:0016787 GO:0003677 GO:0009359
Cluster-22178.78103 GO:0046872
Cluster-22178.78104 GO:0035556 GO:0016055 GO:0008013 GO:0016342
Cluster-22178.78105 GO:0016055 GO:0035556 GO:0008013 GO:0016342
Cluster-22178.78106 GO:0005515
Cluster-22178.78107 GO:0016567 GO:0008152 GO:0061630 GO:0005515
GO:0004842 GO:0016491 GO:0008270 GO:0046872 GO:0005680
GO:0005622
Cluster-22178.78108 GO:0016579 GO:0015940 GO:0008897 GO:0005515
GO:0005096 GO:0000287 GO:0036459
Cluster-22178.78109 GO:0006355 GO:0003700 GO:0005515 GO:0005667
GO:0042025
Cluster-22178.78116 GO:0016787 GO:0005524 GO:0005515 GO:0003677
GO:0016021
Cluster-22178.78119 GO:0006552 GO:0006550 GO:0006568 GO:0015940
GO:0055114 GO:0019521 GO:0006574 GO:0006631 GO:0018874
GO:0006098 GO:0006633 GO:0006554 GO:0046168 GO:0003857
GO:0008677 GO:0004616 GO:0016616 GO:0051287 GO:0016491
Cluster-22178.78121 GO:0051536 GO:0003824
Cluster-22178.78125 GO:0006508 GO:0008152 GO:0008270 GO:0016788
GO:0004181 GO:0005739
Cluster-22178.78126 GO:0055114 GO:0006355 GO:0043631 GO:0006568
GO:0004652 GO:0000334 GO:0005506 GO:0016779 GO:0045735

Cluster-22178.78128 GO:0005515 GO:0003774 GO:0005524 GO:0016459
Cluster-22178.78138 GO:0019217 GO:0005515 GO:0000062
Cluster-22178.78143 GO:0044260 GO:0044238 GO:0005634
Cluster-22178.78145 GO:0006418 GO:0000049 GO:0000166 GO:0005524
GO:0004812
Cluster-22178.78147 GO:0016020
Cluster-22178.78150 GO:0007186 GO:0015684 GO:0007165 GO:0007264
GO:0004871 GO:0005525 GO:0003924 GO:0031683 GO:0015093
GO:0019001 GO:0016021
Cluster-22178.78151 GO:0000154 GO:0006396 GO:0016433
Cluster-22178.78159 GO:0030036 GO:0016043 GO:0015743 GO:0007067
GO:0003779 GO:0016740 GO:0017048 GO:0048037 GO:0072686
GO:0042729
Cluster-22178.78163 GO:0042207 GO:0006570 GO:0006559 GO:0055114
GO:0005515 GO:0004411
Cluster-22178.78165 GO:0006355 GO:0003700 GO:0005515 GO:0043565
GO:0016787 GO:0005667
Cluster-22178.78166 GO:0006560 GO:0006525 GO:0006420 GO:0005524
GO:0004814
Cluster-22178.78171 GO:0006812 GO:0034508 GO:0045893 GO:0007275
GO:0006810 GO:0006874 GO:0015672 GO:0006811 GO:0010508
GO:0008270 GO:0005215 GO:0030234 GO:0005515 GO:0005261
GO:0030176 GO:0016020 GO:0000776
Cluster-22178.78174 GO:0005515 GO:0003677 GO:0019028
Cluster-22178.78175 GO:0006355 GO:0005634
Cluster-22178.78180 GO:0007165 GO:0006468 GO:0004872 GO:0005524
GO:0004672 GO:0016021
Cluster-22178.78184 GO:0007172 GO:0007165 GO:0006468 GO:0004871
GO:0004713 GO:0005524 GO:0004672 GO:0044212 GO:0005925
Cluster-22178.78190 GO:0006810 GO:0007275 GO:0006874 GO:0006812
GO:0006914 GO:0034508 GO:0045893 GO:0010508 GO:0015672
GO:0006811 GO:0008270 GO:0005515 GO:0030234 GO:0005261
GO:0005215 GO:0016020 GO:0030176 GO:0000776

Cluster-22178.78195 GO:0055114 GO:0006544 GO:0006563 GO:0006566
GO:0046653 GO:0046983 GO:0046872 GO:0003950 GO:0005506
GO:0008115 GO:0016705 GO:0020037
Cluster-22178.78196 GO:0019079 GO:0006810 GO:0009234 GO:0016787
GO:0000166 GO:0003677 GO:0008026 GO:0005524 GO:0004386
GO:0003676
Cluster-22178.78198 GO:0006470 GO:0006570 GO:0004725 GO:0008138

Cluster-22178.78202 GO:0055114 GO:0004800
Cluster-22178.78204 GO:0031505 GO:0006817 GO:0007186 GO:0004930
GO:0005315 GO:0016020 GO:0016021
Cluster-22178.78209 GO:0007411
Cluster-22178.78212 GO:0019956 GO:0005198 GO:0019031 GO:0016021
GO:0019028
Cluster-22178.78216 GO:0007165 GO:0007186 GO:0007264 GO:0005525
GO:0004871 GO:0019001 GO:0031683 GO:0003924
Cluster-22178.78221 GO:0042138 GO:0006310
Cluster-22178.78227 GO:0006047
Cluster-22178.78229 GO:0009607 GO:0019901 GO:0016020 GO:0016021
Cluster-22178.78234 GO:0005515 GO:0016020
Cluster-22178.78235 GO:0016787 GO:0005524 GO:0003676 GO:0005515
GO:0003677
Cluster-22178.78236 GO:0046872
Cluster-22178.78237 GO:0046872
Cluster-22178.78240 GO:0042254 GO:0006412 GO:0003735 GO:0005840
GO:0005622
Cluster-22178.78241 GO:0006950 GO:0016310 GO:0009415 GO:0009069
GO:0006468 GO:0005524 GO:0004672 GO:0004674
Cluster-22178.78242 GO:0072519 GO:0015994 GO:0055114 GO:0016567
GO:0042167 GO:0004842 GO:0008270 GO:0004074 GO:0003677
GO:0005634
Cluster-22178.78244 GO:0016567 GO:0005515 GO:0004842
Cluster-22178.78250 GO:0050840 GO:0005576

Cluster-22178.78251 GO:0006024 GO:0015012 GO:0016021

Cluster-22178.78253 GO:0005515 GO:0030695

Cluster-22178.78255 GO:0006468 GO:0034227 GO:0046034 GO:0006144 GO:0002098 GO:0000049 GO:0004017 GO:0003677 GO:0016787 GO:0003676 GO:0046872 GO:0008270 GO:0005524 GO:0004672 GO:0005634

Cluster-22178.78257 GO:0006470 GO:0006570 GO:0004725

Cluster-22178.78263 GO:0042742 GO:0009234 GO:0050832 GO:0046872 GO:0008270 GO:0005515 GO:0005622

Cluster-22178.78264 GO:0005515 GO:0016020

Cluster-22178.78265 GO:0007186 GO:0008152 GO:0016747 GO:0005515 GO:0004930 GO:0016021

Cluster-22178.78268 GO:0004252 GO:0016021 GO:0005886

Cluster-22178.78269 GO:0016567 GO:0006810 GO:0005515 GO:0004842 GO:0046872 GO:0008270 GO:0030288 GO:0005680

Cluster-22178.78273 GO:0030683 GO:0040007 GO:0005515 GO:0003676 GO:0008270 GO:0005524 GO:0008092 GO:0019012 GO:0019898 GO:0016021 GO:0005737

Cluster-22178.78274 GO:0005515

Cluster-22178.78275 GO:0040007 GO:0030683 GO:0002143 GO:0005515 GO:0008270 GO:0005524 GO:0008092 GO:0003676 GO:0019012 GO:0005737 GO:0019898

Cluster-22178.78278 GO:0006122 GO:0008242 GO:0005750

Cluster-22178.78284 GO:0006357 GO:0001104 GO:0016592

Cluster-22178.78287 GO:0006771 GO:0019497 GO:0007339 GO:0046872 GO:0003993 GO:0005515 GO:0005576

Cluster-22178.7829 GO:0007165 GO:0043401 GO:0006355 GO:0030284 GO:0005515 GO:0003677 GO:0005496 GO:0005634

Cluster-22178.78291 GO:0016021

Cluster-22178.78300 GO:0002949 GO:0006260 GO:0008152 GO:0006388 GO:0003724 GO:0005525 GO:0003924 GO:0016887 GO:0005524 GO:0016301 GO:0003677 GO:0003972 GO:0003723 GO:0004003 GO:0005657

Cluster-22178.78301 GO:0046872
Cluster-22178.78304 GO:0006629 GO:0009395 GO:0007165 GO:0006914
GO:0046339 GO:0006606 GO:0035556 GO:0045502 GO:0005515
GO:0008134 GO:0042803 GO:0004435 GO:0008081 GO:0005509
GO:0019028 GO:0005667 GO:0030286
Cluster-22178.78305 GO:0051258 GO:0048870 GO:0009395 GO:0006629
GO:0006355 GO:0046339 GO:0030168 GO:0006914 GO:0006357
GO:0035556 GO:0006606 GO:0007165 GO:0043565 GO:0008081
GO:0003700 GO:0004435 GO:0045502 GO:0008134 GO:0042803
GO:0005515 GO:0001104 GO:0005102 GO:0030674 GO:0005509
GO:0019028 GO:0031514 GO:0030286 GO:0005577 GO:0005667
GO:0016592
Cluster-22178.78306 GO:0003723
Cluster-22178.78307 GO:0006914 GO:0046339 GO:0006606 GO:0035556
GO:0007165 GO:0009395 GO:0006629 GO:0005509 GO:0004435
GO:0008081 GO:0045502 GO:0008134 GO:0042803 GO:0005515
GO:0030286 GO:0005667 GO:0019028
Cluster-22178.78309 GO:0008152 GO:0009878 GO:0034508 GO:0005525
GO:0008168 GO:0046872 GO:0016787 GO:0000776
Cluster-22178.78310 GO:0051775 GO:0006810 GO:0009405 GO:0045892
GO:0035556 GO:0008200 GO:0003677 GO:0005515 GO:0005576
GO:0005634 GO:0005737
Cluster-22178.78311 GO:0055114 GO:0045454 GO:0016972
Cluster-22178.78312 GO:0006665 GO:0006687 GO:0005975 GO:0006807
GO:0004348 GO:0016021
Cluster-22178.78313 GO:0006665 GO:0005975 GO:0006687 GO:0006807
GO:0004348 GO:0016021
Cluster-22178.78314 GO:0006665 GO:0006687 GO:0005975 GO:0006807
GO:0004348 GO:0016021
Cluster-22178.78315 GO:0006807 GO:0005975 GO:0006687 GO:0006665
GO:0004348 GO:0016021
Cluster-22178.78319 GO:0005515
Cluster-22178.78323 GO:0006511 GO:0031625

Cluster-22178.78324 GO:0006468 GO:0055085 GO:0006821 GO:0004672
GO:0046983 GO:0005247 GO:0016020
Cluster-22178.78326 GO:0006355 GO:0006313 GO:0004803 GO:0003700
GO:0003677 GO:0005667 GO:0005622
Cluster-22178.78330 GO:0042207 GO:0006570 GO:0006810 GO:0009072
GO:0015743 GO:0004334 GO:0005886
Cluster-22178.78331 GO:0009069 GO:0000184 GO:0006313 GO:0000087
GO:0006570 GO:0006470 GO:0016310 GO:0008144 GO:0004803
GO:0016773 GO:0004674 GO:0005515 GO:0005198 GO:0008168
GO:0004725 GO:0005622 GO:0019028 GO:0019031
Cluster-22178.78333 GO:0006418 GO:0005524 GO:0004812 GO:0003676
GO:0000166
Cluster-22178.78334 GO:0019684 GO:0016567 GO:0006118 GO:0008270
GO:0046872 GO:0004842 GO:0045156 GO:0005515 GO:0016021
GO:0005680 GO:0030077
Cluster-22178.78339 GO:0016310 GO:0007178 GO:0006468 GO:0009069
GO:0005026 GO:0046872 GO:0003774 GO:0005524 GO:0005515
GO:0016020 GO:0016459
Cluster-22178.78340 GO:0019882 GO:0007165 GO:0006886 GO:0006955
GO:0042289 GO:0042613 GO:0016020
Cluster-22178.78343 GO:0005975 GO:0005515 GO:0004553
Cluster-22178.78349 GO:0006402 GO:0051252 GO:0045454 GO:0046872
GO:0004535 GO:0005515 GO:0004064 GO:0003723 GO:0005737
GO:0005634
Cluster-22178.78351 GO:0032780 GO:0042030 GO:0005543 GO:0005739
Cluster-22178.78357 GO:0006144 GO:0042254 GO:0055114 GO:0006508
GO:0006412 GO:0016491 GO:0050660 GO:0071949 GO:0004197
GO:0017111 GO:0016614 GO:0003735 GO:0003968 GO:0004252
GO:0016020 GO:0031379 GO:0005840
Cluster-22178.78359 GO:0007165 GO:0006144 GO:0005515 GO:0004114

Cluster-22178.78361 GO:0005515
Cluster-22178.78362 GO:0008152 GO:0016491

Cluster-22178.78363 GO:0030244 GO:0005985 GO:0005982 GO:0016567
GO:0006011 GO:0004842 GO:0046872 GO:0008270 GO:0005515
GO:0016760 GO:0005680 GO:0016020
Cluster-22178.78369 GO:0046872 GO:0008289 GO:0005515
Cluster-22178.78374 GO:0006506 GO:0000045 GO:0033617 GO:0016758
GO:0016021 GO:0005737
Cluster-22178.78380 GO:0007009
Cluster-22178.78383 GO:0005975 GO:0031175 GO:0015979 GO:0005982
GO:0005985 GO:0008017 GO:0030507 GO:0005515 GO:0005516
GO:0004134 GO:0008091 GO:0045298 GO:0009539 GO:0009523
GO:0016020
Cluster-22178.78384 GO:0000746 GO:0019058 GO:0005975 GO:0006260
GO:0003677 GO:0003887 GO:0004553 GO:0042575 GO:0019867
Cluster-22178.78388 GO:0005975 GO:0004252 GO:0016773 GO:0016021
Cluster-22178.78392 GO:0016567 GO:0003676 GO:0005515 GO:0004842
GO:0008270 GO:0046872 GO:0005634
Cluster-22178.78394 GO:0016567 GO:0046872 GO:0008270 GO:0003676
GO:0005515 GO:0004842 GO:0005634
Cluster-22178.78397 GO:0005515 GO:0008270
Cluster-22178.78398 GO:0007264 GO:0015684 GO:0015093 GO:0005525
GO:0019028 GO:0016021
Cluster-22178.78399 GO:0006355 GO:0003700 GO:0005667 GO:0042025
Cluster-22178.78405 GO:0042330
Cluster-22178.78408 GO:0005515
Cluster-22178.78409 GO:0005515
Cluster-22178.78413 GO:0006954 GO:0007165 GO:0006812 GO:0006836
GO:0005328 GO:0005125 GO:0005576 GO:0016021
Cluster-22178.78420 GO:0035556
Cluster-22178.78421 GO:0008152 GO:0046413 GO:0018836 GO:0005515
GO:0016491
Cluster-22178.78424 GO:0009395 GO:0015684 GO:0004620 GO:0015093
GO:0005525 GO:0016021
Cluster-22178.78425 GO:0015991 GO:0046677 GO:0015992 GO:0005515

GO:0016779 GO:0015078 GO:0016020 GO:0033179
Cluster-22178.78427 GO:0006468 GO:0004672 GO:0005524
Cluster-22178.78429 GO:0006486 GO:0007016 GO:0016757 GO:0008378
GO:0016010 GO:0016020
Cluster-22178.78432 GO:0031124
Cluster-22178.78438 GO:0016021
Cluster-22178.78441 GO:0006139 GO:0003676 GO:0005524 GO:0003677
GO:0016818 GO:0008026 GO:0016787 GO:0004003 GO:0005657
GO:0005634
Cluster-22178.78444 GO:0006355 GO:0055114 GO:0006914 GO:0005515
GO:0016491 GO:0043565 GO:0003700 GO:0005667
Cluster-22178.78450 GO:0006479 GO:0006355 GO:0006629 GO:0055114
GO:0007165 GO:0034968 GO:0006554 GO:0004252 GO:0016641
GO:0018024 GO:0005507 GO:0003677 GO:0005044 GO:0070008
GO:0016020 GO:0005763 GO:0005694
Cluster-22178.78456 GO:0007606 GO:0007165 GO:0004888 GO:0016021
Cluster-22178.78457 GO:0005515 GO:0000062 GO:0016021
Cluster-22178.78461 GO:0006352 GO:0005634
Cluster-22178.78463 GO:0042254 GO:0006412 GO:0003723 GO:0003735
GO:0005840
Cluster-22178.78464 GO:0005515
Cluster-22178.78470 GO:0016579 GO:0043248 GO:0036459 GO:0016021
Cluster-22178.78474 GO:0006508 GO:0009847 GO:0000166 GO:0003676
GO:0008233
Cluster-22178.78476 GO:0009847 GO:0006508 GO:0008233 GO:0003676

Cluster-22178.78480 GO:0055114 GO:0008152 GO:0006118 GO:0016627
GO:0050660 GO:0003995
Cluster-22178.78481 GO:0006468 GO:0006810 GO:0015696 GO:0016787
GO:0008519 GO:0004672 GO:0005524 GO:0016020 GO:0031224
GO:0016021 GO:0005794
Cluster-22178.78482 GO:0015696 GO:0006468 GO:0006810 GO:0005524
GO:0004672 GO:0008519 GO:0016787 GO:0005794 GO:0016021

GO:0016020
Cluster-22178.78484 GO:0055070 GO:0005507
Cluster-22178.78487 GO:0005515 GO:0016012 GO:0016021
Cluster-22178.78490 GO:0006281 GO:0006310 GO:0005524 GO:0009378
GO:0005515 GO:0009379 GO:0005657
Cluster-22178.78491 GO:0008608 GO:0000049 GO:0042729 GO:0005815
GO:0005876
Cluster-22178.78493 GO:0006468 GO:0004672 GO:0005524 GO:0016773
GO:0016020
Cluster-22178.78496 GO:0009405 GO:0006355 GO:0006811 GO:0005515
GO:0005216 GO:0003700 GO:0043565 GO:0005667 GO:0005576
Cluster-22178.78498 GO:0007165 GO:0005515 GO:0005096
Cluster-22178.78504 GO:0006468 GO:0007169 GO:0005524 GO:0004714
GO:0016020
Cluster-22178.78506 GO:0046008 GO:0007165 GO:0005179 GO:0003677
GO:0004518 GO:0005576
Cluster-22178.78509 GO:0005515
Cluster-22178.78512 GO:0033151 GO:0043565 GO:0005515
Cluster-22178.78514 GO:0045765 GO:0005737
Cluster-22178.78519 GO:0016485 GO:0005506 GO:0016021
Cluster-22178.78520 GO:0006811 GO:0006810 GO:0055085 GO:0005215
GO:0016020
Cluster-22178.78521 GO:0006810 GO:0055085 GO:0016020
Cluster-22178.78524 GO:0009056 GO:0008270
Cluster-22178.78526 GO:0006284 GO:0006281 GO:0006289 GO:0003684
GO:0016799 GO:0003906 GO:0008270
Cluster-22178.78527 GO:0032801 GO:0006812 GO:0019076 GO:0005261
GO:0033644
Cluster-22178.78528 GO:0055114 GO:0016491
Cluster-22178.78530 GO:0046660 GO:0015992 GO:0006810 GO:0006397
GO:0015986 GO:0015078 GO:0003676 GO:0008270 GO:0005643
GO:0000276 GO:0005634
Cluster-22178.78531 GO:0006810 GO:0006891 GO:0005643 GO:0030173

Cluster-22178.78532 GO:0006355 GO:0015991 GO:0015031 GO:0015992
GO:0006891 GO:0006260 GO:0015078 GO:0003676 GO:0045502
GO:0008134 GO:0042803 GO:0008565 GO:0046983 GO:0033179
GO:0030286 GO:0005667 GO:0017119
Cluster-22178.78533 GO:0045893 GO:0005515 GO:0005634
Cluster-22178.78536 GO:0008380 GO:0005681
Cluster-22178.78537 GO:0046872 GO:0005634
Cluster-22178.78552 GO:0007165 GO:0001664 GO:0005789
Cluster-22178.78556 GO:0006303 GO:0016021
Cluster-22178.78557 GO:0006508 GO:0043039 GO:0055114 GO:0005515
GO:0005509 GO:0051536 GO:0016876 GO:0046872 GO:0005524
GO:0008270 GO:0008234 GO:0016020
Cluster-22178.78558 GO:0006520 GO:0055114 GO:0043039 GO:0006508
GO:0051536 GO:0008270 GO:0005524 GO:0005509 GO:0003677
GO:0008234 GO:0003676 GO:0016829 GO:0046872 GO:0016876
GO:0004519 GO:0005515 GO:0016020
Cluster-22178.78559 GO:0006508 GO:0008236 GO:0016787
Cluster-22178.78560 GO:0006508 GO:0016787 GO:0008236
Cluster-22178.78564 GO:0005515
Cluster-22178.78565 GO:0006265 GO:0031175 GO:0005516 GO:0005515
GO:0003677 GO:0030507 GO:0046872 GO:0003916 GO:0008270
GO:0008233 GO:0004222 GO:0005694 GO:0031012 GO:0008091
Cluster-22178.78568 GO:0005975 GO:0003676 GO:0008270 GO:0008184
GO:0003723
Cluster-22178.78569 GO:0005975 GO:0003723 GO:0008184 GO:0008270
GO:0003676
Cluster-22178.78570 GO:0006308 GO:0016888
Cluster-22178.78571 GO:0006260 GO:0000398 GO:0006813 GO:0046872
GO:0005249 GO:0008270 GO:0030955 GO:0016021 GO:0005681
GO:0008076 GO:0005634
Cluster-22178.78574 GO:0006412 GO:0046487 GO:0015991 GO:0008152
GO:0055114 GO:0042254 GO:0009396 GO:0015992 GO:0003824
GO:0015078 GO:0003735 GO:0004488 GO:0050662 GO:0016491

GO:0033179 GO:0005840
Cluster-22178.78576 GO:0051603 GO:0004298 GO:0005839
Cluster-22178.78577 GO:0006355 GO:0043401 GO:0055114 GO:0006613
GO:0016209 GO:0043565 GO:0003700 GO:0016491 GO:0008270
GO:0016757 GO:0005634 GO:0016021 GO:0005667 GO:0016020
GO:0005784 GO:0030176
Cluster-22178.78583 GO:0005515
Cluster-22178.78585 GO:0006352 GO:0005634
Cluster-22178.78590 GO:0046872
Cluster-22178.78592 GO:0006260 GO:0003677 GO:0005515 GO:0005664
Cluster-22178.78595 GO:0007165 GO:0007268 GO:0030550 GO:0005576
Cluster-22178.78596 GO:0055114
Cluster-22178.78602 GO:0007155 GO:0005515 GO:0016021
Cluster-22178.78603 GO:0006807 GO:0016810
Cluster-22178.78605 GO:0006508 GO:0030436 GO:0004190 GO:0005515

Cluster-22178.78610 GO:0006355 GO:0003700 GO:0043565 GO:0005667
Cluster-22178.78614 GO:0098542 GO:0006570 GO:0006470 GO:0008138
GO:0004725
Cluster-22178.78615 GO:0016773
Cluster-22178.78617 GO:0009069 GO:0006468 GO:0007178 GO:0016310
GO:0004675 GO:0005524 GO:0004672 GO:0016020
Cluster-22178.78620 GO:0006525 GO:0071436 GO:0006814 GO:0006560
GO:0007165 GO:0006090 GO:0005515 GO:0008948 GO:0015081
GO:0016020
Cluster-22178.78622 GO:0006457 GO:0000287 GO:0008253 GO:0005509
GO:0005737
Cluster-22178.78623 GO:0007165 GO:0006952 GO:0005576
Cluster-22178.78624 GO:0015991 GO:0009755 GO:0006355 GO:0006119
GO:0009405 GO:0015992 GO:0042967 GO:0007165 GO:0007268
GO:0008080 GO:0003700 GO:0030550 GO:0046961 GO:0005576
GO:0005667 GO:0033178
Cluster-22178.78629 GO:0005975 GO:0004649

Cluster-22178.78637 GO:0005515 GO:0016020
Cluster-22178.78640 GO:0046872 GO:0005515 GO:0016020
Cluster-22178.78641 GO:0045454 GO:0016021 GO:0016020
Cluster-22178.78642 GO:0005515 GO:0046872 GO:0016020
Cluster-22178.78645 GO:0008270
Cluster-22178.78646 GO:0007165 GO:0007186 GO:0004871 GO:0005834
Cluster-22178.78648 GO:0006397 GO:0007155 GO:0008134 GO:0042803
GO:0005198 GO:0005515 GO:0045502 GO:0005882 GO:0005667
GO:0000445 GO:0030286 GO:0009288
Cluster-22178.78651 GO:0006355 GO:0007049 GO:0043565 GO:0003700
GO:0003677 GO:0005667 GO:0005634
Cluster-22178.78653 GO:0008152 GO:0016042 GO:0006816 GO:0006810
GO:0008203 GO:0042030 GO:0016787 GO:0005246 GO:0016298
GO:0016020
Cluster-22178.78659 GO:0006355 GO:0009271
Cluster-22178.78661 GO:0006613 GO:0007165 GO:0006525 GO:0018401
GO:0030168 GO:0006560 GO:0006355 GO:0055114 GO:0051258
GO:0004656 GO:0030674 GO:0005102 GO:0008270 GO:0005515
GO:0003723 GO:0051082 GO:0016702 GO:0005783 GO:0005577
GO:0030176 GO:0005784 GO:0005622
Cluster-22178.78662 GO:0007264 GO:0005525
Cluster-22178.78663 GO:0061630 GO:0046872
Cluster-22178.78664 GO:0006241 GO:0006206 GO:0006144 GO:0019079
GO:0006183 GO:0006228 GO:0045454 GO:0006165 GO:0004550
GO:0005524 GO:0008026 GO:0003677 GO:0005515 GO:0016787
Cluster-22178.78666 GO:0003676
Cluster-22178.78670 GO:0006508
Cluster-22178.78671 GO:0006468 GO:0005975 GO:0035556 GO:0005524
GO:0004672 GO:0033691 GO:0003779 GO:0016773
Cluster-22178.78672 GO:0006468 GO:0035556 GO:0005975 GO:0005524
GO:0004672 GO:0016773 GO:0003779 GO:0033691
Cluster-22178.78678 GO:0006508 GO:0004198 GO:0005509 GO:0005622
Cluster-22178.78682 GO:0016192 GO:0007165 GO:0006891 GO:0000921

GO:0030168 GO:0006886 GO:0051258 GO:0030674 GO:0005102
GO:0005198 GO:0005515 GO:0008375 GO:0016020 GO:0017119
GO:0005577 GO:0005940 GO:0019028 GO:0016021 GO:0005801
GO:0019031 GO:0005789
Cluster-22178.78683 GO:0009231 GO:0005985 GO:0005982 GO:0005991
GO:0005993 GO:0008531 GO:0005509 GO:0004555 GO:0008375
GO:0005737 GO:0016020
Cluster-22178.78686 GO:0006886 GO:0006813 GO:0006811 GO:0004252
GO:0005249 GO:0008076 GO:0005783 GO:0016020 GO:0030176
GO:0016021 GO:0005886
Cluster-22178.78693 GO:0009069 GO:0016310 GO:0004674
Cluster-22178.78695 GO:0015991 GO:0060041 GO:0006119 GO:0006886
GO:0006506 GO:0015992 GO:0016579 GO:0016192 GO:0036459
GO:0016820 GO:0005515 GO:0016746 GO:0046961 GO:0016021
GO:0005789 GO:0030117 GO:0000221
Cluster-22178.78698 GO:0004386
Cluster-22178.78701 GO:0019217 GO:0016192 GO:0006886 GO:0000062
GO:0005515 GO:0030117
Cluster-22178.78702 GO:0007165 GO:0042254 GO:0006412 GO:0007016
GO:0003735 GO:0005158 GO:0005200 GO:0000062 GO:0005925
GO:0001726 GO:0005622 GO:0005856 GO:0005899 GO:0005840
Cluster-22178.78704 GO:0005524 GO:0016787 GO:0003677 GO:0003676
GO:0008017 GO:0045298
Cluster-22178.78706 GO:0051259 GO:0005975 GO:0005515 GO:0016773

Cluster-22178.78707 GO:0006355 GO:0003682 GO:0003677 GO:0005634
GO:0000785 GO:0000775
Cluster-22178.78709 GO:0007186 GO:0004930 GO:0016021
Cluster-22178.78711 GO:0006144 GO:0006355 GO:0007165 GO:0004114
GO:0005515
Cluster-22178.78714 GO:0003677 GO:0005634
Cluster-22178.78715 GO:0007186 GO:0042710 GO:0004930 GO:0016021
Cluster-22178.78716 GO:0006508 GO:0055114 GO:0006510 GO:0016491

GO:0004176 GO:0016829 GO:0004252
Cluster-22178.78718 GO:0006468 GO:0005524 GO:0004672
Cluster-22178.78719 GO:0009405 GO:0006468 GO:0005524 GO:0004672
GO:0005576
Cluster-22178.78720 GO:0006355 GO:0006351 GO:0019521 GO:0055114
GO:0008152 GO:0006098 GO:0006144 GO:0006206 GO:0003899
GO:0005515 GO:0046872 GO:0003700 GO:0051287 GO:0016616
GO:0004616 GO:0003677 GO:0008641 GO:0005634 GO:0005667
GO:0005730
Cluster-22178.78724 GO:0006807 GO:0009252 GO:0003676 GO:0008270
GO:0046872 GO:0004356 GO:0004190 GO:0005923 GO:0016021
GO:0005634
Cluster-22178.78726 GO:0006541 GO:0006508 GO:0008236 GO:0016787

Cluster-22178.78727 GO:0006508 GO:0006541 GO:0016787 GO:0008236

Cluster-22178.78728 GO:0005515 GO:0009288
Cluster-22178.78730 GO:0055114 GO:0006520 GO:0008152 GO:0016491
GO:0016746
Cluster-22178.78731 GO:0051252 GO:0006397 GO:0006468 GO:0004540
GO:0008233 GO:0016773 GO:0005524 GO:0004672 GO:0016020
Cluster-22178.78732 GO:0006621 GO:0046923 GO:0005783 GO:0016021
Cluster-22178.78733 GO:0006118 GO:0045454 GO:0009055 GO:0015035

Cluster-22178.78735 GO:0035556 GO:0019087 GO:0055114 GO:0006468
GO:0005524 GO:0016773 GO:0047134 GO:0003712 GO:0003724
GO:0016818 GO:0004672 GO:0005515 GO:0005667 GO:0016020
GO:0016021 GO:0005634
Cluster-22178.78736 GO:0008013 GO:0008144 GO:0016342
Cluster-22178.78738 GO:0003924
Cluster-22178.78740 GO:0006265 GO:0042157 GO:0006869 GO:0008289
GO:0003917 GO:0003677 GO:0005694 GO:0005576
Cluster-22178.78744 GO:0046935 GO:0005944

Cluster-22178.78745 GO:0009405 GO:0006479 GO:0051252 GO:0006355
GO:0040007 GO:0006274 GO:0008033 GO:0007165 GO:0006364
GO:0008283 GO:0034968 GO:0006379 GO:0006554 GO:0008083
GO:0005515 GO:0005198 GO:0018024 GO:0004540 GO:0003723
GO:0008289 GO:0051015 GO:0003677 GO:0005509 GO:0030674
GO:0016787 GO:0005578 GO:0005615 GO:0005737 GO:0030677
GO:0005694 GO:0000172
Cluster-22178.78746 GO:0006139 GO:0016114 GO:0006694 GO:0006355
GO:0006281 GO:0006206 GO:0002949 GO:0009058 GO:0006310
GO:0007275 GO:0019079 GO:0003678 GO:0003723 GO:0016301
GO:0008134 GO:0004161 GO:0016887 GO:0005524 GO:0004127
GO:0003724 GO:0009378 GO:0005657 GO:0005667 GO:0009379
Cluster-22178.78749 GO:0006468 GO:0016773 GO:0004672 GO:0005524
GO:0016020
Cluster-22178.78755 GO:0000921 GO:0005515 GO:0005940 GO:0016021
Cluster-22178.78757 GO:0005515
Cluster-22178.78759 GO:0000062
Cluster-22178.78762 GO:0004190 GO:0016021
Cluster-22178.78764 GO:0006695 GO:0006694 GO:0004631 GO:0005737
Cluster-22178.78765 GO:0003676
Cluster-22178.78776 GO:0005975 GO:0006687 GO:0006665 GO:0006807
GO:0004348 GO:0016021
Cluster-22178.78777 GO:0016032 GO:0030639 GO:0006886 GO:0006486
GO:0006888 GO:0019872 GO:0009117 GO:0008270 GO:0008830
GO:0016757 GO:0008378 GO:0016020 GO:0030127
Cluster-22178.78780 GO:0051258 GO:0009405 GO:0006412 GO:0006606
GO:0030168 GO:0006810 GO:0007165 GO:0042254 GO:0042803
GO:0008134 GO:0005198 GO:0045502 GO:0005102 GO:0003735
GO:0030674 GO:0005840 GO:0048786 GO:0005577 GO:0005886
GO:0030286 GO:0005622 GO:0005882 GO:0016020 GO:0005667
Cluster-22178.78782 GO:0006260 GO:0006351 GO:0030414 GO:0003887
GO:0003677 GO:0005515 GO:0042575
Cluster-22178.78784 GO:0006096 GO:0006098 GO:0006094 GO:0006012

GO:0006013 GO:0006000 GO:0003872 GO:0005945
Cluster-22178.78789 GO:0055085 GO:0030001 GO:0008146 GO:0046873
GO:0016020
Cluster-22178.78790 GO:0009058 GO:0030170
Cluster-22178.78791 GO:0006355 GO:0043401 GO:0007275 GO:0043565
GO:0008270 GO:0003700 GO:0005515 GO:0042600 GO:0016021
GO:0005634 GO:0005667
Cluster-22178.78792 GO:0006118 GO:0055114 GO:0018342 GO:0009916
GO:0008318
Cluster-22178.78793 GO:0046854 GO:0006000 GO:0015976 GO:0006013
GO:0005975 GO:0006096 GO:0006098 GO:0006094 GO:0042578
GO:0042132
Cluster-22178.78799 GO:0006508 GO:0016787 GO:0008236
Cluster-22178.78804 GO:0055114 GO:0019521 GO:0046487 GO:0009405
GO:0009396 GO:0050792 GO:0006810 GO:0006098 GO:0007264
GO:0003824 GO:0005525 GO:0004518 GO:0008200 GO:0004488
GO:0004616 GO:0005576
Cluster-22178.78809 GO:0005524
Cluster-22178.78811 GO:0046872
Cluster-22178.78816 GO:0007186 GO:0007165 GO:0007264 GO:0004871
GO:0005525 GO:0003924 GO:0019001 GO:0031683
Cluster-22178.78817 GO:0035556 GO:0009190 GO:0042802 GO:0016849

Cluster-22178.78823 GO:0005198 GO:0019031 GO:0016021 GO:0019028
Cluster-22178.78825 GO:0005515
Cluster-22178.78827 GO:0006508 GO:0005515 GO:0004252
Cluster-22178.78828 GO:0046488 GO:0016307
Cluster-22178.78830 GO:0016192 GO:0006886 GO:0005515 GO:0030117
Cluster-22178.78833 GO:0043087 GO:0035023 GO:0005089
Cluster-22178.78835 GO:0015979 GO:0046872
Cluster-22178.78836 GO:0006241 GO:0006206 GO:0006144 GO:0019079
GO:0006183 GO:0045454 GO:0006228 GO:0006165 GO:0005524
GO:0004550 GO:0003677 GO:0008026 GO:0005515 GO:0016787

Cluster-22178.78837 GO:0005515
Cluster-22178.78844 GO:0016757
Cluster-22178.78848 GO:0009116 GO:0009165 GO:0006144 GO:0006098
GO:0004749 GO:0000287
Cluster-22178.78849 GO:0009116 GO:0006098 GO:0006144 GO:0009165
GO:0004749 GO:0000287
Cluster-22178.78855 GO:0006888 GO:0005622 GO:0005801
Cluster-22178.78856 GO:0006811 GO:0005216 GO:0016020
Cluster-22178.78858 GO:0006811 GO:0007165 GO:0007268 GO:0004970
GO:0004867 GO:0016020
Cluster-22178.78859 GO:0006576 GO:0007264 GO:0003723 GO:0003924
GO:0005524 GO:0003724 GO:0005525
Cluster-22178.78863 GO:0006520 GO:0016021
Cluster-22178.78868 GO:0009405 GO:0016117 GO:0016705 GO:0005515
GO:0003677 GO:0003924 GO:0046872 GO:0005525
Cluster-22178.78871 GO:0048193 GO:0007165 GO:0006355 GO:0004871
GO:0003700 GO:0008270 GO:0016020 GO:0005634 GO:0005667
Cluster-22178.78872 GO:0007156 GO:0005509 GO:0005515 GO:0016020
Cluster-22178.78879 GO:0006486 GO:0006952 GO:0016042 GO:0007586
GO:0008373 GO:0008047 GO:0005576
Cluster-22178.78880 GO:0007165 GO:0007155 GO:0051205 GO:0005173
GO:0016021 GO:0016020
Cluster-22178.78883 GO:0006457 GO:0000774 GO:0008092 GO:0051087
GO:0042803 GO:0005515 GO:0019898 GO:0005737
Cluster-22178.78884 GO:0005975 GO:0004553 GO:0005515
Cluster-22178.78887 GO:0006355 GO:0006281 GO:0090263 GO:0006310
GO:0016567 GO:0043531 GO:0003678 GO:0003723 GO:0008134
GO:0005524 GO:0016887 GO:0003724 GO:0009378 GO:0016787
GO:0003677 GO:0098519 GO:0005657 GO:0005667 GO:0009379
Cluster-22178.78889 GO:0000162 GO:0006571 GO:0055114 GO:0009094
GO:0004764
Cluster-22178.78890 GO:0005516 GO:0005515
Cluster-22178.78893 GO:0035556 GO:0005515

Cluster-22178.78896 GO:0045859 GO:0016534 GO:0016533
Cluster-22178.78897 GO:0006486 GO:0008373
Cluster-22178.78898 GO:0006486 GO:0008373
Cluster-22178.78906 GO:0032481 GO:0002218
Cluster-22178.78909 GO:0006432 GO:0000162 GO:0006571 GO:0009094
GO:0005515 GO:0000166 GO:0004826 GO:0005524 GO:0009328
GO:0005737
Cluster-22178.78915 GO:0005509 GO:0005515
Cluster-22178.78917 GO:0006464 GO:0051537 GO:0003677 GO:0016151
GO:0046872
Cluster-22178.78918 GO:0046872
Cluster-22178.78921 GO:0009878 GO:0009058 GO:0046872 GO:0030170

Cluster-22178.78923 GO:0030639 GO:0019872 GO:0043087 GO:0006888
GO:0009117 GO:0016032 GO:0006486 GO:0006886 GO:0035023
GO:0008270 GO:0016757 GO:0008378 GO:0005089 GO:0008830
GO:0030127 GO:0016020
Cluster-22178.78924 GO:0015996 GO:0015994 GO:0051603 GO:0004298
GO:0047746 GO:0005839
Cluster-22178.78925 GO:0042981 GO:0005634
Cluster-22178.78929 GO:0051252 GO:0007010 GO:0003779 GO:0016891

Cluster-22178.78931 GO:0030001 GO:0046872
Cluster-22178.78937 GO:0007218 GO:0007165 GO:0030141
Cluster-22178.78940 GO:0009252 GO:0046436 GO:0043087 GO:0006464
GO:0015992 GO:0035023 GO:0006885 GO:0006814 GO:0005089
GO:0008716 GO:0005515 GO:0015385 GO:0032797 GO:0016020
Cluster-22178.78942 GO:0003774 GO:0016459
Cluster-22178.78944 GO:0046872 GO:0008270
Cluster-22178.78947 GO:0006355 GO:0043565 GO:0003700 GO:0005667
Cluster-22178.78954 GO:0008284 GO:0001819 GO:0010884 GO:0042802
GO:0005515 GO:0016020
Cluster-22178.78955 GO:0006814 GO:0005248 GO:0001518 GO:0005634

Cluster-22178.78956 GO:0001819 GO:0008284 GO:0010884 GO:0042802 GO:0005515 GO:0016020

Cluster-22178.78961 GO:0009245 GO:0006807 GO:0032801 GO:0006812 GO:0019076 GO:0006817 GO:0005315 GO:0008759 GO:0005261 GO:0016021 GO:0033644 GO:0016020

Cluster-22178.78965 GO:0046439 GO:0006534 GO:0019530 GO:0055114 GO:0005506 GO:0017172 GO:0016702

Cluster-22178.78966 GO:0006886 GO:0016192 GO:0005515 GO:0030131 GO:0030117

Cluster-22178.78967 GO:0016192 GO:0006886 GO:0005515 GO:0030117 GO:0030131

Cluster-22178.78971 GO:0005515

Cluster-22178.78972 GO:0055114 GO:0005996 GO:0048029 GO:0051536 GO:0016853

Cluster-22178.78973 GO:0016746 GO:0005634

Cluster-22178.78977 GO:0006352 GO:0055085 GO:0022857 GO:0003677 GO:0016021

Cluster-22178.78978 GO:0045944 GO:0007219 GO:0008440 GO:0003713 GO:0005667 GO:0016607

Cluster-22178.78980 GO:0008152 GO:0006123 GO:0015992 GO:0006351 GO:0004129 GO:0008270 GO:0003677 GO:0016746 GO:0045277 GO:0005751 GO:0005743 GO:0005634

Cluster-22178.78981 GO:0006123 GO:0008152 GO:0015992 GO:0016746 GO:0004129 GO:0045277 GO:0005751 GO:0005743

Cluster-22178.78982 GO:0008152 GO:0006123 GO:0015992 GO:0004129 GO:0016746 GO:0045277 GO:0005743 GO:0005751

Cluster-22178.78986 GO:0009069 GO:0006468 GO:0016310 GO:0055114 GO:0007178 GO:0005515 GO:0004675 GO:0016671 GO:0005524 GO:0003756 GO:0016020 GO:0005783

Cluster-22178.78990 GO:0006355 GO:0043565 GO:0005096 GO:0008270 GO:0046872 GO:0003700 GO:0005667

Cluster-22178.78995 GO:0005515

Cluster-22178.78997 GO:0006281 GO:0006508 GO:0002949 GO:0006510

GO:0019079 GO:0006310 GO:0005525 GO:0004176 GO:0043531

GO:0003924 GO:0004252 GO:0003723 GO:0003724 GO:0005524

GO:0016887 GO:0098519 GO:0009378 GO:0005657 GO:0009379

Cluster-22178.79001 GO:0003676 GO:0003723

Cluster-22178.79004 GO:0000902 GO:0007165 GO:0005179 GO:0005576

Cluster-22178.79005 GO:0006355 GO:0046649 GO:0007165 GO:0046983

GO:0004872 GO:0005515 GO:0009986 GO:0016021

Cluster-22178.79006 GO:0046649 GO:0006355 GO:0007165 GO:0046983

GO:0004872 GO:0005515 GO:0009986 GO:0016021

Cluster-22178.79011 GO:0009415 GO:0006950

Cluster-22178.79012 GO:0007155 GO:0003677 GO:0046872 GO:0016020

Cluster-22178.79014 GO:0045046 GO:0055114 GO:0008152 GO:0016491

GO:0032403 GO:0005779

Cluster-22178.79015 GO:0006118 GO:0006814 GO:0015992 GO:0006144

GO:0006790 GO:0006120 GO:0000103 GO:0006744 GO:0004781

GO:0003954 GO:0008137 GO:0005524 GO:0004020 GO:0005739

Cluster-22178.79023 GO:0003824

Cluster-22178.79024 GO:0006816 GO:0006810 GO:0042030 GO:0005246

GO:0016020

Cluster-22178.79030 GO:0009968 GO:0007165 GO:0005509 GO:0005737

GO:0005578

Cluster-22178.79031 GO:0009968 GO:0005737

Cluster-22178.79033 GO:0006888 GO:0006886 GO:0046872 GO:0008270

GO:0003677 GO:0030127

Cluster-22178.79034 GO:0006468 GO:0004672 GO:0005524 GO:0016773

GO:0016020

Cluster-22178.79038 GO:0007165 GO:0051262 GO:0008289

Cluster-22178.79040 GO:0007165 GO:0005515

Cluster-22178.79042 GO:0006508 GO:0008236

Cluster-22178.79044 GO:0006508 GO:0008236

Cluster-22178.79048 GO:0030246 GO:0005639

Cluster-22178.79054 GO:0006525 GO:0071436 GO:0006814 GO:0006560

GO:0006090 GO:0008948 GO:0015081 GO:0008270 GO:0016020

Cluster-22178.79055 GO:0008270
Cluster-22178.79057 GO:0032259 GO:0003723 GO:0005845
Cluster-22178.79060 GO:0015979 GO:0016020 GO:0009539 GO:0009523
Cluster-22178.79061 GO:0009405 GO:0009094 GO:0006571 GO:0009073
GO:0000162 GO:0004107 GO:0005515 GO:0033644
Cluster-22178.79062 GO:0055114 GO:0051321 GO:0018401 GO:0006525
GO:0006560 GO:0016491 GO:0016702 GO:0004656 GO:0005783
Cluster-22178.79065 GO:0051028 GO:0006355 GO:0005515 GO:0003677
GO:0005856 GO:0005634
Cluster-22178.79066 GO:0006355 GO:0003677 GO:0005515 GO:0005856
GO:0005634
Cluster-22178.79067 GO:0055085 GO:0006810 GO:0006118 GO:0005524
GO:0016887 GO:0003924 GO:0016491 GO:0005525 GO:0009055
GO:0042626 GO:0016301 GO:0005515 GO:0016021 GO:0016020
Cluster-22178.79070 GO:0003924
Cluster-22178.79073 GO:0006260 GO:0007165 GO:0016567 GO:0046872
GO:0008270 GO:0005524 GO:0004842 GO:0005179 GO:0003677
GO:0016818 GO:0005515 GO:0005680 GO:0005576 GO:0005634
Cluster-22178.79080 GO:0005515 GO:0016020
Cluster-22178.79084 GO:0006355 GO:0006468 GO:0030071 GO:0031145
GO:0005515 GO:0004672 GO:0005524 GO:0005634 GO:0019028
GO:0016020 GO:0005680
Cluster-22178.79086 GO:0007067 GO:0003924 GO:0005525 GO:0000775
Cluster-22178.79087 GO:0007067 GO:0005525 GO:0003924 GO:0000775
Cluster-22178.79088 GO:0006508 GO:0004181 GO:0008270
Cluster-22178.79089 GO:0009878 GO:0046872
Cluster-22178.79090 GO:0006355 GO:0003700 GO:0005667
Cluster-22178.79093 GO:0006313 GO:0004803 GO:0003677 GO:0046872

Cluster-22178.79097 GO:0007165 GO:0006468 GO:0004872 GO:0005524
GO:0004672 GO:0016021
Cluster-22178.79102 GO:0006568 GO:0008210 GO:0006552 GO:0006550
GO:0055114 GO:0006574 GO:0006694 GO:0008152 GO:0008207

GO:0006631 GO:0018874 GO:0008209 GO:0006554 GO:0006633
GO:0003857 GO:0003854 GO:0016616 GO:0016491
Cluster-22178.79106 GO:0051090 GO:0005634
Cluster-22178.79107 GO:0006413 GO:0006446 GO:0003723 GO:0003743
GO:0005737 GO:0005840
Cluster-22178.79108 GO:0006310 GO:0006810 GO:0000150 GO:0042626
GO:0005515 GO:0003677
Cluster-22178.7911 GO:0043087 GO:0005089 GO:0005737
Cluster-22178.79112 GO:0008053 GO:0006520 GO:0003924 GO:0046872
GO:0016829 GO:0016021 GO:0005741
Cluster-22178.79115 GO:0006629 GO:0007186 GO:0008270 GO:0003677
GO:0004930 GO:0016021
Cluster-22178.79119 GO:0005634
Cluster-22178.79120 GO:0015012 GO:0006024 GO:0009264 GO:0007283
GO:0008253 GO:0016757 GO:0004252 GO:0003677 GO:0000786
GO:0016021 GO:0005634 GO:0016020
Cluster-22178.79121 GO:0019058 GO:0006810 GO:0046872 GO:0016021
Cluster-22178.79126 GO:0006355 GO:0008152 GO:0055114 GO:0016872
GO:0008270 GO:0016491 GO:0016020 GO:0005634
Cluster-22178.79127 GO:0019076 GO:0006308 GO:0046718 GO:0006812
GO:0006885 GO:0055085 GO:0075521 GO:0015299 GO:0008855
GO:0016021 GO:0019028 GO:0044163 GO:0030430 GO:0009318
GO:0042025
Cluster-22178.79129 GO:0007264 GO:0005525 GO:0003723
Cluster-22178.79134 GO:0055114 GO:0004800
Cluster-22178.79136 GO:0042254 GO:0006412 GO:0008033 GO:0055114
GO:0000105 GO:0009228 GO:0004789 GO:0003735 GO:0017150
GO:0050660 GO:0005622 GO:0005840
Cluster-22178.79139 GO:0042254 GO:0006412 GO:0000105 GO:0055114
GO:0008033 GO:0003735 GO:0050660 GO:0017150 GO:0005840
GO:0005622
Cluster-22178.7914 GO:0006260 GO:0055114 GO:0003677 GO:0016491
GO:0016209

Cluster-22178.79142 GO:0006355 GO:0007165 GO:0051258 GO:0030168 GO:0000921 GO:0030674 GO:0043565 GO:0003700 GO:0005102 GO:0005667 GO:0005940 GO:0005577 GO:0016021

Cluster-22178.79143 GO:0006355 GO:0043565 GO:0003700 GO:0005515 GO:0005667

Cluster-22178.79147 GO:0055036

Cluster-22178.79152 GO:0006508 GO:0008270 GO:0003676 GO:0004222

Cluster-22178.79155 GO:0007165 GO:0007411 GO:0005179 GO:0016021 GO:0005576

Cluster-22178.79159 GO:0006144 GO:0003968 GO:0005524 GO:0031379

Cluster-22178.79162 GO:0006351 GO:0006144 GO:0006206 GO:0003899 GO:0005515 GO:0003677 GO:0005730

Cluster-22178.79163 GO:0016310 GO:0006468 GO:0009069 GO:0010508 GO:0007165 GO:0005096 GO:0003676 GO:0004674 GO:0005515

Cluster-22178.79164 GO:0048193 GO:0015948 GO:0006457 GO:0006308 GO:0055114 GO:0008855 GO:0016817 GO:0008134 GO:0042803 GO:0051082 GO:0045502 GO:0030286 GO:0016272 GO:0016020 GO:0005667 GO:0009318

Cluster-22178.79165 GO:0006310 GO:0006520 GO:0003677 GO:0016597 GO:0016743

Cluster-22178.79170 GO:0003723 GO:0003676

Cluster-22178.79173 GO:0006306 GO:0003677 GO:0008170

Cluster-22178.79177 GO:0009312 GO:0006486 GO:0008455 GO:0008375 GO:0016021 GO:0005795

Cluster-22178.79181 GO:0006508 GO:0016787 GO:0008236

Cluster-22178.79182 GO:0006355 GO:0005515 GO:0005634

Cluster-22178.79185 GO:0000725 GO:0019229 GO:0003677 GO:0046983 GO:0005576 GO:0097196 GO:0005634

Cluster-22178.79193 GO:0006468 GO:0005524 GO:0004672

Cluster-22178.79194 GO:0005515

Cluster-22178.79205 GO:0005509 GO:0005515 GO:0016021

Cluster-22178.79208 GO:0051090 GO:0006886 GO:0008536 GO:0005515

GO:0005634
Cluster-22178.79209 GO:0005525
Cluster-22178.79210 GO:0046872 GO:0003677 GO:0005634
Cluster-22178.79211 GO:0051726 GO:0007165 GO:0006974 GO:0055114
GO:0016491 GO:0046872
Cluster-22178.79212 GO:0000226 GO:0051537 GO:0043231 GO:0015630
Cluster-22178.79215 GO:0005515
Cluster-22178.79224 GO:0005198 GO:0019012
Cluster-22178.79225 GO:0005524 GO:0016887 GO:0003924 GO:0005525
GO:0000166 GO:0003677
Cluster-22178.79226 GO:0006355 GO:0009725 GO:0003677 GO:0005515
GO:0005634
Cluster-22178.79227 GO:0019634 GO:0016567 GO:0016579 GO:0036459
GO:0004842 GO:0005680
Cluster-22178.79230 GO:0051082 GO:0031072 GO:0005198 GO:0019028
Cluster-22178.79233 GO:0016055 GO:0045947 GO:0008190 GO:0008017
GO:0008013 GO:0045298 GO:0016342
Cluster-22178.79234 GO:0005096 GO:0015631 GO:0045298
Cluster-22178.79237 GO:0005198 GO:0012506
Cluster-22178.79240 GO:0007186 GO:0004930 GO:0046983 GO:0016021
Cluster-22178.79244 GO:0008270 GO:0005634
Cluster-22178.79246 GO:0008270 GO:0005515
Cluster-22178.79248 GO:0055085 GO:0006810 GO:0022857 GO:0005515
GO:0005215 GO:0016021 GO:0016020
Cluster-22178.79249 GO:0055085 GO:0006810 GO:0005515 GO:0005215
GO:0016021 GO:0016020
Cluster-22178.79250 GO:0030001 GO:0046872 GO:0031083 GO:0030133
Cluster-22178.79254 GO:0030488 GO:0008033 GO:0055114 GO:0009082
GO:0009451 GO:0042138 GO:0006310 GO:0046872 GO:0016491
GO:0016429 GO:0016021 GO:0031515
Cluster-22178.79256 GO:0008610 GO:0008152 GO:0016747
Cluster-22178.79259 GO:0007031 GO:0006508 GO:0004252 GO:0005509
GO:0005779

Cluster-22178.79261 GO:0008152 GO:0006338 GO:0005515 GO:0003824
GO:0016836 GO:0090544
Cluster-22178.79264 GO:0017038 GO:0006278 GO:0003677 GO:0016787
GO:0003676 GO:0003964 GO:0005524 GO:0016020
Cluster-22178.79265 GO:0006470 GO:0008138 GO:0019028
Cluster-22178.79266 GO:0016740 GO:0051424
Cluster-22178.79269 GO:0006040 GO:0055114 GO:0008762
Cluster-22178.79270 GO:0019684 GO:0015979 GO:0042254 GO:0006412
GO:0006355 GO:0003735 GO:0016021 GO:0005840 GO:0009523
GO:0005622
Cluster-22178.79275 GO:0006554 GO:0006355 GO:0006479 GO:0018024
GO:0046983
Cluster-22178.79276 GO:0055114 GO:0016491
Cluster-22178.79280 GO:0035556 GO:0005515
Cluster-22178.79281 GO:0035556
Cluster-22178.79282 GO:0006508 GO:0004190
Cluster-22178.79286 GO:0007186 GO:0004930 GO:0005515 GO:0016021
Cluster-22178.79291 GO:0006281 GO:0006508 GO:0006099 GO:0006310
GO:0009378 GO:0016301 GO:0005524 GO:0016829 GO:0016887
GO:0004222 GO:0003678 GO:0009379 GO:0005657
Cluster-22178.79292 GO:0005515
Cluster-22178.79293 GO:0006886 GO:0006888 GO:0003677 GO:0008270
GO:0046872 GO:0030127
Cluster-22178.79294 GO:0006323
Cluster-22178.79295 GO:0006260 GO:0006308 GO:0006281 GO:0017108
GO:0003723 GO:0033557 GO:0005634
Cluster-22178.79296 GO:0010951 GO:0007165 GO:0009405 GO:0040007
GO:0008283 GO:0008083 GO:0008376 GO:0051015 GO:0030674
GO:0004866 GO:0032580
Cluster-22178.79298 GO:0016853
Cluster-22178.79302 GO:0006355 GO:0005515 GO:0005634
Cluster-22178.79304 GO:0015696 GO:0006468 GO:0006810 GO:0004672
GO:0005524 GO:0016787 GO:0008519 GO:0016021 GO:0005794

GO:0031224 GO:0016020
Cluster-22178.79305 GO:0006888 GO:0006886 GO:0007010 GO:0003779 GO:0008270 GO:0030127
Cluster-22178.79313 GO:0055114 GO:0006468 GO:0004672 GO:0008270 GO:0005524 GO:0046872 GO:0047134 GO:0003676 GO:0003697 GO:0016787 GO:0003723 GO:0003677 GO:0005515
Cluster-22178.79314 GO:0008152 GO:0003824
Cluster-22178.79316 GO:0006952
Cluster-22178.79317 GO:0051260 GO:0006355 GO:0006352 GO:0015074 GO:0003677 GO:0003700 GO:0005667 GO:0005669
Cluster-22178.79319 GO:0006281 GO:0008270 GO:0046872 GO:0005515

Cluster-22178.79326 GO:0003677 GO:0005634
Cluster-22178.79327 GO:0016020
Cluster-22178.79328 GO:0006260 GO:0051259 GO:0003677 GO:0008270

Cluster-22178.79329 GO:0006605 GO:0007165 GO:0051028 GO:0005634
Cluster-22178.79332 GO:0008270 GO:0005515
Cluster-22178.79333 GO:0006355 GO:0046656 GO:0015948 GO:0043401 GO:0008152 GO:0016746 GO:0005515 GO:0030269 GO:0019031 GO:0016021
Cluster-22178.79335 GO:0007155 GO:0005515 GO:0016021
Cluster-22178.79336 GO:0008146 GO:0019028
Cluster-22178.79337 GO:0006139 GO:0008408 GO:0003676
Cluster-22178.79338 GO:0006139 GO:0003676 GO:0008408
Cluster-22178.79341 GO:0006260 GO:0018142 GO:0006269 GO:0000166 GO:0004672 GO:0003723
Cluster-22178.79344 GO:0016579 GO:0036459 GO:0032183 GO:0070140 GO:0008201
Cluster-22178.79350 GO:0006355 GO:0006281 GO:0006310 GO:0019079 GO:0002949 GO:0003723 GO:0016301 GO:0008134 GO:0003678 GO:0009378 GO:0016887 GO:0005524 GO:0003724 GO:0005667 GO:0009379 GO:0005657

Cluster-22178.79351 GO:0007165 GO:0007264 GO:0005525
Cluster-22178.79352 GO:0006413 GO:0006355 GO:0042254 GO:0005509
GO:0005515 GO:0003700 GO:0005667 GO:0005622
Cluster-22178.79356 GO:0006284 GO:0003905 GO:0003677
Cluster-22178.79357 GO:0055114 GO:0008544 GO:0016491 GO:0005507

Cluster-22178.79358 GO:0007264 GO:0007186 GO:0007165 GO:0015684
GO:0006888 GO:0003924 GO:0015093 GO:0019001 GO:0031683
GO:0004871 GO:0005525 GO:0019033 GO:0005622 GO:0016021
GO:0005801
Cluster-22178.7936 GO:0006260 GO:0018142 GO:0006144 GO:0006308
GO:0016779 GO:0016888 GO:0042624
Cluster-22178.79363 GO:0008283 GO:0040007 GO:0007165 GO:0043087
GO:0005085 GO:0030674 GO:0051015 GO:0005515 GO:0008083
Cluster-22178.79365 GO:0006744 GO:0006120 GO:0008608 GO:0006914
GO:0015992 GO:0006814 GO:0006118 GO:0008137 GO:0003954
GO:0042729 GO:0005739 GO:0072686
Cluster-22178.79366 GO:0008061 GO:0005643
Cluster-22178.79367 GO:0008270
Cluster-22178.79368 GO:0005509 GO:0005515
Cluster-22178.79369 GO:0048870 GO:0006914 GO:0051258 GO:0030168
GO:0000921 GO:0007165 GO:0007155 GO:0005102 GO:0005509
GO:0005515 GO:0030674 GO:0005577 GO:0016021 GO:0005940
GO:0031514
Cluster-22178.79373 GO:0006886 GO:0006355 GO:0008536 GO:0005634
Cluster-22178.79374 GO:0006621 GO:0005525 GO:0003924 GO:0046923
GO:0016021 GO:0005783
Cluster-22178.79377 GO:0032065 GO:0005515 GO:0005543 GO:0005938
Cluster-22178.79380 GO:0006355 GO:0003700 GO:0003677 GO:0043565
GO:0005634 GO:0005667
Cluster-22178.79381 GO:0007268 GO:0007165 GO:0006811 GO:0006123
GO:0055085 GO:0015992 GO:0005515 GO:0005216 GO:0004970
GO:0004129 GO:0016020 GO:0045277

Cluster-22178.79382 GO:0007268 GO:0007165 GO:0006811 GO:0006123 GO:0055085 GO:0015992 GO:0005515 GO:0005216 GO:0004970 GO:0004129 GO:0016020 GO:0045277
Cluster-22178.79387 GO:0035307 GO:0030436 GO:0006508 GO:0004190 GO:0071595
Cluster-22178.79389 GO:0006468 GO:0007165 GO:0007186 GO:0004672 GO:0005524 GO:0004871 GO:0005834
Cluster-22178.79391 GO:0005515 GO:0008168
Cluster-22178.79392 GO:0042147 GO:0003676 GO:0004222 GO:0031012
Cluster-22178.79396 GO:0006355 GO:0003700 GO:0005667
Cluster-22178.79397 GO:0043401 GO:0006355 GO:0006629 GO:0003700 GO:0008270 GO:0043565 GO:0005634 GO:0005667
Cluster-22178.79398 GO:0007156 GO:0005509 GO:0016020
Cluster-22178.79402 GO:0055114 GO:0016702
Cluster-22178.79404 GO:0006357 GO:0055114 GO:0001104 GO:0016702 GO:0008013 GO:0016592 GO:0016342
Cluster-22178.79407 GO:0006771 GO:0019497 GO:0055114 GO:0003677 GO:0005515 GO:0046872 GO:0003993 GO:0005525 GO:0016620
Cluster-22178.79408 GO:0060962 GO:0003712 GO:0005667
Cluster-22178.79409 GO:0018144 GO:0035023 GO:0055114 GO:0019497 GO:0035556 GO:0006144 GO:0043087 GO:0006771 GO:0003968 GO:0046872 GO:0005515 GO:0005198 GO:0005089 GO:0003993 GO:0047134 GO:0008270 GO:0016818 GO:0031379 GO:0005634
Cluster-22178.79411 GO:0046656 GO:0015948 GO:0008270 GO:0030269 GO:0016021
Cluster-22178.79413 GO:0005515
Cluster-22178.79417 GO:0007264 GO:0015684 GO:0015093 GO:0005524 GO:0003924 GO:0016887 GO:0005525 GO:0016021
Cluster-22178.79418 GO:0005515
Cluster-22178.79419 GO:0006355 GO:0003677
Cluster-22178.79420 GO:0006355 GO:0003677
Cluster-22178.79424 GO:0035556 GO:0007165 GO:0046339 GO:0006629 GO:0009395 GO:0005515 GO:0004435

Cluster-22178.79425 GO:0046339 GO:0015992 GO:0035556 GO:0006123
GO:0007165 GO:0042254 GO:0009395 GO:0007411 GO:0006629
GO:0006412 GO:0003735 GO:0008081 GO:0004129 GO:0004435
GO:0005515 GO:0005622 GO:0045277 GO:0005840 GO:0016021
Cluster-22178.79428 GO:0006486 GO:0008373
Cluster-22178.79431 GO:0001503 GO:0007155 GO:0016758 GO:0005789
GO:0005576
Cluster-22178.79433 GO:0006281 GO:0046487 GO:0009396 GO:0006308
GO:0055114 GO:0000077 GO:0004488 GO:0043565 GO:0000014
GO:0003824 GO:0016021
Cluster-22178.79435 GO:0046872
Cluster-22178.79438 GO:0015684 GO:0015093 GO:0005524 GO:0003924
GO:0016887 GO:0005525 GO:0042802 GO:0016021
Cluster-22178.79440 GO:0008270
Cluster-22178.79449 GO:0006468 GO:0004672 GO:0005524 GO:0016773
GO:0016020
Cluster-22178.79450 GO:0016114 GO:0006284 GO:0006694 GO:0006886
GO:0016192 GO:0005515 GO:0005198 GO:0003677 GO:0008661
GO:0005634 GO:0030117
Cluster-22178.79453 GO:0005515
Cluster-22178.79456 GO:0005634
Cluster-22178.79460 GO:0008270 GO:0046872 GO:0005515
Cluster-22178.79461 GO:0006810 GO:0006281 GO:0000723 GO:0003678
GO:0005525 GO:0003924 GO:0016887 GO:0005524 GO:0005657
Cluster-22178.79465 GO:0007165 GO:0007264 GO:0046656 GO:0050853
GO:0050852 GO:0050524 GO:0005525 GO:0003924 GO:0016887
GO:0005524
Cluster-22178.79467 GO:0007156 GO:0005509 GO:0016020
Cluster-22178.79469 GO:0035556
Cluster-22178.79473 GO:0015979 GO:0046872
Cluster-22178.79479 GO:0016226 GO:0005515 GO:0005506 GO:0051536

Cluster-22178.79481 GO:0043044 GO:0001522 GO:0009451 GO:0003723

GO:0009982 GO:0016586
Cluster-22178.79483 GO:0016192 GO:0006886 GO:0005198 GO:0030130
GO:0030132
Cluster-22178.79484 GO:0006886 GO:0016192 GO:0005198 GO:0030130
GO:0030132
Cluster-22178.79491 GO:0003779 GO:0005543
Cluster-22178.79492 GO:0015631 GO:0005515 GO:0045298 GO:0005815
Cluster-22178.79493 GO:0006118 GO:0019684 GO:0009772 GO:0005515
GO:0045156
Cluster-22178.79496 GO:0006099 GO:0008152 GO:0006090 GO:0034477
GO:0004518 GO:0005515 GO:0008924 GO:0016829 GO:0016021
Cluster-22178.79497 GO:0045895 GO:0007531 GO:0008301 GO:0005634
Cluster-22178.79501 GO:0043087 GO:0035023 GO:0046872 GO:0005089

Cluster-22178.79503 GO:0009966 GO:0005576
Cluster-22178.79504 GO:0003677 GO:0005515 GO:0005634
Cluster-22178.79508 GO:0005515
Cluster-22178.79510 GO:0046872
Cluster-22178.79514 GO:0016747
Cluster-22178.79516 GO:0007155 GO:0006355 GO:0003700 GO:0019905
GO:0043565 GO:0005667 GO:0019028
Cluster-22178.79518 GO:0019089 GO:0003676
Cluster-22178.79519 GO:0006886 GO:0006888 GO:0005515 GO:0008270
GO:0046872 GO:0030127
Cluster-22178.79521 GO:0051537 GO:0005515 GO:0043231
Cluster-22178.79526 GO:0016567 GO:0004842 GO:0051537 GO:0005515
GO:0043231
Cluster-22178.79529 GO:0016567 GO:0005515 GO:0051537 GO:0004842
GO:0043231
Cluster-22178.79538 GO:0009395 GO:0035556 GO:0007205 GO:0046486
GO:0004143 GO:0016301
Cluster-22178.79539 GO:0007165 GO:0006144 GO:0005515 GO:0004114

Cluster-22178.79543 GO:0019054 GO:0004857
Cluster-22178.79544 GO:0006308 GO:0006355 GO:0006606 GO:0003700
GO:0045502 GO:0003723 GO:0008134 GO:0042803 GO:0005198
GO:0005524 GO:0016887 GO:0008855 GO:0003724 GO:0009318
GO:0030286 GO:0005667 GO:0005882
Cluster-22178.79545 GO:0006308 GO:0006355 GO:0006606 GO:0019069
GO:0003700 GO:0008134 GO:0042803 GO:0045502 GO:0003723
GO:0003724 GO:0016887 GO:0008855 GO:0005524 GO:0009318
GO:0030286 GO:0005667
Cluster-22178.79546 GO:0008270 GO:0046872 GO:0005515
Cluster-22178.79547 GO:0007165 GO:0000166 GO:0005158 GO:0046872
GO:0005899
Cluster-22178.79549 GO:0006629 GO:0006505 GO:0006886 GO:0016788

Cluster-22178.79555 GO:0019497 GO:0006771 GO:0046872 GO:0005515
GO:0003993
Cluster-22178.79556 GO:0019497 GO:0006771 GO:0003993 GO:0005515
GO:0046872
Cluster-22178.79559 GO:0016310 GO:0009069 GO:0006468 GO:0004674
GO:0005524 GO:0004672
Cluster-22178.79562 GO:0009267 GO:0032099 GO:0016192 GO:0007186
GO:0006886 GO:0008343 GO:0001678 GO:0000186 GO:0005515
GO:0005615
Cluster-22178.79564 GO:0006260 GO:0015979 GO:0016020 GO:0009523
GO:0009539
Cluster-22178.79566 GO:0005975 GO:0016773 GO:0004252 GO:0016021
Cluster-22178.79570 GO:0006355 GO:0007165 GO:0005179 GO:0005515
GO:0003677 GO:0005576
Cluster-22178.79571 GO:0006468 GO:0005524 GO:0004672
Cluster-22178.79579 GO:0005515 GO:0016301 GO:0005524 GO:0003774
GO:0016459
Cluster-22178.79581 GO:0008168
Cluster-22178.79584 GO:0035556 GO:0007165 GO:0004871

Cluster-22178.79586 GO:0055114 GO:0005515
Cluster-22178.79590 GO:0016485 GO:0003333 GO:0009405 GO:0006886
GO:0035556 GO:0006865 GO:0055085 GO:0015031 GO:0043087
GO:0006888 GO:0006810 GO:0019069 GO:0015171 GO:0005515
GO:0008565 GO:0005576 GO:0030127 GO:0016020
Cluster-22178.79591 GO:0007165 GO:0016192 GO:0006886 GO:0008283
GO:0040007 GO:0051015 GO:0008083 GO:0030674 GO:0008092
GO:0030131 GO:0019898 GO:0005737
Cluster-22178.79592 GO:0016579 GO:0006355 GO:0003700 GO:0036459
GO:0005667 GO:0005634
Cluster-22178.79595 GO:0006355 GO:0009405 GO:0006308 GO:0006914
GO:0007049 GO:0003677 GO:0019865 GO:0008855 GO:0043565
GO:0003700 GO:0005667 GO:0005634 GO:0009318 GO:0019814
GO:0016021
Cluster-22178.79596 GO:0055085 GO:0022857 GO:0016021 GO:0016020
Cluster-22178.79597 GO:0055085 GO:0022857 GO:0016020 GO:0016021
Cluster-22178.79598 GO:0006869 GO:0006508 GO:0004222 GO:0008289
GO:0046872 GO:0005576
Cluster-22178.79601 GO:0016192 GO:0006904 GO:0046983 GO:0046914

Cluster-22178.79605 GO:0007165
Cluster-22178.79607 GO:0006468 GO:0016773 GO:0004672 GO:0005524
GO:0016020
Cluster-22178.79611 GO:0043087 GO:0035023 GO:0005089 GO:0005887
Cluster-22178.79612 GO:0035023 GO:0043087 GO:0005089 GO:0005887
Cluster-22178.79613 GO:0030154 GO:0016021
Cluster-22178.79614 GO:0000226 GO:0015630 GO:0016020
Cluster-22178.79616 GO:0042254 GO:0006412 GO:0006629 GO:0006508
GO:0008152 GO:0006230 GO:0006206 GO:0003735 GO:0008236
GO:0016787 GO:0004797 GO:0005524 GO:0016020 GO:0005840
Cluster-22178.79617 GO:0042254 GO:0006412 GO:0006230 GO:0006206
GO:0003735 GO:0004797 GO:0005524 GO:0005840
Cluster-22178.79619 GO:0008152 GO:0006230 GO:0006206 GO:0042254

GO:0006412 GO:0006629 GO:0006508 GO:0005524 GO:0004797
GO:0008236 GO:0003735 GO:0016787 GO:0005840 GO:0016020
Cluster-22178.79622 GO:0003682 GO:0000785
Cluster-22178.79625 GO:0006310 GO:0006281 GO:0008270
Cluster-22178.79627 GO:0007165 GO:0042312 GO:0008217 GO:0003677
GO:0005179 GO:0005576 GO:0005634
Cluster-22178.79630 GO:0006468 GO:0016773 GO:0005524 GO:0004672
GO:0016020
Cluster-22178.79631 GO:0016567 GO:0006915 GO:0032465 GO:0015031
GO:0006464 GO:0005515 GO:0004842 GO:0016020
Cluster-22178.79632 GO:0006535 GO:0006534 GO:0042967 GO:0046872
GO:0009001 GO:0005737
Cluster-22178.79641 GO:0016567 GO:0019856 GO:0046872 GO:0008270
GO:0004842 GO:0005515 GO:0016021 GO:0005680
Cluster-22178.79642 GO:0006508 GO:0003676 GO:0003723 GO:0004190

Cluster-22178.79649 GO:0055114
Cluster-22178.79653 GO:0006571 GO:0009094 GO:0008033 GO:0006432
GO:0006796 GO:0006119 GO:0006508 GO:0000162 GO:0004826
GO:0008234 GO:0000049 GO:0000287 GO:0005524 GO:0004427
GO:0005737 GO:0009328
Cluster-22178.79658 GO:0005515
Cluster-22178.79659 GO:0005515 GO:0008270
Cluster-22178.79660 GO:0006810 GO:0009405 GO:0006952 GO:0055085
GO:0005525 GO:0019855 GO:0042626 GO:0005524 GO:0016887
GO:0003924 GO:0005576 GO:0016021
Cluster-22178.79661 GO:0009405 GO:0006810 GO:0006952 GO:0055085
GO:0098519 GO:0005524 GO:0016887 GO:0003924 GO:0005525
GO:0019855 GO:0042626 GO:0005576 GO:0016021
Cluster-22178.79664 GO:0005515
Cluster-22178.79668 GO:0006355 GO:0003700 GO:0043565 GO:0010181
GO:0005667
Cluster-22178.79669 GO:0007165

Cluster-22178.79670 GO:0006468 GO:0009069 GO:0051262 GO:0055085 GO:0006811 GO:0016310 GO:0005216 GO:0005524 GO:0004674 GO:0016020
Cluster-22178.79671 GO:0007166 GO:0005515 GO:0016020
Cluster-22178.79673 GO:0007304 GO:0005213 GO:0042600 GO:0005576
Cluster-22178.79676 GO:0006508 GO:0016787 GO:0008236
Cluster-22178.79680 GO:0030168 GO:0000921 GO:0051258 GO:0007155 GO:0044237 GO:0007165 GO:0005102 GO:0030674 GO:0005515 GO:0005509 GO:0005940 GO:0016021 GO:0005577
Cluster-22178.79682 GO:0006118 GO:0009055 GO:0016491 GO:0016021 GO:0016020
Cluster-22178.79683 GO:0051260 GO:0046872 GO:0003677 GO:0016020
Cluster-22178.79684 GO:0015979 GO:0008360 GO:0007186 GO:0006886 GO:0016817 GO:0004930 GO:0003677 GO:0042802 GO:0005515 GO:0009539 GO:0009523 GO:0005801 GO:0016021 GO:0016020
Cluster-22178.79687 GO:0006000 GO:0015976 GO:0046854 GO:0005975 GO:0006013 GO:0006096 GO:0006094 GO:0006098 GO:0042578 GO:0042132
Cluster-22178.79689 GO:0035091
Cluster-22178.79692 GO:0008283 GO:0040007 GO:0007165 GO:0006955 GO:0008083 GO:0005137 GO:0005576 GO:0005895
Cluster-22178.79694 GO:0007165 GO:0006955 GO:0008283 GO:0040007 GO:0008083 GO:0005137 GO:0005576 GO:0005895
Cluster-22178.79696 GO:0006486 GO:0008373
Cluster-22178.79699 GO:0005515
Cluster-22178.79702 GO:0005515
Cluster-22178.79704 GO:0046983 GO:0046872 GO:0016021 GO:0019012
Cluster-22178.79707 GO:0007165 GO:0007009 GO:0051087 GO:0019904

Cluster-22178.79708 GO:0006886 GO:0016192 GO:0005515 GO:0005198 GO:0030132 GO:0030130
Cluster-22178.79710 GO:0008063
Cluster-22178.79712 GO:0034314 GO:0030041 GO:0005885 GO:0015629

Cluster-22178.79713 GO:0006810 GO:0006811 GO:0005515 GO:0005230
GO:0016020
Cluster-22178.79714 GO:0005515 GO:0016020
Cluster-22178.79717 GO:0004190 GO:0016021
Cluster-22178.79718 GO:0006457 GO:0016192 GO:0007165 GO:0030521
GO:0009372 GO:0006886 GO:0009405 GO:0006355 GO:0005509
GO:0003677 GO:0005496 GO:0008233 GO:0004882 GO:0008270
GO:0005515 GO:0051082 GO:0005783 GO:0005634 GO:0030117
GO:0016021
Cluster-22178.79719 GO:0007623 GO:0006351 GO:0007155 GO:0005515
GO:0016021 GO:0005634
Cluster-22178.79720 GO:0006522 GO:0006123 GO:0006188 GO:0009152
GO:0006144 GO:0015992 GO:0016567 GO:0006531 GO:0004129
GO:0004842 GO:0004018 GO:0045277 GO:0016020 GO:0005740
Cluster-22178.79722 GO:0055114 GO:0008152 GO:0042254 GO:0006412
GO:0006954 GO:0007165 GO:0005125 GO:0042578 GO:0051287
GO:0003735 GO:0016616 GO:0005840 GO:0005576
Cluster-22178.79723 GO:0003697
Cluster-22178.79731 GO:0006144 GO:0006206 GO:0007165 GO:0015774
GO:0055114 GO:0006351 GO:0015159 GO:0005509 GO:0051920
GO:0003899 GO:0005515 GO:0004888 GO:0016020 GO:0005730
GO:0016021 GO:0005578
Cluster-22178.79740 GO:0042998 GO:0046872 GO:0071253 GO:0005802
Cluster-22178.79742 GO:0006412 GO:0042254 GO:0003735 GO:0005840
GO:0005761
Cluster-22178.79743 GO:0046983 GO:0005515
Cluster-22178.79745 GO:0016055 GO:0007165 GO:0007275 GO:0005102
GO:0005576
Cluster-22178.79748 GO:0016773
Cluster-22178.79749 GO:0006468 GO:0005524 GO:0004672
Cluster-22178.79750 GO:0006886 GO:0000166 GO:0046872 GO:0005783
GO:0016021
Cluster-22178.79752 GO:0055114 GO:0008152 GO:0006355 GO:0006508

GO:0007339 GO:0006118 GO:0016627 GO:0050660 GO:0003700
GO:0003995 GO:0004197 GO:0005515 GO:0005576 GO:0005667
Cluster-22178.79753 GO:0008152 GO:0055114 GO:0006118 GO:0006355
GO:0006508 GO:0050660 GO:0003700 GO:0016627 GO:0004197
GO:0003995 GO:0005667
Cluster-22178.79760 GO:0003676
Cluster-22178.79761 GO:0051028 GO:0006554 GO:0006479 GO:0034968
GO:0008270 GO:0005515 GO:0018024 GO:0005634
Cluster-22178.79764 GO:0006355 GO:0005515 GO:0016021 GO:0005634
Cluster-22178.79765 GO:0043039 GO:0016068 GO:0006606 GO:0015031
GO:0005515 GO:0008565 GO:0016876 GO:0005524 GO:0005634
GO:0016021 GO:0005737
Cluster-22178.79766 GO:0006357 GO:0005515
Cluster-22178.79767 GO:0006355 GO:0008270 GO:0043565 GO:0003700
GO:0005515 GO:0005667 GO:0005634
Cluster-22178.79773 GO:0007178 GO:0006520 GO:0016310 GO:0009069
GO:0006468 GO:0006310 GO:0005524 GO:0016597 GO:0016743
GO:0003677 GO:0004675 GO:0016020
Cluster-22178.79776 GO:0006281 GO:0007186 GO:0004930 GO:0030915
GO:0016021
Cluster-22178.79778 GO:0008152 GO:0016491
Cluster-22178.79779 GO:0019076 GO:0005975 GO:0032801 GO:0006812
GO:0005261 GO:0005515 GO:0004415 GO:0033644
Cluster-22178.79788 GO:0006355 GO:0003712 GO:0005667
Cluster-22178.79789 GO:0006457 GO:0003723 GO:0042803 GO:0005515
GO:0051087 GO:0000774 GO:0008092 GO:0019013 GO:0005737
GO:0019898
Cluster-22178.79791 GO:0055114 GO:0006779 GO:0017022 GO:0016459
Cluster-22178.79793 GO:0007186 GO:0004930 GO:0016021
Cluster-22178.7980 GO:0009399
Cluster-22178.79801 GO:0006810 GO:0055085 GO:0006123 GO:0015992
GO:0006814 GO:0043039 GO:0005524 GO:0008270 GO:0003723
GO:0005215 GO:0004129 GO:0016876 GO:0016020 GO:0005739

GO:0045277 GO:0016021
Cluster-22178.79808 GO:0006144 GO:0006265 GO:0006206 GO:0006351
GO:0003916 GO:0008270 GO:0003676 GO:0003899 GO:0005515
GO:0003677 GO:0005694 GO:0005730
Cluster-22178.79809 GO:0008218 GO:0006118 GO:0008152 GO:0055114
GO:0006836 GO:0003995 GO:0019905 GO:0016491 GO:0016021
Cluster-22178.79812 GO:0010508 GO:0007155 GO:0051015
Cluster-22178.79813 GO:0055085 GO:0006886 GO:0016192 GO:0006810
GO:0022857 GO:0005215 GO:0016021 GO:0030117 GO:0016020
Cluster-22178.79814 GO:0051603 GO:0006511 GO:0004175 GO:0004298
GO:0005839 GO:0019773
Cluster-22178.79815 GO:0015031 GO:0006464 GO:0005515 GO:0003723

Cluster-22178.79821 GO:0007186 GO:0004930 GO:0016021
Cluster-22178.79822 GO:0007186 GO:0004930 GO:0016021
Cluster-22178.79827 GO:0008270 GO:0003723 GO:0005515 GO:0003676

Cluster-22178.79834 GO:0005515
Cluster-22178.79835 GO:0016021
Cluster-22178.79838 GO:0048013 GO:0019497 GO:0006771 GO:0006468
GO:0003993 GO:0005524 GO:0004672 GO:0046872 GO:0016773
GO:0005515 GO:0016020
Cluster-22178.79839 GO:0007186 GO:0051260 GO:0006464 GO:0004930
GO:0016020 GO:0016021
Cluster-22178.79846 GO:0006355 GO:0005634
Cluster-22178.79852 GO:0005515
Cluster-22178.79856 GO:0005198 GO:0005515 GO:0005882
Cluster-22178.79857 GO:0006812 GO:0055085 GO:0008324
Cluster-22178.79859 GO:0005887
Cluster-22178.79860 GO:0005515
Cluster-22178.79861 GO:0005515 GO:0016020
Cluster-22178.79863 GO:0006621 GO:0046923 GO:0005783 GO:0016021
Cluster-22178.79865 GO:0007165 GO:0006948 GO:0005515 GO:0004871

GO:0016020
Cluster-22178.79868 GO:0055114 GO:0016310 GO:0009069 GO:0006303
GO:0006631 GO:0030328 GO:0016491 GO:0005524 GO:0050151
GO:0004677 GO:0071949 GO:0016670 GO:0003677 GO:0005958
GO:0005634
Cluster-22178.79869 GO:0006303 GO:0030328 GO:0006631 GO:0009069
GO:0016310 GO:0055114 GO:0016670 GO:0003677 GO:0071949
GO:0050151 GO:0004677 GO:0016491 GO:0005524 GO:0005634
GO:0005958
Cluster-22178.79870 GO:0016567 GO:0005515 GO:0061630 GO:0004842
GO:0046872 GO:0008270 GO:0005622
Cluster-22178.79875 GO:0009401 GO:0006812 GO:0055085 GO:0008324
GO:0016021 GO:0016020
Cluster-22178.79876 GO:0003723
Cluster-22178.79885 GO:0018342 GO:0005515 GO:0004663 GO:0008270
GO:0008318 GO:0005968
Cluster-22178.79887 GO:0007186 GO:0006629 GO:0000166 GO:0004930
GO:0046872 GO:0016021
Cluster-22178.79889 GO:0005515
Cluster-22178.79891 GO:0006547 GO:0000105 GO:0030154 GO:0009116
GO:0006144 GO:0004635 GO:0004114 GO:0003824 GO:0016021
Cluster-22178.79897 GO:0009435 GO:0008987 GO:0051539 GO:0019804
GO:0016020
Cluster-22178.79900 GO:0009405 GO:0005198 GO:0005515
Cluster-22178.79902 GO:0005524 GO:0016787 GO:0003677
Cluster-22178.79906 GO:0051304 GO:0005515
Cluster-22178.79911 GO:0042967 GO:0016747 GO:0008080
Cluster-22178.79912 GO:0005515
Cluster-22178.79914 GO:0016567 GO:0003723 GO:0005515 GO:0003677
GO:0008270 GO:0046872 GO:0004842 GO:0005680
Cluster-22178.79915 GO:0035556 GO:0005515
Cluster-22178.79917 GO:0005515
Cluster-22178.79925 GO:0008608 GO:0009405 GO:0006118 GO:0009055

GO:0005506 GO:0020037 GO:0005515 GO:0005198 GO:0016021
GO:0072686 GO:0042729
Cluster-22178.79926 GO:0016020 GO:0016021
Cluster-22178.79929 GO:0006468 GO:0005524 GO:0004672
Cluster-22178.79932 GO:0015992 GO:0006123 GO:0004129 GO:0005576
GO:0005739 GO:0045277
Cluster-22178.79933 GO:0019722 GO:0003676
Cluster-22178.79934 GO:0005515
Cluster-22178.79939 GO:0007021 GO:0006355 GO:0006260 GO:0008053
GO:0006357 GO:0006914 GO:0006457 GO:0003700 GO:0043565
GO:0003924 GO:0001104 GO:0051082 GO:0015631 GO:0045298
GO:0016021 GO:0019028 GO:0016272 GO:0005741 GO:0016592
GO:0005667
Cluster-22178.79941 GO:0008152 GO:0006120 GO:0006744 GO:0015992
GO:0019049 GO:0005975 GO:0055114 GO:0006814 GO:0003824
GO:0008137 GO:0030246 GO:0016836
Cluster-22178.79942 GO:0006357 GO:0014068 GO:0050776 GO:0007165
GO:0005102 GO:0043548 GO:0001104 GO:0016020 GO:0016592
Cluster-22178.79946 GO:0006468 GO:0004672 GO:0005524
Cluster-22178.79947 GO:0016567 GO:0006955 GO:0007165 GO:0040007
GO:0008283 GO:0004842 GO:0008083 GO:0005136 GO:0003690
GO:0005737 GO:0016516 GO:0005576
Cluster-22178.79948 GO:0055114 GO:0006950 GO:0016638 GO:0003697
GO:0008270
Cluster-22178.79951 GO:0004386 GO:0005524 GO:0008237 GO:0008270
GO:0003723 GO:0005634
Cluster-22178.79953 GO:0006890 GO:0007165 GO:0006812 GO:0055085
GO:0009607 GO:0008324 GO:0016020 GO:0016021
Cluster-22178.79956 GO:0006725 GO:0016853
Cluster-22178.79958 GO:0035556 GO:0006351 GO:0005515
Cluster-22178.79962 GO:0006886 GO:0006605 GO:0009229 GO:0006772
GO:0030975 GO:0004788 GO:0005524 GO:0016020
Cluster-22178.79966 GO:0006281 GO:0051726 GO:0005096 GO:0003824

GO:0017025 GO:0003677 GO:0004518 GO:0005634
Cluster-22178.7997 GO:0005515
Cluster-22178.79970 GO:0006355 GO:0043093 GO:0000917 GO:0006811
GO:0055085 GO:0005216 GO:0005515 GO:0016020 GO:0005737
Cluster-22178.79972 GO:0006508 GO:0008270 GO:0004222 GO:0008233
GO:0031012
Cluster-22178.79977 GO:0016788
Cluster-22178.79980 GO:0016021
Cluster-22178.79994 GO:0055114 GO:0006412 GO:0042254 GO:0007156
GO:0016491 GO:0005509 GO:0003735 GO:0005840 GO:0005622
GO:0016020
Cluster-22178.79998 GO:0009607 GO:0016021
Cluster-22178.79999 GO:0015991 GO:0043248 GO:0016820 GO:0005515
GO:0000221
Cluster-22178.80011 GO:0035556 GO:0005515
Cluster-22178.80012 GO:0006886 GO:0006367 GO:0048280 GO:0048870
GO:0015031 GO:0008565 GO:0016020 GO:0031514 GO:0005672
GO:0000139 GO:0005737
Cluster-22178.80013 GO:0007186 GO:0004930 GO:0016021 GO:0016020
Cluster-22178.80019 GO:0005515
Cluster-22178.80022 GO:0006457 GO:0042803 GO:0005515 GO:0051087
GO:0004064 GO:0000774 GO:0008092 GO:0019898 GO:0005737
Cluster-22178.80023 GO:0055085 GO:0006810 GO:0006811 GO:0005215
GO:0016020
Cluster-22178.80024 GO:0007186 GO:0004930 GO:0016020
Cluster-22178.80025 GO:0007186 GO:0004930 GO:0043565 GO:0016021
GO:0016020 GO:0005634
Cluster-22178.80026 GO:0043039 GO:0050690 GO:0006418 GO:0008608
GO:0004812 GO:0000049 GO:0000166 GO:0005537 GO:0005524
GO:0003676 GO:0072686 GO:0042729 GO:0005737
Cluster-22178.80030 GO:0007186 GO:0006397 GO:0046660 GO:0034599
GO:0004252 GO:0004930 GO:0005634 GO:0019028 GO:0005886
GO:0016021 GO:0005789

Cluster-22178.80031 GO:0007186 GO:0006397 GO:0046660 GO:0034599 GO:0004252 GO:0004930 GO:0019028 GO:0005634 GO:0016021 GO:0005789 GO:0005886
Cluster-22178.80039 GO:0006355 GO:0010951 GO:0006952 GO:0008270 GO:0004866 GO:0005515
Cluster-22178.80040 GO:0006355 GO:0005515 GO:0008270
Cluster-22178.80041 GO:0006952 GO:0010951 GO:0006355 GO:0004866 GO:0005515 GO:0008270
Cluster-22178.80044 GO:0006355 GO:0003682 GO:0003677 GO:0005634 GO:0000775 GO:0000785
Cluster-22178.80048 GO:0006531 GO:0006522 GO:0006529 GO:0004066

Cluster-22178.80053 GO:0009058 GO:0030170
Cluster-22178.80055 GO:0005524 GO:0016772
Cluster-22178.80056 GO:0009094 GO:0008033 GO:0006571 GO:0000162 GO:0006432 GO:0005524 GO:0008168 GO:0004826 GO:0000287 GO:0000049 GO:0009328
Cluster-22178.80057 GO:0008134 GO:0042803 GO:0045502 GO:0005667 GO:0030286
Cluster-22178.80059 GO:0009307 GO:0006308 GO:0009036 GO:0000287 GO:0003677 GO:0009359
Cluster-22178.80061 GO:0042254 GO:0006412 GO:0006508 GO:0008234 GO:0003735 GO:0005840
Cluster-22178.80064 GO:0003676 GO:0004190 GO:0016021
Cluster-22178.80066 GO:0003676
Cluster-22178.80067 GO:0005515
Cluster-22178.80068 GO:0007017 GO:0007018 GO:0005524 GO:0003777 GO:0008017 GO:0005874 GO:0045298
Cluster-22178.80070 GO:0019497 GO:0006771 GO:0003677 GO:0003993

Cluster-22178.80071 GO:0006355 GO:0043565 GO:0003700 GO:0005634 GO:0005667
Cluster-22178.80072 GO:0015031 GO:0007131 GO:0007155 GO:0007165

GO:0006308 GO:0006904 GO:0006886 GO:0006955 GO:0008270
GO:0008855 GO:0005057 GO:0005125 GO:0005515 GO:0016020
GO:0000145 GO:0005737 GO:0005576 GO:0000794 GO:0009318
Cluster-22178.80073 GO:0055085 GO:0005741
Cluster-22178.80079 GO:0006270 GO:0006198 GO:0055085 GO:0030866
GO:0005198 GO:0004115 GO:0003779 GO:0008092 GO:0016021
GO:0005856
Cluster-22178.80080 GO:0030866 GO:0006198 GO:0008092 GO:0003779
GO:0005198 GO:0004115 GO:0005856
Cluster-22178.80082 GO:0006144 GO:0043161 GO:0015992 GO:0006206
GO:0006289 GO:0015986 GO:0009069 GO:0016310 GO:0007178
GO:0006351 GO:0006468 GO:0006281 GO:0005524 GO:0003684
GO:0004675 GO:0015078 GO:0003899 GO:0000276 GO:0016020
GO:0005730 GO:0005634
Cluster-22178.80083 GO:0009395 GO:0005515 GO:0004620
Cluster-22178.80085 GO:0008270
Cluster-22178.80095 GO:0016570 GO:0006355 GO:0006368 GO:0016593
Cluster-22178.80096 GO:0006886 GO:0003924 GO:0005515 GO:0005047
GO:0005525 GO:0005786 GO:0005785
Cluster-22178.80097 GO:0042967 GO:0006865 GO:0051205 GO:0003333
GO:0008080 GO:0015171 GO:0016021 GO:0019033 GO:0016020
Cluster-22178.80098 GO:0005515
Cluster-22178.80099 GO:0048306
Cluster-22178.80103 GO:0007169 GO:0006468 GO:0004714 GO:0005524
GO:0016020
Cluster-22178.80104 GO:0006470 GO:0006570 GO:0004222 GO:0004725
GO:0008138
Cluster-22178.80106 GO:0009405 GO:0009097 GO:0009099 GO:0055114
GO:0006438 GO:0008152 GO:0009098 GO:0000166 GO:0016706
GO:0005524 GO:0004832 GO:0016758 GO:0016020 GO:0005737
Cluster-22178.80107 GO:0009098 GO:0008152 GO:0006438 GO:0009099
GO:0009097 GO:0009405 GO:0016758 GO:0004832 GO:0005524
GO:0000166 GO:0005737 GO:0016020

Cluster-22178.80109 GO:0006438 GO:0009098 GO:0009405 GO:0009097 GO:0009099 GO:0005524 GO:0004832 GO:0000166 GO:0005737 GO:0016020
Cluster-22178.80114 GO:0016787
Cluster-22178.80117 GO:0005201 GO:0005515 GO:0005578 GO:0005581
Cluster-22178.80118 GO:0005515 GO:0005201 GO:0005581 GO:0005578
Cluster-22178.80119 GO:0005515
Cluster-22178.80120 GO:0006508 GO:0004197 GO:0008233
Cluster-22178.80121 GO:0040007 GO:0008283 GO:0007165 GO:0008083

Cluster-22178.80122 GO:0006355 GO:0007165 GO:0005198 GO:0003677 GO:0042974 GO:0005667 GO:0019013
Cluster-22178.80123 GO:0006278 GO:0003964
Cluster-22178.80128 GO:0055114 GO:0047134
Cluster-22178.80129 GO:0055114 GO:0047134
Cluster-22178.8013 GO:0005515
Cluster-22178.80130 GO:0007165 GO:0006508 GO:0005515 GO:0004197

Cluster-22178.80132 GO:0006032 GO:0007165 GO:0050830 GO:0005515 GO:0008061
Cluster-22178.80133 GO:0016020
Cluster-22178.80143 GO:0006508 GO:0008236 GO:0016787
Cluster-22178.80150 GO:0015994 GO:0004408 GO:0005739
Cluster-22178.80151 GO:0006144 GO:0006206 GO:0006351 GO:0003899 GO:0005730
Cluster-22178.80152 GO:0007155 GO:0016021
Cluster-22178.80155 GO:0042254 GO:0006744 GO:0015992 GO:0006814 GO:0006412 GO:0006118 GO:0006120 GO:0008152 GO:0005975 GO:0003735 GO:0004553 GO:0008137 GO:0003824 GO:0009055 GO:0008484 GO:0016020 GO:0005622 GO:0005840
Cluster-22178.80159 GO:0007155 GO:0032981
Cluster-22178.80160 GO:0046983
Cluster-22178.80167 GO:0055114 GO:0007186 GO:0016702 GO:0004930

GO:0016021
Cluster-22178.80170 GO:0016310 GO:0006468 GO:0009069 GO:0007165
GO:0005524 GO:0004672 GO:0004674 GO:0005179 GO:0003677
GO:0005887 GO:0005576
Cluster-22178.80171 GO:0009069 GO:0007165 GO:0006351 GO:0006206
GO:0006144 GO:0016310 GO:0005179 GO:0003677 GO:0003899
GO:0004674 GO:0005576 GO:0005730 GO:0005887
Cluster-22178.80177 GO:0006355 GO:0005515
Cluster-22178.80180 GO:0006510 GO:0006508 GO:0016567 GO:0004176
GO:0008270 GO:0046872 GO:0004842 GO:0005515 GO:0042802
GO:0061630
Cluster-22178.80183 GO:0006914 GO:0007165 GO:0016301 GO:0005515
GO:0016021
Cluster-22178.80184 GO:0008270
Cluster-22178.80185 GO:0006397 GO:0008270 GO:0004190 GO:0016021
Cluster-22178.80186 GO:0004867
Cluster-22178.80187 GO:0019064 GO:0007034 GO:0019031
Cluster-22178.80190 GO:0007165 GO:0006355 GO:0003677 GO:0005515

Cluster-22178.80191 GO:0007165 GO:0006355 GO:0005515 GO:0003677

Cluster-22178.80198 GO:0006412 GO:0042254 GO:0003735 GO:0015934
GO:0005840
Cluster-22178.80201 GO:0071816 GO:0005515 GO:0016021
Cluster-22178.80202 GO:0071816 GO:0016021
Cluster-22178.80212 GO:0006570 GO:0006470 GO:0055114 GO:0005515
GO:0008138 GO:0050661 GO:0004725 GO:0050660 GO:0004499
Cluster-22178.80213 GO:0005515
Cluster-22178.80216 GO:0016021
Cluster-22178.80219 GO:0007218 GO:0006355 GO:0046872 GO:0005184
GO:0003677 GO:0005198 GO:0019031 GO:0005576
Cluster-22178.80225 GO:0007155 GO:0007165 GO:0006914 GO:0048870
GO:0030168 GO:0000921 GO:0051258 GO:0030674 GO:0005515

GO:0005509 GO:0005102 GO:0031514 GO:0005940 GO:0016021
GO:0005577
Cluster-22178.80226 GO:0006614 GO:0006810 GO:0002949 GO:0006260
GO:0003723 GO:0016887 GO:0003924 GO:0005524 GO:0003724
GO:0003678 GO:0005525 GO:0030288 GO:0005657 GO:0019031
Cluster-22178.80229 GO:0006397 GO:0016788
Cluster-22178.80231 GO:0006260 GO:0006139 GO:0019073 GO:0003676
GO:0003723 GO:0003677 GO:0003887 GO:0005515 GO:0008408
GO:0042575
Cluster-22178.80234 GO:0006468 GO:0004672 GO:0005524 GO:0016773
GO:0016020
Cluster-22178.80239 GO:0019646 GO:0006955 GO:0006118 GO:0000902
GO:0045454 GO:0015035 GO:0009055 GO:0016020
Cluster-22178.80242 GO:0042157 GO:0007093 GO:0006869 GO:0008289
GO:0005576 GO:0000776 GO:0016020
Cluster-22178.80243 GO:0001510 GO:0031167 GO:0006306 GO:0009452
GO:0008152 GO:0008170 GO:0005515 GO:0003677 GO:0008168
GO:0005201 GO:0005578 GO:0019013 GO:0005581 GO:0016021
Cluster-22178.80245 GO:0006412 GO:0042254 GO:0003735 GO:0005840
GO:0005739
Cluster-22178.80251 GO:0006412 GO:0042254 GO:0006281 GO:0035556
GO:0046034 GO:0006464 GO:0006144 GO:0003735 GO:0005515
GO:0004017 GO:0016151 GO:0046872 GO:0008270 GO:0005840
Cluster-22178.80252 GO:0006811 GO:0055085 GO:0048511 GO:0007268
GO:0007165 GO:0006813 GO:0004970 GO:0005267 GO:0005216
GO:0016020
Cluster-22178.80253 GO:0006464 GO:0006144 GO:0035556 GO:0046034
GO:0006281 GO:0042254 GO:0006412 GO:0008270 GO:0046872
GO:0016151 GO:0004017 GO:0003735 GO:0005515 GO:0005840
Cluster-22178.80254 GO:0007094 GO:0042742
Cluster-22178.80263 GO:0005515
Cluster-22178.80268 GO:0007165
Cluster-22178.80271 GO:0006629

Cluster-22178.80272 GO:0008152 GO:0003824 GO:0008270
Cluster-22178.80273 GO:0035556
Cluster-22178.80277 GO:0008168
Cluster-22178.80278 GO:0006888 GO:0005801 GO:0005622
Cluster-22178.80281 GO:0006270 GO:0005096
Cluster-22178.80282 GO:0046872 GO:0004222
Cluster-22178.80287 GO:0006605 GO:0007165 GO:0051539 GO:0005515
GO:0005887 GO:0016020
Cluster-22178.80290 GO:0006952
Cluster-22178.80298 GO:0015946 GO:0055114 GO:0004022
Cluster-22178.80299 GO:0006270 GO:0046935 GO:0032040 GO:0005944
Cluster-22178.80305 GO:0007165 GO:0005509 GO:0005515 GO:0005578
Cluster-22178.80307 GO:0007165 GO:0006812 GO:0006885 GO:0055085
GO:0007218 GO:0005184 GO:0005057 GO:0015299 GO:0005576
GO:0016021
Cluster-22178.80310 GO:0000162 GO:0006571 GO:0009074 GO:0009821
GO:0006103 GO:0009094 GO:0006511 GO:0007275 GO:0006536
GO:0004838 GO:0030170 GO:0005634
Cluster-22178.80311 GO:0035556 GO:0008270
Cluster-22178.80312 GO:0006606 GO:0048870 GO:0006914 GO:0051607
GO:0007155 GO:0008092 GO:0019898 GO:0005737 GO:0009424
GO:0031514
Cluster-22178.80315 GO:0055085 GO:0016021
Cluster-22178.80318 GO:0007218 GO:0007050 GO:0006886 GO:0016192
GO:0043565 GO:0005185 GO:0005515 GO:0005198 GO:0019031
GO:0005576 GO:0030117 GO:0019028
Cluster-22178.80327 GO:0008289
Cluster-22178.80333 GO:0055114 GO:0006779 GO:0015994 GO:0007165
GO:0004109 GO:0005509 GO:0005578
Cluster-22178.80335 GO:0016021
Cluster-22178.80343 GO:0005543
Cluster-22178.80345 GO:0007155 GO:0001503 GO:0046935 GO:0003676
GO:0005944 GO:0005576

Cluster-22178.80348 GO:0046872

Cluster-22178.8035 GO:0006508 GO:0004252

Cluster-22178.80350 GO:0005515

Cluster-22178.80355 GO:0006355 GO:0009405 GO:0005515 GO:0005634

Cluster-22178.80358 GO:0007010 GO:0030001 GO:0005975 GO:0016192 GO:0017004 GO:0007165 GO:0006810 GO:0055085 GO:0006357 GO:0015886 GO:0001104 GO:0005515 GO:0005215 GO:0003779 GO:0016853 GO:0046873 GO:0004872 GO:0043395 GO:0005578 GO:0016021 GO:0019031 GO:0016020 GO:0016592

Cluster-22178.80361 GO:0045454 GO:0006118 GO:0055114 GO:0031412 GO:0005516 GO:0016209 GO:0016491 GO:0015035 GO:0009055 GO:0031411 GO:0031514

Cluster-22178.80364 GO:0006281 GO:0006351 GO:0006464 GO:0006206 GO:0006144 GO:0003677 GO:0005515 GO:0003899 GO:0046872 GO:0008270 GO:0016151 GO:0005730

Cluster-22178.80365 GO:0008608 GO:0042254 GO:0006412 GO:0003735 GO:0005840 GO:0005622 GO:0042729 GO:0072686

Cluster-22178.80367 GO:0042254 GO:0006412 GO:0003735 GO:0005840

Cluster-22178.80370 GO:0015979 GO:0042254 GO:0006412 GO:0003677 GO:0003735 GO:0005515 GO:0005840 GO:0009539 GO:0005622 GO:0009523 GO:0016020

Cluster-22178.80372 GO:0006355 GO:0005524 GO:0003677 GO:0005634

Cluster-22178.80375 GO:0016579 GO:0036459 GO:0005096 GO:0005515

Cluster-22178.80382 GO:0051726 GO:0006281 GO:0003824 GO:0004518 GO:0003677 GO:0017025 GO:0005634

Cluster-22178.80383 GO:0007156 GO:0005509 GO:0004222 GO:0016020

Cluster-22178.80385 GO:0003676 GO:0004190 GO:0016021

Cluster-22178.80387 GO:0006771 GO:0019497 GO:0003993 GO:0046872 GO:0016787

Cluster-22178.80390 GO:0017111

Cluster-22178.80395 GO:0003723 GO:0003676 GO:0019013

Cluster-22178.80398 GO:0006810 GO:0005215 GO:0005887

Cluster-22178.80399 GO:0016226
Cluster-22178.80404 GO:0006270 GO:0046983
Cluster-22178.80407 GO:0006508 GO:0005515 GO:0004252
Cluster-22178.80410 GO:0007165 GO:0006355 GO:0001664 GO:0003677

Cluster-22178.80411 GO:0000902 GO:0006955 GO:0016020
Cluster-22178.80418 GO:0016579 GO:0036459 GO:0070140 GO:0032183

Cluster-22178.80420 GO:0007049 GO:0051082
Cluster-22178.80422 GO:0008641
Cluster-22178.80423 GO:0006952 GO:0006810 GO:0009405 GO:0019855
GO:0005576
Cluster-22178.80424 GO:0000290 GO:0005975 GO:0003824 GO:0043169
GO:0016787
Cluster-22178.80425 GO:0006470 GO:0006570 GO:0004725
Cluster-22178.80426 GO:0000062
Cluster-22178.80429 GO:0003676
Cluster-22178.80431 GO:0030145 GO:0004177 GO:0016021
Cluster-22178.80433 GO:0016020
Cluster-22178.8044 GO:0006120 GO:0051252 GO:0006397 GO:0055114
GO:0004222 GO:0004540
Cluster-22178.80440 GO:0022900 GO:0008152 GO:0046983 GO:0016787
GO:0003677 GO:0005739 GO:0005747
Cluster-22178.80444 GO:0019439 GO:0007165 GO:0006508 GO:0055114
GO:0005975 GO:0006090 GO:0008152 GO:0043169 GO:0008774
GO:0004872 GO:0070008 GO:0008236 GO:0004252 GO:0008168
GO:0046872 GO:0003824 GO:0016020 GO:0016021
Cluster-22178.80446 GO:0006637 GO:0055114 GO:0006260 GO:0006635
GO:0046907 GO:0003997 GO:0003887 GO:0003677 GO:0008408
GO:0005777 GO:0005664 GO:0042575
Cluster-22178.80448 GO:0016020 GO:0016021
Cluster-22178.80455 GO:0055085 GO:0006811 GO:0005216 GO:0016020
Cluster-22178.80456 GO:0007010 GO:0000910 GO:0000226 GO:0006260

GO:0004190 GO:0003723 GO:0003677 GO:0003779 GO:0003676
GO:0008017 GO:0045298 GO:0016021 GO:0005664
Cluster-22178.80457 GO:0006811 GO:0055085 GO:0005216 GO:0016020
Cluster-22178.80458 GO:0000910 GO:0046660 GO:0000226 GO:0006260
GO:0007010 GO:0006397 GO:0003779 GO:0008017 GO:0003676
GO:0003723 GO:0003677 GO:0045298 GO:0005664 GO:0005634
Cluster-22178.80459 GO:0051258 GO:0030168 GO:0007165 GO:0005102
GO:0030674 GO:0005577 GO:0030133 GO:0031083
Cluster-22178.80460 GO:0005515
Cluster-22178.80472 GO:0048193 GO:0006355 GO:0005524 GO:0003774
GO:0003677 GO:0008092 GO:0003700 GO:0005507 GO:0005198
GO:0005515 GO:0019898 GO:0005667 GO:0016459 GO:0016020
GO:0005882 GO:0005737 GO:0005634
Cluster-22178.80474 GO:0050853 GO:0050852 GO:0005515 GO:0005198
GO:0008134 GO:0042803 GO:0045502 GO:0016787 GO:0003774
GO:0005524 GO:0003779 GO:0005882 GO:0016459 GO:0005667
GO:0030286
Cluster-22178.80478 GO:0005525
Cluster-22178.80480 GO:0019079 GO:0006468 GO:0008026 GO:0005524
GO:0004672
Cluster-22178.80482 GO:0016787
Cluster-22178.80484 GO:0055114 GO:0010181 GO:0016491
Cluster-22178.80485 GO:0015992 GO:0006119 GO:0006118 GO:0006813
GO:0009055 GO:0008121 GO:0005242 GO:0016021 GO:0008076
Cluster-22178.80486 GO:0006119 GO:0006118 GO:0006813 GO:0015992
GO:0008121 GO:0005242 GO:0009055 GO:0008076 GO:0016021
Cluster-22178.80489 GO:0046983
Cluster-22178.80490 GO:0006810 GO:0006355 GO:0007049 GO:0003700
GO:0043565 GO:0046983 GO:0005667 GO:0005886
Cluster-22178.80492 GO:0006000 GO:0006144 GO:0006013 GO:0000103
GO:0015976 GO:0006790 GO:0008152 GO:0006099 GO:0006094
GO:0005524 GO:0003873 GO:0003924 GO:0005525 GO:0004020
GO:0004781 GO:0016301 GO:0004612

Cluster-22178.80496 GO:0005515
Cluster-22178.80497 GO:0005543
Cluster-22178.80500 GO:0016787
Cluster-22178.80501 GO:0006366 GO:0016192 GO:0006886 GO:0005515
GO:0005198 GO:0003677 GO:0030117 GO:0005576 GO:0005665
Cluster-22178.80505 GO:0031072 GO:0005198 GO:0051082 GO:0019028
Cluster-22178.80509 GO:0030182 GO:0006144 GO:0006730 GO:0006206
GO:0006351 GO:0021549 GO:0003676 GO:0008168 GO:0003899
GO:0003677 GO:0005730
Cluster-22178.80510 GO:0005515
Cluster-22178.80512 GO:0016567 GO:0004842 GO:0005515 GO:0008270
GO:0046872
Cluster-22178.80520 GO:0016310 GO:0008017 GO:0043015 GO:0042802
GO:0004673 GO:0009365 GO:0045298 GO:0016021 GO:0005634
Cluster-22178.80522 GO:0019079 GO:0007165 GO:0043087 GO:0007264
GO:0005515 GO:0005085
Cluster-22178.80524 GO:0006260 GO:0003677 GO:0019028 GO:0005634
Cluster-22178.80525 GO:0005515
Cluster-22178.80526 GO:0006355 GO:0015074 GO:0003676 GO:0003700
GO:0008270 GO:0005667
Cluster-22178.80529 GO:0006351 GO:0006355 GO:0006206 GO:0006144
GO:0034080 GO:0006869 GO:0007186 GO:0042157 GO:0008289
GO:0003676 GO:0003700 GO:0003899 GO:0005515 GO:0004930
GO:0003677 GO:0000775 GO:0016021 GO:0005576 GO:0005730
GO:0005667
Cluster-22178.80531 GO:0008152 GO:0003824 GO:0016836
Cluster-22178.80537 GO:0016021
Cluster-22178.80538 GO:0042967 GO:0004379
Cluster-22178.80539 GO:0035556 GO:0006144 GO:0055114 GO:0006464
GO:0006206 GO:0006351 GO:0006281 GO:0047134 GO:0016151
GO:0046872 GO:0008270 GO:0003899 GO:0003677 GO:0005730
Cluster-22178.80541 GO:0006810 GO:2001070 GO:0016021
Cluster-22178.80542 GO:0006813 GO:0019354 GO:0046487 GO:0055114

GO:0009396 GO:0006730 GO:0006520 GO:0006555 GO:0006779
GO:0051287 GO:0004013 GO:0043115 GO:0004488 GO:0016491
GO:0003824
Cluster-22178.80543 GO:0006813 GO:0019354 GO:0046487 GO:0055114
GO:0006730 GO:0009396 GO:0006520 GO:0006555 GO:0006779
GO:0051287 GO:0004013 GO:0043115 GO:0004488 GO:0016491
GO:0003824
Cluster-22178.80544 GO:0005515
Cluster-22178.80547 GO:0009401 GO:0006090 GO:0006560 GO:0006412
GO:0006814 GO:0006525 GO:0071436 GO:0042254 GO:0008948
GO:0005515 GO:0015081 GO:0016787 GO:0003735 GO:0005840
GO:0016021 GO:0015934 GO:0016020
Cluster-22178.80548 GO:0006560 GO:0006814 GO:0006412 GO:0006090
GO:0016310 GO:0071436 GO:0042254 GO:0006525 GO:0009069
GO:0005515 GO:0008384 GO:0008948 GO:0003735 GO:0016787
GO:0015081 GO:0015934 GO:0008385 GO:0005840 GO:0016020
Cluster-22178.80552 GO:0006812 GO:0006836 GO:0005328 GO:0016021
Cluster-22178.80553 GO:0006812 GO:0006836 GO:0005328 GO:0016021
Cluster-22178.80554 GO:0006812 GO:0006836 GO:0005328 GO:0016021
Cluster-22178.80556 GO:0030155 GO:0007165 GO:0045995 GO:0006308
GO:0030334 GO:0005515 GO:0005102 GO:0008855 GO:0009318
Cluster-22178.80560 GO:0008270 GO:0005509 GO:0016772 GO:0008134
GO:0008201 GO:0005667 GO:0005634
Cluster-22178.80565 GO:0006810 GO:0006310 GO:0003677 GO:0005515
GO:0042626 GO:0000150
Cluster-22178.80570 GO:0017187 GO:0006313 GO:0046983 GO:0004803
GO:0005198 GO:0003677 GO:0008488
Cluster-22178.80571 GO:0006629 GO:0008270 GO:0005524 GO:0004222
GO:0016021
Cluster-22178.80572 GO:0008152 GO:0005509 GO:0005515 GO:0016787

Cluster-22178.80575 GO:0016567 GO:0008270 GO:0046872 GO:0004842
GO:0005515 GO:0005680

Cluster-22178.80577 GO:0006446 GO:0006270 GO:0016579 GO:0008152
GO:0003743 GO:0010333 GO:0036459 GO:0046872 GO:0016829
GO:0005852 GO:0005737 GO:0005840
Cluster-22178.80584 GO:0006355 GO:0005515 GO:0003677 GO:0005856
GO:0005634
Cluster-22178.8059 GO:0005515 GO:0005509
Cluster-22178.80591 GO:0005634
Cluster-22178.80595 GO:0005515
Cluster-22178.80597 GO:0006118 GO:0017004 GO:0005515 GO:0045158
GO:0009512
Cluster-22178.80609 GO:0000746 GO:0019867
Cluster-22178.80615 GO:0007165 GO:0006397 GO:0016567 GO:0042967
GO:0003723 GO:0005125 GO:0004842 GO:0008080 GO:0005634
GO:0005737 GO:0005680
Cluster-22178.80617 GO:0006355 GO:0007275 GO:0003700 GO:0000975
GO:0042600 GO:0005667
Cluster-22178.80621 GO:0008289 GO:0005515 GO:0016301 GO:0005524

Cluster-22178.80622 GO:0046740 GO:0016021 GO:0016020
Cluster-22178.80623 GO:0006355 GO:0003700 GO:0005815 GO:0005667
Cluster-22178.80624 GO:0006810 GO:0055085 GO:0055114 GO:0016301
GO:0005525 GO:0042626 GO:0005524 GO:0016627 GO:0016887
GO:0003924 GO:0016021
Cluster-22178.80627 GO:0001558 GO:0005520 GO:0016942 GO:0005576
Cluster-22178.80630 GO:0006355 GO:0007165 GO:0016310 GO:0000160
GO:0000155 GO:0003677 GO:0005634 GO:0009365
Cluster-22178.80634 GO:0007021 GO:0051082 GO:0015631 GO:0045298
Cluster-22178.80638 GO:0006352 GO:0004190 GO:0005198 GO:0005634
GO:0005669 GO:0016021
Cluster-22178.80643 GO:0006810 GO:0003333 GO:0015979 GO:0055085
GO:0006865 GO:0015171 GO:0016020 GO:0009538 GO:0009522
Cluster-22178.80644 GO:0016192 GO:0006886 GO:0006914 GO:0005198
GO:0030132 GO:0030130 GO:0030176

Cluster-22178.80649 GO:0016787
Cluster-22178.80650 GO:0016070 GO:0010508 GO:0006308 GO:0031110
GO:0008855 GO:0009318 GO:0016020
Cluster-22178.80655 GO:0006525 GO:0006560 GO:0055114 GO:0051321
GO:0018401 GO:0004656 GO:0016491 GO:0016702 GO:0005783
Cluster-22178.80656 GO:0006560 GO:0006525 GO:0018401 GO:0009401
GO:0055114 GO:0051321 GO:0003677 GO:0004656 GO:0016491
GO:0016702 GO:0005783 GO:0005634
Cluster-22178.80657 GO:0006525 GO:0006560 GO:0055114 GO:0051321
GO:0018401 GO:0004656 GO:0016491 GO:0016702 GO:0005783
Cluster-22178.80658 GO:0009058 GO:0006520 GO:0006729 GO:0019752
GO:0016740 GO:0030170 GO:0008124 GO:0016829 GO:0016831
Cluster-22178.80661 GO:0005886
Cluster-22178.80663 GO:0005524
Cluster-22178.80669 GO:0009405 GO:0006869 GO:0007155 GO:0042157
GO:0070206 GO:0006914 GO:0016301 GO:0005515 GO:0008289
GO:0008270 GO:0046872 GO:0005576 GO:0005622
Cluster-22178.80670 GO:0006914 GO:0070206 GO:0006869 GO:0009405
GO:0007155 GO:0042157 GO:0008289 GO:0008270 GO:0046872
GO:0016301 GO:0005515 GO:0005622 GO:0005576
Cluster-22178.80672 GO:0006269 GO:0006351 GO:0006260 GO:0003896
GO:0005730 GO:0005657
Cluster-22178.80673 GO:0042254 GO:0006412 GO:0008270 GO:0046872
GO:0046983 GO:0003735 GO:0005622 GO:0005840
Cluster-22178.80674 GO:0030336 GO:0010972
Cluster-22178.80676 GO:0006355 GO:0046983
Cluster-22178.80677 GO:0006468 GO:0005524 GO:0004672
Cluster-22178.80678 GO:0006468 GO:0004672 GO:0005524
Cluster-22178.80679 GO:0005525 GO:0005515 GO:0003924
Cluster-22178.80680 GO:0003924 GO:0005515 GO:0005525
Cluster-22178.80682 GO:0006816 GO:0005388 GO:0016529
Cluster-22178.80685 GO:0006816 GO:0046872 GO:0005388 GO:0008270
GO:0000166 GO:0016529

Cluster-22178.80686 GO:0006816 GO:0008270 GO:0046872 GO:0005388 GO:0000166 GO:0016529

Cluster-22178.80691 GO:0005576

Cluster-22178.80692 GO:0006355 GO:0008270 GO:0005515 GO:0003700 GO:0005634 GO:0005667

Cluster-22178.80693 GO:0006364 GO:0008168 GO:0005634

Cluster-22178.80697 GO:0007165 GO:0016192 GO:0006886 GO:0008283 GO:0040007 GO:0051015 GO:0008083 GO:0030674 GO:0008092 GO:0030131 GO:0019898 GO:0005737

Cluster-22178.80702 GO:0006479 GO:0034968 GO:0006554 GO:0005515 GO:0003677 GO:0018024 GO:0008270 GO:0005634

Cluster-22178.80708 GO:0006486 GO:0008373

Cluster-22178.80709 GO:0019856 GO:0007186 GO:0055085 GO:0004930 GO:0008440 GO:0016021

Cluster-22178.80712 GO:0016020

Cluster-22178.80713 GO:0016020

Cluster-22178.80714 GO:0016020

Cluster-22178.80716 GO:0016192 GO:0006886 GO:0008270 GO:0005515

Cluster-22178.80721 GO:0042254 GO:0006412 GO:0006465 GO:0003677 GO:0003735 GO:0008233 GO:0005787 GO:0005622 GO:0005840 GO:0016021

Cluster-22178.80727 GO:0015886 GO:0006820 GO:0006355 GO:0017004 GO:0008509 GO:0003676 GO:0005622 GO:0016021

Cluster-22178.80729 GO:0006355 GO:0005515 GO:0043565 GO:0016021 GO:0005634

Cluster-22178.80730 GO:0003676 GO:0003723 GO:0019013

Cluster-22178.80731 GO:0005975 GO:0006465 GO:0035556 GO:0005515 GO:0008233 GO:0030248 GO:0005787 GO:0016021

Cluster-22178.80732 GO:0006355 GO:0006351 GO:0006144 GO:0003723 GO:0003677 GO:0005524 GO:0008092 GO:0003968 GO:0005737 GO:0031379 GO:0019898

Cluster-22178.80735 GO:0006144 GO:0006351 GO:0006355 GO:0003968

GO:0005524 GO:0008092 GO:0003677 GO:0003723 GO:0019898
GO:0031379 GO:0005737
Cluster-22178.80739 GO:0016567 GO:0008270 GO:0046872 GO:0005515
GO:0004842
Cluster-22178.80741 GO:0006887 GO:0016579 GO:0036459 GO:0000145
Cluster-22178.80743 GO:0051252 GO:0006397 GO:0006468 GO:0016773
GO:0005524 GO:0004672 GO:0004540 GO:0016020
Cluster-22178.80744 GO:0035556 GO:0043087 GO:0007165 GO:0007186
GO:0005089 GO:0004871 GO:0005834 GO:0005737
Cluster-22178.80745 GO:0015992 GO:0006826 GO:0015986 GO:0008849
GO:0015078 GO:0005506 GO:0005737 GO:0045263
Cluster-22178.80747 GO:0042254 GO:0006412 GO:0003723 GO:0003676
GO:0003735 GO:0005622 GO:0005840
Cluster-22178.80749 GO:0006810 GO:0055085 GO:0005215 GO:0022857
GO:0016021 GO:0016020
Cluster-22178.80751 GO:0055085 GO:0006810 GO:0022857 GO:0005215
GO:0004177 GO:0016020 GO:0016021
Cluster-22178.80753 GO:0055085 GO:0006810 GO:0022857 GO:0005215
GO:0016021 GO:0016020
Cluster-22178.80756 GO:0005515
Cluster-22178.80760 GO:0016020
Cluster-22178.80761 GO:0009405 GO:0006468 GO:0006629 GO:0016055
GO:0035091 GO:0005524 GO:0004672 GO:0005576
Cluster-22178.80767 GO:0008146 GO:0016021
Cluster-22178.80770 GO:0016567 GO:0006359 GO:0004842 GO:0004859
GO:0005515 GO:0005680 GO:0005576
Cluster-22178.80771 GO:0032259 GO:0055114 GO:0008270 GO:0008168
GO:0016903 GO:0005515
Cluster-22178.80772 GO:0006364 GO:0015986 GO:0015992 GO:0015074
GO:0015078 GO:0032040 GO:0000276
Cluster-22178.80773 GO:0035556 GO:0005515 GO:0008270
Cluster-22178.80774 GO:0006506 GO:0006260 GO:0017176 GO:0016021
GO:0005634

Cluster-22178.80777 GO:0007156 GO:0005198 GO:0005509 GO:0016020
Cluster-22178.80784 GO:0007165 GO:0005515
Cluster-22178.80785 GO:0005515
Cluster-22178.80790 GO:0004252 GO:0016021
Cluster-22178.80791 GO:0006955 GO:0030153 GO:0046813 GO:0061025
GO:0015643 GO:0019814 GO:0016021 GO:0019031
Cluster-22178.80793 GO:0006535 GO:0042967 GO:0006109 GO:0055114
GO:0008152 GO:0016310 GO:0000160 GO:0006534 GO:0000155
GO:0050662 GO:0051287 GO:0005524 GO:0016491 GO:0009001
GO:0004672 GO:0046872 GO:0003824 GO:0005737 GO:0009365
Cluster-22178.80798 GO:0016567 GO:0046872 GO:0004842
Cluster-22178.80799 GO:0007165 GO:0007264 GO:0005525
Cluster-22178.80800 GO:0007264 GO:0007165 GO:0005525
Cluster-22178.80802 GO:0007017 GO:0007018 GO:0005524 GO:0003777
GO:0008017 GO:0005515 GO:0005874 GO:0045298
Cluster-22178.80805 GO:0006388 GO:0016772 GO:0003950
Cluster-22178.80806 GO:0045893 GO:0006413 GO:0006118 GO:0006446
GO:0007275 GO:0008270 GO:0009055 GO:0003743 GO:0020037
GO:0005515 GO:0016021 GO:0005840
Cluster-22178.80808 GO:0042967 GO:0016747 GO:0047961 GO:0008080
GO:0005739
Cluster-22178.80809 GO:0055114 GO:0005515
Cluster-22178.80810 GO:0031505 GO:0016020
Cluster-22178.80814 GO:0030436 GO:0006397 GO:0007165 GO:0003723
GO:0004190 GO:0005179 GO:0005737 GO:0016021 GO:0042601
GO:0005634 GO:0005576
Cluster-22178.80816 GO:0005515 GO:0008270 GO:0005622
Cluster-22178.80818 GO:0006511 GO:0031625
Cluster-22178.80820 GO:0005975 GO:0006355 GO:0055114 GO:0009271
GO:0030246 GO:0016209 GO:0016491
Cluster-22178.80826 GO:0006446 GO:0006413 GO:0005515 GO:0003743
GO:0031369 GO:0005840 GO:0005852
Cluster-22178.80834 GO:0005509

Cluster-22178.80837 GO:0006098 GO:0009165 GO:0006144 GO:0019825
GO:0000287 GO:0004749 GO:0020037
Cluster-22178.80838 GO:0007186 GO:0009405 GO:0006810 GO:0007165
GO:0003924 GO:0031683 GO:0019001 GO:0004871 GO:0005525
GO:0008200 GO:0003677 GO:0005576
Cluster-22178.80845 GO:0006820 GO:0008509 GO:0016021
Cluster-22178.80848 GO:0006820 GO:0016740 GO:0005524 GO:0008509
GO:0016021
Cluster-22178.80849 GO:0006470 GO:0006120 GO:0006744 GO:0015992
GO:0055114 GO:0006814 GO:0006570 GO:0004725 GO:0005524
GO:0008138 GO:0008137
Cluster-22178.80851 GO:0006508 GO:0004185
Cluster-22178.80856 GO:0002949 GO:0006525 GO:0006310 GO:0006355
GO:0006281 GO:0008295 GO:0006560 GO:0016887 GO:0005524
GO:0003724 GO:0004014 GO:0009378 GO:0003678 GO:0003723
GO:0005515 GO:0008134 GO:0005657 GO:0005667 GO:0009379
Cluster-22178.80860 GO:0006310 GO:0006525 GO:0002949 GO:0008295
GO:0006560 GO:0006281 GO:0006355 GO:0009378 GO:0003724
GO:0004014 GO:0016887 GO:0005524 GO:0008134 GO:0005515
GO:0003723 GO:0003678 GO:0009379 GO:0005667 GO:0005657
Cluster-22178.80867 GO:0016032 GO:0006468 GO:0004672 GO:0005524

Cluster-22178.80868 GO:0006560 GO:0006525 GO:0018401 GO:0055114
GO:0004656 GO:0016491 GO:0016702 GO:0005783
Cluster-22178.80869 GO:0055114 GO:0006631 GO:0016491 GO:0071949
GO:0005515 GO:0050151
Cluster-22178.80870 GO:0005515 GO:0046983
Cluster-22178.80871 GO:0007049 GO:0017053
Cluster-22178.80877 GO:0007186 GO:0007165 GO:0006468 GO:0004871
GO:0005524 GO:0004672 GO:0005834
Cluster-22178.80878 GO:0006471 GO:0019087 GO:0003956 GO:0016021
Cluster-22178.80881 GO:0042254 GO:0005730
Cluster-22178.80882 GO:0006470 GO:0050852 GO:0055114 GO:0006570

GO:0004725 GO:0016972 GO:0005515 GO:0016021
Cluster-22178.80887 GO:0051539
Cluster-22178.80889 GO:0051607
Cluster-22178.80891 GO:0046872 GO:0016020
Cluster-22178.80892 GO:0005515 GO:0043565 GO:0005634
Cluster-22178.80893 GO:0006355 GO:0005634
Cluster-22178.80894 GO:0006270 GO:0046983
Cluster-22178.80896 GO:0006270 GO:0046983
Cluster-22178.80897 GO:0006614 GO:0006270 GO:0008312 GO:0046983 GO:0048500
Cluster-22178.80898 GO:0006123 GO:0015992 GO:0004129 GO:0003774 GO:0005198 GO:0045277 GO:0005747 GO:0005882 GO:0005740 GO:0005739 GO:0016459
Cluster-22178.80899 GO:0006123 GO:0006606 GO:0015992 GO:0005198 GO:0004129 GO:0003774 GO:0005747 GO:0005882 GO:0005740 GO:0005739 GO:0016459 GO:0045277
Cluster-22178.80901 GO:0006367 GO:0010506 GO:0006914 GO:0006464 GO:0010508 GO:0006886 GO:0030001 GO:0006355 GO:0007049 GO:0055114 GO:0008113 GO:0046872 GO:0005783 GO:0005669 GO:0016021 GO:0005615
Cluster-22178.80902 GO:0032968 GO:0015743 GO:0006367 GO:0005975 GO:0000910 GO:0000226 GO:0004190 GO:0003677 GO:0004649 GO:0008017 GO:0005634 GO:0016021 GO:0045298
Cluster-22178.80904 GO:0016579 GO:0006270 GO:0036459 GO:0046872

Cluster-22178.80906 GO:0008168
Cluster-22178.80917 GO:0055114 GO:0009690 GO:0006468 GO:0016773 GO:0004672 GO:0005524 GO:0050660 GO:0019139 GO:0016020
Cluster-22178.80918 GO:0009451 GO:0042254 GO:0006412 GO:0008033 GO:0003723 GO:0003735 GO:0004809 GO:0005622 GO:0005840
Cluster-22178.80920 GO:0006886 GO:0005525 GO:0003924 GO:0046872 GO:0005515 GO:0005047 GO:0044212 GO:0005785 GO:0016021 GO:0005786

Cluster-22178.80922 GO:0019028
Cluster-22178.80925 GO:0046872
Cluster-22178.80926 GO:0042967 GO:0006396 GO:0006397 GO:0008080
GO:0016747 GO:0005515 GO:0005622 GO:0005634
Cluster-22178.80929 GO:0015991 GO:0010508 GO:0006904 GO:0048870
GO:0007264 GO:0006914 GO:0015992 GO:0015031 GO:0006260
GO:0006606 GO:0051082 GO:0019901 GO:0005515 GO:0003924
GO:0005525 GO:0015078 GO:0005509 GO:0031514 GO:0033179
GO:0000145
Cluster-22178.80930 GO:0017111
Cluster-22178.80933 GO:0006508 GO:0004222 GO:0008233 GO:0008270
GO:0031012
Cluster-22178.80940 GO:0019497 GO:0006771 GO:0003993 GO:0005515
GO:0046872 GO:0016021 GO:0005576
Cluster-22178.80942 GO:0005515
Cluster-22178.80944 GO:0006508 GO:0005515 GO:0004197 GO:0005739
Cluster-22178.80948 GO:0006352 GO:0003677 GO:0005634
Cluster-22178.80949 GO:0006468 GO:0004672 GO:0005524 GO:0016773
GO:0016020
Cluster-22178.80951 GO:0006308 GO:0007015 GO:0006886 GO:0006914
GO:0007155 GO:0006897 GO:0046872 GO:0005515 GO:0005198
GO:0008270 GO:0008855 GO:0061630 GO:0009288 GO:0019031
GO:0005801 GO:0016021 GO:0019028 GO:0009318 GO:0031083
GO:0005622 GO:0030133 GO:0016020
Cluster-22178.80953 GO:0016567 GO:0006897 GO:0007155 GO:0006308
GO:0006886 GO:0007015 GO:0004842 GO:0008855 GO:0008270
GO:0061630 GO:0046872 GO:0005198 GO:0005515 GO:0016788
GO:0005680 GO:0005622 GO:0016020 GO:0005801 GO:0016021
GO:0019031 GO:0009318 GO:0019028
Cluster-22178.80954 GO:0005515
Cluster-22178.80955 GO:0006886 GO:0007015 GO:0006308 GO:0016567
GO:0006897 GO:0007155 GO:0005198 GO:0005515 GO:0016788
GO:0046872 GO:0061630 GO:0004842 GO:0008855 GO:0008270

GO:0009318 GO:0019028 GO:0005801 GO:0016021 GO:0019031
GO:0016020 GO:0005680 GO:0005622
Cluster-22178.80957 GO:0016567 GO:0004842 GO:0005515 GO:0046872
GO:0008270 GO:0005680
Cluster-22178.80965 GO:0005515
Cluster-22178.80974 GO:0006260 GO:0031023 GO:0006281 GO:0051225
GO:0003689 GO:0005524 GO:0003911 GO:0005663 GO:0042575
Cluster-22178.80977 GO:0008270 GO:0046872
Cluster-22178.80979 GO:0006810 GO:0006355 GO:0006891 GO:0003677
GO:0003713 GO:0005215 GO:0005524 GO:0016020 GO:0017119
GO:0005634 GO:0005667
Cluster-22178.80980 GO:0006457 GO:0006810 GO:0019042 GO:0006144
GO:0006914 GO:0051607 GO:0006206 GO:0006351 GO:0006355
GO:0007049 GO:0003677 GO:0003899 GO:0051082 GO:0003700
GO:0016817 GO:0043565 GO:0016020 GO:0005667 GO:0005730
GO:0016272 GO:0033644 GO:0005634 GO:0016021
Cluster-22178.80981 GO:0006457 GO:0006810 GO:0006144 GO:0006914
GO:0006206 GO:0051607 GO:0006355 GO:0006351 GO:0007049
GO:0003677 GO:0051082 GO:0003899 GO:0043565 GO:0016817
GO:0003700 GO:0005667 GO:0005730 GO:0016020 GO:0016272
GO:0005634 GO:0016021
Cluster-22178.80984 GO:0055085 GO:0006810 GO:0005525 GO:0042626
GO:0005524 GO:0016887 GO:0016773 GO:0003924 GO:0016021
Cluster-22178.80985 GO:0006813 GO:0009405 GO:0005242 GO:0008270
GO:0003677 GO:0008076 GO:0016021
Cluster-22178.80988 GO:0006355 GO:0005524 GO:0005634
Cluster-22178.80991 GO:0032981
Cluster-22178.80994 GO:0044237 GO:0006810 GO:0006357 GO:0007049
GO:0001104 GO:0005515 GO:0005215 GO:0016592 GO:0016021
Cluster-22178.80997 GO:0055085 GO:0006865 GO:0006118 GO:0006810
GO:0003333 GO:0009055 GO:0020037 GO:0015171 GO:0016020
Cluster-22178.80998 GO:0045892 GO:0005634
Cluster-22178.81001 GO:0016032 GO:0006891 GO:0046872 GO:0017119

GO:0005739
Cluster-22178.81006 GO:0006629 GO:0008081
Cluster-22178.81007 GO:0006367 GO:0055114 GO:0016616 GO:0051287
GO:0005672
Cluster-22178.81008 GO:0006024 GO:0016857 GO:0016021
Cluster-22178.81009 GO:0005198 GO:0019012
Cluster-22178.81010 GO:0005198 GO:0019012
Cluster-22178.81013 GO:0006355 GO:0046983 GO:0005515
Cluster-22178.81017 GO:0005515
Cluster-22178.81018 GO:0006479 GO:0006554 GO:0018024
Cluster-22178.81022 GO:0042157 GO:0009069 GO:0006869 GO:0016310
GO:0008384 GO:0008017 GO:0008289 GO:0008385 GO:0005576
GO:0045298
Cluster-22178.81023 GO:0051382 GO:0000776
Cluster-22178.81028 GO:0006355 GO:0043087 GO:0032012 GO:0005086
GO:0043565 GO:0046872 GO:0003700 GO:0008289 GO:0005667
Cluster-22178.81032 GO:0035091
Cluster-22178.81033 GO:0007010 GO:0003676 GO:0003779
Cluster-22178.81035 GO:0007608 GO:0007187 GO:0006812 GO:0004984
GO:0008324 GO:0005549 GO:0016020
Cluster-22178.81037 GO:0009291 GO:0016787 GO:0005524 GO:0003676
GO:0003677 GO:0016020
Cluster-22178.81041 GO:0009246 GO:0055114 GO:0036065 GO:0005506
GO:0016705 GO:0020037 GO:0008417
Cluster-22178.81044 GO:0055114 GO:0043631 GO:0006355 GO:0006568
GO:0004652 GO:0000334 GO:0005506 GO:0045735 GO:0016779
GO:0005515
Cluster-22178.81045 GO:0006260 GO:0019835 GO:0050829 GO:0016021
Cluster-22178.81046 GO:0006355 GO:0006032 GO:0007049 GO:0006260
GO:0005515 GO:0008061 GO:0043565 GO:0003700 GO:0005667
Cluster-22178.81047 GO:0006260 GO:0015992 GO:0007049 GO:0008152
GO:0006032 GO:0006118 GO:0006119 GO:0006355 GO:0015991
GO:0046961 GO:0016787 GO:0003954 GO:0003700 GO:0016765

GO:0043565 GO:0008061 GO:0005515 GO:0033178 GO:0005667
GO:0005739
Cluster-22178.81048 GO:0006413 GO:0042254 GO:0005509 GO:0005515
GO:0005622
Cluster-22178.81049 GO:0006260 GO:0008408 GO:0003677 GO:0003887
GO:0000166 GO:0042575
Cluster-22178.81053 GO:0000077 GO:0006891 GO:0006457 GO:0006323
GO:0006355 GO:0003700 GO:0043565 GO:0046872 GO:0008270
GO:0005515 GO:0051082 GO:0016272 GO:0005667 GO:0017119
Cluster-22178.81057 GO:0045995 GO:0006357 GO:0030334 GO:0007018
GO:0030155 GO:0007165 GO:0007017 GO:0005102 GO:0003777
GO:0016887 GO:0005524 GO:0001104 GO:0005874 GO:0030286
GO:0016592
Cluster-22178.81058 GO:0006621 GO:0046923 GO:0005783 GO:0016021
Cluster-22178.81061 GO:0008270
Cluster-22178.81068 GO:0015991 GO:0016192 GO:0006886 GO:0006351
GO:0019073 GO:0016820 GO:0005515 GO:0030117 GO:0000221
Cluster-22178.81074 GO:0006811 GO:0006308 GO:0007165 GO:0007268
GO:0009307 GO:0004970 GO:0005234 GO:0009036 GO:0016020
GO:0009359
Cluster-22178.81076 GO:0005975 GO:0004553
Cluster-22178.81078 GO:0005975 GO:0004553
Cluster-22178.81079 GO:0008270
Cluster-22178.81080 GO:0006810 GO:0006811 GO:0005230 GO:0016020
Cluster-22178.81081 GO:0019497 GO:0007155 GO:0046740 GO:0006771
GO:0003993 GO:0046872 GO:0005515 GO:0016021 GO:0016020
Cluster-22178.81090 GO:0044564 GO:0006281 GO:0000723 GO:0003678
GO:0005657
Cluster-22178.81095 GO:0051382 GO:0005515 GO:0000776
Cluster-22178.81098 GO:0003676
Cluster-22178.81101 GO:0009878 GO:0022900 GO:0046872 GO:0016021
Cluster-22178.81102 GO:0007010 GO:0003779
Cluster-22178.81108 GO:0009401 GO:0008643 GO:0008270 GO:0008982

GO:0016020 GO:0009357
Cluster-22178.81113 GO:0005515 GO:0008146 GO:0008270
Cluster-22178.81116 GO:0006508 GO:0006886 GO:0007268 GO:0006605
GO:0005515 GO:0042802 GO:0043495 GO:0004197 GO:0033130
GO:0030695 GO:0005742
Cluster-22178.81120 GO:0006814 GO:0007165 GO:0005515 GO:0005509
GO:0005272 GO:0016020 GO:0005578
Cluster-22178.81128 GO:0006260 GO:0005634
Cluster-22178.81129 GO:0006260 GO:0006144 GO:0006308 GO:0007165
GO:0018142 GO:0016888 GO:0005158 GO:0042624 GO:0016779
GO:0005899
Cluster-22178.81134 GO:0009451 GO:0007186 GO:0006396 GO:0008173
GO:0004930 GO:0003723 GO:0016021
Cluster-22178.81138 GO:0006810 GO:0046872 GO:0005215 GO:0016020
Cluster-22178.81141 GO:0006109 GO:0006355 GO:0016310 GO:0000160
GO:0008134 GO:0016301 GO:0005515 GO:0000155 GO:0004672
GO:0003774 GO:0005524 GO:0016459 GO:0005667 GO:0009365
Cluster-22178.81142 GO:0016567 GO:0004842 GO:0005515 GO:0046872
GO:0008270 GO:0005680
Cluster-22178.81146 GO:0006508 GO:0008152 GO:0055114 GO:0008236
GO:0004867 GO:0016787 GO:0047134 GO:0008270 GO:0005576
Cluster-22178.81149 GO:0018279 GO:0005789
Cluster-22178.81159 GO:0006810 GO:0055085 GO:0005215 GO:0022857
GO:0005515 GO:0016021 GO:0016020
Cluster-22178.81162 GO:0003677
Cluster-22178.81165 GO:0009306 GO:0009405 GO:0016020 GO:0005576
Cluster-22178.81166 GO:0005975 GO:0016901 GO:0048038
Cluster-22178.81175 GO:0009405 GO:0043230 GO:0016021 GO:0009289
Cluster-22178.81176 GO:0016567 GO:0006633 GO:0032922 GO:0042967
GO:0055114 GO:0004318 GO:0005515 GO:0004842 GO:0000976
GO:0005835
Cluster-22178.81178 GO:0016567 GO:0055114 GO:0006633 GO:0042967
GO:0032922 GO:0005515 GO:0004318 GO:0000976 GO:0004842

GO:0005835
Cluster-22178.81180 GO:0006308 GO:0019079 GO:0006260 GO:0008855
GO:0030337 GO:0009318 GO:0042575
Cluster-22178.81182 GO:0006855 GO:0055085 GO:0006950 GO:0006457
GO:0006270 GO:0006810 GO:0015893 GO:0006351 GO:0030001
GO:0005524 GO:0003677 GO:0046873 GO:0015297 GO:0015238
GO:0004190 GO:0051082 GO:0016020 GO:0016021 GO:0005634
Cluster-22178.81185 GO:0003677
Cluster-22178.81188 GO:0006468 GO:0004672
Cluster-22178.81190 GO:0005515
Cluster-22178.81196 GO:0006364 GO:0004672 GO:0032040
Cluster-22178.81203 GO:0007010 GO:0007186 GO:0004930 GO:0003779
GO:0016021
Cluster-22178.81204 GO:0006468 GO:0004672 GO:0005524
Cluster-22178.81208 GO:0051082 GO:0005515 GO:0031072
Cluster-22178.81209 GO:0005515 GO:0031072 GO:0051082
Cluster-22178.81214 GO:0006310 GO:0006281 GO:0006508 GO:0009378
GO:0004222 GO:0003678 GO:0008270 GO:0005524 GO:0016887
GO:0009379 GO:0005657 GO:0016021
Cluster-22178.81215 GO:0006511 GO:0031625
Cluster-22178.81217 GO:0046872 GO:0004222
Cluster-22178.81219 GO:0016125 GO:0006694 GO:0047750 GO:0016021
Cluster-22178.81220 GO:0006694 GO:0016125 GO:0047750 GO:0016021
Cluster-22178.81222 GO:0051082
Cluster-22178.81223 GO:0006525 GO:0006560 GO:0071436 GO:0006814
GO:0007342 GO:0055114 GO:0006090 GO:0008948 GO:0005515
GO:0015081 GO:0016020
Cluster-22178.81229 GO:0005515
Cluster-22178.81230 GO:0006810 GO:0006355 GO:0006376 GO:0006281
GO:0000077 GO:0006308 GO:0003729 GO:0000014 GO:0003700
GO:0008270 GO:0043565 GO:0005667 GO:0016021 GO:0005685
Cluster-22178.81238 GO:0015693 GO:0046872 GO:0000166 GO:0015095
GO:0016021 GO:0016020

Cluster-22178.81239 GO:0042254 GO:0046983 GO:0016020 GO:0016021
Cluster-22178.81240 GO:0042254 GO:0046983 GO:0016021 GO:0016020
Cluster-22178.81241 GO:0015693 GO:0000166 GO:0015095 GO:0046872
GO:0016021 GO:0016020
Cluster-22178.81243 GO:0006260 GO:0005515
Cluster-22178.81245 GO:0015979 GO:0009523 GO:0009539 GO:0016020
Cluster-22178.81248 GO:0006402 GO:0051252 GO:0003676 GO:0046872
GO:0004535 GO:0003723 GO:0005737 GO:0016020 GO:0005634
Cluster-22178.81249 GO:0006506 GO:0016021
Cluster-22178.81253 GO:0005975 GO:0008270 GO:0004560 GO:0003676

Cluster-22178.81255 GO:0046983
Cluster-22178.81261 GO:0007165 GO:0005515
Cluster-22178.81264 GO:0006511 GO:0007275 GO:0005515 GO:0046872
GO:0008270 GO:0005634
Cluster-22178.81267 GO:0016051
Cluster-22178.81268 GO:0005515
Cluster-22178.81269 GO:0008152 GO:0006355 GO:0003700 GO:0043565
GO:0008168 GO:0005515 GO:0003677 GO:0005667 GO:0005634
Cluster-22178.81270 GO:0048306
Cluster-22178.81274 GO:0003677
Cluster-22178.81275 GO:0030255 GO:0006189 GO:0006468 GO:0006355
GO:0035556 GO:0005515 GO:0016773 GO:0004672 GO:0005524
GO:0016020 GO:0005668 GO:0005634
Cluster-22178.81281 GO:0035556 GO:0005515
Cluster-22178.81283 GO:0007264 GO:0003924 GO:0016817 GO:0046872
GO:0005515 GO:0005525
Cluster-22178.81284 GO:0007264 GO:0005525 GO:0005515 GO:0046872
GO:0016817 GO:0003924
Cluster-22178.81290 GO:0007017 GO:0006952 GO:0007018 GO:0005524
GO:0003777 GO:0008017 GO:0005874 GO:0016021 GO:0045298
Cluster-22178.81291 GO:0007017 GO:0007018 GO:0006952 GO:0005524
GO:0008017 GO:0003777 GO:0016021 GO:0045298 GO:0005874

Cluster-22178.81293 GO:0006914 GO:0051082
Cluster-22178.81294 GO:0006914 GO:0051082
Cluster-22178.81295 GO:0006914 GO:0051082
Cluster-22178.81298 GO:0051090 GO:0016192 GO:0006886 GO:0005515
GO:0008536 GO:0030117 GO:0005634
Cluster-22178.81303 GO:0005975 GO:0004553 GO:0003824 GO:0043169

Cluster-22178.81305 GO:0005887
Cluster-22178.81307 GO:0005887
Cluster-22178.81308 GO:0006464 GO:0015031 GO:0007186 GO:0005515
GO:0004930 GO:0016021
Cluster-22178.81317 GO:0007154 GO:0016021
Cluster-22178.81321 GO:0006568 GO:0046487 GO:0006550 GO:0006552
GO:0006574 GO:0055114 GO:0008152 GO:0018874 GO:0006298
GO:0006631 GO:0006554 GO:0009396 GO:0006633 GO:0003857
GO:0003824 GO:0050662 GO:0004488 GO:0005524 GO:0016491
Cluster-22178.81322 GO:0006367 GO:0005515 GO:0048786 GO:0005672
Cluster-22178.81325 GO:0008152 GO:0006118 GO:0055114 GO:0050660
GO:0003995 GO:0016627
Cluster-22178.81326 GO:0016020
Cluster-22178.81331 GO:0045502 GO:0042803 GO:0008134 GO:0030286
GO:0005667
Cluster-22178.81332 GO:0006355 GO:0008233 GO:0003700 GO:0003723
GO:0016791 GO:0004252 GO:0005618 GO:0005667 GO:0005634
GO:0016020
Cluster-22178.81334 GO:0005515
Cluster-22178.81338 GO:0008033 GO:0006888 GO:0006886 GO:0008270
GO:0030127
Cluster-22178.81343 GO:0007276
Cluster-22178.81344 GO:0008201 GO:0009512
Cluster-22178.81347 GO:0005515
Cluster-22178.81350 GO:0015940 GO:0015937 GO:0008152 GO:0006468
GO:0009058 GO:0004140 GO:0016773 GO:0004672 GO:0005524

GO:0003824 GO:0005515 GO:0016301 GO:0016020
Cluster-22178.81351 GO:0031167 GO:0006479 GO:0046500 GO:0001510 GO:0009452 GO:0032259 GO:0006464 GO:0003723 GO:0008168 GO:0004719
Cluster-22178.81352 GO:0005975 GO:0006355 GO:0004415 GO:0003677 GO:0005515
Cluster-22178.81355 GO:0016567 GO:0005515 GO:0004842 GO:0008270 GO:0046872 GO:0005680
Cluster-22178.81356 GO:0000917 GO:0001558 GO:0006355 GO:0007049 GO:0008608 GO:0043093 GO:0006260 GO:0005515 GO:0003700 GO:0043565 GO:0019905 GO:0005520 GO:0042729 GO:0005576 GO:0072686 GO:0005737 GO:0016942 GO:0005667
Cluster-22178.81358 GO:0034080 GO:0000775
Cluster-22178.81359 GO:0006355 GO:0008270 GO:0043565 GO:0003700 GO:0005515 GO:0005667
Cluster-22178.81362 GO:0031175 GO:0006265 GO:0003677 GO:0030507 GO:0005515 GO:0005516 GO:0004222 GO:0008233 GO:0046872 GO:0008270 GO:0003916 GO:0005694 GO:0008091 GO:0031012
Cluster-22178.81364 GO:0006446 GO:0006270 GO:0006810 GO:0006816 GO:0006874 GO:0007264 GO:0015743 GO:0006811 GO:0003743 GO:0005219 GO:0005215 GO:0005525 GO:0004190 GO:0005622 GO:0016020 GO:0016021 GO:0005840 GO:0005737 GO:0005852
Cluster-22178.81366 GO:0006511 GO:0006310 GO:0007010 GO:0032259 GO:0005515 GO:0003677 GO:0004175 GO:0003779 GO:0008168 GO:0019773 GO:0005634
Cluster-22178.81367 GO:0046907 GO:0007010 GO:0003779 GO:0005515

Cluster-22178.81370 GO:0000372 GO:0046740 GO:0016021 GO:0030529
Cluster-22178.81371 GO:0016020
Cluster-22178.81372 GO:0016020
Cluster-22178.81374 GO:0006355 GO:0043401 GO:0007010 GO:0006520 GO:0009415 GO:0006950 GO:0008851 GO:0003779 GO:0043565 GO:0008270 GO:0003700 GO:0005667 GO:0005634 GO:0009350

Cluster-22178.81375 GO:0005515
Cluster-22178.81376 GO:0003676 GO:0046935 GO:0003723 GO:0016021 GO:0005944
Cluster-22178.81377 GO:0006813 GO:0046487 GO:0006730 GO:0019521 GO:0055114 GO:0006098 GO:0019354 GO:0006520 GO:0009396 GO:0006779 GO:0006555 GO:0004013 GO:0043115 GO:0003824 GO:0051287 GO:0004616 GO:0004488 GO:0016491
Cluster-22178.81382 GO:0003676
Cluster-22178.81384 GO:0046872 GO:0003677
Cluster-22178.81386 GO:0030036 GO:0019058 GO:0006355 GO:0003677 GO:0003779 GO:0030054
Cluster-22178.81387 GO:0046872
Cluster-22178.81389 GO:0007165 GO:0015986 GO:0015992 GO:0005515 GO:0015078 GO:0016020 GO:0045263
Cluster-22178.81394 GO:0003723
Cluster-22178.81402 GO:0006807 GO:0016810
Cluster-22178.81403 GO:0006260 GO:0003677 GO:0005524 GO:0016887 GO:0019031 GO:0016020
Cluster-22178.81404 GO:0007155 GO:0042246 GO:0006260 GO:0003677 GO:0005524 GO:0016887 GO:0016020 GO:0019031 GO:0016021
Cluster-22178.81406 GO:0006355 GO:0003700 GO:0043565 GO:0005667 GO:0008537
Cluster-22178.81409 GO:0016114 GO:0006284 GO:0006694 GO:0006886 GO:0016192 GO:0005515 GO:0005198 GO:0003677 GO:0008661 GO:0005634 GO:0030117
Cluster-22178.81413 GO:0006508 GO:0004222
Cluster-22178.81414 GO:0042742 GO:0007165 GO:0005515
Cluster-22178.81417 GO:0021549 GO:0006351 GO:0006144 GO:0006730 GO:0006206 GO:0030182 GO:0003899 GO:0003677 GO:0008168 GO:0003676 GO:0005730
Cluster-22178.81420 GO:0016787
Cluster-22178.81421 GO:0032402 GO:0006886 GO:0007165 GO:0006888 GO:0006265 GO:0035556 GO:0005515 GO:0003677 GO:0003918

GO:0008270 GO:0005524 GO:0042470 GO:0030127
Cluster-22178.81424 GO:0008152 GO:0016491
Cluster-22178.81426 GO:0006470 GO:0006570 GO:0004725 GO:0008138

Cluster-22178.81429 GO:0000103 GO:0006144 GO:0004020 GO:0005524

Cluster-22178.81430 GO:0006265 GO:0003677 GO:0003918 GO:0005524
GO:0005634
Cluster-22178.81431 GO:0042967 GO:0006865 GO:0051205 GO:0003333
GO:0008080 GO:0015171 GO:0016021 GO:0019033 GO:0016020
Cluster-22178.81433 GO:0006508 GO:0008270
Cluster-22178.81437 GO:0006508 GO:0016787 GO:0008236
Cluster-22178.81440 GO:0015992 GO:0006123 GO:0004129 GO:0005739
GO:0045277
Cluster-22178.81443 GO:0042254 GO:0006412 GO:0003735 GO:0005840
GO:0005622
Cluster-22178.81444 GO:0042254 GO:0006412 GO:0008270 GO:0003735
GO:0005622 GO:0005840
Cluster-22178.81447 GO:0005515
Cluster-22178.81448 GO:0006810 GO:0005215 GO:0003677 GO:0016020
Cluster-22178.81449 GO:0006810 GO:0005215 GO:0016020
Cluster-22178.81451 GO:0007264 GO:0005525 GO:0003924
Cluster-22178.81453 GO:0007165 GO:0007186 GO:0007264 GO:0006013
GO:0006000 GO:0003873 GO:0005525 GO:0004871 GO:0031683
GO:0005524 GO:0005125 GO:0019001 GO:0003924
Cluster-22178.81455 GO:0046872
Cluster-22178.81464 GO:0005515 GO:0004867
Cluster-22178.81466 GO:0042325 GO:0004252 GO:0019028 GO:0005737
GO:0055036
Cluster-22178.81468 GO:0006810 GO:0016021
Cluster-22178.81471 GO:0015693 GO:0015979 GO:0006810 GO:0015095
GO:0016021 GO:0009523 GO:0009539 GO:0016020
Cluster-22178.81472 GO:0003677 GO:0003676 GO:0005524 GO:0016787

Cluster-22178.81473 GO:0042254 GO:0006412 GO:0008152 GO:0003824 GO:0003735 GO:0005840 GO:0005739

Cluster-22178.81477 GO:0006281 GO:0046034 GO:0006464 GO:0051276 GO:0006144 GO:0004017 GO:0005515 GO:0016151 GO:0005524 GO:0008270 GO:0046872 GO:0005694

Cluster-22178.81478 GO:0046872

Cluster-22178.81480 GO:0006357 GO:0001104 GO:0005515 GO:0016592

Cluster-22178.81483 GO:0007165 GO:0005096 GO:0005515

Cluster-22178.81485 GO:0006284 GO:0003677

Cluster-22178.81487 GO:0002143 GO:0030683 GO:0008092 GO:0005524 GO:0008270 GO:0003676 GO:0005515 GO:0005737 GO:0019898 GO:0019012

Cluster-22178.81488 GO:0005515

Cluster-22178.81489 GO:0016021

Cluster-22178.81492 GO:0008152 GO:0008484 GO:0016787 GO:0046872 GO:0003824 GO:0003676

Cluster-22178.81494 GO:0007015 GO:0006265 GO:0006897 GO:0003918 GO:0005524 GO:0003677

Cluster-22178.81497 GO:0006355 GO:0006810 GO:0016021

Cluster-22178.81498 GO:0006810 GO:0006508 GO:0004197 GO:0016021

Cluster-22178.81501 GO:0007165 GO:0007275 GO:0016055 GO:0005102 GO:0005576

Cluster-22178.81502 GO:0009306 GO:0008152 GO:0055085 GO:0015031 GO:0055114 GO:0022857 GO:0016491 GO:0008565 GO:0016020 GO:0016021

Cluster-22178.81503 GO:0007275 GO:0015031 GO:0006464 GO:0005515 GO:0005634

Cluster-22178.81505 GO:0005198 GO:0019028

Cluster-22178.81506 GO:0005515

Cluster-22178.81513 GO:0007165 GO:0007009 GO:0051087 GO:0019904

Cluster-22178.81514 GO:0006281 GO:0003677 GO:0003684 GO:0034061

GO:0005634
Cluster-22178.81516 GO:0007155 GO:0007339 GO:0004867 GO:0005515
GO:0004222 GO:0008233 GO:0005576 GO:0031012 GO:0016021
Cluster-22178.81522 GO:0007155 GO:0006468 GO:0005524 GO:0004672
GO:0016020
Cluster-22178.81525 GO:0016055 GO:0005515
Cluster-22178.81530 GO:0019478 GO:0016788 GO:0005737
Cluster-22178.81532 GO:0009089 GO:0055114 GO:0009085 GO:0008839
GO:0016620 GO:0051287
Cluster-22178.81534 GO:0005509 GO:0005515 GO:0009986 GO:0016020
Cluster-22178.81536 GO:0043087 GO:0005515 GO:0016020
Cluster-22178.81537 GO:0046872 GO:0003824
Cluster-22178.81538 GO:0006355 GO:0000982 GO:0003700 GO:0003677
GO:0005515 GO:0000978 GO:0005667 GO:0005634
Cluster-22178.81539 GO:0006281 GO:0051225 GO:0006260 GO:0031023
GO:0005524 GO:0003689 GO:0003911 GO:0005663 GO:0042575
Cluster-22178.8154 GO:0005515
Cluster-22178.81540 GO:0006479 GO:0006554 GO:0034968 GO:0005515
GO:0008270 GO:0018024 GO:0005634
Cluster-22178.81542 GO:0006508 GO:0004198 GO:0005622
Cluster-22178.81545 GO:0016310 GO:0007178 GO:0009069 GO:0006468
GO:0004672 GO:0005524 GO:0004675 GO:0016020
Cluster-22178.81546 GO:0006464 GO:0006281 GO:0035556 GO:0046872
GO:0008270 GO:0003677 GO:0016151
Cluster-22178.81547 GO:0055085 GO:0030001 GO:0019048 GO:0046873
GO:0019031 GO:0016020
Cluster-22178.81548 GO:0006355 GO:0003700 GO:0005634 GO:0005667
Cluster-22178.81550 GO:0055114 GO:0033743
Cluster-22178.81553 GO:0051252 GO:0007010 GO:0006281 GO:0016891
GO:0003779 GO:0030915
Cluster-22178.81554 GO:0016310 GO:0007165 GO:0007010 GO:0000160
GO:0000155 GO:0003779 GO:0009365
Cluster-22178.81556 GO:0046500 GO:0007264 GO:0007186 GO:0007165

GO:0015684 GO:0006464 GO:0006554 GO:0006576 GO:0006479
GO:0008152 GO:0015093 GO:0005524 GO:0016740 GO:0018024
GO:0003924 GO:0008168 GO:0019001 GO:0031683 GO:0004719
GO:0004871 GO:0005525 GO:0016021
Cluster-22178.81559 GO:0051028 GO:0006914 GO:0010508 GO:0007186
GO:0004930 GO:0016021 GO:0031966 GO:0005634 GO:0016020
Cluster-22178.81564 GO:0055114 GO:0004672 GO:0005515 GO:0004174

Cluster-22178.81567 GO:0046872
Cluster-22178.81571 GO:0006402 GO:0006367 GO:0051252 GO:0004535
GO:0046872 GO:0003676 GO:0003723 GO:0005737 GO:0005672
GO:0005634
Cluster-22178.81578 GO:0005975 GO:0016758 GO:0016020
Cluster-22178.8158 GO:0005515
Cluster-22178.81585 GO:0000398 GO:0006334 GO:0005515 GO:0005681
GO:0005634
Cluster-22178.81586 GO:0000398 GO:0006334 GO:0005515 GO:0005634
GO:0005681
Cluster-22178.81588 GO:0003676
Cluster-22178.81589 GO:0051537 GO:0043231
Cluster-22178.81590 GO:0005515
Cluster-22178.81592 GO:0007165 GO:0051537 GO:0043231
Cluster-22178.81593 GO:0007264 GO:0007165 GO:0005515 GO:0005525

Cluster-22178.81594 GO:0016042 GO:0009395 GO:0046339 GO:0007275
GO:0010124 GO:0004435 GO:0046872 GO:0005509
Cluster-22178.81595 GO:0007165 GO:0006898 GO:0005044 GO:0016020
Cluster-22178.81596 GO:0006807 GO:0006935 GO:0050568
Cluster-22178.81597 GO:0005515
Cluster-22178.81599 GO:0005515
Cluster-22178.81601 GO:0006570 GO:0006470 GO:0005515 GO:0008138
GO:0004725
Cluster-22178.81602 GO:0006470 GO:0006570 GO:0004725 GO:0005515

Cluster-22178.81604 GO:0042967 GO:0016765 GO:0004379
Cluster-22178.81606 GO:0009264 GO:0006810 GO:0005515 GO:0004386 GO:0008253 GO:0003676 GO:0043531 GO:0005524 GO:0046872 GO:0016020
Cluster-22178.81607 GO:0006810 GO:0042302 GO:0016020 GO:0016021
Cluster-22178.81608 GO:0005634
Cluster-22178.81612 GO:0035434 GO:0006825 GO:0005375 GO:0016021
Cluster-22178.81613 GO:0006013 GO:0006000 GO:0007264 GO:0007155 GO:0007165 GO:0007186 GO:0019001 GO:0031683 GO:0005524 GO:0003924 GO:0005525 GO:0004871 GO:0003873
Cluster-22178.81615 GO:0016579 GO:0036459
Cluster-22178.81618 GO:0006554 GO:0006479 GO:0007165 GO:0006955 GO:0009058 GO:0005125 GO:0008270 GO:0018024 GO:0030170 GO:0005576
Cluster-22178.81619 GO:0016021
Cluster-22178.81621 GO:0034508 GO:0015031 GO:0042254 GO:0010508 GO:0006904 GO:0008270 GO:0005622 GO:0000145 GO:0000776
Cluster-22178.81622 GO:0006468 GO:0005524 GO:0004672
Cluster-22178.81623 GO:0006874 GO:0006816 GO:0006281 GO:0007165 GO:0006730 GO:0006935 GO:0006555 GO:0004013 GO:0004888 GO:0008270 GO:0046872 GO:0005219 GO:0016020 GO:0005622 GO:0016021
Cluster-22178.81625 GO:0006468 GO:0007165 GO:0016567 GO:0009451 GO:0008033 GO:0009020 GO:0005515 GO:0005524 GO:0004672 GO:0016773 GO:0016020
Cluster-22178.81627 GO:0005515
Cluster-22178.81631 GO:0016021
Cluster-22178.81633 GO:0007339 GO:0019497 GO:0006771 GO:0005515 GO:0003993 GO:0046872 GO:0005576
Cluster-22178.81634 GO:0007339 GO:0006771 GO:0019497 GO:0003993 GO:0005515 GO:0046872 GO:0005576
Cluster-22178.81638 GO:0006888 GO:0007165 GO:0015684 GO:0007186

GO:0007264 GO:0005525 GO:0004871 GO:0031683 GO:0015093
GO:0019001 GO:0003924 GO:0005801 GO:0016021 GO:0005622
GO:0019033
Cluster-22178.81639 GO:0008033 GO:0006725 GO:0055114 GO:0006807
GO:0018580 GO:0016853 GO:0016627 GO:0016491 GO:0017150
GO:0050660 GO:0005737
Cluster-22178.81642 GO:0006260 GO:0008152 GO:0002949 GO:0006388
GO:0005525 GO:0003724 GO:0005524 GO:0003924 GO:0016887
GO:0003972 GO:0003677 GO:0016301 GO:0003723 GO:0004003
GO:0005657
Cluster-22178.81646 GO:0004222
Cluster-22178.81647 GO:0055114 GO:0007186 GO:0046872 GO:0004930
GO:0016972 GO:0016021
Cluster-22178.81648 GO:0016567 GO:0008063 GO:0009245 GO:0005515
GO:0003723 GO:0004842 GO:0008233 GO:0008915 GO:0008270
GO:0046872 GO:0016020 GO:0005680
Cluster-22178.81650 GO:0005515
Cluster-22178.81651 GO:0043044 GO:0016567 GO:0008270 GO:0004842
GO:0003682 GO:0031491 GO:0000785 GO:0005680
Cluster-22178.81660 GO:0006013 GO:0005975 GO:0015976 GO:0006000
GO:0046854 GO:0006094 GO:0006098 GO:0006096 GO:0042578
GO:0042132
Cluster-22178.81661 GO:0006813 GO:0007586 GO:0006811 GO:0016042
GO:0005515 GO:0005249 GO:0008047 GO:0005576 GO:0008076
GO:0016020
Cluster-22178.81662 GO:0055114 GO:0050660 GO:0050661 GO:0016491
GO:0004499
Cluster-22178.81663 GO:0006355 GO:0009607 GO:0046907 GO:0008270
GO:0005515
Cluster-22178.81664 GO:0005515 GO:0005789
Cluster-22178.81665 GO:0006468 GO:0006357 GO:0004672 GO:0005524
GO:0001104 GO:0005515 GO:0016592
Cluster-22178.81673 GO:0006468 GO:0006449 GO:0006886 GO:0006118

GO:0006415 GO:0016192 GO:0020037 GO:0004715 GO:0016149
GO:0009055 GO:0005506 GO:0005524 GO:0005576 GO:0005737
GO:0005840 GO:0018444 GO:0030131 GO:0005622
Cluster-22178.81679 GO:0007165 GO:0007264 GO:0005525
Cluster-22178.81682 GO:0042254 GO:0006412 GO:0003735 GO:0005761
GO:0005840
Cluster-22178.81689 GO:0045454
Cluster-22178.81692 GO:0008299
Cluster-22178.81694 GO:0016192 GO:0006886 GO:0006230 GO:0006206
GO:0005515 GO:0008536 GO:0004797 GO:0005524 GO:0030117
GO:0005576
Cluster-22178.81695 GO:0007165 GO:0005515
Cluster-22178.81698 GO:0045995 GO:0030334 GO:0006914 GO:0006308
GO:0007165 GO:0030155 GO:0005102 GO:0008855 GO:0009318
Cluster-22178.81699 GO:0006308 GO:0030334 GO:0045995 GO:0030155
GO:0007165 GO:0008855 GO:0005102 GO:0005515 GO:0009318
Cluster-22178.81700 GO:0006355 GO:0003700 GO:0003677 GO:0043565
GO:0005634 GO:0005667
Cluster-22178.81701 GO:0006508 GO:0008152 GO:0016787 GO:0008236

Cluster-22178.81706 GO:0015031 GO:0007165 GO:0006904 GO:0005515
GO:0000145
Cluster-22178.81708 GO:0000746 GO:0003677
Cluster-22178.81711 GO:0006118 GO:0005975 GO:0022900 GO:0055114
GO:0008184 GO:0008827 GO:0016021 GO:0009319
Cluster-22178.81716 GO:0016310 GO:0007165 GO:0000160 GO:0005515
GO:0000155 GO:0009365
Cluster-22178.81718 GO:0009069 GO:0016310 GO:0005515 GO:0004674

Cluster-22178.81722 GO:0006310 GO:0019079 GO:0006771 GO:0002949
GO:0006281 GO:0019497 GO:0009378 GO:0003724 GO:0003993
GO:0016887 GO:0005524 GO:0005515 GO:0003723 GO:0003678
GO:0009379 GO:0005657

Cluster-22178.81723 GO:0007631 GO:0007187 GO:0007186 GO:0006313
GO:0022410 GO:0006525 GO:0006355 GO:0015074 GO:0003677
GO:0003700 GO:0016499 GO:0005667 GO:0016021
Cluster-22178.81725 GO:0008152 GO:0003824
Cluster-22178.81726 GO:0008152 GO:0003824
Cluster-22178.81727 GO:0050832 GO:0007165 GO:0042742 GO:0005509
GO:0005515 GO:0005578
Cluster-22178.81738 GO:0000062
Cluster-22178.81739 GO:0006886 GO:0055114 GO:0006888 GO:0016706
GO:0003677 GO:0008168 GO:0046872 GO:0008270 GO:0030127
Cluster-22178.81742 GO:0050765 GO:0009405 GO:0016567 GO:0015979
GO:0019538 GO:0005515 GO:0046872 GO:0008270 GO:0016020
GO:0005576 GO:0016012 GO:0009539 GO:0009523
Cluster-22178.81744 GO:0006308 GO:0008855 GO:0005515 GO:0009318
Cluster-22178.81745 GO:0006508 GO:0042254 GO:0004197 GO:0008234
GO:0005515 GO:0005730
Cluster-22178.81746 GO:0006508 GO:0035556 GO:0008234 GO:0005515
GO:0004197
Cluster-22178.81748 GO:0042998 GO:0071253 GO:0005802
Cluster-22178.81752 GO:0007268 GO:0033130 GO:0043495 GO:0005515

Cluster-22178.81755 GO:0006508 GO:0008233 GO:0004222 GO:0008270
GO:0031012
Cluster-22178.81758 GO:0007010 GO:0005515 GO:0003779
Cluster-22178.81759 GO:0007155 GO:0007165 GO:0015992 GO:0030168
GO:0006914 GO:0006606 GO:0015991 GO:0006886 GO:0051258
GO:0030674 GO:0005102 GO:0045502 GO:0042803 GO:0008134
GO:0005515 GO:0015078 GO:0005667 GO:0005783 GO:0030286
GO:0005815 GO:0005577 GO:0033179 GO:0016021
Cluster-22178.81762 GO:0007264 GO:0005525
Cluster-22178.81767 GO:0005515 GO:0016020
Cluster-22178.81768 GO:0005515 GO:0016020
Cluster-22178.81769 GO:0006810 GO:0006914 GO:0005515 GO:0005794

Cluster-22178.81770 GO:0019058 GO:0006355 GO:0003700 GO:0043565 GO:0019031 GO:0005667 GO:0005634
Cluster-22178.81772 GO:0032297 GO:0006468 GO:0003677 GO:0004672 GO:0005524
Cluster-22178.81773 GO:0003677 GO:0003676 GO:0005524 GO:0016787

Cluster-22178.81777 GO:0005515 GO:0016020
Cluster-22178.81779 GO:0006281 GO:0046983 GO:0008270
Cluster-22178.81782 GO:0005544 GO:0005509 GO:0005198 GO:0005578
Cluster-22178.81785 GO:0050852 GO:0006570 GO:0006470 GO:0035091 GO:0004725
Cluster-22178.81791 GO:0004867 GO:0004222 GO:0005515 GO:0008233 GO:0031012
Cluster-22178.81792 GO:0007029 GO:0004866 GO:0005789 GO:0005615 GO:0005576
Cluster-22178.81793 GO:0009405 GO:0006810 GO:0008200 GO:0005576
Cluster-22178.81796 GO:0033588
Cluster-22178.81797 GO:0008270 GO:0005515
Cluster-22178.81799 GO:0006032 GO:0006355 GO:0005524 GO:0008061 GO:0005515 GO:0008134 GO:0009279 GO:0005667
Cluster-22178.81802 GO:0009401 GO:0055085 GO:0006812 GO:0008324 GO:0016020 GO:0016021
Cluster-22178.81804 GO:0055085 GO:0006812 GO:0006890 GO:0006817 GO:0005315 GO:0008324 GO:0016021 GO:0016020
Cluster-22178.81811 GO:0007165 GO:0000160 GO:0046872 GO:0004871

Cluster-22178.81814 GO:0006355 GO:0003676 GO:0003677 GO:0003723

Cluster-22178.81818 GO:0009405 GO:0015991 GO:0015992 GO:0015078 GO:0033179 GO:0016021
Cluster-22178.81822 GO:0007165
Cluster-22178.81825 GO:0006355 GO:0003700 GO:0003677 GO:0043565 GO:0005667 GO:0005634

Cluster-22178.81826 GO:0000917 GO:0006355 GO:0043093 GO:0007049
GO:0003700 GO:0043565 GO:0005667 GO:0005737
Cluster-22178.81838 GO:0006470 GO:0006570 GO:0004725 GO:0008138

Cluster-22178.81840 GO:0002949 GO:0006310 GO:0019079 GO:0006281
GO:0006355 GO:0016887 GO:0005524 GO:0003724 GO:0009378
GO:0043531 GO:0003678 GO:0016740 GO:0003723 GO:0016301
GO:0008134 GO:0005198 GO:0005657 GO:0009379 GO:0005667
GO:0019028
Cluster-22178.81842 GO:0006810 GO:0006811 GO:0005215 GO:0016020
Cluster-22178.81843 GO:0032981
Cluster-22178.81846 GO:0006275 GO:0006355 GO:0043248 GO:0007224
GO:0016032
Cluster-22178.81849 GO:0006309 GO:0006915 GO:0016787 GO:0005634
GO:0005737 GO:0005622
Cluster-22178.81850 GO:0009245 GO:0008915
Cluster-22178.81852 GO:0044599
Cluster-22178.81853 GO:0008608 GO:0042729 GO:0072686
Cluster-22178.81854 GO:0006359
Cluster-22178.81856 GO:0007264 GO:0006281 GO:0006338 GO:0005525
GO:0005515 GO:0090544
Cluster-22178.81858 GO:0005515
Cluster-22178.81876 GO:0055114 GO:0005506 GO:0016705 GO:0020037

Cluster-22178.81877 GO:0055114 GO:0005506 GO:0016705 GO:0020037

Cluster-22178.81879 GO:0005515
Cluster-22178.81884 GO:0005515
Cluster-22178.81891 GO:0005515
Cluster-22178.81895 GO:0005515
Cluster-22178.81904 GO:0006629 GO:0009395 GO:0004620 GO:0016020
Cluster-22178.81907 GO:0007186 GO:0004930 GO:0016021
Cluster-22178.81908 GO:0006099 GO:0006468 GO:0055114 GO:0016117

GO:0006090 GO:0071949 GO:0016705 GO:0016491 GO:0004672
GO:0005524 GO:0008924
Cluster-22178.81911 GO:0055085 GO:0016021
Cluster-22178.81914 GO:0007165
Cluster-22178.81916 GO:0007165
Cluster-22178.81917 GO:0005515
Cluster-22178.81923 GO:0055085 GO:0006810 GO:0022857 GO:0005215
GO:0016021 GO:0016020
Cluster-22178.81925 GO:0005515
Cluster-22178.81929 GO:0007218 GO:0005576
Cluster-22178.81933 GO:0005515 GO:0008270
Cluster-22178.81934 GO:0005509 GO:0005515
Cluster-22178.81935 GO:0007010 GO:0003779
Cluster-22178.81938 GO:0006281 GO:0004518 GO:0003677 GO:0003824

Cluster-22178.81941 GO:0006355 GO:0006352 GO:0055085 GO:0003677
GO:0003700 GO:0022857 GO:0005667 GO:0016021
Cluster-22178.81942 GO:0006352 GO:0055085 GO:0003677 GO:0022857
GO:0016021
Cluster-22178.81949 GO:0006144 GO:0006206 GO:0006351 GO:0003899
GO:0003677 GO:0005730
Cluster-22178.81950 GO:0007259 GO:0001700
Cluster-22178.81951 GO:0007259 GO:0001700
Cluster-22178.81955 GO:0007165 GO:0016310 GO:0046034 GO:0000160
GO:0006144 GO:0004017 GO:0005515 GO:0000155 GO:0009365
Cluster-22178.81956 GO:0006508 GO:0004252
Cluster-22178.81966 GO:0006886 GO:0005543
Cluster-22178.81967 GO:0036094
Cluster-22178.81968 GO:0007165 GO:0055085 GO:0006885 GO:0006812
GO:0005179 GO:0015299 GO:0005576 GO:0016021
Cluster-22178.81973 GO:0005576
Cluster-22178.81974 GO:0050792 GO:0042025
Cluster-22178.81975 GO:0050792 GO:0042025

Cluster-22178.81976 GO:0055114 GO:0005515 GO:0016706 GO:0008168

Cluster-22178.81977 GO:0003676 GO:0046872
Cluster-22178.81984 GO:0006508 GO:0050790 GO:0004197 GO:0008270

Cluster-22178.81991 GO:0015931 GO:0009405 GO:0033227 GO:0006468
GO:0016773 GO:0005524 GO:0004672 GO:0005515 GO:0005198
GO:0051033 GO:0004190 GO:0016021 GO:0016020
Cluster-22178.81993 GO:0030145 GO:0004177 GO:0016021
Cluster-22178.81997 GO:0042967 GO:0016747 GO:0008080 GO:0005515

Cluster-22178.82002 GO:0006468 GO:0016773 GO:0005524 GO:0004672
GO:0005515 GO:0016020
Cluster-22178.82012 GO:0006935 GO:0015992 GO:0007165 GO:0071973
GO:0006355 GO:0015991 GO:0015078 GO:0004871 GO:0003700
GO:0003774 GO:0000220 GO:0009288 GO:0016020 GO:0033179
GO:0005667
Cluster-22178.82014 GO:0006810 GO:0006508 GO:0046034 GO:0006144
GO:0004017 GO:0004190 GO:0042626 GO:0005524 GO:0046872
GO:0016021
Cluster-22178.82015 GO:0005515
Cluster-22178.82019 GO:0007264 GO:0015743 GO:0006816 GO:0006874
GO:0006270 GO:0004190 GO:0005525 GO:0005219 GO:0005622
GO:0016021
Cluster-22178.82022 GO:0006084 GO:0006552 GO:0008299 GO:0046950
GO:0006550 GO:0006574 GO:0042967 GO:0006633 GO:0004421
GO:0004315 GO:0005835
Cluster-22178.82030 GO:0046854
Cluster-22178.82032 GO:0016567 GO:0001848 GO:0046872 GO:0008270
GO:0004842 GO:0005515 GO:0061630 GO:0005615 GO:0005680
Cluster-22178.82038 GO:0006260 GO:0007186 GO:0043093 GO:0007049
GO:0000917 GO:0030474 GO:0006355 GO:0007606 GO:0004930
GO:0003677 GO:0005200 GO:0003700 GO:0043565 GO:0005823

GO:0005815 GO:0005667 GO:0016021 GO:0005737 GO:0005856
GO:0005634 GO:0019028
Cluster-22178.82042 GO:0016020
Cluster-22178.82043 GO:0006508 GO:0016787 GO:0008236
Cluster-22178.82045 GO:0005515 GO:0003723
Cluster-22178.82047 GO:0006281 GO:0006816 GO:0006874 GO:0006730
GO:0006555 GO:0004013 GO:0008270 GO:0046872 GO:0005219
GO:0005622 GO:0016021
Cluster-22178.82053 GO:0006351 GO:0007165 GO:0006260 GO:0000398
GO:0003677 GO:0003887 GO:0008270 GO:0042575 GO:0016020
GO:0005634 GO:0005681
Cluster-22178.82055 GO:0007029 GO:0005515 GO:0005789
Cluster-22178.82061 GO:0055114 GO:0004499 GO:0071949 GO:0016491
GO:0050661 GO:0005515 GO:0050660
Cluster-22178.82067 GO:0016779 GO:0003677 GO:0034061 GO:0005634
Cluster-22178.82068 GO:0007264 GO:0015684 GO:0016887 GO:0003924
GO:0005524 GO:0015093 GO:0005525 GO:0016021
Cluster-22178.82069 GO:0016598 GO:0004057
Cluster-22178.82070 GO:0005975 GO:0006012 GO:0006687 GO:0006027
GO:0046486 GO:0004565 GO:0009341 GO:0016020
Cluster-22178.82071 GO:0005515 GO:0046872
Cluster-22178.82075 GO:0032981
Cluster-22178.82076 GO:0032981
Cluster-22178.82078 GO:0008380 GO:0003824 GO:0005515 GO:0005681
Cluster-22178.82079 GO:0055114 GO:0033743
Cluster-22178.82080 GO:0007165 GO:0006355 GO:0003700 GO:0004871
GO:0005667 GO:0005634
Cluster-22178.82081 GO:0043087 GO:0007165 GO:0035023 GO:0005089
GO:0005515
Cluster-22178.82082 GO:0003723 GO:0005515
Cluster-22178.82086 GO:0007165 GO:0007186 GO:0007264 GO:0006576
GO:0005525 GO:0004871 GO:0031683 GO:0005524 GO:0019001
GO:0003924

Cluster-22178.82091 GO:0005515 GO:0043565 GO:0005634
Cluster-22178.82092 GO:0009878 GO:0005509 GO:0046872
Cluster-22178.82093 GO:0016020
Cluster-22178.82095 GO:0005198
Cluster-22178.82096 GO:0007165 GO:0009451 GO:0016567 GO:0006468
GO:0008033 GO:0005515 GO:0009020 GO:0005524 GO:0004672
GO:0016773 GO:0016020
Cluster-22178.82099 GO:0006118 GO:0031047 GO:0045454 GO:0009055
GO:0015035
Cluster-22178.82100 GO:0009089 GO:0006355 GO:0008837 GO:0005634
GO:0005737
Cluster-22178.82101 GO:0043087 GO:0035023 GO:0005089 GO:0005515

Cluster-22178.82102 GO:0071704 GO:0016868 GO:0005515
Cluster-22178.82107 GO:0016773 GO:0005525 GO:0003677
Cluster-22178.82112 GO:0003677 GO:0005525
Cluster-22178.82115 GO:0005525
Cluster-22178.82118 GO:0003676
Cluster-22178.82119 GO:0006629 GO:0007154 GO:0046486 GO:0055085
GO:0016042 GO:0004806 GO:0016021
Cluster-22178.82120 GO:0007010 GO:0003779
Cluster-22178.82121 GO:0018149 GO:0042710 GO:0008270 GO:0019031
Cluster-22178.82128 GO:0003723 GO:0005515 GO:0005198 GO:0016020
Cluster-22178.82129 GO:0006265 GO:0005515 GO:0003677 GO:0046872
GO:0003916 GO:0008270 GO:0005694
Cluster-22178.82133 GO:0045116 GO:0016881 GO:0008641 GO:0005524

Cluster-22178.82134 GO:0008270
Cluster-22178.82136 GO:0005515
Cluster-22178.82137 GO:0005515 GO:0008270 GO:0046872
Cluster-22178.82140 GO:0005509
Cluster-22178.82141 GO:0006886 GO:0005543
Cluster-22178.82142 GO:0006468 GO:0004672 GO:0005524

Cluster-22178.82143 GO:0007186 GO:0004930 GO:0016021
Cluster-22178.82144 GO:0006468 GO:0005524 GO:0004672
Cluster-22178.82145 GO:0007018 GO:0007017 GO:0003777 GO:0008017
GO:0005524 GO:0005874 GO:0045298
Cluster-22178.82148 GO:0006508 GO:0016787 GO:0008236
Cluster-22178.82149 GO:0006471 GO:0003950
Cluster-22178.82156 GO:0006886 GO:0008536
Cluster-22178.82164 GO:0046872 GO:0000166
Cluster-22178.82168 GO:0006144 GO:0005524 GO:0003968 GO:0031379
Cluster-22178.82170 GO:0008168 GO:0005515
Cluster-22178.82172 GO:0008168 GO:0005515
Cluster-22178.82176 GO:0015074 GO:0003677 GO:0046872 GO:0005887
Cluster-22178.82179 GO:0006118 GO:0006886 GO:0006468 GO:0006449
GO:0016192 GO:0006415 GO:0020037 GO:0009055 GO:0004715
GO:0016149 GO:0005506 GO:0005524 GO:0005576 GO:0005840
GO:0005737 GO:0030131 GO:0018444 GO:0005622
Cluster-22178.82180 GO:0006879 GO:0016192 GO:0006415 GO:0006886
GO:0006118 GO:0006449 GO:0006468 GO:0005524 GO:0008199
GO:0005506 GO:0009055 GO:0016149 GO:0004715 GO:0020037
GO:0005622 GO:0030131 GO:0018444 GO:0005840 GO:0005737
GO:0005576
Cluster-22178.82188 GO:0006820 GO:0015675 GO:0035444 GO:0046872
GO:0015099 GO:0008509 GO:0016021
Cluster-22178.8219 GO:0035556 GO:0005515
Cluster-22178.82191 GO:0043087 GO:0007264 GO:0019079 GO:0007165
GO:0005085 GO:0005515
Cluster-22178.82192 GO:0030036 GO:0006914 GO:0006886 GO:0016043
GO:0003924 GO:0003779 GO:0017048 GO:0016021 GO:0005783
Cluster-22178.82195 GO:0043087 GO:0035023 GO:0035556 GO:0005089
GO:0005576
Cluster-22178.82197 GO:0003677
Cluster-22178.82203 GO:0006355 GO:0006468 GO:0007165 GO:0016567
GO:0005524 GO:0004672 GO:0016773 GO:0005200 GO:0003677

GO:0005515 GO:0005856 GO:0005634 GO:0005882 GO:0016020
Cluster-22178.82208 GO:0009405 GO:0045735 GO:0005515 GO:0030430
Cluster-22178.82209 GO:0005515
Cluster-22178.82214 GO:0009712 GO:0055114 GO:0042184 GO:0018874
GO:0046232 GO:0032402 GO:0019261 GO:0042203 GO:0018576
GO:0005506 GO:0042470
Cluster-22178.82221 GO:0003677
Cluster-22178.82226 GO:0032509 GO:0043130 GO:0000813
Cluster-22178.82229 GO:0007165 GO:0005179 GO:0005576
Cluster-22178.82233 GO:0042254 GO:0006412 GO:0006629 GO:0003735
GO:0005840 GO:0019031 GO:0005622 GO:0019028
Cluster-22178.82239 GO:0007275 GO:0016021
Cluster-22178.82244 GO:0006355 GO:0009271
Cluster-22178.82245 GO:0006508 GO:0009446 GO:0006807 GO:0016579
GO:0005515 GO:0004843 GO:0036459 GO:0004668
Cluster-22178.82246 GO:0016579 GO:0006807 GO:0006508 GO:0009446
GO:0036459 GO:0004668 GO:0005515 GO:0004843
Cluster-22178.82248 GO:0008033 GO:0006468 GO:0004672 GO:0005524

Cluster-22178.82250 GO:0005515
Cluster-22178.82253 GO:0016021 GO:0016020
Cluster-22178.82262 GO:0006355 GO:0003700 GO:0005515 GO:0005506
GO:0005667
Cluster-22178.82264 GO:0006265 GO:0003677 GO:0005524 GO:0003918
GO:0005634
Cluster-22178.82268 GO:0055085 GO:0006470 GO:0006811 GO:0006570
GO:0004725 GO:0005216 GO:0008138 GO:0019031 GO:0016020
Cluster-22178.82272 GO:0046872
Cluster-22178.82276 GO:0006468 GO:0005524 GO:0004672 GO:0005515

Cluster-22178.82278 GO:0051258 GO:0042157 GO:0006869 GO:0007165
GO:0051262 GO:0019064 GO:0030168 GO:0005515 GO:0004871
GO:0008289 GO:0030674 GO:0005102 GO:0005861 GO:0005576

GO:0019031 GO:0016020 GO:0005577
Cluster-22178.82281 GO:0009103 GO:0042254 GO:0006412 GO:0008918
GO:0005524 GO:0098519 GO:0003735 GO:0005840 GO:0016021
GO:0005783
Cluster-22178.82282 GO:0006308 GO:0009056 GO:0016888 GO:0008270

Cluster-22178.82283 GO:0009056 GO:0006308 GO:0016888 GO:0008270

Cluster-22178.82284 GO:0030255 GO:0006189 GO:0006468 GO:0006355
GO:0035556 GO:0005515 GO:0016773 GO:0004672 GO:0005524
GO:0016020 GO:0005668 GO:0005634
Cluster-22178.82286 GO:0006355 GO:0007165 GO:0006352 GO:0005179
GO:0003677 GO:0003700 GO:0016987 GO:0005667 GO:0005576
GO:0019031
Cluster-22178.82287 GO:0019082 GO:0006351 GO:0016032 GO:0006269
GO:0004386 GO:0003896 GO:0008270 GO:0004252 GO:0005657
GO:0005730
Cluster-22178.82292 GO:0006858
Cluster-22178.82294 GO:0015992 GO:0015986 GO:0015078 GO:0000276
Cluster-22178.82295 GO:0006952 GO:0008284 GO:0006446 GO:0003743
GO:0005737 GO:0005840 GO:0005852
Cluster-22178.82296 GO:0006887 GO:0006888 GO:0006810 GO:0009405
GO:0006886 GO:0019855 GO:0005524 GO:0008270 GO:0003677
GO:0000166 GO:0005515 GO:0030127 GO:0000145 GO:0005576
Cluster-22178.82300 GO:0007010 GO:0007165 GO:0051258 GO:0030168
GO:0005198 GO:0030674 GO:0005102 GO:0008017 GO:0005577
GO:0045298 GO:0005815
Cluster-22178.82301 GO:0007165 GO:0008608 GO:0030168 GO:0051258
GO:0030674 GO:0005102 GO:0042729 GO:0072686 GO:0005815
GO:0005577
Cluster-22178.82302 GO:0007155 GO:0007165 GO:0030168 GO:0051258
GO:0008608 GO:0030674 GO:0005102 GO:0072686 GO:0042729
GO:0005577

Cluster-22178.82303 GO:0005515
Cluster-22178.82309 GO:0003676
Cluster-22178.82310 GO:0003676
Cluster-22178.82311 GO:0005975 GO:0030246 GO:0005515
Cluster-22178.82319 GO:0007165 GO:0005057 GO:0005515 GO:0005737
Cluster-22178.82320 GO:0007186 GO:0004930 GO:0016021
Cluster-22178.82321 GO:0006508 GO:0004252 GO:0005515 GO:0016021
Cluster-22178.82322 GO:0032259 GO:0003723 GO:0005845
Cluster-22178.82323 GO:0046497 GO:0008152 GO:0055114 GO:0009435
GO:0016491 GO:0004514
Cluster-22178.82326 GO:0005515
Cluster-22178.82328 GO:0006508 GO:0006351 GO:0055114 GO:0030494
GO:0009094 GO:0015979 GO:0000162 GO:0006571 GO:0006260
GO:0009073 GO:0003856 GO:0004252 GO:0003677 GO:0016491
GO:0042025
Cluster-22178.82329 GO:0009069 GO:0007165 GO:0006144 GO:0006206
GO:0006468 GO:0006351 GO:0016310 GO:0005179 GO:0003677
GO:0005524 GO:0004674 GO:0003899 GO:0004672 GO:0005730
GO:0005887 GO:0005576
Cluster-22178.82330 GO:0006355 GO:0006351 GO:0006486 GO:0009097
GO:0006418 GO:0009098 GO:0007155 GO:0009099 GO:0006206
GO:0006144 GO:0006438 GO:0009312 GO:0008455 GO:0098599
GO:0000166 GO:0003899 GO:0008375 GO:0002161 GO:0003676
GO:0004812 GO:0016787 GO:0003677 GO:0008270 GO:0005524
GO:0004832 GO:0071203 GO:0005795 GO:0005737 GO:0016021
GO:0005730
Cluster-22178.82335 GO:0006468 GO:0009069 GO:0016310 GO:0005524
GO:0004672 GO:0004674 GO:0016020
Cluster-22178.82336 GO:0006534 GO:0046439 GO:0019530 GO:0055114
GO:0005506 GO:0017172 GO:0016702
Cluster-22178.82338 GO:0003723 GO:0005515
Cluster-22178.82340 GO:0016021
Cluster-22178.82345 GO:0046872 GO:0005515

Cluster-22178.82349 GO:0007165 GO:0006144 GO:0005515 GO:0004114

Cluster-22178.82351 GO:0071973 GO:0030288
Cluster-22178.82353 GO:0007130 GO:0006810 GO:0070193 GO:0007128
GO:0051607 GO:0048870 GO:0042803 GO:0008134 GO:0045502
GO:0031514 GO:0005667 GO:0030286 GO:0005886 GO:0000795
Cluster-22178.82354 GO:0006810 GO:0009405 GO:0008200 GO:0042151
Cluster-22178.82355 GO:0016020
Cluster-22178.82356 GO:0007155 GO:0001503 GO:0015031 GO:0006206
GO:0006144 GO:0009252 GO:0006807 GO:0006351 GO:0009405
GO:0050920 GO:0003677 GO:0004356 GO:0003899 GO:0003824
GO:0005730 GO:0005576 GO:0009288
Cluster-22178.82358 GO:0007155 GO:0006355 GO:0003700 GO:0019905
GO:0043565 GO:0019028 GO:0005667
Cluster-22178.82361 GO:0046339 GO:0009395 GO:0016042 GO:0006355
GO:0008270 GO:0043565 GO:0004435 GO:0003700 GO:0005515
GO:0005509 GO:0005739 GO:0005667 GO:0005747
Cluster-22178.82362 GO:0009395 GO:0046339 GO:0016042 GO:0004435
GO:0005509
Cluster-22178.82367 GO:0005515
Cluster-22178.82368 GO:0005975 GO:0007165 GO:0004571 GO:0008158
GO:0005509 GO:0016020
Cluster-22178.82371 GO:0019069 GO:0005515
Cluster-22178.82373 GO:0006464 GO:0007165 GO:0006298 GO:0005524
GO:0030983 GO:0019901
Cluster-22178.82374 GO:0007010 GO:0003779
Cluster-22178.82379 GO:0005515
Cluster-22178.82386 GO:0005576
Cluster-22178.82392 GO:0015886 GO:0006007 GO:0017004 GO:0006486
GO:0008373 GO:0003824 GO:0046537 GO:0016021
Cluster-22178.82394 GO:0006486 GO:0006007 GO:0046537 GO:0008373
GO:0003824
Cluster-22178.82398 GO:0055114 GO:0005515 GO:0016616

Cluster-22178.8240 GO:0007165 GO:0016020
Cluster-22178.82401 GO:0005515 GO:0042302 GO:0005549 GO:0016021
Cluster-22178.82402 GO:0055114 GO:0015992 GO:0015986 GO:0016702
GO:0046872 GO:0015078 GO:0031083 GO:0045263 GO:0030133
Cluster-22178.82408 GO:0035556
Cluster-22178.82412 GO:0007005 GO:0006098 GO:0008152 GO:0019521
GO:0055114 GO:0004616 GO:0016491 GO:0031305
Cluster-22178.82415 GO:0006298 GO:0007165 GO:0006464 GO:0019901
GO:0030983 GO:0005524
Cluster-22178.82419 GO:0016787
Cluster-22178.82421 GO:0005515
Cluster-22178.82425 GO:0006576 GO:0007264 GO:0005524 GO:0005525

Cluster-22178.82427 GO:0006576 GO:0007264 GO:0005525 GO:0005524

Cluster-22178.82432 GO:0009069 GO:0006468 GO:0016567 GO:0015979
GO:0016310 GO:0005515 GO:0046872 GO:0008270 GO:0005524
GO:0004672 GO:0004842 GO:0004674 GO:0030089 GO:0005680
GO:0005840
Cluster-22178.82436 GO:0007165 GO:0005515
Cluster-22178.82438 GO:0017004 GO:0016192 GO:0015986 GO:0015992
GO:0006118 GO:0006119 GO:0000042 GO:0006886 GO:0006355
GO:0046961 GO:0008270 GO:0046933 GO:0005515 GO:0003723
GO:0003700 GO:0045158 GO:0046872 GO:0045259 GO:0005667
GO:0009512 GO:0042025 GO:0000275
Cluster-22178.82446 GO:0006313 GO:0004803 GO:0003677 GO:0016787

Cluster-22178.82451 GO:0046497 GO:0006769 GO:0055114 GO:0008750
GO:0050661 GO:0016021
Cluster-22178.82455 GO:0007165 GO:0009405 GO:0008283 GO:0040007
GO:0051015 GO:0008083 GO:0030674
Cluster-22178.82458 GO:0006955
Cluster-22178.82459 GO:0071973 GO:0030288

Cluster-22178.82461 GO:0006886 GO:0006508 GO:0048280 GO:0009116
GO:0005515 GO:0071949 GO:0004222 GO:0003824 GO:0008270
GO:0000139 GO:0005737
Cluster-22178.82464 GO:0043123 GO:0043066 GO:0045766
Cluster-22178.82473 GO:0006974 GO:0007166 GO:0007218 GO:0051726
GO:0007165 GO:0046872 GO:0008270 GO:0004871 GO:0005185
GO:0005515 GO:0005509 GO:0005634 GO:0005576
Cluster-22178.82475 GO:0000921 GO:0007049 GO:0006606 GO:0071973
GO:0019905 GO:0045502 GO:0005198 GO:0042803 GO:0008134
GO:0030286 GO:0005940 GO:0019031 GO:0016021 GO:0005667
GO:0019028
Cluster-22178.82476 GO:0007165 GO:0006606 GO:0030168 GO:0051258
GO:0008134 GO:0042803 GO:0045502 GO:0030674 GO:0005102
GO:0005667 GO:0005577 GO:0030286
Cluster-22178.82477 GO:0009058 GO:0006885 GO:0006812 GO:0055085
GO:0009607 GO:0003824 GO:0015299 GO:0016021
Cluster-22178.82481 GO:0009395 GO:0046486 GO:0035556 GO:0007205
GO:0007165 GO:0016301 GO:0004143
Cluster-22178.82482 GO:0006367 GO:0003677 GO:0005669
Cluster-22178.82484 GO:0006457 GO:0008253 GO:0005509 GO:0000287
GO:0005737
Cluster-22178.82485 GO:0005634
Cluster-22178.82486 GO:0006508 GO:0008152 GO:0006090 GO:0055114
GO:0005975 GO:0007165 GO:0019439 GO:0008236 GO:0004252
GO:0003824 GO:0008168 GO:0008774 GO:0043169 GO:0070008
GO:0004872 GO:0016021 GO:0016020
Cluster-22178.82487 GO:0005887
Cluster-22178.82489 GO:0007165 GO:0006468 GO:0006355 GO:0008152
GO:0005515 GO:0005179 GO:0003824 GO:0005524 GO:0004672
GO:0005576 GO:0005634
Cluster-22178.82494 GO:0005515 GO:0035091
Cluster-22178.82495 GO:0006694 GO:0006470 GO:0035556 GO:0007165
GO:0006570 GO:0009058 GO:0008299 GO:0004725 GO:0050518

GO:0005044 GO:0016779 GO:0005515 GO:0019031 GO:0016020
Cluster-22178.82496 GO:0051301 GO:0019079 GO:0007049 GO:0016021
Cluster-22178.82497 GO:0006468 GO:0005524 GO:0004672 GO:0005515

Cluster-22178.82502 GO:0005515
Cluster-22178.82508 GO:0042254 GO:0009396 GO:0015992 GO:0006412
GO:0046487 GO:0015991 GO:0008152 GO:0055114 GO:0003735
GO:0004488 GO:0050662 GO:0016491 GO:0005515 GO:0003824
GO:0015078 GO:0033179 GO:0005840
Cluster-22178.82509 GO:0015991 GO:0006412 GO:0046487 GO:0055114
GO:0008152 GO:0042254 GO:0009396 GO:0015992 GO:0005515
GO:0003824 GO:0015078 GO:0050662 GO:0003735 GO:0004488
GO:0016491 GO:0033179 GO:0005840
Cluster-22178.82512 GO:0009058 GO:0006629 GO:0016788
Cluster-22178.82514 GO:0006468 GO:0005524 GO:0004672 GO:0016773
GO:0016020
Cluster-22178.82517 GO:0090063 GO:0000226 GO:0000922 GO:0005815
Cluster-22178.82518 GO:0006886 GO:0016192 GO:0006486 GO:0005515
GO:0005198 GO:0016757 GO:0008378 GO:0030117 GO:0016020
Cluster-22178.82519 GO:0006355 GO:0003700 GO:0005667 GO:0005634
Cluster-22178.82522 GO:0019033
Cluster-22178.82525 GO:0006744 GO:0005515
Cluster-22178.82530 GO:0006144 GO:0035556 GO:0046034 GO:0046872
GO:0003676 GO:0003677 GO:0004017 GO:0004519
Cluster-22178.82531 GO:0008285 GO:0006144 GO:0006888 GO:0046034
GO:0006915 GO:0007050 GO:0006858 GO:0006886 GO:0046872
GO:0008270 GO:0005506 GO:0003677 GO:0004017 GO:0030127
Cluster-22178.82532 GO:0005525 GO:0003723
Cluster-22178.82539 GO:0006304 GO:0019079 GO:0043154 GO:0043066
GO:0033821 GO:0019012 GO:0005737 GO:0071986
Cluster-22178.82542 GO:0006412 GO:0042254 GO:0003735 GO:0005840
GO:0005622
Cluster-22178.82544 GO:0009116 GO:0009165 GO:0006144 GO:0006098

GO:0004749 GO:0000287
Cluster-22178.82545 GO:0006144 GO:0000103 GO:0009058 GO:0015937
GO:0008152 GO:0015940 GO:0006468 GO:0005524 GO:0016773
GO:0004672 GO:0004140 GO:0004020 GO:0003824 GO:0005515
GO:0016301 GO:0016020
Cluster-22178.82547 GO:0055114 GO:0008152 GO:0050662 GO:0016491
GO:0003824 GO:0005515
Cluster-22178.8255 GO:0031554 GO:0031556
Cluster-22178.82550 GO:0009058 GO:0030170 GO:0003677
Cluster-22178.82552 GO:0007275 GO:0016021
Cluster-22178.82553 GO:0005975 GO:0006013 GO:0008270 GO:0015923
GO:0004559 GO:0004553
Cluster-22178.82562 GO:0007165 GO:0006355 GO:0003677 GO:0005179
GO:0005634 GO:0005576
Cluster-22178.82564 GO:0016788 GO:0005515
Cluster-22178.82571 GO:0016740 GO:0008270
Cluster-22178.82579 GO:0006812 GO:0006836 GO:0005328 GO:0016021
Cluster-22178.82581 GO:0006534 GO:0042967 GO:0006535 GO:0009001
GO:0046872 GO:0005737
Cluster-22178.82583 GO:0007165 GO:0005509 GO:0005515 GO:0005578
Cluster-22178.82593 GO:0006511 GO:0007005 GO:0008134 GO:0042803
GO:0045502 GO:0030286 GO:0031305 GO:0005667
Cluster-22178.82595 GO:0006281 GO:0000012 GO:0003684 GO:0005515
GO:0005634
Cluster-22178.82597 GO:0003676
Cluster-22178.82598 GO:0016787 GO:0045502 GO:0046872 GO:0042803
GO:0008134 GO:0005667 GO:0030286
Cluster-22178.82599 GO:0006355 GO:0003700 GO:0005515 GO:0003677
GO:0005667
Cluster-22178.82600 GO:0008152 GO:0007160 GO:0005509 GO:0008168
GO:0005576
Cluster-22178.82601 GO:0008152 GO:0016491
Cluster-22178.82608 GO:0006955 GO:0007165 GO:0040007 GO:0008283

GO:0005515 GO:0008083 GO:0008237 GO:0005134 GO:0008270
GO:0005576 GO:0005893
Cluster-22178.82612 GO:0016787 GO:0016020
Cluster-22178.82614 GO:0008168 GO:0019028
Cluster-22178.82615 GO:0008168 GO:0019028
Cluster-22178.82617 GO:0035556 GO:0006144 GO:0006171 GO:0009190
GO:0071816 GO:0004016 GO:0016849 GO:0005886
Cluster-22178.82618 GO:0008152 GO:0006534 GO:0042967 GO:0006535
GO:0046872 GO:0016491 GO:0009001 GO:0005737
Cluster-22178.82624 GO:0005515
Cluster-22178.82629 GO:0046034 GO:0006144 GO:0004017
Cluster-22178.82630 GO:0006355 GO:0006468 GO:0003700 GO:0005524
GO:0004672 GO:0008270 GO:0016773 GO:0043565 GO:0016020
GO:0005667 GO:0005794
Cluster-22178.82631 GO:0003676
Cluster-22178.82633 GO:0019031 GO:0000228
Cluster-22178.82634 GO:0055085 GO:0016192 GO:0006886 GO:0006810
GO:0022857 GO:0005215 GO:0016021 GO:0016020 GO:0030117
Cluster-22178.82636 GO:0006357 GO:0001104 GO:0005515 GO:0016592
Cluster-22178.82637 GO:0006047
Cluster-22178.82638 GO:0006047
Cluster-22178.82639 GO:0042967 GO:0047961 GO:0005739
Cluster-22178.82641 GO:0000271 GO:0016020
Cluster-22178.82644 GO:0005515
Cluster-22178.82647 GO:0007165 GO:0007264 GO:0005525
Cluster-22178.82650 GO:0007165
Cluster-22178.82652 GO:0006914 GO:0030168 GO:0019988 GO:0007165
GO:0007155 GO:0006308 GO:0051258 GO:0005102 GO:0008855
GO:0043399 GO:0030674 GO:0005198 GO:0003723 GO:0005577
GO:0019031 GO:0009318 GO:0019028
Cluster-22178.82661 GO:0005515
Cluster-22178.82662 GO:0006810 GO:0006614 GO:0002949 GO:0006260
GO:0003723 GO:0003724 GO:0003678 GO:0005525 GO:0016887

GO:0003924 GO:0005524 GO:0030288 GO:0005657 GO:0019031
Cluster-22178.82665 GO:0006355 GO:0043565 GO:0003700 GO:0005667
Cluster-22178.82668 GO:0005975 GO:0006355 GO:0003677 GO:0005515
GO:0004415
Cluster-22178.82674 GO:0005515
Cluster-22178.82675 GO:0005509 GO:0005515
Cluster-22178.82681 GO:0007186 GO:0004930 GO:0016021
Cluster-22178.82684 GO:0005515 GO:0005198
Cluster-22178.82688 GO:0006468 GO:0016773 GO:0005524 GO:0004672
GO:0016020
Cluster-22178.82690 GO:0055114 GO:0019439 GO:0005506 GO:0051537
GO:0005515 GO:0016708
Cluster-22178.82700 GO:0008152 GO:0042254 GO:0006412 GO:0003824
GO:0003735 GO:0005840 GO:0005739
Cluster-22178.82710 GO:0006810 GO:0055085 GO:0005215 GO:0016020
Cluster-22178.82712 GO:0006413 GO:0003723 GO:0019012
Cluster-22178.82716 GO:0005515
Cluster-22178.82719 GO:0007155 GO:0042246 GO:0016021
Cluster-22178.82725 GO:0016021
Cluster-22178.82726 GO:0016021
Cluster-22178.82732 GO:0042254 GO:0006412 GO:0003735 GO:0005761
GO:0005840
Cluster-22178.82734 GO:0006355 GO:0003700 GO:0043565 GO:0005667
Cluster-22178.82735 GO:0007165 GO:0042742 GO:0005515
Cluster-22178.82739 GO:0055114 GO:0016491 GO:0030151
Cluster-22178.82746 GO:0009116 GO:0003824
Cluster-22178.82750 GO:0006351
Cluster-22178.82751 GO:0006355 GO:0003690 GO:0005739
Cluster-22178.82757 GO:0006260 GO:0019079 GO:0007165 GO:0030337
GO:0005179 GO:0005576 GO:0042575
Cluster-22178.82759 GO:0006771 GO:0019497 GO:0046872 GO:0003993
GO:0005515 GO:0005576
Cluster-22178.82760 GO:0015684 GO:0005525 GO:0015093 GO:0016021

Cluster-22178.82762 GO:0016310 GO:0000160 GO:0006928 GO:0000155 GO:0004673 GO:0009365
Cluster-22178.82763 GO:0015992 GO:0015074 GO:0006364 GO:0015986 GO:0015078 GO:0005515 GO:0032040 GO:0000276
Cluster-22178.82764 GO:0006810 GO:0003723 GO:0005215 GO:0003676 GO:0016020
Cluster-22178.82768 GO:0052855
Cluster-22178.82770 GO:0019069 GO:0031556 GO:0042558 GO:0009086 GO:0031554 GO:0031419 GO:0008705 GO:0046872 GO:0005524 GO:0005622 GO:0005887
Cluster-22178.82773 GO:0016772
Cluster-22178.82778 GO:0006278 GO:0003964 GO:0030623
Cluster-22178.82782 GO:0006357 GO:0001104 GO:0016592 GO:0016020
Cluster-22178.82783 GO:0005515
Cluster-22178.82793 GO:0008152 GO:0055114 GO:0009103 GO:0005201 GO:0016491 GO:0008918 GO:0005581 GO:0005578
Cluster-22178.82797 GO:0006096 GO:0006098 GO:0006094 GO:0046854 GO:0015976 GO:0006000 GO:0006013 GO:0005975 GO:0042132 GO:0042578
Cluster-22178.82800 GO:0006886 GO:0030176
Cluster-22178.82802 GO:0043087 GO:0006952 GO:0032012 GO:0005086 GO:0005515 GO:0005576
Cluster-22178.82803 GO:0055114 GO:0016972 GO:0033905 GO:0016817

Cluster-22178.82810 GO:0042578
Cluster-22178.82811 GO:0006886 GO:0016032 GO:0005622
Cluster-22178.82813 GO:0045490 GO:0047487
Cluster-22178.82816 GO:0006468 GO:0005524 GO:0004672
Cluster-22178.82817 GO:0006468 GO:0004672 GO:0005524 GO:0005515

Cluster-22178.82818 GO:0007131 GO:0009399 GO:0008219 GO:0006869 GO:0042157 GO:0006468 GO:0008289 GO:0004672 GO:0005524 GO:0005515 GO:0005576

Cluster-22178.82821 GO:0046872 GO:0008270 GO:0005515 GO:0003676

Cluster-22178.82823 GO:0005515
Cluster-22178.82826 GO:0055085 GO:0006811 GO:0005216 GO:0016020
Cluster-22178.82829 GO:0005515
Cluster-22178.82830 GO:0008168
Cluster-22178.82832 GO:0006355 GO:0003700 GO:0003677 GO:0003723
GO:0043565 GO:0005667 GO:0005634
Cluster-22178.82835 GO:0008152 GO:0042073 GO:0008168
Cluster-22178.82837 GO:0006812 GO:0005515 GO:0008324 GO:0046872

Cluster-22178.82838 GO:0006144 GO:0015693 GO:0008152 GO:0006351
GO:0016491 GO:0003968 GO:0003723 GO:0015095 GO:0031379
GO:0008023 GO:0016021
Cluster-22178.82840 GO:0005515
Cluster-22178.82841 GO:0055085 GO:0006821 GO:0005247 GO:0016020
Cluster-22178.82842 GO:0045995 GO:0006308 GO:0030334 GO:0030155
GO:0007165 GO:0005102 GO:0008855 GO:0005515 GO:0009318
Cluster-22178.82847 GO:0005524 GO:0016787 GO:0003677 GO:0005515

Cluster-22178.82848 GO:0006355 GO:0016042 GO:0009395 GO:0046339
GO:0005515 GO:0005509 GO:0004435 GO:0003700 GO:0043565
GO:0008270 GO:0005747 GO:0005739 GO:0005667
Cluster-22178.82850 GO:0006120 GO:0055085 GO:0006744 GO:0015992
GO:0006821 GO:0006118 GO:0006814 GO:0005247 GO:0008137
GO:0003954 GO:0016020 GO:0005739
Cluster-22178.82854 GO:0009847 GO:0006260 GO:0055114 GO:0020037
GO:0016705 GO:0005515 GO:0005506 GO:0005634 GO:0016021
Cluster-22178.82855 GO:0055114 GO:0006260 GO:0009847 GO:0005506
GO:0016705 GO:0020037 GO:0005515 GO:0016021 GO:0005634
Cluster-22178.82863 GO:0007165
Cluster-22178.82866 GO:0006418 GO:0000166 GO:0003677 GO:0004518
GO:0005524 GO:0008270 GO:0004812

Cluster-22178.82867 GO:0006779 GO:0055114 GO:0016226 GO:0051536 GO:0005737
Cluster-22178.82874 GO:0006886 GO:0006605 GO:0045040 GO:0055085 GO:0005515 GO:0022857 GO:0046983 GO:0005742 GO:0016021
Cluster-22178.82875 GO:0055085 GO:0006886 GO:0006605 GO:0045040 GO:0022857 GO:0046983 GO:0005515 GO:0016021 GO:0005742
Cluster-22178.82877 GO:0006468 GO:0004672 GO:0005524 GO:0016773 GO:0016020
Cluster-22178.82881 GO:0005515
Cluster-22178.82883 GO:0046497 GO:0055114 GO:0006769 GO:0050661 GO:0008750 GO:0016021
Cluster-22178.82884 GO:0016021
Cluster-22178.82885 GO:0016021
Cluster-22178.82894 GO:0005515
Cluster-22178.82896 GO:0007275
Cluster-22178.82897 GO:0016310 GO:0007178 GO:0009069 GO:0006468 GO:0005524 GO:0004672 GO:0004675 GO:0016020
Cluster-22178.82907 GO:0006270 GO:0006816 GO:0006874 GO:0035556 GO:0003677 GO:0004518 GO:0004190 GO:0005219 GO:0016021 GO:0005622
Cluster-22178.82908 GO:0007010 GO:0000413 GO:0006457 GO:0003755 GO:0008017 GO:0005887 GO:0045298
Cluster-22178.82909 GO:0015696 GO:0008519 GO:0016020
Cluster-22178.82913 GO:0006412 GO:0051252 GO:0000160 GO:0006465 GO:0016310 GO:0008033 GO:0042254 GO:0015743 GO:0005515 GO:0003723 GO:0003676 GO:0003735 GO:0004526 GO:0000155 GO:0008233 GO:0046983 GO:0005787 GO:0005634 GO:0016021 GO:0005840 GO:0009365 GO:0030677 GO:0005622
Cluster-22178.82914 GO:0005515
Cluster-22178.82915 GO:0016747 GO:0005515 GO:0016020
Cluster-22178.82917 GO:0005515
Cluster-22178.82920 GO:0003723 GO:0003774 GO:0016459
Cluster-22178.82921 GO:0007264 GO:0005525

Cluster-22178.82930 GO:0019012 GO:0016021
Cluster-22178.82934 GO:0016567 GO:0003690 GO:0004842 GO:0005737
Cluster-22178.82940 GO:0005524
Cluster-22178.82943 GO:0055114 GO:0006090 GO:0016117 GO:0006099
GO:0016491 GO:0008924 GO:0071949 GO:0016705
Cluster-22178.82948 GO:0003824 GO:0046872
Cluster-22178.82949 GO:0003723 GO:0005515
Cluster-22178.8295 GO:0005515
Cluster-22178.82950 GO:0005515
Cluster-22178.82951 GO:0005515
Cluster-22178.82952 GO:0005815
Cluster-22178.82954 GO:0005515 GO:0046872 GO:0045202 GO:0005737
Cluster-22178.82957 GO:0005515
Cluster-22178.82966 GO:0005515
Cluster-22178.82968 GO:0007186 GO:0009058 GO:0010038 GO:0015979
GO:0004930 GO:0016020 GO:0016021 GO:0009523
Cluster-22178.82970 GO:0009607 GO:0016021
Cluster-22178.82975 GO:0007005 GO:0016192 GO:0008053 GO:0005198
GO:0003924 GO:0005741 GO:0031305 GO:0016021 GO:0019031
Cluster-22178.82976 GO:0006807 GO:0016810
Cluster-22178.82977 GO:0008053 GO:0016192 GO:0005198 GO:0003924
GO:0016021 GO:0019031 GO:0005741
Cluster-22178.82978 GO:0005515
Cluster-22178.82979 GO:0005515
Cluster-22178.82983 GO:0008152 GO:0006694 GO:0016114 GO:0016624
GO:0008661 GO:0030976 GO:0003824 GO:0016021
Cluster-22178.82986 GO:0006508 GO:0030436 GO:0004190
Cluster-22178.82987 GO:0006119 GO:0006118 GO:0015992 GO:0055114
GO:0008121
Cluster-22178.82989 GO:0015012 GO:0006024 GO:0016021
Cluster-22178.82990 GO:0035023 GO:0043087 GO:0015878 GO:0015225
GO:0005515 GO:0005089 GO:0005886
Cluster-22178.82991 GO:0006284 GO:0006289 GO:0006281 GO:0003684

GO:0016799 GO:0003906 GO:0008270

Cluster-22178.82994 GO:0018149 GO:0042710 GO:0008270 GO:0019031

Cluster-22178.82997 GO:0005509

Cluster-22178.83012 GO:0008270 GO:0003677 GO:0005634

Cluster-22178.83018 GO:0044781 GO:0016021 GO:0016020

Cluster-22178.83024 GO:0007186 GO:0004930 GO:0016021

Cluster-22178.83026 GO:0006508 GO:0000245 GO:0006355 GO:0008270 GO:0004222 GO:0005515 GO:0031012 GO:0005634

Cluster-22178.83030 GO:0006355 GO:0003700 GO:0005667

Cluster-22178.83034 GO:0042742 GO:0009069 GO:0007165 GO:0046339 GO:0016042 GO:0035556 GO:0006629 GO:0006468 GO:0006570 GO:0009395 GO:0055114 GO:0016310 GO:0004511 GO:0005509 GO:0005096 GO:0046935 GO:0004674 GO:0005515 GO:0004435 GO:0005944 GO:0005576

Cluster-22178.83038 GO:0000724 GO:0006260 GO:0003676 GO:0003677 GO:0016021 GO:0005634

Cluster-22178.8304 GO:0015689 GO:0009403 GO:0015098 GO:0016021 GO:0016020

Cluster-22178.83044 GO:0007264 GO:0006576 GO:0005525 GO:0005524 GO:0003924

Cluster-22178.83045 GO:0042432 GO:0009058 GO:0016114 GO:0004064 GO:0016844 GO:0005515

Cluster-22178.83047 GO:0005515

Cluster-22178.83051 GO:0006810 GO:0005515 GO:0016021

Cluster-22178.83052 GO:0042254 GO:0006412 GO:0003735 GO:0005622 GO:0005840

Cluster-22178.83054 GO:0055114 GO:0006464 GO:0008152 GO:0007165 GO:0006298 GO:0016491 GO:0005524 GO:0030983 GO:0019901

Cluster-22178.83063 GO:0007165 GO:0005179 GO:0005576

Cluster-22178.83065 GO:0016070 GO:0008270 GO:0046872 GO:0005515

Cluster-22178.83066 GO:0005515 GO:0046872 GO:0008270

Cluster-22178.83067 GO:0003676 GO:0008270 GO:0046872 GO:0005622

Cluster-22178.83068 GO:0006355 GO:0006118 GO:0006367 GO:0006891
GO:0005515 GO:0019901 GO:0043565 GO:0003700 GO:0015035
GO:0005634 GO:0005667 GO:0017119
Cluster-22178.83071 GO:0008270 GO:0005634
Cluster-22178.83072 GO:0003677
Cluster-22178.83080 GO:0005515
Cluster-22178.83082 GO:0005515
Cluster-22178.83084 GO:0032012 GO:0043087 GO:0005086
Cluster-22178.83087 GO:0016042 GO:0007586 GO:0008047 GO:0005576
Cluster-22178.83088 GO:0016042 GO:0007586 GO:0008047 GO:0005576
Cluster-22178.83089 GO:0006606 GO:0006914 GO:0031083 GO:0016021
Cluster-22178.83090 GO:0016747
Cluster-22178.83094 GO:0007165 GO:0007186 GO:0031683 GO:0019001
GO:0003924 GO:0004871
Cluster-22178.83095 GO:0007186 GO:0007165 GO:0007264 GO:0004871
GO:0005525 GO:0003924 GO:0031683 GO:0019001
Cluster-22178.83097 GO:0006486 GO:0006013 GO:0006000 GO:0003873
GO:0005524 GO:0004576 GO:0016020 GO:0008250
Cluster-22178.83101 GO:0006355 GO:0006810 GO:0006886 GO:0003677
GO:0005515 GO:0005524 GO:0042626 GO:0005783 GO:0016021
Cluster-22178.83103 GO:0006118 GO:0009055 GO:0005515 GO:0009512
Cluster-22178.83106 GO:0006334 GO:0003677 GO:0005634 GO:0019028
GO:0000786
Cluster-22178.83111 GO:0055085 GO:0006885 GO:0006812 GO:0015299
GO:0016021
Cluster-22178.83113 GO:0006278 GO:0003964
Cluster-22178.83114 GO:0006950 GO:0009415 GO:0005515
Cluster-22178.83118 GO:0006812 GO:0006835 GO:0006820 GO:0017153
GO:0005887 GO:0016020
Cluster-22178.83126 GO:0006468 GO:0005524 GO:0004672
Cluster-22178.83128 GO:0006352 GO:0006334 GO:0003677 GO:0046982
GO:0000786 GO:0005634
Cluster-22178.83132 GO:0015992 GO:0043985 GO:0006032 GO:0006118

GO:0006119 GO:0010508 GO:0015991 GO:0006355 GO:0046961
GO:0003954 GO:0003700 GO:0043565 GO:0016765 GO:0005515
GO:0008061 GO:0042393 GO:0005739 GO:0005667 GO:0033178
GO:0005634
Cluster-22178.83133 GO:0015948 GO:0007264 GO:0042981 GO:0005515
GO:0005525 GO:0003924
Cluster-22178.83135 GO:0042254 GO:0006412 GO:0003735 GO:0005622
GO:0016020 GO:0005840
Cluster-22178.83139 GO:0009058 GO:0030170 GO:0046983 GO:0005515

Cluster-22178.83143 GO:0005515 GO:0032040
Cluster-22178.83147 GO:0035091
Cluster-22178.83148 GO:0005515
Cluster-22178.83150 GO:0005515 GO:0016021
Cluster-22178.83152 GO:0005515 GO:0003677 GO:0005634 GO:0016021
Cluster-22178.83154 GO:0046413 GO:0046872 GO:0018836
Cluster-22178.83157 GO:0015992 GO:0007017 GO:0015986 GO:0006119
GO:0007018 GO:0005524 GO:0046933 GO:0008017 GO:0003777
GO:0046961 GO:0045298 GO:0005874 GO:0000275 GO:0045259
Cluster-22178.83161 GO:0006355 GO:0006352 GO:0003677 GO:0016987
GO:0003700 GO:0005667
Cluster-22178.83171 GO:0006355 GO:0003700 GO:0043565 GO:0005667
GO:0005634
Cluster-22178.83177 GO:0006470 GO:0006570 GO:0004725 GO:0008138

Cluster-22178.83185 GO:0006144 GO:0046034 GO:0007049 GO:0035556
GO:0006355 GO:0007165 GO:0008270 GO:0004871 GO:0003700
GO:0005515 GO:0004017 GO:0003677 GO:0005667
Cluster-22178.83187 GO:0005515
Cluster-22178.83192 GO:0006879 GO:0015631 GO:0005515 GO:0005576
GO:0045298
Cluster-22178.83193 GO:0043065 GO:0051321 GO:0097193 GO:0009405
GO:0090200 GO:0005515 GO:0005739 GO:0005576

Cluster-22178.83196 GO:0006621 GO:0046923 GO:0005783 GO:0016021

Cluster-22178.83203 GO:0009432 GO:0006468 GO:0006820 GO:0006333 GO:0022611 GO:0005253 GO:0005524 GO:0004672 GO:0005634 GO:0005887

Cluster-22178.83207 GO:0000271 GO:0016020

Cluster-22178.83214 GO:0005515 GO:0046872 GO:0008270 GO:0005622

Cluster-22178.83217 GO:0016779

Cluster-22178.83218 GO:0016779

Cluster-22178.83221 GO:0042967 GO:0015694 GO:0008080 GO:0015097 GO:0005515 GO:0001772 GO:0097197 GO:0016021 GO:0016020

Cluster-22178.83223 GO:0015694 GO:0042967 GO:0015097 GO:0005515 GO:0008080 GO:0016020 GO:0001772 GO:0097197 GO:0016021

Cluster-22178.83227 GO:0008152 GO:0006479 GO:0006554 GO:0005515 GO:0042802 GO:0008168 GO:0018024

Cluster-22178.83233 GO:0006355 GO:0006351

Cluster-22178.83239 GO:0004222 GO:0005524 GO:0008270 GO:0016021

Cluster-22178.83242 GO:0006813 GO:0007049 GO:0016286 GO:0016021

Cluster-22178.83244 GO:0006886 GO:0016192 GO:0005515 GO:0005198 GO:0004789 GO:0030117

Cluster-22178.83245 GO:0016021

Cluster-22178.83250 GO:0007165 GO:0006810 GO:0008643 GO:0005215 GO:0004872 GO:0016020 GO:0042597 GO:0016021

Cluster-22178.83252 GO:0000287 GO:0008253 GO:0005737

Cluster-22178.83255 GO:0055085 GO:0006874 GO:0006816 GO:0006270 GO:0070588 GO:0030001 GO:0006506 GO:0008270 GO:0016746 GO:0046873 GO:0005509 GO:0005219 GO:0005262 GO:0005515 GO:0005622 GO:0016020 GO:0016021 GO:0005789

Cluster-22178.83260 GO:0005515

Cluster-22178.83262 GO:0005515 GO:0048786

Cluster-22178.83266 GO:0006813 GO:0045132 GO:0016337 GO:0007049 GO:0016286 GO:0005634 GO:0016020 GO:0000775 GO:0016021

Cluster-22178.83267 GO:0006464 GO:0009966 GO:0007275 GO:0008270 GO:0046872 GO:0016151 GO:0016020

Cluster-22178.83269 GO:0006412 GO:0055114 GO:0016491 GO:0008168

Cluster-22178.83270 GO:0007165 GO:0030168 GO:0006914 GO:0048193
GO:0009405 GO:0051258 GO:0030674 GO:0005102 GO:0045502
GO:0042803 GO:0008134 GO:0019901 GO:0008092 GO:0005667
GO:0016020 GO:0030286 GO:0005577 GO:0019898 GO:0005737
Cluster-22178.83276 GO:0042254 GO:0006118 GO:0017004 GO:0003735
GO:0045158 GO:0005840 GO:0009512
Cluster-22178.83280 GO:0006355 GO:0046983 GO:0003677
Cluster-22178.83287 GO:0006914
Cluster-22178.83288 GO:0006814 GO:0006813 GO:0005890
Cluster-22178.83291 GO:0016021
Cluster-22178.83293 GO:0009405 GO:0007268 GO:0007165 GO:0030550
GO:0005576
Cluster-22178.83296 GO:0006535 GO:0006308 GO:0006534 GO:0042967
GO:0010181 GO:0009001 GO:0046983 GO:0008855 GO:0009318
GO:0005737
Cluster-22178.83300 GO:0055114 GO:1901031 GO:0051537 GO:0016491
GO:0051920 GO:0005634
Cluster-22178.83301 GO:0006508 GO:0004252 GO:0046872 GO:0045095
Cluster-22178.83303 GO:0006914 GO:0000921 GO:0007264 GO:0010508
GO:0005096 GO:0005509 GO:0005515 GO:0005940 GO:0016021
Cluster-22178.83304 GO:0000921 GO:0007264 GO:0005509 GO:0005515
GO:0005096 GO:0016021 GO:0005940
Cluster-22178.83306 GO:0006886 GO:0005975 GO:0015031 GO:0005509
GO:0016773 GO:0008565 GO:0016020 GO:0005737
Cluster-22178.83310 GO:0005515
Cluster-22178.83312 GO:0005515 GO:0008270 GO:0005634
Cluster-22178.83317 GO:0003824
Cluster-22178.83318 GO:0003676
Cluster-22178.83326 GO:0006355 GO:0043565 GO:0005201 GO:0003700
GO:0003677 GO:0005815 GO:0005578 GO:0005634 GO:0005667
Cluster-22178.83331 GO:0005515

Cluster-22178.83334 GO:0000166
Cluster-22178.83335 GO:0019042 GO:0009611 GO:0004867 GO:0033644
Cluster-22178.83338 GO:0006457 GO:0004064 GO:0051087 GO:0005515
GO:0042803 GO:0008092 GO:0000774 GO:0005737 GO:0019898
Cluster-22178.83340 GO:0008608 GO:0006260 GO:0006310 GO:0006886
GO:0006281 GO:0003910 GO:0003677 GO:0005622 GO:0042729
GO:0072686
Cluster-22178.83341 GO:0016021
Cluster-22178.83344 GO:0006355 GO:0003677 GO:0016872
Cluster-22178.83345 GO:0006810 GO:0016021
Cluster-22178.83346 GO:0055114 GO:0006144 GO:0046034 GO:0006281
GO:0008270 GO:0046872 GO:0047134 GO:0004017
Cluster-22178.83347 GO:0042254 GO:0006412 GO:0006281 GO:0035556
GO:0046034 GO:0006464 GO:0006144 GO:0003735 GO:0005515
GO:0004017 GO:0016151 GO:0046872 GO:0008270 GO:0005840
Cluster-22178.83350 GO:0000166 GO:0046872
Cluster-22178.83352 GO:0006886 GO:0006888 GO:0008270 GO:0030127
Cluster-22178.83354 GO:0046872 GO:0008270 GO:0003676 GO:0005622
Cluster-22178.83356 GO:0006464 GO:0015031
Cluster-22178.83358 GO:0015031 GO:0006464 GO:0005515
Cluster-22178.83362 GO:0005515 GO:0000228
Cluster-22178.83363 GO:0006812 GO:0008324 GO:0005515 GO:0000228
Cluster-22178.83366 GO:0004222
Cluster-22178.83368 GO:0010506 GO:0019064 GO:0007165 GO:0004222
GO:0046789 GO:0005515 GO:0016788 GO:0005615 GO:0019031
GO:0009986
Cluster-22178.83372 GO:0043154 GO:0043066 GO:0019079 GO:0071986
GO:0005737
Cluster-22178.83373 GO:0055114 GO:0016491
Cluster-22178.83376 GO:0005515
Cluster-22178.83377 GO:0007165 GO:0005515
Cluster-22178.83378 GO:0007165 GO:0005515
Cluster-22178.8338 GO:0008152 GO:0007165 GO:0006955 GO:0030247

GO:0003824 GO:0003676 GO:0046872 GO:0008484 GO:0005044
GO:0016787
Cluster-22178.83380 GO:0003677
Cluster-22178.83381 GO:0003677
Cluster-22178.83382 GO:0008033
Cluster-22178.83383 GO:0006810 GO:0055085 GO:0005524 GO:0016747
GO:0042626 GO:0016021
Cluster-22178.83385 GO:0051258 GO:0048870 GO:0007165 GO:0006606
GO:0006260 GO:0008053 GO:0030168 GO:0019901 GO:0042803
GO:0008134 GO:0045502 GO:0003924 GO:0030674 GO:0005102
GO:0031514 GO:0016021 GO:0005741 GO:0005667 GO:0005577
GO:0030286
Cluster-22178.83386 GO:0007165 GO:0006260 GO:0006606 GO:0008053
GO:0030168 GO:0051258 GO:0048870 GO:0030674 GO:0005102
GO:0019901 GO:0008134 GO:0042803 GO:0045502 GO:0003924
GO:0005741 GO:0005667 GO:0005577 GO:0030286 GO:0031514
GO:0016021
Cluster-22178.83391 GO:0015694 GO:0003677 GO:0015097 GO:0016020
Cluster-22178.83395 GO:0007165 GO:0009069 GO:0006468 GO:0007217
GO:0016310 GO:0004674 GO:0004672 GO:0005524 GO:0005576
Cluster-22178.83397 GO:0046872
Cluster-22178.83399 GO:0050920 GO:0005515 GO:0003824 GO:0009288
Cluster-22178.83401 GO:0007186 GO:0006313 GO:0006508 GO:0005509
GO:0004252 GO:0004930 GO:0016020
Cluster-22178.83403 GO:0006811 GO:0055085 GO:0006816 GO:0005262
GO:0005244 GO:0005515 GO:0016021 GO:0016020
Cluster-22178.83404 GO:0055085 GO:0006811 GO:0006816 GO:0005262
GO:0005515 GO:0005244 GO:0016021 GO:0016020
Cluster-22178.83405 GO:0006816 GO:0006811 GO:0055085 GO:0005244
GO:0005515 GO:0005262 GO:0016020 GO:0016021
Cluster-22178.83407 GO:0006897 GO:0005515 GO:0005096 GO:0016020
GO:0016021
Cluster-22178.83409 GO:0006810 GO:0019058 GO:0046872 GO:0016021

Cluster-22178.83410 GO:0006468 GO:0016773 GO:0004672 GO:0005524
GO:0016020
Cluster-22178.83411 GO:0042254 GO:0005509 GO:0005515 GO:0005622
Cluster-22178.83428 GO:0003677 GO:0016787 GO:0005524
Cluster-22178.83431 GO:0046983
Cluster-22178.83433 GO:0005515
Cluster-22178.83434 GO:0006355 GO:0042967 GO:0016573 GO:0004402
GO:0005515 GO:0000123
Cluster-22178.83450 GO:0006468 GO:0004672 GO:0003774 GO:0005524
GO:0005515 GO:0016459
Cluster-22178.83456 GO:0007160 GO:0005515 GO:0005509
Cluster-22178.83460 GO:0006810 GO:0006468 GO:0006099 GO:0006090
GO:0016117 GO:0055114 GO:0016705 GO:0071949 GO:0005215
GO:0008924 GO:0005524 GO:0004672 GO:0016491 GO:0016773
GO:0016020
Cluster-22178.83463 GO:0006359
Cluster-22178.83467 GO:0042981 GO:0015992 GO:0043065 GO:0006119
GO:0015986 GO:0015991 GO:0015078 GO:0046961 GO:0031225
GO:0005737 GO:0045263 GO:0033178
Cluster-22178.83468 GO:0015986 GO:0015991 GO:0006119 GO:0015992
GO:0046961 GO:0015078 GO:0033178 GO:0045263 GO:0031225
Cluster-22178.83469 GO:0048193 GO:0005515 GO:0016020
Cluster-22178.83470 GO:0006355 GO:0043565 GO:0003677 GO:0003700
GO:0005634 GO:0005667
Cluster-22178.83471 GO:0007165 GO:0005102 GO:0005515 GO:0016020
Cluster-22178.83472 GO:0046413 GO:0018836 GO:0046872
Cluster-22178.83474 GO:0040007 GO:0008283 GO:0008406 GO:0007165
GO:0005515 GO:0008083
Cluster-22178.83475 GO:0007165 GO:0006807 GO:0006811 GO:0006672
GO:0055085 GO:0005216 GO:0016811 GO:0004872 GO:0016020
GO:0016021
Cluster-22178.83482 GO:0006508 GO:0008270 GO:0004222 GO:0031012
Cluster-22178.83484 GO:0006118 GO:0005515 GO:0003824 GO:0009055

Cluster-22178.83485 GO:0005515
Cluster-22178.83486 GO:0003677
Cluster-22178.83488 GO:0006413 GO:0006446 GO:0031369 GO:0003743
GO:0005515 GO:0005840 GO:0005852
Cluster-22178.83490 GO:0003676
Cluster-22178.83491 GO:0071704 GO:0005975 GO:0016868
Cluster-22178.83492 GO:0055085 GO:0030001 GO:0046873 GO:0016020
Cluster-22178.83497 GO:0006468 GO:0046983 GO:0004672 GO:0005524

Cluster-22178.83498 GO:0006281 GO:0043087 GO:0032012 GO:0035556
GO:0005086 GO:0008270 GO:0046872 GO:0005201 GO:0005578
Cluster-22178.83502 GO:0016020
Cluster-22178.83503 GO:0007586 GO:0008152 GO:0016042 GO:0005515
GO:0008236 GO:0005509 GO:0016746 GO:0008047 GO:0005576
Cluster-22178.83504 GO:0007264 GO:0005524 GO:0016787 GO:0003677
GO:0005525
Cluster-22178.83505 GO:0006954 GO:0007165 GO:0008152 GO:0055114
GO:0016616 GO:0051287 GO:0042578 GO:0005125 GO:0005576
Cluster-22178.83513 GO:0006468 GO:0006508 GO:0007169 GO:0006777
GO:0004714 GO:0008270 GO:0004672 GO:0005524 GO:0016773
GO:0004222 GO:0016020 GO:0031012
Cluster-22178.83516 GO:0006777 GO:0007169 GO:0006468 GO:0006508
GO:0004222 GO:0004672 GO:0008270 GO:0005524 GO:0016773
GO:0004714 GO:0031012 GO:0016020
Cluster-22178.83518 GO:0006508 GO:0008270 GO:0005515 GO:0003676
GO:0004222
Cluster-22178.8352 GO:0042254 GO:0006412 GO:0003735 GO:0005840
GO:0005622
Cluster-22178.83524 GO:0031412 GO:0006206 GO:0006144 GO:0006914
GO:0006352 GO:0006351 GO:0006355 GO:0016043 GO:0016987
GO:0003677 GO:0003700 GO:0003779 GO:0003899 GO:0005730
GO:0031411 GO:0005667

Cluster-22178.83526 GO:0007165
Cluster-22178.83536 GO:0046740 GO:0006810 GO:0055085 GO:0005215
GO:0016020 GO:0016021
Cluster-22178.83537 GO:0055085 GO:0006858 GO:0006810 GO:0046740
GO:0005215 GO:0016020 GO:0016021
Cluster-22178.83538 GO:0006858 GO:0006810 GO:0055085 GO:0005215
GO:0016020
Cluster-22178.83542 GO:0016192 GO:0016021 GO:0019031
Cluster-22178.83546 GO:0055114 GO:0008152 GO:0016758 GO:0016706

Cluster-22178.83548 GO:0000271 GO:0006821 GO:0055085 GO:0005247
GO:0016020
Cluster-22178.83553 GO:0005524 GO:0004003 GO:0003677 GO:0005515
GO:0005657
Cluster-22178.83555 GO:0003723 GO:0008017 GO:0005515 GO:0045298
Cluster-22178.83559 GO:0042742 GO:0008270 GO:0005576 GO:0005634
Cluster-22178.83566 GO:0045947 GO:0008608 GO:0008190 GO:0072686
GO:0042729
Cluster-22178.83567 GO:0005515
Cluster-22178.83572 GO:0006468 GO:0004672 GO:0005524
Cluster-22178.83574 GO:0007186 GO:0004930 GO:0016021
Cluster-22178.83583 GO:0042138 GO:0006310
Cluster-22178.83587 GO:0009405 GO:0007186 GO:0004930 GO:0005515
GO:0005576 GO:0016021
Cluster-22178.83591 GO:0006468 GO:0005524 GO:0004672
Cluster-22178.83593 GO:0030170 GO:0003676
Cluster-22178.83594 GO:0006744 GO:0006511 GO:0007275 GO:0046872
GO:0008270 GO:0005515 GO:0005634
Cluster-22178.83597 GO:0019042 GO:0033644
Cluster-22178.83601 GO:0007219 GO:0007275 GO:0030154 GO:0005509
GO:0005515 GO:0016021
Cluster-22178.83603 GO:0005509 GO:0005515
Cluster-22178.83605 GO:0005515 GO:0005509

Cluster-22178.83606 GO:0016310 GO:0035023 GO:0019048 GO:0006813 GO:0006468 GO:0006606 GO:0042967 GO:0043087 GO:0007165 GO:0006952 GO:0009069 GO:0046914 GO:0005515 GO:0004674 GO:0016407 GO:0005089 GO:0046983 GO:0005096 GO:0005242 GO:0016021 GO:0005576 GO:0008076

Cluster-22178.83608 GO:0003924 GO:0005525

Cluster-22178.83612 GO:0030150 GO:0006118 GO:0009055 GO:0008270 GO:0046872 GO:0051536 GO:0005515 GO:0061630 GO:0005742

Cluster-22178.83613 GO:0042475 GO:0042742 GO:0042476 GO:0030345 GO:0016020 GO:0005578 GO:0016021 GO:0019867

Cluster-22178.83616 GO:0003677

Cluster-22178.83619 GO:0006508 GO:0009405 GO:0004252 GO:0005576

Cluster-22178.83622 GO:0019064 GO:0030001 GO:0055085 GO:0046873 GO:0016020 GO:0019031

Cluster-22178.83625 GO:0016757

Cluster-22178.83626 GO:0005515

Cluster-22178.83627 GO:0006470 GO:0006811 GO:0055085 GO:0006570 GO:0004725 GO:0008138 GO:0005216 GO:0019031 GO:0016020

Cluster-22178.83629 GO:0020037 GO:0035091 GO:0005524 GO:0016020

Cluster-22178.83632 GO:0007155 GO:0016020

Cluster-22178.83636 GO:0006771 GO:0019497 GO:0003993 GO:0003677

Cluster-22178.83639 GO:0006810 GO:0006568 GO:0055085 GO:0055114 GO:0005506 GO:0005215 GO:0000334 GO:0016020

Cluster-22178.83641 GO:0016757

Cluster-22178.83644 GO:0006811 GO:0005216 GO:0016020

Cluster-22178.83646 GO:0007156 GO:0006357 GO:0001104 GO:0005515 GO:0005509 GO:0016020 GO:0016592

Cluster-22178.83650 GO:0046677 GO:0006139 GO:0006396 GO:0000154 GO:0046740 GO:0008649 GO:0003676 GO:0005524 GO:0003677 GO:0016818 GO:0008026 GO:0004003 GO:0016787 GO:0016021 GO:0005657

Cluster-22178.83651 GO:0003676 GO:0008270

Cluster-22178.83653 GO:0016021
Cluster-22178.83654 GO:0006118 GO:0009405 GO:0030254 GO:0055085
GO:0006811 GO:0003682 GO:0015075 GO:0009055 GO:0016020
GO:0030257 GO:0005634 GO:0000785 GO:0016021
Cluster-22178.83659 GO:0007156 GO:0005509 GO:0016020
Cluster-22178.83660 GO:0006122 GO:0005750 GO:0005743
Cluster-22178.83662 GO:0007156 GO:0005509 GO:0016020
Cluster-22178.83663 GO:0007156 GO:0005509 GO:0016020
Cluster-22178.83669 GO:0007017 GO:0007018 GO:0006810 GO:0003777
GO:0019871 GO:0030286 GO:0005874 GO:0005576
Cluster-22178.83678 GO:0046872
Cluster-22178.83679 GO:0046872
Cluster-22178.83685 GO:0043401 GO:0006355
Cluster-22178.83686 GO:0015991 GO:0006119 GO:0015992 GO:0000287
GO:0005515 GO:0008900 GO:0005524 GO:0016020 GO:0005889
Cluster-22178.83687 GO:0006629 GO:0005515
Cluster-22178.83691 GO:0005515
Cluster-22178.83692 GO:0051276 GO:0015979 GO:0005515 GO:0005524
GO:0005694 GO:0016020 GO:0009523 GO:0009654
Cluster-22178.83697 GO:0006334 GO:0005515 GO:0003677 GO:0000786
GO:0005634
Cluster-22178.83700 GO:0008360 GO:0015979 GO:0007186 GO:0004930
GO:0003677 GO:0042802 GO:0005515 GO:0016021 GO:0009539
GO:0009523 GO:0016020
Cluster-22178.83702 GO:0008152 GO:0007018 GO:0007017 GO:0003777
GO:0008289 GO:0008017 GO:0005524 GO:0016829 GO:0010333
GO:0005874 GO:0045298 GO:0005739
Cluster-22178.83709 GO:0006351 GO:0006269 GO:0003896 GO:0003677
GO:0005515 GO:0005198 GO:0020037 GO:0019825 GO:0005657
GO:0005730 GO:0005634
Cluster-22178.83710 GO:0006351 GO:0006269 GO:0003896 GO:0003677
GO:0020037 GO:0005515 GO:0019825 GO:0005657 GO:0005730
GO:0005634

Cluster-22178.83716 GO:0007165 GO:0009847 GO:0005102
Cluster-22178.83717 GO:0005515
Cluster-22178.83719 GO:0006468 GO:0004672 GO:0005524
Cluster-22178.83731 GO:0005515
Cluster-22178.83734 GO:0006511 GO:0031625 GO:0019028
Cluster-22178.83737 GO:0051252 GO:0006402 GO:0005975 GO:0003723 GO:0016832 GO:0003676 GO:0004535 GO:0046872 GO:0005634 GO:0005737
Cluster-22178.83738 GO:0007165
Cluster-22178.83741 GO:0006468 GO:0005524 GO:0004672 GO:0005515

Cluster-22178.83743 GO:0006355 GO:0003700 GO:0005667
Cluster-22178.83744 GO:0008152 GO:0016788
Cluster-22178.83747 GO:0008270 GO:0016020 GO:0016021
Cluster-22178.83753 GO:0051297 GO:0005813
Cluster-22178.83754 GO:0051297 GO:0005813
Cluster-22178.83755 GO:0030168 GO:0016567 GO:0007165 GO:0051258 GO:0006355 GO:0005102 GO:0004842 GO:0008270 GO:0061630 GO:0030674 GO:0003700 GO:0043565 GO:0046872 GO:0005515 GO:0003723 GO:0005577 GO:0005680 GO:0005622 GO:0005667
Cluster-22178.83763 GO:0055114 GO:0016779 GO:0016491
Cluster-22178.83764 GO:0005515 GO:0035091
Cluster-22178.83765 GO:0006118 GO:0035556 GO:0006508 GO:0004177 GO:0009055 GO:0051536
Cluster-22178.83766 GO:0006508 GO:0035556 GO:0006118 GO:0051536 GO:0009055 GO:0004177
Cluster-22178.83768 GO:0006605 GO:0006886 GO:0005742
Cluster-22178.83776 GO:0016310 GO:0009069 GO:0004674 GO:0005544 GO:0005509
Cluster-22178.83781 GO:0005515
Cluster-22178.83784 GO:0009878 GO:0046872 GO:0005509
Cluster-22178.83791 GO:0007165 GO:0007606 GO:0004888 GO:0016021
Cluster-22178.83792 GO:0007606 GO:0007165 GO:0004888 GO:0016021

Cluster-22178.83797 GO:0007010 GO:0070206 GO:0007049 GO:0042967
GO:0005515 GO:0003779 GO:0005096 GO:0016407
Cluster-22178.83798 GO:0008270 GO:0016020 GO:0016021
Cluster-22178.83799 GO:0016021 GO:0016020
Cluster-22178.83801 GO:0055114 GO:0016117 GO:0016491 GO:0016705

Cluster-22178.83802 GO:0006355 GO:0019048 GO:0016787
Cluster-22178.83810 GO:0015074 GO:0006777 GO:0006412 GO:0042254
GO:0003735 GO:0005622 GO:0005840
Cluster-22178.83815 GO:0005515
Cluster-22178.83817 GO:0006355 GO:0003677 GO:0005515 GO:0003700
GO:0043565 GO:0008168 GO:0005667 GO:0005634
Cluster-22178.83818 GO:0006351 GO:0006206 GO:0006144 GO:0003677
GO:0003899 GO:0005730
Cluster-22178.83819 GO:0046872
Cluster-22178.83820 GO:0006820 GO:0005524 GO:0016740 GO:0008509
GO:0016021
Cluster-22178.83823 GO:0033588
Cluster-22178.83824 GO:0045454
Cluster-22178.83825 GO:0044237 GO:0006412 GO:0055114 GO:0003824
GO:0016491 GO:0008168
Cluster-22178.83828 GO:0006120 GO:0042546 GO:0055114 GO:0005199
GO:0005618 GO:0016021
Cluster-22178.83831 GO:0000724 GO:0006810 GO:0005886
Cluster-22178.83833 GO:0016462 GO:0005509
Cluster-22178.83835 GO:0043087 GO:0035023 GO:0005089 GO:0005515

Cluster-22178.83837 GO:0005515
Cluster-22178.83838 GO:0009069 GO:0006468 GO:0017004 GO:0006118
GO:0016310 GO:0035556 GO:0004672 GO:0005524 GO:0045158
GO:0004674 GO:0009512
Cluster-22178.83840 GO:0045944 GO:0007219 GO:0003713 GO:0005667
GO:0016607

Cluster-22178.83841 GO:0046872

Cluster-22178.83848 GO:0016021

Cluster-22178.83850 GO:0005515

Cluster-22178.83852 GO:0006118 GO:0045454 GO:0006355 GO:0015035 GO:0009055 GO:0046872 GO:0005634

Cluster-22178.83854 GO:0005515

Cluster-22178.83865 GO:0046872

Cluster-22178.83867 GO:0006351 GO:0006355

Cluster-22178.8387 GO:0006024 GO:0016857 GO:0016021

Cluster-22178.83872 GO:0008270 GO:0016021

Cluster-22178.83879 GO:0016192 GO:0006886 GO:0005515 GO:0005198 GO:0030130 GO:0030132

Cluster-22178.83880 GO:0006351 GO:0007275 GO:0006511 GO:0016567 GO:0006464 GO:0006206 GO:0006144 GO:0003899 GO:0005515 GO:0046872 GO:0016151 GO:0003677 GO:0008270 GO:0004842 GO:0005634 GO:0005730 GO:0005680

Cluster-22178.83881 GO:0006464 GO:0046872 GO:0005198 GO:0016151 GO:0019028

Cluster-22178.83883 GO:0009395 GO:0046339 GO:0035556 GO:0006629 GO:0006355 GO:0007165 GO:0003676 GO:0004435 GO:0005515 GO:0005509 GO:0005622

Cluster-22178.83895 GO:0005886

Cluster-22178.83899 GO:0014068 GO:0008152 GO:0050776 GO:0006479 GO:0000154 GO:0006464 GO:0035556 GO:0006396 GO:0006364 GO:0007165 GO:0046872 GO:0008168 GO:0016151 GO:0005515 GO:0005102 GO:0008649 GO:0043548 GO:0005737 GO:0016020

Cluster-22178.83904 GO:0007411 GO:0007186 GO:0016747 GO:0004930 GO:0016021

Cluster-22178.83916 GO:0007186 GO:0019236 GO:0048268 GO:0007212 GO:0032051 GO:0016021

Cluster-22178.83917 GO:0016310 GO:0006468 GO:0009069 GO:0007165 GO:0016773 GO:0004672 GO:0005524 GO:0008601 GO:0004674 GO:0000159 GO:0016020

Cluster-22178.83918 GO:0006886 GO:0016192 GO:0005515 GO:0030117
Cluster-22178.83919 GO:0006412 GO:0042254 GO:0003735 GO:0005840
GO:0016020 GO:0005622
Cluster-22178.83921 GO:0006535 GO:0006308 GO:0042967 GO:0006534
GO:0010181 GO:0009001 GO:0008855 GO:0046983 GO:0009318
GO:0005737
Cluster-22178.83926 GO:0019028
Cluster-22178.83929 GO:0045859 GO:0005515 GO:0004860 GO:0005576
Cluster-22178.83932 GO:0019064 GO:0000413 GO:0007165 GO:0006457
GO:0016788 GO:0003755 GO:0046789 GO:0009986 GO:0019031
Cluster-22178.83935 GO:0008152 GO:0007067 GO:0016491 GO:0042729
GO:0072686
Cluster-22178.83936 GO:0008152 GO:0006464 GO:0055114 GO:0006479
GO:0006813 GO:0046500 GO:0008171 GO:0004719 GO:0046872
GO:0016491 GO:0051287
Cluster-22178.83937 GO:0006464 GO:0055114 GO:0008152 GO:0006813
GO:0046500 GO:0006479 GO:0004719 GO:0046872 GO:0016491
GO:0008171 GO:0051287
Cluster-22178.83949 GO:0043565 GO:0005515 GO:0005634
Cluster-22178.83953 GO:0032922 GO:0000976
Cluster-22178.83964 GO:0007186 GO:0004930 GO:0016021
Cluster-22178.83966 GO:0006606 GO:0008092 GO:0003677 GO:0005515
GO:0005737 GO:0005634 GO:0019898
Cluster-22178.83969 GO:0006771 GO:0006468 GO:0019497 GO:0048013
GO:0005515 GO:0046872 GO:0004672 GO:0005524 GO:0003993
Cluster-22178.83970 GO:0055114 GO:0006505 GO:0006811 GO:0006629
GO:0006570 GO:0006886 GO:0006118 GO:0007165 GO:0007268
GO:0004097 GO:0004970 GO:0098599 GO:0016787 GO:0016788
GO:0016020
Cluster-22178.83971 GO:0030168 GO:0006606 GO:0006457 GO:0007165
GO:0048870 GO:0051258 GO:0010508 GO:0005102 GO:0030674
GO:0030250 GO:0005525 GO:0051082 GO:0005198 GO:0016272
GO:0005577 GO:0016021 GO:0019031 GO:0019028 GO:0005576

GO:0031514
Cluster-22178.83978 GO:0055085 GO:0006811 GO:0006355 GO:0005515
GO:0005216 GO:0016020
Cluster-22178.83979 GO:0006468 GO:0016773 GO:0005524 GO:0004672
GO:0016020
Cluster-22178.83980 GO:0006814 GO:0009405 GO:0006468 GO:0006120
GO:0006744 GO:0015992 GO:0008137 GO:0016788 GO:0003723
GO:0043022 GO:0004672 GO:0005524 GO:0005739
Cluster-22178.83981 GO:0006355 GO:0043565 GO:0003700 GO:0005667
GO:0005634
Cluster-22178.83989 GO:0009890 GO:0043401 GO:0006355 GO:0003700
GO:0043565 GO:0008270 GO:0005515 GO:0005634 GO:0005667
Cluster-22178.83990 GO:0009890 GO:0043401 GO:0006355 GO:0003700
GO:0043565 GO:0008270 GO:0005515 GO:0005667 GO:0005634
Cluster-22178.83994 GO:0006355 GO:0009405 GO:0003700 GO:0005622
GO:0005576 GO:0005667
Cluster-22178.83997 GO:0007165 GO:0005179 GO:0005576
Cluster-22178.83998 GO:0006355 GO:0055114 GO:0005515 GO:0016491
GO:0043565 GO:0003700 GO:0051537 GO:0005667
Cluster-22178.83999 GO:0007165 GO:0050776 GO:0014068 GO:0045735
GO:0005102 GO:0043548 GO:0016020
Cluster-22178.84000 GO:0016310 GO:0009069 GO:0006468 GO:0004674
GO:0005524 GO:0004672
Cluster-22178.84003 GO:0016310 GO:0009069 GO:0006468 GO:0007165
GO:0005524 GO:0004672 GO:0004674 GO:0004190 GO:0016021
Cluster-22178.84006 GO:0043130
Cluster-22178.84009 GO:0051252 GO:0090266 GO:0003676 GO:0005524
GO:0003677 GO:0016891 GO:0016787 GO:0005680
Cluster-22178.84011 GO:0051252 GO:0006396 GO:0003723 GO:0004525
GO:0005515
Cluster-22178.84020 GO:0001558 GO:0008270 GO:0005520 GO:0005515
GO:0005576 GO:0016942
Cluster-22178.84022 GO:0019079 GO:0019048 GO:0008026 GO:0003677

GO:0005509 GO:0004519 GO:0005515 GO:0016787 GO:0003676
GO:0005524 GO:0008270
Cluster-22178.84029 GO:0006355 GO:0006810 GO:0055085 GO:0016755
GO:0005509 GO:0043565 GO:0005524 GO:0042626 GO:0003700
GO:0005667 GO:0016021
Cluster-22178.84032 GO:0006810 GO:0006355 GO:0055085 GO:0005509
GO:0016755 GO:0042626 GO:0003700 GO:0043565 GO:0005524
GO:0005667 GO:0016021
Cluster-22178.84033 GO:0006413 GO:0006446 GO:0003723 GO:0003743
GO:0005737 GO:0005840
Cluster-22178.84039 GO:0006508 GO:0055114 GO:0016491 GO:0008234

Cluster-22178.8404 GO:0006413 GO:0000184 GO:0006415 GO:0006614
GO:0042254 GO:0019083 GO:0006414 GO:0003735 GO:0022627
GO:0005739 GO:0005840
Cluster-22178.84040 GO:0006508 GO:0015992 GO:0006123 GO:0016740
GO:0004197 GO:0016788 GO:0008242 GO:0016887 GO:0016817
GO:0005524 GO:0004129 GO:0045277
Cluster-22178.84046 GO:0005515
Cluster-22178.84048 GO:0006810 GO:0009405 GO:0016567 GO:0019870
GO:0005515 GO:0004842 GO:0008270 GO:0046872 GO:0005576
GO:0005680
Cluster-22178.84050 GO:0032006 GO:0019031
Cluster-22178.84057 GO:0006355 GO:0042967 GO:0004402 GO:0008270
GO:0003712 GO:0005634 GO:0005667 GO:0000123
Cluster-22178.84063 GO:0043065 GO:0051321 GO:0090200 GO:0097193
GO:0009405 GO:0005515 GO:0005739 GO:0005576
Cluster-22178.84065 GO:0008146
Cluster-22178.84067 GO:0007264 GO:0006109 GO:0055114 GO:0016310
GO:0000160 GO:0000155 GO:0005524 GO:0008641 GO:0016491
GO:0003724 GO:0003723 GO:0004672 GO:0043531 GO:0003924
GO:0005525 GO:0009365
Cluster-22178.84068 GO:0007010 GO:0003779 GO:0046872

Cluster-22178.84069 GO:0006118 GO:0005507 GO:0009055
Cluster-22178.84078 GO:0007165
Cluster-22178.84080 GO:0016310 GO:0006511 GO:0009069 GO:0006468
GO:0004674 GO:0005524 GO:0031625
Cluster-22178.84081 GO:0005515
Cluster-22178.84082 GO:0006554 GO:0006479 GO:0018024
Cluster-22178.84085 GO:0002949 GO:0007264 GO:0006355 GO:0005524
GO:0016887 GO:0005525 GO:0003724 GO:0003723 GO:0008134
GO:0005667
Cluster-22178.84089 GO:0005515
Cluster-22178.84090 GO:0015991 GO:0046325 GO:0009069 GO:0016310
GO:0005515 GO:0016820 GO:0004674 GO:0016773 GO:0000221
GO:0005737
Cluster-22178.84094 GO:0005515
Cluster-22178.84096 GO:0055114 GO:0016491 GO:0016616
Cluster-22178.84097 GO:0009306 GO:0042254 GO:0006412 GO:0006810
GO:0006858 GO:0042626 GO:0003735 GO:0003723 GO:0005840
GO:0005622
Cluster-22178.84099 GO:0006183 GO:0006228 GO:0006165 GO:0006241
GO:0006206 GO:0015979 GO:0006144 GO:0004867 GO:0004550
GO:0009522
Cluster-22178.84100 GO:0007586 GO:0008152 GO:0016042 GO:0005509
GO:0008236 GO:0005515 GO:0016746 GO:0008047 GO:0005576
Cluster-22178.84103 GO:0007186 GO:0004930 GO:0016021
Cluster-22178.84118 GO:0008053 GO:0016192 GO:0007005 GO:0003924
GO:0005198 GO:0019031 GO:0016021 GO:0031305 GO:0005741
Cluster-22178.84126 GO:0007067 GO:0019069 GO:0006457 GO:0007165
GO:0006914 GO:0051607 GO:0030168 GO:0031412 GO:0006355
GO:0006886 GO:0051258 GO:0051382 GO:0006352 GO:0030674
GO:0003677 GO:0005102 GO:0016987 GO:0051082 GO:0008376
GO:0008092 GO:0003700 GO:0005667 GO:0032580 GO:0005783
GO:0016272 GO:0005577 GO:0019898 GO:0005737 GO:0016021
Cluster-22178.84128 GO:0046872

Cluster-22178.84131 GO:0051260
Cluster-22178.84132 GO:0006814 GO:0005272 GO:0016020
Cluster-22178.84136 GO:0005515
Cluster-22178.84138 GO:0008152 GO:0003824 GO:0008134 GO:0005634
GO:0005667
Cluster-22178.84141 GO:0006811 GO:0055085 GO:0015075 GO:0003677
GO:0016020
Cluster-22178.84149 GO:0007264 GO:0006357 GO:0001104 GO:0005515
GO:0005525 GO:0003924 GO:0008641 GO:0016592 GO:0070461
Cluster-22178.84150 GO:0030154 GO:0007275 GO:0007219 GO:0005509
GO:0005515 GO:0016021
Cluster-22178.84154 GO:0006357 GO:0007049 GO:0010038 GO:0044237
GO:0006810 GO:0005215 GO:0001104 GO:0005515 GO:0016021
GO:0016592
Cluster-22178.84159 GO:0005515 GO:0003677
Cluster-22178.84160 GO:0055114
Cluster-22178.84167 GO:0046872
Cluster-22178.84168 GO:0007165 GO:0006355 GO:0003713 GO:0005515
GO:0035257 GO:0046983 GO:0005634 GO:0005667
Cluster-22178.84169 GO:0007165 GO:0005515
Cluster-22178.84171 GO:0006535 GO:0008152 GO:0006534 GO:0042967
GO:0009001 GO:0046872 GO:0016491 GO:0005737
Cluster-22178.84177 GO:0005615
Cluster-22178.84179 GO:0016021 GO:0016020
Cluster-22178.84180 GO:0007165 GO:0006952 GO:0007186 GO:0007264
GO:0003677 GO:0000166 GO:0005525 GO:0004871 GO:0019001
GO:0005524 GO:0031683 GO:0003924 GO:0005576
Cluster-22178.84181 GO:0015992 GO:0000128 GO:0006118 GO:0006119
GO:0009055 GO:0008121 GO:0008023
Cluster-22178.84183 GO:0005515
Cluster-22178.84184 GO:0005515
Cluster-22178.84188 GO:0016310 GO:0007165 GO:0006886 GO:0000160
GO:0000155 GO:0009365 GO:0030176

Cluster-22178.84191 GO:0006265 GO:0006629 GO:0003918 GO:0005515 GO:0003677
Cluster-22178.84194 GO:0016020
Cluster-22178.84195 GO:0006979 GO:0055114 GO:0006804 GO:0006749 GO:0043565 GO:0004602 GO:0005515 GO:0005634
Cluster-22178.84196 GO:0009058 GO:0006479 GO:0046500 GO:0008152 GO:0006464 GO:0016742 GO:0008168 GO:0004719 GO:0019867
Cluster-22178.84201 GO:0007165
Cluster-22178.84205 GO:0019049 GO:0008270
Cluster-22178.84209 GO:0007531 GO:0045895 GO:0005515 GO:0008301 GO:0003723 GO:0005634
Cluster-22178.84210 GO:0016567 GO:0046872 GO:0004842
Cluster-22178.84213 GO:0002218 GO:0032481
Cluster-22178.84214 GO:0055114 GO:0008152 GO:0016491
Cluster-22178.84215 GO:0006744 GO:0015992 GO:0006144 GO:0045444 GO:0006120 GO:0018144 GO:0006814 GO:0006508 GO:0008233 GO:0016787 GO:0003968 GO:0008234 GO:0016779 GO:0000166 GO:0008137 GO:0005198 GO:0016740 GO:0005739 GO:0031379 GO:0019012
Cluster-22178.84216 GO:0015992 GO:0006120 GO:0006814 GO:0006744 GO:0045444 GO:0008137 GO:0005739
Cluster-22178.84223 GO:0008152 GO:0006338 GO:0003824 GO:0005515 GO:0016836 GO:0090544
Cluster-22178.84224 GO:0006412 GO:0003676 GO:0008168 GO:0046872 GO:0008270 GO:0016021
Cluster-22178.84227 GO:0007186 GO:0004930 GO:0016020 GO:0016021
Cluster-22178.84228 GO:0016567 GO:0004842 GO:0016020
Cluster-22178.84232 GO:0035091 GO:0003824
Cluster-22178.84238 GO:0046983
Cluster-22178.84239 GO:0046872 GO:0008270 GO:0005634
Cluster-22178.84247 GO:0019867 GO:0016020
Cluster-22178.84249 GO:0008152 GO:0055114 GO:0019521 GO:0006351 GO:0006355 GO:0006144 GO:0006206 GO:0006098 GO:0003700

GO:0046872 GO:0005515 GO:0003899 GO:0008641 GO:0003677
GO:0004616 GO:0016616 GO:0051287 GO:0005634 GO:0005667
GO:0005730
Cluster-22178.84252 GO:0007165 GO:0007154 GO:0005515 GO:0005922
GO:0005737
Cluster-22178.84254 GO:0009405 GO:0007165 GO:0006874 GO:0006816
GO:0015689 GO:0055114 GO:0015098 GO:0005219 GO:0016491
GO:0005576 GO:0016021 GO:0005622
Cluster-22178.84267 GO:0016757
Cluster-22178.84271 GO:0005515
Cluster-22178.84272 GO:0019497 GO:0006771 GO:0009405 GO:0046872
GO:0005515 GO:0003993 GO:0005576
Cluster-22178.84273 GO:0006771 GO:0019497 GO:0009405 GO:0046872
GO:0005515 GO:0003993 GO:0005576
Cluster-22178.84276 GO:0055114 GO:0016491
Cluster-22178.8428 GO:0006779 GO:0006694 GO:0008152 GO:0055114
GO:0008207 GO:0008209 GO:0019354 GO:0008210 GO:0006813
GO:0003824 GO:0016491 GO:0043115 GO:0016616 GO:0003854
GO:0050662
Cluster-22178.84280 GO:0008152 GO:0006355 GO:0006468 GO:0006754
GO:0004672 GO:0005524 GO:0016887 GO:0003824 GO:0005515
GO:0000275 GO:0005634
Cluster-22178.84284 GO:0016021
Cluster-22178.84285 GO:0006281 GO:0004518
Cluster-22178.84287 GO:0030036 GO:0003779 GO:0030054
Cluster-22178.84288 GO:0050790 GO:0030234
Cluster-22178.84289 GO:0007016 GO:0005515 GO:0016010
Cluster-22178.84295 GO:0035556 GO:0005515
Cluster-22178.84303 GO:0006614 GO:0006635 GO:0006118 GO:0055114
GO:0006637 GO:0003995 GO:0003997 GO:0016627 GO:0005525
GO:0005777
Cluster-22178.84305 GO:0006814 GO:0006118 GO:0015992 GO:0006744
GO:0006120 GO:0008152 GO:0016787 GO:0003954 GO:0008137

GO:0005739
Cluster-22178.84311 GO:0006355 GO:0046983 GO:0005515
Cluster-22178.84318 GO:0006118 GO:0006119 GO:0015992 GO:0009055
GO:0008121
Cluster-22178.84324 GO:0008152 GO:0005515 GO:0005509 GO:0016787

Cluster-22178.84325 GO:0005509 GO:0005515 GO:0005576
Cluster-22178.84331 GO:0007016 GO:0007154 GO:0003677 GO:0005509
GO:0005515 GO:0005576 GO:0016010 GO:0016020
Cluster-22178.84345 GO:0008270 GO:0008233
Cluster-22178.84346 GO:0005515
Cluster-22178.84349 GO:0019684 GO:0015979 GO:0042254 GO:0006412
GO:0006355 GO:0003735 GO:0003677 GO:0003682 GO:0016021
GO:0000785 GO:0000775 GO:0005840 GO:0009523 GO:0005622
GO:0005634
Cluster-22178.84354 GO:0007276
Cluster-22178.84358 GO:0043093 GO:0006810 GO:0000917 GO:0006260
GO:0010508 GO:0005886 GO:0005737
Cluster-22178.84359 GO:0005634
Cluster-22178.84361 GO:0006355 GO:0006810 GO:0007165 GO:0001522
GO:0009451 GO:0046983 GO:0005215 GO:0009982 GO:0035257
GO:0003723 GO:0005515 GO:0003713 GO:0016021 GO:0005667
GO:0005634
Cluster-22178.84363 GO:0006954 GO:0007165 GO:0006011 GO:0008168
GO:0005125 GO:0016020 GO:0005576
Cluster-22178.84369 GO:0007186 GO:0004930 GO:0016021
Cluster-22178.84371 GO:0016567 GO:0055114 GO:0005515 GO:0008270
GO:0004842 GO:0047134 GO:0004859 GO:0005576 GO:0005680
Cluster-22178.84373 GO:0006464 GO:0055114 GO:0008113
Cluster-22178.84378 GO:0006334 GO:0016226 GO:0003677 GO:0051536
GO:0005506 GO:0005634 GO:0000786
Cluster-22178.84386 GO:0006308 GO:0006914 GO:0008855 GO:0008092
GO:0008270 GO:0005737 GO:0019898 GO:0005634 GO:0009318

Cluster-22178.84388 GO:0046983

Cluster-22178.84390 GO:0006118 GO:0007165 GO:0055114 GO:0015992 GO:0006123 GO:0009060 GO:0005506 GO:0005515 GO:0020037 GO:0004129 GO:0009055 GO:0016021 GO:0045277

Cluster-22178.84392 GO:0009069 GO:0006468 GO:0016310 GO:0007178 GO:0004675 GO:0003723 GO:0004672 GO:0005524 GO:0016020 GO:0016021

Cluster-22178.84397 GO:0006725 GO:0046872 GO:0016853

Cluster-22178.84399 GO:0009072 GO:0006570 GO:0042207 GO:0004334

Cluster-22178.84402 GO:0005515

Cluster-22178.84408 GO:0007010 GO:0003779

Cluster-22178.84412 GO:0042254 GO:0006412 GO:0006468 GO:0005524 GO:0004672 GO:0016773 GO:0003735 GO:0005840 GO:0005622 GO:0016020

Cluster-22178.84414 GO:0005525 GO:0005515

Cluster-22178.84415 GO:0001503 GO:0007186 GO:0004930 GO:0016021

Cluster-22178.84419 GO:0016310 GO:0004673 GO:0002161 GO:0009365

Cluster-22178.84420 GO:0016032

Cluster-22178.84423 GO:0070206

Cluster-22178.84426 GO:0016579 GO:0016192 GO:0007218 GO:0005185 GO:0036459 GO:0016021 GO:0005576

Cluster-22178.84429 GO:0006352 GO:0051382 GO:0006281 GO:0006366 GO:0003677 GO:0005669

Cluster-22178.84430 GO:0006858

Cluster-22178.84431 GO:0016567 GO:0008270 GO:0046872 GO:0004842 GO:0061630 GO:0005515

Cluster-22178.84432 GO:0055114 GO:0051258 GO:0006560 GO:0006376 GO:0006355 GO:0018401 GO:0030168 GO:0006613 GO:0007165 GO:0006525 GO:0016702 GO:0005515 GO:0003723 GO:0005102 GO:0008270 GO:0004656 GO:0003729 GO:0030674 GO:0005577 GO:0030176 GO:0005685 GO:0005784 GO:0005622 GO:0005783 GO:0031966

Cluster-22178.84435 GO:0006570 GO:0009058 GO:0008299 GO:0007165
GO:0006694 GO:0006470 GO:0035556 GO:0005515 GO:0016779
GO:0005044 GO:0050518 GO:0004725 GO:0016020 GO:0019031
Cluster-22178.84436 GO:0006355 GO:0008270 GO:0003676 GO:0003677

Cluster-22178.84437 GO:0006355 GO:0003677 GO:0003676 GO:0008270

Cluster-22178.84441 GO:0006013 GO:0006000 GO:0007186 GO:0006508
GO:0007155 GO:0004930 GO:0005524 GO:0003873 GO:0005515
GO:0004252 GO:0016020 GO:0031224
Cluster-22178.84443 GO:0006383 GO:0006367 GO:0006270 GO:0006144
GO:0008643 GO:0006206 GO:0006260 GO:0006351 GO:0009401
GO:0008982 GO:0003677 GO:0004190 GO:0003899 GO:0005515
GO:0003887 GO:0005730 GO:0005666 GO:0005672 GO:0009357
GO:0042575 GO:0016021 GO:0019031 GO:0005789 GO:0032040
Cluster-22178.84447 GO:0007010 GO:0003779
Cluster-22178.84448 GO:0019236 GO:0007186 GO:0003697 GO:0016021
Cluster-22178.84452 GO:0015074 GO:0006364 GO:0008270 GO:0003676

Cluster-22178.84453 GO:0015074
Cluster-22178.84454 GO:0000077 GO:0006281 GO:0030896
Cluster-22178.84455 GO:0000077 GO:0006281 GO:0030896
Cluster-22178.84457 GO:0005515
Cluster-22178.84466 GO:0003676
Cluster-22178.84469 GO:0002949 GO:0016887 GO:0003924 GO:0005524
GO:0005525
Cluster-22178.84471 GO:0006783 GO:0015994 GO:0009236 GO:0016852
GO:0004325
Cluster-22178.84474 GO:0007165
Cluster-22178.84475 GO:0007165 GO:0044038 GO:0016757 GO:0016021
GO:0005886
Cluster-22178.84476 GO:0007165
Cluster-22178.84481 GO:0006438 GO:0007067 GO:0009099 GO:0022900

GO:0009098 GO:0006118 GO:0009097 GO:0004832 GO:0005524
GO:0005506 GO:0009055 GO:0005515 GO:0005198 GO:0020037
GO:0000166 GO:0042597 GO:0005737 GO:0072686 GO:0042729
Cluster-22178.84483 GO:0008270
Cluster-22178.84491 GO:0006520 GO:0006629 GO:0016627 GO:0016743
GO:0016597 GO:0005737 GO:0016021
Cluster-22178.84492 GO:0006629 GO:0006520 GO:0016627 GO:0016743
GO:0016597 GO:0005737 GO:0016021
Cluster-22178.84493 GO:0006629 GO:0006520 GO:0016597 GO:0016743
GO:0016627 GO:0016021 GO:0005737
Cluster-22178.84494 GO:0006629 GO:0006520 GO:0016597 GO:0016743
GO:0016627 GO:0016021 GO:0005737
Cluster-22178.84495 GO:0006650 GO:0047396
Cluster-22178.84496 GO:0005515
Cluster-22178.84497 GO:0006468 GO:0009069 GO:0016310 GO:0000287
GO:0004672 GO:0005524 GO:0016773 GO:0004674 GO:0016020
Cluster-22178.84498 GO:0010972 GO:0030336 GO:0005515
Cluster-22178.84500 GO:0006811 GO:0006810 GO:0005230 GO:0016020
Cluster-22178.84501 GO:0015931 GO:0033227 GO:0051033 GO:0016021
Cluster-22178.84503 GO:0051726 GO:0005634
Cluster-22178.84505 GO:0051726 GO:0005634
Cluster-22178.84507 GO:0051726 GO:0005576 GO:0005634
Cluster-22178.84508 GO:0051726 GO:0005634
Cluster-22178.84517 GO:0007283 GO:0003677 GO:0005515 GO:0005634
GO:0000786
Cluster-22178.84521 GO:0006352 GO:0015074 GO:0051260 GO:0006355
GO:0003700 GO:0003677 GO:0005669 GO:0005667
Cluster-22178.84523 GO:0006355 GO:0003677 GO:0008270
Cluster-22178.84524 GO:0008270 GO:0005515 GO:0003677 GO:0005634
Cluster-22178.84531 GO:0005515 GO:0008270
Cluster-22178.84537 GO:0005515 GO:0008289
Cluster-22178.84538 GO:0006308 GO:0006412 GO:0042254 GO:0009307
GO:0009036 GO:0003677 GO:0003735 GO:0005840 GO:0016021

GO:0005622 GO:0019013 GO:0009359
Cluster-22178.84539 GO:0008152 GO:0016491
Cluster-22178.84541 GO:0005975 GO:0006465 GO:0008233 GO:0005515
GO:0030248 GO:0005787 GO:0016021
Cluster-22178.84544 GO:0008146
Cluster-22178.84551 GO:0051252 GO:0008033 GO:0004540
Cluster-22178.84552 GO:0006231 GO:0006206 GO:0004799 GO:0005634
Cluster-22178.84557 GO:0055114 GO:0052855 GO:0016491
Cluster-22178.84558 GO:0006508 GO:0046656 GO:0015948 GO:0030269
GO:0004222 GO:0016020
Cluster-22178.84560 GO:0015031 GO:0008033 GO:0006886 GO:0000042
GO:0050660 GO:0008565 GO:0005515 GO:0005737 GO:0016020
Cluster-22178.84561 GO:0055114 GO:0016209 GO:0016491
Cluster-22178.84566 GO:0007186 GO:0043093 GO:0006260 GO:0000917
GO:0030474 GO:0006355 GO:0007606 GO:0007049 GO:0003677
GO:0004930 GO:0005200 GO:0003700 GO:0043565 GO:0005667
GO:0005815 GO:0005823 GO:0005634 GO:0016021 GO:0005737
GO:0005856
Cluster-22178.84568 GO:0046740 GO:0006139 GO:0016818 GO:0003677
GO:0008026 GO:0016787 GO:0004003 GO:0003676 GO:0005524
GO:0016021 GO:0005657
Cluster-22178.84574 GO:0033198 GO:0098655 GO:0007165 GO:0006812
GO:0004931 GO:0001614 GO:0005887
Cluster-22178.84579 GO:0007165 GO:0007186 GO:0007264 GO:0005525
GO:0004871 GO:0019001 GO:0031683 GO:0003924
Cluster-22178.84582 GO:0008152 GO:0016491 GO:0050662 GO:0003824

Cluster-22178.84586 GO:0006298 GO:0005524 GO:0030983
Cluster-22178.84587 GO:0006298 GO:0030983 GO:0005524
Cluster-22178.84588 GO:0006355 GO:0006914 GO:0007049 GO:0003677
GO:0043565 GO:0005525 GO:0003700 GO:0005634 GO:0005667
GO:0016021
Cluster-22178.84594 GO:0055114 GO:0016491 GO:0016616

Cluster-22178.84599 GO:0005576
Cluster-22178.84600 GO:0006486 GO:0008373
Cluster-22178.84602 GO:0006351 GO:0006269 GO:0046034 GO:0035556
GO:0006144 GO:0004017 GO:0005515 GO:0004386 GO:0003896
GO:0008270 GO:0005730 GO:0005657
Cluster-22178.84603 GO:0005975 GO:0008184
Cluster-22178.84605 GO:0005975 GO:0008184
Cluster-22178.84614 GO:0006090 GO:0055114 GO:0006814 GO:0071436
GO:0006560 GO:0006525 GO:0015081 GO:0005515 GO:0008948
GO:0016020
Cluster-22178.84615 GO:0035023 GO:0043087 GO:0005089 GO:0005634
Cluster-22178.84618 GO:0006357 GO:0001104 GO:0016592
Cluster-22178.84623 GO:0051382 GO:0007067 GO:0003677
Cluster-22178.84624 GO:0005515
Cluster-22178.84625 GO:0006570 GO:0006470 GO:0004725 GO:0008138

Cluster-22178.84626 GO:0042967 GO:0016747 GO:0008080 GO:0047961
GO:0005739
Cluster-22178.84629 GO:0006355 GO:0006352 GO:0003700 GO:0003677
GO:0016987 GO:0005667
Cluster-22178.84631 GO:0007165 GO:0006355 GO:0046983 GO:0003677
GO:0003723 GO:0005179 GO:0016763 GO:0019028 GO:0005634
GO:0005576
Cluster-22178.84632 GO:0007165 GO:0006355 GO:0003677 GO:0016763
GO:0005179 GO:0003723 GO:0046983 GO:0005576 GO:0005634
GO:0019028
Cluster-22178.84636 GO:0006040 GO:0009058 GO:0009254 GO:0015774
GO:0015159 GO:0016773 GO:0005524 GO:0005515 GO:0016020
Cluster-22178.84640 GO:0042254 GO:0006412 GO:0007411 GO:0003735
GO:0005515 GO:0044599 GO:0005840 GO:0005622
Cluster-22178.84641 GO:0055114 GO:0006118 GO:0003697 GO:0003995
GO:0005856 GO:0070188
Cluster-22178.84647 GO:0006487 GO:0003830 GO:0016020

Cluster-22178.84650 GO:0019497 GO:0006771 GO:0003993 GO:0005524

Cluster-22178.84657 GO:0007264 GO:0006351 GO:0042254 GO:0006412
GO:0005524 GO:0005525 GO:0003735 GO:0017111 GO:0005622
GO:0005840
Cluster-22178.84659 GO:0070206 GO:0042967 GO:0006355 GO:0007010
GO:0043565 GO:0003779 GO:0005096 GO:0016407 GO:0003700
GO:0005515 GO:0005667
Cluster-22178.84662 GO:0003676
Cluster-22178.84665 GO:0007165 GO:0006955 GO:0008283 GO:0040007
GO:0008083 GO:0005179 GO:0005134 GO:0005576 GO:0005893
Cluster-22178.84666 GO:0048870 GO:0008092 GO:0045502 GO:0042803
GO:0008134 GO:0005737 GO:0030286 GO:0019898 GO:0005667
GO:0031514
Cluster-22178.84668 GO:0016055 GO:0005515
Cluster-22178.84670 GO:0016117 GO:0006206 GO:0006144 GO:0055114
GO:0006351 GO:0046872 GO:0005524 GO:0016491 GO:0003677
GO:0000166 GO:0003899 GO:0005198 GO:0016705 GO:0003723
GO:0005730
Cluster-22178.84671 GO:0006886 GO:0005543
Cluster-22178.84674 GO:0006886 GO:0005543
Cluster-22178.84676 GO:0035556 GO:0006310 GO:0046872 GO:0003676
GO:0003824 GO:0050662 GO:0003677 GO:0005634
Cluster-22178.84684 GO:0003723
Cluster-22178.84687 GO:0006810 GO:0042254 GO:0006412 GO:0003735
GO:0003924 GO:0005525 GO:0005861 GO:0005840 GO:0016021
Cluster-22178.84688 GO:0019497 GO:0007154 GO:0006771 GO:0046872
GO:0003993 GO:0005515 GO:0051539 GO:0016820 GO:0016021
Cluster-22178.84690 GO:0006886 GO:0006506 GO:0007010 GO:0005515
GO:0003779
Cluster-22178.84694 GO:0003723 GO:0003676
Cluster-22178.84697 GO:0016021
Cluster-22178.84700 GO:0016021

Cluster-22178.84701 GO:0016021
Cluster-22178.84702 GO:0003677
Cluster-22178.84707 GO:0046872
Cluster-22178.84715 GO:0034229 GO:0006355 GO:0034228 GO:0003700
GO:0043565 GO:0003677 GO:0016021 GO:0005667 GO:0005634
Cluster-22178.84720 GO:0005515
Cluster-22178.84724 GO:0006468 GO:0004672 GO:0005524
Cluster-22178.84725 GO:0009405 GO:0042254 GO:0006412 GO:0006468
GO:0005524 GO:0004672 GO:0016773 GO:0045735 GO:0003735
GO:0005840 GO:0015934 GO:0016020
Cluster-22178.84726 GO:0006468 GO:0005524 GO:0004672
Cluster-22178.84729 GO:0005525 GO:0005515
Cluster-22178.84732 GO:0055085 GO:0005515 GO:0046872 GO:0008270
GO:0016021
Cluster-22178.84747 GO:0006464 GO:0003677 GO:0016151 GO:0046872
GO:0005737
Cluster-22178.84749 GO:0007264 GO:0006355 GO:0003723 GO:0008134
GO:0005524 GO:0003924 GO:0016887 GO:0005525 GO:0003724
GO:0005667 GO:0016020
Cluster-22178.84751 GO:0045893 GO:0006260 GO:0006275 GO:0045947
GO:0008283 GO:0040007 GO:0007165 GO:0003688 GO:0003677
GO:0008190 GO:0008083 GO:0046809 GO:0042025
Cluster-22178.84753 GO:0031124
Cluster-22178.84756 GO:0009405 GO:0015743 GO:0007155 GO:0019062
GO:0006265 GO:0003677 GO:0005515 GO:0005524 GO:0003918
Cluster-22178.84757 GO:0006813 GO:0005242 GO:0008076 GO:0016021
Cluster-22178.84759 GO:0042254 GO:0006412 GO:0003735 GO:0005840
GO:0005622
Cluster-22178.84761 GO:0006352 GO:0006355 GO:0003700 GO:0003677
GO:0016987 GO:0005667
Cluster-22178.84762 GO:0006352 GO:0006355 GO:0003677 GO:0016987
GO:0003700 GO:0005667
Cluster-22178.84767 GO:0006886 GO:0006904 GO:0006810 GO:0006308

GO:0015031 GO:0003676 GO:0008855 GO:0000145 GO:0016020
GO:0009318 GO:0016021 GO:0005801
Cluster-22178.84769 GO:0016567 GO:0005515 GO:0004842 GO:0046872
GO:0008270 GO:0005680
Cluster-22178.84771 GO:0006355 GO:0007165 GO:0009405 GO:0004888
GO:0005198 GO:0005515 GO:0005509 GO:0043565 GO:0003700
GO:0005667 GO:0016021
Cluster-22178.84775 GO:0005737 GO:0005634
Cluster-22178.84776 GO:0005515
Cluster-22178.84777 GO:0006807 GO:0007186 GO:0016814 GO:0008270
GO:0004930 GO:0016021
Cluster-22178.84780 GO:0005515
Cluster-22178.84781 GO:0007187 GO:0006812 GO:0007608 GO:0008146
GO:0005549 GO:0008324 GO:0004984 GO:0016020 GO:0016021
Cluster-22178.84782 GO:0035556
Cluster-22178.84783 GO:0006457 GO:0000774 GO:0008092 GO:0004064
GO:0051087 GO:0042803 GO:0005515 GO:0005737 GO:0019898
Cluster-22178.84787 GO:0006457 GO:0000774 GO:0008092 GO:0042803
GO:0005515 GO:0051087 GO:0005737 GO:0019898
Cluster-22178.84788 GO:0007155 GO:0006368 GO:0006406 GO:0005198
GO:0042803 GO:0008134 GO:0045502 GO:0000446 GO:0005667
GO:0019028 GO:0019031 GO:0009288 GO:0030286
Cluster-22178.84792 GO:0007165 GO:0000772 GO:0005515 GO:0016020
Cluster-22178.84793 GO:0007165 GO:0005515
Cluster-22178.84796 GO:0006281 GO:0046983 GO:0008270
Cluster-22178.84797 GO:0016887 GO:0005524
Cluster-22178.84798 GO:0005524 GO:0016887
Cluster-22178.84805 GO:0006816 GO:0006874 GO:0006270 GO:0000166
GO:0046872 GO:0005219 GO:0005634 GO:0005622 GO:0016021
Cluster-22178.84806 GO:0000166 GO:0046872 GO:0016021
Cluster-22178.84810 GO:0006541 GO:0004359
Cluster-22178.84811 GO:0006468 GO:0004672 GO:0005524
Cluster-22178.84814 GO:0019497 GO:0006771 GO:0046872 GO:0003993

GO:0005515 GO:0016021 GO:0016020
Cluster-22178.84815 GO:0006744 GO:0005515
Cluster-22178.84816 GO:0006468 GO:0006471 GO:0005515 GO:0017176
GO:0004672 GO:0005524 GO:0004715 GO:0003950
Cluster-22178.84817 GO:0002949 GO:0006310 GO:0006073 GO:0006508
GO:0006281 GO:0004222 GO:0016887 GO:0005524 GO:0009378
GO:0003678 GO:0016301 GO:0016762 GO:0005618 GO:0005657
GO:0009379 GO:0048046
Cluster-22178.84822 GO:0043066 GO:0006508 GO:0032968 GO:0043123
GO:0045766 GO:0006367 GO:0008242 GO:0016740 GO:0004197
GO:0003677 GO:0016021 GO:0005634 GO:0016020
Cluster-22178.84825 GO:0006364 GO:0003723 GO:0003676
Cluster-22178.84827 GO:0055114 GO:0006810 GO:0055085 GO:0006118
GO:0016655 GO:0005215 GO:0016020
Cluster-22178.84828 GO:0015684 GO:0007264 GO:0006576 GO:0005525
GO:0015093 GO:0005524 GO:0003924 GO:0016021
Cluster-22178.84830 GO:0071816 GO:0005198 GO:0019028 GO:0019033
GO:0019031
Cluster-22178.84838 GO:0005515 GO:0046872 GO:0045202 GO:0016021
Cluster-22178.84839 GO:0005515
Cluster-22178.84840 GO:0042742
Cluster-22178.84843 GO:0006281 GO:0009432 GO:0003697 GO:0005524

Cluster-22178.84845 GO:0016055 GO:0007275 GO:0007165 GO:0005515
GO:0005509 GO:0005102 GO:0005576 GO:0045095
Cluster-22178.84851 GO:0002098 GO:0034227 GO:0046872 GO:0008270
GO:0003676 GO:0003677 GO:0000049 GO:0005634
Cluster-22178.84852 GO:0007018 GO:0007017 GO:0008017 GO:0003777
GO:0005524 GO:0045298 GO:0005874 GO:0019028
Cluster-22178.84856 GO:0018401 GO:0006560 GO:0055114 GO:0006525
GO:0004656 GO:0016702 GO:0005783
Cluster-22178.84857 GO:0046983
Cluster-22178.84858 GO:0003677

Cluster-22178.84870 GO:0006355 GO:0042967 GO:0006357 GO:0005515
GO:0001104 GO:0004402 GO:0003712 GO:0008270 GO:0016592
GO:0005634 GO:0005667 GO:0000123
Cluster-22178.84873 GO:0009405 GO:0005509 GO:0005515 GO:0016020
Cluster-22178.84885 GO:0006355 GO:0003677 GO:0046872 GO:0016787

Cluster-22178.84888 GO:0007031 GO:0005524 GO:0019031 GO:0005777
Cluster-22178.84890 GO:0006357 GO:0001104 GO:0016592
Cluster-22178.84892 GO:0000917 GO:0006355 GO:0043093 GO:0003700
GO:0043565 GO:0005667 GO:0005737
Cluster-22178.84898 GO:0007155 GO:0001503 GO:0006364 GO:0016567
GO:0000398 GO:0006886 GO:0008270 GO:0004842 GO:0005047
GO:0046872 GO:0003924 GO:0005525 GO:0005515 GO:0005680
GO:0005785 GO:0005732 GO:0005786 GO:0032040 GO:0005681
GO:0045202 GO:0005576 GO:0005634 GO:0034457
Cluster-22178.84906 GO:0006771 GO:0009069 GO:0006470 GO:0016310
GO:0019497 GO:0007178 GO:0006570 GO:0006468 GO:0003993
GO:0005524 GO:0004675 GO:0008138 GO:0004725 GO:0046872
GO:0005515 GO:0016020
Cluster-22178.84907 GO:0046740 GO:0019028 GO:0044219
Cluster-22178.84912 GO:0006807 GO:0016151 GO:0005515
Cluster-22178.84913 GO:0005975 GO:0008092 GO:0003774 GO:0004649
GO:0019898 GO:0005737 GO:0016459
Cluster-22178.84915 GO:0006355 GO:0003677 GO:0009512
Cluster-22178.84917 GO:0007411 GO:0006355 GO:0003700 GO:0008270
GO:0016021 GO:0005667
Cluster-22178.84918 GO:0006807 GO:0018101 GO:0004668 GO:0005509
GO:0005737
Cluster-22178.84919 GO:0018101 GO:0006807 GO:0016226 GO:0005509
GO:0005506 GO:0051536 GO:0004668 GO:0019028 GO:0005737
Cluster-22178.84920 GO:0010508 GO:0015979 GO:0005794 GO:0016020
GO:0009539 GO:0009523
Cluster-22178.84924 GO:0030246

Cluster-22178.84925 GO:0030246
Cluster-22178.84927 GO:0046872 GO:0008270 GO:0005515
Cluster-22178.84930 GO:0005515 GO:0005509
Cluster-22178.84932 GO:0006351 GO:0019079 GO:0006144 GO:0005515
GO:0003968 GO:0005509 GO:0031379
Cluster-22178.84935 GO:0009405 GO:0008219 GO:0005515 GO:0005198
GO:0005509 GO:0005576 GO:0019028
Cluster-22178.84940 GO:0006355 GO:0046656 GO:0006281 GO:0006955
GO:0000902 GO:0006760 GO:0019079 GO:0006310 GO:0051016
GO:0005996 GO:0002949 GO:0003723 GO:0008134 GO:0000150
GO:0004150 GO:0003678 GO:0048029 GO:0009378 GO:0003677
GO:0016853 GO:0005524 GO:0046983 GO:0016887 GO:0003724
GO:0005667 GO:0009379 GO:0008290 GO:0016020 GO:0005657
Cluster-22178.84941 GO:0055085 GO:0030150 GO:0015979 GO:0006810
GO:0005215 GO:0005515 GO:0009539 GO:0009523 GO:0016020
GO:0005742
Cluster-22178.84950 GO:0006531 GO:0006188 GO:0009152 GO:0006522
GO:0006144 GO:0016485 GO:0004018 GO:0016021
Cluster-22178.84951 GO:0018279 GO:0055114 GO:0016627 GO:0005789
Cluster-22178.84957 GO:0031175 GO:0005516 GO:0030507 GO:0008091
Cluster-22178.84960 GO:0000228
Cluster-22178.84962 GO:0003677 GO:0005840 GO:0016021
Cluster-22178.84966 GO:0006355 GO:0005515 GO:0005634
Cluster-22178.84968 GO:0042254 GO:0006412 GO:0016055 GO:0006355
GO:0000921 GO:0003735 GO:0003677 GO:0003700 GO:0043565
GO:0005667 GO:0005634 GO:0016021 GO:0005840 GO:0005940
GO:0005622
Cluster-22178.84969 GO:0007186 GO:0004930 GO:0016021
Cluster-22178.84970 GO:0007186 GO:0004930 GO:0016021
Cluster-22178.84971 GO:0006465 GO:0007186 GO:0008233 GO:0004930
GO:0016021 GO:0005787
Cluster-22178.84977 GO:0006090 GO:0034477 GO:0008152 GO:0006099
GO:0008924 GO:0016829 GO:0004518 GO:0005515 GO:0016021

Cluster-22178.84985 GO:0006744 GO:0030494 GO:0015979 GO:0016831 GO:0005886
Cluster-22178.84990 GO:0008152 GO:0003824 GO:0008484
Cluster-22178.84991 GO:0006139 GO:0005515 GO:0003676 GO:0008408 GO:0043395 GO:0016020 GO:0005578
Cluster-22178.84992 GO:0030155 GO:0007165 GO:0006308 GO:0030334 GO:0045995 GO:0005515 GO:0008855 GO:0005102 GO:0009318
Cluster-22178.84993 GO:0016021 GO:0016020
Cluster-22178.84994 GO:0016020 GO:0016021
Cluster-22178.85000 GO:0006488 GO:0004583 GO:0005789
Cluster-22178.85001 GO:0003700 GO:0005667 GO:0005634
Cluster-22178.85002 GO:0097197 GO:0001772 GO:0016021
Cluster-22178.85003 GO:0007165
Cluster-22178.85008 GO:0007186 GO:0007165 GO:0004871 GO:0005515 GO:0005834
Cluster-22178.8501 GO:0005515
Cluster-22178.85010 GO:0005515
Cluster-22178.85012 GO:0006952 GO:0009405 GO:0006810 GO:0007217 GO:0019855 GO:0005576
Cluster-22178.85014 GO:0032957 GO:0042254 GO:0006412 GO:0052725 GO:0052726 GO:0008270 GO:0005524 GO:0003735 GO:0047325 GO:0005515 GO:0000287 GO:0005840 GO:0005622
Cluster-22178.85029 GO:0006313 GO:0015846 GO:1902604 GO:0015814 GO:0006811 GO:0015074 GO:0005515 GO:0003677 GO:0003723 GO:0015558
Cluster-22178.85032 GO:0042998 GO:0071253 GO:0005515 GO:0005802
Cluster-22178.85034 GO:0016462 GO:0005509
Cluster-22178.85036 GO:0015991 GO:0015992 GO:0005515 GO:0003676 GO:0015078 GO:0033179 GO:0016021
Cluster-22178.85041 GO:0007283 GO:0005794
Cluster-22178.85043 GO:0006464 GO:0007067 GO:0016151 GO:0005515 GO:0042729 GO:0072686
Cluster-22178.85044 GO:0061136 GO:0005515

Cluster-22178.85045 GO:0008283 GO:0040007 GO:0000271 GO:0007165
GO:0009405 GO:0030674 GO:0051015 GO:0008083 GO:0016020
Cluster-22178.85048 GO:0007165 GO:0005179 GO:0042393 GO:0005576
Cluster-22178.85052 GO:0000413 GO:0006457 GO:0003677 GO:0003755

Cluster-22178.85056 GO:0006508 GO:0004198 GO:0005622
Cluster-22178.85060 GO:0007219 GO:0007154 GO:0015886 GO:0017004
GO:0007275 GO:0005515 GO:0005509 GO:0016021 GO:0016020
Cluster-22178.85063 GO:0016021
Cluster-22178.85068 GO:0009056 GO:0008270
Cluster-22178.85085 GO:0006508 GO:0004252 GO:0004197
Cluster-22178.85089 GO:0015992 GO:0006914 GO:0006606 GO:0008053
GO:0015986 GO:0007165 GO:0006119 GO:0046933 GO:0003924
GO:0051082 GO:0046961 GO:0016021 GO:0005741 GO:0045259
GO:0045261
Cluster-22178.85092 GO:0006357 GO:0035091 GO:0001104 GO:0016592
Cluster-22178.85094 GO:0006206 GO:0055114 GO:0006144 GO:0016117
GO:0006351 GO:0005524 GO:0046872 GO:0016491 GO:0003723
GO:0000166 GO:0003677 GO:0016705 GO:0005198 GO:0003899
GO:0005730
Cluster-22178.85095 GO:0005515
Cluster-22178.85096 GO:0007186 GO:0006468 GO:0005515 GO:0004930
GO:0005524 GO:0004672 GO:0016021
Cluster-22178.85102 GO:0006464
Cluster-22178.85108 GO:0006468 GO:0016773 GO:0004672 GO:0005524
GO:0016020
Cluster-22178.85110 GO:0055114 GO:0016903
Cluster-22178.85114 GO:0003723
Cluster-22178.85116 GO:0003676 GO:0016021
Cluster-22178.85139 GO:0015979 GO:0016020 GO:0009539 GO:0009523
Cluster-22178.85142 GO:0009116 GO:0004859 GO:0005576
Cluster-22178.85146 GO:0003677
Cluster-22178.85147 GO:0005886

Cluster-22178.85149 GO:0042254 GO:0006412 GO:0003735 GO:0005840 GO:0005622

Cluster-22178.85152 GO:0006470 GO:0006570 GO:0016787 GO:0004725 GO:0008138

Cluster-22178.85155 GO:0006144 GO:0046034 GO:0046872 GO:0008270 GO:0005515 GO:0004017

Cluster-22178.85158 GO:0042254 GO:0005622

Cluster-22178.85159 GO:0090063 GO:0006508 GO:0006810 GO:0000226 GO:0005515 GO:0004197 GO:0016021 GO:0000922 GO:0005815

Cluster-22178.85161 GO:0006886 GO:0016032 GO:0005622

Cluster-22178.85162 GO:0016747

Cluster-22178.85164 GO:0043087 GO:0044419 GO:0035023 GO:0005089 GO:0005515

Cluster-22178.8517 GO:0006413 GO:0019295 GO:0006446 GO:0003743 GO:0005840

Cluster-22178.85170 GO:0048870 GO:0005975 GO:0030178 GO:0001558 GO:0006914 GO:0006606 GO:0007165 GO:0007275 GO:0005515 GO:0005520 GO:0008270 GO:0004553 GO:0030246 GO:0005576 GO:0031514 GO:0016942

Cluster-22178.85171 GO:0015986 GO:0015992 GO:0015078 GO:0000276

Cluster-22178.85176 GO:0008270 GO:0005515

Cluster-22178.8518 GO:0019900

Cluster-22178.85183 GO:0016757 GO:0016820 GO:0003676

Cluster-22178.85185 GO:0016757 GO:0016820 GO:0003676

Cluster-22178.85189 GO:0015992 GO:0006144 GO:0006807 GO:0000256 GO:0006123 GO:0019901 GO:0004037 GO:0004129 GO:0045277

Cluster-22178.85193 GO:0006357 GO:0006308 GO:0006259 GO:0008270 GO:0016765 GO:0001104 GO:0005515 GO:0004531 GO:0016592

Cluster-22178.85199 GO:0006468 GO:0005524 GO:0004672

Cluster-22178.85207 GO:0006468 GO:0016773 GO:0005524 GO:0004672 GO:0016020

Cluster-22178.85215 GO:0042578

Cluster-22178.85217 GO:0007166 GO:0016020

Cluster-22178.85219 GO:0055114 GO:0019439 GO:0051537 GO:0051015
GO:0016708 GO:0005515 GO:0005506
Cluster-22178.85223 GO:0006412 GO:0042254 GO:0003735 GO:0005840
Cluster-22178.85224 GO:0005739
Cluster-22178.85234 GO:0030153 GO:0006955 GO:0006357 GO:0015643
GO:0001104 GO:0008270 GO:0016592 GO:0019814 GO:0016021
Cluster-22178.85237 GO:0006289 GO:0006281 GO:0043161 GO:0007165
GO:0005179 GO:0003684 GO:0005576
Cluster-22178.85238 GO:0007165 GO:0006289 GO:0006281 GO:0043161
GO:0003684 GO:0005179 GO:0005576
Cluster-22178.85240 GO:0051252 GO:0006351 GO:0006388 GO:0000213
GO:0003714 GO:0000214 GO:0005667
Cluster-22178.85242 GO:0006468 GO:0007165 GO:0007186 GO:0004672
GO:0005524 GO:0016773 GO:0004871 GO:0005834 GO:0016020
Cluster-22178.85260 GO:0006355 GO:0003700 GO:0043565 GO:0010181
GO:0005667
Cluster-22178.85271 GO:0016192 GO:0042262 GO:0003690 GO:0019031
GO:0016021
Cluster-22178.85272 GO:0003676 GO:0003723
Cluster-22178.85274 GO:0006355 GO:0006119 GO:0006412 GO:0015986
GO:0042254 GO:0015992 GO:0035556 GO:0003735 GO:0046961
GO:0003677 GO:0008270 GO:0046933 GO:0005840 GO:0045261
GO:0045259 GO:0005622
Cluster-22178.85277 GO:0016567 GO:0006897 GO:0007155 GO:0006308
GO:0007015 GO:0004842 GO:0008855 GO:0008270 GO:0061630
GO:0046872 GO:0005515 GO:0005198 GO:0016788 GO:0005680
GO:0005622 GO:0016021 GO:0019031 GO:0009288 GO:0009318
GO:0019028
Cluster-22178.85278 GO:0006366
Cluster-22178.85281 GO:0046677
Cluster-22178.85287 GO:0015986 GO:0015992 GO:0015078 GO:0000276
Cluster-22178.85288 GO:0007010 GO:0006298 GO:0006364 GO:0006813
GO:0007017 GO:0005524 GO:0005267 GO:0003779 GO:0030983

GO:0005875 GO:0005732 GO:0005634 GO:0034457
Cluster-22178.85292 GO:0016192
Cluster-22178.85294 GO:0006090 GO:0009607 GO:0071436 GO:0006814
GO:0006560 GO:0006525 GO:0006355 GO:0015081 GO:0008948
GO:0016020
Cluster-22178.85295 GO:0019058 GO:0009103 GO:0019915 GO:0006012
GO:0009117 GO:0009225 GO:0009236 GO:0007076 GO:0003824
GO:0003676 GO:0003978 GO:0000796 GO:0019031 GO:0005789
Cluster-22178.85301 GO:0006468 GO:0045859 GO:0007050 GO:0004672
GO:0005524 GO:0004861 GO:0005515 GO:0005634
Cluster-22178.85302 GO:0008757
Cluster-22178.85304 GO:0015074 GO:0006310 GO:0004672 GO:0003677

Cluster-22178.85306 GO:0006886 GO:0005198 GO:0019031 GO:0005622
GO:0019028
Cluster-22178.85310 GO:0005975 GO:0016853
Cluster-22178.85312 GO:0006355 GO:0035556 GO:0009271 GO:0005515

Cluster-22178.85313 GO:0035556 GO:0009271 GO:0006355 GO:0005515

Cluster-22178.85322 GO:0009435 GO:0051539 GO:0008987 GO:0016020
GO:0019804
Cluster-22178.85324 GO:0006468 GO:0004672 GO:0005524 GO:0016773
GO:0016020
Cluster-22178.85326 GO:0006506 GO:0004584 GO:0000136
Cluster-22178.85330 GO:0042981
Cluster-22178.85333 GO:0006457 GO:0010506 GO:0008270 GO:0051082
GO:0016272 GO:0005615
Cluster-22178.85347 GO:0005515 GO:0019028
Cluster-22178.85348 GO:0006605 GO:0006886 GO:0045040 GO:0055085
GO:0005515 GO:0022857 GO:0046983 GO:0005742 GO:0016021
Cluster-22178.85352 GO:0043066 GO:0035444 GO:0015675 GO:0045766
GO:0043123 GO:0015099 GO:0004222 GO:0046872 GO:0016021

Cluster-22178.85353 GO:0043123 GO:0045766 GO:0042742 GO:0035444
GO:0043066 GO:0015675 GO:0015099 GO:0046872 GO:0016021
Cluster-22178.85354 GO:0006303 GO:0006351 GO:0003676 GO:0008270
GO:0003677 GO:0004003 GO:0005657 GO:0005634
Cluster-22178.85355 GO:0006351 GO:0006119 GO:0015986 GO:0015992
GO:0046961 GO:0003676 GO:0008270 GO:0046933 GO:0045259
GO:0045261
Cluster-22178.85356 GO:0006139 GO:0003676 GO:0005515 GO:0008408
GO:0043395 GO:0016020 GO:0005578
Cluster-22178.85357 GO:0006355 GO:0019911 GO:0003677 GO:0043565
GO:0003700 GO:0005634 GO:0005667 GO:0016021
Cluster-22178.85358 GO:0006508 GO:0016787 GO:0008236
Cluster-22178.85360 GO:0006351 GO:0006269 GO:0006260 GO:0006120
GO:0055114 GO:0006357 GO:0001104 GO:0003896 GO:0016592
GO:0005730 GO:0005657
Cluster-22178.85365 GO:0006576 GO:0007264 GO:0055114 GO:0005509
GO:0016616 GO:0005525 GO:0003924 GO:0005524 GO:0031225
Cluster-22178.85367 GO:0007264 GO:0006576 GO:0055114 GO:0016616
GO:0005509 GO:0005524 GO:0003924 GO:0005525 GO:0031225
Cluster-22178.85368 GO:0055114 GO:0016616
Cluster-22178.85376 GO:0007165 GO:0005515 GO:0005509 GO:0005578
Cluster-22178.85378 GO:0007165 GO:0005509 GO:0005515 GO:0005578
Cluster-22178.85382 GO:0046983
Cluster-22178.85389 GO:0045444 GO:0008270 GO:0046872
Cluster-22178.85397 GO:0009069 GO:0007178 GO:0016310 GO:0004675
GO:0016020
Cluster-22178.85398 GO:0006355 GO:0043565 GO:0008270 GO:0046872
GO:0003700 GO:0005667
Cluster-22178.85402 GO:0030071 GO:0007010 GO:0006468 GO:0031145
GO:0004672 GO:0005524 GO:0003779 GO:0016020 GO:0019028
GO:0005680
Cluster-22178.85404 GO:0007275 GO:0003676 GO:0016020
Cluster-22178.85406 GO:0004177 GO:0030145 GO:0016021

Cluster-22178.85408 GO:0007165 GO:0005515 GO:0005856
Cluster-22178.85411 GO:0005515
Cluster-22178.85413 GO:0009405
Cluster-22178.85416 GO:0046983 GO:0003677
Cluster-22178.85418 GO:0019042 GO:0005524 GO:0003677 GO:0000166
GO:0033644
Cluster-22178.85420 GO:0006355 GO:0003677 GO:0003676 GO:0003723

Cluster-22178.85422 GO:0006355 GO:0043565 GO:0005515 GO:0005634
Cluster-22178.85429 GO:0043043 GO:0006412 GO:0042254 GO:0005507
GO:0003735 GO:0005840 GO:0005737 GO:0005622
Cluster-22178.8543 GO:0042254 GO:0006412 GO:0003723 GO:0003735
GO:0005622 GO:0005840
Cluster-22178.85434 GO:0006449 GO:0006415 GO:0003747 GO:0005840
GO:0018444
Cluster-22178.85436 GO:0035556 GO:0006508 GO:0004197 GO:0008234
GO:0005515
Cluster-22178.85437 GO:0006952 GO:0008284 GO:0005515
Cluster-22178.85438 GO:0016310 GO:0002161 GO:0004673 GO:0009365
Cluster-22178.85443 GO:0006468 GO:0004672 GO:0005524
Cluster-22178.85444 GO:0006313 GO:0045454
Cluster-22178.85445 GO:0006525 GO:0006560 GO:0019089 GO:0006420
GO:0006357 GO:0001104 GO:0005524 GO:0004814 GO:0016592
Cluster-22178.85446 GO:0006560 GO:0019089 GO:0006525 GO:0006420
GO:0006357 GO:0001104 GO:0005524 GO:0004814 GO:0016592
Cluster-22178.85447 GO:0006508 GO:0055114 GO:0016491 GO:0008234

Cluster-22178.85449 GO:0055114 GO:0006412 GO:0016491 GO:0008168

Cluster-22178.85450 GO:0055114 GO:0005201 GO:0016491 GO:0005581
GO:0005578
Cluster-22178.85464 GO:0006144 GO:0043065 GO:0007165 GO:0004114
GO:0016021 GO:0005740

Cluster-22178.85465 GO:0015992 GO:0015986 GO:0015078 GO:0016021
GO:0045263
Cluster-22178.85471 GO:0005515
Cluster-22178.85483 GO:0007264 GO:0015684 GO:0016887 GO:0003924
GO:0005524 GO:0015093 GO:0005525 GO:0016021
Cluster-22178.85484 GO:0046656 GO:0019058 GO:0009165 GO:0006545
GO:0055114 GO:0006761 GO:0004146 GO:0005515 GO:0008270
GO:0019031
Cluster-22178.85486 GO:0006355 GO:0003677
Cluster-22178.85489 GO:0006659 GO:0015031 GO:0016020
Cluster-22178.85490 GO:0006260 GO:0005524 GO:0005634 GO:0000808
Cluster-22178.85494 GO:0019685 GO:0030494 GO:0016836
Cluster-22178.85495 GO:0004527 GO:0003676
Cluster-22178.85496 GO:0003676 GO:0004527
Cluster-22178.85502 GO:0018193
Cluster-22178.85503 GO:0018193
Cluster-22178.85504 GO:0006090 GO:0006470 GO:0006525 GO:0006560
GO:0009405 GO:0006570 GO:0006814 GO:0071436 GO:0015081
GO:0004725 GO:0005272 GO:0008948 GO:0016020 GO:0031224
Cluster-22178.85511 GO:0003723
Cluster-22178.85514 GO:0006812 GO:0008324
Cluster-22178.85515 GO:0019013
Cluster-22178.85517 GO:0006508 GO:0045454 GO:0006650 GO:0042981
GO:0009401 GO:0004197 GO:0047396 GO:0016021
Cluster-22178.85520 GO:0046872
Cluster-22178.85522 GO:0006488 GO:0004583 GO:0005789
Cluster-22178.85528 GO:0006355 GO:0009069 GO:0007411 GO:0016310
GO:0003682 GO:0008384 GO:0003677 GO:0005634 GO:0008537
GO:0008385 GO:0000785 GO:0000775
Cluster-22178.85530 GO:0006366 GO:0003677 GO:0005665
Cluster-22178.85532 GO:0007219 GO:0015886 GO:0007154 GO:0007275
GO:0017004 GO:0005509 GO:0005515 GO:0016021 GO:0016020
Cluster-22178.8554 GO:0006355 GO:0042967 GO:0006605 GO:0007165

GO:0004402 GO:0005634 GO:0000123
Cluster-22178.85546 GO:0006468 GO:0016773 GO:0004672 GO:0005524
GO:0016020
Cluster-22178.85547 GO:0007276
Cluster-22178.85552 GO:0016021
Cluster-22178.85553 GO:0005515
Cluster-22178.85554 GO:0016021
Cluster-22178.85555 GO:0035434 GO:0006825 GO:0005375 GO:0019028
GO:0016021
Cluster-22178.85560 GO:0051479 GO:0005524 GO:0008270 GO:0050504
GO:0016818 GO:0005737 GO:0016021 GO:0005739
Cluster-22178.85564 GO:0009252 GO:0043087 GO:0005089 GO:0005515
GO:0016755 GO:0016020 GO:0005737
Cluster-22178.85568 GO:0030036 GO:0003677 GO:0016787 GO:0003676
GO:0005524 GO:0016817 GO:0003779 GO:0030054
Cluster-22178.85569 GO:0006260 GO:0006810 GO:0005524 GO:0003688
GO:0003676 GO:0004386 GO:0016787 GO:0003677 GO:0046809
Cluster-22178.85572 GO:0005509 GO:0005544
Cluster-22178.85574 GO:0032981
Cluster-22178.85575 GO:0006464 GO:0055114 GO:0016226 GO:0046500
GO:0006479 GO:0004719 GO:0008168 GO:0051536 GO:0005506
Cluster-22178.85580 GO:0007165 GO:0005057
Cluster-22178.85581 GO:0007165 GO:0005515
Cluster-22178.85582 GO:0006310 GO:0007155 GO:0003677 GO:0051015
GO:0010181 GO:0005727
Cluster-22178.85586 GO:0055114 GO:0016491 GO:0016616
Cluster-22178.85591 GO:0006771 GO:0019497 GO:0003993 GO:0005515
GO:0046872
Cluster-22178.85593 GO:0008152 GO:0006464 GO:0055114 GO:0006479
GO:0046500 GO:0006813 GO:0008171 GO:0046872 GO:0004719
GO:0016491 GO:0005515 GO:0051287
Cluster-22178.85598 GO:0006544 GO:0006534 GO:0035556 GO:0006563
GO:0006468 GO:0006094 GO:0006355 GO:0006566 GO:0003941

GO:0004672 GO:0005524 GO:0051539 GO:0005515
Cluster-22178.85605 GO:0006468 GO:0004672 GO:0005524
Cluster-22178.85606 GO:0006468 GO:0003676 GO:0016787 GO:0046872
GO:0004672 GO:0005524
Cluster-22178.85608 GO:0006807 GO:0008270 GO:0016814
Cluster-22178.8561 GO:0005198 GO:0019028
Cluster-22178.85619 GO:0007165 GO:0006914 GO:0016301 GO:0005198

Cluster-22178.85620 GO:0006367 GO:0032968 GO:0003677 GO:0016779
GO:0046872 GO:0008270 GO:0005634
Cluster-22178.85621 GO:0006468 GO:0006397 GO:0051252 GO:0016773
GO:0005524 GO:0004672 GO:0008233 GO:0004540 GO:0016020
Cluster-22178.85623 GO:0051252 GO:0006397 GO:0006468 GO:0008233
GO:0005524 GO:0004672 GO:0004540
Cluster-22178.85624 GO:0006915 GO:0006974 GO:0005515
Cluster-22178.85634 GO:0006886 GO:0006888 GO:0008270 GO:0030127
Cluster-22178.85635 GO:0005524
Cluster-22178.85638 GO:0010508 GO:0046872 GO:0016787 GO:0045502
GO:0042803 GO:0008134 GO:0030286 GO:0005667
Cluster-22178.85645 GO:0055085 GO:0006260 GO:0006281 GO:0009082
GO:0046872 GO:0003911 GO:0016787 GO:0016021
Cluster-22178.85647 GO:0055085 GO:0032481 GO:0002218 GO:0016021
Cluster-22178.85650 GO:0006914 GO:0051082
Cluster-22178.85652 GO:0006260 GO:0004252 GO:0005515 GO:0070008
GO:0003887 GO:0042575
Cluster-22178.85659 GO:0055085 GO:0030206 GO:0030001 GO:0006024
GO:0030158 GO:0046873 GO:0016020
Cluster-22178.85660 GO:0016032 GO:0015979 GO:0051276 GO:0005515
GO:0005524 GO:0046872 GO:0005694 GO:0016021 GO:0009523
Cluster-22178.85667 GO:0016020
Cluster-22178.85669 GO:0006357 GO:0007131 GO:0016055 GO:0006310
GO:0006351 GO:0003723 GO:0005515 GO:0001104 GO:0019013
GO:0016592

Cluster-22178.85670 GO:0016055 GO:0006357 GO:0005515 GO:0001104
GO:0016592
Cluster-22178.85675 GO:0003824
Cluster-22178.85677 GO:0008146 GO:0016021
Cluster-22178.85678 GO:0008146 GO:0016021
Cluster-22178.85680 GO:0006886 GO:0015991 GO:0006563 GO:0046653
GO:0006355 GO:0006544 GO:0016192 GO:0006528 GO:0006566
GO:0016820 GO:0005515 GO:0008418 GO:0003677 GO:0008115
GO:0008270 GO:0030117 GO:0000221
Cluster-22178.85687 GO:0006886 GO:0015031 GO:0005515 GO:0008565
GO:0016020 GO:0005634 GO:0005737
Cluster-22178.85689 GO:0005515
Cluster-22178.85690 GO:0006357 GO:0051262 GO:0046983 GO:0001104
GO:0016592 GO:0016021
Cluster-22178.85692 GO:0016032 GO:0019012
Cluster-22178.85695 GO:0008270 GO:0003676
Cluster-22178.85696 GO:0005515
Cluster-22178.85699 GO:0033617 GO:0005739
Cluster-22178.85702 GO:0055114 GO:0008152 GO:0016714 GO:0016597

Cluster-22178.85706 GO:0006351 GO:0006383 GO:0006468 GO:0006206
GO:0006144 GO:0003677 GO:0003899 GO:0004672 GO:0005524
GO:0005730 GO:0005666
Cluster-22178.85707 GO:0019497 GO:0006771 GO:0006468 GO:0003993
GO:0004672 GO:0005524 GO:0046872 GO:0005515
Cluster-22178.85708 GO:0006351 GO:0006355 GO:0006260 GO:0003887
GO:0003677 GO:0008270 GO:0042575 GO:0005634 GO:0032040
Cluster-22178.85716 GO:0006576 GO:0007264 GO:0002949 GO:0003924
GO:0005524 GO:0005525
Cluster-22178.85717 GO:0005515
Cluster-22178.85725 GO:0042254 GO:0006412 GO:0003735 GO:0005622
GO:0005840
Cluster-22178.85727 GO:0007275 GO:0042600

Cluster-22178.85732 GO:0070588 GO:0006816 GO:0005245 GO:0005891
Cluster-22178.85736 GO:0015986 GO:0015992 GO:0015078 GO:0000276
GO:0016021
Cluster-22178.8574 GO:0019069 GO:0003676 GO:0016787 GO:0046872
GO:0005524
Cluster-22178.85745 GO:0006094 GO:0019497 GO:0015976 GO:0008152
GO:0006020 GO:0006771 GO:0006096 GO:0046486 GO:0006013
GO:0006000 GO:0005515 GO:0046872 GO:0004807 GO:0003677
GO:0003993 GO:0016020
Cluster-22178.85746 GO:0006000 GO:0006013 GO:0006771 GO:0006020
GO:0046486 GO:0006096 GO:0015976 GO:0019497 GO:0008152
GO:0006094 GO:0003993 GO:0003677 GO:0046872 GO:0004807
GO:0005515 GO:0016020
Cluster-22178.8575 GO:0016567 GO:0004842 GO:0009289
Cluster-22178.85751 GO:0045454 GO:0042254 GO:0006412 GO:0006270
GO:0006810 GO:0003924 GO:0005525 GO:0003735 GO:0005515
GO:0016021 GO:0005840 GO:0005861
Cluster-22178.85754 GO:0006486 GO:0016757 GO:0008378 GO:0016020
Cluster-22178.85757 GO:0006260 GO:0015684 GO:0006508 GO:0007264
GO:0004222 GO:0005525 GO:0003924 GO:0015093 GO:0005524
GO:0003677 GO:0016021
Cluster-22178.8576 GO:0006355 GO:0003700 GO:0005667
Cluster-22178.85764 GO:0006811 GO:0055085 GO:0005216 GO:0008440
GO:0016020
Cluster-22178.85765 GO:0055085 GO:0006811 GO:0008440 GO:0005216
GO:0016020
Cluster-22178.85769 GO:0046872
Cluster-22178.85773 GO:0015031 GO:0006464 GO:0005515 GO:0003676

Cluster-22178.85777 GO:0045454 GO:0042254 GO:0006412 GO:0006810
GO:0003924 GO:0005525 GO:0005515 GO:0003735 GO:0016021
GO:0005840 GO:0005861
Cluster-22178.85779 GO:0006508 GO:0004252 GO:0005515

Cluster-22178.85783 GO:0009405 GO:0007268 GO:0007165 GO:0006355
GO:0048193 GO:0030550 GO:0003700 GO:0008270 GO:0016020
GO:0005667 GO:0005634 GO:0005576
Cluster-22178.85791 GO:0016042 GO:0005515 GO:0016020
Cluster-22178.85793 GO:0042802
Cluster-22178.85795 GO:0007276 GO:0006744
Cluster-22178.85796 GO:0006955 GO:0000902 GO:0051252 GO:0006364
GO:0008033 GO:0006811 GO:0006379 GO:0003723 GO:0004190
GO:0004540 GO:0016020 GO:0030677 GO:0016021 GO:0000172
Cluster-22178.85797 GO:0000902 GO:0006955 GO:0051252 GO:0006364
GO:0008033 GO:0006379 GO:0006811 GO:0004540 GO:0003723
GO:0004190 GO:0016020 GO:0030677 GO:0016021 GO:0000172
Cluster-22178.85800 GO:0033588
Cluster-22178.85802 GO:0006260 GO:0008408 GO:0000166 GO:0003677
GO:0003887 GO:0001882 GO:0008270 GO:0042575 GO:0005634
Cluster-22178.85807 GO:0030001 GO:0006355 GO:0008608 GO:0015940
GO:0055114 GO:0007155 GO:0016117 GO:0055085 GO:0016705
GO:0008677 GO:0003677 GO:0016614 GO:0005540 GO:0046873
GO:0016787 GO:0050660 GO:0004222 GO:0005524 GO:0016491
GO:0042729 GO:0072686 GO:0016020
Cluster-22178.85809 GO:0005515 GO:0005856
Cluster-22178.85812 GO:0006352 GO:0003677
Cluster-22178.85813 GO:0006355 GO:0007010 GO:0007049 GO:0043565
GO:0003779 GO:0003700 GO:0005667
Cluster-22178.85814 GO:0016567 GO:0005515 GO:0051537 GO:0004842
GO:0043231
Cluster-22178.85816 GO:0034599 GO:0046660 GO:0006397 GO:0007186
GO:0004930 GO:0004252 GO:0005886 GO:0005789 GO:0016021
GO:0019028 GO:0005634
Cluster-22178.85820 GO:0015743 GO:0006810 GO:0055085 GO:0005524
GO:0016747 GO:0042626 GO:0016021
Cluster-22178.85821 GO:0009399 GO:0006807 GO:0055114 GO:0019337
GO:0016163 GO:0016610

Cluster-22178.85824 GO:0042981 GO:0009058 GO:0030170 GO:0005515

Cluster-22178.85825 GO:0008152 GO:0003824
Cluster-22178.85826 GO:0016114 GO:0008152 GO:0006694 GO:0016624
GO:0003824 GO:0008661 GO:0030976
Cluster-22178.85827 GO:0006694 GO:0008152 GO:0016114 GO:0030976
GO:0008661 GO:0003824 GO:0016624
Cluster-22178.85828 GO:0007264 GO:0006355 GO:0002949 GO:0008134
GO:0003723 GO:0003724 GO:0005525 GO:0016887 GO:0005524
GO:0005667
Cluster-22178.85834 GO:0003779
Cluster-22178.85838 GO:0005515
Cluster-22178.85839 GO:0005515 GO:0061630 GO:0046872 GO:0008270

Cluster-22178.85841 GO:0009405 GO:0016310 GO:0007178 GO:0009069
GO:0004675 GO:0016020 GO:0005576
Cluster-22178.85842 GO:0009405 GO:0006468 GO:0009069 GO:0016310
GO:0007178 GO:0004675 GO:0004672 GO:0005524 GO:0016020
GO:0005576
Cluster-22178.85845 GO:0009252 GO:0008152 GO:0003779 GO:0016755
GO:0005198 GO:0008410 GO:0005856
Cluster-22178.85846 GO:0005515
Cluster-22178.85852 GO:0005515
Cluster-22178.85853 GO:0005515
Cluster-22178.85854 GO:0006265 GO:0019062 GO:0007155 GO:0015743
GO:0009405 GO:0003918 GO:0005524 GO:0005515 GO:0003677
Cluster-22178.85857 GO:0005515
Cluster-22178.85859 GO:0006468 GO:0006810 GO:0009405 GO:0016773
GO:0005524 GO:0004672 GO:0019855 GO:0005576 GO:0016020
Cluster-22178.85861 GO:0006260 GO:0007049 GO:0006355 GO:0043093
GO:0006457 GO:0000917 GO:0006810 GO:0043565 GO:0003700
GO:0051082 GO:0005509 GO:0005737 GO:0005667 GO:0016020
GO:0005783

Cluster-22178.85864 GO:0006554 GO:0006367 GO:0006479 GO:0034968
GO:0008270 GO:0005515 GO:0018024 GO:0005634 GO:0005672
Cluster-22178.85866 GO:0042981 GO:0006144 GO:0007165 GO:0044780
GO:0006355 GO:0006508 GO:0008270 GO:0004197 GO:0003677
GO:0005515 GO:0004114 GO:0043189 GO:0016020 GO:0005634
Cluster-22178.85867 GO:0007165 GO:0006355 GO:0006508 GO:0006144
GO:0004197 GO:0003677 GO:0005515 GO:0004114 GO:0008270
GO:0016020 GO:0005634 GO:0043189
Cluster-22178.85870 GO:0005975 GO:0006013 GO:0006000 GO:0015976
GO:0006096 GO:0006094 GO:0006098 GO:0042578 GO:0042132
Cluster-22178.85879 GO:0006355 GO:0009405 GO:0000917 GO:0048870
GO:0007264 GO:0006171 GO:0043093 GO:0006810 GO:0002949
GO:0008134 GO:0016301 GO:0005525 GO:0016787 GO:0008294
GO:0003677 GO:0005524 GO:0016887 GO:0046983 GO:0005576
GO:0031514 GO:0005737 GO:0005667
Cluster-22178.85880 GO:0006144 GO:0006790 GO:0004781
Cluster-22178.85881 GO:0003677
Cluster-22178.85885 GO:0007165 GO:0005179 GO:0031704 GO:0005515
GO:0005737
Cluster-22178.85888 GO:0016301 GO:0016772
Cluster-22178.85894 GO:0045454 GO:0006118 GO:0009055 GO:0015035
GO:0016021
Cluster-22178.85897 GO:0006487 GO:0003830 GO:0016020
Cluster-22178.85905 GO:0006270 GO:0051056 GO:0033617 GO:0005096
GO:0032040
Cluster-22178.85906 GO:0006811 GO:0005216 GO:0046872 GO:0016020
Cluster-22178.85914 GO:0006749 GO:0055114 GO:0006979 GO:0006804
GO:0016491 GO:0004602 GO:0016209
Cluster-22178.85916 GO:0050821 GO:0015979 GO:0042301 GO:0016020
GO:0009523
Cluster-22178.85918 GO:0006021 GO:0006355 GO:0008654 GO:0019872
GO:0003700 GO:0004512 GO:0043565 GO:0005667
Cluster-22178.8592 GO:0042254 GO:0006412 GO:0003735 GO:0005622

GO:0005840
Cluster-22178.85925 GO:0016032 GO:0019012
Cluster-22178.85929 GO:0045893 GO:0005515 GO:0005634
Cluster-22178.85931 GO:0006206 GO:0006230 GO:0007165 GO:0005524
GO:0004797
Cluster-22178.85932 GO:0008152 GO:0003824 GO:0008270
Cluster-22178.85943 GO:0019073 GO:0006886 GO:0016192 GO:0006351
GO:0015991 GO:0005515 GO:0016820 GO:0000221 GO:0030117
Cluster-22178.85944 GO:0019073 GO:0015991 GO:0016192 GO:0006886
GO:0006351 GO:0005515 GO:0016820 GO:0000221 GO:0030117
Cluster-22178.85945 GO:0008152 GO:0055114 GO:0016616 GO:0051287

Cluster-22178.85948 GO:0007275 GO:0008152 GO:0016032 GO:0016787
GO:0016021
Cluster-22178.85949 GO:0007186 GO:0004930 GO:0016021 GO:0016020
Cluster-22178.85950 GO:0007186 GO:0004930 GO:0016020 GO:0016021
Cluster-22178.85955 GO:0006355 GO:0006810 GO:0002949 GO:0008134
GO:0016301 GO:0003774 GO:0005524 GO:0016459 GO:0005667
Cluster-22178.85958 GO:0019082 GO:0006672 GO:0006807 GO:0016032
GO:0016811 GO:0016021
Cluster-22178.85963 GO:0007010 GO:0003779
Cluster-22178.85964 GO:0005515
Cluster-22178.85972 GO:0005198 GO:0019028
Cluster-22178.85979 GO:0006355 GO:0008608 GO:0003677 GO:0072686
GO:0042729
Cluster-22178.85981 GO:0046872 GO:0003677 GO:0005515
Cluster-22178.85984 GO:0005515 GO:0030234 GO:0016020
Cluster-22178.85988 GO:0005515 GO:0030234 GO:0016020
Cluster-22178.85990 GO:0006777 GO:0007165 GO:0007186 GO:0007264
GO:0006468 GO:0006576 GO:0005525 GO:0004871 GO:0019001
GO:0005524 GO:0004672 GO:0031683 GO:0003924 GO:0005515
Cluster-22178.85992 GO:0006310 GO:0006302 GO:0005515 GO:0005509
GO:0003677 GO:0005634 GO:0009424

Cluster-22178.85998 GO:0007264 GO:0006744 GO:0016831 GO:0005525
GO:0005515 GO:0005886
Cluster-22178.85999 GO:0016310 GO:0009069 GO:0004674 GO:0005515
GO:0004185 GO:0005773
Cluster-22178.86006 GO:0006813 GO:0016043 GO:0016032 GO:0006952
GO:0045893 GO:0006811 GO:0055085 GO:0005216 GO:0015269
GO:0003779 GO:0005576 GO:0016020
Cluster-22178.86009 GO:0031388 GO:0006544 GO:0046487 GO:0046486
GO:0006566 GO:0006563 GO:0008887 GO:0019031
Cluster-22178.86012 GO:0009451 GO:0006479 GO:0006355 GO:0008033
GO:0006364 GO:0046500 GO:0035556 GO:0030488 GO:0006464
GO:0006554 GO:0003723 GO:0018024 GO:0003700 GO:0008168
GO:0004719 GO:0016429 GO:0042025 GO:0031515 GO:0005667
Cluster-22178.86013 GO:0035556 GO:0030488 GO:0006464 GO:0006554
GO:0006364 GO:0046500 GO:0008033 GO:0009451 GO:0006479
GO:0006355 GO:0016429 GO:0003700 GO:0008168 GO:0004719
GO:0003723 GO:0018024 GO:0031515 GO:0005667 GO:0042025
Cluster-22178.86016 GO:0006468 GO:0016773 GO:0005524 GO:0004672
GO:0016020
Cluster-22178.86021 GO:0005515
Cluster-22178.86024 GO:0009395 GO:0006629 GO:0046339 GO:0007165
GO:0035556 GO:0004435
Cluster-22178.86025 GO:0009395 GO:0006629 GO:0046339 GO:0007165
GO:0035556 GO:0004435 GO:0005509
Cluster-22178.86028 GO:0046872 GO:0005515
Cluster-22178.86032 GO:0006357 GO:0003723 GO:0005515 GO:0001104
GO:0016592
Cluster-22178.86040 GO:0008242
Cluster-22178.86048 GO:0016020
Cluster-22178.8605 GO:0005515 GO:0005509 GO:0005576 GO:0005886
Cluster-22178.86051 GO:0046677 GO:0005515 GO:0016779 GO:0016020
Cluster-22178.86057 GO:0005515
Cluster-22178.86061 GO:0005515

Cluster-22178.86062 GO:0006810 GO:0009405 GO:0008200 GO:0005576
Cluster-22178.86065 GO:0008152 GO:0016758
Cluster-22178.86067 GO:0051262 GO:0055085 GO:0006811 GO:0016310
GO:0006468 GO:0009069 GO:0005524 GO:0004674 GO:0005216
GO:0016020
Cluster-22178.86068 GO:0051262 GO:0016310 GO:0006811 GO:0055085
GO:0009069 GO:0006468 GO:0005524 GO:0004674 GO:0005216
GO:0016020
Cluster-22178.86072 GO:0006465 GO:0008233 GO:0005787 GO:0016021
Cluster-22178.86078 GO:0030153
Cluster-22178.86080 GO:0015684 GO:0006355 GO:0007264 GO:0005525
GO:0015093 GO:0005524 GO:0003924 GO:0016887 GO:0003677
GO:0005515 GO:0016021 GO:0005622 GO:0016020
Cluster-22178.86082 GO:0005515 GO:0003824 GO:0016020
Cluster-22178.86086 GO:0006520 GO:0016597 GO:0016743
Cluster-22178.86094 GO:0055114 GO:0045454 GO:0016491 GO:0016209

Cluster-22178.86097 GO:0003676
Cluster-22178.86098 GO:0005515
Cluster-22178.86101 GO:0019497 GO:0006771 GO:0007339 GO:0046872
GO:0003993 GO:0005515 GO:0005576 GO:0016020
Cluster-22178.86105 GO:0007049 GO:0000917 GO:0006351 GO:0006355
GO:0006744 GO:0006260 GO:0006914 GO:0006464 GO:0043093
GO:0003700 GO:0016151 GO:0043565 GO:0008813 GO:0003887
GO:0003677 GO:0005737 GO:0042575 GO:0005815 GO:0005667
Cluster-22178.86116 GO:0006355 GO:0003677 GO:0005634
Cluster-22178.86120 GO:0006915
Cluster-22178.86124 GO:0001503 GO:0007155 GO:0005515 GO:0016758
GO:0005789 GO:0005576
Cluster-22178.86127 GO:0005515
Cluster-22178.86129 GO:0019048 GO:0003677
Cluster-22178.86130 GO:0006355 GO:0016032 GO:0016070 GO:0003677
GO:0019012

Cluster-22178.86136 GO:0045893 GO:0006281 GO:0008081 GO:0005634
Cluster-22178.86137 GO:0006355 GO:0045893 GO:0043565 GO:0003677
GO:0005634
Cluster-22178.86138 GO:0006468 GO:0006357 GO:0005524 GO:0004672
GO:0001104 GO:0016592 GO:0019031
Cluster-22178.86145 GO:0055114 GO:0007186 GO:0005506 GO:0020037
GO:0016705 GO:0004930
Cluster-22178.86150 GO:0045947 GO:0008190
Cluster-22178.86154 GO:0006629 GO:0008081
Cluster-22178.86157 GO:0006265 GO:0031175 GO:0046872 GO:0003916
GO:0008270 GO:0005516 GO:0005515 GO:0003677 GO:0030507
GO:0008091 GO:0005694
Cluster-22178.86159 GO:0006525 GO:0006508 GO:0007268 GO:0006560
GO:0007165 GO:0071436 GO:0006814 GO:0006811 GO:0006090
GO:0008948 GO:0008236 GO:0015081 GO:0004970 GO:0016020
Cluster-22178.86160 GO:0030570 GO:0005576
Cluster-22178.86163 GO:0016192 GO:0019497 GO:0006771 GO:0003993
GO:0016021 GO:0019031
Cluster-22178.86165 GO:0006614 GO:0007283 GO:0006139 GO:0006206
GO:0098519 GO:0016301 GO:0008146 GO:0003723 GO:0005525
GO:0003724 GO:0004127 GO:0005524 GO:0003924 GO:0016887
GO:0043531
Cluster-22178.86166 GO:0071766 GO:0006446 GO:0016192 GO:0008608
GO:0006355 GO:0007009 GO:0071816 GO:0003743 GO:0019843
GO:0052636 GO:0043565 GO:0003700 GO:0003723 GO:0005622
GO:0008537 GO:0005667 GO:0005840 GO:0016021 GO:0072686
GO:0042729
Cluster-22178.86170 GO:0006355 GO:0009405 GO:0043401 GO:0005198
GO:0005515 GO:0016021
Cluster-22178.86171 GO:0007165
Cluster-22178.86173 GO:0005515
Cluster-22178.86177 GO:0007049 GO:0008608 GO:0023052 GO:0008270
GO:0042729 GO:0005876

Cluster-22178.86180 GO:0007165 GO:0006812 GO:0098655 GO:0033198
GO:0004931 GO:0001614 GO:0005887
Cluster-22178.86181 GO:0007165 GO:0019079 GO:0043087 GO:0007264
GO:0005515 GO:0005085
Cluster-22178.86188 GO:0006813 GO:0055114 GO:0005506 GO:0016787
GO:0003677 GO:0016705 GO:0020037 GO:0005267 GO:0005524
GO:0016020
Cluster-22178.86193 GO:0005680
Cluster-22178.86199 GO:0007186 GO:0004930 GO:0016021
Cluster-22178.86208 GO:0006835 GO:0006820 GO:0006812 GO:0055085
GO:0030001 GO:0006508 GO:0046873 GO:0017153 GO:0016020
Cluster-22178.86209 GO:0015992 GO:0015991 GO:0006488 GO:0015078
GO:0004583 GO:0005789 GO:0033179
Cluster-22178.86214 GO:0007186 GO:0051082 GO:0003677 GO:0005515
GO:0004930 GO:0003824 GO:0003676 GO:0005634 GO:0016021
Cluster-22178.86218 GO:0006955 GO:0016567 GO:0019882 GO:0004842

Cluster-22178.86219 GO:0005515
Cluster-22178.86223 GO:0019867
Cluster-22178.86228 GO:0019042 GO:0000166 GO:0003677 GO:0005524
GO:0033644
Cluster-22178.86230 GO:0030001 GO:0033014 GO:0055114 GO:0055085
GO:0046873 GO:0050661 GO:0008883 GO:0016020
Cluster-22178.86231 GO:0032065 GO:0005543 GO:0005515 GO:0005938
Cluster-22178.86239 GO:0032012 GO:0043087 GO:0006051 GO:0006040
GO:0047465 GO:0016772 GO:0005086
Cluster-22178.86241 GO:0016772
Cluster-22178.86246 GO:0006508 GO:0008236 GO:0016787
Cluster-22178.86248 GO:0016192 GO:0006606 GO:0046872 GO:0008270
GO:0061630 GO:0005515 GO:0016021
Cluster-22178.86251 GO:0046486 GO:0008654 GO:0008715 GO:0016021
GO:0016020
Cluster-22178.86254 GO:0006357 GO:0001104 GO:0016592

Cluster-22178.86255 GO:0006801 GO:0004784 GO:0016151 GO:0016209

Cluster-22178.86260 GO:0006355 GO:0008643 GO:0008982 GO:0003682
GO:0003677 GO:0008270 GO:0009357 GO:0019028 GO:0005634
GO:0000785 GO:0000775
Cluster-22178.86266 GO:0005515
Cluster-22178.86267 GO:0006351 GO:0006310 GO:0016055 GO:0007131
GO:0006357 GO:0001104 GO:0005515 GO:0016592
Cluster-22178.86273 GO:0043066 GO:0045766 GO:0043123 GO:0009607
GO:0016021
Cluster-22178.86277 GO:0016627
Cluster-22178.86283 GO:0006633 GO:0015031 GO:0016790 GO:0016021
Cluster-22178.86286 GO:0015991 GO:0000281 GO:0015031 GO:0000226
GO:0040001 GO:0006606 GO:0005515 GO:0016820 GO:0008565
GO:0005634 GO:0005819 GO:0005737 GO:0005874 GO:0000221
Cluster-22178.86291 GO:0005515
Cluster-22178.86296 GO:0016567 GO:0006810 GO:0006952 GO:0007165
GO:0008270 GO:0046872 GO:0004842 GO:0019239 GO:0005179
GO:0005515 GO:0030288 GO:0005680 GO:0005576
Cluster-22178.86298 GO:0005975 GO:0003824
Cluster-22178.86305 GO:0008270
Cluster-22178.86310 GO:0003676 GO:0005524
Cluster-22178.86316 GO:0009058 GO:0055114 GO:0006520 GO:0016491
GO:0030170 GO:0016829
Cluster-22178.86318 GO:0006281
Cluster-22178.86319 GO:0006355 GO:0003677 GO:0003700 GO:0005667
Cluster-22178.86320 GO:0005524 GO:0003676
Cluster-22178.86321 GO:0055114 GO:0006694 GO:0016114 GO:0004506
GO:0016787 GO:0071949 GO:0050660 GO:0016021
Cluster-22178.86322 GO:0006520 GO:0016021
Cluster-22178.86326 GO:0008270 GO:0003677
Cluster-22178.86327 GO:0046740 GO:0016021
Cluster-22178.86332 GO:0019031

Cluster-22178.86334 GO:0006412 GO:0042254 GO:0003735 GO:0005622
GO:0005840
Cluster-22178.86338 GO:0055114 GO:0016616 GO:0016491
Cluster-22178.86340 GO:0015992 GO:0006123 GO:0006302 GO:0004129
GO:0005515 GO:0045277 GO:0005739 GO:0005634
Cluster-22178.86342 GO:0006457 GO:0007165 GO:0030168 GO:0006606
GO:0006886 GO:0010508 GO:0048870 GO:0051382 GO:0051258
GO:0007016 GO:0030674 GO:0005102 GO:0051082 GO:0005198
GO:0005200 GO:0005925 GO:0001726 GO:0005783 GO:0016272
GO:0005577 GO:0005815 GO:0019028 GO:0000776 GO:0031514
GO:0005856 GO:0016021 GO:0019031
Cluster-22178.86344 GO:0015986 GO:0015992 GO:0015078 GO:0045263
Cluster-22178.86349 GO:0007264 GO:0005525
Cluster-22178.86350 GO:0006109 GO:0007264 GO:0006355 GO:0016310
GO:0000160 GO:0008134 GO:0000155 GO:0005525 GO:0005524
GO:0004672 GO:0003924 GO:0016887 GO:0016020 GO:0005667
GO:0009365
Cluster-22178.86351 GO:0016310 GO:0000160 GO:0007264 GO:0006109
GO:0004672 GO:0005524 GO:0016887 GO:0003924 GO:0005525
GO:0000155 GO:0009365
Cluster-22178.86354 GO:0042254 GO:0006412 GO:0009082 GO:0015948
GO:0046656 GO:0003735 GO:0005515 GO:0003723 GO:0030269
GO:0016021 GO:0005840
Cluster-22178.86357 GO:0006418 GO:0006606 GO:0043565 GO:0005524
GO:0003723 GO:0004812 GO:0005515 GO:0005634
Cluster-22178.86365 GO:0006885 GO:0006812 GO:0055085 GO:0015299
GO:0016021
Cluster-22178.86383 GO:0009306
Cluster-22178.86384 GO:0016043 GO:0030036 GO:0003779 GO:0017048

Cluster-22178.86388 GO:0007165 GO:0009405 GO:0030168 GO:0051258
GO:0030674 GO:0005102 GO:0016020 GO:0005577
Cluster-22178.86389 GO:0006810 GO:0006914 GO:0048870 GO:0019901

GO:0046983 GO:0005739 GO:0031514 GO:0016021
Cluster-22178.86393 GO:0019013 GO:0016021
Cluster-22178.86395 GO:0007186 GO:0004930 GO:0016021 GO:0019031
Cluster-22178.86398 GO:0008152 GO:0016836 GO:0000062 GO:0003824

Cluster-22178.86401 GO:0055085 GO:0006811 GO:0005216 GO:0016020
Cluster-22178.86402 GO:0005515
Cluster-22178.86403 GO:0005515
Cluster-22178.86405 GO:0046983
Cluster-22178.86408 GO:0008152 GO:0016791 GO:0016021
Cluster-22178.86409 GO:0006659
Cluster-22178.86410 GO:0000160 GO:0016310 GO:0007268 GO:0006355
GO:0003700 GO:0046983 GO:0033130 GO:0005515 GO:0042802
GO:0043495 GO:0000155 GO:0016021 GO:0009365 GO:0005667
Cluster-22178.86414 GO:0009306
Cluster-22178.86418 GO:0035556 GO:0006464 GO:0006355 GO:0005507
GO:0003700 GO:0016151 GO:0008270 GO:0046872 GO:0003677
GO:0005634 GO:0005667
Cluster-22178.86419 GO:0006351 GO:0006464 GO:0003677 GO:0005515
GO:0016151 GO:0003676 GO:0008270 GO:0046872
Cluster-22178.8642 GO:0016567 GO:0004842 GO:0009289
Cluster-22178.86420 GO:0019988 GO:0006570 GO:0006470 GO:0008138
GO:0004725 GO:0043399
Cluster-22178.86423 GO:0007411 GO:0016021 GO:0019031
Cluster-22178.86425 GO:0044564 GO:0016567 GO:0004842
Cluster-22178.86430 GO:0006952 GO:0055085 GO:0006812 GO:0008324
GO:0016817 GO:0016021
Cluster-22178.86431 GO:0016042 GO:0005515 GO:0016020
Cluster-22178.86433 GO:0006338 GO:0006355 GO:0003676 GO:0005524
GO:0008270 GO:0003677 GO:0016818 GO:0016787 GO:0005634
Cluster-22178.86435 GO:0005515
Cluster-22178.86437 GO:0006950 GO:0016021 GO:0016020
Cluster-22178.86444 GO:0019915 GO:0019058 GO:0005524 GO:0003774

GO:0016459 GO:0019031 GO:0005789
Cluster-22178.86445 GO:0007186 GO:0007165 GO:0004871 GO:0005834
Cluster-22178.86448 GO:0005515
Cluster-22178.86450 GO:0005515
Cluster-22178.86455 GO:0030154 GO:0005737 GO:0005634
Cluster-22178.86456 GO:0006364 GO:0043015 GO:0042802 GO:0008017
GO:0008168 GO:0045298 GO:0009424 GO:0005737
Cluster-22178.86459 GO:0042742
Cluster-22178.86464 GO:0006457 GO:0000413 GO:0003677 GO:0003755

Cluster-22178.86469 GO:0055114 GO:0033539 GO:0007018 GO:0006118
GO:0007017 GO:0003777 GO:0008017 GO:0005524 GO:0003995
GO:0005874 GO:0045298
Cluster-22178.86470 GO:0009069 GO:0006468 GO:0016310 GO:0007165
GO:0046983 GO:0005096 GO:0004674
Cluster-22178.86475 GO:0008270 GO:0046872 GO:0005515 GO:0003677

Cluster-22178.86476 GO:0051260 GO:0005515 GO:0019028
Cluster-22178.86478 GO:0007094 GO:0046872 GO:0008270 GO:0005515
GO:0003677
Cluster-22178.86479 GO:0006570 GO:0006470 GO:0004721 GO:0008138
GO:0004725
Cluster-22178.86484 GO:0034227 GO:0002098 GO:0000049 GO:0008270
GO:0005622
Cluster-22178.86485 GO:0061630 GO:0005515 GO:0008270 GO:0046872

Cluster-22178.86486 GO:0007155 GO:0006897 GO:0016567 GO:0006308
GO:0007015 GO:0008270 GO:0008855 GO:0004842 GO:0061630
GO:0046872 GO:0016788 GO:0005515 GO:0005198 GO:0005622
GO:0005680 GO:0009288 GO:0019031 GO:0016021 GO:0019028
GO:0009318
Cluster-22178.86489 GO:0016567 GO:0006270 GO:0006260 GO:0006351
GO:0006468 GO:0000398 GO:0003677 GO:0004842 GO:0008270

GO:0005524 GO:0005515 GO:0003887 GO:0004672 GO:0005680
GO:0042575 GO:0019028 GO:0005634 GO:0005681 GO:0019031
GO:0005737
Cluster-22178.86503 GO:0019467 GO:0004857 GO:0008073
Cluster-22178.86508 GO:0015012 GO:0006024 GO:0016021
Cluster-22178.86517 GO:0046872 GO:0005515
Cluster-22178.86522 GO:0016032 GO:0007224 GO:0043248 GO:0006275
GO:0006355
Cluster-22178.86524 GO:0031554 GO:0007224 GO:0031556
Cluster-22178.86527 GO:0006855 GO:0015893 GO:0015238 GO:0005515
GO:0005886
Cluster-22178.86529 GO:0006464 GO:0051082 GO:0046872 GO:0005515
GO:0031072 GO:0016151
Cluster-22178.86534 GO:0019042 GO:0003677 GO:0000166 GO:0005524
GO:0033644
Cluster-22178.86547 GO:0006810 GO:0030001 GO:0055085 GO:0005515
GO:0046873 GO:0016020 GO:0016021 GO:0005886
Cluster-22178.86549 GO:0043093 GO:0007049 GO:0000917 GO:0051082
GO:0046983 GO:0005737 GO:0005861
Cluster-22178.86553 GO:0010972 GO:0007017 GO:0030336 GO:0005875
Cluster-22178.86555 GO:0042254 GO:0006412 GO:0006265 GO:0003677
GO:0003735 GO:0008270 GO:0003916 GO:0003676 GO:0005694
GO:0015934 GO:0005840
Cluster-22178.86560 GO:0008152 GO:0005975 GO:0005509 GO:0004571
GO:0016829 GO:0016020
Cluster-22178.86561 GO:0006468 GO:0009069 GO:0007165 GO:0016310
GO:0016787 GO:0005515 GO:0003677 GO:0016773 GO:0008601
GO:0004672 GO:0005524 GO:0004674 GO:0016020 GO:0000159
Cluster-22178.86564 GO:0003723 GO:0046935 GO:0003676 GO:0005944
GO:0016021
Cluster-22178.86568 GO:0007154 GO:0016021
Cluster-22178.86570 GO:0006470 GO:0006570 GO:0008138 GO:0004725

Cluster-22178.86571 GO:0006570 GO:0006470 GO:0004725 GO:0008138

Cluster-22178.86574 GO:0045892 GO:0005634

Cluster-22178.86575 GO:0006457 GO:0009116 GO:0005515 GO:0051082 GO:0016020

Cluster-22178.86576 GO:0006418 GO:0045892 GO:0000166 GO:0004812 GO:0005524 GO:0005634

Cluster-22178.86583 GO:0007165

Cluster-22178.86587 GO:0016020

Cluster-22178.86588 GO:0006352 GO:0006355 GO:0008270 GO:0016987 GO:0003700 GO:0003677 GO:0005515 GO:0005667

Cluster-22178.86589 GO:0006352 GO:0006355 GO:0008270 GO:0016987 GO:0003700 GO:0003677 GO:0005515 GO:0005667

Cluster-22178.86592 GO:0006352 GO:0006355 GO:0003700 GO:0016987 GO:0008270 GO:0005515 GO:0003677 GO:0005667

Cluster-22178.86593 GO:0006811 GO:0055085 GO:0015075 GO:0016020

Cluster-22178.86599 GO:0008270

Cluster-22178.86602 GO:0006051 GO:0006040 GO:0047465

Cluster-22178.86604 GO:0019911

Cluster-22178.86607 GO:0006810 GO:0055085 GO:0005525 GO:0042626 GO:0005524 GO:0016773 GO:0003924 GO:0016887 GO:0016021

Cluster-22178.86608 GO:0006869 GO:0016043 GO:0042157 GO:0030036 GO:0031412 GO:0005198 GO:0017048 GO:0008289 GO:0003779 GO:0005576 GO:0019028 GO:0031411 GO:0019031

Cluster-22178.86617 GO:0016020

Cluster-22178.86621 GO:0016310 GO:0009069 GO:0006468 GO:0004674 GO:0000287 GO:0004672 GO:0005524

Cluster-22178.86625 GO:0006090 GO:0006529 GO:0006522 GO:0015976 GO:0055114 GO:0006108 GO:0006457 GO:0006531 GO:0006099 GO:0004066 GO:0004471 GO:0051082 GO:0016272

Cluster-22178.86635 GO:0006570 GO:0006355 GO:0051607 GO:0055114 GO:0005515 GO:0004511 GO:0003700 GO:0005667

Cluster-22178.86640 GO:0007186 GO:0006397 GO:0004930 GO:0016021

GO:0000445

Cluster-22178.86642 GO:0032065 GO:0005543 GO:0005515 GO:0005938

Cluster-22178.86652 GO:0005515

Cluster-22178.86653 GO:0051607 GO:0055114 GO:0006355 GO:0006570 GO:0003700 GO:0004511 GO:0005515 GO:0005667

Cluster-22178.86654 GO:0007166 GO:0007186 GO:0005515 GO:0004930 GO:0016020 GO:0016021

Cluster-22178.86661 GO:0005515

Cluster-22178.86662 GO:0046872 GO:0008270 GO:0005515 GO:0061630 GO:0005622 GO:0019867

Cluster-22178.86665 GO:0007165 GO:0046872 GO:0016020

Cluster-22178.86666 GO:0006468 GO:0008152 GO:0015937 GO:0015940 GO:0009058 GO:0000103 GO:0006144 GO:0016301 GO:0005515 GO:0004020 GO:0003824 GO:0004672 GO:0004140 GO:0005524 GO:0016773 GO:0016020

Cluster-22178.86668 GO:0042254 GO:0006412 GO:0006468 GO:0009069 GO:0016310 GO:0006950 GO:0003735 GO:0004674 GO:0005524 GO:0004672 GO:0005840 GO:0016021 GO:0005622

Cluster-22178.86669 GO:0009069 GO:0006468 GO:0016310 GO:0004672 GO:0005524 GO:0004674

Cluster-22178.86674 GO:0006694 GO:0007165 GO:0008299 GO:0009058 GO:0050518 GO:0016779 GO:0005515 GO:0005044 GO:0019031 GO:0016020

Cluster-22178.86675 GO:0005515

Cluster-22178.86676 GO:0007165 GO:0006144 GO:0005515 GO:0004114

Cluster-22178.86680 GO:0016020

Cluster-22178.86685 GO:0016310 GO:0007218 GO:0009069 GO:0006468 GO:0004674 GO:0046872 GO:0005524 GO:0005515

Cluster-22178.86686 GO:0016310 GO:0006468 GO:0009069 GO:0042254 GO:0006412 GO:0005524 GO:0004674 GO:0003735 GO:0005515 GO:0005622 GO:0005840

Cluster-22178.86692 GO:0007010 GO:0030071 GO:0006468 GO:0031145

GO:0004672 GO:0005524 GO:0003779 GO:0016020 GO:0019028
GO:0005680
Cluster-22178.86694 GO:0006355 GO:0003700 GO:0005634 GO:0005667
Cluster-22178.86695 GO:0055114 GO:0030436 GO:0004064 GO:0016491
GO:0005509 GO:0005515 GO:0042601
Cluster-22178.86699 GO:0005198 GO:0005882
Cluster-22178.86706 GO:0009307 GO:0006355 GO:0005992 GO:0006811
GO:0055085 GO:0006308 GO:0003677 GO:0005515 GO:0005216
GO:0003700 GO:0003824 GO:0009036 GO:0016020 GO:0009359
GO:0005667
Cluster-22178.86711 GO:0006144 GO:0006206 GO:0046034 GO:0006139
GO:0006576 GO:0005524 GO:0004127 GO:0004017
Cluster-22178.86716 GO:0005509 GO:0005515 GO:0004252 GO:0005886
Cluster-22178.86718 GO:0006468 GO:0016773 GO:0005524 GO:0004672
GO:0016020
Cluster-22178.86721 GO:0006807 GO:0005515 GO:0005509 GO:0004668
GO:0005737
Cluster-22178.86722 GO:0032065 GO:0005543 GO:0005515 GO:0005938
Cluster-22178.8674 GO:0006397 GO:0006606 GO:0005198 GO:0003723
GO:0005882 GO:0005634 GO:0016021 GO:0005737
Cluster-22178.86740 GO:0009060 GO:0006123 GO:0015992 GO:0055114
GO:0007186 GO:0006118 GO:0009055 GO:0004930 GO:0004129
GO:0020037 GO:0005506 GO:0045277 GO:0016021
Cluster-22178.86745 GO:0019441 GO:0006568 GO:0020037 GO:0004833

Cluster-22178.86746 GO:0006810 GO:0007165 GO:0005215 GO:0004872
GO:0016020 GO:0016021
Cluster-22178.86747 GO:0008152 GO:0016758
Cluster-22178.86748 GO:0007165 GO:0005509 GO:0005515 GO:0005578
Cluster-22178.86754 GO:0006801 GO:0016209 GO:0016151 GO:0004784

Cluster-22178.8676 GO:0016032 GO:0005515 GO:0046872
Cluster-22178.86772 GO:0006446 GO:0006355 GO:0097034 GO:0006417

GO:0004518 GO:0003677 GO:0003743 GO:0003700 GO:0008270
GO:0046983 GO:0005667 GO:0005743 GO:0005840 GO:0016021
Cluster-22178.86775 GO:0006024 GO:0015012 GO:0016021
Cluster-22178.86778 GO:0007059 GO:0051301 GO:0031110 GO:0007018
GO:0000090 GO:0007067 GO:0007017 GO:0003777 GO:0008017
GO:0005524 GO:0005874 GO:0005876 GO:0045298 GO:0000940
Cluster-22178.86781 GO:0005515
Cluster-22178.86789 GO:0055114 GO:0006412 GO:0044237 GO:0008168
GO:0016491 GO:0003824
Cluster-22178.86795 GO:0006613 GO:0030176 GO:0005784
Cluster-22178.86799 GO:0005198 GO:0003824 GO:0005515 GO:0043657
GO:0019028
Cluster-22178.86815 GO:0006355 GO:0003677 GO:0016872
Cluster-22178.86817 GO:0005515
Cluster-22178.86819 GO:0016310 GO:0009069 GO:0004674 GO:0005515

Cluster-22178.86821 GO:0006629 GO:0003677 GO:0005815 GO:0005634
Cluster-22178.86823 GO:0015979 GO:0009522 GO:0016021 GO:0009579
Cluster-22178.86824 GO:0006412 GO:0042254 GO:0005515 GO:0003735
GO:0005840 GO:0005622
Cluster-22178.86826 GO:0042254 GO:0006412 GO:0003735 GO:0005515
GO:0005840 GO:0005622
Cluster-22178.86833 GO:0016567 GO:0008134 GO:0005515 GO:0004842
GO:0008270 GO:0046872 GO:0005634 GO:0005667 GO:0005680
GO:0005622
Cluster-22178.86837 GO:0051301 GO:0007059 GO:0005515 GO:0008017
GO:0000444 GO:0045298
Cluster-22178.86839 GO:0005515
Cluster-22178.86842 GO:0016021 GO:0042765
Cluster-22178.86844 GO:0006355 GO:0045454 GO:0042254 GO:0006412
GO:0003735 GO:0005622 GO:0005840
Cluster-22178.86847 GO:0006468 GO:0016773 GO:0005524 GO:0004672
GO:0016020

Cluster-22178.86848 GO:0006813 GO:0006687 GO:0009058 GO:0005242
GO:0008146 GO:0046872 GO:0001733 GO:0008076 GO:0005794
GO:0016021 GO:0005840
Cluster-22178.86850 GO:0007517 GO:0006355 GO:0003677 GO:0005515
GO:0005634
Cluster-22178.86853 GO:0005515 GO:0035091
Cluster-22178.86854 GO:0016032 GO:0006813 GO:0016043 GO:0006952
GO:0045893 GO:0055085 GO:0006811 GO:0005216 GO:0003779
GO:0015269 GO:0005576 GO:0016020
Cluster-22178.86858 GO:0005515
Cluster-22178.86866 GO:0014068 GO:0007165 GO:0050776 GO:0005102
GO:0043548 GO:0005515 GO:0016020
Cluster-22178.86875 GO:0003779
Cluster-22178.86883 GO:0007264 GO:0005525
Cluster-22178.86884 GO:0042254 GO:0006412 GO:0016817 GO:0005515
GO:0003735 GO:0005622 GO:0005840
Cluster-22178.86889 GO:0009405 GO:0005576
Cluster-22178.86895 GO:0044419 GO:0006144 GO:0006508 GO:0004197
GO:0003968 GO:0017111 GO:0031379
Cluster-22178.86897 GO:0006914 GO:0006606 GO:0006355 GO:0043565
GO:0003700 GO:0016788 GO:0003677 GO:0005634 GO:0005667
Cluster-22178.86898 GO:0005975 GO:0005515 GO:0004415 GO:0016020
GO:0009986
Cluster-22178.86899 GO:0006810 GO:0016310 GO:0000160 GO:0055114
GO:0006928 GO:0005515 GO:0000155 GO:0004673 GO:0005215
GO:0016491 GO:0016020 GO:0009365
Cluster-22178.86904 GO:0006351 GO:0007059 GO:0045995 GO:0006308
GO:0051301 GO:0007165 GO:0030155 GO:0006269 GO:0007155
GO:0007067 GO:0000090 GO:0030334 GO:0006914 GO:0031110
GO:0005515 GO:0003896 GO:0005102 GO:0008017 GO:0008855
GO:0009318 GO:0045298 GO:0005730 GO:0005657 GO:0005876
GO:0000940
Cluster-22178.86907 GO:0007178 GO:0016310 GO:0009069 GO:0006468

GO:0005524 GO:0004672 GO:0004675 GO:0016020
Cluster-22178.86911 GO:0046872
Cluster-22178.86923 GO:0008152 GO:0019521 GO:0055114 GO:0006098
GO:0016491 GO:0004616 GO:0016616 GO:0051287
Cluster-22178.86924 GO:0005515
Cluster-22178.86927 GO:0015994 GO:0006783 GO:0033014 GO:0004852
GO:0008270
Cluster-22178.86931 GO:0016021
Cluster-22178.86932 GO:0019684 GO:0006118 GO:0004859 GO:0045156
GO:0005515 GO:0016021 GO:0005576 GO:0030077
Cluster-22178.86934 GO:0019684 GO:0016567 GO:0006118 GO:0004842
GO:0046872 GO:0008270 GO:0005515 GO:0045156 GO:0016021
GO:0005680 GO:0030077
Cluster-22178.86941 GO:0006024 GO:0016857 GO:0016021
Cluster-22178.86948 GO:0006915 GO:0005515 GO:0019028
Cluster-22178.86950 GO:0016021
Cluster-22178.86953 GO:0006887 GO:0006888 GO:0006886 GO:0009405
GO:0006810 GO:0019855 GO:0005524 GO:0008270 GO:0005515
GO:0000166 GO:0003677 GO:0000145 GO:0030127 GO:0005576
Cluster-22178.86955 GO:0015979 GO:0046872
Cluster-22178.86956 GO:0019064 GO:0007165 GO:0046789 GO:0016788
GO:0009986 GO:0019031
Cluster-22178.86960 GO:0046872 GO:0003677 GO:0005737
Cluster-22178.86972 GO:0006412 GO:0042254 GO:0003735 GO:0005840
Cluster-22178.86978 GO:0003779
Cluster-22178.86980 GO:0005515
Cluster-22178.86995 GO:0006508 GO:0008236 GO:0016787
Cluster-22178.86999 GO:0007018 GO:0007017 GO:0006366 GO:0003677
GO:0008017 GO:0003777 GO:0005524 GO:0005665 GO:0045298
GO:0005874
Cluster-22178.8700 GO:0051056 GO:0005096
Cluster-22178.87000 GO:0007018 GO:0007017 GO:0006260 GO:0051607
GO:0019901 GO:0008017 GO:0003777 GO:0005524 GO:0016021

GO:0045298 GO:0005874

Cluster-22178.87002 GO:0006366 GO:0007017 GO:0007018 GO:0005524 GO:0008017 GO:0003777 GO:0003677 GO:0045298 GO:0005874 GO:0005665

Cluster-22178.87004 GO:0030170 GO:0008483

Cluster-22178.87021 GO:0006118 GO:0006468 GO:0020037 GO:0005515 GO:0009055 GO:0005524 GO:0004672 GO:0046872 GO:0016773 GO:0016020

Cluster-22178.87022 GO:0006355 GO:0003700 GO:0005634 GO:0005667

Cluster-22178.87025 GO:0006464 GO:0006465 GO:0008152 GO:0046500 GO:0006479 GO:0008168 GO:0004719 GO:0008233 GO:0016021 GO:0005787

Cluster-22178.87031 GO:0030150 GO:0046872 GO:0005742

Cluster-22178.87034 GO:0008033 GO:0016567 GO:0009451 GO:0007165 GO:0006468 GO:0016773 GO:0004672 GO:0005524 GO:0005515 GO:0009020 GO:0016020

Cluster-22178.87036 GO:0035556 GO:0046034 GO:0006464 GO:0006144 GO:0042254 GO:0006412 GO:0006281 GO:0016151 GO:0046872 GO:0008270 GO:0003735 GO:0005515 GO:0004017 GO:0005840

Cluster-22178.87038 GO:0006355 GO:0006260 GO:0046983 GO:0003677

Cluster-22178.87039 GO:0007304 GO:0055114 GO:0033014 GO:0016772 GO:0005213 GO:0008883 GO:0003677 GO:0050661 GO:0042600 GO:0005576

Cluster-22178.87042 GO:0005515 GO:0005856

Cluster-22178.87047 GO:0006144 GO:0006206 GO:0006351 GO:0003899 GO:0003677 GO:0005730

Cluster-22178.87048 GO:0008270 GO:0016021

Cluster-22178.87051 GO:0006869 GO:0042157 GO:0046836 GO:0004045 GO:0051861 GO:0017089 GO:0008289 GO:0019028 GO:0005576 GO:0005737

Cluster-22178.87053 GO:0007186 GO:0019236 GO:0042742 GO:0003677

Cluster-22178.87054 GO:0016042 GO:0016567 GO:0044564 GO:0004842

Cluster-22178.87058 GO:0003677 GO:0005634
Cluster-22178.87061 GO:0007268 GO:0007165 GO:0015992 GO:0006811
GO:0006123 GO:0055085 GO:0005216 GO:0005515 GO:0004970
GO:0004129 GO:0016020 GO:0045277
Cluster-22178.87067 GO:0007264 GO:0042254 GO:0006412 GO:0003735
GO:0003924 GO:0005525 GO:0005622 GO:0005840
Cluster-22178.87072 GO:0060478 GO:0007342 GO:0006897 GO:0035307
GO:0005886 GO:0071595
Cluster-22178.87074 GO:0016021
Cluster-22178.87079 GO:0043065 GO:0016020 GO:0016021 GO:0005740
Cluster-22178.87080 GO:0007165 GO:0005509 GO:0005515 GO:0005578
Cluster-22178.87082 GO:0006030 GO:0008061 GO:0005576
Cluster-22178.87083 GO:0007165 GO:0008201 GO:0050750 GO:0005783
Cluster-22178.87084 GO:0055114 GO:0050660 GO:0004499 GO:0050661
GO:0016491
Cluster-22178.87091 GO:0016192 GO:0006886 GO:0030117 GO:0030131
GO:0016021
Cluster-22178.87095 GO:0005515 GO:0016020
Cluster-22178.87099 GO:0051090 GO:0006886 GO:0008536 GO:0005515
GO:0005634
Cluster-22178.87101 GO:0006122 GO:0005198 GO:0003779 GO:0005743
GO:0005750 GO:0016021 GO:0005856
Cluster-22178.87113 GO:0016567 GO:0016042 GO:0044564 GO:0004842

Cluster-22178.87120 GO:0006629 GO:0006505 GO:0009058 GO:0006886
GO:0016788
Cluster-22178.87136 GO:0007276
Cluster-22178.87137 GO:0006508 GO:0008233 GO:0004222 GO:0008270
GO:0031012
Cluster-22178.87141 GO:0006144 GO:0006351 GO:0003723 GO:0005524
GO:0003968 GO:0031379

Cluster-22178.87142 GO:0005515
Cluster-22178.87144 GO:0016787 GO:0016020
Cluster-22178.87149 GO:0006270 GO:0051782 GO:0009432 GO:0005515
GO:0009276 GO:0019028
Cluster-22178.87150 GO:0009094 GO:0000162 GO:0006571 GO:0006094
GO:0006096 GO:0004634 GO:0000287 GO:0000015
Cluster-22178.87154 GO:0006355 GO:0043565 GO:0003677 GO:0003700
GO:0005634 GO:0005667
Cluster-22178.87161 GO:0008643 GO:0006355 GO:0006508 GO:0008270
GO:0003682 GO:0008982 GO:0004197 GO:0003677 GO:0000775
GO:0000785 GO:0005634 GO:0009357
Cluster-22178.87180 GO:0006886 GO:0007165 GO:0006355 GO:0004871
GO:0003700 GO:0016020 GO:0005667 GO:0005634
Cluster-22178.87181 GO:0006468 GO:0004672 GO:0005524
Cluster-22178.87185 GO:0008544 GO:0004867
Cluster-22178.87186 GO:0046500 GO:0006400 GO:0009451 GO:0006479
GO:0008033 GO:0006464 GO:0006554 GO:0006396 GO:0008152
GO:0006480 GO:0008176 GO:0018024 GO:0008173 GO:0008168
GO:0004719
Cluster-22178.87187 GO:0009451 GO:0006479 GO:0008152 GO:0008033
GO:0046500 GO:0006400 GO:0006396 GO:0006480 GO:0006554
GO:0006464 GO:0008173 GO:0018024 GO:0008168 GO:0004719
GO:0008176 GO:0005856
Cluster-22178.87196 GO:0048478 GO:0006974 GO:0007049 GO:0044237
GO:0003824 GO:0005634
Cluster-22178.87197 GO:0005515 GO:0000774 GO:0005783
Cluster-22178.87199 GO:0005515 GO:0000774 GO:0005783
Cluster-22178.87205 GO:0003723
Cluster-22178.87206 GO:0015940 GO:0015937 GO:0005524 GO:0004594

Cluster-22178.87218 GO:0006508 GO:0004222
Cluster-22178.87222 GO:0006355 GO:0003700 GO:0005667
Cluster-22178.87223 GO:0009395 GO:0007205 GO:0046486 GO:0004143

Cluster-22178.87230 GO:0006468 GO:0009069 GO:0006812 GO:0016310
GO:0000287 GO:0005515 GO:0008324 GO:0004672 GO:0005524
GO:0004674 GO:0016021
Cluster-22178.87236 GO:0006468 GO:0006449 GO:0006118 GO:0006886
GO:0006415 GO:0016192 GO:0020037 GO:0016149 GO:0004715
GO:0009055 GO:0005506 GO:0005524 GO:0005576 GO:0005737
GO:0005840 GO:0018444 GO:0030131 GO:0005622
Cluster-22178.87241 GO:0006281 GO:0004518
Cluster-22178.87246 GO:0007165 GO:0006810 GO:0008643 GO:0005215
GO:0004872 GO:0016020 GO:0016021 GO:0042597
Cluster-22178.87252 GO:0006974 GO:0006281 GO:0051726 GO:0006260
GO:0050840 GO:0003911 GO:0005576
Cluster-22178.87253 GO:0006811 GO:0006813 GO:0005515 GO:0005249
GO:0016020 GO:0008076
Cluster-22178.87254 GO:0007586 GO:0016042 GO:0008047 GO:0005515
GO:0005576
Cluster-22178.87257 GO:0009190 GO:0035556 GO:0016849 GO:0005515

Cluster-22178.87260 GO:0006171 GO:0006182 GO:0009190 GO:0046039
GO:0006144 GO:0035556 GO:0016849 GO:0004383 GO:0004016
GO:0004252 GO:0005886
Cluster-22178.87261 GO:0035556 GO:0009190 GO:0005515 GO:0016849

Cluster-22178.87264 GO:0005515
Cluster-22178.87265 GO:0019013
Cluster-22178.87269 GO:0006397 GO:0007186 GO:0034599 GO:0046660
GO:0004252 GO:0004930 GO:0019028 GO:0005634 GO:0005886
GO:0005789 GO:0016021
Cluster-22178.87275 GO:0006508 GO:0016787 GO:0008236
Cluster-22178.87278 GO:0055114 GO:0008152 GO:0016491 GO:0051537
GO:0003824
Cluster-22178.87289 GO:0006570 GO:0006508 GO:0042207 GO:0009072

GO:0004334 GO:0008237 GO:0008241 GO:0016020

Cluster-22178.87292 GO:0016310 GO:0007165 GO:0006415 GO:0009069
GO:0006449 GO:0006468 GO:0004674 GO:0016149 GO:0004672
GO:0005524 GO:0005840 GO:0005737 GO:0018444

Cluster-22178.87294 GO:0043087 GO:0035023 GO:0008152 GO:0003824
GO:0005089 GO:0017022 GO:0016459

Cluster-22178.87300 GO:0007264 GO:0015684 GO:0006308 GO:0003924
GO:0008855 GO:0016887 GO:0015093 GO:0005524 GO:0005525
GO:0009318 GO:0016021

Cluster-22178.87305 GO:0006206 GO:0008033 GO:0055114 GO:0006231
GO:0006779 GO:0015940 GO:0019354 GO:0008641 GO:0008677
GO:0016491 GO:0050660 GO:0050797 GO:0043115

Cluster-22178.87306 GO:0008033 GO:0055114 GO:0006231 GO:0006206
GO:0015940 GO:0006779 GO:0019354 GO:0016491 GO:0008677
GO:0008641 GO:0050797 GO:0050660 GO:0043115

Cluster-22178.87307 GO:0003676

Cluster-22178.87308 GO:0006281 GO:0046872 GO:0008270

Cluster-22178.87310 GO:0006576 GO:0006139 GO:0046034 GO:0006144
GO:0006206 GO:0004017 GO:0004127 GO:0005524

Cluster-22178.87312 GO:0008608 GO:0006810 GO:0006816 GO:0042030
GO:0005246 GO:0042729 GO:0016020 GO:0072686

Cluster-22178.87314 GO:0006508 GO:0008234

Cluster-22178.87318 GO:0006541 GO:0004359 GO:0005515

Cluster-22178.87332 GO:0007264 GO:0042742 GO:0005525 GO:0008233
GO:0003924

Cluster-22178.87339 GO:0019497 GO:0006771 GO:0048013 GO:0046872
GO:0003993 GO:0005515

Cluster-22178.87343 GO:0016021

Cluster-22178.87345 GO:0006807 GO:0006672 GO:0016811 GO:0016021

Cluster-22178.87351 GO:0030141

Cluster-22178.87356 GO:0005515 GO:0008168

Cluster-22178.87358 GO:0005515

Cluster-22178.87361 GO:0006810 GO:0006811 GO:0005215 GO:0016020

Cluster-22178.87374 GO:0006576 GO:0007264 GO:0005524 GO:0005525

Cluster-22178.87377 GO:0009451 GO:0006479 GO:0006352 GO:0008152
GO:0008033 GO:0046500 GO:0006400 GO:0006396 GO:0006480
GO:0006554 GO:0006464 GO:0030488 GO:0008173 GO:0004190
GO:0018024 GO:0008168 GO:0004719 GO:0008176 GO:0016429
GO:0005634 GO:0005669 GO:0016021 GO:0005856 GO:0031515
Cluster-22178.87378 GO:0009451 GO:0006479 GO:0008033 GO:0008152
GO:0006400 GO:0046500 GO:0006554 GO:0006464 GO:0030488
GO:0006396 GO:0006480 GO:0018024 GO:0008173 GO:0008168
GO:0004719 GO:0008176 GO:0016429 GO:0031515
Cluster-22178.87379 GO:0006479 GO:0009451 GO:0008033 GO:0008152
GO:0006352 GO:0006400 GO:0046500 GO:0006464 GO:0006554
GO:0006480 GO:0006396 GO:0018024 GO:0004190 GO:0008173
GO:0004719 GO:0008168 GO:0008176 GO:0005634 GO:0005856
GO:0016021 GO:0005669
Cluster-22178.87381 GO:0006260 GO:0003887 GO:0008270 GO:0042575
GO:0005634
Cluster-22178.87396 GO:0005509 GO:0005544 GO:0046872 GO:0045202
Cluster-22178.87397 GO:0046008 GO:0007165 GO:0005179 GO:0005576
Cluster-22178.87399 GO:0007156 GO:0005509 GO:0016020
Cluster-22178.87405 GO:0019236 GO:0045893 GO:0006260 GO:0006275
GO:0007186 GO:0003688 GO:0003677 GO:0016021 GO:0042025
GO:0046809
Cluster-22178.87412 GO:0005515
Cluster-22178.87413 GO:0003774 GO:0016459
Cluster-22178.87414 GO:0030150 GO:0005515 GO:0005742
Cluster-22178.87430 GO:0006468 GO:0009405 GO:0004672 GO:0005524
GO:0016773 GO:0004190 GO:0005515 GO:0005198 GO:0016021
GO:0016020
Cluster-22178.87432 GO:0007165 GO:0045227 GO:0006887 GO:0005179
GO:0004252 GO:0005576 GO:0016020 GO:0000145 GO:0016021
Cluster-22178.87433 GO:0005515 GO:0016020 GO:0016021

Cluster-22178.87434 GO:0005515
Cluster-22178.87438 GO:0018189
Cluster-22178.87441 GO:0005515
Cluster-22178.87444 GO:0006814 GO:0071436 GO:0006560 GO:0006525 GO:0006090 GO:0008948 GO:0015081 GO:0016020
Cluster-22178.87446 GO:0005515
Cluster-22178.87461 GO:0006355 GO:0003700 GO:0010181 GO:0043565 GO:0005667
Cluster-22178.87467 GO:0004222 GO:0005615
Cluster-22178.87469 GO:0035556 GO:0007010 GO:0008289 GO:0005515 GO:0003779
Cluster-22178.87472 GO:0006457 GO:0007165 GO:0006260 GO:0030168 GO:0000917 GO:0006355 GO:0007049 GO:0051258 GO:0019069 GO:0043093 GO:0010508 GO:0006886 GO:0051087 GO:0030674 GO:0005102 GO:0000774 GO:0003700 GO:0046872 GO:0061630 GO:0019905 GO:0008270 GO:0042803 GO:0005198 GO:0005515 GO:0051082 GO:0043565 GO:0005882 GO:0016021 GO:0005783 GO:0005667 GO:0005577 GO:0016272 GO:0005737
Cluster-22178.87474 GO:0006812 GO:0006820 GO:0006835 GO:0017153 GO:0016020
Cluster-22178.87475 GO:0019901 GO:0005515
Cluster-22178.87476 GO:0006355 GO:0015986 GO:0043401 GO:0015992 GO:0046856 GO:0008270 GO:0043565 GO:0015078 GO:0034597 GO:0003700 GO:0005634 GO:0005667 GO:0000276
Cluster-22178.87477 GO:0007165 GO:0005158 GO:0005899
Cluster-22178.87478 GO:0006468 GO:0005524 GO:0004672
Cluster-22178.87479 GO:0005515
Cluster-22178.87483 GO:0005975 GO:0004571 GO:0005509 GO:0016020
Cluster-22178.87484 GO:0006810 GO:0009405 GO:0008200 GO:0005576
Cluster-22178.87485 GO:0005515 GO:0016020
Cluster-22178.87493 GO:0005515
Cluster-22178.87496 GO:0003677
Cluster-22178.87498 GO:0006816 GO:0006874 GO:0006281 GO:0006555

GO:0006730 GO:0004013 GO:0005219 GO:0008270 GO:0046872
GO:0016021 GO:0005622
Cluster-22178.87500 GO:0004527 GO:0003676
Cluster-22178.87501 GO:0008608 GO:0000049 GO:0042729 GO:0005815
GO:0005876
Cluster-22178.87505 GO:0007156 GO:0042254 GO:0006412 GO:0003735
GO:0005509 GO:0016020 GO:0005622 GO:0005840
Cluster-22178.87509 GO:0016192 GO:0005515 GO:0016021
Cluster-22178.87510 GO:0016192 GO:0005515 GO:0016021
Cluster-22178.87516 GO:0006508 GO:0015991 GO:0006119 GO:0015992
GO:0000287 GO:0008237 GO:0005515 GO:0008900 GO:0008241
GO:0005524 GO:0016020 GO:0005889
Cluster-22178.87521 GO:0005524
Cluster-22178.87529 GO:0006508 GO:0016787 GO:0008270 GO:0003950
GO:0004181 GO:0005634
Cluster-22178.87536 GO:0007264 GO:0005525
Cluster-22178.87539 GO:0042432 GO:0009058 GO:0016114 GO:0016844
GO:0004064 GO:0005515
Cluster-22178.87541 GO:0015986 GO:0005975 GO:0015992 GO:0042407
GO:0005509 GO:0051082 GO:0015078 GO:0004571 GO:0045263
GO:0016020 GO:0044284 GO:0061617
Cluster-22178.87549 GO:0006605 GO:0006886 GO:0005515 GO:0005742
Cluster-22178.87554 GO:0016310 GO:0000160 GO:0055114 GO:0000155
GO:0016491 GO:0009365 GO:0016021
Cluster-22178.87558 GO:0006281 GO:0016021 GO:0005634
Cluster-22178.87559 GO:0006355 GO:0003700 GO:0005515 GO:0003677
GO:0005667
Cluster-22178.87563 GO:0006914 GO:0031110 GO:0015031 GO:0000921
GO:0007067 GO:0000090 GO:0007264 GO:0071973 GO:0016192
GO:0051301 GO:0006935 GO:0007059 GO:0010508 GO:0006886
GO:0005096 GO:0008565 GO:0003774 GO:0008017 GO:0005198
GO:0005940 GO:0000940 GO:0005876 GO:0005923 GO:0030132
GO:0016020 GO:0005737 GO:0009288 GO:0016021 GO:0045298

GO:0030130
Cluster-22178.87564 GO:0046500 GO:0006479 GO:0006464 GO:0006465
GO:0008152 GO:0008168 GO:0004719 GO:0008233 GO:0005787
GO:0016021
Cluster-22178.87570 GO:0006120 GO:0006744 GO:0008152 GO:0015992
GO:0006118 GO:0006814 GO:0003824 GO:0008137 GO:0003954
GO:0005739
Cluster-22178.87571 GO:0006468 GO:0005524 GO:0004672
Cluster-22178.87572 GO:0006468 GO:0005524 GO:0004672
Cluster-22178.87574 GO:0003677
Cluster-22178.87578 GO:0006468 GO:0016773 GO:0005524 GO:0004672
GO:0016020
Cluster-22178.87581 GO:0046983 GO:0003677
Cluster-22178.87582 GO:0043401 GO:0006355 GO:0003700 GO:0043565
GO:0008270 GO:0005634 GO:0005667
Cluster-22178.87583 GO:0042967 GO:0047961 GO:0005739
Cluster-22178.87585 GO:0042254 GO:0007018 GO:0006412 GO:0007017
GO:0003777 GO:0005524 GO:0016887 GO:0003735 GO:0005840
GO:0005874 GO:0005622 GO:0030286
Cluster-22178.87587 GO:0009245 GO:0008915
Cluster-22178.8759 GO:0006508 GO:0004252
Cluster-22178.87593 GO:0006807 GO:0007186 GO:0007165 GO:0007264
GO:0004871 GO:0005525 GO:0003726 GO:0003924 GO:0031683
GO:0019001 GO:0003723
Cluster-22178.87594 GO:0005515
Cluster-22178.87598 GO:0015991 GO:0006355 GO:0000160 GO:0016310
GO:0055114 GO:0006928 GO:0015992 GO:0005515 GO:0003700
GO:0015078 GO:0005509 GO:0000155 GO:0004673 GO:0016491
GO:0033179 GO:0009365 GO:0005667 GO:0009279
Cluster-22178.87599 GO:0016310 GO:0000160 GO:0055114 GO:0006355
GO:0015991 GO:0015992 GO:0006928 GO:0015078 GO:0003700
GO:0005515 GO:0016491 GO:0005509 GO:0000155 GO:0004673
GO:0009365 GO:0033179 GO:0009279 GO:0005667

Cluster-22178.87607 GO:0016747 GO:0003676
Cluster-22178.8761 GO:0042254 GO:0006412 GO:0003735 GO:2001070
GO:0005840
Cluster-22178.87616 GO:0006886 GO:0006605 GO:0046872 GO:0003677
GO:0016020
Cluster-22178.87626 GO:0022900 GO:0006810 GO:0055085 GO:0005739
GO:0016021 GO:0016020 GO:0005747
Cluster-22178.87630 GO:0005515
Cluster-22178.87631 GO:0005515
Cluster-22178.87637 GO:0007165 GO:0006570 GO:0009058 GO:0008299
GO:0006694 GO:0006470 GO:0035556 GO:0016779 GO:0005515
GO:0005044 GO:0004725 GO:0050518 GO:0016020 GO:0019031
Cluster-22178.87640 GO:0007165
Cluster-22178.87643 GO:0008146
Cluster-22178.87645 GO:0006355 GO:0043565
Cluster-22178.87647 GO:0015074 GO:0006313 GO:0003677
Cluster-22178.87649 GO:0051188 GO:0009236 GO:0043752 GO:0000166
GO:0005515 GO:0005524 GO:0016887 GO:0043531
Cluster-22178.87654 GO:0005515 GO:2001070
Cluster-22178.87660 GO:0006810 GO:0019871 GO:0005576
Cluster-22178.87666 GO:0015986 GO:0009069 GO:0043087 GO:0015992
GO:0006355 GO:0006468 GO:0007178 GO:0035023 GO:0016310
GO:0004675 GO:0003677 GO:0005524 GO:0005089 GO:0004672
GO:0015078 GO:0016020 GO:0000276 GO:0005634
Cluster-22178.87668 GO:0006468 GO:0006030 GO:0055114 GO:0008061
GO:0005509 GO:0005524 GO:0004672 GO:0016491 GO:0016773
GO:0016020 GO:0005576 GO:0019031
Cluster-22178.87678 GO:0007165 GO:0006260
Cluster-22178.87682 GO:0007186 GO:0004930 GO:0016020
Cluster-22178.87688 GO:0005515
Cluster-22178.87689 GO:0005515 GO:0016020
Cluster-22178.87691 GO:0015931 GO:0015992 GO:0006810 GO:0015991
GO:0033227 GO:0015078 GO:0051033 GO:0004190 GO:0016021

GO:0033179
Cluster-22178.87692 GO:0006090 GO:0006308 GO:0006814 GO:0010508
GO:0006560 GO:0033227 GO:0015931 GO:0006914 GO:0071436
GO:0006525 GO:0008948 GO:0051033 GO:0008855 GO:0015081
GO:0016021 GO:0009318 GO:0016020
Cluster-22178.87693 GO:0005515
Cluster-22178.87694 GO:0007155
Cluster-22178.87696 GO:0019605 GO:0047989 GO:0005615
Cluster-22178.87705 GO:0035023 GO:0008608 GO:0043087 GO:0002949
GO:0006812 GO:0007264 GO:0003924 GO:0008324 GO:0005525
GO:0003723 GO:0016301 GO:0005515 GO:0016887 GO:0005524
GO:0003724 GO:0005089 GO:0098519 GO:0072686 GO:0042729
Cluster-22178.87708 GO:0051260 GO:0007186 GO:0004930 GO:0016021
GO:0016020
Cluster-22178.87712 GO:0006118 GO:0045454 GO:0006355 GO:0015035
GO:0009055 GO:0046872 GO:0005634
Cluster-22178.87714 GO:0006570 GO:0006183 GO:0006165 GO:0006771
GO:0006228 GO:0006241 GO:0006470 GO:0006144 GO:0006206
GO:0019497 GO:0005515 GO:0004725 GO:0003993 GO:0004550
GO:0046872
Cluster-22178.87718 GO:0006508 GO:0004198 GO:0005622
Cluster-22178.87727 GO:0007165
Cluster-22178.87733 GO:0005615
Cluster-22178.87736 GO:0008152 GO:0009877 GO:0009312 GO:0008168
GO:0008757
Cluster-22178.87738 GO:0035556 GO:0005515 GO:0003774 GO:0005524
GO:0016459
Cluster-22178.87742 GO:0018342 GO:0005515 GO:0004663 GO:0008270
GO:0008318 GO:0005968
Cluster-22178.87755 GO:0006355 GO:0003700 GO:0043565 GO:0005667
Cluster-22178.87757 GO:0019605 GO:0006355 GO:0043565 GO:0047989
GO:0003700 GO:0005667 GO:0005615
Cluster-22178.87768 GO:0006378 GO:0006313 GO:0004803 GO:0003677

GO:0003729 GO:0016787 GO:0005849
Cluster-22178.87769 GO:0006313 GO:0003677 GO:0004803 GO:0016787

Cluster-22178.87773 GO:0016567 GO:0006376 GO:0061630 GO:0005515
GO:0003729 GO:0004842 GO:0008270 GO:0046872 GO:0005622
GO:0005685
Cluster-22178.87775 GO:0030001 GO:0006355 GO:0007275 GO:0055085
GO:0030154 GO:0007219 GO:0046873 GO:0005515 GO:0003676
GO:0016020 GO:0045095 GO:0005622 GO:0016021
Cluster-22178.87782 GO:0008270 GO:0016740
Cluster-22178.87783 GO:0006040 GO:0005975 GO:0016740 GO:0016977
GO:0008270 GO:0005576
Cluster-22178.87785 GO:0006629 GO:0006508 GO:0006886 GO:0008152
GO:0015994 GO:0015996 GO:0001514 GO:0006448 GO:0006505
GO:0003723 GO:0016788 GO:0047746 GO:0008236 GO:0005525
GO:0016787 GO:0003746 GO:0005576 GO:0005737 GO:0005840
Cluster-22178.87787 GO:0015992 GO:0015986 GO:0015078 GO:0005515
GO:0000276
Cluster-22178.87788 GO:0051090 GO:0005515 GO:0005634
Cluster-22178.87790 GO:0055085 GO:0006810 GO:0005515 GO:0005215
GO:0016020 GO:0016021
Cluster-22178.87791 GO:0055085 GO:0006810 GO:0005515 GO:0005215
GO:0016021 GO:0016020
Cluster-22178.87792 GO:0006810 GO:0055085 GO:0005215 GO:0005515
GO:0005887 GO:0016020 GO:0016021
Cluster-22178.87794 GO:0005515 GO:0046872
Cluster-22178.87811 GO:0035556 GO:0008270
Cluster-22178.87816 GO:0006807 GO:0006123 GO:0015992 GO:0007067
GO:0005507 GO:0004129 GO:0016810 GO:0045277 GO:0042729
GO:0016020 GO:0072686
Cluster-22178.87819 GO:0043085 GO:0005975 GO:0016485 GO:0035091
GO:0031419 GO:0005515 GO:0046872 GO:0016773 GO:0016021
Cluster-22178.87823 GO:0008270

Cluster-22178.87825 GO:0003676
Cluster-22178.87829 GO:0006810 GO:0055114 GO:0016787 GO:0003677
GO:0004497 GO:0005524 GO:0016715 GO:0005507 GO:0003676
Cluster-22178.87830 GO:0005515
Cluster-22178.87832 GO:0006811 GO:0015931 GO:0033227 GO:0051033
GO:0016020 GO:0016021
Cluster-22178.87835 GO:0055114 GO:0006633 GO:0005506 GO:0016491

Cluster-22178.87836 GO:0006810 GO:0005524 GO:0042626 GO:0016021
Cluster-22178.87837 GO:0006810 GO:0005524 GO:0042626 GO:0016021
Cluster-22178.87842 GO:0016310 GO:0009069 GO:0004674
Cluster-22178.87843 GO:0046872
Cluster-22178.87845 GO:0017000 GO:0007021 GO:0015631 GO:0016787
GO:0051082 GO:0042302 GO:0005515 GO:0045298
Cluster-22178.87846 GO:0016772
Cluster-22178.87847 GO:0007021 GO:0051082 GO:0015631 GO:0042302
GO:0045298 GO:0016021
Cluster-22178.8785 GO:0001533
Cluster-22178.87851 GO:0019089 GO:0048870 GO:0005509 GO:0031514
Cluster-22178.87857 GO:0006508 GO:0004252 GO:0016020
Cluster-22178.87858 GO:0071436 GO:0006814 GO:0006560 GO:0007165
GO:0006525 GO:0050776 GO:0006090 GO:0014068 GO:0043548
GO:0008948 GO:0005515 GO:0005102 GO:0015081 GO:0016020
Cluster-22178.87863 GO:0007010 GO:0006397 GO:0003779 GO:0003723
GO:0005737 GO:0005634
Cluster-22178.87869 GO:0008283 GO:0040007 GO:0007165 GO:0008083
GO:0005515
Cluster-22178.87871 GO:0019028
Cluster-22178.87872 GO:0005515
Cluster-22178.87873 GO:0043087 GO:0007264 GO:0005085
Cluster-22178.87880 GO:0008152 GO:0009452 GO:0033539 GO:0055114
GO:0006118 GO:0007018 GO:0001510 GO:0007017 GO:0003777
GO:0008017 GO:0005524 GO:0008168 GO:0003995 GO:0005874

GO:0045298
Cluster-22178.87884 GO:0005887
Cluster-22178.87885 GO:0006144 GO:0045947 GO:0006508 GO:0045765
GO:0008234 GO:0003968 GO:0008190 GO:0031379 GO:0005737
Cluster-22178.87886 GO:0006807 GO:0008270 GO:0016814 GO:0003676

Cluster-22178.87887 GO:0003676
Cluster-22178.87894 GO:0000917 GO:0043093 GO:0006355 GO:0007049
GO:0003677 GO:0003700 GO:0043565 GO:0005634 GO:0005667
GO:0005815 GO:0005737
Cluster-22178.87897 GO:0004789 GO:0005515 GO:0019012
Cluster-22178.87899 GO:0008270
Cluster-22178.87907 GO:0000166 GO:0005509
Cluster-22178.87908 GO:0006355 GO:0003677 GO:0003723
Cluster-22178.87918 GO:0006470 GO:0006570 GO:0004725
Cluster-22178.87924 GO:0008152 GO:0006206 GO:0055114 GO:0006144
GO:0006351 GO:0006270 GO:0006118 GO:0006275 GO:0050660
GO:0016627 GO:0005524 GO:0043565 GO:0003995 GO:0003899
GO:0005730
Cluster-22178.87929 GO:0055114 GO:0020037 GO:0016705 GO:0005506

Cluster-22178.87931 GO:0055114 GO:0000372 GO:0007165 GO:0016616
GO:0005509 GO:0005515 GO:0030529 GO:0005578 GO:0005634
Cluster-22178.87933 GO:0043395 GO:0005515 GO:0005578 GO:0016020
Cluster-22178.87937 GO:0006811 GO:0005216 GO:0016020
Cluster-22178.87941 GO:0006139 GO:0005524 GO:0016818 GO:0008026
GO:0003676
Cluster-22178.87952 GO:0016192 GO:0006904 GO:0046983 GO:0008270

Cluster-22178.87963 GO:0006355 GO:0030001 GO:0055114 GO:0006308
GO:0006289 GO:0000105 GO:0055085 GO:0008168 GO:0046873
GO:0051287 GO:0008270 GO:0008855 GO:0004399 GO:0009318
GO:0016020 GO:0000439

Cluster-22178.87968 GO:0003333 GO:0009405 GO:0007165 GO:0015979
GO:0050821 GO:0006865 GO:0010468 GO:0042301 GO:0005515
GO:0015171 GO:0004872 GO:0016020 GO:0009523 GO:0016021
Cluster-22178.87981 GO:0006891 GO:0048193 GO:0009236 GO:0007212
GO:0007165 GO:0048268 GO:0048472 GO:0016301 GO:0032051
GO:0005198 GO:0019031 GO:0016021 GO:0030173 GO:0016020
GO:0019028
Cluster-22178.87984 GO:0015074
Cluster-22178.87985 GO:0006614 GO:0030071 GO:0009245 GO:0005047
GO:0030942 GO:0005515 GO:0008915 GO:0008312 GO:0005680
GO:0005786
Cluster-22178.87988 GO:0007165 GO:0008283 GO:0040007 GO:0008083

Cluster-22178.87993 GO:0046983 GO:0046872
Cluster-22178.87994 GO:0007165 GO:0006468 GO:0009069 GO:0007217
GO:0016310 GO:0004674 GO:0005524 GO:0004672 GO:0005576
Cluster-22178.87996 GO:0019370 GO:0005524 GO:0008237 GO:0008270
GO:0003723 GO:0004386 GO:0005634
Cluster-22178.88007 GO:0005515
Cluster-22178.88008 GO:0042254 GO:0006412 GO:0003735 GO:0005840
Cluster-22178.88009 GO:0006412 GO:0042254 GO:0003735 GO:0005840
GO:0005622
Cluster-22178.88017 GO:0007165 GO:0007186 GO:0007264 GO:0005525
GO:0004871 GO:0031683 GO:0046872 GO:0019001 GO:0003924
Cluster-22178.88022 GO:0006814 GO:0006886 GO:0006308 GO:0006265
GO:0055114 GO:0006120 GO:0045995 GO:0030155 GO:0006313
GO:0006605 GO:0007165 GO:0015992 GO:0030334 GO:0006744
GO:0008137 GO:0003677 GO:0003918 GO:0008855 GO:0005524
GO:0005102 GO:0009318 GO:0016020
Cluster-22178.88025 GO:0006351 GO:0007165 GO:0007155 GO:0051205
GO:0006144 GO:0006206 GO:0003899 GO:0003677 GO:0005173
GO:0016020 GO:0005730 GO:0016021
Cluster-22178.88026 GO:0030001 GO:0007010 GO:0005975 GO:0006308

GO:0007165 GO:0006810 GO:0016192 GO:0006357 GO:0055085
GO:0005515 GO:0005198 GO:0001104 GO:0003779 GO:0005215
GO:0046873 GO:0016853 GO:0008855 GO:0043395 GO:0004872
GO:0005578 GO:0019028 GO:0009318 GO:0031083 GO:0019031
GO:0016021 GO:0030133 GO:0016592 GO:0016020
Cluster-22178.88031 GO:0016579 GO:0006508 GO:0004843 GO:0016021
Cluster-22178.88034 GO:0005096 GO:0005634
Cluster-22178.88037 GO:0055114 GO:0006118 GO:0003824 GO:0009916

Cluster-22178.88039 GO:0009245 GO:0007165 GO:0008915 GO:0008270
GO:0005515 GO:0005179 GO:0016021 GO:0005737
Cluster-22178.88046 GO:0003779 GO:0005515 GO:0005622
Cluster-22178.88049 GO:0006570 GO:0006470 GO:0008138 GO:0004725

Cluster-22178.88050 GO:0043087 GO:0035023 GO:0005089
Cluster-22178.88051 GO:0006355 GO:0006281 GO:0019079 GO:0006310
GO:0008134 GO:0003723 GO:0003678 GO:0003677 GO:0009378
GO:0003724 GO:0005524 GO:0008270 GO:0016887 GO:0005667
GO:0009379 GO:0005657
Cluster-22178.88056 GO:0006650 GO:0006508 GO:0045454 GO:0009401
GO:0042981 GO:0004197 GO:0047396 GO:0016021
Cluster-22178.88062 GO:0006355 GO:0043565 GO:0003700 GO:0005667
Cluster-22178.88063 GO:0006355 GO:0003700 GO:0043565 GO:0005667
Cluster-22178.88066 GO:0005515 GO:0016020
Cluster-22178.88067 GO:0005975 GO:0006355 GO:0004649 GO:0003677

Cluster-22178.88068 GO:0006811 GO:0055085 GO:0006308 GO:0009307
GO:0006355 GO:0005992 GO:0003700 GO:0003824 GO:0009036
GO:0003677 GO:0005515 GO:0005216 GO:0016020 GO:0009359
GO:0005667
Cluster-22178.88069 GO:0045995 GO:0051258 GO:0006308 GO:0031412
GO:0018401 GO:0030168 GO:0015031 GO:0000921 GO:0051607
GO:0006144 GO:0030155 GO:0055114 GO:0006886 GO:0006606

GO:0048193 GO:0030334 GO:0019079 GO:0048870 GO:0006560
GO:0015991 GO:0006260 GO:0008053 GO:0051262 GO:0007165
GO:0006525 GO:0015992 GO:0006914 GO:0007155 GO:0045502
GO:0005102 GO:0008855 GO:0003968 GO:0003924 GO:0042803
GO:0019905 GO:0003774 GO:0004656 GO:0016702 GO:0008134
GO:0030674 GO:0015078 GO:0005198 GO:0008565 GO:0009318
GO:0033179 GO:0030286 GO:0005940 GO:0019867 GO:0005577
GO:0005783 GO:0019031 GO:0016021 GO:0005801 GO:0031379
GO:0031514 GO:0019028 GO:0005815 GO:0005882 GO:0005737
GO:0005741 GO:0016020 GO:0016459 GO:0005667 GO:0031411
Cluster-22178.88071 GO:0055114 GO:0016616 GO:0005515
Cluster-22178.88076 GO:0007018 GO:0005515 GO:0005868
Cluster-22178.88083 GO:0006355 GO:0009271
Cluster-22178.88091 GO:0006571 GO:0009073 GO:0000162 GO:0009094
GO:0055114 GO:0016491 GO:0003856
Cluster-22178.88092 GO:0003677
Cluster-22178.88093 GO:0003677
Cluster-22178.88094 GO:0016757
Cluster-22178.88098 GO:0005992 GO:0006355 GO:0009307 GO:0006308
GO:0055085 GO:0006811 GO:0005216 GO:0005515 GO:0003677
GO:0009036 GO:0003824 GO:0003700 GO:0005667 GO:0009359
GO:0016020
Cluster-22178.88099 GO:0007009
Cluster-22178.88103 GO:0000398 GO:0046740 GO:0006355 GO:0009271
GO:0030246 GO:0005634 GO:0016021
Cluster-22178.88106 GO:0007268 GO:0043495 GO:0033130
Cluster-22178.88110 GO:0006626 GO:0007165 GO:0004872 GO:0005741
Cluster-22178.88114 GO:0006629 GO:0019439 GO:0055085 GO:0016833
GO:0022857 GO:0016021
Cluster-22178.88121 GO:0005515
Cluster-22178.88129 GO:0016567 GO:0006810 GO:0005515 GO:0004842
GO:0008270 GO:0046872 GO:0030288 GO:0005680
Cluster-22178.88131 GO:0006508 GO:0008236

Cluster-22178.88133 GO:0006508 GO:0008236

Cluster-22178.88137 GO:0016021

Cluster-22178.8814 GO:0006364 GO:0006614 GO:0042254 GO:0042273 GO:0006413 GO:0006415 GO:0000184 GO:0006414 GO:0019083 GO:0003677 GO:0003735 GO:0042803 GO:0003729 GO:0022625 GO:0005840

Cluster-22178.88141 GO:0055114 GO:0006464 GO:0008113

Cluster-22178.88143 GO:0006338 GO:0006355 GO:0005524 GO:0008270 GO:0003676 GO:0016818 GO:0005634 GO:0016020

Cluster-22178.88148 GO:0007017 GO:0001510 GO:0006118 GO:0007018 GO:0033539 GO:0055114 GO:0009452 GO:0003995 GO:0008168 GO:0005524 GO:0008017 GO:0003777 GO:0045298 GO:0005874

Cluster-22178.88150 GO:0006811 GO:0007268 GO:0006810 GO:0007165 GO:0015288 GO:0004970 GO:0005234 GO:0016020

Cluster-22178.88159 GO:0042254 GO:0006412 GO:0003735 GO:0005840

Cluster-22178.88161 GO:0008152 GO:0008134 GO:0003824 GO:0005634 GO:0005667

Cluster-22178.88163 GO:0006468 GO:0006357 GO:0005515 GO:0001104 GO:0004672 GO:0005524 GO:0016592

Cluster-22178.88169 GO:0046439 GO:0006534 GO:0019530 GO:0055114 GO:0005506 GO:0017172

Cluster-22178.88170 GO:0008270 GO:0005515 GO:0003676 GO:0005634

Cluster-22178.88180 GO:0005515 GO:0008270 GO:0005622

Cluster-22178.88184 GO:0008168

Cluster-22178.8819 GO:0006351 GO:0003714 GO:0005667

Cluster-22178.88196 GO:0008152 GO:0003824

Cluster-22178.88197 GO:0046872

Cluster-22178.88207 GO:0001558 GO:0003700 GO:0097351 GO:0005667

Cluster-22178.88213 GO:0043087 GO:0035023 GO:0005515 GO:0005089

Cluster-22178.88226 GO:0007165 GO:0040007 GO:0008283 GO:0008083 GO:0051015 GO:0030674 GO:0005615

Cluster-22178.88227 GO:0007165 GO:0040007 GO:0008283 GO:0008083

GO:0005615
Cluster-22178.88228 GO:0006570 GO:0006470 GO:0008270 GO:0004721
GO:0005515 GO:0004725 GO:0008138
Cluster-22178.88229 GO:0006464 GO:0030488 GO:0006554 GO:0006480
GO:0006396 GO:0046500 GO:0006400 GO:0008033 GO:0008152
GO:0006352 GO:0006479 GO:0009451 GO:0016429 GO:0008176
GO:0004719 GO:0008168 GO:0018024 GO:0008173 GO:0031515
GO:0005856 GO:0005669
Cluster-22178.88230 GO:0006355 GO:0003700 GO:0005667 GO:0005634
Cluster-22178.88234 GO:0003676
Cluster-22178.88242 GO:0006355 GO:0006886 GO:0016192 GO:0006397
GO:0005515 GO:0005198 GO:0005737 GO:0005634 GO:0030117
Cluster-22178.88243 GO:0003677 GO:0016787 GO:0005524
Cluster-22178.88253 GO:0006525 GO:0071436 GO:0006364 GO:0006812
GO:0032801 GO:0006814 GO:0006560 GO:0019076 GO:0006090
GO:0015081 GO:0004222 GO:0005261 GO:0008948 GO:0008168
GO:0016020 GO:0033644 GO:0005737
Cluster-22178.88254 GO:0009307 GO:0006308 GO:0009036 GO:0003677
GO:0009359
Cluster-22178.88255 GO:0006367 GO:0003333 GO:0006355 GO:0005515
GO:0046983 GO:0016020
Cluster-22178.88258 GO:0005975 GO:0016773 GO:0005886 GO:0016021
Cluster-22178.88260 GO:0006446 GO:0006413 GO:0003743 GO:0003723
GO:0005840 GO:0005737
Cluster-22178.88261 GO:0006355 GO:0043565 GO:0003700 GO:0003677
GO:0005634 GO:0005667 GO:0005815
Cluster-22178.88264 GO:0006355 GO:0008270
Cluster-22178.88265 GO:0030246
Cluster-22178.88267 GO:0006099 GO:0006090 GO:0034477 GO:0008152
GO:0004518 GO:0005515 GO:0008924 GO:0016829 GO:0016021
Cluster-22178.88269 GO:0006810 GO:0032259 GO:0055114 GO:0005215
GO:0016972 GO:0008168 GO:0016020
Cluster-22178.88273 GO:0005515

Cluster-22178.88274 GO:0016757 GO:0005515
Cluster-22178.88275 GO:0005515 GO:0016757
Cluster-22178.88294 GO:0008152 GO:0016042 GO:0006810 GO:0008203 GO:0006816 GO:0042030 GO:0016298 GO:0005246 GO:0016787 GO:0016020
Cluster-22178.88296 GO:0016310 GO:0009069 GO:0006303 GO:0005524 GO:0016773 GO:0004677 GO:0003677 GO:0005515 GO:0005958 GO:0005634
Cluster-22178.88297 GO:0016310 GO:0009069 GO:0006303 GO:0005524 GO:0016773 GO:0004677 GO:0003677 GO:0005515 GO:0005958 GO:0005634
Cluster-22178.88300 GO:0009267 GO:0032099 GO:0007186 GO:0006886 GO:0016192 GO:0008343 GO:0001678 GO:0000186 GO:0005515 GO:0005615
Cluster-22178.88304 GO:0016310 GO:0009069 GO:0006468 GO:0004674 GO:0004672 GO:0005524 GO:0016773 GO:0003677 GO:0005515 GO:0016787 GO:0016020
Cluster-22178.88308 GO:0007165 GO:0015979 GO:0004888 GO:0005515 GO:0005509 GO:0016020 GO:0009523 GO:0016021
Cluster-22178.88313 GO:0030170 GO:0003676
Cluster-22178.88314 GO:0008092 GO:0005515 GO:0005737 GO:0019898
Cluster-22178.88316 GO:0016192 GO:0006886 GO:0006351 GO:0006260 GO:0005515 GO:0003677 GO:0003887 GO:0008536 GO:0042575 GO:0030117 GO:0005576
Cluster-22178.8832 GO:0019882 GO:0006886 GO:0006955 GO:0007165 GO:0042289 GO:0042613 GO:0016020
Cluster-22178.88329 GO:0006298 GO:0005515 GO:0030983 GO:0005524

Cluster-22178.88333 GO:0005515
Cluster-22178.88334 GO:0006396 GO:0008270 GO:0042470 GO:0016020 GO:0016021
Cluster-22178.88337 GO:0006508 GO:0004197
Cluster-22178.88341 GO:0006281 GO:0006144 GO:0006464 GO:0046034

GO:0004017 GO:0008270 GO:0046872 GO:0016151
Cluster-22178.88346 GO:0009117 GO:0006012 GO:0007076 GO:0009236
GO:0019058 GO:0019915 GO:0009225 GO:0009103 GO:0003978
GO:0005524 GO:0003774 GO:0003824 GO:0003676 GO:0016459
GO:0019031 GO:0005789 GO:0000796
Cluster-22178.88347 GO:0007264 GO:0005525
Cluster-22178.88348 GO:0010508 GO:0006914 GO:0007264 GO:0005525

Cluster-22178.88349 GO:0007264 GO:0019079 GO:0005525
Cluster-22178.8835 GO:0030153 GO:0006955 GO:0015643 GO:0019814
Cluster-22178.88350 GO:0045765 GO:0045947 GO:0006508 GO:0006144
GO:0008190 GO:0003968 GO:0008234 GO:0005737 GO:0031379
Cluster-22178.88351 GO:0006355 GO:0003700 GO:0043565 GO:0005667
Cluster-22178.88353 GO:0007094 GO:0006606 GO:0006355 GO:0005515
GO:0003700 GO:0042025 GO:0005667
Cluster-22178.88357 GO:0055085 GO:0006812 GO:0006885 GO:0007165
GO:0015299 GO:0005179 GO:0016021 GO:0005576
Cluster-22178.88358 GO:0009059 GO:0030176
Cluster-22178.88360 GO:0007156 GO:0005509 GO:0016020
Cluster-22178.88363 GO:0015628 GO:0040007 GO:0006118 GO:0015031
GO:0008283 GO:0007165 GO:0006810 GO:0006614 GO:0009055
GO:0005525 GO:0003676 GO:0003723 GO:0005515 GO:0008083
GO:0005524 GO:0008565 GO:0004386 GO:0016787 GO:0030145
GO:0015627
Cluster-22178.88366 GO:0006486 GO:0008378 GO:0016020
Cluster-22178.88368 GO:0006486 GO:0008378 GO:0016020
Cluster-22178.88369 GO:0006486 GO:0008378 GO:0016020
Cluster-22178.88370 GO:0035023 GO:0043087 GO:0006355 GO:0005515
GO:0005089 GO:0042393
Cluster-22178.88384 GO:0016567 GO:0008270 GO:0046872 GO:0004842
GO:0005515 GO:0061630 GO:0005680
Cluster-22178.88391 GO:0000077
Cluster-22178.88392 GO:0005515

Cluster-22178.88394 GO:0005739
Cluster-22178.88397 GO:0005515
Cluster-22178.88399 GO:0042254 GO:0006412 GO:0016773 GO:0005515
GO:0003735 GO:0005622 GO:0005840
Cluster-22178.88407 GO:0009405 GO:0006810 GO:0006886 GO:0006887
GO:0006888 GO:0003677 GO:0000166 GO:0005515 GO:0019855
GO:0008270 GO:0005524 GO:0030127 GO:0000145 GO:0005576
Cluster-22178.88408 GO:0005525
Cluster-22178.88410 GO:0006355 GO:0046034 GO:0006144 GO:0006464
GO:0005515 GO:0004017 GO:0003677 GO:0016151 GO:0046872
GO:0008270 GO:0019031
Cluster-22178.88421 GO:0007169 GO:0006468 GO:0004714 GO:0005524
GO:0016020
Cluster-22178.88422 GO:0006468 GO:0016773 GO:0004672 GO:0005524
GO:0016020
Cluster-22178.8844 GO:0007155 GO:0005540
Cluster-22178.88441 GO:0045905 GO:0006452 GO:0008544 GO:0006448
GO:0045901 GO:0003723 GO:0003746 GO:0043022 GO:0005840
Cluster-22178.88442 GO:0045901 GO:0008544 GO:0006448 GO:0045905
GO:0006452 GO:0043022 GO:0003746 GO:0003723 GO:0005840
Cluster-22178.88452 GO:0003774 GO:0016459
Cluster-22178.88453 GO:0007264 GO:0007165 GO:0006952 GO:0007186
GO:0003677 GO:0000166 GO:0019001 GO:0005524 GO:0031683
GO:0003924 GO:0005525 GO:0004871 GO:0005576
Cluster-22178.88462 GO:0005515
Cluster-22178.88471 GO:0007411 GO:0007034 GO:0030001
Cluster-22178.88476 GO:0005515
Cluster-22178.88477 GO:0005515
Cluster-22178.88478 GO:0005515
Cluster-22178.88485 GO:0007010 GO:0006355 GO:0005515 GO:0003779

Cluster-22178.88495 GO:0005515 GO:0016020
Cluster-22178.88504 GO:0042432 GO:0009058 GO:0016114 GO:0016844

GO:0004064
Cluster-22178.88505 GO:0005515
Cluster-22178.8851 GO:0055114 GO:0050660 GO:0004499 GO:0016491
GO:0050661
Cluster-22178.88511 GO:0006508 GO:0043039 GO:0055114 GO:0005515
GO:0005509 GO:0051536 GO:0016876 GO:0046872 GO:0008270
GO:0005524 GO:0008234 GO:0016020
Cluster-22178.88518 GO:0007165 GO:0007340 GO:0007342 GO:0005509
GO:0005515 GO:0005578 GO:0001669
Cluster-22178.88519 GO:0035556 GO:0007218 GO:0016747 GO:0005185
GO:0005576
Cluster-22178.88523 GO:0007009 GO:0006355 GO:0006308 GO:0005975
GO:0051258 GO:0007275 GO:0007165 GO:0030168 GO:0051082
GO:0005515 GO:0005198 GO:0030674 GO:0003677 GO:0016818
GO:0016773 GO:0008855 GO:0005524 GO:0008270 GO:0005102
GO:0005634 GO:0019028 GO:0009318 GO:0016021 GO:0019031
GO:0005741 GO:0005639 GO:0005577
Cluster-22178.88525 GO:0005515
Cluster-22178.88527 GO:0007067 GO:0051382 GO:0003677
Cluster-22178.88529 GO:0006355 GO:0046907 GO:0005515 GO:0009289
Cluster-22178.88532 GO:0005515
Cluster-22178.88534 GO:0009405 GO:0007165 GO:0090063 GO:0048193
GO:0000226 GO:0051258 GO:0030168 GO:0009252 GO:0016755
GO:0030674 GO:0005102 GO:0016020 GO:0005815 GO:0005577
GO:0000922
Cluster-22178.88536 GO:0034599 GO:0005789
Cluster-22178.88539 GO:0015031 GO:0016021
Cluster-22178.88540 GO:0043395 GO:0016020 GO:0005578
Cluster-22178.88544 GO:0009166 GO:0016787
Cluster-22178.88546 GO:0019082 GO:0016032 GO:0016773
Cluster-22178.88547 GO:0045333 GO:0005739 GO:0005634
Cluster-22178.88548 GO:0045333 GO:0005634 GO:0005739
Cluster-22178.88550 GO:0030153

Cluster-22178.88553 GO:0006813 GO:0030436 GO:0005267 GO:0042601
Cluster-22178.88555 GO:0055114 GO:0016616 GO:0016491
Cluster-22178.88562 GO:0007165 GO:0016021
Cluster-22178.88565 GO:0051056 GO:0005096 GO:0005515 GO:0003676

Cluster-22178.88566 GO:0007411 GO:0005515 GO:0044599
Cluster-22178.88575 GO:0006457 GO:0006355 GO:0015074 GO:0005198
GO:0005515 GO:0003700 GO:0005667 GO:0019028
Cluster-22178.88577 GO:0019013
Cluster-22178.88587 GO:0015694 GO:0042967 GO:0015097 GO:0005515
GO:0008080 GO:0016020 GO:0097197 GO:0001772 GO:0016021
Cluster-22178.88590 GO:0043087 GO:0035023 GO:0005089 GO:0005634
Cluster-22178.88597 GO:0007275 GO:0009966 GO:0016020
Cluster-22178.88611 GO:0055114 GO:0016491 GO:0051287 GO:0016620

Cluster-22178.88613 GO:0055114 GO:0016620 GO:0051287 GO:0016491

Cluster-22178.88624 GO:0006812 GO:0016310 GO:0009069 GO:0006468
GO:0008324 GO:0004672 GO:0005524 GO:0004674 GO:0000287
GO:0005515 GO:0016021
Cluster-22178.88625 GO:0006812 GO:0016310 GO:0009069 GO:0006468
GO:0008324 GO:0004672 GO:0005524 GO:0004674 GO:0000287
GO:0005515 GO:0016021
Cluster-22178.88626 GO:0046500 GO:0006400 GO:0006554 GO:0030488
GO:0006464 GO:0006480 GO:0006396 GO:0006479 GO:0009451
GO:0008033 GO:0008152 GO:0006352 GO:0008176 GO:0016429
GO:0018024 GO:0008173 GO:0004719 GO:0008168 GO:0031515
GO:0005856 GO:0005669
Cluster-22178.88628 GO:0015074
Cluster-22178.88631 GO:0016310 GO:0007165 GO:0000160 GO:0000155
GO:0005515 GO:0009365
Cluster-22178.88633 GO:0006396 GO:0042967 GO:0006397 GO:0008080
GO:0016747 GO:0005515 GO:0005622 GO:0005634

Cluster-22178.88635 GO:0006508 GO:0005509 GO:0004198 GO:0016791
GO:0005622
Cluster-22178.88636 GO:0006508 GO:0005509 GO:0004198 GO:0016791
GO:0005622
Cluster-22178.88638 GO:0015986 GO:0015992 GO:0015074 GO:0006364
GO:0015078 GO:0000276 GO:0032040
Cluster-22178.8864 GO:0007186 GO:0030163 GO:0004930 GO:0016021
Cluster-22178.88641 GO:0015986 GO:0015992 GO:0015074 GO:0006364
GO:0015078 GO:0000276 GO:0032040
Cluster-22178.88648 GO:0006948
Cluster-22178.88650 GO:0006274 GO:0007165 GO:0008283 GO:0040007
GO:0003677 GO:0008083 GO:0016787 GO:0005737
Cluster-22178.88660 GO:0006897 GO:0048193 GO:0008219 GO:0007015
GO:0016020
Cluster-22178.88662 GO:0008152 GO:0003824
Cluster-22178.88684 GO:0046872 GO:0045202
Cluster-22178.88690 GO:0016226 GO:0006464 GO:0008199 GO:0046872
GO:0016151
Cluster-22178.88696 GO:0006355 GO:0005515 GO:0005634 GO:0005668
Cluster-22178.88700 GO:0006355 GO:0009190 GO:0007049 GO:0008608
GO:0006171 GO:0035556 GO:0006260 GO:0006914 GO:0006144
GO:0004016 GO:0016849 GO:0003700 GO:0043565 GO:0003677
GO:0042729 GO:0005634 GO:0005667 GO:0005815 GO:0005876
GO:0005886
Cluster-22178.8872 GO:0032922 GO:0000976
Cluster-22178.88726 GO:0007165 GO:0005179 GO:0005576
Cluster-22178.88729 GO:0015991 GO:0015992 GO:0005515 GO:0015078
GO:0016021 GO:0033179
Cluster-22178.88731 GO:0006811 GO:0006810 GO:0055114 GO:0005230
GO:0016020
Cluster-22178.88736 GO:0009401 GO:0008643 GO:0008982 GO:0016020
GO:0009357
Cluster-22178.88737 GO:0006508 GO:0006813 GO:0015269 GO:0005524

GO:0008233 GO:0016021 GO:0016020
Cluster-22178.88738 GO:0006508 GO:0006813 GO:0005524 GO:0015269
GO:0008233 GO:0016021 GO:0016020
Cluster-22178.88739 GO:0006508 GO:0006813 GO:0008233 GO:0015269
GO:0005524 GO:0016020 GO:0016021
Cluster-22178.88748 GO:0008270 GO:0003723
Cluster-22178.88750 GO:0007186 GO:0006508 GO:0035556 GO:0005515
GO:0004252 GO:0004930 GO:0004672 GO:0016021
Cluster-22178.88751 GO:0006508 GO:0004252
Cluster-22178.88754 GO:0005515
Cluster-22178.88756 GO:0006355 GO:0007094 GO:0006606 GO:0005515
GO:0003700 GO:0005667 GO:0042025
Cluster-22178.88762 GO:0005515
Cluster-22178.88763 GO:0006351 GO:0006468 GO:0004672 GO:0005524
GO:0016773 GO:0016020
Cluster-22178.88767 GO:0005515
Cluster-22178.88768 GO:0004386 GO:0005524 GO:0008270 GO:0008237
GO:0003723 GO:0005634
Cluster-22178.88769 GO:0005515
Cluster-22178.88775 GO:0007165 GO:0005515 GO:0008270
Cluster-22178.88789 GO:0035091
Cluster-22178.88793 GO:0007018 GO:0007017 GO:0003777 GO:0008017
GO:0005524 GO:0005874 GO:0045298
Cluster-22178.88796 GO:0005515
Cluster-22178.88797 GO:0016192 GO:0006886 GO:0005198 GO:0005515
GO:0030132 GO:0030130
Cluster-22178.8880 GO:0006231 GO:0006206 GO:0004799
Cluster-22178.88800 GO:0008283 GO:0040007 GO:0007165 GO:0008270
GO:0008083 GO:0003676
Cluster-22178.88801 GO:0005515 GO:0046872
Cluster-22178.88803 GO:0007165 GO:0006811 GO:0007268 GO:0004970
GO:0016020
Cluster-22178.88806 GO:0006355 GO:0043565 GO:0030695

Cluster-22178.88812 GO:0015992 GO:0055085 GO:0006123 GO:0006814
GO:0006810 GO:0008270 GO:0004129 GO:0005215 GO:0003723
GO:0016021 GO:0045277 GO:0005739 GO:0016020
Cluster-22178.88818 GO:0006750 GO:0006749 GO:0004357 GO:0017109
Cluster-22178.88821 GO:0008270
Cluster-22178.88822 GO:0006813 GO:0006508 GO:0006144 GO:0004197
GO:0004252 GO:0017111 GO:0005267 GO:0003968 GO:0016020
GO:0031379
Cluster-22178.88832 GO:0016020 GO:0016021
Cluster-22178.88833 GO:0035556 GO:0006464 GO:0006281 GO:0003676
GO:0016151 GO:0046872 GO:0008270 GO:0004519 GO:0003677
GO:0005737
Cluster-22178.88834 GO:0046983
Cluster-22178.88837 GO:0006400 GO:0008616 GO:0008479
Cluster-22178.88843 GO:0006260 GO:0015992 GO:0006118 GO:0006119
GO:0006355 GO:0015991 GO:0007049 GO:0006032 GO:0046961
GO:0003954 GO:0008061 GO:0005515 GO:0003700 GO:0016765
GO:0043565 GO:0033178 GO:0005667 GO:0005739
Cluster-22178.88847 GO:0006508 GO:0005525 GO:0004190
Cluster-22178.8886 GO:0016567 GO:0009405 GO:0004842 GO:0009289
Cluster-22178.88862 GO:0015743 GO:0030001 GO:1901031 GO:0006914
GO:0006359 GO:0055085 GO:0046873 GO:0008270 GO:0046872
GO:0005634 GO:0016020
Cluster-22178.88865 GO:0046872
Cluster-22178.88869 GO:0042302
Cluster-22178.88871 GO:0016021
Cluster-22178.88889 GO:0007304 GO:0005213 GO:0008270 GO:0046872
GO:0005515 GO:0005794 GO:0042600 GO:0005576
Cluster-22178.88890 GO:0007304 GO:0005515 GO:0005213 GO:0008270
GO:0046872 GO:0005576 GO:0005794 GO:0042600
Cluster-22178.88892 GO:0006260 GO:0006355 GO:0006351 GO:0008270
GO:0003682 GO:0003677 GO:0003887 GO:0005886 GO:0000785
GO:0019031 GO:0000775 GO:0005634 GO:0042575

Cluster-22178.88893 GO:0055114 GO:0016491 GO:0016616
Cluster-22178.88899 GO:0035023 GO:0043087 GO:0005089
Cluster-22178.88903 GO:0000413 GO:0006457 GO:0003755
Cluster-22178.88908 GO:0000910 GO:0000226 GO:0003723 GO:0003676
GO:0008017 GO:0045298
Cluster-22178.88913 GO:0019012
Cluster-22178.88915 GO:0019012
Cluster-22178.88921 GO:0005515
Cluster-22178.88923 GO:0042967 GO:0008080 GO:0016747
Cluster-22178.88925 GO:0022904 GO:0001558 GO:0005520 GO:0008270
GO:0016020 GO:0016942 GO:0005576
Cluster-22178.88928 GO:0007165 GO:0032402 GO:0009307 GO:0006308
GO:0003677 GO:0005515 GO:0009036 GO:0042470 GO:0009359
Cluster-22178.88929 GO:0007165 GO:0032402 GO:0035556 GO:0006265
GO:0005515 GO:0003677 GO:0003918 GO:0005524 GO:0042470
Cluster-22178.8893 GO:0042742
Cluster-22178.88931 GO:0019700 GO:0055085 GO:0042916 GO:0042254
GO:0006412 GO:0006810 GO:0016747 GO:0005524 GO:0016829
GO:0042626 GO:0003735 GO:0005622 GO:0016021 GO:0005840
Cluster-22178.88934 GO:0035091
Cluster-22178.88935 GO:0006886 GO:0006355 GO:0007049 GO:0051382
GO:0019901 GO:0003700 GO:0043565 GO:0005783 GO:0005667
GO:0000776 GO:0016021
Cluster-22178.88937 GO:0035556 GO:0006281 GO:0005201 GO:0008270
GO:0046872 GO:0005578
Cluster-22178.88938 GO:0009772 GO:0019684 GO:0006118 GO:0045156

Cluster-22178.88950 GO:0046872
Cluster-22178.88954 GO:0007186 GO:0007165 GO:0003677 GO:0000166
GO:0004871 GO:0005525 GO:0003924 GO:0019001 GO:0005524
GO:0031683
Cluster-22178.88962 GO:0044780 GO:0006118 GO:0009055 GO:0020037

Cluster-22178.88963 GO:0000917 GO:0000390 GO:0070193 GO:0006355
GO:0015991 GO:0007049 GO:0048870 GO:0000921 GO:0007130
GO:0010508 GO:0048309 GO:0043093 GO:0006606 GO:0007128
GO:0015992 GO:0051028 GO:0006914 GO:0008134 GO:0045502
GO:0003700 GO:0008092 GO:0042803 GO:0005515 GO:0005198
GO:0015078 GO:0043565 GO:0003677 GO:0019905 GO:0003774
GO:0033179 GO:0031514 GO:0016021 GO:0000795 GO:0005882
GO:0071008 GO:0019898 GO:0030286 GO:0005940 GO:0005634
GO:0005737 GO:0016459 GO:0005667
Cluster-22178.88965 GO:0006355 GO:0043531
Cluster-22178.88966 GO:0006520 GO:0009058 GO:0030170 GO:0016829

Cluster-22178.88970 GO:0008608 GO:0006396 GO:0051252 GO:0003676
GO:0000175 GO:0003723 GO:0019867 GO:0042729 GO:0072686
Cluster-22178.88971 GO:0043087 GO:0035023 GO:0006468 GO:0006508
GO:0005524 GO:0004672 GO:0016773 GO:0008234 GO:0005089
GO:0005515 GO:0016020
Cluster-22178.88972 GO:0006508 GO:0006468 GO:0035023 GO:0043087
GO:0005515 GO:0005089 GO:0008234 GO:0016773 GO:0005524
GO:0004672 GO:0016020
Cluster-22178.88973 GO:0035023 GO:0043087 GO:0006265 GO:0003677
GO:0005515 GO:0005089 GO:0003916 GO:0005694
Cluster-22178.88984 GO:0003950
Cluster-22178.88995 GO:0006357 GO:0051262 GO:0046983 GO:0001104
GO:0016592
Cluster-22178.88996 GO:0006446 GO:0007165 GO:0006468 GO:0006413
GO:0043565 GO:0005524 GO:0004672 GO:0005515 GO:0003743
GO:0005840 GO:0016020
Cluster-22178.88997 GO:0006468 GO:0006413 GO:0007165 GO:0006446
GO:0004672 GO:0005524 GO:0043565 GO:0005515 GO:0003743
GO:0005840 GO:0016020
Cluster-22178.88999 GO:0019497 GO:0006771 GO:0016787 GO:0046872
GO:0003993

Cluster-22178.89001 GO:0006633 GO:0042967 GO:0055114 GO:0004318 GO:0005515 GO:0046872 GO:0005835
Cluster-22178.89005 GO:0003676 GO:0008270
Cluster-22178.89007 GO:0006807 GO:0005515 GO:0036361
Cluster-22178.89010 GO:0008152 GO:0006508 GO:0004181 GO:0016788 GO:0008270
Cluster-22178.89011 GO:0046836 GO:0017089 GO:0051861 GO:0005737
Cluster-22178.89024 GO:0008152 GO:0006355 GO:0006468 GO:0007165 GO:0004672 GO:0005524 GO:0003824 GO:0005179 GO:0005515 GO:0005634 GO:0005576
Cluster-22178.89027 GO:0006570 GO:0006470 GO:0008138 GO:0004725

Cluster-22178.89030 GO:0007009 GO:0051726 GO:0019904 GO:0005515 GO:0051087 GO:0005737 GO:0005634
Cluster-22178.89037 GO:0006771 GO:0019497 GO:0016783 GO:0005515 GO:0003993
Cluster-22178.89039 GO:0007165 GO:0006457 GO:0005509 GO:0005578
Cluster-22178.89040 GO:0043087 GO:0005515 GO:0016020
Cluster-22178.89043 GO:0008152 GO:0006508 GO:0016788 GO:0004181 GO:0008270 GO:0046872
Cluster-22178.89048 GO:0007155 GO:0006397 GO:0045502 GO:0008134 GO:0042803 GO:0005198 GO:0005515 GO:0005667 GO:0005882 GO:0030286 GO:0009288 GO:0000445
Cluster-22178.89052 GO:0043087 GO:0035023 GO:0046872 GO:0005089

Cluster-22178.89058 GO:0006397 GO:0006123 GO:0015992 GO:0005515 GO:0004129 GO:0003774 GO:0005524 GO:0016459 GO:0045277
Cluster-22178.89060 GO:0005515
Cluster-22178.89061 GO:0007268 GO:0007165 GO:0006811 GO:0005234 GO:0004970 GO:0016020
Cluster-22178.89062 GO:0007165 GO:0006811 GO:0007268 GO:0004970 GO:0005234 GO:0016020
Cluster-22178.89078 GO:0009395 GO:0007205 GO:0046486 GO:0004143

Cluster-22178.89091 GO:0015937 GO:0008152 GO:0015940 GO:0009058
GO:0006468 GO:0003824 GO:0004672 GO:0005524 GO:0004140
GO:0016773 GO:0005515 GO:0016301 GO:0016020
Cluster-22178.89102 GO:0005975 GO:0005509 GO:0004571 GO:0016020
Cluster-22178.89114 GO:0007517 GO:0006355 GO:0005515 GO:0008289
GO:0003677 GO:0005634
Cluster-22178.89115 GO:0005743 GO:0005747
Cluster-22178.89116 GO:0046486 GO:0009069 GO:0006468 GO:0016310
GO:0016042 GO:0004683 GO:0005516 GO:0003847 GO:0004672
GO:0005524 GO:0008247
Cluster-22178.8912 GO:0006629 GO:0007186 GO:0004930 GO:0016021
Cluster-22178.89125 GO:0005515 GO:0003723
Cluster-22178.89129 GO:0006650 GO:0045454 GO:0006508 GO:0009401
GO:0042981 GO:0004197 GO:0047396 GO:0016021
Cluster-22178.89134 GO:0055085 GO:0006865 GO:0006810 GO:0003333
GO:0015171 GO:0016020
Cluster-22178.89135 GO:0005515 GO:0016757 GO:0016021
Cluster-22178.89136 GO:0005515 GO:0016757 GO:0016021
Cluster-22178.89139 GO:0008270
Cluster-22178.89141 GO:0042254 GO:0006412 GO:0006355 GO:0006729
GO:0003677 GO:0008124 GO:0003735 GO:0005515 GO:0003682
GO:0005634 GO:0000775 GO:0005840 GO:0000785 GO:0016021
GO:0005622
Cluster-22178.89149 GO:0008152 GO:0016791 GO:0005515 GO:0043395
GO:0008270 GO:0016020 GO:0019028 GO:0005578
Cluster-22178.89151 GO:0008152 GO:0043395 GO:0008270 GO:0005515
GO:0016791 GO:0016020 GO:0019028 GO:0005578
Cluster-22178.89152 GO:0008152 GO:0043395 GO:0008270 GO:0016791
GO:0005515 GO:0005578 GO:0016020
Cluster-22178.89161 GO:0006260 GO:0006351 GO:0003887 GO:0003677
GO:0042575
Cluster-22178.89164 GO:0005201 GO:0005581 GO:0005576 GO:0005578

Cluster-22178.89171 GO:0007165 GO:0007186 GO:0007264 GO:0005525 GO:0004871 GO:0031683 GO:0019001 GO:0003924
Cluster-22178.89173 GO:0006397 GO:0006355 GO:0006470 GO:0042981 GO:0003677 GO:0004721 GO:0005507 GO:0003700 GO:0005634 GO:0005667
Cluster-22178.89174 GO:0006626 GO:0007165 GO:0004872 GO:0005741
Cluster-22178.89178 GO:0006464 GO:0042262 GO:0006355 GO:0015986 GO:0007165 GO:0046933 GO:0008270 GO:0005515 GO:0003677 GO:0001664 GO:0045259
Cluster-22178.89187 GO:0016567 GO:0006412 GO:0042254 GO:0005515 GO:0003735 GO:0004842 GO:0005840 GO:0005622
Cluster-22178.89190 GO:0006308 GO:0016310 GO:0006355 GO:0009307 GO:0009069 GO:0006468 GO:0007165 GO:0005096 GO:0009036 GO:0003700 GO:0004674 GO:0003677 GO:0005737 GO:0005667 GO:0009359
Cluster-22178.89198 GO:0004518 GO:0005515 GO:0019867
Cluster-22178.89205 GO:0019033
Cluster-22178.89207 GO:0006118 GO:0006351 GO:0006270 GO:0006275 GO:0008152 GO:0055114 GO:0006144 GO:0006206 GO:0003899 GO:0003995 GO:0050660 GO:0043565 GO:0016627 GO:0005524 GO:0005730
Cluster-22178.89209 GO:0006355 GO:0008270 GO:0005515 GO:0003677 GO:0005634
Cluster-22178.89210 GO:0015886 GO:0017004 GO:0016021
Cluster-22178.89215 GO:0005515
Cluster-22178.89219 GO:0042254 GO:0006412 GO:0051016 GO:0005515 GO:0003735 GO:0005509 GO:0005840 GO:0030479
Cluster-22178.89220 GO:0007275 GO:0007219 GO:0007154 GO:0005515 GO:0005509 GO:0016020 GO:0016021
Cluster-22178.89231 GO:0007165 GO:0007411 GO:0005179 GO:0016021 GO:0005576
Cluster-22178.89234 GO:0006520 GO:0005515
Cluster-22178.89235 GO:0003774 GO:0016459

Cluster-22178.89237 GO:0006357 GO:0005515 GO:0001104 GO:0016592
Cluster-22178.89240 GO:0006355 GO:0007165 GO:0042974 GO:0005667
Cluster-22178.89246 GO:0005515
Cluster-22178.89259 GO:0016192 GO:0006886 GO:0005201 GO:0005581
GO:0005578
Cluster-22178.89264 GO:0051424 GO:0016021
Cluster-22178.89275 GO:0016817 GO:0005198 GO:0019028
Cluster-22178.89276 GO:0055085 GO:0030001 GO:0005198 GO:0005515
GO:0046873 GO:0016020 GO:0019028
Cluster-22178.89278 GO:0016567 GO:0006810 GO:0005515 GO:0046872
GO:0008270 GO:0004842 GO:0030288 GO:0005680
Cluster-22178.89294 GO:0005515
Cluster-22178.89302 GO:0007165 GO:0005179 GO:0005576 GO:0016021
Cluster-22178.89303 GO:0035023 GO:0043087 GO:0006265 GO:0003677
GO:0005515 GO:0005089 GO:0003916 GO:0005694
Cluster-22178.89337 GO:0046500 GO:0006479 GO:0006464 GO:0008152
GO:0006465 GO:0004719 GO:0008168 GO:0008233 GO:0005787
GO:0016021
Cluster-22178.89339 GO:0030168 GO:0051258 GO:0007165 GO:0003723
GO:0030674 GO:0005102 GO:0005577
Cluster-22178.89344 GO:0005737
Cluster-22178.89353 GO:0005515
Cluster-22178.89356 GO:0055114 GO:0046872 GO:0016491
Cluster-22178.89362 GO:0005515 GO:0008270
Cluster-22178.89363 GO:0008270 GO:0005515
Cluster-22178.89365 GO:0051028 GO:0006468 GO:0004672 GO:0005524
GO:0005515 GO:0005634
Cluster-22178.89369 GO:0006812 GO:0005515 GO:0008324 GO:0000228
Cluster-22178.89389 GO:0006886 GO:0008152 GO:0003824 GO:0016836
GO:0016021 GO:0005783
Cluster-22178.89390 GO:0006118 GO:0006119 GO:0006813 GO:0015992
GO:0008121 GO:0005242 GO:0009055 GO:0008076 GO:0016021
Cluster-22178.89403 GO:0006470 GO:0006570 GO:0004725 GO:0008138

GO:0004721 GO:0005515 GO:0008270
Cluster-22178.89410 GO:0007165 GO:0007172 GO:0030168 GO:0006468
GO:0005975 GO:0051258 GO:0030674 GO:0005102 GO:0004713
GO:0005524 GO:0003824 GO:0004871 GO:0004672 GO:0005925
GO:0005577 GO:0016021
Cluster-22178.89426 GO:0006308 GO:0006357 GO:0070206 GO:0006891
GO:0007155 GO:0019069 GO:0045502 GO:0001104 GO:0042803
GO:0008134 GO:0008855 GO:0003950 GO:0016021 GO:0019028
GO:0009318 GO:0030286 GO:0005886 GO:0005667 GO:0017119
GO:0016592
Cluster-22178.89429 GO:0030001 GO:0046872 GO:0003950
Cluster-22178.8945 GO:0007605 GO:0005515
Cluster-22178.89452 GO:0015979 GO:0050821 GO:0042301 GO:0016020
GO:0009523
Cluster-22178.89463 GO:0006468 GO:0004672 GO:0005524
Cluster-22178.89465 GO:0006811 GO:0055085 GO:0046872 GO:0005216
GO:0016020 GO:0045202
Cluster-22178.89477 GO:0006355 GO:0030036 GO:0019058 GO:0003779
GO:0003677 GO:0030054
Cluster-22178.89478 GO:0006355 GO:0019058 GO:0030036 GO:0003779
GO:0003677 GO:0030054
Cluster-22178.89485 GO:0006355 GO:0005634 GO:0043189 GO:0016021
Cluster-22178.89487 GO:0007264 GO:0003924 GO:0016887 GO:0005524
GO:0005525 GO:0016020
Cluster-22178.89490 GO:0005515
Cluster-22178.89496 GO:0006355 GO:0000105 GO:0055114 GO:0006537
GO:0009607 GO:0015930 GO:0016638 GO:0003824 GO:0016491
GO:0016627 GO:0005737
Cluster-22178.89497 GO:0007165 GO:0046486 GO:0035556 GO:0007205
GO:0009395 GO:0005509 GO:0016301 GO:0004143 GO:0005578
Cluster-22178.89502 GO:0051607 GO:0016032 GO:0007049 GO:0003677

Cluster-22178.89504 GO:0006525 GO:0006560 GO:0006814 GO:0071436

GO:0019076 GO:0006090 GO:0006812 GO:0032801 GO:0005261
GO:0008948 GO:0015081 GO:0004222 GO:0016020 GO:0033644
Cluster-22178.89506 GO:0019239 GO:0005615
Cluster-22178.89507 GO:0019239
Cluster-22178.89509 GO:0019239 GO:0005615
Cluster-22178.89518 GO:0005198 GO:0042803 GO:0008134 GO:0045502
GO:0016021 GO:0030286 GO:0005882 GO:0005667
Cluster-22178.89526 GO:0006281 GO:0043240 GO:0016020
Cluster-22178.89530 GO:0007165
Cluster-22178.89534 GO:0006886 GO:0005801 GO:0016020
Cluster-22178.89539 GO:0006355 GO:0010508 GO:0003700 GO:0005667
Cluster-22178.89540 GO:0006355 GO:0010508 GO:0003700 GO:0005667
Cluster-22178.89547 GO:0042254 GO:0006412 GO:0016788 GO:0005515
GO:0003735 GO:0005840 GO:0005622
Cluster-22178.89548 GO:0042254 GO:0006412 GO:0016788 GO:0005515
GO:0003735 GO:0005622 GO:0005840
Cluster-22178.89549 GO:0042254 GO:0006412 GO:0016788 GO:0005515
GO:0003735 GO:0005840 GO:0005622
Cluster-22178.89552 GO:0006355 GO:0005634
Cluster-22178.89555 GO:0016032 GO:0006508 GO:0042967 GO:0006555
GO:0019281 GO:0005975 GO:0008233 GO:0004222 GO:0008899
GO:0008270 GO:0004649 GO:0031012 GO:0005737
Cluster-22178.89560 GO:0006807 GO:0007067 GO:0016810 GO:0042729
GO:0072686
Cluster-22178.89564 GO:0007411
Cluster-22178.89566 GO:0055114 GO:0016491 GO:0016021
Cluster-22178.89580 GO:0006355 GO:0003682 GO:0003677 GO:0005634
GO:0000775 GO:0000785
Cluster-22178.89582 GO:0006355 GO:0003682 GO:0003677 GO:0005634
GO:0000775 GO:0000785
Cluster-22178.89588 GO:0003333 GO:0006355 GO:0005515 GO:0046983
GO:0016020
Cluster-22178.89589 GO:0015684 GO:0007264 GO:0005525 GO:0003924

GO:0016887 GO:0015093 GO:0005524 GO:0016021
Cluster-22178.89592 GO:0055085 GO:0006810 GO:0006508 GO:0004222
GO:0008270 GO:0016021 GO:0031012 GO:0016020
Cluster-22178.89593 GO:0006810 GO:0055085 GO:0016020 GO:0016021
Cluster-22178.89596 GO:0015693 GO:0015095 GO:0016021 GO:0016020
Cluster-22178.89598 GO:0006388 GO:0003950 GO:0016772
Cluster-22178.89611 GO:0006858 GO:0005524 GO:0016887
Cluster-22178.89616 GO:0006470 GO:0006570 GO:0004725 GO:0008138

Cluster-22178.89620 GO:0040007 GO:0008283 GO:0007165 GO:0009405
GO:0030674 GO:0008083 GO:0051015
Cluster-22178.89628 GO:0045454 GO:0006355 GO:0006118 GO:0046872
GO:0015035 GO:0009055 GO:0005634
Cluster-22178.89631 GO:0019058 GO:0051539 GO:0019031
Cluster-22178.89632 GO:0005515
Cluster-22178.89638 GO:0035556
Cluster-22178.89646 GO:0005515
Cluster-22178.89647 GO:0030154 GO:0016021
Cluster-22178.89650 GO:0007165 GO:0004518
Cluster-22178.89661 GO:0055114 GO:0006120 GO:0016021
Cluster-22178.89664 GO:2001256 GO:0046486 GO:0009395 GO:0035556
GO:0007205 GO:0005515 GO:0016301 GO:0004143 GO:0030176
Cluster-22178.89665 GO:0046486 GO:2001256 GO:0035556 GO:0007205
GO:0009395 GO:0016301 GO:0005515 GO:0004143 GO:0030176
Cluster-22178.89667 GO:0046486 GO:2001256 GO:0007205 GO:0035556
GO:0009395 GO:0016301 GO:0005515 GO:0004143 GO:0030176
Cluster-22178.89681 GO:0006355 GO:0035556 GO:0006352 GO:0003700
GO:0005667 GO:0005669 GO:0016020
Cluster-22178.89683 GO:0006355 GO:0043401 GO:0051191 GO:0008270
GO:0043565 GO:0003700 GO:0005667 GO:0005634
Cluster-22178.89688 GO:0007186 GO:0004930 GO:0016020 GO:0019031
Cluster-22178.89701 GO:0006351 GO:0006144 GO:0006206 GO:0003899
GO:0046983 GO:0005730

Cluster-22178.89707 GO:0016310 GO:0006144 GO:0006206 GO:0006351
GO:0009069 GO:0006468 GO:0004674 GO:0016773 GO:0005524
GO:0004672 GO:0003899 GO:0003677 GO:0016020 GO:0005730
Cluster-22178.89708 GO:0005515
Cluster-22178.89710 GO:0035556
Cluster-22178.89716 GO:0006563 GO:0046653 GO:0006566 GO:0006544
GO:0008115 GO:0005515 GO:0016020
Cluster-22178.89717 GO:0006826 GO:0006508 GO:0042742 GO:0006468
GO:0009405 GO:0006827 GO:0016773 GO:0005524 GO:0004672
GO:0004222 GO:0005381 GO:0033573 GO:0005576 GO:0016020
Cluster-22178.89718 GO:0006508 GO:0042742 GO:0006468 GO:0009405
GO:0006827 GO:0006826 GO:0005381 GO:0016773 GO:0004672
GO:0005524 GO:0004222 GO:0005576 GO:0016020 GO:0033573
Cluster-22178.89724 GO:0003676 GO:0016020
Cluster-22178.89727 GO:0006355 GO:0005622
Cluster-22178.89733 GO:0007275 GO:0003676 GO:0016020
Cluster-22178.89735 GO:0042742 GO:0006505 GO:0006629 GO:0009058
GO:0006886 GO:0016788 GO:0005576
Cluster-22178.89739 GO:0030639 GO:0051694 GO:0005523
Cluster-22178.8974 GO:0007165 GO:0006955 GO:0006886 GO:0019882
GO:0042289 GO:0016020 GO:0042613
Cluster-22178.89747 GO:0007155 GO:0005515 GO:0005198 GO:0031012
Cluster-22178.89748 GO:0007165 GO:0007155 GO:0005198 GO:0005515
GO:0005179 GO:0031012 GO:0005576
Cluster-22178.89750 GO:0005515 GO:0005737
Cluster-22178.89756 GO:0005525
Cluster-22178.89766 GO:0000398 GO:0006260 GO:0006270 GO:0006351
GO:0008270 GO:0003887 GO:0003677 GO:0005681 GO:0019031
GO:0019028 GO:0005634 GO:0042575
Cluster-22178.89769 GO:0006606 GO:0006260 GO:0008092 GO:0005737
GO:0019898 GO:0016021
Cluster-22178.89784 GO:0000398 GO:0006355 GO:0006468 GO:0016567
GO:0007165 GO:0003700 GO:0004672 GO:0005515 GO:0004842

GO:0005524 GO:0008270 GO:0003677 GO:0003682 GO:0000785
GO:0005681 GO:0019031 GO:0000775 GO:0005737 GO:0005634
GO:0019028 GO:0005680 GO:0016020 GO:0005667
Cluster-22178.89794 GO:0006284 GO:0016787 GO:0003677 GO:0003905
GO:0016021
Cluster-22178.89798 GO:0035556 GO:0008270
Cluster-22178.89801 GO:0046034 GO:0006144 GO:0006464 GO:0046836
GO:0006281 GO:0017089 GO:0016151 GO:0008270 GO:0046872
GO:0004017 GO:0003677 GO:0051861 GO:0005737
Cluster-22178.89816 GO:0006313 GO:0015074 GO:0003677
Cluster-22178.89824 GO:0008152 GO:0016491
Cluster-22178.89825 GO:0007155 GO:0008152 GO:0016491 GO:0016020
Cluster-22178.89829 GO:0035064
Cluster-22178.89838 GO:0005515
Cluster-22178.89844 GO:0003824
Cluster-22178.89848 GO:0007165 GO:0016567 GO:0000105 GO:0030168
GO:0051258 GO:0055114 GO:0051287 GO:0030674 GO:0004842
GO:0004399 GO:0005102 GO:0008270 GO:0005515 GO:0005198
GO:0046872 GO:0005680 GO:0005577 GO:0005622 GO:0019028
GO:0019031
Cluster-22178.89850 GO:0007186 GO:0004930 GO:0016020 GO:0019031
Cluster-22178.89863 GO:0015743 GO:0030001 GO:1901031 GO:0006914
GO:0006359 GO:0055085 GO:0046873 GO:0008270 GO:0046872
GO:0005634 GO:0016020
Cluster-22178.89866 GO:0009405 GO:0007186 GO:0004930 GO:0005515
GO:0005576 GO:0016021
Cluster-22178.89867 GO:0005515
Cluster-22178.89877 GO:0051301 GO:0032955
Cluster-22178.89878 GO:0007186 GO:0004930 GO:0016021
Cluster-22178.89883 GO:0055114 GO:0016491
Cluster-22178.89894 GO:0009401 GO:0008289 GO:0016021
Cluster-22178.89901 GO:0006352 GO:0006355 GO:0003700 GO:0005515
GO:0003677 GO:0016987 GO:0005667

Cluster-22178.89904 GO:0015986 GO:0015992 GO:0015078 GO:0005515 GO:0000276

Cluster-22178.89907 GO:0016020

Cluster-22178.89915 GO:0016021

Cluster-22178.89918 GO:0016020

Cluster-22178.89920 GO:0006355 GO:0003700 GO:0005198 GO:0019031 GO:0019028 GO:0005667

Cluster-22178.89922 GO:0035556 GO:0019700 GO:0042916 GO:0005524 GO:0016829 GO:0046872

Cluster-22178.89925 GO:0007029 GO:0005789

Cluster-22178.89933 GO:0006265 GO:0006302 GO:0003917 GO:0003677 GO:0005634 GO:0005694

Cluster-22178.89941 GO:0008152 GO:0008171 GO:0008168

Cluster-22178.89944 GO:0006432 GO:0005524 GO:0000287 GO:0003723

Cluster-22178.89946 GO:0030695 GO:0042802 GO:0005515

Cluster-22178.89948 GO:0006508 GO:0006771 GO:0019497 GO:0016740 GO:0005515 GO:0004197 GO:0008242 GO:0046872 GO:0003993

Cluster-22178.89952 GO:0042578

Cluster-22178.89957 GO:0046777 GO:0046330 GO:0046621 GO:0008285 GO:0006921 GO:0008631 GO:0035329 GO:0048679 GO:0001841 GO:0007165 GO:0060215 GO:0060706 GO:0003157 GO:0030336 GO:0016310 GO:0051897 GO:0007417 GO:0071902 GO:0090090 GO:0006468 GO:0009069 GO:0004672 GO:0043539 GO:0004674 GO:0004190 GO:0000287 GO:0005524 GO:0046983 GO:0016021 GO:0005829 GO:0005654 GO:0016020

Cluster-22178.89958 GO:0005525

Cluster-22178.89962 GO:0006298 GO:0007165 GO:0006464 GO:0019901 GO:0030983 GO:0005524

Cluster-22178.89967 GO:0005515

Cluster-22178.89974 GO:0006508 GO:0004252

Cluster-22178.89977 GO:0003723 GO:0003676

Cluster-22178.89979 GO:0006508 GO:0008234

Cluster-22178.89989 GO:0043087 GO:0015992 GO:0015986 GO:0009069
GO:0035023 GO:0016310 GO:0007178 GO:0006355 GO:0006468
GO:0005089 GO:0005524 GO:0003677 GO:0004675 GO:0015078
GO:0004672 GO:0000276 GO:0016020 GO:0005634
Cluster-22178.89995 GO:0005515
Cluster-22178.9000 GO:0006260 GO:0016032 GO:0003887 GO:0003677
GO:0042575
Cluster-22178.90004 GO:0010951 GO:0006351 GO:0007283 GO:0006144
GO:0006206 GO:0003899 GO:0003677 GO:0003824 GO:0004866
GO:0005634 GO:0005730 GO:0000786
Cluster-22178.90010 GO:0055114 GO:0005506 GO:0016705 GO:0020037

Cluster-22178.90016 GO:0009405 GO:0032955 GO:0051301 GO:0005515

Cluster-22178.90025 GO:0016192 GO:0006886 GO:0018193 GO:0005198
GO:0005515 GO:0016020 GO:0030126
Cluster-22178.90032 GO:0007186 GO:0019069 GO:0004930 GO:0016021
Cluster-22178.90036 GO:0006508 GO:0005515 GO:0004252
Cluster-22178.90041 GO:0006468 GO:0009069 GO:0016310 GO:0005524
GO:0004674
Cluster-22178.90042 GO:0007165 GO:0006468 GO:0005515 GO:0004672
GO:0005524
Cluster-22178.90044 GO:0005515
Cluster-22178.90049 GO:0046872 GO:0008270 GO:0005515
Cluster-22178.90052 GO:0006355 GO:0003700 GO:0005515 GO:0005667
GO:0042025
Cluster-22178.90057 GO:0019062 GO:0006869 GO:0009405 GO:0007155
GO:0005319 GO:0016021
Cluster-22178.90070 GO:0007186 GO:0004930 GO:0016020
Cluster-22178.90075 GO:0006470 GO:0030145 GO:0004721 GO:0003824
GO:0000287
Cluster-22178.90076 GO:0006470 GO:0007010 GO:0003779 GO:0000287
GO:0030145 GO:0003824 GO:0004721

Cluster-22178.90081 GO:0006508 GO:0004252
Cluster-22178.90083 GO:0055114 GO:0006144 GO:0006206 GO:0008152 GO:0006275 GO:0006118 GO:0006351 GO:0006270 GO:0043565 GO:0016627 GO:0005524 GO:0050660 GO:0003899 GO:0003995 GO:0005730
Cluster-22178.90084 GO:0016772
Cluster-22178.90091 GO:0046872
Cluster-22178.90097 GO:0006812 GO:0015672 GO:0005261 GO:0016020
Cluster-22178.90103 GO:0006508 GO:0005515 GO:0004252 GO:0005509 GO:0005576
Cluster-22178.90105 GO:0007165 GO:0005509 GO:0005578
Cluster-22178.90109 GO:0007264 GO:0006629 GO:0015979 GO:0016791 GO:0005524 GO:0003924 GO:0016887 GO:0005525 GO:0016020 GO:0009539 GO:0009523
Cluster-22178.90111 GO:0006811 GO:0055085 GO:0006810 GO:0042254 GO:0006412 GO:0005215 GO:0003735 GO:0005840 GO:0016020
Cluster-22178.90118 GO:0016485 GO:0043085 GO:0005975 GO:0016773 GO:0046872 GO:0035091 GO:0031419 GO:0005515 GO:0016021
Cluster-22178.90131 GO:0042254 GO:0006412 GO:0003735 GO:0005840 GO:0015934
Cluster-22178.90137 GO:0055085 GO:0002949 GO:0006810 GO:0003924 GO:0016887 GO:0005524 GO:0042626 GO:0005525 GO:0016021
Cluster-22178.90138 GO:0006810 GO:0002949 GO:0055085 GO:0042626 GO:0005525 GO:0016887 GO:0003924 GO:0005524 GO:0016021
Cluster-22178.90139 GO:0006810 GO:0055085 GO:0005524 GO:0016887 GO:0042626 GO:0016021
Cluster-22178.90149 GO:0005515
Cluster-22178.90150 GO:0000105 GO:0005515
Cluster-22178.90158 GO:0006355 GO:0006270 GO:0043565 GO:0003700 GO:0005667
Cluster-22178.90162 GO:0007029 GO:0035092 GO:0006355 GO:0003677 GO:0046872 GO:0005789 GO:0000228
Cluster-22178.90165 GO:0055085 GO:0016021

Cluster-22178.90169 GO:0005198
Cluster-22178.90174 GO:0006355 GO:0030639 GO:0003700 GO:0005667
GO:0005634
Cluster-22178.90176 GO:0005515
Cluster-22178.9018 GO:0032259 GO:0006914 GO:0003924 GO:0008168
GO:0005737
Cluster-22178.90182 GO:0016787
Cluster-22178.90213 GO:0006260 GO:0010508 GO:0008134 GO:0042803
GO:0045502 GO:0030286 GO:0005667
Cluster-22178.90214 GO:0006260 GO:0045502 GO:0008134 GO:0042803
GO:0030286 GO:0005667
Cluster-22178.90216 GO:0006810 GO:0006886 GO:0015721 GO:0015031
GO:0046982 GO:0005215 GO:0008565 GO:0016020 GO:0016021
GO:0005886 GO:0005737
Cluster-22178.90224 GO:0006486 GO:0006013 GO:0006000 GO:0003873
GO:0005515 GO:0004576 GO:0005524 GO:0008250 GO:0016020
Cluster-22178.90239 GO:0006468 GO:0005524 GO:0004672
Cluster-22178.90241 GO:0005515
Cluster-22178.90261 GO:0055085 GO:0006508 GO:0006810 GO:0008270
GO:0004222 GO:0031012 GO:0016021 GO:0016020
Cluster-22178.90263 GO:0042254 GO:0005622
Cluster-22178.90276 GO:0046872
Cluster-22178.90279 GO:0007275 GO:0016021
Cluster-22178.90280 GO:0016310 GO:0009405 GO:0007186 GO:0007165
GO:0015684 GO:0007264 GO:0009069 GO:0004871 GO:0005525
GO:0003924 GO:0019001 GO:0031683 GO:0000166 GO:0045735
GO:0004386 GO:0004674 GO:0015093 GO:0005524 GO:0003677
GO:0016021
Cluster-22178.90281 GO:0007169 GO:0006777 GO:0006468 GO:0006508
GO:0004222 GO:0005524 GO:0008270 GO:0004672 GO:0016773
GO:0004714 GO:0031012 GO:0016020
Cluster-22178.90283 GO:0016567 GO:0006508 GO:0008152 GO:0061630
GO:0005515 GO:0016788 GO:0004842 GO:0004181 GO:0008270

GO:0046872
Cluster-22178.90285 GO:0055114 GO:0004174 GO:0046332
Cluster-22178.90286 GO:0055114 GO:0004174 GO:0046332
Cluster-22178.90288 GO:0007186 GO:0004930 GO:0016021
Cluster-22178.90293 GO:0005515 GO:0046872 GO:0031225
Cluster-22178.90300 GO:0007156 GO:0005509 GO:0016020
Cluster-22178.90303 GO:0006281 GO:0019079 GO:0016567 GO:0006260
GO:0006811 GO:0030337 GO:0005515 GO:0008270 GO:0046872
GO:0004842 GO:0003911 GO:0042575 GO:0016020 GO:0005680
Cluster-22178.90311 GO:0008152 GO:0006522 GO:0006529 GO:0008033
GO:0006531 GO:0003824 GO:0004066 GO:0004810
Cluster-22178.90316 GO:0055085 GO:0006811 GO:0005216 GO:0016020
Cluster-22178.90319 GO:0007605 GO:0046983
Cluster-22178.90321 GO:0006493 GO:0006355 GO:0000030 GO:0008270
GO:0016020 GO:0000136 GO:0005634
Cluster-22178.90322 GO:0044780 GO:0006118 GO:0016491 GO:0009055
GO:0020037
Cluster-22178.90329 GO:0007165 GO:0003677 GO:0005509 GO:0005515
GO:0005578
Cluster-22178.90361 GO:0007155 GO:0005515 GO:0016021
Cluster-22178.90364 GO:0006355 GO:0003700 GO:0005515 GO:0043565
GO:0008270 GO:0005667
Cluster-22178.90366 GO:0005515 GO:0003676
Cluster-22178.90375 GO:0006955
Cluster-22178.90386 GO:0050821 GO:0015979 GO:0042301 GO:0009523
GO:0016020
Cluster-22178.90388 GO:0006468 GO:0042254 GO:0006412 GO:0009405
GO:0003735 GO:0045735 GO:0016773 GO:0005524 GO:0004672
GO:0016020 GO:0015934 GO:0005840
Cluster-22178.90391 GO:0010508
Cluster-22178.90399 GO:0007165 GO:0008283 GO:0040007 GO:0008083
GO:0016020
Cluster-22178.90402 GO:0006468 GO:0015979 GO:0004672 GO:0005524

GO:0016798 GO:0009522 GO:0016020
Cluster-22178.90404 GO:0005515
Cluster-22178.90410 GO:0007155 GO:0007275 GO:0005198 GO:0031012
GO:0005578
Cluster-22178.90418 GO:0007165 GO:0048268 GO:0007212 GO:0032051
GO:0016021
Cluster-22178.90420 GO:0007165 GO:0016021
Cluster-22178.90422 GO:0007186 GO:0004930 GO:0016021
Cluster-22178.90426 GO:0015846 GO:1902604 GO:0006811 GO:0015814
GO:0003723 GO:0015558 GO:0005515
Cluster-22178.90438 GO:0016310 GO:0009069 GO:0004674 GO:0004185
GO:0005515 GO:0005773
Cluster-22178.90440 GO:0006123 GO:0015992 GO:0004129 GO:0045277
Cluster-22178.90443 GO:0005515
Cluster-22178.90445 GO:0006355 GO:0003700 GO:0042025 GO:0005667
Cluster-22178.90447 GO:0007165 GO:0005044 GO:0016020
Cluster-22178.90450 GO:0006955 GO:0007165 GO:0007155 GO:0006508
GO:0005198 GO:0005164 GO:0004222 GO:0016020
Cluster-22178.90452 GO:0042254 GO:0006412 GO:0006464 GO:0003735
GO:0005515 GO:0016151 GO:0005840 GO:0005622
Cluster-22178.90458 GO:0007411
Cluster-22178.90461 GO:0009396 GO:0006554 GO:0006633 GO:0009058
GO:0006631 GO:0018874 GO:0055114 GO:0006574 GO:0008152
GO:0006568 GO:0006552 GO:0046487 GO:0006550 GO:0016491
GO:0050662 GO:0004488 GO:0003824 GO:0003857
Cluster-22178.90466 GO:0015074 GO:0008270
Cluster-22178.90492 GO:0006807 GO:0006665 GO:0005975 GO:0006683
GO:0006687 GO:0006629 GO:0004336 GO:0004348
Cluster-22178.90493 GO:0006687 GO:0005975 GO:0006683 GO:0006807
GO:0004336
Cluster-22178.90506 GO:0007165 GO:0005515
Cluster-22178.90512 GO:0005515
Cluster-22178.90513 GO:0046656 GO:0015948 GO:0009082 GO:0042254

GO:0006412 GO:0030269 GO:0003723 GO:0003735 GO:0005515
GO:0005840 GO:0016021
Cluster-22178.90515 GO:0006777 GO:0051539 GO:0019008
Cluster-22178.90519 GO:0055114 GO:0031110 GO:0006784 GO:0005096
GO:0016627 GO:0005515 GO:0005737 GO:0016021
Cluster-22178.90524 GO:0006810 GO:0005524 GO:0005515 GO:0042393
GO:0005886
Cluster-22178.90526 GO:0046983 GO:0003677
Cluster-22178.90531 GO:0006468 GO:0005524 GO:0004672
Cluster-22178.90532 GO:0016310 GO:0042157 GO:0009069 GO:0006869
GO:0008017 GO:0008289 GO:0008384 GO:0045298 GO:0008385
GO:0005576
Cluster-22178.90536 GO:0030976 GO:0070403 GO:0000287 GO:0008168

Cluster-22178.90537 GO:0007165 GO:0007186 GO:0005515 GO:0004871
GO:0005834
Cluster-22178.90538 GO:0006468 GO:0016773 GO:0005524 GO:0004672
GO:0016020
Cluster-22178.90541 GO:0007165 GO:0005515 GO:0005509 GO:0005578
Cluster-22178.90543 GO:0035556 GO:0007205 GO:0009395 GO:0007165
GO:0046486 GO:0004143 GO:0005509 GO:0016301 GO:0005578
Cluster-22178.90554 GO:0007165 GO:0005975 GO:0005509 GO:0008158
GO:0004571 GO:0016020
Cluster-22178.90556 GO:0040007 GO:0008283 GO:0007165 GO:0008083

Cluster-22178.90559 GO:0006897 GO:0001527 GO:0016020
Cluster-22178.90566 GO:0055114 GO:0015994 GO:0006788 GO:0004392

Cluster-22178.90567 GO:0007155 GO:0016755 GO:0016020
Cluster-22178.90571 GO:0009308 GO:0055114 GO:0008131 GO:0005507
GO:0048038 GO:0008270
Cluster-22178.90579 GO:0051382 GO:0006281 GO:0006813 GO:0071805
GO:0015079 GO:0003677 GO:0005887

Cluster-22178.90586 GO:0055114 GO:0006633 GO:0042967 GO:0004318
GO:0005835
Cluster-22178.90592 GO:0046656 GO:0042254 GO:0006412 GO:0015948
GO:0009082 GO:0003723 GO:0003735 GO:0030269 GO:0016021
GO:0005840
Cluster-22178.90597 GO:0016485 GO:0043085 GO:0005975 GO:0006355
GO:0016773 GO:0008270 GO:0046872 GO:0035091 GO:0005515
GO:0031419 GO:0016021 GO:0005634
Cluster-22178.90611 GO:0015977 GO:0006355 GO:0019643 GO:0006094
GO:0006099 GO:0008964
Cluster-22178.90616 GO:0006633 GO:0006779 GO:0016117 GO:0006554
GO:0018874 GO:0019354 GO:0006631 GO:0015940 GO:0006574
GO:0008033 GO:0055114 GO:0006550 GO:0006552 GO:0006813
GO:0006568 GO:0050660 GO:0050151 GO:0016491 GO:0016616
GO:0071949 GO:0051287 GO:0008677 GO:0016705 GO:0043115
GO:0003857
Cluster-22178.90620 GO:0006814 GO:0016829
Cluster-22178.90625 GO:0004222 GO:0016020
Cluster-22178.90630 GO:0005515
Cluster-22178.90631 GO:0006914 GO:0003676 GO:0008270 GO:0046872

Cluster-22178.90636 GO:0023052
Cluster-22178.90637 GO:0019956
Cluster-22178.9065 GO:0030001 GO:0055085 GO:0046873 GO:0016020
Cluster-22178.90662 GO:0006750 GO:0006749 GO:0004357 GO:0017109
Cluster-22178.90670 GO:0016020
Cluster-22178.90673 GO:0008270 GO:0046872
Cluster-22178.90674 GO:0046872 GO:0008270
Cluster-22178.90678 GO:0005515 GO:0000228
Cluster-22178.90680 GO:0005515 GO:0000228
Cluster-22178.90686 GO:0010951 GO:0007283 GO:0004866 GO:0003677
GO:0005634 GO:0000786
Cluster-22178.90694 GO:0005515

Cluster-22178.90699 GO:0006771 GO:0019497 GO:0008270 GO:0046872 GO:0005515 GO:0003993
Cluster-22178.90702 GO:0006928 GO:0005198
Cluster-22178.90707 GO:0006260 GO:0006310 GO:0006281 GO:0003724 GO:0016887 GO:0046872 GO:0005524 GO:0003677 GO:0003723 GO:0009378 GO:0005657 GO:0009379
Cluster-22178.90715 GO:0005515
Cluster-22178.90717 GO:0006468 GO:0005524 GO:0004672
Cluster-22178.90725 GO:0006355 GO:0008270 GO:0043565 GO:0003700 GO:0005667
Cluster-22178.90727 GO:0005515
Cluster-22178.90729 GO:0019299 GO:0016857 GO:0005515 GO:0005737
Cluster-22178.90730 GO:0009820 GO:0050364
Cluster-22178.90734 GO:0004222 GO:0005515
Cluster-22178.90738 GO:0009116 GO:0003824
Cluster-22178.90742 GO:0006260 GO:0015991 GO:0015992 GO:0005515 GO:0016853 GO:0015078 GO:0033179
Cluster-22178.90744 GO:0015991 GO:0015992 GO:0006260 GO:0016853 GO:0005515 GO:0015078 GO:0033179
Cluster-22178.90750 GO:0006308 GO:0031047 GO:0009056 GO:0005975 GO:0008270 GO:0016888 GO:0004560
Cluster-22178.90757 GO:0007165 GO:0005515 GO:0005179 GO:0005576
Cluster-22178.90759 GO:0016310 GO:0009069 GO:0006468 GO:0004683 GO:0005516
Cluster-22178.90760 GO:0033227 GO:0015931 GO:0051033 GO:0016021
Cluster-22178.90771 GO:0046486 GO:0042254 GO:0007205 GO:0035556 GO:0006412 GO:0032065 GO:0055114 GO:0009231 GO:0009395 GO:0003735 GO:0005543 GO:0004143 GO:0047134 GO:0016301 GO:0005515 GO:0008686 GO:0005840 GO:0005938
Cluster-22178.90782 GO:0003676 GO:0005515
Cluster-22178.90784 GO:0009405 GO:0007156 GO:0005509 GO:0005198 GO:0016020 GO:0019028 GO:0005576
Cluster-22178.90786 GO:0003677 GO:0005515 GO:0046872

Cluster-22178.90789 GO:0016773
Cluster-22178.90791 GO:0007186 GO:0006397 GO:0046660 GO:0034599
GO:0004252 GO:0004930 GO:0019028 GO:0005634 GO:0016021
GO:0005789 GO:0005886
Cluster-22178.90798 GO:0007165 GO:0007264 GO:0006259 GO:0043087
GO:0006308 GO:0006357 GO:0001104 GO:0005515 GO:0005085
GO:0004531 GO:0016765 GO:0008270 GO:0016592
Cluster-22178.90800 GO:0006560 GO:0071436 GO:0006814 GO:0006525
GO:0006090 GO:0006725 GO:0016853 GO:0008948 GO:0015081
GO:0016020
Cluster-22178.90824 GO:0007010 GO:0009405 GO:0007264 GO:0046718
GO:0046812 GO:0005525 GO:0003779 GO:0003924 GO:0039624
GO:0009986
Cluster-22178.90826 GO:0046872
Cluster-22178.90827 GO:0007186 GO:0004930 GO:0005515
Cluster-22178.90829 GO:0005515
Cluster-22178.90830 GO:0000271 GO:0016020
Cluster-22178.90837 GO:0006955 GO:0007165 GO:0005125 GO:0016021
GO:0005789 GO:0005576
Cluster-22178.9084 GO:0006952 GO:0003677 GO:0005576
Cluster-22178.90855 GO:0006260 GO:0006449 GO:0006415 GO:0006810
GO:0016149 GO:0005525 GO:0005198 GO:0005886 GO:0005737
GO:0019031 GO:0005840 GO:0019028 GO:0018444
Cluster-22178.90868 GO:0005515 GO:0016020
Cluster-22178.90869 GO:0006313 GO:0015074 GO:0003677 GO:0005515
GO:0016020
Cluster-22178.90885 GO:0006814 GO:0006744 GO:0006120 GO:0055114
GO:0015992 GO:0008137
Cluster-22178.90891 GO:0005515 GO:0016757 GO:0016020
Cluster-22178.90896 GO:0005975 GO:0007165 GO:0004571 GO:0008158
GO:0005509 GO:0016020
Cluster-22178.90897 GO:0015743 GO:0006355 GO:0006914 GO:0006886
GO:0005622 GO:0005634

Cluster-22178.90899 GO:0006886 GO:0006914 GO:0015743 GO:0005622
Cluster-22178.90903 GO:0003676
Cluster-22178.90908 GO:0007264 GO:0002949 GO:0005509 GO:0005515
GO:0005525
Cluster-22178.90914 GO:0007342
Cluster-22178.90934 GO:0006351 GO:0030683 GO:0016020 GO:0019012
Cluster-22178.90935 GO:0006206 GO:0006144 GO:0006241 GO:0006228
GO:0006813 GO:0006165 GO:0006814 GO:0006183 GO:0004550
GO:0005890
Cluster-22178.90938 GO:0015992 GO:0006506 GO:0015986 GO:0015078
GO:0016746 GO:0005789 GO:0016021 GO:0000276
Cluster-22178.90950 GO:0007339 GO:0006771 GO:0019497 GO:0005515
GO:0003993 GO:0046872 GO:0016020 GO:0005576
Cluster-22178.90953 GO:0008608 GO:0035023 GO:0007264 GO:0002949
GO:0006812 GO:0043087 GO:0016301 GO:0005515 GO:0003723
GO:0005525 GO:0008324 GO:0003924 GO:0098519 GO:0003724
GO:0005089 GO:0005524 GO:0016887 GO:0042729 GO:0072686
Cluster-22178.9096 GO:0055085 GO:0006810 GO:0022857 GO:0005215
GO:0016020 GO:0016021
Cluster-22178.90960 GO:0035556 GO:0008270
Cluster-22178.90974 GO:0007275 GO:0030494 GO:0019685 GO:0016836
GO:0046983 GO:0005578
Cluster-22178.90981 GO:0040007 GO:0006508 GO:0006144 GO:0007165
GO:0008283 GO:0003968 GO:0016817 GO:0004252 GO:0008083
GO:0070008 GO:0008270 GO:0004197 GO:0017111 GO:0016021
GO:0031379 GO:0016020
Cluster-22178.90982 GO:0005515
Cluster-22178.90987 GO:0006338 GO:0006355 GO:0005524 GO:0008270
GO:0003676 GO:0016818 GO:0003677 GO:0005634
Cluster-22178.90999 GO:0003682 GO:0000785
Cluster-22178.91003 GO:0070008 GO:0004252
Cluster-22178.91004 GO:0007165
Cluster-22178.9101 GO:0008272 GO:0015116 GO:0016021

Cluster-22178.91011 GO:0006260 GO:0045892 GO:0006351 GO:0006367
GO:0006270 GO:0032968 GO:0007217 GO:0003676 GO:0008270
GO:0003887 GO:0003677 GO:0042575 GO:0005634
Cluster-22178.91017 GO:0006355 GO:0005622
Cluster-22178.91029 GO:0005515 GO:0016021
Cluster-22178.91031 GO:0019354 GO:0006779 GO:0055114 GO:0009878
GO:0043115 GO:0046872
Cluster-22178.91040 GO:0034599 GO:0006468 GO:0005524 GO:0004672
GO:0005789 GO:0016020
Cluster-22178.91041 GO:0016020
Cluster-22178.91043 GO:0008233
Cluster-22178.91053 GO:0017004 GO:0015886 GO:0017000 GO:0016787
GO:0016021
Cluster-22178.91074 GO:0009069 GO:0006468 GO:0016310 GO:0004674
GO:0004672 GO:0005524 GO:0016773 GO:0016020
Cluster-22178.91075 GO:0006886 GO:0005622
Cluster-22178.91085 GO:0055085 GO:0016021
Cluster-22178.91094 GO:0005515
Cluster-22178.91112 GO:0022900 GO:0055114 GO:0006118 GO:0008827
GO:0009319 GO:0016021
Cluster-22178.91115 GO:0006355 GO:0005634
Cluster-22178.91116 GO:0006879 GO:0005515 GO:0008199
Cluster-22178.91120 GO:0006468 GO:0009069 GO:0007178 GO:0016310
GO:0004675 GO:0046872 GO:0005524 GO:0005026 GO:0016020
Cluster-22178.91127 GO:0008152 GO:0003824 GO:0016836
Cluster-22178.91128 GO:0008152 GO:0016836 GO:0003824
Cluster-22178.91130 GO:0008152 GO:0003824 GO:0016836
Cluster-22178.91133 GO:0008152 GO:0006508 GO:0008233 GO:0003824
GO:0016836
Cluster-22178.91145 GO:0005515
Cluster-22178.91158 GO:0007264 GO:0006812 GO:0005524 GO:0008324
GO:0005525 GO:0016021
Cluster-22178.91174 GO:0005975 GO:0071704 GO:0016868

Cluster-22178.91185 GO:0006816 GO:0000166 GO:0005388 GO:0046872 GO:0008270 GO:0016529
Cluster-22178.91186 GO:0005515
Cluster-22178.91187 GO:0008643 GO:0006355 GO:0008270 GO:0008982 GO:0003682 GO:0003677 GO:0000775 GO:0000785 GO:0009357 GO:0019028 GO:0005634
Cluster-22178.91195 GO:0006118 GO:0055114 GO:0009496 GO:0005515 GO:0005198 GO:0051537 GO:0008289 GO:0042651 GO:0009512
Cluster-22178.91196 GO:0007049 GO:0016020 GO:0016021 GO:0016012
Cluster-22178.91203 GO:0006270 GO:0005524 GO:0003678 GO:0003677 GO:0005634 GO:0042555 GO:0005657
Cluster-22178.91205 GO:0005198 GO:0019031
Cluster-22178.91209 GO:0007275 GO:0005515 GO:0004867 GO:0005578
Cluster-22178.91223 GO:0046325
Cluster-22178.91224 GO:0046325
Cluster-22178.91230 GO:0007165 GO:0040007 GO:0008283 GO:0008083

Cluster-22178.91242 GO:0006508 GO:0004197
Cluster-22178.91248 GO:0006825 GO:0035434 GO:0006813 GO:0007186 GO:0005267 GO:0004930 GO:0005375 GO:0016021
Cluster-22178.91250 GO:0055114 GO:0020037 GO:0016705 GO:0005506

Cluster-22178.91261 GO:0006508 GO:0004252
Cluster-22178.91266 GO:0006355 GO:0005515 GO:0043565 GO:0005634
Cluster-22178.91267 GO:0023052
Cluster-22178.91268 GO:0005634
Cluster-22178.91270 GO:0008270 GO:0016020
Cluster-22178.91271 GO:0006144 GO:0006914 GO:0006260 GO:0035556 GO:0006171 GO:0008608 GO:0007049 GO:0006355 GO:0009190 GO:0003677 GO:0043565 GO:0003700 GO:0016849 GO:0004016 GO:0005886 GO:0005815 GO:0005876 GO:0005667 GO:0005634 GO:0042729
Cluster-22178.91276 GO:0006144 GO:0006914 GO:0035556 GO:0006260

GO:0043093 GO:0006171 GO:0051301 GO:0008608 GO:0007049
GO:0006355 GO:0009190 GO:0000917 GO:0006886 GO:0003677
GO:0043565 GO:0003700 GO:0016849 GO:0004016 GO:0005886
GO:0005815 GO:0005876 GO:0005667 GO:0005783 GO:0005737
GO:0016021 GO:0005634 GO:0042729
Cluster-22178.91279 GO:0016310 GO:0009069 GO:0004674
Cluster-22178.91282 GO:0008168 GO:0008270
Cluster-22178.91293 GO:0007186 GO:0009372 GO:0009405 GO:0006468
GO:0008233 GO:0004930 GO:0016773 GO:0005524 GO:0004672
GO:0016021 GO:0016020
Cluster-22178.91295 GO:0046486 GO:0007205 GO:0009395 GO:0004143

Cluster-22178.91299 GO:0006505 GO:0006629 GO:0006886 GO:0016787
GO:0016788
Cluster-22178.91304 GO:0019049 GO:0016192 GO:0016021
Cluster-22178.91305 GO:0019049
Cluster-22178.91315 GO:0042254 GO:0006412 GO:0007165 GO:0006308
GO:0006891 GO:0051082 GO:0003735 GO:0005515 GO:0008855
GO:0017119 GO:0009318 GO:0005622 GO:0005840
Cluster-22178.91319 GO:0043093 GO:0007275 GO:0007165 GO:0000917
GO:0005044 GO:0005578 GO:0016020 GO:0005737
Cluster-22178.91320 GO:0006206 GO:0006144 GO:0016310 GO:0009069
GO:0006468 GO:0006351 GO:0004672 GO:0005524 GO:0004674
GO:0003677 GO:0003899 GO:0005730
Cluster-22178.91330 GO:0051301 GO:0007264 GO:0005525 GO:0016020
GO:0005622
Cluster-22178.91347 GO:0006351 GO:0030683 GO:0016020 GO:0019012
Cluster-22178.91353 GO:0042254 GO:0006412 GO:0006810 GO:0009405
GO:0005515 GO:0003735 GO:0008200 GO:0005840 GO:0042151
GO:0005622
Cluster-22178.91354 GO:0006355
Cluster-22178.91367 GO:0006355 GO:0043565
Cluster-22178.91370 GO:0006355 GO:0043565

Cluster-22178.91371 GO:0006355 GO:0043565
Cluster-22178.91374 GO:0006355 GO:0003700 GO:0005515 GO:0008270
GO:0043565 GO:0005634 GO:0005667
Cluster-22178.91375 GO:0007049 GO:0016012 GO:0016020 GO:0016021
Cluster-22178.91376 GO:0007049 GO:0016012 GO:0016020 GO:0016021
Cluster-22178.91386 GO:0006468 GO:0004672 GO:0005524
Cluster-22178.91394 GO:0032801 GO:0006812 GO:0019076 GO:0007016
GO:0005261 GO:0016020 GO:0016010 GO:0033644
Cluster-22178.91398 GO:0019076 GO:0007016 GO:0006812 GO:0032801
GO:0005261 GO:0016010 GO:0016020 GO:0033644
Cluster-22178.91405 GO:0003676
Cluster-22178.91408 GO:0006811 GO:0016020
Cluster-22178.91422 GO:0006449 GO:0006468 GO:0006886 GO:0006118
GO:0006415 GO:0016192 GO:0016149 GO:0004715 GO:0009055
GO:0020037 GO:0005524 GO:0005506 GO:0005737 GO:0005840
GO:0005576 GO:0005622 GO:0018444 GO:0030131
Cluster-22178.91430 GO:0015979 GO:0016020 GO:0009523 GO:0009539
Cluster-22178.91432 GO:0042073 GO:0006355 GO:0006351 GO:0000398
GO:0006260 GO:0003682 GO:0003887 GO:0003677 GO:0008270
GO:0005634 GO:0042575 GO:0000785 GO:0005681 GO:0000775
GO:0032040
Cluster-22178.91438 GO:0019236 GO:0007186 GO:0016021
Cluster-22178.91443 GO:0008152 GO:0016788
Cluster-22178.91444 GO:0055085 GO:0018401 GO:0030168 GO:0007165
GO:0006810 GO:0006525 GO:0008283 GO:0006323 GO:0000160
GO:0051258 GO:0006308 GO:0006560 GO:0051028 GO:0001934
GO:0006869 GO:0015986 GO:0042157 GO:0016310 GO:0042262
GO:0055114 GO:0048309 GO:0009405 GO:0008284 GO:0030001
GO:0040007 GO:0005102 GO:0008855 GO:0046873 GO:0005157
GO:0030674 GO:0008289 GO:0016702 GO:0042626 GO:0046983
GO:0004656 GO:0000155 GO:0003690 GO:0005198 GO:0033178
GO:0009365 GO:0019031 GO:0016021 GO:0009318 GO:0005576
GO:0019028 GO:0005577 GO:0005783 GO:0016020 GO:0005615

Cluster-22178.91446 GO:0006542 GO:0006807 GO:0009252 GO:0004356

Cluster-22178.91452 GO:0006468 GO:0005524 GO:0004672
Cluster-22178.91453 GO:0019031
Cluster-22178.91454 GO:0006355 GO:0043565 GO:0003700 GO:0005667
Cluster-22178.91455 GO:0019911
Cluster-22178.91456 GO:0007165
Cluster-22178.91459 GO:0003677
Cluster-22178.91462 GO:0006355 GO:0043401 GO:0005515 GO:0008270
GO:0043565 GO:0003700 GO:0005634 GO:0005667
Cluster-22178.91469 GO:0007165 GO:0005179 GO:0005576
Cluster-22178.91470 GO:0007165 GO:0005179 GO:0005576
Cluster-22178.91474 GO:0005515
Cluster-22178.91476 GO:1900758
Cluster-22178.91479 GO:0009245 GO:0003774 GO:0008915 GO:0016459
Cluster-22178.91498 GO:0032065 GO:0007165 GO:0000062 GO:0008242
GO:0005543 GO:0003774 GO:0005524 GO:0005515 GO:0005938
GO:0005856 GO:0016459
Cluster-22178.9150 GO:0019856
Cluster-22178.91500 GO:0015979 GO:0009297 GO:0006352 GO:0003723
GO:0003677 GO:0009522 GO:0005669 GO:0019867
Cluster-22178.91511 GO:0006629 GO:0016627 GO:0005737 GO:0016021
Cluster-22178.91524 GO:0006508 GO:0006817 GO:0043123 GO:0007165
GO:0045766 GO:0030436 GO:0043066 GO:0004190 GO:0004871
GO:0005315 GO:0016020 GO:0016021
Cluster-22178.91525 GO:0007165 GO:0045766 GO:0043123 GO:0006817
GO:0043066 GO:0005315 GO:0004871 GO:0016020 GO:0016021
Cluster-22178.91527 GO:0016114 GO:0005515 GO:0008685
Cluster-22178.91533 GO:0005515
Cluster-22178.91538 GO:0006464 GO:0035556 GO:0046836 GO:0046872
GO:0017089 GO:0016151 GO:0051861 GO:0003677 GO:0005737
Cluster-22178.91542 GO:0006351 GO:0046488 GO:0006269 GO:0003896
GO:0016307 GO:0005657 GO:0005730

Cluster-22178.91550 GO:0007264 GO:0006576 GO:0005525 GO:0005524
GO:0003924
Cluster-22178.91554 GO:0005975 GO:0055114 GO:0016491 GO:0016616
GO:0004553
Cluster-22178.91560 GO:0006468 GO:0009069 GO:0016310 GO:0005524
GO:0004672 GO:0016773 GO:0004674 GO:0016020
Cluster-22178.91561 GO:0009405 GO:0006810 GO:0008200 GO:0042151
Cluster-22178.91564 GO:0005524 GO:0016887 GO:0003924 GO:0003723
GO:0005525 GO:0003724
Cluster-22178.91573 GO:0006355
Cluster-22178.91575 GO:0006260 GO:0000079 GO:0003887 GO:0003677
GO:0019901 GO:0005515 GO:0042575
Cluster-22178.91577 GO:0006260 GO:0000079 GO:0019901 GO:0005515
GO:0003677 GO:0003887 GO:0042575
Cluster-22178.91581 GO:0008270
Cluster-22178.91585 GO:0016021
Cluster-22178.91586 GO:0007165 GO:0009069 GO:0009307 GO:0006468
GO:0016310 GO:0006308 GO:0003677 GO:0004674 GO:0009036
GO:0005096 GO:0009359
Cluster-22178.91599 GO:0006605 GO:0007165
Cluster-22178.91602 GO:0015694 GO:0015097 GO:0016020
Cluster-22178.91606 GO:0035556 GO:0005515
Cluster-22178.91607 GO:0006355 GO:0015074 GO:0008270 GO:0003676
GO:0003700 GO:0005667
Cluster-22178.91610 GO:0006486 GO:0008378 GO:0016020
Cluster-22178.91611 GO:0006486 GO:0008378 GO:0016020
Cluster-22178.91624 GO:0007342
Cluster-22178.91626 GO:0046872
Cluster-22178.91628 GO:0007264 GO:0006355 GO:0009271 GO:0003924
GO:0005525
Cluster-22178.91638 GO:0055114 GO:0051920
Cluster-22178.91639 GO:0006310 GO:0006281 GO:0042754 GO:0045892
GO:0009378 GO:0005524 GO:0009379 GO:0005657

Cluster-22178.91641 GO:0046500 GO:0006400 GO:0006479 GO:0009451
GO:0030488 GO:0006464 GO:0008033 GO:0008152 GO:0008176
GO:0004719 GO:0008168 GO:0008171 GO:0016429 GO:0031515
Cluster-22178.91645 GO:0006508 GO:0006144 GO:0005509 GO:0017111
GO:0004197 GO:0016817 GO:0003968 GO:0031379
Cluster-22178.91646 GO:0006508 GO:0006144 GO:0017111 GO:0005509
GO:0004197 GO:0003968 GO:0016817 GO:0031379
Cluster-22178.91647 GO:0005747 GO:0005739
Cluster-22178.9166 GO:0016021
Cluster-22178.91663 GO:0006811 GO:0007268 GO:0007165 GO:0003924
GO:0008270 GO:0046872 GO:0005234 GO:0005525 GO:0004970
GO:0005622 GO:0016020
Cluster-22178.91665 GO:0007268 GO:0007165 GO:0006811 GO:0008270
GO:0046872 GO:0003924 GO:0005525 GO:0004970 GO:0005234
GO:0016020 GO:0005622
Cluster-22178.91666 GO:0006355 GO:0005634
Cluster-22178.91667 GO:0006355 GO:0005634
Cluster-22178.91668 GO:0006355 GO:0005634
Cluster-22178.91670 GO:0046999 GO:0008270 GO:0019867
Cluster-22178.91679 GO:0005515 GO:0016755 GO:0008270
Cluster-22178.91680 GO:0005515
Cluster-22178.91682 GO:0006541 GO:0005515 GO:0004359
Cluster-22178.91684 GO:0000186 GO:0007186 GO:0008343 GO:0001678
GO:0006820 GO:0006835 GO:0006812 GO:0009267 GO:0032099
GO:0017153 GO:0016020 GO:0005615
Cluster-22178.91688 GO:0006468 GO:0004672 GO:0005524
Cluster-22178.91690 GO:0006486 GO:0008373
Cluster-22178.91692 GO:0032006 GO:0005515
Cluster-22178.91697 GO:0005515
Cluster-22178.91708 GO:0007165
Cluster-22178.91709 GO:0007205 GO:0035556 GO:0046486 GO:0009395
GO:0004143 GO:0005515 GO:0016301
Cluster-22178.91710 GO:0005515

Cluster-22178.91716 GO:0007264 GO:0005525 GO:0003924
Cluster-22178.91722 GO:0006355 GO:0003677 GO:0005515 GO:0003700
GO:0005667
Cluster-22178.91744 GO:0014068 GO:0050776 GO:0007165 GO:0043548
GO:0005102 GO:0005515 GO:0016020
Cluster-22178.9175 GO:0003824
Cluster-22178.91751 GO:0007186 GO:0006355 GO:0004930 GO:0003700
GO:0042025 GO:0016021 GO:0005667
Cluster-22178.91758 GO:0015074 GO:0016032 GO:0006308 GO:0042578
GO:0008833
Cluster-22178.91764 GO:0006771 GO:0019497 GO:0003993
Cluster-22178.91768 GO:0006555 GO:0006556 GO:0008283 GO:0007165
GO:0040007 GO:0006355 GO:0006955 GO:0006954 GO:0004478
GO:0043565 GO:0008092 GO:0003700 GO:0005149 GO:0019898
GO:0005667 GO:0045323 GO:0005737
Cluster-22178.91773 GO:0005515
Cluster-22178.91774 GO:0015700 GO:0005515 GO:0005198 GO:0015105
GO:0016021 GO:0019028
Cluster-22178.91776 GO:0015700 GO:0005198 GO:0015105 GO:0019028
GO:0016021
Cluster-22178.91783 GO:0006952
Cluster-22178.91789 GO:0005515
Cluster-22178.91795 GO:0015986 GO:0015992 GO:0015078 GO:0000276
Cluster-22178.91797 GO:0035556 GO:0006464 GO:0016151 GO:0046872

Cluster-22178.91798 GO:0005515
Cluster-22178.91799 GO:0007264 GO:0007165 GO:0007186 GO:0019001
GO:0031683 GO:0003924 GO:0005525 GO:0004871 GO:0032040
Cluster-22178.91801 GO:0006355 GO:0003700 GO:0043565 GO:0005634
GO:0005667
Cluster-22178.91803 GO:0000917 GO:0043093 GO:0005737
Cluster-22178.91805 GO:0007155
Cluster-22178.91811 GO:0016485 GO:0005975 GO:0043085 GO:0031419

GO:0005515 GO:0035091 GO:0016773 GO:0046872 GO:0016021
Cluster-22178.91830 GO:0046486 GO:0007205 GO:0009395 GO:0004143

Cluster-22178.91836 GO:0006313
Cluster-22178.91864 GO:0007165 GO:0003677 GO:0005509 GO:0005515
GO:0005578
Cluster-22178.91870 GO:0042254 GO:0006412 GO:0003735 GO:0015934
GO:0005840
Cluster-22178.91878 GO:0019497 GO:0006771 GO:0005515 GO:0003993
GO:0046872
Cluster-22178.91881 GO:0006811 GO:0006810 GO:0005230 GO:0016020
Cluster-22178.91883 GO:0071630 GO:0031144 GO:0006468 GO:0016773
GO:0004672 GO:0005524 GO:0016020 GO:0005634
Cluster-22178.91885 GO:0000917 GO:0006886 GO:0043093 GO:0006914
GO:0005198 GO:0042803 GO:0008134 GO:0045502 GO:0005882
GO:0016020 GO:0005667 GO:0005801 GO:0030286 GO:0005737
Cluster-22178.91889 GO:0040007 GO:0008283 GO:0007165 GO:0003676
GO:0008083
Cluster-22178.91891 GO:0008270
Cluster-22178.91895 GO:0006355 GO:0003676 GO:0005507 GO:0003700
GO:0008168 GO:0016817 GO:0003677 GO:0005634 GO:0005667
Cluster-22178.91900 GO:0016021
Cluster-22178.91902 GO:0055114 GO:0016491
Cluster-22178.91903 GO:0030833 GO:0034314 GO:0015629 GO:0005885
Cluster-22178.91922 GO:0019051 GO:0042025
Cluster-22178.91925 GO:0010884 GO:0008284 GO:0001819 GO:0005515

Cluster-22178.91927 GO:0005515
Cluster-22178.91928 GO:0022900 GO:0005743 GO:0070469
Cluster-22178.91945 GO:0006810 GO:0005886 GO:0016021
Cluster-22178.91948 GO:0006355 GO:0043565 GO:0008270 GO:0003700
GO:0005515 GO:0005634 GO:0005667
Cluster-22178.91950 GO:0006310 GO:0006308 GO:0006281 GO:0003676

GO:0004520
Cluster-22178.91957 GO:0006260 GO:0005975 GO:0016758 GO:0016020
Cluster-22178.91958 GO:0000746
Cluster-22178.91961 GO:0043087 GO:0035556 GO:0035023 GO:0019825
GO:0005089 GO:0005515
Cluster-22178.91965 GO:0009878 GO:0046872
Cluster-22178.91967 GO:0006355 GO:0003677
Cluster-22178.91974 GO:0006468 GO:0005524 GO:0004672
Cluster-22178.91983 GO:0008152 GO:0008484 GO:0003824
Cluster-22178.91989 GO:0003950
Cluster-22178.91994 GO:0035091
Cluster-22178.91998 GO:0008270 GO:0005524 GO:0046872 GO:0005515
GO:0051087 GO:0005622
Cluster-22178.92001 GO:0015986 GO:0015992 GO:0015078 GO:0005515
GO:0000276
Cluster-22178.92003 GO:0005515
Cluster-22178.92004 GO:0007165 GO:0005201 GO:0005179 GO:0005578
GO:0005576 GO:0005581
Cluster-22178.92005 GO:0007165 GO:0005179 GO:0005201 GO:0005581
GO:0005578 GO:0005576
Cluster-22178.92010 GO:0042742 GO:0005515
Cluster-22178.92011 GO:0046983
Cluster-22178.92013 GO:0042262 GO:0030001 GO:0006606 GO:0006914
GO:0006206 GO:0006869 GO:0042157 GO:0007155 GO:0006383
GO:0006935 GO:0006351 GO:0006355 GO:0055085 GO:0008053
GO:0006144 GO:0071973 GO:0006810 GO:0003924 GO:0005198
GO:0003899 GO:0042803 GO:0003774 GO:0003677 GO:0003690
GO:0008289 GO:0008134 GO:0045502 GO:0046873 GO:0009288
GO:0016020 GO:0005741 GO:0005667 GO:0005730 GO:0005666
GO:0016021 GO:0019031 GO:0019028 GO:0005576 GO:0005886
GO:0030286 GO:0005882
Cluster-22178.92015 GO:0009225 GO:0007076 GO:0009236 GO:0006012
GO:0009117 GO:0009103 GO:0019915 GO:0019058 GO:0003676

GO:0003824 GO:0003978 GO:0019031 GO:0005789 GO:0000796

Cluster-22178.92020 GO:0007155 GO:0006481 GO:0006479 GO:0004671

GO:0005515 GO:0016021

Cluster-22178.92044 GO:0006554 GO:0006574 GO:0055114 GO:0006633

GO:0006568 GO:0018874 GO:0006550 GO:0006552 GO:0006631

GO:0008270 GO:0016491 GO:0051287 GO:0003857 GO:0016616

Cluster-22178.92047 GO:0006508 GO:0008236 GO:0004252 GO:0070008

Cluster-22178.92049 GO:0006508 GO:0070008 GO:0008236 GO:0004252

Cluster-22178.92051 GO:0016567 GO:0005515 GO:0004842 GO:0008270

GO:0046872 GO:0005680

Cluster-22178.92054 GO:0007165 GO:0009405 GO:0030168 GO:0051258

GO:0030674 GO:0005102 GO:0016020 GO:0005577

Cluster-22178.92056 GO:0006952 GO:0005515 GO:0005509 GO:0005576

Cluster-22178.92058 GO:0006811 GO:0055085 GO:0015075 GO:0016020

Cluster-22178.92061 GO:0006310 GO:0006302 GO:0003677 GO:0005634

Cluster-22178.92062 GO:0006302 GO:0006310 GO:0003677 GO:0005634

Cluster-22178.92071 GO:0016567 GO:0008270 GO:0046872 GO:0004842

GO:0005515 GO:0005680

Cluster-22178.92073 GO:0006855 GO:0015893 GO:0005515 GO:0015238

GO:0005886

Cluster-22178.92081 GO:0055114 GO:0043093 GO:0000917 GO:0008270

GO:0047134 GO:0005737

Cluster-22178.92083 GO:0055114 GO:0005506 GO:0020037 GO:0016705

Cluster-22178.92088 GO:0005515 GO:0016020

Cluster-22178.92090 GO:0007165 GO:0005179 GO:0005576

Cluster-22178.92093 GO:0006260 GO:0006811 GO:0055085 GO:0005524

GO:0008092 GO:0003689 GO:0019901 GO:0005216 GO:0016021

GO:0019898 GO:0005737 GO:0042575 GO:0016020 GO:0005663

Cluster-22178.92095 GO:0005576

Cluster-22178.92104 GO:0007178 GO:0016310 GO:0009069 GO:0006468

GO:0007010 GO:0003779 GO:0004672 GO:0005524 GO:0004675
GO:0016020
Cluster-22178.92113 GO:0006694 GO:0009058 GO:0008299 GO:0007165
GO:0050518 GO:0005044 GO:0005515 GO:0016779 GO:0019031
GO:0016020
Cluster-22178.92117 GO:0007264 GO:0005525
Cluster-22178.92131 GO:0006468 GO:0005515 GO:0004672 GO:0005524

Cluster-22178.92132 GO:0006468 GO:0004672 GO:0005524
Cluster-22178.92138 GO:0006547 GO:0000105 GO:0007165 GO:0007186
GO:0004635 GO:0004871 GO:0005834
Cluster-22178.92152 GO:0015074 GO:0006313 GO:0003677
Cluster-22178.92156 GO:0007165 GO:0016021
Cluster-22178.92157 GO:0030474 GO:0005200 GO:0005823 GO:0005856
Cluster-22178.92160 GO:0005515
Cluster-22178.92163 GO:0009089 GO:0009058 GO:0008837 GO:0003824

Cluster-22178.92165 GO:0009058 GO:0003824
Cluster-22178.92170 GO:0006383 GO:0006351 GO:0006144 GO:0006206
GO:0003899 GO:0003677 GO:0005730 GO:0005666
Cluster-22178.92178 GO:0035556 GO:0051262 GO:0005515 GO:0044212

Cluster-22178.92179 GO:0005515
Cluster-22178.92184 GO:0006260 GO:0006351 GO:0006269 GO:0003896
GO:0005657 GO:0005730
Cluster-22178.92185 GO:0071973 GO:0005198 GO:0008270
Cluster-22178.92204 GO:0006694 GO:0006188 GO:0008299 GO:0009058
GO:0007165 GO:0005524 GO:0050518 GO:0000287 GO:0005044
GO:0016879 GO:0005515 GO:0016779 GO:0019031 GO:0016020
Cluster-22178.92206 GO:0030435 GO:0006814 GO:0016829 GO:0031160
GO:0005739
Cluster-22178.92208 GO:0008283 GO:0040007 GO:0007165 GO:0030674
GO:0051015 GO:0008083 GO:0005615

Cluster-22178.92210 GO:0005515
Cluster-22178.92215 GO:0046836 GO:0051861 GO:0017089 GO:0005737
Cluster-22178.92219 GO:0007165
Cluster-22178.92221 GO:0015992 GO:0006119 GO:0015991 GO:0005524
GO:0005515 GO:0008900 GO:0000287 GO:0005889 GO:0016020
Cluster-22178.92228 GO:0006508 GO:0006629 GO:0006144 GO:0017111
GO:0004252 GO:0004197 GO:0070008 GO:0003968 GO:0016817
GO:0031379
Cluster-22178.92231 GO:0007156 GO:0005509 GO:0016020
Cluster-22178.92233 GO:0007186 GO:0003677 GO:0005515 GO:0051082
GO:0004930 GO:0003676 GO:0003824 GO:0005634 GO:0016021
Cluster-22178.92245 GO:0042710 GO:0007411 GO:0007186 GO:0004930
GO:0005515 GO:0016021
Cluster-22178.92248 GO:0055114 GO:0016641 GO:0005515 GO:0005507

Cluster-22178.92249 GO:0016020
Cluster-22178.92250 GO:0006364 GO:0032040
Cluster-22178.92251 GO:0006886 GO:0016192 GO:0009405 GO:0005515
GO:0030117
Cluster-22178.92253 GO:0055114 GO:0008152 GO:0009877 GO:0016491
GO:0050662 GO:0003824
Cluster-22178.92254 GO:0006351 GO:0006355 GO:0003677 GO:0005515

Cluster-22178.92260 GO:0005515
Cluster-22178.92262 GO:0042981 GO:0005515
Cluster-22178.92263 GO:0005515
Cluster-22178.92266 GO:0051260 GO:0008270
Cluster-22178.92269 GO:0006260 GO:0005198 GO:0019031 GO:0019033
GO:0019028
Cluster-22178.92271 GO:0008152 GO:0016787 GO:0005515 GO:0005509

Cluster-22178.92275 GO:0006816 GO:0006874 GO:0006810 GO:0006270
GO:0042030 GO:0005219 GO:0005246 GO:0004190 GO:0005622

GO:0016021 GO:0016020
Cluster-22178.92284 GO:0015937 GO:0015940 GO:0005524 GO:0004140

Cluster-22178.92297 GO:0055114 GO:0006784 GO:0007010 GO:0016627
GO:0005096 GO:0003779 GO:0005515 GO:0016021
Cluster-22178.92303 GO:0006633 GO:0008152 GO:0042967 GO:0016491
GO:0004315 GO:0005835
Cluster-22178.92308 GO:0000122 GO:0007050 GO:0009405 GO:0045859
GO:0043303 GO:0017069 GO:0004861 GO:0005737 GO:0005634
GO:0005576
Cluster-22178.92316 GO:0006270 GO:0010038 GO:0016032 GO:0046938
GO:0015031 GO:0005515 GO:0016756 GO:0005198 GO:0046872
GO:0008565 GO:0016020 GO:0016021
Cluster-22178.92321 GO:0007205 GO:0035556 GO:0009395 GO:0055114
GO:0042254 GO:0046486 GO:0006412 GO:0047134 GO:0004143
GO:0003735 GO:0005515 GO:0016301 GO:0005840
Cluster-22178.92336 GO:0010508
Cluster-22178.92343 GO:0006355 GO:0003700 GO:0003677 GO:0043565
GO:0005667 GO:0005634
Cluster-22178.92344 GO:0003677 GO:0008270
Cluster-22178.92349 GO:0008283 GO:0040007 GO:0007165 GO:0008083

Cluster-22178.92363 GO:0006886 GO:0007186 GO:0006950 GO:0004930
GO:0016021 GO:0005783
Cluster-22178.92364 GO:0006950 GO:0007186 GO:0006886 GO:0004930
GO:0005783 GO:0016021
Cluster-22178.92365 GO:0008152 GO:0006479 GO:0006464 GO:0046500
GO:0004719 GO:0008168
Cluster-22178.92378 GO:0016020
Cluster-22178.92390 GO:0000398 GO:0006270 GO:0005739 GO:0005681
Cluster-22178.92391 GO:0003676
Cluster-22178.92396 GO:0016598 GO:0004057
Cluster-22178.92403 GO:0005515

Cluster-22178.92421 GO:0005515
Cluster-22178.92422 GO:0005515
Cluster-22178.92428 GO:0006355 GO:0007155 GO:0019062 GO:0019058
GO:0003677
Cluster-22178.92440 GO:0007224
Cluster-22178.92447 GO:0006687 GO:0005975 GO:0006027 GO:0006012
GO:0046486 GO:0004565 GO:0009341
Cluster-22178.92448 GO:0006012 GO:0046486 GO:0006687 GO:0005975
GO:0006027 GO:0004565 GO:0009341
Cluster-22178.92453 GO:0006525 GO:0042254 GO:0006310 GO:0006313
GO:0015074 GO:0006352 GO:0006355 GO:0006412 GO:0016987
GO:0003735 GO:0003677 GO:0000150 GO:0043565 GO:0019843
GO:0003700 GO:0005622 GO:0005667 GO:0005840
Cluster-22178.92469 GO:0003824
Cluster-22178.92472 GO:0000910 GO:0000226 GO:0006355 GO:0008017
GO:0003700 GO:0045298 GO:0005667 GO:0042025
Cluster-22178.92474 GO:0005515
Cluster-22178.92498 GO:0055114 GO:0006810 GO:0042254 GO:0006412
GO:0008168 GO:0005215 GO:0016706 GO:0003735 GO:0019031
GO:0005840 GO:0016020
Cluster-22178.92499 GO:0006355 GO:0007267 GO:0046872 GO:0003677

Cluster-22178.92511 GO:0000160 GO:0005515
Cluster-22178.92517 GO:0009069 GO:0006468 GO:0016310 GO:0000287
GO:0005524 GO:0004672 GO:0016773 GO:0004674 GO:0016020
Cluster-22178.92524 GO:0007165 GO:0008283 GO:0006259 GO:0040007
GO:0055114 GO:0003677 GO:0008083 GO:0047134 GO:0003824
GO:0005524 GO:0016627 GO:0005694
Cluster-22178.92525 GO:0019087 GO:0016021
Cluster-22178.92529 GO:0007186 GO:0003677 GO:0004930 GO:0016021
Cluster-22178.92531 GO:0007186 GO:0004930 GO:0016021
Cluster-22178.92533 GO:0006811 GO:0055085 GO:0005216 GO:0016020
GO:0019012 GO:0016021

Cluster-22178.92540 GO:0006355 GO:0003700 GO:0005198 GO:0019031
GO:0019028 GO:0005667
Cluster-22178.92554 GO:0016032 GO:0019082 GO:0016773
Cluster-22178.92555 GO:0016020
Cluster-22178.92564 GO:0035556 GO:0008270
Cluster-22178.92566 GO:0006469 GO:0045859 GO:0004862 GO:0005952
Cluster-22178.92573 GO:0007165 GO:0007186 GO:0007264 GO:0005525
GO:0004871 GO:0019001 GO:0005524 GO:0031683 GO:0003924
GO:0003677 GO:0000166
Cluster-22178.92574 GO:0007049
Cluster-22178.92582 GO:0006508 GO:0008236
Cluster-22178.92583 GO:0005515
Cluster-22178.92584 GO:0006812 GO:0032801 GO:0019076 GO:0005515
GO:0008270 GO:0005261 GO:0033644
Cluster-22178.92594 GO:0007268 GO:0006811 GO:0007165 GO:0004970
GO:0005234 GO:0016020
Cluster-22178.92605 GO:0006468 GO:0035556 GO:0016773 GO:0004672
GO:0005524 GO:0016020
Cluster-22178.92613 GO:0006303 GO:0009069 GO:0016310 GO:0003677
GO:0005515 GO:0004677 GO:0005524 GO:0016773 GO:0005634
GO:0005958
Cluster-22178.92621 GO:0006284 GO:0003677
Cluster-22178.92627 GO:0046656 GO:0042254 GO:0006412 GO:0009082
GO:0015948 GO:0030269 GO:0003723 GO:0003735 GO:0016021
GO:0005840
Cluster-22178.92628 GO:0006486 GO:0016757 GO:0008378 GO:0016020
Cluster-22178.92635 GO:0007155 GO:0006606 GO:0005198 GO:0005882
Cluster-22178.92637 GO:0006402 GO:0055114 GO:0051252 GO:0005507
GO:0004535 GO:0046872 GO:0016641 GO:0003723 GO:0005737
GO:0005634
Cluster-22178.92638 GO:0051252 GO:0055114 GO:0006402 GO:0003723
GO:0016641 GO:0004535 GO:0046872 GO:0005507 GO:0005634
GO:0005737

Cluster-22178.92642 GO:0008272 GO:0006820 GO:0008509 GO:0015116 GO:0016021 GO:0012511

Cluster-22178.92651 GO:0006281 GO:0006310 GO:0006260 GO:0003723 GO:0009378 GO:0003677 GO:0016887 GO:0046872 GO:0005524 GO:0003724 GO:0009379 GO:0005657

Cluster-22178.92656 GO:0008270 GO:0005515

Cluster-22178.92657 GO:0006013 GO:0005975 GO:0015976 GO:0006000 GO:0006096 GO:0006098 GO:0006094 GO:0042578 GO:0042132

Cluster-22178.92663 GO:0007059 GO:0051301 GO:0006486 GO:0006955 GO:0031110 GO:0007165 GO:0007067 GO:0000090 GO:0005125 GO:0008417 GO:0008017 GO:0045298 GO:0005576 GO:0005876 GO:0000940 GO:0016020

Cluster-22178.92665 GO:0006955 GO:0007165 GO:0040007 GO:0008283 GO:0005125 GO:0005138 GO:0005896 GO:0005576

Cluster-22178.92681 GO:0007165 GO:0009231 GO:0005515 GO:0008531

Cluster-22178.92682 GO:0007155 GO:0015684 GO:0015093 GO:0005525 GO:0009289 GO:0016021

Cluster-22178.92683 GO:0015684 GO:0007155 GO:0005525 GO:0015093 GO:0009289 GO:0016021

Cluster-22178.92686 GO:0009405 GO:0006486 GO:0016757 GO:0008378 GO:0008376 GO:0005198 GO:0032580 GO:0016020

Cluster-22178.92696 GO:0007049 GO:0006355 GO:0043093 GO:0000917 GO:0043565 GO:0003700 GO:0003677 GO:0005737 GO:0005667 GO:0005634

Cluster-22178.92701 GO:0007067 GO:0007264 GO:0007186 GO:0007165 GO:0015684 GO:0009245 GO:0006576 GO:0005524 GO:0015093 GO:0003924 GO:0008915 GO:0031683 GO:0019001 GO:0004871 GO:0005525 GO:0005634 GO:0016021 GO:0000775

Cluster-22178.92702 GO:0009245 GO:0007264 GO:0007067 GO:0005525 GO:0008915 GO:0000775 GO:0005634

Cluster-22178.92712 GO:0007049 GO:0048309 GO:0051028 GO:0007165 GO:0051205 GO:0006334 GO:0004872 GO:0051082 GO:0016021

GO:0005634
Cluster-22178.92713 GO:0006810 GO:0005524
Cluster-22178.92722 GO:0007186 GO:0006869 GO:0042157 GO:0004930
GO:0008289 GO:0046872 GO:0008270 GO:0016021 GO:0005576
Cluster-22178.92735 GO:0006355 GO:0003677
Cluster-22178.92737 GO:0005524 GO:0046872 GO:0003824 GO:0016020
Cluster-22178.92738 GO:0006265 GO:0055114 GO:0019337 GO:0009399
GO:0006807 GO:0016163 GO:0003690 GO:0003677 GO:0016610
Cluster-22178.92740 GO:0009405 GO:0006914 GO:0004222 GO:0005515
GO:0008270 GO:0005576
Cluster-22178.92752 GO:0006269 GO:0006351 GO:0008270 GO:0003896
GO:0004386 GO:0005198 GO:0005515 GO:0005657 GO:0005730
GO:0019028
Cluster-22178.92753 GO:0007586 GO:0016042 GO:0008152 GO:0016746
GO:0005509 GO:0005515 GO:0008236 GO:0008047 GO:0005576
Cluster-22178.9276 GO:0007165 GO:0008283 GO:0015979 GO:0040007
GO:0008083
Cluster-22178.92768 GO:0005975 GO:0005515 GO:0004415
Cluster-22178.92772 GO:0030866 GO:0008092 GO:0005856
Cluster-22178.92775 GO:0006810 GO:0005515 GO:0005215 GO:0016020
Cluster-22178.92777 GO:0005515
Cluster-22178.92786 GO:0042157 GO:0006869 GO:0008289 GO:0005576
Cluster-22178.92789 GO:0006457 GO:0006541 GO:0007165 GO:0005509
GO:0004359 GO:0016021 GO:0005578
Cluster-22178.92811 GO:0007186 GO:0004930 GO:0016021
Cluster-22178.92817 GO:0043087 GO:0018342 GO:0035023 GO:0008318
GO:0005089 GO:0016020
Cluster-22178.92825 GO:0051260 GO:0005096 GO:0005515 GO:0019028
Cluster-22178.92831 GO:0007186 GO:0006869 GO:0005515 GO:0005319
GO:0004930 GO:0016021
Cluster-22178.92833 GO:0042254 GO:0046983 GO:0016020 GO:0016021
Cluster-22178.92836 GO:0015992 GO:0008152 GO:0006744 GO:0006120
GO:0006814 GO:0003824 GO:0016836 GO:0008137 GO:0005739

Cluster-22178.92837 GO:0006355
Cluster-22178.92844 GO:0006468 GO:0010215 GO:0016049 GO:0005524
GO:0004672 GO:0031225
Cluster-22178.92846 GO:0006904 GO:0016192
Cluster-22178.92855 GO:0006810 GO:0005215 GO:0016020
Cluster-22178.92865 GO:0005975 GO:0006047 GO:0005524 GO:0016773

Cluster-22178.92875 GO:0008270
Cluster-22178.92884 GO:0007165
Cluster-22178.92886 GO:0006744 GO:0006120 GO:0015992 GO:0006814
GO:0006118 GO:0008137 GO:0003954 GO:0005739
Cluster-22178.92893 GO:0006355 GO:0005515 GO:0016787
Cluster-22178.92903 GO:0005515
Cluster-22178.92909 GO:0009890 GO:0006355 GO:0043401 GO:0008270
GO:0043565 GO:0003700 GO:0005515 GO:0005667 GO:0005634
Cluster-22178.92912 GO:0009405 GO:0051301 GO:0032955 GO:0005515

Cluster-22178.92920 GO:0008272 GO:0015116 GO:0016021
Cluster-22178.92922 GO:0008643 GO:0009401 GO:0008982 GO:0008270
GO:0009357 GO:0016020
Cluster-22178.92924 GO:0009877 GO:0009312 GO:0005515 GO:0016758
GO:0005634
Cluster-22178.92940 GO:0005509 GO:0005515
Cluster-22178.92944 GO:0006030 GO:0008061 GO:0005576
Cluster-22178.9295 GO:0043462 GO:0006813 GO:0008556 GO:0005886
Cluster-22178.92955 GO:0006338 GO:0003677 GO:0090544
Cluster-22178.92966 GO:0005515 GO:0005509
Cluster-22178.92973 GO:0007411 GO:0007186 GO:0004930 GO:0016021
Cluster-22178.92980 GO:0008283 GO:0006355 GO:0040007 GO:0007165
GO:0043401 GO:0007186 GO:0008270 GO:0043565 GO:0004930
GO:0003700 GO:0008083 GO:0005515 GO:0016021 GO:0005667
GO:0005634
Cluster-22178.92983 GO:0005515

Cluster-22178.92987 GO:0016021
Cluster-22178.92992 GO:0006468 GO:0005524 GO:0004672
Cluster-22178.92993 GO:0006468 GO:0005524 GO:0004672
Cluster-22178.92999 GO:0019083 GO:0005525 GO:0016887 GO:0003924
GO:0005524 GO:0016020
Cluster-22178.93009 GO:0007155 GO:0005515 GO:0016020 GO:0016021
Cluster-22178.93020 GO:0006468 GO:0005524 GO:0004672 GO:0016773
GO:0016020
Cluster-22178.93035 GO:0006118 GO:0015035
Cluster-22178.93040 GO:0005515
Cluster-22178.93051 GO:0006468 GO:0004672 GO:0005524
Cluster-22178.93056 GO:0006570 GO:0006470 GO:0008138 GO:0004725

Cluster-22178.93058 GO:0016887 GO:0005524
Cluster-22178.93062 GO:0045892 GO:0006260 GO:0032968 GO:0007217
GO:0006367 GO:0006351 GO:0006270 GO:0008270 GO:0003676
GO:0003887 GO:0003677 GO:0005634 GO:0042575
Cluster-22178.93065 GO:0042254 GO:0006412 GO:0003735 GO:0005840
GO:0005622
Cluster-22178.9307 GO:0003774 GO:0016459
Cluster-22178.93073 GO:0042981 GO:0006914 GO:0006355 GO:0003700
GO:0043565 GO:0008134 GO:0042803 GO:0005515 GO:0045502
GO:0030286 GO:0005667
Cluster-22178.93079 GO:0006355 GO:0005515 GO:0046983
Cluster-22178.9308 GO:0003774 GO:0016459
Cluster-22178.93102 GO:0008289 GO:0005515
Cluster-22178.93111 GO:0007156 GO:0005509 GO:0016020
Cluster-22178.93113 GO:0007156 GO:0005509 GO:0016020
Cluster-22178.93119 GO:0006355 GO:0003700 GO:0043565 GO:0005667
Cluster-22178.93122 GO:0005515 GO:0043565 GO:0005634
Cluster-22178.93124 GO:0007264 GO:0000287 GO:0003924 GO:0005525
GO:0030976
Cluster-22178.93138 GO:0046983

Cluster-22178.93142 GO:0006464 GO:0006144 GO:0006888 GO:0046034
GO:0006886 GO:0008270 GO:0046872 GO:0016151 GO:0003677
GO:0004017 GO:0030127
Cluster-22178.93143 GO:0016773 GO:0046872
Cluster-22178.93160 GO:0006368 GO:0004190 GO:0016021 GO:0008023
Cluster-22178.93195 GO:0006468 GO:0004672 GO:0005524
Cluster-22178.93197 GO:0005515
Cluster-22178.93200 GO:0005515
Cluster-22178.9322 GO:0006471 GO:0006355 GO:0009405 GO:0003956
GO:0043565 GO:0003700 GO:0005576 GO:0005667
Cluster-22178.93222 GO:0042981
Cluster-22178.93224 GO:0006366 GO:0003677 GO:0016787 GO:0046872
GO:0008270 GO:0005634 GO:0005665
Cluster-22178.93226 GO:0006812 GO:0055085 GO:0018149 GO:0009405
GO:0006810 GO:0008324 GO:0003810 GO:0005515 GO:0019870
GO:0016021 GO:0005576
Cluster-22178.93229 GO:0006355
Cluster-22178.93230 GO:0006570 GO:0006470 GO:0005515 GO:0004725

Cluster-22178.9324 GO:0006396
Cluster-22178.93244 GO:0006468 GO:0009069 GO:0006355 GO:0007165
GO:0016310 GO:0005096 GO:0004674 GO:0003700 GO:0005667
GO:0005737
Cluster-22178.93247 GO:0006952
Cluster-22178.93257 GO:0006810 GO:0007165 GO:0003774 GO:0005524
GO:0016459
Cluster-22178.93264 GO:0005201 GO:0005581 GO:0005578
Cluster-22178.93268 GO:0007165 GO:0043087 GO:0007264 GO:0005085

Cluster-22178.9327 GO:0045454 GO:0006118 GO:0016301 GO:0015035
GO:0009055
Cluster-22178.93273 GO:0000413 GO:0006457 GO:0003677 GO:0003755

Cluster-22178.93276 GO:0003676 GO:0016020
Cluster-22178.93279 GO:0046339 GO:0006629 GO:0009395 GO:0035556
GO:0007165 GO:0004435
Cluster-22178.93282 GO:0016192 GO:0006886 GO:0005198 GO:0030130
GO:0030132
Cluster-22178.93283 GO:0006508 GO:0004252
Cluster-22178.93286 GO:0016021
Cluster-22178.93292 GO:0006811 GO:0055085 GO:0003677 GO:0015075
GO:0016020
Cluster-22178.93294 GO:0005515 GO:0005856
Cluster-22178.93300 GO:0006308 GO:0016032 GO:0015074 GO:0042578
GO:0008833
Cluster-22178.93317 GO:0055114 GO:0045454
Cluster-22178.93321 GO:0006355 GO:0010508 GO:0048870 GO:0006308
GO:0007049 GO:0006323 GO:0006810 GO:0070206 GO:0006914
GO:0006606 GO:0010506 GO:0051082 GO:0045502 GO:0042803
GO:0008134 GO:0005198 GO:0003700 GO:0003677 GO:0008855
GO:0005634 GO:0009318 GO:0031514 GO:0005615 GO:0019867
GO:0016021 GO:0005667 GO:0005882 GO:0005886 GO:0030286
Cluster-22178.93336 GO:0019497 GO:0006771 GO:0003993 GO:0005515
GO:0046872
Cluster-22178.93337 GO:0005515
Cluster-22178.93346 GO:0005861
Cluster-22178.93352 GO:0006355 GO:0003677
Cluster-22178.93355 GO:0003677
Cluster-22178.93357 GO:0006144 GO:0007165 GO:0008272 GO:0006508
GO:0004222 GO:0015116 GO:0005515 GO:0005179 GO:0004114
GO:0016021 GO:0005576
Cluster-22178.9336 GO:0035556 GO:0043087 GO:0005515 GO:0030695
Cluster-22178.93367 GO:0031012
Cluster-22178.93375 GO:0008270
Cluster-22178.93391 GO:0007165 GO:0005515
Cluster-22178.93394 GO:0007186 GO:0004930 GO:0016021

Cluster-22178.93433 GO:0016310 GO:0009069 GO:0006468 GO:0004674
GO:0005524
Cluster-22178.93435 GO:0016310 GO:0016197 GO:0009069 GO:0006468
GO:0000724 GO:0005524 GO:0004674 GO:0005515 GO:0016021
GO:0030119
Cluster-22178.93444 GO:0006810 GO:0006270 GO:0006816 GO:0006874
GO:0004190 GO:0005246 GO:0005219 GO:0042030 GO:0016020
GO:0016021 GO:0005622
Cluster-22178.93467 GO:0006012 GO:0009117 GO:0008108
Cluster-22178.93468 GO:0007186 GO:0003723 GO:0005509 GO:0008270
GO:0004930 GO:0016020 GO:0016021 GO:0019031
Cluster-22178.93470 GO:0007304 GO:0005213 GO:0005576 GO:0042600
Cluster-22178.93477 GO:0005515
Cluster-22178.93481 GO:0006468 GO:0005515 GO:0004672 GO:0005524

Cluster-22178.93487 GO:0006355 GO:0030234 GO:0004222 GO:0003677
GO:0005634 GO:0016020
Cluster-22178.93496 GO:0005515
Cluster-22178.93513 GO:0051382 GO:0019882 GO:0016310 GO:0009069
GO:0006468 GO:0006955 GO:0006886 GO:0007165 GO:0005524
GO:0004674 GO:0042289 GO:0042613 GO:0000776 GO:0016020
Cluster-22178.93523 GO:0005515
Cluster-22178.93540 GO:0033227 GO:0042254 GO:0006412 GO:0006810
GO:0006270 GO:0015931 GO:0004190 GO:0051033 GO:0003735
GO:0003924 GO:0005525 GO:0005861 GO:0016021 GO:0005840
Cluster-22178.93545 GO:0003676 GO:0004190 GO:0003723 GO:0016021
Cluster-22178.93546 GO:0042981 GO:0016021 GO:0005634
Cluster-22178.93547 GO:0005515 GO:0016020
Cluster-22178.93550 GO:0007186 GO:0004930 GO:0016021
Cluster-22178.93551 GO:0007186 GO:0004930
Cluster-22178.93558 GO:0019402 GO:0006355 GO:0005515 GO:0046872

Cluster-22178.93564 GO:0015074 GO:0008270 GO:0003676

Cluster-22178.93565 GO:0006810 GO:0018189 GO:0055085 GO:0005524
GO:0005515 GO:0042626 GO:0016021
Cluster-22178.93575 GO:0006629 GO:0006355 GO:0006605 GO:0006886
GO:0043565 GO:0003700 GO:0005742 GO:0005667
Cluster-22178.93582 GO:0048544 GO:0005515
Cluster-22178.93588 GO:0007186 GO:0007165 GO:0007264 GO:0004871
GO:0005525 GO:0003924 GO:0019001 GO:0031683 GO:0032040
Cluster-22178.93589 GO:0006465 GO:0008233 GO:0005515 GO:0016757
GO:0005787 GO:0016021
Cluster-22178.93599 GO:0008033 GO:0035556 GO:0006396 GO:0051252
GO:0003723 GO:0004526 GO:0005515 GO:0030677
Cluster-22178.93600 GO:0055114 GO:0006118 GO:0016655 GO:0005515
GO:0005509 GO:0005525 GO:0019031
Cluster-22178.93601 GO:0005515
Cluster-22178.93603 GO:0007165 GO:0005509 GO:0005515 GO:0005578
Cluster-22178.93613 GO:0055085 GO:0022857 GO:0016021
Cluster-22178.93616 GO:0003677
Cluster-22178.93620 GO:0023052
Cluster-22178.93623 GO:0023052
Cluster-22178.93626 GO:0016020
Cluster-22178.93628 GO:0005515
Cluster-22178.93630 GO:0007186 GO:0004930 GO:0016021
Cluster-22178.93639 GO:0006355
Cluster-22178.93651 GO:0031505 GO:0019064 GO:0007049 GO:0006355
GO:0007165 GO:0043565 GO:0003700 GO:0046789 GO:0009986
GO:0019031 GO:0005667 GO:0005634
Cluster-22178.93652 GO:0003676
Cluster-22178.93653 GO:0007156 GO:0005509 GO:0016020
Cluster-22178.93663 GO:0016310 GO:0009069 GO:0006508 GO:0008270
GO:0004222 GO:0008233 GO:0008384 GO:0031012 GO:0008385
Cluster-22178.93669 GO:0016310 GO:0007178 GO:0009069 GO:0004675
GO:0016020
Cluster-22178.93674 GO:0051252 GO:0016891

Cluster-22178.93679 GO:0005515
Cluster-22178.93681 GO:0005515
Cluster-22178.93684 GO:0019033
Cluster-22178.93685 GO:0007165 GO:0005044 GO:0016020
Cluster-22178.93698 GO:0016226 GO:0006334 GO:0051536 GO:0005506 GO:0003677 GO:0000786 GO:0005634
Cluster-22178.93700 GO:0006351 GO:0008270 GO:0003677 GO:0005515 GO:0005634
Cluster-22178.93708 GO:0006468 GO:0005524 GO:0004672
Cluster-22178.93709 GO:0006810 GO:0005976 GO:0045444 GO:0016779 GO:0005515 GO:0008092 GO:0005886 GO:0005737 GO:0016021 GO:0019898
Cluster-22178.93713 GO:0007165 GO:0006396 GO:0006144 GO:0004114 GO:0016020 GO:0005622
Cluster-22178.93715 GO:0009236 GO:0006355 GO:0048472 GO:0003677 GO:0005515 GO:0046872 GO:0016021
Cluster-22178.9372 GO:0007156 GO:0005509 GO:0016020
Cluster-22178.93722 GO:0015074 GO:0003676 GO:0008270
Cluster-22178.93725 GO:0007067 GO:0006355 GO:0009401 GO:0007049 GO:0005515 GO:0003677 GO:0003700 GO:0005634 GO:0005667 GO:0000775
Cluster-22178.93744 GO:0005524 GO:0005515
Cluster-22178.93749 GO:0005975 GO:0008063 GO:0016773
Cluster-22178.93750 GO:0005975 GO:0008063 GO:0016773
Cluster-22178.93760 GO:0006397 GO:0005515 GO:0005198 GO:0000445 GO:0019031 GO:0019028
Cluster-22178.93764 GO:0007165
Cluster-22178.93766 GO:0006470 GO:0008138
Cluster-22178.93792 GO:0006468 GO:0005524 GO:0004672
Cluster-22178.93801 GO:0006118 GO:0055114 GO:0051537 GO:0005198 GO:0009496 GO:0009512 GO:0042651
Cluster-22178.93802 GO:0006397 GO:0006897 GO:0042967 GO:0046660 GO:0006807 GO:0016814 GO:0008270 GO:0008080 GO:0005634

GO:0016020 GO:0042470 GO:0001527
Cluster-22178.93803 GO:0006397 GO:0005515 GO:0005198 GO:0019028
GO:0000445 GO:0019031
Cluster-22178.93812 GO:0006355 GO:0003677 GO:0005515 GO:0003700
GO:0005667
Cluster-22178.93831 GO:0035064
Cluster-22178.93834 GO:0005509
Cluster-22178.93844 GO:0000413 GO:0006457 GO:0003755
Cluster-22178.93845 GO:0006468 GO:0004672 GO:0005524
Cluster-22178.93859 GO:0005515
Cluster-22178.93865 GO:0007017 GO:0007018 GO:0003777 GO:0030286
GO:0005874
Cluster-22178.93872 GO:0042157 GO:0006869 GO:0007186 GO:0046872
GO:0004930 GO:0008289 GO:0005576 GO:0016021
Cluster-22178.93873 GO:0050909 GO:0007186 GO:0004930 GO:0016021
Cluster-22178.93878 GO:0006629 GO:0006810 GO:0016627 GO:0005215
GO:0005737 GO:0016021
Cluster-22178.93882 GO:0043395 GO:0005578 GO:0016020 GO:0016021
Cluster-22178.93892 GO:0003676 GO:0003677 GO:0016787 GO:0003729
GO:0005524
Cluster-22178.93896 GO:0005887
Cluster-22178.93904 GO:0005515 GO:0016020
Cluster-22178.93908 GO:0005201 GO:0005581 GO:0005578
Cluster-22178.93909 GO:0006357 GO:0030168 GO:0051258 GO:0010508
GO:0007165 GO:0005102 GO:0001104 GO:0030674 GO:0016021
GO:0005577 GO:0016592
Cluster-22178.9391 GO:0005978 GO:0005985 GO:0005982 GO:0004373
Cluster-22178.93910 GO:0051258 GO:0030168 GO:0006357 GO:0007165
GO:0010508 GO:0005102 GO:0001104 GO:0030674 GO:0005577
GO:0016021 GO:0016592
Cluster-22178.93915 GO:0006308 GO:0008855 GO:0009318
Cluster-22178.93919 GO:0007275 GO:0042600
Cluster-22178.93929 GO:0055085 GO:0006811 GO:0003677 GO:0015075

GO:0016020
Cluster-22178.93936 GO:0005524 GO:0016887 GO:0016021
Cluster-22178.93937 GO:0007017 GO:0007165 GO:0005515 GO:0005875
Cluster-22178.93944 GO:0055085 GO:0006821 GO:0006811 GO:0005216
GO:0005247 GO:0016020
Cluster-22178.93960 GO:0007186 GO:0004930 GO:0016021
Cluster-22178.93961 GO:0006885 GO:0006814 GO:0016021
Cluster-22178.93963 GO:0042981 GO:0005515
Cluster-22178.93966 GO:0006355 GO:0043565
Cluster-22178.93981 GO:0006357 GO:0051262 GO:0046983 GO:0001104
GO:0016021 GO:0016592
Cluster-22178.93989 GO:0005975 GO:0004553
Cluster-22178.93993 GO:0006355 GO:0006352 GO:0003677 GO:0016987
GO:0003700 GO:0005667
Cluster-22178.94001 GO:0008152 GO:0016491
Cluster-22178.94002 GO:0008152 GO:0016491
Cluster-22178.94004 GO:0006355 GO:0003677
Cluster-22178.94014 GO:0006468 GO:0004672 GO:0005524
Cluster-22178.94016 GO:0005739
Cluster-22178.94019 GO:0007165 GO:0006355 GO:0006508 GO:0006144
GO:0004197 GO:0003677 GO:0005515 GO:0004114 GO:0008270
GO:0016020 GO:0005634 GO:0043189
Cluster-22178.94033 GO:0019354 GO:0006098 GO:0006555 GO:0006779
GO:0009396 GO:0006520 GO:0046487 GO:0006813 GO:0055114
GO:0019521 GO:0006730 GO:0004488 GO:0004616 GO:0051287
GO:0016491 GO:0043115 GO:0004013 GO:0003824
Cluster-22178.94038 GO:0016310 GO:0009089 GO:0008270 GO:0004673
GO:0008837 GO:0005515 GO:0009365
Cluster-22178.94047 GO:0005515
Cluster-22178.94050 GO:0007165 GO:0030153 GO:0005515 GO:0016757

Cluster-22178.94053 GO:0021987 GO:0005515
Cluster-22178.94083 GO:0005515

Cluster-22178.94085 GO:0016021
Cluster-22178.94103 GO:0006813 GO:0003677 GO:0005242 GO:0008076
GO:0016021
Cluster-22178.94107 GO:0006777 GO:0051539 GO:0019008
Cluster-22178.94126 GO:0042330
Cluster-22178.94127 GO:0006298 GO:0008152 GO:0016491 GO:0005524

Cluster-22178.94133 GO:0005515
Cluster-22178.94139 GO:0007275 GO:0005201 GO:0005581 GO:0005578
Cluster-22178.94150 GO:0005515
Cluster-22178.94156 GO:0006308 GO:0006259 GO:0004531
Cluster-22178.9416 GO:0003677 GO:0005634
Cluster-22178.94170 GO:0002949 GO:0006914 GO:0007018 GO:0007017
GO:0003724 GO:0016887 GO:0005524 GO:0003777 GO:0005525
GO:0003924 GO:0008092 GO:0005515 GO:0016301 GO:0003723
GO:0019898 GO:0030286 GO:0005874 GO:0005737
Cluster-22178.94174 GO:0006355 GO:0007165 GO:0003712 GO:0005667
Cluster-22178.94181 GO:0007186 GO:0004930 GO:0016021
Cluster-22178.94184 GO:0006810 GO:0009405 GO:0008200 GO:0005576
Cluster-22178.94193 GO:0035023 GO:0043087 GO:0005089 GO:0003677
GO:0034061 GO:0005634
Cluster-22178.94196 GO:0007304 GO:0005213 GO:0042600 GO:0005576
Cluster-22178.94198 GO:0006468 GO:0042967 GO:0016747 GO:0004672
GO:0005524 GO:0008080
Cluster-22178.94199 GO:0034061 GO:0005515 GO:0003677 GO:0005634
Cluster-22178.94200 GO:0006122 GO:0034061 GO:0003677 GO:0005515
GO:0005634 GO:0005750 GO:0005743
Cluster-22178.94206 GO:0007186 GO:0004930 GO:0016021
Cluster-22178.94210 GO:0005975 GO:0004553 GO:0008270
Cluster-22178.94230 GO:0015992 GO:0006123 GO:0004129 GO:0045277
Cluster-22178.94249 GO:0017148 GO:0008270 GO:0030598
Cluster-22178.94260 GO:0006260 GO:0061024 GO:0005524 GO:0003676
GO:0003887 GO:0003677 GO:0030176 GO:0042575

Cluster-22178.94264 GO:0003676
Cluster-22178.9429 GO:0055114 GO:0006090 GO:0009098 GO:0006816
GO:0006810 GO:0009099 GO:0009097 GO:0042030 GO:0003852
GO:0005506 GO:0005246 GO:0016705 GO:0020037 GO:0016020
Cluster-22178.94298 GO:0007264 GO:0006955 GO:0043087 GO:0019882
GO:0016579 GO:0005085 GO:0032183 GO:0070140 GO:0008270
GO:0036459 GO:0016020 GO:0042613
Cluster-22178.9431 GO:0016310 GO:0009405 GO:0007178 GO:0009069
GO:0004675 GO:0016020 GO:0005576
Cluster-22178.94314 GO:0006629 GO:0005515 GO:0016627 GO:0016021
GO:0005737
Cluster-22178.94336 GO:0006771 GO:0006468 GO:0048013 GO:0019497
GO:0005515 GO:0003993 GO:0016773 GO:0004672 GO:0005524
GO:0046872 GO:0016020
Cluster-22178.94346 GO:0006270 GO:0006355 GO:0003700 GO:0004252
GO:0003677 GO:0005667 GO:0005886
Cluster-22178.94347 GO:0007186 GO:0004930 GO:0005515 GO:0016020
Cluster-22178.94351 GO:0016020
Cluster-22178.94376 GO:0006506 GO:0006468 GO:0009069 GO:0055085
GO:0006811 GO:0016310 GO:0051262 GO:0005216 GO:0003824
GO:0004674 GO:0005524 GO:0016020 GO:0016021 GO:0005789
Cluster-22178.94379 GO:0016560 GO:0005777 GO:0016021
Cluster-22178.94381 GO:0006771 GO:0019497 GO:0046872 GO:0005515
GO:0003993
Cluster-22178.94396 GO:0005515
Cluster-22178.94404 GO:0006836 GO:0045892 GO:0006812 GO:0042754
GO:0005328 GO:0016021
Cluster-22178.94414 GO:0007186 GO:0004930 GO:0016021
Cluster-22178.94426 GO:0006468 GO:0042619 GO:0005515 GO:0003677
GO:0016773 GO:0005524 GO:0004672 GO:0016020
Cluster-22178.94427 GO:0042619 GO:0006468 GO:0016773 GO:0005524
GO:0004672 GO:0005515 GO:0003677 GO:0016020
Cluster-22178.94433 GO:0006355 GO:0009236 GO:0046872 GO:0005515

GO:0003677 GO:0048472 GO:0016021
Cluster-22178.94438 GO:0006935 GO:0007165 GO:0004888 GO:0016020
GO:0016021
Cluster-22178.94444 GO:0007186 GO:0004930 GO:0016021
Cluster-22178.94446 GO:0007186 GO:0004930 GO:0016021
Cluster-22178.94452 GO:0005982 GO:0006446 GO:0007186 GO:0055085
GO:0015703 GO:0016052 GO:0005985 GO:0051260 GO:0007606
GO:0050790 GO:0006413 GO:0006811 GO:0019236 GO:0003743
GO:0005509 GO:0015109 GO:0005216 GO:0004556 GO:0004932
GO:0030234 GO:0016020 GO:0005840
Cluster-22178.94455 GO:0050790 GO:0030234
Cluster-22178.94471 GO:0055114 GO:0005506 GO:0020037 GO:0016705

Cluster-22178.94475 GO:0005509
Cluster-22178.94479 GO:0008168 GO:0008270
Cluster-22178.94481 GO:0016021
Cluster-22178.94502 GO:0003924 GO:0005525
Cluster-22178.94507 GO:0007264 GO:0019251 GO:0006783 GO:0005525
GO:0016852
Cluster-22178.94509 GO:0008270 GO:0003676
Cluster-22178.94512 GO:0016021
Cluster-22178.94513 GO:0016021
Cluster-22178.94534 GO:0030036 GO:0016043 GO:0005525 GO:0017048
GO:0003779
Cluster-22178.94546 GO:0016042 GO:0006486 GO:0006952 GO:0007586
GO:0008373 GO:0008047 GO:0005576
Cluster-22178.94558 GO:0005515
Cluster-22178.94566 GO:0007264 GO:0006406 GO:0003723 GO:0005525
GO:0005524 GO:0016887 GO:0005634 GO:0005737
Cluster-22178.94567 GO:0007010 GO:0003779 GO:0005515
Cluster-22178.94578 GO:0032780 GO:0042030 GO:0005739
Cluster-22178.94579 GO:0032780 GO:0042030 GO:0005739
Cluster-22178.94585 GO:0006355 GO:0005524 GO:0003774 GO:0005515

GO:0008134 GO:0016301 GO:0016459 GO:0005667
Cluster-22178.94593 GO:0006771 GO:0007165 GO:0007186 GO:0019497
GO:0030154 GO:0005515 GO:0019001 GO:0046872 GO:0031683
GO:0003924 GO:0003993 GO:0004871 GO:0016020 GO:0016021
Cluster-22178.94594 GO:0006771 GO:0007165 GO:0007186 GO:0019497
GO:0005515 GO:0031683 GO:0046872 GO:0019001 GO:0003924
GO:0003993 GO:0004871 GO:0016020
Cluster-22178.94603 GO:0006310 GO:0006302 GO:0003677 GO:0005634
Cluster-22178.94604 GO:0006310 GO:0006302 GO:0003677 GO:0005634
Cluster-22178.94605 GO:0006310 GO:0006302 GO:0003677 GO:0005634
Cluster-22178.94608 GO:0006355 GO:0005515 GO:0016021 GO:0005634
Cluster-22178.94639 GO:0005515
Cluster-22178.94648 GO:0006265 GO:0042981 GO:0005515 GO:0003677
GO:0003918 GO:0005524 GO:0019012 GO:0005634
Cluster-22178.94658 GO:0015684 GO:0005515 GO:0015093 GO:0016021
Cluster-22178.94678 GO:0006479 GO:0046500 GO:0008152 GO:0006465
GO:0006464 GO:0008233 GO:0004719 GO:0008168 GO:0005787
GO:0016021
Cluster-22178.94679 GO:0009405
Cluster-22178.9468 GO:0006415 GO:0000184 GO:0006413 GO:0006412
GO:0042254 GO:0006614 GO:0019083 GO:0006414 GO:0003723
GO:0003735 GO:0022625 GO:0005622 GO:0005840
Cluster-22178.94695 GO:0006508 GO:0008236 GO:0004252 GO:0070008

Cluster-22178.94697 GO:0055085 GO:0016021
Cluster-22178.94699 GO:0006810 GO:0006118 GO:0015979 GO:0020037
GO:0005506 GO:0009055 GO:0042626 GO:0005524 GO:0031361
GO:0016021
Cluster-22178.9470 GO:0042254 GO:0006412 GO:0006355 GO:0003677
GO:0003735 GO:0005840
Cluster-22178.94707 GO:0006468 GO:0006814 GO:0015992 GO:0006120
GO:0006744 GO:0016787 GO:0008137 GO:0005524 GO:0004672
GO:0016773 GO:0005739 GO:0016020

Cluster-22178.94713 GO:0016787
Cluster-22178.94716 GO:0007154 GO:0016021
Cluster-22178.94718 GO:0008219 GO:0005198 GO:0005515 GO:0051082

Cluster-22178.94721 GO:0005515
Cluster-22178.94725 GO:0007304 GO:0007010 GO:0003779 GO:0005213
GO:0042600 GO:0005576
Cluster-22178.94727 GO:0003677
Cluster-22178.94734 GO:0008152 GO:0003824
Cluster-22178.94742 GO:0046872
Cluster-22178.94750 GO:0006355 GO:0005515 GO:0003700 GO:0043565
GO:0005667
Cluster-22178.94762 GO:0006813
Cluster-22178.94763 GO:0006468 GO:0016787 GO:0016773 GO:0005524
GO:0004672 GO:0016020
Cluster-22178.94769 GO:0043087 GO:0035023 GO:0005089 GO:0005515

Cluster-22178.94773 GO:0000042 GO:0005515
Cluster-22178.94800 GO:0008608 GO:0006308 GO:0006914 GO:0008855
GO:0016887 GO:0005524 GO:0008134 GO:0042803 GO:0045502
GO:0019867 GO:0030286 GO:0009318 GO:0042729 GO:0005667
GO:0072686
Cluster-22178.94821 GO:0006687 GO:0006816 GO:0006810 GO:0009058
GO:0042030 GO:0001733 GO:0005246 GO:0004867 GO:0008146
GO:0005639 GO:0016021 GO:0005794 GO:0016020
Cluster-22178.94842 GO:0005515
Cluster-22178.94845 GO:0005515
Cluster-22178.94846 GO:0006308 GO:0016888
Cluster-22178.94847 GO:0005975 GO:0006308 GO:0031047 GO:0009056
GO:0016888 GO:0004560 GO:0008270
Cluster-22178.94848 GO:0031047 GO:0006308 GO:0005975 GO:0004560
GO:0016888
Cluster-22178.94854 GO:0006355 GO:0003677 GO:0005634

Cluster-22178.94855 GO:0005515
Cluster-22178.94856 GO:0045892 GO:0005634
Cluster-22178.94857 GO:0045892 GO:0005634
Cluster-22178.94859 GO:0042981
Cluster-22178.94869 GO:0009405 GO:0005576
Cluster-22178.94890 GO:0006915
Cluster-22178.94891 GO:0006810 GO:0048193 GO:0006914 GO:0006357
GO:0007018 GO:0007017 GO:0006308 GO:0008855 GO:0001104
GO:0019871 GO:0008134 GO:0042803 GO:0045502 GO:0003777
GO:0016020 GO:0016592 GO:0005667 GO:0030286 GO:0009318
GO:0019028 GO:0005576 GO:0016021 GO:0005874
Cluster-22178.94892 GO:0006418 GO:0005524 GO:0004812 GO:0000166

Cluster-22178.94899 GO:0006470 GO:0006570 GO:0016567 GO:0004842
GO:0004725 GO:0008270 GO:0043565 GO:0005515 GO:0005634
Cluster-22178.94901 GO:0006570 GO:0016567 GO:0006470 GO:0005515
GO:0004842 GO:0004725 GO:0043565 GO:0008270 GO:0005634
Cluster-22178.94902 GO:0050909 GO:0007186 GO:0055085 GO:0004930
GO:0005515 GO:0016021
Cluster-22178.94908 GO:0006144 GO:0019083 GO:0003968 GO:0031379
Cluster-22178.9491 GO:0008152 GO:0055114 GO:0019901 GO:0016491
Cluster-22178.94917 GO:0005515 GO:0008270
Cluster-22178.94937 GO:0042981 GO:0005515
Cluster-22178.94945 GO:0003677 GO:0008270
Cluster-22178.94952 GO:0006355 GO:0055114 GO:0046872 GO:0031418
GO:0005506 GO:0016706 GO:0005634
Cluster-22178.94955 GO:0006508 GO:0004222
Cluster-22178.94958 GO:0000226 GO:0006355 GO:0005515 GO:0016020
GO:0015630
Cluster-22178.94978 GO:0003677 GO:0005515 GO:0008270 GO:2001070
GO:0046983 GO:0019867
Cluster-22178.94979 GO:0006508 GO:0008241 GO:0008237 GO:0016020
Cluster-22178.94994 GO:0019058 GO:0051539 GO:0019031

Cluster-22178.94995 GO:0019058 GO:0051539 GO:0019031
Cluster-22178.94999 GO:0007275 GO:0003700 GO:0005634 GO:0005667
Cluster-22178.95003 GO:0004190 GO:0032040 GO:0016021
Cluster-22178.95006 GO:0008146 GO:0016021
Cluster-22178.95007 GO:0007165 GO:0005057
Cluster-22178.95019 GO:0007165 GO:0009231 GO:0008531 GO:0005515

Cluster-22178.95028 GO:0030168 GO:0051258 GO:0006457 GO:0007165 GO:0005102 GO:0000774 GO:0042803 GO:0051087 GO:0030674 GO:0005577
Cluster-22178.95034 GO:0006412 GO:0042254 GO:0003735 GO:0005840
Cluster-22178.95036 GO:0043565 GO:0005634
Cluster-22178.95047 GO:0005201 GO:0005578 GO:0005581
Cluster-22178.95078 GO:0016021
Cluster-22178.95082 GO:0003779
Cluster-22178.95083 GO:0019068 GO:0003779
Cluster-22178.95098 GO:0006355
Cluster-22178.9510 GO:0007165 GO:0040007 GO:0008283 GO:0008083
Cluster-22178.95104 GO:0005515 GO:0016020
Cluster-22178.95116 GO:0008033 GO:0001682 GO:0043087 GO:0009098 GO:0035556 GO:0007264 GO:0051252 GO:0008270 GO:0004526 GO:0005085 GO:0005509 GO:0030677
Cluster-22178.95137 GO:0007268 GO:0007165 GO:0006508 GO:0004197 GO:0004252 GO:0005044 GO:0030550 GO:0070008 GO:0016020 GO:0005576
Cluster-22178.95139 GO:0003677 GO:0046872 GO:0008270 GO:0005634
Cluster-22178.95142 GO:0005515
Cluster-22178.95145 GO:0006355 GO:0003677 GO:0046872 GO:0008270 GO:0005634
Cluster-22178.9515 GO:0006355 GO:0005634
Cluster-22178.95171 GO:0006260 GO:0006308 GO:0006144 GO:0007165 GO:0018142 GO:0016888 GO:0042624 GO:0005158 GO:0016779 GO:0005899

Cluster-22178.95186 GO:0035023 GO:0006355 GO:0043087 GO:0003677 GO:0005089
Cluster-22178.95187 GO:0035023 GO:0043087 GO:0006355 GO:0005089 GO:0003677
Cluster-22178.95210 GO:0016310 GO:0006144 GO:0006206 GO:0006351 GO:0009069 GO:0006468 GO:0004672 GO:0005524 GO:0004683 GO:0003899 GO:0003677 GO:0005516 GO:0005730
Cluster-22178.95225 GO:0009069 GO:0006468 GO:0015979 GO:0016310 GO:0004672 GO:0005524 GO:0016773 GO:0004674 GO:0016020 GO:0009539 GO:0009523
Cluster-22178.95237 GO:0005515
Cluster-22178.95238 GO:0046983
Cluster-22178.95244 GO:0005515
Cluster-22178.95245 GO:0003824 GO:0005515 GO:0016020
Cluster-22178.95263 GO:0008283 GO:0040007 GO:0007165 GO:0008083 GO:0005515
Cluster-22178.95275 GO:0006520 GO:0009058 GO:0030170 GO:0016829 GO:0003824
Cluster-22178.95280 GO:0007165 GO:0006810 GO:0008158 GO:0005215 GO:0016021 GO:0016020
Cluster-22178.95286 GO:0005887
Cluster-22178.95289 GO:0042742
Cluster-22178.95294 GO:0015743
Cluster-22178.95295 GO:0006275 GO:0006270 GO:0043565 GO:0005524

Cluster-22178.95305 GO:0018342 GO:0007010 GO:0008318 GO:0003779 GO:0020037
Cluster-22178.95310 GO:0005515 GO:0005201 GO:0005581 GO:0005578
Cluster-22178.95312 GO:0046872 GO:0008270
Cluster-22178.95317 GO:0009103 GO:0008918
Cluster-22178.95320 GO:0006810 GO:0007165 GO:0004872 GO:0016020
Cluster-22178.95330 GO:0007155 GO:0006508 GO:0007186 GO:0008270 GO:0004222 GO:0004930 GO:0003950 GO:0005634 GO:0016020

GO:0031012 GO:0016021
Cluster-22178.95331 GO:0007186 GO:0006508 GO:0004222 GO:0004930
GO:0003950 GO:0008270 GO:0016021 GO:0031012 GO:0016020
GO:0005634
Cluster-22178.95336 GO:0006486 GO:0008373
Cluster-22178.9536 GO:0006118 GO:0019069 GO:0009055 GO:0016021
Cluster-22178.95366 GO:0009395 GO:0006629 GO:0004620 GO:0005515
GO:0042802 GO:0016020
Cluster-22178.95372 GO:0016021
Cluster-22178.95387 GO:0007165 GO:0007186 GO:0005525 GO:0004871
GO:0031683 GO:0019001 GO:0003924
Cluster-22178.9539 GO:0006457 GO:0007165 GO:0006260 GO:0019882
GO:0006955 GO:0006886 GO:0006355 GO:0005509 GO:0042289
GO:0051082 GO:0003700 GO:0016020 GO:0005783 GO:0005667
GO:0005634 GO:0005789 GO:0042613
Cluster-22178.95393 GO:0006633 GO:0055114 GO:0005506 GO:0016491

Cluster-22178.95412 GO:0006506 GO:0006355 GO:0005515 GO:0046983
GO:0016758 GO:0016021
Cluster-22178.95428 GO:0006810 GO:0009069 GO:0016310 GO:0055085
GO:0015693 GO:0008643 GO:0015095 GO:0005351 GO:0004674
GO:0005524 GO:0016020 GO:0016021 GO:0000139
Cluster-22178.95436 GO:0044237 GO:0042254 GO:0016310 GO:0019748
GO:0000160 GO:0006468 GO:0006109 GO:0006412 GO:0003774
GO:0005524 GO:0016773 GO:0000155 GO:0003735 GO:0004672
GO:0019843 GO:0003924 GO:0005525 GO:0003824 GO:0005515
GO:0005622 GO:0016459 GO:0005840 GO:0009365
Cluster-22178.95437 GO:0006812 GO:0006457 GO:0006836 GO:0005515
GO:0005328 GO:0016021
Cluster-22178.9544 GO:0048193 GO:0055085 GO:0006396 GO:0015992
GO:0016567 GO:0015743 GO:0030001 GO:0015991 GO:0004842
GO:0008270 GO:0008452 GO:0061630 GO:0046873 GO:0015078
GO:0046872 GO:0005515 GO:0005198 GO:0005622 GO:0016020

GO:0033179
Cluster-22178.95444 GO:0006914 GO:0003676 GO:0008270 GO:0046872

Cluster-22178.95448 GO:0022900 GO:0046907 GO:0016651
Cluster-22178.95451 GO:0006486 GO:0008373
Cluster-22178.95457 GO:0016021
Cluster-22178.95458 GO:0008270 GO:0046872 GO:0005515
Cluster-22178.95459 GO:0061630
Cluster-22178.95465 GO:0006814 GO:0006885 GO:0016021
Cluster-22178.95476 GO:0005201 GO:0005581 GO:0005578
Cluster-22178.95484 GO:0006464
Cluster-22178.95507 GO:0005543
Cluster-22178.95509 GO:0007165 GO:0042742 GO:0006355 GO:0003700
GO:0005515 GO:0003677 GO:0005667 GO:0005576
Cluster-22178.9551 GO:0035556 GO:0005506 GO:0046872 GO:0043130
GO:0003677
Cluster-22178.95520 GO:0006412 GO:0042254 GO:0003735 GO:0005840
Cluster-22178.95522 GO:0007275 GO:0005201 GO:0005581 GO:0005578
Cluster-22178.95530 GO:0006486 GO:0016757 GO:0008378 GO:0016020
Cluster-22178.95532 GO:0006486 GO:0008378 GO:0016757 GO:0016020
Cluster-22178.95535 GO:0015992 GO:0006744 GO:0006120 GO:0006810
GO:0006814 GO:0006118 GO:0005215 GO:0003954 GO:0008137
GO:0005737 GO:0019031 GO:0016021 GO:0005739
Cluster-22178.95542 GO:0005515
Cluster-22178.95548 GO:0046872
Cluster-22178.95551 GO:0005515
Cluster-22178.95579 GO:0046983
Cluster-22178.95581 GO:0046983
Cluster-22178.95598 GO:0006355 GO:0003677 GO:0003723
Cluster-22178.95614 GO:0009405 GO:0005615
Cluster-22178.95615 GO:0016567 GO:0008152 GO:0005515 GO:0061630
GO:0008270 GO:0046872 GO:0016491 GO:0004842 GO:0005622
GO:0005680

Cluster-22178.95621 GO:0006810 GO:0006355 GO:0055085 GO:0005215

GO:0003700 GO:0008270 GO:0016020 GO:0005634 GO:0005667

Cluster-22178.95630 GO:0006308 GO:0008855 GO:0046983 GO:0009318

Cluster-22178.95647 GO:0006508 GO:0016032 GO:0005975 GO:0008152

GO:0016832 GO:0004197 GO:0046983 GO:0008168 GO:0008171

Cluster-22178.95661 GO:0006508 GO:0004198 GO:0005622

Cluster-22178.95666 GO:0008168

Cluster-22178.95670 GO:0016021

Cluster-22178.95672 GO:0008146

Cluster-22178.95673 GO:0008146

Cluster-22178.95682 GO:0006355

Cluster-22178.95687 GO:0007596 GO:0010951 GO:0008191

Cluster-22178.95691 GO:0045859 GO:0006508 GO:0015994 GO:0008152

GO:0015996 GO:0008033 GO:0047746 GO:0008236 GO:0016787

GO:0019887 GO:0008168 GO:0005956

Cluster-22178.95716 GO:0005861

Cluster-22178.95721 GO:0008152 GO:0016491

Cluster-22178.95722 GO:0008152 GO:0016491

Cluster-22178.95730 GO:0005515 GO:0016021

Cluster-22178.95734 GO:0019310 GO:0055114 GO:0005506 GO:0050113

GO:0005737

Cluster-22178.95741 GO:0040001 GO:0000226 GO:0006606 GO:0000281

GO:0015031 GO:0005515 GO:0008565 GO:0005634 GO:0005819

GO:0005874 GO:0005737

Cluster-22178.95762 GO:0019069

Cluster-22178.95763 GO:0006813 GO:0090063 GO:0000226 GO:0006814

GO:0005890 GO:0005815 GO:0000922

Cluster-22178.95774 GO:0015994 GO:0055114 GO:0033014 GO:0016491

GO:0004418

Cluster-22178.95775 GO:0015992 GO:0006119 GO:0006118 GO:0009055

GO:0008121 GO:0005634

Cluster-22178.95782 GO:0005515

Cluster-22178.95785 GO:0005524 GO:0016787 GO:0003677 GO:0003676

Cluster-22178.95795 GO:0019069
Cluster-22178.95797 GO:0007165 GO:0002532 GO:0055114 GO:0008152 GO:0050662 GO:0016791 GO:0005515 GO:0016491 GO:0004896 GO:0016021
Cluster-22178.95799 GO:0055114 GO:0008168 GO:0016706 GO:0005515

Cluster-22178.95826 GO:0007154 GO:0016020
Cluster-22178.95831 GO:0006355 GO:0005634
Cluster-22178.95836 GO:0007165 GO:0005515
Cluster-22178.95838 GO:0005739
Cluster-22178.95841 GO:0016758 GO:0005789
Cluster-22178.95855 GO:0006508 GO:0004222
Cluster-22178.95874 GO:0006744 GO:0019836 GO:0005515 GO:0005576
Cluster-22178.95880 GO:0034755 GO:0006826 GO:0005381 GO:0016021
Cluster-22178.95886 GO:0019058 GO:0006118 GO:0017004 GO:0019054 GO:0005515 GO:0004857 GO:0045158 GO:0009512
Cluster-22178.95893 GO:0006302 GO:0006265 GO:0003677 GO:0003917 GO:0005694 GO:0005634
Cluster-22178.95898 GO:0006355 GO:0003677 GO:0009512
Cluster-22178.95909 GO:0005515 GO:0016021
Cluster-22178.95922 GO:0005515
Cluster-22178.95937 GO:0015937 GO:0008152 GO:0006139 GO:0015940 GO:0006206 GO:0016301 GO:0016740 GO:0004127 GO:0005524 GO:0004140
Cluster-22178.95971 GO:0010207 GO:0005525 GO:0046872
Cluster-22178.95972 GO:0006606 GO:0006457 GO:0000774 GO:0008092 GO:0051087 GO:0003677 GO:0042803 GO:0019901 GO:0051082 GO:0019898 GO:0005737 GO:0005634
Cluster-22178.95980 GO:0007156 GO:0005509 GO:0016020
Cluster-22178.95991 GO:0046983 GO:0046872 GO:0016021 GO:0019012
Cluster-22178.96015 GO:0006355 GO:0005515 GO:0003677 GO:0008270

Cluster-22178.96030 GO:0006470 GO:0006570 GO:0055114 GO:0004725 GO:0008138 GO:0004721 GO:0016491
Cluster-22178.96048 GO:0055085 GO:0006811 GO:0003677 GO:0015075 GO:0016020
Cluster-22178.96067 GO:0006457 GO:0006355 GO:0003700 GO:0043565 GO:0003677 GO:0005515 GO:0051082 GO:0016272 GO:0005634 GO:0005667
Cluster-22178.96069 GO:0005515
Cluster-22178.96078 GO:0006144 GO:0018144 GO:0016818 GO:0005198 GO:0003968 GO:0031379
Cluster-22178.96083 GO:0007155 GO:0005515 GO:0016021
Cluster-22178.96113 GO:0006629 GO:0003677 GO:0005815 GO:0005634
Cluster-22178.96115 GO:0015979 GO:0006118 GO:0007155 GO:0009055 GO:0020037 GO:0005515 GO:0005506 GO:0016021 GO:0031361
Cluster-22178.96120 GO:0006508 GO:0000746 GO:0009405 GO:0007165 GO:0004222 GO:0005515
Cluster-22178.96126 GO:0007165 GO:0006955 GO:0006935 GO:0008009 GO:0005576
Cluster-22178.96128 GO:0006260 GO:0006886 GO:0042803 GO:0008134 GO:0005198 GO:0045502 GO:0005801 GO:0030286 GO:0016020 GO:0005667
Cluster-22178.96136 GO:0006807 GO:0007411 GO:0006537 GO:0007224 GO:0055114 GO:0003677 GO:0015930 GO:0046872
Cluster-22178.96147 GO:0042981 GO:0007155 GO:0006817 GO:0042246 GO:0005315 GO:0003677 GO:0016021 GO:0005789 GO:0005634 GO:0016020
Cluster-22178.96151 GO:0042325 GO:0005737
Cluster-22178.9617 GO:0007186 GO:0004930 GO:0016021
Cluster-22178.96182 GO:0055114 GO:0005515 GO:0016620 GO:0051287

Cluster-22178.96195 GO:0055114 GO:0016032 GO:0016491 GO:0019012
Cluster-22178.96196 GO:0003950
Cluster-22178.96216 GO:0008152 GO:0016746 GO:0005856

Cluster-22178.96221 GO:0006094 GO:0007017 GO:0008284 GO:0007018
GO:0015976 GO:0006952 GO:0006096 GO:0006144 GO:0000287
GO:0030955 GO:0005515 GO:0003777 GO:0004743 GO:0016787
GO:0005524 GO:0008017 GO:0005874 GO:0045298
Cluster-22178.96224 GO:0042254 GO:0006412 GO:0003735 GO:0005840
Cluster-22178.96232 GO:0006270 GO:0006275 GO:0043565 GO:0005524

Cluster-22178.96237 GO:0003677 GO:0003676 GO:0005524 GO:0016787

Cluster-22178.96241 GO:0005515
Cluster-22178.96246 GO:0035023 GO:0043087 GO:0005089
Cluster-22178.96256 GO:0006355 GO:0006260 GO:0003700 GO:0005667
GO:0005634 GO:0005622
Cluster-22178.96257 GO:0006260 GO:0006355 GO:0003700 GO:0005622
GO:0005667 GO:0005634
Cluster-22178.96258 GO:0006355 GO:0006260 GO:0003700 GO:0005667
GO:0005634 GO:0005622
Cluster-22178.96287 GO:0006355 GO:0007094 GO:0006308 GO:0051259
GO:0006810 GO:0006914 GO:0045502 GO:0042802 GO:0042803
GO:0008134 GO:0003700 GO:0043015 GO:0008855 GO:0009318
GO:0016021 GO:0045298 GO:0005667 GO:0005886 GO:0030286
GO:0005815
Cluster-22178.96289 GO:0005515
Cluster-22178.96290 GO:0007186 GO:0004930 GO:0016021
Cluster-22178.96292 GO:0005515
Cluster-22178.96293 GO:0005975 GO:0048038 GO:0016901
Cluster-22178.96294 GO:0035556 GO:0007049 GO:0046339 GO:0009395
GO:0007165 GO:0006629 GO:0004435 GO:0005515
Cluster-22178.96295 GO:0005515 GO:0016020
Cluster-22178.96309 GO:0046983 GO:0003723 GO:0016787 GO:0030145

Cluster-22178.96312 GO:0006812 GO:0046983 GO:0008324 GO:0016021
Cluster-22178.96314 GO:0046983

Cluster-22178.96376 GO:0006508 GO:0005515 GO:0008233
Cluster-22178.96379 GO:0055114 GO:0006886 GO:0006106 GO:0005506
GO:0016705 GO:0020037 GO:0016020
Cluster-22178.96392 GO:0051537 GO:0005515
Cluster-22178.96401 GO:0007165 GO:0035556 GO:0005515 GO:0003677

Cluster-22178.96402 GO:0007165 GO:0035556 GO:0005515 GO:0003677

Cluster-22178.96404 GO:0055114 GO:0043087 GO:0035023 GO:0035556
GO:0007186 GO:0005089 GO:0047134 GO:0004930 GO:0005515
GO:0016020
Cluster-22178.96409 GO:0055085
Cluster-22178.96414 GO:0005975 GO:0016773
Cluster-22178.96416 GO:0006629 GO:0005515
Cluster-22178.96418 GO:0006816 GO:0046872 GO:0005388 GO:0000166
GO:0016529
Cluster-22178.96431 GO:0015937 GO:0008152 GO:0015940 GO:0006139
GO:0006206 GO:0016301 GO:0016740 GO:0004127 GO:0005524
GO:0004140
Cluster-22178.96438 GO:0045765 GO:0005737
Cluster-22178.96447 GO:0055114 GO:0006888 GO:0006886 GO:0008270
GO:0046872 GO:0008168 GO:0016706 GO:0003677 GO:0030127
Cluster-22178.96452 GO:0006606 GO:0008360 GO:0008053 GO:0007155
GO:0042157 GO:0006869 GO:0007009 GO:0003924 GO:0008289
GO:0005515 GO:0005198 GO:0005741 GO:0016020 GO:0019031
GO:0016021 GO:0005576 GO:0019028
Cluster-22178.96468 GO:0005515
Cluster-22178.96474 GO:0008152 GO:0003824 GO:0003676 GO:0016787
GO:0008484 GO:0046872
Cluster-22178.96508 GO:0005515
Cluster-22178.96514 GO:0006206 GO:0006144 GO:0016310 GO:0006468
GO:0009069 GO:0006351 GO:0004672 GO:0005524 GO:0005516
GO:0003677 GO:0003899 GO:0004683 GO:0005730

Cluster-22178.96518 GO:0006310 GO:0006281 GO:0006260 GO:0003677 GO:0003723 GO:0009378 GO:0003724 GO:0016887 GO:0046872 GO:0005524 GO:0009379 GO:0005657
Cluster-22178.96519 GO:0006694 GO:0009058 GO:0008299 GO:0016779 GO:0050518
Cluster-22178.96532 GO:0051028 GO:0005634
Cluster-22178.96540 GO:0005515
Cluster-22178.96546 GO:0031145 GO:0030071 GO:0005680
Cluster-22178.96547 GO:0006355 GO:0006886 GO:0015031 GO:0007049 GO:0003677 GO:0008565 GO:0043565 GO:0003700 GO:0005634 GO:0005667 GO:0016020 GO:0005737
Cluster-22178.96557 GO:0055114 GO:0005515 GO:0046422 GO:0016020 GO:0009507
Cluster-22178.96558 GO:0055114 GO:0030246 GO:0046422 GO:0005515 GO:0009507 GO:0016020
Cluster-22178.96567 GO:0055114 GO:0005506 GO:0020037 GO:0016705

Cluster-22178.96573 GO:0006814 GO:0006813 GO:0005242 GO:0008076 GO:0016021 GO:0005890
Cluster-22178.96575 GO:0005515
Cluster-22178.96582 GO:0006260 GO:0005524 GO:0046983 GO:0003677 GO:0031224
Cluster-22178.96587 GO:0046983 GO:0031224
Cluster-22178.96589 GO:0008270 GO:0005622
Cluster-22178.96590 GO:0005515
Cluster-22178.96591 GO:0005515
Cluster-22178.966 GO:0035556 GO:0005515 GO:0008270 GO:0046872
Cluster-22178.9660 GO:0006886 GO:0016020 GO:0005801
Cluster-22178.96607 GO:0007166 GO:0007165 GO:0004871 GO:0005634
Cluster-22178.96611 GO:0005524 GO:0035299
Cluster-22178.96616 GO:0007186 GO:0007010 GO:0003779 GO:0004930 GO:0016021
Cluster-22178.96632 GO:0046872 GO:0016787 GO:0003676

Cluster-22178.96635 GO:0006955 GO:0007165 GO:0008152 GO:0016787
GO:0005044 GO:0008484 GO:0046872 GO:0003824 GO:0003676
GO:0030247
Cluster-22178.96643 GO:0007017 GO:0007018 GO:0005524 GO:0003777
GO:0008017 GO:0005874 GO:0045298
Cluster-22178.96647 GO:0051607
Cluster-22178.96649 GO:0006355 GO:0008270 GO:0046872 GO:0046983
GO:0003677
Cluster-22178.96683 GO:0016192 GO:0006886 GO:0005515 GO:0030117
Cluster-22178.96684 GO:0016310 GO:0009089 GO:0004673 GO:0008270
GO:0005515 GO:0008837 GO:0009365
Cluster-22178.96685 GO:0046872
Cluster-22178.96724 GO:0006118 GO:0055114 GO:0009055 GO:0046872
GO:0051536 GO:0016491
Cluster-22178.96727 GO:0006351 GO:0006468 GO:0000398 GO:0016567
GO:0006270 GO:0006260 GO:0005515 GO:0003887 GO:0004672
GO:0003677 GO:0004842 GO:0008270 GO:0005524 GO:0042575
GO:0019028 GO:0005634 GO:0005681 GO:0019031 GO:0005737
GO:0005680
Cluster-22178.96734 GO:0009247 GO:0006464 GO:0016758
Cluster-22178.96735 GO:0006508 GO:0004222 GO:0008270 GO:0031012
Cluster-22178.96741 GO:0006118 GO:0019684 GO:0046983 GO:0045156
GO:0003677 GO:0030077
Cluster-22178.96742 GO:0006508 GO:0006084 GO:0003824 GO:0004222
GO:0031012
Cluster-22178.96749 GO:0005525
Cluster-22178.96751 GO:0006313 GO:0005515
Cluster-22178.96755 GO:0005742
Cluster-22178.96765 GO:0016021
Cluster-22178.96766 GO:0007186 GO:0022900 GO:0004930 GO:0016020
GO:0005747 GO:0005739
Cluster-22178.96778 GO:0016020
Cluster-22178.96779 GO:0042742 GO:0055114 GO:0004174

Cluster-22178.96783 GO:0042254 GO:0006412 GO:0003735 GO:0005840
GO:0016021 GO:0005622
Cluster-22178.96787 GO:0005975 GO:0001575 GO:0006012 GO:0046486
GO:0004557
Cluster-22178.96793 GO:0009401 GO:0008643 GO:0008982 GO:0008270
GO:0016020 GO:0009357
Cluster-22178.96799 GO:0000079 GO:0030436 GO:0006260 GO:0005515
GO:0019901 GO:0003887 GO:0003677 GO:0042575 GO:0042601
Cluster-22178.96802 GO:0016787 GO:0046872 GO:0005515 GO:0003676
GO:0031966
Cluster-22178.96807 GO:0015074
Cluster-22178.96809 GO:0043093 GO:0006355 GO:0000917 GO:0006457
GO:0007049 GO:0006260 GO:0051082 GO:0005509 GO:0043565
GO:0003700 GO:0005634 GO:0005667 GO:0005783 GO:0005737
GO:0032040
Cluster-22178.96817 GO:0043087 GO:0035556 GO:0035023 GO:0019825
GO:0005089 GO:0005515
Cluster-22178.96820 GO:0003676
Cluster-22178.96830 GO:0006810 GO:0015893 GO:0006855 GO:0055085
GO:0015238 GO:0015297 GO:0016020
Cluster-22178.96837 GO:0006357 GO:0001104 GO:0016592
Cluster-22178.96856 GO:0003950
Cluster-22178.96859 GO:0006486 GO:0008378 GO:0016757 GO:0016020
Cluster-22178.96860 GO:0005198 GO:0019028
Cluster-22178.96863 GO:0035023 GO:0055114 GO:0048870 GO:0000917
GO:0043087 GO:0006816 GO:0033014 GO:0043093 GO:0005515
GO:0050661 GO:0005388 GO:0005089 GO:0008883 GO:0005737
GO:0031514 GO:0005815 GO:0016529
Cluster-22178.96874 GO:0006412 GO:0042254 GO:0003735 GO:0019843
GO:0005840
Cluster-22178.96876 GO:0005515
Cluster-22178.96881 GO:0046872 GO:0005515
Cluster-22178.96927 GO:0051289 GO:0008140 GO:0005667

Cluster-22178.96935 GO:0055114 GO:0007165 GO:0008152 GO:0016491

Cluster-22178.96945 GO:0016021
Cluster-22178.96975 GO:0008152 GO:0016491
Cluster-22178.96980 GO:0003677
Cluster-22178.96981 GO:0003824
Cluster-22178.96982 GO:0007411
Cluster-22178.97042 GO:0006486 GO:0008378 GO:0016020
Cluster-22178.97054 GO:0005515
Cluster-22178.97055 GO:0005515
Cluster-22178.97079 GO:0035023 GO:0043087 GO:0005089
Cluster-22178.97080 GO:0043087 GO:0035023 GO:0005089
Cluster-22178.97084 GO:0007018 GO:0007017 GO:0003777 GO:0008017
GO:0005524 GO:0005515 GO:0005874 GO:0045298
Cluster-22178.97104 GO:0006313 GO:0015074 GO:0003677
Cluster-22178.97110 GO:0006355 GO:0007155 GO:0007165 GO:0051258
GO:0030168 GO:0030674 GO:0016818 GO:0003677 GO:0005524
GO:0008270 GO:0005102 GO:0005634 GO:0005577
Cluster-22178.97123 GO:0055085 GO:0005975 GO:0006814 GO:0006810
GO:0007165 GO:0005102 GO:0005215 GO:0003796 GO:0016021
GO:0016020
Cluster-22178.97131 GO:0048193 GO:0030168 GO:0009252 GO:0006952
GO:0007165 GO:0007155 GO:0051258 GO:0030474 GO:0010508
GO:0005102 GO:0016755 GO:0030674 GO:0005200 GO:0008092
GO:0005198 GO:0005823 GO:0019898 GO:0005577 GO:0009279
GO:0005882 GO:0016020 GO:0019867 GO:0005856 GO:0005737
Cluster-22178.97137 GO:0006270 GO:0032040
Cluster-22178.97154 GO:0006355 GO:0006260 GO:0006352 GO:0003677
GO:0008408 GO:0016987 GO:0003700 GO:0005667
Cluster-22178.97189 GO:0002532 GO:0007165 GO:0008152 GO:0055114
GO:0016791 GO:0005515 GO:0050662 GO:0004896 GO:0016491
GO:0016021
Cluster-22178.97205 GO:0005515

Cluster-22178.97206 GO:0042254 GO:0006412 GO:0008152 GO:0008270
GO:0003824 GO:0003735 GO:0005840
Cluster-22178.97208 GO:0006355 GO:0003677 GO:0008270 GO:0005634
Cluster-22178.97212 GO:0006355 GO:0003677 GO:0003700 GO:0005667
Cluster-22178.97215 GO:0007411
Cluster-22178.97246 GO:0055114 GO:0006118 GO:0016655
Cluster-22178.97247 GO:0006118 GO:0055114 GO:0016655
Cluster-22178.97248 GO:0006506 GO:0016192 GO:0016746 GO:0005789
GO:0016021
Cluster-22178.97252 GO:0006355 GO:0046983 GO:0005515
Cluster-22178.97265 GO:0005515 GO:0046872 GO:0016021
Cluster-22178.97275 GO:0007268 GO:0043495 GO:0033130
Cluster-22178.97278 GO:0006810 GO:0046872 GO:0005215 GO:0016020
Cluster-22178.97281 GO:0006164 GO:0006531 GO:0006522 GO:0015031
GO:0019083 GO:0006144 GO:0005515 GO:0005525 GO:0004019
GO:0005524 GO:0003924 GO:0016887 GO:0005789 GO:0016021
Cluster-22178.9729 GO:0006468 GO:0016773 GO:0004672 GO:0005524
GO:0016020
Cluster-22178.97298 GO:0045454 GO:0010112 GO:0005515
Cluster-22178.97331 GO:0006471 GO:0019825 GO:0003956 GO:0005576
Cluster-22178.97353 GO:0055085 GO:0022857 GO:0016021
Cluster-22178.97359 GO:0005634
Cluster-22178.97361 GO:0005515
Cluster-22178.97366 GO:0005982 GO:0005985 GO:0005975 GO:0004650

Cluster-22178.97374 GO:0003677 GO:0005515 GO:0016021 GO:0005634
Cluster-22178.97382 GO:0009966 GO:0007275 GO:0016020
Cluster-22178.97390 GO:0006260 GO:0006816 GO:0034639 GO:0003677
GO:0005388 GO:0016021 GO:0005664 GO:0016529
Cluster-22178.97395 GO:0008146
Cluster-22178.97398 GO:0008146
Cluster-22178.97407 GO:0042981 GO:0005515
Cluster-22178.97408 GO:0007010 GO:0003779

Cluster-22178.97412 GO:0046872
Cluster-22178.97418 GO:0007186 GO:0004930 GO:0016021 GO:0016020
Cluster-22178.97436 GO:0055114 GO:0005506 GO:0016491
Cluster-22178.97476 GO:0030206 GO:0015018 GO:0016020
Cluster-22178.97486 GO:0003950 GO:0005634 GO:0016020
Cluster-22178.97489 GO:0006468 GO:0035091 GO:0005524 GO:0004672

Cluster-22178.97490 GO:0003779
Cluster-22178.97491 GO:0003779
Cluster-22178.97497 GO:0042157 GO:0006118 GO:0009405 GO:0006869
GO:0006810 GO:0008289 GO:0009055 GO:0016021 GO:0005576
GO:0016020
Cluster-22178.97498 GO:0016567 GO:0004842 GO:0005515 GO:0008270
GO:0046872 GO:0005680
Cluster-22178.97505 GO:0007165 GO:0005515 GO:0016757
Cluster-22178.97511 GO:0046872 GO:0016787 GO:0008146 GO:0015016

Cluster-22178.97533 GO:0006270
Cluster-22178.97539 GO:0003677
Cluster-22178.97546 GO:0006308 GO:0016888 GO:0016787
Cluster-22178.97559 GO:0003677
Cluster-22178.97563 GO:0005515
Cluster-22178.97579 GO:0030036 GO:0017048 GO:0003779 GO:0005737
Cluster-22178.97695 GO:0030436 GO:0042601
Cluster-22178.97699 GO:0008270 GO:0008237
Cluster-22178.97702 GO:0008270 GO:0008237
Cluster-22178.97703 GO:0008237 GO:0008270
Cluster-22178.97705 GO:0008270 GO:0008237
Cluster-22178.97730 GO:0015992 GO:0015986 GO:0022900 GO:0015078
GO:0016021
Cluster-22178.97734 GO:0005515
Cluster-22178.97735 GO:0006816 GO:0006810 GO:0042030 GO:0005246
GO:0016020

Cluster-22178.97746 GO:0019069 GO:0003676 GO:0005524 GO:0046872
GO:0016787
Cluster-22178.97750 GO:0016032 GO:0019012
Cluster-22178.97751 GO:0005515
Cluster-22178.97752 GO:0005515
Cluster-22178.97760 GO:0007186 GO:0004930 GO:0016021
Cluster-22178.97782 GO:0006355 GO:0016787 GO:0003677
Cluster-22178.97793 GO:0005515
Cluster-22178.97807 GO:0005515
Cluster-22178.97829 GO:0001510 GO:0008152 GO:0009452 GO:0000042
GO:0008168 GO:0005515 GO:0008757
Cluster-22178.97842 GO:0003723 GO:0003676
Cluster-22178.97846 GO:0016192 GO:0006508 GO:0006812 GO:0005515
GO:0004190 GO:0004867 GO:0008233 GO:0008324 GO:0016021
GO:0019031
Cluster-22178.97848 GO:0005515
Cluster-22178.97850 GO:0006465 GO:0055085 GO:0008233 GO:0022857
GO:0016021 GO:0005787
Cluster-22178.97856 GO:0007186 GO:0004930 GO:0016021
Cluster-22178.97869 GO:0048309 GO:0006914 GO:0051028 GO:0007049
GO:0005198 GO:0019028 GO:0019031
Cluster-22178.97883 GO:0006468 GO:0005524 GO:0004672 GO:0016773
GO:0016020
Cluster-22178.97889 GO:0006468 GO:0046983 GO:0005524 GO:0004672

Cluster-22178.97890 GO:0006355 GO:0003700 GO:0043565 GO:0005667
Cluster-22178.97895 GO:0045747 GO:0016310 GO:0007049 GO:0043087
GO:0006221 GO:0009069 GO:0004674 GO:0016836 GO:0005085
Cluster-22178.97904 GO:0007186 GO:0009847 GO:0006810 GO:0006869
GO:0004930 GO:0005319 GO:0005886 GO:0016021
Cluster-22178.97913 GO:0046856 GO:0034597 GO:0030246
Cluster-22178.97932 GO:0006118 GO:0016491 GO:0009055 GO:0016020
Cluster-22178.97945 GO:0006270 GO:0005515

Cluster-22178.97946 GO:0005515

Cluster-22178.97947 GO:0005515

Cluster-22178.97958 GO:0006397 GO:0005515 GO:0003723 GO:0005737 GO:0005634

Cluster-22178.97964 GO:0005515

Cluster-22178.97969 GO:0000226 GO:0045454 GO:0042254 GO:0006308 GO:0000910 GO:0006118 GO:0010508 GO:0006412 GO:0008855 GO:0008017 GO:0003735 GO:0015035 GO:0009055 GO:0003723 GO:0005515 GO:0045298 GO:0005840 GO:0019028 GO:0009318

Cluster-22178.97971 GO:0006118 GO:0010508 GO:0045454 GO:0000226 GO:0000910 GO:0006308 GO:0005515 GO:0003723 GO:0008017 GO:0009055 GO:0015035 GO:0008855 GO:0009318 GO:0019028 GO:0045298

Cluster-22178.97977 GO:0007165 GO:0030155 GO:0010508 GO:0045995 GO:0030334 GO:0007155 GO:0005102

Cluster-22178.9798 GO:0042967 GO:0008080

Cluster-22178.98002 GO:0003677

Cluster-22178.98007 GO:0007165 GO:0006955 GO:0006508 GO:0008152 GO:0016788 GO:0004181 GO:0008270 GO:0005125 GO:0005576

Cluster-22178.98027 GO:0045454 GO:0016020

Cluster-22178.98037 GO:0006810 GO:0005524

Cluster-22178.98047 GO:0051258 GO:0006308 GO:0045995 GO:0007094 GO:0030168 GO:0051607 GO:0030334 GO:0006606 GO:0007155 GO:0007165 GO:0030155 GO:0008092 GO:0045502 GO:0005515 GO:0008134 GO:0042803 GO:0008855 GO:0005102 GO:0030674 GO:0005737 GO:0019028 GO:0009318 GO:0030286 GO:0019898 GO:0005577 GO:0005667

Cluster-22178.98058 GO:0006570 GO:0006470 GO:0008138 GO:0004725

Cluster-22178.98059 GO:0006771 GO:0006570 GO:0019497 GO:0006470 GO:0005515 GO:0008138 GO:0046872 GO:0004725 GO:0003993

Cluster-22178.98062 GO:0016032 GO:0050920 GO:0046872 GO:0003824 GO:0005515 GO:0009288 GO:0005615

Cluster-22178.98069 GO:0006144 GO:0007165 GO:0006351 GO:0005524 GO:0003824 GO:0003968 GO:0005179 GO:0050662 GO:0003677 GO:0031379 GO:0005634

Cluster-22178.98084 GO:0006470 GO:0008138

Cluster-22178.98104 GO:0006508 GO:0008233

Cluster-22178.98105 GO:0005515

Cluster-22178.98115 GO:0055114 GO:0016491

Cluster-22178.98135 GO:0006468 GO:0016773 GO:0005524 GO:0004672 GO:0016020

Cluster-22178.98139 GO:0006144 GO:0007165 GO:0006508 GO:0006355 GO:0008270 GO:0005515 GO:0003677 GO:0004197 GO:0004114 GO:0043189 GO:0016020 GO:0005634

Cluster-22178.98146 GO:0006725 GO:0016853

Cluster-22178.98176 GO:0055114 GO:0016491

Cluster-22178.98182 GO:0046500 GO:0006479 GO:0006464 GO:0008152 GO:0006465 GO:0004719 GO:0008168 GO:0008233 GO:0005787 GO:0016021

Cluster-22178.98184 GO:0035091

Cluster-22178.98202 GO:0035556 GO:0007165 GO:0005515

Cluster-22178.98228 GO:0007165

Cluster-22178.98229 GO:0004252 GO:0005886

Cluster-22178.98251 GO:0008152 GO:0015074 GO:0003824 GO:0016021

Cluster-22178.98257 GO:0016567 GO:0006464 GO:0005515 GO:0016151 GO:0004842 GO:0046872 GO:0008270 GO:0005680

Cluster-22178.98266 GO:0006468 GO:0005524 GO:0004672

Cluster-22178.98278 GO:0006118 GO:0055114 GO:0009055 GO:0016625 GO:0016491 GO:0051536

Cluster-22178.98299 GO:0006810 GO:0016192 GO:0004222 GO:0005215 GO:0016020 GO:0016021

Cluster-22178.98302 GO:0007165 GO:0005975 GO:0005515 GO:0004553 GO:0005509 GO:0005578

Cluster-22178.98305 GO:0005515

Cluster-22178.98309 GO:0016068

Cluster-22178.98325 GO:0006265 GO:0003916 GO:0003677 GO:0005694
Cluster-22178.98328 GO:0016579 GO:0036459
Cluster-22178.98336 GO:0005515
Cluster-22178.98338 GO:0007049 GO:0005515
Cluster-22178.98343 GO:0006508 GO:0006260 GO:0004252
Cluster-22178.98344 GO:0006355 GO:0005634 GO:0016021
Cluster-22178.98349 GO:0019087 GO:0035434 GO:0006825 GO:0007186
GO:0005375 GO:0004930 GO:0016021
Cluster-22178.98372 GO:0019497 GO:0006771 GO:0003993 GO:0016012
Cluster-22178.98377 GO:0007165 GO:0005515
Cluster-22178.98415 GO:0006810 GO:0005215 GO:0016020
Cluster-22178.98427 GO:0005515
Cluster-22178.98457 GO:0045893 GO:0006468 GO:0007275 GO:0046983
GO:0005524 GO:0008270 GO:0004672
Cluster-22178.98461 GO:0046872
Cluster-22178.98463 GO:0048870 GO:0030168 GO:0051258 GO:0007165
GO:0007130 GO:0006810 GO:0005102 GO:0030674 GO:0005198
GO:0000795 GO:0005886 GO:0005577 GO:0031514 GO:0005882
Cluster-22178.98483 GO:0005515 GO:0003774 GO:0005524 GO:0016459
GO:0005856
Cluster-22178.98505 GO:0006528 GO:0008418
Cluster-22178.98513 GO:0051252 GO:0004540
Cluster-22178.98518 GO:0006777 GO:0006433 GO:0006511 GO:0006560
GO:0006525 GO:0004827 GO:0004175 GO:0005524 GO:0000166
GO:0005737 GO:0019773
Cluster-22178.9852 GO:0006351 GO:0006269 GO:0008146 GO:0004386
GO:0003896 GO:0008270 GO:0005730 GO:0005657
Cluster-22178.98534 GO:0006270 GO:0005515
Cluster-22178.98539 GO:0006887 GO:0001514 GO:0006448 GO:0020037
GO:0003723 GO:0005525 GO:0003746 GO:0000145 GO:0005840
GO:0005737
Cluster-22178.98557 GO:0006869 GO:0006725 GO:0006629 GO:0016853
GO:0008047 GO:0042627

Cluster-22178.98559 GO:0007165 GO:0005179
Cluster-22178.98589 GO:0016021
Cluster-22178.98596 GO:0006468 GO:0005524 GO:0004672 GO:0005515

Cluster-22178.9860 GO:0005515
Cluster-22178.98616 GO:0006725 GO:0019087 GO:0016853 GO:0016021
Cluster-22178.98667 GO:0006811 GO:0015814 GO:1902604 GO:0015846 GO:0015558
Cluster-22178.98673 GO:0007275 GO:0007186 GO:0004867 GO:0004930 GO:0003824 GO:0051536 GO:0005578 GO:0016021
Cluster-22178.98707 GO:0005515
Cluster-22178.98710 GO:0006886 GO:0006817 GO:0005315 GO:0005524 GO:0016887 GO:0016020 GO:0005622
Cluster-22178.98718 GO:0007186 GO:0004930 GO:0016021
Cluster-22178.98737 GO:0006468 GO:0016773 GO:0005524 GO:0004672 GO:0016020
Cluster-22178.98747 GO:0007186 GO:0004930 GO:0016021
Cluster-22178.98771 GO:0009878 GO:0046872
Cluster-22178.98784 GO:0006816 GO:0006874 GO:0005219 GO:0004190 GO:0016021 GO:0005622
Cluster-22178.98804 GO:0005515
Cluster-22178.98807 GO:0007165 GO:0006812 GO:0006835 GO:0006820 GO:0017153 GO:0005515 GO:0016020
Cluster-22178.98812 GO:0044237 GO:0003824
Cluster-22178.98842 GO:0061630 GO:0005515 GO:0046872 GO:0008270

Cluster-22178.98843 GO:0003723 GO:0008270 GO:0046872 GO:0005515 GO:0061630
Cluster-22178.98845 GO:0009395 GO:0046486 GO:0035556 GO:0007205 GO:0016301 GO:0005515 GO:0004143
Cluster-22178.98859 GO:0044564
Cluster-22178.98872 GO:0005515
Cluster-22178.98880 GO:0003682 GO:0000785

Cluster-22178.9892 GO:0009607 GO:0016021
Cluster-22178.98921 GO:0006468 GO:0016773 GO:0005524 GO:0004672 GO:0016020
Cluster-22178.98948 GO:0009966 GO:0015986 GO:0015992 GO:0015078 GO:0016021 GO:0000276
Cluster-22178.98954 GO:0006355 GO:0046983 GO:0003677
Cluster-22178.98955 GO:0006355 GO:0003677 GO:0046983
Cluster-22178.98979 GO:0005975 GO:0055114 GO:0016491 GO:0016616 GO:0004553
Cluster-22178.9899 GO:0001558 GO:0005520 GO:0008270 GO:0016942 GO:0005576 GO:0005886
Cluster-22178.99003 GO:0007165 GO:0007186 GO:0007606 GO:0005509 GO:0005515 GO:0004888 GO:0004930 GO:0016021
Cluster-22178.99035 GO:0005515 GO:0008270 GO:0005622
Cluster-22178.99038 GO:0006355 GO:0043565 GO:0003700 GO:0005634 GO:0005667 GO:0016020
Cluster-22178.99046 GO:0007049 GO:0006606 GO:0005515 GO:0017053
Cluster-22178.99061 GO:0006771 GO:0019497 GO:0005515 GO:0003993 GO:0046872
Cluster-22178.99095 GO:0015991 GO:0007165 GO:0014068 GO:0043248 GO:0055114 GO:0050776 GO:0043548 GO:0016820 GO:0005515 GO:0033743 GO:0005102 GO:0016020 GO:0000221
Cluster-22178.99116 GO:0005515
Cluster-22178.99128 GO:0030170 GO:0008483
Cluster-22178.99133 GO:0005515 GO:0004252 GO:0005509 GO:0016021
Cluster-22178.99142 GO:0016567 GO:0006508 GO:0006510 GO:0061630 GO:0005515 GO:0004842 GO:0004176 GO:0008270 GO:0046872
Cluster-22178.99143 GO:0016567 GO:0006508 GO:0006510 GO:0005515 GO:0061630 GO:0003723 GO:0004842 GO:0004176 GO:0008270 GO:0046872
Cluster-22178.99165 GO:0007165 GO:0006355 GO:0006289 GO:0000439
Cluster-22178.99181 GO:0009405 GO:0005198
Cluster-22178.99185 GO:0006355 GO:0005634

Cluster-22178.99186 GO:0006355 GO:0043565 GO:0003700 GO:0005667
Cluster-22178.99191 GO:0016567 GO:0004842
Cluster-22178.99218 GO:0006355 GO:0046983 GO:0005515
Cluster-22178.99221 GO:0016020
Cluster-22178.99245 GO:0008270 GO:0046983 GO:0005515
Cluster-22178.99254 GO:0006118 GO:0006119 GO:0015992 GO:0015074 GO:0008121
Cluster-22178.99260 GO:0005515 GO:0030234 GO:0016020
Cluster-22178.99281 GO:0007275 GO:0009966 GO:0016020
Cluster-22178.99313 GO:0019337 GO:0055114 GO:0006807 GO:0009399 GO:0046872 GO:0016163 GO:0016610 GO:0045202
Cluster-22178.99314 GO:0019337 GO:0055114 GO:0035556 GO:0006807 GO:0009399 GO:0046872 GO:0016163 GO:0016610 GO:0045202
Cluster-22178.99325 GO:0006355 GO:0005634
Cluster-22178.99329 GO:0006508 GO:0004869 GO:0008234 GO:0004197

Cluster-22178.99333 GO:0051252 GO:0004523 GO:0003676 GO:0005198 GO:0019028
Cluster-22178.99334 GO:0006470 GO:0008138
Cluster-22178.99343 GO:0006955 GO:0019028
Cluster-22178.99345 GO:0005515
Cluster-22178.99409 GO:0007606 GO:0007186 GO:0005201 GO:0004930 GO:0005578 GO:0016021
Cluster-22178.99411 GO:0019497 GO:0006771 GO:0046872 GO:0005515 GO:0003993
Cluster-22178.99421 GO:0051321 GO:0006468 GO:0004672 GO:0005524 GO:0005515 GO:0016020
Cluster-22178.99433 GO:0007155 GO:0005515 GO:0016021
Cluster-22178.99434 GO:0007155 GO:0005515 GO:0016021
Cluster-22178.99447 GO:0048013 GO:0006771 GO:0019497 GO:0003993 GO:0005515 GO:0046872
Cluster-22178.99449 GO:0005515
Cluster-22178.99474 GO:0003677

Cluster-22178.99480 GO:0005515
Cluster-22178.99510 GO:0006468 GO:0005524 GO:0004672 GO:0016773 GO:0016020
Cluster-22178.99543 GO:0006810 GO:0055085 GO:0022857 GO:0016020 GO:0016021
Cluster-22178.99548 GO:0055085 GO:0022857 GO:0016021
Cluster-22178.99585 GO:0005198 GO:0019028
Cluster-22178.99607 GO:0005515
Cluster-22178.99648 GO:0006486 GO:0006122 GO:0009312 GO:0008375 GO:0008455 GO:0005795 GO:0016021 GO:0005743 GO:0005750
Cluster-22178.9966 GO:0048478 GO:0006974 GO:0055114 GO:0007049 GO:0016717 GO:0005634
Cluster-22178.99662 GO:0006915
Cluster-22178.99664 GO:0055114 GO:0008033 GO:0006779 GO:0016117 GO:0019354 GO:0016491 GO:0004499 GO:0050660 GO:0050661 GO:0071949 GO:0005515 GO:0043115 GO:0016705
Cluster-22178.99667 GO:0006471 GO:0009405 GO:0045893 GO:1902208 GO:0003677 GO:0003956 GO:0005576
Cluster-22178.99668 GO:0005515
Cluster-22178.99674 GO:0005789 GO:0016021
Cluster-22178.99675 GO:0005515 GO:0008270 GO:0005622
Cluster-22178.99682 GO:0008146
Cluster-22178.9969 GO:0055114 GO:0016717
Cluster-22178.9971 GO:0016021
Cluster-22178.99722 GO:0016032 GO:0006672 GO:0019082 GO:0006807 GO:0016811 GO:0016021
Cluster-22178.99734 GO:0006355 GO:0007264 GO:0003700 GO:0005096 GO:0043565 GO:0005667
Cluster-22178.99739 GO:0015979 GO:0009523 GO:0016020
Cluster-22178.99765 GO:0016773
Cluster-22178.99766 GO:0006816 GO:0070588 GO:0005262 GO:0016020
Cluster-22178.99808 GO:0070072
Cluster-22178.99894 GO:0006355 GO:0016567 GO:0006310 GO:0006352

GO:0003677 GO:0000150 GO:0004842 GO:0016987 GO:0003700
GO:0005667 GO:0005622
Cluster-22178.9992 GO:0019497 GO:0006771 GO:0003993 GO:0005515
GO:0046872
Cluster-22178.99926 GO:0007186 GO:0007165 GO:0004871 GO:0005834
Cluster-22178.99942 GO:0015889 GO:0031419 GO:0008270
Cluster-22178.99978 GO:0005783
Cluster-22713.1 GO:0009307 GO:0006308 GO:0003677 GO:0005524
GO:0009036 GO:0009359
Cluster-22963.2 GO:0007165 GO:0005515 GO:0005509 GO:0005578
Cluster-257.0 GO:0005886 GO:0031225
Cluster-26018.0 GO:0007165 GO:0009058 GO:0003824 GO:0005102
Cluster-26582.1 GO:0007218 GO:0005185 GO:0005576
Cluster-27102.0 GO:0006303 GO:0004003 GO:0005515 GO:0003677
GO:0005657
Cluster-28549.0 GO:0005515
Cluster-29748.0 GO:0045502 GO:0008134 GO:0042803 GO:0005667
GO:0030286
Cluster-30092.0 GO:0055114 GO:0010277
Cluster-30311.0 GO:0019031
Cluster-30478.0 GO:0005515 GO:0005509
Cluster-30568.0 GO:0042254 GO:0006412 GO:0006298 GO:0003735
GO:0004519 GO:0005622 GO:0005840
Cluster-30643.0 GO:0019064 GO:0006308 GO:0006914 GO:0048870
GO:0006810 GO:0008092 GO:0008855 GO:0016021 GO:0019898
GO:0019031 GO:0005737 GO:0005886 GO:0031514 GO:0009318
Cluster-30976.1 GO:0007339 GO:0005515 GO:0016021 GO:0016020
GO:0005576
Cluster-31189.0 GO:0015976 GO:0006094 GO:0016310 GO:0006096
GO:0004618
Cluster-31922.0 GO:0016042 GO:0009395 GO:0046339 GO:0004435
GO:0005509 GO:0016021
Cluster-32048.2 GO:0007186 GO:0004930 GO:0008537 GO:0016021

GO:0016020

Cluster-32261.0 GO:0006814 GO:0005272 GO:0016020

Cluster-32876.1 GO:0055114 GO:0008152 GO:0016491

Cluster-33351.0 GO:0006351 GO:0006144 GO:0019083 GO:0006206
GO:0003899 GO:0003677 GO:0005730

Cluster-33421.0 GO:0055085 GO:0006811 GO:0005216 GO:0016020

Cluster-33787.0 GO:0008270

Cluster-33914.0 GO:0042254 GO:0006412 GO:0003735 GO:0015934
GO:0005840

Cluster-34011.1 GO:0009847 GO:0008168 GO:0046983 GO:0016021

Cluster-34534.0 GO:0006820 GO:0008509 GO:0016021

Cluster-34558.0 GO:0005515

Cluster-34580.2 GO:0046872

Cluster-35002.1 GO:0005509

Cluster-35051.0 GO:0006071 GO:0046486 GO:0008152 GO:0016491
GO:0004371

Cluster-35157.0 GO:0004190 GO:0016021

Cluster-35426.0 GO:0042254 GO:0006412 GO:0003735 GO:0005622
GO:0005840

Cluster-35700.0 GO:0006508 GO:0009002

Cluster-36037.0 GO:0006355 GO:0008270 GO:0005524 GO:0016818
GO:0003677 GO:0005634

Cluster-36097.0 GO:0007165
