# Supplementary material for: Transcriptomic analysis of female and male gonads in juvenile snakeskin gourami (Trichopodus pectoralis)
Source: Sci Rep. 2020 Mar 23;10:5240. doi: 10.1038/s41598-020-61738-0 (PMC7090014; doi:10.1038/s41598-020-61738-0)
Supplement: Supplementary file 3 — Table S3. [file 41598_2020_61738_MOESM3_ESM.pdf]

Transcriptomic analysis of female and male gonads in juvenile snakeskin gourami (Trichopodus pectoralis)

Surintorn Boonanuntanasarn1*, Araya Jangprai1, Uthairat Na-Nakorn2*
1 School of Animal Technology and Innovation, Institute of Agricultural Technology, Suranaree University of Technology, 111 University Avenue, Muang, Nakhon Ratchasima, 30000, Thailand
2 Department of Aquaculture, Faculty of Fisheries, Kasetsart University, 50 Paholyothin Road, Chatujak, Bangkok 10900, Thailand

*Corresponding author:
S. Boonanuntanasarn; surinton@sut.ac.th, Tel: +6644224371, Fax: +6644224150
Uthairat Na-Nakorn; e-mail: ffisurn@ku.ac.th

Table S3 GO enrichment of unigenes that were expressed in testis.

Cluster-10049.8 GO:0006508 GO:0004190
Cluster-10102.0 GO:0005634
Cluster-10383.0 GO:0005515
Cluster-10616.1 GO:0005096
Cluster-10835.0 GO:0007186 GO:0004930 GO:0016021
Cluster-11116.1 GO:0007005 GO:0019497 GO:0006771 GO:0003993
GO:0005515 GO:0046872 GO:0032865
Cluster-11509.0 GO:0019087 GO:0006508 GO:0016032 GO:0008233
GO:0004252 GO:0008236
Cluster-11644.0 GO:0005515
Cluster-11709.0 GO:0007156 GO:0005509 GO:0005634 GO:0016020
Cluster-11743.0 GO:0007165 GO:0007268 GO:0009405 GO:0030550
GO:0005576
Cluster-11805.0 GO:0006357 GO:0001104 GO:0008270 GO:0005518
GO:0016592 GO:0005581
Cluster-11899.0 GO:0014068 GO:0050776 GO:0006508 GO:0007165

GO:0005102 GO:0008233 GO:0043548 GO:0016020

Cluster-12098.0 GO:0016020

Cluster-12611.3 GO:0007186 GO:0004930 GO:0016021

Cluster-13872.1 GO:0019236 GO:0009607 GO:0007606 GO:0050909

GO:0007186 GO:0016503 GO:0004930 GO:0016021

Cluster-14367.1 GO:0000045 GO:0008270 GO:0005737

Cluster-14512.0 GO:0006811 GO:0005216 GO:0016020

Cluster-14599.0 GO:0006118 GO:0020037 GO:0009055

Cluster-1502.1 GO:0007166 GO:0007165 GO:0004871 GO:0005634

Cluster-15057.0 GO:0006813 GO:0005516 GO:0015269 GO:0016021

Cluster-15118.0 GO:0007010 GO:0003779

Cluster-15719.0 GO:0005975 GO:0008270 GO:0016832

Cluster-15846.5 GO:0006813 GO:0005242 GO:0016021 GO:0008076

Cluster-15999.0 GO:0006260 GO:0030168 GO:0015031 GO:0051607

GO:0006457 GO:0016567 GO:0007165 GO:0006810 GO:0007021

GO:0048870 GO:0006308 GO:0051258 GO:0006118 GO:0016032

GO:0006606 GO:0006914 GO:0022900 GO:0019089 GO:0006886

GO:0010508 GO:0006397 GO:0005102 GO:0008855 GO:0031386

GO:0030674 GO:0005525 GO:0008134 GO:0045502 GO:0015631

GO:0008565 GO:0003677 GO:0005506 GO:0009055 GO:0003924

GO:0016881 GO:0005198 GO:0042803 GO:0020037 GO:0051082

GO:0005886 GO:0030286 GO:0005882 GO:0016021 GO:0019031

GO:0031514 GO:0009318 GO:0019028 GO:0005577 GO:0016272

GO:0042597 GO:0016020 GO:0005667 GO:0045298 GO:0000445

GO:0005737

Cluster-16299.0 GO:0006144 GO:0006508 GO:0004252 GO:0003968

GO:0017111 GO:0004197 GO:0031379

Cluster-16340.2 GO:0006813 GO:0006820 GO:0008556 GO:0016021

Cluster-16704.0 GO:0016817

Cluster-17026.0 GO:0042254 GO:0001522 GO:0030515 GO:0072588

Cluster-171.2 GO:0009072 GO:0006570 GO:0042207 GO:0016310

GO:0004673 GO:0004334 GO:0009365

Cluster-17144.0 GO:0006260 GO:0007165 GO:0043093 GO:0008406

GO:0008283	GO:0007304	GO:0007049	GO:0006308	GO:0007010
GO:0000917	GO:0040007	GO:0006355	GO:0008855	GO:0003700
GO:0005213	GO:0003779	GO:0008083	GO:0005667	GO:0005737
GO:0042600	GO:0009318	GO:0005576		
Cluster-17269.0	GO:0006355	GO:0005515		
Cluster-17536.0	GO:0007156	GO:0005509	GO:0005515	GO:0016829
GO:0016021	GO:0016020			
Cluster-17658.1	GO:0042254	GO:0006412	GO:0003735	GO:0005840
GO:0005622				
Cluster-17874.0	GO:0006508	GO:0008017	GO:0008236	GO:0016787
GO:0045298				
Cluster-18002.1	GO:0006955	GO:0007165	GO:0040007	GO:0008283
GO:0005134	GO:0008083	GO:0005893	GO:0005576	
Cluster-18326.2	GO:0005515			
Cluster-18414.0	GO:0007264	GO:0006355	GO:0010506	GO:0005509
GO:0003924	GO:0003700	GO:0005525	GO:0005667	GO:0005615
Cluster-18682.0	GO:0006351	GO:0006206	GO:0006144	GO:0006820
GO:0003677	GO:0003899	GO:0005730	GO:0016021	
Cluster-18972.0	GO:0005515	GO:0016787		
Cluster-19169.1	GO:0003723			
Cluster-19799.2	GO:0003950			
Cluster-19827.0	GO:0015696	GO:0009405	GO:0005509	GO:0008519
GO:0016020	GO:0005576			
Cluster-19884.0	GO:0006118	GO:0003954	GO:0005739	
Cluster-19894.0	GO:0005515			
Cluster-20191.0	GO:0016746			
Cluster-20513.0	GO:0051262	GO:0005515		
Cluster-20632.0	GO:0007165	GO:0009416	GO:0005179	GO:0005576
Cluster-20691.0	GO:0015992	GO:0055114	GO:0006119	GO:0006118
GO:0008121				
Cluster-20741.0	GO:0007186	GO:0004930	GO:0016021	
Cluster-20832.0	GO:0008033	GO:0055114	GO:0071949	GO:0016491
GO:0050660				

Cluster-20898.0 GO:0035556 GO:0009190 GO:0006468 GO:0016849
GO:0004672
Cluster-20918.0 GO:0006412 GO:0042254 GO:0003735 GO:0005840
GO:0005622
Cluster-2106.1 GO:0006955 GO:0007165 GO:0006935 GO:0008009
GO:0005576
Cluster-21237.0 GO:0015979 GO:0009538 GO:0009522
Cluster-21244.1 GO:0007165 GO:0008283 GO:0040007 GO:0008083
Cluster-21404.0 GO:0006955 GO:0002474 GO:0005515 GO:0042612
GO:0005576
Cluster-21441.0 GO:0009405 GO:0006810 GO:0055085 GO:0005215
GO:0045735 GO:0016020
Cluster-21498.0 GO:0020037
Cluster-21846.0 GO:0005634
Cluster-21904.0 GO:0034599 GO:0007010 GO:0003779 GO:0005789
Cluster-22062.0 GO:0016020
Cluster-22083.0 GO:0000077 GO:0006355 GO:0006281 GO:0003700
GO:0043565 GO:0005667 GO:0030896
Cluster-22178.100000 GO:0009058 GO:0030170
Cluster-22178.100001 GO:0008289 GO:0016020
Cluster-22178.100002 GO:0016310 GO:0016791 GO:0004673 GO:0009365
Cluster-22178.100003 GO:0016310 GO:0016791 GO:0004673 GO:0009365
Cluster-22178.100005 GO:0006355 GO:0003677 GO:0005515 GO:0003700
GO:0005622 GO:0005667
Cluster-22178.100007 GO:0007130 GO:0000795
Cluster-22178.100008 GO:0006914 GO:0010508 GO:0006886 GO:0006810
GO:0007130 GO:0016021 GO:0000795 GO:0005886 GO:0005783
GO:0005634
Cluster-22178.100010 GO:0005515 GO:0016787
Cluster-22178.100019 GO:0007264 GO:0006310 GO:0006206 GO:0006013
GO:0006000 GO:0006281 GO:0006139 GO:0009378 GO:0003873
GO:0098519 GO:0005524 GO:0016887 GO:0004127 GO:0003724
GO:0003723 GO:0005525 GO:0009379 GO:0005657

Cluster-22178.100021 GO:0005515 GO:0003682 GO:0000785
Cluster-22178.100025 GO:0006355 GO:0007155 GO:0005198 GO:0003700
GO:0008270 GO:0046872 GO:0043565 GO:0005667 GO:0031012
Cluster-22178.100028 GO:0031175 GO:0008017 GO:0030507 GO:0005516
GO:0045298 GO:0008091
Cluster-22178.100030 GO:0003677
Cluster-22178.100031 GO:0055114 GO:0030168 GO:0051258 GO:0006355
GO:0019069 GO:0044237 GO:0007165 GO:0016817 GO:0005102
GO:0051287 GO:0003723 GO:0030674 GO:0016616 GO:0005577
Cluster-22178.100033 GO:0015986 GO:0031175 GO:0015992 GO:0006826
GO:0005516 GO:0005506 GO:0030507 GO:0015078 GO:0008849
GO:0045263 GO:0005737 GO:0008091
Cluster-22178.100034 GO:0006813 GO:0003677 GO:0005242 GO:0016021
GO:0008076
Cluster-22178.100039 GO:0006260 GO:0006351 GO:0006269 GO:0003896
GO:0008270 GO:0046872 GO:0003677 GO:0005515 GO:0005657
GO:0005622 GO:0005730
Cluster-22178.100040 GO:0006269 GO:0006351 GO:0006260 GO:0005515
GO:0003677 GO:0046872 GO:0008270 GO:0003896 GO:0005634
GO:0005730 GO:0005622 GO:0005657
Cluster-22178.100041 GO:0006351 GO:0006269 GO:0006260 GO:0003677
GO:0005515 GO:0003896 GO:0046872 GO:0008270 GO:0005730
GO:0005634 GO:0005657 GO:0005622
Cluster-22178.100042 GO:0019497 GO:0007155 GO:0006771 GO:0009405
GO:0016798 GO:0046872 GO:0003993 GO:0030246 GO:0005515
GO:0005576
Cluster-22178.100044 GO:0007155 GO:0006811 GO:0003729 GO:0005515
GO:0016021 GO:0019012 GO:0016020
Cluster-22178.100045 GO:0006811 GO:0005515 GO:0016020
Cluster-22178.100046 GO:0007155 GO:0006811 GO:0005515 GO:0016020
Cluster-22178.100050 GO:0006952 GO:0004190 GO:0005576 GO:0016021
Cluster-22178.100051 GO:0007165 GO:0006811 GO:0007268 GO:0055085
GO:0004970 GO:0005216 GO:0016020

Cluster-22178.100059 GO:0015031 GO:0046938 GO:0016032 GO:0010038
GO:0006270 GO:0008565 GO:0046872 GO:0005515 GO:0016756
GO:0005198 GO:0016021 GO:0016020
Cluster-22178.100060 GO:0046983
Cluster-22178.100061 GO:0043462 GO:0006355 GO:0006813 GO:0016829
GO:0008556 GO:0005886 GO:0005634
Cluster-22178.100062 GO:0006886 GO:0016310 GO:0008152 GO:0032922
GO:0006457 GO:0015031 GO:0006260 GO:0042803 GO:0016301
GO:0000976 GO:0008168 GO:0016774 GO:0051087 GO:0008270
GO:0008565 GO:0000774 GO:0005737 GO:0016020 GO:0005622
Cluster-22178.100072 GO:0006338 GO:0016818 GO:0003676 GO:0005524
GO:0005634
Cluster-22178.100078 GO:0005887
Cluster-22178.100079 GO:0006810 GO:0006814 GO:0045893 GO:0055085
GO:0015700 GO:0005215 GO:0015105 GO:0016020 GO:0016021
Cluster-22178.100080 GO:0006771 GO:0019497 GO:0046872 GO:0005515
GO:0003993 GO:0019031
Cluster-22178.100083 GO:0006811 GO:0055085 GO:0006352 GO:0019087
GO:0005515 GO:0005216 GO:0016020 GO:0005634 GO:0016021
Cluster-22178.100084 GO:0008092 GO:0005737 GO:0019898
Cluster-22178.100086 GO:0006313 GO:0004803 GO:0003677 GO:0005096
GO:0005615
Cluster-22178.100089 GO:0007275 GO:0019685 GO:0030494 GO:0016836
GO:0005578
Cluster-22178.100090 GO:0003723 GO:0019013
Cluster-22178.100094 GO:0006397 GO:0003723 GO:0005515 GO:0005737
GO:0005634
Cluster-22178.100096 GO:0007264 GO:0000287 GO:0003924 GO:0005525
GO:0030976
Cluster-22178.100097 GO:0015991 GO:0005515 GO:0016820 GO:0000221
Cluster-22178.100099 GO:0045747 GO:0006221 GO:0000917 GO:0007049
GO:0006470 GO:0008138 GO:0019239
Cluster-22178.100100 GO:0005515

Cluster-22178.100103 GO:0045995 GO:0006886 GO:0006412 GO:0030334
GO:0070206 GO:0007165 GO:0030155 GO:0042254 GO:0005198
GO:0005515 GO:0005102 GO:0003735 GO:0005840 GO:0019031
GO:0005801 GO:0019028 GO:0016020
Cluster-22178.100104 GO:0005515
Cluster-22178.100105 GO:0030833 GO:0006298 GO:0034314 GO:0055085
GO:0030983 GO:0005524 GO:0005885 GO:0015629 GO:0016021
Cluster-22178.100106 GO:0006298 GO:0030983 GO:0005524
Cluster-22178.100108 GO:0015979 GO:0009523 GO:0016020
Cluster-22178.100109 GO:0015979 GO:0009523 GO:0016020
Cluster-22178.100110 GO:0007165 GO:0006355 GO:0005515 GO:0003700
GO:0008270 GO:0043565 GO:0005667
Cluster-22178.100113 GO:0006396 GO:0019031 GO:0005622
Cluster-22178.100118 GO:0015979 GO:0006355 GO:0005515 GO:0042651
GO:0009654 GO:0009523
Cluster-22178.100119 GO:0005515
Cluster-22178.10012 GO:0045454 GO:0006118 GO:0016301 GO:0015035
GO:0009055
Cluster-22178.100127 GO:0007264 GO:0006886 GO:0005543 GO:0003924
GO:0005525
Cluster-22178.100129 GO:0007423 GO:0016773 GO:0005212
Cluster-22178.100130 GO:0010826 GO:0006355 GO:0042384 GO:0003700
GO:0005622 GO:0036038 GO:0005667
Cluster-22178.100133 GO:0008430
Cluster-22178.100136 GO:0008033
Cluster-22178.100139 GO:0006810 GO:0016021
Cluster-22178.100144 GO:0006810 GO:0018189 GO:0055085 GO:0005524
GO:0042626 GO:0005515 GO:0016021
Cluster-22178.100145 GO:0006810 GO:0005215 GO:0016021
Cluster-22178.100150 GO:0005515
Cluster-22178.100157 GO:0006270 GO:0006874 GO:0006816 GO:0055085
GO:0007031 GO:0006206 GO:0006144 GO:0006351 GO:0030001
GO:0070588 GO:0006811 GO:0003677 GO:0005509 GO:0046873

GO:0005216 GO:0008270 GO:0003899 GO:0005515 GO:0005262
GO:0005219 GO:0016020 GO:0005730 GO:0005779 GO:0005622
GO:0016021
Cluster-22178.100158 GO:0006470 GO:0055114 GO:0006570 GO:0050660
GO:0004725 GO:0004499 GO:0008138 GO:0050661
Cluster-22178.100175 GO:0042981 GO:0006914 GO:0006807 GO:0045502
GO:0005515 GO:0008134 GO:0042803 GO:0016151 GO:0005667
GO:0030286
Cluster-22178.100177 GO:0019901
Cluster-22178.100178 GO:0019901
Cluster-22178.100179 GO:0015074 GO:0016567 GO:0004842 GO:0016020
Cluster-22178.100181 GO:0042475 GO:0042476 GO:0030345 GO:0005578
Cluster-22178.100183 GO:0006119 GO:0006118 GO:0015992 GO:0055114
GO:0008121
Cluster-22178.100184 GO:0005515
Cluster-22178.100187 GO:0005515
Cluster-22178.100194 GO:0009405 GO:0010506 GO:0045735 GO:0008270
GO:0005615 GO:0016020
Cluster-22178.100195 GO:0007166 GO:0007160 GO:0007165 GO:0016485
GO:0009405 GO:0005509 GO:0004871 GO:0045502 GO:0042803
GO:0008134 GO:0005515 GO:0030286 GO:0005667 GO:0001527
GO:0016021 GO:0005840 GO:0005634
Cluster-22178.100196 GO:0000917
Cluster-22178.100197 GO:0006281 GO:0006139 GO:0006310 GO:0007264
GO:0006000 GO:0006206 GO:0006013 GO:0003723 GO:0005525
GO:0003873 GO:0098519 GO:0009378 GO:0003724 GO:0004127
GO:0016887 GO:0005524 GO:0009379 GO:0005657
Cluster-22178.100201 GO:0009069 GO:0016310 GO:0004674
Cluster-22178.100206 GO:0006508 GO:0006123 GO:0015992 GO:0016788
GO:0004222 GO:0004129 GO:0008270 GO:0005576 GO:0005743
GO:0045277 GO:0031012
Cluster-22178.100207 GO:0006508 GO:0015992 GO:0006123 GO:0016788
GO:0008270 GO:0004129 GO:0004222 GO:0005576 GO:0031012

GO:0045277 GO:0005743
Cluster-22178.100208 GO:0006355 GO:0006308 GO:0051258 GO:0042157
GO:0019069 GO:0006810 GO:0006869 GO:0007165 GO:0030168
GO:0003723 GO:0008289 GO:0030674 GO:0008855 GO:0005102
GO:0005576 GO:0009318 GO:0005886 GO:0005577
Cluster-22178.100209 GO:0007018 GO:0006810 GO:0007017 GO:0003677
GO:0003777 GO:0016887 GO:0005524 GO:0005874 GO:0030286
Cluster-22178.100212 GO:0006520 GO:0042302 GO:0005856
Cluster-22178.100214 GO:0046872
Cluster-22178.100219 GO:0006355 GO:0005515 GO:0003677
Cluster-22178.100220 GO:0003676
Cluster-22178.100229 GO:0003723
Cluster-22178.100230 GO:0006811 GO:0055085 GO:0006813 GO:0007165
GO:0007268 GO:0004970 GO:0005242 GO:0005216 GO:0016021
GO:0008076 GO:0016020
Cluster-22178.100232 GO:0009116 GO:0019058 GO:0007165 GO:0003824
GO:0004871 GO:0043531 GO:0016021 GO:0042470
Cluster-22178.100234 GO:0007275 GO:0005201 GO:0005578 GO:0005581
Cluster-22178.100238 GO:0009399 GO:0006355 GO:0003700 GO:0005667
Cluster-22178.100239 GO:0006891 GO:0016021 GO:0016020 GO:0017119
Cluster-22178.100241 GO:0015991 GO:0005515 GO:0016820 GO:0000221
Cluster-22178.100247 GO:0042407 GO:0010508 GO:0006810 GO:0005215
GO:0061617 GO:0044284
Cluster-22178.100249 GO:0055114 GO:0003676 GO:0016645
Cluster-22178.100250 GO:0032784 GO:0003677
Cluster-22178.100254 GO:0007165 GO:0008158 GO:0016020
Cluster-22178.100255 GO:0045893 GO:0016032 GO:0055114 GO:0016491
GO:0019012 GO:0005634
Cluster-22178.100257 GO:0019825 GO:0020037
Cluster-22178.100269 GO:0006508 GO:0004252
Cluster-22178.100271 GO:0016055 GO:0007165 GO:0007275 GO:0006298
GO:0030983 GO:0098519 GO:0005524 GO:0005102 GO:0005576
Cluster-22178.100273 GO:0008270 GO:0005515

Cluster-22178.100277 GO:0005515 GO:0008289 GO:0008270 GO:0046872

Cluster-22178.100278 GO:0006810 GO:0005515 GO:0008270 GO:0046872
GO:0016021
Cluster-22178.100283 GO:0016598 GO:0004057
Cluster-22178.100287 GO:0005515 GO:0003677
Cluster-22178.100288 GO:0007186 GO:0055085 GO:0006487 GO:0016787
GO:0004930 GO:0008250 GO:0016021
Cluster-22178.100290 GO:0006355 GO:0008270 GO:0043565 GO:0003700
GO:0005667
Cluster-22178.100293 GO:0003333 GO:0006810 GO:0006865 GO:0055085
GO:0015171 GO:0016020
Cluster-22178.100294 GO:0055085 GO:0006814 GO:0006810 GO:0005215
GO:0061630 GO:0005515 GO:0016021 GO:0019867 GO:0016020
Cluster-22178.100299 GO:0007165 GO:0005515 GO:0004872 GO:0046872
GO:0016021
Cluster-22178.100301 GO:0008283 GO:0040007 GO:0007165 GO:0008083
GO:0016020
Cluster-22178.100303 GO:0008654 GO:0006464 GO:0007165 GO:0046486
GO:0008283 GO:0040007 GO:0008715 GO:0008083 GO:0003697
GO:0016020
Cluster-22178.100305 GO:0005509 GO:0016462
Cluster-22178.100306 GO:0006144 GO:0032402 GO:0008152 GO:0016491
GO:0003968 GO:0031379 GO:0042470
Cluster-22178.10031 GO:0006468 GO:0004672 GO:0005524
Cluster-22178.100310 GO:0006118 GO:0019646 GO:0009055
Cluster-22178.100311 GO:0007155 GO:0016755 GO:0016020
Cluster-22178.100325 GO:0016192 GO:0006886 GO:0045444 GO:0019072
GO:0005515 GO:0042025 GO:0030117
Cluster-22178.100328 GO:0060272 GO:0070121 GO:0035143 GO:0007154
GO:0042246 GO:0035050 GO:0007155 GO:0031101 GO:0035162
GO:0008283 GO:0022832 GO:0055077 GO:0016021 GO:0005922
Cluster-22178.100329 GO:0008430

Cluster-22178.100332 GO:0005515
Cluster-22178.100333 GO:0000723 GO:0006281 GO:0007017 GO:0007018
GO:0005524 GO:0003777 GO:0003678 GO:0008017 GO:0005874
GO:0005657 GO:0045298
Cluster-22178.100334 GO:0005524
Cluster-22178.100335 GO:0007160 GO:0006520 GO:0055114
Cluster-22178.100336 GO:0005515 GO:0046872
Cluster-22178.100337 GO:0009117 GO:0019872 GO:0030639 GO:0008830
GO:0043565
Cluster-22178.100345 GO:0015992 GO:0015991 GO:0015078 GO:0033179
Cluster-22178.100346 GO:0043248 GO:0006886 GO:0016192 GO:0015991
GO:0007155 GO:0005515 GO:0016820 GO:0000221 GO:0030117
Cluster-22178.100347 GO:0007165 GO:0006955 GO:0006396 GO:0005126
GO:0005576 GO:0019031 GO:0016021 GO:0001527 GO:0005622
Cluster-22178.100349 GO:0005515
Cluster-22178.100350 GO:0040007 GO:0005737
Cluster-22178.100352 GO:0050909 GO:0007186 GO:0004930 GO:0016021
Cluster-22178.100354 GO:0007165
Cluster-22178.100355 GO:0007165
Cluster-22178.100357 GO:0006355 GO:0043565 GO:0008270 GO:0005634
Cluster-22178.100360 GO:0046872 GO:0008270 GO:0005515 GO:0005622
GO:0031083
Cluster-22178.100363 GO:0003723
Cluster-22178.100370 GO:0006366 GO:0006384 GO:0008017 GO:0003700
GO:0005515 GO:0045298 GO:0016021 GO:0005667 GO:0005634
Cluster-22178.100372 GO:0061630 GO:0005515 GO:0019867
Cluster-22178.100373 GO:0030833 GO:0006298 GO:0034314 GO:0055085
GO:0030983 GO:0005524 GO:0005885 GO:0015629 GO:0016021
Cluster-22178.100374 GO:0042407 GO:0044284 GO:0061617
Cluster-22178.100375 GO:0007165 GO:0005515 GO:0005509 GO:0005578
Cluster-22178.100376 GO:0006406 GO:0007131 GO:0007130 GO:0007283
GO:0003723 GO:0005634 GO:0005737
Cluster-22178.100383 GO:0007049 GO:0048870 GO:0000917 GO:0006468

GO:0006355 GO:0006260 GO:0070206 GO:0043093 GO:0003700
GO:0004672 GO:0043565 GO:0003887 GO:0001882 GO:0005524
GO:0003677 GO:0019867 GO:0005737 GO:0042575 GO:0031514
GO:0005667
Cluster-22178.100384 GO:0009069 GO:0006468 GO:0007165 GO:0016310
GO:0005524 GO:0005102 GO:0004674
Cluster-22178.100385 GO:0043093 GO:0006260 GO:0070206 GO:0000917
GO:0006468 GO:0006355 GO:0007049 GO:0048870 GO:0051382
GO:0003677 GO:0005524 GO:0003887 GO:0001882 GO:0003700
GO:0043565 GO:0004672 GO:0005667 GO:0031514 GO:0042575
GO:0000776 GO:0019867 GO:0005737
Cluster-22178.100386 GO:0006811 GO:0055085 GO:0005216 GO:0016020
Cluster-22178.100390 GO:0006940 GO:0019684 GO:0006118 GO:0045156
GO:0030077 GO:0016021
Cluster-22178.100391 GO:0009252 GO:0051258 GO:0030168 GO:0048193
GO:0000226 GO:0090063 GO:0009405 GO:0007165 GO:0005102
GO:0030674 GO:0016755 GO:0005577 GO:0000922 GO:0005815
GO:0016020
Cluster-22178.100393 GO:0046872
Cluster-22178.100403 GO:0006470 GO:0006570 GO:0004725 GO:0005515

Cluster-22178.100414 GO:0006470 GO:0006570 GO:0008138 GO:0004725

Cluster-22178.100419 GO:0006886 GO:0016192 GO:0005515 GO:0005198
GO:0030130 GO:0030132
Cluster-22178.100421 GO:0006614 GO:0006810 GO:0016887 GO:0003924
GO:0005524 GO:0005525 GO:0005576
Cluster-22178.100422 GO:0035556
Cluster-22178.100423 GO:0035556 GO:0019028
Cluster-22178.100424 GO:0007186 GO:0006869 GO:0005319 GO:0005515
GO:0004930 GO:0016021
Cluster-22178.100428 GO:0007049 GO:0030168 GO:0051258 GO:0048870
GO:0007165 GO:0005102 GO:0005524 GO:0030674 GO:0016021

GO:0005577 GO:0031514
Cluster-22178.100431 GO:0007275 GO:0042254 GO:0003735 GO:0005198
GO:0005201 GO:0005578 GO:0005739 GO:0005581 GO:0005840
Cluster-22178.100434 GO:0006914 GO:0007049 GO:0006260 GO:0006606
GO:0016787 GO:0003723 GO:0005515 GO:0003677 GO:0016887
GO:0005524 GO:0003724
Cluster-22178.100435 GO:0051056 GO:0005096
Cluster-22178.100436 GO:0016192 GO:0006914 GO:0016021
Cluster-22178.100440 GO:0006470 GO:0019497 GO:0006570 GO:0006771
GO:0004725 GO:0003993 GO:0046872 GO:0005515
Cluster-22178.100441 GO:0005515
Cluster-22178.100446 GO:0005515 GO:0016020
Cluster-22178.100448 GO:0007017 GO:0000398 GO:0005681 GO:0005875
Cluster-22178.100450 GO:0007606 GO:0040007 GO:0008283 GO:0050790
GO:0007186 GO:0007165 GO:0019236 GO:0007411 GO:0008083
GO:0030234 GO:0005125 GO:0016503 GO:0004930 GO:0016021
Cluster-22178.100456 GO:0008152 GO:0044564 GO:0008484 GO:0003824

Cluster-22178.100462 GO:0006812 GO:0007165 GO:0006820 GO:0006835
GO:0005102 GO:0017153 GO:0016020
Cluster-22178.100463 GO:0006812 GO:0007165 GO:0006820 GO:0006835
GO:0005102 GO:0017153 GO:0016020
Cluster-22178.100464 GO:0003677
Cluster-22178.100466 GO:0046872
Cluster-22178.10047 GO:0072519 GO:0006508 GO:0006118 GO:0016567
GO:0016491 GO:0008233 GO:0009055 GO:0004842 GO:0004222
GO:0031012 GO:0016020
Cluster-22178.100473 GO:0055114 GO:0005507 GO:0016491
Cluster-22178.100475 GO:0006725 GO:0005515 GO:0004497
Cluster-22178.100476 GO:0006725 GO:0004497 GO:0005515
Cluster-22178.100477 GO:0006886 GO:0010508 GO:0007130 GO:0006810
GO:0006914 GO:0005783 GO:0005634 GO:0016021 GO:0000795
GO:0005886

Cluster-22178.100478 GO:0007130 GO:0000795
Cluster-22178.100482 GO:0016567 GO:0008270 GO:0046872 GO:0005515
GO:0004842 GO:0005680
Cluster-22178.100485 GO:0006118 GO:0009055 GO:0009512
Cluster-22178.100486 GO:0006355 GO:0043565 GO:0008270 GO:0005634
Cluster-22178.100487 GO:0008270
Cluster-22178.100491 GO:0006825 GO:0007186 GO:0035434 GO:0004930
GO:0005375 GO:0016021
Cluster-22178.100492 GO:0005515
Cluster-22178.100493 GO:0005515
Cluster-22178.100495 GO:0019058 GO:0007165 GO:0009116 GO:0043531
GO:0003824 GO:0004871 GO:0042470 GO:0016021
Cluster-22178.100497 GO:0008146
Cluster-22178.100504 GO:0006811 GO:0009395 GO:0006351 GO:0006355
GO:0006629 GO:0055085 GO:0035556 GO:0006260 GO:0007165
GO:0006270 GO:0006810 GO:0050790 GO:0046339 GO:0006874
GO:0006816 GO:0005219 GO:0003887 GO:0030234 GO:0003682
GO:0004435 GO:0005515 GO:0016773 GO:0035091 GO:0003677
GO:0005216 GO:0016021 GO:0019031 GO:0042575 GO:0000785
GO:0000775 GO:0005634 GO:0005622 GO:0016020
Cluster-22178.100510 GO:0006464 GO:0007186 GO:0008270 GO:0016151
GO:0004930
Cluster-22178.100515 GO:0006310 GO:0006281 GO:0006308 GO:0004520
GO:0003676
Cluster-22178.100516 GO:0018149 GO:0004252 GO:0003810 GO:0016020
GO:0005618
Cluster-22178.100517 GO:0030695
Cluster-22178.100518 GO:0006308 GO:0006260 GO:0006281 GO:0017108
GO:0047869 GO:0016021 GO:0005634 GO:0033557
Cluster-22178.100519 GO:0005515
Cluster-22178.100521 GO:0055085 GO:0016021
Cluster-22178.100524 GO:0019901
Cluster-22178.100525 GO:0019901

Cluster-22178.100526 GO:0046872
Cluster-22178.100527 GO:0015946 GO:0043087 GO:0055114 GO:0006355
GO:0003700 GO:0005515 GO:0004022 GO:0005667 GO:0042025
Cluster-22178.100528 GO:0009405 GO:0005576
Cluster-22178.100536 GO:0006468 GO:0007623
Cluster-22178.100540 GO:0007165 GO:0006955 GO:0006396 GO:0005126
GO:0005576 GO:0019031 GO:0001527 GO:0016021 GO:0005622
Cluster-22178.100541 GO:0006955 GO:0007165 GO:0006396 GO:0005126
GO:0005576 GO:0005622 GO:0001527 GO:0016021 GO:0019031
Cluster-22178.100542 GO:0006508 GO:0008152 GO:0046872 GO:0004190
GO:0000166 GO:0003824 GO:0005515
Cluster-22178.100543 GO:0006355 GO:0003700 GO:0008270 GO:0043565
GO:0005667
Cluster-22178.100545 GO:0005515
Cluster-22178.100546 GO:0051536 GO:0003824
Cluster-22178.100551 GO:0008360 GO:0005887
Cluster-22178.100552 GO:0055114 GO:0005506 GO:0016705 GO:0020037

Cluster-22178.100560 GO:0006260 GO:0006269 GO:0006351 GO:0008270
GO:0046872 GO:0003896 GO:0003677 GO:0005515 GO:0005622
GO:0005657 GO:0005730
Cluster-22178.100561 GO:0046872 GO:0008270 GO:0005515 GO:0005622
Cluster-22178.100562 GO:0007018 GO:0007009 GO:0007017 GO:0003777
GO:0005515 GO:0005874 GO:0019031 GO:0030286
Cluster-22178.100563 GO:0055085 GO:0006810 GO:0042626 GO:0005525
GO:0016887 GO:0005524 GO:0016021
Cluster-22178.100564 GO:0005509 GO:0005515 GO:0005544 GO:0016021
Cluster-22178.100565 GO:0005515
Cluster-22178.100570 GO:0006886 GO:0007130 GO:0006810 GO:0006914
GO:0005783 GO:0005634 GO:0016021 GO:0000795 GO:0005886
Cluster-22178.100573 GO:0046849 GO:0005515 GO:0005576 GO:0016021
Cluster-22178.100575 GO:0040007 GO:0008283 GO:0007165 GO:0008083

Cluster-22178.100577 GO:0007165 GO:0006355 GO:0030168 GO:0051258
GO:0003677 GO:0005515 GO:0030674 GO:0005102 GO:0005577
Cluster-22178.100578 GO:0005515 GO:0005198 GO:0019028
Cluster-22178.100582 GO:0006468 GO:0004672 GO:0005524 GO:0005515

Cluster-22178.100583 GO:0045747 GO:0006221 GO:0007049 GO:0019239

Cluster-22178.100584 GO:0051289 GO:0006260 GO:0015031 GO:0006457
GO:0006810 GO:0032922 GO:0007049 GO:0006886 GO:0009405
GO:0008239 GO:0000774 GO:0008565 GO:0008270 GO:0051087
GO:0070009 GO:0008140 GO:0000976 GO:0042803 GO:0051082
GO:0005886 GO:0016020 GO:0005667 GO:0005737
Cluster-22178.100592 GO:0055114 GO:0042157 GO:0006869 GO:0016491
GO:0008289 GO:0005576
Cluster-22178.100593 GO:0006448 GO:0006414 GO:0006810 GO:0005524
GO:0003746 GO:0016887 GO:0003676 GO:0016787 GO:0003677
GO:0005840 GO:0005853
Cluster-22178.100594 GO:0007018 GO:0007017 GO:0003777 GO:0005874
GO:0030286
Cluster-22178.100595 GO:0015031 GO:0006810 GO:0042147 GO:0007018
GO:0007017 GO:0003777 GO:0003676 GO:0005524 GO:0016887
GO:0008565 GO:0003677 GO:0016787 GO:0005874 GO:0030286
GO:0030904
Cluster-22178.100600 GO:0006118 GO:0045454 GO:0015035 GO:0009055
GO:0005515 GO:0003723
Cluster-22178.100604 GO:0005515
Cluster-22178.100610 GO:0015886 GO:0017004 GO:0005515 GO:0016021
GO:0016020
Cluster-22178.100611 GO:0048193 GO:0015031 GO:0042147 GO:0006810
GO:0007018 GO:0007017 GO:0016887 GO:0008565 GO:0005524
GO:0003677 GO:0016787 GO:0003676 GO:0003777 GO:0030286
GO:0016020 GO:0030904 GO:0005874
Cluster-22178.100614 GO:0006355 GO:0003677 GO:0003700 GO:0043565

GO:0005634 GO:0005667
Cluster-22178.100616 GO:0006260 GO:0008017 GO:0005198 GO:0045298
GO:0005882
Cluster-22178.100618 GO:0005515
Cluster-22178.100619 GO:0043066 GO:0003950 GO:0051015 GO:0016788

Cluster-22178.100620 GO:0005515
Cluster-22178.100621 GO:0006355 GO:0003677 GO:0005515 GO:0046872
GO:0005634
Cluster-22178.100626 GO:0007165 GO:0008283 GO:0035556 GO:0043087
GO:0006955 GO:0040007 GO:0035023 GO:0043065 GO:0055114
GO:0047134 GO:0005137 GO:0005089 GO:0005515 GO:0008083
GO:0005740 GO:0005576 GO:0005895 GO:0016021
Cluster-22178.100632 GO:0007186 GO:0004930 GO:0005515 GO:0005509

Cluster-22178.100633 GO:0055114 GO:0006520 GO:0007160
Cluster-22178.100637 GO:0006811 GO:0055085 GO:0005216 GO:0016020
Cluster-22178.100640 GO:0006811 GO:0055085 GO:0005216 GO:0016020
Cluster-22178.100642 GO:0006811 GO:0055085 GO:0005216 GO:0016020
Cluster-22178.100646 GO:0019229 GO:0005576
Cluster-22178.100656 GO:0006810 GO:0007050 GO:0030261 GO:0003677
GO:0019871 GO:0005509 GO:0005515 GO:0005576
Cluster-22178.100657 GO:0006338 GO:0005524 GO:0016818 GO:0003676
GO:0005634
Cluster-22178.100658 GO:0035434 GO:0006508 GO:0006825 GO:0005375
GO:0008237 GO:0043169 GO:0004222 GO:0008270 GO:0016021
GO:0031012
Cluster-22178.100659 GO:0006825 GO:0006364 GO:0006508 GO:0035434
GO:0004222 GO:0008270 GO:0005375 GO:0016021 GO:0031012
Cluster-22178.100665 GO:0055085 GO:0005975 GO:0006487 GO:0007186
GO:0003824 GO:0004930 GO:0043169 GO:0016787 GO:0016021
GO:0008250
Cluster-22178.100674 GO:0019901

Cluster-22178.100675 GO:0046931 GO:0031556 GO:0006810 GO:0031554
GO:0052331 GO:0006812 GO:0015267 GO:0046930
Cluster-22178.100677 GO:0055114 GO:0006769 GO:0046497 GO:0050661
GO:0008750 GO:0016021
Cluster-22178.100679 GO:0015631 GO:0045298
Cluster-22178.100681 GO:0007156 GO:0005509 GO:0016020
Cluster-22178.100685 GO:0010508 GO:0006810 GO:0042407 GO:0005215
GO:0061617 GO:0044284
Cluster-22178.100686 GO:0007224
Cluster-22178.100687 GO:0006310 GO:0006508 GO:0030436 GO:0003677
GO:0005515 GO:0004190 GO:0005634 GO:0016021
Cluster-22178.100688 GO:0006310 GO:0005515 GO:0003677 GO:0005634
Cluster-22178.100689 GO:0006858 GO:0007018 GO:0007017 GO:0006308
GO:0003777 GO:0008855 GO:0009318 GO:0005874 GO:0030286
Cluster-22178.100693 GO:0004867
Cluster-22178.100697 GO:0007165
Cluster-22178.100698 GO:0007165
Cluster-22178.100703 GO:0019497 GO:0006771 GO:0005515 GO:0003993
GO:0046872
Cluster-22178.100704 GO:0007165 GO:0006812 GO:0006835 GO:0006820
GO:0017153 GO:0005102 GO:0016020
Cluster-22178.100708 GO:0007264 GO:0005525 GO:0003924
Cluster-22178.100709 GO:0005515
Cluster-22178.100714 GO:0006886 GO:0016192 GO:0006313 GO:0004803
GO:0005515 GO:0003677
Cluster-22178.100715 GO:0003724 GO:0005515 GO:0003723 GO:0005524

Cluster-22178.100716 GO:0070072 GO:0006955 GO:0000902 GO:0016020
Cluster-22178.100718 GO:0008430
Cluster-22178.100720 GO:0006508 GO:0004252 GO:0019013
Cluster-22178.100721 GO:0006355 GO:0005515 GO:0004222 GO:0003677
GO:0005634
Cluster-22178.100722 GO:0006355 GO:0006560 GO:0005985 GO:0006814

GO:0006090 GO:0006525 GO:0071436 GO:0005982 GO:0006012
GO:0004575 GO:0005515 GO:0008948 GO:0003677 GO:0004564
GO:0015081 GO:0004222 GO:0017177 GO:0005634 GO:0016020
Cluster-22178.100726 GO:0006506 GO:0009069 GO:0006468 GO:0006811
GO:0016310 GO:0055085 GO:0051262 GO:0005216 GO:0004674
GO:0003824 GO:0005524 GO:0016020 GO:0005789 GO:0016021
Cluster-22178.100729 GO:0006298 GO:0030983 GO:0098519 GO:0005524

Cluster-22178.10073 GO:0006355 GO:0008270 GO:0003676 GO:0005634
Cluster-22178.100730 GO:0006298 GO:0005524 GO:0030983
Cluster-22178.100731 GO:0007275 GO:0003700 GO:0005524 GO:0005634
GO:0005667
Cluster-22178.100732 GO:0006886 GO:0006355 GO:0045995 GO:0007049
GO:0048870 GO:0030155 GO:0007165 GO:0006606 GO:0006914
GO:0030334 GO:0019901 GO:0003700 GO:0008092 GO:0043565
GO:0005102 GO:0031514 GO:0016021 GO:0005737 GO:0005783
GO:0005667 GO:0019898
Cluster-22178.100740 GO:0007017 GO:0006817 GO:0007018 GO:0006810
GO:0003723 GO:0005515 GO:0005509 GO:0016887 GO:0005524
GO:0003724 GO:0003777 GO:0005315 GO:0016020 GO:0030286
GO:0005874
Cluster-22178.100742 GO:0006810 GO:0006817 GO:0045892 GO:0007018
GO:0007017 GO:0003724 GO:0005524 GO:0016887 GO:0005509
GO:0005315 GO:0003777 GO:0005515 GO:0003723 GO:0030286
GO:0016020 GO:0005874
Cluster-22178.100743 GO:0006817 GO:0006810 GO:0006355 GO:0007017
GO:0007018 GO:0006308 GO:0005509 GO:0005524 GO:0016887
GO:0008855 GO:0003724 GO:0003723 GO:0008134 GO:0005515
GO:0005315 GO:0003777 GO:0005667 GO:0016020 GO:0030286
GO:0009318 GO:0005874
Cluster-22178.100752 GO:0051252 GO:0003676 GO:0004523
Cluster-22178.100754 GO:0048870 GO:0007165 GO:0006810 GO:0046872
GO:0008270 GO:0004871 GO:0008289 GO:0005515 GO:0016021

GO:0016020 GO:0031514
Cluster-22178.100756 GO:0005515 GO:0008270 GO:0046872
Cluster-22178.100762 GO:0006508 GO:0007165 GO:0004197 GO:0008158
GO:0031225 GO:0016020
Cluster-22178.100767 GO:0048870 GO:0007049 GO:0006810 GO:0008270
GO:0046872 GO:0005515 GO:0016021 GO:0005634 GO:0031514
Cluster-22178.100770 GO:0006357 GO:0001104 GO:0016592
Cluster-22178.100771 GO:0006464
Cluster-22178.100772 GO:0055114 GO:0020037 GO:0016705 GO:0005506

Cluster-22178.100773 GO:0042254 GO:0006412 GO:0019058 GO:0007165
GO:0009116 GO:0003735 GO:0003824 GO:0004871 GO:0042470
GO:0016021 GO:0005840
Cluster-22178.100775 GO:0007018 GO:0007017 GO:0003777 GO:0005874
GO:0030286
Cluster-22178.100782 GO:0019497 GO:0006771 GO:0046872 GO:0005515
GO:0003993
Cluster-22178.100783 GO:0006508 GO:0004252 GO:0019031
Cluster-22178.100786 GO:0005509 GO:0005515 GO:0005544 GO:0016021
Cluster-22178.100791 GO:0007186 GO:0043401 GO:0007165 GO:0040007
GO:0006355 GO:0008283 GO:0008083 GO:0003700 GO:0004930
GO:0043565 GO:0008270 GO:0005634 GO:0005667 GO:0016021
Cluster-22178.100793 GO:0015992 GO:0000921 GO:0030168 GO:0006606
GO:0006123 GO:0006810 GO:0007165 GO:0051258 GO:0051382
GO:0048870 GO:0010508 GO:0005102 GO:0030674 GO:0003677
GO:0004129 GO:0005886 GO:0005940 GO:0005577 GO:0005739
GO:0045277 GO:0016021 GO:0000776 GO:0031514
Cluster-22178.100795 GO:0007017 GO:0007018 GO:0003777 GO:0030286
GO:0005874
Cluster-22178.100796 GO:0005515 GO:0016021
Cluster-22178.100797 GO:0005515 GO:0005509
Cluster-22178.100804 GO:0016579 GO:0003677 GO:0036459 GO:0008270

Cluster-22178.100806 GO:0006355 GO:0006308 GO:0008855 GO:0009318
Cluster-22178.100811 GO:0009058 GO:0030170
Cluster-22178.100812 GO:0006355 GO:0006099 GO:0003677 GO:0016829

Cluster-22178.100814 GO:0005515
Cluster-22178.100817 GO:0006810 GO:0006816 GO:0005246 GO:0042030
GO:0016020
Cluster-22178.100819 GO:0005515 GO:0005198 GO:0042802 GO:0019028
Cluster-22178.100820 GO:0005515 GO:0005198 GO:0019028
Cluster-22178.100822 GO:0007155 GO:0005509 GO:0005515 GO:0005576
GO:0005739 GO:0005747
Cluster-22178.100823 GO:0007155 GO:0005515 GO:0005509 GO:0005576
GO:0005739 GO:0005747
Cluster-22178.100825 GO:0007165 GO:0005509 GO:0005515 GO:0005578
Cluster-22178.100826 GO:0007165 GO:0005509 GO:0005515 GO:0005578
Cluster-22178.100834 GO:0005509
Cluster-22178.100836 GO:0006570 GO:0006470 GO:0005515 GO:0004725

Cluster-22178.100837 GO:0006479 GO:0006464 GO:0046500 GO:0008171
GO:0004719 GO:0008168
Cluster-22178.100839 GO:0005515 GO:0008270
Cluster-22178.100842 GO:0007155 GO:0015986 GO:0015992 GO:0016301
GO:0005515 GO:0005540 GO:0005524 GO:0015078 GO:0000276
Cluster-22178.100848 GO:0007155 GO:0035434 GO:0006606 GO:0006825
GO:0019904 GO:0030246 GO:0005375 GO:0016021
Cluster-22178.100849 GO:0035434 GO:0006606 GO:0006825 GO:0005375
GO:0019904 GO:0016021 GO:0005887
Cluster-22178.100854 GO:0007264 GO:0006576 GO:0015684 GO:2001070
GO:0015093 GO:0005524 GO:0003924 GO:0005525 GO:0016021
Cluster-22178.100856 GO:0008063
Cluster-22178.100866 GO:0002949 GO:0006810 GO:0006415 GO:0006449
GO:0006355 GO:0016887 GO:0005524 GO:0098519 GO:0003678
GO:0016149 GO:0008134 GO:0005657 GO:0018444 GO:0005667

GO:0005840 GO:0005737
Cluster-22178.100867 GO:0006836 GO:0007304 GO:0006812 GO:0005328
GO:0005213 GO:0005576 GO:0042600 GO:0016021
Cluster-22178.100872 GO:0006810 GO:0007165 GO:0006955 GO:0055085
GO:0006935 GO:0008009 GO:0005215 GO:0016020 GO:0005576
Cluster-22178.100873 GO:0005515
Cluster-22178.100874 GO:0043039 GO:0005524 GO:0016876
Cluster-22178.100876 GO:0030234 GO:0005515 GO:0016020
Cluster-22178.100884 GO:0007165 GO:0005515
Cluster-22178.100888 GO:0006355 GO:0009271
Cluster-22178.100890 GO:0003824 GO:0005524
Cluster-22178.100892 GO:0005524 GO:0005515
Cluster-22178.100893 GO:0005524 GO:0005515 GO:0003824
Cluster-22178.100895 GO:0005524 GO:0003824 GO:0005515
Cluster-22178.100899 GO:0007165
Cluster-22178.100900 GO:0019072 GO:0006886 GO:0045444 GO:0016192
GO:0005515 GO:0030117 GO:0042025
Cluster-22178.100901 GO:0045444 GO:0006355 GO:0019072 GO:0043189
GO:0042025 GO:0005634
Cluster-22178.100907 GO:0046332
Cluster-22178.100911 GO:0007155
Cluster-22178.100912 GO:0006468 GO:0004672 GO:0005524
Cluster-22178.100914 GO:0006123 GO:0015992 GO:0004129 GO:0045277
GO:0005739
Cluster-22178.100915 GO:0007264 GO:0006355 GO:0015684 GO:0005524
GO:0015093 GO:0016887 GO:0003924 GO:0005525 GO:0008134
GO:0005515 GO:0016021 GO:0005667
Cluster-22178.10092 GO:0003723 GO:0003676
Cluster-22178.100922 GO:0006807 GO:0046660 GO:0006897 GO:0006397
GO:0008270 GO:0016814 GO:0001527 GO:0016020 GO:0005634
Cluster-22178.100931 GO:0007165 GO:0006810 GO:0006814 GO:0006464
GO:0006750 GO:0055085 GO:0004363 GO:0005524 GO:0016151
GO:0005215 GO:0005102 GO:0016020 GO:0016021

Cluster-22178.100932 GO:0006355 GO:0006955 GO:0006814 GO:0006750
GO:0006464 GO:0055085 GO:0006810 GO:0007165 GO:0005215
GO:0016151 GO:0005524 GO:0005102 GO:0030247 GO:0004363
GO:0005044 GO:0003677 GO:0016021 GO:0016020
Cluster-22178.100938 GO:0006869 GO:0007165 GO:0042157 GO:0007131
GO:0030168 GO:0015031 GO:0009405 GO:0000160 GO:0007021
GO:0016310 GO:0051258 GO:0030674 GO:0000155 GO:0005102
GO:0015631 GO:0046983 GO:0005198 GO:0051082 GO:0008289
GO:0005577 GO:0019028 GO:0000794 GO:0005576 GO:0045298
GO:0016021 GO:0019031 GO:0009365
Cluster-22178.100939 GO:0006811 GO:0006352 GO:0055085 GO:0019087
GO:0005515 GO:0005216 GO:0016021 GO:0016020 GO:0005634
Cluster-22178.100947 GO:0007018 GO:0006810 GO:0007017 GO:0003777
GO:0016887 GO:0005524 GO:0003677 GO:0005874 GO:0030286
Cluster-22178.100951 GO:0003824
Cluster-22178.100954 GO:0055114 GO:0042157 GO:0006869 GO:0016491
GO:0008289 GO:0005576
Cluster-22178.100955 GO:0006810 GO:0006811 GO:0005230 GO:0016020
Cluster-22178.100956 GO:0006355 GO:0005634
Cluster-22178.100967 GO:0008283 GO:0040007 GO:0007165 GO:0008083
GO:0005515
Cluster-22178.100968 GO:0007165 GO:0008283 GO:0040007 GO:0008083
GO:0005515
Cluster-22178.100970 GO:0007205 GO:0046486 GO:0009395 GO:0004143

Cluster-22178.100971 GO:0016567 GO:0004842
Cluster-22178.100972 GO:0046339 GO:0006874 GO:0006816 GO:0050790
GO:0035556 GO:0006260 GO:0055085 GO:0007165 GO:0006270
GO:0006810 GO:0006811 GO:0009395 GO:0006351 GO:0006629
GO:0006355 GO:0016773 GO:0003677 GO:0035091 GO:0005216
GO:0004435 GO:0005515 GO:0003682 GO:0005219 GO:0003887
GO:0030234 GO:0005622 GO:0016020 GO:0000775 GO:0000785
GO:0005634 GO:0019031 GO:0016021 GO:0042575

Cluster-22178.100974 GO:0007017 GO:0006810 GO:0007018 GO:0005524
GO:0016887 GO:0003777 GO:0003677 GO:0030286 GO:0005874
Cluster-22178.100975 GO:0007017 GO:0006810 GO:0007018 GO:0005524
GO:0016887 GO:0003777 GO:0003677 GO:0030286 GO:0005874
Cluster-22178.100977 GO:0006810 GO:0007018 GO:0007017 GO:0003777
GO:0005524 GO:0016887 GO:0003677 GO:0005874 GO:0030286
Cluster-22178.100978 GO:0006810 GO:0007018 GO:0007017 GO:0003777
GO:0005524 GO:0016887 GO:0003677 GO:0005874 GO:0030286
Cluster-22178.100983 GO:0015672 GO:0006812 GO:0005261 GO:0016020
Cluster-22178.100990 GO:0055085 GO:0006810 GO:0005515 GO:0016021
Cluster-22178.100991 GO:0006464
Cluster-22178.100993 GO:0006464
Cluster-22178.100994 GO:0006464 GO:0009247 GO:0016758
Cluster-22178.101003 GO:0007155 GO:0006915 GO:0051090 GO:0008608
GO:0005509 GO:0008242 GO:0005634 GO:0072686 GO:0005576
GO:0042729
Cluster-22178.101004 GO:0008608 GO:0006915 GO:0051090 GO:0009405
GO:0007165 GO:0008242 GO:0016301 GO:0005515 GO:0072686
GO:0005634 GO:0005576 GO:0042729
Cluster-22178.101008 GO:0006396 GO:0006955 GO:0007165 GO:0005126
GO:0016021 GO:0001527 GO:0019031 GO:0005622 GO:0005576
Cluster-22178.101013 GO:0007186 GO:0004930 GO:0016021
Cluster-22178.101017 GO:0055114 GO:0005506 GO:0016705 GO:0020037

Cluster-22178.10102 GO:0006164 GO:0006531 GO:0042254 GO:0006412
GO:0006144 GO:0008152 GO:0006522 GO:0003723 GO:0050662
GO:0003735 GO:0016491 GO:0005525 GO:0003824 GO:0004019
GO:0005840
Cluster-22178.101020 GO:0006570 GO:0050852 GO:0055114 GO:0006470
GO:0005515 GO:0004725 GO:0016972 GO:0016021
Cluster-22178.101021 GO:0005515
Cluster-22178.101027 GO:0006915 GO:0000105 GO:0045735 GO:0005622
Cluster-22178.101028 GO:0045735

Cluster-22178.101032 GO:0016032 GO:0051082 GO:0019028 GO:0016021
Cluster-22178.101037 GO:0006310 GO:0005515 GO:0003677 GO:0005634
Cluster-22178.101039 GO:0006396 GO:0007165 GO:0006955 GO:0005126
GO:0019031 GO:0016021 GO:0001527 GO:0005622 GO:0005576
Cluster-22178.101046 GO:0005524 GO:0003774 GO:0016887 GO:0005515
GO:0016459 GO:0005856
Cluster-22178.101049 GO:0003677
Cluster-22178.101050 GO:0009405 GO:0016973 GO:0005515 GO:0005576
GO:0005643
Cluster-22178.101052 GO:0005198 GO:0012506
Cluster-22178.101053 GO:0007224 GO:0005515
Cluster-22178.101057 GO:0055085 GO:0002949 GO:0030866 GO:0006810
GO:0042626 GO:0005525 GO:0008092 GO:0003924 GO:0016887
GO:0005524 GO:0016021 GO:0005856
Cluster-22178.101061 GO:0006281 GO:0006310 GO:0008270
Cluster-22178.101062 GO:0006464 GO:0007186 GO:0008270 GO:0004930
GO:0016151
Cluster-22178.101078 GO:0003774 GO:0016459
Cluster-22178.101082 GO:0006355 GO:0007165 GO:0051258 GO:0030168
GO:0030674 GO:0005515 GO:0003677 GO:0005102 GO:0005577
Cluster-22178.101083 GO:0006811 GO:0055085 GO:0005216 GO:0016020
GO:0000228
Cluster-22178.101086 GO:0006355 GO:0046872 GO:0003677 GO:0005515
GO:0005634
Cluster-22178.101091 GO:0030694
Cluster-22178.101094 GO:0006614 GO:0006810 GO:0005525 GO:0016887
GO:0003924 GO:0005524 GO:0005576
Cluster-22178.101095 GO:0006310 GO:0009847 GO:0006810 GO:0007155
GO:0002949 GO:0032012 GO:0043087 GO:0030866 GO:0006576
GO:0006355 GO:0006281 GO:0009378 GO:0016887 GO:0005524
GO:0008134 GO:0005086 GO:0008092 GO:0005667 GO:0009379
GO:0005657 GO:0005856
Cluster-22178.101097 GO:0006629 GO:0008081

Cluster-22178.101110 GO:0007165 GO:0006811 GO:0007268 GO:0005234 GO:0004970 GO:0016020
Cluster-22178.101114 GO:0007186 GO:0007165 GO:0004871 GO:0005834
Cluster-22178.101116 GO:0006952 GO:0006310 GO:0046872 GO:0000150 GO:0003677 GO:0005515
Cluster-22178.101118 GO:0005515
Cluster-22178.101123 GO:0019497 GO:0006771 GO:0009405 GO:0046872 GO:0016798 GO:0003993 GO:0005515 GO:0005576
Cluster-22178.101131 GO:0005515
Cluster-22178.101132 GO:0015991 GO:0006118 GO:0006119 GO:0015992 GO:0003954 GO:0046961 GO:0016765 GO:0005739 GO:0033178
Cluster-22178.101135 GO:0009405 GO:0006629 GO:0006468 GO:0035091 GO:0005524 GO:0004672 GO:0005576
Cluster-22178.101136 GO:0006313 GO:0005509
Cluster-22178.101141 GO:0042407 GO:0010508 GO:0006810 GO:0005215 GO:0061617 GO:0044284
Cluster-22178.101144 GO:0006357 GO:0005975 GO:0001104 GO:0004649 GO:0016592
Cluster-22178.101149 GO:0008146
Cluster-22178.101151 GO:0051258 GO:0007130 GO:0006886 GO:0010508 GO:0006355 GO:0006260 GO:0006606 GO:0030168 GO:0015992 GO:0006357 GO:0006914 GO:0007165 GO:0006810 GO:0019068 GO:0005525 GO:0003700 GO:0003924 GO:0043565 GO:0001104 GO:0005102 GO:0030674 GO:0019867 GO:0016021 GO:0000795 GO:0005634 GO:0005577 GO:0005886 GO:0005783 GO:0016592 GO:0005667
Cluster-22178.101157 GO:0016032 GO:0019082
Cluster-22178.101158 GO:0006468 GO:0004672 GO:0005524
Cluster-22178.101161 GO:0009405
Cluster-22178.101166 GO:0006570 GO:0055114 GO:0005515 GO:0004511

Cluster-22178.101168 GO:0006952 GO:0007165 GO:0005126 GO:0005576
Cluster-22178.101169 GO:0016043 GO:0030036 GO:0003779 GO:0017048

GO:0005525
Cluster-22178.101172 GO:0006570 GO:0006470 GO:0019988 GO:0043399
GO:0004725 GO:0008138
Cluster-22178.101175 GO:0046872 GO:0008270 GO:0005515 GO:0005622
GO:0031083
Cluster-22178.101177 GO:0016020
Cluster-22178.101180 GO:0019497 GO:0006771 GO:0007339 GO:0046872
GO:0003993 GO:0005515 GO:0005576
Cluster-22178.101183 GO:0005975 GO:0055114 GO:0009117 GO:0046373
GO:0046556 GO:0016705 GO:0005515
Cluster-22178.101184 GO:0006355 GO:0006099 GO:0016829 GO:0003677

Cluster-22178.101186 GO:0007165 GO:0035556 GO:0043087 GO:0007264
GO:0005509 GO:0005085 GO:0005578
Cluster-22178.101189 GO:0016021
Cluster-22178.101190 GO:0019497 GO:0006771 GO:0009405 GO:0046872
GO:0016798 GO:0003993 GO:0005515 GO:0005576
Cluster-22178.101192 GO:0006820 GO:0006835 GO:0006812 GO:0017153
GO:0016020
Cluster-22178.101195 GO:0006725 GO:0005515 GO:0004497
Cluster-22178.101196 GO:0006725 GO:0004497 GO:0005515
Cluster-22178.101197 GO:0006725 GO:0005515 GO:0004497
Cluster-22178.101198 GO:0006816 GO:0005388 GO:0008233 GO:0016529
Cluster-22178.101201 GO:0007049 GO:0005634
Cluster-22178.101202 GO:0048870 GO:0006355 GO:0007165 GO:0006810
GO:0046872 GO:0008289 GO:0004871 GO:0005515 GO:0008270
GO:0005524 GO:0003677 GO:0016818 GO:0016021 GO:0005634
GO:0031514 GO:0016020
Cluster-22178.101205 GO:0005515
Cluster-22178.101206 GO:0007218 GO:0006810 GO:0007186 GO:0004930
GO:0005525 GO:0042626 GO:0005524 GO:0005184 GO:0016021
GO:0005576 GO:0005861
Cluster-22178.101211 GO:0005509 GO:0005515

Cluster-22178.101217 GO:0006413 GO:0006813 GO:0006446 GO:0005516 GO:0031369 GO:0003743 GO:0015269 GO:0005852 GO:0005840 GO:0016021

Cluster-22178.101221 GO:0006886 GO:0007130 GO:0006914 GO:0048870 GO:0000398 GO:0006260 GO:0005634 GO:0031514 GO:0005783 GO:0000795 GO:0016021

Cluster-22178.101224 GO:0009405

Cluster-22178.101229 GO:0001558 GO:0008270 GO:0005520 GO:0005576 GO:0005634 GO:0016942

Cluster-22178.101230 GO:0008168 GO:0019028

Cluster-22178.101234 GO:0006771 GO:0019497 GO:0046872 GO:0005515 GO:0003993

Cluster-22178.101237 GO:0007155 GO:0005515 GO:0016021

Cluster-22178.101244 GO:0055114 GO:0008152 GO:0016491

Cluster-22178.101245 GO:0055114 GO:0008152 GO:0016491

Cluster-22178.101246 GO:0005515

Cluster-22178.10125 GO:0006810 GO:0016020

Cluster-22178.101252 GO:0006355 GO:0005524 GO:0008134 GO:0005667

Cluster-22178.101255 GO:0005198 GO:0019028

Cluster-22178.101258 GO:0008270

Cluster-22178.101261 GO:0016055 GO:0046849 GO:0005515 GO:0005576 GO:0016021

Cluster-22178.101262 GO:0042254 GO:0006412 GO:0003735 GO:0005840 GO:0005622

Cluster-22178.101269 GO:0005515

Cluster-22178.101274 GO:0007010 GO:0003779 GO:0005515

Cluster-22178.101276 GO:0007017 GO:0006388 GO:0007018 GO:0008270 GO:0005524 GO:0003777 GO:0008017 GO:0016772 GO:0003677 GO:0005874 GO:0045298

Cluster-22178.101277 GO:0010923 GO:0042254 GO:0006412 GO:0019902 GO:0003735 GO:0005622 GO:0005840

Cluster-22178.101283 GO:0006891 GO:0030036 GO:0006810 GO:0015986 GO:0016043 GO:0005525 GO:0046933 GO:0003779 GO:0017048

GO:0016021 GO:0045259 GO:0017119
Cluster-22178.101284 GO:0007264 GO:0006576 GO:0015684 GO:0005524
GO:0015093 GO:0003924 GO:0005525 GO:2001070 GO:0016021
Cluster-22178.101286 GO:0006812 GO:0006820 GO:0006835 GO:0017153
GO:0016020
Cluster-22178.101287 GO:0006820 GO:0006835 GO:0006812 GO:0007165
GO:0005102 GO:0017153 GO:0016020
Cluster-22178.101288 GO:0006771 GO:0019497 GO:0003993
Cluster-22178.101291 GO:0055114 GO:0016114 GO:0006694 GO:0004506
GO:0050660 GO:0016021
Cluster-22178.101299 GO:0043401 GO:0006355 GO:0003700 GO:0008270
GO:0043565 GO:0005634 GO:0005667
Cluster-22178.101307 GO:0006468 GO:0004672 GO:0005524 GO:0016020
Cluster-22178.101310 GO:0006886 GO:0000184 GO:0005515 GO:0016021
GO:0005783
Cluster-22178.101311 GO:0016021 GO:0005886
Cluster-22178.101312 GO:0006811 GO:0055085 GO:0009405 GO:0006810
GO:0008200 GO:0005216 GO:0016020 GO:0005576
Cluster-22178.101320 GO:0005975 GO:0005515 GO:0016773
Cluster-22178.101321 GO:0005975 GO:0016773 GO:0005515
Cluster-22178.101323 GO:0006351 GO:0003677 GO:0005634
Cluster-22178.101325 GO:0006396 GO:0005515 GO:0000176
Cluster-22178.101347 GO:0005515
Cluster-22178.101348 GO:0005975 GO:0045493 GO:0007155 GO:0042274
GO:0046559 GO:0005515 GO:0005198 GO:0019031 GO:0030686
GO:0005576 GO:0019028
Cluster-22178.101349 GO:0045493 GO:0005975 GO:0008152 GO:0008608
GO:0007067 GO:0016597 GO:0005515 GO:0046559 GO:0072686
GO:0005576 GO:0042729
Cluster-22178.101352 GO:0019051 GO:0042025
Cluster-22178.101359 GO:0030206 GO:0006024 GO:0030158
Cluster-22178.101363 GO:0006306 GO:0032775 GO:0009007 GO:0003677

Cluster-22178.101365 GO:0043065 GO:0005515 GO:0016779 GO:0005737
Cluster-22178.101367 GO:0015992 GO:0006914 GO:0006357 GO:0015743
GO:0007155 GO:0051382 GO:0048870 GO:0000160 GO:0015991
GO:0006260 GO:0008053 GO:0006605 GO:0007165 GO:0010508
GO:0006886 GO:0006606 GO:0019079 GO:0000398 GO:0051258
GO:0006308 GO:0006355 GO:0051607 GO:0015031 GO:0030168
GO:0000921 GO:0006144 GO:0006810 GO:0015078 GO:0005515
GO:0001104 GO:0005198 GO:0008565 GO:0003700 GO:0004871
GO:0008134 GO:0030674 GO:0043565 GO:0003924 GO:0003968
GO:0042803 GO:0003774 GO:0045502 GO:0008855 GO:0005102
GO:0005737 GO:0016459 GO:0005667 GO:0005741 GO:0016020
GO:0031379 GO:0019031 GO:0005801 GO:0016021 GO:0019028
GO:0031514 GO:0005742 GO:0016592 GO:0005681 GO:0019867
GO:0000776 GO:0005577 GO:0009318 GO:0033179 GO:0005886
GO:0030286 GO:0005940
Cluster-22178.101368 GO:0005515 GO:0005524 GO:0003774 GO:0005856
GO:0016459
Cluster-22178.101371 GO:0035064
Cluster-22178.101373 GO:0007049 GO:0006308 GO:0019901 GO:0008855
GO:0009318 GO:0005815 GO:0016021
Cluster-22178.101375 GO:0003723 GO:0019013
Cluster-22178.101376 GO:0055114 GO:0016209 GO:0016491
Cluster-22178.101377 GO:0015694 GO:0005515 GO:0015097 GO:0016020
Cluster-22178.101385 GO:0006464 GO:0005524 GO:0046872
Cluster-22178.101395 GO:0046872 GO:0003677 GO:0005634
Cluster-22178.101398 GO:0006886 GO:0005783 GO:0016021
Cluster-22178.101399 GO:0006886 GO:0005515 GO:0016021 GO:0005783
Cluster-22178.101401 GO:0006886 GO:0005783 GO:0016021
Cluster-22178.101402 GO:0006886 GO:0000184 GO:0005515 GO:0016021
GO:0005783
Cluster-22178.101403 GO:0000184 GO:0006886 GO:0005515 GO:0016021
GO:0005783
Cluster-22178.101404 GO:0000184 GO:0006886 GO:0005515 GO:0016021

GO:0005783
Cluster-22178.101422 GO:0005515
Cluster-22178.101423 GO:0005515
Cluster-22178.101425 GO:0019028 GO:0030133 GO:0031083
Cluster-22178.101428 GO:0006810 GO:0007018 GO:0007017 GO:0003777
GO:0005524 GO:0016887 GO:0003677 GO:0005874 GO:0030286
Cluster-22178.101429 GO:0032012 GO:0043087 GO:0005086
Cluster-22178.101438 GO:0043248 GO:0006886 GO:0016192 GO:0005515
GO:0030117
Cluster-22178.101442 GO:0008283 GO:0040007 GO:0007165 GO:0008083
GO:0005515
Cluster-22178.101449 GO:0046983
Cluster-22178.101451 GO:0005515 GO:0008537
Cluster-22178.101452 GO:0005515 GO:0008537
Cluster-22178.101455 GO:0006811 GO:0007165 GO:0050830 GO:0004864
GO:0046872 GO:0016020 GO:0031083
Cluster-22178.101456 GO:0050830 GO:0007165 GO:0006811 GO:0004864
GO:0046872 GO:0031083 GO:0016020
Cluster-22178.101467 GO:0007186 GO:0006487 GO:0005975 GO:0055085
GO:0043169 GO:0016787 GO:0003824 GO:0004930 GO:0008250
GO:0016021
Cluster-22178.101469 GO:0006270 GO:0006355 GO:0003677 GO:0008270

Cluster-22178.101472 GO:0005515
Cluster-22178.101475 GO:0006511 GO:0005515 GO:0004175 GO:0019773
Cluster-22178.101476 GO:0006281 GO:0043240 GO:0016021
Cluster-22178.101477 GO:0019072 GO:0006508 GO:0010508 GO:0031175
GO:0006308 GO:0051276 GO:0006357 GO:0001104 GO:0005515
GO:0004181 GO:0005516 GO:0030507 GO:0004180 GO:0008270
GO:0005524 GO:0008855 GO:0016887 GO:0009318 GO:0042025
GO:0016021 GO:0005694 GO:0016592 GO:0008091
Cluster-22178.101478 GO:0006888 GO:0006886 GO:0030127
Cluster-22178.101480 GO:0006570 GO:0055114 GO:0005515 GO:0004511

Cluster-22178.101481 GO:0042546 GO:0005199 GO:0005515 GO:0009277
GO:0005618
Cluster-22178.101483 GO:0042546 GO:0005199 GO:0005515 GO:0005618
GO:0009277
Cluster-22178.101484 GO:0042546 GO:0005515 GO:0005199 GO:0005618
GO:0009277
Cluster-22178.101485 GO:0030168 GO:0051258 GO:0007165 GO:0007155
GO:0005102 GO:0003723 GO:0030674 GO:0005577
Cluster-22178.101488 GO:0007017 GO:0042147 GO:0007018 GO:0006810
GO:0015031 GO:0016787 GO:0003677 GO:0016887 GO:0008565
GO:0005524 GO:0003676 GO:0003777 GO:0030904 GO:0030286
GO:0005874
Cluster-22178.101489 GO:0006448 GO:0006414 GO:0007017 GO:0007018
GO:0006118 GO:0022900 GO:0005506 GO:0016887 GO:0003746
GO:0005524 GO:0020037 GO:0003777 GO:0009055 GO:0005853
GO:0042597 GO:0030286 GO:0005874 GO:0005840
Cluster-22178.101491 GO:0003774 GO:0016459
Cluster-22178.101499 GO:0019042 GO:0006468 GO:0007186 GO:0005524
GO:0004672 GO:0004930 GO:0033644 GO:0016020
Cluster-22178.101500 GO:0016301
Cluster-22178.101502 GO:0006118 GO:0006814 GO:0006120 GO:0006744
GO:0015992 GO:0008137 GO:0003954 GO:0005739
Cluster-22178.101504 GO:0019031
Cluster-22178.101511 GO:0008152 GO:0055114 GO:0009396 GO:0046487
GO:0007010 GO:0003824 GO:0016491 GO:0003779 GO:0004488
GO:0050662
Cluster-22178.101513 GO:0046872 GO:0016301 GO:0016020
Cluster-22178.101514 GO:0016301 GO:0016020
Cluster-22178.101515 GO:0055085 GO:0006811 GO:0016310 GO:0009069
GO:0004674 GO:0005216 GO:0016020
Cluster-22178.10152 GO:0008654 GO:0046486 GO:0008715 GO:0016020
Cluster-22178.101521 GO:0010181 GO:0016020

Cluster-22178.101523 GO:0009058 GO:0030170
Cluster-22178.101524 GO:0006541 GO:0016787
Cluster-22178.101531 GO:0020037 GO:0019825
Cluster-22178.101532 GO:0051082 GO:0031072
Cluster-22178.101534 GO:0006606 GO:0006914 GO:0070206 GO:0007165
GO:0048870 GO:0051382 GO:0006355 GO:0003700 GO:0043565
GO:0005198 GO:0042803 GO:0008134 GO:0045502 GO:0030286
GO:0005882 GO:0005667 GO:0019031 GO:0031514 GO:0019028
GO:0000776
Cluster-22178.101539 GO:0006810 GO:0055085 GO:0005525 GO:0042626
GO:0005524 GO:0016887 GO:0016021
Cluster-22178.101541 GO:0032324 GO:0015631 GO:0045298
Cluster-22178.101543 GO:0019867
Cluster-22178.101545 GO:0005515
Cluster-22178.101546 GO:0005515
Cluster-22178.101547 GO:0005515
Cluster-22178.101551 GO:0005515
Cluster-22178.101560 GO:0007186 GO:0004930 GO:0016021
Cluster-22178.101561 GO:0016567 GO:0016881 GO:0031386
Cluster-22178.101562 GO:0051382 GO:0006308 GO:0006914 GO:0009401
GO:0015743 GO:0006810 GO:0044237 GO:0016567 GO:0031386
GO:0008855 GO:0016881 GO:0005886 GO:0000776 GO:0009318
Cluster-22178.101563 GO:0006308 GO:0006914 GO:0051382 GO:0009401
GO:0015743 GO:0044237 GO:0006810 GO:0008855 GO:0005886
GO:0000776 GO:0009318
Cluster-22178.101564 GO:0005515
Cluster-22178.101565 GO:0042981
Cluster-22178.101566 GO:0005515
Cluster-22178.101569 GO:0019064 GO:0007165 GO:0018149 GO:0003810
GO:0046789 GO:0009986 GO:0019031
Cluster-22178.101574 GO:0005515
Cluster-22178.101575 GO:0005515
Cluster-22178.101576 GO:0005515

Cluster-22178.101580 GO:0046872 GO:0008270 GO:0005515

Cluster-22178.101584 GO:0007186 GO:0035434 GO:0006825 GO:0005975 GO:0005375 GO:0043169 GO:0004930 GO:0003824 GO:0016021

Cluster-22178.101586 GO:0006508 GO:0051276 GO:0005515 GO:0004180 GO:0016887 GO:0005524 GO:0008270 GO:0004181 GO:0005694

Cluster-22178.101587 GO:0006508 GO:0031175 GO:0051276 GO:0005516 GO:0004180 GO:0005515 GO:0030507 GO:0008270 GO:0005524 GO:0004181 GO:0005694 GO:0008091

Cluster-22178.101590 GO:0005515

Cluster-22178.101598 GO:0006811 GO:0006810 GO:0005230 GO:0016020

Cluster-22178.101600 GO:0006811 GO:0006810 GO:0009607 GO:0005230 GO:0016020 GO:0016021

Cluster-22178.101601 GO:0006952 GO:0005515

Cluster-22178.101602 GO:0019079 GO:0006260 GO:0030337 GO:0042575

Cluster-22178.101607 GO:0042254 GO:0006412 GO:0005515 GO:0003735 GO:0005840

Cluster-22178.101608 GO:0006468 GO:0004672 GO:0005524 GO:0005515

Cluster-22178.101613 GO:0019013 GO:0016021 GO:0016012

Cluster-22178.101619 GO:0005515

Cluster-22178.10162 GO:0006468 GO:0016773 GO:0004672 GO:0005524 GO:0016020

Cluster-22178.101621 GO:0005615

Cluster-22178.101622 GO:0005615

Cluster-22178.101623 GO:0005615

Cluster-22178.101625 GO:0005515

Cluster-22178.101627 GO:0019031

Cluster-22178.101628 GO:0019031

Cluster-22178.101631 GO:0005975 GO:0005515 GO:0004553 GO:0016020 GO:0016021 GO:0042470

Cluster-22178.101633 GO:0005634

Cluster-22178.101635 GO:0007165 GO:0005515 GO:0016020

Cluster-22178.101638 GO:0042254 GO:0006412 GO:0003735 GO:0008270

GO:0005840 GO:0005622
Cluster-22178.101641 GO:0007017 GO:0007018 GO:0006811 GO:0055085
GO:0005216 GO:0003777 GO:0016020 GO:0030286 GO:0005874
Cluster-22178.101644 GO:0040007 GO:0008283 GO:0007165 GO:0008083
GO:0016020
Cluster-22178.101645 GO:0007165 GO:0008283 GO:0007155 GO:0040007
GO:0008083 GO:0005515 GO:0005125 GO:0005524 GO:0003774
GO:0016020 GO:0016459 GO:0016021
Cluster-22178.101646 GO:0005515
Cluster-22178.101657 GO:0007059 GO:0007049 GO:0031110 GO:0051301
GO:0000917 GO:0000090 GO:0007067 GO:0043093 GO:0008017
GO:0045298 GO:0005876 GO:0005737 GO:0000940
Cluster-22178.101661 GO:0015979 GO:0070588 GO:0006816 GO:0005245
GO:0005515 GO:0005509 GO:0005891 GO:0009654 GO:0019898
GO:0009523
Cluster-22178.101663 GO:0055085 GO:0016021
Cluster-22178.101664 GO:0009890
Cluster-22178.101672 GO:0006865 GO:0005515 GO:0016020 GO:0005643
Cluster-22178.101673 GO:0044237 GO:0003824
Cluster-22178.101677 GO:0007165 GO:0030168 GO:0006914 GO:0006606
GO:0010506 GO:0009405 GO:0006955 GO:0051258 GO:0042262
GO:0005126 GO:0003690 GO:0030674 GO:0005102 GO:0045502
GO:0042803 GO:0008134 GO:0005198 GO:0005667 GO:0005882
GO:0016020 GO:0030286 GO:0005577 GO:0005576 GO:0005615
Cluster-22178.101678 GO:0051258 GO:0042262 GO:0009405 GO:0006914
GO:0030168 GO:0010506 GO:0006606 GO:0007165 GO:0045502
GO:0005198 GO:0008134 GO:0042803 GO:0005102 GO:0003690
GO:0030674 GO:0005615 GO:0030286 GO:0005577 GO:0005667
GO:0016020 GO:0005882
Cluster-22178.101679 GO:0009405 GO:0042262 GO:0051258 GO:0007165
GO:0006606 GO:0010506 GO:0030168 GO:0006914 GO:0005198
GO:0008134 GO:0042803 GO:0045502 GO:0003690 GO:0030674
GO:0005102 GO:0005615 GO:0005882 GO:0016020 GO:0005667

GO:0005577 GO:0030286
Cluster-22178.101680 GO:0007165 GO:0030168 GO:0006914 GO:0006606
GO:0010506 GO:0009405 GO:0051258 GO:0042262 GO:0003690
GO:0030674 GO:0005102 GO:0045502 GO:0005198 GO:0008134
GO:0042803 GO:0005667 GO:0005882 GO:0016020 GO:0030286
GO:0005577 GO:0005615
Cluster-22178.101681 GO:0006955 GO:0009405 GO:0051258 GO:0042262
GO:0007165 GO:0006914 GO:0030168 GO:0010506 GO:0006606
GO:0045502 GO:0042803 GO:0008134 GO:0005198 GO:0003690
GO:0030674 GO:0005126 GO:0005102 GO:0005576 GO:0005615
GO:0005667 GO:0016020 GO:0005882 GO:0030286 GO:0005577
Cluster-22178.101682 GO:0007165 GO:0006606 GO:0010506 GO:0030168
GO:0006914 GO:0009405 GO:0006955 GO:0042262 GO:0051258
GO:0005126 GO:0030674 GO:0003690 GO:0005102 GO:0042803
GO:0008134 GO:0005198 GO:0045502 GO:0005882 GO:0016020
GO:0005667 GO:0005577 GO:0030286 GO:0005576 GO:0005615
Cluster-22178.101687 GO:0006281 GO:0006260 GO:0016787 GO:0046872
GO:0005515 GO:0003911
Cluster-22178.101688 GO:0006281 GO:0006260 GO:0046872 GO:0016787
GO:0003911 GO:0005515
Cluster-22178.101690 GO:0006260 GO:0006281 GO:0003911 GO:0005515
GO:0046872 GO:0016787
Cluster-22178.101691 GO:0006281 GO:0006260 GO:0016787 GO:0046872
GO:0005515 GO:0003911
Cluster-22178.101694 GO:0005515 GO:0008537
Cluster-22178.101696 GO:0015991 GO:0006118 GO:0006886 GO:0022900
GO:0007155 GO:0007264 GO:0006914 GO:0015992 GO:0051607
GO:0020037 GO:0008092 GO:0015078 GO:0009055 GO:0005506
GO:0005096 GO:0033179 GO:0005737 GO:0016021 GO:0005783
GO:0042597 GO:0019898
Cluster-22178.101701 GO:0030414 GO:0005515
Cluster-22178.101708 GO:0015991 GO:0015992 GO:0015078 GO:0033179
Cluster-22178.101711 GO:0006364 GO:0000902 GO:0006955 GO:0016818

GO:0003723 GO:0005524 GO:0008168 GO:0016020 GO:0005634
Cluster-22178.101716 GO:0006570 GO:0006771 GO:0006470 GO:0019497
GO:0005515 GO:0008138 GO:0004725 GO:0003993 GO:0046872
Cluster-22178.101721 GO:0043066 GO:0003950 GO:0051015 GO:0016788

Cluster-22178.101724 GO:0007010 GO:0003779
Cluster-22178.101725 GO:0005975 GO:0006446 GO:0016901 GO:0048038
GO:0003743 GO:0005737 GO:0005840 GO:0005852
Cluster-22178.101728 GO:0055114 GO:0016620
Cluster-22178.101731 GO:0009085 GO:0055114 GO:0009089 GO:0051287
GO:0008839 GO:0016620
Cluster-22178.101735 GO:0061024 GO:0030176
Cluster-22178.101736 GO:0006771 GO:0009405 GO:0019497 GO:0006260
GO:0005515 GO:0003689 GO:0046872 GO:0016798 GO:0005524
GO:0003993 GO:0005663 GO:0005576 GO:0042575
Cluster-22178.101737 GO:0008092 GO:0019898 GO:0009512 GO:0005737
Cluster-22178.101738 GO:0046872 GO:0005515
Cluster-22178.101748 GO:0007165 GO:0006955 GO:0005164 GO:0016020
Cluster-22178.101751 GO:0005515 GO:0046872 GO:0008270 GO:0045202
Cluster-22178.101754 GO:0005524 GO:0016887 GO:0016020
Cluster-22178.101756 GO:0006812 GO:0008324 GO:0016021
Cluster-22178.101764 GO:0015074 GO:0008270 GO:0003676
Cluster-22178.101767 GO:0015986 GO:0042254 GO:0015992 GO:0035556
GO:0006355 GO:0006119 GO:0006412 GO:0003677 GO:0003735
GO:0046961 GO:0008270 GO:0046933 GO:0005515 GO:0045259
GO:0045261 GO:0005622 GO:0005840
Cluster-22178.101775 GO:0005515
Cluster-22178.101787 GO:0006355 GO:0003700 GO:0005667
Cluster-22178.101792 GO:0006094 GO:0006098 GO:0006096 GO:0006000
GO:0006013 GO:0006012 GO:0003872 GO:0005945
Cluster-22178.101793 GO:0006915 GO:0045735 GO:0005622
Cluster-22178.101794 GO:0000105 GO:0006915 GO:0045735 GO:0005622
Cluster-22178.101795 GO:0006915 GO:0045735 GO:0005622

Cluster-22178.101796 GO:0045735
Cluster-22178.101800 GO:0016567 GO:0046872 GO:0008270 GO:0004842
GO:0005515 GO:0005680
Cluster-22178.101801 GO:0008270 GO:0046872 GO:0005515
Cluster-22178.101802 GO:0005515
Cluster-22178.101809 GO:0006366 GO:0006384 GO:0008017 GO:0003700
GO:0005515 GO:0045298 GO:0016021 GO:0005667 GO:0005634
Cluster-22178.101816 GO:0005524
Cluster-22178.101818 GO:0005524
Cluster-22178.101820 GO:0005524
Cluster-22178.101822 GO:0043631 GO:0005515 GO:0004652
Cluster-22178.101824 GO:0006355 GO:0043565 GO:0019901 GO:0003700
GO:0005667
Cluster-22178.101826 GO:0009405 GO:0005576
Cluster-22178.101827 GO:0009405 GO:0005576
Cluster-22178.101828 GO:0030414 GO:0000287 GO:0004867 GO:0030976
GO:0005515 GO:0005576
Cluster-22178.101829 GO:0006810 GO:0016310 GO:0004673 GO:0005198
GO:0005882 GO:0009365 GO:0016021
Cluster-22178.101830 GO:0046677 GO:0016779
Cluster-22178.101831 GO:0046677 GO:0016779
Cluster-22178.101835 GO:0006468 GO:0004672 GO:0005524 GO:0016773
GO:0016020
Cluster-22178.101840 GO:0009966 GO:0016798 GO:0005515 GO:0005576
Cluster-22178.101841 GO:0007275 GO:0005578
Cluster-22178.101842 GO:0005515 GO:0016798
Cluster-22178.101846 GO:0010038 GO:0043087 GO:0046938 GO:0032012
GO:0005086 GO:0046872 GO:0016756
Cluster-22178.101849 GO:0016773
Cluster-22178.101850 GO:0007423 GO:0005212 GO:0016773
Cluster-22178.101853 GO:0007018 GO:0007017 GO:0003777 GO:0008017
GO:0005524 GO:0005874 GO:0045298
Cluster-22178.101855 GO:0006355 GO:0006351 GO:0005515

Cluster-22178.101861 GO:0009405
Cluster-22178.101871 GO:0006260 GO:0003677 GO:0003887 GO:0042575
Cluster-22178.101874 GO:0042407 GO:0010508 GO:0006810 GO:0005215
GO:0061617 GO:0044284
Cluster-22178.101876 GO:0048870 GO:0045995 GO:0007049 GO:0006355
GO:0006886 GO:0051252 GO:0006914 GO:0030334 GO:0006606
GO:0030155 GO:0007165 GO:0043565 GO:0008092 GO:0003700
GO:0019901 GO:0005102 GO:0016891 GO:0005737 GO:0016021
GO:0031514 GO:0019898 GO:0005667 GO:0005783
Cluster-22178.101877 GO:0051289 GO:0008140 GO:0005667
Cluster-22178.101879 GO:0015979 GO:0006355 GO:0016021 GO:0009539
GO:0005622 GO:0009523
Cluster-22178.101881 GO:0006869 GO:0005319
Cluster-22178.101887 GO:0055085 GO:0005975 GO:0007186 GO:0006487
GO:0004930 GO:0003824 GO:0043169 GO:0016787 GO:0016021
GO:0008250
Cluster-22178.101889 GO:0006891 GO:0016021 GO:0017119
Cluster-22178.101895 GO:0005515 GO:0003724 GO:0003723 GO:0005524

Cluster-22178.101896 GO:0003723 GO:0005524 GO:0003724 GO:0005515

Cluster-22178.101897 GO:0016192
Cluster-22178.101899 GO:0005515
Cluster-22178.101900 GO:0016567 GO:0007155 GO:0006308 GO:0005515
GO:0005198 GO:0004842 GO:0008855 GO:0046872 GO:0005524
GO:0008270 GO:0009318 GO:0005680 GO:0019031 GO:0009288
GO:0005622
Cluster-22178.101902 GO:0016579 GO:0051087 GO:0005198 GO:0019904
GO:0036459 GO:0019028
Cluster-22178.101907 GO:0008152 GO:0016787
Cluster-22178.101908 GO:0005515
Cluster-22178.101911 GO:0007169 GO:0006468 GO:0004714 GO:0005524
GO:0016020

Cluster-22178.101912 GO:0005515
Cluster-22178.101913 GO:0006384 GO:0006366 GO:0008017 GO:0003700
GO:0005515 GO:0016021 GO:0045298 GO:0005634 GO:0005667
Cluster-22178.101916 GO:0005634
Cluster-22178.101917 GO:0046849 GO:0005515 GO:0005576 GO:0016021
Cluster-22178.101918 GO:0046849 GO:0005515 GO:0016021 GO:0005576
Cluster-22178.101919 GO:0016055 GO:0046849 GO:0005515 GO:0005576
GO:0016021
Cluster-22178.101920 GO:0046849 GO:0005515 GO:0016021 GO:0005576
Cluster-22178.101922 GO:0006508 GO:0046849 GO:0016192 GO:0005524
GO:0004222 GO:0005515 GO:0016021 GO:0005576
Cluster-22178.101923 GO:0006313
Cluster-22178.101926 GO:0008233
Cluster-22178.101927 GO:0007165 GO:0006825 GO:0006470 GO:0016531
GO:0008138 GO:0005179 GO:0005507 GO:0005576 GO:0005758
Cluster-22178.101928 GO:0006825 GO:0006470 GO:0007165 GO:0005507
GO:0005179 GO:0016531 GO:0008138 GO:0005758 GO:0005576
Cluster-22178.101929 GO:0007165 GO:0006470 GO:0006825 GO:0008138
GO:0016531 GO:0005179 GO:0005507 GO:0005576 GO:0005758
Cluster-22178.101932 GO:0007186 GO:0004930 GO:0016021
Cluster-22178.101934 GO:0004252 GO:0005515 GO:0019028 GO:0055036
Cluster-22178.101945 GO:0007155 GO:0016755 GO:0016020
Cluster-22178.101946 GO:0004222
Cluster-22178.101959 GO:0006260 GO:0006281 GO:0005515 GO:0003911
GO:0016787 GO:0046872
Cluster-22178.101961 GO:0051262 GO:0032784 GO:0007165 GO:0042262
GO:0003690 GO:0003677
Cluster-22178.101962 GO:0007165 GO:0042262 GO:0032784 GO:0051262
GO:0003677 GO:0003690
Cluster-22178.101965 GO:0007186 GO:0007165 GO:0005057 GO:0004930
GO:0016021
Cluster-22178.101966 GO:0007165 GO:0005057
Cluster-22178.101970 GO:0030436 GO:0006508 GO:0004190

Cluster-22178.101975 GO:0009117 GO:0030639 GO:0019872 GO:0008830

Cluster-22178.101976 GO:0019872 GO:0030639 GO:0009117 GO:0008830
GO:0043565
Cluster-22178.101977 GO:0019872 GO:0030639 GO:0009117 GO:0008830

Cluster-22178.101978 GO:0005911 GO:0016020
Cluster-22178.101979 GO:0005911 GO:0016020
Cluster-22178.101980 GO:0006355 GO:0003677
Cluster-22178.101983 GO:0055114 GO:0005506 GO:0020037 GO:0016705

Cluster-22178.101984 GO:0048193 GO:0008053 GO:0000921 GO:0030168
GO:0006914 GO:0007165 GO:0007155 GO:0051258 GO:0009405
GO:0006397 GO:0005102 GO:0030674 GO:0046872 GO:0003924
GO:0005198 GO:0005577 GO:0005940 GO:0005741 GO:0016020
GO:0019031 GO:0000445 GO:0016021 GO:0019028
Cluster-22178.101985 GO:0000921 GO:0005940 GO:0016021
Cluster-22178.101986 GO:0007165 GO:0006812 GO:0006835 GO:0006820
GO:0017153 GO:0005102 GO:0016020
Cluster-22178.101989 GO:0007165 GO:0016032 GO:0006508 GO:0004197
GO:0005179 GO:0004672 GO:0005576
Cluster-22178.101990 GO:0016032 GO:0006508 GO:0007165 GO:0005179
GO:0004672 GO:0004197 GO:0005576
Cluster-22178.101996 GO:0030991
Cluster-22178.101998 GO:0006810 GO:0006281 GO:0055085 GO:0006206
GO:0006231 GO:0055114 GO:0047134 GO:0005215 GO:0004799
GO:0046872 GO:0008270 GO:0016020 GO:0016021
Cluster-22178.102002 GO:0007049 GO:0048309 GO:0006914 GO:0051028
GO:0005198 GO:0019031 GO:0019028
Cluster-22178.102004 GO:0007010 GO:0007304 GO:0003779 GO:0005213
GO:0005576 GO:0042600
Cluster-22178.102005 GO:0006810 GO:0006811 GO:0005230 GO:0016020
Cluster-22178.102009 GO:0005515

Cluster-22178.102014 GO:0019229 GO:0005576
Cluster-22178.102016 GO:0006119 GO:0015986 GO:0000921 GO:0015992
GO:0046961 GO:0008092 GO:0046933 GO:0045259 GO:0000275
GO:0016021 GO:0019898 GO:0005737 GO:0005940
Cluster-22178.102017 GO:0030234 GO:0005515 GO:0016020
Cluster-22178.102025 GO:0006352 GO:0055085 GO:0006811 GO:0019087
GO:0005515 GO:0005216 GO:0016021 GO:0016020 GO:0005634
Cluster-22178.102026 GO:0055085 GO:0006811 GO:0005216 GO:0016020
Cluster-22178.102027 GO:0006814 GO:0015986 GO:0055085 GO:0006811
GO:0015992 GO:0051016 GO:0005248 GO:0005216 GO:0015078
GO:0016020 GO:0008290 GO:0045263 GO:0001518 GO:0016021
Cluster-22178.102033 GO:0006468 GO:0007264 GO:0015684 GO:0016887
GO:0003924 GO:0004672 GO:0005524 GO:0015093 GO:0005525
GO:0005515 GO:0016021
Cluster-22178.102035 GO:0006090 GO:0006811 GO:0006814 GO:0006560
GO:0015991 GO:0006508 GO:0071436 GO:0007165 GO:0007268
GO:0006525 GO:0004970 GO:0008236 GO:0008948 GO:0016820
GO:0015081 GO:0000221 GO:0016020
Cluster-22178.102041 GO:0016846
Cluster-22178.102046 GO:0006094 GO:0006099 GO:0015976 GO:0004612
GO:0005524
Cluster-22178.102050 GO:0006825 GO:0009117 GO:0005975 GO:0046373
GO:0007186 GO:0035434 GO:0004930 GO:0003824 GO:0046556
GO:0005375 GO:0043169 GO:0016021
Cluster-22178.102054 GO:0005515 GO:0016020
Cluster-22178.102061 GO:0006260 GO:0015992 GO:0070206 GO:0009252
GO:0006869 GO:0016567 GO:0042157 GO:0007049 GO:0007021
GO:0006308 GO:0015991 GO:0004842 GO:0015631 GO:0008270
GO:0008855 GO:0016755 GO:0004857 GO:0015078 GO:0008289
GO:0046872 GO:0005515 GO:0051082 GO:0005622 GO:0016020
GO:0045298 GO:0009318 GO:0033179 GO:0005576
Cluster-22178.102065 GO:0006123 GO:0015992 GO:0016788 GO:0004129
GO:0045277 GO:0005743

Cluster-22178.102069 GO:0006914 GO:0030334 GO:0006606 GO:0030155
GO:0007165 GO:0048870 GO:0045995 GO:0007049 GO:0006355
GO:0006886 GO:0051252 GO:0005102 GO:0016891 GO:0043565
GO:0008092 GO:0003700 GO:0019901 GO:0019898 GO:0005667
GO:0005783 GO:0005737 GO:0016021 GO:0031514
Cluster-22178.102080 GO:0006468 GO:0004672 GO:0005524 GO:0016773
GO:0016020
Cluster-22178.102081 GO:0006952 GO:0006310 GO:0046872 GO:0000150
GO:0003677 GO:0005515
Cluster-22178.102082 GO:0003723 GO:0019013
Cluster-22178.102084 GO:0016310 GO:0000160 GO:0006355 GO:0007165
GO:0018106 GO:0005524 GO:0043565 GO:0004871 GO:0004673
GO:0005515 GO:0005886 GO:0009365 GO:0016021 GO:0005634
Cluster-22178.102088 GO:0006206 GO:0006144 GO:0006241 GO:0006228
GO:0006165 GO:0019079 GO:0006183 GO:0004550 GO:0008270
GO:0003723
Cluster-22178.102095 GO:0006355 GO:0003677
Cluster-22178.102097 GO:0005515
Cluster-22178.102099 GO:0005515
Cluster-22178.102104 GO:0007165 GO:0015991 GO:0015992 GO:0015078
GO:0001664 GO:0033179
Cluster-22178.102105 GO:0005975 GO:0006508 GO:0043169 GO:0003824
GO:0008236
Cluster-22178.102107 GO:0005975 GO:0043169 GO:0003824
Cluster-22178.102109 GO:0005975 GO:0006508 GO:0043169 GO:0003824
GO:0008236
Cluster-22178.102110 GO:0005975 GO:0006508 GO:0043169 GO:0003824
GO:0008236
Cluster-22178.102111 GO:0006750 GO:0006749 GO:0004357 GO:0017109
Cluster-22178.102113 GO:0035444 GO:0015675 GO:0015099 GO:0003677
GO:0046872 GO:0016021
Cluster-22178.102116 GO:0006355 GO:0003723 GO:0003677
Cluster-22178.102118 GO:0019058 GO:0045859 GO:0016534 GO:0016533

Cluster-22178.102119 GO:0019058 GO:0051028 GO:0045859 GO:0016534
GO:0016533 GO:0005634
Cluster-22178.102122 GO:0007009 GO:0005515
Cluster-22178.102125 GO:0005515
Cluster-22178.102126 GO:0006915 GO:0045735 GO:0005622
Cluster-22178.102127 GO:0055114 GO:0006570 GO:0004511
Cluster-22178.102130 GO:0016032 GO:0016021
Cluster-22178.102132 GO:0006771 GO:0009405 GO:0019497 GO:0005515
GO:0003677 GO:0046872 GO:0016798 GO:0003993 GO:0005634
GO:0005576
Cluster-22178.102133 GO:0005515
Cluster-22178.102135 GO:0006096 GO:0006098 GO:0043093 GO:0006013
GO:0006000 GO:0006071 GO:0000917 GO:0006281 GO:0006094
GO:0015976 GO:0055114 GO:0042132 GO:0008270 GO:0016491
GO:0016209 GO:0005622 GO:0016021 GO:0030915 GO:0005737
Cluster-22178.102136 GO:0006094 GO:0000917 GO:0055114 GO:0015976
GO:0043093 GO:0006098 GO:0006096 GO:0006071 GO:0006000
GO:0006013 GO:0016209 GO:0042132 GO:0016491 GO:0008270
GO:0005737 GO:0016021 GO:0005622
Cluster-22178.102137 GO:0050830 GO:0006810 GO:0045859 GO:0007017
GO:0050829 GO:0007018 GO:0016887 GO:0005524 GO:0004860
GO:0003924 GO:0005215 GO:0005525 GO:0003777 GO:0030286
GO:0016020 GO:0005874 GO:0005576
Cluster-22178.102139 GO:0006771 GO:0019497 GO:0016998 GO:0006032
GO:0004568 GO:0005515 GO:0046872 GO:0003993
Cluster-22178.102141 GO:0006032 GO:0016998 GO:0019497 GO:0006412
GO:0042254 GO:0006771 GO:0003993 GO:0046872 GO:0005515
GO:0003735 GO:0003723 GO:0004568 GO:0005840
Cluster-22178.102144 GO:0007218 GO:0006355 GO:0007010 GO:0003779
GO:0005185 GO:0005509 GO:0005544 GO:0003677 GO:0005634
GO:0005576
Cluster-22178.102145 GO:0007218 GO:0009733 GO:0007010 GO:0007186
GO:0004930 GO:0003779 GO:0005544 GO:0005509 GO:0005185

GO:0016021 GO:0005576
Cluster-22178.102146 GO:0006355 GO:0007264 GO:0007186 GO:0007165
GO:0015684 GO:0043531 GO:0003924 GO:0031683 GO:0019001
GO:0004871 GO:0005525 GO:0003723 GO:0008134 GO:0000166
GO:0016887 GO:0005524 GO:0015093 GO:0003724 GO:0003677
GO:0016021 GO:0005667 GO:0016020
Cluster-22178.102150 GO:0016226 GO:0008199
Cluster-22178.102154 GO:0008270 GO:0008237
Cluster-22178.102155 GO:0006508 GO:0005515 GO:0004252
Cluster-22178.102162 GO:0008270
Cluster-22178.102164 GO:0005515
Cluster-22178.102165 GO:0006144 GO:0007165 GO:0004114
Cluster-22178.102171 GO:0008270 GO:0005515 GO:0004222
Cluster-22178.102173 GO:0015991 GO:0016820 GO:0005515 GO:0000221
Cluster-22178.102174 GO:0009432 GO:0015991 GO:0051782 GO:0016820
GO:0005515 GO:0000221 GO:0009276
Cluster-22178.102175 GO:0015991 GO:0016820 GO:0005515 GO:0000221
Cluster-22178.102179 GO:0006355 GO:0003677 GO:0003700 GO:0005667
Cluster-22178.102180 GO:0007165 GO:0005515
Cluster-22178.102182 GO:0055114 GO:0042773 GO:0016651 GO:0016209
GO:0016491 GO:0008270 GO:0005622
Cluster-22178.102183 GO:0006096 GO:0042773 GO:0006098 GO:0043093
GO:0006000 GO:0006071 GO:0006013 GO:0000917 GO:0006094
GO:0006281 GO:0015976 GO:0055114 GO:0042132 GO:0016651
GO:0016491 GO:0008270 GO:0016209 GO:0005622 GO:0016021
GO:0005737 GO:0030915
Cluster-22178.102185 GO:0006468 GO:0035023 GO:0043087 GO:0005515
GO:0005089 GO:0004672 GO:0005524 GO:0016773 GO:0016020
Cluster-22178.102186 GO:0035023 GO:0043087 GO:0006468 GO:0005089
GO:0016773 GO:0005524 GO:0004672 GO:0005515 GO:0016020
Cluster-22178.102187 GO:0006468 GO:0035023 GO:0043087 GO:0005515
GO:0005089 GO:0005524 GO:0004672 GO:0016773 GO:0016020
Cluster-22178.102188 GO:0035023 GO:0043087 GO:0006468 GO:0005089

GO:0016773 GO:0004672 GO:0005524 GO:0005515 GO:0016020
Cluster-22178.102193 GO:0042802
Cluster-22178.102194 GO:0042802
Cluster-22178.102202 GO:0006810 GO:0042967 GO:0005515 GO:0008811
GO:0003774 GO:0005524 GO:0016459 GO:0005856
Cluster-22178.102206 GO:0035434 GO:0007186 GO:0046373 GO:0005975
GO:0006825 GO:0009117 GO:0005375 GO:0043169 GO:0046556
GO:0004930 GO:0003824 GO:0016021
Cluster-22178.102209 GO:0005975 GO:0043169 GO:0003824
Cluster-22178.102218 GO:0005515 GO:0016021
Cluster-22178.102221 GO:0008033 GO:0006464 GO:0006554 GO:0006480
GO:0008152 GO:0006400 GO:0046500 GO:0009451 GO:0006479
GO:0008168 GO:0004719 GO:0008757 GO:0008171 GO:0008176
GO:0018024
Cluster-22178.102223 GO:0022900 GO:0005747 GO:0005739
Cluster-22178.102225 GO:0009607 GO:0055114 GO:0009405 GO:0047134
GO:0008270 GO:0045735 GO:0016021 GO:0016020
Cluster-22178.102226 GO:0008270 GO:0005887
Cluster-22178.102228 GO:0030234 GO:0005515 GO:0016020
Cluster-22178.102229 GO:0043087 GO:0010038 GO:0046938 GO:0032012
GO:0005086 GO:0046872 GO:0016756
Cluster-22178.102230 GO:0006310 GO:0005515 GO:0003677 GO:0016021
GO:0005634
Cluster-22178.102232 GO:0005516
Cluster-22178.102237 GO:0010508 GO:0007050
Cluster-22178.102240 GO:0007165 GO:0016020 GO:0016021
Cluster-22178.102246 GO:0046983
Cluster-22178.102247 GO:0046656 GO:0006814 GO:0030269 GO:0005737
GO:0012506
Cluster-22178.102249 GO:0016021
Cluster-22178.102251 GO:0022900 GO:0005747 GO:0005739
Cluster-22178.102252 GO:0009236 GO:0007076 GO:0009225 GO:0009117
GO:0006012 GO:0019915 GO:0009103 GO:0019058 GO:0003978

GO:0003824 GO:0005789 GO:0019031 GO:0000796
Cluster-22178.102260 GO:0019901
Cluster-22178.102271 GO:0007186 GO:0006811 GO:0009405 GO:0006355
GO:0004930 GO:0008270 GO:0005216 GO:0003700 GO:0043565
GO:0005515 GO:0005667 GO:0019031 GO:0016021 GO:0031224
GO:0005576 GO:0005634
Cluster-22178.102272 GO:0006355 GO:0009405 GO:0006811 GO:0007186
GO:0005515 GO:0043565 GO:0003700 GO:0005216 GO:0008270
GO:0004930 GO:0005634 GO:0005576 GO:0031224 GO:0016021
GO:0019031 GO:0005667
Cluster-22178.102274 GO:0006355 GO:0009405 GO:0006811 GO:0007186
GO:0005515 GO:0043565 GO:0003700 GO:0005216 GO:0008270
GO:0004930 GO:0005634 GO:0005576 GO:0031224 GO:0016021
GO:0019031 GO:0005667
Cluster-22178.102275 GO:0006811 GO:0006355 GO:0009405 GO:0007186
GO:0043565 GO:0003700 GO:0005515 GO:0008270 GO:0004930
GO:0005216 GO:0019031 GO:0016021 GO:0005576 GO:0005634
GO:0031224 GO:0005667
Cluster-22178.102276 GO:0006811 GO:0009405 GO:0006355 GO:0007186
GO:0003700 GO:0043565 GO:0005515 GO:0004930 GO:0008270
GO:0005216 GO:0019031 GO:0016021 GO:0031224 GO:0005576
GO:0005634 GO:0005667
Cluster-22178.102279 GO:0009405 GO:0007186 GO:0006355 GO:0006811
GO:0005216 GO:0004930 GO:0003700 GO:0008270 GO:0043565
GO:0005576 GO:0005667 GO:0005634 GO:0019031 GO:0016021
Cluster-22178.102280 GO:0007186 GO:0006811 GO:0006355 GO:0009405
GO:0008270 GO:0004930 GO:0005216 GO:0043565 GO:0003700
GO:0005515 GO:0005667 GO:0016021 GO:0019031 GO:0005634
GO:0005576 GO:0031224
Cluster-22178.102283 GO:0034508 GO:0005515 GO:0000776
Cluster-22178.102285 GO:0005515
Cluster-22178.102286 GO:0006869 GO:0007165 GO:0008289 GO:0005524
GO:0003774 GO:0005515 GO:0005856 GO:0016459 GO:0005576

Cluster-22178.102288 GO:0006865 GO:0005515 GO:0016020 GO:0005643
Cluster-22178.102290 GO:0006355 GO:0003700 GO:0005515 GO:0005667
Cluster-22178.102301 GO:0006355 GO:0003700 GO:0043565 GO:0005667
Cluster-22178.102302 GO:0015684 GO:0006468 GO:0007264 GO:0005525 GO:0004672 GO:0005524 GO:0015093 GO:0016887 GO:0003924 GO:0016021
Cluster-22178.102304 GO:0007264 GO:0006468 GO:0015684 GO:0005515 GO:0003924 GO:0016887 GO:0015093 GO:0005524 GO:0004672 GO:0005525 GO:0016021
Cluster-22178.102314 GO:0006024 GO:0030206 GO:0030158
Cluster-22178.102315 GO:0055114 GO:0016491
Cluster-22178.102316 GO:0007186 GO:0005509 GO:0005515 GO:0004930

Cluster-22178.102317 GO:0007186 GO:0005515 GO:0004930
Cluster-22178.102319 GO:0008654 GO:0016780 GO:0016020
Cluster-22178.102323 GO:0006355 GO:0006816 GO:0006874 GO:0006270 GO:0008270 GO:0005219 GO:0003700 GO:0005667 GO:0005634 GO:0005622 GO:0016021
Cluster-22178.102333 GO:0006811 GO:0055085 GO:0005216 GO:0016020
Cluster-22178.102341 GO:0015992 GO:0015991 GO:0015078 GO:0046983 GO:0033180
Cluster-22178.102347 GO:0030234 GO:0005515 GO:0016020
Cluster-22178.102348 GO:0006468 GO:0005515 GO:0004672 GO:0005524

Cluster-22178.102349 GO:0019497 GO:0006771 GO:0006468 GO:0004672 GO:0005524 GO:0046872 GO:0003993 GO:0005515
Cluster-22178.102357 GO:0006807 GO:0009253 GO:0009252 GO:0008745

Cluster-22178.102359 GO:0016567 GO:0004842
Cluster-22178.102362 GO:0007017 GO:0005198 GO:0019028 GO:0005875
Cluster-22178.102364 GO:0051258 GO:0006184 GO:0007018 GO:0000086 GO:0030030 GO:0051084 GO:0007275 GO:0005200 GO:0005525 GO:0003924 GO:0005515 GO:0005874 GO:0005829 GO:0005856

GO:0043025 GO:0042600 GO:0043209 GO:0005886 GO:0005929
GO:0033269
Cluster-22178.102366 GO:0030694
Cluster-22178.102368 GO:0016055 GO:0007165 GO:0007275 GO:0006570
GO:0055114 GO:0004511 GO:0005102 GO:0005576
Cluster-22178.102377 GO:0005515
Cluster-22178.102381 GO:0006771 GO:0019497 GO:0046872 GO:0003993
GO:0005515
Cluster-22178.102383 GO:0007264 GO:0016192 GO:0006886 GO:0005515
GO:0005198 GO:0005525 GO:0030132 GO:0030130
Cluster-22178.102384 GO:0007268 GO:0007165 GO:0006811 GO:0004970
GO:0005234 GO:0016020
Cluster-22178.102390 GO:0006865 GO:0055085 GO:0003333 GO:0006810
GO:0015171 GO:0016020
Cluster-22178.102394 GO:0004222 GO:0016012
Cluster-22178.102397 GO:0004222 GO:0016012
Cluster-22178.102400 GO:0000917 GO:0006355 GO:0043093 GO:0006744
GO:0006260 GO:0007049 GO:0006914 GO:0003700 GO:0043565
GO:0008813 GO:0005667 GO:0005815 GO:0005737
Cluster-22178.102407 GO:0005515
Cluster-22178.102410 GO:0055114 GO:0007165 GO:0006355 GO:0005507
GO:0003677 GO:0016641 GO:0005044 GO:0016020
Cluster-22178.102419 GO:0006508 GO:0008233 GO:0005634
Cluster-22178.102420 GO:0005515
Cluster-22178.102421 GO:0006508 GO:0008233 GO:0005634
Cluster-22178.102423 GO:0006508 GO:0008233 GO:0005634
Cluster-22178.102424 GO:0006508 GO:0008233 GO:0005634
Cluster-22178.102427 GO:0006508 GO:0008233 GO:0005634
Cluster-22178.102428 GO:0006508 GO:0008233 GO:0005634
Cluster-22178.102442 GO:0005615
Cluster-22178.102443 GO:0006270 GO:0031175 GO:0030507 GO:0008017
GO:0005516 GO:0008091 GO:0045298
Cluster-22178.102444 GO:0007049

Cluster-22178.102448 GO:0000746 GO:0007186 GO:0003677 GO:0004930
GO:0016020 GO:0019867 GO:0016021
Cluster-22178.102454 GO:0005975 GO:0055114 GO:0009117 GO:0046373
GO:0046556 GO:0005515 GO:0016705
Cluster-22178.102456 GO:0010923 GO:0019902
Cluster-22178.102459 GO:0046914
Cluster-22178.102460 GO:0035023 GO:0043087 GO:0005515 GO:0005089
GO:0046914
Cluster-22178.102462 GO:0006357 GO:0032784 GO:0071988 GO:0006810
GO:0006006 GO:0006308 GO:2000123 GO:0009405 GO:0006886
GO:0030474 GO:0008855 GO:0008772 GO:0003677 GO:0005200
GO:0003676 GO:0051082 GO:0001104 GO:0016791 GO:0005886
GO:0005823 GO:0016592 GO:0016020 GO:0005737 GO:0005856
GO:0019028 GO:0009318
Cluster-22178.102464 GO:0005515
Cluster-22178.102470 GO:0005975 GO:0006508 GO:0016032 GO:0004197
GO:0005515 GO:0003824
Cluster-22178.102471 GO:0000042 GO:0005515
Cluster-22178.102475 GO:0051262 GO:0015979 GO:0008152 GO:0003824
GO:0016787 GO:0009523 GO:0009539 GO:0016020
Cluster-22178.102476 GO:0006817
Cluster-22178.102477 GO:0006269 GO:0007155 GO:0006351 GO:0006260
GO:0003677 GO:0005540 GO:0005515 GO:0008270 GO:0003896
GO:0005730 GO:0005657
Cluster-22178.102478 GO:0003677 GO:0005515 GO:0004527
Cluster-22178.102479 GO:0005515 GO:0004527 GO:0003677
Cluster-22178.102483 GO:0006355 GO:0003677 GO:0016021
Cluster-22178.102489 GO:0007018 GO:0007009 GO:0007017 GO:0005515
GO:0003777 GO:0019031 GO:0005874 GO:0030286
Cluster-22178.102490 GO:0055114 GO:0048870 GO:0006534 GO:0007049
GO:0019530 GO:0032402 GO:0046439 GO:0007094 GO:0005506
GO:0017172 GO:0042470 GO:0031514
Cluster-22178.102494 GO:0007155 GO:0005515 GO:0016021

Cluster-22178.102498 GO:0006904 GO:0000902 GO:0006955 GO:0009307 GO:0015031 GO:0006308 GO:0048870 GO:0009036 GO:0016020 GO:0000145 GO:0031514 GO:0009359 GO:0016021
Cluster-22178.102499 GO:0005515
Cluster-22178.102506 GO:0005515
Cluster-22178.102519 GO:0005525 GO:0016887 GO:0003924 GO:0005524 GO:0016020
Cluster-22178.102521 GO:0005515
Cluster-22178.102522 GO:0005515
Cluster-22178.102525 GO:0005515
Cluster-22178.102526 GO:0005515
Cluster-22178.102529 GO:0005515
Cluster-22178.102531 GO:0005515
Cluster-22178.102535 GO:0005515
Cluster-22178.102538 GO:0006811 GO:0055085 GO:0005216 GO:0016020
Cluster-22178.102542 GO:0006468 GO:0004672 GO:0005524 GO:0005515

Cluster-22178.102543 GO:0005515
Cluster-22178.102545 GO:0006468 GO:0005198 GO:0004672 GO:0005524 GO:0019012
Cluster-22178.102546 GO:0006468 GO:0005524 GO:0004672
Cluster-22178.102547 GO:0006468 GO:0005524 GO:0004672 GO:0005198 GO:0019012
Cluster-22178.102550 GO:0009451 GO:0009877 GO:0006479 GO:0008033 GO:0008152 GO:0046500 GO:0006400 GO:0032259 GO:0006554 GO:0006464 GO:0009312 GO:0006480 GO:0018024 GO:0008168 GO:0004719 GO:0008757 GO:0008176 GO:0008171
Cluster-22178.102556 GO:0006281 GO:0003824
Cluster-22178.102561 GO:0006355 GO:0005634
Cluster-22178.102562 GO:0007155 GO:0016301 GO:0005540 GO:0005515 GO:0005524
Cluster-22178.102564 GO:0007268 GO:0007165 GO:0006811 GO:0004970 GO:0016020

Cluster-22178.102566 GO:0005515
Cluster-22178.102567 GO:0005515
Cluster-22178.102569 GO:0016567 GO:0032465 GO:0009607 GO:0006915
GO:0004842 GO:0016021
Cluster-22178.102570 GO:0046872 GO:0016021
Cluster-22178.102578 GO:0006397 GO:0005515 GO:0005524 GO:0003774
GO:0016459
Cluster-22178.102581 GO:0006810 GO:0005215 GO:0005515 GO:0003824
GO:0005737 GO:0016021
Cluster-22178.102585 GO:0007165 GO:0007186 GO:0004930 GO:0005057
GO:0016021
Cluster-22178.102588 GO:0019882 GO:0006355 GO:0006955 GO:0042613
GO:0016020
Cluster-22178.102592 GO:0003677 GO:0005525
Cluster-22178.102597 GO:0008033 GO:0008152 GO:0009451 GO:0009877
GO:0006479 GO:0006554 GO:0032259 GO:0006464 GO:0009312
GO:0006480 GO:0046500 GO:0006400 GO:0008168 GO:0004719
GO:0008757 GO:0018024 GO:0008171 GO:0008176
Cluster-22178.102604 GO:0006260 GO:0005634 GO:0042720
Cluster-22178.102607 GO:0006468 GO:0004672 GO:0005524
Cluster-22178.102610 GO:0030261 GO:0003677
Cluster-22178.102613 GO:0008152 GO:0008484
Cluster-22178.102615 GO:0044564 GO:0008152 GO:0008484 GO:0003824

Cluster-22178.102616 GO:0044564 GO:0008152 GO:0008484 GO:0003824

Cluster-22178.10262 GO:0005201 GO:0005581 GO:0005578
Cluster-22178.102621 GO:0006687 GO:0006027 GO:0006012 GO:0046486
GO:0004565 GO:0009341
Cluster-22178.102622 GO:0006012 GO:0046486 GO:0006687 GO:0006027
GO:0004565 GO:0009341
Cluster-22178.102627 GO:0046487 GO:0051603 GO:0055114 GO:0009396
GO:0004488 GO:0003824 GO:0004298 GO:0005839

Cluster-22178.102631 GO:0006118 GO:0008608 GO:0003954 GO:0008270
GO:0008440 GO:0042729 GO:0005739 GO:0072686
Cluster-22178.102632 GO:0006935 GO:0006355 GO:0007165 GO:0006955
GO:0003700 GO:0008009 GO:0005576 GO:0005667
Cluster-22178.102635 GO:0046872 GO:0016787 GO:0005515 GO:0045202
Cluster-22178.102637 GO:0046872
Cluster-22178.102638 GO:0006813 GO:0008556 GO:0005886
Cluster-22178.102640 GO:0006606 GO:0048193 GO:0030334 GO:0015992
GO:0006914 GO:0019079 GO:0007155 GO:0055114 GO:0006260
GO:0008053 GO:0018401 GO:0051607 GO:0000921 GO:0030168
GO:0006144 GO:0007165 GO:0030155 GO:0006525 GO:0045995
GO:0051258 GO:0006308 GO:0048870 GO:0006560 GO:0015991
GO:0003774 GO:0004656 GO:0015078 GO:0003968 GO:0003924
GO:0042803 GO:0005198 GO:0005102 GO:0008855 GO:0030674
GO:0016702 GO:0008134 GO:0045502 GO:0005577 GO:0005741
GO:0005783 GO:0016020 GO:0016459 GO:0005667 GO:0019867
GO:0005815 GO:0030286 GO:0005940 GO:0005882 GO:0019031
GO:0016021 GO:0031379 GO:0009318 GO:0033179 GO:0031514
GO:0019028
Cluster-22178.102642 GO:0046872 GO:0019012
Cluster-22178.102643 GO:0046872 GO:0019012
Cluster-22178.102644 GO:0016055 GO:0046872 GO:0008013 GO:0019012
GO:0016342
Cluster-22178.102646 GO:0007264 GO:0005525 GO:0003723
Cluster-22178.102649 GO:0055114 GO:0046872 GO:0016702
Cluster-22178.102655 GO:0007186 GO:0004930 GO:0016021
Cluster-22178.102656 GO:0008270
Cluster-22178.102657 GO:0008270
Cluster-22178.102660 GO:0005515
Cluster-22178.102663 GO:0006614 GO:0006810 GO:0005524 GO:0003924
GO:0016887 GO:0005525 GO:0005576
Cluster-22178.102664 GO:0006260 GO:0006308 GO:0006869 GO:0009405
GO:0006281 GO:0042157 GO:0008289 GO:0017108 GO:0033557

GO:0005576 GO:0005634
Cluster-22178.102671 GO:0008270
Cluster-22178.102672 GO:0055114 GO:0007165 GO:0005507 GO:0070008
GO:0004252 GO:0016641 GO:0005044 GO:0016020
Cluster-22178.102674 GO:0006355 GO:0051301 GO:0016817 GO:0003677
GO:0005515 GO:0005634
Cluster-22178.102675 GO:0005515
Cluster-22178.102676 GO:0006355 GO:0051301 GO:0016817 GO:0003677
GO:0005515 GO:0005634
Cluster-22178.102678 GO:0055114 GO:0006606 GO:0005506 GO:0020037
GO:0016705 GO:0016021
Cluster-22178.102680 GO:0006355 GO:0005509 GO:0003700 GO:0005515
GO:0005667 GO:0005622
Cluster-22178.102688 GO:0005200 GO:0005856 GO:0005882
Cluster-22178.102694 GO:0003677 GO:0019013
Cluster-22178.102696 GO:0006955
Cluster-22178.102697 GO:0007010 GO:0009069 GO:0006468 GO:0016310
GO:0007178 GO:0004675 GO:0005524 GO:0003779 GO:0016020
Cluster-22178.102700 GO:0005198 GO:0003779 GO:0016021 GO:0005856
GO:0097197 GO:0001772
Cluster-22178.102701 GO:0005515
Cluster-22178.102702 GO:0030866 GO:0003779 GO:0008092 GO:0005198
GO:0005856
Cluster-22178.102703 GO:0030866 GO:0005198 GO:0008092 GO:0003779
GO:0005856
Cluster-22178.102707 GO:0030155 GO:0007165 GO:0007155 GO:0006915
GO:0045995 GO:0001836 GO:0006974 GO:0030334 GO:0006919
GO:0005102 GO:0016020
Cluster-22178.10271 GO:0006813 GO:0055085 GO:0006811 GO:0005216
GO:0005198 GO:0005249 GO:0016020 GO:0019012 GO:0008076
Cluster-22178.102721 GO:0055085 GO:0006811 GO:0005216 GO:0016020
Cluster-22178.102722 GO:0006811 GO:0055085 GO:0005216 GO:0016020
Cluster-22178.102723 GO:0046325 GO:0006269 GO:0006351 GO:0003896

GO:0005515 GO:0005730 GO:0005657

Cluster-22178.102726 GO:0055114 GO:0008033 GO:0006351 GO:0055085

GO:0006260 GO:0006269 GO:0006810 GO:0007165 GO:0003896

GO:0005515 GO:0016491 GO:0008270 GO:0005524 GO:0042626

GO:0050660 GO:0005509 GO:0003677 GO:0016021 GO:0005578

GO:0005657 GO:0005730

Cluster-22178.102729 GO:0002949 GO:0005524 GO:0003774 GO:0016459

Cluster-22178.102730 GO:0016787

Cluster-22178.102732 GO:0015684 GO:0007264 GO:0006468 GO:0005525

GO:0003924 GO:0016887 GO:0015093 GO:0004672 GO:0005524

GO:0016021

Cluster-22178.102734 GO:0005515

Cluster-22178.102735 GO:0016021

Cluster-22178.102736 GO:0006024 GO:0007264 GO:0006810 GO:0009405

GO:0015684 GO:0015012 GO:0019870 GO:0005524 GO:0015093

GO:0005525 GO:0005576 GO:0016021

Cluster-22178.102737 GO:0015031 GO:0006464 GO:0005615

Cluster-22178.102739 GO:0001558 GO:0015031 GO:0006464 GO:0007049

GO:0005515 GO:0005520 GO:0005576 GO:0016942 GO:0017053

Cluster-22178.102743 GO:0006464 GO:0005515 GO:0046872 GO:0005524

GO:0008270

Cluster-22178.102747 GO:0006508 GO:0004252 GO:0019031

Cluster-22178.102748 GO:0006810 GO:0007165 GO:0006750 GO:0006464

GO:0055085 GO:0006355 GO:0006955 GO:0006814 GO:0005044

GO:0004363 GO:0003677 GO:0005524 GO:0030247 GO:0005215

GO:0016151 GO:0016020

Cluster-22178.102757 GO:0006468 GO:0007339 GO:0005515 GO:0005524

GO:0004672 GO:0016773 GO:0005576 GO:0016020

Cluster-22178.102763 GO:0006464

Cluster-22178.102768 GO:0008033 GO:0050660

Cluster-22178.102774 GO:0009405 GO:0006886 GO:0007160 GO:0048193

GO:0005515 GO:0005509 GO:0016020 GO:0005801

Cluster-22178.102781 GO:0005783

Cluster-22178.102782 GO:0055114 GO:0046856 GO:0006779 GO:0016887
GO:0005524 GO:0003774 GO:0034597 GO:0005515 GO:0016459
Cluster-22178.102792 GO:0046983
Cluster-22178.102793 GO:0006355 GO:0003700 GO:0005667 GO:0005622
Cluster-22178.102794 GO:0005515
Cluster-22178.102799 GO:0005515
Cluster-22178.102808 GO:0009405 GO:0019684 GO:0006118 GO:0045156
GO:0030077 GO:0016021
Cluster-22178.102809 GO:0005515
Cluster-22178.102812 GO:0055114 GO:0016706 GO:0008168
Cluster-22178.102819 GO:0030639 GO:0019872 GO:0009117 GO:0008830

Cluster-22178.102821 GO:0007275 GO:0005201 GO:0005198 GO:0005578
GO:0005581
Cluster-22178.102826 GO:0009405 GO:0006869 GO:0055114 GO:0042157
GO:0008289 GO:0016491 GO:0005576
Cluster-22178.102827 GO:0007155 GO:0006465 GO:0005515 GO:0008233
GO:0005787 GO:0016021
Cluster-22178.102828 GO:0015979 GO:0051056 GO:0005096 GO:0009523
GO:0009539 GO:0016020
Cluster-22178.102832 GO:0000921 GO:0051607 GO:0006914 GO:0007155
GO:0006457 GO:0006308 GO:0000077 GO:0006468 GO:0071816
GO:0006886 GO:0005524 GO:0016773 GO:0016888 GO:0019905
GO:0004672 GO:0051082 GO:0016272 GO:0005940 GO:0005783
GO:0016020 GO:0016021
Cluster-22178.102838 GO:0007186 GO:0006869 GO:0009847 GO:0005319
GO:0004930 GO:0016021
Cluster-22178.102840 GO:0005515
Cluster-22178.102841 GO:0019064 GO:0055085 GO:0006810 GO:0006811
GO:0005215 GO:0016020 GO:0019867
Cluster-22178.102843 GO:0043248 GO:0006754 GO:0016887 GO:0005524
GO:0005515 GO:0000275
Cluster-22178.102848 GO:0005515

Cluster-22178.102856 GO:0042254 GO:0006412 GO:0006351 GO:0003735
GO:0005840 GO:0005622
Cluster-22178.102858 GO:0051205 GO:0016021
Cluster-22178.102860 GO:0004386
Cluster-22178.102861 GO:0003677 GO:0008270
Cluster-22178.102877 GO:0006260 GO:0003887 GO:0008270 GO:0042575
GO:0005634
Cluster-22178.102880 GO:0006260 GO:0006352 GO:0006355 GO:0008270
GO:0016987 GO:0003700 GO:0003677 GO:0003887 GO:0005667
GO:0005634 GO:0042575
Cluster-22178.102882 GO:0006508 GO:0039693 GO:0007165 GO:0019784
GO:0005509 GO:0005515 GO:0005578
Cluster-22178.102892 GO:0055085 GO:0015992 GO:0051016 GO:0008643
GO:0015986 GO:0009401 GO:0006811 GO:0006139 GO:0006814
GO:0005524 GO:0008026 GO:0016818 GO:0005216 GO:0008982
GO:0015078 GO:0003676 GO:0005248 GO:0001518 GO:0008290
GO:0016020 GO:0016021 GO:0045263 GO:0009357
Cluster-22178.102894 GO:0006811 GO:0005198 GO:0016020 GO:0019028
Cluster-22178.102898 GO:0006812 GO:0016887 GO:0016021
Cluster-22178.102899 GO:0006886 GO:0043248
Cluster-22178.102909 GO:0009405 GO:0019835 GO:0019867
Cluster-22178.102915 GO:0007165 GO:0005044 GO:0016020 GO:0016021
Cluster-22178.102916 GO:0042254 GO:0006412 GO:0007165 GO:0003735
GO:0005044 GO:0016020 GO:0005840 GO:0005622
Cluster-22178.102918 GO:0055085 GO:0006865 GO:0006810 GO:0003333
GO:0015171 GO:0016020
Cluster-22178.102920 GO:0051276 GO:0006508 GO:0004181 GO:0005524
GO:0008270 GO:0016887 GO:0004180 GO:0005515 GO:0005694
Cluster-22178.102929 GO:0007264 GO:0007186 GO:0007165 GO:0003924
GO:0019001 GO:0031683 GO:0005524 GO:0004871 GO:0005525
Cluster-22178.102934 GO:0005509 GO:0005515
Cluster-22178.102940 GO:0003924
Cluster-22178.102942 GO:0003924

Cluster-22178.102945 GO:0008092 GO:0005737 GO:0019898
Cluster-22178.102948 GO:0007275 GO:0009231 GO:0008686 GO:0005578
Cluster-22178.102949 GO:0055114 GO:0006559 GO:0006570 GO:0042207
GO:0004411
Cluster-22178.102951 GO:0003677
Cluster-22178.102956 GO:0006139 GO:0006281 GO:0006206 GO:0006013
GO:0006000 GO:0007264 GO:0006310 GO:0005525 GO:0003723
GO:0005524 GO:0016887 GO:0003724 GO:0004127 GO:0009378
GO:0003873 GO:0005657 GO:0009379
Cluster-22178.102957 GO:0007264 GO:0006000 GO:0006013 GO:0006206
GO:0006139 GO:0003723 GO:0098519 GO:0003873 GO:0016887
GO:0005524 GO:0004127 GO:0003724 GO:0005525 GO:0016021
Cluster-22178.102959 GO:0005515
Cluster-22178.102969 GO:0045893 GO:0005634 GO:0016021
Cluster-22178.102973 GO:0006810 GO:0010508 GO:0042407 GO:0005215
GO:0044284 GO:0061617
Cluster-22178.102975 GO:0042407 GO:0010508 GO:0006810 GO:0005215
GO:0061617 GO:0044284
Cluster-22178.102977 GO:0006810 GO:0006811 GO:0005230 GO:0016020
Cluster-22178.102986 GO:0006771 GO:0008283 GO:0040007 GO:0007165
GO:0019497 GO:0016788 GO:0005515 GO:0008083 GO:0046872
GO:0003993
Cluster-22178.102991 GO:0005515 GO:0030976 GO:0004867 GO:0000287

Cluster-22178.102992 GO:0046983
Cluster-22178.102996 GO:0006468 GO:0005515 GO:0005524 GO:0004672

Cluster-22178.102998 GO:0008152 GO:0006508 GO:0000166 GO:0005515
GO:0003824 GO:0046872 GO:0004190
Cluster-22178.103000 GO:0006812 GO:0055085 GO:0019497 GO:0006771
GO:0003993 GO:0008324 GO:0046872 GO:0005515 GO:0016021
Cluster-22178.103008 GO:0055085 GO:0006810 GO:0022857 GO:0005215
GO:0016021 GO:0016020

Cluster-22178.103011 GO:0055085 GO:0022857 GO:0016021
Cluster-22178.103012 GO:0007155 GO:0003677 GO:0019028
Cluster-22178.103013 GO:0006858 GO:0007155 GO:0006914 GO:0003677
GO:0005515 GO:0019901 GO:0019028 GO:0016021
Cluster-22178.103014 GO:0042742
Cluster-22178.103017 GO:0007165 GO:0006811 GO:0007268 GO:0004970
GO:0005234 GO:0016020
Cluster-22178.103018 GO:0006886 GO:0001522 GO:0009451 GO:0007165
GO:0007268 GO:0006811 GO:0006888 GO:0003723 GO:0005234
GO:0009982 GO:0004970 GO:0008270 GO:0016020 GO:0030127
GO:0016021
Cluster-22178.103019 GO:0016020
Cluster-22178.103026 GO:0046983
Cluster-22178.103027 GO:0006810 GO:0016020
Cluster-22178.103029 GO:0007165 GO:0008283 GO:0040007 GO:0008083
GO:0016020 GO:0016021
Cluster-22178.103034 GO:0005515
Cluster-22178.103037 GO:0006032 GO:0008061
Cluster-22178.103038 GO:0007154 GO:0016021
Cluster-22178.103039 GO:0006260 GO:0003677 GO:0008270
Cluster-22178.103041 GO:0005515
Cluster-22178.103042 GO:0055085 GO:0022857 GO:0008270 GO:0016021
GO:0005622
Cluster-22178.103058 GO:0007050 GO:0030261 GO:0005509 GO:0005515
GO:0003677
Cluster-22178.103062 GO:0009069 GO:0006468 GO:0016310 GO:0007178
GO:0007154 GO:0004675 GO:0005515 GO:0005524 GO:0016020
Cluster-22178.103064 GO:0019497 GO:0006771 GO:0046872 GO:0005515
GO:0003993
Cluster-22178.103069 GO:0005982 GO:0005985 GO:0009758 GO:0043087
GO:0007264 GO:0050053 GO:0005085
Cluster-22178.103071 GO:0006355 GO:0005515 GO:0003723
Cluster-22178.103075 GO:0055085 GO:0016021

Cluster-22178.103077 GO:0055085 GO:0022857 GO:0016021
Cluster-22178.103083 GO:0016310 GO:0009069 GO:0006468 GO:0004674
GO:0004672 GO:0005524
Cluster-22178.103100 GO:0048870 GO:0006810 GO:0007165 GO:0008289
GO:0004871 GO:0008270 GO:0046872 GO:0005515 GO:0016021
GO:0031514 GO:0016020
Cluster-22178.103101 GO:0006810 GO:0048870 GO:0007049 GO:0005515
GO:0046872 GO:0008270 GO:0005634 GO:0031514 GO:0016021
Cluster-22178.103104 GO:0055114 GO:0007186 GO:0016641 GO:0004930
GO:0005507 GO:0016020
Cluster-22178.103105 GO:0006221 GO:0045747 GO:0007049 GO:0019239

Cluster-22178.103113 GO:0007596 GO:0007165 GO:0006950 GO:0055114
GO:0005198 GO:0020037 GO:0016705 GO:0005179 GO:0005506
GO:0019028 GO:0005576
Cluster-22178.103115 GO:0006355 GO:0008053 GO:0006357 GO:0001104
GO:0003700 GO:0003924 GO:0016592 GO:0005741 GO:0005667
GO:0019031 GO:0016021
Cluster-22178.103125 GO:0006897 GO:0006351 GO:0006144 GO:0006206
GO:0003899 GO:0003677 GO:0016020 GO:0005730 GO:0001527
Cluster-22178.103126 GO:0042967 GO:0006355 GO:0043565 GO:0003700
GO:0004402 GO:0000123 GO:0016021 GO:0005861 GO:0005667
GO:0005634
Cluster-22178.103132 GO:0006099 GO:0006355 GO:0016829 GO:0003677

Cluster-22178.103139 GO:0006836 GO:0006812 GO:0005328 GO:0016021
Cluster-22178.103149 GO:0006144 GO:0006206 GO:0006351 GO:0005515
GO:0003899 GO:0003677 GO:0016021 GO:0005730
Cluster-22178.103150 GO:0005861
Cluster-22178.103151 GO:0006816 GO:0070588 GO:0005262 GO:0005515
GO:0016020
Cluster-22178.103154 GO:0005515
Cluster-22178.103155 GO:0007165 GO:0005515 GO:0005509 GO:0005578

Cluster-22178.103157 GO:0015979 GO:0009523 GO:0016020
Cluster-22178.103162 GO:0007186 GO:0007218 GO:0004930 GO:0016021
Cluster-22178.103163 GO:0007186 GO:0007218 GO:0004930 GO:0016021
Cluster-22178.103165 GO:0055085 GO:0016021
Cluster-22178.103167 GO:0055114 GO:0007165 GO:0005507 GO:0070008 GO:0004252 GO:0005044 GO:0016641 GO:0016020
Cluster-22178.103171 GO:0009820 GO:0004867 GO:0050364
Cluster-22178.103181 GO:0016567 GO:0004842
Cluster-22178.103185 GO:0016042 GO:0007586 GO:0008047 GO:0005576
Cluster-22178.103200 GO:0006351 GO:0003677 GO:0005634
Cluster-22178.103202 GO:0006351 GO:0003677 GO:0005634
Cluster-22178.103206 GO:0042967 GO:0008080 GO:0016747
Cluster-22178.103208 GO:0005982 GO:0005985 GO:0005975 GO:0008810

Cluster-22178.10321 GO:0007165 GO:0008283 GO:0008406 GO:0040007 GO:0008083
Cluster-22178.103211 GO:0007165 GO:0005982 GO:0005985 GO:0006955 GO:0006935 GO:0005975 GO:0008810 GO:0008009 GO:0005576
Cluster-22178.103213 GO:0006811 GO:0055085 GO:0006118 GO:0006816 GO:0006874 GO:0009055 GO:0005219 GO:0051536 GO:0005509 GO:0016884 GO:0005216 GO:0016021 GO:0005622 GO:0016020
Cluster-22178.103216 GO:0051301 GO:0006355 GO:0005515 GO:0003677 GO:0016817 GO:0005634
Cluster-22178.103218 GO:0006891 GO:0016021 GO:0017119
Cluster-22178.10323 GO:0006810 GO:0007165 GO:0005215 GO:0008158 GO:0016021 GO:0016020
Cluster-22178.103236 GO:0051382 GO:0048193 GO:0005515 GO:0000776 GO:0016020
Cluster-22178.103239 GO:0006998 GO:0006406 GO:0009056 GO:0006508 GO:0006611 GO:0008270 GO:0004252 GO:0031965
Cluster-22178.103240 GO:0046872 GO:0016787
Cluster-22178.103241 GO:0030334 GO:0055114 GO:0008033 GO:0045995 GO:0007155 GO:0007165 GO:0030155 GO:0017150 GO:0050660

GO:0005102
Cluster-22178.103243 GO:0030155 GO:0007165 GO:0045995 GO:0055114
GO:0030334 GO:0005102 GO:0016491 GO:0019028
Cluster-22178.103248 GO:0007186 GO:0004930 GO:0005515
Cluster-22178.103249 GO:0007186 GO:0004930 GO:0005515
Cluster-22178.103250 GO:0006355 GO:0006886 GO:0006605 GO:0005515
GO:0016818 GO:0003677 GO:0005524 GO:0008270 GO:0005634
GO:0005742
Cluster-22178.103253 GO:0032922 GO:0008270 GO:0000976
Cluster-22178.103265 GO:0006355 GO:0006886 GO:0006605 GO:0008270
GO:0005524 GO:0005515 GO:0016818 GO:0003677 GO:0005634
GO:0005742
Cluster-22178.103267 GO:0008270 GO:0042393 GO:0005634
Cluster-22178.103270 GO:0016310 GO:0009069 GO:0006468 GO:0004674
GO:0005524
Cluster-22178.103274 GO:0051252 GO:0006869 GO:0008063 GO:0005319
GO:0004540 GO:0019031
Cluster-22178.103279 GO:0006270 GO:0008168 GO:0005515
Cluster-22178.103282 GO:0006813 GO:0004252 GO:0005267 GO:0016021
Cluster-22178.103288 GO:0005515
Cluster-22178.103291 GO:0046872
Cluster-22178.103292 GO:0004867
Cluster-22178.103296 GO:0008270 GO:0005887
Cluster-22178.103297 GO:0006355 GO:0006813 GO:0043462 GO:0008556
GO:0016829 GO:0005634 GO:0005886
Cluster-22178.103299 GO:0007154 GO:0006554 GO:0006479 GO:0018024
GO:0005515 GO:0016021
Cluster-22178.103308 GO:0016021
Cluster-22178.103310 GO:0006355 GO:0005515 GO:0005634 GO:0005668
Cluster-22178.103314 GO:0007009 GO:0005515
Cluster-22178.103317 GO:0032801 GO:0006812 GO:0019076 GO:0005515
GO:0005261 GO:0033644
Cluster-22178.103321 GO:0019072 GO:0045444 GO:0042025

Cluster-22178.103326 GO:0005515
Cluster-22178.103330 GO:0006508 GO:0005515 GO:0004252
Cluster-22178.103331 GO:0006508 GO:0005515 GO:0004252
Cluster-22178.103334 GO:0043631 GO:0005515 GO:0004652
Cluster-22178.103335 GO:0008270 GO:0005515
Cluster-22178.103336 GO:0007165 GO:0005524 GO:0003774 GO:0005515
GO:0005856 GO:0016459
Cluster-22178.103337 GO:0005515
Cluster-22178.103338 GO:0006810 GO:0015858 GO:0005337 GO:0016021
Cluster-22178.103339 GO:0005515 GO:0061630
Cluster-22178.103341 GO:0005515 GO:0042470
Cluster-22178.103343 GO:0016032 GO:0006508 GO:0019082 GO:0004197

Cluster-22178.103344 GO:0015631 GO:0045298
Cluster-22178.103348 GO:0007156 GO:0005509 GO:0003677 GO:0016020
Cluster-22178.103349 GO:0016772
Cluster-22178.103351 GO:0008608 GO:0009405 GO:0007165 GO:0016301
GO:0005515 GO:0042729 GO:0072686 GO:0005576
Cluster-22178.103355 GO:0007155 GO:0005540
Cluster-22178.103359 GO:0006508 GO:0005515 GO:0004252
Cluster-22178.103360 GO:0003723 GO:0005515 GO:0005524 GO:0003774
GO:0016887 GO:0003724 GO:0016459 GO:0005815
Cluster-22178.103365 GO:0007155 GO:0006915 GO:0008608 GO:0005509
GO:0008242 GO:0072686 GO:0005576 GO:0042729
Cluster-22178.103367 GO:0055085 GO:0006811 GO:0006506 GO:0006874
GO:0006816 GO:0005219 GO:0005509 GO:0016746 GO:0005216
GO:0016021 GO:0005789 GO:0005622 GO:0016020
Cluster-22178.103373 GO:0010826 GO:0042384 GO:0036038
Cluster-22178.103374 GO:0004386
Cluster-22178.103377 GO:0005515
Cluster-22178.103378 GO:0016579 GO:0036459
Cluster-22178.103379 GO:0006508 GO:0008233 GO:0005634
Cluster-22178.103383 GO:0006468 GO:0006397 GO:0010508 GO:0006886

GO:0016310 GO:0009069 GO:0019069 GO:0006810 GO:0006457
GO:0051607 GO:0006914 GO:0051082 GO:0042803 GO:0008092
GO:0003924 GO:0005525 GO:0003678 GO:0051087 GO:0005524
GO:0003774 GO:0000774 GO:0004674 GO:0005737 GO:0000445
GO:0016021 GO:0016459 GO:0005783 GO:0005657 GO:0019898
Cluster-22178.103384 GO:0007155 GO:0003774 GO:0016459
Cluster-22178.103392 GO:0007339 GO:0019497 GO:0006771 GO:0003993
GO:0005515 GO:0046872 GO:0005576
Cluster-22178.103398 GO:0010508 GO:0006810 GO:0042407 GO:0005215
GO:0061617 GO:0044284
Cluster-22178.103401 GO:0055085 GO:0022857 GO:0016021
Cluster-22178.103402 GO:0006955 GO:0019882 GO:0016020 GO:0042613
Cluster-22178.103405 GO:0046983
Cluster-22178.103422 GO:0007016 GO:0055085 GO:0006811 GO:0009968
GO:0005216 GO:0005737 GO:0016020 GO:0016010
Cluster-22178.103425 GO:0005515
Cluster-22178.103438 GO:0005515
Cluster-22178.103440 GO:0005201 GO:0005578 GO:0005581
Cluster-22178.103454 GO:0043462 GO:0006813 GO:0006468 GO:0005515
GO:0008556 GO:0004672 GO:0005524 GO:0005886
Cluster-22178.103472 GO:0055085 GO:0006811 GO:0005216 GO:0016020
Cluster-22178.103478 GO:0046413 GO:0005515 GO:0018836
Cluster-22178.103482 GO:0000160 GO:0071555 GO:0016310 GO:0046797
GO:0004673 GO:0000155 GO:0016021 GO:0009365
Cluster-22178.103485 GO:0007165 GO:0006355 GO:0030521 GO:0003677
GO:0003700 GO:0005496 GO:0008270 GO:0004882 GO:0043565
GO:0005634 GO:0005667
Cluster-22178.103486 GO:0019089
Cluster-22178.103491 GO:0055114 GO:0008152 GO:0016491
Cluster-22178.103496 GO:0043631 GO:0005515 GO:0004652
Cluster-22178.103497 GO:0043631 GO:0004652 GO:0005515
Cluster-22178.103498 GO:0043631 GO:0005515 GO:0004652
Cluster-22178.103501 GO:0042254 GO:0015684 GO:0006576 GO:0006412

GO:0016887 GO:0005524 GO:0015093 GO:0003735 GO:0003924
GO:0005525 GO:0005198 GO:0005622 GO:0019013 GO:0016021
GO:0005840 GO:0019031
Cluster-22178.103502 GO:0042254 GO:0006412 GO:0003735 GO:0005198
GO:0005524 GO:0003924 GO:0016887 GO:0005525 GO:0005622
GO:0019031 GO:0005840 GO:0016021
Cluster-22178.103504 GO:0005198 GO:0019028
Cluster-22178.103505 GO:0006505 GO:0006629 GO:0006886 GO:0042967
GO:0008374 GO:0016788
Cluster-22178.103506 GO:0016020
Cluster-22178.103522 GO:0016021 GO:0009279
Cluster-22178.103526 GO:0005515 GO:0005509 GO:0005544
Cluster-22178.103528 GO:0042157 GO:0006281 GO:0009405 GO:0006869
GO:0006308 GO:0006260 GO:0017108 GO:0008289 GO:0005634
GO:0005576 GO:0033557
Cluster-22178.103533 GO:0015992 GO:0006144 GO:0055114 GO:0006351
GO:0006118 GO:0006119 GO:0003968 GO:0008121 GO:0003723
GO:0031379
Cluster-22178.103535 GO:0055114 GO:0008152 GO:0016714 GO:0016746
GO:0016597
Cluster-22178.103541 GO:0005515
Cluster-22178.103544 GO:0035092 GO:0003677 GO:0000228
Cluster-22178.103551 GO:0016021
Cluster-22178.103552 GO:0055085 GO:0006352 GO:0006811 GO:0019087
GO:0005515 GO:0005216 GO:0016020 GO:0005634 GO:0016021
Cluster-22178.103558 GO:0006353 GO:0046983 GO:0003723
Cluster-22178.103562 GO:0009405 GO:0015948 GO:0046656 GO:0030269
GO:0016020 GO:0005576
Cluster-22178.10357 GO:0007186 GO:0004930 GO:0016021
Cluster-22178.103570 GO:0008270
Cluster-22178.103572 GO:0006260 GO:0009214 GO:0003677 GO:0005515
GO:0004113 GO:0016020
Cluster-22178.103573 GO:0016579 GO:0006508 GO:0036459 GO:0005515

GO:0005509 GO:0032183 GO:0070140 GO:0004843
Cluster-22178.103578 GO:0055085 GO:0006811 GO:0019087 GO:0005216
GO:0016020 GO:0016021
Cluster-22178.103582 GO:0005515
Cluster-22178.103586 GO:0006357 GO:0051276 GO:0006508 GO:0010508
GO:0006308 GO:0031175 GO:0005516 GO:0004180 GO:0030507
GO:0008855 GO:0016887 GO:0005524 GO:0008270 GO:0005515
GO:0001104 GO:0004181 GO:0005694 GO:0016592 GO:0008091
GO:0009318 GO:0016021
Cluster-22178.103589 GO:0005515
Cluster-22178.103591 GO:0007165 GO:0006355 GO:0005515 GO:0004871
GO:0003677 GO:0016021 GO:0005634
Cluster-22178.103594 GO:0043087 GO:0035023 GO:0005089
Cluster-22178.103599 GO:0006820 GO:0006835 GO:0006812 GO:0017153
GO:0016020
Cluster-22178.103614 GO:0005634
Cluster-22178.103615 GO:0005634
Cluster-22178.103616 GO:0001848 GO:0005615 GO:0005634
Cluster-22178.103617 GO:0005634
Cluster-22178.103618 GO:0001848 GO:0005634 GO:0005615
Cluster-22178.103619 GO:0005634
Cluster-22178.103620 GO:0005634
Cluster-22178.103621 GO:0015986 GO:0015992 GO:0015078 GO:0016301
GO:0005524 GO:0000276
Cluster-22178.103623 GO:0042773 GO:0055114 GO:0016491 GO:0016209
GO:0016651 GO:0008270 GO:0005622
Cluster-22178.103626 GO:0006811 GO:0006810 GO:0005230 GO:0016020
Cluster-22178.103628 GO:0007186 GO:0004930 GO:0016020
Cluster-22178.10363 GO:0015931 GO:0008360 GO:0006914 GO:0071436
GO:0006525 GO:0007155 GO:0006090 GO:0006560 GO:0006814
GO:0033227 GO:0015081 GO:0051033 GO:0008948 GO:0051082
GO:0016020 GO:0016021 GO:0009288
Cluster-22178.103630 GO:0006810 GO:0005622

Cluster-22178.103631 GO:0006118 GO:0009055 GO:0020037
Cluster-22178.103632 GO:0008270 GO:0005524
Cluster-22178.103633 GO:0051260 GO:0006355 GO:0030639 GO:0006811
GO:0006352 GO:0055085 GO:0003677 GO:0005216 GO:0016987
GO:0003700 GO:0016020 GO:0005667
Cluster-22178.103636 GO:0006468 GO:0006836 GO:0006812 GO:0007218
GO:0005335 GO:0016773 GO:0004672 GO:0005524 GO:0030141
GO:0016020 GO:0005887
Cluster-22178.103645 GO:0007155 GO:0005540
Cluster-22178.103647 GO:0006289 GO:0043161 GO:0006281 GO:0007155
GO:0005524 GO:0003774 GO:0003684 GO:0016459 GO:0016020
Cluster-22178.103654 GO:0035556 GO:0006858 GO:0005544 GO:0005509
GO:0005515
Cluster-22178.103658 GO:0046983
Cluster-22178.103660 GO:0001558 GO:0005520 GO:0016942 GO:0005576
Cluster-22178.103665 GO:0006470 GO:0008138 GO:0019028
Cluster-22178.103676 GO:0007094 GO:0051258 GO:0007165 GO:0030168
GO:0000921 GO:0005198 GO:0008134 GO:0042803 GO:0045502
GO:0003779 GO:0030674 GO:0005516 GO:0005102 GO:0005524
GO:0003774 GO:0032982 GO:0016021 GO:0030016 GO:0005882
GO:0005667 GO:0016459 GO:0005577 GO:0005940 GO:0030286
Cluster-22178.103680 GO:0006570 GO:0042207 GO:0006559 GO:0055114
GO:0004411
Cluster-22178.103682 GO:0006306 GO:0003677 GO:0008170
Cluster-22178.103685 GO:0015992 GO:0015074 GO:0015986 GO:0006119
GO:0046933 GO:0046961 GO:0000275 GO:0045259
Cluster-22178.103691 GO:0006885 GO:0006814 GO:0015992 GO:0015385
GO:0016021
Cluster-22178.103703 GO:0005515
Cluster-22178.103706 GO:0007156 GO:0005509 GO:0016020
Cluster-22178.10371 GO:0006355 GO:0005515 GO:0003700 GO:0005509
GO:0005667 GO:0042025
Cluster-22178.103710 GO:0005515

Cluster-22178.103716 GO:0016021
Cluster-22178.103726 GO:0005515
Cluster-22178.103727 GO:0005515
Cluster-22178.103728 GO:0005515
Cluster-22178.103729 GO:0005985 GO:0005982 GO:0009117 GO:0048040
GO:0005515
Cluster-22178.103730 GO:0005515
Cluster-22178.103734 GO:0006508 GO:0008234
Cluster-22178.103736 GO:0006750 GO:0006749 GO:0017111 GO:0004357
GO:0017109
Cluster-22178.103737 GO:0006750 GO:0006749 GO:0017111 GO:0004357
GO:0017109
Cluster-22178.103738 GO:0017111
Cluster-22178.103739 GO:0051252 GO:0015074 GO:0003676 GO:0008270
GO:0004523
Cluster-22178.103746 GO:0007186 GO:0005515 GO:0004930 GO:0016021
Cluster-22178.10375 GO:0005515
Cluster-22178.103750 GO:0042254 GO:0006412 GO:0016846 GO:0003735
GO:0005198 GO:0005840 GO:0005882
Cluster-22178.103752 GO:0006367 GO:0006281
Cluster-22178.103766 GO:0006811 GO:0016020
Cluster-22178.103768 GO:0046854
Cluster-22178.103770 GO:0006355 GO:0043401 GO:0008270 GO:0043565
GO:0003700 GO:0005667 GO:0005634
Cluster-22178.103774 GO:0006468 GO:0006355 GO:0008270 GO:0004672
GO:0005524 GO:0043565 GO:0016773 GO:0003700 GO:0005667
GO:0016020
Cluster-22178.103776 GO:0055085 GO:0016021
Cluster-22178.103778 GO:0015684 GO:0007264 GO:0005525 GO:0015093
GO:0003924 GO:0016021
Cluster-22178.103781 GO:0006468 GO:0005524 GO:0004672
Cluster-22178.103784 GO:0006468 GO:0004672 GO:0005524
Cluster-22178.103785 GO:0006468 GO:0004672 GO:0005524

Cluster-22178.103786 GO:0006468 GO:0016773 GO:0004672 GO:0005524 GO:0016020
Cluster-22178.103787 GO:0006468 GO:0016773 GO:0005524 GO:0004672 GO:0016020
Cluster-22178.103788 GO:0006468 GO:0016773 GO:0005524 GO:0004672 GO:0016020
Cluster-22178.103789 GO:0006468 GO:0016773 GO:0004672 GO:0005524 GO:0016020
Cluster-22178.103799 GO:0006810 GO:0005215 GO:0005515 GO:0003824 GO:0005737 GO:0016021
Cluster-22178.103801 GO:0019911
Cluster-22178.103807 GO:0007049 GO:0009401 GO:0048870 GO:0006351 GO:0010508 GO:0006206 GO:0006914 GO:0006144 GO:0006869 GO:0006446 GO:0006457 GO:0042157 GO:0008289 GO:0042803 GO:0005198 GO:0003899 GO:0051082 GO:0005524 GO:0000774 GO:0051087 GO:0003743 GO:0005840 GO:0019031 GO:0019867 GO:0016021 GO:0031514 GO:0005576 GO:0019028 GO:0005634 GO:0009424 GO:0005730
Cluster-22178.103810 GO:0006771 GO:0019497 GO:0016998 GO:0006032 GO:0004568 GO:0005515 GO:0046872 GO:0003993
Cluster-22178.103820 GO:0009069 GO:0006468 GO:0006506 GO:0051262 GO:0055085 GO:0006811 GO:0016310 GO:0005216 GO:0005524 GO:0003824 GO:0004674 GO:0016020 GO:0016021 GO:0005789
Cluster-22178.103823 GO:0006281 GO:0006260 GO:0003911
Cluster-22178.103828 GO:0016021 GO:0097197 GO:0001772
Cluster-22178.103829 GO:0005515 GO:0001772 GO:0097197 GO:0016021
Cluster-22178.103836 GO:1901031 GO:0005515 GO:0005634
Cluster-22178.103841 GO:0042921 GO:0006355 GO:0043402 GO:0043401 GO:0007165 GO:0008270 GO:0043565 GO:0004883 GO:0003700 GO:0005496 GO:0003677 GO:0005634 GO:0005667
Cluster-22178.103842 GO:0006355 GO:0006011 GO:0030036 GO:0017048 GO:0043565 GO:0003779 GO:0003700 GO:0005667 GO:0016020
Cluster-22178.103849 GO:0006355 GO:0006816 GO:0070588 GO:0000975

GO:0005262 GO:0003700 GO:0016020 GO:0005667
Cluster-22178.103853 GO:0006811 GO:0051297 GO:0016020 GO:0005813
Cluster-22178.103854 GO:0006811 GO:0006810 GO:0005230 GO:0016020
Cluster-22178.103855 GO:0007304 GO:0006812 GO:0006836 GO:0005213
GO:0005328 GO:0016021 GO:0042600 GO:0005576
Cluster-22178.103857 GO:0015886 GO:0017004 GO:0005515 GO:0016021
Cluster-22178.103861 GO:0005198 GO:0019028
Cluster-22178.103862 GO:0046872
Cluster-22178.103869 GO:0006508 GO:0004252
Cluster-22178.103870 GO:0007186 GO:0004930 GO:0016020
Cluster-22178.103875 GO:0006468 GO:0005524 GO:0004672
Cluster-22178.103879 GO:0005992 GO:0019497 GO:0006771 GO:0003824
GO:0005515 GO:0003993 GO:0046872
Cluster-22178.10388 GO:0055114 GO:0006879 GO:0007010 GO:0016491
GO:0003779 GO:0046872 GO:0008199
Cluster-22178.103881 GO:0006811 GO:0055085 GO:0005216 GO:0016020
Cluster-22178.103883 GO:0005515
Cluster-22178.103885 GO:0006355 GO:0003677 GO:0046983
Cluster-22178.103891 GO:0030866 GO:0005198 GO:0008092 GO:0003779
GO:0005856
Cluster-22178.103894 GO:0055085 GO:0005975 GO:0006487 GO:0007186
GO:0003824 GO:0004930 GO:0016787 GO:0043169 GO:0016021
GO:0008250
Cluster-22178.103899 GO:0007165 GO:0003774 GO:0005524 GO:0005515
GO:0016459 GO:0005856
Cluster-22178.103901 GO:0006820 GO:0006888 GO:0006886 GO:0055085
GO:0008509 GO:0016021 GO:0030127
Cluster-22178.103903 GO:0006355 GO:0043401 GO:0043565 GO:0008270
GO:0003700 GO:0005634 GO:0005667
Cluster-22178.103906 GO:0006144 GO:0006206 GO:0006351 GO:0003899
GO:0003677 GO:0005730
Cluster-22178.103909 GO:0005515
Cluster-22178.103913 GO:0009733 GO:0007218 GO:0007186 GO:0007010

GO:0003779 GO:0004930 GO:0005185 GO:0005509 GO:0005544
GO:0016021 GO:0005576
Cluster-22178.103914 GO:0005515 GO:0030976 GO:0004867 GO:0000287

Cluster-22178.103916 GO:0016787
Cluster-22178.103920 GO:0030521 GO:0055085 GO:0006355 GO:0006810
GO:0007165 GO:0006814 GO:0004882 GO:0005215 GO:0005496
GO:0003677 GO:0016021 GO:0005634 GO:0016020
Cluster-22178.103921 GO:0019497 GO:0006771 GO:0046872 GO:0003993
GO:0005515 GO:0016020
Cluster-22178.103931 GO:0008092 GO:0005737 GO:0009512 GO:0019898
Cluster-22178.103932 GO:0040007 GO:0008283 GO:0007165 GO:0008083

Cluster-22178.103962 GO:0007186 GO:0006144 GO:0003968 GO:0004930
GO:0031379
Cluster-22178.103967 GO:0006298 GO:0005524 GO:0030983
Cluster-22178.103984 GO:0008270
Cluster-22178.103991 GO:0006355 GO:0007165 GO:0006810 GO:0006814
GO:0030521 GO:0055085 GO:0003677 GO:0004882 GO:0005215
GO:0005496 GO:0005634 GO:0016020 GO:0016021
Cluster-22178.103992 GO:0030521 GO:0055085 GO:0006355 GO:0006814
GO:0007165 GO:0006810 GO:0004882 GO:0005496 GO:0005215
GO:0003677 GO:0016021 GO:0005634 GO:0016020
Cluster-22178.103993 GO:0006814 GO:0007165 GO:0006810 GO:0006355
GO:0055085 GO:0030521 GO:0003677 GO:0005496 GO:0005215
GO:0004882 GO:0016020 GO:0005634 GO:0016021
Cluster-22178.103997 GO:0006144 GO:0003968 GO:0016817 GO:0031379
Cluster-22178.104006 GO:0046914
Cluster-22178.104007 GO:0046914
Cluster-22178.10401 GO:0007186 GO:0004930 GO:0016021
Cluster-22178.104012 GO:0019058 GO:0006810 GO:0007165 GO:0030168
GO:0006351 GO:0007049 GO:0006308 GO:0051258 GO:0030674
GO:0005102 GO:0008855 GO:0017053 GO:0005577 GO:0005886

GO:0009318 GO:0019028 GO:0019031
Cluster-22178.104013 GO:0007010 GO:0003779 GO:0005515
Cluster-22178.104019 GO:0006470 GO:0006570 GO:0008138 GO:0004725

Cluster-22178.104026 GO:0006836 GO:0006812 GO:0019058 GO:0006886
GO:0008146 GO:0005328 GO:0016021 GO:0016020
Cluster-22178.104037 GO:0030155 GO:0007165 GO:0007155 GO:0045995
GO:0008033 GO:0055114 GO:0030334 GO:0005102 GO:0017150
GO:0050660
Cluster-22178.104039 GO:0003697
Cluster-22178.104042 GO:0045735
Cluster-22178.104043 GO:0006915 GO:0045735 GO:0005622
Cluster-22178.10405 GO:0007218 GO:0018990 GO:0008255 GO:0016021
Cluster-22178.104050 GO:0055085 GO:0006811 GO:0005216 GO:0016020
Cluster-22178.104057 GO:0006468 GO:0005524 GO:0004672
Cluster-22178.104059 GO:0008152 GO:0016746 GO:0016021
Cluster-22178.104060 GO:0008270 GO:0005887
Cluster-22178.104061 GO:0008654 GO:0043772 GO:0005886
Cluster-22178.104070 GO:0009103 GO:0016192 GO:0005515 GO:0016021
GO:0016020
Cluster-22178.104071 GO:0005515 GO:0061630
Cluster-22178.104081 GO:0007155 GO:0018149 GO:0005515 GO:0016021
Cluster-22178.104082 GO:0007155 GO:0019064 GO:0018149 GO:0005515
GO:0019031 GO:0016021
Cluster-22178.104084 GO:0007155 GO:0017004 GO:0045766 GO:0019064
GO:0015886 GO:0046813 GO:0043066 GO:0043123 GO:0006955
GO:0000902 GO:0018149 GO:0061025 GO:0005515 GO:0016020
GO:0005887 GO:0042470 GO:0016021 GO:0019031
Cluster-22178.104089 GO:0009396 GO:0055114 GO:0046487 GO:0004488
GO:0005515 GO:0003824 GO:0005739
Cluster-22178.104090 GO:0046487 GO:0009396 GO:0055114 GO:0019049
GO:0004488 GO:0005515 GO:0003824 GO:0005739
Cluster-22178.104091 GO:0009396 GO:0055114 GO:0046487 GO:0003824

GO:0016491 GO:0004488 GO:0005515 GO:0005739
Cluster-22178.104092 GO:0019049 GO:0009396 GO:0055114 GO:0046487
GO:0003824 GO:0045502 GO:0042803 GO:0008134 GO:0004488
GO:0005515 GO:0030286 GO:0005667 GO:0005739
Cluster-22178.104095 GO:0055114 GO:0009396 GO:0046487 GO:0005515
GO:0003824 GO:0004488 GO:0005739
Cluster-22178.104100 GO:0005515 GO:0008270 GO:0046872
Cluster-22178.104101 GO:0016887 GO:0005524 GO:0005515
Cluster-22178.104108 GO:0045893 GO:0019836
Cluster-22178.104116 GO:0006955 GO:0007165 GO:0005164 GO:0016020
GO:0019031
Cluster-22178.104118 GO:0006955 GO:0007165 GO:0046983 GO:0008270
GO:0005164 GO:0016020
Cluster-22178.104120 GO:0006955 GO:0007165 GO:0046983 GO:0008270
GO:0005164 GO:0016020
Cluster-22178.104123 GO:0019042 GO:0033644 GO:0016020
Cluster-22178.104128 GO:0006508 GO:0016032 GO:0008233
Cluster-22178.104130 GO:0016032 GO:0006508 GO:0008233
Cluster-22178.104131 GO:0000105 GO:0045735
Cluster-22178.104132 GO:0006886 GO:0005198 GO:0005783 GO:0016021
Cluster-22178.104133 GO:0009117 GO:0019872 GO:0030639 GO:0008830

Cluster-22178.104135 GO:0007018 GO:0006810 GO:0007017 GO:0003777
GO:0016887 GO:0005524 GO:0003677 GO:0005874 GO:0030286
Cluster-22178.104138 GO:0007165 GO:0005179 GO:0005576
Cluster-22178.104140 GO:0016021
Cluster-22178.104141 GO:0016021
Cluster-22178.104144 GO:0015684 GO:0055085 GO:0015093 GO:0016021
Cluster-22178.104146 GO:0006333 GO:0005634
Cluster-22178.104152 GO:0006811 GO:0016020
Cluster-22178.104157 GO:0016772 GO:0016301
Cluster-22178.104167 GO:0006816 GO:0008233 GO:0005388 GO:0016529
Cluster-22178.104171 GO:0007156 GO:0005509 GO:0016020

Cluster-22178.104178 GO:0034508 GO:0005515 GO:0005509 GO:0000776
Cluster-22178.104190 GO:0008270 GO:0003676
Cluster-22178.104192 GO:0055114 GO:0006950 GO:0007165 GO:0005506
GO:0005179 GO:0020037 GO:0016705 GO:0005576
Cluster-22178.104195 GO:0006364 GO:0005730
Cluster-22178.104198 GO:0007034 GO:0042157 GO:0009405 GO:0006869
GO:0008289 GO:0005576 GO:0030430
Cluster-22178.104209 GO:0005515 GO:0016020
Cluster-22178.104214 GO:0006810 GO:0005215 GO:0016020
Cluster-22178.104215 GO:0007264 GO:0006468 GO:0006464 GO:0005515
GO:0016151 GO:0005525 GO:0005524 GO:0004672
Cluster-22178.104216 GO:0006464 GO:0007264 GO:0006468 GO:0016151
GO:0005525 GO:0004672 GO:0005524 GO:0005515
Cluster-22178.104217 GO:0030418 GO:0005515 GO:0030410
Cluster-22178.104218 GO:0030418 GO:0007264 GO:0005515 GO:0005525
GO:0030410
Cluster-22178.104219 GO:0007264 GO:0006468 GO:0006464 GO:0005515
GO:0016151 GO:0005525 GO:0004672 GO:0005524
Cluster-22178.104220 GO:0006468 GO:0007264 GO:0005515 GO:0005525
GO:0005524 GO:0004672
Cluster-22178.104221 GO:0006464 GO:0006468 GO:0007264 GO:0005525
GO:0016151 GO:0004672 GO:0005524 GO:0005515
Cluster-22178.104225 GO:0007264 GO:0043087 GO:0005085 GO:0016021
GO:0016012
Cluster-22178.104229 GO:0005515 GO:0051537 GO:0043231
Cluster-22178.104233 GO:0005515 GO:0015631 GO:0045298
Cluster-22178.104237 GO:0006355 GO:0008270 GO:0005524 GO:0003677
GO:0016818 GO:0005515 GO:0005634
Cluster-22178.104241 GO:0046872 GO:0016787 GO:0005515 GO:0003676

Cluster-22178.104242 GO:0016787 GO:0046872 GO:0003676 GO:0005515

Cluster-22178.104243 GO:0006355 GO:0016747

Cluster-22178.104250 GO:0005515
Cluster-22178.104252 GO:0007165
Cluster-22178.104255 GO:0005515 GO:0016020 GO:0016021
Cluster-22178.104257 GO:0009405 GO:0010506 GO:0045735 GO:0008270
GO:0016020 GO:0005615
Cluster-22178.104259 GO:0030246
Cluster-22178.104261 GO:0007154 GO:0005515 GO:0005509 GO:0016020
Cluster-22178.104262 GO:0030182 GO:0021549
Cluster-22178.104263 GO:0021549 GO:0030182
Cluster-22178.104264 GO:0030182 GO:0007165 GO:0021549 GO:0006955
GO:0005164 GO:0016020
Cluster-22178.104265 GO:0006955 GO:0030182 GO:0007165 GO:0021549
GO:0005164 GO:0016020
Cluster-22178.104266 GO:0006914 GO:0048193 GO:0007049 GO:0007264
GO:0043093 GO:0006355 GO:0010508 GO:0000917 GO:0043565
GO:0005096 GO:0003700 GO:0005737 GO:0005667 GO:0016020
Cluster-22178.104280 GO:0016192 GO:0016021
Cluster-22178.104284 GO:0006260 GO:0006351 GO:0003677 GO:0003887
GO:0032040 GO:0042575
Cluster-22178.104290 GO:0005515
Cluster-22178.104294 GO:0006810 GO:0005643
Cluster-22178.104296 GO:0030474 GO:0005200 GO:0005856 GO:0005823
Cluster-22178.104297 GO:0030474 GO:0005200 GO:0005823 GO:0005856
Cluster-22178.104299 GO:0004190 GO:0016021
Cluster-22178.104302 GO:0007018 GO:0007017 GO:0007264 GO:0042073
GO:0006308 GO:0006914 GO:0003777 GO:0005525 GO:0008855
GO:0016887 GO:0005524 GO:0009318 GO:0016021 GO:0005874
GO:0030286
Cluster-22178.104303 GO:0055085 GO:0006811 GO:0005216 GO:0005198
GO:0008270 GO:0019028 GO:0016020 GO:0019031
Cluster-22178.104307 GO:0042254 GO:0006412 GO:0003735 GO:0005840
GO:0005622
Cluster-22178.104308 GO:0005515

Cluster-22178.104309 GO:0035556
Cluster-22178.104311 GO:0005975 GO:0006508 GO:0016032 GO:0004197
GO:0005515 GO:0003824
Cluster-22178.104319 GO:0006768 GO:0009102 GO:0005198 GO:0005524
GO:0008270 GO:0042410 GO:0019031
Cluster-22178.104323 GO:0008152 GO:0055114 GO:0016491
Cluster-22178.104324 GO:0008152 GO:0055114 GO:0016491
Cluster-22178.104330 GO:0006771 GO:0019497 GO:0007339 GO:0046872
GO:0003993 GO:0005515 GO:0005576
Cluster-22178.104331 GO:0046872 GO:0016787
Cluster-22178.104335 GO:0006355 GO:0006810 GO:0006351 GO:0007275
GO:0008270 GO:0005524 GO:0030250 GO:0046872 GO:0042626
GO:0005576 GO:0045202 GO:0016021
Cluster-22178.104336 GO:0006351 GO:0007275 GO:0006810 GO:0006355
GO:0042626 GO:0030250 GO:0046872 GO:0008270 GO:0005524
GO:0045202 GO:0016021 GO:0005576
Cluster-22178.104338 GO:0008152 GO:0016746
Cluster-22178.104343 GO:0006468 GO:0016773 GO:0005524 GO:0004672
GO:0005515 GO:0016020
Cluster-22178.104345 GO:0006508 GO:0042254 GO:0006412 GO:0016787
GO:0003735 GO:0042802 GO:0008236 GO:0005515 GO:0005622
GO:0005840
Cluster-22178.104346 GO:0016021
Cluster-22178.104352 GO:0006811 GO:0006810 GO:0009607 GO:0005230
GO:0016021 GO:0016020
Cluster-22178.104354 GO:0005515
Cluster-22178.104357 GO:0006397 GO:0051252 GO:0004540
Cluster-22178.104361 GO:0007165 GO:0043093 GO:0006914 GO:0030168
GO:0000921 GO:0006886 GO:0000917 GO:0006308 GO:0051258
GO:0030674 GO:0005102 GO:0008855 GO:0051082 GO:0008092
GO:0005783 GO:0005577 GO:0019898 GO:0005940 GO:0009318
GO:0016021 GO:0005737
Cluster-22178.104366 GO:0006396 GO:0045859 GO:0006355 GO:0006948

GO:0007165 GO:0043401 GO:0005496 GO:0004860 GO:0030284
GO:0005515 GO:0003677 GO:0000176 GO:0005634 GO:0005576
Cluster-22178.104371 GO:0007339 GO:0006412 GO:0042254 GO:0003735
GO:0005515 GO:0005576 GO:0005840
Cluster-22178.104372 GO:0016021
Cluster-22178.104374 GO:0046872 GO:0019012
Cluster-22178.104376 GO:0006810 GO:0009405 GO:0055114 GO:0005506
GO:0008200 GO:0020037 GO:0016705 GO:0005576
Cluster-22178.104377 GO:0015066 GO:0005515
Cluster-22178.104386 GO:0018342 GO:0055114 GO:0008318 GO:0016638
GO:0005515
Cluster-22178.104389 GO:0005515
Cluster-22178.104391 GO:0003676 GO:0003723
Cluster-22178.104395 GO:0005515
Cluster-22178.104397 GO:0005515 GO:0030234 GO:0016020
Cluster-22178.104403 GO:0055114 GO:0055085 GO:0006807 GO:0042128
GO:0006810 GO:0016491 GO:0008942 GO:0046872 GO:0005524
GO:0042626 GO:0051537 GO:0016021
Cluster-22178.104404 GO:0055114 GO:0055085 GO:0006807 GO:0006810
GO:0042128 GO:0016491 GO:0008942 GO:0005524 GO:0046872
GO:0042626 GO:0051537 GO:0016021
Cluster-22178.104405 GO:0006810 GO:0042128 GO:0055085 GO:0006807
GO:0055114 GO:0042626 GO:0051537 GO:0016491 GO:0008942
GO:0005524 GO:0046872 GO:0016021
Cluster-22178.104406 GO:0005515
Cluster-22178.104409 GO:0051252 GO:0006402 GO:0003723 GO:0004535
GO:0046872 GO:0003676 GO:0005634 GO:0005737
Cluster-22178.104412 GO:0008289 GO:0005515 GO:0005516
Cluster-22178.104413 GO:0015684 GO:0007264 GO:0005525 GO:0003924
GO:0015093 GO:0016021
Cluster-22178.104416 GO:0005515 GO:0016020
Cluster-22178.104420 GO:0007165
Cluster-22178.104423 GO:0007165

Cluster-22178.104442 GO:0005515
Cluster-22178.104443 GO:0016301 GO:0016772
Cluster-22178.104448 GO:0016021
Cluster-22178.104449 GO:0019028
Cluster-22178.104450 GO:0019028
Cluster-22178.104455 GO:0006400 GO:0008193 GO:0000287
Cluster-22178.104456 GO:0006400 GO:0000287 GO:0008193
Cluster-22178.104463 GO:0008033 GO:0008152 GO:0015994 GO:0015996 GO:0006508 GO:0045859 GO:0019887 GO:0008168 GO:0016787 GO:0047746 GO:0008236 GO:0005956
Cluster-22178.104472 GO:0008152 GO:0008134 GO:0020037 GO:0003824 GO:0005667 GO:0005634
Cluster-22178.104474 GO:0005634
Cluster-22178.104478 GO:0055085 GO:0022857 GO:0016021
Cluster-22178.104492 GO:0000902 GO:0006955 GO:0016020
Cluster-22178.104495 GO:0009401 GO:0006812 GO:0006835 GO:0006820 GO:0017153 GO:0016020 GO:0016021
Cluster-22178.104497 GO:0006820 GO:0006835 GO:0019073 GO:0006812 GO:0017153 GO:0016020
Cluster-22178.104499 GO:0006812 GO:0019073 GO:0006835 GO:0006820 GO:0017153 GO:0016020
Cluster-22178.104511 GO:0005515
Cluster-22178.104524 GO:0006355 GO:0006468 GO:0005515 GO:0003677 GO:0004672 GO:0005576
Cluster-22178.104528 GO:0030474 GO:0005200 GO:0005856 GO:0005823
Cluster-22178.104531 GO:0006570 GO:0006470 GO:0004725 GO:0008138

Cluster-22178.104536 GO:0006468 GO:0004672 GO:0005524
Cluster-22178.104537 GO:0005975 GO:0016758 GO:0016020
Cluster-22178.104539 GO:0007049 GO:0048870 GO:0000917 GO:0006355 GO:0006468 GO:0006260 GO:0070206 GO:0043093 GO:0003700 GO:0043565 GO:0004672 GO:0003887 GO:0001882 GO:0005524 GO:0003677 GO:0019867 GO:0005737 GO:0031514 GO:0042575

GO:0005667
Cluster-22178.104546 GO:0006811 GO:0006886 GO:0006888 GO:0008270
GO:0005216 GO:0030127 GO:0016020
Cluster-22178.104547 GO:0006888 GO:0006811 GO:0006886 GO:0005216
GO:0008270 GO:0030127 GO:0016020
Cluster-22178.104549 GO:0071988 GO:0007155 GO:0048193 GO:0007094
GO:0030001 GO:0051301 GO:0007165 GO:0006810 GO:0007067
GO:0000090 GO:0055085 GO:0031110 GO:0030168 GO:0070206
GO:0030474 GO:0007059 GO:0006308 GO:0048870 GO:0051258
GO:0008017 GO:0005198 GO:0042803 GO:0030674 GO:0046873
GO:0005102 GO:0008855 GO:0008134 GO:0045502 GO:0016020
GO:0005667 GO:0005876 GO:0005577 GO:0045298 GO:0005882
GO:0000940 GO:0030286 GO:0031514 GO:0009318 GO:0019028
GO:0016021 GO:0019031
Cluster-22178.104550 GO:0007165 GO:0070206 GO:0030168 GO:0007094
GO:0006355 GO:0051258 GO:0030674 GO:0005102 GO:0042803
GO:0008134 GO:0005198 GO:0045502 GO:0003700 GO:0005667
GO:0005577 GO:0030286 GO:0019028 GO:0019031 GO:0016021
Cluster-22178.104553 GO:0006771 GO:0019497 GO:0046872 GO:0003993
GO:0005515
Cluster-22178.104554 GO:0006771 GO:0019497 GO:0046872 GO:0005515
GO:0003993
Cluster-22178.104559 GO:0051252 GO:0003677 GO:0004521 GO:0003779

Cluster-22178.104560 GO:0051252 GO:0003677 GO:0004521 GO:0003779

Cluster-22178.104565 GO:0006886 GO:0006457 GO:0006260 GO:0032922
GO:0015031 GO:0051087 GO:0042803 GO:0000976 GO:0008270
GO:0008565 GO:0008168 GO:0000774 GO:0016020 GO:0005737
Cluster-22178.104566 GO:0030261 GO:0007050 GO:0003677 GO:0005515
GO:0005509
Cluster-22178.104576 GO:0005515
Cluster-22178.104579 GO:0040007 GO:0005737

Cluster-22178.104583 GO:0006468 GO:0016773 GO:0004672 GO:0005524 GO:0016020
Cluster-22178.104585 GO:0006468 GO:0005524 GO:0004672
Cluster-22178.104588 GO:0043065 GO:0016779 GO:0005737
Cluster-22178.104590 GO:0032801 GO:0006812 GO:0019076 GO:0042254 GO:0006412 GO:0003735 GO:0005261 GO:0005840 GO:0033644 GO:0005622
Cluster-22178.104595 GO:0006915 GO:0005515 GO:0005509
Cluster-22178.104599 GO:0006605 GO:0006886 GO:0006355 GO:0005524 GO:0008270 GO:0003677 GO:0016818 GO:0005515 GO:0005742 GO:0005634
Cluster-22178.104602 GO:0019497 GO:0006771 GO:0005992 GO:0003993 GO:0005515 GO:0003824 GO:0046872
Cluster-22178.104603 GO:0005992 GO:0019497 GO:0006771 GO:0005515 GO:0003824 GO:0003993 GO:0046872
Cluster-22178.104605 GO:0019497 GO:0006771 GO:0005992 GO:0046872 GO:0003993 GO:0005515 GO:0003824
Cluster-22178.104606 GO:0006771 GO:0019497 GO:0005992 GO:0003993 GO:0005515 GO:0003824 GO:0046872
Cluster-22178.104607 GO:0006771 GO:0006032 GO:0016998 GO:0019497 GO:0005515 GO:0004568 GO:0003993 GO:0046872
Cluster-22178.104608 GO:0019497 GO:0006032 GO:0016998 GO:0006771 GO:0046872 GO:0003993 GO:0004568 GO:0005515
Cluster-22178.104619 GO:0005543
Cluster-22178.104620 GO:0006412 GO:0042254 GO:0046872 GO:0003735 GO:0005622 GO:0005840
Cluster-22178.104624 GO:0007154 GO:0055085 GO:0016747 GO:0016021
Cluster-22178.104636 GO:0005742
Cluster-22178.104640 GO:0035556 GO:0006468 GO:0005515 GO:0016773 GO:0004672 GO:0005524 GO:0016020
Cluster-22178.104646 GO:0043565 GO:0005515 GO:0005634
Cluster-22178.104647 GO:0006810 GO:0009405 GO:0055085 GO:0005215 GO:0045735 GO:0016020

Cluster-22178.104648 GO:0019497 GO:0006771 GO:0007155 GO:0046872
GO:0003993 GO:0005515 GO:0016021
Cluster-22178.104655 GO:0007165 GO:0051607 GO:0006355 GO:0050778
GO:0007259 GO:0003677 GO:0005125
Cluster-22178.104656 GO:0007165 GO:0051607 GO:0006355 GO:0050778
GO:0007259 GO:0003677 GO:0005125
Cluster-22178.10466 GO:0046849 GO:0005576
Cluster-22178.104662 GO:0055114 GO:0009308 GO:0005507 GO:0048038
GO:0008131
Cluster-22178.104666 GO:0008237 GO:0008270
Cluster-22178.104668 GO:0006355 GO:0016818 GO:0003677 GO:0008270
GO:0005524 GO:0005634
Cluster-22178.104669 GO:0055085 GO:0022857 GO:0016021
Cluster-22178.104673 GO:0006308 GO:0006814 GO:0016787 GO:0016888
GO:0005272 GO:0016020
Cluster-22178.104699 GO:0000746 GO:0015979 GO:0030494 GO:0046872
GO:0000166 GO:0019867
Cluster-22178.104700 GO:0007339 GO:0005515 GO:0005576 GO:0016020
Cluster-22178.104704 GO:0006269 GO:0006351 GO:0003896 GO:0005730
GO:0005657
Cluster-22178.104706 GO:0006471 GO:0003950 GO:0016817
Cluster-22178.10472 GO:0007165
Cluster-22178.104724 GO:0006816 GO:0070588 GO:0005262 GO:0016820
GO:0005515 GO:0016021 GO:0005739 GO:0016020
Cluster-22178.104727 GO:0015992 GO:0006914 GO:0006446 GO:0006415
GO:0007155 GO:0006308 GO:0006858 GO:0006413 GO:0006449
GO:0006355 GO:0015991 GO:0008855 GO:0003677 GO:0003743
GO:0015078 GO:0003700 GO:0016149 GO:0043565 GO:0019901
GO:0018444 GO:0017119 GO:0005667 GO:0005840 GO:0016021
GO:0005737 GO:0009318 GO:0033179
Cluster-22178.104734 GO:0048870 GO:0055114 GO:0006352 GO:0007049
GO:0015991 GO:0030001 GO:0006355 GO:0010508 GO:0007094
GO:0006914 GO:0015992 GO:0055085 GO:0007155 GO:0006810

GO:0007165 GO:0003700 GO:0004871 GO:0015078 GO:0016491
GO:0003774 GO:0016987 GO:0046873 GO:0005509 GO:0003677
GO:0033179 GO:0031514 GO:0005886 GO:0005667 GO:0016459
GO:0016020
Cluster-22178.104735 GO:0007049 GO:0006352 GO:0048870 GO:0055114
GO:0007094 GO:0010508 GO:0030001 GO:0006355 GO:0015991
GO:0055085 GO:0015992 GO:0006914 GO:0006810 GO:0007165
GO:0007155 GO:0015078 GO:0003700 GO:0004871 GO:0016987
GO:0003774 GO:0016491 GO:0003677 GO:0005509 GO:0046873
GO:0031514 GO:0033179 GO:0005886 GO:0016020 GO:0016459
GO:0005667
Cluster-22178.104739 GO:0007155 GO:0006771 GO:0019497 GO:0005515
GO:0003993 GO:0046872 GO:0016021
Cluster-22178.104743 GO:0005515
Cluster-22178.104744 GO:0006835 GO:0006820 GO:0006812 GO:0017153
GO:0016020
Cluster-22178.104745 GO:0005509 GO:0005515
Cluster-22178.104746 GO:0005509 GO:0005515
Cluster-22178.104748 GO:0009405 GO:0005576
Cluster-22178.104751 GO:0006355 GO:0008270 GO:0005524 GO:0003677
GO:0016818 GO:0005634
Cluster-22178.104756 GO:0016021
Cluster-22178.104757 GO:0016021
Cluster-22178.104758 GO:0031224
Cluster-22178.104762 GO:0006357 GO:0006914 GO:0048193 GO:0006308
GO:0045892 GO:0007017 GO:0007018 GO:0008855 GO:0008092
GO:0003777 GO:0003700 GO:0045502 GO:0042803 GO:0008134
GO:0001104 GO:0030286 GO:0019898 GO:0005667 GO:0016592
GO:0016020 GO:0005737 GO:0005874 GO:0016021 GO:0019028
GO:0009318
Cluster-22178.104764 GO:0042384 GO:0006355 GO:0010826 GO:0003700
GO:0005622 GO:0005667 GO:0036038
Cluster-22178.104766 GO:0042476 GO:0042475 GO:0030345 GO:0005578

Cluster-22178.104773 GO:0019049 GO:0008270 GO:0005515
Cluster-22178.104775 GO:0051258 GO:0042262 GO:0009405 GO:0030168 GO:0006914 GO:0006606 GO:0010506 GO:0007165 GO:0045502 GO:0005198 GO:0042803 GO:0008134 GO:0005102 GO:0003690 GO:0030674 GO:0005615 GO:0030286 GO:0005577 GO:0005667 GO:0005882 GO:0016020
Cluster-22178.104783 GO:0016310 GO:0009069 GO:0006468 GO:0004674 GO:0005524 GO:0000287
Cluster-22178.104786 GO:0006310 GO:0007165 GO:0000150 GO:0005515 GO:0003677
Cluster-22178.104787 GO:0006310 GO:0007165 GO:0005515 GO:0003677 GO:0000150
Cluster-22178.104788 GO:0006310 GO:0006886 GO:0007165 GO:0006415 GO:0006449 GO:0048280 GO:0005515 GO:0003677 GO:0000150 GO:0016149 GO:0018444 GO:0000139 GO:0005840 GO:0005737
Cluster-22178.104791 GO:0006310 GO:0007165 GO:0000150 GO:0005515 GO:0003677
Cluster-22178.104794 GO:0006308 GO:0008855 GO:0005515 GO:0005737 GO:0009318 GO:0016021
Cluster-22178.104795 GO:0006308 GO:0046914 GO:0046983 GO:0008855 GO:0005515 GO:0005737 GO:0009318
Cluster-22178.104796 GO:0006308 GO:0005515 GO:0008855 GO:0009318 GO:0005737
Cluster-22178.104800 GO:0015992 GO:0006123 GO:0004129 GO:0045277
Cluster-22178.104801 GO:0070588 GO:0006816 GO:0005262 GO:0016020
Cluster-22178.104802 GO:0005198 GO:0016021
Cluster-22178.104804 GO:0003723 GO:0003676
Cluster-22178.104807 GO:0007264 GO:0015684 GO:0005524 GO:0015093 GO:0016887 GO:0003924 GO:0005525 GO:0016021
Cluster-22178.104812 GO:0005525
Cluster-22178.104814 GO:0006351 GO:0003714 GO:0005667
Cluster-22178.104815 GO:0006351 GO:0003714 GO:0005667
Cluster-22178.104818 GO:0006955 GO:0019882 GO:0016020 GO:0042613

Cluster-22178.104819 GO:0007186 GO:0004930 GO:0016021

Cluster-22178.104832 GO:0005515

Cluster-22178.104841 GO:0007166 GO:0007165 GO:0004888 GO:0016020

Cluster-22178.104850 GO:0046487 GO:0055114 GO:0007093 GO:0009396 GO:0005515 GO:0004488 GO:0003824 GO:0016491 GO:0005739 GO:0000776

Cluster-22178.104854 GO:0019882 GO:0006955 GO:0042613 GO:0016020

Cluster-22178.104857 GO:0007264 GO:0015684 GO:0016887 GO:0003924 GO:0005524 GO:0015093 GO:0005525 GO:0016021

Cluster-22178.104859 GO:0006812 GO:0006820 GO:0006835 GO:0017153 GO:0016021 GO:0016020

Cluster-22178.104861 GO:0019058 GO:0007156 GO:0005509 GO:0016020

Cluster-22178.104862 GO:0007156 GO:0005509 GO:0016020

Cluster-22178.104873 GO:0015074 GO:0003677 GO:0005515

Cluster-22178.104874 GO:0006468 GO:0005524 GO:0004672 GO:0005515

Cluster-22178.10488 GO:0006629 GO:0042254 GO:0006412 GO:0003735 GO:0005622 GO:0005840

Cluster-22178.104882 GO:0007165 GO:0051252 GO:0006396 GO:0003723 GO:0004525 GO:0005515

Cluster-22178.104886 GO:0006886 GO:0006606 GO:0007049 GO:0055114 GO:0016705 GO:0020037 GO:0005506 GO:0016020 GO:0005634

Cluster-22178.104887 GO:0016020

Cluster-22178.104890 GO:0016032 GO:0005515 GO:0019031

Cluster-22178.104896 GO:0007186 GO:0005515 GO:0004930 GO:0016021 GO:0016020

Cluster-22178.104897 GO:0007186 GO:0005515 GO:0004930 GO:0016021 GO:0016020

Cluster-22178.104900 GO:0042967 GO:0006555 GO:0019281 GO:0008899 GO:0005737

Cluster-22178.104904 GO:0006811 GO:0050830 GO:0046872 GO:0016020 GO:0031083

Cluster-22178.104907 GO:0006367 GO:0051252 GO:0004540 GO:0005672

GO:0019028
Cluster-22178.104909 GO:0051252 GO:0004540 GO:0019028
Cluster-22178.104911 GO:0051252 GO:0006367 GO:0004540 GO:0019028
GO:0005672
Cluster-22178.104913 GO:0019028
Cluster-22178.104914 GO:0051252 GO:0004540
Cluster-22178.104926 GO:0007186 GO:0004930 GO:0016021
Cluster-22178.104930 GO:0006144 GO:0006206 GO:0006351 GO:0003723
GO:0003899 GO:0003677 GO:0005730
Cluster-22178.104931 GO:0008643 GO:0006629 GO:0008982 GO:0009357
Cluster-22178.104932 GO:0020037 GO:0019825
Cluster-22178.104933 GO:0008270 GO:0003697
Cluster-22178.104939 GO:0005515
Cluster-22178.104947 GO:0006355 GO:0005668 GO:0005634
Cluster-22178.104951 GO:0006334 GO:0003677 GO:0005634 GO:0000786
Cluster-22178.104956 GO:0007165 GO:0055114 GO:0005507 GO:0016641
GO:0005044 GO:0016020
Cluster-22178.104957 GO:0006825 GO:0032259 GO:0007212 GO:0048268
GO:0035434 GO:0046983 GO:0008168 GO:0032051 GO:0005375
GO:0016021
Cluster-22178.104964 GO:0003677 GO:0005634
Cluster-22178.104982 GO:0005515 GO:0016020
Cluster-22178.104985 GO:0009607 GO:0016021
Cluster-22178.104991 GO:0043683 GO:0005198 GO:0005515 GO:0019028
Cluster-22178.104993 GO:0006812 GO:0009401 GO:0006820 GO:0006835
GO:0017153 GO:0016021 GO:0016020
Cluster-22178.105003 GO:0042254 GO:0006412 GO:0003735 GO:0016020
GO:0005840
Cluster-22178.105006 GO:0007186 GO:0016755 GO:0004930 GO:0005515

Cluster-22178.105011 GO:0007275 GO:0005201 GO:0005578 GO:0005581
Cluster-22178.105013 GO:0006468 GO:0004672 GO:0005524
Cluster-22178.105015 GO:0007018 GO:0007017 GO:0006914 GO:0008017

GO:0003777 GO:0005524 GO:0045298 GO:0005874

Cluster-22178.105016 GO:0007017 GO:0009405 GO:0051252 GO:0007018

GO:0048870 GO:0048309 GO:0007165 GO:0051028 GO:0006914

GO:0045502 GO:0003723 GO:0042803 GO:0008134 GO:0005198

GO:0003777 GO:0004871 GO:0005524 GO:0004521 GO:0008017

GO:0031514 GO:0005874 GO:0045298 GO:0005667 GO:0005882

GO:0016020 GO:0030286

Cluster-22178.105017 GO:0003774 GO:0005515 GO:0016459

Cluster-22178.105018 GO:0006811 GO:0055085 GO:0005216 GO:0016020

Cluster-22178.10503 GO:0006816 GO:0006810 GO:0005246 GO:0042030

GO:0030234 GO:0016020

Cluster-22178.105032 GO:0006935 GO:0007165 GO:0006955 GO:0008009

GO:0005576

Cluster-22178.105037 GO:0030414 GO:0005515

Cluster-22178.105039 GO:0006810 GO:0009405 GO:0030414 GO:0019871

GO:0005576

Cluster-22178.105040 GO:0009405 GO:0006810 GO:0030414 GO:0019871

GO:0005576

Cluster-22178.105042 GO:0016042 GO:0007586 GO:0005515 GO:0030414

GO:0008047 GO:0005576

Cluster-22178.105043 GO:0007586 GO:0016042 GO:0008047 GO:0005576

Cluster-22178.105045 GO:0007586 GO:0016042 GO:0008047 GO:0005515

GO:0005576

Cluster-22178.105052 GO:0004866 GO:0005576 GO:0005615

Cluster-22178.105053 GO:0004866 GO:0005615 GO:0005576

Cluster-22178.105055 GO:0004866 GO:0005576 GO:0005615

Cluster-22178.105057 GO:0004866 GO:0005576 GO:0005615

Cluster-22178.105059 GO:0005576

Cluster-22178.105063 GO:0006771 GO:0006468 GO:0019497 GO:0005515

GO:0003993 GO:0046872 GO:0004672 GO:0005524

Cluster-22178.105065 GO:0019079 GO:0043066 GO:0043154 GO:0008146

GO:0016021 GO:0019031 GO:0005737 GO:0071986

Cluster-22178.105068 GO:0005515

Cluster-22178.105071 GO:0006810 GO:0005215 GO:0016020
Cluster-22178.105080 GO:0006465 GO:0008233 GO:0005515 GO:0016021
GO:0005787
Cluster-22178.105085 GO:0006955 GO:0007165 GO:0040007 GO:0008283
GO:0009098 GO:0008083 GO:0005143 GO:0005515 GO:0042022
GO:0005576
Cluster-22178.105088 GO:0019497 GO:0006771 GO:0046872 GO:0003993
GO:0005515
Cluster-22178.105089 GO:0005515
Cluster-22178.105096 GO:0006144 GO:0006508 GO:0004252 GO:0003968
GO:0017111 GO:0004197 GO:0031379
Cluster-22178.105098 GO:0005515 GO:0008270 GO:0004190 GO:0016021
Cluster-22178.105102 GO:0009069 GO:0006468 GO:0016310 GO:0005524
GO:0004672 GO:0004674
Cluster-22178.105105 GO:0055114 GO:0010277
Cluster-22178.105118 GO:0006118 GO:0055114 GO:0005515 GO:0009916

Cluster-22178.105125 GO:0006508 GO:0004252
Cluster-22178.105131 GO:0007264 GO:0006351 GO:0006206 GO:0006144
GO:0002949 GO:0003677 GO:0003899 GO:0003924 GO:0005525
GO:0005730
Cluster-22178.105138 GO:0006508 GO:0004198 GO:0005509 GO:0005622
Cluster-22178.105149 GO:0009069 GO:0006468 GO:0016310 GO:0007178
GO:0004675 GO:0005524 GO:0004672 GO:0016020
Cluster-22178.105155 GO:0006508 GO:0004252
Cluster-22178.105156 GO:0000079 GO:0019901
Cluster-22178.105158 GO:0046983 GO:0003677
Cluster-22178.105160 GO:0035088 GO:0030855 GO:0007016 GO:0007411
GO:0051017 GO:0022614 GO:0008360 GO:0007159 GO:0008092
GO:0051015 GO:0050839 GO:0016323 GO:0005737 GO:0032587
GO:0005884 GO:0005829 GO:0030863 GO:0001931 GO:0030175
GO:0019898 GO:0016324 GO:0005730 GO:0005925 GO:0031528
Cluster-22178.105171 GO:0016021

Cluster-22178.105173 GO:0007059 GO:0051301 GO:0009405 GO:0006355
GO:0006606 GO:0031110 GO:0007067 GO:0000090 GO:0003700
GO:0043565 GO:0008017 GO:0045298 GO:0005876 GO:0000940
GO:0009424 GO:0016020 GO:0005667
Cluster-22178.105184 GO:0016055 GO:0007275 GO:0007165 GO:0005102
GO:0005576
Cluster-22178.105189 GO:0015074 GO:0003677
Cluster-22178.105203 GO:0016301
Cluster-22178.105210 GO:0005515
Cluster-22178.105212 GO:0031505
Cluster-22178.105221 GO:0008270
Cluster-22178.105223 GO:0008270
Cluster-22178.105224 GO:0008270
Cluster-22178.105225 GO:0006468 GO:0005524 GO:0004672
Cluster-22178.105228 GO:0005515
Cluster-22178.105229 GO:0005515
Cluster-22178.105233 GO:0046983
Cluster-22178.105238 GO:0006744 GO:0006120 GO:0015992 GO:0006814
GO:0006118 GO:0008137 GO:0003954 GO:0005739
Cluster-22178.105239 GO:0006120 GO:0006744 GO:0015992 GO:0006118
GO:0006814 GO:0008137 GO:0003954 GO:0005739
Cluster-22178.105240 GO:0015992 GO:0006120 GO:0006744 GO:0006118
GO:0006814 GO:0003954 GO:0008137 GO:0005739
Cluster-22178.105241 GO:0015992 GO:0006120 GO:0006744 GO:0006118
GO:0006814 GO:0003954 GO:0008137 GO:0005739
Cluster-22178.105243 GO:0005515
Cluster-22178.105248 GO:0000398 GO:0007017 GO:0005681 GO:0005875
Cluster-22178.105249 GO:0007017 GO:0000398 GO:0005681 GO:0005875
Cluster-22178.105250 GO:0000398 GO:0007017 GO:0005681 GO:0005875
Cluster-22178.105252 GO:0007017 GO:0000398 GO:0005875 GO:0005681
Cluster-22178.105253 GO:0008270
Cluster-22178.105254 GO:0006508 GO:0008270 GO:0004222 GO:0031012
Cluster-22178.105269 GO:0010038

Cluster-22178.105270 GO:0005515
Cluster-22178.105271 GO:0005515
Cluster-22178.105275 GO:0005515
Cluster-22178.105279 GO:0008017 GO:0045298
Cluster-22178.105290 GO:0051056 GO:0005096
Cluster-22178.105291 GO:0005515
Cluster-22178.105292 GO:0046931 GO:0031556 GO:0006810 GO:0031554 GO:0052331 GO:0006812 GO:0015267 GO:0046930
Cluster-22178.105296 GO:0007076 GO:0000796
Cluster-22178.105304 GO:0016021
Cluster-22178.105310 GO:0007165 GO:0006955 GO:0006935 GO:0008009 GO:0005576
Cluster-22178.105318 GO:0015074 GO:0015992 GO:0006119 GO:0015986 GO:0046933 GO:0046961 GO:0045259 GO:0000275
Cluster-22178.105324 GO:0005515
Cluster-22178.105327 GO:0009271 GO:0007186 GO:0015684 GO:0007165 GO:0006355 GO:0007264 GO:0004871 GO:0005525 GO:0003924 GO:0031683 GO:0019001 GO:0015093 GO:0016021
Cluster-22178.105328 GO:0007186 GO:0007165 GO:0005057 GO:0004930 GO:0016021
Cluster-22178.105334 GO:0003676
Cluster-22178.105337 GO:0008152 GO:0006771 GO:0006508 GO:0009231 GO:0003919 GO:0008236 GO:0016787
Cluster-22178.105351 GO:0016887 GO:0005524
Cluster-22178.105357 GO:0004222 GO:0008270 GO:0016012
Cluster-22178.105362 GO:0032012 GO:0043087 GO:0005086
Cluster-22178.105369 GO:0006355 GO:0003677 GO:0005634
Cluster-22178.105383 GO:0004651
Cluster-22178.105384 GO:0006351 GO:0006281 GO:0006355 GO:0006206 GO:0006144 GO:0003677 GO:0003899 GO:0008270 GO:0046872 GO:0008168 GO:0005730
Cluster-22178.105386 GO:0005515
Cluster-22178.105389 GO:0006260 GO:0005515 GO:0004190 GO:0016021

GO:0005634
Cluster-22178.105392 GO:0016560 GO:0006468 GO:0004672 GO:0005524
GO:0005777 GO:0016021
Cluster-22178.105393 GO:0007049
Cluster-22178.105394 GO:0055114 GO:0006779 GO:0046856 GO:0016887
GO:0003774 GO:0005524 GO:0034597 GO:0005515 GO:0016459
Cluster-22178.105398 GO:0006508 GO:0008270 GO:0004222 GO:0031012
Cluster-22178.105399 GO:0007165 GO:0006355 GO:0003700 GO:0004871
GO:0005667
Cluster-22178.105400 GO:0007268 GO:0006811 GO:0007165 GO:0005234
GO:0004970 GO:0016020
Cluster-22178.105408 GO:0006508 GO:0008152 GO:0008270 GO:0004181
GO:0016788
Cluster-22178.105411 GO:0006464 GO:0015979 GO:0004867 GO:0046872
GO:0005524 GO:0016020 GO:0009522
Cluster-22178.105418 GO:0007154 GO:0005515 GO:0016020
Cluster-22178.105419 GO:0005198 GO:0019028
Cluster-22178.105421 GO:0007339 GO:0005515 GO:0005576 GO:0016020
Cluster-22178.105426 GO:0055085 GO:0006810 GO:0005215 GO:0016020
Cluster-22178.105428 GO:0005509 GO:0005515 GO:0004064
Cluster-22178.105429 GO:0009405 GO:0005576
Cluster-22178.105432 GO:0006118 GO:0006351 GO:0019684 GO:0006144
GO:0006206 GO:0003899 GO:0003677 GO:0045156 GO:0003779
GO:0030077 GO:0005634 GO:0005730 GO:0016021
Cluster-22178.105433 GO:0006024 GO:0030206 GO:0030158 GO:0008375
GO:0016020
Cluster-22178.105435 GO:0006508 GO:0009245 GO:0008915 GO:0008270
GO:0004222 GO:0031012 GO:0005886
Cluster-22178.105437 GO:0051726
Cluster-22178.105439 GO:0006811 GO:0055085 GO:0051260 GO:0043158
GO:0005216 GO:0003677 GO:0004252 GO:0016020
Cluster-22178.105446 GO:0006812 GO:0006835 GO:0006820 GO:0017153
GO:0016020

Cluster-22178.105461 GO:0006814 GO:0005272 GO:0016020
Cluster-22178.105467 GO:0006891 GO:0016021 GO:0017119
Cluster-22178.105468 GO:0006891 GO:0016021 GO:0017119
Cluster-22178.10547 GO:0005515
Cluster-22178.105471 GO:0044237 GO:0007165 GO:0008601 GO:0003824
GO:0000159
Cluster-22178.105472 GO:0044237 GO:0007165 GO:0003824 GO:0008601
GO:0000159
Cluster-22178.105474 GO:0016070 GO:0006468 GO:0016032 GO:0005524
GO:0004672 GO:0019012
Cluster-22178.105476 GO:0005515
Cluster-22178.105477 GO:0005515
Cluster-22178.105478 GO:0006813 GO:0006508 GO:0006144 GO:0004197
GO:0005515 GO:0017111 GO:0003968 GO:0015269 GO:0016817
GO:0016020 GO:0031379
Cluster-22178.105479 GO:0005515
Cluster-22178.105481 GO:0006486 GO:0008373
Cluster-22178.105485 GO:0006355 GO:0005515
Cluster-22178.105486 GO:0016021 GO:0019028
Cluster-22178.105487 GO:0006355 GO:0005515
Cluster-22178.105489 GO:0006355 GO:0043565 GO:0003700 GO:0003677
GO:0005667
Cluster-22178.105500 GO:0006213 GO:0016763
Cluster-22178.105510 GO:0006810 GO:0009405 GO:0030414 GO:0019871
GO:0005576
Cluster-22178.105512 GO:0055085 GO:0006206 GO:0055114 GO:0006231
GO:0006952 GO:0006810 GO:0006281 GO:0047134 GO:0005215
GO:0004799 GO:0046872 GO:0008270 GO:0016021 GO:0016020
Cluster-22178.105522 GO:0007165 GO:0005515
Cluster-22178.105525 GO:0007186 GO:0004930 GO:0016021
Cluster-22178.105527 GO:0008270 GO:0005515
Cluster-22178.105539 GO:0006810 GO:0005622
Cluster-22178.105545 GO:0055114 GO:0016491

Cluster-22178.105549 GO:0030168 GO:0006935 GO:0051258 GO:0007165
GO:0071973 GO:0005102 GO:0003774 GO:0003677 GO:0051082
GO:0030674 GO:0005577 GO:0009288 GO:0016020
Cluster-22178.105550 GO:0006418 GO:0006355 GO:0009405 GO:0005524
GO:0043565 GO:0005525 GO:0004812 GO:0003677 GO:0005515
GO:0005634 GO:0016020
Cluster-22178.105553 GO:0007165 GO:0030168 GO:0010508 GO:0016032
GO:0006418 GO:0051258 GO:0004812 GO:0030674 GO:0005102
GO:0005524 GO:0005515 GO:0005198 GO:0043565 GO:0005882
GO:0016020 GO:0005815 GO:0005577 GO:0005634
Cluster-22178.105557 GO:0006468 GO:0004672 GO:0005524 GO:0016773
GO:0016020
Cluster-22178.105559 GO:0050790 GO:0007186 GO:0030234 GO:0004930
GO:0004222 GO:0016021
Cluster-22178.105560 GO:0050790 GO:0008283 GO:0040007 GO:0007165
GO:0007186 GO:0005125 GO:0004222 GO:0004930 GO:0030234
GO:0008083 GO:0016021
Cluster-22178.105562 GO:0003676 GO:0008270
Cluster-22178.105566 GO:0007165 GO:0008158 GO:0016020
Cluster-22178.105567 GO:0007165 GO:0008158 GO:0016020
Cluster-22178.105571 GO:0007186 GO:0006118 GO:0004930 GO:0009055
GO:0016020 GO:0016021 GO:0009512
Cluster-22178.105573 GO:0005515 GO:0016021
Cluster-22178.105576 GO:0007186 GO:0004930 GO:0004867 GO:0016021
Cluster-22178.105578 GO:0005544 GO:0005515 GO:0005509 GO:0016021
Cluster-22178.105582 GO:0006355 GO:0003677 GO:0005507 GO:0003700
GO:0005667 GO:0005634
Cluster-22178.105594 GO:0031012
Cluster-22178.105600 GO:0048786
Cluster-22178.105601 GO:0048786
Cluster-22178.105602 GO:0006629 GO:0008081
Cluster-22178.105615 GO:0006811 GO:0016020
Cluster-22178.105617 GO:0007339 GO:0005515 GO:0005576

Cluster-22178.105618 GO:0006468 GO:0005524 GO:0004672
Cluster-22178.105621 GO:0005975 GO:0007155 GO:0005515 GO:0004553
GO:0016021 GO:0016020
Cluster-22178.10563 GO:0008277 GO:0006886 GO:0015031 GO:0008565
GO:0016021
Cluster-22178.105652 GO:0007178 GO:0009069 GO:0006468 GO:0016310
GO:0005524 GO:0004675 GO:0016020
Cluster-22178.105654 GO:0007018 GO:0007017 GO:0003777 GO:0008017
GO:0005524 GO:0005874 GO:0045298
Cluster-22178.105657 GO:0016567 GO:0004842
Cluster-22178.105659 GO:0006810 GO:0019871 GO:0005576
Cluster-22178.105663 GO:0004651
Cluster-22178.105665 GO:0016117 GO:0008033 GO:0055114 GO:0016705
GO:0050661 GO:0071949 GO:0050660 GO:0016491 GO:0004499
Cluster-22178.105670 GO:0007165 GO:0005179 GO:0042393 GO:0005576
Cluster-22178.105673 GO:0055085 GO:0006807 GO:0055114 GO:0042128
GO:0006810 GO:0042626 GO:0051537 GO:0008942 GO:0016491
GO:0046872 GO:0005524 GO:0016021
Cluster-22178.105674 GO:0007165 GO:0006887 GO:0005515 GO:0030667
GO:0005856
Cluster-22178.105675 GO:0006887 GO:0007165 GO:0005515 GO:0005856
GO:0030667
Cluster-22178.105676 GO:0006810 GO:0042967 GO:0008811 GO:0005515
GO:0003774 GO:0005524 GO:0016459 GO:0005856
Cluster-22178.105679 GO:0005515
Cluster-22178.105680 GO:0008270
Cluster-22178.105690 GO:0019497 GO:0006771 GO:0005992 GO:0046872
GO:0003993 GO:0005515 GO:0003824
Cluster-22178.105691 GO:0007275
Cluster-22178.105693 GO:0007275
Cluster-22178.105694 GO:0007186 GO:0006725 GO:0055114 GO:0019236
GO:0016021
Cluster-22178.105704 GO:0048193 GO:0005515 GO:0016020 GO:0016021

Cluster-22178.105716 GO:0006810 GO:0031556 GO:0046931 GO:0052331
GO:0031554 GO:0006812 GO:0015267 GO:0046930
Cluster-22178.105723 GO:0007186 GO:0004930 GO:0016021
Cluster-22178.105730 GO:0005515
Cluster-22178.105731 GO:0043248
Cluster-22178.105732 GO:0006470 GO:0019497 GO:0006570 GO:0006771
GO:0003993 GO:0004725 GO:0046872 GO:0005515 GO:0008138
Cluster-22178.105735 GO:0006810 GO:0003676 GO:0005886 GO:0005789
Cluster-22178.105737 GO:0006231 GO:0006206 GO:0006355 GO:0008270
GO:0005524 GO:0050797 GO:0003676 GO:0050660 GO:0016787
GO:0005515 GO:0016818 GO:0003677 GO:0005634
Cluster-22178.105749 GO:0051056 GO:0008152 GO:0005096 GO:0003824

Cluster-22178.105750 GO:0051056 GO:0008152 GO:0005096 GO:0003824

Cluster-22178.105756 GO:0043087
Cluster-22178.105758 GO:0003779
Cluster-22178.105759 GO:0003779
Cluster-22178.105763 GO:0007154 GO:0005515 GO:0005509 GO:0016020
Cluster-22178.105764 GO:0007154 GO:0005509 GO:0016020
Cluster-22178.105765 GO:0031224
Cluster-22178.105771 GO:0005515
Cluster-22178.105772 GO:0007154 GO:0006144 GO:0007186 GO:0006508
GO:0003968 GO:0004930 GO:0016817 GO:0017111 GO:0004197
GO:0016021 GO:0031379 GO:0016020
Cluster-22178.105791 GO:0006355 GO:0006468 GO:0003700 GO:0016773
GO:0043565 GO:0004672 GO:0005524 GO:0008270 GO:0016020
GO:0005667
Cluster-22178.105796 GO:0016020
Cluster-22178.105799 GO:0055114 GO:0007165 GO:0005044 GO:0016641
GO:0005507 GO:0016020
Cluster-22178.105800 GO:0006812 GO:0055085 GO:0008324 GO:0016021
Cluster-22178.105803 GO:0055114 GO:0006807 GO:0055085 GO:0042128

GO:0006810 GO:0046872 GO:0005524 GO:0016491 GO:0008942
GO:0051537 GO:0042626 GO:0016021
Cluster-22178.105824 GO:0006182 GO:0009190 GO:0046039 GO:0006144
GO:0035556 GO:0016849 GO:0004383 GO:0020037 GO:0008270
GO:0005634 GO:0016021
Cluster-22178.105835 GO:0006570 GO:0006470 GO:0050852 GO:0005515
GO:0004725
Cluster-22178.105848 GO:0008270
Cluster-22178.105863 GO:0016021
Cluster-22178.105865 GO:0003333 GO:0006865 GO:0015171 GO:0016020
Cluster-22178.105878 GO:0009228 GO:0006772 GO:0004417
Cluster-22178.105879 GO:0009228 GO:0006772 GO:0004417
Cluster-22178.105881 GO:0019058 GO:0005198
Cluster-22178.105888 GO:0005515
Cluster-22178.105889 GO:0004866
Cluster-22178.105895 GO:0006811 GO:0055085 GO:0005216 GO:0004190
GO:0016020 GO:0016021
Cluster-22178.105896 GO:0055085 GO:0006811 GO:0005216 GO:0016020
Cluster-22178.105901 GO:0006811 GO:0055085 GO:0006814 GO:0005248
GO:0005216 GO:0001518 GO:0016020
Cluster-22178.105902 GO:0006811 GO:0055085 GO:0005216 GO:0016020
Cluster-22178.105903 GO:0006811 GO:0055085 GO:0000166 GO:0005216
GO:0046872 GO:0016020
Cluster-22178.105904 GO:0031224
Cluster-22178.105905 GO:0006814 GO:0005248 GO:0001518
Cluster-22178.105908 GO:0008289
Cluster-22178.10591 GO:0007165 GO:0001664
Cluster-22178.105910 GO:0006508 GO:0006817 GO:0006810 GO:0007224
GO:0030436 GO:0004190 GO:0005215 GO:0005315 GO:0005525
GO:0016020 GO:0016021
Cluster-22178.105913 GO:0055085 GO:0030436 GO:0006508 GO:0006817
GO:0006810 GO:0005215 GO:0005315 GO:0005525 GO:0004190
GO:0016021 GO:0016020

Cluster-22178.105915 GO:0005887 GO:0016021
Cluster-22178.105917 GO:0006457 GO:0000413 GO:0004859 GO:0003755 GO:0005576 GO:0016021
Cluster-22178.105919 GO:0016310 GO:0005524 GO:0016301
Cluster-22178.105923 GO:0006811 GO:0055085 GO:0016598 GO:0004057 GO:0005216 GO:0016020
Cluster-22178.105927 GO:0007165 GO:0070008 GO:0004252 GO:0005044 GO:0016020
Cluster-22178.105930 GO:0005515
Cluster-22178.105939 GO:0030001 GO:0046872
Cluster-22178.105940 GO:0033926
Cluster-22178.105942 GO:0005515 GO:0016020
Cluster-22178.105946 GO:0006470 GO:0006570 GO:0007275 GO:0007165 GO:0004725 GO:0008138 GO:0005179 GO:0005576
Cluster-22178.105951 GO:0015694 GO:0015097 GO:0005515 GO:0016020
Cluster-22178.105952 GO:0006144 GO:0006182 GO:0046039 GO:0006508 GO:0009234 GO:0004383 GO:0004197
Cluster-22178.105954 GO:0007165 GO:0048268 GO:0007212 GO:0032051 GO:0004888 GO:0016021
Cluster-22178.105959 GO:0006694 GO:0006351 GO:0007623 GO:0006355 GO:0016125 GO:0003700 GO:0047750 GO:0043565 GO:0016021 GO:0005667 GO:0005634
Cluster-22178.105960 GO:0006955 GO:0030182 GO:0007165 GO:0021549 GO:0005164 GO:0016020
Cluster-22178.105964 GO:0006355 GO:0003677
Cluster-22178.105973 GO:0006869 GO:0016032 GO:0008289 GO:0003677 GO:0051082 GO:0016021 GO:0005576 GO:0005634
Cluster-22178.105975 GO:0005515 GO:0005524 GO:0003774 GO:0016459
Cluster-22178.105977 GO:0005515
Cluster-22178.105978 GO:0005515 GO:0005524 GO:0003774 GO:0016459
Cluster-22178.105982 GO:0007165 GO:0051258 GO:0030168 GO:0005102 GO:0030674 GO:0005577
Cluster-22178.105984 GO:0005524 GO:0003774 GO:0005515 GO:0016459

Cluster-22178.106008 GO:0005515
Cluster-22178.106016 GO:0006955 GO:0030153 GO:0015643 GO:0019814
Cluster-22178.106028 GO:0007067 GO:0000090 GO:0007059 GO:0055114
GO:0051301 GO:0031110 GO:0008017 GO:0016627 GO:0005876
GO:0045298 GO:0000940
Cluster-22178.106029 GO:0055114 GO:0016627
Cluster-22178.106034 GO:0003677
Cluster-22178.106036 GO:0003924 GO:0005524 GO:0005515 GO:0005525

Cluster-22178.106037 GO:0005515 GO:0005525 GO:0003924 GO:0005524

Cluster-22178.106038 GO:0005515 GO:0005525 GO:0003924 GO:0005524

Cluster-22178.106039 GO:0005524 GO:0003924 GO:0005525 GO:0005515

Cluster-22178.106041 GO:0005525 GO:0005515 GO:0005524 GO:0003924

Cluster-22178.106043 GO:0005515 GO:0016021
Cluster-22178.106044 GO:0040007 GO:0008283 GO:0006310 GO:0007165
GO:0008083 GO:0003677 GO:0005634
Cluster-22178.106045 GO:0008283 GO:0040007 GO:0007165 GO:0006310
GO:0003677 GO:0008083 GO:0005634
Cluster-22178.106050 GO:0045893 GO:0009271 GO:0007165 GO:0006629
GO:0006355 GO:0005515 GO:0001000 GO:0005509 GO:0005578
Cluster-22178.106054 GO:0007155 GO:0006891 GO:0051015 GO:0017119
Cluster-22178.106059 GO:0043113 GO:0006810 GO:0007213 GO:0005515
GO:0043236 GO:0005215 GO:0016020
Cluster-22178.106069 GO:0006468 GO:0016773 GO:0005524 GO:0004672
GO:0016020
Cluster-22178.106073 GO:0006470 GO:0004721 GO:0030145 GO:0000287

Cluster-22178.106075 GO:0006470 GO:0004721 GO:0030145 GO:0000287

Cluster-22178.106078 GO:0006260 GO:0055114 GO:0006206 GO:0008998
GO:0016491 GO:0005887
Cluster-22178.106084 GO:0019497 GO:0006771 GO:0003993 GO:0005515
GO:0046872
Cluster-22178.106087 GO:0007275 GO:0005578
Cluster-22178.106088 GO:0030155 GO:0007165 GO:0007155 GO:0006915
GO:0045995 GO:0001836 GO:0006974 GO:0055114 GO:0006919
GO:0030334 GO:0051082 GO:0005102 GO:0016491 GO:0046872
Cluster-22178.106090 GO:0008270
Cluster-22178.106092 GO:0007264 GO:0005525 GO:0005515
Cluster-22178.106095 GO:0015991 GO:0006308 GO:0016567 GO:0019058
GO:0019079 GO:0006313 GO:0015992 GO:0005515 GO:0015078
GO:0046872 GO:0004803 GO:0003677 GO:0004842 GO:0008855
GO:0008270 GO:0033179 GO:0009318 GO:0019028 GO:0005680
GO:0005622
Cluster-22178.106097 GO:0003723
Cluster-22178.106098 GO:0008152 GO:0046782 GO:0019060 GO:0008289
GO:0005515 GO:0003824
Cluster-22178.106099 GO:0008152 GO:0005515 GO:0003824
Cluster-22178.106106 GO:0006468 GO:0005515 GO:0005524 GO:0004672
GO:0016020
Cluster-22178.106107 GO:0006468 GO:0004672 GO:0005524 GO:0005515
GO:0016020
Cluster-22178.106117 GO:0005515
Cluster-22178.106118 GO:0005515
Cluster-22178.106124 GO:0008289
Cluster-22178.106125 GO:0005634
Cluster-22178.106131 GO:0005515
Cluster-22178.106132 GO:0005515
Cluster-22178.106135 GO:0006413 GO:0007264 GO:0017148 GO:0005515
GO:0005525 GO:0005845
Cluster-22178.106141 GO:0009755 GO:0009405 GO:0006810 GO:0008200
GO:0005576 GO:0019013

Cluster-22178.106147 GO:0005515
Cluster-22178.10615 GO:0005515
Cluster-22178.106154 GO:0006508 GO:0051276 GO:0031175 GO:0030507
GO:0005515 GO:0004180 GO:0005516 GO:0004181 GO:0008270
GO:0005524 GO:0005694 GO:0008091
Cluster-22178.106155 GO:0007165 GO:0032509 GO:0006355 GO:0043130
GO:0005179 GO:0000813 GO:0005634 GO:0005576
Cluster-22178.106162 GO:0003676
Cluster-22178.106165 GO:0008033 GO:0055114 GO:0020037 GO:0016705
GO:0005506
Cluster-22178.106166 GO:0042128 GO:0006810 GO:0055114 GO:0055085
GO:0006807 GO:0016491 GO:0008942 GO:0046872 GO:0005524
GO:0042626 GO:0051537 GO:0016021
Cluster-22178.10617 GO:0006813 GO:0009103 GO:0008918 GO:0005242
GO:0008076 GO:0016021
Cluster-22178.106177 GO:0006468 GO:0007169 GO:0006807 GO:0004714
GO:0005515 GO:0003933 GO:0005524 GO:0016020
Cluster-22178.106179 GO:0005515 GO:0004252
Cluster-22178.106199 GO:0016070 GO:0016788 GO:0003723 GO:0005737
Cluster-22178.106204 GO:0005515 GO:0003824
Cluster-22178.106207 GO:0005515
Cluster-22178.106208 GO:0005515 GO:0003824
Cluster-22178.106218 GO:0006355 GO:0003677 GO:0003700 GO:0043565
GO:0005634 GO:0005667
Cluster-22178.106222 GO:0005515
Cluster-22178.106225 GO:0015694 GO:0015097 GO:0005515 GO:0016020
Cluster-22178.106233 GO:0040007 GO:0005737
Cluster-22178.106234 GO:0040007 GO:0005737
Cluster-22178.106235 GO:0040007 GO:0005737
Cluster-22178.106236 GO:0040007 GO:0005737
Cluster-22178.106242 GO:0006633 GO:0055114 GO:0016491 GO:0005506

Cluster-22178.106245 GO:0003677

Cluster-22178.106248 GO:0009116 GO:0003824
Cluster-22178.106252 GO:0007166 GO:0005515 GO:0016020
Cluster-22178.106254 GO:0006355 GO:0043565 GO:0003700 GO:0005667 GO:0005815
Cluster-22178.106269 GO:0006355 GO:0043565 GO:0003700 GO:0005634 GO:0005667
Cluster-22178.106270 GO:0006355 GO:0003700 GO:0043565 GO:0005667 GO:0005634
Cluster-22178.106271 GO:0007165 GO:0006811 GO:0007268 GO:0004970 GO:0016020
Cluster-22178.106274 GO:0005515
Cluster-22178.106279 GO:0006771 GO:0019497 GO:0006013 GO:0006000 GO:0003873 GO:0016301 GO:0005524 GO:0003993
Cluster-22178.106280 GO:0005515
Cluster-22178.106282 GO:0015940 GO:0009245 GO:0009103 GO:0009029 GO:0005524
Cluster-22178.106285 GO:0007049 GO:0048870 GO:0000917 GO:0006468 GO:0006355 GO:0006508 GO:0006260 GO:0070206 GO:0043093 GO:0003700 GO:0004672 GO:0043565 GO:0003887 GO:0001882 GO:0008233 GO:0005524 GO:0003677 GO:0019867 GO:0016021 GO:0005737 GO:0042575 GO:0031514 GO:0005667
Cluster-22178.106287 GO:0016032
Cluster-22178.106292 GO:0005515 GO:0005525 GO:0003924 GO:0005524

Cluster-22178.106309 GO:0051258 GO:0006308 GO:0030036 GO:0016043 GO:0015991 GO:0007010 GO:0030168 GO:0015992 GO:0006914 GO:0035556 GO:0007264 GO:0007165 GO:0003779 GO:0015078 GO:0051082 GO:0008855 GO:0005096 GO:0005102 GO:0030674 GO:0017048 GO:0016021 GO:0009318 GO:0033179 GO:0005577
Cluster-22178.106311 GO:0006024 GO:0030206 GO:0008375 GO:0030158 GO:0016020
Cluster-22178.106312 GO:0030206 GO:0006024 GO:0008375 GO:0030158 GO:0016020

Cluster-22178.106323 GO:0008152 GO:0003824
Cluster-22178.106326 GO:0005515
Cluster-22178.106327 GO:0035556 GO:0009405 GO:0019835 GO:0005515
GO:0019867
Cluster-22178.106328 GO:0009116 GO:0003824 GO:0016021
Cluster-22178.106334 GO:0006511
Cluster-22178.106338 GO:0005992 GO:0003824
Cluster-22178.106340 GO:0005992 GO:0006771 GO:0019497 GO:0003824
GO:0005515 GO:0003993 GO:0046872
Cluster-22178.106342 GO:0000087 GO:0006470 GO:0006570 GO:0004725
GO:0005622
Cluster-22178.106345 GO:2001256 GO:0009966 GO:0005515 GO:0005737
GO:0016020 GO:0030176
Cluster-22178.106347 GO:0009966 GO:0005515 GO:0005737 GO:0016020
Cluster-22178.106354 GO:0006814 GO:0055085 GO:0006810 GO:0005215
GO:0016020 GO:0016021
Cluster-22178.106367 GO:0007156 GO:0005515 GO:0005509 GO:0016020
Cluster-22178.106368 GO:0055114 GO:0015992 GO:0006118 GO:0006119
GO:0008121
Cluster-22178.106369 GO:0055114 GO:0015992 GO:0006119 GO:0006118
GO:0008121
Cluster-22178.106381 GO:0005515 GO:0016012
Cluster-22178.106384 GO:0005515
Cluster-22178.106392 GO:0007186 GO:0004930 GO:0016021
Cluster-22178.106397 GO:0005982 GO:0006012 GO:0005985 GO:0004564
GO:0004575 GO:0017177
Cluster-22178.106400 GO:0016020
Cluster-22178.106417 GO:0005515
Cluster-22178.106429 GO:0030036 GO:0003779 GO:0017048 GO:0005737
Cluster-22178.106436 GO:0006123 GO:0015992 GO:0004129 GO:0045277
Cluster-22178.106437 GO:0006123 GO:0015992 GO:0004129 GO:0045277
GO:0033588
Cluster-22178.106442 GO:0007165 GO:0005509 GO:0005578

Cluster-22178.106443 GO:0006351 GO:0003676 GO:0008270
Cluster-22178.106447 GO:0006955 GO:0007165 GO:0005164 GO:0016020
Cluster-22178.106448 GO:0007186 GO:0004930 GO:0016021
Cluster-22178.106449 GO:0006206 GO:0006139 GO:0005524 GO:0003924 GO:0016887 GO:0005525 GO:0004127 GO:0005911 GO:0016020
Cluster-22178.106450 GO:0009058 GO:0003824
Cluster-22178.106466 GO:0007186 GO:0004930 GO:0016021 GO:0016020
Cluster-22178.106472 GO:0016787
Cluster-22178.106476 GO:0005515
Cluster-22178.106509 GO:0007186 GO:0004930 GO:0016021
Cluster-22178.106523 GO:0008209 GO:0008207 GO:0008210 GO:0008152 GO:0006694 GO:0055114 GO:0016616 GO:0003854 GO:0050662 GO:0003824 GO:0016491
Cluster-22178.106530 GO:0006811 GO:0055085 GO:0005216 GO:0016020
Cluster-22178.106531 GO:0019082 GO:0003677
Cluster-22178.106532 GO:0019082 GO:0003677
Cluster-22178.106535 GO:0006811 GO:0055085 GO:0005216 GO:0016020
Cluster-22178.106536 GO:0019082 GO:0003677
Cluster-22178.106537 GO:0006811 GO:0055085 GO:0005216 GO:0016020
Cluster-22178.106539 GO:0006355 GO:0006811 GO:0055085 GO:0005216 GO:0016020
Cluster-22178.106545 GO:0006184 GO:0051258 GO:0007017 GO:0006952 GO:0003924 GO:0005200 GO:0005525 GO:0005856 GO:0005737 GO:0005874 GO:0005576
Cluster-22178.106549 GO:0006206 GO:0006144 GO:0006351 GO:0003677 GO:0003899 GO:0005730
Cluster-22178.106554 GO:0007160 GO:0005509 GO:0005576
Cluster-22178.106570 GO:0006351 GO:0003677 GO:0005634
Cluster-22178.106575 GO:0006144 GO:0006464 GO:0006269 GO:0046034 GO:0006281 GO:0006351 GO:0008270 GO:0004386 GO:0005506 GO:0004017 GO:0046872 GO:0016151 GO:0003896 GO:0005515 GO:0005657 GO:0005730
Cluster-22178.106587 GO:0005515

Cluster-22178.106589 GO:0009236 GO:0043087 GO:0006886 GO:0007264
GO:0051188 GO:0000166 GO:0043752 GO:0005085 GO:0005801
GO:0016020
Cluster-22178.106599 GO:0019087 GO:0055085 GO:0006811 GO:0005216
GO:0005515 GO:0016020 GO:0016021
Cluster-22178.106602 GO:0019911
Cluster-22178.106615 GO:0005515
Cluster-22178.106624 GO:0055085 GO:0016021
Cluster-22178.106625 GO:0055085 GO:0016021
Cluster-22178.106626 GO:0055085 GO:0016021
Cluster-22178.106627 GO:0006520 GO:0055085 GO:0008851 GO:0009350
GO:0016021
Cluster-22178.106628 GO:0055085 GO:0006520 GO:0008851 GO:0016021
GO:0009350
Cluster-22178.106629 GO:0006810 GO:0015858 GO:0055085 GO:0005337
GO:0016021
Cluster-22178.106630 GO:0016020
Cluster-22178.106631 GO:0016192 GO:0005515 GO:0005198 GO:0016021
GO:0019031
Cluster-22178.106635 GO:0006355 GO:0005506 GO:0008270 GO:0003677
GO:0005515
Cluster-22178.106636 GO:0009405 GO:0005576
Cluster-22178.106637 GO:0015694 GO:0015097 GO:0016020
Cluster-22178.106639 GO:0015694 GO:0015097 GO:0016020
Cluster-22178.106641 GO:0015694 GO:0015097 GO:0016020
Cluster-22178.106642 GO:0015694 GO:0015097 GO:0016020
Cluster-22178.106644 GO:0009405 GO:0005576
Cluster-22178.106645 GO:0015694 GO:0015097 GO:0016020
Cluster-22178.106646 GO:0015694 GO:0015097 GO:0016020
Cluster-22178.106653 GO:0008210 GO:0008209 GO:0008207 GO:0055114
GO:0008152 GO:0006694 GO:0003854 GO:0050662 GO:0016616
GO:0016491 GO:0003824
Cluster-22178.106654 GO:0005975 GO:0006396 GO:0080009 GO:0009451

GO:0016758 GO:0008174 GO:0003723 GO:0016020
Cluster-22178.106659 GO:0007010 GO:0008270 GO:0003779
Cluster-22178.106660 GO:0034755 GO:0006826 GO:0005381 GO:0016021
Cluster-22178.106661 GO:0008168 GO:0005515
Cluster-22178.106665 GO:0046872 GO:0008270
Cluster-22178.106668 GO:0043087 GO:0035023 GO:0005089
Cluster-22178.106670 GO:0007275 GO:0005578
Cluster-22178.106672 GO:0046872
Cluster-22178.106676 GO:0008146 GO:0046872
Cluster-22178.106681 GO:0003333 GO:0006810 GO:0006412 GO:0042254
GO:0055085 GO:0006865 GO:0015171 GO:0003735 GO:0016020
GO:0005840 GO:0005622
Cluster-22178.106690 GO:0016192 GO:0006886 GO:0005515 GO:0030117
Cluster-22178.106697 GO:0006355 GO:0003700 GO:0005667
Cluster-22178.106698 GO:0006355 GO:0003700 GO:0005667
Cluster-22178.106703 GO:0007186 GO:0004930 GO:0016020
Cluster-22178.106704 GO:0005515
Cluster-22178.106707 GO:0010181 GO:0016020
Cluster-22178.106716 GO:0016042 GO:0009395 GO:0004629
Cluster-22178.106725 GO:0006355 GO:0007275 GO:0043565 GO:0005201
GO:0005581 GO:0005578
Cluster-22178.106726 GO:0007275 GO:0005201 GO:0005578 GO:0005581
Cluster-22178.106728 GO:0007275 GO:0005201 GO:0005581 GO:0005578
Cluster-22178.106729 GO:0007275 GO:0006355 GO:0005201 GO:0043565
GO:0005581 GO:0005578
Cluster-22178.106733 GO:0015992 GO:0006123 GO:0004129 GO:0045277
Cluster-22178.106734 GO:0006123 GO:0006950 GO:0015992 GO:0004129
GO:0016021 GO:0045277
Cluster-22178.106737 GO:0015992 GO:0006950 GO:0006123 GO:0004129
GO:0045277 GO:0016021
Cluster-22178.106741 GO:0006123 GO:0015992 GO:0004129 GO:0045277
Cluster-22178.106742 GO:0006915 GO:0006974
Cluster-22178.106748 GO:0046983

Cluster-22178.106753 GO:0015979 GO:0006468 GO:0008168 GO:0005524 GO:0004672 GO:0009522 GO:0016020
Cluster-22178.106755 GO:0019497 GO:0006771 GO:0046872 GO:0003993 GO:0016788 GO:0005515
Cluster-22178.106764 GO:0006506 GO:0048193 GO:0051382 GO:0005515 GO:0016746 GO:0016020 GO:0000776 GO:0005789 GO:0016021
Cluster-22178.106765 GO:0051382 GO:0048193 GO:0006506 GO:0016746 GO:0005515 GO:0016021 GO:0005789 GO:0000776 GO:0016020
Cluster-22178.106766 GO:0008270
Cluster-22178.10677 GO:0046854
Cluster-22178.106771 GO:0006364 GO:0055114 GO:0016705 GO:0020037 GO:0008168 GO:0005096 GO:0005506
Cluster-22178.106772 GO:0006355 GO:0003677 GO:0003700 GO:0000976 GO:0005634 GO:0005667
Cluster-22178.106777 GO:0006811 GO:0055085 GO:0005216 GO:0016020
Cluster-22178.106779 GO:0042802 GO:0005515
Cluster-22178.10678 GO:0046854
Cluster-22178.106788 GO:0015074 GO:0016567 GO:0004842 GO:0016020
Cluster-22178.106798 GO:0046983
Cluster-22178.106801 GO:0046983
Cluster-22178.106805 GO:0006468 GO:0005524 GO:0004672 GO:0005515

Cluster-22178.106812 GO:0006364 GO:0006955 GO:0030153 GO:0055114 GO:0016705 GO:0020037 GO:0015643 GO:0005506 GO:0019814 GO:0030688
Cluster-22178.106818 GO:0005515
Cluster-22178.10682 GO:0008152 GO:0016788
Cluster-22178.106822 GO:0008152 GO:0016787
Cluster-22178.106823 GO:0005975 GO:0004553
Cluster-22178.106826 GO:0006260 GO:0003887 GO:0003677 GO:0042575
Cluster-22178.10683 GO:0008152 GO:0016788
Cluster-22178.106832 GO:0042254 GO:0006412 GO:0003735 GO:0005840 GO:0005622

Cluster-22178.106840 GO:0051258 GO:0051382 GO:0000917 GO:0030168
GO:0006914 GO:0043093 GO:0007165 GO:0006457 GO:0042803
GO:0000774 GO:0005102 GO:0030674 GO:0051087 GO:0005737
GO:0000776 GO:0019028 GO:0005577
Cluster-22178.106842 GO:0000917 GO:0051382 GO:0051258 GO:0043093
GO:0006457 GO:0007165 GO:0006914 GO:0030168 GO:0042803
GO:0030674 GO:0051087 GO:0000774 GO:0005102 GO:0019028
GO:0000776 GO:0005737 GO:0005577
Cluster-22178.106843 GO:0007165 GO:0006457 GO:0043093 GO:0030168
GO:0006914 GO:0000917 GO:0051258 GO:0051382 GO:0051087
GO:0030674 GO:0005102 GO:0000774 GO:0042803 GO:0005577
GO:0000776 GO:0019028 GO:0005737
Cluster-22178.106844 GO:0006457 GO:0007165 GO:0019069 GO:0006914
GO:0051258 GO:0030168 GO:0051382 GO:0042803 GO:0051087
GO:0030674 GO:0005102 GO:0000774 GO:0000776 GO:0005577
Cluster-22178.106845 GO:0000917 GO:0051382 GO:0051258 GO:0007165
GO:0006457 GO:0043093 GO:0030168 GO:0006914 GO:0042803
GO:0051087 GO:0030674 GO:0005102 GO:0000774 GO:0000776
GO:0019028 GO:0005737 GO:0005577
Cluster-22178.106846 GO:0030168 GO:0006914 GO:0043093 GO:0007165
GO:0006457 GO:0051382 GO:0051258 GO:0000917 GO:0000774
GO:0005102 GO:0030674 GO:0051087 GO:0042803 GO:0005577
GO:0005737 GO:0000776 GO:0019028
Cluster-22178.106847 GO:0005515
Cluster-22178.10685 GO:0006508 GO:0004252 GO:0005515
Cluster-22178.106850 GO:0006355 GO:0003700 GO:0042025 GO:0005667
Cluster-22178.106856 GO:0006355 GO:0043401
Cluster-22178.106860 GO:0006281 GO:0006051 GO:0015986 GO:0006040
GO:0015992 GO:0000723 GO:0047465 GO:0003678 GO:0015078
GO:0000276 GO:0005657
Cluster-22178.106861 GO:0000723 GO:0015992 GO:0006040 GO:0015986
GO:0006051 GO:0006281 GO:0015078 GO:0003678 GO:0047465
GO:0000276 GO:0005657

Cluster-22178.106862 GO:0006040 GO:0000723 GO:0015992 GO:0015986 GO:0006281 GO:0006051 GO:0003678 GO:0015078 GO:0047465 GO:0000276 GO:0005657
Cluster-22178.106867 GO:0008270
Cluster-22178.106868 GO:0008270
Cluster-22178.106869 GO:0032780 GO:0055114 GO:0046677 GO:0050485 GO:0016779 GO:0008013 GO:0042030 GO:0005739 GO:0005634 GO:0016342
Cluster-22178.106870 GO:0032780 GO:0042030 GO:0005634 GO:0005739
Cluster-22178.106871 GO:0046677 GO:0055114 GO:0032780 GO:0008013 GO:0042030 GO:0016779 GO:0050485 GO:0016342 GO:0005634 GO:0005739
Cluster-22178.106872 GO:0032780 GO:0042030 GO:0005739 GO:0005634
Cluster-22178.106874 GO:0055114 GO:0046677 GO:0032780 GO:0008013 GO:0042030 GO:0050485 GO:0016779 GO:0016342 GO:0005634 GO:0005739
Cluster-22178.106875 GO:0046677 GO:0032780 GO:0042030 GO:0008013 GO:0008270 GO:0016779 GO:0016342 GO:0005739 GO:0005634
Cluster-22178.106877 GO:0032780 GO:0046677 GO:0016779 GO:0008013 GO:0042030 GO:0008270 GO:0005634 GO:0005739 GO:0016342
Cluster-22178.106878 GO:0046677 GO:0032780 GO:0008270 GO:0008013 GO:0042030 GO:0016779 GO:0016342 GO:0005739
Cluster-22178.10688 GO:0005515
Cluster-22178.106886 GO:0006886 GO:0019058 GO:0006836 GO:0006812 GO:0005328 GO:0008146 GO:0016020 GO:0016021
Cluster-22178.106900 GO:0006355 GO:0003677
Cluster-22178.106903 GO:0005515
Cluster-22178.106904 GO:0005515
Cluster-22178.106908 GO:0005515
Cluster-22178.106909 GO:0008207 GO:0008209 GO:0008210 GO:0008152 GO:0006694 GO:0055114 GO:0016616 GO:0003854 GO:0050662 GO:0003824 GO:0016491
Cluster-22178.106913 GO:0051262 GO:0044212

Cluster-22178.106914 GO:0006260 GO:0002949 GO:0004003 GO:0003677 GO:0005524 GO:0016887 GO:0003924 GO:0005525 GO:0016020 GO:0005657
Cluster-22178.106922 GO:0006508 GO:0008270 GO:0004222 GO:0031012
Cluster-22178.106927 GO:0007165 GO:0005509 GO:0005578
Cluster-22178.106928 GO:0008233
Cluster-22178.106932 GO:0005515
Cluster-22178.106941 GO:0006811 GO:0006810 GO:0005230 GO:0016020
Cluster-22178.106959 GO:0009235
Cluster-22178.106961 GO:0007275 GO:0006511 GO:0005634
Cluster-22178.106962 GO:0006508 GO:0016032 GO:0004197
Cluster-22178.106969 GO:0015992 GO:0015991 GO:0015078 GO:0033179
Cluster-22178.106972 GO:0051726 GO:0005515 GO:0008270 GO:0016020 GO:0005634
Cluster-22178.106973 GO:0006950 GO:0051726 GO:0008270 GO:0005515 GO:0005634 GO:0016020 GO:0016021
Cluster-22178.10698 GO:0006891 GO:0006810 GO:0015986 GO:0016043 GO:0046933 GO:0003779 GO:0016021 GO:0045259 GO:0017119
Cluster-22178.106982 GO:0046872
Cluster-22178.106984 GO:0046872
Cluster-22178.106985 GO:0004190 GO:0005515 GO:0016021
Cluster-22178.106986 GO:0006468 GO:0009069 GO:0016310 GO:0006850 GO:0004674 GO:0005524 GO:0004672 GO:0016773 GO:0016020 GO:0005743
Cluster-22178.106992 GO:0005515 GO:0005509
Cluster-22178.106994 GO:0005515 GO:0005509
Cluster-22178.106997 GO:0007172 GO:0007165 GO:0006468 GO:0004871 GO:0004713 GO:0004672 GO:0005524 GO:0005925
Cluster-22178.107002 GO:0031175 GO:0030507 GO:0005516 GO:0008091
Cluster-22178.107004 GO:0008270 GO:0003676
Cluster-22178.107009 GO:0003774 GO:0005524 GO:0016459
Cluster-22178.107012 GO:0006355 GO:0043565 GO:0003700 GO:0005667 GO:0019867

Cluster-22178.107013 GO:0007154 GO:0006952 GO:0005515 GO:0016020
Cluster-22178.107028 GO:0006281 GO:0015986 GO:0015992 GO:0000723
GO:0003678 GO:0015078 GO:0000276 GO:0005657
Cluster-22178.107032 GO:0006468 GO:0004672
Cluster-22178.107033 GO:0006468 GO:0004672
Cluster-22178.107039 GO:0009190 GO:0035556 GO:0008270 GO:0016849

Cluster-22178.107043 GO:0043401 GO:0006355 GO:0003700 GO:0043565
GO:0008270 GO:0031423 GO:0019012 GO:0005667 GO:0005634
Cluster-22178.107045 GO:0006298 GO:0030983 GO:0005524 GO:0008537
Cluster-22178.107053 GO:0040007 GO:0005737
Cluster-22178.107076 GO:0006355 GO:0006525 GO:0003700 GO:0005667
Cluster-22178.107082 GO:0006396 GO:0007165 GO:0006144 GO:0004114
GO:0005622
Cluster-22178.107092 GO:0034508 GO:0005509 GO:0005515 GO:0000776
Cluster-22178.107095 GO:0006396 GO:0006948 GO:0007165 GO:0043401
GO:0045859 GO:0006355 GO:0005496 GO:0004860 GO:0005515
GO:0030284 GO:0003677 GO:0000176 GO:0005634 GO:0005576
Cluster-22178.107101 GO:0008152 GO:0003824 GO:0020037 GO:0008134
GO:0005634 GO:0005667
Cluster-22178.107102 GO:0005515
Cluster-22178.107103 GO:0005515
Cluster-22178.107105 GO:0005515
Cluster-22178.107113 GO:0005861
Cluster-22178.107116 GO:0006771 GO:0002532 GO:0007165 GO:0019497
GO:0005515 GO:0046872 GO:0004896 GO:0003993 GO:0016021
Cluster-22178.107117 GO:0006891 GO:0017119 GO:0016021
Cluster-22178.107118 GO:0016021 GO:0012511
Cluster-22178.107120 GO:0007275 GO:0006355 GO:0005201 GO:0043565
GO:0005578 GO:0005581
Cluster-22178.107121 GO:0007275 GO:0006355 GO:0005201 GO:0043565
GO:0005581 GO:0005578
Cluster-22178.107122 GO:0007275 GO:0006355 GO:0005201 GO:0043565

GO:0005581 GO:0005578
Cluster-22178.107144 GO:0005515 GO:0015002 GO:0016020 GO:0016021
Cluster-22178.107155 GO:0006206 GO:0006139 GO:0003924 GO:0016887
GO:0005524 GO:0004127 GO:0005525 GO:0005515 GO:0016020
GO:0005911
Cluster-22178.107164 GO:0016021
Cluster-22178.107165 GO:0071805 GO:0006813 GO:0015079 GO:0005887
Cluster-22178.107171 GO:0015074 GO:0003677 GO:0005515
Cluster-22178.107188 GO:0006355 GO:0006260 GO:0003700 GO:0005667
GO:0005634
Cluster-22178.107201 GO:0006508 GO:0007034 GO:0030001 GO:0004252

Cluster-22178.107204 GO:0004222 GO:0008233 GO:0031012
Cluster-22178.107222 GO:0032266 GO:0043130 GO:0005515
Cluster-22178.107226 GO:0009271 GO:0006355 GO:0016021
Cluster-22178.107227 GO:0019079 GO:0006525 GO:0006144 GO:0003968
GO:0009015 GO:0016021 GO:0031379
Cluster-22178.107246 GO:0006144 GO:0007165 GO:0004114 GO:0008233
GO:0005515 GO:0004222 GO:0031012
Cluster-22178.107252 GO:0006355 GO:0003700 GO:0003677 GO:0005667
Cluster-22178.107255 GO:0007165 GO:0006508 GO:0008234 GO:0005179
GO:0005576
Cluster-22178.107258 GO:0030474 GO:0071988 GO:0005515
Cluster-22178.107269 GO:0015991 GO:0015992 GO:0046034 GO:0005524
GO:0016820 GO:0033178
Cluster-22178.107270 GO:0006260 GO:0000808 GO:0005634
Cluster-22178.107281 GO:0000398 GO:0007275 GO:0008270 GO:0004222
GO:0005634 GO:0042600 GO:0005681
Cluster-22178.107295 GO:0006412 GO:0042254 GO:0003735 GO:0005840
GO:0005622
Cluster-22178.107296 GO:0006811 GO:0007186 GO:0055085 GO:0004930
GO:0005216 GO:0016021 GO:0016020
Cluster-22178.107297 GO:0015012 GO:0015684 GO:0009405 GO:0006810

GO:0007264 GO:0006024 GO:0005525 GO:0015093 GO:0005524
GO:0019870 GO:0016021 GO:0005576
Cluster-22178.107299 GO:0006810 GO:0009405 GO:0015684 GO:0006024
GO:0007264 GO:0015012 GO:0019870 GO:0005525 GO:0005524
GO:0015093 GO:0005576 GO:0016021
Cluster-22178.10731 GO:0015074 GO:0003676 GO:0008270
Cluster-22178.107310 GO:0005515
Cluster-22178.107311 GO:0008152 GO:0016746 GO:0016021
Cluster-22178.107338 GO:0032797
Cluster-22178.107339 GO:0055085 GO:0006811 GO:0006355 GO:0005515
GO:0005198 GO:0005216 GO:0005887 GO:0016020 GO:0019028
Cluster-22178.107340 GO:0006811 GO:0055085 GO:0006355 GO:0005216
GO:0005515 GO:0005198 GO:0005887 GO:0019028 GO:0016020
Cluster-22178.107341 GO:0046983
Cluster-22178.107346 GO:0016567 GO:0008270 GO:0004842
Cluster-22178.107350 GO:0006779 GO:0046856 GO:0055114 GO:0034597
GO:0004867
Cluster-22178.107353 GO:0016021
Cluster-22178.107354 GO:0000166 GO:0046872
Cluster-22178.107355 GO:0016021
Cluster-22178.107367 GO:0006355 GO:0003677 GO:0003700 GO:0005667
Cluster-22178.107369 GO:0006355 GO:0003677 GO:0003700 GO:0005667
Cluster-22178.107372 GO:0009103 GO:0007283 GO:0016020
Cluster-22178.107391 GO:0019354 GO:0006779 GO:0009878 GO:0055114
GO:0043115 GO:0046872
Cluster-22178.107404 GO:0040007 GO:0005737
Cluster-22178.107406 GO:0040007 GO:0005737
Cluster-22178.107408 GO:0007155 GO:0005515 GO:0016021
Cluster-22178.107432 GO:0007169 GO:0006468 GO:0004714 GO:0004672
GO:0005524 GO:0016020 GO:0000228
Cluster-22178.107436 GO:0006308 GO:0006260 GO:0008855 GO:0009318
Cluster-22178.107452 GO:0006310 GO:0008152 GO:0055114 GO:0003677
GO:0016491 GO:0005634

Cluster-22178.107456 GO:0006310 GO:0006378 GO:0008152 GO:0055114
GO:0003677 GO:0003729 GO:0016491 GO:0005634 GO:0005849
Cluster-22178.107474 GO:0016032 GO:0030001 GO:0051258 GO:0015986
GO:0007165 GO:0015992 GO:0030168 GO:0055085 GO:0005198
GO:0015078 GO:0030674 GO:0046873 GO:0003677 GO:0005102
GO:0005634 GO:0045263 GO:0016020 GO:0005882 GO:0005577
Cluster-22178.107475 GO:0005515
Cluster-22178.107477 GO:0006869 GO:0008289 GO:0005515 GO:0005576
Cluster-22178.10748 GO:0016021
Cluster-22178.10749 GO:0016021
Cluster-22178.107493 GO:0006771 GO:0019497 GO:0003993 GO:0005515
GO:0046872 GO:0016020
Cluster-22178.107495 GO:0007155 GO:0005540 GO:0005515 GO:0005509

Cluster-22178.10750 GO:0005515 GO:0004222 GO:0016021
Cluster-22178.107503 GO:0006506 GO:0051382 GO:0048193 GO:0016746
GO:0005515 GO:0000776 GO:0016020 GO:0016021 GO:0005789
Cluster-22178.107507 GO:0005515
Cluster-22178.107508 GO:0005515
Cluster-22178.107509 GO:0005515
Cluster-22178.107514 GO:0006355 GO:0003700 GO:0005667 GO:0042025
Cluster-22178.107519 GO:0009069 GO:0006468 GO:0016310 GO:0000287
GO:0005524 GO:0004674
Cluster-22178.107529 GO:0005515
Cluster-22178.10753 GO:0006144 GO:0007165 GO:0004114
Cluster-22178.107535 GO:0006357 GO:0001104 GO:0016592
Cluster-22178.107536 GO:0006357 GO:0001104 GO:0016592
Cluster-22178.107537 GO:0006357 GO:0001104 GO:0016592
Cluster-22178.107539 GO:0006357 GO:0001104 GO:0016592 GO:0005634
Cluster-22178.10754 GO:0006144 GO:0007165 GO:0004114
Cluster-22178.107547 GO:0055085 GO:0022857 GO:0016021
Cluster-22178.107550 GO:0007186 GO:0004930 GO:0005515 GO:0016020
Cluster-22178.107560 GO:0015979 GO:0016020 GO:0009523 GO:0009539

Cluster-22178.107565 GO:0005515 GO:0016020
Cluster-22178.107595 GO:0035556
Cluster-22178.107602 GO:0008152 GO:0008484 GO:0003824
Cluster-22178.107604 GO:0007275 GO:0006355 GO:0005201 GO:0043565 GO:0005578 GO:0005581
Cluster-22178.107605 GO:0006355 GO:0007275 GO:0043565 GO:0005201 GO:0005581 GO:0005578
Cluster-22178.107618 GO:0055114 GO:0016705 GO:0020037 GO:0005506

Cluster-22178.107635 GO:0006308 GO:0006412 GO:0006355 GO:0006396 GO:0048193 GO:0042254 GO:0003700 GO:0043565 GO:0008270 GO:0008855 GO:0003735 GO:0005840 GO:0009318 GO:0000176 GO:0005622 GO:0016020 GO:0005667
Cluster-22178.107649 GO:0001558 GO:0005520 GO:0005576 GO:0016942
Cluster-22178.107652 GO:0015858 GO:0006810 GO:0005515 GO:0005337 GO:0016021
Cluster-22178.107655 GO:0008146
Cluster-22178.107658 GO:0035023 GO:0043087 GO:0005089
Cluster-22178.107659 GO:0043087 GO:0035023 GO:0005089
Cluster-22178.107661 GO:0043087 GO:0035023 GO:0005089
Cluster-22178.107663 GO:0035023 GO:0043087 GO:0005089
Cluster-22178.107669 GO:0052331 GO:0006812 GO:0046931 GO:0006810 GO:0015267 GO:0046930
Cluster-22178.107677 GO:0007165
Cluster-22178.107678 GO:0008146 GO:0046872
Cluster-22178.107682 GO:0055085 GO:0006810 GO:0042626 GO:0016887 GO:0005524 GO:0016021
Cluster-22178.107702 GO:0016567 GO:0004842 GO:0005680
Cluster-22178.107715 GO:0051260
Cluster-22178.107721 GO:0046872 GO:0016787
Cluster-22178.107724 GO:0005515
Cluster-22178.107727 GO:0006281 GO:0004518
Cluster-22178.107733 GO:0007186 GO:0004930 GO:0016020 GO:0016021

Cluster-22178.107737 GO:0008616 GO:0033739 GO:0016021

Cluster-22178.107740 GO:0008152 GO:0042998 GO:0007339 GO:0045859 GO:0007155 GO:0019887 GO:0005515 GO:0016787 GO:0071253 GO:0016021 GO:0005956 GO:0005576 GO:0005802

Cluster-22178.107745 GO:0046797

Cluster-22178.107746 GO:0007165 GO:0006355 GO:0003700 GO:0004871 GO:0005634 GO:0005667

Cluster-22178.107755 GO:0006270

Cluster-22178.107758 GO:0008289

Cluster-22178.10776 GO:0006118 GO:0009055 GO:0016491 GO:0016020

Cluster-22178.107762 GO:0055114 GO:0046872 GO:0050660 GO:0016614

Cluster-22178.107763 GO:0055114 GO:0046872 GO:0050660 GO:0016614

Cluster-22178.107764 GO:0006816 GO:0006811 GO:0005244 GO:0005262 GO:0005886 GO:0016021

Cluster-22178.107766 GO:0006468 GO:0005524 GO:0004672

Cluster-22178.107771 GO:0006355 GO:0003677

Cluster-22178.107787 GO:0008360 GO:0009405 GO:0006508 GO:0004197 GO:0016020

Cluster-22178.107788 GO:0008360 GO:0016020

Cluster-22178.107789 GO:0006811 GO:0055085 GO:0003677 GO:0015075 GO:0016020

Cluster-22178.107801 GO:0006754 GO:0016887 GO:0005524 GO:0005515 GO:0000275

Cluster-22178.107821 GO:0046656 GO:0006761 GO:0055114 GO:0042493 GO:0004146

Cluster-22178.107824 GO:0006508 GO:0004252

Cluster-22178.107830 GO:0006355 GO:0005515 GO:0003677 GO:0008270

Cluster-22178.107832 GO:0006355 GO:0055114 GO:0006352 GO:0005509 GO:0003677 GO:0016491 GO:0003700 GO:0016987 GO:0005667

Cluster-22178.107834 GO:0016579 GO:0006465 GO:0008233 GO:0036459

GO:0016021 GO:0005787
Cluster-22178.107835 GO:0006465 GO:0008233 GO:0005787 GO:0016021
Cluster-22178.107837 GO:0016579 GO:0006465 GO:0008233 GO:0005515
GO:0036459 GO:0016021 GO:0005787
Cluster-22178.107842 GO:0019028
Cluster-22178.107843 GO:0006468 GO:0016773 GO:0005524 GO:0004672
GO:0016020
Cluster-22178.107847 GO:0006811 GO:0006878 GO:0007268 GO:0007165
GO:0005507 GO:0005234 GO:0004970 GO:0009279 GO:0016020
Cluster-22178.107849 GO:0006878 GO:0007268 GO:0007165 GO:0006811
GO:0005507 GO:0005234 GO:0004970 GO:0016020 GO:0009279
Cluster-22178.107868 GO:0006355 GO:0003677
Cluster-22178.107873 GO:0005515
Cluster-22178.107874 GO:0009847 GO:0055114 GO:0020037 GO:0016705
GO:0005506 GO:0016021
Cluster-22178.107875 GO:0009847 GO:0055114 GO:0016705 GO:0020037
GO:0005506 GO:0016021
Cluster-22178.107879 GO:0005515 GO:0008440 GO:0016020 GO:0005737
Cluster-22178.107881 GO:0005515 GO:0005509
Cluster-22178.107885 GO:0006952 GO:0007018 GO:0007017 GO:0000077
GO:0005515 GO:0003777 GO:0008017 GO:0005524 GO:0005874
GO:0045298 GO:0016021
Cluster-22178.107886 GO:0000077 GO:0007017 GO:0006952 GO:0007018
GO:0005524 GO:0003777 GO:0008017 GO:0005515 GO:0005874
GO:0016021 GO:0045298
Cluster-22178.107887 GO:0007017 GO:0007018 GO:0000077 GO:0005515
GO:0005524 GO:0008017 GO:0003777 GO:0045298 GO:0005874
Cluster-22178.10789 GO:0006508 GO:0007186 GO:0005515 GO:0004252
GO:0004930 GO:0016020 GO:0016021
Cluster-22178.107892 GO:0007275 GO:0005578
Cluster-22178.107902 GO:0016020
Cluster-22178.107904 GO:0006952 GO:0004869 GO:0005576
Cluster-22178.107913 GO:0007186 GO:0004930 GO:0016020

Cluster-22178.107923 GO:0002218 GO:0032481 GO:0008168
Cluster-22178.107925 GO:0006508 GO:0004252
Cluster-22178.107941 GO:0042981 GO:0005515
Cluster-22178.107942 GO:0006768 GO:0009102 GO:0042981 GO:0042410 GO:0005524
Cluster-22178.107945 GO:0003677 GO:0008270
Cluster-22178.107946 GO:0015992 GO:0055085 GO:0090263 GO:0015986 GO:0016567 GO:0006810 GO:0015078 GO:0000276 GO:0016021 GO:0005789
Cluster-22178.107947 GO:0055085 GO:0090263 GO:0015992 GO:0016567 GO:0006810 GO:0015986 GO:0015078 GO:0016021 GO:0005789 GO:0000276
Cluster-22178.107950 GO:0005515
Cluster-22178.107955 GO:0005515 GO:0005509 GO:0019031
Cluster-22178.107960 GO:0016846 GO:0005509 GO:0005515 GO:0005198

Cluster-22178.107961 GO:0005198 GO:0005515
Cluster-22178.107962 GO:0005515 GO:0005198 GO:0009986 GO:0016020
Cluster-22178.107966 GO:0016579 GO:0006508 GO:0036459 GO:0032183 GO:0070140 GO:0004843 GO:0005515 GO:0005509
Cluster-22178.10797 GO:0016032 GO:0019082 GO:0019239
Cluster-22178.107987 GO:0005515 GO:0046872
Cluster-22178.107996 GO:0006914 GO:0006415 GO:0006449 GO:0051607 GO:0003747 GO:0018444 GO:0005840
Cluster-22178.107997 GO:0051607 GO:0006449 GO:0006914 GO:0006415 GO:0003747 GO:0005840 GO:0018444
Cluster-22178.107998 GO:0006449 GO:0051607 GO:0006914 GO:0006415 GO:0003747 GO:0005840 GO:0018444
Cluster-22178.108008 GO:0007186 GO:0004930 GO:0016021
Cluster-22178.108019 GO:0006886 GO:0006801 GO:0055114 GO:0006888 GO:0004784 GO:0008270 GO:0046872 GO:0030127
Cluster-22178.108028 GO:0015694 GO:0015097 GO:0005515 GO:0016020
Cluster-22178.108049 GO:0008146 GO:0003676 GO:0008270 GO:0016021

Cluster-22178.108050 GO:0007264 GO:0043087 GO:0005085
Cluster-22178.108067 GO:0006879 GO:0005515 GO:0005576
Cluster-22178.108074 GO:0006468 GO:0004672 GO:0008270 GO:0005524
GO:0016021
Cluster-22178.108078 GO:0006810 GO:0008146 GO:0005215 GO:0016020
Cluster-22178.108080 GO:0008146
Cluster-22178.108083 GO:0042919 GO:0006810 GO:0042925 GO:0005515
GO:0016021
Cluster-22178.108088 GO:0006508 GO:0016740 GO:0008234 GO:0048786
Cluster-22178.108092 GO:0007268 GO:0009405 GO:0007165 GO:0046872
GO:0030550 GO:0005576
Cluster-22178.108094 GO:0007165 GO:0042981 GO:0005515
Cluster-22178.10810 GO:0006355 GO:0005515 GO:0003677 GO:0005789
Cluster-22178.108104 GO:0042254 GO:0006412 GO:0046034 GO:0006144
GO:0005515 GO:0003735 GO:0004017 GO:0046872 GO:0005840
GO:0005622
Cluster-22178.108108 GO:0015986 GO:0015992 GO:0016070 GO:0030036
GO:0003779 GO:0015078 GO:0030054 GO:0045263
Cluster-22178.108109 GO:0030036 GO:0016070 GO:0003779 GO:0030054
Cluster-22178.108119 GO:0005515 GO:0005856
Cluster-22178.108123 GO:0006366 GO:0006457 GO:0003677 GO:0005737
GO:0005665
Cluster-22178.108134 GO:0005515
Cluster-22178.108135 GO:0007339 GO:0005515 GO:0005576
Cluster-22178.108137 GO:0007339 GO:0005515 GO:0005576
Cluster-22178.108148 GO:0006351 GO:0006355 GO:0015991 GO:0006308
GO:0007165 GO:0006810 GO:0071973 GO:0008053 GO:0006886
GO:0043039 GO:0006269 GO:0007155 GO:0006606 GO:0048193
GO:0015992 GO:0006914 GO:0003896 GO:0004871 GO:0003700
GO:0016876 GO:0008855 GO:0005198 GO:0005515 GO:0015078
GO:0003924 GO:0043565 GO:0005524 GO:0009318 GO:0031083
GO:0033179 GO:0019028 GO:0019031 GO:0016021 GO:0005801
GO:0005886 GO:0009288 GO:0005737 GO:0030133 GO:0005741

GO:0016020 GO:0005730 GO:0005667 GO:0005657
Cluster-22178.108149 GO:0006771 GO:0019497 GO:0046872 GO:0005515
GO:0003993
Cluster-22178.108150 GO:0015074 GO:0016042 GO:0007586 GO:0006355
GO:0016567 GO:0008047 GO:0043565 GO:0008270 GO:0003700
GO:0004842 GO:0003723 GO:0005667 GO:0005576
Cluster-22178.108152 GO:0016567 GO:0042254 GO:0007586 GO:0016042
GO:0006412 GO:0006355 GO:0015074 GO:0003735 GO:0004842
GO:0008270 GO:0003723 GO:0003700 GO:0043565 GO:0008047
GO:0005667 GO:0005576 GO:0005840
Cluster-22178.108161 GO:0051056 GO:0005096
Cluster-22178.108167 GO:0007050
Cluster-22178.108168 GO:0005515
Cluster-22178.108176 GO:0005515
Cluster-22178.108183 GO:0051252 GO:0007165 GO:0006396 GO:0005515
GO:0004525 GO:0003723
Cluster-22178.108185 GO:0006468 GO:0005524 GO:0004672
Cluster-22178.108189 GO:0006508 GO:0007165 GO:0005515 GO:0004197

Cluster-22178.10819 GO:0042254 GO:0003735 GO:0005840
Cluster-22178.108192 GO:0030436 GO:0042601
Cluster-22178.108196 GO:0007059 GO:0048870 GO:0051301 GO:0006260
GO:0006914 GO:0031110 GO:0007155 GO:0007067 GO:0000090
GO:0019901 GO:0005198 GO:0008017 GO:0019905 GO:0003774
GO:0019031 GO:0045298 GO:0031514 GO:0019028 GO:0005876
GO:0000940 GO:0005882 GO:0016459
Cluster-22178.108218 GO:0007156 GO:0005509 GO:0005515 GO:0016020
Cluster-22178.108222 GO:0055114 GO:0005506 GO:0020037 GO:0016705

Cluster-22178.108226 GO:0007155 GO:0005540
Cluster-22178.108227 GO:0007155 GO:0005540
Cluster-22178.108228 GO:0007155 GO:0005540
Cluster-22178.108235 GO:0016020

Cluster-22178.108238 GO:0006323
Cluster-22178.108244 GO:0019042 GO:0033644 GO:0019028 GO:0031224
Cluster-22178.108245 GO:0019042 GO:0031224 GO:0033644
Cluster-22178.108247 GO:0055114 GO:0016730
Cluster-22178.108251 GO:0055114 GO:0016730
Cluster-22178.108259 GO:0007165 GO:0005044 GO:0019031 GO:0016020
Cluster-22178.108266 GO:0046856 GO:0008152 GO:0006508 GO:0034597
GO:0004181 GO:0008270 GO:0005515 GO:0016788 GO:0003723
Cluster-22178.108271 GO:0018342 GO:0006355 GO:0006813 GO:0006457
GO:0009271 GO:0015269 GO:0005242 GO:0008318 GO:0051082
GO:0005516 GO:0016286 GO:0016272 GO:0016021 GO:0071203
GO:0008076
Cluster-22178.108274 GO:0006811 GO:0016560 GO:0018342 GO:0006355
GO:0050790 GO:0006813 GO:0055085 GO:0009271 GO:0006457
GO:0051082 GO:0030234 GO:0015269 GO:0005242 GO:0005216
GO:0005516 GO:0008318 GO:0016286 GO:0016021 GO:0071203
GO:0016272 GO:0005777 GO:0016020 GO:0008076
Cluster-22178.108276 GO:0006811 GO:0055085 GO:0006813 GO:0015269
GO:0005516 GO:0005216 GO:0005242 GO:0016286 GO:0016021
GO:0008076 GO:0016020
Cluster-22178.108280 GO:0006810 GO:0009405 GO:0006812 GO:0071705
GO:0006836 GO:0005328 GO:0019871 GO:0005576 GO:0016021
Cluster-22178.10830 GO:0007186 GO:0004930 GO:0016798 GO:0016021
Cluster-22178.108303 GO:0042981
Cluster-22178.108315 GO:0055114 GO:0006807 GO:0042128 GO:0008942

Cluster-22178.108328 GO:0006810 GO:0003333 GO:0006865 GO:0055085
GO:0015171 GO:0016020
Cluster-22178.108332 GO:0005515
Cluster-22178.108334 GO:0006888 GO:0006886 GO:0008270 GO:0005515
GO:0030127
Cluster-22178.108346 GO:0006813 GO:0005267
Cluster-22178.108359 GO:0006525 GO:0055114 GO:0006560 GO:0018401

GO:0016702 GO:0004656 GO:0005783
Cluster-22178.108375 GO:0006810 GO:0005215 GO:0003824 GO:0005515
GO:0016021 GO:0005737
Cluster-22178.108377 GO:0006810 GO:0005215 GO:0016020
Cluster-22178.108380 GO:0006810 GO:0005215 GO:0016020
Cluster-22178.108391 GO:0055114 GO:0016705 GO:0020037 GO:0003677
GO:0005506
Cluster-22178.108392 GO:0055114 GO:0003677 GO:0020037 GO:0016705
GO:0005506
Cluster-22178.108395 GO:0055114 GO:0020037 GO:0016705 GO:0005506

Cluster-22178.108400 GO:0005515
Cluster-22178.108407 GO:0006260 GO:0043039 GO:0016876 GO:0005524

Cluster-22178.108417 GO:0055114 GO:0006694 GO:0008152 GO:0008210
GO:0008209 GO:0008207 GO:0016491 GO:0003824 GO:0003854
GO:0050662 GO:0016616
Cluster-22178.108437 GO:0055085 GO:0006811 GO:0003676 GO:0003677
GO:0005216 GO:0016020 GO:0005634
Cluster-22178.108439 GO:0007010 GO:0055085 GO:0006811 GO:0005216
GO:0003677 GO:0003779 GO:0003676 GO:0005634 GO:0016020
Cluster-22178.108442 GO:0030206 GO:0006024 GO:0008375 GO:0030158
GO:0016020
Cluster-22178.108446 GO:0006855 GO:0055085 GO:0006810 GO:0015893
GO:0006355 GO:0015297 GO:0003677 GO:0015238 GO:0016020
Cluster-22178.108461 GO:0006811 GO:0007165 GO:0007268 GO:0004970
GO:0016020
Cluster-22178.108465 GO:0005515 GO:0043565 GO:0008270 GO:0005634
Cluster-22178.108474 GO:0006821 GO:0006508 GO:0004252
Cluster-22178.108484 GO:0046677
Cluster-22178.108485 GO:0006886 GO:0009451 GO:0001522 GO:0006396
GO:0006888 GO:0003723 GO:0009982 GO:0008270 GO:0030127
GO:0016021 GO:0005622

Cluster-22178.108489 GO:0007264 GO:0005525 GO:0003924
Cluster-22178.108490 GO:0030246
Cluster-22178.108496 GO:0008017 GO:0045298
Cluster-22178.108509 GO:0000077 GO:0006281 GO:0005525 GO:0005543
GO:0003924 GO:0030896
Cluster-22178.108510 GO:0005543
Cluster-22178.108511 GO:0005543
Cluster-22178.108519 GO:0007275 GO:0006810 GO:0003700 GO:0005667
GO:0005634
Cluster-22178.108528 GO:0003950
Cluster-22178.108542 GO:0042981
Cluster-22178.108544 GO:0006810 GO:0005524
Cluster-22178.108545 GO:0046488 GO:0016307
Cluster-22178.10855 GO:0005509
Cluster-22178.108578 GO:0015631 GO:0045298
Cluster-22178.108579 GO:0015631 GO:0045298
Cluster-22178.108582 GO:0009069 GO:0006468 GO:0016310 GO:0007178
GO:0004675 GO:0005524 GO:0004672 GO:0016020
Cluster-22178.108590 GO:0000398 GO:0034258 GO:0034257 GO:0019901
GO:0005634 GO:0016020
Cluster-22178.108593 GO:0016020
Cluster-22178.108598 GO:0006118 GO:0009055 GO:0009512
Cluster-22178.108610 GO:0015986 GO:0015991 GO:0015992 GO:0015078
GO:0008289 GO:0045263 GO:0033177 GO:0005743 GO:0016021
Cluster-22178.108627 GO:0015696 GO:0051607 GO:0007186 GO:0007165
GO:0004871 GO:0008519 GO:0005834 GO:0016020
Cluster-22178.108640 GO:0016567 GO:0004842 GO:0008270
Cluster-22178.108657 GO:0071705 GO:0055114 GO:0020037 GO:0016705
GO:0005506
Cluster-22178.108662 GO:0030435 GO:0005515 GO:0031160
Cluster-22178.108663 GO:0030435 GO:0005515 GO:0031160
Cluster-22178.108664 GO:0030036 GO:0016043 GO:0017048 GO:0003779

Cluster-22178.108672 GO:0019882 GO:0006955 GO:0042613 GO:0016020
Cluster-22178.108702 GO:0006869 GO:0006629 GO:0008047 GO:0042627
Cluster-22178.108703 GO:0009878 GO:0046872 GO:0005515
Cluster-22178.108705 GO:0007165 GO:0008283 GO:0006771 GO:0040007
GO:0019497 GO:0016788 GO:0008083 GO:0005515 GO:0003993
GO:0046872
Cluster-22178.108717 GO:0006855 GO:0055085 GO:0050909 GO:0006810
GO:0015893 GO:0007186 GO:0015297 GO:0004930 GO:0015238
GO:0016021 GO:0016020
Cluster-22178.108740 GO:0005515
Cluster-22178.108753 GO:0005201 GO:0005581 GO:0005578
Cluster-22178.108757 GO:0005515
Cluster-22178.108769 GO:0009236 GO:0006824 GO:0015087 GO:0016020
Cluster-22178.108773 GO:0045765 GO:0005737
Cluster-22178.108774 GO:0045765 GO:0005737
Cluster-22178.108777 GO:0005515 GO:0004064
Cluster-22178.108783 GO:0005515
Cluster-22178.108791 GO:0042254 GO:0007275 GO:0005201 GO:0003735
GO:0005198 GO:0005581 GO:0005840 GO:0005739 GO:0005578
Cluster-22178.108792 GO:0007275 GO:0042254 GO:0005198 GO:0003735
GO:0005201 GO:0005578 GO:0005739 GO:0005581 GO:0005840
Cluster-22178.108794 GO:0006812 GO:0006836 GO:0005328 GO:0016021
Cluster-22178.10880 GO:0005515 GO:0042470
Cluster-22178.108818 GO:0005515
Cluster-22178.108819 GO:0055114 GO:0005975 GO:0004553 GO:0016616
GO:0016491
Cluster-22178.10882 GO:0005515 GO:0042470
Cluster-22178.108823 GO:0009069 GO:0006468 GO:0016310 GO:0007178
GO:0004675 GO:0004672 GO:0005524 GO:0016020
Cluster-22178.108825 GO:0006468 GO:0009069 GO:0007178 GO:0016310
GO:0004675 GO:0004672 GO:0005524 GO:0016020
Cluster-22178.108834 GO:0006397 GO:0006355 GO:0008270 GO:0005524
GO:0003677 GO:0016818 GO:0003723 GO:0005737 GO:0005634

Cluster-22178.108835 GO:0006355 GO:0006206 GO:0006231 GO:0016787
GO:0003677 GO:0016818 GO:0005515 GO:0005524 GO:0008270
GO:0050660 GO:0050797 GO:0003676 GO:0005634
Cluster-22178.108844 GO:0006468 GO:0004672 GO:0005524
Cluster-22178.10885 GO:0019867
Cluster-22178.108869 GO:0042246 GO:0007155 GO:0009607 GO:0016021
Cluster-22178.108880 GO:0006812 GO:0007165 GO:0033198 GO:0098655
GO:0001614 GO:0004931 GO:0005887
Cluster-22178.108881 GO:0098655 GO:0033198 GO:0006812 GO:0007165
GO:0001614 GO:0004931 GO:0005887
Cluster-22178.108886 GO:0005515
Cluster-22178.108900 GO:0009405 GO:0006810 GO:0008200 GO:0042151
Cluster-22178.108902 GO:0009405 GO:0006810 GO:0008200 GO:0042151
Cluster-22178.108903 GO:0006206 GO:0006144 GO:0006351 GO:0003677
GO:0003899 GO:0005730
Cluster-22178.108904 GO:0009271 GO:0006355
Cluster-22178.108912 GO:0007154 GO:0016021
Cluster-22178.108917 GO:0007218
Cluster-22178.108923 GO:0046870
Cluster-22178.108925 GO:0055114 GO:0046872 GO:0050660 GO:0016614

Cluster-22178.108931 GO:0005515
Cluster-22178.108932 GO:0005515
Cluster-22178.108948 GO:0007186 GO:0004930 GO:0016020 GO:0016021
Cluster-22178.108952 GO:0009117 GO:0046373 GO:0005975 GO:0046556
GO:0005634
Cluster-22178.108955 GO:0006357 GO:0001104 GO:0003779 GO:0016592
Cluster-22178.10896 GO:0007156 GO:0005515 GO:0005509 GO:0016020
Cluster-22178.108960 GO:0000413 GO:0006457 GO:0003755
Cluster-22178.108965 GO:0019082 GO:0016032
Cluster-22178.108973 GO:0015074
Cluster-22178.108981 GO:0005524 GO:0005515
Cluster-22178.108987 GO:0007165 GO:0015948 GO:0051028 GO:0006914

GO:0009405 GO:0010508 GO:0007018 GO:0007017 GO:0048870
GO:0048309 GO:0055114 GO:0008017 GO:0005524 GO:0042803
GO:0008134 GO:0005198 GO:0045502 GO:0003777 GO:0004871
GO:0005882 GO:0016020 GO:0005667 GO:0030286 GO:0031514
GO:0005874 GO:0016021 GO:0045298
Cluster-22178.108994 GO:0007017 GO:0007018 GO:0003777 GO:0030286
GO:0005874 GO:0016021
Cluster-22178.109008 GO:0055085 GO:0006629 GO:0016021
Cluster-22178.109009 GO:0055085 GO:0006355 GO:0030001 GO:0003700
GO:0046873 GO:0003677 GO:0005667 GO:0016020
Cluster-22178.10901 GO:0046872 GO:0005515
Cluster-22178.109013 GO:0019031
Cluster-22178.109020 GO:0001682 GO:0008033 GO:0051252 GO:0004526
GO:0030677
Cluster-22178.109040 GO:0006355 GO:0043401 GO:0008270 GO:0003677

Cluster-22178.109050 GO:0005515
Cluster-22178.109058 GO:0006355 GO:0006352 GO:0004190 GO:0003700
GO:0005667 GO:0005634 GO:0005669 GO:0016021
Cluster-22178.109067 GO:0071705 GO:0055114 GO:0016705 GO:0020037
GO:0005506
Cluster-22178.109071 GO:0006807 GO:0007411 GO:0055114 GO:0007224
GO:0006537 GO:0046872 GO:0003677 GO:0015930
Cluster-22178.109081 GO:0005198 GO:0003779 GO:0005856 GO:0005576
Cluster-22178.109089 GO:0007165 GO:0005515 GO:0005856
Cluster-22178.109090 GO:0007165 GO:0006955 GO:0000902 GO:0008283
GO:0040007 GO:0008083 GO:0016020
Cluster-22178.109091 GO:0008283 GO:0040007 GO:0007165 GO:0006955
GO:0000902 GO:0008083 GO:0016020
Cluster-22178.109104 GO:0035023 GO:0015979 GO:0050821 GO:0043087
GO:0005089 GO:0042301 GO:0009523 GO:0016020
Cluster-22178.109108 GO:0005515
Cluster-22178.10911 GO:0043087 GO:0007264 GO:0035556 GO:0005085

GO:0005509
Cluster-22178.109121 GO:0015031 GO:0009306 GO:0015450 GO:0005515
GO:0009941 GO:0016021
Cluster-22178.109122 GO:0005515
Cluster-22178.109123 GO:0006334 GO:0005515 GO:0005634
Cluster-22178.109124 GO:0005515
Cluster-22178.109143 GO:0001558 GO:0005520 GO:0005576 GO:0016942
Cluster-22178.109154 GO:0005515
Cluster-22178.109155 GO:0032012 GO:0071973 GO:0043087 GO:0005198
GO:0003774 GO:0005086 GO:0009288
Cluster-22178.109156 GO:0043087 GO:0071973 GO:0032012 GO:0005086
GO:0003774 GO:0005198 GO:0009288
Cluster-22178.109157 GO:0043087 GO:0032012 GO:0071973 GO:0003774
GO:0005086 GO:0005198 GO:0009288
Cluster-22178.109158 GO:0032012 GO:0043087 GO:0071973 GO:0003774
GO:0005198 GO:0005086 GO:0009288 GO:0005634
Cluster-22178.10916 GO:0006355 GO:0009607 GO:0005515 GO:0016021
Cluster-22178.109160 GO:0008053 GO:0006357 GO:0001104 GO:0003924
GO:0016592 GO:0005741 GO:0016021
Cluster-22178.109167 GO:0015858 GO:0006810 GO:0005337 GO:0016021
Cluster-22178.109170 GO:0005515
Cluster-22178.10918 GO:0003824 GO:0035091
Cluster-22178.109180 GO:0055085 GO:0016021
Cluster-22178.109192 GO:0003676 GO:0008270
Cluster-22178.109194 GO:0008270 GO:0003676
Cluster-22178.109197 GO:0005198 GO:0019028 GO:0016020
Cluster-22178.109209 GO:0055114 GO:0005507 GO:0016715 GO:0004497

Cluster-22178.109215 GO:0005515 GO:0005509
Cluster-22178.109224 GO:0055114 GO:0016491 GO:0005515 GO:0005856
Cluster-22178.109226 GO:0007049 GO:0048309 GO:0030474 GO:0007094
GO:0006260 GO:0006606 GO:0006914 GO:0051028 GO:0006810
GO:0005215 GO:0005200 GO:0042803 GO:0008134 GO:0005198

GO:0045502 GO:0016021 GO:0005856 GO:0005823 GO:0030286
GO:0005882 GO:0005667
Cluster-22178.109229 GO:0006094 GO:0006355 GO:0006351 GO:0006144
GO:0006206 GO:0006352 GO:0003899 GO:0003677 GO:0003700
GO:0005525 GO:0016987 GO:0004611 GO:0019028 GO:0005667
GO:0005730
Cluster-22178.109241 GO:0009405
Cluster-22178.109242 GO:0009058 GO:0005524 GO:0016874
Cluster-22178.109261 GO:0005515
Cluster-22178.109264 GO:0005975 GO:0030246 GO:0031224
Cluster-22178.109265 GO:0006811 GO:0007165 GO:0007268 GO:0008283
GO:0040007 GO:0004970 GO:0005125 GO:0008083 GO:0016021
GO:0016020
Cluster-22178.10927 GO:0006769 GO:0046497 GO:0003953
Cluster-22178.109272 GO:0006355 GO:0003700 GO:0005509 GO:0005515
GO:0005622 GO:0009986 GO:0016020 GO:0005667
Cluster-22178.109276 GO:0007165 GO:0006468 GO:0005515 GO:0005125
GO:0004672 GO:0005524
Cluster-22178.109282 GO:0006915 GO:0006974
Cluster-22178.109284 GO:0006281 GO:0000077 GO:0005524 GO:0005515
GO:0042393 GO:0030896
Cluster-22178.109289 GO:0005201 GO:0005578 GO:0005581
Cluster-22178.109302 GO:0055085 GO:0006810 GO:0005215 GO:0016020
Cluster-22178.109304 GO:0006355
Cluster-22178.109305 GO:0055085 GO:0006814 GO:0006810 GO:0005215
GO:0016021 GO:0016020
Cluster-22178.109309 GO:0030168 GO:0015992 GO:0051258 GO:0007155
GO:0007165 GO:0006119 GO:0006118 GO:0009055 GO:0005102
GO:0003723 GO:0030674 GO:0008121 GO:0005577
Cluster-22178.10931 GO:0006281 GO:0008270 GO:0046872
Cluster-22178.109310 GO:0007155 GO:0030168 GO:0051258 GO:0007165
GO:0030674 GO:0005102 GO:0005577
Cluster-22178.109311 GO:0007155 GO:0051258 GO:0030168 GO:0007165

GO:0030674 GO:0005102 GO:0005577

Cluster-22178.109312 GO:0030168 GO:0051258 GO:0007165 GO:0007155

GO:0005102 GO:0003723 GO:0030674 GO:0005577

Cluster-22178.109313 GO:0007155 GO:0030168 GO:0051258 GO:0007165

GO:0030674 GO:0005102 GO:0005577

Cluster-22178.109315 GO:0015991 GO:0015992 GO:0015078 GO:0033179

Cluster-22178.10934 GO:0006508 GO:0008270 GO:0004222

Cluster-22178.109343 GO:0006812 GO:0016887 GO:0016021

Cluster-22178.109348 GO:0009877 GO:0009312 GO:0008757

Cluster-22178.109360 GO:0050792 GO:0007165 GO:0007166 GO:0006827

GO:0045892 GO:0006826 GO:0005509 GO:0005381 GO:0008270

GO:0005515 GO:0004518 GO:0004871 GO:0046872 GO:0016020

GO:0005634 GO:0033573

Cluster-22178.109370 GO:0009405 GO:0005198 GO:0005515

Cluster-22178.109375 GO:0016021

Cluster-22178.109380 GO:0005515

Cluster-22178.109383 GO:0005515

Cluster-22178.109408 GO:0007165 GO:0055114 GO:0005515 GO:0005044

GO:0016020

Cluster-22178.109410 GO:0006084 GO:0006552 GO:0008299 GO:0007165

GO:0046950 GO:0006550 GO:0006574 GO:0005044 GO:0004421

GO:0016020

Cluster-22178.109417 GO:0000160 GO:0016310 GO:0071555 GO:0046797

GO:0004673 GO:0000155 GO:0016021 GO:0009365

Cluster-22178.109418 GO:0046797 GO:0000160 GO:0016310 GO:0071555

GO:0004673 GO:0000155 GO:0016021 GO:0009365

Cluster-22178.109422 GO:0006355 GO:0003677

Cluster-22178.109423 GO:0006355 GO:0003677

Cluster-22178.10944 GO:0009271 GO:0050832 GO:0006355

Cluster-22178.109440 GO:0008146

Cluster-22178.109441 GO:0008146

Cluster-22178.109442 GO:0008146

Cluster-22178.109453 GO:0006886 GO:0016192 GO:0007165 GO:0005515

GO:0005198 GO:0030126
Cluster-22178.109456 GO:0019049 GO:0008270 GO:0005515
Cluster-22178.10946 GO:0015992 GO:0015078 GO:0033644 GO:0055036
Cluster-22178.109460 GO:0015992 GO:0006123 GO:0004129 GO:0045277
Cluster-22178.109461 GO:0009306 GO:0046740 GO:0006915 GO:0003677
GO:0016021
Cluster-22178.109462 GO:0015074 GO:0016567 GO:0004842 GO:0016020
Cluster-22178.109467 GO:0009878 GO:0046872 GO:0005515
Cluster-22178.109468 GO:0009878 GO:0005515 GO:0046872
Cluster-22178.109481 GO:0055114 GO:0006725 GO:0006508 GO:0008199
GO:0008270 GO:0003824 GO:0004181
Cluster-22178.109482 GO:0008152 GO:0006508 GO:0016788 GO:0004181
GO:0008270
Cluster-22178.109487 GO:0003723 GO:0005515
Cluster-22178.109489 GO:0007155 GO:0006771 GO:0042254 GO:0007165
GO:0019497 GO:0014068 GO:0050776 GO:0006468 GO:0006412
GO:0005524 GO:0005102 GO:0003993 GO:0043548 GO:0005509
GO:0003735 GO:0004672 GO:0046872 GO:0005515 GO:0005622
GO:0016020 GO:0005840 GO:0005576
Cluster-22178.10949 GO:0005515
Cluster-22178.109496 GO:0042981 GO:0005515
Cluster-22178.109505 GO:0006508 GO:0004180 GO:0004181 GO:0008270

Cluster-22178.109510 GO:0006952 GO:0005576
Cluster-22178.109528 GO:0042254 GO:0006412 GO:0090266 GO:0003735
GO:0005515 GO:0005680 GO:0005840 GO:0005622
Cluster-22178.10954 GO:0007165 GO:0005179 GO:0005576
Cluster-22178.109541 GO:0035556 GO:0006144 GO:0006182 GO:0009190
GO:0046039 GO:0006468 GO:0005524 GO:0004672 GO:0016849
GO:0004383
Cluster-22178.109547 GO:0007155 GO:0051258 GO:0030168 GO:0007165
GO:0030674 GO:0005102 GO:0005577
Cluster-22178.109558 GO:0016567 GO:0016747 GO:0004842 GO:0005515

GO:0005680
Cluster-22178.109571 GO:0007165 GO:0007268 GO:0006810 GO:0006811
GO:0004970 GO:0005234 GO:0016020 GO:0005622
Cluster-22178.109572 GO:0006810 GO:0005622
Cluster-22178.109573 GO:0006810 GO:0005622
Cluster-22178.109586 GO:0005576
Cluster-22178.109588 GO:0005615
Cluster-22178.109589 GO:0005615 GO:0005576
Cluster-22178.109592 GO:0046872 GO:0005615 GO:0005576
Cluster-22178.109606 GO:0055085 GO:0016021
Cluster-22178.109609 GO:0007165 GO:0000772 GO:0005515 GO:0016020
Cluster-22178.109616 GO:0005515 GO:0005198 GO:0019028
Cluster-22178.109618 GO:0007010 GO:0008270 GO:0003779
Cluster-22178.109620 GO:0005515 GO:0004190 GO:0016021
Cluster-22178.109622 GO:0005515
Cluster-22178.109623 GO:0006694 GO:0008168 GO:0005515
Cluster-22178.109625 GO:0043248 GO:0006754 GO:0005524 GO:0016887
GO:0005515 GO:0000275
Cluster-22178.109626 GO:0043248 GO:0006754 GO:0016887 GO:0005524
GO:0005515 GO:0000275
Cluster-22178.109627 GO:0006754 GO:0043248 GO:0005515 GO:0016887
GO:0005524 GO:0000275
Cluster-22178.109634 GO:0016021
Cluster-22178.109635 GO:0016021
Cluster-22178.109639 GO:0006355 GO:0005515
Cluster-22178.10968 GO:0005524 GO:0016887
Cluster-22178.109687 GO:0006468 GO:0009405 GO:0006810 GO:0004672
GO:0005524 GO:0008200 GO:0005515 GO:0042151 GO:0016021
Cluster-22178.109708 GO:0006508 GO:0008270 GO:0004222 GO:0031012
Cluster-22178.109742 GO:0005515 GO:0005509
Cluster-22178.109743 GO:0009069 GO:0006468 GO:0016310 GO:0005524
GO:0004672 GO:0004674
Cluster-22178.109744 GO:0003723

Cluster-22178.109752 GO:0042981 GO:0016020
Cluster-22178.109753 GO:0042981 GO:0016020
Cluster-22178.109757 GO:0042981
Cluster-22178.109808 GO:0005515 GO:0008440 GO:0016020 GO:0005737
Cluster-22178.109814 GO:0005515
Cluster-22178.109818 GO:0003676 GO:0003723
Cluster-22178.109824 GO:0006118 GO:0009055 GO:0016491 GO:0016020
Cluster-22178.109825 GO:0006118 GO:0007165 GO:0009055 GO:0016491
GO:0016020
Cluster-22178.109849 GO:0007165 GO:0004872 GO:0016021
Cluster-22178.109851 GO:0007165 GO:0004872 GO:0016021
Cluster-22178.109855 GO:0015074 GO:0003677 GO:0005515
Cluster-22178.109856 GO:0015074 GO:0005515 GO:0003677
Cluster-22178.109897 GO:0055085 GO:0006811 GO:0005216 GO:0016020
Cluster-22178.109907 GO:0018149
Cluster-22178.109917 GO:0009234 GO:0046039 GO:0006508 GO:0006144
GO:0006182 GO:0004197 GO:0004383
Cluster-22178.109921 GO:0006308 GO:0006281 GO:0006118 GO:0009405
GO:0006260 GO:0042157 GO:0006810 GO:0006869 GO:0009055
GO:0008289 GO:0017108 GO:0016021 GO:0005634 GO:0005576
GO:0016020 GO:0033557
Cluster-22178.109925 GO:0040007 GO:0005737
Cluster-22178.109926 GO:0005515 GO:0004064
Cluster-22178.109935 GO:0000917 GO:0051301 GO:0007059 GO:0007067
GO:0000090 GO:0043093 GO:0006914 GO:0031110 GO:0070206
GO:0010506 GO:0008017 GO:0005737 GO:0045298 GO:0005615
GO:0000940 GO:0005876
Cluster-22178.109941 GO:0015074 GO:0006355 GO:0008270 GO:0003700
GO:0003676 GO:0005667
Cluster-22178.109942 GO:0015074 GO:0003677 GO:0003676 GO:0005524

Cluster-22178.109943 GO:0006355 GO:0015074 GO:0008270 GO:0003676
GO:0003700 GO:0005667

Cluster-22178.109969 GO:0015979 GO:0009539 GO:0009523 GO:0016020
Cluster-22178.109973 GO:0007268 GO:0006811 GO:0007165 GO:0004970
GO:0016020
Cluster-22178.109998 GO:0051252 GO:0006402 GO:0006470 GO:0008138
GO:0003723 GO:0003676 GO:0046872 GO:0004535 GO:0005634
GO:0005737
Cluster-22178.110000 GO:0030168 GO:0000921 GO:0030334 GO:0007165
GO:0030155 GO:0007155 GO:0045995 GO:0051258 GO:0048870
GO:0007094 GO:0005102 GO:0030674 GO:0008092 GO:0005515
GO:0042803 GO:0008134 GO:0045502 GO:0019898 GO:0005577
GO:0030286 GO:0005940 GO:0005667 GO:0016021 GO:0005737
GO:0031514
Cluster-22178.110009 GO:0007029 GO:0006817 GO:0005315 GO:0016021
GO:0016020 GO:0005789
Cluster-22178.110014 GO:0005515
Cluster-22178.110022 GO:0005509 GO:0005544
Cluster-22178.110023 GO:0005509 GO:0005544
Cluster-22178.110050 GO:0007155 GO:0006508 GO:0050790 GO:0019058
GO:0055114 GO:0019062 GO:0004197 GO:0016706 GO:0008168
Cluster-22178.110051 GO:0003677 GO:0008270
Cluster-22178.11009 GO:0006468 GO:0009069 GO:0016310 GO:0000287
GO:0005524 GO:0004674
Cluster-22178.110090 GO:0042254 GO:0006412 GO:0016192 GO:0003735
GO:0042393 GO:0005634 GO:0016021 GO:0005840
Cluster-22178.110091 GO:0016192 GO:0042393 GO:0016021 GO:0005634
Cluster-22178.110098 GO:0006355 GO:0006508 GO:0006352 GO:0003677
GO:0004180 GO:0008270 GO:0016987 GO:0004181 GO:0003700
GO:0005667
Cluster-22178.110099 GO:0006508 GO:0004181 GO:0008270
Cluster-22178.110104 GO:0006260 GO:0006281 GO:0003676 GO:0003910
GO:0003723
Cluster-22178.110106 GO:0006355 GO:0006468 GO:0006289 GO:0005515
GO:0004672 GO:0005524 GO:0000439

Cluster-22178.110116 GO:0007165 GO:0006950 GO:0007017 GO:0007018
GO:0009405 GO:0008608 GO:0005179 GO:0005524 GO:0008017
GO:0045502 GO:0042803 GO:0008134 GO:0003777 GO:0005667
GO:0016020 GO:0030286 GO:0072686 GO:0005576 GO:0042729
GO:0045298 GO:0005874
Cluster-22178.110117 GO:0005515
Cluster-22178.110125 GO:0007165 GO:0006144 GO:0004114
Cluster-22178.110135 GO:0007186 GO:0004930 GO:0016021
Cluster-22178.110137 GO:0006351 GO:0006144 GO:0006206 GO:0003899
GO:0005634 GO:0005730
Cluster-22178.110144 GO:0007224 GO:0015992 GO:0006123 GO:0007275
GO:0004129 GO:0045277
Cluster-22178.110146 GO:0015992 GO:0007224 GO:0007275 GO:0006123
GO:0004129 GO:0045277
Cluster-22178.110148 GO:0007165 GO:0055114 GO:0051287 GO:0004888
GO:0005515 GO:0005509 GO:0016651 GO:0048038 GO:0016021
Cluster-22178.110162 GO:0015992 GO:0006123 GO:0004129 GO:0045277
Cluster-22178.110167 GO:0035556 GO:0032065 GO:0055114 GO:0009231
GO:0047134 GO:0005543 GO:0008686 GO:0016301 GO:0005515
GO:0005938
Cluster-22178.110176 GO:0005515 GO:0005509 GO:0016020
Cluster-22178.110187 GO:0006935 GO:0007165 GO:0006955 GO:0008009
GO:0005576
Cluster-22178.110198 GO:0006355 GO:0003677
Cluster-22178.110205 GO:0005515
Cluster-22178.110207 GO:0005515
Cluster-22178.110208 GO:0005515
Cluster-22178.110212 GO:0005515
Cluster-22178.110218 GO:0015684 GO:0007264 GO:0005525 GO:0005524
GO:0015093 GO:0003924 GO:0016021
Cluster-22178.110219 GO:0006355 GO:0005515 GO:0003677 GO:0003700
GO:0005507 GO:0004386 GO:0005667 GO:0005634
Cluster-22178.110220 GO:0006355 GO:0005515 GO:0003677 GO:0003700

GO:0005507 GO:0004386 GO:0005667 GO:0005634
Cluster-22178.110222 GO:0006568 GO:0055114 GO:0000334 GO:0005506

Cluster-22178.110223 GO:0055114 GO:0006568 GO:0005506 GO:0000334
GO:0004386
Cluster-22178.110225 GO:0005515
Cluster-22178.110226 GO:0006144 GO:0006536 GO:0006355 GO:0006177
GO:0006164 GO:0003700 GO:0005524 GO:0003922 GO:0005667
Cluster-22178.110229 GO:0016021
Cluster-22178.110230 GO:0006260 GO:0003677 GO:0004003 GO:0005525
GO:0005524 GO:0003924 GO:0016887 GO:0016020 GO:0005657
Cluster-22178.110234 GO:0006355 GO:0003677
Cluster-22178.110247 GO:0046872 GO:0008270
Cluster-22178.110263 GO:0006810 GO:0016021
Cluster-22178.110279 GO:0042998 GO:0071253 GO:0005802
Cluster-22178.110283 GO:0005515
Cluster-22178.110284 GO:0005515
Cluster-22178.110290 GO:0005975 GO:0016868
Cluster-22178.110291 GO:0071973 GO:0016567 GO:0007165 GO:0071704
GO:0030168 GO:0015031 GO:0006886 GO:0006355 GO:0044780
GO:0005975 GO:0051258 GO:0006935 GO:0016868 GO:0030674
GO:0005102 GO:0008565 GO:0031386 GO:0003774 GO:0003700
GO:0019120 GO:0043565 GO:0016881 GO:0016020 GO:0005667
GO:0005577 GO:0005737 GO:0009288
Cluster-22178.110294 GO:0015031 GO:0030168 GO:0007165 GO:0071973
GO:0016567 GO:0006935 GO:0051258 GO:0006355 GO:0044780
GO:0006886 GO:0031386 GO:0003774 GO:0008565 GO:0005102
GO:0030674 GO:0043565 GO:0016881 GO:0003700 GO:0005577
GO:0005667 GO:0016020 GO:0009288 GO:0005737
Cluster-22178.110295 GO:0006935 GO:0051258 GO:0044780 GO:0006355
GO:0010508 GO:0006914 GO:0070206 GO:0030168 GO:0016567
GO:0071973 GO:0007165 GO:0043565 GO:0016881 GO:0003700
GO:0003774 GO:0031386 GO:0005102 GO:0030674 GO:0017048

GO:0009288 GO:0005577 GO:0005667 GO:0016020
Cluster-22178.110296 GO:0006397 GO:0005524 GO:0003774 GO:0016459
Cluster-22178.110298 GO:0055114 GO:0008152 GO:0009058 GO:0016742
GO:0016491
Cluster-22178.110305 GO:0006275 GO:0006355 GO:0003700 GO:0003677
GO:0042025 GO:0005667
Cluster-22178.110333 GO:0005515
Cluster-22178.110336 GO:0055114 GO:0009308 GO:0048038 GO:0005507
GO:0008131
Cluster-22178.110349 GO:0055114 GO:0016972
Cluster-22178.110361 GO:0055114 GO:0005506 GO:0016705 GO:0020037

Cluster-22178.110362 GO:0055114 GO:0005506 GO:0003677 GO:0016705
GO:0020037
Cluster-22178.110395 GO:0055114 GO:0005507 GO:0016491
Cluster-22178.110399 GO:0006260 GO:0009245 GO:0006338 GO:0046487
GO:0015977 GO:0008270 GO:0016747 GO:0016984 GO:0003887
GO:0003677 GO:0009573 GO:0042575 GO:0090544
Cluster-22178.11040 GO:0006633 GO:0042967 GO:0055114 GO:0016772
GO:0005515 GO:0016301 GO:0004318 GO:0005835
Cluster-22178.110400 GO:0006338 GO:0008270 GO:0090544
Cluster-22178.110414 GO:0007155 GO:0005540
Cluster-22178.110421 GO:0009058 GO:0005524
Cluster-22178.11044 GO:0006487 GO:0007186 GO:0004930 GO:0008250
GO:0016021
Cluster-22178.110440 GO:0009116 GO:0003824
Cluster-22178.110442 GO:0006468 GO:0016773 GO:0005524 GO:0004672
GO:0016020
Cluster-22178.110454 GO:0006508 GO:0004197
Cluster-22178.110476 GO:0003774 GO:0005524 GO:0051537 GO:0016459
Cluster-22178.110478 GO:0005515
Cluster-22178.110485 GO:0005978
Cluster-22178.110494 GO:0007154 GO:0055085 GO:0016747 GO:0016021

Cluster-22178.110502 GO:0016787
Cluster-22178.110511 GO:0007165 GO:0006119 GO:0015986 GO:0015992
GO:0046961 GO:0004252 GO:0005044 GO:0070008 GO:0046933
GO:0045259 GO:0045261 GO:0016020
Cluster-22178.110529 GO:0046740 GO:0016021
Cluster-22178.110535 GO:0006563 GO:0006094 GO:0006566 GO:0006544
GO:0006534 GO:0051539 GO:0008270 GO:0003941
Cluster-22178.110548 GO:0010181 GO:0016020
Cluster-22178.110551 GO:0007155 GO:0005515 GO:0016021
Cluster-22178.110560 GO:0006468 GO:0016773 GO:0005524 GO:0004672
GO:0016020
Cluster-22178.110562 GO:0007155 GO:0005515 GO:0016020 GO:0016021
Cluster-22178.110570 GO:0015992 GO:0055114 GO:0006950 GO:0006119
GO:0006118 GO:0008121
Cluster-22178.110571 GO:0006950
Cluster-22178.110572 GO:0015992 GO:0055114 GO:0006118 GO:0006119
GO:0008121
Cluster-22178.110573 GO:0006118 GO:0006119 GO:0015992 GO:0055114
GO:0008121
Cluster-22178.110574 GO:0006119 GO:0006118 GO:0015992 GO:0055114
GO:0008121
Cluster-22178.110585 GO:0019031
Cluster-22178.110586 GO:0007165 GO:0040007 GO:0008283 GO:0008083
GO:0016020
Cluster-22178.110587 GO:0006275 GO:0006270 GO:0043565 GO:0005524

Cluster-22178.110598 GO:0006396 GO:0009451 GO:0055114 GO:0008173
GO:0003723
Cluster-22178.110599 GO:0055114 GO:0007155 GO:0006396 GO:0009451
GO:0003723 GO:0008173 GO:0005540
Cluster-22178.110613 GO:0004222 GO:0008233 GO:0031012
Cluster-22178.110637 GO:0055114 GO:0016117 GO:0016491 GO:0071949
GO:0016817 GO:0016705

Cluster-22178.110642 GO:0016042 GO:0035556 GO:0009395 GO:0046339
GO:0006289 GO:0007165 GO:0006629 GO:0004435 GO:0008270
GO:0005515 GO:0005509 GO:0004003 GO:0000439 GO:0005657
GO:0005634
Cluster-22178.110644 GO:0016042 GO:0046339 GO:0006289 GO:0009395
GO:0005509 GO:0004003 GO:0004435 GO:0008270 GO:0005634
GO:0000439 GO:0005657
Cluster-22178.110660 GO:0055114 GO:0051287
Cluster-22178.11067 GO:0006979 GO:0006804 GO:0006749 GO:0055114
GO:0016491 GO:0016209 GO:0004602
Cluster-22178.110673 GO:0043087 GO:0035023 GO:0005089
Cluster-22178.110675 GO:0006810 GO:0009405 GO:0006952 GO:0019871
GO:0005576
Cluster-22178.110682 GO:0005201 GO:0005578 GO:0005581
Cluster-22178.110695 GO:0006468 GO:0004672 GO:0005524 GO:0008270
GO:0005515
Cluster-22178.110699 GO:0015031 GO:0006886 GO:0008277 GO:0008565
GO:0016021
Cluster-22178.110704 GO:0000166 GO:0046872 GO:0008168
Cluster-22178.110709 GO:0043130
Cluster-22178.11071 GO:0007186 GO:0004930 GO:0016021
Cluster-22178.110714 GO:0016021
Cluster-22178.110720 GO:0005515
Cluster-22178.110729 GO:0006810 GO:0006468 GO:0008200 GO:0004672
GO:0005524 GO:0005576
Cluster-22178.110732 GO:0006468 GO:0007169 GO:0005524 GO:0004714
GO:0005887
Cluster-22178.110744 GO:0006355 GO:0007339 GO:0007268 GO:0007165
GO:0007304 GO:0006811 GO:0003677 GO:0005515 GO:0005213
GO:0004970 GO:0005576 GO:0016020 GO:0042600
Cluster-22178.110754 GO:0006914 GO:0007165 GO:0048870 GO:0007017
GO:0009405 GO:0051252 GO:0007018 GO:0005524 GO:0004521
GO:0008017 GO:0003777 GO:0004871 GO:0045502 GO:0003723

GO:0008134 GO:0042803 GO:0005198 GO:0030286 GO:0005667
GO:0005882 GO:0016020 GO:0005874 GO:0045298 GO:0031514
Cluster-22178.110757 GO:0005515
Cluster-22178.110762 GO:0030474 GO:0071988 GO:0005515
Cluster-22178.110765 GO:0008270 GO:0004222 GO:0005576 GO:0005618
Cluster-22178.110769 GO:0040007 GO:0005737
Cluster-22178.110785 GO:0005515 GO:0003676 GO:0016787 GO:0046872

Cluster-22178.110793 GO:0051726 GO:0046983 GO:0019012 GO:0005634
Cluster-22178.110795 GO:0019054 GO:0005515 GO:0003677 GO:0004857

Cluster-22178.110797 GO:0006810 GO:0009271 GO:0016567 GO:0006606
GO:0070206 GO:0019064 GO:0006355 GO:0006308 GO:0031386
GO:0008855 GO:0003700 GO:0016881 GO:0005667 GO:0005886
GO:0009318 GO:0019031 GO:0016021
Cluster-22178.110798 GO:0008152 GO:0015074 GO:0003824 GO:0016021
Cluster-22178.110806 GO:0003682 GO:0000785
Cluster-22178.110818 GO:0005198 GO:0019031 GO:0019028
Cluster-22178.11082 GO:0015074
Cluster-22178.110822 GO:0003774 GO:0005524 GO:0016459
Cluster-22178.110855 GO:0007268 GO:0006811 GO:0007165 GO:0004970
GO:0016020
Cluster-22178.110866 GO:0016055 GO:0007165 GO:0007275 GO:0005102
GO:0005576
Cluster-22178.110867 GO:0007165 GO:0031704 GO:0005179
Cluster-22178.110871 GO:0006470 GO:0006570 GO:0004725 GO:0008138
GO:0005515
Cluster-22178.110875 GO:0006570 GO:0006470 GO:0005515 GO:0008138
GO:0004725
Cluster-22178.110888 GO:0006355 GO:0005096 GO:0003677 GO:0005515
GO:0005634
Cluster-22178.110894 GO:0008608 GO:0005815 GO:0042729 GO:0072686
Cluster-22178.110907 GO:0007165 GO:0030155 GO:0045995 GO:0030334

GO:0007155 GO:0005102
Cluster-22178.110911 GO:0006935 GO:0007165 GO:0006955 GO:0008009
GO:0005576
Cluster-22178.110924 GO:0055114 GO:0016614 GO:0050660 GO:0046872

Cluster-22178.110927 GO:0006810 GO:0006811 GO:0005230 GO:0016020
Cluster-22178.110930 GO:0051056 GO:0005096
Cluster-22178.110933 GO:0016021
Cluster-22178.110935 GO:0006468 GO:0005515 GO:0004672 GO:0005524

Cluster-22178.110939 GO:0007165 GO:0006449 GO:0006415 GO:0006508
GO:0005515 GO:0008236 GO:0016149 GO:0018444 GO:0005840
GO:0005737
Cluster-22178.110942 GO:0006536 GO:0006144 GO:0006177 GO:0006164
GO:0003922 GO:0005524
Cluster-22178.110945 GO:0005515
Cluster-22178.110977 GO:0055114 GO:0006508 GO:0004197 GO:0016491

Cluster-22178.110980 GO:0005515
Cluster-22178.110984 GO:0007010 GO:0008270 GO:0003779
Cluster-22178.11099 GO:0006811 GO:0016020
Cluster-22178.111011 GO:0006355 GO:0007186 GO:0015684 GO:0007165
GO:0007264 GO:0008134 GO:0000166 GO:0003723 GO:0001671
GO:0004871 GO:0005525 GO:0043531 GO:0003924 GO:0031683
GO:0019001 GO:0051087 GO:0003677 GO:0003724 GO:0016887
GO:0005524 GO:0015093 GO:0016021 GO:0016020 GO:0005667
Cluster-22178.111017 GO:0015074 GO:0005991 GO:0006952 GO:0005985
GO:0005982 GO:0010181 GO:0004555 GO:0005576
Cluster-22178.111019 GO:0006355 GO:0006810 GO:0048870 GO:0006914
GO:0051382 GO:0070206 GO:0007049 GO:0006260 GO:0008270
GO:0003700 GO:0005667 GO:0005634 GO:0000776 GO:0031514
GO:0016021
Cluster-22178.111026 GO:0016567 GO:0004842

Cluster-22178.111044 GO:0007156 GO:0051301 GO:0003677 GO:0005509
GO:0016020
Cluster-22178.111047 GO:0007156 GO:0051301 GO:0003677 GO:0005509
GO:0016020
Cluster-22178.111049 GO:0007156 GO:0005509 GO:0016020
Cluster-22178.111055 GO:0006810 GO:0005643
Cluster-22178.111058 GO:0016787 GO:0005524 GO:0003676 GO:0003677

Cluster-22178.111064 GO:0006457 GO:0006364 GO:0015675 GO:0035444
GO:0055085 GO:0006508 GO:0030001 GO:0008654 GO:0046873
GO:0003677 GO:0008270 GO:0043772 GO:0015099 GO:0004190
GO:0051082 GO:0004252 GO:0005515 GO:0046872 GO:0005732
GO:0016020 GO:0016272 GO:0005622 GO:0005886 GO:0009424
GO:0005634 GO:0034457 GO:0042025 GO:0016021
Cluster-22178.111079 GO:0015074 GO:0003676 GO:0008270
Cluster-22178.111080 GO:0015074 GO:0008270 GO:0003676
Cluster-22178.111081 GO:0015074 GO:0008270 GO:0003676
Cluster-22178.11109 GO:0046486 GO:0009395 GO:0030261 GO:0005975
GO:0007205 GO:0003677 GO:0004553 GO:0004143
Cluster-22178.111095 GO:0000910 GO:0000226 GO:0006355 GO:0008017
GO:0003700 GO:0045298 GO:0005667 GO:0042025
Cluster-22178.111099 GO:0005515
Cluster-22178.111100 GO:0007155 GO:0007165 GO:0005173 GO:0016020
Cluster-22178.111105 GO:0030695
Cluster-22178.11111 GO:0046486 GO:0007205 GO:0009395 GO:0005975
GO:0030261 GO:0003677 GO:0004553 GO:0004143
Cluster-22178.111116 GO:0016021
Cluster-22178.111118 GO:0015031 GO:0019069 GO:0016485 GO:0006418
GO:0006352 GO:0006355 GO:0008565 GO:0005524 GO:0016987
GO:0004812 GO:0005509 GO:0003677 GO:0005544 GO:0003700
GO:0000166 GO:0005667
Cluster-22178.111122 GO:0003676
Cluster-22178.111139 GO:0016032 GO:0006406 GO:0006611 GO:0006998

GO:0046983 GO:0031965 GO:0016021

Cluster-22178.11114 GO:0046486 GO:0009395 GO:0030261 GO:0005975

GO:0007205 GO:0005515 GO:0003677 GO:0004553 GO:0004143

Cluster-22178.111142 GO:0055085 GO:0022857 GO:0016021

Cluster-22178.11115 GO:0046740 GO:0006813 GO:0006811 GO:0005249

GO:0016021 GO:0016020 GO:0008076

Cluster-22178.111157 GO:0005515

Cluster-22178.111159 GO:0007156 GO:0005509 GO:0016020

Cluster-22178.111169 GO:0008270 GO:0003677

Cluster-22178.111188 GO:0006571 GO:0000162 GO:0009094 GO:0006096

GO:0006094 GO:0007264 GO:0005525 GO:0004634 GO:0000287

GO:0000015

Cluster-22178.11120 GO:0006118 GO:0009055 GO:0020037 GO:0005509

Cluster-22178.111204 GO:0070140 GO:0032183

Cluster-22178.111214 GO:0035091 GO:0003824

Cluster-22178.111227 GO:0006355 GO:0043401 GO:0043565 GO:0019900

GO:0008270 GO:0046872 GO:0003700 GO:0005667 GO:0005634

Cluster-22178.111229 GO:0000186 GO:0008343 GO:0007186 GO:0001678

GO:0051276 GO:0009267 GO:0032099 GO:0005634 GO:0005737

GO:0005615

Cluster-22178.11125 GO:0006486 GO:0008378 GO:0016757 GO:0016020

Cluster-22178.11127 GO:0006621 GO:0046923 GO:0016021 GO:0005783

Cluster-22178.111270 GO:0046872 GO:0045202

Cluster-22178.111277 GO:0006810 GO:0006164 GO:0006807 GO:0006188

GO:0055085 GO:0006144 GO:0005525 GO:0003937 GO:0042626

GO:0005524 GO:0016887 GO:0003924 GO:0016021 GO:0042720

Cluster-22178.111279 GO:0005515

Cluster-22178.111298 GO:0006281 GO:0016032 GO:0035556 GO:0006464

GO:0055114 GO:0005506 GO:0016151 GO:0008270 GO:0046872

GO:0016491

Cluster-22178.111305 GO:0050792 GO:0004518 GO:0005515

Cluster-22178.111309 GO:0006952

Cluster-22178.111312 GO:0006260 GO:0006351 GO:0006269 GO:0003896
GO:0008270 GO:0003677 GO:0005657 GO:0005730 GO:0005634
Cluster-22178.111321 GO:0005198 GO:0003677 GO:0019013
Cluster-22178.111341 GO:0035556
Cluster-22178.111342 GO:0006310 GO:0055114 GO:0008152 GO:0006378
GO:0003729 GO:0003677 GO:0016491 GO:0005849 GO:0005634
Cluster-22178.111343 GO:0055085 GO:0006810 GO:0022857 GO:0005215
GO:0016021 GO:0016020
Cluster-22178.111381 GO:0043087 GO:0007165 GO:0019239
Cluster-22178.111399 GO:0003723
Cluster-22178.11140 GO:0007165 GO:0005179 GO:0005576
Cluster-22178.111406 GO:0006355 GO:0008270 GO:0043565 GO:0003700
GO:0005634 GO:0005667
Cluster-22178.111408 GO:0007186 GO:0007165 GO:0035556 GO:0004871
GO:0005834
Cluster-22178.111439 GO:0003677 GO:0005198 GO:0019013
Cluster-22178.111443 GO:0006914
Cluster-22178.111449 GO:0009306 GO:0051382 GO:0005515 GO:0051082
GO:0016020 GO:0000776
Cluster-22178.111450 GO:0051382 GO:0009306 GO:0005515 GO:0000776
GO:0016020
Cluster-22178.111457 GO:0008152 GO:0003824
Cluster-22178.111467 GO:0015992 GO:0009401 GO:0006123 GO:0004129
GO:0045277 GO:0016021
Cluster-22178.111488 GO:0008152 GO:0006508 GO:0004181 GO:0016788
GO:0008270
Cluster-22178.111501 GO:0006098 GO:0006094 GO:0006096 GO:0005985
GO:0005982 GO:0004347
Cluster-22178.111509 GO:0005515
Cluster-22178.111515 GO:0019031
Cluster-22178.111516 GO:0051225 GO:0008092 GO:0046983 GO:0030430
GO:0019898 GO:0070652 GO:0005737
Cluster-22178.111524 GO:0005515

Cluster-22178.111529 GO:0006874 GO:0006816 GO:0007186 GO:0005219
GO:0004930 GO:0005622 GO:0016021
Cluster-22178.111542 GO:0003779
Cluster-22178.111543 GO:0003779
Cluster-22178.111546 GO:0004866 GO:0005576
Cluster-22178.11155 GO:0016021 GO:0016020
Cluster-22178.111553 GO:0003723
Cluster-22178.111555 GO:0003723
Cluster-22178.111558 GO:0030178 GO:0007275 GO:0005576
Cluster-22178.111560 GO:0007275 GO:0030178 GO:0005515 GO:0005576
Cluster-22178.111581 GO:0007155 GO:0005540
Cluster-22178.111584 GO:0008152 GO:0008484 GO:0003824
Cluster-22178.111608 GO:0015031 GO:0016020
Cluster-22178.111609 GO:0015031 GO:0016020
Cluster-22178.111615 GO:0051258 GO:0030168 GO:0007165 GO:0030674
GO:0003677 GO:0005102 GO:0005577
Cluster-22178.11162 GO:0007016 GO:0005515 GO:0016010 GO:0005789
Cluster-22178.111639 GO:0006352 GO:0006355 GO:0016987 GO:0003677
GO:0003700 GO:0009512 GO:0005667
Cluster-22178.11164 GO:0006355 GO:0035556 GO:0003677 GO:0003700
GO:0005667
Cluster-22178.111640 GO:0006355 GO:0006352 GO:0003700 GO:0016987
GO:0003677 GO:0005667 GO:0009512
Cluster-22178.111641 GO:0006355 GO:0006352 GO:0003700 GO:0016987
GO:0003677 GO:0005667 GO:0009512
Cluster-22178.111663 GO:0006355 GO:0005515 GO:0003677 GO:0005507
GO:0004386 GO:0003700 GO:0005667 GO:0005634
Cluster-22178.111665 GO:0005515
Cluster-22178.11167 GO:0007411
Cluster-22178.111714 GO:0022900 GO:0006355 GO:0003677 GO:0005747
GO:0005739
Cluster-22178.111715 GO:0022900 GO:0006355 GO:0003677 GO:0005747
GO:0005739

Cluster-22178.111717 GO:0006629
Cluster-22178.111720 GO:0055085 GO:0007154 GO:0016021
Cluster-22178.111726 GO:0003677 GO:0005515
Cluster-22178.11173 GO:0009847 GO:0005515 GO:0016021
Cluster-22178.111734 GO:0046872
Cluster-22178.111746 GO:0055114 GO:0006761 GO:0046656 GO:0042493 GO:0004146
Cluster-22178.111753 GO:0043123 GO:0043066 GO:0045766
Cluster-22178.111754 GO:0043123 GO:0045766 GO:0043066
Cluster-22178.111769 GO:0016021
Cluster-22178.111792 GO:0046983 GO:0003677
Cluster-22178.111793 GO:0046983 GO:0003677
Cluster-22178.111795 GO:0006355 GO:0015991 GO:0003677 GO:0016820 GO:0005524 GO:0033178
Cluster-22178.111804 GO:0042545 GO:0005985 GO:0005982 GO:0005515 GO:0005509 GO:0030599 GO:0005618
Cluster-22178.111806 GO:0005509 GO:0005515 GO:0016020
Cluster-22178.111815 GO:0006865 GO:0055085 GO:0006810 GO:0003333 GO:0015171 GO:0016020
Cluster-22178.111816 GO:0003333 GO:0006810 GO:0007165 GO:0016310 GO:0000160 GO:0006865 GO:0055085 GO:0015171 GO:0000155 GO:0016020 GO:0009365
Cluster-22178.111832 GO:0007155 GO:0045502 GO:0008134 GO:0042803 GO:0003774 GO:0005667 GO:0016459 GO:0030286
Cluster-22178.11184 GO:0042742
Cluster-22178.111840 GO:0006468 GO:0005515 GO:0005524 GO:0004672

Cluster-22178.111846 GO:0005515
Cluster-22178.111847 GO:0006810 GO:0005215 GO:0016020
Cluster-22178.11185 GO:0042742
Cluster-22178.111866 GO:0006771 GO:0019497 GO:0003993 GO:0005515 GO:0046872
Cluster-22178.111867 GO:0006771 GO:0019497 GO:0046872 GO:0003993

GO:0005515
Cluster-22178.111868 GO:0005515
Cluster-22178.111869 GO:0006771 GO:0019497 GO:0046872 GO:0003993
GO:0005515
Cluster-22178.111870 GO:0005515
Cluster-22178.111875 GO:0007155 GO:0055114 GO:0015031 GO:0006464
GO:0016491 GO:0005540
Cluster-22178.11189 GO:0005515 GO:0016021
Cluster-22178.111892 GO:0006468 GO:0004672 GO:0005524
Cluster-22178.111893 GO:0006468 GO:0004672 GO:0005524
Cluster-22178.111896 GO:0006468 GO:0005524 GO:0004672
Cluster-22178.111904 GO:0006952
Cluster-22178.11193 GO:0005856
Cluster-22178.111930 GO:0005524 GO:0016787 GO:0003677
Cluster-22178.111933 GO:0016787 GO:0005524 GO:0003677
Cluster-22178.111945 GO:0005515 GO:0043565 GO:0005634
Cluster-22178.111946 GO:0005515 GO:0043565 GO:0005634
Cluster-22178.111968 GO:0007186 GO:0004930 GO:0016021
Cluster-22178.111979 GO:0006811 GO:0055085 GO:0005216 GO:0016020
Cluster-22178.112002 GO:0006508 GO:0016032 GO:0004197
Cluster-22178.112012 GO:0006810 GO:0016021 GO:0016020
Cluster-22178.11205 GO:0007034 GO:0005515 GO:0003677 GO:0046872

Cluster-22178.112050 GO:0005201 GO:0005578 GO:0005581
Cluster-22178.112080 GO:0007264 GO:0043087 GO:0035023 GO:0005085
GO:0005515 GO:0005089
Cluster-22178.112107 GO:0017004 GO:0015886 GO:0016021
Cluster-22178.112121 GO:0071973 GO:0006810 GO:0009405 GO:0006308
GO:0006935 GO:0008855 GO:0003774 GO:0019855 GO:0005576
GO:0016020 GO:0009318 GO:0009288
Cluster-22178.112124 GO:0007186 GO:0004930 GO:0016021
Cluster-22178.112130 GO:0007186 GO:0004930 GO:0016020 GO:0016021
Cluster-22178.112131 GO:0007186 GO:0004930 GO:0016021 GO:0016020

Cluster-22178.112133 GO:0016887 GO:0005524 GO:0017111 GO:0000166

Cluster-22178.112139 GO:0015074 GO:0006508 GO:0004190
Cluster-22178.112149 GO:0044237
Cluster-22178.112159 GO:0007186 GO:0004930 GO:0016021
Cluster-22178.112170 GO:0016021
Cluster-22178.112182 GO:0006810 GO:0042967 GO:0005515 GO:0008811
GO:0003676 GO:0003774 GO:0005524 GO:0016459 GO:0005856
Cluster-22178.112198 GO:0035556 GO:0008270 GO:0005634
Cluster-22178.11221 GO:0006813 GO:0015269 GO:0016020
Cluster-22178.112220 GO:0006355 GO:0006260 GO:0003700 GO:0005622
GO:0005667 GO:0005634
Cluster-22178.112221 GO:0006367 GO:0006508 GO:0004252 GO:0005672
Cluster-22178.112222 GO:0009395 GO:0046339 GO:0006629 GO:0035556
GO:0007165 GO:0004435
Cluster-22178.112224 GO:0007165 GO:0035556 GO:0009395 GO:0006629
GO:0046339 GO:0004435 GO:0005515
Cluster-22178.112233 GO:0005515
Cluster-22178.112245 GO:0000724
Cluster-22178.112258 GO:0015991 GO:0006355 GO:0015992 GO:0046034
GO:0016820 GO:0003677 GO:0005524 GO:0033178
Cluster-22178.112272 GO:0006355 GO:0006810 GO:0055085 GO:0016301
GO:0008134 GO:0005524 GO:0016887 GO:0003924 GO:0005525
GO:0042626 GO:0019028 GO:0005667 GO:0016021
Cluster-22178.112285 GO:0005789
Cluster-22178.11229 GO:0006508 GO:0046983 GO:0005525 GO:0005515
GO:0004252 GO:0016020
Cluster-22178.112302 GO:0051603 GO:0004298 GO:0005839
Cluster-22178.112305 GO:0019634 GO:0016021
Cluster-22178.112316 GO:0006355 GO:0008270
Cluster-22178.112338 GO:0006508 GO:0004252
Cluster-22178.112365 GO:0043065 GO:0005737
Cluster-22178.112371 GO:0006508 GO:0015994 GO:0008152 GO:0015996

GO:0006418 GO:0047746 GO:0000166 GO:0008236 GO:0004812
GO:0016787 GO:0005524 GO:0005576
Cluster-22178.112378 GO:0006508 GO:0008233
Cluster-22178.112384 GO:0019069 GO:0048870 GO:0071988 GO:0030474
GO:0031514
Cluster-22178.112385 GO:0030474 GO:0071988 GO:0048870 GO:0019069
GO:0031514
Cluster-22178.112390 GO:0006810 GO:0009405 GO:0019871 GO:0005576
Cluster-22178.112398 GO:0006098 GO:0006096 GO:0019079 GO:0006144
GO:0006000 GO:0006013 GO:0006012 GO:0006094 GO:0000723
GO:0003677 GO:0005524 GO:0003872 GO:0003723 GO:0003968
GO:0005945 GO:0000784 GO:0031379
Cluster-22178.112399 GO:0000723 GO:0006094 GO:0006000 GO:0006144
GO:0006013 GO:0006012 GO:0006098 GO:0006096 GO:0019079
GO:0003968 GO:0003723 GO:0005524 GO:0003872 GO:0003677
GO:0031379 GO:0005945 GO:0000784
Cluster-22178.112400 GO:0006094 GO:0000723 GO:0019079 GO:0006096
GO:0006098 GO:0006012 GO:0006144 GO:0006000 GO:0006013
GO:0003723 GO:0003968 GO:0003677 GO:0003872 GO:0005524
GO:0031379 GO:0005945 GO:0000784
Cluster-22178.112403 GO:0043395 GO:0016020 GO:0005578
Cluster-22178.112407 GO:0004222 GO:0031012
Cluster-22178.112409 GO:0007050
Cluster-22178.112410 GO:0016021
Cluster-22178.112430 GO:0005515
Cluster-22178.112437 GO:0005515
Cluster-22178.11244 GO:0016021
Cluster-22178.112440 GO:0046872
Cluster-22178.112442 GO:0006355 GO:0046872
Cluster-22178.112456 GO:0030155 GO:0045995 GO:0007165 GO:0007155
GO:0030334 GO:0005102
Cluster-22178.112468 GO:0004064 GO:0005515
Cluster-22178.112471 GO:0005515 GO:0004064

Cluster-22178.112478 GO:0042128 GO:0005515

Cluster-22178.112488 GO:0005515

Cluster-22178.112507 GO:0007155 GO:0005515 GO:0016021

Cluster-22178.112514 GO:0046872 GO:0003677 GO:0030430

Cluster-22178.112517 GO:0006465 GO:0008233 GO:0016021 GO:0005787

Cluster-22178.112520 GO:0008209 GO:0008207 GO:0008210 GO:0006694 GO:0055114 GO:0016616 GO:0050662 GO:0003854 GO:0003824

Cluster-22178.112521 GO:0008209 GO:0008207 GO:0008210 GO:0006694 GO:0055114 GO:0016616 GO:0050662 GO:0003854 GO:0003824

Cluster-22178.112537 GO:0006570 GO:0006470 GO:0004725 GO:0008138 GO:0046872

Cluster-22178.112548 GO:0005515

Cluster-22178.112555 GO:0005515 GO:0016020

Cluster-22178.112557 GO:0005525 GO:0005515 GO:0016020

Cluster-22178.112577 GO:0006560 GO:0006525 GO:0018401 GO:0055114 GO:0004656 GO:0003676 GO:0005525 GO:0016702 GO:0003924 GO:0016491 GO:0005783

Cluster-22178.112589 GO:0002949 GO:0005524 GO:0003774 GO:0016459

Cluster-22178.112598 GO:0031224

Cluster-22178.112605 GO:0007165 GO:0005509 GO:0005578

Cluster-22178.112606 GO:0005515

Cluster-22178.112615 GO:0009405 GO:0006810 GO:0008200 GO:0046872 GO:0005576

Cluster-22178.112620 GO:0001881

Cluster-22178.112624 GO:0015631 GO:0045298

Cluster-22178.112638 GO:0006813 GO:0006814 GO:0005890

Cluster-22178.112650 GO:0003677 GO:0016301 GO:0016772

Cluster-22178.112659 GO:0006811 GO:0007165 GO:0007268 GO:0004970 GO:0005234 GO:0016020

Cluster-22178.112663 GO:0006810 GO:0015858 GO:0005337 GO:0005515 GO:0016021

Cluster-22178.11267 GO:0006811 GO:0007165 GO:0007268 GO:0004970 GO:0016020

Cluster-22178.112671 GO:0042254 GO:0006412 GO:0007186 GO:0001558
GO:0005520 GO:0004930 GO:0003735 GO:0005515 GO:0005622
GO:0016021 GO:0005840 GO:0016942 GO:0005576
Cluster-22178.112678 GO:0007264 GO:0007155 GO:0007165 GO:0016192
GO:0071973 GO:0030168 GO:0008053 GO:0000226 GO:0090063
GO:0006886 GO:0051258 GO:0006935 GO:0030674 GO:0003774
GO:0005096 GO:0005102 GO:0005515 GO:0003924 GO:0004871
GO:0005741 GO:0016020 GO:0005815 GO:0005577 GO:0019033
GO:0009288 GO:0005801 GO:0019867 GO:0016021 GO:0000922
Cluster-22178.11268 GO:0007268 GO:0007165 GO:0006811 GO:0005234
GO:0004970 GO:0004222 GO:0008270 GO:0005524 GO:0016020
GO:0016021
Cluster-22178.112685 GO:0005515
Cluster-22178.112689 GO:0007268 GO:0007165 GO:0006811 GO:0004970
GO:0005234 GO:0016020
Cluster-22178.112694 GO:0005201 GO:0005581 GO:0005578
Cluster-22178.112703 GO:0006468 GO:0007165 GO:0016773 GO:0004672
GO:0005524 GO:0004872 GO:0005515 GO:0016021 GO:0016020
Cluster-22178.112728 GO:0004867 GO:0051536 GO:0003824
Cluster-22178.112741 GO:0042254 GO:0006412 GO:0007155 GO:0003677
GO:0005515 GO:0003735 GO:0046983 GO:0005840 GO:0016021
GO:0005622
Cluster-22178.112766 GO:0007156 GO:0005509 GO:0005515 GO:0016020
Cluster-22178.112767 GO:0007156 GO:0005509 GO:0016020
Cluster-22178.112799 GO:0006308 GO:0006259 GO:0004531
Cluster-22178.112809 GO:0006468 GO:0005524 GO:0004672 GO:0005515

Cluster-22178.112819 GO:0033014 GO:0006783 GO:0015994 GO:0008270
GO:0004852
Cluster-22178.112820 GO:0007212 GO:0048268 GO:0006355 GO:0032051
GO:0003677 GO:0008270 GO:0016021
Cluster-22178.112823 GO:0045895 GO:0007268 GO:0009405 GO:0007165
GO:0007531 GO:0030550 GO:0008301 GO:0005634 GO:0005576

Cluster-22178.112834 GO:0005515
Cluster-22178.112858 GO:0042967 GO:0006633 GO:0006508 GO:0004252
GO:0004315 GO:0005835
Cluster-22178.112868 GO:0006096 GO:0006020 GO:0006094 GO:0006098
GO:0015976 GO:0006000 GO:0006013 GO:0004332
Cluster-22178.112870 GO:0019062 GO:0019058 GO:1901031 GO:0007155
GO:0005634
Cluster-22178.112872 GO:0006013 GO:0006000 GO:0015976 GO:0006096
GO:0006098 GO:0006094 GO:0006020 GO:0004332
Cluster-22178.112875 GO:0006013 GO:0015976 GO:0006000 GO:0006096
GO:0006098 GO:0006020 GO:0006094 GO:0004332
Cluster-22178.112876 GO:0006013 GO:0015976 GO:0006000 GO:0006098
GO:0006094 GO:0006020 GO:0006096 GO:0004332
Cluster-22178.112888 GO:0008210 GO:0008207 GO:0008209 GO:0055114
GO:0006694 GO:0003854 GO:0050662 GO:0016616 GO:0003824
Cluster-22178.112889 GO:0003824 GO:0050662
Cluster-22178.112890 GO:0050832
Cluster-22178.112894 GO:0009298 GO:0006525 GO:0006355 GO:0006000
GO:0006013 GO:0003700 GO:0004615 GO:0005667 GO:0005737
Cluster-22178.112924 GO:0035556 GO:0005515
Cluster-22178.112937 GO:0007165 GO:0005158 GO:0005899
Cluster-22178.112950 GO:0016310 GO:0009069 GO:0004674
Cluster-22178.112958 GO:0031492 GO:0000785 GO:0005634
Cluster-22178.112959 GO:0031492 GO:0005634 GO:0000785
Cluster-22178.112966 GO:0007186 GO:0007268 GO:0006810 GO:0007165
GO:0006811 GO:0004970 GO:0004930 GO:0005230 GO:0016020
GO:0016021
Cluster-22178.112970 GO:0007155 GO:0006891 GO:0051015 GO:0017119
Cluster-22178.112980 GO:0019087 GO:0015031 GO:0019068 GO:0005198
GO:0016021 GO:0016020
Cluster-22178.112983 GO:0006470 GO:0007165 GO:0006570 GO:0008138
GO:0004725 GO:0016020
Cluster-22178.112986 GO:0006260 GO:0006265 GO:0003677 GO:0003916

GO:0005727
Cluster-22178.112999 GO:0006952 GO:0008284 GO:0005515 GO:0008270
GO:0046872 GO:0016020 GO:0005622 GO:0019867
Cluster-22178.11301 GO:0006508 GO:0005515 GO:0005509 GO:0004222
GO:0008270 GO:0031012
Cluster-22178.113011 GO:0050792 GO:0005515 GO:0004518
Cluster-22178.113013 GO:0009405 GO:0005515
Cluster-22178.113020 GO:0042967 GO:0006810 GO:0005524 GO:0003774
GO:0008811 GO:0005515 GO:0005856 GO:0016459
Cluster-22178.113024 GO:0009271 GO:0007186 GO:0006355 GO:0004930
GO:0016021
Cluster-22178.113039 GO:0006508 GO:0008234
Cluster-22178.113040 GO:0006355 GO:0043401 GO:0009405 GO:0043565
GO:0008270 GO:0003700 GO:0005634 GO:0005667 GO:0005576
Cluster-22178.113056 GO:0004519 GO:0003677
Cluster-22178.113066 GO:0006813 GO:0006814 GO:0005890
Cluster-22178.113093 GO:0006470 GO:0019497 GO:0006570 GO:0006771
GO:0003993 GO:0004725 GO:0046872 GO:0008138 GO:0005515
Cluster-22178.113105 GO:0007016 GO:0005515 GO:0005789 GO:0016010
Cluster-22178.113117 GO:0035556 GO:0006397 GO:0016788
Cluster-22178.113118 GO:0006486 GO:0006397 GO:0008373 GO:0016788

Cluster-22178.113133 GO:0006886 GO:0006506
Cluster-22178.113137 GO:0046872
Cluster-22178.113141 GO:0043130
Cluster-22178.113142 GO:0006355 GO:0007010 GO:0003779 GO:0003712
GO:0005667
Cluster-22178.113170 GO:0005515 GO:0016021
Cluster-22178.113175 GO:0055114 GO:0047134 GO:0046872
Cluster-22178.11326 GO:0035556 GO:0009190 GO:0016849
Cluster-22178.113264 GO:0006693 GO:0006364 GO:0010207 GO:0006691
GO:0006749 GO:0019530 GO:0015979 GO:0051382 GO:0051082
GO:0005525 GO:0003924 GO:0016758 GO:0003840 GO:0000776

Cluster-22178.113268 GO:0008272 GO:0015116 GO:0016021
Cluster-22178.113287 GO:0015986 GO:0016567 GO:0015992 GO:0055085
GO:0090263 GO:0003677 GO:0015078 GO:0000276 GO:0016021
Cluster-22178.1133 GO:0006812 GO:0000166 GO:0046872 GO:0016887
GO:0016021
Cluster-22178.11330 GO:0006355 GO:0003677
Cluster-22178.113319 GO:0003723
Cluster-22178.113326 GO:0035023 GO:0009877 GO:0009312 GO:0043087
GO:0005089 GO:0016758
Cluster-22178.113327 GO:0009312 GO:0035023 GO:0009877 GO:0043087
GO:0005089 GO:0016758
Cluster-22178.113328 GO:0043087 GO:0009312 GO:0035023 GO:0009877
GO:0016758 GO:0005089
Cluster-22178.113329 GO:0035023 GO:0009877 GO:0009312 GO:0043087
GO:0005089 GO:0016758
Cluster-22178.113332 GO:0055085 GO:0006811 GO:0005216 GO:0016020
GO:0005576
Cluster-22178.113339 GO:0015031 GO:0006812 GO:0010167 GO:0055085
GO:0006771 GO:0006270 GO:0006810 GO:0015706 GO:0019497
GO:0030001 GO:0008565 GO:0003993 GO:0046873 GO:0046872
GO:0008324 GO:0004190 GO:0005515 GO:0016020 GO:0019031
GO:0016021
Cluster-22178.113370 GO:0009405 GO:0005515 GO:0005576
Cluster-22178.113373 GO:0005515
Cluster-22178.113380 GO:0006888 GO:0006886 GO:0008270 GO:0005515
GO:0030127
Cluster-22178.113382 GO:0019079 GO:0005515
Cluster-22178.113417 GO:0008152 GO:0016758 GO:0003824
Cluster-22178.113431 GO:0005515 GO:0043395 GO:0016020 GO:0005578
Cluster-22178.113440 GO:0010508 GO:0006260 GO:0003779 GO:0005543

Cluster-22178.113465 GO:0007172 GO:0007165 GO:0006468 GO:0004713
GO:0004871 GO:0005925

Cluster-22178.113488 GO:0005515 GO:0019013 GO:0016021 GO:0016020
Cluster-22178.113497 GO:0006364 GO:0032040
Cluster-22178.113504 GO:0055114 GO:0042128 GO:0006807 GO:0008942
GO:0016491 GO:0051537
Cluster-22178.113508 GO:0003676
Cluster-22178.113515 GO:0016021
Cluster-22178.113520 GO:0006470 GO:0006570 GO:0008138 GO:0004725

Cluster-22178.113539 GO:0006468 GO:0009190 GO:0035556 GO:0005524
GO:0004672 GO:0016849
Cluster-22178.113550 GO:0006506 GO:0016746 GO:0005789 GO:0016021
GO:0019028
Cluster-22178.113576 GO:0005515
Cluster-22178.113580 GO:0006508 GO:0008234
Cluster-22178.113582 GO:0006508 GO:0008234
Cluster-22178.113607 GO:0016887 GO:0005524
Cluster-22178.113610 GO:0006308 GO:0045444 GO:0008855 GO:0009318
Cluster-22178.113614 GO:0045444 GO:0003677
Cluster-22178.113650 GO:0006810 GO:0005215 GO:0005515 GO:0016020
Cluster-22178.113672 GO:0006355 GO:0005515 GO:0005668 GO:0005634
Cluster-22178.113678 GO:0006836 GO:0006826 GO:0034755 GO:0006812
GO:0005328 GO:0005381 GO:0016021
Cluster-22178.113718 GO:0006891 GO:0007155 GO:0051015 GO:0017119
Cluster-22178.113732 GO:0006270 GO:0006355 GO:0003677
Cluster-22178.113733 GO:0009069 GO:0006468 GO:0016310 GO:0005516
GO:0004683 GO:0016773 GO:0005524 GO:0004672 GO:0016020
Cluster-22178.113735 GO:0055114 GO:0007049 GO:0006606 GO:0006355
GO:0006886 GO:0009271 GO:0005506 GO:0020037 GO:0016705
GO:0005634 GO:0016020
Cluster-22178.113739 GO:0007165 GO:0007606 GO:0004888 GO:0016020
Cluster-22178.113752 GO:0006260 GO:0006355 GO:0003700 GO:0005622
GO:0005634 GO:0005667
Cluster-22178.113753 GO:0006260 GO:0006355 GO:0003700 GO:0005634

GO:0005667
Cluster-22178.113807 GO:0003676
Cluster-22178.113820 GO:0006606 GO:0006260 GO:0055114 GO:0042803
GO:0008134 GO:0045502 GO:0003723 GO:0016903 GO:0005667
GO:0030286
Cluster-22178.113824 GO:0006397
Cluster-22178.113836 GO:0007606 GO:0007165 GO:0007186 GO:0004930
GO:0004888 GO:0005886 GO:0016021 GO:0016020
Cluster-22178.113838 GO:0007606 GO:0007186 GO:0007165 GO:0004930
GO:0004888 GO:0005886 GO:0016021 GO:0016020
Cluster-22178.113847 GO:0016747
Cluster-22178.113849 GO:0016747
Cluster-22178.113858 GO:0019497 GO:0006470 GO:0006771 GO:0006298
GO:0006570 GO:0005524 GO:0046872 GO:0003993 GO:0004725
GO:0030983 GO:0005515 GO:0016020
Cluster-22178.113870 GO:0008270
Cluster-22178.113879 GO:0030001 GO:0019497 GO:0015706 GO:0006810
GO:0006270 GO:0006771 GO:0055085 GO:0006812 GO:0010167
GO:0015031 GO:0005515 GO:0004190 GO:0046872 GO:0008324
GO:0046873 GO:0003993 GO:0008565 GO:0019031 GO:0016021
GO:0016020
Cluster-22178.113887 GO:0005524 GO:0008270
Cluster-22178.113892 GO:0007166 GO:0007186 GO:0004930 GO:0005515
GO:0016021 GO:0016020
Cluster-22178.113893 GO:0005509 GO:0005515 GO:0016846
Cluster-22178.113907 GO:0016747
Cluster-22178.113933 GO:0030474 GO:0006468 GO:0007049 GO:0005200
GO:0003676 GO:0004672 GO:0005524 GO:0016773 GO:0005747
GO:0016020 GO:0005739 GO:0005823 GO:0005856
Cluster-22178.113934 GO:0006468 GO:0030474 GO:0005524 GO:0004672
GO:0016773 GO:0005200 GO:0003676 GO:0005739 GO:0016020
GO:0005747 GO:0005856 GO:0005823
Cluster-22178.113947 GO:0022900 GO:0016032 GO:0006355 GO:0008270

GO:0003682 GO:0003677 GO:0000785 GO:0000775 GO:0005739
GO:0005634 GO:0005747 GO:0019012
Cluster-22178.11396 GO:0006308 GO:0055114 GO:0009307 GO:0016491
GO:0009036 GO:0005515 GO:0003677 GO:0009359
Cluster-22178.113964 GO:0005515
Cluster-22178.113981 GO:0006955 GO:0007165 GO:0006935 GO:0008009
GO:0005576
Cluster-22178.114006 GO:0005515
Cluster-22178.114019 GO:0004867 GO:0005576
Cluster-22178.11403 GO:0009058 GO:0005524
Cluster-22178.114036 GO:0006206 GO:0006231 GO:0006355 GO:0050660
GO:0050797 GO:0003676 GO:0005524 GO:0008270 GO:0016818
GO:0003677 GO:0005515 GO:0016787 GO:0005634
Cluster-22178.114039 GO:0005096
Cluster-22178.114045 GO:0006468 GO:0006810 GO:0009405 GO:0005524
GO:0004672 GO:0008200 GO:0005515 GO:0042151 GO:0016021
Cluster-22178.114072 GO:0007264 GO:0005525
Cluster-22178.114112 GO:0006468 GO:0005524 GO:0004672
Cluster-22178.114118 GO:0055114 GO:0007165 GO:0048038 GO:0016651
GO:0005515 GO:0005509 GO:0004888 GO:0051287 GO:0016021
Cluster-22178.114123 GO:0009405
Cluster-22178.11414 GO:0032955 GO:0051301 GO:0007165 GO:0005544
GO:0005509 GO:0016020
Cluster-22178.114144 GO:0006914 GO:0006260 GO:0010508 GO:0005524
GO:0035299
Cluster-22178.114145 GO:0006260 GO:0010506 GO:0000921 GO:0055114
GO:0006914 GO:0007165 GO:0050750 GO:0016491 GO:0008201
GO:0005615 GO:0016021 GO:0005940 GO:0005783
Cluster-22178.114146 GO:0006457 GO:0006260 GO:0007049 GO:0000921
GO:0006914 GO:0035299 GO:0051082 GO:0005524 GO:0016021
GO:0016272 GO:0005940
Cluster-22178.114150 GO:0006886 GO:0007049 GO:0055114 GO:0007165
GO:0006457 GO:0006260 GO:0010506 GO:0015031 GO:0000921

GO:0006914 GO:0051082 GO:0050750 GO:0008201 GO:0016491
GO:0008565 GO:0005615 GO:0016021 GO:0005737 GO:0005783
GO:0016020 GO:0016272 GO:0005940
Cluster-22178.114160 GO:0005509 GO:0005515 GO:0016020
Cluster-22178.114172 GO:0005515
Cluster-22178.114178 GO:0035023 GO:0043087 GO:0055114 GO:0016972
GO:0005089
Cluster-22178.114179 GO:0035023 GO:0043087 GO:0055114 GO:0016972
GO:0005089
Cluster-22178.114205 GO:0005975 GO:0016773
Cluster-22178.114206 GO:0005975 GO:0016773
Cluster-22178.114207 GO:0005975 GO:0016773
Cluster-22178.114258 GO:0009058 GO:0008152 GO:0016788 GO:0016787
GO:0005576
Cluster-22178.11426 GO:0006570 GO:0006470 GO:0004725 GO:0008138

Cluster-22178.114273 GO:0004252 GO:0070008
Cluster-22178.11428 GO:0006470 GO:0006570 GO:0004725 GO:0008138

Cluster-22178.114301 GO:0006355 GO:0003700 GO:0043565 GO:0005815
GO:0005667
Cluster-22178.114320 GO:0055114 GO:0046872 GO:0016702 GO:0005515

Cluster-22178.114327 GO:0010508 GO:0016042 GO:0046339 GO:0009395
GO:0006308 GO:0005509 GO:0004435 GO:0008855 GO:0009318
Cluster-22178.114356 GO:0007155 GO:0005515 GO:0016021
Cluster-22178.114393 GO:0016491
Cluster-22178.114400 GO:0005515
Cluster-22178.114406 GO:0032324
Cluster-22178.114413 GO:0003779
Cluster-22178.114414 GO:0003779
Cluster-22178.114417 GO:0003779
Cluster-22178.114421 GO:0016787

Cluster-22178.114435 GO:0005515 GO:0016020
Cluster-22178.114444 GO:0006480 GO:0008152 GO:0008168 GO:0008171

Cluster-22178.114447 GO:0006285 GO:0006281 GO:0006284 GO:0006824
GO:0006308 GO:0009236 GO:0006289 GO:0003684 GO:0008534
GO:0003677 GO:0015087 GO:0016020
Cluster-22178.114471 GO:0004190 GO:0016020
Cluster-22178.114473 GO:0006355 GO:0006352 GO:0003700 GO:0003677
GO:0016987 GO:0005667
Cluster-22178.114476 GO:0007131 GO:0003677
Cluster-22178.114518 GO:0031505
Cluster-22178.114527 GO:0016787 GO:0046872 GO:0005886
Cluster-22178.114535 GO:0005515
Cluster-22178.114537 GO:0006468 GO:0007275 GO:0016773 GO:0005524
GO:0004672 GO:0042600 GO:0016020
Cluster-22178.114539 GO:0007411 GO:0005886 GO:0016020 GO:0016021
Cluster-22178.114572 GO:0015693 GO:0015095 GO:0016021
Cluster-22178.114595 GO:0009405 GO:0006810 GO:0055085 GO:0005215
GO:0045735 GO:0016020
Cluster-22178.114603 GO:0007186 GO:0004930 GO:0016021
Cluster-22178.114606 GO:0030001 GO:0007186 GO:0055085 GO:0046873
GO:0004930 GO:0016021 GO:0016020
Cluster-22178.114621 GO:0006869 GO:0005319
Cluster-22178.114622 GO:0006869 GO:0005319
Cluster-22178.114623 GO:0006869 GO:0005319
Cluster-22178.114637 GO:0009306 GO:0016020
Cluster-22178.114642 GO:0005515
Cluster-22178.114654 GO:0003677 GO:0005634
Cluster-22178.114655 GO:0006486 GO:0015979 GO:0008378 GO:0016757
GO:0009522 GO:0016020 GO:0016021
Cluster-22178.114667 GO:0005515
Cluster-22178.114684 GO:0048870 GO:0019069 GO:0030474 GO:0071988
GO:0031514

Cluster-22178.114700 GO:0007186 GO:0004930 GO:0016021
Cluster-22178.114704 GO:0007155 GO:0005540 GO:0016020
Cluster-22178.114725 GO:0006355 GO:0003700 GO:0043565 GO:0005667
Cluster-22178.114727 GO:0006355 GO:0003700 GO:0008270 GO:0005667
GO:0005634
Cluster-22178.114750 GO:0005515
Cluster-22178.114755 GO:0006334 GO:0003677 GO:0000786 GO:0019028
GO:0005634
Cluster-22178.114773 GO:0007186 GO:0004930 GO:0016020
Cluster-22178.114784 GO:0016021
Cluster-22178.114793 GO:0005576
Cluster-22178.114817 GO:0006470 GO:0006465 GO:0006570 GO:0004725
GO:0008233 GO:0008138 GO:0016021 GO:0005787
Cluster-22178.114819 GO:0007186 GO:0004222 GO:0004930 GO:0016021
GO:0016020
Cluster-22178.114827 GO:0009312 GO:0009877 GO:0035023 GO:0043087
GO:0005089 GO:0016758
Cluster-22178.114828 GO:0043087 GO:0009877 GO:0035023 GO:0009312
GO:0016758 GO:0005089
Cluster-22178.114829 GO:0005507
Cluster-22178.114860 GO:0006935 GO:0007165 GO:0006955 GO:0008009
GO:0005576
Cluster-22178.11487 GO:0016021
Cluster-22178.114901 GO:0004222 GO:0008233 GO:0031012
Cluster-22178.114919 GO:0005515
Cluster-22178.114936 GO:0040007 GO:0008283 GO:0007165 GO:0008083
GO:0016020
Cluster-22178.114938 GO:0015031 GO:0009306 GO:0001522 GO:0009451
GO:0007186 GO:0009982 GO:0015450 GO:0004930 GO:0003723
GO:0016021 GO:0009941
Cluster-22178.114945 GO:0016032 GO:0018142 GO:0006260 GO:0006308
GO:0050920 GO:0006144 GO:0005515 GO:0016779 GO:0016888
GO:0003824 GO:0042624 GO:0046872 GO:0005615 GO:0009288

Cluster-22178.114954 GO:0008270
Cluster-22178.114955 GO:0006468 GO:0004867 GO:0004672 GO:0005524
GO:0005515
Cluster-22178.114970 GO:0042254 GO:0006412 GO:0003735 GO:0005622
GO:0005840
Cluster-22178.114973 GO:0008270
Cluster-22178.114984 GO:0006810 GO:0015858 GO:0005337 GO:0005515
GO:0016021
Cluster-22178.114987 GO:0006769 GO:0006486 GO:0046497 GO:0003953
GO:0008378 GO:0016020
Cluster-22178.114991 GO:0008152 GO:0016791
Cluster-22178.114992 GO:0055114 GO:0008033 GO:0016117 GO:0016491
GO:0008168 GO:0050660 GO:0071949 GO:0016705
Cluster-22178.11501 GO:0005525
Cluster-22178.115092 GO:0006468 GO:0005515 GO:0046872 GO:0004672
GO:0005524
Cluster-22178.115116 GO:0016032 GO:0019031
Cluster-22178.115164 GO:0006813 GO:0006814 GO:0005890
Cluster-22178.115177 GO:0042246 GO:0007155 GO:0016021 GO:0009512
Cluster-22178.115182 GO:0005515
Cluster-22178.115195 GO:0030436 GO:0015031 GO:0006508 GO:0004190
GO:0005789 GO:0016021
Cluster-22178.115198 GO:0016020
Cluster-22178.115199 GO:0006508 GO:0016805
Cluster-22178.115227 GO:0007156 GO:0005509 GO:0016020
Cluster-22178.115228 GO:0046872 GO:0005515
Cluster-22178.115237 GO:0055085 GO:0006811 GO:0005216 GO:0016020
Cluster-22178.115243 GO:0008360 GO:0042803 GO:0008134 GO:0045502
GO:0051082 GO:0016020 GO:0005667 GO:0030286
Cluster-22178.115251 GO:0019836 GO:0005515 GO:0003723 GO:0042025
GO:0005576
Cluster-22178.115252 GO:0019836 GO:0003723 GO:0005515 GO:0005576
GO:0042025

Cluster-22178.115253 GO:0019087 GO:0005515 GO:0016021
Cluster-22178.115257 GO:0019836 GO:0005515 GO:0003723 GO:0042025
GO:0005576
Cluster-22178.115258 GO:0019836 GO:0005515 GO:0003723 GO:0042025
GO:0005576
Cluster-22178.115268 GO:0006508 GO:0006367 GO:0004252 GO:0005672
Cluster-22178.11527 GO:0006814 GO:0032006 GO:0005272 GO:0016020
Cluster-22178.115294 GO:0006355 GO:0003700 GO:0003677 GO:0005634
GO:0005667
Cluster-22178.115311 GO:0035092 GO:0003677 GO:0005509 GO:0016020
GO:0000228
Cluster-22178.11534 GO:0007165 GO:0006810 GO:0006606 GO:0015031
GO:0030168 GO:0006914 GO:0006886 GO:0010508 GO:0006355
GO:0007049 GO:0051258 GO:0030674 GO:0005102 GO:0008565
GO:0051082 GO:0008289 GO:0003700 GO:0043565 GO:0005783
GO:0016020 GO:0005667 GO:0005577 GO:0005886 GO:0016021
GO:0005794 GO:0005737
Cluster-22178.115348 GO:0006810 GO:0006811 GO:0003677 GO:0005230
GO:0016020
Cluster-22178.115350 GO:0006508 GO:0006351 GO:0006810 GO:0009405
GO:0043565 GO:0008241 GO:0008237 GO:0008200 GO:0005576
GO:0016020
Cluster-22178.115369 GO:0030168 GO:0000921 GO:0071973 GO:0007165
GO:0007155 GO:0006525 GO:0051258 GO:0006886 GO:0006527
GO:0006560 GO:0005102 GO:0008792 GO:0030674 GO:0004871
GO:0005198 GO:0005577 GO:0005940 GO:0016020 GO:0016021
GO:0005801
Cluster-22178.115377 GO:0006814 GO:0007186 GO:0006744 GO:0006120
GO:0015992 GO:0055114 GO:0008137 GO:0004930 GO:0016021
Cluster-22178.115404 GO:0006810 GO:0005524 GO:0016820 GO:0005215
GO:0043190
Cluster-22178.115405 GO:0006810 GO:0005524 GO:0016820 GO:0005215
GO:0043190

Cluster-22178.115406 GO:0055085 GO:0006811 GO:0006355 GO:0005515
GO:0005216 GO:0016020
Cluster-22178.115430 GO:0071436 GO:0006814 GO:0006560 GO:0006525
GO:0006090 GO:0008948 GO:0015081 GO:0016020
Cluster-22178.115434 GO:0055114 GO:0010309
Cluster-22178.115459 GO:0006801 GO:0016151 GO:0004784 GO:0005515
GO:0016209 GO:0003723
Cluster-22178.115463 GO:0044341 GO:0010923 GO:0006817 GO:0019902
GO:0015321 GO:0016020
Cluster-22178.115467 GO:0007165 GO:0005515
Cluster-22178.115468 GO:0003676
Cluster-22178.115479 GO:0016192 GO:0016021
Cluster-22178.115482 GO:0007165 GO:0006810 GO:0006955 GO:0008283
GO:0040007 GO:0008083 GO:0008200 GO:0005134 GO:0005576
GO:0005893
Cluster-22178.115506 GO:0007186 GO:0004930 GO:0016021 GO:0016020
Cluster-22178.115507 GO:0007186 GO:0006397 GO:0004930 GO:0003723
GO:0016020 GO:0005634 GO:0005737
Cluster-22178.115533 GO:0005515
Cluster-22178.11555 GO:0004190 GO:0016021
Cluster-22178.115573 GO:0051252 GO:0006397 GO:0004540
Cluster-22178.115583 GO:0006811 GO:0055085 GO:0005216 GO:0005887
GO:0016020
Cluster-22178.11559 GO:0005509 GO:0005515
Cluster-22178.115590 GO:0007155 GO:0005540
Cluster-22178.115592 GO:0005515
Cluster-22178.1156 GO:0006629 GO:0007165 GO:0046339 GO:0009395
GO:0035556 GO:0016310 GO:0016042 GO:0000160 GO:0000155
GO:0005509 GO:0005515 GO:0004435 GO:0009365 GO:0016021
Cluster-22178.115641 GO:0006355 GO:0008270 GO:0003700 GO:0005667
GO:0005634
Cluster-22178.115648 GO:0006810 GO:0009405 GO:0019871 GO:0005515
GO:0005576

Cluster-22178.115651 GO:0008134 GO:0003677 GO:0005667 GO:0005634
Cluster-22178.115661 GO:0006811 GO:0055085 GO:0005216 GO:0016020
Cluster-22178.115670 GO:0006508 GO:0006355 GO:0008152 GO:0016788
GO:0043565 GO:0008270 GO:0003700 GO:0004181 GO:0005667
Cluster-22178.115688 GO:0040007 GO:0005737
Cluster-22178.115696 GO:0003950
Cluster-22178.115755 GO:0007156 GO:0005509 GO:0016020
Cluster-22178.115760 GO:0016020
Cluster-22178.115775 GO:0006096 GO:0009058 GO:0006094 GO:0016310
GO:0008152 GO:0055114 GO:0015976 GO:0004618 GO:0016742
GO:0016491
Cluster-22178.115776 GO:0055114 GO:0008152 GO:0016491
Cluster-22178.115790 GO:0045444 GO:0030695
Cluster-22178.115793 GO:0006508 GO:0004252
Cluster-22178.115797 GO:0010038
Cluster-22178.1158 GO:0009405 GO:0006810 GO:0006952 GO:0007165
GO:0019855 GO:0005509 GO:0005515 GO:0005578 GO:0005576
Cluster-22178.115819 GO:0009190 GO:0006144 GO:0006171 GO:0035556
GO:0016849 GO:0004016 GO:0005886
Cluster-22178.115824 GO:0005515
Cluster-22178.115845 GO:0005975 GO:0055114 GO:0016758 GO:0016491
GO:0016020
Cluster-22178.115850 GO:0008270 GO:0005634
Cluster-22178.115874 GO:0005515
Cluster-22178.115879 GO:0009405 GO:0005576
Cluster-22178.115892 GO:0005515
Cluster-22178.115910 GO:0015858 GO:0006810 GO:0005337 GO:0016021
Cluster-22178.115926 GO:0009405 GO:0006952 GO:0005576
Cluster-22178.11597 GO:0006810 GO:0009405 GO:0007094 GO:0007155
GO:0008289 GO:0019905 GO:0016020 GO:0048786 GO:0005794
Cluster-22178.115978 GO:0043087 GO:0007264 GO:0007165 GO:0005085

Cluster-22178.11598 GO:0006810 GO:0007155 GO:0009405 GO:0007094

GO:0006886 GO:0048870 GO:0006308 GO:0019905 GO:0008855
GO:0008200 GO:0008289 GO:0005783 GO:0016020 GO:0009318
GO:0031514 GO:0005576 GO:0030089 GO:0048786 GO:0005794
GO:0016021
Cluster-22178.115986 GO:0006308 GO:0008855 GO:0009318
Cluster-22178.115996 GO:0005515
Cluster-22178.11601 GO:0005515
Cluster-22178.116014 GO:0006556 GO:0006270 GO:0006555 GO:0004478

Cluster-22178.11603 GO:0070836
Cluster-22178.116038 GO:0008270 GO:0017176 GO:0005515 GO:0008376
GO:0032580 GO:0005622 GO:0016020
Cluster-22178.11605 GO:0005515
Cluster-22178.116061 GO:0005525
Cluster-22178.116065 GO:0006468 GO:0004672 GO:0005524 GO:0016020
Cluster-22178.116067 GO:0016032
Cluster-22178.116069 GO:0007606 GO:0007165 GO:0007186 GO:0004888
GO:0004930 GO:0016021 GO:0016020
Cluster-22178.116085 GO:0005515
Cluster-22178.116091 GO:0046232 GO:0042184 GO:0018874 GO:0042203
GO:0006468 GO:0019261 GO:0009712 GO:0055114 GO:0018576
GO:0005506 GO:0016773 GO:0004672 GO:0005524 GO:0016020
Cluster-22178.116114 GO:0006813 GO:0005242 GO:0008076 GO:0016021
Cluster-22178.116148 GO:0008152 GO:0006508 GO:0008236 GO:0016787

Cluster-22178.116162 GO:0006355 GO:0046872 GO:0005634
Cluster-22178.116169 GO:0016021
Cluster-22178.116175 GO:0005887
Cluster-22178.116188 GO:0055085 GO:0030001 GO:0046873 GO:0016020
Cluster-22178.116208 GO:0005515
Cluster-22178.116221 GO:0042262 GO:0051258 GO:0009405 GO:0006606
GO:0010506 GO:0030168 GO:0006914 GO:0007165 GO:0008134
GO:0042803 GO:0005198 GO:0045502 GO:0005102 GO:0003690

GO:0030674 GO:0005615 GO:0005577 GO:0030286 GO:0005882
GO:0016020 GO:0005667
Cluster-22178.116225 GO:0005975 GO:0003824 GO:0005515
Cluster-22178.116234 GO:0006468 GO:0004672 GO:0005524 GO:0005515
GO:0016020
Cluster-22178.116236 GO:0051258 GO:0006468 GO:0006886 GO:0006560
GO:0006527 GO:0000921 GO:0030168 GO:0007155 GO:0006525
GO:0071973 GO:0007165 GO:0004672 GO:0004871 GO:0005198
GO:0016773 GO:0005524 GO:0005102 GO:0008792 GO:0030674
GO:0005801 GO:0016021 GO:0005940 GO:0005577 GO:0016020
Cluster-22178.11624 GO:0005515 GO:0016020
Cluster-22178.11625 GO:0017004 GO:0015886 GO:0005515 GO:0016021
Cluster-22178.116251 GO:0005201 GO:0005581 GO:0005578
Cluster-22178.116252 GO:0005201 GO:0005578 GO:0005581
Cluster-22178.116283 GO:0006816 GO:0006810 GO:0009097 GO:0009099
GO:0055114 GO:0009098 GO:0006090 GO:0003852 GO:0005506
GO:0005246 GO:0020037 GO:0016705 GO:0042030 GO:0016020
Cluster-22178.116285 GO:0070072 GO:0006508 GO:0004197
Cluster-22178.116312 GO:0042254 GO:0006412 GO:0007165 GO:0006508
GO:0006629 GO:0035556 GO:0009395 GO:0046339 GO:0005509
GO:0005515 GO:0003735 GO:0004197 GO:0004435 GO:0005578
GO:0005840
Cluster-22178.116330 GO:0019058 GO:0006461 GO:0005739
Cluster-22178.11634 GO:0005515 GO:0016020
Cluster-22178.116352 GO:0006355 GO:0043401
Cluster-22178.116383 GO:0007094 GO:0005198 GO:0005882
Cluster-22178.116393 GO:0006298 GO:0005524 GO:0030983 GO:0016021
Cluster-22178.116398 GO:0005634
Cluster-22178.116414 GO:0007155 GO:0016567 GO:0061630 GO:0005515
GO:0046872 GO:0008270 GO:0008168 GO:0004842 GO:0005622
Cluster-22178.116423 GO:0007029 GO:0016021 GO:0005789
Cluster-22178.116430 GO:0007155 GO:0006338 GO:0005515 GO:0004527
GO:0003676 GO:0016514 GO:0005634 GO:0016021 GO:0016586

Cluster-22178.116435 GO:0006511 GO:0016567 GO:0007275 GO:0005515 GO:0008270 GO:0046872 GO:0004842 GO:0005634

Cluster-22178.116491 GO:0010508 GO:0070206 GO:0005198 GO:0003774 GO:0005882 GO:0016459

Cluster-22178.116531 GO:0004222 GO:0005524 GO:0008270 GO:0016021

Cluster-22178.116548 GO:0006633 GO:0007186 GO:0006813 GO:0003824 GO:0004930 GO:0016747 GO:0005267 GO:0016021

Cluster-22178.116553 GO:0060048 GO:0048769 GO:0055008 GO:0048739 GO:0003009 GO:0030241 GO:0055003 GO:0030916 GO:0030240 GO:0007512 GO:0001756 GO:0070080 GO:0031432 GO:0008307 GO:0030018

Cluster-22178.11658 GO:0009231 GO:0008531

Cluster-22178.116589 GO:0015074 GO:0005515

Cluster-22178.116592 GO:0035556 GO:0006468 GO:0009190 GO:0005524 GO:0004672 GO:0016849

Cluster-22178.116607 GO:0007165 GO:0005515 GO:0005096 GO:0003729

Cluster-22178.116621 GO:0008146 GO:0016021 GO:0019012

Cluster-22178.116657 GO:0043087 GO:0006355 GO:0005089 GO:0019902 GO:0005737 GO:0005634

Cluster-22178.116658 GO:0019902

Cluster-22178.116679 GO:0007186 GO:0004930 GO:0016021

Cluster-22178.116701 GO:0006468 GO:0005524 GO:0004672

Cluster-22178.116709 GO:0032324

Cluster-22178.116719 GO:0055085 GO:0006260 GO:0022857 GO:0003677 GO:0016021 GO:0005664

Cluster-22178.116731 GO:0005515 GO:0005524

Cluster-22178.116746 GO:0034314 GO:0001558 GO:0030041 GO:0005520 GO:0005515 GO:0015629 GO:0005885 GO:0016942 GO:0005576

Cluster-22178.116747 GO:0015991 GO:0015992 GO:0003723 GO:0005515 GO:0005198 GO:0015078 GO:0019028 GO:0033179

Cluster-22178.116748 GO:0016887 GO:0003774 GO:0005524 GO:0005515 GO:0016459

Cluster-22178.116749 GO:0019058 GO:0004867
Cluster-22178.11677 GO:0006508 GO:0004197
Cluster-22178.11678 GO:0006508 GO:0004197
Cluster-22178.116830 GO:0030246
Cluster-22178.116853 GO:0007186 GO:0004930 GO:0016021 GO:0016020
Cluster-22178.116903 GO:0006801 GO:0055114 GO:0046872
Cluster-22178.116936 GO:0005515
Cluster-22178.116942 GO:0007155 GO:0005540
Cluster-22178.116956 GO:0006468 GO:0007169 GO:0004672 GO:0005524
GO:0004714 GO:0016020
Cluster-22178.116998 GO:0009607 GO:0016021
Cluster-22178.11701 GO:0006811 GO:0055085 GO:0035444 GO:0015675
GO:0005216 GO:0015099 GO:0046872 GO:0016020 GO:0016021
Cluster-22178.117017 GO:0006812 GO:0008324 GO:0016021
Cluster-22178.117020 GO:0030001 GO:0055085 GO:0046873 GO:0016020
Cluster-22178.117041 GO:0006508 GO:0004197
Cluster-22178.117045 GO:0006486 GO:0008378 GO:0016020
Cluster-22178.117095 GO:0016020
Cluster-22178.117109 GO:0006120 GO:0006397 GO:0051252 GO:0055114
GO:0004540
Cluster-22178.117110 GO:0055114 GO:0006120 GO:0051252 GO:0006397
GO:0004540
Cluster-22178.117133 GO:0003677 GO:0008270
Cluster-22178.117146 GO:0006955 GO:0000902 GO:0005515 GO:0016020
GO:0005887
Cluster-22178.117149 GO:0007165 GO:0006144 GO:0005515 GO:0004114

Cluster-22178.117150 GO:0007165 GO:0006144 GO:0005515 GO:0004114

Cluster-22178.117163 GO:0007049 GO:0006914 GO:0006886 GO:0030474
GO:0003676 GO:0005200 GO:0016021 GO:0005823 GO:0005856
GO:0005783
Cluster-22178.117168 GO:0006771 GO:0019497 GO:0046872 GO:0005515

GO:0003993

Cluster-22178.117170 GO:0005515 GO:0016020

Cluster-22178.117185 GO:0055085 GO:0030001 GO:0046873 GO:0016020

Cluster-22178.117186 GO:0006355 GO:0005524 GO:0005634

Cluster-22178.11723 GO:0007165 GO:0006260 GO:0005179 GO:0005524

GO:0003677 GO:0005576 GO:0016020

Cluster-22178.117245 GO:0046983

Cluster-22178.11730 GO:0005515

Cluster-22178.11731 GO:0005515

Cluster-22178.117315 GO:0009451 GO:0051539 GO:0003824

Cluster-22178.117411 GO:0051775 GO:0006508 GO:0045892 GO:0004198

GO:0005509 GO:0005622 GO:0005737

Cluster-22178.117462 GO:0006351 GO:0006144 GO:0006206 GO:0003899

GO:0003677 GO:0003723 GO:0005730

Cluster-22178.117497 GO:0035556 GO:0009190 GO:0006468 GO:0016849

GO:0005524 GO:0004672

Cluster-22178.117498 GO:0009190 GO:0006468 GO:0035556 GO:0016849

GO:0004672 GO:0005524

Cluster-22178.11758 GO:0016032

Cluster-22178.117592 GO:0019069 GO:0007165 GO:0015979 GO:0005509

GO:0005515 GO:0005578 GO:0042025 GO:0009522

Cluster-22178.117611 GO:0051056 GO:0005096

Cluster-22178.117612 GO:0051056 GO:0005096

Cluster-22178.117623 GO:0019068 GO:0019904 GO:0008134 GO:0005667

Cluster-22178.117645 GO:0007186 GO:0004930 GO:0016021 GO:0016020

Cluster-22178.117647 GO:0007186 GO:0004930 GO:0016021 GO:0016020

Cluster-22178.11769 GO:0006355 GO:0009271

Cluster-22178.11771 GO:0070836

Cluster-22178.117757 GO:0015074 GO:0043087 GO:0035023 GO:0008270

GO:0005089

Cluster-22178.11777 GO:0009405 GO:0009279 GO:0005576

Cluster-22178.117793 GO:0005515

Cluster-22178.11785 GO:0055085 GO:0006811 GO:0005216 GO:0016020

Cluster-22178.117863 GO:0003677

Cluster-22178.117875 GO:0006810 GO:0015893 GO:0007186 GO:0006855 GO:0055085 GO:0015238 GO:0015297 GO:0004930 GO:0016020 GO:0016021

Cluster-22178.117932 GO:0070836 GO:0006952 GO:0005576

Cluster-22178.117938 GO:0005515

Cluster-22178.117939 GO:0005515

Cluster-22178.11798 GO:0006308 GO:0006914 GO:0055085 GO:0007067 GO:0030001 GO:0008855 GO:0008270 GO:0046873 GO:0005198 GO:0019031 GO:0072686 GO:0019028 GO:0016020 GO:0009318 GO:0042729

Cluster-22178.11799 GO:0006013 GO:0006004 GO:0006000 GO:0008736 GO:0005515 GO:0005737

Cluster-22178.118031 GO:0019028

Cluster-22178.118070 GO:0006816 GO:0070588 GO:0005515 GO:0005245 GO:0005891

Cluster-22178.118085 GO:0006298 GO:0030983 GO:0005524

Cluster-22178.11809 GO:0006468 GO:0006836 GO:0006812 GO:0004672 GO:0005335 GO:0005887

Cluster-22178.118112 GO:0046872

Cluster-22178.118114 GO:0006355 GO:0043401 GO:0008270 GO:0043565 GO:0003700 GO:0005634 GO:0005667

Cluster-22178.118121 GO:0006811 GO:0007165 GO:0007268 GO:0006306 GO:0005234 GO:0004970 GO:0003677 GO:0008170 GO:0016020

Cluster-22178.118122 GO:0007165 GO:0007268 GO:0006306 GO:0006811 GO:0003677 GO:0008170 GO:0005234 GO:0004970 GO:0016020

Cluster-22178.118124 GO:0006306 GO:0007268 GO:0007165 GO:0006811 GO:0008170 GO:0003677 GO:0005234 GO:0004970 GO:0016020

Cluster-22178.118163 GO:0006468 GO:0004672 GO:0005524

Cluster-22178.118166 GO:0004222 GO:0008233 GO:0031012

Cluster-22178.118171 GO:0016021

Cluster-22178.11818 GO:0006810 GO:0005524

Cluster-22178.11819 GO:0006810 GO:0006164 GO:0006807 GO:0006188

GO:0055085 GO:0006144 GO:0005525 GO:0003937 GO:0042626
GO:0005524 GO:0016887 GO:0003924 GO:0042720 GO:0016021
Cluster-22178.118192 GO:0016021
Cluster-22178.118217 GO:0006471 GO:0009405 GO:0003956 GO:0005576
Cluster-22178.118232 GO:0015986 GO:0015992 GO:0055114 GO:0015078
GO:0016972 GO:0000276
Cluster-22178.118281 GO:0007166 GO:0016020
Cluster-22178.118282 GO:0007186 GO:0004930 GO:0016021
Cluster-22178.118307 GO:0035556 GO:0006811 GO:0005509 GO:0005515
GO:0005216 GO:0016627 GO:0016020
Cluster-22178.118328 GO:0006955 GO:0030153 GO:0043087 GO:0005089
GO:0015643 GO:0019814 GO:0005737
Cluster-22178.118349 GO:0003677
Cluster-22178.118354 GO:0045454
Cluster-22178.118363 GO:0030246 GO:0005921
Cluster-22178.11838 GO:0006355 GO:0006352 GO:0016987 GO:0003677
GO:0005515 GO:0003700 GO:0005667
Cluster-22178.118383 GO:0006355 GO:0005634
Cluster-22178.118384 GO:0006355 GO:0005634
Cluster-22178.118521 GO:0005515
Cluster-22178.118530 GO:0006768 GO:0009102 GO:0005524 GO:0042410

Cluster-22178.118545 GO:0003723
Cluster-22178.118555 GO:0019076 GO:0006812 GO:0032801 GO:0005261
GO:0046935 GO:0005944 GO:0033644
Cluster-22178.118561 GO:0008608 GO:0008033 GO:0006308 GO:0005515
GO:0000166 GO:0016879 GO:0008855 GO:0005096 GO:0005524
GO:0009318 GO:0042729 GO:0005876 GO:0005737
Cluster-22178.118562 GO:0005515 GO:0005096
Cluster-22178.118573 GO:0007156 GO:0005515 GO:0005509 GO:0016021
GO:0016020
Cluster-22178.118587 GO:0008033 GO:0051252 GO:0004540 GO:0015631
GO:0045298

Cluster-22178.118595 GO:0006355 GO:0051258 GO:0007155 GO:0007165
GO:0006914 GO:0030168 GO:0000921 GO:0005198 GO:0043565
GO:0003700 GO:0030674 GO:0003774 GO:0005102 GO:0016021
GO:0005667 GO:0016459 GO:0005882 GO:0005940 GO:0005577
Cluster-22178.118607 GO:0007186 GO:0004930 GO:0016021
Cluster-22178.118612 GO:0007049 GO:0043093 GO:0006355 GO:0000917
GO:0043565 GO:0003700 GO:0003677 GO:0005737 GO:0005815
GO:0005634 GO:0005667 GO:0045095
Cluster-22178.118613 GO:0016192 GO:0006355 GO:0005515 GO:0042393
GO:0016021 GO:0005634
Cluster-22178.118615 GO:0005515
Cluster-22178.11862 GO:0046872
Cluster-22178.118652 GO:0006118 GO:0022904 GO:0009055 GO:0016021
GO:0016020
Cluster-22178.118659 GO:0005515 GO:0042470
Cluster-22178.118672 GO:0007018 GO:0006281 GO:0007017 GO:0000077
GO:0006308 GO:0000014 GO:0003777 GO:0008017 GO:0005524
GO:0005874 GO:0045298
Cluster-22178.118674 GO:0007165 GO:0007275 GO:0055085 GO:0005179
GO:0022857 GO:0019028 GO:0005576 GO:0016021
Cluster-22178.118697 GO:0007165 GO:0005515
Cluster-22178.118699 GO:0005975 GO:0016758 GO:0016020
Cluster-22178.11871 GO:0005856
Cluster-22178.118711 GO:0005506 GO:0046872 GO:0043130
Cluster-22178.118715 GO:0015940 GO:0015937 GO:0006508 GO:0008236
GO:0016787 GO:0004140 GO:0005524
Cluster-22178.118724 GO:0005515
Cluster-22178.118730 GO:0034477 GO:0004518 GO:0005515
Cluster-22178.118739 GO:0006810 GO:0016021
Cluster-22178.11874 GO:0000975
Cluster-22178.118740 GO:0045859 GO:0006469 GO:0008270 GO:0004862
GO:0003676 GO:0005952
Cluster-22178.118742 GO:0006469 GO:0045859 GO:0003676 GO:0008270

GO:0004862 GO:0019028 GO:0005952

Cluster-22178.118757 GO:0005198

Cluster-22178.118758 GO:0016747

Cluster-22178.118763 GO:0006355 GO:0003677 GO:0016021

Cluster-22178.118768 GO:0006364 GO:0007165 GO:0005179 GO:0005576 GO:0005730

Cluster-22178.11878 GO:0006816 GO:0070588 GO:0005975 GO:0004553 GO:0005262 GO:0042597 GO:0016020

Cluster-22178.118787 GO:0009069 GO:0016310 GO:0005524 GO:0004674

Cluster-22178.118806 GO:0006486 GO:0008378 GO:0016020

Cluster-22178.118811 GO:0006687 GO:0006683 GO:0006364 GO:0005975 GO:0006807 GO:0004336 GO:0005515 GO:0032040

Cluster-22178.118812 GO:0005515

Cluster-22178.118818 GO:0007165 GO:0008283 GO:0040007 GO:0008083 GO:0016020

Cluster-22178.118844 GO:0050792 GO:0022900 GO:0008270 GO:0042025 GO:0005747 GO:0005739

Cluster-22178.118857 GO:0006695 GO:0006694 GO:0042254 GO:0006412 GO:0004631 GO:0003735 GO:0005737 GO:0005840

Cluster-22178.11887 GO:0055085 GO:0016021

Cluster-22178.118899 GO:0015992 GO:0055114 GO:0006468 GO:0006118 GO:0006119 GO:0005524 GO:0004672 GO:0016773 GO:0008121 GO:0016020

Cluster-22178.118904 GO:0006771 GO:0019497 GO:0003993 GO:0005515 GO:0008270 GO:0046872

Cluster-22178.118905 GO:0006771 GO:0019497 GO:0003993 GO:0005515 GO:0046872

Cluster-22178.118906 GO:0019497 GO:0006771 GO:0046872 GO:0003993 GO:0005515

Cluster-22178.118910 GO:0019497 GO:0006771 GO:0005515 GO:0003993 GO:0046872

Cluster-22178.118917 GO:0005515 GO:0005576

Cluster-22178.118918 GO:0005515 GO:0005576
Cluster-22178.118939 GO:0046488 GO:0016307
Cluster-22178.118942 GO:0008272 GO:0055085 GO:0015116 GO:0016021
GO:0005741
Cluster-22178.118954 GO:0055114 GO:0016491
Cluster-22178.11896 GO:0006486 GO:0008417 GO:0016020
Cluster-22178.118966 GO:0005515
Cluster-22178.11897 GO:0006355 GO:0003700 GO:0005667 GO:0042025
Cluster-22178.118976 GO:0006812 GO:0055085 GO:0008324 GO:0016021
Cluster-22178.118981 GO:0016021
Cluster-22178.118982 GO:0005882
Cluster-22178.119020 GO:0046872 GO:0008270 GO:0061630 GO:0005515

Cluster-22178.119039 GO:0005515
Cluster-22178.119064 GO:0016021
Cluster-22178.119090 GO:0016020
Cluster-22178.119093 GO:0019497 GO:0006771 GO:0046872 GO:0005515
GO:0003993
Cluster-22178.119100 GO:0006810 GO:0009405 GO:0055085 GO:0005215
GO:0045735 GO:0016020
Cluster-22178.119101 GO:0009405 GO:0006810 GO:0055085 GO:0045735
GO:0005215 GO:0016020
Cluster-22178.119105 GO:0007029 GO:0055085 GO:0005198 GO:0019028
GO:0005789 GO:0016021
Cluster-22178.119119 GO:0006144 GO:0003968 GO:0005524 GO:0031379
Cluster-22178.119125 GO:0019643 GO:0006094 GO:0006099 GO:0015977
GO:0008964 GO:0016021
Cluster-22178.119134 GO:0006468 GO:0042981 GO:0016773 GO:0005524
GO:0004672 GO:0016020
Cluster-22178.119138 GO:0006355 GO:0003700 GO:0043565 GO:0005667
Cluster-22178.119139 GO:0006563 GO:0006566 GO:0006778 GO:0042967
GO:0006544 GO:0006783 GO:0030170 GO:0003870 GO:0005759
Cluster-22178.119140 GO:0006778 GO:0006544 GO:0042967 GO:0006783

GO:0006563 GO:0006566 GO:0030170 GO:0003870 GO:0005759
Cluster-22178.119151 GO:0016021
Cluster-22178.119152 GO:0008033 GO:0005524 GO:0016879 GO:0000166
GO:0005737
Cluster-22178.11916 GO:0008152 GO:0003824 GO:0016491 GO:0050662

Cluster-22178.119167 GO:0006355 GO:0003677 GO:0005515 GO:0005789
Cluster-22178.11917 GO:0008152 GO:0003824 GO:0050662 GO:0016491

Cluster-22178.119194 GO:0042998 GO:0071253 GO:0005515 GO:0005802
Cluster-22178.11921 GO:0009405 GO:0006810 GO:0006811 GO:0055085
GO:0019871 GO:0005515 GO:0005216 GO:0016020 GO:0005576
Cluster-22178.119226 GO:0006508 GO:0008233 GO:0004222 GO:0031012
Cluster-22178.11923 GO:0030420 GO:0006449 GO:0006415 GO:0003747
GO:0005840 GO:0018444
Cluster-22178.119237 GO:0015979 GO:0045892 GO:0005515 GO:0016020
GO:0009539 GO:0005634 GO:0009523
Cluster-22178.119253 GO:0006275
Cluster-22178.119277 GO:0000045 GO:0008270 GO:0005737
Cluster-22178.119292 GO:0006011 GO:0005982 GO:0005985 GO:0030244
GO:0016760 GO:0046872 GO:0008270 GO:0016020
Cluster-22178.119298 GO:0004190 GO:0016021
Cluster-22178.11930 GO:0007156 GO:0005509 GO:0016020
Cluster-22178.11931 GO:0006810 GO:0006811 GO:0005230 GO:0016020
Cluster-22178.119318 GO:0006810 GO:0006816 GO:0030234 GO:0042030
GO:0005246 GO:0016020
Cluster-22178.119319 GO:0006814 GO:0005272 GO:0016627 GO:0016020
Cluster-22178.119329 GO:0009399 GO:0005515
Cluster-22178.11933 GO:0046872
Cluster-22178.119330 GO:0006468 GO:0004672 GO:0005524
Cluster-22178.119332 GO:0006468 GO:0004672 GO:0005524
Cluster-22178.119352 GO:0051536 GO:0003824
Cluster-22178.119359 GO:0005515 GO:0016021 GO:0000228

Cluster-22178.119360 GO:0005515

Cluster-22178.119374 GO:0051603 GO:0004298 GO:0005856 GO:0005839

Cluster-22178.119387 GO:0035556 GO:0006508 GO:0008234 GO:0005515 GO:0008270

Cluster-22178.119390 GO:0006568 GO:0035556 GO:0018874 GO:0042207 GO:0008270 GO:0018822 GO:0005515

Cluster-22178.119395 GO:0007154 GO:0005515 GO:0003824 GO:0016020

Cluster-22178.119435 GO:0007186 GO:0004930 GO:0016021 GO:0016020

Cluster-22178.119438 GO:0046872

Cluster-22178.119439 GO:0030234 GO:0016020

Cluster-22178.119476 GO:0006810 GO:0015858 GO:0005515 GO:0005337 GO:0003774 GO:0005524 GO:0016459 GO:0016021

Cluster-22178.119490 GO:0003676 GO:0003723

Cluster-22178.119511 GO:0006813 GO:0050790 GO:0055085 GO:0006811 GO:0051607 GO:0005198 GO:0030234 GO:0005516 GO:0005216 GO:0015269 GO:0016020 GO:0019028 GO:0016021 GO:0019031

Cluster-22178.119524 GO:0005515

Cluster-22178.119527 GO:0006814 GO:0055085 GO:0006810 GO:0005215 GO:0016021 GO:0016020

Cluster-22178.119541 GO:0000062

Cluster-22178.119542 GO:0000062

Cluster-22178.119593 GO:0071973 GO:0043123 GO:0007165 GO:0006260 GO:0004871 GO:0005198 GO:0005515

Cluster-22178.119597 GO:0007275 GO:0005578

Cluster-22178.119618 GO:0007186 GO:0004930 GO:0016021

Cluster-22178.119641 GO:0003676

Cluster-22178.119647 GO:0030150 GO:0003824 GO:0005742

Cluster-22178.119659 GO:0005515

Cluster-22178.119666 GO:0016021

Cluster-22178.119672 GO:0006171 GO:0009190 GO:0035556 GO:0006144 GO:0004016 GO:0016849 GO:0016020 GO:0005886

Cluster-22178.119688 GO:0006606 GO:0006914 GO:0010508 GO:0009405 GO:0010506 GO:0006260 GO:0030168 GO:0007165 GO:0007264

GO:0051258 GO:0006355 GO:0008017 GO:0005524 GO:0008270
GO:0003677 GO:0016818 GO:0005198 GO:0042803 GO:0005102
GO:0005096 GO:0030674 GO:0008092 GO:0008134 GO:0045502
GO:0005577 GO:0016020 GO:0005667 GO:0045298 GO:0005615
GO:0005737 GO:0005634 GO:0019898 GO:0030286 GO:0005882
GO:0019031 GO:0019028
Cluster-22178.119749 GO:0008152 GO:0016846 GO:0008270
Cluster-22178.119768 GO:0005524
Cluster-22178.119793 GO:0007165 GO:0006144 GO:0005515 GO:0004114

Cluster-22178.119827 GO:0005509 GO:0005515
Cluster-22178.119830 GO:0007155 GO:0005540
Cluster-22178.119842 GO:0007165 GO:0043401 GO:0009405 GO:0006355
GO:0005179 GO:0005576
Cluster-22178.119849 GO:0006308 GO:0000077 GO:0006281 GO:0007017
GO:0007018 GO:0005524 GO:0003777 GO:0000014 GO:0008017
GO:0005874 GO:0045298
Cluster-22178.119878 GO:0046914 GO:0046983
Cluster-22178.119886 GO:0005515 GO:0008270
Cluster-22178.119890 GO:0006816 GO:0006810 GO:0042030 GO:0005246
GO:0016020
Cluster-22178.119898 GO:0009254 GO:0006040 GO:0005524 GO:0016773

Cluster-22178.119900 GO:0019497 GO:0006771 GO:0006468 GO:0003993
GO:0046872 GO:0004672 GO:0005524 GO:0016773 GO:0005515
GO:0016020
Cluster-22178.119904 GO:0019497 GO:0006468 GO:0006771 GO:0016773
GO:0046872 GO:0004672 GO:0005524 GO:0003993 GO:0005515
GO:0016020
Cluster-22178.119911 GO:0003677
Cluster-22178.119918 GO:0009405 GO:0055114 GO:0016702 GO:0046872
GO:0005576
Cluster-22178.11992 GO:0006355 GO:0007517 GO:0003677 GO:0005515

GO:0005634
Cluster-22178.119930 GO:0007186 GO:0006355 GO:0015986 GO:0015992
GO:0004930 GO:0015078 GO:0016020 GO:0000276
Cluster-22178.119934 GO:0035444 GO:0055085 GO:0043066 GO:0015675
GO:0045766 GO:0000902 GO:0043123 GO:0006955 GO:0030001
GO:0015099 GO:0046873 GO:0046872 GO:0004252 GO:0005886
GO:0016020 GO:0016021
Cluster-22178.119969 GO:0019054 GO:0003677 GO:0005515 GO:0004857

Cluster-22178.119978 GO:0015992 GO:0015991 GO:0008270 GO:0015078
GO:0005515 GO:0033177
Cluster-22178.119994 GO:0006508 GO:0005515 GO:0004252
Cluster-22178.120018 GO:0006465 GO:0016579 GO:0036459 GO:0005515
GO:0008233 GO:0005787 GO:0016021
Cluster-22178.120044 GO:0005975 GO:0006687 GO:0006683 GO:0006807
GO:0006364 GO:0004336 GO:0032040
Cluster-22178.120082 GO:0044419 GO:0006144 GO:0006508 GO:0003968
GO:0017111 GO:0004197 GO:0031379
Cluster-22178.120083 GO:0006508 GO:0006144 GO:0044419 GO:0004197
GO:0017111 GO:0003968 GO:0031379
Cluster-22178.120092 GO:0007276 GO:0005198 GO:0005515 GO:0019028
Cluster-22178.120093 GO:0007276 GO:0005515 GO:0005198 GO:0019028
Cluster-22178.120101 GO:0051258 GO:0030168 GO:0007165 GO:0030674
GO:0005102 GO:0048786 GO:0005577
Cluster-22178.120104 GO:0015886 GO:0017004 GO:0005515 GO:0016021
Cluster-22178.120129 GO:0016310 GO:0009069 GO:0005515 GO:0004674
GO:0005524
Cluster-22178.120141 GO:0006771 GO:0019497 GO:0006013 GO:0006000
GO:0003873 GO:0016301 GO:0003993 GO:0005524
Cluster-22178.120144 GO:0051258 GO:0007010 GO:0009405 GO:0030168
GO:0042157 GO:0007067 GO:0007165 GO:0006869 GO:0003779
GO:0008289 GO:0005198 GO:0005515 GO:0005102 GO:0030674
GO:0019031 GO:0005576 GO:0072686 GO:0019028 GO:0042729

GO:0005577 GO:0016020
Cluster-22178.120150 GO:0042438 GO:0046872 GO:0005507
Cluster-22178.120168 GO:0003677 GO:0008270
Cluster-22178.120182 GO:0006120 GO:0006397 GO:0051252 GO:0055114
GO:0004540
Cluster-22178.120197 GO:0055085 GO:0006810 GO:0009405 GO:0005215
GO:0045735 GO:0016020
Cluster-22178.120199 GO:0006260 GO:0006281 GO:0043140 GO:0005657
Cluster-22178.120200 GO:0006281 GO:0006260 GO:0043140 GO:0005657
Cluster-22178.120209 GO:0000154 GO:0006396 GO:0016433
Cluster-22178.12021 GO:0006355 GO:0003700 GO:0005667
Cluster-22178.12022 GO:0007268 GO:0007165 GO:0006508 GO:0006811
GO:0008233 GO:0005234 GO:0004970 GO:0005524 GO:0016020
GO:0016021
Cluster-22178.120221 GO:0055114 GO:0019058 GO:0006468 GO:0009690
GO:0050660 GO:0019139 GO:0004672 GO:0005524 GO:0005515
GO:0019031
Cluster-22178.120227 GO:0001558 GO:0005520 GO:0005576 GO:0016942
Cluster-22178.120242 GO:0000917 GO:0010508 GO:0043093 GO:0007264
GO:0005096 GO:0046983 GO:0019031 GO:0005737
Cluster-22178.120243 GO:0010508 GO:0000917 GO:0043093 GO:0007264
GO:0046983 GO:0005096 GO:0019031 GO:0005737
Cluster-22178.120251 GO:0031110
Cluster-22178.120285 GO:0016021 GO:0016012
Cluster-22178.120292 GO:0051252 GO:0006367 GO:0006402 GO:0003723
GO:0046872 GO:0004535 GO:0003676 GO:0005672 GO:0005634
GO:0005737
Cluster-22178.120350 GO:0006952 GO:0045926
Cluster-22178.120375 GO:0005515
Cluster-22178.120394 GO:0007165 GO:0005179 GO:0005576
Cluster-22178.12040 GO:0007156 GO:0055114 GO:0006260 GO:0005509
GO:0016020
Cluster-22178.120409 GO:0020037 GO:0019825

Cluster-22178.120414 GO:0009103 GO:0016020
Cluster-22178.120416 GO:0005515
Cluster-22178.120417 GO:0043161 GO:0006289 GO:0009405 GO:0009103
GO:0006281 GO:0003684 GO:0005509 GO:0016020 GO:0005576
Cluster-22178.12043 GO:0016021
Cluster-22178.120431 GO:0055114 GO:0016491 GO:0071949
Cluster-22178.12044 GO:0016021
Cluster-22178.120441 GO:0007050
Cluster-22178.120445 GO:0007049 GO:0051301 GO:0007155 GO:0005540
GO:0016021
Cluster-22178.120467 GO:0007165 GO:0006886 GO:0006955 GO:0019882
GO:0042289 GO:0016020 GO:0042613 GO:0005887 GO:0016021
Cluster-22178.120488 GO:0006886 GO:0007186 GO:0006144 GO:0006807
GO:0000256 GO:0004037 GO:0004930 GO:0005741 GO:0016021
Cluster-22178.120506 GO:0006508 GO:0004222 GO:0008233 GO:0008270
GO:0031012
Cluster-22178.120507 GO:0000902 GO:0006955 GO:0046983 GO:0016020
Cluster-22178.120515 GO:0005524 GO:0016301
Cluster-22178.120517 GO:0006139 GO:0006206 GO:0004127 GO:0016301
GO:0005524
Cluster-22178.120578 GO:0006814 GO:0016627 GO:0005272 GO:0016020
Cluster-22178.120585 GO:0005515 GO:0008270
Cluster-22178.120588 GO:0051382 GO:0007067 GO:0007156 GO:0003677
GO:0005509 GO:0016020
Cluster-22178.120592 GO:0016068 GO:0005524 GO:0003774 GO:0016459
Cluster-22178.120596 GO:0005515 GO:0016020
Cluster-22178.120607 GO:0007186 GO:0004930 GO:0016021 GO:0016020
Cluster-22178.120650 GO:0007165 GO:0035556
Cluster-22178.120651 GO:0035556 GO:0007165
Cluster-22178.120682 GO:0048013 GO:0019497 GO:0006468 GO:0006771
GO:0003993 GO:0005524 GO:0004672 GO:0046872 GO:0005515
Cluster-22178.120689 GO:0055114 GO:0016491 GO:0046872
Cluster-22178.120690 GO:0005515

Cluster-22178.120695 GO:0016787
Cluster-22178.120707 GO:0006118 GO:0009055 GO:0019031
Cluster-22178.120709 GO:0006413 GO:0006446 GO:0003743 GO:0005840
Cluster-22178.120714 GO:0005515
Cluster-22178.120718 GO:0006886 GO:0003924 GO:0005047 GO:0005525 GO:0005515 GO:0005786 GO:0005785
Cluster-22178.120722 GO:0007264 GO:0006576 GO:0007165 GO:0007186 GO:0001664 GO:0005524 GO:0031683 GO:0019001 GO:0003924 GO:0005525 GO:0004871
Cluster-22178.120734 GO:0008061 GO:0005737
Cluster-22178.120735 GO:0005515
Cluster-22178.120745 GO:0006950 GO:0005576 GO:0005615
Cluster-22178.120749 GO:0006811 GO:0016020
Cluster-22178.120750 GO:0006811 GO:0006810 GO:0005230 GO:0016020
Cluster-22178.120763 GO:0006952
Cluster-22178.120769 GO:0050832 GO:0005515
Cluster-22178.120799 GO:0006811 GO:0055085 GO:0005216 GO:0016020
Cluster-22178.120864 GO:0019497 GO:0006771 GO:0046872 GO:0005515 GO:0003993
Cluster-22178.120875 GO:0009451 GO:0045454 GO:0006370 GO:0006144 GO:0005509 GO:0003968 GO:0004482 GO:0005524 GO:0019012 GO:0031379
Cluster-22178.120876 GO:0045454 GO:0009451 GO:0006370 GO:0006144 GO:0005509 GO:0005524 GO:0004482 GO:0003968 GO:0019012 GO:0031379
Cluster-22178.12088 GO:0005515
Cluster-22178.12092 GO:0055114 GO:0006118 GO:0009496 GO:0003950 GO:0051537 GO:0009512 GO:0042651
Cluster-22178.120925 GO:0019836 GO:0051205 GO:0005576 GO:0016021
Cluster-22178.120951 GO:0006888 GO:0005622
Cluster-22178.120952 GO:0006952 GO:0046849 GO:0004869 GO:0005576
Cluster-22178.120953 GO:0004869
Cluster-22178.120963 GO:0006298 GO:0005524 GO:0030983

Cluster-22178.120967 GO:0016787 GO:0005515
Cluster-22178.120981 GO:0007165 GO:0008158 GO:0016020
Cluster-22178.120983 GO:0007186 GO:0004930 GO:0016021
Cluster-22178.12099 GO:0055114 GO:0006508 GO:0050792 GO:0004190
GO:0016491 GO:0050662 GO:0042025
Cluster-22178.121016 GO:0006885 GO:0015992 GO:0006814 GO:0005509
GO:0015385 GO:0005515 GO:0016021 GO:0009986 GO:0016020
Cluster-22178.121018 GO:0006814 GO:0015992 GO:0006885 GO:0005509
GO:0015385 GO:0005515 GO:0016020 GO:0009986 GO:0016021
Cluster-22178.121048 GO:0019079
Cluster-22178.121090 GO:0055085 GO:0006811 GO:0005216 GO:0005515
GO:0016021 GO:0016020
Cluster-22178.121094 GO:0006313 GO:0007165 GO:0055114 GO:0005179
GO:0004803 GO:0004174 GO:0003677 GO:0005576
Cluster-22178.12111 GO:0045859 GO:0007165 GO:0007049 GO:0016538
GO:0008158 GO:0016020
Cluster-22178.121118 GO:0005515
Cluster-22178.121126 GO:0045893 GO:0019836
Cluster-22178.121184 GO:0006468 GO:0005524 GO:0004672
Cluster-22178.121230 GO:0005515
Cluster-22178.121241 GO:0005515
Cluster-22178.121275 GO:0007186 GO:0004930 GO:0003677 GO:0034061
GO:0016021 GO:0005634
Cluster-22178.121278 GO:0055085 GO:0071585 GO:0010273 GO:0005507
GO:0046870 GO:0016021
Cluster-22178.121313 GO:0006508 GO:0008270 GO:0004222 GO:0005515
GO:0005509 GO:0031012
Cluster-22178.121319 GO:0007275 GO:0005578
Cluster-22178.121328 GO:0007264 GO:0005525
Cluster-22178.121346 GO:0005515
Cluster-22178.121361 GO:0003824
Cluster-22178.121363 GO:0006816 GO:0070588 GO:0005262 GO:0016020
Cluster-22178.121374 GO:0005515

Cluster-22178.12140 GO:0055114 GO:0016491 GO:0050661 GO:0003723
GO:0003676
Cluster-22178.121411 GO:0005515
Cluster-22178.12143 GO:0003676
Cluster-22178.121447 GO:0005094 GO:0005737
Cluster-22178.121451 GO:0006468 GO:0005524 GO:0004672
Cluster-22178.121457 GO:0006206 GO:0006144 GO:0007275 GO:0042254
GO:0006352 GO:0006351 GO:0006412 GO:0006094 GO:0006355
GO:0016987 GO:0004611 GO:0003677 GO:0003735 GO:0005525
GO:0003700 GO:0003899 GO:0005730 GO:0005667 GO:0005840
GO:0019028
Cluster-22178.121458 GO:0006506 GO:0016021
Cluster-22178.12148 GO:0004190 GO:0016020
Cluster-22178.121536 GO:0003677
Cluster-22178.121539 GO:0005515
Cluster-22178.121567 GO:0006355 GO:0003677
Cluster-22178.121595 GO:0019031
Cluster-22178.121612 GO:0006508 GO:0005515 GO:0004252 GO:0016020
Cluster-22178.121638 GO:0005515 GO:0005509
Cluster-22178.121655 GO:0016020
Cluster-22178.121661 GO:0006574 GO:0055114 GO:0008152 GO:0006568
GO:0046487 GO:0006550 GO:0006552 GO:0006554 GO:0009396
GO:0006633 GO:0018874 GO:0006631 GO:0003824 GO:0003857
GO:0016491 GO:0050662 GO:0004488
Cluster-22178.121667 GO:0006955 GO:0019882 GO:0016020 GO:0042613
Cluster-22178.121703 GO:0005861
Cluster-22178.121705 GO:0005861
Cluster-22178.121707 GO:0005576 GO:0005861
Cluster-22178.12172 GO:0005515
Cluster-22178.12174 GO:0006508 GO:0005515 GO:0004252
Cluster-22178.121768 GO:0015986 GO:0015992 GO:0015078 GO:0000276
Cluster-22178.12182 GO:0006771 GO:0019497 GO:0046872 GO:0008270
GO:0005515 GO:0003993 GO:0005622

Cluster-22178.12183 GO:0006771 GO:0019497 GO:0003993 GO:0005515
GO:0008270 GO:0046872 GO:0005622
Cluster-22178.121831 GO:0016740 GO:0005515 GO:0005509
Cluster-22178.12184 GO:0008285 GO:0019497 GO:0006771 GO:0006915
GO:0007050 GO:0046872 GO:0003993 GO:0005515
Cluster-22178.12192 GO:0006144 GO:0015976 GO:0006096 GO:0006094
GO:0003824 GO:0004743 GO:0030955 GO:0000287
Cluster-22178.121922 GO:0006508 GO:0004181 GO:0008270
Cluster-22178.12193 GO:0015976 GO:0006144 GO:0006094 GO:0006096
GO:0000287 GO:0030955 GO:0004743
Cluster-22178.121933 GO:0008270
Cluster-22178.121957 GO:0010508 GO:0006886 GO:0007094 GO:0006606
GO:0006904 GO:0048870 GO:0051258 GO:0007165 GO:0006302
GO:0030168 GO:0015031 GO:0070206 GO:0042803 GO:0005198
GO:0008565 GO:0008134 GO:0045502 GO:0008092 GO:0030674
GO:0005102 GO:0048786 GO:0005634 GO:0005737 GO:0016020
GO:0005667 GO:0005577 GO:0031514 GO:0019028 GO:0016021
GO:0000145 GO:0019898 GO:0005815 GO:0030286
Cluster-22178.12199 GO:0030420 GO:0006355 GO:0005515 GO:0005509
GO:0005634
Cluster-22178.122015 GO:0007165 GO:0006355 GO:0003700 GO:0004871
GO:0005634 GO:0005667
Cluster-22178.122036 GO:0009069 GO:0006468 GO:0016310 GO:0007178
GO:0005026 GO:0004672 GO:0005524 GO:0046872 GO:0016020
Cluster-22178.122071 GO:0005515
Cluster-22178.122072 GO:0005515
Cluster-22178.12209 GO:0055114
Cluster-22178.12213 GO:0019031
Cluster-22178.122137 GO:0006355 GO:0003677
Cluster-22178.122139 GO:0003677 GO:0008168 GO:0016817
Cluster-22178.12218 GO:0006090 GO:0010508 GO:0009405 GO:0048193
GO:0006633 GO:0006606 GO:0006914 GO:0015992 GO:0007155
GO:0043093 GO:0006308 GO:0051258 GO:0000917 GO:0015991

GO:0006260 GO:0030168 GO:0007165 GO:0006810 GO:0007264
GO:0015078 GO:0003924 GO:0003774 GO:0005525 GO:0005102
GO:0008855 GO:0005096 GO:0003989 GO:0030674 GO:0005737
GO:0005577 GO:0016020 GO:0016459 GO:0016021 GO:0033179
GO:0009318 GO:0009317 GO:0005886
Cluster-22178.122205 GO:0009069 GO:0016310 GO:0004674
Cluster-22178.12223 GO:0003676 GO:0003723
Cluster-22178.122297 GO:0005515
Cluster-22178.122328 GO:0015074
Cluster-22178.122359 GO:0035556
Cluster-22178.122381 GO:0043087 GO:0035023 GO:0005089
Cluster-22178.122392 GO:0006955 GO:0007165 GO:0005126 GO:0005576
Cluster-22178.122430 GO:0016114 GO:0006694 GO:0008152 GO:0003824
GO:0030976 GO:0008661 GO:0016624
Cluster-22178.122462 GO:0019079 GO:0006355 GO:0003700 GO:0043565
GO:0003677 GO:0003723 GO:0005667 GO:0005634
Cluster-22178.122476 GO:0016020
Cluster-22178.12249 GO:0046660 GO:0006694 GO:0006397 GO:0006270
GO:0008168 GO:0003676 GO:0005634
Cluster-22178.12250 GO:0006694 GO:0046660 GO:0006397 GO:0006270
GO:0003676 GO:0008168 GO:0004190 GO:0016021 GO:0005634
Cluster-22178.122526 GO:0003677 GO:0003824 GO:0030151 GO:0030170
GO:0005634
Cluster-22178.122556 GO:0005515 GO:0005198 GO:0019013
Cluster-22178.122595 GO:0003333 GO:0042246 GO:0007155 GO:0006865
GO:0015696 GO:0033617 GO:0015171 GO:0008519 GO:0016020
GO:0016021
Cluster-22178.122610 GO:0006508 GO:0008236 GO:0016787
Cluster-22178.122613 GO:0006508 GO:0008236 GO:0016787
Cluster-22178.122689 GO:0070206 GO:0006406 GO:0003723 GO:0005634
GO:0005737
Cluster-22178.122757 GO:0006355 GO:0005515 GO:0070461
Cluster-22178.122772 GO:0042967 GO:0006633 GO:0004315 GO:0005835

Cluster-22178.12278 GO:0006468 GO:0006397 GO:0051252 GO:0004540
GO:0004672 GO:0005524
Cluster-22178.12280 GO:0019031
Cluster-22178.122806 GO:0019058
Cluster-22178.122829 GO:0035556 GO:0006171 GO:0006144 GO:0009190
GO:0004016 GO:0016849 GO:0005886
Cluster-22178.12287 GO:0042967 GO:0006355 GO:0046872 GO:0008270
GO:0003712 GO:0004402 GO:0000123 GO:0005667 GO:0005634
Cluster-22178.12288 GO:0042967 GO:0006355 GO:0003712 GO:0008270
GO:0046872 GO:0004402 GO:0000123 GO:0005634 GO:0005667
Cluster-22178.122961 GO:0035091
Cluster-22178.122979 GO:0006914 GO:0015992 GO:0030168 GO:0007155
GO:0042157 GO:0006869 GO:0007165 GO:0006308 GO:0051258
GO:0015991 GO:0008855 GO:0005102 GO:0030674 GO:0008289
GO:0015078 GO:0051082 GO:0020037 GO:0005198 GO:0005515
GO:0005577 GO:0019031 GO:0019028 GO:0005576 GO:0033179
GO:0009318
Cluster-22178.12303 GO:0005515
Cluster-22178.123131 GO:0009607 GO:0019236 GO:0007186 GO:0050909
GO:0007606 GO:0004930 GO:0016503 GO:0016021
Cluster-22178.12315 GO:0006810 GO:0015288 GO:0016020
Cluster-22178.1232 GO:0008033 GO:0000166 GO:0005524 GO:0016879
GO:0005737
Cluster-22178.123225 GO:0015986 GO:0015992 GO:0015078 GO:0016820
GO:0016021 GO:0005739 GO:0000276
Cluster-22178.123231 GO:0005515
Cluster-22178.123306 GO:0005515
Cluster-22178.123368 GO:0005515
Cluster-22178.123397 GO:0005515
Cluster-22178.123445 GO:0009306 GO:0015031 GO:0001522 GO:0009451
GO:0007186 GO:0009982 GO:0015450 GO:0004930 GO:0003723
GO:0016021 GO:0009941
Cluster-22178.123466 GO:0008299 GO:0006552 GO:0006550 GO:0046950

GO:0006574 GO:0006084 GO:0004421
Cluster-22178.123482 GO:0007165 GO:0005179
Cluster-22178.123483 GO:0005515
Cluster-22178.123495 GO:0016337 GO:0016020
Cluster-22178.123521 GO:0007165 GO:0005515
Cluster-22178.123537 GO:0017004 GO:0006506 GO:0020037 GO:0016746
GO:0016021 GO:0005789
Cluster-22178.12358 GO:0005515
Cluster-22178.123598 GO:0006810 GO:0043093 GO:0055085 GO:0006914
GO:0051028 GO:0000917 GO:0030001 GO:0048870 GO:0048309
GO:0046873 GO:0003774 GO:0008134 GO:0042803 GO:0045502
GO:0005215 GO:0016020 GO:0005667 GO:0016459 GO:0005886
GO:0030286 GO:0031514 GO:0005737
Cluster-22178.123605 GO:0006935 GO:0007165 GO:0006955 GO:0008009
GO:0005576
Cluster-22178.123640 GO:0005515 GO:0004190 GO:0016021
Cluster-22178.123686 GO:0007165 GO:0005179 GO:0005576
Cluster-22178.123691 GO:0006998 GO:0006406 GO:0006611 GO:0016032
GO:0046983 GO:0051087 GO:0016021 GO:0031965
Cluster-22178.123713 GO:0006355 GO:0006352 GO:0003677 GO:0016987
GO:0003700 GO:0005667
Cluster-22178.123725 GO:0005198 GO:0016021
Cluster-22178.12378 GO:0046983
Cluster-22178.123797 GO:0004867 GO:0005576
Cluster-22178.123798 GO:0004867 GO:0005576
Cluster-22178.123801 GO:0005515
Cluster-22178.123805 GO:0007165
Cluster-22178.123807 GO:0007165
Cluster-22178.123808 GO:0006308 GO:0007165 GO:0008855 GO:0009318
Cluster-22178.12407 GO:0007165 GO:0007155 GO:0051258 GO:0030168
GO:0005102 GO:0030674 GO:0005577
Cluster-22178.12413 GO:0009405 GO:0006810 GO:0007165 GO:0008200
GO:0005509 GO:0005515 GO:0046872 GO:0005578 GO:0005576

Cluster-22178.12415 GO:0007165 GO:0005515 GO:0005509 GO:0005578
Cluster-22178.12433 GO:0006457 GO:0035556 GO:0048193 GO:0051082
GO:0003677 GO:0046872 GO:0019028 GO:0016020 GO:0016272
GO:0016021 GO:0045202
Cluster-22178.12438 GO:0006508 GO:0004190
Cluster-22178.12439 GO:0006508 GO:0004190
Cluster-22178.12440 GO:0006508 GO:0004190
Cluster-22178.12441 GO:0006508 GO:0004190
Cluster-22178.12451 GO:0055114 GO:0042981 GO:0006355 GO:0006955
GO:0016491 GO:0043565 GO:0003700 GO:0051537 GO:0005667
Cluster-22178.12455 GO:0007268 GO:0006811 GO:0007165 GO:0004970
GO:0016020
Cluster-22178.12456 GO:0007165 GO:0006811 GO:0007268 GO:0004970
GO:0005234 GO:0016020
Cluster-22178.12506 GO:0006464 GO:0046500 GO:0006479 GO:0008610
GO:0004719 GO:0008168 GO:0008171
Cluster-22178.12509 GO:0006810 GO:0016021
Cluster-22178.1256 GO:0009058 GO:0003824
Cluster-22178.1260 GO:0005515
Cluster-22178.12604 GO:0007264 GO:0006182 GO:0006744 GO:0015992
GO:0006144 GO:0006814 GO:0006118 GO:0006355 GO:0046039
GO:0006120 GO:0005096 GO:0008137 GO:0004383 GO:0003723
GO:0009055 GO:0016020
Cluster-22178.12614 GO:0050829 GO:0050830 GO:0003676
Cluster-22178.1262 GO:0043087 GO:0007264 GO:0005085
Cluster-22178.12635 GO:0006355 GO:0003677
Cluster-22178.1268 GO:0019068 GO:0005515 GO:0016020
Cluster-22178.12681 GO:0015992 GO:0015078 GO:0033644 GO:0055036
Cluster-22178.12695 GO:0032324 GO:0019236 GO:0007155 GO:0007186
GO:0005540
Cluster-22178.12698 GO:0016773
Cluster-22178.12717 GO:0015986 GO:0006260 GO:0055085 GO:0015992
GO:0006814 GO:0030001 GO:0006355 GO:0006811 GO:0005216

GO:0046873 GO:0005248 GO:0015078 GO:0003700 GO:0022857
GO:0001518 GO:0016020 GO:0005667 GO:0045263 GO:0042025
GO:0016021
Cluster-22178.12724 GO:0006771 GO:0019497 GO:0003993 GO:0005515
GO:0046872 GO:0016021
Cluster-22178.12730 GO:0006629
Cluster-22178.12732 GO:0015979 GO:0016020 GO:0009523 GO:0009539
Cluster-22178.12751 GO:0006308 GO:0006120 GO:0006814 GO:0006118
GO:0015992 GO:0006744 GO:0007264 GO:0009307 GO:0009055
GO:0008137 GO:0005198 GO:0009036 GO:0005096 GO:0019031
GO:0016021 GO:0009359 GO:0019028 GO:0016020
Cluster-22178.12752 GO:0006282 GO:0009307 GO:0006914 GO:0006308
GO:0008855 GO:0009036 GO:0009318 GO:0009359 GO:0016021
Cluster-22178.12765 GO:0048013 GO:0007186 GO:0004930
Cluster-22178.12774 GO:0016567 GO:0004842 GO:0005515 GO:0005507

Cluster-22178.12782 GO:0008152 GO:0016491
Cluster-22178.12783 GO:0008152 GO:0016491
Cluster-22178.12784 GO:0008152 GO:0016491
Cluster-22178.12818 GO:0006817 GO:0005315 GO:0005515 GO:0005524
GO:0016887 GO:0016020
Cluster-22178.1282 GO:0009306 GO:0015031 GO:0015450 GO:0016021
GO:0009941 GO:0019031 GO:0019028 GO:0016012
Cluster-22178.12820 GO:0006817 GO:0005524 GO:0016887 GO:0005315
GO:0005515 GO:0016020
Cluster-22178.12847 GO:0015986 GO:0015992 GO:0015078
Cluster-22178.12858 GO:0005975 GO:0016773
Cluster-22178.12868 GO:0051536 GO:0046872 GO:0045202
Cluster-22178.12869 GO:0010506 GO:0005515 GO:0005615
Cluster-22178.12885 GO:0046872
Cluster-22178.12892 GO:0050909 GO:0007186 GO:0004930 GO:0016021
Cluster-22178.12893 GO:0007186 GO:0004930 GO:0016021
Cluster-22178.12909 GO:0007339 GO:0007155 GO:0005515 GO:0016021

GO:0005576

Cluster-22178.12910 GO:0008233 GO:0004222 GO:0031012

Cluster-22178.12911 GO:0008233 GO:0004222 GO:0031012

Cluster-22178.12914 GO:0007339 GO:0005515 GO:0005576

Cluster-22178.12918 GO:0006952 GO:0055085 GO:0016021 GO:0005576

Cluster-22178.12919 GO:0048544 GO:0009069 GO:0006468 GO:0016310 GO:0004674 GO:0005524 GO:0004672 GO:0016773 GO:0016020

Cluster-22178.1293 GO:0006813 GO:0006814 GO:0005890

Cluster-22178.12950 GO:0005515

Cluster-22178.12965 GO:0006807 GO:0016811

Cluster-22178.12967 GO:0016020 GO:0016021

Cluster-22178.12981 GO:0005515

Cluster-22178.12982 GO:0005515

Cluster-22178.13001 GO:0007186 GO:0004930 GO:0016021

Cluster-22178.13004 GO:0006269 GO:0006260 GO:0006351 GO:0003896 GO:0005730 GO:0005657

Cluster-22178.13005 GO:0005515 GO:0042470

Cluster-22178.13012 GO:0007186 GO:0004930 GO:0016021

Cluster-22178.13018 GO:0006486 GO:0008373

Cluster-22178.13019 GO:0006486 GO:0008373

Cluster-22178.13020 GO:0006486 GO:0008373

Cluster-22178.13022 GO:0006355 GO:0006486 GO:0008373 GO:0003677

Cluster-22178.13036 GO:0046486 GO:0006687 GO:0006027 GO:0006012 GO:0030206 GO:0005975 GO:0015018 GO:0004565 GO:0016020 GO:0009341

Cluster-22178.13038 GO:0005975 GO:0030206 GO:0006012 GO:0006027 GO:0006687 GO:0046486 GO:0004565 GO:0015018 GO:0009341 GO:0016020

Cluster-22178.13045 GO:0000917 GO:0006355 GO:0043093 GO:0003700 GO:0043565 GO:0005198 GO:0019031 GO:0005737 GO:0005882 GO:0019028 GO:0005667

Cluster-22178.13066 GO:0005856

Cluster-22178.13071 GO:0006468 GO:0005524 GO:0004672 GO:0016773 GO:0016020

Cluster-22178.13078 GO:0005509

Cluster-22178.13091 GO:0006468 GO:0016773 GO:0004672 GO:0005524 GO:0016020

Cluster-22178.13092 GO:0005515

Cluster-22178.13107 GO:0005515

Cluster-22178.13108 GO:0016020

Cluster-22178.13160 GO:0006633 GO:0007186 GO:0015940 GO:0008770 GO:0004930 GO:0016021

Cluster-22178.13161 GO:0006633 GO:0007186 GO:0015940 GO:0008770 GO:0004930 GO:0016021

Cluster-22178.13171 GO:0003677

Cluster-22178.13189 GO:0007169 GO:0006807 GO:0006468 GO:0003933 GO:0005524 GO:0004714 GO:0005515 GO:0016020

Cluster-22178.13198 GO:0006468 GO:0005524 GO:0004672 GO:0016773 GO:0003677 GO:0016020

Cluster-22178.1322 GO:0006952 GO:0007165 GO:0005126 GO:0008270 GO:0003676 GO:0005576

Cluster-22178.13220 GO:0007165 GO:0035556

Cluster-22178.13221 GO:0035556 GO:0007165 GO:0005515

Cluster-22178.13245 GO:0046872 GO:0003677

Cluster-22178.13251 GO:0005515

Cluster-22178.13252 GO:0004867 GO:0000228

Cluster-22178.13266 GO:0003824

Cluster-22178.13268 GO:0016192 GO:0003824 GO:0016021

Cluster-22178.13278 GO:0009405 GO:0016020

Cluster-22178.13294 GO:0035556 GO:0007165 GO:0005179 GO:0005515 GO:0005576

Cluster-22178.13295 GO:0009069 GO:0006468 GO:0016310 GO:0016773 GO:0004672 GO:0005524 GO:0004674 GO:0016020

Cluster-22178.13298 GO:0004867

Cluster-22178.13300 GO:0009058 GO:0007186 GO:0004930 GO:0005524

GO:0005783 GO:0016020 GO:0016021
Cluster-22178.13304 GO:0007131 GO:0003677
Cluster-22178.13314 GO:0006260 GO:0006351 GO:0006269 GO:0003896
GO:0008270 GO:0003677 GO:0005657 GO:0005730
Cluster-22178.13325 GO:0006954 GO:0007165 GO:0005125 GO:0005576
Cluster-22178.13326 GO:0006954 GO:0007165 GO:0005125 GO:0005576
Cluster-22178.13339 GO:0007165 GO:0006396 GO:0006144 GO:0004114
GO:0005622
Cluster-22178.13342 GO:0007155 GO:0005198 GO:0019028 GO:0031012
Cluster-22178.13382 GO:0055085 GO:0006811 GO:0005515 GO:0005216
GO:0016020
Cluster-22178.13383 GO:0006811 GO:0055085 GO:0005515 GO:0005216
GO:0016020
Cluster-22178.13408 GO:0006629 GO:0008081
Cluster-22178.13409 GO:0006629 GO:0008081
Cluster-22178.13419 GO:0005515
Cluster-22178.13455 GO:0006144 GO:0007165 GO:0004114 GO:0005515

Cluster-22178.13457 GO:0005887
Cluster-22178.13470 GO:0007154 GO:0005509 GO:0016020
Cluster-22178.13474 GO:0019229 GO:0020037 GO:0005515 GO:0005576
Cluster-22178.13531 GO:0006367 GO:0005672
Cluster-22178.13536 GO:0008242 GO:0005515
Cluster-22178.13539 GO:0003677
Cluster-22178.1354 GO:0042254 GO:0006412 GO:0042981 GO:0006355
GO:0003677 GO:0003735 GO:0005840
Cluster-22178.13542 GO:0006412 GO:0042254 GO:0006508 GO:0003735
GO:0004222 GO:0008270 GO:0005840 GO:0031012 GO:0005622
Cluster-22178.13561 GO:0043248
Cluster-22178.13567 GO:0009231 GO:0006508 GO:0006771 GO:0008152
GO:0016787 GO:0008236 GO:0003919
Cluster-22178.13577 GO:0006865 GO:0003333 GO:0006810 GO:0005215
GO:0015631 GO:0015171 GO:0045298 GO:0016020

Cluster-22178.13589 GO:0005515 GO:0003723
Cluster-22178.13590 GO:0005515 GO:0003723
Cluster-22178.13649 GO:0005515
Cluster-22178.13658 GO:0006313 GO:0003677 GO:0004803
Cluster-22178.13661 GO:0007165 GO:0005179 GO:0005576
Cluster-22178.1367 GO:0008146 GO:0016021
Cluster-22178.13674 GO:0016746
Cluster-22178.13681 GO:0006508 GO:0008237 GO:0008241 GO:0016020 GO:0005615
Cluster-22178.13682 GO:0006508 GO:0008241 GO:0008237 GO:0016020 GO:0005615
Cluster-22178.13685 GO:0006508 GO:0015992 GO:0006123 GO:0008237 GO:0008241 GO:0005507 GO:0004129 GO:0016020 GO:0045277
Cluster-22178.13687 GO:0004866 GO:0003824 GO:0005615
Cluster-22178.13692 GO:0003779
Cluster-22178.13697 GO:0016032 GO:0030246 GO:0016020
Cluster-22178.13725 GO:0019497 GO:0006771 GO:0003993 GO:0005515 GO:0046872
Cluster-22178.13741 GO:0005198 GO:0019028 GO:0019031 GO:0005882
Cluster-22178.13747 GO:0006811 GO:0055085 GO:0006355 GO:0003700 GO:0008270 GO:0043565 GO:0005216 GO:0016020 GO:0005634 GO:0005667
Cluster-22178.13749 GO:0006508 GO:0008270 GO:0004222 GO:0031012
Cluster-22178.13771 GO:0006813 GO:0005242 GO:0016021 GO:0008076
Cluster-22178.13797 GO:0045444
Cluster-22178.13824 GO:0006810 GO:0008152 GO:0006030 GO:0008061 GO:0016788 GO:0005576 GO:0016020 GO:0016021
Cluster-22178.13825 GO:0006810 GO:0008152 GO:0006030 GO:0016788 GO:0008061 GO:0016020 GO:0005576 GO:0016021
Cluster-22178.13826 GO:0008017 GO:0045298
Cluster-22178.13840 GO:0043087 GO:0007264 GO:0005085
Cluster-22178.13842 GO:0015074
Cluster-22178.1385 GO:0006508 GO:0050790 GO:0004197 GO:0019900

Cluster-22178.13856 GO:0006810 GO:0016567 GO:0005515 GO:0005215
GO:0004842 GO:0008270 GO:0046872 GO:0016020 GO:0005680
Cluster-22178.13861 GO:0055114 GO:0016641 GO:0005507 GO:0005634
Cluster-22178.13865 GO:0008654 GO:0046486 GO:0008715 GO:0003697
GO:0016020
Cluster-22178.1387 GO:0030246
Cluster-22178.13878 GO:0016020
Cluster-22178.13880 GO:0042619
Cluster-22178.13884 GO:0009451 GO:0008033 GO:0004809 GO:0003723

Cluster-22178.13887 GO:0006355 GO:0043565 GO:0003677 GO:0003700
GO:0005667 GO:0005634
Cluster-22178.13888 GO:0005515
Cluster-22178.13893 GO:0005515 GO:0016020
Cluster-22178.13911 GO:0044419 GO:0007264 GO:0006508 GO:0006144
GO:0004197 GO:0017111 GO:0005525 GO:0003968 GO:0003924
GO:0031379
Cluster-22178.13930 GO:0015992 GO:0046782 GO:0015991 GO:0007155
GO:0015078 GO:0005515 GO:0033179 GO:0016020
Cluster-22178.13937 GO:0007165 GO:0005509 GO:0005578
Cluster-22178.13938 GO:0008033 GO:0009451 GO:0009020
Cluster-22178.13939 GO:0006355 GO:0003677 GO:0003700 GO:0043565
GO:0005634 GO:0005667
Cluster-22178.13942 GO:0003824 GO:0051536
Cluster-22178.13949 GO:0005094 GO:0005737
Cluster-22178.13951 GO:0005094 GO:0005737
Cluster-22178.13958 GO:0005515
Cluster-22178.13983 GO:0046872
Cluster-22178.13999 GO:0043087 GO:0007264 GO:0005085
Cluster-22178.14006 GO:0007186 GO:0004930 GO:0016021
Cluster-22178.14013 GO:0015986 GO:0015992 GO:0015078 GO:0000276
Cluster-22178.14014 GO:0016192 GO:0006904
Cluster-22178.14036 GO:0042254 GO:0006412 GO:0003735 GO:0005622

GO:0005840
Cluster-22178.14053 GO:0007165 GO:0005515
Cluster-22178.14063 GO:0007165 GO:0055114 GO:0016972
Cluster-22178.14064 GO:0007165 GO:0055114 GO:0015986 GO:0015992
GO:0016972 GO:0015078 GO:0000276
Cluster-22178.14066 GO:0007165 GO:0005515
Cluster-22178.14081 GO:0006355 GO:0000266 GO:0003677 GO:0005739
Cluster-22178.14108 GO:0005516
Cluster-22178.14112 GO:0008289
Cluster-22178.14124 GO:0055085 GO:0006810 GO:0005515 GO:0005215
GO:0016021 GO:0016020
Cluster-22178.14127 GO:0008233
Cluster-22178.14140 GO:0006810 GO:0005215 GO:0016020
Cluster-22178.14146 GO:0005515
Cluster-22178.14152 GO:0016020 GO:0016021
Cluster-22178.14154 GO:0043087 GO:0007264 GO:0005085
Cluster-22178.14176 GO:0051302
Cluster-22178.14177 GO:0010884 GO:0008284 GO:0001819
Cluster-22178.14183 GO:0006812 GO:0015074 GO:0008324 GO:0016021
GO:0005886
Cluster-22178.14188 GO:0016255 GO:0042765
Cluster-22178.14216 GO:0007186 GO:0004930 GO:0016021
Cluster-22178.14217 GO:0006744 GO:0006120 GO:0015992 GO:0006814
GO:0006118 GO:0008270 GO:0008137 GO:0003954 GO:0005739
Cluster-22178.14231 GO:0006355 GO:0005622
Cluster-22178.14232 GO:0006355 GO:0003700 GO:0005622 GO:0005667
Cluster-22178.14233 GO:0006355 GO:0016032 GO:0005622
Cluster-22178.14234 GO:0016032 GO:0006355 GO:0005622
Cluster-22178.14241 GO:0006508 GO:0004197 GO:0008234
Cluster-22178.14247 GO:0005515
Cluster-22178.14288 GO:0005515
Cluster-22178.14311 GO:0030682
Cluster-22178.14323 GO:0043401 GO:0006355 GO:0005515 GO:0003700

GO:0043565 GO:0008270 GO:0005634 GO:0005667
Cluster-22178.14339 GO:0015074 GO:0051252 GO:0004523 GO:0003676

Cluster-22178.14344 GO:0006468 GO:0004672 GO:0005524
Cluster-22178.14347 GO:0005515
Cluster-22178.14351 GO:0005515
Cluster-22178.14364 GO:0006508 GO:0004222 GO:0005515
Cluster-22178.14371 GO:0016192 GO:0016021
Cluster-22178.14407 GO:0016021
Cluster-22178.14423 GO:0042254 GO:0006869 GO:0042157 GO:0015986
GO:0015992 GO:0009405 GO:0042262 GO:0006571 GO:0055114
GO:0009094 GO:0006810 GO:0007165 GO:0000162 GO:0008053
GO:0000105 GO:0030168 GO:0006118 GO:0006412 GO:0006432
GO:0006308 GO:0051258 GO:0003735 GO:0003690 GO:0005506
GO:0005524 GO:0008270 GO:0020037 GO:0005198 GO:0051082
GO:0009055 GO:0015078 GO:0003924 GO:0030674 GO:0051287
GO:0005102 GO:0004399 GO:0008855 GO:0000166 GO:0004826
GO:0008289 GO:0016020 GO:0005741 GO:0005577 GO:0005622
GO:0045263 GO:0005737 GO:0005886 GO:0009318 GO:0019028
GO:0005576 GO:0016021 GO:0005840 GO:0019031 GO:0009328
Cluster-22178.14425 GO:0006367 GO:0006281 GO:0005672
Cluster-22178.14428 GO:0006576 GO:0019497 GO:0006614 GO:0006810
GO:0006771 GO:0002949 GO:0000166 GO:0005515 GO:0003723
GO:0005525 GO:0046872 GO:0003924 GO:0003677 GO:0003993
GO:0003724 GO:0005524 GO:0016887
Cluster-22178.14432 GO:0016226 GO:0005201 GO:0008199 GO:0005581
GO:0005578
Cluster-22178.14441 GO:0006144 GO:0032465 GO:0006915 GO:0016567
GO:0006351 GO:0005524 GO:0003968 GO:0004842 GO:0003723
GO:0005509 GO:0005198 GO:0031379 GO:0019031
Cluster-22178.14446 GO:0005515
Cluster-22178.14449 GO:0007186 GO:0004930 GO:0016021
Cluster-22178.14453 GO:0005515

Cluster-22178.1448 GO:0008152 GO:0006480 GO:0008168 GO:0008171
Cluster-22178.14488 GO:0016032
Cluster-22178.14491 GO:0009405
Cluster-22178.14496 GO:0016021
Cluster-22178.14511 GO:0006835 GO:0006820 GO:0006812 GO:0017153 GO:0016020
Cluster-22178.1452 GO:0055114 GO:0016620
Cluster-22178.14535 GO:0005975 GO:0004553 GO:0030246 GO:0008270 GO:0003723 GO:0005576
Cluster-22178.14539 GO:0007156 GO:0005509 GO:0016020
Cluster-22178.14571 GO:0006568 GO:0009094 GO:0006571 GO:0000162 GO:0016301 GO:0003774 GO:0005524 GO:0016887 GO:0004640 GO:0016459
Cluster-22178.14579 GO:0007166 GO:0007186 GO:0004930 GO:0016021 GO:0016020
Cluster-22178.14586 GO:0008270 GO:0005515 GO:0009295
Cluster-22178.14600 GO:0006118 GO:0055114 GO:0022900 GO:0003677 GO:0008827 GO:0046872 GO:0009319 GO:0016021
Cluster-22178.14617 GO:0043123 GO:0007165 GO:0008272 GO:0015116 GO:0004871 GO:0005515 GO:0016021
Cluster-22178.14632 GO:0003676 GO:0005622
Cluster-22178.14649 GO:0005975 GO:0004559
Cluster-22178.14690 GO:0000041 GO:0016021
Cluster-22178.14691 GO:0000041 GO:0005509 GO:0016021 GO:0016020
Cluster-22178.14706 GO:0007015 GO:0006814 GO:0003785 GO:0005272 GO:0016020
Cluster-22178.14721 GO:0008270
Cluster-22178.14755 GO:0042254 GO:0007275 GO:0006144 GO:0006206 GO:0006094 GO:0006355 GO:0006412 GO:0006351 GO:0006352 GO:0003735 GO:0003677 GO:0004611 GO:0016987 GO:0003899 GO:0003700 GO:0005525 GO:0005667 GO:0005730 GO:0019028 GO:0005840
Cluster-22178.14773 GO:0030212 GO:0004867

Cluster-22178.14785 GO:0005975 GO:0000902 GO:0016773
Cluster-22178.14790 GO:0009297 GO:0030246 GO:0019867 GO:0005634
Cluster-22178.14794 GO:0006412 GO:0006687 GO:0006355 GO:0005975
GO:0042254 GO:0046486 GO:0006027 GO:0006012 GO:0006260
GO:0005198 GO:0003700 GO:0043565 GO:0003735 GO:0004565
GO:0019028 GO:0005840 GO:0019031 GO:0009341 GO:0005667
Cluster-22178.14795 GO:0005515
Cluster-22178.14796 GO:0007264 GO:0005525
Cluster-22178.14819 GO:0005515
Cluster-22178.14820 GO:0005515
Cluster-22178.14822 GO:0000166 GO:0046872 GO:0008168
Cluster-22178.14828 GO:0016020
Cluster-22178.14836 GO:0006508 GO:0008270 GO:0004222 GO:0031012
Cluster-22178.14855 GO:0016021
Cluster-22178.14866 GO:0015946 GO:0055114 GO:0008233 GO:0005515
GO:0004022
Cluster-22178.14867 GO:0043087 GO:0050821 GO:0015979 GO:0035023
GO:0005089 GO:0042301 GO:0009523 GO:0016020
Cluster-22178.14876 GO:0042128 GO:0006807 GO:0055114 GO:0051537
GO:0016491 GO:0008942
Cluster-22178.1488 GO:0015992 GO:0015991 GO:0008270 GO:0015078
GO:0005515 GO:0033177
Cluster-22178.14880 GO:0048544 GO:0009069 GO:0006468 GO:0035556
GO:0016310 GO:0055114 GO:0005515 GO:0004674 GO:0047134
GO:0005524 GO:0004672 GO:0016773 GO:0016020
Cluster-22178.14893 GO:0006810 GO:0005215 GO:0016020
Cluster-22178.14901 GO:0046849 GO:0005576
Cluster-22178.14909 GO:0006357 GO:0001104 GO:0016592
Cluster-22178.14918 GO:0006013 GO:0005975 GO:0006000 GO:0015976
GO:0006098 GO:0006094 GO:0006096 GO:0042578 GO:0042132
Cluster-22178.14950 GO:0016032
Cluster-22178.14958 GO:0005515 GO:0043565 GO:0005634
Cluster-22178.14969 GO:0055114 GO:0005198 GO:0016491 GO:0019028

Cluster-22178.14975 GO:0030168 GO:0042254 GO:0007165 GO:0007155
GO:0051258 GO:0006412 GO:0007094 GO:0006355 GO:0005102
GO:0003735 GO:0030674 GO:0003700 GO:0043565 GO:0005577
GO:0005622 GO:0005667 GO:0005840
Cluster-22178.14979 GO:0055114 GO:0006525 GO:0018401 GO:0006560
GO:0016702 GO:0004656 GO:0005783
Cluster-22178.14983 GO:0030001 GO:0006351 GO:0055085 GO:0006260
GO:0046873 GO:0003887 GO:0017022 GO:0043565 GO:0046872
GO:0008270 GO:0005634 GO:0016459 GO:0016020 GO:0042575
GO:0016021
Cluster-22178.15017 GO:0007275 GO:0005578
Cluster-22178.15023 GO:0007165 GO:0030474 GO:0009069 GO:0006468
GO:0016310 GO:0005179 GO:0004674 GO:0005200 GO:0005524
GO:0005576 GO:0019028 GO:0005823 GO:0005856
Cluster-22178.15042 GO:0016021
Cluster-22178.15051 GO:0005576
Cluster-22178.15053 GO:0005515 GO:0016020
Cluster-22178.15055 GO:0005515 GO:0016020
Cluster-22178.15058 GO:0000062
Cluster-22178.15059 GO:0022904 GO:0005887 GO:0016020
Cluster-22178.15061 GO:0022904 GO:0005887 GO:0016020
Cluster-22178.1507 GO:0000041 GO:0016021
Cluster-22178.15086 GO:0016020
Cluster-22178.15087 GO:0016020
Cluster-22178.15089 GO:0016020
Cluster-22178.15094 GO:0071630 GO:0031144 GO:0009512 GO:0005634
Cluster-22178.15101 GO:0005515
Cluster-22178.15108 GO:0007165 GO:0008284 GO:0008283 GO:0040007
GO:0001934 GO:0005157 GO:0005615
Cluster-22178.15110 GO:0055114 GO:0007165 GO:0016651 GO:0048038
GO:0004888 GO:0051287 GO:0005509 GO:0005515 GO:0016021
Cluster-22178.15120 GO:0003723 GO:0042025
Cluster-22178.15147 GO:0008152 GO:0003824

Cluster-22178.15149 GO:0008152 GO:0003824
Cluster-22178.15191 GO:0007165 GO:0006144 GO:0005515 GO:0004114

Cluster-22178.15192 GO:0007165 GO:0005158 GO:0005899
Cluster-22178.15193 GO:0006886 GO:0016567 GO:0006888 GO:0005515
GO:0004842 GO:0046872 GO:0008270 GO:0030127 GO:0019031
GO:0000228
Cluster-22178.15195 GO:0005515
Cluster-22178.15197 GO:0046034 GO:0015992 GO:0015991 GO:0005525
GO:0003924 GO:0043531 GO:0016887 GO:0005524 GO:0016820
GO:0033178
Cluster-22178.15200 GO:0003723
Cluster-22178.15222 GO:0007304 GO:0005213 GO:0042600 GO:0005576
Cluster-22178.15236 GO:0007165 GO:0004872 GO:0016021
Cluster-22178.15241 GO:0005515 GO:0005096 GO:0004867 GO:0005576
Cluster-22178.15256 GO:0015631 GO:0045298
Cluster-22178.15260 GO:0006914 GO:0016021
Cluster-22178.15308 GO:0007166 GO:0016020
Cluster-22178.15309 GO:0055085 GO:0016021
Cluster-22178.1531 GO:0008146 GO:0016021
Cluster-22178.15316 GO:0055085 GO:0006810 GO:0005525 GO:0042626
GO:0005524 GO:0016887 GO:0003924 GO:0016301 GO:0016021
Cluster-22178.15317 GO:0006508 GO:0004222 GO:0008270 GO:0031012
Cluster-22178.1533 GO:0050776 GO:0007165 GO:0014068 GO:0005102
GO:0043548 GO:0016020
Cluster-22178.15331 GO:0055085 GO:0019370 GO:0008237 GO:0008270
GO:0016021
Cluster-22178.15382 GO:0016772 GO:0016301 GO:0003677
Cluster-22178.15383 GO:0003677 GO:0016772 GO:0016301
Cluster-22178.15389 GO:0005515
Cluster-22178.15394 GO:0005515
Cluster-22178.15397 GO:0006281 GO:0006260 GO:0043140 GO:0005657
Cluster-22178.15410 GO:0016021

Cluster-22178.15419 GO:0005515
Cluster-22178.15448 GO:0030036 GO:0006118 GO:0003779 GO:0017048
GO:0020037 GO:0009055
Cluster-22178.15452 GO:0055114 GO:0019337 GO:0009399 GO:0006807
GO:0016163 GO:0016610
Cluster-22178.15479 GO:0003676
Cluster-22178.1550 GO:0006508 GO:0016032 GO:0007005 GO:0019082
GO:0001558 GO:0005520 GO:0008239 GO:0070009 GO:0004252
GO:0005515 GO:0016942 GO:0031305 GO:0005576
Cluster-22178.15500 GO:0055114 GO:0009060 GO:0001558 GO:0006118
GO:0006412 GO:0015992 GO:0006123 GO:0055085 GO:0042254
GO:0007186 GO:0009055 GO:0004129 GO:0020037 GO:0005515
GO:0005520 GO:0016747 GO:0004930 GO:0005506 GO:0003735
GO:0005840 GO:0045277 GO:0016021 GO:0005576 GO:0005622
GO:0016942
Cluster-22178.15501 GO:0019497 GO:0006771 GO:0046872 GO:0005515
GO:0003993
Cluster-22178.15502 GO:0019497 GO:0006771 GO:0046872 GO:0005515
GO:0003993
Cluster-22178.15503 GO:0019497 GO:0006771 GO:0003993 GO:0005515
GO:0046872
Cluster-22178.15531 GO:0007186 GO:0004930 GO:0016021
Cluster-22178.15542 GO:0003677 GO:0005515 GO:0003676 GO:0005524
GO:0016787
Cluster-22178.15549 GO:0019058 GO:0019031
Cluster-22178.15553 GO:0006144 GO:0006508 GO:0016817 GO:0070008
GO:0003968 GO:0004197 GO:0004252 GO:0031379
Cluster-22178.15555 GO:0005789
Cluster-22178.15566 GO:0006355 GO:0003700 GO:0008270 GO:0043565
GO:0005667 GO:0005634
Cluster-22178.15571 GO:0055114 GO:0005506 GO:0016705 GO:0020037

Cluster-22178.15572 GO:0006935 GO:0007165 GO:0016567 GO:0004888

GO:0004842 GO:0016020
Cluster-22178.15580 GO:0008152 GO:0007155 GO:0005540 GO:0016746

Cluster-22178.15583 GO:0007186 GO:0004930 GO:0016021
Cluster-22178.15584 GO:0007186 GO:0004930 GO:0016021
Cluster-22178.15588 GO:0005515
Cluster-22178.1561 GO:0006486 GO:0000030 GO:0016020 GO:0000136
Cluster-22178.15626 GO:0046872
Cluster-22178.15631 GO:0005515
Cluster-22178.15646 GO:0008270 GO:0005634
Cluster-22178.15654 GO:0055114 GO:0007049 GO:0006281 GO:0002949
GO:0006260 GO:0006310 GO:0003924 GO:0005525 GO:0003723
GO:0016887 GO:0005524 GO:0003724 GO:0004003 GO:0009378
GO:0098519 GO:0016706 GO:0003677 GO:0005634 GO:0005657
GO:0009379
Cluster-22178.15658 GO:0007165 GO:0031704 GO:0005179
Cluster-22178.15688 GO:0030436 GO:0042601
Cluster-22178.15690 GO:0030436 GO:0042601
Cluster-22178.15691 GO:0007165 GO:0006810 GO:0004872 GO:0016020
Cluster-22178.15733 GO:0005201 GO:0005515 GO:0005578 GO:0005581
Cluster-22178.15749 GO:0030414
Cluster-22178.15755 GO:0043565 GO:0005515 GO:0005634
Cluster-22178.15756 GO:0005515 GO:0043565 GO:0005634
Cluster-22178.15757 GO:0005515 GO:0043565 GO:0005634
Cluster-22178.15758 GO:0043565 GO:0005515 GO:0005634
Cluster-22178.15766 GO:0005515
Cluster-22178.15773 GO:0005737
Cluster-22178.15774 GO:0005515 GO:0016020
Cluster-22178.15783 GO:0006810 GO:0007186 GO:0005215 GO:0004930
GO:0016021
Cluster-22178.15839 GO:0006468 GO:0005524 GO:0004672
Cluster-22178.15844 GO:0016021
Cluster-22178.15845 GO:0006355 GO:0003700 GO:0005667

Cluster-22178.15846 GO:0016021
Cluster-22178.15847 GO:0006355 GO:0003700 GO:0005667
Cluster-22178.15858 GO:0015031 GO:0009306 GO:0006412 GO:0042254
GO:0004222 GO:0015450 GO:0003735 GO:0005887 GO:0009941
GO:0005840 GO:0016021
Cluster-22178.15861 GO:0007186 GO:0004930 GO:0016021
Cluster-22178.15863 GO:0007186 GO:0004930 GO:0016021
Cluster-22178.15879 GO:0007186 GO:0004930 GO:0016021
Cluster-22178.15891 GO:0006552 GO:0006550 GO:0006568 GO:0015940
GO:0016114 GO:0006694 GO:0055114 GO:0008033 GO:0006574
GO:0006631 GO:0018874 GO:0006633 GO:0006554 GO:0003857
GO:0004506 GO:0008677 GO:0016616 GO:0071949 GO:0051287
GO:0050660 GO:0016491 GO:0016021
Cluster-22178.15896 GO:0009405 GO:0006810 GO:0019855 GO:0005576
GO:0016021
Cluster-22178.15924 GO:0055085 GO:0016021
Cluster-22178.15925 GO:0055085 GO:0016021
Cluster-22178.15926 GO:0055085 GO:0016021
Cluster-22178.15927 GO:0055085 GO:0016021
Cluster-22178.15931 GO:0046486 GO:0016042 GO:0005524 GO:0004806

Cluster-22178.15958 GO:0008152 GO:0016491
Cluster-22178.15960 GO:0046487 GO:0055114 GO:0009396 GO:0008152
GO:0050662 GO:0004488 GO:0016491 GO:0003824
Cluster-22178.15976 GO:0006468 GO:0000746 GO:0006810 GO:0009405
GO:0005524 GO:0004672 GO:0008200 GO:0005515 GO:0042151
GO:0019867 GO:0016021
Cluster-22178.15988 GO:0007304 GO:0005213 GO:0042600 GO:0005576
Cluster-22178.15996 GO:0009405 GO:0005515 GO:0016020 GO:0016021
Cluster-22178.16025 GO:0005515
Cluster-22178.16027 GO:0015886 GO:0055114 GO:0017004 GO:0005506
GO:0020037 GO:0016705 GO:0016021
Cluster-22178.16052 GO:0007165 GO:0006508 GO:0004252 GO:0070008

GO:0005044 GO:0016020
Cluster-22178.16070 GO:0006820 GO:0015992 GO:0006123 GO:0004129
GO:0016021 GO:0045277 GO:0005740
Cluster-22178.16080 GO:0006813 GO:0005249 GO:0030955 GO:0008076
GO:0016021
Cluster-22178.16081 GO:0008283 GO:0040007 GO:0007165 GO:0000902
GO:0006955 GO:0008083 GO:0016020
Cluster-22178.16094 GO:0005515
Cluster-22178.16126 GO:0061025 GO:0046813 GO:0019031 GO:0016021
Cluster-22178.16163 GO:0007165 GO:0055114 GO:0004888 GO:0051287
GO:0005515 GO:0005509 GO:0016651 GO:0048038 GO:0016021
Cluster-22178.16164 GO:0035091 GO:0005515 GO:0046914
Cluster-22178.1618 GO:0020037 GO:0005515
Cluster-22178.16186 GO:0006355 GO:0003677 GO:0003676
Cluster-22178.16187 GO:0006355 GO:0003676 GO:0003677
Cluster-22178.16189 GO:0003950
Cluster-22178.16192 GO:0006096 GO:0006952 GO:0006144 GO:0007017
GO:0006094 GO:0008284 GO:0007018 GO:0015976 GO:0004743
GO:0016787 GO:0005524 GO:0008017 GO:0000287 GO:0030955
GO:0005515 GO:0003777 GO:0045298 GO:0005874
Cluster-22178.16222 GO:0006614 GO:0030212 GO:0004867 GO:0030942
GO:0004252 GO:0008312 GO:0005886 GO:0005786
Cluster-22178.16242 GO:0055085 GO:0007275 GO:0007165 GO:0022857
GO:0005179 GO:0016021 GO:0019028 GO:0005576
Cluster-22178.16258 GO:0006338 GO:0003676 GO:0016818 GO:0005524
GO:0005634
Cluster-22178.16259 GO:0055085 GO:0019028 GO:0016021
Cluster-22178.16260 GO:0055085 GO:0016021 GO:0019028
Cluster-22178.16272 GO:0009405 GO:0005515 GO:0005576
Cluster-22178.16293 GO:0007275 GO:0003774 GO:0005524 GO:0016459
Cluster-22178.16299 GO:0007275 GO:0006313 GO:0004803 GO:0003677
GO:0016021
Cluster-22178.16307 GO:0007186 GO:0004930 GO:0016021

Cluster-22178.16308 GO:0007304 GO:0006118 GO:0005213 GO:0009055
GO:0051536 GO:0005576 GO:0042600
Cluster-22178.16314 GO:0006090 GO:0015969 GO:0006814 GO:0071436
GO:0006560 GO:0006525 GO:0015081 GO:0008948 GO:0016020
Cluster-22178.16328 GO:0003824
Cluster-22178.16339 GO:0006807 GO:0036361
Cluster-22178.16340 GO:0007186 GO:0004930 GO:0016021
Cluster-22178.16342 GO:0019058 GO:0005198
Cluster-22178.16347 GO:0006448 GO:0006414 GO:0003746 GO:0005840
Cluster-22178.16353 GO:0006355 GO:0003700 GO:0005667
Cluster-22178.16354 GO:0006355 GO:0003700 GO:0005667
Cluster-22178.16357 GO:0005525
Cluster-22178.16363 GO:0006810 GO:0043565 GO:0005515 GO:0005634
GO:0016020
Cluster-22178.16372 GO:0006144 GO:0007165 GO:0004114 GO:0005515

Cluster-22178.16374 GO:0020037 GO:0005515
Cluster-22178.16382 GO:0008270 GO:0005515 GO:0004222
Cluster-22178.16387 GO:0030866 GO:0005198 GO:0008092 GO:0003779
GO:0005856
Cluster-22178.16399 GO:0006811 GO:0055085 GO:0005216 GO:0016817
GO:0016020
Cluster-22178.16401 GO:0005515
Cluster-22178.16425 GO:0019054 GO:0004857
Cluster-22178.16426 GO:0019054 GO:0004857
Cluster-22178.16431 GO:0006122 GO:0005750 GO:0005743
Cluster-22178.16432 GO:0055085 GO:0006811 GO:0009405 GO:0006810
GO:0008200 GO:0005216 GO:0005576 GO:0016020
Cluster-22178.16437 GO:0019497 GO:0006470 GO:0006811 GO:0006771
GO:0006570 GO:0046872 GO:0003993 GO:0004725 GO:0005216
GO:0008138 GO:0005515 GO:0016020
Cluster-22178.16444 GO:0019825 GO:0020037
Cluster-22178.16536 GO:0055114 GO:0008033 GO:0016627 GO:0016491

GO:0050660 GO:0017150 GO:0005737
Cluster-22178.16538 GO:0008643 GO:0055114 GO:0009401 GO:0008982
GO:0016627 GO:0009357 GO:0016020 GO:0005737
Cluster-22178.16552 GO:0006468 GO:0006355 GO:0004672 GO:0005524

Cluster-22178.1656 GO:0007165 GO:0007268 GO:0009405 GO:0030550
GO:0005576
Cluster-22178.16598 GO:0007275 GO:0042254 GO:0005201 GO:0003735
GO:0005198 GO:0005581 GO:0005840 GO:0005578 GO:0005739
Cluster-22178.16621 GO:0015986 GO:0006269 GO:0006351 GO:0015992
GO:0061630 GO:0005515 GO:0046872 GO:0008270 GO:0003896
GO:0015078 GO:0004386 GO:0005730 GO:0000276 GO:0005657
Cluster-22178.16623 GO:0006351 GO:0015986 GO:0006269 GO:0015992
GO:0005515 GO:0061630 GO:0015078 GO:0003896 GO:0004386
GO:0046872 GO:0008270 GO:0005730 GO:0005657 GO:0000276
Cluster-22178.16649 GO:0008270 GO:0003677
Cluster-22178.16651 GO:0003677 GO:0008270
Cluster-22178.16655 GO:0009306 GO:0003824 GO:0016021
Cluster-22178.1666 GO:0006000 GO:0006013 GO:0005524 GO:0016301
GO:0003873
Cluster-22178.16675 GO:0018298 GO:0030076
Cluster-22178.16679 GO:0051536 GO:0003824
Cluster-22178.16680 GO:0008270
Cluster-22178.16696 GO:0008168
Cluster-22178.16732 GO:0019385 GO:0009279
Cluster-22178.16733 GO:0006468 GO:0005524 GO:0004672
Cluster-22178.16735 GO:0006486 GO:0000030 GO:0000136 GO:0016020
Cluster-22178.1674 GO:0006955 GO:0000902 GO:0016021 GO:0016020
Cluster-22178.16754 GO:0042742 GO:0007155 GO:0005540 GO:0030246
GO:0005576
Cluster-22178.16756 GO:0046497 GO:0009435 GO:0004514
Cluster-22178.16757 GO:0016020
Cluster-22178.16771 GO:0008608 GO:0042729 GO:0072686

Cluster-22178.16772 GO:0006281 GO:0006310 GO:0019079 GO:0002949
GO:0003723 GO:0009378 GO:0016301 GO:0016887 GO:0043531
GO:0005524 GO:0003678 GO:0003724 GO:0009379 GO:0005657
Cluster-22178.16774 GO:0006355 GO:0006281 GO:0006576 GO:0006694
GO:0006139 GO:0016114 GO:0019079 GO:0009058 GO:0006310
GO:0002949 GO:0006206 GO:0004161 GO:0008134 GO:0016301
GO:0003723 GO:0003678 GO:0043531 GO:0098519 GO:0003677
GO:0009378 GO:0016787 GO:0003724 GO:0004127 GO:0005524
GO:0016887 GO:0005667 GO:0009379 GO:0005657
Cluster-22178.16775 GO:0030212 GO:0006614 GO:0008312 GO:0004867
GO:0004252 GO:0030942 GO:0005786 GO:0005886
Cluster-22178.16778 GO:0005509 GO:0005515
Cluster-22178.16779 GO:0005515 GO:0005509
Cluster-22178.16780 GO:0005515 GO:0005509
Cluster-22178.16826 GO:0006355 GO:0005515 GO:0005634
Cluster-22178.16827 GO:0006355 GO:0005515 GO:0005634
Cluster-22178.16837 GO:0003676 GO:0008270
Cluster-22178.16838 GO:0007340 GO:0007342 GO:0006355 GO:0003677
GO:0016020 GO:0001669
Cluster-22178.16844 GO:0005515
Cluster-22178.16865 GO:0003676
Cluster-22178.16878 GO:0007186 GO:0004930 GO:0016021
Cluster-22178.16880 GO:0046872
Cluster-22178.16887 GO:0002949 GO:0000723 GO:0006810 GO:0006281
GO:0003678 GO:0003724 GO:0005524 GO:0003774 GO:0098519
GO:0016301 GO:0003723 GO:0005657 GO:0016459
Cluster-22178.16888 GO:0006810 GO:0006816 GO:0003676 GO:0008270
GO:0042030 GO:0005246 GO:0016020 GO:0005634
Cluster-22178.16889 GO:0006810 GO:0006816 GO:0005246 GO:0042030
GO:0016020
Cluster-22178.16890 GO:0007155
Cluster-22178.16891 GO:0007155
Cluster-22178.16897 GO:0006508 GO:0004252

Cluster-22178.16914 GO:0005515
Cluster-22178.16915 GO:0022904 GO:0016020 GO:0005887
Cluster-22178.16936 GO:0006355 GO:0005515 GO:0046983
Cluster-22178.16939 GO:0004252 GO:0005886
Cluster-22178.16945 GO:0006810 GO:0009405 GO:0008200 GO:0005515 GO:0005576
Cluster-22178.16974 GO:0055085 GO:0006811 GO:0005216 GO:0000228 GO:0016020
Cluster-22178.16980 GO:0008134 GO:0005667 GO:0005634
Cluster-22178.16997 GO:0005509
Cluster-22178.16998 GO:0005509 GO:0005515
Cluster-22178.17027 GO:0005515 GO:0008270 GO:0016020 GO:0005622
Cluster-22178.17034 GO:0006810 GO:0007186 GO:0006811 GO:0055085 GO:0005216 GO:0004930 GO:0016020 GO:0016021
Cluster-22178.17051 GO:0007186 GO:0004930 GO:0016020 GO:0016021
Cluster-22178.17056 GO:0006323 GO:0006182 GO:0007165 GO:0006914 GO:0006144 GO:0009252 GO:0046039 GO:0006955 GO:0030474 GO:0016755 GO:0004383 GO:0005164 GO:0005200 GO:0016020 GO:0005823 GO:0005856
Cluster-22178.17057 GO:0007268 GO:0006811 GO:0007165 GO:0004970 GO:0016020
Cluster-22178.17059 GO:0007275 GO:0005578
Cluster-22178.17071 GO:0005525
Cluster-22178.17089 GO:0051726
Cluster-22178.1709 GO:0006606
Cluster-22178.17092 GO:0006270 GO:0032040
Cluster-22178.17096 GO:0006270 GO:0032040
Cluster-22178.17098 GO:0006310 GO:0002949 GO:0006260 GO:0006281 GO:0055114 GO:0007049 GO:0004003 GO:0009378 GO:0016706 GO:0098519 GO:0003677 GO:0016887 GO:0005524 GO:0003724 GO:0003723 GO:0003924 GO:0005525 GO:0009379 GO:0005657 GO:0005634
Cluster-22178.17100 GO:0005515

Cluster-22178.17101 GO:0007165 GO:0005515 GO:0005509 GO:0032797
GO:0005578
Cluster-22178.17111 GO:0004222 GO:0016021
Cluster-22178.17119 GO:0005515
Cluster-22178.17120 GO:0009372 GO:0043768 GO:0005506
Cluster-22178.17127 GO:0005515
Cluster-22178.17137 GO:0020037 GO:0019825
Cluster-22178.17139 GO:0006955 GO:0007165 GO:0005164 GO:0016020
Cluster-22178.17140 GO:0007165 GO:0005515 GO:0005509 GO:0005578
Cluster-22178.17143 GO:0008152 GO:0006508 GO:0004222 GO:0008270
GO:0016491 GO:0005576
Cluster-22178.17145 GO:0005515
Cluster-22178.17161 GO:0007155 GO:0008270 GO:0005540
Cluster-22178.17164 GO:0005198 GO:0019031 GO:0019028
Cluster-22178.17179 GO:0006351 GO:0006269 GO:0015986 GO:0015992
GO:0061630 GO:0005515 GO:0015078 GO:0003896 GO:0004386
GO:0008270 GO:0046872 GO:0005730 GO:0005657 GO:0000276
Cluster-22178.17188 GO:0016032 GO:0006508 GO:0001558 GO:0005520
GO:0008239 GO:0070009 GO:0004252 GO:0005515 GO:0019031
GO:0005576 GO:0016942
Cluster-22178.17190 GO:0007165 GO:0006144 GO:0004114
Cluster-22178.17194 GO:0006810 GO:0006817 GO:0006508 GO:0030436
GO:0004190 GO:0005525 GO:0005215 GO:0005315 GO:0016020
GO:0016021
Cluster-22178.17207 GO:0016021
Cluster-22178.17212 GO:0009405 GO:0005576
Cluster-22178.17229 GO:0051082 GO:0031072
Cluster-22178.17244 GO:0015991 GO:0006508 GO:0016818 GO:0016820
GO:0004222 GO:0003724 GO:0003712 GO:0033178 GO:0005634
GO:0005667
Cluster-22178.17252 GO:0006886 GO:0005198 GO:0005622 GO:0019028
GO:0019031
Cluster-22178.17258 GO:0045444 GO:0044038 GO:0006813 GO:0016757

GO:0005242 GO:0005886 GO:0016021 GO:0008076

Cluster-22178.17280 GO:0007165 GO:0008158 GO:0016020

Cluster-22178.17282 GO:0005515

Cluster-22178.17284 GO:0008152 GO:0042967 GO:0016407

Cluster-22178.17285 GO:0042967 GO:0008152 GO:0016407

Cluster-22178.17288 GO:0019684 GO:0006118 GO:0008270 GO:0045156

GO:0030077 GO:0016021

Cluster-22178.17289 GO:0016020

Cluster-22178.17290 GO:0008270

Cluster-22178.17291 GO:0019684 GO:0006118 GO:0008270 GO:0045156

GO:0030077 GO:0016021

Cluster-22178.17322 GO:0007186 GO:0009405 GO:0008270 GO:0004222

GO:0004930 GO:0005576 GO:0016021

Cluster-22178.17326 GO:0007186 GO:0004930 GO:0016021

Cluster-22178.17334 GO:0006886 GO:0016192 GO:0030131

Cluster-22178.17346 GO:0042254 GO:0006412 GO:0003735 GO:0005840

Cluster-22178.17355 GO:0006812 GO:0006836 GO:0005328 GO:0008146

GO:0016021

Cluster-22178.17356 GO:0016192 GO:0042393 GO:0005515 GO:0016021

GO:0005634

Cluster-22178.17361 GO:0005975 GO:0016773 GO:0005524

Cluster-22178.17368 GO:0008270

Cluster-22178.17371 GO:0008270

Cluster-22178.1739 GO:0006810 GO:0005215 GO:0016020

Cluster-22178.17395 GO:0006446 GO:0006413 GO:0003743 GO:0005840

Cluster-22178.17396 GO:0019031

Cluster-22178.17397 GO:0007155 GO:0042246 GO:0005509 GO:0016021

Cluster-22178.17431 GO:0003676 GO:0019013

Cluster-22178.17432 GO:0003676 GO:0019013

Cluster-22178.17443 GO:0006915

Cluster-22178.17471 GO:0007155 GO:0016021

Cluster-22178.17491 GO:0006355 GO:0043565 GO:0003700 GO:0005667

Cluster-22178.17520 GO:0005515 GO:0043565 GO:0005634

Cluster-22178.17532 GO:0006357 GO:0001104 GO:0016592
Cluster-22178.17536 GO:0016192 GO:0006904
Cluster-22178.17546 GO:0006468 GO:0016773 GO:0005524 GO:0008270
GO:0004672 GO:0005515 GO:0016020
Cluster-22178.17556 GO:0005515 GO:0016020
Cluster-22178.17565 GO:0006333 GO:0005634
Cluster-22178.17582 GO:0006206 GO:0006144 GO:0006281 GO:0006355
GO:0006351 GO:0008270 GO:0046872 GO:0008168 GO:0003677
GO:0003899 GO:0005730
Cluster-22178.17583 GO:0007186 GO:0004930 GO:0016021
Cluster-22178.17586 GO:0005515
Cluster-22178.17595 GO:0006396 GO:0008053 GO:0006869 GO:0042157
GO:0042262 GO:0006308 GO:0008855 GO:0003690 GO:0005525
GO:0008289 GO:0003924 GO:0005198 GO:0051082 GO:0000176
GO:0005741 GO:0030133 GO:0016021 GO:0019031 GO:0031083
GO:0009318 GO:0019028 GO:0005576
Cluster-22178.17596 GO:0009069 GO:0042157 GO:0006869 GO:0006396
GO:0008053 GO:0006308 GO:0016310 GO:0009306 GO:0042262
GO:0003690 GO:0008855 GO:0004674 GO:0005198 GO:0003924
GO:0008289 GO:0005525 GO:0030133 GO:0005741 GO:0000176
GO:0005576 GO:0019028 GO:0009318 GO:0031083 GO:0019031
GO:0016021
Cluster-22178.17602 GO:0006570 GO:0055114 GO:0007156 GO:0005509
GO:0004511 GO:0016020
Cluster-22178.17614 GO:0006144 GO:0035556 GO:0006468 GO:0006182
GO:0046039 GO:0009190 GO:0005524 GO:0004672 GO:0004383
GO:0016849
Cluster-22178.17615 GO:0006468 GO:0006182 GO:0009190 GO:0046039
GO:0006144 GO:0035556 GO:0004383 GO:0016849 GO:0004672
GO:0005524
Cluster-22178.17626 GO:0001558 GO:0019082 GO:0006508 GO:0016032
GO:0005515 GO:0004252 GO:0070009 GO:0008239 GO:0005520
GO:0005576 GO:0016942

Cluster-22178.17632 GO:0006812 GO:0034755 GO:0006826 GO:0006836
GO:0005381 GO:0005328 GO:0016021

Cluster-22178.17657 GO:0016573 GO:0000079 GO:0032801 GO:0006812
GO:0019076 GO:0005975 GO:0019901 GO:0005261 GO:0004553
GO:0019012 GO:0016021 GO:0033644

Cluster-22178.17677 GO:0055114 GO:0047134 GO:0046872

Cluster-22178.17678 GO:0055114 GO:0046872 GO:0047134

Cluster-22178.17680 GO:0045893 GO:0005737

Cluster-22178.17681 GO:0045893 GO:0005737

Cluster-22178.17700 GO:0006570 GO:0006470 GO:0008138 GO:0004725

Cluster-22178.17702 GO:0006570 GO:0006470 GO:0004725 GO:0008138

Cluster-22178.17722 GO:0003697 GO:0005887

Cluster-22178.17726 GO:0016020 GO:0016021 GO:0005886

Cluster-22178.17729 GO:0006812 GO:0015725 GO:0008643 GO:0006820
GO:0046411 GO:0015649 GO:0008767 GO:0016021

Cluster-22178.17733 GO:0003824 GO:0051536

Cluster-22178.17734 GO:0051536 GO:0003824

Cluster-22178.17744 GO:0006260 GO:0008270 GO:0003677

Cluster-22178.17747 GO:0006914 GO:0006357 GO:0070206 GO:0030168
GO:0008053 GO:0006606 GO:0006260 GO:0042157 GO:0007165
GO:0006869 GO:0048870 GO:0051258 GO:0010508 GO:0005102
GO:0030674 GO:0003924 GO:0008289 GO:0005525 GO:0005198
GO:0001104 GO:0019901 GO:0005577 GO:0016592 GO:0005741
GO:0005882 GO:0016021 GO:0005576 GO:0031514

Cluster-22178.17755 GO:0016567 GO:0004842 GO:0005515 GO:0008270
GO:0046872 GO:0005622

Cluster-22178.17781 GO:0009312 GO:0008455 GO:0016021 GO:0005795

Cluster-22178.17783 GO:0005515 GO:0004867

Cluster-22178.17784 GO:0005515 GO:0004867

Cluster-22178.17802 GO:0044237 GO:0007165 GO:0003824 GO:0008601
GO:0000159

Cluster-22178.17807 GO:0040007 GO:0007155 GO:0008283 GO:0007165 GO:0005540 GO:0008083

Cluster-22178.17832 GO:0005198 GO:0019028

Cluster-22178.17853 GO:0051301 GO:0048870 GO:0007059 GO:0007155 GO:0000090 GO:0007067 GO:0031110 GO:0006914 GO:0006260 GO:0005198 GO:0019901 GO:0003774 GO:0019905 GO:0008017 GO:0019028 GO:0031514 GO:0019031 GO:0045298 GO:0016459 GO:0005882 GO:0000940 GO:0005876

Cluster-22178.17857 GO:0016310 GO:0035556 GO:0055114 GO:0048544 GO:0006468 GO:0009069 GO:0047134 GO:0004674 GO:0005524 GO:0004672 GO:0016773 GO:0005515 GO:0016020

Cluster-22178.17874 GO:0055114 GO:0051287 GO:0016491 GO:0016616

Cluster-22178.17881 GO:0000160 GO:0016310 GO:0006109 GO:0007186 GO:0007165 GO:0015684 GO:0007264 GO:0004871 GO:0005525 GO:0003924 GO:0019001 GO:0031683 GO:0004672 GO:0003723 GO:0003724 GO:0016887 GO:0015093 GO:0005524 GO:0000155 GO:0016021 GO:0009365

Cluster-22178.17882 GO:0030682 GO:0004867

Cluster-22178.17884 GO:0016021

Cluster-22178.17892 GO:0042545 GO:0005985 GO:0005982 GO:0030599 GO:0005618

Cluster-22178.17896 GO:0033539 GO:0055114 GO:0006118 GO:0003995

Cluster-22178.17901 GO:0007010 GO:0003779

Cluster-22178.17915 GO:0019497 GO:0006771 GO:0046872 GO:0003993 GO:0005515

Cluster-22178.17916 GO:0019497 GO:0006771 GO:0003993 GO:0005515 GO:0046872

Cluster-22178.17931 GO:0009405

Cluster-22178.17932 GO:0006468 GO:0005524 GO:0004672

Cluster-22178.17940 GO:0007165 GO:0070008 GO:0004252 GO:0005044 GO:0016020

Cluster-22178.17964 GO:0015979 GO:0009523 GO:0009539 GO:0016020
Cluster-22178.17980 GO:0006508 GO:0016032 GO:0004197 GO:0005515

Cluster-22178.1799 GO:0055114 GO:0005506 GO:0016705 GO:0020037
Cluster-22178.17993 GO:0005515
Cluster-22178.17994 GO:0007264 GO:0005524 GO:0003924 GO:0005525
GO:0019013
Cluster-22178.17996 GO:0008168
Cluster-22178.17997 GO:0007165 GO:0005179 GO:0005576
Cluster-22178.18003 GO:0005515
Cluster-22178.18005 GO:0006508 GO:0008270 GO:0004252 GO:0005634
Cluster-22178.18006 GO:0006508 GO:0004252
Cluster-22178.18015 GO:0005515
Cluster-22178.18046 GO:0003723
Cluster-22178.18063 GO:0055114 GO:0015992 GO:0006123 GO:0009060
GO:0006118 GO:0004129 GO:0009055 GO:0005506 GO:0020037
GO:0016021 GO:0045277
Cluster-22178.18067 GO:0006811 GO:0007165 GO:0007268 GO:0004970
GO:0005234 GO:0016020
Cluster-22178.18091 GO:0007165 GO:0008283 GO:0040007 GO:0008083
GO:0016020
Cluster-22178.18102 GO:0007049 GO:0019069 GO:0005515
Cluster-22178.18115 GO:0006355 GO:0030474 GO:0070206 GO:0051607
GO:0007049 GO:0005515 GO:0043565 GO:0005200 GO:0003700
GO:0005667 GO:0005856 GO:0005823
Cluster-22178.18125 GO:0008146
Cluster-22178.18129 GO:0003824 GO:0004867 GO:0051536
Cluster-22178.18133 GO:0006120 GO:0006814 GO:0006744 GO:0015992
GO:0008137 GO:0016020 GO:0005739
Cluster-22178.18148 GO:0006468 GO:0006810 GO:0008200 GO:0005515
GO:0005524 GO:0004672 GO:0005576 GO:0016020
Cluster-22178.18149 GO:0055085 GO:0016021
Cluster-22178.18155 GO:0006468 GO:0005524 GO:0004672 GO:0005515

Cluster-22178.18158 GO:0043401 GO:0006355
Cluster-22178.18159 GO:0005515
Cluster-22178.18164 GO:0006355 GO:0030420 GO:0005509 GO:0005515 GO:0005634
Cluster-22178.18167 GO:0007029 GO:0005789
Cluster-22178.18168 GO:0008152 GO:0016491
Cluster-22178.18179 GO:0042254 GO:0006412 GO:0016887 GO:0003924 GO:0005524 GO:0005525 GO:0003735 GO:0098519 GO:0005840
Cluster-22178.18183 GO:0006355 GO:0003677
Cluster-22178.18222 GO:0006457 GO:0010508 GO:0007165 GO:0006810 GO:0006914 GO:0030168 GO:0051258 GO:0030674 GO:0051082 GO:0005102 GO:0008289 GO:0005794 GO:0005577 GO:0016272
Cluster-22178.18224 GO:0030001 GO:0051258 GO:0006810 GO:0007165 GO:0006457 GO:0055085 GO:0030168 GO:0006914 GO:0051082 GO:0008289 GO:0046873 GO:0030674 GO:0005102 GO:0005794 GO:0016020 GO:0005577 GO:0016272
Cluster-22178.18225 GO:0006457 GO:0007165 GO:0006810 GO:0006914 GO:0030168 GO:0051258 GO:0030674 GO:0051082 GO:0005102 GO:0008289 GO:0016272 GO:0005794 GO:0005577
Cluster-22178.18226 GO:0006810 GO:0000917 GO:0043093 GO:0006308 GO:0008855 GO:0009318 GO:0005794 GO:0005737
Cluster-22178.18228 GO:0006308 GO:0006810 GO:0000917 GO:0043093 GO:0008855 GO:0005794 GO:0005737 GO:0009318
Cluster-22178.18244 GO:0002098 GO:0034227 GO:0008270 GO:0046872 GO:0000049
Cluster-22178.18251 GO:0006144 GO:0005524 GO:0003968 GO:0031379
Cluster-22178.18256 GO:0006810 GO:0009405 GO:0006468 GO:0004672 GO:0005524 GO:0005515 GO:0008200 GO:0016021 GO:0042151
Cluster-22178.18257 GO:0006468 GO:0006810 GO:0009405 GO:0008200 GO:0005515 GO:0016773 GO:0005524 GO:0004672 GO:0016020 GO:0042151 GO:0016021
Cluster-22178.18258 GO:0006468 GO:0006810 GO:0009405 GO:0008200

GO:0005515 GO:0004672 GO:0005524 GO:0016773 GO:0016020
GO:0042151 GO:0016021
Cluster-22178.18260 GO:0006810 GO:0009405 GO:0006468 GO:0005515
GO:0008200 GO:0005524 GO:0004672 GO:0016021 GO:0042151
Cluster-22178.18270 GO:0003677
Cluster-22178.18274 GO:0006355 GO:0043565 GO:0003700 GO:0005634
GO:0005667
Cluster-22178.18307 GO:0005201 GO:0005578 GO:0005581
Cluster-22178.18315 GO:0007165 GO:0009405 GO:0005198 GO:0005515
GO:0016021
Cluster-22178.18326 GO:0016787 GO:0046872
Cluster-22178.18327 GO:0006771 GO:0019497 GO:0005515 GO:0003993
GO:0046872
Cluster-22178.18333 GO:0006355 GO:0030420 GO:0005509 GO:0005515
GO:0005634
Cluster-22178.18342 GO:0005509
Cluster-22178.18345 GO:0006629
Cluster-22178.18354 GO:0005515
Cluster-22178.18368 GO:0035556
Cluster-22178.18372 GO:0016020
Cluster-22178.18375 GO:0030001 GO:0007034
Cluster-22178.18379 GO:0035023 GO:0043087 GO:0005515 GO:0005089

Cluster-22178.18380 GO:0000398 GO:0008270 GO:0005681
Cluster-22178.18383 GO:0042254 GO:0006412 GO:0003735 GO:0005622
GO:0005840
Cluster-22178.18391 GO:0009190 GO:0035556 GO:0016849
Cluster-22178.18392 GO:0009190 GO:0035556 GO:0016849
Cluster-22178.18399 GO:0006355 GO:0046983 GO:0003677
Cluster-22178.18415 GO:0007154 GO:0055085 GO:0016747 GO:0016021
Cluster-22178.18419 GO:0006468 GO:0005524 GO:0004672
Cluster-22178.18432 GO:0016020
Cluster-22178.18434 GO:0015074 GO:0006313 GO:0003677

Cluster-22178.18442 GO:0007156 GO:0050708 GO:0005509 GO:0016020
GO:0005737
Cluster-22178.18448 GO:0007186 GO:0004930 GO:0016021
Cluster-22178.18452 GO:0006457 GO:0006520 GO:0016743
Cluster-22178.18457 GO:0006355 GO:0006352 GO:0006810 GO:0002949
GO:0042967 GO:0016301 GO:0008811 GO:0003700 GO:0003676
GO:0003677 GO:0016987 GO:0016887 GO:0005524 GO:0003774
GO:0005667 GO:0016459
Cluster-22178.18475 GO:0004252 GO:0012511 GO:0016021
Cluster-22178.18494 GO:0007165 GO:0006955 GO:0005125 GO:0005576
Cluster-22178.18507 GO:0006468 GO:0004672 GO:0051082
Cluster-22178.18515 GO:0051382 GO:0030683 GO:0005515 GO:0000776
GO:0044386
Cluster-22178.18521 GO:0007186 GO:0007165 GO:0006260 GO:0004003
GO:0003677 GO:0003924 GO:0019001 GO:0031683 GO:0005524
GO:0004871 GO:0005525 GO:0005657
Cluster-22178.18563 GO:0005515
Cluster-22178.18567 GO:0006265 GO:0007165 GO:0005057 GO:0003918
GO:0003677 GO:0005694
Cluster-22178.18573 GO:0007165 GO:0006144 GO:0004114
Cluster-22178.18574 GO:0016787 GO:0046872
Cluster-22178.18602 GO:0016042 GO:0007586 GO:0006355 GO:0042254
GO:0006412 GO:0043565 GO:0008047 GO:0003700 GO:0003735
GO:0005622 GO:0005840 GO:0005667 GO:0005576
Cluster-22178.18607 GO:0006355 GO:0030420 GO:0005509 GO:0005515
GO:0005634
Cluster-22178.18609 GO:0006355 GO:0030420 GO:0005515 GO:0005509
GO:0005634
Cluster-22178.18616 GO:0009312 GO:0008455 GO:0005795 GO:0016021
Cluster-22178.18628 GO:0030420 GO:0006355 GO:0005515 GO:0005509
GO:0005634
Cluster-22178.18633 GO:0006355 GO:0030420 GO:0005509 GO:0005515
GO:0005634

Cluster-22178.18637 GO:0006357 GO:0001104 GO:0016592
Cluster-22178.18653 GO:0005515 GO:0019031
Cluster-22178.18656 GO:0005515 GO:0019031
Cluster-22178.18668 GO:0006367 GO:0015986 GO:0015992 GO:0015078
GO:0005634
Cluster-22178.18688 GO:0006629
Cluster-22178.18691 GO:0000226 GO:0016020 GO:0015630
Cluster-22178.18694 GO:0006811 GO:0051260 GO:0055085 GO:0005216
GO:0016020
Cluster-22178.18705 GO:0033617 GO:0005515
Cluster-22178.18707 GO:0005515
Cluster-22178.18722 GO:0000746 GO:0005509
Cluster-22178.18723 GO:0005509
Cluster-22178.18724 GO:0005509
Cluster-22178.18756 GO:0006811 GO:0055085 GO:0051262 GO:0005216
GO:0016020
Cluster-22178.18771 GO:0005509 GO:0005515
Cluster-22178.18774 GO:0006750 GO:0006749 GO:0004357 GO:0017109
Cluster-22178.18780 GO:0007165 GO:0040007 GO:0008283 GO:0008083
GO:0051015 GO:0030674
Cluster-22178.18788 GO:0006355 GO:0046872 GO:0005634
Cluster-22178.18789 GO:0007155 GO:0005515 GO:0005540
Cluster-22178.18792 GO:0005201 GO:0005581 GO:0005578
Cluster-22178.18825 GO:0015693 GO:0015095 GO:0016021
Cluster-22178.1883 GO:0006355 GO:0055085 GO:0006811 GO:0005216
GO:0003700 GO:0043565 GO:0008270 GO:0016020 GO:0005634
GO:0005667
Cluster-22178.18830 GO:0007275 GO:0005578
Cluster-22178.18834 GO:0055085 GO:0022857 GO:0016021
Cluster-22178.18835 GO:0005515
Cluster-22178.1885 GO:0007606 GO:0033227 GO:0017004 GO:0007186
GO:0019236 GO:0015886 GO:0007224 GO:0015031 GO:0015931
GO:0051033 GO:0004932 GO:0016020 GO:0016021

Cluster-22178.18857 GO:0006468 GO:0006278 GO:0016773 GO:0003964 GO:0005524 GO:0004672 GO:0016020
Cluster-22178.18864 GO:0008061 GO:0003724 GO:0005524 GO:0003723 GO:0005737
Cluster-22178.18868 GO:0019082 GO:0016032 GO:0006508 GO:0004197 GO:0016021
Cluster-22178.18911 GO:0005515
Cluster-22178.18915 GO:0005515
Cluster-22178.18933 GO:0005509 GO:0005515
Cluster-22178.18944 GO:0055114 GO:0016614 GO:0050660 GO:0046872

Cluster-22178.18947 GO:0055085 GO:0007155 GO:0030193 GO:0022857 GO:0016021 GO:0005576
Cluster-22178.18951 GO:0015074 GO:0003676 GO:0003677 GO:0005524

Cluster-22178.18952 GO:0006355 GO:0015074 GO:0003676 GO:0003700 GO:0008270 GO:0005667
Cluster-22178.18959 GO:0005525 GO:0003924
Cluster-22178.18961 GO:0003924 GO:0005525
Cluster-22178.18972 GO:0035023 GO:0043087 GO:0005089
Cluster-22178.18977 GO:0000287 GO:0030976 GO:0019031
Cluster-22178.18991 GO:0005515 GO:0005509
Cluster-22178.18998 GO:0006508 GO:0004252
Cluster-22178.19002 GO:0005576 GO:0005615
Cluster-22178.19014 GO:0007186 GO:0004930 GO:0016021
Cluster-22178.19016 GO:0007186 GO:0004930 GO:0016021
Cluster-22178.19019 GO:0007186 GO:0004930 GO:0016021
Cluster-22178.19024 GO:0006304 GO:0007165 GO:0005179 GO:0033821 GO:0005576
Cluster-22178.19029 GO:0042981
Cluster-22178.19049 GO:0005515
Cluster-22178.19058 GO:0035556 GO:0009190 GO:0016849
Cluster-22178.19059 GO:0035556 GO:0009190 GO:0016849

Cluster-22178.19060 GO:0009190 GO:0035556 GO:0016849
Cluster-22178.19061 GO:0009190 GO:0006144 GO:0006171 GO:0035556
GO:0016849 GO:0004016 GO:0005886
Cluster-22178.19083 GO:0046983
Cluster-22178.19084 GO:0003723
Cluster-22178.19085 GO:0016310 GO:0009069 GO:0004674 GO:0003723
GO:0019031
Cluster-22178.19088 GO:0003723
Cluster-22178.19104 GO:0006508 GO:0007165 GO:0008270 GO:0005125
GO:0004222 GO:0031012
Cluster-22178.19106 GO:0007264 GO:0071973 GO:0015992 GO:0055085
GO:0030001 GO:0015991 GO:0009405 GO:0006886 GO:0006308
GO:0046873 GO:0003774 GO:0008855 GO:0005096 GO:0005198
GO:0015078 GO:0016020 GO:0009318 GO:0033179 GO:0009288
Cluster-22178.19110 GO:0006355 GO:0003677
Cluster-22178.1913 GO:0006508 GO:0004252
Cluster-22178.19165 GO:0006816 GO:0070588 GO:2001070 GO:0005262
GO:0005515 GO:0016020
Cluster-22178.19169 GO:0007186 GO:0004930 GO:0016021
Cluster-22178.19192 GO:0009405 GO:0019062 GO:0007155 GO:0046870

Cluster-22178.19193 GO:0007268 GO:0007165 GO:0006811 GO:0055085
GO:0005515 GO:0005216 GO:0004970 GO:0016020
Cluster-22178.19194 GO:0006351 GO:0003677 GO:0005634
Cluster-22178.1920 GO:0006955 GO:0007165 GO:0005164 GO:0016020
Cluster-22178.19204 GO:0018279 GO:0006413 GO:0006486 GO:0003723
GO:0004579 GO:0016021 GO:0008250 GO:0019012 GO:0005783
Cluster-22178.19224 GO:0016567 GO:0006355 GO:0004842 GO:0008270
GO:0046872 GO:0061630 GO:0005515 GO:0003677 GO:0005680
GO:0005622
Cluster-22178.19230 GO:0030683 GO:0007264 GO:0005525 GO:0044386
Cluster-22178.19231 GO:0007264 GO:0030683 GO:0007029 GO:0005525
GO:0044386 GO:0005789

Cluster-22178.19241 GO:0006355 GO:0006281 GO:0006351 GO:0006144
GO:0006206 GO:0003899 GO:0003677 GO:0008168 GO:0046872
GO:0008270 GO:0005730
Cluster-22178.19246 GO:0051252 GO:0008033 GO:0015631 GO:0004540
GO:0045298
Cluster-22178.1925 GO:0055085 GO:0016021
Cluster-22178.19254 GO:0006000 GO:0006013 GO:0007186 GO:0007165
GO:0007264 GO:0004871 GO:0005525 GO:0003924 GO:0019001
GO:0005524 GO:0031683 GO:0005515 GO:0003873
Cluster-22178.19265 GO:0006470 GO:0008138
Cluster-22178.193 GO:0007186 GO:0004930 GO:0016021
Cluster-22178.19301 GO:0007165
Cluster-22178.19305 GO:0008270
Cluster-22178.19306 GO:0006355 GO:0003677 GO:0003700 GO:0005515
GO:0005667
Cluster-22178.19319 GO:0005515
Cluster-22178.19340 GO:0005515
Cluster-22178.19341 GO:0006281 GO:0006351 GO:0006144 GO:0006464
GO:0006206 GO:0035556 GO:0003899 GO:0003677 GO:0046872
GO:0008270 GO:0016151 GO:0005730
Cluster-22178.19354 GO:0035556 GO:0005515
Cluster-22178.19355 GO:0035556 GO:0005515
Cluster-22178.19360 GO:0008152 GO:0016791 GO:0008641
Cluster-22178.1937 GO:0005515
Cluster-22178.19374 GO:0007156 GO:0005509 GO:0016020
Cluster-22178.19380 GO:0016055 GO:0006412 GO:0042254 GO:0007165
GO:0007275 GO:0005102 GO:0003735 GO:0005622 GO:0005840
GO:0005576
Cluster-22178.19385 GO:0003729
Cluster-22178.19409 GO:0055085 GO:0006811 GO:0005216 GO:0016020
Cluster-22178.19415 GO:0008643 GO:0009401 GO:0008982 GO:0004190
GO:0009357 GO:0016021
Cluster-22178.19430 GO:0006771 GO:0019497 GO:0005515 GO:0003993

GO:0046872
Cluster-22178.19448 GO:0007155 GO:0005540
Cluster-22178.19454 GO:0005515 GO:0008270
Cluster-22178.19476 GO:0043087 GO:0019882 GO:0006144 GO:0035556
GO:0048193 GO:0007165 GO:0055114 GO:0018144 GO:0035023
GO:0006955 GO:0006886 GO:0008270 GO:0047134 GO:0005089
GO:0042289 GO:0016818 GO:0003968 GO:0005198 GO:0005515
GO:0016020 GO:0031379 GO:0042613
Cluster-22178.19480 GO:0015074 GO:0003676 GO:0008270
Cluster-22178.19481 GO:0015074
Cluster-22178.19494 GO:0007154 GO:0016021
Cluster-22178.19497 GO:0005975 GO:0004553
Cluster-22178.19509 GO:0008289
Cluster-22178.19515 GO:0006355 GO:0003700 GO:0005861 GO:0005667
GO:0005634
Cluster-22178.19520 GO:0005515
Cluster-22178.19524 GO:0005515
Cluster-22178.19542 GO:0005975 GO:0004553
Cluster-22178.19543 GO:0005975 GO:0004553
Cluster-22178.19556 GO:0043087 GO:0007264 GO:0005085
Cluster-22178.19557 GO:0043087 GO:0007264 GO:0005085
Cluster-22178.19558 GO:0006355 GO:0007264 GO:0043087 GO:0016818
GO:0003677 GO:0005085 GO:0005524 GO:0008270 GO:0005634
Cluster-22178.19566 GO:0006355 GO:0016579 GO:0006465 GO:0005515
GO:0008233 GO:0036459 GO:0005787 GO:0005634 GO:0016021
Cluster-22178.19567 GO:0006465 GO:0008233 GO:0005787 GO:0016021
Cluster-22178.19581 GO:0006470 GO:0008138
Cluster-22178.19583 GO:0006570 GO:0006470 GO:0019988 GO:0008138
GO:0004725 GO:0043399
Cluster-22178.19592 GO:0005515
Cluster-22178.19595 GO:0005515
Cluster-22178.19596 GO:0005515
Cluster-22178.19605 GO:0006412 GO:0042254 GO:0003735 GO:0005840

GO:0005622
Cluster-22178.19606 GO:0006144 GO:0006396 GO:0007165 GO:0004114
GO:0005622
Cluster-22178.19607 GO:0006355 GO:0003677
Cluster-22178.19614 GO:0006310 GO:0008152 GO:0055114 GO:0003677
GO:0016491 GO:0005634
Cluster-22178.19631 GO:0006810 GO:0006952 GO:0006281 GO:0055085
GO:0055114 GO:0006231 GO:0006206 GO:0005215 GO:0004799
GO:0047134 GO:0008270 GO:0046872 GO:0016020 GO:0016021
Cluster-22178.19632 GO:0006281 GO:0006810 GO:0006231 GO:0055114
GO:0006206 GO:0055085 GO:0008270 GO:0046872 GO:0004799
GO:0005215 GO:0047134 GO:0016020 GO:0016021
Cluster-22178.19633 GO:0055085 GO:0006206 GO:0055114 GO:0006231
GO:0006810 GO:0006281 GO:0047134 GO:0004799 GO:0005215
GO:0046872 GO:0008270 GO:0016021 GO:0016020
Cluster-22178.19639 GO:0035556 GO:0003950 GO:0005634
Cluster-22178.19645 GO:0006118 GO:0006119 GO:0006468 GO:0015992
GO:0055114 GO:0008121 GO:0004672 GO:0005524 GO:0016773
GO:0016020
Cluster-22178.19646 GO:0006118 GO:0006119 GO:0006468 GO:0015992
GO:0055114 GO:0008121 GO:0004672 GO:0005524 GO:0016773
GO:0016020
Cluster-22178.19650 GO:0004866 GO:0005576 GO:0005615
Cluster-22178.19667 GO:0007165
Cluster-22178.19686 GO:0006865 GO:0055085 GO:0003333 GO:0006810
GO:0015171 GO:0016021 GO:0016020
Cluster-22178.19687 GO:0055085 GO:0006865 GO:0006810 GO:0003333
GO:0015171 GO:0016020
Cluster-22178.19688 GO:0009405 GO:0005576
Cluster-22178.19694 GO:0015992 GO:0006744 GO:0006120 GO:0006355
GO:0006814 GO:0006118 GO:0003954 GO:0008137 GO:0005515
GO:0005198 GO:0005739
Cluster-22178.19703 GO:0006355 GO:0004866 GO:0031423 GO:0005515

GO:0005615 GO:0019012 GO:0005576 GO:0005634

Cluster-22178.19704 GO:0005515 GO:0004866 GO:0031423 GO:0005615

GO:0019012 GO:0005576

Cluster-22178.19705 GO:0005515 GO:0004866 GO:0031423 GO:0005615

GO:0019012 GO:0005576

Cluster-22178.19725 GO:0016310 GO:0007154 GO:0004673 GO:0009365

GO:0016021

Cluster-22178.19735 GO:0007155 GO:0009288

Cluster-22178.19738 GO:0006269 GO:0006351 GO:0007165 GO:0006260

GO:0005509 GO:0005515 GO:0003896 GO:0005578 GO:0005730

GO:0005657

Cluster-22178.19766 GO:0006446 GO:0007165 GO:0006413 GO:0005515

GO:0003743 GO:0005125 GO:0016020 GO:0005840

Cluster-22178.19776 GO:0006486 GO:0008373

Cluster-22178.19777 GO:0006486 GO:0008373

Cluster-22178.19778 GO:0006486 GO:0008373

Cluster-22178.19794 GO:0005515

Cluster-22178.19797 GO:0006952 GO:0048013 GO:0007186 GO:0004930

GO:0005576

Cluster-22178.19799 GO:0006810 GO:0006816 GO:0005246 GO:0003676

GO:0008270 GO:0042030 GO:0016020 GO:0005634

Cluster-22178.19813 GO:0046487 GO:0019643 GO:0006355 GO:0006099

GO:0003700 GO:0003994 GO:0042025 GO:0005667

Cluster-22178.19819 GO:0043401 GO:0006355 GO:0003700 GO:0008270

GO:0043565 GO:0005634 GO:0005667

Cluster-22178.19823 GO:0006468 GO:0005515 GO:0005524 GO:0004672

Cluster-22178.19827 GO:0007264 GO:0005525 GO:0005524

Cluster-22178.19846 GO:0005515

Cluster-22178.19861 GO:0007165 GO:0006508 GO:0004252 GO:0070008

GO:0005044 GO:0016020

Cluster-22178.19869 GO:0006935 GO:0007165 GO:0004888 GO:0005198

GO:0016020 GO:0016021

Cluster-22178.19870 GO:0007165 GO:0006935 GO:0005198 GO:0004888 GO:0016021 GO:0016020
Cluster-22178.19888 GO:0006486 GO:0006413 GO:0018279 GO:0004579 GO:0003723 GO:0016021 GO:0005783 GO:0019012 GO:0008250
Cluster-22178.19892 GO:0008152 GO:0003676 GO:0016788
Cluster-22178.19893 GO:0008152 GO:0016788 GO:0003676
Cluster-22178.19900 GO:0007605 GO:0005515
Cluster-22178.19913 GO:0007275 GO:0005578
Cluster-22178.19916 GO:0007165 GO:0007155 GO:0030168 GO:0051258 GO:0005102 GO:0030674 GO:0005577
Cluster-22178.19922 GO:0050792 GO:0008168 GO:0046872 GO:0000166 GO:0004518
Cluster-22178.19935 GO:0008152 GO:0016746
Cluster-22178.19944 GO:0007186 GO:0004930 GO:0016021
Cluster-22178.19952 GO:0001558 GO:0042998 GO:0005520 GO:0071253 GO:0016942 GO:0005802 GO:0005576
Cluster-22178.19953 GO:0001558 GO:0005520 GO:0016942 GO:0005576
Cluster-22178.19962 GO:0005515 GO:0005576
Cluster-22178.19968 GO:0006433 GO:0006560 GO:0006525 GO:0005515 GO:0000166 GO:0004827 GO:0005524 GO:0046872 GO:0005737
Cluster-22178.1998 GO:0006508 GO:0007165 GO:0005044 GO:0004252 GO:0005515 GO:0046983 GO:0005525 GO:0016020
Cluster-22178.19981 GO:0010038
Cluster-22178.19982 GO:0010038
Cluster-22178.19984 GO:0007155 GO:0005515 GO:0016021
Cluster-22178.19985 GO:0007155 GO:0005515 GO:0016021
Cluster-22178.19989 GO:0023052
Cluster-22178.1999 GO:0006508 GO:0007165 GO:0046983 GO:0005525 GO:0005044 GO:0005515 GO:0004252 GO:0016020
Cluster-22178.19993 GO:0015992 GO:0055114 GO:0006811 GO:0055085 GO:0006123 GO:0006118 GO:0016655 GO:0004129 GO:0005216 GO:0045277 GO:0016020
Cluster-22178.20007 GO:0008152 GO:0016746

Cluster-22178.20008 GO:0006508 GO:0004252
Cluster-22178.20018 GO:0005982 GO:0005985 GO:0005975 GO:0008810

Cluster-22178.20023 GO:0007186 GO:0004930 GO:0016020
Cluster-22178.20024 GO:0007169 GO:0006468 GO:0018342 GO:0005524
GO:0004672 GO:0004714 GO:0008318 GO:0016020
Cluster-22178.20025 GO:0015694 GO:0015097 GO:0016020
Cluster-22178.20031 GO:0035556
Cluster-22178.20049 GO:0005515
Cluster-22178.20064 GO:0009405 GO:0005515 GO:0005198
Cluster-22178.20069 GO:0007165 GO:0005515
Cluster-22178.20083 GO:0006508 GO:0004197
Cluster-22178.20085 GO:0005975 GO:0016820 GO:0005524 GO:0016773

Cluster-22178.20100 GO:0042254 GO:0006412 GO:0006260 GO:0003735
GO:0005622 GO:0005840
Cluster-22178.20107 GO:0005515
Cluster-22178.20125 GO:0000045 GO:0008270 GO:0005737
Cluster-22178.20126 GO:0007094 GO:0008092 GO:0005515 GO:0005737
GO:0019898
Cluster-22178.20129 GO:0007165 GO:0006468 GO:0009069 GO:0016310
GO:0048193 GO:0051607 GO:0015886 GO:0006914 GO:0015232
GO:0004674 GO:0005524 GO:0016020
Cluster-22178.20134 GO:0007010 GO:0003779
Cluster-22178.20136 GO:0007010 GO:0003779
Cluster-22178.20148 GO:0003924 GO:0005525
Cluster-22178.20160 GO:0006508 GO:0009405 GO:0008270 GO:0004222
GO:0031012 GO:0005576
Cluster-22178.20176 GO:0005515
Cluster-22178.20178 GO:0005515
Cluster-22178.20181 GO:2000123
Cluster-22178.20189 GO:0015840 GO:0071918 GO:0015204 GO:0016021
Cluster-22178.20190 GO:0015840 GO:0071918 GO:0015204 GO:0016021

Cluster-22178.20196 GO:0050830
Cluster-22178.20199 GO:0006506 GO:0017176 GO:0016021
Cluster-22178.20206 GO:0007264 GO:0006508 GO:0005096 GO:0005525
GO:0008233 GO:0005515 GO:0005634
Cluster-22178.20210 GO:0007275 GO:0006511 GO:0046872 GO:0005634
Cluster-22178.20226 GO:0006955 GO:0007165 GO:0040007 GO:0008283
GO:0009098 GO:0005515 GO:0005143 GO:0008083 GO:0042022
GO:0005576
Cluster-22178.20231 GO:0005509 GO:0016462
Cluster-22178.20232 GO:0005509 GO:0016462
Cluster-22178.20234 GO:0005515
Cluster-22178.20257 GO:0016787
Cluster-22178.20258 GO:0016787
Cluster-22178.20266 GO:0006810 GO:0005215 GO:0016020
Cluster-22178.20271 GO:0005634
Cluster-22178.2028 GO:0048193 GO:0008168 GO:0005783
Cluster-22178.20280 GO:0005509 GO:0005515 GO:0016020 GO:0016021
Cluster-22178.20281 GO:0005509 GO:0005515
Cluster-22178.20283 GO:0005509 GO:0005515
Cluster-22178.20293 GO:0003677
Cluster-22178.20297 GO:0055114 GO:0005506 GO:0016705 GO:0020037

Cluster-22178.20306 GO:0045892 GO:0006508 GO:0042754 GO:0004222
GO:0031012
Cluster-22178.20327 GO:0003723
Cluster-22178.20328 GO:0017004 GO:0055114 GO:0016020
Cluster-22178.20335 GO:0006367 GO:0005672
Cluster-22178.20341 GO:0005515
Cluster-22178.20357 GO:0007165 GO:0005515
Cluster-22178.20382 GO:0015631 GO:0045298
Cluster-22178.20393 GO:0006955 GO:0004867 GO:0005576
Cluster-22178.20401 GO:0043039 GO:0005524 GO:0016876
Cluster-22178.20407 GO:0007166 GO:0000105 GO:0006547 GO:0003729

GO:0004635 GO:0016020
Cluster-22178.20410 GO:0055114 GO:0006633 GO:0016491 GO:0005506

Cluster-22178.20411 GO:0055114 GO:0006633 GO:0016491 GO:0005506

Cluster-22178.20414 GO:0006950 GO:0004866 GO:0005576 GO:0005615
Cluster-22178.20416 GO:0006950 GO:0004866 GO:0005615 GO:0005576
Cluster-22178.20428 GO:0007186 GO:0004930 GO:0016021
Cluster-22178.20429 GO:0007186 GO:0004930 GO:0016021
Cluster-22178.20439 GO:0006813 GO:0008152 GO:0005516 GO:0016791
GO:0015269 GO:0046872 GO:0003824 GO:0016021
Cluster-22178.20464 GO:0016021
Cluster-22178.20481 GO:0006206 GO:0055114 GO:0006260 GO:0007049
GO:0016491 GO:0008998
Cluster-22178.20482 GO:0071988 GO:0008360 GO:0030474 GO:0005887
Cluster-22178.20483 GO:0006206 GO:0055114 GO:0006260 GO:0016491
GO:0008998 GO:0005887
Cluster-22178.20488 GO:0006468 GO:0005524 GO:0004672
Cluster-22178.20499 GO:0042967 GO:0006555 GO:0019281 GO:0009432
GO:0006281 GO:0016887 GO:0008899 GO:0005524 GO:0003724
GO:0003723 GO:0003697 GO:0005737 GO:0033588
Cluster-22178.20522 GO:0006935 GO:0000160 GO:0005524 GO:0016817
GO:0003676 GO:0000156 GO:0003824 GO:0016787 GO:0008984
GO:0003677 GO:0005737
Cluster-22178.20524 GO:0006355 GO:0046983 GO:0005515
Cluster-22178.20528 GO:0016788 GO:0005515 GO:0005856
Cluster-22178.20535 GO:0006303
Cluster-22178.20540 GO:0006771 GO:0019497 GO:0007339 GO:0046872
GO:0005515 GO:0003993 GO:0005576
Cluster-22178.20548 GO:0000077 GO:0006357 GO:0006281 GO:0005524
GO:0042393 GO:0001104 GO:0005515 GO:0030896 GO:0016592
Cluster-22178.20556 GO:0006367 GO:0005672
Cluster-22178.20558 GO:0006886 GO:0005622

Cluster-22178.20574 GO:0007165 GO:0007275 GO:0042254 GO:0006412 GO:0005044 GO:0003735 GO:0005578 GO:0016020 GO:0005622 GO:0005840

Cluster-22178.20585 GO:0055085 GO:0016021

Cluster-22178.20587 GO:0055085 GO:0022857 GO:0016021

Cluster-22178.20599 GO:0016567 GO:0004842

Cluster-22178.20606 GO:0006323

Cluster-22178.20607 GO:0005515

Cluster-22178.20611 GO:0007218 GO:0030141

Cluster-22178.20613 GO:0007018 GO:0007017 GO:0003777 GO:0005874 GO:0030286

Cluster-22178.20619 GO:0006807 GO:0007169 GO:0018342 GO:0006468 GO:0004668 GO:0004672 GO:0005524 GO:0008318 GO:0005509 GO:0004714 GO:0005737 GO:0016020

Cluster-22178.20621 GO:0007169 GO:0006468 GO:0018342 GO:0005524 GO:0004672 GO:0004714 GO:0008318 GO:0016020

Cluster-22178.20622 GO:0006468 GO:0018342 GO:0007169 GO:0006807 GO:0008318 GO:0004714 GO:0005509 GO:0004672 GO:0005524 GO:0004668 GO:0016020 GO:0005737

Cluster-22178.20627 GO:0006813 GO:0005267

Cluster-22178.20636 GO:0007015 GO:0003785 GO:0003779

Cluster-22178.2064 GO:0005515 GO:0043395 GO:0016020 GO:0005578

Cluster-22178.20641 GO:0031554 GO:0055114 GO:0031556 GO:0016491 GO:0016620

Cluster-22178.20646 GO:0006310 GO:0003677 GO:0005634

Cluster-22178.20649 GO:0006118 GO:0055114 GO:0009055 GO:0004497 GO:0016705 GO:0020037 GO:0005506

Cluster-22178.20651 GO:0005515

Cluster-22178.20652 GO:0005515

Cluster-22178.20666 GO:0017004 GO:0015886 GO:0005515 GO:0016021 GO:0016020

Cluster-22178.20667 GO:0017004 GO:0015886 GO:0005515 GO:0016020 GO:0016021

Cluster-22178.20681 GO:0007186 GO:0007165 GO:0040007 GO:0008283
GO:0008083 GO:0004930 GO:0016020 GO:0016021

Cluster-22178.20689 GO:0008270

Cluster-22178.20694 GO:0006355 GO:0005515

Cluster-22178.20695 GO:0006629

Cluster-22178.20697 GO:0006355 GO:0003700 GO:0043565 GO:0005634
GO:0005667

Cluster-22178.20709 GO:0006355 GO:0003700 GO:0005667

Cluster-22178.2072 GO:0009451 GO:0001522 GO:0007186 GO:0015031
GO:0009306 GO:0003723 GO:0015450 GO:0004930 GO:0009982
GO:0009941 GO:0016021

Cluster-22178.20729 GO:0009966 GO:0005515 GO:0005737 GO:0016020

Cluster-22178.20732 GO:0051252 GO:0007165 GO:0006396 GO:0005515
GO:0003723 GO:0004525

Cluster-22178.20735 GO:0016021

Cluster-22178.20736 GO:0016021

Cluster-22178.20748 GO:0005515 GO:0003676

Cluster-22178.20750 GO:0051252 GO:0006397 GO:0006468 GO:0004540
GO:0005524 GO:0004672

Cluster-22178.20751 GO:0051252 GO:0006397 GO:0006468 GO:0004540
GO:0005524 GO:0004672

Cluster-22178.20754 GO:0005198

Cluster-22178.20761 GO:0035023 GO:0043087 GO:0005089

Cluster-22178.20765 GO:0035023 GO:0043087 GO:0005089

Cluster-22178.20796 GO:0006631 GO:0006468 GO:0035556 GO:0055114
GO:0005515 GO:0045300 GO:0047134 GO:0005524 GO:0004672
GO:0016021

Cluster-22178.20797 GO:0009069 GO:0006468 GO:0016310 GO:0005524
GO:0004672 GO:0004674

Cluster-22178.20801 GO:0006811 GO:0007165 GO:0007268 GO:0004970
GO:0016020

Cluster-22178.20809 GO:0005515

Cluster-22178.20821 GO:0019089 GO:0010508 GO:0006606 GO:0006914

GO:0070206 GO:0005198 GO:0008134 GO:0042803 GO:0045502
GO:0008017 GO:0019028 GO:0045298 GO:0019031 GO:0005882
GO:0005667 GO:0030286
Cluster-22178.20822 GO:0005515
Cluster-22178.20836 GO:0007186 GO:0004930 GO:0016021
Cluster-22178.20838 GO:0043066 GO:0043154 GO:0019079 GO:0008146
GO:0019031 GO:0016021 GO:0071986 GO:0005737
Cluster-22178.20843 GO:0005515
Cluster-22178.20848 GO:0055114 GO:0006725 GO:0008199 GO:0003824

Cluster-22178.20856 GO:0000746 GO:0006468 GO:0005198 GO:0005515
GO:0004672 GO:0005524 GO:0019012
Cluster-22178.20864 GO:0055114 GO:0016491 GO:0005507
Cluster-22178.20871 GO:0006468 GO:0019076 GO:0006357 GO:0006812
GO:0032801 GO:0005261 GO:0001104 GO:0004672 GO:0005524
GO:0016592 GO:0033644
Cluster-22178.20879 GO:0005198 GO:0005882
Cluster-22178.20884 GO:0003677
Cluster-22178.20889 GO:0042981 GO:0005515
Cluster-22178.20895 GO:0005515
Cluster-22178.20900 GO:0006355 GO:0005524 GO:0008270 GO:0016818
GO:0003677 GO:0005634
Cluster-22178.20901 GO:0006355 GO:0008270 GO:0005524 GO:0003677
GO:0016818 GO:0005634
Cluster-22178.20902 GO:0006355 GO:0016818 GO:0003677 GO:0008017
GO:0008270 GO:0005524 GO:0005634 GO:0045298
Cluster-22178.20903 GO:0005509
Cluster-22178.20906 GO:0005515
Cluster-22178.20915 GO:0007017 GO:0007018 GO:0042162 GO:0042803
GO:0005524 GO:0008017 GO:0003777 GO:0045298 GO:0005874
Cluster-22178.20925 GO:0005515
Cluster-22178.20933 GO:0005515
Cluster-22178.20935 GO:0005515

Cluster-22178.20936 GO:0005515
Cluster-22178.20957 GO:0006355
Cluster-22178.2096 GO:0008053 GO:0042157 GO:0006355 GO:0006869
GO:0003924 GO:0008289 GO:0003677 GO:0016021 GO:0005576
GO:0005741
Cluster-22178.20970 GO:0005515
Cluster-22178.20985 GO:0055085 GO:0006810 GO:0005515 GO:0005215
GO:0016021 GO:0016020
Cluster-22178.20996 GO:0019239
Cluster-22178.20999 GO:0015031 GO:0016021 GO:0005887
Cluster-22178.21002 GO:0005515
Cluster-22178.21006 GO:0004222 GO:0005515 GO:0005509
Cluster-22178.21013 GO:0001558 GO:0005515 GO:0005520 GO:0016942
GO:0005576
Cluster-22178.21014 GO:0001558 GO:0005515 GO:0005520 GO:0016942
GO:0005576
Cluster-22178.21015 GO:0006308 GO:0008855 GO:0009318
Cluster-22178.21017 GO:0006355 GO:0046983 GO:0005515
Cluster-22178.21019 GO:0006355 GO:0043565 GO:0003700 GO:0005634
GO:0005667
Cluster-22178.21025 GO:0061630 GO:0005515 GO:0008270 GO:0046872

Cluster-22178.21036 GO:0000045 GO:0008270 GO:0005737
Cluster-22178.21050 GO:0000287 GO:0004867 GO:0030976 GO:0005515

Cluster-22178.21057 GO:0005515
Cluster-22178.21061 GO:0016787 GO:0046872
Cluster-22178.21065 GO:0006396 GO:0051252 GO:0004525 GO:0003723

Cluster-22178.21074 GO:0006486 GO:0008373
Cluster-22178.21076 GO:0014068 GO:0050776 GO:0007155 GO:0007165
GO:0006955 GO:0051249 GO:0005102 GO:0043548 GO:0019031
GO:0016021 GO:0016020

Cluster-22178.21086 GO:0007186 GO:0006629 GO:0006355 GO:0004930
GO:0016021
Cluster-22178.21092 GO:0006560 GO:0006420 GO:0006525 GO:0005524
GO:0004814
Cluster-22178.21100 GO:0006355 GO:0035556 GO:0003700 GO:0005667
GO:0042025
Cluster-22178.21114 GO:0006508 GO:0006144 GO:0044419 GO:0017111
GO:0003968 GO:0004197 GO:0031379
Cluster-22178.21115 GO:0055114 GO:0046656 GO:0006761 GO:0042493
GO:0004146
Cluster-22178.21118 GO:0007155 GO:0009847 GO:0005540
Cluster-22178.21123 GO:0005515
Cluster-22178.21129 GO:0006355 GO:0005622
Cluster-22178.21137 GO:0016020
Cluster-22178.21138 GO:0005887 GO:0016020
Cluster-22178.21160 GO:0005515
Cluster-22178.21171 GO:0006355 GO:0043565 GO:0008270 GO:0003700
GO:0005667
Cluster-22178.21176 GO:0006351 GO:0006446 GO:0006355 GO:0006413
GO:0006269 GO:0003896 GO:0004386 GO:0003700 GO:0008270
GO:0043565 GO:0003743 GO:0005840 GO:0005657 GO:0005730
GO:0005667
Cluster-22178.21178 GO:0043087 GO:0035023 GO:0005089
Cluster-22178.21183 GO:0006118 GO:0006119 GO:0015992 GO:0006468
GO:0008121 GO:0004672 GO:0005524
Cluster-22178.21227 GO:0046872
Cluster-22178.21233 GO:0006308 GO:0007165 GO:0007186 GO:0009307
GO:0007264 GO:0005525 GO:0004871 GO:0031683 GO:0009036
GO:0019001 GO:0003924 GO:0003677 GO:0009359
Cluster-22178.21234 GO:0007264 GO:0007165 GO:0007186 GO:0006013
GO:0006000 GO:0003873 GO:0016301 GO:0019001 GO:0005524
GO:0031683 GO:0003924 GO:0005525 GO:0004871
Cluster-22178.2124 GO:0005525

Cluster-22178.21240 GO:0006508 GO:0004197
Cluster-22178.21245 GO:0006508
Cluster-22178.21247 GO:0006508 GO:0004222
Cluster-22178.21248 GO:0006355 GO:0006508 GO:0003700 GO:0005622
GO:0005667
Cluster-22178.21249 GO:0006508 GO:0004222
Cluster-22178.21250 GO:0006508 GO:0004222 GO:0008270
Cluster-22178.21253 GO:0006281 GO:0004519
Cluster-22178.21255 GO:0006281 GO:0004519
Cluster-22178.21257 GO:0006281 GO:0004519
Cluster-22178.21264 GO:0006470 GO:0006570 GO:0004725 GO:0008138
GO:0005515
Cluster-22178.21265 GO:0006470 GO:0016032 GO:0006570 GO:0004725
GO:0005525 GO:0005515 GO:0008138 GO:0019013
Cluster-22178.21266 GO:0006570 GO:0006470 GO:0016032 GO:0008138
GO:0005525 GO:0004725 GO:0019013
Cluster-22178.21276 GO:0035556 GO:0008270 GO:0005634
Cluster-22178.21277 GO:0050852 GO:0050853
Cluster-22178.21286 GO:0006952
Cluster-22178.21288 GO:0006468 GO:0004672 GO:0005524
Cluster-22178.21290 GO:0005515
Cluster-22178.21297 GO:0005515
Cluster-22178.21311 GO:0007165 GO:0007186 GO:0019001 GO:0031683
GO:0003924 GO:0005525 GO:0004871
Cluster-22178.21314 GO:0005515
Cluster-22178.21327 GO:0007165 GO:0016021
Cluster-22178.21328 GO:0050821 GO:0015979 GO:0042301 GO:0016020
GO:0009523
Cluster-22178.21332 GO:0016043 GO:0030036 GO:0003779 GO:0017048

Cluster-22178.21338 GO:0006814 GO:0007165 GO:0006810 GO:0006355
GO:0055085 GO:0030521 GO:0003677 GO:0005496 GO:0005215
GO:0004882 GO:0016020 GO:0005634 GO:0016021

Cluster-22178.21348 GO:0008643 GO:0008982 GO:0009357

Cluster-22178.21352 GO:0006865 GO:0055085 GO:0006357 GO:0006810

GO:0003333 GO:0001104 GO:0015171 GO:0016020 GO:0016592

Cluster-22178.21353 GO:0006865 GO:0055085 GO:0003333 GO:0006810

GO:0015171 GO:0016020 GO:0016021

Cluster-22178.21357 GO:0007217

Cluster-22178.2136 GO:0008152 GO:0043395 GO:0005515 GO:0016791

GO:0005578 GO:0016020

Cluster-22178.2137 GO:0005515 GO:0043395 GO:0016020 GO:0005578

Cluster-22178.21375 GO:0006811 GO:0016020

Cluster-22178.21379 GO:0046872

Cluster-22178.21391 GO:0005515 GO:0008270 GO:0005622

Cluster-22178.21393 GO:0006810 GO:0010508 GO:0071973 GO:0006935

GO:0048870 GO:0006606 GO:0005198 GO:0003774 GO:0005882

GO:0031514 GO:0016020 GO:0009288 GO:0016021

Cluster-22178.21395 GO:0006260 GO:0006281 GO:0004866 GO:0003911

GO:0005615 GO:0005576

Cluster-22178.21401 GO:0007165

Cluster-22178.21408 GO:0006468 GO:0004672 GO:0005524

Cluster-22178.21411 GO:0006508 GO:0004252

Cluster-22178.21418 GO:0035556

Cluster-22178.21420 GO:0006479 GO:0006481 GO:0004671 GO:0016021

Cluster-22178.21424 GO:0005515

Cluster-22178.21433 GO:0005515

Cluster-22178.21443 GO:0006807 GO:0005515 GO:0016810

Cluster-22178.21450 GO:0005615

Cluster-22178.21458 GO:0008152 GO:0008484 GO:0003824

Cluster-22178.21464 GO:0003682 GO:0000785

Cluster-22178.21466 GO:0055085 GO:0006811 GO:0015992 GO:0015986

GO:0015078 GO:0005216 GO:0000276 GO:0016020

Cluster-22178.21467 GO:0006811 GO:0009058 GO:0055085 GO:0005216

GO:0016020

Cluster-22178.21468 GO:0006811 GO:0009058 GO:0055085 GO:0005216

GO:0016020
Cluster-22178.21469 GO:0006811 GO:0009058 GO:0055085 GO:0005216
GO:0016020
Cluster-22178.21470 GO:0006811 GO:0055085 GO:0005216 GO:0016020
Cluster-22178.21471 GO:0006811 GO:0055085 GO:0005216 GO:0016020
Cluster-22178.21472 GO:0006811 GO:0055085 GO:0005216 GO:0031224
GO:0016020
Cluster-22178.21476 GO:0007155 GO:0018149 GO:0005515 GO:0003677
GO:0016021
Cluster-22178.21482 GO:0055114 GO:0016616 GO:0051287
Cluster-22178.21501 GO:0005515 GO:0005634
Cluster-22178.21515 GO:0055114 GO:0047134
Cluster-22178.21516 GO:0016787
Cluster-22178.21517 GO:0016787
Cluster-22178.21519 GO:0007186 GO:0004930 GO:0016021
Cluster-22178.21526 GO:0005200 GO:0005882 GO:0005856
Cluster-22178.21527 GO:0005515
Cluster-22178.21530 GO:0003677 GO:0042802 GO:0005515
Cluster-22178.21547 GO:0016021
Cluster-22178.21556 GO:0006813 GO:0007165 GO:0006955 GO:0005249
GO:0030955 GO:0005164 GO:0016021 GO:0008076 GO:0016020
Cluster-22178.21557 GO:0003723 GO:0019013
Cluster-22178.21564 GO:0007010 GO:0003779
Cluster-22178.21565 GO:0007010 GO:0003779
Cluster-22178.21583 GO:0005515 GO:0016020
Cluster-22178.21606 GO:0007186 GO:0007165 GO:0006260 GO:0003677
GO:0004003 GO:0004871 GO:0005525 GO:0003924 GO:0031683
GO:0005524 GO:0019001 GO:0005657
Cluster-22178.21608 GO:0007165 GO:0007186 GO:0006260 GO:0004003
GO:0003677 GO:0005524 GO:0031683 GO:0019001 GO:0003924
GO:0005525 GO:0004871 GO:0005657
Cluster-22178.21614 GO:0042254 GO:0006412 GO:0007165 GO:0040007
GO:0008283 GO:0003735 GO:0008083 GO:0005840 GO:0005622

Cluster-22178.21619 GO:0055114 GO:0016730
Cluster-22178.21622 GO:0042254 GO:0006412 GO:0003735 GO:0005622 GO:0005840
Cluster-22178.21625 GO:0006555 GO:0042967 GO:0019281 GO:0006281 GO:0009432 GO:0008899 GO:0016887 GO:0005524 GO:0003724 GO:0003723 GO:0003697 GO:0005737 GO:0033588
Cluster-22178.21636 GO:0030001 GO:0007034
Cluster-22178.21638 GO:0016787 GO:0046872
Cluster-22178.21644 GO:0006869 GO:0009405 GO:0042157 GO:0007034 GO:0008289 GO:0030430 GO:0005576
Cluster-22178.21645 GO:0006508 GO:0044419 GO:0006144 GO:0006811 GO:0055085 GO:0005216 GO:0003723 GO:0004197 GO:0017111 GO:0003968 GO:0016020 GO:0031379
Cluster-22178.21646 GO:0015994 GO:0006811 GO:0006508 GO:0006144 GO:0055085 GO:0033014 GO:0044419 GO:0046872 GO:0003968 GO:0030976 GO:0003723 GO:0005216 GO:0017111 GO:0004655 GO:0004197 GO:0031379 GO:0016020
Cluster-22178.21662 GO:0005515
Cluster-22178.21666 GO:0032040 GO:0016020
Cluster-22178.21669 GO:0009069 GO:0016310 GO:0004674
Cluster-22178.21676 GO:0006570 GO:0006508 GO:0006470 GO:0006144 GO:0004197 GO:0008138 GO:0017111 GO:0004725 GO:0003968 GO:0031379
Cluster-22178.2169 GO:0035556 GO:0046872 GO:0005515 GO:0045202
Cluster-22178.21692 GO:0016818 GO:0003723 GO:0005524 GO:0005634
Cluster-22178.21709 GO:0006811 GO:0055085 GO:0005216 GO:0016020
Cluster-22178.21716 GO:0007165 GO:0005509 GO:0005578
Cluster-22178.21719 GO:0055085 GO:0006811 GO:0007268 GO:0007165 GO:0004970 GO:0005515 GO:0005216 GO:0016020 GO:0019033
Cluster-22178.21720 GO:0006811 GO:0055085 GO:0007268 GO:0007165 GO:0004970 GO:0005216 GO:0005515 GO:0019033 GO:0016020
Cluster-22178.21721 GO:0055085 GO:0006811 GO:0007165 GO:0007268 GO:0004970 GO:0005216 GO:0005515 GO:0019033 GO:0016020

Cluster-22178.21735 GO:0007165 GO:0008283 GO:0040007 GO:0008083
GO:0016020

Cluster-22178.21736 GO:0007076 GO:0000796

Cluster-22178.21738 GO:0007076 GO:0000796

Cluster-22178.21744 GO:0046983

Cluster-22178.21754 GO:0030001 GO:0005985 GO:0006814 GO:0006811
GO:0015986 GO:0005982 GO:0015992 GO:0006012 GO:0055085
GO:0005248 GO:0004575 GO:0015078 GO:0005216 GO:0046873
GO:0004564 GO:0017177 GO:0045263 GO:0016021 GO:0016020
GO:0001518

Cluster-22178.21760 GO:0006468 GO:0016772 GO:0005524 GO:0008270
GO:0004672 GO:0016773 GO:0003677 GO:0016020

Cluster-22178.21766 GO:0006486 GO:0051301 GO:0003677 GO:0008373

Cluster-22178.21775 GO:0030866 GO:0002949 GO:0055085 GO:0006810
GO:0016887 GO:0005096 GO:0003924 GO:0008092 GO:0005524
GO:0042626 GO:0005525 GO:0005856 GO:0016021

Cluster-22178.21777 GO:0046872 GO:0000166

Cluster-22178.21799 GO:0009116 GO:0003824 GO:0016021

Cluster-22178.21800 GO:0006468 GO:0009069 GO:0016310 GO:0005524
GO:0004674 GO:0030089

Cluster-22178.21802 GO:0009069 GO:0006468 GO:0016310 GO:0005524
GO:0004674 GO:0030089

Cluster-22178.21803 GO:0030089

Cluster-22178.21808 GO:0007165 GO:0005179

Cluster-22178.21812 GO:0015986 GO:0015992 GO:0015078 GO:0005515
GO:0000276

Cluster-22178.21823 GO:0042710 GO:0006810 GO:0042626 GO:0005524
GO:0016021

Cluster-22178.21832 GO:0006914 GO:0005737

Cluster-22178.21839 GO:0006814 GO:0005985 GO:0030001 GO:0006811
GO:0005982 GO:0015986 GO:0055085 GO:0006012 GO:0015992
GO:0004575 GO:0005248 GO:0015078 GO:0046873 GO:0005216

GO:0004564 GO:0045263 GO:0017177 GO:0001518 GO:0016020
Cluster-22178.21841 GO:0006626 GO:0043565 GO:0005741
Cluster-22178.21843 GO:0006355 GO:0006308 GO:0005198 GO:0070009
GO:0008239 GO:0003700 GO:0008855 GO:0043565 GO:0009318
GO:0005667
Cluster-22178.21848 GO:0008270
Cluster-22178.21896 GO:0006629 GO:0046849 GO:0006952 GO:0004869
GO:0005576
Cluster-22178.21899 GO:0006952 GO:0004869 GO:0005576
Cluster-22178.21903 GO:0019054 GO:0004857
Cluster-22178.21904 GO:0019054 GO:0004857
Cluster-22178.21905 GO:0019054 GO:0004857
Cluster-22178.21908 GO:0008270
Cluster-22178.21909 GO:0042254 GO:0006412 GO:0006260 GO:0003887
GO:0003735 GO:0001882 GO:0046872 GO:0008270 GO:0042575
GO:0005840 GO:0005622
Cluster-22178.21918 GO:0016055 GO:0006448 GO:0006414 GO:0003746
GO:0005840
Cluster-22178.21919 GO:0033014 GO:0015994 GO:0006783 GO:0004852
GO:0031225
Cluster-22178.21927 GO:0008608 GO:0008152 GO:0046872 GO:0016791
GO:0003824 GO:0072686 GO:0042729
Cluster-22178.21930 GO:0005515 GO:0046332 GO:0016021
Cluster-22178.21931 GO:0005515 GO:0046332 GO:0016021
Cluster-22178.21932 GO:0046332 GO:0005515 GO:0016021
Cluster-22178.21933 GO:0046332 GO:0005515 GO:0016021
Cluster-22178.21941 GO:0055114 GO:0007165 GO:0016972
Cluster-22178.21945 GO:0006810 GO:0055085 GO:0042626 GO:0005524
GO:0016021
Cluster-22178.2196 GO:0006771 GO:0019497 GO:0046872 GO:0003993
GO:0005515
Cluster-22178.21963 GO:0000041 GO:0008270 GO:0005509 GO:0016021
Cluster-22178.21978 GO:0005515

Cluster-22178.21979 GO:0055114 GO:0009395 GO:0016042 GO:0006139 GO:0005509 GO:0004623 GO:0016818 GO:0008026 GO:0051536 GO:0005524 GO:0003676 GO:0005576
Cluster-22178.21985 GO:0006355 GO:0003700 GO:0003677 GO:0005667
Cluster-22178.21993 GO:0016772
Cluster-22178.21996 GO:0005515
Cluster-22178.22007 GO:0046983
Cluster-22178.22008 GO:0015979 GO:0050821 GO:0042301 GO:0016020 GO:0009523
Cluster-22178.22011 GO:0016192 GO:0005515 GO:0042393 GO:0005634 GO:0016021
Cluster-22178.22023 GO:0009190 GO:0035556 GO:0016849
Cluster-22178.22024 GO:0009190 GO:0035556 GO:0016849
Cluster-22178.22025 GO:0006144 GO:0009190 GO:0006171 GO:0035556 GO:0016849 GO:0004016 GO:0005886
Cluster-22178.22030 GO:0006355 GO:0055085 GO:0006811 GO:0005216 GO:0005515 GO:0016020
Cluster-22178.22032 GO:0042302
Cluster-22178.22054 GO:0006508 GO:0005509 GO:0004252 GO:0005515

Cluster-22178.22056 GO:0016310 GO:0006508 GO:0009069 GO:0006468 GO:0004674 GO:0005524 GO:0005509 GO:0005515 GO:0004252
Cluster-22178.22057 GO:0006508 GO:0004252 GO:0005515 GO:0005509

Cluster-22178.22058 GO:0006355 GO:0006508 GO:0003677 GO:0004252 GO:0019013
Cluster-22178.22070 GO:0006570 GO:0007186 GO:0006470 GO:0050852 GO:0004725 GO:0004930 GO:0016020 GO:0016021
Cluster-22178.22072 GO:0005525 GO:0003924
Cluster-22178.22083 GO:0006270 GO:0006355 GO:0003677
Cluster-22178.22084 GO:0006355 GO:0009306 GO:0003677
Cluster-22178.22085 GO:0006355 GO:0009271
Cluster-22178.22089 GO:0008146 GO:0016021

Cluster-22178.22093 GO:0006468 GO:0004672 GO:0005524
Cluster-22178.22108 GO:0006479 GO:0006554 GO:0005515 GO:0018024

Cluster-22178.22111 GO:0005515
Cluster-22178.22114 GO:0019013
Cluster-22178.22116 GO:0007010 GO:0003779
Cluster-22178.22126 GO:0030234 GO:0005515 GO:0016020
Cluster-22178.22128 GO:0006820 GO:0008509 GO:0016021
Cluster-22178.22130 GO:0005975 GO:0046656 GO:0005982 GO:0005985
GO:0015948 GO:0030269 GO:0008810 GO:0016021
Cluster-22178.22138 GO:0005509 GO:0005515
Cluster-22178.22139 GO:0005509 GO:0005515
Cluster-22178.22140 GO:0007160 GO:0005509 GO:0005515
Cluster-22178.22143 GO:0040007 GO:0008283 GO:0007165 GO:0008083
GO:0016020
Cluster-22178.22152 GO:0006811 GO:0055085 GO:0006874 GO:0006816
GO:0006118 GO:0051536 GO:0009055 GO:0005219 GO:0005216
GO:0005509 GO:0005622 GO:0016021 GO:0016020
Cluster-22178.2216 GO:0016021
Cluster-22178.22161 GO:0005515
Cluster-22178.22163 GO:0006457 GO:0000413 GO:0003755
Cluster-22178.22168 GO:0005515
Cluster-22178.22170 GO:0016310 GO:0009069 GO:0006468 GO:0004683
GO:0005516
Cluster-22178.22174 GO:0006811 GO:0016020
Cluster-22178.22181 GO:0006570 GO:0006810 GO:0009405 GO:0019988
GO:0006470 GO:0043399 GO:0019870 GO:0008138 GO:0004725
GO:0005576
Cluster-22178.22196 GO:0055085 GO:0006810 GO:0016887 GO:0005524
GO:0042626 GO:0005525 GO:0016021
Cluster-22178.22197 GO:0055085 GO:0006810 GO:0016887 GO:0005524
GO:0042626 GO:0016021
Cluster-22178.22200 GO:0000154 GO:0006396 GO:0016433 GO:0016021

Cluster-22178.22201 GO:0006396 GO:0000154 GO:0016433 GO:0016021

Cluster-22178.22204 GO:0006470 GO:0006570 GO:0004725 GO:0008138 GO:0044599

Cluster-22178.22205 GO:0019497 GO:0006470 GO:0006771 GO:0006570 GO:0046872 GO:0004725 GO:0003993 GO:0005515 GO:0016020

Cluster-22178.22226 GO:0006486 GO:0008373

Cluster-22178.22227 GO:0051252 GO:0008033 GO:0004526 GO:0030677 GO:0005634

Cluster-22178.22252 GO:0005515

Cluster-22178.22257 GO:0016021

Cluster-22178.22259 GO:0003774 GO:0005524 GO:0016459

Cluster-22178.22260 GO:0003774 GO:0005524 GO:0016459

Cluster-22178.22266 GO:0035556 GO:0005515

Cluster-22178.22271 GO:0055114 GO:0071705 GO:0005506 GO:0016705 GO:0020037

Cluster-22178.22274 GO:0055114 GO:0005506 GO:0016705 GO:0020037

Cluster-22178.22275 GO:0055114 GO:0016705 GO:0020037 GO:0005506

Cluster-22178.22276 GO:0055114 GO:0016705 GO:0020037 GO:0005506 GO:0016021

Cluster-22178.22289 GO:0055114 GO:0008168 GO:0016706

Cluster-22178.22292 GO:0055114 GO:0016706 GO:0008168

Cluster-22178.22296 GO:0007165 GO:0008283 GO:0042381 GO:0040007 GO:0008083 GO:0046872 GO:0005576

Cluster-22178.22303 GO:0016020

Cluster-22178.22309 GO:0042254 GO:0006412 GO:0007186 GO:0008270 GO:0004930 GO:0003735 GO:0005840

Cluster-22178.22310 GO:0008270

Cluster-22178.22320 GO:0006011 GO:0016020

Cluster-22178.22321 GO:0009405 GO:0005198

Cluster-22178.22324 GO:0046872 GO:0000166

Cluster-22178.22329 GO:0006352 GO:0005634

Cluster-22178.22331 GO:0042254 GO:0006412 GO:0007586 GO:0006355
GO:0016042 GO:0003735 GO:0003700 GO:0043565 GO:0008047
GO:0005667 GO:0005576 GO:0005840 GO:0005622
Cluster-22178.22332 GO:0007275
Cluster-22178.22334 GO:0006810 GO:0009405 GO:0019870 GO:0005576
Cluster-22178.22339 GO:0007186 GO:0004930 GO:0016021
Cluster-22178.22340 GO:0007165 GO:0007186 GO:0006771 GO:0019497
GO:0005515 GO:0003993 GO:0004871 GO:0019001 GO:0046872
GO:0031683 GO:0003924 GO:0016020
Cluster-22178.22342 GO:0046872
Cluster-22178.22358 GO:0006355 GO:0046872 GO:0003779 GO:0005622
Cluster-22178.22359 GO:0035556 GO:0006281 GO:0046872 GO:0008270
GO:0005506
Cluster-22178.22360 GO:0006355 GO:0046872 GO:0003779 GO:0005622
Cluster-22178.22361 GO:0006355 GO:0003779 GO:0005622
Cluster-22178.22362 GO:0007275 GO:0005515 GO:0005578
Cluster-22178.22383 GO:0006269 GO:0006260 GO:0006351 GO:0003896
GO:0005730 GO:0005657
Cluster-22178.22407 GO:0007017 GO:0007165 GO:0007275 GO:0007018
GO:0005524 GO:0003777 GO:0008017 GO:0005179 GO:0005874
GO:0045298 GO:0005576
Cluster-22178.22413 GO:0006355 GO:0005634
Cluster-22178.22427 GO:0006771 GO:0019497 GO:0005992 GO:0046872
GO:0003993 GO:0005515 GO:0003824
Cluster-22178.22429 GO:0007586 GO:0016042 GO:0008047 GO:0005576
Cluster-22178.22436 GO:0055114 GO:0016702 GO:0046872
Cluster-22178.22439 GO:0005201 GO:0005515 GO:0005578 GO:0005581
Cluster-22178.22445 GO:0006855 GO:0055085 GO:0006810 GO:0015893
GO:0015297 GO:0015238 GO:0016020
Cluster-22178.22457 GO:0007165 GO:0006355 GO:0003700 GO:0005102
GO:0042025 GO:0005667
Cluster-22178.22466 GO:0035434 GO:0006825 GO:0005375 GO:0016021
Cluster-22178.22468 GO:0005509 GO:0005515

Cluster-22178.22471 GO:0007268 GO:0006811 GO:0007165 GO:0004970
GO:0005234 GO:0016020
Cluster-22178.22473 GO:0006811 GO:0007165 GO:0007268 GO:0004970
GO:0005234 GO:0016020
Cluster-22178.22484 GO:0016887 GO:0005524 GO:0016020
Cluster-22178.22487 GO:0035556 GO:0046339 GO:0009395 GO:0007165
GO:0006629 GO:0004435 GO:0008061 GO:0005515
Cluster-22178.22492 GO:0006508 GO:0004197 GO:0008270 GO:0046332
GO:0005739
Cluster-22178.22493 GO:0006508 GO:0004197 GO:0046332 GO:0008270
GO:0005739
Cluster-22178.22494 GO:0006508 GO:0046332 GO:0008270 GO:0004197
GO:0005739
Cluster-22178.22495 GO:0006123 GO:0015992 GO:0004129 GO:0008289
GO:0045277 GO:0005743
Cluster-22178.22510 GO:0042254 GO:0005634
Cluster-22178.2252 GO:0008033 GO:0005524 GO:0016879 GO:0000166
GO:0005737 GO:0016021
Cluster-22178.22526 GO:0007411
Cluster-22178.22539 GO:0005515
Cluster-22178.22554 GO:0006508 GO:0008270 GO:0004222 GO:0031012
Cluster-22178.22565 GO:0006811 GO:0006810 GO:0005230 GO:0016020
Cluster-22178.22571 GO:0055114 GO:0010277
Cluster-22178.22572 GO:0006886 GO:0007165 GO:0009401 GO:0051258
GO:0030168 GO:0005198 GO:0030674 GO:0005102 GO:0005783
GO:0005577 GO:0016021
Cluster-22178.22586 GO:0055114 GO:0006537 GO:0019988 GO:0006826
GO:0006470 GO:0006807 GO:0034755 GO:0015930 GO:0043399
GO:0008138 GO:0005381 GO:0016021
Cluster-22178.2259 GO:0007275
Cluster-22178.22592 GO:0006783 GO:0015994 GO:0033014 GO:0004852
GO:0031225
Cluster-22178.22593 GO:0015994 GO:0006783 GO:0033014 GO:0004852

GO:0031225
Cluster-22178.22594 GO:0033014 GO:0015994 GO:0006783 GO:0004852
GO:0031225
Cluster-22178.22595 GO:0015994 GO:0006783 GO:0033014 GO:0004852
GO:0031225
Cluster-22178.22597 GO:0030866 GO:0005198 GO:0008092 GO:0003779
GO:0005856
Cluster-22178.22603 GO:0007155 GO:0007156 GO:0042246 GO:0005509
GO:0005887 GO:0016020 GO:0016021
Cluster-22178.22607 GO:0007156 GO:0033227 GO:0015931 GO:0005509
GO:0051033 GO:0016020 GO:0016021
Cluster-22178.22635 GO:0006355 GO:0003677
Cluster-22178.22636 GO:0006355 GO:0003677
Cluster-22178.22644 GO:0006468 GO:0006979 GO:0004672 GO:0019031
Cluster-22178.22663 GO:0005515
Cluster-22178.22671 GO:0030036 GO:0003779 GO:0030054
Cluster-22178.22685 GO:0007186 GO:0004930 GO:0016021
Cluster-22178.22687 GO:0000166 GO:0046872
Cluster-22178.22688 GO:0019058 GO:0046872 GO:0000166
Cluster-22178.22689 GO:0046872 GO:0000166
Cluster-22178.22691 GO:0046872 GO:0000166
Cluster-22178.22692 GO:0019058 GO:0046872 GO:0000166
Cluster-22178.22697 GO:0016021
Cluster-22178.22710 GO:0006836 GO:0006812 GO:0005328 GO:0016021
Cluster-22178.22711 GO:0006508 GO:0008152 GO:0008270 GO:0016788
GO:0004181 GO:0005515
Cluster-22178.22730 GO:0005515
Cluster-22178.22731 GO:0005515
Cluster-22178.22740 GO:0016788
Cluster-22178.22741 GO:0005887
Cluster-22178.22745 GO:0007275 GO:0009966 GO:0016020
Cluster-22178.22755 GO:0005515
Cluster-22178.22757 GO:0005515

Cluster-22178.22766 GO:0015074 GO:0003677 GO:0005515
Cluster-22178.22770 GO:0006355 GO:0005622
Cluster-22178.22771 GO:0006355 GO:0005622
Cluster-22178.22772 GO:0015979 GO:0006355 GO:0016021 GO:0005622 GO:0009539 GO:0009523
Cluster-22178.22776 GO:0006308 GO:0016888
Cluster-22178.22785 GO:0055085 GO:0007275 GO:0051260 GO:0006811 GO:0005216 GO:0016020 GO:0016021
Cluster-22178.22786 GO:0006811 GO:0051260 GO:0007275 GO:0055085 GO:0005216 GO:0016021 GO:0016020
Cluster-22178.22791 GO:0007155 GO:0006508 GO:0050790 GO:0019058 GO:0055114 GO:0019062 GO:0004197 GO:0016706 GO:0008168
Cluster-22178.22807 GO:0005576
Cluster-22178.22808 GO:0005576
Cluster-22178.22810 GO:0008283 GO:0040007 GO:0007165 GO:0006955 GO:0005129 GO:0008083 GO:0005576
Cluster-22178.22825 GO:0005515 GO:0008270
Cluster-22178.22860 GO:0006810 GO:0005515 GO:0016021
Cluster-22178.22861 GO:0006810 GO:0005515 GO:0016021
Cluster-22178.22864 GO:0043087 GO:0006812 GO:0035023 GO:0008324 GO:0005515 GO:0005089
Cluster-22178.22867 GO:0006611 GO:0006508 GO:0009056 GO:0006406 GO:0006998 GO:0004252 GO:0008270 GO:0031965
Cluster-22178.22873 GO:0042981 GO:0006508 GO:0004197
Cluster-22178.22885 GO:0005515
Cluster-22178.22894 GO:0001836 GO:0045995 GO:0030334 GO:0006919 GO:0006974 GO:0007165 GO:0030155 GO:0006915 GO:0007155 GO:0005102 GO:0016020
Cluster-22178.22895 GO:0006508 GO:0004252
Cluster-22178.22897 GO:0005515 GO:0016020
Cluster-22178.22899 GO:0042742 GO:0009069 GO:0006506 GO:0016310 GO:0007049 GO:0005515 GO:0004674 GO:0005789 GO:0005887
Cluster-22178.229 GO:0016037 GO:0031404 GO:0005515 GO:0005198

GO:0019013 GO:0030089
Cluster-22178.22900 GO:0003723
Cluster-22178.22901 GO:0006508 GO:0008239 GO:0003723
Cluster-22178.22909 GO:0003824 GO:0005515
Cluster-22178.22928 GO:0003677
Cluster-22178.22929 GO:0009415 GO:0006950 GO:0006367 GO:0003677 GO:0005672
Cluster-22178.22930 GO:0009415 GO:0006950 GO:0003677
Cluster-22178.22932 GO:0009415 GO:0006950 GO:0003677
Cluster-22178.22933 GO:0006950 GO:0009415 GO:0003677
Cluster-22178.22947 GO:0007186 GO:0004930 GO:0016021
Cluster-22178.2295 GO:0006355 GO:0008134 GO:0005515 GO:0003723 GO:0003724 GO:0005524 GO:0016887 GO:0005667
Cluster-22178.22957 GO:0035556 GO:0006352 GO:0006355 GO:0003700 GO:0016020 GO:0005669 GO:0005667
Cluster-22178.22958 GO:0006352 GO:0035556 GO:0006355 GO:0003700 GO:0005669 GO:0016020 GO:0005667
Cluster-22178.22959 GO:0046034 GO:0006206 GO:0006464 GO:0006144 GO:0006351 GO:0016151 GO:0046872 GO:0003677 GO:0004017 GO:0003899 GO:0005515 GO:0005506 GO:0005730
Cluster-22178.22966 GO:0007165 GO:0006355 GO:0003677 GO:0005515 GO:0004871 GO:0016021 GO:0005634
Cluster-22178.22970 GO:0008152 GO:0016758
Cluster-22178.22980 GO:0006355 GO:0005515
Cluster-22178.22982 GO:0019988 GO:0006826 GO:0055114 GO:0006537 GO:0006807 GO:0006470 GO:0034755 GO:0043399 GO:0008138 GO:0005381 GO:0015930 GO:0016021
Cluster-22178.22983 GO:0034755 GO:0006826 GO:0019988 GO:0006537 GO:0055114 GO:0006470 GO:0006807 GO:0008138 GO:0005381 GO:0043399 GO:0015930 GO:0016021
Cluster-22178.22984 GO:0006470 GO:0006807 GO:0006826 GO:0019988 GO:0055114 GO:0006537 GO:0034755 GO:0043399 GO:0008138 GO:0005381 GO:0015930 GO:0016021

Cluster-22178.22996 GO:0046413 GO:0005515 GO:0018836

Cluster-22178.22999 GO:0007010 GO:0018342 GO:0042393 GO:0020037 GO:0008318 GO:0003779

Cluster-22178.23000 GO:0007010 GO:0018342 GO:0020037 GO:0008318 GO:0003779

Cluster-22178.23001 GO:0051258 GO:0030168 GO:0007165 GO:0005102 GO:0030674 GO:0005198 GO:0005577 GO:0005882

Cluster-22178.23004 GO:0051537 GO:0043231

Cluster-22178.23008 GO:0007186 GO:0006351 GO:0006144 GO:0006206 GO:0003899 GO:0005515 GO:0003677 GO:0004930 GO:0005730 GO:0016021

Cluster-22178.23017 GO:0008270

Cluster-22178.23022 GO:0006144 GO:0006351 GO:0003723 GO:0005524 GO:0003968 GO:0031379

Cluster-22178.23027 GO:0006144 GO:0003968 GO:0031379

Cluster-22178.23030 GO:0019028

Cluster-22178.23031 GO:0055114 GO:0016972

Cluster-22178.23035 GO:0007186 GO:0006351 GO:0003676 GO:0004930 GO:0008270 GO:0016021

Cluster-22178.23037 GO:0055085 GO:0006811 GO:0005216 GO:0016020

Cluster-22178.23038 GO:0005515

Cluster-22178.23050 GO:0035556 GO:0005515

Cluster-22178.23052 GO:0031110

Cluster-22178.2306 GO:0006446 GO:0006413 GO:0003743 GO:0005515 GO:0005840

Cluster-22178.23063 GO:0006465 GO:0048870 GO:0016567 GO:0008233 GO:0004842 GO:0008270 GO:0046872 GO:0005515 GO:0016021 GO:0005680 GO:0005622 GO:0031514 GO:0019028 GO:0005787 GO:0048786

Cluster-22178.23082 GO:0005982 GO:0006012 GO:0005985 GO:0004564 GO:0004575 GO:0017177 GO:0019031

Cluster-22178.23095 GO:0007165 GO:0005515

Cluster-22178.23100 GO:0005515

Cluster-22178.23113 GO:0051301 GO:0007059 GO:0000917 GO:0006914
GO:0031110 GO:0070206 GO:0010506 GO:0007067 GO:0000090
GO:0043093 GO:0008017 GO:0005737 GO:0005615 GO:0045298
GO:0000940 GO:0005876
Cluster-22178.23124 GO:0006355 GO:0003677
Cluster-22178.23135 GO:0050832 GO:0042742
Cluster-22178.23136 GO:0006614 GO:0008312 GO:0048500
Cluster-22178.2314 GO:0006566 GO:0006563 GO:0008654 GO:0046486
GO:0006544 GO:0004609
Cluster-22178.23142 GO:0006355 GO:0045454 GO:0046983 GO:0005515

Cluster-22178.23148 GO:0006904 GO:0000145
Cluster-22178.23149 GO:0006355 GO:0043565 GO:0003700 GO:0005667
Cluster-22178.2315 GO:0006171 GO:0006144 GO:0000287 GO:0004016
Cluster-22178.23155 GO:0006351 GO:0006486 GO:0006206 GO:0006144
GO:0003677 GO:0003899 GO:0008373 GO:0005730
Cluster-22178.23156 GO:0051252 GO:0006351 GO:0008033 GO:0006144
GO:0006206 GO:0003899 GO:0003677 GO:0004526 GO:0030677
GO:0005634 GO:0005730
Cluster-22178.2316 GO:0006544 GO:0046486 GO:0008654 GO:0006563
GO:0006566 GO:0004609
Cluster-22178.23173 GO:0051258 GO:0030168 GO:0007165 GO:0030674
GO:0005102 GO:0005577
Cluster-22178.23184 GO:0019031
Cluster-22178.23189 GO:0005198 GO:0005882
Cluster-22178.23190 GO:0005198 GO:0005882
Cluster-22178.23192 GO:0015986 GO:0015992 GO:0015078 GO:0000276
Cluster-22178.23193 GO:0055114 GO:0015946 GO:0004022 GO:0008233

Cluster-22178.23203 GO:0006182 GO:0009190 GO:0046039 GO:0035556
GO:0006144 GO:0020037 GO:0004383 GO:0016849 GO:0008270
GO:0005634
Cluster-22178.23212 GO:0007264 GO:0005525

Cluster-22178.23222 GO:0030474 GO:0006355 GO:0008608 GO:0048870
GO:0000921 GO:0010508 GO:0006352 GO:0048309 GO:0007155
GO:0006606 GO:0051028 GO:0006914 GO:0008134 GO:0045502
GO:0005200 GO:0003700 GO:0008092 GO:0016987 GO:0042803
GO:0005198 GO:0005507 GO:0003677 GO:0008017 GO:0019905
GO:0003774 GO:0031514 GO:0019028 GO:0072686 GO:0019031
GO:0016021 GO:0005882 GO:0005823 GO:0019898 GO:0030286
GO:0005940 GO:0042729 GO:0005634 GO:0045298 GO:0005737
GO:0005856 GO:0016459 GO:0005667
Cluster-22178.23239 GO:0005515
Cluster-22178.23242 GO:0005515
Cluster-22178.23247 GO:0006260 GO:0007165 GO:0007186 GO:0031683
GO:0005524 GO:0019001 GO:0003924 GO:0005525 GO:0004871
GO:0004003 GO:0003677 GO:0005657
Cluster-22178.23248 GO:0007186 GO:0007165 GO:0006260 GO:0003677
GO:0004003 GO:0004871 GO:0005525 GO:0003924 GO:0019001
GO:0005524 GO:0031683 GO:0005657
Cluster-22178.2326 GO:0007010 GO:0003779
Cluster-22178.23261 GO:0008152 GO:0016746 GO:0016021
Cluster-22178.23263 GO:0009755 GO:0005576
Cluster-22178.23264 GO:0007155 GO:0009069 GO:0006468 GO:0016310
GO:0008134 GO:0042803 GO:0045502 GO:0004674 GO:0005524
GO:0005667 GO:0005856 GO:0030286
Cluster-22178.23265 GO:0005856
Cluster-22178.23268 GO:0055114 GO:0007155 GO:0070072 GO:0005515
GO:0005509 GO:0005576 GO:0016020
Cluster-22178.23276 GO:0007186 GO:0004930 GO:0016020
Cluster-22178.23280 GO:0015992 GO:0006123 GO:0008289 GO:0004129
GO:0005743 GO:0045277
Cluster-22178.23283 GO:0007155 GO:0016020
Cluster-22178.23286 GO:0006950 GO:0006260 GO:0045893 GO:0007186
GO:0006275 GO:0004930 GO:0003688 GO:0003677 GO:0042025
GO:0046809

Cluster-22178.23290 GO:0006355 GO:0007094 GO:0051258 GO:0007165
GO:0070206 GO:0030168 GO:0048193 GO:0045502 GO:0005198
GO:0042803 GO:0008134 GO:0003700 GO:0030674 GO:0005102
GO:0019028 GO:0019031 GO:0016021 GO:0005667 GO:0016020
GO:0030286 GO:0005577

Cluster-22178.23310 GO:0005515

Cluster-22178.23311 GO:0016788 GO:0016881

Cluster-22178.23312 GO:0007165 GO:0005179 GO:0005507 GO:0005576

Cluster-22178.23323 GO:0000917 GO:0006355 GO:0043093 GO:0007049
GO:0003677 GO:0003700 GO:0043565 GO:0045095 GO:0005634
GO:0005667 GO:0005815 GO:0005737

Cluster-22178.23329 GO:0006352

Cluster-22178.23330 GO:0006352

Cluster-22178.23337 GO:0007186 GO:0004930 GO:0016021

Cluster-22178.23341 GO:0006810 GO:0042967 GO:0006633 GO:0055114
GO:0004318 GO:0051082 GO:0005215 GO:0031072 GO:0016020
GO:0005835

Cluster-22178.23342 GO:0055114 GO:0006633 GO:0042967 GO:0051082
GO:0031072 GO:0004318 GO:0005835

Cluster-22178.23343 GO:0006694 GO:0008152 GO:0055114 GO:0008207
GO:0008209 GO:0008210 GO:0003824 GO:0016491 GO:0016616
GO:0050662 GO:0003854 GO:0071949

Cluster-22178.23358 GO:0016485 GO:0019069 GO:0015031 GO:0008565

Cluster-22178.2336 GO:0005515

Cluster-22178.23374 GO:0007165 GO:0006898 GO:0005044 GO:0005515
GO:0016020

Cluster-22178.23378 GO:0055114 GO:0008152 GO:0001503 GO:0016491

Cluster-22178.23379 GO:0055114 GO:0001503 GO:0008152 GO:0016491

Cluster-22178.23387 GO:0015074 GO:0051252 GO:0004523 GO:0003676

Cluster-22178.23389 GO:0015074 GO:0051252 GO:0004523 GO:0008270
GO:0005198 GO:0003676 GO:0019028
Cluster-22178.23390 GO:0015074 GO:0051252 GO:0004523 GO:0008270
GO:0005198 GO:0003676 GO:0019028
Cluster-22178.23394 GO:0015074
Cluster-22178.23396 GO:0015074 GO:0051252 GO:0004523 GO:0008270
GO:0005198 GO:0003676 GO:0019028
Cluster-22178.23397 GO:0051252 GO:0015074 GO:0003676 GO:0004523

Cluster-22178.23413 GO:0045444 GO:0016032 GO:0005515 GO:0004252
GO:0005509 GO:0016021
Cluster-22178.23415 GO:0003676 GO:0016021
Cluster-22178.23416 GO:0006465 GO:0008233 GO:0005787 GO:0016021
Cluster-22178.23417 GO:0004222
Cluster-22178.23418 GO:0005509 GO:0005515
Cluster-22178.23419 GO:0019497 GO:0006771 GO:0003993 GO:0005515
GO:0046872 GO:0005576
Cluster-22178.23423 GO:0015992 GO:0015986 GO:0015078 GO:0005515
GO:0000276
Cluster-22178.23425 GO:0005515
Cluster-22178.23428 GO:0030414 GO:0005515
Cluster-22178.23438 GO:0016042 GO:0007586 GO:0008047 GO:0005576
Cluster-22178.23439 GO:0016021
Cluster-22178.23457 GO:0070403
Cluster-22178.23459 GO:0045859 GO:0006308 GO:0006265 GO:0003677
GO:0005515 GO:0004860 GO:0008270 GO:0005524 GO:0003918
GO:0008855 GO:0009318 GO:0016020 GO:0005576 GO:0005622
Cluster-22178.2346 GO:0042254 GO:0006412 GO:0006810 GO:0055085
GO:0005515 GO:0003735 GO:0005215 GO:0016020 GO:0016021
GO:0005840 GO:0005622
Cluster-22178.23460 GO:0045859 GO:0006308 GO:0006265 GO:0005515
GO:0003677 GO:0004860 GO:0003918 GO:0008855 GO:0005524
GO:0008270 GO:0016020 GO:0009318 GO:0005576 GO:0005622

Cluster-22178.23467 GO:0051258 GO:0030168 GO:0006869 GO:0007165
GO:0007155 GO:0042157 GO:0008289 GO:0005102 GO:0003779
GO:0030674 GO:0005577 GO:0016021 GO:0005622 GO:0005576
Cluster-22178.23470 GO:0006260 GO:0045893 GO:0006275 GO:0003677
GO:0003688 GO:0042025 GO:0046809
Cluster-22178.23471 GO:0006260 GO:0006275 GO:0045893 GO:0003677
GO:0003688 GO:0046809 GO:0042025
Cluster-22178.23486 GO:0007186 GO:0004930 GO:0016021
Cluster-22178.23492 GO:0006629
Cluster-22178.23493 GO:0035556 GO:0046872 GO:0005515
Cluster-22178.23506 GO:0015979 GO:0005525 GO:0009523 GO:0009539
GO:0016020
Cluster-22178.23524 GO:0043565 GO:0005515 GO:0005634
Cluster-22178.23525 GO:0055085 GO:0006812 GO:0006885 GO:0015299
GO:0043565 GO:0005515 GO:0016021 GO:0005634
Cluster-22178.23533 GO:0004222 GO:0008233 GO:0031012
Cluster-22178.23542 GO:0090263 GO:0016567 GO:0005515 GO:0046872
GO:0008270
Cluster-22178.23548 GO:0006811 GO:0055085 GO:0008270 GO:0005216
GO:0005198 GO:0019031 GO:0019028 GO:0016020
Cluster-22178.23551 GO:0006366
Cluster-22178.23552 GO:0005515
Cluster-22178.23554 GO:0042254 GO:0006412 GO:0003735 GO:0005622
GO:0005840
Cluster-22178.23568 GO:0022904 GO:0015740 GO:0015556 GO:0016021
GO:0016020
Cluster-22178.23569 GO:0007165 GO:0007259 GO:0006813 GO:0050778
GO:0051607 GO:0030955 GO:0005249 GO:0005125 GO:0008076
GO:0016021
Cluster-22178.23571 GO:0006122 GO:0003333 GO:0006810 GO:0006367
GO:0006366 GO:0006865 GO:0055085 GO:0006811 GO:0015171
GO:0005215 GO:0005674 GO:0016020 GO:0005750 GO:0005743
Cluster-22178.23579 GO:0008270

Cluster-22178.23587 GO:0006355 GO:0003700 GO:0005634 GO:0005667
Cluster-22178.2359 GO:0016829
Cluster-22178.23592 GO:0055085 GO:0006811 GO:0005216 GO:0016020
Cluster-22178.23607 GO:0046872
Cluster-22178.23608 GO:0034508 GO:0005509 GO:0005515 GO:0000776
Cluster-22178.23609 GO:0034508 GO:0005509 GO:0005515 GO:0000776
Cluster-22178.23611 GO:0006614 GO:0006810 GO:0005525 GO:0005524
GO:0016887 GO:0003924 GO:0005576
Cluster-22178.23615 GO:0007165 GO:0007268 GO:0007186 GO:0006811
GO:0004930 GO:0004970 GO:0016020 GO:0016021
Cluster-22178.23619 GO:0009439
Cluster-22178.23620 GO:0009082
Cluster-22178.23626 GO:0007165 GO:0051258 GO:0030168 GO:0005102
GO:0030674 GO:0005577
Cluster-22178.23629 GO:0007165 GO:0007186 GO:0007606 GO:0004930
GO:0004888 GO:0016021 GO:0016020
Cluster-22178.23631 GO:0006355 GO:0043401
Cluster-22178.23632 GO:0043401 GO:0006355
Cluster-22178.23638 GO:0015708 GO:0007165 GO:0006955 GO:0005125
GO:0008270 GO:0003723 GO:0005576
Cluster-22178.23640 GO:0006915 GO:0045735 GO:0005622
Cluster-22178.23643 GO:0006869 GO:0005319
Cluster-22178.23656 GO:0007010 GO:0005524 GO:0003779 GO:0016887

Cluster-22178.23657 GO:0007010 GO:0005524 GO:0003779 GO:0016887

Cluster-22178.23659 GO:0005524 GO:0016887
Cluster-22178.23676 GO:0005515 GO:0046872
Cluster-22178.23679 GO:0005515
Cluster-22178.23681 GO:0042998 GO:0071253 GO:0005802
Cluster-22178.23690 GO:0006351 GO:0006144 GO:0003968 GO:0003723
GO:0005524 GO:0031379
Cluster-22178.23697 GO:0016021

Cluster-22178.23707 GO:0007186 GO:0004930 GO:0016020
Cluster-22178.23712 GO:0017004 GO:0015886 GO:0016021
Cluster-22178.23714 GO:0017004 GO:0015886 GO:0016021
Cluster-22178.23715 GO:0006811 GO:0016020
Cluster-22178.23727 GO:0007304 GO:0001503 GO:0005213 GO:0005576 GO:0042600
Cluster-22178.23728 GO:0007304 GO:0001503 GO:0005213 GO:0042600 GO:0005576
Cluster-22178.23739 GO:0046872
Cluster-22178.23749 GO:0009405 GO:0005576
Cluster-22178.23752 GO:0030150 GO:0046872 GO:0005742
Cluster-22178.23764 GO:0006810 GO:0019871 GO:0005576
Cluster-22178.23765 GO:0009405 GO:0006810 GO:0008219 GO:0019871 GO:0005576
Cluster-22178.23772 GO:0005096
Cluster-22178.23778 GO:0007155 GO:0003677 GO:0003676 GO:0005515 GO:0005524 GO:0016787 GO:0016020
Cluster-22178.23779 GO:0006355 GO:0005524 GO:0008270 GO:0003677 GO:0016818 GO:0005634
Cluster-22178.23780 GO:0035556
Cluster-22178.23786 GO:0005509 GO:0016021
Cluster-22178.2379 GO:0042742 GO:0005576
Cluster-22178.23803 GO:0006355 GO:0043093 GO:0000917 GO:0070206 GO:0007049 GO:0003677 GO:0043565 GO:0046872 GO:0003700 GO:0005634 GO:0005667 GO:0045095 GO:0005737 GO:0005815
Cluster-22178.23807 GO:0006508 GO:0016740 GO:0008242 GO:0004197

Cluster-22178.23808 GO:0006508 GO:0008242 GO:0016740 GO:0004197

Cluster-22178.23809 GO:0047869 GO:0016020
Cluster-22178.23826 GO:0005515
Cluster-22178.23831 GO:0005615
Cluster-22178.23833 GO:0006576 GO:0007264 GO:0002949 GO:0003924

GO:0005524 GO:0005525
Cluster-22178.23839 GO:0005509
Cluster-22178.23840 GO:0005509 GO:0005515
Cluster-22178.23844 GO:0005509
Cluster-22178.23854 GO:0016114 GO:0006260 GO:0006694 GO:0006816
GO:0009058 GO:0005388 GO:0004161 GO:0003677 GO:0005664
GO:0016529
Cluster-22178.23863 GO:0005515
Cluster-22178.23868 GO:0007155 GO:0000902 GO:0006955 GO:0017004
GO:0018149 GO:0015886 GO:0008061 GO:0003677 GO:0005515
GO:0042470 GO:0016020 GO:0005737 GO:0005887 GO:0019031
GO:0016021
Cluster-22178.23880 GO:0055114 GO:0016491 GO:0005507
Cluster-22178.23885 GO:0008146 GO:0016021
Cluster-22178.23887 GO:0006508 GO:0004252
Cluster-22178.23888 GO:0006508 GO:0009405 GO:0008270 GO:0004222
GO:0031012 GO:0005576
Cluster-22178.23904 GO:0050792 GO:0004518 GO:0005515
Cluster-22178.23909 GO:0008643 GO:0051607 GO:0070206 GO:0015031
GO:0030168 GO:0007155 GO:0007165 GO:0051258 GO:0009306
GO:0007049 GO:0006904 GO:0005102 GO:0008982 GO:0030674
GO:0005198 GO:0005577 GO:0000145 GO:0016021 GO:0019031
GO:0019028 GO:0009357
Cluster-22178.23913 GO:0007165 GO:0006144 GO:0004114
Cluster-22178.23916 GO:0016787
Cluster-22178.23925 GO:0005982 GO:0006012 GO:0005985 GO:0004564
GO:0004575 GO:0017177
Cluster-22178.23929 GO:0005985 GO:0006012 GO:0005982 GO:0004564
GO:0004575 GO:0017177
Cluster-22178.23935 GO:0007186 GO:0004930 GO:0016021
Cluster-22178.23946 GO:0006468 GO:0005524 GO:0004672 GO:0005515

Cluster-22178.23963 GO:0006771 GO:0009069 GO:0015628 GO:0016310

GO:0019497 GO:0015031 GO:0005515 GO:0003993 GO:0004674
GO:0008565 GO:0046872 GO:0015627
Cluster-22178.23964 GO:0019497 GO:0006771 GO:0046872 GO:0005515
GO:0003993
Cluster-22178.23967 GO:0007165 GO:0005509 GO:0005578
Cluster-22178.23971 GO:0055085 GO:0006814 GO:0006810 GO:0005215
GO:0016020 GO:0016021
Cluster-22178.23974 GO:0006144 GO:0035556 GO:0006182 GO:0046039
GO:0009190 GO:0008270 GO:0004383 GO:0016849 GO:0020037
Cluster-22178.23989 GO:0005515
Cluster-22178.23995 GO:0006952 GO:0006508 GO:0008236 GO:0016787
GO:0005576
Cluster-22178.23997 GO:0006508 GO:0006952 GO:0016787 GO:0008236
GO:0005576
Cluster-22178.24005 GO:0007186 GO:0006508 GO:0006144 GO:0007154
GO:0004197 GO:0017111 GO:0004930 GO:0003968 GO:0016817
GO:0016020 GO:0016021 GO:0031379
Cluster-22178.24010 GO:0005543
Cluster-22178.24021 GO:0016567 GO:0046872 GO:0008270 GO:0005515
GO:0004842 GO:0005680
Cluster-22178.24023 GO:0006397 GO:0006370 GO:0004484
Cluster-22178.24026 GO:0006811 GO:0006810 GO:0005230 GO:0016020
Cluster-22178.24031 GO:0030178 GO:0007275 GO:0005576
Cluster-22178.24036 GO:0007165 GO:0005509 GO:0005578
Cluster-22178.24041 GO:0008146 GO:0016021
Cluster-22178.24042 GO:0008146 GO:0016021
Cluster-22178.24044 GO:0008146 GO:0016021
Cluster-22178.24045 GO:0006457 GO:0006366 GO:0003677 GO:0005665
GO:0005737
Cluster-22178.24054 GO:0006118 GO:0006816 GO:0006874 GO:0000226
GO:0055085 GO:0006811 GO:0005509 GO:0005216 GO:0005219
GO:0009055 GO:0051536 GO:0016020 GO:0016021 GO:0005622
Cluster-22178.24062 GO:0005515

Cluster-22178.24067 GO:0046872
Cluster-22178.24068 GO:0046872
Cluster-22178.24069 GO:0007156 GO:0005509 GO:0016020
Cluster-22178.24075 GO:0006547 GO:0016153
Cluster-22178.24076 GO:0006547 GO:0016153
Cluster-22178.24081 GO:0005515 GO:0004064
Cluster-22178.24097 GO:0003677
Cluster-22178.24100 GO:0006260 GO:0006281 GO:0003911 GO:0004866
GO:0005615 GO:0005576
Cluster-22178.24108 GO:0005515
Cluster-22178.2411 GO:0006771 GO:0019497 GO:0007155 GO:0003993
GO:0005515 GO:0046872 GO:0016021
Cluster-22178.2413 GO:0009405 GO:0019865 GO:0019814
Cluster-22178.24132 GO:0015992 GO:0015986 GO:0015078 GO:0045263
Cluster-22178.24139 GO:0008270 GO:0003677
Cluster-22178.24140 GO:0008270
Cluster-22178.24142 GO:0007187 GO:0006506 GO:0007608 GO:0006811
GO:0007165 GO:0019064 GO:0055085 GO:0016740 GO:0046789
GO:0016788 GO:0004984 GO:0005216 GO:0005549 GO:0019031
GO:0005789 GO:0016020 GO:0009986
Cluster-22178.24145 GO:0007165 GO:0019064 GO:0055085 GO:0007187
GO:0006506 GO:0007608 GO:0006811 GO:0005216 GO:0005549
GO:0016740 GO:0046789 GO:0016788 GO:0004984 GO:0016020
GO:0009986 GO:0005789 GO:0019031
Cluster-22178.24146 GO:0055085 GO:0019064 GO:0007165 GO:0007608
GO:0006811 GO:0007187 GO:0006506 GO:0005549 GO:0005216
GO:0004984 GO:0046789 GO:0016788 GO:0016740 GO:0009986
GO:0016020 GO:0005789 GO:0019031
Cluster-22178.24153 GO:0007264 GO:0005525 GO:0005524 GO:0016887

Cluster-22178.24167 GO:0051056 GO:0015979 GO:0005096 GO:0016020
GO:0009523 GO:0009539
Cluster-22178.24168 GO:0007186 GO:0000042 GO:0006508 GO:0004930

GO:0005515 GO:0008233 GO:0016021
Cluster-22178.24188 GO:0005515
Cluster-22178.24189 GO:0005515
Cluster-22178.24205 GO:0006621 GO:0051252 GO:0008033 GO:0004526
GO:0046923 GO:0030677 GO:0005783 GO:0005634 GO:0016021
Cluster-22178.24221 GO:0046872
Cluster-22178.24241 GO:0003723
Cluster-22178.24243 GO:0006355 GO:0003677
Cluster-22178.24247 GO:0004222 GO:0008233 GO:0031012
Cluster-22178.24257 GO:0005887 GO:0005739
Cluster-22178.24266 GO:0005509 GO:0005515 GO:0009986 GO:0016020
Cluster-22178.24268 GO:0006308 GO:0008855 GO:0009318
Cluster-22178.24269 GO:0015991 GO:0006308 GO:0015992 GO:0030488
GO:0008855 GO:0015078 GO:0033179 GO:0031515 GO:0009318
GO:0016021
Cluster-22178.2427 GO:0006355 GO:0042254 GO:0006412 GO:0003735
GO:0005515 GO:0003677 GO:0005622 GO:0005840
Cluster-22178.24273 GO:0007156 GO:0005509 GO:0016020
Cluster-22178.24274 GO:0007156 GO:0003677 GO:0005509 GO:0016020
Cluster-22178.24278 GO:0006955 GO:0000902 GO:0016020
Cluster-22178.24280 GO:0042981 GO:0005515
Cluster-22178.24283 GO:0005515
Cluster-22178.24287 GO:0016042 GO:0007165 GO:0007586 GO:0008047
GO:0005179 GO:0005576
Cluster-22178.24288 GO:0007586 GO:0016042 GO:0008047 GO:0005576
Cluster-22178.24289 GO:0055114 GO:0030246 GO:0046422 GO:0005515
GO:0009507 GO:0016020
Cluster-22178.24292 GO:0000746 GO:0006468 GO:0005524 GO:0004672
GO:0005515
Cluster-22178.24293 GO:0000746 GO:0006468 GO:0005524 GO:0004672
GO:0005515
Cluster-22178.24296 GO:0015031 GO:0005887 GO:0016021
Cluster-22178.24300 GO:0000077 GO:0006308 GO:0006508 GO:0006281

GO:0004252 GO:0000014
Cluster-22178.24318 GO:0006810 GO:0055085 GO:0005525 GO:0042626
GO:0005524 GO:0016887 GO:0016021
Cluster-22178.24319 GO:0055085 GO:0006810 GO:0016887 GO:0005524
GO:0042626 GO:0005525 GO:0016021
Cluster-22178.24322 GO:0006810 GO:0055085 GO:0005525 GO:0042626
GO:0005524 GO:0016887 GO:0016021
Cluster-22178.24326 GO:0005515
Cluster-22178.24330 GO:0006508 GO:0055114 GO:0006313 GO:0008168
GO:0005515 GO:0004252 GO:0016706
Cluster-22178.24331 GO:0043087 GO:0016032 GO:0007094 GO:0005815
Cluster-22178.24332 GO:0043087
Cluster-22178.24343 GO:0005515
Cluster-22178.24356 GO:0006351 GO:0006144 GO:0006206 GO:0003899
GO:0003677 GO:0005730
Cluster-22178.24365 GO:0009451 GO:0001522 GO:0006886 GO:0006810
GO:0006888 GO:0019087 GO:0006396 GO:0003723 GO:0008270
GO:0009982 GO:0005215 GO:0016020 GO:0030127 GO:0005622
GO:0016021
Cluster-22178.24371 GO:0005515
Cluster-22178.24374 GO:0003677 GO:0019013
Cluster-22178.24377 GO:0003677 GO:0019013
Cluster-22178.24379 GO:0003677 GO:0019013
Cluster-22178.24382 GO:0009395 GO:0046486 GO:0007205 GO:0004143

Cluster-22178.24383 GO:0007205 GO:0046486 GO:0009395 GO:0004143

Cluster-22178.24384 GO:0007205 GO:0046486 GO:0009395 GO:0004143

Cluster-22178.24397 GO:0007010 GO:0006355 GO:0003700 GO:0043565
GO:0003779 GO:0005667
Cluster-22178.24401 GO:0003333
Cluster-22178.24412 GO:0007169 GO:0007165 GO:0006468 GO:0004672

GO:0005524 GO:0004714 GO:0005179 GO:0000228 GO:0016020
Cluster-22178.24417 GO:0007264 GO:0005525
Cluster-22178.24421 GO:0006694 GO:0008152 GO:0055114 GO:0008207
GO:0008209 GO:0008210 GO:0003824 GO:0016491 GO:0016616
GO:0003854 GO:0050662 GO:0016021
Cluster-22178.24422 GO:0055114 GO:0008152 GO:0006694 GO:0008210
GO:0008207 GO:0008209 GO:0016491 GO:0003824 GO:0050662
GO:0003854 GO:0016616
Cluster-22178.24429 GO:0019836 GO:0005515 GO:0005576
Cluster-22178.24431 GO:0006355 GO:0019836 GO:0003676 GO:0005515
GO:0005576 GO:0005622
Cluster-22178.24434 GO:0000975
Cluster-22178.24441 GO:0005515
Cluster-22178.24444 GO:0005515 GO:0008270 GO:0016021 GO:0009276
Cluster-22178.24459 GO:0006260 GO:0006364 GO:0006351 GO:0006270
GO:0006814 GO:1901031 GO:0003677 GO:0003887 GO:0005248
GO:0032040 GO:0042575 GO:0001518 GO:0005732 GO:0034457
GO:0005634
Cluster-22178.24460 GO:0046872 GO:0003677 GO:0005634
Cluster-22178.24466 GO:0007155 GO:0005540 GO:0005515
Cluster-22178.24468 GO:0006508 GO:0007017 GO:0007018 GO:0006807
GO:0017070 GO:0005524 GO:0008017 GO:0004222 GO:0003933
GO:0003777 GO:0045298 GO:0005874
Cluster-22178.24470 GO:0006807 GO:0007017 GO:0006508 GO:0007018
GO:0005524 GO:0008017 GO:0003933 GO:0003777 GO:0004222
GO:0017070 GO:0045298 GO:0005874
Cluster-22178.24471 GO:0006807 GO:0006508 GO:0007017 GO:0007018
GO:0005524 GO:0008017 GO:0004222 GO:0003777 GO:0003933
GO:0017070 GO:0045298 GO:0005874
Cluster-22178.24480 GO:0009103 GO:0009245 GO:0015940 GO:0005524
GO:0009029
Cluster-22178.24482 GO:0009245 GO:0015940 GO:0009103 GO:0009029
GO:0005524

Cluster-22178.2449 GO:0017009 GO:0006508 GO:0004252

Cluster-22178.24505 GO:0005515

Cluster-22178.24518 GO:0007165 GO:0006955 GO:0005125 GO:0005576

Cluster-22178.24520 GO:0016021

Cluster-22178.24522 GO:0016021

Cluster-22178.24524 GO:0007155 GO:0005515 GO:0016021

Cluster-22178.24526 GO:0006260 GO:0006355 GO:0003700 GO:0005622 GO:0005667 GO:0005634

Cluster-22178.24541 GO:0050829 GO:0050830

Cluster-22178.24542 GO:0042710 GO:0008152 GO:0006308 GO:0016301 GO:0019825 GO:0005524 GO:0008855 GO:0009318

Cluster-22178.24553 GO:0030521 GO:0055085 GO:0006355 GO:0006810 GO:0007165 GO:0006814 GO:0004882 GO:0005215 GO:0005496 GO:0003677 GO:0016021 GO:0005634 GO:0016020

Cluster-22178.24559 GO:0005515

Cluster-22178.24594 GO:0006364 GO:0005515 GO:0008270 GO:0032040 GO:0005737

Cluster-22178.24598 GO:0006364 GO:0006260 GO:0005515 GO:0003697 GO:0008270 GO:0042025 GO:0019012 GO:0032040 GO:0016021 GO:0005737

Cluster-22178.246 GO:0006820 GO:0006835 GO:0006812 GO:0017153 GO:0016020

Cluster-22178.24612 GO:0006000 GO:0006206 GO:0006013 GO:0007264 GO:0006310 GO:0006139 GO:0006281 GO:0016887 GO:0005524 GO:0003724 GO:0004127 GO:0009378 GO:0003873 GO:0005525 GO:0003723 GO:0005515 GO:0005657 GO:0009379

Cluster-22178.24613 GO:0006506 GO:0004584 GO:0000136

Cluster-22178.24616 GO:0018298 GO:0006355 GO:0009584

Cluster-22178.24617 GO:0005515

Cluster-22178.24619 GO:0009584 GO:0006355 GO:0033227 GO:0018298 GO:0019076 GO:0006812 GO:0015931 GO:0032801 GO:0005261 GO:0051033 GO:0033644 GO:0016021

Cluster-22178.24620 GO:0005515 GO:0042470

Cluster-22178.24624 GO:0005515
Cluster-22178.24630 GO:0006352 GO:0006355 GO:0006310 GO:0016567
GO:0000150 GO:0003700 GO:0016987 GO:0004842 GO:0003677
GO:0005622 GO:0005667
Cluster-22178.24631 GO:0015986 GO:0005982 GO:0015992 GO:0055085
GO:0006012 GO:0030001 GO:0006814 GO:0005985 GO:0006811
GO:0046873 GO:0005216 GO:0004564 GO:0005248 GO:0004575
GO:0015078 GO:0001518 GO:0016020 GO:0017177 GO:0045263
GO:0016021
Cluster-22178.24632 GO:0006355
Cluster-22178.24637 GO:0015991 GO:0006413 GO:0006446 GO:0019089
GO:0006914 GO:0015992 GO:0051082 GO:0003743 GO:0019901
GO:0015078 GO:0017119 GO:0033179 GO:0016021 GO:0005840
Cluster-22178.24642 GO:0005515 GO:0005634
Cluster-22178.24657 GO:0006471 GO:0003950
Cluster-22178.24683 GO:0033588
Cluster-22178.24684 GO:0044419 GO:0006508 GO:0006144 GO:0004197
GO:0017111 GO:0003968 GO:0031379
Cluster-22178.24685 GO:0006813 GO:0008556 GO:0016021
Cluster-22178.24691 GO:0006886 GO:0016192 GO:0006810 GO:0003824
GO:0005215 GO:0005515 GO:0005198 GO:0005737 GO:0016021
GO:0030117
Cluster-22178.24704 GO:0006891 GO:0016021 GO:0017119
Cluster-22178.24705 GO:0006891 GO:0017119 GO:0016021
Cluster-22178.24710 GO:0005515
Cluster-22178.24712 GO:0008152 GO:0016787
Cluster-22178.24713 GO:0008152 GO:0016787
Cluster-22178.24722 GO:0007156 GO:0005509 GO:0016020
Cluster-22178.24745 GO:0055114 GO:0055085 GO:0006807 GO:0006810
GO:0042128 GO:0016491 GO:0008942 GO:0005524 GO:0046872
GO:0042626 GO:0051537 GO:0016021
Cluster-22178.24747 GO:0005515
Cluster-22178.24748 GO:0006479 GO:0006481 GO:0004671 GO:0016021

GO:0016020
Cluster-22178.24753 GO:0006265 GO:0003918 GO:0003677 GO:0005694
Cluster-22178.24755 GO:0006687 GO:0005975 GO:0005515 GO:0004308
GO:0055036 GO:0033644 GO:0016020
Cluster-22178.24759 GO:0005515
Cluster-22178.24761 GO:0003677
Cluster-22178.24774 GO:0005524 GO:0003774 GO:0016459
Cluster-22178.24776 GO:0005524 GO:0003774 GO:0016459
Cluster-22178.24778 GO:0006810 GO:0010508 GO:0042407 GO:0005215
GO:0044284 GO:0061617
Cluster-22178.24801 GO:0006468 GO:0005515 GO:0005524 GO:0004672

Cluster-22178.24802 GO:0016567 GO:0090263
Cluster-22178.24803 GO:0006888 GO:0006886 GO:0030127
Cluster-22178.24804 GO:0005985 GO:0005982 GO:0005975 GO:0008810

Cluster-22178.24805 GO:0003677 GO:0005634
Cluster-22178.24806 GO:0016021
Cluster-22178.24814 GO:0003950
Cluster-22178.24817 GO:0006112 GO:0007010 GO:0007165 GO:0003779
GO:0005179 GO:0005576
Cluster-22178.24818 GO:0055085 GO:0006811 GO:0005216 GO:0016020
Cluster-22178.24822 GO:0015992 GO:0006123 GO:0004129 GO:0005515
GO:0005856 GO:0045277 GO:0005743 GO:0005751
Cluster-22178.24823 GO:0005856
Cluster-22178.24824 GO:0015992 GO:0006123 GO:0004129 GO:0005856
GO:0045277 GO:0005743 GO:0005751
Cluster-22178.24825 GO:0005515
Cluster-22178.24827 GO:0005515
Cluster-22178.24831 GO:0016020
Cluster-22178.24839 GO:0008152 GO:0006412 GO:0042254 GO:0006355
GO:0003824 GO:0008270 GO:0003735 GO:0005840 GO:0005634
Cluster-22178.24842 GO:0006355 GO:0043565 GO:0003700 GO:0005667

Cluster-22178.24844 GO:0006811 GO:0055085 GO:0005216 GO:0016020
Cluster-22178.24851 GO:0005515
Cluster-22178.24852 GO:0005515
Cluster-22178.24858 GO:0006810 GO:0003676 GO:0005215 GO:0016020
Cluster-22178.24865 GO:0007016 GO:0006811 GO:0005216 GO:0005515
GO:0016021 GO:0016020 GO:0016010
Cluster-22178.24868 GO:0008152 GO:0006281 GO:0006355 GO:0003677
GO:0008270 GO:0008168 GO:0016758
Cluster-22178.24877 GO:0005515
Cluster-22178.24883 GO:0006570 GO:0006470 GO:0004725 GO:0000166
GO:0008138 GO:0046872
Cluster-22178.24910 GO:0006468 GO:0004672 GO:0005524
Cluster-22178.24921 GO:0016887 GO:0003774 GO:0005524 GO:0016459
Cluster-22178.24934 GO:0005975 GO:0005515 GO:0016846 GO:0004415

Cluster-22178.24936 GO:0009069 GO:0006468 GO:0006506 GO:0051262
GO:0055085 GO:0006811 GO:0016310 GO:0005216 GO:0005524
GO:0003824 GO:0004674 GO:0016020 GO:0016021 GO:0005789
Cluster-22178.24945 GO:0044038 GO:0016757 GO:0005886 GO:0016021
GO:0016020
Cluster-22178.24946 GO:0008152 GO:0008134 GO:0003824 GO:0005667
GO:0005634
Cluster-22178.24954 GO:0007186 GO:0007631 GO:0015684 GO:0007165
GO:0007264 GO:0007218 GO:0004871 GO:0005525 GO:0004222
GO:0003924 GO:0005524 GO:0031683 GO:0015093 GO:0019001
GO:0016021
Cluster-22178.24959 GO:0005515
Cluster-22178.24963 GO:0055085 GO:0006811 GO:0006813 GO:0007186
GO:0004930 GO:0005216 GO:0005242 GO:0016021 GO:0016020
GO:0008076
Cluster-22178.24972 GO:0006817 GO:0008270 GO:0046872 GO:0000166
GO:0005315 GO:0016020
Cluster-22178.24973 GO:0046872 GO:0008270 GO:0000166

Cluster-22178.24975 GO:0015992 GO:0015986 GO:0015078 GO:0005515
GO:0000276
Cluster-22178.24977 GO:0008152 GO:0006364 GO:0003824 GO:0008168
GO:0005634
Cluster-22178.24978 GO:0042549 GO:0015979 GO:0009539 GO:0009523
Cluster-22178.24985 GO:0055085 GO:0006811 GO:0005216 GO:0016020
Cluster-22178.24989 GO:0006355 GO:0005515 GO:0003677 GO:0016887
GO:0046872 GO:0005524 GO:0008270
Cluster-22178.24990 GO:0006355 GO:0003677 GO:0005515 GO:0005524
GO:0008270 GO:0046872 GO:0016887
Cluster-22178.24993 GO:0006468 GO:0005524 GO:0004672 GO:0003677

Cluster-22178.24995 GO:0030001 GO:0055085 GO:0046873 GO:0016020
Cluster-22178.25003 GO:0043087 GO:0007264 GO:0035023 GO:0005085
GO:0005515 GO:0005089
Cluster-22178.25010 GO:0005975 GO:0007186 GO:0016832 GO:0004930
GO:0016020 GO:0016021
Cluster-22178.25016 GO:0006811 GO:0007186 GO:0055085 GO:0005216
GO:0004930 GO:0016020 GO:0016021
Cluster-22178.25018 GO:0019083 GO:0055085 GO:0009058 GO:0006811
GO:0005216 GO:0016020
Cluster-22178.25030 GO:0019031
Cluster-22178.25031 GO:0007034 GO:0008608 GO:0042157 GO:0009405
GO:0006869 GO:0008289 GO:0051082 GO:0005876 GO:0005576
GO:0042729 GO:0030430
Cluster-22178.25033 GO:0003676
Cluster-22178.25034 GO:0003676
Cluster-22178.25037 GO:0019497 GO:0006771 GO:0003993
Cluster-22178.25043 GO:0071107 GO:0016021
Cluster-22178.25044 GO:0055114 GO:0020037 GO:0016705 GO:0005506

Cluster-22178.25045 GO:0055114 GO:0020037 GO:0016705 GO:0005506

Cluster-22178.25046 GO:0055114 GO:0020037 GO:0016705 GO:0005506

Cluster-22178.25053 GO:0042981 GO:0007016 GO:0020037 GO:0016010

Cluster-22178.25057 GO:0016255 GO:0042765

Cluster-22178.25058 GO:0009415 GO:0007304 GO:0006950 GO:0005213 GO:0005576 GO:0042600

Cluster-22178.25059 GO:0032324

Cluster-22178.25060 GO:0042302

Cluster-22178.25062 GO:0004222

Cluster-22178.25063 GO:0006508 GO:0009405 GO:0008270 GO:0004222 GO:0031012 GO:0005576

Cluster-22178.25068 GO:0050909 GO:0007186 GO:0003779 GO:0004930 GO:0016021

Cluster-22178.25069 GO:0005515 GO:0016020

Cluster-22178.25074 GO:0005515

Cluster-22178.25075 GO:0005515

Cluster-22178.25077 GO:0055085 GO:0006812 GO:0008324 GO:0016021

Cluster-22178.25080 GO:0055114 GO:0016491 GO:0030151

Cluster-22178.25082 GO:0005515

Cluster-22178.25084 GO:0006260 GO:0002949 GO:0004003 GO:0003677 GO:0005524 GO:0016887 GO:0003924 GO:0005525 GO:0016020 GO:0005657

Cluster-22178.25086 GO:0005515

Cluster-22178.25094 GO:0070588 GO:0006816 GO:0006260 GO:0003697 GO:0005515 GO:0005245 GO:0005891

Cluster-22178.25095 GO:0006935 GO:0007165 GO:0004888 GO:0016021 GO:0016020

Cluster-22178.25096 GO:0006560 GO:0006814 GO:0006508 GO:0006090 GO:0006811 GO:0007165 GO:0007268 GO:0046486 GO:0071436 GO:0006525 GO:0016042 GO:0008236 GO:0008948 GO:0004970 GO:0016787 GO:0004806 GO:0015081 GO:0016020

Cluster-22178.25097 GO:0006816 GO:0006811 GO:0005244 GO:0005262 GO:0005886 GO:0016021

Cluster-22178.25098 GO:0005886 GO:0016020 GO:0016021

Cluster-22178.25126 GO:0005515

Cluster-22178.25127 GO:0005515

Cluster-22178.25129 GO:0005515 GO:0005634

Cluster-22178.25132 GO:0006816 GO:0006811 GO:0005262 GO:0005244 GO:0005886 GO:0016021

Cluster-22178.25134 GO:0006144 GO:0014068 GO:0050776 GO:0000256 GO:0007165 GO:0008270 GO:0005102 GO:0050385 GO:0043548 GO:0016021 GO:0016020

Cluster-22178.25135 GO:0014068 GO:0006144 GO:0000256 GO:0050776 GO:0007165 GO:0008270 GO:0005102 GO:0050385 GO:0043548 GO:0016021 GO:0016020

Cluster-22178.25139 GO:0006468 GO:0005524 GO:0004672 GO:0016020

Cluster-22178.25142 GO:0006508 GO:0004252 GO:0005515

Cluster-22178.25147 GO:0016773

Cluster-22178.25148 GO:0016773

Cluster-22178.25157 GO:0005576

Cluster-22178.25161 GO:0006468 GO:0004672 GO:0005524

Cluster-22178.25174 GO:0046872 GO:0000166

Cluster-22178.25176 GO:0007010 GO:0003779 GO:0016817 GO:0005543

Cluster-22178.25180 GO:0016817 GO:0005543

Cluster-22178.25188 GO:0003676

Cluster-22178.25190 GO:0055114 GO:0055085 GO:0006807 GO:0042128 GO:0006810 GO:0008942 GO:0016491 GO:0046872 GO:0005524 GO:0042626 GO:0051537 GO:0016021

Cluster-22178.25203 GO:0000746 GO:0006468 GO:0005524 GO:0004672 GO:0005515

Cluster-22178.25204 GO:0000746 GO:0006468 GO:0004672 GO:0005524 GO:0005515

Cluster-22178.25209 GO:0006771 GO:0019497 GO:0046872 GO:0005515 GO:0003993

Cluster-22178.25224 GO:0007165 GO:0007264 GO:0035556 GO:0043087

GO:0005509 GO:0005085 GO:0003950 GO:0005578 GO:0005634
Cluster-22178.25229 GO:0007156 GO:0005509 GO:0016020
Cluster-22178.25231 GO:0007156 GO:0005509 GO:0016020
Cluster-22178.25233 GO:0016567 GO:0046872 GO:0008270 GO:0005515
GO:0004842 GO:0005680
Cluster-22178.25234 GO:0031224
Cluster-22178.25236 GO:0006508 GO:0004252
Cluster-22178.25237 GO:0007165 GO:0006508 GO:0005515 GO:0004252
GO:0005044 GO:0016020
Cluster-22178.25247 GO:0005515
Cluster-22178.25248 GO:0005515
Cluster-22178.25260 GO:0043395 GO:0005515 GO:0005578 GO:0016020
Cluster-22178.25264 GO:0009405 GO:0006810 GO:0006468 GO:0005524
GO:0004672 GO:0005515 GO:0008200 GO:0016021 GO:0042151
Cluster-22178.25275 GO:1901031 GO:0006814 GO:0006270 GO:0006364
GO:0006351 GO:0006260 GO:0005248 GO:0003887 GO:0003677
GO:0005634 GO:0034457 GO:0005732 GO:0001518 GO:0042575
GO:0032040
Cluster-22178.25278 GO:0006750 GO:0006749 GO:0017111 GO:0004357
GO:0017109
Cluster-22178.25279 GO:0005515
Cluster-22178.25280 GO:0006810 GO:0042147 GO:0015031 GO:0005524
GO:0008565 GO:0030904
Cluster-22178.25281 GO:0006810 GO:0005524
Cluster-22178.25284 GO:0006810 GO:0005524
Cluster-22178.25289 GO:0015992 GO:0006123 GO:0004129 GO:0045277
Cluster-22178.25291 GO:0007186 GO:0006488 GO:0004930 GO:0004583
GO:0005789 GO:0016021
Cluster-22178.25293 GO:0005515
Cluster-22178.25297 GO:0006825 GO:0035434 GO:0005375 GO:0003723
GO:0019013 GO:0016021
Cluster-22178.25309 GO:0046740 GO:0003774 GO:0005524 GO:0005515
GO:0016459 GO:0016021

Cluster-22178.25315 GO:0006355 GO:0003700 GO:0008270 GO:0043565
GO:0005634 GO:0005667
Cluster-22178.25317 GO:0007165 GO:0006355 GO:0004871 GO:0003700
GO:0005667
Cluster-22178.25329 GO:0019013
Cluster-22178.25336 GO:0007268 GO:0007165 GO:0006811 GO:0009236
GO:0006824 GO:0015087 GO:0004970 GO:0005234 GO:0016020
Cluster-22178.25339 GO:0005515
Cluster-22178.25342 GO:0051260
Cluster-22178.25350 GO:0006144 GO:0004114
Cluster-22178.25356 GO:0030308 GO:0060047 GO:0055010 GO:0042694
GO:0055003 GO:0005509 GO:0003785 GO:0030016 GO:0015629
Cluster-22178.25368 GO:0006355 GO:0007165 GO:0035257 GO:0003713
GO:0005634 GO:0005667
Cluster-22178.25370 GO:0006355 GO:0046983 GO:0005515
Cluster-22178.25371 GO:0006355 GO:0046983 GO:0005515
Cluster-22178.25374 GO:0046413 GO:0018836 GO:0005515
Cluster-22178.25385 GO:0007165 GO:0006811 GO:0007268 GO:0004970
GO:0016020
Cluster-22178.25386 GO:0007218 GO:0006811 GO:0007268 GO:0007165
GO:0006878 GO:0004970 GO:0005507 GO:0005234 GO:0009279
GO:0016020
Cluster-22178.25390 GO:0006811 GO:0005216 GO:0016020
Cluster-22178.25396 GO:0006468 GO:0004672 GO:0005524
Cluster-22178.25398 GO:0055114 GO:0046332 GO:0004174
Cluster-22178.25399 GO:0004867
Cluster-22178.25400 GO:0007339 GO:0005515 GO:0016020 GO:0005576
Cluster-22178.25401 GO:0007015 GO:0019069 GO:0006897 GO:0005515

Cluster-22178.25402 GO:0007339 GO:0006897 GO:0007015 GO:0019069
GO:0005515 GO:0016020 GO:0005576
Cluster-22178.25405 GO:0032784 GO:0004222 GO:0003677
Cluster-22178.25406 GO:0007015 GO:0019069 GO:0006897 GO:0007339

GO:0005515 GO:0005576 GO:0016020

Cluster-22178.25420 GO:0006094 GO:0006098 GO:0006096 GO:0005982

GO:0005985 GO:0004347 GO:0016021

Cluster-22178.25421 GO:0006812 GO:0032801 GO:0019076 GO:0046935

GO:0005261 GO:0033644 GO:0005944

Cluster-22178.25422 GO:0006355 GO:0003700 GO:0043565 GO:0005667

Cluster-22178.25434 GO:0006508 GO:0004252

Cluster-22178.25438 GO:0006974 GO:0006919 GO:0006915 GO:0006412

GO:0001836 GO:0008168

Cluster-22178.25443 GO:0016226 GO:0008199

Cluster-22178.25444 GO:0016226 GO:0008199

Cluster-22178.25447 GO:0006749 GO:0055114 GO:0006979 GO:0006804

GO:0004602

Cluster-22178.25470 GO:0046497 GO:0009435 GO:0004514

Cluster-22178.25477 GO:0009245 GO:0008915

Cluster-22178.25485 GO:0004190 GO:0008270 GO:0005515 GO:0016021

Cluster-22178.25487 GO:0055114 GO:0016717 GO:0005634

Cluster-22178.25491 GO:0016021

Cluster-22178.25493 GO:0006810 GO:0009405 GO:0008200 GO:0005576

Cluster-22178.25497 GO:0006144 GO:0005524 GO:0003968 GO:0031379

Cluster-22178.255 GO:0006744 GO:0006120 GO:0016310 GO:0015992

GO:0006814 GO:0010124 GO:0009069 GO:0004674 GO:0008137

GO:0005739

Cluster-22178.25501 GO:0006308 GO:0008855 GO:0005515 GO:0005737

GO:0009318 GO:0016021

Cluster-22178.25503 GO:0016861 GO:0016020

Cluster-22178.25504 GO:0016020

Cluster-22178.25506 GO:0007264 GO:0006024 GO:0015684 GO:0015012

GO:0015093 GO:0005524 GO:0005525 GO:0016021

Cluster-22178.25511 GO:0007165 GO:0055114 GO:0004252 GO:0005044

GO:0016641 GO:0070008 GO:0005507 GO:0016020

Cluster-22178.25516 GO:0005515

Cluster-22178.25521 GO:0005515 GO:0008270 GO:0046872

Cluster-22178.25524 GO:0015979 GO:0005515 GO:0005639 GO:0030089

Cluster-22178.2553 GO:0008152 GO:0000226 GO:0016491

Cluster-22178.25535 GO:0005515

Cluster-22178.25539 GO:0005515

Cluster-22178.25545 GO:0006357 GO:0001104 GO:0005515 GO:0016592

Cluster-22178.25550 GO:0006811 GO:0055085 GO:0051260 GO:0006605 GO:0006886 GO:0008013 GO:0005216 GO:0016342 GO:0016592 GO:0016020

Cluster-22178.25553 GO:0045892 GO:0005634

Cluster-22178.25566 GO:0006508 GO:0004197

Cluster-22178.25573 GO:0044038 GO:0016757 GO:0005886 GO:0016021 GO:0016020

Cluster-22178.25578 GO:0019028

Cluster-22178.25584 GO:0005515

Cluster-22178.25589 GO:0006811 GO:0055085 GO:0006816 GO:0006874 GO:0006118 GO:0051536 GO:0009055 GO:0005219 GO:0005216 GO:0005509 GO:0005622 GO:0016021 GO:0016020

Cluster-22178.25593 GO:0006811 GO:0007165 GO:0007268 GO:0004970 GO:0005234 GO:0016020

Cluster-22178.25594 GO:0007165 GO:0006811 GO:0007268 GO:0004970 GO:0005234 GO:0016020

Cluster-22178.25595 GO:0007165 GO:0007268 GO:0006508 GO:0006811 GO:0004222 GO:0004970 GO:0005234 GO:0016020

Cluster-22178.25603 GO:0007186 GO:0004930 GO:0016021 GO:0016020

Cluster-22178.25604 GO:0005515

Cluster-22178.25609 GO:0006355 GO:0003677

Cluster-22178.25611 GO:0006355 GO:0003677

Cluster-22178.25618 GO:0005515

Cluster-22178.25624 GO:0048544 GO:0006508 GO:0008360 GO:0055114 GO:0005509 GO:0005515 GO:0004197 GO:0010277 GO:0016020

Cluster-22178.25633 GO:0016032 GO:0005515

Cluster-22178.25635 GO:0019049 GO:0005515 GO:0008270

Cluster-22178.25641 GO:0006355 GO:0003677 GO:0043565

Cluster-22178.25651 GO:0006771 GO:0019497 GO:0006303 GO:0046872
GO:0005515 GO:0003993
Cluster-22178.25664 GO:0051258 GO:0007049 GO:0008283 GO:0007165
GO:0030168 GO:0015031 GO:0040007 GO:0006886 GO:0015743
GO:0042157 GO:0007155 GO:0006869 GO:0006606 GO:0048193
GO:0008289 GO:0030674 GO:0005102 GO:0005515 GO:0005198
GO:0008083 GO:0005125 GO:0005524 GO:0003774 GO:0008565
GO:0019905 GO:0005576 GO:0016021 GO:0005882 GO:0005737
GO:0016459 GO:0016020 GO:0005577
Cluster-22178.25665 GO:0006508 GO:0042981 GO:0004197
Cluster-22178.25666 GO:0005515 GO:0046872 GO:0045202
Cluster-22178.25686 GO:0034508 GO:0005509 GO:0005515 GO:0000776
Cluster-22178.25687 GO:0034508 GO:0005515 GO:0000776
Cluster-22178.25689 GO:0018342 GO:0008318 GO:0005515
Cluster-22178.25698 GO:0007156 GO:0005509 GO:0016020
Cluster-22178.25699 GO:0009069 GO:0006468 GO:0006506 GO:0051262
GO:0016310 GO:0006811 GO:0055085 GO:0005216 GO:0005524
GO:0004674 GO:0003824 GO:0016020 GO:0005789 GO:0016021
Cluster-22178.25703 GO:0006771 GO:0055085 GO:0019497 GO:0005515
GO:0003993 GO:0046872 GO:0008270 GO:0016020 GO:0016021
Cluster-22178.25706 GO:0007156 GO:0005509 GO:0016020
Cluster-22178.25707 GO:0007017 GO:0007018 GO:0005524 GO:0008017
GO:0003777 GO:0045298 GO:0005874
Cluster-22178.25719 GO:0006547 GO:0000105 GO:0046332 GO:0004635

Cluster-22178.25720 GO:0006547 GO:0000105 GO:0046332 GO:0004635

Cluster-22178.25723 GO:0010181 GO:0016020
Cluster-22178.25724 GO:0010181 GO:0016020
Cluster-22178.25725 GO:0005887
Cluster-22178.25728 GO:0006412 GO:0042254 GO:0003735 GO:0005840
Cluster-22178.25737 GO:0050790 GO:0030234
Cluster-22178.25739 GO:0016021 GO:0019028

Cluster-22178.25749 GO:0005515

Cluster-22178.25759 GO:0032006 GO:0000724 GO:0016197 GO:0030119

Cluster-22178.25760 GO:0005515

Cluster-22178.25762 GO:0009451 GO:0080009 GO:0006396 GO:0008174 GO:0003723 GO:0016746

Cluster-22178.25763 GO:0016746

Cluster-22178.25764 GO:0015031 GO:0008565 GO:0016746 GO:0016021

Cluster-22178.25768 GO:0008270 GO:0005515

Cluster-22178.25773 GO:0006914 GO:0016021

Cluster-22178.25792 GO:0007165 GO:0006952 GO:0005126 GO:0005576

Cluster-22178.25793 GO:0006468 GO:0005524 GO:0004672

Cluster-22178.25795 GO:0016021

Cluster-22178.25803 GO:0006810 GO:0005524

Cluster-22178.25808 GO:0007186 GO:0004930 GO:0016021 GO:0016020

Cluster-22178.25809 GO:0006446 GO:0006413 GO:0003743 GO:0031369 GO:0005840 GO:0005852

Cluster-22178.25811 GO:0009405

Cluster-22178.25821 GO:0006260 GO:0006351 GO:0003677 GO:0003887 GO:0032040 GO:0042575

Cluster-22178.25822 GO:0009405 GO:0005615

Cluster-22178.25823 GO:0008152 GO:0016491

Cluster-22178.25824 GO:0007156 GO:0005509 GO:0016020

Cluster-22178.25827 GO:0030430

Cluster-22178.2583 GO:0051259 GO:0004866 GO:0034618

Cluster-22178.25833 GO:0006810 GO:0055085 GO:0042407 GO:0005215 GO:0016020 GO:0061617 GO:0016021 GO:0044284

Cluster-22178.25835 GO:0005515 GO:0030414

Cluster-22178.25843 GO:0005515

Cluster-22178.25845 GO:0007165 GO:0006825 GO:0006470 GO:0016531 GO:0008138 GO:0005179 GO:0005507 GO:0005576 GO:0005758

Cluster-22178.25849 GO:0006123 GO:0015992 GO:0004129 GO:0045277 GO:0005739

Cluster-22178.25860 GO:0006368 GO:0004867 GO:0005576 GO:0008023

Cluster-22178.25866 GO:0006935 GO:0006955 GO:0007165 GO:0008009 GO:0036128 GO:0005576

Cluster-22178.25870 GO:0007155 GO:0071918 GO:0015840 GO:0005509 GO:0015204 GO:0005515 GO:0005576 GO:0016021

Cluster-22178.25871 GO:0015840 GO:0071918 GO:0007155 GO:0005509 GO:0015204 GO:0005515 GO:0016021 GO:0005576

Cluster-22178.25876 GO:0007015 GO:0019069 GO:0007339 GO:0006897 GO:0005515 GO:0005576 GO:0016020

Cluster-22178.25877 GO:0008654 GO:0043772 GO:0005886

Cluster-22178.25878 GO:0008270 GO:0003677 GO:0005515

Cluster-22178.25879 GO:0009405 GO:0045502 GO:0008134 GO:0042803 GO:0005667 GO:0030286 GO:0005576

Cluster-22178.25880 GO:0016567 GO:0005515 GO:0004842

Cluster-22178.25889 GO:0005515

Cluster-22178.25900 GO:0005515

Cluster-22178.25905 GO:0005515

Cluster-22178.25917 GO:0019013

Cluster-22178.25920 GO:0009405 GO:0006810 GO:0009755 GO:0008200 GO:0005576

Cluster-22178.25923 GO:0019013

Cluster-22178.25924 GO:0006935 GO:0006955 GO:0007165 GO:0006355 GO:0003700 GO:0008009 GO:0005667 GO:0005576

Cluster-22178.25927 GO:0055085 GO:0022857 GO:0016021

Cluster-22178.25929 GO:0006812 GO:0006820 GO:0006835 GO:0017153 GO:0016020

Cluster-22178.25935 GO:0007165 GO:0032509 GO:0006355 GO:0005179 GO:0043130 GO:0005634 GO:0005576 GO:0000813

Cluster-22178.25938 GO:0005515

Cluster-22178.25940 GO:0006355 GO:0042967 GO:0032259 GO:0003723 GO:0004402 GO:0003712 GO:0008270 GO:0005845 GO:0005667 GO:0005634 GO:0000123

Cluster-22178.25948 GO:0006396 GO:0005515 GO:0000176

Cluster-22178.25950 GO:0016032

Cluster-22178.25961 GO:0005515
Cluster-22178.25968 GO:0055114 GO:0008152 GO:0016491 GO:0051287

Cluster-22178.25969 GO:0008152 GO:0055114 GO:0016491 GO:0051287

Cluster-22178.25970 GO:0007165 GO:0051260 GO:0006811 GO:0055085 GO:0005216 GO:0000772 GO:0016020
Cluster-22178.25973 GO:0000746 GO:0006355 GO:0006468 GO:0005515 GO:0003700 GO:0004672 GO:0005524 GO:0005667
Cluster-22178.25974 GO:0000746 GO:0006468 GO:0005515 GO:0005524 GO:0004672
Cluster-22178.25976 GO:0046034 GO:0035556 GO:0008608 GO:0006144 GO:0046872 GO:0004017 GO:0005876 GO:0042729
Cluster-22178.25979 GO:0008033 GO:0055114 GO:0020037 GO:0016705 GO:0005506
Cluster-22178.25986 GO:0051726 GO:0005634
Cluster-22178.25987 GO:0051726 GO:0005634
Cluster-22178.25992 GO:0055114 GO:0005515 GO:0046422 GO:0016020 GO:0009507
Cluster-22178.2600 GO:0007165 GO:0005515
Cluster-22178.26005 GO:0016310 GO:0006468 GO:0009069 GO:0004674 GO:0004672 GO:0005524
Cluster-22178.26006 GO:0009069 GO:0006468 GO:0016310 GO:0005524 GO:0004672 GO:0004674
Cluster-22178.26007 GO:0005515
Cluster-22178.26021 GO:0006281 GO:0046872 GO:0005506 GO:0008270

Cluster-22178.26022 GO:0007165 GO:0005044 GO:0070008 GO:0004252 GO:0016020
Cluster-22178.26039 GO:0005515
Cluster-22178.26041 GO:0006508 GO:0009405 GO:0008270 GO:0004222 GO:0031012 GO:0005576
Cluster-22178.26043 GO:0007275 GO:0005201 GO:0005578 GO:0005581

Cluster-22178.26046 GO:0006308 GO:0006914 GO:0007264 GO:0042073
GO:0007017 GO:0007018 GO:0005524 GO:0016887 GO:0008855
GO:0005525 GO:0003777 GO:0030286 GO:0005874 GO:0016021
GO:0009318
Cluster-22178.26047 GO:0007186 GO:0005515 GO:0004930
Cluster-22178.26055 GO:0051082 GO:0005515
Cluster-22178.26056 GO:0016020 GO:0016021
Cluster-22178.26057 GO:0005515 GO:0005509
Cluster-22178.26069 GO:0006144 GO:0003968 GO:0005524 GO:0031379
Cluster-22178.26071 GO:0005515
Cluster-22178.26078 GO:0000166 GO:0046872
Cluster-22178.26082 GO:0031556 GO:0031554
Cluster-22178.26093 GO:0061630
Cluster-22178.26105 GO:0006355 GO:0008270 GO:0043565 GO:0003700
GO:0005667 GO:0019031
Cluster-22178.26107 GO:0003950
Cluster-22178.26118 GO:0009094 GO:0000162 GO:0006571 GO:0004664

Cluster-22178.26119 GO:0016485 GO:0007160 GO:0009405 GO:0005509
GO:0005515 GO:0001527 GO:0016021 GO:0005840
Cluster-22178.26120 GO:0009405 GO:0007165 GO:0007160 GO:0007166
GO:0016485 GO:0005509 GO:0005515 GO:0004871 GO:0005634
GO:0005840 GO:0001527 GO:0016021
Cluster-22178.26121 GO:0051258 GO:0030168 GO:0048193 GO:0007165
GO:0005102 GO:0030674 GO:0005577 GO:0016020
Cluster-22178.26125 GO:0006355 GO:0006270 GO:0008270 GO:0003677

Cluster-22178.26127 GO:0042157 GO:0006869 GO:0008289 GO:0005576
Cluster-22178.26131 GO:0016020
Cluster-22178.26140 GO:0055085 GO:0016021
Cluster-22178.26142 GO:0006816 GO:0006811 GO:0005244 GO:0005262
GO:0016021
Cluster-22178.26147 GO:0006807 GO:0019497 GO:0009252 GO:0006771

GO:0003993 GO:0046872 GO:0004356 GO:0005515

Cluster-22178.26155 GO:0005515

Cluster-22178.26157 GO:0051258 GO:0030168 GO:0007165 GO:0030674

GO:0005102 GO:0005577

Cluster-22178.26158 GO:0005515

Cluster-22178.26160 GO:0005515

Cluster-22178.26174 GO:0055114 GO:0000105 GO:0006547 GO:0000287

GO:0016641 GO:0005507 GO:0003879 GO:0005737

Cluster-22178.26176 GO:0006468 GO:0016773 GO:0004672 GO:0005524

GO:0016020

Cluster-22178.26183 GO:0007186 GO:0042246 GO:0007155 GO:0004930

GO:0016021

Cluster-22178.26185 GO:0006811 GO:0055085 GO:0005216 GO:0016020

Cluster-22178.26190 GO:0043087 GO:0032012 GO:0005086

Cluster-22178.26201 GO:0008270 GO:0005921

Cluster-22178.26202 GO:0006355 GO:0003677

Cluster-22178.26203 GO:0032481 GO:0002218

Cluster-22178.26205 GO:0016055 GO:0008013 GO:0016342

Cluster-22178.26208 GO:0019031

Cluster-22178.26209 GO:0019031

Cluster-22178.26211 GO:0008272 GO:0006508 GO:0007165 GO:0006144

GO:0004114 GO:0005515 GO:0015116 GO:0004222 GO:0016020

GO:0016021

Cluster-22178.26212 GO:0009405 GO:0016192 GO:0006886 GO:0006508

GO:0004198 GO:0005509 GO:0030131 GO:0005622

Cluster-22178.26218 GO:0006810 GO:0006355 GO:0006260 GO:0007049

GO:0006914 GO:0016788 GO:0003700 GO:0016881 GO:0043565

GO:0003774 GO:0008270 GO:0005634 GO:0005667 GO:0016459

GO:0016021

Cluster-22178.26221 GO:0007186 GO:0004930 GO:0005515 GO:0016020

Cluster-22178.26223 GO:0046486 GO:0007165 GO:0040007 GO:0008283

GO:0008654 GO:0006464 GO:0008083 GO:0008715 GO:0003697

GO:0016020

Cluster-22178.26224 GO:0008283 GO:0040007 GO:0007165 GO:0046486 GO:0006464 GO:0016226 GO:0008654 GO:0008199 GO:0003697 GO:0008715 GO:0008083 GO:0016020

Cluster-22178.26225 GO:0008654 GO:0006464 GO:0007165 GO:0046486 GO:0008283 GO:0040007 GO:0008715 GO:0008083 GO:0003697 GO:0016020

Cluster-22178.26226 GO:0008654 GO:0006464 GO:0007165 GO:0046486 GO:0008283 GO:0040007 GO:0008715 GO:0008083 GO:0003697 GO:0016020

Cluster-22178.26227 GO:0055114 GO:0006886 GO:0006606 GO:0005506 GO:0016705 GO:0020037 GO:0016020

Cluster-22178.26228 GO:0006606 GO:0055114 GO:0006886 GO:0010508 GO:0020037 GO:0016705 GO:0005506 GO:0016020

Cluster-22178.26229 GO:0006886 GO:0006535 GO:0006606 GO:0006534 GO:0042967 GO:0051607 GO:0055114 GO:0009001 GO:0020037 GO:0016705 GO:0005506 GO:0016020 GO:0005737

Cluster-22178.26233 GO:0006606 GO:0055114 GO:0016705 GO:0020037 GO:0005506

Cluster-22178.26236 GO:0006886 GO:0006606 GO:0055114 GO:0016705 GO:0020037 GO:0005506 GO:0016020

Cluster-22178.26237 GO:0055114 GO:0006886 GO:0006606 GO:0005506 GO:0016705 GO:0020037 GO:0016020

Cluster-22178.26238 GO:0006606 GO:0055114 GO:0020037 GO:0016705 GO:0005506

Cluster-22178.26240 GO:0008152 GO:0016491

Cluster-22178.26249 GO:0055114 GO:0006090 GO:0006120 GO:0006813 GO:0006525 GO:0045859 GO:0006560 GO:0071436 GO:0006814 GO:0015081 GO:0005267 GO:0016534 GO:0008948 GO:0016533 GO:0016020

Cluster-22178.26250 GO:0006260 GO:0007067 GO:0055114 GO:0007049 GO:0006352 GO:0006120 GO:0006355 GO:0006813 GO:0045859 GO:0016987 GO:0003677 GO:0016534 GO:0005267 GO:0003700 GO:0003887 GO:0005667 GO:0016020 GO:0016533 GO:0000775

GO:0005634 GO:0042575
Cluster-22178.26251 GO:0006120 GO:0055114 GO:0045859 GO:0006813
GO:0016534 GO:0005267 GO:0016533
Cluster-22178.26260 GO:0016021
Cluster-22178.26261 GO:0016021
Cluster-22178.26262 GO:0016021
Cluster-22178.26284 GO:0018342 GO:0008318
Cluster-22178.26288 GO:0006816 GO:0005388 GO:0016529
Cluster-22178.26290 GO:0005515
Cluster-22178.26292 GO:0008270
Cluster-22178.26297 GO:0006508 GO:0007165 GO:0005044 GO:0004252
GO:0016020
Cluster-22178.26301 GO:0007165 GO:0006355 GO:0004872 GO:0003700
GO:0016021 GO:0005667
Cluster-22178.26303 GO:0005515 GO:0016021
Cluster-22178.26308 GO:0006260 GO:0003678 GO:0005524 GO:0005657
Cluster-22178.26309 GO:0003333 GO:0015979 GO:0016020 GO:0009539
GO:0009523
Cluster-22178.26317 GO:0006890
Cluster-22178.26323 GO:0006506 GO:0017176 GO:0016021
Cluster-22178.26335 GO:0042312 GO:0008217 GO:0007165 GO:0005179
GO:0005576
Cluster-22178.26341 GO:0006814 GO:0016829 GO:0016020
Cluster-22178.26346 GO:0008152 GO:0015074 GO:0003824 GO:0016021
Cluster-22178.26348 GO:0042998 GO:0007155 GO:0007339 GO:0071253
GO:0005515 GO:0016021 GO:0005576 GO:0005802
Cluster-22178.26351 GO:0006265 GO:0003677 GO:0003916
Cluster-22178.26355 GO:0008608 GO:0008152 GO:0003824 GO:0016791
GO:0046872 GO:0042729 GO:0072686
Cluster-22178.26362 GO:0006355 GO:0003700 GO:0003677 GO:0005667
Cluster-22178.26364 GO:0006355 GO:0005515 GO:0003700 GO:0043565
GO:0005667 GO:0005634
Cluster-22178.26365 GO:0007165 GO:0005179 GO:0005576

Cluster-22178.26385 GO:0006468 GO:0005515 GO:0005524 GO:0004672

Cluster-22178.26390 GO:0006106 GO:0015833 GO:0005524 GO:0008270
GO:0046872 GO:0000166 GO:0005515 GO:0016020
Cluster-22178.26391 GO:0005515 GO:0008270 GO:0046872
Cluster-22178.26393 GO:0003676 GO:0003723
Cluster-22178.26394 GO:0003723 GO:0003676
Cluster-22178.26395 GO:0046872 GO:0003676
Cluster-22178.26399 GO:0006470 GO:0006570 GO:0019988 GO:0043399
GO:0008138 GO:0004725 GO:0016787
Cluster-22178.26404 GO:0015986 GO:0007155 GO:0015992 GO:0005540
GO:0016301 GO:0015078 GO:0005524 GO:0000276
Cluster-22178.26405 GO:0071988 GO:0030474 GO:0005515
Cluster-22178.26406 GO:0015031 GO:0008277 GO:0009372 GO:0006886
GO:0009405 GO:0008233 GO:0008565 GO:0005515 GO:0016021
Cluster-22178.26409 GO:0051252 GO:0015074 GO:0006402 GO:0003723
GO:0046872 GO:0004535 GO:0003676 GO:0005634 GO:0005737
Cluster-22178.26414 GO:0007156 GO:0005509 GO:0016020
Cluster-22178.26422 GO:0046487 GO:0055114 GO:0009396 GO:0003824
GO:0005515 GO:0004488 GO:0005739
Cluster-22178.26423 GO:0003333 GO:0006810 GO:0006865 GO:0055085
GO:0015171 GO:0016020
Cluster-22178.26434 GO:0006811 GO:0055085 GO:0006118 GO:0046325
GO:0009055 GO:0051536 GO:0005216 GO:0016020
Cluster-22178.26435 GO:0008272 GO:0015116 GO:0016021
Cluster-22178.26444 GO:0055114 GO:0050660 GO:0030246 GO:0016491

Cluster-22178.26455 GO:0005515
Cluster-22178.26456 GO:0006508 GO:0042254 GO:0006412 GO:0016787
GO:0003735 GO:0005515 GO:0008236 GO:0042802 GO:0005622
GO:0005840
Cluster-22178.26457 GO:0006508 GO:0042254 GO:0006412 GO:0016787
GO:0005515 GO:0008236 GO:0042802 GO:0003735 GO:0005622

GO:0005840
Cluster-22178.26458 GO:0006508 GO:0042254 GO:0006412 GO:0016787
GO:0005515 GO:0042802 GO:0008236 GO:0003735 GO:0005622
GO:0005840
Cluster-22178.26471 GO:0006355 GO:0003677 GO:0005515 GO:0008270
GO:0046872
Cluster-22178.2648 GO:0006955 GO:0007165 GO:0005164 GO:0016020
Cluster-22178.26480 GO:0005515 GO:0016020
Cluster-22178.26483 GO:0009058 GO:0006687 GO:0001733 GO:0005794
GO:0016021
Cluster-22178.26488 GO:0006281 GO:0000049 GO:0005509 GO:0005515
GO:0005634
Cluster-22178.26489 GO:0046983 GO:0005515 GO:0003677
Cluster-22178.26493 GO:0006308 GO:0007018 GO:0007017 GO:0003777
GO:0008855 GO:0005874 GO:0030286 GO:0009318
Cluster-22178.26495 GO:0015694 GO:0005515 GO:0015097 GO:0016020
Cluster-22178.26496 GO:0015840 GO:0007155 GO:0071918 GO:0005509
GO:0015204 GO:0005515 GO:0016021 GO:0005576
Cluster-22178.26500 GO:0007166 GO:0007186 GO:0004930 GO:0016021
GO:0016020
Cluster-22178.26502 GO:0007165 GO:0016255 GO:0005179 GO:0008270
GO:0005576 GO:0042765
Cluster-22178.26507 GO:0005515
Cluster-22178.26508 GO:0005515
Cluster-22178.26518 GO:0016255 GO:0007304 GO:0009415 GO:0006950
GO:0005213 GO:0005576 GO:0042765 GO:0042600
Cluster-22178.26524 GO:0006468 GO:0016872 GO:0004672 GO:0005524
GO:0003723
Cluster-22178.26531 GO:0016567 GO:0048870 GO:0006465 GO:0005515
GO:0046872 GO:0008270 GO:0008233 GO:0004842 GO:0048786
GO:0019028 GO:0005787 GO:0031514 GO:0005622 GO:0016021
GO:0005680
Cluster-22178.26532 GO:0007165 GO:0005515 GO:0043130 GO:0032266

Cluster-22178.26535 GO:0005515
Cluster-22178.26549 GO:0006817
Cluster-22178.26553 GO:0005515
Cluster-22178.26569 GO:0033617
Cluster-22178.26573 GO:0006810 GO:0016820 GO:0005215 GO:0005524
GO:0043190
Cluster-22178.26574 GO:0006771 GO:0006807 GO:0009252 GO:0019497
GO:0004356 GO:0005515 GO:0003993 GO:0046872
Cluster-22178.26576 GO:0006771 GO:0009252 GO:0019497 GO:0006807
GO:0005515 GO:0004356 GO:0046872 GO:0003993
Cluster-22178.26578 GO:0006415 GO:0006449 GO:0003747 GO:0018444
GO:0005840
Cluster-22178.26585 GO:0022900 GO:0005739 GO:0005747
Cluster-22178.26592 GO:0055114 GO:0008270 GO:0047134 GO:0005622
Cluster-22178.26599 GO:0015931 GO:0033227 GO:0006468 GO:0005524
GO:0004672 GO:0051033 GO:0005515 GO:0016021
Cluster-22178.26604 GO:0016310 GO:0009069 GO:0004674
Cluster-22178.26613 GO:0035299 GO:0005524
Cluster-22178.26616 GO:0006904 GO:0034453 GO:0043130 GO:0005515
GO:0005737 GO:0005815 GO:0000145
Cluster-22178.26626 GO:0006468 GO:0048511 GO:0005524 GO:0004672

Cluster-22178.26627 GO:0005515
Cluster-22178.26631 GO:0055114 GO:0007165 GO:0016972
Cluster-22178.26633 GO:0007186 GO:0005515 GO:0004930
Cluster-22178.26643 GO:0008608 GO:0042729 GO:0005876
Cluster-22178.26644 GO:0042262 GO:0007165 GO:0051262 GO:0032784
GO:0003677 GO:0003690
Cluster-22178.26656 GO:0006508 GO:0004222
Cluster-22178.26657 GO:0016055 GO:0006298 GO:0007165 GO:0007275
GO:0030983 GO:0005524 GO:0005102 GO:0005576
Cluster-22178.26660 GO:0006118 GO:0006814 GO:0015992 GO:0006120

GO:0006744 GO:0003954 GO:0008137 GO:0005739
Cluster-22178.26661 GO:0006744 GO:0006120 GO:0015992 GO:0006814
GO:0006118 GO:0008137 GO:0003954 GO:0005739
Cluster-22178.26664 GO:0006355 GO:0006412 GO:0042254 GO:0006352
GO:0003735 GO:0003677 GO:0003700 GO:0016987 GO:0005667
GO:0005840
Cluster-22178.26665 GO:0005515 GO:0005509
Cluster-22178.26666 GO:0005509 GO:0005515
Cluster-22178.26667 GO:0006355 GO:0046983 GO:0003677 GO:0005515
GO:0003700 GO:0005667 GO:0005634
Cluster-22178.26668 GO:0008152 GO:0016491
Cluster-22178.26676 GO:0009405 GO:0016747 GO:0016020 GO:0016021
GO:0005576
Cluster-22178.26678 GO:0006468 GO:0005524 GO:0004672
Cluster-22178.26680 GO:0007186 GO:0019079 GO:0006260 GO:0030337
GO:0004930 GO:0016020 GO:0042575 GO:0016021
Cluster-22178.26681 GO:0007186 GO:0004930 GO:0016020 GO:0016021
Cluster-22178.26685 GO:0006570 GO:0007186 GO:0006470 GO:0050852
GO:0004725 GO:0004930 GO:0016020 GO:0016021
Cluster-22178.26686 GO:0006570 GO:0007186 GO:0050852 GO:0006470
GO:0004725 GO:0004930 GO:0016020 GO:0016021
Cluster-22178.26687 GO:0006470 GO:0050852 GO:0006570 GO:0007186
GO:0004725 GO:0004930 GO:0016021 GO:0016020
Cluster-22178.26688 GO:0006470 GO:0050852 GO:0006570 GO:0007186
GO:0007156 GO:0004930 GO:0004725 GO:0005509 GO:0016021
GO:0016020
Cluster-22178.26689 GO:0007156 GO:0006570 GO:0007186 GO:0050852
GO:0006470 GO:0005509 GO:0004930 GO:0004725 GO:0016020
GO:0016021
Cluster-22178.26691 GO:0008270 GO:0005887 GO:0005789
Cluster-22178.26713 GO:0016021
Cluster-22178.26719 GO:0007155 GO:0009847 GO:0005540
Cluster-22178.26721 GO:0006468 GO:0004672 GO:0005524

Cluster-22178.26725 GO:0006144 GO:0006206 GO:0006470 GO:0006570
GO:0006351 GO:0004725 GO:0003899 GO:0008138 GO:0005730
Cluster-22178.26726 GO:0009405 GO:0005576
Cluster-22178.26728 GO:0008376 GO:0005515 GO:0017176 GO:0008270
GO:0016020 GO:0032580 GO:0005622
Cluster-22178.26729 GO:0071816 GO:0003677
Cluster-22178.26733 GO:0019050 GO:0008270 GO:0003676 GO:0005622
Cluster-22178.26737 GO:0070836 GO:0055085 GO:0022857 GO:0016021
Cluster-22178.26756 GO:0007186 GO:0004930 GO:0016021
Cluster-22178.26758 GO:0030154 GO:0005509 GO:0005544 GO:0016021
Cluster-22178.26759 GO:0007165 GO:0006810 GO:0009405 GO:0005515
GO:0005509 GO:0019871 GO:0004888 GO:0016021 GO:0005576
Cluster-22178.2676 GO:0006260 GO:0006355 GO:0003700 GO:0005667
GO:0005634
Cluster-22178.26768 GO:0055114 GO:0051920 GO:0016020
Cluster-22178.26770 GO:0055114 GO:0051920
Cluster-22178.26771 GO:0006355 GO:0003677
Cluster-22178.26772 GO:0055114 GO:0051920
Cluster-22178.26781 GO:0035556 GO:0009395 GO:0046339 GO:0007165
GO:0006629 GO:0004435 GO:0005515 GO:0008061
Cluster-22178.26786 GO:0007186 GO:0007166 GO:0006547 GO:0000105
GO:0003729 GO:0004635 GO:0004930 GO:0016020 GO:0016021
Cluster-22178.26789 GO:0006355 GO:0005975 GO:0003682 GO:0043169
GO:0003677 GO:0008270 GO:0003824 GO:0005634 GO:0000785
GO:0000775
Cluster-22178.26793 GO:0033926
Cluster-22178.26794 GO:0033926
Cluster-22178.26796 GO:0006260 GO:0005524 GO:0003678 GO:0005657
Cluster-22178.26799 GO:0006725 GO:0055114 GO:0006265 GO:0019337
GO:0006807 GO:0009399 GO:0003690 GO:0016853 GO:0003677
GO:0016163 GO:0016610
Cluster-22178.26802 GO:0005515
Cluster-22178.26805 GO:0009231 GO:0007275 GO:0008686 GO:0005578

Cluster-22178.26806 GO:0007275 GO:0009231 GO:0008686 GO:0005578
Cluster-22178.26807 GO:0006820 GO:0006835 GO:0006812 GO:0007165
GO:0005102 GO:0017153 GO:0016020
Cluster-22178.26811 GO:0005525 GO:0016887 GO:0005524
Cluster-22178.26818 GO:0055114 GO:0015946 GO:0043087 GO:0006355
GO:0003700 GO:0004022 GO:0005515 GO:0042025 GO:0005667
Cluster-22178.26820 GO:0008270
Cluster-22178.26828 GO:0016337 GO:0016020
Cluster-22178.26830 GO:0007165 GO:0005179 GO:0005576 GO:0009512
Cluster-22178.26834 GO:0009607 GO:0006811 GO:0055085 GO:0005216
GO:0019031 GO:0016021 GO:0016020
Cluster-22178.26845 GO:0006508 GO:0005985 GO:0006887 GO:0005982
GO:0006144 GO:0006075 GO:0004252 GO:0003843 GO:0003968
GO:0017111 GO:0004197 GO:0000148 GO:0031379 GO:0000145
GO:0016020 GO:0030430
Cluster-22178.26847 GO:0008289
Cluster-22178.26849 GO:0008289
Cluster-22178.26854 GO:0006355 GO:0043401 GO:0005515 GO:0043565
GO:0008270 GO:0003700 GO:0005667 GO:0005634
Cluster-22178.26858 GO:0015986 GO:0015992 GO:0015078 GO:0005515
GO:0000276
Cluster-22178.26860 GO:0005634
Cluster-22178.26861 GO:0006825 GO:0007165 GO:0005507 GO:0016531
GO:0005179 GO:0005758 GO:0019028 GO:0005576
Cluster-22178.26866 GO:0006355 GO:0005515 GO:0016021 GO:0005634
Cluster-22178.26867 GO:0006355 GO:0005515 GO:0005634 GO:0016021
Cluster-22178.26878 GO:0005515 GO:0004190 GO:0008270 GO:0016021
Cluster-22178.26881 GO:0007186 GO:0004930 GO:0016020 GO:0016021
Cluster-22178.26884 GO:0003950 GO:0031224
Cluster-22178.26888 GO:0007165 GO:0006144 GO:0004114
Cluster-22178.26892 GO:0007160 GO:0007165 GO:0006004 GO:0006000
GO:0006914 GO:0006013 GO:0007166 GO:0009405 GO:0016485
GO:0048870 GO:0005509 GO:0005515 GO:0008134 GO:0042803

GO:0008736 GO:0045502 GO:0004871 GO:0008092 GO:0005667
GO:0019898 GO:0030286 GO:0031514 GO:0005634 GO:0016021
GO:0001527 GO:0005840 GO:0005737
Cluster-22178.26895 GO:0016032
Cluster-22178.26896 GO:0005515
Cluster-22178.26906 GO:0006144 GO:0004114
Cluster-22178.26909 GO:0006313 GO:0055114 GO:0004803 GO:0003677
GO:0016491
Cluster-22178.26910 GO:0006313 GO:0055114 GO:0003677 GO:0004803
GO:0016491
Cluster-22178.26914 GO:0006812 GO:0006820 GO:0006835 GO:0017153
GO:0016020
Cluster-22178.26930 GO:0046325
Cluster-22178.26932 GO:0009069 GO:0015979 GO:0030494 GO:0016310
GO:0005515 GO:0004674
Cluster-22178.26933 GO:0019058 GO:0006260 GO:0005198 GO:0005515
GO:0019028 GO:0005882 GO:0034464
Cluster-22178.26934 GO:0007165 GO:0005179 GO:0005576
Cluster-22178.26936 GO:0022900 GO:0016651
Cluster-22178.26949 GO:0006260 GO:0006351 GO:0003677 GO:0003887
GO:0042575
Cluster-22178.26951 GO:0006355 GO:0005515 GO:0005634 GO:0005668
Cluster-22178.26952 GO:0006355 GO:0005668 GO:0005634
Cluster-22178.26958 GO:0016787
Cluster-22178.26960 GO:0016787 GO:0005515
Cluster-22178.26965 GO:0046740 GO:0005509 GO:0003677 GO:0033644
GO:0042765 GO:0016021
Cluster-22178.26972 GO:0005515
Cluster-22178.26980 GO:0007016 GO:0006811 GO:0005216 GO:0005515
GO:0016020 GO:0016021 GO:0016010
Cluster-22178.26983 GO:0006468 GO:0004672 GO:0005524 GO:0016773
GO:0016020
Cluster-22178.26989 GO:0003723 GO:0009279

Cluster-22178.26993 GO:0007154 GO:0005509 GO:0016020
Cluster-22178.26996 GO:0005515
Cluster-22178.26999 GO:0032006
Cluster-22178.2700 GO:0006304 GO:0006811 GO:0055085 GO:0005216
GO:0003677 GO:0043565 GO:0003824 GO:0016020
Cluster-22178.27003 GO:0040007 GO:0008283 GO:0007165 GO:0008083
GO:0005515 GO:0005634
Cluster-22178.27013 GO:0006144 GO:0007186 GO:0003968 GO:0005515
GO:0004930 GO:0031379
Cluster-22178.27014 GO:0016032 GO:0019031
Cluster-22178.27028 GO:0016787 GO:0005515
Cluster-22178.27035 GO:0006457 GO:0006355 GO:0003700 GO:0043565
GO:0005515 GO:0003677 GO:0051082 GO:0016272 GO:0005634
GO:0005667
Cluster-22178.27037 GO:0008152 GO:0008134 GO:0003824 GO:0005634
GO:0005667
Cluster-22178.27038 GO:0006744
Cluster-22178.27042 GO:0006811 GO:0055085 GO:0005515 GO:0005216
GO:0016020
Cluster-22178.27050 GO:0007264 GO:0005525
Cluster-22178.27055 GO:0043565 GO:0005515 GO:0005634
Cluster-22178.27076 GO:0040007 GO:0050790 GO:0008283 GO:0007186
GO:0007165 GO:0008083 GO:0030234 GO:0005125 GO:0004222
GO:0004930 GO:0016021
Cluster-22178.27085 GO:0006351 GO:0006281 GO:0006355 GO:0006144
GO:0006206 GO:0003899 GO:0003677 GO:0008168 GO:0008270
GO:0046872 GO:0005730
Cluster-22178.27086 GO:0006206 GO:0006144 GO:0006281 GO:0006355
GO:0006351 GO:0008270 GO:0046872 GO:0008168 GO:0003677
GO:0003899 GO:0005730
Cluster-22178.27088 GO:0019239
Cluster-22178.27089 GO:0019239
Cluster-22178.27090 GO:0005509 GO:0005515 GO:0005544

Cluster-22178.27092 GO:0019069 GO:0009245 GO:0005524 GO:0008915

Cluster-22178.27096 GO:0055085 GO:0006811 GO:0005216 GO:0005515 GO:0016020
Cluster-22178.27106 GO:0006508 GO:0004222 GO:0008270 GO:0031012
Cluster-22178.27107 GO:0003723 GO:0003676
Cluster-22178.2711 GO:0006098 GO:0018874 GO:0006554 GO:0006550 GO:0006694 GO:0016114 GO:0015940 GO:0019354 GO:0006631 GO:0046168 GO:0006633 GO:0006779 GO:0016117 GO:0006813 GO:0006568 GO:0006552 GO:0006574 GO:0019521 GO:0008033 GO:0055114 GO:0051287 GO:0004616 GO:0016491 GO:0050660 GO:0003857 GO:0008677 GO:0071949 GO:0016616 GO:0004506 GO:0016705 GO:0043115 GO:0016021
Cluster-22178.2712 GO:0006888 GO:0051539 GO:0005622
Cluster-22178.27122 GO:0009117 GO:0030639 GO:0019872 GO:0008830

Cluster-22178.27128 GO:0016021
Cluster-22178.27130 GO:0003950 GO:0005634
Cluster-22178.27132 GO:0050909 GO:0007606 GO:0007186 GO:0009607 GO:0019236 GO:0016503 GO:0004930 GO:0016021
Cluster-22178.27136 GO:0008152 GO:0030695 GO:0016746 GO:0005856
Cluster-22178.27145 GO:0051258 GO:0030168 GO:0007165 GO:0006457 GO:0005102 GO:0000774 GO:0051087 GO:0042803 GO:0030674 GO:0005577
Cluster-22178.27147 GO:0007165 GO:0006457 GO:0030168 GO:0051258 GO:0030674 GO:0051087 GO:0042803 GO:0000774 GO:0005102 GO:0005577
Cluster-22178.27151 GO:0007165 GO:0006470 GO:0006825 GO:0016531 GO:0008138 GO:0005179 GO:0005507 GO:0005576 GO:0005758
Cluster-22178.27152 GO:0006825 GO:0007165 GO:0005507 GO:0016531 GO:0005179 GO:0005758 GO:0019028 GO:0005576
Cluster-22178.27157 GO:0005515
Cluster-22178.27158 GO:0009058 GO:0030170

Cluster-22178.27159 GO:0009058 GO:0006355 GO:0003677 GO:0030170

Cluster-22178.27160 GO:0006629 GO:0016627 GO:0016021 GO:0005737
Cluster-22178.27171 GO:0006952 GO:0016747 GO:0005515
Cluster-22178.27172 GO:0005515
Cluster-22178.27173 GO:0006952 GO:0005515 GO:0016747
Cluster-22178.27192 GO:0016310 GO:0009069 GO:0006468 GO:0006629
GO:0007010 GO:0005524 GO:0004672 GO:0003779 GO:0004674
Cluster-22178.27197 GO:0009405 GO:0006955 GO:0051258 GO:0042262
GO:0007165 GO:0030168 GO:0006914 GO:0006606 GO:0010506
GO:0045502 GO:0008134 GO:0042803 GO:0005198 GO:0005126
GO:0030674 GO:0003690 GO:0005102 GO:0005576 GO:0005615
GO:0005667 GO:0005882 GO:0016020 GO:0030286 GO:0005577
Cluster-22178.27211 GO:0006810 GO:0005215 GO:0005515 GO:0003824
GO:0016021 GO:0005737
Cluster-22178.27214 GO:0006886 GO:0016192 GO:0006810 GO:0003824
GO:0005215 GO:0005198 GO:0005515 GO:0016021 GO:0005737
GO:0030117
Cluster-22178.27217 GO:0006810 GO:0050821 GO:0015979 GO:0005515
GO:0042301 GO:0005215 GO:0003824 GO:0016020 GO:0005737
GO:0009523 GO:0016021
Cluster-22178.27221 GO:0005515 GO:0042802
Cluster-22178.27232 GO:0030435 GO:0005515 GO:0031160
Cluster-22178.27237 GO:0005515
Cluster-22178.27251 GO:0007165 GO:0006955 GO:0005044 GO:0030247

Cluster-22178.27252 GO:0008152 GO:0055114 GO:0016491
Cluster-22178.27255 GO:0006351 GO:0006206 GO:0006144 GO:0003677
GO:0003899 GO:0005515 GO:0005730 GO:0016021
Cluster-22178.27258 GO:0035556 GO:0007165 GO:0005509 GO:0016301
GO:0005578
Cluster-22178.27262 GO:0007165 GO:0035556 GO:0016301 GO:0005509
GO:0005578

Cluster-22178.27264 GO:0006810 GO:0009405 GO:0015684 GO:0007264
GO:0005525 GO:0043531 GO:0005524 GO:0015093 GO:0019870
GO:0016021 GO:0005576
Cluster-22178.27268 GO:0007009 GO:0007017 GO:0007018 GO:0003777
GO:0005515 GO:0030286 GO:0005874 GO:0019031
Cluster-22178.27271 GO:0006810 GO:0015986 GO:0042626 GO:0033178
Cluster-22178.27289 GO:0007186 GO:0004930 GO:0016021
Cluster-22178.27294 GO:0008152 GO:0016787
Cluster-22178.27301 GO:0055085 GO:0022857 GO:0016021
Cluster-22178.27306 GO:0055114 GO:0016491
Cluster-22178.27307 GO:0005515
Cluster-22178.27308 GO:0005515
Cluster-22178.27324 GO:0006865 GO:0016020
Cluster-22178.27329 GO:0016192 GO:0006886 GO:0030131 GO:0030117
Cluster-22178.27330 GO:0016192 GO:0006886 GO:0009451 GO:0006370
GO:0004482 GO:0030131 GO:0030117
Cluster-22178.27337 GO:0005515
Cluster-22178.27353 GO:0006355 GO:0005634
Cluster-22178.27357 GO:0007186 GO:0005515 GO:0004930 GO:0016020
Cluster-22178.27358 GO:0008277 GO:0006886 GO:0009372 GO:0009405
GO:0015031 GO:0005515 GO:0008233 GO:0008565 GO:0016021
Cluster-22178.27360 GO:0007186 GO:0004930 GO:0005515 GO:0016021
GO:0016020
Cluster-22178.27363 GO:0007186 GO:0004930 GO:0005515 GO:0016020
GO:0016021
Cluster-22178.27368 GO:0042802
Cluster-22178.27369 GO:0042802
Cluster-22178.27372 GO:0019497 GO:0006771 GO:0046872 GO:0005515
GO:0016788 GO:0003993
Cluster-22178.27373 GO:0006000 GO:0006013 GO:0019299 GO:0008740
GO:0030145
Cluster-22178.27375 GO:0007017 GO:0007018 GO:0005524 GO:0008017
GO:0003777 GO:0045298 GO:0005874

Cluster-22178.27376 GO:0005515 GO:0016021
Cluster-22178.27379 GO:0008233 GO:0004222 GO:0031012
Cluster-22178.27380 GO:0006281 GO:0051382
Cluster-22178.27386 GO:0005515
Cluster-22178.27388 GO:0005515 GO:0008270
Cluster-22178.27392 GO:0005515 GO:0042720
Cluster-22178.27393 GO:0006570 GO:0006470 GO:0006289 GO:0016787 GO:0004003 GO:0004725 GO:0046872 GO:0005634 GO:0000439 GO:0005657
Cluster-22178.27394 GO:0015986 GO:0007155 GO:0015992 GO:0016301 GO:0005540 GO:0015078 GO:0005524 GO:0000276
Cluster-22178.27399 GO:0009405 GO:0019865 GO:0005515 GO:0019814 GO:0016021
Cluster-22178.27411 GO:0016021
Cluster-22178.27412 GO:0006468 GO:0004672 GO:0005524
Cluster-22178.27415 GO:0006355 GO:0003677
Cluster-22178.27419 GO:0055085 GO:0006471 GO:0007154 GO:0006629 GO:0003956 GO:0016021
Cluster-22178.27420 GO:0055085 GO:0006471 GO:0007154 GO:0003956 GO:0016021
Cluster-22178.27422 GO:0007154 GO:0006629 GO:0055085 GO:0006471 GO:0003956 GO:0016021
Cluster-22178.27423 GO:0006629 GO:0007154 GO:0006471 GO:0055085 GO:0003956 GO:0016021
Cluster-22178.27430 GO:0006355 GO:0003677 GO:0003700 GO:0005667
Cluster-22178.27440 GO:0006508 GO:0004222 GO:0008270 GO:0031012
Cluster-22178.27441 GO:0007165 GO:0015740 GO:0015556 GO:0004872 GO:0016021
Cluster-22178.27442 GO:0006508 GO:0015740 GO:0008270 GO:0015556 GO:0004222 GO:0031012 GO:0016021
Cluster-22178.27443 GO:0016021
Cluster-22178.27444 GO:0007275 GO:0005201 GO:0005581 GO:0005578
Cluster-22178.27447 GO:0055114 GO:0042254 GO:0006412 GO:0016491

GO:0046935 GO:0003735 GO:0005622 GO:0005840 GO:0005944
Cluster-22178.27450 GO:0006691 GO:0019530 GO:0006749 GO:0006693
GO:0003840
Cluster-22178.27452 GO:0006693 GO:0006691 GO:0019530 GO:0006749
GO:0003840
Cluster-22178.27455 GO:0006120 GO:0006744 GO:0006814 GO:0015992
GO:0055114 GO:0008137
Cluster-22178.27458 GO:0006771 GO:0006355 GO:0009231 GO:0003919
GO:0000982 GO:0000978 GO:0005634
Cluster-22178.27461 GO:0009607 GO:0055085 GO:0006811 GO:0030976
GO:0005201 GO:0000287 GO:0005216 GO:0005581 GO:0016021
GO:0019031 GO:0005578 GO:0016020
Cluster-22178.27467 GO:0006306 GO:0005515 GO:0003677 GO:0008170

Cluster-22178.27482 GO:0055085 GO:0007154 GO:0016021
Cluster-22178.27483 GO:0007154 GO:0055085 GO:0016021
Cluster-22178.27493 GO:0030991
Cluster-22178.27497 GO:0006606
Cluster-22178.27499 GO:0031110 GO:0006744 GO:0016831 GO:0004519
GO:0003677 GO:0005886
Cluster-22178.27505 GO:0006812 GO:0016887 GO:0016021
Cluster-22178.27517 GO:0006260 GO:0019079 GO:0007186 GO:0004930
GO:0030337 GO:0016021 GO:0042575 GO:0016020
Cluster-22178.27522 GO:0009405 GO:0006260 GO:0010506 GO:0003774
GO:0016459 GO:0005576 GO:0035792 GO:0005615
Cluster-22178.27523 GO:0006260 GO:0005576
Cluster-22178.27533 GO:0010508
Cluster-22178.27537 GO:0005515
Cluster-22178.27545 GO:0016021
Cluster-22178.27552 GO:0055114 GO:0016117 GO:0071949 GO:0005198
GO:0016705 GO:0016491 GO:0016817 GO:0045095
Cluster-22178.27559 GO:0007186 GO:0004930 GO:0016021
Cluster-22178.27560 GO:0007186 GO:0004930 GO:0016021

Cluster-22178.27566 GO:0051262
Cluster-22178.27567 GO:0007016 GO:0042981 GO:0020037 GO:0016010
Cluster-22178.27580 GO:0016021
Cluster-22178.27581 GO:0042384 GO:0010826 GO:0036038
Cluster-22178.27584 GO:0050790 GO:0030234
Cluster-22178.27588 GO:0006412 GO:0042254 GO:0003735 GO:0005840
Cluster-22178.27602 GO:0006464 GO:0015979 GO:0046872 GO:0005524
GO:0004867 GO:0016020 GO:0009522
Cluster-22178.27603 GO:0006464 GO:0005524 GO:0046872
Cluster-22178.27604 GO:0035556 GO:0009190 GO:0055114 GO:0050660
GO:0016491 GO:0016849
Cluster-22178.27605 GO:0008152 GO:0016757 GO:0016021
Cluster-22178.27608 GO:0007017 GO:0005875
Cluster-22178.27610 GO:0008092 GO:0005737 GO:0019898
Cluster-22178.27613 GO:0007304 GO:0043087 GO:0007165 GO:0007010
GO:0007264 GO:0004872 GO:0005213 GO:0003779 GO:0005085
GO:0016021 GO:0042600 GO:0005576
Cluster-22178.27616 GO:0055114 GO:0016020 GO:0016021
Cluster-22178.27617 GO:0009405 GO:0035091
Cluster-22178.27626 GO:0016020
Cluster-22178.27627 GO:0004659 GO:0016020 GO:0016021
Cluster-22178.27637 GO:0007264 GO:0007186 GO:0007165 GO:0003924
GO:0031683 GO:0019001 GO:0004871 GO:0005525 GO:0032040
Cluster-22178.27639 GO:0006811 GO:0006810 GO:0005230 GO:0016020
Cluster-22178.27641 GO:0007165 GO:0007186 GO:0004871 GO:0005834
Cluster-22178.27647 GO:0042147 GO:0000079 GO:0019901 GO:0005856
Cluster-22178.27652 GO:0005515
Cluster-22178.27654 GO:0005515
Cluster-22178.27655 GO:0005515
Cluster-22178.27658 GO:0009250 GO:0046527
Cluster-22178.27668 GO:0055114 GO:0019530 GO:0046439 GO:0006534
GO:0005506 GO:0017172
Cluster-22178.27669 GO:0007049 GO:0042407 GO:0051259 GO:0006886

GO:0006144 GO:0015031 GO:0015743 GO:0003968 GO:0019901
GO:0051082 GO:0008565 GO:0044284 GO:0005737 GO:0031379
GO:0061617 GO:0016020
Cluster-22178.27671 GO:0009103 GO:0007283 GO:0016020
Cluster-22178.27673 GO:0006144 GO:0008092 GO:0003968 GO:0031379
GO:0005737 GO:0019898
Cluster-22178.27676 GO:0003924 GO:0005525
Cluster-22178.27677 GO:0005525 GO:0003924
Cluster-22178.27684 GO:0005515
Cluster-22178.27686 GO:0005515
Cluster-22178.27699 GO:0005515
Cluster-22178.27700 GO:0005515
Cluster-22178.27702 GO:0016598 GO:0004057
Cluster-22178.27706 GO:0006308 GO:0006260 GO:0006281 GO:0042157
GO:0006869 GO:0009405 GO:0006118 GO:0008289 GO:0009055
GO:0017108 GO:0016021 GO:0005576 GO:0005634 GO:0033557
Cluster-22178.27708 GO:0008608 GO:0010508 GO:0042729 GO:0005876
Cluster-22178.27712 GO:0007268 GO:0007165 GO:0006811 GO:0004970
GO:0016020
Cluster-22178.27713 GO:0043039 GO:0006352 GO:0006357 GO:0001104
GO:0016876 GO:0005524 GO:0016592 GO:0005634 GO:0005669
Cluster-22178.27717 GO:0007165 GO:0010506 GO:0006260 GO:0006914
GO:0000921 GO:0035299 GO:0008201 GO:0050750 GO:0005524
GO:0005783 GO:0005615 GO:0016021 GO:0005940
Cluster-22178.27719 GO:0046872
Cluster-22178.27721 GO:0008146
Cluster-22178.27731 GO:0006353 GO:0007218 GO:0007186 GO:0004930
GO:0003723 GO:0016021
Cluster-22178.27732 GO:0046740 GO:0044219 GO:0019028
Cluster-22178.27733 GO:0046740 GO:0044219 GO:0019028
Cluster-22178.27740 GO:0006355 GO:0005992 GO:0003824 GO:0005515
GO:0005634
Cluster-22178.27748 GO:0006749 GO:0019530 GO:0006691 GO:0006693

GO:0003840 GO:0051537 GO:0043231
Cluster-22178.27749 GO:0019530 GO:0006749 GO:0006691 GO:0006693
GO:0003840 GO:0051537 GO:0043231
Cluster-22178.27751 GO:0006468 GO:0016773 GO:0005524 GO:0004672
GO:0016020
Cluster-22178.27753 GO:0006355 GO:0016043 GO:0006351 GO:0006206
GO:0006144 GO:0031412 GO:0006352 GO:0003677 GO:0003899
GO:0003779 GO:0016987 GO:0003700 GO:0005730 GO:0005667
GO:0031411
Cluster-22178.27757 GO:0000166 GO:0046872
Cluster-22178.27763 GO:0042254 GO:0007186 GO:0006412 GO:0004930
GO:0003735 GO:0005622 GO:0016021 GO:0005840
Cluster-22178.27764 GO:0042254 GO:0007186 GO:0006412 GO:0004930
GO:0003735 GO:0005622 GO:0005840 GO:0016021
Cluster-22178.27765 GO:0005198 GO:0005515 GO:0019028
Cluster-22178.27766 GO:0006396 GO:0080009 GO:0009451 GO:0008174
GO:0016746 GO:0003723
Cluster-22178.27769 GO:0016020
Cluster-22178.27775 GO:0042981 GO:0005515 GO:0008270
Cluster-22178.27779 GO:0006508 GO:0004197 GO:0008234
Cluster-22178.27780 GO:0006508 GO:0008234
Cluster-22178.27781 GO:0006508 GO:0016226 GO:0008234 GO:0004197
GO:0051536 GO:0005506
Cluster-22178.27786 GO:0046872 GO:0005515 GO:0045202
Cluster-22178.27788 GO:0006144 GO:0007165 GO:0004114
Cluster-22178.27789 GO:0007165 GO:0006144 GO:0004114
Cluster-22178.27792 GO:0006355 GO:0003700 GO:0005667
Cluster-22178.27805 GO:0048870 GO:0006465 GO:0016567 GO:0046872
GO:0008270 GO:0008233 GO:0004842 GO:0005515 GO:0005622
GO:0016021 GO:0005680 GO:0048786 GO:0005787 GO:0019028
GO:0031514
Cluster-22178.27806 GO:0006465 GO:0048870 GO:0016567 GO:0008233
GO:0004842 GO:0008270 GO:0046872 GO:0005515 GO:0016021

GO:0005680 GO:0005622 GO:0031514 GO:0005787 GO:0019028
GO:0048786
Cluster-22178.27807 GO:0016114 GO:0006465 GO:0016567 GO:0009058
GO:0042432 GO:0008233 GO:0004842 GO:0008270 GO:0016844
GO:0046872 GO:0005515 GO:0016021 GO:0005680 GO:0005622
GO:0005787
Cluster-22178.27824 GO:0005515
Cluster-22178.27826 GO:0031175 GO:0051276 GO:0006807 GO:0006508
GO:0009058 GO:0008270 GO:0005524 GO:0004181 GO:0003933
GO:0005516 GO:0004180 GO:0005515 GO:0030507 GO:0008091
GO:0005694
Cluster-22178.27827 GO:0005515
Cluster-22178.27829 GO:0006935 GO:0006955 GO:0007165 GO:0008009
GO:0005576
Cluster-22178.27830 GO:0006935 GO:0006955 GO:0007165 GO:0008009
GO:0005576
Cluster-22178.27844 GO:0006468 GO:0005524 GO:0004672
Cluster-22178.27854 GO:0005509 GO:0005515 GO:0009986 GO:0016020
Cluster-22178.27856 GO:0005515
Cluster-22178.27857 GO:0006281 GO:0005515 GO:0005509 GO:0005634
Cluster-22178.27858 GO:0006281 GO:0005515 GO:0005509 GO:0000049
GO:0005634
Cluster-22178.27859 GO:0006412 GO:0042254 GO:0007186 GO:0004930
GO:0003735 GO:0005840 GO:0016021 GO:0005622
Cluster-22178.27860 GO:0016021
Cluster-22178.27861 GO:0006470 GO:0006402 GO:0051252 GO:0046872
GO:0004535 GO:0003676 GO:0003723 GO:0008138 GO:0005737
GO:0005634
Cluster-22178.27866 GO:0030246
Cluster-22178.27869 GO:0006355 GO:0003676 GO:0005622
Cluster-22178.27873 GO:0005201 GO:0005581 GO:0005578
Cluster-22178.27879 GO:0008152 GO:0003676 GO:0016787
Cluster-22178.2788 GO:0005739 GO:0005747

Cluster-22178.27889 GO:0046872
Cluster-22178.27892 GO:0015684 GO:0007264 GO:0006576 GO:0006355
GO:0016887 GO:0015093 GO:0005524 GO:0003677 GO:0005525
GO:0003924 GO:0043531 GO:0008134 GO:0000166 GO:0016020
GO:0005667 GO:0016021
Cluster-22178.27898 GO:0006811 GO:0016020
Cluster-22178.27900 GO:0006811 GO:0016020
Cluster-22178.27904 GO:0006810 GO:0005886
Cluster-22178.27913 GO:0015074 GO:0051252 GO:0004523 GO:0003676

Cluster-22178.27924 GO:0006811 GO:0055085 GO:0005216 GO:0016020
Cluster-22178.27926 GO:0005515
Cluster-22178.27928 GO:0016579 GO:0036459
Cluster-22178.27929 GO:0006817 GO:0016887 GO:0005524 GO:0005515
GO:0005315 GO:0016020
Cluster-22178.27936 GO:0016021
Cluster-22178.27941 GO:0006355 GO:0006449 GO:0002949 GO:0006810
GO:0006415 GO:0003678 GO:0016149 GO:0005198 GO:0008134
GO:0005524 GO:0016887 GO:0098519 GO:0005840 GO:0005737
GO:0019028 GO:0005657 GO:0018444 GO:0005667
Cluster-22178.27942 GO:0006464 GO:0016151
Cluster-22178.27943 GO:0008270 GO:0003697
Cluster-22178.27945 GO:0005198 GO:0005096 GO:0019028
Cluster-22178.27952 GO:0009966
Cluster-22178.27955 GO:0006119 GO:0015992 GO:0000921 GO:0015986
GO:0008092 GO:0045502 GO:0042803 GO:0008134 GO:0046933
GO:0046961 GO:0005737 GO:0016021 GO:0000275 GO:0005940
GO:0030286 GO:0019898 GO:0005667 GO:0045259
Cluster-22178.27957 GO:0009847 GO:0016021
Cluster-22178.27961 GO:0007049 GO:0010506 GO:0006355 GO:0008270
GO:0043565 GO:0003700 GO:0005615 GO:0005667
Cluster-22178.27964 GO:0006355 GO:0003677
Cluster-22178.27965 GO:0006355 GO:0003677

Cluster-22178.27968 GO:0006506 GO:0016746 GO:0016021 GO:0005789
Cluster-22178.27970 GO:0005515
Cluster-22178.27972 GO:0016787
Cluster-22178.27984 GO:0006270 GO:0008168 GO:0005515
Cluster-22178.27988 GO:0006813 GO:0006814 GO:0005890 GO:0016021
Cluster-22178.27989 GO:0006813 GO:0006817 GO:0044341 GO:0006814 GO:0015321 GO:0005544 GO:0005509 GO:0016021 GO:0005890 GO:0016020
Cluster-22178.27990 GO:0006817 GO:0044341 GO:0015321 GO:0005544 GO:0005509 GO:0016020
Cluster-22178.27991 GO:0006814 GO:0044341 GO:0006813 GO:0006817 GO:0015321 GO:0005509 GO:0005544 GO:0016021 GO:0016020 GO:0005890
Cluster-22178.27992 GO:0006270
Cluster-22178.27993 GO:0006814 GO:0006817 GO:0006813 GO:0044341 GO:0015321 GO:0005544 GO:0005509 GO:0016021 GO:0016020 GO:0005890
Cluster-22178.27998 GO:0000166 GO:0046872 GO:0016021
Cluster-22178.28002 GO:0007165 GO:0042262 GO:0032784 GO:0051262 GO:0003677 GO:0003690 GO:0046983
Cluster-22178.28006 GO:0005515
Cluster-22178.28007 GO:0030168 GO:0051258 GO:0007165 GO:0030674 GO:0005515 GO:0005102 GO:0005577
Cluster-22178.28010 GO:0006825 GO:0007186 GO:0035434 GO:0004930 GO:0005375 GO:0016021
Cluster-22178.28012 GO:0005515
Cluster-22178.28013 GO:0005515 GO:0016012
Cluster-22178.2802 GO:0048268 GO:0007212 GO:0019905 GO:0032051 GO:0016021
Cluster-22178.28023 GO:0007186 GO:0004930 GO:0016021
Cluster-22178.28025 GO:0015986 GO:0015992 GO:0015078 GO:0000276
Cluster-22178.28028 GO:0055085 GO:0022857 GO:0016021
Cluster-22178.28030 GO:0007165 GO:0004872 GO:0016021

Cluster-22178.28031 GO:0006508 GO:0009405 GO:0046856 GO:0008152 GO:0005515 GO:0016788 GO:0008270 GO:0034597 GO:0004181 GO:0005576

Cluster-22178.28033 GO:0006355 GO:0055114 GO:0016641 GO:0003677 GO:0005507

Cluster-22178.2804 GO:0019905

Cluster-22178.28040 GO:0042407 GO:0061617 GO:0044284

Cluster-22178.28043 GO:0006508 GO:0008270 GO:0004222 GO:0031012

Cluster-22178.28046 GO:0006508 GO:0004197 GO:0016740 GO:0008242

Cluster-22178.28059 GO:0042816 GO:0008615 GO:0055114 GO:0005515 GO:0050570 GO:0051287

Cluster-22178.28066 GO:0009395 GO:0046486 GO:0035556 GO:0007205 GO:0016301 GO:0005509 GO:0004143

Cluster-22178.28070 GO:0055114 GO:0016616 GO:0005507 GO:0016491

Cluster-22178.28071 GO:0055114 GO:0016491 GO:0005507 GO:0016616

Cluster-22178.28074 GO:0055114 GO:0016491 GO:0005507 GO:0016616

Cluster-22178.28075 GO:0055114 GO:0016616 GO:0005507 GO:0016491

Cluster-22178.28076 GO:0055114 GO:0005507 GO:0016616 GO:0016491

Cluster-22178.28086 GO:0008270

Cluster-22178.28094 GO:0007165 GO:0006952 GO:0005126 GO:0005576

Cluster-22178.28095 GO:0006771 GO:0019497 GO:0005515 GO:0003993 GO:0046872

Cluster-22178.28096 GO:0005524

Cluster-22178.28099 GO:0015992 GO:0015986 GO:0015078 GO:0045263 GO:0019031

Cluster-22178.28100 GO:0006355 GO:0015986 GO:0008053 GO:0006357 GO:0015992 GO:0001104 GO:0003700 GO:0015078 GO:0003924

GO:0005741 GO:0016592 GO:0045263 GO:0005667 GO:0016021
GO:0019031
Cluster-22178.28101 GO:0008053 GO:0006357 GO:0006355 GO:0003700
GO:0003924 GO:0001104 GO:0016021 GO:0019031 GO:0016592
GO:0005741 GO:0005667
Cluster-22178.28102 GO:0006308 GO:0015992 GO:0015986 GO:0008855
GO:0015078 GO:0019031 GO:0009318 GO:0045263
Cluster-22178.28108 GO:0005515
Cluster-22178.28112 GO:0005515
Cluster-22178.28117 GO:0006355 GO:0016055 GO:0007275 GO:0007165
GO:0003700 GO:0005102 GO:0005576 GO:0005667 GO:0042025
Cluster-22178.28131 GO:0016746
Cluster-22178.28138 GO:0006260 GO:0006281 GO:0003911 GO:0004866
GO:0005615 GO:0005576
Cluster-22178.28140 GO:0016301
Cluster-22178.28150 GO:0019031
Cluster-22178.28154 GO:0046914 GO:0043565 GO:0005515 GO:0005634
GO:0016020
Cluster-22178.28155 GO:0043565 GO:0046914 GO:0005515 GO:0005634
GO:0016020
Cluster-22178.28156 GO:0046914 GO:0043565 GO:0005515 GO:0005634
GO:0016020
Cluster-22178.28160 GO:0006355 GO:0003723
Cluster-22178.28161 GO:0007623 GO:0006355 GO:0005737 GO:0005634
Cluster-22178.28163 GO:0006355 GO:0007623 GO:0005634 GO:0005737
Cluster-22178.28164 GO:0019031
Cluster-22178.28166 GO:0006508 GO:0042254 GO:0006412 GO:0016787
GO:0005515 GO:0008236 GO:0042802 GO:0003735 GO:0005622
GO:0005840
Cluster-22178.28167 GO:0015031 GO:0007017 GO:0006810 GO:0042147
GO:0007018 GO:0005524 GO:0016887 GO:0008565 GO:0003777
GO:0003676 GO:0016787 GO:0003677 GO:0030286 GO:0005874
GO:0030904

Cluster-22178.28168 GO:0007165 GO:0006470 GO:0008138 GO:0005179
GO:0005576
Cluster-22178.28171 GO:0005515
Cluster-22178.28173 GO:0051262 GO:0015979 GO:0008152 GO:0016787
GO:0003824 GO:0016020 GO:0009523 GO:0009539
Cluster-22178.28174 GO:0006468 GO:0004672 GO:0005524
Cluster-22178.28176 GO:0007165 GO:0006144 GO:0004114
Cluster-22178.28177 GO:0007165 GO:0006144 GO:0004114
Cluster-22178.28178 GO:0006144 GO:0007165 GO:0004114
Cluster-22178.28179 GO:0005975 GO:0015074 GO:0055085 GO:0006811
GO:0016758 GO:0005216 GO:0016020
Cluster-22178.28189 GO:0006468 GO:0006812 GO:0032801 GO:0019076
GO:0005261 GO:0005524 GO:0004672 GO:0033644
Cluster-22178.28193 GO:0007165 GO:0005515 GO:0005102
Cluster-22178.28195 GO:0007165 GO:0005102 GO:0005515
Cluster-22178.28201 GO:0008152 GO:0003824
Cluster-22178.28206 GO:0016787 GO:0005506
Cluster-22178.28215 GO:0044238 GO:0005515
Cluster-22178.28219 GO:0019058 GO:0050790 GO:0006508 GO:0007155
GO:0019062 GO:0055114 GO:0004197 GO:0016706 GO:0008168
Cluster-22178.28220 GO:0019062 GO:0019058 GO:0055114 GO:0007155
GO:0016706 GO:0008168
Cluster-22178.28221 GO:0050790 GO:0006508 GO:0055114 GO:0004197
GO:0016706 GO:0008168
Cluster-22178.28226 GO:0006811 GO:0055085 GO:0005216 GO:0016020
Cluster-22178.28230 GO:0046914
Cluster-22178.28233 GO:0005509
Cluster-22178.28237 GO:0046872
Cluster-22178.28239 GO:0035556
Cluster-22178.28240 GO:0006468 GO:0004672
Cluster-22178.28245 GO:0016021 GO:0016012
Cluster-22178.28249 GO:0005976 GO:0005515 GO:0016779
Cluster-22178.28251 GO:0006123 GO:0006820 GO:0015992 GO:0004129

GO:0016021 GO:0005740 GO:0045277
Cluster-22178.28256 GO:0006457 GO:0007165 GO:0006810 GO:0019882
GO:0006955 GO:0070193 GO:0000917 GO:0015991 GO:0006355
GO:0007049 GO:0009098 GO:0008608 GO:0048870 GO:0043093
GO:0007128 GO:0006914 GO:0051028 GO:0015992 GO:0006886
GO:0016573 GO:0007130 GO:0006397 GO:0051301 GO:0048309
GO:0042289 GO:0008134 GO:0045502 GO:0004871 GO:0003700
GO:0008092 GO:0005198 GO:0042803 GO:0051082 GO:0015078
GO:0043565 GO:0005882 GO:0019898 GO:0030286 GO:0005886
GO:0031514 GO:0033179 GO:0016021 GO:0000795 GO:0042613
GO:0016020 GO:0005667 GO:0005876 GO:0016272 GO:0042729
GO:0000445 GO:0005737
Cluster-22178.28258 GO:0006281 GO:0009069 GO:0016310 GO:0043161
GO:0006289 GO:0019083 GO:0003684 GO:0005515 GO:0004674
GO:0016021
Cluster-22178.28259 GO:0006289 GO:0019083 GO:0043161 GO:0016310
GO:0006281 GO:0009069 GO:0004674 GO:0003684 GO:0005515
GO:0016021
Cluster-22178.28261 GO:0006281 GO:0009069 GO:0006289 GO:0043161
GO:0016310 GO:0003684 GO:0005515 GO:0004674
Cluster-22178.28275 GO:0006260 GO:0006351 GO:0006269 GO:0007155
GO:0003896 GO:0008270 GO:0003677 GO:0005540 GO:0005657
GO:0005730
Cluster-22178.28297 GO:0007165 GO:0005179 GO:0005576
Cluster-22178.28305 GO:0007186 GO:0004930 GO:0016020
Cluster-22178.28310 GO:0006725 GO:0005515 GO:0004497
Cluster-22178.28312 GO:0006725 GO:0004497 GO:0005515
Cluster-22178.28315 GO:0055085 GO:0022857 GO:0008270 GO:0016021
GO:0005622
Cluster-22178.28316 GO:0006810 GO:0003333 GO:0006865 GO:0055085
GO:0015171 GO:0016020
Cluster-22178.28318 GO:0006865 GO:0055085 GO:0006810 GO:0003333
GO:0015171 GO:0016020

Cluster-22178.28323 GO:0016887 GO:0005524
Cluster-22178.28324 GO:0005975 GO:0005524 GO:0016773
Cluster-22178.28329 GO:0005544 GO:0005509
Cluster-22178.28334 GO:0034508 GO:0005509 GO:0005515 GO:0000776
Cluster-22178.28335 GO:0034508 GO:0005509 GO:0005515 GO:0000776
Cluster-22178.28339 GO:0005975 GO:0006446 GO:0016901 GO:0048038
GO:0003743 GO:0005737 GO:0005840 GO:0005852
Cluster-22178.28350 GO:0007165
Cluster-22178.28353 GO:0006506
Cluster-22178.28354 GO:0042710 GO:0005887
Cluster-22178.28356 GO:0009234 GO:0005515
Cluster-22178.28358 GO:0055085 GO:0006811 GO:0005216 GO:0016020
Cluster-22178.28360 GO:0005515
Cluster-22178.28364 GO:0055114 GO:0006779 GO:0046856 GO:0016887
GO:0003774 GO:0005524 GO:0034597 GO:0005515 GO:0016459
Cluster-22178.28372 GO:0009451 GO:0008033 GO:0006357 GO:0001104
GO:0009020 GO:0016592
Cluster-22178.28374 GO:0042384 GO:0034464
Cluster-22178.28380 GO:0016020
Cluster-22178.28383 GO:0015074 GO:0006313 GO:0003725 GO:0003677

Cluster-22178.28385 GO:0051262 GO:0032784 GO:0042262 GO:0007165
GO:0003690 GO:0003677
Cluster-22178.28386 GO:0006355 GO:0007165 GO:0051258 GO:0030168
GO:0030674 GO:0005515 GO:0003677 GO:0005102 GO:0005577
Cluster-22178.28394 GO:0006915 GO:0007155 GO:0051090 GO:0008242
GO:0005509 GO:0005576 GO:0005634
Cluster-22178.28397 GO:0006355 GO:0003700 GO:0005667 GO:0005634
Cluster-22178.28398 GO:0016192 GO:0009405 GO:0006508 GO:0006629
GO:0006355 GO:0003700 GO:0004222 GO:0005667 GO:0005634
Cluster-22178.28399 GO:0016192 GO:0006629
Cluster-22178.28401 GO:0016192
Cluster-22178.28404 GO:0019069

Cluster-22178.28405 GO:0019069
Cluster-22178.28406 GO:0019836 GO:0009069 GO:0006468 GO:0007178
GO:0016310 GO:0004675 GO:0043395 GO:0005524 GO:0005576
GO:0005578 GO:0016020
Cluster-22178.28407 GO:0051258 GO:0030168 GO:0006355 GO:0007165
GO:0005102 GO:0030674 GO:0005515 GO:0003677 GO:0005577
Cluster-22178.28417 GO:0042967 GO:0006355 GO:0008270 GO:0003712
GO:0004402 GO:0000123 GO:0005634 GO:0005667
Cluster-22178.28421 GO:0000398 GO:0006355 GO:0003700 GO:0004222
GO:0000976 GO:0003677 GO:0005667 GO:0005634
Cluster-22178.28422 GO:0000398 GO:0006355 GO:0000976 GO:0004222
GO:0003700 GO:0003677 GO:0005634 GO:0005667
Cluster-22178.28423 GO:0055085 GO:0030001 GO:0006914 GO:0046873
GO:0016020
Cluster-22178.28428 GO:0005515
Cluster-22178.28432 GO:0007165
Cluster-22178.28433 GO:0035556 GO:0046872 GO:0005515
Cluster-22178.28441 GO:0006914 GO:0007128 GO:0006629 GO:0070193
GO:0046486 GO:0007130 GO:0045502 GO:0042803 GO:0008962
GO:0008134 GO:0005198 GO:0000795 GO:0030286 GO:0005667
Cluster-22178.28442 GO:0007130 GO:0007128 GO:0070193 GO:0046486
GO:0006629 GO:0008962 GO:0000795
Cluster-22178.28443 GO:0006629 GO:0007130 GO:0070193 GO:0006914
GO:0007128 GO:0046486 GO:0045502 GO:0008134 GO:0042803
GO:0005198 GO:0003774 GO:0008962 GO:0000795 GO:0030286
GO:0016459 GO:0005667
Cluster-22178.28445 GO:0007130 GO:0070193 GO:0007128 GO:0008608
GO:0003723 GO:0042729 GO:0072686 GO:0000795
Cluster-22178.28453 GO:0006955 GO:0005515
Cluster-22178.28457 GO:0007165 GO:0006633 GO:0015940 GO:0008770
GO:0005509 GO:0005578
Cluster-22178.28464 GO:0008270 GO:0016021
Cluster-22178.28470 GO:0008270

Cluster-22178.28473 GO:0055114 GO:0016705 GO:0020037 GO:0005506

Cluster-22178.28475 GO:0055114 GO:0005506 GO:0016705 GO:0020037

Cluster-22178.28476 GO:0055114 GO:0016705 GO:0020037 GO:0005506

Cluster-22178.28478 GO:0008146 GO:0046872
Cluster-22178.28479 GO:0005515
Cluster-22178.28493 GO:0044238 GO:0051258 GO:0007165 GO:0006606
GO:0010506 GO:0030168 GO:0005198 GO:0008134 GO:0042803
GO:0045502 GO:0030674 GO:0005102 GO:0019028 GO:0019031
GO:0016021 GO:0005615 GO:0005882 GO:0005667 GO:0005577
GO:0030286
Cluster-22178.28495 GO:0006260 GO:0009236 GO:0006810 GO:0015858
GO:0006351 GO:0005337 GO:0003887 GO:0003677 GO:0016021
GO:0042575
Cluster-22178.28499 GO:0006396 GO:0006355 GO:0045859 GO:0043401
GO:0007165 GO:0006948 GO:0005496 GO:0004860 GO:0003677
GO:0005515 GO:0030284 GO:0000176 GO:0005576 GO:0005634
Cluster-22178.28501 GO:0016788 GO:0005515
Cluster-22178.28502 GO:0005515
Cluster-22178.28503 GO:0005515
Cluster-22178.28507 GO:0006888 GO:0006886 GO:0008270 GO:0030127
Cluster-22178.28508 GO:0005515
Cluster-22178.28509 GO:0002949 GO:0005524 GO:0003774 GO:0098519
GO:0016459
Cluster-22178.28511 GO:0016567 GO:0004842 GO:0005680
Cluster-22178.28513 GO:0005515
Cluster-22178.28514 GO:0006582 GO:0006952 GO:0005515 GO:0061630
GO:0030411 GO:0008270 GO:0046872
Cluster-22178.28518 GO:0016020
Cluster-22178.2852 GO:0005515
Cluster-22178.28535 GO:0006259 GO:0005524 GO:0003824 GO:0003677

GO:0005694
Cluster-22178.28541 GO:0016598 GO:0004057
Cluster-22178.28543 GO:0008152 GO:0003824
Cluster-22178.28553 GO:0006206 GO:0006801 GO:0055114 GO:0006144
GO:0006614 GO:0006351 GO:0046872 GO:0005525 GO:0004784
GO:0003677 GO:0003899 GO:0005730
Cluster-22178.28559 GO:0006144 GO:0006206 GO:0006351 GO:0006816
GO:0003899 GO:0003677 GO:0005388 GO:0005921 GO:0016529
GO:0005730
Cluster-22178.28562 GO:0006047 GO:0005975 GO:0016773 GO:0005524

Cluster-22178.28570 GO:0006771 GO:0007155 GO:0019497 GO:0005975
GO:0005515 GO:0003993 GO:0004553 GO:0046872 GO:0016021
Cluster-22178.28573 GO:0019497 GO:0005975 GO:0006771 GO:0007155
GO:0046872 GO:0003993 GO:0004553 GO:0005515 GO:0016021
Cluster-22178.28574 GO:0005975 GO:0019497 GO:0007155 GO:0006771
GO:0046872 GO:0004553 GO:0003993 GO:0005515 GO:0016021
Cluster-22178.28575 GO:0019497 GO:0005975 GO:0007155 GO:0006771
GO:0004553 GO:0003993 GO:0046872 GO:0005515 GO:0016021
Cluster-22178.28577 GO:0006368 GO:0007186 GO:0004930 GO:0008023
GO:0016021
Cluster-22178.28578 GO:0006479 GO:0006554 GO:0018024
Cluster-22178.28580 GO:0006355 GO:0043401
Cluster-22178.28588 GO:0019684 GO:0006118 GO:0004859 GO:0005515
GO:0045156 GO:0016021 GO:0030077 GO:0005576
Cluster-22178.28589 GO:0006118 GO:0019684 GO:0004859 GO:0045156
GO:0016021 GO:0030077 GO:0005576
Cluster-22178.28591 GO:0019064 GO:0006810 GO:0006811 GO:0055085
GO:0005215 GO:0019867 GO:0016020
Cluster-22178.28595 GO:0006825 GO:0006606 GO:0006090 GO:0006525
GO:0035434 GO:0006560 GO:0071436 GO:0006814 GO:0015081
GO:0005375 GO:0019904 GO:0008948 GO:0016021 GO:0016020
Cluster-22178.28597 GO:0006825 GO:0006606 GO:0006090 GO:0006560

GO:0071436 GO:0006814 GO:0006525 GO:0035434 GO:0015081
GO:0019904 GO:0008948 GO:0005375 GO:0016021 GO:0016020
Cluster-22178.28602 GO:0035023 GO:0043087 GO:0006355 GO:0003677
GO:0005089 GO:0005515
Cluster-22178.28603 GO:0055114 GO:0006811 GO:0007268 GO:0007165
GO:0008168 GO:0005234 GO:0004970 GO:0016706 GO:0016020
Cluster-22178.28604 GO:0055114 GO:0016706 GO:0008168
Cluster-22178.28611 GO:0006771 GO:0006570 GO:0019497 GO:0006470
GO:0005515 GO:0046872 GO:0004725 GO:0003993
Cluster-22178.28612 GO:0006470 GO:0019497 GO:0006570 GO:0006771
GO:0003993 GO:0004725 GO:0046872 GO:0005515
Cluster-22178.28617 GO:0005515
Cluster-22178.28618 GO:0005515
Cluster-22178.28619 GO:0005515
Cluster-22178.28620 GO:0005515
Cluster-22178.28621 GO:0005515
Cluster-22178.28624 GO:0007186 GO:0050909 GO:0004930 GO:0016021
Cluster-22178.28625 GO:0007059 GO:0030261 GO:0003677 GO:0005524
GO:0016887 GO:0009295 GO:0019028
Cluster-22178.28626 GO:0016887 GO:0005524
Cluster-22178.28629 GO:0043066 GO:0003950 GO:0016788 GO:0051015

Cluster-22178.28631 GO:0006357 GO:0006355 GO:0001104 GO:0003677
GO:0016592
Cluster-22178.28636 GO:0055114 GO:0019337 GO:0009399 GO:0006807
GO:0046872 GO:0016163 GO:0045202 GO:0016610
Cluster-22178.28644 GO:0000774 GO:0005783
Cluster-22178.28646 GO:0015672 GO:0006812 GO:0005261 GO:0016020
Cluster-22178.28651 GO:0007155 GO:0000902 GO:0006955 GO:0016755
GO:0016020
Cluster-22178.28659 GO:0006355 GO:0006412 GO:0042254 GO:0003677
GO:0003735 GO:0005840
Cluster-22178.28661 GO:0006355 GO:0003700 GO:0043565 GO:0005667

Cluster-22178.28663 GO:0019911
Cluster-22178.28668 GO:0051260 GO:0019497 GO:0006771 GO:0005515
GO:0003993 GO:0046872 GO:0016021
Cluster-22178.28669 GO:0005515
Cluster-22178.28672 GO:0016021
Cluster-22178.28674 GO:0023052
Cluster-22178.28675 GO:0046856 GO:0015031 GO:0034597 GO:0016021
Cluster-22178.28682 GO:0005515 GO:0016020
Cluster-22178.28684 GO:0005515
Cluster-22178.28685 GO:0006355 GO:0003700 GO:0043565 GO:0008270
GO:0005667
Cluster-22178.28690 GO:0006355 GO:0008270 GO:0043565 GO:0003700
GO:0005515 GO:0005667
Cluster-22178.28694 GO:0006811 GO:0055085 GO:0005216 GO:0016020
Cluster-22178.28696 GO:0006744 GO:0031110 GO:0004519 GO:0003677
GO:0016831 GO:0005886
Cluster-22178.28702 GO:0005524 GO:0004386
Cluster-22178.28707 GO:0007165 GO:0010506 GO:0006606 GO:0006914
GO:0030168 GO:0006955 GO:0009405 GO:0042262 GO:0051258
GO:0003690 GO:0030674 GO:0005126 GO:0005102 GO:0008134
GO:0042803 GO:0005198 GO:0045502 GO:0016020 GO:0005882
GO:0005667 GO:0005577 GO:0030286 GO:0005576 GO:0005615
Cluster-22178.28711 GO:0016567 GO:0004842
Cluster-22178.28712 GO:0007165 GO:0043401 GO:0006355 GO:0003677
GO:0005496 GO:0030284 GO:0005634
Cluster-22178.28715 GO:0007165 GO:0006827 GO:0006826 GO:0007166
GO:0005381 GO:0005515 GO:0005509 GO:0004871 GO:0046872
GO:0008270 GO:0016020 GO:0005634 GO:0033573
Cluster-22178.28725 GO:0006351 GO:0019031
Cluster-22178.2873 GO:0006508 GO:0004222 GO:0016020
Cluster-22178.28732 GO:0005515
Cluster-22178.28740 GO:0005524
Cluster-22178.28741 GO:0007009 GO:0016773

Cluster-22178.28742 GO:0006771 GO:0019497 GO:0003993 GO:0005515 GO:0046872 GO:0016020
Cluster-22178.28743 GO:0006771 GO:0019497 GO:0046872 GO:0003993 GO:0005515 GO:0016020
Cluster-22178.28744 GO:0006814 GO:0006885 GO:0015992 GO:0005515 GO:0015385 GO:0016020
Cluster-22178.28754 GO:0005515
Cluster-22178.28757 GO:0006355 GO:0003677 GO:0003723
Cluster-22178.28773 GO:0005515
Cluster-22178.28778 GO:0046488 GO:0016307
Cluster-22178.28786 GO:0016055 GO:0007411 GO:0008013 GO:0005515 GO:0016342
Cluster-22178.28791 GO:0019058 GO:0045859 GO:0016534 GO:0016533
Cluster-22178.28792 GO:0045892 GO:0005634
Cluster-22178.28795 GO:0015631 GO:0005515 GO:0045298
Cluster-22178.28798 GO:0007156 GO:0005509 GO:0016020
Cluster-22178.28800 GO:0006367 GO:0005672
Cluster-22178.28802 GO:0048193 GO:0007049 GO:0006357 GO:0071973 GO:0005515 GO:0019901 GO:0001104 GO:0005198 GO:0019031 GO:0016020 GO:0016592 GO:0019028
Cluster-22178.28803 GO:0005515
Cluster-22178.28805 GO:0055114 GO:0016491
Cluster-22178.28824 GO:0006858 GO:0035556 GO:0005544 GO:0005515 GO:0005509
Cluster-22178.28825 GO:0006858 GO:0035556 GO:0005515 GO:0005509 GO:0005544
Cluster-22178.28826 GO:0007165 GO:0005179 GO:0005576
Cluster-22178.28827 GO:0005975 GO:0006357 GO:0004649 GO:0001104 GO:0016592
Cluster-22178.28829 GO:0006276
Cluster-22178.28830 GO:0009396 GO:0055114 GO:0008152 GO:0007010 GO:0046487 GO:0003779 GO:0016491 GO:0003824 GO:0050662 GO:0004488

Cluster-22178.28831 GO:0046487 GO:0007010 GO:0008152 GO:0055114 GO:0009396 GO:0004488 GO:0050662 GO:0003824 GO:0016491 GO:0003779

Cluster-22178.28832 GO:0007010 GO:0046487 GO:0009396 GO:0055114 GO:0008152 GO:0050662 GO:0004488 GO:0003779 GO:0016491 GO:0003824

Cluster-22178.28842 GO:0030414 GO:0005515 GO:0005509 GO:0005576

Cluster-22178.28844 GO:0006914 GO:0007049 GO:0006355 GO:0043565 GO:0003700 GO:0003677 GO:0005667 GO:0005634

Cluster-22178.28850 GO:0016998 GO:0006032 GO:0004568 GO:0019239

Cluster-22178.28855 GO:0007049 GO:0006355 GO:0000917 GO:0007094 GO:0006914 GO:0051607 GO:0006323 GO:0043093 GO:0043565 GO:0003700 GO:0045502 GO:0008134 GO:0042803 GO:0005515 GO:0008017 GO:0017048 GO:0005737 GO:0045298 GO:0030286 GO:0005887 GO:0005667

Cluster-22178.28856 GO:0006914 GO:0051607 GO:0043093 GO:0051301 GO:0007059 GO:0007049 GO:0006355 GO:0006118 GO:0007094 GO:0000917 GO:0008017 GO:0003954 GO:0017048 GO:0043565 GO:0046872 GO:0003700 GO:0045502 GO:0005515 GO:0008134 GO:0042803 GO:0000444 GO:0030286 GO:0005887 GO:0005667 GO:0005739 GO:0005737 GO:0045298

Cluster-22178.28861 GO:0007186 GO:0007165 GO:0004871 GO:0005834

Cluster-22178.28869 GO:0006465 GO:0048870 GO:0016567 GO:0008233 GO:0004842 GO:0046872 GO:0008270 GO:0005515 GO:0016021 GO:0005680 GO:0005622 GO:0031514 GO:0048786 GO:0019028 GO:0005787

Cluster-22178.28871 GO:0009405 GO:0006355 GO:0051258 GO:0007165 GO:0048193 GO:0000921 GO:0030168 GO:0005198 GO:0016818 GO:0003677 GO:0030674 GO:0005102 GO:0008270 GO:0005524 GO:0005634 GO:0019028 GO:0016021 GO:0019031 GO:0016020 GO:0005577 GO:0005940

Cluster-22178.28873 GO:0005634

Cluster-22178.28875 GO:0000921 GO:0015992 GO:0015986 GO:0006119
GO:0008092 GO:0046933 GO:0046961 GO:0005737 GO:0005940
GO:0016021 GO:0019898 GO:0000275 GO:0045259
Cluster-22178.28880 GO:0007165 GO:0005179 GO:0005576
Cluster-22178.28882 GO:0006308 GO:0051258 GO:0015991 GO:0006479
GO:0030168 GO:0015031 GO:0007165 GO:0007172 GO:0042262
GO:0006468 GO:0006886 GO:0015992 GO:0007131 GO:0042157
GO:0006869 GO:0008168 GO:0004871 GO:0008289 GO:0008855
GO:0005102 GO:0030674 GO:0015078 GO:0005198 GO:0008565
GO:0004713 GO:0003690 GO:0016021 GO:0019031 GO:0019028
GO:0005576 GO:0031083 GO:0009318 GO:0033180 GO:0005737
GO:0000794 GO:0005577 GO:0005925 GO:0016020 GO:0030133
Cluster-22178.28884 GO:0000921 GO:0008092 GO:0005737 GO:0005940
GO:0019898 GO:0016021
Cluster-22178.28887 GO:0008641
Cluster-22178.28888 GO:0043065 GO:0005740 GO:0016021
Cluster-22178.28895 GO:0046872
Cluster-22178.28896 GO:0005515
Cluster-22178.28898 GO:0030154 GO:0005544 GO:0005509 GO:0016021
Cluster-22178.28901 GO:0006013 GO:0015976 GO:0006000 GO:0006098
GO:0006020 GO:0006094 GO:0006096 GO:0004332
Cluster-22178.28902 GO:0006013 GO:0015976 GO:0006000 GO:0006094
GO:0006020 GO:0006098 GO:0006096 GO:0004332
Cluster-22178.28903 GO:0007049 GO:0007067 GO:0006464 GO:0000775
GO:0005634
Cluster-22178.28907 GO:0006357 GO:0001104 GO:0016592
Cluster-22178.28909 GO:0003677
Cluster-22178.28914 GO:0000166 GO:0008270 GO:0046872
Cluster-22178.28923 GO:0016887 GO:0005524 GO:0005515
Cluster-22178.28926 GO:0006384 GO:0006366 GO:0003700 GO:0008017
GO:0005515 GO:0045298 GO:0016021 GO:0005634 GO:0005667
Cluster-22178.28932 GO:0005515
Cluster-22178.28933 GO:0005515

Cluster-22178.28936 GO:0020037
Cluster-22178.2894 GO:0015986 GO:0015992 GO:0015078 GO:0000276
Cluster-22178.28940 GO:0006144 GO:0007165 GO:0004114 GO:0003677

Cluster-22178.28943 GO:0046034 GO:0006144 GO:0006464 GO:0004017 GO:0016151 GO:0046872 GO:0005761
Cluster-22178.28952 GO:0005515 GO:0004527 GO:0003677
Cluster-22178.28953 GO:0005515 GO:0004527 GO:0003677
Cluster-22178.28965 GO:0015074 GO:0007178 GO:0016310 GO:0009069 GO:0006468 GO:0005524 GO:0004675 GO:0016020
Cluster-22178.2898 GO:0006570 GO:0006470 GO:0007165 GO:0004725 GO:0008138
Cluster-22178.28983 GO:0006810 GO:0007018 GO:0007017 GO:0003677 GO:0003777 GO:0005524 GO:0016887 GO:0005874 GO:0030286
Cluster-22178.28990 GO:0006352 GO:0007264 GO:0007186 GO:0007165 GO:0003924 GO:0031683 GO:0019001 GO:0004871 GO:0005525 GO:0003677
Cluster-22178.28992 GO:0005515
Cluster-22178.28993 GO:0033617 GO:0015631 GO:0045298 GO:0005739
Cluster-22178.28994 GO:0033617 GO:0015631 GO:0005739 GO:0045298
Cluster-22178.28999 GO:0007186 GO:0004930 GO:0016021
Cluster-22178.29000 GO:0005976 GO:0016779 GO:0005515
Cluster-22178.29006 GO:0008616 GO:0055114 GO:0006400 GO:0008479 GO:0016020
Cluster-22178.29011 GO:0030261 GO:0007059 GO:0009966 GO:0016798 GO:0005515 GO:0005576 GO:0005737
Cluster-22178.29026 GO:0007155 GO:0005515 GO:0005509 GO:0005747 GO:0005576 GO:0005739
Cluster-22178.29027 GO:0042802
Cluster-22178.29030 GO:0006810 GO:0055085 GO:0016020
Cluster-22178.29040 GO:0006611 GO:0007186 GO:0006914 GO:0006406 GO:0070206 GO:0006998 GO:0006260 GO:0006606 GO:0006355 GO:0006886 GO:0006351 GO:0007094 GO:0007049 GO:0003677

GO:0004930 GO:0045502 GO:0008134 GO:0042803 GO:0005198
GO:0043565 GO:0003700 GO:0005667 GO:0005882 GO:0005783
GO:0030286 GO:0042025 GO:0031965 GO:0016021
Cluster-22178.29051 GO:0005887 GO:0016021
Cluster-22178.29062 GO:0003677
Cluster-22178.29065 GO:0005515 GO:0016020
Cluster-22178.29067 GO:0003677
Cluster-22178.29070 GO:0006144 GO:0003968 GO:0031379
Cluster-22178.29071 GO:0046656 GO:0006508 GO:0006761 GO:0009165
GO:0006545 GO:0055114 GO:0004146 GO:0008236
Cluster-22178.29074 GO:0046872
Cluster-22178.29076 GO:0043565 GO:0005515 GO:0005634
Cluster-22178.29077 GO:0016032 GO:0005515
Cluster-22178.29080 GO:0043087 GO:0009405 GO:0017137 GO:0044161
Cluster-22178.29085 GO:0043087 GO:0006355 GO:0009405 GO:0046983
GO:0003677 GO:0017137 GO:0044161
Cluster-22178.29087 GO:0048870 GO:0006308 GO:0006955 GO:0000902
GO:0009307 GO:0009036 GO:0031514 GO:0016020 GO:0009359
Cluster-22178.29095 GO:0006813 GO:0015979 GO:0006811 GO:0005509
GO:0005249 GO:0046872 GO:0008076 GO:0016020 GO:0009523
GO:0016021
Cluster-22178.29103 GO:0007165 GO:0002532 GO:0004896 GO:0016021
Cluster-22178.29104 GO:0006814 GO:0051016 GO:0006811 GO:0055085
GO:0006139 GO:0005248 GO:0005216 GO:0016818 GO:0008026
GO:0005524 GO:0003676 GO:0001518 GO:0008290 GO:0016020
Cluster-22178.29106 GO:0006468 GO:0005524 GO:0004672 GO:0016773
GO:0016020
Cluster-22178.2911 GO:0006357 GO:0001104 GO:0016592
Cluster-22178.29118 GO:0006355 GO:0003677
Cluster-22178.29120 GO:0006351 GO:0006570 GO:0042254 GO:0006412
GO:0006206 GO:0006144 GO:0006470 GO:0003677 GO:0008138
GO:0003735 GO:0003899 GO:0004725 GO:0005730 GO:0005622
GO:0005840

Cluster-22178.29121 GO:0055114 GO:0016055 GO:0007275 GO:0007165
GO:0006570 GO:0005102 GO:0004511 GO:0005576
Cluster-22178.29126 GO:0019530 GO:0006749 GO:0006691 GO:0006693
GO:0003840
Cluster-22178.29127 GO:0006691 GO:0006749 GO:0019530 GO:0006693
GO:0003840
Cluster-22178.29128 GO:0006693 GO:0006749 GO:0019530 GO:0006691
GO:0003840
Cluster-22178.29129 GO:0006749 GO:0019530 GO:0006691 GO:0006693
GO:0003840
Cluster-22178.29130 GO:0006693 GO:0006749 GO:0019530 GO:0006691
GO:0003840
Cluster-22178.29131 GO:0019530 GO:0006749 GO:0006691 GO:0006693
GO:0005515 GO:0046872 GO:0003840 GO:0008270
Cluster-22178.29135 GO:0051321
Cluster-22178.29138 GO:0006813 GO:0008556 GO:0016021
Cluster-22178.29141 GO:0009405 GO:0016021 GO:0005576
Cluster-22178.29147 GO:0005515
Cluster-22178.29148 GO:0019835 GO:0009405 GO:0019867
Cluster-22178.29150 GO:0006355 GO:0003677 GO:0005634
Cluster-22178.29157 GO:0006357 GO:0005515 GO:0001104 GO:0016592
Cluster-22178.29163 GO:0005201 GO:0005581 GO:0005578
Cluster-22178.29168 GO:0051258 GO:0042262 GO:0009405 GO:0006955
GO:0030168 GO:0006914 GO:0006606 GO:0010506 GO:0007165
GO:0045502 GO:0005198 GO:0042803 GO:0008134 GO:0005102
GO:0005126 GO:0030674 GO:0003690 GO:0005615 GO:0005576
GO:0030286 GO:0005577 GO:0005667 GO:0005882 GO:0016020
Cluster-22178.29169 GO:0009405 GO:0042262 GO:0051258 GO:0007165
GO:0006606 GO:0010506 GO:0030168 GO:0006914 GO:0005198
GO:0008134 GO:0042803 GO:0045502 GO:0030674 GO:0003690
GO:0005102 GO:0005615 GO:0005882 GO:0016020 GO:0005667
GO:0005577 GO:0030286
Cluster-22178.29170 GO:0051258 GO:0042262 GO:0006955 GO:0009405

GO:0006914 GO:0030168 GO:0010506 GO:0006606 GO:0007165
GO:0045502 GO:0005198 GO:0042803 GO:0008134 GO:0005102
GO:0003690 GO:0030674 GO:0005126 GO:0005615 GO:0005576
GO:0030286 GO:0005577 GO:0005667 GO:0016020 GO:0005882
Cluster-22178.29173 GO:0006468 GO:0016773 GO:0005524 GO:0004672
GO:0016020
Cluster-22178.29175 GO:0060962 GO:0003712 GO:0005667
Cluster-22178.29178 GO:0018342 GO:0055114 GO:0008318 GO:0016638
GO:0005515
Cluster-22178.29182 GO:0019836 GO:0045893
Cluster-22178.29185 GO:0007018 GO:0006355 GO:0007017 GO:0006606
GO:0005515 GO:0051082 GO:0003777 GO:0008017 GO:0003700
GO:0005524 GO:0005667 GO:0005874 GO:0045298
Cluster-22178.29187 GO:0055114 GO:0005515 GO:0016638
Cluster-22178.29193 GO:0007005 GO:0031305
Cluster-22178.29195 GO:0007165 GO:0008283 GO:0040007 GO:0008083

Cluster-22178.29197 GO:0006260 GO:0006606 GO:0007049 GO:0006914
GO:0003677 GO:0005515 GO:0016787 GO:0003723 GO:0003724
GO:0005524 GO:0016887
Cluster-22178.29201 GO:0006865 GO:0016787 GO:0016020
Cluster-22178.29207 GO:0043113 GO:0006810 GO:0007213 GO:0005515
GO:0043236 GO:0005215 GO:0016020
Cluster-22178.29214 GO:0048193 GO:0005515 GO:0016021 GO:0016020
Cluster-22178.29215 GO:0050909 GO:0007186 GO:0003779 GO:0004930
GO:0016021
Cluster-22178.29216 GO:0006811 GO:0055085 GO:0005216 GO:0003723
GO:0016020
Cluster-22178.29218 GO:0008233
Cluster-22178.29219 GO:0034477 GO:0006812 GO:0006885 GO:0055085
GO:0004518 GO:0015299 GO:0016021
Cluster-22178.29223 GO:0006308 GO:0016485 GO:0009405 GO:0007166
GO:0006013 GO:0006000 GO:0006914 GO:0007165 GO:0007160

GO:0006004 GO:0004871 GO:0008134 GO:0042803 GO:0005515
GO:0008736 GO:0045502 GO:0008270 GO:0008855 GO:0005509
GO:0005840 GO:0001527 GO:0016021 GO:0005737 GO:0009318
GO:0005634 GO:0030286 GO:0005667
Cluster-22178.29226 GO:0019497 GO:0006013 GO:0006000 GO:0006771
GO:0003993 GO:0005524 GO:0003873 GO:0016301
Cluster-22178.29227 GO:0006464 GO:0046872 GO:0005524
Cluster-22178.29231 GO:0051028 GO:0005634
Cluster-22178.29232 GO:0006355 GO:0003677
Cluster-22178.29233 GO:0007155 GO:0005515 GO:0016021
Cluster-22178.29234 GO:0007155 GO:0005515 GO:0016021
Cluster-22178.29240 GO:0007160 GO:0007165 GO:0009405 GO:0016485
GO:0007166 GO:0005515 GO:0005509 GO:0004871 GO:0005634
GO:0016021 GO:0001527 GO:0005840
Cluster-22178.29243 GO:0050765 GO:0005515
Cluster-22178.29244 GO:0003682 GO:0000785
Cluster-22178.29245 GO:0007165 GO:0007268 GO:0009405 GO:0006468
GO:0030550 GO:0004672 GO:0005524 GO:0005576
Cluster-22178.29247 GO:0006468 GO:0005515 GO:0005524 GO:0004672

Cluster-22178.29254 GO:0006811 GO:0006810 GO:0005230 GO:0016020
Cluster-22178.29268 GO:0005515
Cluster-22178.29270 GO:0007067 GO:0005198 GO:0019028 GO:0072686
GO:0042729 GO:0019031
Cluster-22178.29284 GO:0015672 GO:0006812 GO:0005261 GO:0008289
GO:0016020
Cluster-22178.29289 GO:0006520 GO:0055114 GO:0007160
Cluster-22178.2929 GO:0006813 GO:0007186 GO:0005267 GO:0004930
GO:0016021
Cluster-22178.29293 GO:0016032 GO:0008270 GO:0005634 GO:0042025
Cluster-22178.29294 GO:0035556 GO:0046872 GO:0003677
Cluster-22178.29296 GO:0006118 GO:0006270 GO:0045454 GO:0009055
GO:0015035 GO:0032040

Cluster-22178.29297 GO:0016032 GO:0008270 GO:0042025 GO:0005634
Cluster-22178.29298 GO:0006355 GO:0006886 GO:0048870 GO:0045995
GO:0007049 GO:0030155 GO:0007165 GO:0006914 GO:0030334
GO:0006606 GO:0019901 GO:0008092 GO:0043565 GO:0003700
GO:0005102 GO:0031514 GO:0005737 GO:0016021 GO:0005667
GO:0005783 GO:0019898
Cluster-22178.29301 GO:0007018 GO:0007017 GO:0003677 GO:0003777
GO:0008017 GO:0005524 GO:0008270 GO:0005874 GO:0045298
Cluster-22178.29314 GO:0016032 GO:0045444 GO:0004252 GO:0005515
GO:0005509 GO:0016021
Cluster-22178.29317 GO:0034477 GO:0055085 GO:0006812 GO:0006885
GO:0015299 GO:0004518 GO:0016021
Cluster-22178.29321 GO:0006412 GO:0042254 GO:0019843 GO:0003735
GO:0005840
Cluster-22178.29324 GO:0006260 GO:0006206 GO:0055114 GO:0006144
GO:0009186 GO:0004748 GO:0005524 GO:0005971
Cluster-22178.29325 GO:0019867
Cluster-22178.29334 GO:0005515
Cluster-22178.29335 GO:0046872
Cluster-22178.29336 GO:0046872
Cluster-22178.29341 GO:0006506 GO:0016032 GO:0006508 GO:0008233
GO:0016758 GO:0016021
Cluster-22178.29354 GO:0006355 GO:0006099 GO:0003677 GO:0016829

Cluster-22178.29357 GO:0006355 GO:0000978 GO:0000982 GO:0003700
GO:0003677 GO:0005667 GO:0005634
Cluster-22178.29365 GO:0006308 GO:0005515 GO:0016888
Cluster-22178.29367 GO:0006890 GO:0016763
Cluster-22178.29374 GO:0006355 GO:0007049 GO:0006260 GO:0006606
GO:0006914 GO:0008134 GO:0003677 GO:0003723 GO:0016787
GO:0003724 GO:0016887 GO:0005524 GO:0005667
Cluster-22178.29375 GO:0006508 GO:0045892 GO:0042754 GO:0008270
GO:0004222 GO:0031012

Cluster-22178.29378 GO:0003924 GO:0043531 GO:0016887 GO:0005524
GO:0005525 GO:0016020
Cluster-22178.29379 GO:0005615
Cluster-22178.29396 GO:0008033 GO:0050660
Cluster-22178.29398 GO:0016772
Cluster-22178.29400 GO:0009405 GO:0051258 GO:0000160 GO:0007021
GO:0016310 GO:0042157 GO:0006869 GO:0007165 GO:0015031
GO:0030168 GO:0007131 GO:0051082 GO:0005198 GO:0008289
GO:0030674 GO:0000155 GO:0046983 GO:0005102 GO:0015631
GO:0000794 GO:0019028 GO:0005576 GO:0045298 GO:0016021
GO:0009365 GO:0019031 GO:0005577
Cluster-22178.29401 GO:0005524
Cluster-22178.29404 GO:0006955 GO:0000902 GO:0030155 GO:0007165
GO:0009307 GO:0045995 GO:0048870 GO:0006308 GO:0030334
GO:0005102 GO:0009036 GO:0016020 GO:0031514 GO:0009359
Cluster-22178.29406 GO:0007165 GO:0005179 GO:0005576
Cluster-22178.29409 GO:0016021
Cluster-22178.2941 GO:0003924
Cluster-22178.29418 GO:0008033 GO:0005515
Cluster-22178.29421 GO:0005515
Cluster-22178.29426 GO:0007010 GO:0003779
Cluster-22178.29437 GO:0042254 GO:0006412 GO:0003735 GO:0005515
GO:0005840 GO:0005622
Cluster-22178.29447 GO:0003677
Cluster-22178.29455 GO:0005507
Cluster-22178.29456 GO:0005507
Cluster-22178.29463 GO:0006570 GO:0055114 GO:0050852 GO:0006470
GO:0005515 GO:0016972 GO:0004725 GO:0016021
Cluster-22178.29465 GO:0009966
Cluster-22178.29480 GO:0006606 GO:0006914 GO:0030168 GO:0015992
GO:0051607 GO:0007165 GO:0007155 GO:0051258 GO:0007094
GO:0015991 GO:0005102 GO:0030674 GO:0015078 GO:0008134
GO:0042803 GO:0045502 GO:0005577 GO:0030286 GO:0005667

GO:0016021 GO:0033179
Cluster-22178.29493 GO:0005524 GO:0016887 GO:0003723 GO:0003724

Cluster-22178.29495 GO:0005515
Cluster-22178.29499 GO:0005975 GO:0006260 GO:0006810 GO:0004553
GO:0005886
Cluster-22178.29500 GO:0006355 GO:0006260 GO:0003677 GO:0003887
GO:0008270 GO:0003700 GO:0005576 GO:0005634 GO:0005667
GO:0042575 GO:0016021
Cluster-22178.29501 GO:0006508 GO:0004197 GO:0008234
Cluster-22178.29502 GO:0006269 GO:0006351 GO:0006260 GO:0003677
GO:0005515 GO:0008270 GO:0046872 GO:0003896 GO:0005730
GO:0005622 GO:0005657
Cluster-22178.29504 GO:0006355 GO:0007165 GO:0003700 GO:0004871
GO:0005634 GO:0005667
Cluster-22178.29507 GO:0005515
Cluster-22178.29512 GO:0006352 GO:0006810 GO:0006355 GO:0003676
GO:0003700 GO:0016987 GO:0005215 GO:0003677 GO:0016020
GO:0005667
Cluster-22178.29520 GO:0007165 GO:0005515 GO:0016020
Cluster-22178.29521 GO:0006260 GO:0006914 GO:0006810 GO:0009307
GO:0006308 GO:0048870 GO:0007130 GO:0006886 GO:0009036
GO:0003677 GO:0005198 GO:0005886 GO:0005783 GO:0005882
GO:0016021 GO:0000795 GO:0031514 GO:0009359 GO:0005634
Cluster-22178.29522 GO:0006886 GO:0006810 GO:0007130 GO:0048870
GO:0006914 GO:0031514 GO:0005783 GO:0005634 GO:0016021
GO:0000795 GO:0005886
Cluster-22178.29525 GO:0045995 GO:0030334 GO:0048870 GO:0006308
GO:0007165 GO:0000902 GO:0030155 GO:0006955 GO:0009307
GO:0005102 GO:0009036 GO:0016020 GO:0031514 GO:0009359
Cluster-22178.29533 GO:0006810 GO:0005886
Cluster-22178.29535 GO:0006355 GO:0006457 GO:0006914 GO:0010506
GO:0007049 GO:0006260 GO:0051082 GO:0019901 GO:0043565

GO:0008270 GO:0003700 GO:0005667 GO:0016272 GO:0005615
Cluster-22178.29541 GO:0005515
Cluster-22178.29543 GO:0051252 GO:0007186 GO:0035434 GO:0006825
GO:0016891 GO:0005375 GO:0004930 GO:0016021
Cluster-22178.29544 GO:0022900 GO:0007186 GO:0004930 GO:0016021
Cluster-22178.29546 GO:0007154 GO:0005515 GO:0001533 GO:0016020
Cluster-22178.29547 GO:0006952 GO:0005515
Cluster-22178.29559 GO:0007165 GO:0009401 GO:0009405 GO:0005615
Cluster-22178.29563 GO:0001503 GO:0007304 GO:0005213 GO:0005576
GO:0042600
Cluster-22178.29565 GO:0022904 GO:0006118 GO:0009055 GO:0016747
GO:0016020 GO:0016021
Cluster-22178.29567 GO:0004222
Cluster-22178.29571 GO:0007165 GO:0002532 GO:0006547 GO:0000105
GO:0000287 GO:0003879 GO:0004896 GO:0005737 GO:0016021
Cluster-22178.29573 GO:0016021
Cluster-22178.29577 GO:0045892 GO:0006836 GO:0042754 GO:0006812
GO:0003676 GO:0005328 GO:0005622 GO:0016021
Cluster-22178.29581 GO:0006260 GO:0003677 GO:0046983 GO:0005524
GO:0031224
Cluster-22178.29584 GO:0005515 GO:0016021
Cluster-22178.29588 GO:0005975 GO:0008608 GO:0006118 GO:0008270
GO:0008440 GO:0004553 GO:0030246 GO:0003954 GO:0005576
GO:0005739 GO:0072686 GO:0042729
Cluster-22178.29590 GO:0006144 GO:0006470 GO:0006508 GO:0006570
GO:0003968 GO:0004725 GO:0017111 GO:0008138 GO:0004197
GO:0031379
Cluster-22178.29592 GO:0007165 GO:0055114 GO:0043087 GO:0007264
GO:0016491 GO:0005085 GO:0046872
Cluster-22178.29594 GO:0055114 GO:0043087 GO:0007264 GO:0007165
GO:0016491 GO:0046872 GO:0005085
Cluster-22178.29596 GO:0005634
Cluster-22178.29597 GO:0005515

Cluster-22178.29598 GO:0016567 GO:0004842
Cluster-22178.29602 GO:0005515
Cluster-22178.29609 GO:0010826 GO:0042384 GO:0036038
Cluster-22178.29610 GO:0042384 GO:0010826 GO:0036038
Cluster-22178.29617 GO:0009405 GO:0006810 GO:0046872 GO:0008200 GO:0005576
Cluster-22178.29618 GO:0006810 GO:0009405 GO:0008200 GO:0046872 GO:0005576
Cluster-22178.29622 GO:0019031
Cluster-22178.29623 GO:0019031
Cluster-22178.29626 GO:0005515
Cluster-22178.29627 GO:0006370 GO:0015991 GO:0016192 GO:0006886 GO:0009451 GO:0004482 GO:0016820 GO:0000221 GO:0030117 GO:0030131
Cluster-22178.29635 GO:0009878 GO:0046872
Cluster-22178.29638 GO:0006468 GO:0004672 GO:0005524
Cluster-22178.29648 GO:0016043 GO:0003779
Cluster-22178.29649 GO:0015991 GO:0015992 GO:0015078 GO:0033179
Cluster-22178.29650 GO:0015992 GO:0015991 GO:0015078 GO:0033179
Cluster-22178.29652 GO:0005515
Cluster-22178.29662 GO:0005515
Cluster-22178.29663 GO:0019073 GO:0009451 GO:0001522 GO:0009982 GO:0005515 GO:0003723
Cluster-22178.29665 GO:0045444 GO:0016032 GO:0005509 GO:0004252 GO:0005515 GO:0016021 GO:0005739 GO:0005747
Cluster-22178.29666 GO:0007165 GO:0005515 GO:0016020
Cluster-22178.29668 GO:0006865 GO:0055085 GO:0003333 GO:0006810 GO:0015171 GO:0016020
Cluster-22178.29677 GO:0006118 GO:0009055 GO:0016021
Cluster-22178.29684 GO:0016032 GO:0051082 GO:0016021
Cluster-22178.29689 GO:0005515 GO:0003724 GO:0003723 GO:0005524

Cluster-22178.29691 GO:0005515

Cluster-22178.29692 GO:0008210 GO:0008207 GO:0008209 GO:0055114 GO:0006694 GO:0008152 GO:0050662 GO:0003854 GO:0016616 GO:0016491 GO:0003824

Cluster-22178.29696 GO:0055085 GO:0016021

Cluster-22178.29704 GO:0055114 GO:0008168 GO:0016706

Cluster-22178.29706 GO:0007018 GO:0007017 GO:0003777 GO:0008017 GO:0005524 GO:0005874 GO:0045298

Cluster-22178.29710 GO:0007275 GO:0005578

Cluster-22178.29712 GO:0042407 GO:0016757 GO:0044284 GO:0061617

Cluster-22178.29713 GO:0016032 GO:0044780 GO:0019033

Cluster-22178.29714 GO:0005576

Cluster-22178.29715 GO:0005576

Cluster-22178.29719 GO:0046872 GO:0016020

Cluster-22178.29720 GO:0016020

Cluster-22178.29723 GO:0007186 GO:0004930 GO:0016021

Cluster-22178.29727 GO:0006811 GO:0006352 GO:0055085 GO:0005216 GO:0016021 GO:0016020 GO:0005634

Cluster-22178.29730 GO:0006810 GO:0008200 GO:0005576

Cluster-22178.29731 GO:0005515

Cluster-22178.29738 GO:0051252 GO:0004523 GO:0003676 GO:0005198 GO:0019028

Cluster-22178.29740 GO:0050765

Cluster-22178.29742 GO:0007165 GO:0007275 GO:0006298 GO:0016055 GO:0005102 GO:0005524 GO:0030983 GO:0098519 GO:0005576

Cluster-22178.29743 GO:0007275 GO:0006468 GO:0045893 GO:0008270 GO:0004672 GO:0005524 GO:0046983

Cluster-22178.29747 GO:0016020

Cluster-22178.29754 GO:0030212 GO:0006308 GO:0016888 GO:0004867

Cluster-22178.29760 GO:0003774 GO:0016459

Cluster-22178.29766 GO:0008017 GO:0045298

Cluster-22178.29773 GO:0016020

Cluster-22178.29774 GO:0016020

Cluster-22178.29775 GO:0005515
Cluster-22178.29779 GO:0008544
Cluster-22178.29787 GO:0015672 GO:0006812 GO:0008289 GO:0005261 GO:0016020
Cluster-22178.29788 GO:0006955 GO:0000902 GO:0016020
Cluster-22178.29790 GO:0005515 GO:0008270
Cluster-22178.29793 GO:0019497 GO:0006771 GO:0003993 GO:0005515 GO:0046872 GO:0016020
Cluster-22178.29795 GO:0005515 GO:0016779
Cluster-22178.29796 GO:0007165 GO:0006355 GO:0030168 GO:0051258 GO:0003677 GO:0005515 GO:0030674 GO:0005102 GO:0005577
Cluster-22178.29797 GO:0007093 GO:0042147 GO:0000776
Cluster-22178.29800 GO:0006468 GO:0007031 GO:0005524 GO:0004672 GO:0016773 GO:0005777 GO:0016020
Cluster-22178.29802 GO:0006468 GO:0007031 GO:0004672 GO:0005524 GO:0005777
Cluster-22178.29803 GO:0007031 GO:0006468 GO:0005524 GO:0004672 GO:0005777
Cluster-22178.29804 GO:0006468 GO:0007031 GO:0004672 GO:0005524 GO:0005777
Cluster-22178.29805 GO:0006468 GO:0007031 GO:0004672 GO:0005524 GO:0016773 GO:0005777 GO:0016020
Cluster-22178.29821 GO:0005975 GO:0055085 GO:0006811 GO:0016758 GO:0005216 GO:0016020
Cluster-22178.29824 GO:0031175 GO:0005516 GO:0030507 GO:0005515 GO:0008091
Cluster-22178.29840 GO:0006470 GO:0006570 GO:0004725 GO:0008138 GO:0005515
Cluster-22178.29843 GO:0015074 GO:0006031 GO:0008270 GO:0004100 GO:0005515
Cluster-22178.29847 GO:0007165 GO:0015992 GO:0015991 GO:0015078 GO:0001664 GO:0033179
Cluster-22178.29850 GO:0005515

Cluster-22178.29851 GO:0005515
Cluster-22178.29854 GO:0051260 GO:0016020
Cluster-22178.29857 GO:0006468 GO:0005515 GO:0004672 GO:0005524

Cluster-22178.29861 GO:0005515
Cluster-22178.29862 GO:0005515
Cluster-22178.29863 GO:0045444
Cluster-22178.29865 GO:0006355 GO:0006289 GO:0004867 GO:0000439
Cluster-22178.29868 GO:0006355 GO:0003700 GO:0005667 GO:0005634
Cluster-22178.29869 GO:0006355 GO:0003700 GO:0005667 GO:0005634
Cluster-22178.29871 GO:0006869 GO:0009405 GO:0042157 GO:0007034
GO:0008289 GO:0030430 GO:0005576
Cluster-22178.29876 GO:0051301 GO:0031110 GO:0007059 GO:0007049
GO:0006355 GO:0007067 GO:0000090 GO:0043565 GO:0008017
GO:0003700 GO:0000940 GO:0045298 GO:0005876 GO:0005667
Cluster-22178.29879 GO:0006406 GO:0007130 GO:0007283 GO:0007131
GO:0003723 GO:0005634 GO:0005737
Cluster-22178.29880 GO:0007283 GO:0007130 GO:0007131 GO:0006406
GO:0003723 GO:0005634 GO:0005737
Cluster-22178.29889 GO:0006144 GO:0000256 GO:0008270 GO:0050385

Cluster-22178.29891 GO:0009069 GO:0006468 GO:0006351 GO:0006206
GO:0006144 GO:0016310 GO:0005516 GO:0003677 GO:0003899
GO:0004683 GO:0004672 GO:0005524 GO:0016773 GO:0005730
GO:0016020
Cluster-22178.29894 GO:0003677 GO:0005634
Cluster-22178.29896 GO:0007166 GO:0045892 GO:0007165 GO:0050792
GO:0004871 GO:0046872 GO:0008270 GO:0004518 GO:0005515
GO:0005509 GO:0005634
Cluster-22178.29898 GO:0006260 GO:0006694 GO:0009405 GO:0006351
GO:0016125 GO:0006269 GO:0047750 GO:0003896 GO:0005515
GO:0005198 GO:0016021 GO:0005657 GO:0005730
Cluster-22178.29900 GO:0006820 GO:0016021

Cluster-22178.29907 GO:0006508 GO:0004252 GO:0005515
Cluster-22178.29909 GO:0006886 GO:0005783 GO:0016021
Cluster-22178.29911 GO:0016021
Cluster-22178.29914 GO:0043065 GO:0005515 GO:0005737
Cluster-22178.29917 GO:0008152 GO:0055114 GO:0016491
Cluster-22178.29918 GO:0006508 GO:0008152 GO:0016787 GO:0008236

Cluster-22178.29923 GO:0016310 GO:0034314 GO:0009069 GO:0030833
GO:0006468 GO:0004674 GO:0008239 GO:0005524 GO:0070009
GO:0016021 GO:0015629 GO:0005885 GO:0042765
Cluster-22178.29924 GO:0007165
Cluster-22178.29925 GO:0007165
Cluster-22178.29927 GO:0003824
Cluster-22178.29933 GO:0007186 GO:0004930 GO:0016021
Cluster-22178.2994 GO:0046983
Cluster-22178.29946 GO:0019069 GO:0006367 GO:0005672 GO:0042025
GO:0032040
Cluster-22178.29951 GO:0005524 GO:0003774 GO:0016459
Cluster-22178.29959 GO:0046983
Cluster-22178.29961 GO:0016021
Cluster-22178.29971 GO:0055114 GO:0016491 GO:0030151
Cluster-22178.29977 GO:0005515
Cluster-22178.29978 GO:0005515
Cluster-22178.29982 GO:0006940 GO:0016787
Cluster-22178.29983 GO:0006940 GO:0016787
Cluster-22178.29986 GO:0007411 GO:0016021
Cluster-22178.29987 GO:0000398 GO:0005681
Cluster-22178.29992 GO:0046872 GO:0008270 GO:0000166
Cluster-22178.29998 GO:0007186 GO:0004930 GO:0016021
Cluster-22178.29999 GO:0007186 GO:0004930 GO:0016021
Cluster-22178.30007 GO:0016020
Cluster-22178.30011 GO:0016032 GO:0019031
Cluster-22178.30013 GO:0006810 GO:0055085 GO:0005215 GO:0016020

Cluster-22178.30015 GO:0007165 GO:0040007 GO:0008283 GO:0008083

Cluster-22178.30016 GO:0019087 GO:0016021

Cluster-22178.30029 GO:0008283 GO:0040007 GO:0007165 GO:0005125
GO:0008083 GO:0016021

Cluster-22178.30041 GO:0016021

Cluster-22178.30043 GO:0006814 GO:0006810 GO:0045893 GO:0055085
GO:0015700 GO:0015105 GO:0005215 GO:0016020 GO:0016021

Cluster-22178.30047 GO:0006468 GO:0005524 GO:0004672 GO:0016773
GO:0016020

Cluster-22178.30051 GO:0006813 GO:0005267 GO:0005515 GO:0004252
GO:0008771 GO:0070008

Cluster-22178.30055 GO:0006508 GO:0007186 GO:0004252 GO:0008017
GO:0004930 GO:0045298 GO:0016021

Cluster-22178.30057 GO:0005634

Cluster-22178.30058 GO:0005634

Cluster-22178.30060 GO:0006355 GO:0005515 GO:0003700 GO:0005667

Cluster-22178.30062 GO:0055114 GO:0006118 GO:0016655 GO:0005515

Cluster-22178.30063 GO:0055114 GO:0006118 GO:0016655 GO:0005515

Cluster-22178.30066 GO:0006118 GO:0055114 GO:0005515 GO:0016655

Cluster-22178.30072 GO:0003676

Cluster-22178.30075 GO:0005515

Cluster-22178.30077 GO:0045893 GO:0016021 GO:0005634

Cluster-22178.30079 GO:0005201 GO:0005581 GO:0005578

Cluster-22178.30080 GO:0005201 GO:0005581 GO:0005578

Cluster-22178.30085 GO:0016829 GO:0005576

Cluster-22178.30102 GO:0008146

Cluster-22178.30103 GO:0006886 GO:0007264 GO:0005525 GO:0005543
GO:0003924

Cluster-22178.30104 GO:0003697 GO:0070188

Cluster-22178.30106 GO:0005515 GO:0016020 GO:0016021
Cluster-22178.30108 GO:0016567 GO:0005515 GO:0004842
Cluster-22178.30110 GO:0006355 GO:0003700 GO:0005509 GO:0005515
GO:0005667 GO:0005622
Cluster-22178.30112 GO:0007186 GO:0004930 GO:0016021
Cluster-22178.30113 GO:0055085 GO:0008643 GO:0015992 GO:0051016
GO:0015986 GO:0006139 GO:0009401 GO:0006811 GO:0006814
GO:0005524 GO:0016818 GO:0008026 GO:0008982 GO:0005216
GO:0003676 GO:0015078 GO:0005248 GO:0016020 GO:0008290
GO:0001518 GO:0016021 GO:0045263 GO:0009357
Cluster-22178.30122 GO:0055085 GO:0006811 GO:0007165 GO:0007268
GO:0004970 GO:0005216 GO:0016020
Cluster-22178.30125 GO:0071107
Cluster-22178.30131 GO:0006508 GO:0008152 GO:0008270 GO:0004181
GO:0016788
Cluster-22178.30140 GO:0098542 GO:0006118 GO:0009055 GO:0005515
GO:0005509 GO:0019031 GO:0009512
Cluster-22178.30144 GO:0006468 GO:0004672 GO:0005524 GO:0016773
GO:0016020
Cluster-22178.30147 GO:0015992 GO:0006120 GO:0006744 GO:0006118
GO:0006814 GO:0003954 GO:0008137 GO:0005739
Cluster-22178.30148 GO:0006118 GO:0022900 GO:0020037 GO:0005515
GO:0009055 GO:0005506 GO:0042597
Cluster-22178.30149 GO:0005515
Cluster-22178.3015 GO:0006836 GO:0019905
Cluster-22178.30157 GO:0007049 GO:0006260 GO:0006606 GO:0006914
GO:0003677 GO:0005515 GO:0003723 GO:0016787 GO:0003724
GO:0005524 GO:0016887
Cluster-22178.30158 GO:0006914 GO:0006606 GO:0006260 GO:0016787
GO:0003723 GO:0003677 GO:0005524 GO:0016887 GO:0003724
Cluster-22178.30159 GO:0006914 GO:0007049 GO:0006606 GO:0006260
GO:0005524 GO:0016887 GO:0003724 GO:0003723 GO:0016787
GO:0003677 GO:0005515

Cluster-22178.30165 GO:0055085 GO:0006811 GO:0005216 GO:0016020
Cluster-22178.30168 GO:0005544 GO:0005515 GO:0005509
Cluster-22178.30175 GO:0006470 GO:0019988 GO:0006570 GO:0006810
GO:0009405 GO:0004725 GO:0043399 GO:0019870 GO:0008138
GO:0005576
Cluster-22178.30180 GO:0005515
Cluster-22178.30181 GO:0006355
Cluster-22178.30188 GO:0006952 GO:0016747 GO:0005515
Cluster-22178.30191 GO:0046872
Cluster-22178.30193 GO:0006511 GO:0006771 GO:0019497 GO:0005515
GO:0004175 GO:0003993 GO:0046872 GO:0019773
Cluster-22178.30197 GO:0008152 GO:0016787
Cluster-22178.30199 GO:0016021
Cluster-22178.3020 GO:0007219 GO:0007275 GO:0016021
Cluster-22178.30201 GO:0019064 GO:0055085 GO:0006810 GO:0006811
GO:0005215 GO:0019867 GO:0016020
Cluster-22178.30214 GO:0008270
Cluster-22178.30216 GO:0008270
Cluster-22178.30228 GO:0030436 GO:0006508 GO:0004190
Cluster-22178.30233 GO:0015031 GO:0006464 GO:0005515
Cluster-22178.30234 GO:0006464 GO:0015031 GO:0005515
Cluster-22178.30235 GO:0002098 GO:0034227 GO:0008270 GO:0046872
GO:0003676 GO:0003677 GO:0000049 GO:0005634
Cluster-22178.3024 GO:0007186 GO:0004930 GO:0016021
Cluster-22178.30242 GO:0019722 GO:0007165
Cluster-22178.3025 GO:0007186 GO:0004930 GO:0005742 GO:0016021
GO:0016020
Cluster-22178.30254 GO:0006810 GO:0015992 GO:0055085 GO:0006118
GO:0000902 GO:0006119 GO:0006955 GO:0055114 GO:0006811
GO:0005216 GO:0005509 GO:0005524 GO:0042626 GO:0008121
GO:0016020 GO:0016021
Cluster-22178.30256 GO:0005515
Cluster-22178.30263 GO:0005515 GO:0005509

Cluster-22178.30265 GO:0006355 GO:0043401 GO:0009405 GO:0043565
GO:0008270 GO:0003700 GO:0005634 GO:0005667 GO:0016020
Cluster-22178.30269 GO:0006284 GO:0005515 GO:0003905 GO:0003677
GO:0008270 GO:0005622
Cluster-22178.30274 GO:0006955 GO:0000902 GO:0006364 GO:0008168
GO:0005524 GO:0016818 GO:0003723 GO:0016020 GO:0005634
Cluster-22178.30275 GO:0019064 GO:0018149 GO:0007165 GO:0046789
GO:0003810 GO:0009986 GO:0019031
Cluster-22178.30277 GO:0002949 GO:0006614 GO:0043531 GO:0003924
GO:0005524 GO:0003724 GO:0005525 GO:0003723
Cluster-22178.30280 GO:0005515
Cluster-22178.30288 GO:0004222
Cluster-22178.30289 GO:0006914
Cluster-22178.30293 GO:0007165 GO:0006144 GO:0005198 GO:0004114
GO:0045095
Cluster-22178.30299 GO:0007264 GO:0006355 GO:0043093 GO:0000917
GO:0010508 GO:0006914 GO:0007049 GO:0048193 GO:0043565
GO:0005096 GO:0003700 GO:0005667 GO:0016020 GO:0005737
Cluster-22178.30304 GO:0006468 GO:0005524 GO:0004672
Cluster-22178.30305 GO:0006468 GO:0005524 GO:0004672
Cluster-22178.30306 GO:0006011 GO:0005982 GO:0005985 GO:0030244
GO:0016760 GO:0016020
Cluster-22178.30311 GO:0016032 GO:0055114 GO:0016491 GO:0019012
Cluster-22178.30312 GO:0006525 GO:0006560 GO:0071436 GO:0006814
GO:0007304 GO:0006090 GO:0005509 GO:0008948 GO:0015081
GO:0005213 GO:0005576 GO:0016020 GO:0042600
Cluster-22178.30315 GO:0048870 GO:0031514
Cluster-22178.30316 GO:0048870 GO:0031514
Cluster-22178.30319 GO:0016787
Cluster-22178.30320 GO:0006486 GO:0016757 GO:0008378 GO:0016020
Cluster-22178.30321 GO:0006807 GO:0042549 GO:0015979 GO:0005515
GO:0008270 GO:0016814 GO:0009523 GO:0009539
Cluster-22178.30322 GO:0042549 GO:0015979 GO:0006807 GO:0016814

GO:0008270 GO:0005515 GO:0009523 GO:0009539

Cluster-22178.30323 GO:0042549 GO:0015979 GO:0006807 GO:0005515

GO:0008270 GO:0016814 GO:0009523 GO:0009539

Cluster-22178.30327 GO:0009405 GO:0010506 GO:0045735 GO:0008270

GO:0005615 GO:0016020

Cluster-22178.30329 GO:0015991 GO:0005515 GO:0016820 GO:0000221

Cluster-22178.30330 GO:0009405 GO:0006508 GO:0004222 GO:0008270

GO:0005576 GO:0031012

Cluster-22178.30334 GO:0006260 GO:0003677 GO:0005515

Cluster-22178.30335 GO:0006570 GO:0006470 GO:0005515 GO:0004725

GO:0008138

Cluster-22178.30339 GO:0046872

Cluster-22178.30352 GO:0005515 GO:0003724 GO:0003723 GO:0005524

Cluster-22178.30353 GO:0003724 GO:0005515 GO:0005524 GO:0003723

Cluster-22178.30357 GO:0008152 GO:0003824 GO:0008134 GO:0020037

GO:0005634 GO:0005667

Cluster-22178.30361 GO:0006468 GO:0006807 GO:0007169 GO:0005515

GO:0004714 GO:0005524 GO:0003933 GO:0016020

Cluster-22178.30364 GO:0006308 GO:0071816 GO:0010508 GO:0015992

GO:0006606 GO:0007155 GO:0015986 GO:0015078 GO:0005198

GO:0008855 GO:0005524 GO:0016787 GO:0003677 GO:0009288

GO:0019031 GO:0019028 GO:0009318

Cluster-22178.30369 GO:0030246

Cluster-22178.30372 GO:0005515 GO:0005198 GO:0019028

Cluster-22178.30375 GO:0046497 GO:0055114 GO:0006769 GO:0050661

GO:0008750 GO:0016021

Cluster-22178.30383 GO:0007130 GO:0048870 GO:0051258 GO:0007165

GO:0006810 GO:0030168 GO:0015992 GO:0005198 GO:0030674

GO:0005102 GO:0031514 GO:0000795 GO:0016021 GO:0005882

GO:0005886 GO:0005577

Cluster-22178.30393 GO:0006457 GO:0071973 GO:0006144 GO:0006355

GO:0007009 GO:0015991 GO:0006351 GO:0000917 GO:0006935
GO:0048870 GO:0006308 GO:0007049 GO:0043093 GO:0042157
GO:0006869 GO:0006206 GO:0015992 GO:0006914 GO:0006606
GO:0006886 GO:0051087 GO:0017048 GO:0008855 GO:0000774
GO:0008289 GO:0016818 GO:0003677 GO:0005524 GO:0003774
GO:0008270 GO:0051082 GO:0005198 GO:0003899 GO:0042803
GO:0015078 GO:0005882 GO:0005576 GO:0019028 GO:0009318
GO:0033179 GO:0031514 GO:0019031 GO:0016021 GO:0005730
GO:0055036 GO:0005783 GO:0016020 GO:0009424 GO:0005634
GO:0009288 GO:0005737 GO:0019867
Cluster-22178.30396 GO:0006206 GO:0006144 GO:0055114 GO:0006351
GO:0015991 GO:0033743 GO:0003899 GO:0005515 GO:0016820
GO:0000221 GO:0005730
Cluster-22178.30402 GO:0006355 GO:0043565 GO:0003700 GO:0005667
Cluster-22178.30405 GO:0006508 GO:0004252
Cluster-22178.30408 GO:0018342 GO:0008318 GO:0005515
Cluster-22178.30410 GO:0055085 GO:0006885 GO:0006812 GO:0034477
GO:0004518 GO:0015299 GO:0016021
Cluster-22178.30412 GO:0005515
Cluster-22178.30416 GO:0045893 GO:0006506 GO:0006355 GO:0005515
GO:0016740 GO:0003723 GO:0005789 GO:0016021 GO:0005634
Cluster-22178.30421 GO:0009117 GO:0046373 GO:0005975 GO:0055114
GO:0005515 GO:0016705 GO:0046556
Cluster-22178.30422 GO:0046373 GO:0055114 GO:0005975 GO:0009117
GO:0016705 GO:0005515 GO:0046556 GO:0046872
Cluster-22178.30424 GO:0005576
Cluster-22178.30428 GO:0009966
Cluster-22178.30433 GO:0010972 GO:0030336 GO:0006811 GO:0005216
GO:0016020
Cluster-22178.30434 GO:0007165 GO:0006952 GO:0005126 GO:0005576
Cluster-22178.30436 GO:0046872 GO:0016020
Cluster-22178.30443 GO:0051258 GO:0006090 GO:0030168 GO:0006144
GO:0006633 GO:0006260 GO:0007165 GO:0019058 GO:0004114

GO:0005198 GO:0005515 GO:0003989 GO:0005102 GO:0030674

GO:0019028 GO:0005577 GO:0009317 GO:0005882

Cluster-22178.30444 GO:0006144 GO:0007165 GO:0004114

Cluster-22178.30447 GO:0020037 GO:0019825

Cluster-22178.30448 GO:0019825 GO:0020037

Cluster-22178.30449 GO:0006355 GO:0003723 GO:0016021

Cluster-22178.30454 GO:0003677

Cluster-22178.30457 GO:0006144 GO:0006182 GO:0032509 GO:0046039

GO:0043130 GO:0004383 GO:0020037 GO:0000813

Cluster-22178.30458 GO:0035556 GO:0006144 GO:0006182 GO:0009190

GO:0046039 GO:0046872 GO:0016849 GO:0004383

Cluster-22178.30459 GO:0006144 GO:0032509 GO:0006182 GO:0046039

GO:0043130 GO:0020037 GO:0004383 GO:0000813

Cluster-22178.30461 GO:0006811 GO:0006465 GO:0009451 GO:0001522

GO:0006886 GO:0006396 GO:0006888 GO:0007268 GO:0007165

GO:0004970 GO:0005234 GO:0003723 GO:0008233 GO:0009982

GO:0008270 GO:0016021 GO:0005787 GO:0005622 GO:0030127

GO:0016020

Cluster-22178.30476 GO:0016021

Cluster-22178.30479 GO:0016021 GO:0009279

Cluster-22178.30483 GO:0006355 GO:0042384 GO:0010826 GO:0003700

GO:0005622 GO:0005667 GO:0036038

Cluster-22178.30484 GO:0006098 GO:0019521 GO:0055114 GO:0051287

GO:0016491 GO:0004616 GO:0016620

Cluster-22178.30485 GO:0055114 GO:0019521 GO:0006098 GO:0051287

GO:0004616

Cluster-22178.30486 GO:0055114 GO:0016620 GO:0051287 GO:0016491

Cluster-22178.30491 GO:0006693 GO:0006691 GO:0019530 GO:0006749

GO:0051537 GO:0003840 GO:0043231

Cluster-22178.30492 GO:0006955 GO:0019882 GO:0016020 GO:0042613

Cluster-22178.30493 GO:0019882 GO:0006955 GO:0042613 GO:0016020

Cluster-22178.30501 GO:0046872 GO:0008270 GO:0000166

Cluster-22178.30503 GO:0006355 GO:0015743 GO:0003677
Cluster-22178.30506 GO:0006260 GO:0006351 GO:0003677 GO:0003887
GO:0042575
Cluster-22178.30510 GO:0006807 GO:0007165 GO:0008158 GO:0003933
GO:0016020
Cluster-22178.30513 GO:0006811 GO:0055085 GO:0005216 GO:0016020
Cluster-22178.30517 GO:0006810 GO:0007018 GO:0007017 GO:0003777
GO:0005524 GO:0016887 GO:0003677 GO:0005874 GO:0030286
Cluster-22178.30519 GO:0006508 GO:0004252
Cluster-22178.30524 GO:0016021 GO:0042765
Cluster-22178.30527 GO:0005515
Cluster-22178.30528 GO:0006810 GO:0005515 GO:0008200 GO:0005576
Cluster-22178.30534 GO:0006810 GO:0007130 GO:0007067 GO:0005198
GO:0005886 GO:0000795 GO:0005882 GO:0042729 GO:0072686
Cluster-22178.30535 GO:0007130 GO:0007165 GO:0006810 GO:0048870
GO:0030168 GO:0051258 GO:0005198 GO:0030674 GO:0005102
GO:0031514 GO:0005882 GO:0005577 GO:0000795 GO:0005886
Cluster-22178.30538 GO:0003677
Cluster-22178.30542 GO:0016787
Cluster-22178.30546 GO:0006771 GO:0019497 GO:0055085 GO:0006812
GO:0005515 GO:0046872 GO:0008324 GO:0003993 GO:0016021
Cluster-22178.30547 GO:0007010 GO:0003779
Cluster-22178.30558 GO:0006384 GO:0006366 GO:0005515 GO:0008017
GO:0003700 GO:0005667 GO:0005634 GO:0016021 GO:0045298
Cluster-22178.30559 GO:0006468 GO:0005515 GO:0005524 GO:0004672

Cluster-22178.30561 GO:0007165 GO:0005509 GO:0005578
Cluster-22178.30562 GO:0007165 GO:0005515
Cluster-22178.30564 GO:0007186 GO:0007165 GO:0003924 GO:0031683
GO:0019001 GO:0004871 GO:0005525
Cluster-22178.30565 GO:0055114 GO:0016491
Cluster-22178.30569 GO:0016032 GO:0019031
Cluster-22178.30571 GO:0006310 GO:0003677 GO:0005515 GO:0005634

Cluster-22178.30585 GO:0006414 GO:0006448 GO:0016055 GO:0003746 GO:0005840
Cluster-22178.30587 GO:0015031 GO:0043401 GO:0006355 GO:0006468 GO:0003700 GO:0043565 GO:0004715 GO:0005524 GO:0008270 GO:0030176 GO:0005667 GO:0005634
Cluster-22178.30590 GO:0055085 GO:0006814 GO:0006810 GO:0005215 GO:0061630 GO:0005515 GO:0016021 GO:0019867 GO:0016020
Cluster-22178.30593 GO:0016567 GO:0004842
Cluster-22178.30594 GO:0006355 GO:0019904 GO:0008134 GO:0003700 GO:0043565 GO:0005667
Cluster-22178.30599 GO:0042765 GO:0016021
Cluster-22178.30608 GO:0051252 GO:0004523 GO:0003676 GO:0005198 GO:0019028
Cluster-22178.30609 GO:0005975 GO:0004553
Cluster-22178.30612 GO:0055085 GO:0016021
Cluster-22178.30614 GO:0006810 GO:0006814 GO:0055085 GO:0061630 GO:0005515 GO:0005215 GO:0016020 GO:0016021 GO:0019867
Cluster-22178.30617 GO:0005515
Cluster-22178.30618 GO:0006118 GO:0006468 GO:0009055 GO:0003676 GO:0005524 GO:0004672 GO:0016773 GO:0016020
Cluster-22178.30629 GO:0006541 GO:0004359
Cluster-22178.30634 GO:0006269 GO:0006355 GO:0006351 GO:0003677 GO:0008270 GO:0004386 GO:0003896 GO:0005730 GO:0005657
Cluster-22178.30635 GO:0006355 GO:0003677
Cluster-22178.30637 GO:0046872
Cluster-22178.30639 GO:0008152 GO:0016787 GO:0003676
Cluster-22178.30647 GO:0051252 GO:0003676 GO:0005198 GO:0004523 GO:0019028
Cluster-22178.30651 GO:0043087 GO:0035023 GO:0005089 GO:0005515

Cluster-22178.30652 GO:0006278 GO:0003964 GO:0008168
Cluster-22178.30653 GO:0035023 GO:0043087 GO:0005089 GO:0005515

Cluster-22178.30657 GO:0006355 GO:0043565 GO:0003700 GO:0005667
Cluster-22178.30660 GO:0006508 GO:0006629 GO:0006144 GO:0004252
GO:0017111 GO:0004197 GO:0016817 GO:0003968 GO:0070008
GO:0031379
Cluster-22178.30661 GO:0006506 GO:0000746 GO:0019867 GO:0016021
Cluster-22178.30669 GO:0009395 GO:0046339 GO:0035556 GO:0050790
GO:0006629 GO:0007165 GO:0016773 GO:0004435 GO:0035091
GO:0005515 GO:0030234
Cluster-22178.30670 GO:0006811 GO:0009395 GO:0006351 GO:0006355
GO:0006629 GO:0035556 GO:0006260 GO:0055085 GO:0046339
GO:0006270 GO:0006810 GO:0007165 GO:0006874 GO:0006816
GO:0005219 GO:0004435 GO:0003887 GO:0005515 GO:0016773
GO:0003677 GO:0035091 GO:0005216 GO:0003682 GO:0000775
GO:0019031 GO:0000785 GO:0016021 GO:0042575 GO:0005634
GO:0005622 GO:0016020
Cluster-22178.30675 GO:0050909 GO:0007186 GO:0004930 GO:0016021
Cluster-22178.30677 GO:0016021
Cluster-22178.30688 GO:0003723
Cluster-22178.30690 GO:0009058 GO:0030170
Cluster-22178.30691 GO:0009058 GO:0030170
Cluster-22178.30699 GO:0015992 GO:0006120 GO:0006744 GO:0006118
GO:0006814 GO:0003954 GO:0008137 GO:0005739
Cluster-22178.30701 GO:0006357 GO:0005975 GO:0004649 GO:0001104
GO:0016592
Cluster-22178.30708 GO:0016846 GO:0005515
Cluster-22178.30717 GO:0000226 GO:0019087 GO:0015630 GO:0016021
Cluster-22178.30718 GO:0000226 GO:0019087 GO:0015630 GO:0016021
Cluster-22178.30734 GO:0006914 GO:0007049 GO:0048193 GO:0006355
GO:0043093 GO:0007264 GO:0000917 GO:0010508 GO:0043565
GO:0005096 GO:0003700 GO:0005737 GO:0005667 GO:0016020
Cluster-22178.30735 GO:0046872
Cluster-22178.30750 GO:0006508 GO:0004190
Cluster-22178.30752 GO:0004222

Cluster-22178.30757 GO:0006310 GO:0003677 GO:0005515 GO:0005634
Cluster-22178.30760 GO:0006302 GO:0005634
Cluster-22178.30762 GO:0003677 GO:0000166
Cluster-22178.30768 GO:0006351 GO:0006269 GO:0006260 GO:0005515
GO:0003677 GO:0003896 GO:0008270 GO:0046872 GO:0005730
GO:0005657 GO:0005622
Cluster-22178.30771 GO:0005515 GO:0061630
Cluster-22178.30773 GO:0003677 GO:0005634
Cluster-22178.30775 GO:0003677 GO:0005634
Cluster-22178.30778 GO:0007017 GO:0007018 GO:0006810 GO:0042147
GO:0015031 GO:0048193 GO:0003777 GO:0003676 GO:0016787
GO:0003677 GO:0005524 GO:0016887 GO:0008565 GO:0005874
GO:0030904 GO:0016020 GO:0030286
Cluster-22178.30784 GO:0055114 GO:0016491 GO:0030246 GO:0050660

Cluster-22178.30785 GO:0055114 GO:0016491 GO:0050660
Cluster-22178.30786 GO:0030234 GO:0005515 GO:0016020
Cluster-22178.30796 GO:0015031
Cluster-22178.30797 GO:0019058 GO:0045859 GO:0016534 GO:0016533
Cluster-22178.30800 GO:0015886 GO:0017004 GO:0016021
Cluster-22178.30801 GO:0044564 GO:0008152 GO:0008484 GO:0003824

Cluster-22178.30802 GO:0006508 GO:0008270 GO:0004222 GO:0031012
Cluster-22178.30803 GO:0006260 GO:0006270 GO:0006351 GO:0003887
GO:0003677 GO:0032040 GO:0005789 GO:0019031 GO:0042575
Cluster-22178.30805 GO:0007165 GO:0016020
Cluster-22178.30808 GO:0005975 GO:0004553
Cluster-22178.30811 GO:0019073 GO:0005515
Cluster-22178.30817 GO:0006508 GO:0008236
Cluster-22178.30820 GO:0007596 GO:0007165 GO:0006950 GO:0055114
GO:0016705 GO:0020037 GO:0005198 GO:0005506 GO:0005179
GO:0019028 GO:0005576
Cluster-22178.30821 GO:0051258 GO:0030168 GO:0007165 GO:0030674

GO:0005102 GO:0005515 GO:0005577
Cluster-22178.30824 GO:0007017 GO:0007018 GO:0005524 GO:0008017
GO:0003777 GO:0045298 GO:0005874
Cluster-22178.30827 GO:0005515
Cluster-22178.30828 GO:0019497 GO:0006771 GO:0046872 GO:0003993
GO:0005515
Cluster-22178.30836 GO:0005515
Cluster-22178.30841 GO:0005515
Cluster-22178.30842 GO:0071107
Cluster-22178.30848 GO:0006570 GO:0007186 GO:0006470 GO:0050852
GO:0004930 GO:0004725 GO:0016020 GO:0016021
Cluster-22178.30849 GO:0050852 GO:0006470 GO:0006570 GO:0007186
GO:0004725 GO:0004930 GO:0016021 GO:0016020
Cluster-22178.30851 GO:0055085 GO:0006811 GO:0009607 GO:0030976
GO:0000287 GO:0005216 GO:0016021 GO:0019031 GO:0016020
Cluster-22178.30852 GO:0055085 GO:0006811 GO:0005216 GO:0016020
Cluster-22178.30854 GO:0019049 GO:0005515 GO:0008270
Cluster-22178.30856 GO:0008544 GO:0042157 GO:0016559 GO:0006468
GO:0006869 GO:0004672 GO:0005524 GO:0008289 GO:0005779
GO:0019028 GO:0005576
Cluster-22178.30857 GO:0016817
Cluster-22178.30859 GO:0006811 GO:0006810 GO:0005230 GO:0016020
Cluster-22178.30866 GO:0046872 GO:0003677
Cluster-22178.30867 GO:0006351 GO:0006281 GO:0046034 GO:0006269
GO:0006464 GO:0006144 GO:0005515 GO:0016151 GO:0003896
GO:0046872 GO:0004017 GO:0005506 GO:0004386 GO:0008270
GO:0005730 GO:0005657
Cluster-22178.30868 GO:0046034 GO:0006351 GO:0006281 GO:0006144
GO:0006464 GO:0006269 GO:0016151 GO:0003896 GO:0046872
GO:0005515 GO:0004386 GO:0008270 GO:0004017 GO:0005506
GO:0005657 GO:0005730
Cluster-22178.30871 GO:0006270 GO:0035556 GO:0003677 GO:0005515

Cluster-22178.30872 GO:0055085 GO:0005515 GO:0005634 GO:0016021
Cluster-22178.30880 GO:0006468 GO:0016773 GO:0004672 GO:0005524
GO:0016020
Cluster-22178.30884 GO:0016032 GO:0055114 GO:0016491 GO:0019012
Cluster-22178.30890 GO:0006810 GO:0006351 GO:0015858 GO:0006260
GO:0009236 GO:0003887 GO:0003677 GO:0005337 GO:0042575
GO:0016021
Cluster-22178.30891 GO:0006531 GO:0006188 GO:0009152 GO:0006522
GO:0048193 GO:0006144 GO:0004018 GO:0016020
Cluster-22178.30895 GO:0007275 GO:0005201 GO:0005581 GO:0005578
Cluster-22178.30898 GO:0006629
Cluster-22178.30901 GO:0006955 GO:0007165 GO:0006396 GO:0005126
GO:0005576 GO:0005622 GO:0001527 GO:0019031
Cluster-22178.30910 GO:0008270 GO:0005634
Cluster-22178.30911 GO:0003723 GO:0019013
Cluster-22178.30913 GO:0005515
Cluster-22178.30914 GO:0030145 GO:0016787 GO:0003723
Cluster-22178.30919 GO:0015979 GO:0005515 GO:0009538 GO:0009522
Cluster-22178.30922 GO:0005515
Cluster-22178.30923 GO:0006869 GO:0051252 GO:0008063 GO:0005319
GO:0004540
Cluster-22178.30924 GO:0008063 GO:0051252 GO:0006869 GO:0004540
GO:0005319 GO:0019031
Cluster-22178.30926 GO:0006935 GO:0007165 GO:0004888 GO:0016020
Cluster-22178.30927 GO:0005515
Cluster-22178.3093 GO:0006955 GO:0007165 GO:0035023 GO:0043087
GO:0005515 GO:0005044 GO:0005089 GO:0030247
Cluster-22178.30930 GO:0005515
Cluster-22178.3095 GO:0007165 GO:0006955 GO:0005164 GO:0016020
Cluster-22178.30952 GO:0006508 GO:0030436 GO:0006310 GO:0004190
GO:0003677 GO:0005515 GO:0005634
Cluster-22178.30956 GO:0051258 GO:0006308 GO:0048309 GO:0007049
GO:0015628 GO:0006534 GO:0016032 GO:0006351 GO:0030168

GO:0015031 GO:0051028 GO:0042967 GO:0006535 GO:0006810
GO:0007165 GO:0004866 GO:0009001 GO:0008565 GO:0008855
GO:0005102 GO:0030674 GO:0005737 GO:0015627 GO:0019028
GO:0009318 GO:0005886 GO:0005577 GO:0017053
Cluster-22178.30957 GO:0015031 GO:0015628 GO:0006534 GO:0042967
GO:0006535 GO:0016032 GO:0008565 GO:0004866 GO:0009001
GO:0005737 GO:0015627
Cluster-22178.30958 GO:0015031 GO:0030168 GO:0006810 GO:0007165
GO:0016567 GO:0051258 GO:0006308 GO:0007049 GO:0015628
GO:0016032 GO:0006351 GO:0008565 GO:0008855 GO:0004842
GO:0005102 GO:0030674 GO:0004866 GO:0005886 GO:0005577
GO:0017053 GO:0015627 GO:0019028 GO:0009318
Cluster-22178.30959 GO:0007049 GO:0015628 GO:0051258 GO:0006308
GO:0006351 GO:0016032 GO:0030168 GO:0015031 GO:0006810
GO:0007165 GO:0004866 GO:0005102 GO:0008565 GO:0008855
GO:0030674 GO:0015627 GO:0009318 GO:0019028 GO:0005577
GO:0005886 GO:0017053
Cluster-22178.30960 GO:0006351 GO:0016032 GO:0007049 GO:0015628
GO:0051258 GO:0006308 GO:0007165 GO:0006810 GO:0015031
GO:0030168 GO:0004866 GO:0030674 GO:0005102 GO:0008855
GO:0008565 GO:0009318 GO:0019028 GO:0015627 GO:0017053
GO:0005577 GO:0005886
Cluster-22178.30961 GO:0015628 GO:0006534 GO:0042967 GO:0015031
GO:0016032 GO:0006535 GO:0004866 GO:0008565 GO:0009001
GO:0015627 GO:0005737
Cluster-22178.30962 GO:0006535 GO:0016032 GO:0015031 GO:0015628
GO:0006534 GO:0042967 GO:0009001 GO:0008565 GO:0004866
GO:0005737 GO:0015627
Cluster-22178.30966 GO:0006771 GO:0019497 GO:0046872 GO:0005515
GO:0003993 GO:0016020
Cluster-22178.30968 GO:0004867 GO:0000287 GO:0030976 GO:0005515

Cluster-22178.30969 GO:0006355 GO:0003677

Cluster-22178.3098 GO:0005515 GO:0016020
Cluster-22178.30986 GO:0006406 GO:0007130 GO:0007283 GO:0007131
GO:0003723 GO:0005634 GO:0005737
Cluster-22178.30990 GO:0006468 GO:0004672 GO:0005524
Cluster-22178.30992 GO:0005515
Cluster-22178.30999 GO:0008270
Cluster-22178.31005 GO:0007186 GO:0004930 GO:0016021
Cluster-22178.31006 GO:0008270
Cluster-22178.31015 GO:0005515
Cluster-22178.31018 GO:0006281 GO:0000723 GO:0016887 GO:0005524
GO:0003678 GO:0005657
Cluster-22178.31019 GO:0009245 GO:0005515 GO:0008915
Cluster-22178.31025 GO:0005515 GO:0046872 GO:0045202
Cluster-22178.31038 GO:0005515
Cluster-22178.31039 GO:0007165 GO:0005179 GO:0005576
Cluster-22178.31040 GO:0005201 GO:0005581 GO:0005578
Cluster-22178.31043 GO:0003333 GO:0006810 GO:0055085 GO:0006865
GO:0015171 GO:0005524 GO:0016020
Cluster-22178.31048 GO:0051082 GO:0031072
Cluster-22178.31049 GO:0030170 GO:0003676
Cluster-22178.31052 GO:0046488 GO:0016307
Cluster-22178.31056 GO:0055085 GO:0016021
Cluster-22178.31062 GO:0006357 GO:0005515 GO:0001104 GO:0003723
GO:0016592
Cluster-22178.31065 GO:0006355 GO:0043565
Cluster-22178.31069 GO:0019497 GO:0006771 GO:0046872 GO:0005515
GO:0003993 GO:0005737
Cluster-22178.31075 GO:0006570 GO:0006470 GO:0004725 GO:0008138

Cluster-22178.31080 GO:0005515
Cluster-22178.31081 GO:0016021
Cluster-22178.31082 GO:0015992 GO:0016021
Cluster-22178.31088 GO:0006695 GO:0006694 GO:0032297 GO:0003677

GO:0004631 GO:0005737
Cluster-22178.31090 GO:0006351 GO:0006144 GO:0003968 GO:0005524
GO:0003723 GO:0031379
Cluster-22178.31095 GO:0006508 GO:0004252 GO:0005515
Cluster-22178.31105 GO:0006355 GO:0007155 GO:0003677
Cluster-22178.31106 GO:0007155 GO:0006355 GO:0003677
Cluster-22178.31107 GO:0006570 GO:0006771 GO:0006470 GO:0019497
GO:0005515 GO:0008138 GO:0003993 GO:0004725 GO:0046872
Cluster-22178.31112 GO:0005515
Cluster-22178.31123 GO:0007165 GO:0006144 GO:0004114
Cluster-22178.31137 GO:0009058 GO:0003824
Cluster-22178.31139 GO:0055114 GO:0006633 GO:0005506 GO:0016491
GO:0008375 GO:0016020
Cluster-22178.31144 GO:0005515
Cluster-22178.31153 GO:0050852 GO:0006470 GO:0006570 GO:0004725
GO:0005515
Cluster-22178.31154 GO:0009405 GO:0008270 GO:0003676 GO:0003677

Cluster-22178.31158 GO:0046983
Cluster-22178.31159 GO:0006807 GO:0045182 GO:0004252 GO:0016151
GO:0005886
Cluster-22178.31160 GO:0055114 GO:0015992 GO:0006123 GO:0009060
GO:0006118 GO:0004129 GO:0009055 GO:0005506 GO:0020037
GO:0016021 GO:0045277
Cluster-22178.31173 GO:0005515
Cluster-22178.31186 GO:0006355 GO:0019069 GO:0007165 GO:0030168
GO:0051258 GO:0055114 GO:0051287 GO:0003723 GO:0030674
GO:0016616 GO:0016817 GO:0005102 GO:0005577
Cluster-22178.31191 GO:0006355 GO:0003677
Cluster-22178.31194 GO:0006351 GO:0006260 GO:0003887 GO:0003677
GO:0042575
Cluster-22178.31199 GO:0006355 GO:0006260 GO:0003700 GO:0005667
Cluster-22178.31200 GO:0006260 GO:0006308 GO:0008855 GO:0009318

Cluster-22178.31204 GO:0046856 GO:0006779 GO:0055114 GO:0005515
GO:0034597 GO:0005524 GO:0003774 GO:0016887 GO:0016459
Cluster-22178.31206 GO:0003677
Cluster-22178.31210 GO:0019521 GO:0055114 GO:0006098 GO:0016491
GO:0051287 GO:0016620 GO:0004616
Cluster-22178.31217 GO:0052331 GO:0006812 GO:0007155 GO:0046931
GO:0006810 GO:0015267 GO:0005515 GO:0016021 GO:0046930
GO:0016020
Cluster-22178.31218 GO:0016020
Cluster-22178.31228 GO:0016055 GO:0007165 GO:0007275 GO:0006570
GO:0055114 GO:0004511 GO:0005102 GO:0005576
Cluster-22178.31239 GO:0007264 GO:0004190 GO:0005525 GO:0016020
Cluster-22178.31241 GO:0055114 GO:0008168 GO:0016706
Cluster-22178.31242 GO:0008283 GO:0040007 GO:0007165 GO:0008083

Cluster-22178.31243 GO:0019497 GO:0006013 GO:0006000 GO:0055114
GO:0006771 GO:0005524 GO:0003993 GO:0005507 GO:0016641
GO:0003873 GO:0016301
Cluster-22178.31244 GO:0005515
Cluster-22178.31245 GO:0005515
Cluster-22178.31246 GO:0005515
Cluster-22178.31247 GO:0006886 GO:0006888 GO:0005515 GO:0008270
GO:0046872 GO:0030127
Cluster-22178.31250 GO:0007155 GO:0005509 GO:0005515 GO:0005576
GO:0005739 GO:0005747
Cluster-22178.31252 GO:0007186 GO:0006886 GO:0004930 GO:0005783
GO:0016021
Cluster-22178.31259 GO:0007165 GO:0005515 GO:0005509 GO:0005578
Cluster-22178.31263 GO:0005507
Cluster-22178.31265 GO:0006811 GO:0005216 GO:0016020
Cluster-22178.31266 GO:0007186 GO:0008270 GO:0004930
Cluster-22178.31267 GO:0006303 GO:0003677
Cluster-22178.31279 GO:0008270

Cluster-22178.31283 GO:0046872 GO:0005515
Cluster-22178.31285 GO:0005515 GO:0046872
Cluster-22178.31286 GO:0019385
Cluster-22178.31287 GO:0006468 GO:0005524 GO:0004672
Cluster-22178.31290 GO:0006260 GO:0006310 GO:0006281 GO:0006355
GO:0003724 GO:0008270 GO:0005524 GO:0016887 GO:0003677
GO:0009378 GO:0008191 GO:0016787 GO:0003678 GO:0008134
GO:0005515 GO:0003697 GO:0003723 GO:0005657 GO:0009379
GO:0005667 GO:0042025
Cluster-22178.31294 GO:0015992 GO:0015986 GO:0015078 GO:0000276
Cluster-22178.31295 GO:0015986 GO:0015992 GO:0015078 GO:0000276
Cluster-22178.31302 GO:0015031 GO:0008565
Cluster-22178.31303 GO:0055114 GO:0046856 GO:0006779 GO:0003774
GO:0005524 GO:0016887 GO:0034597 GO:0005515 GO:0016459
Cluster-22178.31304 GO:0030246
Cluster-22178.31305 GO:0007165 GO:0007186 GO:0016755 GO:0004871
GO:0005834
Cluster-22178.31306 GO:0005515
Cluster-22178.31307 GO:0005515
Cluster-22178.31311 GO:0009069 GO:0006468 GO:0016310 GO:0005524
GO:0000287 GO:0004674 GO:0005515
Cluster-22178.31312 GO:0016579 GO:0036459
Cluster-22178.31318 GO:0005515
Cluster-22178.31325 GO:0005515
Cluster-22178.31327 GO:0016772
Cluster-22178.31332 GO:0006260 GO:0006606 GO:0042967 GO:0006914
GO:0003724 GO:0005524 GO:0016887 GO:0003677 GO:0016787
GO:0003723 GO:0004379
Cluster-22178.31333 GO:0034080 GO:0005515 GO:0000775
Cluster-22178.31338 GO:0006355 GO:0003700 GO:0003677 GO:0005667
Cluster-22178.31339 GO:0006355 GO:0003700 GO:0005515 GO:0005667
GO:0042025
Cluster-22178.31341 GO:0005524 GO:0009507

Cluster-22178.31351 GO:0015992 GO:0006260 GO:0006118 GO:0006119
GO:0005524 GO:0003677 GO:0008121
Cluster-22178.31353 GO:0007010 GO:0006506 GO:0006816 GO:0006874
GO:0007304 GO:0005509 GO:0016746 GO:0005213 GO:0005219
GO:0003779 GO:0005576 GO:0005789 GO:0016021 GO:0005622
GO:0042600
Cluster-22178.31356 GO:0005524 GO:0003723 GO:0005515 GO:0003724

Cluster-22178.31357 GO:0008270
Cluster-22178.31359 GO:0008270
Cluster-22178.31361 GO:0008270
Cluster-22178.31364 GO:0007016 GO:0016010
Cluster-22178.31365 GO:0006771 GO:0019497 GO:0046872 GO:0005515
GO:0003993
Cluster-22178.31366 GO:0006771 GO:0019497 GO:0007016 GO:0003993
GO:0005515 GO:0046872 GO:0016010
Cluster-22178.31367 GO:0015979 GO:0005515
Cluster-22178.31368 GO:0005515
Cluster-22178.31370 GO:0009102 GO:0006206 GO:0006144 GO:0006768
GO:0006351 GO:0010508 GO:0003677 GO:0005524 GO:0042410
GO:0003899 GO:0005515 GO:0005198 GO:0005730 GO:0005882
GO:0030133 GO:0019028 GO:0031083 GO:0019031
Cluster-22178.31376 GO:0019042 GO:0006468 GO:0007186 GO:0005524
GO:0004672 GO:0004930 GO:0033644 GO:0016020
Cluster-22178.31377 GO:0006606 GO:0048870 GO:0006308 GO:0010508
GO:0007155 GO:0003774 GO:0008855 GO:0005198 GO:0005815
GO:0009318 GO:0005882 GO:0031514 GO:0016459 GO:0019028
Cluster-22178.31378 GO:0007186 GO:0004930 GO:0051537 GO:0016021
GO:0043231
Cluster-22178.31389 GO:0006811 GO:0055085 GO:0006813 GO:0007268
GO:0007165 GO:0004970 GO:0005216 GO:0005242 GO:0016021
GO:0008076 GO:0016020
Cluster-22178.31392 GO:0007165 GO:0040007 GO:0008283 GO:0008083

Cluster-22178.31403 GO:0006032 GO:0016998 GO:0019239 GO:0004568

Cluster-22178.31404 GO:0023052
Cluster-22178.31407 GO:0042254 GO:0006412 GO:0003723 GO:0003735
GO:0005840
Cluster-22178.31408 GO:0006355 GO:0003700 GO:0005515 GO:0005667
GO:0042025
Cluster-22178.31409 GO:0005515
Cluster-22178.31411 GO:0000160 GO:0006935 GO:0003677 GO:0016787
GO:0008984 GO:0003676 GO:0003824 GO:0000156 GO:0005524
GO:0016817 GO:0005737
Cluster-22178.31414 GO:0007067 GO:0009401 GO:0007049 GO:0005515
GO:0005634 GO:0000775
Cluster-22178.31418 GO:0006351 GO:0006270 GO:0006260 GO:0003677
GO:0003887 GO:0042575 GO:0019031 GO:0005789 GO:0032040
Cluster-22178.31421 GO:0030246
Cluster-22178.31423 GO:0030246
Cluster-22178.31424 GO:0005515 GO:0005634
Cluster-22178.31431 GO:0015992 GO:0007155 GO:0015986 GO:0005524
GO:0015078 GO:0016301 GO:0005540 GO:0005515 GO:0000276
Cluster-22178.31433 GO:0006468 GO:0005524 GO:0004672
Cluster-22178.31435 GO:0009411 GO:0006289 GO:0004519
Cluster-22178.31441 GO:0007186 GO:0007165 GO:0004871 GO:0016755
GO:0005834
Cluster-22178.31442 GO:0006506 GO:0006308 GO:0055114 GO:0051082
GO:0051287 GO:0016616 GO:0017176 GO:0008855 GO:0009318
GO:0016021
Cluster-22178.31444 GO:0006355 GO:0043401 GO:0006886 GO:0009094
GO:0055114 GO:0016310 GO:0006571 GO:0000162 GO:0004673
GO:0008977 GO:0004665 GO:0005622 GO:0009365
Cluster-22178.31450 GO:0016787
Cluster-22178.31455 GO:0005515

Cluster-22178.31457 GO:0007165 GO:0005179 GO:0005576
Cluster-22178.31464 GO:0007165 GO:0009847 GO:0006869 GO:0042157
GO:0007067 GO:0009069 GO:0006260 GO:0006355 GO:0006468
GO:0016310 GO:0007049 GO:0004674 GO:0005096 GO:0005198
GO:0004871 GO:0003700 GO:0008289 GO:0043565 GO:0016020
GO:0005667 GO:0005815 GO:0042729 GO:0072686 GO:0019028
GO:0005576 GO:0019031
Cluster-22178.31465 GO:0007067 GO:0042157 GO:0006869 GO:0006355
GO:0006308 GO:0008608 GO:0003677 GO:0008855 GO:0005198
GO:0008289 GO:0005576 GO:0019028 GO:0072686 GO:0042729
GO:0009318 GO:0019031
Cluster-22178.31466 GO:0006955 GO:0007165 GO:0006396 GO:0005126
GO:0005576 GO:0016021 GO:0001527 GO:0019031 GO:0005622
Cluster-22178.31467 GO:0005525 GO:0003677
Cluster-22178.31470 GO:0009607 GO:0016021
Cluster-22178.31472 GO:0009432 GO:0006281 GO:0005524 GO:0003697

Cluster-22178.31476 GO:0006355 GO:0051301 GO:0016817 GO:0003677
GO:0005515 GO:0005634
Cluster-22178.31480 GO:0045893 GO:0055085 GO:0015700 GO:0006810
GO:0006814 GO:0005215 GO:0015105 GO:0016021 GO:0016020
Cluster-22178.31482 GO:0008270 GO:0003723 GO:0005201 GO:0005515
GO:0005578 GO:0005581
Cluster-22178.31488 GO:0007094 GO:0007165 GO:0007155 GO:0051258
GO:0051607 GO:0030168 GO:0048870 GO:0005515 GO:0030674
GO:0005102 GO:0031514 GO:0005577
Cluster-22178.31496 GO:0008270 GO:0046872 GO:0005515
Cluster-22178.31500 GO:0007186 GO:0004930 GO:0016021
Cluster-22178.31502 GO:0006511 GO:0005515
Cluster-22178.31505 GO:0006355 GO:0005975 GO:0043169 GO:0003682
GO:0003677 GO:0008270 GO:0003824 GO:0005634 GO:0000785
GO:0000775
Cluster-22178.31508 GO:0006811 GO:0055085 GO:0005216 GO:0016020

Cluster-22178.31511 GO:0009069 GO:0006468 GO:0009405 GO:0016310
GO:0005524 GO:0004672 GO:0004674
Cluster-22178.31519 GO:0007165
Cluster-22178.31525 GO:0003676
Cluster-22178.31526 GO:0007155 GO:0005540
Cluster-22178.31532 GO:0005515
Cluster-22178.31537 GO:0006858 GO:0035556 GO:0005544 GO:0005515
GO:0005509
Cluster-22178.31543 GO:0005515
Cluster-22178.31544 GO:0006950 GO:0007186 GO:0006508 GO:0006355
GO:0003700 GO:0004930 GO:0008270 GO:0005198 GO:0004190
GO:0005667 GO:0005634 GO:0019028
Cluster-22178.31545 GO:0006950 GO:0007186 GO:0006508 GO:0006355
GO:0003700 GO:0004930 GO:0008270 GO:0005198 GO:0004190
GO:0019028 GO:0005667 GO:0005634
Cluster-22178.31548 GO:0007165 GO:0006955 GO:0005515 GO:0005125
GO:0005576
Cluster-22178.31550 GO:0006310 GO:0003677 GO:0005515 GO:0005634
Cluster-22178.31552 GO:0006355 GO:0051297 GO:0007049 GO:0051607
GO:0003677 GO:0003700 GO:0043565 GO:0048786 GO:0005667
GO:0005813
Cluster-22178.31560 GO:0006869 GO:0007186 GO:0004930 GO:0005319
GO:0005515 GO:0016021
Cluster-22178.31562 GO:0005201 GO:0005578 GO:0005581
Cluster-22178.31565 GO:0007165 GO:0043401 GO:0006355 GO:0009103
GO:0005496 GO:0008918 GO:0003677 GO:0005515 GO:0030284
GO:0005634
Cluster-22178.31566 GO:0009103 GO:0006355 GO:0043401 GO:0007165
GO:0005515 GO:0030284 GO:0003677 GO:0008918 GO:0005496
GO:0005634
Cluster-22178.31571 GO:0055114 GO:0015992 GO:0006744 GO:0006814
GO:0006120 GO:0042773 GO:0008137
Cluster-22178.31572 GO:0007275 GO:0005515 GO:0004867 GO:0005578

Cluster-22178.31584 GO:0003677
Cluster-22178.31587 GO:0030246
Cluster-22178.31589 GO:0003676
Cluster-22178.31590 GO:0003676
Cluster-22178.31596 GO:0016021
Cluster-22178.31597 GO:0007186 GO:0004930 GO:0016020
Cluster-22178.31612 GO:0005515 GO:0042802
Cluster-22178.31617 GO:0005515
Cluster-22178.31618 GO:0055114 GO:0015886 GO:0005975 GO:0009117 GO:0017004 GO:0046373 GO:0046556 GO:0005515 GO:0016705 GO:0016021
Cluster-22178.31619 GO:0005515
Cluster-22178.31620 GO:0007166 GO:0005515 GO:0016020
Cluster-22178.31635 GO:0031492 GO:0000785 GO:0005634
Cluster-22178.31639 GO:0005515
Cluster-22178.31640 GO:0005515
Cluster-22178.31641 GO:0005515
Cluster-22178.31645 GO:0006468 GO:0005515 GO:0004672 GO:0005524 GO:0016020
Cluster-22178.31653 GO:0005515 GO:0005789
Cluster-22178.31656 GO:0016020
Cluster-22178.31658 GO:0006355 GO:0043401 GO:0043565 GO:0019900 GO:0008270 GO:0046872 GO:0003700 GO:0005634 GO:0005667
Cluster-22178.31660 GO:0006457 GO:0006810 GO:0006260 GO:0006914 GO:0006355 GO:0007049 GO:0051087 GO:0000774 GO:0008270 GO:0042803 GO:0016788 GO:0003700 GO:0043565 GO:0016881 GO:0005667 GO:0005634 GO:0016021
Cluster-22178.31665 GO:0009405 GO:0045735
Cluster-22178.31666 GO:0009405 GO:0019835 GO:0019867
Cluster-22178.31668 GO:0006144 GO:0003968 GO:0016817 GO:0031379
Cluster-22178.31671 GO:0007186 GO:0004930
Cluster-22178.31675 GO:0008283 GO:0040007 GO:0007165 GO:0008083

Cluster-22178.31676 GO:0006448 GO:0006414 GO:0005525 GO:0003924 GO:0003746 GO:0005840 GO:0005737

Cluster-22178.31684 GO:0016114 GO:0006465 GO:0042432 GO:0009058 GO:0008270 GO:0016844 GO:0008233 GO:0005515 GO:0005622 GO:0016021 GO:0005787

Cluster-22178.31685 GO:0009405

Cluster-22178.31696 GO:0000723 GO:0051252 GO:0006281 GO:0006355 GO:0019079 GO:0006310 GO:0003678 GO:0008134 GO:0003723 GO:0003724 GO:0005524 GO:0008270 GO:0016887 GO:0003677 GO:0016891 GO:0009378 GO:0016787 GO:0005657 GO:0009379 GO:0005667

Cluster-22178.31706 GO:0009405 GO:0006471 GO:0003956 GO:0005576

Cluster-22178.31707 GO:0006471 GO:0009405 GO:0003956 GO:0005576

Cluster-22178.31708 GO:0006508 GO:0004197 GO:0008234

Cluster-22178.31709 GO:0007165 GO:0009405 GO:0006810 GO:0008200 GO:0005515 GO:0005576

Cluster-22178.31710 GO:0033617 GO:0005198 GO:0019028

Cluster-22178.31711 GO:0055085 GO:0006355 GO:0006810 GO:0006814 GO:0009271 GO:0005215 GO:0005515 GO:0016020

Cluster-22178.31713 GO:0009271 GO:0006814 GO:0006810 GO:0006355 GO:0055085 GO:0005515 GO:0005215 GO:0016020

Cluster-22178.31715 GO:0006810 GO:0005215 GO:0016020

Cluster-22178.31716 GO:0003676 GO:0005515

Cluster-22178.31720 GO:0006323 GO:0030089

Cluster-22178.31725 GO:0055114 GO:0006570 GO:0004511

Cluster-22178.31729 GO:0006448 GO:0006414 GO:0005525 GO:0003924 GO:0003746 GO:0005840 GO:0005737

Cluster-22178.31732 GO:0008233 GO:0016791 GO:0004252 GO:0005618 GO:0016020

Cluster-22178.31734 GO:0007165 GO:0040007 GO:0008283 GO:0008083

Cluster-22178.31742 GO:0006955 GO:0019882 GO:0016020 GO:0042613

Cluster-22178.31744 GO:0006955 GO:0019882 GO:0016020 GO:0042613

Cluster-22178.31745 GO:0019882 GO:0006955 GO:0042613 GO:0016020
Cluster-22178.31747 GO:0019882 GO:0006955 GO:0042613 GO:0016020
Cluster-22178.31757 GO:0032801 GO:0015931 GO:0006812 GO:0019076
GO:0033227 GO:0051033 GO:0005261 GO:0016021 GO:0033644
Cluster-22178.31765 GO:0006508 GO:0008270 GO:0004222 GO:0031012
GO:0016020
Cluster-22178.31766 GO:0003950 GO:0005634
Cluster-22178.31769 GO:0006355 GO:0007017 GO:0003723 GO:0005875
GO:0016021
Cluster-22178.31774 GO:0006355 GO:0005515 GO:0003700 GO:0003677
GO:0005667
Cluster-22178.31776 GO:0006352 GO:0003677
Cluster-22178.31779 GO:0030246 GO:0005639
Cluster-22178.3178 GO:0016021
Cluster-22178.31785 GO:0005861
Cluster-22178.31788 GO:0001503 GO:0006355 GO:0003677
Cluster-22178.31789 GO:0016310 GO:0006118 GO:0045454 GO:0009069
GO:0009055 GO:0004674 GO:0015035 GO:0005524 GO:0003677
Cluster-22178.31791 GO:0035556 GO:0009190 GO:0016849
Cluster-22178.31793 GO:0007186 GO:0004930 GO:0016020 GO:0016021
Cluster-22178.31795 GO:0009878 GO:0046872
Cluster-22178.31798 GO:0005515
Cluster-22178.31807 GO:0006508 GO:0006915 GO:0004222 GO:0008270

Cluster-22178.31812 GO:0005515
Cluster-22178.31817 GO:0006298 GO:0030983 GO:0005524
Cluster-22178.31821 GO:0007049
Cluster-22178.31823 GO:0070072
Cluster-22178.31826 GO:0007165
Cluster-22178.31828 GO:0006606 GO:0007018 GO:0006355 GO:0007017
GO:0003777 GO:0003700 GO:0008017 GO:0005524 GO:0005515
GO:0051082 GO:0005874 GO:0045298 GO:0005667
Cluster-22178.31830 GO:0005515

Cluster-22178.31837 GO:0010923 GO:0019902
Cluster-22178.31840 GO:0006355 GO:0007049 GO:0006260 GO:0043565
GO:0003700 GO:0005667
Cluster-22178.31841 GO:0006468 GO:0004672 GO:0005524
Cluster-22178.31848 GO:0003677
Cluster-22178.31850 GO:0007275 GO:0009231 GO:0008686 GO:0005578
Cluster-22178.31852 GO:0007156 GO:0006357 GO:0005509 GO:0001104
GO:0016592 GO:0016020
Cluster-22178.31853 GO:0006810 GO:0006811 GO:0006915 GO:0005230
GO:0016020
Cluster-22178.31854 GO:0007034
Cluster-22178.31856 GO:0006570 GO:0006470 GO:0004725 GO:0008138

Cluster-22178.31858 GO:0042742 GO:0008270
Cluster-22178.31861 GO:0008168 GO:0019028
Cluster-22178.31865 GO:0006470 GO:0008138
Cluster-22178.31875 GO:0006270 GO:0005515 GO:0008168
Cluster-22178.3188 GO:0006164 GO:0006531 GO:0006144 GO:0006522
GO:0005525 GO:0004019
Cluster-22178.31884 GO:0006260 GO:0009069 GO:0042157 GO:0007067
GO:0007165 GO:0006869 GO:0009847 GO:0016310 GO:0007049
GO:0006355 GO:0006468 GO:0005096 GO:0004674 GO:0043565
GO:0008289 GO:0003700 GO:0005198 GO:0005815 GO:0005667
GO:0016020 GO:0019031 GO:0005576 GO:0072686 GO:0019028
GO:0042729
Cluster-22178.31886 GO:0008270
Cluster-22178.3189 GO:0006522 GO:0006144 GO:0006531 GO:0006164
GO:0004019 GO:0005525
Cluster-22178.31893 GO:0001558 GO:0005515 GO:0005520 GO:0016942
GO:0005576
Cluster-22178.31923 GO:0006260 GO:0007275 GO:0003677 GO:0016787
GO:0005578
Cluster-22178.31926 GO:0006144 GO:0007165 GO:0004114

Cluster-22178.31929 GO:0007155 GO:0016755 GO:0016020
Cluster-22178.31938 GO:0019064 GO:0019031 GO:0005739
Cluster-22178.31939 GO:0016192 GO:0006886 GO:0030117
Cluster-22178.31964 GO:0004866 GO:0005576 GO:0005615
Cluster-22178.31966 GO:0007156 GO:0005509 GO:0016020
Cluster-22178.31987 GO:0005515
Cluster-22178.31989 GO:0051016 GO:0007264 GO:0003924 GO:0005525
GO:0008290
Cluster-22178.31992 GO:0009584 GO:0006355 GO:0018298
Cluster-22178.31994 GO:0006464 GO:0055114 GO:0035556 GO:0046872
GO:0047134 GO:0016151
Cluster-22178.31995 GO:0016310 GO:0004673 GO:0009365
Cluster-22178.320 GO:0007165 GO:0005509 GO:0005515 GO:0005578
Cluster-22178.32003 GO:0006468 GO:0016773 GO:0008270 GO:0005524
GO:0004672 GO:0016772 GO:0003677 GO:0016020
Cluster-22178.32004 GO:0006468 GO:0016773 GO:0005524 GO:0008270
GO:0004672 GO:0003677 GO:0016020
Cluster-22178.32011 GO:0016032 GO:0006355 GO:0007010 GO:0006816
GO:0006874 GO:0003779 GO:0005219 GO:0003700 GO:0003682
GO:0003677 GO:0005634 GO:0019028 GO:0016012 GO:0016021
GO:0000785 GO:0000775 GO:0032040 GO:0005667 GO:0005622
Cluster-22178.32013 GO:0046983
Cluster-22178.3202 GO:0046907
Cluster-22178.32022 GO:0006468 GO:0006118 GO:0016491 GO:0004672
GO:0005524 GO:0009055 GO:0016020
Cluster-22178.32024 GO:0006547 GO:0000105 GO:0002532 GO:0007165
GO:0004896 GO:0003879 GO:0000287 GO:0005737 GO:0016021
Cluster-22178.32039 GO:0005515
Cluster-22178.32049 GO:0005887
Cluster-22178.32051 GO:0006836 GO:0006812 GO:0005328 GO:0016021
Cluster-22178.32053 GO:0019836 GO:0005576
Cluster-22178.32058 GO:0043087 GO:0035023 GO:0007517 GO:0006355
GO:0003700 GO:0005089 GO:0005515 GO:0003677 GO:0005667

GO:0005634 GO:0042025
Cluster-22178.32063 GO:0019042 GO:0006468 GO:0007186 GO:0005524
GO:0004672 GO:0004930 GO:0033644 GO:0016020
Cluster-22178.32071 GO:0006887 GO:0000145
Cluster-22178.32078 GO:0007186 GO:0004930 GO:0005515 GO:0016021
Cluster-22178.32079 GO:0007411 GO:0007186 GO:0004930 GO:0016021
Cluster-22178.32082 GO:0055114 GO:0016714
Cluster-22178.32102 GO:0005515
Cluster-22178.32105 GO:0005515
Cluster-22178.32106 GO:0006810 GO:0042919 GO:0042925 GO:0005515
GO:0016021
Cluster-22178.32107 GO:0008270
Cluster-22178.32114 GO:0000746
Cluster-22178.32115 GO:0000746
Cluster-22178.32124 GO:0019058 GO:0042025
Cluster-22178.32132 GO:0008061
Cluster-22178.32139 GO:0005515
Cluster-22178.32146 GO:0006144 GO:0006816 GO:0070588 GO:0009089
GO:0009113 GO:0008837 GO:0005262 GO:0004637 GO:0005515
GO:0016020
Cluster-22178.32147 GO:0006351 GO:0031556 GO:0042254 GO:0006412
GO:0006816 GO:0070588 GO:0031554 GO:0003735 GO:0005515
GO:0003714 GO:0005262 GO:0016020 GO:0005667 GO:0005840
GO:0005622
Cluster-22178.32174 GO:0006310 GO:0006260 GO:0006281 GO:0006355
GO:0003677 GO:0008191 GO:0016787 GO:0009378 GO:0003724
GO:0016887 GO:0005524 GO:0008270 GO:0008134 GO:0005515
GO:0003723 GO:0003697 GO:0003678 GO:0009379 GO:0005667
GO:0005657 GO:0042025
Cluster-22178.32189 GO:0007264 GO:0003924 GO:0005525
Cluster-22178.32191 GO:0000975
Cluster-22178.32199 GO:0006355 GO:0005634
Cluster-22178.32206 GO:0008270

Cluster-22178.3221 GO:0030001 GO:0055085 GO:0046873 GO:0016020
Cluster-22178.32211 GO:0006265 GO:0008270 GO:0005524 GO:0003918
GO:0003677 GO:0005634
Cluster-22178.32216 GO:0005525 GO:0005524 GO:0016887 GO:0003924
GO:0016020 GO:0019028
Cluster-22178.32217 GO:0008033 GO:0008152 GO:0016787 GO:0008168
GO:0005576
Cluster-22178.32221 GO:0006879 GO:0005576
Cluster-22178.32235 GO:0006886 GO:0008536
Cluster-22178.32252 GO:0006413 GO:0006355 GO:0017148 GO:0006914
GO:0010506 GO:0006260 GO:0045502 GO:0051082 GO:0008134
GO:0042803 GO:0003700 GO:0005516 GO:0031514 GO:0005845
GO:0005615 GO:0005667 GO:0030286 GO:0005815
Cluster-22178.32254 GO:0006260 GO:0006281 GO:0003911 GO:0004222
GO:0008270 GO:0005524 GO:0016021
Cluster-22178.32255 GO:0005515 GO:0016788
Cluster-22178.32268 GO:0007165 GO:0006955 GO:0006412 GO:0042254
GO:0030247 GO:0003735 GO:0005044 GO:0005840 GO:0005622
Cluster-22178.32270 GO:0042254 GO:0006412 GO:0003735 GO:0005622
GO:0005840
Cluster-22178.32279 GO:0008152 GO:0003824
Cluster-22178.32280 GO:0008152 GO:0003824
Cluster-22178.32282 GO:0008608 GO:0005876 GO:0042729
Cluster-22178.32285 GO:0006260 GO:0006606 GO:0006914 GO:0003724
GO:0016887 GO:0005524 GO:0003677 GO:0003723 GO:0016787
Cluster-22178.32286 GO:0006260 GO:0006606 GO:0007049 GO:0006914
GO:0003724 GO:0016887 GO:0005524 GO:0005515 GO:0003677
GO:0016787 GO:0003723
Cluster-22178.32301 GO:0006355 GO:0003677
Cluster-22178.32303 GO:0042981 GO:0005515
Cluster-22178.32318 GO:0016020
Cluster-22178.32322 GO:0008201 GO:0046914
Cluster-22178.32328 GO:0003950

Cluster-22178.32329 GO:0046740 GO:0009252 GO:0006807 GO:0006542 GO:0004356 GO:0044219 GO:0019028
Cluster-22178.32339 GO:0006388 GO:0003950 GO:0016772
Cluster-22178.32346 GO:0015696 GO:0008519 GO:0016020
Cluster-22178.32349 GO:0006281 GO:0004519
Cluster-22178.32351 GO:0007156 GO:0005509 GO:0016020
Cluster-22178.32355 GO:0006281 GO:0009432 GO:0005524 GO:0003697

Cluster-22178.32369 GO:0006508 GO:0016787 GO:0008236
Cluster-22178.32371 GO:0006206 GO:0006144 GO:0006352 GO:0006355 GO:0006351 GO:0016987 GO:0003700 GO:0003677 GO:0005515 GO:0003899 GO:0005730 GO:0005667
Cluster-22178.32372 GO:0001514 GO:0006887 GO:0006448 GO:0005515 GO:0020037 GO:0003723 GO:0005525 GO:0003746 GO:0000145 GO:0005840 GO:0005737
Cluster-22178.32376 GO:0006508 GO:0008270 GO:0004222 GO:0031012
Cluster-22178.32378 GO:0006355 GO:0003700 GO:0005667
Cluster-22178.32389 GO:0033227 GO:0015931 GO:0051033 GO:0004222 GO:0016021
Cluster-22178.32391 GO:0000287
Cluster-22178.32399 GO:0006355 GO:0046872 GO:0005524 GO:0008270 GO:0016887 GO:0003677 GO:0005515
Cluster-22178.32401 GO:0009878 GO:0006355 GO:0046872
Cluster-22178.32419 GO:0055114 GO:0016020
Cluster-22178.32422 GO:2001256 GO:0030176
Cluster-22178.32425 GO:0015671 GO:0020037 GO:0019825 GO:0005344 GO:0005506
Cluster-22178.32426 GO:0005975 GO:0004553
Cluster-22178.32431 GO:0016021
Cluster-22178.32440 GO:0006355
Cluster-22178.32441 GO:0015991 GO:0015992 GO:0015078 GO:0033179
Cluster-22178.3245 GO:0006310 GO:0006281 GO:0048255 GO:0009378 GO:0005524 GO:0016887 GO:0009379 GO:0005657

Cluster-22178.32469 GO:0007411 GO:0016055 GO:0005515 GO:0008013 GO:0016342

Cluster-22178.3247 GO:0006281 GO:0048255 GO:0006310 GO:0009378 GO:0008061 GO:0005524 GO:0016887 GO:0009379 GO:0005657

Cluster-22178.32480 GO:0019530 GO:0055114 GO:0006534 GO:0046439 GO:0017172 GO:0005506

Cluster-22178.32489 GO:0003677 GO:0005739

Cluster-22178.32492 GO:0008270

Cluster-22178.32500 GO:0006816 GO:0005388 GO:0016529

Cluster-22178.32514 GO:0003677

Cluster-22178.32518 GO:0006281 GO:0006139 GO:0007264 GO:0006310 GO:0006013 GO:0006206 GO:0006000 GO:0003723 GO:0005525 GO:0009378 GO:0003873 GO:0005524 GO:0016887 GO:0003724 GO:0004127 GO:0009379 GO:0005657

Cluster-22178.32520 GO:0015992 GO:0006120 GO:0006744 GO:0006355 GO:0006118 GO:0006814 GO:0003954 GO:0008137 GO:0005515 GO:0005739

Cluster-22178.32525 GO:0019042 GO:0033644 GO:0016020

Cluster-22178.32529 GO:0003677

Cluster-22178.32549 GO:0005515

Cluster-22178.32551 GO:0055114 GO:0016491 GO:0046872

Cluster-22178.32555 GO:0006396

Cluster-22178.32556 GO:0007186 GO:0004930 GO:0016021

Cluster-22178.32557 GO:0005525 GO:0005524 GO:0003924 GO:0016020

Cluster-22178.32569 GO:0042967 GO:0006633 GO:0055114 GO:0004318 GO:0005835

Cluster-22178.32570 GO:0035556 GO:0009405 GO:0019835 GO:0005515 GO:0019867

Cluster-22178.32575 GO:0016020

Cluster-22178.32580 GO:0001522 GO:0009451 GO:0003723 GO:0009982

Cluster-22178.32597 GO:0007049 GO:0006355 GO:0043093 GO:0000917 GO:0043565 GO:0003700 GO:0003677 GO:0005737 GO:0005634

GO:0005667
Cluster-22178.32605 GO:0030414 GO:0005509
Cluster-22178.32624 GO:0016579 GO:0006470 GO:0008138 GO:0036459 GO:0070140 GO:0032183
Cluster-22178.32629 GO:0035556 GO:0005515 GO:0046872
Cluster-22178.32635 GO:0006310 GO:0006281 GO:0006508 GO:0004181 GO:0008270
Cluster-22178.32638 GO:0007018 GO:0006412 GO:0006355 GO:0007017 GO:0042254 GO:0002949 GO:0008134 GO:0003777 GO:0003735 GO:0005524 GO:0016887 GO:0005840 GO:0005874 GO:0005667 GO:0005622 GO:0030286
Cluster-22178.32640 GO:0006810 GO:0042626 GO:0005524 GO:0016021
Cluster-22178.32642 GO:0015886 GO:0019497 GO:0006771 GO:0017004 GO:0046872 GO:0003993 GO:0005515 GO:0016021
Cluster-22178.32653 GO:0005515 GO:0043565 GO:0005634
Cluster-22178.32657 GO:0000012 GO:0006281 GO:0006814 GO:0015992 GO:0006885 GO:0003684 GO:0005515 GO:0015385 GO:0003677 GO:0005634 GO:0016021
Cluster-22178.32666 GO:0009395 GO:0046486 GO:0031124 GO:0007205 GO:0004143
Cluster-22178.32697 GO:0000166 GO:0046872
Cluster-22178.32720 GO:0005515
Cluster-22178.32722 GO:0046854
Cluster-22178.32728 GO:0055114 GO:0016972
Cluster-22178.32730 GO:0006355 GO:0003700 GO:0042302 GO:0000975 GO:0005667
Cluster-22178.32731 GO:0006355 GO:0003700 GO:0042302 GO:0005667
Cluster-22178.32732 GO:0006355 GO:0000975 GO:0042302 GO:0003700 GO:0005667
Cluster-22178.32740 GO:0006355 GO:0003700 GO:0043565 GO:0005634 GO:0005667
Cluster-22178.32744 GO:0007264 GO:0005525 GO:0003924 GO:0005524

Cluster-22178.32749 GO:0005515
Cluster-22178.32751 GO:0055085 GO:0022857 GO:0005887 GO:0016021
GO:0036128
Cluster-22178.32752 GO:0055085 GO:0016021
Cluster-22178.32754 GO:0055085 GO:0022857 GO:0036128 GO:0016021
Cluster-22178.32758 GO:0005737
Cluster-22178.32765 GO:0009847 GO:0055114 GO:0020037 GO:0016705
GO:0005506 GO:0016021
Cluster-22178.32776 GO:0005515
Cluster-22178.32777 GO:0005515
Cluster-22178.32779 GO:0006508 GO:0005515 GO:0004252 GO:0005525
GO:0046983 GO:0016020
Cluster-22178.32780 GO:0007217
Cluster-22178.32782 GO:0003950
Cluster-22178.32784 GO:0003676
Cluster-22178.3280 GO:0055085 GO:0006811 GO:0051260 GO:0005216
GO:0016020
Cluster-22178.32804 GO:0007275 GO:0007165 GO:0009966 GO:0014068
GO:0045444 GO:0050776 GO:0043548 GO:0005102 GO:0016020
Cluster-22178.32805 GO:0009966 GO:0007275 GO:0045444 GO:0016020
Cluster-22178.32819 GO:0006810 GO:0042407 GO:0055085 GO:0005215
GO:0016020 GO:0016021 GO:0061617 GO:0044284
Cluster-22178.32823 GO:0009890
Cluster-22178.32828 GO:0008270 GO:0005515 GO:0042802
Cluster-22178.32831 GO:0006047 GO:0005975 GO:0016773 GO:0005524

Cluster-22178.32832 GO:0016301 GO:0046872 GO:0016020
Cluster-22178.32843 GO:0005515
Cluster-22178.32848 GO:0006570 GO:0006118 GO:0050855 GO:0006470
GO:2000401 GO:0008138 GO:0004725 GO:0015035 GO:0016020
GO:0019033
Cluster-22178.32850 GO:0006470 GO:2000401 GO:0006118 GO:0006570
GO:0050855 GO:0015035 GO:0004725 GO:0008138 GO:0016020

GO:0019033
Cluster-22178.32855 GO:0006811 GO:0006367 GO:0006810 GO:0006366
GO:0005215 GO:0016020 GO:0005674
Cluster-22178.32856 GO:0006366 GO:0006865 GO:0055085 GO:0006811
GO:0006122 GO:0006367 GO:0003333 GO:0006810 GO:0005215
GO:0015171 GO:0005750 GO:0005743 GO:0016020 GO:0005674
Cluster-22178.32868 GO:0005515
Cluster-22178.32870 GO:0006810 GO:0016021
Cluster-22178.32872 GO:0006506 GO:0006874 GO:0006816 GO:0006811
GO:0055085 GO:0005509 GO:0005216 GO:0016746 GO:0005219
GO:0016020 GO:0005789 GO:0016021 GO:0005622
Cluster-22178.32874 GO:0005515
Cluster-22178.32883 GO:0007165 GO:0001664
Cluster-22178.32895 GO:0006810 GO:0000917 GO:0006355 GO:0043093
GO:0003700 GO:0043565 GO:0005667 GO:0005886 GO:0005737
Cluster-22178.32908 GO:0030246 GO:0005639
Cluster-22178.32917 GO:0032509 GO:0006308 GO:0008855 GO:0043130
GO:0000813 GO:0009318
Cluster-22178.3292 GO:0006560 GO:0006814 GO:0071436 GO:0006525
GO:0006811 GO:0006090 GO:0055085 GO:0008948 GO:0005216
GO:0015081 GO:0016020
Cluster-22178.32925 GO:0007205 GO:0035556 GO:0046486 GO:0009395
GO:0004143 GO:0005509 GO:0016301
Cluster-22178.32932 GO:0005515
Cluster-22178.32935 GO:0007212 GO:0048268 GO:0032051 GO:0008270
GO:0016021
Cluster-22178.32945 GO:0006108 GO:0006810 GO:0006099 GO:0006090
GO:0015976 GO:0055114 GO:0004471 GO:0005471 GO:0051287
GO:0005524 GO:0016021
Cluster-22178.32946 GO:0006099 GO:0006108 GO:0055114 GO:0015976
GO:0006090 GO:0051287 GO:0004471 GO:0005515
Cluster-22178.32954 GO:0008152 GO:0030695 GO:0016746
Cluster-22178.32955 GO:0008152 GO:0016746 GO:0005856

Cluster-22178.32956 GO:0008152 GO:0030695 GO:0016746 GO:0005856
Cluster-22178.32959 GO:0006270 GO:0005515 GO:0008168
Cluster-22178.32975 GO:0016021
Cluster-22178.32982 GO:0043401 GO:0006355
Cluster-22178.32987 GO:0007186 GO:0009372 GO:0009405 GO:0006468
GO:0008233 GO:0004930 GO:0005524 GO:0004672 GO:0016021
Cluster-22178.33 GO:0007015 GO:0006865 GO:0003333 GO:0003785
GO:0015171 GO:0016020
Cluster-22178.33007 GO:0055114 GO:0008033 GO:0016117 GO:0004499
GO:0016491 GO:0050660 GO:0071949 GO:0050661 GO:0016705
Cluster-22178.33012 GO:0004222
Cluster-22178.33024 GO:0006351 GO:0006355 GO:0006508 GO:0006352
GO:0006206 GO:0006144 GO:0003677 GO:0003899 GO:0005515
GO:0016987 GO:0003700 GO:0008233 GO:0005730 GO:0005667
GO:0005634
Cluster-22178.33026 GO:0006468 GO:0016773 GO:0004672 GO:0005524
GO:0016020
Cluster-22178.33028 GO:0042967 GO:0047961 GO:0005739
Cluster-22178.33032 GO:0007165 GO:0004252 GO:0019028 GO:0055036
Cluster-22178.33033 GO:0005515 GO:0005634
Cluster-22178.33036 GO:0008191
Cluster-22178.33038 GO:0055114 GO:0006525 GO:0006118 GO:0016655
GO:0009015 GO:0005886
Cluster-22178.33041 GO:0007165 GO:0006508 GO:0004197 GO:0008158
GO:0031225 GO:0016020
Cluster-22178.33044 GO:0007165 GO:0006508 GO:0004197 GO:0008158
GO:0031225 GO:0016020
Cluster-22178.33054 GO:0009968 GO:0005737
Cluster-22178.33056 GO:0007186 GO:0004930 GO:0016021
Cluster-22178.33061 GO:0006836 GO:0007304 GO:0006812 GO:0005328
GO:0005213 GO:0005576 GO:0042600 GO:0016021
Cluster-22178.33064 GO:0009298 GO:0006000 GO:0006013 GO:0004615
GO:0005737

Cluster-22178.33067 GO:0006816 GO:0006810 GO:0006814 GO:0015992
GO:0006744 GO:0006120 GO:0005246 GO:0008137 GO:0046872
GO:0042030 GO:0016020 GO:0005743

Cluster-22178.33070 GO:0006333 GO:0005634

Cluster-22178.33072 GO:0006629 GO:0016627 GO:0005737 GO:0016021

Cluster-22178.33074 GO:0046872

Cluster-22178.33081 GO:0005515

Cluster-22178.33083 GO:0006508 GO:0007186 GO:0004930 GO:0004252
GO:0016020

Cluster-22178.33091 GO:0035556 GO:0005515

Cluster-22178.33094 GO:0006950 GO:0006508 GO:0007186 GO:0008270
GO:0004930 GO:0004190 GO:0005198 GO:0005634 GO:0019028

Cluster-22178.33095 GO:0006950 GO:0007186 GO:0006355 GO:0006508
GO:0004930 GO:0003700 GO:0008270 GO:0005198 GO:0004190
GO:0019028 GO:0005667 GO:0005634

Cluster-22178.3310 GO:0003676 GO:0008270

Cluster-22178.33107 GO:0006144 GO:0035556 GO:2000123 GO:0009190
GO:0006171 GO:0016849 GO:0004016 GO:0005886

Cluster-22178.33114 GO:0007606 GO:0016021

Cluster-22178.33133 GO:0007169 GO:0006468 GO:0004714 GO:0005524
GO:0004672 GO:0016020 GO:0005887

Cluster-22178.33137 GO:0008152 GO:0006468 GO:0004672 GO:0005524
GO:0016491

Cluster-22178.33138 GO:0019042 GO:0006468 GO:0007186 GO:0005524
GO:0004672 GO:0004930 GO:0033644 GO:0016020

Cluster-22178.33139 GO:0007186 GO:0006468 GO:0019042 GO:0004930
GO:0005524 GO:0004672 GO:0016020 GO:0033644

Cluster-22178.33140 GO:0005509

Cluster-22178.33141 GO:0003676 GO:0003723

Cluster-22178.33149 GO:0031412 GO:0009405 GO:0005524 GO:0003774
GO:0005515 GO:0031411 GO:0016459 GO:0016020

Cluster-22178.33153 GO:0007165 GO:0005509 GO:0005578

Cluster-22178.33160 GO:0005887

Cluster-22178.33161 GO:0016301
Cluster-22178.33170 GO:0007155 GO:0005509 GO:0005576
Cluster-22178.33171 GO:0007049 GO:0017053
Cluster-22178.33174 GO:0006814 GO:0006813 GO:0005890
Cluster-22178.33177 GO:0006807 GO:0019082 GO:0006672 GO:0016032
GO:0016811 GO:0016021
Cluster-22178.33178 GO:0005515
Cluster-22178.33183 GO:0006629 GO:0042967 GO:0008374
Cluster-22178.33184 GO:0005515 GO:0005509
Cluster-22178.33188 GO:0006448 GO:0006414 GO:0003746 GO:0005515
GO:0005840
Cluster-22178.33189 GO:0003676
Cluster-22178.33196 GO:0006367 GO:0051252 GO:0006506 GO:0006402
GO:0003723 GO:0046872 GO:0004535 GO:0003676 GO:0005634
GO:0005672 GO:0005737 GO:0016021
Cluster-22178.33197 GO:0006506 GO:0003676 GO:0016021
Cluster-22178.33207 GO:0006118 GO:0006814 GO:0006120 GO:0006744
GO:0015992 GO:0008137 GO:0003954 GO:0005739
Cluster-22178.33215 GO:0043044 GO:0007010 GO:0031491 GO:0003779
GO:0000785
Cluster-22178.33217 GO:0000002
Cluster-22178.33222 GO:0006355 GO:0008270 GO:0003700 GO:0005515
GO:0005634 GO:0005667
Cluster-22178.33224 GO:0006355 GO:0005515 GO:0003700 GO:0008270
GO:0005634 GO:0005667
Cluster-22178.33228 GO:0006810 GO:0019083 GO:0055085 GO:0003924
GO:0016887 GO:0005524 GO:0042626 GO:0005525 GO:0016021
Cluster-22178.33229 GO:0055085 GO:0019083 GO:0006810 GO:0005525
GO:0042626 GO:0005524 GO:0003924 GO:0016887 GO:0016301
GO:0016021
Cluster-22178.33235 GO:0006629 GO:0009058 GO:0005524 GO:0016874

Cluster-22178.33243 GO:0006355 GO:0006281 GO:0070836 GO:0008270

GO:0008168 GO:0003677
Cluster-22178.33245 GO:0005783
Cluster-22178.33248 GO:0016021
Cluster-22178.33254 GO:0009069 GO:0006468 GO:0016310 GO:0006807
GO:0005509 GO:0005524 GO:0004672 GO:0004668 GO:0016773
GO:0016151 GO:0004674 GO:0016020 GO:0005737
Cluster-22178.33256 GO:0006468 GO:0009069 GO:0016310 GO:0006807
GO:0005509 GO:0016151 GO:0004674 GO:0016773 GO:0004668
GO:0005524 GO:0004672 GO:0016020 GO:0005737
Cluster-22178.33257 GO:0006807 GO:0016310 GO:0006468 GO:0009069
GO:0016773 GO:0004668 GO:0005524 GO:0004672 GO:0004674
GO:0016151 GO:0005509 GO:0005737 GO:0016020
Cluster-22178.33259 GO:0007155 GO:0005515 GO:0016021
Cluster-22178.33263 GO:0046914 GO:0005515
Cluster-22178.33271 GO:0071107
Cluster-22178.33272 GO:0071107
Cluster-22178.33277 GO:0055114 GO:0019521 GO:0006098 GO:0051287
GO:0004616
Cluster-22178.33290 GO:0043087 GO:0035023 GO:0005089 GO:0005515

Cluster-22178.33293 GO:0006511 GO:0007186 GO:0007218 GO:0004930
GO:0016021 GO:0005576
Cluster-22178.33300 GO:0007156 GO:0005509 GO:0016020
Cluster-22178.33311 GO:0006810 GO:0005215 GO:0005515 GO:0016020
Cluster-22178.33339 GO:0005198 GO:0019028
Cluster-22178.33340 GO:0015031 GO:0009306 GO:0046836 GO:0017089
GO:0015450 GO:0051861 GO:0005515 GO:0005737 GO:0016021
GO:0009941
Cluster-22178.33343 GO:0006457 GO:0000413 GO:0003755 GO:0004859
GO:0016021 GO:0005576
Cluster-22178.33352 GO:0031505 GO:0006817 GO:0005315 GO:0016020
Cluster-22178.33354 GO:0008152 GO:0006606 GO:0010508 GO:0003824
GO:0045502 GO:0008134 GO:0042803 GO:0030286 GO:0005667

Cluster-22178.33360 GO:0031047 GO:0006308 GO:0005975 GO:0004560
GO:0016888

Cluster-22178.33361 GO:0009056 GO:0031047 GO:0006308 GO:0005975
GO:0004560 GO:0016888 GO:0008270

Cluster-22178.33364 GO:0006355 GO:0005975 GO:0003677 GO:0003682
GO:0043169 GO:0003824 GO:0008270 GO:0005634 GO:0000785
GO:0000775

Cluster-22178.33368 GO:0006810 GO:0046034 GO:0006144 GO:0005515
GO:0004017 GO:0042626 GO:0046872 GO:0005524 GO:0016021

Cluster-22178.33370 GO:0006355 GO:0006313 GO:0015074 GO:0006352
GO:0003677 GO:0043565 GO:0016987 GO:0003700 GO:0005667

Cluster-22178.33373 GO:0032065 GO:0005543 GO:0005096 GO:0005515
GO:0005938

Cluster-22178.33376 GO:0003723

Cluster-22178.33377 GO:0055114 GO:0016641 GO:0005507

Cluster-22178.33379 GO:0008152 GO:0015979 GO:0006013 GO:0050821
GO:0015976 GO:0006000 GO:0006096 GO:0046486 GO:0006020
GO:0006094 GO:0004807 GO:0042301 GO:0009523 GO:0016020

Cluster-22178.33382 GO:0006810 GO:0016020

Cluster-22178.33390 GO:0005515 GO:0005509

Cluster-22178.33391 GO:0005515 GO:0005509

Cluster-22178.33401 GO:0005975 GO:0004553

Cluster-22178.33406 GO:0007165 GO:0008283 GO:0040007 GO:0008083

Cluster-22178.33420 GO:0007155 GO:0016021

Cluster-22178.33425 GO:0043087 GO:0035023 GO:0005089

Cluster-22178.33426 GO:0043087 GO:0035023 GO:0005089

Cluster-22178.33439 GO:0006468 GO:0000746 GO:0005524 GO:0004672
GO:0005515

Cluster-22178.33457 GO:0016788

Cluster-22178.33460 GO:0006281 GO:0006260 GO:0016787 GO:0046872
GO:0005515 GO:0003911

Cluster-22178.33461 GO:0035091

Cluster-22178.33467 GO:0008270
Cluster-22178.33479 GO:0035556 GO:0046034 GO:0006464 GO:0006144
GO:0006281 GO:0016151 GO:0046872 GO:0008270 GO:0004017
Cluster-22178.33493 GO:0006470 GO:0006570 GO:0004725 GO:0005515

Cluster-22178.33498 GO:0002098 GO:0034227 GO:0000049
Cluster-22178.33518 GO:0005516
Cluster-22178.33530 GO:0006470 GO:0006570 GO:0004725 GO:0008138
GO:0005515
Cluster-22178.33533 GO:0055114 GO:0016645 GO:0003676
Cluster-22178.33541 GO:0006468 GO:0005524 GO:0004672
Cluster-22178.33549 GO:0016787
Cluster-22178.33557 GO:0000975
Cluster-22178.33566 GO:0006355 GO:0003700 GO:0008270 GO:0043565
GO:0005634 GO:0005667
Cluster-22178.3357 GO:0030639 GO:0019872 GO:0009117 GO:0008830
Cluster-22178.33581 GO:0008270
Cluster-22178.33591 GO:0007155 GO:0005540
Cluster-22178.33595 GO:0055114 GO:0005507 GO:0016641
Cluster-22178.33618 GO:0006810 GO:0008270 GO:0016021
Cluster-22178.33626 GO:0015074
Cluster-22178.33634 GO:0055114 GO:0032922 GO:0016491 GO:0000976

Cluster-22178.33636 GO:0007165 GO:0007186 GO:0005057 GO:0004930
GO:0016021
Cluster-22178.33650 GO:0005975 GO:0016901 GO:0048038
Cluster-22178.33651 GO:0046488 GO:0016307
Cluster-22178.33663 GO:0030150 GO:0005742
Cluster-22178.33666 GO:0016567 GO:0008270 GO:0046872 GO:0005515
GO:0004842 GO:0005680
Cluster-22178.33676 GO:0006265 GO:0003677 GO:0003916
Cluster-22178.33681 GO:0005515
Cluster-22178.33692 GO:0046813 GO:0061025 GO:0007165 GO:0006355

GO:0043565 GO:0005515 GO:0016021 GO:0019031 GO:0005634
Cluster-22178.33693 GO:0007165 GO:0005515
Cluster-22178.33698 GO:0042330
Cluster-22178.33707 GO:0055085 GO:0016021
Cluster-22178.33708 GO:0009405 GO:0042254 GO:0006412 GO:0005198
GO:0003735 GO:0005840
Cluster-22178.33726 GO:0006412 GO:0042254 GO:0003735 GO:0005622
GO:0005840
Cluster-22178.33727 GO:0005515
Cluster-22178.33731 GO:0046486 GO:0006351 GO:0007165 GO:0006144
GO:0006071 GO:0006206 GO:0005509 GO:0003899 GO:0005515
GO:0004371 GO:0008168 GO:0005730 GO:0005578
Cluster-22178.33737 GO:0005515
Cluster-22178.33739 GO:0019911
Cluster-22178.33764 GO:0006310 GO:0007155 GO:0005198 GO:0051015
GO:0003677 GO:0003824 GO:0003779 GO:0005882 GO:0005727
Cluster-22178.33779 GO:0035556 GO:0005515
Cluster-22178.33781 GO:0006468 GO:0005524 GO:0004672 GO:0003677

Cluster-22178.33788 GO:0006820 GO:0016021
Cluster-22178.33790 GO:0005975 GO:0030036 GO:0004649 GO:0003779
GO:0005515 GO:0017048
Cluster-22178.33797 GO:0006468 GO:0004672 GO:0005524
Cluster-22178.33798 GO:0006013 GO:0006000 GO:0009298 GO:0004615
GO:0005737
Cluster-22178.33807 GO:0005982 GO:0006012 GO:0005985 GO:0004575
GO:0004564 GO:0016021 GO:0017177
Cluster-22178.3381 GO:0015991 GO:0005975 GO:0006338 GO:0006816
GO:0006874 GO:0015992 GO:0005219 GO:0015078 GO:0003779
GO:0003677 GO:0004553 GO:0033179 GO:0016021 GO:0090544
GO:0005886 GO:0005622
Cluster-22178.33829 GO:0005515
Cluster-22178.33840 GO:0001558 GO:0005520 GO:0005515 GO:0016942

GO:0005576
Cluster-22178.33842 GO:0009395 GO:0016042 GO:0004629
Cluster-22178.33855 GO:0005198 GO:0016787
Cluster-22178.33856 GO:0006508 GO:0016787 GO:0005198 GO:0008236

Cluster-22178.33861 GO:0003774 GO:0005506 GO:0005524 GO:0005515
GO:0016459
Cluster-22178.33865 GO:0006355 GO:0015684 GO:0015093 GO:0005622
GO:0016021
Cluster-22178.33874 GO:0008152 GO:0003824 GO:0003676 GO:0008484
GO:0016787 GO:0046872
Cluster-22178.33875 GO:0008152 GO:0006955 GO:0007165 GO:0008484
GO:0046872 GO:0003676 GO:0003824 GO:0030247 GO:0016787
GO:0005044
Cluster-22178.33879 GO:0016567 GO:0009405 GO:0004842 GO:0005576
GO:0005680
Cluster-22178.33883 GO:0043248 GO:0006506 GO:0006886
Cluster-22178.33884 GO:0019069 GO:0006355 GO:0005524 GO:0003677

Cluster-22178.33898 GO:0030168 GO:0007165 GO:0051258 GO:0006355
GO:0007094 GO:0003774 GO:0005102 GO:0030674 GO:0043565
GO:0003700 GO:0045502 GO:0005198 GO:0008134 GO:0042803
GO:0030286 GO:0005577 GO:0005667 GO:0016459 GO:0005882
Cluster-22178.33899 GO:0006355 GO:0019048
Cluster-22178.33903 GO:0019083 GO:0003676
Cluster-22178.33905 GO:0003676
Cluster-22178.33923 GO:0006270 GO:0016567 GO:0006260 GO:0006355
GO:0006351 GO:0003682 GO:0003677 GO:0008270 GO:0004842
GO:0003887 GO:0005515 GO:0005680 GO:0005634 GO:0042575
GO:0005737 GO:0000775 GO:0000785
Cluster-22178.33933 GO:0032509 GO:0006531 GO:0042254 GO:0019941
GO:0006144 GO:0006289 GO:0006188 GO:0006285 GO:0016559
GO:0006281 GO:0006412 GO:0006308 GO:0006522 GO:0010498

GO:0009152 GO:0003684 GO:0003735 GO:0008855 GO:0043130
GO:0008534 GO:0008168 GO:0019843 GO:0004018 GO:0005779
GO:0000813 GO:0005622 GO:0009318 GO:0005840
Cluster-22178.33944 GO:0016021
Cluster-22178.3395 GO:0035556 GO:0007165
Cluster-22178.3396 GO:0007165 GO:0035556
Cluster-22178.33962 GO:0016301
Cluster-22178.33963 GO:0006810 GO:0005215 GO:0046872 GO:0016020
Cluster-22178.33970 GO:0006118 GO:0003950 GO:0009055 GO:0005507

Cluster-22178.33973 GO:0042254 GO:0005622
Cluster-22178.33981 GO:0006468 GO:0008033 GO:0005524 GO:0004672

Cluster-22178.33982 GO:0006468 GO:0006979 GO:0004672 GO:0005524
GO:0019031
Cluster-22178.33983 GO:0006979 GO:0006468 GO:0004672 GO:0005524
GO:0019031
Cluster-22178.33985 GO:0006810 GO:0055085 GO:0016020
Cluster-22178.33988 GO:0007264 GO:0005096
Cluster-22178.33991 GO:0007264 GO:0005096 GO:0008270
Cluster-22178.33992 GO:0008270
Cluster-22178.33994 GO:0009245 GO:0008915 GO:0005515
Cluster-22178.33996 GO:0007224 GO:0005515 GO:0061630 GO:0008270
GO:0046872 GO:0005622
Cluster-22178.340 GO:0003677 GO:0016021
Cluster-22178.34000 GO:0006355 GO:0003700 GO:0043565 GO:0005667
Cluster-22178.34003 GO:0006508 GO:0007165 GO:0008158 GO:0004197
GO:0016020 GO:0031225
Cluster-22178.34006 GO:0055085 GO:0030001 GO:0046873 GO:0008270
GO:0016020
Cluster-22178.34013 GO:0070206 GO:0006260 GO:0007049 GO:0005198
GO:0005882
Cluster-22178.34014 GO:0042254 GO:0001522 GO:0005515 GO:0030515

GO:0072588
Cluster-22178.34016 GO:0055114 GO:0016491 GO:0016209
Cluster-22178.34019 GO:0007186 GO:0004930 GO:0016021
Cluster-22178.34020 GO:0019076 GO:0006812 GO:0015931 GO:0032801
GO:0033227 GO:0006355 GO:0009584 GO:0018298 GO:0005261
GO:0051033 GO:0033644 GO:0016021
Cluster-22178.34029 GO:0071805 GO:0006813 GO:0003677 GO:0015079
GO:0016020 GO:0005634
Cluster-22178.34030 GO:0030435 GO:0006351 GO:0005509 GO:0043565

Cluster-22178.34034 GO:0009058 GO:0006144 GO:0006508 GO:0004252
GO:0030170 GO:0003877
Cluster-22178.34037 GO:0051249 GO:0006955 GO:0005515
Cluster-22178.34039 GO:0003677
Cluster-22178.34051 GO:0006508 GO:0004222
Cluster-22178.34052 GO:0043093 GO:0000917 GO:0010508 GO:0006886
GO:0006606 GO:0005783 GO:0005737 GO:0019867 GO:0016021
Cluster-22178.34063 GO:0051087
Cluster-22178.34064 GO:0005515
Cluster-22178.34070 GO:0009405 GO:0016192 GO:0006629 GO:0006508
GO:0004222
Cluster-22178.34072 GO:0016301
Cluster-22178.34080 GO:0016567 GO:0046872 GO:0008270 GO:0005515
GO:0004842 GO:0005680
Cluster-22178.34086 GO:0030234 GO:0016020
Cluster-22178.34089 GO:0016021
Cluster-22178.34090 GO:0006506 GO:0006355 GO:0005515 GO:0016758
GO:0046983 GO:0016021
Cluster-22178.34093 GO:0005861
Cluster-22178.34094 GO:0006470 GO:2000401 GO:0006570 GO:0006118
GO:0050855 GO:0004725 GO:0015035 GO:0008138 GO:0016020
Cluster-22178.34095 GO:0006570 GO:0006118 GO:0050855 GO:0006470
GO:2000401 GO:0008138 GO:0015035 GO:0004725 GO:0016020

GO:0019033
Cluster-22178.34096 GO:0043087 GO:0035023 GO:0005089 GO:0005515

Cluster-22178.34105 GO:0007187 GO:0006468 GO:0009069 GO:0007608
GO:0016310 GO:0007178 GO:0005549 GO:0004675 GO:0004984
GO:0004672 GO:0005524 GO:0016020
Cluster-22178.34106 GO:0007156 GO:0005509 GO:0016020
Cluster-22178.34107 GO:0003723
Cluster-22178.34108 GO:0007155 GO:0005515 GO:0016021
Cluster-22178.34121 GO:0008191
Cluster-22178.34125 GO:2001070 GO:0046983 GO:0008270 GO:0005515
GO:0003677 GO:0019867
Cluster-22178.34128 GO:0005515
Cluster-22178.34129 GO:0007165 GO:0006508 GO:0004197 GO:0008234
GO:0005179 GO:0005576
Cluster-22178.34132 GO:0006355 GO:0005634
Cluster-22178.34145 GO:0043353 GO:0015671 GO:0007507 GO:0042542
GO:0009725 GO:0001666 GO:0050873 GO:0031444 GO:0005506
GO:0005344 GO:0019825 GO:0020037
Cluster-22178.34156 GO:0007186 GO:0004930 GO:0030246 GO:0016020
GO:0016021
Cluster-22178.34166 GO:0015992 GO:0015991 GO:0006260 GO:0015078
GO:0016853 GO:0005515 GO:0033179
Cluster-22178.34167 GO:0009405
Cluster-22178.34182 GO:0009968 GO:0005737
Cluster-22178.34198 GO:0007186 GO:0030246 GO:0004930 GO:0016021
GO:0016020
Cluster-22178.34199 GO:0007155 GO:0009405 GO:0019062
Cluster-22178.3420 GO:0008152 GO:0016791 GO:0003682 GO:0000785
Cluster-22178.34202 GO:0006308 GO:0009307 GO:0003677 GO:0009036
GO:0009359
Cluster-22178.34203 GO:0043087 GO:0007264 GO:0005085 GO:0016021
GO:0016012

Cluster-22178.34210 GO:0006814 GO:0042773 GO:0055114 GO:0015992 GO:0006120 GO:0006744 GO:0008137 GO:0016651
Cluster-22178.34215 GO:0005524 GO:0003774 GO:0016459
Cluster-22178.34219 GO:0006486 GO:0003677 GO:0008373
Cluster-22178.3422 GO:0007165 GO:0040007 GO:0008283 GO:0008083
Cluster-22178.34222 GO:0016021
Cluster-22178.34228 GO:0006672 GO:0006807 GO:0016811 GO:0005524 GO:0016021
Cluster-22178.34230 GO:0030234 GO:0016020
Cluster-22178.34232 GO:0008270 GO:0005634
Cluster-22178.34234 GO:0005515
Cluster-22178.34237 GO:0005515
Cluster-22178.34238 GO:0005515
Cluster-22178.3424 GO:0016055 GO:0007275 GO:0007165 GO:0005102 GO:0005576
Cluster-22178.34242 GO:0072519 GO:0016567 GO:0008270 GO:0004842

Cluster-22178.3426 GO:0007165 GO:0007275 GO:0006281 GO:0016055 GO:0004518 GO:0005102 GO:0005576
Cluster-22178.34272 GO:0005515 GO:0003677 GO:0008270
Cluster-22178.34276 GO:0042254 GO:0006412 GO:0003735 GO:0005622 GO:0005840
Cluster-22178.34278 GO:0006118 GO:0009055 GO:0020037 GO:0005515

Cluster-22178.34285 GO:0042981 GO:0043065 GO:0016192 GO:0006886 GO:0005198 GO:0005737 GO:0030130 GO:0030132
Cluster-22178.34287 GO:0019825 GO:0020037
Cluster-22178.34290 GO:0006355 GO:0003677
Cluster-22178.34291 GO:0006457 GO:0006366 GO:0003677 GO:0005737 GO:0005665
Cluster-22178.34292 GO:0006366 GO:0006457 GO:0003677 GO:0005665 GO:0005737
Cluster-22178.34298 GO:0006629 GO:0006508 GO:0008218 GO:0006886

GO:0055114 GO:0008152 GO:0046486 GO:0009058 GO:0006505
GO:0016042 GO:0016788 GO:0008236 GO:0016787 GO:0016491
GO:0003847 GO:0008247
Cluster-22178.3430 GO:0006812 GO:0006836 GO:0005328 GO:0016021
Cluster-22178.34301 GO:0004252 GO:0055036 GO:0019028
Cluster-22178.34303 GO:0005515
Cluster-22178.34311 GO:0007165 GO:0016020
Cluster-22178.34334 GO:0016043 GO:0003779
Cluster-22178.34337 GO:0055114 GO:0009308 GO:0003676 GO:0005524
GO:0016638 GO:0042597
Cluster-22178.34338 GO:0003676 GO:0005524
Cluster-22178.34346 GO:0006464
Cluster-22178.34347 GO:0007067 GO:0009401 GO:0007049 GO:0005515
GO:0005634 GO:0000775
Cluster-22178.34348 GO:0006506 GO:0042742 GO:0007067 GO:0009069
GO:0016310 GO:0007049 GO:0009401 GO:0005515 GO:0004674
GO:0005634 GO:0005789 GO:0000775
Cluster-22178.34356 GO:0007050 GO:0045859 GO:0000122 GO:0004861
GO:0017069 GO:0005634 GO:0005737
Cluster-22178.34361 GO:0006952 GO:0055085 GO:0030001 GO:0046873
GO:0005815 GO:0016020 GO:0009279
Cluster-22178.34364 GO:0016021
Cluster-22178.34370 GO:0006820 GO:0008509 GO:0016021
Cluster-22178.34372 GO:0006820 GO:0008509 GO:0016021
Cluster-22178.34379 GO:0019079 GO:0003723 GO:0005515
Cluster-22178.34380 GO:0007067 GO:0006810 GO:0015992 GO:0006813
GO:0015991 GO:0006308 GO:0005516 GO:0015269 GO:0008855
GO:0015078 GO:0005886 GO:0072686 GO:0042729 GO:0009318
GO:0033179 GO:0016021
Cluster-22178.34381 GO:0005856
Cluster-22178.34391 GO:0007010 GO:0007304 GO:0005213 GO:0003779
GO:0005576 GO:0042600
Cluster-22178.34396 GO:0005515

Cluster-22178.34400 GO:0007186 GO:0007606 GO:0005201 GO:0004930
GO:0016021 GO:0005578
Cluster-22178.34402 GO:0007186 GO:0007606 GO:0004930 GO:0016021
Cluster-22178.34404 GO:0007186 GO:0007606 GO:0005515 GO:0005201
GO:0004930 GO:0016021 GO:0005578
Cluster-22178.34414 GO:0035556
Cluster-22178.34416 GO:0006570 GO:0006493 GO:0006470 GO:0008138
GO:0000030 GO:0004725 GO:0016020 GO:0000136
Cluster-22178.34420 GO:0006118 GO:0015992 GO:0055114 GO:0009060
GO:0006123 GO:0005506 GO:0020037 GO:0009055 GO:0004129
GO:0045277 GO:0016021
Cluster-22178.34422 GO:0006811 GO:0006813 GO:0005249 GO:0008076
GO:0016020
Cluster-22178.34427 GO:0044564 GO:0006468 GO:0035091 GO:0016773
GO:0004672 GO:0005524 GO:0016020
Cluster-22178.34431 GO:0003677 GO:0005198
Cluster-22178.34432 GO:0005198 GO:0003677
Cluster-22178.34441 GO:0007155 GO:0005515 GO:0005509 GO:0005747
GO:0005739 GO:0005576
Cluster-22178.34443 GO:0000917 GO:0006355 GO:0043093 GO:0007049
GO:0055085 GO:0003700 GO:0046983 GO:0043565 GO:0016020
GO:0005667 GO:0016021 GO:0005737
Cluster-22178.34445 GO:0043401 GO:0006355
Cluster-22178.34448 GO:0005524 GO:0003774 GO:0005515 GO:0016459
Cluster-22178.34450 GO:0007165 GO:0016020
Cluster-22178.34454 GO:0046872
Cluster-22178.34457 GO:0031110 GO:0009069 GO:0000090 GO:0007067
GO:0051301 GO:0016310 GO:0007059 GO:0006468 GO:0005524
GO:0004674 GO:0008017 GO:0003677 GO:0004672 GO:0000940
GO:0005876 GO:0045298
Cluster-22178.34459 GO:0030246
Cluster-22178.34461 GO:0005515
Cluster-22178.34462 GO:0005515

Cluster-22178.34463 GO:0005515
Cluster-22178.34473 GO:0015031 GO:0008277 GO:0006886 GO:0008565
GO:0004222 GO:0005515 GO:0016021
Cluster-22178.34481 GO:0005525
Cluster-22178.34488 GO:0048268 GO:0035434 GO:0006825 GO:0032259
GO:0007212 GO:0032051 GO:0005375 GO:0046983 GO:0008168
GO:0016021
Cluster-22178.34502 GO:0005515
Cluster-22178.34503 GO:0042254 GO:0006412 GO:0009058 GO:0003824
GO:0003735 GO:0005622 GO:0005840
Cluster-22178.34504 GO:0016579 GO:0036459
Cluster-22178.3451 GO:0020037 GO:0019825
Cluster-22178.34514 GO:0043087 GO:0007264 GO:0005085 GO:0016012
GO:0016021
Cluster-22178.34520 GO:0006355 GO:0043401 GO:0008270 GO:0043565
GO:0003700 GO:0005667 GO:0005634
Cluster-22178.34523 GO:0005515
Cluster-22178.34527 GO:0005515
Cluster-22178.34529 GO:0006260 GO:0016032 GO:0005975 GO:0016758
GO:0016020
Cluster-22178.34534 GO:0007186 GO:0006869 GO:0005319 GO:0005515
GO:0004930 GO:0016021
Cluster-22178.34535 GO:0007186 GO:0004930 GO:0005515 GO:0016021
Cluster-22178.34547 GO:0005515
Cluster-22178.34554 GO:0006570 GO:0006470 GO:0007165 GO:0004721
GO:0004725 GO:0008138
Cluster-22178.34557 GO:0008152 GO:0006508 GO:0004197 GO:0003824
GO:0003676
Cluster-22178.34559 GO:0006355 GO:0003700 GO:0000975 GO:0005667
GO:0005622
Cluster-22178.3456 GO:0015886 GO:0015992 GO:0006119 GO:0006118
GO:0017004 GO:0008121 GO:0016021
Cluster-22178.34563 GO:0046488 GO:0016307

Cluster-22178.34567 GO:0006313 GO:0004803 GO:0003677

Cluster-22178.34572 GO:0003950

Cluster-22178.34575 GO:0005856

Cluster-22178.34587 GO:0000398 GO:0055114 GO:0015693 GO:0045454
GO:0016491 GO:0016209 GO:0015095 GO:0016021 GO:0005681

Cluster-22178.34592 GO:0019028

Cluster-22178.34595 GO:0007268 GO:0051260 GO:0007165 GO:0006508
GO:0055085 GO:0006811 GO:0006144 GO:0017111 GO:0004197
GO:0005216 GO:0003968 GO:0004970 GO:0016817 GO:0016020
GO:0031379

Cluster-22178.34605 GO:0007165 GO:0006955 GO:0005125 GO:0005576

Cluster-22178.34617 GO:0008270 GO:0046872 GO:0005515

Cluster-22178.34622 GO:0006118 GO:0008608 GO:0005975 GO:0030246
GO:0003954 GO:0004553 GO:0008270 GO:0008440 GO:0042729
GO:0005576 GO:0072686 GO:0005739

Cluster-22178.34624 GO:0008608 GO:0005975 GO:0006118 GO:0004553
GO:0008440 GO:0008270 GO:0003954 GO:0030246 GO:0042729
GO:0072686 GO:0005739 GO:0005576

Cluster-22178.34626 GO:0016032 GO:0055114 GO:0016491 GO:0019012

Cluster-22178.34627 GO:0016567 GO:0090263 GO:0008270 GO:0046872
GO:0005515

Cluster-22178.34628 GO:0000746 GO:0019867

Cluster-22178.34637 GO:0009264 GO:0051260 GO:0008253 GO:0016020

Cluster-22178.34640 GO:0070206

Cluster-22178.34645 GO:0006366 GO:0003677 GO:0005665

Cluster-22178.34649 GO:0006355 GO:0003677

Cluster-22178.34651 GO:0005783 GO:0016021

Cluster-22178.34652 GO:0006468 GO:0000746 GO:0004672 GO:0005524
GO:0005515

Cluster-22178.34654 GO:0070008 GO:0004252

Cluster-22178.34657 GO:0019239

Cluster-22178.34658 GO:0006479 GO:0006481 GO:0006629 GO:0016627
GO:0004671 GO:0016021 GO:0005737 GO:0016020

Cluster-22178.34662 GO:0016787
Cluster-22178.34673 GO:0005515 GO:0008270
Cluster-22178.34683 GO:0007165 GO:0005044 GO:0016020
Cluster-22178.34690 GO:0030036 GO:0003779 GO:0030054
Cluster-22178.34691 GO:0006508 GO:0004198 GO:0005515 GO:0005622
Cluster-22178.34696 GO:0006813 GO:0005249 GO:0008076
Cluster-22178.34697 GO:0006355 GO:0003682 GO:0043565 GO:0008270
GO:0003700 GO:0005667 GO:0000785
Cluster-22178.3470 GO:0007165 GO:0005509 GO:0005515 GO:0005578
Cluster-22178.34700 GO:0008168 GO:0019028
Cluster-22178.34701 GO:0008168 GO:0019028
Cluster-22178.34702 GO:0005515
Cluster-22178.34703 GO:0046039 GO:0009190 GO:0006182 GO:0006144
GO:0035556 GO:0004383 GO:0016849 GO:0020037
Cluster-22178.3471 GO:0009249 GO:0055085 GO:0042158 GO:0006810
GO:0009405 GO:0016757 GO:0005215 GO:0045735 GO:0016020
Cluster-22178.34712 GO:0019051 GO:0006508 GO:0004252 GO:0042025
GO:0016020
Cluster-22178.34717 GO:0007264 GO:0006351 GO:0015684 GO:0006144
GO:0006206 GO:0003899 GO:0003677 GO:0003924 GO:0015093
GO:0005525 GO:0005730 GO:0016020 GO:0005615 GO:0016021
Cluster-22178.3472 GO:0006355 GO:0003677 GO:0046872
Cluster-22178.34721 GO:0009058 GO:0005524 GO:0016874 GO:0016021
Cluster-22178.34722 GO:0008152 GO:0016310 GO:0007178 GO:0019836
GO:0006468 GO:0006260 GO:0006270 GO:0009069 GO:0003887
GO:0016791 GO:0005515 GO:0005524 GO:0043395 GO:0004675
GO:0042575 GO:0005578 GO:0005576 GO:0005727 GO:0016020
Cluster-22178.34723 GO:0005515
Cluster-22178.34734 GO:0008253 GO:0000287 GO:0005737
Cluster-22178.34736 GO:0008253 GO:0000287 GO:0005737
Cluster-22178.34738 GO:0016773
Cluster-22178.34739 GO:0007304 GO:0043087 GO:0007165 GO:0007264
GO:0005213 GO:0004872 GO:0005085 GO:0016021 GO:0042600

GO:0005576
Cluster-22178.34743 GO:0006351 GO:0030001 GO:0006260 GO:0055085
GO:0003887 GO:0017022 GO:0046873 GO:0046872 GO:0008270
GO:0043565 GO:0042575 GO:0016020 GO:0016459 GO:0005634
GO:0016021
Cluster-22178.34750 GO:0007165 GO:0004872 GO:0016021
Cluster-22178.34756 GO:0006508 GO:0005509 GO:0004198 GO:0005622
Cluster-22178.34760 GO:0007304 GO:0005213 GO:0005576 GO:0042600
Cluster-22178.34761 GO:0007304 GO:0005213 GO:0005576 GO:0042600
Cluster-22178.34763 GO:0019028
Cluster-22178.34766 GO:0006468 GO:0005515 GO:0005524 GO:0004672

Cluster-22178.34768 GO:0006468 GO:0005515 GO:0005524 GO:0004672

Cluster-22178.34771 GO:0006464 GO:0046872 GO:0005524
Cluster-22178.34772 GO:0006355 GO:0006270 GO:0008270 GO:0005515
GO:0005634 GO:0032040
Cluster-22178.34779 GO:0006468 GO:0016773 GO:0008270 GO:0005524
GO:0004672 GO:0005515 GO:0016020
Cluster-22178.34783 GO:0006355 GO:0006397 GO:0016192 GO:0006886
GO:0005515 GO:0005198 GO:0005737 GO:0030117 GO:0005634
Cluster-22178.34795 GO:0006397 GO:0009069 GO:0006468 GO:0016310
GO:0006260 GO:0035556 GO:0055114 GO:0003677 GO:0005515
GO:0047134 GO:0004674 GO:0005524 GO:0004672 GO:0005634
GO:0005664
Cluster-22178.34800 GO:0030991
Cluster-22178.34801 GO:0019497 GO:0006771 GO:0046872 GO:0003993
GO:0005515
Cluster-22178.34803 GO:0046488 GO:0016307
Cluster-22178.34813 GO:0008270 GO:0005622
Cluster-22178.34815 GO:0055114 GO:0006940 GO:0006952 GO:0016620
GO:0005515
Cluster-22178.34820 GO:0006364 GO:0006123 GO:0015992 GO:0055114

GO:0016705 GO:0020037 GO:0005506 GO:0004129 GO:0005096
GO:0008168 GO:0005739 GO:0045277
Cluster-22178.34833 GO:0006355
Cluster-22178.34836 GO:0007186 GO:0006950 GO:0055114 GO:0004930
GO:0004800
Cluster-22178.34838 GO:0006508 GO:0006811 GO:0006810 GO:0016805
GO:0005215 GO:0016020
Cluster-22178.34843 GO:0007186 GO:0055085 GO:0050909 GO:0006629
GO:0004930 GO:0016021
Cluster-22178.34844 GO:0006508 GO:0008270 GO:0004222 GO:0031012
Cluster-22178.34854 GO:0006355 GO:0003700 GO:0005667 GO:0005634
Cluster-22178.34865 GO:0007186 GO:0004930 GO:0016020 GO:0016021
Cluster-22178.34871 GO:0006269 GO:0006351 GO:0006260 GO:0003677
GO:0008270 GO:0003896 GO:0005730 GO:0005657
Cluster-22178.34877 GO:0003676
Cluster-22178.34880 GO:0046836 GO:0017089 GO:0051861 GO:0046872
GO:0005737
Cluster-22178.34882 GO:0046836 GO:0046872 GO:0051861 GO:0017089
GO:0005737
Cluster-22178.34886 GO:0005515
Cluster-22178.34887 GO:0006810 GO:0005886
Cluster-22178.34889 GO:0046486 GO:0005975 GO:0046168 GO:0055114
GO:0006072 GO:0051287 GO:0016616 GO:0004367 GO:0009331
Cluster-22178.34892 GO:0006811 GO:0005515 GO:0005198 GO:0016020
GO:0019028
Cluster-22178.34897 GO:0006810 GO:0006629 GO:0005215 GO:0016627
GO:0016021 GO:0005737
Cluster-22178.34905 GO:0005515
Cluster-22178.34906 GO:0006304 GO:0055114 GO:0003677 GO:0005515
GO:0003824 GO:0016702
Cluster-22178.34907 GO:0007155 GO:0006144 GO:0046034 GO:0046872
GO:0004017 GO:0016021
Cluster-22178.34911 GO:0007165 GO:0006807 GO:0016811 GO:0005515

Cluster-22178.34924 GO:0000226 GO:0006355 GO:0005515 GO:0015630
GO:0016020
Cluster-22178.34925 GO:0006508 GO:0035556 GO:0008242 GO:0016740
GO:0004197 GO:0016020 GO:0016021
Cluster-22178.34927 GO:0006000 GO:0006013 GO:0003873 GO:0005524

Cluster-22178.34929 GO:0006000 GO:0006013 GO:0061630 GO:0003873
GO:0005524
Cluster-22178.34930 GO:0006000 GO:0006013 GO:0006614 GO:0005524
GO:0016301 GO:0003873 GO:0005525
Cluster-22178.34933 GO:0042246 GO:0007155 GO:0005515 GO:0008270
GO:0046872 GO:0016021
Cluster-22178.34935 GO:0005515 GO:0016846 GO:0016817
Cluster-22178.34941 GO:0006457 GO:0016226 GO:0005515 GO:0005506
GO:0051536
Cluster-22178.34942 GO:0005515
Cluster-22178.34945 GO:0006629
Cluster-22178.34948 GO:0007275 GO:0005578
Cluster-22178.34950 GO:0016020
Cluster-22178.34951 GO:0016020
Cluster-22178.34953 GO:0001503 GO:0007155 GO:0005515 GO:0008270
GO:0046872 GO:0005576
Cluster-22178.34954 GO:0007155 GO:0001503 GO:0046872 GO:0008270
GO:0005515 GO:0005576
Cluster-22178.34956 GO:0016567 GO:0008270 GO:0046872 GO:0005515
GO:0004842 GO:0005680
Cluster-22178.34957 GO:0042967 GO:0047961 GO:0005739
Cluster-22178.34965 GO:0009405 GO:0016020
Cluster-22178.34967 GO:0006621 GO:0046923 GO:0016021 GO:0005783
Cluster-22178.34974 GO:0008219 GO:0009405 GO:0007339 GO:0005509
GO:0005515 GO:0005576
Cluster-22178.34980 GO:0007165 GO:0006955 GO:0005164 GO:0016020

Cluster-22178.34984 GO:0005515
Cluster-22178.34986 GO:0005515
Cluster-22178.34990 GO:0009405 GO:0005576
Cluster-22178.34991 GO:0006629
Cluster-22178.34995 GO:0006355 GO:0046872 GO:0005634
Cluster-22178.35012 GO:0043401 GO:0006355 GO:0007264 GO:0055114
GO:0005525 GO:0003924 GO:0016491
Cluster-22178.35013 GO:0005515 GO:0016020
Cluster-22178.35015 GO:0008152 GO:0005515 GO:0016746 GO:0043565
GO:0005634
Cluster-22178.35018 GO:0019062 GO:0019058 GO:0007155 GO:0006355
GO:0005515 GO:0043565 GO:0005634
Cluster-22178.35020 GO:0007186 GO:0004930 GO:0016020
Cluster-22178.35025 GO:0006367 GO:0006508 GO:0004252 GO:0005672
Cluster-22178.35029 GO:0006355 GO:0003700 GO:0003677 GO:0005634
GO:0005667
Cluster-22178.35039 GO:0006811 GO:0007186 GO:0055085 GO:0004930
GO:0005216 GO:0016021 GO:0016020
Cluster-22178.35052 GO:0009058 GO:0019079 GO:0005524 GO:0003676
GO:0016787 GO:0005509 GO:0005515 GO:0003677 GO:0008026
Cluster-22178.35063 GO:0019068
Cluster-22178.35070 GO:0007165 GO:0000917 GO:0009271 GO:0006355
GO:0043093 GO:0007049 GO:0005179 GO:0005576 GO:0005737
Cluster-22178.35071 GO:0030908 GO:0019867 GO:0016020
Cluster-22178.35082 GO:0042754 GO:0006812 GO:0045892 GO:0006836
GO:0005328 GO:0003676 GO:0016021 GO:0005622
Cluster-22178.35084 GO:0007165 GO:0005044 GO:0016020
Cluster-22178.35086 GO:0007165 GO:0005044 GO:0016020
Cluster-22178.35090 GO:0061024 GO:0006260 GO:0003677 GO:0003887
GO:0003676 GO:0005524 GO:0042575 GO:0030176
Cluster-22178.35091 GO:0019354 GO:0006779 GO:0035023 GO:0043087
GO:0055114 GO:0043115 GO:0005089
Cluster-22178.35107 GO:0043065 GO:0005515 GO:0005737

Cluster-22178.35114 GO:0006807 GO:0007018 GO:0006508 GO:0007017
GO:0003777 GO:0003933 GO:0004222 GO:0008017 GO:0005524
GO:0017070 GO:0005874 GO:0045298
Cluster-22178.35119 GO:0006479 GO:0006481 GO:0006629 GO:0016627
GO:0004671 GO:0016021 GO:0005737 GO:0016020
Cluster-22178.35120 GO:0006481 GO:0006629 GO:0006479 GO:0004671
GO:0016627 GO:0016020 GO:0005737 GO:0016021
Cluster-22178.35127 GO:0043087 GO:0019904 GO:0005515 GO:0008134
GO:0005667
Cluster-22178.35131 GO:0000917 GO:0006397 GO:0051258 GO:0048870
GO:0035307 GO:0007165 GO:0043093 GO:0006260 GO:0007218
GO:0002949 GO:0030168 GO:0005184 GO:0003723 GO:0003677
GO:0030674 GO:0016787 GO:0003724 GO:0005102 GO:0005524
GO:0016887 GO:0031514 GO:0005576 GO:0000445 GO:0005737
GO:0005577 GO:0071595
Cluster-22178.35136 GO:0006508 GO:0004252
Cluster-22178.35143 GO:0008270 GO:0005515
Cluster-22178.35146 GO:0007165 GO:0004252 GO:0019028 GO:0055036
Cluster-22178.35149 GO:0005515
Cluster-22178.35151 GO:0070403
Cluster-22178.35158 GO:0005515 GO:0008270
Cluster-22178.35164 GO:0016021
Cluster-22178.35166 GO:0000390 GO:0016787 GO:0019239 GO:0071008
Cluster-22178.35171 GO:0006506 GO:0017176 GO:0016021
Cluster-22178.35179 GO:0007304 GO:0005213 GO:0042600 GO:0005576
Cluster-22178.35181 GO:0006810 GO:0009405 GO:0000917 GO:0043093
GO:0006355 GO:0019871 GO:0042803 GO:0008134 GO:0045502
GO:0003700 GO:0005576 GO:0005667 GO:0030286 GO:0005737
Cluster-22178.35193 GO:0005887
Cluster-22178.35200 GO:0055085 GO:0006811 GO:0055114 GO:0015992
GO:0006118 GO:0006119 GO:0006810 GO:0042626 GO:0005524
GO:0008121 GO:0005509 GO:0005216 GO:0016021 GO:0016020
Cluster-22178.35202 GO:0035023 GO:0043087 GO:0005089

Cluster-22178.35209 GO:0006351 GO:0003714 GO:0005667
Cluster-22178.35213 GO:0004112 GO:0005515
Cluster-22178.35214 GO:0004112 GO:0005515
Cluster-22178.35216 GO:0006468 GO:0008152 GO:0004672 GO:0005524
GO:0016773 GO:0016791 GO:0016020
Cluster-22178.35219 GO:0008289
Cluster-22178.35233 GO:0005515
Cluster-22178.35236 GO:0009878 GO:0046872
Cluster-22178.35237 GO:0007155 GO:0042998 GO:0045859 GO:0006508
GO:0007339 GO:0008033 GO:0015994 GO:0008152 GO:0015996
GO:0016787 GO:0071253 GO:0047746 GO:0005515 GO:0008236
GO:0019887 GO:0008168 GO:0005802 GO:0005576 GO:0016021
GO:0005956
Cluster-22178.35241 GO:0046935 GO:0005944
Cluster-22178.35244 GO:0002949 GO:0006576 GO:0007264 GO:0015684
GO:0016887 GO:0003924 GO:0005524 GO:0015093 GO:0005525
GO:0016021
Cluster-22178.35246 GO:0002949 GO:0015684 GO:0006576 GO:0007264
GO:0005525 GO:0016887 GO:0003924 GO:0005524 GO:0015093
GO:0016021
Cluster-22178.35249 GO:0005515 GO:0008537
Cluster-22178.35250 GO:0007286 GO:0005739 GO:0005929 GO:0044297
GO:0097225
Cluster-22178.35251 GO:0007165
Cluster-22178.35254 GO:0006508 GO:0007186 GO:0006144 GO:0004197
GO:0017111 GO:0004252 GO:0004930 GO:0003968 GO:0031379
GO:0016021
Cluster-22178.35256 GO:0006355 GO:0046872 GO:0003677
Cluster-22178.35258 GO:0051289 GO:0008140 GO:0005667
Cluster-22178.3526 GO:0006355 GO:0006119 GO:0006118 GO:0006914
GO:0055114 GO:0015992 GO:0007049 GO:0008121 GO:0003677
GO:0043565 GO:0003700 GO:0005667 GO:0005634 GO:0016021
Cluster-22178.35260 GO:0007165 GO:0005179 GO:0005576

Cluster-22178.35264 GO:0050909 GO:0007156 GO:0007186 GO:0004930
GO:0005509 GO:0019031 GO:0016021 GO:0016020
Cluster-22178.35269 GO:0019028
Cluster-22178.35284 GO:0007165 GO:0040007 GO:0008283 GO:0008083

Cluster-22178.35286 GO:0007165 GO:0006955 GO:0006935 GO:0008009
GO:0005576
Cluster-22178.35292 GO:0045859 GO:0006771 GO:0006000 GO:0006013
GO:0019497 GO:0007049 GO:0016538 GO:0003873 GO:0005524
GO:0003993
Cluster-22178.35299 GO:0046983
Cluster-22178.35306 GO:0008544 GO:0006468 GO:0005524 GO:0004672
GO:0005515
Cluster-22178.35307 GO:0008544 GO:0006468 GO:0005524 GO:0004672
GO:0005515
Cluster-22178.35309 GO:0006355 GO:0003700 GO:0005667
Cluster-22178.35310 GO:0006183 GO:0019079 GO:0006165 GO:0006228
GO:0006241 GO:0006144 GO:0006206 GO:0004550
Cluster-22178.35311 GO:0006355 GO:0043565 GO:0003700 GO:0005667
Cluster-22178.35312 GO:0007166 GO:0005515 GO:0016020
Cluster-22178.35319 GO:0005198 GO:0005515 GO:0019028
Cluster-22178.35320 GO:0005198 GO:0005515 GO:0019028
Cluster-22178.35336 GO:0007268 GO:0007165 GO:0006811 GO:0004970
GO:0016020
Cluster-22178.35348 GO:0005515 GO:0016020
Cluster-22178.35351 GO:0042967 GO:0006633 GO:0055114 GO:0031072
GO:0004318 GO:0051082 GO:0016021 GO:0005835
Cluster-22178.35354 GO:0005515 GO:0003676
Cluster-22178.35361 GO:0016755
Cluster-22178.35362 GO:0016755
Cluster-22178.35364 GO:0016755
Cluster-22178.35365 GO:0016755
Cluster-22178.35368 GO:0055085 GO:0022857 GO:0016021

Cluster-22178.35395 GO:0006468 GO:0005524 GO:0004672
Cluster-22178.35397 GO:0055085 GO:0006811 GO:0007165 GO:0009416
GO:0003676 GO:0003677 GO:0005216 GO:0005179 GO:0016020
GO:0005634 GO:0005576
Cluster-22178.35399 GO:0005515
Cluster-22178.35400 GO:0008152 GO:0046849 GO:0008484 GO:0003824
GO:0005576
Cluster-22178.35403 GO:0006508 GO:0004222
Cluster-22178.35409 GO:0006355 GO:0003700 GO:0005667 GO:0005634
Cluster-22178.35412 GO:0006355 GO:0003700 GO:0005634 GO:0005667
Cluster-22178.35416 GO:0007165 GO:0005509 GO:0005515 GO:0005578
Cluster-22178.35420 GO:0042981
Cluster-22178.35427 GO:0055114 GO:0016702
Cluster-22178.35434 GO:0005515 GO:0016757 GO:0016020
Cluster-22178.35435 GO:0005515
Cluster-22178.35436 GO:0006308 GO:0006367 GO:0006810 GO:0009405
GO:0008855 GO:0008200 GO:0005576 GO:0005672 GO:0009318
Cluster-22178.35441 GO:0005096 GO:0005515
Cluster-22178.35443 GO:0006810 GO:0007213 GO:0043113 GO:0005215
GO:0005515 GO:0043236 GO:0016020
Cluster-22178.35455 GO:0006355 GO:0003677 GO:0005886
Cluster-22178.35461 GO:0055114 GO:0051536
Cluster-22178.35472 GO:0003677
Cluster-22178.35473 GO:0003677
Cluster-22178.35474 GO:0016020
Cluster-22178.35475 GO:0006468 GO:0009069 GO:0006807 GO:0016310
GO:0005509 GO:0004674 GO:0016151 GO:0004672 GO:0005524
GO:0004668 GO:0016773 GO:0016020 GO:0005737
Cluster-22178.35476 GO:0006468 GO:0009069 GO:0019069 GO:0006807
GO:0016310 GO:0005509 GO:0016151 GO:0004674 GO:0005524
GO:0004672 GO:0016773 GO:0004668 GO:0016020 GO:0005737
Cluster-22178.35477 GO:0005515 GO:0005524 GO:0042470
Cluster-22178.35482 GO:0005515

Cluster-22178.35486 GO:0061024 GO:0015948 GO:0046656 GO:0030269
GO:0046872 GO:0003677 GO:0045202 GO:0016021 GO:0030176
Cluster-22178.35494 GO:0006493 GO:0000030 GO:0000136 GO:0016020
Cluster-22178.35496 GO:0051289 GO:0008140 GO:0005667
Cluster-22178.35499 GO:0007224 GO:0016310 GO:0042967 GO:0009069
GO:0006468 GO:0005524 GO:0008270 GO:0004674 GO:0008080
Cluster-22178.35504 GO:0042967 GO:0016310 GO:0007224 GO:0009069
GO:0006468 GO:0008080 GO:0004674 GO:0005524 GO:0008270
Cluster-22178.35505 GO:0031225
Cluster-22178.3551 GO:0006508 GO:0008270 GO:0004222
Cluster-22178.35515 GO:0005515 GO:0016020
Cluster-22178.35516 GO:0042967 GO:0007015 GO:0047961 GO:0003785
GO:0005739
Cluster-22178.35524 GO:0005576 GO:0016021
Cluster-22178.35527 GO:0005515
Cluster-22178.35531 GO:0005515 GO:0008270
Cluster-22178.35534 GO:0007186 GO:0004930 GO:0030246 GO:0016020
GO:0016021
Cluster-22178.35538 GO:0015986 GO:0015992 GO:0015078 GO:0000276
Cluster-22178.35542 GO:0008643 GO:0006206 GO:0006367 GO:0006874
GO:0006816 GO:0006383 GO:0009401 GO:0030001 GO:0055085
GO:0006260 GO:0006144 GO:0006270 GO:0006351 GO:0003677
GO:0008982 GO:0005515 GO:0003899 GO:0004190 GO:0046873
GO:0005219 GO:0003887 GO:0005622 GO:0016020 GO:0005666
GO:0005730 GO:0005789 GO:0005672 GO:0009357 GO:0016021
GO:0032040 GO:0019031 GO:0042575
Cluster-22178.35547 GO:0007165 GO:0006955 GO:0005125 GO:0005576
Cluster-22178.35549 GO:0046872
Cluster-22178.35553 GO:0007009 GO:0051082
Cluster-22178.35554 GO:0008152 GO:0016746
Cluster-22178.35555 GO:0000398
Cluster-22178.35557 GO:0006810 GO:0042407 GO:0055085 GO:0005215
GO:0016020 GO:0044284 GO:0061617 GO:0016021

Cluster-22178.35558 GO:0007165 GO:0008289
Cluster-22178.35560 GO:0008270
Cluster-22178.35564 GO:0035556 GO:0006511 GO:0004175 GO:0005515
GO:0019773
Cluster-22178.35569 GO:0006915 GO:0006355 GO:0001836 GO:0006919
GO:0006974 GO:0003677 GO:0003700 GO:0005667
Cluster-22178.35574 GO:0002949
Cluster-22178.35575 GO:0042754 GO:0045892
Cluster-22178.35578 GO:0043087 GO:0032012 GO:0005086 GO:0005515

Cluster-22178.35579 GO:0043087 GO:0032012 GO:0005086 GO:0005515

Cluster-22178.35584 GO:0008270
Cluster-22178.35589 GO:0009451 GO:0007264 GO:0006400 GO:0035023
GO:0008033 GO:0006231 GO:0006206 GO:0043087 GO:0003677
GO:0008176 GO:0005085 GO:0050797 GO:0005089 GO:0050660
Cluster-22178.35599 GO:0006281 GO:0003908
Cluster-22178.35600 GO:0035556 GO:0006281 GO:0003908
Cluster-22178.35614 GO:0051262 GO:0044212 GO:0005515
Cluster-22178.35615 GO:0016579 GO:0036459
Cluster-22178.35618 GO:0051603 GO:0004298 GO:0005839
Cluster-22178.35623 GO:0006807 GO:0055114 GO:0006730 GO:0010181
GO:0005525 GO:0016491 GO:0004089 GO:0008270
Cluster-22178.35643 GO:0006508 GO:0008237 GO:0008241 GO:0016020
Cluster-22178.35645 GO:0015074 GO:0016310 GO:0004673 GO:0003677
GO:0008907 GO:0009365
Cluster-22178.35648 GO:0055085 GO:0006811 GO:0005216 GO:0016020
Cluster-22178.35657 GO:0006810 GO:0015893 GO:0006855 GO:0055085
GO:0015297 GO:0015238 GO:0016020
Cluster-22178.35661 GO:0006355 GO:0043401
Cluster-22178.35666 GO:0055114 GO:0040007 GO:0006259 GO:0008283
GO:0007165 GO:0016627 GO:0005524 GO:0003824 GO:0047134
GO:0008083 GO:0003677 GO:0005694

Cluster-22178.35671 GO:0006450 GO:0005515

Cluster-22178.35675 GO:0003677 GO:0046872 GO:0046983 GO:0016021 GO:0019012

Cluster-22178.35679 GO:0006508 GO:0004222 GO:0008270

Cluster-22178.3568 GO:0006810 GO:0006816 GO:0005246 GO:0042030 GO:0016020

Cluster-22178.35681 GO:0006265 GO:0008152 GO:0008092 GO:0016491 GO:0003916 GO:0003677 GO:0005737 GO:0019898

Cluster-22178.35688 GO:0006810 GO:0015721 GO:0005215 GO:0005515 GO:0046982 GO:0005886

Cluster-22178.35694 GO:0006468 GO:0004672 GO:0005524

Cluster-22178.35698 GO:0005515

Cluster-22178.35699 GO:0042981

Cluster-22178.35706 GO:0003950

Cluster-22178.35707 GO:0003950

Cluster-22178.35714 GO:0055085 GO:0019120 GO:0016021

Cluster-22178.35715 GO:0055085 GO:0019120 GO:0016021

Cluster-22178.35720 GO:0006886 GO:0016192 GO:0005515 GO:0030117

Cluster-22178.35721 GO:0007264 GO:0003924 GO:0016887 GO:0005524 GO:0005525

Cluster-22178.35735 GO:0006351 GO:0003714 GO:0005667

Cluster-22178.35736 GO:0006351 GO:0003714 GO:0005667

Cluster-22178.35742 GO:0007186 GO:0007165 GO:0007264 GO:0004871 GO:0005525 GO:0003924 GO:0031683 GO:0019001 GO:0032040

Cluster-22178.35743 GO:0016192 GO:0006886 GO:0005515 GO:0030117

Cluster-22178.35744 GO:0005509

Cluster-22178.35753 GO:0007165 GO:0043565 GO:0005515 GO:0016020

Cluster-22178.35760 GO:0003723 GO:0016021

Cluster-22178.35762 GO:0003924

Cluster-22178.35763 GO:0003924 GO:0005615

Cluster-22178.35764 GO:0032509 GO:0043130 GO:0000813

Cluster-22178.35773 GO:0006355 GO:0043401 GO:0008270 GO:0043565 GO:0003700 GO:0005634 GO:0005667

Cluster-22178.35779 GO:0051260 GO:0005515 GO:0016020

Cluster-22178.3578 GO:0006265 GO:0003918 GO:0003677 GO:0005694

Cluster-22178.35781 GO:0009890 GO:0043401 GO:0006355 GO:0003700 GO:0043565 GO:0008270 GO:0005515 GO:0005634 GO:0005667

Cluster-22178.35784 GO:0005515

Cluster-22178.35797 GO:0005515 GO:0046872 GO:0045202

Cluster-22178.35800 GO:0031110 GO:0005096 GO:0005515 GO:0005737

Cluster-22178.35802 GO:0006810 GO:0009405 GO:0019855 GO:0005576

Cluster-22178.35805 GO:0051321 GO:0046983

Cluster-22178.35806 GO:0051321 GO:0046983

Cluster-22178.35826 GO:0005515

Cluster-22178.35833 GO:0006955 GO:0007165 GO:0005164 GO:0016020

Cluster-22178.35837 GO:0000746 GO:0007186 GO:0003677 GO:0004930 GO:0016021 GO:0016020 GO:0019867

Cluster-22178.35842 GO:0006269 GO:0019354 GO:0006260 GO:0006779 GO:0016117 GO:0006351 GO:0055114 GO:0008033 GO:0071949 GO:0008270 GO:0016491 GO:0050660 GO:0016705 GO:0005515 GO:0043115 GO:0003896 GO:0005730 GO:0005657

Cluster-22178.35850 GO:0006508 GO:0004222

Cluster-22178.35852 GO:0006508 GO:0004222

Cluster-22178.35861 GO:0006817 GO:0005315 GO:0016020

Cluster-22178.35862 GO:0006308 GO:0008855 GO:0051082 GO:0009318

Cluster-22178.35869 GO:0007016 GO:0016010

Cluster-22178.35871 GO:0006355 GO:0003677 GO:0008270 GO:0046872 GO:0046983

Cluster-22178.35887 GO:0005975 GO:0016773

Cluster-22178.3590 GO:0005515

Cluster-22178.35902 GO:0006310 GO:0006281 GO:0005515 GO:0009378 GO:0005524 GO:0005657 GO:0009379

Cluster-22178.35908 GO:0007165 GO:0007275 GO:0000917 GO:0043093 GO:0005044 GO:0016020 GO:0005578 GO:0005737

Cluster-22178.35912 GO:0043085 GO:0016485 GO:0016021

Cluster-22178.35918 GO:0046872

Cluster-22178.3592 GO:0005515

Cluster-22178.35928 GO:0007165 GO:0006955 GO:0005125 GO:0005576

Cluster-22178.35940 GO:0016021

Cluster-22178.35942 GO:0032922 GO:0000976

Cluster-22178.35953 GO:0006950

Cluster-22178.35955 GO:0006506 GO:0017176 GO:0016021

Cluster-22178.35960 GO:0006355 GO:0003677

Cluster-22178.35963 GO:0046677 GO:0006270 GO:0003677

Cluster-22178.35972 GO:0007165 GO:0008158 GO:0005515 GO:0016020

Cluster-22178.35973 GO:0007165 GO:0005515 GO:0008158 GO:0016020

Cluster-22178.35977 GO:0009755 GO:0005576

Cluster-22178.35989 GO:0019079 GO:0006979 GO:0006804 GO:0006749
GO:0055114 GO:0003677 GO:0004602

Cluster-22178.36000 GO:0009235

Cluster-22178.36001 GO:0009235

Cluster-22178.36017 GO:0005509 GO:0005544

Cluster-22178.36020 GO:0003774 GO:0005524 GO:0005515 GO:0016459

Cluster-22178.36023 GO:0007017 GO:0007018 GO:0003777 GO:0030286
GO:0005874

Cluster-22178.36024 GO:0005515

Cluster-22178.36025 GO:0005515

Cluster-22178.36028 GO:0005515 GO:0061630 GO:0046872 GO:0008270

Cluster-22178.36035 GO:0009405 GO:0008360 GO:0006508 GO:0004197
GO:0016020

Cluster-22178.36036 GO:0005515

Cluster-22178.36042 GO:0005515

Cluster-22178.36050 GO:0019856 GO:0055114 GO:0016702 GO:0016020

Cluster-22178.36053 GO:0006448 GO:0006414 GO:0006184 GO:0005525
GO:0003924 GO:0003746 GO:0005840

Cluster-22178.36056 GO:0005789

Cluster-22178.36057 GO:0005789

Cluster-22178.36069 GO:0007165 GO:0005096

Cluster-22178.36071 GO:0032065 GO:0007165 GO:0005543 GO:0005096 GO:0005515 GO:0005938
Cluster-22178.36083 GO:0007016 GO:0007154 GO:0015813 GO:0015846 GO:0006814 GO:0015501 GO:0003677 GO:0005515 GO:0005509 GO:0016021 GO:0005576 GO:0016010 GO:0016020
Cluster-22178.36084 GO:0051252 GO:0006396 GO:0008608 GO:0003723 GO:0003676 GO:0000175 GO:0042729 GO:0072686
Cluster-22178.36089 GO:0005515
Cluster-22178.36093 GO:0008033 GO:0000166 GO:0005524 GO:0016879 GO:0005737
Cluster-22178.36099 GO:0005515
Cluster-22178.36103 GO:0008270 GO:0043565 GO:0005515 GO:0005634
Cluster-22178.36113 GO:0007165
Cluster-22178.36115 GO:0005515 GO:0016020
Cluster-22178.36117 GO:0005515 GO:0003774 GO:0005524 GO:0016459
Cluster-22178.36118 GO:0006606 GO:0003774 GO:0005198 GO:0016459 GO:0005882
Cluster-22178.36124 GO:0005524 GO:0005515
Cluster-22178.36125 GO:0006355 GO:0003700 GO:0043565 GO:0005667
Cluster-22178.36126 GO:0009405 GO:0051603 GO:0004298 GO:0005839 GO:0005576
Cluster-22178.36127 GO:0009405 GO:0051603 GO:0004298 GO:0005576 GO:0005839
Cluster-22178.36129 GO:0055114 GO:0016972
Cluster-22178.36134 GO:0006364 GO:0051252 GO:0006955 GO:0000902 GO:0006379 GO:0006811 GO:0008033 GO:0004540 GO:0003723 GO:0004190 GO:0016020 GO:0030677 GO:0000172 GO:0016021
Cluster-22178.36138 GO:0016887 GO:0005524
Cluster-22178.36139 GO:0005515
Cluster-22178.36141 GO:0005515
Cluster-22178.36144 GO:0003779 GO:0005622
Cluster-22178.36148 GO:0016567 GO:0005515 GO:0004842
Cluster-22178.3615 GO:0007186 GO:0006351 GO:0009069 GO:0006468

GO:0016310 GO:0006144 GO:0006206 GO:0003899 GO:0003677
GO:0004674 GO:0004930 GO:0005524 GO:0005634 GO:0005730
GO:0016021
Cluster-22178.36155 GO:0006355 GO:0051382 GO:0006281 GO:0003677
GO:0046872
Cluster-22178.36156 GO:0009069 GO:0000746 GO:0007165 GO:0015031
GO:0006914 GO:0006606 GO:0008360 GO:0006355 GO:0006468
GO:0006886 GO:0016573 GO:0007049 GO:0016310 GO:0005524
GO:0008565 GO:0004674 GO:0051082 GO:0046872 GO:0043565
GO:0003700 GO:0005667 GO:0016020 GO:0005815 GO:0005737
GO:0016021
Cluster-22178.36157 GO:0008219 GO:0005976 GO:0006886 GO:0016779
GO:0017119 GO:0000775
Cluster-22178.36163 GO:0003723 GO:0070461
Cluster-22178.36167 GO:0019069
Cluster-22178.36172 GO:0016032 GO:0005198 GO:0016020
Cluster-22178.36173 GO:0016032 GO:0005198 GO:0016020
Cluster-22178.36181 GO:0007165 GO:0005515 GO:0016020
Cluster-22178.36182 GO:0046034 GO:0006090 GO:0006144 GO:0071436
GO:0006814 GO:0006560 GO:0006525 GO:0015081 GO:0046872
GO:0004017 GO:0008948 GO:0016020
Cluster-22178.36183 GO:0007165 GO:0005515
Cluster-22178.36209 GO:0007186 GO:0004930 GO:0005515
Cluster-22178.36211 GO:0007264 GO:0005525
Cluster-22178.36222 GO:0016573 GO:0006270 GO:0006397 GO:0006355
GO:0042967 GO:0004402 GO:0003723 GO:0003712 GO:0008270
GO:0005667 GO:0005634 GO:0005737 GO:0000123
Cluster-22178.36227 GO:0006508 GO:0008270 GO:0004222 GO:0008233
GO:0031012
Cluster-22178.36234 GO:0046872
Cluster-22178.36235 GO:0046872
Cluster-22178.36238 GO:0000917 GO:0043093 GO:0006355 GO:0003677
GO:0030234 GO:0003700 GO:0043565 GO:0016020 GO:0005634

GO:0005667 GO:0005737
Cluster-22178.36240 GO:0010024 GO:0015886 GO:0055114 GO:0015232
GO:0050897 GO:0016636
Cluster-22178.36241 GO:0015994 GO:0006788 GO:0055114 GO:0004392
GO:0000975 GO:0008270
Cluster-22178.36244 GO:0006771 GO:0009405 GO:0019497 GO:0003677
GO:0005515 GO:0046872 GO:0016798 GO:0003993 GO:0005576
GO:0005634
Cluster-22178.36246 GO:0016579 GO:0036459 GO:0070140 GO:0032183
GO:0016021
Cluster-22178.36254 GO:0008270
Cluster-22178.36259 GO:0005201 GO:0005578 GO:0005581
Cluster-22178.36271 GO:0055085 GO:0016021
Cluster-22178.36272 GO:0008270 GO:0046872
Cluster-22178.36279 GO:0007165 GO:0055114 GO:0004252 GO:0005044
GO:0016641 GO:0070008 GO:0005507 GO:0016020
Cluster-22178.36281 GO:0006771 GO:0006468 GO:0048013 GO:0019497
GO:0005515 GO:0003993 GO:0046872 GO:0005524 GO:0004672
Cluster-22178.36286 GO:0051382 GO:0048870 GO:0015991 GO:0015031
GO:0000921 GO:0006260 GO:0006810 GO:0005975 GO:0048309
GO:0006397 GO:0006886 GO:0051028 GO:0015992 GO:0006914
GO:0006606 GO:0008092 GO:0045502 GO:0042802 GO:0008134
GO:0015078 GO:0051082 GO:0042803 GO:0016758 GO:0008565
GO:0003677 GO:0043015 GO:0016021 GO:0031514 GO:0033179
GO:0030286 GO:0005886 GO:0005940 GO:0019898 GO:0005737
GO:0000445 GO:0045298 GO:0000776 GO:0005667 GO:0016020
Cluster-22178.36292 GO:0006355 GO:0003677 GO:0003682 GO:0000775
GO:0000785 GO:0005634
Cluster-22178.36293 GO:0006355 GO:0003682 GO:0003677 GO:0005634
GO:0000775 GO:0000785
Cluster-22178.36294 GO:0007166 GO:0007186 GO:0005515 GO:0004930
GO:0016021 GO:0016020
Cluster-22178.36296 GO:0006629

Cluster-22178.36297 GO:0006629
Cluster-22178.36298 GO:0006629
Cluster-22178.36299 GO:0006629
Cluster-22178.36301 GO:0006486 GO:0008378 GO:0016020
Cluster-22178.36309 GO:0005515
Cluster-22178.36310 GO:0006122 GO:0008146 GO:0005750
Cluster-22178.36329 GO:0055114 GO:0046872 GO:0016702 GO:0005515

Cluster-22178.3633 GO:0022900 GO:0055114 GO:0006118 GO:0007596
GO:0010951 GO:0008827 GO:0008191 GO:0005515 GO:0004252
GO:0009319 GO:0016021 GO:0019012
Cluster-22178.36333 GO:0006811 GO:0055085 GO:0008061 GO:0005216
GO:0016020 GO:0005737
Cluster-22178.36334 GO:0008270
Cluster-22178.36358 GO:0007186 GO:0051537 GO:0004930 GO:0016021
GO:0043231
Cluster-22178.36362 GO:0007166 GO:0016020
Cluster-22178.36365 GO:0006355 GO:0090527 GO:0043565 GO:0003700
GO:0005667
Cluster-22178.36367 GO:0005515
Cluster-22178.36372 GO:0007049 GO:0051258 GO:0030168 GO:0006357
GO:0031110 GO:0007165 GO:0006457 GO:0005102 GO:0001104
GO:0051082 GO:0030674 GO:0005577 GO:0016272 GO:0016592
Cluster-22178.36374 GO:0000746 GO:0007186 GO:0003677 GO:0004930
GO:0016021 GO:0016020 GO:0019867
Cluster-22178.3638 GO:0006955 GO:0007165 GO:0035023 GO:0043087
GO:0005515 GO:0005044 GO:0005089 GO:0030247
Cluster-22178.36387 GO:0006471 GO:0003956 GO:0019825 GO:0005576
Cluster-22178.36388 GO:0055085 GO:0030154 GO:0007219 GO:0030001
GO:0007275 GO:0046873 GO:0005515 GO:0016021 GO:0016020
GO:0045095
Cluster-22178.36395 GO:0000975
Cluster-22178.36402 GO:0006355 GO:0005634

Cluster-22178.36405 GO:0005515
Cluster-22178.36406 GO:0005515
Cluster-22178.36409 GO:0006468 GO:0006355 GO:0003677 GO:0005515
GO:0004672 GO:0005576
Cluster-22178.36411 GO:0055085 GO:0006811 GO:0003676 GO:0003677
GO:0005216 GO:0016020 GO:0005634
Cluster-22178.36412 GO:0006955 GO:0051249
Cluster-22178.36413 GO:0051249 GO:0006955 GO:0005515
Cluster-22178.36414 GO:0051249 GO:0006955 GO:0005515
Cluster-22178.36419 GO:0004252 GO:0016021
Cluster-22178.36422 GO:0044030 GO:0005634
Cluster-22178.36424 GO:0007016 GO:0019031 GO:0016010
Cluster-22178.36426 GO:0044030 GO:0005634
Cluster-22178.36429 GO:0003676 GO:0005739
Cluster-22178.36430 GO:0003676
Cluster-22178.36431 GO:0006402 GO:0051252 GO:0004535 GO:0046872
GO:0003676 GO:0003723 GO:0005737 GO:0005634
Cluster-22178.36434 GO:0048511 GO:0055085 GO:0019684 GO:0006811
GO:0015979 GO:0007268 GO:0007165 GO:0006813 GO:0004970
GO:0005267 GO:0005216 GO:0016021 GO:0009523 GO:0016020
Cluster-22178.36438 GO:0051259
Cluster-22178.36453 GO:0006570 GO:0006470 GO:0042754 GO:0045892
GO:0008138 GO:0005515 GO:0004725 GO:0016020
Cluster-22178.36467 GO:0003676
Cluster-22178.36468 GO:0008270
Cluster-22178.36478 GO:0003677
Cluster-22178.36497 GO:0016310 GO:0031047 GO:0009069 GO:0006468
GO:0004674 GO:0004672 GO:0005524
Cluster-22178.36500 GO:0007186 GO:0006869 GO:0005319 GO:0005515
GO:0004930 GO:0016021
Cluster-22178.36505 GO:0016788
Cluster-22178.36508 GO:0033539 GO:0055114 GO:0016998 GO:0007017
GO:0007018 GO:0006118 GO:0052862 GO:0005524 GO:0008017

GO:0003777 GO:0052861 GO:0003995 GO:0045298 GO:0005874
Cluster-22178.36512 GO:0016310 GO:0006468 GO:0009069 GO:0009405
GO:0004672 GO:0005524 GO:0004674 GO:0045735
Cluster-22178.36513 GO:0046872 GO:0016820 GO:0000166
Cluster-22178.36525 GO:0055114 GO:0005506 GO:0000287 GO:0030976
GO:0020037 GO:0016705
Cluster-22178.36534 GO:0003676 GO:0003723
Cluster-22178.36541 GO:0006771 GO:0006355 GO:0019497 GO:0048013
GO:0001104 GO:0005515 GO:0046872 GO:0003993 GO:0016592
Cluster-22178.36543 GO:0006508 GO:0004197
Cluster-22178.36550 GO:0005515
Cluster-22178.36564 GO:0005515
Cluster-22178.36565 GO:0005515
Cluster-22178.36568 GO:0019497 GO:0006771 GO:0005515 GO:0003993
GO:0046872
Cluster-22178.36572 GO:0032922 GO:0000976
Cluster-22178.36587 GO:0006355 GO:0045893 GO:0043565 GO:0003677
GO:0005634
Cluster-22178.36592 GO:0007165 GO:0005057
Cluster-22178.36601 GO:0003676
Cluster-22178.36604 GO:0007275 GO:0005201 GO:0005578 GO:0005581
Cluster-22178.36613 GO:0007165 GO:0006566 GO:0006563 GO:0006783
GO:0042967 GO:0006544 GO:0006778 GO:0003870 GO:0005179
GO:0030170 GO:0005576 GO:0005759
Cluster-22178.36614 GO:0005515
Cluster-22178.36616 GO:0015991 GO:0015992 GO:0015078 GO:0033179
Cluster-22178.36617 GO:0015991 GO:0015992 GO:0015078 GO:0033179
Cluster-22178.36618 GO:0015991 GO:0009069 GO:0015992 GO:0016310
GO:0015078 GO:0004674 GO:0033179
Cluster-22178.36619 GO:0015991 GO:0015992 GO:0015078 GO:0033179
Cluster-22178.36626 GO:0006508 GO:0008152 GO:0016042 GO:0046486
GO:0016787 GO:0004806 GO:0008236
Cluster-22178.36631 GO:0019058 GO:0005198

Cluster-22178.36634 GO:0006811 GO:0006357 GO:0006810 GO:0006508
GO:0004222 GO:0005215 GO:0005515 GO:0001104 GO:0016592
GO:0016020
Cluster-22178.36638 GO:0009252 GO:0006807 GO:0004356
Cluster-22178.36640 GO:0016114 GO:0006694 GO:0055114 GO:0050660
GO:0004506 GO:0016021
Cluster-22178.36645 GO:0003723
Cluster-22178.36649 GO:0019049 GO:0042998 GO:0006355 GO:0043565
GO:0046872 GO:0008270 GO:0003700 GO:0071253 GO:0005515
GO:0005634 GO:0005667 GO:0005802
Cluster-22178.36654 GO:0005515
Cluster-22178.36655 GO:0006935 GO:0007165 GO:0005198 GO:0004888
GO:0016021 GO:0019031 GO:0016020 GO:0019028
Cluster-22178.36659 GO:0006355 GO:0001104 GO:0005515 GO:0016592
Cluster-22178.36665 GO:0031110 GO:0006457 GO:0051082 GO:0016272
Cluster-22178.36669 GO:0016020 GO:0016021
Cluster-22178.36674 GO:0007186 GO:0004930 GO:0016021
Cluster-22178.36678 GO:0035556 GO:0005515
Cluster-22178.36680 GO:0007165 GO:0098655 GO:0006812 GO:0033198
GO:0001614 GO:0008270 GO:0004931 GO:0005887
Cluster-22178.36684 GO:0006508 GO:0004197 GO:0005515 GO:0005739
Cluster-22178.36686 GO:0006119 GO:0006118 GO:0015992 GO:0008121
GO:0009055
Cluster-22178.36690 GO:0006355 GO:0019072 GO:0003677 GO:0043565
GO:0003700 GO:0005634 GO:0005667 GO:0042025
Cluster-22178.36700 GO:0046872 GO:0016787 GO:0005515 GO:0003676
GO:0031966
Cluster-22178.36705 GO:0016021
Cluster-22178.36706 GO:0005515
Cluster-22178.36710 GO:0006265 GO:0005524 GO:0003918 GO:0003677

Cluster-22178.36718 GO:0006814 GO:0005272 GO:0016020
Cluster-22178.36725 GO:0005515

Cluster-22178.36727 GO:0007264 GO:0005525
Cluster-22178.36733 GO:0005515 GO:0016817
Cluster-22178.36735 GO:0016310 GO:0009069 GO:0006468 GO:0004683
GO:0005516
Cluster-22178.36742 GO:0006508 GO:0004222 GO:0005515 GO:0005509
GO:0008270 GO:0031012
Cluster-22178.3675 GO:0051537
Cluster-22178.36752 GO:0006904 GO:0016192
Cluster-22178.36754 GO:0000226 GO:0008608 GO:0000910 GO:0006886
GO:0008017 GO:0008536 GO:0005515 GO:0045298 GO:0042729
GO:0072686
Cluster-22178.36756 GO:0019872 GO:0008654 GO:0055085 GO:0030001
GO:0006021 GO:0016817 GO:0004512 GO:0046873 GO:0003677
GO:0005515 GO:0016020
Cluster-22178.36764 GO:0009401 GO:0042981 GO:0006650 GO:0045454
GO:0006508 GO:0047396 GO:0004197 GO:0003677 GO:0005509
GO:0016021
Cluster-22178.36769 GO:0005515
Cluster-22178.36771 GO:0006355 GO:0003677
Cluster-22178.36777 GO:0006355 GO:0005975 GO:0016773 GO:0005524
GO:0003677
Cluster-22178.36783 GO:0007165 GO:0005509 GO:0005515 GO:0005578
Cluster-22178.36784 GO:0007165 GO:0005515 GO:0005509 GO:0005578
Cluster-22178.36786 GO:0005515
Cluster-22178.36792 GO:0007219 GO:0045944 GO:0008440 GO:0003713
GO:0005667 GO:0016607
Cluster-22178.36799 GO:0006810 GO:2001070 GO:0016021
Cluster-22178.36804 GO:0006355 GO:0016021
Cluster-22178.36805 GO:0006355 GO:0043565 GO:0003700 GO:0005667
GO:0005634
Cluster-22178.36812 GO:0006810 GO:0005471 GO:0005524 GO:0016021
GO:0005634
Cluster-22178.36814 GO:0006810 GO:0005471 GO:0005524 GO:0016021

GO:0005634
Cluster-22178.36819 GO:0070836 GO:0006952 GO:0005576
Cluster-22178.36823 GO:0006457 GO:0007165 GO:0005509 GO:0005578
Cluster-22178.36825 GO:0042254 GO:0006412 GO:0003735 GO:0005622
GO:0005840
Cluster-22178.36828 GO:0007264 GO:0005525
Cluster-22178.36846 GO:0009245 GO:0008915
Cluster-22178.36852 GO:0005524 GO:0003774 GO:0016887 GO:0005515
GO:0016459
Cluster-22178.3686 GO:0005515
Cluster-22178.36861 GO:0006355 GO:0006457 GO:0051082 GO:0005515
GO:0003677 GO:0043565 GO:0003700 GO:0005667 GO:0005634
GO:0016272
Cluster-22178.36865 GO:0006470 GO:0006570 GO:0004725
Cluster-22178.36867 GO:0007186 GO:0004930 GO:0016817 GO:0016021
Cluster-22178.36870 GO:0005515
Cluster-22178.36873 GO:0006260 GO:0003677 GO:0008270 GO:0019033
Cluster-22178.36877 GO:0006355
Cluster-22178.36880 GO:0007165 GO:0043130 GO:0032266 GO:0005515

Cluster-22178.36887 GO:0008643 GO:0015693 GO:0055085 GO:0016310
GO:0009069 GO:0006810 GO:0005524 GO:0004674 GO:0005351
GO:0015095 GO:0016021 GO:0000139 GO:0016020
Cluster-22178.3689 GO:0005515
Cluster-22178.36892 GO:0009452 GO:0008152 GO:0001510 GO:0006306
GO:0031167 GO:0008168 GO:0008170 GO:0003677 GO:0016021
GO:0019013
Cluster-22178.36893 GO:0042256 GO:0043022
Cluster-22178.36894 GO:0007165 GO:0008158 GO:0005515 GO:0016020
Cluster-22178.36901 GO:0043565 GO:0005515 GO:0005634
Cluster-22178.36902 GO:0005515
Cluster-22178.36909 GO:0019064 GO:0019031 GO:0005739
Cluster-22178.3691 GO:0055114 GO:0003824

Cluster-22178.36910 GO:0006570 GO:0006470 GO:0008138 GO:0004725

Cluster-22178.36912 GO:0008360 GO:0006260 GO:0016020
Cluster-22178.36913 GO:0003723
Cluster-22178.36915 GO:0006807 GO:0005509 GO:0004668 GO:0005737
Cluster-22178.36918 GO:0006144 GO:0035556 GO:0006182 GO:0046039
GO:0009190 GO:0006468 GO:0004672 GO:0005524 GO:0004383
GO:0016849
Cluster-22178.36919 GO:0006470 GO:0006570 GO:0008138 GO:0004725

Cluster-22178.36922 GO:0007165 GO:0006810 GO:0007268 GO:0006811
GO:0015288 GO:0004970 GO:0005234 GO:0016020
Cluster-22178.36923 GO:0016787
Cluster-22178.36926 GO:0016567 GO:0004842 GO:0008270 GO:0046872
GO:0061630 GO:0005515 GO:0005680 GO:0005622
Cluster-22178.36928 GO:0006355 GO:0016032 GO:0048870 GO:0003700
GO:0043565 GO:0031514 GO:0005667
Cluster-22178.3693 GO:0055114 GO:0008152 GO:0016829 GO:0016491
GO:0003824
Cluster-22178.36932 GO:0019354 GO:0006269 GO:0016117 GO:0006779
GO:0006260 GO:0006351 GO:0055114 GO:0008033 GO:0071949
GO:0050660 GO:0016491 GO:0008270 GO:0043115 GO:0005515
GO:0016705 GO:0003896 GO:0005730 GO:0005657
Cluster-22178.36935 GO:0006397 GO:0003723 GO:0005515 GO:0005634
GO:0005737
Cluster-22178.36938 GO:0008270 GO:0005886
Cluster-22178.36944 GO:0055114 GO:0006355 GO:0006413 GO:0006351
GO:0045893 GO:0006269 GO:0006446 GO:0043565 GO:0003700
GO:0003896 GO:0008270 GO:0004386 GO:0047134 GO:0003743
GO:0003677 GO:0005840 GO:0005634 GO:0005657 GO:0005667
GO:0005730
Cluster-22178.36957 GO:0006351 GO:0005515
Cluster-22178.36958 GO:0006810 GO:0005215 GO:0016020

Cluster-22178.36961 GO:0006570 GO:0006771 GO:0006470 GO:0019497 GO:0005198 GO:0005515 GO:0004222 GO:0003993 GO:0004725 GO:0046872

Cluster-22178.36969 GO:0003676 GO:0008376 GO:0032580

Cluster-22178.36978 GO:0030153 GO:0006955 GO:0015643 GO:0019814

Cluster-22178.3698 GO:0007186 GO:0004930 GO:0008270 GO:0016021

Cluster-22178.36981 GO:0046872

Cluster-22178.36982 GO:0005515 GO:0008270 GO:0046872

Cluster-22178.36983 GO:0006511 GO:0006886 GO:0007275 GO:0000910 GO:0000226 GO:0008608 GO:0005515 GO:0008270 GO:0008017 GO:0008536 GO:0005634 GO:0072686 GO:0042729 GO:0045298

Cluster-22178.36985 GO:0006351 GO:0006206 GO:0006144 GO:0003677 GO:0003899 GO:0005515 GO:0005730

Cluster-22178.36993 GO:0006468 GO:0004672 GO:0005524 GO:0016773 GO:0016020

Cluster-22178.37009 GO:0051258 GO:0030168 GO:0007165 GO:0030674 GO:0005102 GO:0005577

Cluster-22178.37011 GO:0006355 GO:0005515 GO:0003700 GO:0008270 GO:0043565 GO:0005747 GO:0005739 GO:0005667

Cluster-22178.37013 GO:0009405 GO:0005515 GO:0003682 GO:0000785 GO:0005576

Cluster-22178.37019 GO:0016021 GO:0016020

Cluster-22178.37020 GO:0055085 GO:0046872 GO:0016020 GO:0016021 GO:0045202

Cluster-22178.37021 GO:0055085 GO:0046872 GO:0016020 GO:0016021 GO:0045202

Cluster-22178.37023 GO:0035556 GO:0009190 GO:0020037 GO:0016849 GO:0019825

Cluster-22178.37033 GO:0006812 GO:0006885 GO:0055085 GO:0015299 GO:0016021

Cluster-22178.37051 GO:0006508 GO:0045893 GO:1902208 GO:0016579 GO:0004843 GO:0003677 GO:0008270 GO:0051536 GO:0036459

Cluster-22178.37065 GO:0006275 GO:0006270 GO:0005524 GO:0043565

Cluster-22178.37067 GO:0005515
Cluster-22178.37072 GO:0006605 GO:0007165 GO:0006811 GO:0004190 GO:0016021 GO:0016020
Cluster-22178.37074 GO:0006468 GO:0006355 GO:0003700 GO:0004672 GO:0005524 GO:0008270 GO:0043565 GO:0016773 GO:0016020 GO:0005667 GO:0005794
Cluster-22178.37075 GO:0006468 GO:0006355 GO:0004672 GO:0005524 GO:0008270 GO:0043565 GO:0003700 GO:0005794 GO:0005667
Cluster-22178.37076 GO:0045859 GO:0016055 GO:0004860
Cluster-22178.37097 GO:0006355 GO:0046983
Cluster-22178.37101 GO:0006259 GO:0003824 GO:0003677 GO:0005524 GO:0005694
Cluster-22178.37110 GO:0007165 GO:0005515
Cluster-22178.37118 GO:0004252 GO:0070008
Cluster-22178.37119 GO:0006810 GO:0006811 GO:0003677 GO:0005230 GO:0016020
Cluster-22178.37122 GO:0006433 GO:0006511 GO:0006560 GO:0006525 GO:0006777 GO:0051539 GO:0000166 GO:0003824 GO:0004827 GO:0004175 GO:0051536 GO:0005524 GO:0019773 GO:0005737 GO:0019008
Cluster-22178.37132 GO:0015979 GO:0016020 GO:0009523
Cluster-22178.37134 GO:0006310 GO:0019079 GO:0055085 GO:0006260 GO:0006206 GO:0006281 GO:0006355 GO:0006139 GO:0003677 GO:0009378 GO:0003724 GO:0004127 GO:0016887 GO:0005524 GO:0008134 GO:0016301 GO:0003723 GO:0022857 GO:0003678 GO:0009379 GO:0005667 GO:0005657 GO:0016021
Cluster-22178.37139 GO:0015886 GO:0017004 GO:0005515 GO:0016021 GO:0016020
Cluster-22178.37143 GO:0045859 GO:0007050 GO:0004861 GO:0005634
Cluster-22178.37153 GO:0046872
Cluster-22178.37154 GO:0009069 GO:0006468 GO:0007165 GO:0010508 GO:0016310 GO:0005515 GO:0005096 GO:0004674 GO:0003676

Cluster-22178.37156 GO:0006355 GO:0003700 GO:0008270 GO:0003682
GO:0043565 GO:0000785 GO:0005667
Cluster-22178.37166 GO:0007165 GO:0009058 GO:0008299 GO:0006694
GO:0016779 GO:0005515 GO:0005044 GO:0050518 GO:0016020
Cluster-22178.37169 GO:0046836 GO:0051861 GO:0017089 GO:0005737
Cluster-22178.37175 GO:0005515 GO:0008270
Cluster-22178.37179 GO:0006306 GO:0008170 GO:0003677 GO:0005515

Cluster-22178.37186 GO:0009069 GO:0006468 GO:0016310 GO:0005515
GO:0004674 GO:0005524 GO:0004672 GO:0016020 GO:0016021
Cluster-22178.37192 GO:0005515
Cluster-22178.37196 GO:0006810 GO:0006811 GO:0005230 GO:0016020
Cluster-22178.37198 GO:0006284 GO:0016787 GO:0003677 GO:0003905
GO:0016021
Cluster-22178.37199 GO:0001503 GO:0005515 GO:0005509
Cluster-22178.37210 GO:0005515
Cluster-22178.37213 GO:0015992 GO:0015986 GO:0015078 GO:0000276
Cluster-22178.37219 GO:0005515
Cluster-22178.37222 GO:0001503 GO:0007155 GO:0005515 GO:0005634
Cluster-22178.37224 GO:0001503 GO:0007155 GO:0005515 GO:0005634
Cluster-22178.37225 GO:0042802 GO:0005515
Cluster-22178.3723 GO:0007155 GO:0006928 GO:0005198 GO:0005515
GO:0016021
Cluster-22178.37232 GO:0007156 GO:0005509 GO:0016020
Cluster-22178.37238 GO:0007018 GO:0006388 GO:0007017 GO:0008017
GO:0016772 GO:0003777 GO:0008270 GO:0005524 GO:0003677
GO:0045298 GO:0005874
Cluster-22178.37239 GO:0006470 GO:0050852 GO:0055114 GO:0006570
GO:0004725 GO:0016972 GO:0005515 GO:0016021
Cluster-22178.3724 GO:0007186 GO:0004930 GO:0016021
Cluster-22178.37243 GO:0005515
Cluster-22178.37247 GO:0055085 GO:0006865 GO:0006811 GO:0006366
GO:0006367 GO:0003333 GO:0006810 GO:0006122 GO:0005215

GO:0015171 GO:0005743 GO:0005750 GO:0016020 GO:0005674
Cluster-22178.37249 GO:0005515 GO:0005576
Cluster-22178.37257 GO:0005515 GO:0016020
Cluster-22178.37259 GO:0007165 GO:0006629 GO:0005524 GO:0005179
GO:0016627 GO:0016021 GO:0005737
Cluster-22178.37261 GO:0006629 GO:0007165 GO:0005524 GO:0005179
GO:0016627 GO:0005737 GO:0016021
Cluster-22178.37263 GO:0005515
Cluster-22178.37264 GO:0008289 GO:0005515
Cluster-22178.37278 GO:0006397 GO:0006897 GO:0006807 GO:0046660
GO:0016814 GO:0008270 GO:0005634 GO:0016020 GO:0001527
Cluster-22178.37286 GO:0006352 GO:0006355 GO:0003700 GO:0019904
GO:0003677 GO:0016987 GO:0005667
Cluster-22178.37305 GO:0006144 GO:0006206 GO:0006351 GO:0046872
GO:0008270 GO:0003899 GO:0003677 GO:0005730
Cluster-22178.37308 GO:0007186 GO:0007155 GO:0006508 GO:0004930
GO:0004222 GO:0008270 GO:0016020 GO:0016021 GO:0031012
Cluster-22178.37309 GO:0006508 GO:0007186 GO:0008270 GO:0004222
GO:0004930 GO:0003950 GO:0031012 GO:0016021 GO:0005634
GO:0016020
Cluster-22178.37314 GO:0005515
Cluster-22178.37316 GO:0017148 GO:0019835 GO:0009405 GO:0042710
GO:0009372 GO:0019867
Cluster-22178.37317 GO:0006468 GO:0006836 GO:0006812 GO:0005335
GO:0016773 GO:0004672 GO:0005524 GO:0016020 GO:0005887
Cluster-22178.37319 GO:0006508 GO:0004222
Cluster-22178.37332 GO:0005515 GO:0016020
Cluster-22178.37339 GO:0007186 GO:0004930 GO:0016021
Cluster-22178.37345 GO:0006811 GO:0006816 GO:0005244 GO:0005262
GO:0016021
Cluster-22178.37351 GO:0007304 GO:0046872 GO:0005213 GO:0005576
GO:0042600
Cluster-22178.37352 GO:0007304 GO:0005213 GO:0046872 GO:0005576

GO:0042600
Cluster-22178.37354 GO:0046872
Cluster-22178.37355 GO:0006879 GO:0008199
Cluster-22178.37363 GO:0006935 GO:0007154 GO:0007165 GO:0009451
GO:0006955 GO:0001522 GO:0009982 GO:0003723 GO:0005509
GO:0008009 GO:0005515 GO:0005576 GO:0016020
Cluster-22178.37365 GO:0007165 GO:0009451 GO:0006955 GO:0001522
GO:0006935 GO:0007154 GO:0003723 GO:0005515 GO:0008009
GO:0005509 GO:0009982 GO:0005576 GO:0016020
Cluster-22178.37366 GO:0051726 GO:0005634
Cluster-22178.37369 GO:0007165 GO:0031122 GO:0006811 GO:0035058
GO:0007052 GO:0055085 GO:0004888 GO:0005216 GO:0005515
GO:0097546 GO:0016020 GO:0005813 GO:0016021
Cluster-22178.37370 GO:0031122 GO:0006811 GO:0035058 GO:0055085
GO:0007052 GO:0007165 GO:0008270 GO:0004888 GO:0005216
GO:0005515 GO:0005813 GO:0016021 GO:0097546 GO:0016020
Cluster-22178.37373 GO:0006352 GO:0006606 GO:0006355 GO:0003700
GO:0016987 GO:0019904 GO:0003677 GO:0005667
Cluster-22178.37374 GO:0006355 GO:0006352 GO:0016987 GO:0003677
GO:0019904 GO:0003700 GO:0005667
Cluster-22178.37377 GO:0046872
Cluster-22178.37378 GO:0006355 GO:0005515 GO:0005634 GO:0016021
Cluster-22178.37388 GO:0007186 GO:0004930 GO:0016021
Cluster-22178.37397 GO:0008270
Cluster-22178.37398 GO:0008270
Cluster-22178.37403 GO:0033617
Cluster-22178.37407 GO:0005515
Cluster-22178.37413 GO:0016032 GO:0019012
Cluster-22178.37414 GO:0006811 GO:0006810 GO:0005230 GO:0016020
Cluster-22178.37416 GO:0007339 GO:0005515 GO:0016020 GO:0005576
Cluster-22178.37417 GO:0030153 GO:0006955 GO:0015643 GO:0019814
Cluster-22178.37420 GO:0007264 GO:0051016 GO:0005525 GO:0003924
GO:0008290

Cluster-22178.37425 GO:0055114 GO:0000398 GO:0035556 GO:0046872
GO:0047134 GO:0005681
Cluster-22178.37426 GO:0030001 GO:0007155 GO:0055085 GO:0051015
GO:0016853 GO:0046873 GO:0003682 GO:0016020 GO:0000785
Cluster-22178.37428 GO:0005975 GO:0006355 GO:0005524 GO:0016773
GO:0003677
Cluster-22178.37430 GO:0008608 GO:0006950 GO:0005515 GO:0072686
GO:0042729
Cluster-22178.37437 GO:0006570 GO:0009405 GO:0031412 GO:0006470
GO:0050852 GO:0005515 GO:0004725 GO:0003774 GO:0005524
GO:0016020 GO:0031411 GO:0016459
Cluster-22178.37442 GO:0007154 GO:0006935 GO:0007219 GO:0006955
GO:0007165 GO:0007275 GO:0005515 GO:0008009 GO:0016021
GO:0005576 GO:0016020
Cluster-22178.37443 GO:0008289
Cluster-22178.37456 GO:0005515
Cluster-22178.37459 GO:0090263 GO:0016567
Cluster-22178.3746 GO:0005515
Cluster-22178.37463 GO:0019299 GO:0016857 GO:0005515 GO:0005737
Cluster-22178.37464 GO:0005737
Cluster-22178.37465 GO:0005737
Cluster-22178.37469 GO:0005515
Cluster-22178.37474 GO:0006139 GO:0006206 GO:0006614 GO:0006813
GO:0005525 GO:0004127 GO:0005524 GO:0005267 GO:0043531
GO:0098519 GO:0016301 GO:0045095
Cluster-22178.37481 GO:0006807 GO:0009399 GO:0019337 GO:0055114
GO:0007165 GO:0006355 GO:0016163 GO:0004872 GO:0003677
GO:0016020 GO:0016610
Cluster-22178.37482 GO:0019013
Cluster-22178.37484 GO:0042254 GO:0006412 GO:0003735 GO:0005840
GO:0005622
Cluster-22178.37487 GO:0016998 GO:0006032 GO:0004568 GO:0005737
Cluster-22178.37489 GO:0006355 GO:0005515 GO:0005634 GO:0005668

Cluster-22178.37492 GO:0030435 GO:0005515

Cluster-22178.37494 GO:0030435 GO:0071816 GO:0042803 GO:0008134 GO:0005515 GO:0045502 GO:0051082 GO:0030286 GO:0005667

Cluster-22178.37495 GO:0030435 GO:0005515

Cluster-22178.37500 GO:0006810 GO:0007165 GO:0042626 GO:0005524 GO:0005158 GO:0016021 GO:0005899

Cluster-22178.37504 GO:0009116

Cluster-22178.37518 GO:0034080 GO:0008270 GO:0000775

Cluster-22178.37519 GO:0055114 GO:0010024 GO:0009058 GO:0050897 GO:0005524 GO:0016779 GO:0016636

Cluster-22178.37521 GO:0043085 GO:0000290 GO:0034220 GO:0006811 GO:0008047 GO:0015075 GO:0016021

Cluster-22178.37531 GO:0000105 GO:0005515

Cluster-22178.37533 GO:0005515

Cluster-22178.37535 GO:0005515

Cluster-22178.37538 GO:0005515

Cluster-22178.37553 GO:0055085 GO:0006810 GO:0007165 GO:0043531 GO:0016887 GO:0003924 GO:0005524 GO:0042626 GO:0005525 GO:0016021 GO:0016020

Cluster-22178.37558 GO:0015979 GO:0016310 GO:0006468 GO:0009069 GO:0016773 GO:0004672 GO:0005524 GO:0004674 GO:0009523 GO:0009539 GO:0016020

Cluster-22178.37560 GO:0005887

Cluster-22178.37562 GO:0019028

Cluster-22178.37565 GO:0015992 GO:0007156 GO:0006118 GO:0006119 GO:0005509 GO:0008121 GO:0016020

Cluster-22178.37568 GO:0016567 GO:0004842 GO:0008270 GO:0046872

Cluster-22178.37572 GO:0043039 GO:0006432 GO:0008033 GO:0009094 GO:0006418 GO:0000162 GO:0006571 GO:0000287 GO:0004812 GO:0000049 GO:0000166 GO:0005524 GO:0004826 GO:0009328 GO:0005737

Cluster-22178.37573 GO:0055085 GO:0006629 GO:0016021

Cluster-22178.37577 GO:0016012 GO:0016021
Cluster-22178.37579 GO:0016012 GO:0016021
Cluster-22178.37580 GO:0009405 GO:0006886 GO:0006355 GO:0016032
GO:0006810 GO:0016192 GO:0016070 GO:0006812 GO:0016779
GO:0005198 GO:0005515 GO:0019871 GO:0003677 GO:0016887
GO:0019012 GO:0030117 GO:0005576 GO:0016021
Cluster-22178.37581 GO:0006811 GO:0006810 GO:0009405 GO:0005216
GO:0016020 GO:0016021
Cluster-22178.37582 GO:0006355 GO:0055114 GO:0003677 GO:0003723
GO:0003700 GO:0046872 GO:0008270 GO:0043565 GO:0016702
GO:0005667
Cluster-22178.37584 GO:0006397 GO:0005634
Cluster-22178.37592 GO:0051726
Cluster-22178.37597 GO:0006355 GO:0043565 GO:0003700 GO:0005667
Cluster-22178.37602 GO:0008152 GO:0019521 GO:0055114 GO:0006098
GO:0004616 GO:0016616 GO:0051287
Cluster-22178.37604 GO:0016310 GO:0006508 GO:0009069 GO:0008233
GO:0004222 GO:0008270 GO:0008384 GO:0031012 GO:0008385
Cluster-22178.37605 GO:0006470 GO:0006570 GO:0004725 GO:0008138

Cluster-22178.37606 GO:0006470 GO:0006570 GO:0004725
Cluster-22178.37613 GO:0046813 GO:0061025 GO:0007165 GO:0006355
GO:0005515 GO:0019031 GO:0016021 GO:0005634
Cluster-22178.37615 GO:0055114 GO:0009308 GO:0007067 GO:0006355
GO:0043565 GO:0003700 GO:0048038 GO:0005507 GO:0008131
GO:0019901 GO:0072686 GO:0005667 GO:0042729
Cluster-22178.37616 GO:0006506 GO:0017176 GO:0016021
Cluster-22178.37619 GO:0042967 GO:0006355 GO:0003677 GO:0008080
GO:0005634
Cluster-22178.37622 GO:0006357 GO:0007165 GO:0005179 GO:0042393
GO:0001104 GO:0005576 GO:0016592
Cluster-22178.37623 GO:0007165 GO:0006357 GO:0042393 GO:0001104
GO:0005179 GO:0016592 GO:0005576

Cluster-22178.37627 GO:0055114 GO:0005515 GO:0016491
Cluster-22178.37633 GO:0007059 GO:0051301 GO:0048870 GO:0000090
GO:0007155 GO:0007067 GO:0006260 GO:0031110 GO:0006914
GO:0005198 GO:0019901 GO:0019905 GO:0008017 GO:0003774
GO:0031514 GO:0019028 GO:0019031 GO:0045298 GO:0005882
GO:0016459 GO:0005876 GO:0000940
Cluster-22178.37634 GO:0005515
Cluster-22178.37636 GO:0006355 GO:0007010 GO:0003779 GO:0005634
Cluster-22178.37649 GO:0005525 GO:0003924
Cluster-22178.37651 GO:0030154 GO:0007219 GO:0007275 GO:0016021
Cluster-22178.37653 GO:0016020
Cluster-22178.37661 GO:0003724 GO:0005524 GO:0003723 GO:0016887

Cluster-22178.37664 GO:0006885 GO:0006814 GO:0016021 GO:0019028
Cluster-22178.37665 GO:0006885 GO:0006814 GO:0019028 GO:0016021
Cluster-22178.37671 GO:0042802 GO:0005515
Cluster-22178.37674 GO:0006355 GO:0007155 GO:0005198 GO:0046872
GO:0008270 GO:0043565 GO:0003700 GO:0005667 GO:0031012
Cluster-22178.37675 GO:0006118 GO:0016567 GO:0019646 GO:0005515
GO:0004842 GO:0009055 GO:0046872 GO:0008270 GO:0005680
Cluster-22178.37681 GO:0006955 GO:0030153 GO:0006357 GO:0001104
GO:0015643 GO:0008270 GO:0016592 GO:0019814 GO:0016021
Cluster-22178.37683 GO:0003677 GO:0003824 GO:0030151 GO:0030170
GO:0005634
Cluster-22178.37685 GO:0055114 GO:0047134 GO:0046872
Cluster-22178.37686 GO:0006810 GO:0046849 GO:0006355 GO:0006352
GO:0030420 GO:0003677 GO:0008200 GO:0016987 GO:0003700
GO:0005576 GO:0005667
Cluster-22178.37687 GO:0030683 GO:0044386
Cluster-22178.37688 GO:0005515
Cluster-22178.37691 GO:0055085 GO:0006811 GO:0005975 GO:0016857
GO:0005216 GO:0016020
Cluster-22178.37695 GO:0005975 GO:0004553 GO:0030248 GO:0005576

Cluster-22178.37705 GO:0005515
Cluster-22178.37708 GO:0006355 GO:0005515 GO:0003700 GO:0043565
GO:0051082 GO:0005737 GO:0005667
Cluster-22178.37713 GO:0008440 GO:0005515 GO:0005737 GO:0016020
Cluster-22178.37722 GO:0003677 GO:0005515 GO:0046983
Cluster-22178.37735 GO:0006355 GO:0009271 GO:0016021
Cluster-22178.37751 GO:0051301 GO:0016021
Cluster-22178.37768 GO:0006355 GO:0003677 GO:0005515 GO:0003700
GO:0005667
Cluster-22178.37769 GO:0005515 GO:0035091
Cluster-22178.37776 GO:0004252 GO:0005886 GO:0016021
Cluster-22178.37777 GO:0055114 GO:0006570 GO:0006470 GO:0016491
GO:0004725
Cluster-22178.37779 GO:0015031 GO:0030176
Cluster-22178.37791 GO:0007275 GO:0045893 GO:0008270
Cluster-22178.37792 GO:0007165 GO:0006810 GO:0007155 GO:0055085
GO:0006914 GO:0015992 GO:0010508 GO:0007094 GO:0015991
GO:0006355 GO:0030001 GO:0006352 GO:0007049 GO:0055114
GO:0048870 GO:0005509 GO:0003677 GO:0046873 GO:0016987
GO:0016491 GO:0003774 GO:0004871 GO:0003700 GO:0015078
GO:0016020 GO:0005667 GO:0016459 GO:0005886 GO:0033179
GO:0031514
Cluster-22178.37795 GO:0006778 GO:0042967 GO:0006544 GO:0006783
GO:0006563 GO:0006468 GO:0006566 GO:0016773 GO:0004672
GO:0005524 GO:0030170 GO:0003870 GO:0005759 GO:0016020
Cluster-22178.37808 GO:0051262 GO:0006357 GO:0001104 GO:0046983
GO:0016021 GO:0016592
Cluster-22178.37817 GO:0071203
Cluster-22178.37821 GO:0006144 GO:0003968 GO:0031379
Cluster-22178.37827 GO:0055085 GO:0006811 GO:0006554 GO:0006367
GO:0006479 GO:0034968 GO:0016817 GO:0008270 GO:0005515
GO:0005216 GO:0018024 GO:0019015 GO:0016020 GO:0005672
GO:0005634

Cluster-22178.37828 GO:0042254 GO:0006412 GO:0004527 GO:0005515
GO:0003735 GO:0003676 GO:0005840
Cluster-22178.37831 GO:0055114 GO:0015992 GO:0006744 GO:0006120
GO:0006814 GO:0046872 GO:0000166 GO:0008137
Cluster-22178.37833 GO:0005515 GO:0005634
Cluster-22178.37837 GO:0006468 GO:0004672 GO:0005524
Cluster-22178.37862 GO:0003676 GO:0005515 GO:0046872 GO:0016787
GO:0031966
Cluster-22178.37863 GO:0035556 GO:0009190 GO:0016849 GO:0016021
Cluster-22178.37866 GO:0008608 GO:0005096 GO:0005515 GO:0042729
GO:0005876
Cluster-22178.37867 GO:0005096 GO:0005515
Cluster-22178.37872 GO:0003950 GO:0005515
Cluster-22178.37874 GO:0033617 GO:0015631 GO:0005739 GO:0045298
Cluster-22178.37875 GO:0071816 GO:0016765 GO:0005515
Cluster-22178.37878 GO:0042381 GO:0003676 GO:0005515 GO:0005576
Cluster-22178.37888 GO:0009253 GO:0006807 GO:0009252 GO:0008745

Cluster-22178.37893 GO:0019836 GO:0006744 GO:0005515 GO:0005576
Cluster-22178.37894 GO:0005515 GO:0061630
Cluster-22178.37896 GO:0007275 GO:0016747 GO:0016021
Cluster-22178.37902 GO:0006955 GO:0007165 GO:0006355 GO:0003700
GO:0005179 GO:0005667 GO:0005576
Cluster-22178.37907 GO:0015876 GO:0008521 GO:0016021
Cluster-22178.37920 GO:0006566 GO:0006468 GO:0006563 GO:0006778
GO:0006783 GO:0042967 GO:0006544 GO:0003870 GO:0030170
GO:0005524 GO:0004672 GO:0016773 GO:0016020 GO:0005759
Cluster-22178.37927 GO:0016757
Cluster-22178.37928 GO:0016757
Cluster-22178.37935 GO:0004252 GO:0016021
Cluster-22178.37938 GO:0007017 GO:0045859 GO:0007018 GO:0006810
GO:0016887 GO:0003924 GO:0005524 GO:0004860 GO:0005215
GO:0005525 GO:0003777 GO:0030286 GO:0005874 GO:0005576

GO:0016020
Cluster-22178.37940 GO:0015979 GO:0005524 GO:0009522
Cluster-22178.37942 GO:0007275 GO:0005515 GO:0008440 GO:0042600
Cluster-22178.37950 GO:0008440
Cluster-22178.37957 GO:0007155 GO:0005515 GO:0016020 GO:0016021
Cluster-22178.37961 GO:0015979 GO:0006355 GO:0005515 GO:0046983
GO:0042651 GO:0009654 GO:0009523
Cluster-22178.37969 GO:0016301
Cluster-22178.37972 GO:0005515 GO:0046872 GO:0008270 GO:0003723

Cluster-22178.37979 GO:0007275 GO:0019685 GO:0030494 GO:0046983
GO:0016836 GO:0005578
Cluster-22178.37985 GO:0007165 GO:0040007 GO:0008283 GO:0009069
GO:0006468 GO:0016310 GO:0007178 GO:0008083 GO:0004675
GO:0005524 GO:0016020
Cluster-22178.37988 GO:0006811 GO:0055085 GO:0005216 GO:0016020
Cluster-22178.37989 GO:0005515
Cluster-22178.37990 GO:0005515
Cluster-22178.37992 GO:0004659 GO:0016021
Cluster-22178.37993 GO:0004659 GO:0016021
Cluster-22178.37997 GO:0006807 GO:0004252 GO:0016151 GO:0045182
GO:0005886
Cluster-22178.37998 GO:0006355 GO:0006144 GO:0006464 GO:0046034
GO:0004017 GO:0005515 GO:0003677 GO:0008270 GO:0046872
GO:0016151 GO:0019031
Cluster-22178.37999 GO:0046034 GO:0006144 GO:0006464 GO:0006355
GO:0016151 GO:0008270 GO:0046872 GO:0004017 GO:0003677
GO:0019031
Cluster-22178.3800 GO:0042981
Cluster-22178.38000 GO:0005576
Cluster-22178.38005 GO:0006032 GO:0003723 GO:0016887 GO:0005524
GO:0005515 GO:0003724 GO:0008061
Cluster-22178.38006 GO:0005515 GO:2001070

Cluster-22178.38009 GO:0051260 GO:0016020
Cluster-22178.38010 GO:0051260 GO:0016020
Cluster-22178.38011 GO:0016192 GO:0006886 GO:0006355 GO:0003676
GO:0003677 GO:0005198 GO:0005515 GO:0005622 GO:0030117
Cluster-22178.38014 GO:0006338 GO:0006281 GO:0006355 GO:0006308
GO:0000077 GO:0016818 GO:0008270 GO:0005524 GO:0003676
GO:0000014 GO:0005634
Cluster-22178.38019 GO:0006355 GO:0005622
Cluster-22178.38027 GO:0008219 GO:0009405 GO:0005515 GO:0005198
GO:0005509 GO:0019028 GO:0005576
Cluster-22178.38031 GO:0006915 GO:0006919 GO:0006974 GO:0001836
GO:0006412 GO:0008168
Cluster-22178.38032 GO:0006412 GO:0001836 GO:0006974 GO:0006919
GO:0006915 GO:0008168
Cluster-22178.38035 GO:0001836 GO:0006412 GO:0006915 GO:0006919
GO:0006974 GO:0008168
Cluster-22178.38037 GO:0055114 GO:0006563 GO:0006566 GO:0006544
GO:0006546 GO:0004375
Cluster-22178.38038 GO:0007264 GO:0007186 GO:0007165 GO:0003924
GO:0031683 GO:0019001 GO:0004871 GO:0005525
Cluster-22178.38046 GO:0006511 GO:0005201 GO:0005581 GO:0005578
Cluster-22178.38060 GO:0006825 GO:0016531 GO:0005507 GO:0005758
Cluster-22178.38067 GO:0006810 GO:0005515 GO:0016021
Cluster-22178.38083 GO:0042742 GO:0016301 GO:0005576
Cluster-22178.38098 GO:0007165 GO:0005515
Cluster-22178.38099 GO:0007165 GO:0006812 GO:0006835 GO:0006820
GO:0017153 GO:0005515 GO:0016020
Cluster-22178.38100 GO:0006812 GO:0007165 GO:0006820 GO:0006835
GO:0005515 GO:0017153 GO:0016020
Cluster-22178.38102 GO:0006144 GO:0007165 GO:0004114
Cluster-22178.38109 GO:0055114 GO:0016209 GO:0051920 GO:0016491
GO:0005198 GO:0019028
Cluster-22178.38122 GO:0006626 GO:0005741

Cluster-22178.38123 GO:0006814 GO:0006183 GO:0006813 GO:0006228
GO:0006165 GO:0006241 GO:0006206 GO:0006144 GO:0004550
GO:0005890
Cluster-22178.38124 GO:0006183 GO:0006814 GO:0006813 GO:0006228
GO:0006165 GO:0006241 GO:0006206 GO:0006144 GO:0004550
GO:0005890
Cluster-22178.38142 GO:0005515
Cluster-22178.38145 GO:0007049 GO:0005198 GO:0004045 GO:0005882
Cluster-22178.38150 GO:0005515 GO:0016755
Cluster-22178.38158 GO:0005198 GO:0005515 GO:0019028
Cluster-22178.38167 GO:0007186 GO:0019087 GO:0004930 GO:0016021
Cluster-22178.38169 GO:0030976 GO:0016757
Cluster-22178.38170 GO:0016757
Cluster-22178.38182 GO:0006810 GO:0006955 GO:0000902 GO:0006817
GO:0006508 GO:0030436 GO:0002143 GO:0007224 GO:0004190
GO:0005525 GO:0005315 GO:0005215 GO:0016020 GO:0016021
GO:0005737
Cluster-22178.38187 GO:0007165 GO:0016020
Cluster-22178.38207 GO:0007130 GO:0000795
Cluster-22178.38209 GO:0006355 GO:0003677 GO:0005515 GO:0004252
GO:0005886 GO:0005634
Cluster-22178.38211 GO:0015074
Cluster-22178.38247 GO:0030001 GO:0007155 GO:0055085 GO:0051015
GO:0046873 GO:0003682 GO:0016020 GO:0000785
Cluster-22178.38248 GO:0055085 GO:0007155 GO:0030001 GO:0008270
GO:0016853 GO:0051015 GO:0003682 GO:0046873 GO:0000785
GO:0016020
Cluster-22178.38249 GO:0006555 GO:0006556 GO:0004478
Cluster-22178.38256 GO:0007155 GO:0006810 GO:0005215 GO:0005737
GO:0016021
Cluster-22178.38257 GO:0006464 GO:0055114 GO:0008152 GO:0007165
GO:0006298 GO:0005524 GO:0016491 GO:0030983 GO:0019901
Cluster-22178.38260 GO:0006284 GO:0007165 GO:0004872 GO:0003905

GO:0003677 GO:0016021
Cluster-22178.38261 GO:0005515
Cluster-22178.38276 GO:0005515
Cluster-22178.38290 GO:0005515
Cluster-22178.38292 GO:0005515
Cluster-22178.38294 GO:0005515
Cluster-22178.38296 GO:0055114 GO:0015992 GO:0006744 GO:0006814
GO:0006120 GO:0008137
Cluster-22178.38298 GO:0000917 GO:0006355 GO:0007015 GO:0051258
GO:0006308 GO:0048870 GO:0006897 GO:0006810 GO:0007165
GO:0030168 GO:0006397 GO:0010508 GO:0043093 GO:0006914
GO:0003700 GO:0008092 GO:0030674 GO:0005102 GO:0008855
GO:0051082 GO:0043565 GO:0008017 GO:0009318 GO:0031514
GO:0019898 GO:0005886 GO:0000445 GO:0045298 GO:0005737
GO:0005783 GO:0005667 GO:0005577 GO:0009424
Cluster-22178.3830 GO:0090063 GO:0008643 GO:0000226 GO:0009401
GO:0008982 GO:0009357 GO:0016020 GO:0000922 GO:0005815
Cluster-22178.38302 GO:0005515
Cluster-22178.38303 GO:0006355 GO:0003700 GO:0000975 GO:0005667
GO:0005622
Cluster-22178.38311 GO:0032968 GO:0007217 GO:0006367 GO:0006351
GO:0006270 GO:0045892 GO:0006260 GO:0003887 GO:0003677
GO:0008270 GO:0003676 GO:0005634 GO:0042575
Cluster-22178.38317 GO:0055114 GO:0016817 GO:0033905 GO:0016972

Cluster-22178.38324 GO:0006308 GO:0009307 GO:0003677 GO:0009036
GO:0009359
Cluster-22178.38331 GO:0006886 GO:0046653 GO:0006355 GO:0006563
GO:0015991 GO:0006544 GO:0006528 GO:0016192 GO:0006566
GO:0016820 GO:0005515 GO:0008418 GO:0003677 GO:0008115
GO:0008270 GO:0030117 GO:0000221
Cluster-22178.38338 GO:0008270 GO:0005525 GO:0005515
Cluster-22178.38339 GO:0005887

Cluster-22178.38340 GO:0008270 GO:0003677
Cluster-22178.38346 GO:0020037 GO:0016021
Cluster-22178.38347 GO:0005515
Cluster-22178.38348 GO:0006351 GO:0006206 GO:0006144 GO:0003677
GO:0003899 GO:0008242 GO:0005730
Cluster-22178.38349 GO:0005856
Cluster-22178.38354 GO:0005975 GO:0006687 GO:0006665 GO:0006807
GO:0004348 GO:0016021
Cluster-22178.38356 GO:0006807 GO:0006665 GO:0006687 GO:0005975
GO:0004348 GO:0016021
Cluster-22178.38357 GO:0016021
Cluster-22178.38359 GO:0045892 GO:0048870 GO:0006935 GO:0000160
GO:0016310 GO:0007049 GO:0015991 GO:0010508 GO:0000917
GO:0006914 GO:0015992 GO:0043093 GO:0007165 GO:0003924
GO:0015078 GO:0005525 GO:0005515 GO:0004673 GO:0000155
GO:0003677 GO:0005737 GO:0009365 GO:0031514 GO:0033179
GO:0005815
Cluster-22178.38360 GO:0000160 GO:0016310 GO:0007049 GO:0045892
GO:0048870 GO:0006935 GO:0010508 GO:0000917 GO:0015991
GO:0006355 GO:0006914 GO:0015992 GO:0007165 GO:0043093
GO:0003700 GO:0015078 GO:0005525 GO:0003924 GO:0005515
GO:0003677 GO:0000155 GO:0004673 GO:0009365 GO:0005737
GO:0031514 GO:0033179 GO:0005815 GO:0005667
Cluster-22178.38366 GO:0008033 GO:0009451 GO:0009020 GO:0005515

Cluster-22178.38367 GO:0006118 GO:0020037 GO:0009055
Cluster-22178.38368 GO:0055114 GO:0016491 GO:0005515
Cluster-22178.38369 GO:0055114 GO:0016491 GO:0005515
Cluster-22178.38374 GO:0051301 GO:0007059 GO:0005515 GO:0008017
GO:0000444 GO:0045298
Cluster-22178.38379 GO:0005515
Cluster-22178.38380 GO:0006461 GO:0044780 GO:0005739
Cluster-22178.38383 GO:0006804 GO:0019079 GO:0006749 GO:0006979

GO:0055114 GO:0005515 GO:0003723 GO:0004602
Cluster-22178.38386 GO:0008053 GO:0009069 GO:0007165 GO:0016310
GO:0006468 GO:0005096 GO:0015631 GO:0004674 GO:0003924
GO:0045502 GO:0051082 GO:0042803 GO:0008134 GO:0030286
GO:0005667 GO:0005741 GO:0016021 GO:0045298
Cluster-22178.38392 GO:0055114 GO:0006364 GO:0005506 GO:0008168
GO:0005096 GO:0016705 GO:0020037
Cluster-22178.38395 GO:0007155 GO:0007165
Cluster-22178.38397 GO:0019087 GO:0007009 GO:0005515 GO:0016021
Cluster-22178.38401 GO:0006812 GO:0032801 GO:0006090 GO:0019076
GO:0071436 GO:0006814 GO:0006560 GO:0006525 GO:0004222
GO:0015081 GO:0008948 GO:0005261 GO:0033644 GO:0016020
Cluster-22178.38410 GO:0005198 GO:0008270 GO:0003779 GO:0005856
Cluster-22178.38416 GO:0006144 GO:0006206 GO:0006351 GO:0003899
GO:0005515 GO:0003677 GO:0016021 GO:0005730
Cluster-22178.38417 GO:0008270 GO:0003676
Cluster-22178.38418 GO:0003723 GO:0005515
Cluster-22178.38421 GO:0006355 GO:0003723 GO:0016887 GO:0005524
GO:0008134 GO:0003724 GO:0005667
Cluster-22178.38423 GO:0006570 GO:0000105 GO:0006470 GO:0006547
GO:0004725 GO:0003879 GO:0005737
Cluster-22178.38436 GO:0007178 GO:0016310 GO:0009069 GO:0006468
GO:0004672 GO:0005524 GO:0004675 GO:0016020
Cluster-22178.38437 GO:0005515 GO:0016020
Cluster-22178.38443 GO:0007264 GO:0005525
Cluster-22178.38447 GO:0009271 GO:0007264 GO:0006355 GO:0005525
GO:0003924
Cluster-22178.38450 GO:0071705
Cluster-22178.38452 GO:0005515
Cluster-22178.38457 GO:0006468 GO:0006270 GO:0005975 GO:0035556
GO:0004672 GO:0005524 GO:0016773 GO:0016020 GO:0032040
Cluster-22178.38461 GO:0042254 GO:0006412 GO:0003735 GO:0005840
GO:0005622

Cluster-22178.38463 GO:0006412 GO:0042254 GO:0003735 GO:0005622
GO:0005840
Cluster-22178.38468 GO:0007165 GO:0002532 GO:0006771 GO:0007411
GO:0019497 GO:0007166 GO:0005515 GO:0003993 GO:0004896
GO:0046872 GO:0016020 GO:0016021
Cluster-22178.38471 GO:0048013 GO:0019497 GO:0006771 GO:0005515
GO:0003993 GO:0046872
Cluster-22178.38474 GO:0019497 GO:0048013 GO:0006771 GO:0007186
GO:0046872 GO:0004930 GO:0003993 GO:0005515
Cluster-22178.38479 GO:0006508 GO:0006468 GO:0003676 GO:0008270
GO:0004672 GO:0005524 GO:0005515 GO:0005634
Cluster-22178.38480 GO:0006468 GO:0006508 GO:0003676 GO:0004672
GO:0008270 GO:0005524 GO:0005515 GO:0005634
Cluster-22178.38481 GO:0006508 GO:0006468 GO:0005515 GO:0003676
GO:0005524 GO:0008270 GO:0004672 GO:0005634
Cluster-22178.38490 GO:0016042 GO:0007586 GO:0006810 GO:0009405
GO:0007275 GO:0030178 GO:0008047 GO:0008200 GO:0005576
Cluster-22178.38493 GO:0016020
Cluster-22178.38495 GO:0006298 GO:0030983 GO:0005524
Cluster-22178.38511 GO:0015991 GO:0015992 GO:0015078 GO:0033177
Cluster-22178.38512 GO:0006771 GO:0019497 GO:0046872 GO:0005515
GO:0003993
Cluster-22178.38513 GO:0006090 GO:0071436 GO:0006814 GO:0006560
GO:0006525 GO:0015081 GO:0008948 GO:0016020
Cluster-22178.38514 GO:0007275 GO:0035091
Cluster-22178.38515 GO:0005975 GO:0046008 GO:0007165 GO:0005179
GO:0004553 GO:0005576
Cluster-22178.38516 GO:0005515
Cluster-22178.38520 GO:0005975 GO:0006308 GO:0031047 GO:0016888
GO:0004560
Cluster-22178.38521 GO:0006308 GO:0031047 GO:0005975 GO:0004560
GO:0016888
Cluster-22178.38522 GO:0042981

Cluster-22178.38525 GO:0006355 GO:0005622
Cluster-22178.38529 GO:0006313 GO:0019901
Cluster-22178.38535 GO:0006468 GO:0006355 GO:0003677 GO:0004672
GO:0005524 GO:0016773 GO:0005200 GO:0005634 GO:0005882
GO:0016020 GO:0005856
Cluster-22178.38538 GO:0007156 GO:0005509 GO:0005515 GO:0016020
Cluster-22178.38539 GO:0006811 GO:0055085 GO:0005515 GO:0005216
GO:0016020 GO:0005634
Cluster-22178.38545 GO:0016567 GO:0046872 GO:0008270 GO:0004842
GO:0005515 GO:0005680 GO:0042470
Cluster-22178.3855 GO:0019076 GO:0006812 GO:0032801 GO:0005261
GO:0046935 GO:0005944 GO:0033644
Cluster-22178.38552 GO:0003677 GO:0003676 GO:0008270 GO:0016021
Cluster-22178.38553 GO:0006355 GO:0007049 GO:0006914 GO:0003677
GO:0003700 GO:0043565 GO:0005667 GO:0005634
Cluster-22178.38557 GO:0009405 GO:0005615
Cluster-22178.38561 GO:0006355 GO:0003677
Cluster-22178.38565 GO:0042981 GO:0006914 GO:0006355 GO:0003700
GO:0043565 GO:0042803 GO:0008134 GO:0005515 GO:0045502
GO:0030286 GO:0005667
Cluster-22178.38568 GO:0006366 GO:0046872 GO:0008270 GO:0016787
GO:0003677 GO:0005665 GO:0005634
Cluster-22178.38572 GO:0006810 GO:0007165 GO:0003682 GO:0005215
GO:0008158 GO:0046935 GO:0005944 GO:0016020 GO:0000785
GO:0016021
Cluster-22178.38573 GO:0055085 GO:0006810 GO:0006820 GO:0008509
GO:0005215 GO:0016021 GO:0016020
Cluster-22178.38574 GO:0006090 GO:0006811 GO:0071436 GO:0006814
GO:0006560 GO:0006525 GO:0015081 GO:0008948 GO:0005515
GO:0005198 GO:0016020 GO:0019028
Cluster-22178.38582 GO:0046500 GO:0006464 GO:0006479 GO:0008168
GO:0004719
Cluster-22178.38584 GO:0006468 GO:0009069 GO:0016310 GO:0004672

GO:0005524 GO:0004674
Cluster-22178.38597 GO:0006807 GO:0016811
Cluster-22178.38608 GO:0001503 GO:0005515 GO:0005509
Cluster-22178.38610 GO:0007186 GO:0006355 GO:0004930 GO:0016021
GO:0005622
Cluster-22178.38619 GO:0033014 GO:0055114 GO:0015994 GO:0004418
GO:0016491
Cluster-22178.38622 GO:0006914 GO:0008270 GO:0046872 GO:0003676

Cluster-22178.38624 GO:0055114 GO:0007264 GO:0008272 GO:0016491
GO:0003924 GO:0005525 GO:0015116 GO:0016021
Cluster-22178.38626 GO:0005515
Cluster-22178.38628 GO:0043401 GO:0006355 GO:0003700 GO:0008270
GO:0043565 GO:0005667 GO:0005634
Cluster-22178.38629 GO:0003677
Cluster-22178.38630 GO:0046677
Cluster-22178.38633 GO:0007156 GO:0005509 GO:0016020
Cluster-22178.38640 GO:0009405 GO:0008219 GO:0005515 GO:0005198
GO:0005509 GO:0005576 GO:0019028
Cluster-22178.38641 GO:0055114 GO:0016032 GO:0016491 GO:0019012
Cluster-22178.38650 GO:0016020
Cluster-22178.38651 GO:0007018 GO:0005515 GO:0005868
Cluster-22178.38653 GO:0019031
Cluster-22178.38655 GO:0016055 GO:0008013 GO:0016342
Cluster-22178.38656 GO:0006084 GO:0006508 GO:0004222 GO:0003824
GO:0031012
Cluster-22178.38658 GO:0042128 GO:0008270 GO:0005515 GO:0003677
GO:0005634
Cluster-22178.38660 GO:0016820 GO:0000166 GO:0046872
Cluster-22178.38675 GO:0055114 GO:0016491 GO:0034464 GO:0016021
Cluster-22178.38677 GO:0006355 GO:0034477 GO:0004518
Cluster-22178.38681 GO:0006090 GO:0006560 GO:0009405 GO:0006814
GO:0071436 GO:0006525 GO:0015081 GO:0008948 GO:0016020

Cluster-22178.38682 GO:0006355 GO:0003700 GO:0003682 GO:0043565
GO:0008270 GO:0000785 GO:0005667
Cluster-22178.38689 GO:0006807 GO:0007411 GO:0016055 GO:0003824
GO:0046914 GO:0008013 GO:0005515 GO:0016342
Cluster-22178.38691 GO:0005515
Cluster-22178.38692 GO:0006814 GO:0006813 GO:0005890
Cluster-22178.38704 GO:0016567 GO:0005515 GO:0004842 GO:0046872
GO:0008270 GO:0005680
Cluster-22178.38709 GO:0005515
Cluster-22178.38713 GO:0005515
Cluster-22178.38723 GO:0016032 GO:0055114 GO:0016491 GO:0019012
Cluster-22178.38727 GO:0043401 GO:0006355 GO:0003700 GO:0043565
GO:0008270 GO:0005634 GO:0005667
Cluster-22178.38734 GO:0006886 GO:0055114 GO:0006888 GO:0006801
GO:0008270 GO:0046872 GO:0004784 GO:0030127
Cluster-22178.38735 GO:0040007 GO:0008283 GO:0007165 GO:0046983
GO:0008083
Cluster-22178.38742 GO:0006468 GO:0016773 GO:0004672 GO:0005524
GO:0016020
Cluster-22178.38744 GO:0006771 GO:0019497 GO:0007154 GO:0016820
GO:0005515 GO:0051539 GO:0003993 GO:0046872 GO:0016021
Cluster-22178.38745 GO:0006323 GO:0006810 GO:0006914 GO:0070206
GO:0010506 GO:0006606 GO:0006355 GO:0010508 GO:0048870
GO:0006308 GO:0007049 GO:0003677 GO:0008855 GO:0045502
GO:0051082 GO:0008134 GO:0042803 GO:0005198 GO:0003700
GO:0005667 GO:0005882 GO:0030286 GO:0005886 GO:0005634
GO:0031514 GO:0009318 GO:0016021 GO:0005615 GO:0019867
Cluster-22178.38751 GO:0016567 GO:0004842 GO:0005515 GO:0008270
GO:0046872 GO:0005680
Cluster-22178.38764 GO:0016042 GO:0009395 GO:0046339 GO:0009405
GO:0030001 GO:0004435 GO:0046872 GO:0005509
Cluster-22178.38769 GO:0009451 GO:0008033 GO:0006879 GO:0008199
GO:0009020

Cluster-22178.38773 GO:0008608 GO:0006118 GO:0008440 GO:0008270
GO:0003954 GO:0072686 GO:0005739 GO:0042729
Cluster-22178.38774 GO:0000077 GO:0030420 GO:0030896
Cluster-22178.38779 GO:0005515
Cluster-22178.38780 GO:0005515 GO:0016020
Cluster-22178.38781 GO:0030435 GO:0005515 GO:0016020
Cluster-22178.38787 GO:0016310 GO:0015074 GO:0003677 GO:0008907
GO:0004673 GO:0009365 GO:0005576
Cluster-22178.38791 GO:0004190 GO:0005515 GO:0016021
Cluster-22178.38792 GO:0003676
Cluster-22178.38801 GO:0005515
Cluster-22178.38802 GO:0007275 GO:0005201 GO:0005581 GO:0005578
Cluster-22178.38804 GO:0042254 GO:0006412 GO:0003735 GO:0005840
Cluster-22178.38811 GO:0005515
Cluster-22178.38813 GO:0019064 GO:0007165 GO:0005515 GO:0046789
GO:0016788 GO:0009986 GO:0019031
Cluster-22178.38814 GO:0008152 GO:0046872 GO:0008484 GO:0003824

Cluster-22178.38820 GO:0051297 GO:0009405 GO:0051056 GO:0005096
GO:0005813
Cluster-22178.38822 GO:0006629 GO:0003677 GO:0005634
Cluster-22178.38827 GO:0046782 GO:0006486 GO:0008373 GO:0005198
GO:0046983 GO:0019012
Cluster-22178.38828 GO:0051782 GO:0009432 GO:0006270 GO:0005515
GO:0019028 GO:0009276
Cluster-22178.38829 GO:0009372
Cluster-22178.38845 GO:0006066 GO:0042967 GO:0005525 GO:0004026

Cluster-22178.38846 GO:0005634
Cluster-22178.38847 GO:0005515
Cluster-22178.38848 GO:0015031 GO:0015992 GO:0015991 GO:0006355
GO:0003676 GO:0015078 GO:0008565 GO:0008134 GO:0042803
GO:0045502 GO:0030286 GO:0033179 GO:0005667

Cluster-22178.38853 GO:0005975 GO:0004553
Cluster-22178.38860 GO:0006865 GO:0003333 GO:0015171 GO:0016020
GO:0005634
Cluster-22178.38862 GO:0016042 GO:0008203 GO:0016298
Cluster-22178.38872 GO:0006508 GO:0016032 GO:0006506 GO:0016758
GO:0008233 GO:0016021
Cluster-22178.38875 GO:0007155 GO:0005515 GO:0005509 GO:0005576
Cluster-22178.3888 GO:0035556
Cluster-22178.38881 GO:0007165 GO:0005515 GO:0000772 GO:0016020
Cluster-22178.38884 GO:0007166 GO:0005515 GO:0016021 GO:0016020
Cluster-22178.38886 GO:0008360 GO:0051082 GO:0016020
Cluster-22178.38887 GO:0016567 GO:0004842 GO:0005680
Cluster-22178.38889 GO:0046872 GO:0005515
Cluster-22178.38891 GO:0046872 GO:0003677
Cluster-22178.38894 GO:0015886 GO:0015232
Cluster-22178.38899 GO:0022900 GO:0008270 GO:0005739 GO:0005747
Cluster-22178.38900 GO:0006744 GO:0006812 GO:0006120 GO:0015992
GO:0042773 GO:0006351 GO:0006814 GO:0008324 GO:0003677
GO:0008137 GO:0005743 GO:0016021 GO:0005634
Cluster-22178.38906 GO:0016032 GO:0005515 GO:0042025
Cluster-22178.38907 GO:0055114 GO:0016671 GO:0003756 GO:0005783
Cluster-22178.38912 GO:0005576
Cluster-22178.38913 GO:0006468 GO:0005524 GO:0004672
Cluster-22178.38919 GO:0006260 GO:0042742 GO:0006276 GO:0005576
Cluster-22178.38925 GO:0019646 GO:0016567 GO:0006118 GO:0009055
GO:0004842 GO:0008270 GO:0046872 GO:0005515 GO:0005680
Cluster-22178.38930 GO:0018149 GO:0003810
Cluster-22178.38933 GO:0016020
Cluster-22178.38934 GO:0016020
Cluster-22178.38942 GO:0006508 GO:0008270 GO:0004222
Cluster-22178.38950 GO:0035091
Cluster-22178.38964 GO:0008376 GO:0016021 GO:0032580
Cluster-22178.38968 GO:0006508 GO:0008270 GO:0008233 GO:0004222

GO:0031012
Cluster-22178.38972 GO:0008152 GO:0055114 GO:0003824 GO:0016836
GO:0016491
Cluster-22178.38974 GO:0016055 GO:0005515 GO:0008013 GO:0016342
Cluster-22178.38975 GO:0016055 GO:0006355 GO:0005515 GO:0043565
GO:0008013 GO:0003700 GO:0005667 GO:0016342
Cluster-22178.38981 GO:0006811 GO:0055085 GO:0015992 GO:0051260
GO:0015986 GO:0015078 GO:0005216 GO:0016020
Cluster-22178.38990 GO:0007165 GO:0005057
Cluster-22178.38993 GO:0006260
Cluster-22178.38994 GO:0016020
Cluster-22178.38995 GO:0007165 GO:0015858 GO:0006810 GO:0006955
GO:0005337 GO:0005125 GO:0005576 GO:0016021
Cluster-22178.38997 GO:0007165 GO:0006955 GO:0005164 GO:0016020
Cluster-22178.39000 GO:0008033 GO:0055114 GO:0016117 GO:0006779
GO:0019354 GO:0006397 GO:0016491 GO:0050660 GO:0071949
GO:0003723 GO:0016705 GO:0043115 GO:0005515 GO:0005737
GO:0005634
Cluster-22178.39004 GO:0003333 GO:0006865 GO:0015171 GO:0016020
Cluster-22178.39005 GO:0030154 GO:0007165 GO:0005515 GO:0005509
GO:0016021 GO:0005578
Cluster-22178.39007 GO:0042742 GO:0005515
Cluster-22178.39008 GO:0046983
Cluster-22178.39009 GO:0006813 GO:0015269 GO:0046983 GO:0016020
Cluster-22178.39010 GO:0006144 GO:0035556 GO:0006182 GO:0046039
GO:0009190 GO:0008270 GO:0016849 GO:0004383 GO:0020037
GO:0016021 GO:0005634
Cluster-22178.39016 GO:0019310 GO:0055114 GO:0050113 GO:0005506
GO:0005737
Cluster-22178.39017 GO:0007165 GO:0005515
Cluster-22178.39023 GO:0007154 GO:0016021
Cluster-22178.39025 GO:0006412 GO:0042254 GO:0006810 GO:0003735
GO:0016021 GO:0005840

Cluster-22178.39027 GO:0016020
Cluster-22178.39029 GO:0016020
Cluster-22178.39036 GO:0002949 GO:0015948 GO:0015684 GO:0006810
GO:0007264 GO:0055114 GO:0006576 GO:0006355 GO:0003724
GO:0016887 GO:0015093 GO:0005524 GO:0003677 GO:0005525
GO:0003924 GO:0008134 GO:0000166 GO:0003723 GO:0005667
GO:0016021
Cluster-22178.39037 GO:0015948 GO:0015684 GO:0006810 GO:0006614
GO:0007264 GO:0002949 GO:0007018 GO:0006355 GO:0007017
GO:0006576 GO:0055114 GO:0003677 GO:0008017 GO:0003724
GO:0015093 GO:0005524 GO:0016887 GO:0000166 GO:0008134
GO:0003723 GO:0003777 GO:0005525 GO:0003924 GO:0016020
GO:0005667 GO:0005874 GO:0016021 GO:0045298
Cluster-22178.39042 GO:0055085 GO:0019083 GO:0006298 GO:0006810
GO:0042626 GO:0016887 GO:0005524 GO:0030983 GO:0016021
Cluster-22178.39044 GO:0006468 GO:0016773 GO:0004672 GO:0005524
GO:0016020
Cluster-22178.39056 GO:0055114 GO:0016706 GO:0008168
Cluster-22178.3906 GO:0006568 GO:0006631 GO:0006552 GO:0006550
GO:0018874 GO:0055114 GO:0006574 GO:0006554 GO:0008152
GO:0006633 GO:0003857 GO:0050662 GO:0016491 GO:0003824
Cluster-22178.39064 GO:0071973 GO:0007165 GO:0030168 GO:0048870
GO:0051258 GO:0030674 GO:0008270 GO:0015631 GO:0005102
GO:0005198 GO:0004871 GO:0009428 GO:0016020 GO:0005882
GO:0005577 GO:0031514 GO:0045298 GO:0016021 GO:0030288
Cluster-22178.39066 GO:0006468 GO:0005524 GO:0004672
Cluster-22178.39070 GO:0003723 GO:0019013
Cluster-22178.39077 GO:0007165 GO:0009446 GO:0040007 GO:0006508
GO:0008283 GO:0016579 GO:0006807 GO:0005515 GO:0008083
GO:0004843 GO:0004668 GO:0036459 GO:0005125 GO:0016021
Cluster-22178.39083 GO:0006629 GO:0006605 GO:0006886 GO:0005742
Cluster-22178.39084 GO:0006865 GO:0003333 GO:0015171 GO:0019867
GO:0016020

Cluster-22178.39089 GO:0055114 GO:0016641 GO:0005507
Cluster-22178.39098 GO:0006355 GO:0055114 GO:0015940 GO:0042262 GO:0015986 GO:0007165 GO:0006464 GO:0006260 GO:0005515 GO:0001664 GO:0008677 GO:0003677 GO:0046983 GO:0008270 GO:0046933 GO:0045259
Cluster-22178.39100 GO:0003779
Cluster-22178.39113 GO:0051321 GO:0005515
Cluster-22178.39117 GO:0006560 GO:0006412 GO:0006814 GO:0006090 GO:0019076 GO:0042254 GO:0071436 GO:0006525 GO:0032801 GO:0006812 GO:0008948 GO:0005261 GO:0003735 GO:0015081 GO:0005840 GO:0016020 GO:0033644 GO:0005622
Cluster-22178.39121 GO:0006468 GO:0004672 GO:0005524
Cluster-22178.39127 GO:0006355 GO:0003700 GO:0043565 GO:0005634 GO:0005667
Cluster-22178.39136 GO:0005861
Cluster-22178.39142 GO:0016055 GO:0007165 GO:0007275 GO:0005102 GO:0005576
Cluster-22178.39143 GO:0006355 GO:0007165 GO:0042974 GO:0005667
Cluster-22178.39145 GO:0007165 GO:0006355 GO:0042974 GO:0005667
Cluster-22178.39146 GO:0007165 GO:0006355 GO:0042974 GO:0005667
Cluster-22178.39148 GO:0009878 GO:0046872
Cluster-22178.39149 GO:0006468 GO:0006355 GO:0003677 GO:0004672 GO:0005524 GO:0016773 GO:0016020
Cluster-22178.39155 GO:0005515
Cluster-22178.39157 GO:0019722 GO:0003676
Cluster-22178.39161 GO:0016055 GO:0008013 GO:0005515 GO:0016342
Cluster-22178.39164 GO:0000724 GO:0003676
Cluster-22178.39165 GO:0000724 GO:0003676
Cluster-22178.39166 GO:0008283 GO:0040007 GO:0007165 GO:0006810 GO:0005215 GO:0008083 GO:0005737 GO:0016021 GO:0016020
Cluster-22178.39168 GO:0007605 GO:0005515
Cluster-22178.39170 GO:0051262 GO:0006811 GO:0055085 GO:0005216 GO:0016020

Cluster-22178.39178 GO:0051260
Cluster-22178.39183 GO:0006355 GO:0007049 GO:0043565 GO:0003700
GO:0003677 GO:0005634 GO:0005667
Cluster-22178.39184 GO:0005515
Cluster-22178.39187 GO:0055114 GO:0016705 GO:0020037 GO:0005506

Cluster-22178.39188 GO:0006112 GO:0005515
Cluster-22178.39189 GO:0006112 GO:0005515
Cluster-22178.39191 GO:0006355 GO:0005975 GO:0003677 GO:0005524
GO:0016773
Cluster-22178.39195 GO:0006298 GO:0009405 GO:0005524 GO:0030983
GO:0033644
Cluster-22178.39205 GO:0007165 GO:0005509 GO:0005578
Cluster-22178.39208 GO:0006402 GO:0051252 GO:0046872 GO:0004535
GO:0003676 GO:0003723 GO:0005198 GO:0005737 GO:0005634
Cluster-22178.39211 GO:0007586 GO:0016042 GO:0006952 GO:0006486
GO:0008047 GO:0008373 GO:0005576
Cluster-22178.39212 GO:0007049
Cluster-22178.39228 GO:0006810 GO:0005215 GO:0016020
Cluster-22178.39232 GO:0016021
Cluster-22178.39236 GO:0006542 GO:0006807 GO:0009252 GO:0004356

Cluster-22178.39246 GO:0006520 GO:0055114 GO:0006779 GO:0044564
GO:0008152 GO:0019354 GO:0016491 GO:0051287 GO:0043115
Cluster-22178.39247 GO:0006355 GO:0016055 GO:0003677 GO:0005198
GO:0008017 GO:0008242 GO:0019028 GO:0019031 GO:0045298
Cluster-22178.39248 GO:0006355 GO:0016055 GO:0003677 GO:0005198
GO:0008017 GO:0008242 GO:0019028 GO:0019031 GO:0045298
Cluster-22178.39249 GO:0006355 GO:0016055 GO:0003677 GO:0005198
GO:0008017 GO:0008242 GO:0019028 GO:0019031 GO:0045298
Cluster-22178.39254 GO:0007165 GO:0008283 GO:0008406 GO:0040007
GO:0005515 GO:0008083
Cluster-22178.39260 GO:0006355 GO:0030234 GO:0004222 GO:0003677

GO:0005634 GO:0016020
Cluster-22178.3927 GO:0006869 GO:0009405 GO:0042157 GO:0008289
GO:0016020 GO:0005576
Cluster-22178.39272 GO:0019752 GO:0016831 GO:0030170
Cluster-22178.39276 GO:0007010 GO:0003779
Cluster-22178.39279 GO:0015946 GO:0055114 GO:0004022
Cluster-22178.39287 GO:0042330
Cluster-22178.39290 GO:0006357 GO:0001104 GO:0016592
Cluster-22178.39291 GO:0015031 GO:0046856 GO:0034597 GO:0016021
Cluster-22178.39293 GO:0007276 GO:0043093 GO:0000917 GO:0007165
GO:0006310 GO:0051082 GO:0005179 GO:0003677 GO:0005515
GO:0005737 GO:0005576 GO:0005634
Cluster-22178.39307 GO:0016567 GO:0004842 GO:0019031
Cluster-22178.39308 GO:0006269 GO:0019354 GO:0016117 GO:0006260
GO:0006779 GO:0006351 GO:0008033 GO:0055114 GO:0071949
GO:0016491 GO:0008270 GO:0050660 GO:0016705 GO:0043115
GO:0005515 GO:0003896 GO:0005730 GO:0005657
Cluster-22178.39313 GO:0005515 GO:0004252 GO:0005509 GO:0005886
Cluster-22178.39324 GO:0005515
Cluster-22178.39326 GO:0005515
Cluster-22178.39333 GO:0055114 GO:0030151 GO:0016491
Cluster-22178.39334 GO:0048870 GO:0031514
Cluster-22178.39336 GO:0006412 GO:0042254 GO:0007339 GO:0003735
GO:0005515 GO:0005576 GO:0005840
Cluster-22178.39337 GO:0009117 GO:0008270 GO:0000287 GO:0000166
GO:0008253 GO:0005737
Cluster-22178.39364 GO:0003677 GO:0005515
Cluster-22178.39365 GO:0006935 GO:0007165 GO:0004888 GO:0016020
Cluster-22178.39373 GO:0006810 GO:0055085 GO:0005215 GO:0016020
Cluster-22178.39380 GO:0007165 GO:0019064 GO:0016788 GO:0005515
GO:0046789 GO:0019031 GO:0009986
Cluster-22178.39381 GO:0007165 GO:0006810 GO:0055085 GO:0006744
GO:0005179 GO:0008813 GO:0003676 GO:0005576 GO:0016020

GO:0005737 GO:0016021
Cluster-22178.39385 GO:0000266 GO:0008270 GO:0005739
Cluster-22178.3939 GO:0046872
Cluster-22178.39392 GO:0007186 GO:0004930 GO:0016021
Cluster-22178.39393 GO:0016021 GO:0005886
Cluster-22178.39397 GO:0032780 GO:0042030 GO:0005739
Cluster-22178.39404 GO:0007339 GO:0005515 GO:0005576
Cluster-22178.39408 GO:0006914 GO:0009405
Cluster-22178.39410 GO:0006629 GO:0006505 GO:0006886 GO:0016787
GO:0016788
Cluster-22178.39411 GO:0005887
Cluster-22178.39413 GO:0055114 GO:0007283 GO:0051287 GO:0003677
GO:0000786 GO:0005634
Cluster-22178.39419 GO:0016567 GO:0006355 GO:0004842 GO:0003700
GO:0046872 GO:0008270 GO:0005515 GO:0005622 GO:0005667
Cluster-22178.39422 GO:0006355 GO:0003677 GO:0016787
Cluster-22178.39424 GO:0006355 GO:0006396 GO:0042176 GO:0003723
GO:0030234 GO:0043565 GO:0003700 GO:0005667 GO:0000502
Cluster-22178.39425 GO:0042176 GO:0006396 GO:0006355 GO:0043565
GO:0003700 GO:0016787 GO:0003723 GO:0030234 GO:0005667
GO:0000502
Cluster-22178.39427 GO:0008270 GO:0005634
Cluster-22178.39428 GO:0034599 GO:0005783 GO:0005789
Cluster-22178.39435 GO:0005739
Cluster-22178.39436 GO:0007186 GO:0007165 GO:0007264 GO:0003677
GO:0000166 GO:0004871 GO:0005525 GO:0003924 GO:0019001
GO:0031683 GO:0005524
Cluster-22178.3944 GO:0006810 GO:0006355 GO:0005215 GO:0003700
GO:0043565 GO:0005198 GO:0016020 GO:0005667
Cluster-22178.39440 GO:0006935 GO:0007165 GO:0004888 GO:0016020
Cluster-22178.39443 GO:0008033 GO:0006771 GO:0019497 GO:0046872
GO:0005515 GO:0003993 GO:0016021
Cluster-22178.39446 GO:0006281 GO:0009058 GO:0006310 GO:0016742

Cluster-22178.39447 GO:0006310 GO:0009058 GO:0006281 GO:0016742

Cluster-22178.39457 GO:0006120 GO:0055114
Cluster-22178.39461 GO:0003774 GO:0016459
Cluster-22178.39463 GO:0016579 GO:0006508 GO:0045893 GO:1902208
GO:0003677 GO:0004843 GO:0036459
Cluster-22178.39466 GO:0005515 GO:0016021
Cluster-22178.39468 GO:0042254 GO:0006412 GO:0003735 GO:0005840
Cluster-22178.39473 GO:0007010 GO:0003779
Cluster-22178.39476 GO:0007165 GO:0006355 GO:0003677 GO:0005515
GO:0005179 GO:0005576
Cluster-22178.39480 GO:0006508 GO:0015996 GO:0008152 GO:0015994
GO:0016787 GO:0008236 GO:0047746 GO:0005576
Cluster-22178.39484 GO:0008152 GO:0016042 GO:0007586 GO:0008047
GO:0016746 GO:0005509 GO:0008236 GO:0005515 GO:0005576
Cluster-22178.39492 GO:0006355 GO:0043565 GO:0003700 GO:0005667
Cluster-22178.39493 GO:0055114 GO:0006694 GO:0008210 GO:0008209
GO:0008207 GO:0003824 GO:0050662 GO:0003854 GO:0016616
Cluster-22178.39494 GO:0008209 GO:0008207 GO:0008210 GO:0006694
GO:0055114 GO:0016616 GO:0050662 GO:0003854 GO:0003824
Cluster-22178.39499 GO:0005515
Cluster-22178.39504 GO:0009103 GO:0008918 GO:0005198 GO:0005882
Cluster-22178.39505 GO:0033905 GO:0005198 GO:0005882
Cluster-22178.39508 GO:0009069 GO:0006355 GO:0016310 GO:0070009
GO:0003700 GO:0008239 GO:0004674 GO:0005667 GO:0005634
Cluster-22178.39512 GO:0006118 GO:0017004 GO:0045158 GO:0003950
GO:0009512
Cluster-22178.39513 GO:0006118 GO:0017004 GO:0045158 GO:0003950
GO:0009512
Cluster-22178.39514 GO:0006118 GO:0017004 GO:0003950 GO:0045158
GO:0009512
Cluster-22178.39515 GO:0003950

Cluster-22178.39522 GO:0007186 GO:0004930 GO:0016021
Cluster-22178.39526 GO:0003779
Cluster-22178.39529 GO:0006270 GO:0006351 GO:0006260 GO:0003677
GO:0003887 GO:0008270 GO:0005634 GO:0042575
Cluster-22178.39533 GO:0006260 GO:0007165 GO:0006869 GO:0009847
GO:0042157 GO:0007067 GO:0009069 GO:0016310 GO:0007049
GO:0006355 GO:0006468 GO:0004674 GO:0005096 GO:0004871
GO:0003700 GO:0008289 GO:0043565 GO:0005815 GO:0016020
GO:0005667 GO:0042729 GO:0072686 GO:0005576
Cluster-22178.39534 GO:0006260 GO:0016310 GO:0007049 GO:0009847
GO:0007165 GO:0009069 GO:0006468 GO:0006355 GO:0004674
GO:0003700 GO:0043565 GO:0005096 GO:0005815 GO:0016020
GO:0005667
Cluster-22178.39542 GO:0006812 GO:0008270 GO:0016887 GO:0045735
GO:0003676 GO:0016021
Cluster-22178.39544 GO:0007059 GO:0051301 GO:0031175 GO:0031110
GO:0006810 GO:0000090 GO:0007067 GO:0005515 GO:0008017
GO:0030507 GO:0005516 GO:0016021 GO:0045298 GO:0005876
GO:0008091 GO:0000940
Cluster-22178.39546 GO:0016125 GO:0006355 GO:0006351 GO:0007623
GO:0006694 GO:0043565 GO:0047750 GO:0003700 GO:0005667
GO:0005634 GO:0016021
Cluster-22178.39548 GO:0006355 GO:0006352 GO:0006606 GO:0019904
GO:0003677 GO:0003700 GO:0016987 GO:0005667
Cluster-22178.39555 GO:0016020
Cluster-22178.39557 GO:0006810 GO:0006811 GO:0005216 GO:0016021
Cluster-22178.39558 GO:0021549 GO:0030182
Cluster-22178.39559 GO:0006468 GO:0042619 GO:0016773 GO:0005524
GO:0004672 GO:0004222 GO:0005515 GO:0016020
Cluster-22178.39562 GO:0008270
Cluster-22178.39563 GO:0005515 GO:0016020
Cluster-22178.39568 GO:0055085 GO:0035556 GO:0016021 GO:0019028
Cluster-22178.39569 GO:0055085 GO:0035556 GO:0016021 GO:0019028

Cluster-22178.39570 GO:0016627
Cluster-22178.39571 GO:0007165 GO:0005096 GO:0003729
Cluster-22178.39575 GO:0005515
Cluster-22178.39578 GO:0015986 GO:0015992 GO:0015078 GO:0000276
Cluster-22178.39583 GO:0006486 GO:0016757 GO:0008378 GO:0016020
Cluster-22178.39585 GO:0006811 GO:0055085 GO:0005216 GO:0005515
GO:0016020
Cluster-22178.39593 GO:0055114 GO:0042981 GO:0016714
Cluster-22178.39594 GO:0010884 GO:0008284 GO:0001819
Cluster-22178.39611 GO:0005515
Cluster-22178.39612 GO:0009069 GO:0006468 GO:0006355 GO:0015986
GO:0016310 GO:0007178 GO:0015992 GO:0003677 GO:0004675
GO:0015078 GO:0005524 GO:0004672 GO:0016020 GO:0005634
GO:0000276
Cluster-22178.39615 GO:0019064 GO:0019031
Cluster-22178.39618 GO:0008033 GO:0006355 GO:0003700 GO:0008270
GO:0005524 GO:0000166 GO:0003697 GO:0016879 GO:0016021
GO:0005737 GO:0042025 GO:0005667
Cluster-22178.39619 GO:0006351 GO:0006269 GO:0006260 GO:0003677
GO:0003896 GO:0008270 GO:0005730 GO:0005657
Cluster-22178.39620 GO:0055085 GO:0006811 GO:0008440 GO:0005216
GO:0016020
Cluster-22178.39622 GO:0055114 GO:0016491 GO:0051287
Cluster-22178.39623 GO:0015992 GO:0007094 GO:0009405 GO:0010508
GO:0006376 GO:0006508 GO:0015991 GO:0055114 GO:0006308
GO:0016787 GO:0003729 GO:0008233 GO:0005524 GO:0008855
GO:0005515 GO:0015078 GO:0046872 GO:0016714 GO:0008092
GO:0016020 GO:0019898 GO:0005685 GO:0009318 GO:0033179
GO:0005634 GO:0005737
Cluster-22178.39626 GO:0005515 GO:0003682 GO:0000785
Cluster-22178.39632 GO:0035556
Cluster-22178.39637 GO:0009236 GO:0006891 GO:0048472 GO:0030173
GO:0016021

Cluster-22178.39643 GO:0006865 GO:0003333 GO:0016747 GO:0015171 GO:0005634 GO:0016020

Cluster-22178.39645 GO:0005515

Cluster-22178.39653 GO:0005515

Cluster-22178.39656 GO:0003677

Cluster-22178.39658 GO:0006486 GO:0009405 GO:0008376 GO:0005198 GO:0008378 GO:0016757 GO:0032580 GO:0016020

Cluster-22178.39666 GO:0032065 GO:0006355 GO:0003700 GO:0005543 GO:0043565 GO:0003677 GO:0005515 GO:0005938 GO:0005667 GO:0005634

Cluster-22178.39667 GO:0032065 GO:0005515 GO:0005543 GO:0005938

Cluster-22178.39668 GO:0006468 GO:0051252 GO:0004672 GO:0005524 GO:0016891

Cluster-22178.39674 GO:0006355 GO:0003677

Cluster-22178.39676 GO:0006468 GO:0005524 GO:0004672

Cluster-22178.39678 GO:0008615 GO:0055114 GO:0010181 GO:0004733

Cluster-22178.39679 GO:0007165 GO:0005057

Cluster-22178.39689 GO:0016567 GO:0042167 GO:0055114 GO:0072519 GO:0015994 GO:0003677 GO:0004074 GO:0008270 GO:0004842 GO:0005634

Cluster-22178.3969 GO:0016567 GO:0004842

Cluster-22178.39690 GO:0055114 GO:0006694 GO:0015936 GO:0050662 GO:0004420

Cluster-22178.39695 GO:0007186 GO:0004930 GO:0016021

Cluster-22178.39696 GO:0006810 GO:0007186 GO:0004930 GO:0005215 GO:0016020 GO:0016021

Cluster-22178.39697 GO:0007186 GO:0004930 GO:0016021

Cluster-22178.39699 GO:0043087 GO:0035023 GO:0005089 GO:0005634

Cluster-22178.39711 GO:0030154 GO:0016021

Cluster-22178.39714 GO:0009405 GO:0042998 GO:0071253 GO:0005802 GO:0005576

Cluster-22178.39718 GO:0005515 GO:0046872 GO:0008270

Cluster-22178.3972 GO:0016567 GO:0070206 GO:0005515 GO:0004842
GO:0046872 GO:0008270 GO:0019028 GO:0005622
Cluster-22178.39723 GO:0055085 GO:0006810 GO:0019083 GO:0042626
GO:0016887 GO:0005524 GO:0016021
Cluster-22178.39727 GO:0009878 GO:0008270 GO:0046872
Cluster-22178.39728 GO:0046872 GO:0046935 GO:0005944 GO:0005576
Cluster-22178.39729 GO:0046872
Cluster-22178.39730 GO:0005515
Cluster-22178.39733 GO:0006470 GO:0006570 GO:0004725 GO:0005515

Cluster-22178.39734 GO:0006402 GO:0055114 GO:0051252 GO:0005507
GO:0004535 GO:0046872 GO:0016641 GO:0003723 GO:0005737
GO:0005634
Cluster-22178.39736 GO:0006886 GO:0055114 GO:0007049 GO:0006606
GO:0005506 GO:0020037 GO:0016705 GO:0005634 GO:0016020
Cluster-22178.3974 GO:0055114 GO:0045892 GO:0019836 GO:0006118
GO:0046872 GO:0051536 GO:0016491 GO:0009055 GO:0005576
GO:0005634
Cluster-22178.39741 GO:0006810 GO:0006270 GO:0008168 GO:0005515
GO:0003676 GO:0005783 GO:0005794
Cluster-22178.39744 GO:0006313 GO:0015074 GO:0005515 GO:0003677

Cluster-22178.39746 GO:0006355 GO:0000917 GO:0009405 GO:0007049
GO:0043093 GO:0006914 GO:0006744 GO:0006260 GO:0043565
GO:0008813 GO:0003700 GO:0003677 GO:0005576 GO:0005737
GO:0005667 GO:0005815
Cluster-22178.3975 GO:0006118 GO:0019836 GO:0045892 GO:0055114
GO:0009055 GO:0046872 GO:0051536 GO:0016491 GO:0005576
GO:0005634
Cluster-22178.39750 GO:0007283 GO:0005515
Cluster-22178.39751 GO:0006355 GO:0007267 GO:0046872 GO:0003677

Cluster-22178.39755 GO:0007267 GO:0006355 GO:0003677 GO:0005515

GO:0046872
Cluster-22178.39758 GO:0009405 GO:0034061 GO:0003677 GO:0005515
GO:0005576 GO:0005634
Cluster-22178.39762 GO:0040007 GO:0008283 GO:0006355 GO:0007186
GO:0007165 GO:0043401 GO:0043565 GO:0008270 GO:0003700
GO:0004930 GO:0008083 GO:0005515 GO:0016021 GO:0005667
GO:0005634
Cluster-22178.39767 GO:0043087 GO:0042619
Cluster-22178.39773 GO:0008152 GO:0055114 GO:0016491
Cluster-22178.39774 GO:0055114 GO:0008152 GO:0016491
Cluster-22178.39776 GO:0006811 GO:0006508 GO:0004197 GO:0016020
Cluster-22178.39783 GO:0006396 GO:0000154 GO:0016433
Cluster-22178.39784 GO:0000921 GO:0006468 GO:0005524 GO:0004672
GO:0005940 GO:0019028 GO:0016021
Cluster-22178.39786 GO:0007018 GO:0007017 GO:0005515 GO:0008017
GO:0003777 GO:0005524 GO:0045298 GO:0005874
Cluster-22178.39787 GO:0007018 GO:0007017 GO:0005515 GO:0008017
GO:0003777 GO:0005524 GO:0045298 GO:0005874
Cluster-22178.39788 GO:0044564 GO:0006468 GO:0035091 GO:0005524
GO:0004672 GO:0016773 GO:0016020
Cluster-22178.39791 GO:0055085 GO:0016021 GO:0016020 GO:0019031
Cluster-22178.39795 GO:0007029 GO:0005789
Cluster-22178.39798 GO:0019752 GO:0030170 GO:0016831
Cluster-22178.39800 GO:0006914 GO:0015743 GO:0006355 GO:0006886
GO:0007165 GO:0004871 GO:0005198 GO:0005622 GO:0019031
GO:0019028 GO:0005634 GO:0016020
Cluster-22178.39801 GO:0006405 GO:0006812 GO:0008324 GO:0005515
GO:0042025
Cluster-22178.39802 GO:0006935 GO:0007165 GO:0006955 GO:0008009
GO:0005576
Cluster-22178.39809 GO:0016787 GO:0005524 GO:0003676 GO:0003677
GO:0005634
Cluster-22178.39810 GO:0019497 GO:0006771 GO:0005515 GO:0003993

GO:0046872
Cluster-22178.39818 GO:0006812 GO:0006629 GO:0008081 GO:0008324

Cluster-22178.39820 GO:0006508 GO:0007186 GO:0004930 GO:0004252
GO:0016020
Cluster-22178.39825 GO:0007067 GO:0005975 GO:0004553 GO:0072686
GO:0042729
Cluster-22178.39827 GO:0006810 GO:0009405 GO:0019870 GO:0005576
Cluster-22178.39828 GO:0006355 GO:0005515 GO:0005634
Cluster-22178.39833 GO:0019031
Cluster-22178.39838 GO:0007264 GO:0005525
Cluster-22178.39854 GO:0006260 GO:0000398 GO:0006351 GO:0042073
GO:0006355 GO:0008270 GO:0003887 GO:0003677 GO:0003682
GO:0032040 GO:0000775 GO:0005681 GO:0000785 GO:0042575
GO:0005634
Cluster-22178.39855 GO:0005737
Cluster-22178.39859 GO:0006744 GO:0005515
Cluster-22178.39865 GO:0007165 GO:0007186 GO:0005525 GO:0004871
GO:0031683 GO:0019001 GO:0003924
Cluster-22178.39868 GO:0002949 GO:0009058 GO:0006614 GO:0007264
GO:0016114 GO:0006694 GO:0007018 GO:0006576 GO:0007017
GO:0006355 GO:0003724 GO:0016887 GO:0005524 GO:0003676
GO:0005525 GO:0003777 GO:0003924 GO:0016301 GO:0008134
GO:0004161 GO:0003723 GO:0030286 GO:0005667 GO:0005874
Cluster-22178.39879 GO:0005509 GO:0005515
Cluster-22178.39880 GO:0005515
Cluster-22178.39884 GO:0046836 GO:0035556 GO:0051861 GO:0046872
GO:0017089 GO:0003677 GO:0005737
Cluster-22178.39885 GO:0006355 GO:0016032 GO:0009405 GO:0006810
GO:0016070 GO:0016779 GO:0003677 GO:0005515 GO:0019871
GO:0005576 GO:0019012
Cluster-22178.39913 GO:0046935 GO:0005944
Cluster-22178.39915 GO:0009451 GO:0006879 GO:0008033 GO:0008199

GO:0009020
Cluster-22178.39919 GO:0007018 GO:0007017 GO:0008017 GO:0003777
GO:0005524 GO:0045298 GO:0005874
Cluster-22178.39923 GO:0007165 GO:0006355 GO:0003677 GO:0005515
GO:0005179 GO:0005576
Cluster-22178.39933 GO:0005515
Cluster-22178.39935 GO:0006508 GO:0004222 GO:0003779 GO:0005622
Cluster-22178.39937 GO:0055085 GO:0006885 GO:0006812 GO:0015299
GO:0016747 GO:0016021
Cluster-22178.39938 GO:0016757
Cluster-22178.39946 GO:0006355 GO:0043087 GO:0035023 GO:0003677
GO:0005089
Cluster-22178.39952 GO:0006144 GO:0003968 GO:0005515 GO:0031379
Cluster-22178.39958 GO:0006468 GO:0042742 GO:0006508 GO:0006827
GO:0006826 GO:0005381 GO:0004672 GO:0005524 GO:0016773
GO:0004222 GO:0005576 GO:0016020 GO:0033573
Cluster-22178.39964 GO:0008289
Cluster-22178.39966 GO:0006470 GO:0006570 GO:0008138 GO:0004725
GO:0004721
Cluster-22178.39971 GO:0006265 GO:0003677 GO:0005524 GO:0003918
GO:0005634
Cluster-22178.39975 GO:0005515
Cluster-22178.39977 GO:0015979 GO:0005515 GO:0009507 GO:0009523
Cluster-22178.39979 GO:0015979 GO:0005515 GO:0009523 GO:0009507
Cluster-22178.39997 GO:0006468 GO:0009069 GO:0016310 GO:0005516
GO:0004683 GO:0016773 GO:0004672 GO:0005524 GO:0016020
Cluster-22178.40003 GO:0016567 GO:0008270 GO:0046872 GO:0005515
GO:0004842
Cluster-22178.40005 GO:0016567 GO:0008270 GO:0046872 GO:0004842

Cluster-22178.40006 GO:0016567 GO:0004842 GO:0008270 GO:0046872

Cluster-22178.40013 GO:0005737

Cluster-22178.40014 GO:0007186 GO:0006810 GO:0008200 GO:0004930 GO:0005576 GO:0016020
Cluster-22178.40015 GO:0055114 GO:0000723 GO:0007017 GO:0006281 GO:0007018 GO:0005524 GO:0008270 GO:0016972 GO:0003678 GO:0008017 GO:0003777 GO:0005515 GO:0045298 GO:0005874 GO:0005657
Cluster-22178.40023 GO:0006508 GO:0004222
Cluster-22178.40024 GO:0006508 GO:0004222
Cluster-22178.40033 GO:0006812 GO:0055085 GO:0008324 GO:0016021
Cluster-22178.40034 GO:0043039 GO:0016876 GO:0005524 GO:0003676

Cluster-22178.40035 GO:0008152 GO:0016757
Cluster-22178.40036 GO:0006813 GO:0005242 GO:0003677 GO:0016021 GO:0008076
Cluster-22178.40037 GO:0006813 GO:0005242 GO:0003677 GO:0008076 GO:0016021
Cluster-22178.40038 GO:0007165 GO:0035556 GO:0007186 GO:0019001 GO:0031683 GO:0003924 GO:0004871
Cluster-22178.40041 GO:0046872
Cluster-22178.40043 GO:0006355 GO:0006814 GO:0006118 GO:0015992 GO:0006744 GO:0006120 GO:0003954 GO:0005515 GO:0008137 GO:0005739
Cluster-22178.40044 GO:0042113 GO:0051260 GO:0002764
Cluster-22178.40046 GO:0051726 GO:0006813 GO:0015269 GO:0016020 GO:0005634
Cluster-22178.40049 GO:0006570 GO:0045892 GO:0006470 GO:0042754 GO:0008138 GO:0005515 GO:0004725 GO:0016020
Cluster-22178.40050 GO:0006570 GO:0006470 GO:0008138 GO:0004725

Cluster-22178.40057 GO:0030474 GO:0005200 GO:0005823 GO:0005856
Cluster-22178.40067 GO:0051016 GO:0007264 GO:0005525 GO:0003924 GO:0008290
Cluster-22178.40073 GO:0006508 GO:0004190

Cluster-22178.40092 GO:0008152 GO:0006260 GO:0006123 GO:0015992
GO:0005975 GO:0016624 GO:0004129 GO:0004649 GO:0005524
GO:0003689 GO:0042575 GO:0005663 GO:0045277
Cluster-22178.40099 GO:0051252 GO:0016891
Cluster-22178.40100 GO:0019031
Cluster-22178.40108 GO:0005515 GO:0003723
Cluster-22178.40109 GO:0006468 GO:0005524 GO:0004672
Cluster-22178.40110 GO:0006468 GO:0005524 GO:0004672
Cluster-22178.40113 GO:0043401 GO:0006355 GO:0003700 GO:0019900
GO:0043565 GO:0046872 GO:0008270 GO:0005634 GO:0005667
Cluster-22178.40121 GO:0003723
Cluster-22178.40125 GO:0006464 GO:0046872 GO:0005524 GO:0016020
Cluster-22178.40130 GO:0055114 GO:0006570 GO:0004511
Cluster-22178.40131 GO:0006470 GO:0006570 GO:0055114 GO:0008138
GO:0004725 GO:0033743
Cluster-22178.40140 GO:0055114 GO:0016671 GO:0003756 GO:0005783
Cluster-22178.40150 GO:0005515 GO:0008270
Cluster-22178.40151 GO:0016255 GO:0042765
Cluster-22178.40157 GO:0005515
Cluster-22178.40161 GO:0007264 GO:0005525
Cluster-22178.40163 GO:0009405
Cluster-22178.40164 GO:0005815
Cluster-22178.40168 GO:0006814 GO:0006886 GO:0018149 GO:0006560
GO:0007411 GO:0006090 GO:0006525 GO:0071436 GO:0006810
GO:0006888 GO:0005515 GO:0008948 GO:0015081 GO:0003810
GO:0016020 GO:0030127
Cluster-22178.40173 GO:0016773
Cluster-22178.40175 GO:0019058 GO:0005198
Cluster-22178.40176 GO:0006887 GO:0000145
Cluster-22178.40191 GO:0046486 GO:0009395 GO:0009231 GO:0055114
GO:0032065 GO:0035556 GO:0007205 GO:0016301 GO:0005515
GO:0005543 GO:0008686 GO:0004143 GO:0047134 GO:0005938
Cluster-22178.40193 GO:0006468 GO:0004672 GO:0005524 GO:0043565

GO:0005515 GO:0005634
Cluster-22178.40195 GO:0007165 GO:0005057
Cluster-22178.40196 GO:0007165 GO:0005057
Cluster-22178.40198 GO:0006811 GO:0005198 GO:0005515 GO:0019028
GO:0016020
Cluster-22178.40200 GO:0046872 GO:0005887
Cluster-22178.40203 GO:0055085 GO:0007155 GO:0030001 GO:0003682
GO:0046873 GO:0016853 GO:0051015 GO:0000785 GO:0016020
Cluster-22178.40214 GO:0006505 GO:0006629 GO:0006886 GO:0042967
GO:0016788 GO:0008374 GO:0005739
Cluster-22178.40216 GO:0006351 GO:0019079 GO:0006144 GO:0003968
GO:0005515 GO:0005509 GO:0031379
Cluster-22178.40228 GO:0005887
Cluster-22178.40229 GO:0046872 GO:0008270 GO:0016021
Cluster-22178.40234 GO:0006486 GO:0003677 GO:0008373
Cluster-22178.40240 GO:0042981 GO:0006955 GO:0005515
Cluster-22178.40246 GO:0006810 GO:0055085 GO:0016021 GO:0016020
Cluster-22178.40249 GO:0007165
Cluster-22178.40250 GO:0003676
Cluster-22178.40252 GO:0006811 GO:0055085 GO:0050790 GO:0006813
GO:0015269 GO:0016286 GO:0030234 GO:0005216 GO:0005516
GO:0016021 GO:0016020
Cluster-22178.40254 GO:0031554 GO:0031556
Cluster-22178.40261 GO:0006479 GO:0046500 GO:0008152 GO:0006464
GO:0003723 GO:0003724 GO:0004719 GO:0008168
Cluster-22178.40263 GO:0007156 GO:0005509 GO:0005515 GO:0016020
Cluster-22178.40267 GO:0005515
Cluster-22178.40268 GO:0016192 GO:0005515 GO:0016021
Cluster-22178.4027 GO:0006367 GO:0032968 GO:0045892 GO:0003677
GO:0005515 GO:0005634
Cluster-22178.40272 GO:0005515
Cluster-22178.40274 GO:0016070 GO:0006355 GO:0016032 GO:0003677
GO:0019012

Cluster-22178.40276 GO:0022900 GO:0005747 GO:0005739
Cluster-22178.40278 GO:0005515
Cluster-22178.40281 GO:0005515
Cluster-22178.40287 GO:0043093 GO:0006457 GO:0000917 GO:0045502
GO:0051082 GO:0005198 GO:0042803 GO:0008134 GO:0005667
GO:0005882 GO:0005737 GO:0016272 GO:0030286
Cluster-22178.40294 GO:0000287 GO:0008253 GO:0005737
Cluster-22178.40302 GO:0006813 GO:0005249 GO:0008076
Cluster-22178.40311 GO:0006418 GO:0045892 GO:0004812 GO:0005524
GO:0000166 GO:0005634
Cluster-22178.40313 GO:0007165 GO:0005179 GO:0005576
Cluster-22178.40315 GO:0006355 GO:0043087 GO:0035023 GO:0003677
GO:0005089
Cluster-22178.40317 GO:0043087 GO:0019904 GO:0005515 GO:0008134
GO:0005667
Cluster-22178.40319 GO:0033926
Cluster-22178.40328 GO:0009069 GO:0042157 GO:0007165 GO:0006869
GO:0009847 GO:0006260 GO:0006355 GO:0006468 GO:0016310
GO:0007049 GO:0005096 GO:0004674 GO:0005198 GO:0043565
GO:0008289 GO:0003700 GO:0005667 GO:0016020 GO:0005815
GO:0005576 GO:0019028 GO:0019031
Cluster-22178.40329 GO:0009986 GO:0016020
Cluster-22178.40332 GO:0006144 GO:0015940 GO:0000103 GO:0008152
GO:0015937 GO:0016887 GO:0004140 GO:0005524 GO:0004020
GO:0016740 GO:0016301
Cluster-22178.40333 GO:0051537 GO:0043231
Cluster-22178.40355 GO:0006810 GO:0005215 GO:0016020
Cluster-22178.40357 GO:0006952 GO:0015940 GO:0005515 GO:0008897
GO:0003677 GO:0004190 GO:0000287 GO:0016021
Cluster-22178.40360 GO:0016020
Cluster-22178.40364 GO:0007049 GO:0051301 GO:0006355 GO:0003700
GO:0016021 GO:0005667 GO:0005634
Cluster-22178.40369 GO:0046836 GO:0051861 GO:0017089 GO:0005737

Cluster-22178.4037 GO:0016021
Cluster-22178.40384 GO:0006506 GO:0006874 GO:0006816 GO:0006355
GO:0055085 GO:0006811 GO:0005509 GO:0005216 GO:0016746
GO:0005219 GO:0016020 GO:0016021 GO:0005789 GO:0005622
Cluster-22178.40386 GO:0005515
Cluster-22178.40387 GO:0007275 GO:0008152 GO:0016032 GO:0016787
GO:0016021
Cluster-22178.40388 GO:0006468 GO:0009069 GO:0007187 GO:0007178
GO:0007608 GO:0016310 GO:0005549 GO:0004675 GO:0005524
GO:0004672 GO:0004984 GO:0016020
Cluster-22178.40389 GO:0006465 GO:0008233 GO:0005515 GO:0016757
GO:0005787 GO:0016021
Cluster-22178.40390 GO:0007165 GO:0005096 GO:0003729
Cluster-22178.40398 GO:0046872 GO:0008270
Cluster-22178.4040 GO:0006278 GO:0003964 GO:0005515 GO:0005509
Cluster-22178.40402 GO:0055085 GO:0019953 GO:0000105 GO:0006308
GO:0055114 GO:0030001 GO:0006355 GO:0004399 GO:0008855
GO:0008270 GO:0051287 GO:0046873 GO:0016021 GO:0016020
GO:0009318
Cluster-22178.40403 GO:0070206 GO:0006891 GO:0008608 GO:0045502
GO:0008134 GO:0042803 GO:0003950 GO:0005667 GO:0072686
GO:0017119 GO:0019028 GO:0042729 GO:0030286 GO:0005886
GO:0016021
Cluster-22178.40409 GO:0042981
Cluster-22178.40411 GO:0009117 GO:0000166 GO:0008253 GO:0000287
GO:0005737
Cluster-22178.40412 GO:0016787
Cluster-22178.40413 GO:0030170 GO:0003676
Cluster-22178.40423 GO:0006418 GO:0004812 GO:0005524
Cluster-22178.40424 GO:0007165 GO:0009372 GO:0005515 GO:0005509
GO:0005578
Cluster-22178.40425 GO:0008168 GO:0019028
Cluster-22178.40428 GO:0006260 GO:0019054 GO:0005975 GO:0004857

GO:0016758 GO:0016020
Cluster-22178.40432 GO:0005509 GO:0005544
Cluster-22178.40435 GO:0043565 GO:0005634
Cluster-22178.40444 GO:0006355 GO:0016773 GO:0003700 GO:0005667
GO:0042025
Cluster-22178.40451 GO:0006508 GO:0008236 GO:0016787
Cluster-22178.40453 GO:0006537 GO:0055114 GO:0007224 GO:0006807
GO:0007411 GO:0046872 GO:0003677 GO:0015930
Cluster-22178.40456 GO:0016310 GO:0000160 GO:0007165 GO:0006281
GO:0008270 GO:0046872 GO:0000155 GO:0009365 GO:0016021
Cluster-22178.40458 GO:0050792 GO:0042025 GO:0016021
Cluster-22178.40467 GO:0009451 GO:0008033 GO:0006879 GO:0070072
GO:0009020 GO:0008199
Cluster-22178.40471 GO:0045893 GO:0006269 GO:0006446 GO:0006413
GO:0006355 GO:0006351 GO:0005096 GO:0008270 GO:0004386
GO:0003743 GO:0003677 GO:0043565 GO:0003700 GO:0003896
GO:0005657 GO:0005667 GO:0005730 GO:0005840 GO:0005634
Cluster-22178.40483 GO:0016881 GO:0005524
Cluster-22178.40488 GO:0006629 GO:0006505 GO:0006886 GO:0016787
GO:0016788
Cluster-22178.40489 GO:0035023 GO:0007165 GO:0043087 GO:0005515
GO:0005089 GO:0005057
Cluster-22178.40490 GO:0043087 GO:0007165 GO:0035023 GO:0005057
GO:0005089 GO:0005515
Cluster-22178.40494 GO:0006950 GO:0009415 GO:0005515
Cluster-22178.40495 GO:0009415 GO:0006950 GO:0005515
Cluster-22178.40500 GO:0006355 GO:0043565 GO:0005515 GO:0005634
GO:0016021
Cluster-22178.40502 GO:0009103 GO:0055085 GO:0005515 GO:0016021
GO:0016020
Cluster-22178.40504 GO:0006886 GO:0005515
Cluster-22178.40505 GO:0034968 GO:0006479 GO:0042254 GO:0006412
GO:0006554 GO:0018024 GO:0003735 GO:0005515 GO:0008270

GO:0005634 GO:0005840
Cluster-22178.40510 GO:0007009 GO:0051082 GO:0003779
Cluster-22178.40517 GO:0007186 GO:0016598 GO:0004930 GO:0004057
GO:0016021
Cluster-22178.40524 GO:0006355 GO:0006260 GO:0003700 GO:0005667
Cluster-22178.40527 GO:0006508 GO:0004198 GO:0005509 GO:0005622
Cluster-22178.40529 GO:0006807 GO:0006355 GO:0003700 GO:0000982
GO:0003677 GO:0016812 GO:0005515 GO:0000978 GO:0005634
GO:0005667
Cluster-22178.40530 GO:0006914 GO:0009405 GO:0008270 GO:0005515
GO:0004222 GO:0005576
Cluster-22178.40531 GO:0007067 GO:0005515 GO:0000775
Cluster-22178.40535 GO:0006123 GO:0022900 GO:0015992 GO:0005507
GO:0004129 GO:0016021 GO:0045277 GO:0016020
Cluster-22178.40537 GO:0003824
Cluster-22178.40538 GO:0009405 GO:0019538 GO:0046872 GO:0005576
GO:0016012
Cluster-22178.40543 GO:0007586 GO:0016042 GO:0008047 GO:0005576
Cluster-22178.40545 GO:0007049
Cluster-22178.40551 GO:0048870 GO:0031505 GO:0007049 GO:0006355
GO:0043565 GO:0003700 GO:0019905 GO:0004252 GO:0003677
GO:0005667 GO:0005634 GO:0031514
Cluster-22178.40553 GO:0016192 GO:0006904
Cluster-22178.40556 GO:0015992 GO:0006123 GO:0030436 GO:0006810
GO:0009297 GO:0006355 GO:0003774 GO:0005524 GO:0004129
GO:0005515 GO:0008134 GO:0005667 GO:0016459 GO:0019867
GO:0042601 GO:0045277
Cluster-22178.40557 GO:0008152 GO:0005515 GO:0043565 GO:0016746
GO:0005634
Cluster-22178.40559 GO:0006468 GO:0055114 GO:0016706 GO:0016773
GO:0008168 GO:0004672 GO:0005524 GO:0016020
Cluster-22178.40573 GO:0008152 GO:0016746 GO:0005524
Cluster-22178.40580 GO:0046836 GO:0051861 GO:0017089 GO:0005737

GO:0019028

Cluster-22178.40582 GO:0007059 GO:0051301 GO:0000444

Cluster-22178.40590 GO:0055114 GO:0007165 GO:0008283 GO:0040007 GO:0006259 GO:0047134 GO:0003824 GO:0016627 GO:0005524 GO:0003677 GO:0008083 GO:0005694

Cluster-22178.40593 GO:0006355 GO:0005524 GO:0003700 GO:0003676 GO:0005667 GO:0042025

Cluster-22178.40603 GO:0042254 GO:0006412 GO:0006355 GO:0003700 GO:0043565 GO:0003735 GO:0005840 GO:0005622 GO:0005667

Cluster-22178.40607 GO:0045444

Cluster-22178.40610 GO:0004064

Cluster-22178.40627 GO:0006355 GO:0003677

Cluster-22178.40628 GO:0008146

Cluster-22178.40632 GO:0051252 GO:0055114 GO:0006402 GO:0016641 GO:0003723 GO:0046872 GO:0004535 GO:0005507 GO:0005634 GO:0005737

Cluster-22178.40640 GO:0005515

Cluster-22178.40642 GO:0042254 GO:0006412 GO:0046872 GO:0005515 GO:0003735 GO:0005622 GO:0005840

Cluster-22178.40643 GO:0005515

Cluster-22178.40644 GO:0006468 GO:0005524 GO:0004672

Cluster-22178.40645 GO:0036065 GO:0009246 GO:0055114 GO:0020037 GO:0016705 GO:0008417 GO:0005506

Cluster-22178.40649 GO:0015979 GO:0000062 GO:0005515 GO:0009522 GO:0016021 GO:0009579

Cluster-22178.40651 GO:0001819 GO:0008284 GO:0007165 GO:0010884 GO:0005509 GO:0005515 GO:0005578

Cluster-22178.40652 GO:0015684 GO:0015093 GO:0016021

Cluster-22178.40655 GO:0007165 GO:0005515 GO:0032266 GO:0043130

Cluster-22178.40656 GO:0000122 GO:0007050 GO:0009405 GO:0045859 GO:0043303 GO:0017069 GO:0004861 GO:0005737 GO:0005634 GO:0005576

Cluster-22178.40658 GO:0055114 GO:0005515 GO:0010277

Cluster-22178.40659 GO:0006508 GO:0045444 GO:0005515 GO:0004198 GO:0005622

Cluster-22178.40661 GO:0007165

Cluster-22178.40670 GO:0005634

Cluster-22178.40672 GO:0006888 GO:0071436 GO:0006810 GO:0006525 GO:0007411 GO:0006090 GO:0018149 GO:0006886 GO:0006814 GO:0006560 GO:0003810 GO:0015081 GO:0005515 GO:0008948 GO:0016020 GO:0030127

Cluster-22178.40673 GO:0016310 GO:0009069 GO:0008384 GO:0008385

Cluster-22178.40674 GO:0006858 GO:0006468 GO:0004672 GO:0005524 GO:0016773 GO:0016020

Cluster-22178.40675 GO:0006544 GO:0046486 GO:0008654 GO:0006563 GO:0006566 GO:0004609

Cluster-22178.40683 GO:0055085 GO:0006811 GO:0005992 GO:0006355 GO:0003700 GO:0003824 GO:0005515 GO:0005216 GO:0016020 GO:0005667

Cluster-22178.40684 GO:0006355 GO:0005992 GO:0006811 GO:0055085 GO:0005515 GO:0005216 GO:0003700 GO:0003824 GO:0016020 GO:0005667

Cluster-22178.40688 GO:0042476 GO:0042475 GO:0000287 GO:0030345 GO:0008253 GO:0005737 GO:0005578

Cluster-22178.40689 GO:0051016 GO:0006468 GO:0007165 GO:0007172 GO:0004713 GO:0004871 GO:0030479 GO:0005925

Cluster-22178.40690 GO:0048544 GO:0008270 GO:0046872 GO:0005515 GO:0061630 GO:0005622

Cluster-22178.40696 GO:0055114 GO:0019064 GO:0006814 GO:0006118 GO:0016655 GO:0019031

Cluster-22178.40703 GO:0007067 GO:0000090 GO:0006468 GO:0007059 GO:0031110 GO:0051301 GO:0008017 GO:0005524 GO:0004672 GO:0045298 GO:0005876 GO:0000940

Cluster-22178.40706 GO:0051382 GO:0051301 GO:0031110 GO:0007059 GO:0006468 GO:0000090 GO:0007067 GO:0004672 GO:0005524

GO:0008017 GO:0000940 GO:0045298 GO:0005876 GO:0000776

Cluster-22178.40710 GO:0042967 GO:0047961 GO:0005739

Cluster-22178.40711 GO:0042967 GO:0047961 GO:0005739

Cluster-22178.40712 GO:0042967 GO:0047961 GO:0005739

Cluster-22178.40718 GO:0035556 GO:0005515 GO:0019013 GO:0016021

Cluster-22178.40719 GO:0035556 GO:0005515

Cluster-22178.40722 GO:0015992 GO:0015986 GO:0015078 GO:0000276

Cluster-22178.40729 GO:0035556 GO:0006144 GO:0006182 GO:0046039

GO:0009190 GO:0046872 GO:0020037 GO:0016849 GO:0004383

Cluster-22178.40732 GO:0055114 GO:0009395 GO:0016310 GO:0006629

GO:0006468 GO:0006570 GO:0046339 GO:0016042 GO:0035556

GO:0042742 GO:0009069 GO:0007165 GO:0004435 GO:0005515

GO:0005096 GO:0004674 GO:0004511 GO:0005509 GO:0005576

Cluster-22178.40739 GO:0055114 GO:0046872 GO:0016491

Cluster-22178.40741 GO:0007154 GO:0005509 GO:0016020

Cluster-22178.40743 GO:0006355 GO:0000917 GO:0043093 GO:0006457

GO:0006914 GO:0006260 GO:0010506 GO:0051082 GO:0005515

GO:0043565 GO:0003700 GO:0005507 GO:0005506 GO:0003677

GO:0008270 GO:0008017 GO:0005634 GO:0005737 GO:0045298

GO:0005615 GO:0005667 GO:0016272

Cluster-22178.40744 GO:0006355 GO:0010506 GO:0006260 GO:0005515

GO:0003677 GO:0043565 GO:0008270 GO:0005507 GO:0003700

GO:0008017 GO:0005667 GO:0005634 GO:0005615 GO:0045298

Cluster-22178.40755 GO:0035307 GO:0048870 GO:0006260 GO:0007218

GO:0016787 GO:0003723 GO:0003677 GO:0005184 GO:0016887

GO:0005524 GO:0003724 GO:0005576 GO:0031514 GO:0071595

Cluster-22178.40760 GO:0006955 GO:0007165 GO:0040007 GO:0008283

GO:0008083 GO:0005179 GO:0005134 GO:0005576 GO:0005893

Cluster-22178.40770 GO:0008376 GO:0032580

Cluster-22178.40771 GO:0016021 GO:0019012

Cluster-22178.40775 GO:0006914 GO:0005515

Cluster-22178.40776 GO:0006914 GO:0005515

Cluster-22178.40782 GO:0005515

Cluster-22178.40787 GO:0042254 GO:0006412 GO:0008270 GO:0005524 GO:0003735 GO:0005622 GO:0005840

Cluster-22178.40788 GO:0006412 GO:0042254 GO:0005524 GO:0008270 GO:0003735 GO:0005622 GO:0005840

Cluster-22178.40790 GO:0006310 GO:0006281 GO:0006139 GO:0006206 GO:0005515 GO:0009378 GO:0004127 GO:0005524 GO:0009379 GO:0005657

Cluster-22178.40791 GO:0006281 GO:0006310 GO:0009378 GO:0005515 GO:0009379 GO:0005657

Cluster-22178.40792 GO:0016887 GO:0005524

Cluster-22178.40800 GO:0006865 GO:0055085 GO:0006810 GO:0003333 GO:0015171 GO:0016020

Cluster-22178.40801 GO:0006352 GO:0003677

Cluster-22178.40814 GO:0007165 GO:0008283 GO:0040007 GO:0008083

Cluster-22178.40817 GO:0008017 GO:0045298

Cluster-22178.40821 GO:0009405

Cluster-22178.40822 GO:0006370 GO:0009451 GO:0016192 GO:0006886 GO:0015991 GO:0004482 GO:0016820 GO:0000221 GO:0030131 GO:0030117

Cluster-22178.40829 GO:0006915 GO:0005622

Cluster-22178.40837 GO:0007606 GO:0007165 GO:0004888 GO:0016020

Cluster-22178.40838 GO:0000975

Cluster-22178.4084 GO:0006811 GO:0016020

Cluster-22178.40841 GO:0009089 GO:0007186 GO:0016310 GO:0005515 GO:0004673 GO:0004930 GO:0008837 GO:0008270 GO:0009365 GO:0016021

Cluster-22178.40848 GO:0006355 GO:0003700 GO:0008270 GO:0005667 GO:0005634

Cluster-22178.40851 GO:0006508 GO:0008236 GO:0016787

Cluster-22178.40853 GO:0005975 GO:0016901 GO:0048038

Cluster-22178.40857 GO:0007156 GO:0015706 GO:0006357 GO:0010167 GO:0005509 GO:0005515 GO:0001104 GO:0016020 GO:0016592

Cluster-22178.40860 GO:0015992 GO:0015986 GO:0005515 GO:0003676
GO:0015078 GO:0008270 GO:0019013
Cluster-22178.40864 GO:0098542
Cluster-22178.40872 GO:0006275 GO:0046872
Cluster-22178.40874 GO:0055114 GO:0016491 GO:0016021
Cluster-22178.40876 GO:0055114 GO:0016491 GO:0016021
Cluster-22178.40878 GO:0008152 GO:0008484 GO:0003824
Cluster-22178.40881 GO:0051726 GO:0005634
Cluster-22178.40883 GO:0015074 GO:0016310 GO:0004673 GO:0008907
GO:0003677 GO:0009365
Cluster-22178.40891 GO:0016021
Cluster-22178.40893 GO:0005515
Cluster-22178.40895 GO:0016020
Cluster-22178.40897 GO:0032801 GO:0006812 GO:0019076 GO:0005515
GO:0005261 GO:0033644
Cluster-22178.40898 GO:0005515
Cluster-22178.40904 GO:0005515
Cluster-22178.40907 GO:0055085 GO:0006810 GO:0005215 GO:0043565
GO:0016020 GO:0016021 GO:0005634
Cluster-22178.40910 GO:0016032 GO:0007304 GO:0005213 GO:0005576
GO:0042600
Cluster-22178.40915 GO:0019028
Cluster-22178.40923 GO:0005515
Cluster-22178.40924 GO:0005515
Cluster-22178.40925 GO:0005515
Cluster-22178.40927 GO:0006464 GO:0005515 GO:0016151
Cluster-22178.40930 GO:0006355 GO:0046983 GO:0003677
Cluster-22178.40932 GO:0005515
Cluster-22178.40937 GO:0006308 GO:0055114 GO:0010508 GO:0006570
GO:0009405 GO:0007094 GO:0006508 GO:0015991 GO:0006376
GO:0015992 GO:0015078 GO:0008092 GO:0016714 GO:0046872
GO:0005515 GO:0008233 GO:0008855 GO:0005524 GO:0003729
GO:0016787 GO:0004511 GO:0005737 GO:0033179 GO:0009318

GO:0005634 GO:0019898 GO:0005685 GO:0016020
Cluster-22178.40941 GO:0016021
Cluster-22178.40943 GO:0006355 GO:0042393 GO:0003677 GO:0005515
GO:0003723 GO:0003700 GO:0008270 GO:0046872 GO:0043565
GO:0005667 GO:0016021
Cluster-22178.40944 GO:0006355 GO:0042393 GO:0003677 GO:0005515
GO:0003700 GO:0008270 GO:0046872 GO:0043565 GO:0005667
GO:0016021
Cluster-22178.40947 GO:0004866
Cluster-22178.40952 GO:0030153 GO:0007165 GO:0005515 GO:0016757

Cluster-22178.40956 GO:0019079 GO:0005515 GO:0005509
Cluster-22178.40957 GO:0005515 GO:0005737
Cluster-22178.40964 GO:0008270 GO:0003676 GO:0016021
Cluster-22178.40965 GO:0016192 GO:0019079 GO:0006260 GO:0005515
GO:0003677 GO:0016787 GO:0003676 GO:0003678 GO:0005524
GO:0016021 GO:0005657
Cluster-22178.40966 GO:0016021 GO:0005886
Cluster-22178.40968 GO:0006826 GO:0055114 GO:0006827 GO:0006468
GO:0006508 GO:0046168 GO:0042742 GO:0004672 GO:0004222
GO:0005524 GO:0016773 GO:0005381 GO:0016616 GO:0051287
GO:0033573 GO:0005576 GO:0016020
Cluster-22178.40977 GO:0004222
Cluster-22178.40981 GO:0016310 GO:0015979 GO:0006468 GO:0009069
GO:0004674 GO:0004672 GO:0005524 GO:0009523 GO:0009539
GO:0016020
Cluster-22178.40997 GO:0009405 GO:0005515 GO:0003682 GO:0000785
GO:0005576
Cluster-22178.40998 GO:0016055 GO:0007165 GO:0007411 GO:0008289
GO:0005515 GO:0005576 GO:0016021
Cluster-22178.41000 GO:0006468 GO:0005515 GO:0004672 GO:0005524

Cluster-22178.41009 GO:0006811 GO:0006355 GO:0003677 GO:0005216

GO:0016020 GO:0005634
Cluster-22178.41010 GO:0006811 GO:0005216 GO:0016020
Cluster-22178.41019 GO:0006810 GO:0055085 GO:0003723 GO:0005524
GO:0016887 GO:0003724 GO:0042626 GO:0016021
Cluster-22178.41023 GO:0042246 GO:0007155 GO:0005515 GO:0008270
GO:0046872 GO:0016021
Cluster-22178.41024 GO:0042246 GO:0007155 GO:0005515 GO:0008270
GO:0046872 GO:0016021
Cluster-22178.41030 GO:0006464 GO:0006412 GO:0042254 GO:0016151
GO:0046872 GO:0008270 GO:0003735 GO:0005840 GO:0005622
Cluster-22178.41037 GO:0005737
Cluster-22178.41038 GO:0016192 GO:0070008 GO:0004252 GO:0016021
Cluster-22178.41040 GO:0009405 GO:0017176
Cluster-22178.41050 GO:0005515 GO:0003682 GO:0000785
Cluster-22178.41053 GO:0008270 GO:0005515 GO:0005783
Cluster-22178.41054 GO:0008270 GO:0005515 GO:0005783
Cluster-22178.41055 GO:0015700 GO:0005198 GO:0005515 GO:0015105
GO:0019028 GO:0016021
Cluster-22178.41058 GO:0016020
Cluster-22178.41060 GO:0005783
Cluster-22178.41063 GO:0071436 GO:0046486 GO:0007268 GO:0007165
GO:0006525 GO:0016042 GO:0006370 GO:0006814 GO:0006560
GO:0006397 GO:0006508 GO:0006090 GO:0006811 GO:0016787
GO:0004806 GO:0015081 GO:0008948 GO:0008236 GO:0004484
GO:0004970 GO:0016020 GO:0016021
Cluster-22178.41067 GO:0051259
Cluster-22178.41072 GO:0006520 GO:0009058 GO:0030170 GO:0016829
GO:0008483
Cluster-22178.41074 GO:0009058 GO:0008483 GO:0030170
Cluster-22178.41079 GO:0006310 GO:0006810 GO:0006302 GO:0005215
GO:0005515 GO:0005509 GO:0003677 GO:0016021 GO:0005737
GO:0005634
Cluster-22178.41087 GO:0019089 GO:0006886 GO:0006606 GO:0006807

GO:0051258 GO:0006118 GO:0051607 GO:0030168 GO:0006891
GO:0008053 GO:0019551 GO:0007165 GO:0004352 GO:0003924
GO:0042803 GO:0005198 GO:0005524 GO:0008092 GO:0045502
GO:0008134 GO:0019901 GO:0005102 GO:0030674 GO:0005737
GO:0030173 GO:0005577 GO:0005667 GO:0005741 GO:0016020
GO:0019031 GO:0016021 GO:0019028 GO:0030286 GO:0005815
GO:0019898 GO:0017119
Cluster-22178.41089 GO:0006886 GO:0006118 GO:0006807 GO:0019551
GO:0009405 GO:0004352 GO:0016020
Cluster-22178.41097 GO:0005515 GO:0016020
Cluster-22178.41099 GO:0006807 GO:0006672 GO:0019082 GO:0016032
GO:0016811 GO:0005515 GO:0016021
Cluster-22178.41100 GO:0045892 GO:2001256 GO:0030176 GO:0005634
Cluster-22178.41102 GO:0001522 GO:0009451 GO:0009982 GO:0003723

Cluster-22178.41111 GO:0006886 GO:0016192 GO:0006508 GO:0004198
GO:0005509 GO:0005515 GO:0030131 GO:0005622
Cluster-22178.41118 GO:0007264 GO:0007165 GO:0007186 GO:0019001
GO:0031683 GO:0003924 GO:0005525 GO:0004871
Cluster-22178.41123 GO:0045454
Cluster-22178.41125 GO:0051304 GO:0005515
Cluster-22178.41129 GO:0005515
Cluster-22178.41132 GO:0007186 GO:0004930 GO:0016021
Cluster-22178.41133 GO:0007018 GO:0006412 GO:0006355 GO:0007017
GO:0042254 GO:0007264 GO:0002949 GO:0000166 GO:0008134
GO:0003723 GO:0005525 GO:0003777 GO:0003677 GO:0003735
GO:0003724 GO:0005524 GO:0016887 GO:0005840 GO:0005874
GO:0005667 GO:0005622 GO:0030286
Cluster-22178.41138 GO:0006357 GO:0001104 GO:0016592
Cluster-22178.41141 GO:0006139 GO:0004003 GO:0016787 GO:0003677
GO:0016818 GO:0008026 GO:0005524 GO:0003676 GO:0005634
GO:0005657
Cluster-22178.41150 GO:0005515 GO:0005856

Cluster-22178.41160 GO:0006313 GO:0007275 GO:0004803 GO:0003677
GO:0016021
Cluster-22178.41167 GO:0044419 GO:0006144 GO:0006508 GO:0004197
GO:0003968 GO:0017111 GO:0031379
Cluster-22178.41178 GO:0004222
Cluster-22178.41182 GO:0016020
Cluster-22178.41185 GO:0055114 GO:0046872
Cluster-22178.41188 GO:0009405 GO:0019835 GO:0019867
Cluster-22178.41189 GO:0008270 GO:0005515
Cluster-22178.41191 GO:0006468 GO:0005524 GO:0004672
Cluster-22178.41193 GO:0046487 GO:0007010 GO:0008152 GO:0055114
GO:0009396 GO:0004488 GO:0050662 GO:0003824 GO:0016491
GO:0003779
Cluster-22178.41202 GO:0006418 GO:0005524 GO:0004812 GO:0003676
GO:0000166
Cluster-22178.41203 GO:0006418 GO:0019079 GO:0003676 GO:0005524
GO:0000166 GO:0003677 GO:0008026 GO:0016787 GO:0004812
Cluster-22178.41214 GO:0006486 GO:0016757 GO:0008378 GO:0016020
Cluster-22178.41221 GO:0006302 GO:0015992 GO:0006123 GO:0005515
GO:0004129 GO:0005634 GO:0005739 GO:0045277
Cluster-22178.41222 GO:0006302 GO:0006123 GO:0015992 GO:0005515
GO:0004129 GO:0005739 GO:0005634 GO:0045277
Cluster-22178.41225 GO:0006302 GO:0006123 GO:0015992 GO:0005515
GO:0004129 GO:0005739 GO:0005634 GO:0045277
Cluster-22178.41226 GO:0007009 GO:0007165
Cluster-22178.41232 GO:0055085 GO:0006811 GO:0004064 GO:0005216
GO:0016020
Cluster-22178.41238 GO:0055114 GO:0016616
Cluster-22178.41245 GO:0055114 GO:0008152 GO:0016491 GO:0051287

Cluster-22178.41248 GO:0006355 GO:0003682 GO:0003677 GO:0005634
GO:0000775 GO:0000785
Cluster-22178.41253 GO:0005515

Cluster-22178.41255 GO:0005515
Cluster-22178.41261 GO:0019229 GO:0003676 GO:0005576
Cluster-22178.41264 GO:0006508 GO:0006811 GO:0006810 GO:0016805
GO:0005215 GO:0016020
Cluster-22178.41265 GO:0006811 GO:0009405 GO:0006810 GO:0055085
GO:0005215 GO:0045735 GO:0016020
Cluster-22178.41266 GO:0006508 GO:0006810 GO:0006811 GO:0005215
GO:0016805 GO:0016020
Cluster-22178.4127 GO:0009755 GO:0005576
Cluster-22178.41274 GO:0006468 GO:0005524 GO:0004672
Cluster-22178.41275 GO:0042967 GO:0008080
Cluster-22178.41279 GO:0005515
Cluster-22178.41280 GO:0006448 GO:0006414 GO:0005525 GO:0003924
GO:0003746 GO:0005840 GO:0005737
Cluster-22178.41283 GO:0009405 GO:0007623 GO:0019835 GO:0006351
GO:0006355 GO:0003700 GO:0043565 GO:0005667 GO:0005634
GO:0019867
Cluster-22178.41291 GO:0007264 GO:0015684 GO:0015093 GO:0005524
GO:0003924 GO:0005525 GO:0016021
Cluster-22178.41301 GO:0000266 GO:0018149 GO:0003810 GO:0005739
Cluster-22178.41303 GO:0005515 GO:0003682 GO:0000785
Cluster-22178.41305 GO:0006810 GO:0009405 GO:0005515 GO:0019870
GO:0046872 GO:0008270 GO:0005576
Cluster-22178.41306 GO:0055114 GO:0051287 GO:0016620
Cluster-22178.41307 GO:0055114 GO:0016620
Cluster-22178.41310 GO:0000398 GO:0034258 GO:0034257 GO:0019901
GO:0005634 GO:0016020
Cluster-22178.41314 GO:0006468 GO:0005515 GO:0005524 GO:0004672

Cluster-22178.41316 GO:0006468 GO:0005515 GO:0004672 GO:0005524

Cluster-22178.41328 GO:0006771 GO:0042254 GO:0015992 GO:0009396
GO:0015991 GO:0046487 GO:0006412 GO:0019497 GO:0055114

GO:0008152 GO:0050662 GO:0004488 GO:0003735 GO:0016491
GO:0003993 GO:0005515 GO:0046872 GO:0015078 GO:0003824
GO:0033179 GO:0005840
Cluster-22178.41329 GO:0006810 GO:0005471 GO:0005524 GO:0005634
GO:0016021
Cluster-22178.41330 GO:0008608 GO:0019236 GO:0042710 GO:0007186
GO:0034258 GO:0007606 GO:0004930 GO:0004932 GO:0034257
GO:0016787 GO:0016021 GO:0042729 GO:0016020 GO:0042765
GO:0072686
Cluster-22178.41333 GO:0006468 GO:0004672
Cluster-22178.41336 GO:0006355 GO:0006338 GO:0016818 GO:0003676
GO:0005524 GO:0005634
Cluster-22178.41339 GO:0019882 GO:0006955 GO:0042613 GO:0016020
Cluster-22178.41342 GO:0006457 GO:0051082 GO:0005509 GO:0005783
Cluster-22178.41344 GO:0030141
Cluster-22178.41345 GO:0030141
Cluster-22178.41346 GO:0006810 GO:0009405 GO:0008200 GO:0005576
Cluster-22178.41348 GO:0043401 GO:0006355 GO:0003700 GO:0008270
GO:0043565 GO:0005634 GO:0005667
Cluster-22178.41352 GO:0008270 GO:0046872 GO:0061630 GO:0005515
GO:0005622 GO:0019867
Cluster-22178.41356 GO:0006397 GO:0016043 GO:0030036 GO:0017048
GO:0003723 GO:0051082 GO:0003779 GO:0005634 GO:0005737
Cluster-22178.41361 GO:0006810 GO:0007186 GO:0007218 GO:0005184
GO:0005524 GO:0005525 GO:0004930 GO:0042626 GO:0005576
GO:0016021
Cluster-22178.41364 GO:0006468 GO:0005515 GO:0005524 GO:0004672

Cluster-22178.41368 GO:0005515
Cluster-22178.41373 GO:0006355 GO:0003700 GO:0043565 GO:0005667
Cluster-22178.41374 GO:0007283 GO:0003677 GO:0000786 GO:0005634
Cluster-22178.41378 GO:0006810 GO:0007218 GO:0008610 GO:0006741
GO:0015947 GO:0046497 GO:0008152 GO:0055114 GO:0006308

GO:0006769 GO:0008855 GO:0016747 GO:0019871 GO:0016301
GO:0003951 GO:0046332 GO:0030058 GO:0042597 GO:0005886
GO:0009318 GO:0005576
Cluster-22178.41392 GO:0006506 GO:0000045 GO:0033617 GO:0016758
GO:0016021 GO:0005737
Cluster-22178.41393 GO:0006470 GO:0006570 GO:0008270 GO:0004725
GO:0008138 GO:0005515 GO:0004721
Cluster-22178.41407 GO:0005515
Cluster-22178.41409 GO:0006508 GO:0008236 GO:0003950 GO:0016787
GO:0005634
Cluster-22178.4141 GO:0007275 GO:0005578
Cluster-22178.41410 GO:0006508 GO:0016787 GO:0008236 GO:0003950
GO:0005634
Cluster-22178.41411 GO:0006508 GO:0008236
Cluster-22178.41417 GO:0035556 GO:0005515
Cluster-22178.41428 GO:0006468 GO:0030150 GO:0005524 GO:0004672
GO:0005742
Cluster-22178.41429 GO:0048870 GO:0030246 GO:0003774 GO:0005524
GO:0005515 GO:0016459 GO:0031514
Cluster-22178.41432 GO:0006355 GO:0006281 GO:0042128 GO:0055114
GO:0006807 GO:0003677 GO:0008270 GO:0008942 GO:0016491
GO:0008168 GO:0051537
Cluster-22178.41436 GO:0003677
Cluster-22178.41437 GO:0043093 GO:0006260 GO:0000917 GO:0006810
GO:0010508 GO:0005886 GO:0005737
Cluster-22178.41438 GO:0042254
Cluster-22178.41442 GO:0015074 GO:0003677 GO:0046872 GO:0005887
Cluster-22178.41448 GO:0043087 GO:0007165 GO:0005057 GO:0030695
GO:0005089 GO:0005737
Cluster-22178.41449 GO:0043087 GO:0000723 GO:0007165 GO:0005057
GO:0030695 GO:0005089 GO:0005515 GO:0003677 GO:0005737
GO:0000784
Cluster-22178.41450 GO:0005515

Cluster-22178.41452 GO:0006355 GO:0035064 GO:0005634
Cluster-22178.41455 GO:0005515 GO:0016020
Cluster-22178.41459 GO:0055114 GO:0006810 GO:0016491 GO:0016788
GO:0050660 GO:0016020
Cluster-22178.41460 GO:0006144 GO:0046034 GO:0046872 GO:0004017

Cluster-22178.41463 GO:0007165 GO:0003676 GO:0005509 GO:0005578
Cluster-22178.41465 GO:0055114 GO:0047134 GO:0005515
Cluster-22178.41468 GO:0046872
Cluster-22178.41472 GO:0019079 GO:0006144 GO:0003968 GO:0031379
GO:0019028
Cluster-22178.41475 GO:0019031
Cluster-22178.41477 GO:0006355 GO:0055085 GO:0007049 GO:0043565
GO:0046983 GO:0003700 GO:0005667 GO:0016020 GO:0016021
Cluster-22178.41480 GO:0055114 GO:0008168 GO:0016706
Cluster-22178.41481 GO:0003774 GO:0016459
Cluster-22178.41492 GO:0006357 GO:0008152 GO:0046872 GO:0003824
GO:0001104 GO:0016791 GO:0016021 GO:0016592
Cluster-22178.41497 GO:0005515
Cluster-22178.41498 GO:0003676
Cluster-22178.41499 GO:0003824
Cluster-22178.41500 GO:0009607 GO:0016021
Cluster-22178.41501 GO:0006118 GO:0003824 GO:0005515 GO:0009055

Cluster-22178.41503 GO:0009405 GO:0006810 GO:0008200 GO:0005576
Cluster-22178.41505 GO:0006468 GO:0005524 GO:0004672
Cluster-22178.41509 GO:0005515
Cluster-22178.41514 GO:0051726 GO:0005634
Cluster-22178.41531 GO:0005515 GO:0004867 GO:0030414
Cluster-22178.41538 GO:0050852 GO:0050853 GO:0016310 GO:0016772

Cluster-22178.41546 GO:0016747 GO:0016787 GO:0008270 GO:0005515

Cluster-22178.41547 GO:0043248 GO:0006892 GO:0006278 GO:0006605
GO:0003964 GO:0051018 GO:0019901 GO:0005886 GO:0012505
GO:0005829 GO:0005802 GO:0045211 GO:0030054

Cluster-22178.41560 GO:0005524

Cluster-22178.41565 GO:0006858

Cluster-22178.41570 GO:0006352 GO:0009236 GO:0015979 GO:0006270
GO:0006468 GO:0004672 GO:0005524 GO:0005669

Cluster-22178.41573 GO:0006378 GO:0003729 GO:0016787 GO:0005849

Cluster-22178.41578 GO:0006955 GO:0007165 GO:0006935 GO:0008009
GO:0005576

Cluster-22178.4158 GO:0006886 GO:0006505 GO:0016788 GO:0008270

Cluster-22178.41594 GO:0008152 GO:0003824

Cluster-22178.41595 GO:0042967 GO:0016747 GO:0008080 GO:0016021

Cluster-22178.41596 GO:0042967 GO:0008080 GO:0016747 GO:0016021

Cluster-22178.41597 GO:0006310 GO:0006281 GO:0006508 GO:0004181
GO:0004180 GO:0008270

Cluster-22178.41598 GO:0005856

Cluster-22178.41599 GO:0006418 GO:0002161

Cluster-22178.41601 GO:0015992 GO:0006744 GO:0006120 GO:0006468
GO:0006814 GO:0016773 GO:0004672 GO:0005524 GO:0016787
GO:0008137 GO:0005739 GO:0016020

Cluster-22178.41602 GO:0016310 GO:0009069 GO:0006468 GO:0004674
GO:0004672 GO:0005524 GO:0016773 GO:0016020

Cluster-22178.41604 GO:0007264 GO:0005525 GO:0005524 GO:0016887

Cluster-22178.41605 GO:0007165 GO:0005515

Cluster-22178.41606 GO:0007165 GO:0005515

Cluster-22178.41610 GO:0032481 GO:0002218

Cluster-22178.41611 GO:0003824

Cluster-22178.41615 GO:0019068

Cluster-22178.41619 GO:0007268 GO:0033130 GO:0005515 GO:0042802
GO:0043495

Cluster-22178.41621 GO:0006355 GO:0009405 GO:0007165 GO:0043565

GO:0003700 GO:0004888 GO:0005509 GO:0005198 GO:0005515
GO:0016021 GO:0005667
Cluster-22178.41624 GO:0006144 GO:0006351 GO:0007165 GO:0003968
GO:0003824 GO:0005524 GO:0003677 GO:0050662 GO:0005179
GO:0031379 GO:0005634
Cluster-22178.41627 GO:0005515
Cluster-22178.41628 GO:0016310 GO:0009069 GO:0006468 GO:0005515
GO:0004674 GO:0000287 GO:0005524
Cluster-22178.41630 GO:0005985 GO:0005982 GO:0005978 GO:0005515
GO:0004373
Cluster-22178.41633 GO:0032775 GO:0031167 GO:0007165 GO:0007275
GO:0006306 GO:0006298 GO:0016055 GO:0030983 GO:0098519
GO:0030414 GO:0005102 GO:0009007 GO:0008168 GO:0005524
GO:0005576
Cluster-22178.41635 GO:0008152 GO:0055114 GO:0003824 GO:0050662
GO:0016491
Cluster-22178.41656 GO:0042742 GO:0006468 GO:0016872 GO:0003723
GO:0005524 GO:0004672
Cluster-22178.41657 GO:0007128 GO:0007130 GO:0070193 GO:0005515
GO:0000795
Cluster-22178.41658 GO:0007155 GO:0007156 GO:0007218 GO:0005509
GO:0016020
Cluster-22178.41660 GO:0006355 GO:0005515
Cluster-22178.41664 GO:0005515 GO:0016020
Cluster-22178.41665 GO:0030001 GO:0055085 GO:0046873 GO:0016020
Cluster-22178.41667 GO:0030001 GO:0055085 GO:0046873 GO:0016020
Cluster-22178.41670 GO:0016192 GO:0006886 GO:0004789 GO:0005198
GO:0005515 GO:0030117
Cluster-22178.41671 GO:0005515
Cluster-22178.41672 GO:0046740 GO:0016021
Cluster-22178.41673 GO:0046740 GO:0016021
Cluster-22178.41674 GO:0006338 GO:0006355 GO:0003676 GO:0016818
GO:0005524 GO:0005634

Cluster-22178.41676 GO:0006355 GO:0042742 GO:0007165 GO:0003700
GO:0003677 GO:0005515 GO:0005576 GO:0005667
Cluster-22178.41686 GO:0003924 GO:0005615
Cluster-22178.41687 GO:0004672 GO:0005515
Cluster-22178.41690 GO:0005515
Cluster-22178.41692 GO:0072519 GO:0016567 GO:0042254 GO:0006412
GO:0007165 GO:0004842 GO:0003735 GO:0005179 GO:0005840
GO:0005622 GO:0005576
Cluster-22178.41699 GO:0016021
Cluster-22178.41717 GO:0019079 GO:0005524 GO:0016787 GO:0003677
GO:0008026 GO:0003676
Cluster-22178.41718 GO:0006418 GO:0019079 GO:0005524 GO:0003676
GO:0004812 GO:0016787 GO:0000166 GO:0008026 GO:0003677
Cluster-22178.41722 GO:0016070 GO:0016032 GO:0006355 GO:0003677
GO:0019012
Cluster-22178.41725 GO:0006355 GO:0006468 GO:0003677 GO:0004672
GO:0005524 GO:0016773 GO:0016020
Cluster-22178.41730 GO:0006810 GO:0007165 GO:0005215 GO:0008158
GO:0016020
Cluster-22178.41733 GO:0007021 GO:0015631 GO:0051082 GO:0005783
GO:0045298
Cluster-22178.41740 GO:0007165 GO:0009446 GO:0040007 GO:0006508
GO:0008283 GO:0016579 GO:0006807 GO:0005515 GO:0008083
GO:0004843 GO:0004668 GO:0036459 GO:0005125 GO:0016021
Cluster-22178.41743 GO:0007165 GO:0007218 GO:0006260 GO:0030168
GO:0051258 GO:0048870 GO:0005184 GO:0030674 GO:0005102
GO:0031514 GO:0005576 GO:0005577
Cluster-22178.41744 GO:0006397 GO:0048870 GO:0051258 GO:0035307
GO:0007165 GO:0030168 GO:0006260 GO:0003723 GO:0005198
GO:0030674 GO:0016787 GO:0003677 GO:0016887 GO:0005524
GO:0005102 GO:0003724 GO:0031514 GO:0000445 GO:0005882
GO:0071595 GO:0005577
Cluster-22178.41745 GO:0051258 GO:0048870 GO:0035307 GO:0007165

GO:0030168 GO:0006260 GO:0007218 GO:0003723 GO:0005184
GO:0016787 GO:0030674 GO:0003677 GO:0005524 GO:0016887
GO:0003724 GO:0005102 GO:0005576 GO:0031514 GO:0005577
GO:0071595
Cluster-22178.41748 GO:0030570 GO:0005576
Cluster-22178.41749 GO:0016032 GO:0019012
Cluster-22178.41750 GO:0016032 GO:0019012
Cluster-22178.41751 GO:0016032 GO:0019012
Cluster-22178.41759 GO:0006281 GO:0006310 GO:0005524 GO:0043565
GO:0009378 GO:0003677 GO:0005657 GO:0009379
Cluster-22178.41762 GO:0006825 GO:0035434 GO:0005375 GO:0016021
Cluster-22178.41772 GO:0006457 GO:0000413 GO:0003755 GO:0003677

Cluster-22178.41773 GO:0042710
Cluster-22178.41775 GO:0006310 GO:0035556 GO:0003677 GO:0050662
GO:0003824 GO:0003676 GO:0046872 GO:0005634
Cluster-22178.41776 GO:0007165 GO:0007155 GO:0005173 GO:0016020
Cluster-22178.41781 GO:0006468 GO:0005524 GO:0004672
Cluster-22178.41784 GO:0006355 GO:0005515 GO:0003700 GO:0005667
Cluster-22178.41790 GO:0070403
Cluster-22178.41795 GO:0070072 GO:0006486 GO:0008373
Cluster-22178.41796 GO:0007165 GO:0035023 GO:0043087 GO:0005089
GO:0005515
Cluster-22178.41797 GO:0006468 GO:0005524 GO:0004672 GO:0005515

Cluster-22178.4180 GO:0006486 GO:0008373
Cluster-22178.41802 GO:0006508 GO:0039693 GO:0019784
Cluster-22178.41803 GO:0005515
Cluster-22178.41804 GO:0019497 GO:0006771 GO:0046872 GO:0005515
GO:0003993
Cluster-22178.41805 GO:0006281
Cluster-22178.41806 GO:0015986 GO:0015992 GO:0015078 GO:0000276
Cluster-22178.41814 GO:0005515

Cluster-22178.41821 GO:0004867 GO:0003723 GO:0005525 GO:0003724 GO:0035064 GO:0005524 GO:0043531 GO:0016887 GO:0003924 GO:0005576
Cluster-22178.41823 GO:0035556 GO:0006468 GO:0006355 GO:0030255 GO:0005524 GO:0004672 GO:0016773 GO:0005634 GO:0005668 GO:0016020
Cluster-22178.41829 GO:0009069 GO:0006468 GO:0016310 GO:0005524 GO:0005515 GO:0004674
Cluster-22178.41830 GO:0000077 GO:0030896 GO:0019012
Cluster-22178.41831 GO:0006355 GO:0003677 GO:0005507 GO:0003700 GO:0005667 GO:0005634
Cluster-22178.41837 GO:0016055 GO:0006298 GO:0006306 GO:0007275 GO:0007165 GO:0031167 GO:0032775 GO:0005524 GO:0008168 GO:0009007 GO:0005102 GO:0030414 GO:0030983 GO:0005576
Cluster-22178.41839 GO:0051260 GO:0003677 GO:0046872 GO:0016020
Cluster-22178.41845 GO:0006446 GO:0006355 GO:0003743 GO:0003677 GO:0003700 GO:0005507 GO:0003676 GO:0016817 GO:0008168 GO:0005852 GO:0005634 GO:0005667 GO:0005840 GO:0005737
Cluster-22178.41846 GO:0006508 GO:0006810 GO:0006144 GO:0046034 GO:0004190 GO:0004017 GO:0046872 GO:0005524 GO:0042626 GO:0016021
Cluster-22178.41849 GO:0050690 GO:0005537
Cluster-22178.41854 GO:0005515
Cluster-22178.41855 GO:0006891 GO:0009878 GO:0046872 GO:0030173
Cluster-22178.41861 GO:0042967 GO:0046660 GO:0006807 GO:0006397 GO:0006897 GO:0016814 GO:0008270 GO:0008080 GO:0001527 GO:0005634 GO:0016020 GO:0042470
Cluster-22178.41862 GO:0006897 GO:0006397 GO:0046660 GO:0042967 GO:0006807 GO:0008080 GO:0008270 GO:0016814 GO:0042470 GO:0016020 GO:0005634 GO:0001527
Cluster-22178.41863 GO:0006807 GO:0042967 GO:0006897 GO:0008080 GO:0008270 GO:0016814 GO:0001527 GO:0042470 GO:0016020
Cluster-22178.41877 GO:0007165 GO:0019079 GO:0007264 GO:0043087

GO:0005515 GO:0005085
Cluster-22178.41878 GO:0016598 GO:0004057
Cluster-22178.41880 GO:0003676
Cluster-22178.41890 GO:0048193 GO:0007165 GO:0003729 GO:0005096
GO:0005737 GO:0005783
Cluster-22178.41891 GO:0007165 GO:0003729 GO:0005096
Cluster-22178.41906 GO:0002949 GO:0007264 GO:0006576 GO:0098519
GO:0005525 GO:0005524 GO:0003924
Cluster-22178.41911 GO:0016020
Cluster-22178.41916 GO:0019082 GO:0016032
Cluster-22178.41919 GO:0005515
Cluster-22178.41924 GO:0035556 GO:0006508 GO:0005515 GO:0008234
GO:0004197
Cluster-22178.41927 GO:0006270 GO:0006355 GO:0003677 GO:0005789
GO:0016021
Cluster-22178.41931 GO:0008152 GO:0016836 GO:0003824
Cluster-22178.41932 GO:0003677
Cluster-22178.41934 GO:0009401 GO:0008289 GO:0016021
Cluster-22178.41936 GO:0007165
Cluster-22178.41937 GO:0006570 GO:0006470 GO:0055114 GO:0004725
GO:0016730 GO:0005680
Cluster-22178.41941 GO:0006955
Cluster-22178.41942 GO:0009405 GO:0006886 GO:0051382 GO:0051258
GO:0006308 GO:0007165 GO:0006869 GO:0006457 GO:0007155
GO:0006606 GO:0030168 GO:0006357 GO:0001104 GO:0051082
GO:0008289 GO:0004871 GO:0030674 GO:0005102 GO:0008855
GO:0009318 GO:0005576 GO:0000776 GO:0016021 GO:0005801
GO:0016592 GO:0016020 GO:0005577 GO:0016272
Cluster-22178.41950 GO:0035556 GO:0016579 GO:0043087 GO:0007264
GO:0008270 GO:0036459 GO:0005085 GO:0032183 GO:0070140
Cluster-22178.41952 GO:0007264 GO:0005525
Cluster-22178.41956 GO:0006260 GO:0006810 GO:0003887 GO:0001882
GO:0042575 GO:0016020 GO:0016021

Cluster-22178.41960 GO:0005515 GO:0016021
Cluster-22178.41961 GO:0009307 GO:0006308 GO:0055114 GO:0003677
GO:0004497 GO:0016715 GO:0005507 GO:0009036 GO:0009359
Cluster-22178.41965 GO:0008270
Cluster-22178.41970 GO:0009058 GO:0019079 GO:0008152 GO:0003677
GO:0008026 GO:0016787 GO:0003824 GO:0003676 GO:0005524
Cluster-22178.41971 GO:0003676 GO:0008270 GO:0005622
Cluster-22178.41973 GO:0007165 GO:0005509 GO:0005515 GO:0005578
GO:0016020
Cluster-22178.41974 GO:0007165 GO:0005509 GO:0005515 GO:0005578
Cluster-22178.41994 GO:0006508 GO:0004252
Cluster-22178.42003 GO:0007411
Cluster-22178.42004 GO:0007411
Cluster-22178.42005 GO:0006144 GO:0002949 GO:0006260 GO:0007264
GO:0006355 GO:0016887 GO:0046983 GO:0003924 GO:0005524
GO:0046914 GO:0005525 GO:0004114 GO:0008134 GO:0003677
GO:0005667
Cluster-22178.42006 GO:0006144 GO:0006260 GO:0002949 GO:0007264
GO:0006355 GO:0005524 GO:0008270 GO:0046983 GO:0016887
GO:0003677 GO:0046914 GO:0003924 GO:0005525 GO:0004114
GO:0008134 GO:0005667
Cluster-22178.42008 GO:0006118 GO:0045454 GO:0005515 GO:0015035
GO:0009055
Cluster-22178.42018 GO:0006468 GO:0016773 GO:0004672 GO:0005524
GO:0005515 GO:0016020 GO:0019031
Cluster-22178.42019 GO:0046872
Cluster-22178.4202 GO:0055114 GO:0003824 GO:0016020
Cluster-22178.42021 GO:0006998 GO:0006406 GO:0006611 GO:0031965
Cluster-22178.42022 GO:0051537 GO:0005515 GO:0005509 GO:0043231
Cluster-22178.42028 GO:0006355 GO:0007165 GO:0043248 GO:0035257
GO:0005515 GO:0003713 GO:0046983 GO:0005634 GO:0005667
Cluster-22178.42029 GO:0009512 GO:0016021
Cluster-22178.4203 GO:0055114 GO:0003824 GO:0016020

Cluster-22178.42030 GO:0016310 GO:0000160 GO:0005975 GO:0007259
GO:0001700 GO:0015992 GO:0015986 GO:0015078 GO:0030248
GO:0004553 GO:0046983 GO:0000155 GO:0009365 GO:0016021
GO:0045263 GO:0005576
Cluster-22178.42032 GO:0006886 GO:0042967 GO:0006505 GO:0006629
GO:0016788 GO:0008374
Cluster-22178.42033 GO:0007165 GO:0055114 GO:0005515 GO:0051920
GO:0008270 GO:0019013
Cluster-22178.42034 GO:0007165
Cluster-22178.42036 GO:0006486 GO:0008378 GO:0016021 GO:0016020
Cluster-22178.42039 GO:0006486 GO:0008378 GO:0016020 GO:0016021
Cluster-22178.42041 GO:0016021
Cluster-22178.42049 GO:0006811 GO:0055085 GO:0005216 GO:0005515
GO:0016020
Cluster-22178.42050 GO:0006886 GO:0016192 GO:0005515 GO:0008270

Cluster-22178.42054 GO:0006118 GO:0006810 GO:0015979 GO:0005506
GO:0020037 GO:0005524 GO:0042626 GO:0009055 GO:0031361
GO:0016021
Cluster-22178.42064 GO:0042981
Cluster-22178.42065 GO:0006355 GO:0008270 GO:0043565 GO:0003682
GO:0003700 GO:0005667 GO:0000785
Cluster-22178.42067 GO:0004190 GO:0016021
Cluster-22178.42068 GO:0006570 GO:0006470 GO:0005515 GO:0004725
GO:0008138 GO:0005634
Cluster-22178.42070 GO:0016485 GO:0005975 GO:0043085 GO:0035091
GO:0031419 GO:0005515 GO:0016773 GO:0046872 GO:0016021
Cluster-22178.42077 GO:0006355 GO:0003676 GO:0005622
Cluster-22178.42079 GO:0005515 GO:0016020
Cluster-22178.42080 GO:0006355 GO:0006289 GO:0000439
Cluster-22178.42081 GO:0005515
Cluster-22178.42083 GO:0000160 GO:0016310 GO:0006935 GO:0005515
GO:0004673 GO:0000155 GO:0009365 GO:0005737

Cluster-22178.42086 GO:0043087 GO:0035023 GO:0005089
Cluster-22178.42087 GO:0035023 GO:0043087 GO:0005089
Cluster-22178.42088 GO:0006468 GO:0005524 GO:0004672
Cluster-22178.42100 GO:0005515 GO:0000228
Cluster-22178.42104 GO:0006351 GO:0000917 GO:0035556 GO:0043093
GO:0005515 GO:0008270 GO:0005737
Cluster-22178.42112 GO:0006355 GO:0003677
Cluster-22178.42117 GO:0007165 GO:0007275 GO:0030168 GO:0006606
GO:0030178 GO:0001558 GO:0048870 GO:0005975 GO:0051258
GO:0030674 GO:0030246 GO:0005520 GO:0008270 GO:0004553
GO:0005102 GO:0051082 GO:0005515 GO:0016942 GO:0005577
GO:0005576 GO:0031514 GO:0016021
Cluster-22178.42121 GO:0005515
Cluster-22178.42127 GO:0008270
Cluster-22178.42131 GO:0007166 GO:0003677 GO:0005515 GO:0046983
GO:0016020
Cluster-22178.42133 GO:0006928 GO:0015992 GO:0015991 GO:0006355
GO:0055114 GO:0000160 GO:0016310 GO:0004673 GO:0000155
GO:0005509 GO:0016491 GO:0005515 GO:0003700 GO:0015078
GO:0005667 GO:0009279 GO:0033179 GO:0009365
Cluster-22178.42135 GO:0008152 GO:0003824
Cluster-22178.42136 GO:0006096 GO:0006094 GO:0006144 GO:0015976
GO:0004743 GO:0030955 GO:0000287 GO:0046872
Cluster-22178.42137 GO:0015976 GO:0006144 GO:0006096 GO:0006094
GO:0046872 GO:0000287 GO:0030955 GO:0004743
Cluster-22178.42139 GO:0019058 GO:0019915 GO:0003676 GO:0005524
GO:0003774 GO:0019031 GO:0005789 GO:0016459
Cluster-22178.42143 GO:0007156 GO:0005509 GO:0016020
Cluster-22178.42146 GO:0007165 GO:0008283 GO:0040007 GO:0005515
GO:0008083 GO:0004386 GO:0019031
Cluster-22178.42161 GO:0006525 GO:0019089 GO:0006560 GO:0006357
GO:0006420 GO:0001104 GO:0004814 GO:0005524 GO:0016592
Cluster-22178.42162 GO:0008152 GO:0055114 GO:0003824 GO:0016491

GO:0016836
Cluster-22178.42167 GO:0045490 GO:0047487
Cluster-22178.42170 GO:0007268 GO:0007165 GO:0015992 GO:0006123
GO:0055085 GO:0006811 GO:0005216 GO:0005515 GO:0004129
GO:0004970 GO:0016020 GO:0045277
Cluster-22178.42172 GO:0006629
Cluster-22178.42175 GO:0006357 GO:0006811 GO:0006813 GO:0006446
GO:0005249 GO:0001104 GO:0003743 GO:0005737 GO:0005840
GO:0016592 GO:0005852 GO:0008076 GO:0016020
Cluster-22178.42177 GO:0005515
Cluster-22178.42179 GO:0007165 GO:0009069 GO:0006914 GO:0006468
GO:0016310 GO:0048870 GO:0004674 GO:0005096 GO:0005198
GO:0042803 GO:0008134 GO:0045502 GO:0008092 GO:0005882
GO:0005667 GO:0019898 GO:0030286 GO:0031514 GO:0019028
GO:0019031 GO:0005737
Cluster-22178.42181 GO:0006406 GO:0007264 GO:0005524 GO:0016887
GO:0005525 GO:0003723 GO:0005737 GO:0005634
Cluster-22178.42183 GO:0016192 GO:0006904
Cluster-22178.42184 GO:0006955 GO:0030153 GO:0015643 GO:0019814
Cluster-22178.42188 GO:0071630 GO:0031144 GO:0030212 GO:0003677
GO:0004867 GO:0008270 GO:0005634
Cluster-22178.42189 GO:0007186 GO:0004930 GO:0016021
Cluster-22178.42199 GO:0006355 GO:0005515 GO:0016021 GO:0005634
Cluster-22178.42209 GO:0005509 GO:0016020
Cluster-22178.42210 GO:0035091
Cluster-22178.42216 GO:0006468 GO:0004672 GO:0005524 GO:0016773
GO:0016020
Cluster-22178.42217 GO:0006313 GO:0046872 GO:0008270 GO:0004867
GO:0003677 GO:0005515 GO:0004803
Cluster-22178.42220 GO:0006468 GO:0005524 GO:0004672
Cluster-22178.42224 GO:0019058 GO:0051539 GO:0019031
Cluster-22178.42228 GO:0008152 GO:0003824
Cluster-22178.42232 GO:0045490 GO:0005515 GO:0047487

Cluster-22178.42236 GO:0006355 GO:0007623 GO:0005737 GO:0005634
Cluster-22178.42238 GO:0001522 GO:0009451 GO:0003723 GO:0009982

Cluster-22178.42250 GO:0008270 GO:0003676
Cluster-22178.42251 GO:0008270 GO:0003676
Cluster-22178.42252 GO:0008270 GO:0003676
Cluster-22178.42257 GO:0006570 GO:0006470 GO:0008138 GO:0004725

Cluster-22178.42260 GO:0006013 GO:0005975 GO:0006886 GO:0008270
GO:0008536 GO:0004553 GO:0003824 GO:0004559 GO:0015923
Cluster-22178.42264 GO:0016032 GO:0006508 GO:0004197
Cluster-22178.42267 GO:0006694 GO:0006304 GO:0030001 GO:0016125
GO:0047750 GO:0046872 GO:0003677 GO:0016021
Cluster-22178.42271 GO:0006355 GO:0003700 GO:0003677 GO:0043565
GO:0016021 GO:0005634 GO:0005667
Cluster-22178.42272 GO:0006355 GO:0043565 GO:0003677 GO:0003700
GO:0005667 GO:0005634 GO:0016021
Cluster-22178.42273 GO:0007218 GO:0007165 GO:0006355 GO:0003700
GO:0005102 GO:0043565 GO:0005184 GO:0003677 GO:0016021
GO:0005667 GO:0005634
Cluster-22178.42274 GO:0046872
Cluster-22178.42278 GO:0005515
Cluster-22178.42281 GO:0015031 GO:0016021
Cluster-22178.42282 GO:0035556 GO:0005515
Cluster-22178.42285 GO:0048193 GO:0005515 GO:0005783
Cluster-22178.42286 GO:0009878 GO:0005515 GO:0008270 GO:0046872
GO:0046983
Cluster-22178.42289 GO:0006269 GO:0006351 GO:0005506 GO:0046872
GO:0008270 GO:0004386 GO:0003896 GO:0005730 GO:0005657
Cluster-22178.42290 GO:0009271 GO:0006355 GO:0016021
Cluster-22178.42291 GO:0006950 GO:0007186 GO:0004930
Cluster-22178.42294 GO:0055114 GO:0006464 GO:0008113
Cluster-22178.42296 GO:0055114 GO:0008152 GO:0016491

Cluster-22178.42299 GO:0006914 GO:0000921 GO:0051607 GO:0007155
GO:0006457 GO:0006308 GO:0000077 GO:0071816 GO:0006468
GO:0006886 GO:0016773 GO:0005524 GO:0016888 GO:0019905
GO:0004672 GO:0051082 GO:0016272 GO:0005940 GO:0016020
GO:0005783 GO:0016021
Cluster-22178.42306 GO:0006355 GO:0003700 GO:0043565 GO:0005667
GO:0005634
Cluster-22178.42308 GO:0035556
Cluster-22178.42311 GO:0030036 GO:0005975 GO:0005515 GO:0017048
GO:0003779 GO:0004649
Cluster-22178.42314 GO:0016787
Cluster-22178.42315 GO:0006814 GO:0006120 GO:0007031 GO:0006744
GO:0055114 GO:0015992 GO:0008137 GO:0005524 GO:0019031
GO:0005777
Cluster-22178.42316 GO:0005515
Cluster-22178.42317 GO:0015031 GO:0016021
Cluster-22178.42321 GO:0006351 GO:0055114 GO:0008033 GO:0006810
GO:0007165 GO:0006269 GO:0006260 GO:0055085 GO:0005515
GO:0003896 GO:0003677 GO:0005509 GO:0050660 GO:0042626
GO:0005524 GO:0008270 GO:0016491 GO:0005578 GO:0016021
GO:0005730 GO:0005657
Cluster-22178.42323 GO:0006935 GO:0055114 GO:0006468 GO:0035556
GO:0030472 GO:0071973 GO:0004672 GO:0016779 GO:0005515
GO:0047134 GO:0005524 GO:0003774 GO:0016773 GO:0009288
GO:0042729 GO:0072686 GO:0016020
Cluster-22178.42325 GO:0051726 GO:0005634
Cluster-22178.42333 GO:0046872 GO:0000166
Cluster-22178.42336 GO:0006260 GO:0005975 GO:0006397 GO:0006810
GO:0016758 GO:0008092 GO:0019898 GO:0000445 GO:0005737
GO:0005886 GO:0016020
Cluster-22178.42338 GO:0017004 GO:0006118 GO:0019054 GO:0009405
GO:0007155 GO:0019062 GO:0005515 GO:0004857 GO:0045158
GO:0009512

Cluster-22178.42339 GO:0006118 GO:0017004 GO:0019058 GO:0005515
GO:0045158 GO:0009512
Cluster-22178.42342 GO:0006355 GO:0042073 GO:0003677 GO:0003682
GO:0008270 GO:0005634 GO:0000775 GO:0032040 GO:0000785
Cluster-22178.42343 GO:0046486 GO:0009058 GO:0008152 GO:0016042
GO:0016787 GO:0016788 GO:0003847 GO:0008247
Cluster-22178.42346 GO:0006109 GO:0006402 GO:0003723
Cluster-22178.42347 GO:0015979 GO:0008270 GO:0009523 GO:0016021
Cluster-22178.42348 GO:0006109 GO:0006402 GO:0003723
Cluster-22178.42360 GO:0032979 GO:0042578 GO:0005743
Cluster-22178.42364 GO:0005515
Cluster-22178.42365 GO:0003682 GO:0005515 GO:0000785
Cluster-22178.42371 GO:0016788 GO:0016881
Cluster-22178.42374 GO:0009058 GO:0055114 GO:0006520 GO:0016491
GO:0030170 GO:0016829
Cluster-22178.42378 GO:0016567 GO:0006810 GO:0004842 GO:0046872
GO:0008270 GO:0005515 GO:0030288 GO:0005680
Cluster-22178.42380 GO:0007264 GO:0006406 GO:0003723 GO:0016887
GO:0005524 GO:0005525 GO:0005634 GO:0005737
Cluster-22178.42388 GO:0007165 GO:0005158 GO:0005899
Cluster-22178.42390 GO:0006576 GO:0015684 GO:0015889 GO:0031419
GO:0016887 GO:0003924 GO:0005524 GO:0015093 GO:0005525
GO:0016021
Cluster-22178.42395 GO:0007186 GO:0004930 GO:0046872 GO:0016021
Cluster-22178.42396 GO:0007224
Cluster-22178.42397 GO:0007186 GO:0046872 GO:0004930 GO:0016021
Cluster-22178.42398 GO:0007224
Cluster-22178.42399 GO:0007224
Cluster-22178.42400 GO:0007224
Cluster-22178.42402 GO:0000917 GO:0043093 GO:0005737
Cluster-22178.4242 GO:0055085 GO:0071585 GO:0010273 GO:0005507
GO:0046870 GO:0016021
Cluster-22178.42420 GO:0006355

Cluster-22178.42422 GO:0006281 GO:0006144 GO:0006464 GO:0046034
GO:0035556 GO:0004017 GO:0008270 GO:0046872 GO:0016151
Cluster-22178.42423 GO:0006355 GO:0042254 GO:0046872 GO:0043565
GO:0003700 GO:0003735 GO:0005840 GO:0005667 GO:0005763
Cluster-22178.42426 GO:0005515
Cluster-22178.42427 GO:0007165
Cluster-22178.4243 GO:0010273 GO:0071585 GO:0055085 GO:0046870
GO:0005507 GO:0016021
Cluster-22178.42435 GO:0006816 GO:0000746 GO:0006810 GO:0005246
GO:0042030 GO:0003677 GO:0016020
Cluster-22178.42436 GO:0006810 GO:0006816 GO:0005246 GO:0042030
GO:0016020
Cluster-22178.42452 GO:0016757
Cluster-22178.42454 GO:0006835 GO:0006820 GO:0006812 GO:0017153
GO:0016020 GO:0016021
Cluster-22178.42457 GO:0016021
Cluster-22178.42458 GO:0016021
Cluster-22178.42459 GO:0016021
Cluster-22178.42460 GO:0007049
Cluster-22178.42462 GO:0007155
Cluster-22178.42467 GO:0016567 GO:0019058 GO:0006464 GO:0005198
GO:0005515 GO:0004842 GO:0016151 GO:0046872 GO:0008270
GO:0005680
Cluster-22178.42468 GO:0007165 GO:0006468 GO:0005515 GO:0003677
GO:0016787 GO:0016773 GO:0004672 GO:0008601 GO:0005524
GO:0016020 GO:0000159
Cluster-22178.42470 GO:0006464 GO:0008152 GO:0046500 GO:0006479
GO:0004719 GO:0008168 GO:0003724 GO:0003723
Cluster-22178.42471 GO:0006355 GO:0006351 GO:0007623 GO:0043565
GO:0003700 GO:0003677 GO:0005667 GO:0005634
Cluster-22178.42476 GO:0006508 GO:0007339 GO:0009405 GO:0006928
GO:0006144 GO:0007155 GO:0007165 GO:0005173 GO:0003968
GO:0005198 GO:0005515 GO:0017111 GO:0004197 GO:0031379

GO:0005634 GO:0005576 GO:0016020
Cluster-22178.42480 GO:0010181
Cluster-22178.42481 GO:0010181
Cluster-22178.42484 GO:0008610 GO:0008152 GO:0016747
Cluster-22178.42488 GO:0015031 GO:0006206 GO:0006139 GO:0006606
GO:0008565 GO:0016887 GO:0005524 GO:0004127 GO:0005737
GO:0005634
Cluster-22178.42495 GO:0055114 GO:0008615 GO:0010181 GO:0004733

Cluster-22178.42496 GO:0007010 GO:0006479 GO:0006481 GO:0004671
GO:0003779 GO:0016021
Cluster-22178.42497 GO:0003676 GO:0003677 GO:0016787 GO:0005524

Cluster-22178.42499 GO:0007339 GO:0005515 GO:0005576 GO:0016020
Cluster-22178.42500 GO:0007339 GO:0005515 GO:0005576 GO:0016020
Cluster-22178.42504 GO:0006355 GO:0003677
Cluster-22178.42505 GO:0008270 GO:0005515 GO:0003677
Cluster-22178.42507 GO:0005515
Cluster-22178.42510 GO:0008152 GO:0042254 GO:0006412 GO:0006355
GO:0003824 GO:0008270 GO:0003735 GO:0005840 GO:0005634
Cluster-22178.42514 GO:0003677 GO:0046872
Cluster-22178.42526 GO:0003677 GO:0046872
Cluster-22178.42528 GO:0042967 GO:0007156 GO:0003677 GO:0008811
GO:0005515 GO:0005509 GO:0016020
Cluster-22178.42529 GO:0046872 GO:0046983 GO:0019012 GO:0016021
Cluster-22178.4253 GO:0051252 GO:0006260 GO:0003887 GO:0004523
GO:0042575
Cluster-22178.42533 GO:0016020
Cluster-22178.42535 GO:0004222
Cluster-22178.42543 GO:0046740 GO:0016021
Cluster-22178.42544 GO:0030414 GO:0005576
Cluster-22178.42546 GO:0006812 GO:0055085 GO:0008324 GO:0016021
Cluster-22178.42551 GO:0008017 GO:0045298

Cluster-22178.42554 GO:0016791
Cluster-22178.42559 GO:0007034
Cluster-22178.42560 GO:0008209 GO:0008207 GO:0008210 GO:0006694
GO:0008152 GO:0055114 GO:0016616 GO:0003854 GO:0050662
GO:0003824 GO:0016491
Cluster-22178.42561 GO:0000398 GO:0003677 GO:0005681
Cluster-22178.42562 GO:0005515 GO:0003824 GO:0016020
Cluster-22178.42565 GO:0000372 GO:0007005 GO:0003677 GO:0016787
GO:0003676 GO:0005524 GO:0016887 GO:0032865 GO:0030529
Cluster-22178.42572 GO:0016192 GO:0005515 GO:0016021
Cluster-22178.42573 GO:0016021
Cluster-22178.42582 GO:0051260 GO:0005515 GO:0016020
Cluster-22178.42584 GO:0006355 GO:0043130 GO:0003700 GO:0005622
GO:0005667
Cluster-22178.42587 GO:0020037 GO:0005515
Cluster-22178.42591 GO:0005515
Cluster-22178.42596 GO:0006879 GO:0046872 GO:0008199
Cluster-22178.42598 GO:0016310 GO:0009069 GO:0004674
Cluster-22178.42599 GO:0016032
Cluster-22178.42604 GO:0046872
Cluster-22178.42607 GO:0006464 GO:0006888 GO:0006886 GO:0008270
GO:0046872 GO:0016151 GO:0030127
Cluster-22178.42612 GO:0006810 GO:0019031 GO:0016020
Cluster-22178.42613 GO:0031412 GO:0006606 GO:0006914 GO:0019064
GO:0051382 GO:0010508 GO:0003677 GO:0019901 GO:0008134
GO:0042803 GO:0045502 GO:0005815 GO:0030286 GO:0005667
GO:0031411 GO:0019031 GO:0000776
Cluster-22178.42618 GO:0016032 GO:0016020 GO:0016021
Cluster-22178.42622 GO:0005515
Cluster-22178.42627 GO:0055085 GO:0006811 GO:0006000 GO:0005975
GO:0006013 GO:0008270 GO:0005216 GO:0004476 GO:0004064
GO:0019031 GO:0016020
Cluster-22178.42639 GO:0006144 GO:0004114 GO:0016020

Cluster-22178.42640 GO:0005515
Cluster-22178.42641 GO:0008152 GO:0016746
Cluster-22178.42642 GO:0055070 GO:0005507
Cluster-22178.42647 GO:0046034 GO:0035556 GO:0006144 GO:0006464
GO:0016151 GO:0046872 GO:0008270 GO:0004017
Cluster-22178.42651 GO:0015994 GO:0033014 GO:0005515 GO:0004418

Cluster-22178.42669 GO:0005576
Cluster-22178.42670 GO:0016021
Cluster-22178.42674 GO:0004252 GO:0005515 GO:0070008
Cluster-22178.42675 GO:0006351 GO:0008270 GO:0003677 GO:0005634
Cluster-22178.42678 GO:0055036
Cluster-22178.42681 GO:0030472 GO:0005515 GO:0003677 GO:0042729
GO:0072686
Cluster-22178.42683 GO:0000723 GO:0006281 GO:0055085 GO:0007165
GO:0006810 GO:0003924 GO:0043531 GO:0003676 GO:0003678
GO:0005525 GO:0005515 GO:0016301 GO:0000166 GO:0016887
GO:0005524 GO:0008270 GO:0042626 GO:0003677 GO:0098519
GO:0016021 GO:0005657 GO:0016020
Cluster-22178.42687 GO:0006260 GO:0006308 GO:0016567 GO:0006281
GO:0005525 GO:0004842 GO:0017108 GO:0008270 GO:0005515
GO:0003723 GO:0033557 GO:0005634
Cluster-22178.42691 GO:0006260 GO:0003677 GO:0005634
Cluster-22178.42692 GO:0005515
Cluster-22178.42693 GO:0006418 GO:0043039 GO:0003676 GO:0005524
GO:0000166 GO:0000049 GO:0004812 GO:0005737
Cluster-22178.42698 GO:0048870 GO:0048309 GO:0007049 GO:0006355
GO:0015991 GO:0000917 GO:0010508 GO:0015992 GO:0051028
GO:0070206 GO:0006914 GO:0006260 GO:0006606 GO:0043093
GO:0006364 GO:0043565 GO:0015078 GO:0003700 GO:0051082
GO:0016853 GO:0005737 GO:0000775 GO:0016021 GO:0005634
GO:0031514 GO:0033179 GO:0005815 GO:0005667
Cluster-22178.42705 GO:0009405 GO:0006468 GO:0005198 GO:0005515

GO:0004190 GO:0004672 GO:0005524 GO:0016773 GO:0016020
GO:0016021
Cluster-22178.42709 GO:0050829 GO:0019835 GO:0006260 GO:0016021
Cluster-22178.42711 GO:0019497 GO:0006508 GO:0006771 GO:0008242
GO:0046872 GO:0003993 GO:0016740 GO:0005515 GO:0004197
Cluster-22178.42713 GO:0007186 GO:0004930 GO:0016021
Cluster-22178.42718 GO:0006811 GO:0007165 GO:0007268 GO:0006508
GO:0005234 GO:0008233 GO:0004970 GO:0005524 GO:0016021
GO:0016020
Cluster-22178.42727 GO:0006032 GO:0005515 GO:0008061
Cluster-22178.42730 GO:0030683 GO:0044386 GO:0005783
Cluster-22178.42731 GO:0006397 GO:0006355 GO:0006470 GO:0042981
GO:0003677 GO:0005507 GO:0003700 GO:0004721 GO:0005667
GO:0005634
Cluster-22178.42732 GO:0005515
Cluster-22178.42737 GO:0009058 GO:0006813 GO:0006687 GO:0001733
GO:0046872 GO:0008146 GO:0005242 GO:0005794 GO:0016021
GO:0005840 GO:0008076
Cluster-22178.42743 GO:0035556 GO:0009082 GO:0006468 GO:0005515
GO:0005524 GO:0004672
Cluster-22178.42748 GO:0007186 GO:0035023 GO:0035556 GO:0055114
GO:0043087 GO:0005515 GO:0005089 GO:0047134 GO:0004930
GO:0016020
Cluster-22178.42749 GO:0035556 GO:0035023 GO:0043087 GO:0055114
GO:0007186 GO:0047134 GO:0004930 GO:0005089 GO:0005515
GO:0016020
Cluster-22178.42750 GO:0043087 GO:0055114 GO:0035556 GO:0035023
GO:0007186 GO:0004930 GO:0047134 GO:0005089 GO:0005515
GO:0016020
Cluster-22178.42754 GO:0007186 GO:0035023 GO:0035556 GO:0055114
GO:0043087 GO:0005515 GO:0005089 GO:0047134 GO:0004930
GO:0016020
Cluster-22178.42758 GO:0006352 GO:0006470 GO:0008138 GO:0005861

GO:0005669
Cluster-22178.42759 GO:0023052
Cluster-22178.42763 GO:0055085 GO:0006810 GO:0005524 GO:0042626
GO:0016021
Cluster-22178.42764 GO:0006614 GO:0016301 GO:0003723 GO:0003724
GO:0005525 GO:0003924 GO:0016887 GO:0005524
Cluster-22178.42765 GO:0005515
Cluster-22178.42766 GO:0005515
Cluster-22178.42767 GO:0030420
Cluster-22178.42771 GO:0007165 GO:0007186 GO:0006468 GO:0007264
GO:0006576 GO:0006777 GO:0005515 GO:0005525 GO:0004871
GO:0004672 GO:0005524 GO:0031683 GO:0019001 GO:0003924
Cluster-22178.42774 GO:0006310 GO:0006260 GO:0006281 GO:0006355
GO:0003677 GO:0009378 GO:0008191 GO:0016787 GO:0003724
GO:0005524 GO:0008270 GO:0016887 GO:0008134 GO:0005515
GO:0003697 GO:0003723 GO:0003678 GO:0009379 GO:0005667
GO:0005657 GO:0042025
Cluster-22178.42776 GO:0007165
Cluster-22178.4278 GO:0007155 GO:0006298 GO:0005524 GO:0030983
Cluster-22178.42781 GO:0006468 GO:0005524 GO:0004672
Cluster-22178.42782 GO:0006396 GO:0003723 GO:0016779
Cluster-22178.42783 GO:0008270
Cluster-22178.42784 GO:0042254 GO:0006412 GO:0005515 GO:0003735
GO:0005840 GO:0005622
Cluster-22178.42785 GO:0034080 GO:0008270 GO:0000775
Cluster-22178.42794 GO:0007165 GO:0005179 GO:0005887 GO:0005576
Cluster-22178.42796 GO:0042254 GO:0006570 GO:0006412 GO:0006470
GO:0003735 GO:0004725 GO:0005840
Cluster-22178.42799 GO:0006486 GO:0003677 GO:0008373
Cluster-22178.42801 GO:0042254 GO:0006412 GO:0003735 GO:0046872
GO:0005840 GO:0005622
Cluster-22178.42803 GO:0005975
Cluster-22178.42806 GO:0006807 GO:0006683 GO:0006687 GO:0005975

GO:0004336
Cluster-22178.42809 GO:0007268 GO:0005515 GO:0043495 GO:0033130

Cluster-22178.42810 GO:0055114 GO:0015992 GO:0006123 GO:0009060
GO:0006118 GO:0010508 GO:0004129 GO:0009055 GO:0005506
GO:0020037 GO:0016021 GO:0045277
Cluster-22178.42812 GO:0005509 GO:0005515
Cluster-22178.42815 GO:0006914 GO:0007155 GO:0010508
Cluster-22178.42818 GO:0008270
Cluster-22178.42824 GO:0005515 GO:0046872 GO:0008270
Cluster-22178.42828 GO:0042754 GO:0006508 GO:0045892 GO:0004222
GO:0008270 GO:0031012
Cluster-22178.42832 GO:0008152 GO:0016746 GO:0019028
Cluster-22178.42834 GO:0006355 GO:0043401 GO:0008270 GO:0043565
GO:0003700 GO:0005667 GO:0005634
Cluster-22178.42835 GO:0006355 GO:0043401 GO:0043565 GO:0008270
GO:0003700 GO:0005667 GO:0005634
Cluster-22178.42836 GO:0006355 GO:0043401 GO:0043565 GO:0008270
GO:0003700 GO:0005634 GO:0005667
Cluster-22178.42838 GO:0009252 GO:0046436 GO:0006750 GO:0004363
GO:0008716 GO:0046872 GO:0005524
Cluster-22178.42841 GO:0006468 GO:0003677 GO:0008270 GO:0005524
GO:0004672 GO:0016773 GO:0016772 GO:0016020
Cluster-22178.42843 GO:0005515
Cluster-22178.42845 GO:0015986 GO:0042254 GO:0015992 GO:0035556
GO:0006355 GO:0006119 GO:0006412 GO:0003677 GO:0046961
GO:0003735 GO:0008270 GO:0046933 GO:0045259 GO:0045261
GO:0005622 GO:0005840
Cluster-22178.42854 GO:0016021
Cluster-22178.42855 GO:0016021
Cluster-22178.42865 GO:0015994 GO:0006779 GO:0055114 GO:0004109
GO:0005856
Cluster-22178.42867 GO:0030071 GO:0051028 GO:0005515 GO:0005680

GO:0005634
Cluster-22178.42868 GO:0006412 GO:0042254 GO:0003735 GO:0005622
GO:0005840
Cluster-22178.42872 GO:0043123 GO:0043066 GO:0045766
Cluster-22178.42876 GO:0006465 GO:0008233 GO:0005787 GO:0016021
Cluster-22178.42879 GO:0006810 GO:0006816 GO:0005246 GO:0042030
GO:0016020
Cluster-22178.42885 GO:0019497 GO:0006771 GO:0046872 GO:0003993
GO:0005515
Cluster-22178.42887 GO:0055085 GO:0022857 GO:0016021
Cluster-22178.4289 GO:0006508 GO:0004252
Cluster-22178.42891 GO:0042157 GO:0019915 GO:0007165 GO:0006869
GO:0008289 GO:0005576 GO:0008537 GO:0005789
Cluster-22178.42892 GO:0008537
Cluster-22178.42894 GO:0023052
Cluster-22178.42899 GO:0007186 GO:0004930 GO:0016021
Cluster-22178.42901 GO:0007165 GO:0008601 GO:0000159 GO:0019031
Cluster-22178.42915 GO:0006206 GO:0006144 GO:0006351 GO:0003677
GO:0003899 GO:0005730
Cluster-22178.42917 GO:0006355 GO:0005515 GO:0046983
Cluster-22178.42919 GO:0005515 GO:0016020
Cluster-22178.42920 GO:0006954 GO:0031167 GO:0007165 GO:0008168
GO:0005125 GO:0005576
Cluster-22178.42936 GO:0006835 GO:0006820 GO:0006812 GO:0017153
GO:0005515 GO:0016020
Cluster-22178.42947 GO:0015684 GO:0006576 GO:0007264 GO:0005525
GO:0003924 GO:0005524 GO:0015093 GO:0016021
Cluster-22178.42949 GO:0006576 GO:0007264 GO:0015684 GO:0003924
GO:0005524 GO:0015093 GO:0005525 GO:0016021
Cluster-22178.42953 GO:0007165 GO:0006955 GO:0005164 GO:0016020
Cluster-22178.42958 GO:0055085 GO:0007606 GO:0006810 GO:0007165
GO:0005524 GO:0042626 GO:0004888 GO:0016021 GO:0016020
Cluster-22178.42960 GO:0043087 GO:0005089 GO:0005737

Cluster-22178.42962 GO:0006468 GO:0004672 GO:0005524 GO:0016773
GO:0016020
Cluster-22178.42964 GO:0006357 GO:0006811 GO:0006813 GO:0006446
GO:0005249 GO:0003743 GO:0001104 GO:0005737 GO:0005840
GO:0016020 GO:0005852 GO:0008076 GO:0016592
Cluster-22178.42967 GO:0005515
Cluster-22178.42974 GO:0015992 GO:0015991 GO:0015078 GO:0005515
GO:0016020 GO:0033179
Cluster-22178.42977 GO:0043248 GO:0006355
Cluster-22178.42981 GO:0030420
Cluster-22178.42984 GO:0015031 GO:0005515
Cluster-22178.42987 GO:0008270 GO:0003676
Cluster-22178.42993 GO:0015992 GO:0005515 GO:0016021
Cluster-22178.43002 GO:0015693 GO:0004222 GO:0015095 GO:0005515
GO:0008233 GO:0016021 GO:0031012
Cluster-22178.43003 GO:0005982 GO:0005985 GO:0006075 GO:0003843
GO:0005515 GO:0016020 GO:0000148
Cluster-22178.43004 GO:0005509
Cluster-22178.43008 GO:0006955
Cluster-22178.43010 GO:0006563 GO:0006566 GO:0046486 GO:0046487
GO:0031388 GO:0006544 GO:0035091 GO:0008887
Cluster-22178.43014 GO:0006810 GO:0006260 GO:0055085 GO:0005524
GO:0016887 GO:0003924 GO:0005525 GO:0003678 GO:0042626
GO:0003676 GO:0005657 GO:0016021
Cluster-22178.43017 GO:0007275 GO:0005201 GO:0005578 GO:0005581
Cluster-22178.43021 GO:0005975 GO:0008270 GO:0004560 GO:0003676

Cluster-22178.43030 GO:0006353 GO:0005739
Cluster-22178.43031 GO:0006450 GO:0005515
Cluster-22178.43032 GO:0016021
Cluster-22178.43033 GO:0046872
Cluster-22178.43037 GO:0042981 GO:0008152 GO:0016757
Cluster-22178.4304 GO:0046872

Cluster-22178.43040 GO:0051382 GO:0006281 GO:0005515 GO:0003712 GO:0005667 GO:0000124
Cluster-22178.43043 GO:0032012 GO:0043087 GO:0005086
Cluster-22178.43049 GO:0003676 GO:0016020
Cluster-22178.43050 GO:0008134 GO:0005667 GO:0005634
Cluster-22178.43054 GO:0007165 GO:0006955 GO:0008283 GO:0040007 GO:0005137 GO:0008083 GO:0005895 GO:0005576
Cluster-22178.43055 GO:0016021
Cluster-22178.4306 GO:0006508 GO:0004198 GO:0005622
Cluster-22178.43070 GO:0006629 GO:0006812 GO:0008324 GO:0008081

Cluster-22178.43071 GO:0006629 GO:0009607 GO:0006812 GO:0008324 GO:0008081 GO:0016021
Cluster-22178.43075 GO:0000160 GO:0016310 GO:0016192 GO:0006886 GO:0000155 GO:0016021 GO:0009365 GO:0005622 GO:0030131
Cluster-22178.43083 GO:0003950
Cluster-22178.43101 GO:0006357 GO:0001104 GO:0016592
Cluster-22178.43105 GO:0007186 GO:0004930 GO:0019031 GO:0016020
Cluster-22178.43107 GO:0007304 GO:0005213 GO:0042600 GO:0005576
Cluster-22178.43117 GO:0006810 GO:0042626 GO:0005524 GO:0016021
Cluster-22178.43133 GO:0009228 GO:0004789
Cluster-22178.43135 GO:0006814 GO:0042998 GO:0015992 GO:0055114 GO:0006744 GO:0006120 GO:0071253 GO:0008137 GO:0003824 GO:0005802
Cluster-22178.43141 GO:0043087 GO:0007165 GO:0040007 GO:0008283 GO:0008083 GO:0005515 GO:0051015 GO:0030674 GO:0005085
Cluster-22178.43143 GO:0006213 GO:0016763
Cluster-22178.43145 GO:0006213 GO:0016763
Cluster-22178.43150 GO:0055085 GO:0016021 GO:0055036
Cluster-22178.43151 GO:0006955 GO:0019882 GO:0016020 GO:0042613
Cluster-22178.4316 GO:0009069 GO:0006468 GO:0016310 GO:0035556 GO:0005515 GO:0016773 GO:0005524 GO:0004672 GO:0004674 GO:0016020

Cluster-22178.43160 GO:0006820 GO:0016021
Cluster-22178.43167 GO:0010508
Cluster-22178.43171 GO:0005515
Cluster-22178.43177 GO:0055114 GO:0016491 GO:0051537 GO:0003676

Cluster-22178.43182 GO:0005975 GO:0008270 GO:0005198 GO:0019028
GO:0005634
Cluster-22178.43184 GO:0051252 GO:0004523 GO:0003676 GO:0003824

Cluster-22178.43186 GO:0046872 GO:0003824
Cluster-22178.43190 GO:0015833 GO:0000166 GO:0005524 GO:0016021
Cluster-22178.43191 GO:0015833 GO:0000166 GO:0005524 GO:0016021
Cluster-22178.43192 GO:0006807 GO:0006396 GO:0006144 GO:0004000
GO:0003723
Cluster-22178.43193 GO:0005515 GO:0005509
Cluster-22178.43196 GO:0006694 GO:0006529 GO:0006522 GO:0006531
GO:0008299 GO:0006118 GO:0017004 GO:0009058 GO:0071771
GO:0045158 GO:0050518 GO:0016779 GO:0004066 GO:0009512
Cluster-22178.43198 GO:0009058 GO:0006952 GO:0043565 GO:0003824
GO:0008289 GO:0005634 GO:0009279
Cluster-22178.43204 GO:0003723 GO:0003676
Cluster-22178.43208 GO:0007165 GO:0005509 GO:0005515 GO:0005578
Cluster-22178.43219 GO:0005515 GO:0015631 GO:0045298
Cluster-22178.43220 GO:0007165 GO:0006468 GO:0006531 GO:0035556
GO:0006522 GO:0042967 GO:0004672 GO:0005524 GO:0005057
GO:0047200 GO:0016773 GO:0016020 GO:0016021 GO:0005856
Cluster-22178.43224 GO:0005975 GO:0016901 GO:0048038
Cluster-22178.43225 GO:0034755 GO:0055114 GO:0006826 GO:0005381
GO:0016706 GO:0008168 GO:0016021
Cluster-22178.43226 GO:0006281 GO:0006310 GO:0006206 GO:0006139
GO:0002949 GO:0009378 GO:0016301 GO:0016791 GO:0005524
GO:0016887 GO:0004127 GO:0009379 GO:0005657
Cluster-22178.43227 GO:0055114 GO:0016616 GO:0005515

Cluster-22178.43230 GO:0071203
Cluster-22178.43231 GO:0007009 GO:0006281 GO:0071203
Cluster-22178.43234 GO:0046872
Cluster-22178.43235 GO:0046872
Cluster-22178.43238 GO:0006367 GO:0046983
Cluster-22178.43239 GO:0046872
Cluster-22178.43240 GO:0007165 GO:0006355 GO:0048193 GO:0004871
GO:0003700 GO:0008270 GO:0016020 GO:0005667 GO:0005634
Cluster-22178.43241 GO:0006468 GO:0005524 GO:0004672 GO:0016773
GO:0016020
Cluster-22178.43242 GO:0006508 GO:0006144 GO:0009058 GO:0003877
GO:0030170 GO:0004252
Cluster-22178.43246 GO:0008270
Cluster-22178.43249 GO:0015727 GO:0007156 GO:0006629 GO:0005509
GO:0004857 GO:0015129 GO:0016020 GO:0042627 GO:0005887
Cluster-22178.43257 GO:0006383 GO:0007155 GO:0042157 GO:0006869
GO:0006206 GO:0006606 GO:0010508 GO:0042262 GO:0006810
GO:0006144 GO:0008053 GO:0006355 GO:0006351 GO:0003690
GO:0003677 GO:0042803 GO:0005198 GO:0003899 GO:0003924
GO:0045502 GO:0008134 GO:0008289 GO:0005730 GO:0005666
GO:0005667 GO:0005741 GO:0005882 GO:0005886 GO:0030286
GO:0005576 GO:0019028 GO:0019031 GO:0016021
Cluster-22178.43260 GO:0005515
Cluster-22178.43261 GO:0055114 GO:0017148 GO:0006355 GO:0006413
GO:0006260 GO:0010506 GO:0006206 GO:0006914 GO:0008998
GO:0003700 GO:0051082 GO:0016491 GO:0005516 GO:0005615
GO:0005845 GO:0031514 GO:0005667
Cluster-22178.43265 GO:0006357 GO:0001104 GO:0016592
Cluster-22178.43266 GO:0004867 GO:0031224
Cluster-22178.43267 GO:0006629 GO:0008081
Cluster-22178.43278 GO:0015992 GO:0015074 GO:0015986 GO:0006364
GO:0015078 GO:0000276 GO:0032040
Cluster-22178.43281 GO:0046983 GO:0003677

Cluster-22178.43283 GO:0007259 GO:0016310 GO:0000160 GO:0005975
GO:0015986 GO:0001700 GO:0015992 GO:0015078 GO:0030248
GO:0000155 GO:0004553 GO:0046983 GO:0045263 GO:0005576
GO:0009365 GO:0016021
Cluster-22178.43284 GO:0008270
Cluster-22178.43285 GO:0061024 GO:0003677 GO:0046872 GO:0008270
GO:0030176
Cluster-22178.43286 GO:0006308 GO:0009056 GO:0008270 GO:0016888

Cluster-22178.43294 GO:0006468 GO:0005524 GO:0004672
Cluster-22178.43295 GO:0006468 GO:0005524 GO:0004672 GO:0016773
GO:0016020
Cluster-22178.43297 GO:0007165 GO:0005515 GO:0032266 GO:0043130

Cluster-22178.43298 GO:0016787 GO:0046872 GO:0015016 GO:0008146

Cluster-22178.43299 GO:0016070 GO:0016032 GO:0006355 GO:0006886
GO:0016192 GO:0006810 GO:0009405 GO:0005198 GO:0005515
GO:0019871 GO:0003677 GO:0016779 GO:0005576 GO:0030117
GO:0019012
Cluster-22178.43300 GO:0005515
Cluster-22178.43301 GO:0019836 GO:0022904 GO:0005576 GO:0016020
GO:0016021
Cluster-22178.43306 GO:0055114 GO:0008152 GO:0050662 GO:0016491
GO:0003824
Cluster-22178.43307 GO:0005515
Cluster-22178.43321 GO:0005524 GO:0016773 GO:0016020
Cluster-22178.43327 GO:0000186 GO:0001678 GO:0032099 GO:0007186
GO:0008343 GO:0009267 GO:0005615
Cluster-22178.43329 GO:0006468 GO:0005524 GO:0004672
Cluster-22178.43331 GO:0016192 GO:0006886 GO:0005515 GO:0030117
Cluster-22178.43334 GO:0006633 GO:0016790
Cluster-22178.43337 GO:0005975 GO:0008608 GO:0006118 GO:0008270

GO:0008440 GO:0004553 GO:0030246 GO:0003954 GO:0005576
GO:0005739 GO:0072686 GO:0042729
Cluster-22178.43342 GO:0005515
Cluster-22178.43345 GO:0006952 GO:0005515
Cluster-22178.43347 GO:0045502 GO:0042803 GO:0008134 GO:0005667
GO:0030286 GO:0016020
Cluster-22178.43350 GO:0006955 GO:0007165 GO:0040007 GO:0008283
GO:0008083 GO:0005179 GO:0005134 GO:0005576 GO:0005893
Cluster-22178.43351 GO:0006338 GO:0005524 GO:0016818 GO:0003676
GO:0005634
Cluster-22178.43352 GO:0000774 GO:0005515 GO:0005783
Cluster-22178.43355 GO:0005515
Cluster-22178.43358 GO:0005515
Cluster-22178.43360 GO:0006355 GO:0009271
Cluster-22178.43365 GO:0007010 GO:0006270 GO:0006355 GO:0005515
GO:0003779
Cluster-22178.43381 GO:0006468 GO:0016773 GO:0005524 GO:0004672
GO:0016020
Cluster-22178.4339 GO:0006355 GO:0003700 GO:0005667 GO:0042025
Cluster-22178.43391 GO:0009058 GO:0006144 GO:0006508 GO:0004252
GO:0030170 GO:0003877
Cluster-22178.43392 GO:0006508 GO:0006144 GO:0004252 GO:0003877

Cluster-22178.43397 GO:0006353 GO:0005739
Cluster-22178.43402 GO:0006525 GO:0055114 GO:0006560 GO:0018401
GO:0016702 GO:0004656 GO:0005783
Cluster-22178.43404 GO:0015992 GO:0015991 GO:0015078 GO:0033177
Cluster-22178.43409 GO:0006470 GO:0006570 GO:0008270 GO:0004725
GO:0008138 GO:0004721 GO:0005515
Cluster-22178.4341 GO:0046983
Cluster-22178.43410 GO:0030154 GO:0016021
Cluster-22178.43411 GO:0007067 GO:0072686 GO:0042729
Cluster-22178.43412 GO:0006811 GO:0007016 GO:0005216 GO:0016010

GO:0016020
Cluster-22178.43418 GO:0007165 GO:0019073 GO:0005515 GO:0016021
Cluster-22178.43423 GO:0019684 GO:0008152 GO:0006118 GO:0016758
GO:0005198 GO:0045156 GO:0019031 GO:0016021 GO:0030077
Cluster-22178.43426 GO:0006570 GO:0055114 GO:0004511
Cluster-22178.43430 GO:0005515 GO:0003677 GO:0046872
Cluster-22178.43435 GO:0042254 GO:0006412 GO:0003735 GO:0019843
GO:0005840 GO:0005622
Cluster-22178.43436 GO:0051028 GO:0048309 GO:0007049 GO:0042262
GO:0006355 GO:0043093 GO:0007623 GO:0000917 GO:0043565
GO:0003700 GO:0003690 GO:0003677 GO:0005737 GO:0005667
GO:0005634
Cluster-22178.43445 GO:0006525 GO:0006433 GO:0006560 GO:0005515
GO:0000166 GO:0046872 GO:0005524 GO:0004827 GO:0005737
Cluster-22178.43449 GO:0015948 GO:0008168 GO:0005515
Cluster-22178.43453 GO:0008270
Cluster-22178.43455 GO:0000160 GO:0005515
Cluster-22178.43456 GO:0008152 GO:0016791 GO:0016021
Cluster-22178.43457 GO:0008152 GO:0016491 GO:0005515
Cluster-22178.43458 GO:0055085 GO:0022900 GO:0006810 GO:0016021
GO:0016020 GO:0005747 GO:0005739 GO:0071203
Cluster-22178.43460 GO:0007224 GO:0046872 GO:0008270 GO:0061630
GO:0005515 GO:0005622
Cluster-22178.43463 GO:0005515 GO:0003677 GO:0046872 GO:0019031
Cluster-22178.43467 GO:0019684 GO:0006904 GO:0006118 GO:0016192
GO:0005515 GO:0045156 GO:0016021 GO:0030077
Cluster-22178.43470 GO:0003677 GO:0003676 GO:0005524 GO:0003729
GO:0016787
Cluster-22178.43471 GO:0003729 GO:0016787 GO:0005524 GO:0003676
GO:0003677
Cluster-22178.43475 GO:0006281 GO:0042254 GO:0006412 GO:0006310
GO:0009378 GO:0098519 GO:0003735 GO:0005524 GO:0016887
GO:0003678 GO:0009379 GO:0005657 GO:0005840

Cluster-22178.43479 GO:0032012 GO:0043087 GO:0003924 GO:0005086

Cluster-22178.43481 GO:0006355 GO:0005634

Cluster-22178.43487 GO:0006953 GO:0034364 GO:0005576

Cluster-22178.43493 GO:0045490 GO:0045892 GO:0047487

Cluster-22178.43496 GO:0042967 GO:0008080

Cluster-22178.43500 GO:0008152 GO:0016746

Cluster-22178.43501 GO:0005515 GO:0005509 GO:0005576

Cluster-22178.43510 GO:0007186 GO:0001503 GO:0004930 GO:0016021

Cluster-22178.43521 GO:0006468 GO:0005524 GO:0004672

Cluster-22178.43527 GO:0005515 GO:0008270

Cluster-22178.43528 GO:0006807 GO:0006672 GO:0016811 GO:0016021

Cluster-22178.43529 GO:0005096 GO:0005515

Cluster-22178.43531 GO:0005515 GO:0005096

Cluster-22178.43532 GO:0005096 GO:0005515

Cluster-22178.43533 GO:0005096 GO:0005515

Cluster-22178.43537 GO:0019497 GO:0006771 GO:0008270 GO:0046872 GO:0005515 GO:0003993

Cluster-22178.43538 GO:0006771 GO:0019497 GO:0003993 GO:0005515 GO:0046872 GO:0008270

Cluster-22178.43546 GO:0006836 GO:0055114 GO:0006812 GO:0007165 GO:0006118 GO:0006954 GO:0005125 GO:0009916 GO:0005328 GO:0016021 GO:0005576

Cluster-22178.43549 GO:0051252 GO:0006396 GO:0008608 GO:0003723 GO:0003676 GO:0000175 GO:0042729 GO:0072686

Cluster-22178.43554 GO:0005887

Cluster-22178.43557 GO:0007186 GO:0004930 GO:0016020

Cluster-22178.43559 GO:0055114 GO:0016491 GO:0030151

Cluster-22178.43561 GO:0005982 GO:0005985 GO:0006118 GO:0030683 GO:0030245 GO:0055114 GO:0015031 GO:0005515 GO:0008810 GO:0046983 GO:0016655 GO:0016020 GO:0019012 GO:0016021 GO:0019031

Cluster-22178.43566 GO:0051726 GO:0006813 GO:0015269 GO:0016020

GO:0005634
Cluster-22178.43568 GO:0005515
Cluster-22178.43571 GO:0006629 GO:0009607 GO:0006812 GO:0008324
GO:0008081 GO:0016021
Cluster-22178.43572 GO:0016579 GO:0036459
Cluster-22178.43577 GO:0046836 GO:0006508 GO:0017089 GO:0008236
GO:0051861 GO:0005737
Cluster-22178.43580 GO:0005524
Cluster-22178.43587 GO:0006814 GO:0006744 GO:0006120 GO:0008152
GO:0015992 GO:0008137 GO:0016836 GO:0003824 GO:0005739
Cluster-22178.43588 GO:0007165 GO:0005509 GO:0005578
Cluster-22178.43592 GO:0006811 GO:0006810 GO:0005230 GO:0016020
Cluster-22178.43595 GO:0006810 GO:0006811 GO:0005230 GO:0016020
Cluster-22178.43596 GO:0008283 GO:0040007 GO:0007165 GO:0008083

Cluster-22178.43600 GO:0006094 GO:0006096 GO:0006144 GO:0055114
GO:0015976 GO:0004743 GO:0030955 GO:0000287 GO:0003824
Cluster-22178.43605 GO:0008237 GO:0008270
Cluster-22178.43608 GO:0008152 GO:0008168
Cluster-22178.43610 GO:0006486 GO:0016757 GO:0008378 GO:0016020
Cluster-22178.43613 GO:0007264 GO:0043087 GO:0016485 GO:0007165
GO:0005085 GO:0005515 GO:0016021
Cluster-22178.43614 GO:0050909 GO:0010038 GO:0007186 GO:0009058
GO:0004930 GO:0016021
Cluster-22178.43620 GO:0006144 GO:0019079 GO:0006351 GO:0006303
GO:0016817 GO:0003968 GO:0004003 GO:0003677 GO:0031379
GO:0005657
Cluster-22178.43625 GO:0007165 GO:0005057
Cluster-22178.43626 GO:0003676 GO:0005515
Cluster-22178.43628 GO:0006351 GO:0003714 GO:0005667
Cluster-22178.43629 GO:0006355 GO:0008134 GO:0005524 GO:0005667
Cluster-22178.43630 GO:0006281 GO:0000012 GO:0005515 GO:0003684
GO:0005634

Cluster-22178.43633 GO:0055085 GO:0007155 GO:0030001 GO:0016853
GO:0051015 GO:0003682 GO:0046873 GO:0000785 GO:0016020
Cluster-22178.43634 GO:0055085 GO:0030001 GO:0007155 GO:0003779
GO:0051015 GO:0016853 GO:0046873 GO:0003682 GO:0000785
GO:0016020
Cluster-22178.43636 GO:0005515 GO:0016020
Cluster-22178.43642 GO:0007009 GO:0008608 GO:0051082 GO:0072686
GO:0042729
Cluster-22178.43643 GO:0008608 GO:0007009 GO:0051082 GO:0042729
GO:0072686
Cluster-22178.43653 GO:0035556 GO:0005515 GO:0016020
Cluster-22178.43656 GO:0042967 GO:0008080 GO:0016747
Cluster-22178.43657 GO:0006355 GO:0043565 GO:0003924 GO:0005525
GO:0003700 GO:0005667 GO:0012511 GO:0016021
Cluster-22178.43658 GO:0016021 GO:0005789
Cluster-22178.43659 GO:0007186 GO:0009405 GO:0004930 GO:0016021
Cluster-22178.43661 GO:0005576
Cluster-22178.43664 GO:0016310 GO:0009069 GO:0004674 GO:0008239
GO:0070009
Cluster-22178.43665 GO:0009069 GO:0016310 GO:0070009 GO:0008239
GO:0004674
Cluster-22178.43666 GO:0007165 GO:0016043 GO:0000921 GO:0016301
GO:0003779 GO:0016021 GO:0005940
Cluster-22178.43673 GO:0008641
Cluster-22178.43675 GO:0007264 GO:0005525 GO:0005524 GO:0003924
GO:0016887
Cluster-22178.43676 GO:0042254 GO:0006412 GO:0003735 GO:0005622
GO:0005840
Cluster-22178.43677 GO:0006891 GO:0030173
Cluster-22178.43678 GO:0007165 GO:0008033 GO:0000166 GO:0005524
GO:0016879 GO:0005737
Cluster-22178.43686 GO:0045892 GO:0042919 GO:0006486 GO:0006810
GO:0042925 GO:0008373 GO:0016021 GO:0005634

Cluster-22178.43688 GO:0006486 GO:0008373
Cluster-22178.43690 GO:0006486 GO:0070072 GO:0008373
Cluster-22178.43691 GO:0030234 GO:0016020
Cluster-22178.43695 GO:0007059 GO:0008218 GO:0006260 GO:0008152
GO:0008168 GO:0005509 GO:0005737
Cluster-22178.43698 GO:0006891 GO:0030173
Cluster-22178.43700 GO:0006468 GO:0016773 GO:0005524 GO:0004672
GO:0016020
Cluster-22178.43701 GO:0006352 GO:0006470 GO:0008138 GO:0005861
GO:0005669
Cluster-22178.43710 GO:0006118 GO:0008152 GO:0055114 GO:0050660
GO:0003995 GO:0016627
Cluster-22178.43711 GO:0006118 GO:0008152 GO:0055114 GO:0050660
GO:0003995 GO:0016627
Cluster-22178.43719 GO:0043123 GO:0007165 GO:0045766 GO:0006508
GO:0006817 GO:0030436 GO:0043066 GO:0004190 GO:0004871
GO:0005315 GO:0016020 GO:0016021
Cluster-22178.43726 GO:0043087 GO:0042742 GO:0035023 GO:0005089

Cluster-22178.43728 GO:0005515
Cluster-22178.43733 GO:0015031 GO:0016021 GO:0005789
Cluster-22178.43734 GO:0007165 GO:0017000 GO:0030168 GO:0051258
GO:0007021 GO:0016787 GO:0030674 GO:0051082 GO:0042302
GO:0005515 GO:0015631 GO:0005102 GO:0045298 GO:0005577
Cluster-22178.43736 GO:0009245 GO:0008915
Cluster-22178.43737 GO:0006508 GO:0006260 GO:0006940 GO:0008270
GO:0005515 GO:0004222
Cluster-22178.43745 GO:0019872 GO:0055085 GO:0008654 GO:0006021
GO:0030001 GO:0016817 GO:0004512 GO:0046873 GO:0005515
GO:0016020
Cluster-22178.43750 GO:0016020
Cluster-22178.43751 GO:0006811 GO:0007165 GO:0055085 GO:0005216
GO:0016020

Cluster-22178.43754 GO:0009267 GO:0032099 GO:0000186 GO:0007186
GO:0008343 GO:0001678 GO:0045502 GO:0005198 GO:0008134
GO:0042803 GO:0030286 GO:0005615 GO:0005667 GO:0005882
Cluster-22178.43756 GO:0016567 GO:0006355 GO:0090263 GO:0007049
GO:0003677 GO:0003700 GO:0005667 GO:0009424
Cluster-22178.43760 GO:0006508 GO:0016032 GO:0007186 GO:0008241
GO:0003676 GO:0004930 GO:0008237 GO:0016021 GO:0016020
Cluster-22178.43770 GO:0016021
Cluster-22178.43788 GO:0008152 GO:0016757
Cluster-22178.43798 GO:0043087 GO:0006265 GO:0035023 GO:0005524
GO:0003918 GO:0005089 GO:0003677 GO:0019028
Cluster-22178.43802 GO:0017025 GO:0005096 GO:0005634
Cluster-22178.43803 GO:0055114 GO:0019064 GO:0006814 GO:0006118
GO:0016655 GO:0019031
Cluster-22178.43805 GO:0006915
Cluster-22178.43813 GO:0006289 GO:0006284 GO:0006810 GO:0042254
GO:0006281 GO:0006412 GO:0008270 GO:0003906 GO:0003684
GO:0003735 GO:0003924 GO:0005525 GO:0016799 GO:0005840
GO:0016021 GO:0005861
Cluster-22178.43815 GO:0006355 GO:0003677
Cluster-22178.43821 GO:0018193 GO:0016192 GO:0006886 GO:0008061
GO:0005198 GO:0005515 GO:0016020 GO:0030126
Cluster-22178.43822 GO:0016020
Cluster-22178.43825 GO:0042981
Cluster-22178.43828 GO:0050776 GO:0014068 GO:0006118 GO:0007275
GO:0007165 GO:0006259 GO:0003824 GO:0009055 GO:0020037
GO:0005102 GO:0005524 GO:0043548 GO:0003677 GO:0016021
GO:0016020 GO:0005694
Cluster-22178.43829 GO:0016055 GO:0031167 GO:0032775 GO:0006298
GO:0006306 GO:0007165 GO:0007275 GO:0030414 GO:0030983
GO:0098519 GO:0008168 GO:0009007 GO:0005524 GO:0005102
GO:0005576
Cluster-22178.43830 GO:0010468 GO:0016021

Cluster-22178.43832 GO:0006771 GO:0009103 GO:0019497 GO:0005515
GO:0046872 GO:0008918 GO:0008168 GO:0016817 GO:0003993
Cluster-22178.43834 GO:0006040 GO:0055114 GO:0008762
Cluster-22178.43835 GO:0009405 GO:0006810 GO:0008200 GO:0005576
Cluster-22178.43838 GO:0006355 GO:0008270 GO:0043565 GO:0003700
GO:0005667 GO:0005634
Cluster-22178.43842 GO:0000154 GO:0006396 GO:0004252 GO:0016433
GO:0055036 GO:0019028
Cluster-22178.43844 GO:0008152 GO:0008033 GO:0055114 GO:0005975
GO:0009451 GO:0005985 GO:0006479 GO:0006396 GO:0006464
GO:0005982 GO:0006400 GO:0046500 GO:0008757 GO:0008168
GO:0004719 GO:0008173 GO:0008810 GO:0008176
Cluster-22178.43845 GO:0008152 GO:0005985 GO:0005982 GO:0005975
GO:0008810 GO:0008168
Cluster-22178.43846 GO:0019087 GO:0016021
Cluster-22178.43847 GO:0007165 GO:0005057
Cluster-22178.43848 GO:0006260 GO:0003677 GO:0005664
Cluster-22178.43855 GO:0006269 GO:0018142 GO:0009058 GO:0006260
GO:0003723
Cluster-22178.43862 GO:0006506 GO:0017176 GO:0016021
Cluster-22178.43866 GO:0006633 GO:0008152 GO:0006574 GO:0006554
GO:0055114 GO:0018874 GO:0006550 GO:0006552 GO:0006631
GO:0006568 GO:0003824 GO:0016491 GO:0050662 GO:0051287
GO:0003857
Cluster-22178.43868 GO:0007186 GO:0007165 GO:0007264 GO:0004871
GO:0005525 GO:0003924 GO:0019001 GO:0031683
Cluster-22178.43871 GO:0006351 GO:0006144 GO:0006464 GO:0006206
GO:0003899 GO:0003677 GO:0008270 GO:0003676 GO:0016151
GO:0005730
Cluster-22178.43873 GO:0007165 GO:0007264 GO:0005525
Cluster-22178.43874 GO:0003824
Cluster-22178.43875 GO:0003824
Cluster-22178.43882 GO:0007165 GO:0019012

Cluster-22178.43883 GO:0005515
Cluster-22178.43884 GO:0006570 GO:0006355 GO:0051607 GO:0055114
GO:0005515 GO:0004511 GO:0003700 GO:0005667
Cluster-22178.43885 GO:0006355 GO:0006570 GO:0055114 GO:0004511
GO:0005515 GO:0003700 GO:0005576 GO:0005667
Cluster-22178.43886 GO:0005515
Cluster-22178.43895 GO:0019497 GO:0007339 GO:0006771 GO:0003993
GO:0046872 GO:0005515 GO:0016020 GO:0005576
Cluster-22178.43899 GO:0008270 GO:0005515
Cluster-22178.43901 GO:0006303 GO:0009069 GO:0016310 GO:0005515
GO:0003677 GO:0004677 GO:0016773 GO:0005524 GO:0005634
GO:0005958
Cluster-22178.43909 GO:0007010 GO:0003779
Cluster-22178.43916 GO:0005515
Cluster-22178.43921 GO:0005509
Cluster-22178.43922 GO:0005509
Cluster-22178.43928 GO:0006771 GO:0019497 GO:0046872 GO:0003993
GO:0005515
Cluster-22178.43938 GO:0005515
Cluster-22178.43942 GO:0006457
Cluster-22178.43943 GO:0030420
Cluster-22178.43944 GO:0005515
Cluster-22178.43949 GO:0009058 GO:0008152 GO:0006508 GO:0006629
GO:0008236 GO:0016788 GO:0016787
Cluster-22178.43952 GO:0006810 GO:0005215 GO:0016020
Cluster-22178.43953 GO:0005515 GO:0019901
Cluster-22178.43957 GO:0006355 GO:0003700 GO:0005634 GO:0005667
Cluster-22178.43960 GO:0006886 GO:0016192 GO:0016021 GO:0030131
GO:0030117
Cluster-22178.43966 GO:0005515 GO:0003677 GO:0005737
Cluster-22178.43968 GO:0006355
Cluster-22178.43970 GO:0006351 GO:0006260 GO:0003887 GO:0003677
GO:0042575

Cluster-22178.43971 GO:0006298 GO:0005524 GO:0030983 GO:0016021
Cluster-22178.43975 GO:0035556
Cluster-22178.43977 GO:0006810 GO:0006351 GO:0006468 GO:0055085
GO:0007166 GO:0005515 GO:0005215 GO:0022857 GO:0005524
GO:0004672 GO:0016773 GO:0016020 GO:0016021
Cluster-22178.43979 GO:0007155 GO:0048870 GO:0008608 GO:0072686
GO:0042729 GO:0031514
Cluster-22178.43981 GO:0006118 GO:0045454 GO:0009055 GO:0015035

Cluster-22178.43985 GO:0006094 GO:0006096 GO:0055114 GO:0006144
GO:0015976 GO:0004743 GO:0030955 GO:0000287 GO:0003824
Cluster-22178.43987 GO:0005198 GO:0046872
Cluster-22178.4399 GO:0006355 GO:0005515
Cluster-22178.43991 GO:0003677
Cluster-22178.43993 GO:0019538 GO:0003677
Cluster-22178.43994 GO:0005515
Cluster-22178.440 GO:0006355 GO:0035556 GO:0051694 GO:0003700
GO:0005523 GO:0005515 GO:0005667
Cluster-22178.44001 GO:0009058 GO:0006144 GO:0006508 GO:0004252
GO:0003877 GO:0030170
Cluster-22178.44003 GO:0046500 GO:0006400 GO:0006396 GO:0006480
GO:0006554 GO:0030488 GO:0006464 GO:0009451 GO:0006479
GO:0008152 GO:0008033 GO:0008176 GO:0016429 GO:0008173
GO:0018024 GO:0008168 GO:0004719 GO:0031515 GO:0005856
Cluster-22178.44006 GO:0015979 GO:0016020 GO:0009539 GO:0009523
Cluster-22178.44012 GO:0008270
Cluster-22178.44019 GO:0006355 GO:0008270 GO:0043565 GO:0005515
GO:0003700 GO:0005634 GO:0005667
Cluster-22178.44028 GO:0008270
Cluster-22178.44030 GO:0072519 GO:0016567 GO:0004842 GO:0008270

Cluster-22178.44032 GO:0006355 GO:0016787 GO:0005507 GO:0003700
GO:0003677 GO:0005667 GO:0005634

Cluster-22178.44034 GO:0007218 GO:0005185 GO:0005576
Cluster-22178.44035 GO:0007218 GO:0005185 GO:0005576
Cluster-22178.44037 GO:0009058 GO:0006144 GO:0006508 GO:0004252
GO:0030170 GO:0003877
Cluster-22178.44042 GO:0006771 GO:0019497 GO:0016192 GO:0003993
GO:0019031 GO:0016021
Cluster-22178.44046 GO:0006508 GO:0016787 GO:0008236
Cluster-22178.44048 GO:0005198 GO:0016021
Cluster-22178.44050 GO:0007165 GO:0005179 GO:0005576
Cluster-22178.44051 GO:0006308 GO:0007264 GO:0015684 GO:0005524
GO:0015093 GO:0016887 GO:0008855 GO:0003924 GO:0005525
GO:0016021 GO:0009318
Cluster-22178.44056 GO:0055114
Cluster-22178.44060 GO:0006508 GO:0030436 GO:0004190
Cluster-22178.44062 GO:0017004 GO:0006118 GO:0042254 GO:0003735
GO:0045158 GO:0009512 GO:0005840
Cluster-22178.44063 GO:0006355 GO:0003682 GO:0043565 GO:0008270
GO:0003700 GO:0005667 GO:0000785
Cluster-22178.44070 GO:0003676 GO:0016787 GO:0046872
Cluster-22178.44075 GO:0006874 GO:0006816 GO:0005219 GO:0046872
GO:0004252 GO:0016021 GO:0005622 GO:0005886
Cluster-22178.44077 GO:0055114 GO:0016714
Cluster-22178.44089 GO:0008283 GO:0042742 GO:0040007 GO:0007165
GO:0008083 GO:0005198 GO:0008270 GO:0019013
Cluster-22178.44107 GO:0006812 GO:0015672 GO:0055114 GO:0016638
GO:0005261 GO:0016020
Cluster-22178.4411 GO:0006633 GO:0055114 GO:0003677 GO:0016491
GO:0005506
Cluster-22178.44110 GO:0006508 GO:0008236 GO:0016787
Cluster-22178.44112 GO:0006206 GO:0006144 GO:0006241 GO:0006228
GO:0045454 GO:0006165 GO:0019079 GO:0006183 GO:0004550
GO:0005524 GO:0016787 GO:0008026 GO:0003677 GO:0005515
Cluster-22178.44123 GO:0005515

Cluster-22178.44126 GO:0007049 GO:0006355 GO:0006914 GO:0003700
GO:0005198 GO:0043565 GO:0005667
Cluster-22178.44127 GO:0007165 GO:0006355 GO:0006914 GO:0005198
GO:0016301 GO:0003700 GO:0043565 GO:0005667
Cluster-22178.44128 GO:0006355 GO:0007165 GO:0006914 GO:0005198
GO:0016301 GO:0043565 GO:0003700 GO:0005667
Cluster-22178.44136 GO:0007165 GO:0001819 GO:0008284 GO:0010884
GO:0005509 GO:0005515 GO:0005578
Cluster-22178.44138 GO:0008284 GO:0001819 GO:0007165 GO:0010884
GO:0005515 GO:0005509 GO:0005578
Cluster-22178.44142 GO:0007218
Cluster-22178.44150 GO:0055114 GO:0006940 GO:0006952 GO:0016620
GO:0005515
Cluster-22178.44153 GO:0005515
Cluster-22178.44159 GO:0030071 GO:0046983 GO:0042802 GO:0005515
GO:0005680
Cluster-22178.44160 GO:0006810 GO:0006771 GO:0015986 GO:0019497
GO:0003677 GO:0030674 GO:0042626 GO:0003993 GO:0043130
GO:0016887 GO:0005524 GO:0042802 GO:0005515 GO:0000166
GO:0005525 GO:0008092 GO:0003924 GO:0046872 GO:0033178
Cluster-22178.44164 GO:0006351 GO:0007275 GO:0006206 GO:0006144
GO:0003677 GO:0003899 GO:0005515 GO:0003774 GO:0005524
GO:0005730 GO:0016459
Cluster-22178.44165 GO:0003774 GO:0005524 GO:0005515 GO:0016459
Cluster-22178.44166 GO:0005198 GO:0003682 GO:0000785
Cluster-22178.44167 GO:0042254 GO:0006412 GO:0003735 GO:0005840
GO:0005622
Cluster-22178.44170 GO:0016055 GO:0005515
Cluster-22178.44172 GO:0006355 GO:0003677 GO:0046872 GO:0005634
Cluster-22178.44174 GO:0007010 GO:0003779 GO:0008168
Cluster-22178.44186 GO:0006754 GO:0017004 GO:0055114 GO:0006811
GO:0005216 GO:0005509 GO:0005515 GO:0005524 GO:0016887
GO:0000275 GO:0016020

Cluster-22178.44189 GO:0006355 GO:0008270 GO:0005515
Cluster-22178.44191 GO:0005815
Cluster-22178.44193 GO:0005515
Cluster-22178.44195 GO:0007016 GO:0005515 GO:0016010
Cluster-22178.44196 GO:0006355 GO:0061024 GO:0007264 GO:0007186 GO:0007165 GO:0010033 GO:0043531 GO:0003924 GO:0031683 GO:0019001 GO:0004871 GO:0005525 GO:0008134 GO:0000166 GO:0016887 GO:0005524 GO:0003677 GO:0019867 GO:0005667 GO:0016020
Cluster-22178.44199 GO:0000045 GO:0005737
Cluster-22178.44207 GO:0006396 GO:0006807 GO:0006144 GO:0004000 GO:0003723
Cluster-22178.44209 GO:0006807 GO:0006396 GO:0006144 GO:0004000 GO:0003723
Cluster-22178.44210 GO:0006144 GO:0006396 GO:0006807 GO:0003723 GO:0004000
Cluster-22178.44214 GO:0090266 GO:0006810 GO:0006351 GO:0006260 GO:0005515 GO:0003677 GO:0003887 GO:0008270 GO:0005634 GO:0016020 GO:0042575 GO:0032040 GO:0005680 GO:0019031
Cluster-22178.44218 GO:0006123 GO:0006807 GO:0015992 GO:0007067 GO:0004129 GO:0005507 GO:0016810 GO:0045277 GO:0016020 GO:0042729 GO:0072686
Cluster-22178.44219 GO:0015992 GO:0015031 GO:0006260 GO:0006891 GO:0015991 GO:0006355 GO:0046983 GO:0008565 GO:0045502 GO:0042803 GO:0008134 GO:0003676 GO:0015078 GO:0017119 GO:0005667 GO:0030286 GO:0033179
Cluster-22178.44220 GO:0009103 GO:0055085 GO:0005515 GO:0016021 GO:0016020
Cluster-22178.44222 GO:0016887 GO:0005524 GO:0005525
Cluster-22178.44226 GO:0055114 GO:0006801 GO:0046872 GO:0004784

Cluster-22178.44227 GO:0042493 GO:0006761 GO:0046656 GO:0055114 GO:0004146

Cluster-22178.44233 GO:0008152 GO:0008134 GO:0003824 GO:0005634 GO:0005667

Cluster-22178.44237 GO:0006468 GO:0007165 GO:0055114 GO:0035556 GO:0005057 GO:0005524 GO:0004672 GO:0047134

Cluster-22178.44238 GO:0006468 GO:0016773 GO:0004672 GO:0005524 GO:0016020

Cluster-22178.44242 GO:0055085 GO:0006810 GO:0005525 GO:0042626 GO:0005524 GO:0016887 GO:0003924 GO:0016301 GO:0016021

Cluster-22178.44246 GO:0006090 GO:0006725 GO:0006814 GO:0071436 GO:0006560 GO:0006525 GO:0015081 GO:0008948 GO:0016853 GO:0016020

Cluster-22178.44250 GO:0051260

Cluster-22178.44252 GO:0055114 GO:0006308 GO:0000105 GO:0019953 GO:0055085 GO:0006355 GO:0030001 GO:0008270 GO:0008855 GO:0004399 GO:0046873 GO:0051287 GO:0016021 GO:0009318 GO:0016020

Cluster-22178.44258 GO:0030472 GO:0008608 GO:0005509 GO:0072686 GO:0042729

Cluster-22178.44259 GO:0030472 GO:0008608 GO:0005509 GO:0072686 GO:0042729

Cluster-22178.44260 GO:0003723 GO:0003676 GO:0016021

Cluster-22178.44261 GO:0005198 GO:0019028

Cluster-22178.44263 GO:0006468 GO:0006413 GO:0007165 GO:0006446 GO:0003743 GO:0005515 GO:0005524 GO:0004672 GO:0043565 GO:0016020 GO:0005840

Cluster-22178.44264 GO:0007165 GO:0042981 GO:0005515 GO:0003677

Cluster-22178.44266 GO:0006355 GO:0005634

Cluster-22178.44274 GO:0005515

Cluster-22178.44276 GO:0000746 GO:0019867

Cluster-22178.44279 GO:0006118 GO:0022904 GO:0009055 GO:0016020 GO:0016021

Cluster-22178.44280 GO:0006270 GO:0006446 GO:0006886 GO:0004190

GO:0003743 GO:0005852 GO:0005737 GO:0030176 GO:0005840
GO:0016021
Cluster-22178.44281 GO:0005515
Cluster-22178.44282 GO:0019033
Cluster-22178.44296 GO:0003677
Cluster-22178.44297 GO:0006281 GO:0006310 GO:0005524 GO:0009378
GO:0003677 GO:0009379 GO:0005657
Cluster-22178.44299 GO:0016021
Cluster-22178.4430 GO:0006760 GO:0046656 GO:0016788 GO:0004150
Cluster-22178.44301 GO:0042312 GO:0008217 GO:0000245 GO:0006355
GO:0006508 GO:0007165 GO:0008270 GO:0004222 GO:0005179
GO:0005515 GO:0031012 GO:0005576 GO:0005634
Cluster-22178.44303 GO:0007165 GO:0007186 GO:0007264 GO:0005525
GO:0004871 GO:0031683 GO:0019001 GO:0003924
Cluster-22178.44311 GO:0009607 GO:0016021
Cluster-22178.44314 GO:0005515
Cluster-22178.44317 GO:0005515
Cluster-22178.44318 GO:0046872
Cluster-22178.44319 GO:0046872
Cluster-22178.44320 GO:0046872
Cluster-22178.44325 GO:0005515
Cluster-22178.44329 GO:0046872 GO:0005515 GO:0016021
Cluster-22178.44331 GO:0042578
Cluster-22178.44333 GO:0016310 GO:0009069 GO:0006468 GO:0004674
GO:0000287 GO:0005524 GO:0004672
Cluster-22178.44334 GO:0006468 GO:0016773 GO:0004672 GO:0005524
GO:0016020
Cluster-22178.44337 GO:0016757
Cluster-22178.44339 GO:0009405 GO:0005576
Cluster-22178.44340 GO:0035556
Cluster-22178.44342 GO:0006355 GO:0003700 GO:0008270 GO:0043565
GO:0005667
Cluster-22178.44348 GO:0000917 GO:0043093 GO:0000002 GO:0007049

GO:0070206 GO:0003677 GO:0016787 GO:0003723 GO:0003724
GO:0005524 GO:0016887 GO:0045095 GO:0000262 GO:0005737
Cluster-22178.44352 GO:0005515
Cluster-22178.44355 GO:0007617 GO:0005576
Cluster-22178.44359 GO:0016567 GO:0005515 GO:0004842 GO:0008270
GO:0046872
Cluster-22178.44360 GO:0015992 GO:0006820 GO:0006744 GO:0006120
GO:0006814 GO:0008137 GO:0005743 GO:0016021
Cluster-22178.44361 GO:0006413 GO:0003723 GO:0019012
Cluster-22178.44362 GO:0006413 GO:0003723 GO:0019012
Cluster-22178.44363 GO:0005515
Cluster-22178.44364 GO:0005515
Cluster-22178.44367 GO:0005515
Cluster-22178.44371 GO:0006508 GO:0050790 GO:0009405 GO:0004197
GO:0005576
Cluster-22178.44372 GO:0005515 GO:0008270
Cluster-22178.44373 GO:0008270 GO:0005515
Cluster-22178.44375 GO:0016773
Cluster-22178.44378 GO:0007411
Cluster-22178.44380 GO:0006955 GO:0006412 GO:0042254 GO:0003735
GO:0005622 GO:0005840
Cluster-22178.44385 GO:0006355 GO:0043565 GO:0008270 GO:0005515
GO:0003700 GO:0005667 GO:0005634
Cluster-22178.44388 GO:0016021
Cluster-22178.4439 GO:0005515
Cluster-22178.44391 GO:0003723
Cluster-22178.44392 GO:0003723
Cluster-22178.44393 GO:0003723
Cluster-22178.44395 GO:0003723
Cluster-22178.44398 GO:0006915 GO:0008285 GO:0007050 GO:0005515

Cluster-22178.44401 GO:0008270 GO:0046872 GO:0003676
Cluster-22178.44402 GO:0003676 GO:0008270 GO:0046872

Cluster-22178.44406 GO:0019497 GO:0006508 GO:0006771 GO:0008242 GO:0046872 GO:0003993 GO:0016740 GO:0005515 GO:0004197

Cluster-22178.44408 GO:0015992 GO:0006123 GO:0004129 GO:0005739 GO:0005576 GO:0045277

Cluster-22178.44416 GO:0055114 GO:0016491 GO:0016616

Cluster-22178.44420 GO:0008270 GO:0046872 GO:0005515

Cluster-22178.44422 GO:0005509

Cluster-22178.44424 GO:0007034 GO:0051259

Cluster-22178.44425 GO:0007156 GO:0005509 GO:0005515 GO:0016020

Cluster-22178.44427 GO:0007156 GO:0005509 GO:0016020

Cluster-22178.44428 GO:0016021 GO:0016020

Cluster-22178.4443 GO:0006955 GO:0007165 GO:0005164 GO:0016020

Cluster-22178.44440 GO:0006139 GO:0005515 GO:0003676 GO:0008408 GO:0043395 GO:0016020 GO:0005578

Cluster-22178.44442 GO:0016032 GO:0005198 GO:0016020

Cluster-22178.44446 GO:0043087 GO:0035023 GO:0006397 GO:0046872 GO:0005089 GO:0003723 GO:0005737 GO:0005634

Cluster-22178.44454 GO:0007156 GO:0005509 GO:0016020

Cluster-22178.44460 GO:0016192 GO:0006886 GO:0005515 GO:0008270

Cluster-22178.44464 GO:0009246 GO:0055114 GO:0036065 GO:0020037 GO:0016705 GO:0008417 GO:0005506

Cluster-22178.44466 GO:0006334 GO:0000398 GO:0005515 GO:0005681 GO:0005634

Cluster-22178.44467 GO:0043087 GO:0046339 GO:0009395 GO:0035556 GO:0007264 GO:0006629 GO:0007165 GO:0008081 GO:0004435 GO:0005085 GO:0005515 GO:0016020

Cluster-22178.44469 GO:0006468 GO:0016773 GO:0005524 GO:0004672 GO:0016020

Cluster-22178.44473 GO:0006812 GO:0006836 GO:0016032 GO:0005328 GO:0016021

Cluster-22178.44484 GO:0008270 GO:0005515 GO:0005576

Cluster-22178.44488 GO:0009307 GO:0006308 GO:0009036 GO:0003677

GO:0005515 GO:0009359
Cluster-22178.44493 GO:0043065 GO:0006144 GO:0007165 GO:0004114
GO:0016021 GO:0005740
Cluster-22178.44496 GO:0035556 GO:0045333 GO:0005739 GO:0005634
Cluster-22178.44498 GO:0007165 GO:0045893 GO:0005102 GO:0005634
Cluster-22178.44499 GO:0003779
Cluster-22178.44510 GO:0007067 GO:0051382 GO:0003677
Cluster-22178.44515 GO:0005515
Cluster-22178.44523 GO:0006412 GO:0042254 GO:0006355 GO:0003700
GO:0043565 GO:0003735 GO:0005840 GO:0005622 GO:0005667
Cluster-22178.44531 GO:0002949 GO:0032065 GO:0007165 GO:0000062
GO:0016887 GO:0005543 GO:0003774 GO:0005524 GO:0005515
GO:0005938 GO:0005856 GO:0016459
Cluster-22178.44533 GO:0005524
Cluster-22178.44537 GO:0006470 GO:0006570 GO:0019988 GO:0008138
GO:0004725 GO:0043399
Cluster-22178.44540 GO:0042254 GO:0006412 GO:0010923 GO:0003735
GO:0019902 GO:0005840 GO:0005622
Cluster-22178.44544 GO:0006355 GO:0006260 GO:0003700 GO:0005667
GO:0005634
Cluster-22178.44546 GO:0016068 GO:0015031 GO:0006606 GO:0008565
GO:0005634 GO:0005737 GO:0016021
Cluster-22178.44550 GO:0016485 GO:0005975 GO:0043085 GO:0016773
GO:0046872 GO:0035091 GO:0031419 GO:0005515 GO:0016021
Cluster-22178.44551 GO:0005515 GO:0016817
Cluster-22178.44552 GO:0007165 GO:0006289 GO:0006281 GO:0043161
GO:0003684 GO:0005179 GO:0005576
Cluster-22178.44554 GO:0006355 GO:0006357 GO:0003712 GO:0001104
GO:0005667 GO:0016592
Cluster-22178.44559 GO:0006511 GO:0007275 GO:0008270 GO:0005515
GO:0005634
Cluster-22178.44563 GO:0016192 GO:0006904
Cluster-22178.44565 GO:0009405 GO:0005576

Cluster-22178.44571 GO:0055114 GO:0004499 GO:0050661 GO:0016491 GO:0050660
Cluster-22178.44574 GO:0006811 GO:0006351 GO:0006813 GO:0006260 GO:0006810 GO:0006269 GO:0005215 GO:0003896 GO:0005267 GO:0004190 GO:0008270 GO:0003677 GO:0016021 GO:0005657 GO:0016020 GO:0005730
Cluster-22178.44577 GO:0006508 GO:0005515 GO:0004252 GO:0005509 GO:0005576
Cluster-22178.44579 GO:0006260 GO:0000724 GO:0003677 GO:0003676 GO:0016021 GO:0005634
Cluster-22178.44581 GO:0006508 GO:0005515 GO:0004252 GO:0005509 GO:0005576
Cluster-22178.44590 GO:0006259 GO:0003824 GO:0003677 GO:0005524 GO:0005694
Cluster-22178.44591 GO:0006259 GO:0040007 GO:0008283 GO:0007165 GO:0055114 GO:0008083 GO:0003677 GO:0005524 GO:0016627 GO:0003824 GO:0047134 GO:0005694
Cluster-22178.44592 GO:0008283 GO:0006259 GO:0040007 GO:0007165 GO:0055114 GO:0003677 GO:0008083 GO:0005524 GO:0016627 GO:0047134 GO:0003824 GO:0005694
Cluster-22178.44593 GO:0040007 GO:0006259 GO:0008283 GO:0007165 GO:0055114 GO:0008083 GO:0003677 GO:0016627 GO:0005524 GO:0003824 GO:0047134 GO:0005694
Cluster-22178.44594 GO:0006118 GO:0016126 GO:0055114 GO:0006040 GO:0016491 GO:0004128 GO:0016021
Cluster-22178.44597 GO:0051260 GO:0007010 GO:0003779
Cluster-22178.44599 GO:0005515
Cluster-22178.44600 GO:0006122 GO:0030866 GO:0005198 GO:0003779 GO:0008092 GO:0005856 GO:0005743 GO:0005750 GO:0016021
Cluster-22178.44603 GO:0005978 GO:0005982 GO:0005985 GO:0004373 GO:0005515
Cluster-22178.44604 GO:0006313
Cluster-22178.44608 GO:0030976 GO:0000287

Cluster-22178.44610 GO:0046872
Cluster-22178.44611 GO:0005515
Cluster-22178.44614 GO:0005515
Cluster-22178.44617 GO:0007165 GO:0006355 GO:0005515 GO:0003713
GO:0004519 GO:0035257 GO:0046983 GO:0005634 GO:0005667
GO:0019031
Cluster-22178.44618 GO:0051258 GO:0006508 GO:0006355 GO:0030168
GO:0016567 GO:0007165 GO:0003700 GO:0043565 GO:0046872
GO:0008236 GO:0005515 GO:0005102 GO:0004842 GO:0008270
GO:0061630 GO:0030674 GO:0005577 GO:0005680 GO:0005622
GO:0005667
Cluster-22178.44619 GO:0055085 GO:0006811 GO:0006355 GO:0046325
GO:0043565 GO:0005216 GO:0016020
Cluster-22178.44620 GO:0005524 GO:0016887
Cluster-22178.44621 GO:0016887 GO:0005524
Cluster-22178.44625 GO:0009059 GO:0030176
Cluster-22178.44626 GO:0048309 GO:0051028 GO:0006396 GO:0000176
Cluster-22178.44627 GO:0055114 GO:0047134
Cluster-22178.44633 GO:0046872 GO:0016021 GO:0016020
Cluster-22178.44634 GO:0004252 GO:0016021
Cluster-22178.44635 GO:0019229 GO:0003676 GO:0005576
Cluster-22178.44636 GO:0030036 GO:0003779 GO:0030054
Cluster-22178.44641 GO:0016787
Cluster-22178.44643 GO:0007050 GO:0045859 GO:0004861 GO:0005634
Cluster-22178.44644 GO:0045859 GO:0007050 GO:0004861 GO:0005634
Cluster-22178.44646 GO:0006457 GO:0005509 GO:0008253 GO:0000287
GO:0005737
Cluster-22178.44648 GO:0015031 GO:0006606 GO:0016787 GO:0003677
GO:0008565 GO:0005524 GO:0003676 GO:0005634 GO:0005737
Cluster-22178.44651 GO:0016787
Cluster-22178.44652 GO:0055085 GO:0030001 GO:0008272 GO:0015116
GO:0046873 GO:0016020 GO:0016021
Cluster-22178.44653 GO:0030001 GO:0055085 GO:0046873 GO:0016020

Cluster-22178.44656 GO:0003676
Cluster-22178.44657 GO:0003676
Cluster-22178.44659 GO:0006260 GO:0006281 GO:0003676 GO:0043140
GO:0016874 GO:0005524 GO:0003677 GO:0016818 GO:0016787
GO:0010181 GO:0005657 GO:0005622 GO:0016020 GO:0005634
Cluster-22178.44660 GO:0019882 GO:0006955 GO:0005515 GO:0042613
GO:0016020
Cluster-22178.44664 GO:0006355 GO:0043093 GO:0007165 GO:0000917
GO:0030521 GO:0051056 GO:0005515 GO:0003677 GO:0004882
GO:0005096 GO:0005496 GO:0005634 GO:0005737
Cluster-22178.44665 GO:0030521 GO:0051056 GO:0006260 GO:0006355
GO:0043093 GO:0007165 GO:0000917 GO:0005096 GO:0004882
GO:0005496 GO:0005515 GO:0003677 GO:0005737 GO:0005634
Cluster-22178.44668 GO:0005515
Cluster-22178.44670 GO:0009877 GO:0005515 GO:0016746 GO:0005829
Cluster-22178.44673 GO:0007165 GO:0000917 GO:0007172 GO:0043093
GO:0006468 GO:0004871 GO:0004713 GO:0005925 GO:0005737
Cluster-22178.44674 GO:0007172 GO:0000917 GO:0007165 GO:0006468
GO:0043093 GO:0004713 GO:0004871 GO:0005515 GO:0005737
GO:0005925
Cluster-22178.44676 GO:0030154 GO:0016021
Cluster-22178.44679 GO:0007010 GO:0005524 GO:0016787 GO:0003779
GO:0003677 GO:0003676
Cluster-22178.44685 GO:0016192 GO:0005515 GO:0016021
Cluster-22178.44688 GO:0046486 GO:0042967 GO:0004144
Cluster-22178.44689 GO:0006269 GO:0006351 GO:0006260 GO:0003677
GO:0008270 GO:0003896 GO:0005730 GO:0005657
Cluster-22178.44697 GO:0007264 GO:0000398 GO:0005524 GO:0003924
GO:0005525
Cluster-22178.44699 GO:0006914 GO:0007049 GO:0043093 GO:0006355
GO:0000917 GO:0043565 GO:0003700 GO:0003677 GO:0005515
GO:0005737 GO:0005667 GO:0005634
Cluster-22178.44703 GO:0055114 GO:0016616 GO:0016491

Cluster-22178.44707 GO:0005515
Cluster-22178.44712 GO:0006260 GO:0006276 GO:0005515 GO:0016020
Cluster-22178.44719 GO:0008242
Cluster-22178.44721 GO:0005515
Cluster-22178.44722 GO:0006858
Cluster-22178.44723 GO:0006310 GO:0003677 GO:0000150
Cluster-22178.44724 GO:0003676 GO:0003677 GO:0016787 GO:0005524

Cluster-22178.44726 GO:0043401 GO:0006355
Cluster-22178.44728 GO:0006355 GO:0003700 GO:0008270 GO:0043565
GO:0005634 GO:0005667
Cluster-22178.44729 GO:0006355 GO:0043401
Cluster-22178.44732 GO:0006886 GO:0006888 GO:0006464 GO:0016151
GO:0008270 GO:0046872 GO:0030127
Cluster-22178.44733 GO:0019028
Cluster-22178.44734 GO:0003723 GO:0004867 GO:0005524 GO:0003924
GO:0043531 GO:0005525 GO:0035064 GO:0003724 GO:0005576
Cluster-22178.44739 GO:0006260 GO:0005524 GO:0005634 GO:0000808
Cluster-22178.44743 GO:0005515
Cluster-22178.4475 GO:0005975 GO:0006468 GO:0005524 GO:0004672
GO:0016773 GO:0030248 GO:0004553 GO:0005576 GO:0016020
Cluster-22178.44752 GO:0022904 GO:0003676 GO:0008017 GO:0045298
GO:0016020
Cluster-22178.44756 GO:0006950 GO:0005515 GO:0016020
Cluster-22178.44757 GO:0015979 GO:0005515 GO:0005509 GO:0009654
GO:0019898 GO:0009523
Cluster-22178.44764 GO:0007275 GO:0009966 GO:0003723 GO:0032266
GO:0043130 GO:0034464 GO:0016020
Cluster-22178.44765 GO:0006468 GO:0005524 GO:0004672 GO:0016773
GO:0016020
Cluster-22178.44766 GO:0006506 GO:0005789 GO:0016021
Cluster-22178.44770 GO:0006260 GO:0006914 GO:0015992 GO:0000921
GO:0007049 GO:0006886 GO:0015991 GO:0003774 GO:0015078

GO:0005887 GO:0005940 GO:0005783 GO:0016459 GO:0019867
GO:0016021 GO:0033179
Cluster-22178.44771 GO:0015992 GO:0006357 GO:0006914 GO:0015743
GO:0007155 GO:0051382 GO:0048870 GO:0000160 GO:0015991
GO:0006260 GO:0008053 GO:0006605 GO:0007165 GO:0006886
GO:0010508 GO:0006606 GO:0019079 GO:0000398 GO:0051258
GO:0006308 GO:0006355 GO:0030168 GO:0015031 GO:0000921
GO:0051607 GO:0006144 GO:0006810 GO:0015078 GO:0005515
GO:0005198 GO:0001104 GO:0008565 GO:0004871 GO:0003700
GO:0008134 GO:0030674 GO:0003924 GO:0043565 GO:0003968
GO:0042803 GO:0003774 GO:0045502 GO:0008855 GO:0005102
GO:0005737 GO:0016459 GO:0005667 GO:0005741 GO:0016020
GO:0031379 GO:0019031 GO:0016021 GO:0005801 GO:0019028
GO:0031514 GO:0005742 GO:0016592 GO:0019867 GO:0005681
GO:0000776 GO:0005577 GO:0009318 GO:0033179 GO:0030286
GO:0005886 GO:0005940
Cluster-22178.44780 GO:0006355 GO:0005525 GO:0003700 GO:0003924
GO:0043565 GO:0016021 GO:0012511 GO:0005667
Cluster-22178.44781 GO:0007264 GO:0007010 GO:0009405 GO:0046718
GO:0003779 GO:0003924 GO:0005525 GO:0046812 GO:0039624
GO:0009986
Cluster-22178.44787 GO:0055114 GO:0016705 GO:0020037 GO:0005506

Cluster-22178.44788 GO:0042073 GO:0006355 GO:0008270 GO:0003682
GO:0003677 GO:0000775 GO:0032040 GO:0000785 GO:0005634
Cluster-22178.44789 GO:0006355 GO:0042073 GO:0003677 GO:0003682
GO:0008270 GO:0005634 GO:0000785 GO:0032040 GO:0000775
Cluster-22178.44790 GO:0006355 GO:0042073 GO:0000398 GO:0003677
GO:0003682 GO:0008270 GO:0005634 GO:0000775 GO:0032040
GO:0000785 GO:0005681
Cluster-22178.44801 GO:0008152 GO:0016747 GO:0016746
Cluster-22178.44802 GO:0005515
Cluster-22178.44806 GO:0003723 GO:0003676

Cluster-22178.44809 GO:0043123 GO:0045766 GO:0043066 GO:0005515

Cluster-22178.44813 GO:0005509
Cluster-22178.44814 GO:0006355 GO:0046983 GO:0005509 GO:0005515 GO:0051015
Cluster-22178.44817 GO:0016746
Cluster-22178.44819 GO:0007049 GO:0051082
Cluster-22178.44827 GO:0003824 GO:0005198 GO:0046872 GO:0008270 GO:0051536 GO:0019028
Cluster-22178.44828 GO:0006355 GO:0003700 GO:0043565 GO:0046872 GO:0008270 GO:0005667
Cluster-22178.44830 GO:0001503 GO:0007186 GO:0006814 GO:0015992 GO:0006885 GO:0015385 GO:0004930 GO:0016021
Cluster-22178.44833 GO:0008152 GO:0006468 GO:0003824 GO:0005524 GO:0004672
Cluster-22178.44841 GO:0006570 GO:0006814 GO:0006744 GO:0006120 GO:0006470 GO:0055114 GO:0015992 GO:0008138 GO:0008137 GO:0004725 GO:0005524 GO:0016020
Cluster-22178.44843 GO:0035556
Cluster-22178.44848 GO:0006144 GO:0006629 GO:0006508 GO:0003968 GO:0070008 GO:0016817 GO:0004197 GO:0004252 GO:0017111 GO:0031379
Cluster-22178.44855 GO:0006816 GO:0006874 GO:0006270 GO:0035556 GO:0004190 GO:0004518 GO:0003677 GO:0005515 GO:0005219 GO:0005622 GO:0016021
Cluster-22178.44862 GO:0006811 GO:0035058 GO:0007052 GO:0055085 GO:0031122 GO:0007165 GO:0005515 GO:0004888 GO:0005216 GO:0016021 GO:0005813 GO:0016020 GO:0097546
Cluster-22178.44863 GO:0007165 GO:0031122 GO:0055085 GO:0035058 GO:0007052 GO:0006811 GO:0004888 GO:0005216 GO:0005515 GO:0097546 GO:0016020 GO:0005813 GO:0016021
Cluster-22178.44864 GO:0006811 GO:0007052 GO:0035058 GO:0055085 GO:0031122 GO:0007165 GO:0005515 GO:0005216 GO:0004888

GO:0016021 GO:0005813 GO:0016020 GO:0097546

Cluster-22178.44865 GO:0009847 GO:0006508 GO:0003676 GO:0008233

GO:0000166 GO:0003723

Cluster-22178.44869 GO:0006886 GO:0006260 GO:0008134 GO:0042803

GO:0005198 GO:0045502 GO:0016020 GO:0005667 GO:0005801

GO:0030286

Cluster-22178.44871 GO:0006355 GO:0003676 GO:0005622

Cluster-22178.44873 GO:0015992 GO:0006123 GO:0016579 GO:0036459

GO:0005515 GO:0004129 GO:0045277

Cluster-22178.44878 GO:0005515

Cluster-22178.44885 GO:0016021

Cluster-22178.44886 GO:0046872 GO:0008270 GO:0003677

Cluster-22178.44893 GO:0006355 GO:0048870 GO:0005515 GO:0003700

GO:0043565 GO:0031514 GO:0019028 GO:0005667

Cluster-22178.44898 GO:0016567 GO:0004842

Cluster-22178.4490 GO:0006313 GO:0007275

Cluster-22178.44900 GO:0043066 GO:0045766 GO:0043123 GO:0005515

Cluster-22178.44902 GO:0015940 GO:0019521 GO:0055114 GO:0006574

GO:0006552 GO:0006550 GO:0006568 GO:0006633 GO:0006554

GO:0006631 GO:0018874 GO:0006098 GO:0008677 GO:0003857

GO:0016491 GO:0004616 GO:0051287

Cluster-22178.44903 GO:0006000 GO:0015976 GO:0006013 GO:0051289

GO:0006020 GO:0006094 GO:0006098 GO:0006096 GO:0004332

GO:0005515

Cluster-22178.44905 GO:0006811 GO:0006813 GO:0005249 GO:0008076

GO:0016020

Cluster-22178.44906 GO:0051205 GO:0016021

Cluster-22178.44907 GO:0019643 GO:0019069 GO:0006094 GO:0009069

GO:0006468 GO:0006099 GO:0015977 GO:0007165 GO:0006308

GO:0016310 GO:0008964 GO:0008855 GO:0005096 GO:0004674

GO:0009318

Cluster-22178.44910 GO:0006355 GO:0009271 GO:0016021

Cluster-22178.44911 GO:0046872 GO:0016021
Cluster-22178.44912 GO:0046872 GO:0016021
Cluster-22178.44913 GO:0046872 GO:0016021
Cluster-22178.44920 GO:0006355 GO:0003677
Cluster-22178.44923 GO:0006468 GO:0005524 GO:0004672 GO:0016773
GO:0016020
Cluster-22178.44925 GO:0022900 GO:0016021
Cluster-22178.44931 GO:0055085 GO:0016021
Cluster-22178.44936 GO:0007050
Cluster-22178.44938 GO:0003779
Cluster-22178.44947 GO:0051258 GO:0009405 GO:0010508 GO:0006260
GO:0006606 GO:0030168 GO:0006357 GO:0051262 GO:0007165
GO:0019058 GO:0007067 GO:0007155 GO:0004871 GO:0001104
GO:0005198 GO:0005515 GO:0005102 GO:0030674 GO:0000775
GO:0019031 GO:0016021 GO:0019867 GO:0042729 GO:0019028
GO:0072686 GO:0005577 GO:0005882 GO:0016592 GO:0016020
Cluster-22178.44950 GO:0051258 GO:0009405 GO:0006904 GO:0006886
GO:0030168 GO:0015031 GO:0006810 GO:0007165 GO:0019871
GO:0005198 GO:0005102 GO:0030674 GO:0005801 GO:0005576
GO:0005577 GO:0005882 GO:0016020 GO:0000145
Cluster-22178.44955 GO:0007264 GO:0098519 GO:0005525 GO:0003924
GO:0016887 GO:0005524
Cluster-22178.44956 GO:0003677
Cluster-22178.44958 GO:0046872 GO:0005515
Cluster-22178.44959 GO:0016192 GO:0006886 GO:0005515 GO:0030117
Cluster-22178.44962 GO:0003677
Cluster-22178.44966 GO:0005975 GO:0006355 GO:0008270 GO:0003824
GO:0003682 GO:0043169 GO:0003677 GO:0000785 GO:0000775
GO:0005634
Cluster-22178.44976 GO:0006816 GO:0006874 GO:0005219 GO:0046872
GO:0004252 GO:0016021 GO:0005622 GO:0005886
Cluster-22178.44977 GO:0009058 GO:0006508 GO:0006144 GO:0004252
GO:0030170 GO:0003877

Cluster-22178.44980 GO:0005515
Cluster-22178.44982 GO:0015940 GO:0015937 GO:0004140 GO:0005524
GO:0005921
Cluster-22178.44989 GO:0007165 GO:0005515 GO:0005509 GO:0005578
Cluster-22178.44991 GO:0008152 GO:0015074 GO:0003824 GO:0016021
Cluster-22178.44995 GO:0006139 GO:0031167 GO:0008168
Cluster-22178.44996 GO:0031167 GO:0006139 GO:0008168
Cluster-22178.44999 GO:0009405
Cluster-22178.45001 GO:0005515 GO:0008270
Cluster-22178.45002 GO:0008152 GO:0003824
Cluster-22178.45005 GO:0042981
Cluster-22178.45006 GO:0030071 GO:0005515 GO:0046983 GO:0005680
Cluster-22178.45007 GO:0008152 GO:0008168
Cluster-22178.45012 GO:0008152 GO:0016491
Cluster-22178.45015 GO:0016021
Cluster-22178.45018 GO:0006406 GO:0007264 GO:0016887 GO:0005524
GO:0005525 GO:0003723 GO:0005737 GO:0005634
Cluster-22178.45020 GO:0015628 GO:0006118 GO:0040007 GO:0015031
GO:0006810 GO:0006614 GO:0007165 GO:0008283 GO:0005525
GO:0009055 GO:0003676 GO:0008083 GO:0005515 GO:0004386
GO:0005524 GO:0016887 GO:0008565 GO:0015627
Cluster-22178.45025 GO:0006694 GO:0016114 GO:0006351 GO:0006886
GO:0006284 GO:0006206 GO:0006144 GO:0016192 GO:0006383
GO:0003899 GO:0005515 GO:0005198 GO:0008661 GO:0003677
GO:0030117 GO:0005634 GO:0005730 GO:0005666
Cluster-22178.45027 GO:0005515
Cluster-22178.45029 GO:0006397 GO:0005634
Cluster-22178.45030 GO:0006811 GO:0006813 GO:0005249 GO:0008270
GO:0008076 GO:0016020
Cluster-22178.45031 GO:0006813 GO:0006811 GO:0008270 GO:0005249
GO:0016020 GO:0008076
Cluster-22178.45033 GO:0055085 GO:0022857 GO:0016021
Cluster-22178.45035 GO:0016192 GO:0006886 GO:0018193 GO:0008061

GO:0005515 GO:0005198 GO:0030126 GO:0016020
Cluster-22178.45036 GO:0016020
Cluster-22178.45037 GO:0046872
Cluster-22178.45039 GO:0006886 GO:0006888 GO:0003677 GO:0008270
GO:0046872 GO:0045202 GO:0030127
Cluster-22178.45043 GO:0016021 GO:0016020
Cluster-22178.45047 GO:0015979 GO:0009523 GO:0009539 GO:0016020
Cluster-22178.45050 GO:0007018 GO:0005515 GO:0005868
Cluster-22178.45051 GO:0035556 GO:0005515 GO:0003677
Cluster-22178.45053 GO:0031047 GO:0045454 GO:0006118 GO:0015035
GO:0009055
Cluster-22178.45054 GO:0006355 GO:0003700 GO:0043565 GO:0005667
Cluster-22178.45056 GO:0016310 GO:0009069 GO:0006468 GO:0004674
GO:0005524 GO:0004672
Cluster-22178.45057 GO:0007059 GO:0051301 GO:0005515 GO:0008017
GO:0045298 GO:0000444
Cluster-22178.45061 GO:0035556 GO:0009279
Cluster-22178.45063 GO:0007268 GO:0006811 GO:0007165 GO:0005234
GO:0004970 GO:0016020
Cluster-22178.45067 GO:0009424
Cluster-22178.45068 GO:0042742 GO:0005515
Cluster-22178.45071 GO:0043087 GO:0032012 GO:0005086
Cluster-22178.45073 GO:0006813 GO:0015269 GO:0016020
Cluster-22178.45076 GO:0005515
Cluster-22178.45079 GO:0006479 GO:0009451 GO:0046500 GO:0008152
GO:0006464 GO:0030488 GO:0008033 GO:0016429 GO:0004719
GO:0008168 GO:0031515
Cluster-22178.45080 GO:0007165 GO:0005515
Cluster-22178.45088 GO:0006355 GO:0008270 GO:0003677 GO:0005634
Cluster-22178.45090 GO:0051028 GO:0005975 GO:0048309 GO:0019281
GO:0006555 GO:0042967 GO:0008899 GO:0004553 GO:0042302
GO:0005737
Cluster-22178.45096 GO:0022904 GO:0016651 GO:0005743

Cluster-22178.45101 GO:0006810 GO:0055085 GO:0005215 GO:0005515
GO:0016021 GO:0016020
Cluster-22178.45102 GO:0006810 GO:0055085 GO:0005215 GO:0005515
GO:0016021 GO:0016020
Cluster-22178.45106 GO:0042967 GO:0016747 GO:0008080 GO:0016021
Cluster-22178.45114 GO:0046677 GO:0008152 GO:0070569
Cluster-22178.45125 GO:0002098 GO:0034227 GO:0000049 GO:0003677
GO:0046872 GO:0008270 GO:0003676 GO:0005634
Cluster-22178.45129 GO:0006230 GO:0006470 GO:0006888 GO:0006206
GO:0006886 GO:0006570 GO:0003824 GO:0004725 GO:0051536
GO:0008270 GO:0004797 GO:0005524 GO:0008138 GO:0030127
Cluster-22178.45130 GO:0035556 GO:0032065 GO:0055114 GO:0016301
GO:0005515 GO:0047134 GO:0005543 GO:0005938
Cluster-22178.45133 GO:0006525 GO:0055114 GO:0006560 GO:0018401
GO:0016702 GO:0004656 GO:0005783
Cluster-22178.45134 GO:0006355 GO:0008191 GO:0043565
Cluster-22178.45135 GO:0006355 GO:0005634
Cluster-22178.45145 GO:0009395 GO:0035556 GO:0008654 GO:0006506
GO:0046486 GO:0004143 GO:0016746 GO:0005515 GO:0005789
GO:0016021 GO:0016020
Cluster-22178.45148 GO:0046486 GO:0006506 GO:0008654 GO:0035556
GO:0009395 GO:0005515 GO:0016746 GO:0004143 GO:0016020
GO:0016021 GO:0005789
Cluster-22178.45152 GO:0005515
Cluster-22178.45155 GO:0042203 GO:0032402 GO:0019261 GO:0046232
GO:0018874 GO:0042184 GO:0055114 GO:0009712 GO:0005506
GO:0018576 GO:0042470
Cluster-22178.45156 GO:0006308 GO:0016888
Cluster-22178.45157 GO:0006308 GO:0016888
Cluster-22178.45159 GO:0005515
Cluster-22178.45162 GO:0003924 GO:0005525
Cluster-22178.45165 GO:0006544 GO:0008654 GO:0046486 GO:0006563
GO:0006566 GO:0008270 GO:0046872 GO:0005515 GO:0004609

Cluster-22178.45166 GO:0016070 GO:0043087 GO:0016021 GO:0019013
Cluster-22178.45167 GO:0006357 GO:0006470 GO:0006570 GO:0004725
GO:0008138 GO:0001104 GO:0016592
Cluster-22178.45174 GO:0042438 GO:0005507
Cluster-22178.45175 GO:0005509 GO:0005515
Cluster-22178.45178 GO:0007165 GO:0006869 GO:0042157 GO:0019915
GO:0008289 GO:0005789 GO:0005576 GO:0008537
Cluster-22178.45180 GO:0006144 GO:0046034 GO:0016055 GO:0032968
GO:0006367 GO:0008013 GO:0046872 GO:0004017 GO:0003677
GO:0016342 GO:0005634
Cluster-22178.45183 GO:0042254 GO:0005975 GO:0003735 GO:0004553
GO:0005840
Cluster-22178.45184 GO:0005515
Cluster-22178.45187 GO:0007165 GO:0005515 GO:0005509 GO:0005578
Cluster-22178.45188 GO:0006821 GO:0055085 GO:0005247 GO:0020037
GO:0016020
Cluster-22178.45189 GO:0055114 GO:0051287
Cluster-22178.45191 GO:0006094 GO:0006118 GO:0017004 GO:0006096
GO:0009094 GO:0000162 GO:0006571 GO:0000287 GO:0004634
GO:0045158 GO:0000015 GO:0009512
Cluster-22178.45200 GO:0003677
Cluster-22178.45202 GO:0008146 GO:0016021
Cluster-22178.45203 GO:0046983 GO:0036128
Cluster-22178.45205 GO:0008152 GO:0016788
Cluster-22178.45207 GO:0042262 GO:0006090 GO:0006357 GO:0006914
GO:0006633 GO:0048870 GO:0051258 GO:0030168 GO:0070206
GO:0006260 GO:0007165 GO:0042803 GO:0005198 GO:0001104
GO:0008017 GO:0003690 GO:0045502 GO:0008134 GO:0003989
GO:0005102 GO:0030674 GO:0045298 GO:0005577 GO:0005667
GO:0016021 GO:0019031 GO:0019028 GO:0031514 GO:0030286
GO:0009317 GO:0005882 GO:0016592
Cluster-22178.45208 GO:0005515
Cluster-22178.45214 GO:0016021

Cluster-22178.45215 GO:0005975 GO:0048870 GO:0001558 GO:0030178
GO:0006914 GO:0006606 GO:0007275 GO:0007165 GO:0005515
GO:0008270 GO:0005520 GO:0004553 GO:0030246 GO:0005576
GO:0031514 GO:0016942
Cluster-22178.45216 GO:0030178 GO:0001558 GO:0005975 GO:0048870
GO:0007165 GO:0007275 GO:0006606 GO:0006914 GO:0005515
GO:0030246 GO:0004553 GO:0008270 GO:0005520 GO:0031514
GO:0005576 GO:0016942
Cluster-22178.45223 GO:0007165 GO:0009372 GO:0005515 GO:0005509
GO:0005578
Cluster-22178.45224 GO:0006355 GO:0009271
Cluster-22178.45225 GO:0006119 GO:0006118 GO:0006351 GO:0055114
GO:0006144 GO:0015992 GO:0008121 GO:0003723 GO:0003968
GO:0019033 GO:0031379
Cluster-22178.45230 GO:0006355 GO:0043565 GO:0003700 GO:0005667
Cluster-22178.45233 GO:0006468 GO:0005524 GO:0003774 GO:0004672
GO:0016459
Cluster-22178.45235 GO:0007165
Cluster-22178.45236 GO:0043087 GO:0035023 GO:0035556 GO:0008092
GO:0005089 GO:0005576 GO:0005737 GO:0019898
Cluster-22178.4524 GO:0016788
Cluster-22178.45240 GO:0003676 GO:0008270 GO:0046872
Cluster-22178.45248 GO:0006284 GO:0008725 GO:0008440
Cluster-22178.45253 GO:0006308 GO:0055114 GO:0016114 GO:0006694
GO:0006810 GO:0003924 GO:0005525 GO:0003723 GO:0004506
GO:0016887 GO:0008855 GO:0005524 GO:0003724 GO:0050660
GO:0098519 GO:0016021 GO:0009318 GO:0016020
Cluster-22178.45254 GO:0007049 GO:0006260 GO:0048870 GO:0006914
GO:0006810 GO:0005886 GO:0031514 GO:0005634
Cluster-22178.45256 GO:0007165 GO:0005179 GO:0005576
Cluster-22178.45257 GO:0035064
Cluster-22178.45259 GO:0046872
Cluster-22178.45260 GO:0007264 GO:0005525 GO:0003924

Cluster-22178.45262 GO:0007275 GO:0035091
Cluster-22178.45267 GO:0019497 GO:0006771 GO:0007165 GO:0002532
GO:0046872 GO:0003993 GO:0004896 GO:0005515 GO:0016021
Cluster-22178.45272 GO:0032979 GO:0042578 GO:0005743
Cluster-22178.45276 GO:0006289 GO:0055085 GO:0000105 GO:0055114
GO:0006308 GO:0006355 GO:0030001 GO:0008855 GO:0008270
GO:0004399 GO:0051287 GO:0046873 GO:0008168 GO:0000439
GO:0016020 GO:0009318
Cluster-22178.45278 GO:0008152 GO:0019684 GO:0006118 GO:0045156
GO:0016758 GO:0030077 GO:0016021
Cluster-22178.45279 GO:0008033 GO:0009451 GO:0009020 GO:0005515

Cluster-22178.45282 GO:0006816 GO:0070588 GO:0015002 GO:0005515
GO:0005262 GO:0016020
Cluster-22178.45283 GO:0008643 GO:0055085 GO:0005351 GO:0000139
GO:0016020 GO:0016021
Cluster-22178.45287 GO:0016787
Cluster-22178.45291 GO:0030246
Cluster-22178.45293 GO:0005515
Cluster-22178.4530 GO:0006449 GO:0006415 GO:0007155 GO:0005540
GO:0005515 GO:0003747 GO:0018444 GO:0005840
Cluster-22178.45301 GO:0007275 GO:0006468 GO:0045893 GO:0008270
GO:0005524 GO:0004672 GO:0046983
Cluster-22178.45305 GO:0015937 GO:0015940 GO:0005524 GO:0004594

Cluster-22178.45307 GO:0007165 GO:0005509 GO:0005515 GO:0005578
Cluster-22178.45308 GO:0016788
Cluster-22178.45310 GO:0006144 GO:0002098 GO:0034227 GO:0046034
GO:0006468 GO:0046872 GO:0005524 GO:0008270 GO:0004672
GO:0003676 GO:0016787 GO:0000049 GO:0004017 GO:0003677
GO:0005634
Cluster-22178.45318 GO:0030153
Cluster-22178.45322 GO:0006470 GO:0006570 GO:0008138 GO:0004725

GO:0005515 GO:0004721 GO:0008270
Cluster-22178.45323 GO:0006486 GO:0008417 GO:0016020
Cluster-22178.45324 GO:0006355 GO:0006486 GO:0003682 GO:0003677
GO:0008270 GO:0008417 GO:0005634 GO:0016020 GO:0005789
GO:0000775 GO:0000785
Cluster-22178.45325 GO:0006486 GO:0008417 GO:0008270 GO:0005789
GO:0016020
Cluster-22178.45326 GO:0055085 GO:0006812 GO:0006835 GO:0006820
GO:0030001 GO:0006508 GO:0017153 GO:0046873 GO:0016020
Cluster-22178.45328 GO:0003677
Cluster-22178.45330 GO:0046872
Cluster-22178.45334 GO:0016757
Cluster-22178.45336 GO:0005515
Cluster-22178.45337 GO:0005515
Cluster-22178.45340 GO:0006508 GO:0008152 GO:0008270 GO:0005515
GO:0004181 GO:0016788 GO:0005576
Cluster-22178.45347 GO:0035556 GO:0005515 GO:0019013 GO:0016021
Cluster-22178.45349 GO:0007264 GO:0003924 GO:0016887 GO:0005524
GO:0005525 GO:0016020
Cluster-22178.45357 GO:0008152 GO:0016757
Cluster-22178.45358 GO:0008152 GO:0016757
Cluster-22178.45363 GO:0005515
Cluster-22178.45365 GO:0055114 GO:0042773 GO:0016651 GO:0016020
GO:0016021 GO:0019031
Cluster-22178.45366 GO:0042773 GO:0055114 GO:0016651 GO:0019031
GO:0016021 GO:0016020
Cluster-22178.45372 GO:0045892 GO:0006508 GO:0042754 GO:0008270
GO:0004222 GO:0031012
Cluster-22178.45375 GO:0006355 GO:0007165 GO:0006955 GO:0005179
GO:0003700 GO:0005576 GO:0005667
Cluster-22178.45376 GO:0006955 GO:0007165 GO:0006355 GO:0003700
GO:0005179 GO:0005667 GO:0005576
Cluster-22178.45377 GO:0006355 GO:0007165 GO:0006955 GO:0005179

GO:0003700 GO:0005576 GO:0005667
Cluster-22178.45378 GO:0006355 GO:0007165 GO:0006955 GO:0005179
GO:0003700 GO:0005576 GO:0005667
Cluster-22178.45383 GO:0006691 GO:0019530 GO:0006749 GO:0006693
GO:0006486 GO:0051252 GO:0003840 GO:0008373 GO:0004540
Cluster-22178.45385 GO:0006355 GO:0006629 GO:0006508 GO:0009405
GO:0016192 GO:0004222 GO:0003700 GO:0005667 GO:0005634
Cluster-22178.45387 GO:0009069 GO:0042254 GO:0006412 GO:0016310
GO:0003735 GO:0004674 GO:0005622 GO:0005840
Cluster-22178.45390 GO:0008270 GO:0005515 GO:0005509
Cluster-22178.45392 GO:0005975 GO:0004571 GO:0005509 GO:0016020
Cluster-22178.45396 GO:0046983 GO:0005515
Cluster-22178.45397 GO:0006629
Cluster-22178.45399 GO:0036065 GO:0009246 GO:0055114 GO:0020037
GO:0016705 GO:0008417 GO:0005506
Cluster-22178.45402 GO:0006000 GO:0006013 GO:0007155 GO:0006508
GO:0007186 GO:0005524 GO:0004930 GO:0004252 GO:0005515
GO:0003873 GO:0031224 GO:0016020
Cluster-22178.45409 GO:0006508 GO:0004197 GO:0004252
Cluster-22178.45411 GO:0006554 GO:0006811 GO:0055085 GO:0034968
GO:0006479 GO:0006367 GO:0008270 GO:0018024 GO:0005216
GO:0005515 GO:0019015 GO:0005634 GO:0005672 GO:0016020
Cluster-22178.45417 GO:0006355 GO:0007155 GO:0046872 GO:0008270
GO:0043565 GO:0003700 GO:0005198 GO:0005515 GO:0009288
GO:0019031 GO:0031225 GO:0005667 GO:0019028
Cluster-22178.45418 GO:0000226 GO:0000910 GO:0008017 GO:0019031
GO:0045298
Cluster-22178.45419 GO:0005515
Cluster-22178.45420 GO:0003824 GO:0051536
Cluster-22178.45425 GO:0005515 GO:0005737
Cluster-22178.45429 GO:0006118 GO:0009055
Cluster-22178.45433 GO:0006508 GO:0015031 GO:0008236 GO:0016787

Cluster-22178.45440 GO:0006412 GO:0042254 GO:0016226 GO:0045893
GO:0005506 GO:0003735 GO:0051536 GO:0005634 GO:0005622
GO:0005840
Cluster-22178.45442 GO:0005515
Cluster-22178.45448 GO:0009405 GO:0006351 GO:0051716 GO:0006810
GO:0006364 GO:0006383 GO:0006260 GO:0006206 GO:0009236
GO:0006144 GO:0003887 GO:0003899 GO:0005515 GO:0008200
GO:0004190 GO:0016817 GO:0003677 GO:0042575 GO:0005576
GO:0005634 GO:0034457 GO:0032040 GO:0005789 GO:0016021
GO:0046658 GO:0016020 GO:0005730 GO:0005666 GO:0005732
Cluster-22178.45449 GO:0009405 GO:0006508 GO:0050790 GO:0004197
GO:0005576
Cluster-22178.45452 GO:0003950 GO:0005515
Cluster-22178.45453 GO:0009082
Cluster-22178.45455 GO:0006468 GO:0005524 GO:0004672 GO:0016773
GO:0016020
Cluster-22178.45459 GO:0008033 GO:0009451 GO:0046872 GO:0009020

Cluster-22178.4546 GO:0006486 GO:0008378 GO:0016757 GO:0016020
Cluster-22178.45460 GO:0007165
Cluster-22178.45466 GO:0003677
Cluster-22178.45468 GO:0034080 GO:0008270 GO:0000775
Cluster-22178.45470 GO:0034080 GO:0008270 GO:0000775
Cluster-22178.45484 GO:0043087 GO:0042742 GO:0035023 GO:0005089

Cluster-22178.45485 GO:0043087 GO:0042742 GO:0035023 GO:0005089

Cluster-22178.45486 GO:0035023 GO:0042742 GO:0043087 GO:0005089

Cluster-22178.45487 GO:0042742 GO:0043087 GO:0035023 GO:0005089

Cluster-22178.45488 GO:0016049 GO:0006468 GO:0010215 GO:0004672
GO:0005524 GO:0031225

Cluster-22178.45489 GO:0016049 GO:0006468 GO:0010215 GO:0005524
GO:0004672 GO:0031225
Cluster-22178.45490 GO:0016049 GO:0006468 GO:0010215 GO:0004672
GO:0005524 GO:0031225
Cluster-22178.45491 GO:0016049 GO:0010215 GO:0006468 GO:0005524
GO:0004672 GO:0031225
Cluster-22178.45500 GO:0007264 GO:0005525
Cluster-22178.45506 GO:0005515
Cluster-22178.45507 GO:0061024 GO:0030176 GO:0016020
Cluster-22178.45508 GO:0008270 GO:0046872 GO:0005515
Cluster-22178.45513 GO:0003924
Cluster-22178.45514 GO:0061025 GO:0006376 GO:0046813 GO:0003729
GO:0005685 GO:0016021 GO:0019031
Cluster-22178.45517 GO:0005975 GO:0005509 GO:0004571 GO:0016020
Cluster-22178.45518 GO:0005975 GO:0004571 GO:0005509 GO:0016020
Cluster-22178.45525 GO:0015858 GO:0007165 GO:0006810 GO:0040007
GO:0015991 GO:0008283 GO:0015992 GO:0008083 GO:0005337
GO:0015078 GO:0005524 GO:0003774 GO:0033179 GO:0016459
GO:0016021
Cluster-22178.45529 GO:0005515
Cluster-22178.45532 GO:0006508 GO:0003676 GO:0008233
Cluster-22178.45533 GO:0016055 GO:0032780 GO:0007165 GO:0006914
GO:0030168 GO:0006260 GO:0006355 GO:0016573 GO:0006308
GO:0048870 GO:0051258 GO:0030674 GO:0008013 GO:0008855
GO:0005102 GO:0005515 GO:0005198 GO:0043565 GO:0042030
GO:0003700 GO:0005667 GO:0005739 GO:0016342 GO:0005577
GO:0031514 GO:0009318
Cluster-22178.45536 GO:0003677
Cluster-22178.45537 GO:0006888 GO:0006886 GO:0008536 GO:0005622
Cluster-22178.45538 GO:0006886 GO:0008536
Cluster-22178.45540 GO:0007224 GO:0016032 GO:0006275 GO:0006355

Cluster-22178.45545 GO:2001256 GO:0030176

Cluster-22178.45549 GO:0046656 GO:0006457 GO:0015948 GO:0030269
GO:0016021
Cluster-22178.45551 GO:0005975 GO:0046486 GO:0001575 GO:0006012
GO:0004557
Cluster-22178.45554 GO:0006281 GO:0003824 GO:0003908
Cluster-22178.45563 GO:0005515
Cluster-22178.45571 GO:0006468 GO:0004672 GO:0005524
Cluster-22178.45574 GO:0006355 GO:0003677
Cluster-22178.45575 GO:0035556 GO:0046872
Cluster-22178.45578 GO:0005515
Cluster-22178.45579 GO:0019988 GO:0006470 GO:0006570 GO:0004725
GO:0008138 GO:0043399
Cluster-22178.45580 GO:0007031 GO:0005524 GO:0019031 GO:0005777
Cluster-22178.45586 GO:0008152 GO:0016491
Cluster-22178.45589 GO:0005509
Cluster-22178.45590 GO:0005515
Cluster-22178.45591 GO:0006260 GO:0016192 GO:0019079 GO:0005524
GO:0003678 GO:0003676 GO:0016787 GO:0005515 GO:0003677
GO:0016021 GO:0005657
Cluster-22178.45593 GO:0005515 GO:0008270
Cluster-22178.45597 GO:0005515
Cluster-22178.45600 GO:0005515
Cluster-22178.45601 GO:0003677 GO:0046872 GO:0008270
Cluster-22178.45605 GO:0055085 GO:0016021 GO:0055036
Cluster-22178.45607 GO:0008152 GO:0008484 GO:0003824
Cluster-22178.45608 GO:0005515
Cluster-22178.45609 GO:0006310 GO:0003677 GO:0005515 GO:0005634
Cluster-22178.45613 GO:0048870 GO:0045502 GO:0008134 GO:0042803
GO:0008092 GO:0005667 GO:0031514 GO:0005737 GO:0030286
GO:0019898
Cluster-22178.45615 GO:0006308 GO:0006891 GO:0042254 GO:0006412
GO:0007165 GO:0008855 GO:0051082 GO:0005515 GO:0003735
GO:0005622 GO:0005840 GO:0017119 GO:0009318

Cluster-22178.45616 GO:0042254 GO:0006412 GO:0007165 GO:0006891 GO:0005515 GO:0003735 GO:0016817 GO:0017119 GO:0005840 GO:0005622
Cluster-22178.45617 GO:0006886 GO:0007186 GO:0050909 GO:0007031 GO:0000921 GO:0051276 GO:0005198 GO:0005515 GO:0000166 GO:0003677 GO:0008773 GO:0004930 GO:0005524 GO:0019028 GO:0005801 GO:0016021 GO:0019031 GO:0016020 GO:0005694 GO:0005940 GO:0005777
Cluster-22178.45619 GO:0007165 GO:0043087 GO:0007264 GO:0016485 GO:0005515 GO:0005085 GO:0016021
Cluster-22178.45621 GO:0020037 GO:0003676 GO:0003723 GO:0046872 GO:0005576
Cluster-22178.45626 GO:0046872
Cluster-22178.45628 GO:0005515
Cluster-22178.45639 GO:0055114 GO:0008168 GO:0005515 GO:0016706

Cluster-22178.45641 GO:0055114 GO:0008168 GO:0016706 GO:0005515

Cluster-22178.45644 GO:0006508 GO:0008152 GO:0008270 GO:0016788 GO:0004181
Cluster-22178.45647 GO:0008152 GO:0006886 GO:0016836 GO:0003824 GO:0005783 GO:0016021
Cluster-22178.45649 GO:0003697
Cluster-22178.45652 GO:0003697
Cluster-22178.45665 GO:0015684 GO:0007264 GO:0005525 GO:0015093 GO:0016021 GO:0019028
Cluster-22178.45666 GO:0005975 GO:0006355 GO:0016763 GO:0003677 GO:0030248 GO:0005634 GO:0019028
Cluster-22178.45667 GO:0005975 GO:0006355 GO:0003677 GO:0030248 GO:0016763 GO:0005634 GO:0019028
Cluster-22178.45672 GO:0008270 GO:0003676
Cluster-22178.45677 GO:0042254 GO:0006412 GO:0051252 GO:0008033 GO:0003735 GO:0004540 GO:0015934 GO:0005840

Cluster-22178.45681 GO:0006886 GO:0006531 GO:0006188 GO:0009152
GO:0006522 GO:0006144 GO:0004018 GO:0005737
Cluster-22178.45682 GO:0005515 GO:0061630 GO:0046872 GO:0008270

Cluster-22178.45686 GO:0003677 GO:0005634
Cluster-22178.45690 GO:0007268 GO:0009405 GO:0007165 GO:0006355
GO:0048193 GO:0030550 GO:0003700 GO:0008270 GO:0016020
GO:0005667 GO:0005634 GO:0005576
Cluster-22178.45691 GO:0005515
Cluster-22178.45692 GO:0008210 GO:0008207 GO:0046487 GO:0008209
GO:0009396 GO:0055114 GO:0008152 GO:0006694 GO:0003854
GO:0050662 GO:0004488 GO:0016616 GO:0016491 GO:0003824
Cluster-22178.45694 GO:0009058 GO:0006144 GO:0006508 GO:0004252
GO:0003877 GO:0030170
Cluster-22178.45697 GO:0006508 GO:0016579 GO:0006511 GO:0004843
GO:0005622
Cluster-22178.45700 GO:0043565 GO:0005515 GO:0005634
Cluster-22178.45701 GO:0003677
Cluster-22178.45702 GO:0006816 GO:0006810 GO:0005246 GO:0042030
GO:0005515 GO:0016020
Cluster-22178.45703 GO:0006810 GO:0006816 GO:0005515 GO:0042030
GO:0005246 GO:0016020
Cluster-22178.45704 GO:0007155 GO:0006508 GO:0006412 GO:0042254
GO:0004222 GO:0003735 GO:0005622 GO:0005840 GO:0016020
Cluster-22178.45705 GO:0006412 GO:0042254 GO:0007155 GO:0006508
GO:0003735 GO:0051537 GO:0004222 GO:0016020 GO:0043231
GO:0005840 GO:0005622
Cluster-22178.45706 GO:0006508 GO:0007155 GO:0042254 GO:0006412
GO:0003735 GO:0004222 GO:0016020 GO:0005622 GO:0005840
Cluster-22178.45707 GO:0016021
Cluster-22178.45708 GO:0005515
Cluster-22178.45709 GO:0016021
Cluster-22178.45711 GO:0006810 GO:0015893 GO:0006855 GO:0055085

GO:0015238 GO:0015297 GO:0016020
Cluster-22178.45721 GO:0016567 GO:0046872 GO:0008270 GO:0005515
GO:0004842 GO:0005680
Cluster-22178.45733 GO:0006508 GO:0004252
Cluster-22178.45734 GO:0008270
Cluster-22178.45735 GO:1901031 GO:0055114 GO:0004190 GO:0005634
GO:0016021
Cluster-22178.45736 GO:0042254 GO:0006412 GO:0003723 GO:0003735
GO:0005622 GO:0005840
Cluster-22178.45741 GO:0007165 GO:0005515
Cluster-22178.45744 GO:0006506 GO:0007009 GO:0005515 GO:0005509
GO:0003676 GO:0017176 GO:0016021 GO:0005737
Cluster-22178.45745 GO:0019684 GO:0006904 GO:0006118 GO:0016192
GO:0045156 GO:0005515 GO:0016021 GO:0030077
Cluster-22178.45746 GO:0016310 GO:0007178 GO:0009069 GO:0006468
GO:0005524 GO:0004672 GO:0004675 GO:0016020
Cluster-22178.45748 GO:0003676 GO:0003723
Cluster-22178.45753 GO:0015979 GO:0009523 GO:0009539 GO:0016020
Cluster-22178.45756 GO:0006605 GO:0006886 GO:0005515 GO:0005742
Cluster-22178.45758 GO:0006493 GO:0000030 GO:0016020 GO:0000136
Cluster-22178.45761 GO:0006508 GO:0008236
Cluster-22178.45763 GO:0006481 GO:0006479 GO:0004671 GO:0016020
GO:0016021
Cluster-22178.45764 GO:0007224 GO:0015074
Cluster-22178.45777 GO:0030182 GO:0006281 GO:0021549 GO:0006310
GO:0005524 GO:0009378 GO:0005657 GO:0009379
Cluster-22178.45780 GO:0055114 GO:0009308 GO:0005507 GO:0048038
GO:0008131 GO:0008270
Cluster-22178.45789 GO:0005515
Cluster-22178.45790 GO:0005515 GO:0046872 GO:0031225
Cluster-22178.45800 GO:0005515
Cluster-22178.45806 GO:0005515 GO:0016021
Cluster-22178.45811 GO:0006858

Cluster-22178.45813 GO:0007264 GO:0005525
Cluster-22178.45814 GO:0005198 GO:0005882
Cluster-22178.45815 GO:0015992 GO:0015986 GO:0015078 GO:0000276
Cluster-22178.45819 GO:0006811 GO:0005216 GO:0016020
Cluster-22178.45820 GO:0003677
Cluster-22178.45821 GO:0005975 GO:0006013 GO:0006886 GO:0008536
GO:0004553 GO:0003824 GO:0004559 GO:0008270 GO:0015923
Cluster-22178.45822 GO:0005515 GO:0035091
Cluster-22178.45826 GO:0034599 GO:0005789
Cluster-22178.45828 GO:0009271 GO:0006355
Cluster-22178.45833 GO:0005515
Cluster-22178.45834 GO:0055085 GO:0030001 GO:0045040 GO:0007268
GO:0033130 GO:0046873 GO:0043495 GO:0005198 GO:0005515
GO:0042802 GO:0005742 GO:0019028 GO:0016020
Cluster-22178.45836 GO:0016192 GO:0006886 GO:0008536 GO:0005515
GO:0030117 GO:0005856
Cluster-22178.45839 GO:0006471 GO:0003956
Cluster-22178.4584 GO:0006449 GO:0007155 GO:0006415 GO:0003747
GO:0005540 GO:0005840 GO:0018444
Cluster-22178.45843 GO:0006940
Cluster-22178.45844 GO:0055114 GO:0008152 GO:0015991 GO:0006412
GO:0007010 GO:0046487 GO:0009396 GO:0051607 GO:0015992
GO:0042254 GO:0003779 GO:0003824 GO:0015078 GO:0051082
GO:0016491 GO:0050662 GO:0004488 GO:0003735 GO:0005840
GO:0033179
Cluster-22178.45845 GO:0046488 GO:0005515 GO:0016307
Cluster-22178.45846 GO:0005515
Cluster-22178.45849 GO:0005515
Cluster-22178.45851 GO:0006816 GO:0006874 GO:0006397 GO:0046660
GO:0006811 GO:0004190 GO:0005219 GO:0005634 GO:0016020
GO:0005622 GO:0016021
Cluster-22178.45859 GO:0005515
Cluster-22178.45860 GO:0016567 GO:0046872 GO:0008270 GO:0005515

GO:0004842 GO:0005680
Cluster-22178.45863 GO:0000184 GO:0008270 GO:0005524 GO:0003677
GO:0004386 GO:0005737 GO:0005634
Cluster-22178.45864 GO:0000184 GO:0008270 GO:0005524 GO:0003677
GO:0004386 GO:0005634 GO:0005737
Cluster-22178.45865 GO:0043087 GO:0019904 GO:0005515 GO:0008134
GO:0005667
Cluster-22178.45873 GO:0005515
Cluster-22178.45874 GO:0005515
Cluster-22178.45877 GO:0015074 GO:0006364 GO:0008270 GO:0003676

Cluster-22178.45879 GO:0005515
Cluster-22178.45880 GO:0006570 GO:0006470 GO:0005515 GO:0004725
GO:0008138 GO:0016021
Cluster-22178.45881 GO:0008152 GO:0003824
Cluster-22178.45884 GO:0045765 GO:0045947 GO:0006508 GO:0006144
GO:0008190 GO:0008234 GO:0003968 GO:0031379 GO:0005737
Cluster-22178.45885 GO:0006810 GO:0009405 GO:0008200 GO:0051537
GO:0005515 GO:0042151 GO:0005643
Cluster-22178.45896 GO:0007172 GO:0007165 GO:0006468 GO:0051258
GO:0030168 GO:0030674 GO:0004713 GO:0005102 GO:0004871
GO:0004672 GO:0005524 GO:0005925 GO:0005577 GO:0016021
Cluster-22178.45899 GO:0003677
Cluster-22178.45900 GO:0006397 GO:0008033 GO:0055114 GO:0019354
GO:0016117 GO:0006779 GO:0003723 GO:0016705 GO:0005515
GO:0043115 GO:0071949 GO:0016491 GO:0008270 GO:0050660
GO:0005634 GO:0005737
Cluster-22178.45902 GO:0015979 GO:0009522 GO:0009538
Cluster-22178.45904 GO:0008152 GO:0007160 GO:0008168 GO:0005509
GO:0005576
Cluster-22178.45906 GO:0007165 GO:0007186 GO:0055114 GO:0008643
GO:0005351 GO:0016641 GO:0005525 GO:0004871 GO:0005507
GO:0019001 GO:0031683 GO:0003924 GO:0016021 GO:0000139

Cluster-22178.45908 GO:0006771 GO:0019497 GO:0005515 GO:0003993 GO:0046872
Cluster-22178.45909 GO:0006771 GO:0019497 GO:0046872 GO:0005515 GO:0003993
Cluster-22178.45912 GO:0006144 GO:0055114 GO:0006206 GO:0016117 GO:0006351 GO:0016491 GO:0005524 GO:0046872 GO:0003723 GO:0016705 GO:0005198 GO:0003899 GO:0000166 GO:0003677 GO:0005730
Cluster-22178.45914 GO:0005515
Cluster-22178.45915 GO:0005515 GO:0016020
Cluster-22178.45917 GO:0090266 GO:0006270 GO:0006351 GO:0009236 GO:0006260 GO:0005515 GO:0003677 GO:0003887 GO:0008270 GO:0005634 GO:0042575 GO:0005789 GO:0032040 GO:0005680
Cluster-22178.45918 GO:0007224
Cluster-22178.45920 GO:0006359
Cluster-22178.45924 GO:0006313 GO:0007165 GO:0009069 GO:0000184 GO:0006470 GO:0016310 GO:0000087 GO:0006570 GO:0004674 GO:0016773 GO:0004803 GO:0008144 GO:0004725 GO:0008168 GO:0005198 GO:0005515 GO:0005622 GO:0019031 GO:0019028
Cluster-22178.45929 GO:0007264 GO:0007165 GO:0007186 GO:0006355 GO:0003677 GO:0005524 GO:0016887 GO:0003724 GO:0003723 GO:0000166 GO:0008134 GO:0031683 GO:0019001 GO:0043531 GO:0003924 GO:0005525 GO:0004871 GO:0005667 GO:0016020
Cluster-22178.45933 GO:0015992 GO:0006119 GO:0006118 GO:0008121 GO:0009055 GO:0004222
Cluster-22178.45943 GO:0005515
Cluster-22178.45944 GO:0006771 GO:0019497 GO:0046872 GO:0005515 GO:0003993
Cluster-22178.45948 GO:0005515
Cluster-22178.45954 GO:0055114 GO:0007049 GO:0007067 GO:0006412 GO:0008168 GO:0016491 GO:0000775 GO:0005634
Cluster-22178.45960 GO:0009069 GO:0006468 GO:0016310 GO:0007178 GO:0005026 GO:0005524 GO:0046872 GO:0016020

Cluster-22178.45964 GO:0006468 GO:0006351 GO:0004672 GO:0005524
GO:0016773 GO:0016020
Cluster-22178.45965 GO:0000160 GO:0006935 GO:0000156 GO:0005515
GO:0008984 GO:0005737
Cluster-22178.45969 GO:0008152 GO:0003824
Cluster-22178.45970 GO:0016020 GO:0019012
Cluster-22178.45974 GO:0035556 GO:0007218 GO:0055085 GO:0005185
GO:0016747 GO:0016021 GO:0005576
Cluster-22178.45983 GO:0006397 GO:0008270
Cluster-22178.45986 GO:0006468 GO:0006351 GO:0006144 GO:0003723
GO:0005524 GO:0004672 GO:0003968 GO:0031379
Cluster-22178.45991 GO:0003676
Cluster-22178.45994 GO:0007165 GO:0005179 GO:0005634 GO:0005576
Cluster-22178.45996 GO:0006355 GO:0003677
Cluster-22178.45998 GO:0003723 GO:0003676
Cluster-22178.45999 GO:0005515
Cluster-22178.46 GO:0050829 GO:0019835 GO:0016021
Cluster-22178.46000 GO:0006241 GO:0006206 GO:0006144 GO:0007010
GO:0006183 GO:0006228 GO:0006165 GO:0004550 GO:0003779
Cluster-22178.46007 GO:0006096 GO:0006094 GO:0015976 GO:0055114
GO:0006144 GO:0000287 GO:0030955 GO:0004743 GO:0003824
Cluster-22178.46008 GO:0006144 GO:0003968 GO:0031379
Cluster-22178.46009 GO:0016192 GO:0071973 GO:0007264 GO:0007067
GO:0000090 GO:0000921 GO:0015031 GO:0006914 GO:0031110
GO:0010508 GO:0006886 GO:0007059 GO:0006935 GO:0051301
GO:0008017 GO:0003774 GO:0008565 GO:0005096 GO:0005198
GO:0016020 GO:0030132 GO:0005923 GO:0005876 GO:0005940
GO:0000940 GO:0030130 GO:0016021 GO:0045298 GO:0009288
GO:0005737
Cluster-22178.46011 GO:0006468 GO:0005524 GO:0004672
Cluster-22178.46012 GO:0006468 GO:0004672 GO:0005524
Cluster-22178.46013 GO:0007264 GO:0007186 GO:0007165 GO:0003924
GO:0019001 GO:0031683 GO:0004871 GO:0005525

Cluster-22178.46021 GO:0005515
Cluster-22178.46022 GO:0006771 GO:0006468 GO:0048013 GO:0019497
GO:0005515 GO:0003993 GO:0004672 GO:0005524 GO:0046872
GO:0019028
Cluster-22178.46026 GO:0006810 GO:0006812 GO:0030474 GO:0007018
GO:0015991 GO:0007017 GO:0006355 GO:0007049 GO:0048870
GO:0006606 GO:0006807 GO:0006914 GO:0015992 GO:0010508
GO:0006886 GO:0008134 GO:0045502 GO:0003700 GO:0003777
GO:0005200 GO:0008092 GO:0016814 GO:0008017 GO:0016887
GO:0003774 GO:0008270 GO:0042803 GO:0005515 GO:0005198
GO:0051082 GO:0015078 GO:0043565 GO:0005882 GO:0019898
GO:0005815 GO:0005823 GO:0005886 GO:0030286 GO:0031514
GO:0033179 GO:0019028 GO:0005801 GO:0016021 GO:0005874
GO:0019031 GO:0016020 GO:0005667 GO:0016459 GO:0048786
GO:0045298 GO:0005856 GO:0005737
Cluster-22178.46027 GO:0005982 GO:0005985 GO:0005986 GO:0003677
GO:0050307
Cluster-22178.46028 GO:0005985 GO:0005982 GO:0005986 GO:0050307
GO:0003677
Cluster-22178.46031 GO:0009306 GO:0016020
Cluster-22178.46037 GO:0006914
Cluster-22178.46038 GO:0006260 GO:0006323 GO:0019012 GO:0005634
Cluster-22178.46039 GO:0009966 GO:0007275 GO:0016020
Cluster-22178.46040 GO:0007155 GO:0030155 GO:0006810 GO:0007165
GO:0030334 GO:0000921 GO:0048193 GO:0055085 GO:0006260
GO:0030001 GO:0045995 GO:0046873 GO:0005102 GO:0004871
GO:0016020 GO:0005940 GO:0005886 GO:0019028 GO:0005634
GO:0016021
Cluster-22178.46041 GO:0009451 GO:0007155 GO:0006260 GO:0030488
GO:0008033 GO:0016429 GO:0031515 GO:0005634
Cluster-22178.46042 GO:0006260 GO:0005634
Cluster-22178.46043 GO:0006281 GO:0006412 GO:0042254 GO:0006310
GO:0009378 GO:0098519 GO:0003735 GO:0005524 GO:0016887

GO:0003924 GO:0005525 GO:0003678 GO:0009379 GO:0005657
GO:0005840
Cluster-22178.46049 GO:0003824
Cluster-22178.46053 GO:0006355 GO:0003677 GO:0004252 GO:0005515
GO:0005886 GO:0005634
Cluster-22178.46060 GO:0015074 GO:0006313 GO:0003677
Cluster-22178.46066 GO:0008616 GO:0006281 GO:0046034 GO:0006144
GO:0006366 GO:0016853 GO:0004017 GO:0003677 GO:0004867
GO:0016740 GO:0046872 GO:0008270 GO:0005576 GO:0005665
Cluster-22178.4607 GO:0020037 GO:0004185 GO:0016021 GO:0005773
Cluster-22178.46072 GO:0006012 GO:0055085 GO:0006465 GO:0009117
GO:0006811 GO:0008233 GO:0008108 GO:0005216 GO:0016021
GO:0005787 GO:0016020
Cluster-22178.46074 GO:0007264 GO:0005525 GO:0005524 GO:0016887
GO:0003924
Cluster-22178.46075 GO:0007264 GO:0005525 GO:0016887 GO:0003924
GO:0005524
Cluster-22178.46076 GO:0007264 GO:0005525 GO:0003924 GO:0016887
GO:0005524
Cluster-22178.46079 GO:0008152 GO:0042967 GO:0016407 GO:0008811

Cluster-22178.46082 GO:0042254 GO:0006412 GO:0007264 GO:0003735
GO:0005525 GO:0003924 GO:0005840 GO:0005622
Cluster-22178.46086 GO:0006000 GO:0006013 GO:0006012 GO:0006470
GO:0006094 GO:0006098 GO:0006570 GO:0006096 GO:0005096
GO:0003872 GO:0004721 GO:0004725 GO:0016301 GO:0005945
Cluster-22178.46090 GO:0006355 GO:0005975 GO:0003682 GO:0043169
GO:0003677 GO:0008270 GO:0003824 GO:0005634 GO:0000785
GO:0000775
Cluster-22178.46091 GO:0006313 GO:0015074 GO:0003677
Cluster-22178.46092 GO:0015979 GO:0005198 GO:0016020 GO:0009522
Cluster-22178.46093 GO:0005515
Cluster-22178.46097 GO:0055114 GO:0030976 GO:0050660 GO:0003824

GO:0016491 GO:0000287
Cluster-22178.46100 GO:0055114 GO:0047134 GO:0008270 GO:0070403

Cluster-22178.46101 GO:0035556 GO:0005515 GO:0016021
Cluster-22178.46102 GO:0010024 GO:0055114 GO:0008152 GO:0016491
GO:0050897 GO:0016636
Cluster-22178.46103 GO:0008641
Cluster-22178.46105 GO:0008152 GO:0007264 GO:0000062 GO:0003824
GO:0005525 GO:0016836
Cluster-22178.46107 GO:0005515 GO:0046872 GO:0008270
Cluster-22178.46111 GO:0007016 GO:0007154 GO:0006355 GO:0007268
GO:0009405 GO:0007165 GO:0030550 GO:0003677 GO:0005515
GO:0005576 GO:0005634 GO:0016010 GO:0016020
Cluster-22178.46112 GO:0003676
Cluster-22178.46113 GO:0006206 GO:0006144 GO:0016310 GO:0009069
GO:0006468 GO:0006351 GO:0005524 GO:0004672 GO:0004674
GO:0003677 GO:0003899 GO:0005730
Cluster-22178.46114 GO:0006351 GO:0006468 GO:0006144 GO:0006206
GO:0003899 GO:0003677 GO:0016773 GO:0005524 GO:0004672
GO:0016020 GO:0005730
Cluster-22178.46115 GO:0006464 GO:0051537 GO:0016151 GO:0008270
GO:0046872 GO:0070403 GO:0003677 GO:0005634
Cluster-22178.46118 GO:0010508 GO:0006886 GO:0006915 GO:0043093
GO:0015992 GO:0000917 GO:0015991 GO:0006355 GO:0006308
GO:0051258 GO:0006919 GO:0051382 GO:0006457 GO:0007165
GO:0030168 GO:0042803 GO:0005515 GO:0051082 GO:0015078
GO:0003700 GO:0019904 GO:0051087 GO:0030674 GO:0005102
GO:0000774 GO:0008855 GO:0000776 GO:0005737 GO:0005783
GO:0005739 GO:0005667 GO:0005577 GO:0016272 GO:0033179
GO:0009318 GO:0016021
Cluster-22178.46129 GO:0055114 GO:0016491 GO:0016616
Cluster-22178.46137 GO:0042254 GO:0006412 GO:0006915 GO:0005515
GO:0003735 GO:0005509 GO:0005840 GO:0005622

Cluster-22178.46141 GO:0032065 GO:0005515 GO:0005543 GO:0005938
Cluster-22178.46143 GO:0016020
Cluster-22178.46144 GO:0016020
Cluster-22178.46145 GO:0006807 GO:0005515 GO:0016812
Cluster-22178.46147 GO:0006355 GO:0003700 GO:0043565 GO:0005667
Cluster-22178.46148 GO:0006355 GO:0003700 GO:0043565 GO:0005667
Cluster-22178.46152 GO:0006270
Cluster-22178.46157 GO:0005515
Cluster-22178.46159 GO:0005515
Cluster-22178.46160 GO:0005515 GO:0035091 GO:0005737
Cluster-22178.46161 GO:0007283 GO:0007130 GO:0006468 GO:0044780
GO:0007131 GO:0006308 GO:0006914 GO:0004672 GO:0005524
GO:0008855 GO:0009318
Cluster-22178.46162 GO:0006118 GO:0006814 GO:0006120 GO:0006744
GO:0008608 GO:0006914 GO:0015992 GO:0008137 GO:0003954
GO:0042729 GO:0072686 GO:0005739
Cluster-22178.46163 GO:0006468 GO:0004672 GO:0005524
Cluster-22178.46165 GO:0006468 GO:0009069 GO:0006807 GO:0016310
GO:0005509 GO:0016151 GO:0004674 GO:0004668 GO:0016773
GO:0005524 GO:0004672 GO:0016020 GO:0005737
Cluster-22178.46167 GO:0005198 GO:0016021
Cluster-22178.46168 GO:0005198 GO:0016021
Cluster-22178.46174 GO:0006470 GO:0019988 GO:0006570 GO:0007165
GO:0003676 GO:0004725 GO:0016829 GO:0005515 GO:0008138
GO:0043399 GO:0005179 GO:0009424 GO:0005576
Cluster-22178.46175 GO:0006355 GO:0003700 GO:0005667 GO:0005634
Cluster-22178.46178 GO:0008152 GO:0006508 GO:0004190 GO:0016491

Cluster-22178.46181 GO:0008152 GO:0055114 GO:0006508 GO:0016491
GO:0004190 GO:0051287
Cluster-22178.46182 GO:0055114 GO:0006508 GO:0008152 GO:0051287
GO:0004190 GO:0016491
Cluster-22178.46183 GO:0046983

Cluster-22178.46184 GO:0005509 GO:0005576
Cluster-22178.46186 GO:0005975 GO:0008063 GO:0016773
Cluster-22178.46187 GO:0046872 GO:0008270 GO:0005515
Cluster-22178.46189 GO:0003723
Cluster-22178.46192 GO:0031556 GO:0035556 GO:0031554 GO:0006468
GO:0019087 GO:0055114 GO:0016818 GO:0047134 GO:0003712
GO:0003724 GO:0005524 GO:0016773 GO:0005515 GO:0004672
GO:0016020 GO:0005667 GO:0005634 GO:0016021
Cluster-22178.46194 GO:0005975 GO:0004553
Cluster-22178.46201 GO:0045454 GO:0042254 GO:0006412 GO:0006810
GO:0003735 GO:0005515 GO:0003924 GO:0005525 GO:0005861
GO:0016021 GO:0005840
Cluster-22178.46204 GO:0006531 GO:0006522 GO:0006529 GO:0004066

Cluster-22178.46209 GO:0007186 GO:0031505 GO:0006817 GO:0005315
GO:0004930 GO:0016020 GO:0016021
Cluster-22178.46212 GO:0006468 GO:0007165 GO:0016773 GO:0004672
GO:0005524 GO:0005179 GO:0005840 GO:0005576 GO:0016020
Cluster-22178.46214 GO:0006508 GO:0004197 GO:0008234
Cluster-22178.46216 GO:0006351 GO:0006357 GO:0016055 GO:0005515
GO:0001104 GO:0016592
Cluster-22178.46223 GO:0005515
Cluster-22178.46226 GO:0055085 GO:0006470 GO:0006811 GO:0006570
GO:0004725 GO:0008138 GO:0003677 GO:0005216 GO:0019031
GO:0016020
Cluster-22178.46227 GO:0055114 GO:0006629 GO:0006355 GO:0007165
GO:0005507 GO:0016641 GO:0005044 GO:0003677 GO:0005763
GO:0016020
Cluster-22178.46233 GO:0006281
Cluster-22178.46234 GO:0042157 GO:0006869 GO:0010508 GO:0008289
GO:0005576
Cluster-22178.46240 GO:0019076 GO:0006812 GO:0032801 GO:0006508
GO:0016032 GO:0008233 GO:0005261 GO:0033644

Cluster-22178.46244 GO:0006281 GO:0006310
Cluster-22178.46251 GO:0055085 GO:0006810 GO:0006355 GO:0003700
GO:0005215 GO:0008270 GO:0016020 GO:0005667 GO:0005634
Cluster-22178.46252 GO:0005515
Cluster-22178.46253 GO:0009117 GO:0000287 GO:0008270 GO:0008253
GO:0000166 GO:0005737
Cluster-22178.46256 GO:0003723 GO:0003676
Cluster-22178.46258 GO:0006269 GO:0006351 GO:0006357 GO:0055114
GO:0006260 GO:0006120 GO:0001104 GO:0003896 GO:0005730
GO:0016592 GO:0005657
Cluster-22178.46259 GO:0006351 GO:0006269 GO:0046677 GO:0006120
GO:0042967 GO:0006260 GO:0006357 GO:0055114 GO:0001104
GO:0046353 GO:0003896 GO:0016592 GO:0005730 GO:0005657
Cluster-22178.46262 GO:0055114 GO:0016491 GO:0005525 GO:0005515

Cluster-22178.46267 GO:0015992 GO:0015991 GO:0015078 GO:0005198
GO:0019028 GO:0016021 GO:0033179
Cluster-22178.46269 GO:0015992 GO:0015991 GO:0015078 GO:0005198
GO:0033179
Cluster-22178.46271 GO:0051056 GO:0006284 GO:0006144 GO:0043547
GO:0006508 GO:0003968 GO:0003676 GO:0005096 GO:0016817
GO:0008270 GO:0005515 GO:0017111 GO:0004197 GO:0003677
GO:0031379
Cluster-22178.46276 GO:0000226 GO:0043085 GO:0008033 GO:0016485
GO:0000910 GO:0016226 GO:0008017 GO:0005524 GO:0000166
GO:0016879 GO:0045298 GO:0016021 GO:0005737
Cluster-22178.46277 GO:0008152 GO:0016757
Cluster-22178.46279 GO:0005515
Cluster-22178.46281 GO:0003779 GO:0005622
Cluster-22178.46283 GO:0008654 GO:0046486 GO:0009395 GO:0004143
GO:0003676 GO:0005524 GO:0016020
Cluster-22178.46284 GO:0009395 GO:0046486 GO:0008654 GO:0004143
GO:0016020

Cluster-22178.46290 GO:0007165 GO:0019012
Cluster-22178.46292 GO:2000123 GO:0055085 GO:0006812 GO:0006885
GO:0015299 GO:0016021
Cluster-22178.46294 GO:0007218 GO:0007165 GO:0005057 GO:0005184
GO:0005576
Cluster-22178.46298 GO:0010508 GO:0009405 GO:0007049 GO:0016020
Cluster-22178.46303 GO:0006310 GO:0006281 GO:0005515 GO:0005509
GO:0009378 GO:0005524 GO:0009379 GO:0005657
Cluster-22178.46305 GO:0018342 GO:0008318 GO:0008270 GO:0005515
GO:0004663 GO:0005968
Cluster-22178.46306 GO:0055085 GO:0030001 GO:0006813 GO:0006904
GO:0016192 GO:0005249 GO:0046873 GO:0005515 GO:0008076
GO:0016020
Cluster-22178.46307 GO:0008033 GO:0006418 GO:0009094 GO:0000162
GO:0006571 GO:0006432 GO:0043039 GO:0005524 GO:0004826
GO:0000287 GO:0004812 GO:0000049 GO:0000166 GO:0009328
GO:0005737
Cluster-22178.46309 GO:0006260 GO:0000917 GO:0043093 GO:0045095
GO:0005737
Cluster-22178.46314 GO:0042157 GO:0006869 GO:0008289 GO:0005576
Cluster-22178.46315 GO:0043087 GO:0006051 GO:0006040 GO:0032012
GO:0005086 GO:0047465
Cluster-22178.46317 GO:0055114 GO:0016616
Cluster-22178.46318 GO:0005515
Cluster-22178.46323 GO:0006397 GO:0000445
Cluster-22178.46324 GO:0006397 GO:0000445
Cluster-22178.46325 GO:0046872
Cluster-22178.46331 GO:0005515
Cluster-22178.46332 GO:0005515
Cluster-22178.46333 GO:0006886 GO:0005515 GO:0008536
Cluster-22178.46335 GO:0006886 GO:0008536 GO:0005515
Cluster-22178.46339 GO:0030150 GO:0006118 GO:0009055 GO:0046872
GO:0008270 GO:0051536 GO:0061630 GO:0005515 GO:0005742

Cluster-22178.46341 GO:0015031 GO:0051607 GO:0055085 GO:0006260
GO:0006457 GO:0048870 GO:0015991 GO:0006355 GO:0042384
GO:0006904 GO:0000917 GO:0006914 GO:0015992 GO:0048193
GO:0008360 GO:0006606 GO:0007155 GO:0043093 GO:0030001
GO:0046873 GO:0003700 GO:0045502 GO:0008134 GO:0000975
GO:0003677 GO:0043565 GO:0015078 GO:0051082 GO:0005198
GO:0042803 GO:0030286 GO:0034464 GO:0000145 GO:0016021
GO:0019028 GO:0033179 GO:0031514 GO:0016272 GO:0005667
GO:0016020 GO:0005737
Cluster-22178.46342 GO:0003676
Cluster-22178.46344 GO:0008270 GO:0046872
Cluster-22178.46353 GO:0005576
Cluster-22178.46355 GO:0006470 GO:0006570 GO:0004725
Cluster-22178.46359 GO:0008152 GO:0009452 GO:0006306 GO:0031167
GO:0001510 GO:0005201 GO:0008168 GO:0003677 GO:0005515
GO:0008170 GO:0016021 GO:0005581 GO:0019013 GO:0005578
Cluster-22178.46362 GO:0006310 GO:0006508 GO:0006281 GO:0006260
GO:0008152 GO:0003677 GO:0016788 GO:0004181 GO:0008270
GO:0005634
Cluster-22178.46372 GO:0006813
Cluster-22178.46373 GO:0006886 GO:0006468 GO:0007264 GO:0006606
GO:0006914 GO:0005096 GO:0016773 GO:0005524 GO:0004672
GO:0016020 GO:0005783 GO:0016021
Cluster-22178.46374 GO:0006118 GO:0015986 GO:0045454 GO:0015992
GO:0015078 GO:0009055 GO:0015035 GO:0000276
Cluster-22178.46376 GO:0007165 GO:0010508
Cluster-22178.46382 GO:0046872 GO:0008270 GO:0005515 GO:0005622
Cluster-22178.46383 GO:0007010 GO:0006355 GO:0005515 GO:0003779

Cluster-22178.46399 GO:0000226 GO:0016310 GO:0000910 GO:0009069
GO:0006468 GO:0008017 GO:0004674 GO:0005524 GO:0004672
GO:0045298
Cluster-22178.46403 GO:0006260 GO:0015979 GO:0006468 GO:0004715

GO:0005524 GO:0005515 GO:0005198 GO:0019031 GO:0009523
GO:0009539 GO:0016020 GO:0019028
Cluster-22178.46406 GO:0016032 GO:0046872
Cluster-22178.46407 GO:0007155 GO:0042246 GO:0008270 GO:0046872
GO:0005515 GO:0016021
Cluster-22178.46408 GO:0008152 GO:0055114 GO:0016491
Cluster-22178.46412 GO:0005515
Cluster-22178.46423 GO:0046856 GO:0015031 GO:0034597 GO:0016021
Cluster-22178.46424 GO:0006560 GO:0009405 GO:0006814 GO:0006570
GO:0006090 GO:0006470 GO:0006525 GO:0071436 GO:0015031
GO:0046856 GO:0005272 GO:0008948 GO:0004725 GO:0034597
GO:0015081 GO:0031224 GO:0016021 GO:0016020
Cluster-22178.46429 GO:0005524 GO:0016787 GO:0003677
Cluster-22178.46430 GO:0070588 GO:0006816 GO:0005245 GO:0005515
GO:0005891
Cluster-22178.46431 GO:0015743
Cluster-22178.46432 GO:0035444 GO:0015675 GO:0015099 GO:0046872
GO:0016021
Cluster-22178.46434 GO:0007586 GO:0016042 GO:0008047 GO:0005576
Cluster-22178.46435 GO:0016567 GO:0004842 GO:0005515
Cluster-22178.46436 GO:0006260 GO:0003887 GO:0042575
Cluster-22178.46437 GO:0006694 GO:0009058 GO:0008299 GO:0016779
GO:0050518
Cluster-22178.46438 GO:0016192 GO:0006886 GO:0006284 GO:0016114
GO:0006694 GO:0005198 GO:0005515 GO:0003677 GO:0008661
GO:0005634 GO:0030117
Cluster-22178.46439 GO:0039693 GO:0006914 GO:0006606 GO:0006508
GO:0019784 GO:0005198 GO:0019031 GO:0019028
Cluster-22178.46443 GO:0019048 GO:0019031
Cluster-22178.46445 GO:0006032 GO:0016998 GO:0004568
Cluster-22178.46448 GO:0006468 GO:0004672 GO:0005524
Cluster-22178.4645 GO:0007156 GO:0005509 GO:0016020 GO:0016021
Cluster-22178.46451 GO:0007165

Cluster-22178.46453 GO:0006355 GO:0007165 GO:0003723 GO:0016763 GO:0005179 GO:0003677 GO:0046983 GO:0005634 GO:0019028 GO:0005576

Cluster-22178.46456 GO:0006281 GO:0006952 GO:0006464 GO:0035556 GO:0008270 GO:0046872 GO:0016151 GO:0005576

Cluster-22178.46460 GO:0006367 GO:0005672

Cluster-22178.46462 GO:0042802 GO:0043015 GO:0008017 GO:0045298

Cluster-22178.46465 GO:0032065 GO:0005515 GO:0005543 GO:0005938

Cluster-22178.46466 GO:0043039 GO:0006418 GO:0000166 GO:0000049 GO:0004812 GO:0003676 GO:0005524 GO:0019031 GO:0005737

Cluster-22178.46470 GO:0006355 GO:0009069 GO:0016310 GO:0070009 GO:0004674 GO:0003700 GO:0008239 GO:0005667 GO:0005634

Cluster-22178.46471 GO:0009069 GO:0016310 GO:0070009 GO:0004674 GO:0008239

Cluster-22178.46473 GO:0008201 GO:0009512

Cluster-22178.46477 GO:0055085 GO:0016021

Cluster-22178.46478 GO:0050840 GO:0005576

Cluster-22178.46479 GO:0005515

Cluster-22178.46487 GO:0019054 GO:0005509 GO:0004857

Cluster-22178.46489 GO:0006777 GO:0004222 GO:0051539 GO:0019008

Cluster-22178.46490 GO:0006886 GO:0006351 GO:0005515 GO:0008536

Cluster-22178.46491 GO:0006886 GO:0006351 GO:0008536 GO:0005515

Cluster-22178.46492 GO:0006468 GO:0055114 GO:0004497 GO:0016706 GO:0008168 GO:0005524 GO:0004672 GO:0016715 GO:0005507

Cluster-22178.46495 GO:0003676 GO:0005515

Cluster-22178.46496 GO:0003677 GO:0003676 GO:0005515

Cluster-22178.46497 GO:0003676 GO:0005515

Cluster-22178.46501 GO:0008152 GO:0008610 GO:0016747

Cluster-22178.46502 GO:0008152 GO:0008610 GO:0016747

Cluster-22178.46503 GO:0009117 GO:0000287 GO:0008270 GO:0008253 GO:0000166 GO:0005737

Cluster-22178.46504 GO:0009117 GO:0000166 GO:0008253 GO:0008270 GO:0000287 GO:0005737
Cluster-22178.46505 GO:0034477 GO:0004518 GO:0005515
Cluster-22178.46506 GO:0006098 GO:0046168 GO:0009094 GO:0055114 GO:0019521 GO:0006571 GO:0015940 GO:0000162 GO:0051287 GO:0004616 GO:0016616 GO:0008977 GO:0008677 GO:0004665
Cluster-22178.46507 GO:0051056 GO:0043547 GO:0003676 GO:0005515 GO:0008270 GO:0005096
Cluster-22178.46508 GO:0051056 GO:0006284 GO:0006144 GO:0043547 GO:0006508 GO:0003676 GO:0003968 GO:0005096 GO:0016817 GO:0008270 GO:0005515 GO:0017111 GO:0004197 GO:0003677 GO:0031379
Cluster-22178.46509 GO:0008270 GO:0046872 GO:0061630 GO:0005515

Cluster-22178.46510 GO:0005515 GO:0061630 GO:0008270 GO:0046872

Cluster-22178.46512 GO:0015979 GO:0022900 GO:0009539 GO:0005739 GO:0009523 GO:0016020 GO:0005747
Cluster-22178.46513 GO:0005515
Cluster-22178.46515 GO:0005515
Cluster-22178.46521 GO:0006396 GO:0006807 GO:0006144 GO:0004000 GO:0003723
Cluster-22178.46522 GO:0007186 GO:0007606 GO:0019236 GO:0005515 GO:0004932 GO:0016020
Cluster-22178.46524 GO:0042619
Cluster-22178.46525 GO:0007165 GO:0005515
Cluster-22178.46526 GO:0002098 GO:0034227 GO:0000049
Cluster-22178.46528 GO:0006355 GO:0000226 GO:0005515 GO:0015630 GO:0016020
Cluster-22178.46529 GO:0006810 GO:0016021
Cluster-22178.46534 GO:0006355 GO:0055114 GO:0046872 GO:0047134 GO:0003677 GO:0008289
Cluster-22178.46539 GO:0055114 GO:0016702

Cluster-22178.4654 GO:0019497 GO:0006771 GO:0003993 GO:0005515 GO:0046872 GO:0019867

Cluster-22178.46541 GO:0005515

Cluster-22178.46543 GO:0016884

Cluster-22178.46549 GO:0003677 GO:0046872

Cluster-22178.46550 GO:0006313 GO:0006355 GO:0006352 GO:0015074 GO:0000049 GO:0003677 GO:0003700 GO:0016987 GO:0005667

Cluster-22178.46552 GO:0007165 GO:0019953 GO:0000723 GO:0043047 GO:0003677 GO:0003697 GO:0003676 GO:0000772 GO:0005576 GO:0000784

Cluster-22178.46553 GO:0046983

Cluster-22178.46554 GO:0005515 GO:0008061

Cluster-22178.46555 GO:0003676 GO:0003723

Cluster-22178.46558 GO:0006486 GO:0070072 GO:0008373

Cluster-22178.46561 GO:0000723 GO:0019953 GO:0007165 GO:0000772 GO:0003676 GO:0003697 GO:0003677 GO:0043047 GO:0000784 GO:0005576

Cluster-22178.46562 GO:0046872 GO:0003677

Cluster-22178.46565 GO:0006914 GO:0051082

Cluster-22178.46567 GO:0008270 GO:0003676

Cluster-22178.46568 GO:0090266 GO:0051252 GO:0005524 GO:0003676 GO:0016787 GO:0003677 GO:0016891 GO:0005680

Cluster-22178.46573 GO:0000413 GO:0006457 GO:0003755 GO:0003676

Cluster-22178.46575 GO:0006281 GO:0004518

Cluster-22178.46576 GO:0055085 GO:0006810 GO:0016021 GO:0016020

Cluster-22178.46577 GO:0016114 GO:0008685 GO:0005515

Cluster-22178.46579 GO:0005515

Cluster-22178.46580 GO:0009405

Cluster-22178.46581 GO:0009405 GO:0005576

Cluster-22178.46583 GO:0005201 GO:0008168 GO:0005581 GO:0005578

Cluster-22178.46587 GO:2001070 GO:0005515

Cluster-22178.46591 GO:0009228 GO:0004789

Cluster-22178.46592 GO:0006891 GO:0030173
Cluster-22178.46601 GO:0005515
Cluster-22178.46602 GO:0006886 GO:0015031 GO:0005975 GO:0005509
GO:0008565 GO:0016773 GO:0016020 GO:0005737
Cluster-22178.46603 GO:0016573
Cluster-22178.46607 GO:0005515
Cluster-22178.46609 GO:0006886 GO:0005622
Cluster-22178.46613 GO:0006531 GO:0006468 GO:0007165 GO:0006522
GO:0042967 GO:0035556 GO:0016773 GO:0047200 GO:0005057
GO:0005524 GO:0004672 GO:0016020 GO:0005856 GO:0016021
Cluster-22178.46614 GO:0008289
Cluster-22178.46615 GO:0006810 GO:0042626
Cluster-22178.46619 GO:0009058 GO:0015979 GO:0005515 GO:0016779
GO:0016772 GO:0016020 GO:0009523 GO:0009539
Cluster-22178.46625 GO:0005524 GO:0016773 GO:0016020
Cluster-22178.46626 GO:0019082 GO:0016032 GO:0006508 GO:0000723
GO:0006879 GO:0005515 GO:0004252 GO:0003677 GO:0070009
GO:0008239 GO:0005576 GO:0000784
Cluster-22178.46627 GO:0006355 GO:0046983 GO:0008270 GO:0005515
GO:0005634
Cluster-22178.46629 GO:0005515 GO:0046872 GO:0016021 GO:0045202
Cluster-22178.46630 GO:0006810 GO:0008152 GO:0009405 GO:0008200
GO:0004867 GO:0005576 GO:0042151
Cluster-22178.46633 GO:0045947 GO:0008608 GO:0008190 GO:0003677
GO:0072686 GO:0042729
Cluster-22178.46635 GO:0007156 GO:0006886 GO:0016192 GO:0005515
GO:0005509 GO:0030117 GO:0016020
Cluster-22178.46638 GO:0035023 GO:0043087 GO:0005089 GO:0003677
GO:0034061 GO:0005634
Cluster-22178.46640 GO:0006355 GO:0003700 GO:0005667
Cluster-22178.46642 GO:0006355 GO:0003677
Cluster-22178.46645 GO:0005515
Cluster-22178.46646 GO:0031110 GO:0030168 GO:0015031 GO:0006891

GO:0007067	GO:0000090	GO:0042157	GO:0006869	GO:0007165
GO:0051301	GO:0051258	GO:0007059	GO:0006813	GO:0006904
GO:0015269	GO:0008017	GO:0005102	GO:0030674	GO:0005516
GO:0008289	GO:0000940	GO:0005577	GO:0005876	GO:0017119
GO:0000145	GO:0045298	GO:0016021	GO:0005576
Cluster-22178.46648	GO:0003676	GO:0003723
Cluster-22178.46651	GO:0006914	GO:0008270
Cluster-22178.46652	GO:0003774	GO:0008270	GO:0019901	GO:0016459
Cluster-22178.46653	GO:0008270
Cluster-22178.46654	GO:0008152	GO:0008134	GO:0003824	GO:0005667
GO:0005634
Cluster-22178.46657	GO:0019068	GO:0043565	GO:0005198	GO:0005515
GO:0005634
Cluster-22178.46660	GO:0007049	GO:0051301	GO:0006355	GO:0003700
GO:0016021	GO:0005634	GO:0005667
Cluster-22178.46663	GO:0090263	GO:0016567	GO:0005515	GO:0008270

Cluster-22178.46664	GO:0015948	GO:0007264	GO:0042981	GO:0005525
GO:0005515	GO:0003924
Cluster-22178.4667	GO:0051056	GO:0005096	GO:0005634
Cluster-22178.46670	GO:0035556	GO:0006355	GO:0003677	GO:0005515
GO:0003700	GO:0005667
Cluster-22178.46678	GO:0032801	GO:0006812	GO:0019076	GO:0008270
GO:0005515	GO:0005261	GO:0033644	GO:0005634
Cluster-22178.46679	GO:0009405	GO:0005515	GO:0003682	GO:0000785
GO:0005576
Cluster-22178.46685	GO:0042254	GO:0006412	GO:0003735	GO:0005840
Cluster-22178.46686	GO:0051276	GO:0007031	GO:0051082	GO:0008773
GO:0005515	GO:0005524	GO:0005694	GO:0005777
Cluster-22178.46687	GO:0006541	GO:0042823	GO:0042819	GO:0016887
GO:0005524	GO:0004359
Cluster-22178.4669	GO:0007186	GO:0004930	GO:0016021
Cluster-22178.46691	GO:0006412	GO:0042254	GO:0008270	GO:0046872

GO:0005515 GO:0003735 GO:0004867 GO:0005840 GO:0005622
Cluster-22178.46692 GO:0008270 GO:0046872 GO:0005634
Cluster-22178.46693 GO:0006858
Cluster-22178.46696 GO:0016021
Cluster-22178.46697 GO:0016021
Cluster-22178.46698 GO:0006308 GO:0010508 GO:0006397 GO:0008855
GO:0016021 GO:0009318 GO:0000445
Cluster-22178.46699 GO:0006351 GO:0007034 GO:0006269 GO:0003896
GO:0005657 GO:0005730
Cluster-22178.46700 GO:0006351 GO:0006269 GO:0003896 GO:0005657
GO:0005730
Cluster-22178.46701 GO:0003677 GO:0005634
Cluster-22178.46702 GO:0016032 GO:0006807 GO:0006672 GO:0019082
GO:0016811 GO:0016021
Cluster-22178.46705 GO:0009069 GO:0006468 GO:0016310 GO:0005524
GO:0004672 GO:0003677 GO:0004674
Cluster-22178.46713 GO:0008217 GO:0042312 GO:0007165 GO:0006355
GO:0000245 GO:0006508 GO:0004222 GO:0008270 GO:0005515
GO:0005179 GO:0031012 GO:0005576 GO:0005634
Cluster-22178.46714 GO:0035434 GO:0018149 GO:0006825 GO:0005375
GO:0005515 GO:0008270 GO:0003810 GO:0016021
Cluster-22178.46716 GO:0016310 GO:0006468 GO:0009069 GO:0004672
GO:0005524 GO:0003774 GO:0004674 GO:0017048 GO:0005198
GO:0016459 GO:0005882
Cluster-22178.46719 GO:0015031 GO:0015979 GO:0055114 GO:0030145
GO:0008270 GO:0008565 GO:0016817 GO:0016972 GO:0009523
GO:0016021
Cluster-22178.46726 GO:0042981 GO:0016788 GO:0051499 GO:0005634
Cluster-22178.46728 GO:0051276 GO:0005515 GO:0005524 GO:0005694
Cluster-22178.46729 GO:0019083 GO:0019229 GO:0006144 GO:0003968
GO:0005576 GO:0031379
Cluster-22178.46732 GO:2001256 GO:0030176
Cluster-22178.46733 GO:0006355 GO:0005515 GO:0008270

Cluster-22178.46735 GO:0006355 GO:0006771 GO:0019497 GO:0008152
GO:0000287 GO:0016791 GO:0008134 GO:0005524 GO:0003993
GO:0008253 GO:0005667 GO:0005737
Cluster-22178.46737 GO:0005515
Cluster-22178.46740 GO:0006468 GO:0005524 GO:0004672
Cluster-22178.46742 GO:0000012 GO:0006281 GO:0003684 GO:0005515
GO:0005634
Cluster-22178.46744 GO:0042981 GO:0007264 GO:0015948 GO:0003924
GO:0005515 GO:0005525
Cluster-22178.46746 GO:0007264 GO:0007165 GO:0007186 GO:0031683
GO:0005524 GO:0019001 GO:0003924 GO:0005525 GO:0004871
GO:0000166 GO:0003677
Cluster-22178.46747 GO:0008270
Cluster-22178.46748 GO:0008270
Cluster-22178.46749 GO:0033014 GO:0007304 GO:0055114 GO:0003677
GO:0050661 GO:0016772 GO:0005213 GO:0008883 GO:0005576
GO:0042600
Cluster-22178.46751 GO:0048193 GO:0006914 GO:0005515 GO:0016020
Cluster-22178.46754 GO:0006281 GO:0016567 GO:0006308 GO:0006260
GO:0003723 GO:0005515 GO:0008270 GO:0017108 GO:0004842
GO:0005525 GO:0005634 GO:0033557
Cluster-22178.46757 GO:0006094 GO:0006098 GO:0006096 GO:0006364
GO:0006000 GO:0006013 GO:0006012 GO:0003872 GO:0004222
GO:0005945
Cluster-22178.46759 GO:0005975 GO:0006367 GO:0008184 GO:0005634
Cluster-22178.46760 GO:0005515 GO:0061630 GO:0008270 GO:0046872

Cluster-22178.46761 GO:0046872 GO:0008270 GO:0005515 GO:0061630

Cluster-22178.46762 GO:0005515 GO:0061630 GO:0046872 GO:0008270

Cluster-22178.46766 GO:0008270
Cluster-22178.46767 GO:0006468 GO:0009069 GO:0007067 GO:0000090

GO:0007059 GO:0016310 GO:0051301 GO:0031110 GO:0004674
GO:0008017 GO:0004672 GO:0005524 GO:0045298 GO:0005876
GO:0000940
Cluster-22178.46768 GO:0006914
Cluster-22178.46769 GO:0090063 GO:0007096 GO:0000226 GO:0004190
GO:0003723 GO:0005515 GO:0008146 GO:0005634 GO:0016020
GO:0000922 GO:0005815
Cluster-22178.46773 GO:0007156 GO:0005515 GO:0005509 GO:0016020
Cluster-22178.46774 GO:0019497 GO:0006771 GO:0009103 GO:0046872
GO:0008918 GO:0008168 GO:0016817 GO:0003993 GO:0005515
Cluster-22178.46779 GO:0005975 GO:0004553 GO:0005515
Cluster-22178.46782 GO:0006355 GO:0003677
Cluster-22178.46785 GO:0007155 GO:0005515 GO:0016021
Cluster-22178.46787 GO:0016491
Cluster-22178.46791 GO:0006139 GO:0005515 GO:0003676 GO:0008408
GO:0043395 GO:0016020 GO:0005578
Cluster-22178.46792 GO:0045040 GO:0005515 GO:0005742
Cluster-22178.46794 GO:0015992 GO:0006123 GO:0004129 GO:0005746
GO:0045277
Cluster-22178.46798 GO:0035556 GO:0007264 GO:0005525
Cluster-22178.46804 GO:0007618 GO:0006468 GO:0016773 GO:0004672
GO:0005524 GO:0016020 GO:0005576
Cluster-22178.46808 GO:0042967 GO:0016747 GO:0008080
Cluster-22178.46809 GO:0042475 GO:0006520 GO:0042476 GO:0005515
GO:0030170 GO:0016829 GO:0008483 GO:0030345 GO:0005578
Cluster-22178.46810 GO:0042476 GO:0006520 GO:0042475 GO:0030345
GO:0008483 GO:0016829 GO:0005515 GO:0030170 GO:0005578
Cluster-22178.46811 GO:0006520 GO:0005515 GO:0008483 GO:0016829
GO:0030170
Cluster-22178.46812 GO:0006351 GO:0015016 GO:0003677 GO:0016787
GO:0005634
Cluster-22178.46815 GO:0006955 GO:0019882 GO:0016020 GO:0042613
Cluster-22178.46823 GO:0006725 GO:0016853 GO:0055036

Cluster-22178.46824 GO:0005515 GO:0035091
Cluster-22178.46825 GO:0007017 GO:0006364 GO:0007010 GO:0006298
GO:0003779 GO:0005524 GO:0030983 GO:0005634 GO:0034457
GO:0005732 GO:0005875
Cluster-22178.46827 GO:0003677
Cluster-22178.46828 GO:0016579 GO:0006511 GO:0006508 GO:0004843
GO:0005622
Cluster-22178.46832 GO:0007224 GO:0045182
Cluster-22178.46833 GO:0007186 GO:0004930 GO:0016020 GO:0016021
Cluster-22178.46839 GO:0000160 GO:0016310 GO:0008152 GO:0055114
GO:0006412 GO:0046487 GO:0015991 GO:0030001 GO:0055085
GO:0048193 GO:0009396 GO:0015992 GO:0042254 GO:0003824
GO:0015078 GO:0005515 GO:0051082 GO:0016491 GO:0046983
GO:0003735 GO:0004488 GO:0050662 GO:0046873 GO:0000155
GO:0016021 GO:0009365 GO:0005840 GO:0033179 GO:0005634
GO:0016020
Cluster-22178.46841 GO:0006468 GO:0004672
Cluster-22178.46844 GO:0006464 GO:0035556 GO:0006355 GO:0046872
GO:0008270 GO:0016151 GO:0005507 GO:0003700 GO:0003677
GO:0005634 GO:0005667
Cluster-22178.46846 GO:0006464 GO:0006281 GO:0006355 GO:0006351
GO:0008270 GO:0046872 GO:0016151 GO:0003700 GO:0005507
GO:0003676 GO:0003677 GO:0005515 GO:0005667 GO:0005634
Cluster-22178.46848 GO:0006355 GO:0003677 GO:0005515 GO:0004252
GO:0005886 GO:0005634
Cluster-22178.46851 GO:0003677
Cluster-22178.46853 GO:0043087 GO:0007264 GO:0015074 GO:0005085
GO:0003677
Cluster-22178.46859 GO:0007165 GO:0042742 GO:0040007 GO:0008283
GO:0008083 GO:0008270
Cluster-22178.46862 GO:0008270 GO:0046872
Cluster-22178.46868 GO:0045454 GO:0006118 GO:0005515 GO:0009055
GO:0015035

Cluster-22178.46870 GO:0005515
Cluster-22178.46871 GO:0055114 GO:0016705 GO:0020037 GO:0005506

Cluster-22178.46875 GO:0006355 GO:0007165 GO:0006357 GO:0051262
GO:0003723 GO:0001104 GO:0005515 GO:0045182 GO:0003700
GO:0004871 GO:0019028 GO:0005667 GO:0005634 GO:0016592
Cluster-22178.46878 GO:0021987 GO:0005515
Cluster-22178.46882 GO:0006606 GO:0007017 GO:0007018 GO:0005524
GO:0008017 GO:0003777 GO:0045298 GO:0005874
Cluster-22178.46883 GO:0005515
Cluster-22178.46884 GO:0005515
Cluster-22178.46887 GO:0015994 GO:0033014 GO:0005515 GO:0004418

Cluster-22178.46892 GO:0006213 GO:0006090 GO:0006525 GO:0007155
GO:0006560 GO:0006814 GO:0007186 GO:0071436 GO:0015081
GO:0016817 GO:0004930 GO:0016763 GO:0008948 GO:0016021
GO:0016020
Cluster-22178.46894 GO:0015979 GO:0046872 GO:0009523 GO:0016021
Cluster-22178.46896 GO:0045454 GO:0055114 GO:0016972
Cluster-22178.46899 GO:0006631 GO:0018874 GO:0006298 GO:0009396
GO:0006554 GO:0006633 GO:0006568 GO:0006552 GO:0046487
GO:0006550 GO:0055114 GO:0006574 GO:0008152 GO:0050662
GO:0004488 GO:0016491 GO:0005524 GO:0003857 GO:0003824
Cluster-22178.46900 GO:0035556 GO:0005515
Cluster-22178.46901 GO:0016773
Cluster-22178.46903 GO:0009405 GO:0000184 GO:0004386 GO:0005524
GO:0008270 GO:0003677 GO:0019867 GO:0005737
Cluster-22178.46908 GO:0006355 GO:0003700 GO:0043565 GO:0005667
GO:0005634
Cluster-22178.46910 GO:0055114 GO:0016491
Cluster-22178.46911 GO:0055114 GO:0051287 GO:0016491 GO:0016616

Cluster-22178.46914 GO:0008544

Cluster-22178.46918 GO:0006810 GO:0006576 GO:0006355 GO:0007264
GO:0002949 GO:0008134 GO:0098519 GO:0003723 GO:0003724
GO:0005525 GO:0016887 GO:0003924 GO:0005524 GO:0005667
Cluster-22178.46921 GO:0006352 GO:0051382 GO:0006366 GO:0006281
GO:0003677 GO:0005669
Cluster-22178.46922 GO:0006955 GO:0007165 GO:0003723 GO:0005125
GO:0003676 GO:0005576
Cluster-22178.46923 GO:0005887
Cluster-22178.46929 GO:0006355 GO:0006032 GO:0006730 GO:0008134
GO:0005515 GO:0008061 GO:0008168 GO:0005524 GO:0005667
GO:0005576 GO:0009279
Cluster-22178.46942 GO:0030036 GO:0003676 GO:0016817 GO:0003779
GO:0005524 GO:0003677 GO:0016787 GO:0030054
Cluster-22178.46944 GO:0005515
Cluster-22178.46945 GO:0009405 GO:0016791 GO:0005515
Cluster-22178.46959 GO:0051205 GO:0016021
Cluster-22178.46961 GO:0055114 GO:0047134
Cluster-22178.46965 GO:0003677 GO:0003676 GO:0005524 GO:0016787
GO:0019031
Cluster-22178.46979 GO:0003676 GO:0016020
Cluster-22178.46980 GO:0006486 GO:0008373
Cluster-22178.46981 GO:0006486 GO:0008373
Cluster-22178.46982 GO:0006486 GO:0008373
Cluster-22178.46985 GO:0016567 GO:0005515 GO:0004842 GO:0008270
GO:0046872 GO:0005680
Cluster-22178.46986 GO:0019988 GO:0006470 GO:0008152 GO:0006570
GO:0008168 GO:0004725 GO:0043399 GO:0008138
Cluster-22178.46988 GO:0006886 GO:0005737
Cluster-22178.46989 GO:0006810 GO:0016567 GO:0006606 GO:0006914
GO:0010508 GO:0016032 GO:0007049 GO:0048870 GO:0004842
GO:0042803 GO:0008134 GO:0045502 GO:0005667 GO:0005886
GO:0030286 GO:0031514 GO:0019867
Cluster-22178.46990 GO:0000902 GO:0042254 GO:0006412 GO:0003735

GO:0005840 GO:0005622
Cluster-22178.46991 GO:0016032 GO:0016567 GO:0006886 GO:0015031
GO:0042262 GO:0003690 GO:0008565 GO:0004842 GO:0016020
GO:0005737
Cluster-22178.46992 GO:0007186 GO:0005975 GO:0004930 GO:0003676
GO:0016853 GO:0003723 GO:0016021
Cluster-22178.46994 GO:0005515
Cluster-22178.46996 GO:0005515
Cluster-22178.47001 GO:0042254 GO:0006412 GO:0005515 GO:0003735
GO:0016817 GO:0005840 GO:0005622
Cluster-22178.47002 GO:0034080 GO:0008270 GO:0000775
Cluster-22178.47003 GO:0006810 GO:0006260 GO:0003677 GO:0016787
GO:0004386 GO:0003676 GO:0003688 GO:0005524 GO:0046809
Cluster-22178.47004 GO:0006260 GO:0006810 GO:0003688 GO:0004386
GO:0003676 GO:0005524 GO:0003677 GO:0016787 GO:0046809
Cluster-22178.47006 GO:0046872
Cluster-22178.47008 GO:0008641
Cluster-22178.47011 GO:0006886 GO:0005737
Cluster-22178.47023 GO:0007186 GO:0004930 GO:0016021
Cluster-22178.47025 GO:0098542 GO:0006470 GO:0008138
Cluster-22178.47028 GO:0007186 GO:0007165 GO:0007264 GO:0004871
GO:0005525 GO:0003924 GO:0019001 GO:0031683
Cluster-22178.47030 GO:0005515
Cluster-22178.47031 GO:0006554 GO:0006479 GO:0007165 GO:0006355
GO:0034968 GO:0005515 GO:0005509 GO:0018024 GO:0005578
GO:0005694
Cluster-22178.47032 GO:0005515
Cluster-22178.47034 GO:0000226 GO:0016226 GO:0016485 GO:0000910
GO:0008033 GO:0043085 GO:0008017 GO:0005524 GO:0000166
GO:0016879 GO:0045298 GO:0016021 GO:0005737
Cluster-22178.47038 GO:0030036 GO:0015684 GO:0007264 GO:0030976
GO:0005525 GO:0003924 GO:0003779 GO:0015093 GO:0000287
GO:0016021 GO:0030054

Cluster-22178.47039 GO:0007094 GO:0055114 GO:0016620 GO:0051287

Cluster-22178.47041 GO:0009405 GO:0000184 GO:0003677 GO:0004386
GO:0008270 GO:0005524 GO:0005576 GO:0005737
Cluster-22178.47044 GO:0019836 GO:0005525 GO:0005524 GO:0016887
GO:0005576
Cluster-22178.47045 GO:0055085 GO:0006812 GO:0009607 GO:0007165
GO:0006890 GO:0008324 GO:0016021 GO:0016020
Cluster-22178.47053 GO:0006694 GO:0009058 GO:0008299 GO:0007165
GO:0050518 GO:0005044 GO:0005515 GO:0016779 GO:0019031
GO:0016020
Cluster-22178.47059 GO:0016021
Cluster-22178.47067 GO:0006511 GO:0005201 GO:0005578 GO:0005581
Cluster-22178.47069 GO:0016055 GO:0007275 GO:0007165 GO:0005102
GO:0005515 GO:0005509 GO:0005576 GO:0045095
Cluster-22178.47075 GO:0007165 GO:0019012
Cluster-22178.47076 GO:0055114 GO:0006355 GO:0005515 GO:0016491

Cluster-22178.47079 GO:0055114 GO:0044237 GO:0006412 GO:0008168
GO:0016491 GO:0003824
Cluster-22178.4708 GO:0055114 GO:0008152 GO:0050662 GO:0051287
GO:0016491 GO:0003824
Cluster-22178.47085 GO:0006418 GO:0000166 GO:0004812 GO:0005524

Cluster-22178.47086 GO:0008152 GO:0006694 GO:0016114 GO:0008661
GO:0030976 GO:0003824 GO:0016624
Cluster-22178.47090 GO:0007156 GO:0005509 GO:0016020
Cluster-22178.47093 GO:0031505 GO:0016020
Cluster-22178.47096 GO:0006468 GO:0005524 GO:0004672 GO:0016020
Cluster-22178.47097 GO:0005515
Cluster-22178.47098 GO:0016192 GO:0006886 GO:0005515 GO:0030117
Cluster-22178.47100 GO:0006418 GO:0043039 GO:0005524 GO:0003676
GO:0004812 GO:0000166 GO:0000049 GO:0005737 GO:0019031

Cluster-22178.47102 GO:0046872
Cluster-22178.47103 GO:0046872
Cluster-22178.47104 GO:0008270
Cluster-22178.47106 GO:0006955 GO:0009058 GO:0007165 GO:0006479
GO:0006554 GO:0030170 GO:0018024 GO:0008270 GO:0005125
GO:0005576
Cluster-22178.47113 GO:0009878 GO:0046872 GO:0008270
Cluster-22178.47114 GO:0007165 GO:0019087 GO:0005509 GO:0003676
GO:0016021 GO:0005578
Cluster-22178.47115 GO:0007067 GO:0006457 GO:0007165 GO:0006914
GO:0051607 GO:0030168 GO:0031412 GO:0006355 GO:0006886
GO:0051258 GO:0051382 GO:0006352 GO:0030674 GO:0003677
GO:0005102 GO:0016987 GO:0051082 GO:0008376 GO:0008092
GO:0003700 GO:0005667 GO:0032580 GO:0005783 GO:0016272
GO:0005577 GO:0019898 GO:0005737 GO:0016021
Cluster-22178.47117 GO:0006355 GO:0006351 GO:0006090 GO:0006144
GO:0006357 GO:0030908 GO:0031412 GO:0006260 GO:0006633
GO:0003723 GO:0008092 GO:0003968 GO:0003677 GO:0003989
GO:0005737 GO:0031379 GO:0031411 GO:0016020 GO:0019898
GO:0009317
Cluster-22178.47118 GO:0006468 GO:0016773 GO:0005524 GO:0004672
GO:0019028 GO:0016020
Cluster-22178.47119 GO:0006468 GO:0016773 GO:0004672 GO:0005524
GO:0016020
Cluster-22178.47123 GO:0006886 GO:0008152 GO:0006605 GO:0016491
GO:0016020
Cluster-22178.47124 GO:0008270 GO:0005515 GO:0005783
Cluster-22178.47125 GO:0006265 GO:0003677 GO:0003918 GO:0005524
GO:0005815 GO:0005634
Cluster-22178.47127 GO:0007186 GO:0004930 GO:0016021
Cluster-22178.47129 GO:0006825 GO:0003676 GO:0016531 GO:0005507
GO:0005758
Cluster-22178.47134 GO:0000154 GO:0006396 GO:0016433 GO:0004252

GO:0055036 GO:0019028
Cluster-22178.47135 GO:0043065 GO:0051321 GO:0090200 GO:0009405
GO:0097193 GO:0005515 GO:0005739 GO:0005576
Cluster-22178.47140 GO:0035556 GO:0005515
Cluster-22178.47142 GO:0006298 GO:0007165 GO:0005515 GO:0030983
GO:0005524 GO:0005096
Cluster-22178.47146 GO:0016021
Cluster-22178.47147 GO:0016021
Cluster-22178.4715 GO:0007165 GO:0005515 GO:0005789
Cluster-22178.47154 GO:0006869 GO:0005319
Cluster-22178.47159 GO:0006571 GO:0000162 GO:0009094 GO:0008033
GO:0006432 GO:0004826 GO:0005524 GO:0008168 GO:0000049
GO:0000287 GO:0009328
Cluster-22178.47160 GO:0005515
Cluster-22178.47161 GO:0008643 GO:0055085 GO:0005351 GO:0000139
GO:0016021 GO:0016020
Cluster-22178.47162 GO:0055085 GO:0006313 GO:0022857 GO:0003677
GO:0016020 GO:0016021
Cluster-22178.47163 GO:0015992 GO:0055085 GO:0006811 GO:0015986
GO:0051260 GO:0015078 GO:0005216 GO:0016020
Cluster-22178.47171 GO:0006814 GO:0007275 GO:0015846 GO:0015813
GO:0007219 GO:0007154 GO:0007016 GO:0005509 GO:0005515
GO:0003677 GO:0015501 GO:0016020 GO:0016010 GO:0005576
GO:0016021
Cluster-22178.47172 GO:0006357 GO:0006508 GO:0004252 GO:0001104
GO:0016592
Cluster-22178.47174 GO:0004252 GO:0070008
Cluster-22178.47176 GO:0016032 GO:0019082
Cluster-22178.47181 GO:0006886 GO:0006955 GO:0006468 GO:0008152
GO:0006402 GO:0007165 GO:0042254 GO:0016192 GO:1902209
GO:0042802 GO:0005198 GO:0005515 GO:0003824 GO:0046872
GO:0005125 GO:0004672 GO:0008484 GO:0048027 GO:0030117
GO:0005576 GO:0005730

Cluster-22178.47182 GO:0006886 GO:0006955 GO:0006468 GO:0008152
GO:0007165 GO:0042254 GO:0016192 GO:0005515 GO:0005198
GO:0003824 GO:0046872 GO:0005125 GO:0004672 GO:0008484
GO:0030117 GO:0005576 GO:0005730
Cluster-22178.47183 GO:0006468 GO:0042254 GO:0016192 GO:0006886
GO:0008152 GO:0005515 GO:0005198 GO:0046872 GO:0004672
GO:0008484 GO:0003824 GO:0030117 GO:0005730
Cluster-22178.47184 GO:0055114 GO:0006355 GO:0016491 GO:0003824
GO:0042393
Cluster-22178.47192 GO:0006465 GO:0008233 GO:0016021 GO:0005787
Cluster-22178.47197 GO:0003723
Cluster-22178.47198 GO:0007010 GO:0004222 GO:0003779 GO:0031012
Cluster-22178.47204 GO:0006807 GO:0009252 GO:0003676 GO:0004356
GO:0008270 GO:0005923 GO:0005634
Cluster-22178.47205 GO:0005524 GO:0003774 GO:0016459
Cluster-22178.47209 GO:0042254 GO:0006412 GO:0003735 GO:0005840
Cluster-22178.47210 GO:0055114 GO:0016616 GO:0016491
Cluster-22178.47211 GO:0055114 GO:0016616 GO:0016491
Cluster-22178.47218 GO:0005515
Cluster-22178.47219 GO:0006308 GO:0009307 GO:0005515 GO:0003677
GO:0009036 GO:0009359
Cluster-22178.47223 GO:0005515
Cluster-22178.47224 GO:0042254 GO:0006412 GO:0003735 GO:0019843
GO:0005840 GO:0005622
Cluster-22178.47230 GO:0006810 GO:0016114 GO:0006694 GO:0006308
GO:0055114 GO:0003724 GO:0050660 GO:0016887 GO:0008855
GO:0005524 GO:0098519 GO:0005525 GO:0003924 GO:0003723
GO:0004506 GO:0016020 GO:0016021 GO:0009318
Cluster-22178.47231 GO:0006308 GO:0007264 GO:0015684 GO:0015093
GO:0005524 GO:0003924 GO:0008855 GO:0016887 GO:0005525
GO:0016021 GO:0009318
Cluster-22178.47233 GO:0007154 GO:0005509 GO:0016020
Cluster-22178.47234 GO:0005515

Cluster-22178.47236 GO:0006914 GO:0019028 GO:0005737

Cluster-22178.47241 GO:0019835 GO:0050829 GO:0016021

Cluster-22178.47248 GO:0005509

Cluster-22178.47251 GO:0007186 GO:0007165 GO:0003924 GO:0019001 GO:0031683 GO:0004871 GO:0005525

Cluster-22178.47252 GO:0005525 GO:0005515 GO:0008270

Cluster-22178.47254 GO:0030001 GO:0046872 GO:0008270 GO:0005515 GO:0000166 GO:0019956

Cluster-22178.47257 GO:0006457 GO:0005515

Cluster-22178.47260 GO:0046872 GO:0005634

Cluster-22178.47261 GO:0006886 GO:0016192 GO:0030117 GO:0016021 GO:0030131

Cluster-22178.47262 GO:0006355 GO:0008270 GO:0003677 GO:0003682 GO:0000775 GO:0000785 GO:0005783 GO:0005634

Cluster-22178.47263 GO:0009405 GO:0005576

Cluster-22178.47266 GO:0055085 GO:0016021 GO:0055036

Cluster-22178.47271 GO:0006629 GO:0046486 GO:0008152 GO:0016042 GO:0016787 GO:0003847 GO:0019028 GO:0008247

Cluster-22178.47272 GO:0008152 GO:0009252 GO:0005198 GO:0008410 GO:0003779 GO:0016755 GO:0005856

Cluster-22178.47273 GO:0006629 GO:0046486 GO:0008152 GO:0016042 GO:0016787 GO:0003847 GO:0019028 GO:0008247

Cluster-22178.47276 GO:0005634

Cluster-22178.47286 GO:0009435 GO:0051539 GO:0008987 GO:0016020 GO:0019804

Cluster-22178.47287 GO:0009069 GO:0006468 GO:0016032 GO:0007165 GO:0010508 GO:0016310 GO:0007131 GO:0005515 GO:0005096 GO:0004674 GO:0003676 GO:0000794

Cluster-22178.47288 GO:0006355 GO:0042981 GO:0008152 GO:0005515 GO:0016757 GO:0003677

Cluster-22178.47293 GO:0046872

Cluster-22178.47298 GO:0006508 GO:0009847 GO:0000166 GO:0003676 GO:0008233

Cluster-22178.47313 GO:0005515 GO:0046872 GO:0008270 GO:0003723
GO:0005622
Cluster-22178.47314 GO:0016757
Cluster-22178.47316 GO:0005515
Cluster-22178.47317 GO:0032968 GO:0007165 GO:0006367 GO:0050750
GO:0008201 GO:0003677 GO:0005634 GO:0005783
Cluster-22178.47321 GO:0006367 GO:0005672
Cluster-22178.47324 GO:0006367 GO:0005672
Cluster-22178.47325 GO:0016226 GO:0006464 GO:0046872 GO:0008199
GO:0016151
Cluster-22178.47329 GO:0003924 GO:0046872 GO:0005525
Cluster-22178.4733 GO:0009190 GO:0035556 GO:0016849
Cluster-22178.47342 GO:0006779 GO:0016117 GO:0055114 GO:0008033
GO:0019354 GO:0050660 GO:0008270 GO:0016491 GO:0016705
GO:0043115 GO:0005515 GO:0071949
Cluster-22178.47344 GO:0006508 GO:0007165 GO:0007268 GO:0006811
GO:0005524 GO:0004970 GO:0005234 GO:0008233 GO:0016020
GO:0016021
Cluster-22178.47348 GO:0043087 GO:0035023 GO:0005089
Cluster-22178.47349 GO:0006278 GO:0017038 GO:0005524 GO:0003964
GO:0003676 GO:0016787 GO:0003677 GO:0016020
Cluster-22178.47355 GO:0022904 GO:0003676 GO:0008017 GO:0045298
GO:0016020
Cluster-22178.47362 GO:0034453 GO:0005815
Cluster-22178.47363 GO:0008152 GO:0006614 GO:0005524 GO:0005525
GO:0016301
Cluster-22178.47364 GO:0008152 GO:0006614 GO:0005525 GO:0016301
GO:0005524
Cluster-22178.47368 GO:0006351 GO:0007034 GO:0006269 GO:0003896
GO:0005657 GO:0005730
Cluster-22178.47369 GO:0043401 GO:0006355 GO:0003700 GO:0008270
GO:0043565 GO:0005667 GO:0005634
Cluster-22178.47371 GO:0009308 GO:0055114 GO:0003676 GO:0016638

GO:0003723 GO:0042597
Cluster-22178.47376 GO:0008033 GO:0004810 GO:0016437 GO:0008270
GO:0003676 GO:0016779
Cluster-22178.47377 GO:0008033 GO:0004810 GO:0016437 GO:0008270
GO:0003676 GO:0016779
Cluster-22178.47383 GO:0006355 GO:0005634
Cluster-22178.47384 GO:0006118 GO:0030001 GO:0015991 GO:0051258
GO:0006308 GO:0007165 GO:0019069 GO:0055085 GO:0030168
GO:0015992 GO:0020037 GO:0015078 GO:0009055 GO:0046873
GO:0005506 GO:0030674 GO:0005102 GO:0008855 GO:0009318
GO:0033179 GO:0016020 GO:0005577
Cluster-22178.47386 GO:0006886 GO:0006468 GO:0007264 GO:0006606
GO:0006914 GO:0004672 GO:0005524 GO:0005096 GO:0016773
GO:0005783 GO:0016020 GO:0016021
Cluster-22178.47388 GO:0042254 GO:0006412 GO:0003735 GO:0046872
GO:0005840 GO:0005622
Cluster-22178.47393 GO:0006118 GO:0055114 GO:0051537 GO:0009496
GO:0009512 GO:0042651
Cluster-22178.47395 GO:0019079 GO:0009058 GO:0008152 GO:0016787
GO:0003677 GO:0008026 GO:0005515 GO:0005509 GO:0005524
GO:0003824 GO:0003676
Cluster-22178.47400 GO:0006355 GO:0003700 GO:0005515 GO:0008270
GO:0043565 GO:0005634 GO:0005667
Cluster-22178.47405 GO:0046872
Cluster-22178.47407 GO:0009058 GO:0030170
Cluster-22178.47409 GO:0035091
Cluster-22178.47410 GO:0015689 GO:0055114 GO:0009405 GO:0007165
GO:0006816 GO:0006874 GO:0005219 GO:0016491 GO:0015098
GO:0016021 GO:0005622 GO:0005576
Cluster-22178.47413 GO:0016310 GO:0009069 GO:0004674 GO:0005515

Cluster-22178.47414 GO:0005515
Cluster-22178.47419 GO:0007165

Cluster-22178.47420 GO:0007165 GO:0000917 GO:0007172 GO:0043093 GO:0006468 GO:0005515 GO:0004871 GO:0004713 GO:0005096 GO:0005925 GO:0005737

Cluster-22178.47421 GO:0005515 GO:0003677 GO:0005737

Cluster-22178.47423 GO:0009432 GO:0051782 GO:0006270 GO:0005515 GO:0019028 GO:0009276

Cluster-22178.47425 GO:0003676

Cluster-22178.47428 GO:0006687 GO:0009058 GO:0001733 GO:0008146 GO:0005794 GO:0016021

Cluster-22178.47430 GO:0009058 GO:0006687 GO:0008146 GO:0001733 GO:0016021 GO:0005794

Cluster-22178.47441 GO:0005634

Cluster-22178.47442 GO:0008270

Cluster-22178.47443 GO:0008270

Cluster-22178.47449 GO:0055085 GO:0006811 GO:0046983 GO:0005216 GO:0005515 GO:0016020

Cluster-22178.47456 GO:0035556 GO:0005515

Cluster-22178.47462 GO:0008152 GO:0006338 GO:0005515 GO:0003824 GO:0090544

Cluster-22178.47463 GO:0007165 GO:0005096

Cluster-22178.47465 GO:0006206 GO:0006144 GO:0006351 GO:0003677 GO:0003899 GO:0005730

Cluster-22178.47466 GO:0016020 GO:0016021

Cluster-22178.47467 GO:0016020 GO:0016021

Cluster-22178.47469 GO:0005515 GO:0003677 GO:0046983

Cluster-22178.47474 GO:0032797

Cluster-22178.47477 GO:0008483 GO:0030170

Cluster-22178.47478 GO:0006260 GO:0006351 GO:0007165 GO:0006269 GO:0003896 GO:0005657 GO:0005730

Cluster-22178.47479 GO:0008219 GO:0009399 GO:0007131 GO:0006468 GO:0042157 GO:0006869 GO:0005524 GO:0004672 GO:0008289 GO:0005515 GO:0005576

Cluster-22178.4748 GO:0009405 GO:0006810 GO:0055085 GO:0045735

GO:0005215 GO:0016020
Cluster-22178.47481 GO:0006869 GO:0006468 GO:0042157 GO:0009399
GO:0007131 GO:0008219 GO:0005515 GO:0008289 GO:0004672
GO:0005524 GO:0005576
Cluster-22178.47483 GO:0016310 GO:0009069 GO:0006303 GO:0005524
GO:0004677 GO:0003677 GO:0005958 GO:0005634
Cluster-22178.47485 GO:0007283 GO:0003677 GO:0046983 GO:0008270
GO:0000786 GO:0005634
Cluster-22178.47486 GO:0006270 GO:0009236 GO:0006359 GO:0006260
GO:0006355 GO:0034755 GO:0006351 GO:0006826 GO:0006352
GO:0003677 GO:0005381 GO:0016987 GO:0003887 GO:0003700
GO:0005667 GO:0042575 GO:0016021 GO:0005789
Cluster-22178.47487 GO:0016021 GO:0016020
Cluster-22178.47488 GO:0042998 GO:0005198 GO:0071253 GO:0016021
GO:0019031 GO:0005802
Cluster-22178.47496 GO:0009607 GO:0019069 GO:0016021
Cluster-22178.47497 GO:0043087 GO:0051321 GO:0061136 GO:0035023
GO:0043531 GO:0005089 GO:0005737
Cluster-22178.47498 GO:0043087 GO:0051321 GO:0035023 GO:0043531
GO:0005089 GO:0005737
Cluster-22178.47499 GO:0006506 GO:0006468 GO:0003676 GO:0004715
GO:0005524 GO:0017176 GO:0005515 GO:0005509 GO:0016021
GO:0005737
Cluster-22178.47500 GO:0006338 GO:0005524 GO:0046872 GO:0016818
GO:0003676 GO:0005634
Cluster-22178.47501 GO:0006355 GO:0005515 GO:0061630 GO:0042393
GO:0003677 GO:0043565 GO:0008270 GO:0046872 GO:0003700
GO:0005667 GO:0016021
Cluster-22178.47504 GO:0019058
Cluster-22178.47505 GO:0004672
Cluster-22178.47506 GO:0016021
Cluster-22178.47508 GO:0008152 GO:0006206 GO:0055114 GO:0006144
GO:0006351 GO:0006270 GO:0006118 GO:0006275 GO:0050660

GO:0016627 GO:0005524 GO:0043565 GO:0003995 GO:0003899
GO:0005730
Cluster-22178.47510 GO:0003924 GO:0005515 GO:0005525
Cluster-22178.47511 GO:0006468 GO:0004672 GO:0005524 GO:0016773
GO:0005515 GO:0016020
Cluster-22178.47512 GO:0006308 GO:0055114 GO:0010508 GO:0006397
GO:0007155 GO:0016491 GO:0008855 GO:0051082 GO:0016021
GO:0000445 GO:0009318 GO:0019028
Cluster-22178.47515 GO:0071973 GO:0006606 GO:0006935 GO:0042803
GO:0008134 GO:0005515 GO:0045502 GO:0003774 GO:0016020
GO:0005667 GO:0030286 GO:0009288
Cluster-22178.47516 GO:0016055 GO:0005515
Cluster-22178.47517 GO:0016055 GO:0005515
Cluster-22178.47518 GO:0042254 GO:0006412 GO:0003735 GO:0005840
Cluster-22178.47526 GO:0019012 GO:0016021
Cluster-22178.47529 GO:0035556 GO:0005515
Cluster-22178.47530 GO:0007018 GO:0000077 GO:0005515 GO:0005868
Cluster-22178.47531 GO:0004064 GO:0005515
Cluster-22178.47532 GO:0006357 GO:0006468 GO:0006508 GO:0007010
GO:0005524 GO:0004672 GO:0016773 GO:0003779 GO:0016805
GO:0001104 GO:0016592 GO:0016020
Cluster-22178.47534 GO:0043087 GO:0006051 GO:0006040 GO:0032012
GO:0005086 GO:0047465 GO:0016772
Cluster-22178.47535 GO:0045116 GO:0016881 GO:0005524 GO:0008641

Cluster-22178.47536 GO:0042325 GO:0005737
Cluster-22178.47539 GO:0006355 GO:0003700 GO:0005525 GO:0043565
GO:0016020 GO:0005667
Cluster-22178.47540 GO:0005996 GO:0055114 GO:0051536 GO:0048029
GO:0016853
Cluster-22178.47545 GO:0005515
Cluster-22178.47549 GO:0030155 GO:0006886 GO:0007165 GO:0007155
GO:0045995 GO:0006914 GO:0055114 GO:0019049 GO:0030334

GO:0005102 GO:0016702 GO:0031224 GO:0016020 GO:0005801
Cluster-22178.47551 GO:0045995 GO:0051258 GO:0055114 GO:0006886
GO:0030334 GO:0019049 GO:0030168 GO:0006914 GO:0007165
GO:0030155 GO:0007155 GO:0016702 GO:0005102 GO:0030674
GO:0005801 GO:0031224 GO:0005577 GO:0016020
Cluster-22178.47554 GO:0003774 GO:0005524 GO:0005515 GO:0016459
Cluster-22178.47555 GO:0005524 GO:0003774 GO:0005515 GO:0016459
Cluster-22178.47556 GO:0006366 GO:0003677 GO:0016787 GO:0046872
GO:0008270 GO:0005634 GO:0005665
Cluster-22178.47564 GO:0030168 GO:0051258 GO:0006308 GO:0055085
GO:0030001 GO:0007165 GO:0008855 GO:0005102 GO:0046873
GO:0030674 GO:0005515 GO:0005577 GO:0009318 GO:0016020
Cluster-22178.47565 GO:0006468 GO:0009069 GO:0016310 GO:0005524
GO:0004674
Cluster-22178.47566 GO:0006357 GO:0001104 GO:0016592
Cluster-22178.47572 GO:0015992 GO:0006123 GO:0004129 GO:0045277
GO:0005751 GO:0005743
Cluster-22178.47573 GO:0046872
Cluster-22178.47575 GO:0005515
Cluster-22178.47576 GO:0005515
Cluster-22178.47577 GO:0033617 GO:0005739
Cluster-22178.47578 GO:0033617 GO:0005739
Cluster-22178.47580 GO:0016787
Cluster-22178.47586 GO:0016579 GO:0032324 GO:0008270 GO:0036459

Cluster-22178.47589 GO:0015074 GO:0016042 GO:0006629 GO:0006468
GO:0006313 GO:0046486 GO:0005524 GO:0004672 GO:0004806
GO:0003677 GO:0005515
Cluster-22178.47590 GO:0008270 GO:0005634
Cluster-22178.47593 GO:0051259 GO:0005975 GO:0016773 GO:0005515

Cluster-22178.47594 GO:0051259
Cluster-22178.47596 GO:0051259 GO:0005975 GO:0005515 GO:0016773

Cluster-22178.47599 GO:0008152 GO:0008168
Cluster-22178.47600 GO:0006355 GO:0043401 GO:0043565 GO:0008270 GO:0003700 GO:0005667 GO:0005634
Cluster-22178.47601 GO:0006355 GO:0043401 GO:0008270 GO:0043565 GO:0003700 GO:0003677 GO:0005634 GO:0005667
Cluster-22178.47602 GO:0055114 GO:0008152 GO:0006811 GO:0006694 GO:0008210 GO:0051260 GO:0046487 GO:0009396 GO:0055085 GO:0008207 GO:0008209 GO:0003824 GO:0016491 GO:0050662 GO:0003854 GO:0005216 GO:0004488 GO:0016616 GO:0016020
Cluster-22178.47603 GO:0043401 GO:0006355 GO:0003700 GO:0008270 GO:0043565 GO:0005634 GO:0005667
Cluster-22178.47612 GO:0006412 GO:0042254 GO:0003735 GO:0005840 GO:0005622
Cluster-22178.47614 GO:0000917 GO:0006355 GO:0043093 GO:0003700 GO:0043565 GO:0003677 GO:0005737 GO:0005667 GO:0005634
Cluster-22178.47617 GO:0042176 GO:0000245 GO:0030234 GO:0000502 GO:0005634
Cluster-22178.47622 GO:0042254 GO:0006412 GO:0006915 GO:0005515 GO:0003735 GO:0005509 GO:0005840 GO:0005622
Cluster-22178.47623 GO:0008152 GO:0003824
Cluster-22178.47625 GO:0030001 GO:0046872
Cluster-22178.47626 GO:0016021
Cluster-22178.47631 GO:0016787
Cluster-22178.47633 GO:0006260 GO:0006351 GO:0007165 GO:0006269 GO:0003896 GO:0005657 GO:0005730
Cluster-22178.47634 GO:0046983
Cluster-22178.47636 GO:0050790 GO:0030234
Cluster-22178.47638 GO:0005515
Cluster-22178.47639 GO:0016310 GO:0009069 GO:0006468 GO:0004674 GO:0008270 GO:0005524 GO:0004672
Cluster-22178.47641 GO:0003676 GO:0003723
Cluster-22178.47643 GO:0006814 GO:0006813 GO:0016787 GO:0005890

Cluster-22178.47644 GO:0006144 GO:0006468 GO:0006171 GO:0000287 GO:0005524 GO:0004672 GO:0004016
Cluster-22178.47645 GO:0008270 GO:0046872 GO:0003677
Cluster-22178.47646 GO:0016567 GO:0004842 GO:0016020
Cluster-22178.47650 GO:0046488 GO:0016307
Cluster-22178.47651 GO:0055085 GO:0035556 GO:0006811 GO:0006816 GO:0006810 GO:0042030 GO:0005246 GO:0005216 GO:0005515 GO:0016020
Cluster-22178.47652 GO:0006284 GO:0016787 GO:0003677 GO:0003905 GO:0016021
Cluster-22178.47655 GO:0035091 GO:0005515
Cluster-22178.47658 GO:0005198 GO:0005882
Cluster-22178.47659 GO:0006464 GO:0006560 GO:0008295 GO:0006886 GO:0016192 GO:0006525 GO:0004014 GO:0005515 GO:0030117
Cluster-22178.47663 GO:0006470 GO:0008138 GO:0070403
Cluster-22178.47666 GO:0005515
Cluster-22178.47667 GO:0005515
Cluster-22178.47668 GO:0007166 GO:0016020
Cluster-22178.47669 GO:0031554 GO:0009086 GO:0019069 GO:0042558 GO:0031556 GO:0005524 GO:0046872 GO:0008705 GO:0031419 GO:0005622 GO:0005887
Cluster-22178.47673 GO:0046872 GO:0003950
Cluster-22178.47678 GO:0006355 GO:0006816 GO:0070588 GO:0006352 GO:0003677 GO:0005515 GO:0016987 GO:0003700 GO:0005262 GO:0005667 GO:0016020
Cluster-22178.47680 GO:0006355 GO:0032065 GO:0016579 GO:0005515 GO:0003677 GO:0043565 GO:0036459 GO:0005543 GO:0003700 GO:0005667 GO:0005634 GO:0005938
Cluster-22178.47681 GO:0006355 GO:0003690 GO:0005739
Cluster-22178.47683 GO:0006952 GO:0005515
Cluster-22178.47687 GO:0006807 GO:0007067 GO:0016810 GO:0042729 GO:0072686
Cluster-22178.47691 GO:0006810 GO:0009405 GO:0006310 GO:0006051

GO:0006040 GO:0003677 GO:0005515 GO:0047465 GO:0008200
GO:0019905 GO:0005576 GO:0005886
Cluster-22178.47692 GO:0017025
Cluster-22178.47695 GO:0046872 GO:0043130
Cluster-22178.47698 GO:0006355 GO:0003690 GO:0005739
Cluster-22178.47699 GO:0016567 GO:0015991 GO:0015992 GO:0005515
GO:0015078 GO:0004842 GO:0008270 GO:0046872 GO:0033179
GO:0005680 GO:0005622
Cluster-22178.47700 GO:0005515
Cluster-22178.47701 GO:0008033 GO:0055114 GO:0004499 GO:0016491
GO:0050661 GO:0050660
Cluster-22178.47705 GO:0051259 GO:0007034
Cluster-22178.47707 GO:0005515
Cluster-22178.47708 GO:0007155 GO:0042246 GO:0046872 GO:0008270
GO:0005515 GO:0016021
Cluster-22178.47711 GO:0006508 GO:0004222 GO:0019902 GO:0008270
GO:0005576
Cluster-22178.47715 GO:0006355 GO:0003700 GO:0003677 GO:0005667
Cluster-22178.47717 GO:0006355 GO:0003700 GO:0003677 GO:0005667
Cluster-22178.47718 GO:0055114 GO:0016705 GO:0020037 GO:0005506

Cluster-22178.47720 GO:0005524
Cluster-22178.47725 GO:0000746 GO:0004252 GO:0055036 GO:0019028
GO:0019867
Cluster-22178.47732 GO:0007186 GO:0007165 GO:0015684 GO:0007264
GO:0004871 GO:0005525 GO:0003924 GO:0016887 GO:0015093
GO:0019001 GO:0031683 GO:0005524 GO:0016021
Cluster-22178.47734 GO:0015991 GO:0015992 GO:0015078 GO:0033177
Cluster-22178.47737 GO:0055114 GO:0006260 GO:0032266 GO:0043130

Cluster-22178.47741 GO:0030036 GO:0003779 GO:0005515 GO:0030054
Cluster-22178.47750 GO:0005515
Cluster-22178.47751 GO:0042254 GO:0006412 GO:0005515 GO:0003735

GO:0005622 GO:0005840
Cluster-22178.47754 GO:0006744 GO:0006120 GO:0015992 GO:0006412
GO:0006814 GO:0042254 GO:0042773 GO:2001256 GO:0003735
GO:0008137 GO:0005743 GO:0030176 GO:0005840 GO:0005622
Cluster-22178.47756 GO:0006351 GO:0003677 GO:0003676 GO:0008270
GO:0005634
Cluster-22178.47757 GO:0015979 GO:0008146 GO:0030145 GO:0009523
GO:0016021
Cluster-22178.47760 GO:0030150 GO:0007017 GO:0007018 GO:0005524
GO:0003777 GO:0008017 GO:0005874 GO:0045298 GO:0005742
Cluster-22178.47769 GO:0006013 GO:0006000 GO:0005524 GO:0003873
GO:0016301
Cluster-22178.47775 GO:0005515
Cluster-22178.47777 GO:0007165 GO:0007186 GO:0043934 GO:0015937
GO:0008152 GO:0015940 GO:0016301 GO:0004871 GO:0019001
GO:0031683 GO:0005524 GO:0004140 GO:0003924 GO:0016887
GO:0016020
Cluster-22178.47780 GO:0006351 GO:0003677 GO:0005634
Cluster-22178.47781 GO:0006355 GO:0016021 GO:0005634
Cluster-22178.47784 GO:0045947 GO:0008190
Cluster-22178.47786 GO:0006323 GO:0003677 GO:0005515 GO:0019012
Cluster-22178.47787 GO:0005975 GO:0008270 GO:0008184 GO:0003723
GO:0003676
Cluster-22178.47793 GO:0006260 GO:0008270 GO:0003677
Cluster-22178.47794 GO:0019497 GO:0006771 GO:0055114 GO:0003756
GO:0016671 GO:0003993 GO:0005783
Cluster-22178.47796 GO:0016787
Cluster-22178.47797 GO:0006259 GO:0007165 GO:0007275 GO:0006118
GO:0014068 GO:0050776 GO:0003677 GO:0043548 GO:0005524
GO:0005102 GO:0020037 GO:0009055 GO:0003824 GO:0005694
GO:0016020 GO:0016021
Cluster-22178.47798 GO:0043087 GO:0006051 GO:0006040 GO:0032012
GO:0005086 GO:0016772 GO:0047465

Cluster-22178.47802 GO:0043401 GO:0007275 GO:0006355 GO:0003700
GO:0043565 GO:0008270 GO:0005515 GO:0016021 GO:0042600
GO:0005634 GO:0005667
Cluster-22178.47805 GO:0005515
Cluster-22178.47806 GO:0005515
Cluster-22178.47808 GO:0045892 GO:0042754 GO:0006281 GO:0006310
GO:0005524 GO:0009378 GO:0005657 GO:0009379
Cluster-22178.47809 GO:0005515
Cluster-22178.47813 GO:0007005 GO:0006265 GO:0003917 GO:0003677
GO:0003918 GO:0005694 GO:0031305
Cluster-22178.47817 GO:0007165 GO:0005515 GO:0004871 GO:0016021
Cluster-22178.47821 GO:0006355 GO:0016791 GO:0043565
Cluster-22178.47822 GO:0006470 GO:0006570 GO:0004725 GO:0008138

Cluster-22178.47823 GO:0055085 GO:0022857 GO:0016021
Cluster-22178.47825 GO:0030334 GO:0015031 GO:0030155 GO:0007165
GO:0045995 GO:0008033 GO:0006904 GO:0051252 GO:0006355
GO:0005102 GO:0003677 GO:0005507 GO:0003700 GO:0004540
GO:0000145 GO:0005667 GO:0016021 GO:0005634
Cluster-22178.47831 GO:0006351 GO:0006260 GO:0006269 GO:0003896
GO:0005657 GO:0005730
Cluster-22178.47833 GO:0016747
Cluster-22178.47834 GO:0003676 GO:0005515 GO:0008270 GO:0046872

Cluster-22178.47839 GO:0006464 GO:0046872 GO:0016151 GO:0003677
GO:0005737
Cluster-22178.47841 GO:0003682 GO:0000785
Cluster-22178.47842 GO:0006303 GO:0005198 GO:0016021
Cluster-22178.47843 GO:0055085 GO:0006810 GO:0016887 GO:0003924
GO:0005524 GO:0042626 GO:0005525 GO:0016301 GO:0016021
Cluster-22178.47846 GO:0007186 GO:0030246 GO:0004930 GO:0016020
GO:0016021
Cluster-22178.47849 GO:0007049 GO:0006260 GO:0006355 GO:0043093

GO:0006457 GO:0006810 GO:0000917 GO:0043565 GO:0003700
GO:0051082 GO:0005509 GO:0005737 GO:0005667 GO:0016020
GO:0005783
Cluster-22178.47853 GO:0003676
Cluster-22178.47855 GO:0009235
Cluster-22178.47859 GO:0043087 GO:0035023 GO:0007165 GO:0005057
GO:0005089
Cluster-22178.47860 GO:0006810 GO:0005215 GO:0016021 GO:0016020
Cluster-22178.47861 GO:0005198 GO:0019028
Cluster-22178.47862 GO:0009405 GO:0005576
Cluster-22178.47863 GO:0005515 GO:0016021
Cluster-22178.47865 GO:0051252 GO:0006396 GO:0008608 GO:0003723
GO:0003676 GO:0000175 GO:0042729 GO:0072686 GO:0019867
Cluster-22178.47866 GO:0051252 GO:0008608 GO:0006396 GO:0003723
GO:0003676 GO:0000175 GO:0042729 GO:0072686
Cluster-22178.47868 GO:0008608 GO:0006396 GO:0051252 GO:0003676
GO:0000175 GO:0003723 GO:0042729 GO:0072686
Cluster-22178.47869 GO:0006396 GO:0008608 GO:0051252 GO:0000175
GO:0003676 GO:0003723 GO:0072686 GO:0042729
Cluster-22178.47870 GO:0040007 GO:0008283 GO:0008406 GO:0007165
GO:0008083
Cluster-22178.47872 GO:0016114 GO:0019385 GO:0006694 GO:0003824
GO:0030976 GO:0008661 GO:0000287
Cluster-22178.47874 GO:0006468 GO:0004672 GO:0005524 GO:0016773
GO:0016020
Cluster-22178.47875 GO:0006468 GO:0016773 GO:0004672 GO:0005524
GO:0016020
Cluster-22178.47876 GO:0046872 GO:0020037 GO:0016020
Cluster-22178.47881 GO:0000162 GO:0006571 GO:0043248 GO:0009094
GO:0051090 GO:0005515 GO:0004664 GO:0005634
Cluster-22178.47882 GO:0051260
Cluster-22178.47883 GO:0006470 GO:0006570 GO:0004725 GO:0008138
GO:0016787

Cluster-22178.47884 GO:0006470 GO:0006570 GO:0008138 GO:0004725

Cluster-22178.47885 GO:0046872
Cluster-22178.47887 GO:0008168 GO:0005515
Cluster-22178.47889 GO:0006351 GO:0006468 GO:0006144 GO:0006206
GO:0003899 GO:0005524 GO:0004672 GO:0005634 GO:0005730
Cluster-22178.47895 GO:0016052 GO:0005985 GO:0005982 GO:0004556
GO:0005509
Cluster-22178.47898 GO:0055114 GO:0005506 GO:0020037 GO:0016705

Cluster-22178.47900 GO:0003824
Cluster-22178.47903 GO:0007166 GO:0016020
Cluster-22178.47904 GO:0006355 GO:0043402 GO:0043401 GO:0007165
GO:0042921 GO:0003677 GO:0043565 GO:0008270 GO:0004883
GO:0005496 GO:0003700 GO:0005667 GO:0005634
Cluster-22178.47905 GO:0043401 GO:0006355
Cluster-22178.47908 GO:0005615
Cluster-22178.47926 GO:0030001 GO:0006813 GO:0006355 GO:0015940
GO:0008608 GO:0055114 GO:0007155 GO:0016117 GO:0055085
GO:0015979 GO:0016705 GO:0008677 GO:0016614 GO:0005540
GO:0003677 GO:0016787 GO:0046873 GO:0050660 GO:0004222
GO:0016491 GO:0005524 GO:0042729 GO:0072686 GO:0016020
Cluster-22178.47928 GO:0035556 GO:0007165 GO:0003677 GO:0005515

Cluster-22178.47929 GO:0019028
Cluster-22178.47931 GO:0007165 GO:0007172 GO:0006468 GO:0030168
GO:0005975 GO:0051258 GO:0030674 GO:0004713 GO:0004871
GO:0003824 GO:0005102 GO:0004672 GO:0005524 GO:0005925
GO:0005577
Cluster-22178.47936 GO:0035023 GO:0043087 GO:0006355 GO:0005089
GO:0005515 GO:0042393
Cluster-22178.47938 GO:0005985 GO:0005982 GO:0005975 GO:0004045
GO:0004650

Cluster-22178.47948 GO:0016462 GO:0005509
Cluster-22178.47949 GO:0043087 GO:0032012 GO:0005086 GO:0046872 GO:0008289
Cluster-22178.47950 GO:0043087 GO:0030639 GO:0032012 GO:0005086

Cluster-22178.47951 GO:0022904 GO:0016020
Cluster-22178.47953 GO:0030246
Cluster-22178.47959 GO:0006144 GO:0006355 GO:0006508 GO:0003700 GO:0003968 GO:0043565 GO:0004197 GO:0003677 GO:0017111 GO:0031379 GO:0005634 GO:0005667
Cluster-22178.47960 GO:0016020 GO:0016021
Cluster-22178.47964 GO:0009405 GO:0007155 GO:0005515 GO:0005509 GO:0003824 GO:0051536 GO:0016020 GO:0005576
Cluster-22178.47967 GO:0022904 GO:0016651 GO:0005743
Cluster-22178.47969 GO:0008152 GO:0003723 GO:0008168
Cluster-22178.47971 GO:0007264 GO:0006338 GO:0005525 GO:0003676 GO:0005524 GO:0003924 GO:0016818 GO:0005634
Cluster-22178.47972 GO:0006338 GO:0007264 GO:0003676 GO:0005525 GO:0003924 GO:0005524 GO:0016818 GO:0005634
Cluster-22178.47973 GO:0019354 GO:0006779 GO:0035023 GO:0043087 GO:0055114 GO:0005089 GO:0043115
Cluster-22178.47974 GO:0055114 GO:0043087 GO:0035023 GO:0019354 GO:0006779 GO:0043115 GO:0005089
Cluster-22178.47977 GO:0042254 GO:0006123 GO:0015992 GO:0005507 GO:0004129 GO:0016020 GO:0005730 GO:0045277
Cluster-22178.47979 GO:0055085 GO:0006885 GO:0006812 GO:0015299 GO:0016021
Cluster-22178.47980 GO:0006206 GO:0006464 GO:0055114 GO:0006230 GO:0007010 GO:0005524 GO:0008270 GO:0004797 GO:0046872 GO:0003779 GO:0050660 GO:0016151 GO:0016614
Cluster-22178.47983 GO:0006810 GO:0006144 GO:0015992 GO:0006396 GO:0006807 GO:0051252 GO:0009405 GO:0016891 GO:0004000 GO:0003723 GO:0003726 GO:0019855 GO:0015078 GO:0055036

GO:0033644 GO:0005576
Cluster-22178.47990 GO:0042254 GO:0006412 GO:0003735 GO:0005622
GO:0005840
Cluster-22178.47999 GO:0006468 GO:0005524 GO:0004672
Cluster-22178.48000 GO:0051252 GO:0016891
Cluster-22178.48001 GO:0008284 GO:0001819 GO:0010884 GO:0005515
GO:0042802 GO:0016020
Cluster-22178.48002 GO:0006118 GO:0007186 GO:0046983 GO:0009055
GO:0004930 GO:0009512 GO:0016021
Cluster-22178.48004 GO:0007264 GO:0005525
Cluster-22178.48007 GO:0006508 GO:0009002
Cluster-22178.48008 GO:0007186 GO:0006526 GO:0042967 GO:0015979
GO:0004358 GO:0004930 GO:0016020 GO:0016021 GO:0009523
GO:0009539
Cluster-22178.48016 GO:0008152 GO:0016746 GO:0005524
Cluster-22178.48017 GO:0006468 GO:0005524 GO:0004672 GO:0016773
GO:0016020
Cluster-22178.48018 GO:0008152 GO:0005524 GO:0016746
Cluster-22178.48020 GO:0006468 GO:0004672 GO:0005524
Cluster-22178.48030 GO:0005515
Cluster-22178.48037 GO:0005515
Cluster-22178.48038 GO:0007617 GO:0006355 GO:0003700 GO:0008270
GO:0009289 GO:0005667 GO:0005634 GO:0005576
Cluster-22178.48040 GO:0042967 GO:0008080 GO:0016747
Cluster-22178.48042 GO:0042981 GO:0006355 GO:0003700 GO:0043565
GO:0005667 GO:0005634
Cluster-22178.48046 GO:0016021
Cluster-22178.48048 GO:0005515 GO:0005576
Cluster-22178.4805 GO:0015948 GO:0007264 GO:0042981 GO:0005525
GO:0003924
Cluster-22178.48053 GO:0005509 GO:0005515
Cluster-22178.48054 GO:0000105 GO:0055114 GO:0006537 GO:0009607
GO:0006355 GO:0003824 GO:0016491 GO:0016627 GO:0015930

GO:0003723 GO:0016638 GO:0005737

Cluster-22178.48055 GO:0006355 GO:0000105 GO:0009607 GO:0006537 GO:0055114 GO:0015930 GO:0016638 GO:0003824 GO:0016627 GO:0016491 GO:0005737

Cluster-22178.48057 GO:0007165 GO:0007186 GO:0035556 GO:0043087 GO:0015979 GO:0004871 GO:0005089 GO:0009579 GO:0005834 GO:0016021 GO:0009522 GO:0005737

Cluster-22178.48061 GO:0016032 GO:0005096 GO:0019031

Cluster-22178.48062 GO:0006144 GO:0055114 GO:0046034 GO:0005506 GO:0005515 GO:0004017 GO:0020037 GO:0016705

Cluster-22178.48065 GO:0051028 GO:0048309 GO:0030430

Cluster-22178.48066 GO:0006355 GO:0003700 GO:0005667 GO:0005622

Cluster-22178.48072 GO:0007165 GO:0007186 GO:0000166 GO:0003677 GO:0031683 GO:0005524 GO:0019001 GO:0003924 GO:0005525 GO:0004871 GO:0016020

Cluster-22178.48075 GO:0016310 GO:0004673 GO:0043015 GO:0042802 GO:0008017 GO:0005634 GO:0045298 GO:0016021 GO:0009365

Cluster-22178.48083 GO:0016310 GO:0000160 GO:0000155 GO:0046983 GO:0009365 GO:0016021

Cluster-22178.48085 GO:0016021

Cluster-22178.48086 GO:0016567 GO:0008270 GO:0046872 GO:0003677 GO:0004842 GO:0005515 GO:0005680

Cluster-22178.48087 GO:0006464 GO:0007186 GO:0004930 GO:0016020

Cluster-22178.48094 GO:0007049

Cluster-22178.48097 GO:0048193 GO:0005198 GO:0005882 GO:0016020

Cluster-22178.48099 GO:0005515

Cluster-22178.48100 GO:0042381 GO:0042742 GO:0016032 GO:0008152 GO:0007275 GO:0016787 GO:0016021

Cluster-22178.48101 GO:0006457 GO:0009116 GO:0005515 GO:0051082 GO:0016020

Cluster-22178.48102 GO:0046983

Cluster-22178.48106 GO:0005515

Cluster-22178.48120 GO:0050790 GO:0006355 GO:0055114 GO:0016491

GO:0030234 GO:0003677

Cluster-22178.48128 GO:0006508 GO:0009002

Cluster-22178.48135 GO:0006935 GO:0051258 GO:0048024 GO:0010508 GO:0006886 GO:0030168 GO:0048193 GO:0007165 GO:0071973 GO:0003774 GO:0019905 GO:0005102 GO:0008017 GO:0030674 GO:0009288 GO:0045298 GO:0005634 GO:0005577 GO:0016020

Cluster-22178.48136 GO:0005515 GO:0005509

Cluster-22178.48146 GO:0007130 GO:0000795

Cluster-22178.48150 GO:0005515 GO:0003677 GO:0005737

Cluster-22178.48162 GO:0006352 GO:0006355 GO:0008270 GO:0003700 GO:0016987 GO:0005515 GO:0003677 GO:0005667

Cluster-22178.48164 GO:0043093 GO:0006260 GO:0000917 GO:0005737 GO:0045095

Cluster-22178.48166 GO:0006260 GO:0015979 GO:0016020 GO:0009523 GO:0009539

Cluster-22178.48174 GO:0003824

Cluster-22178.48178 GO:0006012 GO:0006470 GO:0019497 GO:0006570 GO:0005982 GO:0005985 GO:0006771 GO:0004725 GO:0003993 GO:0046872 GO:0004564 GO:0005515 GO:0004575 GO:0008138 GO:0017177

Cluster-22178.48179 GO:0006470 GO:0006570 GO:0004725 GO:0005515

Cluster-22178.48180 GO:0019497 GO:0006470 GO:0006012 GO:0006771 GO:0005982 GO:0005985 GO:0006570 GO:0004564 GO:0046872 GO:0003993 GO:0004725 GO:0008138 GO:0004575 GO:0005515 GO:0017177

Cluster-22178.48184 GO:0003824

Cluster-22178.48186 GO:0005515 GO:0019028

Cluster-22178.48188 GO:0007304 GO:0007165 GO:0006355 GO:0003700 GO:0005213 GO:0043565 GO:0008270 GO:0005515 GO:0005179 GO:0042600 GO:0005667 GO:0005576

Cluster-22178.48193 GO:0005198 GO:0019028

Cluster-22178.48194 GO:0006810 GO:0055085 GO:0005215 GO:0016021

GO:0016020
Cluster-22178.48195 GO:0055085 GO:0006810 GO:0005215 GO:0016020
GO:0016021
Cluster-22178.48196 GO:0006260 GO:0009405 GO:0003677 GO:0008270
GO:0005664
Cluster-22178.48197 GO:0000917 GO:0010508 GO:0043093 GO:0005737
Cluster-22178.48198 GO:0005515
Cluster-22178.48199 GO:0007268 GO:0005515 GO:0043495 GO:0033130

Cluster-22178.48201 GO:0006412 GO:0008168
Cluster-22178.48208 GO:0043248 GO:0055085 GO:0022857 GO:0016021
Cluster-22178.48225 GO:0006355 GO:0043565 GO:0003700 GO:0005667
Cluster-22178.48228 GO:0055114 GO:0005515 GO:0047134
Cluster-22178.48232 GO:0005515
Cluster-22178.48235 GO:0015012 GO:0006810 GO:0006024 GO:0042626
GO:0005524 GO:0005515 GO:0003723 GO:0016021
Cluster-22178.48236 GO:0006468 GO:0006446 GO:0004672 GO:0003743
GO:0035091 GO:0005737 GO:0005840 GO:0005852
Cluster-22178.48238 GO:0020037 GO:0019825
Cluster-22178.4824 GO:0007264 GO:0007165 GO:0007186 GO:0006013
GO:0006000 GO:0003873 GO:0005524 GO:0031683 GO:0019001
GO:0003924 GO:0005525 GO:0004871
Cluster-22178.48240 GO:0007165 GO:0006811 GO:0007268 GO:0055085
GO:0005216 GO:0004970 GO:0016020
Cluster-22178.48242 GO:0005515 GO:0016020
Cluster-22178.48244 GO:0008152 GO:0006260 GO:0016787 GO:0003677
GO:0003887 GO:0042575
Cluster-22178.48245 GO:0016255 GO:0003723 GO:0003676 GO:0042765
Cluster-22178.48246 GO:0001558 GO:0005515 GO:0005520 GO:0016942
GO:0005576
Cluster-22178.48248 GO:0007165 GO:0006955 GO:0006935 GO:0008009
GO:0005576
Cluster-22178.48250 GO:0055114 GO:0008033 GO:0006397 GO:0016117

GO:0006779 GO:0019354 GO:0016705 GO:0005515 GO:0043115
GO:0003723 GO:0050660 GO:0016491 GO:0008270 GO:0071949
GO:0005737 GO:0005634
Cluster-22178.48252 GO:0016757
Cluster-22178.48253 GO:0006351 GO:0017111 GO:0005515 GO:0005524

Cluster-22178.48256 GO:0006468 GO:0004672 GO:0005524 GO:0016773
GO:0005515 GO:0016020
Cluster-22178.48258 GO:0043130
Cluster-22178.48259 GO:0042254 GO:0006412 GO:0003735 GO:0005622
GO:0005840
Cluster-22178.48260 GO:0016021
Cluster-22178.48261 GO:0006508 GO:0005515 GO:0004252
Cluster-22178.48263 GO:0030001 GO:0015743 GO:0006816 GO:0006874
GO:0055085 GO:0046873 GO:0005219 GO:0016020 GO:0005634
GO:0016021 GO:0005622
Cluster-22178.48265 GO:0006355 GO:0010024 GO:0006270 GO:0055114
GO:0003677 GO:0004252 GO:0050897 GO:0016636 GO:0003700
GO:0005634 GO:0005667 GO:0005886
Cluster-22178.48267 GO:0006355 GO:0005515 GO:0016021 GO:0005634
Cluster-22178.48271 GO:0006355 GO:0003700 GO:0003677 GO:0043565
GO:0005634 GO:0005667
Cluster-22178.48277 GO:0007275 GO:0006511 GO:0008053 GO:0003677
GO:0005515 GO:0016787 GO:0005524 GO:0008270 GO:0046872
GO:0003924 GO:0005741 GO:0005634 GO:0016021
Cluster-22178.48279 GO:0051252 GO:0006351 GO:0006396 GO:0007049
GO:0005515 GO:0003723 GO:0004525 GO:0017053 GO:0005887
Cluster-22178.48280 GO:0006396 GO:0007049 GO:0051252 GO:0006351
GO:0004525 GO:0005509 GO:0004252 GO:0005515 GO:0005544
GO:0003723 GO:0005886 GO:0017053
Cluster-22178.48282 GO:0006807 GO:0006396 GO:0006144 GO:0004000
GO:0003723
Cluster-22178.48284 GO:0046872

Cluster-22178.48285 GO:0046872 GO:0008270 GO:0005634
Cluster-22178.48288 GO:0006355 GO:0003700 GO:0043565 GO:0005667
Cluster-22178.48289 GO:0006468 GO:0009405 GO:0006810 GO:0004672
GO:0005524 GO:0016773 GO:0019855 GO:0005576 GO:0016020
Cluster-22178.48291 GO:0008360 GO:0015979 GO:0007186 GO:0004930
GO:0003677 GO:0042802 GO:0005515 GO:0016021 GO:0009539
GO:0009523 GO:0016020
Cluster-22178.48293 GO:0006468 GO:0004672 GO:0005524
Cluster-22178.48294 GO:0006904 GO:0000145
Cluster-22178.48297 GO:0003676 GO:0003723 GO:0016021
Cluster-22178.48299 GO:0007264 GO:0006355 GO:0010033 GO:0061024
GO:0008134 GO:0000166 GO:0003677 GO:0043531 GO:0016887
GO:0003924 GO:0005524 GO:0005525 GO:0005667 GO:0016020
GO:0019867
Cluster-22178.48300 GO:0010033 GO:0007264 GO:0006355 GO:0061024
GO:0000166 GO:0003677 GO:0008134 GO:0005525 GO:0005524
GO:0016887 GO:0043531 GO:0003924 GO:0016020 GO:0005667
GO:0019867
Cluster-22178.48301 GO:0007165 GO:0007264 GO:0006629 GO:0035556
GO:0043087 GO:0046339 GO:0009395 GO:0005515 GO:0005085
GO:0008081 GO:0004435
Cluster-22178.48307 GO:0046872
Cluster-22178.48308 GO:0019836 GO:0005576
Cluster-22178.48313 GO:0045859 GO:0004860
Cluster-22178.48314 GO:0055085 GO:0030001 GO:0006813 GO:0005249
GO:0046873 GO:0005515 GO:0016021 GO:0016020 GO:0008076
Cluster-22178.48316 GO:0007264 GO:0007186 GO:0007165 GO:0003924
GO:0031683 GO:0019001 GO:0004871 GO:0005525
Cluster-22178.48321 GO:0003676 GO:0005515 GO:0008270 GO:0005634
Cluster-22178.48323 GO:0007219 GO:0007275 GO:0016021
Cluster-22178.48324 GO:0007219 GO:0007165 GO:0007275 GO:0005179
GO:0016021 GO:0005576
Cluster-22178.48325 GO:0006468 GO:0005524 GO:0004672 GO:0016773

GO:0016020
Cluster-22178.48327 GO:0009103 GO:0008918
Cluster-22178.48328 GO:0004252 GO:0016021
Cluster-22178.48332 GO:0007094 GO:0030154 GO:0016021
Cluster-22178.48333 GO:0030154 GO:0018189 GO:0016021
Cluster-22178.48348 GO:0005515
Cluster-22178.48353 GO:0005515
Cluster-22178.48354 GO:0005515
Cluster-22178.48356 GO:0006355 GO:0006281 GO:0008270 GO:0008168
GO:0003677 GO:0005515 GO:0016020
Cluster-22178.48357 GO:0005198 GO:0005882
Cluster-22178.48360 GO:0000902 GO:0006955 GO:0016020
Cluster-22178.48364 GO:0008283 GO:0040007 GO:0042742 GO:0007165
GO:0008270 GO:0008083
Cluster-22178.48366 GO:0006355 GO:0003677 GO:0003700 GO:0043565
GO:0016021 GO:0005634 GO:0005667
Cluster-22178.48369 GO:0015684 GO:0007264 GO:0000287 GO:0030976
GO:0005525 GO:0015093 GO:0003924 GO:0016021
Cluster-22178.48370 GO:0016310 GO:0009069 GO:0006468 GO:0004674
GO:0004672 GO:0005524 GO:0000287
Cluster-22178.48372 GO:0046034 GO:0006139 GO:0006461 GO:0019627
GO:0006144 GO:0006206 GO:0004127 GO:0016151 GO:0005524
GO:0004017
Cluster-22178.48374 GO:0006334 GO:0007165 GO:0006955 GO:0005044
GO:0030247 GO:0005634
Cluster-22178.48375 GO:0006468 GO:0006813 GO:0006814 GO:0004672
GO:0005524 GO:0016773 GO:0005890 GO:0016020
Cluster-22178.48378 GO:0009878 GO:0008152 GO:0003924 GO:0016787
GO:0008168 GO:0046872 GO:0005525
Cluster-22178.48381 GO:0055114 GO:0008270 GO:0047134
Cluster-22178.48388 GO:0007165 GO:0005179 GO:0005576
Cluster-22178.48389 GO:0006064 GO:0006118 GO:0016491 GO:0008927
GO:0009055 GO:0016020 GO:0016021

Cluster-22178.48396 GO:0005515

Cluster-22178.48397 GO:0007165 GO:0006950 GO:0005179 GO:0005576

Cluster-22178.48403 GO:0045046 GO:0055085 GO:0006886 GO:0006810
GO:0005524 GO:0032403 GO:0042626 GO:0016021 GO:0030176
GO:0005779 GO:0016020

Cluster-22178.48405 GO:0005524 GO:0008270 GO:0003676 GO:0005515

Cluster-22178.48413 GO:0042967 GO:0006633 GO:0006574 GO:0006552
GO:0008299 GO:0046950 GO:0006550 GO:0006084 GO:0004421
GO:0004315 GO:0005835

Cluster-22178.48415 GO:0005515

Cluster-22178.48416 GO:0007186 GO:0005975 GO:0004930 GO:0003676
GO:0016853 GO:0046983 GO:0016021

Cluster-22178.48417 GO:0043087 GO:0007264 GO:0031412 GO:0035556
GO:0005085 GO:0005509 GO:0005815

Cluster-22178.48422 GO:0009877 GO:0009312 GO:0016758 GO:0016020

Cluster-22178.48423 GO:0005515

Cluster-22178.48430 GO:0006139 GO:0003676 GO:0016818 GO:0008026
GO:0005524

Cluster-22178.48432 GO:0008289

Cluster-22178.48438 GO:0006468 GO:0005524 GO:0004672 GO:0016773
GO:0016020

Cluster-22178.48439 GO:0006570 GO:0006470 GO:0019988 GO:0043399
GO:0004725 GO:0008138 GO:0008270

Cluster-22178.48441 GO:0007155 GO:0005515 GO:0009289

Cluster-22178.48454 GO:0055085 GO:0022857 GO:0016021

Cluster-22178.48458 GO:0055114 GO:0006813 GO:0045116 GO:0005524
GO:0008641 GO:0016881 GO:0016491 GO:0051287 GO:0016616

Cluster-22178.48460 GO:0007010 GO:0008270 GO:0003779 GO:0005515

Cluster-22178.48462 GO:0046677 GO:0008152 GO:0070569

Cluster-22178.48464 GO:0005515 GO:0008270 GO:0046872

Cluster-22178.48465 GO:0055114 GO:0016714

Cluster-22178.48466 GO:0055114 GO:0016714
Cluster-22178.48471 GO:0051191
Cluster-22178.48476 GO:0005515
Cluster-22178.48477 GO:0004222
Cluster-22178.48478 GO:0035091
Cluster-22178.48480 GO:0005515
Cluster-22178.48482 GO:0006468 GO:0005524 GO:0004672
Cluster-22178.48483 GO:0008152 GO:0050662 GO:0016491 GO:0003824 GO:0005515
Cluster-22178.48484 GO:0006886 GO:0006810 GO:0009405 GO:0006888 GO:0006887 GO:0005515 GO:0003677 GO:0000166 GO:0005524 GO:0008270 GO:0019855 GO:0005576 GO:0000145 GO:0030127
Cluster-22178.48485 GO:0006308 GO:0007049 GO:0008855 GO:0005634 GO:0009318
Cluster-22178.48488 GO:0005515
Cluster-22178.4849 GO:0005515
Cluster-22178.48491 GO:0007021 GO:0046872 GO:0051082 GO:0015631 GO:0045298
Cluster-22178.48492 GO:0007021 GO:0015631 GO:0051082 GO:0046872 GO:0045298
Cluster-22178.48496 GO:0007411 GO:0016055 GO:0005576
Cluster-22178.48501 GO:0006281 GO:0045893 GO:0008081 GO:0005634
Cluster-22178.48503 GO:0016021
Cluster-22178.48505 GO:0003723
Cluster-22178.48506 GO:0006633 GO:0009396 GO:0006554 GO:0009058 GO:0006631 GO:0018874 GO:0008152 GO:0055114 GO:0006574 GO:0006552 GO:0046487 GO:0006550 GO:0006568 GO:0016491 GO:0004488 GO:0050662 GO:0003824 GO:0003857
Cluster-22178.48507 GO:0006552 GO:0006550 GO:0046487 GO:0006568 GO:0008152 GO:0055114 GO:0006574 GO:0006631 GO:0009058 GO:0018874 GO:0006633 GO:0009396 GO:0006554 GO:0003857 GO:0003824 GO:0004488 GO:0050662 GO:0016491
Cluster-22178.48511 GO:0006388 GO:0051252 GO:0000213 GO:0000214

Cluster-22178.48514 GO:0006729 GO:0035556 GO:0016567 GO:0042254
GO:0006412 GO:0008270 GO:0004842 GO:0008124 GO:0003735
GO:0005515 GO:0005622 GO:0016021 GO:0032040 GO:0005840
GO:0005680
Cluster-22178.48515 GO:0045859 GO:0005515 GO:0004860 GO:0005576
Cluster-22178.48516 GO:0016021
Cluster-22178.48520 GO:0006355 GO:0005198 GO:0003700 GO:0005667
GO:0019028 GO:0019031
Cluster-22178.48521 GO:0055114 GO:0006308 GO:0015948 GO:0006457
GO:0048193 GO:0051082 GO:0045502 GO:0042803 GO:0008134
GO:0016817 GO:0008855 GO:0009318 GO:0005667 GO:0016020
GO:0030286 GO:0016272
Cluster-22178.48522 GO:0005515
Cluster-22178.48523 GO:0009069 GO:0006468 GO:0016310 GO:0005516
GO:0004683
Cluster-22178.48524 GO:0005515 GO:0046872
Cluster-22178.48530 GO:0006814 GO:0016829
Cluster-22178.48534 GO:0006310 GO:0006281 GO:0003676 GO:0003677
GO:0009378 GO:0016787 GO:0005657 GO:0009379
Cluster-22178.48536 GO:0005515
Cluster-22178.48538 GO:0006355 GO:0003676 GO:0005622
Cluster-22178.4854 GO:0030001 GO:0006816 GO:0006874 GO:0055085
GO:0005509 GO:0046873 GO:0005219 GO:0016020 GO:0016021
GO:0005622
Cluster-22178.48541 GO:0046849 GO:0008152 GO:0008484 GO:0003824
GO:0005576
Cluster-22178.48550 GO:0008270
Cluster-22178.48555 GO:0008152 GO:0006355 GO:0006506 GO:0000917
GO:0006260 GO:0043093 GO:0006457 GO:0008484 GO:0043565
GO:0003824 GO:0003700 GO:0051082 GO:0045502 GO:0016740
GO:0005515 GO:0008134 GO:0042803 GO:0005737 GO:0005789
GO:0005861 GO:0030286 GO:0016272 GO:0005667
Cluster-22178.48556 GO:0006508 GO:0008233

Cluster-22178.48561 GO:0048786
Cluster-22178.48564 GO:0006468 GO:0006816 GO:0006810 GO:0009607
GO:0034508 GO:0005246 GO:0003677 GO:0005524 GO:0004672
GO:0042030 GO:0000776 GO:0016020 GO:0016021
Cluster-22178.48584 GO:0055114 GO:0006508 GO:0004197 GO:0020037
GO:0019825 GO:0016702
Cluster-22178.4859 GO:0007165
Cluster-22178.48595 GO:0005509 GO:0005544
Cluster-22178.48596 GO:0019083 GO:0055085 GO:0006810 GO:0016887
GO:0005524 GO:0042626 GO:0003724 GO:0005525 GO:0003723
GO:0016021
Cluster-22178.48598 GO:0006468 GO:0005524 GO:0004672 GO:0016773
GO:0016020
Cluster-22178.48600 GO:0006260 GO:0006629 GO:0016627 GO:0003677
GO:0003887 GO:0016021 GO:0005737 GO:0042575
Cluster-22178.48601 GO:0006260 GO:0006629 GO:0016627 GO:0003677
GO:0003887 GO:0005737 GO:0016021 GO:0042575
Cluster-22178.48609 GO:1900025 GO:0019069 GO:0048203 GO:0043093
GO:0006914 GO:0010508 GO:0006886 GO:0034315 GO:0006457
GO:0006810 GO:0007165 GO:0006260 GO:0030168 GO:0000917
GO:2000146 GO:0006355 GO:0007049 GO:0051258 GO:0061630
GO:0008017 GO:0019905 GO:0008270 GO:0042803 GO:0005515
GO:0005198 GO:0051082 GO:0043565 GO:0035650 GO:0017048
GO:0051087 GO:0030674 GO:0005102 GO:0000774 GO:0003700
GO:0046872 GO:0005783 GO:0005667 GO:0005577 GO:0016272
GO:0045298 GO:0019867 GO:0005737 GO:0005882 GO:0005886
GO:0016021
Cluster-22178.4861 GO:0007165 GO:0008134 GO:0005634 GO:0005667
Cluster-22178.48613 GO:0042254 GO:0006412 GO:0003677 GO:0003735
GO:0005840
Cluster-22178.4862 GO:0007165
Cluster-22178.48626 GO:0007165 GO:0042254 GO:0016192 GO:1902209
GO:0006955 GO:0006886 GO:0006468 GO:0008152 GO:0006402

GO:0048027 GO:0005515 GO:0042802 GO:0005198 GO:0003824
GO:0004672 GO:0005125 GO:0046872 GO:0008484 GO:0005730
GO:0030117 GO:0005576
Cluster-22178.48628 GO:0006955 GO:0006886 GO:0006468 GO:0008152
GO:0006402 GO:0007165 GO:0042254 GO:0016192 GO:1902209
GO:0005515 GO:0005198 GO:0003824 GO:0004672 GO:0005125
GO:0046872 GO:0008484 GO:0048027 GO:0030117 GO:0005576
GO:0005730
Cluster-22178.48629 GO:0042254 GO:0016192 GO:0007165 GO:0006468
GO:0006955 GO:0006886 GO:0008152 GO:0005515 GO:0005198
GO:0008484 GO:0005125 GO:0004672 GO:0046872 GO:0003824
GO:0005730 GO:0005576 GO:0030117
Cluster-22178.4863 GO:0007165 GO:0008134 GO:0005634 GO:0005667
Cluster-22178.48632 GO:0006468 GO:0004672 GO:0005524
Cluster-22178.48633 GO:0008152 GO:0006508 GO:0006629 GO:0008236
GO:0016787
Cluster-22178.48635 GO:0005515
Cluster-22178.48636 GO:0007623 GO:0006351 GO:0006355 GO:0003677
GO:0003700 GO:0043565 GO:0005634 GO:0005667
Cluster-22178.48642 GO:0007264 GO:0005096
Cluster-22178.48647 GO:0016887 GO:0005524
Cluster-22178.48649 GO:0019684 GO:0006118 GO:0005515 GO:0042802
GO:0045156 GO:0030077 GO:0016021
Cluster-22178.48650 GO:0006355 GO:0005515 GO:0016021 GO:0005634
Cluster-22178.48651 GO:0006355 GO:0005515 GO:0005634 GO:0016021
Cluster-22178.48652 GO:0006355 GO:0005515 GO:0005634 GO:0016021
Cluster-22178.48656 GO:0006506 GO:0017176 GO:0016021
Cluster-22178.48658 GO:0009069 GO:0006468 GO:0006810 GO:0007178
GO:0016310 GO:0005515 GO:0046872 GO:0003774 GO:0005524
GO:0005026 GO:0016459 GO:0016020
Cluster-22178.48660 GO:0006520 GO:0016829 GO:0046872
Cluster-22178.48661 GO:0009451 GO:0001522 GO:0003723 GO:0009982

Cluster-22178.48665 GO:0007224 GO:0000077 GO:0003824 GO:0030896
Cluster-22178.48667 GO:0016310 GO:0000160 GO:0051252 GO:0008171
GO:0004673 GO:0000155 GO:0004540 GO:0009365 GO:0005737
GO:0016020
Cluster-22178.48668 GO:0051726 GO:0006281 GO:0004518 GO:0003677
GO:0017025 GO:0003824 GO:0005096 GO:0005634
Cluster-22178.48670 GO:0006355 GO:0008270 GO:0046872 GO:0005634
Cluster-22178.48673 GO:0016787 GO:0046872
Cluster-22178.48674 GO:0055085 GO:0016021
Cluster-22178.48675 GO:0007283 GO:0003677 GO:0000786 GO:0005634
Cluster-22178.48677 GO:0005515
Cluster-22178.48678 GO:0005515
Cluster-22178.48685 GO:0008146 GO:0016021
Cluster-22178.48689 GO:0051056 GO:0003676 GO:0005096
Cluster-22178.48690 GO:0035556 GO:0007186 GO:0046872 GO:0008270
GO:0004930 GO:0043130 GO:0003677 GO:0016021
Cluster-22178.48694 GO:0006265 GO:0007165 GO:0003918 GO:0004864
GO:0005524 GO:0003677
Cluster-22178.48696 GO:0003676 GO:0019031 GO:0016020
Cluster-22178.48700 GO:0006120 GO:0055114 GO:0042546 GO:0005199
GO:0005618 GO:0016021
Cluster-22178.48703 GO:0055114 GO:0006520 GO:0009058 GO:0016491
GO:0030170 GO:0016829
Cluster-22178.48710 GO:0042475 GO:0070175 GO:0003677 GO:0005198

Cluster-22178.48711 GO:0007165 GO:0005096 GO:0005515
Cluster-22178.48712 GO:0005515
Cluster-22178.48715 GO:0006672 GO:0015931 GO:0006807 GO:0033227
GO:0051033 GO:0016811 GO:0016021
Cluster-22178.48717 GO:0016755 GO:0008270 GO:0005515
Cluster-22178.48718 GO:0051028 GO:0005515 GO:0005634
Cluster-22178.48719 GO:0033198 GO:0000077 GO:0006812 GO:0098655
GO:0007165 GO:0004931 GO:0001614 GO:0030896 GO:0005887

GO:0019012
Cluster-22178.48724 GO:0005515
Cluster-22178.48727 GO:0005515 GO:0008270
Cluster-22178.48729 GO:0006355 GO:0006886 GO:0006955 GO:0019882
GO:0030521 GO:0007165 GO:0003700 GO:0004882 GO:0005496
GO:0042289 GO:0003677 GO:0042613 GO:0005634 GO:0042025
GO:0005667 GO:0016020
Cluster-22178.48731 GO:0008152 GO:0007050 GO:0045859 GO:0004861
GO:0016491 GO:0005634
Cluster-22178.48732 GO:0006771 GO:0019497 GO:0003993 GO:0016021
Cluster-22178.48733 GO:0006144 GO:0004114 GO:0016020
Cluster-22178.48736 GO:0007010 GO:0006508 GO:0006468 GO:0006357
GO:0001104 GO:0016805 GO:0003779 GO:0016773 GO:0005524
GO:0004672 GO:0016020 GO:0016592
Cluster-22178.48740 GO:0015992 GO:0070206 GO:0042157 GO:0006869
GO:0016567 GO:0007049 GO:0015991 GO:0008270 GO:0004842
GO:0046872 GO:0015078 GO:0008289 GO:0005515 GO:0005622
GO:0005576 GO:0033179
Cluster-22178.48741 GO:0016021
Cluster-22178.48745 GO:0015937 GO:0015940 GO:0005524 GO:0004140
GO:0005921
Cluster-22178.48747 GO:0006078 GO:0000910 GO:0000226 GO:0042546
GO:0008017 GO:0016021 GO:0045298 GO:0009276
Cluster-22178.48748 GO:0006468 GO:0004672 GO:0005524
Cluster-22178.48750 GO:0006309 GO:0006915 GO:0016787 GO:0005634
GO:0005737 GO:0005622
Cluster-22178.48752 GO:0042254 GO:0006412 GO:0003735 GO:0005622
GO:0005840
Cluster-22178.48754 GO:0055114 GO:0042981 GO:0016714
Cluster-22178.48761 GO:0016559 GO:0016779 GO:0005779
Cluster-22178.48763 GO:0051262 GO:0006357 GO:0007165 GO:0006355
GO:0003700 GO:0004871 GO:0045182 GO:0005515 GO:0001104
GO:0003723 GO:0016592 GO:0019028 GO:0005634 GO:0005667

Cluster-22178.48770 GO:0005515
Cluster-22178.48771 GO:0008233 GO:0008270
Cluster-22178.48772 GO:0008233 GO:0008270
Cluster-22178.48774 GO:0006024 GO:0015012 GO:0005634 GO:0016021
Cluster-22178.48775 GO:0006024 GO:0015012 GO:0005634 GO:0016021
Cluster-22178.48778 GO:0007218 GO:0016192 GO:0016579 GO:0005185
GO:0036459 GO:0005576 GO:0016021
Cluster-22178.48784 GO:0009405 GO:0006810 GO:0008200 GO:0042151
Cluster-22178.48785 GO:0009405 GO:0006810 GO:0008200 GO:0042151
Cluster-22178.48787 GO:0007186 GO:0004930 GO:0003729 GO:0046872
GO:0016021
Cluster-22178.48788 GO:0009116
Cluster-22178.48789 GO:0005515
Cluster-22178.48790 GO:0016021
Cluster-22178.48792 GO:0005515
Cluster-22178.48795 GO:0048193 GO:0005783
Cluster-22178.48796 GO:0042381 GO:0005515 GO:0003676 GO:0005576
Cluster-22178.48799 GO:0006810 GO:0046872 GO:0005622
Cluster-22178.48801 GO:0016310 GO:0009405 GO:0009069 GO:0006468
GO:0004674 GO:0004672 GO:0005524 GO:0045735
Cluster-22178.48805 GO:0007186 GO:0007165 GO:0007155 GO:0007264
GO:0004871 GO:0005525 GO:0003924 GO:0031683 GO:0019001
GO:0005540
Cluster-22178.48806 GO:0043395 GO:0005515 GO:0005578 GO:0016020
Cluster-22178.48810 GO:0009452 GO:0008152 GO:0033539 GO:0055114
GO:0007018 GO:0006118 GO:0007017 GO:0001510 GO:0008017
GO:0003777 GO:0008168 GO:0005524 GO:0003995 GO:0045298
GO:0005874
Cluster-22178.48811 GO:0055114 GO:0033539 GO:0009452 GO:0007017
GO:0001510 GO:0007018 GO:0006118 GO:0008168 GO:0005524
GO:0008017 GO:0003777 GO:0003995 GO:0045298 GO:0005874
Cluster-22178.48812 GO:0006464 GO:0008152 GO:0046500 GO:0006479
GO:0009058 GO:0004719 GO:0008168 GO:0016742 GO:0019867

Cluster-22178.48823 GO:0015992 GO:0006120 GO:0006814 GO:0006744
GO:0016836 GO:0008137 GO:0005739
Cluster-22178.48824 GO:0008152 GO:0003824 GO:0016836
Cluster-22178.48828 GO:0043087 GO:0055114 GO:0035023 GO:0005506
GO:0005089 GO:0016705 GO:0020037
Cluster-22178.48832 GO:0003824
Cluster-22178.48833 GO:0006468 GO:0016192 GO:0005524 GO:0004672
GO:0016021
Cluster-22178.48840 GO:0005515
Cluster-22178.48841 GO:0046500 GO:0006400 GO:0006479 GO:0009451
GO:0030488 GO:0006464 GO:0008033 GO:0008152 GO:0008176
GO:0004719 GO:0008168 GO:0008171 GO:0016429 GO:0031515
Cluster-22178.48845 GO:0007186 GO:0004930 GO:0016021
Cluster-22178.48846 GO:0016310 GO:0006118 GO:0045454 GO:0009069
GO:0015035 GO:0004674 GO:0009055 GO:0005524 GO:0003677
Cluster-22178.48847 GO:0051016 GO:0016192 GO:0007165 GO:0005515
GO:0030414 GO:0016021 GO:0019031 GO:0030479 GO:0016020
Cluster-22178.48850 GO:0051276 GO:0015979 GO:0005524 GO:0005515
GO:0009654 GO:0009523 GO:0016020 GO:0005694
Cluster-22178.48854 GO:0006281 GO:0006468 GO:0006351 GO:0007178
GO:0016310 GO:0009069 GO:0006206 GO:0006289 GO:0043161
GO:0006144 GO:0003899 GO:0004675 GO:0003684 GO:0005524
GO:0005634 GO:0005730 GO:0016020
Cluster-22178.48858 GO:0006355 GO:0055085 GO:0006811 GO:0005216
GO:0005515 GO:0016020
Cluster-22178.48860 GO:0006525 GO:0006560 GO:0006814 GO:0071436
GO:0006090 GO:0008948 GO:0015081 GO:0016020
Cluster-22178.48864 GO:0046332 GO:0016817
Cluster-22178.48871 GO:0019051 GO:0042025
Cluster-22178.48873 GO:0006525 GO:0071436 GO:0006814 GO:0006560
GO:0007165 GO:0014068 GO:0050776 GO:0006090 GO:0043548
GO:0008948 GO:0005515 GO:0015081 GO:0005102 GO:0016020
Cluster-22178.48874 GO:0019497 GO:0006979 GO:0006771 GO:0007155

GO:0006804 GO:0046872 GO:0004601 GO:0003993 GO:0005515
GO:0016021
Cluster-22178.48875 GO:0009821 GO:0006103 GO:0009094 GO:0000162
GO:0006571 GO:0009074 GO:0006536 GO:0004838 GO:0030170
Cluster-22178.48876 GO:0005515
Cluster-22178.48877 GO:0006810 GO:0006816 GO:0006508 GO:0006468
GO:0055085 GO:0006811 GO:0035556 GO:0005515 GO:0004190
GO:0005246 GO:0005216 GO:0042030 GO:0004672 GO:0016020
Cluster-22178.48879 GO:0098542
Cluster-22178.48880 GO:0055085 GO:0006811 GO:0005216 GO:0016020
Cluster-22178.48883 GO:0046856 GO:0015992 GO:0043401 GO:0006355
GO:0015986 GO:0015078 GO:0003700 GO:0034597 GO:0008270
GO:0043565 GO:0000276 GO:0005667 GO:0005634
Cluster-22178.48891 GO:0006338 GO:0006493 GO:0000030 GO:0016818
GO:0003676 GO:0005524 GO:0016020 GO:0005634 GO:0000136
Cluster-22178.48892 GO:0006914 GO:0007275 GO:0009966 GO:0016020
Cluster-22178.48901 GO:0009306 GO:0005886 GO:0016021 GO:0016020
Cluster-22178.48902 GO:0009405 GO:0006810 GO:0019871 GO:0005576
Cluster-22178.48907 GO:0055114 GO:0008152 GO:0016491
Cluster-22178.48911 GO:0006904 GO:0006891 GO:0006887 GO:0015031
GO:0000145 GO:0017119
Cluster-22178.48919 GO:0019497 GO:0006771 GO:0048013 GO:0046872
GO:0005515 GO:0003993
Cluster-22178.48927 GO:0006275 GO:0005515 GO:0016020 GO:0016021
Cluster-22178.48934 GO:0015031 GO:0009306 GO:0008270 GO:0015450
GO:0016021 GO:0009941
Cluster-22178.48935 GO:0007049 GO:0006032 GO:0006118 GO:0006119
GO:0006355 GO:0015991 GO:0006260 GO:0015992 GO:0003700
GO:0016765 GO:0043565 GO:0008061 GO:0005515 GO:0046961
GO:0003954 GO:0033178 GO:0005739 GO:0005667
Cluster-22178.48940 GO:0006468 GO:0043093 GO:0000917 GO:0007172
GO:0007165 GO:0004713 GO:0004871 GO:0005515 GO:0005737
GO:0005925

Cluster-22178.48943 GO:0046436 GO:0009252 GO:0006090 GO:0006633
GO:0008716 GO:0003989 GO:0046872 GO:0005524 GO:0009317
Cluster-22178.48945 GO:0090266 GO:0006351 GO:0051016 GO:0006260
GO:0004190 GO:0003677 GO:0003887 GO:0008270 GO:0005634
GO:0019028 GO:0042575 GO:0030479 GO:0016021 GO:0005680
GO:0005789
Cluster-22178.48950 GO:0031110 GO:2001256 GO:0042470 GO:0030176
Cluster-22178.48953 GO:0016021
Cluster-22178.48954 GO:0042254 GO:0006412 GO:0003735 GO:0005840
GO:0005622
Cluster-22178.48955 GO:0046872
Cluster-22178.48956 GO:0007186 GO:0004930 GO:0016021
Cluster-22178.48957 GO:0005515
Cluster-22178.48958 GO:0005515
Cluster-22178.48959 GO:0007156 GO:0005509 GO:0016020
Cluster-22178.48961 GO:0006468 GO:0005524 GO:0004672 GO:0005198

Cluster-22178.48967 GO:0005515 GO:0003677
Cluster-22178.48969 GO:0007186 GO:0001503 GO:0004930 GO:0016021
Cluster-22178.48970 GO:0007606 GO:0007186 GO:0004930 GO:0016021
Cluster-22178.48974 GO:0006508 GO:0008270 GO:0004222 GO:0031012
Cluster-22178.48975 GO:0006355 GO:0006508 GO:0006144 GO:0003677
GO:0004197 GO:0017111 GO:0043565 GO:0003968 GO:0003700
GO:0005667 GO:0005634 GO:0031379
Cluster-22178.48983 GO:0005985 GO:0005982 GO:0006769 GO:0046854
GO:0006012 GO:0009267 GO:0046497 GO:0003953 GO:0004575
GO:0004564 GO:0017177 GO:0016012 GO:0019033 GO:0016020
GO:0016021
Cluster-22178.48984 GO:0055085 GO:0016021
Cluster-22178.48988 GO:0051028 GO:0048309 GO:0030430
Cluster-22178.48990 GO:0006464 GO:0008270 GO:0046872 GO:0016151
GO:0005515
Cluster-22178.48991 GO:0006355 GO:0009405 GO:0005515 GO:0005634

Cluster-22178.48992 GO:0006771 GO:0019497 GO:0003993
Cluster-22178.48995 GO:0009098 GO:0006418 GO:0006438 GO:0006486
GO:0009099 GO:0009097 GO:0007155 GO:0005524 GO:0004832
GO:0002161 GO:0000166 GO:0008375 GO:0004812 GO:0005737
Cluster-22178.48997 GO:0008270
Cluster-22178.48999 GO:0008233
Cluster-22178.490 GO:0050832 GO:0006338 GO:0016818 GO:0003676
GO:0005524 GO:0005634
Cluster-22178.49003 GO:0005515
Cluster-22178.49005 GO:0005515
Cluster-22178.49006 GO:0005515
Cluster-22178.49008 GO:0007010 GO:0051260 GO:0003779
Cluster-22178.49016 GO:0005515 GO:0016021
Cluster-22178.49017 GO:0006694 GO:0006284 GO:0016114 GO:0016192
GO:0006886 GO:0003677 GO:0008661 GO:0005198 GO:0005515
GO:0030117 GO:0005634
Cluster-22178.49019 GO:0006355 GO:0003677
Cluster-22178.49021 GO:0005789
Cluster-22178.49023 GO:0016020
Cluster-22178.49024 GO:0016020 GO:0016021
Cluster-22178.49025 GO:0016020
Cluster-22178.49029 GO:0006355 GO:0008270 GO:0003677 GO:0003682
GO:0000775 GO:0000785 GO:0005783 GO:0005634
Cluster-22178.49031 GO:0005524 GO:0003677
Cluster-22178.49033 GO:0051382 GO:0007049 GO:0006355 GO:0042073
GO:0000917 GO:0000921 GO:0043093 GO:0006810 GO:0043565
GO:0003700 GO:0005737 GO:0016021 GO:0000776 GO:0005940
GO:0005886 GO:0005815 GO:0005667
Cluster-22178.49041 GO:0006144 GO:0002098 GO:0034227 GO:0046034
GO:0006468 GO:0005524 GO:0008270 GO:0004017 GO:0003677
GO:0016787 GO:0003676 GO:0046872 GO:0004672 GO:0020037
GO:0000049 GO:0005634
Cluster-22178.49042 GO:0006810 GO:0009405 GO:0008200 GO:0005576

Cluster-22178.49044 GO:0009847 GO:0005576 GO:0005634 GO:0016020
GO:0016021
Cluster-22178.49045 GO:0005634 GO:0005576
Cluster-22178.49047 GO:0006771 GO:0019497 GO:0046872 GO:0003993
GO:0005515
Cluster-22178.49051 GO:0018279 GO:0005789
Cluster-22178.49056 GO:0016021
Cluster-22178.49057 GO:0016021
Cluster-22178.49058 GO:0016021
Cluster-22178.49063 GO:0006886 GO:0015031 GO:0008565 GO:0016020
GO:0005737
Cluster-22178.49065 GO:0009271 GO:0055114 GO:0006355 GO:0005975
GO:0016491 GO:0016209 GO:0030246
Cluster-22178.49072 GO:0015992 GO:0015991 GO:0006355 GO:0015078
GO:0003677 GO:0019031 GO:0005886 GO:0033179
Cluster-22178.49077 GO:0003676
Cluster-22178.49078 GO:0007517 GO:0006355 GO:0005515 GO:0008289
GO:0003677 GO:0005634
Cluster-22178.49081 GO:0009059 GO:0019073 GO:0008654 GO:0043772
GO:0005886 GO:0030176
Cluster-22178.49083 GO:0006810 GO:0005515 GO:0005509 GO:0016021
Cluster-22178.49084 GO:0005515
Cluster-22178.49085 GO:0005515
Cluster-22178.49087 GO:0016740 GO:0016021
Cluster-22178.49089 GO:0070072 GO:0007186 GO:0007165 GO:0007264
GO:0004871 GO:0005525 GO:0003924 GO:0019001 GO:0031683
Cluster-22178.49091 GO:0055085
Cluster-22178.49092 GO:0016846 GO:0003723
Cluster-22178.49094 GO:0055085 GO:0006810 GO:0005524 GO:0042626
GO:0016021
Cluster-22178.49095 GO:0006464 GO:0008152 GO:0046500 GO:0006479
GO:0008168 GO:0004719 GO:0003724 GO:0003723
Cluster-22178.49099 GO:0045892 GO:0019856 GO:0005515 GO:0003700

GO:0005667 GO:0016021 GO:0019012

Cluster-22178.49101 GO:0002949 GO:0019079 GO:0006310 GO:0006355

GO:0006281 GO:0005524 GO:0016887 GO:0003724 GO:0009378

GO:0003678 GO:0003723 GO:0016301 GO:0008134 GO:0005657

GO:0005667 GO:0009379

Cluster-22178.49104 GO:0015992 GO:0006119 GO:0006118 GO:0008121

GO:0009055

Cluster-22178.49112 GO:0030206 GO:0015018 GO:0031225 GO:0016020

Cluster-22178.49114 GO:0042967 GO:0006355 GO:0008270 GO:0003712

GO:0004402 GO:0000123 GO:0005634 GO:0005667

Cluster-22178.49115 GO:0005515

Cluster-22178.49117 GO:0006355 GO:0006914 GO:0043565 GO:0003700

GO:0005667

Cluster-22178.49121 GO:0005515 GO:0016755 GO:0008270

Cluster-22178.49125 GO:0009214 GO:0006468 GO:0004113 GO:0005524

GO:0004672 GO:0016020 GO:0019031

Cluster-22178.49139 GO:0006260 GO:0009966 GO:0007264 GO:0005096

GO:0005576

Cluster-22178.49142 GO:0008270

Cluster-22178.49143 GO:0006071 GO:0046486 GO:0004371

Cluster-22178.49147 GO:0006355 GO:0008270 GO:0003700 GO:0003676

GO:0005634 GO:0005667

Cluster-22178.49150 GO:0006955 GO:0007165 GO:0005125 GO:0003676

GO:0005576

Cluster-22178.49152 GO:0006260 GO:0007275 GO:0016787 GO:0003677

GO:0005578

Cluster-22178.49154 GO:0019497 GO:0006771 GO:0055114 GO:0016706

GO:0003993 GO:0008168 GO:0016021

Cluster-22178.49157 GO:0055114 GO:0033743 GO:0005515 GO:0016021

Cluster-22178.49159 GO:0009307 GO:0045454 GO:0055114 GO:0006308

GO:0003677 GO:0016790 GO:0009036 GO:0016491 GO:0016209

GO:0009359

Cluster-22178.49160 GO:0019031

Cluster-22178.49163 GO:0006535 GO:0042967 GO:0006534 GO:0009001
GO:0005737
Cluster-22178.49166 GO:0070206
Cluster-22178.49167 GO:0019058
Cluster-22178.49168 GO:0006470 GO:0006570 GO:0004725 GO:0005515

Cluster-22178.49169 GO:0006820 GO:0008509 GO:0016021
Cluster-22178.49171 GO:0042254 GO:0006412 GO:0003735 GO:0005739
GO:0005840
Cluster-22178.49175 GO:0016310 GO:0015991 GO:0006468 GO:0006886
GO:0006914 GO:0015992 GO:0015031 GO:0006260 GO:0009069
GO:0015078 GO:0005515 GO:0008565 GO:0005524 GO:0004674
GO:0005737 GO:0033179 GO:0016020
Cluster-22178.49176 GO:0006563 GO:0006566 GO:0046653 GO:0006544
GO:0008115 GO:0005515 GO:0016020
Cluster-22178.49177 GO:0006563 GO:0006566 GO:0046653 GO:0006544
GO:0008115 GO:0005515
Cluster-22178.49179 GO:0055114 GO:0042128 GO:0030151 GO:0016491

Cluster-22178.49183 GO:0008270 GO:0003677 GO:0005515
Cluster-22178.49188 GO:0006820 GO:0016021
Cluster-22178.49189 GO:0016032
Cluster-22178.49194 GO:0000921 GO:0006812 GO:0006355 GO:0015991
GO:0030474 GO:0006308 GO:0015992 GO:0051028 GO:0006914
GO:0006606 GO:0006807 GO:0010508 GO:0006886 GO:0048309
GO:0008855 GO:0045502 GO:0008134 GO:0016814 GO:0008092
GO:0005200 GO:0003700 GO:0008270 GO:0016887 GO:0008017
GO:0051082 GO:0042803 GO:0005515 GO:0043565 GO:0015078
GO:0030286 GO:0005940 GO:0005815 GO:0005823 GO:0019898
GO:0009318 GO:0033179 GO:0019031 GO:0005801 GO:0016021
GO:0005667 GO:0016020 GO:0005737 GO:0005856 GO:0045298
Cluster-22178.49196 GO:0015986 GO:0015991 GO:0006119 GO:0015992
GO:0042981 GO:0043065 GO:0046961 GO:0015078 GO:0033178

GO:0045263 GO:0005737 GO:0031225
Cluster-22178.49203 GO:0007218 GO:0046677 GO:0009116 GO:0007631
GO:0008270
Cluster-22178.49208 GO:0006351 GO:0006144 GO:0006206 GO:0003899
GO:0005634 GO:0005730
Cluster-22178.49210 GO:0055114 GO:0016491
Cluster-22178.49211 GO:0055114 GO:0016491
Cluster-22178.49215 GO:0003677
Cluster-22178.49217 GO:0005515
Cluster-22178.49219 GO:0046872
Cluster-22178.49220 GO:0046872
Cluster-22178.49221 GO:0046872
Cluster-22178.49222 GO:0008289
Cluster-22178.49223 GO:0006412 GO:0042254 GO:0008289 GO:0003735
GO:0005739 GO:0005840
Cluster-22178.49225 GO:0006470 GO:0006570 GO:0050852 GO:0004725

Cluster-22178.49229 GO:0017004 GO:0020037
Cluster-22178.49230 GO:0005515
Cluster-22178.49231 GO:0006570 GO:0006470 GO:0004725 GO:0008138

Cluster-22178.49233 GO:0007264 GO:0005524 GO:0016887 GO:0003924
GO:0005525
Cluster-22178.49235 GO:0006468 GO:0009103 GO:0009245 GO:0015940
GO:0009029 GO:0005524 GO:0004672 GO:0016773 GO:0016020
Cluster-22178.49238 GO:0009245 GO:0008915 GO:0046872 GO:0045202
Cluster-22178.49240 GO:0006351 GO:0003677 GO:0008270 GO:0005634
Cluster-22178.49241 GO:0016787
Cluster-22178.49250 GO:0035556
Cluster-22178.49252 GO:0045893 GO:0007275 GO:0008270
Cluster-22178.49254 GO:0006816 GO:0005388 GO:0016529
Cluster-22178.49257 GO:0006355 GO:0006606 GO:0007094 GO:0003700
GO:0005515 GO:0005667 GO:0042025

Cluster-22178.49261 GO:0016032 GO:0046872 GO:0008270 GO:0005515

Cluster-22178.49262 GO:0006355 GO:0005515 GO:0003700 GO:0008270
GO:0043565 GO:0005634 GO:0005667
Cluster-22178.49264 GO:0006355 GO:0005506 GO:0003700 GO:0005515
GO:0005667
Cluster-22178.49265 GO:0007186 GO:0004930 GO:0016021
Cluster-22178.4927 GO:0006357 GO:0055114 GO:0042803 GO:0008134
GO:0001104 GO:0045502 GO:0003723 GO:0016903 GO:0016592
GO:0005667 GO:0030286
Cluster-22178.49272 GO:0016787
Cluster-22178.49273 GO:0015074 GO:0006904 GO:0015031 GO:0006260
GO:0042742 GO:0001503 GO:0007165 GO:0005515 GO:0005198
GO:0001664 GO:0003887 GO:0003677 GO:0019031 GO:0019028
GO:0042575 GO:0005887 GO:0000145
Cluster-22178.49278 GO:0005515
Cluster-22178.49284 GO:0006118 GO:0006412 GO:0042254 GO:0045454
GO:0006355 GO:0003735 GO:0003700 GO:0015035 GO:0009055
GO:0005667 GO:0016021 GO:0005840 GO:0005622
Cluster-22178.49286 GO:0006355 GO:0006260 GO:0046034 GO:0051028
GO:0006144 GO:0048309 GO:0005515 GO:0004017 GO:0003676
GO:0003700 GO:0046872 GO:0008270 GO:0043565 GO:0005667
Cluster-22178.49288 GO:0046872
Cluster-22178.49290 GO:0050920 GO:0003824 GO:0009288
Cluster-22178.49300 GO:0006952
Cluster-22178.49307 GO:0035556 GO:0006812 GO:0055085 GO:0005515
GO:0008324
Cluster-22178.49310 GO:0042981 GO:0000902
Cluster-22178.49314 GO:0005515
Cluster-22178.49315 GO:0030683 GO:0051382 GO:0005515 GO:0044386
GO:0000776
Cluster-22178.49318 GO:0000902
Cluster-22178.49319 GO:0035556 GO:0016787 GO:0005515 GO:0003677

GO:0005524 GO:0003676 GO:0005507 GO:0016020 GO:0016021
Cluster-22178.49321 GO:0005515
Cluster-22178.49323 GO:0006355 GO:0006281 GO:0055114 GO:0006725
GO:0019079 GO:0006310 GO:0006260 GO:0003723 GO:0005515
GO:0008134 GO:0003678 GO:0003824 GO:0009378 GO:0003677
GO:0005524 GO:0016887 GO:0003724 GO:0005667 GO:0009379
GO:0005657
Cluster-22178.49327 GO:0006886
Cluster-22178.49331 GO:0000062
Cluster-22178.49333 GO:0006355 GO:0005515 GO:0008270
Cluster-22178.49340 GO:0006574 GO:0055114 GO:0019521 GO:0015940
GO:0006568 GO:0006550 GO:0006552 GO:0006554 GO:0006633
GO:0006098 GO:0018874 GO:0006631 GO:0008677 GO:0003857
GO:0016491 GO:0051287 GO:0004616
Cluster-22178.49342 GO:0008152 GO:0016787 GO:0005515
Cluster-22178.49343 GO:0007165 GO:0004872 GO:0016021
Cluster-22178.49345 GO:0035556 GO:0007165 GO:0003774 GO:0005524
GO:0005515 GO:0016459
Cluster-22178.49350 GO:0006468 GO:0009069 GO:0017004 GO:0006118
GO:0035556 GO:0016310 GO:0004672 GO:0005524 GO:0004674
GO:0045158 GO:0009512
Cluster-22178.49351 GO:0006468 GO:0005524 GO:0004672
Cluster-22178.49354 GO:0006811 GO:0055085 GO:0006812 GO:0006816
GO:0070588 GO:0005262 GO:0008324 GO:0005216 GO:0016021
GO:0016020
Cluster-22178.49355 GO:0006281 GO:0009432 GO:0005524 GO:0003697

Cluster-22178.49357 GO:0007186 GO:0004930 GO:0016021
Cluster-22178.49360 GO:0003676 GO:0003677 GO:0016787 GO:0005524

Cluster-22178.49361 GO:0007130 GO:0007283 GO:0007131 GO:0006520
GO:0008851 GO:0009350
Cluster-22178.49364 GO:0005515 GO:0008270 GO:0046872

Cluster-22178.49371 GO:0009405
Cluster-22178.49372 GO:0015996 GO:0015994 GO:0008270 GO:0005515
GO:0047746
Cluster-22178.49373 GO:0019076 GO:0006812 GO:0032801 GO:0005261
GO:0005515 GO:0033644
Cluster-22178.49376 GO:0006357 GO:0001104 GO:0016592
Cluster-22178.49378 GO:0006418 GO:0043039 GO:0005524 GO:0003676
GO:0004812 GO:0000166 GO:0000049 GO:0005737 GO:0019031
Cluster-22178.49381 GO:0000917 GO:0006457 GO:0006886 GO:0043093
GO:0006355 GO:0005515 GO:0005198 GO:0051082 GO:0003700
GO:0043565 GO:0005783 GO:0005667 GO:0016021 GO:0016272
GO:0005737
Cluster-22178.49384 GO:0005515
Cluster-22178.49387 GO:0008360 GO:0033588
Cluster-22178.49388 GO:0005515 GO:0046872 GO:0031225
Cluster-22178.49390 GO:0016021
Cluster-22178.49392 GO:0006396 GO:0048870 GO:0016787 GO:0031514
GO:0005622
Cluster-22178.49395 GO:0007186 GO:0004930 GO:0046872 GO:0008270
GO:0016021
Cluster-22178.49397 GO:0006955 GO:0007165 GO:0040007 GO:0008283
GO:0008083 GO:0005515 GO:0008237 GO:0005134 GO:0008270
GO:0005576 GO:0005893
Cluster-22178.49399 GO:0000917 GO:0015991 GO:0006355 GO:0007049
GO:0043093 GO:0006914 GO:0015992 GO:0005515 GO:0003700
GO:0015078 GO:0043565 GO:0003677 GO:0033179 GO:0005634
GO:0005737 GO:0005667
Cluster-22178.49404 GO:0007049 GO:0008608 GO:0023052 GO:0008270
GO:0042729 GO:0005876
Cluster-22178.49409 GO:0006355 GO:0008270 GO:0043565 GO:0005515
GO:0003700 GO:0005634 GO:0005667
Cluster-22178.49413 GO:0008152 GO:0008610 GO:0016747
Cluster-22178.49415 GO:0003723 GO:0008017 GO:0005515 GO:0045298

Cluster-22178.49416 GO:0003723 GO:0030170 GO:0005515
Cluster-22178.49418 GO:0006629
Cluster-22178.49420 GO:0005515
Cluster-22178.49422 GO:0007165 GO:0003677 GO:0005515 GO:0005509
GO:0016021 GO:0005578 GO:0005634
Cluster-22178.49423 GO:0003824
Cluster-22178.49426 GO:0005515
Cluster-22178.49427 GO:0005515
Cluster-22178.49428 GO:0006355 GO:0005515 GO:0016021 GO:0005634
Cluster-22178.49429 GO:0005515
Cluster-22178.49430 GO:0006144 GO:0000398 GO:0045454 GO:0006118
GO:0003968 GO:0015035 GO:0009055 GO:0031379 GO:0005681
Cluster-22178.49436 GO:0003677 GO:0003676 GO:0005524 GO:0016787

Cluster-22178.49438 GO:0007165 GO:0006804 GO:0006355 GO:0006749
GO:0006979 GO:0006352 GO:0055114 GO:0003677 GO:0005515
GO:0005509 GO:0016987 GO:0003700 GO:0004602 GO:0005578
GO:0005667
Cluster-22178.49440 GO:0015992 GO:0005975 GO:0006260 GO:0008152
GO:0006123 GO:0016624 GO:0005524 GO:0004649 GO:0003689
GO:0004129 GO:0005663 GO:0042575 GO:0045277
Cluster-22178.49443 GO:0006123 GO:0006260 GO:0008152 GO:0005975
GO:0015992 GO:0004129 GO:0003689 GO:0004649 GO:0005524
GO:0016624 GO:0045277 GO:0042575 GO:0005663
Cluster-22178.49444 GO:0055114 GO:0016972 GO:0005515
Cluster-22178.49445 GO:0055114 GO:0016972 GO:0005515
Cluster-22178.49446 GO:0055114 GO:0005515 GO:0016972
Cluster-22178.49448 GO:0040007 GO:0015991 GO:0015992 GO:0055085
GO:0008283 GO:0007165 GO:0015858 GO:0006810 GO:0015078
GO:0008083 GO:0003774 GO:0005524 GO:0005337 GO:0016021
GO:0033179 GO:0016459
Cluster-22178.49450 GO:0005975 GO:0005515 GO:0098599 GO:0016832

Cluster-22178.49451 GO:0005975 GO:0098599 GO:0016832 GO:0005515

Cluster-22178.49460 GO:0005524 GO:0003677 GO:0000166 GO:0005525

Cluster-22178.49463 GO:0019882 GO:0006955 GO:0042613 GO:0016020
Cluster-22178.49465 GO:0046872 GO:0003677
Cluster-22178.49470 GO:0016787
Cluster-22178.49477 GO:0008033 GO:0015992 GO:0015991 GO:0015078
GO:0008134 GO:0042803 GO:0045502 GO:0030286 GO:0033179
GO:0005667
Cluster-22178.49479 GO:0006446 GO:0006413 GO:0003743 GO:0003723
GO:0005840 GO:0005737
Cluster-22178.49487 GO:0008053 GO:0000921 GO:0019882 GO:0006914
GO:0043093 GO:0007049 GO:0006308 GO:0000917 GO:0007018
GO:0010508 GO:0006955 GO:0007017 GO:0008017 GO:0005524
GO:0008855 GO:0003777 GO:0003924 GO:0051082 GO:0005940
GO:0005741 GO:0016020 GO:0031966 GO:0005874 GO:0016021
GO:0045298 GO:0042613 GO:0005737 GO:0009318 GO:0019028
Cluster-22178.49491 GO:0006508 GO:0008270 GO:0004222 GO:0016788

Cluster-22178.49493 GO:0006508 GO:0016788 GO:0004222 GO:0008270

Cluster-22178.49497 GO:0006687 GO:0009058 GO:0001733 GO:0005515
GO:0005794 GO:0016021
Cluster-22178.4950 GO:0007264 GO:0005525
Cluster-22178.49500 GO:0005515
Cluster-22178.49504 GO:0016021
Cluster-22178.49508 GO:0006270 GO:0005515
Cluster-22178.49510 GO:0007264 GO:0005525
Cluster-22178.49512 GO:0005515
Cluster-22178.49514 GO:0043087 GO:0035023 GO:0008017 GO:0005089
GO:0005515 GO:0045298
Cluster-22178.49515 GO:0007165 GO:0015893 GO:0006308 GO:0006855

GO:0015238 GO:0008855 GO:0009318 GO:0005886

Cluster-22178.49518 GO:0005543

Cluster-22178.49521 GO:0006935 GO:0006308 GO:0006869 GO:0071973 GO:0042157 GO:0008289 GO:0003774 GO:0008855 GO:0009288 GO:0009318 GO:0016020 GO:0005576

Cluster-22178.49526 GO:0009415 GO:0006950 GO:0005515

Cluster-22178.49528 GO:0035556 GO:0009395 GO:0043087 GO:0046339 GO:0007165 GO:0007264 GO:0006629 GO:0004435 GO:0008081 GO:0005515 GO:0005085

Cluster-22178.49531 GO:0007165 GO:0005515 GO:0005509 GO:0005578

Cluster-22178.49541 GO:0071479 GO:0000077 GO:0005515 GO:0005634

Cluster-22178.49542 GO:0007165 GO:0016032 GO:0046740 GO:0003676 GO:0008270 GO:0046872 GO:0016020 GO:0016021

Cluster-22178.49544 GO:0006352 GO:0003723 GO:0003676 GO:0003677

Cluster-22178.49549 GO:0006511 GO:0031625

Cluster-22178.49553 GO:0000921 GO:0006355 GO:0042254 GO:0006412 GO:0043565 GO:0003700 GO:0003735 GO:0003677 GO:0005940 GO:0005622 GO:0016021 GO:0005840 GO:0005667 GO:0005634

Cluster-22178.49558 GO:0007165 GO:0005179 GO:0005576

Cluster-22178.49562 GO:0003676 GO:0005515 GO:0005524

Cluster-22178.49564 GO:0006269 GO:0006351 GO:0003896 GO:0005730 GO:0005657

Cluster-22178.49565 GO:0003677

Cluster-22178.49566 GO:0004518 GO:0005515 GO:0019867

Cluster-22178.49568 GO:0007165 GO:0006955 GO:0030153 GO:0015643 GO:0016021 GO:0019814

Cluster-22178.49571 GO:0035556 GO:0007165 GO:0031704 GO:0005179

Cluster-22178.49572 GO:0046718 GO:0005515

Cluster-22178.49573 GO:0007186 GO:0004930 GO:0016021

Cluster-22178.49576 GO:0035091

Cluster-22178.49579 GO:0006144 GO:0046034 GO:0046872 GO:0008270

GO:0005515 GO:0004017
Cluster-22178.49585 GO:0012511 GO:0016021
Cluster-22178.49586 GO:0007165 GO:0015684 GO:0007186 GO:0007264
GO:0005515 GO:0005525 GO:0004871 GO:0015093 GO:0019001
GO:0031683 GO:0003924 GO:0016021
Cluster-22178.49597 GO:0046983 GO:0003677 GO:0005634
Cluster-22178.49599 GO:0005515
Cluster-22178.49602 GO:0008270 GO:0005622
Cluster-22178.49603 GO:0008270 GO:0005622
Cluster-22178.49605 GO:0042254 GO:0006412 GO:0003735 GO:0005515
GO:0005840
Cluster-22178.49607 GO:0008152 GO:0006412 GO:0042254 GO:0016846
GO:0003735 GO:0005622 GO:0005840
Cluster-22178.49609 GO:0035064
Cluster-22178.49612 GO:0035023 GO:0043087 GO:0005515 GO:0005089

Cluster-22178.49616 GO:0007165 GO:0007009 GO:0006471 GO:0005515
GO:0019904 GO:0003950 GO:0016021 GO:0005737
Cluster-22178.49619 GO:0019076 GO:0022900 GO:0032801 GO:0006812
GO:0005261 GO:0033644 GO:0016021
Cluster-22178.49622 GO:0006468 GO:0005524 GO:0004672
Cluster-22178.49623 GO:0006886 GO:0003333 GO:0009405 GO:0016485
GO:0006810 GO:0019069 GO:0006865 GO:0055085 GO:0035556
GO:0006888 GO:0015031 GO:0043087 GO:0005515 GO:0015171
GO:0008565 GO:0005576 GO:0016020 GO:0030127
Cluster-22178.49625 GO:0005975 GO:0042254 GO:0003735 GO:0004553
GO:0005840
Cluster-22178.49626 GO:0006464
Cluster-22178.49627 GO:0006914 GO:0000921 GO:0006606 GO:0009069
GO:0007165 GO:0048870 GO:0006308 GO:0016310 GO:0006468
GO:0008855 GO:0005096 GO:0004674 GO:0045502 GO:0008134
GO:0042803 GO:0019901 GO:0005940 GO:0030286 GO:0005667
GO:0016021 GO:0031514 GO:0009318

Cluster-22178.49634 GO:0055085 GO:0019083 GO:0006810 GO:0042626
GO:0003724 GO:0005525 GO:0016887 GO:0005524 GO:0003723
GO:0016021
Cluster-22178.49636 GO:0007165 GO:0006508 GO:0005515 GO:0016820
GO:0016021 GO:0016020 GO:0005739
Cluster-22178.49637 GO:0003682 GO:0000785
Cluster-22178.49638 GO:0003676
Cluster-22178.49640 GO:0006468 GO:0004672 GO:0005515
Cluster-22178.49644 GO:0016021
Cluster-22178.49645 GO:0006886 GO:0035091
Cluster-22178.49648 GO:0005975 GO:0004415
Cluster-22178.49649 GO:0006888 GO:0006260 GO:0045859 GO:0006886
GO:0019887 GO:0008270 GO:0005515 GO:0005956 GO:0005634
GO:0030127
Cluster-22178.49651 GO:0007212 GO:0006281 GO:0048268 GO:0044237
GO:0003824 GO:0032051 GO:0016021
Cluster-22178.49652 GO:0005515 GO:0042802
Cluster-22178.49656 GO:0007034 GO:0030001 GO:0007411
Cluster-22178.49663 GO:0046034 GO:0006144 GO:0006464 GO:0016151
GO:0046872 GO:0008270 GO:0004017 GO:0005634
Cluster-22178.49664 GO:0006144 GO:0006464 GO:0046034 GO:0004017
GO:0046872 GO:0008270 GO:0016151 GO:0005634
Cluster-22178.49667 GO:0006468 GO:0006355 GO:0006754 GO:0008152
GO:0005515 GO:0016887 GO:0005524 GO:0004672 GO:0003824
GO:0000275 GO:0005634
Cluster-22178.49669 GO:0019882 GO:0006886 GO:0006955 GO:0007165
GO:0042289 GO:0042613 GO:0016020
Cluster-22178.49673 GO:2000401 GO:0050855 GO:0005515
Cluster-22178.49674 GO:0007130 GO:0000795
Cluster-22178.49675 GO:0035556 GO:0005515
Cluster-22178.49676 GO:0035556 GO:0005515
Cluster-22178.49679 GO:0055085 GO:0016021
Cluster-22178.49682 GO:0009252 GO:0006807 GO:0019068 GO:0016567

GO:0009253 GO:0046872 GO:0005524 GO:0008270 GO:0004842
GO:0008745 GO:0005515 GO:0003677 GO:0000166 GO:0005680
GO:0019028
Cluster-22178.49683 GO:0019068 GO:0009253 GO:0016567 GO:0009252
GO:0006807 GO:0008745 GO:0003677 GO:0000166 GO:0005515
GO:0046872 GO:0008270 GO:0005524 GO:0004842 GO:0019028
GO:0005680
Cluster-22178.49684 GO:0015684 GO:0055085 GO:0006812 GO:0006885
GO:0015093 GO:0015299 GO:0016021
Cluster-22178.49687 GO:0005515
Cluster-22178.49688 GO:0055114 GO:0005515 GO:0016491
Cluster-22178.49692 GO:0055114 GO:0043087 GO:0035023 GO:0006779
GO:0019354 GO:0043115 GO:0005089
Cluster-22178.49694 GO:0042254 GO:0006412 GO:0004222 GO:0005524
GO:0008270 GO:0046872 GO:0005096 GO:0003677 GO:0003735
GO:0005840 GO:0045202 GO:0016021 GO:0005622
Cluster-22178.49700 GO:0006355 GO:0003677
Cluster-22178.49702 GO:0006560 GO:0006525 GO:0018401 GO:0051321
GO:0055114 GO:0004656 GO:0005515 GO:0016702 GO:0005783
Cluster-22178.49703 GO:0016462 GO:0005509
Cluster-22178.49704 GO:0016462 GO:0005509
Cluster-22178.49705 GO:0005096
Cluster-22178.49706 GO:0008152 GO:0003677 GO:0070569
Cluster-22178.49708 GO:0016192 GO:0019051 GO:0005515 GO:0042025
Cluster-22178.49714 GO:0006281 GO:0004518 GO:0003677 GO:0003824

Cluster-22178.49717 GO:0045893 GO:1902208 GO:0016579 GO:0006508
GO:0051536 GO:0036459 GO:0008270 GO:0004843 GO:0003677
Cluster-22178.49718 GO:0005515
Cluster-22178.49719 GO:0005515
Cluster-22178.49720 GO:0007165 GO:0007186 GO:0004871 GO:0005834
Cluster-22178.49721 GO:0032780 GO:0006355 GO:0003700 GO:0042030
GO:0008270 GO:0005667 GO:0005739 GO:0005634

Cluster-22178.49722 GO:0009403 GO:0016021 GO:0016020
Cluster-22178.49723 GO:0022904 GO:0016020 GO:0016021
Cluster-22178.49724 GO:0022904 GO:0016021 GO:0016020
Cluster-22178.49727 GO:0000226 GO:0000910 GO:0007186 GO:0004930
GO:0008017 GO:0046983 GO:0003677 GO:0045298 GO:0016021
Cluster-22178.49728 GO:0042981
Cluster-22178.49730 GO:0016310 GO:0007275 GO:0009069 GO:0006468
GO:0004674 GO:0004672 GO:0005524 GO:0046872 GO:0016773
GO:0045202 GO:0042600 GO:0016020
Cluster-22178.49735 GO:0007049 GO:0043093 GO:0006355 GO:0000917
GO:0043565 GO:0003700 GO:0003677 GO:0005737 GO:0005634
GO:0005667
Cluster-22178.49739 GO:0005509
Cluster-22178.49740 GO:0000398 GO:0007165 GO:0005681
Cluster-22178.49744 GO:0006811 GO:0006810 GO:0005216 GO:0016021
Cluster-22178.49746 GO:0006614 GO:0006118 GO:0006508 GO:0020037
GO:0004252 GO:0005525 GO:0009055 GO:0005524 GO:0016887
GO:0003924
Cluster-22178.4975 GO:0006508 GO:0008234 GO:0016021
Cluster-22178.49750 GO:0006308 GO:0006281 GO:0008852 GO:0019901
GO:0005515
Cluster-22178.49751 GO:0006308 GO:0006281 GO:0008852 GO:0005515
GO:0019901
Cluster-22178.49756 GO:0006811 GO:0055085 GO:0005216 GO:0016020
Cluster-22178.49757 GO:0051382 GO:0006308 GO:0048870 GO:0055114
GO:0006352 GO:0006355 GO:0009690 GO:0010508 GO:0035092
GO:0000921 GO:0070206 GO:0006914 GO:0006260 GO:0010506
GO:0019139 GO:0005524 GO:0008855 GO:0050660 GO:0016787
GO:0003677 GO:0016021 GO:0005615 GO:0000776 GO:0009318
GO:0031514 GO:0000228 GO:0005940 GO:0005815
Cluster-22178.49770 GO:0007264 GO:0007186 GO:0015684 GO:0007165
GO:0006810 GO:0055085 GO:0003924 GO:0015093 GO:0019001
GO:0031683 GO:0004871 GO:0005525 GO:0005215 GO:0016020

GO:0016021
Cluster-22178.49772 GO:0008270
Cluster-22178.49773 GO:0016192 GO:0016021
Cluster-22178.49775 GO:0006935 GO:0006955 GO:0007165 GO:0008009
GO:0005576
Cluster-22178.49777 GO:0008152 GO:0003824
Cluster-22178.49781 GO:0005515 GO:0003677
Cluster-22178.49788 GO:0006810 GO:0006508 GO:0046034 GO:0006144
GO:0004017 GO:0004190 GO:0042626 GO:0005524 GO:0046872
GO:0016021
Cluster-22178.49789 GO:0003677
Cluster-22178.49791 GO:0006355 GO:0055085 GO:0003700 GO:0005667
GO:0016021
Cluster-22178.49794 GO:0005515
Cluster-22178.49795 GO:0006412 GO:0042254 GO:0003735 GO:0005763
GO:0005840 GO:0005622
Cluster-22178.49796 GO:0051252 GO:0006468 GO:0003676 GO:0016773
GO:0005524 GO:0004672 GO:0004523 GO:0016020
Cluster-22178.49797 GO:0042254 GO:0006412 GO:0003735 GO:0005622
GO:0005840 GO:0005763
Cluster-22178.49798 GO:0046740 GO:0019028 GO:0044219
Cluster-22178.49804 GO:0006355 GO:0003700 GO:0005515 GO:0008270
GO:0005634 GO:0005667
Cluster-22178.49806 GO:0007018 GO:0007017 GO:0010207 GO:0003777
GO:0005515 GO:0005874 GO:0030286 GO:0005737
Cluster-22178.49809 GO:0035064
Cluster-22178.49813 GO:0006412 GO:0042254 GO:0003735 GO:0005840
GO:0005622
Cluster-22178.49818 GO:0005515 GO:0019031
Cluster-22178.49819 GO:0009058 GO:0015979 GO:0016772 GO:0016779
GO:0016020 GO:0009523 GO:0009539
Cluster-22178.49823 GO:0006144 GO:0009165 GO:0006098 GO:0009116
GO:0000287 GO:0004749

Cluster-22178.49825 GO:0008152 GO:0055114 GO:0007275 GO:0009103
GO:0005201 GO:0008918 GO:0016491 GO:0005581 GO:0005578
Cluster-22178.49826 GO:0006486 GO:0008378 GO:0016020
Cluster-22178.49829 GO:0006508 GO:0004198 GO:0005509 GO:0005622
Cluster-22178.49832 GO:0006355 GO:0003700 GO:0005667
Cluster-22178.49837 GO:0006308 GO:0040007 GO:0006355 GO:0006955
GO:0007010 GO:0008283 GO:0007165 GO:0003779 GO:0003700
GO:0005515 GO:0008083 GO:0008855 GO:0005134 GO:0005893
GO:0005576 GO:0009318 GO:0005667
Cluster-22178.49840 GO:0055114 GO:0016903
Cluster-22178.49845 GO:0008152 GO:0055114 GO:0016491
Cluster-22178.49846 GO:0015986 GO:0015992 GO:0015078 GO:0000276
Cluster-22178.49847 GO:0006355 GO:0003676 GO:0003677 GO:0005634
Cluster-22178.49848 GO:0006355 GO:0003676 GO:0003677 GO:0005634
Cluster-22178.49849 GO:0007049 GO:0006351 GO:0070176
Cluster-22178.49850 GO:0055085 GO:0006030 GO:0006821 GO:0005247
GO:0008061 GO:0016020 GO:0005576
Cluster-22178.49852 GO:0006355 GO:0005515 GO:0003700 GO:0043565
GO:0005524 GO:0005667
Cluster-22178.49855 GO:0006364 GO:0008152 GO:0016758 GO:0032040
Cluster-22178.49863 GO:0016310 GO:0009069 GO:0000184 GO:0008380
GO:0004674 GO:0005681
Cluster-22178.49865 GO:0019882 GO:0006955 GO:0006886 GO:0007165
GO:0042289 GO:0042613 GO:0016020
Cluster-22178.49867 GO:0016021
Cluster-22178.49868 GO:0043087 GO:0007264 GO:0005085
Cluster-22178.49872 GO:0055114 GO:0019497 GO:0006771 GO:0016671
GO:0003756 GO:0003993 GO:0005783
Cluster-22178.49875 GO:0005515
Cluster-22178.49878 GO:0006355 GO:0007165 GO:0006367 GO:0046983
GO:0005509 GO:0005515 GO:0005578
Cluster-22178.49879 GO:0015991 GO:0009069 GO:0046325 GO:0016310
GO:0016820 GO:0005515 GO:0016773 GO:0004674 GO:0005737

GO:0000221
Cluster-22178.49887 GO:0006355 GO:0005634
Cluster-22178.49894 GO:0015991 GO:0015992 GO:0015078 GO:0033177
Cluster-22178.49900 GO:0006486 GO:0008378 GO:0016757 GO:0016020
Cluster-22178.49903 GO:0007264 GO:0005525
Cluster-22178.49905 GO:0055085 GO:0051607 GO:0030168 GO:0006810
GO:0007165 GO:0006308 GO:0051258 GO:0030001 GO:0005102
GO:0008855 GO:0030674 GO:0046873 GO:0005577 GO:0016020
GO:0016021 GO:0009318
Cluster-22178.49906 GO:0019087 GO:0016021
Cluster-22178.49908 GO:0042254 GO:0006412 GO:0006355 GO:0006729
GO:0008124 GO:0003735 GO:0005515 GO:0003677 GO:0003682
GO:0005634 GO:0016021 GO:0000785 GO:0000775 GO:0005840
GO:0005622
Cluster-22178.49909 GO:0035556 GO:0006144 GO:0006206 GO:0006270
GO:0006351 GO:0003899 GO:0005515 GO:0003677 GO:0032040
GO:0005730
Cluster-22178.49915 GO:0006810 GO:0009451 GO:0001522 GO:0006355
GO:0005515 GO:0003723 GO:0005215 GO:0009982 GO:0046983
GO:0016021
Cluster-22178.49916 GO:0006810 GO:0001522 GO:0009451 GO:0006355
GO:0005515 GO:0003723 GO:0005215 GO:0009982 GO:0046983
GO:0016021
Cluster-22178.49920 GO:0005975 GO:0016773
Cluster-22178.49921 GO:0016627
Cluster-22178.49924 GO:0042254 GO:0001522
Cluster-22178.49926 GO:0006355 GO:0043093 GO:0000917 GO:0006260
GO:0043565 GO:0003700 GO:0005667 GO:0005737
Cluster-22178.49927 GO:0006260 GO:0006355 GO:0043093 GO:0000917
GO:0043565 GO:0003700 GO:0005737 GO:0005667
Cluster-22178.49931 GO:0007186 GO:0006629 GO:0004930 GO:0000166
GO:0046872 GO:0016021
Cluster-22178.49934 GO:0006464 GO:0008608 GO:0051082 GO:0016151

GO:0031072 GO:0072686 GO:0005634 GO:0042729
Cluster-22178.49935 GO:0009086 GO:0031554 GO:0019069 GO:0042558
GO:0031556 GO:0046872 GO:0005524 GO:0008705 GO:0031419
GO:0005622 GO:0005887
Cluster-22178.49938 GO:0055114 GO:0006479 GO:0006486 GO:0006355
GO:0006629 GO:0006554 GO:0007165 GO:0034968 GO:0005507
GO:0008375 GO:0018024 GO:0016641 GO:0003677 GO:0051920
GO:0005044 GO:0005622 GO:0016020 GO:0005763 GO:0005694
Cluster-22178.49940 GO:0004252 GO:0016021
Cluster-22178.49946 GO:0006554 GO:0008152 GO:0006479 GO:0008168
GO:0018024 GO:0042802 GO:0005515
Cluster-22178.49947 GO:0051260
Cluster-22178.49950 GO:0008270
Cluster-22178.49952 GO:0005515
Cluster-22178.49955 GO:0006355 GO:0005634
Cluster-22178.49957 GO:0007010 GO:0003779
Cluster-22178.49958 GO:0003676
Cluster-22178.49961 GO:0042147
Cluster-22178.49962 GO:0003924 GO:0005525
Cluster-22178.49965 GO:0007224 GO:0000077 GO:0003824 GO:0030896
Cluster-22178.49968 GO:0007017 GO:0007018 GO:0030150 GO:0005524
GO:0003777 GO:0008017 GO:0005742 GO:0005874 GO:0045298
Cluster-22178.49969 GO:0007017 GO:0007018 GO:0006457 GO:0030150
GO:0051082 GO:0017048 GO:0005524 GO:0008017 GO:0003777
GO:0005742 GO:0016272 GO:0045298 GO:0005874
Cluster-22178.49970 GO:0005515
Cluster-22178.49973 GO:0055085 GO:0032007 GO:0048011 GO:0005515
GO:0046872 GO:0016021
Cluster-22178.49974 GO:0003723
Cluster-22178.49975 GO:0043087 GO:0007165 GO:0007010 GO:0007264
GO:0004872 GO:0003779 GO:0005085 GO:0016021
Cluster-22178.49976 GO:0006508 GO:0008270 GO:0003676 GO:0005515
GO:0004222

Cluster-22178.49978 GO:0006886 GO:0008536
Cluster-22178.49982 GO:0005515 GO:0005198 GO:0019028
Cluster-22178.49984 GO:0018149 GO:0008270 GO:0019031 GO:0005886
Cluster-22178.49986 GO:0006096 GO:0000162 GO:0006357 GO:0006144
GO:0009405 GO:0006397 GO:0006094 GO:0006571 GO:0009094
GO:0005524 GO:0004634 GO:0005198 GO:0001104 GO:0000287
GO:0003723 GO:0003968 GO:0016592 GO:0000015 GO:0005576
GO:0019028 GO:0005634 GO:0019031 GO:0031379 GO:0005737
Cluster-22178.49987 GO:0016021
Cluster-22178.49988 GO:0006605 GO:0006886 GO:0005515 GO:0005742
Cluster-22178.49989 GO:0006950 GO:0051087 GO:0001671
Cluster-22178.49990 GO:0009069 GO:0006468 GO:0016310 GO:0015979
GO:0004674 GO:0005524 GO:0004672 GO:0016020 GO:0009539
GO:0009523
Cluster-22178.49991 GO:0006308 GO:0031047 GO:0005975 GO:0016888
GO:0004560
Cluster-22178.49993 GO:0015074 GO:0051537
Cluster-22178.49995 GO:0016787
Cluster-22178.5000 GO:0005789
Cluster-22178.50000 GO:0016772 GO:0005524
Cluster-22178.50001 GO:0016772
Cluster-22178.50002 GO:0055114 GO:0009405 GO:0051920 GO:0003677

Cluster-22178.50003 GO:0009082 GO:0006306 GO:0032775 GO:0006914
GO:0070009 GO:0005515 GO:0009007 GO:0008239
Cluster-22178.50005 GO:0046856 GO:0015031 GO:0034597 GO:0016021
Cluster-22178.50006 GO:0006355 GO:0003677
Cluster-22178.50009 GO:0007186 GO:0004930 GO:0016021
Cluster-22178.50010 GO:0051258 GO:0030168 GO:0000226 GO:0090063
GO:0007165 GO:0005102 GO:0030674 GO:0003677 GO:0005815
GO:0005577 GO:0000922
Cluster-22178.50011 GO:0016310 GO:0009069 GO:0007186 GO:0007165
GO:0003924 GO:0019001 GO:0031683 GO:0004871 GO:0016772

GO:0004674 GO:0005525 GO:0005515 GO:0016021
Cluster-22178.50016 GO:0008152 GO:0016192 GO:0006886 GO:0005515
GO:0003824 GO:0030117
Cluster-22178.50022 GO:0000226 GO:0051537 GO:0043231 GO:0015630
Cluster-22178.50023 GO:0006950
Cluster-22178.50024 GO:0019497 GO:0006771 GO:0055114 GO:0003993
GO:0003756 GO:0016671 GO:0005783
Cluster-22178.50025 GO:0008270
Cluster-22178.50026 GO:0005634
Cluster-22178.50027 GO:0055085 GO:0006811 GO:0006810 GO:0005215
GO:0016020
Cluster-22178.50029 GO:0009405 GO:0009098 GO:0005198 GO:0005882
Cluster-22178.50032 GO:0008616 GO:0016192 GO:0006886 GO:0043248
GO:0016740 GO:0005515 GO:0016853 GO:0030117
Cluster-22178.50035 GO:0055114 GO:0009308 GO:0048038 GO:0005507
GO:0008131
Cluster-22178.5004 GO:0007165 GO:0005515
Cluster-22178.50041 GO:0003676
Cluster-22178.50042 GO:0017004 GO:0006118 GO:0008610 GO:0008152
GO:0045158 GO:0016747 GO:0009512
Cluster-22178.50044 GO:0008207 GO:0006631 GO:0008209 GO:0018874
GO:0006633 GO:0006554 GO:0006552 GO:0006550 GO:0006568
GO:0008210 GO:0006813 GO:0006694 GO:0008152 GO:0055114
GO:0006574 GO:0016616 GO:0003854 GO:0050662 GO:0016491
GO:0003857 GO:0003824
Cluster-22178.50047 GO:0006812 GO:0051191 GO:0070836 GO:0006836
GO:0005328 GO:0016021 GO:0005634
Cluster-22178.50050 GO:0003676 GO:0005515 GO:0003723 GO:0008270

Cluster-22178.50055 GO:0006813 GO:0015269 GO:0016020
Cluster-22178.50056 GO:0006813 GO:0015269 GO:0016020
Cluster-22178.50058 GO:0015979 GO:0046872
Cluster-22178.50059 GO:0046872 GO:0008270 GO:0003723 GO:0005515

Cluster-22178.50064 GO:0007304 GO:0019684 GO:0016567 GO:0006118
GO:0046872 GO:0008270 GO:0004842 GO:0005213 GO:0045156
GO:0005515 GO:0042600 GO:0016021 GO:0005680 GO:0005576
GO:0030077
Cluster-22178.50069 GO:0006571 GO:0009094 GO:0009405 GO:0006094
GO:0000162 GO:0006144 GO:0006357 GO:0006096 GO:0003968
GO:0001104 GO:0005198 GO:0000287 GO:0004634 GO:0005524
GO:0019031 GO:0031379 GO:0019028 GO:0005576 GO:0016592
GO:0000015
Cluster-22178.50074 GO:0009399 GO:0005515 GO:0008270 GO:0016021
Cluster-22178.50075 GO:0070403 GO:0000287 GO:0030976 GO:0005515

Cluster-22178.50076 GO:0005515 GO:0008061
Cluster-22178.50077 GO:0005515
Cluster-22178.50078 GO:0005515
Cluster-22178.50080 GO:0009245 GO:0005515 GO:0061630 GO:0046872
GO:0008270 GO:0008915
Cluster-22178.50081 GO:0009245 GO:0008270 GO:0008915 GO:0046872
GO:0061630 GO:0005515
Cluster-22178.50085 GO:0007005 GO:0006508 GO:0004222 GO:0031305
Cluster-22178.50086 GO:0006355 GO:0005515 GO:0005634
Cluster-22178.50091 GO:0046872
Cluster-22178.50093 GO:0007010 GO:0015031 GO:0006464 GO:0003779
GO:0005615
Cluster-22178.50095 GO:0007155 GO:0006606 GO:0051276 GO:0003677
GO:0000166 GO:0005515 GO:0003774 GO:0005524 GO:0016459
GO:0005694
Cluster-22178.50097 GO:0005524 GO:0035091 GO:0020037 GO:0016020
Cluster-22178.50098 GO:0005634
Cluster-22178.50099 GO:0005634
Cluster-22178.50104 GO:0005515
Cluster-22178.50110 GO:0019467 GO:0008073 GO:0004857

Cluster-22178.50111 GO:0009396 GO:0015992 GO:0006771 GO:0042254
GO:0055114 GO:0019497 GO:0008152 GO:0015991 GO:0006412
GO:0046487 GO:0016491 GO:0003993 GO:0050662 GO:0004488
GO:0003735 GO:0046872 GO:0003824 GO:0015078 GO:0005515
GO:0005840 GO:0033179
Cluster-22178.50113 GO:0005515 GO:0016020
Cluster-22178.50114 GO:0006694 GO:0008152 GO:0000226 GO:0016114
GO:0008661 GO:0016624 GO:0030976 GO:0003824 GO:0015630
Cluster-22178.50116 GO:0070588 GO:0006816 GO:0006812 GO:0055085
GO:0006811 GO:0005216 GO:0005262 GO:0008324 GO:0016020
GO:0016021
Cluster-22178.50117 GO:0051258 GO:0042157 GO:0007165 GO:0006869
GO:0019064 GO:0051262 GO:0030168 GO:0005515 GO:0004871
GO:0008289 GO:0030674 GO:0005102 GO:0005861 GO:0019028
GO:0005576 GO:0019031 GO:0016020 GO:0005577
Cluster-22178.50121 GO:0000105 GO:0055085 GO:0006289 GO:0055114
GO:0006308 GO:0006355 GO:0030001 GO:0004399 GO:0008270
GO:0008855 GO:0046873 GO:0051287 GO:0008168 GO:0000439
GO:0016020 GO:0009318
Cluster-22178.50122 GO:0006030 GO:0006355 GO:0008092 GO:0008061
GO:0019898 GO:0005737 GO:0005634 GO:0005576
Cluster-22178.50126 GO:0008152 GO:0008168
Cluster-22178.50132 GO:0003676
Cluster-22178.50137 GO:0042254 GO:0003735 GO:0005739 GO:0005840
Cluster-22178.50139 GO:0035091 GO:0005515
Cluster-22178.50141 GO:0008152 GO:0016757
Cluster-22178.50152 GO:0006825 GO:0035434 GO:0005375 GO:0016021
Cluster-22178.50156 GO:0006468 GO:0005524 GO:0004672
Cluster-22178.50157 GO:0006468 GO:0006355 GO:0005524 GO:0004672
GO:0043565
Cluster-22178.50158 GO:0006468 GO:0004672 GO:0005524
Cluster-22178.50159 GO:0006355 GO:0005622
Cluster-22178.50164 GO:0005515 GO:0046872 GO:0045202 GO:0005737

Cluster-22178.50165 GO:0046872 GO:0005515 GO:0005737 GO:0045202
Cluster-22178.50166 GO:0000045 GO:0005737
Cluster-22178.50167 GO:0006355 GO:0003700 GO:0005198 GO:0019028
GO:0005667
Cluster-22178.50169 GO:0006355 GO:0003700 GO:0005667
Cluster-22178.50171 GO:0007264 GO:0010506 GO:0006260 GO:0009966
GO:0005096 GO:0005576 GO:0005615
Cluster-22178.50173 GO:0006118 GO:0018149 GO:0003950 GO:0009055
GO:0003810 GO:0005507
Cluster-22178.50177 GO:0016310 GO:0006468 GO:0009069 GO:0001558
GO:0005520 GO:0008270 GO:0004672 GO:0005524 GO:0004674
GO:0016942 GO:0005576
Cluster-22178.50185 GO:0006351 GO:0006355 GO:0003677 GO:0015016
GO:0016787 GO:0003700 GO:0005634 GO:0005667
Cluster-22178.50186 GO:0006351 GO:0016787 GO:0015016 GO:0003677
GO:0005634
Cluster-22178.50187 GO:0018193 GO:0005515 GO:0046872
Cluster-22178.50191 GO:0005515
Cluster-22178.50198 GO:0008210 GO:0008209 GO:0008207 GO:0055114
GO:0006694 GO:0050662 GO:0003854 GO:0016616 GO:0016491
GO:0003824
Cluster-22178.50200 GO:0055114 GO:0010309 GO:0005515
Cluster-22178.50201 GO:0005515
Cluster-22178.50202 GO:0005515
Cluster-22178.50203 GO:0006606 GO:0031110 GO:0006869 GO:0007155
GO:0009405 GO:0008270 GO:0008289 GO:0005198 GO:0042803
GO:0008134 GO:0045502 GO:0030286 GO:0016020 GO:0005882
GO:0005667 GO:0019031 GO:0019028 GO:0005576
Cluster-22178.50207 GO:0006310 GO:0000917 GO:0006302 GO:0006355
GO:0043093 GO:0003677 GO:0003700 GO:0043565 GO:0005634
GO:0005667 GO:0005737
Cluster-22178.50210 GO:0007017 GO:0005875
Cluster-22178.50211 GO:0003677

Cluster-22178.50214 GO:0006355 GO:0006810 GO:0034229 GO:0003677 GO:0043565 GO:0005215 GO:0034228 GO:0003700 GO:0005634 GO:0005667 GO:0016020 GO:0016021

Cluster-22178.50219 GO:0016567 GO:0006810 GO:0005515 GO:0004842 GO:0008270 GO:0046872 GO:0016020 GO:0005680

Cluster-22178.50223 GO:0006810 GO:0055085 GO:0042626 GO:0005524 GO:0016021

Cluster-22178.50225 GO:0016192 GO:0016021

Cluster-22178.50226 GO:0016192 GO:0016021

Cluster-22178.50227 GO:0016192 GO:0016021

Cluster-22178.50228 GO:0008152 GO:0055114 GO:0003824 GO:0005515 GO:0016491 GO:0050662

Cluster-22178.50229 GO:0055114 GO:0042773 GO:0016651

Cluster-22178.50230 GO:0008380 GO:0003824 GO:0005515 GO:0005681

Cluster-22178.50231 GO:0008380 GO:0005515 GO:0003824 GO:0005681

Cluster-22178.50232 GO:0008380 GO:0003824 GO:0005515 GO:0005681

Cluster-22178.50236 GO:0005515

Cluster-22178.5024 GO:0006446 GO:0006413 GO:0005515 GO:0003743 GO:0005840

Cluster-22178.50240 GO:0071816 GO:0005975 GO:0016740 GO:0004649

Cluster-22178.50241 GO:0004177 GO:0030145 GO:0016021

Cluster-22178.50245 GO:0055114 GO:0006570 GO:0004511 GO:0005515

Cluster-22178.50247 GO:0055114 GO:0016616

Cluster-22178.50250 GO:0003676

Cluster-22178.50252 GO:0016462 GO:0005737

Cluster-22178.50255 GO:0010468 GO:0015992 GO:0015991 GO:0022900 GO:0015078 GO:0033179 GO:0016021

Cluster-22178.50257 GO:0055085 GO:0005515

Cluster-22178.50268 GO:0000398 GO:0019064 GO:0019031 GO:0005681

Cluster-22178.50273 GO:0015946 GO:0006481 GO:0055114 GO:0006479 GO:0004671 GO:0004022 GO:0016021

Cluster-22178.50274 GO:0016310 GO:0015979 GO:0006468 GO:0009069
GO:0004674 GO:0004672 GO:0005524 GO:0009523 GO:0009539
GO:0016020
Cluster-22178.50275 GO:0005515 GO:0046872 GO:0045202 GO:0005737
Cluster-22178.50278 GO:0016579 GO:0036459 GO:0008270
Cluster-22178.50280 GO:0043087 GO:0005085
Cluster-22178.50287 GO:0006886 GO:0016192 GO:0019013
Cluster-22178.50291 GO:0007165 GO:0006355 GO:0005515 GO:0003677
GO:0005179 GO:0005576
Cluster-22178.50293 GO:0004252 GO:0016021
Cluster-22178.50294 GO:0055114 GO:0047134
Cluster-22178.5030 GO:0009877 GO:0051304 GO:0008360 GO:0009312
GO:0070836 GO:0016758 GO:0016020
Cluster-22178.50305 GO:0042254 GO:0006396 GO:0009451 GO:0006412
GO:0015940 GO:0015937 GO:0003735 GO:0005524 GO:0004594
GO:0004252 GO:0008173 GO:0003723 GO:0055036 GO:0005622
GO:0019028 GO:0005840
Cluster-22178.50308 GO:0015991 GO:0005515 GO:0016820 GO:0000221
Cluster-22178.50309 GO:0005515 GO:0008270 GO:0043565 GO:0005634
Cluster-22178.5031 GO:0008360 GO:0070836 GO:0016020
Cluster-22178.50310 GO:0006206 GO:0015992 GO:0006289 GO:0043161
GO:0006144 GO:0009069 GO:0015986 GO:0016310 GO:0007178
GO:0006351 GO:0006281 GO:0006468 GO:0005524 GO:0004675
GO:0003684 GO:0015078 GO:0003899 GO:0000276 GO:0016020
GO:0005730 GO:0005634
Cluster-22178.50311 GO:0006810 GO:0042254 GO:0006412 GO:0071816
GO:0003735 GO:0005840 GO:0016021 GO:0005622
Cluster-22178.50316 GO:0000062 GO:0005515 GO:0016021
Cluster-22178.50318 GO:0007155 GO:0030246
Cluster-22178.5032 GO:0070836 GO:0008360 GO:0016020
Cluster-22178.50321 GO:0035556 GO:0046872 GO:0008270
Cluster-22178.50322 GO:0003678 GO:0005524 GO:0046983 GO:0005657
Cluster-22178.50324 GO:0005515

Cluster-22178.50330 GO:0006313 GO:0003677 GO:0004803 GO:0008537
Cluster-22178.50331 GO:0009069 GO:0006468 GO:0016310 GO:0005524
GO:0004672 GO:0004674
Cluster-22178.50332 GO:0006355 GO:0006810 GO:0002949 GO:0008134
GO:0003774 GO:0005524 GO:0016459 GO:0005667
Cluster-22178.50340 GO:0016567 GO:0004842 GO:0005515 GO:0046872
GO:0008270 GO:0005680
Cluster-22178.50341 GO:0006355 GO:0008270 GO:0043565 GO:0003700
GO:0005667 GO:0005634
Cluster-22178.50342 GO:0007016 GO:0042254 GO:0006412 GO:0007165
GO:0003779 GO:0005158 GO:0000062 GO:0005200 GO:0003735
GO:0005856 GO:0005899 GO:0005622 GO:0005840 GO:0001726
GO:0005925
Cluster-22178.50343 GO:0007016 GO:0042254 GO:0006412 GO:0007165
GO:0003779 GO:0005158 GO:0000062 GO:0005200 GO:0003735
GO:0005899 GO:0005856 GO:0005622 GO:0005840 GO:0001726
GO:0005925
Cluster-22178.50345 GO:0005515
Cluster-22178.50347 GO:0006418 GO:0005524 GO:0004812 GO:0000166

Cluster-22178.5035 GO:0006139 GO:0006206 GO:0000921 GO:0004127
GO:0005524 GO:0016021 GO:0005940
Cluster-22178.50353 GO:0055114 GO:0016491 GO:0016616
Cluster-22178.50354 GO:0007165 GO:0007264 GO:0005525 GO:0005509
GO:0005524 GO:0016887 GO:0005578
Cluster-22178.50356 GO:0008608 GO:0030472 GO:0005509 GO:0042729
GO:0072686
Cluster-22178.50358 GO:0006367 GO:0030001 GO:0008608 GO:0055085
GO:0030472 GO:0005509 GO:0046873 GO:0004190 GO:0042729
GO:0016020 GO:0072686 GO:0005672 GO:0016021
Cluster-22178.50362 GO:0006351 GO:0006281 GO:0006144 GO:0006206
GO:0006464 GO:0003899 GO:0005515 GO:0003677 GO:0016151
GO:0008270 GO:0046872 GO:0005730

Cluster-22178.50363 GO:0003677 GO:0046872

Cluster-22178.50364 GO:0003677 GO:0046872

Cluster-22178.50367 GO:0055114 GO:0052855 GO:0016491

Cluster-22178.50368 GO:0003723 GO:0016021

Cluster-22178.50370 GO:0042802 GO:0005515 GO:0030695

Cluster-22178.50372 GO:0071704 GO:0005515 GO:0016868

Cluster-22178.50373 GO:0006281 GO:0008081 GO:0005634

Cluster-22178.50374 GO:0008146

Cluster-22178.50375 GO:0055114 GO:0006576 GO:0007264 GO:0005525
GO:0003924 GO:0005524 GO:0005509 GO:0016616 GO:0031225

Cluster-22178.50380 GO:0005515

Cluster-22178.50382 GO:0007165 GO:0005179 GO:0005576

Cluster-22178.50383 GO:0055114 GO:0009399 GO:0015694 GO:0050660
GO:0015097 GO:0003677 GO:0016614 GO:0016020

Cluster-22178.50384 GO:0006811 GO:0055085 GO:0006813 GO:0008270
GO:0003677 GO:0005216 GO:0005242 GO:0016021 GO:0008076
GO:0016020

Cluster-22178.50388 GO:0008191 GO:0004190 GO:0019031 GO:0016021

Cluster-22178.50389 GO:0006508 GO:0016788 GO:0004222 GO:0008270

Cluster-22178.50390 GO:0006384 GO:0006366 GO:0003700 GO:0005667
GO:0005634

Cluster-22178.50395 GO:0015031 GO:0005886 GO:0016021

Cluster-22178.50396 GO:0007155 GO:0035556 GO:0005515

Cluster-22178.50397 GO:0005198 GO:0016787 GO:0019028

Cluster-22178.50400 GO:0016567 GO:0005515 GO:0004842 GO:0008270
GO:0046872 GO:0005680

Cluster-22178.50401 GO:0008033 GO:0030488 GO:0019076 GO:0032801
GO:0006812 GO:0006468 GO:0009451 GO:0004672 GO:0005524
GO:0016429 GO:0005261 GO:0033644 GO:0031515

Cluster-22178.50405 GO:0019634 GO:0015716 GO:0019058 GO:0046872
GO:0005887

Cluster-22178.50415 GO:0043154 GO:0043066 GO:0019079 GO:0005515

GO:0071986 GO:0005737
Cluster-22178.50416 GO:0015693 GO:0015095 GO:0016020 GO:0016021
Cluster-22178.50425 GO:0046872 GO:0016020
Cluster-22178.50426 GO:0003677 GO:0046872
Cluster-22178.50430 GO:0006313 GO:0015074 GO:0003677 GO:0019028
Cluster-22178.50433 GO:0005975 GO:0004571 GO:0005509 GO:0016020
Cluster-22178.50436 GO:0006814 GO:0005272 GO:0016020
Cluster-22178.50447 GO:0015684 GO:0046487 GO:0009396 GO:0055114
GO:0004488 GO:0016491 GO:0005524 GO:0015093 GO:0003824
GO:0004329 GO:0016021
Cluster-22178.50452 GO:0005515
Cluster-22178.50455 GO:0006281 GO:0051726 GO:0017025 GO:0003677
GO:0004518 GO:0005096 GO:0003824 GO:0005634
Cluster-22178.50457 GO:0009847 GO:0016021
Cluster-22178.50458 GO:0007155 GO:0051607 GO:0006914 GO:0048870
GO:0006606 GO:0008092 GO:0031514 GO:0009424 GO:0005737
GO:0019898
Cluster-22178.50459 GO:0055085 GO:0018189 GO:0006914 GO:0006821
GO:0007005 GO:0006810 GO:0042626 GO:0005247 GO:0005524
GO:0005515 GO:0016021 GO:0016020 GO:0031305
Cluster-22178.50460 GO:0006810 GO:0007005 GO:0006821 GO:0018189
GO:0055085 GO:0006914 GO:0005515 GO:0005247 GO:0042626
GO:0005524 GO:0016020 GO:0031305 GO:0016021
Cluster-22178.50461 GO:0006810 GO:0006821 GO:0007005 GO:0006914
GO:0018189 GO:0055085 GO:0005524 GO:0042626 GO:0005247
GO:0031305 GO:0016020 GO:0016021
Cluster-22178.50462 GO:0006355 GO:0003677 GO:0005507 GO:0003700
GO:0046872 GO:0005667 GO:0005634
Cluster-22178.50463 GO:0008152 GO:0055114 GO:0003824 GO:0005515
GO:0050662 GO:0016491
Cluster-22178.50466 GO:0006308 GO:0008239 GO:0070009 GO:0008855
GO:0009318
Cluster-22178.50467 GO:0006308 GO:0008239 GO:0008855 GO:0070009

GO:0009318
Cluster-22178.50469 GO:0006355 GO:0003676 GO:0005515 GO:0005634
GO:0019028
Cluster-22178.50474 GO:0016021
Cluster-22178.50475 GO:0005515
Cluster-22178.50480 GO:0006464
Cluster-22178.50481 GO:0006810 GO:0015986 GO:0042626 GO:0033178
Cluster-22178.50482 GO:0042254 GO:0006412 GO:0006355 GO:0045454
GO:0003700 GO:0003735 GO:0005840 GO:0005622 GO:0005667
Cluster-22178.50483 GO:0045454 GO:0006355 GO:0006412 GO:0042254
GO:0003700 GO:0003735 GO:0005622 GO:0005840 GO:0005667
Cluster-22178.50484 GO:0009432 GO:0051782 GO:0003824 GO:0009276
Cluster-22178.50485 GO:0051782 GO:0009432 GO:0003824 GO:0009276
Cluster-22178.50487 GO:0007165 GO:0007186 GO:0007264 GO:0005525
GO:0004871 GO:0008270 GO:0031683 GO:0046872 GO:0019001
GO:0003924 GO:0005515 GO:0005622
Cluster-22178.50489 GO:0006206 GO:0006144 GO:0016570 GO:0006368
GO:0006816 GO:0006874 GO:0006270 GO:0006351 GO:0005219
GO:0003899 GO:0005622 GO:0016021 GO:0005730 GO:0005634
GO:0016593
Cluster-22178.50490 GO:0006144 GO:0006206 GO:0016570 GO:0006816
GO:0006874 GO:0006368 GO:0006351 GO:0005219 GO:0003899
GO:0005622 GO:0016021 GO:0005634 GO:0005730 GO:0016593
Cluster-22178.50491 GO:0019031
Cluster-22178.50492 GO:0007166 GO:0005515 GO:0016020
Cluster-22178.50495 GO:0006820 GO:0015992 GO:0006120 GO:0006744
GO:0006814 GO:0043565 GO:0008509 GO:0008137 GO:0016021
GO:0005743 GO:0005634
Cluster-22178.50496 GO:0006468 GO:0006355 GO:0006298 GO:0043565
GO:0016773 GO:0005524 GO:0004672 GO:0003700 GO:0030983
GO:0005667 GO:0016020
Cluster-22178.50497 GO:0046486 GO:0042967 GO:0045017 GO:0004144

Cluster-22178.50502 GO:0006629 GO:0016627 GO:0016021 GO:0005737
Cluster-22178.50503 GO:0005524
Cluster-22178.50505 GO:0048280 GO:0006886 GO:0003676 GO:0005737
GO:0000139
Cluster-22178.50507 GO:0009159 GO:0006206 GO:0050144 GO:0070694

Cluster-22178.50508 GO:0006259 GO:0005515 GO:0003824 GO:0003677
GO:0005524 GO:0005694
Cluster-22178.50510 GO:0006955 GO:0007165 GO:0006508 GO:0007155
GO:0005164 GO:0005198 GO:0004222 GO:0016020
Cluster-22178.50511 GO:0009306 GO:0005515 GO:0016020
Cluster-22178.50515 GO:0006468 GO:0005524 GO:0004672
Cluster-22178.50519 GO:0002949 GO:0019079 GO:0006310 GO:0006771
GO:0019497 GO:0006281 GO:0003993 GO:0003724 GO:0005524
GO:0016887 GO:0009378 GO:0003678 GO:0005515 GO:0003723
GO:0005657 GO:0009379
Cluster-22178.50522 GO:0003924 GO:0005525 GO:0005861
Cluster-22178.50524 GO:0005515
Cluster-22178.50526 GO:0007165 GO:0007275 GO:0000917 GO:0043093
GO:0005044 GO:0016020 GO:0005578 GO:0005737
Cluster-22178.50530 GO:0007059 GO:0051301 GO:0031110 GO:0006310
GO:0007155 GO:0007067 GO:0000090 GO:0008017 GO:0003677
GO:0051015 GO:0045298 GO:0005876 GO:0000940 GO:0005727
Cluster-22178.50532 GO:0006397 GO:0006370 GO:0005515 GO:0004484
GO:0016020
Cluster-22178.50536 GO:0006355 GO:0035091 GO:0003700 GO:0005515
GO:0016773 GO:0042025 GO:0005667
Cluster-22178.50539 GO:0005515
Cluster-22178.5054 GO:0016021
Cluster-22178.50543 GO:0006744 GO:0055114 GO:0005515
Cluster-22178.50546 GO:0032040
Cluster-22178.50547 GO:0008033 GO:0007165 GO:0005524 GO:0016879
GO:0000166 GO:0005737

Cluster-22178.50549 GO:0005615
Cluster-22178.50550 GO:0006366 GO:0006384 GO:0016226 GO:0001503
GO:0051536 GO:0003700 GO:0005506 GO:0005634 GO:0005667
Cluster-22178.50551 GO:0018149 GO:0009069 GO:0006468 GO:0016310
GO:0007178 GO:0004675 GO:0003810 GO:0005524 GO:0016020
Cluster-22178.50554 GO:0006468 GO:0003677 GO:0005515 GO:0005524
GO:0004672
Cluster-22178.50555 GO:0044419 GO:0006144 GO:0006508 GO:0004197
GO:0003968 GO:0017111 GO:0031379
Cluster-22178.50557 GO:0042254 GO:0006412 GO:0003735 GO:0005840
GO:0015934
Cluster-22178.50559 GO:0005515
Cluster-22178.50560 GO:0015992 GO:0015991 GO:0015078 GO:0033177
Cluster-22178.50561 GO:0015991 GO:0007165 GO:0007186 GO:0015992
GO:0019001 GO:0031683 GO:0003924 GO:0015078 GO:0004871
GO:0033177
Cluster-22178.50563 GO:0009966 GO:0007275 GO:0016020
Cluster-22178.50564 GO:0005198 GO:0045095
Cluster-22178.50565 GO:0005975 GO:0005982 GO:0005985 GO:0004650
GO:0004045
Cluster-22178.50566 GO:0005515
Cluster-22178.50570 GO:0007276 GO:0007165 GO:0006310 GO:0003677
GO:0005515 GO:0005179 GO:0005576 GO:0005634
Cluster-22178.50571 GO:0008544 GO:0000166 GO:0046872 GO:0008168

Cluster-22178.50573 GO:0051015
Cluster-22178.50574 GO:0051015
Cluster-22178.50575 GO:0007018 GO:0000398 GO:0005515 GO:0005868
GO:0005681
Cluster-22178.50577 GO:0006508 GO:0055085 GO:0004197 GO:0016021
Cluster-22178.50579 GO:0051537 GO:0043231
Cluster-22178.50581 GO:0006355 GO:0005634
Cluster-22178.50584 GO:0007264 GO:0007165 GO:0007186 GO:0031683

GO:0005524 GO:0019001 GO:0003924 GO:0005525 GO:0004871
GO:0016021
Cluster-22178.50585 GO:0016560 GO:0007264 GO:0007186 GO:0007165
GO:0003924 GO:0019001 GO:0031683 GO:0005524 GO:0004871
GO:0005525 GO:0005777 GO:0016021 GO:0005887
Cluster-22178.50586 GO:0006260 GO:0000723 GO:0003677 GO:0003676
GO:0000784 GO:0005634
Cluster-22178.50589 GO:0006355 GO:0003700 GO:0005667
Cluster-22178.50593 GO:0032040
Cluster-22178.50597 GO:0006810 GO:0003333 GO:0006865 GO:0055085
GO:0015171 GO:0016020
Cluster-22178.50600 GO:0005515
Cluster-22178.50602 GO:0048193 GO:0055114 GO:0007165 GO:0007186
GO:0005525 GO:0004871 GO:0031683 GO:0019001 GO:0016491
GO:0003924 GO:0050661 GO:0005783
Cluster-22178.50603 GO:0006886 GO:0008536
Cluster-22178.50607 GO:0006308 GO:0009307 GO:0005515 GO:0003677
GO:0009036 GO:0009359
Cluster-22178.50614 GO:0055085 GO:0006811 GO:0008270 GO:0005216
GO:0016020
Cluster-22178.50618 GO:0007165 GO:0018106 GO:0000160 GO:0016310
GO:0035556 GO:0004673 GO:0046872 GO:0005524 GO:0016020
GO:0009365
Cluster-22178.50619 GO:0009117 GO:0006190 GO:0050483 GO:0000287

Cluster-22178.50620 GO:0006355 GO:0005515
Cluster-22178.50622 GO:0006313 GO:0004803 GO:0003677
Cluster-22178.50624 GO:0008152 GO:0003824
Cluster-22178.50625 GO:0008152 GO:0003824
Cluster-22178.50629 GO:0006807 GO:0042967 GO:0006537 GO:0055114
GO:0016407 GO:0003676 GO:0015930 GO:0005515
Cluster-22178.50632 GO:0006486 GO:0008373
Cluster-22178.50633 GO:0006486 GO:0008373

Cluster-22178.50636 GO:0003677 GO:0005515 GO:0005737

Cluster-22178.50637 GO:0007050 GO:0006468 GO:0045859 GO:0004861 GO:0005524 GO:0004672 GO:0005634

Cluster-22178.50638 GO:0050821 GO:0020027 GO:0006468 GO:0030097 GO:0006457 GO:0005524 GO:0004672 GO:0030492 GO:0005833

Cluster-22178.50639 GO:0009607 GO:0055085 GO:0030001 GO:0009405 GO:0046873 GO:0005515 GO:0005198 GO:0016021 GO:0016020

Cluster-22178.50640 GO:0006355 GO:0016791 GO:0004252 GO:0003723 GO:0008233 GO:0003700 GO:0016020 GO:0005667 GO:0005634 GO:0005618

Cluster-22178.50641 GO:0006351 GO:0005975 GO:0005515 GO:0008270 GO:0004553 GO:0003676 GO:0033925 GO:0005737

Cluster-22178.50647 GO:0005515

Cluster-22178.50654 GO:0030001 GO:0000166 GO:0019956 GO:0005515 GO:0003682 GO:0046872 GO:0008270 GO:0000785

Cluster-22178.50656 GO:0006468 GO:0004672 GO:0005524

Cluster-22178.50659 GO:0003824

Cluster-22178.50660 GO:0007155 GO:0006886 GO:0030155 GO:0007165 GO:0055114 GO:0006914 GO:0030334 GO:0019049 GO:0045995 GO:0016702 GO:0005102 GO:0016020 GO:0031224 GO:0005801

Cluster-22178.50662 GO:0035556

Cluster-22178.50664 GO:0006367 GO:0032968 GO:0003677 GO:0005634

Cluster-22178.50666 GO:0006418 GO:0004812 GO:0005524 GO:0008270 GO:0004518 GO:0003677 GO:0000166

Cluster-22178.50669 GO:0007186 GO:0004930 GO:0016021

Cluster-22178.50674 GO:0005525 GO:0016887 GO:0005524

Cluster-22178.50677 GO:0033014 GO:0055114 GO:0015994 GO:0004418 GO:0016491

Cluster-22178.50678 GO:0005509

Cluster-22178.50679 GO:0046983

Cluster-22178.50680 GO:0007034 GO:0006308 GO:0008855 GO:0009318

Cluster-22178.50687 GO:0042254 GO:0006412 GO:0019843 GO:0003735 GO:0005622 GO:0005840

Cluster-22178.50698 GO:0008152
Cluster-22178.50700 GO:0006298 GO:0005524 GO:0030983
Cluster-22178.50705 GO:0009235 GO:0006576 GO:0019079 GO:0005524
GO:0005515 GO:0043657
Cluster-22178.50706 GO:0006281 GO:0004518
Cluster-22178.50709 GO:0055114 GO:0008152 GO:0016491
Cluster-22178.50710 GO:0042967 GO:0008080 GO:0005198 GO:0019028
Cluster-22178.50711 GO:0046740 GO:0007155 GO:0005515 GO:0016021
GO:0016020
Cluster-22178.50718 GO:0008270 GO:0005515 GO:0042765 GO:0016021
Cluster-22178.50720 GO:0009405 GO:0043303 GO:0046872 GO:0005576
Cluster-22178.50721 GO:0043303 GO:0009405 GO:0046872 GO:0005198
GO:0005576
Cluster-22178.50722 GO:0008152 GO:0006090 GO:0006099 GO:0005515
GO:0008924 GO:0016829 GO:0016021
Cluster-22178.50725 GO:0007275 GO:0005515 GO:0016021
Cluster-22178.50729 GO:0016021
Cluster-22178.50730 GO:0005515 GO:0016021
Cluster-22178.50731 GO:0016021
Cluster-22178.50733 GO:0022900 GO:0005515 GO:0016021
Cluster-22178.50734 GO:0022900 GO:0005515 GO:0016021
Cluster-22178.50735 GO:0006810 GO:0005215 GO:0016021
Cluster-22178.50737 GO:0016480
Cluster-22178.50739 GO:0007165 GO:0006355 GO:0003677 GO:0005515
GO:0005179 GO:0005576
Cluster-22178.50740 GO:0051028 GO:0006464 GO:0015031 GO:0008643
GO:0048309 GO:0008982 GO:0005515 GO:0009357
Cluster-22178.50742 GO:0046488 GO:0016307 GO:0008270 GO:0005515

Cluster-22178.50743 GO:0006468 GO:0004672 GO:0005524
Cluster-22178.50744 GO:0008152 GO:0003824 GO:0016836
Cluster-22178.50746 GO:0006144 GO:0006351 GO:0003723 GO:0003968
GO:0031379

Cluster-22178.50749 GO:0015991 GO:0055085 GO:0015992 GO:0005515
GO:0015078 GO:0008270 GO:0046872 GO:0033179 GO:0016021
Cluster-22178.5075 GO:0055085 GO:0022857 GO:0016021
Cluster-22178.50750 GO:0055085 GO:0006821 GO:0008270 GO:0005247
GO:0016020
Cluster-22178.50752 GO:0006488 GO:0004583 GO:0005789
Cluster-22178.50757 GO:0045859 GO:0006468 GO:0016310 GO:0048870
GO:0007050 GO:0007165 GO:0009069 GO:0006606 GO:0006914
GO:0005198 GO:0019901 GO:0051082 GO:0008092 GO:0004861
GO:0004674 GO:0005096 GO:0031514 GO:0005634 GO:0019028
GO:0019031 GO:0005737 GO:0005882 GO:0019898
Cluster-22178.50764 GO:0009058 GO:0016740 GO:0030170
Cluster-22178.50765 GO:0016021
Cluster-22178.50766 GO:0016021
Cluster-22178.50771 GO:0055114 GO:0008641 GO:0051287
Cluster-22178.50772 GO:0055114 GO:0008641 GO:0051287
Cluster-22178.50775 GO:0007010 GO:0008270 GO:0003779 GO:0005515

Cluster-22178.50776 GO:0003676
Cluster-22178.50778 GO:0071705 GO:0004190 GO:0003676 GO:0045095
GO:0016021
Cluster-22178.50779 GO:0071705 GO:0003676 GO:0045095
Cluster-22178.50781 GO:0046872 GO:0032797
Cluster-22178.50783 GO:0005096
Cluster-22178.50786 GO:0006508 GO:0055114 GO:0006144 GO:0004197
GO:0017111 GO:0035091 GO:0016491 GO:0003968 GO:0031379
Cluster-22178.50789 GO:0006468 GO:0005515 GO:0005524 GO:0004672

Cluster-22178.50793 GO:0006308 GO:0009307 GO:0006355 GO:0009036
GO:0003700 GO:0005515 GO:0003677 GO:0005667 GO:0009359
Cluster-22178.50797 GO:0046872
Cluster-22178.50800 GO:0006629 GO:0009058 GO:0016788
Cluster-22178.50802 GO:0016567 GO:0007165 GO:0007166 GO:0005515

GO:0005509 GO:0004871 GO:0004842 GO:0046872 GO:0008270
GO:0005634 GO:0005680
Cluster-22178.50804 GO:0009058 GO:0008152 GO:0016747 GO:0005524
GO:0016874
Cluster-22178.50805 GO:0006090 GO:0006525 GO:0006814 GO:0071436
GO:0006560 GO:0004672 GO:0015081 GO:0008948 GO:0016020
Cluster-22178.50807 GO:0009097 GO:0009099 GO:0009098 GO:0006438
GO:0000166 GO:0005524 GO:0004832 GO:0005737
Cluster-22178.50808 GO:0016337 GO:0045132 GO:0006813 GO:0007049
GO:0016286 GO:0016020 GO:0005634 GO:0016021 GO:0000775
Cluster-22178.50812 GO:0006606 GO:0007094 GO:0000042 GO:0005515
GO:0005789
Cluster-22178.50815 GO:0005198 GO:0016021 GO:0019028
Cluster-22178.50817 GO:0006468 GO:0016773 GO:0004672 GO:0005524
GO:0016020
Cluster-22178.50824 GO:0045859 GO:0005515 GO:0004860 GO:0005576
Cluster-22178.50826 GO:0006629 GO:0006810 GO:0016787 GO:0005524
GO:0042626 GO:0016021
Cluster-22178.50829 GO:0005515
Cluster-22178.5083 GO:0006508 GO:0004252 GO:0005515 GO:0016020
Cluster-22178.50830 GO:0006355 GO:0003676 GO:0005515 GO:0043565
GO:0005634
Cluster-22178.50833 GO:0006810 GO:0009405 GO:0019871 GO:0005576
Cluster-22178.50834 GO:0006887 GO:0008289 GO:0000145
Cluster-22178.50835 GO:0055085 GO:0006821 GO:0005247 GO:0016020
Cluster-22178.50837 GO:0003677 GO:0005515 GO:0046872
Cluster-22178.50842 GO:0006270 GO:0006323 GO:0016020
Cluster-22178.50843 GO:0006355 GO:0006260 GO:0003700 GO:0005634
GO:0005667
Cluster-22178.50844 GO:0006355 GO:0006260 GO:0003700 GO:0005667
GO:0005634
Cluster-22178.50845 GO:0006323 GO:0006270 GO:0016020
Cluster-22178.50846 GO:0007049

Cluster-22178.50848 GO:0007283 GO:0055114 GO:0003677 GO:0051287
GO:0005634 GO:0000786
Cluster-22178.50849 GO:0007283 GO:0003677 GO:0005634 GO:0000786
Cluster-22178.50850 GO:0007283 GO:0003677 GO:0005634 GO:0000786
Cluster-22178.50851 GO:0006470 GO:0006570 GO:0004725 GO:0008138

Cluster-22178.50852 GO:0005515
Cluster-22178.50853 GO:0003677 GO:0008270 GO:0046872
Cluster-22178.50856 GO:0006206 GO:0006144 GO:0006352 GO:0006355
GO:0006351 GO:0016987 GO:0003700 GO:0003677 GO:0003899
GO:0005730 GO:0005667
Cluster-22178.50859 GO:0006396 GO:0009451 GO:0001522 GO:0009982
GO:0003723
Cluster-22178.50860 GO:0008033 GO:0008168
Cluster-22178.50866 GO:0009396 GO:0055114 GO:0015684 GO:0046487
GO:0016491 GO:0005524 GO:0015093 GO:0003824 GO:0004329
GO:0004488 GO:0016021
Cluster-22178.50867 GO:0007218
Cluster-22178.50870 GO:0008152 GO:0006508 GO:0008236 GO:0016787

Cluster-22178.50871 GO:0007186 GO:0007165 GO:0004871 GO:0005834
Cluster-22178.50873 GO:0007264 GO:0006119 GO:0006118 GO:0015992
GO:0055114 GO:0035556 GO:0008121 GO:0005525 GO:0005507
Cluster-22178.50875 GO:0006310 GO:0055085 GO:0009396 GO:0015031
GO:0046487 GO:0006355 GO:0006281 GO:0008152 GO:0055114
GO:0004488 GO:0009378 GO:0003724 GO:0008565 GO:0016491
GO:0016887 GO:0005524 GO:0008134 GO:0003723 GO:0003824
GO:0003678 GO:0022857 GO:0005667 GO:0009379 GO:0005657
GO:0016021
Cluster-22178.50880 GO:0006355 GO:0043565 GO:0003700 GO:0005634
GO:0005667
Cluster-22178.50887 GO:0000062
Cluster-22178.50891 GO:0006367 GO:0006355 GO:0008152 GO:0048870

GO:0017048 GO:0003677 GO:0003682 GO:0016491 GO:0008440
GO:0031514 GO:0005634 GO:0005672 GO:0000785 GO:0000775
Cluster-22178.50895 GO:0008643 GO:0055085 GO:0005351 GO:0000139
GO:0016020 GO:0016021
Cluster-22178.50898 GO:0042254 GO:0005515 GO:0005622
Cluster-22178.50899 GO:0006413 GO:0005515 GO:0005509
Cluster-22178.50900 GO:0007010 GO:0003779
Cluster-22178.50901 GO:0006955 GO:0030153 GO:0006531 GO:0006529
GO:0006522 GO:0004066 GO:0015643 GO:0019814
Cluster-22178.50902 GO:0055114 GO:0016972 GO:0005515
Cluster-22178.50907 GO:0008146
Cluster-22178.50910 GO:0006468 GO:0007169 GO:0004672 GO:0005524
GO:0005515 GO:0004714 GO:0016020
Cluster-22178.50916 GO:0006544 GO:0046486 GO:0008654 GO:0006563
GO:0006566 GO:0004609
Cluster-22178.50917 GO:0046486 GO:0006566 GO:0006563 GO:0008654
GO:0006544 GO:0004609 GO:0005515 GO:0008270 GO:0046872
Cluster-22178.50926 GO:0008616 GO:0006886 GO:0016192 GO:0043248
GO:0016740 GO:0005515 GO:0016853 GO:0030117
Cluster-22178.50929 GO:0005515
Cluster-22178.50930 GO:0006355 GO:0003677 GO:0003682 GO:0000775
GO:0000785 GO:0005634
Cluster-22178.50931 GO:0006355 GO:0003677 GO:0003682 GO:0000775
GO:0000785 GO:0005634
Cluster-22178.50932 GO:0007165 GO:0005057
Cluster-22178.50934 GO:0006468 GO:0006118 GO:0046983 GO:0016773
GO:0004672 GO:0005524 GO:0003676 GO:0009055 GO:0016020
Cluster-22178.50935 GO:0006118 GO:0006468 GO:0009055 GO:0003676
GO:0004672 GO:0005524 GO:0046983 GO:0016773 GO:0016020
Cluster-22178.50936 GO:0006118 GO:0006468 GO:0009055 GO:0003676
GO:0004672 GO:0005524 GO:0046983 GO:0016773 GO:0016020
Cluster-22178.50940 GO:0016787 GO:0005524 GO:0004386 GO:0003676
GO:0003677

Cluster-22178.50941 GO:0005515 GO:0016020
Cluster-22178.50943 GO:0043093 GO:0006886 GO:0000917 GO:0007094
GO:0006914 GO:0015031 GO:0005515 GO:0008565 GO:0003774
GO:0016459 GO:0016020 GO:0005783 GO:0005737 GO:0016021
Cluster-22178.50944 GO:0016787
Cluster-22178.50949 GO:0046872
Cluster-22178.50951 GO:0009878 GO:0046872
Cluster-22178.50955 GO:0006811 GO:0007165 GO:0007268 GO:0005234
GO:0004970 GO:0016020
Cluster-22178.50956 GO:0007049 GO:0017053
Cluster-22178.50962 GO:0017176
Cluster-22178.50965 GO:0016788 GO:0005515
Cluster-22178.50968 GO:0006814 GO:0006118 GO:0006744 GO:0006120
GO:0015992 GO:0008137 GO:0009055 GO:0016020
Cluster-22178.50972 GO:0006260 GO:0009966 GO:0007264 GO:0005096
GO:0005576
Cluster-22178.50973 GO:0000226 GO:0006914 GO:0007165 GO:0008283
GO:0000910 GO:0048870 GO:0009405 GO:0040007 GO:0006355
GO:0015631 GO:0008017 GO:0005096 GO:0003700 GO:0043565
GO:0005198 GO:0008083 GO:0042803 GO:0008134 GO:0045502
GO:0051082 GO:0030286 GO:0016020 GO:0005882 GO:0005667
GO:0045298 GO:0031514
Cluster-22178.50974 GO:0019836 GO:0005525 GO:0016887 GO:0005524
GO:0005576
Cluster-22178.50977 GO:0008033 GO:0006351 GO:0009451 GO:0006269
GO:0003896 GO:0009020 GO:0005657 GO:0005730
Cluster-22178.50979 GO:0006629 GO:0008081
Cluster-22178.50981 GO:0007186 GO:0004930 GO:0016021
Cluster-22178.5099 GO:0033014 GO:0007165 GO:0046413 GO:0006783
GO:0015994 GO:0004852 GO:0005515 GO:0018836
Cluster-22178.50991 GO:0009058 GO:0008201 GO:0030170 GO:0009512
Cluster-22178.50992 GO:0009058 GO:0030170 GO:0008201 GO:0009512
Cluster-22178.50994 GO:0019012

Cluster-22178.50995 GO:0042254 GO:0006412 GO:0005515 GO:0003735 GO:0005840
Cluster-22178.51000 GO:0055114 GO:0006355 GO:0016491 GO:0005515

Cluster-22178.51004 GO:0006144 GO:0019079 GO:0006351 GO:0005509 GO:0003968 GO:0005515 GO:0031379
Cluster-22178.51006 GO:0019068 GO:0003676 GO:0008233 GO:0008168 GO:0008047
Cluster-22178.51007 GO:0006367 GO:0006486 GO:0006355 GO:0018279 GO:0004579 GO:0046983 GO:0005783 GO:0016020 GO:0008250 GO:0005672 GO:0016021
Cluster-22178.51008 GO:0009058 GO:0008483 GO:0030170
Cluster-22178.51015 GO:0005096
Cluster-22178.51018 GO:0007264 GO:0005096 GO:0005525
Cluster-22178.51021 GO:0019497 GO:1901031 GO:0006771 GO:0003993 GO:0008270 GO:0046872 GO:0005515 GO:0005634
Cluster-22178.51022 GO:0016020
Cluster-22178.51024 GO:0016020
Cluster-22178.51026 GO:0006955 GO:0005201 GO:0005578 GO:0005581
Cluster-22178.51032 GO:0006468 GO:0008168 GO:0004672 GO:0005524 GO:0005515
Cluster-22178.51033 GO:0006468 GO:0005515 GO:0005524 GO:0004672 GO:0008168
Cluster-22178.51038 GO:0007165 GO:0005509 GO:0005515 GO:0005578
Cluster-22178.51040 GO:0006364 GO:0004222
Cluster-22178.51043 GO:0006313 GO:0004803 GO:0003677
Cluster-22178.51047 GO:0009245 GO:0016567 GO:0008915 GO:0008270 GO:0046872 GO:0008233 GO:0004842 GO:0003723 GO:0005515 GO:0005680 GO:0016020
Cluster-22178.51049 GO:0043087 GO:0005085 GO:0005515
Cluster-22178.51051 GO:0030153 GO:0016192 GO:0006886 GO:0006955 GO:0015643 GO:0005515 GO:0005198 GO:0030117 GO:0019814
Cluster-22178.51056 GO:0042254 GO:0006412 GO:0003735 GO:0005515

GO:0046872 GO:0005840 GO:0005622
Cluster-22178.51057 GO:0016192 GO:0006886 GO:0005515 GO:0042802

Cluster-22178.51060 GO:0006309 GO:0016787 GO:0005737 GO:0005634
Cluster-22178.51062 GO:0006313 GO:0004803 GO:0003677
Cluster-22178.51063 GO:0048870 GO:0006935 GO:0006357 GO:0000921
GO:0006606 GO:0071973 GO:0008092 GO:0016817 GO:0005198
GO:0001104 GO:0003774 GO:0005737 GO:0009288 GO:0016021
GO:0031514 GO:0005940 GO:0019898 GO:0016020 GO:0016592
GO:0005882
Cluster-22178.51064 GO:0006355 GO:0003677 GO:0003700 GO:0005667
Cluster-22178.51065 GO:0035023 GO:0035556 GO:0055114 GO:0043087
GO:0006955 GO:0007165 GO:0040007 GO:0008283 GO:0005089
GO:0047134 GO:0005137 GO:0005515 GO:0008083 GO:0005895
GO:0005576
Cluster-22178.51067 GO:0006118 GO:0022900 GO:0055114 GO:0008827
GO:0016021 GO:0009319
Cluster-22178.51068 GO:0006820 GO:0006835 GO:0006812 GO:0017153
GO:0016020
Cluster-22178.51069 GO:0006820 GO:0006835 GO:0006812 GO:0005515
GO:0017153 GO:0016020
Cluster-22178.51073 GO:0007304 GO:0005213 GO:0005576 GO:0042600
Cluster-22178.51077 GO:0007268 GO:0007165 GO:0042742 GO:0030550
GO:0005576
Cluster-22178.51079 GO:0007275 GO:0005578
Cluster-22178.51081 GO:0055114 GO:0010309 GO:0005515
Cluster-22178.51082 GO:0019087 GO:0016021
Cluster-22178.51083 GO:0006468 GO:0005524 GO:0004672
Cluster-22178.51085 GO:0006355 GO:0009772 GO:0006118 GO:0019684
GO:0045156 GO:0003676 GO:0003700 GO:0005667
Cluster-22178.51086 GO:0055114 GO:0051920
Cluster-22178.51095 GO:0042254 GO:0006412 GO:0003735 GO:0000774
GO:0005783 GO:0005840 GO:0005622

Cluster-22178.51097 GO:0042254 GO:0006412 GO:0003924 GO:0003735
GO:0005525 GO:0005622 GO:0005840
Cluster-22178.51098 GO:0007264 GO:0015684 GO:0015093 GO:0005524
GO:0003924 GO:0016887 GO:0005525 GO:0016021
Cluster-22178.5110 GO:0006886 GO:0015031 GO:0008565 GO:0016020
GO:0005737
Cluster-22178.51100 GO:0033925 GO:0005515 GO:0005737
Cluster-22178.51102 GO:0006468 GO:0006118 GO:0005524 GO:0004672
GO:0016773 GO:0046983 GO:0009055 GO:0003676 GO:0016020
Cluster-22178.51107 GO:0007010 GO:0005975 GO:0007165 GO:0006810
GO:0016192 GO:0006357 GO:0005198 GO:0001104 GO:0005515
GO:0003779 GO:0005215 GO:0016853 GO:0043395 GO:0004872
GO:0005578 GO:0019028 GO:0019031 GO:0016021 GO:0016592
GO:0016020
Cluster-22178.51109 GO:0009405 GO:0005576
Cluster-22178.51111 GO:0008152 GO:0008610 GO:0016747
Cluster-22178.51113 GO:0006468 GO:0005515 GO:0005524 GO:0004672
GO:0016773 GO:0016020
Cluster-22178.51114 GO:0006468 GO:0005515 GO:0016773 GO:0004672
GO:0005524 GO:0016020
Cluster-22178.51117 GO:0008152 GO:0007165 GO:0006955 GO:0030247
GO:0003676 GO:0003824 GO:0046872 GO:0008484 GO:0005044
GO:0016787
Cluster-22178.51119 GO:0009405 GO:0033644
Cluster-22178.51120 GO:0050909 GO:0006506 GO:0016021 GO:0005789
Cluster-22178.51124 GO:0006355 GO:0003690 GO:0005739
Cluster-22178.51126 GO:0006355 GO:0003700 GO:0005622 GO:0005667
Cluster-22178.51127 GO:0006355 GO:0005622
Cluster-22178.51131 GO:0006351 GO:0006814 GO:0006260 GO:0003677
GO:0003887 GO:0005248 GO:0042575 GO:0001518
Cluster-22178.51133 GO:0055114 GO:0006479 GO:0006486 GO:0006355
GO:0006554 GO:0007165 GO:0034968 GO:0005507 GO:0008375
GO:0018024 GO:0016641 GO:0003677 GO:0005044 GO:0051920

GO:0005622 GO:0005763 GO:0016020 GO:0005694

Cluster-22178.51138 GO:0007049 GO:0000917 GO:0006355 GO:0043093

GO:0003700 GO:0043565 GO:0003677 GO:0005815 GO:0005737

GO:0005634 GO:0005667

Cluster-22178.51139 GO:0016032 GO:0019031

Cluster-22178.51140 GO:0015684 GO:0007264 GO:0005525 GO:0005509

GO:0005515 GO:0015093 GO:0016021

Cluster-22178.51141 GO:0006508 GO:0004198 GO:0005509 GO:0005622

Cluster-22178.51142 GO:0016020

Cluster-22178.51143 GO:0006047

Cluster-22178.51144 GO:0046872 GO:0008270 GO:0003676

Cluster-22178.51145 GO:0009058 GO:0016772 GO:0016779 GO:0031224

Cluster-22178.51150 GO:0007165 GO:0006313 GO:0005515 GO:0005509

GO:0005578

Cluster-22178.51152 GO:0007049 GO:0005515

Cluster-22178.51153 GO:0006955 GO:0030153 GO:0015643 GO:0019814

Cluster-22178.51154 GO:0005515

Cluster-22178.51155 GO:0016567 GO:0004842 GO:0005515 GO:0008270

GO:0046872

Cluster-22178.51158 GO:0019076 GO:0032801 GO:0006812 GO:0006468

GO:0007155 GO:0001503 GO:0004672 GO:0005524 GO:0016773

GO:0005261 GO:0033644 GO:0005576 GO:0016020

Cluster-22178.51165 GO:0045859 GO:0046677 GO:0004860

Cluster-22178.51169 GO:0009405 GO:0008270 GO:0003723 GO:0004222

GO:0005576

Cluster-22178.51170 GO:0007205 GO:0035556 GO:0007165 GO:0046486

GO:0009395 GO:0004143 GO:0016301

Cluster-22178.51171 GO:0008152 GO:0051252 GO:0008033 GO:0004540

GO:0016491

Cluster-22178.51174 GO:0051225 GO:0071973 GO:0006935 GO:0003774

GO:0016020 GO:0009288 GO:0070652

Cluster-22178.51175 GO:0006351 GO:0008270 GO:0003677 GO:0005634

Cluster-22178.51178 GO:0005515

Cluster-22178.51180 GO:0006310 GO:0006281
Cluster-22178.51183 GO:0005515
Cluster-22178.51185 GO:0007186 GO:0004930 GO:0000975 GO:0046872
GO:0016021
Cluster-22178.51187 GO:0016021
Cluster-22178.51189 GO:0016579 GO:0006511 GO:0006508 GO:0004843
GO:0005622
Cluster-22178.51190 GO:0005515
Cluster-22178.51195 GO:0042254 GO:0003735 GO:0005840 GO:0005739
Cluster-22178.51199 GO:0046872
Cluster-22178.51200 GO:0008033 GO:0006379 GO:0006364 GO:0051252
GO:0003723 GO:0004540 GO:0000172 GO:0030677
Cluster-22178.51203 GO:0016197 GO:0000724 GO:0006270 GO:0030119
Cluster-22178.51206 GO:0008152 GO:0005975 GO:0005509 GO:0004571
GO:0016829 GO:0016020
Cluster-22178.51208 GO:0019478 GO:0016788 GO:0005737
Cluster-22178.51210 GO:0055085 GO:0006355 GO:0006270 GO:0019058
GO:0022857 GO:0003700 GO:0003677 GO:0004252 GO:0005886
GO:0019031 GO:0016021 GO:0042765 GO:0005667 GO:0016020
Cluster-22178.51212 GO:0004222
Cluster-22178.51213 GO:0004222
Cluster-22178.51214 GO:0005525
Cluster-22178.51219 GO:0046872 GO:0043130
Cluster-22178.51220 GO:0046872 GO:0043130
Cluster-22178.51222 GO:0006260 GO:0003677 GO:0005664
Cluster-22178.51224 GO:0042967 GO:0019497 GO:0006771 GO:0007155
GO:0003993 GO:0008080 GO:0046872 GO:0016747 GO:0005515
GO:0016021 GO:0016020
Cluster-22178.51225 GO:0030168 GO:0051258 GO:0016192 GO:0006886
GO:0007165 GO:0005102 GO:0005198 GO:0005515 GO:0030674
GO:0005577 GO:0030117
Cluster-22178.51228 GO:0055114 GO:0006694 GO:0008210 GO:0008207
GO:0008209 GO:0016491 GO:0003824 GO:0050662 GO:0003854

GO:0016616
Cluster-22178.51229 GO:0055114 GO:0006694 GO:0008152 GO:0008210 GO:0008207 GO:0008209 GO:0016491 GO:0003824 GO:0003854 GO:0050662 GO:0016616
Cluster-22178.51236 GO:0003723 GO:0000178
Cluster-22178.51247 GO:0005515 GO:0008270
Cluster-22178.51248 GO:0008270 GO:0005515
Cluster-22178.51249 GO:0005515
Cluster-22178.51252 GO:0071203
Cluster-22178.51253 GO:0016567 GO:0005515 GO:0004842 GO:0008270 GO:0046872 GO:0005680
Cluster-22178.51255 GO:0006355 GO:0009271
Cluster-22178.51256 GO:0006270 GO:0005515
Cluster-22178.51260 GO:0005739
Cluster-22178.51265 GO:0016579 GO:0036459 GO:0032183 GO:0070140

Cluster-22178.51267 GO:0006355 GO:0008270 GO:0005515
Cluster-22178.51269 GO:0007264 GO:0006576 GO:0005524 GO:0003924 GO:0005525
Cluster-22178.51273 GO:0006629 GO:0016627 GO:0005737 GO:0016021
Cluster-22178.51275 GO:0006139 GO:0003676 GO:0008408
Cluster-22178.51277 GO:0007010 GO:0003779
Cluster-22178.51288 GO:0007165 GO:0005515 GO:0005179 GO:0008270 GO:0005576
Cluster-22178.51290 GO:0006139 GO:0043395 GO:0003676 GO:0008408 GO:0005515 GO:0005578 GO:0016020
Cluster-22178.51291 GO:0009069 GO:0006468 GO:0016310 GO:0005524 GO:0004674
Cluster-22178.51293 GO:0005543
Cluster-22178.51296 GO:0006260 GO:0055085 GO:0015074 GO:0006810 GO:0005525 GO:0003676 GO:0003678 GO:0042626 GO:0005524 GO:0003924 GO:0016887 GO:0005657 GO:0016021
Cluster-22178.51298 GO:0006468 GO:0004672 GO:0005524 GO:0008134

GO:0042803 GO:0045502 GO:0030286 GO:0005856 GO:0005667
Cluster-22178.51299 GO:0055085 GO:0005741
Cluster-22178.51301 GO:0005515
Cluster-22178.51304 GO:0016310 GO:0000160 GO:0000155 GO:0046983
GO:0009365 GO:0016021
Cluster-22178.51306 GO:0007049 GO:0006260 GO:0070206 GO:0006355
GO:0000002 GO:0003724 GO:0005524 GO:0016887 GO:0003677
GO:0008134 GO:0003723 GO:0016787 GO:0000262 GO:0045095
GO:0005667
Cluster-22178.51307 GO:0000002 GO:0003723 GO:0005524 GO:0016887
GO:0003924 GO:0005525 GO:0003724 GO:0045095 GO:0000262
Cluster-22178.51308 GO:0006534 GO:0046439 GO:0055114 GO:0019530
GO:0016702 GO:0005506 GO:0017172
Cluster-22178.51312 GO:0043093 GO:0006468 GO:0007165 GO:0007172
GO:0000917 GO:0005515 GO:0005096 GO:0004871 GO:0004713
GO:0005925 GO:0005737
Cluster-22178.51313 GO:0005515
Cluster-22178.51318 GO:0005975 GO:0008152 GO:0006508 GO:0006631
GO:0009058 GO:0016787 GO:0016746 GO:0008184 GO:0008236
GO:0016788
Cluster-22178.51319 GO:0045893 GO:0046872 GO:0005634
Cluster-22178.51322 GO:0006510 GO:0006310 GO:0006508 GO:0006281
GO:0016887 GO:0005524 GO:0003724 GO:0009378 GO:0003924
GO:0004176 GO:0003678 GO:0005525 GO:0003723 GO:0004252
GO:0005657 GO:0009379
Cluster-22178.51324 GO:0016021
Cluster-22178.51325 GO:0016021
Cluster-22178.51328 GO:0006013 GO:0006000 GO:0006012 GO:0006094
GO:0006098 GO:0006096 GO:0003872 GO:0005945
Cluster-22178.51329 GO:0006310 GO:0006281 GO:0003724 GO:0016887
GO:0005524 GO:0016829 GO:0005515 GO:0003723 GO:0009378
GO:0005657 GO:0042597 GO:0009379
Cluster-22178.51331 GO:0006541 GO:0004359

Cluster-22178.51332 GO:0007165 GO:0006541 GO:0006457 GO:0004359 GO:0042302 GO:0005509 GO:0016021 GO:0005578
Cluster-22178.51333 GO:0007212 GO:0048268 GO:0044237 GO:0032051 GO:0003824 GO:0016021
Cluster-22178.51337 GO:0035023 GO:0043087 GO:0005089
Cluster-22178.51339 GO:0009069 GO:0016192 GO:0006886 GO:0016310 GO:0005515 GO:0004674 GO:0030117
Cluster-22178.51341 GO:0015986 GO:0017038 GO:0006511 GO:0031625 GO:0005198 GO:0046933 GO:0045259 GO:0016020 GO:0019031
Cluster-22178.51346 GO:0000398 GO:0006298 GO:0007264 GO:0005525 GO:0016887 GO:0003924 GO:0005524 GO:0008270 GO:0004519
Cluster-22178.51347 GO:0000398 GO:0007264 GO:0006298 GO:0007010 GO:0016887 GO:0003779 GO:0003924 GO:0008270 GO:0005524 GO:0005525 GO:0004519 GO:0019028
Cluster-22178.51349 GO:0055114 GO:0016817 GO:0033743 GO:0042302

Cluster-22178.51353 GO:0055114 GO:0048870 GO:0000160 GO:0016310 GO:0015991 GO:0006355 GO:0006928 GO:0015992 GO:0003700 GO:0015078 GO:0005515 GO:0005198 GO:0016491 GO:0016620 GO:0000155 GO:0004673 GO:0005509 GO:0016021 GO:0009365 GO:0031514 GO:0033179 GO:0009279 GO:0005667 GO:0005882
Cluster-22178.51357 GO:0006974 GO:0006260 GO:0006281 GO:0007067 GO:0051726 GO:0003911 GO:0050840 GO:0005576 GO:0072686 GO:0042729
Cluster-22178.51362 GO:0005515
Cluster-22178.51364 GO:0015979 GO:0008270 GO:0016020 GO:0009523 GO:0009539
Cluster-22178.51367 GO:0042476 GO:0042475 GO:0030345 GO:0005578 GO:0019867
Cluster-22178.51368 GO:0055114 GO:0009396 GO:0007608 GO:0007187 GO:0046487 GO:0004984 GO:0005524 GO:0003824 GO:0004329 GO:0004488 GO:0005549 GO:0016020
Cluster-22178.51369 GO:0006310 GO:0003677 GO:0005515 GO:0005634

Cluster-22178.51371 GO:0006310 GO:0003677 GO:0005515 GO:0005634
Cluster-22178.51372 GO:0030334 GO:0006265 GO:0030001 GO:0006449
GO:0019064 GO:0055085 GO:0008053 GO:0006415 GO:0071973
GO:0030155 GO:0016567 GO:0007165 GO:0006935 GO:0045995
GO:0008270 GO:0003774 GO:0003677 GO:0003924 GO:0016881
GO:0046789 GO:0005198 GO:0003917 GO:0003747 GO:0003918
GO:0031386 GO:0005102 GO:0046873 GO:0009986 GO:0005694
GO:0016020 GO:0005741 GO:0009288 GO:0018444 GO:0016021
GO:0019031 GO:0005840 GO:0019028
Cluster-22178.51373 GO:0005524
Cluster-22178.51374 GO:0007155 GO:0006310 GO:0010181 GO:0051015
GO:0003677 GO:0005727
Cluster-22178.51376 GO:0004252 GO:0016021
Cluster-22178.51378 GO:0043401 GO:0006355 GO:0003700 GO:0008270
GO:0043565 GO:0005667 GO:0005634 GO:0016021
Cluster-22178.51385 GO:0007165 GO:0005509 GO:0005578
Cluster-22178.51387 GO:0055114 GO:0016491 GO:0005506
Cluster-22178.51388 GO:0006355 GO:0005198 GO:0003700 GO:0043565
GO:0019031 GO:0005667 GO:0019028
Cluster-22178.51392 GO:0046872
Cluster-22178.51398 GO:0016021 GO:0019012
Cluster-22178.51401 GO:0046907 GO:0006367 GO:0005672
Cluster-22178.51402 GO:0006367 GO:0046907 GO:0005672
Cluster-22178.51403 GO:0046907 GO:0006367 GO:0005672
Cluster-22178.51407 GO:0051205 GO:0016021
Cluster-22178.51408 GO:0007165 GO:0005515 GO:0005509 GO:0005578
Cluster-22178.5141 GO:0008270
Cluster-22178.51410 GO:0006412 GO:0042254 GO:0006827 GO:0006836
GO:0006826 GO:0006812 GO:0005328 GO:0003735 GO:0005381
GO:0016020 GO:0005622 GO:0016021 GO:0005840 GO:0033573
Cluster-22178.51412 GO:0016021
Cluster-22178.51413 GO:0006629
Cluster-22178.51414 GO:0007165 GO:0005509 GO:0005515 GO:0005578

Cluster-22178.51416 GO:0006310 GO:0006260 GO:0006281 GO:0003677
GO:0005634
Cluster-22178.51419 GO:0055114 GO:0005506 GO:0020037 GO:0016705

Cluster-22178.51420 GO:0006000 GO:0006013 GO:0007186 GO:0007165
GO:0007264 GO:0004871 GO:0005525 GO:0003924 GO:0005524
GO:0031683 GO:0019001 GO:0003873
Cluster-22178.51422 GO:0006457 GO:0007165 GO:0005509
Cluster-22178.51423 GO:0005515
Cluster-22178.51426 GO:0070588 GO:0006816 GO:0015002 GO:0005515
GO:0005262 GO:0016020
Cluster-22178.51427 GO:0006355 GO:0005515 GO:0003700 GO:0005667
Cluster-22178.51429 GO:0006357 GO:0006810 GO:0007018 GO:0007017
GO:0003777 GO:0019871 GO:0001104 GO:0005874 GO:0030286
GO:0016592 GO:0005576 GO:0019028
Cluster-22178.51430 GO:0042981 GO:0006606 GO:0005515
Cluster-22178.51433 GO:0045046 GO:0016579 GO:0008270 GO:0036459
GO:0032403 GO:0070140 GO:0032183 GO:0005515 GO:0005779
Cluster-22178.51435 GO:0050832 GO:0008376 GO:0016757 GO:0016020
GO:0032580 GO:0005576
Cluster-22178.5144 GO:0000079 GO:0019901
Cluster-22178.51440 GO:0005515
Cluster-22178.51441 GO:0005515
Cluster-22178.51444 GO:0005515
Cluster-22178.51446 GO:0000002 GO:0005524 GO:0003924 GO:0016887
GO:0005525 GO:0003724 GO:0016787 GO:0003723 GO:0003677
GO:0000262 GO:0045095
Cluster-22178.51449 GO:0005515
Cluster-22178.5145 GO:0000079 GO:0019901
Cluster-22178.51454 GO:0006355 GO:0007155 GO:0043565 GO:0005515
GO:0003700 GO:0005667
Cluster-22178.51455 GO:0006825 GO:0007186 GO:0004930 GO:0016531
GO:0005507 GO:0005758 GO:0016021

Cluster-22178.51457 GO:0055114 GO:0005975 GO:0016491
Cluster-22178.51458 GO:0019069 GO:0015743 GO:0006570 GO:0006810
GO:0009072 GO:0042207 GO:0004334 GO:0005886
Cluster-22178.51465 GO:0006811 GO:0042254 GO:0006412 GO:0006816
GO:0005262 GO:0003735 GO:0005244 GO:0016021 GO:0005840
GO:0005622
Cluster-22178.51469 GO:0007165 GO:0006308 GO:0008855 GO:0009318
Cluster-22178.51472 GO:0007165 GO:0019905 GO:0016021
Cluster-22178.51473 GO:0007165 GO:0006955 GO:0006281 GO:0006355
GO:0003677 GO:0016818 GO:0042803 GO:0005044 GO:0042162
GO:0030247 GO:0008270 GO:0005524 GO:0005634
Cluster-22178.51474 GO:0006744 GO:0005515
Cluster-22178.51475 GO:0006486 GO:0006811 GO:0008378 GO:0032797
GO:0016020 GO:0016021
Cluster-22178.51476 GO:0006303 GO:0030328 GO:0006631 GO:0009069
GO:0016310 GO:0055114 GO:0016670 GO:0003677 GO:0071949
GO:0050151 GO:0004677 GO:0016491 GO:0005524 GO:0005634
GO:0005958
Cluster-22178.51477 GO:0009405 GO:0007186 GO:0004930 GO:0005515
GO:0016021 GO:0016020 GO:0005576
Cluster-22178.51478 GO:0042254 GO:0006412 GO:0006508 GO:0003735
GO:0005509 GO:0004198 GO:0005840 GO:0005622
Cluster-22178.51480 GO:0042254 GO:0006412 GO:0006508 GO:0004198
GO:0005509 GO:0003735 GO:0005840 GO:0005622
Cluster-22178.51482 GO:0050840 GO:0005515 GO:0005576
Cluster-22178.51489 GO:0006260 GO:0006606 GO:0006914 GO:0003677
GO:0003723 GO:0016787 GO:0003724 GO:0016887 GO:0005524
Cluster-22178.51490 GO:0046872
Cluster-22178.51493 GO:0055085 GO:0009405 GO:0006811 GO:0005216
GO:0005576 GO:0016020 GO:0016021
Cluster-22178.51495 GO:0006811 GO:0055085 GO:0005216 GO:0016021
GO:0016020
Cluster-22178.51500 GO:0006511 GO:0007275 GO:0008053 GO:0016787

GO:0005515 GO:0003677 GO:0003924 GO:0005524 GO:0008270
GO:0046872 GO:0005634 GO:0005741 GO:0016021
Cluster-22178.51501 GO:0008134 GO:0042803 GO:0045502 GO:0005667
GO:0030286
Cluster-22178.51503 GO:0019953 GO:0007165 GO:0045893 GO:0003676
GO:0008430 GO:0000772 GO:0005576
Cluster-22178.51506 GO:0009058 GO:0000103 GO:0006144 GO:0006468
GO:0015940 GO:0015937 GO:0008152 GO:0016773 GO:0005524
GO:0005515 GO:0016301 GO:0003824 GO:0004020 GO:0004140
GO:0004672 GO:0016020
Cluster-22178.51508 GO:0006470 GO:0042981 GO:0006397 GO:0006355
GO:0004721 GO:0003700 GO:0005507 GO:0003677 GO:0005667
GO:0005634
Cluster-22178.51509 GO:0006397 GO:0006355 GO:0006470 GO:0042981
GO:0003677 GO:0005507 GO:0003700 GO:0004721 GO:0005634
GO:0005667
Cluster-22178.51510 GO:0008152 GO:0008168 GO:0003723
Cluster-22178.51511 GO:0005515 GO:0005509 GO:0019028
Cluster-22178.51515 GO:0007009 GO:0005515
Cluster-22178.51518 GO:0008270 GO:0005515
Cluster-22178.51521 GO:0061024 GO:0016020 GO:0030176
Cluster-22178.51522 GO:0061024 GO:0016020 GO:0030176
Cluster-22178.51529 GO:0005515
Cluster-22178.51531 GO:0005975 GO:0006013 GO:0004553 GO:0004559
GO:0015923 GO:0008270
Cluster-22178.51532 GO:0016310 GO:0007165 GO:0009069 GO:0006468
GO:0004674 GO:0016773 GO:0004672 GO:0005524 GO:0008601
GO:0005515 GO:0003677 GO:0016787 GO:0000159 GO:0016020
Cluster-22178.51538 GO:0003824 GO:0005615
Cluster-22178.51539 GO:0003824 GO:0005615
Cluster-22178.51542 GO:0006468 GO:0005524 GO:0004672
Cluster-22178.51543 GO:0006508 GO:0006511 GO:0016579 GO:0004843
GO:0005622

Cluster-22178.51546 GO:0006468 GO:0004672 GO:0005524
Cluster-22178.51547 GO:0005975 GO:0005985 GO:0005982 GO:0007411
GO:0004650 GO:0004045
Cluster-22178.51549 GO:0006508
Cluster-22178.5155 GO:0010506 GO:0000077 GO:0005515 GO:0005615
Cluster-22178.51550 GO:0006270 GO:0035556 GO:0004518 GO:0003677
GO:0004190 GO:0016021
Cluster-22178.51557 GO:0042967 GO:0006355 GO:0003712 GO:0008270
GO:0004402 GO:0000123 GO:0005667 GO:0005634
Cluster-22178.51558 GO:0016192 GO:0006904 GO:0046983 GO:0008270

Cluster-22178.51559 GO:0016192 GO:0006904 GO:0046983 GO:0008270

Cluster-22178.51560 GO:0016192 GO:0006904
Cluster-22178.51561 GO:0006904 GO:0016192 GO:0008270 GO:0046983

Cluster-22178.51568 GO:0019904 GO:0005515 GO:0005737
Cluster-22178.51571 GO:0055114 GO:0007165 GO:0016817 GO:0033743
GO:0005179 GO:0005576
Cluster-22178.51572 GO:0006810 GO:0006479 GO:0046500 GO:0008152
GO:0006464 GO:0008757 GO:0042626 GO:0008168 GO:0005524
GO:0004719 GO:0016021
Cluster-22178.51575 GO:0055114 GO:0016616
Cluster-22178.51577 GO:0016021
Cluster-22178.51579 GO:0046497 GO:0006769 GO:0055114 GO:0008750
GO:0050661 GO:0016021
Cluster-22178.51580 GO:0007264 GO:0003924 GO:0005525 GO:0016021
Cluster-22178.51581 GO:0007264 GO:0003924 GO:0005525 GO:0016021
Cluster-22178.51583 GO:0003676
Cluster-22178.51584 GO:0005515 GO:0016021
Cluster-22178.51586 GO:0006298 GO:0006887 GO:0030983 GO:0005198
GO:0005524 GO:0000145 GO:0019028 GO:0019031
Cluster-22178.51590 GO:0006811 GO:0055085 GO:0015031 GO:0006810

GO:0005215 GO:0008565 GO:0016021 GO:0016020
Cluster-22178.51591 GO:0015833 GO:0046872 GO:0005524 GO:0000166

Cluster-22178.51593 GO:0008152 GO:0003824
Cluster-22178.51594 GO:0019058
Cluster-22178.51595 GO:0007218
Cluster-22178.51597 GO:0006281 GO:0006310 GO:0005737
Cluster-22178.51599 GO:0030682 GO:0042147
Cluster-22178.51600 GO:0006123 GO:0015992 GO:0004129 GO:0045277
GO:0005751 GO:0005743
Cluster-22178.51601 GO:0007264 GO:0007186 GO:0015684 GO:0007165
GO:0003924 GO:0031683 GO:0019001 GO:0015093 GO:0005102
GO:0004871 GO:0005525 GO:0016021
Cluster-22178.51610 GO:0006284 GO:0016114 GO:0006694 GO:0016192
GO:0006886 GO:0005198 GO:0005515 GO:0008661 GO:0003677
GO:0005634 GO:0030117
Cluster-22178.51611 GO:0006812 GO:0016887 GO:0008270 GO:0003676
GO:0016021
Cluster-22178.51613 GO:0005515 GO:0016020
Cluster-22178.51616 GO:0008063 GO:0005975 GO:0016773
Cluster-22178.51619 GO:0003824
Cluster-22178.51624 GO:0006352 GO:0006355 GO:0006334 GO:0016987
GO:0003700 GO:0003677 GO:0005667 GO:0005634
Cluster-22178.51629 GO:0006508 GO:0004198 GO:0005622
Cluster-22178.51636 GO:0009306
Cluster-22178.51638 GO:0005515
Cluster-22178.51645 GO:0006260 GO:0004190 GO:0003677 GO:0016021
GO:0005664
Cluster-22178.51651 GO:0007165
Cluster-22178.51652 GO:0030001 GO:0006801 GO:0055114 GO:0046872

Cluster-22178.51654 GO:0003824
Cluster-22178.51656 GO:0007186 GO:0007166 GO:0005515 GO:0004930

GO:0016020 GO:0016021
Cluster-22178.51658 GO:0005515 GO:0008270
Cluster-22178.51662 GO:0015986 GO:0006355 GO:0006367 GO:0007165
GO:0015992 GO:0005515 GO:0005509 GO:0046983 GO:0015078
GO:0005578 GO:0000276
Cluster-22178.51663 GO:0015986 GO:0006355 GO:0007165 GO:0015992
GO:0005509 GO:0005515 GO:0046983 GO:0015078 GO:0005578
GO:0000276
Cluster-22178.51664 GO:0006576 GO:0005525 GO:0003924 GO:0005524

Cluster-22178.51666 GO:0000077 GO:0006281 GO:0030896
Cluster-22178.51667 GO:0016310 GO:0006468 GO:0006355 GO:0015991
GO:0010508 GO:0015992 GO:0051607 GO:0000921 GO:0006914
GO:0006260 GO:0009069 GO:0007155 GO:0015078 GO:0005198
GO:0005524 GO:0008270 GO:0004674 GO:0016818 GO:0003677
GO:0016021 GO:0019867 GO:0005634 GO:0033179 GO:0005940
GO:0005882
Cluster-22178.51669 GO:0006570 GO:0006470 GO:0005515 GO:0004725
GO:0016021
Cluster-22178.5167 GO:0006814 GO:0006118 GO:0006744 GO:0006120
GO:0015992 GO:0005515 GO:0008137 GO:0003954 GO:0043565
GO:0005634 GO:0005739
Cluster-22178.51672 GO:0006810 GO:0032259 GO:0055114 GO:0005215
GO:0016972 GO:0008168 GO:0016020
Cluster-22178.51675 GO:0005515
Cluster-22178.51679 GO:0003677
Cluster-22178.51680 GO:0035023 GO:0007165 GO:0043087 GO:0005089
GO:0005515
Cluster-22178.51688 GO:0007165 GO:0005515
Cluster-22178.51691 GO:0007165 GO:0005515
Cluster-22178.51692 GO:0006355 GO:0003700 GO:0003676 GO:0008270
GO:0043565 GO:0005622 GO:0005667
Cluster-22178.51693 GO:0006308 GO:0008855 GO:0009318

Cluster-22178.51694 GO:0015743 GO:0045859 GO:0006308 GO:0004860
GO:0008855 GO:0009318 GO:0005576
Cluster-22178.51696 GO:0007165
Cluster-22178.51699 GO:0071704 GO:0005975 GO:0016868
Cluster-22178.51702 GO:0015979 GO:0009579 GO:0016021 GO:0009522
Cluster-22178.51703 GO:0016055 GO:0007165 GO:0007275 GO:0006298
GO:0006306 GO:0032775 GO:0031167 GO:0030414 GO:0030983
GO:0005524 GO:0008168 GO:0009007 GO:0005102 GO:0005576
Cluster-22178.51709 GO:0005515
Cluster-22178.51711 GO:0006144 GO:0006464 GO:0046034 GO:0004017
GO:0003677 GO:0046872 GO:0008270 GO:0016151
Cluster-22178.51721 GO:0005509 GO:0005515
Cluster-22178.51722 GO:0006771 GO:0006281 GO:0019497 GO:0005515
GO:0003677 GO:0000166 GO:0005525 GO:0003993 GO:0003924
GO:0016887 GO:0046872 GO:0005524
Cluster-22178.51723 GO:0007017 GO:0007018 GO:0030150 GO:0005524
GO:0008017 GO:0003777 GO:0005742 GO:0045298 GO:0005874
Cluster-22178.51724 GO:0051015
Cluster-22178.51726 GO:0042742 GO:0003779 GO:0005543 GO:0005576
Cluster-22178.51727 GO:0006508 GO:0007264 GO:0007186 GO:0007165
GO:0003924 GO:0005524 GO:0031683 GO:0019001 GO:0004871
GO:0005525 GO:0000166 GO:0003677 GO:0004197
Cluster-22178.51728 GO:0046872
Cluster-22178.51729 GO:0016020
Cluster-22178.51731 GO:0007264 GO:0003924 GO:0016887 GO:0005524
GO:0005525
Cluster-22178.51732 GO:0007264 GO:0005524 GO:0016887 GO:0003924
GO:0005525 GO:0016020
Cluster-22178.51733 GO:0007264 GO:0005525 GO:0003924 GO:0016887
GO:0005524
Cluster-22178.51735 GO:0006886 GO:0015031 GO:0008565 GO:0016740
GO:0016021 GO:0016020 GO:0005737
Cluster-22178.51737 GO:0007165 GO:0006468 GO:0004872 GO:0004672

GO:0005524 GO:0016021
Cluster-22178.51739 GO:0006812 GO:0050909 GO:0005515 GO:0008324
GO:0016021
Cluster-22178.51742 GO:0008608 GO:0072686 GO:0042729
Cluster-22178.51746 GO:0009052 GO:0006098 GO:0015976 GO:0004751
GO:0030246
Cluster-22178.51747 GO:0015940 GO:0015937 GO:0005524 GO:0004594

Cluster-22178.51749 GO:0005515
Cluster-22178.51754 GO:0006633 GO:0055114 GO:0016491 GO:0005506

Cluster-22178.51756 GO:0008152 GO:0006400 GO:0008616 GO:0003824
GO:0008479
Cluster-22178.51757 GO:0006508 GO:0004252 GO:0005515 GO:0005509
GO:0019013 GO:0005576
Cluster-22178.51761 GO:0045444 GO:0006418 GO:0000166 GO:0004812
GO:0005524
Cluster-22178.51762 GO:0016559 GO:0005779
Cluster-22178.51763 GO:0015684 GO:0015093 GO:0005525 GO:0016021
Cluster-22178.51764 GO:0015684 GO:0015093 GO:0005525 GO:0016021
Cluster-22178.51768 GO:0006355 GO:0009607 GO:0003677 GO:0016021
Cluster-22178.51769 GO:0006355 GO:0009607 GO:0003677 GO:0005739
GO:0005747 GO:0016021
Cluster-22178.51771 GO:0006744 GO:0005515 GO:0008270 GO:0046872

Cluster-22178.51772 GO:0016020 GO:0016021
Cluster-22178.51774 GO:0006289 GO:0043130 GO:0004003 GO:0000439
GO:0005657 GO:0005634
Cluster-22178.51775 GO:0055085 GO:0006811 GO:0005216 GO:0016020
Cluster-22178.51777 GO:0016021
Cluster-22178.51778 GO:0016021
Cluster-22178.51784 GO:0006259 GO:0003824 GO:0005515 GO:0003677
GO:0005524 GO:0005694

Cluster-22178.51795 GO:0007165 GO:0007130 GO:0004252 GO:0005096 GO:0016021 GO:0000795

Cluster-22178.51796 GO:0009236 GO:0015992 GO:0055114 GO:0006119 GO:0006118 GO:0016627 GO:0008121 GO:0051073 GO:0008818 GO:0003697 GO:0070188 GO:0005856

Cluster-22178.51798 GO:0055114 GO:0016972 GO:0016627 GO:0003697 GO:0070188 GO:0005856

Cluster-22178.51799 GO:0005789

Cluster-22178.51800 GO:0006635 GO:0006637 GO:0055114 GO:0003997 GO:0005777

Cluster-22178.51801 GO:0046740 GO:0016021

Cluster-22178.51803 GO:0055085 GO:0005741

Cluster-22178.51806 GO:0006886 GO:0008536 GO:0005515

Cluster-22178.51810 GO:0030145 GO:0004177 GO:0016021

Cluster-22178.51812 GO:0006367 GO:0005672

Cluster-22178.51815 GO:0007017 GO:0007018 GO:0005524 GO:0008017 GO:0003777 GO:0045298 GO:0005874

Cluster-22178.51816 GO:0006281 GO:0006310

Cluster-22178.51817 GO:0006310 GO:0006281

Cluster-22178.51821 GO:0007165 GO:0005856

Cluster-22178.51826 GO:0005515

Cluster-22178.51828 GO:0005515 GO:0016021

Cluster-22178.51829 GO:0005509

Cluster-22178.51830 GO:0005509

Cluster-22178.51831 GO:0005515

Cluster-22178.51833 GO:0046872

Cluster-22178.51834 GO:0006629 GO:0005515 GO:0004222 GO:0008270 GO:0005524 GO:0016021

Cluster-22178.51843 GO:0006355 GO:0005634

Cluster-22178.51845 GO:0010508 GO:0008284 GO:0015991 GO:0007009 GO:0001819 GO:0006308 GO:0007264 GO:0008053 GO:0015992 GO:0010884 GO:0000166 GO:0005525 GO:0015078 GO:0003924 GO:0003677 GO:0005524 GO:0016887 GO:0008855 GO:0009318

GO:0033179 GO:0016021 GO:0005741
Cluster-22178.51846 GO:0007165 GO:0005179 GO:0005576
Cluster-22178.51847 GO:0006468 GO:0055085 GO:0004672 GO:0005524
GO:0016773 GO:0022857 GO:0016020 GO:0016021
Cluster-22178.51852 GO:0007017 GO:0006281 GO:0007018 GO:0006260
GO:0006310 GO:0003777 GO:0005515 GO:0003887 GO:0016887
GO:0005524 GO:0008017 GO:0016787 GO:0009378 GO:0016818
GO:0003677 GO:0045298 GO:0005874 GO:0005634 GO:0042575
GO:0009360 GO:0005657 GO:0009379
Cluster-22178.51853 GO:0006352 GO:0006355 GO:0007165 GO:0003700
GO:0016987 GO:0005179 GO:0003677 GO:0019031 GO:0005667
GO:0005576
Cluster-22178.51854 GO:0005515
Cluster-22178.51855 GO:0005985 GO:0005982 GO:0016157
Cluster-22178.51856 GO:0006397 GO:0008152 GO:0003723 GO:0016788
GO:0005515 GO:0005737 GO:0005634
Cluster-22178.51863 GO:0007264 GO:0003924 GO:0005525
Cluster-22178.51864 GO:0042967 GO:0008080 GO:0016747
Cluster-22178.51868 GO:0030683 GO:0055085 GO:0006355 GO:0022857
GO:0043565 GO:0016021 GO:0019012
Cluster-22178.51872 GO:0015979 GO:0005515 GO:0009522 GO:0009538
Cluster-22178.51873 GO:0016310 GO:0009069 GO:0006468 GO:0004674
GO:0004672 GO:0005524
Cluster-22178.51874 GO:0001510 GO:0006306 GO:0031167 GO:0008152
GO:0009452 GO:0008170 GO:0003677 GO:0005515 GO:0008168
GO:0005201 GO:0005578 GO:0019013 GO:0005581 GO:0016021
Cluster-22178.51875 GO:0006886 GO:0006888 GO:0008270 GO:0030127
Cluster-22178.51877 GO:0006570 GO:0006470 GO:0005515 GO:0008138
GO:0004725 GO:0016021
Cluster-22178.51878 GO:0006355 GO:0003676 GO:0003700 GO:0005524
GO:0042025 GO:0005667
Cluster-22178.51879 GO:0005515
Cluster-22178.51880 GO:0006144 GO:0003968 GO:0031379

Cluster-22178.51883 GO:0007186 GO:0007606 GO:0019236 GO:0006629
GO:0004932 GO:0016020
Cluster-22178.51884 GO:0007165 GO:0008601 GO:0000159
Cluster-22178.51886 GO:0007165 GO:0008270 GO:0008601 GO:0000159
Cluster-22178.51887 GO:0007165 GO:0008270 GO:0008601 GO:0000159
Cluster-22178.51889 GO:0005515
Cluster-22178.51891 GO:0006570 GO:0006470 GO:0055114 GO:0004725
GO:0008138 GO:0016491 GO:0016021
Cluster-22178.51892 GO:0006570 GO:0030639 GO:0006470 GO:0009117
GO:0019872 GO:0008138 GO:0004725 GO:0008830 GO:0016021
Cluster-22178.51893 GO:0006508 GO:0008233
Cluster-22178.51894 GO:0001558 GO:0005520 GO:0005515 GO:0005576
GO:0016942
Cluster-22178.51895 GO:0055114 GO:0042157 GO:0006869 GO:0015694
GO:0008289 GO:0050660 GO:0015097 GO:0003677 GO:0016614
GO:0005576 GO:0016020
Cluster-22178.51899 GO:0006508 GO:0005524 GO:0008233 GO:0016021
Cluster-22178.51903 GO:0005515
Cluster-22178.51910 GO:0001558 GO:0006468 GO:0009069 GO:0016310
GO:0004674 GO:0005520 GO:0004672 GO:0005524 GO:0008270
GO:0016942 GO:0005576
Cluster-22178.51912 GO:0043087 GO:0009069 GO:0006952 GO:0007165
GO:0019048 GO:0048870 GO:0016310 GO:0035023 GO:0006468
GO:0005096 GO:0046983 GO:0004674 GO:0005089 GO:0046914
GO:0003779 GO:0005515 GO:0005576 GO:0031514
Cluster-22178.51916 GO:0016567 GO:0008270 GO:0046872 GO:0004842
GO:0005515 GO:0005680
Cluster-22178.51917 GO:0019439 GO:0016833
Cluster-22178.51918 GO:0007160 GO:0005509 GO:0005576
Cluster-22178.51920 GO:0046872
Cluster-22178.51929 GO:0007165 GO:0005515 GO:0008270
Cluster-22178.51931 GO:0006468 GO:0016773 GO:0004672 GO:0005524
GO:0016020

Cluster-22178.51940 GO:0042470
Cluster-22178.51942 GO:0046849 GO:0005576
Cluster-22178.51944 GO:0003950 GO:0005515
Cluster-22178.51945 GO:0005515 GO:0003950
Cluster-22178.51950 GO:0006486 GO:0009097 GO:0009098 GO:0006418
GO:0009099 GO:0007155 GO:0009312 GO:0006438 GO:0000166
GO:0008375 GO:0008455 GO:0098599 GO:0003676 GO:0002161
GO:0003677 GO:0004812 GO:0016787 GO:0005524 GO:0004832
GO:0005795 GO:0016021 GO:0005737
Cluster-22178.51951 GO:0055114 GO:0052855 GO:0016491
Cluster-22178.51953 GO:0009405 GO:0008289
Cluster-22178.51956 GO:0003677 GO:0008270
Cluster-22178.51957 GO:0003676
Cluster-22178.51960 GO:0006355 GO:0005622
Cluster-22178.51961 GO:0007018 GO:0005515 GO:0005868
Cluster-22178.51963 GO:0006355 GO:0000245 GO:0006508 GO:0007165
GO:0008217 GO:0042312 GO:0005179 GO:0005515 GO:0008270
GO:0004222 GO:0005576 GO:0005634 GO:0031012
Cluster-22178.51965 GO:0005515 GO:0008270
Cluster-22178.51967 GO:0016567 GO:0005515 GO:0004842
Cluster-22178.51968 GO:0015991 GO:0015992 GO:0015078 GO:0033180
Cluster-22178.51973 GO:0016579 GO:0036459
Cluster-22178.51974 GO:0006310 GO:0006281 GO:0005515
Cluster-22178.51976 GO:0016579 GO:0036459 GO:0003677
Cluster-22178.51977 GO:0046872 GO:0035091 GO:0005515
Cluster-22178.51978 GO:0006810 GO:0042626
Cluster-22178.51980 GO:0006119 GO:0006118 GO:0000128 GO:0015992
GO:0008121 GO:0009055 GO:0008023
Cluster-22178.51981 GO:0016032 GO:0005096 GO:0019031
Cluster-22178.51983 GO:0006470 GO:0008138
Cluster-22178.51986 GO:0006810 GO:0019871 GO:0003676 GO:0005576
Cluster-22178.51987 GO:0006468 GO:0016773 GO:0005524 GO:0004672
GO:0016020

Cluster-22178.51990 GO:0005975 GO:0004553
Cluster-22178.51994 GO:0005543
Cluster-22178.51998 GO:0008643 GO:0018401 GO:0006525 GO:0015948
GO:0055114 GO:0046656 GO:0006355 GO:0006560 GO:0030269
GO:0005351 GO:0004656 GO:0043565 GO:0016702 GO:0003700
GO:0000139 GO:0005667 GO:0016020 GO:0005783 GO:0016021
Cluster-22178.51999 GO:0016310 GO:0009069 GO:0006468 GO:0004674
GO:0005524 GO:0004672
Cluster-22178.52006 GO:0006468 GO:0019911 GO:0004672 GO:0005524

Cluster-22178.52009 GO:0005515
Cluster-22178.52010 GO:0035023 GO:0015992 GO:0043087 GO:0015986
GO:0015078 GO:0005089 GO:0003677 GO:0000276
Cluster-22178.52011 GO:0006276 GO:0006355 GO:0043565 GO:0003700
GO:0005515 GO:0005667 GO:0016020
Cluster-22178.52012 GO:0015992 GO:0006811 GO:0009405 GO:0006118
GO:0006119 GO:0009055 GO:0005216 GO:0008121 GO:0019904
GO:0005515 GO:0005737 GO:0005576
Cluster-22178.52015 GO:0006813 GO:0006687 GO:0009058 GO:0005242
GO:0008146 GO:0046872 GO:0001733 GO:0008076 GO:0005840
GO:0005794 GO:0016021
Cluster-22178.52016 GO:0006687 GO:0009058 GO:0008146 GO:0001733
GO:0016021 GO:0005794
Cluster-22178.52019 GO:0003676
Cluster-22178.52020 GO:0004386 GO:0003676 GO:0005524
Cluster-22178.52021 GO:0006810 GO:0005524 GO:0016887 GO:0003676
GO:0004386
Cluster-22178.52023 GO:0006955 GO:0030683 GO:0000902 GO:0006811
GO:0005515 GO:0005216 GO:0016020 GO:0019012
Cluster-22178.52024 GO:0000902 GO:0030683 GO:0006955 GO:0007010
GO:0006811 GO:0005216 GO:0005515 GO:0003779 GO:0019012
GO:0016020
Cluster-22178.52027 GO:0019012 GO:0016021

Cluster-22178.52029 GO:0006810 GO:0016021
Cluster-22178.52031 GO:0006821 GO:0055085 GO:0005247 GO:0020037
GO:0016020
Cluster-22178.52032 GO:0009069 GO:0007186 GO:0007165 GO:0016310
GO:0005515 GO:0003924 GO:0031683 GO:0019001 GO:0016772
GO:0004871 GO:0005525 GO:0004674 GO:0016021
Cluster-22178.52033 GO:0016068 GO:0015031 GO:0006606 GO:0008565
GO:0005737 GO:0005634 GO:0016021
Cluster-22178.52034 GO:0051607 GO:0051258 GO:0030168 GO:0007165
GO:0005102 GO:0005525 GO:0030674 GO:0051082 GO:0005577
Cluster-22178.52035 GO:0052331 GO:0006812 GO:0046931 GO:0006952
GO:0007165 GO:0006810 GO:0015267 GO:0005126 GO:0046930
GO:0005576
Cluster-22178.52036 GO:0005975 GO:0004560 GO:0003676 GO:0008270

Cluster-22178.52038 GO:0022904 GO:0016651 GO:0005743
Cluster-22178.52039 GO:0005515 GO:0016020
Cluster-22178.52040 GO:0022904 GO:0016651 GO:0005743
Cluster-22178.52041 GO:0022904 GO:0016651 GO:0005743
Cluster-22178.52042 GO:0000077
Cluster-22178.52043 GO:0007010 GO:0003779
Cluster-22178.52044 GO:0007010 GO:0003779
Cluster-22178.52045 GO:0006213 GO:0006090 GO:0007155 GO:0006525
GO:0071436 GO:0007186 GO:0006814 GO:0006560 GO:0016817
GO:0015081 GO:0004930 GO:0016763 GO:0008948 GO:0016021
GO:0016020
Cluster-22178.52047 GO:0018149 GO:0042710 GO:0008270
Cluster-22178.52048 GO:0042710 GO:0018149 GO:0008270 GO:0019031
Cluster-22178.52049 GO:0042710 GO:0018149 GO:0008270 GO:0019031
Cluster-22178.52051 GO:0019058 GO:0007283 GO:0007165 GO:0008601
GO:0000159
Cluster-22178.52052 GO:0008270 GO:0046872
Cluster-22178.52054 GO:0006812 GO:0007165 GO:0033198 GO:0098655

GO:0001614 GO:0004931 GO:0005887
Cluster-22178.52056 GO:0007165 GO:0006812 GO:0033198 GO:0098655
GO:0004931 GO:0001614 GO:0005887
Cluster-22178.52060 GO:0022904 GO:0046983 GO:0016651 GO:0005743
Cluster-22178.52061 GO:0007275 GO:0016021
Cluster-22178.52064 GO:0003676 GO:0008270 GO:0046872
Cluster-22178.52065 GO:0016567 GO:0008270 GO:0046872 GO:0004842
GO:0003723 GO:0005515 GO:0005887 GO:0005680
Cluster-22178.52069 GO:0007264 GO:0015684 GO:0015093 GO:0003924
GO:0005525 GO:0016021
Cluster-22178.5207 GO:0055085 GO:0007165 GO:0005515 GO:0005509
GO:0004888 GO:0016021
Cluster-22178.52070 GO:0008152 GO:0006338 GO:0003824 GO:0005515
GO:0016836 GO:0090544
Cluster-22178.52073 GO:0000105 GO:0005201 GO:0005581 GO:0005578
Cluster-22178.52074 GO:0010024 GO:0055114 GO:0017022 GO:0003677
GO:0016636 GO:0046983 GO:0050897 GO:0016459
Cluster-22178.52077 GO:0006468 GO:0005524 GO:0004672 GO:0016773
GO:0016020
Cluster-22178.52078 GO:0008270 GO:0005515
Cluster-22178.52082 GO:0006810 GO:0007268 GO:0015893 GO:0007165
GO:0006811 GO:0006855 GO:0055085 GO:0015238 GO:0015297
GO:0004970 GO:0005234 GO:0016020
Cluster-22178.52084 GO:0016117 GO:0006206 GO:0006144 GO:0055114
GO:0006351 GO:0005524 GO:0046872 GO:0016491 GO:0000166
GO:0003677 GO:0016705 GO:0003899 GO:0005198 GO:0003723
GO:0005730
Cluster-22178.52086 GO:0006206 GO:0006144 GO:0055114 GO:0016117
GO:0006351 GO:0005524 GO:0046872 GO:0016491 GO:0003723
GO:0000166 GO:0003677 GO:0016705 GO:0005198 GO:0003899
GO:0005730
Cluster-22178.52092 GO:0010508 GO:0000921 GO:0005515 GO:0005509
GO:0016021 GO:0005940

Cluster-22178.52093 GO:0008615 GO:0055114 GO:0010181 GO:0004733

Cluster-22178.52094 GO:0015876 GO:0006810 GO:0055085 GO:0008521
GO:0005215 GO:0016021 GO:0016020
Cluster-22178.52095 GO:0015876 GO:0055085 GO:0006810 GO:0008521
GO:0005215 GO:0016021 GO:0016020
Cluster-22178.52097 GO:0005975 GO:0005515 GO:0004553
Cluster-22178.52103 GO:0005524 GO:0005515
Cluster-22178.52104 GO:0001503 GO:0007155 GO:0005515 GO:0005524
GO:0005576
Cluster-22178.52105 GO:0005524 GO:0005515
Cluster-22178.52106 GO:0006355 GO:0007165 GO:0006605 GO:0007268
GO:0043495 GO:0005515 GO:0033130 GO:0043565 GO:0030695
Cluster-22178.52111 GO:0015979 GO:0006468 GO:0005524 GO:0004672
GO:0016773 GO:0009539 GO:0009523 GO:0016020
Cluster-22178.52114 GO:0005515
Cluster-22178.52116 GO:0006364 GO:0005515 GO:0032040
Cluster-22178.52117 GO:0016021
Cluster-22178.52119 GO:0006438 GO:0009098 GO:0009097 GO:0009099
GO:0004832 GO:0005524 GO:0000166 GO:0005737
Cluster-22178.52129 GO:0006355 GO:0007617 GO:0003700 GO:0008270
GO:0005576 GO:0005667 GO:0005634
Cluster-22178.52135 GO:0003676 GO:0004190 GO:0005923 GO:0016021
Cluster-22178.52137 GO:0006457 GO:0000413 GO:0003755
Cluster-22178.52138 GO:0006468 GO:0019911 GO:0004672 GO:0005524
GO:0016773 GO:0016020
Cluster-22178.52141 GO:0000226 GO:0000910 GO:0006367 GO:0015743
GO:0032968 GO:0008017 GO:0003677 GO:0004190 GO:0045298
GO:0016021 GO:0005634
Cluster-22178.52142 GO:0019872 GO:0006021 GO:0008654 GO:0004512

Cluster-22178.52143 GO:0016055 GO:0007275 GO:0007165 GO:0005102
GO:0005515 GO:0005509 GO:0005576 GO:0045095

Cluster-22178.52146 GO:0055085 GO:0006865 GO:0003333 GO:0006810
GO:0015171 GO:0016021 GO:0016020
Cluster-22178.52147 GO:0006605 GO:0007165 GO:0006468 GO:0005515
GO:0005524 GO:0004672
Cluster-22178.52148 GO:0005515
Cluster-22178.52153 GO:0006850 GO:0055114 GO:0016491 GO:0031418
GO:0005506 GO:0016706 GO:0005743
Cluster-22178.52158 GO:0031047 GO:0003723 GO:0019012 GO:0016021
Cluster-22178.52160 GO:0006310 GO:0000150 GO:0003677
Cluster-22178.52161 GO:0055085 GO:0006351 GO:0006810 GO:0006468
GO:0005215 GO:0005524 GO:0004672 GO:0016773 GO:0005515
GO:0016021 GO:0016020
Cluster-22178.52163 GO:0000077 GO:0071479 GO:0005515 GO:0005634
Cluster-22178.52164 GO:0000077 GO:0071479 GO:0005515 GO:0005634
Cluster-22178.52166 GO:0006355 GO:0003677 GO:0046872
Cluster-22178.52169 GO:0006355 GO:0007165 GO:0042974 GO:0005667
Cluster-22178.52170 GO:0007165 GO:0006281 GO:0006464 GO:0005179
GO:0016151 GO:0046872 GO:0008270 GO:0005576 GO:0005742
Cluster-22178.52172 GO:0015991 GO:0006351 GO:0055114 GO:0006090
GO:0006206 GO:0015992 GO:0015031 GO:0006144 GO:0006633
GO:0003899 GO:0015078 GO:0003677 GO:0003989 GO:0047134
GO:0033179 GO:0016021 GO:0005730 GO:0016020 GO:0009317
Cluster-22178.52173 GO:0006090 GO:0055114 GO:0006351 GO:0015991
GO:0006633 GO:0006144 GO:0006206 GO:0015992 GO:0015031
GO:0015078 GO:0003899 GO:0047134 GO:0003989 GO:0003677
GO:0016021 GO:0033179 GO:0009317 GO:0016020 GO:0005730
Cluster-22178.52175 GO:0006812 GO:0016747 GO:0008324 GO:0005515

Cluster-22178.52177 GO:0055114 GO:0020037 GO:0016705 GO:0005506

Cluster-22178.52181 GO:0016310 GO:0006468 GO:0009069 GO:0001558
GO:0005520 GO:0005524 GO:0004672 GO:0008270 GO:0004674
GO:0016942 GO:0005576 GO:0016020

Cluster-22178.52189 GO:0006554 GO:0034968 GO:0007165 GO:0055114
GO:0006355 GO:0006629 GO:0006479 GO:0006486 GO:0051920
GO:0005044 GO:0003677 GO:0005507 GO:0018024 GO:0016641
GO:0008375 GO:0005622 GO:0005694 GO:0016020 GO:0005763
Cluster-22178.52190 GO:0055114 GO:0006479 GO:0006486 GO:0006629
GO:0006355 GO:0006554 GO:0007165 GO:0034968 GO:0005507
GO:0008375 GO:0018024 GO:0016641 GO:0003677 GO:0051920
GO:0005044 GO:0005622 GO:0016020 GO:0005763 GO:0005694
Cluster-22178.52193 GO:0055085 GO:0034314 GO:0030833 GO:0006821
GO:0006364 GO:0005247 GO:0015629 GO:0005730 GO:0016020
GO:0005885
Cluster-22178.52196 GO:0005975 GO:0004415 GO:0005515
Cluster-22178.52209 GO:0006554 GO:0016310 GO:0035556 GO:0006468
GO:0009069 GO:0006479 GO:0005524 GO:0004672 GO:0004674
GO:0018024 GO:0017048 GO:0005615
Cluster-22178.52214 GO:0006260 GO:0015684 GO:0006508 GO:0007264
GO:0005525 GO:0004222 GO:0003924 GO:0005524 GO:0015093
GO:0003677 GO:0016021
Cluster-22178.52215 GO:0003676
Cluster-22178.52217 GO:0006801 GO:0055114 GO:0004784 GO:0046872

Cluster-22178.52218 GO:0003676 GO:0003723
Cluster-22178.52219 GO:0006281 GO:0006355 GO:0006308 GO:0000077
GO:0005515 GO:0008270 GO:0000014 GO:0003700 GO:0003676
GO:0005634 GO:0005667
Cluster-22178.52220 GO:0006259 GO:0000166 GO:0003677 GO:0003824
GO:0005524 GO:0005694
Cluster-22178.52223 GO:0015992 GO:0043066 GO:0015986 GO:0045766
GO:0016567 GO:0043123 GO:0043130 GO:0004842 GO:0015078
GO:0003723 GO:0000276
Cluster-22178.52225 GO:0002062 GO:0042246 GO:0035988 GO:0003676
GO:0005634
Cluster-22178.52229 GO:0006810 GO:0006351 GO:0006468 GO:0055085

GO:0005515 GO:0005215 GO:0005524 GO:0004672 GO:0016020
GO:0016021
Cluster-22178.52231 GO:0045893 GO:0005515 GO:0005634
Cluster-22178.52232 GO:0007217
Cluster-22178.52233 GO:0006355 GO:0006810 GO:0055085 GO:0008270
GO:0003700 GO:0005215 GO:0005634 GO:0005667 GO:0016020
Cluster-22178.52234 GO:0003677
Cluster-22178.52236 GO:0006355 GO:0003700 GO:0005525 GO:0003924
GO:0043565 GO:0005667 GO:0016021 GO:0012511
Cluster-22178.52237 GO:0006355 GO:0043565 GO:0003924 GO:0005525
GO:0003700 GO:0005667 GO:0012511 GO:0016021
Cluster-22178.52238 GO:0006771 GO:0006000 GO:0015074 GO:0019497
GO:0006013 GO:0003873 GO:0005524 GO:0003993
Cluster-22178.52239 GO:0008152 GO:0016788
Cluster-22178.52240 GO:0007218 GO:0005576
Cluster-22178.52242 GO:0005515
Cluster-22178.52246 GO:0007186 GO:0005975 GO:0003723 GO:0016853
GO:0046983 GO:0004930 GO:0003676 GO:0016021
Cluster-22178.52248 GO:0016032 GO:0016887 GO:0005524 GO:0005525
GO:0019013
Cluster-22178.52250 GO:0015031 GO:0016021
Cluster-22178.52251 GO:0005515
Cluster-22178.52253 GO:0007165 GO:0004872 GO:0016021
Cluster-22178.52254 GO:0006144 GO:0006508 GO:0019229 GO:0016817
GO:0070008 GO:0003968 GO:0017111 GO:0004252 GO:0004197
GO:0031379 GO:0005576
Cluster-22178.52257 GO:0019076 GO:0006352 GO:0006355 GO:0006886
GO:0006505 GO:0046718 GO:0075521 GO:0007165 GO:0003700
GO:0098599 GO:0016788 GO:0016987 GO:0005179 GO:0016787
GO:0003677 GO:0019031 GO:0005576 GO:0019028 GO:0042025
GO:0005667 GO:0044163 GO:0030430
Cluster-22178.52259 GO:0005975 GO:0004571 GO:0005509 GO:0016020
Cluster-22178.52260 GO:0005524 GO:0016787 GO:0003677 GO:0008017

GO:0003676 GO:0045298
Cluster-22178.52261 GO:0051260 GO:0005515
Cluster-22178.52262 GO:0007186 GO:0007165 GO:0007264 GO:0004871
GO:0005525 GO:0003924 GO:0019001 GO:0031683
Cluster-22178.52263 GO:0009228 GO:0033617 GO:0004789 GO:0005739
Cluster-22178.52265 GO:0055114 GO:0008152 GO:0051287 GO:0016616

Cluster-22178.52266 GO:0005975 GO:0004553
Cluster-22178.52267 GO:0006812 GO:0006278 GO:0006836 GO:0030623
GO:0005328 GO:0003964 GO:0016021
Cluster-22178.52269 GO:0030474 GO:0006886 GO:0000724 GO:0016197
GO:0006308 GO:0006457 GO:0006810 GO:0071988 GO:0007155
GO:0006914 GO:0019064 GO:0042803 GO:0003676 GO:0051087
GO:0008855 GO:0000774 GO:0030119 GO:0009318 GO:0016021
GO:0019031 GO:0009288 GO:0005783
Cluster-22178.52275 GO:0007275 GO:0005634
Cluster-22178.52278 GO:0007411 GO:0000045 GO:0005737
Cluster-22178.52279 GO:0007264 GO:0005515 GO:0005525 GO:0005096

Cluster-22178.52283 GO:0015979 GO:0046872
Cluster-22178.52284 GO:0055114 GO:0006006 GO:0006749 GO:0006098
GO:0004345 GO:0050661
Cluster-22178.52285 GO:0006493 GO:0000030 GO:0000136 GO:0016020
Cluster-22178.52286 GO:0002682 GO:0030246
Cluster-22178.52288 GO:0016772 GO:0005524
Cluster-22178.52293 GO:0008152 GO:0055114 GO:0016491
Cluster-22178.52295 GO:0055085 GO:0016020 GO:0016021
Cluster-22178.52296 GO:0003723 GO:0005515
Cluster-22178.52297 GO:0019872 GO:0008654 GO:0006355 GO:0006021
GO:0043565 GO:0004512 GO:0003700 GO:0005667
Cluster-22178.52304 GO:0061630 GO:0005515 GO:0003723 GO:0008270
GO:0046872
Cluster-22178.52307 GO:0007264 GO:0005525

Cluster-22178.52309 GO:0000902
Cluster-22178.52312 GO:0006814 GO:0006118 GO:0007165 GO:0015992 GO:0006744 GO:0006120 GO:0003954 GO:0008137 GO:0005739
Cluster-22178.52314 GO:0006508 GO:0006629 GO:0006886 GO:0015996 GO:0001514 GO:0015994 GO:0006505 GO:0006448 GO:0003723 GO:0008236 GO:0016788 GO:0047746 GO:0005525 GO:0016787 GO:0003746 GO:0005576 GO:0005737 GO:0005840
Cluster-22178.52316 GO:0007165 GO:0030155 GO:0055085 GO:0008053 GO:0030334 GO:0019064 GO:0030001 GO:0045995 GO:0006265 GO:0003677 GO:0046873 GO:0005102 GO:0003918 GO:0003917 GO:0046789 GO:0005198 GO:0003924 GO:0005741 GO:0016020 GO:0005694 GO:0009986 GO:0019028 GO:0019031 GO:0016021
Cluster-22178.52328 GO:0007155 GO:0042157 GO:0006869 GO:0006367 GO:0015684 GO:0051726 GO:0006281 GO:0009432 GO:0005524 GO:0015093 GO:0016887 GO:0003697 GO:0017025 GO:0005525 GO:0008289 GO:0009289 GO:0005576 GO:0005672 GO:0005634 GO:0016021
Cluster-22178.52329 GO:0006281 GO:0008270 GO:0046872 GO:0005815
Cluster-22178.52330 GO:0016310 GO:0009401 GO:0030474 GO:0071988 GO:0007165 GO:0009069 GO:0006468 GO:0004674 GO:0005096 GO:0008376 GO:0051082 GO:0005815 GO:0032580
Cluster-22178.52331 GO:0055085 GO:0006468 GO:0006810 GO:0006351 GO:0016773 GO:0005524 GO:0004672 GO:0005215 GO:0005515 GO:0016021 GO:0016020
Cluster-22178.52334 GO:0006888 GO:0006810 GO:0009405 GO:0006886 GO:0008270 GO:0005524 GO:0019855 GO:0003677 GO:0000166 GO:0005576 GO:0030127
Cluster-22178.52335 GO:0006508 GO:0008270 GO:0004222 GO:0031012 GO:0016020
Cluster-22178.52337 GO:0016310 GO:0009069 GO:0004674 GO:0004185 GO:0005515 GO:0005773
Cluster-22178.52339 GO:0005515
Cluster-22178.52342 GO:0055114 GO:0016491

Cluster-22178.52348 GO:0006260 GO:0006914 GO:0003887 GO:0001882
GO:0008092 GO:0042575 GO:0019898 GO:0005737
Cluster-22178.52349 GO:0055085 GO:0016021
Cluster-22178.52351 GO:0015979 GO:0006260 GO:0009539 GO:0009523
GO:0016020
Cluster-22178.52354 GO:0006281 GO:0006310 GO:0005515
Cluster-22178.52355 GO:0006310 GO:0006281 GO:0005515
Cluster-22178.52356 GO:0005515
Cluster-22178.52360 GO:0008283 GO:0040007 GO:0007165 GO:0008083
GO:0003676
Cluster-22178.52362 GO:0045227 GO:0004252 GO:0016020 GO:0016021
Cluster-22178.52364 GO:0035556 GO:0016055 GO:0008013 GO:0016342
Cluster-22178.52368 GO:0006355 GO:0008134 GO:0005524 GO:0044599
GO:0005667
Cluster-22178.52370 GO:0006388 GO:0003950 GO:0016772
Cluster-22178.52372 GO:0003676
Cluster-22178.52374 GO:0006355 GO:0005515
Cluster-22178.52375 GO:0043087 GO:0007264 GO:0005085
Cluster-22178.52376 GO:0006123 GO:0015992 GO:0005515 GO:0004129
GO:0045277
Cluster-22178.52385 GO:0055085 GO:0046872 GO:0008270 GO:0005515
GO:0016021
Cluster-22178.52387 GO:0006144 GO:0002098 GO:0034227 GO:0046034
GO:0006468 GO:0005524 GO:0008270 GO:0004017 GO:0003677
GO:0016787 GO:0003676 GO:0046872 GO:0004672 GO:0020037
GO:0000049 GO:0005634
Cluster-22178.52389 GO:0006144 GO:0007165 GO:0004114 GO:0003677

Cluster-22178.52390 GO:0006825 GO:0035434 GO:0005375 GO:0016021
Cluster-22178.52392 GO:0008152 GO:0003824
Cluster-22178.52395 GO:0016020
Cluster-22178.52396 GO:0042254 GO:0006412 GO:0051726 GO:0006281
GO:0003735 GO:0003677 GO:0004518 GO:0017025 GO:0003824

GO:0005096 GO:0005634 GO:0005840 GO:0005622
Cluster-22178.52397 GO:0007264 GO:0043087 GO:0005085
Cluster-22178.52398 GO:0005975 GO:0071704 GO:0016868 GO:0005515

Cluster-22178.52409 GO:0035556
Cluster-22178.52410 GO:0035556
Cluster-22178.52411 GO:0008033 GO:0007165 GO:0016879 GO:0005524
GO:0000166 GO:0005737
Cluster-22178.52412 GO:0006952 GO:0009405 GO:0006810 GO:0019855
GO:0033897 GO:0003723 GO:0016020 GO:0005576
Cluster-22178.52421 GO:0050852 GO:0050853 GO:0048193 GO:0016787
GO:0045502 GO:0008134 GO:0042803 GO:0005198 GO:0005515
GO:0003774 GO:0016459 GO:0005667 GO:0005882 GO:0016020
GO:0030286
Cluster-22178.52424 GO:0006355 GO:0005198 GO:0005515 GO:0043565
GO:0005524 GO:0003774 GO:0003700 GO:0005667 GO:0016459
GO:0005882
Cluster-22178.52429 GO:0048193 GO:0050853 GO:0050852 GO:0003774
GO:0005524 GO:0016787 GO:0003779 GO:0005198 GO:0005515
GO:0042803 GO:0008134 GO:0045502 GO:0030286 GO:0016020
GO:0005882 GO:0005667 GO:0016459
Cluster-22178.52430 GO:0009405 GO:0005198
Cluster-22178.52431 GO:0008283 GO:0040007 GO:0007165 GO:0006955
GO:0005138 GO:0005125 GO:0005576 GO:0005896
Cluster-22178.52433 GO:0055114 GO:0008033 GO:0004499 GO:0016491
GO:0050661 GO:0050660
Cluster-22178.52438 GO:0008270
Cluster-22178.52440 GO:0008270
Cluster-22178.52443 GO:0007155 GO:0042967 GO:0016747 GO:0005515
GO:0008080 GO:0016021
Cluster-22178.52445 GO:0006508 GO:0004197
Cluster-22178.52453 GO:0006368 GO:0005975 GO:0006406 GO:0005524
GO:0016773 GO:0000446

Cluster-22178.52456 GO:0000917 GO:0015991 GO:0006308 GO:0007165
GO:0006810 GO:0043093 GO:0006606 GO:0008053 GO:0015992
GO:0042802 GO:0015078 GO:0003924 GO:0043015 GO:0008855
GO:0009318 GO:0033179 GO:0019028 GO:0016021 GO:0045298
GO:0005737 GO:0005741 GO:0005886
Cluster-22178.52458 GO:0006952 GO:0009058 GO:0043565 GO:0003824
GO:0009279 GO:0005634
Cluster-22178.52463 GO:0006355 GO:0003700 GO:0043565 GO:0005667
GO:0005634
Cluster-22178.52465 GO:0003682 GO:0005515 GO:0000785
Cluster-22178.52470 GO:0006468 GO:0055114 GO:0004497 GO:0016706
GO:0005507 GO:0016715 GO:0008168 GO:0004672 GO:0005524
Cluster-22178.52471 GO:0005975 GO:0055114 GO:0016491
Cluster-22178.52482 GO:0006269 GO:0006351 GO:0006260 GO:0003896
GO:0005730 GO:0005657
Cluster-22178.52485 GO:0015743
Cluster-22178.52487 GO:0016310 GO:0006886 GO:0006468 GO:0015991
GO:0006260 GO:0015992 GO:0015031 GO:0006914 GO:0009069
GO:0015078 GO:0005515 GO:0003723 GO:0004674 GO:0005524
GO:0008565 GO:0005737 GO:0033179 GO:0016020
Cluster-22178.52488 GO:0005515
Cluster-22178.52492 GO:0006508 GO:0035556 GO:0004197 GO:0005515
GO:0008234
Cluster-22178.52495 GO:0005515
Cluster-22178.52500 GO:0051259 GO:0007034 GO:0006457 GO:0047869
GO:0000774 GO:0051087 GO:0042803
Cluster-22178.52501 GO:0018142 GO:0006269 GO:0009058 GO:0007165
GO:0006810 GO:0006260 GO:0003723 GO:0005215 GO:0016020
Cluster-22178.52502 GO:0006260 GO:0009231 GO:0003678 GO:0008686
GO:0005524 GO:0016887 GO:0005657 GO:0033588
Cluster-22178.52505 GO:0016021
Cluster-22178.52506 GO:0007165 GO:0005179 GO:0005576 GO:0016021
Cluster-22178.52508 GO:0046999 GO:0009451 GO:0051536 GO:0051539

GO:0003824 GO:0019867
Cluster-22178.52509 GO:0046999 GO:0009451 GO:0051536 GO:0003824
GO:0051539 GO:0019867
Cluster-22178.52511 GO:0006464
Cluster-22178.52512 GO:0006464
Cluster-22178.52516 GO:0042967 GO:0009401 GO:0008080
Cluster-22178.52519 GO:0043066 GO:0035444 GO:0015675 GO:0045766
GO:0043123 GO:0004222 GO:0015099 GO:0046872 GO:0016021
Cluster-22178.52524 GO:0040007 GO:0008283 GO:0007165 GO:0008083
GO:0003676
Cluster-22178.52526 GO:0007154 GO:0006812 GO:0016887 GO:0016021
Cluster-22178.52533 GO:0006144 GO:0006206 GO:0006241 GO:0006508
GO:0006165 GO:0006228 GO:0006355 GO:0006183 GO:0006886
GO:0004550 GO:0008536 GO:0004222 GO:0005634
Cluster-22178.52534 GO:0015979 GO:0031175 GO:0008017 GO:0030507
GO:0005516 GO:0045298 GO:0008091 GO:0009523 GO:0009539
GO:0016020
Cluster-22178.52537 GO:0006144 GO:0009190 GO:0006171 GO:0035556
GO:0016849 GO:0004016 GO:0005886
Cluster-22178.52539 GO:0005515 GO:0046872 GO:0045202 GO:0016021
Cluster-22178.52540 GO:0006510 GO:0006508 GO:0055114 GO:0004252
GO:0016829 GO:0004176 GO:0016491
Cluster-22178.52542 GO:0007264 GO:0005525
Cluster-22178.52543 GO:0009306 GO:0016192 GO:0016020
Cluster-22178.52544 GO:0016192 GO:0009306 GO:0016020
Cluster-22178.52546 GO:0006355 GO:0043565 GO:0046872
Cluster-22178.52547 GO:0046339 GO:0009395 GO:0016042 GO:0007021
GO:0010124 GO:0007275 GO:0046872 GO:0004435 GO:0015631
GO:0051082 GO:0005509 GO:0045298
Cluster-22178.52548 GO:0007275 GO:0007165 GO:0006886 GO:0016192
GO:0016055 GO:0015991 GO:0015992 GO:0005515 GO:0015078
GO:0005102 GO:0033179 GO:0005576 GO:0016021
Cluster-22178.52553 GO:0006979 GO:0055114 GO:0006804 GO:0006118

GO:0006749 GO:0016655 GO:0004602 GO:0071203
Cluster-22178.52560 GO:0042742 GO:0005515
Cluster-22178.52561 GO:0006144 GO:0006206 GO:0006383 GO:0006351
GO:0003899 GO:0003677 GO:0005730 GO:0005666
Cluster-22178.52562 GO:0006508 GO:0006144 GO:0004197 GO:0017111
GO:0004252 GO:0003968 GO:0031379
Cluster-22178.52566 GO:0006468 GO:0005524 GO:0004672
Cluster-22178.52567 GO:0006468 GO:0005524 GO:0004672
Cluster-22178.52568 GO:0005975 GO:0005515 GO:0004415
Cluster-22178.52576 GO:0051028 GO:0006406 GO:0050765 GO:0006810
GO:0005515 GO:0003723 GO:0005622 GO:0005737 GO:0005634
Cluster-22178.52577 GO:0007165 GO:0006479 GO:0034968 GO:0019953
GO:0006554 GO:0005515 GO:0018024 GO:0008270 GO:0000772
GO:0005634 GO:0005576
Cluster-22178.52578 GO:0007009 GO:0005515 GO:0005737
Cluster-22178.52585 GO:0006355 GO:0003924 GO:0043565 GO:0003700
GO:0005525 GO:0005667 GO:0016021 GO:0012511
Cluster-22178.52587 GO:0006094 GO:0006096 GO:0009094 GO:0006571
GO:0000162 GO:0000287 GO:0004634 GO:0000015
Cluster-22178.52592 GO:0006886 GO:0008536
Cluster-22178.52593 GO:0006886 GO:0008536
Cluster-22178.52594 GO:0000398 GO:0005681
Cluster-22178.52597 GO:0031556 GO:0031554
Cluster-22178.52598 GO:0007165 GO:0019856 GO:0006955 GO:0034229
GO:0005164 GO:0034228 GO:0016020 GO:0016021
Cluster-22178.52601 GO:0000723 GO:0003697 GO:0003676
Cluster-22178.52604 GO:0007186 GO:0004930 GO:0003677 GO:0016021
Cluster-22178.52605 GO:0007186 GO:0004930 GO:0016021
Cluster-22178.52606 GO:0006468 GO:0007186 GO:0055114 GO:0016614
GO:0005524 GO:0004672 GO:0004930 GO:0016021
Cluster-22178.52607 GO:0006338 GO:0008152 GO:0016836 GO:0005515
GO:0003824 GO:0090544
Cluster-22178.52608 GO:0006468 GO:0005524 GO:0004672 GO:0005515

Cluster-22178.5261 GO:0007165 GO:0005515 GO:0005737
Cluster-22178.52610 GO:0006725 GO:0016853 GO:0055036
Cluster-22178.52611 GO:0006725 GO:0016853 GO:0055036
Cluster-22178.52613 GO:0007275 GO:0006511 GO:0016787 GO:0003677 GO:0005515 GO:0046872 GO:0005524 GO:0008270 GO:0005634
Cluster-22178.52617 GO:0005515
Cluster-22178.52618 GO:0046497 GO:0006769 GO:0055114 GO:0008750 GO:0050661 GO:0016021
Cluster-22178.52622 GO:0042802 GO:0043015 GO:0008017 GO:0045298
Cluster-22178.52628 GO:0006810 GO:0005524 GO:0042626 GO:0016021
Cluster-22178.52631 GO:0006364
Cluster-22178.52636 GO:0016310 GO:0009069 GO:0006468 GO:0004674 GO:0005524 GO:0004672
Cluster-22178.52644 GO:0006810 GO:0042626 GO:0005524 GO:0016021
Cluster-22178.52646 GO:0006869 GO:0005319 GO:0016831
Cluster-22178.52648 GO:0051382 GO:0006281 GO:0016055
Cluster-22178.52652 GO:0006886 GO:0016192 GO:0005515 GO:0008270 GO:0030117
Cluster-22178.52655 GO:0043165 GO:0005509 GO:0019867
Cluster-22178.52656 GO:0009405
Cluster-22178.52659 GO:0007165 GO:0008289
Cluster-22178.52660 GO:0007165 GO:0008289
Cluster-22178.52662 GO:0016820 GO:0005739 GO:0016021
Cluster-22178.52665 GO:0030833 GO:0034314 GO:0015629 GO:0005885
Cluster-22178.52668 GO:0005515
Cluster-22178.52669 GO:0005515
Cluster-22178.52670 GO:0051056 GO:0019048 GO:0006355 GO:0005515 GO:0003677 GO:0005096
Cluster-22178.52679 GO:0045454 GO:0005515
Cluster-22178.52681 GO:0006355 GO:0003700 GO:0005622 GO:0005667
Cluster-22178.52685 GO:0007339 GO:0006771 GO:0019497 GO:0005515 GO:0003993 GO:0046872 GO:0016020 GO:0005576

Cluster-22178.52686 GO:0019049 GO:0005515
Cluster-22178.52695 GO:0045454 GO:0006118 GO:0015035 GO:0009055

Cluster-22178.52696 GO:0051382 GO:0048870 GO:0006355 GO:0009405
GO:0000917 GO:0010508 GO:0055085 GO:0043093 GO:0006810
GO:0003700 GO:0045502 GO:0008134 GO:0042803 GO:0019871
GO:0005737 GO:0016021 GO:0000776 GO:0005576 GO:0031514
GO:0030286 GO:0005667 GO:0016020
Cluster-22178.52705 GO:0007049 GO:0008171 GO:0005634
Cluster-22178.52707 GO:0008152 GO:0008168 GO:0005634
Cluster-22178.52709 GO:0008171 GO:0005634
Cluster-22178.52711 GO:0008152 GO:0008168
Cluster-22178.52712 GO:0006355 GO:0007049 GO:0003700 GO:0005667
Cluster-22178.52714 GO:0016567 GO:0007165 GO:0006952 GO:0006810
GO:0046872 GO:0008270 GO:0004842 GO:0019239 GO:0005179
GO:0005515 GO:0030288 GO:0005680 GO:0005576
Cluster-22178.52715 GO:0006810 GO:0016567 GO:0008270 GO:0046872
GO:0004842 GO:0005515 GO:0005680 GO:0030288
Cluster-22178.52717 GO:0006810 GO:0016567 GO:0005515 GO:0046872
GO:0008270 GO:0004842 GO:0005680 GO:0030288
Cluster-22178.52719 GO:0055114 GO:0016491 GO:0016209
Cluster-22178.52722 GO:0042254 GO:0006412 GO:0035556 GO:0005515
GO:0003735 GO:0015934 GO:0005840
Cluster-22178.52723 GO:0046872
Cluster-22178.52724 GO:0071705 GO:0003676 GO:0045095
Cluster-22178.52726 GO:0051016 GO:0008290
Cluster-22178.52728 GO:0009405 GO:0016567 GO:0004842 GO:0005515

Cluster-22178.52729 GO:0006468 GO:0004672 GO:0005524
Cluster-22178.52730 GO:0000103 GO:0055085 GO:0006144 GO:0006810
GO:0006355 GO:0006468 GO:0042626 GO:0004020 GO:0016887
GO:0005524 GO:0004672 GO:0008134 GO:0016301 GO:0016021
GO:0005667

Cluster-22178.52733 GO:0006576 GO:0005525 GO:0005524 GO:0003924

Cluster-22178.52736 GO:0032183 GO:0070140
Cluster-22178.52737 GO:0016579 GO:0032183 GO:0070140 GO:0036459

Cluster-22178.52738 GO:0042254 GO:0005515 GO:0005730 GO:0016020 GO:0016021
Cluster-22178.52740 GO:0055114 GO:0006357 GO:0008013 GO:0016702 GO:0001104 GO:0016342 GO:0016592
Cluster-22178.52742 GO:0035023 GO:0043087 GO:0005089 GO:0003677 GO:0034061 GO:0005634
Cluster-22178.52746 GO:0051301 GO:0005515 GO:0003677
Cluster-22178.52747 GO:0006090 GO:0008152 GO:0006099 GO:0008924 GO:0005515 GO:0016829 GO:0016021
Cluster-22178.52748 GO:0006090 GO:0008152 GO:0006099 GO:0005515 GO:0008924 GO:0016829 GO:0016021
Cluster-22178.52750 GO:0016310 GO:0006260 GO:0007131 GO:0007165 GO:0010508 GO:0006468 GO:0009069 GO:0016032 GO:0004674 GO:0003676 GO:0005096 GO:0005515 GO:0005815 GO:0000794
Cluster-22178.52752 GO:0032402 GO:0042470
Cluster-22178.52754 GO:0055114 GO:0003676 GO:0016491 GO:0003723

Cluster-22178.52755 GO:0003723 GO:0003676
Cluster-22178.52759 GO:0005515
Cluster-22178.52761 GO:0030168 GO:0006606 GO:0055085 GO:0006810 GO:0007165 GO:0006457 GO:0051258 GO:0007049 GO:0009405 GO:0010508 GO:0005102 GO:0030674 GO:0005215 GO:0051082 GO:0045502 GO:0042803 GO:0008134 GO:0005198 GO:0030286 GO:0016272 GO:0005577 GO:0005667 GO:0005882 GO:0016020 GO:0019031 GO:0005578 GO:0019028
Cluster-22178.52762 GO:0055085 GO:0006810 GO:0022857 GO:0005215 GO:0005525 GO:0003924 GO:0046872 GO:0005515 GO:0004045 GO:0016021 GO:0016020 GO:0016012

Cluster-22178.52763 GO:0005515
Cluster-22178.52764 GO:0015979 GO:0005515 GO:0009522
Cluster-22178.52766 GO:0000079 GO:0019901
Cluster-22178.52767 GO:0051382 GO:0006914 GO:0006606 GO:0000776
GO:0031083 GO:0016021
Cluster-22178.52770 GO:0005515
Cluster-22178.52772 GO:0015979 GO:0009539 GO:0009523
Cluster-22178.52773 GO:0006189 GO:0030255 GO:0006468 GO:0006355
GO:0035556 GO:0003677 GO:0005515 GO:0004672 GO:0005524
GO:0016773 GO:0005668 GO:0016020 GO:0005634
Cluster-22178.52779 GO:0005576
Cluster-22178.52780 GO:0006480 GO:0006118 GO:0045454 GO:0009055
GO:0015035 GO:0005515 GO:0008168
Cluster-22178.52781 GO:0006468 GO:0004672 GO:0005524 GO:0008168
GO:0005515
Cluster-22178.52784 GO:0003824
Cluster-22178.52785 GO:0003824 GO:0005515
Cluster-22178.52788 GO:0006032 GO:0008061
Cluster-22178.52793 GO:0006508 GO:0008233 GO:0004222 GO:0008270
GO:0031012
Cluster-22178.52795 GO:0005515
Cluster-22178.52796 GO:0006353 GO:0016758
Cluster-22178.52798 GO:0006508 GO:0004222 GO:0004659 GO:0005515
GO:0008270 GO:0016021 GO:0031012
Cluster-22178.52802 GO:0009190 GO:0035556 GO:0016849 GO:0042802

Cluster-22178.52813 GO:0046486 GO:0016042 GO:0003924 GO:0004806

Cluster-22178.52815 GO:0016579 GO:0036459
Cluster-22178.52816 GO:0009451 GO:0008033 GO:0046872 GO:0009020

Cluster-22178.52817 GO:0008033 GO:0009451 GO:0046872 GO:0009020

Cluster-22178.52818 GO:0042254 GO:0006413 GO:0005515 GO:0005509
GO:0005622
Cluster-22178.52822 GO:0006367 GO:0000398 GO:0005681 GO:0005672
Cluster-22178.52823 GO:0006118 GO:0019836 GO:0009055 GO:0009512
GO:0005576
Cluster-22178.52825 GO:0015979 GO:0015031 GO:0055114 GO:0030145
GO:0016972 GO:0008270 GO:0016817 GO:0008565 GO:0016021
GO:0009523
Cluster-22178.52827 GO:0009271 GO:0006355 GO:0003677 GO:0005515
GO:0005524 GO:0016787 GO:0005639
Cluster-22178.52829 GO:0008152 GO:0003824
Cluster-22178.52833 GO:0006400 GO:0046500 GO:0000154 GO:0006364
GO:0006479 GO:0009451 GO:0006464 GO:0008033 GO:0006396
GO:0008176 GO:0003723 GO:0004719 GO:0008168 GO:0008649
GO:0005737
Cluster-22178.52834 GO:0006464 GO:0008033 GO:0006396 GO:0006400
GO:0046500 GO:0000154 GO:0006479 GO:0006364 GO:0009451
GO:0004719 GO:0008168 GO:0008649 GO:0008176 GO:0003723
GO:0005737
Cluster-22178.52835 GO:0016310 GO:0007178 GO:0006468 GO:0009069
GO:0005524 GO:0004672 GO:0016773 GO:0004675 GO:0016020
Cluster-22178.52836 GO:0022904 GO:0003676 GO:0008017 GO:0016020
GO:0045298
Cluster-22178.52838 GO:0022904 GO:0003676 GO:0008017 GO:0045298
GO:0016020
Cluster-22178.52841 GO:0016567 GO:0008270 GO:0046872 GO:0005515
GO:0061630 GO:0004842 GO:0005622
Cluster-22178.52846 GO:0006355 GO:0005524 GO:0003774 GO:0008134
GO:0016301 GO:0005515 GO:0016459 GO:0005667
Cluster-22178.52848 GO:0055114 GO:0006118 GO:0016627 GO:0003995

Cluster-22178.52850 GO:0006355 GO:0005524 GO:0003774 GO:0005515
GO:0005856 GO:0016459 GO:0009289

Cluster-22178.52853 GO:0006465 GO:0015992 GO:0042254 GO:0006412
GO:0008233 GO:0003735 GO:0003677 GO:0016021 GO:0005840
GO:0005622 GO:0005787
Cluster-22178.52855 GO:0009405 GO:0008146 GO:0005576
Cluster-22178.52856 GO:0007165 GO:0046008 GO:0005179 GO:0005576
Cluster-22178.52857 GO:0008289
Cluster-22178.52858 GO:0046008 GO:0007165 GO:0005179 GO:0005576
Cluster-22178.52863 GO:0042254 GO:0006412 GO:0046872 GO:0003735
GO:0005622 GO:0005840
Cluster-22178.52864 GO:0042549 GO:0015979 GO:0051205 GO:0016772
GO:0016301 GO:0009523 GO:0009539 GO:0016021
Cluster-22178.52865 GO:0006352 GO:0006355 GO:0006270 GO:0003700
GO:0016987 GO:0003677 GO:0019031 GO:0005667
Cluster-22178.52871 GO:0007165 GO:0005125 GO:0016020
Cluster-22178.52873 GO:0035556 GO:0008270 GO:0005515 GO:0045735
GO:0003677
Cluster-22178.52875 GO:0006784 GO:0055114 GO:0016627 GO:0016021
Cluster-22178.52877 GO:0006303 GO:0005515
Cluster-22178.52880 GO:0046332 GO:0016817
Cluster-22178.52881 GO:0006139 GO:0008408 GO:0003676 GO:0005515
GO:0019033
Cluster-22178.52884 GO:0016788
Cluster-22178.52885 GO:0007178 GO:0009069 GO:0006468 GO:0016310
GO:0005524 GO:0004675 GO:0016020
Cluster-22178.52886 GO:0007224 GO:0016310 GO:0042967 GO:0006468
GO:0009069 GO:0005524 GO:0008270 GO:0004674 GO:0008080
Cluster-22178.52887 GO:0009069 GO:0006468 GO:0007224 GO:0016310
GO:0005524 GO:0008270 GO:0004674
Cluster-22178.52890 GO:0006355 GO:0003700 GO:0003677 GO:0043565
GO:0005634 GO:0005667
Cluster-22178.52891 GO:0007264 GO:0045040 GO:0005515 GO:0042802
GO:0005525 GO:0046983 GO:0003924 GO:0005742 GO:0016021
Cluster-22178.52901 GO:0006259 GO:0005524 GO:0005515 GO:0003824

GO:0003677 GO:0005694
Cluster-22178.52902 GO:0006813 GO:0005242 GO:0003677 GO:0008076
GO:0016021
Cluster-22178.52903 GO:0042246 GO:0007155 GO:0005515 GO:0016021
Cluster-22178.52905 GO:0006468 GO:0004672 GO:0005524
Cluster-22178.52910 GO:0006812 GO:0032801 GO:0019076 GO:0005515
GO:0005261 GO:0008270 GO:0033644
Cluster-22178.52913 GO:0019076 GO:0006812 GO:0032801 GO:0008270
GO:0005261 GO:0005515 GO:0033644
Cluster-22178.52914 GO:0019076 GO:0006812 GO:0032801 GO:0008270
GO:0005261 GO:0005515 GO:0033644 GO:0005634
Cluster-22178.52915 GO:0032801 GO:0006812 GO:0019076 GO:0005515
GO:0008270 GO:0005261 GO:0033644
Cluster-22178.52921 GO:0015948 GO:0046656 GO:0003677 GO:0030269
GO:0016020 GO:0005576 GO:0005634
Cluster-22178.52922 GO:0006771 GO:0019497 GO:0046872 GO:0003993
GO:0005515 GO:0016021
Cluster-22178.52924 GO:0006270
Cluster-22178.52926 GO:0015992 GO:0006886 GO:0051301 GO:0007067
GO:0000090 GO:0007165 GO:0006810 GO:0030168 GO:0031110
GO:0006891 GO:0015991 GO:0006351 GO:0051382 GO:0051258
GO:0007059 GO:0007049 GO:0008017 GO:0005198 GO:0015078
GO:0030674 GO:0005102 GO:0005783 GO:0005876 GO:0005577
GO:0000776 GO:0005634 GO:0045298 GO:0017119 GO:0017053
GO:0005886 GO:0000940 GO:0033179 GO:0016021
Cluster-22178.52929 GO:0031966
Cluster-22178.52931 GO:0046872
Cluster-22178.52933 GO:0006118 GO:0008237 GO:0008270 GO:0051536
GO:0009055 GO:0001533
Cluster-22178.52934 GO:0007304 GO:0055114 GO:0033014 GO:0005213
GO:0016772 GO:0008883 GO:0003677 GO:0050661 GO:0042600
GO:0005576
Cluster-22178.52935 GO:0005515

Cluster-22178.52939 GO:0006891 GO:0006355 GO:0006118 GO:0043565
GO:0015035 GO:0003700 GO:0019901 GO:0005515 GO:0017119
GO:0005667
Cluster-22178.5294 GO:0016820
Cluster-22178.52941 GO:0042981 GO:0008270
Cluster-22178.52943 GO:0006418 GO:0005524 GO:0004812 GO:0005515
GO:0000166
Cluster-22178.52946 GO:0006355 GO:0003700 GO:0005667
Cluster-22178.52947 GO:0046903 GO:0006807 GO:0009405 GO:0016151
GO:0019867
Cluster-22178.52948 GO:0005515 GO:0005509
Cluster-22178.52950 GO:0032968 GO:0006270 GO:0006367 GO:0004064
GO:0004190 GO:0003677 GO:0005509 GO:0005515 GO:0005634
GO:0016021
Cluster-22178.52951 GO:0048193 GO:0006914 GO:0010508 GO:0016192
GO:0006886 GO:0007155 GO:0005515 GO:0005801 GO:0016021
GO:0009288 GO:0016020
Cluster-22178.52952 GO:0007264 GO:0005525
Cluster-22178.52954 GO:0005515
Cluster-22178.52959 GO:0016310 GO:0000160 GO:0005509 GO:0000155
GO:0046983 GO:0009365 GO:0016021
Cluster-22178.52963 GO:0008270
Cluster-22178.52966 GO:0006355 GO:0007165 GO:0006352 GO:0005179
GO:0003677 GO:0016987 GO:0003700 GO:0005576 GO:0005667
GO:0019031
Cluster-22178.52967 GO:0005975 GO:0030168 GO:0051258 GO:0006468
GO:0007165 GO:0007172 GO:0005524 GO:0004672 GO:0004871
GO:0003824 GO:0005102 GO:0004713 GO:0030674 GO:0005577
GO:0005925
Cluster-22178.52972 GO:0015992 GO:0006810 GO:0015858 GO:0015991
GO:0005337 GO:0015078 GO:0016021 GO:0033179
Cluster-22178.52973 GO:0055114 GO:0008152 GO:0016491
Cluster-22178.52974 GO:0046872

Cluster-22178.52976 GO:0042254 GO:0006412 GO:0003735 GO:0005622 GO:0005840
Cluster-22178.52986 GO:0005515 GO:0000228
Cluster-22178.52989 GO:0051016 GO:0042254 GO:0006412 GO:0030414 GO:0003735 GO:0005515 GO:0005509 GO:0030479 GO:0005840
Cluster-22178.52991 GO:0005515
Cluster-22178.52994 GO:0006122 GO:0030866 GO:0005198 GO:0003779 GO:0008092 GO:0005856 GO:0005743 GO:0005750 GO:0016021
Cluster-22178.52996 GO:0007165 GO:0006811 GO:0007268 GO:0004970 GO:0016020
Cluster-22178.52998 GO:0015991 GO:0006810 GO:0009405 GO:0008200 GO:0016820 GO:0005515 GO:0005576 GO:0000221
Cluster-22178.52999 GO:0006886 GO:0042720 GO:0005737
Cluster-22178.53000 GO:0005515 GO:0016021
Cluster-22178.53001 GO:0008152 GO:0007186 GO:0004930 GO:0003824 GO:0016021
Cluster-22178.53002 GO:0006284 GO:0016114 GO:0006694 GO:0006886 GO:0016192 GO:0005515 GO:0005198 GO:0003677 GO:0008661 GO:0005634 GO:0030117
Cluster-22178.53003 GO:0006508 GO:0016787 GO:0008236
Cluster-22178.53007 GO:0006470 GO:0006754 GO:0006570 GO:0005524 GO:0016887 GO:0004725 GO:0008138 GO:0005515 GO:0000275
Cluster-22178.53008 GO:0009069 GO:0006771 GO:0016310 GO:0006470 GO:0019497 GO:0007178 GO:0006570 GO:0006468 GO:0004222 GO:0003993 GO:0005524 GO:0004675 GO:0008138 GO:0004725 GO:0046872 GO:0005515 GO:0016020
Cluster-22178.53009 GO:0006470 GO:0006570 GO:0006754 GO:0004725 GO:0016887 GO:0005524 GO:0005515 GO:0008138 GO:0000275
Cluster-22178.5301 GO:0007186 GO:0007166 GO:0004930 GO:0016021 GO:0016020
Cluster-22178.53011 GO:0006771 GO:0006570 GO:0019497 GO:0006470 GO:0005515 GO:0008138 GO:0046872 GO:0003993 GO:0004725
Cluster-22178.53013 GO:0006470 GO:0006570 GO:0004725 GO:0008138

GO:0003676

Cluster-22178.53019 GO:0006468 GO:0005524 GO:0004672

Cluster-22178.53020 GO:0006470 GO:0006570 GO:0004725 GO:0008138

GO:0005515 GO:0016021

Cluster-22178.53021 GO:0006084 GO:0006550 GO:0046950 GO:0008299

GO:0006552 GO:0006574 GO:0006633 GO:0042967 GO:0004315

GO:0004421 GO:0005835

Cluster-22178.53028 GO:0007275 GO:0003700 GO:0005667 GO:0005634

Cluster-22178.5303 GO:0030170 GO:0008483

Cluster-22178.53036 GO:0008270 GO:0046872 GO:0061630 GO:0005515

GO:0016021

Cluster-22178.53037 GO:0006508 GO:0006886 GO:0009116 GO:0048280

GO:0071949 GO:0005515 GO:0003824 GO:0004222 GO:0005737

GO:0000139

Cluster-22178.53041 GO:0008270 GO:0005515

Cluster-22178.53042 GO:0030178 GO:0007165 GO:0007275 GO:0009878

GO:0016579 GO:0005179 GO:0005515 GO:0003677 GO:0036459

GO:0008270 GO:0046872 GO:0005576

Cluster-22178.53044 GO:0006508 GO:0004197 GO:0008234

Cluster-22178.53046 GO:0046872

Cluster-22178.53049 GO:0005634

Cluster-22178.53052 GO:0042720

Cluster-22178.53055 GO:0048870 GO:0009058 GO:0016787 GO:0046872

GO:0016788 GO:0031514

Cluster-22178.53058 GO:0016787

Cluster-22178.53059 GO:0016021

Cluster-22178.53065 GO:0006468 GO:0004672 GO:0005524

Cluster-22178.53066 GO:0005975 GO:0003824

Cluster-22178.53067 GO:0019497 GO:0006013 GO:0006000 GO:0006771

GO:0003993 GO:0005524 GO:0003873 GO:0016301

Cluster-22178.53071 GO:0007049 GO:0005515

Cluster-22178.53075 GO:0006810 GO:0009405 GO:0019870 GO:0005515

GO:0061630 GO:0008270 GO:0046872 GO:0005576

Cluster-22178.53076 GO:0009395 GO:0046486 GO:0008654 GO:0005524 GO:0003676 GO:0004143 GO:0016020

Cluster-22178.53077 GO:0000226 GO:0090063 GO:0016192 GO:0051090 GO:0006886 GO:0005515 GO:0005815 GO:0000922 GO:0030117 GO:0005634

Cluster-22178.53083 GO:0051726 GO:0000736 GO:0030414 GO:0046872 GO:0070336 GO:0003677 GO:0005634

Cluster-22178.53086 GO:0006468 GO:0006479 GO:0006554 GO:0008152 GO:0018024 GO:0005515 GO:0004672 GO:0005524 GO:0008168

Cluster-22178.53088 GO:0051603 GO:0006511 GO:0004175 GO:0004298 GO:0005839 GO:0019773

Cluster-22178.53089 GO:0015031 GO:0016021

Cluster-22178.53095 GO:0006508 GO:0008236

Cluster-22178.53097 GO:0015996 GO:0015994 GO:0047746

Cluster-22178.53101 GO:0055114 GO:0045046 GO:0008152 GO:0016491 GO:0032403 GO:0005779

Cluster-22178.53102 GO:0055114 GO:0004800 GO:0016021

Cluster-22178.53103 GO:0004252 GO:0016021

Cluster-22178.53108 GO:0016020 GO:0016021

Cluster-22178.53109 GO:0050909 GO:0006566 GO:0006563 GO:0006778 GO:0006783 GO:0006812 GO:0006544 GO:0042967 GO:0003870 GO:0030170 GO:0005515 GO:0008324 GO:0005759 GO:0016021

Cluster-22178.53110 GO:0005515

Cluster-22178.53111 GO:0016020

Cluster-22178.53114 GO:0000921 GO:0007049 GO:0016310 GO:0006468 GO:0009069 GO:0005524 GO:0004672 GO:0003774 GO:0004674 GO:0005198 GO:0005940 GO:0016021 GO:0016459 GO:0005882

Cluster-22178.53115 GO:0042176 GO:0005515 GO:0030234 GO:0000502

Cluster-22178.53118 GO:0007275 GO:0006040 GO:0009435 GO:0009607 GO:0006807 GO:0000105 GO:0006355 GO:0006051 GO:0008033 GO:0055114 GO:0006537 GO:0046497 GO:0007219 GO:0008152 GO:0016638 GO:0018580 GO:0015930 GO:0016491 GO:0050660 GO:0004514 GO:0047465 GO:0016627 GO:0016829 GO:0003824

GO:0017150 GO:0005737 GO:0016021
Cluster-22178.53120 GO:0006355 GO:0006817 GO:0006810 GO:0019087
GO:0055085 GO:0000978 GO:0000982 GO:0005315 GO:0005634
GO:0016020 GO:0016021
Cluster-22178.53124 GO:0006260 GO:0005515 GO:0003677 GO:0003887
GO:0016021 GO:0042575 GO:0009360
Cluster-22178.53128 GO:0055085 GO:0006355 GO:0055114 GO:0003700
GO:0016491 GO:0005741 GO:0005667
Cluster-22178.53129 GO:0007156 GO:0005509 GO:0016020
Cluster-22178.53130 GO:0055114 GO:0042128 GO:0016491 GO:0030151

Cluster-22178.53131 GO:0005515
Cluster-22178.53135 GO:0005515
Cluster-22178.53138 GO:0006355 GO:0005634
Cluster-22178.53139 GO:0046872
Cluster-22178.53140 GO:0006508 GO:0019079 GO:0004190 GO:0003678
GO:0005657 GO:0016020
Cluster-22178.53144 GO:0009069 GO:0006468 GO:0016310 GO:0004674
GO:0004672 GO:0005524 GO:0016773 GO:0016020
Cluster-22178.53145 GO:0009069 GO:0006468 GO:0016310 GO:0004674
GO:0005524 GO:0004672 GO:0016773 GO:0016020
Cluster-22178.53147 GO:0016310 GO:0009069 GO:0006468 GO:0005524
GO:0004672 GO:0016773 GO:0004674 GO:0016020
Cluster-22178.53150 GO:0006355 GO:0016787 GO:0003677 GO:0003700
GO:0005507 GO:0005634 GO:0005667
Cluster-22178.53151 GO:0009968 GO:0005737
Cluster-22178.53152 GO:0016787 GO:0003725 GO:0008270 GO:0003676

Cluster-22178.53153 GO:0006457 GO:0000413 GO:0003755
Cluster-22178.53154 GO:0007264 GO:0007186 GO:0015684 GO:0007165
GO:0006888 GO:0003924 GO:0031683 GO:0019001 GO:0015093
GO:0004871 GO:0005525 GO:0019033 GO:0005622 GO:0005801
GO:0016021

Cluster-22178.53155 GO:0006888 GO:0007264 GO:0015684 GO:0007165
GO:0007186 GO:0015093 GO:0019001 GO:0031683 GO:0003924
GO:0005525 GO:0004871 GO:0005622 GO:0016021 GO:0005801
GO:0019033
Cluster-22178.53162 GO:0006355 GO:0032324
Cluster-22178.53164 GO:0005515
Cluster-22178.53165 GO:0008270
Cluster-22178.53166 GO:0006886 GO:0016192 GO:0005515 GO:0030117
Cluster-22178.53167 GO:0016192 GO:0006886 GO:0005515 GO:0030117
Cluster-22178.53169 GO:0019478 GO:0016788 GO:0005737
Cluster-22178.53170 GO:0010468 GO:0005515 GO:0016021 GO:0005634
Cluster-22178.53172 GO:0006614 GO:0006810 GO:0006260 GO:0003677
GO:0000166 GO:0016787 GO:0003688 GO:0005525 GO:0003676
GO:0004386 GO:0005524 GO:0046809
Cluster-22178.53173 GO:0003677 GO:0005515 GO:0003676 GO:0005507
GO:0005524 GO:0016787
Cluster-22178.53174 GO:0005515 GO:0004222
Cluster-22178.53175 GO:0042254 GO:0006412 GO:0007264 GO:0043248
GO:0005509 GO:0003735 GO:0005515 GO:0005525 GO:0005840
GO:0005622
Cluster-22178.53176 GO:0005515 GO:0008270 GO:0005921
Cluster-22178.53177 GO:0016021
Cluster-22178.53180 GO:0003924 GO:0003723 GO:0005525
Cluster-22178.53181 GO:0005525 GO:0003723
Cluster-22178.53182 GO:0005525 GO:0003723
Cluster-22178.53183 GO:0005525 GO:0003723
Cluster-22178.53193 GO:0016032 GO:0005524
Cluster-22178.53194 GO:0009069 GO:0006260 GO:0015031 GO:0015992
GO:0006914 GO:0006886 GO:0006468 GO:0015991 GO:0016310
GO:0004674 GO:0005524 GO:0008565 GO:0005515 GO:0003723
GO:0015078 GO:0016020 GO:0033179 GO:0005737
Cluster-22178.53195 GO:0016310 GO:0015991 GO:0006468 GO:0006886
GO:0006914 GO:0015031 GO:0015992 GO:0006260 GO:0009069

GO:0015078 GO:0005515 GO:0008565 GO:0005524 GO:0004674
GO:0005737 GO:0033179 GO:0016020
Cluster-22178.53198 GO:0005515
Cluster-22178.53199 GO:0000917 GO:0006355 GO:0043093 GO:0003677
GO:0003700 GO:0043565 GO:0005634 GO:0005667 GO:0005737
Cluster-22178.53212 GO:0006313 GO:0016787 GO:0004803 GO:0003677

Cluster-22178.53213 GO:0006313 GO:0006378 GO:0003677 GO:0004803
GO:0016787 GO:0003729 GO:0005849
Cluster-22178.53215 GO:0006470 GO:0030145 GO:0004721 GO:0003824
GO:0000287
Cluster-22178.53218 GO:0016310 GO:0006468 GO:0009069 GO:0005524
GO:0004672 GO:0016773 GO:0004674 GO:0016020
Cluster-22178.53219 GO:0006508 GO:0004185
Cluster-22178.53223 GO:0015979 GO:0015693 GO:0030436 GO:0006508
GO:0015684 GO:0006952 GO:0015093 GO:0004190 GO:0015095
GO:0009523 GO:0009539 GO:0016021 GO:0005576 GO:0016020
Cluster-22178.53227 GO:0006355 GO:0007165 GO:0004871 GO:0003700
GO:0005667
Cluster-22178.53228 GO:0006396 GO:0003723 GO:0003676
Cluster-22178.53232 GO:0006606 GO:0016192 GO:0005515 GO:0061630
GO:0046872 GO:0008270 GO:0016021
Cluster-22178.53238 GO:0016192 GO:0006886 GO:0015031 GO:0008565
GO:0030117
Cluster-22178.53242 GO:0006144 GO:0015992 GO:0006206 GO:0006260
GO:0006270 GO:0055114 GO:0016032 GO:0006119 GO:0006118
GO:0006351 GO:0003677 GO:0003899 GO:0008121 GO:0003887
GO:0005730 GO:0032040 GO:0042575
Cluster-22178.53243 GO:0001514 GO:0006448 GO:0005525 GO:0003723
GO:0003746 GO:0005840 GO:0005737
Cluster-22178.53245 GO:0006281 GO:0006310
Cluster-22178.53248 GO:0006468 GO:0006412 GO:0055114 GO:0042254
GO:0007165 GO:0035556 GO:0005515 GO:0016788 GO:0005057

GO:0004672 GO:0003735 GO:0005524 GO:0008270 GO:0047134
GO:0005840 GO:0005622
Cluster-22178.53250 GO:0006464 GO:0006144 GO:0035556 GO:0006313
GO:0006810 GO:0048870 GO:0006308 GO:0008608 GO:0046034
GO:0006355 GO:0000917 GO:0006914 GO:0043093 GO:0008855
GO:0046872 GO:0003700 GO:0045502 GO:0008134 GO:0008270
GO:0003677 GO:0004803 GO:0004017 GO:0043565 GO:0016151
GO:0003676 GO:0051082 GO:0005515 GO:0042803 GO:0030286
GO:0005886 GO:0016021 GO:0072686 GO:0019028 GO:0009318
GO:0031514 GO:0005667 GO:0005737 GO:0042729
Cluster-22178.53251 GO:0006144 GO:0046034 GO:0003723 GO:0046872
GO:0004017 GO:0003676
Cluster-22178.53252 GO:0019722 GO:0003676
Cluster-22178.53255 GO:0006869 GO:0042157 GO:0055114 GO:0050662
GO:0008289 GO:0016491 GO:0031083 GO:0005576
Cluster-22178.53258 GO:0006355 GO:0043401 GO:0043565 GO:0046872
GO:0008270 GO:0003700 GO:0005667 GO:0005634
Cluster-22178.53259 GO:0046486 GO:0006313 GO:0006468 GO:0006629
GO:0016042 GO:0006260 GO:0015074 GO:0005515 GO:0003677
GO:0003887 GO:0004806 GO:0005524 GO:0004672 GO:0042575
Cluster-22178.5326 GO:0035556 GO:0007165 GO:0004871
Cluster-22178.53261 GO:0055114 GO:0006836 GO:0003333 GO:0006118
GO:0006954 GO:0055085 GO:0006865 GO:0006812 GO:0007165
GO:0005125 GO:0009916 GO:0015171 GO:0005328 GO:0016021
GO:0005576 GO:0016020
Cluster-22178.53263 GO:0007165 GO:0007275 GO:0006954 GO:0007219
GO:0030154 GO:0055085 GO:0006812 GO:0006836 GO:0005328
GO:0005125 GO:0005576 GO:0016021
Cluster-22178.53265 GO:0005198 GO:0003676 GO:0016787 GO:0046872
GO:0019028 GO:0005622
Cluster-22178.53271 GO:0009405 GO:0003676 GO:0005576
Cluster-22178.53272 GO:0042330
Cluster-22178.53274 GO:0007264 GO:0005096 GO:0005525

Cluster-22178.53277 GO:0006886 GO:0006605 GO:0005742
Cluster-22178.53278 GO:0007187 GO:0050912 GO:0033227 GO:0015931 GO:0006811 GO:0007154 GO:0007607 GO:0005509 GO:0051033 GO:0008527 GO:0005515 GO:0016020 GO:0016021
Cluster-22178.53279 GO:0007607 GO:0007154 GO:0050912 GO:0007187 GO:0005515 GO:0008527 GO:0005509 GO:0016021 GO:0016020
Cluster-22178.53282 GO:0010508
Cluster-22178.53284 GO:0006355 GO:0016817
Cluster-22178.53288 GO:0006486 GO:0008378 GO:0016020
Cluster-22178.53289 GO:0030414 GO:0005515
Cluster-22178.53290 GO:0006486 GO:0016757 GO:0008378 GO:0016020
Cluster-22178.53292 GO:0006355 GO:0042254 GO:0006412 GO:0008270 GO:0003676 GO:0003700 GO:0003735 GO:0016818 GO:0005840 GO:0005667 GO:0005634
Cluster-22178.53294 GO:0003677
Cluster-22178.53296 GO:0006811 GO:0055085 GO:0015075 GO:0016020
Cluster-22178.53297 GO:0000271 GO:0007186 GO:0005515 GO:0004930 GO:0016021 GO:0016020
Cluster-22178.53298 GO:0043401 GO:0006364 GO:0006355 GO:0003700 GO:0008270 GO:0043565 GO:0005732 GO:0005634 GO:0005667 GO:0034457
Cluster-22178.53301 GO:0015992 GO:0006120 GO:0006744 GO:2001256 GO:0006814 GO:0042254 GO:0006412 GO:0042773 GO:0003735 GO:0008137 GO:0005622 GO:0005840 GO:0005743 GO:0030176
Cluster-22178.53302 GO:0008270 GO:0016020
Cluster-22178.53304 GO:0003677
Cluster-22178.53305 GO:0005515
Cluster-22178.53306 GO:0007165 GO:0031704 GO:0005179
Cluster-22178.53310 GO:0019028
Cluster-22178.53312 GO:0008152 GO:0047355 GO:0016624 GO:0016020
Cluster-22178.53313 GO:0005515
Cluster-22178.53314 GO:0003723 GO:0005524 GO:0003724 GO:0003677 GO:0000166 GO:0005525

Cluster-22178.53320 GO:0031224
Cluster-22178.53324 GO:0005515 GO:0003774 GO:0005524 GO:0016459
Cluster-22178.53326 GO:0055114 GO:0004800
Cluster-22178.53327 GO:0019058 GO:0015992 GO:0006914 GO:0015991
GO:0006139 GO:0006308 GO:0008270 GO:0008855 GO:0005515
GO:0051082 GO:0015078 GO:0008168 GO:0055036 GO:0033644
GO:0009318 GO:0033179 GO:0019031
Cluster-22178.53328 GO:0033925 GO:0005737
Cluster-22178.53334 GO:0009069 GO:0006468 GO:0016310 GO:0005516
GO:0004683
Cluster-22178.53335 GO:0016787
Cluster-22178.53344 GO:0043087 GO:0035023 GO:0035556 GO:0005089
GO:0005515 GO:0005576
Cluster-22178.53345 GO:0008063 GO:0005515 GO:0019901 GO:0008270
GO:0005634
Cluster-22178.53348 GO:0016740
Cluster-22178.53349 GO:0006560 GO:0006814 GO:0071436 GO:0006525
GO:0006090 GO:0008948 GO:0015081 GO:0016020
Cluster-22178.53350 GO:0005515
Cluster-22178.53351 GO:0007264 GO:0005525
Cluster-22178.53355 GO:0051302
Cluster-22178.53358 GO:0006206 GO:0008152 GO:0006230 GO:0006629
GO:0006508 GO:0042254 GO:0006412 GO:0005524 GO:0004797
GO:0016787 GO:0003735 GO:0008236 GO:0005840 GO:0016020
Cluster-22178.53360 GO:0043565 GO:0005515 GO:0005634 GO:0005615
Cluster-22178.53363 GO:0006465 GO:0019087 GO:0008233 GO:0008270
GO:0016021 GO:0005787
Cluster-22178.53368 GO:0006836 GO:0055114 GO:0008152 GO:0006118
GO:0008218 GO:0016491 GO:0019905 GO:0003995 GO:0016021
Cluster-22178.53371 GO:0006508 GO:0045947 GO:0045765 GO:0006144
GO:0008190 GO:0003968 GO:0008234 GO:0005737 GO:0031379
Cluster-22178.53372 GO:0016020
Cluster-22178.53373 GO:0005739

Cluster-22178.53374 GO:0008270
Cluster-22178.53375 GO:0008270
Cluster-22178.53376 GO:0022904 GO:0003676 GO:0008017 GO:0045298
GO:0016020
Cluster-22178.53378 GO:0022904 GO:0003676 GO:0008017 GO:0045298
GO:0016020
Cluster-22178.53381 GO:0008152 GO:0006508 GO:0003676 GO:0008236
GO:0005524 GO:0016787
Cluster-22178.53382 GO:0008152 GO:0006508 GO:0008236 GO:0003676
GO:0016787 GO:0005524
Cluster-22178.53383 GO:0017004 GO:0015886 GO:0006914 GO:0007411
GO:0006260 GO:0015232 GO:0005198 GO:0008092 GO:0019028
GO:0016020 GO:0005737 GO:0019898 GO:0019031
Cluster-22178.53384 GO:0006886 GO:0016020
Cluster-22178.53385 GO:0006308 GO:0006948 GO:0006886 GO:0008855
GO:0005615 GO:0005634 GO:0009318 GO:0016020
Cluster-22178.53386 GO:0016567 GO:0042167 GO:0072519 GO:0015994
GO:0055114 GO:0003677 GO:0004842 GO:0004074 GO:0008270
GO:0005634
Cluster-22178.53387 GO:0006468 GO:0005524 GO:0004672 GO:0046983

Cluster-22178.53388 GO:0019069
Cluster-22178.53392 GO:0006355 GO:0006334 GO:0042967 GO:0003677
GO:0005515 GO:0016747 GO:0008080 GO:0005634 GO:0019028
GO:0000786
Cluster-22178.53393 GO:0006281 GO:0009432 GO:0051782 GO:0006310
GO:0007165 GO:0006260 GO:0051016 GO:0003723 GO:0005509
GO:0098519 GO:0009378 GO:0003724 GO:0003689 GO:0016887
GO:0005524 GO:0042575 GO:0005663 GO:0005578 GO:0009276
GO:0009379 GO:0005657 GO:0030479
Cluster-22178.53394 GO:0042967 GO:0006334 GO:0006355 GO:0008080
GO:0016747 GO:0005515 GO:0003677 GO:0000786 GO:0019028
GO:0005634

Cluster-22178.53395 GO:0003676
Cluster-22178.53397 GO:0051252 GO:0006402 GO:0004535 GO:0046872
GO:0003723 GO:0005634 GO:0005737
Cluster-22178.53399 GO:0005515
Cluster-22178.53401 GO:0019634 GO:0016567 GO:0016579 GO:0036459
GO:0004842 GO:0005680
Cluster-22178.53404 GO:0019605 GO:0008152 GO:0070569 GO:0047989
GO:0005615
Cluster-22178.53408 GO:0003723 GO:0003676 GO:0046935 GO:0005944
GO:0016021
Cluster-22178.53410 GO:0005887
Cluster-22178.53416 GO:0006886 GO:0016192 GO:0005515 GO:0030117
Cluster-22178.53417 GO:0005515
Cluster-22178.53419 GO:0007010 GO:0006813 GO:0030955 GO:0005249
GO:0003779 GO:0008076 GO:0016020 GO:0019033 GO:0016021
Cluster-22178.53423 GO:0043065 GO:0005740 GO:0016021
Cluster-22178.53425 GO:0019064 GO:0006914 GO:0007155 GO:0006457
GO:0006810 GO:0071988 GO:0006308 GO:0006886 GO:0030474
GO:0008855 GO:0000774 GO:0051087 GO:0003676 GO:0042803
GO:0005783 GO:0009288 GO:0016021 GO:0019031 GO:0009318
Cluster-22178.53427 GO:0043123 GO:0045766 GO:0043066
Cluster-22178.53429 GO:0006807 GO:0003824 GO:0003723 GO:0046914

Cluster-22178.53430 GO:0006468 GO:0016773 GO:0005524 GO:0004672
GO:0016020
Cluster-22178.53431 GO:0006468 GO:0004672 GO:0005524 GO:0016773
GO:0016020
Cluster-22178.53436 GO:0005515
Cluster-22178.53437 GO:0019856 GO:0017038 GO:0016787 GO:0003723
GO:0005515 GO:0003677 GO:0008270 GO:0005524 GO:0003676
GO:0016020
Cluster-22178.53438 GO:0017038 GO:0003676 GO:0008270 GO:0005524
GO:0005515 GO:0003677 GO:0016787 GO:0003723 GO:0016020

Cluster-22178.53439 GO:0070588 GO:0006816 GO:0005515 GO:0005245
GO:0005891
Cluster-22178.53442 GO:0006032 GO:0008061 GO:0005869
Cluster-22178.53444 GO:0006952 GO:0006810 GO:0009405 GO:0019855
GO:0005576
Cluster-22178.53448 GO:0006396 GO:0006364 GO:0000154 GO:0008649
GO:0005737
Cluster-22178.53449 GO:0035556
Cluster-22178.53450 GO:0006886 GO:0003924 GO:0005525 GO:0005047
GO:0005786 GO:0005785
Cluster-22178.53454 GO:0005515
Cluster-22178.53455 GO:0005515
Cluster-22178.53458 GO:0008152
Cluster-22178.53459 GO:0055114 GO:0006694 GO:0006813 GO:0008210
GO:0008207 GO:0008209 GO:0003824 GO:0050662 GO:0003854
GO:0016616
Cluster-22178.53461 GO:0008152
Cluster-22178.53462 GO:0009405 GO:0006810 GO:0019871 GO:0005576
Cluster-22178.53463 GO:0008210 GO:0006813 GO:0008209 GO:0008207
GO:0055114 GO:0006694 GO:0050662 GO:0003854 GO:0016616
GO:0003824
Cluster-22178.53465 GO:0009405 GO:0009372 GO:0008233 GO:0016021
Cluster-22178.53467 GO:0016021
Cluster-22178.53468 GO:0006810 GO:0003333 GO:0006865 GO:0055085
GO:0015171 GO:0016020 GO:0016021
Cluster-22178.53469 GO:0006865 GO:0055085 GO:0006810 GO:0003333
GO:0015171 GO:0016020
Cluster-22178.53470 GO:0032324 GO:0006468 GO:0005524 GO:0004672

Cluster-22178.53471 GO:0005515
Cluster-22178.53473 GO:0043039 GO:0006531 GO:0016055 GO:0006522
GO:0006419 GO:0000166 GO:0003676 GO:0005524 GO:0016876
GO:0004813

Cluster-22178.53475 GO:0055085 GO:0005515 GO:0016021
Cluster-22178.53476 GO:0046872 GO:0005515 GO:0016020
Cluster-22178.53478 GO:0042254 GO:0006310 GO:0006810 GO:0009069
GO:0002949 GO:0006206 GO:0006412 GO:0006281 GO:0006139
GO:0016310 GO:0003735 GO:0098519 GO:0009378 GO:0003724
GO:0004127 GO:0004674 GO:0016887 GO:0005524 GO:0016301
GO:0003723 GO:0005525 GO:0003924 GO:0009379 GO:0005657
GO:0005622 GO:0005840
Cluster-22178.53479 GO:0007155 GO:0042157 GO:0006869 GO:0009405
GO:0006914 GO:0008289 GO:0005576 GO:0016020 GO:0019867
Cluster-22178.53482 GO:0016310 GO:0006139 GO:0006281 GO:0006412
GO:0006206 GO:0002949 GO:0009069 GO:0006810 GO:0042254
GO:0006310 GO:0003924 GO:0005525 GO:0003723 GO:0016301
GO:0005524 GO:0016887 GO:0004674 GO:0003724 GO:0004127
GO:0009378 GO:0098519 GO:0003735 GO:0005840 GO:0005622
GO:0005657 GO:0009379
Cluster-22178.53483 GO:0006281 GO:0006412 GO:0016310 GO:0006139
GO:0009069 GO:0006810 GO:0042254 GO:0006310 GO:0006206
GO:0002949 GO:0003723 GO:0016301 GO:0003924 GO:0005525
GO:0009378 GO:0098519 GO:0003735 GO:0005524 GO:0016887
GO:0004674 GO:0003724 GO:0004127 GO:0005840 GO:0009379
GO:0005622 GO:0005657
Cluster-22178.53484 GO:0007155 GO:0042157 GO:0009405 GO:0006869
GO:0008289 GO:0005576 GO:0016020
Cluster-22178.53485 GO:0006955 GO:0007165 GO:0006869 GO:0009405
GO:0040007 GO:0042157 GO:0007155 GO:0008283 GO:0005143
GO:0008083 GO:0008289 GO:0016020 GO:0042022 GO:0005576
Cluster-22178.53487 GO:0046497 GO:0006012 GO:0009267 GO:0046854
GO:0006769 GO:0005982 GO:0005985 GO:0004564 GO:0004575
GO:0003953 GO:0016021 GO:0016020 GO:0019033 GO:0016012
GO:0017177
Cluster-22178.53488 GO:0016579 GO:0009228 GO:0036459 GO:0004789

Cluster-22178.53493 GO:0055114 GO:0015992 GO:0055085 GO:0006811 GO:0006119 GO:0006118 GO:0006810 GO:0005524 GO:0042626 GO:0005216 GO:0008121 GO:0005509 GO:0016021 GO:0016020

Cluster-22178.53495 GO:0006777 GO:0007165 GO:0005515 GO:0003779

Cluster-22178.53499 GO:0006508 GO:0008152 GO:0005524 GO:0016787 GO:0008236 GO:0003676

Cluster-22178.53500 GO:0009405 GO:0051607 GO:0016021

Cluster-22178.53507 GO:0005515 GO:0046872 GO:0008270

Cluster-22178.53508 GO:0005515 GO:0046872 GO:0008270

Cluster-22178.53510 GO:0042157 GO:0006869 GO:0005515 GO:0008289 GO:0005576

Cluster-22178.53511 GO:0016763 GO:0005515

Cluster-22178.53513 GO:0046872 GO:0008144 GO:0003676

Cluster-22178.53514 GO:0009507

Cluster-22178.53521 GO:0046872 GO:0005515

Cluster-22178.53525 GO:0008408 GO:0000166 GO:0003677

Cluster-22178.53528 GO:0008033 GO:0055114 GO:0033151 GO:0046872 GO:0043565 GO:0050660 GO:0017150

Cluster-22178.53532 GO:0006355 GO:0042981 GO:0006810 GO:0016887 GO:0005524 GO:0008134 GO:0005667

Cluster-22178.53533 GO:0008152 GO:0016787 GO:0005198 GO:0019028 GO:0016021

Cluster-22178.53534 GO:0006355 GO:0043565 GO:0003677 GO:0003700 GO:0005667 GO:0005634

Cluster-22178.53535 GO:0008152 GO:0003824 GO:0003677

Cluster-22178.53536 GO:0046872

Cluster-22178.53537 GO:0046872

Cluster-22178.53540 GO:0006935 GO:0000723 GO:0019048 GO:0050920 GO:0000160 GO:0006281 GO:0007017 GO:0009405 GO:0007018 GO:0008270 GO:0005524 GO:0008017 GO:0000156 GO:0003777 GO:0003824 GO:0003678 GO:0008984 GO:0005515 GO:0005657 GO:0016020 GO:0009288 GO:0005737 GO:0019031 GO:0005874

GO:0045298
Cluster-22178.53543 GO:0019497 GO:0006771 GO:0046872 GO:0008270
GO:0003993 GO:0051082 GO:0005515 GO:0005622
Cluster-22178.53544 GO:0006508 GO:0004252
Cluster-22178.53545 GO:0035556 GO:0005975 GO:0006954 GO:0007165
GO:0006952 GO:0004553 GO:0046872 GO:0005125 GO:0005515
GO:0003677 GO:0005576
Cluster-22178.53546 GO:0005198 GO:0019028
Cluster-22178.53547 GO:0005198 GO:0019028
Cluster-22178.53548 GO:0006355 GO:0005515 GO:0046872
Cluster-22178.53549 GO:0006109 GO:0009058 GO:0008033 GO:0006694
GO:0016310 GO:0016114 GO:0000160 GO:0000155 GO:0003723
GO:0004161 GO:0005524 GO:0004672 GO:0016887 GO:0003724
GO:0009365
Cluster-22178.53551 GO:0008152 GO:0015948 GO:0046656 GO:0030269
GO:0005509 GO:0005515 GO:0016746 GO:0016021 GO:0019031
Cluster-22178.53554 GO:0005515
Cluster-22178.53559 GO:0007076 GO:0000796
Cluster-22178.53560 GO:0007275 GO:0006570 GO:0006355 GO:0006470
GO:0007219 GO:0030154 GO:0008138 GO:0005515 GO:0004725
GO:0003700 GO:0005667 GO:0016021
Cluster-22178.53561 GO:0032012 GO:0006952 GO:0043087 GO:0005086
GO:0005576
Cluster-22178.53563 GO:0007165 GO:0030414 GO:0005509 GO:0005578
Cluster-22178.53564 GO:0051321 GO:0055114 GO:0009401 GO:0018401
GO:0006525 GO:0006560 GO:0016491 GO:0016702 GO:0003677
GO:0004656 GO:0005634 GO:0005783
Cluster-22178.53565 GO:0007283
Cluster-22178.53567 GO:0008152 GO:0003824 GO:0016817
Cluster-22178.53568 GO:0031554 GO:0006355 GO:0031556 GO:0003677

Cluster-22178.53569 GO:0006260 GO:0006281 GO:0006310 GO:0005524
GO:0046872 GO:0016887 GO:0003724 GO:0009378 GO:0003723

GO:0003677 GO:0005657 GO:0009379
Cluster-22178.53572 GO:0006281 GO:0006260 GO:0006310 GO:0003677
GO:0003910
Cluster-22178.53574 GO:0006313 GO:0035438 GO:0003677 GO:0046872

Cluster-22178.53575 GO:0016020
Cluster-22178.53578 GO:0005515 GO:0043565 GO:0005634
Cluster-22178.53581 GO:0006760 GO:0046656 GO:0043565 GO:0004150
GO:0005515 GO:0005634
Cluster-22178.53584 GO:0009405 GO:0015631 GO:0005576 GO:0045298
Cluster-22178.53586 GO:0006508 GO:0006144 GO:0044419 GO:0017111
GO:0003968 GO:0004197 GO:0031379
Cluster-22178.53588 GO:0007186 GO:0006468 GO:0055114 GO:0016614
GO:0004930 GO:0004672 GO:0005524 GO:0016021
Cluster-22178.53591 GO:0006355 GO:0005634
Cluster-22178.53595 GO:0008152 GO:0003824
Cluster-22178.53596 GO:0008270 GO:0046872 GO:0003676
Cluster-22178.53597 GO:0044237 GO:0006810 GO:0010038 GO:0007049
GO:0006357 GO:0005515 GO:0001104 GO:0005215 GO:0016592
GO:0016021
Cluster-22178.53600 GO:0006508 GO:0005509 GO:0005515 GO:0004252
GO:0005576
Cluster-22178.53601 GO:0006508 GO:0005515 GO:0004252 GO:0005509
GO:0005576 GO:0019013
Cluster-22178.53604 GO:0006144 GO:0009116 GO:0030154 GO:0000105
GO:0006547 GO:0003824 GO:0004114 GO:0004635 GO:0016021
Cluster-22178.53605 GO:0016310 GO:0006470 GO:0007178 GO:0019497
GO:0006570 GO:0006468 GO:0006771 GO:0009069 GO:0004725
GO:0046872 GO:0005515 GO:0003993 GO:0005524 GO:0008138
GO:0004675 GO:0016020
Cluster-22178.53609 GO:0006886 GO:0006281 GO:0006888 GO:0055114
GO:0006464 GO:0003677 GO:0016972 GO:0016151 GO:0008270
GO:0046872 GO:0030127

Cluster-22178.53610 GO:0005515 GO:0003677 GO:0034061 GO:0005634
Cluster-22178.53611 GO:0016021
Cluster-22178.53612 GO:0003697 GO:0005515
Cluster-22178.53613 GO:0016021
Cluster-22178.53614 GO:0009451 GO:0008033 GO:0009020 GO:0046872

Cluster-22178.53623 GO:0009245 GO:0008915
Cluster-22178.53627 GO:0006281 GO:0034061 GO:0003677 GO:0003684
GO:0005634
Cluster-22178.53628 GO:0006355 GO:0003700 GO:0043565 GO:0005667
Cluster-22178.53630 GO:0055114 GO:0016491
Cluster-22178.53631 GO:0042254 GO:0006412 GO:0003735 GO:0005840
GO:0005622
Cluster-22178.53632 GO:0046660 GO:0006397 GO:0005634
Cluster-22178.53634 GO:0006508 GO:0055085 GO:0004197 GO:0016021
Cluster-22178.53635 GO:0006355 GO:0006886 GO:0005515 GO:0005622
Cluster-22178.53636 GO:0055114 GO:0016714
Cluster-22178.53642 GO:0015684 GO:0006576 GO:0007264 GO:0005525
GO:0003924 GO:0005524 GO:0015093 GO:0016021
Cluster-22178.53644 GO:0016032 GO:0016973 GO:0006508 GO:0004197
GO:0005643 GO:0016021
Cluster-22178.53646 GO:0030178 GO:0007165 GO:0007275 GO:0005515
GO:0035091 GO:0005576
Cluster-22178.53647 GO:0006886 GO:0016192 GO:0030131
Cluster-22178.53648 GO:0016881 GO:0016788
Cluster-22178.53649 GO:0006260 GO:0030168 GO:0007165 GO:0006457
GO:0007049 GO:0051258 GO:0000917 GO:0006355 GO:0043093
GO:0019069 GO:0006886 GO:0010508 GO:0005102 GO:0000774
GO:0051087 GO:0030674 GO:0003700 GO:0046872 GO:0019905
GO:0008270 GO:0061630 GO:0043565 GO:0005515 GO:0005198
GO:0042803 GO:0051082 GO:0005882 GO:0016021 GO:0005577
GO:0016272 GO:0005783 GO:0005667 GO:0005737
Cluster-22178.53650 GO:0006355 GO:0006099 GO:0006479 GO:0006108

GO:0055114 GO:0015976 GO:0006090 GO:0034968 GO:0022904
GO:0006554 GO:0018024 GO:0004471 GO:0046872 GO:0051287
GO:0016651 GO:0005743 GO:0005694
Cluster-22178.53651 GO:0006525 GO:0006560 GO:0006511 GO:0006433
GO:0006777 GO:0000166 GO:0051539 GO:0005524 GO:0051536
GO:0004175 GO:0004827 GO:0003824 GO:0019773 GO:0019008
GO:0005737
Cluster-22178.53654 GO:0006260 GO:0005515
Cluster-22178.53655 GO:0007165
Cluster-22178.53656 GO:0007165
Cluster-22178.53657 GO:0005515
Cluster-22178.53658 GO:0006396 GO:0006260 GO:0019079 GO:0030337
GO:0016021 GO:0000176 GO:0019028 GO:0019013 GO:0042575
Cluster-22178.53659 GO:0005515
Cluster-22178.5366 GO:0046872
Cluster-22178.53660 GO:0016579 GO:0055114 GO:0036459 GO:0016491

Cluster-22178.53661 GO:0016579 GO:0008270 GO:0036459
Cluster-22178.53663 GO:0016579 GO:0036459
Cluster-22178.53668 GO:0000271 GO:0016020
Cluster-22178.53672 GO:0006139 GO:0015940 GO:0008152 GO:0015937
GO:0007186 GO:0006614 GO:0006810 GO:0007165 GO:0006206
GO:0016301 GO:0004140 GO:0003924 GO:0031683 GO:0019001
GO:0004871 GO:0005525 GO:0005524 GO:0004127 GO:0042597
Cluster-22178.53677 GO:0016757
Cluster-22178.53678 GO:0006260 GO:0006486 GO:0006270 GO:0016757
GO:0046983 GO:0003980 GO:0042802 GO:0005515 GO:0003887
GO:0042575 GO:0005727
Cluster-22178.5368 GO:0008270
Cluster-22178.53680 GO:0005515
Cluster-22178.53681 GO:0030639 GO:0019872 GO:0009117 GO:0008830

Cluster-22178.53685 GO:0035556

Cluster-22178.53687 GO:0006541 GO:0004359 GO:0005515
Cluster-22178.53688 GO:0009069 GO:0016310 GO:0004674
Cluster-22178.53692 GO:0007304 GO:0008063 GO:0005213 GO:0005576
GO:0042600
Cluster-22178.53693 GO:0007304 GO:0008063 GO:0005213 GO:0005515
GO:0008270 GO:0005576 GO:0042600
Cluster-22178.53695 GO:0006820 GO:0006355 GO:0008509 GO:0003676
GO:0005622 GO:0016021
Cluster-22178.53698 GO:0006270 GO:0046935 GO:0032040 GO:0005944
Cluster-22178.53700 GO:0006904 GO:0000145
Cluster-22178.53701 GO:0019354 GO:0055114 GO:0006779 GO:0071949
GO:0005515 GO:0043115 GO:0016491 GO:0005576 GO:0016020
Cluster-22178.53707 GO:0015031 GO:0006904 GO:0007165 GO:0005515
GO:0000145
Cluster-22178.53711 GO:0009245 GO:0016747
Cluster-22178.53712 GO:0046872
Cluster-22178.53713 GO:0050853 GO:0050852 GO:0007165 GO:0007264
GO:0006813 GO:0005525 GO:0003924 GO:0015269 GO:0016020
Cluster-22178.53714 GO:0016114 GO:0006355 GO:0006468 GO:0055085
GO:0009058 GO:0042432 GO:0003700 GO:0004672 GO:0005515
GO:0016773 GO:0016844 GO:0005524 GO:0004064 GO:0016021
GO:0016020 GO:0005667
Cluster-22178.53715 GO:0006355 GO:0003700 GO:0005667
Cluster-22178.53716 GO:0007165 GO:0046008 GO:0005179 GO:0005576
Cluster-22178.53718 GO:0016787 GO:0016020
Cluster-22178.53719 GO:0033925 GO:0005737
Cluster-22178.53720 GO:0046872
Cluster-22178.53723 GO:0006508 GO:0042254 GO:0006412 GO:0005509
GO:0004198 GO:0003735 GO:0005622 GO:0005840
Cluster-22178.53724 GO:0046983
Cluster-22178.53725 GO:0008270
Cluster-22178.53726 GO:0006096 GO:0006144 GO:0006357 GO:0000162
GO:0006094 GO:0006397 GO:0009405 GO:0009094 GO:0006571

GO:0004634 GO:0005524 GO:0003723 GO:0000287 GO:0001104
GO:0005198 GO:0003968 GO:0000015 GO:0016592 GO:0005634
GO:0019028 GO:0005576 GO:0005737 GO:0031379 GO:0019031
Cluster-22178.53727 GO:0015977 GO:0006351 GO:0006270 GO:0006099
GO:0006355 GO:0019643 GO:0006094 GO:0006260 GO:0008964
GO:0003677 GO:0003887 GO:0042575
Cluster-22178.53729 GO:0016310 GO:0006468 GO:0009069 GO:0004674
GO:0004672 GO:0005524
Cluster-22178.53731 GO:0006123 GO:0030436 GO:0015992 GO:0009297
GO:0005515 GO:0004129 GO:0005524 GO:0003774 GO:0016459
GO:0019867 GO:0042601 GO:0045277
Cluster-22178.53732 GO:0030036 GO:0006396 GO:0003779 GO:0003723
GO:0003676 GO:0030054
Cluster-22178.53738 GO:0006470 GO:0006402 GO:0051252 GO:0006468
GO:0003676 GO:0004672 GO:0005524 GO:0004535 GO:0046872
GO:0008138 GO:0003723 GO:0005737 GO:0005634
Cluster-22178.53744 GO:0005515
Cluster-22178.53747 GO:0023052 GO:0007049 GO:0008608 GO:0005876
GO:0042729
Cluster-22178.53749 GO:0007283 GO:0005515 GO:0003677 GO:0005634
GO:0000786
Cluster-22178.53751 GO:0055085 GO:0006885 GO:0006812 GO:0007165
GO:0015299 GO:0005179 GO:0016021 GO:0005576
Cluster-22178.53753 GO:0006904 GO:0006891 GO:0006887 GO:0015031
GO:0000145 GO:0017119
Cluster-22178.53761 GO:0007165 GO:0070008 GO:0005509 GO:0004252
GO:0005578
Cluster-22178.53763 GO:0015031 GO:0008277 GO:0006886 GO:0006313
GO:0008565 GO:0003676 GO:0004803 GO:0003677 GO:0016021
Cluster-22178.53768 GO:0006367 GO:0019069 GO:0004190 GO:0016021
GO:0005672
Cluster-22178.53769 GO:0046660 GO:0006397 GO:0005634
Cluster-22178.53770 GO:0016567 GO:0006457 GO:0055085 GO:0007034

GO:0030001 GO:0006308 GO:0046873 GO:0008855 GO:0031386
GO:0051082 GO:0003824 GO:0016881 GO:0016020 GO:0016272
GO:0009318 GO:0019028
Cluster-22178.53774 GO:0055114 GO:0016020
Cluster-22178.53776 GO:0009405 GO:0006886 GO:0051258 GO:0006308
GO:0007155 GO:0007165 GO:0006869 GO:0006457 GO:0030168
GO:0006357 GO:0006606 GO:0051082 GO:0005198 GO:0001104
GO:0008289 GO:0004871 GO:0030674 GO:0008855 GO:0005102
GO:0005576 GO:0019028 GO:0009318 GO:0019031 GO:0016021
GO:0005801 GO:0016592 GO:0016020 GO:0016272 GO:0005577
Cluster-22178.53779 GO:0016567 GO:0005515 GO:0004842
Cluster-22178.53782 GO:0008152 GO:0016491
Cluster-22178.53783 GO:0045182
Cluster-22178.53787 GO:0009966 GO:0005515 GO:0005576 GO:0016021
Cluster-22178.53788 GO:0006470 GO:0050852 GO:0009231 GO:0009058
GO:0006570 GO:0006771 GO:0004725 GO:0003824 GO:0003919
Cluster-22178.53789 GO:0055114 GO:0020037 GO:0016705 GO:0005506

Cluster-22178.53790 GO:0016020
Cluster-22178.53792 GO:0010038 GO:0046938 GO:0046872 GO:0016756

Cluster-22178.53793 GO:0006355 GO:0006468 GO:0016567 GO:0007165
GO:0005515 GO:0004672 GO:0003682 GO:0003677 GO:0005524
GO:0008270 GO:0004842 GO:0005634 GO:0019028 GO:0005737
GO:0000785 GO:0000775 GO:0016020 GO:0005680
Cluster-22178.53794 GO:0006355 GO:0005622
Cluster-22178.53795 GO:0008152 GO:0006633 GO:0042967 GO:0008610
GO:0016747 GO:0004315 GO:0005835
Cluster-22178.53798 GO:0008152 GO:0008610 GO:0016747
Cluster-22178.53804 GO:0055114 GO:0006952 GO:0008017 GO:0047134
GO:0046872 GO:0005515 GO:0045298 GO:0005576
Cluster-22178.53806 GO:0046872
Cluster-22178.53807 GO:0016740 GO:0003676 GO:0005634

Cluster-22178.53809 GO:0006555 GO:0030494 GO:0006556 GO:0019685 GO:0004478 GO:0016836

Cluster-22178.53814 GO:0055085 GO:0030001 GO:0005975 GO:0016758 GO:0046873 GO:0016020

Cluster-22178.53819 GO:0006198 GO:0004115

Cluster-22178.53823 GO:0006576 GO:0006614 GO:0002949 GO:0016787 GO:0003677 GO:0016887 GO:0003924 GO:0005524 GO:0003676 GO:0005525

Cluster-22178.53825 GO:0015684 GO:0007165 GO:0007264 GO:0005525 GO:0005102 GO:0015093 GO:0003924 GO:0016021

Cluster-22178.53827 GO:0006560 GO:0006433 GO:0006511 GO:0006525 GO:0006777 GO:0000166 GO:0051539 GO:0004175 GO:0004827 GO:0003824 GO:0005524 GO:0051536 GO:0019773 GO:0019008 GO:0005737

Cluster-22178.53829 GO:0006119 GO:0006118 GO:0015992 GO:0055114 GO:0008121 GO:0051537 GO:0016491 GO:0051536

Cluster-22178.53837 GO:0006355 GO:0006032 GO:0008061 GO:0005515 GO:0008134 GO:0005524 GO:0005667 GO:0009279

Cluster-22178.53838 GO:0006355 GO:0006730 GO:0006032 GO:0008061 GO:0005515 GO:0008134 GO:0005524 GO:0008168 GO:0005576 GO:0005667 GO:0009279

Cluster-22178.53839 GO:0006355 GO:0009405 GO:0006914 GO:0006308 GO:0007049 GO:0003677 GO:0008855 GO:0019865 GO:0043565 GO:0003700 GO:0005634 GO:0005667 GO:0009318 GO:0019814 GO:0016021

Cluster-22178.53840 GO:0016567 GO:0008270 GO:0046872 GO:0004842 GO:0005515 GO:0005680

Cluster-22178.53841 GO:0005639

Cluster-22178.53842 GO:0007217

Cluster-22178.53843 GO:0000921 GO:0005515 GO:0016021 GO:0005940

Cluster-22178.53845 GO:0005515

Cluster-22178.53851 GO:0005515

Cluster-22178.53852 GO:0005515

Cluster-22178.53853 GO:0043248 GO:0004252 GO:0016021
Cluster-22178.53855 GO:0007186 GO:0004930 GO:0016021
Cluster-22178.53856 GO:0006479 GO:0006367 GO:0034968 GO:0055085
GO:0006811 GO:0006554 GO:0005515 GO:0005216 GO:0018024
GO:0008270 GO:0016020 GO:0005672 GO:0005634
Cluster-22178.53859 GO:0006355 GO:0007165 GO:0043248 GO:0035257
GO:0003713 GO:0005515 GO:0046983 GO:0005667 GO:0005634
Cluster-22178.5386 GO:0007275 GO:0005578
Cluster-22178.53861 GO:0005515 GO:0005516
Cluster-22178.53864 GO:0051028 GO:0007186 GO:0004930 GO:0005634
GO:0031966 GO:0016020 GO:0016021
Cluster-22178.53865 GO:0006355 GO:0009271
Cluster-22178.53867 GO:0009103 GO:0006771 GO:0019497 GO:0005515
GO:0003993 GO:0008918 GO:0046872
Cluster-22178.53871 GO:0008643 GO:0005351 GO:0000139 GO:0016021
Cluster-22178.53872 GO:0046872 GO:0008270
Cluster-22178.53873 GO:0008061 GO:0005515
Cluster-22178.53874 GO:0008061 GO:0005515
Cluster-22178.53875 GO:0005515
Cluster-22178.53879 GO:0055114 GO:0006779 GO:0019354 GO:0051287
GO:0008641 GO:0043115
Cluster-22178.53885 GO:0008270 GO:0005515 GO:0005921
Cluster-22178.53889 GO:0043565 GO:0046872 GO:0008270 GO:0005515
GO:0005634 GO:0005622
Cluster-22178.53890 GO:0006352 GO:0051382 GO:0006366 GO:0006281
GO:0003677 GO:0005669
Cluster-22178.53893 GO:0055114 GO:0006368 GO:0016570 GO:0016491
GO:0030151 GO:0035091 GO:0016593
Cluster-22178.53894 GO:0005634
Cluster-22178.53898 GO:0060478 GO:0007342 GO:0006897 GO:0005886
Cluster-22178.53905 GO:0019882 GO:0006955 GO:0042613 GO:0016020
Cluster-22178.53906 GO:0006955 GO:0005201 GO:0005581 GO:0005578
Cluster-22178.53910 GO:0016579 GO:0036459

Cluster-22178.53912 GO:0007165 GO:0004864
Cluster-22178.53931 GO:0009069 GO:0016310 GO:0016192 GO:0006886
GO:0004674 GO:0005515 GO:0030117
Cluster-22178.53932 GO:0045947 GO:0008190
Cluster-22178.53934 GO:0006812 GO:0008324
Cluster-22178.53943 GO:0006260 GO:0006351 GO:0006886 GO:0016192
GO:0008536 GO:0003887 GO:0003677 GO:0005515 GO:0042575
GO:0005576 GO:0030117
Cluster-22178.53950 GO:0042254 GO:0006412 GO:0006508 GO:0004197
GO:0003735 GO:0005840 GO:0005622
Cluster-22178.53952 GO:0043039 GO:0016876 GO:0005524
Cluster-22178.53955 GO:0006259 GO:0003677
Cluster-22178.53957 GO:0055114 GO:0019530 GO:0006534 GO:0046439
GO:0016702 GO:0017172 GO:0005506
Cluster-22178.53958 GO:0042254 GO:0006412 GO:0003735 GO:0005840
GO:0005622
Cluster-22178.53959 GO:0015992 GO:0006120 GO:0006744 GO:0006355
GO:0006118 GO:0006814 GO:0003954 GO:0005515 GO:0008137
GO:0005739
Cluster-22178.53960 GO:0006771 GO:0006260 GO:0019497 GO:0008408
GO:0000166 GO:0003887 GO:0003677 GO:0003993 GO:0042575
Cluster-22178.53962 GO:0006691 GO:0006749 GO:0019530 GO:0006281
GO:0006693 GO:0006289 GO:0006284 GO:0016787 GO:0003906
GO:0005516 GO:0003684 GO:0016799 GO:0008270 GO:0003840
Cluster-22178.53963 GO:0019530 GO:0006749 GO:0006281 GO:0006691
GO:0006693 GO:0006289 GO:0006284 GO:0005516 GO:0016787
GO:0003906 GO:0003684 GO:0016799 GO:0008270 GO:0003840
Cluster-22178.53964 GO:0006812 GO:0006836 GO:0005515 GO:0005328
GO:0016021
Cluster-22178.5397 GO:0035556
Cluster-22178.53970 GO:0006810 GO:0043093 GO:0006606 GO:0055085
GO:0006144 GO:0000917 GO:0006351 GO:0006355 GO:0003677
GO:0005524 GO:0003723 GO:0003968 GO:0008092 GO:0016020

GO:0019898 GO:0005886 GO:0031379 GO:0005737
Cluster-22178.53972 GO:0006468 GO:0005524 GO:0004672
Cluster-22178.53973 GO:0006468 GO:0004672 GO:0005524
Cluster-22178.53974 GO:0055114 GO:0009236 GO:0015994 GO:0016994

Cluster-22178.53975 GO:0051260 GO:0005515 GO:0016021
Cluster-22178.53976 GO:0015992 GO:0015986 GO:0051082 GO:0003723
GO:0015078 GO:0016020
Cluster-22178.53978 GO:0006355 GO:0007165 GO:0061025 GO:0046813
GO:0005515 GO:0043565 GO:0005634 GO:0016021 GO:0019031
Cluster-22178.53979 GO:0005515
Cluster-22178.53980 GO:0006241 GO:0006144 GO:0006206 GO:0006886
GO:0006183 GO:0006165 GO:0006508 GO:0006355 GO:0006228
GO:0008536 GO:0004222 GO:0004550 GO:0005634
Cluster-22178.53981 GO:0006241 GO:0006206 GO:0006144 GO:0006183
GO:0006886 GO:0006228 GO:0006355 GO:0006165 GO:0006508
GO:0004222 GO:0008536 GO:0004550 GO:0005634
Cluster-22178.53983 GO:0006998 GO:0006406 GO:0006611 GO:0031965
GO:0005576
Cluster-22178.53987 GO:0007186 GO:0008270 GO:0004930 GO:0016021
Cluster-22178.53988 GO:0006887 GO:0000145
Cluster-22178.53989 GO:0007186 GO:0004930 GO:0016021
Cluster-22178.5399 GO:0003677 GO:0008270 GO:0046872
Cluster-22178.53991 GO:0006887 GO:0006886 GO:0005801 GO:0000145
GO:0016020
Cluster-22178.53997 GO:0051252 GO:0006402 GO:0046872 GO:0004535
GO:0003723 GO:0005634 GO:0005737
Cluster-22178.53999 GO:0005515
Cluster-22178.54002 GO:0000077 GO:0006914 GO:0006144 GO:0006206
GO:0010508 GO:0006351 GO:0003899 GO:0005515 GO:0003677
GO:0005730
Cluster-22178.54003 GO:0006570 GO:0007165 GO:0019988 GO:0006470
GO:0005179 GO:0005515 GO:0043399 GO:0008138 GO:0016829

GO:0003676 GO:0004725 GO:0005576 GO:0009424
Cluster-22178.54006 GO:0006687 GO:0006953 GO:0050290 GO:0005576
Cluster-22178.54012 GO:0006457 GO:0007165 GO:0005509 GO:0005578
Cluster-22178.54013 GO:0006541 GO:0006457 GO:0007165 GO:0004359
GO:0005509 GO:0005578 GO:0016021
Cluster-22178.54014 GO:0007165 GO:0042710 GO:0040007 GO:0008283
GO:0005975 GO:0008083 GO:0051015 GO:0043169 GO:0030674
GO:0003824 GO:0003676 GO:0008270
Cluster-22178.54015 GO:0006481 GO:0006383 GO:0006468 GO:0006351
GO:0006479 GO:0006206 GO:0006144 GO:0004671 GO:0003677
GO:0003899 GO:0005524 GO:0004672 GO:0005730 GO:0005666
GO:0016021
Cluster-22178.54021 GO:0055114 GO:0016620 GO:0016491 GO:0051287

Cluster-22178.54024 GO:0005515
Cluster-22178.54027 GO:0042254 GO:0006412 GO:0003735 GO:0008289
GO:0005840 GO:0005739
Cluster-22178.54031 GO:0043039 GO:0003676 GO:0016876 GO:0005524
GO:0016021
Cluster-22178.54034 GO:0007186 GO:0004930 GO:0005634 GO:0016021
Cluster-22178.54037 GO:0009058 GO:0016114 GO:0042432 GO:0005515
GO:0005509 GO:0016844
Cluster-22178.54038 GO:0016114 GO:0009058 GO:0042432 GO:0005509
GO:0005515 GO:0016844 GO:0004064
Cluster-22178.54039 GO:0016114 GO:0009058 GO:0042432 GO:0005509
GO:0005515 GO:0016844
Cluster-22178.5404 GO:0006814 GO:0016627 GO:0005272 GO:0016020
Cluster-22178.54041 GO:0006508 GO:0008152 GO:0005524 GO:0016787
GO:0003676 GO:0008236
Cluster-22178.54042 GO:0005515
Cluster-22178.54043 GO:0005515
Cluster-22178.54045 GO:0005515
Cluster-22178.54046 GO:0007034

Cluster-22178.54050 GO:0005515
Cluster-22178.54052 GO:0005515
Cluster-22178.54054 GO:0009103 GO:0042967 GO:0055085 GO:0008811
GO:0005515 GO:0016020 GO:0016021
Cluster-22178.54055 GO:0055085 GO:0016021 GO:0005576
Cluster-22178.54057 GO:0016567 GO:0046872 GO:0008270 GO:0004842
GO:0005515 GO:0061630 GO:0005622
Cluster-22178.54058 GO:0005515
Cluster-22178.54061 GO:0006260 GO:0042254 GO:0006412 GO:0005524
GO:0003735 GO:0005840 GO:0005622 GO:0000808 GO:0005634
Cluster-22178.54062 GO:0006367 GO:0005672
Cluster-22178.54065 GO:0006621 GO:0046923 GO:0005783 GO:0016021
Cluster-22178.54067 GO:0007017 GO:0007018 GO:0003777 GO:0030286
GO:0005874
Cluster-22178.54068 GO:0030001 GO:0055085 GO:0007186 GO:0046873
GO:0004930 GO:0016020 GO:0016021
Cluster-22178.54069 GO:0007165 GO:0019882 GO:0055085 GO:0007017
GO:0030001 GO:0006955 GO:0006886 GO:0007018 GO:0016787
GO:0042289 GO:0046873 GO:0004190 GO:0046872 GO:0003777
GO:0016020 GO:0030286 GO:0042613 GO:0005874
Cluster-22178.54071 GO:0016021 GO:0005887
Cluster-22178.54072 GO:0006886 GO:0006464 GO:0006888 GO:0016151
GO:0046872 GO:0008270 GO:0030127
Cluster-22178.54074 GO:0005515
Cluster-22178.54075 GO:0043531 GO:0016887 GO:0003924 GO:0005524
GO:0003724 GO:0035064 GO:0005525 GO:0004867 GO:0003723
GO:0005576
Cluster-22178.54076 GO:0031224
Cluster-22178.54077 GO:0007631 GO:0007218 GO:0005094 GO:0005737
Cluster-22178.54079 GO:0048870 GO:0031514
Cluster-22178.54081 GO:0051539
Cluster-22178.54090 GO:0004064 GO:0005515
Cluster-22178.54091 GO:0005515

Cluster-22178.54092 GO:0007186 GO:0004930 GO:0016021
Cluster-22178.54095 GO:0045040 GO:0005515 GO:0005742
Cluster-22178.54099 GO:0005515
Cluster-22178.54100 GO:0006508 GO:0008234 GO:0004197
Cluster-22178.54101 GO:0006508 GO:0008234
Cluster-22178.54102 GO:0005515
Cluster-22178.54105 GO:0046872 GO:0004222
Cluster-22178.54112 GO:0006260 GO:0003677 GO:0005634
Cluster-22178.54118 GO:0006355 GO:0005515 GO:0005634
Cluster-22178.54121 GO:0009451 GO:0006118 GO:0008033 GO:0006418
GO:0045454 GO:0035556 GO:0006464 GO:0030488 GO:0005515
GO:0000166 GO:0009055 GO:0016151 GO:0015035 GO:0004812
GO:0016429 GO:0005524 GO:0031515
Cluster-22178.54122 GO:0051297 GO:0005813
Cluster-22178.54127 GO:0071816 GO:0031492 GO:0000785 GO:0005634
Cluster-22178.54131 GO:0016598 GO:0004057
Cluster-22178.54132 GO:0046080 GO:0016787
Cluster-22178.54134 GO:0017025
Cluster-22178.54143 GO:0009451 GO:0001522 GO:0003723 GO:0009982
GO:0008017 GO:0045298
Cluster-22178.54146 GO:0006886 GO:0006887 GO:0006891 GO:0048278
GO:0005737 GO:0017119
Cluster-22178.54148 GO:0006355 GO:0005634
Cluster-22178.5415 GO:0009405 GO:0006471 GO:0000398 GO:0003956
GO:0005576 GO:0005634
Cluster-22178.54153 GO:0006355 GO:0016310 GO:0008270 GO:0016817
GO:0003700 GO:0016772 GO:0005634 GO:0005667
Cluster-22178.54155 GO:0051603 GO:0006511 GO:0004175 GO:0004298
GO:0005839 GO:0019773
Cluster-22178.54157 GO:0005515
Cluster-22178.54158 GO:0007186 GO:0004930 GO:0008270 GO:0016021
GO:0005622
Cluster-22178.54159 GO:0055085 GO:0048024 GO:0030001 GO:0008017

GO:0019901 GO:0046873 GO:0045298 GO:0016020 GO:0005634
Cluster-22178.54162 GO:0008152 GO:0046034 GO:0006464 GO:0008033
GO:0006144 GO:0051252 GO:0016151 GO:0046872 GO:0016846
GO:0004017 GO:0004540 GO:0016787
Cluster-22178.54163 GO:0032259 GO:0015946 GO:0055114 GO:0006364
GO:0008168 GO:0004022 GO:0005515 GO:0005634
Cluster-22178.54165 GO:0016567 GO:0006915 GO:0006400 GO:0008616
GO:0032465 GO:0004842 GO:0008479
Cluster-22178.54166 GO:0006400 GO:0008616 GO:0008479
Cluster-22178.54169 GO:0006139 GO:0004003 GO:0016787 GO:0008026
GO:0016818 GO:0003677 GO:0005524 GO:0003676 GO:0005634
GO:0005657
Cluster-22178.54170 GO:0006850 GO:0005743
Cluster-22178.54171 GO:0006744 GO:0006120 GO:0006825 GO:0015886
GO:0015992 GO:0006814 GO:0006118 GO:0017004 GO:0005507
GO:0016531 GO:0008137 GO:0003954 GO:0016021 GO:0005758
GO:0005739
Cluster-22178.54174 GO:0006570 GO:0006470 GO:0004725
Cluster-22178.54176 GO:0000228
Cluster-22178.54177 GO:0006629 GO:0008081
Cluster-22178.54179 GO:2001070
Cluster-22178.5418 GO:0007156 GO:0007165 GO:0050821 GO:0015979
GO:0005509 GO:0042301 GO:0001664 GO:0016020 GO:0009523
Cluster-22178.54180 GO:0005515
Cluster-22178.54183 GO:0006355 GO:0046983 GO:0016020
Cluster-22178.54185 GO:0006355 GO:0003824
Cluster-22178.54186 GO:0006355 GO:0003824
Cluster-22178.54188 GO:0006810 GO:0009405 GO:0019855 GO:0005576
Cluster-22178.54189 GO:0006508 GO:0004252 GO:0005515
Cluster-22178.54195 GO:0015991 GO:0042967 GO:0006555 GO:0019281
GO:0048309 GO:0051028 GO:0005975 GO:0042302 GO:0016820
GO:0004553 GO:0008899 GO:0005524 GO:0033178 GO:0005737
Cluster-22178.54199 GO:0005886

Cluster-22178.54201 GO:0005515 GO:0046872 GO:0045202 GO:0016021
Cluster-22178.54203 GO:0006886 GO:0005543
Cluster-22178.54205 GO:0007275 GO:0007304 GO:0005213 GO:0042600
Cluster-22178.54207 GO:0006468 GO:0016773 GO:0004672 GO:0005524
GO:0016020
Cluster-22178.54209 GO:0007275 GO:0005634
Cluster-22178.54210 GO:0008270 GO:0046872
Cluster-22178.54211 GO:0008270 GO:0046872
Cluster-22178.54214 GO:0031023 GO:0051225
Cluster-22178.54215 GO:0035556 GO:0005515
Cluster-22178.54218 GO:0006468 GO:0004672 GO:0005524 GO:0005515

Cluster-22178.54220 GO:0004386
Cluster-22178.54221 GO:0016818 GO:0008270 GO:0005524
Cluster-22178.54222 GO:0031175 GO:0006914 GO:0006355 GO:0009271
GO:0008270 GO:0005516 GO:0030507 GO:0005815 GO:0008091
Cluster-22178.54224 GO:0015992 GO:0015991 GO:0015078 GO:0016772
GO:0016301 GO:0033179
Cluster-22178.54225 GO:0016567 GO:0006308 GO:0006914 GO:0035091
GO:0005515 GO:0004842 GO:0008855 GO:0008270 GO:0046872
GO:0009318 GO:0005680 GO:0005622
Cluster-22178.54226 GO:0006779 GO:0055114 GO:0006464 GO:0019354
GO:0016151 GO:0016491 GO:0046872 GO:0016829 GO:0008270
GO:0043115 GO:0005515 GO:0003677 GO:0016616 GO:0051287
Cluster-22178.54227 GO:0007264 GO:0005525
Cluster-22178.54229 GO:0006820 GO:0016021
Cluster-22178.54230 GO:0016740 GO:0016021
Cluster-22178.54231 GO:0042578
Cluster-22178.54239 GO:0006810 GO:0006812 GO:0046931 GO:0052331
GO:0015267 GO:0035091 GO:0046930
Cluster-22178.54241 GO:0006468 GO:0006449 GO:0006886 GO:0006118
GO:0006415 GO:0016192 GO:0020037 GO:0004715 GO:0016149
GO:0009055 GO:0005506 GO:0005524 GO:0005737 GO:0005840

GO:0018444 GO:0030131 GO:0005622

Cluster-22178.54245 GO:0007275 GO:0006511 GO:0008270 GO:0005515

GO:0005634

Cluster-22178.54248 GO:0006144 GO:0006522 GO:0006531 GO:0006164

GO:0006955 GO:0006810 GO:0007165 GO:0015858 GO:0005125

GO:0005337 GO:0004019 GO:0005525 GO:0016021 GO:0005576

Cluster-22178.54249 GO:0005739

Cluster-22178.54251 GO:0006511 GO:0007005 GO:0006810 GO:0045502

GO:0042803 GO:0008134 GO:0005509 GO:0005622 GO:0030286

GO:0005667 GO:0031305

Cluster-22178.54255 GO:0042254 GO:0006413 GO:0005509 GO:0005515

GO:0005622

Cluster-22178.54256 GO:0032259 GO:0008168

Cluster-22178.54257 GO:0008270 GO:0005516 GO:0016787

Cluster-22178.54262 GO:0016310 GO:0035023 GO:0009069 GO:0043087

GO:0004674 GO:0005515 GO:0005089

Cluster-22178.54266 GO:0005524 GO:0016773 GO:0016020

Cluster-22178.54268 GO:0046983

Cluster-22178.54271 GO:0005515 GO:0033588

Cluster-22178.54278 GO:0015992 GO:0016052 GO:0005975 GO:0055114

GO:0015986 GO:0016491 GO:0015078 GO:0004553 GO:0030246

GO:0000276

Cluster-22178.54279 GO:0005509 GO:0019012

Cluster-22178.54280 GO:0006355 GO:0008270 GO:0046872 GO:0003700

GO:0005667 GO:0042025

Cluster-22178.54281 GO:0022900 GO:0005747 GO:0019031 GO:0005739

Cluster-22178.54283 GO:0006357 GO:0001104 GO:0016592 GO:0071203

Cluster-22178.54286 GO:0006886 GO:0005515 GO:0005763 GO:0005801

GO:0016020

Cluster-22178.54288 GO:0035023 GO:0043087 GO:0005089

Cluster-22178.54290 GO:0005515

Cluster-22178.54293 GO:0006508 GO:0008270 GO:0004222

Cluster-22178.54294 GO:0006869 GO:0015694 GO:0042157 GO:0055114

GO:0003677 GO:0016614 GO:0015097 GO:0008289 GO:0050660
GO:0016020 GO:0005576
Cluster-22178.54295 GO:0042157 GO:0006869 GO:0015694 GO:0055114
GO:0015097 GO:0003677 GO:0016614 GO:0008289 GO:0050660
GO:0005576 GO:0016020
Cluster-22178.54297 GO:0042254 GO:0006412 GO:0003735 GO:0005840
Cluster-22178.54299 GO:0007016 GO:0016010
Cluster-22178.54303 GO:0006955 GO:0009405 GO:0005515 GO:0043022
GO:0016788 GO:0003723 GO:0016020
Cluster-22178.54304 GO:0030150 GO:0005742
Cluster-22178.54309 GO:0006276
Cluster-22178.54310 GO:0007165 GO:0005975 GO:0005509 GO:0004571
GO:0008158 GO:0016020
Cluster-22178.54311 GO:0005515
Cluster-22178.54313 GO:0030001 GO:0007034 GO:0007411
Cluster-22178.54314 GO:0006886 GO:0016192 GO:0004789 GO:0005515
GO:0005198 GO:0019012 GO:0030117
Cluster-22178.54317 GO:0000042 GO:0007094 GO:0006606 GO:0005515
GO:0005789
Cluster-22178.5432 GO:0006355 GO:0005515 GO:0003700 GO:0003677
GO:0005667
Cluster-22178.54324 GO:0019722 GO:0003676
Cluster-22178.54326 GO:0006811 GO:0006810 GO:0005230 GO:0016020
Cluster-22178.54327 GO:0006468 GO:0055114 GO:0004497 GO:0016706
GO:0008168 GO:0004672 GO:0005524 GO:0016715 GO:0005507
Cluster-22178.54335 GO:0046872
Cluster-22178.54336 GO:0009405 GO:0016021
Cluster-22178.54337 GO:0006897 GO:0007015 GO:0003677 GO:0008270
GO:0046872
Cluster-22178.5434 GO:0009405 GO:0006468 GO:0019748 GO:0003774
GO:0005524 GO:0004672 GO:0016773 GO:0005576 GO:0016459
Cluster-22178.54343 GO:0005525 GO:0003924
Cluster-22178.54349 GO:0006659

Cluster-22178.54354 GO:0006313 GO:0004803 GO:0003677
Cluster-22178.54355 GO:0005515
Cluster-22178.54356 GO:0007186 GO:0004930 GO:0016021
Cluster-22178.54361 GO:0016579 GO:0036459
Cluster-22178.54362 GO:0000226 GO:0090063 GO:0005815 GO:0000922
Cluster-22178.54363 GO:0009415 GO:0006470 GO:0006950 GO:0006508
GO:0006570 GO:0000087 GO:0008270 GO:0004222 GO:0004725
GO:0031012 GO:0005622
Cluster-22178.54364 GO:0008270
Cluster-22178.54366 GO:0034314 GO:0030041 GO:0005885 GO:0015629
Cluster-22178.54369 GO:0005509 GO:0005544
Cluster-22178.54372 GO:0046740 GO:0006810 GO:0042254 GO:0006412
GO:0006260 GO:0051082 GO:0003735 GO:0016798 GO:0016020
GO:0005886 GO:0005622 GO:0005840 GO:0016021
Cluster-22178.54379 GO:0010506 GO:0005615
Cluster-22178.54382 GO:0006468 GO:0005524 GO:0004672 GO:0016773
GO:0016020
Cluster-22178.54383 GO:0005634
Cluster-22178.54384 GO:0007165 GO:0050830 GO:0006032 GO:0008061
GO:0005515
Cluster-22178.54385 GO:0035556 GO:0005515
Cluster-22178.54389 GO:0006355 GO:0003700 GO:0005622 GO:0005667
Cluster-22178.54392 GO:0043087 GO:0003677 GO:0003676 GO:0005089
GO:0005524 GO:0016787 GO:0005737
Cluster-22178.54393 GO:0009395 GO:0016042 GO:0006355 GO:0043093
GO:0000917 GO:0006364 GO:0043565 GO:0003700 GO:0003677
GO:0005515 GO:0004629 GO:0005737 GO:0005667 GO:0005634
Cluster-22178.54399 GO:0005515
Cluster-22178.54407 GO:0019497 GO:0006771 GO:0046872 GO:0005515
GO:0003993 GO:0005576
Cluster-22178.54408 GO:0051028 GO:0009236 GO:0006144 GO:0006260
GO:0006355 GO:0048309 GO:0015994 GO:0046034 GO:0016993
GO:0004017 GO:0008270 GO:0005515 GO:0046872 GO:0043565

GO:0003676 GO:0003700 GO:0005667

Cluster-22178.5441 GO:0005515

Cluster-22178.54414 GO:0006468 GO:0004672 GO:0005524

Cluster-22178.54415 GO:0006355 GO:0003700 GO:0003677 GO:0043565 GO:0005667 GO:0005634

Cluster-22178.54416 GO:0000160 GO:0016310 GO:0005975 GO:0046983 GO:0046872 GO:0016868 GO:0000155 GO:0016021 GO:0009365

Cluster-22178.54417 GO:0043093 GO:0006260 GO:0010506 GO:0008053 GO:0006355 GO:0000917 GO:0007049 GO:0043565 GO:0003924 GO:0003700 GO:0005667 GO:0005741 GO:0005634 GO:0005737 GO:0016021 GO:0005615

Cluster-22178.54418 GO:0035556

Cluster-22178.54419 GO:0042246 GO:0006508 GO:0007155 GO:0008239 GO:0004252 GO:0005515 GO:0070009 GO:0016021

Cluster-22178.5442 GO:0007165 GO:0006771 GO:0006260 GO:0019497 GO:0003677 GO:0005515 GO:0003993 GO:0000772 GO:0046872 GO:0016020 GO:0005634 GO:0005664

Cluster-22178.54420 GO:0006464 GO:0006144 GO:0046034 GO:0046872 GO:0016151 GO:0004017

Cluster-22178.54421 GO:0035556 GO:0007165 GO:0003774 GO:0005524 GO:0005515 GO:0016021 GO:0097197 GO:0001772 GO:0016459

Cluster-22178.54424 GO:0006801 GO:0055114 GO:0046872

Cluster-22178.54425 GO:0042967 GO:0006355 GO:0045132 GO:0003676 GO:0008080 GO:0005515 GO:0004402 GO:0000775 GO:0000123 GO:0005634

Cluster-22178.54426 GO:0030153 GO:0006955 GO:0015643 GO:0046872 GO:0000166 GO:0019814

Cluster-22178.54427 GO:0006468 GO:0005524 GO:0004672 GO:0005515

Cluster-22178.54428 GO:0006468 GO:0005524 GO:0004672 GO:0005515

Cluster-22178.54429 GO:0051056 GO:0005096

Cluster-22178.54430 GO:0042254 GO:0006412 GO:0008152 GO:0003735

GO:0003824 GO:0005840 GO:0005739
Cluster-22178.54432 GO:0006508 GO:0030436 GO:0004190
Cluster-22178.54433 GO:0005634
Cluster-22178.54434 GO:0046872
Cluster-22178.54436 GO:0015979 GO:0042549 GO:0005515 GO:0009523
GO:0009539
Cluster-22178.54439 GO:0007186 GO:0004930 GO:0016021
Cluster-22178.54440 GO:0007186 GO:0007010 GO:0004930 GO:0003779
GO:0016021
Cluster-22178.54445 GO:0008537
Cluster-22178.54447 GO:0009966 GO:0007275 GO:0016020
Cluster-22178.54449 GO:0016021
Cluster-22178.54451 GO:0045454
Cluster-22178.54453 GO:0016021
Cluster-22178.54455 GO:0042262 GO:0003690 GO:0008092 GO:0005737
GO:0019898
Cluster-22178.54457 GO:0006886 GO:0007264 GO:0006468 GO:0006606
GO:0006914 GO:0004672 GO:0005524 GO:0016773 GO:0005096
GO:0005783 GO:0016020 GO:0016021
Cluster-22178.54458 GO:0046486 GO:0008654 GO:0008715 GO:0016020
Cluster-22178.54461 GO:0006511 GO:0005515
Cluster-22178.54462 GO:0046813 GO:0061025 GO:0016021 GO:0019031
Cluster-22178.54463 GO:0009405 GO:0016560 GO:0005515 GO:0005096
GO:0016021 GO:0005777
Cluster-22178.54464 GO:0006355 GO:0003677 GO:0005515 GO:0003700
GO:0008270 GO:0043565 GO:0005634 GO:0005667 GO:0005815
Cluster-22178.54468 GO:0006013 GO:0006144 GO:0006000 GO:0000103
GO:0006614 GO:0015976 GO:0008152 GO:0006094 GO:0006099
GO:0005524 GO:0016887 GO:0098519 GO:0003873 GO:0003924
GO:0004020 GO:0005525 GO:0004612 GO:0016301
Cluster-22178.54470 GO:0000103 GO:0006144 GO:0004020 GO:0005524

Cluster-22178.54472 GO:0005634

Cluster-22178.54473 GO:0005634
Cluster-22178.54477 GO:0006820 GO:0009432 GO:0006333 GO:0022611
GO:0005253 GO:0005634 GO:0005887
Cluster-22178.54482 GO:0015031 GO:0042147 GO:0007165 GO:0008565
GO:0005198 GO:0005515 GO:0019031 GO:0019028 GO:0030904
Cluster-22178.54483 GO:0006470 GO:0006570 GO:0008138 GO:0004725
GO:0005515 GO:0004721 GO:0008270
Cluster-22178.54484 GO:0006276 GO:0009236 GO:0055085 GO:0048472
GO:0016021
Cluster-22178.54485 GO:0055085 GO:0009236 GO:0006276 GO:0048472
GO:0016021
Cluster-22178.54486 GO:0007166 GO:0007010 GO:0003779 GO:0005515
GO:0016020
Cluster-22178.54487 GO:0007156 GO:0005509 GO:0016020
Cluster-22178.54490 GO:0000398 GO:0006355 GO:0008270 GO:0005515
GO:0003677 GO:0003682 GO:0000785 GO:0005681 GO:0000775
GO:0005634
Cluster-22178.54491 GO:0005515
Cluster-22178.54493 GO:0007275 GO:0034477 GO:0004518 GO:0005515
GO:0003700 GO:0046872 GO:0005634 GO:0005667
Cluster-22178.54494 GO:0016021
Cluster-22178.54495 GO:0019087 GO:0055085 GO:0016021
Cluster-22178.54496 GO:0005982 GO:0005985 GO:0005978 GO:0005515
GO:0004373
Cluster-22178.54500 GO:0008284 GO:0006952 GO:0003676 GO:0046872

Cluster-22178.54505 GO:0006510 GO:0006508 GO:0004176
Cluster-22178.54506 GO:0030153 GO:0006955 GO:0015643 GO:0003676
GO:0019814
Cluster-22178.54507 GO:0006144 GO:0006508 GO:0004197 GO:0003968
GO:0004252 GO:0017111 GO:0031379
Cluster-22178.54508 GO:0006355 GO:0006810 GO:0035556 GO:0017048
GO:0005198 GO:0003774 GO:0005215 GO:0003700 GO:0016459

GO:0005667 GO:0005882 GO:0016020
Cluster-22178.54509 GO:0000921 GO:0006355 GO:0003700 GO:0043565
GO:0005198 GO:0016021 GO:0005940 GO:0005882 GO:0005667
Cluster-22178.54510 GO:0006811 GO:0019439 GO:0006813 GO:0005249
GO:0003677 GO:0016833 GO:0016020 GO:0008076
Cluster-22178.54514 GO:0006308 GO:0006260 GO:0006281 GO:0017108
GO:0047869 GO:0005515 GO:0005634 GO:0033557
Cluster-22178.54517 GO:0008654 GO:0055085 GO:0006812 GO:0016780
GO:0008324 GO:0016021 GO:0016020
Cluster-22178.54518 GO:0006338 GO:0046983 GO:0005524 GO:0000049
GO:0003676 GO:0016818 GO:0005634
Cluster-22178.54522 GO:0006418 GO:0005524 GO:0004812 GO:0000166
GO:0003676
Cluster-22178.54525 GO:0006418 GO:0004812 GO:0005524 GO:0000166
GO:0003676
Cluster-22178.54526 GO:0006468 GO:0004672 GO:0005524 GO:0016773
GO:0016020
Cluster-22178.5453 GO:0005515
Cluster-22178.54531 GO:0051252 GO:0006402 GO:0005975 GO:0003723
GO:0016832 GO:0003676 GO:0046872 GO:0004535 GO:0005634
GO:0005737
Cluster-22178.54533 GO:0006355 GO:0003723 GO:0005515 GO:0005634
Cluster-22178.54535 GO:0016021
Cluster-22178.54538 GO:0016021
Cluster-22178.54539 GO:0016197 GO:0000724 GO:0005515 GO:0030119
Cluster-22178.54541 GO:0006355 GO:0003700 GO:0008270 GO:0043565
GO:0005667
Cluster-22178.54542 GO:0008152 GO:0006614 GO:0003824 GO:0005525
GO:0016491 GO:0005198 GO:0050662 GO:0019028
Cluster-22178.54543 GO:0042254 GO:0006412 GO:0003735 GO:0005840
GO:0005622
Cluster-22178.54544 GO:0008270
Cluster-22178.54545 GO:0006633 GO:0006554 GO:0018874 GO:0006631

GO:0006098 GO:0015940 GO:0006574 GO:0019521 GO:0055114
GO:0006550 GO:0006552 GO:0006813 GO:0006568 GO:0016491
GO:0004616 GO:0051287 GO:0008677 GO:0003857
Cluster-22178.54546 GO:0051287
Cluster-22178.54547 GO:0006574 GO:0055114 GO:0008152 GO:0006020
GO:0006550 GO:0008218 GO:0006118 GO:0006552 GO:0016491
GO:0004491 GO:0003995
Cluster-22178.54548 GO:0055114 GO:0015992 GO:0006123 GO:0009060
GO:0006118 GO:0005524 GO:0004129 GO:0009055 GO:0005506
GO:0020037 GO:0035299 GO:0016021 GO:0045277
Cluster-22178.54549 GO:0070206 GO:0006406 GO:0006998 GO:0006611
GO:0051082 GO:0005198 GO:0019031 GO:0019028 GO:0031965
Cluster-22178.54551 GO:0006544 GO:0006546 GO:0006566 GO:0055114
GO:0006563 GO:0016616 GO:0004375
Cluster-22178.54553 GO:0006265 GO:0003677 GO:0003918 GO:0005524
GO:0016020
Cluster-22178.54554 GO:0008152 GO:0016491
Cluster-22178.54555 GO:0030036 GO:0006351 GO:0006396 GO:0006144
GO:0006206 GO:0006270 GO:0006816 GO:0006874 GO:0006383
GO:0003676 GO:0005219 GO:0003779 GO:0003899 GO:0003723
GO:0003677 GO:0016021 GO:0030054 GO:0005622 GO:0005730
GO:0005666
Cluster-22178.54556 GO:0016310 GO:0015012 GO:0006024 GO:0009069
GO:0006468 GO:0004674 GO:0005524 GO:0016021 GO:0045095
Cluster-22178.54557 GO:0008152 GO:0016746
Cluster-22178.54558 GO:0046872 GO:0045202
Cluster-22178.54559 GO:0006693 GO:0006691 GO:0006281 GO:0006749
GO:0019530 GO:0006284 GO:0006289 GO:0003684 GO:0016799
GO:0003906 GO:0016787 GO:0005516 GO:0008270 GO:0003840
Cluster-22178.54560 GO:0032099 GO:0009267 GO:0001678 GO:0006693
GO:0008343 GO:0007186 GO:0006749 GO:0019530 GO:0000186
GO:0006691 GO:0003840 GO:0008270 GO:0005516 GO:0016787
GO:0005615

Cluster-22178.54561 GO:0007050 GO:0006355 GO:0045859 GO:0000122
GO:0004861 GO:0003700 GO:0017069 GO:0005667 GO:0005634
GO:0005737
Cluster-22178.54563 GO:0007017 GO:0007018 GO:0005524 GO:0003777
GO:0008017 GO:0005874 GO:0045298
Cluster-22178.54565 GO:0007017 GO:0007018 GO:0005524 GO:0003777
GO:0008017 GO:0005874 GO:0045298
Cluster-22178.54567 GO:0005515 GO:0003950
Cluster-22178.54568 GO:0003950 GO:0005515
Cluster-22178.54569 GO:0035091 GO:0005515
Cluster-22178.54571 GO:0006364 GO:0004222
Cluster-22178.54572 GO:0006355 GO:0003677 GO:0005634
Cluster-22178.54573 GO:0007165 GO:0006606 GO:0006955 GO:0005044
GO:0030247
Cluster-22178.54575 GO:0003824 GO:0005515
Cluster-22178.54576 GO:0019354 GO:0055114 GO:0006464 GO:0006779
GO:0051287 GO:0043115 GO:0005515 GO:0016616 GO:0003677
GO:0016491 GO:0016829 GO:0008270 GO:0046872 GO:0016151
Cluster-22178.54578 GO:0008270
Cluster-22178.54579 GO:0007217 GO:0007275 GO:0007268 GO:0004867
GO:0005578
Cluster-22178.54580 GO:0035556 GO:0055114 GO:0007165 GO:0006468
GO:0047134 GO:0008270 GO:0004672 GO:0005524 GO:0005057
GO:0005515
Cluster-22178.54581 GO:0055114 GO:0035556 GO:0006468 GO:0007165
GO:0005057 GO:0005524 GO:0008270 GO:0004672 GO:0047134
GO:0005515
Cluster-22178.54583 GO:0005515
Cluster-22178.54584 GO:0051607 GO:0051082
Cluster-22178.54586 GO:0016787
Cluster-22178.54589 GO:0006355 GO:0016192 GO:0006886 GO:0005198
GO:0005515 GO:0030126
Cluster-22178.54592 GO:0044419 GO:0006144 GO:0006508 GO:0003968

GO:0017111 GO:0004197 GO:0031379
Cluster-22178.54593 GO:0005515
Cluster-22178.54595 GO:0006555 GO:0006556 GO:0004478
Cluster-22178.54596 GO:0006810 GO:0009405 GO:0006952 GO:0006556
GO:0006555 GO:0004478 GO:0019855 GO:0046872 GO:0005576
GO:0045202
Cluster-22178.54597 GO:0006629 GO:0009755 GO:0006556 GO:0006810
GO:0009405 GO:0006952 GO:0006730 GO:0006555 GO:0016740
GO:0004478 GO:0046872 GO:0019855 GO:0005576 GO:0045202
Cluster-22178.54602 GO:0006338 GO:0006493 GO:0005524 GO:0003676
GO:0000030 GO:0016818 GO:0000136 GO:0005634 GO:0016020
Cluster-22178.54604 GO:0006260 GO:0005524 GO:0003678 GO:0030288
GO:0019031 GO:0005657
Cluster-22178.54606 GO:0019031
Cluster-22178.54607 GO:0006260 GO:0019079 GO:0005524 GO:0003678
GO:0003676 GO:0016787 GO:0003677 GO:0005657
Cluster-22178.54609 GO:0006355 GO:0005622
Cluster-22178.54610 GO:0016788
Cluster-22178.54612 GO:0006355 GO:0005515 GO:0005634
Cluster-22178.54613 GO:0007186 GO:0030246 GO:0004930 GO:0016020
Cluster-22178.54615 GO:0051260 GO:0007186 GO:0004930 GO:0016021
GO:0016020
Cluster-22178.54616 GO:0055114 GO:1901031 GO:0051537 GO:0051920
GO:0016491 GO:0005634
Cluster-22178.54617 GO:0006886 GO:0022900 GO:0006888 GO:0008270
GO:0005747 GO:0030127 GO:0005739
Cluster-22178.54621 GO:0043085 GO:0000290 GO:0003779 GO:0008047

Cluster-22178.54625 GO:0006206 GO:0006221 GO:0006541 GO:0016787
GO:0003883
Cluster-22178.54626 GO:0006357 GO:0006508 GO:0008237 GO:0001104
GO:0005515 GO:0016805 GO:0005618 GO:0005634 GO:0016592
Cluster-22178.54628 GO:0006468 GO:0007010 GO:0003779 GO:0004672

GO:0005524 GO:0019028 GO:0016020
Cluster-22178.54629 GO:0006468 GO:0005524 GO:0004672
Cluster-22178.54638 GO:0055085 GO:0006821 GO:0008270 GO:0005247
GO:0016020
Cluster-22178.54639 GO:0006313 GO:0055085 GO:0003677 GO:0022857
GO:0016020 GO:0016021
Cluster-22178.54643 GO:0006098 GO:0009165 GO:0006144 GO:0004749
GO:0020037 GO:0019825 GO:0000287
Cluster-22178.54645 GO:0005198 GO:0019028
Cluster-22178.54648 GO:0005861
Cluster-22178.54649 GO:0009264 GO:0008253
Cluster-22178.54652 GO:0006355 GO:0016032 GO:0007017 GO:0007018
GO:0003777 GO:0045502 GO:0042803 GO:0008134 GO:0003774
GO:0005524 GO:0008017 GO:0003677 GO:0005874 GO:0045298
GO:0005634 GO:0030286 GO:0016459 GO:0005667
Cluster-22178.54654 GO:0006352 GO:0006355 GO:0003700 GO:0016987
GO:0003677 GO:0005667
Cluster-22178.54658 GO:0043087 GO:0006914 GO:0032012 GO:0008053
GO:0003924 GO:0005086 GO:0016021 GO:0005741
Cluster-22178.54659 GO:0006355 GO:0006281 GO:0002949 GO:0006310
GO:0003678 GO:0043531 GO:0008134 GO:0016301 GO:0003723
GO:0003724 GO:0016887 GO:0005524 GO:0009378 GO:0005657
GO:0005667 GO:0009379
Cluster-22178.54661 GO:0007165 GO:0042254 GO:0015992 GO:0070206
GO:0006914 GO:0007034 GO:0006606 GO:0007218 GO:0015991
GO:0010508 GO:0030474 GO:0006412 GO:0006308 GO:0005179
GO:0003677 GO:0003735 GO:0008855 GO:0005198 GO:0005200
GO:0015078 GO:0005622 GO:0005823 GO:0005576 GO:0009318
GO:0033179 GO:0005856 GO:0005840
Cluster-22178.54669 GO:0016773
Cluster-22178.54670 GO:0015031 GO:0030176
Cluster-22178.54671 GO:0005515
Cluster-22178.54672 GO:0005515

Cluster-22178.54673 GO:0007264 GO:0006357 GO:0005515 GO:0001104
GO:0003924 GO:0008641 GO:0005525 GO:0016592
Cluster-22178.54674 GO:0005198 GO:0019028
Cluster-22178.54675 GO:0005198 GO:0019028
Cluster-22178.54677 GO:0007186 GO:0004930 GO:0016021
Cluster-22178.54681 GO:0006468 GO:0006118 GO:0055114 GO:0009496
GO:0005524 GO:0004672 GO:0051537 GO:0042651 GO:0009512
Cluster-22178.54684 GO:0006687 GO:0009058 GO:0003677 GO:0008146
GO:0001733 GO:0016021 GO:0005794
Cluster-22178.5469 GO:0046872
Cluster-22178.54690 GO:0055114 GO:0016491
Cluster-22178.54691 GO:0051289 GO:0006914 GO:0070206 GO:0007165
GO:0006506 GO:0008140 GO:0005515 GO:0005509 GO:0016740
GO:0016021 GO:0005789 GO:0005886 GO:0005667 GO:0016012
GO:0005578
Cluster-22178.54693 GO:0006355 GO:0018279 GO:0006506 GO:0006810
GO:0006260 GO:0004579 GO:0003677 GO:0017176 GO:0005524
GO:0003678 GO:0005634 GO:0008250 GO:0005657 GO:0016021
Cluster-22178.54694 GO:0006270 GO:0006810 GO:0006260 GO:0006355
GO:0018279 GO:0006506 GO:0004579 GO:0003677 GO:0005524
GO:0017176 GO:0000062 GO:0003678 GO:0008250 GO:0005657
GO:0005634 GO:0016021
Cluster-22178.54695 GO:0006606 GO:0048870 GO:0031514
Cluster-22178.54697 GO:0006468 GO:0009405 GO:0051082 GO:0005515
GO:0004672 GO:0005524 GO:0019865 GO:0019814
Cluster-22178.54702 GO:0030244 GO:0061136 GO:0005982 GO:0005985
GO:0016567 GO:0019915 GO:0006011 GO:0004842 GO:0008270
GO:0046872 GO:0005515 GO:0016760 GO:0005789 GO:0005680
GO:0016020
Cluster-22178.54703 GO:0030244 GO:0006011 GO:0005985 GO:0016567
GO:0005982 GO:0008270 GO:0046872 GO:0004842 GO:0016760
GO:0005515 GO:0005680 GO:0016020
Cluster-22178.54706 GO:0006351 GO:0006206 GO:0006144 GO:0003677

GO:0003899 GO:0008270 GO:0046872 GO:0003676 GO:0005730
Cluster-22178.54708 GO:0007165 GO:0007155 GO:0005515 GO:0005198
GO:0005179 GO:0005576 GO:0031012
Cluster-22178.54710 GO:0006825 GO:0035434 GO:0005375 GO:0016021
GO:0019031 GO:0005887
Cluster-22178.54712 GO:0019031 GO:0030288
Cluster-22178.54718 GO:0042981
Cluster-22178.54719 GO:0046872
Cluster-22178.54720 GO:0005576
Cluster-22178.54721 GO:0015031 GO:0006464 GO:0005515
Cluster-22178.54722 GO:0015031 GO:0006464 GO:0005515
Cluster-22178.54723 GO:0032040
Cluster-22178.54726 GO:0042981
Cluster-22178.54727 GO:0046872 GO:0003676
Cluster-22178.54729 GO:0006260 GO:0006084 GO:0006810 GO:0006614
GO:0005524 GO:0003688 GO:0005525 GO:0003676 GO:0003824
GO:0004386 GO:0016787 GO:0003677 GO:0000166 GO:0046809
Cluster-22178.54734 GO:0007275 GO:0008270 GO:0046872 GO:0005515
GO:0061630 GO:0042600
Cluster-22178.54737 GO:0007156 GO:0005509 GO:0016020
Cluster-22178.54738 GO:0007156 GO:0005509 GO:0005634 GO:0016020
Cluster-22178.5474 GO:0051603 GO:0004298 GO:0005839
Cluster-22178.54740 GO:0007156 GO:0005509 GO:0005634 GO:0016020
Cluster-22178.54743 GO:0048203 GO:0034315 GO:1900025 GO:2000146
GO:0035650
Cluster-22178.54746 GO:0006629 GO:0035434 GO:0006825 GO:0009252
GO:0003677 GO:0005375 GO:0046983 GO:0008963 GO:0016021
Cluster-22178.54750 GO:0030153 GO:0006955 GO:0015643 GO:0019814
Cluster-22178.54751 GO:0006468 GO:0009069 GO:0055114 GO:0016310
GO:0035556 GO:0008152 GO:0005515 GO:0005524 GO:0004672
GO:0016846 GO:0047134 GO:0004674
Cluster-22178.54758 GO:0003677 GO:0005515
Cluster-22178.54759 GO:0055114 GO:0051920

Cluster-22178.54760 GO:0003677 GO:0019901
Cluster-22178.54761 GO:0003824 GO:0051537 GO:0043231
Cluster-22178.54762 GO:0046872
Cluster-22178.54763 GO:0042157 GO:0006869 GO:0008289 GO:0005515
GO:0005576
Cluster-22178.54767 GO:0015074 GO:0055085 GO:0006260 GO:0006810
GO:0003924 GO:0016887 GO:0005524 GO:0042626 GO:0003678
GO:0003676 GO:0005525 GO:0016021 GO:0005657
Cluster-22178.54768 GO:0006308 GO:0006355 GO:0003677 GO:0008855
GO:0009318
Cluster-22178.54770 GO:0006744 GO:0042207 GO:0006120 GO:0055114
GO:0015992 GO:0006814 GO:0006570 GO:0006559 GO:0004411
GO:0016817 GO:0005515 GO:0008137 GO:0005739
Cluster-22178.54772 GO:0016055 GO:0006281 GO:0051382
Cluster-22178.54773 GO:0015992 GO:0015986 GO:0015078 GO:0016020
Cluster-22178.54774 GO:0051301 GO:0032955 GO:0005515
Cluster-22178.54776 GO:0006659
Cluster-22178.54777 GO:0006659
Cluster-22178.54778 GO:0006260 GO:0005515 GO:0003677 GO:0008270

Cluster-22178.54781 GO:0008270 GO:0005515
Cluster-22178.54786 GO:0051260 GO:0046872 GO:0003677 GO:0016020
Cluster-22178.54788 GO:0006810 GO:0009405 GO:0051775 GO:0035556
GO:0045892 GO:0003677 GO:0005515 GO:0008200 GO:0005576
GO:0005634 GO:0005737
Cluster-22178.54789 GO:0006810 GO:0009405 GO:0035556 GO:0003677
GO:0005515 GO:0008200 GO:0005576 GO:0005634
Cluster-22178.54790 GO:0006817
Cluster-22178.54791 GO:0006488 GO:0004583 GO:0005789
Cluster-22178.54792 GO:0004867 GO:0005524 GO:0005576
Cluster-22178.54800 GO:0043087 GO:0035023 GO:0008152 GO:0003824
GO:0005089 GO:0017022 GO:0016459
Cluster-22178.54801 GO:0015979 GO:0015931 GO:0006352 GO:0033227

GO:0003677 GO:0051033 GO:0005515 GO:0009539 GO:0009523
GO:0005669 GO:0016021 GO:0016020
Cluster-22178.54803 GO:0008152 GO:0016787
Cluster-22178.54804 GO:0005515 GO:0046872 GO:0016021
Cluster-22178.54806 GO:0006508 GO:0006816 GO:0070588 GO:0006355
GO:0007165 GO:0005509 GO:0005515 GO:0043565 GO:0003700
GO:0005262 GO:0019033 GO:0005667 GO:0005578 GO:0016020
Cluster-22178.54807 GO:0006506 GO:0005789
Cluster-22178.54809 GO:0006270 GO:0007010 GO:0006367 GO:0006629
GO:0032968 GO:0003677 GO:0003779 GO:0005634 GO:0032040
Cluster-22178.54811 GO:0006355 GO:0005515 GO:0003723 GO:0005634
Cluster-22178.54812 GO:0032797
Cluster-22178.54816 GO:0006508 GO:0008233
Cluster-22178.54817 GO:0006508 GO:0004197 GO:0008233
Cluster-22178.54824 GO:0046500 GO:0006810 GO:0032259 GO:0006464
GO:0001510 GO:0031167 GO:0006306 GO:0006479 GO:0009452
GO:0008170 GO:0003677 GO:0008270 GO:0003723 GO:0019871
GO:0005515 GO:0008168 GO:0004719 GO:0046872 GO:0003676
GO:0005576
Cluster-22178.54825 GO:0007017 GO:0007018 GO:0005524 GO:0003777
GO:0008017 GO:0005874 GO:0045298 GO:0019028
Cluster-22178.54828 GO:0016021
Cluster-22178.54832 GO:0007165
Cluster-22178.54833 GO:0007165 GO:0005515
Cluster-22178.54836 GO:0022900 GO:0006397 GO:0003723 GO:0005634
GO:0005739 GO:0005737 GO:0005747
Cluster-22178.54840 GO:0042254 GO:0006412 GO:0007411 GO:0003735
GO:0005515 GO:0044599 GO:0005622 GO:0005840
Cluster-22178.54841 GO:0051260 GO:0005515
Cluster-22178.54842 GO:0003677 GO:0003676 GO:0005524 GO:0016787

Cluster-22178.54847 GO:0007165 GO:0042254 GO:0006412 GO:0003735
GO:0005125 GO:0005840 GO:0005622

Cluster-22178.54851 GO:0003723 GO:0003676 GO:0016021

Cluster-22178.54859 GO:0016192 GO:0006886 GO:0005515 GO:0005198 GO:0030130 GO:0030132 GO:0030117

Cluster-22178.54860 GO:0007165 GO:0008601 GO:0000159

Cluster-22178.54868 GO:0006886 GO:0005515 GO:0008536

Cluster-22178.54869 GO:0006811 GO:0006614 GO:0008312 GO:0003723 GO:0005216 GO:0005515 GO:0048500 GO:0016020

Cluster-22178.54871 GO:0046872

Cluster-22178.54875 GO:0016567 GO:0006915 GO:0032465 GO:0015031 GO:0006464 GO:0005515 GO:0004842 GO:0016020

Cluster-22178.54876 GO:0050832

Cluster-22178.54878 GO:0005515 GO:0005198 GO:0019028

Cluster-22178.54879 GO:0006355 GO:0003700 GO:0005515 GO:0005634 GO:0005667

Cluster-22178.54883 GO:0005525 GO:0005515

Cluster-22178.54886 GO:0042254 GO:0006412 GO:0003735 GO:0005840

Cluster-22178.54887 GO:0015948 GO:0008152 GO:0046656 GO:0030269 GO:0016746 GO:0016021

Cluster-22178.54888 GO:0016032 GO:0006541 GO:0042819 GO:0009236 GO:0042823 GO:0016070 GO:0016787 GO:0004359 GO:0003824 GO:0019012

Cluster-22178.5489 GO:0003697

Cluster-22178.54891 GO:0006396 GO:0051537 GO:0043231

Cluster-22178.54892 GO:0008152 GO:0006508 GO:0004181 GO:0016788 GO:0008270 GO:0005739

Cluster-22178.54894 GO:0042981 GO:0005515

Cluster-22178.54895 GO:0042981 GO:0005515

Cluster-22178.54896 GO:0042981 GO:0005515

Cluster-22178.54903 GO:0006310 GO:0042138 GO:0003824

Cluster-22178.54906 GO:0007283 GO:0003677 GO:0005515 GO:0000786 GO:0005634

Cluster-22178.54907 GO:0006508 GO:0007031 GO:0005509 GO:0004252 GO:0005779

Cluster-22178.54910 GO:0006355 GO:0003677 GO:0008270 GO:0016021
Cluster-22178.54917 GO:0035556 GO:0042967 GO:0046339 GO:0009395
GO:0007165 GO:0006629 GO:0008080 GO:0004435 GO:0005515
Cluster-22178.54918 GO:0008152 GO:0003824
Cluster-22178.54919 GO:0006139 GO:0006206 GO:0006614 GO:0006810
GO:0005525 GO:0003724 GO:0004127 GO:0005524 GO:0016887
GO:0003924 GO:0000166 GO:0003677 GO:0098519 GO:0003723
Cluster-22178.54920 GO:0008152 GO:0003824
Cluster-22178.54926 GO:0051537 GO:0043231
Cluster-22178.54929 GO:0006814 GO:0006886 GO:0006265 GO:0055114
GO:0006308 GO:0045995 GO:0006120 GO:0007165 GO:0006605
GO:0006313 GO:0030155 GO:0015992 GO:0030334 GO:0006744
GO:0008137 GO:0005515 GO:0003677 GO:0005524 GO:0008855
GO:0003918 GO:0005102 GO:0009318 GO:0016020
Cluster-22178.5493 GO:0006771 GO:0019497 GO:0003993 GO:0005515
GO:0046872
Cluster-22178.54930 GO:0006465 GO:0008233 GO:0016021 GO:0005887
GO:0005787
Cluster-22178.54931 GO:0015876 GO:0008521 GO:0016021
Cluster-22178.54932 GO:0015876 GO:0008521 GO:0016021
Cluster-22178.54933 GO:0007264 GO:0042254 GO:0006144 GO:0006206
GO:0006576 GO:0006351 GO:0055114 GO:0008152 GO:0051287
GO:0003677 GO:0016491 GO:0005524 GO:0003724 GO:0003723
GO:0003899 GO:0005525 GO:0005730 GO:0005634
Cluster-22178.54934 GO:0007009
Cluster-22178.54940 GO:0006024 GO:0030206 GO:0006470 GO:0006570
GO:0004725 GO:0008138 GO:0030158
Cluster-22178.54941 GO:0005515 GO:0005198 GO:0005882
Cluster-22178.54942 GO:0007165 GO:0006468 GO:0016773 GO:0004672
GO:0005524 GO:0005179 GO:0005840 GO:0016020 GO:0005576
Cluster-22178.54946 GO:0006542 GO:0006807 GO:0006355 GO:0009252
GO:0003677 GO:0004356 GO:0005622
Cluster-22178.54947 GO:0008270 GO:0005515 GO:0042765 GO:0016021

Cluster-22178.54956 GO:0031124 GO:0006468 GO:0030976 GO:0000287
GO:0005524 GO:0004672
Cluster-22178.54957 GO:0031124 GO:0006468 GO:0030976 GO:0005524
GO:0004672 GO:0000287
Cluster-22178.54958 GO:0009451 GO:0006879 GO:0008033 GO:0009020
GO:0008199
Cluster-22178.54961 GO:0006355 GO:0009307 GO:0006308 GO:0005515
GO:0003677 GO:0009036 GO:0003700 GO:0005667 GO:0009359
Cluster-22178.54963 GO:0015994 GO:0015995 GO:0046406
Cluster-22178.54965 GO:0006468 GO:0004672 GO:0005524 GO:0016773
GO:0016020
Cluster-22178.54966 GO:0006891 GO:0007165 GO:0005515 GO:0017119
Cluster-22178.54973 GO:0005975 GO:0007165 GO:0004553 GO:0005509
GO:0005578
Cluster-22178.54974 GO:0009186 GO:0055114 GO:0006144 GO:0006206
GO:0006260 GO:0005524 GO:0004748 GO:0005971
Cluster-22178.54975 GO:0016125 GO:0006694 GO:0047750 GO:0016021
Cluster-22178.54977 GO:0016787 GO:0005524 GO:0003676 GO:0003677

Cluster-22178.54980 GO:0005515
Cluster-22178.54981 GO:0005515
Cluster-22178.54984 GO:0042773 GO:0006814 GO:0015992 GO:0055114
GO:0008152 GO:0006744 GO:0055085 GO:0006120 GO:0006812
GO:0008137 GO:0008324 GO:0008168 GO:0016021
Cluster-22178.54987 GO:0055114 GO:0016491 GO:0010181
Cluster-22178.54990 GO:0006816 GO:0070588 GO:0015992 GO:0005262
GO:0016021 GO:0016020
Cluster-22178.54991 GO:0015948 GO:0046656 GO:0030269 GO:0016021
Cluster-22178.54992 GO:0046872 GO:0005515 GO:0003677
Cluster-22178.54993 GO:0006084 GO:0015947 GO:0005515 GO:0018492

Cluster-22178.54996 GO:0006118 GO:0006570 GO:0055114 GO:0004097

Cluster-22178.55000 GO:0007264 GO:0007186 GO:0007165 GO:0003924
GO:0019001 GO:0031683 GO:0004871 GO:0005525
Cluster-22178.55002 GO:0006508 GO:0042981 GO:0004197 GO:0005506

Cluster-22178.55004 GO:0042981 GO:0006508 GO:0004197 GO:0005506

Cluster-22178.55005 GO:0000226 GO:0043085 GO:0016226 GO:0008033
GO:0000910 GO:0016485 GO:0000166 GO:0016879 GO:0008017
GO:0005524 GO:0045298 GO:0016021 GO:0005737
Cluster-22178.55007 GO:0006281 GO:0003684 GO:0016020
Cluster-22178.55008 GO:0009405 GO:0007165 GO:0007186 GO:0006355
GO:0005509 GO:0005515 GO:0005198 GO:0004888 GO:0004930
GO:0003700 GO:0043565 GO:0005667 GO:0016021
Cluster-22178.55014 GO:0006270 GO:0005515
Cluster-22178.55017 GO:0017004 GO:0007165 GO:0015886 GO:0004871
GO:2001070 GO:0016021
Cluster-22178.55018 GO:0008270 GO:0008233 GO:0005634
Cluster-22178.55019 GO:0007275 GO:0046872 GO:0061630 GO:0042600
Cluster-22178.55028 GO:0046034 GO:0006144 GO:0006464 GO:0004017
GO:0016151 GO:0008270 GO:0046872
Cluster-22178.55030 GO:0005515 GO:0005509
Cluster-22178.55033 GO:0005509 GO:0005515
Cluster-22178.55034 GO:0005509 GO:0005515
Cluster-22178.55036 GO:0005975 GO:0030682 GO:0008270 GO:0004553

Cluster-22178.55038 GO:0005515
Cluster-22178.55041 GO:0005576 GO:0030133 GO:0031083
Cluster-22178.55042 GO:0035556 GO:0005524 GO:0046872
Cluster-22178.55044 GO:0015979 GO:0046872 GO:0016021 GO:0009523
Cluster-22178.55045 GO:0045444 GO:0006000 GO:0006013 GO:0007186
GO:0007165 GO:0016255 GO:0007264 GO:0004871 GO:0005525
GO:0003924 GO:0019001 GO:0005524 GO:0031683 GO:0003873
GO:0042765

Cluster-22178.55046 GO:0007165 GO:0010506 GO:0005515 GO:0005615
Cluster-22178.55047 GO:0010506 GO:0007165 GO:0005515 GO:0005615
Cluster-22178.55054 GO:0030695 GO:0005515 GO:0042802
Cluster-22178.55058 GO:0003676 GO:0031224
Cluster-22178.55066 GO:0007275 GO:0006511 GO:0006744 GO:0008270
GO:0046872 GO:0005515 GO:0005634
Cluster-22178.55067 GO:0006468 GO:0004672 GO:0005524
Cluster-22178.55071 GO:0006355 GO:0046872
Cluster-22178.55074 GO:0006351
Cluster-22178.55079 GO:0006508 GO:0016579 GO:0008270 GO:0004843
GO:0036459
Cluster-22178.55080 GO:0008270 GO:0046872 GO:0005634
Cluster-22178.55085 GO:0016021
Cluster-22178.55086 GO:0006904 GO:0003779 GO:0000145
Cluster-22178.55088 GO:0008537
Cluster-22178.55089 GO:0006556 GO:0019685 GO:0030494 GO:0006555
GO:0004478 GO:0016836
Cluster-22178.55090 GO:0006357 GO:0019051 GO:0001104 GO:0042025
GO:0016592
Cluster-22178.55091 GO:0070403 GO:0000287 GO:0030976 GO:0005515

Cluster-22178.55092 GO:0016567 GO:0009271 GO:0006355 GO:0006744
GO:0055114 GO:0005515 GO:0004842 GO:0008270 GO:0046872
Cluster-22178.55095 GO:0005515
Cluster-22178.55098 GO:0008152 GO:0006633 GO:0042967 GO:0004315
GO:0016747 GO:0005835
Cluster-22178.55099 GO:0006351 GO:0006468 GO:0006281 GO:0016310
GO:0007178 GO:0009069 GO:0006206 GO:0006289 GO:0043161
GO:0006144 GO:0003899 GO:0004675 GO:0003684 GO:0005524
GO:0005634 GO:0016020 GO:0005730
Cluster-22178.55104 GO:0051260 GO:0003677 GO:0046872
Cluster-22178.55105 GO:0051260 GO:0046872 GO:0003677
Cluster-22178.55110 GO:0005515

Cluster-22178.55113 GO:0005975 GO:0071704 GO:0016868

Cluster-22178.55116 GO:0007275 GO:0045893 GO:0008270

Cluster-22178.55122 GO:0007264 GO:0005525 GO:0003924

Cluster-22178.55130 GO:0006275 GO:0006270 GO:0005524 GO:0043565

Cluster-22178.55132 GO:0017004 GO:0006560 GO:0006525 GO:0015886 GO:0006418 GO:0006420 GO:0000166 GO:0004812 GO:0004814 GO:0005524 GO:0016021

Cluster-22178.55134 GO:0006355 GO:0003677

Cluster-22178.55135 GO:0006468 GO:0016773 GO:0005524 GO:0004672 GO:0016020

Cluster-22178.55136 GO:0042254 GO:0006412 GO:0015979 GO:0003735 GO:0005515 GO:0003677 GO:0016020 GO:0009523 GO:0005622 GO:0009539 GO:0005840

Cluster-22178.55140 GO:0046486 GO:0006071 GO:0005515 GO:0004371 GO:0008270 GO:0016829 GO:0005524 GO:0043565 GO:0005783 GO:0005634

Cluster-22178.55151 GO:0006047

Cluster-22178.55153 GO:0035556 GO:0046339 GO:0009395 GO:0007165 GO:0006629 GO:0004435 GO:0005509 GO:0005515 GO:0005578

Cluster-22178.55155 GO:0006118 GO:0051536 GO:0009055

Cluster-22178.55156 GO:0003676 GO:0016020

Cluster-22178.55157 GO:0055085 GO:0006811 GO:0005216 GO:0016020

Cluster-22178.55158 GO:0006570 GO:0006470 GO:0019988 GO:0008138 GO:0043399 GO:0005515 GO:0004725 GO:0005634

Cluster-22178.55161 GO:0006817 GO:0008152 GO:0003824

Cluster-22178.55162 GO:0007165 GO:0007155 GO:0005102 GO:0005515 GO:0016021

Cluster-22178.55163 GO:0006144 GO:0007601 GO:0004114 GO:0030553

Cluster-22178.55164 GO:0016973 GO:0006355 GO:0048870 GO:0003700 GO:0005667 GO:0031514 GO:0005643 GO:0016021

Cluster-22178.55166 GO:0055085 GO:0016020 GO:0016021

Cluster-22178.55167 GO:0006351 GO:0006355 GO:0003712 GO:0003676
GO:0008270 GO:0003677 GO:0005634 GO:0005667
Cluster-22178.55169 GO:0006914 GO:0000921 GO:0070206 GO:0010506
GO:0006260 GO:0006308 GO:0048870 GO:0055114 GO:0051382
GO:0006352 GO:0009690 GO:0006355 GO:0010508 GO:0035092
GO:0008855 GO:0005524 GO:0050660 GO:0016787 GO:0003677
GO:0019139 GO:0000228 GO:0005940 GO:0005815 GO:0016021
GO:0005615 GO:0000776 GO:0031514 GO:0009318
Cluster-22178.55170 GO:0044780 GO:0006468 GO:0007283 GO:0007130
GO:0006308 GO:0006914 GO:0007131 GO:0005524 GO:0004672
GO:0008855 GO:0009318
Cluster-22178.55171 GO:0016020
Cluster-22178.55174 GO:0005515
Cluster-22178.55176 GO:0030976 GO:0016787 GO:0000287 GO:0046872
GO:0070403
Cluster-22178.55177 GO:0007165 GO:0006886 GO:0005515 GO:0005783
GO:0016021
Cluster-22178.55184 GO:0051252 GO:0006355 GO:0001682 GO:0008033
GO:0004526 GO:0003700 GO:0008270 GO:0016747 GO:0030677
GO:0005667 GO:0005634
Cluster-22178.55185 GO:0008152 GO:0003824
Cluster-22178.55186 GO:0035556 GO:0009395 GO:0046339 GO:0007165
GO:0006629 GO:0004435 GO:0005509 GO:0005515 GO:0005578
Cluster-22178.55192 GO:0000226 GO:0051537 GO:0015630 GO:0043231
Cluster-22178.55193 GO:0006468 GO:0016773 GO:0005524 GO:0004672
GO:0016020
Cluster-22178.55196 GO:0015937 GO:0015940 GO:0006412 GO:0042254
GO:0005524 GO:0004594 GO:0003735 GO:0004252 GO:0005622
GO:0005840 GO:0019028 GO:0055036
Cluster-22178.55197 GO:0006120 GO:0006744 GO:0015992 GO:0006118
GO:0006814 GO:0008137 GO:0003954 GO:0005739
Cluster-22178.55198 GO:0015992 GO:0006744 GO:0006120 GO:0006310
GO:0006814 GO:0006118 GO:0046872 GO:0003954 GO:0003677

GO:0008137 GO:0000166 GO:0005739
Cluster-22178.55199 GO:0021549 GO:0006351 GO:0030182 GO:0006730
GO:0006144 GO:0006206 GO:0003899 GO:0003677 GO:0003676
GO:0008168 GO:0005730
Cluster-22178.55200 GO:0009058 GO:0006631 GO:0006508 GO:0008152
GO:0005975 GO:0008236 GO:0016788 GO:0016787 GO:0016746
GO:0008184
Cluster-22178.55203 GO:0005515
Cluster-22178.55205 GO:0003676 GO:0005515 GO:0046872 GO:0008270

Cluster-22178.55208 GO:0003824 GO:0052855
Cluster-22178.55209 GO:0055085 GO:0035556 GO:0032007 GO:0048011
GO:0008270 GO:0046872 GO:0005515 GO:0016021
Cluster-22178.55213 GO:0003824
Cluster-22178.55216 GO:0043087 GO:0032012 GO:0005086
Cluster-22178.55217 GO:0006810 GO:0005215
Cluster-22178.55218 GO:0019089
Cluster-22178.55219 GO:0019089
Cluster-22178.55222 GO:0005515 GO:0005737
Cluster-22178.55223 GO:0008063 GO:0008270 GO:0005515 GO:0019901
GO:0005634
Cluster-22178.55226 GO:0006206 GO:0006144 GO:0006351 GO:0046983
GO:0003899 GO:0005730
Cluster-22178.55235 GO:0006529 GO:0006522 GO:0006560 GO:0006526
GO:0006531 GO:0004055 GO:0005524 GO:0004066
Cluster-22178.55236 GO:0006468 GO:0006355 GO:0007264 GO:0006457
GO:0035556 GO:0007031 GO:0051082 GO:0042803 GO:0016820
GO:0005515 GO:0004672 GO:0046872 GO:0003924 GO:0005525
GO:0003700 GO:0051087 GO:0003677 GO:0005524 GO:0000774
GO:0004222 GO:0016021 GO:0005667 GO:0005779 GO:0005739
GO:0016272
Cluster-22178.55237 GO:0042967 GO:0008152 GO:0006633 GO:0016491
GO:0046983 GO:0004315 GO:0005835

Cluster-22178.55238 GO:0055114 GO:0032922 GO:0042967 GO:0006633
GO:0016567 GO:0000976 GO:0004842 GO:0004318 GO:0005515
GO:0005835
Cluster-22178.55239 GO:0006633 GO:0008152 GO:0042967 GO:0046983
GO:0016491 GO:0004315 GO:0005835
Cluster-22178.55241 GO:0016567 GO:0032922 GO:0000976 GO:0005515
GO:0004842
Cluster-22178.55243 GO:0016055 GO:0006468 GO:0006629 GO:0035091
GO:0005524 GO:0004672
Cluster-22178.55246 GO:0006171 GO:0006144 GO:0009190 GO:0035556
GO:0016849 GO:0004016 GO:0005886
Cluster-22178.55248 GO:0007275 GO:0016021
Cluster-22178.55250 GO:0007275 GO:0016021
Cluster-22178.55252 GO:0010508 GO:0006369 GO:0019064 GO:0006378
GO:0006260 GO:0006379 GO:0016758 GO:0008270 GO:0005849
GO:0019031 GO:0005815 GO:0005789
Cluster-22178.55253 GO:0006378 GO:0051258 GO:0006369 GO:0006308
GO:0007165 GO:0006379 GO:0006260 GO:0030168 GO:0019064
GO:0051082 GO:0030674 GO:0005102 GO:0008855 GO:0016758
GO:0009318 GO:0005849 GO:0019031 GO:0005789 GO:0005815
GO:0005577
Cluster-22178.55259 GO:0019497 GO:0006771 GO:0046872 GO:0030414
GO:0003993 GO:0005515 GO:0005576
Cluster-22178.55261 GO:0006355 GO:0009252 GO:0006807 GO:0004356
GO:0005515 GO:0043565 GO:0003700 GO:0055036 GO:0005667
GO:0005634
Cluster-22178.55264 GO:0005515
Cluster-22178.55268 GO:0007021 GO:0051082 GO:0015631 GO:0045298
Cluster-22178.55270 GO:0006355 GO:0003677 GO:0003676 GO:0003723

Cluster-22178.55273 GO:0003676
Cluster-22178.55284 GO:0010508 GO:0006355 GO:0048870 GO:0051258
GO:0007165 GO:0006555 GO:0006914 GO:0030168 GO:0042803

GO:0008134 GO:0045502 GO:0003700 GO:0008092 GO:0030674
GO:0005102 GO:0031514 GO:0019028 GO:0016021 GO:0005737
GO:0005667 GO:0005577 GO:0019898 GO:0030286
Cluster-22178.55288 GO:0006952 GO:0055085 GO:0006812 GO:0008324
GO:0016817 GO:0016021
Cluster-22178.55289 GO:0008270 GO:0005622
Cluster-22178.55290 GO:0006812 GO:0008324 GO:0016747 GO:0005515

Cluster-22178.55291 GO:0006812 GO:0005515 GO:0008324 GO:0016747

Cluster-22178.55294 GO:0055114 GO:0016972 GO:0005515
Cluster-22178.55297 GO:0003676 GO:0008270
Cluster-22178.55298 GO:0009117 GO:0005985 GO:0005982 GO:0007186
GO:0007165 GO:0048040 GO:0003824 GO:0004871 GO:0050662
GO:0005834
Cluster-22178.55299 GO:0015940 GO:0008897 GO:0016829 GO:0000287

Cluster-22178.55300 GO:0007034 GO:0008654 GO:0006544 GO:0006566
GO:0006563 GO:0046486 GO:0008270 GO:0004609 GO:0005515
GO:0019028
Cluster-22178.55301 GO:0003723 GO:0005515 GO:0016887 GO:0003924
GO:0005524 GO:0003774 GO:0003724 GO:0005525 GO:0016459
Cluster-22178.55303 GO:0006771 GO:0006000 GO:0006013 GO:0019497
GO:0016301 GO:0003873 GO:0003993 GO:0005524
Cluster-22178.55304 GO:0055114 GO:0016491 GO:0046872
Cluster-22178.55305 GO:0046872 GO:0005515 GO:0032040
Cluster-22178.55306 GO:0008270
Cluster-22178.55307 GO:0046872
Cluster-22178.55309 GO:0007050 GO:0045859 GO:0004861 GO:0016020
GO:0005634
Cluster-22178.55310 GO:0007186 GO:0006890 GO:0004930 GO:0016021
GO:0019028
Cluster-22178.55316 GO:0005525 GO:0035064 GO:0003724 GO:0005524

GO:0003924 GO:0016887 GO:0043531 GO:0003723 GO:0004867
GO:0005576
Cluster-22178.55317 GO:0006511 GO:0031625
Cluster-22178.55320 GO:0006457 GO:0006955 GO:0007094 GO:0007155
GO:0019882 GO:0005198 GO:0051082 GO:0019028 GO:0019031
GO:0016272 GO:0009288
Cluster-22178.55323 GO:0006897 GO:0007094 GO:0006955 GO:0006457
GO:0042254 GO:0007155 GO:0007015 GO:0019882 GO:0005198
GO:0051082 GO:0019028 GO:0019031 GO:0005622 GO:0009288
GO:0016272
Cluster-22178.55324 GO:0006955 GO:0042254 GO:0006457 GO:0006897
GO:0007094 GO:0007155 GO:0007015 GO:0019882 GO:0005198
GO:0051082 GO:0019028 GO:0019031 GO:0016272 GO:0005622
GO:0009288
Cluster-22178.55325 GO:0016559 GO:0016779 GO:0005779
Cluster-22178.55326 GO:0006807 GO:0008033 GO:0006725 GO:0055114
GO:0050660 GO:0017150 GO:0016491 GO:0016627 GO:0016853
GO:0018580 GO:0005737
Cluster-22178.55334 GO:0007156 GO:0005509 GO:0016020
Cluster-22178.55335 GO:0007156 GO:0005515 GO:0005509 GO:0016020
Cluster-22178.55337 GO:0003676 GO:0008270
Cluster-22178.55339 GO:0007165 GO:0006367 GO:0006886 GO:0016192
GO:0032968 GO:0007259 GO:0051607 GO:0050778 GO:0003677
GO:0005515 GO:0005125 GO:0030117 GO:0005634
Cluster-22178.55343 GO:0043087 GO:0007264 GO:0007165 GO:0005085

Cluster-22178.55344 GO:0006810 GO:0006816 GO:0015994 GO:0006779
GO:0004853 GO:0005246 GO:0042030 GO:0016020
Cluster-22178.55345 GO:0006090 GO:0006108 GO:0055114 GO:0006099
GO:0015976 GO:0004471 GO:0005515
Cluster-22178.55349 GO:0009405 GO:0006810 GO:0019871 GO:0005576
Cluster-22178.55352 GO:0007186 GO:0019069 GO:0006836 GO:0004930
GO:0019905 GO:0004190 GO:0016021

Cluster-22178.55353 GO:0046813 GO:0061025 GO:0006355 GO:0005524
GO:0003677 GO:0016787 GO:0019031 GO:0016021 GO:0005634
Cluster-22178.55354 GO:0015992 GO:0015991 GO:0016567 GO:0046872
GO:0008270 GO:0015078 GO:0004842 GO:0005515 GO:0061630
GO:0005680 GO:0033179
Cluster-22178.55355 GO:0006355 GO:0046813 GO:0061025 GO:0003677
GO:0016787 GO:0005524 GO:0005634 GO:0016021 GO:0019031
Cluster-22178.55356 GO:0046813 GO:0061025 GO:0006355 GO:0005524
GO:0003677 GO:0016787 GO:0016021 GO:0019031 GO:0005634
Cluster-22178.55357 GO:0006464 GO:0015031 GO:0022900 GO:0005515
GO:0005743 GO:0070469
Cluster-22178.55358 GO:0009252 GO:0006807 GO:0008270 GO:0004356
GO:0003676 GO:0005634
Cluster-22178.55359 GO:0005515
Cluster-22178.55360 GO:0005524
Cluster-22178.55361 GO:0032259 GO:0008168
Cluster-22178.55362 GO:0042742 GO:0035556 GO:0005515
Cluster-22178.55363 GO:0016021
Cluster-22178.55365 GO:0015992 GO:0006123 GO:0004129 GO:0005515
GO:0005739 GO:0045277
Cluster-22178.55366 GO:0007165 GO:0006270
Cluster-22178.55368 GO:0006351 GO:0017111 GO:0003700 GO:0005515
GO:0005524 GO:0005667
Cluster-22178.55369 GO:0005515
Cluster-22178.55373 GO:0005515
Cluster-22178.55374 GO:0006355 GO:0043565 GO:0003700 GO:0005667
Cluster-22178.55377 GO:0005524 GO:0016772
Cluster-22178.55381 GO:0000398 GO:0007009 GO:0005634
Cluster-22178.55383 GO:0002949 GO:0006260 GO:0035988 GO:0006310
GO:0002062 GO:0006576 GO:0006281 GO:0006355 GO:0042246
GO:0016887 GO:0005524 GO:0016787 GO:0009378 GO:0003677
GO:0003924 GO:0003678 GO:0005525 GO:0008134 GO:0016301
GO:0005657 GO:0009379 GO:0005667 GO:0005634

Cluster-22178.55384 GO:0016765
Cluster-22178.55385 GO:0031505 GO:0009229 GO:0030975 GO:0019031
Cluster-22178.55386 GO:0003676
Cluster-22178.55387 GO:0006779 GO:0019354 GO:0055114 GO:0043115
GO:0008641 GO:0051287
Cluster-22178.55389 GO:0006811 GO:0005216 GO:0016020
Cluster-22178.55391 GO:0005515
Cluster-22178.55395 GO:0006351 GO:0043565 GO:0003677
Cluster-22178.55404 GO:0006412 GO:0042254 GO:0003735 GO:0005515
GO:0005622 GO:0005840
Cluster-22178.55405 GO:0006571 GO:0006694 GO:0015940 GO:0009094
GO:0019521 GO:0055114 GO:0008210 GO:0000162 GO:0046168
GO:0008209 GO:0008207 GO:0006098 GO:0004665 GO:0003824
GO:0008677 GO:0004616 GO:0016616 GO:0008977 GO:0003854
GO:0051287 GO:0050662 GO:0016021
Cluster-22178.55407 GO:0008152 GO:0003824
Cluster-22178.55409 GO:0006470 GO:0006402 GO:0006468 GO:0051252
GO:0004535 GO:0046872 GO:0005524 GO:0004672 GO:0003676
GO:0003723 GO:0008138 GO:0005737 GO:0005634
Cluster-22178.5541 GO:0030261 GO:0003677
Cluster-22178.55411 GO:0005515
Cluster-22178.55412 GO:0006810 GO:0055085 GO:0030150 GO:0005215
GO:0005515 GO:0016020 GO:0005742
Cluster-22178.55413 GO:0003677 GO:0046983
Cluster-22178.55414 GO:0015893 GO:0030436 GO:0006855 GO:0015238
GO:0005886 GO:0042601
Cluster-22178.55415 GO:0005515
Cluster-22178.55416 GO:0007219 GO:0045944 GO:0003713 GO:0005667
GO:0016607
Cluster-22178.55419 GO:0006468 GO:0006465 GO:0016773 GO:0005524
GO:0004672 GO:0008233 GO:0016757 GO:0005787 GO:0016020
GO:0016021
Cluster-22178.55421 GO:0007165 GO:0040007 GO:0008283 GO:0008083

GO:0005515 GO:0016787 GO:0004190 GO:0016021
Cluster-22178.55424 GO:0042254 GO:0006412 GO:0003735 GO:0005622
GO:0005783 GO:0005840
Cluster-22178.55426 GO:0006270
Cluster-22178.55428 GO:0022900 GO:0006955 GO:0007165 GO:0016032
GO:0006355 GO:0005164 GO:0003677 GO:0003682 GO:0000785
GO:0000775 GO:0016020 GO:0019012 GO:0005747 GO:0005634
GO:0005739
Cluster-22178.55429 GO:0043165 GO:0005509 GO:0019867
Cluster-22178.55430 GO:0043165 GO:0005509 GO:0019867
Cluster-22178.55431 GO:0016787 GO:0019028
Cluster-22178.55436 GO:0005524 GO:0005515 GO:0003676 GO:0005634
Cluster-22178.55437 GO:0005515 GO:0003676 GO:0005524 GO:0005634
Cluster-22178.55438 GO:0007049 GO:0007015 GO:0006397 GO:0010508
GO:0051607 GO:0000921 GO:0006357 GO:0006914 GO:0009252
GO:0007155 GO:0006897 GO:0006457 GO:0008092 GO:0051082
GO:0042803 GO:0001104 GO:0003774 GO:0000774 GO:0016755
GO:0051087 GO:0003677 GO:0009288 GO:0005737 GO:0000445
GO:0016021 GO:0016272 GO:0005940 GO:0005815 GO:0019898
GO:0016459 GO:0016592
Cluster-22178.55439 GO:0009451 GO:0006298 GO:0006400 GO:0008033
GO:0030983 GO:0008176 GO:0019955 GO:0005524 GO:0016020
Cluster-22178.55440 GO:0005515 GO:0008146 GO:0015016 GO:0016787

Cluster-22178.55441 GO:0006952 GO:0005509
Cluster-22178.55442 GO:0055114 GO:0006520 GO:0008152 GO:0016491

Cluster-22178.55443 GO:0055114
Cluster-22178.55445 GO:0005515
Cluster-22178.55458 GO:0040007 GO:0008283 GO:0006955 GO:0007165
GO:0006464 GO:0019752 GO:0030170 GO:0008083 GO:0016831
GO:0005134 GO:0016151 GO:0005576 GO:0005893
Cluster-22178.55460 GO:0042967 GO:0006355 GO:0003712 GO:0008270

GO:0004402 GO:0000123 GO:0005667 GO:0005634

Cluster-22178.55462 GO:0006479 GO:0006351 GO:0006468 GO:0006481 GO:0006383 GO:0006144 GO:0006206 GO:0003899 GO:0003677 GO:0004671 GO:0005524 GO:0004672 GO:0005730 GO:0005666 GO:0016021

Cluster-22178.55463 GO:0006550 GO:0006552 GO:0006813 GO:0006568 GO:0006574 GO:0008033 GO:0055114 GO:0018874 GO:0019354 GO:0006631 GO:0006633 GO:0006779 GO:0016117 GO:0006554 GO:0016705 GO:0043115 GO:0003857 GO:0050661 GO:0071949 GO:0050660 GO:0050151 GO:0008270 GO:0004499 GO:0016491

Cluster-22178.55465 GO:0016043 GO:0000398 GO:0003779 GO:0005681

Cluster-22178.55466 GO:0016579 GO:0036459

Cluster-22178.55467 GO:0006413 GO:0006446 GO:0003743 GO:0005515 GO:0005840

Cluster-22178.55472 GO:0016020

Cluster-22178.55476 GO:0048870 GO:0006914 GO:0051607 GO:0006606 GO:0008092 GO:0045502 GO:0008134 GO:0042803 GO:0005737 GO:0009424 GO:0030286 GO:0019898 GO:0005667 GO:0031514

Cluster-22178.55484 GO:0007224

Cluster-22178.55490 GO:0003712 GO:0003724 GO:0016818 GO:0005667 GO:0005634

Cluster-22178.55491 GO:0006479 GO:0006464 GO:0046500 GO:0004719 GO:0008168

Cluster-22178.55497 GO:0005515

Cluster-22178.55498 GO:0006508 GO:0005515 GO:0004252

Cluster-22178.55499 GO:0005515

Cluster-22178.55500 GO:0019089

Cluster-22178.55504 GO:0015986 GO:0006508 GO:0015992 GO:0006308 GO:0051082 GO:0004252 GO:0005515 GO:0008855 GO:0015078 GO:0009318 GO:0045263 GO:0016021

Cluster-22178.55508 GO:0007224

Cluster-22178.55510 GO:0005515

Cluster-22178.55511 GO:0016579 GO:0036459

Cluster-22178.55512 GO:0046740 GO:0006771 GO:0006810 GO:0015992
GO:0006123 GO:0055085 GO:0006570 GO:0006814 GO:0019497
GO:0006470 GO:0008138 GO:0003677 GO:0008270 GO:0003993
GO:0003723 GO:0005515 GO:0046872 GO:0005215 GO:0004725
GO:0004129 GO:0005739 GO:0016020 GO:0033644 GO:0045277
GO:0016021
Cluster-22178.55513 GO:0006771 GO:0046740 GO:0006810 GO:0015992
GO:0055085 GO:0006123 GO:0006814 GO:0006570 GO:0019497
GO:0006470 GO:0003677 GO:0008138 GO:0008270 GO:0003993
GO:0003723 GO:0005515 GO:0046872 GO:0004129 GO:0005215
GO:0004725 GO:0005739 GO:0016020 GO:0033644 GO:0016021
GO:0045277
Cluster-22178.55514 GO:0006814 GO:0006810 GO:0006123 GO:0055085
GO:0015992 GO:0003723 GO:0004129 GO:0005215 GO:0008270
GO:0016020 GO:0005739 GO:0016021 GO:0045277
Cluster-22178.55520 GO:0016310 GO:0006811 GO:0006468 GO:0006855
GO:0055085 GO:0009069 GO:0007268 GO:0006810 GO:0007165
GO:0015893 GO:0005234 GO:0015297 GO:0004970 GO:0015238
GO:0005524 GO:0004674 GO:0016020
Cluster-22178.55521 GO:0006855 GO:0055085 GO:0009069 GO:0006810
GO:0007268 GO:0007165 GO:0015893 GO:0016310 GO:0006811
GO:0006468 GO:0005524 GO:0004674 GO:0004970 GO:0015297
GO:0005234 GO:0015238 GO:0016020
Cluster-22178.55523 GO:0007186 GO:0004190 GO:0005515 GO:0004930
GO:0016021
Cluster-22178.55526 GO:0005515
Cluster-22178.55527 GO:0046034 GO:0006139 GO:0006461 GO:0019627
GO:0006144 GO:0006206 GO:0004127 GO:0016151 GO:0005524
GO:0004017
Cluster-22178.55528 GO:0032040
Cluster-22178.55529 GO:0008033 GO:0051252 GO:0007165 GO:0003676
GO:0005179 GO:0004526 GO:0030677 GO:0005576 GO:0005634
Cluster-22178.55536 GO:0005515

Cluster-22178.55537 GO:0030472 GO:0006891 GO:0005515 GO:0042729
GO:0072686 GO:0017119
Cluster-22178.55538 GO:0015631 GO:0045298
Cluster-22178.55542 GO:0016226 GO:0016559 GO:0006355 GO:0016567
GO:0008270 GO:0046872 GO:0043565 GO:0051536 GO:0004842
GO:0005515 GO:0005737 GO:0005779 GO:0016020
Cluster-22178.55543 GO:0018279 GO:0005789
Cluster-22178.55547 GO:0071705 GO:0003676 GO:0045095
Cluster-22178.55548 GO:0006376 GO:0003729 GO:0005685
Cluster-22178.55549 GO:0007186 GO:0004930 GO:0016020 GO:0016021
Cluster-22178.55551 GO:0006281 GO:0008270 GO:0046872
Cluster-22178.55556 GO:0005515 GO:0005509 GO:0004064
Cluster-22178.55560 GO:0008152 GO:0016791 GO:0003824 GO:0046872
GO:0016021
Cluster-22178.55561 GO:0008152 GO:0003824 GO:0016791 GO:0046872
GO:0016021
Cluster-22178.55562 GO:0032183 GO:0070140
Cluster-22178.55563 GO:0003724 GO:0005525 GO:0016887 GO:0003924
GO:0005524 GO:0003774 GO:0005515 GO:0003723 GO:0016459
Cluster-22178.55565 GO:0015074 GO:0042967 GO:0050832 GO:0042742
GO:0003676 GO:0008080 GO:0019239 GO:0003677 GO:0031224
Cluster-22178.55567 GO:0050832 GO:0042967 GO:0015074 GO:0042742
GO:0008080 GO:0003676 GO:0003677 GO:0019239 GO:0031224
Cluster-22178.55568 GO:0006298 GO:0005524 GO:0030983
Cluster-22178.55571 GO:0007049 GO:0006260 GO:0015992 GO:0006810
GO:0006355 GO:0015986 GO:0015078 GO:0003700 GO:0043565
GO:0003677 GO:0045263 GO:0005634 GO:0005667
Cluster-22178.55573 GO:0007264 GO:0005525
Cluster-22178.55574 GO:0006397 GO:0005634
Cluster-22178.55575 GO:0005634
Cluster-22178.55576 GO:0016055 GO:0006281 GO:0051382
Cluster-22178.55577 GO:0015992 GO:0055114 GO:0006744 GO:0006814
GO:0006120 GO:0008137

Cluster-22178.55579 GO:0016787
Cluster-22178.55583 GO:0003676
Cluster-22178.55586 GO:0005515
Cluster-22178.55587 GO:0019521 GO:0055114 GO:0008152 GO:0006098
GO:0007005 GO:0016491 GO:0004616 GO:0031305
Cluster-22178.55588 GO:0006879 GO:0046872 GO:0008199
Cluster-22178.5559 GO:0006606 GO:0006260 GO:0051028 GO:0048309
GO:0055114 GO:0051382 GO:0019072 GO:0016903 GO:0008134
GO:0042803 GO:0045502 GO:0003723 GO:0005815 GO:0030286
GO:0005667 GO:0042025 GO:0000776
Cluster-22178.55593 GO:0008063 GO:0008270 GO:0005515
Cluster-22178.55595 GO:0016567 GO:0016032 GO:0090263 GO:0005515
GO:0004842 GO:0046872 GO:0008270 GO:0005680
Cluster-22178.55596 GO:0009236 GO:0006824 GO:0006355 GO:0015087
GO:0003676 GO:0003700 GO:0005667 GO:0016020
Cluster-22178.55597 GO:0006824 GO:0009236 GO:0006355 GO:0003700
GO:0003676 GO:0015087 GO:0005667 GO:0016020
Cluster-22178.55606 GO:0006886 GO:0016192 GO:0005515 GO:0030117
Cluster-22178.55607 GO:0006886 GO:0016192 GO:0005515 GO:0030117
Cluster-22178.55612 GO:0047355 GO:0003677 GO:0016020
Cluster-22178.55614 GO:0004672
Cluster-22178.55618 GO:0042254 GO:0006412 GO:0019843 GO:0003735
GO:0005840
Cluster-22178.55620 GO:0009405 GO:0006811 GO:0005216 GO:0008375
GO:0005576 GO:0016020
Cluster-22178.55624 GO:0006260 GO:0003677 GO:0005664
Cluster-22178.55626 GO:0007016 GO:0007165 GO:0042254 GO:0006412
GO:0005158 GO:0003779 GO:0005200 GO:0000062 GO:0003735
GO:0005622 GO:0005856 GO:0005899 GO:0005840 GO:0001726
GO:0005925
Cluster-22178.55627 GO:0046931 GO:0006952 GO:0007165 GO:0006810
GO:0006812 GO:0052331 GO:0005126 GO:0015267 GO:0005576
GO:0046930

Cluster-22178.55629 GO:0006771 GO:0019497 GO:0006260 GO:0008408
GO:0000166 GO:0003887 GO:0003677 GO:0003993 GO:0042575
Cluster-22178.55632 GO:0005525 GO:0003924 GO:0005861
Cluster-22178.55633 GO:0045892 GO:0005515 GO:0005634
Cluster-22178.55637 GO:0006508 GO:0008234
Cluster-22178.55639 GO:0005515 GO:0008270 GO:0005622
Cluster-22178.5564 GO:0030154 GO:0016021
Cluster-22178.55640 GO:0005515 GO:0008270 GO:0005622
Cluster-22178.55641 GO:0006334 GO:0007283 GO:0008270 GO:0005524
GO:0046872 GO:0003677 GO:0005515 GO:0000786 GO:0005634
Cluster-22178.55642 GO:0006468 GO:0055114 GO:0004497 GO:0016706
GO:0016715 GO:0005507 GO:0008168 GO:0004672 GO:0005524
Cluster-22178.55643 GO:0051258 GO:0070206 GO:0030168 GO:0016973
GO:0007165 GO:0005102 GO:0030674 GO:0003677 GO:0005643
GO:0005577
Cluster-22178.55644 GO:0007264 GO:0015684 GO:0015093 GO:0005525
GO:0019028 GO:0016021
Cluster-22178.55648 GO:0042254 GO:0006904 GO:0015031 GO:0005515
GO:0000145
Cluster-22178.55652 GO:0006813 GO:0006351 GO:0006412 GO:0006886
GO:0042254 GO:0016567 GO:0006464 GO:0006206 GO:0006144
GO:0006888 GO:0030955 GO:0005515 GO:0003899 GO:0004519
GO:0046872 GO:0005249 GO:0016151 GO:0003676 GO:0003677
GO:0003735 GO:0008270 GO:0004842 GO:0005737 GO:0005840
GO:0016021 GO:0005730 GO:0030127 GO:0008076 GO:0005622
GO:0005680
Cluster-22178.55655 GO:0035556 GO:0006144 GO:0006206 GO:0016567
GO:0055114 GO:0006351 GO:0006813 GO:0004842 GO:0047134
GO:0008270 GO:0003677 GO:0046872 GO:0005249 GO:0005515
GO:0003899 GO:0030955 GO:0005680 GO:0008076 GO:0005730
GO:0016021 GO:0005737
Cluster-22178.55657 GO:0006144 GO:0006888 GO:0006464 GO:0006206
GO:0042254 GO:0016567 GO:0006886 GO:0006412 GO:0006351

GO:0008270 GO:0004842 GO:0003735 GO:0003677 GO:0046872
GO:0003676 GO:0016151 GO:0003899 GO:0005515 GO:0004519
GO:0005622 GO:0005680 GO:0005730 GO:0030127 GO:0005737
GO:0005840
Cluster-22178.55658 GO:0006206 GO:0006144 GO:0035556 GO:0016567
GO:0055114 GO:0006813 GO:0006351 GO:0008270 GO:0047134
GO:0004842 GO:0003677 GO:0046872 GO:0005249 GO:0030955
GO:0003899 GO:0005515 GO:0005680 GO:0005730 GO:0008076
GO:0005737 GO:0016021
Cluster-22178.55659 GO:0006351 GO:0006144 GO:0006206 GO:0005515
GO:0003899 GO:0003677 GO:0008270 GO:0046872 GO:0005730
Cluster-22178.55661 GO:0006464 GO:0006206 GO:0006144 GO:0042254
GO:0006281 GO:0006351 GO:0006412 GO:0008270 GO:0003677
GO:0003735 GO:0046872 GO:0016151 GO:0003676 GO:0003899
GO:0004519 GO:0005622 GO:0005730 GO:0005840
Cluster-22178.55665 GO:0019370 GO:0008270 GO:0008237
Cluster-22178.55669 GO:0006810 GO:0009405 GO:0006281 GO:0000077
GO:0055085 GO:0003723 GO:0004222 GO:0008270 GO:0016020
GO:0005576 GO:0030896
Cluster-22178.5567 GO:0042720 GO:0019031
Cluster-22178.55670 GO:0008376 GO:0016757 GO:0032580 GO:0016020
Cluster-22178.55672 GO:0006355 GO:0043565 GO:0005515 GO:0003700
GO:0005667
Cluster-22178.55674 GO:0033588
Cluster-22178.55677 GO:0006260 GO:0055114
Cluster-22178.55678 GO:0008033 GO:0006269 GO:0009451 GO:0006351
GO:0003896 GO:0009020 GO:0005657 GO:0005730
Cluster-22178.55681 GO:0003676
Cluster-22178.55685 GO:0006351 GO:0006412 GO:0006265 GO:0042254
GO:0006464 GO:0006206 GO:0006144 GO:0003899 GO:0016151
GO:0003676 GO:0003677 GO:0003735 GO:0003916 GO:0008270
GO:0005840 GO:0005694 GO:0005730 GO:0005622
Cluster-22178.55686 GO:0008033 GO:0009451 GO:0008175 GO:0005515

Cluster-22178.55688 GO:0006260 GO:0000166 GO:0003887 GO:0003677
GO:0008408 GO:0001882 GO:0008270 GO:0042575 GO:0005634
Cluster-22178.55689 GO:0045892 GO:0008270 GO:0005634
Cluster-22178.55692 GO:0006355 GO:0006955 GO:0007165 GO:0005515
GO:0003677 GO:0005125 GO:0005634 GO:0005576
Cluster-22178.55695 GO:0008643 GO:0006820 GO:0015725 GO:0006812
GO:0046411 GO:0015649 GO:0016021
Cluster-22178.55696 GO:0006820 GO:0008643 GO:0015725 GO:0006812
GO:0046411 GO:0015649 GO:0016021
Cluster-22178.55698 GO:0045893 GO:0034508 GO:0015672 GO:0006812
GO:0007275 GO:0010508 GO:0008270 GO:0005261 GO:0005515
GO:0000776 GO:0016020
Cluster-22178.55699 GO:0007165 GO:0006955 GO:0046872 GO:0005164
GO:0016020
Cluster-22178.55700 GO:0006508 GO:0008270 GO:0046872 GO:0004252
GO:0008236 GO:0005618
Cluster-22178.55701 GO:0007165 GO:0006955 GO:0005164 GO:0016020
Cluster-22178.55703 GO:0006355 GO:0005515 GO:0005634
Cluster-22178.55704 GO:0055085 GO:0006810 GO:0005524 GO:0016887
GO:0005215 GO:0042626 GO:0003676 GO:0000166 GO:0003677
GO:0016021
Cluster-22178.55707 GO:0007264 GO:0006811 GO:0055114 GO:0010181
GO:0005216 GO:0005525 GO:0016491 GO:0016020 GO:0005615
Cluster-22178.55708 GO:0031175 GO:0030507 GO:0005516 GO:0008091
Cluster-22178.55709 GO:0006281 GO:0046872 GO:0008270
Cluster-22178.55710 GO:0003676
Cluster-22178.55712 GO:0000271 GO:0055085 GO:0006821 GO:0005247
GO:0016020
Cluster-22178.55713 GO:0005515 GO:0016773 GO:0005737
Cluster-22178.55714 GO:0006144 GO:0015992 GO:0006123 GO:0000256
GO:0006807 GO:0004037 GO:0004129 GO:0019901 GO:0045277
Cluster-22178.55716 GO:0009395 GO:0035556 GO:0007205 GO:0046486

GO:0004143 GO:0016301
Cluster-22178.55720 GO:0046872
Cluster-22178.55726 GO:0046872
Cluster-22178.55731 GO:0051016 GO:0006508 GO:0008152 GO:0016787
GO:0008236 GO:0008290
Cluster-22178.55732 GO:0006368 GO:0008023
Cluster-22178.55733 GO:0003950 GO:0046872
Cluster-22178.55734 GO:0006144 GO:0007165 GO:0004114 GO:0005515

Cluster-22178.55735 GO:0003950
Cluster-22178.55736 GO:0005975 GO:0004415 GO:0005515
Cluster-22178.55738 GO:0006355 GO:0010508 GO:0051028 GO:0048309
GO:0006606 GO:0008134 GO:0005524 GO:0003700 GO:0005667
Cluster-22178.55740 GO:0005515
Cluster-22178.55741 GO:0005515
Cluster-22178.55743 GO:0006468 GO:0004672 GO:0005524 GO:0045502
GO:0008134 GO:0042803 GO:0005856 GO:0030286 GO:0005667
Cluster-22178.55744 GO:0006576 GO:0007264 GO:0015684 GO:0003924
GO:0015093 GO:0005524 GO:0005525 GO:0016021
Cluster-22178.55745 GO:0007264 GO:0006576 GO:0015684 GO:0015093
GO:0005524 GO:0003924 GO:0005525 GO:0016021
Cluster-22178.55751 GO:0006367 GO:0007010 GO:0006270 GO:0032968
GO:0003677 GO:0003779 GO:0005634 GO:0032040
Cluster-22178.55752 GO:0015992 GO:0015986 GO:0015078 GO:0000276
Cluster-22178.55758 GO:0000002 GO:0016887 GO:0003924 GO:0005524
GO:0003724 GO:0005525 GO:0003723 GO:0016787 GO:0003677
GO:0000262 GO:0045095
Cluster-22178.55760 GO:0005515
Cluster-22178.55762 GO:0009877 GO:0009312 GO:0008757
Cluster-22178.55763 GO:0022904 GO:0016651 GO:0046983 GO:0005743
Cluster-22178.55765 GO:0042254 GO:0006412 GO:0055114 GO:0015074
GO:0003735 GO:0003824 GO:0050660 GO:0016491 GO:0005840
GO:0005622

Cluster-22178.55766 GO:0055114 GO:0015074 GO:0042254 GO:0006412
GO:0003824 GO:0050660 GO:0016491 GO:0003735 GO:0005840
GO:0005622
Cluster-22178.55769 GO:0046983
Cluster-22178.55770 GO:0008152 GO:0003824 GO:0016836
Cluster-22178.55773 GO:0016579 GO:0016192 GO:0006886 GO:0006506
GO:0015991 GO:0036459 GO:0005515 GO:0016820 GO:0016746
GO:0016021 GO:0000221 GO:0005789 GO:0030117
Cluster-22178.55775 GO:0051252 GO:0004540 GO:0003677
Cluster-22178.55776 GO:0005515
Cluster-22178.55778 GO:0006310 GO:0006281
Cluster-22178.55781 GO:0017038 GO:0007165 GO:0005158 GO:0008270
GO:0005524 GO:0003676 GO:0016787 GO:0003677 GO:0005899
GO:0005634 GO:0016020
Cluster-22178.55788 GO:0006470 GO:0006570 GO:0006355 GO:0004725
GO:0003700 GO:0043565 GO:0008138 GO:0016020 GO:0005667
Cluster-22178.55795 GO:0006468 GO:0016192 GO:0005524 GO:0004672
GO:0016021
Cluster-22178.55798 GO:0016746
Cluster-22178.55805 GO:0005737
Cluster-22178.55811 GO:0006144 GO:0006508 GO:0003968 GO:0017111
GO:0004197 GO:0016817 GO:0031379
Cluster-22178.55813 GO:0006886 GO:0006367 GO:0006888 GO:0008270
GO:0016021 GO:0030127 GO:0005672
Cluster-22178.55816 GO:0006355 GO:0006468 GO:0006298 GO:0004672
GO:0005524 GO:0016773 GO:0043565 GO:0003700 GO:0030983
GO:0005667 GO:0016020
Cluster-22178.55817 GO:0055114 GO:0006631 GO:0071949 GO:0016491
GO:0050151
Cluster-22178.55818 GO:0006269 GO:0006351 GO:0003896 GO:0019825
GO:0005515 GO:0005198 GO:0020037 GO:0003677 GO:0005657
GO:0005634 GO:0019028 GO:0005730
Cluster-22178.55819 GO:0006269 GO:0006351 GO:0003896 GO:0019825

GO:0005515 GO:0005198 GO:0020037 GO:0003677 GO:0005657
GO:0005634 GO:0005730
Cluster-22178.55821 GO:0016579 GO:0036459
Cluster-22178.55822 GO:0043123 GO:0045766 GO:0043066
Cluster-22178.55823 GO:0045766 GO:0043066 GO:0043123
Cluster-22178.55824 GO:0006308 GO:0000077 GO:0006376 GO:0006281
GO:0006355 GO:0006810 GO:0043565 GO:0000014 GO:0003700
GO:0003729 GO:0005685 GO:0016021 GO:0005739 GO:0005667
Cluster-22178.55826 GO:0046872 GO:0005515 GO:0032040
Cluster-22178.55828 GO:0006355 GO:0007165 GO:0035257 GO:0005515
GO:0003713 GO:0046983 GO:0005667 GO:0005634
Cluster-22178.55829 GO:0006355 GO:0005515 GO:0005634
Cluster-22178.55833 GO:0055085 GO:0032007 GO:0048011 GO:0005515
GO:0046872 GO:0008270 GO:0016021
Cluster-22178.55834 GO:0055114
Cluster-22178.55836 GO:0003824 GO:0030151 GO:0030170
Cluster-22178.55839 GO:0005509 GO:0003676
Cluster-22178.55841 GO:0005509 GO:0003676 GO:0005515
Cluster-22178.55842 GO:0003676 GO:0005515 GO:0005509
Cluster-22178.55844 GO:0015986 GO:0006508 GO:0007165 GO:0015992
GO:0005044 GO:0004197 GO:0004252 GO:0008270 GO:0015078
GO:0016020
Cluster-22178.55845 GO:0015992 GO:0007165 GO:0006952 GO:0006508
GO:0015986 GO:0015078 GO:0008270 GO:0004252 GO:0003677
GO:0004197 GO:0005044 GO:0016020
Cluster-22178.55846 GO:0006367 GO:0006123 GO:0015992 GO:0004129
GO:0046872 GO:0008270 GO:0005634 GO:0005672 GO:0045277
Cluster-22178.55848 GO:0019068 GO:0043565 GO:0005515 GO:0005198
GO:0005634
Cluster-22178.55850 GO:0006289 GO:0006355 GO:0016192 GO:0016021
GO:0000439
Cluster-22178.55851 GO:0003677 GO:0005515
Cluster-22178.55853 GO:0005515 GO:0003677

Cluster-22178.55859 GO:0006468 GO:0006606 GO:0016773 GO:0004672
GO:0005524 GO:0016020
Cluster-22178.55860 GO:0006367 GO:0032968 GO:0003677 GO:0005634
Cluster-22178.55861 GO:0006359
Cluster-22178.55866 GO:0022900 GO:0006904 GO:0006118 GO:0006260
GO:0006891 GO:0015031 GO:0006457 GO:0007067 GO:0009055
GO:0020037 GO:0051082 GO:0005506 GO:0000775 GO:0005634
GO:0042597 GO:0016272 GO:0000145 GO:0017119
Cluster-22178.55867 GO:0022900 GO:0006904 GO:0006118 GO:0006891
GO:0006260 GO:0015031 GO:0006457 GO:0007067 GO:0009055
GO:0020037 GO:0051082 GO:0005506 GO:0000775 GO:0005634
GO:0042597 GO:0016272 GO:0000145 GO:0017119
Cluster-22178.55870 GO:0006351 GO:0006810 GO:0006468 GO:0055085
GO:0005515 GO:0005215 GO:0016773 GO:0004672 GO:0005524
GO:0016020 GO:0016021
Cluster-22178.55871 GO:0006468 GO:0006351 GO:0006810 GO:0055085
GO:0005515 GO:0005524 GO:0004672 GO:0016773 GO:0005215
GO:0016020 GO:0016021
Cluster-22178.55872 GO:0006355 GO:0005524 GO:0043565 GO:0005515
GO:0003700 GO:0005667
Cluster-22178.55873 GO:0019722 GO:0006352 GO:0006402 GO:0006730
GO:0007018 GO:0051252 GO:0007017 GO:0006355 GO:0008017
GO:0016987 GO:0005524 GO:0003677 GO:0003676 GO:0003700
GO:0003777 GO:0046872 GO:0004535 GO:0005515 GO:0003723
GO:0016740 GO:0005667 GO:0045298 GO:0005874 GO:0005737
GO:0005634
Cluster-22178.55875 GO:0043093 GO:0000917 GO:0008376 GO:0005737
GO:0032580
Cluster-22178.55876 GO:0006570 GO:0006470 GO:0004725 GO:0008138

Cluster-22178.55877 GO:0046500 GO:0006260 GO:0035556 GO:0030488
GO:0006464 GO:0006554 GO:0006479 GO:0006351 GO:0009451
GO:0008033 GO:0003677 GO:0016429 GO:0003887 GO:0018024

GO:0004719 GO:0031515 GO:0042575
Cluster-22178.55878 GO:0035023 GO:0043087 GO:0005089 GO:0005515

Cluster-22178.5588 GO:0006355 GO:0007186 GO:0015684 GO:0007165
GO:0006810 GO:0007264 GO:0008134 GO:0000166 GO:0003723
GO:0001671 GO:0004871 GO:0005525 GO:0003924 GO:0043531
GO:0019001 GO:0031683 GO:0003677 GO:0051087 GO:0003724
GO:0016887 GO:0015093 GO:0005524 GO:0016021 GO:0016020
GO:0005667
Cluster-22178.55881 GO:0006952 GO:0008284 GO:0005515
Cluster-22178.55884 GO:0042254 GO:0006412 GO:0007165 GO:0006281
GO:0006355 GO:0003735 GO:0003677 GO:0004872 GO:0008168
GO:0008270 GO:0005840 GO:0005622
Cluster-22178.55885 GO:0030153 GO:0006367 GO:0006955 GO:0006357
GO:0015643 GO:0004252 GO:0001104 GO:0005672 GO:0016592
GO:0019814 GO:0005886
Cluster-22178.55886 GO:0006570 GO:0006470 GO:0004725 GO:0008138
GO:0046872
Cluster-22178.55891 GO:0007165 GO:0005509 GO:0005578
Cluster-22178.55894 GO:0006810 GO:0006811 GO:0005230 GO:0016020
Cluster-22178.55896 GO:0006464 GO:0015031 GO:0005515
Cluster-22178.55899 GO:0014068 GO:0050776 GO:0006351 GO:0007165
GO:0008270 GO:0005102 GO:0003676 GO:0043548 GO:0016020
Cluster-22178.55901 GO:0016114 GO:0006694 GO:0055114 GO:0003676
GO:0050660 GO:0004506 GO:0016021
Cluster-22178.55902 GO:0006952 GO:0005576
Cluster-22178.55905 GO:0019082 GO:0016032 GO:0006281 GO:0007049
GO:0030150 GO:0005515 GO:0003677 GO:0003824 GO:0005096
GO:0005742 GO:0034464
Cluster-22178.55906 GO:0016192 GO:0019217 GO:0006886 GO:0015992
GO:0006123 GO:0005515 GO:0000062 GO:0004129 GO:0030117
GO:0045277
Cluster-22178.55907 GO:0007049 GO:0030150 GO:0019082 GO:0006281

GO:0016032 GO:0003824 GO:0005096 GO:0005515 GO:0034464
GO:0005742
Cluster-22178.55909 GO:0045892 GO:0005634
Cluster-22178.55910 GO:0032065 GO:0005543 GO:0005515 GO:0005938
Cluster-22178.55914 GO:0032780 GO:0016820 GO:0042030 GO:0046872
GO:0005739
Cluster-22178.55915 GO:0006506 GO:0016758 GO:0016021
Cluster-22178.55918 GO:0006418 GO:0005524 GO:0004812 GO:0000166
GO:0005515
Cluster-22178.55923 GO:0008152 GO:0008134 GO:0003824 GO:0005667
GO:0005634
Cluster-22178.55924 GO:0007094 GO:0006355 GO:0005515 GO:0003700
GO:0042025 GO:0005667
Cluster-22178.55925 GO:0003677 GO:0008270
Cluster-22178.55930 GO:0009405 GO:0006810 GO:0007049 GO:0019855
GO:0017053 GO:0005576
Cluster-22178.55935 GO:0006355 GO:0043401 GO:0043565 GO:0008270
GO:0003700 GO:0016021 GO:0005667 GO:0005634
Cluster-22178.55940 GO:0008610 GO:0006396 GO:0016747 GO:0008452

Cluster-22178.55942 GO:0005978 GO:0005982 GO:0005985 GO:0004373

Cluster-22178.55943 GO:0006396 GO:0008610 GO:0008452 GO:0016747

Cluster-22178.55945 GO:0032259 GO:0055114 GO:0006810 GO:0008168
GO:0005215 GO:0016972 GO:0016020
Cluster-22178.55950 GO:0003676
Cluster-22178.55952 GO:0005634
Cluster-22178.55954 GO:0006555 GO:0006457 GO:0006355 GO:0003700
GO:0008270 GO:0005198 GO:0008134 GO:0042803 GO:0051082
GO:0045502 GO:0030286 GO:0016272 GO:0005882 GO:0005667
GO:0048786
Cluster-22178.55955 GO:0015992 GO:0006123 GO:0006508 GO:0005524

GO:0008242 GO:0016817 GO:0016887 GO:0004129 GO:0016740
GO:0016788 GO:0004197 GO:0045277 GO:0005680
Cluster-22178.55957 GO:0016788
Cluster-22178.55958 GO:0055114 GO:0006468 GO:0016616 GO:0051287
GO:0005524 GO:0004672
Cluster-22178.55959 GO:0006270 GO:0003723 GO:0003676
Cluster-22178.55960 GO:0055114 GO:0007186 GO:0046872 GO:0016972
GO:0004930 GO:0016021
Cluster-22178.55963 GO:0019497 GO:0006771 GO:0005975 GO:0046872
GO:0004553 GO:0005515 GO:0003993
Cluster-22178.55964 GO:0042262 GO:0006464 GO:0007165 GO:0006355
GO:0015986 GO:0046933 GO:0008270 GO:0005515 GO:0003677
GO:0001664 GO:0045259
Cluster-22178.55965 GO:0006308 GO:0016310 GO:0019643 GO:0019069
GO:0009069 GO:0006094 GO:0006468 GO:0006099 GO:0007165
GO:0015977 GO:0008855 GO:0005096 GO:0004674 GO:0008964
GO:0009318
Cluster-22178.55966 GO:0042981
Cluster-22178.55967 GO:0007264 GO:0007186 GO:0007165 GO:0015684
GO:0003924 GO:0015093 GO:0019001 GO:0031683 GO:0004871
GO:0005525 GO:0016021
Cluster-22178.55969 GO:0050829 GO:0006260 GO:0019835 GO:0016021
Cluster-22178.55972 GO:0005634
Cluster-22178.55974 GO:0006813 GO:0006355 GO:0009252 GO:0006542
GO:0006811 GO:0006807 GO:0003677 GO:0004356 GO:0005249
GO:0008076 GO:0016020
Cluster-22178.55976 GO:0016042 GO:0019497 GO:0007586 GO:0006771
GO:0003993 GO:0008047 GO:0046872 GO:0005515 GO:0005576
Cluster-22178.55977 GO:0006351 GO:0006479 GO:0031167 GO:0006206
GO:0006144 GO:0008152 GO:0003677 GO:0003899 GO:0008168
GO:0005730
Cluster-22178.55979 GO:0005975 GO:0003824
Cluster-22178.55981 GO:0006811 GO:0055085 GO:0006355 GO:0005515

GO:0005216 GO:0005761 GO:0016020
Cluster-22178.55982 GO:0003677
Cluster-22178.55985 GO:0005515
Cluster-22178.55986 GO:0046872
Cluster-22178.55987 GO:0005515
Cluster-22178.55988 GO:0009271 GO:0006355 GO:0003676 GO:0005524
GO:0003677 GO:0016787 GO:0004003 GO:0005657
Cluster-22178.55990 GO:0006508 GO:0004222
Cluster-22178.55992 GO:0003824
Cluster-22178.55995 GO:0016887 GO:0005524
Cluster-22178.55998 GO:0006468 GO:0005515 GO:0005524 GO:0004672
GO:0016021
Cluster-22178.55999 GO:0006810 GO:0055114 GO:0055085 GO:0016301
GO:0016627 GO:0005524 GO:0003924 GO:0016887 GO:0005525
GO:0042626 GO:0016021
Cluster-22178.56002 GO:0006355 GO:0003700 GO:0005667
Cluster-22178.56004 GO:0007169 GO:0050830 GO:0006468 GO:0005524
GO:0016817 GO:0004714 GO:0005515 GO:0016020
Cluster-22178.56005 GO:0015992 GO:0015986 GO:0015078 GO:0000276
Cluster-22178.56007 GO:0006508 GO:0006144 GO:0004197 GO:0017111
GO:0003968 GO:0004252 GO:0031379
Cluster-22178.56008 GO:0006998 GO:0006406 GO:0006611 GO:0031965
GO:0005576
Cluster-22178.56010 GO:0005515
Cluster-22178.56011 GO:0006355 GO:0001104 GO:0016592
Cluster-22178.56012 GO:0048193 GO:0005887 GO:0005783
Cluster-22178.56013 GO:0006260 GO:0007165 GO:0005179 GO:0000808
GO:0005634 GO:0005576
Cluster-22178.56014 GO:0005515
Cluster-22178.56017 GO:0051694 GO:0005523
Cluster-22178.56020 GO:0009405
Cluster-22178.56022 GO:0055114 GO:0046872 GO:0016972 GO:0019028
Cluster-22178.56023 GO:0006511 GO:0031625

Cluster-22178.56024 GO:0046870
Cluster-22178.56029 GO:0046872
Cluster-22178.56030 GO:0006144 GO:0046034 GO:0046872 GO:0004017

Cluster-22178.56031 GO:0046872
Cluster-22178.56033 GO:0015693 GO:0015979 GO:0015095 GO:0016020
GO:0016021 GO:0009523 GO:0009539
Cluster-22178.56034 GO:0035556 GO:0005815
Cluster-22178.56035 GO:0006464 GO:0015031 GO:0032465 GO:0006915
GO:0016567 GO:0004842 GO:0005515 GO:0016020
Cluster-22178.56037 GO:0006206 GO:0006541 GO:0006221 GO:0016787
GO:0003883
Cluster-22178.56038 GO:0006206 GO:0006221 GO:0006541 GO:0016787
GO:0003883
Cluster-22178.56040 GO:0006355 GO:0007264 GO:0009271 GO:0005525

Cluster-22178.56041 GO:0009271 GO:0007264 GO:0006355 GO:0005525

Cluster-22178.56046 GO:0050840 GO:0005515 GO:0005576 GO:0019028
Cluster-22178.56047 GO:0055085 GO:0016021 GO:0016020
Cluster-22178.56049 GO:0006270 GO:0006275 GO:0005524 GO:0043565

Cluster-22178.56050 GO:0005515
Cluster-22178.56051 GO:0090063 GO:0000226 GO:0005634 GO:0005815
GO:0000922
Cluster-22178.56056 GO:0006810 GO:0006468 GO:0015696 GO:0008519
GO:0016787 GO:0004672 GO:0005524 GO:0016020 GO:0031224
GO:0005794 GO:0016021
Cluster-22178.56058 GO:0019062 GO:0019058 GO:0007155 GO:0006355
GO:0003677
Cluster-22178.56059 GO:0016021
Cluster-22178.56061 GO:0035556 GO:0055114 GO:0006468 GO:0047134
GO:0005524 GO:0004672 GO:0016773 GO:0005515 GO:0016020

Cluster-22178.56062 GO:0005515
Cluster-22178.56063 GO:0005515
Cluster-22178.56065 GO:0006355 GO:0009271
Cluster-22178.56066 GO:0006412 GO:0006351 GO:0006144 GO:0006206
GO:0045116 GO:0007264 GO:0042254 GO:0003924 GO:0016881
GO:0005525 GO:0003899 GO:0005515 GO:0005524 GO:0003735
GO:0003677 GO:0005840 GO:0005622 GO:0005730
Cluster-22178.56067 GO:0030145 GO:0004177 GO:0019031
Cluster-22178.56068 GO:0006629 GO:0045892 GO:0003677
Cluster-22178.56075 GO:0006606 GO:0010506 GO:0006355 GO:0003700
GO:0005615 GO:0005667
Cluster-22178.56076 GO:0006144 GO:0006464 GO:0006206 GO:0006281
GO:0006351 GO:0046872 GO:0008270 GO:0003676 GO:0016151
GO:0003899 GO:0003677 GO:0005730
Cluster-22178.5608 GO:0008270
Cluster-22178.56084 GO:0006468 GO:0016773 GO:0004672 GO:0005524
GO:0016020
Cluster-22178.56085 GO:0006468 GO:0042254 GO:0006412 GO:0005524
GO:0004672 GO:0003735 GO:0005622 GO:0005840
Cluster-22178.56086 GO:0005515
Cluster-22178.56087 GO:0006302 GO:0006310 GO:0005515 GO:0003677
GO:0005634
Cluster-22178.56088 GO:0005515
Cluster-22178.56092 GO:0043085 GO:0000290 GO:0008047
Cluster-22178.56094 GO:0015992 GO:0055114 GO:0006120 GO:0006744
GO:0006122 GO:0006810 GO:0042773 GO:0006814 GO:0005215
GO:0008137 GO:0005750
Cluster-22178.56098 GO:0051321 GO:0055114 GO:0009401 GO:0018401
GO:0006525 GO:0006560 GO:0016491 GO:0016702 GO:0003677
GO:0004656 GO:0005634 GO:0005783
Cluster-22178.561 GO:0006355 GO:0006948
Cluster-22178.56105 GO:0006771 GO:0019497 GO:0005515 GO:0003993
GO:0046872

Cluster-22178.56107 GO:0007049 GO:0016310 GO:0000160 GO:0006886
GO:0006468 GO:0006260 GO:0006914 GO:0009069 GO:0005515
GO:0016757 GO:0004674 GO:0003774 GO:0005524 GO:0046983
GO:0000155 GO:0009365 GO:0016021 GO:0005801 GO:0016020
GO:0016459
Cluster-22178.56114 GO:0006811 GO:0007186 GO:0007165 GO:0006810
GO:0007268 GO:0004930 GO:0004970 GO:0005230 GO:0016021
GO:0016020
Cluster-22178.56115 GO:0006811 GO:0007186 GO:0007165 GO:0007268
GO:0004930 GO:0004970 GO:0016021 GO:0016020
Cluster-22178.56116 GO:0005515
Cluster-22178.56117 GO:0018189 GO:0030154 GO:0016021
Cluster-22178.56119 GO:0005975 GO:0004553
Cluster-22178.56121 GO:0000166 GO:0046872
Cluster-22178.56124 GO:0006886 GO:0046872 GO:0000166 GO:0005783
GO:0016021
Cluster-22178.56125 GO:0006810 GO:0005215 GO:0016021
Cluster-22178.56126 GO:0006886 GO:0000166 GO:0046872 GO:0005783
GO:0016021
Cluster-22178.56129 GO:0006629 GO:0009252 GO:0003677 GO:0046983
GO:0008963 GO:0016021
Cluster-22178.56130 GO:0006468 GO:0016773 GO:0005524 GO:0004672
GO:0016020
Cluster-22178.56138 GO:0006629 GO:0006355 GO:0043401 GO:0008270
GO:0043565 GO:0003700 GO:0005667 GO:0005634
Cluster-22178.56139 GO:0043401 GO:0006355 GO:0006629 GO:0003700
GO:0043565 GO:0008270 GO:0005667 GO:0005634
Cluster-22178.56141 GO:0043065 GO:0046983 GO:0003677 GO:0016021
GO:0005740
Cluster-22178.56143 GO:0051252 GO:0016891
Cluster-22178.56147 GO:0006355 GO:0016032 GO:0043565 GO:0003700
GO:0005667 GO:0005634
Cluster-22178.56148 GO:0006397 GO:0006570 GO:0006310 GO:0006281

GO:0006807 GO:0006470 GO:0006260 GO:0006935 GO:0006370
GO:0050568 GO:0008138 GO:0004484 GO:0004725 GO:0003910
GO:0005524
Cluster-22178.56149 GO:0006570 GO:0006397 GO:0006370 GO:0006935
GO:0006807 GO:0006470 GO:0004484 GO:0050568 GO:0008138
GO:0004725
Cluster-22178.56150 GO:0006468 GO:0004672 GO:0005524
Cluster-22178.56151 GO:0006355 GO:0006486 GO:0006811 GO:0004576
GO:0016020 GO:0008250
Cluster-22178.56152 GO:0006811 GO:0007165 GO:0051225 GO:0006486
GO:0007155 GO:0006355 GO:0004871 GO:0004576 GO:0003677
GO:0003723 GO:0009288 GO:0070652 GO:0008250 GO:0016020
GO:0005634
Cluster-22178.56156 GO:0009271 GO:0006355 GO:0005975 GO:0055114
GO:0030246 GO:0016491 GO:0016209
Cluster-22178.56157 GO:0055085 GO:0006810 GO:0006118 GO:0009055
GO:0005525 GO:0042626 GO:0005524 GO:0003924 GO:0016491
GO:0016887 GO:0016301 GO:0016021 GO:0016020
Cluster-22178.56158 GO:0006464 GO:0016151 GO:0031072 GO:0051082

Cluster-22178.56159 GO:0006450 GO:0005515
Cluster-22178.56160 GO:0043087 GO:0071929 GO:0042967 GO:0019799
GO:0005089 GO:0008080 GO:0005515 GO:0005737 GO:0005874
GO:0045298 GO:0016021
Cluster-22178.56161 GO:0035556 GO:0007165 GO:0003774 GO:0005524
GO:0005515 GO:0016021 GO:0001772 GO:0097197 GO:0016459
Cluster-22178.56162 GO:0006468 GO:0004672 GO:0005524
Cluster-22178.56163 GO:0006493 GO:0000030 GO:0016020 GO:0000136
Cluster-22178.56166 GO:0005515
Cluster-22178.56167 GO:0003725
Cluster-22178.56168 GO:0006470 GO:0000287 GO:0003924 GO:0005525
GO:0030145 GO:0004721 GO:0003824
Cluster-22178.56169 GO:0016020

Cluster-22178.56171 GO:0006464 GO:0015031 GO:0005515 GO:0005634
Cluster-22178.56172 GO:0016787
Cluster-22178.56174 GO:0019058 GO:0006886 GO:0005515 GO:0019031
GO:0005622
Cluster-22178.56175 GO:0006508 GO:0008654 GO:0008270 GO:0016780
GO:0004181 GO:0016020
Cluster-22178.56179 GO:0006508 GO:0004252
Cluster-22178.56180 GO:0007186 GO:0006629 GO:0004930 GO:0003677
GO:0008270 GO:0016021
Cluster-22178.56182 GO:0006260 GO:0003887 GO:0042575
Cluster-22178.56187 GO:0051260 GO:0005515 GO:0016021
Cluster-22178.56189 GO:0006355 GO:0003676 GO:0005515 GO:0043565
GO:0005634
Cluster-22178.56190 GO:0006457 GO:0005515
Cluster-22178.56191 GO:0008152 GO:0019087 GO:0006508 GO:0008236
GO:0008168
Cluster-22178.56192 GO:0015992 GO:0016567 GO:0015986 GO:0004842
GO:0015078 GO:0043130 GO:0003723 GO:0000276
Cluster-22178.56195 GO:0006118 GO:0009055 GO:0005515
Cluster-22178.56197 GO:0009405 GO:0005576
Cluster-22178.56198 GO:0005515
Cluster-22178.56199 GO:0005515 GO:0008168
Cluster-22178.56200 GO:0006801 GO:0055114 GO:0006914 GO:0045502
GO:0042803 GO:0008134 GO:0046872 GO:0005667 GO:0030286
Cluster-22178.56202 GO:0006914 GO:0055114 GO:0006801 GO:0008134
GO:0042803 GO:0045502 GO:0046872 GO:0005667 GO:0030286
Cluster-22178.56205 GO:0006013 GO:0006000 GO:0005524 GO:0003873

Cluster-22178.56207 GO:0006310 GO:0006281 GO:0009378 GO:0003723
GO:0003678 GO:0003724 GO:0005524 GO:0016887 GO:0009379
GO:0005657
Cluster-22178.56214 GO:0006144 GO:0006508 GO:0006355 GO:0003968
GO:0003700 GO:0043565 GO:0017111 GO:0004197 GO:0003677

GO:0031379 GO:0005667 GO:0005634
Cluster-22178.56215 GO:0006468 GO:0005524 GO:0004672
Cluster-22178.56216 GO:0003924 GO:0005198 GO:0019028
Cluster-22178.56218 GO:0007275 GO:0009966 GO:0016020
Cluster-22178.56221 GO:0051028 GO:0005634
Cluster-22178.56222 GO:0019089 GO:0006886 GO:0006355 GO:0008608
GO:0007049 GO:0048870 GO:0009271 GO:0051607 GO:0003700
GO:0043565 GO:0031514 GO:0042729 GO:0016021 GO:0005783
GO:0005667 GO:0005876
Cluster-22178.56223 GO:0006813 GO:0051726 GO:0015269 GO:0005634
GO:0016020
Cluster-22178.56227 GO:0006508 GO:0055085 GO:0022857 GO:0004197
GO:0016021
Cluster-22178.56231 GO:0006614 GO:0046907 GO:0006355 GO:0006281
GO:0000723 GO:0003677 GO:0005515 GO:0016787 GO:0005525
GO:0003678 GO:0003676 GO:0005524 GO:0005657 GO:0009289
Cluster-22178.56232 GO:0003779 GO:0005198 GO:0005856 GO:0016021
Cluster-22178.56233 GO:0015031 GO:0055114 GO:0016491 GO:0008565
GO:0004190 GO:0005515 GO:0016021
Cluster-22178.56235 GO:0007017 GO:0007018 GO:0005524 GO:0008017
GO:0003777 GO:0045298 GO:0005874 GO:0005576
Cluster-22178.56236 GO:0016021
Cluster-22178.56244 GO:0006508 GO:0055085 GO:0046813 GO:0035556
GO:0061025 GO:0016805 GO:0016021 GO:0019031
Cluster-22178.56247 GO:0055085 GO:0015979 GO:0030150 GO:0006810
GO:0005215 GO:0005515 GO:0009539 GO:0009523 GO:0016020
GO:0005742
Cluster-22178.56248 GO:0007186 GO:0015684 GO:0007165 GO:0007264
GO:0004871 GO:0005525 GO:0003924 GO:0019001 GO:0015093
GO:0008270 GO:0031683 GO:0016021
Cluster-22178.56250 GO:0016567 GO:0055114 GO:0006633 GO:0042967
GO:0004318 GO:0005515 GO:0004842 GO:0005835
Cluster-22178.56253 GO:0005515 GO:0043565 GO:0008270 GO:0005634

Cluster-22178.56254 GO:0008152 GO:0006397 GO:0003723 GO:0004064
GO:0005515 GO:0016788 GO:0005737 GO:0005634
Cluster-22178.56257 GO:0030168 GO:0051258 GO:0007165 GO:0030674
GO:0005515 GO:0005102 GO:0005577
Cluster-22178.56259 GO:0006118 GO:0005515 GO:0015035
Cluster-22178.5626 GO:0003779
Cluster-22178.56260 GO:0019370 GO:0045926 GO:0006508 GO:0006952
GO:0008270 GO:0004222 GO:0005525 GO:0008237
Cluster-22178.56263 GO:0005515
Cluster-22178.56264 GO:0019069 GO:0007165 GO:0006914 GO:0051258
GO:0030168 GO:0007049 GO:0030674 GO:0005102 GO:0005577
Cluster-22178.56266 GO:0048309 GO:0051028 GO:0030430
Cluster-22178.56268 GO:0007156 GO:0008152 GO:0016791 GO:0005509
GO:0016020
Cluster-22178.56272 GO:0000166 GO:0046872
Cluster-22178.56273 GO:0032259 GO:0046872 GO:0003723 GO:0005845
Cluster-22178.56274 GO:0006606 GO:0048870 GO:0007094 GO:0005198
GO:0008134 GO:0042803 GO:0045502 GO:0030286 GO:0005882
GO:0031514 GO:0005667
Cluster-22178.56278 GO:0006511 GO:0016747
Cluster-22178.56279 GO:0006511 GO:0016747
Cluster-22178.56282 GO:0007165
Cluster-22178.56283 GO:0019645 GO:0016560 GO:0005515 GO:0016021
GO:0005777
Cluster-22178.56284 GO:0019521 GO:0055114 GO:0046168 GO:0009094
GO:0015940 GO:0000162 GO:0006571 GO:0006098 GO:0008677
GO:0004665 GO:0051287 GO:0008977 GO:0004616 GO:0016616
Cluster-22178.56285 GO:0008152 GO:0055114 GO:0046487 GO:0006355
GO:0006281 GO:0055085 GO:0015031 GO:0009396 GO:0006310
GO:0003678 GO:0022857 GO:0003824 GO:0008134 GO:0003723
GO:0003724 GO:0005524 GO:0016491 GO:0008565 GO:0016887
GO:0004488 GO:0009378 GO:0016021 GO:0005657 GO:0005667
GO:0009379

Cluster-22178.56287 GO:0007219 GO:0055085 GO:0030154 GO:0006355
GO:0030001 GO:0007275 GO:0003676 GO:0046873 GO:0005622
GO:0016021 GO:0045095 GO:0016020
Cluster-22178.56288 GO:0006355 GO:0030001 GO:0007275 GO:0007219
GO:0055085 GO:0030154 GO:0046873 GO:0005515 GO:0003676
GO:0045095 GO:0016020 GO:0005622 GO:0016021
Cluster-22178.56290 GO:0006687 GO:0006027 GO:0046486 GO:0005975
GO:0006012 GO:0005515 GO:0046872 GO:0004565 GO:0009341
Cluster-22178.56291 GO:0042546 GO:0009664 GO:0007275 GO:0005199
GO:0005578 GO:0005618
Cluster-22178.56294 GO:0030246
Cluster-22178.56295 GO:0005509 GO:0005515
Cluster-22178.56296 GO:0005515
Cluster-22178.56297 GO:0005515
Cluster-22178.56299 GO:0008152 GO:0005198 GO:0016746 GO:0019028
Cluster-22178.56300 GO:0008152 GO:0016746 GO:0005198 GO:0019028
Cluster-22178.56301 GO:0005198 GO:0019028
Cluster-22178.56305 GO:0006468 GO:0005524 GO:0004672
Cluster-22178.56306 GO:0005509 GO:0005515
Cluster-22178.56308 GO:0016772 GO:0005739
Cluster-22178.56313 GO:0006606 GO:0006914 GO:0006355 GO:0003700
GO:0043565 GO:0003677 GO:0016788 GO:0005634 GO:0005667
Cluster-22178.56316 GO:0006355 GO:0043401 GO:0043565 GO:0008270
GO:0003700 GO:0005634 GO:0005667
Cluster-22178.56318 GO:0006418 GO:0000166 GO:0003676 GO:0005524
GO:0004812
Cluster-22178.56320 GO:0046740 GO:0016021
Cluster-22178.56321 GO:0006355 GO:0008270 GO:0043565 GO:0003682
GO:0003700 GO:0005667 GO:0000785
Cluster-22178.56322 GO:0016567 GO:0061630 GO:0005515 GO:0003677
GO:0004842 GO:0043565 GO:0046872 GO:0008270 GO:0005634
GO:0005680
Cluster-22178.56327 GO:0055114 GO:0009089 GO:0006508 GO:0009085

GO:0008839 GO:0004222 GO:0008270 GO:0005509 GO:0005515
GO:0031012
Cluster-22178.56328 GO:1900758
Cluster-22178.56329 GO:0005515
Cluster-22178.56334 GO:0030866 GO:0016192 GO:0006886 GO:0008092
GO:0005515 GO:0005856 GO:0030117
Cluster-22178.56336 GO:0006886 GO:0016192 GO:0030866 GO:0005515
GO:0008092 GO:0005856 GO:0030117
Cluster-22178.56337 GO:0005515 GO:0005739
Cluster-22178.56338 GO:0006355 GO:0006357 GO:0001104 GO:0016592
Cluster-22178.56340 GO:0003677
Cluster-22178.56343 GO:0006754 GO:0005524 GO:0016887 GO:0000275
Cluster-22178.56345 GO:0019491 GO:0033990 GO:0005515 GO:0016021
Cluster-22178.56346 GO:0006308 GO:0007049 GO:0043093 GO:0006355
GO:0000917 GO:0008855 GO:0043565 GO:0003700 GO:0005737
GO:0005667 GO:0009318
Cluster-22178.56347 GO:0007049 GO:0006308 GO:0000917 GO:0006355
GO:0043093 GO:0003700 GO:0043565 GO:0008855 GO:0005737
GO:0009318 GO:0005667
Cluster-22178.56349 GO:0070206 GO:0005975 GO:0048870 GO:0016857
GO:0005198 GO:0005515 GO:0005882 GO:0031514
Cluster-22178.56350 GO:0006355 GO:0007049 GO:0070206 GO:0005975
GO:0048870 GO:0003677 GO:0005198 GO:0003700 GO:0016857
GO:0043565 GO:0031514 GO:0005667
Cluster-22178.56351 GO:0005975 GO:0070206 GO:0048870 GO:0016857
GO:0005198 GO:0005515 GO:0005882 GO:0031514
Cluster-22178.56352 GO:0009405 GO:0010508 GO:0051258 GO:0007165
GO:0019058 GO:0007067 GO:0007155 GO:0006260 GO:0006606
GO:0030168 GO:0006357 GO:0051262 GO:0005515 GO:0001104
GO:0005198 GO:0004871 GO:0046872 GO:0030674 GO:0005102
GO:0042729 GO:0019028 GO:0072686 GO:0000775 GO:0019031
GO:0019867 GO:0016021 GO:0005882 GO:0016592 GO:0016020
GO:0005577

Cluster-22178.56353 GO:0006013 GO:0006000 GO:0006144 GO:0006260 GO:0000103 GO:0015976 GO:0006790 GO:0008152 GO:0006094 GO:0006099 GO:0005524 GO:0016887 GO:0003873 GO:0003677 GO:0003924 GO:0005525 GO:0004020 GO:0004781 GO:0004612 GO:0016301
Cluster-22178.56355 GO:0046034 GO:0006144 GO:0016567 GO:0004842 GO:0046872 GO:0008270 GO:0005515 GO:0061630 GO:0004017 GO:0005622
Cluster-22178.56356 GO:0019867
Cluster-22178.56358 GO:0005515
Cluster-22178.56368 GO:0009069 GO:0016310 GO:0004674 GO:0005515 GO:0019028
Cluster-22178.56370 GO:0006270 GO:0006367 GO:0015684 GO:0007155 GO:0005525 GO:0015093 GO:0016021 GO:0032040 GO:0009289 GO:0005672
Cluster-22178.56371 GO:0006820 GO:0008509 GO:0016021
Cluster-22178.56373 GO:0005525 GO:0003924 GO:0005861
Cluster-22178.56374 GO:0006810 GO:0005524 GO:0003676 GO:0004386

Cluster-22178.56375 GO:0006810 GO:0016787 GO:0005524 GO:0003676 GO:0004386 GO:0003677 GO:0000166
Cluster-22178.56376 GO:0006554 GO:0034968 GO:0006479 GO:0008270 GO:0018024 GO:0005515 GO:0016021 GO:0005634
Cluster-22178.56380 GO:0006810 GO:0007165 GO:0006744 GO:0055085 GO:0005179 GO:0008813 GO:0003676 GO:0005576 GO:0016020 GO:0005737 GO:0016021
Cluster-22178.56381 GO:0006810 GO:0007165 GO:0055085 GO:0006744 GO:0005179 GO:0003676 GO:0008813 GO:0016020 GO:0005576 GO:0016021 GO:0005737
Cluster-22178.56382 GO:0015675 GO:0035444 GO:0046872 GO:0015099 GO:0016021
Cluster-22178.56383 GO:0035444 GO:0015675 GO:0015099 GO:0046872 GO:0016021

Cluster-22178.56385 GO:0006260 GO:0008608 GO:0015992 GO:0015991 GO:0006355 GO:0003700 GO:0015078 GO:0005876 GO:0033179 GO:0042729 GO:0005667
Cluster-22178.56386 GO:0006506 GO:0016746 GO:0005789 GO:0016021
Cluster-22178.56387 GO:0042967 GO:0006355 GO:0008080 GO:0016747

Cluster-22178.56391 GO:0002949 GO:0006914 GO:0007018 GO:0007017 GO:0003724 GO:0016887 GO:0005524 GO:0005525 GO:0003777 GO:0003924 GO:0008092 GO:0016301 GO:0005515 GO:0003723 GO:0019898 GO:0030286 GO:0005874 GO:0005737
Cluster-22178.56392 GO:0046872 GO:0000166
Cluster-22178.56393 GO:0007264 GO:0006355 GO:0003677 GO:0005515 GO:0005524 GO:0003924 GO:0016887 GO:0005525 GO:0016020 GO:0005622
Cluster-22178.56396 GO:0005515
Cluster-22178.56399 GO:0016579 GO:0036459
Cluster-22178.56400 GO:0009405
Cluster-22178.56403 GO:0006629 GO:0009405 GO:0005524 GO:0003677 GO:0000166 GO:0008081 GO:0005576
Cluster-22178.56404 GO:0019068 GO:0008283 GO:0007264 GO:0007186 GO:0006270 GO:0007165 GO:0015684 GO:0043087 GO:0035556 GO:0006576 GO:0040007 GO:0035023 GO:0016887 GO:0005524 GO:0015093 GO:0005089 GO:0016757 GO:0008083 GO:0005198 GO:0005515 GO:0003924 GO:0031683 GO:0046872 GO:0019001 GO:0004871 GO:0005525 GO:0016020 GO:0016021
Cluster-22178.56405 GO:0003676
Cluster-22178.56409 GO:0007049 GO:0000917 GO:0006355 GO:0043093 GO:0003700 GO:0043565 GO:0003677 GO:0005815 GO:0005737 GO:0005634 GO:0005667
Cluster-22178.56410 GO:0048193 GO:0006357 GO:0016192 GO:0006869 GO:0006886 GO:0030001 GO:0006891 GO:0007165 GO:0030474 GO:0051225 GO:0005179 GO:0042803 GO:0005515 GO:0005198 GO:0001104 GO:0005200 GO:0046872 GO:0005319 GO:0008134

GO:0045502 GO:0016020 GO:0005667 GO:0019867 GO:0005856
GO:0070652 GO:0005823 GO:0030286 GO:0016592 GO:0017119
GO:0005801 GO:0016021 GO:0019031 GO:0019033 GO:0019028
GO:0005576
Cluster-22178.56415 GO:0070072
Cluster-22178.56416 GO:0006260 GO:0005515
Cluster-22178.56418 GO:0009405 GO:0097193 GO:0090200 GO:0051321
GO:0043065 GO:0005515 GO:0005576 GO:0005739
Cluster-22178.56419 GO:0097193 GO:0009405 GO:0090200 GO:0051321
GO:0043065 GO:0005515 GO:0005576 GO:0005739
Cluster-22178.56428 GO:1900758
Cluster-22178.56429 GO:0006289 GO:0004003 GO:0000439 GO:0005657
GO:0005634
Cluster-22178.56430 GO:0055114
Cluster-22178.56431 GO:0005515 GO:0005509
Cluster-22178.56432 GO:0015991 GO:0005975 GO:0051028 GO:0048309
GO:0019281 GO:0042967 GO:0006555 GO:0042302 GO:0016820
GO:0005524 GO:0008899 GO:0004553 GO:0033178 GO:0005737
Cluster-22178.56437 GO:0035556 GO:0006378 GO:0006206 GO:0006144
GO:0006351 GO:0046872 GO:0008270 GO:0003677 GO:0003899
GO:0003729 GO:0005849 GO:0005730
Cluster-22178.56439 GO:0006090 GO:0006525 GO:0006560 GO:0006814
GO:0007186 GO:0071436 GO:0015081 GO:0004930 GO:0008948
GO:0016020
Cluster-22178.56440 GO:0006468 GO:0016192 GO:0005524 GO:0004672
GO:0016021
Cluster-22178.56441 GO:0006952 GO:0019013 GO:0016021
Cluster-22178.56446 GO:0016255 GO:0042765
Cluster-22178.56447 GO:0007165 GO:0098655 GO:0006812 GO:0033198
GO:0001614 GO:0004931 GO:0008270 GO:0005887
Cluster-22178.56449 GO:0006011 GO:0005982 GO:0005985 GO:0030244
GO:0016760 GO:0016020
Cluster-22178.56453 GO:0055114 GO:0033743

Cluster-22178.56454 GO:0055114 GO:0033743
Cluster-22178.56457 GO:0055085 GO:0030001 GO:0046873 GO:0016020
Cluster-22178.56459 GO:0006446 GO:0006468 GO:0006413 GO:0003743
GO:0004672 GO:0005524 GO:0005840
Cluster-22178.56461 GO:0016021
Cluster-22178.56462 GO:0008270
Cluster-22178.56463 GO:0008270
Cluster-22178.56466 GO:0000917 GO:0006355 GO:0043093 GO:0007049
GO:0006260 GO:0008053 GO:0051259 GO:0003700 GO:0034618
GO:0003924 GO:0005741 GO:0005667 GO:0005634 GO:0016021
GO:0005737
Cluster-22178.56467 GO:0055114 GO:0006807 GO:0006281 GO:0006355
GO:0042128 GO:0008270 GO:0008168 GO:0008942 GO:0016491
GO:0051537 GO:0003677
Cluster-22178.56468 GO:0051424 GO:0016021
Cluster-22178.56471 GO:0004672
Cluster-22178.56475 GO:0006071 GO:0046486 GO:0043565 GO:0016829
GO:0005524 GO:0005515 GO:0003677 GO:0004371 GO:0005783
GO:0005634
Cluster-22178.56476 GO:0006397 GO:0051252 GO:0006468 GO:0004540
GO:0004672 GO:0005524 GO:0016773 GO:0016020
Cluster-22178.56477 GO:0006355 GO:0003677 GO:0016021
Cluster-22178.56478 GO:0005515 GO:0003676
Cluster-22178.56480 GO:0006334 GO:0003677 GO:0005634 GO:0000786
Cluster-22178.56485 GO:0006810 GO:0009405 GO:0005515 GO:0019871
GO:0005576
Cluster-22178.56487 GO:0015684 GO:0007264 GO:0005525 GO:0003924
GO:0015093 GO:0001527 GO:0016021
Cluster-22178.56490 GO:0003677 GO:0005524 GO:0016787
Cluster-22178.56492 GO:0006606 GO:0042981 GO:0005515
Cluster-22178.56493 GO:0006807 GO:0009252 GO:0006355 GO:0003700
GO:0043565 GO:0004356 GO:0005634 GO:0005667 GO:0055036
Cluster-22178.56494 GO:0007155 GO:0051015 GO:0098599

Cluster-22178.56495 GO:0006269 GO:0006351 GO:0035556 GO:0006260 GO:0003677 GO:0046872 GO:0003896 GO:0005730 GO:0005657
Cluster-22178.56496 GO:0098599
Cluster-22178.56497 GO:0019087 GO:0005515 GO:0016021
Cluster-22178.56498 GO:0006886 GO:0009847 GO:0006508 GO:0008233 GO:0005515
Cluster-22178.56499 GO:0006310 GO:0006355 GO:0003700 GO:0003677 GO:0000150 GO:0005667 GO:0005737
Cluster-22178.56500 GO:0000077 GO:0051082
Cluster-22178.56501 GO:0055085 GO:0008643 GO:0005351 GO:0000139 GO:0016020 GO:0016021
Cluster-22178.56502 GO:0016787
Cluster-22178.56503 GO:0035023 GO:0006886 GO:0006486 GO:0016032 GO:0009117 GO:0006888 GO:0043087 GO:0019872 GO:0030639 GO:0008830 GO:0005089 GO:0008378 GO:0016757 GO:0008270 GO:0016020 GO:0030127
Cluster-22178.56504 GO:0000723 GO:0006260 GO:0008270 GO:0003676 GO:0003677 GO:0005634 GO:0000784
Cluster-22178.56507 GO:0007165
Cluster-22178.56511 GO:0007096 GO:0007165 GO:0040007 GO:0008283 GO:0008083 GO:0016020 GO:0005634
Cluster-22178.56513 GO:0055114 GO:0008152 GO:0016491
Cluster-22178.56518 GO:0008152 GO:0016624
Cluster-22178.56519 GO:0007156 GO:0005509 GO:0016020
Cluster-22178.56520 GO:0005515
Cluster-22178.56521 GO:0006355 GO:0003700 GO:0008270 GO:0042025 GO:0016020 GO:0005667 GO:0005634
Cluster-22178.56523 GO:0006955 GO:0006635 GO:0055114 GO:0006637 GO:0019882 GO:0005515 GO:0030234 GO:0003997 GO:0016020 GO:0005777
Cluster-22178.56527 GO:0015991 GO:0006810 GO:0009405 GO:0008200 GO:0005515 GO:0016820 GO:0005576 GO:0000221
Cluster-22178.56529 GO:0006468 GO:0004672 GO:0005524

Cluster-22178.56533 GO:0005515 GO:0003723
Cluster-22178.56535 GO:0042254 GO:0006412 GO:0003735 GO:0046872
GO:0005840 GO:0005622
Cluster-22178.56542 GO:0042254 GO:0006412 GO:0009103 GO:0003735
GO:0098519 GO:0005096 GO:0005524 GO:0008918 GO:0005783
GO:0016021 GO:0005840
Cluster-22178.56543 GO:0003676
Cluster-22178.56544 GO:0006355 GO:0003677 GO:0005634
Cluster-22178.56545 GO:0006032 GO:0005524 GO:0016887 GO:0003723
GO:0008061 GO:0003724 GO:0005515
Cluster-22178.56548 GO:0016567 GO:0005515 GO:0061630 GO:0043565
GO:0046872 GO:0008270 GO:0004842 GO:0005634 GO:0005680
Cluster-22178.56550 GO:0007165 GO:0015936 GO:0006694 GO:0055114
GO:0050662 GO:0004420 GO:0008158 GO:0016020
Cluster-22178.56551 GO:0016567 GO:0004842
Cluster-22178.56554 GO:0000077 GO:0071479 GO:0005515 GO:0005634
Cluster-22178.56555 GO:0000077 GO:0071479 GO:0005515 GO:0005634
Cluster-22178.56556 GO:0000077 GO:0071479 GO:0005515 GO:0005634
Cluster-22178.56558 GO:0005515 GO:0016020
Cluster-22178.56559 GO:0046677 GO:0007631 GO:0007218 GO:0008270

Cluster-22178.56561 GO:0006890
Cluster-22178.56563 GO:0005515
Cluster-22178.56564 GO:0055114 GO:0006631 GO:0071949 GO:0016491
GO:0050151
Cluster-22178.56566 GO:0006338 GO:0006886 GO:0090544 GO:0030176
Cluster-22178.56568 GO:0007186 GO:0055114 GO:0016972 GO:0004930
GO:0046872 GO:0016021
Cluster-22178.56569 GO:0008654 GO:0016780 GO:0016020
Cluster-22178.56570 GO:0016772 GO:0016301
Cluster-22178.56571 GO:0006355 GO:0045454 GO:0006118 GO:0009401
GO:0051087 GO:0043565 GO:0003700 GO:0009055 GO:0015035
GO:0005667 GO:0016021

Cluster-22178.56574 GO:0035023 GO:0006885 GO:0046436 GO:0009252 GO:0043087 GO:0006464 GO:0015992 GO:0006814 GO:0005089 GO:0005515 GO:0015385 GO:0008716 GO:0016020

Cluster-22178.56577 GO:0016567 GO:0007165 GO:0006260 GO:0005179 GO:0005515 GO:0016818 GO:0003677 GO:0046872 GO:0008270 GO:0005524 GO:0004842 GO:0005634 GO:0005576 GO:0005680

Cluster-22178.5658 GO:0035556 GO:0007268 GO:0007165 GO:0030550 GO:0005576 GO:0016020

Cluster-22178.56580 GO:0030153 GO:0006955 GO:0015643 GO:0046872 GO:0000166 GO:0019814

Cluster-22178.56582 GO:0042742 GO:0008270 GO:0005634 GO:0005576

Cluster-22178.56583 GO:0055114 GO:0003756 GO:0016671 GO:0005783

Cluster-22178.56584 GO:0016787

Cluster-22178.56585 GO:0003676

Cluster-22178.56586 GO:0006771 GO:0019497 GO:0015979 GO:0005515 GO:0046872 GO:0003993 GO:0016020 GO:0009523

Cluster-22178.56588 GO:0003677 GO:0005515 GO:0003676 GO:0005524 GO:0016787

Cluster-22178.56589 GO:0046080 GO:0016787

Cluster-22178.5659 GO:0050909 GO:0007186 GO:0004930 GO:0005886 GO:0016021

Cluster-22178.56590 GO:0003676 GO:0005515 GO:0005634

Cluster-22178.56591 GO:0016310 GO:0000160 GO:0006109 GO:0006355 GO:0005524 GO:0004672 GO:0003774 GO:0008134 GO:0005515 GO:0000155 GO:0009365 GO:0016020 GO:0016459 GO:0005667

Cluster-22178.56592 GO:0055114 GO:0016491

Cluster-22178.56593 GO:0055085 GO:0006810 GO:0005515 GO:0005215 GO:0016021 GO:0016020

Cluster-22178.56596 GO:0045454 GO:0042254 GO:0006412 GO:0006810 GO:0003924 GO:0005525 GO:0003735 GO:0005515 GO:0016021 GO:0005840 GO:0005861

Cluster-22178.56598 GO:0007264 GO:0003676 GO:0005515 GO:0005525 GO:0008270

Cluster-22178.56600 GO:0016567 GO:0005515 GO:0004842 GO:0008270 GO:0046872 GO:0005680
Cluster-22178.56601 GO:0005544 GO:0005509
Cluster-22178.56602 GO:0008237 GO:0008270
Cluster-22178.56610 GO:0046872 GO:0035091
Cluster-22178.56612 GO:0055114 GO:0043401 GO:0006355 GO:0003700 GO:0008270 GO:0016491 GO:0043565 GO:0005634 GO:0005667
Cluster-22178.56613 GO:0055114 GO:0006118 GO:0009496 GO:0051537 GO:0009512 GO:0042651
Cluster-22178.56614 GO:0046872
Cluster-22178.56615 GO:0040007 GO:0008283 GO:0007165 GO:0008083

Cluster-22178.56616 GO:0040007 GO:0005737
Cluster-22178.56617 GO:0005515
Cluster-22178.56618 GO:0005515
Cluster-22178.56619 GO:0006119 GO:0006118 GO:0015992 GO:0008121

Cluster-22178.56620 GO:0009069 GO:0006825 GO:0006744 GO:0006123 GO:0015992 GO:0006814 GO:0006118 GO:0016310 GO:0006120 GO:0016531 GO:0003954 GO:0004674 GO:0008137 GO:0004129 GO:0005507 GO:0005739 GO:0005758 GO:0045277
Cluster-22178.56621 GO:0003676 GO:0019013
Cluster-22178.56623 GO:0006388 GO:0051252 GO:0006351 GO:0000213 GO:0003714 GO:0005667 GO:0000214
Cluster-22178.56624 GO:0005515
Cluster-22178.56633 GO:0030001 GO:0005515 GO:0046872
Cluster-22178.56634 GO:0006810 GO:0055085 GO:0006260 GO:0015074 GO:0003678 GO:0003676 GO:0042626 GO:0005525 GO:0003924 GO:0016887 GO:0005524 GO:0016021 GO:0005657
Cluster-22178.56637 GO:0016301 GO:0016772
Cluster-22178.56638 GO:0005525
Cluster-22178.56640 GO:0003676
Cluster-22178.56642 GO:0003676 GO:0016820 GO:0016757

Cluster-22178.56644 GO:0017134
Cluster-22178.56646 GO:0005515
Cluster-22178.56647 GO:0008643 GO:0005351 GO:0016021 GO:0016020
GO:0000139
Cluster-22178.56648 GO:0016310 GO:0000160 GO:0048870 GO:0007165
GO:0006810 GO:0000103 GO:0006144 GO:0005515 GO:0008134
GO:0042803 GO:0045502 GO:0004020 GO:0000155 GO:0005524
GO:0031514 GO:0009365 GO:0016021 GO:0005667 GO:0030286
GO:0005886
Cluster-22178.56651 GO:0006123 GO:0035023 GO:0015992 GO:0043087
GO:0005515 GO:0005089 GO:0004129 GO:0045277
Cluster-22178.56652 GO:0043087 GO:0015992 GO:0035023 GO:0006123
GO:0004129 GO:0005089 GO:0005515 GO:0045277
Cluster-22178.56655 GO:0016310 GO:0043248 GO:0006886 GO:0016192
GO:0009069 GO:0004674 GO:0005515 GO:0030117
Cluster-22178.56660 GO:0006357 GO:0007264 GO:0008641 GO:0003924
GO:0005525 GO:0005515 GO:0001104 GO:0016592
Cluster-22178.56661 GO:0002949 GO:0006260 GO:0035988 GO:0006310
GO:0002062 GO:0042246 GO:0006576 GO:0006281 GO:0006355
GO:0016887 GO:0005524 GO:0003677 GO:0016787 GO:0009378
GO:0003678 GO:0005525 GO:0003924 GO:0008134 GO:0016301
GO:0005657 GO:0009379 GO:0005667 GO:0005634
Cluster-22178.56662 GO:0043093 GO:0007165 GO:0006914 GO:0015992
GO:0015991 GO:0006355 GO:0010508 GO:0000917 GO:0048870
GO:0045892 GO:0006935 GO:0000160 GO:0007049 GO:0016310
GO:0000155 GO:0004673 GO:0003677 GO:0005515 GO:0003924
GO:0003700 GO:0005525 GO:0015078 GO:0005667 GO:0005815
GO:0033179 GO:0031514 GO:0005737 GO:0009365
Cluster-22178.56663 GO:0006486 GO:0035429 GO:0015725 GO:0015128
GO:0016787 GO:0008373 GO:0046872 GO:0016020
Cluster-22178.56665 GO:0007264 GO:0006614 GO:0042254 GO:0006412
GO:0005524 GO:0016887 GO:0003924 GO:0005525 GO:0003724
GO:0003723 GO:0098519 GO:0003735 GO:0005622 GO:0005840

Cluster-22178.56666 GO:0005991 GO:0005985 GO:0005982 GO:0007186
GO:0004930 GO:0005509 GO:0004555
Cluster-22178.56669 GO:0005991 GO:0007186 GO:0005985 GO:0005982
GO:0005509 GO:0004555 GO:0004930
Cluster-22178.56670 GO:0007186 GO:0005985 GO:0005982 GO:0005991
GO:0004555 GO:0005509 GO:0004930
Cluster-22178.56676 GO:0003676
Cluster-22178.56677 GO:0003676
Cluster-22178.56678 GO:0003676 GO:0005515
Cluster-22178.56681 GO:0006468 GO:0004672 GO:0005524
Cluster-22178.56682 GO:0003676
Cluster-22178.56685 GO:0000746 GO:0004252 GO:0019028 GO:0055036
GO:0019867
Cluster-22178.56686 GO:0000746 GO:0004252 GO:0019028 GO:0055036
GO:0019867
Cluster-22178.56688 GO:0000746 GO:0004252 GO:0019867 GO:0019028
GO:0055036
Cluster-22178.56689 GO:0005515
Cluster-22178.56692 GO:0007157 GO:0009405 GO:0008270 GO:0046872
GO:0016021
Cluster-22178.56695 GO:0007264 GO:0007165 GO:0007186 GO:0031683
GO:0019001 GO:0003924 GO:0005525 GO:0004871
Cluster-22178.56697 GO:0009214 GO:0006468 GO:0004113 GO:0005524
GO:0004672 GO:0016020 GO:0019031
Cluster-22178.56699 GO:0007165 GO:0005179 GO:0005576 GO:0031224
Cluster-22178.56700 GO:0006351 GO:0006144 GO:0006206 GO:0051603
GO:0003899 GO:0003677 GO:0004298 GO:0005730 GO:0005839
Cluster-22178.56701 GO:0005509 GO:0005515
Cluster-22178.56705 GO:0005515
Cluster-22178.56706 GO:0097193 GO:0007165 GO:0090200 GO:0043065
GO:0016020 GO:0005739
Cluster-22178.56710 GO:0015991 GO:0006119 GO:0007264 GO:0015684
GO:0007165 GO:0007186 GO:0015992 GO:0005515 GO:0019001

GO:0031683 GO:0003924 GO:0005525 GO:0004871 GO:0046961
GO:0015093 GO:0005634 GO:0016021 GO:0033178
Cluster-22178.56712 GO:0016021
Cluster-22178.56715 GO:0055114 GO:0006508 GO:0006886 GO:0006118
GO:0008270 GO:0016655 GO:0004181 GO:0008536 GO:0005886
Cluster-22178.56716 GO:0016020
Cluster-22178.56718 GO:0019497 GO:0006470 GO:0006771 GO:0006570
GO:0046872 GO:0003993 GO:0004725 GO:0004222 GO:0005515
Cluster-22178.56719 GO:0006464 GO:0032259 GO:0006554 GO:0008152
GO:0046500 GO:0006479 GO:0008168 GO:0004719 GO:0018024
Cluster-22178.56722 GO:0042438 GO:0007264 GO:0007165 GO:0015684
GO:0007186 GO:0030036 GO:0015093 GO:0098519 GO:0019001
GO:0031683 GO:0003924 GO:0003779 GO:0005525 GO:0004871
GO:0005507 GO:0009295 GO:0016021 GO:0030054
Cluster-22178.56725 GO:0016787
Cluster-22178.56730 GO:0006270
Cluster-22178.56735 GO:0045893 GO:1902208 GO:0046856 GO:0003677
GO:0034597
Cluster-22178.56737 GO:0006810 GO:0003774 GO:0005524 GO:0016459
Cluster-22178.56740 GO:0005515
Cluster-22178.56741 GO:0006355 GO:0006351 GO:0005634
Cluster-22178.56745 GO:0009058 GO:0006687 GO:0001733 GO:0008146
GO:0043130 GO:0005794 GO:0016021
Cluster-22178.56749 GO:0007165 GO:0006355 GO:0005515 GO:0016787
GO:0003700 GO:0043565 GO:0016817 GO:0005524 GO:0008270
GO:0005667 GO:0005737
Cluster-22178.56750 GO:0006355 GO:0010508 GO:0007165 GO:0016787
GO:0005515 GO:0043565 GO:0016817 GO:0008270 GO:0005524
GO:0003700 GO:0005667 GO:0005737
Cluster-22178.56755 GO:0006397 GO:0006508 GO:0006606 GO:0039693
GO:0006914 GO:0005198 GO:0003682 GO:0003723 GO:0019784
GO:0019028 GO:0005634 GO:0019031 GO:0000785 GO:0005737
Cluster-22178.56756 GO:0030866 GO:0008092 GO:0005856

Cluster-22178.56757 GO:0042176 GO:0005515 GO:0030234 GO:0000502
Cluster-22178.56759 GO:0006886 GO:0008536
Cluster-22178.56763 GO:0008168 GO:0019028
Cluster-22178.56764 GO:0006400 GO:0000287 GO:0008193
Cluster-22178.56766 GO:0000723 GO:0006281 GO:0005524 GO:0046872 GO:0003678 GO:0003824 GO:0016787 GO:0003677 GO:0005657 GO:0016020
Cluster-22178.56769 GO:0006359 GO:0008270 GO:0005634
Cluster-22178.56770 GO:0005515
Cluster-22178.56773 GO:0006396 GO:0003723
Cluster-22178.56777 GO:0006119 GO:0006796 GO:0004427 GO:0000287 GO:0005737
Cluster-22178.56779 GO:0042773 GO:0006351 GO:0006814 GO:0006744 GO:0006120 GO:0015992 GO:0006206 GO:0006144 GO:0003677 GO:0008137 GO:0003899 GO:0005730 GO:0005743
Cluster-22178.56780 GO:0006118 GO:0005515 GO:0009055 GO:0046872 GO:0008270 GO:0009512
Cluster-22178.56781 GO:0006821 GO:0055085 GO:0020037 GO:0005247 GO:0016020
Cluster-22178.56782 GO:0006355 GO:0046872
Cluster-22178.56783 GO:0006887 GO:0006891 GO:0006308 GO:0006886 GO:0008855 GO:0005801 GO:0000145 GO:0016020 GO:0009318 GO:0017119
Cluster-22178.56784 GO:0007155 GO:0006468 GO:0004672 GO:0005524 GO:0016020
Cluster-22178.56785 GO:0006090 GO:0006525 GO:0006814 GO:0071436 GO:0006560 GO:0015081 GO:0008948 GO:0016020
Cluster-22178.56790 GO:0006123 GO:0015992 GO:0055114 GO:0004129 GO:0016645 GO:0045277
Cluster-22178.56794 GO:0003676 GO:0008408
Cluster-22178.56799 GO:0016884 GO:0016874
Cluster-22178.56800 GO:0008152 GO:0055114 GO:0016620 GO:0016491

Cluster-22178.56802 GO:0007165 GO:0003824 GO:0031704 GO:0005179

Cluster-22178.56805 GO:0051276 GO:0006606 GO:0007155 GO:0005524 GO:0003774 GO:0000166 GO:0003677 GO:0005515 GO:0005694 GO:0016459

Cluster-22178.56806 GO:0007165 GO:0005179 GO:0005576

Cluster-22178.56809 GO:0009878 GO:0022900 GO:0046872 GO:0016021

Cluster-22178.56814 GO:0006468 GO:0005524 GO:0004672

Cluster-22178.56815 GO:0009607 GO:0019069 GO:0016021

Cluster-22178.56817 GO:0016310 GO:0006919 GO:0006915 GO:0006468 GO:0009069 GO:0004674 GO:0004672 GO:0005524 GO:0016020 GO:0005739

Cluster-22178.56822 GO:0007186 GO:0006355 GO:0003700 GO:0004930 GO:0043565 GO:0008270 GO:0003713 GO:0003677 GO:0016021 GO:0005667

Cluster-22178.56824 GO:0005515

Cluster-22178.56830 GO:0031225

Cluster-22178.56832 GO:0006281 GO:0008270 GO:0046872

Cluster-22178.56833 GO:0006468 GO:0005524 GO:0004672

Cluster-22178.56837 GO:0009267 GO:0005515 GO:0003723 GO:0016020

Cluster-22178.56839 GO:0006144 GO:0006206 GO:0006098 GO:0008152 GO:0019521 GO:0055114 GO:0006351 GO:0006355 GO:0008641 GO:0003677 GO:0016616 GO:0004616 GO:0051287 GO:0003700 GO:0046872 GO:0003899 GO:0005515 GO:0005667 GO:0005730 GO:0005634

Cluster-22178.56840 GO:0003677 GO:0003824

Cluster-22178.56841 GO:0005515

Cluster-22178.56843 GO:0016773 GO:0005524 GO:0016020

Cluster-22178.56846 GO:0005515 GO:0008270

Cluster-22178.56849 GO:0019087 GO:0016021

Cluster-22178.56850 GO:0006413 GO:0006446 GO:0003743 GO:0005840

Cluster-22178.56852 GO:0006465 GO:0008233 GO:0016021 GO:0005787

Cluster-22178.56853 GO:0043093 GO:0042254 GO:0006897 GO:0006606

GO:0007015 GO:0006412 GO:0000917 GO:0006465 GO:0007049
GO:0003735 GO:0003677 GO:0008233 GO:0008017 GO:0005622
GO:0005787 GO:0019028 GO:0005737 GO:0019867 GO:0045298
GO:0016021 GO:0005840
Cluster-22178.56854 GO:0030163 GO:0008914
Cluster-22178.56858 GO:0005515
Cluster-22178.56860 GO:0008152 GO:0016787 GO:0005198 GO:0019028
GO:0016021
Cluster-22178.56861 GO:0016567 GO:0004842 GO:0005515 GO:0008270
GO:0046872 GO:0005680
Cluster-22178.56862 GO:0008608 GO:0016787 GO:0042729 GO:0072686
Cluster-22178.56864 GO:0000042 GO:0005515
Cluster-22178.56866 GO:0006144 GO:0008033 GO:0006464 GO:0046034
GO:0008152 GO:0051252 GO:0016846 GO:0046872 GO:0016151
GO:0004540 GO:0016787 GO:0004017
Cluster-22178.56867 GO:0008152 GO:0003824
Cluster-22178.56869 GO:0006364 GO:0005730
Cluster-22178.56873 GO:0042967 GO:0006633 GO:0055114 GO:0004318
GO:0005835
Cluster-22178.56875 GO:0008270
Cluster-22178.56876 GO:0006351 GO:0006206 GO:0006144 GO:0003677
GO:0003899 GO:0005730
Cluster-22178.56882 GO:0006270 GO:0006869 GO:0006367 GO:0015684
GO:0051726 GO:0007155 GO:0042157 GO:0015093 GO:0017025
GO:0005525 GO:0008289 GO:0009289 GO:0005576 GO:0005672
GO:0005634 GO:0032040 GO:0016021
Cluster-22178.56885 GO:0008152 GO:0008168
Cluster-22178.56886 GO:0006265 GO:0009755 GO:0006364 GO:0003918
GO:0005515 GO:0003677 GO:0032040 GO:0005576 GO:0005694
Cluster-22178.56887 GO:0009755 GO:0006364 GO:0005515 GO:0005576
GO:0032040
Cluster-22178.56894 GO:0005515
Cluster-22178.56898 GO:0055114 GO:0008152 GO:0016491 GO:0005515

Cluster-22178.56899 GO:0009401 GO:0006812 GO:0055085 GO:0008324 GO:0016020 GO:0016021
Cluster-22178.56900 GO:0015992 GO:0006120 GO:0006814 GO:0006744 GO:0008137 GO:0005743
Cluster-22178.56901 GO:0042254 GO:0006412 GO:0007342 GO:0003735 GO:0046872 GO:0019843 GO:0005840 GO:0005622
Cluster-22178.56905 GO:0006886 GO:0007165 GO:0005515 GO:0016021 GO:0005783
Cluster-22178.56909 GO:0006613 GO:0055114 GO:0006355 GO:0043401 GO:0008270 GO:0016491 GO:0016757 GO:0043565 GO:0016209 GO:0003700 GO:0005784 GO:0030176 GO:0005667 GO:0016020 GO:0016021 GO:0005634
Cluster-22178.56910 GO:0005524
Cluster-22178.56912 GO:0007264 GO:0035556 GO:0006468 GO:0006355 GO:0003677 GO:0005524 GO:0004222 GO:0016820 GO:0005515 GO:0004672 GO:0046872 GO:0003700 GO:0005525 GO:0005667 GO:0005739 GO:0016021
Cluster-22178.56913 GO:0006355 GO:0006468 GO:0007031 GO:0035556 GO:0006457 GO:0007264 GO:0003700 GO:0005525 GO:0003924 GO:0046872 GO:0004672 GO:0005515 GO:0042803 GO:0016820 GO:0051082 GO:0004222 GO:0000774 GO:0005524 GO:0003677 GO:0051087 GO:0016021 GO:0016272 GO:0005739 GO:0005667 GO:0005779
Cluster-22178.56914 GO:0007031 GO:0035556 GO:0007264 GO:0006457 GO:0006355 GO:0006468 GO:0000774 GO:0005524 GO:0003677 GO:0051087 GO:0003924 GO:0046872 GO:0004672 GO:0003700 GO:0005525 GO:0051082 GO:0005515 GO:0042803 GO:0016820 GO:0016272 GO:0005779 GO:0005667 GO:0005739 GO:0016021
Cluster-22178.56915 GO:0007155 GO:0006310 GO:0019079 GO:0002949 GO:0006355 GO:0006281 GO:0007049 GO:0009378 GO:0016887 GO:0005524 GO:0003724 GO:0003723 GO:0008134 GO:0003678 GO:0005667 GO:0009379 GO:0005657

Cluster-22178.56918 GO:0016787
Cluster-22178.56919 GO:0042967 GO:0016407
Cluster-22178.56920 GO:0005515
Cluster-22178.56921 GO:0005525
Cluster-22178.56922 GO:0005515
Cluster-22178.56923 GO:0003723 GO:0003774 GO:0016459
Cluster-22178.56925 GO:0005515
Cluster-22178.56926 GO:0006355 GO:0005515 GO:0004252 GO:0003677
GO:0005634 GO:0005886
Cluster-22178.56932 GO:0046436 GO:0009252 GO:0006090 GO:0006633
GO:0019370 GO:0046872 GO:0003989 GO:0008270 GO:0005524
GO:0008237 GO:0008716 GO:0009317
Cluster-22178.56933 GO:0006355 GO:0046487 GO:0055114 GO:0009396
GO:0008152 GO:0050662 GO:0004488 GO:0003677 GO:0016491
GO:0003824
Cluster-22178.56934 GO:0008152 GO:0006355 GO:0003824 GO:0003677
GO:0050662 GO:0016491
Cluster-22178.56938 GO:0007017 GO:0007018 GO:0005524 GO:0008017
GO:0003777 GO:0019028 GO:0045298 GO:0005874
Cluster-22178.56939 GO:0007017 GO:0007018 GO:0005524 GO:0003777
GO:0008017 GO:0019028 GO:0005874 GO:0045298
Cluster-22178.56940 GO:0015979 GO:0009523 GO:0009539
Cluster-22178.56945 GO:0046836 GO:0017089 GO:0003677 GO:0046872
GO:0051861 GO:0005737
Cluster-22178.56946 GO:0046983
Cluster-22178.56947 GO:0006506 GO:0005515 GO:0016746 GO:0016021
GO:0005789
Cluster-22178.56948 GO:0019069 GO:0005524
Cluster-22178.56951 GO:0005515
Cluster-22178.56952 GO:0007165 GO:0005515
Cluster-22178.56953 GO:0008152 GO:0005975 GO:0005509 GO:0004571
GO:0016829 GO:0016020
Cluster-22178.56954 GO:0008152 GO:0005975 GO:0005509 GO:0004571

GO:0016829 GO:0016020
Cluster-22178.56955 GO:0006449 GO:0006415 GO:0006013 GO:0016226
GO:0006000 GO:0003873 GO:0016301 GO:0005506 GO:0016740
GO:0005524 GO:0016149 GO:0051536 GO:0018444 GO:0005840
GO:0005737
Cluster-22178.56957 GO:0006887 GO:0046488 GO:0016307 GO:0008289
GO:0019031 GO:0000145
Cluster-22178.56960 GO:0016192 GO:0016567 GO:0090263 GO:0016021
GO:0005634
Cluster-22178.56961 GO:0008152 GO:0003824
Cluster-22178.56962 GO:0005515 GO:0043395 GO:0008191 GO:0016020
GO:0005578
Cluster-22178.56963 GO:0008152 GO:0003677 GO:0003824 GO:0008134
GO:0005667 GO:0005634
Cluster-22178.56964 GO:0042742 GO:0007165 GO:0001503 GO:0045502
GO:0001664 GO:0042803 GO:0008134 GO:0005515 GO:0030286
GO:0005887 GO:0005667
Cluster-22178.56965 GO:0005515
Cluster-22178.56966 GO:0032324 GO:0019033
Cluster-22178.56969 GO:0006935 GO:0051301 GO:0007059 GO:0010508
GO:0006886 GO:0000921 GO:0015031 GO:0006914 GO:0031110
GO:0007264 GO:0000090 GO:0007067 GO:0071973 GO:0003774
GO:0005096 GO:0008565 GO:0008017 GO:0009288 GO:0005737
GO:0016021 GO:0045298 GO:0000940 GO:0005940 GO:0005876
GO:0005923 GO:0016020
Cluster-22178.56971 GO:0042254 GO:0006412 GO:0003735 GO:0005515
GO:0005622 GO:0005840
Cluster-22178.56972 GO:0006886 GO:0030244 GO:0005047 GO:0005515
GO:0016866 GO:0005525 GO:0003924 GO:0005785 GO:0005786
Cluster-22178.56975 GO:0030244 GO:0006886 GO:0003924 GO:0005525
GO:0016866 GO:0005047 GO:0005515 GO:0005786 GO:0005785
Cluster-22178.56978 GO:0006355 GO:0043565 GO:0003700 GO:0005515
GO:0005667

Cluster-22178.56979 GO:0006955 GO:0000902 GO:0016020
Cluster-22178.56983 GO:0007016 GO:0016010
Cluster-22178.56985 GO:0008219 GO:0051262 GO:0005515 GO:0044212

Cluster-22178.56987 GO:0006355 GO:0007275 GO:0000398 GO:0006357 GO:0003682 GO:0003677 GO:0001104 GO:0008270 GO:0005634 GO:0016592 GO:0042600 GO:0000775 GO:0005681 GO:0000785 GO:0016021
Cluster-22178.56988 GO:0005515 GO:0008270
Cluster-22178.56995 GO:0005515
Cluster-22178.56996 GO:0006812 GO:0006820 GO:0006835 GO:0017153 GO:0016020
Cluster-22178.56997 GO:0045947 GO:0008190
Cluster-22178.56999 GO:0015979 GO:0006480 GO:0008152 GO:0008168 GO:0009523 GO:0016021
Cluster-22178.57001 GO:0008201
Cluster-22178.57003 GO:0042254 GO:0006412 GO:0006810 GO:0005515 GO:0003735 GO:0003924 GO:0005525 GO:0005861 GO:0016021 GO:0005840
Cluster-22178.57004 GO:0033227 GO:0006412 GO:0015931 GO:0045454 GO:0006810 GO:0006270 GO:0042254 GO:0003924 GO:0005525 GO:0004190 GO:0051033 GO:0005515 GO:0003735 GO:0005840 GO:0016021 GO:0005861
Cluster-22178.57006 GO:0015684 GO:0007264 GO:0005525 GO:0005524 GO:0015093 GO:0016887 GO:0003924 GO:0016021
Cluster-22178.57007 GO:0003723 GO:0003676
Cluster-22178.57008 GO:0003676 GO:0003723 GO:0046872 GO:0005576
Cluster-22178.5701 GO:0015031 GO:0008277 GO:0006886 GO:0008565 GO:0016021
Cluster-22178.57013 GO:0046740 GO:0016567 GO:0046872 GO:0008270 GO:0004842 GO:0005515 GO:0005680 GO:0044156
Cluster-22178.57016 GO:0005515
Cluster-22178.57017 GO:0005515

Cluster-22178.57020 GO:0003677
Cluster-22178.57021 GO:0020037 GO:0003677
Cluster-22178.57022 GO:0006886 GO:0005801 GO:0016020
Cluster-22178.57023 GO:0006310 GO:0006281
Cluster-22178.57024 GO:0051607
Cluster-22178.57025 GO:0043087 GO:0005085 GO:0005515
Cluster-22178.57027 GO:0000723 GO:0042254 GO:0006412 GO:0003735
GO:0003677 GO:0005840 GO:0005622 GO:0000784
Cluster-22178.57030 GO:0005975 GO:0030001 GO:0055085 GO:0016758
GO:0046873 GO:0016020
Cluster-22178.57031 GO:0009116 GO:0007631 GO:0046677 GO:0007218
GO:0008270
Cluster-22178.57035 GO:0019882 GO:0007165 GO:0006955 GO:0016567
GO:0004842 GO:0005515
Cluster-22178.57038 GO:0006952 GO:0001558 GO:0003677 GO:0005515
GO:0005520 GO:0005576 GO:0016942
Cluster-22178.57039 GO:0001558 GO:0005515 GO:0005520 GO:0005576
GO:0016942
Cluster-22178.57040 GO:0001558 GO:0006952 GO:0005520 GO:0003677
GO:0005515 GO:0005576 GO:0016942
Cluster-22178.57041 GO:0016311 GO:0035023 GO:0043087 GO:0009252
GO:0005515 GO:0005089 GO:0050380 GO:0016020
Cluster-22178.57042 GO:0016311 GO:0009252 GO:0043087 GO:0035023
GO:0005515 GO:0050380 GO:0005089 GO:0016020
Cluster-22178.57043 GO:0051539
Cluster-22178.57044 GO:0046872 GO:0003723 GO:0005515 GO:0016021
Cluster-22178.57046 GO:0005975 GO:0000902 GO:0016773 GO:0016787

Cluster-22178.57048 GO:0006468 GO:0016773 GO:0005524 GO:0004672
GO:0016020
Cluster-22178.57049 GO:0006139 GO:0008408 GO:0003676
Cluster-22178.57050 GO:0006139 GO:0008408 GO:0003676
Cluster-22178.57051 GO:0015031 GO:0051262 GO:0051082

Cluster-22178.57055 GO:0006814 GO:0006820 GO:0015992 GO:0006744
GO:0006120 GO:0008137 GO:0043565 GO:0008509 GO:0005634
GO:0016021 GO:0005743
Cluster-22178.57056 GO:0006367 GO:0006260 GO:0003676 GO:0005634
GO:0005672
Cluster-22178.57058 GO:0006468 GO:0009069 GO:0016310 GO:0004672
GO:0005524 GO:0004674
Cluster-22178.57059 GO:0016310 GO:0009069 GO:0006468 GO:0004674
GO:0005524 GO:0004672
Cluster-22178.57060 GO:0055085 GO:0030001 GO:0046983 GO:0046873
GO:0016020
Cluster-22178.57061 GO:0055085 GO:0007165 GO:0007268 GO:0030639
GO:0030550 GO:0005576
Cluster-22178.57062 GO:0009058 GO:0030170
Cluster-22178.57063 GO:0006464
Cluster-22178.57064 GO:0005634
Cluster-22178.57065 GO:0008270 GO:0005515
Cluster-22178.57066 GO:0006468 GO:0004672 GO:0005524 GO:0016773
GO:0016020
Cluster-22178.57068 GO:0006414 GO:0006448 GO:0055114 GO:0003677
GO:0016706 GO:0005515 GO:0003746 GO:0005853 GO:0005840
Cluster-22178.5707 GO:0006886 GO:0005622
Cluster-22178.57071 GO:0006120 GO:0006814 GO:0006744 GO:0015992
GO:0008137 GO:0016773 GO:0005743
Cluster-22178.57073 GO:0046688 GO:0006118 GO:0009055 GO:0005515
GO:0020037 GO:0005507 GO:0042597
Cluster-22178.57075 GO:0048511 GO:0048870 GO:0006810 GO:0006355
GO:0003700 GO:0043565 GO:0005515 GO:0005198 GO:0016021
GO:0031514 GO:0019028 GO:0005667
Cluster-22178.57076 GO:0046688 GO:0006118 GO:0009055 GO:0005507
GO:0020037 GO:0005515 GO:0042597
Cluster-22178.57077 GO:0006351 GO:0043565
Cluster-22178.57078 GO:0015979 GO:0005515 GO:0008270 GO:0016020

GO:0009539 GO:0009523
Cluster-22178.57079 GO:0006413 GO:0006446 GO:0003723 GO:0003743
GO:0005737 GO:0005840
Cluster-22178.57082 GO:0006479 GO:0006554 GO:0018024 GO:0046872

Cluster-22178.57083 GO:0003676
Cluster-22178.57084 GO:0006281
Cluster-22178.57089 GO:0035556 GO:0055114 GO:0047134
Cluster-22178.57090 GO:0019079 GO:0006468 GO:0008026 GO:0005524
GO:0004672
Cluster-22178.57091 GO:0046872 GO:0030991
Cluster-22178.57092 GO:0008152 GO:0003824
Cluster-22178.57094 GO:0034061 GO:0016779 GO:0003677 GO:0005634
Cluster-22178.57097 GO:0016021
Cluster-22178.5710 GO:0006144 GO:0006464 GO:0046034 GO:0006281
GO:0046872 GO:0008270 GO:0016151 GO:0004017
Cluster-22178.57100 GO:0016021
Cluster-22178.57101 GO:0006545 GO:0009165 GO:0055114 GO:0046656
GO:0006761 GO:0004146
Cluster-22178.57102 GO:0006334 GO:0003677 GO:0005634 GO:0000786
Cluster-22178.57107 GO:0015886 GO:0006357 GO:0007165 GO:0006810
GO:0016192 GO:0017004 GO:0005975 GO:0007010 GO:0004872
GO:0043395 GO:0016853 GO:0005215 GO:0003779 GO:0001104
GO:0005198 GO:0005515 GO:0016592 GO:0016020 GO:0019031
GO:0016021 GO:0005578 GO:0019028
Cluster-22178.57109 GO:0004866
Cluster-22178.57110 GO:0035556 GO:0055085 GO:0046813 GO:0061025
GO:0006508 GO:0016805 GO:0019031 GO:0016021
Cluster-22178.57111 GO:0006508 GO:0007186 GO:0061025 GO:0055114
GO:0035556 GO:0046813 GO:0006120 GO:0016805 GO:0004930
GO:0019031 GO:0016021
Cluster-22178.57114 GO:0005515 GO:0008270
Cluster-22178.57115 GO:0005515

Cluster-22178.57117 GO:0007411 GO:0006914 GO:0006308 GO:0006814
GO:0006813 GO:0019069 GO:0016055 GO:0008239 GO:0008855
GO:0004190 GO:0070009 GO:0016021 GO:0009318 GO:0005576
GO:0005890
Cluster-22178.57118 GO:0007009
Cluster-22178.57119 GO:0006355 GO:0003700 GO:0042025 GO:0005667
Cluster-22178.57122 GO:0051028 GO:0045893 GO:0007275 GO:0008270
GO:0031012 GO:0005634
Cluster-22178.57125 GO:0005515
Cluster-22178.57126 GO:0010508 GO:0006355 GO:0006914 GO:0003677
GO:0003700 GO:0043565 GO:0005634 GO:0005667
Cluster-22178.57131 GO:0051252 GO:0007276 GO:0016891
Cluster-22178.57133 GO:0006511 GO:0007275 GO:0005515 GO:0008270
GO:0005634
Cluster-22178.57135 GO:0008152 GO:0055114 GO:0016616 GO:0051287

Cluster-22178.57136 GO:0055114 GO:0030151 GO:0016491 GO:0005515
GO:0035091
Cluster-22178.57139 GO:0055114 GO:0016616 GO:0016491
Cluster-22178.57143 GO:0007187 GO:0006812 GO:0007608 GO:0005549
GO:0008324 GO:0004984 GO:0016020
Cluster-22178.57145 GO:0006359 GO:0006260 GO:0015992 GO:0006351
GO:0015991 GO:0090266 GO:0015078 GO:0005524 GO:0003677
GO:0003887 GO:0032040 GO:0005680 GO:0019031 GO:0033179
GO:0042575
Cluster-22178.57150 GO:0006952
Cluster-22178.57152 GO:0006508 GO:0007155 GO:0009058 GO:0006952
GO:0007165 GO:0008013 GO:0003824 GO:0004222 GO:0005126
GO:0008134 GO:0016342 GO:0019028 GO:0005667 GO:0005634
GO:0005576
Cluster-22178.57153 GO:0016020
Cluster-22178.57155 GO:0045892 GO:0005515 GO:0005634
Cluster-22178.57156 GO:0046872

Cluster-22178.57157 GO:0032259 GO:0015946 GO:0055114 GO:0006364
GO:0008168 GO:0005515 GO:0004022 GO:0005634
Cluster-22178.57159 GO:0005975 GO:0006890 GO:0015926 GO:0008270
GO:0003676 GO:0004252 GO:0005515 GO:0016021
Cluster-22178.57160 GO:0055114 GO:0042546 GO:0006120 GO:0005199
GO:0016021 GO:0005618
Cluster-22178.57161 GO:0006338 GO:0006355 GO:0003677 GO:0016818
GO:0003676 GO:0005524 GO:0008270 GO:0005634
Cluster-22178.57162 GO:0009405
Cluster-22178.57163 GO:0003677
Cluster-22178.57167 GO:0006468 GO:0005524 GO:0004672 GO:0003677

Cluster-22178.57168 GO:0008152 GO:0008168 GO:0016021
Cluster-22178.57169 GO:0000160 GO:0006935 GO:0006904 GO:0016559
GO:0006396 GO:0055085 GO:0015031 GO:0006614 GO:0016310
GO:0009405 GO:0051252 GO:0006468 GO:0030001 GO:0006869
GO:0042157 GO:0008289 GO:0004672 GO:0042802 GO:0000175
GO:0004673 GO:0046873 GO:0005515 GO:0003723 GO:0046983
GO:0008312 GO:0005524 GO:0000155 GO:0016021 GO:0009365
GO:0048500 GO:0005576 GO:0000145 GO:0005737 GO:0016020
GO:0005779
Cluster-22178.57170 GO:0006355 GO:0019882 GO:0006955 GO:0042613
GO:0016020
Cluster-22178.57171 GO:0006955 GO:0019882 GO:0016020 GO:0042613
Cluster-22178.57172 GO:0006955
Cluster-22178.57176 GO:0006508 GO:0004197
Cluster-22178.57181 GO:0006606 GO:0006807 GO:0006812 GO:0030474
GO:0008017 GO:0003774 GO:0008270 GO:0016887 GO:0005200
GO:0016814 GO:0008092 GO:0005198 GO:0005515 GO:0005815
GO:0005823 GO:0019898 GO:0005882 GO:0016459 GO:0019031
GO:0045298 GO:0016021 GO:0005856 GO:0005737
Cluster-22178.57182 GO:0005975 GO:0007165 GO:0004553 GO:0005509
GO:0005578

Cluster-22178.57184 GO:0016787 GO:0046872 GO:0003676

Cluster-22178.57185 GO:0035556 GO:0006508 GO:0008239 GO:0005515 GO:0004252 GO:0070009

Cluster-22178.57188 GO:0007157 GO:0006367 GO:0009405 GO:0032968 GO:0046872 GO:0008270 GO:0003677 GO:0016021 GO:0005634 GO:0005672

Cluster-22178.5719 GO:0042981 GO:0006952 GO:0005515

Cluster-22178.57193 GO:0055114 GO:0006118 GO:0016627 GO:0003995

Cluster-22178.57196 GO:0006457 GO:0000774 GO:0051087 GO:0042803

Cluster-22178.57200 GO:0006357 GO:0006367 GO:0006810 GO:0009405 GO:0006468 GO:0046872 GO:0005524 GO:0004672 GO:0019870 GO:0005515 GO:0001104 GO:0016592 GO:0005576 GO:0005672

Cluster-22178.57203 GO:0006355 GO:0003700 GO:0008270 GO:0043565 GO:0005667

Cluster-22178.57205 GO:0006886 GO:0016192 GO:0005515 GO:0030117

Cluster-22178.57208 GO:0007165 GO:0007186 GO:0007264 GO:0005525 GO:0004871 GO:0019001 GO:0031683 GO:0003924

Cluster-22178.57209 GO:0006886 GO:0016192

Cluster-22178.57210 GO:0006886 GO:0016192

Cluster-22178.57211 GO:0006470 GO:0006570 GO:0004725 GO:0008138

Cluster-22178.57215 GO:0007186 GO:0035556 GO:0035023 GO:0043087 GO:0055114 GO:0005515 GO:0004930 GO:0047134 GO:0005089 GO:0016020

Cluster-22178.57216 GO:0009116 GO:0003824

Cluster-22178.57217 GO:0006886 GO:0005622

Cluster-22178.57219 GO:0006869 GO:0042157 GO:0005515 GO:0008289 GO:0005576

Cluster-22178.57224 GO:0006355 GO:0003700 GO:0005667 GO:0005634

Cluster-22178.57225 GO:0006355 GO:0003677 GO:0005515 GO:0005634

Cluster-22178.57226 GO:0007165 GO:0005044 GO:0016020

Cluster-22178.5723 GO:0005515
Cluster-22178.57231 GO:0006614 GO:0008312 GO:0005515 GO:0048500
Cluster-22178.57232 GO:0055085 GO:0006810 GO:0005215 GO:0016020
Cluster-22178.57239 GO:0006486 GO:0009312 GO:0008375 GO:0008455
GO:0005795 GO:0016021
Cluster-22178.57240 GO:0016310 GO:0000160 GO:0000155 GO:0046983
GO:0009365 GO:0016021
Cluster-22178.57241 GO:0006270 GO:0006412 GO:0042254 GO:0006198
GO:0030866 GO:0003735 GO:0005198 GO:0008092 GO:0003779
GO:0004115 GO:0005622 GO:0005856 GO:0005840
Cluster-22178.57243 GO:0005515 GO:0008270
Cluster-22178.57245 GO:0005515
Cluster-22178.57249 GO:0006779 GO:0015994 GO:0004853
Cluster-22178.57250 GO:0006508 GO:0008242 GO:0016740 GO:0004197

Cluster-22178.57251 GO:0006355 GO:0043565 GO:0003677 GO:0003700
GO:0005634 GO:0005667 GO:0005815
Cluster-22178.57253 GO:0019836 GO:0005576
Cluster-22178.57259 GO:0006383 GO:0006629 GO:0006351 GO:0006144
GO:0006206 GO:0055085 GO:0003899 GO:0003677 GO:0005730
GO:0005666 GO:0016021
Cluster-22178.57262 GO:0042254 GO:0006412 GO:0006310 GO:0006281
GO:0003678 GO:0005524 GO:0016887 GO:0098519 GO:0003735
GO:0009378 GO:0005840 GO:0005657 GO:0009379
Cluster-22178.57263 GO:0005515
Cluster-22178.57264 GO:0048037
Cluster-22178.57265 GO:0005975 GO:0005509 GO:0004571 GO:0016020
Cluster-22178.57267 GO:0015986 GO:0005975 GO:0015992 GO:0042407
GO:0051082 GO:0005509 GO:0015078 GO:0004571 GO:0045263
GO:0016020 GO:0044284 GO:0061617
Cluster-22178.5727 GO:0042981 GO:0005515
Cluster-22178.57271 GO:0005515
Cluster-22178.57273 GO:0019836 GO:0005576

Cluster-22178.57276 GO:0006118 GO:0006119 GO:0007264 GO:0035556
GO:0055114 GO:0015992 GO:0008121 GO:0005507 GO:0005525
Cluster-22178.57281 GO:0016021
Cluster-22178.57287 GO:0006694 GO:0055114 GO:0015936 GO:0007165
GO:0008158 GO:0004420 GO:0050662 GO:0016020
Cluster-22178.57288 GO:0006355 GO:0003677
Cluster-22178.57289 GO:0003677 GO:0005515
Cluster-22178.57293 GO:0015743 GO:0005515 GO:0003774 GO:0005524
GO:0016459
Cluster-22178.57295 GO:0015992 GO:0006935 GO:0006120 GO:0006744
GO:0007165 GO:0006955 GO:0006118 GO:0006814 GO:0003954
GO:0008137 GO:0008009 GO:0005576 GO:0005739
Cluster-22178.57299 GO:0016579 GO:0070140 GO:0032183 GO:0036459

Cluster-22178.5730 GO:0007165 GO:0040007 GO:0008283 GO:0008083
Cluster-22178.57300 GO:0006810 GO:0006355 GO:0055085 GO:0000103
GO:0006144 GO:0008134 GO:0016301 GO:0042626 GO:0004020
GO:0016887 GO:0005524 GO:0008270 GO:0005667 GO:0016021
Cluster-22178.57301 GO:0006522 GO:0048193 GO:0006419 GO:0043039
GO:0006531 GO:0003676 GO:0016876 GO:0004813 GO:0005524
GO:0005198 GO:0000166 GO:0016020
Cluster-22178.57305 GO:0043087 GO:0035023 GO:0005089
Cluster-22178.57308 GO:0008033 GO:0009452 GO:0008152 GO:0001510
GO:0006400 GO:0009451 GO:0006306 GO:0008168 GO:0016429
GO:0051537 GO:0003723 GO:0008176 GO:0008170 GO:0003677
GO:0043231
Cluster-22178.57309 GO:0006355 GO:0008270 GO:0005634
Cluster-22178.57310 GO:0006355 GO:0008270 GO:0005634
Cluster-22178.57311 GO:0019058 GO:0007165 GO:0046789 GO:0005515
GO:0043565 GO:0005634 GO:0019031 GO:0009986
Cluster-22178.57312 GO:0046872
Cluster-22178.57313 GO:0046872 GO:0003676
Cluster-22178.57314 GO:0006886 GO:0006888 GO:0008270 GO:0030127

Cluster-22178.57315 GO:0016192 GO:0006886 GO:0006486 GO:0015991
GO:0043248 GO:0016820 GO:0008378 GO:0016020 GO:0030117
GO:0000221
Cluster-22178.57319 GO:0075521 GO:0006308 GO:0019076 GO:0006885
GO:0006812 GO:0055085 GO:0046718 GO:0008855 GO:0015299
GO:0019028 GO:0044163 GO:0042025 GO:0030430 GO:0009318
GO:0016021
Cluster-22178.57320 GO:0006812 GO:0006885 GO:0055085 GO:0006308
GO:0015299 GO:0008855 GO:0009318 GO:0016021
Cluster-22178.57321 GO:0075521 GO:0006812 GO:0006885 GO:0055085
GO:0046718 GO:0006308 GO:0019076 GO:0008855 GO:0015299
GO:0042025 GO:0030430 GO:0009318 GO:0019028 GO:0044163
GO:0016021
Cluster-22178.57326 GO:0007264 GO:0003723 GO:0005525
Cluster-22178.57334 GO:0051289 GO:0006506 GO:0007165 GO:0008140
GO:0016740 GO:0005515 GO:0005509 GO:0005886 GO:0016021
GO:0005789 GO:0005667 GO:0016012 GO:0005578
Cluster-22178.57336 GO:0019521 GO:0055114 GO:0006574 GO:0015940
GO:0008152 GO:0006508 GO:0006568 GO:0006813 GO:0006552
GO:0006550 GO:0009403 GO:0006520 GO:0006554 GO:0006633
GO:0006098 GO:0006631 GO:0006810 GO:0018874 GO:0008677
GO:0003824 GO:0005215 GO:0003857 GO:0016743 GO:0016836
GO:0016491 GO:0008233 GO:0016597 GO:0051287 GO:0004616
GO:0016616 GO:0016020
Cluster-22178.57337 GO:0015031 GO:0006659 GO:0016020
Cluster-22178.57340 GO:0006605 GO:0007165 GO:0051539 GO:0005515
GO:0016020 GO:0005887
Cluster-22178.57341 GO:0005515
Cluster-22178.57343 GO:0007275 GO:0006836 GO:0051539 GO:0005515
GO:0019905 GO:0016020 GO:0016021 GO:0005887
Cluster-22178.57344 GO:0006605 GO:0007165 GO:0051539 GO:0005515
GO:0016020 GO:0005887
Cluster-22178.57345 GO:0007165 GO:0005515 GO:0000166 GO:0046872

GO:0045202
Cluster-22178.57348 GO:0006914 GO:0006525 GO:0071436 GO:0048870
GO:0006090 GO:0006355 GO:0006886 GO:0006814 GO:0006560
GO:0015081 GO:0043565 GO:0003700 GO:0008948 GO:0005667
GO:0016020 GO:0005783 GO:0005801 GO:0016021 GO:0031514
Cluster-22178.57350 GO:0016567 GO:0061630 GO:0005515 GO:0004842
GO:0008270 GO:0046872 GO:0043565 GO:0005634 GO:0005680
Cluster-22178.57353 GO:0032012 GO:0043087 GO:0008289 GO:0005086
GO:0046872
Cluster-22178.57361 GO:0003779
Cluster-22178.57362 GO:0004386 GO:0005515 GO:0005524 GO:0031083
Cluster-22178.57363 GO:0006355 GO:0043565 GO:0008270 GO:0003700
GO:0005515 GO:0005667 GO:0005634
Cluster-22178.57364 GO:0048278 GO:0006887 GO:0006633 GO:0006090
GO:0051082 GO:0005198 GO:0003989 GO:0005737 GO:0009317
Cluster-22178.57367 GO:0006914 GO:0006206 GO:0006606 GO:0042157
GO:0007155 GO:0006383 GO:0006869 GO:0042262 GO:0030001
GO:0006144 GO:0008053 GO:0055085 GO:0071973 GO:0006810
GO:0006935 GO:0006355 GO:0006351 GO:0003774 GO:0003690
GO:0003677 GO:0003924 GO:0005198 GO:0003899 GO:0042803
GO:0046873 GO:0008289 GO:0045502 GO:0008134 GO:0005667
GO:0005730 GO:0005666 GO:0016020 GO:0005741 GO:0009288
GO:0030286 GO:0005886 GO:0005882 GO:0016021 GO:0019031
GO:0019028 GO:0005576
Cluster-22178.57368 GO:0055114 GO:0006836 GO:0008152 GO:0016491
GO:0019905 GO:0016021
Cluster-22178.57369 GO:0009966 GO:0043565 GO:0005515 GO:0005634
GO:0005737 GO:0016020
Cluster-22178.57370 GO:0055114 GO:0009058 GO:0016491 GO:0030170

Cluster-22178.57374 GO:0009451 GO:0001522 GO:0006810 GO:0007165
GO:0006355 GO:0009982 GO:0005215 GO:0046983 GO:0003713
GO:0005515 GO:0003723 GO:0035257 GO:0016021 GO:0005667

GO:0005634
Cluster-22178.57376 GO:0003677
Cluster-22178.57377 GO:0003677
Cluster-22178.57383 GO:0006784 GO:0055114 GO:0006508 GO:0004252
GO:0016627 GO:0016020 GO:0016021
Cluster-22178.57384 GO:0005515
Cluster-22178.57385 GO:0005515
Cluster-22178.57387 GO:0035556 GO:0009190 GO:0016849
Cluster-22178.57390 GO:0017000 GO:0007021 GO:0016787 GO:0051082
GO:0015631 GO:0042302 GO:0045298
Cluster-22178.57393 GO:0008152 GO:0005524 GO:0016301 GO:0005634
GO:0042597
Cluster-22178.57396 GO:0016567 GO:0005515 GO:0004842 GO:0046872
GO:0008270 GO:0005680
Cluster-22178.57397 GO:0005985 GO:0006012 GO:0005982 GO:0004564
GO:0004575 GO:0017177
Cluster-22178.57398 GO:0007186 GO:0006310 GO:0006367 GO:0006281
GO:0006106 GO:0007411 GO:0016788 GO:0000287 GO:0000400
GO:0004930 GO:0016020 GO:0005672 GO:0016021
Cluster-22178.57401 GO:0009058 GO:0055114 GO:0006520 GO:0016491
GO:0030170 GO:0016829
Cluster-22178.57402 GO:0006281 GO:0003824 GO:0004518 GO:0003677

Cluster-22178.57403 GO:0006810 GO:0051252 GO:0004386 GO:0003676
GO:0016887 GO:0005524 GO:0000166 GO:0003677 GO:0016891
Cluster-22178.57404 GO:0051252 GO:0006810 GO:0003677 GO:0016891
GO:0000166 GO:0003676 GO:0004386 GO:0005524 GO:0016887
Cluster-22178.57405 GO:0016757
Cluster-22178.57406 GO:0046034 GO:0006144 GO:0004017 GO:0003677
GO:0046872
Cluster-22178.57407 GO:0042967 GO:0008080 GO:0016747
Cluster-22178.57409 GO:0006241 GO:0006206 GO:0006144 GO:0006886
GO:0006183 GO:0006355 GO:0006228 GO:0006508 GO:0006165

GO:0004222 GO:0008536 GO:0004550 GO:0005634
Cluster-22178.57412 GO:0005982 GO:0005985 GO:0007601 GO:0006012
GO:0006144 GO:0004575 GO:0030553 GO:0005515 GO:0004114
GO:0004564 GO:0017177 GO:0005576
Cluster-22178.57415 GO:0006468 GO:0006182 GO:0009190 GO:0046039
GO:0035556 GO:0006144 GO:0016849 GO:0004383 GO:0005524
GO:0004672
Cluster-22178.57418 GO:0007010 GO:0003779 GO:0033926
Cluster-22178.57419 GO:0006810 GO:0055085 GO:0005524 GO:0016887
GO:0042626 GO:0016021
Cluster-22178.57422 GO:0000413 GO:0006457 GO:0003755 GO:0003676

Cluster-22178.57423 GO:0055085 GO:0006810 GO:0005215 GO:0016020
GO:0016021
Cluster-22178.57426 GO:0006886 GO:0007165 GO:0006355 GO:0003700
GO:0004871 GO:0016020 GO:0005634 GO:0005667
Cluster-22178.57429 GO:0007049 GO:0017053
Cluster-22178.57431 GO:0006576 GO:0006281 GO:0006777 GO:0006310
GO:0007264 GO:0000103 GO:0002949 GO:0006144 GO:0003723
GO:0004020 GO:0005525 GO:0003924 GO:0043531 GO:0098519
GO:0009378 GO:0003724 GO:0016887 GO:0005524 GO:0009379
GO:0005657
Cluster-22178.57432 GO:0006468 GO:0055114 GO:0019087 GO:0035556
GO:0005515 GO:0004672 GO:0016818 GO:0003712 GO:0003724
GO:0047134 GO:0016773 GO:0005524 GO:0005634 GO:0016021
GO:0016020 GO:0005667
Cluster-22178.57437 GO:0046436 GO:0009252 GO:0006750 GO:0019836
GO:0006541 GO:0046872 GO:0005524 GO:0016787 GO:0008716
GO:0004363 GO:0005576
Cluster-22178.5744 GO:0006886 GO:0006355 GO:0007059 GO:0051301
GO:0000090 GO:0007067 GO:0015833 GO:0031110 GO:0000166
GO:0005515 GO:0003700 GO:0043565 GO:0008017 GO:0005524
GO:0005801 GO:0045298 GO:0016020 GO:0005667 GO:0005876

GO:0000940
Cluster-22178.57440 GO:0006468 GO:0005524 GO:0004672 GO:0016773
GO:0016020
Cluster-22178.57442 GO:0007264 GO:0043087 GO:0005085
Cluster-22178.57443 GO:0006351 GO:0006479 GO:0006383 GO:0006481
GO:0006468 GO:0006144 GO:0006206 GO:0003899 GO:0003677
GO:0004671 GO:0004672 GO:0005524 GO:0005730 GO:0005666
GO:0016021
Cluster-22178.57444 GO:0005515
Cluster-22178.57445 GO:0055070 GO:0005507
Cluster-22178.57447 GO:0042967 GO:0007018 GO:0007017 GO:0003824
GO:0008080 GO:0008017 GO:0003777 GO:0051536 GO:0005524
GO:0045298 GO:0005874
Cluster-22178.57449 GO:0006355 GO:0005524 GO:0003774 GO:0016887
GO:0003924 GO:0005525 GO:0003724 GO:0003723 GO:0008134
GO:0005515 GO:0016459 GO:0005667
Cluster-22178.57450 GO:0005515 GO:0005524 GO:0003774 GO:0016459
Cluster-22178.57451 GO:0006355 GO:0003723 GO:0008134 GO:0005515
GO:0016887 GO:0003924 GO:0003774 GO:0005524 GO:0003724
GO:0005525 GO:0005667 GO:0016459
Cluster-22178.57455 GO:0019953 GO:0007165 GO:0045893 GO:0003676
GO:0008430 GO:0000772 GO:0005576
Cluster-22178.57456 GO:0006119 GO:0006412 GO:0006355 GO:0042254
GO:0015986 GO:0035556 GO:0015992 GO:0003735 GO:0046961
GO:0003677 GO:0008270 GO:0046933 GO:0005840 GO:0045261
GO:0045259 GO:0005622
Cluster-22178.57458 GO:0043565
Cluster-22178.57459 GO:0016020
Cluster-22178.57463 GO:0006260 GO:0003677 GO:0003924 GO:0005615
GO:0005634
Cluster-22178.57467 GO:0042998 GO:0007186 GO:0071253 GO:0004930
GO:0005802
Cluster-22178.57473 GO:0009058 GO:0005515 GO:0004866

Cluster-22178.57474 GO:0006810 GO:0005622
Cluster-22178.57475 GO:0003676 GO:0016021
Cluster-22178.57478 GO:0006812 GO:0055085 GO:0008324 GO:0016021
Cluster-22178.57482 GO:0003723
Cluster-22178.57484 GO:0005515
Cluster-22178.57487 GO:0008063
Cluster-22178.57488 GO:0015992 GO:0034508 GO:0015991 GO:0015078
GO:0008270 GO:0003723 GO:0033179 GO:0000776
Cluster-22178.57489 GO:0005975 GO:0046373 GO:0009117 GO:0046556

Cluster-22178.5749 GO:0009103 GO:0007275 GO:0007165 GO:0055085
GO:0005179 GO:0005576 GO:0019028 GO:0016020 GO:0016021
Cluster-22178.57491 GO:0002949 GO:0006310 GO:0006281 GO:0006355
GO:0003724 GO:0003678 GO:0016887 GO:0005524 GO:0008134
GO:0003723 GO:0009378 GO:0005657 GO:0009379 GO:0005667
Cluster-22178.57493 GO:0006355 GO:0006464 GO:0035556 GO:0003677
GO:0008270 GO:0046872 GO:0003700 GO:0005507 GO:0016151
GO:0005634 GO:0005667
Cluster-22178.57494 GO:0005975 GO:0006013 GO:0034639 GO:0004553
GO:0004559 GO:0008270 GO:0046872 GO:0015923 GO:0016021
Cluster-22178.57498 GO:0006541 GO:0005515 GO:0004359
Cluster-22178.57501 GO:0003676 GO:0003723
Cluster-22178.57503 GO:0006821 GO:0055085 GO:0005247 GO:0046983
GO:0016020
Cluster-22178.57505 GO:0006974 GO:0006915 GO:0051260 GO:0005515

Cluster-22178.57506 GO:0055114 GO:0006098 GO:0005975 GO:0006749
GO:0006006 GO:0050661 GO:0004345
Cluster-22178.57507 GO:0006811 GO:0019439 GO:0006813 GO:0005249
GO:0008270 GO:0005515 GO:0003677 GO:0016833 GO:0005622
GO:0016020 GO:0008076
Cluster-22178.57508 GO:0006886 GO:0008144 GO:0046872 GO:0003676

Cluster-22178.57512 GO:0007186 GO:0007165 GO:0007264 GO:0004871
GO:0005525 GO:0003924 GO:0019001 GO:0031683
Cluster-22178.57516 GO:0006465 GO:0016032 GO:0008233 GO:0016021
GO:0005787
Cluster-22178.57517 GO:0007005 GO:0006508 GO:0004222 GO:0008270
GO:0031012 GO:0031305
Cluster-22178.57518 GO:0007005 GO:0006508 GO:0004222 GO:0008270
GO:0031305 GO:0031012
Cluster-22178.57519 GO:0009451 GO:0015986 GO:0015992 GO:0030488
GO:0008033 GO:0016429 GO:0015078 GO:0008168 GO:0031515
GO:0000276
Cluster-22178.57523 GO:0019073 GO:0006139 GO:0006260 GO:0003676
GO:0003723 GO:0008408 GO:0005515 GO:0003887 GO:0003677
GO:0042575
Cluster-22178.57525 GO:0005515 GO:0005509
Cluster-22178.57528 GO:0019856 GO:0017038 GO:0005515 GO:0003677
GO:0003723 GO:0016787 GO:0003676 GO:0008270 GO:0005524
GO:0016020
Cluster-22178.57529 GO:0009069 GO:0006468 GO:0016310 GO:0004672
GO:0005524 GO:0004674
Cluster-22178.57530 GO:0016310 GO:0009069 GO:0006468 GO:0004674
GO:0004672 GO:0005524
Cluster-22178.57532 GO:0006570 GO:0042207 GO:0006810 GO:0009072
GO:0015743 GO:0004334 GO:0005886
Cluster-22178.57533 GO:0003677
Cluster-22178.57534 GO:0005198 GO:0019028
Cluster-22178.57540 GO:0016787 GO:0004222
Cluster-22178.57541 GO:0005515
Cluster-22178.57543 GO:0006886 GO:0005515 GO:0005763 GO:0016020
GO:0005801
Cluster-22178.57548 GO:0006468 GO:0035556 GO:0005524 GO:0004672
GO:0016773 GO:0005515 GO:0016020
Cluster-22178.57550 GO:0005515 GO:0046872

Cluster-22178.57551 GO:0005515
Cluster-22178.57552 GO:0005515
Cluster-22178.57553 GO:0015031 GO:0055114 GO:0016491 GO:0008565 GO:0005515 GO:0016021
Cluster-22178.57555 GO:0006260 GO:0032259 GO:0006370 GO:0006397 GO:0006310 GO:0006281 GO:0003676 GO:0003910 GO:0005524 GO:0008168 GO:0005515 GO:0004484
Cluster-22178.57556 GO:0032259 GO:0006370 GO:0006260 GO:0006281 GO:0006397 GO:0006310 GO:0005524 GO:0008168 GO:0003910 GO:0003676 GO:0004484 GO:0005515 GO:0005887
Cluster-22178.57558 GO:0006464
Cluster-22178.57559 GO:0035556 GO:0031167 GO:0009451 GO:0006400 GO:0008033 GO:0008168 GO:0008176
Cluster-22178.57561 GO:0008146 GO:0016021
Cluster-22178.57562 GO:0051016 GO:0050840 GO:0016021 GO:0005576 GO:0030479
Cluster-22178.57565 GO:0015940 GO:0055114 GO:0003677 GO:0008677

Cluster-22178.57567 GO:0005515 GO:0019904 GO:0030246 GO:0005737
Cluster-22178.57568 GO:0030246
Cluster-22178.57569 GO:0030246
Cluster-22178.57573 GO:0005515
Cluster-22178.57574 GO:0003723 GO:0005515
Cluster-22178.57576 GO:0010207 GO:0007017 GO:0007018 GO:0003777 GO:0005515 GO:0005737 GO:0030286 GO:0005874
Cluster-22178.57577 GO:0015684 GO:0006576 GO:0007264 GO:0005525 GO:0003924 GO:0015093 GO:0005524 GO:0016021
Cluster-22178.57578 GO:0006810 GO:0055085 GO:0006888 GO:0005515 GO:0022857 GO:0005215 GO:0016020 GO:0005801 GO:0019867 GO:0016021 GO:0005622
Cluster-22178.57581 GO:0003676
Cluster-22178.57583 GO:0009405 GO:0003723 GO:0046872 GO:0003676 GO:0016788 GO:0043022 GO:0005634

Cluster-22178.57586 GO:0006555 GO:0009405 GO:0006810 GO:0006952
GO:0006629 GO:0009755 GO:0006556 GO:0019855 GO:0046872
GO:0004478 GO:0045202 GO:0005576
Cluster-22178.57587 GO:0009306 GO:0016021
Cluster-22178.57589 GO:0007186 GO:0004930 GO:0016021
Cluster-22178.57590 GO:0046872 GO:0045202
Cluster-22178.57593 GO:0030168 GO:0006810 GO:0007165 GO:0016567
GO:0071973 GO:0007067 GO:0007049 GO:0008608 GO:0051258
GO:0006935 GO:0006308 GO:0030474 GO:0015991 GO:0006606
GO:0015992 GO:0006914 GO:0006869 GO:0071988 GO:0009405
GO:0010508 GO:0005102 GO:0031386 GO:0008855 GO:0030674
GO:0008289 GO:0003774 GO:0005215 GO:0015078 GO:0016881
GO:0005198 GO:0051082 GO:0019031 GO:0016021 GO:0009318
GO:0033179 GO:0005576 GO:0019028 GO:0072686 GO:0005577
GO:0016020 GO:0009288 GO:0042729
Cluster-22178.57594 GO:0045892 GO:0003676 GO:0005634
Cluster-22178.57598 GO:0050708 GO:0005515 GO:0051537 GO:0005737
Cluster-22178.57599 GO:0016567 GO:0001510 GO:0009452 GO:0032922
GO:0055114 GO:0005515 GO:0004842 GO:0051537 GO:0046872
GO:0000976 GO:0008270 GO:0008168 GO:0016491
Cluster-22178.57601 GO:0005515
Cluster-22178.57602 GO:0005515 GO:0070403 GO:0008168
Cluster-22178.57606 GO:0006281 GO:0003824 GO:0004518 GO:0003677

Cluster-22178.57607 GO:0006570 GO:0006470 GO:0004725 GO:0008138

Cluster-22178.57609 GO:0006468 GO:0005198 GO:0005524 GO:0004672
GO:0016773 GO:0016020
Cluster-22178.57610 GO:0042254 GO:0006412 GO:0003735 GO:0005856
GO:0005622 GO:0005840
Cluster-22178.57613 GO:0055085 GO:0030206 GO:0030001 GO:0006024
GO:0030158 GO:0046873 GO:0016020
Cluster-22178.57615 GO:0006355 GO:0005634

Cluster-22178.57616 GO:0015992 GO:0006119 GO:0015991 GO:0006355
GO:0003700 GO:0046961 GO:0005667 GO:0033178
Cluster-22178.57618 GO:0044780 GO:0006886 GO:0005801 GO:0016020
Cluster-22178.57619 GO:0007264 GO:0005525
Cluster-22178.57621 GO:0005515
Cluster-22178.57622 GO:0016117 GO:0009405 GO:0046872 GO:0003924
GO:0005525 GO:0003677 GO:0016705 GO:0005515
Cluster-22178.57629 GO:0009395 GO:0006955 GO:0030153 GO:0006629
GO:0006606 GO:0035556 GO:0006914 GO:0046339 GO:0007165
GO:0004435 GO:0008081 GO:0005515 GO:0042803 GO:0008134
GO:0045502 GO:0015643 GO:0005509 GO:0019814 GO:0019028
GO:0030286 GO:0005667
Cluster-22178.57635 GO:0006810 GO:0018279 GO:0006355 GO:0004579
GO:0008270 GO:0008250 GO:0005634 GO:0016021 GO:0005622
Cluster-22178.57636 GO:0000398 GO:0005975 GO:0004560 GO:0003676
GO:0008270 GO:0005681
Cluster-22178.57639 GO:0006811 GO:0005509 GO:0005216 GO:0016020
GO:0005576
Cluster-22178.57641 GO:0046872 GO:0008270 GO:0003677
Cluster-22178.57645 GO:0005515
Cluster-22178.57646 GO:0006355 GO:0006313 GO:0004803 GO:0003677

Cluster-22178.57652 GO:0046856 GO:0034597 GO:0004190 GO:0016021
Cluster-22178.57656 GO:0007165 GO:0007186 GO:0007264 GO:0006508
GO:0000166 GO:0004197 GO:0003677 GO:0005525 GO:0004871
GO:0031683 GO:0005524 GO:0019001 GO:0003924
Cluster-22178.57657 GO:0006812 GO:0000166 GO:0046872 GO:0016887
GO:0016021
Cluster-22178.57658 GO:0007165 GO:0006506 GO:0006508 GO:0008236
GO:0005179 GO:0005576
Cluster-22178.57660 GO:0046872 GO:0000166
Cluster-22178.57661 GO:0032259 GO:0008168
Cluster-22178.57663 GO:0046872

Cluster-22178.57664 GO:0006123 GO:0015992 GO:0004129 GO:0045277
GO:0005739
Cluster-22178.57668 GO:0009113 GO:0006144 GO:0009252 GO:0046436
GO:0008716 GO:0070140 GO:0032183 GO:0004637 GO:0005524
GO:0046872
Cluster-22178.57672 GO:0009396 GO:0000226 GO:0043093 GO:0055114
GO:0008152 GO:0045132 GO:0046487 GO:0000917 GO:0016787
GO:0004488 GO:0005509 GO:0008092 GO:0003824 GO:0005515
GO:0019898 GO:0005737 GO:0016021 GO:0000775 GO:0005634
GO:0015630
Cluster-22178.57674 GO:0007264 GO:0005525 GO:0003924
Cluster-22178.57675 GO:0006629 GO:0000413 GO:0006457 GO:0003755

Cluster-22178.57676 GO:0007010 GO:0000413 GO:0006457 GO:0008017
GO:0003755 GO:0005887 GO:0045298
Cluster-22178.57677 GO:0006352
Cluster-22178.57678 GO:0006355 GO:0006281 GO:0006952 GO:0046872
GO:0008270 GO:0043565 GO:0003700 GO:0005667
Cluster-22178.57679 GO:0055114 GO:0016787
Cluster-22178.57682 GO:0003677 GO:0005515 GO:0008017 GO:0034061
GO:0045298 GO:0005634
Cluster-22178.57683 GO:0016055 GO:0006270 GO:0006418 GO:0006423
GO:0006534 GO:0007411 GO:0006811 GO:0004812 GO:0005515
GO:0004817 GO:0000166 GO:0005524 GO:0005576 GO:0016020
GO:0005737
Cluster-22178.57684 GO:0006260 GO:0006281 GO:0003911
Cluster-22178.57686 GO:0019439 GO:0055114 GO:0016708 GO:0005515
GO:0051015 GO:0051537 GO:0005506
Cluster-22178.57692 GO:0007165 GO:0007186 GO:0009069 GO:0016310
GO:0005515 GO:0005525 GO:0004674 GO:0016772 GO:0004871
GO:0031683 GO:0019001 GO:0003924 GO:0016021
Cluster-22178.57695 GO:0008270 GO:0005515 GO:0005634
Cluster-22178.57696 GO:0006470 GO:0006570 GO:0008138 GO:0004725

Cluster-22178.57698 GO:0000079 GO:0019901
Cluster-22178.57699 GO:0006914 GO:0048870 GO:0051607 GO:0006606
GO:0008092 GO:0045502 GO:0042803 GO:0008134 GO:0005737
GO:0030286 GO:0009424 GO:0019898 GO:0005667 GO:0031514
Cluster-22178.57700 GO:0006897 GO:0003676 GO:0016020
Cluster-22178.57702 GO:0005515
Cluster-22178.57703 GO:0019236 GO:0006955 GO:0006118 GO:0006412
GO:0015708 GO:0007606 GO:0009772 GO:0019684 GO:0042254
GO:0007186 GO:0007165 GO:0005125 GO:0046872 GO:0005515
GO:0003723 GO:0045156 GO:0004932 GO:0008270 GO:0003735
GO:0003677 GO:0016021 GO:0005840 GO:0005634 GO:0005576
GO:0005622
Cluster-22178.57705 GO:0006310 GO:0015743 GO:0006302 GO:0003677
GO:0005515 GO:0005789 GO:0005634
Cluster-22178.57707 GO:0006468 GO:0016773 GO:0046872 GO:0008270
GO:0004672 GO:0005524 GO:0005515 GO:0016020
Cluster-22178.57708 GO:0006308 GO:0008380 GO:0007155 GO:0008855
GO:0005200 GO:0005856 GO:0005681 GO:0005882 GO:0009318
Cluster-22178.57715 GO:0007017 GO:0007018 GO:0005975 GO:0006811
GO:0006810 GO:0055085 GO:0005515 GO:0046872 GO:0005215
GO:0003777 GO:0005216 GO:0016868 GO:0005524 GO:0008270
GO:0008017 GO:0005874 GO:0045298 GO:0016020
Cluster-22178.57725 GO:0008152 GO:0016836 GO:0003824
Cluster-22178.57729 GO:0009405 GO:0045747 GO:0006221 GO:0007049

Cluster-22178.57730 GO:0007049 GO:0006221 GO:0045747 GO:0009405
GO:0004867
Cluster-22178.57732 GO:0006367 GO:0006355 GO:0008270 GO:0046872
GO:0003677 GO:0005515 GO:0003713 GO:0005672 GO:0005667
Cluster-22178.57734 GO:0007165 GO:0006355 GO:0005515 GO:0004871
GO:0003700 GO:0005667 GO:0071203
Cluster-22178.57736 GO:0046677 GO:0006810 GO:0006816 GO:0042030

GO:0005246 GO:0046872 GO:0016020

Cluster-22178.57739 GO:0007186 GO:0007165 GO:0005515 GO:0004930

GO:0008601 GO:0016021 GO:0000159

Cluster-22178.57744 GO:0007264 GO:0035556 GO:0006506 GO:0017176

GO:0005525 GO:0016021

Cluster-22178.57746 GO:0006687 GO:0009058 GO:0008146 GO:0001733

GO:0005794 GO:0016021

Cluster-22178.57747 GO:0005515

Cluster-22178.57750 GO:0005515 GO:0061630 GO:0008270 GO:0046872

GO:0016817

Cluster-22178.57751 GO:0007186 GO:0006825 GO:0005507 GO:0016531

GO:0004930 GO:0016021 GO:0005758

Cluster-22178.57756 GO:0042254 GO:0006412 GO:0006576 GO:0007264

GO:0005525 GO:0005524 GO:0003735 GO:0005840 GO:0005622

Cluster-22178.57761 GO:0015979 GO:0030071 GO:0005515 GO:0005680

GO:0009579 GO:0016021 GO:0009522

Cluster-22178.57762 GO:0015979 GO:0030071 GO:0006952 GO:0005515

GO:0005680 GO:0016021 GO:0009522 GO:0009579 GO:0005576

Cluster-22178.57764 GO:0015992 GO:0006123 GO:0007165 GO:0007005

GO:0016567 GO:0008270 GO:0004842 GO:0004129 GO:0016788

GO:0005515 GO:0005887 GO:0045277

Cluster-22178.57768 GO:0032780 GO:0006355 GO:0003700 GO:0042030

GO:0008270 GO:0005515 GO:0016020 GO:0005634 GO:0005667

GO:0005739

Cluster-22178.5777 GO:0048870 GO:0007049 GO:0006355 GO:0009271

GO:0003677 GO:0019028 GO:0071203 GO:0031514

Cluster-22178.57770 GO:0016021

Cluster-22178.57771 GO:0008152 GO:0055114 GO:0016491

Cluster-22178.57773 GO:0008152 GO:0055114 GO:0016491

Cluster-22178.57774 GO:0055114 GO:0015031 GO:0005515 GO:0016706

GO:0008168 GO:0008565

Cluster-22178.57775 GO:0008152 GO:0009234 GO:0005198 GO:0005515

GO:0050662 GO:0003824 GO:0016491 GO:0008270 GO:0046872

GO:0019028 GO:0005622
Cluster-22178.57776 GO:0007165 GO:0016973 GO:0030168 GO:0051258
GO:0030674 GO:0005102 GO:0005577 GO:0005643
Cluster-22178.57777 GO:0006260 GO:0055114 GO:0005198 GO:0008270
GO:0016020 GO:0019028 GO:0019031 GO:0005622
Cluster-22178.57778 GO:0004867 GO:0005515 GO:0016020 GO:0019013
Cluster-22178.57779 GO:0006869 GO:0042157 GO:0006265 GO:0003917
GO:0003677 GO:0008289 GO:0005694 GO:0005576
Cluster-22178.57780 GO:0006396 GO:0006260 GO:0008270 GO:0005198
GO:0019031 GO:0005622 GO:0016020 GO:0019028
Cluster-22178.57781 GO:0006869 GO:0042157 GO:0016125 GO:0006694
GO:0006265 GO:0003917 GO:0005515 GO:0003677 GO:0047750
GO:0008289 GO:0005694 GO:0005576 GO:0016021
Cluster-22178.57784 GO:0007165
Cluster-22178.57785 GO:0030001 GO:0015992 GO:0006123 GO:0000166
GO:0046872 GO:0004129 GO:0045277 GO:0005743 GO:0005751
Cluster-22178.57787 GO:0032801 GO:0006812 GO:0019076 GO:0005261
GO:0005887 GO:0033644
Cluster-22178.57788 GO:0031554 GO:0006468 GO:0031556 GO:0016773
GO:0005524 GO:0004672 GO:0016020
Cluster-22178.57790 GO:0000723 GO:0003697 GO:0003676
Cluster-22178.57791 GO:0000723 GO:0003676 GO:0003697
Cluster-22178.57792 GO:0006468 GO:0007165 GO:0004672 GO:0005524
GO:0005515
Cluster-22178.57794 GO:0007264 GO:0005525 GO:0016020 GO:0005622
Cluster-22178.57796 GO:0006118 GO:0008270 GO:0046872 GO:0009055
GO:0020037 GO:0005198 GO:0005622 GO:0019031
Cluster-22178.57797 GO:0006118 GO:0005198 GO:0020037 GO:0046872
GO:0008270 GO:0009055 GO:0005622 GO:0019031
Cluster-22178.57800 GO:0007186 GO:0004930 GO:0016021
Cluster-22178.57801 GO:0008152 GO:0016042 GO:0005975 GO:0009058
GO:0046486 GO:0006508 GO:0004806 GO:0016788 GO:0008236
GO:0008184 GO:0016787

Cluster-22178.57802 GO:0006813 GO:0009405 GO:0016567 GO:0005249 GO:0004842 GO:0030955 GO:0042802 GO:0005515 GO:0016021 GO:0008076
Cluster-22178.57804 GO:0055114 GO:0008033 GO:0046872 GO:0016491 GO:0071949 GO:0050660
Cluster-22178.57805 GO:0045892 GO:0005634
Cluster-22178.57806 GO:0003677 GO:0005634
Cluster-22178.57809 GO:0008641
Cluster-22178.57811 GO:0006468 GO:0019079 GO:0004672 GO:0005524 GO:0008026
Cluster-22178.57812 GO:0003676
Cluster-22178.57815 GO:0006269 GO:0006260 GO:0006351 GO:0046872 GO:0003896 GO:0005730 GO:0005657
Cluster-22178.57817 GO:0005515
Cluster-22178.57820 GO:0004672
Cluster-22178.57823 GO:0006606
Cluster-22178.57827 GO:0016310 GO:0009069 GO:0006468 GO:0004674 GO:0005524 GO:0004672
Cluster-22178.57829 GO:0006813 GO:0031124 GO:0055114 GO:0030955 GO:0003676 GO:0016491 GO:0005249 GO:0008076 GO:0016021
Cluster-22178.57832 GO:0008283 GO:0040007 GO:0007165 GO:0005096 GO:0008083
Cluster-22178.57833 GO:0017004 GO:0006810 GO:0055114 GO:0016020 GO:0005886
Cluster-22178.57834 GO:0045893 GO:0006351 GO:0008270 GO:0005515 GO:0003700 GO:0005634 GO:0005667
Cluster-22178.57835 GO:0055085 GO:0006118 GO:0016758 GO:0005515 GO:0009055 GO:0016021
Cluster-22178.57836 GO:0003676
Cluster-22178.57840 GO:0007049 GO:0006351 GO:0017053
Cluster-22178.57841 GO:0055114 GO:0007049 GO:0006351 GO:0016491 GO:0050660 GO:0017053
Cluster-22178.57844 GO:0055114 GO:0000921 GO:0033743 GO:0016021

GO:0005887 GO:0005940
Cluster-22178.57845 GO:0030036 GO:0015743 GO:0016043 GO:0007067
GO:0003779 GO:0048037 GO:0017048 GO:0016740 GO:0042729
GO:0072686
Cluster-22178.57846 GO:0055085 GO:0006812 GO:0035556 GO:0005515
GO:0008324
Cluster-22178.5785 GO:0016020 GO:0016021
Cluster-22178.57850 GO:0055114 GO:0010181 GO:0016491
Cluster-22178.57851 GO:0003677
Cluster-22178.57853 GO:0008270
Cluster-22178.57854 GO:0006952 GO:0006810 GO:0009405 GO:0006556
GO:0009755 GO:0006629 GO:0006555 GO:0006730 GO:0004478
GO:0016740 GO:0019855 GO:0046872 GO:0005576 GO:0045202
Cluster-22178.57855 GO:0006418 GO:0006420 GO:0007049 GO:0006260
GO:0006525 GO:0006560 GO:0005524 GO:0004814 GO:0004812
GO:0003677 GO:0000166 GO:0005737 GO:0005815 GO:0005794
Cluster-22178.57859 GO:0005515
Cluster-22178.57861 GO:0006468 GO:0016773 GO:0005524 GO:0004672
GO:0016020
Cluster-22178.57864 GO:0009094 GO:0006103 GO:0009821 GO:0009074
GO:0006571 GO:0000162 GO:0006536 GO:0007275 GO:0006511
GO:0046872 GO:0030170 GO:0004838 GO:0005634
Cluster-22178.57866 GO:0008017 GO:0045298 GO:0016591
Cluster-22178.57868 GO:0042254 GO:0006412 GO:0003735 GO:0005840
Cluster-22178.57869 GO:0006771 GO:0006164 GO:0006144 GO:0019497
GO:0006807 GO:0050830 GO:0005515 GO:0046872 GO:0031423
GO:0004643 GO:0003937 GO:0003993 GO:0005576 GO:0019012
GO:0042720
Cluster-22178.57871 GO:0016192 GO:0006886 GO:0008608 GO:0005515
GO:0042729 GO:0030117 GO:0005634 GO:0072686
Cluster-22178.57873 GO:0005515 GO:0043565 GO:0005634
Cluster-22178.57877 GO:0046500 GO:0006400 GO:0044419 GO:0006810
GO:0006952 GO:0030488 GO:0006464 GO:0009451 GO:0009405

GO:0006479 GO:0008033 GO:0008152 GO:0008176 GO:0016429
GO:0008168 GO:0004719 GO:0019855 GO:0031515 GO:0005576
Cluster-22178.57878 GO:0004867 GO:0005515
Cluster-22178.57879 GO:0020037 GO:0005515
Cluster-22178.57881 GO:0006508 GO:0005975 GO:0034599 GO:0016787
GO:0008184 GO:0008236 GO:0005789
Cluster-22178.57882 GO:0006886 GO:0005622
Cluster-22178.57887 GO:0030414 GO:0005515
Cluster-22178.57889 GO:0006446 GO:0006413 GO:0003743 GO:0003677
GO:0003723 GO:0005840 GO:0019031 GO:0005737
Cluster-22178.57894 GO:0051028 GO:0005634
Cluster-22178.57896 GO:0051260 GO:0006915 GO:0006974 GO:0005515

Cluster-22178.57897 GO:0006508 GO:0060478 GO:0006281 GO:0007342
GO:0006310 GO:0006897 GO:0009378 GO:0004252 GO:0003677
GO:0046872 GO:0009379 GO:0005886 GO:0005657
Cluster-22178.57904 GO:0000917 GO:0043093 GO:0048870 GO:0031514
GO:0005737
Cluster-22178.57905 GO:0051028 GO:0005515 GO:0005634
Cluster-22178.57906 GO:0009052 GO:0006098 GO:0015976 GO:0004751
GO:0030246
Cluster-22178.57907 GO:0006313 GO:0004803 GO:0003677
Cluster-22178.57910 GO:0006355 GO:0051726 GO:0006352 GO:0017025
GO:0003677 GO:0016987 GO:0003700 GO:0005667 GO:0005634
Cluster-22178.57911 GO:0016310 GO:0044237 GO:0016772 GO:0005515

Cluster-22178.57913 GO:0007165 GO:0001664 GO:0016874
Cluster-22178.57916 GO:0005525 GO:0008270 GO:0046872 GO:0005634
Cluster-22178.57920 GO:0015979 GO:0009654 GO:0016020 GO:0009523
Cluster-22178.57922 GO:0006810 GO:0055114 GO:0016787 GO:0004497
GO:0003677 GO:0005524 GO:0003676 GO:0005507 GO:0016715
Cluster-22178.57923 GO:0006614 GO:0008615 GO:0030942 GO:0033856
GO:0020037 GO:0019825 GO:0008312 GO:0005737 GO:0005786

Cluster-22178.57927 GO:0009069 GO:0006468 GO:0006508 GO:0016310
GO:0007178 GO:0004675 GO:0004252 GO:0005524 GO:0016020
Cluster-22178.57928 GO:0006118 GO:0006119 GO:0006536 GO:0007617
GO:0006571 GO:0009821 GO:0055114 GO:0009094 GO:0006103
GO:0035435 GO:0006817 GO:0000162 GO:0009074 GO:0015992
GO:0008121 GO:0030170 GO:0015114 GO:0004838 GO:0005576
GO:0016021
Cluster-22178.57931 GO:0046413 GO:0008152 GO:0016491 GO:0005515
GO:0018836
Cluster-22178.57932 GO:0005515 GO:0046872 GO:0031225
Cluster-22178.57933 GO:0046872 GO:0005515 GO:0031225
Cluster-22178.57934 GO:0006508 GO:0004190 GO:0005515
Cluster-22178.57937 GO:0004190 GO:0003677 GO:0016021
Cluster-22178.57938 GO:0006811 GO:0005216 GO:0016020
Cluster-22178.57942 GO:0016788
Cluster-22178.57944 GO:0006810 GO:0005515 GO:0005215 GO:0016020
Cluster-22178.57945 GO:0006810 GO:0005215 GO:0005515 GO:0016020
Cluster-22178.57946 GO:0055085 GO:0006810 GO:0022857 GO:0005515
GO:0005215 GO:0016021 GO:0016020
Cluster-22178.57947 GO:0006810 GO:0005515 GO:0005215 GO:0016020
Cluster-22178.57948 GO:0055085 GO:0006810 GO:0022857 GO:0005515
GO:0005215 GO:0016021 GO:0016020
Cluster-22178.57949 GO:0016021
Cluster-22178.5795 GO:0007186 GO:0004930 GO:0016021
Cluster-22178.57951 GO:0055114 GO:0006633 GO:0009058 GO:0005506
GO:0016833 GO:0016491
Cluster-22178.57953 GO:0009405 GO:0006355 GO:0005515
Cluster-22178.57954 GO:0016021
Cluster-22178.57955 GO:0005515
Cluster-22178.57957 GO:0006118 GO:0006468 GO:0009055 GO:0005524
GO:0004672
Cluster-22178.57958 GO:0006606 GO:0043248 GO:0015031 GO:0006886
GO:0016192 GO:0015991 GO:0004222 GO:0008565 GO:0005515

GO:0016820 GO:0016021 GO:0000221 GO:0005737 GO:0005634
GO:0030117
Cluster-22178.57962 GO:0050778 GO:0051607 GO:0016192 GO:0006886
GO:0007165 GO:0006367 GO:0007259 GO:0032968 GO:0005125
GO:0005515 GO:0003677 GO:0005634 GO:0030117
Cluster-22178.57963 GO:0005515
Cluster-22178.57964 GO:0006470 GO:0005515 GO:0004721 GO:0008270
GO:0005634
Cluster-22178.57968 GO:0006260 GO:0030168 GO:0006914 GO:0007165
GO:0006810 GO:0051258 GO:0006355 GO:0005102 GO:0030674
GO:0003700 GO:0008092 GO:0043565 GO:0019898 GO:0005577
GO:0005667 GO:0005794 GO:0005737
Cluster-22178.57972 GO:0009405 GO:0019079 GO:0045735
Cluster-22178.57976 GO:0017013 GO:0006313 GO:0016567 GO:0000398
GO:0003677 GO:0005515 GO:0004803 GO:0046872 GO:0008270
GO:0004842 GO:0005634 GO:0005622
Cluster-22178.57977 GO:0046872
Cluster-22178.57978 GO:0015979 GO:0009523 GO:0009539 GO:0016020
Cluster-22178.57979 GO:0006355 GO:0042254 GO:0006412 GO:0006367
GO:0043565 GO:0003700 GO:0005515 GO:0003735 GO:0005622
GO:0005840 GO:0005672 GO:0005667
Cluster-22178.57980 GO:0051424 GO:0016021
Cluster-22178.57982 GO:0005515 GO:0042802
Cluster-22178.57985 GO:0019079 GO:0006310 GO:0002949 GO:0006355
GO:0006281 GO:0098519 GO:0009378 GO:0003724 GO:0005524
GO:0016887 GO:0016301 GO:0008134 GO:0003723 GO:0003678
GO:0043531 GO:0005667 GO:0009379 GO:0005657
Cluster-22178.57987 GO:0006629 GO:0008081
Cluster-22178.57988 GO:0006629 GO:0008081
Cluster-22178.57991 GO:0007015 GO:0006265 GO:0006897 GO:0003918
GO:0005524 GO:0003677
Cluster-22178.57992 GO:0006541 GO:0009252 GO:0046436 GO:0005524
GO:0046872 GO:0008716 GO:0016787

Cluster-22178.57994 GO:0006468 GO:0019079 GO:0006260 GO:0030337
GO:0016773 GO:0005524 GO:0004672 GO:0016020 GO:0042575
Cluster-22178.57995 GO:0005515 GO:0046872
Cluster-22178.57996 GO:0006955 GO:0007165 GO:0005164 GO:0016020
Cluster-22178.57997 GO:0007165 GO:0006955 GO:0005164 GO:0016020
Cluster-22178.58 GO:0006468 GO:0035556 GO:0005524 GO:0004672
GO:0016773 GO:0016020
Cluster-22178.58000 GO:0005515
Cluster-22178.58003 GO:0006887 GO:0007165 GO:0048278 GO:0006810
GO:0016887 GO:0005524 GO:0003774 GO:0005737 GO:0016459
Cluster-22178.58004 GO:0006471 GO:0042967 GO:0003950 GO:0008080
GO:0005515 GO:0016747
Cluster-22178.58005 GO:0006511 GO:0031625
Cluster-22178.58006 GO:0007018 GO:0007017 GO:0003777 GO:0008017
GO:0005524 GO:0016787 GO:0005874 GO:0045298
Cluster-22178.58007 GO:0015684 GO:0005525 GO:0015093 GO:0003924
GO:0016021
Cluster-22178.58010 GO:0006357 GO:0007165 GO:0005509 GO:0001104
GO:0005578 GO:0016592
Cluster-22178.58012 GO:0016310 GO:0009069 GO:0004674
Cluster-22178.58015 GO:0006525 GO:0046740 GO:0006560 GO:0071436
GO:0006814 GO:0006090 GO:0008948 GO:0015081 GO:0016020
GO:0016021
Cluster-22178.58023 GO:0016579 GO:0006508 GO:0009405 GO:0007268
GO:0006952 GO:0007165 GO:0036459 GO:0008270 GO:0004843
GO:0030550 GO:0005576
Cluster-22178.58031 GO:0005515
Cluster-22178.58034 GO:0034314 GO:0006355 GO:0030833 GO:0007165
GO:0007186 GO:0043565 GO:0004871 GO:0003700 GO:0005198
GO:0005856 GO:0005834 GO:0019867 GO:0005667 GO:0005885
GO:0005882
Cluster-22178.58035 GO:0007165 GO:0055085 GO:0000772 GO:0016020
GO:0016021

Cluster-22178.58036 GO:0006629 GO:0016787
Cluster-22178.58037 GO:0007165 GO:0043158 GO:0003677 GO:0004252
GO:0005044 GO:0016020 GO:0005634
Cluster-22178.58039 GO:0006357 GO:0043565 GO:0005515 GO:0001104
GO:0005634 GO:0016592
Cluster-22178.58040 GO:0015992 GO:0006123 GO:0004129 GO:0045277
Cluster-22178.58041 GO:0007165 GO:0046008 GO:0005179 GO:0005576
Cluster-22178.58042 GO:0007165 GO:0046008 GO:0005179 GO:0005576
Cluster-22178.58043 GO:0006468 GO:0051016 GO:0016773 GO:0004672
GO:0005524 GO:0008290 GO:0016020
Cluster-22178.58044 GO:0048544 GO:0005515
Cluster-22178.58050 GO:0006914 GO:0015992 GO:0019058 GO:0006308
GO:0006139 GO:0015991 GO:0008855 GO:0008270 GO:0008168
GO:0015078 GO:0051082 GO:0005515 GO:0033644 GO:0055036
GO:0019031 GO:0033179 GO:0009318
Cluster-22178.58053 GO:0055114 GO:0042967 GO:0006633 GO:0046872
GO:0004318 GO:0005515 GO:0005835
Cluster-22178.58054 GO:0016226 GO:0051536 GO:0005737
Cluster-22178.58055 GO:0007018 GO:0007017 GO:0030150 GO:0008017
GO:0003777 GO:0005524 GO:0005742 GO:0045298 GO:0005874
Cluster-22178.58057 GO:0008168
Cluster-22178.58059 GO:0006506 GO:0016740 GO:0005789
Cluster-22178.58061 GO:0005975 GO:0005515 GO:0004553 GO:2001070

Cluster-22178.58063 GO:0006355 GO:0003723 GO:0005515
Cluster-22178.58064 GO:0006468 GO:0006355 GO:0007165 GO:0030521
GO:0003677 GO:0005524 GO:0004672 GO:0004882 GO:0016773
GO:0005496 GO:0005634 GO:0016020
Cluster-22178.58069 GO:0009404 GO:0016746 GO:0005737
Cluster-22178.58071 GO:0007186 GO:0004930 GO:0000975 GO:0046872
GO:0016021
Cluster-22178.58074 GO:0005739
Cluster-22178.58075 GO:0008654 GO:0019872 GO:0006355 GO:0006021

GO:0004512 GO:0003700 GO:0043565 GO:0005667
Cluster-22178.58076 GO:0006355 GO:0006281 GO:0006310 GO:0005978
GO:0006260 GO:0002949 GO:0009378 GO:0008134 GO:0016301
GO:0005524 GO:0016887 GO:0003678 GO:0005667 GO:0009379
GO:0005657
Cluster-22178.58078 GO:0009451 GO:0030488 GO:0008033 GO:0016429
GO:0008168 GO:0031515
Cluster-22178.58080 GO:0006525 GO:0071436 GO:0006814 GO:0006560
GO:0006090 GO:0008948 GO:0015081 GO:0016020 GO:0016021
Cluster-22178.58081 GO:0060478 GO:0006897 GO:0023052 GO:0007342
GO:0003950 GO:0005886 GO:0005634
Cluster-22178.58082 GO:0016070 GO:0043087 GO:0019013 GO:0016021
Cluster-22178.58083 GO:0006260 GO:0004867 GO:0005515 GO:0061630
GO:0003689 GO:0046872 GO:0005524 GO:0008270 GO:0005663
GO:0042575 GO:0005622
Cluster-22178.58085 GO:0000398 GO:0046983 GO:0019031 GO:0005681
Cluster-22178.58086 GO:0046983
Cluster-22178.58090 GO:0005515
Cluster-22178.58091 GO:0006260 GO:0006810 GO:0006310 GO:0006309
GO:0006281 GO:0003724 GO:0005524 GO:0016887 GO:0003677
GO:0098519 GO:0009378 GO:0016787 GO:0003676 GO:0003887
GO:0000166 GO:0003723 GO:0005657 GO:0009360 GO:0009379
GO:0005737 GO:0042575 GO:0005634
Cluster-22178.58094 GO:0006397 GO:0006810 GO:0015986 GO:0046660
GO:0015992 GO:0003676 GO:0015078 GO:0008270 GO:0005634
GO:0000276 GO:0005643
Cluster-22178.58096 GO:0000281 GO:0000226 GO:0040001 GO:0030245
GO:0006353 GO:0016192 GO:0005985 GO:0005982 GO:0008810
GO:0005874 GO:0016021 GO:0005819
Cluster-22178.58102 GO:0010508
Cluster-22178.58103 GO:0007010 GO:0003779
Cluster-22178.58104 GO:0042254 GO:0006412 GO:0006827 GO:0006812
GO:0006836 GO:0006826 GO:0005328 GO:0003735 GO:0005381

GO:0016020 GO:0016021 GO:0005840 GO:0033573 GO:0005622
Cluster-22178.58105 GO:0042254 GO:0006412 GO:0006812 GO:0006836
GO:0003735 GO:0005328 GO:0016021 GO:0005840 GO:0005622
Cluster-22178.58108 GO:0006771 GO:0019497 GO:0051082 GO:0005515
GO:0046872 GO:0008270 GO:0003993 GO:0005622
Cluster-22178.58109 GO:0006355 GO:0043565 GO:0008270 GO:0003700
GO:0005667 GO:0005634
Cluster-22178.58112 GO:0006351 GO:0006144 GO:0006206 GO:0003899
GO:0005730
Cluster-22178.58113 GO:0006606 GO:0015031 GO:0005515 GO:0008565
GO:0005737 GO:0005634
Cluster-22178.58114 GO:0006412 GO:0042254 GO:0006464 GO:0005515
GO:0003735 GO:0016151 GO:0005840 GO:0005622
Cluster-22178.58115 GO:0006468 GO:0042811 GO:0007218 GO:0005184
GO:0016746 GO:0005524 GO:0004672 GO:0016773 GO:0016020
Cluster-22178.58118 GO:0006769 GO:0055114 GO:0046497 GO:0070403
GO:0008750 GO:0030976 GO:0050661 GO:0000287 GO:0016021
Cluster-22178.58119 GO:0005201 GO:0005581 GO:0005578
Cluster-22178.58120 GO:0006886 GO:0016192 GO:0030866 GO:0005515
GO:0008092 GO:0030117 GO:0005856
Cluster-22178.58124 GO:0008152 GO:0016226 GO:0008168 GO:0051536
GO:0005737
Cluster-22178.58125 GO:0006400 GO:0016226 GO:0008033 GO:0009451
GO:0051536 GO:0008176 GO:0005737
Cluster-22178.58127 GO:0016226 GO:0008152 GO:0008168 GO:0051536
GO:0005737
Cluster-22178.5813 GO:0031412 GO:0005887
Cluster-22178.58130 GO:0016020
Cluster-22178.58132 GO:0006955 GO:0007156 GO:0005509 GO:0016020
Cluster-22178.58133 GO:0055085 GO:0016021
Cluster-22178.58134 GO:0009451 GO:0008610 GO:0006479 GO:0008033
GO:0046486 GO:0046500 GO:0042967 GO:0006396 GO:0006464
GO:0030488 GO:0008168 GO:0004719 GO:0016627 GO:0004144

GO:0016429 GO:0031515 GO:0005622

Cluster-22178.58135 GO:0006402 GO:0051252 GO:0003723 GO:0004535

GO:0046872 GO:0005634 GO:0005737

Cluster-22178.58137 GO:0055085 GO:0005741

Cluster-22178.58138 GO:0055085 GO:0005741

Cluster-22178.58145 GO:0042254 GO:0006412 GO:0006355 GO:0019069

GO:0003700 GO:0003735 GO:0003677 GO:0005840 GO:0042025

GO:0005634 GO:0005667

Cluster-22178.58146 GO:0005515

Cluster-22178.58148 GO:0005515

Cluster-22178.58149 GO:0019290 GO:0006351 GO:0006144 GO:0006206

GO:0006826 GO:0003899 GO:0005515 GO:0003677 GO:0015343

GO:0005730

Cluster-22178.58153 GO:0000079 GO:0019901

Cluster-22178.58155 GO:0006886 GO:0006888 GO:0008270 GO:0030127

Cluster-22178.58157 GO:0005515

Cluster-22178.58158 GO:0016192

Cluster-22178.58159 GO:0006357 GO:0001104 GO:0016592

Cluster-22178.58160 GO:0005515

Cluster-22178.58163 GO:0015979 GO:0006621 GO:0046923 GO:0009523

GO:0009539 GO:0016021 GO:0016020 GO:0005783

Cluster-22178.58164 GO:0007165 GO:0007275 GO:0016055 GO:0005102

GO:0005576

Cluster-22178.58165 GO:0040001 GO:0000226 GO:0006606 GO:0071705

GO:0000281 GO:0015031 GO:0015991 GO:0008565 GO:0003677

GO:0016820 GO:0005515 GO:0000221 GO:0005874 GO:0005737

GO:0005634 GO:0005819

Cluster-22178.58167 GO:0051260 GO:0005515

Cluster-22178.58169 GO:0043401 GO:0006355 GO:0003700 GO:0043565

GO:0008270 GO:0005634 GO:0005667

Cluster-22178.58174 GO:0006281 GO:0006810 GO:0016787 GO:0003677

GO:0005524 GO:0008270 GO:0016817 GO:0003676

Cluster-22178.58175 GO:0006270 GO:0046983

Cluster-22178.58176 GO:0005515
Cluster-22178.58178 GO:0016226 GO:0008199
Cluster-22178.58181 GO:0009058 GO:0006546 GO:0006566 GO:0006563
GO:0006544 GO:0055114 GO:0004375 GO:0030170
Cluster-22178.58185 GO:0055114 GO:0006940 GO:0006952 GO:0016620
GO:0005515
Cluster-22178.58186 GO:0007050 GO:0006468 GO:0045859 GO:0004861
GO:0005524 GO:0004672 GO:0005634
Cluster-22178.58192 GO:0006629 GO:0006481 GO:0007165 GO:0006479
GO:0035556 GO:0004671 GO:0016627 GO:0016020 GO:0005737
GO:0016021
Cluster-22178.58194 GO:0006813 GO:0055114 GO:0003677 GO:0020037
GO:0016705 GO:0005506 GO:0016787 GO:0005524 GO:0005267
GO:0016020
Cluster-22178.58197 GO:0016255 GO:0042765
Cluster-22178.58198 GO:0006468 GO:0005524 GO:0004672 GO:0016773
GO:0016020
Cluster-22178.58199 GO:0006468 GO:0016773 GO:0005524 GO:0004672
GO:0035091 GO:0016020
Cluster-22178.58200 GO:0006554 GO:0006355 GO:0034968 GO:0006479
GO:0007165 GO:0018024 GO:0005515 GO:0005509 GO:0005578
GO:0005694
Cluster-22178.58201 GO:0005515
Cluster-22178.58202 GO:0005515
Cluster-22178.58204 GO:0001522 GO:0009451 GO:0009982 GO:0003676
GO:0046872 GO:0016787 GO:0003723 GO:0019031
Cluster-22178.58205 GO:0006355 GO:0009306 GO:0003682 GO:0043565
GO:0008270 GO:0003700 GO:0005667 GO:0016020 GO:0000785
Cluster-22178.58206 GO:0050832 GO:0046872 GO:0005576
Cluster-22178.58208 GO:0006479 GO:0032259 GO:0006464 GO:0046500
GO:0016787 GO:0008168 GO:0004719
Cluster-22178.58209 GO:0008643 GO:0005351 GO:0005507 GO:0000139
GO:0016021 GO:0016020

Cluster-22178.58210 GO:0006189 GO:0006355 GO:0003700 GO:0003824
GO:0043565 GO:0008270 GO:0005667
Cluster-22178.58214 GO:0006376 GO:0003729 GO:0016021 GO:0005685
Cluster-22178.58217 GO:0005515
Cluster-22178.58219 GO:0035556 GO:0009306 GO:0006464 GO:0051090
GO:0016151 GO:0046872 GO:0005515 GO:0003677 GO:0005634
Cluster-22178.58220 GO:0006310 GO:0006024 GO:0002949 GO:0006570
GO:0006281 GO:0006470 GO:0030206 GO:0008138 GO:0009378
GO:0003724 GO:0030158 GO:0016887 GO:0005524 GO:0016301
GO:0003723 GO:0003678 GO:0004725 GO:0009379 GO:0005657
Cluster-22178.58222 GO:0055085 GO:0006812 GO:0006885 GO:0015299
GO:0016021
Cluster-22178.58224 GO:0005509
Cluster-22178.58226 GO:0045947 GO:0008608 GO:0008190 GO:0072686
GO:0042729
Cluster-22178.58227 GO:0008608 GO:0045947 GO:0008190 GO:0003677
GO:0042729 GO:0072686
Cluster-22178.58231 GO:0006955 GO:0009405 GO:0005515 GO:0016020
Cluster-22178.58233 GO:0007275 GO:0016021
Cluster-22178.58234 GO:0045454 GO:0055114 GO:0016972
Cluster-22178.58236 GO:0007275 GO:0003676 GO:0042600
Cluster-22178.58239 GO:0007165 GO:0046008 GO:0030474 GO:0005200
GO:0005057 GO:0008270 GO:0005179 GO:0005823 GO:0005856
GO:0005576 GO:0005634
Cluster-22178.58240 GO:0006621 GO:0046923 GO:0005783 GO:0016021
Cluster-22178.58241 GO:0006402 GO:0051252 GO:0003676 GO:0004535
GO:0046872 GO:0003723 GO:0005737 GO:0005634
Cluster-22178.58243 GO:0016020
Cluster-22178.58245 GO:0003676
Cluster-22178.58246 GO:0015986 GO:0015992 GO:0007049 GO:0015078
GO:0000276
Cluster-22178.58248 GO:0003824
Cluster-22178.58249 GO:0043158 GO:0007165 GO:0003677 GO:0004252

GO:0005044 GO:0016020 GO:0005634
Cluster-22178.58250 GO:0051603 GO:0004298 GO:0005839
Cluster-22178.58251 GO:0006260 GO:0000723 GO:0003676 GO:0003677
GO:0005634 GO:0000784
Cluster-22178.58253 GO:0006281
Cluster-22178.58256 GO:0006355 GO:0005515 GO:0003676 GO:0043565
GO:0005634
Cluster-22178.58257 GO:0006810 GO:0006816 GO:0005515 GO:0042030
GO:0005246 GO:0016020
Cluster-22178.58258 GO:0000103 GO:0006144 GO:0048870 GO:0006810
GO:0004020 GO:0005524 GO:0005515 GO:0042803 GO:0008134
GO:0045502 GO:0016021 GO:0030286 GO:0005886 GO:0031514
GO:0005667
Cluster-22178.58259 GO:0016747
Cluster-22178.58262 GO:0006597 GO:0008295 GO:0006560 GO:0006525
GO:0004014
Cluster-22178.58263 GO:0006118 GO:0006119 GO:0006950 GO:0015992
GO:0008121 GO:0016021
Cluster-22178.58264 GO:0006914 GO:0006891 GO:0006355 GO:0006397
GO:0043565 GO:0003700 GO:0003723 GO:0005737 GO:0017119
GO:0005667 GO:0005634
Cluster-22178.58266 GO:0006508 GO:0004177 GO:0008270
Cluster-22178.58267 GO:0000160 GO:0015031 GO:0005515 GO:0008270
GO:0008565
Cluster-22178.58268 GO:0043565
Cluster-22178.58269 GO:0015992 GO:0015986 GO:0015078 GO:0000276
Cluster-22178.58270 GO:0006351 GO:0006144 GO:0006206 GO:0003899
GO:0003677 GO:0005730
Cluster-22178.58274 GO:0008270
Cluster-22178.58275 GO:0006810 GO:0006412 GO:0042254 GO:0003735
GO:0005840 GO:0016020
Cluster-22178.58276 GO:0009069 GO:0006468 GO:0006357 GO:0016310
GO:0006260 GO:0001104 GO:0005524 GO:0004674 GO:0016592

Cluster-22178.58277 GO:0007017 GO:0004869 GO:0005875

Cluster-22178.58278 GO:0006629 GO:0051252 GO:0055114 GO:0006402 GO:0016114 GO:0006694 GO:0003723 GO:0004506 GO:0046872 GO:0004535 GO:0003676 GO:0050660 GO:0005634 GO:0005737 GO:0016021

Cluster-22178.58281 GO:0015031 GO:0016020

Cluster-22178.58284 GO:0006357 GO:0001104 GO:0016020 GO:0016592

Cluster-22178.58286 GO:0003677 GO:0016787

Cluster-22178.58287 GO:0005198 GO:0019028 GO:0019031

Cluster-22178.58288 GO:0035023 GO:0006260 GO:0043087 GO:0005515 GO:0005089

Cluster-22178.58289 GO:0006468 GO:0042742 GO:0004672 GO:0005524 GO:0016773 GO:0016020

Cluster-22178.58291 GO:0006807 GO:0016810

Cluster-22178.58292 GO:0009847 GO:0003333 GO:0006508 GO:0004185 GO:0016021

Cluster-22178.58294 GO:0006508 GO:0005515 GO:0004222

Cluster-22178.58295 GO:0007034

Cluster-22178.58297 GO:0000774 GO:0005515 GO:0005783

Cluster-22178.58300 GO:0005515

Cluster-22178.58301 GO:0006813 GO:0005249 GO:0008076

Cluster-22178.58302 GO:0006098 GO:0055114 GO:0019521 GO:0016491 GO:0051287 GO:0004616

Cluster-22178.58305 GO:0044564 GO:0007165 GO:0043123 GO:0004871 GO:0005515

Cluster-22178.58306 GO:0043123 GO:0044564 GO:0007165 GO:0004871 GO:0005515

Cluster-22178.58307 GO:0008654 GO:0006021 GO:0019872 GO:0004512

Cluster-22178.58308 GO:0008654 GO:0006021 GO:0019872 GO:0004512

Cluster-22178.58309 GO:0006468 GO:0016773 GO:0004672 GO:0005524 GO:0005515 GO:0016020

Cluster-22178.58313 GO:0055114 GO:0006281 GO:0006310 GO:0007165
GO:0015774 GO:0005515 GO:0004888 GO:0015159 GO:0005524
GO:0005509 GO:0051920 GO:0009378 GO:0016021 GO:0005578
GO:0005657 GO:0016020 GO:0009379
Cluster-22178.58315 GO:0006468 GO:0009058 GO:0015937 GO:0008152
GO:0015940 GO:0005515 GO:0016301 GO:0005524 GO:0004672
GO:0016773 GO:0004140 GO:0003824 GO:0016020
Cluster-22178.58316 GO:0019497 GO:0006771 GO:0003993 GO:0005515
GO:0046872
Cluster-22178.58317 GO:0016567 GO:0008270 GO:0046872 GO:0005515
GO:0004842 GO:0005680
Cluster-22178.58318 GO:0035556 GO:0046872 GO:0005524
Cluster-22178.58319 GO:0035556 GO:0046872 GO:0005524
Cluster-22178.58320 GO:0016820
Cluster-22178.58322 GO:0009405 GO:0005198 GO:0008376 GO:0032580
Cluster-22178.58323 GO:0032065 GO:0007165 GO:0005158 GO:0005543
GO:0005515 GO:0005899 GO:0005938
Cluster-22178.58324 GO:0005887
Cluster-22178.58325 GO:0006571 GO:0000162 GO:0009094 GO:0008033
GO:0006432 GO:0004826 GO:0005524 GO:0000049 GO:0000287
GO:0009328
Cluster-22178.58330 GO:0006952 GO:0006810 GO:0009405 GO:0019855
GO:0005576
Cluster-22178.58331 GO:0005524 GO:0016887
Cluster-22178.58333 GO:0050830 GO:0051016 GO:0005509 GO:0030479
Cluster-22178.58334 GO:0045116 GO:0016881 GO:0008641 GO:0005524

Cluster-22178.58335 GO:0006486 GO:0008378 GO:0016757 GO:0016020
Cluster-22178.58336 GO:0042254 GO:0006412 GO:0003735 GO:0003824
GO:0005622 GO:0005840
Cluster-22178.58337 GO:0006531 GO:0009058 GO:0006520 GO:0006144
GO:0055114 GO:0006188 GO:0009152 GO:0006522 GO:0030170
GO:0004018 GO:0016829 GO:0010277

Cluster-22178.58338 GO:0008270 GO:0003676 GO:0005622
Cluster-22178.58339 GO:0055114 GO:0051536 GO:0016491 GO:0020037
GO:0003676
Cluster-22178.58341 GO:0005996 GO:0016853 GO:0048029
Cluster-22178.58342 GO:0048870 GO:0006355 GO:0016973 GO:0003700
GO:0005643 GO:0016021 GO:0005667 GO:0031514
Cluster-22178.58344 GO:0008270 GO:0046872 GO:0003677
Cluster-22178.58346 GO:0006807 GO:0008033 GO:0006725 GO:0055114
GO:0050660 GO:0017150 GO:0016627 GO:0016491 GO:0016853
GO:0018580 GO:0005737
Cluster-22178.58349 GO:0005515
Cluster-22178.5835 GO:0055085 GO:0006811 GO:0006357 GO:0001104
GO:0005198 GO:0005216 GO:0016020 GO:0016592 GO:0019028
GO:0019031
Cluster-22178.58356 GO:0006730 GO:0008270 GO:0016740
Cluster-22178.58358 GO:0008033 GO:0031167 GO:0009451 GO:0004809
GO:0046872 GO:0003723 GO:0008168
Cluster-22178.58360 GO:0005515
Cluster-22178.58361 GO:0055085 GO:0006812 GO:0008324 GO:0016021
Cluster-22178.58362 GO:0008641
Cluster-22178.58364 GO:0006810 GO:0005515 GO:0042626 GO:0005524
GO:0016021
Cluster-22178.58365 GO:0042254 GO:0006412 GO:0007264 GO:0045040
GO:0005525 GO:0046983 GO:0003924 GO:0003735 GO:0042802
GO:0005515 GO:0005840 GO:0005622 GO:0005742
Cluster-22178.58366 GO:0007156 GO:0005509 GO:0016020
Cluster-22178.58367 GO:0007165 GO:0006508 GO:0006355 GO:0008234
GO:0003677
Cluster-22178.58370 GO:0015979 GO:0009523 GO:0009539
Cluster-22178.58371 GO:0008152 GO:0016787
Cluster-22178.58372 GO:0043631 GO:0004652 GO:0003676
Cluster-22178.58373 GO:0008152 GO:0016787
Cluster-22178.58374 GO:0015986 GO:0015992 GO:0015078 GO:0016020

Cluster-22178.58378 GO:0016114 GO:0008152 GO:0006694 GO:0003824
GO:0008661 GO:0030976 GO:0016624
Cluster-22178.58380 GO:0006694 GO:0008152 GO:0016114 GO:0016624
GO:0030976 GO:0008661 GO:0003824
Cluster-22178.58382 GO:0007165 GO:0051225 GO:0006355 GO:0007155
GO:0004871 GO:0003677 GO:0003723 GO:0009288 GO:0070652
GO:0016020 GO:0005634
Cluster-22178.58384 GO:0046872
Cluster-22178.58385 GO:0043087 GO:0007264 GO:0005085
Cluster-22178.58387 GO:0007165 GO:0006810 GO:0006310 GO:0014068
GO:0050776 GO:0006281 GO:0009405 GO:0005524 GO:0005102
GO:0005179 GO:0009378 GO:0043548 GO:0019871 GO:0005657
GO:0009379 GO:0016020 GO:0005576
Cluster-22178.58391 GO:0007186 GO:0005515 GO:0004930 GO:0042470
GO:0016021
Cluster-22178.58392 GO:0006810 GO:0055085 GO:0042626 GO:0005515
GO:0005524 GO:0016021
Cluster-22178.58393 GO:0008152 GO:0046849 GO:0003824 GO:0008484
GO:0005576
Cluster-22178.58394 GO:0015031 GO:0016021
Cluster-22178.58395 GO:0005509
Cluster-22178.58396 GO:0006525 GO:0006433 GO:0006560 GO:0007165
GO:0005179 GO:0000166 GO:0005524 GO:0004827 GO:0005576
GO:0005737
Cluster-22178.58398 GO:0003774 GO:0016459
Cluster-22178.58400 GO:0007165 GO:0005515 GO:0008270
Cluster-22178.58401 GO:0006366 GO:0006281 GO:0051382 GO:0006352
GO:0003677 GO:0005669
Cluster-22178.58405 GO:0006355 GO:0006807 GO:0000105 GO:0009607
GO:0006537 GO:0055114 GO:0015930 GO:0018580 GO:0016638
GO:0003824 GO:0016627 GO:0016491 GO:0005737
Cluster-22178.58406 GO:0003676
Cluster-22178.58407 GO:0003676

Cluster-22178.58408 GO:0005985 GO:0005982 GO:0006096 GO:0006094 GO:0006098 GO:0005515 GO:0005198 GO:0004347 GO:0005737
Cluster-22178.58411 GO:0055114 GO:0005506 GO:0016705 GO:0020037

Cluster-22178.58412 GO:0071585 GO:0010273 GO:0005507 GO:0046870

Cluster-22178.58415 GO:0000077 GO:0051082
Cluster-22178.58416 GO:0006351 GO:0006355 GO:0006144 GO:0006206 GO:0003899 GO:0003677 GO:0003700 GO:0046983 GO:0005667 GO:0005730
Cluster-22178.58417 GO:0044564 GO:0000723 GO:0006281 GO:0003678 GO:0005657
Cluster-22178.58418 GO:0009451 GO:0006396 GO:0008173 GO:0003723 GO:0008168
Cluster-22178.58419 GO:0007275 GO:0009966 GO:0016020
Cluster-22178.58420 GO:0008171
Cluster-22178.58422 GO:0006355 GO:0005634
Cluster-22178.58426 GO:0006260 GO:0006355 GO:0005524 GO:0016887 GO:0000166 GO:0003677 GO:0008134 GO:0005667
Cluster-22178.58427 GO:0007165 GO:0035556 GO:0005515 GO:0005524 GO:0003774 GO:0016459 GO:0097197 GO:0001772 GO:0016021
Cluster-22178.58428 GO:0006465 GO:0008233 GO:0016021 GO:0005787
Cluster-22178.58431 GO:0016787
Cluster-22178.58433 GO:0006914 GO:0006355 GO:0019082 GO:0043565 GO:0003700 GO:0003677 GO:0005667
Cluster-22178.58435 GO:0006144 GO:0006508 GO:0070008 GO:0003968 GO:0016817 GO:0004197 GO:0017111 GO:0005515 GO:0004252 GO:0031379
Cluster-22178.58437 GO:0006810 GO:0006355 GO:0016301 GO:0008134 GO:0005524 GO:0005667
Cluster-22178.58438 GO:0006355 GO:0006810 GO:0005524 GO:0016301 GO:0008134 GO:0005667
Cluster-22178.58440 GO:0045947 GO:0008190

Cluster-22178.58441 GO:0015986 GO:0015992 GO:0006144 GO:0015078
GO:0003877 GO:0000276
Cluster-22178.58442 GO:0006355 GO:0006810 GO:0005524 GO:0008134
GO:0016301 GO:0005667
Cluster-22178.58445 GO:0009117 GO:0006012 GO:0008108
Cluster-22178.58447 GO:0008053 GO:0006869 GO:0016192 GO:0042157
GO:0009405 GO:0006886 GO:0004185 GO:0005524 GO:0016887
GO:0008289 GO:0005525 GO:0003924 GO:0005198 GO:0005773
GO:0005741 GO:0016020 GO:0019031 GO:0016021 GO:0005576
GO:0019028
Cluster-22178.58448 GO:0003677 GO:0005515
Cluster-22178.58456 GO:0006355 GO:0008270 GO:0046872 GO:0003723

Cluster-22178.58458 GO:0035023 GO:0006885 GO:0006464 GO:0043087
GO:0015992 GO:0046436 GO:0009252 GO:0006814 GO:0005089
GO:0003774 GO:0005515 GO:0015385 GO:0008716 GO:0016020
GO:0016459
Cluster-22178.58463 GO:0006357 GO:0006810 GO:0009405 GO:0006367
GO:0006468 GO:0005524 GO:0004672 GO:0046872 GO:0001104
GO:0005515 GO:0019870 GO:0016592 GO:0005672 GO:0005576
Cluster-22178.58469 GO:0000062 GO:0005515 GO:0016021
Cluster-22178.58474 GO:0005515
Cluster-22178.58475 GO:0005515
Cluster-22178.58479 GO:0003824
Cluster-22178.58482 GO:0002949 GO:0006260 GO:0006310 GO:0006810
GO:0007264 GO:0006576 GO:0006355 GO:0006281 GO:0003724
GO:0016887 GO:0008270 GO:0005524 GO:0003677 GO:0009378
GO:0003678 GO:0005525 GO:0008168 GO:0003924 GO:0016301
GO:0008134 GO:0003723 GO:0005657 GO:0005667 GO:0009379
Cluster-22178.58484 GO:0055114 GO:0006355 GO:0016887 GO:0016491
GO:0005524 GO:0003677
Cluster-22178.58487 GO:0003677
Cluster-22178.58488 GO:0009308 GO:0055114 GO:0003677 GO:0016787

GO:0008131 GO:0003676 GO:0005507 GO:0048038 GO:0005524
Cluster-22178.58489 GO:0005975 GO:0042967 GO:0046486 GO:0004571
GO:0005509 GO:0004144 GO:0016020
Cluster-22178.58490 GO:0045454 GO:0006508 GO:0055114 GO:0008236
GO:0016491 GO:0005576
Cluster-22178.58491 GO:0006446 GO:0006886 GO:0003743 GO:0030176
GO:0005852 GO:0005840 GO:0005737
Cluster-22178.58494 GO:0016192 GO:0006886 GO:0042981 GO:0005515
GO:0030117
Cluster-22178.58495 GO:0042981 GO:0006886 GO:0016192 GO:0005515
GO:0030117
Cluster-22178.58497 GO:0046740 GO:0005515 GO:0016021
Cluster-22178.58501 GO:0006952 GO:0008284 GO:0003676 GO:0046872

Cluster-22178.58502 GO:0003677
Cluster-22178.58503 GO:0007165 GO:0006506 GO:0051289 GO:0016740
GO:0005509 GO:0005515 GO:0008140 GO:0005667 GO:0005578
GO:0016012 GO:0005886 GO:0016021 GO:0005789
Cluster-22178.58504 GO:0007049 GO:0000902 GO:0005975 GO:0005515
GO:0016773 GO:0016021 GO:0005886
Cluster-22178.58505 GO:0009069 GO:0006468 GO:0016310 GO:0019087
GO:0007178 GO:0003677 GO:0005026 GO:0005524 GO:0046872
GO:0016020 GO:0016021
Cluster-22178.58507 GO:0016021
Cluster-22178.58508 GO:0005615 GO:0005576
Cluster-22178.5851 GO:0007165 GO:0007131 GO:0005515 GO:0005509
GO:0005578 GO:0000794
Cluster-22178.58513 GO:0006313 GO:0015074 GO:0003677 GO:0019028
Cluster-22178.58516 GO:0046872
Cluster-22178.58518 GO:0043087 GO:0035023 GO:0005089
Cluster-22178.58522 GO:0031505 GO:0006817 GO:0007186 GO:0004930
GO:0005315 GO:0016021 GO:0016020
Cluster-22178.58524 GO:0007264 GO:0006886 GO:0007186 GO:0007165

GO:0008152 GO:0016836 GO:0003924 GO:0031683 GO:0019001
GO:0003824 GO:0004871 GO:0005525 GO:0005783 GO:0016021
Cluster-22178.58525 GO:0006810 GO:0006508 GO:0006816 GO:0004222
GO:0042030 GO:0008168 GO:0005246 GO:0016020
Cluster-22178.58526 GO:0016773 GO:0003676 GO:0016020
Cluster-22178.58531 GO:0007165 GO:0006508 GO:0004252 GO:0004197
GO:0005044 GO:0070008 GO:0008270 GO:0016020
Cluster-22178.58533 GO:0035091 GO:0005515
Cluster-22178.58535 GO:0042312 GO:0008217 GO:0007165 GO:0006508
GO:0000245 GO:0006355 GO:0004222 GO:0008270 GO:0005515
GO:0005179 GO:0031012 GO:0005634 GO:0005576
Cluster-22178.58537 GO:0006108 GO:0006118 GO:0006099 GO:0006694
GO:0006090 GO:0006529 GO:0006522 GO:0015976 GO:0055114
GO:0017004 GO:0008299 GO:0006457 GO:0009058 GO:0006531
GO:0016779 GO:0004471 GO:0051082 GO:0045158 GO:0050518
GO:0004066 GO:0009512 GO:0016272
Cluster-22178.58539 GO:0006310 GO:0006302 GO:0003677 GO:0005634
Cluster-22178.58540 GO:0006888 GO:0009306 GO:0006886 GO:0008270
GO:0030127 GO:0016020
Cluster-22178.58541 GO:0051056 GO:0005096
Cluster-22178.58542 GO:0051056 GO:0005096
Cluster-22178.58544 GO:0006915
Cluster-22178.58545 GO:0030163 GO:0008270
Cluster-22178.58548 GO:0016192 GO:0006289 GO:0006355 GO:0000439
GO:0016021
Cluster-22178.58550 GO:0006355 GO:0006289 GO:0000439
Cluster-22178.58552 GO:0008152 GO:0016491
Cluster-22178.58555 GO:0008152 GO:0016491
Cluster-22178.58556 GO:0007275 GO:0007219 GO:0016021
Cluster-22178.58558 GO:0006886 GO:0005622
Cluster-22178.58560 GO:0001514 GO:0006448 GO:0005515 GO:0003723
GO:0005525 GO:0003746 GO:0005840 GO:0005737
Cluster-22178.58561 GO:0008152 GO:0009878 GO:0005525 GO:0046872

GO:0016787 GO:0008168
Cluster-22178.58563 GO:0006265 GO:0016579 GO:0006355 GO:0003916
GO:0036459 GO:0003700 GO:0003677 GO:0005515 GO:0005694
GO:0005667
Cluster-22178.58564 GO:0005515 GO:0003824
Cluster-22178.58565 GO:0016020
Cluster-22178.58566 GO:0045116 GO:0006468 GO:0016773 GO:0016881
GO:0005524 GO:0004672 GO:0016020
Cluster-22178.58568 GO:0005975 GO:0006355 GO:0016746 GO:0004649
GO:0005515 GO:0003677
Cluster-22178.58571 GO:0007264 GO:0005525
Cluster-22178.58572 GO:0006508 GO:0055085 GO:0022857 GO:0016805
GO:0016021
Cluster-22178.58575 GO:0007165 GO:0007186 GO:0007264 GO:0005525
GO:0004871 GO:0031683 GO:0019001 GO:0003924
Cluster-22178.58576 GO:0007165 GO:0007186 GO:0004871 GO:0019001
GO:0031683 GO:0003924
Cluster-22178.58577 GO:0006144 GO:0003968 GO:0005524 GO:0031379
Cluster-22178.58578 GO:0006323 GO:0006355 GO:0003700 GO:0005667
GO:0005622
Cluster-22178.58581 GO:0006468 GO:0004672 GO:0005524
Cluster-22178.58582 GO:0006118 GO:0019684 GO:0045156 GO:0016021
GO:0030077
Cluster-22178.58584 GO:0005975 GO:0004553
Cluster-22178.58585 GO:0042330 GO:0006508 GO:0016579 GO:0006511
GO:0004843 GO:0005622
Cluster-22178.58587 GO:0007018 GO:0007017 GO:0008017 GO:0003777
GO:0005524 GO:0045298 GO:0005874 GO:0019028
Cluster-22178.58588 GO:0006888 GO:0007165 GO:0006886 GO:0007606
GO:0008270 GO:0005515 GO:0004888 GO:0003723 GO:0030127
GO:0016020
Cluster-22178.58591 GO:0007031 GO:0051276 GO:0000921 GO:0007186
GO:0050909 GO:0006886 GO:0004930 GO:0005524 GO:0003677

GO:0008773 GO:0005198 GO:0005515 GO:0000166 GO:0005940
GO:0005777 GO:0016020 GO:0005694 GO:0005801 GO:0016021
GO:0019031 GO:0019028
Cluster-22178.58592 GO:0019497 GO:0007154 GO:0006771 GO:0046872
GO:0003993 GO:0016820 GO:0051539 GO:0005515 GO:0016021
Cluster-22178.58593 GO:0006952
Cluster-22178.58595 GO:0016021
Cluster-22178.58597 GO:0006633 GO:0009396 GO:0006554 GO:0006631
GO:0006298 GO:0018874 GO:0008152 GO:0055114 GO:0006574
GO:0006552 GO:0006550 GO:0046487 GO:0006568 GO:0016491
GO:0005524 GO:0004488 GO:0050662 GO:0003824 GO:0003857
Cluster-22178.58598 GO:0006144 GO:0004114 GO:0005515 GO:0016020
Cluster-22178.58602 GO:0035556
Cluster-22178.58603 GO:0006464 GO:0006144 GO:0042157 GO:0006869
GO:0046034 GO:0006355 GO:0008270 GO:0004017 GO:0046872
GO:0043565 GO:0016151 GO:0008289 GO:0003700 GO:0051082
GO:0005815 GO:0005667 GO:0005576
Cluster-22178.58604 GO:0008270 GO:0003676
Cluster-22178.58605 GO:0006351 GO:0043565
Cluster-22178.58606 GO:0006412 GO:0042254 GO:0003735 GO:0005840
Cluster-22178.58607 GO:0045116 GO:0008641 GO:0005524 GO:0016881

Cluster-22178.58608 GO:0005515
Cluster-22178.58609 GO:0006355 GO:0043161 GO:0006281 GO:0006289
GO:0043401 GO:0003684
Cluster-22178.58610 GO:0006810 GO:0034227 GO:0002098 GO:0042626
GO:0000049 GO:0005524 GO:0016021
Cluster-22178.58611 GO:0005975 GO:0004415 GO:0005515
Cluster-22178.58612 GO:0008270
Cluster-22178.58613 GO:0008270
Cluster-22178.58614 GO:0006874 GO:0006816 GO:0005219 GO:0016021
GO:0005622
Cluster-22178.58615 GO:0015992 GO:0043066 GO:0015986 GO:0045766

GO:0016567 GO:0043123 GO:0043130 GO:0004842 GO:0015078
GO:0003723 GO:0000276
Cluster-22178.58616 GO:0048870 GO:0051258 GO:0007165 GO:0030168
GO:0008053 GO:0006606 GO:0006260 GO:0045502 GO:0019901
GO:0008134 GO:0042803 GO:0003924 GO:0030674 GO:0005102
GO:0031514 GO:0016021 GO:0005667 GO:0005741 GO:0030286
GO:0005577
Cluster-22178.58617 GO:0006260 GO:0006950 GO:0007154 GO:0006313
GO:0007165 GO:0006281 GO:0003911 GO:0005179 GO:0016021
GO:0005576
Cluster-22178.58623 GO:0006457 GO:0005515
Cluster-22178.58624 GO:0005515
Cluster-22178.58627 GO:0006468 GO:0016055 GO:0007275 GO:0007165
GO:0004672 GO:0005524 GO:0005102 GO:0005576
Cluster-22178.58629 GO:0007165 GO:0055114 GO:0020037 GO:0016705
GO:0005158 GO:0005506 GO:0005899
Cluster-22178.58631 GO:0055114 GO:0016972
Cluster-22178.58632 GO:0009399 GO:0005515 GO:0008270 GO:0016021
Cluster-22178.58634 GO:0007166 GO:0016567 GO:0007165 GO:0004871
GO:0004842 GO:0008270 GO:0046872 GO:0005509 GO:0005515
GO:0005680 GO:0005634
Cluster-22178.58636 GO:0006024 GO:0030206 GO:0006470 GO:0006570
GO:0030158 GO:0004725 GO:0008138
Cluster-22178.58637 GO:0002949 GO:0006310 GO:0006024 GO:0006470
GO:0030206 GO:0006570 GO:0006281 GO:0030158 GO:0003724
GO:0005524 GO:0016887 GO:0008138 GO:0009378 GO:0004725
GO:0003678 GO:0016301 GO:0003723 GO:0005657 GO:0009379
Cluster-22178.58638 GO:0045454 GO:0006118 GO:0009055 GO:0015035

Cluster-22178.58639 GO:0015996 GO:0015994 GO:0006811 GO:0006508
GO:0015991 GO:0006629 GO:0006355 GO:0006118 GO:0015992
GO:0016042 GO:0045454 GO:0046486 GO:0046872 GO:0015078
GO:0009055 GO:0015035 GO:0008236 GO:0047746 GO:0003847

GO:0016787 GO:0003677 GO:0019031 GO:0033179 GO:0005886
GO:0016020 GO:0008247
Cluster-22178.58640 GO:0006355 GO:0003700 GO:0005515 GO:0005667
GO:0042025
Cluster-22178.58642 GO:0042254 GO:0006412 GO:0003735 GO:0005840
Cluster-22178.58643 GO:0006891 GO:0000184 GO:0006914 GO:0005815
GO:0017119
Cluster-22178.58644 GO:0006355 GO:0051726 GO:0000736 GO:0043565
GO:0046872 GO:0003700 GO:0070336 GO:0003677 GO:0005634
GO:0005667
Cluster-22178.58646 GO:0046872
Cluster-22178.58647 GO:0007275 GO:0005634
Cluster-22178.58648 GO:0006355 GO:0045893 GO:0003677 GO:0043565
GO:0005634
Cluster-22178.5865 GO:0055085 GO:0016021
Cluster-22178.58650 GO:0009253 GO:0006807 GO:0009252 GO:0005198
GO:0008745 GO:0005882
Cluster-22178.58651 GO:0006308 GO:0008855 GO:0009318
Cluster-22178.58655 GO:0005515
Cluster-22178.58656 GO:0015684 GO:0007264 GO:0006576 GO:0005525
GO:0015093 GO:0005524 GO:0003924 GO:0016021
Cluster-22178.58657 GO:0006904 GO:0000145
Cluster-22178.58660 GO:0007076 GO:0000796
Cluster-22178.58661 GO:0007049 GO:0005524 GO:0005515 GO:0009507
GO:0031011 GO:0017053
Cluster-22178.58663 GO:0006144 GO:0015074 GO:0003968 GO:0003677
GO:0005524 GO:0031379
Cluster-22178.58665 GO:0006468 GO:0005524 GO:0004672
Cluster-22178.58667 GO:0006302 GO:0006310 GO:0005515 GO:0003677
GO:0005634
Cluster-22178.58668 GO:0008152 GO:0003824 GO:0048037
Cluster-22178.58671 GO:0055085 GO:0006952 GO:0009405 GO:0006810
GO:0006821 GO:0019855 GO:0005247 GO:0008270 GO:0016020

GO:0005576
Cluster-22178.58674 GO:0006355 GO:0005524 GO:0005634
Cluster-22178.58675 GO:0006811 GO:0006355 GO:0009405 GO:0043565
GO:0003700 GO:0005216 GO:0005515 GO:0005576 GO:0005667
Cluster-22178.58676 GO:0046740 GO:0006457 GO:0016021
Cluster-22178.58677 GO:0007165 GO:0098655 GO:0006812 GO:0033198
GO:0001614 GO:0004931 GO:0008270 GO:0005887
Cluster-22178.58678 GO:0005975 GO:0042325 GO:0004553 GO:0005737
Cluster-22178.58679 GO:0016021 GO:0012511
Cluster-22178.58685 GO:0016779
Cluster-22178.58686 GO:0009234 GO:0006810 GO:0019079 GO:0005524
GO:0016887 GO:0004386 GO:0003676 GO:0016787 GO:0000166
GO:0008026 GO:0003677
Cluster-22178.58687 GO:0006096 GO:0005982 GO:0051156 GO:0006012
GO:0042207 GO:0032259 GO:0006289 GO:0019872 GO:0043087
GO:0043161 GO:0009072 GO:0018149 GO:0005985 GO:0006570
GO:0006281 GO:0006094 GO:0045040 GO:0003684 GO:0004334
GO:0003810 GO:0005089 GO:0005524 GO:0004340 GO:0005515
GO:0008168 GO:0005742
Cluster-22178.58689 GO:0055114 GO:0047134
Cluster-22178.58692 GO:0006814 GO:0032775 GO:0071436 GO:0006306
GO:0006560 GO:0006525 GO:0006090 GO:0005515 GO:0008948
GO:0003677 GO:0009007 GO:0015081 GO:0016020
Cluster-22178.58693 GO:0016114 GO:0006694 GO:0006886 GO:0006351
GO:0006284 GO:0006144 GO:0006206 GO:0016192 GO:0006383
GO:0003899 GO:0005198 GO:0005515 GO:0008661 GO:0003677
GO:0005634 GO:0030117 GO:0005730 GO:0005666
Cluster-22178.58694 GO:0006886 GO:0016192 GO:0006284 GO:0003677
GO:0005198 GO:0005515 GO:0030117 GO:0005634
Cluster-22178.58697 GO:0006811 GO:0055085 GO:0055114 GO:0006810
GO:0007264 GO:0005215 GO:0005525 GO:0016491 GO:0005216
GO:0010181 GO:0005615 GO:0016021 GO:0016020
Cluster-22178.58699 GO:0005525 GO:0003924

Cluster-22178.58700 GO:0005524 GO:0003676 GO:0005634
Cluster-22178.58703 GO:0006355 GO:0006486 GO:0008270 GO:0008417
GO:0003682 GO:0003677 GO:0005789 GO:0000775 GO:0000785
GO:0005634 GO:0016020
Cluster-22178.58705 GO:0007264 GO:0016887 GO:0003924 GO:0005524
GO:0005525 GO:0016020
Cluster-22178.58707 GO:0009405 GO:0003676
Cluster-22178.58710 GO:0007156 GO:0005509 GO:0016020 GO:0005634
Cluster-22178.58712 GO:0070206 GO:0048870 GO:0005515 GO:0031514
GO:0005639
Cluster-22178.58714 GO:0015991 GO:0015992 GO:0015078 GO:0033179
Cluster-22178.58716 GO:0003677
Cluster-22178.58718 GO:0006955
Cluster-22178.58720 GO:0034227 GO:0005737
Cluster-22178.58721 GO:0009405 GO:0005515 GO:0003676 GO:0015485
GO:0008270 GO:0003723
Cluster-22178.58722 GO:0031175 GO:0030474 GO:0015031 GO:0042254
GO:0071988 GO:0007268 GO:0008017 GO:0033130 GO:0003735
GO:0030507 GO:0043495 GO:0005516 GO:0045298 GO:0005840
GO:0008091 GO:0005739
Cluster-22178.58724 GO:0007010 GO:0005515 GO:0003779
Cluster-22178.58725 GO:0006367 GO:0005672
Cluster-22178.58727 GO:0006260 GO:0019079 GO:0003677 GO:0030337
GO:0042575 GO:0005634
Cluster-22178.58729 GO:0005515
Cluster-22178.58730 GO:0006554 GO:0006479 GO:0006886 GO:0018024
GO:0030176
Cluster-22178.58731 GO:0007017 GO:0007018 GO:0005524 GO:0008017
GO:0003777 GO:0045298 GO:0005874
Cluster-22178.58734 GO:0008152 GO:0007186 GO:0004930 GO:0008168
GO:0016021
Cluster-22178.58736 GO:0006468 GO:0005524 GO:0004672 GO:0005789
Cluster-22178.58737 GO:0006144 GO:0006531 GO:0006526 GO:0015074

GO:0008033 GO:0008152 GO:0006529 GO:0006522 GO:0006164
GO:0006177 GO:0006536 GO:0006560 GO:0006541 GO:0005524
GO:0016787 GO:0004066 GO:0004055 GO:0003824 GO:0003922
GO:0004810
Cluster-22178.58740 GO:0006090 GO:0015976 GO:0055114 GO:0006108
GO:0006099 GO:0004471 GO:0008201 GO:0051287
Cluster-22178.58744 GO:0006367 GO:0008270 GO:0005672
Cluster-22178.58746 GO:0006464 GO:0015031 GO:0006508 GO:0006468
GO:0016773 GO:0005524 GO:0004672 GO:0004252 GO:0016021
GO:0016020
Cluster-22178.58748 GO:0007186 GO:0007165 GO:0006810 GO:0055085
GO:0003924 GO:0016887 GO:0019001 GO:0005524 GO:0031683
GO:0042626 GO:0004871 GO:0016021
Cluster-22178.58750 GO:0016310 GO:0007178 GO:0009069 GO:0006468
GO:0005524 GO:0004672 GO:0004675 GO:0016020
Cluster-22178.58752 GO:0007155
Cluster-22178.58753 GO:0006508 GO:0006629 GO:0008152 GO:0016787
GO:0008236
Cluster-22178.58754 GO:0019058
Cluster-22178.58755 GO:0006886 GO:0007165 GO:0019079 GO:0006888
GO:0005515 GO:0016021 GO:0030127
Cluster-22178.58756 GO:0005515
Cluster-22178.58757 GO:0005509
Cluster-22178.58758 GO:0035556 GO:0035023 GO:0043087 GO:0005515
GO:0005089
Cluster-22178.58763 GO:0055085 GO:0016021
Cluster-22178.58766 GO:0007186 GO:0007165 GO:0004871 GO:0005834
Cluster-22178.58768 GO:0048870 GO:0008152 GO:0006355 GO:0006367
GO:0016491 GO:0008440 GO:0003682 GO:0003677 GO:0017048
GO:0000775 GO:0000785 GO:0005634 GO:0005672 GO:0031514
Cluster-22178.58770 GO:0048870 GO:0008152 GO:0006355 GO:0006367
GO:0016491 GO:0008440 GO:0003682 GO:0017048 GO:0003677
GO:0000785 GO:0000775 GO:0005634 GO:0005672 GO:0031514

Cluster-22178.58771 GO:0008152 GO:0000398 GO:0006351 GO:0006355
GO:0006260 GO:0006367 GO:0032968 GO:0008440 GO:0003887
GO:0016491 GO:0008270 GO:0003677 GO:0003682 GO:0005681
GO:0000785 GO:0000775 GO:0042575 GO:0005672 GO:0005634
Cluster-22178.58773 GO:0006508 GO:0004190 GO:0005515
Cluster-22178.58774 GO:0006508 GO:0008234 GO:0004197
Cluster-22178.58777 GO:0019497 GO:0006771 GO:0003993 GO:0005515
GO:0046872
Cluster-22178.58778 GO:0005515 GO:0003924
Cluster-22178.58779 GO:0016032 GO:0015991 GO:0006118 GO:0055114
GO:0015992 GO:0008152 GO:0016740 GO:0003995 GO:0016627
GO:0046872 GO:0005524 GO:0015078 GO:0050660 GO:0005525
GO:0033179
Cluster-22178.58780 GO:0006281 GO:0007186 GO:0006310 GO:0006367
GO:0000287 GO:0000400 GO:0003677 GO:0016788 GO:0004930
GO:0005672 GO:0016021
Cluster-22178.58781 GO:0006355 GO:0005515 GO:0004252 GO:0003677
GO:0005634 GO:0005886
Cluster-22178.58782 GO:0043666 GO:0009878 GO:0009966 GO:0046872
GO:0004864
Cluster-22178.58786 GO:0006570 GO:0006470 GO:0004725 GO:0008138

Cluster-22178.58787 GO:0008608 GO:0003723 GO:0005876 GO:0042729
Cluster-22178.58789 GO:0007264 GO:0006576 GO:0007165 GO:0007186
GO:0098519 GO:0031683 GO:0005524 GO:0019001 GO:0016887
GO:0003924 GO:0005525 GO:0004871
Cluster-22178.58791 GO:0006355 GO:0003700 GO:0005667
Cluster-22178.58792 GO:0016746
Cluster-22178.58793 GO:0009405 GO:0006810 GO:0045444 GO:0008200
GO:0005576
Cluster-22178.58794 GO:0008270
Cluster-22178.58795 GO:0006396
Cluster-22178.58796 GO:0006886 GO:0035091 GO:0016020

Cluster-22178.58797 GO:0004222
Cluster-22178.58799 GO:0006364 GO:0019089 GO:0007034 GO:0008168
GO:0005737
Cluster-22178.58800 GO:0009512
Cluster-22178.58805 GO:0005515
Cluster-22178.58806 GO:0009405 GO:0005515 GO:0045735
Cluster-22178.58809 GO:0005515
Cluster-22178.58811 GO:0009405 GO:0006810 GO:0008200 GO:0042151
Cluster-22178.58814 GO:0055114 GO:0006520 GO:0008152 GO:0009245
GO:0044564 GO:0016747 GO:0016491
Cluster-22178.58816 GO:0051260
Cluster-22178.58817 GO:0008152 GO:0016310 GO:0009069 GO:0006468
GO:0007165 GO:0005096 GO:0004674 GO:0016746 GO:0016787
GO:0015016 GO:0005515
Cluster-22178.58818 GO:0006355 GO:0019048
Cluster-22178.58821 GO:0006468 GO:0005524 GO:0004672 GO:0008168
GO:0005515
Cluster-22178.58822 GO:0006744 GO:0006120 GO:0015992 GO:0055114
GO:0006814 GO:0006468 GO:0005524 GO:0004672 GO:0016491
GO:0008137 GO:0005515 GO:0005743
Cluster-22178.58825 GO:0016567 GO:0009245 GO:0005515 GO:0003723
GO:0008233 GO:0004842 GO:0046872 GO:0008915 GO:0008270
GO:0016020 GO:0005680
Cluster-22178.58826 GO:0006281 GO:0008270
Cluster-22178.58830 GO:0007275 GO:0016021
Cluster-22178.58832 GO:0006629 GO:0006355 GO:0015991 GO:0006886
GO:0016192 GO:0051276 GO:0015031 GO:0043248 GO:0006606
GO:0005515 GO:0016820 GO:0046872 GO:0003677 GO:0008270
GO:0005524 GO:0008565 GO:0016887 GO:0004222 GO:0030117
GO:0005634 GO:0005737 GO:0005694 GO:0005622 GO:0000221
Cluster-22178.58835 GO:0016567 GO:0009069 GO:0016310 GO:0005515
GO:0004842 GO:0004674 GO:0003676 GO:0046872 GO:0008270
GO:0005680

Cluster-22178.58839 GO:0007224 GO:0006813 GO:0008063 GO:0006814
GO:0008270 GO:0005515 GO:0016021 GO:0005890
Cluster-22178.58844 GO:0016579 GO:0044237 GO:0016567 GO:0019634
GO:0004842 GO:0003824 GO:0036459 GO:0005680
Cluster-22178.58849 GO:0007165 GO:0005515
Cluster-22178.58854 GO:0006457 GO:0000413 GO:0004190 GO:0003755
GO:0030430 GO:0016021
Cluster-22178.58859 GO:0022900 GO:0006118 GO:0006511 GO:0005506
GO:0020037 GO:0009055 GO:0042597
Cluster-22178.5886 GO:0006310 GO:0006281 GO:0006308 GO:0004520
GO:0033588
Cluster-22178.58863 GO:0006355 GO:0009966 GO:0003677 GO:0005576
Cluster-22178.58866 GO:0055114 GO:0016491
Cluster-22178.58868 GO:0005524
Cluster-22178.58870 GO:0005515
Cluster-22178.58871 GO:0007186 GO:0006464 GO:0015031 GO:0004930
GO:0005515 GO:0016021
Cluster-22178.58873 GO:0006935 GO:0000160 GO:0006355 GO:0000156
GO:0008984 GO:0005515 GO:0003677 GO:0005737
Cluster-22178.58874 GO:0006355 GO:0000160 GO:0006935 GO:0005515
GO:0003677 GO:0008984 GO:0000156 GO:0005737
Cluster-22178.58875 GO:0007155 GO:0006508 GO:0007186 GO:0006000
GO:0006013 GO:0005515 GO:0004252 GO:0003873 GO:0005524
GO:0004930 GO:0031224 GO:0016020
Cluster-22178.58879 GO:0009405 GO:0005576
Cluster-22178.58880 GO:0007264 GO:0015684 GO:0015093 GO:0005525
GO:0016021
Cluster-22178.58881 GO:0005515
Cluster-22178.58882 GO:0007154 GO:0051301 GO:0016021 GO:0016020
Cluster-22178.58883 GO:0006388 GO:0051252 GO:0006351 GO:0003714
GO:0000213 GO:0005667 GO:0000214
Cluster-22178.58885 GO:0006355 GO:0003677
Cluster-22178.58886 GO:0006576 GO:0003924 GO:0005524 GO:0005525

GO:0003677
Cluster-22178.58888 GO:0035556 GO:0030163 GO:0005515 GO:0008914

Cluster-22178.58892 GO:0006508 GO:0004222 GO:0016020
Cluster-22178.58893 GO:0016567 GO:0006771 GO:0006508 GO:0019497
GO:0004197 GO:0005515 GO:0016740 GO:0004842 GO:0003993
GO:0046872 GO:0008270 GO:0008242 GO:0005680 GO:0005622
Cluster-22178.58895 GO:0006576 GO:0007264 GO:0015684 GO:0055114
GO:0005524 GO:0016627 GO:0015093 GO:0005525 GO:0005737
GO:0016021
Cluster-22178.58896 GO:0055114 GO:0010309 GO:0005515
Cluster-22178.58897 GO:0005515 GO:0016773 GO:0030414 GO:0046872
GO:0005576
Cluster-22178.58898 GO:0005515 GO:0016773 GO:0046872 GO:0030414
GO:0005576
Cluster-22178.58906 GO:0019068 GO:0006310 GO:0005515 GO:0005198
GO:0003677 GO:0005634
Cluster-22178.58907 GO:0006355 GO:0006531 GO:0006419 GO:0006522
GO:0000166 GO:0005524 GO:0004813 GO:0032039 GO:0005622
GO:0016021
Cluster-22178.58910 GO:0006531 GO:0006355 GO:0006419 GO:0006522
GO:0000166 GO:0004813 GO:0005524 GO:0032039 GO:0005622
Cluster-22178.58915 GO:0051205 GO:0016021
Cluster-22178.58916 GO:0019856 GO:0045892 GO:0003700 GO:0005515
GO:0019012 GO:0005667
Cluster-22178.58917 GO:0006470 GO:0006570 GO:0004725 GO:0008138

Cluster-22178.58918 GO:0003676
Cluster-22178.58920 GO:0016021
Cluster-22178.58921 GO:0046813 GO:0061025 GO:0006955 GO:0030153
GO:0015643 GO:0019031 GO:0016021 GO:0019814
Cluster-22178.58922 GO:0000087 GO:0006570 GO:0006508 GO:0009415
GO:0006470 GO:0006950 GO:0004222 GO:0004725 GO:0008270

GO:0005622 GO:0031012
Cluster-22178.58923 GO:0006508 GO:0004252 GO:0004197
Cluster-22178.58925 GO:0019497 GO:0006355 GO:0007264 GO:0006771
GO:0007165 GO:0003924 GO:0046872 GO:0004871 GO:0003700
GO:0005525 GO:0005515 GO:0016887 GO:0005524 GO:0003993
GO:0005634 GO:0005667
Cluster-22178.58926 GO:0006508 GO:0008236
Cluster-22178.58928 GO:0006935 GO:0006955 GO:0006303 GO:0007165
GO:0008009 GO:0005576
Cluster-22178.58929 GO:0006508 GO:0016579 GO:0004843 GO:0005515

Cluster-22178.58930 GO:0006486 GO:0004576 GO:0016021 GO:0008250
GO:0016020
Cluster-22178.58931 GO:0007218 GO:0005184 GO:0005576
Cluster-22178.58934 GO:0055114 GO:0003697 GO:0016638
Cluster-22178.58935 GO:0000105 GO:0042823 GO:0042819 GO:0008033
GO:0055114 GO:0017150 GO:0050660
Cluster-22178.58936 GO:0045859 GO:0019887 GO:0005956
Cluster-22178.58944 GO:0006351 GO:0009405 GO:0006144 GO:0006464
GO:0006206 GO:0016567 GO:0006511 GO:0007275 GO:0046872
GO:0016151 GO:0003899 GO:0005515 GO:0005198 GO:0008270
GO:0004842 GO:0003677 GO:0005634 GO:0005680 GO:0005730
Cluster-22178.58948 GO:0006355 GO:0006508 GO:0034968 GO:0006479
GO:0006554 GO:0018024 GO:0005515 GO:0004252 GO:0005694
Cluster-22178.58949 GO:0006479 GO:0034968 GO:0006508 GO:0006355
GO:0006554 GO:0004252 GO:0005515 GO:0018024 GO:0005694
Cluster-22178.58953 GO:0016567 GO:0005515 GO:0004842 GO:0008270

Cluster-22178.58956 GO:0003676
Cluster-22178.58961 GO:0010508 GO:0006810 GO:0007264 GO:0008053
GO:0003723 GO:0003724 GO:0005525 GO:0016887 GO:0003924
GO:0005524 GO:0005741 GO:0016021
Cluster-22178.58965 GO:0005985 GO:0005982 GO:0009117 GO:0048040

Cluster-22178.58968 GO:0016032 GO:0005515 GO:0019031
Cluster-22178.58970 GO:0055114 GO:0045454 GO:0016972
Cluster-22178.58973 GO:0006464 GO:0015031 GO:0005515
Cluster-22178.58974 GO:0005515
Cluster-22178.58975 GO:0007155 GO:0016020
Cluster-22178.58977 GO:0016567 GO:0008270 GO:0046872 GO:0004842 GO:0005515 GO:0005680
Cluster-22178.58978 GO:0007631 GO:0007218 GO:0055114 GO:0016730

Cluster-22178.58980 GO:0005515
Cluster-22178.58982 GO:0006914 GO:0007155 GO:0007165 GO:0019058 GO:0004871 GO:0003723 GO:0005198 GO:0019031 GO:0019867 GO:0019028 GO:0016020
Cluster-22178.58983 GO:0003723 GO:0005198 GO:0019028 GO:0019867 GO:0019031
Cluster-22178.58984 GO:0001503 GO:0006760 GO:0046656 GO:0004150 GO:0008270
Cluster-22178.58985 GO:0001503 GO:0006760 GO:0046656 GO:0004150 GO:0008270
Cluster-22178.58989 GO:0055114 GO:0006468 GO:0005524 GO:0004672 GO:0016614
Cluster-22178.58990 GO:0007186 GO:0006952 GO:0007165 GO:0007264 GO:0004871 GO:0005525 GO:0003924 GO:0019001 GO:0031683 GO:0005576
Cluster-22178.58991 GO:0007165 GO:0007186 GO:0007264 GO:0006013 GO:0006000 GO:0003873 GO:0005515 GO:0005525 GO:0004871 GO:0019001 GO:0031683 GO:0005524 GO:0003924
Cluster-22178.58994 GO:0005515
Cluster-22178.58996 GO:0015948 GO:0051028 GO:0006914 GO:0046656 GO:0006886 GO:0048309 GO:0016310 GO:0015031 GO:0006260 GO:0048870 GO:0008608 GO:0007049 GO:0000160 GO:0000155 GO:0003774 GO:0005524 GO:0030269 GO:0008565 GO:0046983

GO:0016757 GO:0005198 GO:0005515 GO:0042803 GO:0045502
GO:0008134 GO:0008092 GO:0016459 GO:0005667 GO:0016020
GO:0042729 GO:0005737 GO:0005882 GO:0030286 GO:0019898
GO:0072686 GO:0031514 GO:0009365 GO:0016021 GO:0005801
Cluster-22178.58997 GO:0006118 GO:0051603 GO:0020037 GO:0004298
GO:0009055 GO:0005839
Cluster-22178.58998 GO:0007076 GO:0000796
Cluster-22178.58999 GO:0003676
Cluster-22178.59000 GO:0003950 GO:0003723
Cluster-22178.59001 GO:0010112 GO:0015684 GO:0007264 GO:0005525
GO:0003924 GO:0015093 GO:0016021
Cluster-22178.59004 GO:0006144 GO:0002098 GO:0006468 GO:0046034
GO:0034227 GO:0016787 GO:0003677 GO:0004017 GO:0008270
GO:0005524 GO:0000049 GO:0020037 GO:0004672 GO:0046872
GO:0003676 GO:0005634
Cluster-22178.59005 GO:0003774 GO:0005524 GO:0016459
Cluster-22178.59006 GO:0003774 GO:0005524 GO:0016459
Cluster-22178.59008 GO:0042254 GO:0006412 GO:0003735 GO:0005622
GO:0005840
Cluster-22178.59009 GO:0016310 GO:0009069 GO:0007186 GO:0007165
GO:0003924 GO:0019001 GO:0031683 GO:0004871 GO:0016772
GO:0004674 GO:0005525 GO:0005515 GO:0016021
Cluster-22178.59010 GO:0019497 GO:0006771 GO:0003993 GO:0005515
GO:0046872 GO:0016020
Cluster-22178.59011 GO:0006888 GO:0019079 GO:0007165 GO:0006886
GO:0005515 GO:0030127 GO:0016021
Cluster-22178.59013 GO:0007130 GO:0000795
Cluster-22178.59014 GO:0006368 GO:0008023
Cluster-22178.59019 GO:0006355 GO:0006270 GO:0019058 GO:0055085
GO:0003677 GO:0004252 GO:0003700 GO:0022857 GO:0005667
GO:0016020 GO:0005886 GO:0019031 GO:0016021
Cluster-22178.59024 GO:0071705 GO:0003676 GO:0045095
Cluster-22178.59025 GO:0006807 GO:0007031 GO:0016814 GO:0008270

GO:0005779
Cluster-22178.59027 GO:0050830 GO:0050829 GO:0007009 GO:0003779
GO:0051082
Cluster-22178.59028 GO:0006355 GO:0003700 GO:0030695 GO:0003677
GO:0043565 GO:0005634 GO:0005667
Cluster-22178.59030 GO:0006012 GO:0006013 GO:0006000 GO:0006096
GO:0006094 GO:0006098 GO:0003872 GO:0016301 GO:0005945
Cluster-22178.59035 GO:0035556 GO:0008152 GO:0016746 GO:0005515

Cluster-22178.59036 GO:0046856 GO:0034597
Cluster-22178.5904 GO:0012511 GO:0016021
Cluster-22178.59040 GO:0006366 GO:0006281 GO:0051382 GO:0006352
GO:0003677 GO:0005669
Cluster-22178.59041 GO:0006275 GO:0006270 GO:0005524 GO:0043565
GO:0005515
Cluster-22178.59044 GO:0005515
Cluster-22178.59046 GO:0000921 GO:0016055 GO:0006355 GO:0042254
GO:0006412 GO:0043565 GO:0003700 GO:0003735 GO:0003677
GO:0005940 GO:0005622 GO:0016021 GO:0005840 GO:0005667
GO:0005634
Cluster-22178.59049 GO:0006355 GO:0003723 GO:0046872 GO:0008270

Cluster-22178.59052 GO:0006525 GO:0006560 GO:0043039 GO:0006433
GO:0006418 GO:0004812 GO:0000166 GO:0016876 GO:0005524
GO:0004827 GO:0005737
Cluster-22178.59053 GO:0030420 GO:0008152 GO:0019497 GO:0006355
GO:0006771 GO:0008253 GO:0003993 GO:0005524 GO:0016791
GO:0008134 GO:0000287 GO:0005737 GO:0005667
Cluster-22178.59056 GO:0008270
Cluster-22178.59057 GO:0008270
Cluster-22178.59058 GO:0005515
Cluster-22178.59060 GO:0008270 GO:0016787
Cluster-22178.59061 GO:0055114 GO:0051536

Cluster-22178.59063 GO:0006355 GO:0006351 GO:0000390 GO:0009405
GO:0000917 GO:0006886 GO:0006144 GO:0019064 GO:0006260
GO:0043093 GO:0019835 GO:0003968 GO:0003723 GO:0003677
GO:0005737 GO:0031379 GO:0019031 GO:0019867 GO:0005801
GO:0071008 GO:0016020
Cluster-22178.59065 GO:0008270
Cluster-22178.59066 GO:0055114 GO:0016020
Cluster-22178.59067 GO:0055114 GO:0016020
Cluster-22178.59068 GO:0006511 GO:0005515
Cluster-22178.59069 GO:0006952 GO:0005576
Cluster-22178.59070 GO:0006351 GO:0008270 GO:0003676 GO:0005515
GO:0005639
Cluster-22178.59071 GO:0006351 GO:0008270 GO:0003676 GO:0005515
GO:0005639
Cluster-22178.59072 GO:0003676
Cluster-22178.59073 GO:0005515
Cluster-22178.59076 GO:0006821 GO:0055085 GO:0005247 GO:0016020
Cluster-22178.59077 GO:0006357 GO:0007165 GO:0006817 GO:0006468
GO:0016773 GO:0005524 GO:0004672 GO:0005515 GO:0001104
GO:0005856 GO:0016020 GO:0016592
Cluster-22178.59078 GO:0055114 GO:0046872 GO:0016491 GO:0005576
Cluster-22178.59080 GO:0055114 GO:0016491 GO:0005507
Cluster-22178.59081 GO:0006281 GO:0006915 GO:0032465 GO:0016567
GO:0006310 GO:0009378 GO:0003723 GO:0005515 GO:0005524
GO:0016887 GO:0004842 GO:0003724 GO:0003678 GO:0009379
GO:0005657
Cluster-22178.59082 GO:0005978
Cluster-22178.59084 GO:0006355 GO:0043401
Cluster-22178.59090 GO:0006270 GO:0006397 GO:0006886 GO:0018342
GO:0006396 GO:0005515 GO:0008318 GO:0005634 GO:0005789
GO:0005622
Cluster-22178.59091 GO:0018101 GO:0006807 GO:0004668 GO:0016787
GO:0005509 GO:0005737

Cluster-22178.59092 GO:0030154 GO:0016021
Cluster-22178.59093 GO:0006508 GO:0003676 GO:0008233
Cluster-22178.59094 GO:0006357 GO:0007165 GO:0005057 GO:0001104 GO:0016592
Cluster-22178.59095 GO:0003676 GO:0035091 GO:0005887
Cluster-22178.59097 GO:0005515
Cluster-22178.59099 GO:0003676 GO:0035091 GO:0005887
Cluster-22178.59100 GO:0006461 GO:0010508 GO:0019901 GO:0005739
Cluster-22178.59101 GO:0007340 GO:0007165 GO:0007342 GO:0046872 GO:0003676 GO:0005179 GO:0003677 GO:0005887 GO:0005576 GO:0001669 GO:0045095
Cluster-22178.59103 GO:0003677
Cluster-22178.59104 GO:0006275 GO:0045893 GO:0006260 GO:0019073 GO:0003677 GO:0005515 GO:0003688 GO:0046809 GO:0042025
Cluster-22178.59107 GO:0006355 GO:0008152 GO:0015937 GO:0015940 GO:0006139 GO:0006465 GO:0006206 GO:0016301 GO:0016740 GO:0003676 GO:0004140 GO:0004127 GO:0008233 GO:0005524 GO:0005787 GO:0016021 GO:0005622
Cluster-22178.59111 GO:0005975 GO:0006352 GO:0004553 GO:0003677

Cluster-22178.59112 GO:0007165 GO:0006355 GO:0042974 GO:0003677 GO:0005198 GO:0019013 GO:0005667
Cluster-22178.59113 GO:0006351 GO:0003677 GO:0005634
Cluster-22178.59114 GO:0003676
Cluster-22178.59115 GO:0006412 GO:0042254 GO:0034508 GO:0005975 GO:0003735 GO:0004553 GO:0030248 GO:0000776 GO:0005576 GO:0015934 GO:0005840
Cluster-22178.59116 GO:0006464 GO:0008152 GO:0009452 GO:0001510 GO:0046500 GO:0006479 GO:0008168 GO:0004719 GO:0008171 GO:0031224
Cluster-22178.59118 GO:0033588
Cluster-22178.59120 GO:0009405 GO:0019835 GO:0035023 GO:0035556 GO:0043087 GO:0005515 GO:0005089 GO:0019867

Cluster-22178.59121 GO:0035023 GO:0043087 GO:0005089
Cluster-22178.59124 GO:0007165 GO:0005515
Cluster-22178.59126 GO:0046872 GO:0003677
Cluster-22178.59128 GO:0006468 GO:0042803 GO:0008134 GO:0045502 GO:0005524 GO:0004672 GO:0005667 GO:0019028 GO:0030286 GO:0005856
Cluster-22178.59129 GO:0071107 GO:0042254 GO:0006206 GO:0006144 GO:0006355 GO:0006351 GO:0006412 GO:0006352 GO:0003677 GO:0003735 GO:0016987 GO:0003899 GO:0003700 GO:0003676 GO:0005730 GO:0005667 GO:0016020 GO:0005622 GO:0005840
Cluster-22178.59131 GO:0006412 GO:0042254 GO:0003735 GO:0005622 GO:0005840
Cluster-22178.59135 GO:0031144 GO:0006308 GO:0006412 GO:0030474 GO:0015991 GO:0070206 GO:0007165 GO:0071630 GO:0048309 GO:0010508 GO:0040007 GO:0006606 GO:0007218 GO:0007034 GO:0006914 GO:0051028 GO:0015992 GO:0042254 GO:0005200 GO:0008855 GO:0015078 GO:0005198 GO:0003735 GO:0003677 GO:0005179 GO:0005840 GO:0033179 GO:0009318 GO:0005576 GO:0005823 GO:0005856 GO:0005737 GO:0005634 GO:0005622
Cluster-22178.59136 GO:0051252 GO:0046034 GO:0008152 GO:0008033 GO:0006144 GO:0006464 GO:0004017 GO:0016787 GO:0004540 GO:0016151 GO:0016846 GO:0046872
Cluster-22178.59137 GO:0006804 GO:0006749 GO:0006400 GO:0008616 GO:0006979 GO:0055114 GO:0008479 GO:0004602
Cluster-22178.59140 GO:0016021
Cluster-22178.59141 GO:0030246
Cluster-22178.59142 GO:0007154 GO:0005515 GO:0004252 GO:0005509 GO:0005886 GO:0016020
Cluster-22178.59143 GO:0006570 GO:0006470 GO:0008138 GO:0004725

Cluster-22178.59144 GO:0006470 GO:0006570 GO:0004725
Cluster-22178.59146 GO:0006265 GO:0006302 GO:0003917 GO:0003677 GO:0005634 GO:0005694

Cluster-22178.59149 GO:0007165 GO:0005515 GO:0016020 GO:0016012
Cluster-22178.59150 GO:0007165 GO:0006468 GO:0009069 GO:0007217
GO:0016310 GO:0004674 GO:0005524 GO:0004672 GO:0005576
Cluster-22178.59151 GO:0055114 GO:0016787
Cluster-22178.59152 GO:0000413 GO:0006457 GO:0007010 GO:0008017
GO:0003755 GO:0045298 GO:0005887
Cluster-22178.59154 GO:0006278 GO:0004190 GO:0003964 GO:0016021
Cluster-22178.59155 GO:0009966 GO:0005515 GO:0005576
Cluster-22178.59156 GO:0031412 GO:0006260 GO:0006144 GO:0006281
GO:0003968 GO:0003911 GO:0046872 GO:0008270 GO:0005515
GO:0031379 GO:0031411
Cluster-22178.59159 GO:0015991 GO:0009069 GO:0016192 GO:0006886
GO:0051252 GO:0043248 GO:0006850 GO:0016310 GO:0005515
GO:0016820 GO:0004521 GO:0004674 GO:0030117 GO:0005743
GO:0000221
Cluster-22178.59160 GO:0015694 GO:0042967 GO:0015097 GO:0005515
GO:0008080 GO:0016020 GO:0097197 GO:0001772 GO:0016021
Cluster-22178.59161 GO:0007165 GO:0006891 GO:0008053 GO:0030168
GO:0051258 GO:0006308 GO:0030674 GO:0005102 GO:0003924
GO:0008855 GO:0009318 GO:0005741 GO:0017119 GO:0016021
GO:0005577
Cluster-22178.59164 GO:0006771 GO:0006508 GO:0019497 GO:0016740
GO:0004197 GO:0005515 GO:0046872 GO:0008242 GO:0003993
Cluster-22178.59168 GO:0008152 GO:0006480 GO:0055114 GO:0008171
GO:0008168 GO:0016491
Cluster-22178.59172 GO:0055114 GO:0008152 GO:0051287 GO:0016616

Cluster-22178.59173 GO:0055114 GO:0019521 GO:0006098 GO:0008152
GO:0051287 GO:0016616 GO:0004616
Cluster-22178.59174 GO:0005515 GO:0003723 GO:0046872 GO:0016021
Cluster-22178.59175 GO:0009307 GO:0007268 GO:0007165 GO:0006308
GO:0006811 GO:0009036 GO:0005234 GO:0004970 GO:0009359
GO:0016020

Cluster-22178.59176 GO:0035556 GO:0046907 GO:0006469 GO:0045859 GO:0004862 GO:0005952

Cluster-22178.59183 GO:0016567 GO:0008152 GO:0008168

Cluster-22178.59186 GO:0006886 GO:0005622

Cluster-22178.59189 GO:0006836 GO:0051694 GO:0006412 GO:0042254 GO:0019905 GO:0003723 GO:0003735 GO:0005523 GO:0005622 GO:0005840 GO:0016020

Cluster-22178.59190 GO:0042254 GO:0006412 GO:0051694 GO:0003723 GO:0003735 GO:0005523 GO:0016020 GO:0005622 GO:0005840

Cluster-22178.59192 GO:0006468 GO:0003677 GO:0004672 GO:0005524 GO:0005634

Cluster-22178.59193 GO:0006310 GO:0006511 GO:0007010 GO:0032259 GO:0005515 GO:0003677 GO:0008168 GO:0003779 GO:0004175 GO:0019773 GO:0005634

Cluster-22178.59194 GO:0009405 GO:0006810 GO:0016567 GO:0008200 GO:0004842 GO:0005576

Cluster-22178.59195 GO:0007264 GO:0006576 GO:0015684 GO:0005509 GO:0005524 GO:0015093 GO:0016887 GO:0003924 GO:0005525 GO:0016021

Cluster-22178.59196 GO:0035556 GO:0005515

Cluster-22178.59197 GO:0005515 GO:0008270

Cluster-22178.59198 GO:0008033 GO:0015743 GO:0051252 GO:0006412 GO:0042254 GO:0003676 GO:0003723 GO:0004526 GO:0003735 GO:0005622 GO:0005840 GO:0005634 GO:0030677

Cluster-22178.59203 GO:0031412 GO:0007034 GO:0006457 GO:0051297 GO:0004672 GO:0003677 GO:0051082 GO:0005813 GO:0016272 GO:0019028

Cluster-22178.59206 GO:0019497 GO:0006914 GO:0006810 GO:0006771 GO:0003993 GO:0046872 GO:0005515 GO:0005794

Cluster-22178.59207 GO:0055114 GO:0008168 GO:0016706

Cluster-22178.59210 GO:0005515

Cluster-22178.59211 GO:0006629 GO:0005515

Cluster-22178.59212 GO:0005739

Cluster-22178.59216 GO:0008152 GO:0019049 GO:0005975 GO:0003824
GO:0030246 GO:0016836
Cluster-22178.59217 GO:0006120 GO:0006744 GO:0008152 GO:0055114
GO:0015992 GO:0019049 GO:0005975 GO:0006814 GO:0003824
GO:0008137 GO:0016836 GO:0030246
Cluster-22178.59218 GO:0006367 GO:0032968 GO:0003677 GO:0005634
Cluster-22178.59219 GO:0015979 GO:0005515 GO:0016021
Cluster-22178.59221 GO:0006810 GO:0055085 GO:0005515 GO:0005525
GO:0005215 GO:0022857 GO:0046872 GO:0003924 GO:0016020
GO:0016012 GO:0016021
Cluster-22178.59223 GO:0034968 GO:0006479 GO:0042128 GO:0006554
GO:0018024 GO:0003677 GO:0005515 GO:0008270 GO:0000062
GO:0005634
Cluster-22178.59224 GO:0016021
Cluster-22178.59226 GO:0043631 GO:0004652
Cluster-22178.59229 GO:0055114 GO:0009058 GO:0003676 GO:0005524
GO:0016491 GO:0003677 GO:0010181 GO:0016787
Cluster-22178.59231 GO:0006810 GO:0009405 GO:0019870 GO:0005515
GO:0005576
Cluster-22178.59232 GO:0003677
Cluster-22178.59233 GO:0016021
Cluster-22178.59235 GO:0006468 GO:0016773 GO:0004672 GO:0005524
GO:0016020
Cluster-22178.59236 GO:0006468 GO:0051259 GO:0016773 GO:0005524
GO:0004672 GO:0016020
Cluster-22178.59237 GO:0005198 GO:0019013
Cluster-22178.59241 GO:0006554 GO:0019953 GO:0034968 GO:0006479
GO:0007165 GO:0000772 GO:0008270 GO:0018024 GO:0005515
GO:0005634 GO:0005576
Cluster-22178.59242 GO:0007304 GO:0005213 GO:0046872 GO:0005515
GO:0045202 GO:0016021 GO:0042600 GO:0005576
Cluster-22178.59243 GO:0007304 GO:0005213 GO:0046872 GO:0005515
GO:0045202 GO:0016021 GO:0042600 GO:0005576

Cluster-22178.59245 GO:0006810 GO:0005215 GO:0019028 GO:0016020

Cluster-22178.59246 GO:0005515

Cluster-22178.59247 GO:0007017 GO:0005875

Cluster-22178.59248 GO:0019835 GO:0009405 GO:0016567 GO:0055114 GO:0016114 GO:0046872 GO:0008685 GO:0008270 GO:0016491 GO:0004842 GO:0019867

Cluster-22178.59249 GO:0007018 GO:0007017 GO:0008017 GO:0003777 GO:0005524 GO:0005515 GO:0003723 GO:0045298 GO:0005874

Cluster-22178.59250 GO:0005515 GO:0016020

Cluster-22178.59251 GO:0006508 GO:0061025 GO:0055085 GO:0046813 GO:0035556 GO:0016805 GO:0016021 GO:0019031

Cluster-22178.59253 GO:0006633 GO:0042967 GO:0055114 GO:0004318 GO:0046872 GO:0005835

Cluster-22178.59259 GO:0043087 GO:0005085

Cluster-22178.59263 GO:0009058 GO:0016788 GO:0019015

Cluster-22178.59266 GO:0033925 GO:0005737 GO:0016021

Cluster-22178.5927 GO:0005515

Cluster-22178.59271 GO:0006457 GO:0000413 GO:0003755

Cluster-22178.59272 GO:0006457 GO:0000413 GO:0003755

Cluster-22178.59277 GO:0043087 GO:0005515 GO:0005089 GO:0005737 GO:0016021

Cluster-22178.59278 GO:0043087 GO:0005089 GO:0005515 GO:0016021 GO:0005737

Cluster-22178.59279 GO:0042254 GO:0005515 GO:0016020 GO:0016021 GO:0005730

Cluster-22178.59284 GO:0016021

Cluster-22178.59286 GO:0007165 GO:0005125 GO:0016020

Cluster-22178.59287 GO:0003676 GO:0005524

Cluster-22178.59290 GO:0006413 GO:0006446 GO:0003723 GO:0003743 GO:0005737 GO:0005840

Cluster-22178.59291 GO:0008237 GO:0008270 GO:0005783

Cluster-22178.59292 GO:0043087 GO:0035023 GO:0035556 GO:0009405 GO:0019835 GO:0005089 GO:0005515 GO:0019867

Cluster-22178.59294 GO:0006810 GO:0015991 GO:0015992 GO:0015078 GO:0030288 GO:0033177
Cluster-22178.59295 GO:0030570 GO:0005576
Cluster-22178.59297 GO:0006351 GO:0006260 GO:0006269 GO:0003896 GO:0005515 GO:0005657 GO:0005730
Cluster-22178.59299 GO:0046856 GO:0034597
Cluster-22178.59302 GO:0030246
Cluster-22178.59303 GO:0035091
Cluster-22178.59305 GO:0006508 GO:0016032 GO:0006351 GO:0006206 GO:0006144 GO:0003677 GO:0003899 GO:0005524 GO:0004222 GO:0005730
Cluster-22178.59306 GO:0005515
Cluster-22178.59307 GO:0006281 GO:0006355 GO:0019079 GO:0006310 GO:0002949 GO:0016301 GO:0008134 GO:0003723 GO:0003678 GO:0043531 GO:0098519 GO:0009378 GO:0003724 GO:0005524 GO:0016887 GO:0009379 GO:0005667 GO:0005657
Cluster-22178.59309 GO:0019722 GO:0006402 GO:0051252 GO:0046872 GO:0004535 GO:0003676 GO:0003723 GO:0005515 GO:0005737 GO:0005634
Cluster-22178.59310 GO:0003676
Cluster-22178.59312 GO:0035023 GO:0005976 GO:0043087 GO:0016779 GO:0005089 GO:0043565
Cluster-22178.59316 GO:0000105 GO:0055114 GO:0008033 GO:0050660 GO:0017150
Cluster-22178.59317 GO:0007010 GO:0035023 GO:0043087 GO:0005089 GO:0003779 GO:0043565
Cluster-22178.59318 GO:0007212 GO:0048268 GO:0019236 GO:0007186 GO:0032051 GO:0016021
Cluster-22178.5932 GO:0006281 GO:0045132 GO:0019054 GO:0005975 GO:0006260 GO:0043169 GO:0004857 GO:0003911 GO:0003824 GO:0004553 GO:0005634 GO:0000775
Cluster-22178.59324 GO:0035556 GO:0005975 GO:0006468 GO:0033691 GO:0003779 GO:0016773 GO:0005524 GO:0004672

Cluster-22178.59325 GO:0009405 GO:0050765 GO:0016567 GO:0015979
GO:0019538 GO:0005515 GO:0008270 GO:0046872 GO:0016012
GO:0005576 GO:0016020 GO:0009539 GO:0009523
Cluster-22178.59327 GO:0040007 GO:0008283 GO:0006631 GO:0007165
GO:0055114 GO:0045300 GO:0008083 GO:0045735 GO:0005576
Cluster-22178.59329 GO:0043087 GO:0005089 GO:0005515 GO:0016021
GO:0005737
Cluster-22178.59331 GO:0016021
Cluster-22178.59332 GO:0016310 GO:0009069 GO:0006468 GO:0004683
GO:0005516
Cluster-22178.59334 GO:0007131 GO:0055114 GO:0016705 GO:0000794
Cluster-22178.59337 GO:0005515
Cluster-22178.59338 GO:0055085 GO:0006397 GO:0006810 GO:0003924
GO:0016887 GO:0005524 GO:0042626 GO:0005525 GO:0016301
GO:0016021
Cluster-22178.59340 GO:0016567 GO:0061630 GO:0005515 GO:0004842
GO:0043565 GO:0046872 GO:0008270 GO:0005634 GO:0005680
Cluster-22178.59342 GO:0006950 GO:0005515
Cluster-22178.59346 GO:0006810 GO:0006614 GO:0006144 GO:0000103
GO:0002949 GO:0006260 GO:0016887 GO:0005524 GO:0003724
GO:0003723 GO:0016301 GO:0003924 GO:0043531 GO:0016765
GO:0003678 GO:0004020 GO:0005525 GO:0005657
Cluster-22178.59347 GO:0006355 GO:0003700 GO:0043565 GO:0005667
Cluster-22178.59353 GO:0006810 GO:0016021
Cluster-22178.59354 GO:0034599 GO:0005789
Cluster-22178.59356 GO:0006353 GO:0005739
Cluster-22178.59360 GO:0006468 GO:0016773 GO:0005524 GO:0004672
GO:0016020
Cluster-22178.59361 GO:0006468 GO:0006260 GO:0004672 GO:0005524
GO:0016773 GO:0016020
Cluster-22178.59363 GO:0015992 GO:0006118 GO:0006119 GO:0008121

Cluster-22178.59365 GO:0043087

Cluster-22178.59366 GO:0005515
Cluster-22178.59367 GO:0035556 GO:0005524 GO:0046983 GO:0003676
GO:0005507 GO:0016787 GO:0003677 GO:0005515 GO:0016021
GO:0016020
Cluster-22178.59368 GO:0006260 GO:0055085 GO:0000226 GO:0006810
GO:0006308 GO:0030001 GO:0007010 GO:0005524 GO:0008855
GO:0042626 GO:0003677 GO:0046872 GO:0003779 GO:0005515
GO:0005664 GO:0016021 GO:0009318 GO:0015630
Cluster-22178.59369 GO:0055085 GO:0000226 GO:0006260 GO:0006810
GO:0006308 GO:0030001 GO:0007010 GO:0008855 GO:0005524
GO:0042626 GO:0003677 GO:0003779 GO:0046872 GO:0005515
GO:0016021 GO:0005664 GO:0015630 GO:0009318
Cluster-22178.5937 GO:0007165 GO:0005515
Cluster-22178.59370 GO:0006448 GO:0006414 GO:0055114 GO:0016706
GO:0003677 GO:0005515 GO:0003746 GO:0005853 GO:0005840
Cluster-22178.59374 GO:0006807 GO:0016810
Cluster-22178.59376 GO:0006730 GO:0006555 GO:0004013
Cluster-22178.59377 GO:0035556 GO:0005515
Cluster-22178.59378 GO:0003677
Cluster-22178.59379 GO:0016021
Cluster-22178.59380 GO:0006464 GO:0046500 GO:0006479 GO:0008152
GO:0031167 GO:0004719 GO:0008168
Cluster-22178.59382 GO:0006468 GO:0004672 GO:0005524 GO:0016773
GO:0016020
Cluster-22178.59383 GO:0006468 GO:0004672 GO:0005524 GO:0016773
GO:0016020
Cluster-22178.59384 GO:0006351 GO:0006206 GO:0006144 GO:0003677
GO:0003899 GO:0005730
Cluster-22178.59385 GO:0006812 GO:0015672 GO:0005261 GO:0016020
Cluster-22178.59386 GO:0009405 GO:0003676 GO:0043022 GO:0016788
GO:0003723 GO:0046872 GO:0005634
Cluster-22178.59387 GO:0009451 GO:0001522 GO:0009982 GO:0003723

Cluster-22178.59388 GO:0009451 GO:0001522 GO:0009982 GO:0003723

Cluster-22178.59390 GO:0046872
Cluster-22178.59392 GO:0009512
Cluster-22178.59394 GO:0006508 GO:0004198 GO:0005509 GO:0005622
Cluster-22178.59396 GO:0005515
Cluster-22178.59397 GO:0005201 GO:0005515 GO:0005578 GO:0005581
Cluster-22178.594 GO:0006476 GO:0006355 GO:0006342 GO:0006807
GO:0007304 GO:0016811 GO:0051287 GO:0017136 GO:0005213
GO:0008270 GO:0000118 GO:0005576 GO:0042600
Cluster-22178.59400 GO:0006094 GO:0006096 GO:0055114 GO:0006144
GO:0015976 GO:0004743 GO:0000287 GO:0030955 GO:0003824
Cluster-22178.59401 GO:0006352
Cluster-22178.59402 GO:0005515
Cluster-22178.59404 GO:0015948 GO:0043087 GO:0046656 GO:0030269
GO:0016020 GO:0005576
Cluster-22178.59407 GO:0006355 GO:0042967 GO:0004402 GO:0008080
GO:0005515 GO:0005634 GO:0000123
Cluster-22178.59408 GO:0006508 GO:0004252
Cluster-22178.5941 GO:0055114 GO:0016491 GO:0050660 GO:0005515
Cluster-22178.59411 GO:0003723
Cluster-22178.59415 GO:0015694 GO:0006364 GO:0015097 GO:0046872
GO:0008270 GO:0016020 GO:0005730
Cluster-22178.59418 GO:0019251 GO:0009877 GO:0008152 GO:0006783
GO:0009312 GO:0016852 GO:0008168 GO:0008757
Cluster-22178.59419 GO:0042254 GO:0006412 GO:0005515 GO:0003735
GO:0005622 GO:0005840
Cluster-22178.59420 GO:0006308 GO:0016888
Cluster-22178.59422 GO:0005515
Cluster-22178.59425 GO:0055085 GO:0006260 GO:0006810 GO:0003678
GO:0003676 GO:0042626 GO:0005525 GO:0003924 GO:0016887
GO:0005524 GO:0016021 GO:0005657
Cluster-22178.59426 GO:0005515

Cluster-22178.59427 GO:0055085 GO:0006260 GO:0015074 GO:0006810 GO:0042626 GO:0003678 GO:0003676 GO:0005525 GO:0016887 GO:0003924 GO:0005524 GO:0016021 GO:0005657

Cluster-22178.59428 GO:0008295 GO:0010508 GO:0006560 GO:0006525 GO:0006357 GO:0001104 GO:0004014 GO:0008092 GO:0016592 GO:0019898 GO:0016021 GO:0005737

Cluster-22178.59430 GO:0042742 GO:0006281 GO:0006974 GO:0005975 GO:0004553 GO:0030248 GO:0005634 GO:0005576 GO:0070876

Cluster-22178.59431 GO:0006554 GO:0034968 GO:0006479 GO:0008270 GO:0018024 GO:0005515 GO:0016021 GO:0005634

Cluster-22178.59432 GO:0006486 GO:0016192 GO:0006886 GO:0005198 GO:0005515 GO:0016757 GO:0008378 GO:0030117 GO:0016020

Cluster-22178.59433 GO:0007264 GO:0016787 GO:0005525 GO:0005515

Cluster-22178.59434 GO:0006471 GO:0003950

Cluster-22178.59438 GO:0007218 GO:0005576

Cluster-22178.59439 GO:0031167 GO:0006351 GO:0006479 GO:0008152 GO:0006144 GO:0006206 GO:0003899 GO:0003677 GO:0008168 GO:0005730

Cluster-22178.59440 GO:0055114 GO:0008152 GO:0006694 GO:0008210 GO:0008209 GO:0008207 GO:0016491 GO:0003824 GO:0050662 GO:0003854 GO:0016616

Cluster-22178.59442 GO:0016310 GO:0019087 GO:0007178 GO:0009069 GO:0006468 GO:0005026 GO:0005524 GO:0046872 GO:0003677 GO:0016021 GO:0016020

Cluster-22178.59444 GO:0046872

Cluster-22178.59445 GO:0042254 GO:0006412 GO:0006810 GO:0003735 GO:0005622 GO:0016021 GO:0005840

Cluster-22178.59446 GO:0006508 GO:0009446 GO:0016579 GO:0006807 GO:0005515 GO:0004843 GO:0036459 GO:0004668

Cluster-22178.59449 GO:0006506 GO:0006468 GO:0005515 GO:0004386 GO:0005524 GO:0004672 GO:0016773 GO:0016020 GO:0031083 GO:0005789

Cluster-22178.59450 GO:0030001 GO:0055085 GO:0046873 GO:0016020
Cluster-22178.59451 GO:0055085 GO:0006506 GO:0006468 GO:0004386
GO:0016773 GO:0004672 GO:0005524 GO:0005515 GO:0005789
GO:0016020 GO:0031083
Cluster-22178.59453 GO:0051287 GO:0008191
Cluster-22178.59454 GO:0006355 GO:0006281 GO:0006310 GO:0019079
GO:0002949 GO:0016301 GO:0008134 GO:0003723 GO:0003678
GO:0009378 GO:0003724 GO:0016887 GO:0005524 GO:0005667
GO:0009379 GO:0005657
Cluster-22178.59456 GO:0016746
Cluster-22178.59459 GO:0016192 GO:0006886 GO:0016567 GO:0005515
GO:0004842 GO:0046872 GO:0008270 GO:0005680
Cluster-22178.59460 GO:0006812 GO:0008324
Cluster-22178.59461 GO:0006508 GO:0004222
Cluster-22178.59464 GO:0008285 GO:0006396 GO:0045859 GO:0006915
GO:0007050 GO:0004860 GO:0005515 GO:0016021 GO:0000176
GO:0005576
Cluster-22178.59467 GO:0000166 GO:0046872
Cluster-22178.59468 GO:0006144 GO:0006508 GO:0006614 GO:0016817
GO:0008312 GO:0003968 GO:0004197 GO:0017111 GO:0048500
GO:0031379
Cluster-22178.59469 GO:0006508 GO:0004198 GO:0005515 GO:0005622
Cluster-22178.59471 GO:0007049 GO:0051382 GO:0044780 GO:0008239
GO:0070009 GO:0016021 GO:0000776 GO:0005634
Cluster-22178.59473 GO:0006508 GO:0016805
Cluster-22178.59474 GO:0003677 GO:0046983
Cluster-22178.59476 GO:0016567 GO:0006118 GO:0005515 GO:0020037
GO:0003723 GO:0009055 GO:0004842 GO:0046872 GO:0008270
GO:0005680
Cluster-22178.59477 GO:0010972 GO:0030336 GO:0016579 GO:0036459

Cluster-22178.59478 GO:0016773
Cluster-22178.59480 GO:0016567 GO:0004842 GO:0005515 GO:0004867

Cluster-22178.59482 GO:0005515
Cluster-22178.59483 GO:0005515
Cluster-22178.59488 GO:0005975 GO:0040007 GO:0008283 GO:0042710 GO:0007165 GO:0008270 GO:0003676 GO:0003824 GO:0030674 GO:0043169 GO:0008083 GO:0051015
Cluster-22178.59489 GO:0007186 GO:0007165 GO:0015684 GO:0007264 GO:0004871 GO:0005525 GO:0003924 GO:0031683 GO:0008270 GO:0015093 GO:0019001 GO:0016021
Cluster-22178.59490 GO:0005975 GO:0003824 GO:0043169
Cluster-22178.59492 GO:0006122 GO:0008242 GO:0005750
Cluster-22178.59494 GO:0007076 GO:0000796
Cluster-22178.59496 GO:0006508 GO:0008152 GO:0016787 GO:0008236

Cluster-22178.59497 GO:0006508 GO:0008152 GO:0016787 GO:0008236

Cluster-22178.59499 GO:0003824
Cluster-22178.59500 GO:0030246
Cluster-22178.59501 GO:0019904 GO:0005515 GO:0030246 GO:0005737
Cluster-22178.59502 GO:0007349 GO:0048598 GO:0072669 GO:0016020 GO:0005737
Cluster-22178.59504 GO:0006464 GO:0006554 GO:0008033 GO:0008152 GO:0046500 GO:0006400 GO:0006479 GO:0006886 GO:0016192 GO:0009451 GO:0004719 GO:0008168 GO:0018024 GO:0008176 GO:0030117
Cluster-22178.59508 GO:0007165 GO:0009069 GO:0006468 GO:0035556 GO:0016310 GO:0005198 GO:0017048 GO:0004674 GO:0005524 GO:0004672 GO:0005882
Cluster-22178.59509 GO:0003779 GO:0005515 GO:0005622
Cluster-22178.59510 GO:0034453 GO:0016021 GO:0005815
Cluster-22178.59511 GO:0006629 GO:0055085 GO:0006891 GO:0022857 GO:0005515 GO:0017119 GO:0016021
Cluster-22178.59514 GO:0020037

Cluster-22178.59515 GO:0009401 GO:0007186 GO:0006629 GO:0004930
GO:0005515 GO:0016021
Cluster-22178.59516 GO:0030250 GO:0005576
Cluster-22178.59517 GO:0004867
Cluster-22178.59518 GO:0030866 GO:0008092 GO:0005856
Cluster-22178.59519 GO:0006520 GO:0005515
Cluster-22178.59520 GO:0016567 GO:0061630 GO:0005515 GO:0043565
GO:0046872 GO:0008270 GO:0004842 GO:0005634 GO:0005680
Cluster-22178.59521 GO:0006694 GO:0008152 GO:0016114 GO:0030976
GO:0008661 GO:0003824 GO:0016624
Cluster-22178.59523 GO:0007165 GO:0007186 GO:0019001 GO:0031683
GO:0003924 GO:0005525 GO:0004871
Cluster-22178.59525 GO:0055114 GO:0006633 GO:0005506 GO:0016491

Cluster-22178.59526 GO:0006139 GO:0006465 GO:0006206 GO:0008233
GO:0004127 GO:0005525 GO:0016887 GO:0003924 GO:0005524
GO:0016020 GO:0005911 GO:0005787 GO:0016021
Cluster-22178.59527 GO:0006465 GO:0008233 GO:0016021 GO:0005787
Cluster-22178.59528 GO:0043087
Cluster-22178.59529 GO:0043087
Cluster-22178.59532 GO:0008643 GO:0055085 GO:0005351 GO:0000139
GO:0016020 GO:0016021
Cluster-22178.59534 GO:0006355 GO:0007275 GO:0043565 GO:0003700
GO:0005667
Cluster-22178.59535 GO:0043934 GO:0007264 GO:0005525 GO:0003924
GO:0016887 GO:0005524
Cluster-22178.59536 GO:0006355 GO:0007264 GO:0003924 GO:0016887
GO:0043531 GO:0005524 GO:0003724 GO:0005525 GO:0003723
GO:0005515 GO:0003677 GO:0005622 GO:0016020
Cluster-22178.59537 GO:0007264 GO:0006355 GO:0003723 GO:0005515
GO:0003677 GO:0043531 GO:0016887 GO:0003924 GO:0005524
GO:0003724 GO:0005525 GO:0016020 GO:0005622
Cluster-22178.59539 GO:0006468 GO:0005524 GO:0004672

Cluster-22178.59542 GO:0006955 GO:0007165 GO:0006606 GO:0030247
GO:0005044
Cluster-22178.59543 GO:0042330 GO:0006508 GO:0006511 GO:0016579
GO:0004843 GO:0005622
Cluster-22178.59545 GO:0006886 GO:0006888 GO:0030127
Cluster-22178.59547 GO:0006355 GO:0006098 GO:0055114 GO:0019521
GO:0004616 GO:0050661 GO:0051287 GO:0003676 GO:0005739
GO:0005622
Cluster-22178.5955 GO:0005515
Cluster-22178.59550 GO:0005515
Cluster-22178.59552 GO:0042254 GO:0006412 GO:0008152 GO:0003735
GO:0005515 GO:0005509 GO:0016746 GO:0016747 GO:0005840
GO:0005622
Cluster-22178.59553 GO:0007018 GO:0007017 GO:0003777 GO:0005874
GO:0030286
Cluster-22178.59555 GO:0048309 GO:0009405 GO:0006886 GO:0030001
GO:0006606 GO:0030334 GO:0051028 GO:0006914 GO:0007155
GO:0045995 GO:0051258 GO:0006308 GO:0048870 GO:0000390
GO:0006355 GO:0055085 GO:0015031 GO:0000921 GO:0030168
GO:0006810 GO:0007165 GO:0019835 GO:0030155 GO:0042803
GO:0005198 GO:0015631 GO:0008565 GO:0003677 GO:0004871
GO:0008134 GO:0045502 GO:0005201 GO:0005102 GO:0008855
GO:0046873 GO:0030674 GO:0019867 GO:0045298 GO:0005737
GO:0005577 GO:0016020 GO:0005667 GO:0019031 GO:0016021
GO:0009318 GO:0031514 GO:0005578 GO:0019028 GO:0030286
GO:0005886 GO:0005581 GO:0005940 GO:0005882 GO:0071008
Cluster-22178.59557 GO:0015684 GO:0007264 GO:0005525 GO:0015093
GO:0016021 GO:0019028
Cluster-22178.59559 GO:0042184 GO:0018874 GO:0046232 GO:0019261
GO:0006508 GO:0042203 GO:0009712 GO:0042981 GO:0055114
GO:0018576 GO:0004197 GO:0005515 GO:0005506
Cluster-22178.59560 GO:0009712 GO:0042981 GO:0055114 GO:0042184
GO:0018874 GO:0046232 GO:0019261 GO:0006508 GO:0042203

GO:0018576 GO:0004197 GO:0005515 GO:0005506
Cluster-22178.59561 GO:0009228 GO:0016579 GO:0004789 GO:0036459

Cluster-22178.59562 GO:0006541 GO:0009252 GO:0046436 GO:0016787
GO:0008716 GO:0046872 GO:0005524
Cluster-22178.59563 GO:0016021 GO:0005886
Cluster-22178.59565 GO:0046872
Cluster-22178.59566 GO:0046436 GO:0009252 GO:0006541 GO:0005524
GO:0046872 GO:0008716 GO:0016787
Cluster-22178.59569 GO:0006508 GO:0015992 GO:0006123 GO:0016740
GO:0004197 GO:0016788 GO:0005524 GO:0008242 GO:0016817
GO:0016887 GO:0004129 GO:0005680 GO:0045277
Cluster-22178.59570 GO:0006355 GO:0003677
Cluster-22178.59572 GO:0006355 GO:0006281 GO:0008168 GO:0008270
GO:0005515 GO:0003677 GO:0016020
Cluster-22178.59574 GO:0019217 GO:0006508 GO:0000062
Cluster-22178.59576 GO:0006508 GO:0019217 GO:0000062
Cluster-22178.59577 GO:0006508 GO:0019217 GO:0000062
Cluster-22178.59578 GO:0006508 GO:0019217 GO:0000062
Cluster-22178.59579 GO:0005515
Cluster-22178.59583 GO:0000910 GO:0000226 GO:0008017 GO:0019028
GO:0045298
Cluster-22178.59584 GO:0016579 GO:0005515 GO:0036459
Cluster-22178.59587 GO:0019058 GO:0007165 GO:0007283 GO:0008601
GO:0000159
Cluster-22178.59588 GO:0003677
Cluster-22178.59589 GO:0006357 GO:0006308 GO:0046983 GO:0008855
GO:0001104 GO:0009318 GO:0016592 GO:0016020
Cluster-22178.5959 GO:0005515
Cluster-22178.59590 GO:0048786
Cluster-22178.59591 GO:0048786
Cluster-22178.59592 GO:0048786
Cluster-22178.59594 GO:0045893 GO:0005515 GO:0001000 GO:0003677

GO:0008270
Cluster-22178.59595 GO:0030414
Cluster-22178.59596 GO:0035444 GO:0015675 GO:0015099 GO:0046872
GO:0016021
Cluster-22178.59599 GO:0016020
Cluster-22178.59600 GO:0007010 GO:0008017 GO:0045298
Cluster-22178.59601 GO:0005515
Cluster-22178.59604 GO:0055085 GO:0016021
Cluster-22178.59606 GO:0043085 GO:0006897 GO:0000290 GO:0008047
GO:0016020
Cluster-22178.59608 GO:0006260
Cluster-22178.59609 GO:0005515
Cluster-22178.59610 GO:0007275 GO:0006810 GO:0006508 GO:0005215
GO:0005515 GO:0004198 GO:0005622 GO:0005576
Cluster-22178.59611 GO:0005515
Cluster-22178.59612 GO:0006810 GO:0007275 GO:0006508 GO:0004198
GO:0005515 GO:0005215 GO:0005576 GO:0005622
Cluster-22178.59617 GO:0008134 GO:0042803 GO:0045502 GO:0005667
GO:0030286
Cluster-22178.59618 GO:0003333 GO:0009405
Cluster-22178.59619 GO:0055085 GO:0006270 GO:0030001 GO:0003676
GO:0005524 GO:0046873 GO:0004190 GO:0019031 GO:0016021
GO:0016020
Cluster-22178.59622 GO:0008061 GO:0005643
Cluster-22178.59625 GO:0008152 GO:0003824
Cluster-22178.59626 GO:0019836 GO:0005576
Cluster-22178.59628 GO:0019836 GO:0005576
Cluster-22178.59630 GO:0051191 GO:0016042 GO:0006260 GO:0007586
GO:0006355 GO:0003700 GO:0043565 GO:0008047 GO:0003677
GO:0003887 GO:0009507 GO:0042575 GO:0005667 GO:0005576
Cluster-22178.59631 GO:0046872 GO:0000166 GO:0016820
Cluster-22178.59636 GO:0050909 GO:0005515 GO:0016021
Cluster-22178.59637 GO:0006810 GO:0005794

Cluster-22178.59638 GO:0016020
Cluster-22178.59639 GO:0005921
Cluster-22178.59641 GO:0005643
Cluster-22178.59642 GO:0006470 GO:0042967 GO:0019988 GO:0006570
GO:0006355 GO:0004725 GO:0008080 GO:0016747 GO:0043399
GO:0008138
Cluster-22178.59645 GO:0006413 GO:0007275 GO:0006446 GO:0006118
GO:0055114 GO:0045893 GO:0016706 GO:0005515 GO:0003743
GO:0020037 GO:0008270 GO:0008168 GO:0009055 GO:0005840
GO:0016021
Cluster-22178.59646 GO:0006413 GO:0007275 GO:0006118 GO:0006446
GO:0045893 GO:0003743 GO:0020037 GO:0005515 GO:0008270
GO:0009055 GO:0005840 GO:0016021
Cluster-22178.59647 GO:0007049 GO:0006308 GO:0006397 GO:0007094
GO:0006355 GO:0006555 GO:0070206 GO:0006457 GO:0003700
GO:0005525 GO:0003924 GO:0042803 GO:0008134 GO:0045502
GO:0051082 GO:0008855 GO:0003677 GO:0000445 GO:0009318
GO:0019028 GO:0016272 GO:0030286 GO:0005667
Cluster-22178.59648 GO:0046872
Cluster-22178.59649 GO:0006457 GO:0006555 GO:0006606 GO:0006886
GO:0006355 GO:0008270 GO:0008134 GO:0042803 GO:0005198
GO:0045502 GO:0051082 GO:0003700 GO:0016020 GO:0005882
GO:0005667 GO:0016272 GO:0030286 GO:0048786 GO:0005801
Cluster-22178.59650 GO:0010167 GO:0015706 GO:0005886 GO:0016021
Cluster-22178.59652 GO:0008152 GO:0016791
Cluster-22178.59654 GO:0015979 GO:0005515 GO:0016021
Cluster-22178.59655 GO:0006144 GO:0006508 GO:0006813 GO:0003968
GO:0005267 GO:0004252 GO:0017111 GO:0004197 GO:0031379
GO:0016020
Cluster-22178.59657 GO:0016579 GO:0008270 GO:0036459
Cluster-22178.59659 GO:0016579 GO:0045046 GO:0005515 GO:0032183
GO:0070140 GO:0032403 GO:0036459 GO:0008270 GO:0005779
Cluster-22178.59660 GO:0007155 GO:0010508 GO:0051015

Cluster-22178.59661 GO:0071766 GO:0016192 GO:0006446 GO:0008608
GO:0007009 GO:0071816 GO:0006355 GO:0003743 GO:0043565
GO:0019843 GO:0052636 GO:0003700 GO:0003723 GO:0005622
GO:0005667 GO:0008537 GO:0016021 GO:0005840 GO:0072686
GO:0042729
Cluster-22178.59663 GO:0071766 GO:0007009 GO:0016192 GO:0052636
GO:0003723 GO:0016021
Cluster-22178.59664 GO:0006355 GO:0004518 GO:0003677
Cluster-22178.59666 GO:0007165 GO:0006355 GO:0035091 GO:0000981
GO:0008270 GO:0005634
Cluster-22178.59668 GO:0055114 GO:0046872 GO:0005515 GO:0008017
GO:0047134 GO:0045298
Cluster-22178.59669 GO:0006744 GO:0055114 GO:0046872 GO:0016491

Cluster-22178.59670 GO:0015031 GO:0021986 GO:0005789 GO:0016021
GO:0016020
Cluster-22178.59672 GO:0006508 GO:0000910 GO:0000226 GO:0008017
GO:0004252 GO:0045298 GO:0016021
Cluster-22178.59673 GO:0042546 GO:0000226 GO:0006078 GO:0000910
GO:0008017 GO:0009276 GO:0016021 GO:0045298
Cluster-22178.59675 GO:0007186 GO:0006614 GO:0006304 GO:0007165
GO:0007264 GO:0006000 GO:0006013 GO:0004871 GO:0005525
GO:0003924 GO:0031683 GO:0019001 GO:0003873 GO:0033821
GO:0005524 GO:0019012
Cluster-22178.5968 GO:0055114 GO:0005515 GO:0046872 GO:0016702
Cluster-22178.59680 GO:0006355 GO:0008270 GO:0043565 GO:0003700
GO:0003677 GO:0005515 GO:0005815 GO:0005667 GO:0005634
Cluster-22178.59681 GO:0042742 GO:0005515 GO:0005576
Cluster-22178.59682 GO:0009405 GO:0006206 GO:0006231 GO:0004799
GO:0005576
Cluster-22178.59684 GO:0006397 GO:0043571 GO:0003774 GO:0005524
GO:0005515 GO:0004519 GO:0016459
Cluster-22178.59685 GO:0002949 GO:0055114 GO:0016301 GO:0016972

GO:0003774 GO:0005524 GO:0016459
Cluster-22178.59686 GO:0043571 GO:0006397 GO:0005524 GO:0003774
GO:0005515 GO:0004519 GO:0016459
Cluster-22178.59688 GO:0008152 GO:0016829
Cluster-22178.59689 GO:0006744 GO:0006508 GO:0007276 GO:0004252

Cluster-22178.59690 GO:0009847 GO:0016021
Cluster-22178.59691 GO:0009190 GO:0006171 GO:0035556 GO:0006007
GO:0006144 GO:0015833 GO:0000166 GO:0004016 GO:0046537
GO:0016849 GO:0003824 GO:0005524 GO:0005886
Cluster-22178.59695 GO:0006479 GO:0034968 GO:0006554 GO:0005515
GO:0003677 GO:0018024 GO:0008270 GO:0005634
Cluster-22178.59696 GO:0042254 GO:0043093 GO:0006260 GO:0006357
GO:0006412 GO:0051252 GO:0000917 GO:0006355 GO:0007049
GO:0003735 GO:0001104 GO:0003887 GO:0004523 GO:0003700
GO:0043565 GO:0016592 GO:0005667 GO:0005815 GO:0005622
GO:0042575 GO:0005840 GO:0005737
Cluster-22178.59699 GO:0006367 GO:0006366 GO:0005674
Cluster-22178.5970 GO:0055114 GO:0046872 GO:0016702 GO:0005515
Cluster-22178.59700 GO:0007169 GO:0006468 GO:0004714 GO:0005524
GO:0016020
Cluster-22178.59701 GO:0032183 GO:0070140
Cluster-22178.59703 GO:0008152 GO:0006098 GO:0055114 GO:0019521
GO:0016616 GO:0004616 GO:0051287
Cluster-22178.59704 GO:0016032 GO:0006874 GO:0006508 GO:0004197
GO:0030234 GO:0030176
Cluster-22178.59705 GO:0051028 GO:0008152 GO:0008272 GO:0008484
GO:0003824 GO:0015116 GO:0016021 GO:0005634
Cluster-22178.59706 GO:0046813 GO:0061025 GO:0005515 GO:0019031
GO:0016021
Cluster-22178.59708 GO:0005515
Cluster-22178.5971 GO:0055114 GO:0005515 GO:0016702 GO:0046872
Cluster-22178.59710 GO:0005515

Cluster-22178.59714 GO:0006817 GO:0006270 GO:0005315 GO:0016020
GO:0005887
Cluster-22178.59715 GO:0007186 GO:0006890 GO:0004930 GO:0019028
GO:0016021
Cluster-22178.59717 GO:0005515
Cluster-22178.59719 GO:0006144 GO:0007165 GO:0009405 GO:0004114
GO:0005515 GO:0019028
Cluster-22178.59720 GO:0007157 GO:0009405 GO:0016021
Cluster-22178.59721 GO:0006406 GO:0006998 GO:0006470 GO:0006611
GO:0006570 GO:0004725 GO:0008138 GO:0031965
Cluster-22178.59722 GO:0034220
Cluster-22178.59725 GO:0051260 GO:0004190 GO:0016021
Cluster-22178.59726 GO:0000079 GO:0019901
Cluster-22178.59727 GO:0006629
Cluster-22178.59728 GO:0006886 GO:0008092 GO:0019898 GO:0017119
GO:0005737
Cluster-22178.59729 GO:0006525 GO:0006560 GO:0043039 GO:0006433
GO:0006418 GO:0031412 GO:0004812 GO:0000166 GO:0005524
GO:0016876 GO:0004827 GO:0005737
Cluster-22178.59731 GO:0030474 GO:0071988 GO:0051082
Cluster-22178.59736 GO:0007264 GO:0002949 GO:0005509 GO:0005515
GO:0005525
Cluster-22178.59737 GO:0030163 GO:0006118 GO:0017004 GO:0008616
GO:0045158 GO:0008914 GO:0033739 GO:0005515 GO:0009512
Cluster-22178.59739 GO:0006886 GO:0007010 GO:0006506 GO:0003779
GO:0005515
Cluster-22178.59741 GO:0006298 GO:0006306 GO:0031167 GO:0032775
GO:0005524 GO:0008168 GO:0009007 GO:0030414 GO:0030983
GO:0005576
Cluster-22178.59745 GO:0006621 GO:0005525 GO:0003924 GO:0046923
GO:0016021 GO:0005783
Cluster-22178.59746 GO:0043044 GO:0003676 GO:0046872 GO:0016787
GO:0016586

Cluster-22178.59748 GO:0015992 GO:0055114 GO:0006120 GO:0006744
GO:0030154 GO:0006814 GO:0008137 GO:0016021
Cluster-22178.5975 GO:0008146
Cluster-22178.59750 GO:0030001 GO:0008146 GO:0046872
Cluster-22178.59752 GO:0055114 GO:0016491
Cluster-22178.59754 GO:0009755 GO:0008152 GO:0006364 GO:0016758
GO:0005515 GO:0005576 GO:0032040
Cluster-22178.59755 GO:0004869
Cluster-22178.59759 GO:0040007 GO:0008283 GO:0006955 GO:0007165
GO:0006396 GO:0008083 GO:0005143 GO:0005576 GO:0042022
Cluster-22178.59760 GO:0016192 GO:0006886 GO:0015031 GO:0008565
GO:0030117
Cluster-22178.59761 GO:0006355 GO:0044780 GO:0015991 GO:0000917
GO:0048870 GO:0006457 GO:0000921 GO:0006260 GO:0007094
GO:0010508 GO:0043093 GO:0007155 GO:0015992 GO:0006914
GO:0006606 GO:0048193 GO:0045502 GO:0008134 GO:0003700
GO:0017048 GO:0051082 GO:0005198 GO:0042803 GO:0043565
GO:0015078 GO:0016818 GO:0003677 GO:0008270 GO:0005524
GO:0019028 GO:0031514 GO:0016021 GO:0005882 GO:0030286
GO:0005940 GO:0033180 GO:0005634 GO:0005737 GO:0019867
GO:0005667 GO:0016020 GO:0016272
Cluster-22178.59763 GO:0006952 GO:0019069 GO:0042025
Cluster-22178.59764 GO:0055114 GO:0008152 GO:0006813 GO:0016491
GO:0016620 GO:0016746 GO:0071949 GO:0016021
Cluster-22178.59765 GO:0050912 GO:0007187 GO:0007607 GO:0008527
GO:0016020 GO:0016021
Cluster-22178.59767 GO:0006468 GO:0006771 GO:0019497 GO:0048013
GO:0005515 GO:0046872 GO:0004672 GO:0005524 GO:0016773
GO:0003993 GO:0016020
Cluster-22178.59775 GO:0006468 GO:0005515 GO:0005524 GO:0004672
GO:0016020
Cluster-22178.59777 GO:0006486 GO:0016567 GO:0046872 GO:0005524
GO:0008270 GO:0004842 GO:0016757 GO:0003676 GO:0016787

GO:0016818 GO:0003677 GO:0005515 GO:0005680
Cluster-22178.59778 GO:0006402 GO:0009405 GO:0051252 GO:0004535
GO:0046872 GO:0003723 GO:0005737 GO:0005634
Cluster-22178.59779 GO:0006368 GO:0006406 GO:0000446
Cluster-22178.59781 GO:0000723 GO:0006281 GO:0006614 GO:0019079
GO:0006810 GO:0016887 GO:0008270 GO:0005524 GO:0003678
GO:0003676 GO:0005525 GO:0016787 GO:0008026 GO:0003677
GO:0005657
Cluster-22178.59783 GO:0006471 GO:0007049 GO:0055114 GO:0006351
GO:0050660 GO:0003950 GO:0008270 GO:0016491 GO:0003677
GO:0017053 GO:0005634
Cluster-22178.59784 GO:0007186 GO:0006810 GO:0009307 GO:0006308
GO:0015074 GO:0003677 GO:0008200 GO:0004930 GO:0009036
GO:0016020 GO:0009359 GO:0005576
Cluster-22178.59785 GO:0006313 GO:0046872 GO:0003824
Cluster-22178.59787 GO:0009116
Cluster-22178.59788 GO:0009116
Cluster-22178.59789 GO:0008270 GO:0046872 GO:0005515 GO:0003677

Cluster-22178.59790 GO:0016567 GO:0004842 GO:0008270 GO:0005680
Cluster-22178.59791 GO:0006508 GO:0017148 GO:0007165 GO:0055085
GO:0005515 GO:0043565 GO:0008234 GO:0030598 GO:0005634
GO:0016021
Cluster-22178.59792 GO:0055114 GO:0015994 GO:0007165 GO:0006779
GO:0004109 GO:0005509 GO:0005578
Cluster-22178.59794 GO:0006367 GO:0006352 GO:0003677 GO:0005634
Cluster-22178.59796 GO:0005515
Cluster-22178.59798 GO:0015031 GO:0048193 GO:0006886 GO:0008565
GO:0005198 GO:0005737 GO:0016020 GO:0005882
Cluster-22178.59799 GO:0003677 GO:0005634
Cluster-22178.59800 GO:0003677 GO:0005634
Cluster-22178.59801 GO:0003677 GO:0005634
Cluster-22178.59803 GO:0046413 GO:0008152 GO:0016491 GO:0018836

GO:0005515 GO:0016020
Cluster-22178.59804 GO:0005515
Cluster-22178.59806 GO:0008152 GO:0051028 GO:0006897 GO:0008272
GO:0015116 GO:0003824 GO:0008484 GO:0016021 GO:0016020
GO:0005634
Cluster-22178.59807 GO:0051028 GO:0008152 GO:0008272 GO:0006897
GO:0008484 GO:0003824 GO:0015116 GO:0016021 GO:0005634
GO:0016020
Cluster-22178.59808 GO:0019087 GO:0005515 GO:0016021
Cluster-22178.59809 GO:0007160 GO:0005509 GO:0005576
Cluster-22178.59815 GO:0007155 GO:0006457 GO:0006858 GO:0055114
GO:0006260 GO:0051082 GO:0016972 GO:0005634 GO:0016272
Cluster-22178.59816 GO:0007155 GO:0006457 GO:0006858 GO:0055114
GO:0006260 GO:0051082 GO:0016972 GO:0005634 GO:0016272
Cluster-22178.59819 GO:0016567 GO:0005515 GO:0004842 GO:0008270
GO:0046872
Cluster-22178.59821 GO:0008152 GO:0016746
Cluster-22178.59823 GO:0009403 GO:0016021 GO:0016020
Cluster-22178.59824 GO:0006119 GO:0006118 GO:0055114 GO:0015992
GO:0008121 GO:0005515
Cluster-22178.59825 GO:0006508 GO:0008234
Cluster-22178.59826 GO:0000902 GO:0006308 GO:0009307 GO:0003677
GO:0009036 GO:0009359
Cluster-22178.59828 GO:0007186 GO:0015684 GO:0007165 GO:0006576
GO:0007264 GO:0004871 GO:0005525 GO:0003924 GO:0031683
GO:0005524 GO:0019001 GO:0015093 GO:0003723 GO:0016021
Cluster-22178.59829 GO:0016192 GO:0042262 GO:0003690 GO:0016021
GO:0019031
Cluster-22178.5983 GO:0007165 GO:0030168 GO:0051258 GO:0005515
GO:0005102 GO:0030674 GO:0005577
Cluster-22178.59830 GO:0009405
Cluster-22178.59833 GO:0050790 GO:0006508 GO:0004197 GO:0008234

Cluster-22178.59836 GO:0005515 GO:0005201 GO:0005581 GO:0005578
Cluster-22178.59837 GO:0006351 GO:0006269 GO:0003677 GO:0020037
GO:0005515 GO:0005198 GO:0019825 GO:0003896 GO:0005730
GO:0005634 GO:0005657
Cluster-22178.59839 GO:0006355 GO:0046872 GO:0003677 GO:0005634
Cluster-22178.59844 GO:0042254 GO:0006412 GO:0003735 GO:0005761
GO:0005840
Cluster-22178.59845 GO:0004867
Cluster-22178.59846 GO:0042384 GO:0010826 GO:0050821 GO:0015979
GO:0042301 GO:0036038 GO:0016020 GO:0005737 GO:0009523
Cluster-22178.59849 GO:0007218 GO:0009733 GO:0007186 GO:0007010
GO:0004930 GO:0003779 GO:0005509 GO:0005544 GO:0005185
GO:0016021 GO:0005576
Cluster-22178.59850 GO:0005524
Cluster-22178.59852 GO:0006281 GO:0006308 GO:0008855 GO:0004518
GO:0003677 GO:0005515 GO:0009318
Cluster-22178.59854 GO:0003723 GO:0046935 GO:0003676 GO:0005944
GO:0016021
Cluster-22178.59855 GO:0016021
Cluster-22178.59860 GO:0006464 GO:0051082 GO:0005515 GO:0016151
GO:0031072
Cluster-22178.59861 GO:0006397 GO:0030163 GO:0035556 GO:0003723
GO:0005515 GO:0008914 GO:0005634 GO:0005737
Cluster-22178.59862 GO:0006486 GO:0004576 GO:0016020 GO:0008250
GO:0016021 GO:0036128
Cluster-22178.59868 GO:0007165 GO:0043065 GO:0006144 GO:0004114
GO:0005740 GO:0016021
Cluster-22178.59869 GO:0005201 GO:0005515 GO:0005578 GO:0005581
Cluster-22178.59870 GO:0015012 GO:0006024 GO:0016021
Cluster-22178.59873 GO:0006570 GO:0006470 GO:0055114 GO:0004725
GO:0008138 GO:0033743
Cluster-22178.59874 GO:0006355 GO:0003723 GO:0003677 GO:0003676

Cluster-22178.59875 GO:0006355 GO:0003723 GO:0003676 GO:0003677

Cluster-22178.59877 GO:0006260 GO:0055085 GO:0006281 GO:0003911
GO:0046872 GO:0016787 GO:0016021
Cluster-22178.59879 GO:0019079 GO:0000045 GO:0043066 GO:0015979
GO:0043154 GO:0046872 GO:0016021 GO:0005737 GO:0071986
GO:0009523
Cluster-22178.59881 GO:0006260 GO:0006270 GO:0006355 GO:0006816
GO:0006874 GO:0003700 GO:0005219 GO:0000062 GO:0016884
GO:0016021 GO:0005622 GO:0005667
Cluster-22178.59886 GO:0007186 GO:0006536 GO:0009821 GO:0006103
GO:0009094 GO:0000162 GO:0006571 GO:0009074 GO:0004838
GO:0030170 GO:0004930 GO:0016021
Cluster-22178.59887 GO:0016226 GO:0005506 GO:0051536
Cluster-22178.59891 GO:0043401 GO:0006355 GO:0003700 GO:0008270
GO:0043565 GO:0005667 GO:0005634
Cluster-22178.59896 GO:0000917 GO:0006310 GO:0006302 GO:0043093
GO:0006355 GO:0003677 GO:0003700 GO:0043565 GO:0005634
GO:0005667 GO:0005737
Cluster-22178.59898 GO:0006355 GO:0016573 GO:0006303 GO:0000917
GO:0007155 GO:0043093 GO:0006302 GO:0006310 GO:0001503
GO:0020012 GO:0006810 GO:0043565 GO:0003700 GO:0003677
GO:0005634 GO:0005576 GO:0005737 GO:0005667
Cluster-22178.59899 GO:0000413 GO:0006457 GO:0005515 GO:0003755

Cluster-22178.59900 GO:0006468 GO:0005524 GO:0004672 GO:0016773
GO:0016020
Cluster-22178.59901 GO:0009405
Cluster-22178.59902 GO:0010506 GO:0055085 GO:0016070 GO:0006415
GO:0010508 GO:0006886 GO:0030001 GO:0006449 GO:0006376
GO:0003729 GO:0046873 GO:0016149 GO:0005515 GO:0019901
GO:0003723 GO:0051082 GO:0005685 GO:0018444 GO:0016020
GO:0005783 GO:0005615 GO:0016021 GO:0005840 GO:0005737

Cluster-22178.59904 GO:0006281 GO:0006308 GO:0008855 GO:0003677
GO:0004518 GO:0005515 GO:0009318
Cluster-22178.59906 GO:0006355 GO:0005198 GO:0003700 GO:0005667
GO:0019028
Cluster-22178.59907 GO:0042254 GO:0006412 GO:0019069 GO:0006355
GO:0003735 GO:0003677 GO:0042025 GO:0005840
Cluster-22178.59908 GO:0042254 GO:0006412 GO:0006810 GO:0046740
GO:0006260 GO:0003735 GO:0051082 GO:0016798 GO:0016020
GO:0016021 GO:0005840 GO:0005886 GO:0005622
Cluster-22178.59909 GO:0046740 GO:0042254 GO:0006412 GO:0006810
GO:0006260 GO:0051082 GO:0003735 GO:0005515 GO:0016798
GO:0016020 GO:0005622 GO:0005886 GO:0016021 GO:0005840
Cluster-22178.59912 GO:0070836 GO:0007155 GO:0005515 GO:0016021
Cluster-22178.59913 GO:0005975 GO:0004553
Cluster-22178.59916 GO:0008152 GO:0006836 GO:0055114 GO:0008218
GO:0006118 GO:0019905 GO:0016491 GO:0003995 GO:0016021
Cluster-22178.59917 GO:0006952 GO:0005576
Cluster-22178.59922 GO:0042710 GO:0016787
Cluster-22178.59923 GO:0005515
Cluster-22178.59924 GO:0003779 GO:0005198 GO:0005856
Cluster-22178.59925 GO:0003779 GO:0005198 GO:0005856
Cluster-22178.59926 GO:0006396 GO:0019058 GO:0003676 GO:0003723

Cluster-22178.59928 GO:0019521 GO:0055114 GO:0009094 GO:0046168
GO:0015940 GO:0000162 GO:0006571 GO:0006098 GO:0008677
GO:0004665 GO:0051287 GO:0008977 GO:0016616 GO:0004616
Cluster-22178.59929 GO:0016020
Cluster-22178.59931 GO:0016570 GO:0006368 GO:0003779 GO:0016593
GO:0005622
Cluster-22178.59932 GO:0007264 GO:0005525 GO:0016021 GO:0042765
Cluster-22178.59933 GO:0000184 GO:0005975 GO:0045493 GO:0006260
GO:0003677 GO:0046559 GO:0003676 GO:0046935 GO:0005576
GO:0005944 GO:0005664 GO:0032040 GO:0016021

Cluster-22178.59934 GO:0006412 GO:0006814 GO:0006120 GO:0042254
GO:0006744 GO:0006820 GO:0015992 GO:0008137 GO:0008509
GO:0043565 GO:0003735 GO:0005634 GO:0016021 GO:0005743
GO:0005840 GO:0005622
Cluster-22178.59936 GO:0007264 GO:0006576 GO:0015684 GO:0005524
GO:0015093 GO:0016887 GO:0003924 GO:0005525 GO:0016021
Cluster-22178.59937 GO:0006468 GO:0016773 GO:0005524 GO:0004672
GO:0016020
Cluster-22178.59939 GO:0006614 GO:0008312 GO:0019825 GO:0020037
GO:0030942 GO:0005786
Cluster-22178.59940 GO:0007275 GO:0009405 GO:0006511 GO:0000921
GO:0005515 GO:0061630 GO:0046872 GO:0008270 GO:0005634
GO:0016020 GO:0005940 GO:0016021
Cluster-22178.59943 GO:0006468 GO:0009069 GO:0016567 GO:0015979
GO:0016310 GO:0005515 GO:0008270 GO:0005524 GO:0004672
GO:0046872 GO:0004674 GO:0004842 GO:0030089 GO:0005840
GO:0005680
Cluster-22178.59944 GO:0006323 GO:0005515 GO:0008270 GO:0019012
Cluster-22178.59945 GO:0016310 GO:0015979 GO:0016567 GO:0009069
GO:0006468 GO:0004674 GO:0004842 GO:0005524 GO:0008270
GO:0004672 GO:0046872 GO:0005515 GO:0005840 GO:0005680
GO:0030089
Cluster-22178.59947 GO:0008152 GO:0016491
Cluster-22178.59948 GO:0007172 GO:0007165 GO:0006468 GO:0004713
GO:0004871 GO:0004672 GO:0005524 GO:0005925
Cluster-22178.59952 GO:0007010 GO:0003779
Cluster-22178.59953 GO:0043248 GO:0008616 GO:0006886 GO:0016192
GO:0016740 GO:0016853 GO:0005515 GO:0030117
Cluster-22178.59957 GO:0006355 GO:0003676
Cluster-22178.59958 GO:0006396 GO:0007186 GO:0009451 GO:0003723
GO:0004930 GO:0008173 GO:0016021
Cluster-22178.59959 GO:0007275 GO:0006614 GO:0000184 GO:0006400
GO:0048280 GO:0007218 GO:0045893 GO:0006886 GO:0008616

GO:0006281 GO:0000723 GO:0003677 GO:0016787 GO:0004386
GO:0005524 GO:0008270 GO:0005184 GO:0008479 GO:0003676
GO:0003678 GO:0005507 GO:0005525 GO:0000139 GO:0005657
GO:0005576 GO:0005737
Cluster-22178.59960 GO:0000166 GO:0046872
Cluster-22178.59961 GO:0005524
Cluster-22178.59963 GO:0016757
Cluster-22178.59965 GO:0051028 GO:0042254 GO:0005634
Cluster-22178.59966 GO:0009306 GO:0035091
Cluster-22178.59968 GO:0030001 GO:0007034
Cluster-22178.59969 GO:0016567 GO:0005515 GO:0061630 GO:0004842
GO:0008270 GO:0046872 GO:0005680
Cluster-22178.59973 GO:0046488 GO:0016307
Cluster-22178.59975 GO:0007029 GO:0016021 GO:0005789
Cluster-22178.59977 GO:0044030 GO:0005634
Cluster-22178.59978 GO:0007423 GO:0005212
Cluster-22178.59979 GO:0006508 GO:0008236
Cluster-22178.59980 GO:0003950 GO:0004190 GO:0016020 GO:0016021
GO:0005634
Cluster-22178.59983 GO:0034314 GO:0030833 GO:0030041 GO:0005885
GO:0015629
Cluster-22178.59984 GO:0015979
Cluster-22178.59987 GO:0006278 GO:0005515 GO:0004672 GO:0003964

Cluster-22178.59990 GO:0005515
Cluster-22178.59991 GO:0006355 GO:0006032 GO:0008061 GO:0008134
GO:0005515 GO:0005524 GO:0005667 GO:0009279
Cluster-22178.59993 GO:0042254 GO:0006412 GO:0015937 GO:0015940
GO:0003735 GO:0004252 GO:0004594 GO:0005524 GO:0055036
GO:0019028 GO:0005622 GO:0005840
Cluster-22178.59996 GO:0005515
Cluster-22178.59998 GO:0035556 GO:0006144 GO:0006357 GO:0006182
GO:0008152 GO:0006468 GO:0009190 GO:0046039 GO:0008270

GO:0005524 GO:0003824 GO:0004672 GO:0001104 GO:0003723
GO:0004383 GO:0016849 GO:0016592
Cluster-22178.59999 GO:0019867
Cluster-22178.60000 GO:0003676 GO:0005622
Cluster-22178.60001 GO:0006241 GO:0006206 GO:0006144 GO:0006183
GO:0006228 GO:0007155 GO:0006165 GO:0003676 GO:0004550
GO:0005515 GO:0003723
Cluster-22178.60002 GO:0006810 GO:0015858 GO:0005337 GO:0005789
GO:0016021
Cluster-22178.60003 GO:0008616 GO:0006400 GO:0008479
Cluster-22178.60004 GO:0006260 GO:0019953 GO:0006351 GO:0006364
GO:0003887 GO:0003677 GO:0005198 GO:0005664 GO:0042575
GO:0030141 GO:0005576 GO:0030688
Cluster-22178.60005 GO:0016579 GO:0036459 GO:0005515
Cluster-22178.60007 GO:0006144 GO:0007165 GO:0016567 GO:0004114
GO:0004857 GO:0004842 GO:0032040
Cluster-22178.60008 GO:0002218 GO:0032481
Cluster-22178.6001 GO:0006355 GO:0016485 GO:0006418 GO:0006352
GO:0019069 GO:0015031 GO:0000166 GO:0003700 GO:0004812
GO:0005509 GO:0005544 GO:0003677 GO:0008565 GO:0005524
GO:0016987 GO:0005667
Cluster-22178.60011 GO:0007186 GO:0007165 GO:0048870 GO:0004871
GO:0031514 GO:0005834
Cluster-22178.60014 GO:0007165 GO:0006914 GO:0046339 GO:0035556
GO:0006606 GO:0006629 GO:0009395 GO:0005509 GO:0045502
GO:0042803 GO:0008134 GO:0005515 GO:0004435 GO:0008081
GO:0005667 GO:0030286 GO:0019028
Cluster-22178.60015 GO:0006355 GO:0016787 GO:0003677 GO:0016818
GO:0008270 GO:0005524 GO:0003676 GO:0005634
Cluster-22178.60017 GO:0006750 GO:0005524 GO:0016787 GO:0004363

Cluster-22178.60018 GO:0015991 GO:0006508 GO:0006355 GO:0006629
GO:0006118 GO:0015996 GO:0015994 GO:0006811 GO:0045454

GO:0046486 GO:0015992 GO:0016042 GO:0008236 GO:0047746
GO:0046872 GO:0009055 GO:0015078 GO:0015035 GO:0016787
GO:0003677 GO:0003847 GO:0033179 GO:0019031 GO:0016020
GO:0008247 GO:0005886
Cluster-22178.60021 GO:0015991 GO:0030474 GO:0006547 GO:0006810
GO:0071988 GO:0016567 GO:0015992 GO:0045502 GO:0005515
GO:0008134 GO:0042803 GO:0046872 GO:0015078 GO:0003676
GO:0008270 GO:0004398 GO:0004842 GO:0033179 GO:0005667
GO:0030286 GO:0005886 GO:0005680
Cluster-22178.60023 GO:0000746 GO:0019867
Cluster-22178.60025 GO:0006355 GO:0007275 GO:0003700 GO:0000975
GO:0005667 GO:0042600
Cluster-22178.60029 GO:0006606 GO:0019904 GO:0005515 GO:0005737
Cluster-22178.60031 GO:0045454 GO:0006118 GO:0005515 GO:0009055
GO:0015035
Cluster-22178.60034 GO:0006396 GO:0003723
Cluster-22178.60037 GO:0006269 GO:0006952 GO:0006351 GO:0055114
GO:0008152 GO:0016846 GO:0008270 GO:0033743 GO:0004386
GO:0003896 GO:0005730 GO:0005657
Cluster-22178.60038 GO:0035556 GO:0009405 GO:0046488 GO:0046872
GO:0005524 GO:0016307 GO:0005198
Cluster-22178.60039 GO:0003676
Cluster-22178.60040 GO:0006534 GO:0042967 GO:0006535 GO:0005515
GO:0009001 GO:0016757 GO:0005737
Cluster-22178.60042 GO:0000226 GO:0006814 GO:0090063 GO:0006813
GO:0000922 GO:0005815 GO:0005890
Cluster-22178.60044 GO:0009405 GO:0006355 GO:0005515 GO:0005634
Cluster-22178.60045 GO:0004045
Cluster-22178.60049 GO:0009276 GO:0016021
Cluster-22178.60053 GO:0042147 GO:0015031 GO:0003723 GO:0008565
GO:0030904
Cluster-22178.60054 GO:0006396 GO:0006260 GO:0006807 GO:0006144
GO:0055114 GO:0004000 GO:0003723

Cluster-22178.60056 GO:0003676 GO:0005515
Cluster-22178.60057 GO:0006468 GO:0005524 GO:0004672 GO:0005789
Cluster-22178.60058 GO:0009451 GO:0031167 GO:0001510 GO:0009452
GO:0006396 GO:0008033 GO:0008173 GO:0003723 GO:0008270
GO:0004809 GO:0008168 GO:0016021
Cluster-22178.60059 GO:0003723
Cluster-22178.60061 GO:0004672
Cluster-22178.60063 GO:0006096 GO:0046486 GO:0006094 GO:0006020
GO:0008152 GO:0006013 GO:0006000 GO:0015976 GO:0004807
Cluster-22178.60065 GO:0006511 GO:0016747
Cluster-22178.60066 GO:0006357 GO:0001104 GO:0016592
Cluster-22178.60069 GO:0042967 GO:0016747 GO:0008080 GO:0005515

Cluster-22178.60073 GO:0005515
Cluster-22178.60079 GO:0005515
Cluster-22178.60080 GO:0006357 GO:0006468 GO:0007172 GO:0007165
GO:0004713 GO:0004871 GO:0005198 GO:0001104 GO:0019031
GO:0019028 GO:0005925 GO:0016592 GO:0031083
Cluster-22178.60081 GO:0006511 GO:0051603 GO:0004298 GO:0004175
GO:0019773 GO:0005839
Cluster-22178.60082 GO:0006812 GO:0015948 GO:0046656 GO:0005515
GO:0008324 GO:0030269 GO:0016021
Cluster-22178.60083 GO:0003779
Cluster-22178.60084 GO:0016817 GO:0008168
Cluster-22178.60085 GO:0055114 GO:0003677 GO:0016706 GO:0005515

Cluster-22178.60086 GO:0009058 GO:0008152 GO:0006629 GO:0016788
GO:0016787
Cluster-22178.60087 GO:0007264 GO:0055114 GO:0006811 GO:0005216
GO:0010181 GO:0016491 GO:0005525 GO:0016020 GO:0005615
Cluster-22178.60088 GO:0055114 GO:0045454
Cluster-22178.60090 GO:0006359 GO:0046872 GO:0008270
Cluster-22178.60091 GO:0016021

Cluster-22178.60092 GO:0009069 GO:0016310 GO:0004674 GO:0005515
GO:0016021
Cluster-22178.60094 GO:0016787
Cluster-22178.60097 GO:0007155 GO:0042157 GO:0006869 GO:0008270
GO:0005515 GO:0008289 GO:0005576
Cluster-22178.60099 GO:0003824
Cluster-22178.6010 GO:0006813 GO:0006814 GO:0005890
Cluster-22178.60100 GO:0050790 GO:0006508 GO:0007165 GO:0006955
GO:0030247 GO:0016772 GO:0008234 GO:0030414 GO:0005044
GO:0004197 GO:0016301
Cluster-22178.60102 GO:0016567 GO:0004842 GO:0005515 GO:0046872
GO:0008270 GO:0003723 GO:0005680
Cluster-22178.60103 GO:0035556 GO:0005515
Cluster-22178.60107 GO:0005515
Cluster-22178.60110 GO:0016192 GO:0006886 GO:0015979 GO:0010207
GO:0004045
Cluster-22178.60111 GO:0016192 GO:0006886 GO:0005198 GO:0005515
GO:0030117
Cluster-22178.60112 GO:0006629
Cluster-22178.60114 GO:0000921 GO:0030168 GO:0051262 GO:0006817
GO:0043093 GO:0007165 GO:0051258 GO:0006355 GO:0000917
GO:0005102 GO:0030674 GO:0004871 GO:0005198 GO:0005940
GO:0005577 GO:0016020 GO:0005737 GO:0019031 GO:0016021
GO:0019028 GO:0005911
Cluster-22178.60116 GO:0043401 GO:0006355
Cluster-22178.60118 GO:0006355 GO:0043401
Cluster-22178.60119 GO:1900758 GO:0003779
Cluster-22178.60121 GO:0006817 GO:0008654 GO:0055085 GO:0006812
GO:0008324 GO:0005315 GO:0016780 GO:0016020 GO:0016021
Cluster-22178.60122 GO:0007186 GO:0015684 GO:0007165 GO:0007264
GO:0004871 GO:0005525 GO:0003924 GO:0031683 GO:0008270
GO:0015093 GO:0019001 GO:0016021
Cluster-22178.60123 GO:0046488 GO:0016307 GO:0019867

Cluster-22178.60124 GO:0035556 GO:0016567 GO:0046872 GO:0008270
GO:0004842 GO:0005515 GO:0005680
Cluster-22178.60128 GO:0045881 GO:0032183 GO:0070140 GO:0005515

Cluster-22178.60129 GO:0045881 GO:0070140 GO:0032183 GO:0005515

Cluster-22178.60132 GO:0006260 GO:0007264 GO:0007165 GO:0007186
GO:0006310 GO:0006355 GO:0006281 GO:0006576 GO:0005524
GO:0016887 GO:0003724 GO:0009378 GO:0003677 GO:0098519
GO:0019001 GO:0031683 GO:0003924 GO:0043531 GO:0005525
GO:0004871 GO:0003723 GO:0008134 GO:0005657 GO:0005667
GO:0009379
Cluster-22178.60133 GO:0016070 GO:0009271 GO:0006355 GO:0003677
GO:0005515 GO:0003723
Cluster-22178.60135 GO:0006308 GO:0048870 GO:0007049 GO:0006355
GO:0030474 GO:0070206 GO:0008053 GO:0006511 GO:0007275
GO:0006606 GO:0071988 GO:0046872 GO:0003700 GO:0045502
GO:0008134 GO:0008855 GO:0003924 GO:0003723 GO:0005198
GO:0005515 GO:0042803 GO:0005524 GO:0008270 GO:0004386
GO:0016021 GO:0019031 GO:0019028 GO:0071203 GO:0031514
GO:0009318 GO:0030286 GO:0005634 GO:0005667 GO:0005741
Cluster-22178.60137 GO:0071203
Cluster-22178.60138 GO:0007283 GO:0005515
Cluster-22178.60140 GO:0009405 GO:0033644
Cluster-22178.60141 GO:0006468 GO:0004672 GO:0005524
Cluster-22178.60142 GO:0006355 GO:0043565 GO:0008270 GO:0003700
GO:0005667
Cluster-22178.60143 GO:0045454 GO:0006355 GO:0006118 GO:0009401
GO:0051087 GO:0043565 GO:0015035 GO:0009055 GO:0003700
GO:0005667 GO:0016021
Cluster-22178.60144 GO:0005515 GO:0005737
Cluster-22178.60145 GO:0009058 GO:0042432 GO:0006355 GO:0016114
GO:0005515 GO:0004064 GO:0003676 GO:0016844

Cluster-22178.60146 GO:0009405 GO:0006886 GO:0009069 GO:0016310
GO:0005515 GO:0004674 GO:0008536 GO:0005576
Cluster-22178.60148 GO:0043087 GO:0016070 GO:0019013 GO:0016021
Cluster-22178.60150 GO:0008610 GO:0006633 GO:0008152 GO:0042967
GO:0004315 GO:0005524 GO:0016747 GO:0005835
Cluster-22178.60151 GO:0005856
Cluster-22178.60153 GO:0006357 GO:0001104 GO:0016592
Cluster-22178.60155 GO:0008643 GO:0042597
Cluster-22178.60158 GO:0006118 GO:0009055 GO:0020037
Cluster-22178.60161 GO:0006952 GO:0016772 GO:0005576
Cluster-22178.60162 GO:0009306 GO:0035091
Cluster-22178.60164 GO:0006810 GO:0042254 GO:0006310 GO:0009069
GO:0002949 GO:0006206 GO:0006412 GO:0006281 GO:0016310
GO:0006139 GO:0098519 GO:0003735 GO:0009378 GO:0004674
GO:0004127 GO:0003724 GO:0005524 GO:0016887 GO:0016301
GO:0003723 GO:0005525 GO:0003924 GO:0009379 GO:0005657
GO:0005622 GO:0005840
Cluster-22178.60165 GO:0008092 GO:0019898 GO:0005737
Cluster-22178.60169 GO:0045859 GO:0006468 GO:0005524 GO:0004672
GO:0004860 GO:0005515 GO:0005576
Cluster-22178.60171 GO:0051252 GO:0006810 GO:0007264 GO:0006396
GO:0003677 GO:0016787 GO:0003723 GO:0005525 GO:0004525
GO:0005524 GO:0005576
Cluster-22178.60172 GO:0006468 GO:0005524 GO:0004672
Cluster-22178.60173 GO:0008270 GO:0003676
Cluster-22178.60175 GO:0000226 GO:0000910 GO:0008017 GO:0045298
GO:0019028
Cluster-22178.60176 GO:0005515
Cluster-22178.60177 GO:0010508 GO:0015991 GO:0007264 GO:0008053
GO:0015992 GO:0005525 GO:0015078 GO:0016887 GO:0003924
GO:0005524 GO:0033179 GO:0005741 GO:0019013 GO:0016021
Cluster-22178.60178 GO:0006313 GO:0015031 GO:0006464 GO:0015074
GO:0005515 GO:0003677 GO:0005634

Cluster-22178.6018 GO:0016021
Cluster-22178.60182 GO:0016192 GO:0007165 GO:0030168 GO:0000921
GO:0048193 GO:0006891 GO:0006886 GO:0051258 GO:0030674
GO:0005102 GO:0008375 GO:0005515 GO:0005198 GO:0005507
GO:0017119 GO:0016020 GO:0005940 GO:0005577 GO:0019028
GO:0005801 GO:0016021 GO:0019031
Cluster-22178.60183 GO:0043087 GO:0006260 GO:0006269 GO:0007096
GO:0006351 GO:0003896 GO:0005515 GO:0005657 GO:0005634
GO:0005730
Cluster-22178.60185 GO:0055114 GO:0006779 GO:0017022 GO:0016459
Cluster-22178.60188 GO:2001070
Cluster-22178.60189 GO:0007275 GO:0005515 GO:0061630 GO:0008270
GO:0046872 GO:0042600
Cluster-22178.60191 GO:0045454 GO:0006355 GO:0006118 GO:0006955
GO:0000902 GO:0009401 GO:0051087 GO:0043565 GO:0003700
GO:0015035 GO:0009055 GO:0005667 GO:0016020 GO:0016021
Cluster-22178.60193 GO:0019031
Cluster-22178.60199 GO:0006897 GO:0003676 GO:0016020
Cluster-22178.60202 GO:0016757
Cluster-22178.60204 GO:0016757 GO:0046872
Cluster-22178.60206 GO:0045859 GO:0006355 GO:0007264 GO:0006468
GO:0007050 GO:0005096 GO:0043565 GO:0005524 GO:0004672
GO:0003700 GO:0005515 GO:0004861 GO:0005667 GO:0005634
Cluster-22178.60212 GO:0006810 GO:0005215 GO:0016020
Cluster-22178.60213 GO:0005737 GO:0005634
Cluster-22178.60215 GO:0015979 GO:0005515 GO:0016773
Cluster-22178.60216 GO:0000226 GO:0006914 GO:0007165 GO:0008283
GO:0048870 GO:0000910 GO:0009405 GO:0006355 GO:0040007
GO:0008017 GO:0015631 GO:0005096 GO:0003700 GO:0043565
GO:0008083 GO:0042803 GO:0008134 GO:0005198 GO:0051082
GO:0045502 GO:0030286 GO:0005882 GO:0016020 GO:0005667
GO:0045298 GO:0031514
Cluster-22178.60217 GO:0008270

Cluster-22178.60218 GO:0006367 GO:0015979 GO:0032968 GO:0003677
GO:0005634
Cluster-22178.60221 GO:0055114 GO:0070403 GO:0008270 GO:0047134
GO:0005515
Cluster-22178.60222 GO:0006629 GO:0046339 GO:0009395 GO:0007165
GO:0035556 GO:0004435
Cluster-22178.60223 GO:0007165 GO:0009069 GO:0042742 GO:0035556
GO:0016042 GO:0046339 GO:0006570 GO:0006468 GO:0006629
GO:0016310 GO:0009395 GO:0055114 GO:0005509 GO:0004511
GO:0004674 GO:0046935 GO:0005096 GO:0005515 GO:0004435
GO:0005944 GO:0005576
Cluster-22178.60224 GO:0005515
Cluster-22178.60225 GO:0055114 GO:0006231 GO:0006206 GO:0006118
GO:0016491 GO:0051536 GO:0016625 GO:0009055 GO:0004799
GO:0005634
Cluster-22178.60226 GO:0007186 GO:0004930 GO:0016021
Cluster-22178.60227 GO:0005975 GO:0003676 GO:0016773 GO:0008184

Cluster-22178.60230 GO:0007166 GO:0007165 GO:0008270 GO:0046872
GO:0004871 GO:0016787 GO:0005509 GO:0005515 GO:0005634
Cluster-22178.60231 GO:0006810 GO:0005622
Cluster-22178.60232 GO:0005515
Cluster-22178.60235 GO:0005515
Cluster-22178.60238 GO:0006811 GO:0006355 GO:0009405 GO:0043565
GO:0003700 GO:0005216 GO:0005515 GO:0005667 GO:0005576
Cluster-22178.60240 GO:0015992 GO:0007264 GO:0015684 GO:0007165
GO:0007186 GO:0015991 GO:0006119 GO:0015093 GO:0046961
GO:0019001 GO:0031683 GO:0003924 GO:0005525 GO:0004871
GO:0005515 GO:0033178 GO:0016021 GO:0005634
Cluster-22178.60242 GO:0046907
Cluster-22178.60243 GO:0006123 GO:0009060 GO:0055114 GO:0015992
GO:0007186 GO:0006118 GO:0004129 GO:0009055 GO:0004930
GO:0020037 GO:0005506 GO:0016021 GO:0045277

Cluster-22178.60245 GO:0007165 GO:0005509 GO:0005578
Cluster-22178.60246 GO:0006122 GO:0006508 GO:0004197 GO:0005515
GO:0005743 GO:0005750
Cluster-22178.60249 GO:0006468 GO:0005515 GO:0005524 GO:0004672

Cluster-22178.60250 GO:0000917 GO:0016192 GO:0015986 GO:0043093
GO:0005975 GO:0016052 GO:0015992 GO:0055114 GO:0030246
GO:0015078 GO:0004553 GO:0016491 GO:0005737 GO:0000276
Cluster-22178.60252 GO:0006351 GO:0042128 GO:0006206 GO:0006144
GO:0055114 GO:0006807 GO:0003677 GO:0003899 GO:0005515
GO:0008942 GO:0005730
Cluster-22178.60253 GO:0006397 GO:0006470 GO:0051260 GO:0032006
GO:0004721 GO:0005515 GO:0005634
Cluster-22178.60254 GO:0006807 GO:0016814 GO:0008270
Cluster-22178.60255 GO:0006367 GO:0005672
Cluster-22178.60256 GO:0005515
Cluster-22178.60257 GO:0055114 GO:0006351 GO:0042254 GO:0006412
GO:0008270 GO:0033743 GO:0046872 GO:0003676 GO:0003735
GO:0005840
Cluster-22178.60261 GO:0048193 GO:0016020
Cluster-22178.60262 GO:0016192 GO:0042262 GO:0003690 GO:0019031
GO:0016021
Cluster-22178.60265 GO:0071805 GO:0006813 GO:0015079 GO:0016020
Cluster-22178.60267 GO:0033014 GO:0015994 GO:0046872 GO:0004655

Cluster-22178.60269 GO:0046660 GO:0006397 GO:0003723 GO:0003676
GO:0005634
Cluster-22178.60270 GO:0005515
Cluster-22178.60271 GO:0006013 GO:0006000 GO:0006012 GO:0006098
GO:0006094 GO:0006096 GO:0003872 GO:0005945
Cluster-22178.60272 GO:0008152 GO:0016042 GO:0008203 GO:0006810
GO:0006816 GO:0042030 GO:0016298 GO:0005246 GO:0016787
GO:0016020

Cluster-22178.60273 GO:0043087 GO:0044419 GO:0035023 GO:0005515 GO:0005089 GO:0005887
Cluster-22178.60275 GO:0006355 GO:0000271 GO:0003700 GO:0016020 GO:0005622 GO:0005667
Cluster-22178.60276 GO:0008017 GO:0005515 GO:0003677 GO:0034061 GO:0045298 GO:0005634
Cluster-22178.60277 GO:0005515 GO:0042470
Cluster-22178.60278 GO:0006508 GO:0045947 GO:0045765 GO:0006144 GO:0008190 GO:0003968 GO:0008234 GO:0005737 GO:0031379
Cluster-22178.60279 GO:0009058 GO:0009878 GO:0030170 GO:0046872

Cluster-22178.60280 GO:0006355 GO:0009725 GO:0006412 GO:0042254 GO:0003677 GO:0003735 GO:0005515 GO:0005634 GO:0015934 GO:0005840
Cluster-22178.60282 GO:0006355 GO:0003700 GO:0005667 GO:0042025
Cluster-22178.60283 GO:0006771 GO:0019497 GO:0046872 GO:0003993 GO:0005515
Cluster-22178.60286 GO:0006310 GO:0005515 GO:0003677 GO:0005634
Cluster-22178.60288 GO:0015696 GO:0006468 GO:0005524 GO:0004672 GO:0008519 GO:0016021 GO:0031224 GO:0016020
Cluster-22178.60289 GO:0006281 GO:0003677 GO:0003684 GO:0034061 GO:0005634
Cluster-22178.60291 GO:0007049 GO:0051301 GO:0005509 GO:0016021
Cluster-22178.60292 GO:0006614 GO:0005525 GO:0003724 GO:0005524 GO:0003723 GO:0016887
Cluster-22178.60300 GO:0035429 GO:0015725 GO:0006486 GO:0046872 GO:0008373 GO:0016787 GO:0015128 GO:0016020
Cluster-22178.60302 GO:0009306 GO:0015031 GO:0009403 GO:0015450 GO:0008092 GO:0016020 GO:0009941 GO:0016021 GO:0019898 GO:0005737
Cluster-22178.60303 GO:0006397 GO:0008152 GO:0016788 GO:0005515 GO:0004064 GO:0003723 GO:0005634 GO:0005737
Cluster-22178.60308 GO:0005198 GO:0019028

Cluster-22178.60312 GO:0006355 GO:0009271 GO:0016070 GO:0003723
GO:0005515 GO:0003677
Cluster-22178.60313 GO:0006351 GO:0006269 GO:0003677 GO:0005515
GO:0020037 GO:0019825 GO:0003896 GO:0005730 GO:0005634
GO:0005657
Cluster-22178.60315 GO:0042981 GO:0005515
Cluster-22178.60316 GO:0006355 GO:0043093 GO:0000917 GO:0003677
GO:0043565 GO:0003700 GO:0005667 GO:0005634 GO:0005737
Cluster-22178.60317 GO:0016021
Cluster-22178.60318 GO:0030145 GO:0004177 GO:0005515
Cluster-22178.60319 GO:0007264 GO:0006355 GO:0043565 GO:0003924
GO:0005525 GO:0003700 GO:0005667
Cluster-22178.60321 GO:0055085 GO:0042998 GO:0071253 GO:0016021
GO:0005802
Cluster-22178.60323 GO:0042254 GO:0006412 GO:0003735 GO:0005840
Cluster-22178.60326 GO:0006810 GO:0009733 GO:0005515 GO:0005622
Cluster-22178.60328 GO:0046486 GO:0006027 GO:0006687 GO:0006012
GO:0006812 GO:0006820 GO:0005975 GO:0006835 GO:0017153
GO:0004565 GO:0016020 GO:0005856 GO:0009341
Cluster-22178.60329 GO:0055114 GO:0009308 GO:0023052 GO:0007165
GO:0005507 GO:0048038 GO:0008131 GO:0005125
Cluster-22178.60330 GO:0008146 GO:0046983 GO:0016021 GO:0016020
Cluster-22178.60331 GO:0046983 GO:0008146 GO:0016021
Cluster-22178.60332 GO:0006468 GO:0051016 GO:0005524 GO:0004672
GO:0016773 GO:0016020 GO:0008290
Cluster-22178.60334 GO:0007275
Cluster-22178.60335 GO:0009102 GO:0051090 GO:0006768 GO:0042410
GO:0005524 GO:0005515 GO:0005634
Cluster-22178.60342 GO:0004651
Cluster-22178.60344 GO:0006281 GO:0006260 GO:0016787 GO:0010181
GO:0003677 GO:0016818 GO:0005524 GO:0043140 GO:0003676
GO:0016874 GO:0005634 GO:0016020 GO:0005622 GO:0005657
Cluster-22178.60347 GO:0006260

Cluster-22178.60349 GO:0006470 GO:0006810 GO:0009405 GO:0006952
GO:0006570 GO:0004725 GO:0019855 GO:0046983 GO:0008138
GO:0005576
Cluster-22178.60351 GO:0045017 GO:0000160 GO:0042967 GO:0046486
GO:0005524 GO:0000156 GO:0003677 GO:0004144
Cluster-22178.60353 GO:0006486 GO:0009312 GO:0008455 GO:0008375
GO:0005795 GO:0016021
Cluster-22178.60355 GO:0006952 GO:0006313 GO:0006412 GO:0006310
GO:0015074 GO:0006471 GO:0003677 GO:0005515 GO:0008168
GO:0000150 GO:0003950
Cluster-22178.60357 GO:0016598 GO:0004057
Cluster-22178.60362 GO:0005509
Cluster-22178.60365 GO:0007155 GO:0006869 GO:0015992 GO:0006606
GO:0010508 GO:0007165 GO:0030150 GO:0030168 GO:0070206
GO:0006891 GO:0015991 GO:0044238 GO:0051258 GO:0042803
GO:0005515 GO:0005198 GO:0015078 GO:0030674 GO:0005102
GO:0045502 GO:0008134 GO:0008289 GO:0005667 GO:0005577
GO:0017119 GO:0005742 GO:0005882 GO:0030286 GO:0019028
GO:0005576 GO:0033179 GO:0016021 GO:0019031
Cluster-22178.60366 GO:0005739
Cluster-22178.60368 GO:0006508 GO:0019087 GO:0008152 GO:0008168
GO:0008236
Cluster-22178.60370 GO:0006310 GO:0006810 GO:0005515 GO:0042626
GO:0003677 GO:0000150
Cluster-22178.60372 GO:0005515
Cluster-22178.60373 GO:0006355 GO:0006351 GO:0005634
Cluster-22178.60374 GO:0006351 GO:0006355 GO:0005634
Cluster-22178.60375 GO:0008152 GO:0042967 GO:0016829 GO:0010333
GO:0016747 GO:0008080
Cluster-22178.60376 GO:0016310 GO:0006468 GO:0009069 GO:0004674
GO:0005524 GO:0004672 GO:0003774 GO:0042803 GO:0008134
GO:0005198 GO:0045502 GO:0030286 GO:0005882 GO:0005667
GO:0016459

Cluster-22178.60377 GO:0031175 GO:0030507 GO:0016818 GO:0005516
GO:0008270 GO:0005524 GO:0008091
Cluster-22178.60378 GO:0016020
Cluster-22178.60379 GO:0042803 GO:0008134 GO:0045502 GO:0016020
GO:0005667 GO:0030286
Cluster-22178.60380 GO:0015986 GO:0015992 GO:0005543 GO:0015078

Cluster-22178.60382 GO:0015031 GO:0006464 GO:0005515
Cluster-22178.60384 GO:0000160 GO:0008608 GO:0006955 GO:0019064
GO:0007165 GO:0007172 GO:0006415 GO:0008283 GO:0016310
GO:0040007 GO:0006468 GO:0006449 GO:0048193 GO:0004871
GO:0016149 GO:0008083 GO:0004713 GO:0046983 GO:0008270
GO:0005126 GO:0000155 GO:0016021 GO:0019031 GO:0009365
GO:0005840 GO:0005576 GO:0018444 GO:0005737 GO:0042729
GO:0005876 GO:0016020 GO:0005925
Cluster-22178.60386 GO:0006396 GO:0003723
Cluster-22178.60387 GO:0007275 GO:0006355 GO:0003700 GO:0000975
GO:0005667 GO:0042600
Cluster-22178.60395 GO:0006355 GO:0043093 GO:0000917 GO:0016817
GO:0043565 GO:0003700 GO:0003677 GO:0005737 GO:0005667
GO:0005634
Cluster-22178.60398 GO:0007606 GO:0007005 GO:0043565 GO:0005515
GO:0005634 GO:0031305 GO:0016021
Cluster-22178.60399 GO:0006446 GO:0005515 GO:0003743 GO:0005737
GO:0005852 GO:0005840
Cluster-22178.60400 GO:1901031 GO:0055114 GO:0051920 GO:0005634
Cluster-22178.60401 GO:0006464 GO:0015031 GO:0007186 GO:0004930
GO:0005515 GO:0016021
Cluster-22178.60402 GO:0007165 GO:0005509 GO:0005578
Cluster-22178.60403 GO:0007017 GO:0007010 GO:0006298 GO:0006364
GO:0003779 GO:0005524 GO:0030983 GO:0005634 GO:0034457
GO:0005732 GO:0005875
Cluster-22178.60405 GO:0009451 GO:0003824 GO:0051539 GO:0051536

Cluster-22178.60406 GO:0019058 GO:0005975 GO:0003824 GO:0016857
GO:0008289 GO:0051536 GO:0019031
Cluster-22178.60407 GO:0007275 GO:0016021
Cluster-22178.60408 GO:0004252 GO:0016021 GO:0005886
Cluster-22178.60409 GO:0055085 GO:0015743 GO:0030001 GO:0046873
GO:0004190 GO:0016020 GO:0016021
Cluster-22178.60410 GO:0008152 GO:0070569
Cluster-22178.60411 GO:0055114 GO:0009117 GO:0000287 GO:0016491
GO:0000166 GO:0008253 GO:0005737
Cluster-22178.60413 GO:0006355 GO:0003677
Cluster-22178.60414 GO:0000103 GO:0006144 GO:0006000 GO:0006013
GO:0006614 GO:0006777 GO:0006576 GO:0008312 GO:0005524
GO:0003677 GO:0003873 GO:0016787 GO:0004020 GO:0005525
GO:0016301 GO:0048500
Cluster-22178.60416 GO:0006570 GO:0006470 GO:0005515 GO:0008138
GO:0004725
Cluster-22178.60417 GO:0006570 GO:0006470 GO:0005515 GO:0008138
GO:0004725
Cluster-22178.60418 GO:0007165 GO:0035556 GO:0006810 GO:0005515
GO:0005215 GO:0016020 GO:0016012
Cluster-22178.60419 GO:0001522 GO:0043044 GO:0009451 GO:0009982
GO:0003723 GO:0016586
Cluster-22178.60420 GO:0009451 GO:0001522 GO:0043044 GO:0003723
GO:0009982 GO:0016586
Cluster-22178.60421 GO:0006355 GO:0003700 GO:0005515 GO:0005634
GO:0005667
Cluster-22178.60423 GO:0007165 GO:0004871 GO:0016021
Cluster-22178.60427 GO:0006508 GO:0005509 GO:0005515 GO:0004252
GO:0005576
Cluster-22178.60429 GO:0006383 GO:0006351 GO:0006206 GO:0006144
GO:0004190 GO:0003677 GO:0003899 GO:0046872 GO:0005730
GO:0005666 GO:0016021

Cluster-22178.60431 GO:0006351 GO:0055114 GO:0015774 GO:0007165
GO:0016192 GO:0006206 GO:0006144 GO:0005515 GO:0003899
GO:0051920 GO:0005509 GO:0015159 GO:0005578 GO:0016021
GO:0005730 GO:0016020
Cluster-22178.60433 GO:0030246
Cluster-22178.60435 GO:0015074 GO:0006260 GO:0055085 GO:0006810
GO:0005524 GO:0016887 GO:0003924 GO:0005525 GO:0003676
GO:0042626 GO:0003678 GO:0005657 GO:0016021
Cluster-22178.60437 GO:0006554 GO:0006479 GO:0006886 GO:0018024
GO:0030176
Cluster-22178.60440 GO:0015992 GO:0015986 GO:0003723 GO:0051082
GO:0015078 GO:0016020
Cluster-22178.60441 GO:0006810 GO:0004386 GO:0003676 GO:0005524

Cluster-22178.60443 GO:0045893 GO:0016567 GO:0004842 GO:0003676
GO:0005515 GO:0042025
Cluster-22178.60444 GO:0005515
Cluster-22178.60445 GO:0042254 GO:0006412 GO:0003735 GO:0005840
Cluster-22178.60446 GO:0007034 GO:0006952 GO:0071973 GO:0006355
GO:0000982 GO:0005198 GO:0000978 GO:0030288 GO:0005576
GO:0005634 GO:0009428
Cluster-22178.60450 GO:0055114 GO:0016491
Cluster-22178.60451 GO:0046872
Cluster-22178.60452 GO:0006144 GO:0000103 GO:0008270 GO:0005524
GO:0046872 GO:0004020 GO:0016301 GO:0005515
Cluster-22178.60453 GO:0006270 GO:0046872
Cluster-22178.60454 GO:0050765 GO:0005515
Cluster-22178.60456 GO:0007165 GO:0009405 GO:0090063 GO:0000226
GO:0048193 GO:0030168 GO:0051258 GO:0009252 GO:0016755
GO:0030674 GO:0005102 GO:0016020 GO:0005815 GO:0000922
GO:0005577
Cluster-22178.60457 GO:0034061 GO:0003677 GO:0016779 GO:0005634
Cluster-22178.60459 GO:0051056 GO:0006355 GO:0019048 GO:0003677

GO:0005515 GO:0005096

Cluster-22178.60461 GO:0009058 GO:0008152 GO:0003824 GO:0048037

Cluster-22178.60464 GO:0008152 GO:0009058 GO:0048037 GO:0003824

Cluster-22178.60465 GO:0006816 GO:0016042 GO:0006811 GO:0005262 GO:0005244 GO:0016021 GO:0019031

Cluster-22178.60467 GO:0006397 GO:0003676 GO:0016021

Cluster-22178.60470 GO:0006621 GO:0046923 GO:0005783 GO:0016021

Cluster-22178.60476 GO:0005975 GO:0004553

Cluster-22178.60479 GO:0006812 GO:0006835 GO:0006820 GO:0017153 GO:0005515 GO:0016020

Cluster-22178.60480 GO:0015992 GO:0015986 GO:0015078 GO:0000276

Cluster-22178.60481 GO:0043087 GO:0006308 GO:0006302 GO:0007155 GO:0008855 GO:0005515 GO:0009288 GO:0005634 GO:0009318

Cluster-22178.60482 GO:0003677 GO:0005524 GO:0016787

Cluster-22178.60484 GO:0006402 GO:0051252 GO:0003676 GO:0046872 GO:0004535 GO:0003723 GO:0005737 GO:0016020 GO:0005634

Cluster-22178.60485 GO:0009058 GO:0003824

Cluster-22178.60486 GO:0006810 GO:0009069 GO:0006468 GO:0035556 GO:0016310 GO:0005198 GO:0017048 GO:0004674 GO:0005215 GO:0004672 GO:0005524 GO:0003774 GO:0005882 GO:0016020 GO:0016459

Cluster-22178.60489 GO:0006098 GO:0006144 GO:0009165 GO:0004749 GO:0000287

Cluster-22178.60491 GO:0051287

Cluster-22178.60493 GO:0051287

Cluster-22178.60496 GO:0000062

Cluster-22178.60497 GO:0016567 GO:0006511 GO:0006915 GO:0001836 GO:0008608 GO:0006974 GO:0006919 GO:0034450 GO:0004842 GO:0042729 GO:0000151 GO:0005876

Cluster-22178.6050 GO:0018149 GO:0007155 GO:0017111 GO:0005515 GO:0016021

Cluster-22178.60503 GO:0016043 GO:0000398 GO:0003779 GO:0005681
Cluster-22178.60504 GO:0007010 GO:0006508 GO:0055114 GO:0016972
GO:0008233 GO:0003779 GO:0046872 GO:0005634
Cluster-22178.60505 GO:0035556 GO:0005515
Cluster-22178.60508 GO:0042254 GO:0006412 GO:0006508 GO:0004198
GO:0005509 GO:0003735 GO:0005840 GO:0005622
Cluster-22178.60512 GO:0005515 GO:0046872
Cluster-22178.60513 GO:0006813 GO:0006814 GO:0005890
Cluster-22178.60514 GO:0046983 GO:0005515
Cluster-22178.60515 GO:0007186 GO:0004930 GO:0016021
Cluster-22178.60516 GO:0030168 GO:0008360 GO:0043093 GO:0007165
GO:0051258 GO:0007049 GO:0006355 GO:0000917 GO:0005102
GO:0030674 GO:0003677 GO:0043565 GO:0003700 GO:0005577
GO:0005667 GO:0016020 GO:0005737 GO:0005634
Cluster-22178.60521 GO:0016310 GO:0009069 GO:0006468 GO:0004674
GO:0004672 GO:0005524 GO:0016773 GO:0016020
Cluster-22178.60522 GO:0006810 GO:0006024 GO:0015012 GO:0005515
GO:0003723 GO:0042626 GO:0005524 GO:0016021
Cluster-22178.60524 GO:0055085 GO:0030234 GO:0016020 GO:0016021
Cluster-22178.60525 GO:0008270 GO:0005515
Cluster-22178.60526 GO:0003676 GO:0003723
Cluster-22178.60527 GO:0003723 GO:0003676
Cluster-22178.60530 GO:0042981 GO:0005515
Cluster-22178.60532 GO:0006508 GO:0004197
Cluster-22178.60533 GO:0006508 GO:0004197
Cluster-22178.60536 GO:0006144 GO:0006807 GO:0006396 GO:0003723
GO:0004000
Cluster-22178.60537 GO:0006144 GO:0006396 GO:0006807 GO:0003723
GO:0004000
Cluster-22178.60538 GO:0006687 GO:0050290
Cluster-22178.60539 GO:0007186 GO:0006144 GO:0006807 GO:0006396
GO:0003723 GO:0004000 GO:0004930 GO:0016020
Cluster-22178.6054 GO:0019031

Cluster-22178.60542 GO:0005515
Cluster-22178.60544 GO:0006570 GO:0042967 GO:0006470 GO:0005515
GO:0008080 GO:0004725 GO:0008138
Cluster-22178.60547 GO:0055114 GO:0016616 GO:0016491
Cluster-22178.60548 GO:0043547 GO:0005096 GO:0046983
Cluster-22178.60549 GO:0007264 GO:0007186 GO:0006614 GO:0007165
GO:0006576 GO:0006355 GO:0003677 GO:0016887 GO:0005524
GO:0008270 GO:0005515 GO:0003924 GO:0031683 GO:0019001
GO:0004871 GO:0005525
Cluster-22178.60550 GO:0043547 GO:0046983 GO:0005096
Cluster-22178.60551 GO:0000746 GO:0006118 GO:0005515 GO:0003677
GO:0016491 GO:0009055 GO:0005634 GO:0016020
Cluster-22178.60552 GO:0043547 GO:0005096 GO:0046983
Cluster-22178.60553 GO:0005524 GO:0003774 GO:0016301 GO:0005515
GO:0016459
Cluster-22178.60554 GO:0055114 GO:0008152 GO:0016491 GO:0005515

Cluster-22178.60555 GO:0055114 GO:0004659 GO:0016021 GO:0016020
Cluster-22178.60561 GO:0003677
Cluster-22178.60562 GO:0006369 GO:0006378 GO:0016310 GO:0006379
GO:0009069 GO:0016773 GO:0016765 GO:0004674 GO:0008144
GO:0005515 GO:0003677 GO:0005849
Cluster-22178.60563 GO:0006914 GO:0006355 GO:0003700 GO:0043565
GO:0005667
Cluster-22178.60567 GO:0006974 GO:0006915 GO:0006919 GO:0001836

Cluster-22178.60569 GO:0019082 GO:0008270 GO:0003676 GO:0003677

Cluster-22178.60574 GO:0055114 GO:0016114 GO:0006694 GO:0004506
GO:0050660 GO:0016021
Cluster-22178.60578 GO:0035556 GO:0046872 GO:0005515 GO:0045202
Cluster-22178.60583 GO:0055114 GO:0006357 GO:0005506 GO:0001104
GO:0020037 GO:0016705 GO:0016592

Cluster-22178.60584 GO:0006368 GO:0006357 GO:0055114 GO:0006406 GO:0005506 GO:0020037 GO:0016705 GO:0001104 GO:0016592 GO:0000446
Cluster-22178.60587 GO:0016746
Cluster-22178.60589 GO:0008152 GO:0015066 GO:0008168
Cluster-22178.60590 GO:0055114 GO:0008168 GO:0016491 GO:0016706 GO:0003676
Cluster-22178.60591 GO:0008152 GO:0008168 GO:0015066
Cluster-22178.60592 GO:0006144 GO:0006526 GO:0006531 GO:0006529 GO:0006522 GO:0008152 GO:0008033 GO:0006541 GO:0006536 GO:0006560 GO:0006164 GO:0006177 GO:0005524 GO:0004066 GO:0016787 GO:0004055 GO:0003824 GO:0003922 GO:0004810
Cluster-22178.60595 GO:0043565
Cluster-22178.60596 GO:0035556 GO:0015979 GO:0005515 GO:0016020 GO:0009523 GO:0009539
Cluster-22178.60597 GO:0003677 GO:0005634
Cluster-22178.60598 GO:0006511 GO:0007275 GO:0046872 GO:0005634
Cluster-22178.60599 GO:0007275 GO:0006536 GO:0006511 GO:0006571 GO:0009074 GO:0000162 GO:0006103 GO:0009094 GO:0009821 GO:0004838 GO:0030170 GO:0046872 GO:0005634
Cluster-22178.60601 GO:0005515
Cluster-22178.60606 GO:0007186 GO:0055114 GO:0004930 GO:0016972 GO:0046872 GO:0016021
Cluster-22178.60612 GO:0006355 GO:0006468 GO:0008152 GO:0005515 GO:0003824 GO:0004672 GO:0005524 GO:0005634
Cluster-22178.60614 GO:0008152 GO:0006468 GO:0006355 GO:0004672 GO:0005524 GO:0003824 GO:0005515 GO:0005634
Cluster-22178.60615 GO:0006308 GO:0008855 GO:0009318
Cluster-22178.60616 GO:0006357 GO:0007304 GO:0005213 GO:0001104 GO:0005576 GO:0042600 GO:0016592
Cluster-22178.60617 GO:0006468 GO:0006118 GO:0005524 GO:0004672 GO:0046872 GO:0016773 GO:0009055 GO:0020037 GO:0005515 GO:0016020

Cluster-22178.60620 GO:0005515
Cluster-22178.60621 GO:0005515 GO:0005509
Cluster-22178.60624 GO:0035556 GO:0046872
Cluster-22178.60625 GO:0007049 GO:0006355 GO:0030474 GO:0046983 GO:0043565 GO:0005200 GO:0003700 GO:0005198 GO:0005856 GO:0005823 GO:0005667 GO:0005882
Cluster-22178.60627 GO:0006355 GO:0003700 GO:0000062 GO:0016884 GO:0005667
Cluster-22178.60629 GO:0015979 GO:0042549 GO:0051205 GO:0016021 GO:0009539 GO:0009523
Cluster-22178.60630 GO:0006260 GO:0003677 GO:0005664
Cluster-22178.60631 GO:0032780 GO:0006355 GO:0003700 GO:0042030 GO:0008270 GO:0005739 GO:0005667 GO:0005634
Cluster-22178.60632 GO:0030168 GO:0051607 GO:0051258 GO:0007165 GO:0005525 GO:0005102 GO:0051082 GO:0030674 GO:0005577
Cluster-22178.60633 GO:0003676
Cluster-22178.60634 GO:0000902 GO:0006270 GO:0003676 GO:0008270 GO:0016021 GO:0032040 GO:0005634
Cluster-22178.60635 GO:0016021
Cluster-22178.60636 GO:0006886 GO:0000921 GO:0003774 GO:0016459 GO:0016020 GO:0005940 GO:0016021 GO:0005801
Cluster-22178.60637 GO:0005515 GO:0005201 GO:0003723 GO:0008168 GO:0008270 GO:0005581 GO:0005578
Cluster-22178.60638 GO:0043571 GO:0006397 GO:0003774 GO:0005524 GO:0004519 GO:0005515 GO:0016459
Cluster-22178.60639 GO:0005515 GO:0008270 GO:0016021 GO:0042765
Cluster-22178.60640 GO:0007165 GO:0040007 GO:0008283 GO:0051028 GO:0008083 GO:0005634
Cluster-22178.60641 GO:0007010 GO:0007165 GO:0006629 GO:0035556 GO:0046339 GO:0032065 GO:0009395 GO:0003677 GO:0005515 GO:0004435 GO:0005543 GO:0034061 GO:0003779 GO:0005634 GO:0005938
Cluster-22178.60643 GO:0045859 GO:0019887 GO:0005956

Cluster-22178.60645 GO:0006260 GO:0019079 GO:0005524 GO:0003676
GO:0003723 GO:0005198 GO:0016818 GO:0003887 GO:0003677
GO:0008026 GO:0019028 GO:0005634 GO:0042575
Cluster-22178.60647 GO:0006139 GO:0008408 GO:0003676
Cluster-22178.60649 GO:0008270 GO:0016021
Cluster-22178.60650 GO:0016021 GO:0042765
Cluster-22178.60653 GO:0005515
Cluster-22178.60654 GO:0003677 GO:0005634
Cluster-22178.60656 GO:0050776 GO:0014068 GO:0007165 GO:0006446
GO:0006413 GO:0005102 GO:0003743 GO:0043548 GO:0003723
GO:0005840 GO:0005737 GO:0016020
Cluster-22178.60658 GO:0046034 GO:0006144 GO:0006366 GO:0008616
GO:0006281 GO:0046872 GO:0008270 GO:0004017 GO:0016853
GO:0003677 GO:0016740 GO:0004867 GO:0005576 GO:0005665
Cluster-22178.60659 GO:0003676 GO:0005515 GO:0003677 GO:0016787
GO:0005524
Cluster-22178.60660 GO:0005515
Cluster-22178.60662 GO:0007411 GO:0042742 GO:0005515 GO:0005576
Cluster-22178.60665 GO:0006276 GO:0005975 GO:0003697 GO:0042302
GO:0004553
Cluster-22178.60667 GO:0006355 GO:0005198 GO:0003700 GO:0005667
GO:0019028
Cluster-22178.60669 GO:0007165 GO:0008270
Cluster-22178.60672 GO:0006368 GO:0008023
Cluster-22178.60673 GO:0003677 GO:0046872 GO:0008270
Cluster-22178.60674 GO:0048193 GO:0006457 GO:0051082 GO:0016020
GO:0016021 GO:0016272
Cluster-22178.60679 GO:0015979 GO:0046872
Cluster-22178.6068 GO:0006355 GO:0003677
Cluster-22178.60680 GO:0005515
Cluster-22178.60681 GO:0009306 GO:0055114 GO:0006118 GO:0045454
GO:0009055 GO:0015035 GO:0016491 GO:0016209 GO:0005509
GO:0005788

Cluster-22178.60682 GO:0006355 GO:0015994 GO:0006783 GO:0019079
GO:0033014 GO:0004852 GO:0003700 GO:0043565 GO:0003677
GO:0003682 GO:0004386 GO:0008270 GO:0005634 GO:0000785
GO:0000775 GO:0005667
Cluster-22178.60683 GO:0006355 GO:0006915 GO:0006323 GO:0042254
GO:0006412 GO:0006974 GO:0005515 GO:0003735 GO:0003700
GO:0005667 GO:0005840
Cluster-22178.60686 GO:0005515
Cluster-22178.60688 GO:0008544 GO:0048870 GO:0007165 GO:0005515
GO:0031514
Cluster-22178.60690 GO:0009405 GO:0016301 GO:0016772 GO:0016020
Cluster-22178.60691 GO:0006024 GO:0007283 GO:0009264 GO:0015012
GO:0003677 GO:0004252 GO:0016757 GO:0008253 GO:0005634
GO:0016020 GO:0000786 GO:0016021
Cluster-22178.60693 GO:0007049 GO:0006897 GO:0007342 GO:0060478
GO:0051301 GO:0016021 GO:0005886
Cluster-22178.60698 GO:0015986 GO:0006355 GO:0015992 GO:0046872
GO:0008270 GO:0043565 GO:0015078 GO:0003700 GO:0005667
GO:0000276
Cluster-22178.60699 GO:0042254 GO:0006412 GO:0005515 GO:0003735
GO:0005622 GO:0005840
Cluster-22178.6070 GO:0006355 GO:0008270 GO:0005634
Cluster-22178.60700 GO:0006468 GO:0005524 GO:0004672
Cluster-22178.60701 GO:0006836 GO:0070836 GO:0006812 GO:0051191
GO:0005328 GO:0005634 GO:0016021
Cluster-22178.60703 GO:0001522 GO:0042254 GO:0006508 GO:0008239

Cluster-22178.60705 GO:0046872 GO:0008376 GO:0005515 GO:0032580
Cluster-22178.60708 GO:0008270
Cluster-22178.60709 GO:0008152 GO:0007275 GO:0007165 GO:0008270
GO:0047355 GO:0005179 GO:0016624 GO:0005576 GO:0016020
Cluster-22178.6071 GO:0006355 GO:0008270 GO:0005634
Cluster-22178.60712 GO:0007275 GO:0007304 GO:0006313 GO:0005515

GO:0005213 GO:0042600
Cluster-22178.60713 GO:0019073 GO:0007165 GO:0005515 GO:0003677
GO:0016021
Cluster-22178.60714 GO:0006886 GO:0006888 GO:0008536 GO:0005622
Cluster-22178.60715 GO:0006260 GO:0015992 GO:0042254 GO:0006470
GO:0006351 GO:0009405 GO:0006412 GO:0006570 GO:0015991
GO:0003677 GO:0008138 GO:0003735 GO:0015078 GO:0004725
GO:0003887 GO:0005622 GO:0005840 GO:0016021 GO:0042575
GO:0033179
Cluster-22178.60717 GO:0042254 GO:0001522 GO:0005515 GO:0030515
GO:0072588
Cluster-22178.60720 GO:0006508 GO:0042742 GO:0008152 GO:0016836
GO:0003824 GO:0008233 GO:0005576
Cluster-22178.60721 GO:0016049 GO:0010215 GO:0031225
Cluster-22178.60722 GO:0007165 GO:0005515
Cluster-22178.60723 GO:0007186 GO:0004930 GO:0016787 GO:0016021
Cluster-22178.60725 GO:0010508 GO:0042254 GO:0006412 GO:0003723
GO:0003735 GO:0005622 GO:0005840
Cluster-22178.60727 GO:0006281 GO:0006886 GO:0016192 GO:0043161
GO:0006289 GO:0003684 GO:0003676 GO:0030117
Cluster-22178.60729 GO:0055114 GO:0008152 GO:0006480 GO:0010181
GO:0008168 GO:0016491 GO:0008171
Cluster-22178.60731 GO:0003677 GO:0008270
Cluster-22178.60732 GO:0007165 GO:0006810 GO:0044237 GO:0010038
GO:0007049 GO:0006357 GO:0006308 GO:0001104 GO:0005515
GO:0005215 GO:0004871 GO:0008855 GO:0009318 GO:0016592
GO:0016020
Cluster-22178.60733 GO:0006508 GO:0008234
Cluster-22178.60735 GO:0006281 GO:0009432 GO:0006367 GO:0015684
GO:0051726 GO:0007155 GO:0005525 GO:0003697 GO:0017025
GO:0005524 GO:0015093 GO:0016887 GO:0016021 GO:0005672
GO:0005634 GO:0009289
Cluster-22178.60736 GO:0006118 GO:0015991 GO:0016032 GO:0008152

GO:0055114 GO:0015992 GO:0003995 GO:0016740 GO:0050660
GO:0005525 GO:0015078 GO:0005524 GO:0046872 GO:0016627
GO:0033179
Cluster-22178.60737 GO:0009405 GO:0009878 GO:0005198 GO:0046872

Cluster-22178.60738 GO:0009878 GO:0046872
Cluster-22178.60739 GO:0000398 GO:0008270 GO:0005681
Cluster-22178.60740 GO:0046488 GO:0016307
Cluster-22178.60742 GO:0003676 GO:0005515 GO:0003677 GO:0016787
GO:0005524
Cluster-22178.60743 GO:0006310 GO:0006355 GO:0035556 GO:0003677
GO:0050662 GO:0003824 GO:0003676 GO:0003700 GO:0046872
GO:0008270 GO:0043565 GO:0005634 GO:0005667
Cluster-22178.60748 GO:0007165 GO:0030155 GO:0030334 GO:0006308
GO:0045995 GO:0005515 GO:0008855 GO:0005102 GO:0009318
Cluster-22178.60749 GO:0055114 GO:0008152 GO:0051287 GO:0016616

Cluster-22178.60750 GO:0051260
Cluster-22178.60754 GO:0000413 GO:0006457 GO:0003755
Cluster-22178.60755 GO:0006457 GO:0000413 GO:0003755
Cluster-22178.60756 GO:0008270
Cluster-22178.60757 GO:0015992 GO:0006123 GO:0008270 GO:0046872
GO:0004129 GO:0005634 GO:0045277
Cluster-22178.60761 GO:0006355 GO:0043565 GO:0008289 GO:0003677
GO:0003700 GO:0005667 GO:0005634
Cluster-22178.60762 GO:0006313 GO:0006355 GO:0004803 GO:0003700
GO:0003677 GO:0005667 GO:0005622
Cluster-22178.60764 GO:0006505 GO:0008152 GO:0006508 GO:0006886
GO:0016787 GO:0016788 GO:0008236 GO:0005576
Cluster-22178.60765 GO:0030206 GO:0006024 GO:0030158 GO:0008233
GO:0008375 GO:0016020
Cluster-22178.60766 GO:0008610 GO:0006118 GO:0017004 GO:0008152
GO:0016747 GO:0045158 GO:0009512

Cluster-22178.60768 GO:0006508 GO:0044419 GO:0007275 GO:0006144
GO:0007219 GO:0030154 GO:0004197 GO:0017111 GO:0003700
GO:0003968 GO:0005634 GO:0005667 GO:0031379 GO:0016021
Cluster-22178.60769 GO:0031175 GO:0005516 GO:0030507 GO:0008091
Cluster-22178.60771 GO:0006520 GO:0055114 GO:0006144 GO:0009152
GO:0006188 GO:0006522 GO:0006531 GO:0009058 GO:0004018
GO:0016829 GO:0010277 GO:0030170
Cluster-22178.60772 GO:0006412 GO:0042254 GO:0003735 GO:0005840
Cluster-22178.60773 GO:0007010 GO:0035556 GO:0003779 GO:0005515
GO:0008289
Cluster-22178.60774 GO:0042254 GO:0006412 GO:0006810 GO:0003735
GO:0016020 GO:0005840
Cluster-22178.60776 GO:0035556 GO:0016310 GO:0006468 GO:0009069
GO:0004674 GO:0004672 GO:0005524 GO:0016773 GO:0016020
Cluster-22178.60778 GO:0016032 GO:0006508 GO:0007155 GO:0019082
GO:0001558 GO:0042246 GO:0005520 GO:0008239 GO:0070009
GO:0005515 GO:0004252 GO:0016021 GO:0016942 GO:0005576
Cluster-22178.60779 GO:0006508 GO:0007606 GO:0007165 GO:0016787
GO:0005509 GO:0008236 GO:0005578 GO:0016021
Cluster-22178.60780 GO:0006816 GO:0006874 GO:0005515 GO:0005219
GO:0016021 GO:0005622
Cluster-22178.60781 GO:0016779
Cluster-22178.60782 GO:0006888 GO:0006886 GO:0008270 GO:0030127
Cluster-22178.60783 GO:0003677 GO:0005524 GO:0016787
Cluster-22178.60788 GO:0030168 GO:0055085 GO:0007165 GO:0051258
GO:0048870 GO:0030001 GO:0008270 GO:0005102 GO:0046873
GO:0030674 GO:0046872 GO:0005515 GO:0005622 GO:0005577
GO:0016020 GO:0016021 GO:0031514
Cluster-22178.60791 GO:0016567 GO:0008270 GO:0005515 GO:0004842
GO:0003677 GO:0009279 GO:0005680
Cluster-22178.60792 GO:0006576 GO:0006508 GO:0007264 GO:0007186
GO:0007165 GO:0015684 GO:0003924 GO:0031683 GO:0019001
GO:0004871 GO:0005525 GO:0016887 GO:0005524 GO:0015093

GO:0004222 GO:0005509 GO:0016021
Cluster-22178.60793 GO:0003723
Cluster-22178.60796 GO:0005515
Cluster-22178.60797 GO:0006090 GO:0009401 GO:0006412 GO:0006814
GO:0006560 GO:0006525 GO:0042254 GO:0071436 GO:0008948
GO:0005515 GO:0015081 GO:0016787 GO:0003735 GO:0016021
GO:0005840 GO:0015934 GO:0016020
Cluster-22178.60799 GO:0046935 GO:0005515 GO:0005944
Cluster-22178.60800 GO:0008152 GO:0016491
Cluster-22178.60803 GO:0007342 GO:0007340 GO:0003676 GO:0001669
Cluster-22178.60804 GO:0035091 GO:0005515 GO:0003676 GO:0005887
Cluster-22178.60805 GO:0006260 GO:0003887 GO:0005515 GO:0042575
Cluster-22178.60806 GO:0016579 GO:0005515 GO:0036459
Cluster-22178.60809 GO:0006355 GO:0006030 GO:0008061 GO:0008092
GO:0005634 GO:0005576 GO:0005737 GO:0019898
Cluster-22178.60810 GO:0006508 GO:0016788 GO:0004197 GO:0016740
GO:0016817 GO:0008242 GO:0005634
Cluster-22178.60812 GO:0000902 GO:0006955 GO:0006351 GO:0009405
GO:0006355 GO:0006811 GO:0006464 GO:0005515 GO:0003676
GO:0003700 GO:0016790 GO:0016151 GO:0043565 GO:0046872
GO:0003677 GO:0008270 GO:0045202 GO:0016020 GO:0005667
GO:0005887
Cluster-22178.60813 GO:0051726 GO:0005634
Cluster-22178.60815 GO:0016020
Cluster-22178.60816 GO:0016560 GO:0006468 GO:0005524 GO:0004672
GO:0016021 GO:0005777
Cluster-22178.60817 GO:0006468 GO:0005524 GO:0004672
Cluster-22178.60819 GO:0006281 GO:0030170 GO:0046872 GO:0008483
GO:0003824 GO:0000166
Cluster-22178.60823 GO:0055114 GO:0016887 GO:0005524 GO:0016616

Cluster-22178.60824 GO:0042254 GO:0006412 GO:0003735 GO:0005840
GO:0005622

Cluster-22178.60825 GO:0006281 GO:0046872 GO:0003824 GO:0000166

Cluster-22178.60827 GO:0005515 GO:0016020
Cluster-22178.60828 GO:0009405 GO:0055085 GO:0015689 GO:0022857
GO:0015098 GO:0016021
Cluster-22178.60830 GO:0006206 GO:0006144 GO:0007165 GO:0006351
GO:0015774 GO:0015159 GO:0005515 GO:0003899 GO:0016791
GO:0005509 GO:0016020 GO:0005730 GO:0005578
Cluster-22178.60832 GO:0015031 GO:0016021
Cluster-22178.60833 GO:0019217 GO:0016567 GO:0005515 GO:0046872
GO:0008270 GO:0004842 GO:0000062 GO:0005680
Cluster-22178.60835 GO:0042803 GO:0003723 GO:0008270 GO:0042162

Cluster-22178.60836 GO:0051726 GO:0005634
Cluster-22178.60837 GO:0030328 GO:0055114 GO:0016670 GO:0016491

Cluster-22178.60839 GO:0006298 GO:0007010 GO:0007264 GO:0000398
GO:0004519 GO:0005525 GO:0008270 GO:0005524 GO:0016887
GO:0003779 GO:0003924 GO:0019028
Cluster-22178.60843 GO:0043461 GO:0055085 GO:0016020
Cluster-22178.60844 GO:0006470 GO:0070403 GO:0008138 GO:0005515

Cluster-22178.60848 GO:0016021
Cluster-22178.60850 GO:0006508 GO:0019825 GO:0020037 GO:0004197

Cluster-22178.60851 GO:0006123 GO:0015992 GO:0004129 GO:0045277
Cluster-22178.60852 GO:0015074 GO:0006352 GO:0006355 GO:0051260
GO:0003700 GO:0003677 GO:0005669 GO:0005667
Cluster-22178.60854 GO:0006887 GO:0008289 GO:0000145
Cluster-22178.60858 GO:0006355 GO:0046854 GO:0042967 GO:0004402
GO:0016787 GO:0008270 GO:0003712 GO:0019033 GO:0005667
GO:0005634 GO:0000123
Cluster-22178.60859 GO:0048193 GO:0055114 GO:0007165 GO:0007186

GO:0005525 GO:0004871 GO:0019001 GO:0031683 GO:0016491
GO:0003924 GO:0050661 GO:0005783
Cluster-22178.60860 GO:0030071 GO:0005680
Cluster-22178.60861 GO:0006508 GO:0008233 GO:0003677
Cluster-22178.60862 GO:0006886 GO:0006118 GO:0055114 GO:0008536
GO:0016655 GO:0005886
Cluster-22178.60863 GO:0008616 GO:0006400 GO:0008479
Cluster-22178.60864 GO:1903818 GO:0090063 GO:0030431 GO:0032222
GO:0000226 GO:0034235 GO:0005634 GO:0005815 GO:0000922
Cluster-22178.60865 GO:0006206 GO:0006144 GO:0006351 GO:0003677
GO:0003899 GO:0005730
Cluster-22178.60866 GO:0006355 GO:0006531 GO:0006419 GO:0006522
GO:0000166 GO:0004813 GO:0005524 GO:0032039 GO:0005622
GO:0016021
Cluster-22178.60868 GO:0006419 GO:0006522 GO:0006531 GO:0006355
GO:0004813 GO:0005524 GO:0000166 GO:0005622 GO:0016021
GO:0032039
Cluster-22178.60869 GO:0018990 GO:0007218 GO:0008255
Cluster-22178.60870 GO:0006464 GO:0015031 GO:0005515
Cluster-22178.60875 GO:0007264 GO:0005525
Cluster-22178.60876 GO:0006396 GO:0006807 GO:0006144 GO:0004000
GO:0003723
Cluster-22178.60878 GO:0055085 GO:0030001 GO:0009607 GO:0005515
GO:0046873 GO:0016021 GO:0016020
Cluster-22178.60881 GO:0007607 GO:0006402 GO:0009405 GO:0050912
GO:0051252 GO:0007187 GO:0046872 GO:0004535 GO:0003723
GO:0008527 GO:0005737 GO:0016021 GO:0005634
Cluster-22178.60882 GO:0045766 GO:0016567 GO:0043123 GO:0015986
GO:0043066 GO:0015992 GO:0003723 GO:0043130 GO:0004842
GO:0015078 GO:0000276
Cluster-22178.60883 GO:0006886 GO:0006570 GO:0006470 GO:0050852
GO:0005047 GO:0005515 GO:0044212 GO:0004725 GO:0005525
GO:0046872 GO:0003924 GO:0005785 GO:0016021 GO:0005786

Cluster-22178.60884 GO:0046872
Cluster-22178.60887 GO:0008152 GO:0055114 GO:0016491
Cluster-22178.60888 GO:0007275 GO:0009103 GO:0008152 GO:0055114
GO:0005201 GO:0016491 GO:0008918 GO:0005578 GO:0005581
Cluster-22178.60889 GO:0006357 GO:0001104 GO:0016592
Cluster-22178.60890 GO:0016192 GO:0006886 GO:0030131 GO:0016021
GO:0030117
Cluster-22178.60891 GO:0048870 GO:0019905 GO:0005515 GO:0031514
GO:0019028
Cluster-22178.60893 GO:0005515
Cluster-22178.60894 GO:0007155 GO:0001503 GO:0019028 GO:0005576
Cluster-22178.60895 GO:0016020
Cluster-22178.60896 GO:0003924
Cluster-22178.60897 GO:0003924
Cluster-22178.60899 GO:0015992 GO:0016567 GO:0006119 GO:0007005
GO:0015986 GO:0004842 GO:0008289 GO:0046933 GO:0046961
GO:0045259 GO:0000275
Cluster-22178.60900 GO:0015986 GO:0016567 GO:0006119 GO:0007005
GO:0015992 GO:0046961 GO:0046933 GO:0008289 GO:0004842
GO:0000275 GO:0045259
Cluster-22178.60901 GO:0006281 GO:0004518 GO:0005515
Cluster-22178.60902 GO:0006281 GO:0004518 GO:0005515
Cluster-22178.60903 GO:0045454 GO:0055114 GO:0016209 GO:0016491

Cluster-22178.60904 GO:0046487 GO:0042128 GO:0016117 GO:0006807
GO:0009396 GO:0008033 GO:0055114 GO:0016705 GO:0004488
GO:0003824 GO:0050660 GO:0051537 GO:0008942 GO:0016491
Cluster-22178.60906 GO:0015979 GO:0030494
Cluster-22178.60907 GO:0006355 GO:0006812 GO:0008324 GO:0003677
GO:0016021
Cluster-22178.60908 GO:0009405 GO:0005198 GO:0005882
Cluster-22178.60909 GO:0046872
Cluster-22178.60910 GO:0006888 GO:0055085 GO:0006810 GO:0022857

GO:0005215 GO:0005515 GO:0005622 GO:0019867 GO:0016021
GO:0005801 GO:0016020
Cluster-22178.60911 GO:0008168 GO:0070403
Cluster-22178.60913 GO:0009089 GO:0006355 GO:0008837 GO:0005634
GO:0005737
Cluster-22178.60915 GO:0016032 GO:0006874 GO:0006508 GO:0004197
GO:0030234 GO:0030176
Cluster-22178.60917 GO:0006874 GO:0030234 GO:0030176
Cluster-22178.60918 GO:0006355 GO:0003677 GO:0016021
Cluster-22178.60919 GO:0035556 GO:0006260 GO:0006269 GO:0006351
GO:0046872 GO:0003896 GO:0003677 GO:0005657 GO:0005730
Cluster-22178.60921 GO:0005975 GO:0033925 GO:0004553 GO:0005737
Cluster-22178.60922 GO:0003676 GO:0005515 GO:0008270 GO:0005634
Cluster-22178.60923 GO:0009298 GO:0007219 GO:0042351 GO:0006012
GO:0019673 GO:0006000 GO:0008446 GO:0050662 GO:0070401
GO:0003824 GO:0005622
Cluster-22178.60925 GO:0035444 GO:0007264 GO:0015675 GO:0015099
GO:0005525 GO:0046872 GO:0016021
Cluster-22178.60926 GO:0007165 GO:0006614 GO:0007186 GO:0007264
GO:0006355 GO:0006576 GO:0008270 GO:0005524 GO:0016887
GO:0003677 GO:0005525 GO:0004871 GO:0019001 GO:0031683
GO:0003924 GO:0005515
Cluster-22178.60929 GO:0005975 GO:0030246
Cluster-22178.60930 GO:0046486 GO:0001575 GO:0006012 GO:0005975
GO:0004553 GO:0004557
Cluster-22178.60932 GO:0006367 GO:0006355 GO:0003677 GO:0003713
GO:0046872 GO:0005667 GO:0005672
Cluster-22178.60933 GO:0007165 GO:0016567 GO:0005179 GO:0004842
GO:0005576
Cluster-22178.60937 GO:0016579 GO:0036459
Cluster-22178.60938 GO:0016192 GO:0006364 GO:0005730 GO:0016021
GO:0019031
Cluster-22178.60946 GO:0006571 GO:0000162 GO:0000413 GO:0009094

GO:0006096 GO:0006457 GO:0006094 GO:0004634 GO:0003755
GO:0000287 GO:0000015
Cluster-22178.60947 GO:0043087 GO:0007264 GO:0005085
Cluster-22178.60951 GO:0016567 GO:0004842 GO:0003677 GO:0005515
GO:0005634
Cluster-22178.60952 GO:0006506 GO:0015840 GO:0019752 GO:0006729
GO:0006520 GO:0071918 GO:0009058 GO:0016829 GO:0008124
GO:0016740 GO:0030170 GO:0016831 GO:0015204 GO:0061630
GO:0016746 GO:0016021 GO:0005789
Cluster-22178.60953 GO:0016021
Cluster-22178.60954 GO:0009966 GO:0007275 GO:0016020
Cluster-22178.60955 GO:0008608 GO:0003723 GO:0005876 GO:0042729
Cluster-22178.60959 GO:0046872 GO:0008270 GO:0005515
Cluster-22178.60960 GO:0005515
Cluster-22178.60961 GO:0006468 GO:0006886 GO:0016192 GO:0005524
GO:0004672 GO:0005515 GO:0030117
Cluster-22178.60963 GO:0043087 GO:0006260 GO:0006269 GO:0006351
GO:0007096 GO:0003896 GO:0005515 GO:0005657 GO:0005730
GO:0005634
Cluster-22178.60965 GO:0055114 GO:0016706 GO:0005506 GO:0031418

Cluster-22178.60966 GO:0006355 GO:0043565 GO:0003700 GO:0005667
Cluster-22178.60969 GO:0055085 GO:0016567 GO:0006810 GO:0046872
GO:0008270 GO:0005524 GO:0042626 GO:0004842 GO:0005515
GO:0016021 GO:0005680
Cluster-22178.60972 GO:0016853
Cluster-22178.60975 GO:0006119 GO:0006118 GO:0015992 GO:0055114
GO:0008121 GO:0005515
Cluster-22178.60976 GO:0007275 GO:0042600
Cluster-22178.60979 GO:0055085 GO:0046872 GO:0016021
Cluster-22178.60981 GO:0006508 GO:0004198 GO:0005509 GO:0005622
Cluster-22178.60985 GO:0009405 GO:0046487 GO:0019521 GO:0055114
GO:0050792 GO:0006810 GO:0006098 GO:0007264 GO:0009396

GO:0004518 GO:0008200 GO:0003824 GO:0005525 GO:0004488
GO:0004616 GO:0005576
Cluster-22178.60988 GO:0032006 GO:0019031
Cluster-22178.60990 GO:0055085 GO:0006811 GO:0015992 GO:0051260
GO:0015986 GO:0015078 GO:0005216 GO:0016020
Cluster-22178.60991 GO:0008616 GO:0006400 GO:0008479
Cluster-22178.60993 GO:0009435 GO:0042742 GO:0046497 GO:0016763
GO:0004514 GO:0019901 GO:0005515
Cluster-22178.60994 GO:0006144 GO:0015992 GO:0006206 GO:0015991
GO:0007155 GO:0006351 GO:0015078 GO:0003899 GO:0005515
GO:0003677 GO:0005730 GO:0016020 GO:0033179
Cluster-22178.60995 GO:0007155 GO:0015992 GO:0006206 GO:0006144
GO:0006888 GO:0006351 GO:0006886 GO:0015991 GO:0003677
GO:0032183 GO:0070140 GO:0008270 GO:0003899 GO:0005515
GO:0015078 GO:0030127 GO:0016020 GO:0005730 GO:0033179
Cluster-22178.60996 GO:0006206 GO:0006144 GO:0006888 GO:0007155
GO:0006508 GO:0006351 GO:0006886 GO:0008270 GO:0003677
GO:0004197 GO:0017111 GO:0003968 GO:0003899 GO:0005515
GO:0005730 GO:0030127 GO:0016020 GO:0031379
Cluster-22178.60997 GO:0007155 GO:0006206 GO:0015992 GO:0006144
GO:0006888 GO:0006351 GO:0006886 GO:0015991 GO:0003677
GO:0008270 GO:0003899 GO:0005515 GO:0015078 GO:0030127
GO:0016020 GO:0005730 GO:0033179
Cluster-22178.60998 GO:0040001 GO:0000226 GO:0042254 GO:0000281
GO:0005874 GO:0005819 GO:0005634
Cluster-22178.610 GO:0007186 GO:0004930 GO:0016021
Cluster-22178.61000 GO:0003676
Cluster-22178.61001 GO:0015979 GO:0009507 GO:0009523
Cluster-22178.61011 GO:0006508 GO:0008152 GO:0016042 GO:0046486
GO:0016787 GO:0004806 GO:0008236
Cluster-22178.61014 GO:0005543
Cluster-22178.61015 GO:0006355 GO:0003676 GO:0005622
Cluster-22178.61016 GO:0055114 GO:0050662 GO:0016491 GO:0003824

Cluster-22178.61017 GO:0006412 GO:0042254 GO:0003735 GO:0005622 GO:0005840
Cluster-22178.61018 GO:0016627
Cluster-22178.61020 GO:0055114 GO:0052855 GO:0016491 GO:0016021 GO:0016012
Cluster-22178.61021 GO:0005515
Cluster-22178.61023 GO:0055114 GO:0052855 GO:0016491
Cluster-22178.61024 GO:0008033 GO:0006888 GO:0006554 GO:0008152 GO:0006400 GO:0006886 GO:0009451 GO:0006479 GO:0008168 GO:0008270 GO:0008176 GO:0018024 GO:0019955 GO:0016020 GO:0030127
Cluster-22178.61026 GO:0007049 GO:0051301 GO:0005515 GO:0005887 GO:0016021
Cluster-22178.61029 GO:0003676
Cluster-22178.61032 GO:0006355 GO:0005515 GO:0003677 GO:0005634
Cluster-22178.61034 GO:0006730 GO:0008270 GO:0016740
Cluster-22178.61035 GO:0006730 GO:0016740 GO:0008270
Cluster-22178.61037 GO:0016197 GO:0006308 GO:0030474 GO:0006886 GO:0000724 GO:0006914 GO:0019064 GO:0006457 GO:0071988 GO:0006810 GO:0007155 GO:0003676 GO:0042803 GO:0008855 GO:0000774 GO:0051087 GO:0016021 GO:0019031 GO:0009288 GO:0009318 GO:0030119 GO:0005783
Cluster-22178.61038 GO:0006364 GO:0003676
Cluster-22178.61039 GO:0006364
Cluster-22178.61040 GO:0070072
Cluster-22178.61041 GO:0006144 GO:0006206 GO:0016567 GO:0005975 GO:0006687 GO:0006351 GO:0008270 GO:0004842 GO:0003677 GO:0046872 GO:0004308 GO:0005515 GO:0003899 GO:0033644 GO:0055036 GO:0005730 GO:0016020 GO:0005634
Cluster-22178.61044 GO:0009405 GO:0006811 GO:0005216 GO:0005576
Cluster-22178.61045 GO:0006206 GO:0002949 GO:0006139 GO:0005524 GO:0004127

Cluster-22178.61047 GO:0008152 GO:0005542 GO:0016740
Cluster-22178.61048 GO:0003779
Cluster-22178.61053 GO:0008270 GO:0016779 GO:0003676
Cluster-22178.61055 GO:0016567 GO:0046872 GO:0004842 GO:0005515

Cluster-22178.61056 GO:0007017 GO:0010207 GO:0007018 GO:0005515
GO:0003777 GO:0030286 GO:0005737 GO:0005874
Cluster-22178.61058 GO:0034508 GO:0007059
Cluster-22178.61060 GO:0006576 GO:0007264 GO:0003723 GO:0016301
GO:0098519 GO:0003924 GO:0016887 GO:0005524 GO:0003724
GO:0005525
Cluster-22178.61063 GO:0005515 GO:0005737
Cluster-22178.61064 GO:0043401 GO:0006355 GO:0003700 GO:0008270
GO:0043565 GO:0005634 GO:0005667
Cluster-22178.61066 GO:0006508 GO:0004252 GO:0003676 GO:0016020
Cluster-22178.61067 GO:0006886 GO:0006313 GO:0035307 GO:0006888
GO:0003677 GO:0003676 GO:0008270 GO:0016020 GO:0030127
GO:0071595
Cluster-22178.61068 GO:0006888 GO:0006886 GO:0035307 GO:0006313
GO:0008270 GO:0003676 GO:0003677 GO:0071595 GO:0030127
Cluster-22178.61069 GO:0006778 GO:0042967 GO:0006544 GO:0006783
GO:0006563 GO:0006566 GO:0009058 GO:0016740 GO:0030170
GO:0003870 GO:0005759 GO:0005576
Cluster-22178.61071 GO:0016310 GO:0009069 GO:0009405 GO:0006886
GO:0004674 GO:0008536 GO:0005515 GO:0005576
Cluster-22178.61072 GO:0007264 GO:0006465 GO:0005525 GO:0008233
GO:0005787 GO:0016021
Cluster-22178.61075 GO:0030001 GO:0055085 GO:0046873 GO:0016020
GO:0019867
Cluster-22178.61079 GO:0007264 GO:0003924 GO:0005525 GO:0005634
Cluster-22178.61080 GO:0048870 GO:0007017 GO:0006560 GO:0007018
GO:0006420 GO:0006260 GO:0006525 GO:0004814 GO:0003777
GO:0045502 GO:0000166 GO:0042803 GO:0008134 GO:0005198

GO:0005524 GO:0008017 GO:0005737 GO:0005874 GO:0019031
GO:0045298 GO:0019028 GO:0031514 GO:0030286 GO:0005815
GO:0005667 GO:0005882
Cluster-22178.61082 GO:0005515
Cluster-22178.61083 GO:0006464
Cluster-22178.61088 GO:0000774 GO:0005515 GO:0005783
Cluster-22178.61089 GO:0006955 GO:0030153 GO:0015643 GO:0019814
Cluster-22178.61094 GO:0006281 GO:0006749 GO:0006508 GO:0006118
GO:0006804 GO:0055114 GO:0006979 GO:0016579 GO:0004843
GO:0004602 GO:0036459 GO:0016655 GO:0071203 GO:0016020
Cluster-22178.61095 GO:0005515
Cluster-22178.61097 GO:0000166 GO:0003677 GO:0008408
Cluster-22178.61098 GO:0000166 GO:0003677 GO:0008408
Cluster-22178.61102 GO:0006313 GO:0007005 GO:0016567 GO:0003677
GO:0004842 GO:0004803
Cluster-22178.61104 GO:0042254 GO:0006412 GO:0008270 GO:0003735
GO:0003677 GO:0005840 GO:0005622 GO:0005634
Cluster-22178.61107 GO:0006457
Cluster-22178.61109 GO:0009401 GO:0008643 GO:0005515 GO:0005509
GO:0008982 GO:0005737 GO:0009357
Cluster-22178.61110 GO:0005515 GO:0016020
Cluster-22178.61111 GO:0008152 GO:0016491
Cluster-22178.61112 GO:0007067 GO:0000090 GO:0031110 GO:0051301
GO:0007059 GO:0008017 GO:0000940 GO:0045298 GO:0005876
Cluster-22178.61113 GO:0007067 GO:0000090 GO:0051301 GO:0031110
GO:0007059 GO:0008017 GO:0000940 GO:0005876 GO:0045298
Cluster-22178.61114 GO:0019058 GO:0007165 GO:0043565 GO:0005515
GO:0046789 GO:0019031 GO:0009986 GO:0005634
Cluster-22178.61115 GO:0006468 GO:0010215 GO:0016049 GO:0004672
GO:0005524 GO:0031225
Cluster-22178.61116 GO:0006144 GO:0007156 GO:0009058 GO:0009113
GO:0005524 GO:0046872 GO:0016742 GO:0005509 GO:0051539
GO:0004637 GO:0016020

Cluster-22178.61117 GO:0006915 GO:0005622
Cluster-22178.61118 GO:0016192 GO:0006904 GO:0008144
Cluster-22178.61119 GO:0000902 GO:0005975 GO:0003676 GO:0016773 GO:0005524 GO:0032040
Cluster-22178.61122 GO:0005515
Cluster-22178.61125 GO:0016567 GO:0006886 GO:0016192 GO:0008270 GO:0046872 GO:0004842 GO:0005515 GO:0000775 GO:0005680
Cluster-22178.61126 GO:0015992 GO:0006123 GO:0003774 GO:0004129 GO:0005198 GO:0045277 GO:0016459 GO:0005739 GO:0005882 GO:0005740 GO:0005747
Cluster-22178.61127 GO:0042546 GO:0001575 GO:0009405 GO:0009247 GO:0005975 GO:0008107 GO:0005576 GO:0016020
Cluster-22178.61129 GO:0015992 GO:0019049 GO:0005975 GO:0055114 GO:0008152 GO:0006120 GO:0006744 GO:0006814 GO:0003824 GO:0030246 GO:0016836 GO:0008137
Cluster-22178.61132 GO:0015946 GO:0055114 GO:0005515 GO:0004022 GO:0008233
Cluster-22178.61134 GO:0015992 GO:0006123 GO:0004129 GO:0036128 GO:0005743 GO:0005751 GO:0045277
Cluster-22178.61135 GO:0006364 GO:0005515 GO:0032040
Cluster-22178.61141 GO:0006357 GO:0001104 GO:0016592
Cluster-22178.61142 GO:0005515
Cluster-22178.61143 GO:0005515
Cluster-22178.61144 GO:0005515
Cluster-22178.61145 GO:0005515
Cluster-22178.61146 GO:0006508 GO:0055114 GO:0008152 GO:0008270 GO:0016787 GO:0047134 GO:0008236
Cluster-22178.61147 GO:0035556 GO:0005515
Cluster-22178.61148 GO:0046034 GO:0035556 GO:0006144 GO:0006281 GO:0046872 GO:0008270 GO:0004017 GO:0003677
Cluster-22178.61149 GO:0055114 GO:0008152 GO:0050832 GO:0006694 GO:0008210 GO:0008207 GO:0008209 GO:0016491 GO:0003824 GO:0003854 GO:0050662 GO:0016616

Cluster-22178.61151 GO:0019722
Cluster-22178.61152 GO:0015631 GO:0045298
Cluster-22178.61153 GO:0055114 GO:0016491
Cluster-22178.61154 GO:0055114 GO:0016491
Cluster-22178.61156 GO:0016055 GO:0008013 GO:0046872 GO:0016342
Cluster-22178.61157 GO:0042302
Cluster-22178.61158 GO:0006281 GO:0006310 GO:0016887 GO:0005524
GO:0003678 GO:0009378 GO:0016021 GO:0005657 GO:0009379
Cluster-22178.61160 GO:0009252 GO:0046436 GO:0006633 GO:0006090
GO:0008716 GO:0005524 GO:0046872 GO:0003989 GO:0009317
Cluster-22178.61161 GO:0005515 GO:0005524
Cluster-22178.61162 GO:0055114 GO:0008168 GO:0016706 GO:0019028
Cluster-22178.61164 GO:0003676 GO:0003723 GO:0019013
Cluster-22178.61166 GO:0006470 GO:0006570 GO:0004725 GO:0008138

Cluster-22178.61169 GO:0007155 GO:0006144 GO:0005094 GO:0003877
GO:0005737
Cluster-22178.61172 GO:0005515
Cluster-22178.61176 GO:0006260 GO:0006351 GO:0006269 GO:0003896
GO:0005657 GO:0005730
Cluster-22178.61177 GO:0008270
Cluster-22178.61178 GO:0006468 GO:0005524 GO:0004672
Cluster-22178.61180 GO:0006144 GO:0035556 GO:0009190 GO:0046039
GO:0006182 GO:0046872 GO:0003676 GO:0003723 GO:0016849
GO:0004383 GO:0020037 GO:0005576
Cluster-22178.61182 GO:0035556 GO:0006144 GO:0006182 GO:0009190
GO:0046039 GO:0003676 GO:0046872 GO:0020037 GO:0004383
GO:0016849 GO:0003723 GO:0005576
Cluster-22178.61184 GO:0006281 GO:0003684
Cluster-22178.61185 GO:0007165 GO:0007186 GO:0031683 GO:0019001
GO:0003924 GO:0005525 GO:0004871
Cluster-22178.61186 GO:0009271 GO:0006508 GO:0006355 GO:0004198
GO:0008270 GO:0005622

Cluster-22178.61188 GO:0005887
Cluster-22178.61191 GO:0005515
Cluster-22178.61193 GO:0005515 GO:0008270
Cluster-22178.61196 GO:0006811 GO:0006813 GO:0005249 GO:0008076
GO:0016020 GO:0016021
Cluster-22178.61197 GO:0005515
Cluster-22178.61198 GO:0005515
Cluster-22178.61199 GO:0000390 GO:0071008
Cluster-22178.61200 GO:0006355 GO:0005515 GO:0005634
Cluster-22178.61208 GO:0006446 GO:0006413 GO:0003743 GO:0005840
Cluster-22178.61209 GO:0016192 GO:0006886 GO:0005198 GO:0030130
GO:0030132
Cluster-22178.61211 GO:0006098 GO:0055114 GO:0019521 GO:0008152
GO:0051287 GO:0016616 GO:0004616
Cluster-22178.61212 GO:0006098 GO:0055114 GO:0019521 GO:0008152
GO:0051287 GO:0016616 GO:0004616
Cluster-22178.61214 GO:0006468 GO:0005515 GO:0004672
Cluster-22178.61215 GO:0006508 GO:0070008 GO:0008236 GO:0004252

Cluster-22178.61216 GO:0007264 GO:0016887 GO:0003924 GO:0005524
GO:0005525 GO:0016020
Cluster-22178.61218 GO:0006388 GO:0051252 GO:0006351 GO:0003714
GO:0000213 GO:0005667 GO:0000214
Cluster-22178.61219 GO:0006144 GO:0006206 GO:0015992 GO:0015979
GO:0006123 GO:0050790 GO:0006351 GO:0003677 GO:0004129
GO:0005507 GO:0003899 GO:0030234 GO:0005730 GO:0016020
GO:0009523 GO:0009539 GO:0045277
Cluster-22178.61220 GO:0016052 GO:0005975 GO:0015992 GO:0055114
GO:0015986 GO:0043093 GO:0000917 GO:0016192 GO:0016491
GO:0015078 GO:0004553 GO:0030246 GO:0005737 GO:0000276
Cluster-22178.61221 GO:0019497 GO:0048013 GO:0006468 GO:0006771
GO:0016773 GO:0046872 GO:0005524 GO:0004672 GO:0003993
GO:0005515 GO:0016020

Cluster-22178.61223 GO:0055114
Cluster-22178.61224 GO:0006914 GO:0007165 GO:0004871 GO:0016020
Cluster-22178.61225 GO:0006260 GO:0005515
Cluster-22178.61226 GO:0042254 GO:0006412 GO:0006364 GO:0003735
GO:0005622 GO:0005840
Cluster-22178.61227 GO:0009405 GO:0045735 GO:0005515 GO:0030430
Cluster-22178.61229 GO:0007186 GO:0007275 GO:0007165 GO:0070206
GO:0006260 GO:0005179 GO:0004930 GO:0005576 GO:0016021
Cluster-22178.61230 GO:0006123 GO:0015992 GO:0004129 GO:0045277
GO:0005743
Cluster-22178.61232 GO:0006338 GO:0003677 GO:0016818 GO:0000049
GO:0003676 GO:0005524 GO:0046983 GO:0005634
Cluster-22178.61234 GO:0050708 GO:0005515 GO:0051537 GO:0005737
Cluster-22178.61235 GO:0006139 GO:0008168
Cluster-22178.61240 GO:0006118 GO:0007165 GO:0035556 GO:0004871
GO:0020037 GO:0009055 GO:0008270
Cluster-22178.61241 GO:0006355 GO:0005515 GO:0003677 GO:0008270
GO:0046872
Cluster-22178.61242 GO:0006464 GO:0007186 GO:0004930 GO:0016020
Cluster-22178.61245 GO:0008440
Cluster-22178.61246 GO:0007411 GO:0016055 GO:0008092 GO:0019898
GO:0005576 GO:0005737
Cluster-22178.61247 GO:0006120 GO:0006744 GO:0015992 GO:0006118
GO:0006814 GO:0006629 GO:0016627 GO:0008137 GO:0003954
GO:0016021 GO:0005737 GO:0005739
Cluster-22178.61248 GO:0061024 GO:0046935 GO:0005944 GO:0030176
Cluster-22178.61249 GO:0000917 GO:0043093 GO:0006355 GO:0007049
GO:0006260 GO:0010506 GO:0051016 GO:0003700 GO:0043565
GO:0008290 GO:0005667 GO:0005634 GO:0005615 GO:0005737
Cluster-22178.61250 GO:0043065 GO:0016020 GO:0005737
Cluster-22178.61251 GO:0005975 GO:0006886 GO:0016832 GO:0005741
GO:0016020 GO:0016021
Cluster-22178.61252 GO:0006470 GO:0006570 GO:0008138 GO:0004725

Cluster-22178.61254 GO:0006464 GO:0006898 GO:0007165 GO:0005044
GO:0016020
Cluster-22178.61256 GO:0043093 GO:0006171 GO:0006810 GO:0002949
GO:0000917 GO:0009405 GO:0048870 GO:0016787 GO:0008294
GO:0003677 GO:0005524 GO:0016887 GO:0046983 GO:0003678
GO:0005657 GO:0005576 GO:0031514 GO:0005737
Cluster-22178.61257 GO:0015992 GO:0055114 GO:0055085 GO:0015986
GO:0006119 GO:0046933 GO:0016638 GO:0046961 GO:0005515
GO:0016021 GO:0045259 GO:0045261
Cluster-22178.61258 GO:0006468 GO:0005515 GO:0016773 GO:0004672
GO:0005524 GO:0016020
Cluster-22178.61262 GO:0019497 GO:0006013 GO:0006000 GO:0006771
GO:0003993 GO:0005524 GO:0003873 GO:0016301
Cluster-22178.61263 GO:0006355 GO:0009405 GO:0005515 GO:0005634
Cluster-22178.61265 GO:0006119 GO:0015991 GO:0015992 GO:0046961
GO:0033178
Cluster-22178.61266 GO:0009297 GO:0006376 GO:0043087 GO:0003729
GO:0005685 GO:0019867
Cluster-22178.61268 GO:0006508 GO:0004252
Cluster-22178.61270 GO:0055114 GO:0016491 GO:0071949 GO:0003950
GO:0005634
Cluster-22178.61273 GO:0006446 GO:0006413 GO:0003743 GO:0003723
GO:0005840 GO:0005737
Cluster-22178.61274 GO:0055085 GO:0006468 GO:0022857 GO:0005524
GO:0004672 GO:0016021
Cluster-22178.61275 GO:0006355 GO:0008270 GO:0016820 GO:0005634
GO:0005739 GO:0016021
Cluster-22178.61276 GO:0006468 GO:0055085 GO:0015689 GO:0015098
GO:0022857 GO:0016773 GO:0005524 GO:0004672 GO:0016020
GO:0016021
Cluster-22178.61279 GO:0019370 GO:0034453 GO:0000042 GO:0006508
GO:0004222 GO:0008270 GO:0005515 GO:0008237 GO:0005815

GO:0016021
Cluster-22178.61280 GO:0034453 GO:0000042 GO:0019370 GO:0005515
GO:0008237 GO:0008270 GO:0005815 GO:0016021
Cluster-22178.61284 GO:0055085 GO:0030001 GO:0046873 GO:0016020
Cluster-22178.61285 GO:0055085 GO:0030001 GO:0046873 GO:0016020
Cluster-22178.61288 GO:0005515
Cluster-22178.61289 GO:0005515
Cluster-22178.61291 GO:0008643 GO:0055085 GO:0005351 GO:0022857
GO:0000139 GO:0016021 GO:0016020
Cluster-22178.61293 GO:0005975
Cluster-22178.61294 GO:0006351 GO:0008152 GO:0055114 GO:0006144
GO:0006206 GO:0003899 GO:0003677 GO:0016491 GO:0005730
Cluster-22178.61295 GO:0055085 GO:0006888 GO:0006810 GO:0022857
GO:0005215 GO:0005515 GO:0005801 GO:0019867 GO:0016021
GO:0005622 GO:0016020
Cluster-22178.61299 GO:0046656 GO:0055114 GO:0015948 GO:0006511
GO:0031625 GO:0047068 GO:0018537
Cluster-22178.61307 GO:0007264 GO:0006576 GO:0005524 GO:0005525

Cluster-22178.61309 GO:0016310 GO:0006352 GO:0005975 GO:0009069
GO:0006468 GO:0003824 GO:0005524 GO:0004672 GO:0016773
GO:0005515 GO:0008384 GO:0005669 GO:0016020 GO:0008385
Cluster-22178.61310 GO:0005515
Cluster-22178.61311 GO:0006952 GO:0016787
Cluster-22178.61314 GO:0015886 GO:0055085 GO:0015232 GO:0022857
GO:0016021
Cluster-22178.61315 GO:0008152 GO:0005515 GO:0003824
Cluster-22178.61317 GO:0016020
Cluster-22178.61319 GO:0004672
Cluster-22178.61320 GO:0016567 GO:0004842 GO:0016787 GO:0005680
Cluster-22178.61321 GO:0006879 GO:0008199
Cluster-22178.61323 GO:0009116 GO:0006144 GO:0009165 GO:0006098
GO:0004749 GO:0000287

Cluster-22178.61324 GO:0006406 GO:0055085 GO:0006998 GO:0035556
GO:0009584 GO:0006611 GO:0006355 GO:0018298 GO:0006810
GO:0005215 GO:0005515 GO:0016020 GO:0031965
Cluster-22178.61325 GO:0051252 GO:0006468 GO:0006470 GO:0006402
GO:0008138 GO:0003723 GO:0003676 GO:0004672 GO:0005524
GO:0046872 GO:0004535 GO:0005634 GO:0005737
Cluster-22178.61326 GO:0006468 GO:0016560 GO:0005524 GO:0004672
GO:0016021 GO:0005777
Cluster-22178.61327 GO:0006470 GO:0006402 GO:0051252 GO:0006468
GO:0003676 GO:0004535 GO:0046872 GO:0005524 GO:0004672
GO:0008138 GO:0003723 GO:0005737 GO:0005634
Cluster-22178.61329 GO:0003676
Cluster-22178.61330 GO:0017025 GO:0005634
Cluster-22178.61331 GO:0017025 GO:0005634
Cluster-22178.61332 GO:0006446 GO:0003743 GO:0005852 GO:0005840
GO:0005737
Cluster-22178.61333 GO:0042981 GO:0006468 GO:0043565 GO:0004672
GO:0005524
Cluster-22178.61335 GO:0007010 GO:0055085 GO:0046872 GO:0003779
GO:0016021
Cluster-22178.61336 GO:0005515 GO:0003682 GO:0000785
Cluster-22178.61337 GO:0006413 GO:0006270 GO:0006886 GO:0006446
GO:0003723 GO:0004190 GO:0003743 GO:0005741 GO:0005840
GO:0016021
Cluster-22178.61341 GO:0005515
Cluster-22178.61342 GO:0055085 GO:0006811 GO:0000917 GO:0043093
GO:0006355 GO:0005515 GO:0005216 GO:0005737 GO:0016020
Cluster-22178.61343 GO:0005515
Cluster-22178.61345 GO:0009432 GO:0015991 GO:0051258 GO:0051782
GO:0007165 GO:0030168 GO:0051607 GO:0015992 GO:0005515
GO:0015078 GO:0030674 GO:0005096 GO:0005102 GO:0033179
GO:0005737 GO:0009276 GO:0005577
Cluster-22178.61346 GO:0003677

Cluster-22178.61347 GO:0005515
Cluster-22178.61349 GO:0007016 GO:0016010
Cluster-22178.61350 GO:0003676
Cluster-22178.61351 GO:0055114 GO:0042254 GO:0006412 GO:0006457 GO:0003735 GO:0005515 GO:0005840 GO:0005622
Cluster-22178.61352 GO:0006355 GO:0006950 GO:0009415 GO:0043565 GO:0003700 GO:0005667
Cluster-22178.61353 GO:0006338 GO:0016818 GO:0005515 GO:0003676 GO:0005524 GO:0005634
Cluster-22178.61356 GO:0016055 GO:0003676 GO:0008270 GO:0008013 GO:0016342
Cluster-22178.61357 GO:0071918 GO:0006468 GO:0009069 GO:0016310 GO:0015840 GO:0007178 GO:0015204 GO:0004675 GO:0004672 GO:0005524 GO:0016020 GO:0016021
Cluster-22178.61358 GO:0006508 GO:0005515 GO:0004252
Cluster-22178.61360 GO:0006508 GO:0030436 GO:0035307 GO:0004190 GO:0071595
Cluster-22178.61364 GO:0003676
Cluster-22178.61368 GO:0006118 GO:0006119 GO:0016192 GO:0015992 GO:0008121 GO:0016021
Cluster-22178.61369 GO:0006119 GO:0006118 GO:0015992 GO:0008121

Cluster-22178.61370 GO:0046034 GO:0006144 GO:0004017
Cluster-22178.61371 GO:0051252 GO:0006396 GO:0003723 GO:0004525

Cluster-22178.61374 GO:0007165 GO:0006886 GO:0005515 GO:0005783 GO:0016021
Cluster-22178.61376 GO:0006464 GO:0046872
Cluster-22178.61377 GO:0005524 GO:0003676
Cluster-22178.61378 GO:0006468 GO:0005524 GO:0004672 GO:0005515

Cluster-22178.61379 GO:0006468 GO:0042981 GO:0005524 GO:0004672 GO:0005515

Cluster-22178.61380 GO:0007156 GO:0006396 GO:0051252 GO:0003723
GO:0004525 GO:0005509 GO:0016020
Cluster-22178.61382 GO:0006891 GO:0031110 GO:0015031 GO:0030168
GO:0007165 GO:0006869 GO:0042157 GO:0000090 GO:0007067
GO:0007059 GO:0051301 GO:0051258 GO:0006904 GO:0006813
GO:0008017 GO:0005102 GO:0015269 GO:0030674 GO:0005516
GO:0008289 GO:0005876 GO:0005577 GO:0000940 GO:0000145
GO:0017119 GO:0045298 GO:0016021 GO:0005576
Cluster-22178.61384 GO:0006355 GO:0046983
Cluster-22178.61385 GO:0006810 GO:0009405 GO:0019871 GO:0005576
Cluster-22178.61387 GO:0005518 GO:0005581
Cluster-22178.61388 GO:0009405 GO:0006810 GO:0019871 GO:0005576
Cluster-22178.61392 GO:0009235 GO:0006355 GO:0005515 GO:0046872
GO:0005634
Cluster-22178.61394 GO:0005515 GO:0003677
Cluster-22178.61395 GO:0006508 GO:0004222
Cluster-22178.61399 GO:0016787
Cluster-22178.61400 GO:0016787
Cluster-22178.61404 GO:0008643 GO:0055085 GO:0006885 GO:0006812
GO:0009401 GO:0008982 GO:0005198 GO:0015299 GO:0009357
GO:0005737 GO:0016021
Cluster-22178.61406 GO:0042254 GO:0006412 GO:0005515 GO:0003735
GO:0015934 GO:0005840
Cluster-22178.61409 GO:0003676
Cluster-22178.61410 GO:0008270 GO:0046872 GO:0005515 GO:0005622
GO:0031224
Cluster-22178.61411 GO:0005515
Cluster-22178.61412 GO:0006351 GO:0006144 GO:0006206 GO:0004519
GO:0003899 GO:0003677 GO:0005730 GO:0030880
Cluster-22178.61413 GO:0006260 GO:0006352 GO:0051479 GO:0006355
GO:0016987 GO:0003724 GO:0008270 GO:0005524 GO:0016887
GO:0003677 GO:0005525 GO:0003700 GO:0046872 GO:0003924
GO:0050504 GO:0005515 GO:0003723 GO:0005667 GO:0005737

Cluster-22178.61416 GO:0015675 GO:0035444 GO:0030474 GO:0071988
GO:0007165 GO:0046872 GO:0015099 GO:0051082 GO:0005179
GO:0016021 GO:0005887 GO:0005576
Cluster-22178.61418 GO:0003676 GO:0016021
Cluster-22178.61420 GO:0008152 GO:0005515 GO:0016829 GO:0010333

Cluster-22178.61421 GO:0005515
Cluster-22178.61423 GO:0005515 GO:0016020
Cluster-22178.61429 GO:0006308 GO:0009307 GO:0006281 GO:0009036
GO:0003677 GO:0016020 GO:0009359 GO:0005634
Cluster-22178.61431 GO:0003333 GO:0051205 GO:0042967 GO:0006865
GO:0015171 GO:0008080 GO:0016020 GO:0019033 GO:0016021
Cluster-22178.61433 GO:0016021
Cluster-22178.61434 GO:0006355 GO:0003700 GO:0005667
Cluster-22178.61436 GO:0006629 GO:0005515 GO:0005509 GO:0008081

Cluster-22178.61438 GO:0018401 GO:0055114 GO:0015992 GO:0016567
GO:0006560 GO:0006525 GO:0015986 GO:0015078 GO:0004842
GO:0046983 GO:0016702 GO:0008270 GO:0004656 GO:0000276
GO:0005783
Cluster-22178.61439 GO:0016020 GO:0016021
Cluster-22178.61440 GO:0005634
Cluster-22178.61441 GO:0007165 GO:0042254 GO:0006412 GO:0007016
GO:0003735 GO:0005158 GO:0003779 GO:0005200 GO:0000062
GO:0001726 GO:0005925 GO:0005622 GO:0005899 GO:0005856
GO:0005840
Cluster-22178.61442 GO:0055114 GO:0016972
Cluster-22178.61444 GO:0008210 GO:0006568 GO:0006550 GO:0006552
GO:0006574 GO:0055114 GO:0008152 GO:0006694 GO:0018874
GO:0008209 GO:0006631 GO:0008207 GO:0006554 GO:0006633
GO:0003857 GO:0003854 GO:0016616 GO:0016491
Cluster-22178.61447 GO:0006364 GO:0006270 GO:0006355 GO:0003676
GO:0008270 GO:0005515 GO:0005732 GO:0005634 GO:0034457

Cluster-22178.61448 GO:0019054 GO:0009058 GO:0042742 GO:0004857
GO:0016740 GO:0008191
Cluster-22178.61449 GO:0034220
Cluster-22178.6145 GO:0043087 GO:0035023 GO:0009306 GO:0046872
GO:0005089
Cluster-22178.61450 GO:0007165
Cluster-22178.61451 GO:0006886 GO:0008152 GO:0015074 GO:0015979
GO:0055114 GO:0035091 GO:0016491 GO:0016020 GO:0019028
GO:0019031 GO:0009539 GO:0009523
Cluster-22178.61452 GO:0006886 GO:0055114 GO:0006308 GO:0015074
GO:0008152 GO:0006457 GO:0015979 GO:0042803 GO:0035091
GO:0051087 GO:0008855 GO:0016491 GO:0000774 GO:0019028
GO:0009318 GO:0009523 GO:0009539 GO:0016020
Cluster-22178.61453 GO:0008152 GO:0015979 GO:0006886 GO:0035091
GO:0009523 GO:0009539 GO:0016020 GO:0019028
Cluster-22178.61454 GO:0003676 GO:0008270 GO:0005622
Cluster-22178.61455 GO:0008270 GO:0003676 GO:0015066 GO:0005622
Cluster-22178.61456 GO:0006810 GO:0005622
Cluster-22178.61458 GO:0008152 GO:0006118 GO:0055114 GO:0050660
GO:0003995 GO:0016627
Cluster-22178.61460 GO:0006144 GO:0055114 GO:0000103 GO:0008152
GO:0016032 GO:0006118 GO:0046872 GO:0016627 GO:0005524
GO:0004020 GO:0050660 GO:0005525 GO:0016740 GO:0003995
Cluster-22178.61461 GO:0015977 GO:0046487 GO:0006950 GO:0016984
GO:0005680 GO:0009573 GO:0016021
Cluster-22178.61465 GO:0008152 GO:0016491
Cluster-22178.61466 GO:0006950 GO:0003677
Cluster-22178.61467 GO:0007275 GO:0006511 GO:0016567 GO:0006464
GO:0006206 GO:0006144 GO:0006351 GO:0009405 GO:0003677
GO:0008270 GO:0004842 GO:0003899 GO:0005198 GO:0005515
GO:0046872 GO:0016151 GO:0005730 GO:0005680 GO:0005634
Cluster-22178.6147 GO:0007275 GO:0005578
Cluster-22178.61470 GO:0055085 GO:0006810 GO:0006820 GO:0005215

GO:0016020 GO:0016021
Cluster-22178.61471 GO:0051537 GO:0043231
Cluster-22178.61474 GO:0007268 GO:0006508 GO:0030695 GO:0033130
GO:0004197 GO:0043495 GO:0042802 GO:0005515
Cluster-22178.61475 GO:0007165 GO:0014068 GO:0016226 GO:0035556
GO:0050776 GO:0043548 GO:0051536 GO:0005102 GO:0016020
GO:0005737
Cluster-22178.61478 GO:0055114 GO:0006725 GO:0006810 GO:0008199
GO:0003824 GO:0016021
Cluster-22178.61481 GO:0005515 GO:0005509 GO:0005576
Cluster-22178.61482 GO:0005509 GO:0005515 GO:0005576
Cluster-22178.61483 GO:0006144 GO:0046034 GO:0006355 GO:0043565
GO:0046872 GO:0003700 GO:0004017 GO:0005667
Cluster-22178.61485 GO:0006810 GO:0006629 GO:0042626 GO:0016787
GO:0005524 GO:0016021
Cluster-22178.61486 GO:0051301 GO:0007049 GO:0005509 GO:0016021
Cluster-22178.61488 GO:0006270 GO:0006816 GO:0006874 GO:0006383
GO:0006396 GO:0006144 GO:0006206 GO:0006351 GO:0030036
GO:0003677 GO:0003899 GO:0003723 GO:0005219 GO:0003676
GO:0003779 GO:0005730 GO:0005666 GO:0005622 GO:0030054
GO:0016021
Cluster-22178.61489 GO:0006468 GO:0006813 GO:0016032 GO:0006508
GO:0006118 GO:0055114 GO:0007219 GO:0008152 GO:0007275
GO:0006511 GO:0009236 GO:0035556 GO:0003995 GO:0020037
GO:0046872 GO:0016627 GO:0004672 GO:0048472 GO:0051287
GO:0071949 GO:0016616 GO:0008641 GO:0005524 GO:0008270
GO:0016491 GO:0016773 GO:0050660 GO:0008233 GO:0005634
GO:0016021 GO:0016020
Cluster-22178.61493 GO:0035091
Cluster-22178.61494 GO:0006355
Cluster-22178.61495 GO:0006260 GO:0006914 GO:0006370 GO:0006310
GO:0006397 GO:0006281 GO:0003910 GO:0005524 GO:0003677
GO:0004484

Cluster-22178.61498 GO:0055114 GO:0008168 GO:0016706 GO:0019028
Cluster-22178.61499 GO:0035556 GO:0007165 GO:0005515
Cluster-22178.61500 GO:0006810 GO:0019079 GO:0003676 GO:0004386
GO:0008026 GO:0043531 GO:0005524
Cluster-22178.61502 GO:0005515
Cluster-22178.61503 GO:0015031 GO:0006606 GO:0015991 GO:0006886
GO:0016192 GO:0008565 GO:0016820 GO:0005515 GO:0005737
GO:0000221 GO:0005634 GO:0030117
Cluster-22178.61504 GO:0006508 GO:0006810 GO:0005524 GO:0004386
GO:0003676 GO:0016787 GO:0003677 GO:0004197
Cluster-22178.61505 GO:0015684 GO:0007264 GO:0005525 GO:0015093
GO:0005524 GO:0003924 GO:0016021
Cluster-22178.61507 GO:0043039 GO:0016055 GO:0006531 GO:0006522
GO:0006419 GO:0000166 GO:0003676 GO:0016876 GO:0004813
GO:0005524
Cluster-22178.61508 GO:0006413 GO:0006446 GO:0031369 GO:0003743
GO:0005515 GO:0005852 GO:0005840
Cluster-22178.61511 GO:0006886 GO:0042254 GO:0006412 GO:0006888
GO:0003735 GO:0003824 GO:0008270 GO:0030127 GO:0005840
GO:0005622
Cluster-22178.61517 GO:0006810 GO:0016567 GO:0008270 GO:0046872
GO:0004842 GO:0005515 GO:0005680 GO:0016020
Cluster-22178.61519 GO:0032065 GO:0042998 GO:0007186 GO:0005543
GO:0004930 GO:0071253 GO:0005515 GO:0005938 GO:0005802
Cluster-22178.61521 GO:0015694 GO:0006367 GO:0015097 GO:0046872
GO:0016020 GO:0005634
Cluster-22178.61525 GO:0003723
Cluster-22178.61526 GO:0006522 GO:0006188 GO:0009152 GO:0006144
GO:0055114 GO:0006520 GO:0009058 GO:0006531 GO:0010277
GO:0004018 GO:0016829 GO:0030170
Cluster-22178.6153 GO:0006508 GO:0003723 GO:0008239
Cluster-22178.61531 GO:0006950 GO:0009415 GO:0061630
Cluster-22178.61532 GO:0016226 GO:0005515 GO:0051536 GO:0005506

Cluster-22178.61533 GO:0048870 GO:0031514
Cluster-22178.61534 GO:0006357 GO:0006308 GO:0008855 GO:0046983
GO:0001104 GO:0009318 GO:0016592 GO:0016020
Cluster-22178.61535 GO:0005515
Cluster-22178.61536 GO:0000917 GO:0043093 GO:0015876 GO:0008521
GO:0016021 GO:0005737
Cluster-22178.61539 GO:0009451 GO:0006886 GO:0006479 GO:0006400
GO:0008152 GO:0008033 GO:0006888 GO:0006554 GO:0008176
GO:0019955 GO:0018024 GO:0008168 GO:0008270 GO:0016020
GO:0030127
Cluster-22178.61542 GO:0015886 GO:0017004 GO:0015232 GO:0016020
Cluster-22178.61543 GO:0055085 GO:0019953 GO:0043248 GO:0022857
GO:0005198 GO:0016021 GO:0030141 GO:0005576
Cluster-22178.61545 GO:0006351 GO:0006355 GO:0007049 GO:0006206
GO:0006144 GO:0003677 GO:0003899 GO:0003700 GO:0043565
GO:0005730 GO:0005667 GO:0005634
Cluster-22178.61546 GO:0016301
Cluster-22178.61547 GO:0009069 GO:0006260 GO:0006914 GO:0006886
GO:0006468 GO:0016310 GO:0007049 GO:0000160 GO:0000155
GO:0016757 GO:0004674 GO:0003774 GO:0005524 GO:0046983
GO:0005515 GO:0016020 GO:0016459 GO:0009365 GO:0016021
GO:0005801
Cluster-22178.61549 GO:0005515
Cluster-22178.61550 GO:0006811 GO:0055085 GO:0006816 GO:0006874
GO:0005219 GO:0005509 GO:0005515 GO:0005216 GO:0004064
GO:0016021 GO:0005622 GO:0016020
Cluster-22178.61552 GO:0008270
Cluster-22178.61554 GO:0008152 GO:0055114 GO:0003824 GO:0050662
GO:0016491
Cluster-22178.61555 GO:0005515
Cluster-22178.61556 GO:0006206 GO:0006144 GO:0008152 GO:0006351
GO:0006479 GO:0031167 GO:0008168 GO:0003677 GO:0003899

GO:0005730

Cluster-22178.61558 GO:0005634

Cluster-22178.61561 GO:0006744 GO:0055114 GO:0009271 GO:0016567

GO:0006355 GO:0004842 GO:0046872 GO:0008270 GO:0005515

Cluster-22178.61563 GO:0015979 GO:0015031 GO:0006582 GO:0007155

GO:0006904 GO:0006457 GO:0000774 GO:0030411 GO:0051087

GO:0003677 GO:0042803 GO:0009522 GO:0009538 GO:0000145

Cluster-22178.61565 GO:0016021

Cluster-22178.61566 GO:0006418 GO:0000166 GO:0005524 GO:0004812

Cluster-22178.61567 GO:0008152 GO:0003824

Cluster-22178.61568 GO:0006457 GO:0005509 GO:0051082 GO:0032040

GO:0005783 GO:0019031

Cluster-22178.6157 GO:0007156 GO:0006813 GO:0006811 GO:0030955

GO:0005216 GO:0005509 GO:0005249 GO:0008270 GO:0016020

GO:0008076 GO:0016021

Cluster-22178.61570 GO:0006098 GO:0006813 GO:0055114 GO:0019521

GO:0016616 GO:0004616 GO:0051287

Cluster-22178.61573 GO:0003676

Cluster-22178.61574 GO:0018279 GO:0006486 GO:0004579 GO:0008250

GO:0016021 GO:0005783

Cluster-22178.61579 GO:0007186 GO:0008360 GO:0015979 GO:0003677

GO:0042802 GO:0005515 GO:0004930 GO:0016020 GO:0016021

GO:0009539 GO:0009523

Cluster-22178.61580 GO:0006633 GO:0055114 GO:0016491 GO:0005506

Cluster-22178.61581 GO:0016055 GO:0008013 GO:0008270 GO:0003676

GO:0016342

Cluster-22178.61583 GO:0031124 GO:0005515

Cluster-22178.61585 GO:0050909 GO:0007186 GO:0004930 GO:0016021

Cluster-22178.61586 GO:0055114 GO:0015940 GO:0008677 GO:0016491

GO:0071949

Cluster-22178.61587 GO:0004672

Cluster-22178.61588 GO:0006355 GO:0003700 GO:0004190 GO:0016021
GO:0005667
Cluster-22178.61589 GO:0035556 GO:0019087 GO:0055114 GO:0006468
GO:0005524 GO:0016773 GO:0047134 GO:0003724 GO:0003712
GO:0016818 GO:0004672 GO:0005515 GO:0005667 GO:0016020
GO:0016021 GO:0005634
Cluster-22178.61590 GO:0006457 GO:0005515
Cluster-22178.61592 GO:0008643 GO:0008982 GO:0016787 GO:0015016
GO:0008146 GO:0009357
Cluster-22178.61596 GO:0005515
Cluster-22178.61597 GO:0006278 GO:0004672 GO:0003964 GO:0005515

Cluster-22178.61598 GO:0006355 GO:0006281 GO:0006525 GO:0006310
GO:0003677 GO:0000166 GO:0009015 GO:0008168 GO:0046872
GO:0008270 GO:0005634
Cluster-22178.61600 GO:0018342 GO:0008318
Cluster-22178.61604 GO:0006378 GO:0006313 GO:0003729 GO:0016787
GO:0004803 GO:0003677 GO:0005849
Cluster-22178.61608 GO:0035091
Cluster-22178.61609 GO:0042254 GO:0006412 GO:0003735 GO:0005622
GO:0005840
Cluster-22178.61611 GO:0005515
Cluster-22178.61613 GO:0051205 GO:0055114 GO:0015948 GO:0016021
GO:0016020
Cluster-22178.61614 GO:0009116
Cluster-22178.61615 GO:0007165 GO:0005515 GO:0005096
Cluster-22178.61616 GO:0015979 GO:2001070 GO:0030145 GO:0005515
GO:0008146 GO:0009523 GO:0016021
Cluster-22178.61619 GO:0016973 GO:0006355 GO:0048870 GO:0003700
GO:0031514 GO:0005667 GO:0016021 GO:0005643
Cluster-22178.61621 GO:0042254 GO:0003735 GO:0005840
Cluster-22178.61622 GO:0051252 GO:0006388 GO:0000213 GO:0000214
Cluster-22178.61627 GO:0006952 GO:0006313 GO:0006412 GO:0006310

GO:0006471 GO:0015074 GO:0003677 GO:0005515 GO:0003950
GO:0000150 GO:0008168
Cluster-22178.61629 GO:0043093 GO:0006355 GO:0000917 GO:0003677
GO:0043565 GO:0003700 GO:0005667 GO:0005634 GO:0005737
Cluster-22178.61630 GO:0005576
Cluster-22178.61631 GO:0016567 GO:0004842 GO:0005515
Cluster-22178.61632 GO:0007165 GO:0007186 GO:0006886 GO:0005525
GO:0004871 GO:0003924 GO:0005047 GO:0005834 GO:0005785
GO:0005786
Cluster-22178.61633 GO:0006468 GO:0042254 GO:0006412 GO:0055114
GO:0051287 GO:0003735 GO:0016491 GO:0004672 GO:0016798
GO:0005524 GO:0005840
Cluster-22178.61634 GO:0006508 GO:0004222 GO:0005515
Cluster-22178.61638 GO:0030474 GO:0019069 GO:0005200 GO:0005515
GO:0005823 GO:0005856
Cluster-22178.61639 GO:0055114 GO:0006118 GO:0008152 GO:0016627
GO:0050660 GO:0003995
Cluster-22178.61640 GO:0019069 GO:0030474 GO:0019089 GO:0005200
GO:0005515 GO:0005856 GO:0005823
Cluster-22178.61641 GO:0016021
Cluster-22178.61644 GO:0007165 GO:0005179 GO:0005576
Cluster-22178.61645 GO:0006182 GO:0046039 GO:0009190 GO:0006468
GO:0035556 GO:0006144 GO:0004383 GO:0016849 GO:0004672
GO:0005524
Cluster-22178.61646 GO:0005975 GO:0006013 GO:0008270 GO:0046872
GO:0034639 GO:0004553 GO:0004559 GO:0015923 GO:0016021
Cluster-22178.61647 GO:0003676 GO:0004386 GO:0003677 GO:0003723
GO:0016787 GO:0005524 GO:0005634
Cluster-22178.61649 GO:0006367 GO:0005515 GO:0046872 GO:0008270
GO:0005672
Cluster-22178.61650 GO:0071555 GO:0030288
Cluster-22178.61652 GO:0000160 GO:0016310 GO:0046983 GO:0000155
GO:0016021 GO:0009365

Cluster-22178.61653 GO:0007186 GO:0000271 GO:0004930 GO:0005515 GO:0016021 GO:0016020
Cluster-22178.61654 GO:0000271 GO:0007186 GO:0005515 GO:0004930 GO:0016020 GO:0016021
Cluster-22178.61655 GO:0006310 GO:0006206 GO:0002949 GO:0006281 GO:0006355 GO:0006576 GO:0006139 GO:0006352 GO:0009378 GO:0005524 GO:0016887 GO:0051537 GO:0004127 GO:0003724 GO:0003723 GO:0008134 GO:0005515 GO:0003678 GO:0043231 GO:0009379 GO:0005667 GO:0005657 GO:0005669
Cluster-22178.61656 GO:0006576 GO:0006355 GO:0030001 GO:0006281 GO:0006352 GO:0006139 GO:0006310 GO:0006206 GO:0055085 GO:0002949 GO:0003723 GO:0005515 GO:0008134 GO:0000166 GO:0003678 GO:0046873 GO:0009378 GO:0098519 GO:0003677 GO:0016887 GO:0005524 GO:0004127 GO:0003724 GO:0051537 GO:0005669 GO:0005667 GO:0009379 GO:0043231 GO:0016020 GO:0005657
Cluster-22178.61657 GO:0005515
Cluster-22178.61659 GO:0003676
Cluster-22178.6166 GO:0006355 GO:0007165 GO:0006955 GO:0000902 GO:0007166 GO:0004888 GO:0005515 GO:0043565 GO:0003700 GO:0005667 GO:0016020
Cluster-22178.61661 GO:0055114 GO:0006749 GO:0006804 GO:0006979 GO:0016209 GO:0004602 GO:0016491
Cluster-22178.61662 GO:0003723
Cluster-22178.61670 GO:0009405 GO:0006810 GO:0046486 GO:0006071 GO:0004371 GO:0008200 GO:0042151
Cluster-22178.61672 GO:0003676 GO:0016021
Cluster-22178.61673 GO:0008033 GO:0006400 GO:0006886 GO:0016192 GO:0009451 GO:0007165 GO:0046872 GO:0008176 GO:0005515 GO:0016301 GO:0016021 GO:0030117
Cluster-22178.61677 GO:0005515
Cluster-22178.61678 GO:0007165 GO:0005179 GO:0005576
Cluster-22178.61679 GO:0042476 GO:0046740 GO:0006355 GO:0042475

GO:0030345 GO:0046914 GO:0003700 GO:0008201 GO:0003677
GO:0016021 GO:0005667 GO:0005578
Cluster-22178.61683 GO:0006270 GO:0005198 GO:0019031 GO:0019028
Cluster-22178.61686 GO:0006457 GO:0008013 GO:0051082 GO:0016342
GO:0016272
Cluster-22178.61687 GO:0006457 GO:0051082 GO:0008013 GO:0016342
GO:0016272
Cluster-22178.61688 GO:0007017 GO:0006412 GO:0042254 GO:0007018
GO:0005524 GO:0016887 GO:0003777 GO:0003735 GO:0030286
GO:0005622 GO:0005840 GO:0005874
Cluster-22178.61691 GO:0005515
Cluster-22178.61693 GO:0048193 GO:0070273
Cluster-22178.61694 GO:0005634
Cluster-22178.61695 GO:0055085 GO:0005741
Cluster-22178.61696 GO:0042254 GO:0006412 GO:0006506 GO:0016746
GO:0003735 GO:0016021 GO:0005840 GO:0005789 GO:0005739
Cluster-22178.61698 GO:0000281 GO:0000226 GO:0040001 GO:0005819
GO:0005874
Cluster-22178.61699 GO:0007186 GO:0004930 GO:0016021
Cluster-22178.61701 GO:0006508 GO:0008234
Cluster-22178.61702 GO:0055114 GO:0040007 GO:0006259 GO:0008283
GO:0007165 GO:0016627 GO:0005524 GO:0003824 GO:0047134
GO:0008083 GO:0003677 GO:0005694
Cluster-22178.61704 GO:0006357 GO:0001104 GO:0016592
Cluster-22178.61705 GO:0006541 GO:0006221 GO:0006206 GO:0003883
GO:0016787
Cluster-22178.61707 GO:0043039 GO:0016876 GO:0005524 GO:0003676
GO:0016021
Cluster-22178.61708 GO:0006123 GO:0006812 GO:0006820 GO:0055114
GO:0015992 GO:0006835 GO:0017153 GO:0016638 GO:0004129
GO:0016020 GO:0045277
Cluster-22178.61709 GO:0006364
Cluster-22178.61714 GO:0000398 GO:0019439 GO:0016833 GO:0019031

GO:0005681
Cluster-22178.61717 GO:0050912 GO:0007187 GO:0007607 GO:0008527
GO:0016020 GO:0016021
Cluster-22178.61720 GO:0055114 GO:0015946 GO:0004022
Cluster-22178.61721 GO:0009405 GO:0006629 GO:0006508 GO:0008236
GO:0005515 GO:0005198
Cluster-22178.61722 GO:0006241 GO:0006183 GO:0006144 GO:0006165
GO:0006228 GO:0006206 GO:0004550
Cluster-22178.61723 GO:0005515 GO:0005747 GO:0005739
Cluster-22178.61725 GO:0005515
Cluster-22178.61726 GO:0006355 GO:0006614 GO:0006810 GO:0019079
GO:0016787 GO:0003677 GO:0008026 GO:0005524 GO:0016887
GO:0005525 GO:0003676 GO:0004386
Cluster-22178.61727 GO:0005515
Cluster-22178.61728 GO:0007049 GO:0017053
Cluster-22178.61729 GO:0005515
Cluster-22178.6173 GO:0006810 GO:0016020
Cluster-22178.61730 GO:0003676
Cluster-22178.61731 GO:0003676
Cluster-22178.61734 GO:0006885 GO:0006812 GO:0055085 GO:0009401
GO:0008643 GO:0005198 GO:0008982 GO:0015299 GO:0009357
GO:0016021 GO:0005737
Cluster-22178.61735 GO:0016567 GO:0046983 GO:0004842
Cluster-22178.61737 GO:0006464 GO:0016151 GO:0031072 GO:0051082

Cluster-22178.61739 GO:0003333 GO:0006810 GO:0055085 GO:0006865
GO:0015171 GO:0016020
Cluster-22178.61740 GO:0051258 GO:0016310 GO:0006468 GO:0007094
GO:0006397 GO:0030168 GO:0048193 GO:0009069 GO:0006457
GO:0007165 GO:0008092 GO:0051082 GO:0005515 GO:0005524
GO:0005102 GO:0004674 GO:0030674 GO:0005737 GO:0000445
GO:0016272 GO:0005577 GO:0019898 GO:0016020
Cluster-22178.61741 GO:0007165 GO:0035556

Cluster-22178.61744 GO:0006206 GO:0006013 GO:0045893 GO:0009451
GO:0001510 GO:0009452 GO:0006230 GO:0005975 GO:0008033
GO:0003677 GO:0015923 GO:0004553 GO:0008270 GO:0005524
GO:0003723 GO:0004559 GO:0004809 GO:0004797 GO:0046872
GO:0008168 GO:0005634
Cluster-22178.61748 GO:0007275 GO:0007165 GO:0008152 GO:0016624
GO:0005179 GO:0047355 GO:0008270 GO:0016020 GO:0005576
Cluster-22178.61749 GO:0016568 GO:0042967 GO:0004402 GO:0008080
GO:0000123
Cluster-22178.61751 GO:0006950 GO:0001671 GO:0051087
Cluster-22178.61752 GO:0008270 GO:0030246 GO:0005515
Cluster-22178.61753 GO:0030246 GO:0008270 GO:0005515
Cluster-22178.61755 GO:0009069 GO:0016310 GO:0004674 GO:0005515

Cluster-22178.61756 GO:0007178 GO:0016310 GO:0015840 GO:0009069
GO:0006468 GO:0071918 GO:0004672 GO:0005524 GO:0015204
GO:0004675 GO:0016021 GO:0016020
Cluster-22178.61757 GO:0006508 GO:0005515 GO:0004252
Cluster-22178.61758 GO:0005515 GO:0046872 GO:0045202 GO:0016021
Cluster-22178.61762 GO:0006351 GO:0005515 GO:0003676 GO:0008270
GO:0005639
Cluster-22178.61764 GO:0006952 GO:0006810 GO:0009405 GO:0019855
GO:0005576 GO:0019012
Cluster-22178.61765 GO:0035556 GO:0016567 GO:0004842 GO:0005515
GO:0046872 GO:0008270 GO:0005680
Cluster-22178.61768 GO:0046656 GO:0015948 GO:0030269 GO:0016020
Cluster-22178.61770 GO:0006777 GO:0006810 GO:0008152 GO:0005524
GO:0016301 GO:0005525
Cluster-22178.61771 GO:0008270
Cluster-22178.61772 GO:0055114 GO:0009395 GO:0006260 GO:0016042
GO:0051536 GO:0004623 GO:0005509 GO:0005576
Cluster-22178.61773 GO:0005509 GO:0005515
Cluster-22178.61774 GO:0032324 GO:0009245 GO:0007165 GO:0008270

GO:0008915 GO:0005179 GO:0005515 GO:0005737 GO:0005615
GO:0016021
Cluster-22178.61775 GO:0009245 GO:0032324 GO:0007165 GO:0008270
GO:0008915 GO:0005515 GO:0005179 GO:0005615 GO:0016021
GO:0005737
Cluster-22178.61776 GO:0046938 GO:0042754 GO:0010038 GO:0045892
GO:0005515 GO:0016756 GO:0046872
Cluster-22178.61777 GO:0016567 GO:0006414 GO:0006448 GO:0051302
GO:0005515 GO:0008270 GO:0046872 GO:0003746 GO:0004842
GO:0005853 GO:0005622 GO:0005840
Cluster-22178.61778 GO:0006260 GO:0009966 GO:0005634 GO:0005783
Cluster-22178.61779 GO:0008033 GO:0006396 GO:0009452 GO:0008152
GO:0001510 GO:0006629 GO:0006400 GO:0031167 GO:0009451
GO:0008168 GO:0008176 GO:0008173 GO:0005515
Cluster-22178.61780 GO:0007165 GO:0005515
Cluster-22178.61781 GO:0006367 GO:0006270 GO:0005672
Cluster-22178.61783 GO:0030430
Cluster-22178.61787 GO:0016021
Cluster-22178.61788 GO:0042254 GO:0006412 GO:0005515 GO:0003735
GO:0005622 GO:0005840
Cluster-22178.61789 GO:0008033 GO:0032259 GO:0006364 GO:0008168
GO:0003723
Cluster-22178.61790 GO:0005515
Cluster-22178.61791 GO:0046872 GO:0008270 GO:0005515
Cluster-22178.61793 GO:0006650 GO:0047396
Cluster-22178.61794 GO:0006351 GO:0006886 GO:0015031 GO:0006464
GO:0006888 GO:0005515 GO:0046872 GO:0008270 GO:0046983
GO:0005921 GO:0030127
Cluster-22178.61795 GO:0006281 GO:0004518 GO:0005515
Cluster-22178.61798 GO:0043395 GO:0005515 GO:0005578 GO:0016020
Cluster-22178.61799 GO:0006298 GO:0005524 GO:0030983
Cluster-22178.61800 GO:0030036 GO:0007165 GO:0015684 GO:0007186
GO:0007264 GO:0042438 GO:0005525 GO:0004871 GO:0005507

GO:0019001 GO:0031683 GO:0003924 GO:0003779 GO:0098519
GO:0015093 GO:0030054 GO:0016021 GO:0009295
Cluster-22178.61801 GO:0015031 GO:0006464 GO:0006351 GO:0046983
GO:0005515 GO:0005921
Cluster-22178.61802 GO:0042254 GO:0006412 GO:0015979 GO:0005515
GO:0003735 GO:0003677 GO:0016020 GO:0005840 GO:0009523
GO:0009539 GO:0005622
Cluster-22178.61805 GO:0005515 GO:0016788
Cluster-22178.61807 GO:0005515
Cluster-22178.61809 GO:0006278 GO:0005515 GO:0004672 GO:0003964

Cluster-22178.61810 GO:0055114 GO:0016491 GO:0050661 GO:0016020
Cluster-22178.61811 GO:0051726 GO:0006974 GO:0003677
Cluster-22178.61812 GO:0008270
Cluster-22178.61813 GO:0006468 GO:0004672 GO:0005524
Cluster-22178.61814 GO:0007029 GO:0022900 GO:0005515 GO:0016651
GO:0005789
Cluster-22178.61815 GO:0008270
Cluster-22178.61816 GO:0015991 GO:0015992 GO:0006119 GO:0046961
GO:0035091 GO:0033178
Cluster-22178.61817 GO:0008270 GO:0005515
Cluster-22178.61819 GO:0006355 GO:0009755 GO:0043401 GO:0019281
GO:0006555 GO:0042967 GO:0008899 GO:0005576 GO:0005737
Cluster-22178.61820 GO:0000062
Cluster-22178.61821 GO:0008270
Cluster-22178.61823 GO:0006468 GO:0005524 GO:0004672
Cluster-22178.61825 GO:0006749 GO:0007264 GO:0015684 GO:0006804
GO:0055114 GO:0006979 GO:0015093 GO:0005524 GO:0003924
GO:0004602 GO:0005525 GO:0016021
Cluster-22178.61826 GO:0043087 GO:0005515 GO:0016020
Cluster-22178.61828 GO:0008152 GO:0009452 GO:0008033 GO:0006306
GO:0006479 GO:0009451 GO:0031167 GO:0001510 GO:0006396
GO:0042549 GO:0015979 GO:0006464 GO:0006400 GO:0046500

GO:0003676 GO:0004719 GO:0008168 GO:0008173 GO:0005515
GO:0003677 GO:0008170 GO:0008176 GO:0009539 GO:0009523
Cluster-22178.61829 GO:0009452 GO:0008152 GO:0008033 GO:0006479
GO:0006306 GO:0009451 GO:0031167 GO:0001510 GO:0006396
GO:0006464 GO:0042549 GO:0015979 GO:0046500 GO:0006400
GO:0003676 GO:0004719 GO:0008168 GO:0005515 GO:0008173
GO:0003677 GO:0008170 GO:0008176 GO:0009539 GO:0009523
Cluster-22178.61831 GO:0035556 GO:0006508 GO:0005515 GO:0004252
GO:0004672
Cluster-22178.61832 GO:0005515 GO:0003677
Cluster-22178.61833 GO:0015986 GO:0015992 GO:0015078 GO:0000276
Cluster-22178.61836 GO:0006281 GO:0003684
Cluster-22178.61838 GO:0005515
Cluster-22178.61839 GO:0046907 GO:0006635 GO:0006260 GO:0055114
GO:0006637 GO:0008408 GO:0003887 GO:0003677 GO:0003997
GO:0042575 GO:0005634 GO:0005664 GO:0005777
Cluster-22178.61841 GO:0034227 GO:0046034 GO:0006144 GO:0002098
GO:0006468 GO:0003676 GO:0046872 GO:0008270 GO:0004672
GO:0005524 GO:0004017 GO:0000049 GO:0003677 GO:0016787
GO:0005634
Cluster-22178.61842 GO:0006810 GO:0042626 GO:0005515 GO:0005524
GO:0016021
Cluster-22178.61845 GO:0005515
Cluster-22178.61847 GO:0008219
Cluster-22178.61848 GO:0006355 GO:0008270 GO:0005096 GO:0005544
GO:0005509 GO:0005515 GO:0008023
Cluster-22178.61850 GO:0006260 GO:0007165 GO:0005179 GO:0000808
GO:0005634 GO:0005576
Cluster-22178.61851 GO:0009116 GO:0006098 GO:0006144 GO:0009165
GO:0004749 GO:0000287
Cluster-22178.61852 GO:0007264 GO:0016817 GO:0003924 GO:0046872
GO:0005515 GO:0005525
Cluster-22178.61854 GO:0006281 GO:0000723 GO:0044564 GO:0003678

GO:0005657
Cluster-22178.61855 GO:0044564 GO:0006281 GO:0000723 GO:0003678
GO:0005657
Cluster-22178.61856 GO:0006449 GO:0006415 GO:0003677 GO:0005515
GO:0008270 GO:0016149 GO:0003747 GO:0042025 GO:0018444
GO:0005840 GO:0005737
Cluster-22178.61857 GO:0006629 GO:0006468 GO:0004672 GO:0005524
GO:0016787
Cluster-22178.61858 GO:0006206 GO:0006144 GO:0031144 GO:0071630
GO:0006351 GO:0009405 GO:0010508 GO:0006355 GO:0006468
GO:0005524 GO:0016773 GO:0003677 GO:0003700 GO:0004672
GO:0043565 GO:0003899 GO:0005815 GO:0016020 GO:0005730
GO:0005667 GO:0005634
Cluster-22178.61859 GO:0007165 GO:0008601 GO:0000159
Cluster-22178.61860 GO:0019058 GO:0007165 GO:0007283 GO:0008601
GO:0000159
Cluster-22178.61862 GO:0006508 GO:0007031 GO:0004252 GO:0005509
GO:0005779
Cluster-22178.61863 GO:0006144 GO:0006206 GO:0006351 GO:0003899
GO:0005515 GO:0003677 GO:0005737 GO:0005730
Cluster-22178.61865 GO:0005975 GO:0000902 GO:0016787 GO:0016773

Cluster-22178.61866 GO:0042742 GO:0008152 GO:0016836 GO:0003824
GO:0005576
Cluster-22178.61872 GO:0030833 GO:0034314 GO:0005856 GO:0005885
Cluster-22178.61873 GO:0016746
Cluster-22178.61874 GO:0005506 GO:0016791
Cluster-22178.61877 GO:0035556 GO:0003723 GO:0005515 GO:0000166
GO:0003677 GO:0005524 GO:0046872 GO:0003724 GO:0005525
Cluster-22178.61879 GO:0005515
Cluster-22178.61881 GO:0055114 GO:0015994 GO:0006810 GO:0042167
GO:0008270 GO:0004074 GO:0042597 GO:0016021
Cluster-22178.61882 GO:0005869

Cluster-22178.61885 GO:0006284 GO:0015833 GO:0003677 GO:0000166
GO:0003905 GO:0005524 GO:0016020
Cluster-22178.61886 GO:0016021 GO:0016020
Cluster-22178.61888 GO:0003677 GO:0005634
Cluster-22178.61892 GO:0015947 GO:0006568 GO:0055114 GO:0006979
GO:0006804 GO:0004096 GO:0020037
Cluster-22178.61893 GO:0006367 GO:0005672
Cluster-22178.61894 GO:0003697 GO:0046872
Cluster-22178.61895 GO:0046983
Cluster-22178.61896 GO:0042981 GO:0007165 GO:0005515
Cluster-22178.61898 GO:0006811 GO:0055085 GO:0045735 GO:0005216
GO:0016020
Cluster-22178.61902 GO:0005524
Cluster-22178.61905 GO:0006631 GO:0055114 GO:0008033 GO:0016117
GO:0071949 GO:0051287 GO:0016705 GO:0005515 GO:0016491
GO:0050660 GO:0050151
Cluster-22178.61907 GO:0006355 GO:0043565 GO:0003676 GO:0005515
GO:0005634
Cluster-22178.61908 GO:0007217
Cluster-22178.61910 GO:0030261 GO:0009058 GO:0006614 GO:0007059
GO:0006777 GO:0000723 GO:0006281 GO:0005524 GO:0003677
GO:0016787 GO:0003678 GO:0003824 GO:0005525 GO:0005657
GO:0019031 GO:0009295
Cluster-22178.61911 GO:0046034 GO:0006144 GO:0008033 GO:0006464
GO:0051252 GO:0016151 GO:0046872 GO:0004017 GO:0004540
GO:0016787
Cluster-22178.61912 GO:0005509
Cluster-22178.61919 GO:0051603 GO:0004298 GO:0005839
Cluster-22178.61920 GO:0006810 GO:0006888 GO:0055085 GO:0005515
GO:0022857 GO:0005215 GO:0016020 GO:0005622 GO:0005801
GO:0019867 GO:0016021
Cluster-22178.61922 GO:0022900 GO:0006355 GO:0046872 GO:0005524
GO:0043565 GO:0003676 GO:0016787 GO:0003677 GO:0005515

GO:0005743 GO:0070469
Cluster-22178.61923 GO:0006281 GO:0006810 GO:0006310 GO:0010506
GO:0000400 GO:0000287 GO:0000166 GO:0016788 GO:0003677
GO:0005524 GO:0016887 GO:0005615
Cluster-22178.61926 GO:0035556 GO:0007165
Cluster-22178.61928 GO:0006118 GO:0006814 GO:0006120 GO:0006744
GO:0015992 GO:0008137 GO:0003954 GO:0005739
Cluster-22178.61931 GO:0005515 GO:0005509 GO:0004064
Cluster-22178.61932 GO:0005789
Cluster-22178.61936 GO:0006629 GO:0005515
Cluster-22178.61940 GO:0046500 GO:0006400 GO:0006367 GO:0006464
GO:0006260 GO:0006355 GO:0006351 GO:0006479 GO:0031167
GO:0009451 GO:0008033 GO:0008152 GO:0003682 GO:0008176
GO:0003677 GO:0008270 GO:0008171 GO:0003887 GO:0004719
GO:0008168 GO:0005634 GO:0005672 GO:0042575 GO:0032040
GO:0000775 GO:0000785
Cluster-22178.61942 GO:0005515
Cluster-22178.61944 GO:0006811 GO:0006118 GO:0055085 GO:0015035
GO:0015075 GO:0016020
Cluster-22178.61946 GO:0009058 GO:0055114 GO:0003824 GO:0016209
GO:0051920 GO:0016491
Cluster-22178.61947 GO:0055114 GO:0009058 GO:0016209 GO:0016491
GO:0051920 GO:0003824
Cluster-22178.61948 GO:0006810 GO:0055085 GO:0006511 GO:0005215
GO:0031625 GO:0016020
Cluster-22178.6195 GO:0006139 GO:0006206 GO:0000921 GO:0004127
GO:0005524 GO:0016021 GO:0005940
Cluster-22178.61950 GO:0006355 GO:0003700 GO:0043565 GO:0008270
GO:0005667
Cluster-22178.61952 GO:0006425 GO:0055085 GO:0004819 GO:0000166
GO:0005524 GO:0022857 GO:0005737 GO:0016021
Cluster-22178.61956 GO:0005515
Cluster-22178.61957 GO:0005515

Cluster-22178.61958 GO:0005515
Cluster-22178.61959 GO:0003676 GO:0005515 GO:0046872 GO:0008270

Cluster-22178.61961 GO:0046872
Cluster-22178.61966 GO:0007217 GO:0006260 GO:0003887 GO:0042575
Cluster-22178.61967 GO:0006144 GO:0006614 GO:0006508 GO:0005525
GO:0003968 GO:0005524 GO:0004197 GO:0003677 GO:0017111
GO:0016787 GO:0031379
Cluster-22178.61971 GO:0006605 GO:0006457 GO:0006886 GO:0000413
GO:0003755 GO:0005515 GO:0005742
Cluster-22178.61972 GO:0019058 GO:0005515
Cluster-22178.61974 GO:0006364 GO:0032259 GO:0008033 GO:0008168
GO:0003723
Cluster-22178.61975 GO:0006468 GO:0006816 GO:0006874 GO:0006397
GO:0051252 GO:0005524 GO:0004672 GO:0046872 GO:0005219
GO:0004540 GO:0005622 GO:0016021
Cluster-22178.61977 GO:0001558 GO:0005520 GO:0005515 GO:0005576
GO:0016942
Cluster-22178.61978 GO:0030436
Cluster-22178.61979 GO:0001558 GO:0005515 GO:0005520 GO:0016942
GO:0005576
Cluster-22178.61984 GO:0007156 GO:0005509 GO:0016020
Cluster-22178.61985 GO:0015991 GO:0006265 GO:0016567 GO:0015992
GO:0005515 GO:0003723 GO:0015078 GO:0046872 GO:0003677
GO:0061630 GO:0004842 GO:0005524 GO:0008270 GO:0003918
GO:0033179 GO:0019028 GO:0005680
Cluster-22178.61986 GO:0003677
Cluster-22178.61991 GO:0006357 GO:0001104 GO:0016592
Cluster-22178.61992 GO:0006508 GO:0008236
Cluster-22178.61993 GO:0006310 GO:0006281
Cluster-22178.61995 GO:0007165 GO:0007186 GO:0007264 GO:0005525
GO:0004871 GO:0031683 GO:0019001 GO:0003924
Cluster-22178.61997 GO:0006810 GO:0009405 GO:0006355 GO:0005515

GO:0019871 GO:0000166 GO:0043565 GO:0005576
Cluster-22178.61998 GO:0005515
Cluster-22178.61999 GO:0015979 GO:0016020 GO:0016021 GO:0009523
Cluster-22178.6200 GO:0005515
Cluster-22178.62001 GO:0016192 GO:0006904 GO:0043395 GO:0005578
GO:0016020
Cluster-22178.62002 GO:0006047 GO:0005975 GO:0016773 GO:0005524

Cluster-22178.62003 GO:0006265 GO:0003677 GO:0003918 GO:0005634
GO:0005694
Cluster-22178.62004 GO:0006629 GO:0005515
Cluster-22178.62006 GO:0008152 GO:0006118 GO:0055114 GO:0003995
GO:0050660 GO:0016627
Cluster-22178.62007 GO:0010508 GO:0030001 GO:0006323 GO:0055085
GO:0006914 GO:0048870 GO:0005198 GO:0005516 GO:0051082
GO:0046873 GO:0031514 GO:0016020 GO:0019028 GO:0019031
GO:0016021
Cluster-22178.62008 GO:0016567 GO:0008270 GO:0046872 GO:0005515
GO:0061630 GO:0004842 GO:0005680
Cluster-22178.62010 GO:0008152 GO:0016747 GO:0016746
Cluster-22178.62011 GO:0007155 GO:0042398 GO:0006750 GO:0006749
GO:0004357 GO:0017109 GO:0016021
Cluster-22178.62013 GO:0006750 GO:0006749 GO:0007155 GO:0042398
GO:0004357 GO:0017109 GO:0016021
Cluster-22178.62014 GO:0009432 GO:0051782 GO:0009276
Cluster-22178.62015 GO:0005509
Cluster-22178.62017 GO:0016567 GO:0006308 GO:0006914 GO:0005515
GO:0035091 GO:0004842 GO:0046872 GO:0008270 GO:0008855
GO:0009318 GO:0005680 GO:0005622
Cluster-22178.62018 GO:0005515 GO:0008270
Cluster-22178.62019 GO:0006355 GO:0007165 GO:0043087 GO:0000723
GO:0003677 GO:0005515 GO:0005057 GO:0030695 GO:0003700
GO:0005089 GO:0005667 GO:0042025 GO:0000784 GO:0005737

Cluster-22178.6202 GO:0015074

Cluster-22178.62022 GO:0007631 GO:0007218 GO:0016817 GO:0046872

Cluster-22178.62025 GO:0007264 GO:0006576 GO:0007165 GO:0015684 GO:0019064 GO:0046789 GO:0005524 GO:0015093 GO:0003924 GO:0005525 GO:0042765 GO:0009986 GO:0019031 GO:0016021

Cluster-22178.62026 GO:0016020

Cluster-22178.6203 GO:0009069 GO:0006468 GO:0016310 GO:0004672 GO:0005524 GO:0004674

Cluster-22178.62033 GO:0016021

Cluster-22178.62035 GO:0005515 GO:0016853

Cluster-22178.62037 GO:0005515

Cluster-22178.62039 GO:0006355 GO:0042967 GO:0004402 GO:0005515 GO:0008080 GO:0005634 GO:0000123

Cluster-22178.62042 GO:0016579 GO:0009228 GO:0004789 GO:0036459

Cluster-22178.62044 GO:0007165 GO:0019079 GO:0006260 GO:0005179 GO:0030337 GO:0005576 GO:0042575

Cluster-22178.62046 GO:0019062 GO:0006120 GO:0006355 GO:0006814 GO:0030521 GO:0015992 GO:0006744 GO:0042773 GO:0007165 GO:0008137 GO:0004882 GO:0005496 GO:0003677 GO:0005743 GO:0005634 GO:0019028

Cluster-22178.62047 GO:0008063 GO:0006355 GO:0043565 GO:0008270 GO:0003700 GO:0005515 GO:0005667

Cluster-22178.62049 GO:0016020

Cluster-22178.62050 GO:0032465 GO:0006915 GO:0016567 GO:0003676 GO:0004842

Cluster-22178.62051 GO:0015031 GO:0006464 GO:0006355 GO:0043565 GO:0003700 GO:0003676 GO:0005515 GO:0005667

Cluster-22178.62052 GO:0005515

Cluster-22178.62053 GO:0042254 GO:0006412 GO:0006629 GO:0006007 GO:0005515 GO:0003735 GO:0046537 GO:0003824 GO:0005840 GO:0005622

Cluster-22178.62055 GO:0006812 GO:0032801 GO:0019076 GO:0016567
GO:0004842 GO:0046872 GO:0008270 GO:0005515 GO:0005261
GO:0005680 GO:0033644
Cluster-22178.62057 GO:0007049 GO:0006810 GO:0009405 GO:0019855
GO:0005576 GO:0017053
Cluster-22178.62060 GO:0043087 GO:0015031 GO:0007186 GO:0019058
GO:0007165 GO:0006364 GO:0007264 GO:0008277 GO:0006886
GO:0007606 GO:0004930 GO:0043130 GO:0005096 GO:0008565
GO:0030674 GO:0008092 GO:0005515 GO:0042802 GO:0004888
GO:0016020 GO:0016021 GO:0019031
Cluster-22178.62061 GO:0003676
Cluster-22178.62062 GO:0000290 GO:0043085 GO:0008047
Cluster-22178.62063 GO:0003779
Cluster-22178.62064 GO:0046872
Cluster-22178.62065 GO:0005634 GO:0005737
Cluster-22178.62066 GO:0006260 GO:0006355 GO:0006351 GO:0008270
GO:0003682 GO:0003677 GO:0003887 GO:0000785 GO:0000775
GO:0005634 GO:0042575
Cluster-22178.62068 GO:0006260 GO:0006355 GO:0006351 GO:0008270
GO:0003682 GO:0003677 GO:0003887 GO:0000785 GO:0000775
GO:0005634 GO:0042575
Cluster-22178.62072 GO:0015684 GO:0007264 GO:0005525 GO:0015093
GO:0003924 GO:0016021
Cluster-22178.62073 GO:0005525
Cluster-22178.62075 GO:0019370 GO:0008270 GO:0008237 GO:0005515

Cluster-22178.62078 GO:0003677 GO:0005515
Cluster-22178.62079 GO:0009405 GO:0003723 GO:0008270 GO:0015485
GO:0003676 GO:0005515
Cluster-22178.62080 GO:0007165 GO:0035556
Cluster-22178.62081 GO:0055085 GO:0005515 GO:0022857 GO:0003677
GO:0016021
Cluster-22178.62083 GO:0015846 GO:1902604 GO:0006811 GO:0015814

GO:0015558 GO:0003723 GO:0005515
Cluster-22178.62084 GO:0006869 GO:0042157 GO:0006260 GO:0006396
GO:0055085 GO:0030001 GO:0055114 GO:0006308 GO:0046873
GO:0008270 GO:0008855 GO:0005198 GO:0008289 GO:0016020
GO:0005622 GO:0009318 GO:0005576 GO:0019028 GO:0019031
Cluster-22178.62085 GO:0003676 GO:0005515
Cluster-22178.62086 GO:0006886 GO:0055114 GO:0016740 GO:0005047
GO:0016301 GO:0005515 GO:0005524 GO:0051536 GO:0003924
GO:0016491 GO:0005525 GO:0016625 GO:0005786 GO:0005785
Cluster-22178.62088 GO:0005975 GO:0008270 GO:0008184 GO:0003676

Cluster-22178.62090 GO:0090263 GO:0046856 GO:0016567 GO:0034597

Cluster-22178.62091 GO:0042742
Cluster-22178.62094 GO:0042981
Cluster-22178.62095 GO:0006606 GO:0048193 GO:0015992 GO:0051028
GO:0006914 GO:0006357 GO:0007094 GO:0006886 GO:0048309
GO:0016567 GO:0006457 GO:0010506 GO:0051607 GO:0007018
GO:0006355 GO:0015991 GO:0007017 GO:0016032 GO:0007049
GO:0030154 GO:0006308 GO:0048870 GO:0008017 GO:0005524
GO:0042803 GO:0005515 GO:0005198 GO:0001104 GO:0051082
GO:0015078 GO:0016881 GO:0051087 GO:0031386 GO:0000774
GO:0008855 GO:0008134 GO:0019901 GO:0045502 GO:0003777
GO:0003700 GO:0016020 GO:0005667 GO:0016272 GO:0005634
GO:0005615 GO:0045298 GO:0005737 GO:0005882 GO:0016592
GO:0005815 GO:0030286 GO:0009318 GO:0033179 GO:0031514
GO:0019028 GO:0005874 GO:0005801 GO:0016021
Cluster-22178.62099 GO:0033151 GO:0008033 GO:0055114 GO:0017150
GO:0050660 GO:0043565 GO:0046872
Cluster-22178.621 GO:0015994 GO:0015996 GO:0008270 GO:0047746
Cluster-22178.62100 GO:0006468 GO:0016773 GO:0005524 GO:0004672
GO:0016020
Cluster-22178.62101 GO:0043085 GO:0000290 GO:0008047

Cluster-22178.62102 GO:0006468 GO:0016773 GO:0005524 GO:0004672
GO:0016020
Cluster-22178.62103 GO:0006537 GO:0055114 GO:0006807 GO:0016773
GO:0016972 GO:0035091 GO:0015930 GO:0005515
Cluster-22178.62104 GO:0022900 GO:0005739 GO:0005747 GO:0019031
Cluster-22178.62106 GO:0005515
Cluster-22178.62110 GO:0016055 GO:0006355 GO:0006886 GO:0008536
GO:0003700 GO:0000975 GO:0005667
Cluster-22178.62113 GO:0042157 GO:0009405 GO:0006810 GO:0006869
GO:0015031 GO:0008565 GO:0008289 GO:0005576 GO:0016020
Cluster-22178.62114 GO:0008270 GO:0003729
Cluster-22178.62115 GO:0005515
Cluster-22178.62118 GO:0006824 GO:0009236 GO:0003676 GO:0015087
GO:0016020
Cluster-22178.62119 GO:0006886 GO:0016192 GO:0005515 GO:0016021
GO:0030117
Cluster-22178.62120 GO:0016829 GO:0005576
Cluster-22178.62121 GO:0006094 GO:0006020 GO:0046486 GO:0006096
GO:0006000 GO:0015976 GO:0006013 GO:0008152 GO:0004807
Cluster-22178.62122 GO:0005515 GO:0008270 GO:0030246
Cluster-22178.62124 GO:0003676
Cluster-22178.62125 GO:0006355 GO:0043565 GO:0003700 GO:0042765
GO:0005667 GO:0005634 GO:0016021
Cluster-22178.62126 GO:0055114 GO:0005509 GO:0016616
Cluster-22178.62128 GO:0007264 GO:0008270 GO:0003676 GO:0005515
GO:0005525
Cluster-22178.62133 GO:0006281 GO:0006310
Cluster-22178.62136 GO:0006396 GO:0003723
Cluster-22178.62141 GO:0016829
Cluster-22178.62146 GO:0006260 GO:0006914 GO:0015031 GO:0015992
GO:0009069 GO:0016310 GO:0006886 GO:0015991 GO:0006468
GO:0004674 GO:0008565 GO:0005524 GO:0015078 GO:0005515
GO:0003723 GO:0016020 GO:0005737 GO:0033179

Cluster-22178.62149 GO:0008152 GO:0055114 GO:0003824 GO:0008484 GO:0016020 GO:0016021
Cluster-22178.62150 GO:0008152 GO:0055114 GO:0003824 GO:0008484 GO:0016020 GO:0016021
Cluster-22178.62151 GO:0043087 GO:0035023 GO:0044419 GO:0005515 GO:0005089 GO:0005887
Cluster-22178.62152 GO:0007264 GO:0015684 GO:0006260 GO:0003677 GO:0005524 GO:0015093 GO:0016887 GO:0003924 GO:0005525 GO:0016021
Cluster-22178.62153 GO:0046872
Cluster-22178.62154 GO:0008270
Cluster-22178.62156 GO:0003723
Cluster-22178.62157 GO:0005515
Cluster-22178.62159 GO:0007596
Cluster-22178.62160 GO:0006118 GO:0017004 GO:0008616 GO:0030163 GO:0033739 GO:0005515 GO:0045158 GO:0008914 GO:0009512
Cluster-22178.62161 GO:0015031 GO:0005975 GO:0006886 GO:0008565 GO:0016773 GO:0005509 GO:0005737 GO:0016020
Cluster-22178.62162 GO:0016773
Cluster-22178.62163 GO:0006522 GO:0006188 GO:0009152 GO:0006144 GO:0055114 GO:0006520 GO:0009058 GO:0006531 GO:0010277 GO:0016829 GO:0004018 GO:0030170
Cluster-22178.62167 GO:0007283 GO:0009264 GO:0006024 GO:0015012 GO:0003677 GO:0004252 GO:0016757 GO:0008253 GO:0016020 GO:0005634 GO:0016021 GO:0000786
Cluster-22178.62171 GO:0043087 GO:0006508 GO:0007264 GO:0009847 GO:0005096 GO:0030250 GO:0008233 GO:0005515 GO:0005576
Cluster-22178.62177 GO:0007264 GO:0007165 GO:0015684 GO:0007186 GO:0031683 GO:0008270 GO:0019001 GO:0015093 GO:0003924 GO:0005525 GO:0004871 GO:0016021
Cluster-22178.62178 GO:0007264 GO:0015684 GO:0007165 GO:0007186 GO:0031683 GO:0008270 GO:0015093 GO:0019001 GO:0003924 GO:0005525 GO:0004871 GO:0016021

Cluster-22178.62179 GO:0031556 GO:0031554 GO:0016021
Cluster-22178.62180 GO:0016598 GO:0004057 GO:0046983
Cluster-22178.62181 GO:0005515
Cluster-22178.62182 GO:0006260 GO:0006357 GO:0031110 GO:0070206 GO:0007186 GO:0007165 GO:0007275 GO:0000090 GO:0007067 GO:0007059 GO:0051301 GO:0008017 GO:0004930 GO:0005179 GO:0001104 GO:0005876 GO:0000940 GO:0016592 GO:0016021 GO:0045298 GO:0005576
Cluster-22178.62183 GO:0015979 GO:0042967 GO:0006629 GO:0009058 GO:0016788 GO:0008374 GO:0009523 GO:0009539
Cluster-22178.62184 GO:0007049 GO:0005515
Cluster-22178.62185 GO:0042254 GO:0006412 GO:0009968 GO:0003735 GO:0005622 GO:0005737 GO:0005840
Cluster-22178.62187 GO:0006886 GO:0016192 GO:0005515 GO:0008270

Cluster-22178.62188 GO:0016192 GO:0006886 GO:0008270 GO:0005515

Cluster-22178.62189 GO:0005515 GO:0016757 GO:0016020
Cluster-22178.6219 GO:0008146
Cluster-22178.62190 GO:0016192 GO:0006886 GO:0005515 GO:0008536 GO:0030117
Cluster-22178.62191 GO:0005515
Cluster-22178.62192 GO:0042549 GO:0015979 GO:0009539 GO:0009523
Cluster-22178.62193 GO:0031167 GO:0008152 GO:0008168
Cluster-22178.62194 GO:0006511 GO:0005515
Cluster-22178.62201 GO:0015991 GO:0016192 GO:0006886 GO:0015031 GO:0006606 GO:0005515 GO:0016820 GO:0046872 GO:0008270 GO:0008565 GO:0030117 GO:0005634 GO:0005622 GO:0005737 GO:0000221
Cluster-22178.62202 GO:0016192 GO:0006886 GO:0015991 GO:0006606 GO:0015031 GO:0005515 GO:0016820 GO:0008565 GO:0046872 GO:0008270 GO:0005634 GO:0030117 GO:0000221 GO:0005737 GO:0005622

Cluster-22178.62203 GO:0048278 GO:0006810 GO:0007165 GO:0006887
GO:0003774 GO:0005524 GO:0016459 GO:0005737
Cluster-22178.62205 GO:0007186 GO:0016787 GO:0005524 GO:0003676
GO:0004930 GO:0003677 GO:0016021
Cluster-22178.62206 GO:0003677
Cluster-22178.62209 GO:0022900 GO:0005515 GO:0005747 GO:0005739
Cluster-22178.62210 GO:0007165 GO:0005509 GO:0005515 GO:0005578
Cluster-22178.62212 GO:0006771 GO:0019497 GO:0003993 GO:0005515
GO:0046872
Cluster-22178.62213 GO:0006771 GO:0019497 GO:0005515 GO:0005509
GO:0003993 GO:0046872
Cluster-22178.62214 GO:0019497 GO:0006771 GO:0007165 GO:0046872
GO:0003993 GO:0005509 GO:0005515 GO:0005578
Cluster-22178.62215 GO:0019497 GO:0007165 GO:0006508 GO:0006771
GO:0008233 GO:0003993 GO:0046872 GO:0005515 GO:0005509
GO:0005578
Cluster-22178.62216 GO:0019497 GO:0006771 GO:0007165 GO:0046872
GO:0003993 GO:0005515 GO:0005509 GO:0005578
Cluster-22178.62217 GO:0006508 GO:0008152 GO:0016787 GO:0008236

Cluster-22178.62220 GO:0019497 GO:0007165 GO:0006771 GO:0003993
GO:0046872 GO:0005515 GO:0005509 GO:0005578
Cluster-22178.62222 GO:0006334 GO:0000398 GO:0005515 GO:0005681
GO:0005634
Cluster-22178.62226 GO:0008270
Cluster-22178.62227 GO:0006810 GO:0006814 GO:0055085 GO:0005215
GO:0016020
Cluster-22178.62228 GO:0043248
Cluster-22178.62229 GO:0009069 GO:0006468 GO:0016310 GO:0004674
GO:0004672 GO:0005524 GO:0016773 GO:0016020
Cluster-22178.62231 GO:0016310 GO:0006468 GO:0009069 GO:0004674
GO:0005524 GO:0004672
Cluster-22178.62232 GO:0009069 GO:0006468 GO:0016310 GO:0005524

GO:0004672 GO:0004674
Cluster-22178.62233 GO:0008152 GO:0035556 GO:0016746 GO:0005515

Cluster-22178.62234 GO:0006355 GO:0043565 GO:0003700 GO:0005667
Cluster-22178.62236 GO:0045454
Cluster-22178.62242 GO:0006812 GO:0008324
Cluster-22178.62243 GO:0055114 GO:0006355 GO:0006281 GO:0006351
GO:0051016 GO:0009236 GO:0006260 GO:0003887 GO:0008270
GO:0016491 GO:0003677 GO:0019031 GO:0005634 GO:0042575
GO:0030479 GO:0016020
Cluster-22178.62244 GO:0006414 GO:0006448 GO:0055114 GO:0016706
GO:0005515 GO:0003746 GO:0005853 GO:0005840
Cluster-22178.62245 GO:0007186 GO:0008360 GO:0015979 GO:0003677
GO:0042802 GO:0005515 GO:0004930 GO:0016020 GO:0016021
GO:0009539 GO:0009523
Cluster-22178.62247 GO:0051260 GO:0006468 GO:0016773 GO:0005524
GO:0008270 GO:0004672 GO:0005515 GO:0016020 GO:0005634
Cluster-22178.62248 GO:0006468 GO:0004672 GO:0005524
Cluster-22178.62251 GO:0006508 GO:0004198 GO:0005622
Cluster-22178.62252 GO:0006144 GO:0004114
Cluster-22178.62253 GO:0006094 GO:0006098 GO:0006096 GO:0005982
GO:0005985 GO:0004347
Cluster-22178.62256 GO:0006629 GO:0004252
Cluster-22178.62257 GO:0016757
Cluster-22178.62258 GO:0005634
Cluster-22178.62259 GO:0021549 GO:0006351 GO:0006144 GO:0006730
GO:0006206 GO:0030182 GO:0003899 GO:0003677 GO:0008168
GO:0003676 GO:0005730
Cluster-22178.62260 GO:0006468 GO:0006886 GO:0004672 GO:0005524
GO:0030176
Cluster-22178.62261 GO:0006807 GO:0006606 GO:0006886 GO:0019089
GO:0008053 GO:0006891 GO:0030168 GO:0051607 GO:0007165
GO:0019551 GO:0051258 GO:0006118 GO:0005524 GO:0004352

GO:0003924 GO:0005198 GO:0042803 GO:0005102 GO:0030674
GO:0008092 GO:0019901 GO:0008134 GO:0045502 GO:0005577
GO:0030173 GO:0016020 GO:0005741 GO:0005667 GO:0005737
GO:0019898 GO:0030286 GO:0017119 GO:0016021 GO:0019031
GO:0019028
Cluster-22178.62262 GO:0007165 GO:0015684 GO:0006355 GO:0007264
GO:0006576 GO:0005509 GO:0005515 GO:0005525 GO:0005524
GO:0015093 GO:0016887 GO:0005578 GO:0005634 GO:0016021
Cluster-22178.62264 GO:0006974 GO:0051726 GO:0003677
Cluster-22178.62265 GO:0008654 GO:0016780 GO:0016020
Cluster-22178.62266 GO:0055114 GO:0031145 GO:0030071 GO:0016567
GO:0046872 GO:0008270 GO:0047134 GO:0004842 GO:0061630
GO:0005515 GO:0005680
Cluster-22178.62268 GO:0046656 GO:0006760 GO:0043565 GO:0004150
GO:0005515 GO:0005634
Cluster-22178.62271 GO:0043087 GO:0035023 GO:0005089
Cluster-22178.62272 GO:0007165 GO:0007218 GO:0030141
Cluster-22178.62275 GO:0005515 GO:0003676 GO:0005634
Cluster-22178.62276 GO:0006144 GO:0006464 GO:0006206 GO:0006281
GO:0006351 GO:0046872 GO:0008270 GO:0016151 GO:0003899
GO:0005515 GO:0003677 GO:0005730
Cluster-22178.62277 GO:0006470 GO:0006570 GO:0008138 GO:0004725
GO:0004721
Cluster-22178.62278 GO:0003676 GO:0005515 GO:0005634
Cluster-22178.62281 GO:0009291 GO:0006811 GO:0006865 GO:0055085
GO:0003333 GO:0006810 GO:0005215 GO:0015171 GO:0016020
Cluster-22178.62283 GO:0005515
Cluster-22178.62287 GO:0007264 GO:0043087 GO:0005085
Cluster-22178.62291 GO:0006807 GO:0009252 GO:0004356 GO:0004190
GO:0003676 GO:0008270 GO:0046872 GO:0005634 GO:0016021
GO:0005923
Cluster-22178.62294 GO:0006606 GO:0006260 GO:0015031 GO:0006370
GO:0006397 GO:0006310 GO:0006281 GO:0003910 GO:0005524

GO:0008565 GO:0005515 GO:0004484 GO:0005737 GO:0005634
Cluster-22178.62295 GO:0046872 GO:0005509 GO:0005515
Cluster-22178.62296 GO:0003677 GO:0003676 GO:0005515 GO:0005524
GO:0016787
Cluster-22178.62297 GO:0006886 GO:0003677 GO:0004003 GO:0005524
GO:0016020 GO:0005657
Cluster-22178.62298 GO:0051603 GO:0004298 GO:0005839
Cluster-22178.62299 GO:0055114 GO:0003723 GO:0016491 GO:0050661

Cluster-22178.62303 GO:0006511 GO:0031625
Cluster-22178.62304 GO:0007264 GO:0005525 GO:0003924
Cluster-22178.62305 GO:0006511 GO:0006418 GO:0000166 GO:0005524
GO:0031625 GO:0004812
Cluster-22178.62306 GO:0005634
Cluster-22178.62307 GO:0006950 GO:0001671 GO:0051087
Cluster-22178.62308 GO:0005515 GO:0008270 GO:0005634
Cluster-22178.62310 GO:0003677
Cluster-22178.62312 GO:0055085 GO:0008643 GO:0005351 GO:0000139
GO:0016020 GO:0016021
Cluster-22178.62314 GO:0006470 GO:0042967 GO:0019988 GO:0006570
GO:0006355 GO:0004725 GO:0008080 GO:0016747 GO:0043399
GO:0008138
Cluster-22178.62316 GO:0009405 GO:0006886 GO:0008053 GO:0006869
GO:0016192 GO:0042157 GO:0005525 GO:0008289 GO:0003924
GO:0005198 GO:0004185 GO:0005524 GO:0016887 GO:0019031
GO:0016021 GO:0005576 GO:0019028 GO:0005741 GO:0005773
GO:0016020
Cluster-22178.62317 GO:0016192 GO:0006886 GO:0007264 GO:0006260
GO:0055114 GO:0015031 GO:0005515 GO:0047134 GO:0005096
GO:0016491 GO:0008565 GO:0070403 GO:0008270 GO:0016020
GO:0005737
Cluster-22178.62319 GO:0009405 GO:0007049 GO:0031505 GO:0006355
GO:0003700 GO:0043565 GO:0005667

Cluster-22178.6232 GO:0006357 GO:0055114 GO:0003723 GO:0001104
GO:0016903 GO:0016592
Cluster-22178.62320 GO:0008168
Cluster-22178.62321 GO:0010508 GO:0015979 GO:0005794 GO:0016020
GO:0009539 GO:0009523
Cluster-22178.62323 GO:0006810 GO:0004659 GO:0016021
Cluster-22178.62324 GO:0006508 GO:0005515 GO:0004190 GO:0005861
Cluster-22178.62330 GO:0007169 GO:0035023 GO:0043087 GO:0006468
GO:0006508 GO:0005089 GO:0004185 GO:0004672 GO:0005524
GO:0004714 GO:0005515 GO:0016020
Cluster-22178.62332 GO:0007165 GO:0006302 GO:0004519 GO:0030145
GO:0016787 GO:0005125 GO:0005634
Cluster-22178.62335 GO:0000226 GO:0000910 GO:0007186 GO:0004930
GO:0008017 GO:0046983 GO:0003677 GO:0045298 GO:0016021
Cluster-22178.62340 GO:0006281 GO:0010447 GO:0003677 GO:0004518
GO:0003824 GO:0042597
Cluster-22178.62343 GO:0005737
Cluster-22178.62344 GO:0008152 GO:0003824 GO:0008484
Cluster-22178.62345 GO:0007264 GO:0006119 GO:0006118 GO:0055114
GO:0015992 GO:0035556 GO:0008121 GO:0005507 GO:0005525
Cluster-22178.62347 GO:0007275 GO:0009966 GO:0016020
Cluster-22178.62348 GO:0019033
Cluster-22178.62351 GO:0007264 GO:0007165 GO:0007186 GO:0006013
GO:0006000 GO:0003873 GO:0005515 GO:0005524 GO:0031683
GO:0019001 GO:0003924 GO:0005525 GO:0004871
Cluster-22178.62352 GO:0006631 GO:0007304 GO:0005515 GO:0071949
GO:0005213 GO:0050151 GO:0046872 GO:0005576 GO:0045202
GO:0016021 GO:0042600
Cluster-22178.62354 GO:0003723
Cluster-22178.62355 GO:0005515
Cluster-22178.62357 GO:0000902
Cluster-22178.62358 GO:0006810 GO:0009405 GO:0008200 GO:0051537
GO:0005515 GO:0042151

Cluster-22178.62359 GO:0006351 GO:0006144 GO:0006265 GO:0006206
GO:0005515 GO:0003899 GO:0003677 GO:0003676 GO:0008270
GO:0003916 GO:0005730 GO:0005694
Cluster-22178.62360 GO:0006355 GO:0008270 GO:0046872 GO:0003677

Cluster-22178.62361 GO:0016310 GO:0000160 GO:0000155 GO:0046983
GO:0009365 GO:0016021
Cluster-22178.62363 GO:0030153 GO:0006955 GO:0006367 GO:0006357
GO:0015643 GO:0004252 GO:0001104 GO:0005672 GO:0019814
GO:0016592 GO:0005886
Cluster-22178.62366 GO:0006357 GO:0009847 GO:0048193 GO:0001104
GO:0016592 GO:0005783 GO:0016020
Cluster-22178.62372 GO:0016779
Cluster-22178.62375 GO:0015074 GO:0007224
Cluster-22178.62376 GO:0006285 GO:0042384 GO:0006281 GO:0006289
GO:0006308 GO:0003684 GO:0008534 GO:0019900 GO:0034464
Cluster-22178.62377 GO:0015991 GO:0006508 GO:0004222 GO:0003712
GO:0003724 GO:0016818 GO:0016820 GO:0033178 GO:0005634
GO:0005667
Cluster-22178.62378 GO:0003723
Cluster-22178.62379 GO:0006303 GO:0006508 GO:0004222 GO:0003677
GO:0004003 GO:0005815 GO:0005657
Cluster-22178.62380 GO:0006355 GO:0006275 GO:0016032 GO:0006952
GO:0005515
Cluster-22178.62381 GO:0021549 GO:0030182 GO:0008270
Cluster-22178.62382 GO:0043087 GO:0035023 GO:0005089
Cluster-22178.62383 GO:0035023 GO:0016310 GO:0048870 GO:0019048
GO:0006468 GO:0043087 GO:0006952 GO:0007165 GO:0009069
GO:0003779 GO:0046914 GO:0005515 GO:0005089 GO:0004674
GO:0046983 GO:0005096 GO:0031514 GO:0005576
Cluster-22178.62384 GO:0006298 GO:0007010 GO:0006364 GO:0007017
GO:0006813 GO:0003779 GO:0005524 GO:0005267 GO:0030983
GO:0005875 GO:0034457 GO:0005634 GO:0005732

Cluster-22178.62386 GO:0055114 GO:0008152 GO:0006118 GO:0046872
GO:0016627 GO:0050660 GO:0016787 GO:0003995
Cluster-22178.62387 GO:0006118 GO:0055114 GO:0008152 GO:0016787
GO:0003995 GO:0046872 GO:0016627 GO:0050660 GO:0005887
Cluster-22178.62388 GO:0016567 GO:0004842 GO:0005515
Cluster-22178.6239 GO:0009245 GO:0016747
Cluster-22178.62391 GO:0015991 GO:0030474 GO:0010508 GO:0048309
GO:0006308 GO:0031144 GO:0071630 GO:0007165 GO:0051028
GO:0070206 GO:0015992 GO:0007034 GO:0006914 GO:0006606
GO:0007218 GO:0005198 GO:0015078 GO:0005200 GO:0005179
GO:0003677 GO:0008855 GO:0005576 GO:0005634 GO:0009318
GO:0033179 GO:0005856 GO:0005823
Cluster-22178.62392 GO:0055085 GO:0022857 GO:0016021
Cluster-22178.62394 GO:0006555 GO:0030154 GO:0006556 GO:0019722
GO:0004478 GO:0016021
Cluster-22178.62395 GO:0016567 GO:0090263 GO:0043565
Cluster-22178.6240 GO:0009245 GO:0016747
Cluster-22178.62401 GO:0018101 GO:0006807 GO:0004668 GO:0005509
GO:0005737
Cluster-22178.62402 GO:0005515
Cluster-22178.62407 GO:0048870 GO:0008608 GO:0007155 GO:0016567
GO:0006511 GO:0007275 GO:0005515 GO:0016881 GO:0046872
GO:0061630 GO:0008270 GO:0031386 GO:0004842 GO:0005634
GO:0031514 GO:0042729 GO:0005876 GO:0005680
Cluster-22178.62408 GO:0006555 GO:0006730 GO:0009405 GO:0006810
GO:0006952 GO:0009755 GO:0006556 GO:0006629 GO:0019855
GO:0046872 GO:0016740 GO:0004478 GO:0045202 GO:0005576
Cluster-22178.62409 GO:0004222 GO:0005524 GO:0008270 GO:0016021
Cluster-22178.6241 GO:0009245 GO:0016747
Cluster-22178.62410 GO:0009058 GO:0055114 GO:0010181 GO:0016787
GO:0003677 GO:0016491 GO:0005524 GO:0003676
Cluster-22178.62411 GO:0006531 GO:0006529 GO:0006522 GO:0008270
GO:0046872 GO:0004066 GO:0005515

Cluster-22178.62414 GO:0040007 GO:0008283 GO:0044179 GO:0006955
GO:0007165 GO:0009405 GO:0005515 GO:0008083 GO:0043565
GO:0005134 GO:0005739 GO:0005576 GO:0005747 GO:0005893
Cluster-22178.62416 GO:0006260 GO:0006281 GO:0006308 GO:0017108
GO:0033557 GO:0005634
Cluster-22178.62417 GO:0016773
Cluster-22178.62418 GO:0007049 GO:0017053
Cluster-22178.62419 GO:0007049 GO:0017053
Cluster-22178.62421 GO:0008233 GO:0008270 GO:0005634
Cluster-22178.62422 GO:0016020
Cluster-22178.62423 GO:0006811 GO:0055085 GO:0006812 GO:0046677
GO:0008324 GO:0005216 GO:0016021 GO:0016020
Cluster-22178.62424 GO:0050832 GO:0042967 GO:0015074 GO:0042742
GO:0008080 GO:0003676 GO:0003677 GO:0019239 GO:0031224
Cluster-22178.62432 GO:0006890
Cluster-22178.62433 GO:0006412 GO:0042254 GO:0003735 GO:0005622
GO:0005840
Cluster-22178.62436 GO:0006260 GO:0006637 GO:0055114 GO:0046907
GO:0006635 GO:0003997 GO:0003677 GO:0005664 GO:0005777
Cluster-22178.62437 GO:0016787
Cluster-22178.62438 GO:0046497 GO:0008152 GO:0006769 GO:0006741
GO:0016301 GO:0003677 GO:0003951
Cluster-22178.62440 GO:0007411 GO:0006886 GO:0016192 GO:0005198
GO:0030130 GO:0030132
Cluster-22178.62441 GO:0006468 GO:0009069 GO:0000910 GO:0000226
GO:0016310 GO:0005524 GO:0004672 GO:0008017 GO:0004674
GO:0045298
Cluster-22178.62444 GO:0019087 GO:0016021
Cluster-22178.62445 GO:0055114 GO:0019521 GO:0009094 GO:0006574
GO:0015940 GO:0006571 GO:0006568 GO:0006813 GO:0006552
GO:0006550 GO:0006554 GO:0000162 GO:0006633 GO:0006098
GO:0006631 GO:0018874 GO:0008677 GO:0004665 GO:0003857
GO:0016491 GO:0051287 GO:0008977 GO:0016616 GO:0004616

Cluster-22178.62446 GO:0006810 GO:0016021 GO:0005886
Cluster-22178.62447 GO:0042254 GO:0006412 GO:0003735 GO:0005840
Cluster-22178.62448 GO:0006118 GO:0009069 GO:0045454 GO:0016310
GO:0003677 GO:0009055 GO:0004674 GO:0015035 GO:0005524
Cluster-22178.62450 GO:0030866 GO:0008092 GO:0005856
Cluster-22178.62457 GO:0007165 GO:0007186 GO:0007264 GO:0005525
GO:0004871 GO:0019001 GO:0031683 GO:0003924
Cluster-22178.62458 GO:0007165 GO:0007186 GO:0006508 GO:0008152
GO:0016746 GO:0004190 GO:0003723 GO:0004871 GO:0003676
GO:0005524 GO:0004672 GO:0008270 GO:0031683 GO:0019001
GO:0003924
Cluster-22178.62459 GO:0007165 GO:0007186 GO:0006508 GO:0008152
GO:0016746 GO:0003723 GO:0004190 GO:0004871 GO:0003676
GO:0019001 GO:0005524 GO:0031683 GO:0008270 GO:0004672
GO:0003924
Cluster-22178.62460 GO:0015979 GO:0016020 GO:0009539 GO:0009523
Cluster-22178.62461 GO:0006508 GO:0008242 GO:0016817 GO:0016740
GO:0004197 GO:0016788 GO:0005634
Cluster-22178.62462 GO:0006367 GO:0006351 GO:0006144 GO:0006206
GO:0006366 GO:0003899 GO:0005674 GO:0005634 GO:0005730
Cluster-22178.62466 GO:0019031
Cluster-22178.62467 GO:0055085 GO:0022857 GO:0016021
Cluster-22178.62468 GO:0005515
Cluster-22178.62469 GO:0006281 GO:0007165 GO:0034061 GO:0008601
GO:0003684 GO:0003677 GO:0000159 GO:0005634
Cluster-22178.62470 GO:0006260 GO:0005509
Cluster-22178.62471 GO:0005515
Cluster-22178.62472 GO:0043087 GO:0050790 GO:0035023 GO:0030234
GO:0005089
Cluster-22178.62478 GO:0016021
Cluster-22178.62481 GO:0006814 GO:0055114 GO:0015992 GO:0006120
GO:0006744 GO:0008137 GO:0003677 GO:0008270
Cluster-22178.62483 GO:0006397 GO:0046983 GO:0005515

Cluster-22178.62484 GO:0006355 GO:0008270 GO:0005634
Cluster-22178.62485 GO:0035091
Cluster-22178.62487 GO:0006468 GO:0005524 GO:0004672
Cluster-22178.62489 GO:0007165 GO:0005509 GO:0005578
Cluster-22178.62490 GO:0005515
Cluster-22178.62493 GO:0016021
Cluster-22178.62494 GO:0006468 GO:0004672 GO:0005524
Cluster-22178.62497 GO:0043154 GO:0043066 GO:0019079 GO:0005515
GO:0005737 GO:0071986
Cluster-22178.62499 GO:0008270
Cluster-22178.62500 GO:0003676
Cluster-22178.62502 GO:0003723 GO:0003676
Cluster-22178.62503 GO:0006464 GO:0007275 GO:0009966 GO:0046872
GO:0016151 GO:0016020
Cluster-22178.62505 GO:0006464 GO:0007275 GO:0009966 GO:0046872
GO:0008270 GO:0016151 GO:0016020
Cluster-22178.62507 GO:0042254 GO:0006412 GO:0003735 GO:0005840
GO:0005622
Cluster-22178.62511 GO:0005509
Cluster-22178.62514 GO:0006468 GO:0005524 GO:0004672 GO:0016773
GO:0016020
Cluster-22178.62517 GO:0005509
Cluster-22178.62519 GO:0006270 GO:0015684 GO:0008270 GO:0015093
GO:0003924 GO:0005525 GO:0005634 GO:0032040 GO:0016021
Cluster-22178.62520 GO:0006098 GO:0006631 GO:0018874 GO:0006554
GO:0046168 GO:0006633 GO:0006099 GO:0006568 GO:0006552
GO:0006550 GO:0055114 GO:0019521 GO:0051321 GO:0006574
GO:0008152 GO:0051287 GO:0004616 GO:0016616 GO:0016491
GO:0003857 GO:0005515 GO:0016829
Cluster-22178.62521 GO:0007050 GO:0006508 GO:0006915 GO:0007264
GO:0008285 GO:0006144 GO:0043087 GO:0017111 GO:0004252
GO:0004197 GO:0005085 GO:0003968 GO:0031379
Cluster-22178.62523 GO:0003924

Cluster-22178.62525 GO:0005524 GO:0016787 GO:0003677 GO:0003676
GO:0005515
Cluster-22178.62526 GO:0003676 GO:0005515 GO:0003677 GO:0016787
GO:0005524
Cluster-22178.62527 GO:0010508
Cluster-22178.62528 GO:0016787 GO:0005524 GO:0003676 GO:0005515
GO:0003677
Cluster-22178.62529 GO:0003676
Cluster-22178.62531 GO:0003723
Cluster-22178.62533 GO:0003723 GO:0008270
Cluster-22178.62534 GO:0006144 GO:0006206 GO:0006351 GO:0007618
GO:0006468 GO:0016773 GO:0005524 GO:0004672 GO:0046872
GO:0003899 GO:0003677 GO:0016787 GO:0016020 GO:0005730
GO:0005576
Cluster-22178.62535 GO:0043087 GO:0005515 GO:0016020
Cluster-22178.62537 GO:0006468 GO:0004672 GO:0005524
Cluster-22178.62538 GO:0055085 GO:0007156 GO:0030001 GO:0005509
GO:0046983 GO:0046873 GO:0016020
Cluster-22178.62539 GO:0045454
Cluster-22178.62541 GO:0005634
Cluster-22178.62542 GO:0016773
Cluster-22178.62544 GO:0006629 GO:0005515
Cluster-22178.62547 GO:0055114 GO:0008152 GO:0006118 GO:0016627
GO:0008440 GO:0050660 GO:0003995
Cluster-22178.62548 GO:0006886 GO:0016192 GO:0046872 GO:0030131
Cluster-22178.62549 GO:0006351 GO:0006355 GO:0006352 GO:0006144
GO:0006206 GO:0003899 GO:0003677 GO:0003700 GO:0016987
GO:0046872 GO:0005667 GO:0005730
Cluster-22178.62550 GO:0055114 GO:0008152 GO:0006468 GO:0006118
GO:0006357 GO:0015979 GO:0006480 GO:0017004 GO:0008168
GO:0004672 GO:0045158 GO:0001104 GO:0005524 GO:0047134
GO:0009523 GO:0016021 GO:0009512 GO:0005815 GO:0016592
Cluster-22178.62551 GO:0007165 GO:0015684 GO:0006355 GO:0007264

GO:0006576 GO:0005525 GO:0015093 GO:0005524 GO:0016887
GO:0005515 GO:0005509 GO:0016021 GO:0005578 GO:0005634
Cluster-22178.62553 GO:0006614 GO:0005524 GO:0043531 GO:0005525
GO:0016301
Cluster-22178.62554 GO:0006457 GO:0007165 GO:0006807 GO:0006914
GO:0016301 GO:0051082 GO:0016810 GO:0016272
Cluster-22178.62556 GO:0006446 GO:0006413 GO:0003743 GO:0005840
Cluster-22178.62557 GO:0016311 GO:0030001 GO:0055085 GO:0035023
GO:0009252 GO:0043087 GO:0005515 GO:0046873 GO:0005089
GO:0050380 GO:0016020 GO:0005737
Cluster-22178.62558 GO:0016192 GO:0006886 GO:0030117 GO:0030131
Cluster-22178.62559 GO:0006281 GO:0008270
Cluster-22178.62560 GO:0046983 GO:0003677
Cluster-22178.62561 GO:0055114 GO:0003676 GO:0003677 GO:0016787
GO:0016491 GO:0046872 GO:0005524
Cluster-22178.62562 GO:0016032 GO:0006118 GO:0006119 GO:0006270
GO:0006351 GO:0006144 GO:0055114 GO:0015992 GO:0006206
GO:0008121 GO:0003899 GO:0003677 GO:0005730 GO:0032040
Cluster-22178.62564 GO:0003676
Cluster-22178.62565 GO:0042254 GO:0006412 GO:0003735 GO:0005622
GO:0005840
Cluster-22178.62566 GO:0018149 GO:0003723 GO:0003810 GO:0005515

Cluster-22178.62567 GO:0009228 GO:0006464 GO:0006772 GO:0015031
GO:0006886 GO:0005525 GO:0052855 GO:0008270 GO:0003924
GO:0005047 GO:0004417 GO:0005515 GO:0005785 GO:0005786
Cluster-22178.62568 GO:0005515
Cluster-22178.62570 GO:0016020
Cluster-22178.62572 GO:0008299
Cluster-22178.62574 GO:0007165 GO:0007186 GO:0007264 GO:0006508
GO:0000166 GO:0004197 GO:0003677 GO:0005525 GO:0004871
GO:0031683 GO:0005524 GO:0019001 GO:0003924
Cluster-22178.62576 GO:0006118 GO:0009055 GO:0020037

Cluster-22178.62577 GO:0016021
Cluster-22178.62578 GO:0008152 GO:0055114 GO:0006520 GO:0016491

Cluster-22178.62579 GO:0016787 GO:0046872 GO:0005515 GO:0016012
GO:0016021
Cluster-22178.62580 GO:0008152 GO:0046872 GO:0016866 GO:0031419

Cluster-22178.62582 GO:0007165 GO:0005515 GO:0004872 GO:0016020
GO:0016021
Cluster-22178.62584 GO:0042254 GO:0007009 GO:0005515 GO:0003735
GO:0051082 GO:0043565 GO:0005634 GO:0005840
Cluster-22178.62586 GO:0006355 GO:0003700 GO:0043565 GO:0008537
GO:0005667
Cluster-22178.62587 GO:0006355 GO:0043565 GO:0003700 GO:0005667
Cluster-22178.62590 GO:0048511
Cluster-22178.62591 GO:0007165 GO:0006955 GO:0006118 GO:0006814
GO:0015992 GO:0006120 GO:0006744 GO:0005179 GO:0003954
GO:0008137 GO:0005164 GO:0005576 GO:0005739 GO:0016020
Cluster-22178.62594 GO:0006355 GO:0009252 GO:0006807 GO:0006542
GO:0003677 GO:0004356 GO:0005622
Cluster-22178.62596 GO:0006508 GO:0004190
Cluster-22178.62597 GO:0006810 GO:0006281 GO:0007264 GO:0006355
GO:0000723 GO:0008134 GO:0003677 GO:0016787 GO:0003678
GO:0005525 GO:0005524 GO:0005667 GO:0005657
Cluster-22178.62598 GO:0031554 GO:0031556
Cluster-22178.62599 GO:0005975 GO:0042254 GO:0006412 GO:0009405
GO:0004553 GO:0019843 GO:0003735 GO:0005515 GO:0005509
GO:0005840 GO:0005576
Cluster-22178.62600 GO:0016579 GO:0005515 GO:0036459 GO:0032183
GO:0070140
Cluster-22178.62602 GO:0007010 GO:0003677 GO:0005524 GO:0003779
GO:0004003 GO:0005657
Cluster-22178.62603 GO:0004003 GO:0005524 GO:0003677 GO:0005657

Cluster-22178.62604 GO:0006270 GO:0006874 GO:0006816 GO:0006355
GO:0006260 GO:0016884 GO:0003700 GO:0005219 GO:0000062
GO:0005667 GO:0016021 GO:0005622
Cluster-22178.62607 GO:0006479 GO:0008168
Cluster-22178.62608 GO:0006817 GO:0055085 GO:0005315 GO:0016020
GO:0016021
Cluster-22178.62609 GO:0006302 GO:0006310 GO:0003677 GO:0005634
Cluster-22178.62610 GO:0015031 GO:0006464 GO:0005515
Cluster-22178.62611 GO:0016740 GO:0016887 GO:0005524 GO:0005515
GO:0016301
Cluster-22178.62612 GO:0007010 GO:0051252 GO:0016891 GO:0003779

Cluster-22178.62613 GO:0051225 GO:0007165 GO:0009058 GO:0005179
GO:0016740 GO:0070652 GO:0005576
Cluster-22178.62614 GO:0003676
Cluster-22178.62615 GO:0007155 GO:0030155 GO:0007165 GO:0006308
GO:0030334 GO:0045995 GO:0005515 GO:0008855 GO:0046872
GO:0005102 GO:0009318
Cluster-22178.62616 GO:0055114 GO:0008152 GO:0006313 GO:0016491
GO:0003677 GO:0004803
Cluster-22178.62617 GO:0006308 GO:0007049 GO:0008270 GO:0046872
GO:0008855 GO:0003723 GO:0005515 GO:0005634 GO:0009318
Cluster-22178.62619 GO:0016301 GO:0005515 GO:0005524
Cluster-22178.62620 GO:0006139 GO:0016310 GO:0008152 GO:0006281
GO:0002949 GO:0006206 GO:0006310 GO:0006810 GO:0009069
GO:0005525 GO:0003924 GO:0016301 GO:0003723 GO:0004127
GO:0003724 GO:0004674 GO:0016887 GO:0005524 GO:0098519
GO:0009378 GO:0005657 GO:0009379
Cluster-22178.62623 GO:0006955 GO:0031342 GO:0015675 GO:0009636
GO:0035444 GO:0015643 GO:0046872 GO:0015099 GO:0019814
GO:0016021
Cluster-22178.62626 GO:0006355 GO:0043565 GO:0008270 GO:0003700
GO:0005667

Cluster-22178.62627 GO:0006955 GO:0009405 GO:0031342 GO:0006629 GO:0050920 GO:0009636 GO:0015643 GO:0003824 GO:0019814 GO:0009288
Cluster-22178.62629 GO:0005515
Cluster-22178.62631 GO:0003676
Cluster-22178.62633 GO:0006397 GO:0003676 GO:0016021
Cluster-22178.62634 GO:0004190 GO:0016787 GO:0003676 GO:0015016 GO:0016021
Cluster-22178.62635 GO:0005515
Cluster-22178.62637 GO:0008152 GO:0016747
Cluster-22178.62638 GO:0046872 GO:0000166
Cluster-22178.62639 GO:0006811 GO:0055085 GO:0005216 GO:0016020
Cluster-22178.62641 GO:0006468 GO:0005524 GO:0004672
Cluster-22178.62643 GO:0016310 GO:0000160 GO:0006109 GO:0006355 GO:0005524 GO:0003774 GO:0004672 GO:0008134 GO:0005515 GO:0000155 GO:0009365 GO:0016020 GO:0016459 GO:0005667
Cluster-22178.62645 GO:0016192 GO:0005515 GO:0016021
Cluster-22178.62647 GO:0005515
Cluster-22178.62648 GO:0006468 GO:0004672 GO:0005524
Cluster-22178.62652 GO:0051726 GO:0006974 GO:0003677
Cluster-22178.62653 GO:0006118 GO:0055114 GO:0004190 GO:0005515 GO:0004252 GO:0016655 GO:0005886 GO:0016021
Cluster-22178.62654 GO:0046907 GO:0005515 GO:0008270
Cluster-22178.62655 GO:0051287
Cluster-22178.62656 GO:0051287
Cluster-22178.62657 GO:0070403 GO:0000287 GO:0030976 GO:0005515

Cluster-22178.62658 GO:0005515
Cluster-22178.62660 GO:0055114
Cluster-22178.62661 GO:0046034 GO:0006464 GO:0006144 GO:0004017 GO:0016151 GO:0046872 GO:0008270 GO:0005634
Cluster-22178.62662 GO:0042998 GO:0005198 GO:0071253 GO:0019031 GO:0016021 GO:0005802

Cluster-22178.62663 GO:0006355 GO:0006626 GO:0004518 GO:0003700 GO:0005622 GO:0005667 GO:0005741
Cluster-22178.62664 GO:0000184 GO:0006281 GO:0006355 GO:0055114 GO:0003677 GO:0004386 GO:0016972 GO:0047134 GO:0005524 GO:0008270 GO:0046872 GO:0005737
Cluster-22178.62665 GO:0006270 GO:0005515 GO:0008242
Cluster-22178.62667 GO:0045454 GO:0009306 GO:0019013 GO:0005788
Cluster-22178.62669 GO:0006570 GO:0006952 GO:0006810 GO:0009405 GO:0006470 GO:0008138 GO:0046983 GO:0019855 GO:0004725 GO:0005576
Cluster-22178.62670 GO:0006413 GO:0006265 GO:0006446 GO:0007186 GO:0015684 GO:0007165 GO:0007264 GO:0004871 GO:0005525 GO:0003924 GO:0031683 GO:0019001 GO:0003743 GO:0003690 GO:0015093 GO:0016021 GO:0005840
Cluster-22178.62672 GO:0015031 GO:0006464 GO:0006351 GO:0046983 GO:0005515 GO:0005921
Cluster-22178.62673 GO:0006351 GO:0015031 GO:0006464 GO:0005515 GO:0046983 GO:0005921
Cluster-22178.62674 GO:0005515
Cluster-22178.62676 GO:0016567 GO:0006260 GO:0005524 GO:0003678 GO:0004842 GO:0005657
Cluster-22178.62677 GO:0007050 GO:0005515 GO:0004672 GO:0005887 GO:0016020
Cluster-22178.62678 GO:0007050 GO:0005515 GO:0004672 GO:0005887 GO:0016020
Cluster-22178.62681 GO:0007165 GO:0006221 GO:0030155 GO:0007049 GO:0045747 GO:0045995 GO:0030334 GO:0005515 GO:0051082 GO:0005102 GO:0005777
Cluster-22178.62682 GO:0007050 GO:0005515 GO:0004672 GO:0016020 GO:0005887
Cluster-22178.62684 GO:0006351 GO:0042254 GO:0006412 GO:0003735 GO:0046983 GO:0005840 GO:0005622
Cluster-22178.62686 GO:0000398 GO:0005681

Cluster-22178.62687 GO:0000398 GO:0005681

Cluster-22178.62689 GO:0005634

Cluster-22178.62690 GO:0006260 GO:0010508 GO:0006355 GO:0035091 GO:0003700 GO:0043565 GO:0005667

Cluster-22178.62692 GO:0006313

Cluster-22178.62693 GO:0006570 GO:0006470 GO:0004725 GO:0008138

Cluster-22178.62695 GO:0006355 GO:0003677 GO:0034061 GO:0005634

Cluster-22178.62696 GO:0004867 GO:0005515

Cluster-22178.62698 GO:0006811 GO:0006470 GO:0055085 GO:0006570 GO:0004725 GO:0005216 GO:0003677 GO:0008138 GO:0019031 GO:0016020

Cluster-22178.62699 GO:0006886 GO:0015991 GO:0043248 GO:0016820 GO:0005515 GO:0008536 GO:0005783 GO:0032040 GO:0000221 GO:0019031

Cluster-22178.627 GO:0007165 GO:0005186 GO:0005634

Cluster-22178.62700 GO:0051287

Cluster-22178.62701 GO:0007165 GO:0040007 GO:0008283 GO:0008083 GO:0005096

Cluster-22178.62702 GO:0006355 GO:0003700 GO:0003677 GO:0043565 GO:0016021 GO:0005667 GO:0005634

Cluster-22178.62703 GO:0005515 GO:0035091

Cluster-22178.62704 GO:0043161 GO:0006281 GO:0006289 GO:0003684

Cluster-22178.62705 GO:0005975 GO:0006807 GO:0006355 GO:0043093 GO:0000917 GO:0009058 GO:0008270 GO:0043565 GO:0003933 GO:0004553 GO:0005737

Cluster-22178.62706 GO:0016310 GO:0007178 GO:0009069 GO:0004675 GO:0016020

Cluster-22178.62709 GO:0010884 GO:0008284 GO:0001819

Cluster-22178.62710 GO:0006334 GO:0006355 GO:0006352 GO:0003677 GO:0003700 GO:0016987 GO:0005667 GO:0005634

Cluster-22178.62714 GO:0006810 GO:0016787 GO:0005524 GO:0003676

GO:0004386 GO:0003677
Cluster-22178.62715 GO:0007165 GO:0007606 GO:0003676 GO:0008270
GO:0004888 GO:0016020
Cluster-22178.62716 GO:0005975 GO:0055114 GO:0042254 GO:0006412
GO:0043631 GO:0004553 GO:0004652 GO:0016706 GO:0003677
GO:0003735 GO:0005515 GO:0005840 GO:0005622
Cluster-22178.62719 GO:0007049 GO:0000917 GO:0043093 GO:0006355
GO:0003700 GO:0005667 GO:0005737
Cluster-22178.62721 GO:0006810 GO:0016021
Cluster-22178.62723 GO:0006367 GO:0007165 GO:0005515 GO:0046983
GO:0019031 GO:0005669
Cluster-22178.62727 GO:0007165 GO:0006955 GO:0005125 GO:0003723
GO:0003676 GO:0005576
Cluster-22178.62729 GO:0006144 GO:0006351 GO:0019079 GO:0006303
GO:0003968 GO:0016817 GO:0003677 GO:0004003 GO:0005657
GO:0031379
Cluster-22178.62730 GO:0006506 GO:0017176 GO:0008270 GO:0016021
Cluster-22178.62733 GO:0006508 GO:0006351 GO:0055114 GO:0015979
GO:0009094 GO:0030494 GO:0000162 GO:0009073 GO:0006260
GO:0006571 GO:0003856 GO:0004252 GO:0003677 GO:0016491
GO:0042025
Cluster-22178.62734 GO:0055085 GO:0016021
Cluster-22178.62736 GO:0006351 GO:0006270 GO:0006260 GO:0000398
GO:0003887 GO:0003677 GO:0042575 GO:0005681 GO:0032040
Cluster-22178.62738 GO:0005515
Cluster-22178.62740 GO:0046487 GO:0006355 GO:0015940 GO:0008152
GO:0055114 GO:0006418 GO:0019354 GO:0006779 GO:0009396
GO:0043115 GO:0000166 GO:0003824 GO:0008677 GO:0004488
GO:0051287 GO:0050662 GO:0004812 GO:0016757 GO:0016491
GO:0005524 GO:0016020
Cluster-22178.62741 GO:0006810 GO:0005622
Cluster-22178.62742 GO:0006144 GO:0009165 GO:0009116 GO:0006098
GO:0006397 GO:0004749 GO:0003723 GO:0000287 GO:0005737

GO:0005634
Cluster-22178.62744 GO:0005515 GO:0003824 GO:0046872 GO:0008270

Cluster-22178.62746 GO:0005198 GO:0003924 GO:0019028
Cluster-22178.62748 GO:0009234 GO:0008299
Cluster-22178.62751 GO:0003723 GO:0005515 GO:0003676
Cluster-22178.62752 GO:0009451 GO:0001522 GO:0003723 GO:0009982

Cluster-22178.62753 GO:0042393 GO:0005515
Cluster-22178.62760 GO:0008289 GO:0020037 GO:0005783
Cluster-22178.62761 GO:0006360 GO:0001168
Cluster-22178.62764 GO:0019354 GO:0055114 GO:0016117 GO:0006779
GO:0071949 GO:0043115 GO:0016705 GO:0016491
Cluster-22178.62765 GO:0042254 GO:0006412 GO:0003735 GO:0005840
Cluster-22178.62766 GO:0005515 GO:0005509
Cluster-22178.62767 GO:0006269 GO:0006351 GO:0019825 GO:0005515
GO:0005198 GO:0020037 GO:0003677 GO:0003896 GO:0005634
GO:0019028 GO:0005730 GO:0005657
Cluster-22178.62768 GO:0006351 GO:0007050 GO:0006915 GO:0016579
GO:0006206 GO:0019083 GO:0006144 GO:0008285 GO:0055114
GO:0003677 GO:0003899 GO:0008270 GO:0036459 GO:0016491
GO:0005730
Cluster-22178.62769 GO:0006355 GO:0003682 GO:0016747 GO:0000785
Cluster-22178.6277 GO:0051424 GO:0016021
Cluster-22178.62770 GO:0003677 GO:0003676 GO:0005524 GO:0016787

Cluster-22178.62771 GO:0006879 GO:0042742 GO:0008199 GO:0005576
Cluster-22178.62774 GO:0005515
Cluster-22178.62776 GO:0032012 GO:0043087 GO:0043248 GO:0007160
GO:0005509 GO:0005086 GO:0005783 GO:0005576 GO:0005634
Cluster-22178.62777 GO:0007160 GO:0043248 GO:0043087 GO:0032012
GO:0005086 GO:0005509 GO:0005634 GO:0005576 GO:0005783
Cluster-22178.62778 GO:0005515

Cluster-22178.62779 GO:0006355 GO:0005515 GO:0003677 GO:0008270

Cluster-22178.62782 GO:0009307 GO:0035556 GO:0030488 GO:0006464
GO:0009451 GO:0006308 GO:0008033 GO:0006418 GO:0003677
GO:0004812 GO:0016429 GO:0009036 GO:0005524 GO:0000166
GO:0016151 GO:0031515 GO:0009359
Cluster-22178.62785 GO:0042742 GO:0007094 GO:0016021
Cluster-22178.62788 GO:0006464 GO:0006665 GO:0006807 GO:0006396
GO:0019058 GO:0016567 GO:0005975 GO:0006687 GO:0006468
GO:0005524 GO:0008270 GO:0016773 GO:0004842 GO:0004348
GO:0046872 GO:0004672 GO:0016151 GO:0003676 GO:0003723
GO:0005515 GO:0005680 GO:0016020
Cluster-22178.62789 GO:0019058 GO:0007165 GO:0007283 GO:0008601
GO:0000159
Cluster-22178.62791 GO:0006886 GO:0015991 GO:0006629 GO:0016192
GO:0006606 GO:0043248 GO:0051276 GO:0015031 GO:0005515
GO:0016820 GO:0046872 GO:0004222 GO:0008565 GO:0016887
GO:0005524 GO:0008270 GO:0005634 GO:0030117 GO:0005737
GO:0005694 GO:0000221 GO:0005622
Cluster-22178.62792 GO:0016773 GO:0003676
Cluster-22178.62793 GO:0046872 GO:0008270
Cluster-22178.62794 GO:0007049 GO:0006275 GO:0007155 GO:0020037

Cluster-22178.62796 GO:0005515
Cluster-22178.62797 GO:0006334 GO:0003677 GO:0016021 GO:0000786
GO:0005634
Cluster-22178.62798 GO:0006810 GO:0016021
Cluster-22178.62800 GO:0006465 GO:0007264 GO:0005525 GO:0008233
GO:0003924 GO:0016021 GO:0005787
Cluster-22178.62801 GO:0007165 GO:0006810 GO:0055085 GO:0006898
GO:0005044 GO:0042626 GO:0016887 GO:0005524 GO:0016020
GO:0016021
Cluster-22178.62803 GO:0009186 GO:0055114 GO:0030246

Cluster-22178.62806 GO:0006812 GO:0055085 GO:0008324 GO:0016021
Cluster-22178.62808 GO:0004859 GO:0005576
Cluster-22178.62815 GO:0006096 GO:0006094 GO:0000162 GO:0006571
GO:0009094 GO:0000287 GO:0004634 GO:0000015
Cluster-22178.62817 GO:0045227 GO:0004252 GO:0016021 GO:0016020
Cluster-22178.62819 GO:0003779 GO:0005198 GO:0005856
Cluster-22178.62820 GO:0007165 GO:0016032 GO:0004871 GO:0019867
GO:0016021
Cluster-22178.62821 GO:0007165 GO:0016032 GO:0004871 GO:0019867
GO:0016021
Cluster-22178.62822 GO:0006313 GO:0055114 GO:0004803 GO:0003677
GO:0016702
Cluster-22178.62824 GO:0006468 GO:0004672 GO:0005524
Cluster-22178.62825 GO:0007264 GO:0003924 GO:0005509 GO:0005515
GO:0005525
Cluster-22178.62827 GO:0009395 GO:0008654 GO:0042157 GO:0016567
GO:0046486 GO:0006869 GO:0005198 GO:0005515 GO:0046872
GO:0008289 GO:0061630 GO:0046983 GO:0008270 GO:0004143
GO:0004842 GO:0019028 GO:0005576 GO:0019031 GO:0016020
GO:0005622
Cluster-22178.62828 GO:0006119 GO:0006412 GO:0006355 GO:0035556
GO:0015992 GO:0042254 GO:0015986 GO:0008270 GO:0046933
GO:0003677 GO:0003735 GO:0046961 GO:0005840 GO:0005622
GO:0045259 GO:0045261
Cluster-22178.62829 GO:0043066 GO:0045766 GO:0043123
Cluster-22178.62832 GO:0009069 GO:0006468 GO:0016310 GO:0035556
GO:0005515 GO:0005524 GO:0004672 GO:0004674
Cluster-22178.62833 GO:0006486 GO:0006355 GO:0003700 GO:0008417
GO:0003677 GO:0016020 GO:0005667
Cluster-22178.62836 GO:0005515
Cluster-22178.62839 GO:0003676 GO:0003723
Cluster-22178.62840 GO:0016021
Cluster-22178.62841 GO:0016021

Cluster-22178.62842 GO:0006355 GO:0006465 GO:0008233 GO:0003676
GO:0005787 GO:0005622 GO:0016021
Cluster-22178.62843 GO:0003723 GO:0003676
Cluster-22178.62845 GO:0016021
Cluster-22178.62847 GO:0005515
Cluster-22178.6285 GO:0004222
Cluster-22178.62850 GO:0006000 GO:0006013 GO:0019497 GO:0015074
GO:0006771 GO:0003993 GO:0005524 GO:0003873
Cluster-22178.62855 GO:0006886 GO:0006779 GO:0015994 GO:0055114
GO:0008536 GO:0004109
Cluster-22178.62857 GO:0005543
Cluster-22178.62859 GO:0005515 GO:0043657
Cluster-22178.62860 GO:0007018 GO:0007017 GO:0008017 GO:0003777
GO:0005524 GO:0005515 GO:0045298 GO:0005874 GO:0043657
Cluster-22178.62861 GO:0007283 GO:0007018 GO:0007017 GO:0005515
GO:0003777 GO:0008017 GO:0005524 GO:0005874 GO:0043657
GO:0005794 GO:0045298
Cluster-22178.62862 GO:0007018 GO:0006355 GO:0007017 GO:0003777
GO:0008017 GO:0005524 GO:0008270 GO:0016818 GO:0003677
GO:0005874 GO:0045298 GO:0005634
Cluster-22178.62865 GO:0007017 GO:0006355 GO:0007018 GO:0005524
GO:0008270 GO:0008017 GO:0003777 GO:0016818 GO:0003677
GO:0045298 GO:0005874 GO:0005634
Cluster-22178.62867 GO:0008440
Cluster-22178.62868 GO:0006270 GO:0006605 GO:0006367 GO:0006886
GO:0032968 GO:0003676 GO:0003677 GO:0005515 GO:0016021
GO:0016020 GO:0005742 GO:0005634
Cluster-22178.62869 GO:0007264 GO:0005525 GO:0003924
Cluster-22178.62872 GO:0046488 GO:1901031 GO:0016307 GO:0005634
Cluster-22178.62873 GO:0006370 GO:0006260 GO:0006281 GO:0006310
GO:0006397 GO:0005524 GO:0003910 GO:0004484 GO:0003677
Cluster-22178.62874 GO:0006298 GO:0005524
Cluster-22178.62875 GO:0043087 GO:0005089 GO:0005737

Cluster-22178.62876 GO:0043087 GO:0005089 GO:0005737

Cluster-22178.62878 GO:0016021

Cluster-22178.62880 GO:0009435 GO:0006040 GO:0009607 GO:0000105

GO:0006807 GO:0007275 GO:0006537 GO:0055114 GO:0008033

GO:0046497 GO:0008152 GO:0007219 GO:0006051 GO:0006355

GO:0016491 GO:0050660 GO:0016638 GO:0018580 GO:0015930

GO:0016829 GO:0016627 GO:0003824 GO:0017150 GO:0004514

GO:0047465 GO:0005737 GO:0016021

Cluster-22178.62882 GO:0007050 GO:0045859 GO:0004861 GO:0016462

GO:0005737 GO:0005634

Cluster-22178.62884 GO:0006355 GO:0005622

Cluster-22178.62885 GO:0007165 GO:0042981 GO:0005515

Cluster-22178.62886 GO:0007015 GO:0003785

Cluster-22178.62887 GO:0006438 GO:0009098 GO:0008152 GO:0009099

GO:0009097 GO:0009405 GO:0016758 GO:0004832 GO:0005524

GO:0000166 GO:0005737 GO:0016020 GO:0005783

Cluster-22178.62889 GO:0043039 GO:0005524 GO:0016876 GO:0016021

Cluster-22178.62890 GO:0015991 GO:0015992 GO:0015078 GO:0033179

Cluster-22178.62891 GO:0008218 GO:0006118 GO:0007017 GO:0030036

GO:0055114 GO:0006406 GO:0003995 GO:0017048 GO:0003723

GO:0003779 GO:0005875 GO:0005634 GO:0005737

Cluster-22178.62894 GO:0006355 GO:0006810 GO:0051537 GO:0003713

GO:0003677 GO:0005886 GO:0005667 GO:0005634 GO:0043231

Cluster-22178.62896 GO:0019087 GO:0006769 GO:0015031 GO:0055085

GO:0046497 GO:0008152 GO:0035556 GO:0006741 GO:0006810

GO:0008565 GO:0005525 GO:0003951 GO:0016021 GO:0016020

Cluster-22178.62897 GO:0016787

Cluster-22178.62899 GO:0006810 GO:0007218 GO:0008610 GO:0015947

GO:0006741 GO:0046497 GO:0008152 GO:0006308 GO:0055114

GO:0006769 GO:0016747 GO:0008855 GO:0019871 GO:0016301

GO:0003951 GO:0046332 GO:0030058 GO:0042597 GO:0005886

GO:0009318 GO:0005576

Cluster-22178.62900 GO:0006364 GO:0032259 GO:0008033 GO:0003723

GO:0008168

Cluster-22178.62901 GO:0006359 GO:0006260 GO:0015992 GO:0006351 GO:0090266 GO:0015991 GO:0015078 GO:0005524 GO:0003887 GO:0003677 GO:0005680 GO:0019031 GO:0032040 GO:0033179 GO:0042575

Cluster-22178.62902 GO:0009877 GO:0009312 GO:0008152 GO:0008757 GO:0008168 GO:0005634

Cluster-22178.62905 GO:0007165 GO:0007186 GO:0004871 GO:0005834

Cluster-22178.62906 GO:0006606 GO:0006914 GO:0039693 GO:0006397 GO:0006508 GO:0019784 GO:0005198 GO:0003682 GO:0003723 GO:0000785 GO:0019031 GO:0005737 GO:0005634 GO:0019028

Cluster-22178.62909 GO:0004867

Cluster-22178.62911 GO:0015992 GO:0006123 GO:0046872 GO:0004129 GO:0045277

Cluster-22178.62912 GO:0006418 GO:0002161 GO:0005515

Cluster-22178.62913 GO:0007165 GO:0005515

Cluster-22178.62917 GO:0003723

Cluster-22178.62918 GO:0006508 GO:0050790 GO:0008234 GO:0004197

Cluster-22178.62919 GO:0046677 GO:0009116 GO:0007631 GO:0007218 GO:0008270

Cluster-22178.62920 GO:0007165 GO:0006355 GO:0003677 GO:0005515 GO:0005179 GO:0005576

Cluster-22178.62921 GO:0005634

Cluster-22178.62926 GO:0006637 GO:0055114 GO:0006260 GO:0006635 GO:0046907 GO:0003997 GO:0003677 GO:0003887 GO:0008408 GO:0005777 GO:0005664 GO:0005634 GO:0042575

Cluster-22178.62927 GO:0006508 GO:0006813 GO:0006355 GO:0001522 GO:0042254 GO:0055114 GO:0051287 GO:0016616 GO:0008239 GO:0003700 GO:0005667 GO:0005622

Cluster-22178.62928 GO:0006144 GO:0035556 GO:0009190 GO:0006171 GO:0051537 GO:0016849 GO:0005515 GO:0004016 GO:0005886

Cluster-22178.62929 GO:0006355 GO:0003677 GO:0016021

Cluster-22178.62932 GO:0030866 GO:0005198 GO:0003779 GO:0008092 GO:0005856
Cluster-22178.62936 GO:0003723
Cluster-22178.62937 GO:0006281 GO:0008270 GO:0046983
Cluster-22178.62938 GO:0061024 GO:0010033 GO:0019867
Cluster-22178.62939 GO:0005515
Cluster-22178.62940 GO:0005509 GO:0005515 GO:0019028
Cluster-22178.62943 GO:0043066 GO:0043027
Cluster-22178.62944 GO:0015937 GO:0015940 GO:0042254 GO:0006412 GO:0005524 GO:0004594 GO:0003735 GO:0004252 GO:0005622 GO:0005840 GO:0019028 GO:0055036
Cluster-22178.62946 GO:0005975 GO:0004553 GO:0016020
Cluster-22178.62947 GO:0007267 GO:0046872
Cluster-22178.62948 GO:0006914 GO:0006357 GO:0006206 GO:0006606 GO:0006886 GO:0006457 GO:0007165 GO:0006144 GO:0030168 GO:0015031 GO:0006260 GO:0006355 GO:0006351 GO:0051258 GO:0007049 GO:0003677 GO:0008565 GO:0051082 GO:0003899 GO:0001104 GO:0043565 GO:0030674 GO:0005102 GO:0003700 GO:0005667 GO:0005730 GO:0016020 GO:0016272 GO:0005577 GO:0005737 GO:0016592 GO:0019028
Cluster-22178.62950 GO:0015940 GO:0007186 GO:0006952 GO:0015937 GO:0004930 GO:0005524 GO:0004594
Cluster-22178.62952 GO:0009058 GO:0019054 GO:0004857 GO:0016740 GO:0008191
Cluster-22178.62953 GO:0042742 GO:0009435 GO:0046497 GO:0004514 GO:0016763 GO:0005515 GO:0019901
Cluster-22178.62957 GO:0006352
Cluster-22178.62958 GO:0008270
Cluster-22178.62959 GO:0007165 GO:0005057
Cluster-22178.62961 GO:0030168 GO:0051258 GO:0007165 GO:0005102 GO:0005515 GO:0030674 GO:0042720 GO:0005577
Cluster-22178.62964 GO:0006281 GO:0006144 GO:0006366 GO:0046034 GO:0004867 GO:0004017 GO:0003677 GO:0046872 GO:0008270

GO:0005576 GO:0005665

Cluster-22178.62968 GO:0006508 GO:0004252

Cluster-22178.62969 GO:0006468 GO:0006560 GO:0006814 GO:0055114 GO:0006090 GO:0006525 GO:0019354 GO:0071436 GO:0006779 GO:0005515 GO:0043115 GO:0008948 GO:0004672 GO:0008641 GO:0015081 GO:0005524 GO:0016021 GO:0016020

Cluster-22178.62970 GO:0005515

Cluster-22178.62971 GO:0006508 GO:0006886 GO:0016192 GO:0006506 GO:0005515 GO:0005509 GO:0004198 GO:0030131 GO:0005622

Cluster-22178.62972 GO:0006570 GO:0006470 GO:0008138 GO:0004725

Cluster-22178.62976 GO:0042254 GO:0006412 GO:0003735 GO:0005840

Cluster-22178.62977 GO:0006355 GO:0009966 GO:0003677 GO:0005576

Cluster-22178.62978 GO:0006355 GO:0043565

Cluster-22178.62981 GO:0055114 GO:0006278 GO:0016579 GO:0003964 GO:0036459 GO:0004800

Cluster-22178.62984 GO:0031124 GO:0046872

Cluster-22178.62985 GO:0006418 GO:0004812 GO:0016627 GO:0005524 GO:0000166

Cluster-22178.62986 GO:0006824 GO:0009236 GO:0003676 GO:0015087 GO:0016020

Cluster-22178.62987 GO:0005515 GO:0005201 GO:0005581 GO:0005578

Cluster-22178.62988 GO:0006811 GO:0005216 GO:0016020

Cluster-22178.62989 GO:0009235 GO:0006355 GO:0005515 GO:0046872 GO:0005634

Cluster-22178.6299 GO:0043130 GO:0005515

Cluster-22178.62992 GO:0005515

Cluster-22178.62993 GO:0008092 GO:0005886 GO:0005737 GO:0019898

Cluster-22178.62994 GO:0003676

Cluster-22178.62995 GO:0006464 GO:0015031 GO:0015992 GO:0015986 GO:0015689 GO:0006886 GO:0052855 GO:0008270 GO:0005047 GO:0015078 GO:0005525 GO:0003924 GO:0005515 GO:0015098 GO:0005785 GO:0000276 GO:0016021 GO:0005786

Cluster-22178.62997 GO:0016042 GO:0008152 GO:0008203 GO:0016298 GO:0016787

Cluster-22178.62998 GO:0005515

Cluster-22178.62999 GO:0005515 GO:0008270 GO:0046872

Cluster-22178.63000 GO:0006355 GO:0006810 GO:0005524 GO:0008134 GO:0016301 GO:0005667

Cluster-22178.63001 GO:0007340 GO:0007165 GO:0007342 GO:0046872 GO:0003676 GO:0005179 GO:0003677 GO:0005887 GO:0005576 GO:0045095 GO:0001669

Cluster-22178.63003 GO:0006810 GO:0005215 GO:0016020

Cluster-22178.63004 GO:0006355 GO:0007165 GO:0003700 GO:0004871 GO:0005667

Cluster-22178.63006 GO:0005515

Cluster-22178.63008 GO:0006412 GO:0042254 GO:0009372 GO:0017148 GO:0042710 GO:0016032 GO:0004672 GO:0005524 GO:0003735 GO:0005840 GO:0005763

Cluster-22178.63009 GO:0016567 GO:0004842

Cluster-22178.63010 GO:0006412 GO:0042254 GO:0003735 GO:0005840 GO:0005622

Cluster-22178.63011 GO:0006397 GO:0035023 GO:0043087 GO:0003723 GO:0005089 GO:0046872 GO:0005634 GO:0005737

Cluster-22178.63012 GO:0006468 GO:0051016 GO:0005524 GO:0004672 GO:0030479

Cluster-22178.63013 GO:0005515

Cluster-22178.63015 GO:0070206 GO:0007049 GO:0006355 GO:0043565 GO:0003700 GO:0003677 GO:0005667 GO:0005634

Cluster-22178.63017 GO:0004518 GO:0003677

Cluster-22178.63018 GO:0006825 GO:0016531 GO:0005507 GO:0005758

Cluster-22178.63021 GO:0007049 GO:0006450 GO:0007067 GO:0051082 GO:0000775 GO:0005634

Cluster-22178.63022 GO:0042967 GO:0006479 GO:0007165 GO:0006481 GO:0008080 GO:0016747 GO:0005198 GO:0004671 GO:0019031 GO:0016021 GO:0019028

Cluster-22178.63024 GO:0005515
Cluster-22178.63025 GO:0016020
Cluster-22178.63027 GO:0008152 GO:0006355 GO:0003824 GO:0005634
Cluster-22178.63028 GO:0021987 GO:0016021
Cluster-22178.63030 GO:0005515 GO:0008270
Cluster-22178.63031 GO:0019835 GO:0009405 GO:0030976 GO:0003824
GO:0005515 GO:0019867
Cluster-22178.63032 GO:0005515
Cluster-22178.63034 GO:0007264 GO:0006355 GO:0006576 GO:0015684
GO:0015093 GO:0005524 GO:0003924 GO:0016887 GO:0005525
GO:0003677 GO:0016021
Cluster-22178.63035 GO:0006418 GO:0000166 GO:0003676 GO:0005524
GO:0004812
Cluster-22178.63036 GO:0005515
Cluster-22178.63039 GO:0006367 GO:0015684 GO:0007155 GO:0017025
GO:0005525 GO:0015093 GO:0005672 GO:0016021 GO:0009289
Cluster-22178.63040 GO:0045017 GO:0007155 GO:0006914 GO:0030334
GO:0042967 GO:0048870 GO:0051258 GO:0045995 GO:0071973
GO:0046486 GO:0030155 GO:0007165 GO:0030168 GO:0000921
GO:0051607 GO:0005515 GO:0005198 GO:0042803 GO:0045502
GO:0008134 GO:0008092 GO:0030674 GO:0004144 GO:0005102
GO:0005737 GO:0005667 GO:0005577 GO:0019028 GO:0031514
GO:0016021 GO:0005940 GO:0030286 GO:0019898
Cluster-22178.63042 GO:0003676
Cluster-22178.63044 GO:0016020
Cluster-22178.63045 GO:0055085 GO:0016021
Cluster-22178.63047 GO:0016567 GO:0046872 GO:0004842
Cluster-22178.63048 GO:0006412 GO:0008168 GO:0003676
Cluster-22178.63049 GO:0016567 GO:0006457 GO:0007165 GO:0051607
GO:0030168 GO:0000921 GO:0015031 GO:0007017 GO:0006355
GO:0070193 GO:0007018 GO:0006904 GO:0048870 GO:0051258
GO:0006914 GO:0006357 GO:0051028 GO:0048193 GO:0006606
GO:0007128 GO:0010508 GO:0007130 GO:0007094 GO:0048309

GO:0030674 GO:0051087 GO:0000774 GO:0031386 GO:0005102
GO:0045502 GO:0008134 GO:0019901 GO:0003700 GO:0003777
GO:0005524 GO:0008017 GO:0051082 GO:0042803 GO:0001104
GO:0005198 GO:0005515 GO:0016881 GO:0000145 GO:0005882
GO:0016592 GO:0005940 GO:0030286 GO:0005815 GO:0019028
GO:0031514 GO:0000795 GO:0016021 GO:0005874 GO:0019031
GO:0005667 GO:0016020 GO:0016272 GO:0005577 GO:0005634
GO:0045298
Cluster-22178.63050 GO:0006771 GO:0009231 GO:0042128 GO:0009058
GO:0003919 GO:0035091 GO:0003824
Cluster-22178.63051 GO:0007276 GO:0055085 GO:0006355 GO:0007155
GO:0009725 GO:0003677 GO:0016021 GO:0005634
Cluster-22178.63052 GO:0016192 GO:0006570 GO:0007304 GO:0006470
GO:0008138 GO:0005213 GO:0004725 GO:0005576 GO:0042600
Cluster-22178.63054 GO:0006364 GO:0042254 GO:0006412 GO:0005515
GO:0003735 GO:0005622 GO:0005840
Cluster-22178.63057 GO:0006281 GO:0043093 GO:0000917 GO:0007165
GO:0005125 GO:0008270 GO:0043130 GO:0016020 GO:0005737
Cluster-22178.63059 GO:0006807 GO:0008270 GO:0016814
Cluster-22178.63060 GO:0005515
Cluster-22178.63061 GO:0007018 GO:0030071 GO:0007017 GO:0005515
GO:0008017 GO:0003777 GO:0005524 GO:0045298 GO:0005874
GO:0005680
Cluster-22178.63063 GO:0046413 GO:0008152 GO:0005515 GO:0018836
GO:0016491
Cluster-22178.63064 GO:0006013 GO:0016226 GO:0006000 GO:0006449
GO:0006415 GO:0005524 GO:0016149 GO:0051536 GO:0005506
GO:0016740 GO:0003873 GO:0016301 GO:0005737 GO:0005840
GO:0018444
Cluster-22178.63067 GO:0006468 GO:0005515 GO:0005524 GO:0004672

Cluster-22178.63070 GO:0006118 GO:0006355 GO:0003677 GO:0003682
GO:0015035 GO:0046872 GO:0016020 GO:0005634 GO:0000785

GO:0000775
Cluster-22178.63074 GO:0016021
Cluster-22178.63075 GO:0051726 GO:0006355 GO:0006352 GO:0003677
GO:0017025 GO:0016987 GO:0003700 GO:0005667 GO:0005634
Cluster-22178.63076 GO:0005975 GO:0042254 GO:0006412 GO:0006948
GO:0004553 GO:0003735 GO:0005840 GO:0005622 GO:0019028
Cluster-22178.63077 GO:0016021
Cluster-22178.63078 GO:0055085 GO:0006811 GO:0001522 GO:0042254
GO:0007165 GO:0004872 GO:0005216 GO:0030515 GO:0072588
GO:0016021 GO:0016020
Cluster-22178.63081 GO:0005515
Cluster-22178.63084 GO:0006508 GO:0016757 GO:0004198 GO:0016020
GO:0005622
Cluster-22178.63089 GO:0042254 GO:0006412 GO:0042981 GO:0003735
GO:0005515 GO:0008270 GO:0019843 GO:0005634 GO:0005840
Cluster-22178.63090 GO:0005515 GO:0004519
Cluster-22178.63091 GO:0007601 GO:0006144 GO:0006813 GO:0030553
GO:0005515 GO:0004114 GO:0005242 GO:0016021 GO:0008076
Cluster-22178.63092 GO:0008152 GO:0006230 GO:0006206 GO:0042254
GO:0006412 GO:0006629 GO:0006508 GO:0005524 GO:0004797
GO:0003735 GO:0008236 GO:0016787 GO:0005840 GO:0016020
Cluster-22178.63093 GO:0006310 GO:0003676 GO:0003677
Cluster-22178.63095 GO:0006614 GO:0042147 GO:0006814 GO:0006120
GO:0006744 GO:0015992 GO:0055114 GO:0008137 GO:0005047
GO:0030942 GO:0008312 GO:0005786
Cluster-22178.63097 GO:0003723
Cluster-22178.63100 GO:0042254 GO:0006412 GO:0003735 GO:0005840
Cluster-22178.63103 GO:0005515
Cluster-22178.63104 GO:0003676
Cluster-22178.63105 GO:0006448 GO:0001514 GO:0003723 GO:0005515
GO:0003746 GO:0005525 GO:0005737 GO:0005840
Cluster-22178.63106 GO:0035092 GO:0003677 GO:0000228
Cluster-22178.6311 GO:0006355 GO:0003700 GO:0005667

Cluster-22178.63110 GO:0006508 GO:0015031 GO:0016787 GO:0008236

Cluster-22178.63112 GO:0051252 GO:0006402 GO:0003723 GO:0003676 GO:0004535 GO:0046872 GO:0005634 GO:0005737

Cluster-22178.63113 GO:0006051 GO:0006355 GO:0006537 GO:0008033 GO:0055114 GO:0046497 GO:0008152 GO:0007219 GO:0007275 GO:0009435 GO:0006040 GO:0009607 GO:0000105 GO:0006807 GO:0004514 GO:0047465 GO:0016829 GO:0016627 GO:0003824 GO:0017150 GO:0016638 GO:0015930 GO:0018580 GO:0016491 GO:0050660 GO:0005737 GO:0016021

Cluster-22178.63115 GO:0015684 GO:0015093 GO:0003924 GO:0005525 GO:0016021

Cluster-22178.63116 GO:0009058 GO:0055114 GO:0010024 GO:0016779 GO:0016636 GO:0050897 GO:0005524

Cluster-22178.63118 GO:0007218 GO:0006629 GO:0016021 GO:0005576 GO:0019033

Cluster-22178.63120 GO:0008207 GO:0008209 GO:0008210 GO:0006694 GO:0055114 GO:0016616 GO:0003854 GO:0050662 GO:0003824 GO:0016491 GO:0005634

Cluster-22178.63121 GO:0007165 GO:0006468 GO:0009069 GO:0016310 GO:0035556 GO:0017048 GO:0005198 GO:0004674 GO:0005524 GO:0004672 GO:0005882

Cluster-22178.63122 GO:0046983

Cluster-22178.63128 GO:0006355 GO:0003700 GO:0005667

Cluster-22178.63129 GO:0006355 GO:0003700 GO:0043565 GO:0005634 GO:0005667

Cluster-22178.63130 GO:0006633 GO:0006554 GO:0006631 GO:0018874 GO:0045454 GO:0055114 GO:0008033 GO:0006574 GO:0006552 GO:0006550 GO:0006508 GO:0006568 GO:0050660 GO:0016491 GO:0016616 GO:0071949 GO:0051287 GO:0008236 GO:0003857 GO:0005576

Cluster-22178.63132 GO:0042254 GO:0006412 GO:0003735 GO:0005622 GO:0005840

Cluster-22178.63133 GO:0016192 GO:0006886 GO:0006810 GO:0016787
GO:0005515 GO:0005198 GO:0003677 GO:0005524 GO:0003676
GO:0005634 GO:0030117
Cluster-22178.63134 GO:0006357 GO:0001104 GO:0016592
Cluster-22178.63136 GO:0005975 GO:0006012 GO:0000917 GO:0046486
GO:0001575 GO:0004553 GO:0004557 GO:0016021
Cluster-22178.63137 GO:0043093 GO:0006355 GO:0000917 GO:0006260
GO:0043565 GO:0003700 GO:0005667 GO:0005737
Cluster-22178.63138 GO:0061630
Cluster-22178.63139 GO:0008152 GO:0006554 GO:0006479 GO:0006468
GO:0008168 GO:0004672 GO:0005524 GO:0005515 GO:0018024
Cluster-22178.63140 GO:0005515
Cluster-22178.63143 GO:0006412 GO:0042254 GO:0003735 GO:0005840
GO:0005622
Cluster-22178.63144 GO:0006355 GO:0005515 GO:0003677 GO:0046872
GO:0005634
Cluster-22178.63145 GO:0006810 GO:0006418 GO:0000166 GO:0004812
GO:0005524 GO:0005886
Cluster-22178.63147 GO:0006418 GO:0006810 GO:0005524 GO:0004812
GO:0000166 GO:0005886
Cluster-22178.63149 GO:0006412 GO:0042254 GO:0003735 GO:0005761
GO:0005840
Cluster-22178.63150 GO:0006412 GO:0042254 GO:0003735 GO:0005840
Cluster-22178.63151 GO:0003924
Cluster-22178.63153 GO:0007264 GO:0007165 GO:0007186 GO:0006886
GO:0008152 GO:0016836 GO:0031683 GO:0019001 GO:0003924
GO:0005525 GO:0003824 GO:0004871 GO:0005783 GO:0016021
Cluster-22178.63154 GO:0007165 GO:0008158 GO:0016020
Cluster-22178.63156 GO:0006313 GO:0009069 GO:0000184 GO:0006470
GO:0016310 GO:0000087 GO:0006570 GO:0004674 GO:0016773
GO:0004803 GO:0008144 GO:0004725 GO:0008168 GO:0005198
GO:0005515 GO:0005622 GO:0019031 GO:0019028
Cluster-22178.63157 GO:0007264 GO:0009082 GO:0005525

Cluster-22178.63158 GO:0009712 GO:0055114 GO:0042981 GO:0046232
GO:0018874 GO:0042184 GO:0042203 GO:0006508 GO:0019261
GO:0005515 GO:0004197 GO:0018576 GO:0005506
Cluster-22178.63162 GO:0005975 GO:0004553
Cluster-22178.63163 GO:0005515
Cluster-22178.63166 GO:0006413 GO:0006446 GO:0003743 GO:0005840
Cluster-22178.63167 GO:0003676
Cluster-22178.63168 GO:0006812 GO:0032801 GO:0019076 GO:0006355
GO:0003677 GO:0005261 GO:0033644 GO:0005634
Cluster-22178.63169 GO:0046872
Cluster-22178.63170 GO:0005515
Cluster-22178.63171 GO:0030168 GO:0007165 GO:0032780 GO:0007049
GO:0051258 GO:0006886 GO:0010508 GO:0008017 GO:0005102
GO:0030674 GO:0042030 GO:0005577 GO:0005783 GO:0005739
GO:0045298 GO:0016021
Cluster-22178.63172 GO:0016037 GO:0031404 GO:0005515 GO:0030089
Cluster-22178.63173 GO:0006888 GO:0006886 GO:0008270 GO:0046872
GO:0005515 GO:0030127
Cluster-22178.63175 GO:0009403 GO:0006886 GO:0006605 GO:0005886
GO:0005742 GO:0019867 GO:0016020
Cluster-22178.63176 GO:0051252 GO:0016891
Cluster-22178.63177 GO:0004252 GO:0016021
Cluster-22178.63182 GO:0051382 GO:0007067 GO:0003677
Cluster-22178.63183 GO:0006508 GO:0000902 GO:0005515
Cluster-22178.63186 GO:0000079 GO:0019901
Cluster-22178.63187 GO:0055114 GO:0008270 GO:0004190 GO:0016706
GO:0003677 GO:0005515 GO:0016021
Cluster-22178.63188 GO:0007018 GO:0007017 GO:0008017 GO:0003777
GO:0005524 GO:0045298 GO:0005874
Cluster-22178.63189 GO:0055114 GO:0006810 GO:0055085 GO:0006118
GO:0016655 GO:0005215 GO:0016020
Cluster-22178.63190 GO:0016773 GO:0016020
Cluster-22178.63192 GO:0005515 GO:0061630 GO:0008270 GO:0046872

Cluster-22178.63195 GO:0008053 GO:0006810 GO:0010508 GO:0007264 GO:0005525 GO:0003724 GO:0005524 GO:0003924 GO:0016887 GO:0003723 GO:0016021 GO:0005741

Cluster-22178.63196 GO:0003950

Cluster-22178.63198 GO:0006418 GO:0000166 GO:0005524 GO:0004812

Cluster-22178.63199 GO:0007264 GO:0005515 GO:0005525 GO:0016887 GO:0003924 GO:0005524

Cluster-22178.63200 GO:0055114 GO:0009058 GO:0005524 GO:0016491 GO:0003676 GO:0016787 GO:0010181 GO:0003677 GO:0005634

Cluster-22178.63201 GO:0006355 GO:0003723 GO:0003676 GO:0005634

Cluster-22178.63204 GO:0005634

Cluster-22178.63207 GO:0035556 GO:0005515

Cluster-22178.63208 GO:0006465 GO:0008233 GO:0046872 GO:0016021 GO:0005787

Cluster-22178.63210 GO:0005515 GO:0005096

Cluster-22178.63211 GO:0005515 GO:0005516

Cluster-22178.63212 GO:0006508 GO:0004222

Cluster-22178.63213 GO:0005515

Cluster-22178.63214 GO:0005096 GO:0005515

Cluster-22178.63220 GO:0006355 GO:0006338 GO:0005524 GO:0008270 GO:0003676 GO:0016787 GO:0003677 GO:0016818 GO:0005856 GO:0005739 GO:0005634

Cluster-22178.63223 GO:0006364 GO:0016192 GO:0019031 GO:0016021 GO:0005730

Cluster-22178.63224 GO:0003824

Cluster-22178.63225 GO:0003824

Cluster-22178.63227 GO:0006629 GO:0007186 GO:0009401 GO:0005515 GO:0004930 GO:0016021

Cluster-22178.63228 GO:0005515 GO:0016020

Cluster-22178.63229 GO:0007018 GO:0007017 GO:0008017 GO:0003777 GO:0005524 GO:0045298 GO:0005874

Cluster-22178.63230 GO:0055114 GO:0016627
Cluster-22178.63231 GO:0048511 GO:0048870 GO:0006810 GO:0006355
GO:0003700 GO:0043565 GO:0005198 GO:0005515 GO:0016021
GO:0031514 GO:0005667 GO:0019028
Cluster-22178.63232 GO:0048511 GO:0048870 GO:0006810 GO:0006355
GO:0003700 GO:0043565 GO:0005198 GO:0005515 GO:0016021
GO:0031514 GO:0019028 GO:0005667
Cluster-22178.63234 GO:0006510 GO:0006508 GO:0016567 GO:0042710
GO:0008270 GO:0004176 GO:0046872 GO:0004842 GO:0061630
GO:0005515
Cluster-22178.63236 GO:0045454 GO:0005515
Cluster-22178.6324 GO:0005515
Cluster-22178.63240 GO:0006886 GO:0016192 GO:0030131 GO:0005622
Cluster-22178.63242 GO:0006144 GO:0030494 GO:0006206 GO:0061024
GO:0019685 GO:0007165 GO:0006351 GO:0016836 GO:0005179
GO:0003899 GO:0003677 GO:0030176 GO:0005576 GO:0005730
Cluster-22178.63246 GO:0006468 GO:0004672 GO:0005524 GO:0016773
GO:0016020
Cluster-22178.63247 GO:0045454 GO:0055114 GO:0016972
Cluster-22178.63248 GO:0006950 GO:0051087 GO:0001671
Cluster-22178.63256 GO:0016567 GO:0004842 GO:0005515 GO:0003676

Cluster-22178.63257 GO:0006888 GO:0016032 GO:0006508 GO:0003676
GO:0008233 GO:0005622 GO:0005801
Cluster-22178.63258 GO:0006479 GO:0007165 GO:0006481 GO:0042967
GO:0005198 GO:0004671 GO:0008080 GO:0016747 GO:0019028
GO:0019031 GO:0016021
Cluster-22178.63261 GO:0006355 GO:0004112
Cluster-22178.63262 GO:0005515
Cluster-22178.63263 GO:0007283
Cluster-22178.63264 GO:0019835 GO:0009405 GO:0006122 GO:0035556
GO:0055114 GO:0005515 GO:0016491 GO:0005750 GO:0019867
Cluster-22178.63266 GO:0006508 GO:0015675 GO:0035444 GO:0004252

GO:0046872 GO:0015099 GO:0030288 GO:0016021
Cluster-22178.63269 GO:0005509
Cluster-22178.63270 GO:0006355 GO:0005515 GO:0003677 GO:0046872
GO:0031083 GO:0030133 GO:0005634
Cluster-22178.63271 GO:0006505 GO:0005975 GO:0045454 GO:0006886
GO:0005985 GO:0005982 GO:0008810 GO:0016788 GO:0005509
Cluster-22178.63272 GO:0016032
Cluster-22178.63273 GO:0006468 GO:0016773 GO:0005524 GO:0004672
GO:0016020
Cluster-22178.63275 GO:0006886 GO:0016192 GO:0005198 GO:0005515
GO:0030117
Cluster-22178.63276 GO:0016021
Cluster-22178.63277 GO:0008643 GO:0055085 GO:0005351 GO:0022857
GO:0000139 GO:0016021
Cluster-22178.63278 GO:0006281 GO:0006355 GO:0051276 GO:0005515
GO:0003677 GO:0008168 GO:0005524 GO:0008270 GO:0005694
Cluster-22178.63280 GO:0008270
Cluster-22178.63282 GO:0006098 GO:0006094 GO:0006096 GO:0006013
GO:0006000 GO:0006012 GO:0016301 GO:0003872 GO:0005945
Cluster-22178.63283 GO:0008270
Cluster-22178.63284 GO:0009186 GO:0055114 GO:0030246
Cluster-22178.63285 GO:0006351 GO:0007155 GO:0015991 GO:0015992
GO:0006206 GO:0006144 GO:0003677 GO:0003899 GO:0005515
GO:0015078 GO:0033179 GO:0016020 GO:0005730
Cluster-22178.63287 GO:0006355 GO:0006508 GO:0043087 GO:0003677
GO:0008233 GO:0005524 GO:0005634 GO:0016021
Cluster-22178.63288 GO:0001522 GO:0051090 GO:0042254 GO:0030515
GO:0005515 GO:0005524 GO:0005634 GO:0072588
Cluster-22178.63289 GO:0006810 GO:0006310 GO:0000150 GO:0005525
GO:0003677 GO:0042626 GO:0005515
Cluster-22178.63290 GO:0006310 GO:0006810 GO:0000150 GO:0005515
GO:0042626 GO:0005525 GO:0003677
Cluster-22178.63293 GO:0006402 GO:0019722 GO:0051252 GO:0003676

GO:0004535 GO:0046872 GO:0005515 GO:0003723 GO:0005737
GO:0005634
Cluster-22178.63295 GO:0009228 GO:0033617 GO:0004789 GO:0005739
Cluster-22178.63296 GO:0006355 GO:0006810 GO:0022900 GO:0016301
GO:0008134 GO:0005524 GO:0005739 GO:0005667 GO:0005747
Cluster-22178.63297 GO:0042967 GO:0046486 GO:0004144
Cluster-22178.63298 GO:0006355 GO:0003700 GO:0042025 GO:0005667
Cluster-22178.63299 GO:0005982 GO:0006096 GO:0051156 GO:0006012
GO:0042207 GO:0009072 GO:0032259 GO:0006289 GO:0019872
GO:0043087 GO:0043161 GO:0005985 GO:0018149 GO:0006570
GO:0045040 GO:0006281 GO:0006094 GO:0003684 GO:0004334
GO:0005089 GO:0003810 GO:0004340 GO:0005524 GO:0005515
GO:0008168 GO:0005742
Cluster-22178.6330 GO:0006812 GO:0006820 GO:0006835 GO:0017153
GO:0016020
Cluster-22178.63301 GO:0016021
Cluster-22178.63304 GO:0006260 GO:0006144 GO:0006206 GO:0015979
GO:0016567 GO:0009186 GO:0055114 GO:0006886 GO:0004748
GO:0004842 GO:0005524 GO:0046872 GO:0005198 GO:0000166
GO:0005680 GO:0016020 GO:0009538 GO:0005801 GO:0005971
GO:0009522 GO:0019031 GO:0019028
Cluster-22178.63305 GO:0035307 GO:0030436 GO:0006508 GO:0004190
GO:0071595
Cluster-22178.63306 GO:0006260 GO:0006914 GO:0003887 GO:0001882
GO:0008092 GO:0042575 GO:0019898 GO:0005737
Cluster-22178.63307 GO:0006468 GO:0051301 GO:0016310 GO:0009069
GO:0006914 GO:0006606 GO:0048193 GO:0006355 GO:0051258
GO:0048870 GO:0007059 GO:0007067 GO:0000090 GO:0007165
GO:0006614 GO:0030168 GO:0031110 GO:0005198 GO:0042803
GO:0003677 GO:0005524 GO:0008017 GO:0045502 GO:0008134
GO:0005525 GO:0003700 GO:0030674 GO:0005096 GO:0004674
GO:0016872 GO:0005102 GO:0045298 GO:0005667 GO:0016020
GO:0005876 GO:0005577 GO:0031514 GO:0005840 GO:0016021

GO:0005882 GO:0030286 GO:0000940

Cluster-22178.63310 GO:0009236 GO:0042819 GO:0016070 GO:0042823

GO:0016032 GO:0006541 GO:0004359 GO:0003824 GO:0016787

GO:0005515 GO:0019012

Cluster-22178.63311 GO:0007076 GO:0000796

Cluster-22178.63315 GO:0003676

Cluster-22178.63316 GO:0003723

Cluster-22178.63317 GO:0005201 GO:0005578 GO:0005581

Cluster-22178.63318 GO:0055114 GO:0030328 GO:0016491 GO:0016670

Cluster-22178.6332 GO:0007016 GO:0005515 GO:0005789 GO:0016010

Cluster-22178.63320 GO:0007165 GO:0007275 GO:0006468 GO:0016055

GO:0005102 GO:0004672 GO:0005524 GO:0005576

Cluster-22178.63321 GO:0006468 GO:0005524 GO:0004672 GO:0005515

Cluster-22178.63322 GO:0055114 GO:0008152 GO:0051287 GO:0050662

GO:0016491 GO:0003824

Cluster-22178.63323 GO:0008210 GO:0008207 GO:0008209 GO:0055114

GO:0006694 GO:0050662 GO:0003854 GO:0016616 GO:0003824

Cluster-22178.63325 GO:0030150 GO:0006144 GO:0015976 GO:0016579

GO:0006094 GO:0006096 GO:0008270 GO:0036459 GO:0003824

GO:0004743 GO:0000287 GO:0030955 GO:0005742

Cluster-22178.63326 GO:0045947 GO:0008190

Cluster-22178.63328 GO:0005515

Cluster-22178.63330 GO:0007264 GO:0043087 GO:0007165 GO:0005085

Cluster-22178.63332 GO:0051090 GO:0005634

Cluster-22178.63333 GO:0015675 GO:0035444 GO:0008152 GO:0046872

GO:0003824 GO:0015099 GO:0016021

Cluster-22178.63337 GO:0006276 GO:0005515

Cluster-22178.63338 GO:0007275

Cluster-22178.63339 GO:0006508 GO:0004185

Cluster-22178.63340 GO:0051028 GO:0006554 GO:0034968 GO:0006479

GO:0018024 GO:0008270 GO:0005634
Cluster-22178.63341 GO:0009069 GO:0042742 GO:0006388 GO:0009401
GO:0016310 GO:0003972 GO:0005524 GO:0004674
Cluster-22178.63342 GO:0031342 GO:0006955 GO:0009636 GO:0015675
GO:0035444 GO:0015643 GO:0046872 GO:0015099 GO:0019814
GO:0016021
Cluster-22178.63344 GO:0009405 GO:0006468 GO:0019865 GO:0003779
GO:0005524 GO:0004672 GO:0005515 GO:0051082 GO:0019814
Cluster-22178.63345 GO:0003676
Cluster-22178.63346 GO:0055085 GO:0006811 GO:0005216 GO:0016020
GO:0016021
Cluster-22178.63347 GO:0008152 GO:0016829 GO:0010333 GO:0005515

Cluster-22178.63348 GO:0046034 GO:0034227 GO:0006468 GO:0006144
GO:0002098 GO:0004672 GO:0046872 GO:0003676 GO:0000049
GO:0020037 GO:0005524 GO:0008270 GO:0016787 GO:0003677
GO:0004017 GO:0005634
Cluster-22178.63349 GO:0007264 GO:0051087 GO:0005096
Cluster-22178.63350 GO:0008270
Cluster-22178.63352 GO:0006351 GO:0003676 GO:0005515 GO:0008270
GO:0005639
Cluster-22178.63354 GO:0016579 GO:0034477 GO:0007276 GO:0006364
GO:0006508 GO:0003676 GO:0004222 GO:0036459 GO:0008270
GO:0004518 GO:0004857 GO:0032183 GO:0070140 GO:0030246
GO:0031012
Cluster-22178.63355 GO:0007186 GO:0007166 GO:0004930 GO:0008017
GO:0045298 GO:0016020 GO:0016021
Cluster-22178.63357 GO:0005515
Cluster-22178.63360 GO:0006810 GO:0005622
Cluster-22178.63361 GO:0006090 GO:0006633 GO:0005198 GO:0016874
GO:0003989 GO:0009317
Cluster-22178.63364 GO:0006351 GO:0006952 GO:0009405 GO:0006810
GO:0006334 GO:0006260 GO:0003677 GO:0003887 GO:0019855

GO:0042575 GO:0005576 GO:0005634
Cluster-22178.63365 GO:0006139 GO:0003676 GO:0008408
Cluster-22178.63366 GO:0006576 GO:0006413 GO:0007264 GO:0006446
GO:0015684 GO:0003743 GO:0005515 GO:0046983 GO:0003924
GO:0005524 GO:0015093 GO:0005525 GO:0005576 GO:0016021
GO:0005840
Cluster-22178.63367 GO:0006468 GO:0005524 GO:0004672
Cluster-22178.63368 GO:0005515 GO:0003677
Cluster-22178.63369 GO:0006260
Cluster-22178.63370 GO:0008270
Cluster-22178.63371 GO:0006367 GO:0005515 GO:0005672
Cluster-22178.63372 GO:0006352 GO:0005634
Cluster-22178.63374 GO:0055114 GO:0009058 GO:0003676 GO:0016491
GO:0005524 GO:0003677 GO:0010181 GO:0016787
Cluster-22178.63375 GO:0007165 GO:0006955 GO:0006935 GO:0008009
GO:0005576
Cluster-22178.63376 GO:0055114 GO:0006508 GO:0007010 GO:0046872
GO:0003779 GO:0016972 GO:0008233 GO:0005634
Cluster-22178.63377 GO:0070403 GO:0046872 GO:0016787 GO:0000287
GO:0030976
Cluster-22178.63379 GO:0016772
Cluster-22178.63383 GO:0006481 GO:0006479 GO:0004671 GO:0016021
Cluster-22178.63384 GO:0006260 GO:0055085 GO:0006364 GO:0006270
GO:0030001 GO:0005515 GO:0046873 GO:0032040 GO:0016020
GO:0005732 GO:0034457 GO:0005634
Cluster-22178.63385 GO:0006886 GO:0016192 GO:0004789 GO:0005515
GO:0005198 GO:0030117
Cluster-22178.63386 GO:0006355 GO:0003677
Cluster-22178.63388 GO:0035556 GO:0009190 GO:0016849
Cluster-22178.63389 GO:0009399 GO:0006040 GO:0032509 GO:0006051
GO:0043130 GO:0033926 GO:0047465 GO:0005515 GO:0030151
GO:0016021 GO:0000813
Cluster-22178.63390 GO:0009056 GO:0006308 GO:0016888 GO:0008270

Cluster-22178.63391 GO:0006468 GO:0005524 GO:0004672

Cluster-22178.63392 GO:0006351 GO:0006355 GO:0006260 GO:0003887 GO:0003677 GO:0003682 GO:0008270 GO:0042575 GO:0005634 GO:0000785 GO:0000775

Cluster-22178.63393 GO:0007155 GO:0005540 GO:0016021

Cluster-22178.63395 GO:0003723

Cluster-22178.63396 GO:0007264 GO:0006576 GO:0005525 GO:0008201 GO:0005524 GO:0003924 GO:0016021

Cluster-22178.63400 GO:0000045 GO:0005737

Cluster-22178.63401 GO:0008152 GO:0031419 GO:0016866 GO:0046872

Cluster-22178.63403 GO:0006811 GO:0006888 GO:0006810 GO:0007172 GO:0007165 GO:0006886 GO:0006468 GO:0004713 GO:0004871 GO:0005524 GO:0004672 GO:0005230 GO:0030127 GO:0016020 GO:0005925

Cluster-22178.63404 GO:0000041 GO:0016021

Cluster-22178.63415 GO:0019058 GO:0055114 GO:0000398 GO:0045454 GO:0016209 GO:0016491 GO:0005681

Cluster-22178.63416 GO:0035556 GO:0005525 GO:0003724 GO:0005524 GO:0046872 GO:0000166 GO:0003677 GO:0005515 GO:0003723

Cluster-22178.63417 GO:0006810 GO:0003333 GO:0006355 GO:0055085 GO:0006865 GO:0015171 GO:0003700 GO:0008270 GO:0016020 GO:0005634 GO:0005667

Cluster-22178.63419 GO:0019497 GO:0006144 GO:0006807 GO:0050830 GO:0006771 GO:0006164 GO:0031423 GO:0046872 GO:0004643 GO:0003937 GO:0003993 GO:0005515 GO:0042720 GO:0005576 GO:0019012

Cluster-22178.63426 GO:0042578

Cluster-22178.63427 GO:0006886 GO:0048870 GO:0051258 GO:0045995 GO:0007155 GO:0030155 GO:0006810 GO:0007165 GO:0030334 GO:0030168 GO:0000921 GO:0006606 GO:0045502 GO:0042803 GO:0008134 GO:0008092 GO:0030674 GO:0005102 GO:0031514

GO:0005737 GO:0005801 GO:0005794 GO:0016021 GO:0005667
GO:0016020 GO:0005940 GO:0030286 GO:0005886 GO:0005577
GO:0019898
Cluster-22178.63428 GO:0006000 GO:0019497 GO:0006013 GO:0006771
GO:0005524 GO:0003993 GO:0016301 GO:0003873
Cluster-22178.63429 GO:0007067 GO:0007049 GO:0006367 GO:0005672
GO:0005634 GO:0000775
Cluster-22178.63430 GO:0003676
Cluster-22178.63431 GO:0005515
Cluster-22178.63433 GO:0006355 GO:0000917 GO:0006119 GO:0015986
GO:0043093 GO:0015992 GO:0016817 GO:0043565 GO:0003700
GO:0003677 GO:0046961 GO:0046933 GO:0005634 GO:0005737
GO:0005667 GO:0045259 GO:0045261
Cluster-22178.63434 GO:0006119 GO:0000917 GO:0006355 GO:0043093
GO:0015986 GO:0015992 GO:0003700 GO:0043565 GO:0046961
GO:0003677 GO:0046933 GO:0005634 GO:0005737 GO:0045261
GO:0045259 GO:0005667
Cluster-22178.63436 GO:0015992 GO:0006123 GO:0004129 GO:0005739
GO:0045277
Cluster-22178.63438 GO:0042254 GO:0006412 GO:0003735 GO:0005840
Cluster-22178.63440 GO:0042254 GO:0006412 GO:0003735 GO:0005840
GO:0005622
Cluster-22178.63441 GO:0006118 GO:0006119 GO:0055114 GO:0015992
GO:0008121
Cluster-22178.63445 GO:0042254 GO:0006412 GO:0003735 GO:0003677
GO:0005840 GO:0005622
Cluster-22178.63447 GO:0015986 GO:0046933 GO:0045259
Cluster-22178.63451 GO:0042254 GO:0006412 GO:0006355 GO:0003700
GO:0008270 GO:0046872 GO:0043565 GO:0042393 GO:0003677
GO:0005515 GO:0061630 GO:0003735 GO:0005840 GO:0005622
GO:0005667
Cluster-22178.63452 GO:0006281 GO:0006260 GO:0016787 GO:0010181
GO:0003677 GO:0016818 GO:0005524 GO:0016874 GO:0003676

GO:0043140 GO:0005634 GO:0016020 GO:0005622 GO:0005657
Cluster-22178.63457 GO:0042254 GO:0006412 GO:0000723 GO:0003735
GO:0003677 GO:0005840 GO:0005622 GO:0000784
Cluster-22178.63459 GO:0007017 GO:0007018 GO:0005515 GO:0035091
GO:0005524 GO:0003777 GO:0008017 GO:0005874 GO:0045298
Cluster-22178.63460 GO:0006355 GO:0005515 GO:0003677 GO:0003700
GO:0008168 GO:0043565 GO:0005667 GO:0005634
Cluster-22178.63461 GO:0008207 GO:0008209 GO:0006813 GO:0008210
GO:0008152 GO:0006694 GO:0055114 GO:0016616 GO:0050662
GO:0003854 GO:0003824 GO:0016491
Cluster-22178.63464 GO:0005515 GO:0005509
Cluster-22178.63465 GO:0005515 GO:0005509
Cluster-22178.63466 GO:0005509 GO:0005515
Cluster-22178.63467 GO:0007264 GO:0005525 GO:0005524 GO:0003924
GO:0016887 GO:0016020
Cluster-22178.63468 GO:0008152 GO:0006886 GO:0046872 GO:0008484
GO:0003824 GO:0030176
Cluster-22178.63469 GO:0008612
Cluster-22178.63472 GO:0006950 GO:0001671 GO:0051087
Cluster-22178.63474 GO:0005975 GO:0006810 GO:0009405 GO:0016192
GO:0006886 GO:0004553 GO:0019870 GO:0005198 GO:0005515
GO:0030117 GO:0005576 GO:0019028
Cluster-22178.63475 GO:0007165 GO:0006955 GO:0005125 GO:0016817
GO:0005576 GO:0005789 GO:0016021
Cluster-22178.63485 GO:0003676
Cluster-22178.63486 GO:0055114 GO:0030246 GO:0050485
Cluster-22178.63487 GO:0009116
Cluster-22178.63489 GO:0008033 GO:0006364 GO:0003723 GO:0008168
GO:0005515
Cluster-22178.6349 GO:0006355 GO:0003700 GO:0043565 GO:0005667
Cluster-22178.63490 GO:0006355 GO:0008134 GO:0005524 GO:0044599
GO:0005667
Cluster-22178.63496 GO:0009186 GO:0055114 GO:0030246 GO:0016021

Cluster-22178.63497 GO:0055114 GO:0009186 GO:0030246

Cluster-22178.63498 GO:0016021

Cluster-22178.63500 GO:0055085 GO:0016021

Cluster-22178.63502 GO:0015992 GO:0046034 GO:0003723 GO:0003924 GO:0016887 GO:0043531 GO:0005524 GO:0003724 GO:0005525

Cluster-22178.63503 GO:0034508 GO:0004867 GO:0005515 GO:0000776

Cluster-22178.63506 GO:0009058 GO:0055114 GO:0016787 GO:0010181 GO:0003677 GO:0016491 GO:0005524 GO:0003676

Cluster-22178.63507 GO:0005975 GO:0003796

Cluster-22178.63508 GO:0004672 GO:0019028

Cluster-22178.63512 GO:0006265 GO:0003677 GO:0003917 GO:0005694

Cluster-22178.63515 GO:0008152 GO:0016846

Cluster-22178.63516 GO:0006508 GO:0006468 GO:0005515 GO:0008233 GO:0004672 GO:0005524 GO:0045182 GO:0005634

Cluster-22178.63517 GO:0007275 GO:0006355 GO:0003700 GO:0005667 GO:0042600

Cluster-22178.63518 GO:0008152 GO:0016846

Cluster-22178.63519 GO:0005515

Cluster-22178.63520 GO:0006468 GO:0006508 GO:0004672 GO:0005524 GO:0045182 GO:0008233 GO:0005515 GO:0005634

Cluster-22178.63521 GO:0006468 GO:0006508 GO:0009405 GO:0005515 GO:0004672 GO:0005524 GO:0045182 GO:0008233 GO:0005634

Cluster-22178.63524 GO:0015992 GO:0006260 GO:0042254 GO:0006470 GO:0015991 GO:0006570 GO:0006412 GO:0009405 GO:0006351 GO:0003735 GO:0008138 GO:0003677 GO:0004725 GO:0015078 GO:0003887 GO:0005622 GO:0016021 GO:0005840 GO:0033179 GO:0042575

Cluster-22178.63526 GO:0006633 GO:0008152 GO:0042967 GO:0008610 GO:0005524 GO:0016747 GO:0004315 GO:0005835

Cluster-22178.63532 GO:0006464 GO:0006144 GO:0046034 GO:0006281 GO:0046872 GO:0005524 GO:0008270 GO:0016151 GO:0004017

Cluster-22178.63534 GO:0006281 GO:0006144 GO:0006464 GO:0046034 GO:0004017 GO:0046872 GO:0008270 GO:0016151

Cluster-22178.63535 GO:0016020
Cluster-22178.63537 GO:0016757
Cluster-22178.63538 GO:0006376 GO:0003729 GO:0005685
Cluster-22178.63539 GO:0006554 GO:0008152 GO:0006468 GO:0006479
GO:0008168 GO:0005524 GO:0004672 GO:0018024 GO:0005515
Cluster-22178.63540 GO:0005515
Cluster-22178.63541 GO:0007264 GO:0042254 GO:0006412 GO:0006614
GO:0003723 GO:0003735 GO:0098519 GO:0016887 GO:0003924
GO:0005524 GO:0003724 GO:0005525 GO:0005622 GO:0005840
Cluster-22178.63542 GO:0015991 GO:0006412 GO:0006119 GO:0015992
GO:0042254 GO:0003924 GO:0005525 GO:0051082 GO:0003723
GO:0005524 GO:0016887 GO:0003724 GO:0046961 GO:0003735
GO:0005840 GO:0005622 GO:0033178
Cluster-22178.63545 GO:0006284 GO:0006576 GO:0005525 GO:0003924
GO:0005524 GO:0003905 GO:0003677 GO:0030246
Cluster-22178.63546 GO:0008152 GO:0003677 GO:0008168 GO:0016021
Cluster-22178.63547 GO:0006816 GO:0006874 GO:0018279 GO:0006355
GO:0006506 GO:0006810 GO:0004579 GO:0003677 GO:0017176
GO:0000062 GO:0005219 GO:0005634 GO:0008250 GO:0005622
GO:0016021
Cluster-22178.63550 GO:0006817 GO:0008654 GO:0055085 GO:0006812
GO:0005315 GO:0016780 GO:0008324 GO:0016020 GO:0016021
Cluster-22178.63551 GO:0042254 GO:0006412 GO:0007218 GO:0003735
GO:0005185 GO:0005576 GO:0005840 GO:0005622
Cluster-22178.63554 GO:0006270 GO:0006355 GO:0003700 GO:0043565
GO:0005667
Cluster-22178.63558 GO:0006000 GO:0015976 GO:0006013 GO:0005975
GO:0006096 GO:0006094 GO:0006098 GO:0042578 GO:0042132
Cluster-22178.63559 GO:0016567 GO:0004842 GO:0005515 GO:0008270
GO:0046872 GO:0005680
Cluster-22178.63562 GO:0004672
Cluster-22178.63563 GO:0006094 GO:0006098 GO:0006096 GO:0006000
GO:0006013 GO:0006012 GO:0003872 GO:0005945 GO:0019031

Cluster-22178.63564 GO:0006576 GO:0006351 GO:0055114 GO:0008152
GO:0007264 GO:0042254 GO:0006144 GO:0006206 GO:0003723
GO:0003899 GO:0005525 GO:0003677 GO:0016491 GO:0005524
GO:0003724 GO:0005634 GO:0005730
Cluster-22178.63565 GO:0006397 GO:0003723 GO:0005634 GO:0005737
Cluster-22178.63569 GO:0006940
Cluster-22178.63570 GO:0006366 GO:0006265 GO:0005524 GO:0003918
GO:0003677
Cluster-22178.63571 GO:0006418 GO:0000166 GO:0005524 GO:0004812
GO:0003723
Cluster-22178.63572 GO:0006118 GO:0055114 GO:0005515 GO:0003677
GO:0016655
Cluster-22178.63575 GO:0016773 GO:0046872 GO:0030414 GO:0008270
GO:0005515 GO:0005509 GO:0005576
Cluster-22178.63576 GO:0006807 GO:0070403 GO:0005524 GO:0003933

Cluster-22178.63577 GO:0042254 GO:0006412 GO:0003735 GO:0005840
GO:0005622
Cluster-22178.63583 GO:0046872
Cluster-22178.63584 GO:0042967 GO:0008080 GO:0016747
Cluster-22178.63587 GO:0006270 GO:0009306 GO:0005515
Cluster-22178.63588 GO:0005524
Cluster-22178.63590 GO:0006144 GO:0006807 GO:0006396 GO:0003723
GO:0004000
Cluster-22178.63592 GO:0005524
Cluster-22178.63593 GO:0005524
Cluster-22178.63595 GO:0006811 GO:0006418 GO:0007016 GO:0000166
GO:0005216 GO:0016020 GO:0016010
Cluster-22178.63599 GO:0006446 GO:0006413 GO:0003743 GO:0005840
Cluster-22178.63601 GO:0009235
Cluster-22178.63602 GO:0044237 GO:0005524 GO:0003676
Cluster-22178.63603 GO:0008152 GO:0005524 GO:0003824
Cluster-22178.63605 GO:0055114 GO:0006554 GO:0006574 GO:0006633

GO:0008152 GO:0006568 GO:0006552 GO:0006631 GO:0018874
GO:0006550 GO:0016491 GO:0003824 GO:0016836 GO:0003857
Cluster-22178.63607 GO:0030244 GO:0005515 GO:0019867
Cluster-22178.63610 GO:0008210 GO:0008209 GO:0008207 GO:0055114
GO:0008152 GO:0006694 GO:0003854 GO:0050662 GO:0016616
GO:0005515 GO:0046872 GO:0016491 GO:0003824
Cluster-22178.63612 GO:0016567 GO:0051082 GO:0008270 GO:0046872
GO:0004842 GO:0005515 GO:0005680
Cluster-22178.63613 GO:0090200 GO:0043065 GO:0097193 GO:0007165
GO:0005739 GO:0016020
Cluster-22178.63615 GO:0015628 GO:0040007 GO:0015031 GO:0008283
GO:0006614 GO:0006810 GO:0007165 GO:0005525 GO:0003676
GO:0008083 GO:0005515 GO:0005524 GO:0016887 GO:0008565
GO:0004386 GO:0015627
Cluster-22178.63616 GO:0003676 GO:0016020
Cluster-22178.63618 GO:0006468 GO:0005524 GO:0004672
Cluster-22178.63619 GO:0006396 GO:0016779 GO:0003723
Cluster-22178.63620 GO:0016020
Cluster-22178.63622 GO:0015074 GO:0006810 GO:0007186 GO:0008200
GO:0004930 GO:0005576 GO:0016020
Cluster-22178.63623 GO:0007165 GO:0006606 GO:0007218 GO:0007034
GO:0006914 GO:0070206 GO:0051028 GO:0015992 GO:0030474
GO:0010508 GO:0015991 GO:0031144 GO:0071630 GO:0006308
GO:0048309 GO:0003677 GO:0005179 GO:0008855 GO:0005198
GO:0005200 GO:0015078 GO:0005823 GO:0033179 GO:0009318
GO:0005634 GO:0005576 GO:0005856
Cluster-22178.63624 GO:0008283 GO:0040007 GO:0007165 GO:0008083

Cluster-22178.63625 GO:0006118 GO:0055114 GO:0003995 GO:0003697
GO:0070188 GO:0005856
Cluster-22178.63626 GO:0006355 GO:0003700 GO:0005515 GO:0042025
GO:0005667
Cluster-22178.63627 GO:0007049 GO:0017053

Cluster-22178.63628 GO:0005515 GO:0016301 GO:0003774 GO:0005524 GO:0016459
Cluster-22178.63629 GO:0008152 GO:0006508 GO:0004181 GO:0016788 GO:0008270 GO:0005739
Cluster-22178.63630 GO:0005515 GO:0051539 GO:0016020 GO:0005887
Cluster-22178.63632 GO:0006810 GO:0055085 GO:0005215 GO:0022857 GO:0016021 GO:0016020
Cluster-22178.63635 GO:0043087 GO:0035023 GO:0005089 GO:0005515

Cluster-22178.63636 GO:0006813 GO:0005249 GO:0008076
Cluster-22178.63637 GO:0006605 GO:0006886 GO:0048193 GO:0009297 GO:0005515 GO:0016020 GO:0005742 GO:0019867
Cluster-22178.63638 GO:0015991 GO:0006814 GO:0042773 GO:0055114 GO:0015992 GO:0006120 GO:0006744 GO:0008137 GO:0015078 GO:0033177
Cluster-22178.63640 GO:0015031 GO:0006260 GO:0008375 GO:0003677 GO:0016021 GO:0016020 GO:0005664
Cluster-22178.63641 GO:0008033 GO:0051252 GO:0006364 GO:0004540 GO:0008168
Cluster-22178.63642 GO:0006508 GO:0006771 GO:0006355 GO:0019497 GO:0004252 GO:0005515 GO:0008270 GO:0003993 GO:0005634
Cluster-22178.63643 GO:0006468 GO:0006817 GO:0005524 GO:0004672

Cluster-22178.63645 GO:0005515
Cluster-22178.63646 GO:0015992 GO:0055114 GO:0019069 GO:0015991 GO:0009405 GO:0008270 GO:0004800 GO:0015078 GO:0003723 GO:0005515 GO:0033179 GO:0016020
Cluster-22178.63648 GO:0015031 GO:0006355 GO:0008270 GO:0008565 GO:0016021 GO:0005634
Cluster-22178.63649 GO:0006355 GO:0005198 GO:0003700 GO:0005667 GO:0019028 GO:0042025
Cluster-22178.63651 GO:0006351 GO:0043565
Cluster-22178.63652 GO:0006338 GO:0003677 GO:0090544

Cluster-22178.63653 GO:0042254 GO:0006412 GO:0016773 GO:0005515
GO:0003735 GO:0005622 GO:0005840
Cluster-22178.63654 GO:0006355 GO:0006468 GO:0030071 GO:0031145
GO:0005515 GO:0004672 GO:0005524 GO:0005634 GO:0019028
GO:0016020 GO:0005680
Cluster-22178.63657 GO:0006355 GO:0006468 GO:0030071 GO:0007010
GO:0031145 GO:0005515 GO:0005524 GO:0004672 GO:0003779
GO:0005634 GO:0019028 GO:0016020 GO:0005680
Cluster-22178.63658 GO:0006468 GO:0007010 GO:0005524 GO:0004672
GO:0003779 GO:0019028
Cluster-22178.63659 GO:0007009
Cluster-22178.63661 GO:0006123 GO:0015992 GO:0004129 GO:0045277
GO:0005739
Cluster-22178.63663 GO:0006270 GO:0000398 GO:0003677 GO:0005681
GO:0032040
Cluster-22178.63668 GO:0006508 GO:0004197
Cluster-22178.63670 GO:0006260 GO:0007217 GO:0003887 GO:0042575
Cluster-22178.63672 GO:0005198 GO:0003779 GO:0005856
Cluster-22178.63673 GO:0006950 GO:0051087 GO:0003677 GO:0001671

Cluster-22178.63674 GO:0006351 GO:0045893 GO:0003700 GO:0005515
GO:0008270 GO:0005667 GO:0005634
Cluster-22178.63675 GO:0046872
Cluster-22178.63677 GO:0005515
Cluster-22178.63678 GO:0009214 GO:0051537 GO:0005515 GO:0004113
GO:0016020 GO:0043231
Cluster-22178.63679 GO:0009214 GO:0004113 GO:0005515 GO:0016020
Cluster-22178.63680 GO:0005509
Cluster-22178.63681 GO:0006032 GO:0006270 GO:0006397 GO:0006367
GO:0003723 GO:0004190 GO:0008061 GO:0005737 GO:0016021
GO:0005634
Cluster-22178.63683 GO:0006470 GO:0006570 GO:0042967 GO:0004725
GO:0008138 GO:0005515 GO:0008080

Cluster-22178.63685 GO:0046660 GO:0006397 GO:0005634
Cluster-22178.63686 GO:0006397 GO:0046660 GO:0005634
Cluster-22178.63687 GO:0006355 GO:0003700 GO:0043565 GO:0008270
GO:0005634 GO:0005667
Cluster-22178.63692 GO:0006779 GO:0008033 GO:0055114 GO:0015992
GO:0006119 GO:0006118 GO:0019354 GO:0045454 GO:0050660
GO:0016491 GO:0043115 GO:0008121 GO:0071949
Cluster-22178.63693 GO:0007165 GO:0009069 GO:0006468 GO:0016310
GO:0008152 GO:0005515 GO:0015016 GO:0016746 GO:0016787
GO:0004674 GO:0005096
Cluster-22178.63694 GO:0003723
Cluster-22178.63695 GO:0006807 GO:0050830 GO:0006144 GO:0019497
GO:0006771 GO:0006164 GO:0003937 GO:0003993 GO:0046872
GO:0031423 GO:0004643 GO:0005515 GO:0042720 GO:0019012
GO:0005576
Cluster-22178.63696 GO:0006164 GO:0019497 GO:0006771 GO:0006952
GO:0006144 GO:0006807 GO:0050830 GO:0005515 GO:0046872
GO:0004643 GO:0003937 GO:0031423 GO:0003993 GO:0005576
GO:0019012 GO:0042720
Cluster-22178.63697 GO:0008152 GO:0006508 GO:0016788 GO:0004181
GO:0008270 GO:0005739
Cluster-22178.63698 GO:0006511 GO:0031625 GO:0005515
Cluster-22178.63699 GO:0005515
Cluster-22178.63701 GO:0007165 GO:0006468 GO:0005515 GO:0005524
GO:0004672 GO:0005125
Cluster-22178.63704 GO:0006468 GO:0004672 GO:0005524
Cluster-22178.63707 GO:0006355 GO:0016310 GO:0004673 GO:0003700
GO:0016791 GO:0005667 GO:0009365
Cluster-22178.63708 GO:0006355 GO:0046983 GO:0003700 GO:0005634
GO:0005667
Cluster-22178.63709 GO:0006308 GO:0006281 GO:0006260 GO:0017108
GO:0005634 GO:0033557
Cluster-22178.63710 GO:0015991 GO:0006810 GO:0009405 GO:0008200

GO:0005515 GO:0016820 GO:0005576 GO:0000221

Cluster-22178.63711 GO:0019843 GO:0003723 GO:0005622

Cluster-22178.63713 GO:0006468 GO:0005524 GO:0004672

Cluster-22178.63714 GO:0008053 GO:0010508 GO:0003924 GO:0016021 GO:0005741

Cluster-22178.63716 GO:0016310 GO:0009069 GO:0006468 GO:0004674 GO:0005524 GO:0004672

Cluster-22178.63718 GO:0006629 GO:0006508 GO:0006144 GO:0004197 GO:0017111 GO:0004252 GO:0003968 GO:0031379

Cluster-22178.63719 GO:0008152 GO:0006508 GO:0008236 GO:0016787

Cluster-22178.63720 GO:0055114 GO:0000372 GO:0007165 GO:0005509 GO:0005515 GO:0016616 GO:0030529 GO:0005634 GO:0005578

Cluster-22178.63721 GO:0055085 GO:0046872 GO:0016021 GO:0016020

Cluster-22178.63722 GO:0005515

Cluster-22178.63723 GO:0005975 GO:0007165 GO:0042710 GO:0008283 GO:0040007 GO:0003824 GO:0003676 GO:0008270 GO:0051015 GO:0008083 GO:0043169 GO:0030674

Cluster-22178.63724 GO:0051016 GO:0005515 GO:0030479

Cluster-22178.63725 GO:0007275 GO:0007186 GO:0004252 GO:0005515 GO:0004930 GO:0070008 GO:0003676 GO:0042600

Cluster-22178.63726 GO:0042967 GO:0008152 GO:0006633 GO:0016747 GO:0004315 GO:0005835

Cluster-22178.63728 GO:0009405 GO:0006221 GO:0005985 GO:0005982 GO:0007049 GO:0045747 GO:0006012 GO:0004575 GO:0004190 GO:0004222 GO:0004564 GO:0017177 GO:0016021

Cluster-22178.63733 GO:0006470 GO:0009231 GO:0050852 GO:0006570 GO:0009058 GO:0042128 GO:0006771 GO:0003824 GO:0004725 GO:0003919

Cluster-22178.63734 GO:0008270 GO:0005515

Cluster-22178.63735 GO:0045893 GO:0016032 GO:0007165 GO:0005158 GO:0004866 GO:0005899

Cluster-22178.63737 GO:0006470 GO:0051260 GO:0006397 GO:0032006

GO:0004721 GO:0005515 GO:0005634
Cluster-22178.63738 GO:0006486 GO:0008378 GO:0016020
Cluster-22178.63740 GO:0055114 GO:0042967 GO:0008152 GO:0000902
GO:0006412 GO:0042254 GO:0006351 GO:0016491 GO:0008080
GO:0003714 GO:0003735 GO:0005622 GO:0005840 GO:0005667
GO:0005761
Cluster-22178.63741 GO:0042157 GO:0008284 GO:0006869 GO:0006952
GO:0010333 GO:0016829 GO:0008289 GO:0000287 GO:0005576
Cluster-22178.63744 GO:0051276 GO:0006281 GO:0006355 GO:0008168
GO:0008270 GO:0005524 GO:0005515 GO:0003677 GO:0005694
Cluster-22178.63745 GO:0006383 GO:0006351 GO:0006144 GO:0006206
GO:0003899 GO:0003677 GO:0005666 GO:0005730
Cluster-22178.63746 GO:0009069 GO:0006468 GO:0035556 GO:0016310
GO:0005515 GO:0004674 GO:0005524 GO:0004672
Cluster-22178.63751 GO:0043044 GO:0006338 GO:0055085 GO:0031491
GO:0016818 GO:0003677 GO:0016787 GO:0003676 GO:0005524
GO:0008270 GO:0005634 GO:0000785 GO:0016021
Cluster-22178.63754 GO:0006355 GO:0007165 GO:0046872 GO:0004871
GO:0003700 GO:0016787 GO:0005667 GO:0005634
Cluster-22178.63757 GO:0005783
Cluster-22178.63758 GO:0003677 GO:0005634
Cluster-22178.63759 GO:0003677 GO:0005634
Cluster-22178.63763 GO:0006725 GO:0016853 GO:0046872
Cluster-22178.63765 GO:0005515
Cluster-22178.63766 GO:0006508 GO:0008234
Cluster-22178.63769 GO:0006355 GO:0006368 GO:0007155 GO:0055114
GO:0050660 GO:0003824 GO:0003700 GO:0008270 GO:0016491
GO:0043565 GO:0005667 GO:0008023
Cluster-22178.63770 GO:0006396 GO:0005789
Cluster-22178.63771 GO:0006396 GO:0005789
Cluster-22178.63772 GO:0007186 GO:0007165 GO:0004871 GO:0005834
Cluster-22178.63774 GO:0030168 GO:0000921 GO:0051607 GO:0007165
GO:0006457 GO:0008608 GO:0051258 GO:0048870 GO:0006412

GO:0006355 GO:0042254 GO:0007155 GO:0005102 GO:0030674
GO:0003700 GO:0008134 GO:0045502 GO:0003735 GO:0005515
GO:0042803 GO:0051082 GO:0030286 GO:0001772 GO:0005940
GO:0005840 GO:0016021 GO:0097197 GO:0031514 GO:0072686
GO:0005577 GO:0005622 GO:0016272 GO:0005667 GO:0042729
Cluster-22178.63775 GO:0005515 GO:0008270
Cluster-22178.63776 GO:0019058 GO:0006260 GO:0000166 GO:0003887
GO:0003677 GO:0008408 GO:0042575 GO:0019031
Cluster-22178.63777 GO:0008053 GO:0046872 GO:0008270 GO:0003924
GO:0005515 GO:0005741 GO:0016021
Cluster-22178.63779 GO:0006879 GO:0008199
Cluster-22178.63780 GO:0006351 GO:0006508 GO:0006206 GO:0006144
GO:0003677 GO:0008236 GO:0003899 GO:0016787 GO:0005576
GO:0005730
Cluster-22178.63786 GO:0006355 GO:0006270 GO:0003682 GO:0003677
GO:0005515 GO:0008270 GO:0005634 GO:0000775 GO:0000785
Cluster-22178.63788 GO:0005634
Cluster-22178.63789 GO:0005634
Cluster-22178.63790 GO:0006355 GO:0043093 GO:0000917 GO:0006914
GO:0010506 GO:0003677 GO:0043565 GO:0003700 GO:0005634
GO:0005667 GO:0005737 GO:0005615
Cluster-22178.63792 GO:0006468 GO:0003676 GO:0005524 GO:0004672
GO:0005634
Cluster-22178.63793 GO:0009236 GO:0003676 GO:0005634
Cluster-22178.63794 GO:0006468 GO:0003676 GO:0004672 GO:0005524
GO:0005789 GO:0005634
Cluster-22178.63795 GO:0019051 GO:0005515 GO:0042025
Cluster-22178.63796 GO:0043565
Cluster-22178.63797 GO:0040007 GO:0008283 GO:0007165 GO:0008083
GO:0005615
Cluster-22178.63798 GO:0009847 GO:0016021
Cluster-22178.63799 GO:0005975 GO:0000902 GO:0016773 GO:0005524
GO:0003676 GO:0032040

Cluster-22178.63802 GO:0006419 GO:0006522 GO:0006531 GO:0016055
GO:0043039 GO:0005524 GO:0016876 GO:0004813 GO:0003676
GO:0000166
Cluster-22178.63803 GO:0006419 GO:0006522 GO:0006531 GO:0043039
GO:0004813 GO:0016876 GO:0005524 GO:0003676 GO:0000166
Cluster-22178.63804 GO:0006308 GO:0055114 GO:0006260 GO:0006281
GO:0017108 GO:0016021 GO:0005634 GO:0016020 GO:0033557
Cluster-22178.63805 GO:0016579 GO:0045046 GO:0032403 GO:0036459
GO:0005515 GO:0032183 GO:0070140 GO:0005779
Cluster-22178.63806 GO:0005515 GO:0005869
Cluster-22178.63807 GO:0009056 GO:0006308 GO:0008270 GO:0016888

Cluster-22178.63809 GO:0006468 GO:0004672 GO:0005524 GO:0005515
GO:0016021
Cluster-22178.63812 GO:0006270 GO:0021987 GO:0051028 GO:0005515
GO:0016021 GO:0016020 GO:0005634
Cluster-22178.63814 GO:0007165 GO:0005515
Cluster-22178.63816 GO:0020037 GO:0005515
Cluster-22178.63818 GO:0019722 GO:0003676 GO:0003723
Cluster-22178.63825 GO:0006309 GO:0007155 GO:0006810 GO:0007165
GO:0005173 GO:0016787 GO:0005737 GO:0005634 GO:0043190
GO:0016020
Cluster-22178.63829 GO:0042254 GO:0006412 GO:0032957 GO:0047325
GO:0003735 GO:0005515 GO:0000287 GO:0052725 GO:0052726
GO:0046872 GO:0008270 GO:0005524 GO:0005840 GO:0005622
Cluster-22178.63830 GO:0043087 GO:0005085
Cluster-22178.63831 GO:0006886 GO:0016192 GO:0005515 GO:0051537
GO:0030131 GO:0043231
Cluster-22178.63832 GO:0006468 GO:0004672 GO:0005524
Cluster-22178.63833 GO:0006468 GO:0006817 GO:0005524 GO:0004672

Cluster-22178.63836 GO:0006891 GO:0006355 GO:0005524 GO:0003713
GO:0003677 GO:0017119 GO:0005634 GO:0005667

Cluster-22178.63839 GO:0005515 GO:0008270
Cluster-22178.63842 GO:0006378 GO:0003729 GO:0003677 GO:0005849
Cluster-22178.63845 GO:0005198 GO:0005515 GO:0019028
Cluster-22178.63846 GO:0006576 GO:0008608 GO:0007264 GO:0007165
GO:0007186 GO:0006457 GO:0006950 GO:0051082 GO:0019001
GO:0031683 GO:0003924 GO:0005525 GO:0004871 GO:0005524
GO:0016887 GO:0072686 GO:0042729 GO:0016021 GO:0016272
Cluster-22178.63847 GO:0006810 GO:0002949 GO:0016787 GO:0003677
GO:0005524 GO:0016887 GO:0046983 GO:0003678 GO:0005657
Cluster-22178.63848 GO:0007049 GO:0006144 GO:0006206 GO:0006351
GO:0006355 GO:0003700 GO:0043565 GO:0003899 GO:0003677
GO:0005667 GO:0005634 GO:0005730
Cluster-22178.63849 GO:0006468 GO:0016773 GO:0004672 GO:0005524
GO:0016020
Cluster-22178.63850 GO:0007096 GO:0005634
Cluster-22178.63851 GO:0007165 GO:0034227 GO:0006308 GO:0005179
GO:0008855 GO:0005096 GO:0009318 GO:0005634 GO:0005576
GO:0005737
Cluster-22178.63854 GO:0006355 GO:0016051 GO:0005515 GO:0005634
GO:0016021
Cluster-22178.63855 GO:0046872
Cluster-22178.63857 GO:0019058
Cluster-22178.63859 GO:0055085 GO:0007186 GO:0006812 GO:0008324
GO:0004930 GO:0005886 GO:0016021
Cluster-22178.63860 GO:0009405
Cluster-22178.63861 GO:0015931 GO:0033227 GO:0016055 GO:0006457
GO:0008013 GO:0051082 GO:0051033 GO:0005509 GO:0016342
GO:0005789 GO:0016021 GO:0005783 GO:0016020
Cluster-22178.63862 GO:0006914 GO:0006606 GO:0009069 GO:0051301
GO:0016310 GO:0006468 GO:0030168 GO:0031110 GO:0007067
GO:0000090 GO:0006614 GO:0007165 GO:0051258 GO:0048870
GO:0007059 GO:0005524 GO:0008017 GO:0003677 GO:0042803
GO:0005198 GO:0005096 GO:0004674 GO:0005102 GO:0030674

GO:0005525 GO:0045502 GO:0008134 GO:0005577 GO:0005876
GO:0005667 GO:0045298 GO:0030286 GO:0000940 GO:0005882
GO:0005840 GO:0016021 GO:0031514
Cluster-22178.63863 GO:0006457 GO:0005515
Cluster-22178.63866 GO:0042967 GO:0008811 GO:0005515 GO:0005856
Cluster-22178.63867 GO:0007264 GO:0005525
Cluster-22178.63869 GO:0045454 GO:0019354 GO:0008033 GO:0055114
GO:0006779 GO:0071949 GO:0043115 GO:0016491 GO:0046872
GO:0050660
Cluster-22178.63871 GO:0046872
Cluster-22178.63872 GO:0016567 GO:0007165 GO:0006260 GO:0005515
GO:0016818 GO:0003677 GO:0005179 GO:0004842 GO:0005524
GO:0008270 GO:0046872 GO:0005634 GO:0005576 GO:0005680
Cluster-22178.63873 GO:0006338 GO:0006487 GO:0005524 GO:0003676
GO:0003677 GO:0016818 GO:0016021 GO:0005634 GO:0008250
Cluster-22178.63874 GO:0003676
Cluster-22178.63875 GO:0055085 GO:0006811 GO:0005216 GO:0016020
Cluster-22178.63876 GO:0007186 GO:0042998 GO:0004930 GO:0071253
GO:0005802
Cluster-22178.63877 GO:0016740 GO:0003676 GO:0005634
Cluster-22178.63878 GO:0042254 GO:0006412 GO:0009103 GO:0098519
GO:0003735 GO:0005524 GO:0008918 GO:0005783 GO:0005840
GO:0016021
Cluster-22178.63879 GO:0020037
Cluster-22178.6388 GO:0007186 GO:0004930 GO:0016021
Cluster-22178.63880 GO:0006376 GO:0003729 GO:0016021 GO:0005685
Cluster-22178.63881 GO:0006355 GO:0043565 GO:0003700 GO:0005515
GO:0005667 GO:0005634
Cluster-22178.63882 GO:0006464
Cluster-22178.63884 GO:0005525 GO:0003924
Cluster-22178.63885 GO:0008270 GO:0003676
Cluster-22178.63887 GO:0000902
Cluster-22178.63888 GO:0090263 GO:0016567 GO:0005515

Cluster-22178.63889 GO:0007155 GO:0005515 GO:0016021
Cluster-22178.63890 GO:0005634
Cluster-22178.63891 GO:0009186 GO:0006260 GO:0006206 GO:0055114 GO:0006144 GO:0004748 GO:0005524 GO:0005971
Cluster-22178.63892 GO:0042981
Cluster-22178.63893 GO:0019905
Cluster-22178.63894 GO:0006886 GO:0006605 GO:0008201 GO:0004867 GO:0046914 GO:0016021 GO:0005742
Cluster-22178.63898 GO:0042710 GO:0090063 GO:0035556 GO:0009890 GO:0000226 GO:0005515 GO:0003774 GO:0005524 GO:0016459 GO:0005815 GO:0000922
Cluster-22178.639 GO:0006144 GO:0006182 GO:0046039 GO:0009190 GO:0035556 GO:0016849 GO:0004383
Cluster-22178.63901 GO:0009069 GO:0006468 GO:0006979 GO:0016310 GO:0004674 GO:0016773 GO:0005524 GO:0016020
Cluster-22178.63902 GO:0005515 GO:0003723
Cluster-22178.63905 GO:0015031 GO:0006464 GO:0005515
Cluster-22178.63906 GO:0003723
Cluster-22178.63907 GO:0042157 GO:0006869 GO:0008270 GO:0003677 GO:0008289 GO:0005576 GO:0019028
Cluster-22178.63908 GO:0042158 GO:0009249 GO:0016757 GO:0003677 GO:0016020
Cluster-22178.63910 GO:0007155 GO:0008152 GO:0042246 GO:0016491 GO:0016021
Cluster-22178.63911 GO:0007186 GO:0006890 GO:0004930 GO:0019028 GO:0016021
Cluster-22178.63912 GO:0019722 GO:0006556 GO:0006555 GO:0030154 GO:0004478 GO:0016021
Cluster-22178.63915 GO:0007049 GO:0006355 GO:0003700 GO:0046872 GO:0043565 GO:0019901 GO:0042803 GO:0008134 GO:0045502 GO:0030286 GO:0005667
Cluster-22178.63916 GO:0055114 GO:0006144 GO:0009308 GO:0046034 GO:0006351 GO:0046872 GO:0008270 GO:0003676 GO:0016638

GO:0004017 GO:0003677 GO:0042597
Cluster-22178.63920 GO:0042742
Cluster-22178.63922 GO:0006741 GO:0006769 GO:0008152 GO:0046497
GO:0003951 GO:0016301
Cluster-22178.63923 GO:0007165 GO:0008601 GO:0005515 GO:0000159
Cluster-22178.63924 GO:0007165 GO:0007186 GO:0008601 GO:0004930
GO:0005515 GO:0000159 GO:0016021
Cluster-22178.63929 GO:0008201 GO:0009512
Cluster-22178.63932 GO:0045893 GO:0005515 GO:0005634
Cluster-22178.63933 GO:0005515
Cluster-22178.63936 GO:0006412 GO:0042254 GO:0003735 GO:0005622
GO:0005840
Cluster-22178.63937 GO:0000045 GO:0046486 GO:0006071 GO:0004371
GO:0005737
Cluster-22178.63938 GO:0006772 GO:0006886 GO:0009229 GO:0006605
GO:0030975 GO:0005524 GO:0004788 GO:0016020
Cluster-22178.63939 GO:0008152 GO:0016491
Cluster-22178.63940 GO:0009405 GO:0006508 GO:0006629 GO:0005198
GO:0008236 GO:0005515
Cluster-22178.63942 GO:0009712 GO:0055114 GO:0046232 GO:0042184
GO:0018874 GO:0042203 GO:0019261 GO:0032402 GO:0018576
GO:0005506 GO:0042470
Cluster-22178.63943 GO:0046872 GO:0005515 GO:0003677
Cluster-22178.63946 GO:0006355 GO:0003677
Cluster-22178.63947 GO:0016021
Cluster-22178.63948 GO:0015992 GO:0015991 GO:0015078 GO:0033177
Cluster-22178.63949 GO:0006541 GO:0009252 GO:0046436 GO:0008716
GO:0016787 GO:0005524 GO:0046872
Cluster-22178.63950 GO:0006541 GO:0009252 GO:0046436 GO:0008716
GO:0016787 GO:0005524 GO:0046872
Cluster-22178.63952 GO:0006206 GO:0006144 GO:0006351 GO:0003677
GO:0003899 GO:0005730
Cluster-22178.63954 GO:0042254 GO:0006412 GO:0003735 GO:0005622

GO:0005840 GO:0005789
Cluster-22178.63955 GO:0006468 GO:0042254 GO:0006412 GO:0005524
GO:0004672 GO:0003735 GO:0005622 GO:0005840
Cluster-22178.63956 GO:0006338 GO:0009405 GO:0090544
Cluster-22178.63957 GO:0042254 GO:0006412 GO:0003735 GO:0046872
GO:0005840 GO:0005622
Cluster-22178.63959 GO:0042254 GO:0008615 GO:0055114 GO:0010181
GO:0016638 GO:0004733 GO:0016021 GO:0005622
Cluster-22178.63962 GO:0055114 GO:0005507 GO:0005515
Cluster-22178.63964 GO:0006122 GO:0006355 GO:0006508 GO:0008270
GO:0043565 GO:0004222 GO:0003700 GO:0005515 GO:0031012
GO:0005743 GO:0005750 GO:0005634 GO:0005667
Cluster-22178.63966 GO:0039694 GO:0006144 GO:0003723 GO:0003924
GO:0046872 GO:0003968 GO:0005525 GO:0031379
Cluster-22178.63968 GO:0006470 GO:0006570 GO:0004725 GO:0008138

Cluster-22178.63970 GO:0007264 GO:0005525
Cluster-22178.63971 GO:0006355 GO:0006813 GO:0009252 GO:0006807
GO:0006811 GO:0006542 GO:0004356 GO:0003677 GO:0005249
GO:0016020 GO:0008076
Cluster-22178.63972 GO:0055114 GO:0006351 GO:0006118 GO:0006570
GO:0004097 GO:0003677 GO:0005634
Cluster-22178.63973 GO:0006090 GO:0016310 GO:0006814 GO:0006954
GO:0006560 GO:0006525 GO:0007186 GO:0071436 GO:0007165
GO:0005125 GO:0008948 GO:0015081 GO:0004930 GO:0004673
GO:0009365 GO:0005576 GO:0016020
Cluster-22178.63974 GO:0003723 GO:0003676
Cluster-22178.63976 GO:0008270
Cluster-22178.63977 GO:0006468 GO:0004672 GO:0005524 GO:0016773
GO:0016020
Cluster-22178.63978 GO:0006886 GO:0006397 GO:0015991 GO:0048193
GO:0006914 GO:0015992 GO:0007155 GO:0015078 GO:0005525
GO:0003924 GO:0042802 GO:0003774 GO:0043015 GO:0045298

GO:0016021 GO:0005801 GO:0033179 GO:0005861 GO:0016020
GO:0016459
Cluster-22178.63980 GO:0007165 GO:0007186 GO:0007264 GO:0005525
GO:0004871 GO:0031683 GO:0019001 GO:0003924
Cluster-22178.63981 GO:0016055 GO:0008013 GO:0016020 GO:0016342
Cluster-22178.63983 GO:0006919 GO:0006915 GO:0006974 GO:0001836

Cluster-22178.63984 GO:0006950 GO:0005515
Cluster-22178.63986 GO:0009451 GO:0001522 GO:0009982 GO:0003723

Cluster-22178.63987 GO:0005524 GO:0016787 GO:0003677 GO:0003676
GO:0005515
Cluster-22178.63990 GO:0006952 GO:0045454 GO:0055114 GO:0005509
GO:0016491 GO:0016209
Cluster-22178.63993 GO:0006672 GO:0006139 GO:0006807 GO:0003676
GO:0005524 GO:0008270 GO:0016818 GO:0008026 GO:0016811
GO:0016021
Cluster-22178.63994 GO:0006265 GO:0006366 GO:0003677 GO:0003918
GO:0005524
Cluster-22178.63995 GO:0055085 GO:0015992 GO:0055114 GO:0006119
GO:0015986 GO:0046933 GO:0046961 GO:0005515 GO:0016638
GO:0016021 GO:0045259 GO:0045261
Cluster-22178.63996 GO:0005515
Cluster-22178.63997 GO:0019491 GO:0005515 GO:0033990 GO:0016021
Cluster-22178.63998 GO:0008299
Cluster-22178.63999 GO:0046080 GO:0016787
Cluster-22178.640 GO:0035556 GO:0009190 GO:0016849
Cluster-22178.64003 GO:0008270
Cluster-22178.64004 GO:0005515
Cluster-22178.64005 GO:0042254 GO:0001522 GO:0005515 GO:0030515
GO:0072588
Cluster-22178.64007 GO:0005515
Cluster-22178.64008 GO:0042742 GO:0005576

Cluster-22178.64010 GO:0006468 GO:0055114 GO:0016787 GO:0003723
GO:0003697 GO:0061630 GO:0005515 GO:0003677 GO:0008270
GO:0005524 GO:0004672 GO:0046872 GO:0003676 GO:0047134
Cluster-22178.64011 GO:0005789 GO:0032040
Cluster-22178.64012 GO:0006659 GO:0042967 GO:0016747 GO:0008080

Cluster-22178.64013 GO:0006468 GO:0005524 GO:0004672 GO:0016773
GO:0016020
Cluster-22178.64014 GO:0015031 GO:0008152 GO:0042254 GO:0006412
GO:0008565 GO:0016747 GO:0016746 GO:0003735 GO:0005509
GO:0005622 GO:0016021 GO:0005840
Cluster-22178.64015 GO:0008152 GO:0042254 GO:0006412 GO:0016747
GO:0003735 GO:0005509 GO:0016746 GO:0005840 GO:0005622
Cluster-22178.64016 GO:0006355 GO:0005634
Cluster-22178.64018 GO:0006810 GO:0002949 GO:0003677 GO:0016787
GO:0003678 GO:0016887 GO:0046983 GO:0005524 GO:0005657
Cluster-22178.6402 GO:0006810 GO:0009405 GO:0008200 GO:0046872
GO:0042151
Cluster-22178.64020 GO:0006355 GO:0009405 GO:0008092 GO:0005198
GO:0005737 GO:0019898
Cluster-22178.64024 GO:0007165 GO:0007606 GO:0003676 GO:0008270
GO:0004888 GO:0016020
Cluster-22178.64025 GO:0006952 GO:0007165 GO:0005576
Cluster-22178.64026 GO:0007165 GO:0005515 GO:0003677 GO:0005179
GO:0005576
Cluster-22178.64027 GO:0042254 GO:0006412 GO:0003735 GO:0005840
GO:0005622
Cluster-22178.64030 GO:0016567 GO:0008270 GO:0046872 GO:0004842
GO:0005515 GO:0061630 GO:0005680
Cluster-22178.64031 GO:0005515
Cluster-22178.64033 GO:0006338 GO:0000228
Cluster-22178.64036 GO:0042742 GO:0006352 GO:0051191 GO:0005515
GO:0005576 GO:0005669

Cluster-22178.64037 GO:0003677
Cluster-22178.64038 GO:0006378 GO:0003729 GO:0046872 GO:0008270 GO:0003677 GO:0005849
Cluster-22178.64039 GO:0016042 GO:0015992 GO:0046486 GO:0045454 GO:0015994 GO:0006811 GO:0015996 GO:0006118 GO:0006629 GO:0006355 GO:0015991 GO:0006508 GO:0003847 GO:0003677 GO:0016787 GO:0015035 GO:0015078 GO:0009055 GO:0046872 GO:0047746 GO:0008236 GO:0005886 GO:0008247 GO:0016020 GO:0019031 GO:0033179
Cluster-22178.64040 GO:0006378 GO:0003677 GO:0008270 GO:0046872 GO:0003729 GO:0005849
Cluster-22178.64041 GO:0003723 GO:0003676
Cluster-22178.64044 GO:0006468 GO:0004672 GO:0005524 GO:0016020
Cluster-22178.64045 GO:0008270 GO:0008168
Cluster-22178.64047 GO:0016032 GO:0005515 GO:0016021
Cluster-22178.64049 GO:0015940 GO:0019521 GO:0055114 GO:0006574 GO:0006552 GO:0006550 GO:0006568 GO:0006813 GO:0006633 GO:0006779 GO:0046168 GO:0006554 GO:0006631 GO:0019354 GO:0018874 GO:0006098 GO:0008677 GO:0043115 GO:0003857 GO:0016491 GO:0004616 GO:0016616 GO:0051287
Cluster-22178.64050 GO:0006355 GO:0007275 GO:0043565 GO:0003700 GO:0005667
Cluster-22178.64051 GO:0006886 GO:0017119
Cluster-22178.64053 GO:0005515
Cluster-22178.64054 GO:0006206 GO:0009972 GO:0006807 GO:0046087 GO:0008270 GO:0004126
Cluster-22178.64055 GO:0051289 GO:0007165 GO:0006506 GO:0008140 GO:0005509 GO:0005515 GO:0016740 GO:0005789 GO:0016021 GO:0005886 GO:0005578 GO:0016012 GO:0005667
Cluster-22178.64056 GO:0006955 GO:0030153 GO:0005515 GO:0015643 GO:0019814
Cluster-22178.64057 GO:0055114 GO:0006570 GO:0006470 GO:0016730 GO:0004725 GO:0005680

Cluster-22178.64058 GO:0006804 GO:0006979 GO:0005515 GO:0004601

Cluster-22178.64059 GO:0005515 GO:0005509
Cluster-22178.64060 GO:0045893 GO:0016567 GO:0004842 GO:0005515
GO:0003676 GO:0042025
Cluster-22178.64061 GO:0004252 GO:0016021
Cluster-22178.64062 GO:0016021 GO:0005886
Cluster-22178.64063 GO:0015992 GO:0009297 GO:0006123 GO:0030436
GO:0003774 GO:0005524 GO:0004129 GO:0005515 GO:0019867
GO:0042601 GO:0045277 GO:0016459
Cluster-22178.64067 GO:0055114 GO:0050661 GO:0016491 GO:0016020
Cluster-22178.64068 GO:0046872
Cluster-22178.64069 GO:0016192 GO:0006904 GO:0046983 GO:0046914

Cluster-22178.64074 GO:0006355 GO:0009098 GO:0003677 GO:0005515

Cluster-22178.64077 GO:0015992 GO:0006123 GO:0016310 GO:0009069
GO:0016192 GO:0006886 GO:0003676 GO:0004129 GO:0004674
GO:0005515 GO:0045277 GO:0030117
Cluster-22178.64080 GO:0005515
Cluster-22178.64081 GO:0055114 GO:0006355 GO:0010309 GO:0045735

Cluster-22178.64082 GO:0019497 GO:0006771 GO:0005515 GO:0003993
GO:0046872 GO:0016020
Cluster-22178.64083 GO:0003723
Cluster-22178.64084 GO:0006412 GO:0042254 GO:0007067 GO:0051087
GO:0003735 GO:0005515 GO:0005509 GO:0005840 GO:0005622
GO:0042729 GO:0072686
Cluster-22178.64085 GO:0006367 GO:0006364 GO:0006270 GO:0009236
GO:0006260 GO:0006633 GO:0006355 GO:0006351 GO:0006352
GO:0015940 GO:0003677 GO:0016987 GO:0005515 GO:0003887
GO:0008770 GO:0003700 GO:0005667 GO:0005732 GO:0034457
GO:0005634 GO:0005672 GO:0042575 GO:0032040

Cluster-22178.64086 GO:0006355 GO:0007165 GO:0042254 GO:0006412 GO:0005179 GO:0003677 GO:0003735 GO:0005840
Cluster-22178.64087 GO:0006508 GO:0030436 GO:0004190
Cluster-22178.64088 GO:0046034 GO:0015991 GO:0015992 GO:0016820 GO:0005524 GO:0033178
Cluster-22178.64090 GO:0007017 GO:0007018 GO:0006351 GO:0006144 GO:0006914 GO:0006206 GO:0003899 GO:0003677 GO:0005524 GO:0008017 GO:0003777 GO:0005730 GO:0045298 GO:0005874
Cluster-22178.64091 GO:0007218 GO:0030436 GO:0006206 GO:0006144 GO:0006383 GO:0001682 GO:0008033 GO:0051252 GO:0006351 GO:0003677 GO:0004526 GO:0003899 GO:0005515 GO:0030677 GO:0005730 GO:0005666 GO:0042601 GO:0005576
Cluster-22178.64092 GO:0000398
Cluster-22178.64093 GO:0006560 GO:0016567 GO:0015986 GO:0006525 GO:0018401 GO:0015992 GO:0055114 GO:0004656 GO:0015078 GO:0004842 GO:0008270 GO:0046983 GO:0016702 GO:0005783 GO:0000276
Cluster-22178.64094 GO:0003924
Cluster-22178.64095 GO:0006914 GO:0007155 GO:0007165 GO:0019058 GO:0004871 GO:0003723 GO:0005198 GO:0019031 GO:0019867 GO:0019028 GO:0016020
Cluster-22178.64096 GO:0055085 GO:0007165 GO:0000772 GO:0016021 GO:0016020
Cluster-22178.64099 GO:0006139 GO:0005524 GO:0003676 GO:0016787 GO:0004003 GO:0016818 GO:0008026 GO:0003677 GO:0005657
Cluster-22178.64101 GO:0006420 GO:0006418 GO:0006260 GO:0007049 GO:0006525 GO:0006560 GO:0004814 GO:0005524 GO:0004812 GO:0000166 GO:0003677 GO:0005737 GO:0005794 GO:0005815
Cluster-22178.64102 GO:0007049 GO:0006260 GO:0006418 GO:0006420 GO:0006560 GO:0006525 GO:0008289 GO:0005524 GO:0004814 GO:0003677 GO:0000166 GO:0004812 GO:0005815 GO:0005794 GO:0005737
Cluster-22178.64103 GO:0055114 GO:0000160 GO:0008152 GO:0016310

GO:0006109 GO:0016491 GO:0004672 GO:0005524 GO:0003824
GO:0050662 GO:0051287 GO:0000155 GO:0005198 GO:0009365
GO:0019028
Cluster-22178.64104 GO:0042981 GO:0043531 GO:0005515
Cluster-22178.64108 GO:0042157 GO:0006869 GO:0008289 GO:0005576
GO:0016021
Cluster-22178.64109 GO:0006334 GO:0003677 GO:0005634 GO:0000786
Cluster-22178.64110 GO:0005515 GO:0008270
Cluster-22178.64113 GO:0006886 GO:0048280 GO:0015031 GO:0048870
GO:0008565 GO:0031514 GO:0016020 GO:0000139 GO:0005737
Cluster-22178.64114 GO:0055114 GO:0008152 GO:0006355 GO:0043401
GO:0006511 GO:0016491 GO:0003824 GO:0016872 GO:0031625
GO:0050662 GO:0016020
Cluster-22178.64115 GO:0055114 GO:0016638
Cluster-22178.64117 GO:0042254 GO:0006412 GO:0005515 GO:0003735
GO:0005840 GO:0005622
Cluster-22178.64119 GO:0003723 GO:0003676
Cluster-22178.6412 GO:0006352 GO:0046656 GO:0006355 GO:0015948
GO:0006310 GO:0000150 GO:0016987 GO:0003700 GO:0050524
GO:0003677 GO:0005667
Cluster-22178.64124 GO:0007264 GO:0046872 GO:0005525 GO:0031419

Cluster-22178.64126 GO:0006468 GO:0004672 GO:0005524
Cluster-22178.64129 GO:0035556 GO:0006313
Cluster-22178.64130 GO:0006397 GO:0006367 GO:0006270 GO:0006032
GO:0008061 GO:0004190 GO:0003723 GO:0033691 GO:0005634
GO:0016021 GO:0005737
Cluster-22178.64131 GO:0015992 GO:0015986 GO:0000902 GO:0015078

Cluster-22178.64133 GO:0018101 GO:0006570 GO:0006807 GO:0006470
GO:0008138 GO:0005509 GO:0004668 GO:0004725 GO:0005737
Cluster-22178.64134 GO:0055085 GO:0022857 GO:0016021
Cluster-22178.64135 GO:0006810 GO:0009405 GO:0006952 GO:0019855

GO:0005576
Cluster-22178.64136 GO:0006468 GO:0005524 GO:0004672
Cluster-22178.64137 GO:0017004 GO:0017003 GO:0006468 GO:0005515
GO:0005524 GO:0004672 GO:0005886
Cluster-22178.64138 GO:0015031 GO:0043087 GO:0019069 GO:0016485
GO:0035023 GO:0008565 GO:0005089
Cluster-22178.64139 GO:0003779 GO:0005543
Cluster-22178.64140 GO:0006508 GO:0004185
Cluster-22178.64141 GO:0006457 GO:0006952 GO:0006351 GO:0006950
GO:0006260 GO:0009236 GO:0003677 GO:0003887 GO:0051082
GO:0005524 GO:0008270 GO:0042575 GO:0005634
Cluster-22178.64142 GO:0008270 GO:0016021
Cluster-22178.64144 GO:0006468 GO:0005515 GO:0005524 GO:0004672

Cluster-22178.64145 GO:0005515
Cluster-22178.64147 GO:0005515
Cluster-22178.64149 GO:0007018 GO:0007017 GO:0000921 GO:0003777
GO:0008017 GO:0005524 GO:0005874 GO:0045298 GO:0016021
GO:0005940
Cluster-22178.64151 GO:0006260 GO:0046983 GO:0003887 GO:0005515
GO:0042575
Cluster-22178.64153 GO:0006904 GO:0016874 GO:0000145
Cluster-22178.64154 GO:0006446 GO:0006413 GO:0003743 GO:0005515
GO:0031369 GO:0005852 GO:0005840
Cluster-22178.64157 GO:0051205 GO:0005887 GO:0016021
Cluster-22178.64158 GO:0005524 GO:0008270 GO:0003676 GO:0004222
GO:0016021
Cluster-22178.64159 GO:0051258 GO:0009432 GO:0006281 GO:0008053
GO:0030168 GO:0007165 GO:0006310 GO:0006457 GO:0006302
GO:0003824 GO:0003924 GO:0008092 GO:0003697 GO:0051082
GO:0005102 GO:0005524 GO:0003677 GO:0030674 GO:0016021
GO:0005737 GO:0005634 GO:0019898 GO:0005577 GO:0033588
GO:0016272 GO:0005741

Cluster-22178.64161 GO:0015992 GO:0006119 GO:0015986 GO:0008289 GO:0046933 GO:0046961 GO:0045259 GO:0000275
Cluster-22178.64162 GO:0006464 GO:0006914 GO:0010506 GO:0006084 GO:0006367 GO:0055114 GO:0007049 GO:0030001 GO:0006355 GO:0010508 GO:0006886 GO:0046872 GO:0003824 GO:0008113 GO:0005783 GO:0005615 GO:0005669 GO:0016021
Cluster-22178.64164 GO:2001070 GO:0005515
Cluster-22178.64168 GO:0006886 GO:0006355 GO:0046034 GO:0006464 GO:0006888 GO:0006144 GO:0004017 GO:0016151 GO:0046872 GO:0008270 GO:0030127 GO:0005634
Cluster-22178.64169 GO:0006260 GO:0006468 GO:0003677 GO:0004672 GO:0005524 GO:0016773 GO:0016020
Cluster-22178.64170 GO:0006355 GO:0003824 GO:0003700 GO:0004252 GO:0016021 GO:0005886 GO:0005882 GO:0042025 GO:0005667
Cluster-22178.64171 GO:0010884 GO:0006810 GO:0006270 GO:0019079 GO:0001819 GO:0008284 GO:0006355 GO:0004386 GO:0005524 GO:0016887 GO:0008026 GO:0003676 GO:0008134 GO:0004190 GO:0005667 GO:0016021
Cluster-22178.64172 GO:0042254 GO:0005622
Cluster-22178.64173 GO:0006355 GO:0004252 GO:0003824 GO:0003700 GO:0042025 GO:0005882 GO:0005667 GO:0016021 GO:0005886
Cluster-22178.64174 GO:0055114 GO:0003729 GO:0016491 GO:0008270

Cluster-22178.64176 GO:0001819 GO:0006355 GO:0008284 GO:0010884 GO:0006810 GO:0019079 GO:0006270 GO:0003676 GO:0004190 GO:0008134 GO:0016887 GO:0005524 GO:0004386 GO:0008026 GO:0016021 GO:0005667
Cluster-22178.64179 GO:0006508 GO:0008270 GO:0008233 GO:0004222 GO:0031012
Cluster-22178.64180 GO:0007059 GO:0031110 GO:0051301 GO:0000090 GO:0007067 GO:0008017 GO:0004867 GO:0045298 GO:0005876 GO:0000940
Cluster-22178.64183 GO:0015992 GO:0006308 GO:0006914 GO:0015991

GO:0006810 GO:0006886 GO:0008855 GO:0015078 GO:0016021
GO:0005783 GO:0009318 GO:0033179
Cluster-22178.64184 GO:0046872 GO:0005509 GO:0005515
Cluster-22178.64185 GO:0003924 GO:0005525 GO:0012511 GO:0016021
Cluster-22178.64190 GO:0006508 GO:0005515 GO:0004222
Cluster-22178.64192 GO:0008152 GO:0009190 GO:0046039 GO:0006468
GO:0006357 GO:0006144 GO:0035556 GO:0006182 GO:0004672
GO:0003824 GO:0016849 GO:0004383 GO:0003723 GO:0001104
GO:0008270 GO:0005524 GO:0016592
Cluster-22178.64193 GO:0006355 GO:0009271 GO:0016567 GO:0005515
GO:0046872 GO:0008270 GO:0004842 GO:0005680
Cluster-22178.64195 GO:0006144 GO:0007156 GO:0009113 GO:0005524
GO:0046872 GO:0005509 GO:0004637 GO:0016020
Cluster-22178.64197 GO:0005515
Cluster-22178.64199 GO:0043401 GO:0006355 GO:0003700 GO:0008270
GO:0043565 GO:0005667 GO:0005634
Cluster-22178.64200 GO:0005198 GO:0005882
Cluster-22178.64201 GO:0006520 GO:0055114 GO:0009058 GO:0045454
GO:0016829 GO:0016209 GO:0016491 GO:0005509 GO:0030170
GO:0032040
Cluster-22178.64202 GO:0045893 GO:0016567 GO:0003676 GO:0005515
GO:0004842 GO:0042025
Cluster-22178.64203 GO:0043039 GO:0006531 GO:0006522 GO:0048193
GO:0006419 GO:0000166 GO:0003676 GO:0004813 GO:0016876
GO:0005524 GO:0016020
Cluster-22178.64205 GO:0006144 GO:0055114 GO:0006807 GO:0006260
GO:0006396 GO:0003723 GO:0004000
Cluster-22178.64206 GO:0016567 GO:0008270 GO:0046872 GO:0004842
GO:0005515 GO:0005680
Cluster-22178.64207 GO:0007264 GO:0005525
Cluster-22178.64208 GO:0043087 GO:0007165 GO:0009405 GO:0035023
GO:0005515 GO:0005089
Cluster-22178.64209 GO:0009733 GO:0005515

Cluster-22178.64210 GO:0006631 GO:0055114 GO:0050151 GO:0016491
GO:0071949
Cluster-22178.64211 GO:0006810 GO:0005515 GO:0005622 GO:0016021
Cluster-22178.64213 GO:0006355 GO:0003676 GO:0008134 GO:0046872
GO:0005524 GO:0005667
Cluster-22178.64214 GO:0016567 GO:0004842 GO:0046872 GO:0008270
GO:0005524 GO:0005515 GO:0061630 GO:0005680
Cluster-22178.64215 GO:0006606 GO:0006357 GO:0006914 GO:0015992
GO:0051028 GO:0048309 GO:0010508 GO:0006260 GO:0030168
GO:0006457 GO:0071973 GO:0006810 GO:0007165 GO:0006811
GO:0048870 GO:0051258 GO:0015991 GO:0003774 GO:0015078
GO:0042803 GO:0005198 GO:0001104 GO:0005102 GO:0000774
GO:0051087 GO:0030674 GO:0008092 GO:0005577 GO:0016020
GO:0005737 GO:0009288 GO:0019898 GO:0005886 GO:0005882
GO:0016592 GO:0016021 GO:0033179 GO:0031514
Cluster-22178.64217 GO:0042967 GO:0047961 GO:0005515 GO:0005739
Cluster-22178.64219 GO:0046872
Cluster-22178.64225 GO:0015977 GO:0043039 GO:0019643 GO:0006094
GO:0006099 GO:0006418 GO:0000166 GO:0008964 GO:0000049
GO:0004812 GO:0005524 GO:0031225 GO:0005737
Cluster-22178.64227 GO:0007165 GO:0005179 GO:0031704
Cluster-22178.64229 GO:0006355 GO:0006090 GO:0006633 GO:0003989
GO:0005524 GO:0005634 GO:0009317 GO:0005737
Cluster-22178.64231 GO:0043065 GO:0046983 GO:0005515 GO:0005740
GO:0016021
Cluster-22178.64232 GO:0006123 GO:0015992 GO:0004129 GO:0005743
GO:0005751 GO:0045277
Cluster-22178.64233 GO:0007009 GO:0043087 GO:0019087 GO:0005515
GO:0016021 GO:0005737
Cluster-22178.64236 GO:0055114 GO:0016972
Cluster-22178.64237 GO:0006397 GO:0046872
Cluster-22178.64238 GO:0032968 GO:0007259 GO:0007165 GO:0006367
GO:0016192 GO:0006886 GO:0051607 GO:0050778 GO:0003677

GO:0005515 GO:0005125 GO:0030117 GO:0005634
Cluster-22178.64239 GO:0016772
Cluster-22178.64240 GO:0006935 GO:0007165 GO:0006270 GO:0006955
GO:0008009 GO:0005576
Cluster-22178.64243 GO:0006470 GO:0006570 GO:0008138 GO:0004725

Cluster-22178.64245 GO:0009069 GO:0008608 GO:0007049 GO:0016310
GO:0048870 GO:0009405 GO:0006468 GO:0004674 GO:0005524
GO:0005198 GO:0005815 GO:0016020 GO:0019031 GO:0042729
GO:0031514 GO:0019028 GO:0072686
Cluster-22178.64246 GO:0003723 GO:0003676
Cluster-22178.64247 GO:0006810 GO:0000917 GO:0043093 GO:0006334
GO:0042262 GO:0003690 GO:0019012 GO:0005634 GO:0016021
GO:0005737
Cluster-22178.64248 GO:0000917 GO:0042262 GO:0016310 GO:0006810
GO:0006334 GO:0009069 GO:0043093 GO:0008053 GO:0051082
GO:0003924 GO:0003690 GO:0004674 GO:0005634 GO:0016021
GO:0005737 GO:0005741
Cluster-22178.64249 GO:0005515 GO:0008270
Cluster-22178.6425 GO:0006887 GO:0000145
Cluster-22178.64250 GO:0006614 GO:0006520 GO:0000921 GO:0006396
GO:0046856 GO:0006606 GO:0006355 GO:0008312 GO:0003723
GO:0045502 GO:0008134 GO:0042803 GO:0005515 GO:0043565
GO:0003700 GO:0034597 GO:0005667 GO:0005940 GO:0030286
GO:0048500 GO:0016021
Cluster-22178.64251 GO:0055085 GO:0030234 GO:0016021 GO:0016020
Cluster-22178.64252 GO:0006633 GO:0008152 GO:0042967 GO:0008610
GO:0005524 GO:0016747 GO:0004315 GO:0005835
Cluster-22178.64253 GO:0004252 GO:0016021 GO:0012511
Cluster-22178.64254 GO:0043039 GO:0005524 GO:0016876
Cluster-22178.64255 GO:0005096
Cluster-22178.64256 GO:0030488 GO:0006464 GO:0032259 GO:0008033
GO:0046500 GO:0006364 GO:0006479 GO:0009451 GO:0004719

GO:0008168 GO:0016429 GO:0003723 GO:0031515
Cluster-22178.64257 GO:0006260 GO:0006269 GO:0006351 GO:0008270
GO:0003896 GO:0003677 GO:0005657 GO:0005730
Cluster-22178.64258 GO:0009094 GO:0006103 GO:0009821 GO:0009074
GO:0006571 GO:0000162 GO:0006536 GO:0007165 GO:0004872
GO:0030170 GO:0004838 GO:0005515 GO:0016021
Cluster-22178.64259 GO:0007186 GO:0004930 GO:0016021
Cluster-22178.64262 GO:0007165 GO:0005179 GO:0005576
Cluster-22178.64264 GO:0007264 GO:0007165 GO:0007186 GO:0002949
GO:0003723 GO:0019001 GO:0031683 GO:0003924 GO:0005525
GO:0004871 GO:0003724
Cluster-22178.64265 GO:0006281 GO:0006308 GO:0005515 GO:0003677
GO:0004518 GO:0003723 GO:0008855 GO:0009318
Cluster-22178.64266 GO:0006281 GO:0006308 GO:0003677 GO:0004518
GO:0005515 GO:0008855 GO:0009318
Cluster-22178.64268 GO:0005515
Cluster-22178.64271 GO:0006914 GO:0007165 GO:0031423 GO:0005198
GO:0019028 GO:0019012
Cluster-22178.64273 GO:0007165 GO:0005102
Cluster-22178.64274 GO:0006629 GO:0051321 GO:0005515
Cluster-22178.64275 GO:0003723
Cluster-22178.64277 GO:0008053 GO:0006810 GO:0010508 GO:0007264
GO:0005525 GO:0003724 GO:0005524 GO:0016887 GO:0003924
GO:0003723 GO:0016021 GO:0005741
Cluster-22178.64278 GO:0006355 GO:0016567 GO:0009271 GO:0006144
GO:0055114 GO:0046034 GO:0006744 GO:0004017 GO:0005515
GO:0008270 GO:0046872 GO:0004842
Cluster-22178.64279 GO:0006351 GO:0006468 GO:0007618 GO:0006206
GO:0006144 GO:0003677 GO:0003899 GO:0016787 GO:0046872
GO:0004672 GO:0005524 GO:0016773 GO:0016020 GO:0005576
GO:0005730
Cluster-22178.64280 GO:0007165 GO:0016255 GO:0005515 GO:0016021
GO:0042765

Cluster-22178.64281 GO:0006366 GO:0006265 GO:0005524 GO:0003918 GO:0003677

Cluster-22178.64284 GO:0042254 GO:0006412 GO:0006355 GO:0003700 GO:0043565 GO:0008270 GO:0003735 GO:0005840 GO:0005667

Cluster-22178.64285 GO:0042998 GO:0019049 GO:0071253 GO:0005802

Cluster-22178.64286 GO:0006811 GO:0004190 GO:0051424 GO:0016021 GO:0016020

Cluster-22178.64287 GO:0042967 GO:0006886 GO:0006629 GO:0006505 GO:0016788 GO:0008374 GO:0016787

Cluster-22178.64291 GO:0006355 GO:0045132 GO:0006576 GO:0007264 GO:0015684 GO:0043565 GO:0003924 GO:0005525 GO:0003700 GO:0003676 GO:0016787 GO:0003677 GO:0005524 GO:0015093 GO:0016887 GO:0005634 GO:0000775 GO:0016021 GO:0005667

Cluster-22178.64293 GO:0007276 GO:0006744

Cluster-22178.64296 GO:0046500 GO:0030488 GO:0006464 GO:0032259 GO:0006355 GO:0006479 GO:0006412 GO:0009451 GO:0008033 GO:0008152 GO:0016491 GO:0016429 GO:0004719 GO:0008168 GO:0043565 GO:0003700 GO:0005667 GO:0031515 GO:0005634

Cluster-22178.64297 GO:0043066 GO:0015992 GO:0016567 GO:0043123 GO:0045766 GO:0015986 GO:0043130 GO:0015078 GO:0004842 GO:0003723 GO:0000276

Cluster-22178.64299 GO:0006364 GO:0032040 GO:0005732 GO:0005634 GO:0034457

Cluster-22178.64301 GO:0006508 GO:0006468 GO:0006144 GO:0017111 GO:0004252 GO:0004197 GO:0016773 GO:0004672 GO:0005524 GO:0003968 GO:0016020 GO:0031379

Cluster-22178.64304 GO:0006470 GO:0006570 GO:0004725 GO:0008138 GO:0003677 GO:0004527

Cluster-22178.64305 GO:0009116 GO:0006506 GO:0042393 GO:0016746 GO:0016021 GO:0005789 GO:0005634

Cluster-22178.64306 GO:0008152 GO:0016791

Cluster-22178.64307 GO:0006506 GO:0007165 GO:0051289 GO:0016740 GO:0005515 GO:0005509 GO:0008140 GO:0016012 GO:0005578

GO:0005667 GO:0005886 GO:0005789 GO:0016021
Cluster-22178.64308 GO:0007165 GO:0005509 GO:0005578
Cluster-22178.64311 GO:0030259 GO:0055114 GO:0005975 GO:0042773
GO:0016651 GO:0016758 GO:0005515
Cluster-22178.64313 GO:0006281 GO:0006144 GO:0006974 GO:0015074
GO:0005975 GO:0003677 GO:0030248 GO:0004553 GO:0003968
GO:0005524 GO:0005634 GO:0005576 GO:0070876 GO:0031379
Cluster-22178.64314 GO:0006468 GO:0005515 GO:0005524 GO:0004672

Cluster-22178.64318 GO:0006817 GO:0006468 GO:0005524 GO:0004672

Cluster-22178.6432 GO:0006355 GO:0006144 GO:0006807 GO:0000256
GO:0006948 GO:0004037
Cluster-22178.64321 GO:0006468 GO:0005515 GO:0005524 GO:0004672

Cluster-22178.64323 GO:0006457 GO:0016192 GO:0007165 GO:0030521
GO:0006355 GO:0006886 GO:0009372 GO:0009405 GO:0005509
GO:0003677 GO:0004882 GO:0008270 GO:0008233 GO:0005496
GO:0051082 GO:0005515 GO:0005783 GO:0005634 GO:0030117
GO:0016021
Cluster-22178.64325 GO:0005515
Cluster-22178.64326 GO:0006468 GO:0005524 GO:0004672 GO:0016773
GO:0016020
Cluster-22178.64327 GO:0005515
Cluster-22178.64328 GO:0006886 GO:0006888 GO:0008270 GO:0030127
Cluster-22178.64329 GO:0006510 GO:0006508 GO:0006355 GO:0004176
GO:0003677 GO:0003682 GO:0000785 GO:0000775 GO:0005634
Cluster-22178.64332 GO:0015992 GO:0006123 GO:0004129 GO:0045277
Cluster-22178.64334 GO:0005515 GO:0016020
Cluster-22178.64335 GO:0016021
Cluster-22178.64336 GO:0006508 GO:0006355 GO:0006144 GO:0005198
GO:0004252 GO:0017111 GO:0004197 GO:0003968 GO:0003700
GO:0005667 GO:0019028 GO:0019031 GO:0031379

Cluster-22178.64337 GO:0006355 GO:0006260 GO:0003677 GO:0005664

Cluster-22178.64341 GO:0006260 GO:0005634

Cluster-22178.64342 GO:0043093 GO:0048870 GO:0000917 GO:0005737 GO:0031514

Cluster-22178.64343 GO:0046983

Cluster-22178.64345 GO:0055114 GO:0005515

Cluster-22178.64347 GO:0042967 GO:0016747 GO:0008080

Cluster-22178.64351 GO:0051028 GO:0042254 GO:0005515 GO:0005634

Cluster-22178.64352 GO:0006570 GO:0018101 GO:0006807 GO:0006470 GO:0005509 GO:0008138 GO:0004668 GO:0004725 GO:0005737

Cluster-22178.64354 GO:0005515

Cluster-22178.64355 GO:0016021

Cluster-22178.64358 GO:0006260 GO:0048870 GO:0042254 GO:0006412 GO:0006355 GO:0003700 GO:0043565 GO:0005515 GO:0003735 GO:0003723 GO:0005840 GO:0005622 GO:0031514 GO:0005667

Cluster-22178.64359 GO:0006118 GO:0055114 GO:0005515 GO:0003677 GO:0016655

Cluster-22178.64361 GO:0006355 GO:0005515 GO:0005634 GO:0016021

Cluster-22178.64368 GO:0008201 GO:0046914

Cluster-22178.64370 GO:0006935 GO:0000160 GO:0016310 GO:0004673 GO:0000155 GO:0005737 GO:0009365

Cluster-22178.64371 GO:0016567 GO:0046872 GO:0004842

Cluster-22178.64372 GO:0009228 GO:0046497 GO:0006206 GO:0005975 GO:0009435 GO:0006207 GO:0004789 GO:0004514 GO:0016857 GO:0004590

Cluster-22178.64376 GO:0007010 GO:0003779

Cluster-22178.64380 GO:0042254 GO:0006412 GO:0003676 GO:0003723 GO:0003735 GO:0005622 GO:0005840 GO:0019013

Cluster-22178.64382 GO:0006886 GO:0016192 GO:0008608 GO:0005515 GO:0030117 GO:0005634 GO:0072686 GO:0042729

Cluster-22178.64385 GO:0008092 GO:0019898 GO:0005737

Cluster-22178.64388 GO:0005975 GO:0019058 GO:0006886 GO:0008536 GO:0005515 GO:0004553 GO:0042025

Cluster-22178.64390 GO:0006744 GO:0019836 GO:0005515 GO:0005576
Cluster-22178.64391 GO:0006099 GO:0016829
Cluster-22178.64392 GO:0042254 GO:0006412 GO:0003723 GO:0003735
GO:0003676 GO:0005622 GO:0005840
Cluster-22178.64394 GO:0016021
Cluster-22178.64395 GO:0006480 GO:0008152 GO:0055114 GO:0015979
GO:0006118 GO:0017004 GO:0006468 GO:0045158 GO:0047134
GO:0008168 GO:0004672 GO:0005524 GO:0009512 GO:0016021
GO:0009523
Cluster-22178.64397 GO:0007015 GO:0003785
Cluster-22178.64398 GO:0005507
Cluster-22178.64402 GO:0007165 GO:0005515
Cluster-22178.64403 GO:0006355 GO:0007165 GO:0016787 GO:0005515
GO:0005524 GO:0008270 GO:0043565 GO:0003700 GO:0005667
GO:0005737
Cluster-22178.64404 GO:0006355 GO:0003700 GO:0043565 GO:0005667
Cluster-22178.64405 GO:0016559 GO:0005779
Cluster-22178.64406 GO:0045227 GO:0007165 GO:0006887 GO:0005179
GO:0004252 GO:0005576 GO:0000145 GO:0016020 GO:0016021
Cluster-22178.64407 GO:0006629 GO:0006355 GO:0008270 GO:0005634
Cluster-22178.64409 GO:0046872 GO:0008270
Cluster-22178.64410 GO:0090307 GO:0005515
Cluster-22178.64411 GO:0006397 GO:0006366 GO:0006914 GO:0005515
GO:0000445
Cluster-22178.64412 GO:0008152 GO:0016817 GO:0003824
Cluster-22178.64413 GO:0007165 GO:0006955 GO:0006886 GO:0006355
GO:0019882 GO:0030521 GO:0003677 GO:0042289 GO:0005496
GO:0004882 GO:0016020 GO:0005634 GO:0042613
Cluster-22178.64415 GO:0007018 GO:0007017 GO:0008017 GO:0003777
GO:0005524 GO:0045298 GO:0005874 GO:0005576
Cluster-22178.64416 GO:0007017 GO:0007018 GO:0005524 GO:0003777
GO:0008017 GO:0005576 GO:0005874 GO:0045298
Cluster-22178.64417 GO:0043565 GO:0005515

Cluster-22178.64418 GO:0009405 GO:0030908 GO:0005615
Cluster-22178.64420 GO:0006468 GO:0005524 GO:0004672
Cluster-22178.64422 GO:0006260 GO:0006270 GO:0006355 GO:0003678
GO:0005524 GO:0016887 GO:0003677 GO:0008134 GO:0005657
GO:0042555 GO:0005634 GO:0005667
Cluster-22178.64423 GO:0006412 GO:0042254 GO:0003735 GO:0005622
GO:0005840
Cluster-22178.64424 GO:0006338 GO:0000228
Cluster-22178.64425 GO:0003676 GO:0003677 GO:0016787 GO:0005524

Cluster-22178.64427 GO:0016567 GO:0004842 GO:0005515 GO:0008270
GO:0046872 GO:0005680
Cluster-22178.64428 GO:0003676 GO:0003723
Cluster-22178.64431 GO:0009214 GO:0006605 GO:0006886 GO:0006013
GO:0006000 GO:0003873 GO:0005515 GO:0042802 GO:0008641
GO:0005524 GO:0004113 GO:0016887 GO:0005742 GO:0016020
Cluster-22178.64432 GO:0005524
Cluster-22178.64433 GO:0003723
Cluster-22178.64434 GO:0007155 GO:0055114 GO:0016491 GO:0016616

Cluster-22178.64436 GO:0015031 GO:0030176
Cluster-22178.64437 GO:0046872
Cluster-22178.64439 GO:0009405 GO:0005515
Cluster-22178.64440 GO:0035556 GO:0006281 GO:0006464 GO:0003677
GO:0016151 GO:0008270 GO:0046872
Cluster-22178.64441 GO:0015931 GO:0033227 GO:0051033 GO:0016021
Cluster-22178.64443 GO:0006303 GO:0005515
Cluster-22178.64445 GO:0006355 GO:0007165 GO:0005179 GO:0003677
GO:0016818 GO:0004252 GO:0005524 GO:0008270 GO:0005576
GO:0005634 GO:0005886
Cluster-22178.64446 GO:0005634
Cluster-22178.64447 GO:0005198 GO:0005515 GO:0019028
Cluster-22178.64448 GO:0019058 GO:0005515

Cluster-22178.64449 GO:0006629 GO:0046339 GO:0009395 GO:0007165 GO:0035556 GO:0004435 GO:0005515
Cluster-22178.64450 GO:0006270 GO:0000746 GO:0006816 GO:0006874 GO:0006383 GO:0055085 GO:0006144 GO:0006206 GO:0006351 GO:1901031 GO:0030001 GO:0006352 GO:0003677 GO:0046873 GO:0003899 GO:0005515 GO:0004190 GO:0005219 GO:0046872 GO:0016020 GO:0005666 GO:0005730 GO:0005622 GO:0005634 GO:0019867 GO:0005669 GO:0016021 GO:0019031
Cluster-22178.64451 GO:0015684 GO:0006446 GO:0007264 GO:0006413 GO:0006576 GO:0005525 GO:0015093 GO:0005524 GO:0003924 GO:0046983 GO:0005515 GO:0003743 GO:0005840 GO:0016021 GO:0005576
Cluster-22178.64453 GO:0016567 GO:0046872 GO:0008270 GO:0004842 GO:0061630 GO:0005515 GO:0005622 GO:0005680
Cluster-22178.64455 GO:0006570 GO:0006470 GO:0005515 GO:0008138 GO:0004725
Cluster-22178.64457 GO:0060478 GO:0007342 GO:0023052 GO:0006897 GO:0003950 GO:0005634 GO:0005886
Cluster-22178.64458 GO:0050992 GO:0019288 GO:0046872 GO:0005515 GO:0051745 GO:0005634
Cluster-22178.64460 GO:0019079 GO:0015994 GO:0006783 GO:0033014 GO:0004386 GO:0004852 GO:0008270
Cluster-22178.64462 GO:0007010 GO:0003779
Cluster-22178.64463 GO:0019370 GO:0055114 GO:0008237 GO:0008270 GO:0016491
Cluster-22178.64465 GO:0006118 GO:0015035
Cluster-22178.64467 GO:0008242
Cluster-22178.64468 GO:0007165 GO:0004190 GO:0005515 GO:0004872 GO:0016021 GO:0016020
Cluster-22178.64469 GO:0006368 GO:0016570 GO:0016593
Cluster-22178.64472 GO:0006468 GO:0055085 GO:0015689 GO:0015098 GO:0022857 GO:0016773 GO:0004672 GO:0005524 GO:0016020 GO:0016021

Cluster-22178.64473 GO:0003677 GO:0046872 GO:0046983
Cluster-22178.64474 GO:0006811 GO:0007186 GO:0006813 GO:0004930
GO:0005249 GO:0016021 GO:0008076 GO:0016020
Cluster-22178.64477 GO:0015992 GO:0015986 GO:0015078 GO:0005543

Cluster-22178.64480 GO:0009395 GO:0008654 GO:0042157 GO:0006869
GO:0016567 GO:0046486 GO:0005515 GO:0005198 GO:0046872
GO:0008289 GO:0061630 GO:0008270 GO:0046983 GO:0004143
GO:0004842 GO:0005576 GO:0019028 GO:0019031 GO:0016020
GO:0005622
Cluster-22178.64482 GO:0016567 GO:0006355 GO:0004842 GO:0046872
GO:0008270 GO:0003677 GO:0005515 GO:0003682 GO:0005680
GO:0000775 GO:0000785 GO:0005634
Cluster-22178.64483 GO:0003676
Cluster-22178.64485 GO:0006886 GO:0016192 GO:0005515
Cluster-22178.64486 GO:0006357 GO:0001104 GO:0008270 GO:0016592
Cluster-22178.64487 GO:0015986 GO:0009405 GO:0016226 GO:0006357
GO:0015992 GO:0001104 GO:0051536 GO:0015078 GO:0016020
GO:0016592 GO:0005737 GO:0000276
Cluster-22178.64488 GO:0042254 GO:0006412 GO:0003735 GO:0005840
GO:0005622
Cluster-22178.64489 GO:0046872 GO:0045202
Cluster-22178.6449 GO:0055085 GO:0016021 GO:0019031
Cluster-22178.64491 GO:0043093 GO:0010508 GO:0006364 GO:0000917
GO:0048870 GO:0070206 GO:0031514 GO:0005737
Cluster-22178.64493 GO:0003676 GO:0016021
Cluster-22178.64495 GO:0007186 GO:0004930 GO:0016021
Cluster-22178.64496 GO:0006355 GO:0007186 GO:0005524 GO:0008134
GO:0004930 GO:0005667 GO:0016021
Cluster-22178.64497 GO:0022904 GO:0008270 GO:0016020
Cluster-22178.64502 GO:0006096 GO:0006098 GO:0006020 GO:0006094
GO:0006013 GO:0015976 GO:0006000 GO:0004332
Cluster-22178.64503 GO:0008270

Cluster-22178.64506 GO:0006531 GO:0016567 GO:0015992 GO:0006144
GO:0006188 GO:0009152 GO:0006522 GO:0006123 GO:0004018
GO:0004842 GO:0004129 GO:0005740 GO:0045277
Cluster-22178.64510 GO:0006816 GO:0006874 GO:0005219 GO:0005622
GO:0016021
Cluster-22178.64511 GO:0043248
Cluster-22178.64513 GO:0000245 GO:0005634
Cluster-22178.64516 GO:0006364 GO:0046500 GO:0030488 GO:0015031
GO:0006464 GO:0009451 GO:0007010 GO:0006479 GO:0006468
GO:0055114 GO:0008033 GO:0006725 GO:0016772 GO:0016429
GO:0016491 GO:0010277 GO:0005524 GO:0016301 GO:0008198
GO:0003723 GO:0003779 GO:0008168 GO:0004719 GO:0004672
GO:0031515
Cluster-22178.64517 GO:0015684 GO:0015093 GO:0003924 GO:0005525
GO:0016021
Cluster-22178.64518 GO:0015684 GO:0015093 GO:0003924 GO:0005525
GO:0016021
Cluster-22178.64521 GO:0006355 GO:0005975 GO:0003677 GO:0016773
GO:0005524
Cluster-22178.64522 GO:0006810 GO:0006281 GO:0000723 GO:0004386
GO:0003678 GO:0003676 GO:0005525 GO:0016887 GO:0003924
GO:0005524 GO:0005657
Cluster-22178.64524 GO:0007264 GO:0005524 GO:0005525
Cluster-22178.64527 GO:0006355 GO:0032968 GO:0006367 GO:0003682
GO:0003677 GO:0003700 GO:0005667 GO:0005634 GO:0000775
GO:0000785
Cluster-22178.64528 GO:0006351 GO:0006260 GO:0003887 GO:0003677
GO:0042575 GO:0016021 GO:0005634
Cluster-22178.64529 GO:0007165 GO:0040007 GO:0008033 GO:0008283
GO:0008083
Cluster-22178.64530 GO:0007264 GO:0006576 GO:0007165 GO:0007186
GO:0031683 GO:0005524 GO:0019001 GO:0016887 GO:0003924
GO:0005525 GO:0004871 GO:0098519

Cluster-22178.64531 GO:0006468 GO:0009405 GO:0006605 GO:0006886
GO:0005524 GO:0004672 GO:0016773 GO:0005742 GO:0016020
Cluster-22178.64532 GO:0006355 GO:0019058 GO:0043565 GO:0003700
GO:0005634 GO:0005667 GO:0019031
Cluster-22178.64533 GO:0006355 GO:0006265 GO:0006464 GO:0003677
GO:0008270 GO:0003916 GO:0016151 GO:0005694
Cluster-22178.64534 GO:0055114 GO:0009690 GO:0050660 GO:0019139

Cluster-22178.64537 GO:0007059 GO:0051258 GO:0051301 GO:0007018
GO:0007017 GO:0030168 GO:0031110 GO:0007165 GO:0007067
GO:0000090 GO:0003777 GO:0008017 GO:0005102 GO:0005524
GO:0030674 GO:0005874 GO:0045298 GO:0005577 GO:0005876
GO:0000940
Cluster-22178.64538 GO:0006298 GO:0006810 GO:0019083 GO:0055085
GO:0007021 GO:0051082 GO:0030983 GO:0005524 GO:0016887
GO:0015631 GO:0042626 GO:0045298 GO:0016021
Cluster-22178.64540 GO:0006771 GO:0019497 GO:0046872 GO:0005515
GO:0003993
Cluster-22178.64542 GO:0006813 GO:0005242 GO:0008076 GO:0016021
Cluster-22178.64543 GO:0055114 GO:0016491 GO:0051920 GO:0016209

Cluster-22178.64545 GO:0016020
Cluster-22178.64546 GO:0046872 GO:0005515 GO:0008376 GO:0032580
Cluster-22178.64548 GO:0043087 GO:0008283 GO:0040007 GO:0007165
GO:0005085 GO:0030674 GO:0051015 GO:0005515 GO:0008083
Cluster-22178.64549 GO:0016117 GO:0000413 GO:0008033 GO:0055114
GO:0006457 GO:0050660 GO:0016817 GO:0016491 GO:0004174
GO:0016705 GO:0003755 GO:0005515 GO:0071949
Cluster-22178.64550 GO:0007186 GO:0004930 GO:0016020 GO:0016021
Cluster-22178.64552 GO:0006810 GO:0016021
Cluster-22178.64554 GO:0008152 GO:0007165 GO:0007275 GO:0008270
GO:0047355 GO:0016624 GO:0005179 GO:0005576 GO:0016020
Cluster-22178.64558 GO:0046872 GO:0005515 GO:0003677

Cluster-22178.64561 GO:0009396 GO:0055114 GO:0046487 GO:0004488
GO:0003824
Cluster-22178.64563 GO:0016226 GO:0008199
Cluster-22178.64564 GO:0015940 GO:0019482 GO:0004592
Cluster-22178.64566 GO:0006418 GO:0043039 GO:0006425 GO:0005524
GO:0016876 GO:0000166 GO:0004819 GO:0004812 GO:0005737
Cluster-22178.64567 GO:0019722 GO:0006402 GO:0006817 GO:0044341
GO:0051252 GO:0008270 GO:0015321 GO:0046872 GO:0004535
GO:0003676 GO:0003723 GO:0005737 GO:0005634 GO:0016020
Cluster-22178.64568 GO:0008152 GO:0016787 GO:0005198 GO:0019028
GO:0016021
Cluster-22178.64569 GO:0006486 GO:0016757 GO:0008378 GO:0016020
Cluster-22178.64570 GO:2001256 GO:0042710 GO:0008270 GO:0030176
Cluster-22178.64571 GO:0008270 GO:0003677
Cluster-22178.64572 GO:0005515
Cluster-22178.64575 GO:0015979 GO:0005515 GO:0008270 GO:0016020
GO:0009539 GO:0009523
Cluster-22178.64576 GO:0030682 GO:0015979 GO:0005515 GO:0008270
GO:0016020 GO:0009539 GO:0009523 GO:0019031
Cluster-22178.64578 GO:0006508 GO:0006950 GO:0004197 GO:0004222
GO:0003700 GO:0008270 GO:0005667 GO:0031012
Cluster-22178.64579 GO:0000012 GO:0006468 GO:0006281 GO:0009069
GO:0006397 GO:0016310 GO:0003723 GO:0003684 GO:0005524
GO:0004674 GO:0005634 GO:0005737
Cluster-22178.64580 GO:0051716 GO:0006284 GO:0003906 GO:0003676
GO:0016799 GO:0046658
Cluster-22178.64581 GO:0006468 GO:0005524 GO:0004672
Cluster-22178.64583 GO:0006556 GO:0006555 GO:0006144 GO:0006206
GO:0006414 GO:0006448 GO:0006351 GO:1901031 GO:0055114
GO:0051920 GO:0004478 GO:0003746 GO:0003899 GO:0005515
GO:0003676 GO:0005730 GO:0005634 GO:0005840
Cluster-22178.64585 GO:0043395 GO:0005515 GO:0005578 GO:0016020
Cluster-22178.64586 GO:0006464 GO:0015031 GO:0005515

Cluster-22178.64592 GO:0006338 GO:0007049 GO:0005524 GO:0016818 GO:0005515 GO:0003676 GO:0005634
Cluster-22178.64593 GO:0006412 GO:0042254 GO:0003735 GO:0005622 GO:0005840
Cluster-22178.64594 GO:0006355 GO:0006807 GO:0009252 GO:0004356 GO:0003700 GO:0043565 GO:0005634 GO:0005667 GO:0055036
Cluster-22178.64595 GO:0042967 GO:0018193 GO:0008080 GO:0016747

Cluster-22178.64597 GO:0007018 GO:0007017 GO:0003677 GO:0003777 GO:0008017 GO:0005524 GO:0005874 GO:0045298
Cluster-22178.64598 GO:0007018 GO:0007017 GO:0003777 GO:0008017 GO:0005524 GO:0003677 GO:0005874 GO:0045298
Cluster-22178.64599 GO:0006099 GO:0006108 GO:0015976 GO:0055114 GO:0006090 GO:0051287 GO:0004471 GO:0005524 GO:0005856
Cluster-22178.6460 GO:0019054 GO:0004857
Cluster-22178.64601 GO:0006468 GO:0016773 GO:0004672 GO:0005524 GO:0016020
Cluster-22178.64602 GO:0040007 GO:0008283 GO:0007165 GO:0008083

Cluster-22178.64604 GO:0015689 GO:0009405 GO:0055085 GO:0015098 GO:0022857 GO:0016021
Cluster-22178.64606 GO:0046740 GO:0000398 GO:0030246 GO:0005634 GO:0016021
Cluster-22178.64609 GO:0016758
Cluster-22178.64610 GO:0000724 GO:0017004 GO:0016197 GO:0015886 GO:0030119 GO:0005783 GO:0016021
Cluster-22178.64611 GO:0006531 GO:0006144 GO:0006164 GO:0006522 GO:0004019 GO:0005525
Cluster-22178.64614 GO:0006807 GO:0051252 GO:0009245 GO:0008759 GO:0004521 GO:0003723
Cluster-22178.64619 GO:0016042 GO:0045454 GO:0006468 GO:0007586 GO:0004672 GO:0005524 GO:0008047 GO:0005576 GO:0019028
Cluster-22178.6462 GO:0016020

Cluster-22178.64620 GO:0009058 GO:0016740 GO:0030170 GO:0019012
Cluster-22178.64626 GO:0006446 GO:0006413 GO:0045881 GO:0005515
GO:0003743 GO:0032183 GO:0070140 GO:0003723 GO:0005840
GO:0005737
Cluster-22178.64627 GO:0006888 GO:0003676 GO:0019900 GO:0005801
GO:0005622
Cluster-22178.64628 GO:0055114 GO:0006118 GO:0009496 GO:0051537
GO:0009512 GO:0042651
Cluster-22178.64629 GO:0046486 GO:0045454 GO:0016042 GO:0015992
GO:0006118 GO:0006355 GO:0006629 GO:0015991 GO:0006508
GO:0015994 GO:0006811 GO:0015996 GO:0003677 GO:0016787
GO:0003847 GO:0047746 GO:0008236 GO:0015035 GO:0009055
GO:0015078 GO:0046872 GO:0008247 GO:0016020 GO:0005886
GO:0033179 GO:0019031
Cluster-22178.64631 GO:0016772 GO:0008134 GO:0005509 GO:0008270
GO:0005667 GO:0005634
Cluster-22178.64634 GO:0030334 GO:0051607 GO:0030168 GO:0007165
GO:0030155 GO:0007155 GO:0045995 GO:0051258 GO:0006355
GO:0005102 GO:0030674 GO:0003700 GO:0008134 GO:0042803
GO:0045502 GO:0005577 GO:0030286 GO:0001772 GO:0005667
GO:0016021 GO:0097197
Cluster-22178.64644 GO:0006614 GO:0006520 GO:0000921 GO:0046856
GO:0006606 GO:0006396 GO:0006355 GO:0008312 GO:0003723
GO:0045502 GO:0042803 GO:0008134 GO:0005515 GO:0043565
GO:0003700 GO:0034597 GO:0005667 GO:0030286 GO:0005940
GO:0048500 GO:0016021
Cluster-22178.64645 GO:0006355 GO:0016055 GO:0006886 GO:0000975
GO:0008536 GO:0003700 GO:0005667
Cluster-22178.64647 GO:0006886 GO:0006355 GO:0016055 GO:0000975
GO:0008536 GO:0003700 GO:0005667
Cluster-22178.64648 GO:0007165 GO:0006355 GO:0005515 GO:0004871
GO:0003700 GO:0005667 GO:0071203
Cluster-22178.64649 GO:0008063 GO:0005515 GO:0019901 GO:0008270

GO:0005634
Cluster-22178.64651 GO:0006807 GO:0006633 GO:0042967 GO:0006725
GO:0055114 GO:0008033 GO:0006508 GO:0050660 GO:0016491
GO:0018580 GO:0016853 GO:0017150 GO:0016627 GO:0004315
GO:0004252 GO:0005835 GO:0005737
Cluster-22178.64654 GO:0042254 GO:0006412 GO:0003735 GO:0005622
GO:0005840
Cluster-22178.64655 GO:0042254 GO:0006412 GO:0003735 GO:0005622
GO:0005840
Cluster-22178.64658 GO:0016773
Cluster-22178.64659 GO:0006566 GO:0006563 GO:0006546 GO:0006520
GO:0055114 GO:0006544 GO:0006352 GO:0003677 GO:0004375
GO:0016829
Cluster-22178.64660 GO:0005509 GO:0003676 GO:0005515
Cluster-22178.64663 GO:0005515
Cluster-22178.64664 GO:0006886 GO:0006888 GO:0055114 GO:0016706
GO:0003677 GO:0008168 GO:0046872 GO:0008270 GO:0030127
Cluster-22178.64665 GO:0051252 GO:0006402 GO:0003723 GO:0004535
GO:0046872 GO:0003676 GO:0005634 GO:0005737
Cluster-22178.64666 GO:0008152 GO:0016624
Cluster-22178.64668 GO:0055114 GO:0015994 GO:0006470 GO:0006355
GO:0000087 GO:0006570 GO:0006260 GO:0006788 GO:0004725
GO:0004392 GO:0008134 GO:0005524 GO:0016887 GO:0003677
GO:0005622 GO:0005667
Cluster-22178.64669 GO:0007155 GO:0006275 GO:0007049
Cluster-22178.64670 GO:0006260
Cluster-22178.64671 GO:0000746 GO:0006118 GO:0003677 GO:0005515
GO:0016491 GO:0009055 GO:0005634 GO:0016020
Cluster-22178.64672 GO:0042254 GO:0015986 GO:0035556 GO:0015992
GO:0006119 GO:0006412 GO:0006355 GO:0003677 GO:0046961
GO:0003735 GO:0008270 GO:0046933 GO:0045259 GO:0045261
GO:0005622 GO:0005840
Cluster-22178.64673 GO:0006614 GO:0006810 GO:0019079 GO:0006355

GO:0008026 GO:0003677 GO:0016787 GO:0005525 GO:0004386
GO:0003676 GO:0005524
Cluster-22178.64675 GO:0006486 GO:0055114 GO:0006725 GO:0016757

Cluster-22178.64676 GO:0006355 GO:0009405 GO:0043565 GO:0003700
GO:0005515 GO:0005198 GO:0005509 GO:0005667
Cluster-22178.6468 GO:0006355 GO:0003690 GO:0005739
Cluster-22178.64680 GO:0015992 GO:0055085 GO:0006123 GO:0006811
GO:0007165 GO:0007268 GO:0004129 GO:0004970 GO:0005216
GO:0005515 GO:0045277 GO:0016020
Cluster-22178.64681 GO:0035556 GO:0016567 GO:0008270 GO:0046872
GO:0004842 GO:0005515 GO:0005680
Cluster-22178.64682 GO:0006879 GO:0055114 GO:0008199 GO:0046872
GO:0016491
Cluster-22178.64685 GO:0005524 GO:0003676 GO:0000228
Cluster-22178.64686 GO:0007165 GO:0006355 GO:0051262 GO:0006357
GO:0005515 GO:0001104 GO:0003723 GO:0004871 GO:0003700
GO:0045182 GO:0016592 GO:0005667 GO:0005634 GO:0019028
Cluster-22178.64687 GO:0008652 GO:0000398 GO:0009086 GO:0035556
GO:0003871 GO:0005515 GO:0008270 GO:0005681
Cluster-22178.64688 GO:0035556 GO:0005515
Cluster-22178.6469 GO:0006144 GO:0006508 GO:0070008 GO:0003968
GO:0016817 GO:0004197 GO:0004252 GO:0031379
Cluster-22178.64690 GO:0042254 GO:0006412 GO:0003735 GO:0005840
Cluster-22178.64691 GO:0006096 GO:0006094 GO:0000162 GO:0006571
GO:0009094 GO:0000287 GO:0004634 GO:0000015
Cluster-22178.64692 GO:0005515
Cluster-22178.64693 GO:0000012 GO:0006281 GO:0003684 GO:0005634
Cluster-22178.64694 GO:0042254 GO:0006412 GO:0005515 GO:0003735
GO:0019843 GO:0005840 GO:0005622
Cluster-22178.64695 GO:0006352 GO:0006355 GO:0016987 GO:0003677
GO:0003700 GO:0005667
Cluster-22178.64698 GO:0006118 GO:0051536 GO:0009055

Cluster-22178.6470 GO:0006144 GO:0006508 GO:0016817 GO:0070008
GO:0003968 GO:0004252 GO:0004197 GO:0031379
Cluster-22178.64700 GO:0005525 GO:0003723
Cluster-22178.64702 GO:0030246
Cluster-22178.64703 GO:0016567 GO:0005524 GO:0004842
Cluster-22178.64706 GO:0006886 GO:0015991 GO:0016192 GO:0006606
GO:0000226 GO:0040001 GO:0015031 GO:0043248 GO:0000281
GO:0005515 GO:0016820 GO:0008565 GO:0030117 GO:0005819
GO:0005634 GO:0005874 GO:0005737 GO:0000221
Cluster-22178.64708 GO:0007165 GO:0005515 GO:0005509 GO:0005578
GO:0019028
Cluster-22178.64709 GO:0016020
Cluster-22178.6471 GO:0006144 GO:0006508 GO:0016817 GO:0070008
GO:0003968 GO:0004197 GO:0004252 GO:0031379
Cluster-22178.64711 GO:0015991 GO:0006886 GO:0048280 GO:0005515
GO:0016820 GO:0004869 GO:0005737 GO:0000139 GO:0000221
Cluster-22178.64714 GO:0006118 GO:0007186 GO:0055114 GO:0015992
GO:0006123 GO:0009060 GO:0005506 GO:0020037 GO:0004129
GO:0009055 GO:0004930 GO:0016021 GO:0045277
Cluster-22178.64715 GO:0042274 GO:0003677 GO:0030686
Cluster-22178.64716 GO:0003677
Cluster-22178.64717 GO:0008289
Cluster-22178.64718 GO:0043248 GO:0016310 GO:0009069 GO:0006886
GO:0016192 GO:0004674 GO:0005515 GO:0030117
Cluster-22178.64720 GO:0005509 GO:0005544
Cluster-22178.64724 GO:0006886 GO:0016192 GO:0005515 GO:0005198
GO:0030117
Cluster-22178.64726 GO:0006418 GO:0004812 GO:0005524 GO:0000166

Cluster-22178.64728 GO:0006396
Cluster-22178.64729 GO:0005515
Cluster-22178.64730 GO:0015979 GO:0042254 GO:0006412 GO:0003677
GO:0003735 GO:0005515 GO:0009539 GO:0005622 GO:0009523

GO:0005840 GO:0016020
Cluster-22178.64734 GO:0006813 GO:0006811 GO:0005249 GO:0016020
GO:0008076
Cluster-22178.64735 GO:0007268 GO:0003723 GO:0033130 GO:0005515
GO:0043495 GO:0019867
Cluster-22178.64736 GO:0006355 GO:0042254 GO:0006412 GO:0003735
GO:0003677 GO:0003700 GO:0005667 GO:0005622 GO:0005840
Cluster-22178.64737 GO:0048870 GO:0006914 GO:0034508 GO:0007093
GO:0000776 GO:0019028 GO:0031514
Cluster-22178.64739 GO:0006457 GO:0000413 GO:0003755 GO:0005516

Cluster-22178.64740 GO:0006308 GO:0006412 GO:0007018 GO:0007017
GO:0006355 GO:0006812 GO:0042254 GO:0007165 GO:0003700
GO:0003777 GO:0043565 GO:0005515 GO:0035257 GO:0008017
GO:0008855 GO:0016887 GO:0005524 GO:0003735 GO:0003713
GO:0016021 GO:0045298 GO:0005874 GO:0005840 GO:0009318
GO:0005634 GO:0005622 GO:0005667
Cluster-22178.64741 GO:0006353 GO:0007275 GO:0006355 GO:0007219
GO:0008608 GO:0030154 GO:0003677 GO:0003723 GO:0042729
GO:0072686 GO:0016021
Cluster-22178.64742 GO:0006631 GO:0055114 GO:0050151 GO:0016491
GO:0071949
Cluster-22178.64743 GO:0015684 GO:0007264 GO:0005525 GO:0003924
GO:0015093 GO:0016021
Cluster-22178.64746 GO:0055114 GO:0033014 GO:0008883 GO:0050661

Cluster-22178.64749 GO:0006508 GO:0004177 GO:0005622
Cluster-22178.64750 GO:0035556 GO:0009890 GO:0000226 GO:0042710
GO:0090063 GO:0005524 GO:0003774 GO:0005515 GO:0005815
GO:0000922 GO:0016459
Cluster-22178.64751 GO:0006481 GO:0006629 GO:0007165 GO:0006479
GO:0035556 GO:0004671 GO:0016627 GO:0016020 GO:0005737
GO:0016021

Cluster-22178.64752 GO:0006357 GO:0008270 GO:0001104 GO:0003676
GO:0016592
Cluster-22178.64753 GO:0006807 GO:0016811
Cluster-22178.64755 GO:0008152 GO:0003824 GO:0003677
Cluster-22178.64756 GO:0007264 GO:0006281 GO:0015684 GO:0055114
GO:0005515 GO:0015093 GO:0008270 GO:0003924 GO:0016491
GO:0005525 GO:0016021
Cluster-22178.64757 GO:0043065 GO:0046983 GO:0003677 GO:0005740
GO:0016021
Cluster-22178.64758 GO:0005975 GO:0042742 GO:0007094 GO:0016853

Cluster-22178.64759 GO:0031124 GO:0000398 GO:0006406 GO:0006369
GO:0000166 GO:0005515 GO:0003723 GO:0003714 GO:0003676
GO:0005667 GO:0016605 GO:0005681 GO:0016607
Cluster-22178.64760 GO:0046872
Cluster-22178.64761 GO:0031047
Cluster-22178.64763 GO:0019217 GO:0016567 GO:0046872 GO:0008270
GO:0000062 GO:0004842 GO:0005515 GO:0005680
Cluster-22178.64764 GO:0006260 GO:0000921 GO:0006457 GO:0048870
GO:0000917 GO:0044780 GO:0006355 GO:0015991 GO:0006606
GO:0048193 GO:0015992 GO:0006914 GO:0043093 GO:0007155
GO:0007094 GO:0010508 GO:0017048 GO:0003700 GO:0008134
GO:0045502 GO:0005524 GO:0008270 GO:0003677 GO:0016818
GO:0015078 GO:0043565 GO:0042803 GO:0005198 GO:0051082
GO:0033180 GO:0030286 GO:0005940 GO:0005882 GO:0016021
GO:0031514 GO:0019028 GO:0016272 GO:0016020 GO:0005667
GO:0019867 GO:0005737 GO:0005634
Cluster-22178.64765 GO:0006281 GO:0006308 GO:0006260 GO:0017108
GO:0005634 GO:0033557
Cluster-22178.64766 GO:0006206 GO:0006144 GO:0006351 GO:0006355
GO:0003700 GO:0046983 GO:0003677 GO:0003899 GO:0005730
GO:0005667
Cluster-22178.64767 GO:0006206 GO:0006144 GO:0006351 GO:0046983

GO:0003899 GO:0005730
Cluster-22178.64769 GO:0006355 GO:0003700 GO:0005667
Cluster-22178.64771 GO:0035023 GO:0043087 GO:0005089 GO:0005515
GO:0005509
Cluster-22178.64772 GO:0006355 GO:0005515 GO:0005634
Cluster-22178.64773 GO:0043401 GO:0006355 GO:0003700 GO:0043565
GO:0008270 GO:0005667 GO:0005634
Cluster-22178.64774 GO:0046872
Cluster-22178.64775 GO:0005975 GO:0055114 GO:0042254 GO:0006412
GO:0043631 GO:0004553 GO:0004652 GO:0016706 GO:0003677
GO:0003735 GO:0005515 GO:0005840 GO:0005622
Cluster-22178.64777 GO:0016567 GO:0004842 GO:0005515 GO:0046872
GO:0008270 GO:0005680
Cluster-22178.64778 GO:0055114 GO:0006520 GO:0044564 GO:0009245
GO:0008152 GO:0016491 GO:0016747
Cluster-22178.64779 GO:0055114 GO:0016052 GO:0005975 GO:0015992
GO:0043093 GO:0015986 GO:0016192 GO:0000917 GO:0016491
GO:0004553 GO:0015078 GO:0030246 GO:0005737 GO:0000276
Cluster-22178.64782 GO:0005515
Cluster-22178.64783 GO:0007155 GO:0001503 GO:0046935 GO:0003676
GO:0005944 GO:0005576
Cluster-22178.64786 GO:0006355 GO:0055114 GO:0055085 GO:0016491
GO:0003700 GO:0005667 GO:0005741
Cluster-22178.64788 GO:0000398 GO:0005681
Cluster-22178.64791 GO:0046656 GO:0015948 GO:0006119 GO:0006118
GO:0043087 GO:0015992 GO:0008121 GO:0030269 GO:0009055
GO:0005576 GO:0016020
Cluster-22178.64792 GO:0006810 GO:0005215 GO:0016020
Cluster-22178.64793 GO:0005515
Cluster-22178.64794 GO:0006508 GO:0005515 GO:0003676 GO:0008233

Cluster-22178.64795 GO:0007165 GO:0008270
Cluster-22178.64797 GO:0006886 GO:0007165 GO:0006614 GO:0006352

GO:0004888 GO:0005047 GO:0003677 GO:0098519 GO:0003924
GO:0005525 GO:0005786 GO:0016021 GO:0005785
Cluster-22178.64798 GO:0006508 GO:0006144 GO:0004197 GO:0005515
GO:0004252 GO:0017111 GO:0008289 GO:0003968 GO:0005887
GO:0031379
Cluster-22178.64799 GO:0007165 GO:0030695 GO:0005057
Cluster-22178.64802 GO:0005515
Cluster-22178.64804 GO:0007275 GO:0007165 GO:0006468 GO:0016055
GO:0005102 GO:0004672 GO:0005524 GO:0005576
Cluster-22178.64808 GO:0016567 GO:0006511 GO:0007018 GO:0007017
GO:0004842 GO:0003777 GO:0008017 GO:0005524 GO:0034450
GO:0005515 GO:0005874 GO:0045298 GO:0000151
Cluster-22178.64809 GO:0006810 GO:0009405 GO:0019855 GO:0005576
Cluster-22178.64810 GO:0009405 GO:0016055 GO:0006629 GO:0006468
GO:0044564 GO:0035091 GO:0016773 GO:0004672 GO:0005524
GO:0016020 GO:0005576
Cluster-22178.64811 GO:0015986 GO:0015992 GO:0006364 GO:0015078
GO:0000276 GO:0032040 GO:0016021
Cluster-22178.64812 GO:0006886 GO:0016192 GO:0005515 GO:0030131
GO:0030117
Cluster-22178.64813 GO:0006206 GO:0009405 GO:0006230 GO:0004797
GO:0005524 GO:0005198
Cluster-22178.64814 GO:0004867 GO:0005576
Cluster-22178.64815 GO:0055114 GO:0015684 GO:0007264 GO:0005525
GO:0016491 GO:0003924 GO:0005524 GO:0015093 GO:0000166
GO:0003677 GO:0016021
Cluster-22178.64816 GO:0006355 GO:0005515 GO:0003723
Cluster-22178.64817 GO:0006355 GO:0005515 GO:0003723
Cluster-22178.64818 GO:0006486 GO:0016567 GO:0016787 GO:0016818
GO:0003677 GO:0005515 GO:0005524 GO:0008270 GO:0046872
GO:0004842 GO:0016757 GO:0003676 GO:0005680
Cluster-22178.64819 GO:0043248 GO:0006850 GO:0016310 GO:0009069
GO:0015991 GO:0051252 GO:0016192 GO:0006886 GO:0004674

GO:0004521 GO:0016820 GO:0005515 GO:0000221 GO:0005743
GO:0030117
Cluster-22178.64821 GO:0001522 GO:0051090 GO:0042254 GO:0008270
GO:0005524 GO:0030515 GO:0005515 GO:0072588 GO:0005634
Cluster-22178.64822 GO:0006810 GO:0005643
Cluster-22178.64823 GO:0006123 GO:0015992 GO:0004129 GO:0045277
Cluster-22178.64824 GO:0030001 GO:0009405 GO:0010508 GO:0006886
GO:0043093 GO:0007155 GO:0006357 GO:0006914 GO:0006606
GO:0000917 GO:0000398 GO:0051258 GO:0006935 GO:0048870
GO:0006308 GO:0006810 GO:0007165 GO:0071973 GO:0030168
GO:0015031 GO:0000921 GO:0006260 GO:0055085 GO:0042803
GO:0001104 GO:0005198 GO:0016817 GO:0005524 GO:0003774
GO:0008565 GO:0042626 GO:0045502 GO:0008376 GO:0008134
GO:0008092 GO:0046873 GO:0030674 GO:0008855 GO:0005102
GO:0005634 GO:0009288 GO:0005737 GO:0032580 GO:0005667
GO:0016020 GO:0005577 GO:0019028 GO:0009318 GO:0031514
GO:0019031 GO:0016021 GO:0005882 GO:0016592 GO:0030286
GO:0005940 GO:0005815 GO:0019898
Cluster-22178.64827 GO:0015979 GO:0005515 GO:0016773
Cluster-22178.64829 GO:0031047 GO:0003723 GO:0019012 GO:0016021
Cluster-22178.64830 GO:0005524 GO:0016787 GO:0003677
Cluster-22178.64831 GO:0006351 GO:0007165 GO:0007186 GO:0004871
GO:0019001 GO:0031683 GO:0003924
Cluster-22178.64832 GO:0009607 GO:0016021
Cluster-22178.64833 GO:0055114 GO:0016972
Cluster-22178.64834 GO:0006275 GO:0007155 GO:0007049
Cluster-22178.64836 GO:0042254 GO:0006412 GO:0042981 GO:0003735
GO:0005515 GO:0008270 GO:0019843 GO:0005634 GO:0005840
Cluster-22178.64837 GO:0006468 GO:0046486 GO:0000160 GO:0045017
GO:0042967 GO:0004144 GO:0003677 GO:0005524 GO:0004672
GO:0000156
Cluster-22178.64839 GO:0005515
Cluster-22178.64840 GO:0005515 GO:0005763

Cluster-22178.64841 GO:0006814 GO:0006464 GO:0043087 GO:0015992 GO:0046436 GO:0009252 GO:0035023 GO:0006885 GO:0008716 GO:0005515 GO:0015385 GO:0005089 GO:0016020

Cluster-22178.64843 GO:0006814 GO:0006885 GO:0035023 GO:0009252 GO:0046436 GO:0015992 GO:0043087 GO:0006464 GO:0015385 GO:0005515 GO:0008716 GO:0005089 GO:0016020

Cluster-22178.64844 GO:0009405 GO:0007165 GO:0035023 GO:0043087 GO:0005515 GO:0005089

Cluster-22178.64845 GO:0008270

Cluster-22178.64846 GO:0048544 GO:0005515

Cluster-22178.64849 GO:0022900 GO:0006397 GO:0006355 GO:0035438 GO:0003723 GO:0003682 GO:0000785 GO:0005737 GO:0005747 GO:0005634 GO:0005739

Cluster-22178.64852 GO:0042157 GO:0006952 GO:0006810 GO:0009405 GO:0019855 GO:0005576

Cluster-22178.64853 GO:0032968 GO:0006270 GO:0006367 GO:0006605 GO:0006886 GO:0003676 GO:0003677 GO:0005509 GO:0005515 GO:0005742 GO:0005634 GO:0016020

Cluster-22178.64854 GO:0006355 GO:0006468 GO:0005524 GO:0004672

Cluster-22178.64855 GO:0007169 GO:0006468 GO:0006508 GO:0006397 GO:0005524 GO:0003723 GO:0004714 GO:0004252 GO:0005737 GO:0005634 GO:0030430 GO:0016020

Cluster-22178.64856 GO:0006568 GO:0006550 GO:0018874 GO:0006631 GO:0006552 GO:0006574 GO:0006554 GO:0055114 GO:0008152 GO:0006633 GO:0003857 GO:0016836 GO:0016491 GO:0003824

Cluster-22178.64857 GO:0016310 GO:0009069 GO:0006468 GO:0016773 GO:0004672 GO:0005524 GO:0004674 GO:0000287 GO:0016020

Cluster-22178.64859 GO:0006355 GO:0007010 GO:0003779 GO:0043565 GO:0008270 GO:0003700 GO:0005667

Cluster-22178.64861 GO:0042254 GO:0006412 GO:0003735 GO:0005622 GO:0005840

Cluster-22178.64862 GO:0042254 GO:0006412 GO:0003735 GO:0005622

GO:0005840
Cluster-22178.64863 GO:0055114 GO:0006118 GO:0008152 GO:0016627
GO:0050660 GO:0003995
Cluster-22178.64865 GO:0016579 GO:0006265 GO:0006355 GO:0003700
GO:0003916 GO:0036459 GO:0003677 GO:0005515 GO:0005694
GO:0005667
Cluster-22178.64866 GO:0006352 GO:0006355 GO:0008270 GO:0003700
GO:0016987 GO:0005515 GO:0003677 GO:0005667
Cluster-22178.6487 GO:0055085
Cluster-22178.64870 GO:0051297 GO:0005813
Cluster-22178.64871 GO:0008152 GO:0008168 GO:0019028
Cluster-22178.64872 GO:0005524 GO:0003676
Cluster-22178.64873 GO:0007264 GO:0006576 GO:0008201 GO:0005524
GO:0003924 GO:0005525 GO:0005886 GO:0016021
Cluster-22178.64874 GO:0006206 GO:0043087 GO:0006144 GO:0006351
GO:0007264 GO:0003677 GO:0003899 GO:0005085 GO:0005730
Cluster-22178.64876 GO:0007049 GO:0008608 GO:0006606 GO:0005515
GO:0004222 GO:0072686 GO:0042729
Cluster-22178.64877 GO:0006955 GO:0007165 GO:0006606 GO:0030247
GO:0005044
Cluster-22178.64878 GO:0006693 GO:0006486 GO:0006298 GO:0006691
GO:0019530 GO:0006749 GO:0008373 GO:0003840 GO:0005524
GO:0030983
Cluster-22178.64880 GO:0006749 GO:0006098 GO:0006313 GO:0046486
GO:0046168 GO:0006355 GO:0005975 GO:0006006 GO:0055114
GO:0006072 GO:0051287 GO:0003677 GO:0016616 GO:0004803
GO:0004367 GO:0016645 GO:0050661 GO:0004345 GO:0003676
GO:0009331 GO:0005622
Cluster-22178.64881 GO:0006072 GO:0005975 GO:0006006 GO:0055114
GO:0006355 GO:0046168 GO:0006313 GO:0046486 GO:0006098
GO:0006749 GO:0003676 GO:0016645 GO:0050661 GO:0004345
GO:0004367 GO:0003677 GO:0016616 GO:0004803 GO:0051287
GO:0005622 GO:0009331

Cluster-22178.64882 GO:0015031 GO:0006464 GO:0005198 GO:0005515

Cluster-22178.64883 GO:0006813 GO:0005515 GO:0005242 GO:0016021
GO:0008076
Cluster-22178.64885 GO:0043565
Cluster-22178.64890 GO:0000162 GO:0006571 GO:0016052 GO:0005975
GO:0009094 GO:0006568 GO:0004553 GO:0004834 GO:0030246
Cluster-22178.64893 GO:0007165 GO:0007606 GO:0003676 GO:0005524
GO:0005515 GO:0042393 GO:0003677 GO:0016787 GO:0004888
GO:0016020
Cluster-22178.64896 GO:0007268 GO:0008152 GO:0007217 GO:0003824

Cluster-22178.64897 GO:0006511 GO:0007275 GO:0008053 GO:0005515
GO:0003677 GO:0016787 GO:0003924 GO:0046872 GO:0008270
GO:0005524 GO:0005741 GO:0005634 GO:0016021
Cluster-22178.64898 GO:0006355 GO:0005634
Cluster-22178.64900 GO:0006189 GO:0006355 GO:0003700 GO:0003824
GO:0043565 GO:0008270 GO:0005667
Cluster-22178.64901 GO:0005198 GO:0070140 GO:0032183 GO:0019028
Cluster-22178.64902 GO:0009089 GO:0055114 GO:0009085 GO:0016620
GO:0008839 GO:0016817
Cluster-22178.64905 GO:0006355 GO:0019058 GO:0003723 GO:0005515
GO:0008270 GO:0046872 GO:0003700 GO:0005667 GO:0042025
Cluster-22178.64907 GO:0015992 GO:0006744 GO:0006120 GO:0007165
GO:0006814 GO:0006118 GO:0006955 GO:0005179 GO:0003954
GO:0008137 GO:0005164 GO:0005576 GO:0005739 GO:0016020
Cluster-22178.64908 GO:0016787
Cluster-22178.64909 GO:0006468 GO:0004672 GO:0005524 GO:0016773
GO:0016020
Cluster-22178.64913 GO:0006810 GO:0005515 GO:0016021
Cluster-22178.64915 GO:0006886 GO:0009405 GO:0009069 GO:0016310
GO:0005515 GO:0008536 GO:0004674 GO:0005576
Cluster-22178.64917 GO:0006801 GO:0055114 GO:0046872 GO:0004784

Cluster-22178.64918 GO:0007050 GO:0006355 GO:0007264 GO:0006468
GO:0045859 GO:0004861 GO:0005515 GO:0003700 GO:0004672
GO:0005524 GO:0005096 GO:0043565 GO:0005667 GO:0005634
Cluster-22178.64920 GO:0007264 GO:0051607 GO:0006357 GO:0007049
GO:0051082 GO:0003677 GO:0001104 GO:0005524 GO:0005525
GO:0019028 GO:0016592 GO:0016021
Cluster-22178.64922 GO:0005515
Cluster-22178.64923 GO:0000398
Cluster-22178.64924 GO:0006810 GO:0009264 GO:0005515 GO:0046872
GO:0005524 GO:0043531 GO:0003676 GO:0004386 GO:0008253
GO:0016020
Cluster-22178.64926 GO:0009395 GO:0046339 GO:0006629 GO:0035556
GO:0007165 GO:0005515 GO:0004435
Cluster-22178.64927 GO:0004222 GO:0003676 GO:0005524 GO:0008270
GO:0016021
Cluster-22178.64929 GO:0007264 GO:0007165 GO:0007186 GO:0019001
GO:0031683 GO:0003924 GO:0005525 GO:0004871
Cluster-22178.64930 GO:0048306
Cluster-22178.64931 GO:0046656 GO:0007586 GO:0007165 GO:0006457
GO:0006730 GO:0006807 GO:0000413 GO:0016042 GO:0004888
GO:0003755 GO:0018759 GO:0008047 GO:0005576 GO:0016021
Cluster-22178.64932 GO:0009234 GO:0008152 GO:0050662 GO:0005515
GO:0005198 GO:0008270 GO:0046872 GO:0016491 GO:0003824
GO:0019028 GO:0005622
Cluster-22178.64934 GO:0005515
Cluster-22178.64935 GO:0006265 GO:0015031 GO:0008277 GO:0006886
GO:0008565 GO:0003917 GO:0003677 GO:0016021 GO:0005694
Cluster-22178.64936 GO:0006886 GO:0006605 GO:0008270 GO:0005515
GO:0016020
Cluster-22178.64937 GO:0007264 GO:0007010 GO:0007165 GO:0007186
GO:0005515 GO:0031683 GO:0019001 GO:0031423 GO:0003779
GO:0003924 GO:0005525 GO:0004871 GO:0019012

Cluster-22178.64938 GO:0006629 GO:0006308 GO:0031110 GO:0043565
GO:0008855 GO:0008081 GO:0009318
Cluster-22178.64939 GO:0055114 GO:0015031 GO:0000902 GO:0016645
GO:0008270 GO:0016021 GO:0005789
Cluster-22178.64941 GO:0035556
Cluster-22178.64944 GO:0006457 GO:0006886 GO:0008219 GO:0007304
GO:0051087 GO:0000774 GO:0008270 GO:0005198 GO:0005515
GO:0042803 GO:0005213 GO:0016020 GO:0005783 GO:0019028
GO:0005576 GO:0042600 GO:0016021 GO:0005801
Cluster-22178.64945 GO:0006355 GO:0006281 GO:0006576 GO:0006412
GO:0002949 GO:0007165 GO:0006810 GO:0042254 GO:0007186
GO:0006310 GO:0046914 GO:0019001 GO:0031683 GO:0003924
GO:0004871 GO:0008134 GO:0005524 GO:0046983 GO:0016887
GO:0009378 GO:0003735 GO:0005840 GO:0016021 GO:0005622
GO:0005657 GO:0005667 GO:0009379
Cluster-22178.64947 GO:0005515
Cluster-22178.64948 GO:0006820 GO:0019033 GO:0016021
Cluster-22178.64949 GO:0046872
Cluster-22178.64950 GO:0005509 GO:0005515
Cluster-22178.64951 GO:0042254 GO:0006412 GO:0003735 GO:0005622
GO:0005840
Cluster-22178.64953 GO:0005634
Cluster-22178.64957 GO:0015992 GO:0015986 GO:0015078
Cluster-22178.64958 GO:0055085 GO:0006811 GO:0005216 GO:0016020
Cluster-22178.64959 GO:0008152 GO:0005515 GO:0016746 GO:0019031
Cluster-22178.6496 GO:0045859 GO:0007050 GO:0005515 GO:0004861
GO:0005634
Cluster-22178.64960 GO:0015986 GO:0006119 GO:0006810 GO:0015992
GO:0046961 GO:0045182 GO:0046933 GO:0015288 GO:0016020
GO:0045261 GO:0045259
Cluster-22178.64961 GO:0006979 GO:0016579 GO:0055114 GO:0006118
GO:0006804 GO:0006281 GO:0006749 GO:0006508 GO:0036459
GO:0004602 GO:0016655 GO:0004843 GO:0016020 GO:0071203

Cluster-22178.64967 GO:0006351 GO:0006269 GO:0003896 GO:0005657 GO:0005730
Cluster-22178.64968 GO:0006412 GO:0042254 GO:0003723 GO:0003735 GO:0005840
Cluster-22178.64969 GO:0006468 GO:0032259 GO:0005524 GO:0004672 GO:0008168
Cluster-22178.64970 GO:0055114 GO:0006633 GO:0042967 GO:0046872 GO:0004318 GO:0005835
Cluster-22178.64971 GO:0006468 GO:0004672 GO:0005524 GO:0045502 GO:0008134 GO:0042803 GO:0005856 GO:0030286 GO:0005667
Cluster-22178.64972 GO:0006520 GO:0055114 GO:0016491
Cluster-22178.64973 GO:0006541 GO:0004359
Cluster-22178.64974 GO:0003676
Cluster-22178.64975 GO:0006378 GO:0003676 GO:0008143 GO:0005737 GO:0005634
Cluster-22178.64977 GO:0046872 GO:0008270 GO:0005515 GO:0003676

Cluster-22178.64979 GO:0048870 GO:0007165 GO:0005515 GO:0031514
Cluster-22178.64980 GO:0005515
Cluster-22178.64981 GO:0005515
Cluster-22178.64982 GO:0009405 GO:0005198
Cluster-22178.64983 GO:0008152 GO:0006508 GO:0004197 GO:0003824 GO:0005524 GO:0008242 GO:0016740
Cluster-22178.64985 GO:0006779 GO:0055114 GO:0016226 GO:0051536 GO:0005737
Cluster-22178.64986 GO:0005515
Cluster-22178.64987 GO:0005515
Cluster-22178.6499 GO:0005515
Cluster-22178.64990 GO:0070836 GO:0006468 GO:0016773 GO:0004672 GO:0005524 GO:0016020
Cluster-22178.64991 GO:0018279 GO:0005524 GO:0004579 GO:0016021 GO:0008250
Cluster-22178.64993 GO:0009236 GO:0006811 GO:0004190 GO:0016021

GO:0016020
Cluster-22178.64995 GO:0003677
Cluster-22178.64998 GO:0006940
Cluster-22178.64999 GO:0006886 GO:0016192 GO:0005198 GO:0005515
GO:0030126 GO:0030117
Cluster-22178.65001 GO:0070403 GO:0005515
Cluster-22178.65002 GO:0008270 GO:0003676
Cluster-22178.65003 GO:0008270 GO:0003676
Cluster-22178.65004 GO:0006810 GO:0008152 GO:0016866 GO:0005215
GO:0031419 GO:0005737 GO:0016021
Cluster-22178.65007 GO:0006446 GO:0007218 GO:0006464 GO:0006144
GO:0006413 GO:0006355 GO:0046034 GO:0005975 GO:0006808
GO:0003677 GO:0003743 GO:0004017 GO:0004553 GO:0008270
GO:0030234 GO:0004519 GO:0005515 GO:0003723 GO:0016151
GO:0003700 GO:0003676 GO:0004672 GO:0046872 GO:0043565
GO:0005667 GO:0005840
Cluster-22178.65008 GO:0006278 GO:0005515 GO:0004672 GO:0003964

Cluster-22178.65011 GO:0005515
Cluster-22178.65012 GO:0055114 GO:0015992 GO:0008152 GO:0006355
GO:0006118 GO:0006119 GO:0016491 GO:0003824 GO:0050662
GO:0008121 GO:0003677
Cluster-22178.65013 GO:0019031
Cluster-22178.65016 GO:0007155 GO:0042254 GO:0051607 GO:0000921
GO:0006412 GO:0043015 GO:0003735 GO:0045502 GO:0042803
GO:0008134 GO:0042802 GO:0005667 GO:0030286 GO:0005622
GO:0005940 GO:0001772 GO:0019028 GO:0097197 GO:0005840
GO:0016021 GO:0045298
Cluster-22178.65021 GO:0007165 GO:0005515
Cluster-22178.65022 GO:0016787 GO:0005882
Cluster-22178.65023 GO:0006096 GO:0006094 GO:0006020 GO:0006098
GO:0015976 GO:0006000 GO:0006013 GO:0004332
Cluster-22178.65024 GO:0042967 GO:0016573 GO:0006355 GO:0008270

GO:0008134 GO:0003713 GO:0003677 GO:0004402 GO:0003682
GO:0000785 GO:0000123 GO:0005667 GO:0005634
Cluster-22178.65025 GO:0016787
Cluster-22178.65026 GO:0005515
Cluster-22178.65027 GO:0006812 GO:0016887 GO:0032040 GO:0016021
Cluster-22178.65028 GO:0006412 GO:0042254 GO:0003735 GO:0005840
GO:0005622
Cluster-22178.6503 GO:0007156 GO:0007067 GO:0051382 GO:0005509
GO:0003677 GO:0016020
Cluster-22178.65032 GO:0006470 GO:0006355 GO:0006570 GO:0043565
GO:0003700 GO:0004725 GO:0008138 GO:0005667 GO:0016020
Cluster-22178.65033 GO:0003676
Cluster-22178.65035 GO:0007165 GO:0042967 GO:0005975 GO:0008811
GO:0003723 GO:0008184 GO:0003676 GO:0008270
Cluster-22178.65036 GO:0045132 GO:0000917 GO:0046487 GO:0055114
GO:0008152 GO:0043093 GO:0009396 GO:0000226 GO:0005515
GO:0008092 GO:0003824 GO:0016787 GO:0004488 GO:0005509
GO:0005634 GO:0015630 GO:0005737 GO:0000775 GO:0016021
GO:0019898
Cluster-22178.65037 GO:0042254 GO:0005524
Cluster-22178.65039 GO:0051028 GO:0003677 GO:0008270 GO:0005634
Cluster-22178.65040 GO:0006570 GO:0042207 GO:0006810 GO:0009072
GO:0015743 GO:0004334 GO:0005886
Cluster-22178.65041 GO:0006511 GO:0031625
Cluster-22178.65042 GO:0005515 GO:0009512
Cluster-22178.65043 GO:0005515
Cluster-22178.65044 GO:0016020
Cluster-22178.65046 GO:0006464 GO:0032259 GO:0046500 GO:0006810
GO:0009452 GO:0001510 GO:0031167 GO:0006479 GO:0006306
GO:0008270 GO:0008170 GO:0003677 GO:0008168 GO:0046872
GO:0004719 GO:0003676 GO:0003723 GO:0005515 GO:0019871
GO:0005576
Cluster-22178.65048 GO:0015991 GO:0016192 GO:0006886 GO:0005515

GO:0016820 GO:0030117 GO:0000221
Cluster-22178.65049 GO:0006260 GO:0006355 GO:0006270 GO:0005524
GO:0016887 GO:0003678 GO:0003677 GO:0008134 GO:0005657
GO:0005667 GO:0005634 GO:0042555
Cluster-22178.65050 GO:0006506 GO:0006412 GO:0042254 GO:0003735
GO:0016746 GO:0005739 GO:0005840 GO:0005789 GO:0016021
Cluster-22178.65051 GO:0006364 GO:0032259 GO:0006370 GO:0006144
GO:0006396 GO:0000154 GO:0006479 GO:0031167 GO:0009451
GO:0008152 GO:0017111 GO:0004482 GO:0070008 GO:0008649
GO:0004252 GO:0016817 GO:0008168 GO:0004483 GO:0003968
GO:0031379 GO:0005737
Cluster-22178.65052 GO:0009405 GO:0006338 GO:0090544
Cluster-22178.65054 GO:0055085 GO:0005741
Cluster-22178.65057 GO:0034968 GO:0006355 GO:0006554 GO:0006479
GO:0018024 GO:0005515 GO:0005694
Cluster-22178.65058 GO:0007275 GO:0006511 GO:0005634
Cluster-22178.65061 GO:0006352 GO:0006355 GO:0016987 GO:0003677
GO:0003700 GO:0005667
Cluster-22178.65063 GO:0043248 GO:0006886 GO:0016192 GO:0015991
GO:0016820 GO:0005515 GO:0000221 GO:0030117
Cluster-22178.65064 GO:0016310 GO:0009069 GO:0005515 GO:0004674

Cluster-22178.65066 GO:0008380 GO:0006308 GO:0007155 GO:0008855
GO:0005200 GO:0005856 GO:0005681 GO:0009318 GO:0005882
Cluster-22178.65067 GO:0032922 GO:0009452 GO:0055114 GO:0016567
GO:0001510 GO:0004842 GO:0051537 GO:0016491 GO:0008168
GO:0000976 GO:0046872 GO:0008270 GO:0005515
Cluster-22178.65068 GO:0006013 GO:0005975 GO:0007165 GO:0004553
GO:0008158 GO:0004559 GO:0008270 GO:0015923 GO:0016020
Cluster-22178.65069 GO:0042254 GO:0006412 GO:0006629 GO:0006007
GO:0005515 GO:0003735 GO:0046537 GO:0003824 GO:0005840
GO:0005622
Cluster-22178.65071 GO:0008608 GO:0048870 GO:0007094 GO:0006355

GO:0003700 GO:0031514 GO:0042729 GO:0072686 GO:0005667
Cluster-22178.65072 GO:0008210 GO:0019836 GO:0008207 GO:0008209
GO:0055114 GO:0006694 GO:0008152 GO:0003854 GO:0050662
GO:0005515 GO:0016616 GO:0016491 GO:0003824 GO:0005576
Cluster-22178.65073 GO:0016117 GO:0055114 GO:0006355 GO:0016705
GO:0016491 GO:0071949 GO:0005634
Cluster-22178.65076 GO:0006487 GO:0005975 GO:0004553 GO:0019013
GO:0016021 GO:0008250
Cluster-22178.65077 GO:0009878 GO:0046872
Cluster-22178.65078 GO:0006412 GO:0042254 GO:0006260 GO:0003677
GO:0003887 GO:0003735 GO:0001882 GO:0008270 GO:0046872
GO:0042575 GO:0042025 GO:0005840 GO:0005622
Cluster-22178.65079 GO:0007050 GO:0045859 GO:0000122 GO:0004861
GO:0017069 GO:0005737 GO:0005634
Cluster-22178.65081 GO:0005515
Cluster-22178.65082 GO:0000917 GO:0043093 GO:0006355 GO:0007049
GO:0005515 GO:0003723 GO:0003700 GO:0008092 GO:0043565
GO:0046872 GO:0008270 GO:0005667 GO:0019898 GO:0005815
GO:0005737
Cluster-22178.65085 GO:0055114 GO:0006730 GO:0006813 GO:0006555
GO:0051287 GO:0004013
Cluster-22178.65086 GO:0016787 GO:0016765
Cluster-22178.65088 GO:0055114 GO:0048038 GO:0016151 GO:0016651
GO:0051287
Cluster-22178.65089 GO:0046935 GO:0003676 GO:0003723 GO:0016021
GO:0005944
Cluster-22178.65090 GO:0006260 GO:0005509
Cluster-22178.65091 GO:0006260 GO:0005509
Cluster-22178.65092 GO:0000413 GO:0006457 GO:0003755
Cluster-22178.65093 GO:0000917 GO:0043093 GO:0006355 GO:0007049
GO:0006260 GO:0051259 GO:0003700 GO:0034618 GO:0005667
GO:0005634 GO:0005737
Cluster-22178.65094 GO:0022900 GO:0016021

Cluster-22178.65096 GO:0042254 GO:0006412 GO:0003735 GO:0005840
GO:0005622
Cluster-22178.65098 GO:0009312 GO:0008455 GO:0016021 GO:0005795
Cluster-22178.65099 GO:0005982 GO:0005985 GO:0006096 GO:0006098
GO:0006094 GO:0004347
Cluster-22178.65100 GO:0006468 GO:0016773 GO:0005524 GO:0004672
GO:0016020
Cluster-22178.65101 GO:0003676
Cluster-22178.65102 GO:0007165 GO:0006118 GO:0005509 GO:0016787
GO:0009055 GO:0016491 GO:0016020 GO:0005578 GO:0016021
Cluster-22178.65103 GO:0007186 GO:0007165 GO:0004871 GO:0005834
Cluster-22178.65104 GO:0016559 GO:0005779
Cluster-22178.65108 GO:0048193 GO:0046872 GO:0008270 GO:0003676
GO:0016020
Cluster-22178.65110 GO:0055114 GO:0006511 GO:0007275 GO:0006468
GO:0016627 GO:0046872 GO:0004672 GO:0008270 GO:0005524
GO:0005634
Cluster-22178.65111 GO:0016192 GO:0006886 GO:0006468 GO:0005524
GO:0004672 GO:0016773 GO:0016020
Cluster-22178.65116 GO:0009405 GO:0016114 GO:0006694 GO:0008152
GO:0016624 GO:0008661 GO:0003824 GO:0030976
Cluster-22178.65118 GO:0015684 GO:0007264 GO:0015886 GO:0006281
GO:0046497 GO:0006769 GO:0006310 GO:0002949 GO:0007131
GO:0055114 GO:0008033 GO:0009378 GO:0051287 GO:0050660
GO:0003724 GO:0008750 GO:0016491 GO:0050661 GO:0005525
GO:0003678 GO:0098519 GO:0015232 GO:0005524 GO:0015093
GO:0016887 GO:0016301 GO:0003723 GO:0003924 GO:0009379
GO:0016021 GO:0005657 GO:0000794
Cluster-22178.6512 GO:0009405 GO:0007155 GO:0006260 GO:0008219
GO:0003677 GO:0005515 GO:0016020 GO:0005576
Cluster-22178.65120 GO:0006633 GO:0046436 GO:0009252 GO:0006479
GO:0006481 GO:0006813 GO:0006090 GO:0004671 GO:0005524
GO:0015269 GO:0003989 GO:0008716 GO:0046872 GO:0016020

GO:0009317 GO:0016021
Cluster-22178.65123 GO:0006144 GO:0015992 GO:0006206 GO:0015991
GO:0007155 GO:0006351 GO:0015078 GO:0070140 GO:0032183
GO:0005515 GO:0003899 GO:0003677 GO:0005730 GO:0016020
GO:0033179
Cluster-22178.65124 GO:0003676 GO:0046872
Cluster-22178.65126 GO:0031145 GO:0030071 GO:0007010 GO:0006355
GO:0006468 GO:0004672 GO:0005524 GO:0003779 GO:0005515
GO:0005680 GO:0016020 GO:0005634 GO:0019028
Cluster-22178.65128 GO:0005515
Cluster-22178.65129 GO:0046439 GO:0006534 GO:0055114 GO:0019530
GO:0005506 GO:0017172
Cluster-22178.65130 GO:0007178 GO:0016310 GO:0009069 GO:0006468
GO:0006413 GO:0006446 GO:0005524 GO:0004675 GO:0003743
GO:0005840 GO:0016021 GO:0005576 GO:0016020
Cluster-22178.65131 GO:0043087 GO:0035556 GO:0007264 GO:0008283
GO:0019068 GO:0007165 GO:0015684 GO:0006270 GO:0007186
GO:0035023 GO:0040007 GO:0006576 GO:0015093 GO:0005524
GO:0016887 GO:0016757 GO:0005089 GO:0005179 GO:0046872
GO:0019001 GO:0031683 GO:0003924 GO:0005525 GO:0004871
GO:0005198 GO:0005515 GO:0008083 GO:0016020 GO:0016021
GO:0005576
Cluster-22178.65132 GO:0006355 GO:0006810 GO:0051537 GO:0003677
GO:0003713 GO:0005886 GO:0043231 GO:0005667
Cluster-22178.65133 GO:0005975 GO:0006012 GO:0006027 GO:0006687
GO:0046486 GO:0046872 GO:0004565 GO:0005515 GO:0009341
Cluster-22178.65134 GO:0008283 GO:0040007 GO:0007155 GO:0001558
GO:0007165 GO:0005515 GO:0008083 GO:0005520 GO:0005576
GO:0016942
Cluster-22178.65135 GO:0006364 GO:0005515 GO:0016021 GO:0032040
Cluster-22178.65137 GO:0007165 GO:0030695 GO:0005057
Cluster-22178.65138 GO:0008616 GO:0016192 GO:0006886 GO:0043248
GO:0016740 GO:0005515 GO:0016853 GO:0003676 GO:0030117

Cluster-22178.65140 GO:0007165
Cluster-22178.65141 GO:0035091 GO:0031083
Cluster-22178.65144 GO:0019058 GO:0051536 GO:0008289 GO:0003824
GO:0019031
Cluster-22178.65147 GO:0007275 GO:0042600
Cluster-22178.65150 GO:0005509 GO:0005515
Cluster-22178.65151 GO:0016020
Cluster-22178.65153 GO:0046907 GO:0000413 GO:0006457 GO:0004519
GO:0003755
Cluster-22178.65154 GO:0006811 GO:0055085 GO:0005216 GO:0016020
Cluster-22178.65155 GO:0003723
Cluster-22178.65157 GO:0006109 GO:0055114 GO:0008152 GO:0016310
GO:0000160 GO:0000155 GO:0050662 GO:0005524 GO:0004672
GO:0016491 GO:0003824 GO:0009365
Cluster-22178.65158 GO:0006508 GO:0006511 GO:0016579 GO:0004843
GO:0005622
Cluster-22178.65160 GO:0005515
Cluster-22178.65161 GO:0015889 GO:0031419
Cluster-22178.65162 GO:0009235 GO:0006355 GO:0046872 GO:0005515
GO:0005634
Cluster-22178.65163 GO:0003676
Cluster-22178.65165 GO:0007264 GO:0005524 GO:0003924 GO:0005525

Cluster-22178.65166 GO:0003723
Cluster-22178.65168 GO:0055114 GO:0006508 GO:0006281 GO:0043039
GO:0051536 GO:0046872 GO:0016876 GO:0005524 GO:0008270
GO:0008234 GO:0005515 GO:0005509 GO:0016020
Cluster-22178.65173 GO:0005515
Cluster-22178.65174 GO:0008406 GO:0008283 GO:0040007 GO:0007165
GO:0008083
Cluster-22178.65178 GO:0006568 GO:0006355 GO:0015947 GO:0006804
GO:0055114 GO:0006979 GO:0004096 GO:0020037 GO:0003700
GO:0005667 GO:0005634

Cluster-22178.65179 GO:0006508 GO:0005515 GO:0004252

Cluster-22178.65182 GO:0006323 GO:0005198 GO:0005882

Cluster-22178.65184 GO:0031047 GO:0005515

Cluster-22178.65186 GO:0046500 GO:0001510 GO:0006479 GO:0006306 GO:0031167 GO:0006464 GO:0032259 GO:0009452 GO:0008170 GO:0003723 GO:0003677 GO:0004719 GO:0008270 GO:0008168 GO:0003676

Cluster-22178.65187 GO:0035556 GO:0005515

Cluster-22178.65189 GO:0006468 GO:0006508 GO:0043087 GO:0007169 GO:0035023 GO:0004714 GO:0005515 GO:0005524 GO:0004672 GO:0004185 GO:0005089 GO:0016020

Cluster-22178.65191 GO:0007186 GO:0004930 GO:0016021

Cluster-22178.65192 GO:0055114 GO:0007165 GO:0035556 GO:0016717

Cluster-22178.65195 GO:0003676

Cluster-22178.65196 GO:0006021 GO:0019872 GO:0008654 GO:0004512

Cluster-22178.65197 GO:0035556 GO:0007264 GO:0005525

Cluster-22178.65198 GO:0006282 GO:0006355 GO:0008270 GO:0005096 GO:0005544 GO:0005509 GO:0005515 GO:0008023

Cluster-22178.65200 GO:0019079 GO:0043248 GO:0003676 GO:0003677 GO:0008026 GO:0016787 GO:0005524

Cluster-22178.65201 GO:0006281 GO:0007165 GO:0006144 GO:0006464 GO:0046034 GO:0005179 GO:0004017 GO:0008270 GO:0046872 GO:0016151 GO:0005742 GO:0005576

Cluster-22178.65203 GO:0003677 GO:0003676 GO:0005524 GO:0016787

Cluster-22178.65204 GO:0055114 GO:0006281 GO:0006355 GO:0000184 GO:0047134 GO:0004386 GO:0016972 GO:0005524 GO:0008270 GO:0046872 GO:0003677 GO:0005737

Cluster-22178.65206 GO:0006810 GO:0005515 GO:0016021

Cluster-22178.65207 GO:0007155 GO:0043093 GO:0006914 GO:0015992 GO:0048193 GO:0006606 GO:0010508 GO:0007094 GO:0006457

GO:0000921 GO:0006260 GO:0015991 GO:0044780 GO:0006355
GO:0000917 GO:0048870 GO:0016818 GO:0003677 GO:0008270
GO:0005524 GO:0051082 GO:0042803 GO:0005198 GO:0043565
GO:0015078 GO:0017048 GO:0045502 GO:0008134 GO:0003700
GO:0005667 GO:0016020 GO:0016272 GO:0005634 GO:0005737
GO:0019867 GO:0005882 GO:0005940 GO:0030286 GO:0033180
GO:0019028 GO:0031514 GO:0016021
Cluster-22178.65212 GO:0015992 GO:0015986 GO:0015078 GO:0000276
Cluster-22178.65214 GO:0006397 GO:0003723 GO:0046872 GO:0005515

Cluster-22178.65215 GO:0006397 GO:0046872
Cluster-22178.65216 GO:0051694 GO:0046782 GO:0005523
Cluster-22178.65217 GO:0006807 GO:0055114 GO:0006520 GO:0006118
GO:0019551 GO:0016491 GO:0004352 GO:0051287
Cluster-22178.65218 GO:0042254 GO:0006412 GO:0055114 GO:0061630
GO:0003735 GO:0005515 GO:0042393 GO:0016972 GO:0008270
GO:0046872 GO:0005840 GO:0005622
Cluster-22178.65219 GO:0006633 GO:0008152 GO:0042967 GO:0016747
GO:0004315 GO:0005835
Cluster-22178.65221 GO:0007155 GO:0007165 GO:0030155 GO:0019049
GO:0030334 GO:0030168 GO:0006914 GO:0006886 GO:0051258
GO:0055114 GO:0045995 GO:0030674 GO:0005102 GO:0016702
GO:0016020 GO:0005577 GO:0031224 GO:0005801
Cluster-22178.65222 GO:0005509 GO:0005544
Cluster-22178.65225 GO:0019211 GO:0005515 GO:0016746
Cluster-22178.65229 GO:0016757
Cluster-22178.65232 GO:0009405 GO:0005198
Cluster-22178.65234 GO:0006414 GO:0006206 GO:0006448 GO:0006144
GO:0006555 GO:0006556 GO:0055114 GO:1901031 GO:0006351
GO:0003746 GO:0004478 GO:0051920 GO:0003676 GO:0005515
GO:0003899 GO:0005730 GO:0005840 GO:0005634
Cluster-22178.65235 GO:0003676
Cluster-22178.65236 GO:0007218 GO:0007264 GO:0007165 GO:0009847

GO:0006468 GO:0006118 GO:0018990 GO:0005524 GO:0016491
GO:0016887 GO:0004672 GO:0003924 GO:0008255 GO:0009055
GO:0005525 GO:0005515 GO:0005198 GO:0005739 GO:0016020
GO:0016021
Cluster-22178.65237 GO:0001503 GO:0030683
Cluster-22178.65240 GO:0016192 GO:0006886 GO:0005515 GO:0005198
GO:0003824 GO:0030126 GO:0016020 GO:0016021
Cluster-22178.65243 GO:0045766 GO:0043123 GO:0016567 GO:0015986
GO:0043066 GO:0015992 GO:0003723 GO:0043130 GO:0015078
GO:0004842 GO:0000276
Cluster-22178.65244 GO:0005198 GO:0019028
Cluster-22178.65245 GO:0015992 GO:0043066 GO:0015986 GO:0045766
GO:0016567 GO:0043123 GO:0043130 GO:0004842 GO:0015078
GO:0003723 GO:0000276
Cluster-22178.65247 GO:0006355 GO:0003677
Cluster-22178.65248 GO:0055114 GO:0000051 GO:0008652 GO:0005525
GO:0016620 GO:0046983 GO:0003942 GO:0005737
Cluster-22178.65249 GO:0008152 GO:0006468 GO:0046039 GO:0009190
GO:0006144 GO:0006357 GO:0035556 GO:0006182 GO:0004672
GO:0003824 GO:0004383 GO:0016849 GO:0003723 GO:0001104
GO:0008270 GO:0005524 GO:0016592
Cluster-22178.6525 GO:0006355 GO:0003700 GO:0019033 GO:0005667
GO:0005634
Cluster-22178.65250 GO:0000226 GO:0090063 GO:0000922 GO:0005815
Cluster-22178.65251 GO:0006270 GO:0006367 GO:0006605 GO:0006886
GO:0032968 GO:0003677 GO:0005515 GO:0003676 GO:0016020
GO:0005742 GO:0005634
Cluster-22178.65253 GO:0070836 GO:0007165 GO:0051252 GO:0042254
GO:0006412 GO:0006355 GO:0003676 GO:0005515 GO:0003735
GO:0004523 GO:0005840 GO:0005622
Cluster-22178.65255 GO:0006807 GO:0006811 GO:0006672 GO:0055085
GO:0007165 GO:0006810 GO:0007186 GO:0004930 GO:0004872
GO:0005216 GO:0016811 GO:0016021 GO:0016020

Cluster-22178.65256 GO:0055114 GO:0006480 GO:0008152 GO:0008168 GO:0016491 GO:0010181 GO:0008171
Cluster-22178.65257 GO:0016740
Cluster-22178.65258 GO:0042254 GO:0006412 GO:0051252 GO:0007165 GO:0070836 GO:0004523 GO:0003735 GO:0005515 GO:0003676 GO:0005622 GO:0005840
Cluster-22178.65259 GO:0009401 GO:0006810 GO:0005886
Cluster-22178.65260 GO:0006468 GO:0019076 GO:0006812 GO:0032801 GO:0005261 GO:0004672 GO:0005524 GO:0016773 GO:0016020 GO:0033644
Cluster-22178.65261 GO:0015986 GO:0016567 GO:0006119 GO:0007005 GO:0015992 GO:0046961 GO:0046933 GO:0008289 GO:0004842 GO:0000275 GO:0045259
Cluster-22178.65263 GO:0007275 GO:0009966 GO:0005515 GO:0016020
Cluster-22178.65264 GO:0046872
Cluster-22178.65265 GO:0090063 GO:0000226 GO:0004190 GO:0008146 GO:0005815 GO:0016020 GO:0000922
Cluster-22178.65266 GO:0008270 GO:0005634
Cluster-22178.65267 GO:0006465 GO:0008233 GO:0004659 GO:0016021 GO:0005787
Cluster-22178.65268 GO:0019064 GO:0006369 GO:0006378 GO:0006379 GO:0006260 GO:0010508 GO:0016758 GO:0005789 GO:0005815 GO:0019031 GO:0005849
Cluster-22178.65274 GO:0030154 GO:0016021
Cluster-22178.65275 GO:0010508 GO:0006369 GO:0019064 GO:0006379 GO:0006260 GO:0006378 GO:0016758 GO:0005849 GO:0019031 GO:0005789 GO:0005815
Cluster-22178.65276 GO:0050776 GO:0014068 GO:0009405 GO:0006281 GO:0006310 GO:0006810 GO:0007165 GO:0019871 GO:0005102 GO:0005524 GO:0043548 GO:0009378 GO:0005179 GO:0005576 GO:0005657 GO:0016020 GO:0009379
Cluster-22178.65277 GO:0007186 GO:0004930
Cluster-22178.65278 GO:0042254 GO:0006412 GO:0003735 GO:0005761

GO:0005840
Cluster-22178.65279 GO:0007165 GO:0006629 GO:0006355 GO:0055114
GO:0003677 GO:0051920 GO:0005044 GO:0016641 GO:0005507
GO:0016020 GO:0005763 GO:0005622
Cluster-22178.65281 GO:0007049 GO:0000917 GO:0006355 GO:0010506
GO:0008053 GO:0006260 GO:0043093 GO:0003700 GO:0043565
GO:0003924 GO:0016021 GO:0005615 GO:0005737 GO:0005634
GO:0005741 GO:0005667
Cluster-22178.65282 GO:0042254 GO:0006412 GO:0003735 GO:0005622
GO:0005840
Cluster-22178.65286 GO:0042254 GO:0006412 GO:0003735 GO:0005622
GO:0005840
Cluster-22178.65289 GO:0006007 GO:0006629 GO:0042254 GO:0006412
GO:0003824 GO:0046537 GO:0003735 GO:0005515 GO:0005622
GO:0005840
Cluster-22178.65290 GO:0009231 GO:0005985 GO:0005982 GO:0005991
GO:0005993 GO:0008531 GO:0008375 GO:0005509 GO:0004555
GO:0005737 GO:0016020
Cluster-22178.65291 GO:0006886 GO:0005975 GO:0016832 GO:0005741
GO:0016020 GO:0016021
Cluster-22178.65293 GO:0006811 GO:0005515 GO:0016020
Cluster-22178.65295 GO:0070206 GO:0048870 GO:0005515 GO:0005639
GO:0031514
Cluster-22178.65297 GO:0046486 GO:0009058 GO:0006508 GO:0008152
GO:0016042 GO:0005975 GO:0016788 GO:0008236 GO:0008184
GO:0016787 GO:0004806
Cluster-22178.65300 GO:0042254 GO:0006412 GO:0003735 GO:0005840
GO:0005622
Cluster-22178.65302 GO:0005524
Cluster-22178.65303 GO:0016192 GO:0006886 GO:0055114 GO:0005515
GO:0047134 GO:0070403 GO:0008270 GO:0016491
Cluster-22178.65304 GO:0006468 GO:0005524 GO:0004672
Cluster-22178.65305 GO:0004527 GO:0003676

Cluster-22178.65307 GO:0006270 GO:0030001 GO:0055085 GO:0019064
GO:0046873 GO:0004190 GO:0003676 GO:0046872 GO:0016020
GO:0016021 GO:0019867
Cluster-22178.65309 GO:0019354 GO:0006779 GO:0055114 GO:0043115
GO:0008641 GO:0016491
Cluster-22178.65310 GO:0030150 GO:0005742
Cluster-22178.65312 GO:0045227 GO:0004252 GO:0016020 GO:0016021
Cluster-22178.65313 GO:0042254 GO:0006412 GO:0003735 GO:0005840
Cluster-22178.65315 GO:0046872 GO:0008270
Cluster-22178.65317 GO:0006522 GO:0006419 GO:0043039 GO:0006531
GO:0016055 GO:0003676 GO:0005524 GO:0016876 GO:0004813
GO:0000166
Cluster-22178.65318 GO:0043039 GO:0006531 GO:0016055 GO:0006522
GO:0006419 GO:0000166 GO:0003676 GO:0005524 GO:0004813
GO:0016876
Cluster-22178.65319 GO:0006694 GO:0055114 GO:0000917 GO:0008299
GO:0043093 GO:0047134 GO:0005525 GO:0050518 GO:0005524
GO:0046983 GO:0016887 GO:0005737
Cluster-22178.65322 GO:0035556 GO:0005515
Cluster-22178.65323 GO:0006397 GO:0006355 GO:0022900 GO:0003723
GO:0003682 GO:0035438 GO:0005747 GO:0005739 GO:0005634
GO:0000785 GO:0005737
Cluster-22178.65324 GO:0046487 GO:0015977 GO:0003677 GO:0016984
GO:0009573
Cluster-22178.65325 GO:0006260 GO:0006281 GO:0003910 GO:0003676
GO:0003723
Cluster-22178.65327 GO:0006118 GO:0055114 GO:0016625 GO:0009055
GO:0051536 GO:0016491
Cluster-22178.65328 GO:0055114 GO:0005975 GO:0016491 GO:0004553
GO:0016616
Cluster-22178.65329 GO:0016042 GO:0008203 GO:0008152 GO:0016298
GO:0016787
Cluster-22178.65330 GO:0006633 GO:0055114 GO:0005506 GO:0016491

Cluster-22178.65332 GO:0007165 GO:0007186 GO:0004871 GO:0005834
Cluster-22178.65333 GO:0016020
Cluster-22178.65334 GO:0007155 GO:0006355 GO:0042246 GO:0005622
GO:0016021
Cluster-22178.65337 GO:0004064 GO:0005515
Cluster-22178.65338 GO:0005524 GO:0016787 GO:0003677 GO:0003676
GO:0005515
Cluster-22178.65340 GO:0055114 GO:0007010 GO:0046872 GO:0003779
GO:0016972
Cluster-22178.65341 GO:0042254 GO:0055114 GO:0008615 GO:0010181
GO:0016638 GO:0004733 GO:0005622 GO:0016021
Cluster-22178.65342 GO:0042254 GO:0008615 GO:0055114 GO:0016638
GO:0010181 GO:0004733 GO:0016021 GO:0005622
Cluster-22178.65343 GO:0007165 GO:0040007 GO:0008283 GO:0008083
GO:0051015 GO:0030674
Cluster-22178.65344 GO:0006508 GO:0009085 GO:0009089 GO:0055114
GO:0005509 GO:0005515 GO:0008270 GO:0008839 GO:0004222
GO:0031012
Cluster-22178.65346 GO:0045893 GO:0006351 GO:0008270 GO:0005515
GO:0003700 GO:0005634 GO:0005667
Cluster-22178.65348 GO:0006355 GO:0015986 GO:0006260 GO:0007049
GO:0015992 GO:0003677 GO:0051082 GO:0015078 GO:0003700
GO:0043565 GO:0045263 GO:0005667 GO:0005634
Cluster-22178.65349 GO:0008210 GO:0006694 GO:0055114 GO:0043154
GO:0008207 GO:0019079 GO:0008209 GO:0043066 GO:0003824
GO:0032266 GO:0016616 GO:0051287 GO:0003854 GO:0050662
GO:0016620 GO:0043130 GO:0080019 GO:0005737 GO:0071986
Cluster-22178.65351 GO:0007264 GO:0007186 GO:0007165 GO:0003924
GO:0031683 GO:0019001 GO:0004871 GO:0005525
Cluster-22178.65352 GO:0003676
Cluster-22178.65354 GO:0006606 GO:0015031 GO:0042742 GO:0015991
GO:0008565 GO:0016820 GO:0005515 GO:0000221 GO:0005737

GO:0005634
Cluster-22178.65356 GO:0006355 GO:0042254 GO:0006412 GO:0008270
GO:0043565 GO:0003700 GO:0003735 GO:0005840 GO:0005667
Cluster-22178.65357 GO:0005515
Cluster-22178.65358 GO:0008270
Cluster-22178.65359 GO:0055085 GO:0005741
Cluster-22178.65360 GO:0006817 GO:0005315 GO:0016020
Cluster-22178.65361 GO:0006118 GO:0009451 GO:0006418 GO:0008033
GO:0006308 GO:0045454 GO:0009307 GO:0035556 GO:0006464
GO:0030488 GO:0000166 GO:0005515 GO:0016151 GO:0009055
GO:0015035 GO:0003677 GO:0004812 GO:0016429 GO:0005524
GO:0009036 GO:0009359 GO:0031515
Cluster-22178.65362 GO:0005515
Cluster-22178.65363 GO:0006352 GO:0008152 GO:0055114 GO:0016491
GO:0005634
Cluster-22178.65364 GO:0005515
Cluster-22178.65366 GO:0006886 GO:0009308 GO:0055114 GO:0007264
GO:0007165 GO:0007186 GO:0006888 GO:0019001 GO:0031683
GO:0003924 GO:0005525 GO:0004871 GO:0005507 GO:0008270
GO:0008131 GO:0048038 GO:0030127
Cluster-22178.65368 GO:0006355 GO:0005524 GO:0016887 GO:0005515
GO:0008134 GO:0005667
Cluster-22178.65369 GO:0006886 GO:0006118 GO:0030001 GO:0007338
GO:0022900 GO:0006869 GO:0055085 GO:0020037 GO:0009055
GO:0008289 GO:0046873 GO:0005506 GO:0031083 GO:0005576
GO:0016021 GO:0016020 GO:0042597
Cluster-22178.6537 GO:0007094 GO:0005198 GO:0005882
Cluster-22178.65372 GO:0016310 GO:0008152 GO:0007178 GO:0009069
GO:0006468 GO:0005524 GO:0004675 GO:0016020
Cluster-22178.65373 GO:0015992 GO:0006123 GO:0008270 GO:0004129
GO:0005515 GO:0005740 GO:0045277
Cluster-22178.65374 GO:0006886 GO:0016192 GO:0005198 GO:0030132
GO:0030130

Cluster-22178.65375 GO:0006364 GO:0003676 GO:0008168 GO:0003723

Cluster-22178.65376 GO:0006606 GO:0000921 GO:0006914 GO:0003774 GO:0005198 GO:0019867 GO:0016021 GO:0005940 GO:0005882 GO:0016459

Cluster-22178.65377 GO:0006364 GO:0008168 GO:0003723 GO:0003676

Cluster-22178.65379 GO:0045905 GO:0006452 GO:0006448 GO:0045901 GO:0003723 GO:0005198 GO:0003746 GO:0043022 GO:0019031 GO:0005840

Cluster-22178.65380 GO:0006144 GO:0006464 GO:0006206 GO:0006511 GO:0016567 GO:0007275 GO:0006351 GO:0009405 GO:0008270 GO:0004842 GO:0003677 GO:0046872 GO:0016151 GO:0003899 GO:0005198 GO:0005515 GO:0005680 GO:0005730 GO:0005634

Cluster-22178.65382 GO:0008152 GO:0016747

Cluster-22178.65383 GO:0055085 GO:0006810 GO:0016020 GO:0016021

Cluster-22178.65384 GO:0005515

Cluster-22178.65385 GO:0006308 GO:0009307 GO:0016787 GO:0000287 GO:0009036 GO:0003677 GO:0009359

Cluster-22178.65388 GO:0016020

Cluster-22178.65390 GO:0009399 GO:0006807 GO:0006265 GO:0055114 GO:0019337 GO:0016163 GO:0003677 GO:0003690 GO:0016610

Cluster-22178.65391 GO:0003676 GO:0003723

Cluster-22178.65392 GO:0046938 GO:0010038 GO:0016756 GO:0046872

Cluster-22178.65393 GO:0035023 GO:0043087 GO:0006810 GO:0006355 GO:0005089 GO:0003676 GO:0004386 GO:0043531 GO:0008270 GO:0005524 GO:0005515

Cluster-22178.65396 GO:0042254 GO:0006412 GO:0003735 GO:0003677 GO:0005096 GO:0008270 GO:0005524 GO:0046872 GO:0004222 GO:0005622 GO:0045202 GO:0016021 GO:0005840

Cluster-22178.65398 GO:0006814 GO:0006118 GO:0006744 GO:0006120 GO:0015992 GO:0008137 GO:0009055 GO:0016020

Cluster-22178.65399 GO:0008289 GO:0005515 GO:0005869
Cluster-22178.6540 GO:0007156 GO:0005509 GO:0016020
Cluster-22178.65400 GO:0005515
Cluster-22178.65403 GO:0016787
Cluster-22178.65405 GO:0016579 GO:0070140 GO:0032183 GO:0036459

Cluster-22178.65406 GO:0015979 GO:0019538 GO:0016567 GO:0009405
GO:0050765 GO:0008270 GO:0046872 GO:0005515 GO:0009523
GO:0009539 GO:0016020 GO:0016012 GO:0005576
Cluster-22178.65408 GO:0016579 GO:0036459
Cluster-22178.65409 GO:0007264 GO:0015684 GO:0003924 GO:0015093
GO:0005525 GO:0001527 GO:0016021
Cluster-22178.65413 GO:0006813 GO:0009405 GO:0006812 GO:0016051
GO:0005516 GO:0000166 GO:0016887 GO:0015269 GO:0046872
GO:0016840 GO:0043230 GO:0016021 GO:0009289
Cluster-22178.65415 GO:0006446 GO:0006313 GO:0006413 GO:0004803
GO:0003743 GO:0003677 GO:0003723 GO:0005840
Cluster-22178.65417 GO:0009432 GO:0051782 GO:0005515 GO:0005509
GO:0046983 GO:0009276
Cluster-22178.6542 GO:0007156 GO:0005509 GO:0016020
Cluster-22178.65420 GO:0008152 GO:0007165 GO:0007275 GO:0008270
GO:0047355 GO:0005179 GO:0016624 GO:0005576 GO:0016020
Cluster-22178.65423 GO:0006355 GO:0006098 GO:0019521 GO:0055114
GO:0050661 GO:0051287 GO:0004616 GO:0003676 GO:0005739
GO:0005622
Cluster-22178.65424 GO:0005975 GO:0004571 GO:0005509 GO:0016020
Cluster-22178.65425 GO:0055085 GO:0019087 GO:0016021
Cluster-22178.65426 GO:0015684 GO:0007264 GO:0005525 GO:0003924
GO:0005524 GO:0015093 GO:0016021
Cluster-22178.65427 GO:0051603 GO:0006511 GO:0004175 GO:0004298
GO:0008658 GO:0005839 GO:0019773
Cluster-22178.65428 GO:0030168 GO:0007165 GO:0007155 GO:0006415
GO:0008608 GO:0006352 GO:0051258 GO:0006449 GO:0005102

GO:0003677 GO:0030674 GO:0016149 GO:0005577 GO:0018444
GO:0005840 GO:0005669 GO:0005737 GO:0042729 GO:0019028
GO:0072686
Cluster-22178.65429 GO:0005515
Cluster-22178.65431 GO:0043093 GO:0006171 GO:0006810 GO:0002949
GO:0000917 GO:0009405 GO:0048870 GO:0016787 GO:0008294
GO:0003677 GO:0005524 GO:0016887 GO:0046983 GO:0003678
GO:0005657 GO:0005576 GO:0031514 GO:0005737
Cluster-22178.65432 GO:0001558 GO:0003677 GO:0005515 GO:0016787
GO:0003676 GO:0005524 GO:0005520 GO:0005576 GO:0016942
Cluster-22178.65433 GO:0006310 GO:0006355 GO:0006525 GO:0006281
GO:0003677 GO:0000166 GO:0009015 GO:0008168 GO:0046872
GO:0008270 GO:0005634
Cluster-22178.65434 GO:0006228 GO:0007155 GO:0006165 GO:0006183
GO:0006206 GO:0006144 GO:0006241 GO:0003723 GO:0005515
GO:0004550 GO:0003676
Cluster-22178.65435 GO:0003824
Cluster-22178.65436 GO:0042176 GO:0030234 GO:0000502
Cluster-22178.65437 GO:0009058 GO:0009113 GO:0007156 GO:0006144
GO:0051539 GO:0004637 GO:0005509 GO:0016742 GO:0046872
GO:0005524 GO:0016020
Cluster-22178.65438 GO:0007264 GO:0015684 GO:0015093 GO:0003924
GO:0005525 GO:0016021 GO:0001527
Cluster-22178.65440 GO:0006810 GO:0009401 GO:0005886
Cluster-22178.65441 GO:0009401 GO:0006810 GO:0005886
Cluster-22178.65442 GO:0046872
Cluster-22178.65445 GO:0006265 GO:0003677 GO:0003918 GO:0005694
Cluster-22178.65446 GO:0007156 GO:0005509 GO:0016020
Cluster-22178.65448 GO:0006281 GO:0016192 GO:0006886 GO:0043161
GO:0006289 GO:0003684 GO:0003676 GO:0030117
Cluster-22178.6545 GO:0006486 GO:0008373
Cluster-22178.65452 GO:0006886 GO:0006270 GO:0008536
Cluster-22178.65454 GO:0006270 GO:0006886 GO:0008536

Cluster-22178.65456 GO:0007010 GO:0006281 GO:0006289 GO:0043161 GO:0003684 GO:0003779
Cluster-22178.65457 GO:0006355 GO:0043565 GO:0003682 GO:0008270 GO:0003700 GO:0005667 GO:0000785
Cluster-22178.65459 GO:0005887
Cluster-22178.65460 GO:0006576 GO:0006355 GO:0006281 GO:0006310 GO:0006511 GO:0019079 GO:0002949 GO:0003723 GO:0008134 GO:0016301 GO:0043531 GO:0003678 GO:0016787 GO:0009378 GO:0003677 GO:0016887 GO:0005524 GO:0003724 GO:0005667 GO:0009379 GO:0005657
Cluster-22178.65461 GO:0008152 GO:0016746 GO:0005509
Cluster-22178.65462 GO:0048280 GO:0016192 GO:0006886 GO:0005515 GO:0000139 GO:0030117 GO:0005737
Cluster-22178.65464 GO:0000746 GO:0019058 GO:0005975 GO:0006260 GO:0003677 GO:0003887 GO:0004553 GO:0042575 GO:0019867
Cluster-22178.65465 GO:0005975 GO:0006260 GO:0000746 GO:0019058 GO:0004553 GO:0003887 GO:0003677 GO:0019867 GO:0042575
Cluster-22178.65468 GO:0031124 GO:0005525
Cluster-22178.65470 GO:0006418 GO:0000166 GO:0004812 GO:0005524

Cluster-22178.65471 GO:0006508 GO:0006468 GO:0006144 GO:0004252 GO:0017111 GO:0004197 GO:0016773 GO:0004672 GO:0005524 GO:0003968 GO:0016020 GO:0031379
Cluster-22178.65472 GO:0005515
Cluster-22178.65473 GO:0005509 GO:0008168
Cluster-22178.65474 GO:0046872 GO:0003677
Cluster-22178.65477 GO:0001510 GO:0031167 GO:0009451 GO:0032775 GO:0006306 GO:0008033 GO:0006396 GO:0009452 GO:0003723 GO:0008173 GO:0035299 GO:0008168 GO:0009007 GO:0005524 GO:0004809
Cluster-22178.65478 GO:0004064 GO:0005515
Cluster-22178.65479 GO:0003676 GO:0005886
Cluster-22178.65480 GO:0005515 GO:0005739 GO:0005576

Cluster-22178.65481 GO:0006364 GO:0032040
Cluster-22178.65482 GO:0009755 GO:0006364 GO:0008152 GO:0016758
GO:0005515 GO:0005576 GO:0032040
Cluster-22178.65486 GO:0005515
Cluster-22178.65493 GO:0006355 GO:0005515 GO:0005634
Cluster-22178.65495 GO:0006402 GO:0019722 GO:0051252 GO:0006817
GO:0044341 GO:0003676 GO:0015321 GO:0008270 GO:0046872
GO:0004535 GO:0003723 GO:0005737 GO:0016020 GO:0005634
Cluster-22178.65496 GO:0006511 GO:0031625
Cluster-22178.65497 GO:0046938 GO:0007049 GO:0010038 GO:0016756
GO:0005515 GO:0046872 GO:0017053
Cluster-22178.65498 GO:0000184
Cluster-22178.65499 GO:0015031 GO:0043248 GO:0006606 GO:0015991
GO:0016192 GO:0006886 GO:0008565 GO:0000166 GO:0016820
GO:0005515 GO:0005737 GO:0000221 GO:0030117 GO:0005634
Cluster-22178.65500 GO:0008283 GO:0040007 GO:0007165 GO:0008083

Cluster-22178.65501 GO:0006396 GO:0003723
Cluster-22178.65502 GO:0042254 GO:0006412 GO:0003735 GO:0005840
Cluster-22178.65504 GO:0007165 GO:0005509 GO:0005578
Cluster-22178.65506 GO:0015991 GO:0042742 GO:0015031 GO:0006606
GO:0016820 GO:0005515 GO:0008565 GO:0005634 GO:0005737
GO:0000221
Cluster-22178.65507 GO:0006886 GO:0006270 GO:0008536
Cluster-22178.65509 GO:0055114 GO:0045454 GO:0016972
Cluster-22178.65511 GO:0009298 GO:0006013 GO:0006000 GO:0004615
GO:0005737
Cluster-22178.65513 GO:0000045 GO:0005737
Cluster-22178.65514 GO:0006812 GO:0006820 GO:0006835 GO:0017153
GO:0016020
Cluster-22178.65515 GO:0016192 GO:0051090 GO:0006886 GO:0042742
GO:0009069 GO:0016310 GO:0005515 GO:0004674 GO:0005634
GO:0030117

Cluster-22178.65518 GO:0006810 GO:0005215 GO:0016020
Cluster-22178.65520 GO:0006554 GO:0006633 GO:0016117 GO:0018874
GO:0006631 GO:0006574 GO:0055114 GO:0006090 GO:0008152
GO:0006813 GO:0006099 GO:0006568 GO:0006550 GO:0006552
GO:0016491 GO:0016620 GO:0016746 GO:0071949 GO:0008924
GO:0003857 GO:0016705 GO:0016021
Cluster-22178.65523 GO:0006457 GO:0009058 GO:0008299 GO:0017004
GO:0006531 GO:0006108 GO:0006118 GO:0006099 GO:0006522
GO:0006529 GO:0006694 GO:0006090 GO:0055114 GO:0015976
GO:0004066 GO:0004471 GO:0016779 GO:0051082 GO:0045158
GO:0050518 GO:0009512 GO:0016272
Cluster-22178.65524 GO:0006810 GO:0016021
Cluster-22178.65525 GO:0055114 GO:0006915 GO:0006629 GO:0032465
GO:0009252 GO:0033014 GO:0006511 GO:0016567 GO:0003924
GO:0003676 GO:0003723 GO:0050661 GO:0008883 GO:0008963
GO:0004842 GO:0016021
Cluster-22178.65528 GO:0055114 GO:0016616
Cluster-22178.65531 GO:0006351 GO:0006508 GO:0019083 GO:0006206
GO:0006144 GO:0003677 GO:0005515 GO:0003899 GO:0003723
GO:0008234 GO:0005730
Cluster-22178.65532 GO:0009058 GO:0006520 GO:0019752 GO:0016740
GO:0030170 GO:0016829 GO:0016831 GO:0019012
Cluster-22178.65533 GO:0006955 GO:0006457 GO:0007165 GO:0005164
GO:0016020
Cluster-22178.65534 GO:0005515
Cluster-22178.65535 GO:0003676
Cluster-22178.65537 GO:0015992 GO:0006123 GO:0004129 GO:0045277
Cluster-22178.65540 GO:0002949 GO:0007049 GO:0006310 GO:0019079
GO:0006281 GO:0003724 GO:0003678 GO:0016887 GO:0005524
GO:0003723 GO:0009378 GO:0005657 GO:0009379
Cluster-22178.65543 GO:0015992 GO:0007275 GO:0006123 GO:0030154
GO:0004129 GO:0045277
Cluster-22178.65544 GO:0000226 GO:0007264 GO:0006576 GO:0005525

GO:0005524 GO:0015630 GO:0016020
Cluster-22178.65545 GO:0006520 GO:0055114 GO:0008152 GO:0016491

Cluster-22178.65547 GO:0006260 GO:0005524 GO:0003677
Cluster-22178.65549 GO:0055114 GO:0051920 GO:0016491 GO:0016209

Cluster-22178.65550 GO:0006570 GO:0006810 GO:0009405 GO:0006952
GO:0006470 GO:0008138 GO:0046983 GO:0019855 GO:0004725
GO:0005576
Cluster-22178.65551 GO:0016310 GO:0044237 GO:0006886 GO:0006397
GO:0003924 GO:0016772 GO:0005525 GO:0005515 GO:0005047
GO:0005786 GO:0005785 GO:0005634
Cluster-22178.65552 GO:0005886 GO:0016021
Cluster-22178.65553 GO:0006352 GO:0003677 GO:0005669
Cluster-22178.65555 GO:0006574 GO:0042967 GO:0006633 GO:0006084
GO:0006552 GO:0008299 GO:0046950 GO:0006550 GO:0004421
GO:0004315 GO:0005835
Cluster-22178.65556 GO:0019843 GO:0003723 GO:0005622
Cluster-22178.65558 GO:0032297 GO:0003677
Cluster-22178.65559 GO:0005975 GO:0042254 GO:0006412 GO:0004553
GO:0003735 GO:0042597 GO:0005840
Cluster-22178.65560 GO:0009451 GO:0001522 GO:0009982 GO:0003723
GO:0016021 GO:0016012
Cluster-22178.65565 GO:0008283 GO:0040007 GO:0007165 GO:0016567
GO:0006464 GO:0015031 GO:0055114 GO:0009245 GO:0016616
GO:0008083 GO:0008915 GO:0016491 GO:0004842 GO:0005576
GO:0019028
Cluster-22178.65566 GO:0016567 GO:0007165 GO:0040007 GO:0008283
GO:0009245 GO:0055114 GO:0006464 GO:0015031 GO:0008083
GO:0016616 GO:0004842 GO:0016491 GO:0008915 GO:0019028
GO:0005576
Cluster-22178.65568 GO:0006694 GO:0008168
Cluster-22178.65571 GO:0007349 GO:0016020 GO:0005737

Cluster-22178.65572 GO:0006771 GO:0051301 GO:0019497 GO:0007049
GO:0005515 GO:0043565 GO:0003993 GO:0005634 GO:0016021
Cluster-22178.65573 GO:0007165 GO:0005158 GO:0004252 GO:0005886
GO:0005899
Cluster-22178.65574 GO:0042254 GO:0006412 GO:0003735 GO:0005840
Cluster-22178.65577 GO:0019902 GO:0005515
Cluster-22178.65578 GO:0030154 GO:0006418 GO:0000166 GO:0004812
GO:0005524 GO:0016021
Cluster-22178.65580 GO:0006811 GO:0005515 GO:0016020
Cluster-22178.65581 GO:0045454 GO:0055114 GO:0009306 GO:0005509
GO:0008270 GO:0016209 GO:0016491 GO:0005634 GO:0005788
Cluster-22178.65583 GO:0006508 GO:0004177 GO:0005622
Cluster-22178.65585 GO:0008152 GO:0016758
Cluster-22178.65586 GO:0031072 GO:0051082
Cluster-22178.65588 GO:0006144 GO:0006206 GO:0006351 GO:0006508
GO:0003899 GO:0008236 GO:0003677 GO:0016787 GO:0005730
Cluster-22178.65589 GO:0006396 GO:0005515 GO:0005622
Cluster-22178.65593 GO:0006811 GO:0006351 GO:0006260 GO:0006367
GO:0032968 GO:0006816 GO:0003676 GO:0005262 GO:0003887
GO:0008270 GO:0003677 GO:0005244 GO:0032040 GO:0016021
GO:0042575 GO:0005634
Cluster-22178.65596 GO:0005525 GO:0046914 GO:0046983 GO:0003924

Cluster-22178.65597 GO:0034061 GO:0003677 GO:0016779 GO:0005634
Cluster-22178.65598 GO:0055085 GO:0006260 GO:0006914 GO:0070206
GO:0015992 GO:0007165 GO:0007049 GO:0006308 GO:0051382
GO:0015991 GO:0030001 GO:0008855 GO:0046873 GO:0004871
GO:0015078 GO:0005198 GO:0016020 GO:0033179 GO:0009318
GO:0000776
Cluster-22178.65599 GO:0009165 GO:0006144 GO:0006098 GO:0009116
GO:0000287 GO:0004749
Cluster-22178.65600 GO:0015991 GO:0006886 GO:0016192 GO:0005515
GO:0016820 GO:0030117 GO:0000221

Cluster-22178.65603 GO:0007275 GO:0007165 GO:0008152 GO:0005179 GO:0016624 GO:0047355 GO:0008270 GO:0005576 GO:0016020
Cluster-22178.65604 GO:0006886 GO:0016192 GO:0008152 GO:0003824 GO:0005515 GO:0030117
Cluster-22178.65605 GO:0006629 GO:0008081
Cluster-22178.65606 GO:0004518
Cluster-22178.65608 GO:0000398 GO:0030623 GO:0005515 GO:0008270 GO:0017070 GO:0003723
Cluster-22178.65609 GO:0006471 GO:0008270 GO:0003950 GO:0003677 GO:0005634
Cluster-22178.65610 GO:0009405 GO:0006355 GO:0003723 GO:0003700 GO:0031423 GO:0008270 GO:0043565 GO:0019012 GO:0005667 GO:0005634
Cluster-22178.65611 GO:0005515 GO:0003924
Cluster-22178.65613 GO:0006357 GO:0006446 GO:0003743 GO:0005515 GO:0001104 GO:0016021 GO:0005840 GO:0005737 GO:0005852 GO:0016592
Cluster-22178.65614 GO:0009058 GO:0045454 GO:0006520 GO:0055114 GO:0005509 GO:0030170 GO:0016829 GO:0016209 GO:0016491 GO:0032040
Cluster-22178.65616 GO:0006094 GO:0005525 GO:0004611
Cluster-22178.65622 GO:0003779 GO:0005622
Cluster-22178.65623 GO:0008270 GO:0046872 GO:0003676
Cluster-22178.65624 GO:0006351 GO:0006270 GO:0009236 GO:0006260 GO:0005515 GO:0003887 GO:0003677 GO:0008270 GO:0005634 GO:0042575 GO:0005789 GO:0032040
Cluster-22178.65626 GO:0006468 GO:0016773 GO:0004672 GO:0008270 GO:0005524 GO:0003676 GO:0005515 GO:0016020
Cluster-22178.65628 GO:0005515
Cluster-22178.65629 GO:0003723
Cluster-22178.65631 GO:0006355 GO:0051087
Cluster-22178.65633 GO:0007165 GO:0019058 GO:0042254 GO:0006412 GO:0008152 GO:0046789 GO:0003735 GO:0016758 GO:0005840

GO:0019031 GO:0009986
Cluster-22178.65635 GO:0007165 GO:0051258 GO:0030168 GO:0005975
GO:0006914 GO:0030674 GO:0005102 GO:0004553 GO:0030248
GO:0005576 GO:0005577
Cluster-22178.65637 GO:0006811 GO:0005216 GO:0046872 GO:0016020
Cluster-22178.65639 GO:0030474 GO:0071988 GO:0006270
Cluster-22178.65640 GO:0005515 GO:0003677 GO:0046872 GO:0008270

Cluster-22178.65642 GO:0040007 GO:0008283 GO:0007165 GO:0008083

Cluster-22178.65644 GO:0006012 GO:0005975 GO:0018149 GO:0046486
GO:0006687 GO:0006122 GO:0006027 GO:0004565 GO:0008270
GO:0046872 GO:0005743 GO:0005750 GO:0009341
Cluster-22178.65648 GO:0003824
Cluster-22178.65649 GO:0016773
Cluster-22178.65651 GO:0010508 GO:0015858 GO:0006810 GO:0019076
GO:0032801 GO:0006812 GO:0007049 GO:0016787 GO:0005261
GO:0005337 GO:0016020 GO:0033644 GO:0016021
Cluster-22178.65652 GO:0009250 GO:0003676 GO:0046527
Cluster-22178.65653 GO:0001522 GO:0009451 GO:0003723 GO:0016787
GO:0008270 GO:0046872 GO:0009982 GO:0003676 GO:0005634
GO:0019031
Cluster-22178.65656 GO:0006810 GO:0006465 GO:0008233 GO:0005622
GO:0005787 GO:0016021
Cluster-22178.65657 GO:0042254 GO:0006412 GO:0003735 GO:0005840
GO:0005622
Cluster-22178.65658 GO:0055114 GO:0006508 GO:0015947 GO:0016192
GO:0006886 GO:0030058 GO:0070008 GO:0005515 GO:0004252
GO:0005198 GO:0042597 GO:0030117
Cluster-22178.65659 GO:0006352 GO:0003677 GO:0005669
Cluster-22178.65660 GO:0006106 GO:0016020
Cluster-22178.65661 GO:0006468 GO:0016773 GO:0005524 GO:0004672
GO:0016020

Cluster-22178.65663 GO:0006355 GO:0005982 GO:0005985 GO:0005978
GO:0004373 GO:0005634
Cluster-22178.65664 GO:0009058 GO:0006260 GO:0006269 GO:0018142
GO:0003723 GO:0004672
Cluster-22178.65665 GO:0006355 GO:0006139 GO:0006260 GO:0006914
GO:0005515 GO:0003700 GO:0003676 GO:0043565 GO:0008026
GO:0016818 GO:0003677 GO:0004003 GO:0016787 GO:0005524
GO:0005634 GO:0005667 GO:0005657
Cluster-22178.65666 GO:0008152 GO:0003824 GO:0005524
Cluster-22178.65668 GO:0006334 GO:0006810 GO:0003676 GO:0005634
GO:0005643
Cluster-22178.65669 GO:0006468 GO:0005524 GO:0004672
Cluster-22178.65670 GO:0045454 GO:0009451 GO:0006118 GO:0055114
GO:0006396 GO:0003723 GO:0008173 GO:0016209 GO:0016491
GO:0015035 GO:0009055
Cluster-22178.65673 GO:0042262 GO:0030001 GO:0008053 GO:0031412
GO:0055085 GO:0006073 GO:0003924 GO:0008289 GO:0005198
GO:0016762 GO:0003690 GO:0046873 GO:0016021 GO:0019031
GO:0019028 GO:0005618 GO:0048046 GO:0016020 GO:0005741
Cluster-22178.65675 GO:0042967 GO:0008270 GO:0047961 GO:0003677
GO:0005739
Cluster-22178.65679 GO:0005515
Cluster-22178.65680 GO:0016485 GO:0016021
Cluster-22178.65681 GO:0016020
Cluster-22178.65684 GO:0003723 GO:0003824
Cluster-22178.65685 GO:0046677 GO:0006811 GO:0016020
Cluster-22178.65689 GO:0006508 GO:0006281 GO:0006308 GO:0006260
GO:0008236 GO:0004252 GO:0046872 GO:0008270 GO:0017108
GO:0005634 GO:0033557 GO:0005618
Cluster-22178.65692 GO:0008152 GO:0016791
Cluster-22178.65693 GO:0005515 GO:0016021
Cluster-22178.65695 GO:0006355 GO:0006810 GO:0003677 GO:0005215
GO:0005634 GO:0016020

Cluster-22178.65696 GO:0006613 GO:0007165 GO:0006465 GO:0004872 GO:0008233 GO:0005787 GO:0016021 GO:0030176 GO:0005784
Cluster-22178.65697 GO:0003824
Cluster-22178.65698 GO:0030154 GO:0007219 GO:0006351 GO:0007275 GO:0005515 GO:0016788 GO:0003677 GO:0016021 GO:0005634
Cluster-22178.65699 GO:0007283 GO:0003677 GO:0000786 GO:0005634
Cluster-22178.65701 GO:0006544 GO:0006352 GO:0055114 GO:0006520 GO:0006546 GO:0006563 GO:0006566 GO:0016829 GO:0004375 GO:0003677
Cluster-22178.65702 GO:0000910 GO:0008033 GO:0000226 GO:0005524 GO:0008017 GO:0016879 GO:0000166 GO:0005737 GO:0045298
Cluster-22178.65705 GO:0006364 GO:0006206 GO:0006144 GO:0006351 GO:0006355 GO:0003677 GO:0005524 GO:0016773 GO:0003899 GO:0003700 GO:0046872 GO:0016020 GO:0005732 GO:0005730 GO:0005667 GO:0034457 GO:0005634 GO:0019028
Cluster-22178.65706 GO:0006355 GO:0003677 GO:0003682 GO:0000775 GO:0000785 GO:0005634
Cluster-22178.65707 GO:0007264 GO:0007165 GO:0005515 GO:0005525

Cluster-22178.65708 GO:0000917 GO:0043093 GO:0006355 GO:0003700 GO:0043565 GO:0003677 GO:0005737 GO:0005634 GO:0005667
Cluster-22178.65709 GO:0045454
Cluster-22178.65710 GO:0006418 GO:0006886 GO:0004812 GO:0005524 GO:0002161 GO:0000166 GO:0005741
Cluster-22178.65711 GO:0006810 GO:0009405 GO:0006418 GO:0019871 GO:0000166 GO:0004812 GO:0005524 GO:0005576
Cluster-22178.65712 GO:0005509 GO:0005515
Cluster-22178.65713 GO:0007165 GO:0006468 GO:0009069 GO:0035556 GO:0016310 GO:0007178 GO:0004675 GO:0005515 GO:0005524 GO:0016020 GO:0016012
Cluster-22178.65714 GO:0006464 GO:0015031 GO:0005515
Cluster-22178.65717 GO:0007017 GO:0007018 GO:0006357 GO:0001104 GO:0005198 GO:0005524 GO:0008017 GO:0003777 GO:0019028

GO:0016592 GO:0045298 GO:0005874 GO:0019031
Cluster-22178.65720 GO:0007165 GO:0035556 GO:0003774 GO:0005524
GO:0005515 GO:0016459
Cluster-22178.65722 GO:0050790 GO:0030234
Cluster-22178.65723 GO:0005515 GO:0005509
Cluster-22178.65725 GO:0006446 GO:0045892 GO:0006914 GO:0006413
GO:0003743 GO:0005840 GO:0005634
Cluster-22178.65727 GO:0007165 GO:0006479 GO:0006481 GO:0042967
GO:0004671 GO:0008080 GO:0016747 GO:0016021
Cluster-22178.65728 GO:0006413 GO:0006446 GO:0019079 GO:0016787
GO:0003743 GO:0003677 GO:0008026 GO:0005524 GO:0003676
GO:0005840
Cluster-22178.65729 GO:0015992 GO:0006119 GO:0006118 GO:0006891
GO:0008121 GO:0009055 GO:0030173
Cluster-22178.65731 GO:0006281 GO:0051382
Cluster-22178.65736 GO:0055114 GO:0045454 GO:0016209 GO:0016491

Cluster-22178.65740 GO:0003723 GO:0005515 GO:0003676
Cluster-22178.65742 GO:0007015 GO:0003785
Cluster-22178.65743 GO:0006464 GO:0015031 GO:0030154 GO:0007275
GO:0005515
Cluster-22178.65744 GO:0005634
Cluster-22178.65749 GO:0006629 GO:0006508 GO:0016787 GO:0004252

Cluster-22178.6575 GO:0005515 GO:0005509 GO:0004190 GO:0016021
Cluster-22178.65751 GO:0015031 GO:0008375 GO:0016021 GO:0016020
Cluster-22178.65752 GO:0006810 GO:0006355 GO:0003677 GO:0003713
GO:0051537 GO:0043231 GO:0005667 GO:0005634 GO:0005886
Cluster-22178.65754 GO:0006576 GO:0007264 GO:0008201 GO:0005525
GO:0003924 GO:0005524 GO:0016021
Cluster-22178.65756 GO:0006355 GO:0008270 GO:0003676 GO:0003700
GO:0005667 GO:0005634
Cluster-22178.65758 GO:0007186 GO:0004930 GO:0016021 GO:0016020

Cluster-22178.65759 GO:0016570 GO:0006368 GO:0003779 GO:0016593
GO:0005622
Cluster-22178.65762 GO:0006355 GO:0006468 GO:0019497 GO:0006771
GO:0048013 GO:0005515 GO:0003700 GO:0046872 GO:0004672
GO:0043565 GO:0003993 GO:0005524 GO:0008270 GO:0016773
GO:0005634 GO:0016020 GO:0005667
Cluster-22178.65766 GO:0055085 GO:0006810 GO:0006506 GO:0006468
GO:0004386 GO:0005215 GO:0016773 GO:0005524 GO:0004672
GO:0005515 GO:0005789 GO:0031083 GO:0016020
Cluster-22178.65768 GO:0006955 GO:0007165 GO:0006606 GO:0030247
GO:0005044
Cluster-22178.65773 GO:0009405 GO:0006355 GO:0003700 GO:0043565
GO:0005515 GO:0051539 GO:0004867 GO:0005667
Cluster-22178.65774 GO:0007165 GO:0048870 GO:0005515 GO:0031514
Cluster-22178.65776 GO:0006412 GO:0042254 GO:0003735 GO:0005840
Cluster-22178.65777 GO:0009252 GO:0055114 GO:0046436 GO:0006814
GO:0006118 GO:0003824 GO:0016655 GO:0046872 GO:0005524
GO:0008716
Cluster-22178.65778 GO:0006367 GO:0005524 GO:0003682 GO:0000785
GO:0005672
Cluster-22178.65779 GO:0007165 GO:0016567 GO:0006260 GO:0016818
GO:0003677 GO:0005515 GO:0005179 GO:0004842 GO:0008270
GO:0005524 GO:0046872 GO:0005576 GO:0005634 GO:0005680
Cluster-22178.65781 GO:0005515
Cluster-22178.65785 GO:0007154 GO:0007607 GO:0007187 GO:0050912
GO:0005515 GO:0005509 GO:0008527 GO:0016021 GO:0016020
Cluster-22178.65786 GO:0006508 GO:0006486 GO:0004576 GO:0003676
GO:0004222 GO:0003677 GO:0008250 GO:0016020
Cluster-22178.65789 GO:0006886 GO:0006888 GO:0008270 GO:0030127
Cluster-22178.65790 GO:0006270 GO:0033014 GO:0006629 GO:0055114
GO:0050661 GO:0003723 GO:0003676 GO:0008883
Cluster-22178.65791 GO:0048870 GO:0015031 GO:0010508 GO:0006904
GO:0005515 GO:0005509 GO:0000145 GO:0031514

Cluster-22178.65793 GO:0006367 GO:0005524 GO:0003682 GO:0005672
GO:0000785
Cluster-22178.65794 GO:0006555 GO:0006556 GO:0006508 GO:0008234
GO:0004478
Cluster-22178.65796 GO:0006810 GO:0016787 GO:0005524 GO:0042626
GO:0016021
Cluster-22178.65797 GO:0006813 GO:0008152 GO:0009306 GO:0055114
GO:0009089 GO:0009085 GO:0015031 GO:0003824 GO:0015450
GO:0008839 GO:0051287 GO:0050662 GO:0016620 GO:0016491
GO:0016021 GO:0009941
Cluster-22178.65798 GO:0007165 GO:0016567 GO:0008283 GO:0040007
GO:0004842 GO:0008083 GO:0005576
Cluster-22178.65799 GO:0008152 GO:0016491
Cluster-22178.65802 GO:0000245 GO:0005515 GO:0005634
Cluster-22178.65803 GO:0006118 GO:0015992 GO:0055114 GO:0009060
GO:0006123 GO:0005506 GO:0020037 GO:0009055 GO:0004129
GO:0045277 GO:0016021
Cluster-22178.65804 GO:0006359
Cluster-22178.65805 GO:0006355 GO:0042254 GO:0006412 GO:0043565
GO:0008270 GO:0003700 GO:0003735 GO:0005840 GO:0005667
Cluster-22178.65806 GO:0005515
Cluster-22178.65807 GO:0055114
Cluster-22178.65813 GO:0008270 GO:0046872 GO:0005515
Cluster-22178.65815 GO:0030001 GO:0055085 GO:0046873 GO:0016020
Cluster-22178.65816 GO:0006570 GO:0006470 GO:0000087 GO:0004725
GO:0005622
Cluster-22178.65817 GO:0006355 GO:0055085 GO:0003700 GO:0022857
GO:0005667 GO:0016021
Cluster-22178.65819 GO:0016567 GO:0003824
Cluster-22178.65820 GO:0006810 GO:0003676 GO:0005622
Cluster-22178.65821 GO:0006412 GO:0042254 GO:0006355 GO:0003700
GO:0043565 GO:0003735 GO:0005840 GO:0005622 GO:0005667
Cluster-22178.65825 GO:0001522 GO:0007596 GO:0042254

Cluster-22178.65829 GO:0016817 GO:0005789 GO:0016021
Cluster-22178.65830 GO:0000398
Cluster-22178.65832 GO:0003824 GO:0004866 GO:0005576 GO:0005615
Cluster-22178.65833 GO:0006465 GO:0008270 GO:0046872 GO:0008233
GO:0005787 GO:0016021
Cluster-22178.65834 GO:0006355 GO:0042710 GO:0017148 GO:0009372
GO:0005975 GO:0042967 GO:0004402 GO:0016787 GO:0008270
GO:0003824 GO:0003712 GO:0005667 GO:0005634 GO:0000123
Cluster-22178.65835 GO:0016567 GO:0004842 GO:0046872 GO:0008270
GO:0005515 GO:0061630 GO:0005680 GO:0005622
Cluster-22178.65836 GO:0055114 GO:0016491
Cluster-22178.65837 GO:0016567 GO:0004842
Cluster-22178.65839 GO:0006412 GO:0042254 GO:0003735 GO:0005622
GO:0005840
Cluster-22178.65840 GO:0043066 GO:0008207 GO:0008209 GO:0019079
GO:0006694 GO:0055114 GO:0043154 GO:0008210 GO:0016620
GO:0080019 GO:0043130 GO:0016616 GO:0032266 GO:0050662
GO:0051287 GO:0003854 GO:0003824 GO:0071986 GO:0005737
Cluster-22178.65846 GO:0006281 GO:0006260 GO:0016787 GO:0003677
GO:0005524 GO:0043140 GO:0003676 GO:0005657
Cluster-22178.65848 GO:0070072 GO:0043087 GO:0032012 GO:0005086

Cluster-22178.65849 GO:0055114 GO:0000051 GO:0008652 GO:0046983
GO:0005525 GO:0016620 GO:0003942 GO:0005737
Cluster-22178.65851 GO:0006850 GO:0005743
Cluster-22178.65852 GO:0006725 GO:0016853
Cluster-22178.65853 GO:0006725 GO:0016853
Cluster-22178.65854 GO:0006694 GO:0008152 GO:0055114 GO:0008207
GO:0008209 GO:0008210 GO:0003824 GO:0016491 GO:0016616
GO:0003854 GO:0050662
Cluster-22178.65855 GO:0001514 GO:0006355 GO:0006351 GO:0006464
GO:0006448 GO:0006260 GO:0006269 GO:0005525 GO:0003896
GO:0016151 GO:0003723 GO:0008134 GO:0016887 GO:0003746

GO:0005524 GO:0003677 GO:0005737 GO:0005840 GO:0005657
GO:0005667 GO:0005730
Cluster-22178.65856 GO:0035988 GO:0006310 GO:0006260 GO:0002949
GO:0006281 GO:0006629 GO:0006355 GO:0006576 GO:0042246
GO:0002062 GO:0009378 GO:0016787 GO:0003677 GO:0005524
GO:0016887 GO:0016301 GO:0008134 GO:0003924 GO:0005525
GO:0003678 GO:0009379 GO:0005667 GO:0005657 GO:0005634
Cluster-22178.65857 GO:0009069 GO:0006468 GO:0006413 GO:0006446
GO:0007178 GO:0016310 GO:0004675 GO:0003743 GO:0005524
GO:0005576 GO:0016020 GO:0005840 GO:0016021
Cluster-22178.65858 GO:0016567 GO:0006915 GO:0032465 GO:0004842
GO:0003676
Cluster-22178.65859 GO:0051258 GO:0006308 GO:0015991 GO:0009405
GO:0010508 GO:0015992 GO:0030168 GO:0006914 GO:0006357
GO:0006606 GO:0008053 GO:0007165 GO:0003924 GO:0015078
GO:0051082 GO:0001104 GO:0008855 GO:0005102 GO:0030674
GO:0016021 GO:0009318 GO:0033179 GO:0005577 GO:0005923
GO:0005741 GO:0016592 GO:0016020
Cluster-22178.65860 GO:0008643 GO:0055085 GO:0005351 GO:0000139
GO:0016021 GO:0016020
Cluster-22178.65861 GO:0055114 GO:0003676 GO:0005515 GO:0005634
Cluster-22178.65862 GO:0006914 GO:0019497 GO:0006810 GO:0006771
GO:0003993 GO:0046872 GO:0005515 GO:0005794
Cluster-22178.65864 GO:0006629
Cluster-22178.65865 GO:0003676 GO:0005524
Cluster-22178.65866 GO:0006508 GO:0008152 GO:0016836 GO:0003824
GO:0008233
Cluster-22178.65870 GO:0005739
Cluster-22178.65871 GO:0015992 GO:0006811 GO:0006355 GO:0045454
GO:0015991 GO:0006118 GO:0046872 GO:0009055 GO:0015078
GO:0015035 GO:0003677 GO:0005886 GO:0019031 GO:0016020
GO:0033179
Cluster-22178.65872 GO:0005515

Cluster-22178.65873 GO:0030001 GO:0006355 GO:0009451 GO:0055114 GO:0006352 GO:0006270 GO:0006396 GO:0055085 GO:0003723 GO:0008173 GO:0046873 GO:0003677 GO:0016491 GO:0008270 GO:0005634 GO:0016020
Cluster-22178.65875 GO:0007165 GO:0000772 GO:0046872 GO:0016020
Cluster-22178.65876 GO:0005525 GO:0031012
Cluster-22178.65878 GO:0005525 GO:0031012
Cluster-22178.65879 GO:0005975 GO:0006030 GO:0008061 GO:0004553 GO:0005576
Cluster-22178.65880 GO:0046872 GO:0003950 GO:0005634
Cluster-22178.65881 GO:0007186 GO:0004930
Cluster-22178.65882 GO:0007131 GO:0051598 GO:0005198 GO:0005634
Cluster-22178.65884 GO:0003677 GO:0004386 GO:0005524
Cluster-22178.65890 GO:0016032 GO:0006508 GO:0004197
Cluster-22178.65893 GO:0005975 GO:0003723 GO:0004553
Cluster-22178.65895 GO:0006614 GO:0008312 GO:0005515 GO:0048500
Cluster-22178.65896 GO:1901031 GO:0005634
Cluster-22178.65897 GO:0006508 GO:0044419 GO:0006144 GO:0017111 GO:0004197 GO:0003968 GO:0019028 GO:0031379
Cluster-22178.65898 GO:0016491
Cluster-22178.65899 GO:0007186 GO:0007165 GO:0007264 GO:0004871 GO:0005525 GO:0003924 GO:0031683 GO:0019001
Cluster-22178.65901 GO:0005515
Cluster-22178.65902 GO:0005201 GO:0005578 GO:0005581
Cluster-22178.65904 GO:0030153 GO:0006355 GO:0006955 GO:0015643 GO:0003677 GO:0016021 GO:0019814
Cluster-22178.65906 GO:0006810 GO:0034227 GO:0002098 GO:0042626 GO:0000049 GO:0005524 GO:0016021
Cluster-22178.65908 GO:0006886 GO:0030001 GO:0051258 GO:0007165 GO:0055085 GO:0006891 GO:0000921 GO:0030168 GO:0008375 GO:0005198 GO:0005515 GO:0005507 GO:0004871 GO:0030674 GO:0046873 GO:0005102 GO:0019028 GO:0005801 GO:0016021 GO:0019031 GO:0016020 GO:0017119 GO:0005577 GO:0005940

Cluster-22178.65909 GO:0006468 GO:0004672 GO:0005524
Cluster-22178.65911 GO:0016829 GO:0016020
Cluster-22178.65913 GO:0006554 GO:0006479 GO:0046872 GO:0018024

Cluster-22178.65914 GO:0006508 GO:0006355 GO:0003682 GO:0003677 GO:0008270 GO:0046872 GO:0003676 GO:0004222 GO:0005634 GO:0000785 GO:0000775
Cluster-22178.65918 GO:0007165 GO:0005102
Cluster-22178.65919 GO:0051252 GO:0016891
Cluster-22178.65920 GO:0006952 GO:0007165 GO:0005515
Cluster-22178.65921 GO:0016021 GO:0016020
Cluster-22178.65923 GO:0006974 GO:0051726 GO:0003677
Cluster-22178.65925 GO:0018342 GO:0006270 GO:0006397 GO:0006886 GO:0006811 GO:0006396 GO:0008318 GO:0005515 GO:0005634 GO:0016020 GO:0005622 GO:0005789
Cluster-22178.65928 GO:0006952 GO:0006810 GO:0009405 GO:0019855 GO:0005576 GO:0005634
Cluster-22178.65929 GO:0016757
Cluster-22178.65930 GO:0007010 GO:0005515 GO:0016817 GO:0003779

Cluster-22178.65931 GO:0019048 GO:0006355
Cluster-22178.65934 GO:0016817
Cluster-22178.65936 GO:0003677
Cluster-22178.65938 GO:0006355 GO:0005524 GO:0008134 GO:0005634 GO:0005667
Cluster-22178.65940 GO:0015991 GO:0015992 GO:0046034 GO:0016820 GO:0005524 GO:0003924 GO:0043531 GO:0016887 GO:0005525 GO:0033178
Cluster-22178.65944 GO:0009085 GO:0009089 GO:0015031 GO:0006813 GO:0055114 GO:0009306 GO:0008152 GO:0050662 GO:0051287 GO:0016491 GO:0016620 GO:0015450 GO:0008839 GO:0003824 GO:0009941 GO:0016021
Cluster-22178.65945 GO:0007264 GO:0035556 GO:0005525

Cluster-22178.65946 GO:0009405 GO:0006810 GO:0006952 GO:0006334
GO:0019855 GO:0005576 GO:0005634
Cluster-22178.65947 GO:0006118 GO:0017004 GO:0015684 GO:0016818
GO:0003924 GO:0015093 GO:0003724 GO:0045158 GO:0003712
GO:0005525 GO:0005634 GO:0005667 GO:0009512 GO:0016021
Cluster-22178.65950 GO:0009405 GO:0006810 GO:0006952 GO:0006334
GO:0019855 GO:0005634 GO:0005576
Cluster-22178.65951 GO:0005515 GO:0005509
Cluster-22178.65952 GO:0005515
Cluster-22178.65954 GO:0019645 GO:0016560 GO:0009405 GO:0006810
GO:0008200 GO:0005515 GO:0005777 GO:0016021 GO:0005576
Cluster-22178.65955 GO:0046008 GO:0007165 GO:0005179 GO:0005576
Cluster-22178.65956 GO:0015031 GO:0016020
Cluster-22178.65957 GO:0003824
Cluster-22178.65959 GO:0055114 GO:0009308 GO:0006886 GO:0006888
GO:0007186 GO:0007165 GO:0007264 GO:0004871 GO:0005507
GO:0005525 GO:0003924 GO:0019001 GO:0031683 GO:0048038
GO:0008131 GO:0008270 GO:0030127
Cluster-22178.65960 GO:0006446 GO:0003743 GO:0005840 GO:0005852
GO:0016020 GO:0005737
Cluster-22178.65965 GO:0006486 GO:0004576 GO:0046983 GO:0016020
GO:0008250
Cluster-22178.65968 GO:0006886 GO:0008536
Cluster-22178.65969 GO:0000087 GO:0006470 GO:0006570 GO:0004725
GO:0005622
Cluster-22178.65972 GO:0055114 GO:0006574 GO:0008152 GO:0006568
GO:0006552 GO:0006550 GO:0046487 GO:0009396 GO:0006554
GO:0006633 GO:0006631 GO:0006298 GO:0018874 GO:0003824
GO:0003857 GO:0016491 GO:0005524 GO:0050662 GO:0004488
Cluster-22178.65975 GO:0007016 GO:0005200 GO:0003779 GO:0001726
GO:0005925 GO:0005856
Cluster-22178.65976 GO:0006486 GO:0004576 GO:0008250 GO:0016020
GO:0036128

Cluster-22178.65977 GO:0006355 GO:0006260 GO:0016887 GO:0005524
GO:0008134 GO:0003677 GO:0005667
Cluster-22178.65979 GO:0005515
Cluster-22178.65980 GO:0006812 GO:0055114 GO:0006836 GO:0001522
GO:0006118 GO:0009451 GO:0009982 GO:0005328 GO:0005515
GO:0009916 GO:0003723 GO:0016021
Cluster-22178.65981 GO:0055114 GO:0006520 GO:0006807 GO:0006118
GO:0019551 GO:0016491 GO:0004352 GO:0051287
Cluster-22178.65983 GO:0055114 GO:0035556 GO:0006122 GO:0009405
GO:0019835 GO:0016491 GO:0005515 GO:0005750 GO:0019867
Cluster-22178.65984 GO:0006260 GO:0006355 GO:0008270 GO:0003676
GO:0003682 GO:0008408 GO:0003887 GO:0003677 GO:0000166
GO:0008201 GO:0000785 GO:0000775 GO:0005634 GO:0042575
Cluster-22178.65985 GO:0015979 GO:0019538 GO:0009878 GO:0009405
GO:0050765 GO:0016567 GO:0008270 GO:0046872 GO:0005515
GO:0009539 GO:0009523 GO:0005576 GO:0016012 GO:0016020
Cluster-22178.65986 GO:0006013 GO:0006000 GO:0015976 GO:0006020
GO:0006094 GO:0006098 GO:0006096 GO:0009405 GO:0004332
GO:0016020
Cluster-22178.65987 GO:0055114 GO:0006508 GO:0015631 GO:0030699
GO:0050485 GO:0016779 GO:0004197 GO:0030700 GO:0045298
Cluster-22178.65988 GO:0006260 GO:0006355 GO:0008134 GO:0003677
GO:0016887 GO:0005524 GO:0005667
Cluster-22178.65989 GO:0055085 GO:0006865 GO:0006810 GO:0003333
GO:0015171 GO:0016021 GO:0016020
Cluster-22178.65990 GO:0016567 GO:0008270 GO:0046872 GO:0003723
GO:0004842 GO:0005515 GO:0005680
Cluster-22178.65992 GO:0006308 GO:0008239 GO:0008855 GO:0004252
GO:0051082 GO:0070009 GO:0005886 GO:0016592 GO:0009318
Cluster-22178.65994 GO:0006281 GO:0003677 GO:0003676 GO:0008270
GO:0005524 GO:0016787 GO:0016817
Cluster-22178.65995 GO:0006886 GO:0016192 GO:0005198 GO:0005515
GO:0030117

Cluster-22178.65998 GO:0046034 GO:0005975 GO:0006808 GO:0006355
GO:0006413 GO:0007218 GO:0006464 GO:0006144 GO:0006446
GO:0016151 GO:0003676 GO:0003700 GO:0046872 GO:0004672
GO:0043565 GO:0030234 GO:0005515 GO:0004519 GO:0003723
GO:0004553 GO:0003724 GO:0008270 GO:0005524 GO:0003677
GO:0004017 GO:0003743 GO:0005840 GO:0005667
Cluster-22178.66001 GO:0009058 GO:0042967 GO:0015696 GO:0008152
GO:0006633 GO:0055114 GO:0017111 GO:0004315 GO:0016788
GO:0008519 GO:0016491 GO:0016747 GO:0016020 GO:0005835
Cluster-22178.66002 GO:0042254 GO:0006412 GO:0003735 GO:0005840
Cluster-22178.66004 GO:0003924
Cluster-22178.66006 GO:0007267 GO:0006508 GO:0006355 GO:0008270
GO:0008233 GO:0003700 GO:0005667 GO:0005634
Cluster-22178.66007 GO:0003676 GO:0003950
Cluster-22178.66009 GO:0005515
Cluster-22178.66010 GO:0007049 GO:0005515
Cluster-22178.66011 GO:0030150 GO:0005515 GO:0005744
Cluster-22178.66013 GO:0006396 GO:0005515 GO:0003677 GO:0003723
GO:0019013
Cluster-22178.66014 GO:0006629
Cluster-22178.66015 GO:0043066 GO:0043027
Cluster-22178.66016 GO:0046034 GO:0006144 GO:0000042 GO:0006281
GO:0008270 GO:0046872 GO:0004017 GO:0005515 GO:0003677
Cluster-22178.66019 GO:0006468 GO:0005515 GO:0004672 GO:0005524

Cluster-22178.66020 GO:0003924
Cluster-22178.66021 GO:0016192 GO:0006886 GO:0015986 GO:0015992
GO:0015078 GO:0005515 GO:0000276
Cluster-22178.66023 GO:0009058 GO:0030170
Cluster-22178.66025 GO:0030182 GO:0021549 GO:0008270
Cluster-22178.66026 GO:0006744 GO:0015992 GO:0042773 GO:0000290
GO:0006120 GO:0043085 GO:0005975 GO:0055114 GO:0000902
GO:0006814 GO:0016773 GO:0016787 GO:0005525 GO:0008047

GO:0008137 GO:0005515 GO:0016020
Cluster-22178.66027 GO:0005201 GO:0005515 GO:0016020 GO:0005578
Cluster-22178.66029 GO:0006367 GO:0006506 GO:0006355 GO:0016746
GO:0016021 GO:0005789 GO:0005739
Cluster-22178.66030 GO:0051205 GO:0016021
Cluster-22178.66031 GO:0005515
Cluster-22178.66032 GO:0005515 GO:0016020
Cluster-22178.66033 GO:0009245 GO:0008915 GO:0003824
Cluster-22178.66035 GO:0005515
Cluster-22178.66037 GO:0016579 GO:0042254 GO:0006811 GO:0006352
GO:0009405 GO:0006412 GO:0006355 GO:0007017 GO:0016987
GO:0003677 GO:0003735 GO:0005216 GO:0003700 GO:0036459
GO:0005622 GO:0019013 GO:0005667 GO:0005840 GO:0005875
GO:0005576
Cluster-22178.66038 GO:0007165 GO:0050660 GO:0005515 GO:0003824

Cluster-22178.66039 GO:0006281
Cluster-22178.66040 GO:0015693 GO:0015979 GO:0015095 GO:0016020
GO:0016021 GO:0009523 GO:0009539
Cluster-22178.66041 GO:0015992 GO:0015991 GO:0046034 GO:0005524
GO:0016820 GO:0033178
Cluster-22178.66042 GO:0007155 GO:0006508 GO:0003677 GO:0004197
GO:0051015
Cluster-22178.66043 GO:0006357 GO:0001104 GO:0016592
Cluster-22178.66048 GO:0055114 GO:0035556 GO:0006122 GO:0019835
GO:0009405 GO:0016491 GO:0005515 GO:0019867 GO:0005750
Cluster-22178.66049 GO:0006000 GO:0006013 GO:0042254 GO:0006412
GO:0006004 GO:0003676 GO:0003735 GO:0003723 GO:0008736
GO:0005840 GO:0005737 GO:0005622 GO:0005634
Cluster-22178.66051 GO:0016567 GO:0008270 GO:0046872 GO:0004842
GO:0005515 GO:0005680
Cluster-22178.66052 GO:0006355 GO:0008033 GO:0055114 GO:0071949
GO:0003677 GO:0016491 GO:0050660 GO:0003700 GO:0005507

GO:0005667 GO:0005634
Cluster-22178.66053 GO:0006355 GO:0003700 GO:0005667
Cluster-22178.66056 GO:0009432 GO:0006281 GO:0007155 GO:0006367
GO:0015684 GO:0051726 GO:0005525 GO:0003697 GO:0017025
GO:0005524 GO:0015093 GO:0016887 GO:0016021 GO:0005672
GO:0005634 GO:0009289
Cluster-22178.66058 GO:0006468 GO:0016773 GO:0005524 GO:0004672
GO:0016020
Cluster-22178.66060 GO:0016579 GO:0036459
Cluster-22178.66063 GO:0006631 GO:0018874 GO:0045454 GO:0006633
GO:0006554 GO:0006552 GO:0006550 GO:0006508 GO:0006568
GO:0055114 GO:0008033 GO:0006574 GO:0016616 GO:0071949
GO:0051287 GO:0050660 GO:0016491 GO:0008236 GO:0003857
GO:0005576
Cluster-22178.66064 GO:0030474 GO:0071988 GO:0007165 GO:0035444
GO:0015675 GO:0051082 GO:0005179 GO:0015099 GO:0046872
GO:0005576 GO:0016021 GO:0005887
Cluster-22178.66065 GO:0005634
Cluster-22178.66066 GO:0006013 GO:0006000 GO:0015976 GO:0006098
GO:0006094 GO:0006020 GO:0006096 GO:0009405 GO:0004332
GO:0016020
Cluster-22178.66067 GO:0006402 GO:0051252 GO:0003676 GO:0046872
GO:0004535 GO:0003723 GO:0005737 GO:0005634
Cluster-22178.66070 GO:0006888 GO:0006464 GO:0015031 GO:0006886
GO:0006351 GO:0046983 GO:0008270 GO:0046872 GO:0005515
GO:0030127 GO:0005921
Cluster-22178.66071 GO:0016567 GO:0019835 GO:0009405 GO:0055114
GO:0016114 GO:0016491 GO:0046872 GO:0008685 GO:0008270
GO:0004842 GO:0019867
Cluster-22178.66072 GO:0007186 GO:0016310 GO:0009411 GO:0006289
GO:0004519 GO:0004930 GO:0005524 GO:0046917 GO:0016021
Cluster-22178.66075 GO:0016032 GO:0019012
Cluster-22178.66077 GO:0006468 GO:0016310 GO:0006811 GO:0007165

GO:0015893 GO:0006810 GO:0007268 GO:0009069 GO:0055085
GO:0006855 GO:0015238 GO:0015297 GO:0004970 GO:0005234
GO:0004674 GO:0005524 GO:0016020
Cluster-22178.66081 GO:0055114 GO:0019497 GO:0007165 GO:0006771
GO:0016620 GO:0004872 GO:0005525 GO:0003993 GO:0046872
GO:0005515 GO:0003677 GO:0004190 GO:0016021 GO:0016020
Cluster-22178.66084 GO:0006886 GO:0016192 GO:0005507 GO:0005515
GO:0005198 GO:0030117 GO:0030126
Cluster-22178.66087 GO:0006468 GO:0005524 GO:0004672
Cluster-22178.66088 GO:0015991 GO:0015992 GO:0015078 GO:0033177
Cluster-22178.66089 GO:0055114 GO:0016972 GO:0003676 GO:0005634
Cluster-22178.66090 GO:0005509 GO:0005515
Cluster-22178.66091 GO:0033014 GO:0055114 GO:0050661 GO:0008883
GO:0005515
Cluster-22178.66092 GO:0006355 GO:0000978 GO:0000982 GO:0005515
GO:0005634
Cluster-22178.66093 GO:0006629
Cluster-22178.66095 GO:0016021
Cluster-22178.66097 GO:0006334 GO:0003677 GO:0005634 GO:0000786
Cluster-22178.66098 GO:0042254 GO:0006412 GO:0006355 GO:0003700
GO:0003735 GO:0005840 GO:0005622 GO:0005667
Cluster-22178.66099 GO:0007264 GO:0016817 GO:0003924 GO:0046872
GO:0005515 GO:0005525
Cluster-22178.66101 GO:0055114 GO:0016192 GO:0006886 GO:0006817
GO:0044341 GO:0015321 GO:0010309 GO:0005515 GO:0005198
GO:0016020 GO:0030117
Cluster-22178.66104 GO:0030154 GO:0007275 GO:0016567 GO:0004842
GO:0008270 GO:0046872 GO:0005515 GO:0005680
Cluster-22178.66105 GO:0051090 GO:0000390 GO:0006914 GO:0005515
GO:0042803 GO:0008134 GO:0045502 GO:0071008 GO:0005667
GO:0005634 GO:0030286
Cluster-22178.66107 GO:0006508 GO:0045859 GO:0016817 GO:0008242
GO:0004860 GO:0016740 GO:0016788 GO:0004197 GO:0005576

Cluster-22178.66108 GO:0015940 GO:0006571 GO:0019521 GO:0055114
GO:0009094 GO:0009089 GO:0009085 GO:0006098 GO:0000162
GO:0046168 GO:0004665 GO:0003824 GO:0008839 GO:0008677
GO:0008977 GO:0004616 GO:0016616 GO:0050662 GO:0051287
GO:0016491 GO:0016021
Cluster-22178.66109 GO:0008152 GO:0007275 GO:0007165 GO:0047355
GO:0008270 GO:0005179 GO:0016624 GO:0005576 GO:0016020
Cluster-22178.66110 GO:0006457 GO:0046656 GO:0007586 GO:0006807
GO:0016042 GO:0000413 GO:0006730 GO:0018759 GO:0003755
GO:0008047 GO:0005576
Cluster-22178.66111 GO:0008207 GO:0008209 GO:0008210 GO:0006694
GO:0055114 GO:0016616 GO:0050662 GO:0003854 GO:0003824
GO:0016491 GO:0016021
Cluster-22178.66114 GO:0006468 GO:0005515 GO:0005524 GO:0004672
GO:0016773 GO:0016020
Cluster-22178.66116 GO:0022900 GO:0003676 GO:0005739 GO:0005747
Cluster-22178.66117 GO:0003676
Cluster-22178.66118 GO:0032007 GO:0048011
Cluster-22178.66119 GO:0007165
Cluster-22178.66120 GO:0008047 GO:0008233
Cluster-22178.66121 GO:0019722 GO:0006730 GO:0006352 GO:0006402
GO:0007017 GO:0006355 GO:0007018 GO:0051252 GO:0005524
GO:0008017 GO:0016987 GO:0003677 GO:0004535 GO:0046872
GO:0003676 GO:0003700 GO:0003777 GO:0003723 GO:0016740
GO:0005515 GO:0005667 GO:0005737 GO:0045298 GO:0005874
GO:0005634
Cluster-22178.66122 GO:0006334 GO:0003677 GO:0005634 GO:0000786
Cluster-22178.66123 GO:0006508 GO:0008270
Cluster-22178.66124 GO:0055114 GO:0016301 GO:0005515 GO:0016740
GO:0016625 GO:0051536 GO:0016491 GO:0005524
Cluster-22178.66125 GO:0006468 GO:0005524 GO:0004672 GO:0016773
GO:0016020
Cluster-22178.66127 GO:0034314 GO:0015992 GO:0055114 GO:0006119

GO:0006118 GO:0030833 GO:0008121 GO:0015629 GO:0005885
Cluster-22178.66129 GO:0008616 GO:0034639 GO:0005515 GO:0033739
GO:0016021
Cluster-22178.66131 GO:0042981 GO:0009069 GO:0016310 GO:0005509
GO:0004674 GO:0005544
Cluster-22178.66132 GO:0051252 GO:0006351 GO:0007049 GO:0006396
GO:0005544 GO:0004252 GO:0005515 GO:0005509 GO:0003723
GO:0004525 GO:0017053 GO:0005887 GO:0005886
Cluster-22178.66133 GO:0006810 GO:0005215
Cluster-22178.66135 GO:0030833 GO:0034314 GO:0005856 GO:0005885
Cluster-22178.66136 GO:0006418 GO:0004812 GO:0005524 GO:0000166
GO:0005737
Cluster-22178.66137 GO:0008270 GO:0046872
Cluster-22178.66140 GO:0055114 GO:0008152 GO:0006940 GO:0006118
GO:0016627 GO:0050660 GO:0003995
Cluster-22178.66142 GO:0005515
Cluster-22178.66143 GO:0005515
Cluster-22178.66144 GO:0005515 GO:0008270
Cluster-22178.66145 GO:0006099 GO:0031412 GO:0016829 GO:0031411
Cluster-22178.66146 GO:0016020
Cluster-22178.66147 GO:0015684 GO:0007264 GO:0005525 GO:0015093
GO:0003924 GO:0016021
Cluster-22178.66148 GO:0006355 GO:0043565 GO:0003677 GO:0008236
GO:0016021
Cluster-22178.66149 GO:0005515
Cluster-22178.66150 GO:0006810 GO:0004519 GO:0003677 GO:0005622
Cluster-22178.66151 GO:0007264 GO:0005525 GO:0003924
Cluster-22178.66152 GO:0004386 GO:0003676 GO:0003677 GO:0003723
GO:0016787 GO:0005524 GO:0005634
Cluster-22178.66153 GO:0006171 GO:0006816 GO:0006810 GO:0006144
GO:0046677 GO:0000287 GO:0005246 GO:0004016 GO:0046872
GO:0042030 GO:0016020
Cluster-22178.66155 GO:0005515 GO:0003924

Cluster-22178.66156 GO:0006869 GO:0005319 GO:0016831
Cluster-22178.66158 GO:0017187 GO:0008488
Cluster-22178.66159 GO:0006260 GO:0005524 GO:0003677
Cluster-22178.66160 GO:0006508 GO:0006886 GO:0006605 GO:0007268
GO:0005515 GO:0042802 GO:0004197 GO:0043495 GO:0033130
GO:0030695 GO:0005742
Cluster-22178.66161 GO:0006468 GO:0005524 GO:0004672 GO:0019012
Cluster-22178.66164 GO:0006810 GO:0016021
Cluster-22178.66165 GO:0008152 GO:0008757 GO:0008168
Cluster-22178.66167 GO:0046849 GO:0005515 GO:0005576
Cluster-22178.66168 GO:0046849 GO:0006511 GO:0005515 GO:0005576
Cluster-22178.66170 GO:0006950 GO:0018342 GO:0006270 GO:0006351
GO:0006457 GO:0005524 GO:0051082 GO:0008318 GO:0003677
GO:0005634
Cluster-22178.66171 GO:0006013 GO:0006355 GO:0005975 GO:0006000
GO:0008270 GO:0004476
Cluster-22178.66172 GO:0007186 GO:0007165 GO:0007010 GO:0004871
GO:0005525 GO:0003924 GO:0003779 GO:0019001 GO:0031683
GO:0008270
Cluster-22178.66174 GO:0009059 GO:0031072 GO:0051082 GO:0030176
Cluster-22178.66175 GO:0015684 GO:0007165 GO:0007186 GO:0007264
GO:0006749 GO:0030150 GO:0006804 GO:0006979 GO:0055114
GO:0015093 GO:0004602 GO:0005525 GO:0004871 GO:0031683
GO:0019001 GO:0003924 GO:0005742 GO:0016021
Cluster-22178.66179 GO:0006508 GO:0004185
Cluster-22178.66180 GO:0042254 GO:0006412 GO:0003735 GO:0005840
GO:0005622
Cluster-22178.66181 GO:0006468 GO:0006355 GO:0006886 GO:0000921
GO:0008053 GO:0019684 GO:0007165 GO:0009767 GO:0003924
GO:0004672 GO:0003700 GO:0004871 GO:0016168 GO:0005524
GO:0016021 GO:0005940 GO:0005667 GO:0009521 GO:0016020
GO:0005741
Cluster-22178.66182 GO:0006570 GO:0006118 GO:0006119 GO:0046740

GO:0006470 GO:0055114 GO:0015992 GO:0008121 GO:0008138
GO:0016787 GO:0004725 GO:0016021
Cluster-22178.66183 GO:0006351 GO:0006144 GO:0006206 GO:0003899
GO:0005634 GO:0005730
Cluster-22178.66184 GO:0006461 GO:0010508 GO:0019901 GO:0005739
Cluster-22178.66185 GO:0016032 GO:0005198 GO:0005882
Cluster-22178.66186 GO:0005975 GO:0043169 GO:0003824
Cluster-22178.66187 GO:0006355 GO:0005515 GO:0016818 GO:0003677
GO:0016817 GO:0008270 GO:0005524 GO:0005634
Cluster-22178.66188 GO:0003723 GO:0003824
Cluster-22178.66190 GO:0006950 GO:0018342 GO:0006270 GO:0006351
GO:0006457 GO:0005524 GO:0051082 GO:0008318 GO:0003677
GO:0005634
Cluster-22178.66191 GO:0006123 GO:0015992 GO:0004129 GO:0045277
Cluster-22178.66192 GO:0015031 GO:0055085 GO:0042157 GO:0006869
GO:0015948 GO:0042254 GO:0006457 GO:0048870 GO:0046656
GO:0030001 GO:0010508 GO:0006886 GO:0008565 GO:0030269
GO:0046873 GO:0003735 GO:0008289 GO:0005739 GO:0016020
GO:0005737 GO:0005840 GO:0032040 GO:0016021 GO:0005576
GO:0031514
Cluster-22178.66193 GO:0042312 GO:0008217 GO:0007165 GO:0005179
GO:0005515 GO:0005576
Cluster-22178.66194 GO:0006629 GO:0006412 GO:0042254 GO:0006007
GO:0046537 GO:0005515 GO:0003735 GO:0003824 GO:0005622
GO:0005840
Cluster-22178.66195 GO:0005509 GO:0019012
Cluster-22178.66196 GO:0006508 GO:0006364 GO:0007276 GO:0016579
GO:0034477 GO:0004857 GO:0030246 GO:0032183 GO:0070140
GO:0004518 GO:0036459 GO:0008270 GO:0003676 GO:0004222
GO:0031012
Cluster-22178.66200 GO:0007186 GO:0007165 GO:0003676 GO:0004930
GO:0005125 GO:0016020 GO:0016021
Cluster-22178.66201 GO:0000105

Cluster-22178.66203 GO:0031047 GO:0005515
Cluster-22178.66205 GO:0006265 GO:0042742 GO:0003916 GO:0005524 GO:0003677 GO:0019901 GO:0005515 GO:0005694
Cluster-22178.66207 GO:0006338 GO:0016818 GO:0003676 GO:0005524 GO:0005634
Cluster-22178.66210 GO:0061630 GO:0005515 GO:0046872 GO:0008270

Cluster-22178.66211 GO:0005515 GO:0005201 GO:0005581 GO:0005578
Cluster-22178.66212 GO:0055114 GO:1901031 GO:0051920 GO:0005634
Cluster-22178.66213 GO:0007165 GO:0007186 GO:0004871 GO:0005834
Cluster-22178.66215 GO:0043093 GO:0000917 GO:0007049 GO:0008092 GO:0051082 GO:0005737 GO:0019898
Cluster-22178.66219 GO:0030488 GO:0006352 GO:0006355 GO:0003700 GO:0016987 GO:0003677 GO:0005667 GO:0031515
Cluster-22178.66220 GO:0046872
Cluster-22178.66222 GO:0000045 GO:0005737
Cluster-22178.66224 GO:0006355 GO:0008270 GO:0043565 GO:0003700 GO:0005634 GO:0005667
Cluster-22178.66226 GO:0055114 GO:0016491 GO:0046983
Cluster-22178.66227 GO:0009395 GO:0016042 GO:0006355 GO:0043093 GO:0006364 GO:0000917 GO:0043565 GO:0003700 GO:0004629 GO:0005515 GO:0003677 GO:0005737 GO:0005634 GO:0005667
Cluster-22178.66228 GO:0006629 GO:0006270 GO:0003723 GO:0003676

Cluster-22178.66229 GO:0007155 GO:0006355 GO:0006289 GO:0005515 GO:0016020 GO:0000439
Cluster-22178.66231 GO:0055114 GO:0016491 GO:0000287 GO:0003824 GO:0030976 GO:0050660
Cluster-22178.66234 GO:0006355 GO:0006359 GO:0043565 GO:0008270 GO:0003700 GO:0005667 GO:0005634
Cluster-22178.66235 GO:0007165 GO:0005509 GO:0005578
Cluster-22178.66236 GO:0042254 GO:0006412 GO:0003735 GO:0005840
Cluster-22178.66237 GO:0005975 GO:0000902 GO:0006811 GO:0016773

GO:0005216 GO:0016020
Cluster-22178.66238 GO:0000902 GO:0005975 GO:0003676 GO:0005524
GO:0016773 GO:0032040
Cluster-22178.66239 GO:0016787
Cluster-22178.66241 GO:0006950
Cluster-22178.66242 GO:0005975 GO:0016773 GO:0005615
Cluster-22178.66243 GO:0046983
Cluster-22178.66246 GO:0005515
Cluster-22178.66247 GO:0006118 GO:0000902 GO:0055114 GO:0000287
GO:0016651 GO:0051536 GO:0016491 GO:0009055 GO:0030976
Cluster-22178.66250 GO:0007017 GO:0007018 GO:0005524 GO:0003777
GO:0008017 GO:0005874 GO:0045298
Cluster-22178.66251 GO:0016117 GO:0006355 GO:0055114 GO:0016705
GO:0071949 GO:0016491 GO:0005634
Cluster-22178.66252 GO:0006352 GO:0006355 GO:0007186 GO:0007165
GO:0004871 GO:0003700 GO:0016987 GO:0003677 GO:0005834
GO:0005667
Cluster-22178.66255 GO:0007005 GO:0016567 GO:0006313 GO:0004803
GO:0003677 GO:0004842
Cluster-22178.66256 GO:0055114 GO:0015992 GO:0018401 GO:0006525
GO:0015986 GO:0016567 GO:0006560 GO:0016702 GO:0046983
GO:0008270 GO:0015078 GO:0004842 GO:0004656 GO:0000276
GO:0005783
Cluster-22178.66258 GO:0005515
Cluster-22178.6626 GO:0003676
Cluster-22178.66261 GO:0046677 GO:0006396 GO:0000154 GO:0008649

Cluster-22178.66263 GO:0006118 GO:0006814 GO:0009252 GO:0055114
GO:0046436 GO:0008716 GO:0016655 GO:0005524 GO:0046872
Cluster-22178.66265 GO:0055114 GO:0006508 GO:0015631 GO:0050485
GO:0030699 GO:0016779 GO:0004197 GO:0030700 GO:0045298
Cluster-22178.66266 GO:0051603 GO:0030494 GO:0015979 GO:0004298
GO:0005839

Cluster-22178.66267 GO:0030071 GO:0007018 GO:0007017 GO:0005515 GO:0003777 GO:0008017 GO:0005524 GO:0005680 GO:0005874 GO:0045298
Cluster-22178.66269 GO:0016567 GO:0004842 GO:0005515
Cluster-22178.66270 GO:0009069 GO:0006886 GO:0016192 GO:0006351 GO:0006260 GO:0016310 GO:0005515 GO:0003677 GO:0003887 GO:0004674 GO:0030117 GO:0042575
Cluster-22178.66272 GO:0003723 GO:0005515
Cluster-22178.66273 GO:0006446 GO:0007034 GO:0006413 GO:0003743 GO:0005840
Cluster-22178.66275 GO:0006576 GO:0007155 GO:0007264 GO:0007186 GO:0007165 GO:0002949 GO:0005198 GO:0003924 GO:0019001 GO:0005524 GO:0031683 GO:0004871 GO:0005525 GO:0031012
Cluster-22178.66276 GO:0007165 GO:0042254 GO:0007186 GO:0019684 GO:0015708 GO:0006412 GO:0006955 GO:0006118 GO:0009772 GO:0007606 GO:0019236 GO:0003677 GO:0003735 GO:0004932 GO:0008270 GO:0005515 GO:0045156 GO:0003723 GO:0046872 GO:0005125 GO:0005622 GO:0005576 GO:0005634 GO:0005840 GO:0016021
Cluster-22178.66277 GO:0022900 GO:0016651 GO:0005634
Cluster-22178.66278 GO:0006812 GO:0016051 GO:0009405 GO:0046872 GO:0016887 GO:0016840 GO:0000166 GO:0009289 GO:0016021 GO:0043230
Cluster-22178.66281 GO:0051301 GO:0006468 GO:0032955 GO:0016773 GO:0005524 GO:0004672 GO:0016020
Cluster-22178.66283 GO:0006184 GO:0007165 GO:0007186 GO:0007264 GO:0005525 GO:0004871 GO:0019001 GO:0031683 GO:0003924 GO:0016020
Cluster-22178.66284 GO:0016070 GO:0010506 GO:0055085 GO:0006415 GO:0010508 GO:0006886 GO:0006449 GO:0006376 GO:0006355 GO:0003729 GO:0003700 GO:0046872 GO:0016149 GO:0043565 GO:0019901 GO:0005515 GO:0051082 GO:0003723 GO:0005685 GO:0005783 GO:0018444 GO:0005667 GO:0005840 GO:0016021

GO:0005615 GO:0005737
Cluster-22178.66290 GO:0005515 GO:0003677 GO:0005634
Cluster-22178.66292 GO:0004866 GO:0003824 GO:0005576 GO:0005615
Cluster-22178.66293 GO:0007264 GO:0006119 GO:0006118 GO:0055114
GO:0015992 GO:0008121 GO:0005507 GO:0005525
Cluster-22178.66294 GO:0006260 GO:0005634
Cluster-22178.66295 GO:0007186 GO:0005515 GO:0004252 GO:0070008
GO:0004930
Cluster-22178.66296 GO:0016567 GO:0019058 GO:0006464 GO:0006665
GO:0006807 GO:0006396 GO:0006687 GO:0006468 GO:0005975
GO:0004348 GO:0008270 GO:0005524 GO:0016773 GO:0004842
GO:0003723 GO:0005515 GO:0004672 GO:0046872 GO:0016151
GO:0003676 GO:0016020 GO:0005680
Cluster-22178.66299 GO:0006413 GO:0006270 GO:0006446 GO:0019087
GO:0003743 GO:0008270 GO:0019028 GO:0005840 GO:0016021
Cluster-22178.6630 GO:0007165 GO:0006887 GO:0005179 GO:0005576
GO:0000145
Cluster-22178.66300 GO:0006468 GO:0005524 GO:0004672
Cluster-22178.66301 GO:0043039 GO:0006418 GO:0016787 GO:0004812
GO:0016746 GO:0000049 GO:0000166 GO:0005524 GO:0005737
GO:0005680
Cluster-22178.66303 GO:0015031 GO:0006464 GO:0005515
Cluster-22178.66304 GO:0030908
Cluster-22178.66305 GO:0044237 GO:0005524 GO:0003676
Cluster-22178.66307 GO:0006118 GO:0008270 GO:0005506 GO:0005515
GO:0020037 GO:0009055
Cluster-22178.66310 GO:0016567 GO:0008270 GO:0008017 GO:0005515
GO:0004842 GO:0045298
Cluster-22178.66311 GO:0009405 GO:0006508 GO:0004197
Cluster-22178.66312 GO:0043093 GO:0000917 GO:0046983 GO:0019901
GO:0005737
Cluster-22178.66313 GO:0016032 GO:0005524 GO:0005515
Cluster-22178.66314 GO:0006525 GO:0043039 GO:0006433 GO:0006560

GO:0006418 GO:0004812 GO:0000166 GO:0005524 GO:0016876
GO:0004827 GO:0005737
Cluster-22178.66315 GO:0008270
Cluster-22178.66316 GO:0008152 GO:0008168 GO:0008270 GO:0008237

Cluster-22178.66317 GO:0006313 GO:0006118 GO:0008152 GO:0006940
GO:0015074 GO:0055114 GO:0003677 GO:0003995 GO:0050660
GO:0016627
Cluster-22178.66318 GO:0005975 GO:0016758 GO:0016020
Cluster-22178.66319 GO:0051252 GO:0006402 GO:0004535 GO:0046872
GO:0003723 GO:0005737 GO:0005634
Cluster-22178.66320 GO:0006418 GO:0030154 GO:0005524 GO:0004812
GO:0000166 GO:0016021
Cluster-22178.66321 GO:0006470 GO:0003924 GO:0000287 GO:0030145
GO:0005525 GO:0003824 GO:0004721
Cluster-22178.66322 GO:0006260 GO:0005515
Cluster-22178.66323 GO:0016579 GO:0042254 GO:0006352 GO:0006811
GO:0006412 GO:0009405 GO:0007017 GO:0006355 GO:0016987
GO:0003735 GO:0003677 GO:0005216 GO:0003700 GO:0036459
GO:0005622 GO:0019013 GO:0005667 GO:0005840 GO:0005875
GO:0005576
Cluster-22178.66325 GO:0006874 GO:0006816 GO:0005219 GO:0016021
GO:0005622
Cluster-22178.66326 GO:0003723 GO:0019843 GO:0005622
Cluster-22178.66328 GO:0007218 GO:0016310 GO:0006468 GO:0009069
GO:0004674 GO:0005524 GO:0046872 GO:0003677 GO:0005515
Cluster-22178.66329 GO:0006260 GO:0000077 GO:0006275 GO:0006281
GO:0008270 GO:0003677 GO:0030896 GO:0005634
Cluster-22178.66330 GO:0005737 GO:0005634
Cluster-22178.66332 GO:0005975
Cluster-22178.66333 GO:0003723 GO:0005515
Cluster-22178.66335 GO:0005515
Cluster-22178.66336 GO:0008033 GO:0016879 GO:0005524 GO:0000166

GO:0005515 GO:0005737
Cluster-22178.66338 GO:0016310 GO:0007178 GO:0006468 GO:0009069
GO:0005026 GO:0005524 GO:0004672 GO:0046872 GO:0004675
GO:0003723 GO:0016021 GO:0016020
Cluster-22178.66339 GO:0006886 GO:0008536 GO:0005515
Cluster-22178.66340 GO:0015940 GO:0015937 GO:0005524 GO:0004594

Cluster-22178.66342 GO:0006364 GO:0008168 GO:0005515
Cluster-22178.66343 GO:0006886 GO:0016192 GO:0005198 GO:0005515
GO:0003824 GO:0016020 GO:0030126 GO:0016021
Cluster-22178.66344 GO:0006511 GO:0007275 GO:0005634
Cluster-22178.66349 GO:0019079
Cluster-22178.66350 GO:0006355 GO:0006098 GO:0015940 GO:0055114
GO:0019521 GO:0004616 GO:0051287 GO:0050661 GO:0003676
GO:0008677 GO:0005739 GO:0005622
Cluster-22178.66351 GO:0006629 GO:0006814 GO:0006118 GO:0015992
GO:0006744 GO:0006120 GO:0003954 GO:0008137 GO:0016627
GO:0005739 GO:0005737 GO:0016021
Cluster-22178.66352 GO:0005524 GO:0008270 GO:0016787 GO:0003677

Cluster-22178.66353 GO:0006468 GO:0007264 GO:0005525 GO:0004715
GO:0003924 GO:0005524
Cluster-22178.66356 GO:0006355 GO:0005524 GO:0008270 GO:0016817
GO:0003677 GO:0016818 GO:0005515 GO:0005634
Cluster-22178.66358 GO:0006470 GO:0006260 GO:0006570 GO:0042254
GO:0006412 GO:0006351 GO:0004725 GO:0003735 GO:0003887
GO:0003677 GO:0008138 GO:0005840 GO:0005622 GO:0042575
Cluster-22178.66363 GO:0009059 GO:0031072 GO:0051082 GO:0030176
Cluster-22178.66365 GO:0006364 GO:0005730
Cluster-22178.66366 GO:0005975 GO:0004553
Cluster-22178.66367 GO:0007050 GO:0016192 GO:0006886 GO:0007218
GO:0005515 GO:0005198 GO:0005185 GO:0005576 GO:0030117
GO:0019028 GO:0019031

Cluster-22178.66369 GO:0008152 GO:0008168
Cluster-22178.6637 GO:0030866 GO:0003779 GO:0008092 GO:0005198
GO:0005856
Cluster-22178.66371 GO:0016567 GO:0004842 GO:0005515 GO:0008270
GO:0046872 GO:0005680
Cluster-22178.66372 GO:0006629 GO:0007264 GO:0007165 GO:0046339
GO:0043087 GO:0009395 GO:0035556 GO:0005085 GO:0005515
GO:0008081 GO:0004435
Cluster-22178.66376 GO:0008152 GO:0005515 GO:0016787
Cluster-22178.66378 GO:0008652 GO:0055114 GO:0000051 GO:0003942
GO:0046983 GO:0016620 GO:0005525 GO:0005737
Cluster-22178.66379 GO:0005515
Cluster-22178.66381 GO:0006448 GO:0045901 GO:0006452 GO:0045905
GO:0003746 GO:0043022 GO:0003723 GO:0005198 GO:0005840
GO:0019031
Cluster-22178.66385 GO:0006508 GO:0055114 GO:0050485 GO:0030699
GO:0016779 GO:0004197 GO:0015631 GO:0030700 GO:0045298
Cluster-22178.66388 GO:0055085 GO:0018189 GO:0006914 GO:0006821
GO:0007005 GO:0006810 GO:0042626 GO:0005247 GO:0005524
GO:0016021 GO:0016020 GO:0031305
Cluster-22178.66389 GO:0016773
Cluster-22178.66393 GO:0003779 GO:0005622
Cluster-22178.66394 GO:0019354 GO:0006468 GO:0006779 GO:0055114
GO:0005515 GO:0043115 GO:0004672 GO:0005524 GO:0008641
GO:0016021
Cluster-22178.66395 GO:0006351 GO:0006206 GO:0006144 GO:0003677
GO:0003899 GO:0005730
Cluster-22178.66396 GO:0015876 GO:0051016 GO:0006281 GO:0006412
GO:0042254 GO:0008521 GO:0046983 GO:0003676 GO:0003735
GO:0005622 GO:0030479 GO:0005840 GO:0016021
Cluster-22178.66397 GO:0006820 GO:0016021
Cluster-22178.66398 GO:0006629 GO:0045454 GO:0005515
Cluster-22178.66400 GO:0015979 GO:0019538 GO:0050765 GO:0009405

GO:0016567 GO:0046872 GO:0008270 GO:0005515 GO:0009539
GO:0009523 GO:0016020 GO:0016012 GO:0005576
Cluster-22178.66401 GO:0019538 GO:0015979 GO:0050765 GO:0009405
GO:0016567 GO:0046872 GO:0008270 GO:0005515 GO:0009539
GO:0009523 GO:0016020 GO:0016012 GO:0005576
Cluster-22178.66404 GO:0055114 GO:0020037 GO:0016705 GO:0005506

Cluster-22178.66405 GO:0045454 GO:0006118 GO:0009055 GO:0015035
GO:0005515
Cluster-22178.66406 GO:0007606 GO:0007165 GO:0008270 GO:0004888
GO:0003676 GO:0016020
Cluster-22178.66407 GO:0019033
Cluster-22178.66408 GO:0006098 GO:0008152 GO:0055114 GO:0019521
GO:0016616 GO:0004616 GO:0051287 GO:0016491
Cluster-22178.66409 GO:0019521 GO:0055114 GO:0008152 GO:0006098
GO:0016491 GO:0051287 GO:0004616 GO:0016616
Cluster-22178.66411 GO:0006357 GO:0016787 GO:0001104 GO:0016592
Cluster-22178.66412 GO:0003676
Cluster-22178.66413 GO:0051252 GO:0055114 GO:0006402 GO:0016114
GO:0006694 GO:0003723 GO:0004506 GO:0004535 GO:0046872
GO:0003676 GO:0050660 GO:0005634 GO:0005737 GO:0016021
Cluster-22178.66415 GO:0007268 GO:0006955 GO:0030153 GO:0033130
GO:0043495 GO:0005515 GO:0015643 GO:0003723 GO:0019867
GO:0019814
Cluster-22178.66418 GO:0015992 GO:0015976 GO:0016310 GO:0006123
GO:0006094 GO:0006096 GO:0004129 GO:0004618 GO:0005746
GO:0045277
Cluster-22178.6642 GO:0030866 GO:0005198 GO:0008092 GO:0003779
GO:0005856
Cluster-22178.66420 GO:0003677
Cluster-22178.66421 GO:0005975 GO:0003824
Cluster-22178.66422 GO:0006810 GO:0006355 GO:0005215 GO:0003677
GO:0046872 GO:0016020

Cluster-22178.66423 GO:0006260 GO:0005634
Cluster-22178.66425 GO:0006270 GO:0006260 GO:0006351 GO:0003677
GO:0003887 GO:0005515 GO:0042575
Cluster-22178.66429 GO:0006265 GO:0003918 GO:0005524 GO:0003677
GO:0005634
Cluster-22178.66431 GO:0055085 GO:0006468 GO:0006506 GO:0006810
GO:0004672 GO:0005524 GO:0016773 GO:0005215 GO:0004386
GO:0005515 GO:0005789 GO:0016020 GO:0031083
Cluster-22178.66432 GO:0006511 GO:0006310 GO:0019079 GO:0002949
GO:0006576 GO:0006281 GO:0006355 GO:0016787 GO:0009378
GO:0003677 GO:0016887 GO:0005524 GO:0003724 GO:0003723
GO:0016301 GO:0008134 GO:0043531 GO:0003678 GO:0009379
GO:0005667 GO:0005657
Cluster-22178.66433 GO:0006397 GO:0046660 GO:0005634
Cluster-22178.66434 GO:0006355 GO:0006109 GO:0000160 GO:0016310
GO:0000155 GO:0005515 GO:0008134 GO:0003774 GO:0005524
GO:0004672 GO:0005667 GO:0016459 GO:0016020 GO:0009365
Cluster-22178.66435 GO:0055085 GO:0030001 GO:0046873 GO:0016020
Cluster-22178.66436 GO:0015992 GO:0006814 GO:0006885 GO:0015385
GO:0005634 GO:0016020
Cluster-22178.66437 GO:0001514 GO:0006351 GO:0006355 GO:0006260
GO:0006464 GO:0006448 GO:0006269 GO:0003896 GO:0016151
GO:0005525 GO:0008134 GO:0003723 GO:0005524 GO:0003746
GO:0016887 GO:0003677 GO:0005840 GO:0005737 GO:0005657
GO:0005730 GO:0005667
Cluster-22178.66438 GO:0008152 GO:0003824
Cluster-22178.66444 GO:0007276 GO:0016579 GO:0034477 GO:0006508
GO:0006364 GO:0036459 GO:0008270 GO:0003676 GO:0004222
GO:0004857 GO:0030246 GO:0032183 GO:0070140 GO:0004518
GO:0031012
Cluster-22178.66447 GO:0006357 GO:0008270 GO:0003676 GO:0001104
GO:0016592
Cluster-22178.66450 GO:0015937 GO:0015940 GO:0005524 GO:0004140

Cluster-22178.66456 GO:0015986 GO:0006464 GO:0015031 GO:0015992 GO:0006886 GO:0015689 GO:0005047 GO:0008270 GO:0052855 GO:0015098 GO:0005515 GO:0003924 GO:0015078 GO:0005525 GO:0000276 GO:0005785 GO:0005786 GO:0016021

Cluster-22178.66457 GO:0006886 GO:0016192 GO:0005515 GO:0008536 GO:0030117

Cluster-22178.66458 GO:0006915 GO:0006118 GO:0020037 GO:0009055 GO:0019028 GO:0005622

Cluster-22178.66459 GO:0016579 GO:0036459

Cluster-22178.66462 GO:0003723

Cluster-22178.66465 GO:0006508 GO:0004197 GO:0008234

Cluster-22178.66468 GO:0055114 GO:0043093 GO:0000917 GO:0046872 GO:0008289 GO:0047134 GO:0005737

Cluster-22178.66469 GO:0006464 GO:0016151 GO:0005198 GO:0046872 GO:0019028

Cluster-22178.66470 GO:0006468 GO:0005524 GO:0004672

Cluster-22178.66472 GO:0006164 GO:0006531 GO:0006144 GO:0006522 GO:0005525 GO:0004019

Cluster-22178.66473 GO:0006446 GO:0005515 GO:0003743 GO:0005737 GO:0005840 GO:0005852

Cluster-22178.66474 GO:0005515

Cluster-22178.66476 GO:0005515

Cluster-22178.66477 GO:0006468 GO:0005524 GO:0004672 GO:0046872 GO:0016787 GO:0016773 GO:0016020

Cluster-22178.66479 GO:0035023 GO:0009405 GO:0043087 GO:0005089 GO:0005576

Cluster-22178.66480 GO:0042811 GO:0006468 GO:0007218 GO:0016746 GO:0005184 GO:0016773 GO:0004672 GO:0005524 GO:0016020

Cluster-22178.66482 GO:0008152 GO:0055114 GO:0046487 GO:0000917 GO:0045132 GO:0000226 GO:0009396 GO:0043093 GO:0003824 GO:0008092 GO:0005515 GO:0004488 GO:0005509 GO:0016787 GO:0016021 GO:0000775 GO:0005737 GO:0015630 GO:0005634

GO:0019898
Cluster-22178.66483 GO:0006357 GO:0007165 GO:0005057 GO:0001104
GO:0016592
Cluster-22178.66489 GO:0003676
Cluster-22178.66491 GO:0006397
Cluster-22178.66493 GO:0015992 GO:0006119 GO:0015986 GO:0008289
GO:0046933 GO:0046961 GO:0045259 GO:0000275
Cluster-22178.66494 GO:0006810 GO:0006355 GO:0005215 GO:0003677
GO:0046872 GO:0016020
Cluster-22178.66495 GO:0055085 GO:0006811 GO:0005216 GO:0016020
GO:0016021
Cluster-22178.66496 GO:0006659
Cluster-22178.66498 GO:0006355 GO:0005634
Cluster-22178.66499 GO:0006260 GO:0055114
Cluster-22178.66500 GO:0019354 GO:0006468 GO:0006779 GO:0055114
GO:0005515 GO:0043115 GO:0005524 GO:0004672 GO:0008641
GO:0016021
Cluster-22178.66503 GO:0018342 GO:0006886 GO:0006270 GO:0006397
GO:0006396 GO:0008318 GO:0005515 GO:0005634 GO:0005622
GO:0005789
Cluster-22178.66504 GO:0019867
Cluster-22178.66505 GO:0006811 GO:0006396 GO:0018342 GO:0006397
GO:0006270 GO:0006886 GO:0008318 GO:0005515 GO:0005622
GO:0005789 GO:0005634 GO:0016020
Cluster-22178.66508 GO:0002949 GO:0007059 GO:0030261 GO:0003724
GO:0005525 GO:0003924 GO:0016887 GO:0005524 GO:0005515
GO:0003677 GO:0003723 GO:0009295
Cluster-22178.66510 GO:0006461 GO:0005739
Cluster-22178.66511 GO:0006397 GO:0080019 GO:0000445
Cluster-22178.66514 GO:0006298 GO:0008270 GO:0005524 GO:0030983
GO:0005634
Cluster-22178.66515 GO:0009307 GO:0051276 GO:0006308 GO:0003677
GO:0009036 GO:0009359 GO:0005634 GO:0005737

Cluster-22178.66516 GO:0008270 GO:0005515 GO:0005198 GO:0003677
GO:0016021
Cluster-22178.66518 GO:0005524 GO:0005856
Cluster-22178.66519 GO:0055085 GO:0016020 GO:0016021
Cluster-22178.66521 GO:0055085 GO:0006811 GO:0005216 GO:0016020
GO:0016021
Cluster-22178.66522 GO:0005524 GO:0005856
Cluster-22178.66523 GO:0045947 GO:0008190
Cluster-22178.66525 GO:0006334 GO:0006952 GO:0009405 GO:0006810
GO:0019855 GO:0005576 GO:0005634
Cluster-22178.66526 GO:0006355 GO:0000917 GO:0007049 GO:0043093
GO:0007186 GO:0006914 GO:0006464 GO:0006744 GO:0006260
GO:0043565 GO:0008813 GO:0003700 GO:0016151 GO:0004930
GO:0005737 GO:0016021 GO:0005667 GO:0005815
Cluster-22178.66528 GO:0006952 GO:0007165 GO:0055114 GO:0016491
GO:0046872 GO:0005576
Cluster-22178.66529 GO:0007010 GO:0003779
Cluster-22178.66530 GO:0005515
Cluster-22178.66531 GO:0006412 GO:0042254 GO:0003735 GO:0005840
GO:0005622
Cluster-22178.66532 GO:0006468 GO:0004672 GO:0005524
Cluster-22178.66533 GO:0055085 GO:0006811 GO:0005216 GO:0016020
Cluster-22178.66534 GO:0005525
Cluster-22178.66537 GO:0006525 GO:0042254 GO:0071436 GO:0006412
GO:0006814 GO:0006560 GO:0006090 GO:0009401 GO:0016787
GO:0003735 GO:0015081 GO:0008948 GO:0005515 GO:0016020
GO:0015934 GO:0016021 GO:0005840
Cluster-22178.66539 GO:0005783
Cluster-22178.66540 GO:0016021
Cluster-22178.66541 GO:0006886 GO:0006468 GO:0006355 GO:0009767
GO:0007165 GO:0019684 GO:0008053 GO:0000921 GO:0003700
GO:0004871 GO:0004672 GO:0003924 GO:0005524 GO:0016168
GO:0016021 GO:0005741 GO:0016020 GO:0005667 GO:0009521

GO:0005940
Cluster-22178.66542 GO:0006812 GO:0006820 GO:0006835 GO:0017153
GO:0016020
Cluster-22178.66543 GO:0006144 GO:0006464 GO:0007165 GO:0004114
GO:0016151
Cluster-22178.66545 GO:0006886 GO:0006810 GO:0006355 GO:0005515
GO:0003677 GO:0016788 GO:0042626 GO:0005524 GO:0005783
GO:0016021
Cluster-22178.66547 GO:0055114 GO:0008615 GO:0042254 GO:0004733
GO:0010181 GO:0016638 GO:0005622 GO:0016021
Cluster-22178.6655 GO:0035444 GO:0015675 GO:0005515 GO:0015099
GO:0046983 GO:0046872 GO:0016021
Cluster-22178.66550 GO:0042254 GO:0006412 GO:0003735 GO:0005622
GO:0005840
Cluster-22178.66552 GO:0045893 GO:0006413 GO:0007275 GO:0006118
GO:0006446 GO:0008270 GO:0009055 GO:0003743 GO:0020037
GO:0005515 GO:0005840 GO:0016021
Cluster-22178.66553 GO:0006260 GO:0006468 GO:0008641 GO:0005524
GO:0008270 GO:0004672 GO:0003677 GO:0005515 GO:0016021
Cluster-22178.66556 GO:0042254 GO:0006412 GO:0006810 GO:0045454
GO:0005515 GO:0003735 GO:0005525 GO:0003924 GO:0005861
GO:0016021 GO:0005840
Cluster-22178.66559 GO:0042254 GO:0006412 GO:0003735 GO:0005622
GO:0005840
Cluster-22178.66560 GO:0015992 GO:0015991 GO:0015078 GO:0033177
Cluster-22178.66562 GO:0006508 GO:0006468 GO:0035023 GO:0007169
GO:0043087 GO:0005515 GO:0004714 GO:0004185 GO:0005089
GO:0004672 GO:0005524 GO:0016020
Cluster-22178.66566 GO:0042998 GO:0015074 GO:0003677 GO:0008270
GO:0071253 GO:0005802
Cluster-22178.66567 GO:0006457 GO:0005515
Cluster-22178.66569 GO:0031554 GO:0031556
Cluster-22178.66571 GO:0006810 GO:0016021 GO:0005886

Cluster-22178.66572 GO:0006955 GO:0007165 GO:0005164 GO:0046872
GO:0016020
Cluster-22178.66574 GO:0046008 GO:0007165 GO:0005179 GO:0005576
Cluster-22178.66575 GO:0006412 GO:0042254 GO:0003735 GO:0005622
GO:0005840
Cluster-22178.66576 GO:0006886 GO:0006531 GO:0006522 GO:0009152
GO:0006188 GO:0006144 GO:0004018 GO:0005737
Cluster-22178.66578 GO:0007275 GO:0006810 GO:0055085 GO:0005524
GO:0016887 GO:0005525 GO:0042626 GO:0042600 GO:0016021
Cluster-22178.66579 GO:0007218 GO:0005185 GO:0005576
Cluster-22178.66580 GO:0035556 GO:0005524 GO:0005515
Cluster-22178.66582 GO:0003950
Cluster-22178.66585 GO:0042254 GO:0006412 GO:0003735 GO:0005840
GO:0015934
Cluster-22178.66587 GO:0006468 GO:0051301 GO:0032955 GO:0016773
GO:0005524 GO:0004672 GO:0003676 GO:0016020
Cluster-22178.66588 GO:0007264 GO:0007186 GO:0007165 GO:0006614
GO:0006000 GO:0006013 GO:0043087 GO:0006355 GO:0035023
GO:0003873 GO:0005524 GO:0005089 GO:0005515 GO:0003924
GO:0019001 GO:0031683 GO:0004871 GO:0005525
Cluster-22178.66591 GO:0015979 GO:0009539 GO:0009523 GO:0016020
Cluster-22178.66592 GO:0032012 GO:0043248 GO:0043087 GO:0007160
GO:0005509 GO:0005086 GO:0005783 GO:0005634 GO:0005576
Cluster-22178.66596 GO:0008146 GO:0016021
Cluster-22178.66597 GO:0006355 GO:0007049 GO:0046983 GO:0043565
GO:0003700 GO:0005667
Cluster-22178.66599 GO:0006357 GO:0001104 GO:0016592
Cluster-22178.66600 GO:0006388 GO:0051252 GO:0006351 GO:0003714
GO:0000213 GO:0005667 GO:0000214
Cluster-22178.66601 GO:0005975
Cluster-22178.66602 GO:0005975
Cluster-22178.66604 GO:0005524 GO:0005856
Cluster-22178.66605 GO:0055114 GO:0019646 GO:0015886 GO:0006118

GO:0015232 GO:0016491 GO:0009055
Cluster-22178.66608 GO:0055114 GO:0016117 GO:0016491 GO:0016705

Cluster-22178.66609 GO:0007017 GO:0005875
Cluster-22178.66610 GO:0008270
Cluster-22178.66612 GO:0005515 GO:0008270
Cluster-22178.66616 GO:0006355 GO:0008134 GO:0003677 GO:0016787
GO:0005524 GO:0005667
Cluster-22178.66617 GO:0005982 GO:0006012 GO:0005985 GO:0004564
GO:0005515 GO:0004575 GO:0017177
Cluster-22178.66619 GO:0004659 GO:0016021
Cluster-22178.66622 GO:0006810 GO:0003676 GO:0004386 GO:0003677
GO:0000166 GO:0016787 GO:0005524
Cluster-22178.66623 GO:0003676
Cluster-22178.66628 GO:0005515
Cluster-22178.66630 GO:0006914 GO:0007165 GO:0051082 GO:0016301
GO:0005515 GO:0016021 GO:0032039
Cluster-22178.66631 GO:0008219
Cluster-22178.66632 GO:0006144 GO:0046034 GO:0046872 GO:0004017
GO:0003676
Cluster-22178.66633 GO:0005975 GO:0006260 GO:0043169 GO:0003887
GO:0003824 GO:0042575
Cluster-22178.66636 GO:0006886 GO:0007094 GO:0006420 GO:0006606
GO:0044780 GO:0006355 GO:0006560 GO:0006308 GO:0051258
GO:0006302 GO:0006525 GO:0006457 GO:0007165 GO:0030168
GO:0042803 GO:0005515 GO:0005198 GO:0043565 GO:0005524
GO:0004814 GO:0003700 GO:0030674 GO:0051087 GO:0000774
GO:0008855 GO:0005102 GO:0005634 GO:0005667 GO:0005783
GO:0005577 GO:0009318 GO:0016021
Cluster-22178.66637 GO:0090307 GO:0006508 GO:0006355 GO:0043565
GO:0003700 GO:0004222 GO:0051082 GO:0033588 GO:0005667
Cluster-22178.66639 GO:0055114 GO:0007165 GO:0005506 GO:0005158
GO:0016705 GO:0020037 GO:0005899

Cluster-22178.66641 GO:0046872 GO:0004867
Cluster-22178.66642 GO:0051607 GO:0050778 GO:0007259 GO:0007165
GO:0005125
Cluster-22178.66644 GO:0006144 GO:0006206 GO:0016042 GO:0007586
GO:0009069 GO:0016310 GO:0006468 GO:0006351 GO:0005524
GO:0004674 GO:0003677 GO:0008047 GO:0004672 GO:0003899
GO:0005730 GO:0005576
Cluster-22178.66646 GO:0019076 GO:0075521 GO:0046718 GO:0019028
GO:0044163 GO:0042025 GO:0030430
Cluster-22178.66648 GO:0005634
Cluster-22178.66649 GO:0030833 GO:0006119 GO:0006118 GO:0055114
GO:0015992 GO:0034314 GO:0008121 GO:0005885 GO:0015629
Cluster-22178.66652 GO:0006811 GO:0055085 GO:0015075 GO:0005515
GO:0005886 GO:0016020
Cluster-22178.66654 GO:0006098 GO:0018874 GO:0006631 GO:0006554
GO:0006633 GO:0006568 GO:0006550 GO:0006552 GO:0006574
GO:0019521 GO:0055114 GO:0015940 GO:0051287 GO:0004616
GO:0016491 GO:0003857 GO:0008677
Cluster-22178.66655 GO:0006468 GO:0045454 GO:0007586 GO:0016042
GO:0003697 GO:0004672 GO:0005524 GO:0008047 GO:0005576
GO:0019028
Cluster-22178.66657 GO:0006355 GO:0006284 GO:0003905 GO:0003677
GO:0046983 GO:0003700 GO:0005634 GO:0005667 GO:0016021
Cluster-22178.66658 GO:0007005 GO:0016567 GO:0005515 GO:0016758
GO:0008270 GO:0004842 GO:0005783 GO:0016021
Cluster-22178.66659 GO:0055085 GO:0006812 GO:0006885 GO:0015299
GO:0016021
Cluster-22178.66660 GO:0006355 GO:0005515 GO:0005634
Cluster-22178.66661 GO:0008270 GO:0005096
Cluster-22178.66662 GO:0008643 GO:0009401 GO:0006950 GO:0006457
GO:0005524 GO:0008982 GO:0051082 GO:0016021 GO:0009357
Cluster-22178.66664 GO:0033014 GO:0006783 GO:0015994 GO:0008270
GO:0004852 GO:0004386

Cluster-22178.66665 GO:0016579 GO:0030150 GO:0006144 GO:0015976
GO:0006096 GO:0006094 GO:0003824 GO:0036459 GO:0004743
GO:0030955 GO:0000287 GO:0005742
Cluster-22178.66669 GO:0006813 GO:0009405 GO:0016567 GO:0030955
GO:0042802 GO:0005515 GO:0005249 GO:0004842 GO:0008076
GO:0016021
Cluster-22178.66670 GO:0006118 GO:0009451 GO:0045454 GO:0006396
GO:0055114 GO:0008173 GO:0003723 GO:0015035 GO:0009055
GO:0016209 GO:0016491 GO:0008168
Cluster-22178.66671 GO:0006123 GO:0006812 GO:0055114 GO:0006820
GO:0006835 GO:0015992 GO:0017153 GO:0016638 GO:0004129
GO:0016020 GO:0045277
Cluster-22178.66674 GO:0006352 GO:0004190 GO:0005634 GO:0005669
GO:0016021
Cluster-22178.66675 GO:0015991 GO:0015992 GO:0015078 GO:0033177
Cluster-22178.66677 GO:0006355 GO:0003677
Cluster-22178.66680 GO:0006935 GO:0007165 GO:0006955 GO:0008009
GO:0005576
Cluster-22178.66681 GO:0043087 GO:0048193 GO:0007264 GO:0008283
GO:0007165 GO:0006869 GO:0042981 GO:0040007 GO:0006955
GO:0005134 GO:0005085 GO:0005515 GO:0005319 GO:0008083
GO:0005887 GO:0016020 GO:0005893 GO:0005576
Cluster-22178.66683 GO:0016021
Cluster-22178.66685 GO:0032007 GO:0030682 GO:0048011 GO:0043176

Cluster-22178.66689 GO:0007276
Cluster-22178.66690 GO:0006820 GO:0016021
Cluster-22178.66691 GO:0006355 GO:0003677
Cluster-22178.66692 GO:0048598 GO:0005515 GO:0072669
Cluster-22178.66693 GO:0005515
Cluster-22178.66694 GO:0006144 GO:0006206 GO:0000162 GO:0006432
GO:0006351 GO:0009094 GO:0006571 GO:0003677 GO:0003723
GO:0004519 GO:0003899 GO:0046872 GO:0003676 GO:0004826

GO:0005730 GO:0009328
Cluster-22178.66697 GO:0008152 GO:0031110 GO:0006308 GO:0006629
GO:0003824 GO:0008081 GO:0008855 GO:0043565 GO:0009318
Cluster-22178.66698 GO:0005515 GO:0008270
Cluster-22178.66701 GO:0001514 GO:0006355 GO:0006351 GO:0006464
GO:0006448 GO:0006260 GO:0006269 GO:0005525 GO:0016151
GO:0003896 GO:0003723 GO:0008134 GO:0005524 GO:0003746
GO:0016887 GO:0003677 GO:0005737 GO:0005840 GO:0005657
GO:0005730 GO:0005667
Cluster-22178.66702 GO:0005515 GO:0016020
Cluster-22178.66704 GO:0006771 GO:0019497 GO:0007339 GO:0046872
GO:0005515 GO:0003993 GO:0005576
Cluster-22178.66705 GO:0006811 GO:0055085 GO:0005216 GO:0005515
GO:0012511 GO:0016021 GO:0016020
Cluster-22178.66706 GO:0046487 GO:0055114 GO:0009396 GO:0004329
GO:0003824 GO:0004488 GO:0005524
Cluster-22178.66707 GO:0071630 GO:0031144 GO:0005634
Cluster-22178.66708 GO:0008152 GO:0055114 GO:0016491
Cluster-22178.66709 GO:0005515
Cluster-22178.66711 GO:0042384 GO:0034464
Cluster-22178.66712 GO:0009405 GO:0006352 GO:0005576 GO:0070461
GO:0005669
Cluster-22178.66713 GO:0007264 GO:0006576 GO:0015684 GO:0015093
GO:0005524 GO:0003924 GO:0016887 GO:0005525 GO:0016021
Cluster-22178.66714 GO:0006355 GO:0003712 GO:0005515 GO:0005667
Cluster-22178.66715 GO:0006298 GO:0007010 GO:0006364 GO:0007017
GO:0006813 GO:0030983 GO:0003779 GO:0005524 GO:0005267
GO:0005875 GO:0005634 GO:0034457 GO:0005732
Cluster-22178.66717 GO:0006355 GO:0003712 GO:0005515 GO:0005667
Cluster-22178.66718 GO:0008152 GO:0016491
Cluster-22178.6672 GO:0009236 GO:0006508 GO:0008270 GO:0051073
GO:0008818 GO:0004222 GO:0031012
Cluster-22178.66720 GO:0010024 GO:0055114 GO:0016636 GO:0050897

Cluster-22178.66724 GO:0042254 GO:0006412 GO:0003735 GO:2001070
GO:0005840
Cluster-22178.66725 GO:0042254 GO:0006412 GO:2001070 GO:0003735
GO:0005840
Cluster-22178.66728 GO:0051028 GO:0006508 GO:0005634
Cluster-22178.66729 GO:0048870 GO:0000917 GO:0044780 GO:0006355
GO:0015991 GO:0006260 GO:0000921 GO:0006457 GO:0007094
GO:0010508 GO:0006606 GO:0048193 GO:0015992 GO:0006914
GO:0043093 GO:0007155 GO:0003700 GO:0008134 GO:0045502
GO:0017048 GO:0015078 GO:0043565 GO:0042803 GO:0005198
GO:0051082 GO:0005524 GO:0008270 GO:0003677 GO:0016818
GO:0016021 GO:0031514 GO:0019028 GO:0033180 GO:0030286
GO:0005940 GO:0005882 GO:0019867 GO:0005737 GO:0005634
GO:0016272 GO:0016020 GO:0005667
Cluster-22178.66730 GO:0055114 GO:0035556 GO:0006122 GO:0009405
GO:0019835 GO:0016491 GO:0005515 GO:0005750 GO:0019867
Cluster-22178.66731 GO:0015979 GO:0046872
Cluster-22178.66733 GO:0008299
Cluster-22178.66734 GO:0019069 GO:0006816 GO:0006874 GO:0006260
GO:0000049 GO:0005219 GO:0016020 GO:0071203 GO:0005815
GO:0016021 GO:0005622
Cluster-22178.66735 GO:0007264 GO:0051087 GO:0005096
Cluster-22178.66736 GO:0006811 GO:0006468 GO:0006260 GO:0019069
GO:0006874 GO:0006816 GO:0005262 GO:0005219 GO:0004672
GO:0000049 GO:0005524 GO:0005244 GO:0016021 GO:0071203
GO:0005815 GO:0005622 GO:0016020
Cluster-22178.66737 GO:0005515
Cluster-22178.66738 GO:0046914 GO:0003924 GO:0046983 GO:0005525
GO:0005509
Cluster-22178.66739 GO:0006777 GO:0008152 GO:0006810 GO:0005524
GO:0005525 GO:0016301
Cluster-22178.66741 GO:0005515

Cluster-22178.66742 GO:0003950 GO:0004190 GO:0016020 GO:0016021
GO:0005634
Cluster-22178.66743 GO:0006355 GO:0006508 GO:0043565 GO:0004197
GO:0003700 GO:0005667
Cluster-22178.66744 GO:0006355 GO:0003700 GO:0005515 GO:0008270
GO:0043565 GO:0005667
Cluster-22178.66748 GO:0003950 GO:0016020 GO:0005634
Cluster-22178.66752 GO:0009405 GO:0016772 GO:0016301 GO:0016020
Cluster-22178.66756 GO:0016567 GO:0045893 GO:0004842 GO:0005515
GO:0003676 GO:0042025
Cluster-22178.66757 GO:0006810 GO:0055085 GO:0005215 GO:0016020
Cluster-22178.66758 GO:0055114 GO:0006810 GO:0055085 GO:0006118
GO:0016655 GO:0005215 GO:0016020
Cluster-22178.66760 GO:0006355 GO:0009607 GO:0015979 GO:0046872
GO:0003700 GO:0005667 GO:0042025 GO:0009523 GO:0016021
Cluster-22178.66761 GO:0006470 GO:0006570 GO:0004725 GO:0008138

Cluster-22178.66762 GO:0006284 GO:0006576 GO:0005525 GO:0003924
GO:0005524 GO:0003905 GO:0003677 GO:0030246
Cluster-22178.66763 GO:0016021
Cluster-22178.66764 GO:0006914
Cluster-22178.66766 GO:0006508 GO:0004222 GO:0003676 GO:0005515
GO:0008270
Cluster-22178.66767 GO:0006914 GO:0070206 GO:0051289 GO:0007165
GO:0006506 GO:0008140 GO:0016740 GO:0005515 GO:0005509
GO:0005886 GO:0016021 GO:0005789 GO:0005667 GO:0016012
GO:0005578
Cluster-22178.66768 GO:0015992 GO:0015991 GO:0015078 GO:0005198
GO:0033180 GO:0019031 GO:0009424 GO:0019028
Cluster-22178.66769 GO:0006364 GO:0008168 GO:0005634
Cluster-22178.66771 GO:0009190 GO:0035556 GO:0016849
Cluster-22178.66772 GO:0045892 GO:0003677 GO:0051082
Cluster-22178.66774 GO:0055114 GO:0006570 GO:0004511 GO:0019028

Cluster-22178.66775 GO:0006351 GO:0003676 GO:0005515 GO:0008270 GO:0005639

Cluster-22178.66777 GO:0043087 GO:0032012 GO:0005086

Cluster-22178.66778 GO:0006687 GO:0006027 GO:0046486 GO:0006835 GO:0005975 GO:0006820 GO:0006812 GO:0006012 GO:0017153 GO:0004565 GO:0016020 GO:0009341

Cluster-22178.66779 GO:0006810 GO:0003333 GO:0055085 GO:0006865 GO:0006811 GO:0007016 GO:0015171 GO:0005200 GO:0005215 GO:0016020 GO:0005925 GO:0001726 GO:0005856

Cluster-22178.66780 GO:0007218 GO:0007165 GO:0030141

Cluster-22178.66781 GO:0051382 GO:0005515 GO:0000776

Cluster-22178.66783 GO:0006260 GO:0005975 GO:0019058 GO:0000746 GO:0004553 GO:0003677 GO:0003887 GO:0019867 GO:0042575

Cluster-22178.66787 GO:0006508 GO:0006486 GO:0003677 GO:0004576 GO:0004222 GO:0003676 GO:0008250 GO:0016020

Cluster-22178.66788 GO:0031556 GO:0031554

Cluster-22178.66789 GO:0043065 GO:0046983 GO:0005515 GO:0016021 GO:0005740

Cluster-22178.66790 GO:0005515

Cluster-22178.66792 GO:0046872

Cluster-22178.66793 GO:0050830 GO:0006811 GO:0046872 GO:0031083 GO:0016020

Cluster-22178.66794 GO:0006810 GO:0051205 GO:0004386 GO:0003676 GO:0005524 GO:0003677 GO:0016787 GO:0016021

Cluster-22178.66795 GO:0003723

Cluster-22178.66796 GO:0006457 GO:0006807 GO:0016810 GO:0051082 GO:0016272

Cluster-22178.66797 GO:0006810 GO:0009733 GO:0005515 GO:0005622

Cluster-22178.66798 GO:0005515

Cluster-22178.66799 GO:0010506 GO:0007049 GO:0006260 GO:0006355 GO:0043093 GO:0000917 GO:0043565 GO:0003700 GO:0005737 GO:0005615 GO:0005667 GO:0005634

Cluster-22178.66801 GO:0006355 GO:0006468 GO:0016114 GO:0042432

GO:0009058 GO:0055085 GO:0005515 GO:0004672 GO:0003700
GO:0004064 GO:0016844 GO:0005524 GO:0016773 GO:0016021
GO:0005667 GO:0016020
Cluster-22178.66803 GO:0005515
Cluster-22178.66804 GO:0006355 GO:0006777 GO:0003700 GO:0003824
GO:0005525 GO:0043565 GO:0003924 GO:0005667 GO:0012511
GO:0016021
Cluster-22178.66805 GO:0019079 GO:0005509 GO:0005515
Cluster-22178.66806 GO:0003723
Cluster-22178.66808 GO:0006144 GO:0006470 GO:0006508 GO:0006570
GO:0004725 GO:0003968 GO:0008138 GO:0004197 GO:0017111
GO:0031379
Cluster-22178.66810 GO:0016020
Cluster-22178.66812 GO:0042254 GO:0006412 GO:0003735 GO:0005622
GO:0005840
Cluster-22178.66813 GO:0007218 GO:0007165 GO:0030141
Cluster-22178.66814 GO:0006118 GO:0003954 GO:0005739
Cluster-22178.66815 GO:0019836 GO:0005576
Cluster-22178.66816 GO:0015992 GO:0016021
Cluster-22178.66817 GO:0006364 GO:0003676 GO:0003723
Cluster-22178.66818 GO:0006378 GO:0003677 GO:0008270 GO:0046872
GO:0003729 GO:0005849
Cluster-22178.66819 GO:0045454 GO:0006508 GO:0008234 GO:0016491

Cluster-22178.66820 GO:0006508 GO:0055114 GO:0050485 GO:0030699
GO:0004197 GO:0016779 GO:0015631 GO:0030700 GO:0045298
Cluster-22178.66824 GO:0006139 GO:0003676 GO:0008408
Cluster-22178.66825 GO:0042254 GO:0006412 GO:0006457 GO:0055114
GO:0003735 GO:0005515 GO:0005840 GO:0005622
Cluster-22178.66826 GO:0006974 GO:0051726 GO:0003677
Cluster-22178.66828 GO:0006355 GO:0000736 GO:0051726 GO:0046872
GO:0043565 GO:0003700 GO:0003677 GO:0070336 GO:0005667
GO:0005634

Cluster-22178.66830 GO:0003676
Cluster-22178.66831 GO:0010508 GO:0000917 GO:0043093 GO:0006355 GO:0006914 GO:0003677 GO:0003700 GO:0043565 GO:0005634 GO:0005667 GO:0005737
Cluster-22178.66832 GO:0045859 GO:0006396 GO:0055114 GO:0005515 GO:0047134 GO:0004860 GO:0005576 GO:0000176 GO:0016021
Cluster-22178.66833 GO:0005515
Cluster-22178.66835 GO:0006470 GO:0006570 GO:0004725 GO:0008138

Cluster-22178.66837 GO:0042254 GO:0006412 GO:0003735 GO:0005840 GO:0005622
Cluster-22178.66838 GO:0006355 GO:0003700 GO:0043565 GO:0005667 GO:0005634
Cluster-22178.66839 GO:0006355 GO:0007275 GO:0006353 GO:0008608 GO:0007219 GO:0030154 GO:0003723 GO:0003677 GO:0072686 GO:0042729 GO:0016021
Cluster-22178.66840 GO:0006468 GO:0006413 GO:0006446 GO:0005524 GO:0004672 GO:0003743 GO:0005840
Cluster-22178.66843 GO:0006508 GO:0008654 GO:0008270 GO:0016780 GO:0004181 GO:0016020
Cluster-22178.66845 GO:0030570 GO:0005576
Cluster-22178.66846 GO:0006750 GO:0006749 GO:0007155 GO:0042398 GO:0004357 GO:0017109 GO:0016021
Cluster-22178.66847 GO:0006206 GO:0006139 GO:0005524 GO:0004127

Cluster-22178.66848 GO:0008283 GO:0040007 GO:0007165 GO:0006955 GO:0043087 GO:0035023 GO:0005126 GO:0003723 GO:0008083 GO:0005089 GO:0005576
Cluster-22178.66849 GO:0007165 GO:0006955 GO:0008283 GO:0040007 GO:0035023 GO:0043087 GO:0008083 GO:0005126 GO:0003723 GO:0005089 GO:0005576
Cluster-22178.66850 GO:0006449 GO:0006415 GO:0003747 GO:0005840 GO:0018444

Cluster-22178.66854 GO:0051205 GO:0016021
Cluster-22178.66857 GO:0016567 GO:0005515 GO:0004842 GO:0008270
GO:0051082 GO:0046872 GO:0005680
Cluster-22178.66858 GO:0003676
Cluster-22178.66859 GO:0006886 GO:0006508 GO:0006629 GO:0015996
GO:0001514 GO:0008152 GO:0015994 GO:0006448 GO:0006505
GO:0008236 GO:0016788 GO:0047746 GO:0003723 GO:0004190
GO:0005525 GO:0016787 GO:0003746 GO:0005576 GO:0005840
GO:0005737
Cluster-22178.66860 GO:0008152 GO:0003824
Cluster-22178.66861 GO:0015979 GO:0005515 GO:0016773
Cluster-22178.66862 GO:0006935 GO:0007165 GO:0006810 GO:0004888
GO:0005643 GO:0016020
Cluster-22178.66863 GO:0006508 GO:0005516 GO:0004197
Cluster-22178.66865 GO:0005515
Cluster-22178.66866 GO:0042967 GO:0006355 GO:0003712 GO:0008270
GO:0004402 GO:0016787 GO:0000123 GO:0005667 GO:0005634
Cluster-22178.66867 GO:0042254 GO:0006412 GO:0003735 GO:0005622
GO:0005840
Cluster-22178.66869 GO:0009451 GO:0001522 GO:0044419 GO:0006508
GO:0006144 GO:0017111 GO:0004197 GO:0016787 GO:0003723
GO:0003968 GO:0003676 GO:0009982 GO:0046872 GO:0019031
GO:0031379
Cluster-22178.6687 GO:0042742
Cluster-22178.66870 GO:0005840
Cluster-22178.66872 GO:0006289 GO:0016310 GO:0009411 GO:0007186
GO:0005524 GO:0046917 GO:0004930 GO:0004519 GO:0016021
Cluster-22178.66876 GO:0000746 GO:0003676 GO:0003677
Cluster-22178.66877 GO:0000746 GO:0003676 GO:0003677
Cluster-22178.6688 GO:0042742
Cluster-22178.66880 GO:0018279 GO:0004579 GO:0008250 GO:0016021
Cluster-22178.66881 GO:0006777
Cluster-22178.66882 GO:0006118 GO:0045454 GO:0015035 GO:0009055

Cluster-22178.66883 GO:0016787 GO:0005524
Cluster-22178.66885 GO:0007275 GO:0007165 GO:0008270 GO:0004888
GO:0016021
Cluster-22178.66888 GO:0006183 GO:0006228 GO:0006165 GO:0006241
GO:0006206 GO:0006144 GO:0005515 GO:0003723 GO:0003676
GO:0004550
Cluster-22178.6689 GO:0009405 GO:0007268 GO:0007165 GO:0030550
GO:0005576
Cluster-22178.66890 GO:0006355 GO:0042254 GO:0006412 GO:0043565
GO:0008270 GO:0003700 GO:0003735 GO:0005840 GO:0005667
Cluster-22178.66891 GO:0007049 GO:0000917 GO:0043093 GO:0006355
GO:0003700 GO:0043565 GO:0003677 GO:0005198 GO:0019031
GO:0005737 GO:0019028 GO:0005634 GO:0005667
Cluster-22178.66892 GO:0003677
Cluster-22178.66893 GO:0006281 GO:0046872 GO:0008270
Cluster-22178.66894 GO:0046872 GO:0000166
Cluster-22178.66896 GO:0006508 GO:0005509 GO:0004198 GO:0005622
Cluster-22178.66897 GO:0042254 GO:0006412 GO:0003735 GO:0005622
GO:0005840
Cluster-22178.66899 GO:0015991 GO:0045893 GO:0015992 GO:0015078
GO:0009512 GO:0033179
Cluster-22178.66901 GO:0005096 GO:0017025 GO:0005634
Cluster-22178.66909 GO:0006355 GO:0008168 GO:0016817 GO:0003676
GO:0003700 GO:0005507 GO:0003677 GO:0005667 GO:0005634
Cluster-22178.66910 GO:0006020 GO:0006094 GO:0046486 GO:0006096
GO:0006000 GO:0015976 GO:0006013 GO:0008152 GO:0004807
Cluster-22178.66911 GO:0003676 GO:0003713 GO:0005667 GO:0005634
Cluster-22178.66914 GO:0006511 GO:0006313 GO:0015074 GO:0005975
GO:0004560 GO:0005515 GO:0003677 GO:0046872
Cluster-22178.66916 GO:0055114 GO:0006809 GO:0010181 GO:0016491
GO:0004517 GO:0005515 GO:0016717
Cluster-22178.66919 GO:0016567 GO:0019058 GO:0015986 GO:0006396

GO:0006807 GO:0006665 GO:0006464 GO:0015992 GO:0006687
GO:0006468 GO:0005975 GO:0004348 GO:0004842 GO:0016773
GO:0005524 GO:0008270 GO:0005515 GO:0003723 GO:0003676
GO:0015078 GO:0016151 GO:0046872 GO:0004672 GO:0016020
GO:0005680 GO:0000276
Cluster-22178.66920 GO:0000105 GO:0006547 GO:0004635
Cluster-22178.66921 GO:0006265 GO:0043248 GO:0006886 GO:0016192
GO:0005524 GO:0003918 GO:0003677 GO:0005515 GO:0030117
GO:0005634
Cluster-22178.66922 GO:0006355 GO:0008233 GO:0003700 GO:0003723
GO:0004252 GO:0016791 GO:0005618 GO:0005634 GO:0005667
GO:0016020
Cluster-22178.66923 GO:0055114 GO:0016620
Cluster-22178.66925 GO:0042254 GO:0006412 GO:0003735 GO:0005622
GO:0005840
Cluster-22178.66926 GO:0005975 GO:0003824 GO:0043169 GO:0030089
Cluster-22178.66927 GO:0006355 GO:0004252 GO:0003700 GO:0003824
GO:0042025 GO:0005882 GO:0005667 GO:0016021 GO:0005886
Cluster-22178.66928 GO:0005739
Cluster-22178.66929 GO:0006560 GO:0006814 GO:0071436 GO:0006525
GO:0006771 GO:0006090 GO:0019497 GO:0003677 GO:0008948
GO:0005515 GO:0003993 GO:0046872 GO:0015081 GO:0016020
Cluster-22178.66930 GO:0006464
Cluster-22178.66931 GO:0006998 GO:0006470 GO:0006406 GO:0006570
GO:0006611 GO:0004725 GO:0008138 GO:0031965
Cluster-22178.66932 GO:0006886 GO:0006605 GO:0005515 GO:0008270
GO:0016020
Cluster-22178.66933 GO:0051028 GO:0005634
Cluster-22178.66934 GO:0006820 GO:0009236 GO:0006355 GO:0006270
GO:0019051 GO:0008509 GO:0003723 GO:0005515 GO:0003677
GO:0016021 GO:0042025
Cluster-22178.66935 GO:0051259 GO:0005515 GO:0017119
Cluster-22178.66936 GO:0006626 GO:0003824 GO:0005741

Cluster-22178.66937 GO:0045122 GO:0006351 GO:0006355 GO:0006206
GO:0051262 GO:0006144 GO:0046872 GO:0008134 GO:0005515
GO:0003899 GO:0044212 GO:0003677 GO:0019904 GO:0005634
GO:0005730 GO:0005667
Cluster-22178.66938 GO:0009058 GO:0006687 GO:0001733 GO:0008146
GO:0003677 GO:0016021 GO:0005794
Cluster-22178.66939 GO:0006687 GO:0009058 GO:0008146 GO:0001733
GO:0016021 GO:0005794
Cluster-22178.66940 GO:0007018 GO:0007017 GO:0009069 GO:0016310
GO:0005515 GO:0008017 GO:0003777 GO:0004674 GO:0016773
GO:0005524 GO:0045298 GO:0005874
Cluster-22178.66944 GO:0006351 GO:0006825 GO:0006144 GO:0006206
GO:0016531 GO:0003899 GO:0003677 GO:0005507 GO:0005634
GO:0005730 GO:0005758
Cluster-22178.66945 GO:0006355 GO:0005515 GO:0005634
Cluster-22178.66946 GO:0006364 GO:0003723 GO:0008168 GO:0003676

Cluster-22178.66947 GO:0019058
Cluster-22178.66948 GO:0006468 GO:0004672 GO:0005524
Cluster-22178.66949 GO:0009847
Cluster-22178.66950 GO:0015694 GO:0046872 GO:0008270 GO:0015097
GO:0003677 GO:0005515 GO:0016020
Cluster-22178.66951 GO:0006813 GO:0055114 GO:0051287 GO:0016616

Cluster-22178.66952 GO:0016021
Cluster-22178.66953 GO:0006810 GO:0005622
Cluster-22178.66954 GO:0006418 GO:0005524 GO:0004812 GO:0000166

Cluster-22178.66955 GO:0006418 GO:0005524 GO:0004812 GO:0000166

Cluster-22178.66956 GO:0006687 GO:0006468 GO:0005975 GO:0019058
GO:0016567 GO:0006396 GO:0006807 GO:0006665 GO:0006464
GO:0005515 GO:0003723 GO:0003676 GO:0016151 GO:0046872

GO:0004672 GO:0004348 GO:0004842 GO:0016773 GO:0008270
GO:0005524 GO:0016020 GO:0005680
Cluster-22178.66957 GO:0006468 GO:0005524 GO:0004672 GO:0016773
GO:0016020
Cluster-22178.66958 GO:0015992 GO:0046660 GO:0015986 GO:0006397
GO:0006810 GO:0008270 GO:0003676 GO:0015078 GO:0000276
GO:0005643 GO:0005634
Cluster-22178.66959 GO:0042254 GO:0005634
Cluster-22178.66962 GO:0006468 GO:0004672 GO:0005524 GO:0016773
GO:0016020
Cluster-22178.66963 GO:0019087 GO:0032259 GO:0008168 GO:0016021
Cluster-22178.66964 GO:0046856 GO:0034597 GO:0003677
Cluster-22178.66965 GO:0042254 GO:0006412 GO:0003735 GO:0019843
GO:0005840
Cluster-22178.66966 GO:0006508 GO:0006629 GO:0006144 GO:0004252
GO:0017111 GO:0004197 GO:0003968 GO:0070008 GO:0016817
GO:0031379
Cluster-22178.66968 GO:0015031 GO:0030176
Cluster-22178.66970 GO:0021549 GO:0030182 GO:0008270
Cluster-22178.66971 GO:0045893 GO:0016567 GO:0003676 GO:0005515
GO:0004842 GO:0042025
Cluster-22178.66972 GO:0055085 GO:0006811 GO:0006355 GO:0005515
GO:0005216 GO:0016020 GO:0005761
Cluster-22178.66973 GO:0008270
Cluster-22178.66975 GO:0009405 GO:0019835 GO:0019867
Cluster-22178.66977 GO:0001678 GO:0007186 GO:0008343 GO:0000186
GO:0007049 GO:0032099 GO:0009267 GO:0005634 GO:0005615
Cluster-22178.66978 GO:0006139 GO:0003676 GO:0005524 GO:0016818
GO:0003677 GO:0008026 GO:0016787 GO:0004003 GO:0005657
GO:0005634
Cluster-22178.66981 GO:0006308 GO:0006281 GO:0008852 GO:0016021
Cluster-22178.66982 GO:0006281 GO:0006308 GO:0045766 GO:0043066
GO:0043123 GO:0008852 GO:0016021

Cluster-22178.66983 GO:0007264 GO:0005525
Cluster-22178.66984 GO:0006364 GO:0009451 GO:0030488 GO:0008033
GO:0003723 GO:0008168 GO:0016429 GO:0031515
Cluster-22178.66985 GO:0006520 GO:0000921 GO:0046856 GO:0006606
GO:0006396 GO:0006614 GO:0006355 GO:0008312 GO:0043565
GO:0003700 GO:0034597 GO:0003723 GO:0045502 GO:0008134
GO:0042803 GO:0005515 GO:0030286 GO:0005940 GO:0005667
GO:0048500 GO:0016021
Cluster-22178.66987 GO:0051568 GO:0006479 GO:0000902 GO:0006508
GO:0003779 GO:0042800 GO:0005515 GO:0003682 GO:0072357
GO:0000785 GO:0048188
Cluster-22178.66989 GO:0055114 GO:0016705 GO:0020037 GO:0005506

Cluster-22178.66990 GO:0055114 GO:0020037 GO:0016705 GO:0005506

Cluster-22178.66991 GO:0006886 GO:0008536
Cluster-22178.66996 GO:0006182 GO:0035556 GO:0006357 GO:0006144
GO:0006468 GO:0009190 GO:0046039 GO:0008152 GO:0008270
GO:0005524 GO:0001104 GO:0004383 GO:0016849 GO:0003723
GO:0003824 GO:0004672 GO:0016592
Cluster-22178.66998 GO:0071436 GO:0006814 GO:0006560 GO:0006525
GO:0006090 GO:0005515 GO:0008948 GO:0015081 GO:0008270
GO:0046872 GO:0016020 GO:0019013
Cluster-22178.66999 GO:0055114 GO:0007165 GO:0006810 GO:0005215
GO:0005158 GO:0016705 GO:0020037 GO:0005506 GO:0005899
GO:0016020
Cluster-22178.67000 GO:0006364 GO:0019722 GO:0003676 GO:0003723
GO:0008168
Cluster-22178.67001 GO:0055114 GO:0007165 GO:0005506 GO:0005158
GO:0020037 GO:0016705 GO:0005899
Cluster-22178.67003 GO:0006470 GO:0006400 GO:0008616 GO:0000087
GO:0006570 GO:0015694 GO:0004725 GO:0015097 GO:0008479
GO:0005622 GO:0019031 GO:0016020

Cluster-22178.67004 GO:0006457 GO:0006886 GO:0008219 GO:0007304
GO:0051087 GO:0000774 GO:0008270 GO:0042803 GO:0005515
GO:0005198 GO:0005213 GO:0016020 GO:0005783 GO:0019028
GO:0005576 GO:0042600 GO:0005801 GO:0016021
Cluster-22178.67005 GO:0006308 GO:0051258 GO:0051382 GO:0006886
GO:0009405 GO:0030472 GO:0006606 GO:0006357 GO:0030168
GO:0006457 GO:0007165 GO:0007155 GO:0004871 GO:0005198
GO:0001104 GO:0051082 GO:0005102 GO:0008855 GO:0030674
GO:0005801 GO:0016021 GO:0019031 GO:0042729 GO:0009318
GO:0019028 GO:0072686 GO:0000776 GO:0005577 GO:0016272
GO:0016020 GO:0016592
Cluster-22178.67006 GO:0006412 GO:0042254 GO:0003735 GO:0005840
GO:0005622
Cluster-22178.67007 GO:0006468 GO:0006118 GO:0016773 GO:0004672
GO:0005524 GO:0003676 GO:0009055 GO:0016020
Cluster-22178.67008 GO:0045859 GO:0007049 GO:0016538
Cluster-22178.67009 GO:0055114 GO:0016491
Cluster-22178.67010 GO:0006260 GO:0018142 GO:0006269
Cluster-22178.67011 GO:0042254 GO:0006412 GO:0003735 GO:0005840
GO:0005622
Cluster-22178.67012 GO:0003723
Cluster-22178.67013 GO:0003723
Cluster-22178.67014 GO:0051082 GO:0031072
Cluster-22178.67017 GO:0006396 GO:0051252 GO:0008033 GO:0003676
GO:0004526 GO:0030677
Cluster-22178.67018 GO:0006886 GO:0008277 GO:0006355 GO:0007606
GO:0007165 GO:0007186 GO:0019058 GO:0007264 GO:0043087
GO:0015031 GO:0005515 GO:0042802 GO:0004888 GO:0003700
GO:0008092 GO:0043565 GO:0030674 GO:0004930 GO:0043130
GO:0008565 GO:0005096 GO:0019031 GO:0016021 GO:0016020
GO:0005667
Cluster-22178.67020 GO:0006123 GO:0015992 GO:0004129 GO:0045277
Cluster-22178.67023 GO:0006508 GO:0006355 GO:0004197

Cluster-22178.67025 GO:0005634
Cluster-22178.67026 GO:0007275 GO:0006351 GO:0030154 GO:0007219 GO:0005515 GO:0016788 GO:0003677 GO:0005634 GO:0016021
Cluster-22178.67029 GO:0006836 GO:0006812 GO:0005335 GO:0003676 GO:0005887
Cluster-22178.67030 GO:0009405
Cluster-22178.67033 GO:0006879 GO:0005576
Cluster-22178.67035 GO:0061630 GO:0005515 GO:0008270 GO:0046872

Cluster-22178.67036 GO:0016055 GO:0006531 GO:0043039 GO:0006419 GO:0006522 GO:0000166 GO:0004813 GO:0016876 GO:0005524 GO:0003676
Cluster-22178.67037 GO:0030336 GO:0010972 GO:0016579 GO:0036459

Cluster-22178.67038 GO:0015946 GO:0055114 GO:0030908 GO:0004022

Cluster-22178.67041 GO:0006351 GO:0006269 GO:0006260 GO:0035556 GO:0003677 GO:0003896 GO:0046872 GO:0005730 GO:0005657
Cluster-22178.67042 GO:0006118 GO:0045454 GO:0006144 GO:0000398 GO:0005515 GO:0003968 GO:0015035 GO:0009055 GO:0005681 GO:0016021 GO:0031379
Cluster-22178.67044 GO:0006260 GO:0006355 GO:0006400 GO:0008616 GO:0003700 GO:0008479 GO:0005667 GO:0005634
Cluster-22178.67046 GO:0008616 GO:0006355 GO:0006400 GO:0006260 GO:0008479 GO:0003700 GO:0005667 GO:0005634
Cluster-22178.67047 GO:0023052 GO:0007165 GO:0005125
Cluster-22178.67049 GO:0004672
Cluster-22178.67050 GO:0022008 GO:0005764
Cluster-22178.67053 GO:0016567 GO:0007005 GO:0005515 GO:0008270 GO:0016758 GO:0004842 GO:0005783 GO:0016021
Cluster-22178.67054 GO:0055114 GO:0008152 GO:0006118 GO:0016627 GO:0050660 GO:0003995
Cluster-22178.67055 GO:0005515 GO:0008270

Cluster-22178.67056 GO:0006355 GO:0000921 GO:0006520 GO:0006396
GO:0046856 GO:0006606 GO:0006614 GO:0043565 GO:0034597
GO:0003700 GO:0003723 GO:0045502 GO:0005515 GO:0042803
GO:0008134 GO:0008312 GO:0048500 GO:0016021 GO:0005940
GO:0030286 GO:0005667
Cluster-22178.67058 GO:0006355 GO:0007165 GO:0046983 GO:0035257
GO:0003713 GO:0005667 GO:0005634
Cluster-22178.67059 GO:0006355 GO:0043565 GO:0003700 GO:0005515
GO:0005667
Cluster-22178.67060 GO:0055085 GO:0006810 GO:0005215 GO:0016020
Cluster-22178.67061 GO:0045859 GO:0007050 GO:0004861 GO:0005634
Cluster-22178.67062 GO:0006355 GO:0046983
Cluster-22178.67063 GO:0045454 GO:0000398 GO:0007067 GO:0005681
Cluster-22178.67065 GO:0042254 GO:0006412 GO:0003735 GO:0005622
GO:0005840
Cluster-22178.6707 GO:0006310 GO:0015991 GO:0048870 GO:0015992
GO:0008134 GO:0042803 GO:0045502 GO:0015078 GO:0031514
GO:0033179 GO:0005667 GO:0030286
Cluster-22178.67071 GO:0055114 GO:0016209 GO:0016491 GO:0008270
GO:0016021
Cluster-22178.67072 GO:0005515
Cluster-22178.67074 GO:0008152 GO:0003824 GO:0008484
Cluster-22178.67075 GO:0008270 GO:0005524 GO:0004222 GO:0016021
Cluster-22178.67077 GO:0008152 GO:0016746
Cluster-22178.67080 GO:0007049 GO:0006355 GO:0003677 GO:0003700
GO:0043565 GO:0005667 GO:0005634
Cluster-22178.67081 GO:0006412 GO:0042254 GO:0003735 GO:0005840
GO:0005622
Cluster-22178.67087 GO:0008152 GO:0016787
Cluster-22178.67088 GO:0045132 GO:0005515 GO:0005634 GO:0000775
Cluster-22178.67090 GO:0019058 GO:0007283 GO:0007165 GO:0008601
GO:0000159
Cluster-22178.67091 GO:0042742 GO:0007165 GO:0009395 GO:0008654

GO:0016310 GO:0016042 GO:0000160 GO:0000155 GO:0004623
GO:0005509 GO:0005515 GO:0043772 GO:0005576 GO:0005886
GO:0009365
Cluster-22178.67094 GO:0055114 GO:0006879 GO:0016491 GO:0046872
GO:0008199
Cluster-22178.67095 GO:0006508 GO:0004198 GO:0016757 GO:0005622
GO:0016020
Cluster-22178.67097 GO:0006412 GO:0042254 GO:0003735 GO:0005515
GO:0005840
Cluster-22178.67099 GO:0009252 GO:0006807 GO:0006811 GO:0006542
GO:0006355 GO:0006813 GO:0005249 GO:0004356 GO:0003677
GO:0016020 GO:0008076
Cluster-22178.67100 GO:0005975 GO:0016773 GO:0005615
Cluster-22178.67101 GO:0055114 GO:0051920 GO:0016491 GO:0016209

Cluster-22178.67105 GO:0006355 GO:0005515 GO:0003676 GO:0043565
GO:0005634
Cluster-22178.67106 GO:0051382 GO:0006914 GO:0007049 GO:0006355
GO:0009405 GO:0043565 GO:0019905 GO:0003700 GO:0051082
GO:0000776 GO:0005667 GO:0016020
Cluster-22178.67107 GO:0020037
Cluster-22178.67108 GO:0005524 GO:0016787 GO:0003677 GO:0003676

Cluster-22178.67110 GO:0006165 GO:0007155 GO:0006228 GO:0006183
GO:0006144 GO:0006206 GO:0006241 GO:0003723 GO:0005515
GO:0004550 GO:0003676
Cluster-22178.67111 GO:0006820 GO:0016021
Cluster-22178.67113 GO:0006511 GO:0007275 GO:0008270 GO:0005515
GO:0005634
Cluster-22178.67114 GO:0043248 GO:0015991 GO:0006486 GO:0016192
GO:0006886 GO:0008378 GO:0016820 GO:0000221 GO:0030117
GO:0016020
Cluster-22178.67115 GO:0006189

Cluster-22178.67118 GO:0006457 GO:0008270 GO:0042803 GO:0008134 GO:0045502 GO:0051082 GO:0016272 GO:0030286 GO:0005667
Cluster-22178.67120 GO:0022900 GO:0003676 GO:0005524 GO:0046872 GO:0003677 GO:0005515 GO:0016787 GO:0005743 GO:0070469
Cluster-22178.67121 GO:0006998 GO:0035556 GO:0006611 GO:0006406 GO:0005515 GO:0031965
Cluster-22178.67122 GO:0035023 GO:0043087 GO:0005089
Cluster-22178.67123 GO:0015992 GO:0006120 GO:0006744 GO:0006814 GO:0008137 GO:0005743
Cluster-22178.67124 GO:0006144 GO:0006508 GO:0004197 GO:0003968 GO:0005515 GO:0017111 GO:0031379
Cluster-22178.67127 GO:0005515
Cluster-22178.67128 GO:0016070
Cluster-22178.67129 GO:0007156 GO:0005509 GO:0016020
Cluster-22178.67130 GO:0015991 GO:0005524 GO:0016820 GO:0033178
Cluster-22178.67133 GO:0042138 GO:0006310 GO:0003824
Cluster-22178.67135 GO:0042138 GO:0006310 GO:0003824
Cluster-22178.67137 GO:0042981
Cluster-22178.67140 GO:0006310 GO:0006281 GO:0002949 GO:0003723 GO:0009378 GO:0003724 GO:0016887 GO:0005524 GO:0009379 GO:0005657
Cluster-22178.67141 GO:0005515
Cluster-22178.67143 GO:0019049
Cluster-22178.67144 GO:0030151 GO:0030170 GO:0003824
Cluster-22178.67145 GO:0016192 GO:0006886 GO:0003824 GO:0005515 GO:0005198 GO:0016021 GO:0030126 GO:0016020
Cluster-22178.67147 GO:0006397 GO:0016567 GO:0006508 GO:0004252 GO:0003723 GO:0008233 GO:0030430 GO:0005634 GO:0005737
Cluster-22178.67149 GO:0006813 GO:0071805 GO:0007165 GO:0015079 GO:0016787 GO:0005509 GO:0016021 GO:0005887 GO:0005578
Cluster-22178.67151 GO:0016567 GO:0006397 GO:0006508 GO:0004252 GO:0003723 GO:0008233 GO:0030430 GO:0005634 GO:0005737
Cluster-22178.67153 GO:0035556 GO:0006810 GO:0009405 GO:0008200

GO:0005515 GO:0003677 GO:0005634 GO:0005576
Cluster-22178.67154 GO:0006979 GO:0055114 GO:0006804 GO:0006749
GO:0006400 GO:0008616 GO:0016491 GO:0004602 GO:0016209
GO:0008479
Cluster-22178.67155 GO:0003677 GO:0005576
Cluster-22178.67158 GO:0007275 GO:0007165 GO:0006468 GO:0016055
GO:0005102 GO:0004672 GO:0005524 GO:0005576
Cluster-22178.67162 GO:0006952
Cluster-22178.67164 GO:0006810 GO:0005622
Cluster-22178.67165 GO:0015694 GO:0003677 GO:0015097 GO:0016020
Cluster-22178.67166 GO:0006468 GO:0005524 GO:0004672 GO:0016773
GO:0016020
Cluster-22178.67167 GO:0007165 GO:0006457 GO:0000774 GO:0051087
GO:0016853 GO:0042803 GO:0035091
Cluster-22178.67170 GO:0015684 GO:0007264 GO:0005525 GO:0003924
GO:0015093 GO:0016021
Cluster-22178.67175 GO:0006396 GO:0042967 GO:0030488 GO:0006464
GO:0046486 GO:0046500 GO:0008033 GO:0008610 GO:0009451
GO:0006479 GO:0016429 GO:0004144 GO:0008168 GO:0016627
GO:0004719 GO:0005622 GO:0031515
Cluster-22178.67178 GO:0019236 GO:0007212 GO:0048268 GO:0007186
GO:0032051 GO:0016021
Cluster-22178.67179 GO:0007586 GO:0016042 GO:0008152 GO:0016758
GO:0008047 GO:0005576
Cluster-22178.67183 GO:0055085 GO:0006744 GO:0007411 GO:0006355
GO:0030001 GO:0016055 GO:0003677 GO:0005198 GO:0005515
GO:0046873 GO:0019013 GO:0016020 GO:0005576
Cluster-22178.67187 GO:0042967 GO:0016747 GO:0005515 GO:0008080

Cluster-22178.67188 GO:0003824
Cluster-22178.67189 GO:0006810 GO:0006310 GO:0006309 GO:0006260
GO:0006281 GO:0098519 GO:0003677 GO:0009378 GO:0016787
GO:0003724 GO:0005524 GO:0016887 GO:0003887 GO:0000166

GO:0003723 GO:0003676 GO:0009379 GO:0005657 GO:0009360
GO:0042575 GO:0005634 GO:0005737
Cluster-22178.67190 GO:0046486 GO:0035556 GO:0000226 GO:0006506
GO:0000725 GO:0090063 GO:0008654 GO:0007021 GO:0009395
GO:0016746 GO:0004143 GO:0015631 GO:0005515 GO:0051082
GO:0016020 GO:0005815 GO:0005634 GO:0005789 GO:0016021
GO:0045298 GO:0000922 GO:0097196
Cluster-22178.67191 GO:0045947 GO:0008190
Cluster-22178.67192 GO:0051603 GO:0006511 GO:0004298 GO:0004175
GO:0005515 GO:0003677 GO:0019773 GO:0005839
Cluster-22178.67194 GO:0006888 GO:0003676 GO:0005801 GO:0005622
Cluster-22178.67195 GO:0006886 GO:0008536
Cluster-22178.67196 GO:0042981
Cluster-22178.67199 GO:0006260 GO:0006281 GO:0070206 GO:0003824
GO:0005515 GO:0005096 GO:0034464
Cluster-22178.67200 GO:0006807 GO:0005975 GO:0009058 GO:0000917
GO:0006355 GO:0043093 GO:0004553 GO:0003933 GO:0043565
GO:0008270 GO:0005737
Cluster-22178.67201 GO:0006260 GO:0006914 GO:0045454 GO:0005509
GO:0016491 GO:0005815
Cluster-22178.67202 GO:0006281 GO:0007017 GO:0007018 GO:0006310
GO:0006260 GO:0003887 GO:0005515 GO:0003777 GO:0009378
GO:0016787 GO:0016818 GO:0003677 GO:0005524 GO:0016887
GO:0008017 GO:0005634 GO:0042575 GO:0005874 GO:0045298
GO:0009379 GO:0009360 GO:0005657
Cluster-22178.67203 GO:0051258 GO:0030168 GO:0007172 GO:0007165
GO:0006468 GO:0005102 GO:0004871 GO:0004713 GO:0005524
GO:0004672 GO:0030674 GO:0005577 GO:0005925
Cluster-22178.67204 GO:0015992 GO:0006308 GO:0015991 GO:0015078
GO:0003676 GO:0008855 GO:0009318 GO:0033179
Cluster-22178.67205 GO:0055114 GO:0005515
Cluster-22178.67206 GO:0055114 GO:0005515
Cluster-22178.67208 GO:0006376 GO:0015743 GO:0003729 GO:0005685

Cluster-22178.67209 GO:0006376 GO:0003729 GO:0005685
Cluster-22178.67210 GO:0043565 GO:0005634
Cluster-22178.67211 GO:0005515
Cluster-22178.67215 GO:0006898 GO:0007165 GO:0006464 GO:0005044
GO:0016020
Cluster-22178.67216 GO:0006118 GO:0009055 GO:0051536
Cluster-22178.67217 GO:0006952 GO:0007163 GO:0033206 GO:0016482
GO:0051321 GO:0006928 GO:0008356 GO:0051653 GO:0030036
GO:0005524 GO:0003779 GO:0005576 GO:0042995 GO:0005885
GO:0030478
Cluster-22178.67218 GO:0006928 GO:0008356 GO:0051321 GO:0051653
GO:0030036 GO:0033206 GO:0016482 GO:0007163 GO:0006952
GO:0003779 GO:0005524 GO:0030478 GO:0042995 GO:0005576
GO:0005885
Cluster-22178.67220 GO:0006412 GO:0042254 GO:0003735 GO:0005622
GO:0005840
Cluster-22178.67222 GO:0034968 GO:0006554 GO:0006355 GO:0006479
GO:0018024 GO:0019901 GO:0005694
Cluster-22178.67223 GO:0006230 GO:0016032 GO:0006206 GO:0006310
GO:0007268 GO:0007165 GO:0006302 GO:0015743 GO:0004797
GO:0005515 GO:0030550 GO:0005524 GO:0003677 GO:0005789
GO:0005634 GO:0005576
Cluster-22178.67224 GO:0008152 GO:0055114 GO:0006355 GO:0003700
GO:0016491 GO:0005667
Cluster-22178.67226 GO:0005524
Cluster-22178.67227 GO:0000077 GO:0007224 GO:0003824 GO:0030896
Cluster-22178.67228 GO:0009231 GO:0006771 GO:0003919 GO:0008270
GO:0016021
Cluster-22178.6723 GO:0005198 GO:0005515 GO:0019028
Cluster-22178.67230 GO:1901031 GO:0006413 GO:0005515 GO:0003723
GO:0016020 GO:0019012 GO:0005634 GO:0019028
Cluster-22178.67231 GO:0006810 GO:0009405 GO:0005515 GO:0019870
GO:0005576

Cluster-22178.67232 GO:0006468 GO:0005524 GO:0004672 GO:0016773
GO:0016020
Cluster-22178.67233 GO:0006810 GO:0055085 GO:0005524 GO:0016887
GO:0042626 GO:0016021
Cluster-22178.67234 GO:0006508 GO:0006122 GO:0005515 GO:0004197
GO:0003677 GO:0008270 GO:0005743 GO:0005750
Cluster-22178.67235 GO:0006270 GO:0019079 GO:0006310 GO:0006816
GO:0006874 GO:0007155 GO:0002949 GO:0006355 GO:0006281
GO:0007049 GO:0009378 GO:0003724 GO:0005524 GO:0016887
GO:0005515 GO:0004252 GO:0008134 GO:0003723 GO:0004190
GO:0003678 GO:0005219 GO:0005667 GO:0009379 GO:0005657
GO:0005622 GO:0005886 GO:0016021
Cluster-22178.67238 GO:0035556 GO:0000226 GO:0046486 GO:0009395
GO:0008654 GO:0007021 GO:0090063 GO:0006506 GO:0000725
GO:0004143 GO:0015631 GO:0016746 GO:0051082 GO:0005515
GO:0005815 GO:0016020 GO:0097196 GO:0005789 GO:0016021
GO:0045298 GO:0000922 GO:0005634
Cluster-22178.67239 GO:0006355 GO:0006289 GO:0005515 GO:0016020
GO:0000439
Cluster-22178.67243 GO:0019491 GO:0007034 GO:0033990
Cluster-22178.67245 GO:0006812 GO:0052331 GO:0046931 GO:0006952
GO:0007165 GO:0006810 GO:0015267 GO:0005126 GO:0046930
GO:0005576
Cluster-22178.67248 GO:0005515
Cluster-22178.6725 GO:0006955 GO:0007165 GO:0006935 GO:0008009
GO:0005576
Cluster-22178.67252 GO:0006281 GO:0016032 GO:0006508 GO:0006351
GO:0006206 GO:0006144 GO:0006310 GO:0003678 GO:0003899
GO:0005524 GO:0008270 GO:0016887 GO:0004222 GO:0009378
GO:0003677 GO:0016021 GO:0005657 GO:0005730 GO:0009379
Cluster-22178.67253 GO:0046872
Cluster-22178.67254 GO:0046740 GO:0005515 GO:0016021
Cluster-22178.67255 GO:0008152 GO:0046872 GO:0016746

Cluster-22178.67256 GO:0018101 GO:0006807 GO:0005509 GO:0004668 GO:0005737
Cluster-22178.67258 GO:0015696 GO:0006468 GO:0006810 GO:0005524 GO:0004672 GO:0008519 GO:0016787 GO:0016021 GO:0005794 GO:0016020 GO:0031224
Cluster-22178.67259 GO:0046983 GO:0003677
Cluster-22178.67260 GO:0046983 GO:0003677
Cluster-22178.67267 GO:0007165 GO:0035556 GO:0005515 GO:0003774 GO:0005524 GO:0016459
Cluster-22178.67268 GO:0006355 GO:0016055 GO:0006886 GO:0000975 GO:0008536 GO:0003700 GO:0005667
Cluster-22178.67269 GO:0006508 GO:0006260 GO:0019073 GO:0006139 GO:0003677 GO:0003887 GO:0008408 GO:0008270 GO:0004222 GO:0003676 GO:0042575 GO:0031012
Cluster-22178.67270 GO:0055085 GO:0006811 GO:0006898 GO:0007165 GO:0007010 GO:0003779 GO:0005044 GO:0005216 GO:0016020
Cluster-22178.67271 GO:0005515
Cluster-22178.67274 GO:0006606 GO:0006914 GO:0031083 GO:0016021
Cluster-22178.67277 GO:0055085 GO:0003677 GO:0022857 GO:0005515 GO:0016021
Cluster-22178.67278 GO:0006486 GO:0008373
Cluster-22178.67279 GO:0042981
Cluster-22178.67281 GO:0016579 GO:0005515 GO:0036459 GO:0005096

Cluster-22178.67282 GO:0016310 GO:0006511 GO:0009069 GO:0006468 GO:0004674 GO:0005524 GO:0031625
Cluster-22178.67283 GO:0005515
Cluster-22178.67285 GO:0006810 GO:0009264 GO:0005515 GO:0005524 GO:0046872 GO:0043531 GO:0008253 GO:0004386 GO:0003676 GO:0016020
Cluster-22178.67286 GO:0006144 GO:0000398 GO:0045454 GO:0006118 GO:0003968 GO:0015035 GO:0009055 GO:0005515 GO:0031379 GO:0005681

Cluster-22178.67287 GO:0016021
Cluster-22178.67288 GO:0042254 GO:0006412 GO:0003735 GO:0005840
GO:0005622
Cluster-22178.67289 GO:0050992 GO:0019288 GO:0046872 GO:0051745
GO:0005515
Cluster-22178.67291 GO:0007018 GO:0005515 GO:0005868
Cluster-22178.67292 GO:0006810 GO:0005622
Cluster-22178.67293 GO:0007165 GO:0043401 GO:0006355 GO:0003700
GO:0005186 GO:0043565 GO:0008270 GO:0005615 GO:0005634
GO:0005667
Cluster-22178.67294 GO:0055114 GO:0010277 GO:0005515
Cluster-22178.67295 GO:0055114 GO:0019478 GO:0006355 GO:0003676
GO:0003700 GO:0016491 GO:0043565 GO:0016788 GO:0005515
GO:0050662 GO:0005737 GO:0016020 GO:0005667
Cluster-22178.67296 GO:0006886 GO:0019904 GO:0035091 GO:0019028
Cluster-22178.67298 GO:0030193 GO:0007155 GO:0005515 GO:0005639
GO:0005576
Cluster-22178.6730 GO:0055085 GO:0007154 GO:0016747 GO:0016021
Cluster-22178.67301 GO:0006687 GO:0050290
Cluster-22178.67302 GO:0006260 GO:0016192 GO:0019079 GO:0005524
GO:0003678 GO:0003676 GO:0016787 GO:0005515 GO:0003677
GO:0016021 GO:0005657
Cluster-22178.67303 GO:0050830 GO:0006807 GO:0006144 GO:0019497
GO:0006771 GO:0006164 GO:0003993 GO:0003937 GO:0004643
GO:0031423 GO:0046872 GO:0005515 GO:0042720 GO:0019012
GO:0005576
Cluster-22178.67306 GO:0005515
Cluster-22178.67307 GO:0006486 GO:0004576 GO:0016020 GO:0008250
Cluster-22178.67312 GO:0006606 GO:0071988 GO:0006308 GO:0048870
GO:0006355 GO:0030474 GO:0070206 GO:0008053 GO:0006511
GO:0007275 GO:0003924 GO:0003723 GO:0042803 GO:0005198
GO:0005515 GO:0008270 GO:0005524 GO:0004386 GO:0046872
GO:0003700 GO:0045502 GO:0008134 GO:0008855 GO:0005634

GO:0005667 GO:0005741 GO:0016021 GO:0019031 GO:0019028
GO:0071203 GO:0031514 GO:0009318 GO:0030286
Cluster-22178.67315 GO:0006468 GO:0005524 GO:0004672 GO:0016773
GO:0016020
Cluster-22178.67317 GO:0006260 GO:0003677 GO:0005664
Cluster-22178.67319 GO:0006164 GO:0006177 GO:0006541 GO:0006536
GO:0006560 GO:0008033 GO:0015074 GO:0006522 GO:0006529
GO:0008152 GO:0006531 GO:0006526 GO:0006144 GO:0004810
GO:0003922 GO:0003824 GO:0004055 GO:0016787 GO:0004066
GO:0005524
Cluster-22178.67320 GO:0006508 GO:0008234 GO:0005742
Cluster-22178.67321 GO:0006468 GO:0005524 GO:0004672 GO:0016773
GO:0016020
Cluster-22178.67323 GO:0045893 GO:1902208 GO:0005975 GO:0006810
GO:0008270 GO:0005215 GO:0003950 GO:0016832 GO:0005515
GO:0003677 GO:0005737 GO:0016021 GO:0005634
Cluster-22178.67326 GO:0006432 GO:0000162 GO:0006571 GO:0009094
GO:0005515 GO:0003723 GO:0004826 GO:0009328
Cluster-22178.67327 GO:0051603 GO:0006511 GO:0004175 GO:0004298
GO:0005839 GO:0019773 GO:0016021
Cluster-22178.67328 GO:0005634
Cluster-22178.67329 GO:0006807 GO:0000256 GO:0006144 GO:0004037
GO:0008168 GO:0005634
Cluster-22178.67330 GO:0006413 GO:0045881 GO:0006446 GO:0032183
GO:0070140 GO:0003723 GO:0005515 GO:0003743 GO:0005737
GO:0005840
Cluster-22178.67331 GO:0016021
Cluster-22178.67332 GO:0002949 GO:0006614 GO:0016887 GO:0003924
GO:0005524 GO:0005525
Cluster-22178.67333 GO:0005515
Cluster-22178.67334 GO:0007229 GO:0007155 GO:0007275 GO:0007165
GO:0046982 GO:0001968 GO:0004872 GO:0016021
Cluster-22178.67336 GO:0006412 GO:0042254 GO:0003735 GO:0005840

Cluster-22178.67338 GO:0009966
Cluster-22178.67339 GO:0008152
Cluster-22178.67340 GO:0005524 GO:0008289 GO:0005515 GO:0016301

Cluster-22178.67341 GO:0007264 GO:0005525 GO:0003924
Cluster-22178.67343 GO:0007165 GO:0004872 GO:0016020
Cluster-22178.67345 GO:0006810 GO:0003677 GO:0003676 GO:0004386 GO:0005524 GO:0016787
Cluster-22178.67348 GO:0005515 GO:0046872 GO:0008270
Cluster-22178.67349 GO:0055085 GO:0006810 GO:0005215 GO:0016021 GO:0016020
Cluster-22178.67350 GO:0005515 GO:0003677
Cluster-22178.67351 GO:0016579 GO:0006276 GO:0005975 GO:0008657 GO:0004553 GO:0008270 GO:0036459
Cluster-22178.67352 GO:0007049 GO:0006355 GO:0009245 GO:0006260 GO:0010506 GO:0032324 GO:0007264 GO:0003700 GO:0008915 GO:0043565 GO:0005515 GO:0008017 GO:0008270 GO:0005096 GO:0005615 GO:0045298 GO:0016021 GO:0005815 GO:0005667
Cluster-22178.67353 GO:0006418 GO:0043039 GO:0004866 GO:0003676 GO:0005524 GO:0000049 GO:0000166 GO:0004812 GO:0005737
Cluster-22178.67354 GO:0006418 GO:0043039 GO:0005524 GO:0003676 GO:0004866 GO:0004812 GO:0000049 GO:0000166 GO:0005737
Cluster-22178.67355 GO:0007165 GO:0008601 GO:0000159
Cluster-22178.67356 GO:0005509
Cluster-22178.67357 GO:0008270 GO:0042162 GO:0003723 GO:0042803

Cluster-22178.67358 GO:0000290 GO:0042773 GO:0006744 GO:0015992 GO:0006814 GO:0000902 GO:0006120 GO:0055114 GO:0043085 GO:0005975 GO:0016787 GO:0016773 GO:0005515 GO:0008137 GO:0005525 GO:0008047 GO:0016020
Cluster-22178.67362 GO:0046660 GO:0006397 GO:0005634
Cluster-22178.67364 GO:0015940 GO:0006571 GO:0006694 GO:0019521 GO:0055114 GO:0009094 GO:0008210 GO:0000162 GO:0046168

GO:0017038 GO:0008207 GO:0008209 GO:0006098 GO:0004665
GO:0003824 GO:0008677 GO:0016491 GO:0008977 GO:0004616
GO:0016616 GO:0003854 GO:0050662 GO:0051287 GO:0016020
Cluster-22178.67366 GO:0006396 GO:0003677 GO:0005515 GO:0003723
GO:0019013
Cluster-22178.67370 GO:0051205 GO:0016021
Cluster-22178.67371 GO:0016020
Cluster-22178.67373 GO:0016020
Cluster-22178.67375 GO:0006813 GO:0005515 GO:0005242 GO:0016021
GO:0008076
Cluster-22178.67376 GO:0005515
Cluster-22178.67380 GO:0006355 GO:0005634
Cluster-22178.67381 GO:0006355 GO:0009252 GO:0046436 GO:0003700
GO:0008716 GO:0005622 GO:0005667
Cluster-22178.67382 GO:0016579 GO:0016567 GO:0044237 GO:0019634
GO:0004842 GO:0003824 GO:0036459 GO:0005680
Cluster-22178.67384 GO:0007264 GO:0006357 GO:0001104 GO:0005515
GO:0005525 GO:0003924 GO:0008641 GO:0016592
Cluster-22178.67385 GO:0007264 GO:0006357 GO:0005515 GO:0001104
GO:0005525 GO:0008641 GO:0003924 GO:0016592
Cluster-22178.67386 GO:0005975 GO:0004553
Cluster-22178.67388 GO:0007165 GO:0005179 GO:0005576
Cluster-22178.67389 GO:0007275 GO:0007219 GO:0016021
Cluster-22178.67390 GO:1901031 GO:0006364 GO:0030688 GO:0005634
Cluster-22178.67391 GO:0019028
Cluster-22178.67392 GO:0006508 GO:0004181 GO:0008270
Cluster-22178.67394 GO:0007606 GO:0005515 GO:0016021
Cluster-22178.67395 GO:0006810 GO:0006816 GO:0042967 GO:0016573
GO:0006355 GO:0003713 GO:0004402 GO:0003712 GO:0008270
GO:0005515 GO:0005246 GO:0042030 GO:0016020 GO:0005667
GO:0000123 GO:0005634
Cluster-22178.67396 GO:0006950 GO:0042967 GO:0006270 GO:0016573
GO:0006457 GO:0006355 GO:0003712 GO:0008270 GO:0005524

GO:0051082 GO:0004402 GO:0000123 GO:0005667 GO:0005634
Cluster-22178.67397 GO:0005975 GO:0015926
Cluster-22178.67398 GO:0015979 GO:0007067 GO:0003677 GO:0009522
GO:0016020 GO:0042729 GO:0072686 GO:0005634
Cluster-22178.67400 GO:0006122 GO:0006508 GO:0003677 GO:0004197
GO:0005515 GO:0008270 GO:0005750 GO:0005743
Cluster-22178.67401 GO:0006260 GO:0003887 GO:0005515 GO:0042575
Cluster-22178.67403 GO:0006118 GO:0020037 GO:0009055 GO:0016021
Cluster-22178.67405 GO:0006281 GO:0006289 GO:0043161 GO:0015031
GO:0005515 GO:0003684 GO:0030176
Cluster-22178.67406 GO:0050792 GO:0042025
Cluster-22178.67408 GO:0046872
Cluster-22178.67409 GO:0006874 GO:0006816 GO:0006468 GO:0046872
GO:0005524 GO:0004672 GO:0005219 GO:0005622 GO:0016021
Cluster-22178.67413 GO:0006355 GO:0042173 GO:0005509 GO:0003676
GO:0003700 GO:0008270 GO:0005667 GO:0005737
Cluster-22178.67414 GO:0042254 GO:0003735 GO:0005840 GO:0005739
Cluster-22178.67415 GO:0042254 GO:0003735 GO:0005739 GO:0005840
Cluster-22178.67416 GO:0006098 GO:0007264 GO:0050792 GO:0006810
GO:0009396 GO:0046487 GO:0009405 GO:0055114 GO:0019521
GO:0004488 GO:0004616 GO:0008200 GO:0004518 GO:0003824
GO:0005525 GO:0005576
Cluster-22178.67417 GO:0042967 GO:0016573 GO:0006355 GO:0003713
GO:0004402 GO:0000123 GO:0005634 GO:0005667
Cluster-22178.67419 GO:0006626 GO:0016746 GO:0005741
Cluster-22178.67421 GO:0008152 GO:0007165 GO:0003676 GO:0003824
GO:0005179 GO:0005576
Cluster-22178.67423 GO:0015031 GO:0030176
Cluster-22178.67424 GO:0006820 GO:0006835 GO:0006812 GO:0017153
GO:0016020
Cluster-22178.67425 GO:0005515
Cluster-22178.67427 GO:0003677 GO:0046872 GO:0008270 GO:0016021
Cluster-22178.67428 GO:0005524 GO:0005515 GO:0003676 GO:0005634

Cluster-22178.67430 GO:0007186 GO:0022904 GO:0004930 GO:0016021 GO:0016020 GO:0005741
Cluster-22178.67431 GO:0046872 GO:0045202
Cluster-22178.67432 GO:0055114 GO:0016714
Cluster-22178.67433 GO:0006468 GO:0005524 GO:0004672
Cluster-22178.67436 GO:0009405 GO:0006952 GO:0007275 GO:0016567 GO:0006511 GO:0008270 GO:0046872 GO:0004842 GO:0005515 GO:0005680 GO:0005576 GO:0005634
Cluster-22178.67437 GO:0006950 GO:0005515
Cluster-22178.67438 GO:0006457 GO:0006810 GO:0006904 GO:0007155 GO:0006582 GO:0015031 GO:0042803 GO:0051087 GO:0003677 GO:0030411 GO:0000774 GO:0015288 GO:0016020 GO:0000145
Cluster-22178.67439 GO:0006807 GO:0006396 GO:0006144 GO:0004000 GO:0003723
Cluster-22178.67440 GO:0007283 GO:0003676 GO:0003677 GO:0008270 GO:0019013 GO:0000786 GO:0005634
Cluster-22178.67441 GO:0003723
Cluster-22178.67442 GO:0016192 GO:0006886 GO:0003824 GO:0005515 GO:0005198 GO:0016021 GO:0030126 GO:0016020
Cluster-22178.67443 GO:0005515
Cluster-22178.67444 GO:0003723
Cluster-22178.67446 GO:0006506 GO:0006468 GO:0005515 GO:0004386 GO:0016773 GO:0004672 GO:0005524 GO:0016020 GO:0031083 GO:0005789
Cluster-22178.67447 GO:0016301
Cluster-22178.67448 GO:0007165 GO:0008270 GO:0004888 GO:0016021
Cluster-22178.67449 GO:0007264 GO:0015684 GO:0015093 GO:0003924 GO:0005525 GO:0016021
Cluster-22178.67451 GO:0016787
Cluster-22178.67452 GO:0032259 GO:0008168
Cluster-22178.67453 GO:0040007 GO:0006955 GO:0042981 GO:0007264 GO:0008283 GO:0007165 GO:0006869 GO:0043087 GO:0048193 GO:0005515 GO:0008083 GO:0005319 GO:0005085 GO:0005134

GO:0005576 GO:0005893 GO:0016020 GO:0005887
Cluster-22178.67454 GO:0006629 GO:0046486 GO:0016042 GO:0008152
GO:0016787 GO:0003847 GO:0019028 GO:0008247
Cluster-22178.67455 GO:0006355 GO:0006352 GO:0003700 GO:0003677
GO:0016987 GO:0005667
Cluster-22178.67456 GO:0005634
Cluster-22178.67457 GO:0006974 GO:0006118 GO:0051726 GO:0007275
GO:0006355 GO:0003700 GO:0009055 GO:0051536 GO:0043565
GO:0008270 GO:0005634 GO:0005667
Cluster-22178.67458 GO:0004869
Cluster-22178.67459 GO:0006468 GO:0016773 GO:0004672 GO:0005524
GO:0016020
Cluster-22178.67460 GO:0015992 GO:0015986 GO:0015078 GO:0000276
Cluster-22178.67461 GO:0008033 GO:0009094 GO:0000162 GO:0006571
GO:0006432 GO:0008168 GO:0005524 GO:0004826 GO:0000287
GO:0000049 GO:0009328
Cluster-22178.67464 GO:0015991 GO:0007165 GO:0015992 GO:0046034
GO:0004888 GO:0016820 GO:0005524 GO:0033178 GO:0016021
Cluster-22178.67465 GO:0043087 GO:0005976 GO:0035023 GO:0043565
GO:0005089 GO:0016779
Cluster-22178.67467 GO:0005515 GO:0046872
Cluster-22178.67468 GO:0006457 GO:0005515 GO:0005615
Cluster-22178.67470 GO:0006448 GO:0006414 GO:0007005 GO:0003746
GO:0003950 GO:0032865 GO:0005840
Cluster-22178.67472 GO:0006813 GO:0006508 GO:0015269 GO:0005524
GO:0008233 GO:0016020 GO:0016021
Cluster-22178.67474 GO:0034968 GO:0006479 GO:0006554 GO:0018024
GO:0003677 GO:0005515 GO:0008270 GO:0005634
Cluster-22178.67475 GO:0055085 GO:0006811 GO:0005198 GO:0005216
GO:0016020 GO:0019028
Cluster-22178.67480 GO:0005515
Cluster-22178.67481 GO:0006508 GO:0055114 GO:0008152 GO:0008270
GO:0016787 GO:0047134 GO:0008236

Cluster-22178.67482 GO:0055114 GO:0042254 GO:0006412 GO:0006118
GO:0051537 GO:0003735 GO:0009496 GO:0005840 GO:0009512
GO:0005622 GO:0042651 GO:0005739
Cluster-22178.67483 GO:0009968 GO:0005737
Cluster-22178.67484 GO:0015694 GO:0042967 GO:0015097 GO:0005515
GO:0008080 GO:0016020 GO:0001772 GO:0097197 GO:0016021
Cluster-22178.67487 GO:0046872
Cluster-22178.67488 GO:0006486 GO:0006355 GO:0003677 GO:0008417
GO:0003700 GO:0016020 GO:0005667
Cluster-22178.67489 GO:0006355 GO:0006275 GO:0007224 GO:0016032

Cluster-22178.67493 GO:0006313 GO:0045444 GO:0003677 GO:0004803

Cluster-22178.67495 GO:0006508 GO:0004190
Cluster-22178.67496 GO:0006310 GO:0006281
Cluster-22178.67497 GO:0016021
Cluster-22178.67498 GO:0008152 GO:0009877 GO:0009312 GO:0008168
GO:0008757 GO:0005634
Cluster-22178.6750 GO:0005515 GO:0097228 GO:0036128
Cluster-22178.67503 GO:0006139 GO:0003676 GO:0008408
Cluster-22178.67505 GO:0006355 GO:0003700 GO:0005667
Cluster-22178.67507 GO:0007165 GO:0008601 GO:0008270 GO:0000159
Cluster-22178.67510 GO:0006364 GO:0005515 GO:0005730
Cluster-22178.67512 GO:0007165 GO:0009405 GO:0010951 GO:0008283
GO:0040007 GO:0051015 GO:0008376 GO:0008083 GO:0030674
GO:0004866 GO:0032580
Cluster-22178.67514 GO:0007275 GO:0007165 GO:0008152 GO:0016624
GO:0005179 GO:0047355 GO:0008270 GO:0016020 GO:0005576
Cluster-22178.67515 GO:0006355 GO:0003677
Cluster-22178.67516 GO:0045444
Cluster-22178.67519 GO:0007267 GO:0006508 GO:0006355 GO:0008270
GO:0008233 GO:0003700 GO:0005634 GO:0005667
Cluster-22178.67523 GO:0007264 GO:0005525

Cluster-22178.67524 GO:0006270 GO:0016020
Cluster-22178.67525 GO:0006397 GO:0046872
Cluster-22178.67527 GO:0006810 GO:0009405 GO:0008200 GO:0005515
GO:0042151
Cluster-22178.67528 GO:0006468 GO:0004672 GO:0005524 GO:0016020
Cluster-22178.67529 GO:0006574 GO:0008033 GO:0055114 GO:0008152
GO:0006813 GO:0006568 GO:0006550 GO:0006552 GO:0006554
GO:0046168 GO:0006779 GO:0006633 GO:0016117 GO:0018874
GO:0019354 GO:0006631 GO:0003824 GO:0003857 GO:0043115
GO:0016705 GO:0016491 GO:0050660 GO:0051287 GO:0071949
GO:0016616
Cluster-22178.67530 GO:0005509 GO:0005515
Cluster-22178.67535 GO:0006464 GO:0015031
Cluster-22178.67536 GO:0051603 GO:0006511 GO:0004175 GO:0004298
GO:0005839 GO:0019773
Cluster-22178.67537 GO:0008033 GO:0055114 GO:0006914 GO:0006308
GO:0019049 GO:0006886 GO:0006605 GO:0008855 GO:0016491
GO:0050660 GO:0071949 GO:0005742 GO:0009318
Cluster-22178.67538 GO:0005515
Cluster-22178.67539 GO:0006935 GO:0042407 GO:0006351 GO:0006886
GO:0006206 GO:0006144 GO:0090263 GO:0016567 GO:0071973
GO:0016192 GO:0046872 GO:0005515 GO:0003899 GO:0003774
GO:0003677 GO:0009288 GO:0044284 GO:0016021 GO:0030117
GO:0061617 GO:0005730 GO:0016020
Cluster-22178.67542 GO:0003676
Cluster-22178.67543 GO:0051258 GO:0006886 GO:0030168 GO:0000921
GO:0048193 GO:0006606 GO:0006891 GO:0007165 GO:0005198
GO:0003774 GO:0019905 GO:0005102 GO:0030674 GO:0005801
GO:0016021 GO:0019031 GO:0019028 GO:0005940 GO:0005577
GO:0017119 GO:0016459 GO:0016020 GO:0005882
Cluster-22178.67547 GO:0005515
Cluster-22178.67548 GO:0006123 GO:0015992 GO:0004129 GO:0045277
Cluster-22178.67549 GO:0006468 GO:0046039 GO:0009190 GO:0008152

GO:0006182 GO:0035556 GO:0006144 GO:0006357 GO:0001104
GO:0004383 GO:0016849 GO:0003723 GO:0003824 GO:0004672
GO:0008270 GO:0005524 GO:0016592
Cluster-22178.67552 GO:0006810 GO:0005622
Cluster-22178.67553 GO:0006308 GO:0006289 GO:0006285 GO:0042384
GO:0006281 GO:0019900 GO:0003684 GO:0008534 GO:0034464
Cluster-22178.67554 GO:0006289 GO:0006281 GO:0006284 GO:0008270
GO:0003906 GO:0016799 GO:0003684
Cluster-22178.67555 GO:0007165 GO:0005975 GO:0003676 GO:0008270
GO:0008184 GO:0003723
Cluster-22178.67556 GO:0008152 GO:0006468 GO:0046039 GO:0009190
GO:0035556 GO:0006357 GO:0006144 GO:0006182 GO:0003824
GO:0004672 GO:0001104 GO:0003723 GO:0016849 GO:0004383
GO:0008270 GO:0005524 GO:0016592
Cluster-22178.67559 GO:0043087 GO:0035023 GO:0043565 GO:0005089

Cluster-22178.67560 GO:0006397 GO:0003723 GO:0005634 GO:0005737
Cluster-22178.67563 GO:0006811 GO:0005515 GO:0016020
Cluster-22178.67564 GO:0046872
Cluster-22178.67565 GO:0005198 GO:0005515
Cluster-22178.67567 GO:0018342 GO:0055114 GO:0016491 GO:0008318

Cluster-22178.67568 GO:0018342 GO:0008318
Cluster-22178.67569 GO:0009636 GO:0006955 GO:0031342 GO:0015643
GO:0019814
Cluster-22178.67571 GO:0006355 GO:0043565 GO:0046872
Cluster-22178.67573 GO:0015031 GO:0006464 GO:0030488 GO:0046500
GO:0006364 GO:0008033 GO:0055114 GO:0006725 GO:0007010
GO:0006479 GO:0009451 GO:0008270 GO:0010277 GO:0016491
GO:0016429 GO:0016772 GO:0004719 GO:0003779 GO:0008168
GO:0003723 GO:0016301 GO:0008198 GO:0031515 GO:0005615
Cluster-22178.67575 GO:0006771 GO:0042546 GO:0009247 GO:0001575
GO:0005975 GO:0019497 GO:0003723 GO:0008107 GO:0005515

GO:0046872 GO:0003993 GO:0016020
Cluster-22178.67576 GO:0005515
Cluster-22178.67578 GO:0005515
Cluster-22178.67581 GO:0006397 GO:0003723 GO:0008270 GO:0005634
GO:0005737
Cluster-22178.67585 GO:0006260 GO:0006144 GO:0019064 GO:0043093
GO:0000390 GO:0006351 GO:0000917 GO:0006886 GO:0008270
GO:0005096 GO:0003968 GO:0003723 GO:0016020 GO:0071008
GO:0019031 GO:0005801 GO:0005737 GO:0031379
Cluster-22178.67587 GO:0009190 GO:0035556 GO:0007283 GO:0016849
GO:0003677 GO:0005634 GO:0000786
Cluster-22178.67589 GO:0006508 GO:0004197 GO:0008242 GO:0016740

Cluster-22178.67590 GO:0006355 GO:0003677
Cluster-22178.67591 GO:0042254 GO:0006412 GO:0003735 GO:0005622
GO:0005840
Cluster-22178.67592 GO:0055114 GO:0016567 GO:0016706 GO:0004842
GO:0005515
Cluster-22178.67593 GO:0006412 GO:0042254 GO:0003735 GO:0005840
GO:0005576 GO:0005622
Cluster-22178.67594 GO:0048870 GO:0019904 GO:0031514
Cluster-22178.67595 GO:0006265 GO:0005524 GO:0003918 GO:0003677
GO:0005634
Cluster-22178.67596 GO:0008270 GO:0046872
Cluster-22178.67598 GO:0071436 GO:0006525 GO:0006260 GO:0006914
GO:0006560 GO:0006814 GO:0006886 GO:0006355 GO:0006090
GO:0048870 GO:0015081 GO:0008948 GO:0003700 GO:0043565
GO:0005783 GO:0016020 GO:0005667 GO:0031514 GO:0005801
GO:0016021
Cluster-22178.67599 GO:0006013 GO:0006000 GO:0006012 GO:0006094
GO:0006098 GO:0006096 GO:0003872 GO:0016301 GO:0005945
Cluster-22178.67601 GO:0055114 GO:0008033 GO:0045454 GO:0006355
GO:0016491 GO:0050660 GO:0003677

Cluster-22178.67603 GO:0006812 GO:0032801 GO:0019076 GO:0005261 GO:0033644
Cluster-22178.67605 GO:0006122 GO:0003676 GO:0005750
Cluster-22178.67610 GO:0015979 GO:0016021
Cluster-22178.67611 GO:0043248 GO:0006886 GO:0015991 GO:0008536 GO:0016820 GO:0005515 GO:0000221 GO:0032040 GO:0019031 GO:0005783
Cluster-22178.67612 GO:0002098 GO:0006144 GO:0006468 GO:0046034 GO:0034227 GO:0016787 GO:0004017 GO:0003677 GO:0008270 GO:0005524 GO:0000049 GO:0020037 GO:0004672 GO:0046872 GO:0003676 GO:0005634
Cluster-22178.67614 GO:0016310 GO:0009069 GO:0006468 GO:0004674 GO:0004672 GO:0005524 GO:0016773 GO:0016020
Cluster-22178.67615 GO:0006725 GO:0008033 GO:0055114 GO:0009451 GO:0007010 GO:0006479 GO:0006468 GO:0006464 GO:0030488 GO:0015031 GO:0006364 GO:0046500 GO:0003779 GO:0008168 GO:0004719 GO:0004672 GO:0016301 GO:0008198 GO:0003723 GO:0016772 GO:0016429 GO:0016491 GO:0010277 GO:0005524 GO:0031515
Cluster-22178.67616 GO:0019058 GO:0019634 GO:0015716 GO:0046872 GO:0005887
Cluster-22178.67619 GO:0006952 GO:0005576
Cluster-22178.67620 GO:0003676 GO:0003677 GO:0016787 GO:0005524

Cluster-22178.67622 GO:0000166 GO:0046872
Cluster-22178.67624 GO:0006886 GO:0016032 GO:0000160 GO:0016310 GO:0006888 GO:0003677 GO:0000155 GO:0046983 GO:0008270 GO:0030127 GO:0019028 GO:0016021 GO:0009365
Cluster-22178.67625 GO:0006810 GO:0005215
Cluster-22178.67626 GO:0016579 GO:0016567 GO:0044237 GO:0019634 GO:0004842 GO:0003824 GO:0036459 GO:0005680
Cluster-22178.67629 GO:0006000 GO:0043087 GO:0006013 GO:0006012 GO:0006098 GO:0006457 GO:0006096 GO:0006470 GO:0006094

GO:0006570 GO:0003872 GO:0004725 GO:0051082 GO:0005515
GO:0005945 GO:0016272 GO:0005639 GO:0016020
Cluster-22178.67630 GO:0006281 GO:0006355 GO:0051276 GO:0003677
GO:0005515 GO:0005524 GO:0008270 GO:0008168 GO:0005694
Cluster-22178.67632 GO:0006352 GO:0042254 GO:0006412 GO:0006468
GO:0005524 GO:0004672 GO:0003677 GO:0003735 GO:0005840
GO:0005669 GO:0005622
Cluster-22178.67633 GO:0006486 GO:0006508 GO:0003676 GO:0004222
GO:0004576 GO:0003677 GO:0008250 GO:0016020
Cluster-22178.67634 GO:0009069 GO:0006468 GO:0016310 GO:0007178
GO:0055114 GO:0004675 GO:0005515 GO:0005524 GO:0003756
GO:0016671 GO:0005783 GO:0016020
Cluster-22178.67635 GO:0006468 GO:0006886 GO:0007094 GO:0016310
GO:0009069 GO:0006606 GO:0006355 GO:0048870 GO:0006457
GO:0006810 GO:0032259 GO:0051607 GO:0015031 GO:0010506
GO:0003723 GO:0051082 GO:0042803 GO:0043565 GO:0008565
GO:0005524 GO:0045502 GO:0008134 GO:0003700 GO:0004674
GO:0005737 GO:0005615 GO:0005667 GO:0016020 GO:0016272
GO:0031514 GO:0005845 GO:0005801 GO:0016021 GO:0030286
GO:0005886
Cluster-22178.67637 GO:0006694 GO:0043093 GO:0000917 GO:0006869
GO:0008299 GO:0005524 GO:0016887 GO:0046983 GO:0005525
GO:0050518 GO:0005319 GO:0005737
Cluster-22178.67638 GO:0048193 GO:0003676 GO:0008270 GO:0046872
GO:0016020
Cluster-22178.67639 GO:0046488 GO:0006887 GO:0016307 GO:0008289
GO:0000145 GO:0019031
Cluster-22178.67640 GO:0005515
Cluster-22178.67643 GO:0051015 GO:0030674
Cluster-22178.67644 GO:0030674 GO:0051015
Cluster-22178.67646 GO:0005525 GO:0031012
Cluster-22178.67647 GO:0030150 GO:0055114 GO:0008033 GO:0045454
GO:0006118 GO:0016491 GO:0009055 GO:0050660 GO:0015035

GO:0005515 GO:0005742
Cluster-22178.67650 GO:0006914
Cluster-22178.67652 GO:0045132 GO:0000917 GO:0046487 GO:0007094
GO:0055114 GO:0008152 GO:0007049 GO:0043093 GO:0009396
GO:0000226 GO:0005515 GO:0008092 GO:0003824 GO:0016787
GO:0004488 GO:0005634 GO:0015630 GO:0005737 GO:0016021
GO:0000775 GO:0019898
Cluster-22178.67653 GO:0016787
Cluster-22178.67655 GO:0003676 GO:0008270
Cluster-22178.67656 GO:0006810 GO:0007165 GO:0006935 GO:0004888
GO:0016020 GO:0005643
Cluster-22178.67657 GO:0005515
Cluster-22178.67658 GO:0006357 GO:0001104 GO:0016592
Cluster-22178.67659 GO:0015979 GO:0016020 GO:0009523 GO:0009539
Cluster-22178.67662 GO:0043066 GO:0016310 GO:0043123 GO:0045766
GO:0007165 GO:0009069 GO:0070008 GO:0016772 GO:0004674
GO:0004252 GO:0005515 GO:0005044 GO:0016020
Cluster-22178.67664 GO:0055114 GO:0016972 GO:0005515
Cluster-22178.67665 GO:0005975 GO:0006886 GO:0019058 GO:0004553
GO:0005515 GO:0008536 GO:0042025
Cluster-22178.67666 GO:0055085 GO:0006888 GO:0006810 GO:0005215
GO:0022857 GO:0005515 GO:0005801 GO:0019867 GO:0016021
GO:0005622 GO:0016020
Cluster-22178.67668 GO:0006359 GO:0030036 GO:0007275 GO:0016043
GO:0017048 GO:0003779
Cluster-22178.67670 GO:0008152 GO:0006974 GO:0006915 GO:0016491
GO:0000228
Cluster-22178.67671 GO:0006396 GO:0051252 GO:0008033 GO:0003676
GO:0004526 GO:0030677
Cluster-22178.67675 GO:0006357 GO:0035091 GO:0001104 GO:0016592
Cluster-22178.67676 GO:0055114 GO:0006000 GO:0006013 GO:0005515
GO:0016301 GO:0003873 GO:0016740 GO:0016625 GO:0016491
GO:0051536 GO:0005524

Cluster-22178.67677 GO:0006270 GO:0006816 GO:0006874 GO:0005219
GO:0005524 GO:0005515 GO:0004252 GO:0004190 GO:0016021
GO:0005622 GO:0005886
Cluster-22178.67679 GO:0015031 GO:0006772 GO:0006464 GO:0009228
GO:0006886 GO:0003924 GO:0008270 GO:0052855 GO:0005525
GO:0005515 GO:0005047 GO:0004417 GO:0005786 GO:0005785
Cluster-22178.6768 GO:0006812 GO:0016887 GO:0016021 GO:0016020
Cluster-22178.67681 GO:0006813 GO:0006811 GO:0055085 GO:0005515
GO:0005216 GO:0005242 GO:0008076 GO:0016020 GO:0016021
Cluster-22178.67682 GO:0016757
Cluster-22178.67685 GO:0016192 GO:0006886 GO:0015991 GO:0055114
GO:0016820 GO:0005515 GO:0016491 GO:0030117 GO:0000221
Cluster-22178.67687 GO:0055114 GO:0006537 GO:0042967 GO:0006807
GO:0005515 GO:0015930 GO:0003676 GO:0016407
Cluster-22178.67688 GO:0042967 GO:0006807 GO:0055114 GO:0006537
GO:0015930 GO:0005515 GO:0003676 GO:0016407
Cluster-22178.67691 GO:0050832 GO:0042742 GO:0019239
Cluster-22178.67694 GO:0003677 GO:0005634
Cluster-22178.67695 GO:0019028
Cluster-22178.67696 GO:0042254 GO:0006412 GO:0019843 GO:0003735
GO:0005622 GO:0005840
Cluster-22178.67697 GO:0006355 GO:0006464 GO:0006265 GO:0003677
GO:0016151 GO:0003916 GO:0008270 GO:0005694
Cluster-22178.67698 GO:0008270 GO:0003676
Cluster-22178.67700 GO:0006810 GO:0009401 GO:0005886
Cluster-22178.67701 GO:0006810 GO:0009401 GO:0005886
Cluster-22178.67702 GO:0006810 GO:0007165 GO:0007186 GO:0000166
GO:0003677 GO:0016787 GO:0005525 GO:0004871 GO:0031683
GO:0005524 GO:0019001 GO:0016887 GO:0003924 GO:0005747
GO:0005739
Cluster-22178.67704 GO:0006457 GO:0051082 GO:0016272
Cluster-22178.67705 GO:0006468 GO:0016773 GO:0005524 GO:0004672
GO:0016020

Cluster-22178.67708 GO:0045892 GO:0051775 GO:0035556 GO:0005515
GO:0003677 GO:0005737 GO:0005634
Cluster-22178.67710 GO:0006231 GO:0006206 GO:0009405 GO:0004799
GO:0005576
Cluster-22178.67711 GO:0006955 GO:0006412 GO:0006000 GO:0006013
GO:0006004 GO:0042254 GO:0007165 GO:0005125 GO:0003676
GO:0008736 GO:0003723 GO:0003735 GO:0005737 GO:0005840
GO:0005634 GO:0005576 GO:0005622
Cluster-22178.67712 GO:0009069 GO:0016310 GO:0005524 GO:0004674

Cluster-22178.67714 GO:0006511 GO:0016567 GO:0007017 GO:0007018
GO:0034450 GO:0005524 GO:0008017 GO:0004842 GO:0005515
GO:0005198 GO:0003777 GO:0019028 GO:0000151 GO:0016021
GO:0045298 GO:0005874
Cluster-22178.67716 GO:0009405 GO:0016301 GO:0016772 GO:0016020
Cluster-22178.67717 GO:0006811 GO:0071816 GO:0006355 GO:0006486
GO:0051225 GO:0006357 GO:0007155 GO:0007165 GO:0004576
GO:0004871 GO:0003723 GO:0001104 GO:0042393 GO:0003677
GO:0009288 GO:0070652 GO:0005634 GO:0008250 GO:0016020
GO:0016592
Cluster-22178.67718 GO:0006811 GO:0007155 GO:0006355 GO:0007165
GO:0051225 GO:0004871 GO:0003723 GO:0003677 GO:0009288
GO:0070652 GO:0005634 GO:0016020
Cluster-22178.67719 GO:0009405 GO:0045735 GO:0046872
Cluster-22178.67721 GO:0006508 GO:0006303 GO:0004003 GO:0004222
GO:0003677 GO:0005815 GO:0005657
Cluster-22178.67725 GO:0009405 GO:0005515
Cluster-22178.67729 GO:0009236 GO:0019236 GO:0016310 GO:0007606
GO:0007186 GO:0004932 GO:0003824 GO:0016772 GO:0016020
Cluster-22178.6773 GO:0050821 GO:0015979 GO:0005515 GO:0042301
GO:0046872 GO:0016020 GO:0009523
Cluster-22178.67731 GO:0015940 GO:0006139 GO:0015937 GO:0008152
GO:0030474 GO:0009405 GO:0030254 GO:0006206 GO:0006810

GO:0005200 GO:0004140 GO:0005515 GO:0016301 GO:0004127
GO:0005524 GO:0005856 GO:0005823 GO:0042597 GO:0030257
Cluster-22178.67732 GO:0006810 GO:0006206 GO:0009405 GO:0030474
GO:0030254 GO:0008152 GO:0015937 GO:0015940 GO:0006139
GO:0004127 GO:0005524 GO:0016301 GO:0005515 GO:0005200
GO:0004140 GO:0030257 GO:0005823 GO:0042597 GO:0005856
Cluster-22178.67734 GO:0055114 GO:0016032 GO:0008152 GO:0016491
GO:0019033
Cluster-22178.67735 GO:0016032 GO:0055114 GO:0008152 GO:0016491
GO:0019033
Cluster-22178.67736 GO:0006631 GO:0016310 GO:0009069 GO:0005515
GO:0004674 GO:0016746 GO:0005524
Cluster-22178.67737 GO:0006810 GO:0006206 GO:0030254 GO:0009405
GO:0030474 GO:0008152 GO:0015937 GO:0006139 GO:0015940
GO:0005524 GO:0004127 GO:0016301 GO:0005515 GO:0004140
GO:0005200 GO:0030257 GO:0042597 GO:0005823 GO:0005856
Cluster-22178.67739 GO:0006886 GO:0016192 GO:0005515 GO:0030117
GO:0030131
Cluster-22178.67741 GO:0009058 GO:0030170
Cluster-22178.67743 GO:0007304 GO:0006470 GO:0006570 GO:0016192
GO:0005213 GO:0004725 GO:0008138 GO:0042600 GO:0005576
Cluster-22178.67745 GO:0003824
Cluster-22178.67746 GO:0003824
Cluster-22178.67749 GO:0001522 GO:0009451 GO:0003723 GO:0005515
GO:0009982
Cluster-22178.67750 GO:0016579 GO:0006508 GO:0008270 GO:0004843
GO:0036459
Cluster-22178.67751 GO:0019229 GO:0000725 GO:0046983 GO:0003677
GO:0005634 GO:0097196 GO:0005576
Cluster-22178.67752 GO:0006096 GO:0006094 GO:0016579 GO:0030150
GO:0015976 GO:0006144 GO:0030955 GO:0000287 GO:0004743
GO:0003824 GO:0008270 GO:0036459 GO:0005742
Cluster-22178.67753 GO:0006144 GO:0015976 GO:0030150 GO:0016579

GO:0006094 GO:0006096 GO:0036459 GO:0003824 GO:0004743
GO:0030955 GO:0000287 GO:0005742
Cluster-22178.67754 GO:0006915 GO:0006974 GO:0008152 GO:0016491
GO:0000228
Cluster-22178.67755 GO:0015979 GO:0005515 GO:0009539 GO:0009523
GO:0016020
Cluster-22178.67756 GO:0003723 GO:0005515 GO:0005198
Cluster-22178.67757 GO:0005515
Cluster-22178.67760 GO:0016021
Cluster-22178.67761 GO:0007219 GO:0030154 GO:0007275 GO:0006351
GO:0016788 GO:0003677 GO:0005515 GO:0016021 GO:0005634
Cluster-22178.67763 GO:0035556 GO:0007165 GO:0004871
Cluster-22178.67764 GO:0006355 GO:0006457 GO:0046872 GO:0043565
GO:0003700 GO:0051082 GO:0016272 GO:0019867 GO:0005667
Cluster-22178.67766 GO:0008270 GO:0005515
Cluster-22178.67767 GO:0006118 GO:0006570 GO:0006886 GO:0008033
GO:0055114 GO:0015031 GO:0050660 GO:0008565 GO:0004097
GO:0016020 GO:0005737
Cluster-22178.67769 GO:0055114 GO:0008270 GO:0016491
Cluster-22178.67771 GO:0007049 GO:0006260 GO:0000917 GO:0006355
GO:0043093 GO:0003700 GO:0043565 GO:0003677 GO:0005737
GO:0005667 GO:0005634
Cluster-22178.67772 GO:0070206 GO:0051607 GO:0006606 GO:0009405
GO:0005515 GO:0019028 GO:0016020
Cluster-22178.67776 GO:0046487 GO:0009405 GO:0055114 GO:0019521
GO:0006098 GO:0007264 GO:0050792 GO:0006810 GO:0009396
GO:0008200 GO:0004518 GO:0003824 GO:0005525 GO:0004488
GO:0004616 GO:0005576
Cluster-22178.67777 GO:0090063 GO:0000226 GO:0005815 GO:0000922
Cluster-22178.67778 GO:0007049 GO:0006904 GO:0006123 GO:0015992
GO:0015031 GO:0006914 GO:0004129 GO:0008092 GO:0005198
GO:0003774 GO:0045277 GO:0005737 GO:0005740 GO:0019898
GO:0005882 GO:0000145 GO:0005747 GO:0016459 GO:0005739

Cluster-22178.67779 GO:0006820 GO:0015031 GO:0015992 GO:0015991 GO:0008565 GO:0046872 GO:0008509 GO:0015078 GO:0004190 GO:0005515 GO:0016021 GO:0016020 GO:0033179

Cluster-22178.67781 GO:0046912 GO:0005515 GO:0008270 GO:0008144 GO:0005622

Cluster-22178.67782 GO:0042254 GO:0006412 GO:0043395 GO:0005515 GO:0003735 GO:0005840 GO:0016020 GO:0005578

Cluster-22178.67783 GO:0015031 GO:0006769 GO:0046497 GO:0016567 GO:0004842 GO:0003953 GO:0016021 GO:0005789

Cluster-22178.67784 GO:0046872

Cluster-22178.67785 GO:0006355 GO:0051260 GO:0008270 GO:0003700 GO:0003676 GO:0005634 GO:0005667

Cluster-22178.67786 GO:0044237 GO:0006810 GO:0006351 GO:0006355 GO:0006144 GO:0006206 GO:0003899 GO:0003677 GO:0005730 GO:0016021 GO:0005639

Cluster-22178.67787 GO:0042254 GO:0006412 GO:0005096 GO:0046872 GO:0003735 GO:0003677 GO:0005622 GO:0045202 GO:0005840

Cluster-22178.67788 GO:0009306 GO:0009403 GO:0015031 GO:0015450 GO:0008092 GO:0016020 GO:0016021 GO:0019898 GO:0009941 GO:0005737

Cluster-22178.67791 GO:0015031 GO:0006464 GO:0003723 GO:0005515

Cluster-22178.67792 GO:0008270 GO:0005198 GO:0005515 GO:0003677 GO:0016021

Cluster-22178.67793 GO:0006771 GO:0009231 GO:0003919 GO:0008270 GO:0016021

Cluster-22178.67796 GO:0006813 GO:0006098 GO:0019521 GO:0055114 GO:0051287 GO:0016616 GO:0004616

Cluster-22178.67797 GO:0006470 GO:0006570 GO:0008138 GO:0004725 GO:0016021

Cluster-22178.67799 GO:0008152 GO:0006355 GO:0008168 GO:0043565 GO:0003700 GO:0005515 GO:0003677 GO:0005667 GO:0005634

Cluster-22178.67803 GO:0043087 GO:0006308 GO:0006302 GO:0007155

GO:0008855 GO:0005515 GO:0009288 GO:0009318 GO:0005634
Cluster-22178.67807 GO:0006810 GO:0005515 GO:0019901 GO:0005886
GO:0016020
Cluster-22178.67810 GO:0031505 GO:0006886 GO:0008536 GO:0008771
GO:0005515
Cluster-22178.67811 GO:0006816 GO:0006810 GO:0005246 GO:0042030
GO:0016020 GO:0005887
Cluster-22178.67812 GO:0005515 GO:0043565 GO:0005634
Cluster-22178.67813 GO:0006810 GO:0016567 GO:0015743 GO:0033014
GO:0006783 GO:0015994 GO:0006915 GO:0004842 GO:0008270
GO:0061630 GO:0005215 GO:0046872 GO:0004852 GO:0005515
GO:0005680 GO:0016020 GO:0005634
Cluster-22178.67814 GO:0055085 GO:0005741
Cluster-22178.67816 GO:0016567 GO:0005515 GO:0004842 GO:0008270
GO:0046872 GO:0005680
Cluster-22178.67817 GO:0051321 GO:0043065 GO:0097193 GO:0009405
GO:0090200 GO:0005515 GO:0005576 GO:0005739
Cluster-22178.67818 GO:0006468 GO:0046872 GO:0005524 GO:0004672
GO:0016773 GO:0016787 GO:0016020
Cluster-22178.67819 GO:0046872
Cluster-22178.67820 GO:0007165 GO:0008270
Cluster-22178.67821 GO:0033588
Cluster-22178.67822 GO:0043087 GO:0035023 GO:0055085 GO:0006812
GO:0006885 GO:0007165 GO:0015299 GO:0005057 GO:0005089
GO:0016021
Cluster-22178.67823 GO:0055085 GO:0006810 GO:0005515 GO:0005215
GO:0016021 GO:0016020
Cluster-22178.67824 GO:0055085 GO:0005741
Cluster-22178.67826 GO:0046872 GO:0045202
Cluster-22178.67828 GO:0008643 GO:0008982 GO:0016787 GO:0008146
GO:0015016 GO:0009357
Cluster-22178.67829 GO:0009165 GO:0006144 GO:0009116 GO:0006098
GO:0006397 GO:0004749 GO:0000287 GO:0003723 GO:0005737

GO:0005634
Cluster-22178.67832 GO:0033206 GO:0016482 GO:0007163 GO:0030036
GO:0051653 GO:0051321 GO:0008356 GO:0006928 GO:0005524
GO:0003779 GO:0005885 GO:0042995 GO:0030478
Cluster-22178.67833 GO:0050821 GO:0015979 GO:0006357 GO:0001104
GO:0042301 GO:0009523 GO:0016592 GO:0016020
Cluster-22178.67836 GO:0046872
Cluster-22178.67840 GO:0006260 GO:0005524 GO:0016887 GO:0003677

Cluster-22178.67842 GO:0046656 GO:0006367 GO:0006506 GO:0042493
GO:0006761 GO:0055114 GO:0004146 GO:0005515 GO:0046872
GO:0008270 GO:0017176 GO:0005672 GO:0016021
Cluster-22178.67846 GO:0006813 GO:0009405 GO:0005242 GO:0008270
GO:0003677 GO:0008076 GO:0016021
Cluster-22178.67847 GO:0009405 GO:0006351 GO:0006206 GO:0006464
GO:0006144 GO:0007275 GO:0006511 GO:0016567 GO:0016151
GO:0046872 GO:0005198 GO:0005515 GO:0003899 GO:0004842
GO:0008270 GO:0003677 GO:0005634 GO:0005680 GO:0005730
Cluster-22178.67848 GO:0005739
Cluster-22178.67849 GO:0006355 GO:0016787 GO:0016818 GO:0003677
GO:0008270 GO:0005524 GO:0003676 GO:0005634
Cluster-22178.67850 GO:0005515
Cluster-22178.67852 GO:0007283 GO:0046872 GO:0000062 GO:0003677
GO:0005634 GO:0000786
Cluster-22178.67855 GO:0006468 GO:0005524 GO:0004672 GO:0016773
GO:0016020
Cluster-22178.67856 GO:0005515
Cluster-22178.67859 GO:0000398 GO:0008270 GO:0005681
Cluster-22178.67860 GO:0019028
Cluster-22178.67861 GO:0000902 GO:0006955 GO:0016020
Cluster-22178.67862 GO:0042256 GO:0043022
Cluster-22178.67865 GO:0003676 GO:0005515
Cluster-22178.67866 GO:0006508 GO:0016192 GO:0006886 GO:0009847

GO:0005515 GO:0008233 GO:0030117
Cluster-22178.67867 GO:0005975 GO:0004553
Cluster-22178.67868 GO:0042254 GO:0006412 GO:0003735 GO:0005840
GO:0005739 GO:0005622
Cluster-22178.67869 GO:0043087 GO:0007165 GO:0035023 GO:0005057
GO:0005089
Cluster-22178.67870 GO:0007268 GO:0033130 GO:0003723 GO:0043495
GO:0005515 GO:0019867
Cluster-22178.67872 GO:0009405 GO:0009058 GO:0055114 GO:0016787
GO:0010181 GO:0003677 GO:0005524 GO:0019865 GO:0016491
GO:0003676 GO:0019814
Cluster-22178.67873 GO:0006468 GO:0005524 GO:0004672 GO:0005515

Cluster-22178.67874 GO:0006457 GO:0005737
Cluster-22178.67875 GO:0006606 GO:0009069 GO:0016310 GO:0006468
GO:0006886 GO:0007094 GO:0032259 GO:0051607 GO:0015031
GO:0010506 GO:0006457 GO:0006810 GO:0048870 GO:0006355
GO:0008565 GO:0005524 GO:0043565 GO:0003723 GO:0051082
GO:0042803 GO:0004674 GO:0003700 GO:0045502 GO:0008134
GO:0016272 GO:0005667 GO:0016020 GO:0005737 GO:0005615
GO:0030286 GO:0005886 GO:0005801 GO:0016021 GO:0031514
GO:0005845
Cluster-22178.67876 GO:0032259 GO:0003723 GO:0005845
Cluster-22178.67877 GO:0006352 GO:0006355 GO:0006412 GO:0006351
GO:0006144 GO:0006206 GO:0071107 GO:0042254 GO:0003676
GO:0003700 GO:0003899 GO:0016987 GO:0003735 GO:0003677
GO:0005840 GO:0005622 GO:0005667 GO:0005730 GO:0016020
Cluster-22178.67878 GO:0016482 GO:0033206 GO:0007163 GO:0006928
GO:0008356 GO:0051321 GO:0051653 GO:0030036 GO:0003779
GO:0005524 GO:0042995 GO:0005885 GO:0030478
Cluster-22178.67879 GO:0003676
Cluster-22178.67880 GO:0008356 GO:0006928 GO:0051321 GO:0030036
GO:0051653 GO:0033206 GO:0016482 GO:0007163 GO:0003779

GO:0005524 GO:0030478 GO:0042995 GO:0005885
Cluster-22178.67883 GO:0007018 GO:0007017 GO:0008017 GO:0003777
GO:0005524 GO:0003677 GO:0045298 GO:0005874
Cluster-22178.67885 GO:0006351 GO:0008270 GO:0003676
Cluster-22178.67888 GO:0005198 GO:0005094 GO:0005737 GO:0019028
Cluster-22178.67889 GO:0006355 GO:0006281 GO:0006310 GO:0016887
GO:0005524 GO:0003678 GO:0003724 GO:0003723 GO:0009378
GO:0008134 GO:0005657 GO:0005667 GO:0009379
Cluster-22178.67892 GO:0006144 GO:0006206 GO:0006241 GO:0007155
GO:0006165 GO:0006228 GO:0006183 GO:0004550 GO:0003676
GO:0003723 GO:0005515
Cluster-22178.67893 GO:0016567 GO:0008270 GO:0046872 GO:0004842
GO:0005198 GO:0005515 GO:0005622 GO:0019031 GO:0005680
GO:0019028
Cluster-22178.67897 GO:0006351 GO:0007165 GO:0003676 GO:0016817
GO:0005515 GO:0005509 GO:0004190 GO:0003714 GO:0016021
GO:0005667 GO:0005578
Cluster-22178.67898 GO:0007264 GO:0005525
Cluster-22178.67899 GO:0016567 GO:0006508 GO:0006771 GO:0019497
GO:0005515 GO:0004197 GO:0016740 GO:0004842 GO:0003993
GO:0008242 GO:0046872 GO:0008270 GO:0005622
Cluster-22178.67901 GO:0006402 GO:0006109 GO:0003723
Cluster-22178.67905 GO:0006144 GO:0015976 GO:0006094 GO:0006096
GO:0003824 GO:0004743 GO:0000287 GO:0030955 GO:0005515
Cluster-22178.67906 GO:0006820 GO:0008509 GO:0016021
Cluster-22178.67907 GO:0006351 GO:0006260 GO:0003677 GO:0003887
GO:0042575
Cluster-22178.67909 GO:0008270
Cluster-22178.67910 GO:0006886 GO:0006367 GO:0006605 GO:0006270
GO:0032968 GO:0005515 GO:0005509 GO:0003677 GO:0003676
GO:0016020 GO:0005634 GO:0005742
Cluster-22178.67911 GO:0051016 GO:0008290
Cluster-22178.67912 GO:0046740 GO:0051205 GO:0006810 GO:0042254

GO:0006412 GO:0006260 GO:0051082 GO:0003735 GO:0016798
GO:0016020 GO:0005886 GO:0005622 GO:0005840 GO:0016021
Cluster-22178.67914 GO:0030474 GO:0006270 GO:0071988
Cluster-22178.67915 GO:0006508 GO:0006281 GO:0006412 GO:0006397
GO:0006289 GO:0006284 GO:0019058 GO:0042254 GO:0016740
GO:0003723 GO:0016799 GO:0008242 GO:0008270 GO:0003906
GO:0003735 GO:0003684 GO:0004197 GO:0005840 GO:0000445
GO:0005622
Cluster-22178.67916 GO:0006206 GO:0019083 GO:0055114 GO:0006144
GO:0008285 GO:0016579 GO:0006915 GO:0006351 GO:0007050
GO:0008270 GO:0036459 GO:0016491 GO:0003677 GO:0003899
GO:0005730
Cluster-22178.67917 GO:0008270
Cluster-22178.6792 GO:0005515
Cluster-22178.67920 GO:0006351 GO:0006486 GO:0018279 GO:0006915
GO:0008285 GO:0055114 GO:0019083 GO:0007050 GO:0016579
GO:0006144 GO:0006206 GO:0003899 GO:0036459 GO:0003677
GO:0004579 GO:0016491 GO:0008270 GO:0016021 GO:0008250
GO:0005783 GO:0005739 GO:0005730
Cluster-22178.67924 GO:0009229 GO:0030975
Cluster-22178.67927 GO:0008270 GO:0003676 GO:0015066 GO:0005622
Cluster-22178.67930 GO:0006270 GO:0008033 GO:0006508 GO:0004177
GO:0008168 GO:0016021 GO:0005622
Cluster-22178.67932 GO:0007010 GO:0003779
Cluster-22178.67934 GO:0007010 GO:0016567 GO:0003779 GO:0004857
GO:0004842
Cluster-22178.67935 GO:0006275 GO:0019073 GO:0006260 GO:0045893
GO:0005515 GO:0003677 GO:0003688 GO:0042025 GO:0046809
Cluster-22178.67936 GO:2000123 GO:0006508 GO:0004190
Cluster-22178.67937 GO:0005198 GO:0005094 GO:0005737 GO:0019028
Cluster-22178.67938 GO:0006118 GO:0019646 GO:0006629 GO:0009055

Cluster-22178.67939 GO:0007165 GO:0031704 GO:0005179

Cluster-22178.67940 GO:0055114 GO:0016620
Cluster-22178.67942 GO:0032040
Cluster-22178.67943 GO:0032040
Cluster-22178.67944 GO:0007165 GO:0006810 GO:0016192 GO:0046486 GO:0006633 GO:0006071 GO:0016573 GO:0006090 GO:0004371 GO:0003989 GO:0005515 GO:0004871 GO:0016020 GO:0009317 GO:0005886 GO:0016021
Cluster-22178.67945 GO:0000902 GO:0005975 GO:0016773
Cluster-22178.67947 GO:0007155 GO:0005515 GO:0016021
Cluster-22178.67948 GO:0016192 GO:0006886 GO:0030414 GO:0005506 GO:0030131 GO:0005576
Cluster-22178.67949 GO:0006357 GO:0001104 GO:0016592
Cluster-22178.67950 GO:0006779 GO:0055114 GO:0016567 GO:0016559 GO:0006355 GO:0004842 GO:0043565 GO:0008270 GO:0046872 GO:0005515 GO:0016020 GO:0005779
Cluster-22178.67951 GO:0042254 GO:0006412 GO:0003735 GO:0005622 GO:0005840
Cluster-22178.67952 GO:0015725 GO:0035429 GO:0006486 GO:0008373 GO:0046872 GO:0016787 GO:0015128 GO:0016020
Cluster-22178.67955 GO:0007264 GO:0005525 GO:0003924
Cluster-22178.67956 GO:0006886 GO:0046740 GO:0008536 GO:0016021
Cluster-22178.67957 GO:0007155 GO:0000917 GO:0007050 GO:0046872 GO:0005509 GO:0005515
Cluster-22178.67961 GO:0005869
Cluster-22178.67962 GO:0042967 GO:0008152 GO:0008270 GO:0016407

Cluster-22178.67963 GO:0006139 GO:0003676 GO:0008408
Cluster-22178.67964 GO:0007010 GO:0007018 GO:0007017 GO:0008152 GO:0003777 GO:0008289 GO:0008017 GO:0005524 GO:0016829 GO:0003779 GO:0010333 GO:0005739 GO:0005874 GO:0045298
Cluster-22178.67965 GO:0006364 GO:0016192 GO:0019031 GO:0016021 GO:0005730
Cluster-22178.67967 GO:0009405 GO:0006810 GO:0007186 GO:0005515

GO:0008200 GO:0004930 GO:0005576 GO:0016021
Cluster-22178.67970 GO:0005515
Cluster-22178.67972 GO:0016310 GO:0009069 GO:0005515 GO:0004674

Cluster-22178.67973 GO:0034314 GO:0006355 GO:0030833 GO:0003700
GO:0043565 GO:0005198 GO:0019867 GO:0005856 GO:0005882
GO:0005885 GO:0005667
Cluster-22178.67977 GO:0016070 GO:0006260 GO:0003677 GO:0003887
GO:0005515 GO:0003723 GO:0042575 GO:0009360
Cluster-22178.67980 GO:0007411 GO:0019031 GO:0016021
Cluster-22178.67982 GO:0006886 GO:0006570 GO:0006576 GO:0006470
GO:2000123 GO:0007165 GO:0007186 GO:0007264 GO:0005525
GO:0004725 GO:0004871 GO:0019001 GO:0031683 GO:0003924
GO:0005524 GO:0005543 GO:0016887
Cluster-22178.67983 GO:0046813 GO:0061025 GO:0016021 GO:0019031
Cluster-22178.67984 GO:0055114 GO:0008152 GO:0006954 GO:0007165
GO:0005125 GO:0042578 GO:0051287 GO:0016616 GO:0005576
Cluster-22178.67985 GO:0007005 GO:0006511 GO:0042803 GO:0008134
GO:0005509 GO:0045502 GO:0030286 GO:0005667 GO:0031305
Cluster-22178.67988 GO:0007217
Cluster-22178.67992 GO:0007186 GO:0004930 GO:0016021
Cluster-22178.67994 GO:0006886 GO:0005525 GO:0005047 GO:0003924
GO:0005785 GO:0005786
Cluster-22178.67995 GO:0006260 GO:0015684 GO:0007264 GO:0006576
GO:0005525 GO:0005524 GO:0015093 GO:0016887 GO:0003924
GO:0003677 GO:0016021
Cluster-22178.67996 GO:0005524 GO:0016887 GO:0005515
Cluster-22178.67997 GO:0046872 GO:0005515
Cluster-22178.67998 GO:0006281 GO:0006355 GO:0006310 GO:0009378
GO:0003723 GO:0016301 GO:0008134 GO:0005515 GO:0005524
GO:0016887 GO:0003724 GO:0009379 GO:0005667 GO:0005657
Cluster-22178.67999 GO:0008033 GO:0055114 GO:0006355 GO:0016491
GO:0050660 GO:0005507 GO:0003700 GO:0071949 GO:0003677

GO:0005634 GO:0005667
Cluster-22178.68000 GO:0007178 GO:0019064 GO:0016310 GO:0006468
GO:0009069 GO:0004672 GO:0005524 GO:0004675 GO:0016020
Cluster-22178.68003 GO:0055114 GO:0046872 GO:0047134
Cluster-22178.68004 GO:0006118 GO:0009055 GO:0005515 GO:0009512
Cluster-22178.68005 GO:0006118 GO:0009055 GO:0005515 GO:0009512
Cluster-22178.68006 GO:0006118 GO:0009055 GO:0005515 GO:0009512
Cluster-22178.68008 GO:0048193 GO:0005515 GO:0016020 GO:0016021
Cluster-22178.68009 GO:0005975 GO:0004553
Cluster-22178.68010 GO:0048193 GO:0005515 GO:0016021 GO:0016020
Cluster-22178.68012 GO:0006355 GO:0008654 GO:0006260 GO:0000166
GO:0003677 GO:0008134 GO:0005524 GO:0016887 GO:0016780
GO:0005667 GO:0016020
Cluster-22178.68014 GO:0002949 GO:0007264 GO:0006614 GO:0007165
GO:0015684 GO:0007186 GO:0008152 GO:0006777 GO:0006576
GO:0005524 GO:0015093 GO:0016887 GO:0003677 GO:0098519
GO:0031683 GO:0019001 GO:0043531 GO:0003924 GO:0005525
GO:0004871 GO:0000166 GO:0016301 GO:0016021
Cluster-22178.68017 GO:0009405 GO:0005515
Cluster-22178.68018 GO:0042325 GO:0005789 GO:0005737
Cluster-22178.68020 GO:0016559 GO:0006355 GO:0007165 GO:0016567
GO:0055114 GO:0006779 GO:0005179 GO:0005515 GO:0008270
GO:0046872 GO:0043565 GO:0004842 GO:0005779 GO:0016020
Cluster-22178.68022 GO:0055114 GO:0009186 GO:0030246 GO:0016021
Cluster-22178.68026 GO:0005515 GO:0004867
Cluster-22178.68027 GO:0006367 GO:0006506 GO:0006355 GO:0016746
GO:0005789 GO:0016021 GO:0005739
Cluster-22178.68028 GO:0009186 GO:0055114 GO:0030246 GO:0016021
Cluster-22178.68031 GO:0006468 GO:0003677 GO:0016773 GO:0005524
GO:0004672 GO:0016020
Cluster-22178.68032 GO:0005515
Cluster-22178.68033 GO:0003677 GO:0004518
Cluster-22178.68034 GO:0004190 GO:0016021

Cluster-22178.68035 GO:0043044 GO:0003676 GO:0016787 GO:0046872
GO:0016586
Cluster-22178.68036 GO:0006950 GO:0006508 GO:0008270 GO:0004222
GO:0003700 GO:0004197 GO:0031012 GO:0005667
Cluster-22178.68037 GO:0042967 GO:0008152 GO:0006633 GO:0016747
GO:0004315 GO:0005835
Cluster-22178.68038 GO:0008210 GO:0006694 GO:0043154 GO:0055114
GO:0008209 GO:0019079 GO:0008207 GO:0043066 GO:0003824
GO:0016616 GO:0032266 GO:0050662 GO:0003854 GO:0051287
GO:0080019 GO:0043130 GO:0016620 GO:0005737 GO:0071986
Cluster-22178.68041 GO:0043044 GO:0046872 GO:0016787 GO:0003676
GO:0016586
Cluster-22178.68042 GO:0015992 GO:0006744 GO:0006120 GO:2001256
GO:0006814 GO:0042254 GO:0006412 GO:0042773 GO:0003735
GO:0008137 GO:0005622 GO:0030176 GO:0005743 GO:0005840
Cluster-22178.68044 GO:0043044 GO:0046872 GO:0016787 GO:0003676
GO:0016586
Cluster-22178.68046 GO:0019076 GO:0006308 GO:0046718 GO:0055085
GO:0006885 GO:0006812 GO:0075521 GO:0015299 GO:0008855
GO:0016021 GO:0044163 GO:0019028 GO:0009318 GO:0030430
GO:0042025
Cluster-22178.68047 GO:0055085 GO:0006811 GO:0005216 GO:0016020
Cluster-22178.68048 GO:0007186 GO:0007165 GO:0007010 GO:0003924
GO:0003779 GO:0019001 GO:0008270 GO:0031683 GO:0004871
GO:0005525
Cluster-22178.68050 GO:0006310 GO:0019079 GO:0006760 GO:0002949
GO:0006206 GO:0051016 GO:0006955 GO:0000902 GO:0006355
GO:0046656 GO:0006281 GO:0006139 GO:0003677 GO:0009378
GO:0003724 GO:0004127 GO:0046983 GO:0016887 GO:0005524
GO:0008134 GO:0003723 GO:0003678 GO:0004150 GO:0000150
GO:0043531 GO:0016020 GO:0008290 GO:0005667 GO:0009379
GO:0005657
Cluster-22178.68051 GO:0032183 GO:0070140

Cluster-22178.68053 GO:0005515 GO:0061630 GO:0008270 GO:0046872

Cluster-22178.68054 GO:0005515
Cluster-22178.68056 GO:0003676 GO:0003723
Cluster-22178.68057 GO:0006730 GO:0030163 GO:0005515 GO:0016740 GO:0008270
Cluster-22178.68058 GO:0007224
Cluster-22178.68059 GO:0046836 GO:0017089 GO:0051861 GO:0005737
Cluster-22178.68060 GO:0007224
Cluster-22178.68063 GO:0042254 GO:0006412 GO:0003735 GO:0005840
Cluster-22178.68066 GO:0016787
Cluster-22178.68068 GO:0008033 GO:0006508 GO:0006468 GO:0004222 GO:0008270 GO:0004672 GO:0005524 GO:0016437 GO:0004810 GO:0005576
Cluster-22178.68070 GO:0042254 GO:0006412 GO:0003735 GO:0005840 GO:0005622
Cluster-22178.68071 GO:0042254 GO:0006412 GO:0003735 GO:0005840 GO:0005622
Cluster-22178.68072 GO:0006465 GO:0008233 GO:0005787 GO:0016021
Cluster-22178.68073 GO:0006144 GO:0000103 GO:0005524 GO:0046872 GO:0004020 GO:0016301 GO:0005515
Cluster-22178.68074 GO:0000103 GO:0006144 GO:0004020 GO:0016301 GO:0005515 GO:0005524 GO:0046872
Cluster-22178.68076 GO:0015991 GO:0007155 GO:0006351 GO:0015992 GO:0006206 GO:0006144 GO:0070140 GO:0032183 GO:0003677 GO:0003899 GO:0005515 GO:0015078 GO:0005730 GO:0033179 GO:0016020
Cluster-22178.68077 GO:0055085 GO:0006811 GO:0005216 GO:0016020
Cluster-22178.68080 GO:0006355 GO:0005515 GO:0005634
Cluster-22178.68081 GO:0006355 GO:0007165 GO:0043565 GO:0008270 GO:0005524 GO:0003700 GO:0016787 GO:0005515 GO:0005737 GO:0005667
Cluster-22178.68082 GO:0005515

Cluster-22178.68083 GO:0005515
Cluster-22178.68084 GO:0007186 GO:0006030 GO:0006468 GO:0030416
GO:0008152 GO:0008270 GO:0005524 GO:0016491 GO:0016773
GO:0004930 GO:0008061 GO:0005515 GO:0004672 GO:0016020
GO:0005576 GO:0016021
Cluster-22178.68086 GO:0016567 GO:0006915 GO:0032465 GO:0033014
GO:0055114 GO:0050661 GO:0004842 GO:0008883 GO:0016021
Cluster-22178.68088 GO:0015074 GO:0050832 GO:0042967 GO:0042742
GO:0008080 GO:0003676 GO:0019239 GO:0003677 GO:0031224
Cluster-22178.68089 GO:0009058 GO:0005515 GO:0004866
Cluster-22178.68090 GO:0007264 GO:0031419 GO:0005525 GO:0003924
GO:0046872
Cluster-22178.68092 GO:0006013 GO:0005975 GO:0015923
Cluster-22178.68093 GO:0015684 GO:0003924 GO:0015093 GO:0005515
GO:0005525 GO:0019033 GO:0016021
Cluster-22178.68094 GO:0006120 GO:0006744 GO:0055085 GO:0015992
GO:0055114 GO:0042773 GO:0006814 GO:0015986 GO:0015078
GO:0016651 GO:0008137 GO:0015002 GO:0009512 GO:0016021
GO:0016020
Cluster-22178.68095 GO:0006412 GO:0042254 GO:0003735 GO:0008289
GO:0005840 GO:0005739
Cluster-22178.68096 GO:0005515
Cluster-22178.68097 GO:0006974 GO:0006914 GO:0042254 GO:0009271
GO:0007155 GO:0007094 GO:0006886 GO:0006915 GO:0030001
GO:0006260 GO:0055085 GO:0030168 GO:0015031 GO:0007165
GO:0006810 GO:0006302 GO:0051258 GO:0048870 GO:0006412
GO:0030474 GO:0006355 GO:0008017 GO:0008565 GO:0003735
GO:0043565 GO:0005515 GO:0005198 GO:0042803 GO:0005102
GO:0046873 GO:0030674 GO:0005200 GO:0003700 GO:0008134
GO:0045502 GO:0005577 GO:0016020 GO:0005667 GO:0045298
GO:0005737 GO:0005856 GO:0005634 GO:0048786 GO:0005823
GO:0030286 GO:0005886 GO:0005840 GO:0031514
Cluster-22178.68098 GO:0006974 GO:0042254 GO:0009271 GO:0006886

GO:0006915 GO:0030001 GO:0055085 GO:0030168 GO:0015031
GO:0007165 GO:0006302 GO:0051258 GO:0048870 GO:0030474
GO:0006412 GO:0006355 GO:0008565 GO:0003735 GO:0005515
GO:0005198 GO:0042803 GO:0005102 GO:0046873 GO:0030674
GO:0005200 GO:0003700 GO:0008134 GO:0045502 GO:0005577
GO:0016020 GO:0005667 GO:0005737 GO:0005856 GO:0005634
GO:0005823 GO:0030286 GO:0005840 GO:0031514
Cluster-22178.68099 GO:0055114 GO:0006118 GO:0008152 GO:0008440
GO:0016627 GO:0003995 GO:0050660
Cluster-22178.68100 GO:0051539
Cluster-22178.68103 GO:0003824
Cluster-22178.68104 GO:0005515 GO:0016021
Cluster-22178.68105 GO:0006886 GO:0015991 GO:0006468 GO:0016310
GO:0009069 GO:0006260 GO:0006914 GO:0015992 GO:0015031
GO:0005515 GO:0015078 GO:0004674 GO:0008565 GO:0005524
GO:0033179 GO:0005737 GO:0016020
Cluster-22178.68106 GO:0042254 GO:0051090 GO:0006352 GO:0003735
GO:0003677 GO:0005763 GO:0005634 GO:0005840
Cluster-22178.68108 GO:0055114 GO:0006118 GO:0008152 GO:0016746
GO:0009916
Cluster-22178.68109 GO:0045454 GO:0055114 GO:0016491 GO:0016209

Cluster-22178.68110 GO:0007275 GO:0042600
Cluster-22178.68113 GO:0006771 GO:0019497 GO:0005515 GO:0003993
GO:0046872
Cluster-22178.68114 GO:0005525
Cluster-22178.68117 GO:0055114 GO:0016114 GO:0006402 GO:0006694
GO:0006629 GO:0051252 GO:0046872 GO:0004535 GO:0003676
GO:0050660 GO:0003723 GO:0004506 GO:0005737 GO:0016021
GO:0005634
Cluster-22178.68118 GO:0006464 GO:0051082 GO:0016151 GO:0031072

Cluster-22178.68119 GO:0051694 GO:0005523

Cluster-22178.68121 GO:0055085 GO:0055036 GO:0016021
Cluster-22178.68123 GO:0055085 GO:0007005 GO:0006821 GO:0006914
GO:0005247 GO:0016020 GO:0031305
Cluster-22178.68125 GO:0006144 GO:0006508 GO:0044419 GO:0004197
GO:0003968 GO:0017111 GO:0031379
Cluster-22178.68126 GO:0006355 GO:0006508 GO:0090307 GO:0051082
GO:0004222 GO:0003700 GO:0043565 GO:0005667 GO:0033588
Cluster-22178.68127 GO:0000105 GO:0006807 GO:0000162 GO:0006013
GO:0009607 GO:0006040 GO:0006000 GO:0006004 GO:0006571
GO:0008152 GO:0009094 GO:0055114 GO:0008033 GO:0006537
GO:0006355 GO:0006051 GO:0050660 GO:0004425 GO:0016491
GO:0018580 GO:0015930 GO:0016638 GO:0017150 GO:0003824
GO:0016627 GO:0016829 GO:0047465 GO:0008736 GO:0005737
Cluster-22178.68128 GO:0005515
Cluster-22178.68130 GO:0055114 GO:0030001 GO:0006801 GO:0046872

Cluster-22178.68132 GO:0005739
Cluster-22178.68133 GO:0042254 GO:0006412 GO:0046740 GO:0003735
GO:0005840 GO:0044156 GO:0005622
Cluster-22178.68135 GO:0016021
Cluster-22178.68136 GO:0042742 GO:0070588 GO:0006816 GO:0006260
GO:0003677 GO:0005245 GO:0016887 GO:0005524 GO:0005891
Cluster-22178.68139 GO:0016192 GO:0006886 GO:0005515 GO:0030117
Cluster-22178.68140 GO:0006457 GO:0016192 GO:0007165 GO:0030521
GO:0006355 GO:0006886 GO:0009372 GO:0009405 GO:0005509
GO:0003677 GO:0004882 GO:0008270 GO:0008233 GO:0005496
GO:0051082 GO:0005515 GO:0005783 GO:0005634 GO:0030117
GO:0016021
Cluster-22178.68142 GO:0005515
Cluster-22178.68144 GO:0006364 GO:0009755 GO:0005515 GO:0032040
GO:0005576
Cluster-22178.68145 GO:0000723 GO:0006281 GO:0003678 GO:0005524
GO:0003677 GO:0016787 GO:0005657 GO:0016020

Cluster-22178.68146 GO:0006412 GO:0006355 GO:0006281 GO:0006576
GO:0002949 GO:0007165 GO:0006810 GO:0042254 GO:0006310
GO:0007186 GO:0004871 GO:0046914 GO:0019001 GO:0031683
GO:0003924 GO:0008134 GO:0005524 GO:0016887 GO:0046983
GO:0003735 GO:0009378 GO:0005840 GO:0016021 GO:0005657
GO:0005622 GO:0005667 GO:0009379
Cluster-22178.68148 GO:0051603 GO:0004298 GO:0005839
Cluster-22178.68149 GO:0006897 GO:0005515 GO:0005096 GO:0016020
GO:0016021
Cluster-22178.68150 GO:0015684 GO:0007264 GO:0005525 GO:0003924
GO:0015093 GO:0016021
Cluster-22178.68151 GO:0006897 GO:0005096 GO:0005515 GO:0016020
GO:0016021
Cluster-22178.68152 GO:0006144 GO:0009190 GO:0006171 GO:0035556
GO:0016849 GO:0004016 GO:0005886
Cluster-22178.68153 GO:0008270
Cluster-22178.68154 GO:0009395 GO:0006629 GO:0004620 GO:0016020
Cluster-22178.68155 GO:0005515 GO:0031224
Cluster-22178.68156 GO:0003677 GO:0003676 GO:0005524 GO:0016787

Cluster-22178.68157 GO:0045892 GO:0006308 GO:0046685 GO:0071816
GO:0008855 GO:0003677 GO:0009318 GO:0005634
Cluster-22178.68158 GO:0006811 GO:0006810 GO:0005230 GO:0016020
Cluster-22178.68160 GO:0006914
Cluster-22178.68162 GO:0055114 GO:0010309 GO:0005515
Cluster-22178.68163 GO:0016567 GO:0006144 GO:0006376 GO:0006508
GO:0006118 GO:0003729 GO:0004197 GO:0003677 GO:0017111
GO:0061630 GO:0008270 GO:0051536 GO:0004842 GO:0070008
GO:0004252 GO:0005515 GO:0046872 GO:0016817 GO:0009055
GO:0003968 GO:0005685 GO:0005680 GO:0031379 GO:0045202
Cluster-22178.68165 GO:0046872
Cluster-22178.68166 GO:0006614 GO:0005515 GO:0003723 GO:0003724
GO:0005525 GO:0003924 GO:0016887 GO:0005524

Cluster-22178.68169 GO:0017119
Cluster-22178.68170 GO:0007015 GO:0003785
Cluster-22178.68171 GO:0001575 GO:0006012 GO:0046486 GO:0005975
GO:0004553 GO:0004557
Cluster-22178.68172 GO:0009089 GO:0008207 GO:0008209 GO:0009085
GO:0008210 GO:0006694 GO:0055114 GO:0016616 GO:0003854
GO:0051287 GO:0050662 GO:0016620 GO:0003824 GO:0008839
GO:0016491
Cluster-22178.68174 GO:0009607 GO:0016021
Cluster-22178.68176 GO:0032259 GO:0046872 GO:0003723 GO:0005845
Cluster-22178.68177 GO:0007165 GO:0005158 GO:0003723 GO:0005515
GO:0005899
Cluster-22178.68178 GO:0045859 GO:0004860 GO:0005576
Cluster-22178.68179 GO:0042967 GO:0004379 GO:0008080 GO:0003676

Cluster-22178.68182 GO:0009405 GO:0004859 GO:0005576
Cluster-22178.68183 GO:0006468 GO:0016773 GO:0004672 GO:0005524
GO:0016020
Cluster-22178.68184 GO:0004859 GO:0005576
Cluster-22178.68186 GO:0007224
Cluster-22178.68187 GO:0007186 GO:0006355 GO:0004930 GO:0003700
GO:0008270 GO:0043565 GO:0016021 GO:0005667
Cluster-22178.68188 GO:0003676 GO:0005921
Cluster-22178.68189 GO:0006397 GO:0046660 GO:0005634
Cluster-22178.68191 GO:0007018 GO:0006511 GO:0016567 GO:0007017
GO:0005515 GO:0034450 GO:0008017 GO:0004842 GO:0003777
GO:0005524 GO:0000151 GO:0045298 GO:0005874
Cluster-22178.68192 GO:0031167 GO:0009451 GO:0008033 GO:0004809
GO:0008168 GO:0003723
Cluster-22178.68193 GO:0005515
Cluster-22178.68195 GO:0045892 GO:0007186 GO:0051082 GO:0003677
GO:0004930 GO:0016021
Cluster-22178.68197 GO:0015031 GO:0006464 GO:0005515

Cluster-22178.68198 GO:0006952 GO:0006367 GO:0005669 GO:0009279
Cluster-22178.68199 GO:0016055 GO:0006531 GO:0043039 GO:0006419
GO:0006522 GO:0000166 GO:0004813 GO:0016876 GO:0005524
GO:0003676
Cluster-22178.68200 GO:0030145 GO:0004177
Cluster-22178.68203 GO:0007165 GO:0009451 GO:0006886 GO:0016192
GO:0006400 GO:0008033 GO:0016301 GO:0005515 GO:0008176
GO:0046872 GO:0030117 GO:0016021
Cluster-22178.68204 GO:0055085 GO:0006814 GO:0006810 GO:0005215
GO:0016020
Cluster-22178.68205 GO:0030212 GO:0019068 GO:0008168 GO:0008047
GO:0003676 GO:0008233 GO:0004867 GO:0005515
Cluster-22178.68206 GO:0007264 GO:0007186 GO:0007165 GO:0003924
GO:0031683 GO:0019001 GO:0004871 GO:0005525
Cluster-22178.68207 GO:0006457 GO:0000413 GO:0003755
Cluster-22178.68209 GO:0019087 GO:0003676 GO:0016021
Cluster-22178.68210 GO:0006007 GO:0006629 GO:0006412 GO:0042254
GO:0003824 GO:0046537 GO:0005515 GO:0003735 GO:0005622
GO:0005840
Cluster-22178.68211 GO:0007186 GO:0004930 GO:0019031 GO:0016021
Cluster-22178.68213 GO:0008152 GO:0016787 GO:0005198 GO:0019028
GO:0016021
Cluster-22178.68214 GO:0051258 GO:0006308 GO:0007155 GO:0007165
GO:0000921 GO:0030168 GO:0051607 GO:0006914 GO:0006606
GO:0045502 GO:0005515 GO:0042803 GO:0008134 GO:0005179
GO:0030674 GO:0008855 GO:0005102 GO:0005576 GO:0009318
GO:0016021 GO:0005667 GO:0030286 GO:0005940 GO:0005577
Cluster-22178.68215 GO:0051205 GO:0000042 GO:0005515 GO:0016021
GO:0016020
Cluster-22178.68216 GO:0019497 GO:0055114 GO:0007339 GO:0006118
GO:0006771 GO:0007155 GO:0003993 GO:0046872 GO:0003995
GO:0005515 GO:0016021 GO:0016020 GO:0005576
Cluster-22178.68217 GO:0006886 GO:0007067 GO:0005515 GO:0016020

GO:0042729 GO:0072686 GO:0005801 GO:0005737
Cluster-22178.68219 GO:0046872 GO:0008270 GO:0005515
Cluster-22178.68220 GO:0016020
Cluster-22178.68222 GO:0008270 GO:0005921
Cluster-22178.68223 GO:0008270 GO:0005921
Cluster-22178.68225 GO:0042254 GO:0006412 GO:0003735 GO:0005840
GO:0000228 GO:0005622
Cluster-22178.68227 GO:0006302 GO:0007155 GO:0043087 GO:0006308
GO:0005515 GO:0008855 GO:0009318 GO:0005634 GO:0009288
Cluster-22178.68228 GO:0006810 GO:0009058 GO:0006687 GO:0006816
GO:0001733 GO:0042030 GO:0008146 GO:0004867 GO:0005246
GO:0005794 GO:0016021 GO:0005639 GO:0016020
Cluster-22178.68229 GO:0015743 GO:0006265 GO:0003677 GO:0005524
GO:0003918 GO:0004190 GO:0016021
Cluster-22178.68231 GO:0030150 GO:0016579 GO:0008270 GO:0036459
GO:0005742
Cluster-22178.68232 GO:0006096 GO:0006094 GO:0006144 GO:0015976
GO:0005515 GO:0004743 GO:0000287 GO:0030955 GO:0003824
Cluster-22178.68233 GO:0009405 GO:0005887 GO:0016020
Cluster-22178.68235 GO:0006144 GO:0035556 GO:0006171 GO:0009190
GO:0071816 GO:0016849 GO:0004016 GO:0005886
Cluster-22178.68237 GO:0005515
Cluster-22178.68238 GO:0007186 GO:0006355 GO:0004930 GO:0003700
GO:0008270 GO:0043565 GO:0005667 GO:0016021
Cluster-22178.68239 GO:0042157 GO:0006869 GO:0008270 GO:0003677
GO:0008289 GO:0019028 GO:0005576
Cluster-22178.68240 GO:0007186 GO:0007165 GO:0008601 GO:0005515
GO:0004930 GO:0016021 GO:0000159
Cluster-22178.68241 GO:0016255 GO:0016627 GO:0030414 GO:0042765
GO:0005576
Cluster-22178.68242 GO:0015992 GO:0006123 GO:0004129 GO:0005739
GO:0045277
Cluster-22178.68245 GO:0055114 GO:0008152 GO:0016491 GO:0005515

Cluster-22178.68247 GO:0044237 GO:0003676 GO:0005524
Cluster-22178.68249 GO:0006606 GO:0043087 GO:0006357 GO:0006302
GO:0006308 GO:0009405 GO:0006397 GO:0006355 GO:0008855
GO:0003700 GO:0001104 GO:0016592 GO:0016020 GO:0005667
GO:0009318 GO:0005634
Cluster-22178.68250 GO:0006457 GO:0006405 GO:0000921 GO:0006260
GO:0006606 GO:0006355 GO:0019089 GO:0010508 GO:0051382
GO:0045892 GO:0003677 GO:0051082 GO:0043565 GO:0003700
GO:0005667 GO:0016272 GO:0005940 GO:0005815 GO:0000776
GO:0005634 GO:0019028 GO:0042025 GO:0016021
Cluster-22178.68254 GO:0055114 GO:0005506 GO:0016705 GO:0020037

Cluster-22178.68255 GO:0016021
Cluster-22178.68256 GO:0007217
Cluster-22178.68257 GO:0006355 GO:0003677 GO:0003700 GO:0005667
Cluster-22178.68258 GO:0005515
Cluster-22178.68259 GO:0008270 GO:0046872 GO:0005515 GO:0005622
GO:0005634
Cluster-22178.68260 GO:0005515 GO:0046872 GO:0008270 GO:0005634
GO:0005622
Cluster-22178.68261 GO:0006629
Cluster-22178.68262 GO:0006511 GO:0031625
Cluster-22178.68263 GO:0007165 GO:0008053 GO:0015031 GO:0030168
GO:0006464 GO:0048024 GO:0000917 GO:0006355 GO:0015991
GO:0007049 GO:0051258 GO:0006308 GO:0043093 GO:0007155
GO:0015992 GO:0006914 GO:0030674 GO:0005102 GO:0008855
GO:0003700 GO:0004871 GO:0008092 GO:0005198 GO:0051082
GO:0015078 GO:0043565 GO:0003924 GO:0019898 GO:0009318
GO:0033179 GO:0019028 GO:0016021 GO:0005741 GO:0016020
GO:0005667 GO:0005577 GO:0005634 GO:0005737
Cluster-22178.68264 GO:0006629
Cluster-22178.68266 GO:0006508 GO:0004198 GO:0016757 GO:0016020

GO:0005622
Cluster-22178.68267 GO:0007165
Cluster-22178.68268 GO:0005975 GO:0008270 GO:0004560 GO:0003676

Cluster-22178.68269 GO:0009405 GO:0006810 GO:0019871 GO:0005576
Cluster-22178.68270 GO:0016043 GO:0030036 GO:0003924 GO:0003779
GO:0017048
Cluster-22178.68271 GO:0003924
Cluster-22178.68272 GO:0005515 GO:2001070
Cluster-22178.68273 GO:0055114 GO:0007156 GO:0006412 GO:0042254
GO:0016491 GO:0003735 GO:0005509 GO:0005622 GO:0005840
GO:0016020
Cluster-22178.68276 GO:0006355 GO:0003700 GO:0005515 GO:0043565
GO:0003723 GO:0005667
Cluster-22178.68277 GO:0043565 GO:0004252 GO:0005515 GO:0019028
GO:0055036 GO:0005634
Cluster-22178.68279 GO:0007009 GO:0030001 GO:0006355 GO:0046872
GO:0005515 GO:0005743 GO:0005737 GO:0005747 GO:0005634
Cluster-22178.68280 GO:0005634
Cluster-22178.68283 GO:0055114 GO:0016705 GO:0020037 GO:0005506

Cluster-22178.68284 GO:0015979 GO:0009539 GO:0009523
Cluster-22178.68285 GO:0006298 GO:0006412 GO:0042254 GO:0003735
GO:0004519 GO:0008146 GO:0005840 GO:0016021 GO:0005622
Cluster-22178.68287 GO:0006355 GO:0007186 GO:0043565 GO:0008270
GO:0003700 GO:0004930 GO:0016021 GO:0005667
Cluster-22178.68288 GO:0007010 GO:0003779
Cluster-22178.68290 GO:0005515
Cluster-22178.68293 GO:0009245 GO:0044564 GO:0008152 GO:0006520
GO:0055114 GO:0016491 GO:0016747
Cluster-22178.68294 GO:0006531 GO:0006529 GO:0006522 GO:0008270
GO:0046872 GO:0004066 GO:0005515
Cluster-22178.68295 GO:0030494 GO:0015979 GO:0016032 GO:0006355

GO:0003700 GO:0043565 GO:0005515 GO:0005667
Cluster-22178.68300 GO:0006508 GO:0006281 GO:0006412 GO:0042254
GO:0015876 GO:0051016 GO:0003735 GO:0008521 GO:0046983
GO:0003676 GO:0008239 GO:0030479 GO:0005622 GO:0016021
GO:0005840
Cluster-22178.68302 GO:0006355 GO:0043039 GO:0006418 GO:0006820
GO:0004812 GO:0003677 GO:0000166 GO:0016876 GO:0005524
GO:0043565 GO:0008509 GO:0003700 GO:0005667 GO:0016021
Cluster-22178.68303 GO:0044419 GO:0006508 GO:0006144 GO:0004197
GO:0017111 GO:0003968 GO:0031379
Cluster-22178.68304 GO:0003677 GO:0004859 GO:0005576 GO:0005634
Cluster-22178.68307 GO:0007264 GO:0005525
Cluster-22178.68309 GO:0006412 GO:0042254 GO:0006298 GO:0003735
GO:0004519 GO:0008146 GO:0005622 GO:0016021 GO:0005840
Cluster-22178.68311 GO:0016032 GO:0006355 GO:0007094 GO:0048870
GO:0008608 GO:0003700 GO:0072686 GO:0005667 GO:0031514
GO:0042729
Cluster-22178.68314 GO:0006694 GO:0008152 GO:0055114 GO:0008209
GO:0008207 GO:0008210 GO:0019836 GO:0003824 GO:0016491
GO:0016616 GO:0005515 GO:0050662 GO:0003854 GO:0005576
Cluster-22178.68315 GO:0016567 GO:0005515 GO:0004842
Cluster-22178.68316 GO:0016746 GO:0005634
Cluster-22178.68317 GO:0042254 GO:0006412 GO:0003735 GO:0005840
Cluster-22178.68319 GO:0006355 GO:0043565 GO:0005515 GO:0005634
Cluster-22178.68322 GO:0015994 GO:0033014 GO:0005515 GO:0004418

Cluster-22178.68323 GO:0051568 GO:0000902 GO:0006479 GO:0006508
GO:0003779 GO:0005515 GO:0042800 GO:0003682 GO:0000785
GO:0072357 GO:0048188
Cluster-22178.68324 GO:0006511 GO:0031625
Cluster-22178.68326 GO:0023052 GO:0007165 GO:0005125
Cluster-22178.68327 GO:0019079 GO:0006810 GO:0004386 GO:0003676
GO:0008026 GO:0043531 GO:0005524

Cluster-22178.68332 GO:0005886
Cluster-22178.68333 GO:0006355 GO:0043565 GO:0003700 GO:0005667
Cluster-22178.68334 GO:0008152 GO:0046872 GO:0008270 GO:0003824
GO:0016836 GO:0005515 GO:0061630 GO:0032040
Cluster-22178.68335 GO:0006464 GO:0051260 GO:0007186 GO:0004930
GO:0016020 GO:0016021
Cluster-22178.68336 GO:0005515
Cluster-22178.68337 GO:0006511 GO:0016567 GO:0033014 GO:0009252
GO:0032465 GO:0006915 GO:0006629 GO:0055114 GO:0004842
GO:0008963 GO:0008883 GO:0050661 GO:0003723 GO:0003676
GO:0003924 GO:0016021
Cluster-22178.68338 GO:0006891 GO:0008144 GO:0017119
Cluster-22178.68340 GO:0007155
Cluster-22178.68343 GO:0006270 GO:0003676
Cluster-22178.68344 GO:0043395 GO:0016020 GO:0005578
Cluster-22178.68346 GO:0006308 GO:0042254 GO:0006412 GO:0009307
GO:0009036 GO:0003735 GO:0003677 GO:0016021 GO:0005840
GO:0005622 GO:0019013 GO:0009359
Cluster-22178.68348 GO:0005515
Cluster-22178.68349 GO:0006397 GO:0000012 GO:0009069 GO:0006468
GO:0006281 GO:0016310 GO:0003684 GO:0003723 GO:0004674
GO:0005524 GO:0005634 GO:0005737
Cluster-22178.68351 GO:0006396 GO:0006807 GO:0006144 GO:0004000
GO:0003723
Cluster-22178.68353 GO:0007264 GO:0006351 GO:0006144 GO:0006206
GO:0043087 GO:0005085 GO:0003899 GO:0003677 GO:0005730
Cluster-22178.68354 GO:0006355 GO:0016055 GO:0006886 GO:0000975
GO:0008536 GO:0003700 GO:0005667
Cluster-22178.68355 GO:0016020
Cluster-22178.68356 GO:0022008 GO:0005764
Cluster-22178.68357 GO:0016020
Cluster-22178.68363 GO:0007224
Cluster-22178.68364 GO:0016021

Cluster-22178.68366 GO:0007049 GO:0055114 GO:0008033 GO:0048870 GO:0006118 GO:0030150 GO:0045454 GO:0009055 GO:0015035 GO:0005515 GO:0050660 GO:0016491 GO:0019904 GO:0016021 GO:0005737 GO:0031514 GO:0005634 GO:0005742

Cluster-22178.68367 GO:0005524 GO:0003676

Cluster-22178.68368 GO:0051782 GO:0045444 GO:0006281 GO:0009432 GO:0006260 GO:0006310 GO:0003723 GO:0005524 GO:0003689 GO:0016887 GO:0003724 GO:0009378 GO:0098519 GO:0016021 GO:0005663 GO:0042575 GO:0005657 GO:0009379 GO:0009276 GO:0016020

Cluster-22178.68369 GO:0000917 GO:0043093 GO:0006355 GO:0010506 GO:0006260 GO:0007049 GO:0051016 GO:0003700 GO:0043565 GO:0008290 GO:0005634 GO:0005667 GO:0005615 GO:0005737

Cluster-22178.68370 GO:0006468 GO:0016773 GO:0005524 GO:0004672 GO:0016020

Cluster-22178.68371 GO:0016192 GO:0007009 GO:0071766 GO:0052636 GO:0019843 GO:0003723 GO:0016021 GO:0005622

Cluster-22178.68373 GO:0007156 GO:0005509 GO:0016020

Cluster-22178.68374 GO:0006260 GO:0016255 GO:0003677 GO:0005524 GO:0042765

Cluster-22178.68376 GO:0006352 GO:0006206 GO:0006144 GO:0006351 GO:0016043 GO:0006355 GO:0016987 GO:0003700 GO:0003779 GO:0003677 GO:0003899 GO:0005730 GO:0005667

Cluster-22178.68377 GO:0034227 GO:0002098 GO:0000049

Cluster-22178.68379 GO:0005515

Cluster-22178.68381 GO:0016020

Cluster-22178.68383 GO:0006355 GO:0003700 GO:0005667 GO:0005634

Cluster-22178.68385 GO:0051016 GO:0006508 GO:0008152 GO:0016787 GO:0008236 GO:0008290

Cluster-22178.68386 GO:0006368 GO:0008023

Cluster-22178.68387 GO:0006397 GO:0015992 GO:0000398 GO:0015986 GO:0005515 GO:0015078 GO:0005634

Cluster-22178.68391 GO:0006420 GO:0006525 GO:0006560 GO:0004814

GO:0005524 GO:0009986 GO:0016020
Cluster-22178.68398 GO:0042967 GO:0004379
Cluster-22178.68401 GO:0016117 GO:0006779 GO:0055114 GO:0008033
GO:0019354 GO:0050660 GO:0016491 GO:0008270 GO:0043115
GO:0005515 GO:0016705 GO:0071949
Cluster-22178.68402 GO:0005515
Cluster-22178.68403 GO:0009399 GO:0006355 GO:0006468 GO:0003700
GO:0005524 GO:0004672 GO:0005667
Cluster-22178.68404 GO:0043161 GO:0006281 GO:0006289 GO:0007010
GO:0003779 GO:0003684
Cluster-22178.68405 GO:0046907 GO:0006446 GO:0003743 GO:0005840
GO:0005852 GO:0005737
Cluster-22178.68407 GO:0006310 GO:0019079 GO:0007155 GO:0002949
GO:0006355 GO:0006281 GO:0007049 GO:0009378 GO:0003724
GO:0016887 GO:0005524 GO:0005515 GO:0008134 GO:0003723
GO:0003678 GO:0005667 GO:0009379 GO:0005657
Cluster-22178.68408 GO:0008270
Cluster-22178.68409 GO:0008152 GO:0016491
Cluster-22178.68413 GO:0015979 GO:0046872 GO:0008270 GO:0009523
GO:0009539
Cluster-22178.68414 GO:0006098 GO:0006094 GO:0005985 GO:0005982
GO:0006096 GO:0006352 GO:0004347 GO:0005669
Cluster-22178.68418 GO:0016757 GO:0016020
Cluster-22178.68420 GO:0006817 GO:0051082
Cluster-22178.68423 GO:0008092 GO:0019898 GO:0005737
Cluster-22178.68425 GO:0007156 GO:0007155 GO:0019082 GO:0003677
GO:0005198 GO:0005509 GO:0008270 GO:0016020 GO:0031012
Cluster-22178.68427 GO:0006412 GO:0042254 GO:0005515 GO:0003735
GO:0005840 GO:0016021 GO:0019867
Cluster-22178.68429 GO:0010181 GO:0005759
Cluster-22178.68431 GO:0055114 GO:0009058 GO:0016209 GO:0051920
GO:0016491 GO:0003824
Cluster-22178.68432 GO:0006351 GO:0003677 GO:0005634

Cluster-22178.68433 GO:0006886 GO:0016192 GO:0007218 GO:0005515 GO:0005198 GO:0005185 GO:0043565 GO:0016798 GO:0019028 GO:0030117 GO:0005576 GO:0019031
Cluster-22178.68434 GO:0007218 GO:0006886 GO:0016192 GO:0043565 GO:0016798 GO:0005198 GO:0005515 GO:0005185 GO:0019031 GO:0019028 GO:0005576 GO:0030117
Cluster-22178.68435 GO:0006886 GO:0016192 GO:0005198 GO:0019867 GO:0030126
Cluster-22178.68439 GO:0016192 GO:0006886 GO:0005198 GO:0030126 GO:0019867
Cluster-22178.68440 GO:0034080 GO:0008270 GO:0000775
Cluster-22178.68442 GO:0055114 GO:0055085 GO:0006811 GO:0016209 GO:0016491 GO:0015075 GO:0016020
Cluster-22178.68443 GO:0006890 GO:0005975 GO:0005515 GO:0004252 GO:0003676 GO:0015926 GO:0008270 GO:0016021
Cluster-22178.68444 GO:0016773 GO:0005515
Cluster-22178.68445 GO:0006397 GO:0005515 GO:0003774 GO:0005524 GO:0016459
Cluster-22178.68446 GO:0009755 GO:0006364 GO:0005515 GO:0005576 GO:0032040
Cluster-22178.68448 GO:0046500 GO:0006400 GO:0006464 GO:0030488 GO:0001522 GO:0009451 GO:0006479 GO:0008033 GO:0008152 GO:0008176 GO:0009982 GO:0016429 GO:0008171 GO:0003723 GO:0008168 GO:0004719 GO:0031515
Cluster-22178.68450 GO:0007264 GO:0005525
Cluster-22178.68451 GO:0007165 GO:0006869 GO:0006955 GO:0008283 GO:0042157 GO:0040007 GO:0008289 GO:0005138 GO:0005896 GO:0005576
Cluster-22178.68452 GO:0055114 GO:0016491 GO:0046872
Cluster-22178.68453 GO:0070072
Cluster-22178.68456 GO:0016021
Cluster-22178.68459 GO:0055114 GO:0006355 GO:0019478 GO:0043565 GO:0016491 GO:0003700 GO:0003676 GO:0050662 GO:0005515

GO:0016788 GO:0005737 GO:0005667 GO:0016020
Cluster-22178.68460 GO:0006308 GO:0006914 GO:0010508 GO:0008092
GO:0008855 GO:0003723 GO:0051082 GO:0005515 GO:0005737
GO:0019898 GO:0019013 GO:0009318
Cluster-22178.68461 GO:0055114 GO:0006779 GO:0006355 GO:0016559
GO:0016567 GO:0046872 GO:0008270 GO:0043565 GO:0004842
GO:0005515 GO:0005779 GO:0016020
Cluster-22178.68462 GO:0055114 GO:0016491
Cluster-22178.68463 GO:0046486 GO:0006468 GO:0045017 GO:0000160
GO:0042967 GO:0004144 GO:0003677 GO:0000156 GO:0005524
GO:0004672
Cluster-22178.68465 GO:0016310 GO:0019089 GO:0007155 GO:0004673
GO:0009365 GO:0009288
Cluster-22178.68466 GO:0007264 GO:0005525
Cluster-22178.68467 GO:0043087 GO:0035023 GO:0005089
Cluster-22178.68468 GO:0006464 GO:0030488 GO:0046500 GO:0006400
GO:0008152 GO:0008033 GO:0006479 GO:0001522 GO:0009451
GO:0016429 GO:0008171 GO:0009982 GO:0008176 GO:0004719
GO:0008168 GO:0003723 GO:0031515
Cluster-22178.68469 GO:0007283 GO:0006024 GO:0015012 GO:0003677
GO:0004252 GO:0016757 GO:0016020 GO:0005634 GO:0016021
GO:0000786
Cluster-22178.68471 GO:0006915 GO:0006355 GO:0006919 GO:0008608
GO:0001836 GO:0006511 GO:0016567 GO:0006974 GO:0003700
GO:0034450 GO:0004842 GO:0000151 GO:0042729 GO:0005667
GO:0005876 GO:0005815
Cluster-22178.68473 GO:0006571 GO:0008033 GO:0009094 GO:0006796
GO:0006432 GO:0006119 GO:0006508 GO:0000162 GO:0004826
GO:0008234 GO:0000049 GO:0000287 GO:0005524 GO:0004427
GO:0005737 GO:0009328
Cluster-22178.68478 GO:0006355 GO:0042967 GO:0016747 GO:0008080

Cluster-22178.68479 GO:0006468 GO:0042254 GO:0006412 GO:0055114

GO:0051287 GO:0003735 GO:0016773 GO:0005524 GO:0004672
GO:0016020 GO:0005840
Cluster-22178.68480 GO:0005515 GO:0046935 GO:0046872 GO:0008270
GO:0005944
Cluster-22178.68481 GO:0006493 GO:0006338 GO:0000030 GO:0016818
GO:0003676 GO:0005524 GO:0016020 GO:0005634 GO:0000136
Cluster-22178.68483 GO:0007304 GO:0005515 GO:0005213 GO:0046872
GO:0005576 GO:0016021 GO:0045202 GO:0042600
Cluster-22178.68484 GO:0006865 GO:0055085 GO:0006810 GO:0003333
GO:0015171 GO:0016020
Cluster-22178.68485 GO:0006813 GO:0007601 GO:0006144 GO:0005515
GO:0030553 GO:0005242 GO:0004114 GO:0008076 GO:0016021
Cluster-22178.68487 GO:0016192 GO:0006886 GO:0005515 GO:0008536
GO:0030117
Cluster-22178.68489 GO:0006397 GO:0006914 GO:0006366 GO:0005515
GO:0000445
Cluster-22178.68490 GO:0007411 GO:0006355 GO:0006281 GO:0008270
GO:0008168 GO:0003677 GO:0005515 GO:0016020
Cluster-22178.68491 GO:0007031 GO:0035556 GO:0007264 GO:0006457
GO:0006468 GO:0006355 GO:0000774 GO:0005524 GO:0004222
GO:0051087 GO:0003677 GO:0003924 GO:0004672 GO:0046872
GO:0003700 GO:0005525 GO:0051082 GO:0042803 GO:0016820
GO:0005515 GO:0016272 GO:0005667 GO:0005779 GO:0005739
GO:0016021
Cluster-22178.68492 GO:0016310 GO:0009069 GO:0006355 GO:0004674
GO:0034061 GO:0016779 GO:0003677 GO:0005634
Cluster-22178.68494 GO:0003723 GO:0005856
Cluster-22178.68495 GO:0005515
Cluster-22178.68496 GO:0055085 GO:0006351 GO:0006810 GO:0006468
GO:0005215 GO:0016773 GO:0005524 GO:0004672 GO:0005515
GO:0016021 GO:0016020
Cluster-22178.68497 GO:0007264 GO:0005525 GO:0005524 GO:0003924
GO:0016887 GO:0016020

Cluster-22178.68500 GO:0033691
Cluster-22178.68501 GO:0009372 GO:0004867
Cluster-22178.68502 GO:0006468 GO:0055114 GO:0003677 GO:0061630
GO:0005515 GO:0003697 GO:0003723 GO:0016787 GO:0047134
GO:0003676 GO:0005524 GO:0004672 GO:0008270 GO:0046872
Cluster-22178.68504 GO:0006260 GO:0007049 GO:0000917 GO:0006355
GO:0043093 GO:0003700 GO:0043565 GO:0003677 GO:0005737
GO:0005667 GO:0005634
Cluster-22178.68505 GO:0008152 GO:0006554 GO:0006464 GO:0008033
GO:0006479 GO:0009451 GO:0016192 GO:0006886 GO:0046500
GO:0006400 GO:0004719 GO:0008168 GO:0018024 GO:0008176
GO:0030117
Cluster-22178.68506 GO:0006260 GO:0006284 GO:0003906 GO:0016799

Cluster-22178.68507 GO:0006397 GO:0003682 GO:0003723 GO:0005737
GO:0005634 GO:0000785
Cluster-22178.68509 GO:0016020
Cluster-22178.68510 GO:0046872 GO:0003677
Cluster-22178.68513 GO:0006260 GO:0016567 GO:0007165 GO:0046872
GO:0005524 GO:0008270 GO:0004842 GO:0005179 GO:0005515
GO:0016818 GO:0003677 GO:0005680 GO:0005634 GO:0005576
Cluster-22178.68514 GO:0006525 GO:0006560 GO:0055114 GO:0051321
GO:0018401 GO:0005515 GO:0004656 GO:0016702 GO:0005783
Cluster-22178.68515 GO:0009451 GO:0006886 GO:0008152 GO:0008033
GO:0016192 GO:0007165 GO:0006400 GO:0016301 GO:0005515
GO:0003723 GO:0008168 GO:0004809 GO:0046872 GO:0008176
GO:0030117 GO:0016021
Cluster-22178.68517 GO:0009252 GO:0006542 GO:0006807 GO:0004356

Cluster-22178.68518 GO:0006355 GO:0007155 GO:0042246 GO:0003677
GO:0005634 GO:0016021
Cluster-22178.68519 GO:0009252 GO:0006542 GO:0006807 GO:0004356

Cluster-22178.68523 GO:0006144 GO:0006206 GO:0006355 GO:0044237 GO:0006351 GO:0006810 GO:0003899 GO:0003677 GO:0005639 GO:0016021 GO:0005730

Cluster-22178.68525 GO:0007411

Cluster-22178.68526 GO:0000160 GO:0016310 GO:0071555 GO:0005515 GO:0004673 GO:0000155 GO:0016021 GO:0009365

Cluster-22178.68527 GO:0007165 GO:0019079 GO:0006260 GO:0005179 GO:0030337 GO:0005576 GO:0042575

Cluster-22178.68528 GO:0034477 GO:0016579 GO:0007276 GO:0006364 GO:0006508 GO:0004222 GO:0003676 GO:0008270 GO:0036459 GO:0004518 GO:0030246 GO:0032183 GO:0070140 GO:0004857 GO:0031012

Cluster-22178.68529 GO:0007160 GO:0043087 GO:0043248 GO:0032012 GO:0005086 GO:0005509 GO:0005576 GO:0005634 GO:0005783

Cluster-22178.68530 GO:0003677

Cluster-22178.68532 GO:0007186 GO:0004930 GO:0016020 GO:0016021

Cluster-22178.68533 GO:0007165 GO:0005515 GO:0005737

Cluster-22178.68534 GO:0030153 GO:0007165 GO:0006955 GO:0005524 GO:0015643 GO:0016787 GO:0005515 GO:0005737 GO:0016021 GO:0019814

Cluster-22178.68537 GO:0006355 GO:0016747

Cluster-22178.68539 GO:0007264 GO:0005525

Cluster-22178.68540 GO:0006396 GO:0051252 GO:0006810 GO:0007264 GO:0005525 GO:0004525 GO:0005524 GO:0003677 GO:0003723 GO:0016787

Cluster-22178.68541 GO:0006810 GO:0006281 GO:0007264 GO:0006355 GO:0000723 GO:0008134 GO:0003677 GO:0016787 GO:0003678 GO:0005525 GO:0005524 GO:0005667 GO:0005657

Cluster-22178.68542 GO:0005515

Cluster-22178.68544 GO:0055114 GO:0016209 GO:0016491 GO:0051920

Cluster-22178.68545 GO:0006413 GO:0006446 GO:0003723 GO:0003743 GO:0005737 GO:0005840

Cluster-22178.68548 GO:0006779 GO:0006633 GO:0016117 GO:0006554 GO:0046168 GO:0018874 GO:0019354 GO:0006631 GO:0008152 GO:0006574 GO:0008033 GO:0055114 GO:0006550 GO:0006552 GO:0006813 GO:0006568 GO:0050660 GO:0016491 GO:0016616 GO:0051287 GO:0071949 GO:0003824 GO:0043115 GO:0016705 GO:0003857

Cluster-22178.68549 GO:0007165 GO:0009252 GO:0006935 GO:0006542 GO:0006807 GO:0004888 GO:0004356 GO:0016020

Cluster-22178.68550 GO:0032922 GO:0000976

Cluster-22178.68551 GO:0006508 GO:0004222 GO:0008270 GO:0031012

Cluster-22178.68553 GO:0006487 GO:0005975 GO:0004553 GO:0016021 GO:0008250 GO:0019013

Cluster-22178.68554 GO:0006886 GO:0015031 GO:0006464 GO:0005047 GO:0005515 GO:0008270 GO:0003924 GO:0005525 GO:0052855 GO:0005786 GO:0005785

Cluster-22178.68555 GO:0006355 GO:0005515 GO:0003700 GO:0003677 GO:0046872 GO:0005667

Cluster-22178.68557 GO:0007165 GO:0048870 GO:0005515 GO:0031514

Cluster-22178.68559 GO:0006412 GO:0042254 GO:0003735 GO:0005622 GO:0005840

Cluster-22178.68560 GO:0016192 GO:0006904 GO:0042742 GO:0003677 GO:0046872

Cluster-22178.68561 GO:0006531 GO:0009058 GO:0006520 GO:0055114 GO:0006144 GO:0009152 GO:0006188 GO:0006522 GO:0030170 GO:0016829 GO:0004018 GO:0010277

Cluster-22178.68562 GO:0005515 GO:0016021

Cluster-22178.68564 GO:0046983

Cluster-22178.68568 GO:0010506 GO:0005515 GO:0005615

Cluster-22178.68570 GO:0007155 GO:0005509 GO:0005515 GO:0005747 GO:0005739 GO:0005576

Cluster-22178.68572 GO:0005515

Cluster-22178.68573 GO:0006144 GO:0006470 GO:0006508 GO:0006570 GO:0003968 GO:0004725 GO:0017111 GO:0008138 GO:0004197

GO:0031379
Cluster-22178.68574 GO:0006468 GO:0004672 GO:0005524 GO:0004190
GO:0016021
Cluster-22178.68575 GO:0005515 GO:0030250 GO:0005576
Cluster-22178.68576 GO:0042967 GO:0016407
Cluster-22178.68577 GO:0042967 GO:0016407
Cluster-22178.68579 GO:0005643
Cluster-22178.68580 GO:0016192 GO:0007304 GO:0005213 GO:0042600
GO:0005576
Cluster-22178.68581 GO:0005975 GO:0008152 GO:0016042 GO:0006508
GO:0046486 GO:0009058 GO:0004806 GO:0008184 GO:0016787
GO:0016788 GO:0008236
Cluster-22178.68583 GO:0015979 GO:0009507 GO:0009523
Cluster-22178.68586 GO:0019530 GO:0006749 GO:0006691 GO:0051252
GO:0006486 GO:0006693 GO:0004540 GO:0003840 GO:0008373
Cluster-22178.68588 GO:0007224
Cluster-22178.68589 GO:0008152 GO:0005975 GO:0005509 GO:0004571
GO:0016829 GO:0016020
Cluster-22178.6859 GO:0006508 GO:0016301 GO:0004222 GO:0008270
GO:0031012
Cluster-22178.68591 GO:0006308 GO:0009307 GO:0042254 GO:0006412
GO:0009036 GO:0003735 GO:0003677 GO:0005840 GO:0009359
Cluster-22178.68592 GO:0006508 GO:0005975 GO:0016787 GO:0008184
GO:0008236
Cluster-22178.68593 GO:0009312 GO:0008455 GO:0016021 GO:0005795
Cluster-22178.68594 GO:0051603 GO:0006511 GO:0004175 GO:0004298
GO:0003677 GO:0005515 GO:0005839 GO:0019773
Cluster-22178.68595 GO:0006468 GO:0006886 GO:0015979 GO:0042967
GO:0005524 GO:0004672 GO:0008080 GO:0016020 GO:0009523
GO:0009539 GO:0030176
Cluster-22178.68596 GO:0016579 GO:0005515 GO:0036459
Cluster-22178.68597 GO:0005509 GO:0005544
Cluster-22178.68598 GO:0015979 GO:0042967 GO:0006468 GO:0006886

GO:0004672 GO:0005524 GO:0008080 GO:0009523 GO:0009539
GO:0030176 GO:0016020
Cluster-22178.68599 GO:0019073
Cluster-22178.6860 GO:0008233
Cluster-22178.68600 GO:0003677 GO:0005524 GO:0016787
Cluster-22178.68601 GO:0008289
Cluster-22178.68602 GO:0043039 GO:0005524 GO:0016876 GO:0016021
Cluster-22178.68603 GO:0048024 GO:0010508 GO:0006886 GO:0051258
GO:0006935 GO:0007165 GO:0071973 GO:0048193 GO:0030168
GO:0030674 GO:0019905 GO:0005102 GO:0008017 GO:0003774
GO:0005634 GO:0045298 GO:0009288 GO:0016020 GO:0005577
Cluster-22178.68605 GO:0048024 GO:0030001 GO:0055085 GO:0019901
GO:0046873 GO:0008017 GO:0016020 GO:0005634 GO:0045298
Cluster-22178.68606 GO:0006955 GO:0030153 GO:0008152 GO:0006516
GO:0015643 GO:0016747 GO:0019814 GO:0005737
Cluster-22178.68607 GO:0009405 GO:0042254 GO:0006570 GO:0006412
GO:0006470 GO:0008138 GO:0003735 GO:0008270 GO:0004725
GO:0005576 GO:0005622 GO:0005840
Cluster-22178.68608 GO:0042254 GO:0006412 GO:0003735 GO:0005840
Cluster-22178.68610 GO:0032968 GO:0006367 GO:0006270 GO:0003676
GO:0004064 GO:0005515 GO:0005509 GO:0003677 GO:0005634
Cluster-22178.68611 GO:0045881 GO:0070175 GO:0042475 GO:0003723
GO:0005515 GO:0019028
Cluster-22178.68612 GO:0006355 GO:0006012 GO:0009117 GO:0003700
GO:0008108 GO:0005667 GO:0005634
Cluster-22178.68614 GO:0007165 GO:0035556 GO:0005515 GO:0016012
GO:0016020
Cluster-22178.68616 GO:0006281 GO:0009307 GO:0006308 GO:0003677
GO:0009036 GO:0005634 GO:0009359 GO:0016020
Cluster-22178.68617 GO:0007165 GO:0035556 GO:0005515 GO:0016012
GO:0016020
Cluster-22178.68620 GO:0006351 GO:0090266 GO:0006270 GO:0006260
GO:0051016 GO:0003887 GO:0004190 GO:0003677 GO:0008270

GO:0042575 GO:0005634 GO:0019028 GO:0005789 GO:0016021
GO:0005680 GO:0030479
Cluster-22178.68621 GO:0035556 GO:0042254 GO:0006412 GO:0032183
GO:0070140 GO:0005515 GO:0003735 GO:0005840 GO:0015934
Cluster-22178.68626 GO:0007275 GO:0007219 GO:0030154 GO:0005509
GO:0005515 GO:0016021
Cluster-22178.68627 GO:0006400 GO:0007172 GO:0007165 GO:0006487
GO:0006468 GO:0008616 GO:0006397 GO:0004713 GO:0004871
GO:0008479 GO:0005515 GO:0005925 GO:0008250 GO:0016021
GO:0019028 GO:0005634
Cluster-22178.68628 GO:0055114 GO:0008152 GO:0016491
Cluster-22178.68630 GO:0005515
Cluster-22178.68631 GO:0003677 GO:0016787
Cluster-22178.68632 GO:0006508 GO:0004185
Cluster-22178.68633 GO:0017038 GO:0019856 GO:0003723 GO:0016787
GO:0003677 GO:0005515 GO:0005524 GO:0008270 GO:0003676
GO:0016020
Cluster-22178.68634 GO:0009405 GO:0006486 GO:0007049 GO:0006270
GO:0007067 GO:0006260 GO:0005515 GO:0042802 GO:0003887
GO:0003980 GO:0016757 GO:0046983 GO:0042575 GO:0005634
GO:0005727 GO:0016021 GO:0000775 GO:0016020
Cluster-22178.68636 GO:0042967 GO:0006355 GO:0008270 GO:0003712
GO:0004402 GO:0005515 GO:0000123 GO:0005634 GO:0005667
Cluster-22178.68638 GO:0006118 GO:0006119 GO:0055114 GO:0015992
GO:0008121
Cluster-22178.68640 GO:0051252 GO:0004523 GO:0003676
Cluster-22178.68642 GO:0006412 GO:0006270 GO:0006614 GO:0042254
GO:0007264 GO:0005525 GO:0043531 GO:0003924 GO:0003723
GO:0003724 GO:0005524 GO:0016887 GO:0098519 GO:0003735
GO:0005840 GO:0005622
Cluster-22178.68644 GO:2000123 GO:0005515 GO:0016021
Cluster-22178.68646 GO:0006144 GO:0032801 GO:0006812 GO:0035556
GO:0006182 GO:0009271 GO:0019076 GO:0008152 GO:0046039

GO:0009190 GO:0006508 GO:0006355 GO:0006468 GO:0005524
GO:0016787 GO:0003677 GO:0004672 GO:0004383 GO:0016849
GO:0005261 GO:0008236 GO:0033644 GO:0005887
Cluster-22178.68647 GO:0006779 GO:0008033 GO:0055114 GO:0019354
GO:0045454 GO:0050660 GO:0016491 GO:0043115 GO:0071949
Cluster-22178.68648 GO:0006182 GO:0006508 GO:0046039 GO:0009190
GO:0006468 GO:0008152 GO:0035556 GO:0006144 GO:0008236
GO:0004383 GO:0016849 GO:0016787 GO:0004672 GO:0005524
Cluster-22178.68651 GO:0006144 GO:0035556 GO:0008152 GO:0006508
GO:0046039 GO:0009190 GO:0006182 GO:0006468 GO:0004672
GO:0005524 GO:0016849 GO:0004383 GO:0016787 GO:0008236
Cluster-22178.68652 GO:0035556 GO:0006144 GO:0006182 GO:0008152
GO:0046039 GO:0009190 GO:0006508 GO:0006468 GO:0005524
GO:0003677 GO:0016787 GO:0004672 GO:0008236 GO:0004383
GO:0016849 GO:0005887
Cluster-22178.68655 GO:0051603 GO:0004298 GO:0005839
Cluster-22178.68657 GO:0006810 GO:0005622
Cluster-22178.68660 GO:0042254 GO:0006412 GO:0005515 GO:0003735
GO:0005840 GO:0005622
Cluster-22178.68663 GO:0009253 GO:0016567 GO:0019068 GO:0006807
GO:0009252 GO:0000166 GO:0005515 GO:0008745 GO:0003676
GO:0046872 GO:0003677 GO:0004842 GO:0008270 GO:0005524
GO:0019028 GO:0005680
Cluster-22178.68665 GO:0016787 GO:0005524 GO:0003676 GO:0003677
GO:0005615
Cluster-22178.68666 GO:0015992 GO:0006144 GO:0006188 GO:0009152
GO:0006522 GO:0006123 GO:0006531 GO:0016567 GO:0004018
GO:0004842 GO:0004129 GO:0045277 GO:0005740
Cluster-22178.68667 GO:0006522 GO:0006123 GO:0006188 GO:0009152
GO:0006144 GO:0015992 GO:0016567 GO:0006531 GO:0004129
GO:0004842 GO:0004018 GO:0045277 GO:0005740
Cluster-22178.68668 GO:0006508 GO:0009847 GO:0003333 GO:0004185
GO:0016021

Cluster-22178.68669 GO:0006508 GO:0003333 GO:0009847 GO:0004185
GO:0016021
Cluster-22178.68671 GO:0007264 GO:0005525 GO:0003924
Cluster-22178.68672 GO:0000045 GO:0005737
Cluster-22178.68673 GO:0006357 GO:0001104 GO:0008270 GO:0016592
Cluster-22178.68675 GO:0051252 GO:0006396 GO:0008033 GO:0003676
GO:0004526 GO:0030677
Cluster-22178.68677 GO:0019497 GO:0055114 GO:0007165 GO:0006771
GO:0003993 GO:0005525 GO:0016620 GO:0004872 GO:0046872
GO:0003677 GO:0005515 GO:0004190 GO:0016021 GO:0016020
Cluster-22178.68678 GO:0006265 GO:0003918 GO:0005524 GO:0003677

Cluster-22178.68679 GO:0035091
Cluster-22178.68680 GO:0007049 GO:0006935 GO:0048870 GO:0006355
GO:0000921 GO:0071973 GO:0016567 GO:0051259 GO:0048309
GO:0007094 GO:0006886 GO:0051028 GO:0006914 GO:0007155
GO:0003700 GO:0046872 GO:0008092 GO:0008134 GO:0045502
GO:0004842 GO:0031386 GO:0043565 GO:0016881 GO:0005515
GO:0005198 GO:0042803 GO:0008270 GO:0003774 GO:0016021
GO:0005801 GO:0031514 GO:0019898 GO:0030286 GO:0005940
GO:0005882 GO:0009288 GO:0005737 GO:0005680 GO:0005783
GO:0016020 GO:0005667
Cluster-22178.68681 GO:0006950 GO:0006508 GO:0004222 GO:0003700
GO:0008270 GO:0004197 GO:0031012 GO:0005667
Cluster-22178.68682 GO:0006040 GO:0009435 GO:0009607 GO:0006807
GO:0000105 GO:0007275 GO:0006537 GO:0008033 GO:0055114
GO:0046497 GO:0008152 GO:0007219 GO:0006051 GO:0006355
GO:0016491 GO:0050660 GO:0016638 GO:0018580 GO:0015930
GO:0016829 GO:0016627 GO:0003824 GO:0017150 GO:0004514
GO:0047465 GO:0005737 GO:0016021
Cluster-22178.68683 GO:0007275 GO:0000105 GO:0006807 GO:0009607
GO:0006040 GO:0009435 GO:0006355 GO:0006051 GO:0007219
GO:0008152 GO:0046497 GO:0055114 GO:0008033 GO:0006537

GO:0018580 GO:0015930 GO:0016638 GO:0050660 GO:0016491
GO:0047465 GO:0004514 GO:0017150 GO:0003824 GO:0016627
GO:0016829 GO:0016021 GO:0005737
Cluster-22178.68685 GO:0000226 GO:0046872 GO:0000166 GO:0016020
GO:0015630
Cluster-22178.68687 GO:0015743 GO:0006816 GO:0006874 GO:0007165
GO:0006270 GO:0006810 GO:0006260 GO:0055085 GO:0000226
GO:0030001 GO:0007010 GO:0007187 GO:0010508 GO:0007608
GO:0005179 GO:0003677 GO:0005549 GO:0005524 GO:0042626
GO:0005515 GO:0046872 GO:0004984 GO:0003779 GO:0005219
GO:0016020 GO:0005622 GO:0005576 GO:0015630 GO:0005664
GO:0016021
Cluster-22178.68688 GO:0032259 GO:0006511 GO:0006310 GO:0007010
GO:0004175 GO:0003779 GO:0008168 GO:0005515 GO:0003677
GO:0019773 GO:0005634
Cluster-22178.68692 GO:0007165 GO:0005515 GO:0016820 GO:0005739
GO:0016020 GO:0016021
Cluster-22178.68693 GO:0007165 GO:0003723 GO:0005158 GO:0005515
GO:0005899
Cluster-22178.68694 GO:0005515
Cluster-22178.68695 GO:0055085 GO:0022857 GO:0016021
Cluster-22178.68696 GO:0016192 GO:0005985 GO:0005982 GO:0030245
GO:0006353 GO:0000281 GO:0000226 GO:0040001 GO:0008810
GO:0005819 GO:0016021 GO:0005874
Cluster-22178.68697 GO:0007186 GO:0007165 GO:0007264 GO:0002949
GO:0003723 GO:0004871 GO:0003724 GO:0005525 GO:0003924
GO:0019001 GO:0031683
Cluster-22178.68698 GO:0006355 GO:0005515
Cluster-22178.68699 GO:0005515 GO:0003723
Cluster-22178.68700 GO:0006886 GO:0051258 GO:0048309 GO:0006308
GO:0042157 GO:0006869 GO:0007165 GO:0016567 GO:0016192
GO:0030168 GO:0051028 GO:0006260 GO:0005515 GO:0046872
GO:0008289 GO:0030674 GO:0008270 GO:0008855 GO:0004842

GO:0005102 GO:0019905 GO:0005576 GO:0005634 GO:0009318
GO:0016021 GO:0005783 GO:0005680 GO:0005577
Cluster-22178.68701 GO:0007165 GO:0005515 GO:0003723 GO:0005158
GO:0005899
Cluster-22178.68702 GO:0006355 GO:0006468 GO:0004672 GO:0003700
GO:0005634 GO:0005667
Cluster-22178.68705 GO:0006260 GO:0003677 GO:0003887 GO:0005515
GO:0042575 GO:0016021 GO:0009360
Cluster-22178.68707 GO:0006355 GO:0006338 GO:0003677 GO:0016818
GO:0005524 GO:0008270 GO:0003676 GO:0005634 GO:0005739
Cluster-22178.68709 GO:0042578
Cluster-22178.68711 GO:0016887 GO:0005524 GO:0005515 GO:0005634
Cluster-22178.68713 GO:0090063 GO:0000725 GO:0006351 GO:0007021
GO:0006144 GO:0006206 GO:0000226 GO:0051082 GO:0003899
GO:0003677 GO:0015631 GO:0005634 GO:0097196 GO:0045298
GO:0000922 GO:0005730 GO:0005815
Cluster-22178.68715 GO:0051537 GO:0005509 GO:0005515 GO:0043231
Cluster-22178.68717 GO:0046413 GO:0045444 GO:0018836 GO:0005515

Cluster-22178.68718 GO:0006260 GO:0019953 GO:0006351 GO:0006886
GO:0003887 GO:0003677 GO:0005515 GO:0005198 GO:0005664
GO:0005576 GO:0042575 GO:0030141
Cluster-22178.68719 GO:0007049 GO:0006355 GO:0046872 GO:0043565
GO:0003700 GO:0045502 GO:0019901 GO:0042803 GO:0008134
GO:0030286 GO:0005667
Cluster-22178.68721 GO:0000090 GO:0007067 GO:0007059 GO:0051301
GO:0031110 GO:0008017 GO:0005876 GO:0045298 GO:0000940
Cluster-22178.68722 GO:0051225 GO:0006281 GO:0006260 GO:0031023
GO:0003911 GO:0005524 GO:0046983 GO:0003689 GO:0042575
GO:0005663
Cluster-22178.68723 GO:0045995 GO:0045017 GO:0051258 GO:0048870
GO:0006308 GO:0042967 GO:0030168 GO:0000921 GO:0051607
GO:0030334 GO:0006914 GO:0007165 GO:0006810 GO:0046486

GO:0030155 GO:0007155 GO:0008134 GO:0042803 GO:0045502
GO:0005102 GO:0008855 GO:0004144 GO:0030674 GO:0016021
GO:0005794 GO:0009318 GO:0031514 GO:0005577 GO:0030286
GO:0005940 GO:0005667
Cluster-22178.68728 GO:0015976 GO:0006144 GO:0006096 GO:0006094
GO:0003824 GO:0005515 GO:0000287 GO:0030955 GO:0004743
Cluster-22178.68729 GO:0003676
Cluster-22178.68732 GO:0006164 GO:0006531 GO:0006810 GO:0007165
GO:0015858 GO:0006955 GO:0006144 GO:0006522 GO:0005125
GO:0005525 GO:0005337 GO:0004019 GO:0005576 GO:0016021
Cluster-22178.68733 GO:0005515
Cluster-22178.68735 GO:0006816 GO:0022900 GO:0005388 GO:0005747
GO:0016020 GO:0016529 GO:0005739
Cluster-22178.68738 GO:0016310 GO:0006090 GO:0006525 GO:0006560
GO:0006814 GO:0007186 GO:0071436 GO:0015081 GO:0004930
GO:0004673 GO:0008948 GO:0009365 GO:0016021 GO:0016020
Cluster-22178.68740 GO:0006284 GO:0006289 GO:0042254 GO:0019058
GO:0006412 GO:0006397 GO:0006508 GO:0006281 GO:0008242
GO:0008270 GO:0003684 GO:0003735 GO:0004197 GO:0003906
GO:0016799 GO:0003723 GO:0016740 GO:0005622 GO:0000445
GO:0005840
Cluster-22178.68745 GO:0051603 GO:0006511 GO:0004175 GO:0004298
GO:0005839 GO:0019773
Cluster-22178.68746 GO:0006511 GO:0051603 GO:0004298 GO:0004175
GO:0019773 GO:0005839
Cluster-22178.68748 GO:0035556 GO:0006469 GO:0046907 GO:0045859
GO:0004862 GO:0005952
Cluster-22178.68750 GO:0006260 GO:0005515 GO:0003887 GO:0005615
GO:0042575
Cluster-22178.68751 GO:0035556 GO:0009395 GO:0046339 GO:0007165
GO:0006629 GO:0004435 GO:0046872 GO:0005515
Cluster-22178.68753 GO:0006950 GO:0006508 GO:0008270 GO:0004222
GO:0003700 GO:0004197 GO:0031012 GO:0005667

Cluster-22178.68757 GO:0006309 GO:0006915 GO:0016787 GO:0005634
GO:0005737 GO:0005622
Cluster-22178.68758 GO:0043065 GO:0042981 GO:0015992 GO:0006119
GO:0015991 GO:0015986 GO:0015078 GO:0046961 GO:0031225
GO:0005737 GO:0045263 GO:0033178
Cluster-22178.68759 GO:0006914 GO:0043093 GO:0006355 GO:0006118
GO:0000917 GO:0043565 GO:0003700 GO:0015035 GO:0045502
GO:0008134 GO:0042803 GO:0019901 GO:0005198 GO:0005515
GO:0030286 GO:0005667 GO:0005737
Cluster-22178.68761 GO:0003723 GO:0003676
Cluster-22178.68762 GO:0007264 GO:0043093 GO:0042254 GO:0006457
GO:0006310 GO:0010506 GO:0006260 GO:0006281 GO:0006355
GO:0006412 GO:0000917 GO:0003735 GO:0005096 GO:0000287
GO:0051082 GO:0005515 GO:0043565 GO:0003700 GO:0005667
GO:0016272 GO:0005737 GO:0005615 GO:0005840
Cluster-22178.68763 GO:0006281 GO:0006355 GO:0006412 GO:0000917
GO:0043093 GO:0007264 GO:0042254 GO:0006310 GO:0006457
GO:0010506 GO:0006260 GO:0000287 GO:0051082 GO:0005515
GO:0043565 GO:0003700 GO:0003735 GO:0005096 GO:0005737
GO:0005615 GO:0005840 GO:0005667 GO:0016272
Cluster-22178.68764 GO:0000921 GO:0007096 GO:0007165 GO:0008092
GO:0004871 GO:0005515 GO:0005737 GO:0005940 GO:0005886
GO:0016021 GO:0019898 GO:0005634 GO:0016020
Cluster-22178.68766 GO:0019370 GO:0034453 GO:0000042 GO:0008270
GO:0008237 GO:0005515 GO:0005815 GO:0016021
Cluster-22178.68769 GO:0051252 GO:0000160 GO:0016310 GO:0004540
GO:0004673 GO:0000155 GO:0008171 GO:0016020 GO:0005737
GO:0009365
Cluster-22178.68770 GO:0051252 GO:0000160 GO:0016310 GO:0004540
GO:0000155 GO:0004673 GO:0008171 GO:0016020 GO:0005737
GO:0009365
Cluster-22178.68771 GO:0006810 GO:0005215 GO:0016020
Cluster-22178.68774 GO:0006570 GO:0007165 GO:0006470 GO:0005515

GO:0004721 GO:0004725 GO:0008138
Cluster-22178.68775 GO:0007264 GO:0005525
Cluster-22178.68776 GO:0016055 GO:0007165 GO:0007275 GO:0006570
GO:0006470 GO:0008138 GO:0005515 GO:0004725 GO:0004721
GO:0005102 GO:0005576
Cluster-22178.68778 GO:0003924
Cluster-22178.68779 GO:0006418 GO:0004812 GO:0008270 GO:0005524
GO:0000166
Cluster-22178.68781 GO:0035556 GO:0046836 GO:0017089 GO:0003677
GO:0051861 GO:0046872 GO:0005737
Cluster-22178.68784 GO:0006915 GO:0019028 GO:0005622
Cluster-22178.68789 GO:0006396 GO:0005622
Cluster-22178.68790 GO:0006825 GO:0016531 GO:0005507 GO:0005758
Cluster-22178.68791 GO:0044564 GO:0006468 GO:0005515 GO:0005524
GO:0004672
Cluster-22178.68792 GO:0046872
Cluster-22178.68793 GO:0043093 GO:0006355 GO:0006364 GO:0000917
GO:0009395 GO:0016042 GO:0005515 GO:0004629 GO:0003677
GO:0043565 GO:0003700 GO:0005634 GO:0005667 GO:0005737
Cluster-22178.68794 GO:0005515
Cluster-22178.68795 GO:0005515
Cluster-22178.68796 GO:0005515
Cluster-22178.68798 GO:0032008 GO:0007040 GO:0043410 GO:0032439
GO:0071230 GO:0001919 GO:0042632 GO:0031902 GO:0071986
GO:0045121
Cluster-22178.68799 GO:0006810 GO:0006397 GO:0051252 GO:0006402
GO:0016788 GO:0003723 GO:0003676 GO:0005215 GO:0046872
GO:0004535 GO:0005634 GO:0005576 GO:0005737
Cluster-22178.68800 GO:0051252 GO:0006810 GO:0006402 GO:0003723
GO:0003676 GO:0005215 GO:0004535 GO:0046872 GO:0005634
GO:0005576 GO:0005737
Cluster-22178.68801 GO:0006801 GO:0016765 GO:0016209 GO:0004784
GO:0016151

Cluster-22178.68802 GO:0007160 GO:0008152 GO:0003824 GO:0009976

Cluster-22178.68805 GO:0006364 GO:0006468 GO:0005515 GO:0005524 GO:0004672

Cluster-22178.68809 GO:0004867

Cluster-22178.68812 GO:0006281 GO:0007059 GO:0006777 GO:0000723 GO:0006614 GO:0009089 GO:0009058 GO:0030261 GO:0005525 GO:0003678 GO:0003824 GO:0008837 GO:0003677 GO:0016787 GO:0005524 GO:0019031 GO:0009295 GO:0005657

Cluster-22178.68814 GO:0006508 GO:0046872 GO:0004252 GO:0008236 GO:0005618

Cluster-22178.68816 GO:0008152 GO:0007160 GO:0005509 GO:0008168 GO:0005576

Cluster-22178.68817 GO:0008152 GO:0046912 GO:0003824

Cluster-22178.68818 GO:0022900 GO:0046872 GO:0003676 GO:0005515 GO:0070469 GO:0005743

Cluster-22178.68819 GO:0035023 GO:0006807 GO:0005975 GO:0043087 GO:0005515 GO:0005089 GO:0016810 GO:0005737

Cluster-22178.68820 GO:0005975 GO:0043087 GO:0006807 GO:0035023 GO:0016810 GO:0005089 GO:0005515 GO:0005737

Cluster-22178.68821 GO:0035023 GO:0006807 GO:0043087 GO:0005975 GO:0005515 GO:0005089 GO:0016810 GO:0005737

Cluster-22178.68824 GO:0043093 GO:0006260 GO:0030474 GO:0000917 GO:0006355 GO:0007049 GO:0003677 GO:0003700 GO:0005200 GO:0043565 GO:0005667 GO:0005815 GO:0005823 GO:0019028 GO:0005634 GO:0005856 GO:0005737

Cluster-22178.68825 GO:0006508 GO:0008236

Cluster-22178.68826 GO:0006355 GO:0030695 GO:0003700 GO:0043565 GO:0003677 GO:0016021 GO:0005634 GO:0005667

Cluster-22178.68827 GO:0005783

Cluster-22178.68828 GO:0003723 GO:0005515

Cluster-22178.68831 GO:0005515 GO:0008270

Cluster-22178.68832 GO:0006508 GO:0005515 GO:0004190 GO:0008270

GO:0005861
Cluster-22178.68836 GO:0008283 GO:0040007 GO:0007165 GO:0008284
GO:0005975 GO:0001934 GO:0005157 GO:0005509 GO:0004571
GO:0016020 GO:0005615
Cluster-22178.68839 GO:0009405 GO:0007165 GO:0006629 GO:0035556
GO:0046339 GO:0009395 GO:0005515 GO:0004435 GO:0046872
Cluster-22178.6884 GO:0071704 GO:0006807 GO:0003726 GO:0003723
GO:0016868
Cluster-22178.68840 GO:0006352 GO:0006308 GO:0048870 GO:0055114
GO:0051382 GO:0010508 GO:0035092 GO:0009690 GO:0006355
GO:0010506 GO:0006260 GO:0006914 GO:0000921 GO:0070206
GO:0019139 GO:0050660 GO:0008855 GO:0005524 GO:0003677
GO:0016787 GO:0016021 GO:0005615 GO:0031514 GO:0009318
GO:0000776 GO:0005815 GO:0000228 GO:0005940
Cluster-22178.68844 GO:0009401 GO:0006812 GO:0006885 GO:0055085
GO:0008643 GO:0005198 GO:0008982 GO:0015299 GO:0009357
GO:0016021 GO:0005737
Cluster-22178.68848 GO:0002949 GO:0006310 GO:0006073 GO:0006508
GO:0006281 GO:0004222 GO:0016887 GO:0005524 GO:0009378
GO:0003678 GO:0016762 GO:0016301 GO:0005618 GO:0005657
GO:0009379 GO:0048046
Cluster-22178.68851 GO:0019048 GO:0005634 GO:0019031
Cluster-22178.68853 GO:0005515
Cluster-22178.68854 GO:0006813 GO:0006814 GO:0005890
Cluster-22178.68855 GO:0006508 GO:0006144 GO:0004197 GO:0005515
GO:0004252 GO:0017111 GO:0003968 GO:0070008 GO:0016817
GO:0031379
Cluster-22178.68856 GO:0007275 GO:0006355 GO:0003700 GO:0042600
GO:0005667
Cluster-22178.68857 GO:0006470 GO:0006570 GO:0019988 GO:0043399
GO:0004725 GO:0008138
Cluster-22178.68859 GO:0019370 GO:0000042 GO:0034453 GO:0008270
GO:0008237 GO:0005515 GO:0016021 GO:0005815

Cluster-22178.68861 GO:0046034 GO:0006139 GO:0006144 GO:0006206
GO:0004127 GO:0004017 GO:0005524
Cluster-22178.68863 GO:0006118 GO:0045454 GO:0015035 GO:0009055

Cluster-22178.68864 GO:0006357 GO:0001104 GO:0005515 GO:0003723
GO:0016592
Cluster-22178.68867 GO:0006355 GO:0006810 GO:0003677 GO:0005215
GO:0005634 GO:0016020
Cluster-22178.68869 GO:0055114 GO:0005515 GO:0016706
Cluster-22178.68870 GO:0016567 GO:0004842
Cluster-22178.68871 GO:0006511 GO:0031625
Cluster-22178.68873 GO:0042254 GO:0006412 GO:2001070 GO:0003735
GO:0005840
Cluster-22178.68874 GO:0009405 GO:0016117 GO:0005515 GO:0016705
GO:0003677 GO:0005525 GO:0003924 GO:0046872
Cluster-22178.68875 GO:0009405 GO:0016117 GO:0046872 GO:0003677
GO:0005515 GO:0016705
Cluster-22178.68878 GO:0005887
Cluster-22178.68879 GO:0006531 GO:0016567 GO:0006144 GO:0015992
GO:0006522 GO:0006123 GO:0006188 GO:0009152 GO:0004018
GO:0004129 GO:0004842 GO:0005740 GO:0045277
Cluster-22178.68881 GO:0006955 GO:0030153 GO:0006355 GO:0003677
GO:0015643 GO:0019814 GO:0016021
Cluster-22178.68882 GO:0006364 GO:0016579 GO:0036459 GO:0005730
Cluster-22178.68884 GO:0006810 GO:0016021
Cluster-22178.68885 GO:0006446 GO:0003743 GO:0003676 GO:0003723
GO:0005840 GO:0005850
Cluster-22178.68886 GO:0008152 GO:0003824
Cluster-22178.68887 GO:0005515 GO:0016853
Cluster-22178.68888 GO:0006298 GO:0019073 GO:0019087 GO:0030983
GO:0005524 GO:0016021
Cluster-22178.68890 GO:0007067 GO:0006073 GO:0016762 GO:0005618
GO:0042729 GO:0072686 GO:0048046

Cluster-22178.68891 GO:0055114 GO:0006779 GO:0006468 GO:0019354
GO:0008641 GO:0004672 GO:0005524 GO:0043115 GO:0005515
GO:0016021
Cluster-22178.68893 GO:0005975 GO:0071704 GO:0005515 GO:0016868

Cluster-22178.68894 GO:0006357 GO:0005515 GO:0001104 GO:0003723
GO:0016592
Cluster-22178.68896 GO:0016114 GO:0006694 GO:0008661 GO:0016020
Cluster-22178.68899 GO:0006606 GO:0006891 GO:0006914 GO:0006308
GO:0042246 GO:0007165 GO:0007155 GO:0008855 GO:0005515
GO:0016021 GO:0009318 GO:0017119
Cluster-22178.68903 GO:0006687 GO:0006468 GO:0006355 GO:0016114
GO:0046486 GO:0009058 GO:0006027 GO:0042432 GO:0006012
GO:0055085 GO:0005515 GO:0003700 GO:0004672 GO:0004064
GO:0004565 GO:0005524 GO:0016844 GO:0016773 GO:0016021
GO:0009341 GO:0016020 GO:0005667
Cluster-22178.68905 GO:0006810 GO:0016567 GO:0006915 GO:0033014
GO:0006783 GO:0015994 GO:0004852 GO:0005515 GO:0005215
GO:0004842 GO:0008270 GO:0046872 GO:0016020 GO:0005680
Cluster-22178.68908 GO:0055085 GO:0006811 GO:0005216 GO:0016020
Cluster-22178.68911 GO:0006355 GO:0055114 GO:0008152 GO:0015696
GO:0006352 GO:0009058 GO:0006633 GO:0042967 GO:0004315
GO:0016788 GO:0003700 GO:0008519 GO:0003677 GO:0017111
GO:0016491 GO:0016747 GO:0016987 GO:0005835 GO:0005667
GO:0016020
Cluster-22178.68913 GO:0015031 GO:0008565 GO:0016021
Cluster-22178.68914 GO:0048598 GO:0005515 GO:0072669
Cluster-22178.68916 GO:0006355 GO:0043565 GO:0003700 GO:0005667
Cluster-22178.68918 GO:0005515
Cluster-22178.68919 GO:0006915
Cluster-22178.68925 GO:0070836 GO:0007165 GO:0051252 GO:0006412
GO:0042254 GO:0006355 GO:0003676 GO:0005515 GO:0003735
GO:0004523 GO:0005840 GO:0005622

Cluster-22178.68926 GO:0006355 GO:0035556 GO:0008270 GO:0046872
GO:0005515 GO:0005634
Cluster-22178.68927 GO:0030163 GO:0000398 GO:0006144 GO:0045454
GO:0006118 GO:0015035 GO:0009055 GO:0003968 GO:0005515
GO:0031379 GO:0005681 GO:0016021
Cluster-22178.68929 GO:0006051 GO:0006355 GO:0006537 GO:0008033
GO:0055114 GO:0008152 GO:0007219 GO:0046497 GO:0007275
GO:0009607 GO:0006040 GO:0009435 GO:0000105 GO:0006807
GO:0004514 GO:0047465 GO:0016829 GO:0016627 GO:0017150
GO:0003824 GO:0016638 GO:0018580 GO:0015930 GO:0016491
GO:0050660 GO:0005737 GO:0016021
Cluster-22178.68931 GO:0008033 GO:0016779 GO:0003676 GO:0016437
GO:0004810 GO:0008270
Cluster-22178.68934 GO:0016567 GO:0004842
Cluster-22178.68936 GO:0007264 GO:0007165 GO:0007186 GO:0002949
GO:0003723 GO:0019001 GO:0031683 GO:0003924 GO:0005525
GO:0004871 GO:0003724
Cluster-22178.68937 GO:0046740 GO:0006810 GO:0042254 GO:0006412
GO:0006260 GO:0051082 GO:0003735 GO:0016798 GO:0016020
GO:0005886 GO:0005622 GO:0005840 GO:0016021
Cluster-22178.68940 GO:0016787
Cluster-22178.68942 GO:0016567 GO:0015684 GO:0007264 GO:0006812
GO:0006284 GO:0032801 GO:0006289 GO:0006576 GO:0006355
GO:0006281 GO:0019076 GO:0003684 GO:0003677 GO:0003906
GO:0004842 GO:0016887 GO:0015093 GO:0005524 GO:0008270
GO:0016799 GO:0005261 GO:0005525 GO:0003924 GO:0005680
GO:0033644 GO:0016021
Cluster-22178.68944 GO:0006570 GO:0006470 GO:0004725 GO:0008138

Cluster-22178.68947 GO:0016032 GO:0006891 GO:0046872 GO:0017119
GO:0005739
Cluster-22178.68948 GO:0006396 GO:0003723
Cluster-22178.68950 GO:0007010 GO:0003779

Cluster-22178.68951 GO:0019082 GO:0035556 GO:0007010 GO:0003779 GO:0008270 GO:0005515 GO:0003677
Cluster-22178.68952 GO:0015740 GO:0015556 GO:0005789 GO:0032040 GO:0016021
Cluster-22178.68953 GO:0008270 GO:0003723
Cluster-22178.68957 GO:0006270 GO:0007005 GO:0016884 GO:0000062 GO:0032865 GO:0016021
Cluster-22178.68959 GO:0008643 GO:0005351 GO:0016021 GO:0016020 GO:0000139
Cluster-22178.68960 GO:0006813 GO:0030001 GO:0055085 GO:0046873 GO:0005515 GO:0005249 GO:0016020 GO:0008076 GO:0016021
Cluster-22178.68961 GO:0016787
Cluster-22178.68963 GO:0006357 GO:0051262 GO:0001104 GO:0046983 GO:0016592 GO:0016021
Cluster-22178.68964 GO:0030001 GO:0006355 GO:0007009 GO:0046872 GO:0005515 GO:0005743 GO:0005737 GO:0005747 GO:0005634
Cluster-22178.68970 GO:0000166 GO:0046872
Cluster-22178.68971 GO:0005515 GO:0061630 GO:0046872 GO:0008270 GO:0003723
Cluster-22178.68972 GO:0007049 GO:0016021 GO:0005634
Cluster-22178.68973 GO:0019058 GO:0019031
Cluster-22178.68978 GO:0006207 GO:0006206 GO:0009116 GO:0016310 GO:0004590 GO:0004673 GO:0009365
Cluster-22178.6898 GO:0005975 GO:0004553
Cluster-22178.68980 GO:0006388 GO:0051252 GO:0000213 GO:0000214
Cluster-22178.68981 GO:0019028
Cluster-22178.68982 GO:0042967 GO:0006396 GO:0006397 GO:0008080 GO:0016747 GO:0005515 GO:0005622 GO:0005634
Cluster-22178.68985 GO:0008270
Cluster-22178.68987 GO:0015675 GO:0035444 GO:0006508 GO:0046872 GO:0015099 GO:0004252 GO:0030288 GO:0016021
Cluster-22178.68988 GO:0046872 GO:0000166
Cluster-22178.68989 GO:0006260 GO:0016021

Cluster-22178.6899 GO:0055085 GO:0005975 GO:0005515 GO:0004553
GO:0016021
Cluster-22178.68990 GO:0008152 GO:0016491
Cluster-22178.68992 GO:0006468 GO:0004672 GO:0005524
Cluster-22178.68997 GO:0018101 GO:0006807 GO:0005509 GO:0004668
GO:0016787 GO:0005737
Cluster-22178.68998 GO:0016820 GO:0005739 GO:0016021
Cluster-22178.69001 GO:0006446 GO:0006413 GO:0003743 GO:0003723
GO:0005840
Cluster-22178.69007 GO:0006508 GO:0004185
Cluster-22178.69010 GO:0005515
Cluster-22178.69012 GO:0006260 GO:0006355 GO:0006351 GO:0008270
GO:0003682 GO:0003677 GO:0003887 GO:0000775 GO:0000785
GO:0005634 GO:0042575
Cluster-22178.69013 GO:0000245 GO:0055114 GO:0008033 GO:0016117
GO:0071949 GO:0051287 GO:0016705 GO:0005515 GO:0008270
GO:0016491 GO:0050660 GO:0005634
Cluster-22178.69014 GO:0000245 GO:0008033 GO:0055114 GO:0016117
GO:0071949 GO:0051287 GO:0016705 GO:0005515 GO:0008270
GO:0016491 GO:0050660 GO:0005634
Cluster-22178.69017 GO:0006355 GO:0003700 GO:0005667
Cluster-22178.69018 GO:0016021
Cluster-22178.69019 GO:0005515
Cluster-22178.69020 GO:0045444 GO:0016020
Cluster-22178.69021 GO:0016787
Cluster-22178.69022 GO:1900758
Cluster-22178.69024 GO:0000002 GO:0000262
Cluster-22178.69029 GO:0006270 GO:0006260 GO:0009236 GO:0006351
GO:0006355 GO:0006352 GO:0000398 GO:0003677 GO:0016987
GO:0008270 GO:0005515 GO:0003887 GO:0003700 GO:0005667
GO:0042575 GO:0005634 GO:0005681 GO:0032040
Cluster-22178.69034 GO:0019051 GO:0005515 GO:0042025
Cluster-22178.69036 GO:0006448 GO:0008544 GO:0045901 GO:0006452

GO:0045905 GO:0003746 GO:0043022 GO:0003723 GO:0005840
Cluster-22178.69037 GO:0006820 GO:0055085 GO:0006508 GO:0006810
GO:0008509 GO:0005215 GO:0004190 GO:0016021 GO:0016020
Cluster-22178.69038 GO:0007218 GO:0045893 GO:0007275 GO:0003676
GO:0008270 GO:0005524 GO:0005184 GO:0005515 GO:0005576
Cluster-22178.69039 GO:0005515
Cluster-22178.69041 GO:0006355 GO:0006265 GO:0016579 GO:0003677
GO:0005515 GO:0003916 GO:0036459 GO:0003700 GO:0005694
GO:0005667
Cluster-22178.69042 GO:0006367 GO:0005524 GO:0003682 GO:0000785
GO:0005672
Cluster-22178.69043 GO:0043683 GO:0007275 GO:0009966 GO:0051382
GO:0006914 GO:0007049 GO:0000776 GO:0016020 GO:0016021
Cluster-22178.69046 GO:0006338 GO:0003677 GO:0090544
Cluster-22178.69048 GO:0006355 GO:0003677 GO:0016020
Cluster-22178.69049 GO:0015991 GO:0006811 GO:0048870 GO:0051258
GO:0006457 GO:0071973 GO:0007165 GO:0006810 GO:0006606
GO:0006914 GO:0030168 GO:0015992 GO:0005198 GO:0042803
GO:0015078 GO:0008092 GO:0051087 GO:0030674 GO:0005102
GO:0000774 GO:0003774 GO:0033179 GO:0031514 GO:0005737
GO:0009288 GO:0016020 GO:0005882 GO:0019898 GO:0005577
GO:0005886
Cluster-22178.69050 GO:0019722 GO:0035091 GO:0005515 GO:0003676
GO:0005887 GO:0016021
Cluster-22178.69051 GO:0042967 GO:0005515 GO:0008080 GO:0071203
Cluster-22178.69053 GO:0005975 GO:1902208 GO:0045893 GO:0006810
GO:0008270 GO:0003950 GO:0005215 GO:0016832 GO:0003677
GO:0005515 GO:0005737 GO:0016021 GO:0005634
Cluster-22178.69054 GO:0006446 GO:0003743 GO:0005737 GO:0008023
GO:0005852 GO:0005840
Cluster-22178.69055 GO:0015992 GO:0015986 GO:0015078 GO:0000276
Cluster-22178.69056 GO:0016032 GO:0005515 GO:0005576
Cluster-22178.69058 GO:0007264 GO:0005515 GO:0005525 GO:0005576

Cluster-22178.69059 GO:0016032 GO:0005515 GO:0005576
Cluster-22178.69061 GO:0015031 GO:0030176
Cluster-22178.69062 GO:0006568 GO:0055114 GO:0015947 GO:0006804
GO:0006979 GO:0004096 GO:0020037
Cluster-22178.69063 GO:0006879 GO:0008199
Cluster-22178.69064 GO:0009052 GO:0006098 GO:0015976 GO:0004751

Cluster-22178.69066 GO:0008152 GO:0055114 GO:0016491
Cluster-22178.69072 GO:0006810 GO:0007264 GO:0006396 GO:0051252
GO:0006355 GO:0006281 GO:0000723 GO:0003677 GO:0016787
GO:0005524 GO:0008134 GO:0003723 GO:0005525 GO:0003678
GO:0004525 GO:0005667 GO:0005657
Cluster-22178.69073 GO:0006468 GO:0006118 GO:0018990 GO:0007218
GO:0007264 GO:0007165 GO:0009847 GO:0003924 GO:0008255
GO:0004672 GO:0009055 GO:0005525 GO:0005515 GO:0005198
GO:0016491 GO:0016887 GO:0005524 GO:0016021 GO:0005739
GO:0016020
Cluster-22178.69074 GO:0031110 GO:0051301 GO:0007059 GO:0000090
GO:0007155 GO:0007067 GO:0006310 GO:0003824 GO:0008017
GO:0003677 GO:0051015 GO:0000940 GO:0045298 GO:0005876
GO:0005727
Cluster-22178.69075 GO:0043565 GO:0005515 GO:0005634 GO:0016020
Cluster-22178.69076 GO:0006355 GO:0006614 GO:0000921 GO:0006520
GO:0006606 GO:0046856 GO:0006396 GO:0045502 GO:0003723
GO:0005515 GO:0042803 GO:0008134 GO:0043565 GO:0034597
GO:0003700 GO:0008312 GO:0048500 GO:0016021 GO:0005667
GO:0030286 GO:0005940
Cluster-22178.69077 GO:0019031
Cluster-22178.69078 GO:0006281 GO:0003824 GO:0000166 GO:0046872

Cluster-22178.69079 GO:0006879 GO:0008199
Cluster-22178.69080 GO:0006886 GO:0016192 GO:0006810 GO:0055085
GO:0022857 GO:0005215 GO:0030117 GO:0016020 GO:0016021

Cluster-22178.69081 GO:0006367 GO:0005672
Cluster-22178.69082 GO:0006468 GO:0016773 GO:0005524 GO:0004672 GO:0016020
Cluster-22178.69084 GO:0006886 GO:0005975 GO:0016832 GO:0005741 GO:0016021 GO:0016020
Cluster-22178.69085 GO:0010508 GO:0008295 GO:0006560 GO:0006525 GO:0006357 GO:0001104 GO:0004014 GO:0008092 GO:0016592 GO:0016021 GO:0019898 GO:0005737
Cluster-22178.69087 GO:0006367 GO:0005672
Cluster-22178.69091 GO:0008152 GO:0016491
Cluster-22178.69093 GO:0006420 GO:0055085 GO:0006260 GO:0006525 GO:0007017 GO:0007018 GO:0006560 GO:0005524 GO:0008017 GO:0004814 GO:0022857 GO:0003777 GO:0000166 GO:0005737 GO:0045298 GO:0016021 GO:0005874
Cluster-22178.69094 GO:0045454 GO:0006118 GO:0030150 GO:0008033 GO:0055114 GO:0048870 GO:0005515 GO:0016491 GO:0009055 GO:0015035 GO:0050660 GO:0005742 GO:0031514 GO:0005737 GO:0016021
Cluster-22178.69095 GO:0006260 GO:0000184 GO:0046935 GO:0003677 GO:0005664 GO:0005944
Cluster-22178.69096 GO:0009058 GO:0006520 GO:0019752 GO:0016740 GO:0030170 GO:0016831 GO:0016829 GO:0019012
Cluster-22178.69098 GO:0007165 GO:0032780 GO:0006807 GO:0030168 GO:0010508 GO:0006886 GO:0007049 GO:0051258 GO:0030674 GO:0003933 GO:0008017 GO:0005102 GO:0042030 GO:0005783 GO:0005739 GO:0005577 GO:0045298 GO:0016021
Cluster-22178.69102 GO:0007165 GO:0005515 GO:0008270 GO:0005179 GO:0005576
Cluster-22178.69103 GO:0007165 GO:0005515 GO:0005179 GO:0008270 GO:0005576
Cluster-22178.69104 GO:0009877 GO:0007005 GO:0006448 GO:0006414 GO:0016746 GO:0003950 GO:0003746 GO:0005840 GO:0005829 GO:0032865

Cluster-22178.69105 GO:0007186 GO:0004930 GO:0016021 GO:0016020
Cluster-22178.69108 GO:0009250 GO:0046527 GO:0003676
Cluster-22178.69109 GO:0006771 GO:0019497 GO:0046872 GO:0005515 GO:0003993
Cluster-22178.69111 GO:0007186 GO:0019069 GO:0006836 GO:0004930 GO:0019905 GO:0004190 GO:0016021
Cluster-22178.69112 GO:0006400 GO:0007275 GO:0006614 GO:0045893 GO:0048280 GO:0007218 GO:0006281 GO:0008616 GO:0006886 GO:0000723 GO:0016787 GO:0003677 GO:0005524 GO:0008270 GO:0008479 GO:0005184 GO:0005525 GO:0003678 GO:0003676 GO:0005507 GO:0005657 GO:0000139 GO:0005576 GO:0005737
Cluster-22178.69113 GO:0008270 GO:0005515
Cluster-22178.69114 GO:0009297 GO:0006376 GO:0043087 GO:0003729 GO:0019867 GO:0005685
Cluster-22178.69115 GO:0006810 GO:0006777 GO:0005525 GO:0005524

Cluster-22178.69116 GO:0043631 GO:0006355 GO:0006568 GO:0055114 GO:0005506 GO:0003723 GO:0045735 GO:0016779 GO:0004652 GO:0000334
Cluster-22178.69119 GO:0006468 GO:0016773 GO:0004672 GO:0005524 GO:0016020
Cluster-22178.69120 GO:0042981 GO:0005515
Cluster-22178.69123 GO:0043547 GO:0006508 GO:0006144 GO:0051056 GO:0006284 GO:0004197 GO:0003677 GO:0005515 GO:0017111 GO:0008270 GO:0005096 GO:0016817 GO:0003676 GO:0003968 GO:0031379
Cluster-22178.69124 GO:0006508 GO:0043547 GO:0006144 GO:0006284 GO:0051056 GO:0017111 GO:0005515 GO:0003677 GO:0004197 GO:0016817 GO:0005096 GO:0003968 GO:0031379
Cluster-22178.69125 GO:0006355 GO:0043565 GO:0003700 GO:0005515 GO:0003677 GO:0005667 GO:0005634
Cluster-22178.69126 GO:0046500 GO:0006400 GO:0009451 GO:0006479 GO:0008033 GO:0006464 GO:0006554 GO:0006396 GO:0008152

GO:0006480 GO:0008176 GO:0018024 GO:0008173 GO:0008168
GO:0004719
Cluster-22178.69127 GO:0006260 GO:0061024 GO:0005524 GO:0003676
GO:0003887 GO:0003677 GO:0030176 GO:0042575
Cluster-22178.69129 GO:0007186 GO:0008152 GO:0005515 GO:0004930
GO:0016747 GO:0016021
Cluster-22178.69133 GO:0006511 GO:0043401 GO:0006355 GO:0008152
GO:0055114 GO:0050662 GO:0031625 GO:0016872 GO:0003824
GO:0016491 GO:0016020
Cluster-22178.69137 GO:0051028 GO:0071973 GO:0003774 GO:0005515
GO:0009425 GO:0005634
Cluster-22178.69138 GO:0030168 GO:0051258 GO:0006352 GO:0007155
GO:0007165 GO:0005102 GO:0030674 GO:0003677 GO:0005669
GO:0005577 GO:0019028
Cluster-22178.69139 GO:0015684 GO:0005525 GO:0005524 GO:0015093
GO:0046872 GO:0003924 GO:0016021
Cluster-22178.69141 GO:0003676
Cluster-22178.69144 GO:0006118 GO:0055114 GO:0003995 GO:0016627

Cluster-22178.69145 GO:0042254 GO:0006412 GO:0003676 GO:0003735
GO:0008270 GO:0005840 GO:0005622
Cluster-22178.69147 GO:0005515
Cluster-22178.69148 GO:0000166 GO:0046872
Cluster-22178.69149 GO:0019015
Cluster-22178.69150 GO:0042981
Cluster-22178.69151 GO:0005515 GO:0042802
Cluster-22178.69152 GO:0022900 GO:0007010 GO:0035556 GO:0046872
GO:0003779 GO:0003723 GO:0016021
Cluster-22178.69153 GO:0000041 GO:0042802 GO:0005515 GO:0016021
Cluster-22178.69154 GO:0042981
Cluster-22178.69157 GO:0016337 GO:0005515 GO:0003724 GO:0003723
GO:0016887 GO:0005524 GO:0016020
Cluster-22178.69161 GO:0016021

Cluster-22178.69162 GO:0007165 GO:0005179 GO:0005576
Cluster-22178.69163 GO:0055085 GO:0030001 GO:0008146 GO:0046873
GO:0016020
Cluster-22178.69165 GO:0006281 GO:0010447 GO:0003677 GO:0004518
GO:0003824 GO:0042597
Cluster-22178.69166 GO:0016482 GO:0033206 GO:0007163 GO:0030036
GO:0051653 GO:0008356 GO:0006928 GO:0051321 GO:0003779
GO:0005524 GO:0005885 GO:0042995 GO:0030478
Cluster-22178.69168 GO:0006281 GO:0006139 GO:0006310 GO:0006810
GO:0007165 GO:0002949 GO:0006206 GO:0016301 GO:0005215
GO:0008158 GO:0009378 GO:0004127 GO:0016887 GO:0005524
GO:0016020 GO:0009379 GO:0005657
Cluster-22178.69169 GO:0006206 GO:0002949 GO:0006139 GO:0006281
GO:0006310 GO:0005524 GO:0016887 GO:0004127 GO:0009378
GO:0016791 GO:0016301 GO:0005657 GO:0009379
Cluster-22178.69171 GO:0008152 GO:0015940 GO:0042967 GO:0007034
GO:0055114 GO:0016573 GO:0006355 GO:0003824 GO:0016491
GO:0008677 GO:0008484 GO:0071949 GO:0004402 GO:0000123
Cluster-22178.69172 GO:0006810 GO:0005515 GO:0042626 GO:0005524
GO:0016021
Cluster-22178.69175 GO:0043039 GO:0006771 GO:0019497 GO:0005515
GO:0003993 GO:0016876 GO:0046872 GO:0005524
Cluster-22178.69178 GO:0006468 GO:0016310 GO:0005515 GO:0004673
GO:0005524 GO:0004672 GO:0034464 GO:0009365
Cluster-22178.69179 GO:0006468 GO:0006886 GO:0045667 GO:0016773
GO:0005524 GO:0004672 GO:0016020 GO:0030176
Cluster-22178.69181 GO:0006355 GO:0016579 GO:0032065 GO:0003677
GO:0005515 GO:0003700 GO:0005543 GO:0043565 GO:0036459
GO:0005667 GO:0005634 GO:0005938
Cluster-22178.69183 GO:0015979 GO:0016757
Cluster-22178.69186 GO:0004190 GO:0016021
Cluster-22178.6919 GO:0015074
Cluster-22178.69190 GO:0007050 GO:0045859 GO:0004861 GO:0016020

GO:0005634
Cluster-22178.69192 GO:0009089 GO:0009085 GO:0055114 GO:0016620
GO:0008839 GO:0051287
Cluster-22178.69193 GO:0006771 GO:0019497 GO:0005515 GO:0003993
GO:0030976 GO:0046872
Cluster-22178.69194 GO:0016579 GO:0006278 GO:0055114 GO:0004800
GO:0036459 GO:0003964
Cluster-22178.69195 GO:0005515 GO:0016020
Cluster-22178.69196 GO:0005515
Cluster-22178.69197 GO:0005515
Cluster-22178.69198 GO:0006570 GO:0000087 GO:0006355 GO:0006260
GO:0006470 GO:0008134 GO:0003677 GO:0004725 GO:0016887
GO:0005524 GO:0005667 GO:0005622
Cluster-22178.69199 GO:0007165
Cluster-22178.69200 GO:0006370 GO:0006260 GO:0006281 GO:0006310
GO:0006397 GO:0005524 GO:0008270 GO:0003910 GO:0004484
GO:0003677
Cluster-22178.69202 GO:0015693 GO:0046872 GO:0015095 GO:0000166
GO:0016021 GO:0016020
Cluster-22178.69203 GO:0000184 GO:0006813 GO:0003924 GO:0016887
GO:0046872 GO:0008270 GO:0015269 GO:0005524 GO:0005525
GO:0005242 GO:0005516 GO:0016286 GO:0016021 GO:0008076
Cluster-22178.69205 GO:0006508 GO:0006355 GO:0006122 GO:0005515
GO:0043565 GO:0008270 GO:0003700 GO:0004222 GO:0005634
GO:0005667 GO:0031012 GO:0005743 GO:0005750
Cluster-22178.69206 GO:0006298 GO:0005524 GO:0030983
Cluster-22178.69207 GO:0042742 GO:0006355 GO:0016192 GO:0007165
GO:0006904 GO:0003677 GO:0046872 GO:0042974 GO:0005667
Cluster-22178.69208 GO:0015991 GO:0006355 GO:0006886 GO:0016192
GO:0006528 GO:0008418 GO:0005515 GO:0016820 GO:0003677
GO:0008270 GO:0030117 GO:0000221
Cluster-22178.69209 GO:0006355 GO:0006508 GO:0006118 GO:0015694
GO:0055114 GO:0008152 GO:0015097 GO:0003995 GO:0004197

GO:0005515 GO:0016627 GO:0050660 GO:0003700 GO:0005667
GO:0016020
Cluster-22178.69210 GO:0005515
Cluster-22178.69211 GO:0006508 GO:0007275 GO:0006810 GO:0005515
GO:0004198 GO:0005215 GO:0005576 GO:0005622
Cluster-22178.69212 GO:0007165 GO:0005179 GO:0042393 GO:0005576
Cluster-22178.69213 GO:0019236 GO:0007606 GO:0009772 GO:0015708
GO:0006118 GO:0006955 GO:0006412 GO:0019684 GO:0007165
GO:0042254 GO:0007186 GO:0005125 GO:0046872 GO:0045156
GO:0003723 GO:0005515 GO:0004932 GO:0008270 GO:0003677
GO:0003735 GO:0005840 GO:0016021 GO:0005576 GO:0005634
GO:0005622
Cluster-22178.69214 GO:0006357 GO:0006308 GO:0001104 GO:0008855
GO:0016592 GO:0009318
Cluster-22178.69216 GO:0005515
Cluster-22178.69220 GO:0006772 GO:0006886 GO:0009229 GO:0006605
GO:0030975 GO:0005524 GO:0004788 GO:0016020
Cluster-22178.69221 GO:0008219
Cluster-22178.69222 GO:0006464 GO:0000902 GO:0006955 GO:0006351
GO:0009405 GO:0006355 GO:0006811 GO:0003677 GO:0008270
GO:0005515 GO:0003676 GO:0016790 GO:0003700 GO:0016151
GO:0043565 GO:0046872 GO:0016020 GO:0005667 GO:0005887
GO:0045202
Cluster-22178.69223 GO:0055114 GO:0016620
Cluster-22178.69229 GO:0007010 GO:0003779
Cluster-22178.69233 GO:0009245 GO:0016567 GO:0008233 GO:0004842
GO:0046872 GO:0008915 GO:0008270 GO:0005515 GO:0003723
GO:0005680 GO:0016020
Cluster-22178.69234 GO:0006888 GO:0006281 GO:0006886 GO:0008270
GO:0046872 GO:0005515 GO:0003677 GO:0045202 GO:0030127
Cluster-22178.69235 GO:0035556 GO:0005515
Cluster-22178.69236 GO:0005515 GO:0003723
Cluster-22178.69237 GO:0016567 GO:0008270 GO:0046872 GO:0004842

GO:0005515 GO:0061630 GO:0005622 GO:0005680

Cluster-22178.69240 GO:0042254 GO:0006412 GO:0003735 GO:0005840

Cluster-22178.69241 GO:0010972 GO:0030336 GO:0016579 GO:0036459

Cluster-22178.69242 GO:0003676

Cluster-22178.69243 GO:0016567 GO:0009451 GO:0007165 GO:0042742 GO:0006468 GO:0008033 GO:0005515 GO:0009020 GO:0016773 GO:0005524 GO:0004672 GO:0016020 GO:0005576

Cluster-22178.69247 GO:0006338 GO:0043044 GO:0055085 GO:0016787 GO:0031491 GO:0003677 GO:0016818 GO:0005524 GO:0008270 GO:0003676 GO:0005634 GO:0000785 GO:0016021

Cluster-22178.69248 GO:0007155 GO:0005515 GO:0016021

Cluster-22178.69249 GO:0007165 GO:0006351 GO:0009405 GO:0040007 GO:0008283 GO:0006144 GO:0006206 GO:0003899 GO:0008083 GO:0003677 GO:0005730

Cluster-22178.69251 GO:0005515

Cluster-22178.69253 GO:0007010 GO:0016337 GO:0003779 GO:0016020

Cluster-22178.69256 GO:0006522 GO:0009152 GO:0006188 GO:0006531 GO:0006144 GO:0004018 GO:0005742

Cluster-22178.69257 GO:0016021

Cluster-22178.69258 GO:0006810 GO:0005215 GO:0016020

Cluster-22178.69259 GO:0005515

Cluster-22178.69260 GO:0032922 GO:0016567 GO:0005515 GO:0004842 GO:0000976

Cluster-22178.69264 GO:0032402 GO:0008289 GO:0042470

Cluster-22178.69265 GO:0006364 GO:0006810 GO:0042626 GO:0005524 GO:0032040 GO:0016021

Cluster-22178.69267 GO:0046872 GO:0005515

Cluster-22178.69268 GO:0006810 GO:0002949 GO:0005525 GO:0016301 GO:0005524 GO:0016887 GO:0003924

Cluster-22178.69269 GO:0043565 GO:0005634

Cluster-22178.69270 GO:0046487 GO:0006550 GO:0015940 GO:0006694 GO:0008207 GO:0008209 GO:0018874 GO:0009396 GO:0006554

GO:0006552 GO:0006568 GO:0008210 GO:0006813 GO:0055114
GO:0005975 GO:0006574 GO:0006631 GO:0006633 GO:0003857
GO:0003824 GO:0030976 GO:0008677 GO:0004488 GO:0051287
GO:0003854 GO:0050662 GO:0016620 GO:0016491 GO:0000287
GO:0016616 GO:0070402 GO:0004553
Cluster-22178.69272 GO:0005515 GO:0016021
Cluster-22178.69274 GO:0016310 GO:0009069 GO:0006468 GO:0004674
GO:0005515 GO:0005524 GO:0004672
Cluster-22178.69275 GO:0016021
Cluster-22178.69281 GO:0042254 GO:0005515 GO:0016020 GO:0016021
GO:0005730
Cluster-22178.69283 GO:0019079 GO:0019048 GO:0016787 GO:0008026
GO:0003677 GO:0004519 GO:0005509 GO:0005515 GO:0005524
GO:0008270 GO:0003676
Cluster-22178.69286 GO:0019521 GO:0055114 GO:0008152 GO:0006355
GO:0006351 GO:0006206 GO:0006144 GO:0006098 GO:0046872
GO:0003700 GO:0005515 GO:0003899 GO:0008641 GO:0051287
GO:0004616 GO:0003677 GO:0016616 GO:0005634 GO:0005730
GO:0005667
Cluster-22178.69287 GO:0006529 GO:0006522 GO:0007218 GO:0006955
GO:0007268 GO:0006531 GO:0007217 GO:0030153 GO:0004066
GO:0015643 GO:0019814
Cluster-22178.69288 GO:0042981
Cluster-22178.69289 GO:0042981 GO:0005515
Cluster-22178.69290 GO:0006508 GO:0055114 GO:0006510 GO:0004252
GO:0016491 GO:0016829 GO:0004176
Cluster-22178.69291 GO:0016567 GO:0004842 GO:0004857
Cluster-22178.69296 GO:0015986 GO:0015992 GO:0015078 GO:0005543

Cluster-22178.69297 GO:0015992 GO:0015986 GO:0005543 GO:0015078

Cluster-22178.69298 GO:0050852 GO:0000226 GO:0006470 GO:0006570
GO:0004725 GO:0005515 GO:0005509 GO:0015630

Cluster-22178.69302 GO:0015992 GO:0006744 GO:0006098 GO:0055114
GO:0019521 GO:0008152 GO:0006120 GO:0006814 GO:0008641
GO:0051287 GO:0004616 GO:0016616 GO:0046872 GO:0008137
GO:0005515 GO:0005742 GO:0005743
Cluster-22178.69304 GO:0006355 GO:0019048 GO:0003677 GO:0016020
Cluster-22178.69305 GO:0006355 GO:0003700 GO:0005667
Cluster-22178.69307 GO:0009094 GO:0000162 GO:0000413 GO:0006571
GO:0006094 GO:0006457 GO:0006096 GO:0004634 GO:0000287
GO:0003755 GO:0000015
Cluster-22178.69309 GO:0005515 GO:0019867 GO:0016021
Cluster-22178.69313 GO:0055114 GO:0019521 GO:0008152 GO:0006098
GO:0016491 GO:0051287 GO:0004616 GO:0016616
Cluster-22178.69314 GO:0007165 GO:0005515 GO:0005096 GO:0032040
Cluster-22178.69317 GO:0016746
Cluster-22178.69318 GO:0015979 GO:0007275 GO:0007219 GO:0009539
GO:0009523 GO:0016021 GO:0016020
Cluster-22178.69319 GO:0007275 GO:0007219 GO:0016021
Cluster-22178.69320 GO:0006355 GO:0003700 GO:0043565 GO:0005667
Cluster-22178.69321 GO:0008152 GO:0003723 GO:0008168
Cluster-22178.69322 GO:0016579 GO:0036459 GO:0005783
Cluster-22178.69323 GO:0016579 GO:0036459 GO:0005783
Cluster-22178.69324 GO:0017004 GO:0006118 GO:0006952 GO:0009405
GO:0006869 GO:0042157 GO:0045158 GO:0008289 GO:0009512
GO:0016020 GO:0005576
Cluster-22178.69325 GO:0006914 GO:0006308 GO:0008855 GO:0008092
GO:0046935 GO:0005737 GO:0019898 GO:0005944 GO:0009318
Cluster-22178.69328 GO:0006468 GO:0004672 GO:0005524 GO:0016773
GO:0020037 GO:0016020
Cluster-22178.69331 GO:0006468 GO:0005524 GO:0004672
Cluster-22178.69333 GO:0006629 GO:0007154 GO:0016042 GO:0046486
GO:0055085 GO:0004806 GO:0016021
Cluster-22178.69334 GO:0006351 GO:0006269 GO:0034508 GO:0003896
GO:0004386 GO:0008270 GO:0000776 GO:0005730 GO:0005657

Cluster-22178.69335 GO:0007165 GO:0006950 GO:0005509 GO:0005515
GO:0005578
Cluster-22178.69338 GO:0006418 GO:0004812 GO:0005524 GO:0000166

Cluster-22178.69339 GO:0005515 GO:0042470
Cluster-22178.69340 GO:0006397 GO:0016567 GO:0046872 GO:0003723
GO:0004842 GO:0003676
Cluster-22178.69341 GO:0006355 GO:0016820 GO:0008270 GO:0016021
GO:0005634 GO:0005739
Cluster-22178.69342 GO:0006811 GO:0006418 GO:0015992 GO:0007016
GO:0006119 GO:0015986 GO:0046933 GO:0046961 GO:0000166
GO:0005216 GO:0016020 GO:0045259 GO:0016010 GO:0000275
Cluster-22178.69344 GO:0055085 GO:0006810 GO:0003924 GO:0046872
GO:0022857 GO:0005215 GO:0005525 GO:0005515 GO:0016021
GO:0016020
Cluster-22178.69345 GO:0006355 GO:0003677 GO:0005515 GO:0003700
GO:0005667
Cluster-22178.69347 GO:0005515 GO:0003676
Cluster-22178.69349 GO:0015886 GO:0055114 GO:0019646 GO:0006118
GO:0015232 GO:0016491 GO:0009055
Cluster-22178.69350 GO:0019882 GO:0042254 GO:0006412 GO:0006886
GO:0006955 GO:0007165 GO:0005125 GO:0042289 GO:0003735
GO:0042613 GO:0005622 GO:0005840 GO:0016020
Cluster-22178.69351 GO:0007049 GO:0005515 GO:0008270
Cluster-22178.69352 GO:0055085 GO:0006898 GO:0006811 GO:0007010
GO:0007165 GO:0003779 GO:0005216 GO:0005044 GO:0016020
Cluster-22178.69353 GO:0006260 GO:0035556 GO:0006310 GO:0006281
GO:0016887 GO:0005524 GO:0016787 GO:0009378 GO:0003677
GO:0003678 GO:0003676 GO:0005515 GO:0003887 GO:0009360
GO:0005657 GO:0009379 GO:0042575
Cluster-22178.69354 GO:0006281 GO:0006310 GO:0006260 GO:0035556
GO:0003887 GO:0005515 GO:0003678 GO:0003676 GO:0003677
GO:0009378 GO:0016787 GO:0005524 GO:0016887 GO:0042575

GO:0009379 GO:0005657 GO:0009360
Cluster-22178.69356 GO:0006260 GO:0018142 GO:0006269 GO:0000166
GO:0004672 GO:0003723
Cluster-22178.69358 GO:0006468 GO:0005524 GO:0004672
Cluster-22178.69361 GO:0006313 GO:0006464 GO:0015031 GO:0055114
GO:0003677 GO:0004803 GO:0005515
Cluster-22178.69362 GO:0006144 GO:0035556 GO:0006171 GO:0009190
GO:0051537 GO:0016849 GO:0004016 GO:0005515 GO:0005886
Cluster-22178.69364 GO:0009069 GO:0006468 GO:0007178 GO:0016310
GO:0004675 GO:0003677 GO:0004672 GO:0005524 GO:0016020
Cluster-22178.69368 GO:0034314 GO:0006355 GO:0030833 GO:0003700
GO:0043565 GO:0005856 GO:0005885 GO:0005667
Cluster-22178.69369 GO:0007049 GO:0055114 GO:0006006 GO:0018990
GO:0006355 GO:0006260 GO:0007218 GO:0006098 GO:0006749
GO:0003700 GO:0008255 GO:0003887 GO:0050661 GO:0004345
GO:0008270 GO:0003677 GO:0016021 GO:0042575 GO:0005634
GO:0005576 GO:0005667
Cluster-22178.69370 GO:0007186 GO:0007165 GO:0007264 GO:0006000
GO:0006013 GO:0003873 GO:0004871 GO:0005525 GO:0003924
GO:0019001 GO:0005524 GO:0031683
Cluster-22178.69371 GO:0046872 GO:0016021
Cluster-22178.69372 GO:0006687 GO:0005975 GO:0006665 GO:0006807
GO:0004348 GO:0016021
Cluster-22178.69374 GO:0055085 GO:0022857 GO:0016021
Cluster-22178.69375 GO:0006013 GO:0005975 GO:0015923 GO:0008270
GO:0004553 GO:0004559
Cluster-22178.69376 GO:0006013 GO:0005975 GO:0008270 GO:0015923
GO:0004559 GO:0004553
Cluster-22178.69377 GO:0006351 GO:0006206 GO:0006144 GO:0003677
GO:0003899 GO:0005730 GO:0005634
Cluster-22178.69378 GO:0006804 GO:0006979 GO:0055114 GO:0006749
GO:0016209 GO:0004602 GO:0016491
Cluster-22178.69381 GO:0030246

Cluster-22178.69383 GO:0003824
Cluster-22178.69385 GO:0007155 GO:0003677
Cluster-22178.69386 GO:0006355 GO:0006334 GO:0006352 GO:0003677
GO:0016987 GO:0003700 GO:0005667 GO:0005634
Cluster-22178.69389 GO:0008242
Cluster-22178.69394 GO:0007264 GO:0043087 GO:0005085
Cluster-22178.69396 GO:0006355 GO:0003677 GO:0003700 GO:0016020
GO:0005667 GO:0005634
Cluster-22178.69399 GO:0003723 GO:0042162 GO:0008270 GO:0042803

Cluster-22178.69400 GO:0006355 GO:0006810 GO:0006614 GO:0019079
GO:0016787 GO:0003677 GO:0008026 GO:0005524 GO:0016887
GO:0005525 GO:0003676 GO:0004386
Cluster-22178.69402 GO:0008289
Cluster-22178.69403 GO:0046907
Cluster-22178.69406 GO:0009231 GO:0006771 GO:0003919 GO:0008270
GO:0016021
Cluster-22178.69410 GO:0055114 GO:0008270 GO:0016491 GO:0003729
GO:0016020 GO:0016021
Cluster-22178.69411 GO:0005975 GO:0004553 GO:0016021
Cluster-22178.69414 GO:0009878 GO:0008152 GO:0046872 GO:0003924
GO:0008168 GO:0016787 GO:0005525
Cluster-22178.69415 GO:0006886 GO:0005515 GO:0005525 GO:0005741
Cluster-22178.69416 GO:0005975 GO:0006890 GO:0008270 GO:0015926
GO:0003676 GO:0005515 GO:0004252 GO:0016021
Cluster-22178.69417 GO:0006890 GO:0005975 GO:0005515 GO:0004252
GO:0015926 GO:0008270 GO:0003676 GO:0016021
Cluster-22178.69418 GO:0006890 GO:0005975 GO:0005515 GO:0004252
GO:0008270 GO:0015926 GO:0003676 GO:0016021
Cluster-22178.69421 GO:0006919 GO:0006915 GO:0005739
Cluster-22178.69426 GO:0006270 GO:0046935 GO:0005944 GO:0032040
Cluster-22178.69427 GO:0046872 GO:0016787 GO:0045502 GO:0008134
GO:0042803 GO:0030286 GO:0005667

Cluster-22178.6943 GO:0007165 GO:0040007 GO:0000746 GO:0008283
GO:0008083 GO:0003677 GO:0016020
Cluster-22178.69431 GO:0006260
Cluster-22178.69432 GO:0006260
Cluster-22178.69438 GO:0003924 GO:0005615
Cluster-22178.69439 GO:0030212 GO:0019068 GO:0003676 GO:0008233
GO:0008168 GO:0008047 GO:0005515 GO:0004867
Cluster-22178.6944 GO:0007165 GO:0040007 GO:0008283 GO:0008083
GO:0009512 GO:0016020
Cluster-22178.69440 GO:0016310 GO:0005975 GO:0007178 GO:0005982
GO:0005985 GO:0006468 GO:0009069 GO:0016773 GO:0004672
GO:0005524 GO:0004556 GO:0005509 GO:0004675 GO:0016020
Cluster-22178.69442 GO:0007165 GO:0007186 GO:0004871 GO:0005515
GO:0005834
Cluster-22178.69444 GO:0015694 GO:0005975 GO:0030150 GO:0015097
GO:0004553 GO:0005742 GO:0016020 GO:0016021
Cluster-22178.69446 GO:0006457 GO:0000413 GO:0016117 GO:0008033
GO:0055114 GO:0003755 GO:0005515 GO:0016705 GO:0004174
GO:0071949 GO:0050660 GO:0016491
Cluster-22178.69447 GO:0007018 GO:0006355 GO:0007017 GO:0003777
GO:0008017 GO:0005524 GO:0005515 GO:0005874 GO:0043657
GO:0045298 GO:0005634
Cluster-22178.69448 GO:0034453 GO:0005515 GO:0005815
Cluster-22178.69451 GO:0007186 GO:0007165 GO:0004871 GO:0005834
Cluster-22178.69453 GO:0005976 GO:0035023 GO:0043087 GO:0005089
GO:0016779 GO:0043565
Cluster-22178.69454 GO:0016032 GO:0055085 GO:0005506 GO:0045735
GO:0016021
Cluster-22178.69456 GO:0006915 GO:0006974 GO:0005515
Cluster-22178.69457 GO:0006118 GO:0000746 GO:0009055 GO:0016491
GO:0003677 GO:0005515 GO:0016020 GO:0005634
Cluster-22178.69458 GO:0042167 GO:0006397 GO:0055114 GO:0015994
GO:0003723 GO:0003677 GO:0005515 GO:0008270 GO:0004074

GO:0005634 GO:0005737
Cluster-22178.69459 GO:0046034 GO:0034227 GO:0006468 GO:0002098
GO:0006144 GO:0004672 GO:0046872 GO:0003676 GO:0000049
GO:0020037 GO:0008270 GO:0005524 GO:0016787 GO:0003677
GO:0004017 GO:0005634
Cluster-22178.69460 GO:0000746 GO:0006118 GO:0016491 GO:0009055
GO:0005515 GO:0003677 GO:0005634 GO:0016020
Cluster-22178.69461 GO:0045893 GO:0005634
Cluster-22178.69462 GO:0006313 GO:0030866 GO:0015979 GO:0006914
GO:0048870 GO:0003677 GO:0004803 GO:0005198 GO:0008134
GO:0042803 GO:0030414 GO:0045502 GO:0008092 GO:0005882
GO:0016020 GO:0005667 GO:0030286 GO:0031514 GO:0009539
GO:0005856 GO:0009523
Cluster-22178.69463 GO:0005509
Cluster-22178.69464 GO:0006508 GO:0008234
Cluster-22178.69466 GO:0003677
Cluster-22178.69469 GO:0016567
Cluster-22178.69471 GO:0006891 GO:0006355 GO:0005524 GO:0003713
GO:0003677 GO:0005634 GO:0005667 GO:0017119
Cluster-22178.69472 GO:0042254 GO:0006412 GO:0007264 GO:0045040
GO:0005515 GO:0003735 GO:0042802 GO:0005525 GO:0046983
GO:0003924 GO:0005742 GO:0005840 GO:0016021 GO:0005622
Cluster-22178.69475 GO:0006118 GO:0016567 GO:0019684 GO:0006448
GO:0006414 GO:0005515 GO:0045156 GO:0003676 GO:0046872
GO:0004842 GO:0003746 GO:0008270 GO:0030077 GO:0016021
GO:0005840 GO:0005853
Cluster-22178.69476 GO:0007165 GO:0016874 GO:0001664
Cluster-22178.69477 GO:0006118 GO:0016567 GO:0006448 GO:0006414
GO:0019684 GO:0045156 GO:0005515 GO:0046872 GO:0003676
GO:0008270 GO:0003746 GO:0004842 GO:0030077 GO:0005840
GO:0016021 GO:0005853
Cluster-22178.69478 GO:0005515
Cluster-22178.69479 GO:0016567 GO:0006414 GO:0006448 GO:0019684

GO:0006118 GO:0003746 GO:0008270 GO:0004842 GO:0045156
GO:0005515 GO:0046872 GO:0003676 GO:0005853 GO:0030077
GO:0016021 GO:0005840
Cluster-22178.69482 GO:0007050 GO:0045859 GO:0004861 GO:0005634
Cluster-22178.69483 GO:0006694 GO:0008152 GO:0055114 GO:0008209
GO:0008207 GO:0008210 GO:0019836 GO:0003824 GO:0016491
GO:0016616 GO:0005515 GO:0050662 GO:0003854 GO:0005576
Cluster-22178.69484 GO:0015074 GO:0007017 GO:0007018 GO:0005524
GO:0008017 GO:0003777 GO:0008907 GO:0003677 GO:0045298
GO:0005874
Cluster-22178.69485 GO:0005515 GO:0016021
Cluster-22178.69486 GO:0004386
Cluster-22178.69487 GO:0005515
Cluster-22178.69488 GO:0006281 GO:0008270 GO:0046872 GO:0005815
Cluster-22178.69490 GO:0009405 GO:0005515 GO:0003779 GO:0005622
GO:0005576
Cluster-22178.69492 GO:0051252 GO:0006396 GO:0001682 GO:0008033
GO:0004526 GO:0030677
Cluster-22178.69494 GO:0007165 GO:0005515 GO:0005179 GO:0008270
GO:0005576
Cluster-22178.69495 GO:0007165 GO:0007050 GO:0045859 GO:0005515
GO:0004861 GO:0005179 GO:0008270 GO:0005634 GO:0005576
Cluster-22178.69496 GO:0004651
Cluster-22178.69498 GO:0015992 GO:0015031 GO:0006464 GO:0015986
GO:0006772 GO:0015689 GO:0009228 GO:0006886 GO:0008270
GO:0052855 GO:0005047 GO:0003924 GO:0005525 GO:0015078
GO:0015098 GO:0004417 GO:0005515 GO:0000276 GO:0005785
GO:0005786 GO:0016021
Cluster-22178.69499 GO:0008092 GO:0019898 GO:0005737
Cluster-22178.69500 GO:0006541 GO:0016787
Cluster-22178.69504 GO:0006310 GO:0006281 GO:0005515 GO:0009378
GO:0003723 GO:0003724 GO:0016829 GO:0005524 GO:0016887
GO:0009379 GO:0005657 GO:0042597

Cluster-22178.69505 GO:0006397 GO:0008152 GO:0005515 GO:0016788 GO:0003723 GO:0004064 GO:0005634 GO:0005737
Cluster-22178.69507 GO:0055114 GO:0034599 GO:0005524 GO:0016887 GO:0016491 GO:0005789
Cluster-22178.69509 GO:0006355 GO:0006914 GO:0055114 GO:0005515 GO:0043565 GO:0016491 GO:0003700 GO:0005667
Cluster-22178.69511 GO:0007165 GO:0035556
Cluster-22178.69512 GO:0006406 GO:0055085 GO:0007155 GO:0006368 GO:0030001 GO:0046873 GO:0045502 GO:0042803 GO:0008134 GO:0005198 GO:0030286 GO:0005667 GO:0016020 GO:0009288 GO:0019031 GO:0019028 GO:0000446
Cluster-22178.69513 GO:0000090 GO:0007067 GO:0007165 GO:0030168 GO:0031110 GO:0007017 GO:0007018 GO:0051258 GO:0051301 GO:0007059 GO:0030674 GO:0005524 GO:0005102 GO:0008017 GO:0003777 GO:0000940 GO:0005577 GO:0005876 GO:0005874 GO:0045298
Cluster-22178.69514 GO:0005515 GO:0046872
Cluster-22178.69516 GO:0006464 GO:0042254 GO:0006412 GO:0051536 GO:0016151 GO:0003735 GO:0005622 GO:0005840
Cluster-22178.69519 GO:0006464 GO:0006144 GO:0046034 GO:0006281 GO:0008270 GO:0046872 GO:0016151 GO:0004017
Cluster-22178.69523 GO:0005524
Cluster-22178.69524 GO:0016887 GO:0005524
Cluster-22178.69526 GO:0035091
Cluster-22178.69527 GO:0007165 GO:0007186 GO:0007264 GO:0005525 GO:0004871 GO:0031683 GO:0019001 GO:0003924
Cluster-22178.69530 GO:0006281 GO:0006464 GO:0015992 GO:0055114 GO:0015672 GO:0046872 GO:0008270 GO:0047134 GO:0016151 GO:0005451
Cluster-22178.69531 GO:0055114 GO:0006281 GO:0006464 GO:0008270 GO:0046872 GO:0047134 GO:0016151
Cluster-22178.69532 GO:0006412 GO:0042254 GO:0003735 GO:0005840
Cluster-22178.69533 GO:0043087 GO:0035023 GO:0006412 GO:0042254

GO:0046872 GO:0005089 GO:0003735 GO:0005840 GO:0015934
Cluster-22178.69535 GO:0008152 GO:0008168
Cluster-22178.69536 GO:0006298 GO:0005524 GO:0030983
Cluster-22178.69537 GO:0009405 GO:0006231 GO:0006206 GO:0004799
GO:0005576
Cluster-22178.69542 GO:0006298 GO:0030983 GO:0005524 GO:0030430
Cluster-22178.69543 GO:0031412 GO:0006396 GO:0006139 GO:0008408
GO:0003676 GO:0031411 GO:0005622 GO:0000176
Cluster-22178.69546 GO:0005515 GO:0003677 GO:0008270 GO:0046872
GO:0005622
Cluster-22178.69547 GO:0006351 GO:0005975 GO:0003676 GO:0046872
GO:0008270
Cluster-22178.69548 GO:0006355 GO:0006508 GO:0003682 GO:0003677
GO:0008270 GO:0046872 GO:0004222 GO:0003676 GO:0005634
GO:0000775 GO:0000785
Cluster-22178.69551 GO:0016021
Cluster-22178.69553 GO:0007268 GO:0006468 GO:0016773 GO:0033130
GO:0005524 GO:0004672 GO:0043495 GO:0016020
Cluster-22178.69554 GO:0006570 GO:0016192 GO:0007304 GO:0006470
GO:0008138 GO:0004725 GO:0005213 GO:0005576 GO:0042600
Cluster-22178.69555 GO:0005515
Cluster-22178.69556 GO:0030435 GO:0046872 GO:0031160
Cluster-22178.69557 GO:0006281 GO:0010447 GO:0003824 GO:0003677
GO:0004518 GO:0042597
Cluster-22178.69558 GO:0006367 GO:0006270 GO:0005672
Cluster-22178.69559 GO:0016310 GO:0006468 GO:0009069 GO:0004674
GO:0004672 GO:0005524 GO:0016773 GO:0016020
Cluster-22178.6956 GO:0008152 GO:0055114 GO:0016491
Cluster-22178.69562 GO:0006355 GO:0007049 GO:0008134 GO:0042803
GO:0019901 GO:0045502 GO:0003700 GO:0043565 GO:0046872
GO:0005667 GO:0030286
Cluster-22178.69563 GO:0006355 GO:0046983 GO:0005509 GO:0005515

Cluster-22178.69565 GO:0006260 GO:0006241 GO:0006206 GO:0006144
GO:0006886 GO:0006183 GO:0006355 GO:0006228 GO:0006281
GO:0006508 GO:0006165 GO:0004222 GO:0003911 GO:0008536
GO:0004550 GO:0005634
Cluster-22178.69567 GO:0016021
Cluster-22178.69568 GO:0005975 GO:0032065 GO:0001575 GO:0006412
GO:0006012 GO:0035556 GO:0046486 GO:0042254 GO:0005515
GO:0004557 GO:0004553 GO:0008242 GO:0005543 GO:0003735
GO:0005840 GO:0005938 GO:0005622
Cluster-22178.69570 GO:0007165
Cluster-22178.69572 GO:0006355 GO:0007155 GO:0006508 GO:0008270
GO:0016747 GO:0004222 GO:0003700 GO:0005667 GO:0005634
GO:0016020
Cluster-22178.69573 GO:0006468 GO:0007264 GO:0004715 GO:0003924
GO:0005524 GO:0005525
Cluster-22178.69577 GO:0006260 GO:0005515 GO:0005634
Cluster-22178.69578 GO:0005515 GO:0008270 GO:0043565 GO:0005634
Cluster-22178.69579 GO:0030683 GO:0019012
Cluster-22178.69580 GO:0007010 GO:0016579 GO:0009878 GO:0046872
GO:0036459 GO:0003779
Cluster-22178.69581 GO:0006769 GO:0006741 GO:0008152 GO:0046497
GO:0003677 GO:0003951 GO:0016301
Cluster-22178.69583 GO:0019064 GO:0010508 GO:0006260 GO:0016758
GO:0005789 GO:0005815 GO:0019031
Cluster-22178.69584 GO:0010508 GO:0019064 GO:0006369 GO:0006379
GO:0006260 GO:0006378 GO:0016758 GO:0005849 GO:0005789
GO:0005815 GO:0019031
Cluster-22178.69585 GO:0035023 GO:0007165 GO:0043087 GO:0005089
GO:0016874 GO:0001664
Cluster-22178.69587 GO:0003723
Cluster-22178.69588 GO:0006355 GO:0006412 GO:0009451 GO:0006479
GO:0008033 GO:0008152 GO:0046500 GO:0006464 GO:0030488
GO:0032259 GO:0008168 GO:0043565 GO:0004719 GO:0003700

GO:0016491 GO:0016429 GO:0005634 GO:0005667 GO:0031515
Cluster-22178.69589 GO:0006119 GO:0006810 GO:0015986 GO:0015992
GO:0046961 GO:0015288 GO:0045182 GO:0046933 GO:0045261
GO:0016020 GO:0045259
Cluster-22178.69590 GO:0016192 GO:0016021
Cluster-22178.69593 GO:0003677 GO:0005515
Cluster-22178.69594 GO:0015996 GO:0015994 GO:0006811 GO:0015991
GO:0006508 GO:0006355 GO:0006629 GO:0006118 GO:0015992
GO:0016042 GO:0045454 GO:0046486 GO:0046872 GO:0009055
GO:0015078 GO:0015035 GO:0008236 GO:0047746 GO:0003847
GO:0016787 GO:0003677 GO:0019031 GO:0033179 GO:0005886
GO:0016020 GO:0008247
Cluster-22178.69597 GO:0009098 GO:0006418 GO:0006438 GO:0009099
GO:0009097 GO:0005524 GO:0004832 GO:0002161 GO:0000166
GO:0004812 GO:0005737
Cluster-22178.69599 GO:0016567 GO:0006810 GO:0000917 GO:0043093
GO:0048870 GO:0006914 GO:0005515 GO:0004842 GO:0008270
GO:0046872 GO:0031514 GO:0005680 GO:0005737 GO:0005886
Cluster-22178.69600 GO:0043087 GO:0007165 GO:0035023 GO:0005057
GO:0030170 GO:0005515 GO:0005089
Cluster-22178.69604 GO:0006505 GO:0006448 GO:0006508 GO:0006629
GO:0006886 GO:0001514 GO:0015996 GO:0015994 GO:0008152
GO:0016787 GO:0003746 GO:0003723 GO:0008236 GO:0047746
GO:0016788 GO:0005525 GO:0005576 GO:0005737 GO:0005840
Cluster-22178.69605 GO:0006281 GO:0043240
Cluster-22178.69606 GO:0006415 GO:0006449 GO:0016149 GO:0003747
GO:0008270 GO:0005515 GO:0003677 GO:0005737 GO:0005840
GO:0018444 GO:0042025
Cluster-22178.69607 GO:0006508 GO:0006468 GO:0015031 GO:0006464
GO:0004252 GO:0016773 GO:0005524 GO:0004672 GO:0016020
GO:0016021
Cluster-22178.69608 GO:0006464 GO:0015031 GO:0006468 GO:0006508
GO:0004672 GO:0005524 GO:0016773 GO:0004252 GO:0016021

GO:0016020
Cluster-22178.69609 GO:0015031 GO:0006464
Cluster-22178.69613 GO:0006629 GO:0006355 GO:0007165 GO:0046339
GO:0009395 GO:0035556 GO:0005509 GO:0005515 GO:0003676
GO:0004435 GO:0005622
Cluster-22178.69614 GO:0001522 GO:0009451 GO:0007049 GO:0003723
GO:0005515 GO:0005524 GO:0009982 GO:0031011 GO:0017053
GO:0009507
Cluster-22178.69615 GO:0005515
Cluster-22178.69619 GO:0005543
Cluster-22178.69620 GO:0015992 GO:0015986 GO:0015078 GO:0000276
Cluster-22178.69621 GO:0015992 GO:0015986 GO:0015078 GO:0000276
Cluster-22178.69625 GO:0008152 GO:0008484 GO:0016788
Cluster-22178.69627 GO:0005515
Cluster-22178.69628 GO:0006744 GO:0015992 GO:0042254 GO:0008152
GO:0006120 GO:0005975 GO:0006412 GO:0006814 GO:0006118
GO:0004553 GO:0003735 GO:0009055 GO:0003824 GO:0008484
GO:0008137 GO:0005622 GO:0016020 GO:0005840
Cluster-22178.69629 GO:0006821 GO:0046653 GO:0015991 GO:0006555
GO:0055085 GO:0055114 GO:0016820 GO:0005247 GO:0004489
GO:0016020 GO:0000221
Cluster-22178.69630 GO:0006555 GO:0055085 GO:0055114 GO:0006821
GO:0015991 GO:0046653 GO:0005247 GO:0004489 GO:0016820
GO:0000221 GO:0016020
Cluster-22178.69631 GO:0019076 GO:0006812 GO:0032801 GO:0005261
GO:0005515 GO:0008270 GO:0005634 GO:0033644
Cluster-22178.69633 GO:0042742 GO:0006468 GO:0016773 GO:0005524
GO:0004672 GO:0005515 GO:0016020
Cluster-22178.69636 GO:0030494 GO:0015979 GO:0006355 GO:0016032
GO:0003700 GO:0043565 GO:0005515 GO:0005667
Cluster-22178.69638 GO:0007268 GO:0005515 GO:0043495 GO:0043395
GO:0033130 GO:0005578 GO:0016020 GO:0016021
Cluster-22178.69640 GO:0016192 GO:0006886 GO:0030117 GO:0030131

Cluster-22178.69641 GO:0055085 GO:0006811 GO:0006554 GO:0006367
GO:0006479 GO:0034968 GO:0016817 GO:0008270 GO:0005515
GO:0005216 GO:0018024 GO:0019015 GO:0016020 GO:0005672
GO:0005634
Cluster-22178.69644 GO:0016310 GO:0004673 GO:0009365
Cluster-22178.69645 GO:0010508 GO:0045502 GO:0016787 GO:0042803
GO:0008134 GO:0046872 GO:0005667 GO:0030286
Cluster-22178.69646 GO:0009405 GO:0006659 GO:0005198 GO:0004222
GO:0008289
Cluster-22178.69648 GO:0055114 GO:0015992 GO:0019049 GO:0005975
GO:0006120 GO:0006744 GO:0008152 GO:0006814 GO:0003824
GO:0016836 GO:0030246 GO:0008137
Cluster-22178.69649 GO:0009069 GO:0006814 GO:0005975 GO:0015992
GO:0019049 GO:0055114 GO:0016310 GO:0008152 GO:0006744
GO:0006120 GO:0030246 GO:0016836 GO:0008137 GO:0004674
GO:0003824
Cluster-22178.69650 GO:0003676
Cluster-22178.69651 GO:0006270 GO:0000736 GO:0006364 GO:0070336
GO:0008270 GO:0005732 GO:0005634 GO:0034457
Cluster-22178.69653 GO:0007264 GO:0005525
Cluster-22178.69654 GO:0006355 GO:0051260 GO:0008270 GO:0003700
GO:0003676 GO:0005634 GO:0005667
Cluster-22178.69656 GO:0030154 GO:0018189 GO:0016021
Cluster-22178.69657 GO:0008152 GO:0006508 GO:0006412 GO:0042254
GO:0016787 GO:0008236 GO:0003735 GO:0005198 GO:0005622
GO:0005840 GO:0019028
Cluster-22178.69661 GO:0000917 GO:0006306 GO:0007059 GO:0007049
GO:0048870 GO:0051382 GO:0000090 GO:0007067 GO:0006260
GO:0031110 GO:0030036 GO:0051301 GO:0043093 GO:0048193
GO:0006914 GO:0008134 GO:0045502 GO:0017048 GO:0042803
GO:0005515 GO:0003779 GO:0003677 GO:0008170 GO:0008017
GO:0046983 GO:0031514 GO:0031083 GO:0016021 GO:0017119
GO:0005815 GO:0000940 GO:0030286 GO:0000776 GO:0045298

GO:0005737 GO:0016020 GO:0030133 GO:0005667 GO:0005876
Cluster-22178.69662 GO:0006308 GO:0006355 GO:0007017 GO:0007018
GO:0006812 GO:0007165 GO:0003777 GO:0035257 GO:0005524
GO:0016887 GO:0008855 GO:0008017 GO:0003713 GO:0005874
GO:0016021 GO:0045298 GO:0005634 GO:0009318 GO:0005667
Cluster-22178.69663 GO:0006687 GO:0005975 GO:0004308 GO:0033644
GO:0055036 GO:0016020
Cluster-22178.69665 GO:0006355 GO:0006281 GO:0006397 GO:0000917
GO:0006412 GO:0007264 GO:0043093 GO:0006310 GO:0042254
GO:0006457 GO:0006260 GO:0010506 GO:0051082 GO:0000287
GO:0005515 GO:0043565 GO:0003700 GO:0003735 GO:0005096
GO:0005737 GO:0000445 GO:0005840 GO:0005615 GO:0005667
GO:0016272
Cluster-22178.69667 GO:0003676
Cluster-22178.69670 GO:0006352 GO:0000398 GO:0006351 GO:0006355
GO:0006260 GO:0000736 GO:0003700 GO:0070336 GO:0003887
GO:0046935 GO:0016987 GO:0008270 GO:0003677 GO:0003682
GO:0005681 GO:0000785 GO:0005789 GO:0032040 GO:0000775
GO:0042575 GO:0005634 GO:0019028 GO:0016020 GO:0005667
GO:0005944
Cluster-22178.69673 GO:0008610 GO:0008152 GO:0016747
Cluster-22178.69674 GO:0006206 GO:0006144 GO:0046034 GO:0006139
GO:0005524 GO:0004017 GO:0004127
Cluster-22178.69675 GO:0006570 GO:0016192 GO:0007304 GO:0006470
GO:0008138 GO:0005213 GO:0004725 GO:0005576 GO:0042600
Cluster-22178.69678 GO:0005975 GO:0004553 GO:0019013 GO:0016021
Cluster-22178.69679 GO:0055114 GO:0006788 GO:0015994 GO:0004392

Cluster-22178.69680 GO:0030246
Cluster-22178.69681 GO:0009186 GO:0055114 GO:0030246 GO:0016021
Cluster-22178.69684 GO:0016579 GO:0036459 GO:0008270 GO:0032183
GO:0070140
Cluster-22178.69685 GO:0016579 GO:0036459 GO:0008270 GO:0070140

GO:0032183

Cluster-22178.69687 GO:0019062 GO:0019058 GO:0007155

Cluster-22178.69688 GO:0019058 GO:0019062 GO:0007155

Cluster-22178.69691 GO:0006364 GO:0006383 GO:0006206 GO:0009236 GO:0009405 GO:0006810 GO:0006260 GO:0006144 GO:0006351 GO:0006355 GO:0051716 GO:0003677 GO:0003899 GO:0005515 GO:0004190 GO:0016817 GO:0003887 GO:0008200 GO:0003700 GO:0016020 GO:0005666 GO:0005730 GO:0005667 GO:0034457 GO:0005634 GO:0005789 GO:0005732 GO:0042575 GO:0005576 GO:0032040 GO:0016021 GO:0046658

Cluster-22178.69692 GO:0015948 GO:0046656 GO:0003677 GO:0030269 GO:0016020 GO:0005634 GO:0005576

Cluster-22178.69696 GO:0061025 GO:0046813 GO:0005634 GO:0005737 GO:0016021 GO:0019031

Cluster-22178.69699 GO:0002949 GO:0007264 GO:0007165 GO:0007186 GO:0031683 GO:0019001 GO:0003924 GO:0005525 GO:0003724 GO:0004871 GO:0003723

Cluster-22178.69700 GO:0007165 GO:0046740 GO:0035556 GO:0043087 GO:0006814 GO:0006955 GO:0007010 GO:0003333 GO:0006508 GO:0006741 GO:0046656 GO:0046497 GO:0035023 GO:0008152 GO:0006769 GO:0005089 GO:0030269 GO:0008236 GO:0016301 GO:0003951 GO:0005133 GO:0003779 GO:0046872 GO:0012506 GO:0019028 GO:0005576 GO:0005737 GO:0044219 GO:0005643

Cluster-22178.69701 GO:0006351 GO:0005975 GO:0005515 GO:0008270 GO:0003676 GO:0004553 GO:0033925 GO:0005737

Cluster-22178.69702 GO:0005975 GO:0006351 GO:0004553 GO:0003676 GO:0033925 GO:0008270 GO:0005515 GO:0005737

Cluster-22178.69705 GO:0045132 GO:0006355 GO:0042967 GO:0004402 GO:0005515 GO:0003676 GO:0008080 GO:0005634 GO:0000123 GO:0000775

Cluster-22178.69706 GO:0042967 GO:0007218 GO:0042546 GO:0045132 GO:0006355 GO:0008080 GO:0005515 GO:0005199 GO:0004402 GO:0005618 GO:0000775 GO:0000123 GO:0005634 GO:0005576

Cluster-22178.69708 GO:0006810 GO:0009264 GO:0003676 GO:0004386 GO:0008253 GO:0046872 GO:0005524 GO:0043531 GO:0005515 GO:0016020
Cluster-22178.69709 GO:0055085 GO:0006811 GO:0006554 GO:0006479 GO:0034968 GO:0016817 GO:0008270 GO:0005515 GO:0018024 GO:0005216 GO:0016020 GO:0005634
Cluster-22178.69710 GO:0046034 GO:0006144 GO:0006366 GO:0008616 GO:0006281 GO:0046872 GO:0008270 GO:0016853 GO:0004017 GO:0003677 GO:0004867 GO:0016740 GO:0005576 GO:0005665
Cluster-22178.69711 GO:0003723
Cluster-22178.69713 GO:0005515
Cluster-22178.69715 GO:0006479 GO:0006481 GO:0004671 GO:0016021
Cluster-22178.69717 GO:0004222 GO:0016020 GO:0016021
Cluster-22178.69718 GO:0015684 GO:0007264 GO:0005525 GO:0005515 GO:0015093 GO:0016021
Cluster-22178.69719 GO:0007165 GO:0008152 GO:0003824 GO:0003676 GO:0005179 GO:0005576
Cluster-22178.69720 GO:0030436 GO:0042601
Cluster-22178.69721 GO:0006269 GO:0018142 GO:0006260 GO:0004672 GO:0003723 GO:0000166
Cluster-22178.69722 GO:0055114 GO:0006464 GO:0008113 GO:0009279
Cluster-22178.69724 GO:0006613 GO:0051276 GO:0007029 GO:0005515 GO:0005524 GO:0005694 GO:0005784 GO:0016021 GO:0030176 GO:0005789
Cluster-22178.69728 GO:0016021
Cluster-22178.69729 GO:0008152 GO:0007165 GO:0005179 GO:0003824 GO:0003676 GO:0005576
Cluster-22178.69732 GO:0015684 GO:0006508 GO:0004222 GO:0005525 GO:0015093 GO:0005524 GO:0003924 GO:0016887 GO:0042802 GO:0016021
Cluster-22178.69734 GO:0006511 GO:0051603 GO:0015979 GO:0005515 GO:0003677 GO:0004298 GO:0004175 GO:0016020 GO:0019773 GO:0005839 GO:0009522

Cluster-22178.69736 GO:0006464 GO:0005975 GO:0016868
Cluster-22178.69740 GO:0000162 GO:0006571 GO:0009094 GO:0006096
GO:0006094 GO:0004634 GO:0000287 GO:0000015
Cluster-22178.69742 GO:0006402 GO:0019722 GO:0051252 GO:0006810
GO:0003676 GO:0046872 GO:0004535 GO:0008270 GO:0003723
GO:0005737 GO:0005634
Cluster-22178.69743 GO:0006508 GO:0016757 GO:0004198 GO:0016020
GO:0005622
Cluster-22178.69744 GO:0007268 GO:0033130 GO:0042802 GO:0005515
GO:0030695 GO:0043495
Cluster-22178.69746 GO:0015992 GO:0055114 GO:0008152 GO:0006355
GO:0006118 GO:0006119 GO:0016491 GO:0003677 GO:0008121
Cluster-22178.69747 GO:0042254 GO:0006412 GO:0006298 GO:0003735
GO:0004519 GO:0008146 GO:0016021 GO:0005840 GO:0005622
Cluster-22178.69749 GO:0003676 GO:0042393 GO:0005634
Cluster-22178.69750 GO:0055114 GO:0016491
Cluster-22178.69751 GO:0055085 GO:0006810 GO:0016192 GO:0006886
GO:0005215 GO:0022857 GO:0016021 GO:0016020 GO:0030117
Cluster-22178.69754 GO:0006886 GO:0016192 GO:0005515 GO:0005198
GO:0030130 GO:0030132 GO:0030117
Cluster-22178.69756 GO:0016310 GO:0006468 GO:0006351 GO:0006206
GO:0006144 GO:0009069 GO:0007165 GO:0004672 GO:0003899
GO:0005524 GO:0004674 GO:0005179 GO:0003677 GO:0005576
GO:0005887 GO:0005730
Cluster-22178.69759 GO:0006525 GO:0006420 GO:0006560 GO:0005524
GO:0004814
Cluster-22178.69762 GO:0007264 GO:0007186 GO:0007165 GO:0015684
GO:0003924 GO:0031683 GO:0008270 GO:0015093 GO:0019001
GO:0004871 GO:0005525 GO:0016021
Cluster-22178.69764 GO:0006468 GO:0004672 GO:0005524 GO:0005515

Cluster-22178.69765 GO:0006352 GO:0005669
Cluster-22178.69766 GO:0006376 GO:0003729 GO:0005685

Cluster-22178.69768 GO:0006260 GO:0016567 GO:0006869 GO:0042157
GO:0006352 GO:0006308 GO:0006351 GO:0006355 GO:0004842
GO:0016987 GO:0008855 GO:0008270 GO:0061630 GO:0003677
GO:0003700 GO:0008289 GO:0046872 GO:0005515 GO:0003887
GO:0004190 GO:0005680 GO:0005622 GO:0016020 GO:0005667
GO:0016021 GO:0009318 GO:0042575 GO:0019028 GO:0005576
Cluster-22178.69770 GO:0006886 GO:0015031 GO:0005515 GO:0008565
GO:0016021 GO:0016020 GO:0005737
Cluster-22178.69771 GO:0006914 GO:0015031 GO:0006886 GO:0006298
GO:0008565 GO:0005524 GO:0030983 GO:0005815 GO:0005737
GO:0016020
Cluster-22178.69773 GO:0006470 GO:0006570 GO:0004725 GO:0043130
GO:0032266 GO:0008138
Cluster-22178.69774 GO:0007264 GO:0006576 GO:0008201 GO:0005525
GO:0005524 GO:0003924 GO:0016021
Cluster-22178.69776 GO:0003723
Cluster-22178.69777 GO:0051260 GO:0006355 GO:0006352 GO:0015074
GO:0003677 GO:0003700 GO:0005667 GO:0005669
Cluster-22178.69780 GO:0006119 GO:0015992 GO:0015986 GO:0046961
GO:0046933 GO:0045259 GO:0045261
Cluster-22178.69781 GO:0008152 GO:0016746
Cluster-22178.69784 GO:0030244 GO:0061136 GO:0019915 GO:0006011
GO:0005985 GO:0005982 GO:0016567 GO:0008270 GO:0046872
GO:0004842 GO:0005515 GO:0016760 GO:0005789 GO:0005680
GO:0016020
Cluster-22178.69786 GO:0006629 GO:0042254 GO:0042967 GO:0006412
GO:0003735 GO:0008374 GO:0005840
Cluster-22178.69787 GO:0006355 GO:0005515 GO:0005634
Cluster-22178.69788 GO:0006357 GO:0042254 GO:0006412 GO:0001104
GO:0003735 GO:0005840 GO:0005622 GO:0016592
Cluster-22178.69789 GO:0006886 GO:0016020
Cluster-22178.69791 GO:0006308 GO:0006281 GO:0009307 GO:0009036
GO:0003677 GO:0009359 GO:0005634 GO:0016020

Cluster-22178.69795 GO:0019491 GO:0033990 GO:0005515 GO:0016021
Cluster-22178.69796 GO:0006355 GO:0006418 GO:0004812 GO:0000166
GO:0016818 GO:0003677 GO:0008270 GO:0005524 GO:0002161
GO:0005634
Cluster-22178.69798 GO:0009405 GO:0006468 GO:0019865 GO:0005524
GO:0004672 GO:0005515 GO:0051082 GO:0019814
Cluster-22178.69799 GO:0016020
Cluster-22178.69800 GO:0030212 GO:0005515 GO:0004867
Cluster-22178.69801 GO:0015986 GO:0006810 GO:0006119 GO:0015992
GO:0046961 GO:0015288 GO:0046933 GO:0045182 GO:0045259
GO:0045261 GO:0016020
Cluster-22178.69803 GO:0008654 GO:0006278 GO:0016780 GO:0003964
GO:0016020
Cluster-22178.69804 GO:0031110 GO:0006260 GO:0007067 GO:0000090
GO:0051382 GO:0048870 GO:0007049 GO:0007059 GO:0006306
GO:0000917 GO:0006914 GO:0048193 GO:0043093 GO:0051301
GO:0030036 GO:0017048 GO:0045502 GO:0008134 GO:0046983
GO:0008017 GO:0008170 GO:0003677 GO:0003779 GO:0005515
GO:0042803 GO:0030286 GO:0000940 GO:0005815 GO:0017119
GO:0016021 GO:0031083 GO:0031514 GO:0005876 GO:0005667
GO:0030133 GO:0016020 GO:0005737 GO:0045298 GO:0000776
Cluster-22178.69805 GO:0006412 GO:0042254 GO:0003735 GO:0005622
GO:0005840
Cluster-22178.69806 GO:0007165 GO:0004872 GO:0016021
Cluster-22178.69807 GO:0006355 GO:0005515 GO:0046983
Cluster-22178.69808 GO:0006355 GO:0003333 GO:0046983 GO:0005515
GO:0016020
Cluster-22178.69809 GO:0006606 GO:0005515 GO:0019904 GO:0005737
Cluster-22178.69810 GO:0000921 GO:0042254 GO:0006412 GO:0016055
GO:0006355 GO:0003700 GO:0043565 GO:0003735 GO:0003677
GO:0016021 GO:0005840 GO:0005940 GO:0005622 GO:0005667
GO:0005634
Cluster-22178.69811 GO:0006355 GO:0006289 GO:0005515 GO:0000439

GO:0016020
Cluster-22178.69812 GO:0006412 GO:0042254 GO:0000723 GO:0003735
GO:0003677 GO:0005840 GO:0005622 GO:0000784
Cluster-22178.69813 GO:0000398 GO:0006298 GO:0007264 GO:0005525
GO:0003924 GO:0016887 GO:0005524 GO:0008270 GO:0004519
Cluster-22178.69814 GO:0005515
Cluster-22178.69819 GO:0009069 GO:0016192 GO:0006886 GO:0016310
GO:0005515 GO:0004674 GO:0030117
Cluster-22178.69820 GO:0005515
Cluster-22178.69822 GO:0055114 GO:0016491
Cluster-22178.69823 GO:0003677 GO:0016020
Cluster-22178.69825 GO:0005576
Cluster-22178.69828 GO:0005524 GO:0016787 GO:0003677 GO:0003676

Cluster-22178.69829 GO:0016020
Cluster-22178.69833 GO:0055085 GO:0016020 GO:0016021
Cluster-22178.69834 GO:0006468 GO:0016192 GO:0006886 GO:0005524
GO:0004672 GO:0005515 GO:0030117
Cluster-22178.69835 GO:0030001 GO:0055085 GO:0009306 GO:0046873
GO:0016020
Cluster-22178.69837 GO:0006412 GO:0042254 GO:0003735 GO:0005622
GO:0005840
Cluster-22178.69838 GO:0006888 GO:0006508 GO:0007606 GO:0006886
GO:0007165 GO:0008270 GO:0003723 GO:0004888 GO:0005515
GO:0004197 GO:0016020 GO:0030127
Cluster-22178.69840 GO:0032324 GO:0019033
Cluster-22178.69843 GO:0006508 GO:0015684 GO:0042802 GO:0005524
GO:0008270 GO:0015093 GO:0016887 GO:0003924 GO:0005525
GO:0004222 GO:0016021
Cluster-22178.69844 GO:0006935 GO:0007165 GO:0004888 GO:0016020
Cluster-22178.69846 GO:0006516 GO:0008152 GO:0030153 GO:0006955
GO:0016747 GO:0015643 GO:0003677 GO:0005737 GO:0005634
GO:0019814

Cluster-22178.69847 GO:0008283 GO:0040007 GO:0007165 GO:0008083

Cluster-22178.69848 GO:0006516 GO:0030153 GO:0006955 GO:0015643
GO:0005737 GO:0019814
Cluster-22178.69850 GO:0006357 GO:0001104 GO:0016592
Cluster-22178.69851 GO:0046872 GO:0005524 GO:0016787 GO:0003677
GO:0003676
Cluster-22178.69855 GO:0006810 GO:0005215 GO:0016020
Cluster-22178.69856 GO:0008152 GO:0005515 GO:0003824
Cluster-22178.69857 GO:0046854 GO:0006000 GO:0015976 GO:0006013
GO:0005975 GO:0006096 GO:0006098 GO:0006094 GO:0042578
GO:0042132
Cluster-22178.69866 GO:0005759
Cluster-22178.69867 GO:0007165 GO:0007155 GO:0048193 GO:0006606
GO:0035556 GO:0006357 GO:0006914 GO:0046339 GO:0006629
GO:0009395 GO:0005509 GO:0042803 GO:0008134 GO:0005515
GO:0001104 GO:0045502 GO:0004435 GO:0008081 GO:0016020
GO:0016592 GO:0005667 GO:0030286 GO:0019028
Cluster-22178.69868 GO:0006118 GO:0000917 GO:0006629 GO:0048870
GO:0009395 GO:0007165 GO:0035556 GO:0022900 GO:0043093
GO:0006606 GO:0006914 GO:0006357 GO:0046339 GO:0008134
GO:0045502 GO:0005509 GO:0042803 GO:0020037 GO:0005515
GO:0001104 GO:0004435 GO:0008081 GO:0009055 GO:0005506
GO:0019905 GO:0031514 GO:0019028 GO:0016592 GO:0030286
GO:0005737 GO:0005667 GO:0042597
Cluster-22178.69869 GO:0043531 GO:0005524
Cluster-22178.69870 GO:0042254 GO:0006412 GO:0003735 GO:0005622
GO:0005840
Cluster-22178.69871 GO:0006368 GO:0055114 GO:0016570 GO:0030151
GO:0035091 GO:0005515 GO:0016491 GO:0016593
Cluster-22178.69872 GO:0016567 GO:0006531 GO:0006123 GO:0006522
GO:0009152 GO:0006188 GO:0006144 GO:0015992 GO:0004129
GO:0004842 GO:0004018 GO:0005740 GO:0045277

Cluster-22178.69873 GO:0005515
Cluster-22178.69876 GO:0055114 GO:0007264 GO:0015684 GO:0015093 GO:0005524 GO:0016491 GO:0003924 GO:0005525 GO:0003677 GO:0000166 GO:0016021
Cluster-22178.69877 GO:0017004 GO:0006118 GO:0006468 GO:0008152 GO:0006480 GO:0015979 GO:0055114 GO:0047134 GO:0045158 GO:0004672 GO:0005524 GO:0008168 GO:0016021 GO:0009512 GO:0009523
Cluster-22178.69878 GO:0016579 GO:0036459
Cluster-22178.69880 GO:0006118 GO:0019684 GO:0006274 GO:0045156 GO:0003677 GO:0003676 GO:0030077 GO:0005737 GO:0016021
Cluster-22178.69882 GO:0007034 GO:0030001 GO:0005887
Cluster-22178.69884 GO:0007067 GO:0015743 GO:0016043 GO:0030036 GO:0048037 GO:0017048 GO:0016740 GO:0003779 GO:0042729 GO:0072686
Cluster-22178.69887 GO:0042967 GO:0004379 GO:0008080
Cluster-22178.69888 GO:0008152 GO:0009058 GO:0006886 GO:0006629 GO:0006505 GO:0016788 GO:0016787
Cluster-22178.69889 GO:0006189
Cluster-22178.69890 GO:0006811 GO:0006813 GO:0005249 GO:0016020 GO:0008076
Cluster-22178.69891 GO:0006412 GO:0000921 GO:0051607 GO:0042254 GO:0007155 GO:0005515 GO:0042803 GO:0008134 GO:0045502 GO:0003735 GO:0005840 GO:0016021 GO:0097197 GO:0019028 GO:0005622 GO:0030286 GO:0001772 GO:0005940 GO:0005667
Cluster-22178.69892 GO:0006886 GO:0000921 GO:0051276 GO:0007031 GO:0008773 GO:0051082 GO:0005515 GO:0005524 GO:0005694 GO:0016020 GO:0005777 GO:0005940 GO:0016021 GO:0005801
Cluster-22178.69893 GO:0006412 GO:0042254 GO:0003735 GO:0005840 GO:0005622
Cluster-22178.69894 GO:0006810 GO:0006816 GO:0006508 GO:0006811 GO:0035556 GO:0055085 GO:0005515 GO:0005216 GO:0004190 GO:0005246 GO:0042030 GO:0016020

Cluster-22178.69895 GO:0006139 GO:0016787 GO:0004003 GO:0003677
GO:0008026 GO:0016818 GO:0005524 GO:0003676 GO:0005634
GO:0019013 GO:0005657
Cluster-22178.69896 GO:0007264 GO:0006810 GO:0016787 GO:0005524
GO:0005525 GO:0003677
Cluster-22178.69899 GO:0005975 GO:0016192 GO:0006886 GO:0004553
GO:0005198 GO:0005515 GO:0019028 GO:0030117
Cluster-22178.69900 GO:0045893 GO:0006355 GO:0003677 GO:0043565
GO:0016021 GO:0005634
Cluster-22178.69901 GO:0043087 GO:0035556 GO:0035023 GO:0005515
GO:0005089
Cluster-22178.69903 GO:0004190 GO:0016021
Cluster-22178.69904 GO:0007264 GO:0019079 GO:0006614 GO:0007165
GO:0006304 GO:0007186 GO:0006013 GO:0006000 GO:0043066
GO:0043154 GO:0003873 GO:0033821 GO:0005524 GO:0031683
GO:0019001 GO:0003924 GO:0005525 GO:0004871 GO:0071986
GO:0019012 GO:0005737
Cluster-22178.69905 GO:0043087 GO:0003676 GO:0005089 GO:0003677
GO:0016787 GO:0005524 GO:0005737
Cluster-22178.69906 GO:0006813 GO:0009405 GO:0008152 GO:0007157
GO:0016787 GO:0003677 GO:0016301 GO:0005524 GO:0005267
GO:0003676 GO:0016020 GO:0016021
Cluster-22178.69910 GO:0006144 GO:0006206 GO:0006351 GO:0003899
GO:0003677 GO:0005730
Cluster-22178.69911 GO:0055085 GO:0006811 GO:0005216 GO:0012511
GO:0016021 GO:0016020
Cluster-22178.69913 GO:0015694 GO:0046872 GO:0008270 GO:0015097
GO:0003677 GO:0005515 GO:0016020
Cluster-22178.69914 GO:0006355 GO:0005515 GO:0003723
Cluster-22178.69915 GO:0003824 GO:0030151 GO:0030170
Cluster-22178.69916 GO:0003676
Cluster-22178.69918 GO:0003676
Cluster-22178.69919 GO:0046660 GO:0006397 GO:0003676 GO:0005634

Cluster-22178.69920 GO:0008270 GO:0016021
Cluster-22178.69921 GO:0051191 GO:0006352 GO:0042742 GO:0005515
GO:0005669 GO:0005576
Cluster-22178.69922 GO:0042742 GO:0051191 GO:0006352 GO:0005515
GO:0005576 GO:0005669
Cluster-22178.69923 GO:0006352 GO:0051191 GO:0042742 GO:0005515
GO:0005669 GO:0005576
Cluster-22178.69925 GO:0007186 GO:0004930 GO:0046872 GO:0016021
Cluster-22178.69926 GO:0032780 GO:0042030 GO:0046872 GO:0016820
GO:0005739
Cluster-22178.69930 GO:0055114 GO:0006508 GO:0006886 GO:0015031
GO:0006260 GO:0007264 GO:0016192 GO:0005515 GO:0070403
GO:0008270 GO:0016491 GO:0005096 GO:0008565 GO:0004222
GO:0047134 GO:0005737 GO:0031012 GO:0016020
Cluster-22178.69931 GO:0005515
Cluster-22178.69935 GO:0006468 GO:0031124 GO:0000287 GO:0004672
GO:0005524 GO:0030976
Cluster-22178.69937 GO:0042176 GO:0030234 GO:0005515 GO:0000502
Cluster-22178.69938 GO:0015979 GO:0008152 GO:0016829 GO:0009523
GO:0009539 GO:0016020
Cluster-22178.69939 GO:0003950 GO:0005515
Cluster-22178.69942 GO:0006355 GO:0045132 GO:0003676 GO:0003700
GO:0005524 GO:0043565 GO:0003677 GO:0016787 GO:0000775
GO:0005634 GO:0005667
Cluster-22178.69943 GO:0006886 GO:0016192 GO:0005198 GO:0005515
GO:0030117
Cluster-22178.69944 GO:0016021
Cluster-22178.69946 GO:0007155 GO:0006468 GO:0004672 GO:0005524
GO:0016020
Cluster-22178.69949 GO:0006804 GO:0006979 GO:0055114 GO:0006568
GO:0015947 GO:0020037 GO:0004096
Cluster-22178.69951 GO:0007264 GO:0006468 GO:0005525 GO:0005524
GO:0003924 GO:0004715

Cluster-22178.69953 GO:0030154 GO:0016021
Cluster-22178.69954 GO:0055085 GO:0008053 GO:0015992 GO:0019722 GO:0006308 GO:0006355 GO:0030001 GO:0015991 GO:0008855 GO:0046873 GO:0015078 GO:0003700 GO:0003924 GO:0043565 GO:0005198 GO:0005741 GO:0016020 GO:0005667 GO:0019031 GO:0016021 GO:0009318 GO:0033179 GO:0019028
Cluster-22178.69955 GO:0006672 GO:0055085 GO:0006807 GO:0006811 GO:0007186 GO:0007165 GO:0006810 GO:0004872 GO:0004930 GO:0005216 GO:0016811 GO:0016021 GO:0016020
Cluster-22178.69958 GO:0006744 GO:0055114 GO:0046872 GO:0016491

Cluster-22178.6996 GO:0005515
Cluster-22178.69960 GO:0050690 GO:0006554 GO:0006289 GO:0043161 GO:0000910 GO:0000226 GO:0006281 GO:0006479 GO:0005537 GO:0046872 GO:0008017 GO:0018024 GO:0005515 GO:0003684 GO:0045298
Cluster-22178.69961 GO:0005515
Cluster-22178.69964 GO:0003723
Cluster-22178.69965 GO:0008053 GO:0007264 GO:0010508 GO:0006810 GO:0003924 GO:0016887 GO:0005524 GO:0003724 GO:0005525 GO:0003723 GO:0016021 GO:0005741
Cluster-22178.69967 GO:0006570 GO:0006470 GO:0008138 GO:0004725

Cluster-22178.69969 GO:0055114 GO:0005996 GO:0005525 GO:0016491 GO:0016853 GO:0048029 GO:0050661 GO:0019867
Cluster-22178.69970 GO:0005515
Cluster-22178.69971 GO:0005515
Cluster-22178.69973 GO:0007017 GO:0030001 GO:0007018 GO:0055085 GO:0004190 GO:0016787 GO:0046873 GO:0046872 GO:0003777 GO:0016020 GO:0030286 GO:0005874
Cluster-22178.69974 GO:0007018 GO:0007017 GO:0030001 GO:0055085 GO:0004190 GO:0016787 GO:0046873 GO:0003777 GO:0046872 GO:0016020 GO:0005874 GO:0030286

Cluster-22178.69976 GO:0042967 GO:0007155 GO:0005515 GO:0008080
GO:0016747 GO:0016021
Cluster-22178.69979 GO:0015992 GO:0006123 GO:0004129 GO:0005739
GO:0045277
Cluster-22178.69981 GO:0006955 GO:0006954 GO:0007165 GO:0040007
GO:0008283 GO:0005509 GO:0005515 GO:0005149 GO:0045323
Cluster-22178.69984 GO:0006749 GO:0006804 GO:0006118 GO:0055114
GO:0006979 GO:0016655 GO:0004602 GO:0071203
Cluster-22178.69986 GO:0000413 GO:0006457 GO:0003755
Cluster-22178.69989 GO:0051252 GO:0006402 GO:0004535 GO:0046872
GO:0003723 GO:0005634 GO:0005737
Cluster-22178.69990 GO:0051082 GO:0005515 GO:0031072
Cluster-22178.69991 GO:0006811 GO:0006486 GO:0051225 GO:0007165
GO:0007155 GO:0006355 GO:0004871 GO:0004576 GO:0003677
GO:0003723 GO:0009288 GO:0070652 GO:0008250 GO:0016020
GO:0005634
Cluster-22178.69993 GO:0006260 GO:0003677 GO:0016818 GO:0005524
GO:0016021 GO:0005634
Cluster-22178.69995 GO:0006812 GO:0007304 GO:0008324 GO:0005213
GO:0042600 GO:0005886 GO:0016021 GO:0005576
Cluster-22178.69996 GO:0006812 GO:0006352 GO:0007304 GO:0006457
GO:0005213 GO:0008324 GO:0005669 GO:0016021 GO:0042600
GO:0005886 GO:0005576
Cluster-22178.69998 GO:0006357 GO:0009405 GO:0006171 GO:0046983
GO:0001104 GO:0008294 GO:0016592 GO:0005576
Cluster-22178.69999 GO:0045454
Cluster-22178.7000 GO:0003774 GO:0005524 GO:0016459
Cluster-22178.70000 GO:0003677 GO:0005515
Cluster-22178.70002 GO:0005515 GO:0003677
Cluster-22178.70004 GO:0005515
Cluster-22178.70007 GO:0005515
Cluster-22178.70008 GO:0007049 GO:0006355 GO:0003677 GO:0003682
GO:0000785 GO:0000775 GO:0005634

Cluster-22178.70011 GO:0030001 GO:0006355 GO:0046872 GO:0005634
Cluster-22178.70015 GO:0006351 GO:0015031 GO:0006464 GO:0005515 GO:0046983
Cluster-22178.70018 GO:0009069 GO:0016310 GO:0008608 GO:0007049 GO:0048870 GO:0009405 GO:0006468 GO:0004674 GO:0005524 GO:0005198 GO:0005815 GO:0016020 GO:0019031 GO:0042729 GO:0031514 GO:0019028 GO:0072686
Cluster-22178.70019 GO:0006281 GO:0006260 GO:0006310 GO:0046907 GO:0003677 GO:0005634
Cluster-22178.70020 GO:0009069 GO:0009405 GO:0006468 GO:0007049 GO:0016310 GO:0008608 GO:0048870 GO:0004674 GO:0005524 GO:0005198 GO:0016020 GO:0005815 GO:0042729 GO:0031514 GO:0019028 GO:0072686 GO:0019031
Cluster-22178.70021 GO:0009058 GO:0008483 GO:0030170
Cluster-22178.70022 GO:0006510 GO:0006508 GO:0006355 GO:0016567 GO:0008270 GO:0004176 GO:0046872 GO:0004842 GO:0061630 GO:0005515 GO:0005634
Cluster-22178.70023 GO:0043565 GO:0005515 GO:0019028 GO:0005634 GO:0016020
Cluster-22178.70024 GO:0043565 GO:0005515 GO:0005634
Cluster-22178.70025 GO:0043565 GO:0005515 GO:0005634 GO:0016020
Cluster-22178.70026 GO:0035444 GO:0015675 GO:0006508 GO:0015099 GO:0046872 GO:0004252 GO:0030288 GO:0016021
Cluster-22178.70027 GO:0008270 GO:0005515
Cluster-22178.70028 GO:0006418 GO:0019643 GO:0006094 GO:0006099 GO:0015977 GO:0043039 GO:0005524 GO:0004222 GO:0004812 GO:0000166 GO:0008964 GO:0000049 GO:0005737 GO:0031225
Cluster-22178.70030 GO:0006816 GO:0006099 GO:0006097 GO:0006090 GO:0005388 GO:0004474 GO:0000166 GO:0046872 GO:0008270 GO:0016529
Cluster-22178.70031 GO:0016757
Cluster-22178.70033 GO:0006260 GO:0003887 GO:0005515 GO:0042575
Cluster-22178.70034 GO:0019079 GO:0006810 GO:0008026 GO:0003676

GO:0004386 GO:0005524
Cluster-22178.70036 GO:0008237 GO:0008270
Cluster-22178.70037 GO:0048511 GO:0006118 GO:0045454 GO:0006357
GO:0015035 GO:0001104 GO:0016592
Cluster-22178.70039 GO:0005515
Cluster-22178.70040 GO:0005515 GO:0003677 GO:0019028
Cluster-22178.70041 GO:0042254 GO:0001522 GO:0030515 GO:0072588
Cluster-22178.70045 GO:0006118 GO:0006886 GO:0006449 GO:0006813
GO:0007165 GO:0016192 GO:0006415 GO:0009055 GO:0004871
GO:0005249 GO:0016149 GO:0020037 GO:0030955 GO:0005506
GO:0005840 GO:0016021 GO:0005737 GO:0005622 GO:0008076
GO:0030131 GO:0018444 GO:0016020
Cluster-22178.70046 GO:0006810 GO:0005215 GO:0005737 GO:0016021
Cluster-22178.70048 GO:0016021
Cluster-22178.70049 GO:0006886 GO:0009229 GO:0006605 GO:0006772
GO:0005524 GO:0004788 GO:0030975 GO:0016020
Cluster-22178.70051 GO:0016310 GO:0006468 GO:0015031 GO:0015992
GO:0009069 GO:0004672 GO:0015078 GO:0008384 GO:0005515
GO:0016773 GO:0005524 GO:0016021 GO:0005789 GO:0008385
GO:0033644 GO:0055036 GO:0016020
Cluster-22178.70054 GO:0006412 GO:0042254 GO:0003735 GO:0005622
GO:0005840
Cluster-22178.70056 GO:0045040 GO:0006281 GO:0006289 GO:0043161
GO:0003684 GO:0005515 GO:0005742
Cluster-22178.70057 GO:0005515
Cluster-22178.70060 GO:0015031 GO:0016020
Cluster-22178.70062 GO:0006807 GO:0006811 GO:0055085 GO:0006672
GO:0007165 GO:0004872 GO:0016811 GO:0005216 GO:0016021
GO:0016020
Cluster-22178.70063 GO:0006629 GO:0015991 GO:0006886 GO:0051276
GO:0015031 GO:0043248 GO:0006606 GO:0016192 GO:0046872
GO:0016820 GO:0005515 GO:0008270 GO:0005524 GO:0016887
GO:0008565 GO:0004222 GO:0061630 GO:0005737 GO:0030117

GO:0005634 GO:0005622 GO:0000221 GO:0005694
Cluster-22178.70064 GO:0006355 GO:0043401 GO:0008270 GO:0003677

Cluster-22178.70069 GO:0060478 GO:0006897 GO:0007342 GO:0023052
GO:0003950 GO:0005886 GO:0005634
Cluster-22178.70070 GO:0006606 GO:0015031 GO:0051276 GO:0043248
GO:0016192 GO:0055114 GO:0006397 GO:0006886 GO:0006629
GO:0015991 GO:0004222 GO:0008270 GO:0005524 GO:0008565
GO:0016491 GO:0016887 GO:0051087 GO:0046872 GO:0005515
GO:0016820 GO:0000221 GO:0005622 GO:0005694 GO:0000445
GO:0005737 GO:0030117 GO:0005634 GO:0019028
Cluster-22178.70072 GO:0006886 GO:0006629 GO:0015991 GO:0006606
GO:0015031 GO:0051276 GO:0043248 GO:0016192 GO:0046872
GO:0016820 GO:0005515 GO:0004222 GO:0008270 GO:0005524
GO:0016887 GO:0008565 GO:0016021 GO:0005737 GO:0030117
GO:0005634 GO:0000221 GO:0005622 GO:0005694
Cluster-22178.70073 GO:0003676
Cluster-22178.70079 GO:0008219 GO:0005739
Cluster-22178.70080 GO:0016055 GO:0007165 GO:0007411 GO:0008289
GO:0005515 GO:0005576 GO:0016021
Cluster-22178.70081 GO:0006351 GO:0050790 GO:0006123 GO:0006144
GO:0015992 GO:0015979 GO:0006206 GO:0005507 GO:0004129
GO:0003899 GO:0030234 GO:0003677 GO:0045277 GO:0009523
GO:0009539 GO:0016020 GO:0005730
Cluster-22178.70082 GO:0050790 GO:0006351 GO:0006144 GO:0006206
GO:0015992 GO:0015979 GO:0006123 GO:0004129 GO:0005507
GO:0003899 GO:0030234 GO:0003677 GO:0009523 GO:0009539
GO:0045277 GO:0005730 GO:0016020
Cluster-22178.70084 GO:0045995 GO:0006308 GO:0030334 GO:0030155
GO:0007165 GO:0007155 GO:0005102 GO:0008855 GO:0046872
GO:0005515 GO:0009318
Cluster-22178.70086 GO:0006352 GO:0015031 GO:0006886 GO:0008565
GO:0003677 GO:0005515 GO:0005737 GO:0016020

Cluster-22178.70088 GO:0008152 GO:0003824
Cluster-22178.70090 GO:0006355 GO:0006412 GO:0042254 GO:0003735
GO:0003677 GO:0003700 GO:0005667 GO:0005634 GO:0005840
Cluster-22178.70092 GO:0055114 GO:0045454 GO:0005509 GO:0016209
GO:0016491
Cluster-22178.70093 GO:0006464 GO:0016151
Cluster-22178.70094 GO:0042967 GO:0016568 GO:0008080 GO:0004402
GO:0000123
Cluster-22178.70095 GO:0005975 GO:0003697 GO:0042302 GO:0004553

Cluster-22178.70096 GO:0006097 GO:0006099 GO:0006090 GO:0006418
GO:0055114 GO:0000166 GO:0004812 GO:0004474 GO:0016627
GO:0005524
Cluster-22178.70097 GO:0007154 GO:0016020
Cluster-22178.70099 GO:0005515
Cluster-22178.70100 GO:0007275 GO:0007165 GO:0008152 GO:0016624
GO:0005179 GO:0047355 GO:0008270 GO:0016020 GO:0005576
Cluster-22178.70102 GO:0015986 GO:0006119 GO:0055114 GO:0015992
GO:0046961 GO:0016491 GO:0046933 GO:0000275 GO:0045259
Cluster-22178.70103 GO:0005515
Cluster-22178.70106 GO:0006468 GO:0005524 GO:0004672 GO:0016021
Cluster-22178.70107 GO:0006281 GO:0043087 GO:0035556 GO:0032012
GO:0005086 GO:0046872 GO:0008270 GO:0005201 GO:0005578
Cluster-22178.70109 GO:0006470 GO:0006570 GO:0006412 GO:0042254
GO:0009405 GO:0008270 GO:0004725 GO:0003735 GO:0008138
GO:0005622 GO:0005840 GO:0005576
Cluster-22178.70110 GO:0042254 GO:0006412 GO:0008033 GO:0055114
GO:0000105 GO:0003735 GO:0017150 GO:0050660 GO:0005622
GO:0005840
Cluster-22178.70111 GO:0006355 GO:0007264 GO:0051289 GO:0043565
GO:0005096 GO:0008140 GO:0003700 GO:0005667
Cluster-22178.70112 GO:0006470 GO:0006570 GO:0006094 GO:0006012
GO:0006000 GO:0043087 GO:0006013 GO:0006457 GO:0006096

GO:0006098 GO:0004725 GO:0005515 GO:0051082 GO:0003872
GO:0005945 GO:0016272 GO:0005639 GO:0016020
Cluster-22178.70115 GO:0016310 GO:0009405 GO:0006886 GO:0009069
GO:0004674 GO:0008536 GO:0005515 GO:0005576
Cluster-22178.70116 GO:0009405 GO:0051252 GO:0006402 GO:0003723
GO:0046872 GO:0004535 GO:0005634 GO:0005737
Cluster-22178.70118 GO:0006355 GO:0030833 GO:0034314 GO:0005198
GO:0043565 GO:0003700 GO:0005667 GO:0005885 GO:0005882
GO:0005856 GO:0019867
Cluster-22178.70120 GO:0005515
Cluster-22178.70121 GO:0005515
Cluster-22178.70123 GO:0030163 GO:0008270 GO:0005515
Cluster-22178.70124 GO:0030163 GO:0008270 GO:0005515
Cluster-22178.70126 GO:0055114 GO:0016114 GO:0009405 GO:0019835
GO:0016567 GO:0008270 GO:0046872 GO:0008685 GO:0016491
GO:0004842 GO:0019867
Cluster-22178.70128 GO:0070403 GO:0008168 GO:0000287 GO:0030976

Cluster-22178.70131 GO:0051082 GO:0005639
Cluster-22178.70132 GO:0035556 GO:0046872
Cluster-22178.70133 GO:0016973 GO:0071973 GO:0016192 GO:0006886
GO:0006935 GO:0005515 GO:0005198 GO:0003774 GO:0030117
GO:0005882 GO:0016020 GO:0009288 GO:0005643 GO:0016021
Cluster-22178.70134 GO:0006090 GO:0006633 GO:0051607 GO:0016973
GO:0003989 GO:0016021 GO:0009317 GO:0005643 GO:0019028
Cluster-22178.70136 GO:0006468 GO:0016773 GO:0005524 GO:0004672
GO:0016020
Cluster-22178.70138 GO:0046983
Cluster-22178.70140 GO:0005515 GO:0035091
Cluster-22178.70141 GO:0006364 GO:0007165 GO:0040007 GO:0008283
GO:0005515 GO:0008083 GO:0016021 GO:0032040 GO:0016020
Cluster-22178.70142 GO:0009271 GO:0006355 GO:0005198 GO:0005882
GO:0019028

Cluster-22178.70145 GO:0003950
Cluster-22178.70146 GO:0006412 GO:0042254 GO:0003735 GO:0005622
GO:0005840
Cluster-22178.70147 GO:0006468 GO:0004672 GO:0005524
Cluster-22178.70151 GO:0045116 GO:0016881 GO:0005524 GO:0008641

Cluster-22178.70152 GO:0008152 GO:0003824 GO:0051536 GO:0010181

Cluster-22178.70153 GO:0008152 GO:0051536 GO:0010181 GO:0003824

Cluster-22178.70155 GO:0090063 GO:0042710 GO:0009890 GO:0000226
GO:0035556 GO:0005515 GO:0005198 GO:0003774 GO:0005524
GO:0019028 GO:0016459 GO:0000922 GO:0019031 GO:0005815
Cluster-22178.70156 GO:0042981
Cluster-22178.70157 GO:0006811 GO:0045859 GO:0007268 GO:0007165
GO:0006397 GO:0004970 GO:0004860 GO:0005515 GO:0005576
GO:0005634 GO:0016020
Cluster-22178.70158 GO:0003676
Cluster-22178.70160 GO:0006355 GO:0005975 GO:0006006 GO:0055114
GO:0006072 GO:0006098 GO:0006749 GO:0006313 GO:0046486
GO:0046168 GO:0016645 GO:0050661 GO:0004345 GO:0003676
GO:0051287 GO:0016616 GO:0003677 GO:0004803 GO:0004367
GO:0009331 GO:0005622
Cluster-22178.70163 GO:0005515 GO:0042470 GO:0016020
Cluster-22178.70164 GO:0006570 GO:0055114 GO:0006470 GO:0050661
GO:0008138 GO:0005515 GO:0004499 GO:0050660 GO:0004725
Cluster-22178.70165 GO:0009405 GO:0005515 GO:0005576
Cluster-22178.70166 GO:0006357 GO:0003723 GO:0001104 GO:0005515
GO:0016592
Cluster-22178.70167 GO:0016567 GO:0004842
Cluster-22178.70168 GO:0008152 GO:0016491
Cluster-22178.70169 GO:0006955 GO:0007165 GO:0006334 GO:0030247
GO:0005044 GO:0005634

Cluster-22178.70171 GO:0006811 GO:0055085 GO:0005216 GO:0005515
GO:0012511 GO:0016021 GO:0016020
Cluster-22178.70173 GO:0045454 GO:0055114 GO:0016209 GO:0016491
GO:0016790
Cluster-22178.70175 GO:0007268 GO:0007275 GO:0007217 GO:0004867
GO:0005578
Cluster-22178.70176 GO:0006464 GO:0055114 GO:0006779 GO:0019354
GO:0008270 GO:0046872 GO:0016491 GO:0016151 GO:0051287
GO:0016616 GO:0003677 GO:0043115 GO:0005515
Cluster-22178.70177 GO:0000917 GO:0007049 GO:0006355 GO:0043093
GO:0003700 GO:0005667 GO:0005737
Cluster-22178.70179 GO:0015992 GO:0006412 GO:0042254 GO:0006118
GO:0006119 GO:0009055 GO:0030246 GO:0003735 GO:0008121
GO:0005622 GO:0005840
Cluster-22178.70180 GO:0048478 GO:0006974 GO:0007049 GO:0005634
Cluster-22178.70184 GO:0006355 GO:0006122 GO:0006508 GO:0005515
GO:0008270 GO:0043565 GO:0004222 GO:0003700 GO:0005634
GO:0005667 GO:0031012 GO:0005743 GO:0005750
Cluster-22178.70185 GO:0010508
Cluster-22178.70186 GO:0006888 GO:0006886 GO:0007165 GO:0007606
GO:0008270 GO:0005515 GO:0003723 GO:0004888 GO:0016020
GO:0030127
Cluster-22178.70188 GO:0006886 GO:0042720 GO:0005737
Cluster-22178.70190 GO:0006814 GO:0005248 GO:0005634 GO:0001518
Cluster-22178.70191 GO:0015684 GO:0006270 GO:0006810 GO:0019082
GO:0015721 GO:0070140 GO:0032183 GO:0046982 GO:0015093
GO:0004252 GO:0003924 GO:0005525 GO:0005215 GO:0005783
GO:0016020 GO:0005886 GO:0005634 GO:0016021
Cluster-22178.70192 GO:0005515
Cluster-22178.70193 GO:0016787
Cluster-22178.70197 GO:0007016 GO:0003779 GO:0005200 GO:0005925
GO:0005856 GO:0001726
Cluster-22178.70198 GO:0016787 GO:0046872

Cluster-22178.70199 GO:0046486 GO:0007205 GO:0035556 GO:0006260
GO:0009395 GO:0005515 GO:0016301 GO:0004143 GO:0005634
Cluster-22178.70200 GO:0005524 GO:0016887
Cluster-22178.70201 GO:0003676
Cluster-22178.70202 GO:0009396 GO:0055114 GO:0046487 GO:0009755
GO:0003824 GO:0016491 GO:0004488 GO:0005576
Cluster-22178.70203 GO:0007049
Cluster-22178.70204 GO:0019076 GO:0046718 GO:0006885 GO:0006812
GO:0055085 GO:0075521 GO:0015299 GO:0016021 GO:0019028
GO:0044163 GO:0030430 GO:0042025
Cluster-22178.70205 GO:0046488 GO:0016307 GO:0019867
Cluster-22178.70208 GO:0006820 GO:0022611 GO:0006333 GO:0006468
GO:0009432 GO:0005524 GO:0004672 GO:0005253 GO:0005887
GO:0005634
Cluster-22178.70209 GO:0016021
Cluster-22178.70210 GO:0006446 GO:0006413 GO:0005515 GO:0003743
GO:0031369 GO:0005852 GO:0005840
Cluster-22178.70213 GO:0046740 GO:0006810 GO:0042254 GO:0006412
GO:0006260 GO:0051082 GO:0003735 GO:0016798 GO:0016020
GO:0005886 GO:0005622 GO:0005840 GO:0016021
Cluster-22178.70215 GO:0006810 GO:0042254 GO:0006412 GO:0046740
GO:0006260 GO:0005515 GO:0003735 GO:0051082 GO:0005840
GO:0016021 GO:0005622 GO:0005886
Cluster-22178.70216 GO:0004222
Cluster-22178.70217 GO:0019211 GO:0005515 GO:0016746
Cluster-22178.70218 GO:0006811 GO:0006206 GO:0006144 GO:0006351
GO:0006816 GO:0006874 GO:0003676 GO:0005219 GO:0008270
GO:0003899 GO:0016021 GO:0005622 GO:0016020 GO:0005730
GO:0005634
Cluster-22178.70220 GO:0007049 GO:0006367 GO:0007067 GO:0000775
GO:0005672 GO:0005634
Cluster-22178.70222 GO:0042254 GO:0006412 GO:0003735 GO:0005840
GO:0005622

Cluster-22178.70224 GO:0006810 GO:0006886 GO:0006355 GO:0042967
GO:0003677 GO:0005515 GO:0042626 GO:0008080 GO:0005524
GO:0005783 GO:0016021
Cluster-22178.70225 GO:0006412 GO:0006281 GO:0006355 GO:0048870
GO:0042254 GO:0006457 GO:0006310 GO:0007264 GO:0010506
GO:0008053 GO:0006260 GO:0005515 GO:0000287 GO:0051082
GO:0003700 GO:0043565 GO:0003924 GO:0003735 GO:0005096
GO:0031514 GO:0005615 GO:0016021 GO:0005840 GO:0005741
GO:0005667 GO:0016272
Cluster-22178.70227 GO:0006508 GO:0051260 GO:0008234
Cluster-22178.70228 GO:0005515
Cluster-22178.70229 GO:0043044 GO:0003676 GO:0046872 GO:0016787
GO:0016586
Cluster-22178.70231 GO:0018342 GO:0030001 GO:0055085 GO:0046873
GO:0008318 GO:0016020
Cluster-22178.70232 GO:0042254 GO:0006412 GO:0016773 GO:0005515
GO:0003735 GO:0005622 GO:0005840
Cluster-22178.70234 GO:0006412 GO:0042254 GO:0046872 GO:0008270
GO:0003735 GO:0005515 GO:0061630 GO:0005840
Cluster-22178.70235 GO:0005515 GO:0035091
Cluster-22178.70236 GO:0051252 GO:0006810 GO:0007264 GO:0006281
GO:0006396 GO:0000723 GO:0003677 GO:0003723 GO:0016787
GO:0003678 GO:0005525 GO:0004525 GO:0005524 GO:0005657
Cluster-22178.70237 GO:0000723 GO:0006810 GO:0006281 GO:0006355
GO:0007264 GO:0005525 GO:0003678 GO:0005524 GO:0003677
GO:0008134 GO:0016787 GO:0005657 GO:0005667
Cluster-22178.70238 GO:0009636 GO:0015675 GO:0035444 GO:0006955
GO:0031342 GO:0046872 GO:0015099 GO:0015643 GO:0016021
GO:0019814
Cluster-22178.70240 GO:0006810 GO:0006418 GO:0000166 GO:0005524
GO:0004812 GO:0005886
Cluster-22178.70242 GO:0006355 GO:0008270 GO:0005096 GO:0005544
GO:0005515 GO:0005509 GO:0008023

Cluster-22178.70245 GO:0051603 GO:0006888 GO:0004298 GO:0005801 GO:0005839 GO:0005622
Cluster-22178.70247 GO:0006888 GO:0006260 GO:0051603 GO:0003887 GO:0003677 GO:0004298 GO:0005839 GO:0042575 GO:0005622 GO:0005801
Cluster-22178.70249 GO:0016787
Cluster-22178.70253 GO:0006351 GO:0006260 GO:0003677 GO:0003887 GO:0042575
Cluster-22178.70254 GO:0043085 GO:0016485 GO:0016021
Cluster-22178.70255 GO:0006811 GO:0043085 GO:0006914 GO:0009252 GO:0016485 GO:0006810 GO:0008092 GO:0016755 GO:0019898 GO:0016021 GO:0005737 GO:0016020
Cluster-22178.70256 GO:0043085 GO:0016485 GO:0016021
Cluster-22178.70258 GO:0007165 GO:0008283 GO:0008406 GO:0040007 GO:0005515 GO:0008083
Cluster-22178.70259 GO:0016192 GO:0016021
Cluster-22178.7026 GO:0016021
Cluster-22178.70260 GO:0006464 GO:0051082 GO:0016151 GO:0031072 GO:0005515
Cluster-22178.70262 GO:0006520 GO:0019752 GO:0016829 GO:0030170 GO:0016831
Cluster-22178.70266 GO:0006144 GO:0005515 GO:0003968 GO:0031379
Cluster-22178.70267 GO:0019079 GO:0006355 GO:0007264 GO:0035023 GO:0043087 GO:0003677 GO:0008026 GO:0005085 GO:0005089 GO:0005524
Cluster-22178.70268 GO:0019079 GO:0007264 GO:0006355 GO:0035023 GO:0043087 GO:0003677 GO:0008026 GO:0005085 GO:0005089 GO:0005524
Cluster-22178.70269 GO:0006511
Cluster-22178.70270 GO:0010951 GO:0007283 GO:0004866 GO:0003677 GO:0000786 GO:0005634
Cluster-22178.70271 GO:0005515
Cluster-22178.70272 GO:0006355 GO:0005634

Cluster-22178.70274 GO:0007186 GO:0007165 GO:0004871 GO:0005834
Cluster-22178.70277 GO:0016192 GO:0006886 GO:0051252 GO:0015991
GO:0009069 GO:0016310 GO:0043248 GO:0005198 GO:0005515
GO:0016820 GO:0004521 GO:0004674 GO:0045095 GO:0030117
GO:0000221
Cluster-22178.70280 GO:0042254 GO:0009058 GO:0006412 GO:0003735
GO:0030170 GO:0015934 GO:0005840
Cluster-22178.70281 GO:0008152 GO:0055114 GO:0000917 GO:0046487
GO:0045132 GO:0000226 GO:0009396 GO:0043093 GO:0003824
GO:0008092 GO:0005515 GO:0005509 GO:0004488 GO:0016787
GO:0000775 GO:0016021 GO:0005737 GO:0015630 GO:0005634
GO:0019898
Cluster-22178.70282 GO:0017004 GO:0016192 GO:0006810 GO:0007165
GO:0006357 GO:0015886 GO:0007010 GO:0005975 GO:0016853
GO:0043395 GO:0004872 GO:0005515 GO:0005198 GO:0001104
GO:0003779 GO:0005215 GO:0016020 GO:0016592 GO:0019028
GO:0005578 GO:0016021 GO:0019031
Cluster-22178.70283 GO:0003824 GO:0005515
Cluster-22178.70284 GO:0006355 GO:0046034 GO:0006869 GO:0042157
GO:0006464 GO:0006144 GO:0051082 GO:0008289 GO:0016151
GO:0003700 GO:0046872 GO:0043565 GO:0004017 GO:0008270
GO:0005576 GO:0005667 GO:0005815
Cluster-22178.70285 GO:0006508 GO:0006886 GO:0009116 GO:0048280
GO:0071949 GO:0005515 GO:0003824 GO:0004222 GO:0005737
GO:0000139
Cluster-22178.70287 GO:0006355 GO:0006479 GO:0006351 GO:0009451
GO:0031167 GO:0008033 GO:0008152 GO:0006400 GO:0046500
GO:0006367 GO:0006554 GO:0006464 GO:0006260 GO:0018024
GO:0003887 GO:0004719 GO:0008168 GO:0008176 GO:0003682
GO:0003677 GO:0008270 GO:0008171 GO:0005672 GO:0005634
GO:0042575 GO:0000775 GO:0032040 GO:0000785
Cluster-22178.70288 GO:0006355 GO:0006351 GO:0006479 GO:0009451
GO:0031167 GO:0008033 GO:0008152 GO:0046500 GO:0006400

GO:0006367 GO:0006554 GO:0006464 GO:0006260 GO:0018024
GO:0003887 GO:0004719 GO:0008168 GO:0008176 GO:0003682
GO:0003677 GO:0008270 GO:0008171 GO:0005672 GO:0005634
GO:0042575 GO:0032040 GO:0000775 GO:0000785
Cluster-22178.70289 GO:0030001 GO:0042254 GO:0055085 GO:0046873
GO:0005730 GO:0016020
Cluster-22178.7029 GO:0090063 GO:0000226 GO:0005815 GO:0000922
Cluster-22178.70291 GO:0005515
Cluster-22178.70295 GO:0006367 GO:0006084 GO:0010506 GO:0006464
GO:0006914 GO:0006886 GO:0010508 GO:0006355 GO:0030001
GO:0007049 GO:0055114 GO:0008113 GO:0003824 GO:0046872
GO:0005783 GO:0005669 GO:0016021 GO:0005615
Cluster-22178.70297 GO:0006355 GO:0015991 GO:0045859 GO:0007017
GO:0050829 GO:0007018 GO:0015992 GO:0050830 GO:0006810
GO:0003924 GO:0005215 GO:0005525 GO:0015078 GO:0003777
GO:0008134 GO:0005198 GO:0005524 GO:0016887 GO:0004860
GO:0005874 GO:0019031 GO:0005576 GO:0019028 GO:0033179
GO:0030286 GO:0005667 GO:0016020
Cluster-22178.70298 GO:0019058 GO:0046872 GO:0005887
Cluster-22178.70301 GO:0051603 GO:0004298 GO:0005839
Cluster-22178.70303 GO:0006397 GO:0046660 GO:0005634
Cluster-22178.70307 GO:0006073 GO:0016762 GO:0048046 GO:0005618
Cluster-22178.70310 GO:0003723 GO:0000178
Cluster-22178.70311 GO:0008152
Cluster-22178.70312 GO:0008152
Cluster-22178.70313 GO:0000226 GO:0030168 GO:0051258 GO:0007165
GO:0090063 GO:0005102 GO:0030674 GO:0000922 GO:0005577
GO:0005815
Cluster-22178.70314 GO:0005515
Cluster-22178.70315 GO:0042742
Cluster-22178.70316 GO:0019955 GO:0016020
Cluster-22178.70317 GO:0006886 GO:0016032 GO:0000160 GO:0016310
GO:0006888 GO:0003677 GO:0000155 GO:0046983 GO:0008270

GO:0030127 GO:0019028 GO:0016021 GO:0009365
Cluster-22178.70318 GO:0006886 GO:0016032 GO:0000160 GO:0016310
GO:0006888 GO:0003677 GO:0000155 GO:0046983 GO:0008270
GO:0030127 GO:0019028 GO:0016021 GO:0009365
Cluster-22178.70320 GO:0005515 GO:0016740
Cluster-22178.70321 GO:0017025
Cluster-22178.70322 GO:0006355 GO:0006281 GO:0003677 GO:0046872
GO:0008270
Cluster-22178.70323 GO:0007165 GO:0051028 GO:0070206 GO:0015992
GO:0007034 GO:0006914 GO:0006606 GO:0007218 GO:0015991
GO:0030474 GO:0010508 GO:0006308 GO:0048309 GO:0031144
GO:0071630 GO:0005179 GO:0003677 GO:0008855 GO:0005198
GO:0005200 GO:0015078 GO:0005823 GO:0005576 GO:0005634
GO:0019028 GO:0009318 GO:0033179 GO:0005856 GO:0019031
Cluster-22178.70326 GO:0007165 GO:0042254 GO:0006357 GO:0007169
GO:0051056 GO:0006355 GO:0006468 GO:0006412 GO:0004714
GO:0008201 GO:0003735 GO:0005524 GO:0005096 GO:0004872
GO:0042393 GO:0001104 GO:0046872 GO:0016592 GO:0005622
GO:0005887 GO:0005840
Cluster-22178.70327 GO:0005524 GO:0005515 GO:0016301 GO:0008289

Cluster-22178.70329 GO:0006955 GO:0006367 GO:0030153 GO:0006357
GO:0004252 GO:0001104 GO:0015643 GO:0019814 GO:0016592
GO:0005672 GO:0005886
Cluster-22178.70330 GO:0006357 GO:0051056 GO:0007169 GO:0042254
GO:0007165 GO:0006468 GO:0006355 GO:0006412 GO:0005096
GO:0005524 GO:0004872 GO:0003735 GO:0008201 GO:0004714
GO:0046872 GO:0001104 GO:0042393 GO:0005622 GO:0005887
GO:0016592 GO:0005840
Cluster-22178.70331 GO:0006468 GO:0005524 GO:0004672
Cluster-22178.70334 GO:0006955 GO:0006367 GO:0030153 GO:0006357
GO:0001104 GO:0004252 GO:0015643 GO:0019814 GO:0016592
GO:0005672 GO:0005886

Cluster-22178.70336 GO:0006144 GO:0006820 GO:0006835 GO:0006812
GO:0007155 GO:0003877 GO:0005094 GO:0017153 GO:0005737
GO:0016020
Cluster-22178.70338 GO:0007155 GO:0006812 GO:0006835 GO:0006820
GO:0006144 GO:0017153 GO:0005094 GO:0003877 GO:0016020
GO:0005737
Cluster-22178.70342 GO:0042254 GO:0006412 GO:0003735 GO:0005622
GO:0005840
Cluster-22178.70343 GO:0031110 GO:0006308 GO:0006448 GO:0006414
GO:0008855 GO:0003746 GO:0009318 GO:0005840
Cluster-22178.70348 GO:0045333 GO:0008643 GO:0004659 GO:0008982
GO:0016021 GO:0009357 GO:0005634 GO:0005739
Cluster-22178.70349 GO:0006915 GO:0006118 GO:0009055 GO:0020037
GO:0005622 GO:0019028
Cluster-22178.70350 GO:0046872
Cluster-22178.70353 GO:0016255 GO:0042765
Cluster-22178.70354 GO:0015986 GO:0006118 GO:0006364 GO:0015992
GO:0051536 GO:0009055 GO:0015078 GO:0000276 GO:0016021
GO:0032040
Cluster-22178.70355 GO:0016567 GO:0006914 GO:0006308 GO:0035091
GO:0005515 GO:0008855 GO:0008270 GO:0046872 GO:0004842
GO:0009318 GO:0005622 GO:0005680
Cluster-22178.70356 GO:0006355
Cluster-22178.70357 GO:0055114 GO:0016491
Cluster-22178.70358 GO:0006850 GO:0005743
Cluster-22178.70359 GO:0016310 GO:0009069 GO:0004674 GO:0005515

Cluster-22178.70361 GO:0007165 GO:0005179 GO:0005576
Cluster-22178.70364 GO:0006308 GO:0009307 GO:0003677 GO:0016787
GO:0000287 GO:0009036 GO:0009359
Cluster-22178.70365 GO:0019079 GO:0006310 GO:0006771 GO:0002949
GO:0006281 GO:0019497 GO:0009378 GO:0003993 GO:0003724
GO:0005524 GO:0016887 GO:0005515 GO:0003723 GO:0003678

GO:0009379 GO:0005657
Cluster-22178.70366 GO:0006412 GO:0042254 GO:0008152 GO:0003735
GO:0016491 GO:0005840 GO:0005622
Cluster-22178.70368 GO:0005515
Cluster-22178.70369 GO:0016579 GO:0036459
Cluster-22178.70370 GO:0016579 GO:0036459
Cluster-22178.70372 GO:0007165 GO:0006810 GO:0030168 GO:0006355
GO:0048870 GO:0051258 GO:0048511 GO:0030674 GO:0005102
GO:0005515 GO:0005198 GO:0043565 GO:0003700 GO:0005667
GO:0005577 GO:0019028 GO:0031514 GO:0016021
Cluster-22178.70376 GO:0045892 GO:0005515 GO:0005634
Cluster-22178.70377 GO:0045892 GO:0005515 GO:0005634
Cluster-22178.70378 GO:0045892 GO:0005515 GO:0005634
Cluster-22178.70380 GO:0003677 GO:0003676 GO:0005524 GO:0016787

Cluster-22178.70381 GO:0005615
Cluster-22178.70387 GO:0000226 GO:0046872 GO:0000166 GO:0015630
GO:0016020
Cluster-22178.70389 GO:0009405 GO:0006351 GO:0006807 GO:0006206
GO:0015031 GO:0006144 GO:0009252 GO:0003677 GO:0003899
GO:0004356 GO:0005730
Cluster-22178.70391 GO:0006351 GO:0006144 GO:0006206 GO:0006825
GO:0003899 GO:0016531 GO:0003677 GO:0005507 GO:0005634
GO:0005730 GO:0005758
Cluster-22178.70392 GO:0006810 GO:0006811 GO:0055085 GO:0005216
GO:0005215 GO:0022857 GO:0016020 GO:0016021
Cluster-22178.70398 GO:0015986 GO:0001522 GO:0009451 GO:0015992
GO:0000045 GO:0003723 GO:0005515 GO:0015078 GO:0009982
GO:0045263 GO:0005737
Cluster-22178.70399 GO:0007186 GO:0043395 GO:0005515 GO:0004930
GO:0005578 GO:0016021 GO:0016020
Cluster-22178.70400 GO:0000045 GO:0009451 GO:0001522 GO:0003723
GO:0005515 GO:0009982 GO:0005737

Cluster-22178.70402 GO:0006206 GO:0006144 GO:0006241 GO:0006355 GO:0006228 GO:0006508 GO:0006165 GO:0006886 GO:0006183 GO:0004550 GO:0004222 GO:0008536 GO:0005634

Cluster-22178.70409 GO:0003924

Cluster-22178.70411 GO:0009069 GO:0006468 GO:0016310 GO:0004672 GO:0005524 GO:0004674

Cluster-22178.70412 GO:0006355 GO:0009405 GO:0006397 GO:0006308 GO:0006302 GO:0006357 GO:0043087 GO:0006606 GO:0005515 GO:0001104 GO:0003700 GO:0008855 GO:0005634 GO:0009318 GO:0005667 GO:0016020 GO:0016592

Cluster-22178.70413 GO:0006355 GO:0005634

Cluster-22178.70414 GO:0006468 GO:0005524 GO:0004672 GO:0005515

Cluster-22178.70415 GO:0015979 GO:0005515 GO:0009523 GO:0009539 GO:0016020

Cluster-22178.70416 GO:0006355 GO:0043565 GO:0003700 GO:0005667 GO:0005634

Cluster-22178.70417 GO:0007156 GO:0051252 GO:0006396 GO:0003723 GO:0004525 GO:0005509 GO:0016020

Cluster-22178.70419 GO:0006355 GO:0046983

Cluster-22178.70421 GO:0006355 GO:0006265 GO:0016579 GO:0003677 GO:0005515 GO:0003916 GO:0036459 GO:0003700 GO:0005694 GO:0005667

Cluster-22178.70422 GO:0006206 GO:0006464 GO:0006144 GO:0006281 GO:0006351 GO:0008270 GO:0046872 GO:0016151 GO:0003677 GO:0003899 GO:0005515 GO:0005730

Cluster-22178.70424 GO:0007165 GO:0008283 GO:0040007 GO:0008083

Cluster-22178.70425 GO:0008270

Cluster-22178.70426 GO:0007165 GO:0008283 GO:0040007 GO:0008083

Cluster-22178.70427 GO:0006511 GO:0031625 GO:0005515

Cluster-22178.70431 GO:0035556 GO:0046872 GO:0005515 GO:0003677

Cluster-22178.70433 GO:0006351 GO:0006144 GO:0006206 GO:0003899 GO:0003677 GO:0005730
Cluster-22178.70434 GO:0035556 GO:0046872 GO:0003677 GO:0005515

Cluster-22178.70435 GO:0003676 GO:0005921
Cluster-22178.70436 GO:0003676
Cluster-22178.70438 GO:0006468 GO:0004672 GO:0005524
Cluster-22178.7044 GO:0006285 GO:0006281 GO:0009236 GO:0006289 GO:0006824 GO:0006308 GO:0006284 GO:0003677 GO:0003684 GO:0008534 GO:0015087 GO:0016020
Cluster-22178.70440 GO:0006886 GO:0016192 GO:0005515 GO:0005198 GO:0030117 GO:0030126
Cluster-22178.70442 GO:0016567 GO:0006486 GO:0003676 GO:0004842 GO:0016757 GO:0046872 GO:0005524 GO:0008270 GO:0005515 GO:0003677 GO:0016818 GO:0016787 GO:0005680
Cluster-22178.70444 GO:0009190 GO:0006171 GO:0035556 GO:0006144 GO:0005515 GO:0004016 GO:0016849 GO:0051537 GO:0005886
Cluster-22178.70452 GO:0046872 GO:0005515
Cluster-22178.70454 GO:0007067 GO:0007165 GO:0035091 GO:0005515 GO:0072686 GO:0042729
Cluster-22178.70456 GO:0043087 GO:0005515 GO:0016020
Cluster-22178.70457 GO:0043039 GO:0016876 GO:0005524 GO:0008270

Cluster-22178.70458 GO:0043039 GO:0016876 GO:0005524 GO:0008270

Cluster-22178.70461 GO:0022900 GO:0005739 GO:0005747
Cluster-22178.70462 GO:0006355 GO:0003700 GO:0003677 GO:0005667
Cluster-22178.70463 GO:0051028 GO:0019049 GO:0048309 GO:0005515 GO:0005096
Cluster-22178.70464 GO:0006281 GO:0006310 GO:0005524 GO:0016887 GO:0003678 GO:0009378 GO:0005657 GO:0016021 GO:0009379
Cluster-22178.70470 GO:0006260 GO:0006270 GO:0000398 GO:0006351

GO:0006468 GO:0005524 GO:0008270 GO:0003677 GO:0004672
GO:0003887 GO:0005681 GO:0019031 GO:0005737 GO:0042575
GO:0019028 GO:0005634
Cluster-22178.70471 GO:0000746 GO:0003676 GO:0003677
Cluster-22178.70472 GO:0000042 GO:0006281 GO:0046034 GO:0006144
GO:0004017 GO:0005515 GO:0003677 GO:0008270 GO:0046872
Cluster-22178.70473 GO:0007155 GO:0042398 GO:0006750 GO:0006749
GO:0004357 GO:0017109 GO:0016021
Cluster-22178.70474 GO:0051653 GO:0030036 GO:0006928 GO:0008356
GO:0051321 GO:0033206 GO:0016482 GO:0007163 GO:0003779
GO:0005524 GO:0030478 GO:0005885 GO:0042995
Cluster-22178.70477 GO:0035556 GO:0008270 GO:0005506 GO:0005515
GO:0003677
Cluster-22178.70478 GO:0035556 GO:0008270 GO:0005506 GO:0005515
GO:0003677
Cluster-22178.70479 GO:0035556 GO:0005506 GO:0008270 GO:0005515
GO:0003677
Cluster-22178.70480 GO:0016567 GO:0004842 GO:0046872 GO:0008270

Cluster-22178.70481 GO:0055085 GO:0006367 GO:0008063 GO:0030001
GO:0046872 GO:0046873 GO:0016020 GO:0005672
Cluster-22178.70485 GO:0007018 GO:0007017 GO:0008017 GO:0003777
GO:0005524 GO:0045298 GO:0005874 GO:0030430
Cluster-22178.70486 GO:0090200 GO:0043065 GO:0097193 GO:0009405
GO:0005515 GO:0005576 GO:0005739
Cluster-22178.70488 GO:0003774 GO:0005524 GO:0005506 GO:0005515
GO:0016459
Cluster-22178.70492 GO:0007155 GO:0005515 GO:0016021
Cluster-22178.70493 GO:0046034 GO:0006144 GO:0006888 GO:0006464
GO:0006886 GO:0006355 GO:0016151 GO:0046872 GO:0008270
GO:0004017 GO:0030127 GO:0005634
Cluster-22178.70494 GO:0006979 GO:0006804 GO:0015947 GO:0055114
GO:0006568 GO:0020037 GO:0004096

Cluster-22178.70495 GO:0006468 GO:0006355 GO:0030255 GO:0035556 GO:0004672 GO:0005524 GO:0016773 GO:0005634 GO:0005668 GO:0016020
Cluster-22178.70496 GO:0007264 GO:0015684 GO:0006626 GO:0000287 GO:0016829 GO:0015093 GO:0003924 GO:0005525 GO:0030976 GO:0005741 GO:0016021
Cluster-22178.70497 GO:0015684 GO:0007264 GO:0006626 GO:0000287 GO:0005525 GO:0030976 GO:0016829 GO:0015093 GO:0003924 GO:0005741 GO:0016021
Cluster-22178.70498 GO:0009058 GO:0030170
Cluster-22178.70502 GO:0006189 GO:0030255 GO:0006355 GO:0006468 GO:0035556 GO:0005515 GO:0004672 GO:0005524 GO:0016773 GO:0005668 GO:0016020 GO:0005634
Cluster-22178.70503 GO:0042981 GO:0040007 GO:0006955 GO:0043087 GO:0048193 GO:0008283 GO:0007264 GO:0006869 GO:0007165 GO:0005319 GO:0008083 GO:0005515 GO:0005134 GO:0005085 GO:0005893 GO:0005576 GO:0005887 GO:0016020
Cluster-22178.70504 GO:0042981 GO:0040007 GO:0006955 GO:0043087 GO:0048193 GO:0008283 GO:0007264 GO:0006869 GO:0007165 GO:0008083 GO:0005319 GO:0005515 GO:0005134 GO:0005085 GO:0005893 GO:0005576 GO:0005887 GO:0016020
Cluster-22178.70505 GO:0042981 GO:0040007 GO:0006955 GO:0043087 GO:0048193 GO:0008283 GO:0007264 GO:0006869 GO:0007165 GO:0005319 GO:0008083 GO:0005515 GO:0005134 GO:0005085 GO:0005893 GO:0005576 GO:0005887 GO:0016020
Cluster-22178.70507 GO:0007131 GO:0005515 GO:0003677 GO:0004867

Cluster-22178.70508 GO:0000226 GO:0016114 GO:0006694 GO:0008152 GO:0016624 GO:0008661 GO:0003824 GO:0030976 GO:0015630
Cluster-22178.7051 GO:0000398 GO:0042254 GO:0006412 GO:0003735 GO:0005634 GO:0015934 GO:0005840
Cluster-22178.70510 GO:0055114 GO:0006950 GO:0008270 GO:0016638 GO:0003697

Cluster-22178.70512 GO:0006355 GO:0003700 GO:0005667
Cluster-22178.70513 GO:0046907 GO:0006367 GO:0005672
Cluster-22178.70514 GO:0007186 GO:0007165 GO:0015986 GO:0055085
GO:0015992 GO:0004871 GO:0015078 GO:0003924 GO:0031683
GO:0019001 GO:0000276
Cluster-22178.70516 GO:0030001 GO:0055085 GO:0046873 GO:0016021
GO:0016020
Cluster-22178.70518 GO:0003676 GO:0031224
Cluster-22178.70520 GO:0006144 GO:0055114 GO:0009308 GO:0046034
GO:0046872 GO:0016638 GO:0004017 GO:0003677 GO:0042597
Cluster-22178.70521 GO:0046034 GO:0055114 GO:0006144 GO:0009308
GO:0006351 GO:0003676 GO:0046872 GO:0008270 GO:0004017
GO:0003677 GO:0016638 GO:0042597
Cluster-22178.70522 GO:0006281 GO:0008270 GO:0046872
Cluster-22178.70523 GO:0016021
Cluster-22178.70524 GO:0009847 GO:0007264 GO:0006508 GO:0043087
GO:0005515 GO:0008233 GO:0030250 GO:0005096 GO:0005576
Cluster-22178.70527 GO:0006281 GO:0006308 GO:0008852 GO:0003677
GO:0005515
Cluster-22178.70530 GO:0051302 GO:0006448 GO:0006414 GO:0006355
GO:0003746 GO:0003677 GO:0005840 GO:0005853
Cluster-22178.70531 GO:0016773 GO:0003676
Cluster-22178.70533 GO:0016773
Cluster-22178.70534 GO:0006260 GO:0006508 GO:0016032 GO:0000398
GO:0004197 GO:0000808 GO:0005634
Cluster-22178.70535 GO:0000398 GO:0016032 GO:0006508 GO:0006260
GO:0004197 GO:0005634 GO:0000808
Cluster-22178.70538 GO:0016310 GO:0006096 GO:0046856 GO:0015976
GO:0006094 GO:0034597 GO:0004618
Cluster-22178.70539 GO:0006260 GO:0006351 GO:0003677 GO:0003887
GO:0042575
Cluster-22178.70540 GO:0006810 GO:0006950 GO:0016021 GO:0005921
Cluster-22178.70541 GO:0006885 GO:0006812 GO:0055085 GO:0015299

GO:0016021
Cluster-22178.70542 GO:0006355 GO:0009725 GO:0003677 GO:0005515
GO:0005634
Cluster-22178.70543 GO:0005515 GO:0042302 GO:0005549
Cluster-22178.70545 GO:0043401 GO:0006355 GO:0003700 GO:0008270
GO:0043565 GO:0016021 GO:0005634 GO:0005667
Cluster-22178.70546 GO:0006633 GO:0031412 GO:0019482 GO:0050080
GO:0031411
Cluster-22178.70549 GO:0006355 GO:0006281 GO:0000184 GO:0055114
GO:0003677 GO:0046872 GO:0005524 GO:0008270 GO:0047134
GO:0016972 GO:0004386 GO:0005737
Cluster-22178.70551 GO:0003723 GO:0000178
Cluster-22178.70552 GO:0051603 GO:0006511 GO:0004175 GO:0004298
GO:0005839 GO:0019773
Cluster-22178.70553 GO:0006568 GO:0006552 GO:0006631 GO:0018874
GO:0006550 GO:0055114 GO:0006554 GO:0006574 GO:0015074
GO:0008152 GO:0006633 GO:0003857 GO:0003677 GO:0016491
Cluster-22178.70554 GO:0005975 GO:0004553 GO:0042597
Cluster-22178.70555 GO:0005975 GO:0004553 GO:0042597
Cluster-22178.70556 GO:0006355 GO:0006352 GO:0003677 GO:0005515
GO:0016987 GO:0003700 GO:0008270 GO:0005667
Cluster-22178.70557 GO:0006525 GO:0006560 GO:0055114 GO:0018401
GO:0004656 GO:0016491 GO:0016702 GO:0005783
Cluster-22178.70559 GO:0043087 GO:0035023 GO:0005089
Cluster-22178.70561 GO:0006570 GO:0006298 GO:0009405 GO:0006355
GO:0006470 GO:0005515 GO:0004725 GO:0005524 GO:0005634
Cluster-22178.70563 GO:0006468 GO:0006810 GO:0015696 GO:0016787
GO:0008519 GO:0005524 GO:0004672 GO:0016020 GO:0031224
GO:0016021 GO:0005794
Cluster-22178.70566 GO:0006355 GO:0008270 GO:0003677
Cluster-22178.70567 GO:0015986 GO:0045766 GO:0043123 GO:0016567
GO:0015992 GO:0043066 GO:0003723 GO:0043130 GO:0004842
GO:0015078 GO:0000276

Cluster-22178.70570 GO:0006357 GO:0001104 GO:0016592 GO:0016020
Cluster-22178.70572 GO:0042254 GO:0001522
Cluster-22178.70573 GO:0006260 GO:0006744 GO:0006914 GO:0007186
GO:0043093 GO:0007049 GO:0006351 GO:0000917 GO:0006355
GO:0004930 GO:0003677 GO:0003700 GO:0008813 GO:0043565
GO:0003887 GO:0005815 GO:0005667 GO:0016021 GO:0005737
GO:0042575
Cluster-22178.70575 GO:0042254 GO:0006412 GO:0003735 GO:0015934
GO:0005840
Cluster-22178.70576 GO:0006810 GO:0055085 GO:0015979 GO:0005215
GO:0009579 GO:0016020 GO:0009522 GO:0016021
Cluster-22178.70577 GO:0006355 GO:0006351 GO:0000398 GO:0006260
GO:0003682 GO:0005515 GO:0003677 GO:0003887 GO:0008270
GO:0005634 GO:0042575 GO:0005681 GO:0000785 GO:0000775
Cluster-22178.70580 GO:0042302 GO:0005515 GO:0005549
Cluster-22178.70581 GO:0019530 GO:0055114 GO:0046439 GO:0006534
GO:0017172 GO:0005506
Cluster-22178.70582 GO:0006508 GO:0035444 GO:0015675 GO:0004252
GO:0015099 GO:0046872 GO:0030288 GO:0016021
Cluster-22178.70584 GO:0006825 GO:0016531 GO:0005507 GO:0005758
Cluster-22178.70585 GO:0007165 GO:0009069 GO:0006468 GO:0008152
GO:0016310 GO:0015016 GO:0005515 GO:0016787 GO:0016746
GO:0004674 GO:0005096
Cluster-22178.70586 GO:0007067 GO:0051382 GO:0003677
Cluster-22178.70587 GO:0005515
Cluster-22178.70588 GO:0005515 GO:0016021
Cluster-22178.70589 GO:0016310 GO:0006886 GO:0015991 GO:0006468
GO:0006260 GO:0006914 GO:0015031 GO:0015992 GO:0009069
GO:0015078 GO:0004674 GO:0008565 GO:0005524 GO:0005737
GO:0033179 GO:0016020
Cluster-22178.70590 GO:0000746 GO:0009512
Cluster-22178.70591 GO:0000746
Cluster-22178.70592 GO:0005515

Cluster-22178.70593 GO:0046487 GO:0055114 GO:0009396 GO:0003824
GO:0004488 GO:0004672
Cluster-22178.70595 GO:0005515
Cluster-22178.70596 GO:0005515
Cluster-22178.70597 GO:0006468 GO:0006355 GO:0004672 GO:0003700
GO:0005634 GO:0005667
Cluster-22178.70598 GO:0006355 GO:0042393
Cluster-22178.70599 GO:0015675 GO:0035444 GO:0046872 GO:0015099
GO:0016021
Cluster-22178.70602 GO:0045893 GO:0042025
Cluster-22178.70603 GO:0035556 GO:0008053 GO:0003924 GO:0005741
GO:0016021
Cluster-22178.70604 GO:0016021
Cluster-22178.70605 GO:0006886 GO:0015031 GO:0005975 GO:0005509
GO:0008565 GO:0016773 GO:0016020 GO:0005737
Cluster-22178.70607 GO:0042254 GO:0006412 GO:0006814 GO:0042773
GO:0006744 GO:0006120 GO:0015992 GO:0003735 GO:0008137
GO:0005743 GO:0005840 GO:0005622
Cluster-22178.70610 GO:0006520 GO:0055114 GO:0009058 GO:0016829
GO:0030170 GO:0016491
Cluster-22178.70612 GO:0006888 GO:0055085 GO:0006810 GO:0005215
GO:0022857 GO:0005515 GO:0005622 GO:0019867 GO:0016021
GO:0005801 GO:0016020
Cluster-22178.70613 GO:0055085 GO:0006888 GO:0006810 GO:0022857
GO:0005215 GO:0005515 GO:0016021 GO:0019867 GO:0005801
GO:0005622 GO:0016020
Cluster-22178.70614 GO:0003677 GO:0005515
Cluster-22178.70617 GO:0006270 GO:0019028
Cluster-22178.70620 GO:0006836 GO:0006457 GO:0006812 GO:0005515
GO:0005328 GO:0016021
Cluster-22178.70622 GO:0005515
Cluster-22178.70623 GO:0005515
Cluster-22178.70624 GO:0016012 GO:0016021

Cluster-22178.70626 GO:0005515
Cluster-22178.70628 GO:0055114 GO:0008152 GO:0016491
Cluster-22178.70629 GO:0019752 GO:0006520 GO:0006729 GO:0009058
GO:0016829 GO:0016831 GO:0008124 GO:0016740 GO:0030170
Cluster-22178.70632 GO:0007156 GO:0005509 GO:0016020
Cluster-22178.70634 GO:0006418 GO:0043039 GO:0016876 GO:0004812
GO:0005524 GO:0000166
Cluster-22178.70635 GO:0003676
Cluster-22178.70637 GO:0005515
Cluster-22178.70638 GO:0015992 GO:0006777 GO:0006123 GO:0006810
GO:0005524 GO:0005525 GO:0004129 GO:0045277 GO:0005743
Cluster-22178.70640 GO:0016021
Cluster-22178.70641 GO:0006506 GO:0009058 GO:0019752 GO:0006520
GO:0006729 GO:0008124 GO:0016746 GO:0016740 GO:0030170
GO:0016829 GO:0016831 GO:0005789 GO:0016021
Cluster-22178.70642 GO:0015031 GO:0006606 GO:0016068 GO:0008565
GO:0005515 GO:0005737 GO:0016021 GO:0005634
Cluster-22178.70645 GO:0008152 GO:0008168
Cluster-22178.70646 GO:0030866 GO:0006198 GO:0003779 GO:0008092
GO:0004115 GO:0005198 GO:0005856
Cluster-22178.70647 GO:0005509
Cluster-22178.70650 GO:0005515
Cluster-22178.70655 GO:0005515 GO:0008201
Cluster-22178.70658 GO:0019354 GO:0006464 GO:0055114 GO:0006779
GO:0051287 GO:0003677 GO:0016616 GO:0043115 GO:0005515
GO:0046872 GO:0008270 GO:0016829 GO:0016491 GO:0016151
Cluster-22178.70660 GO:0000287 GO:0070403 GO:0005515 GO:0030976

Cluster-22178.70661 GO:0007165 GO:0006886 GO:0005515 GO:0005783
GO:0016021
Cluster-22178.70664 GO:0045490 GO:0005515 GO:0047487
Cluster-22178.70667 GO:0015694 GO:0006955 GO:0031342 GO:0035444
GO:0009636 GO:0015675 GO:0015097 GO:0015643 GO:0015099

GO:0046872 GO:0016020 GO:0019814 GO:0016021
Cluster-22178.70668 GO:0006355 GO:0007165 GO:0006810 GO:0001522
GO:0009451 GO:0035257 GO:0003723 GO:0003713 GO:0005515
GO:0046983 GO:0005215 GO:0009982 GO:0005667 GO:0005634
GO:0016021
Cluster-22178.70669 GO:0006955 GO:0031342 GO:0015675 GO:0009636
GO:0035444 GO:0015643 GO:0046872 GO:0015099 GO:0019814
GO:0016021
Cluster-22178.70671 GO:0030976 GO:0070403 GO:0008168 GO:0000287

Cluster-22178.70673 GO:0006468 GO:0045893 GO:0007275 GO:0008270
GO:0005524 GO:0004672 GO:0046983
Cluster-22178.70674 GO:0006306 GO:0031167 GO:0032775 GO:0003677
GO:0008170 GO:0008168 GO:0009007
Cluster-22178.70680 GO:0006397 GO:0003676
Cluster-22178.70681 GO:0019031
Cluster-22178.70682 GO:0006626 GO:0003824 GO:0005741
Cluster-22178.70683 GO:0006024 GO:0015012 GO:0016021
Cluster-22178.70686 GO:0030150 GO:0006144 GO:0015976 GO:0016579
GO:0006094 GO:0006096 GO:0008270 GO:0036459 GO:0003824
GO:0004743 GO:0000287 GO:0030955 GO:0005742
Cluster-22178.70687 GO:0051260
Cluster-22178.70688 GO:0000045 GO:0005737
Cluster-22178.70689 GO:0006955 GO:0007165 GO:0005164 GO:0016020
Cluster-22178.70690 GO:0061630
Cluster-22178.70692 GO:0007264 GO:0030623 GO:0005525
Cluster-22178.70693 GO:0043547 GO:0005096
Cluster-22178.70694 GO:0007264 GO:0003924 GO:0005525 GO:0005634
Cluster-22178.70699 GO:0006812 GO:0055085 GO:0008324 GO:0005886
GO:0016021
Cluster-22178.70700 GO:0006468 GO:0005975 GO:0055114 GO:0034080
GO:0016638 GO:0016868 GO:0005198 GO:0046872 GO:0005524
GO:0004672 GO:0046983 GO:0000775

Cluster-22178.70703 GO:0006468 GO:0006816 GO:0006874 GO:0006397
GO:0051252 GO:0005524 GO:0004672 GO:0046872 GO:0005219
GO:0004540 GO:0005622 GO:0016021
Cluster-22178.70706 GO:0006541 GO:0005515 GO:0004359
Cluster-22178.70708 GO:0043087 GO:0009297 GO:0006376 GO:0003729
GO:0005685 GO:0019867
Cluster-22178.70710 GO:0046486 GO:0006071 GO:0004371 GO:0003677
GO:0005515 GO:0016829 GO:0008270 GO:0005524 GO:0043565
GO:0005634 GO:0005783
Cluster-22178.70712 GO:0006355 GO:0043401 GO:0043565 GO:0008270
GO:0003700 GO:0005667 GO:0005634
Cluster-22178.70714 GO:0071805 GO:0019089 GO:0009405 GO:0006904
GO:0006508 GO:0006687 GO:0006813 GO:0009401 GO:0006754
GO:0007067 GO:0006891 GO:0015031 GO:0050290 GO:0015079
GO:0004197 GO:0004867 GO:0046912 GO:0016887 GO:0005524
GO:0042729 GO:0000275 GO:0072686 GO:0005576 GO:0016021
GO:0000145 GO:0017119 GO:0005887
Cluster-22178.70715 GO:0006891 GO:0015031 GO:0006754 GO:0007067
GO:0009401 GO:0009405 GO:0006904 GO:0071805 GO:0019089
GO:0006687 GO:0006813 GO:0006508 GO:0005524 GO:0016887
GO:0004197 GO:0004867 GO:0050290 GO:0015079 GO:0005887
GO:0000145 GO:0017119 GO:0042729 GO:0005576 GO:0000275
GO:0072686
Cluster-22178.70716 GO:0007165 GO:0019087 GO:0005102 GO:0016021
Cluster-22178.70717 GO:0006289 GO:0006865 GO:0055085 GO:0009411
GO:0006397 GO:0006810 GO:0003333 GO:0015171 GO:0004519
GO:0005634 GO:0016020
Cluster-22178.70718 GO:0007165
Cluster-22178.70720 GO:0008152 GO:0008484 GO:0003824
Cluster-22178.70724 GO:0006144 GO:0009165 GO:0006098 GO:0020037
GO:0004749 GO:0000287 GO:0019825
Cluster-22178.70725 GO:0000921 GO:0006355 GO:0003700 GO:0043565
GO:0016021 GO:0005940 GO:0005667

Cluster-22178.70726 GO:0042773 GO:0000290 GO:0006744 GO:0015992
GO:0006814 GO:0000902 GO:0006120 GO:0005975 GO:0043085
GO:0055114 GO:0016787 GO:0016773 GO:0008137 GO:0005515
GO:0005525 GO:0008047 GO:0016020
Cluster-22178.70728 GO:0007165 GO:0007186 GO:0006508 GO:0008152
GO:0016746 GO:0004190 GO:0003723 GO:0004871 GO:0003676
GO:0019001 GO:0008270 GO:0031683 GO:0005524 GO:0004672
GO:0003924
Cluster-22178.70732 GO:0006396 GO:0006807 GO:0006144 GO:0004000
GO:0003723
Cluster-22178.70735 GO:0006144 GO:0006396 GO:0006807 GO:0003723
GO:0004000
Cluster-22178.70736 GO:0005515 GO:0043565 GO:0005634
Cluster-22178.70737 GO:0003676 GO:0003677 GO:0016787 GO:0005524
GO:0046872
Cluster-22178.70739 GO:0006506 GO:0006468 GO:0055085 GO:0005515
GO:0004386 GO:0004672 GO:0005524 GO:0016773 GO:0031083
GO:0016020 GO:0005789 GO:0016021
Cluster-22178.70740 GO:0046872
Cluster-22178.70741 GO:0005515
Cluster-22178.70743 GO:0006812 GO:0055085 GO:0008324 GO:0016021
Cluster-22178.70746 GO:0055114 GO:0016491 GO:0010181
Cluster-22178.70747 GO:0006886 GO:0016192 GO:0005515 GO:0005198
GO:0030132 GO:0030130 GO:0030117
Cluster-22178.70748 GO:0006606 GO:0007094 GO:0000042 GO:0005515
GO:0005789
Cluster-22178.70750 GO:0040007 GO:0008283 GO:0007165 GO:0008083

Cluster-22178.70751 GO:0006281 GO:0046872 GO:0000166 GO:0003824

Cluster-22178.70752 GO:0007165 GO:0006886 GO:0006508 GO:0007606
GO:0035556 GO:0006888 GO:0004197 GO:0005515 GO:0004888
GO:0003723 GO:0046872 GO:0008270 GO:0030127 GO:0016020

Cluster-22178.70755 GO:0006807 GO:0006298 GO:0005524 GO:0030983
GO:0016151
Cluster-22178.70757 GO:0006810 GO:0016021
Cluster-22178.70760 GO:0015031 GO:0006464 GO:0006281 GO:0016151
GO:0005515 GO:0008270
Cluster-22178.70762 GO:0005515 GO:0008270
Cluster-22178.70763 GO:0016787
Cluster-22178.70764 GO:0090063 GO:0000226 GO:0004190 GO:0008146
GO:0005815 GO:0016020 GO:0000922
Cluster-22178.70769 GO:0007165 GO:0000166 GO:0005515 GO:0046872
GO:0045202
Cluster-22178.70770 GO:0006260 GO:0007218 GO:0006098 GO:0006749
GO:0007049 GO:0006006 GO:0055114 GO:0018990 GO:0006355
GO:0008270 GO:0003677 GO:0003700 GO:0008255 GO:0003887
GO:0004345 GO:0050661 GO:0005667 GO:0016021 GO:0042575
GO:0005576 GO:0005634
Cluster-22178.70772 GO:0005515 GO:0016773
Cluster-22178.70774 GO:0005515
Cluster-22178.70776 GO:0009878 GO:0046872
Cluster-22178.70778 GO:0006123 GO:0015992 GO:0006397 GO:0004129
GO:0005524 GO:0003774 GO:0005515 GO:0045277 GO:0016459
Cluster-22178.70780 GO:0043565 GO:0005515 GO:0005634
Cluster-22178.70782 GO:0050832 GO:0006621 GO:0042742 GO:0046923
GO:0005783 GO:0016021
Cluster-22178.70784 GO:0006886 GO:0006888 GO:0008270 GO:0030127
Cluster-22178.70785 GO:0006457 GO:0005509 GO:0051082 GO:0005783
Cluster-22178.70786 GO:0006576 GO:0006777 GO:0006614 GO:0000103
GO:0006000 GO:0006144 GO:0006013 GO:0016301 GO:0005525
GO:0004020 GO:0003873 GO:0003677 GO:0016787 GO:0008312
GO:0005524 GO:0048500
Cluster-22178.70789 GO:0006468 GO:0006397 GO:0051252 GO:0004540
GO:0016773 GO:0004672 GO:0005524 GO:0016020
Cluster-22178.70790 GO:0042742 GO:0005515

Cluster-22178.70793 GO:0055114 GO:0004190 GO:0016491 GO:0016021
Cluster-22178.70794 GO:0007165 GO:0015893 GO:0006308 GO:0006855
GO:0015238 GO:0008855 GO:0009318 GO:0005886
Cluster-22178.70795 GO:0006260 GO:0045893 GO:0006308 GO:0006355
GO:0006281 GO:0017108 GO:0003700 GO:0043565 GO:0003677
GO:0005515 GO:0033557 GO:0005634 GO:0005667
Cluster-22178.70796 GO:0042254 GO:0006412 GO:0003735 GO:0003677
GO:0005096 GO:0046872 GO:0005622 GO:0045202 GO:0005840
Cluster-22178.70798 GO:0006506 GO:0007165 GO:0005179 GO:0005576
Cluster-22178.70799 GO:0015977 GO:0046487 GO:0016984 GO:0003677
GO:0009573
Cluster-22178.70800 GO:0030168 GO:0051262 GO:0006357 GO:0006606
GO:0006260 GO:0007067 GO:0007155 GO:0007165 GO:0019058
GO:0051258 GO:0009405 GO:0010508 GO:0005102 GO:0030674
GO:0004871 GO:0005198 GO:0001104 GO:0005515 GO:0005577
GO:0005882 GO:0016592 GO:0016020 GO:0000775 GO:0019031
GO:0016021 GO:0019867 GO:0019028 GO:0072686 GO:0042729
Cluster-22178.70801 GO:0015992 GO:0055114 GO:0019069 GO:0015991
GO:0009405 GO:0008270 GO:0004800 GO:0015078 GO:0003723
GO:0005515 GO:0033179 GO:0016020
Cluster-22178.70802 GO:0000226 GO:0051537 GO:0015630 GO:0043231
Cluster-22178.70803 GO:0007156 GO:0005509 GO:0016020
Cluster-22178.70805 GO:0006468 GO:0016773 GO:0005524 GO:0004672
GO:0005515 GO:0016020
Cluster-22178.70811 GO:0031047
Cluster-22178.70812 GO:0006888 GO:0055085 GO:0006810 GO:0005215
GO:0022857 GO:0005515 GO:0005622 GO:0005801 GO:0016021
GO:0019867 GO:0016020
Cluster-22178.70813 GO:0006810 GO:0055085 GO:0006888 GO:0005515
GO:0005215 GO:0022857 GO:0016020 GO:0005801 GO:0019867
GO:0016021 GO:0005622
Cluster-22178.70816 GO:0015992 GO:0015986 GO:0017004 GO:0016192
GO:0006355 GO:0006119 GO:0006118 GO:0000042 GO:0006886

GO:0008270 GO:0046933 GO:0046961 GO:0046872 GO:0003700
GO:0045158 GO:0003723 GO:0005515 GO:0009512 GO:0005667
GO:0045259 GO:0000275 GO:0042025
Cluster-22178.70817 GO:0009059 GO:0006468 GO:0004672 GO:0005524
GO:0004519 GO:0003677 GO:0030176
Cluster-22178.70818 GO:0006555 GO:0006730 GO:0006816 GO:0006874
GO:0006281 GO:0005219 GO:0008270 GO:0046872 GO:0004013
GO:0016021 GO:0005622
Cluster-22178.70820 GO:0006367 GO:0000398 GO:0046872 GO:0005681
GO:0005672
Cluster-22178.70822 GO:0016021
Cluster-22178.70825 GO:0007155 GO:0051726 GO:0006367 GO:0015684
GO:0006281 GO:0009432 GO:0016887 GO:0005524 GO:0015093
GO:0017025 GO:0003697 GO:0005525 GO:0009289 GO:0005672
GO:0005634 GO:0016021
Cluster-22178.70826 GO:0005515 GO:0016887 GO:0005524 GO:0005634
Cluster-22178.70827 GO:0006811 GO:0006810 GO:0005230 GO:0016020
Cluster-22178.70830 GO:0006355 GO:0007186 GO:0008270 GO:0004930
GO:0003700 GO:0005667 GO:0005634 GO:0016020
Cluster-22178.70831 GO:0005515
Cluster-22178.70832 GO:0006260 GO:0005515
Cluster-22178.70833 GO:0006470 GO:0000226 GO:0050852 GO:0006570
GO:0004725 GO:0005509 GO:0005515 GO:0015630
Cluster-22178.70834 GO:0055114 GO:0006351 GO:0006206 GO:0006144
GO:0007165 GO:0015774 GO:0005515 GO:0003899 GO:0004888
GO:0015159 GO:0005509 GO:0051920 GO:0016021 GO:0005578
GO:0009289 GO:0016020 GO:0005730
Cluster-22178.70836 GO:0016787
Cluster-22178.70838 GO:0000398 GO:0006813 GO:0005249 GO:0030955
GO:0016021 GO:0005681 GO:0005886 GO:0008076
Cluster-22178.70839 GO:0035556 GO:0009086 GO:0000398 GO:0008652
GO:0008270 GO:0005515 GO:0003871 GO:0005681
Cluster-22178.70842 GO:0009405 GO:0035556 GO:0019835 GO:0005515

GO:0019867
Cluster-22178.70843 GO:0006260 GO:0009405 GO:0005198 GO:0005515
GO:0003887 GO:0003677 GO:0009360 GO:0016021 GO:0042575
Cluster-22178.70844 GO:0009405 GO:0005515 GO:0005198
Cluster-22178.70845 GO:0035091
Cluster-22178.70847 GO:0008063 GO:0005515 GO:0008270
Cluster-22178.70848 GO:0019068 GO:0007165 GO:0015992 GO:0055085
GO:0030001 GO:0015991 GO:0046849 GO:0046873 GO:0045502
GO:0008134 GO:0042803 GO:0015078 GO:0005667 GO:0016020
GO:0030286 GO:0005576 GO:0033179 GO:0016021
Cluster-22178.70849 GO:0006351 GO:0006508 GO:0000162 GO:0009073
GO:0006260 GO:0006571 GO:0055114 GO:0015979 GO:0009094
GO:0030494 GO:0004252 GO:0003677 GO:0003856 GO:0016491
GO:0042025
Cluster-22178.70850 GO:0045493 GO:0006355 GO:0030001 GO:0007009
GO:0006508 GO:0005975 GO:0055114 GO:0008237 GO:0046559
GO:0005515 GO:0046872 GO:0016627 GO:0008241 GO:0016020
GO:0005747 GO:0005576 GO:0005634 GO:0005743 GO:0005737
Cluster-22178.70851 GO:0015994 GO:0006811 GO:0015996 GO:0006118
GO:0006629 GO:0006355 GO:0015991 GO:0006508 GO:0016042
GO:0015992 GO:0046486 GO:0045454 GO:0015035 GO:0009055
GO:0015078 GO:0046872 GO:0047746 GO:0008236 GO:0003847
GO:0003677 GO:0016787 GO:0019031 GO:0033179 GO:0005886
GO:0008247 GO:0016020
Cluster-22178.70852 GO:0015074 GO:0006310 GO:0006313 GO:0000150
GO:0003677
Cluster-22178.70855 GO:0006118 GO:0045454 GO:0015035 GO:0009055
GO:0005515 GO:0005634
Cluster-22178.70857 GO:0042384 GO:0034464
Cluster-22178.70859 GO:0006144 GO:0006206 GO:0050852 GO:0006470
GO:0006570 GO:0006351 GO:0046872 GO:0004725 GO:0044212
GO:0005515 GO:0003899 GO:0003677 GO:0005730
Cluster-22178.70860 GO:0034314 GO:0007165 GO:0006955 GO:0030041

GO:0003676 GO:0005125 GO:0003723 GO:0015629 GO:0005885
GO:0005576
Cluster-22178.70861 GO:0003676 GO:0003723
Cluster-22178.70862 GO:0008152 GO:0046872 GO:0008484 GO:0003824

Cluster-22178.70863 GO:0008152 GO:0003824 GO:0046872 GO:0008484

Cluster-22178.70865 GO:0007264 GO:0003924 GO:0005525 GO:0005634
Cluster-22178.70866 GO:0006813 GO:0015886 GO:0015232 GO:0016286
GO:0016021
Cluster-22178.70867 GO:0007165 GO:0035556 GO:0005509 GO:0046872
GO:0016787 GO:0005578
Cluster-22178.70868 GO:0006123 GO:0016310 GO:0015976 GO:0015992
GO:0006096 GO:0006094 GO:0004129 GO:0004618 GO:0045277
GO:0005746
Cluster-22178.70872 GO:0007165 GO:0007067 GO:0000090 GO:0009069
GO:0006606 GO:0031110 GO:0006914 GO:0030168 GO:0006468
GO:0016310 GO:0007059 GO:0051301 GO:0048870 GO:0051258
GO:0030674 GO:0005102 GO:0008017 GO:0004674 GO:0005096
GO:0005198 GO:0042803 GO:0008134 GO:0045502 GO:0005882
GO:0005667 GO:0005876 GO:0005577 GO:0000940 GO:0030286
GO:0031514 GO:0045298
Cluster-22178.70873 GO:0006470 GO:0006570 GO:0004725 GO:0008138

Cluster-22178.70874 GO:0005515 GO:0016020
Cluster-22178.70875 GO:0006470 GO:0006570 GO:0004725
Cluster-22178.70876 GO:0007264 GO:0007165 GO:0007186 GO:0006013
GO:0043087 GO:0006000 GO:0006355 GO:0035023 GO:0003873
GO:0005524 GO:0005089 GO:0005515 GO:0019001 GO:0031683
GO:0003924 GO:0005525 GO:0004871
Cluster-22178.70878 GO:0003723 GO:0003676 GO:0005515
Cluster-22178.70879 GO:0005515
Cluster-22178.70882 GO:0005515

Cluster-22178.70883 GO:0015992 GO:0015986 GO:0015078 GO:0003676
GO:0003723 GO:0071203 GO:0000276
Cluster-22178.70886 GO:0006397 GO:0006812 GO:0046660 GO:0003676
GO:0016887 GO:0016021 GO:0005634
Cluster-22178.70891 GO:0006814 GO:0006570 GO:0015992 GO:0055114
GO:0006470 GO:0006744 GO:0006120 GO:0008138 GO:0008137
GO:0005524 GO:0004725
Cluster-22178.70892 GO:0006470 GO:0006120 GO:0006744 GO:0015992
GO:0055114 GO:0006814 GO:0006570 GO:0004725 GO:0005524
GO:0008137 GO:0008138 GO:0016020
Cluster-22178.70893 GO:0046034 GO:0006139 GO:0006144 GO:0006206
GO:0007186 GO:0050909 GO:0004127 GO:0004930 GO:0005524
GO:0004017 GO:0016021
Cluster-22178.70895 GO:0007165 GO:0005515
Cluster-22178.70901 GO:0042157 GO:0007186 GO:0006457 GO:0006869
GO:0034080 GO:0006355 GO:0051301 GO:0007059 GO:0003677
GO:0005524 GO:0004930 GO:0051082 GO:0035299 GO:0003700
GO:0003676 GO:0008289 GO:0005667 GO:0016272 GO:0000444
GO:0005576 GO:0016021 GO:0000775
Cluster-22178.70904 GO:0016310 GO:0006123 GO:0015992 GO:0006886
GO:0016192 GO:0009069 GO:0004674 GO:0004129 GO:0003676
GO:0005515 GO:0045277 GO:0030117
Cluster-22178.70905 GO:0006412 GO:0042254 GO:0003735 GO:0005515
GO:0005840 GO:0005622
Cluster-22178.70906 GO:0006351 GO:0043565
Cluster-22178.70907 GO:0006351 GO:0006144 GO:0005198 GO:0003723
GO:0003676 GO:0003968 GO:0019028 GO:0031379
Cluster-22178.70908 GO:0008152 GO:0016836 GO:0061630 GO:0005515
GO:0008270 GO:0046872 GO:0003824 GO:0032040
Cluster-22178.70909 GO:0007165 GO:0005179 GO:0005576
Cluster-22178.70910 GO:0006231 GO:0006206 GO:0009405 GO:0004799
GO:0045735 GO:0005576
Cluster-22178.70911 GO:0006351 GO:0006355 GO:0003677 GO:0005515

GO:0016021 GO:0005634
Cluster-22178.70912 GO:0005515 GO:0016021
Cluster-22178.70913 GO:0019722 GO:0003676 GO:0003723
Cluster-22178.70914 GO:0005515 GO:0043395 GO:0016020 GO:0005578
Cluster-22178.70915 GO:0042254 GO:0006412 GO:0043395 GO:0003735
GO:0005515 GO:0005840 GO:0016020 GO:0005578
Cluster-22178.70917 GO:0006139 GO:0006206 GO:0007009 GO:0005515
GO:0004127 GO:0005524 GO:0005887
Cluster-22178.70918 GO:0003723 GO:0005515
Cluster-22178.70923 GO:0006355 GO:0003700 GO:0003677 GO:0043565
GO:0005667
Cluster-22178.70924 GO:0010508 GO:0006886 GO:0030001 GO:0006355
GO:0007049 GO:0055114 GO:0006367 GO:0010506 GO:0006914
GO:0006464 GO:0008113 GO:0046872 GO:0005669 GO:0005615
GO:0016021 GO:0005783
Cluster-22178.70926 GO:0006468 GO:0005524 GO:0004672
Cluster-22178.70927 GO:0005515 GO:0016021
Cluster-22178.70928 GO:0019236 GO:0007186
Cluster-22178.70933 GO:0006355 GO:0006813 GO:0006811 GO:0006807
GO:0006542 GO:0009252 GO:0004356 GO:0003677 GO:0046872
GO:0005249 GO:0016020 GO:0008076
Cluster-22178.70935 GO:0009512
Cluster-22178.70938 GO:0030418 GO:0006355 GO:0045892 GO:0030410
GO:0003677
Cluster-22178.70939 GO:0008152 GO:0008168
Cluster-22178.70940 GO:0009099 GO:0009097 GO:0009405 GO:0006438
GO:0009098 GO:0008152 GO:0000166 GO:0016758 GO:0004832
GO:0005524 GO:0016020 GO:0005783 GO:0005737
Cluster-22178.70941 GO:0006367 GO:0006952 GO:0006352 GO:0003677
GO:0005634 GO:0009279 GO:0005669
Cluster-22178.70942 GO:0042254 GO:0006412 GO:0046872 GO:0005515
GO:0003735 GO:0005622 GO:0005840
Cluster-22178.70944 GO:0003677 GO:0005634

Cluster-22178.70945 GO:0007015 GO:0007155 GO:0007094 GO:0006897 GO:0006457 GO:0006955 GO:0019882 GO:0051082 GO:0005198 GO:0019028 GO:0009288 GO:0016272 GO:0019031
Cluster-22178.70948 GO:0007186 GO:0005515 GO:0004930 GO:0004190 GO:0016021
Cluster-22178.70953 GO:0055114 GO:0006260 GO:0051536 GO:0016651 GO:0008270 GO:0005634
Cluster-22178.70954 GO:0046740 GO:0005515 GO:0008270 GO:0016021
Cluster-22178.70955 GO:0005515
Cluster-22178.70956 GO:0006605 GO:0007268 GO:0006886 GO:0006508 GO:0004197 GO:0043495 GO:0042802 GO:0005515 GO:0030695 GO:0033130 GO:0005742
Cluster-22178.70959 GO:0007217 GO:0007268 GO:0007275 GO:0004867 GO:0005578
Cluster-22178.70961 GO:0006260 GO:0005524 GO:0016301 GO:0003887 GO:0003677 GO:0009360 GO:0042575
Cluster-22178.70965 GO:0042967 GO:0006886 GO:0006505 GO:0006629 GO:0008374 GO:0016788 GO:0005515
Cluster-22178.70968 GO:0006511 GO:0051603 GO:0004298 GO:0004175 GO:0019773 GO:0005839
Cluster-22178.70972 GO:0007186 GO:0007165 GO:0004871 GO:0016829 GO:0005834 GO:0005576
Cluster-22178.70973 GO:0045454 GO:0006118 GO:0000398 GO:0006144 GO:0005515 GO:0009055 GO:0015035 GO:0003968 GO:0031379 GO:0005681 GO:0016021
Cluster-22178.70974 GO:0005515 GO:0046872
Cluster-22178.70975 GO:0006310 GO:0002949 GO:0006886 GO:0006281 GO:0048870 GO:0009378 GO:0003724 GO:0005524 GO:0016887 GO:0003723 GO:0003678 GO:0046872 GO:0005783 GO:0009379 GO:0005657 GO:0031514 GO:0016021
Cluster-22178.70976 GO:0006378 GO:0003729 GO:0016787 GO:0005849
Cluster-22178.70977 GO:0009451 GO:0001522 GO:0003723 GO:0009982 GO:0016012 GO:0016021

Cluster-22178.70979 GO:0045046 GO:0032403 GO:0005779 GO:0008537
Cluster-22178.7098 GO:0005515 GO:0016021
Cluster-22178.70980 GO:0006541 GO:0004359
Cluster-22178.70981 GO:0005634
Cluster-22178.70982 GO:0003924 GO:0005525
Cluster-22178.70984 GO:0006275 GO:0003924 GO:0005525
Cluster-22178.70985 GO:0006470 GO:0006402 GO:0051252 GO:0006468
GO:0003676 GO:0004672 GO:0005524 GO:0046872 GO:0004535
GO:0008138 GO:0003723 GO:0005737 GO:0005634
Cluster-22178.70987 GO:0006355 GO:0006118 GO:0055114 GO:0004190
GO:0005515 GO:0004252 GO:0016655 GO:0005886 GO:0016021
Cluster-22178.7099 GO:0006816 GO:0006810 GO:0006364 GO:0042030
GO:0005246 GO:0016020
Cluster-22178.70992 GO:0010333 GO:0000287 GO:0016829
Cluster-22178.70993 GO:0008270
Cluster-22178.70996 GO:0003676 GO:0046872
Cluster-22178.70997 GO:0006400 GO:0009451 GO:0031167 GO:0016226
GO:0008033 GO:0008176 GO:0051536 GO:0008168 GO:0005737
Cluster-22178.70998 GO:0005515 GO:0003824 GO:0008270 GO:0046872

Cluster-22178.71001 GO:0051015
Cluster-22178.71003 GO:0006260 GO:0003677 GO:0005664
Cluster-22178.71007 GO:0006355 GO:0046854 GO:0042967 GO:0016787
GO:0004402 GO:0008270 GO:0003712 GO:0005667 GO:0005634
GO:0019033 GO:0000123
Cluster-22178.71008 GO:0007165 GO:0006810 GO:0005158 GO:0005524
GO:0042626 GO:0005899 GO:0016021
Cluster-22178.71009 GO:0005515
Cluster-22178.71010 GO:0000917 GO:0006355 GO:0043093 GO:0007049
GO:0006260 GO:0003700 GO:0005667 GO:0005737
Cluster-22178.71011 GO:0007049 GO:0006825 GO:0007186 GO:0051252
GO:0000917 GO:0035434 GO:0043093 GO:0006355 GO:0003700
GO:0004930 GO:0016891 GO:0005375 GO:0016021 GO:0005737

GO:0005667

Cluster-22178.71017 GO:0006629 GO:0005515 GO:0009424

Cluster-22178.71020 GO:0015996 GO:0015994 GO:0006811 GO:0006118 GO:0006508 GO:0015991 GO:0006629 GO:0006355 GO:0016042 GO:0015992 GO:0046486 GO:0045454 GO:0015078 GO:0009055 GO:0015035 GO:0046872 GO:0008236 GO:0047746 GO:0003847 GO:0003677 GO:0016787 GO:0019031 GO:0033179 GO:0005886 GO:0016020 GO:0008247

Cluster-22178.71022 GO:0006418 GO:0006139 GO:0005524 GO:0003676 GO:0004812 GO:0008026 GO:0016818 GO:0000166

Cluster-22178.71023 GO:0046872

Cluster-22178.71025 GO:0009405 GO:0007165 GO:0006355 GO:0003700 GO:0043565 GO:0005509 GO:0005515 GO:0005198 GO:0004888 GO:0016021 GO:0005667

Cluster-22178.71027 GO:0007049 GO:0000917 GO:0006355 GO:0043093 GO:0003700 GO:0005737 GO:0005667

Cluster-22178.71028 GO:0005515

Cluster-22178.71029 GO:0006537 GO:0055114 GO:0009607 GO:0000105 GO:0006355 GO:0016491 GO:0016627 GO:0003824 GO:0016638 GO:0015930 GO:0005737

Cluster-22178.7103 GO:0008203 GO:0016042 GO:0016298

Cluster-22178.71030 GO:0005515

Cluster-22178.71032 GO:0006468 GO:0016627 GO:0005524 GO:0004672

Cluster-22178.71034 GO:0006468 GO:0016627 GO:0005524 GO:0004672

Cluster-22178.71035 GO:0006310 GO:0007131 GO:0002949 GO:0008033 GO:0055114 GO:0015684 GO:0007264 GO:0015886 GO:0006281 GO:0046497 GO:0006769 GO:0098519 GO:0015232 GO:0016887 GO:0015093 GO:0005524 GO:0016301 GO:0003723 GO:0003924 GO:0051287 GO:0009378 GO:0003724 GO:0050660 GO:0016491 GO:0008750 GO:0050661 GO:0003678 GO:0005525 GO:0005657 GO:0000794 GO:0009379 GO:0016021

Cluster-22178.71038 GO:0006119 GO:0015986 GO:0015992 GO:0046961
GO:0008289 GO:0046933 GO:0045259 GO:0000275
Cluster-22178.71042 GO:0042254 GO:0006412 GO:0003735 GO:0005840
Cluster-22178.71043 GO:0007165 GO:0005515 GO:0003677 GO:0005179
GO:0005576
Cluster-22178.71048 GO:0006520 GO:0019752 GO:0016831 GO:0030170
GO:0016829
Cluster-22178.71049 GO:0007275 GO:0005515 GO:0042600
Cluster-22178.71050 GO:0007275 GO:0005515 GO:0042600
Cluster-22178.71051 GO:0007165 GO:0005515
Cluster-22178.71052 GO:0005515 GO:0005509 GO:0010181
Cluster-22178.71053 GO:0007165 GO:0004888 GO:0010181 GO:0005515
GO:0005509 GO:0016021
Cluster-22178.71054 GO:0005509 GO:0005515 GO:0010181
Cluster-22178.71055 GO:0016192 GO:0006886 GO:0005975 GO:0005515
GO:0005198 GO:0004553 GO:0030117 GO:0019028
Cluster-22178.71057 GO:0046872
Cluster-22178.71059 GO:0006355 GO:0006281 GO:0003677 GO:0030915
Cluster-22178.71060 GO:0006357 GO:0035091 GO:0001104 GO:0016592
Cluster-22178.71061 GO:0006357 GO:0035091 GO:0001104 GO:0016592
Cluster-22178.71062 GO:0055114 GO:0000166 GO:0016616 GO:0051287
GO:0016491 GO:0008270 GO:0046872 GO:0016020
Cluster-22178.71063 GO:0006812 GO:0032801 GO:0007049 GO:0019076
GO:0019058 GO:0006351 GO:0005198 GO:0005261 GO:0070176
GO:0000228 GO:0033644
Cluster-22178.71064 GO:0019497 GO:0006281 GO:0002949 GO:0006310
GO:0019079 GO:0006771 GO:0003678 GO:0005515 GO:0003723
GO:0003724 GO:0003993 GO:0016887 GO:0005524 GO:0009378
GO:0005657 GO:0009379
Cluster-22178.71065 GO:0006281 GO:0019497 GO:0006771 GO:0006310
GO:0019079 GO:0002949 GO:0003723 GO:0005515 GO:0003678
GO:0009378 GO:0016887 GO:0005524 GO:0003724 GO:0003993
GO:0009379 GO:0005657

Cluster-22178.71066 GO:0003779
Cluster-22178.71067 GO:0009069 GO:0006468 GO:0048870 GO:0016310
GO:0005198 GO:0005524 GO:0004674 GO:0031514
Cluster-22178.71068 GO:0007264 GO:0006810 GO:0055114 GO:0006811
GO:0055085 GO:0005216 GO:0010181 GO:0016491 GO:0005525
GO:0005215 GO:0016020 GO:0005615 GO:0016021
Cluster-22178.71071 GO:0006313 GO:0004803 GO:0003677
Cluster-22178.71076 GO:0045490 GO:0047487 GO:0005515
Cluster-22178.71079 GO:0006886 GO:0005741
Cluster-22178.71080 GO:0005524
Cluster-22178.71081 GO:0008270 GO:0005515
Cluster-22178.71083 GO:0007264 GO:0005525
Cluster-22178.71084 GO:0022900 GO:0003677 GO:0005515 GO:0016787
GO:0003676 GO:0046872 GO:0005524 GO:0070469 GO:0005743
Cluster-22178.71087 GO:0006808 GO:0005975 GO:0046034 GO:0006413
GO:0006355 GO:0006506 GO:0006144 GO:0006464 GO:0007218
GO:0006446 GO:0043565 GO:0004672 GO:0046872 GO:0003700
GO:0003676 GO:0016151 GO:0003723 GO:0004519 GO:0005515
GO:0030234 GO:0008270 GO:0005524 GO:0004553 GO:0004017
GO:0003743 GO:0003677 GO:0005789 GO:0005840 GO:0005667
Cluster-22178.71095 GO:0048870 GO:0007165 GO:0005515 GO:0031514
Cluster-22178.71096 GO:0006810 GO:0007218 GO:0006741 GO:0015947
GO:0046497 GO:0008152 GO:0055114 GO:0006308 GO:0006769
GO:0008855 GO:0019871 GO:0016301 GO:0003951 GO:0046332
GO:0030058 GO:0005886 GO:0042597 GO:0009318 GO:0005576
Cluster-22178.71100 GO:0016567 GO:0005515 GO:0004842 GO:0051537
GO:0017119 GO:0043231
Cluster-22178.71102 GO:0016579 GO:0036459 GO:0005783
Cluster-22178.71103 GO:0043087 GO:0007169 GO:0035023 GO:0006468
GO:0006508 GO:0005524 GO:0004672 GO:0004185 GO:0005089
GO:0004714 GO:0005515 GO:0016020
Cluster-22178.71104 GO:0006468 GO:0007169 GO:0004672 GO:0005524
GO:0004714 GO:0005515 GO:0016020

Cluster-22178.71106 GO:0008152 GO:0016226 GO:0051536 GO:0008168
GO:0005737
Cluster-22178.71108 GO:0042967 GO:0016747 GO:0008080
Cluster-22178.71109 GO:0005515
Cluster-22178.71111 GO:0051726 GO:0006974
Cluster-22178.71113 GO:0055114 GO:0015675 GO:0035444 GO:0016491
GO:0046872 GO:0015099 GO:0005506 GO:0031418 GO:0016706
GO:0016021
Cluster-22178.71114 GO:0006471 GO:0003950 GO:0016817
Cluster-22178.71116 GO:0055114 GO:0015992 GO:0015991 GO:0019069
GO:0009405 GO:0004800 GO:0008270 GO:0015078 GO:0003723
GO:0005515 GO:0033179 GO:0016020
Cluster-22178.71117 GO:0015992 GO:0055114 GO:0019069 GO:0015991
GO:0009405 GO:0008270 GO:0004800 GO:0015078 GO:0003723
GO:0005515 GO:0033179 GO:0016020
Cluster-22178.71120 GO:0042254 GO:0006412 GO:0003735 GO:0005840
GO:0005622
Cluster-22178.71121 GO:0019835 GO:0009405 GO:0019867
Cluster-22178.71122 GO:0003723 GO:0005515
Cluster-22178.71123 GO:0006355 GO:0019478 GO:0055114 GO:0016788
GO:0005515 GO:0050662 GO:0003676 GO:0003700 GO:0016491
GO:0043565 GO:0016020 GO:0005667 GO:0005737
Cluster-22178.71124 GO:0006265 GO:0003916 GO:0003677
Cluster-22178.71125 GO:0003712 GO:0005667 GO:0000124
Cluster-22178.71126 GO:0006812 GO:0008324
Cluster-22178.71131 GO:0006605 GO:0006886 GO:0005515 GO:0005742
Cluster-22178.71132 GO:0006412 GO:0006281 GO:0006139 GO:0016310
GO:0042254 GO:0006310 GO:0006810 GO:0009069 GO:0002949
GO:0006206 GO:0016301 GO:0003723 GO:0005525 GO:0003924
GO:0003735 GO:0098519 GO:0009378 GO:0003724 GO:0004127
GO:0004674 GO:0016887 GO:0005524 GO:0005840 GO:0009379
GO:0005657 GO:0005622
Cluster-22178.71133 GO:0016310 GO:0006139 GO:0006412 GO:0006281

GO:0002949 GO:0006206 GO:0006810 GO:0006310 GO:0042254
GO:0009069 GO:0005525 GO:0003924 GO:0016301 GO:0003723
GO:0004674 GO:0003724 GO:0004127 GO:0005524 GO:0016887
GO:0098519 GO:0003735 GO:0009378 GO:0005840 GO:0005657
GO:0005622 GO:0009379
Cluster-22178.71135 GO:0005515 GO:0004252 GO:0043565 GO:0005634
GO:0055036 GO:0019028
Cluster-22178.71137 GO:0009405 GO:0009094 GO:0009073 GO:0006571
GO:0000162 GO:0004107 GO:0005515 GO:0033644
Cluster-22178.71138 GO:0055085 GO:0006812 GO:0008324 GO:0005886
GO:0016021
Cluster-22178.71139 GO:0006576 GO:0005524 GO:0003924 GO:0005525

Cluster-22178.71141 GO:0006779 GO:0009396 GO:0019354 GO:0008152
GO:0015940 GO:0055114 GO:0046487 GO:0016757 GO:0016491
GO:0004488 GO:0050662 GO:0051287 GO:0003824 GO:0008677
GO:0043115 GO:0016020
Cluster-22178.71142 GO:0008152 GO:0006508 GO:0008236 GO:0016787

Cluster-22178.71145 GO:0005515
Cluster-22178.71146 GO:0042967 GO:0006810 GO:0006816 GO:0016573
GO:0006355 GO:0003712 GO:0008270 GO:0003713 GO:0004402
GO:0042030 GO:0005515 GO:0005246 GO:0000123 GO:0016020
GO:0005667 GO:0005634
Cluster-22178.71147 GO:0005975 GO:0043169 GO:0003824
Cluster-22178.71148 GO:0016787
Cluster-22178.71149 GO:0005515
Cluster-22178.71150 GO:0044237
Cluster-22178.71151 GO:0005515
Cluster-22178.71153 GO:0006508 GO:0016853 GO:0008233 GO:0046872
GO:0009507
Cluster-22178.71154 GO:0018342 GO:0019079 GO:0016787 GO:0008318
GO:0003677 GO:0008026 GO:0005524 GO:0003676

Cluster-22178.71155 GO:0006099 GO:0019643 GO:0006355 GO:0006094 GO:0015977 GO:0006270 GO:0008964
Cluster-22178.71159 GO:0008152 GO:0005515 GO:0008168
Cluster-22178.71160 GO:0006814 GO:0000902 GO:0055114 GO:0043085 GO:0005975 GO:0006120 GO:0000290 GO:0042773 GO:0015992 GO:0006744 GO:0005515 GO:0008137 GO:0008047 GO:0005525 GO:0016787 GO:0016773 GO:0016020
Cluster-22178.71163 GO:0005525
Cluster-22178.71164 GO:0006418 GO:0016567 GO:0006886 GO:0002161 GO:0005524 GO:0004842 GO:0004812 GO:0000166 GO:0005741
Cluster-22178.71165 GO:0006810 GO:0009405 GO:0006418 GO:0019871 GO:0000166 GO:0004812 GO:0005524 GO:0005576
Cluster-22178.71166 GO:0030089
Cluster-22178.71168 GO:0006887 GO:0007165 GO:0045227 GO:0005179 GO:0004252 GO:0016021 GO:0005576 GO:0016020 GO:0000145
Cluster-22178.71170 GO:0007049 GO:0006275 GO:0007155
Cluster-22178.71172 GO:0009405 GO:0006810 GO:0008270 GO:0046872 GO:0019870 GO:0005515 GO:0005576
Cluster-22178.71174 GO:0008270 GO:0005515
Cluster-22178.71176 GO:0005524 GO:0005856
Cluster-22178.71177 GO:0006260
Cluster-22178.71178 GO:0015979 GO:0019684 GO:0003723 GO:0009523 GO:0016021 GO:0019012
Cluster-22178.71179 GO:0005634
Cluster-22178.71180 GO:0043158 GO:0007165 GO:0003677 GO:0004252 GO:0005044 GO:0016020 GO:0005634
Cluster-22178.71182 GO:0008033 GO:0006508 GO:0004810 GO:0016437 GO:0008270 GO:0004222 GO:0005576
Cluster-22178.71184 GO:0016559 GO:0006355 GO:0016567 GO:0055114 GO:0006779 GO:0005515 GO:0043565 GO:0008270 GO:0046872 GO:0004842 GO:0005779 GO:0016020
Cluster-22178.71186 GO:0016192 GO:0006886 GO:0005515 GO:0005198 GO:0004789 GO:0030117

Cluster-22178.71187 GO:0042254 GO:0006412 GO:0003735 GO:0005840
GO:0005622
Cluster-22178.71189 GO:0016310 GO:0033617 GO:0003677 GO:0016787
GO:0004673 GO:0003676 GO:0005524 GO:0008270 GO:0009365
Cluster-22178.71190 GO:0046872
Cluster-22178.71192 GO:0006013 GO:0006000 GO:0007165 GO:0007186
GO:0007264 GO:0007155 GO:0005525 GO:0004871 GO:0019001
GO:0031683 GO:0005524 GO:0003924 GO:0003873
Cluster-22178.71193 GO:0006400 GO:0008193 GO:0000287
Cluster-22178.71194 GO:0006281 GO:0006260 GO:0002949 GO:0006031
GO:0006310 GO:0005525 GO:0003678 GO:0043531 GO:0003924
GO:0003887 GO:0016301 GO:0004100 GO:0005524 GO:0016887
GO:0003689 GO:0003677 GO:0098519 GO:0009378 GO:0042575
GO:0005663 GO:0005657 GO:0009360 GO:0009379
Cluster-22178.71197 GO:0042254 GO:0006412 GO:0000902 GO:0042967
GO:0008152 GO:0055114 GO:0003735 GO:0008080 GO:0016491
GO:0005761 GO:0005840 GO:0005622
Cluster-22178.71198 GO:0055114 GO:0015979 GO:0008152 GO:0006480
GO:0006468 GO:0017004 GO:0006118 GO:0008168 GO:0004672
GO:0005524 GO:0045158 GO:0047134 GO:0009523 GO:0009512
GO:0016021
Cluster-22178.71199 GO:0006308 GO:0006955 GO:0006886 GO:0007131
GO:0007155 GO:0007165 GO:0005125 GO:0005057 GO:0005515
GO:0008855 GO:0008270 GO:0005737 GO:0000794 GO:0005576
GO:0009318 GO:0016020
Cluster-22178.71202 GO:0005515
Cluster-22178.71203 GO:0006120 GO:0042207 GO:0006744 GO:0015992
GO:0055114 GO:0006814 GO:0006570 GO:0006559 GO:0016817
GO:0004411 GO:0008137 GO:0005515 GO:0005739
Cluster-22178.71204 GO:0005975 GO:0006886 GO:0016192 GO:0004553
GO:0005198 GO:0005515 GO:0019028 GO:0030117
Cluster-22178.71205 GO:0005975 GO:0006886 GO:0016192 GO:0004553
GO:0005198 GO:0005515 GO:0030117 GO:0019028

Cluster-22178.71206 GO:0008152 GO:0016310 GO:0006120 GO:0006744
GO:0015992 GO:0019049 GO:0005975 GO:0055114 GO:0006814
GO:0009069 GO:0004674 GO:0003824 GO:0008137 GO:0030246
GO:0016836
Cluster-22178.71207 GO:0009405 GO:0006355 GO:0005515 GO:0005634
Cluster-22178.71208 GO:0006355 GO:0009405 GO:0005515 GO:0005634
Cluster-22178.71210 GO:0035435 GO:0006817 GO:0015992 GO:0000162
GO:0009074 GO:0007617 GO:0006119 GO:0006118 GO:0006536
GO:0055114 GO:0009821 GO:0006103 GO:0009094 GO:0006571
GO:0004838 GO:0015114 GO:0030170 GO:0008121 GO:0005576
GO:0016021
Cluster-22178.71211 GO:0015992 GO:0055114 GO:0006817 GO:0035435
GO:0006119 GO:0006118 GO:0008121 GO:0015114 GO:0016021
Cluster-22178.71215 GO:0071973 GO:0008270 GO:0005198
Cluster-22178.71218 GO:0006144 GO:0007156 GO:0009058 GO:0009113
GO:0046872 GO:0005524 GO:0016742 GO:0004637 GO:0051539
GO:0005509 GO:0016020
Cluster-22178.71220 GO:0019856 GO:0009405 GO:0032481 GO:0002218
GO:0005198 GO:0005515 GO:0008270 GO:0016021
Cluster-22178.71221 GO:0005515 GO:0033588
Cluster-22178.71222 GO:0019684 GO:0007165 GO:0042254 GO:0007186
GO:0019236 GO:0015708 GO:0006955 GO:0006118 GO:0006412
GO:0007606 GO:0009772 GO:0004932 GO:0008270 GO:0003677
GO:0003735 GO:0005125 GO:0046872 GO:0005515 GO:0045156
GO:0003723 GO:0005622 GO:0005840 GO:0016021 GO:0005576
GO:0005634
Cluster-22178.71223 GO:0006811 GO:0006813 GO:0005249 GO:0008076
GO:0016020
Cluster-22178.71224 GO:0006888 GO:0006886 GO:0006270 GO:0008270
GO:0030127
Cluster-22178.71225 GO:0007017 GO:0006511 GO:0016567 GO:0007018
GO:0034450 GO:0005515 GO:0005524 GO:0004842 GO:0003777
GO:0008017 GO:0000151 GO:0005874 GO:0045298 GO:0016021

Cluster-22178.71227 GO:0016021
Cluster-22178.71228 GO:0042176 GO:0005515 GO:0030234 GO:0000502
Cluster-22178.71230 GO:0006351 GO:0006813 GO:0016567 GO:0006144
GO:0006206 GO:0005515 GO:0003899 GO:0030955 GO:0046872
GO:0005249 GO:0003677 GO:0004842 GO:0008270 GO:0016021
GO:0005737 GO:0008076 GO:0005730 GO:0005680
Cluster-22178.71236 GO:0006508 GO:0055085 GO:0005515 GO:0008236
GO:0016021
Cluster-22178.71238 GO:0007165 GO:0005515 GO:0016020
Cluster-22178.71239 GO:0055085 GO:0006811 GO:0007010 GO:0003779
GO:0005216 GO:0016020
Cluster-22178.71241 GO:0006955 GO:0007165 GO:0040007 GO:0008283
GO:0005134 GO:0008083 GO:0035091 GO:0005515 GO:0005893
GO:0005576
Cluster-22178.71242 GO:0003677 GO:0046872
Cluster-22178.71246 GO:0046872
Cluster-22178.71247 GO:0008654 GO:0006508 GO:0016780 GO:0004181
GO:0008270 GO:0016020
Cluster-22178.71248 GO:0006886 GO:0016192 GO:0005198 GO:0030130
GO:0030132
Cluster-22178.71249 GO:0016567 GO:0006486 GO:0005515 GO:0016818
GO:0003677 GO:0016787 GO:0003676 GO:0004842 GO:0016757
GO:0005524 GO:0008270 GO:0046872 GO:0005680
Cluster-22178.71250 GO:0016567 GO:0006355 GO:0006486 GO:0008270
GO:0005524 GO:0016757 GO:0004842 GO:0016818 GO:0003677
GO:0046872 GO:0003700 GO:0003676 GO:0008134 GO:0005515
GO:0005680 GO:0005667
Cluster-22178.71251 GO:0006414 GO:0006448 GO:0019684 GO:0016567
GO:0006118 GO:0003746 GO:0008270 GO:0004842 GO:0046872
GO:0003676 GO:0045156 GO:0005515 GO:0005853 GO:0016021
GO:0005840 GO:0030077
Cluster-22178.71253 GO:0007165 GO:0030695 GO:0005057
Cluster-22178.71255 GO:0008033

Cluster-22178.71256 GO:0016192 GO:0006570 GO:0007304 GO:0006470
GO:0008138 GO:0005213 GO:0004725 GO:0005576 GO:0042600
Cluster-22178.71258 GO:0001510 GO:0006306 GO:0031167 GO:0009452
GO:0008152 GO:0008170 GO:0003677 GO:0005515 GO:0008168
GO:0005201 GO:0005578 GO:0019013 GO:0005581 GO:0016021
Cluster-22178.71260 GO:0035556 GO:0005515
Cluster-22178.71262 GO:0000226 GO:0000910 GO:0006554 GO:0006289
GO:0050690 GO:0043161 GO:0006479 GO:0006281 GO:0008017
GO:0005537 GO:0046872 GO:0005515 GO:0003684 GO:0018024
GO:0045298
Cluster-22178.71266 GO:0005515 GO:0046872 GO:0008270
Cluster-22178.71268 GO:0003676
Cluster-22178.71269 GO:0006886 GO:0005515 GO:0005525 GO:0005047
GO:0003924 GO:0005785 GO:0005786
Cluster-22178.71274 GO:0006687 GO:0009058 GO:0005515 GO:0001733
GO:0005794 GO:0016021
Cluster-22178.71275 GO:0000160 GO:0016310 GO:0006886 GO:0016192
GO:0009405 GO:0000155 GO:0016021 GO:0009365 GO:0005622
GO:0030131 GO:0005576
Cluster-22178.71276 GO:0006508 GO:0004197
Cluster-22178.71279 GO:0006508 GO:0004197 GO:0008234
Cluster-22178.71280 GO:0006323
Cluster-22178.71282 GO:0006278 GO:0007165 GO:0003964 GO:0005179
GO:0005576
Cluster-22178.71284 GO:0015992 GO:0015694 GO:0006400 GO:0015986
GO:0006470 GO:0006570 GO:0000087 GO:0008616 GO:0004725
GO:0015078 GO:0008479 GO:0015097 GO:0000276 GO:0005622
GO:0016020 GO:0019031
Cluster-22178.71286 GO:0006508 GO:0006412 GO:0055114 GO:0042254
GO:0006144 GO:0004252 GO:0003968 GO:0071949 GO:0003735
GO:0017111 GO:0016614 GO:0004197 GO:0016491 GO:0050660
GO:0031379 GO:0005840 GO:0016020
Cluster-22178.71287 GO:0042254 GO:0006144 GO:0006508 GO:0006412

GO:0055114 GO:0071949 GO:0004197 GO:0016614 GO:0017111
GO:0003735 GO:0016491 GO:0050660 GO:0004252 GO:0003968
GO:0016020 GO:0031379 GO:0005840
Cluster-22178.71288 GO:0007218 GO:0006144 GO:0042254 GO:0055114
GO:0006412 GO:0006508 GO:0050660 GO:0016491 GO:0004197
GO:0017111 GO:0016614 GO:0003735 GO:0071949 GO:0003968
GO:0004252 GO:0016020 GO:0005840 GO:0031379
Cluster-22178.71292 GO:0008283 GO:0040007 GO:0007165 GO:0008083
GO:0005515 GO:0016020 GO:0016021
Cluster-22178.71293 GO:0006364 GO:0009058 GO:0042432 GO:0016114
GO:0005515 GO:0016844 GO:0032040 GO:0016021
Cluster-22178.71294 GO:0005515
Cluster-22178.71296 GO:0005515
Cluster-22178.71297 GO:0006260 GO:0046983 GO:0003887 GO:0003677
GO:0005515 GO:0042802 GO:0042575
Cluster-22178.71299 GO:0006468 GO:0010215 GO:0016049 GO:0004672
GO:0005524 GO:0031225
Cluster-22178.71303 GO:0016310 GO:0009069 GO:0005515 GO:0004674

Cluster-22178.71304 GO:0016310 GO:0009069 GO:0005515 GO:0004674

Cluster-22178.71306 GO:0006412 GO:0042254 GO:0006364 GO:0003735
GO:0005515 GO:0005840 GO:0005622
Cluster-22178.71308 GO:0007218 GO:0006468 GO:0042811 GO:0004672
GO:0005524 GO:0016773 GO:0016746 GO:0005184 GO:0016020
Cluster-22178.71309 GO:0042811 GO:0006468 GO:0007218 GO:0005184
GO:0016746 GO:0016773 GO:0004672 GO:0005524 GO:0016020
Cluster-22178.71312 GO:0006464 GO:0032259 GO:0009452 GO:0046500
GO:0001510 GO:0031167 GO:0006479 GO:0008168 GO:0004719
GO:0003723
Cluster-22178.71314 GO:0006470 GO:0006570 GO:0008138 GO:0004725

Cluster-22178.71316 GO:0005515

Cluster-22178.71319 GO:0000413 GO:0000162 GO:0006571 GO:0009094 GO:0006457 GO:0006096 GO:0006094 GO:0004634 GO:0003755 GO:0000287 GO:0000015
Cluster-22178.71320 GO:0005515 GO:0016021
Cluster-22178.71321 GO:0006351 GO:0051252 GO:0006388 GO:0000213 GO:0003714 GO:0000214 GO:0005667
Cluster-22178.71322 GO:0006388 GO:0006351 GO:0051252 GO:0000213 GO:0003714 GO:0005667 GO:0000214
Cluster-22178.71324 GO:0006508 GO:0008233 GO:0005634
Cluster-22178.71325 GO:0006886 GO:0006605 GO:0009214 GO:0006000 GO:0006013 GO:0005515 GO:0042802 GO:0003873 GO:0004113 GO:0016887 GO:0008641 GO:0005524 GO:0016020 GO:0005742
Cluster-22178.7133 GO:0019497 GO:0006771 GO:0005515 GO:0003993 GO:0046872
Cluster-22178.71330 GO:0006820 GO:0015675 GO:0035444 GO:0055085 GO:0006810 GO:0046872 GO:0015099 GO:0005215 GO:0016021 GO:0016020
Cluster-22178.71336 GO:0016032 GO:0009405 GO:0044179 GO:0042254 GO:0006412 GO:0043087 GO:0035023 GO:0003735 GO:0005515 GO:0005089 GO:0019012 GO:0005737 GO:0005840
Cluster-22178.71338 GO:0016310 GO:0000160 GO:0007268 GO:0033130 GO:0043495 GO:0005515 GO:0000155 GO:0003723 GO:0009365 GO:0016021 GO:0019867
Cluster-22178.71339 GO:0009308 GO:0055114 GO:0003676 GO:0016638 GO:0003723 GO:0042597
Cluster-22178.71342 GO:0051016 GO:0006468 GO:0016773 GO:0005524 GO:0004672 GO:0008290 GO:0016020
Cluster-22178.71343 GO:0046813 GO:0061025 GO:0005515 GO:0016021 GO:0019031
Cluster-22178.71346 GO:0007291 GO:0006520 GO:0016597 GO:0016743

Cluster-22178.71349 GO:0051424 GO:0005515
Cluster-22178.71353 GO:0004789 GO:0005515 GO:0019012

Cluster-22178.71354 GO:0006260 GO:0019073 GO:0006139 GO:0006508
GO:0004222 GO:0003676 GO:0008270 GO:0003887 GO:0003677
GO:0008408 GO:0031012 GO:0042575
Cluster-22178.71355 GO:0006260 GO:0006139 GO:0019073 GO:0006508
GO:0008270 GO:0004222 GO:0003676 GO:0003677 GO:0003887
GO:0008408 GO:0031012 GO:0042575
Cluster-22178.71357 GO:0006560 GO:0006270 GO:0071436 GO:0006814
GO:0006525 GO:0045116 GO:0006090 GO:0008948 GO:0015081
GO:0005524 GO:0016881 GO:0016020
Cluster-22178.71358 GO:0035023 GO:0006308 GO:0006468 GO:0006508
GO:0007169 GO:0043087 GO:0007165 GO:0015743 GO:0004672
GO:0005515 GO:0005089 GO:0004872 GO:0004185 GO:0005524
GO:0008855 GO:0004714 GO:0009318 GO:0016020
Cluster-22178.71359 GO:0005515
Cluster-22178.71360 GO:0010468 GO:0016021
Cluster-22178.71363 GO:0009405 GO:0005198
Cluster-22178.71365 GO:0006950 GO:0006457 GO:0005524 GO:0051082

Cluster-22178.71366 GO:0009405 GO:0005198
Cluster-22178.71367 GO:0006493 GO:0000030 GO:0000136 GO:0016020
Cluster-22178.71371 GO:0008270
Cluster-22178.71373 GO:0006508 GO:0008270 GO:0004222 GO:0031012
Cluster-22178.71374 GO:0015992 GO:0006118 GO:0006119 GO:0008121

Cluster-22178.71375 GO:0006396 GO:0001522 GO:0009451 GO:0003723
GO:0009982
Cluster-22178.71376 GO:0006281 GO:0051382 GO:0003677
Cluster-22178.71378 GO:0007021 GO:0000160 GO:0016310 GO:0051258
GO:0009405 GO:0007131 GO:0015031 GO:0030168 GO:0007165
GO:0006869 GO:0042157 GO:0003676 GO:0008289 GO:0005198
GO:0003723 GO:0051082 GO:0015631 GO:0005102 GO:0046983
GO:0030674 GO:0000155 GO:0045298 GO:0016021 GO:0009365
GO:0019031 GO:0019028 GO:0000794 GO:0005576 GO:0005577

Cluster-22178.71380 GO:0006355 GO:0006858 GO:0046983
Cluster-22178.71381 GO:0005515
Cluster-22178.71382 GO:0006468 GO:0004672 GO:0005524
Cluster-22178.71383 GO:0002682 GO:0030246
Cluster-22178.71384 GO:0007018 GO:0007017 GO:0008017 GO:0003777 GO:0005524 GO:0045298 GO:0005874 GO:0005576
Cluster-22178.71387 GO:0006508 GO:0004222
Cluster-22178.71388 GO:0045893 GO:0005515 GO:0001000 GO:0003677 GO:0008270
Cluster-22178.71389 GO:0046436 GO:0009252 GO:0008716 GO:0005524 GO:0046872
Cluster-22178.71390 GO:0007017 GO:0007018 GO:0005524 GO:0008017 GO:0003777 GO:0003723 GO:0005515 GO:0045298 GO:0005874
Cluster-22178.71395 GO:0016032 GO:0016567 GO:0090263 GO:0005515 GO:0046872 GO:0008270 GO:0004842 GO:0005680
Cluster-22178.71397 GO:0016567 GO:0090263 GO:0005515
Cluster-22178.71398 GO:0008033
Cluster-22178.71402 GO:0016787 GO:0005524 GO:0003676 GO:0003677

Cluster-22178.71403 GO:0008270 GO:0046872 GO:0005515
Cluster-22178.71405 GO:0051252 GO:0006402 GO:0003723 GO:0003676 GO:0046872 GO:0004535 GO:0005634 GO:0005737
Cluster-22178.71406 GO:0007165 GO:0007275 GO:0016055 GO:0006468 GO:0005102 GO:0004672 GO:0005524 GO:0005576
Cluster-22178.71413 GO:0042254 GO:0006412 GO:0006355 GO:0003735 GO:0003677 GO:0005840 GO:0005622
Cluster-22178.71415 GO:0003676
Cluster-22178.71416 GO:0019042 GO:0006270 GO:0008201 GO:0004867 GO:0046914 GO:0033644
Cluster-22178.71419 GO:0005515
Cluster-22178.71421 GO:0055085 GO:0016032 GO:0005506 GO:0016021
Cluster-22178.71423 GO:0055085 GO:0016032 GO:0005506 GO:0016021
Cluster-22178.71424 GO:0006468 GO:0006471 GO:0005515 GO:0004715

GO:0004672 GO:0005524 GO:0017176 GO:0003950

Cluster-22178.71425 GO:0006355 GO:0005515 GO:0003700 GO:0043565
GO:0005667 GO:0005634

Cluster-22178.71426 GO:0030150 GO:0046872 GO:0005742

Cluster-22178.71428 GO:0006468 GO:0005524 GO:0004672

Cluster-22178.71429 GO:0005515

Cluster-22178.71431 GO:0007264 GO:0006810 GO:0051252 GO:0006396
GO:0003723 GO:0016787 GO:0003677 GO:0004525 GO:0005524
GO:0005525 GO:0005576

Cluster-22178.71432 GO:0006396 GO:0006810 GO:0051252 GO:0007264
GO:0005525 GO:0005524 GO:0004525 GO:0003677 GO:0003723
GO:0016787

Cluster-22178.71433 GO:0009966 GO:0006260 GO:0005783 GO:0005634

Cluster-22178.71434 GO:0006486 GO:0008373

Cluster-22178.71435 GO:0009452 GO:0006396 GO:0008033 GO:0009451
GO:0031167 GO:0001510 GO:0004809 GO:0008270 GO:0008168
GO:0008173 GO:0003723 GO:0016021

Cluster-22178.71436 GO:0006888 GO:0007606 GO:0006508 GO:0006886
GO:0007165 GO:0008270 GO:0003723 GO:0004888 GO:0005515
GO:0004197 GO:0016020 GO:0030127

Cluster-22178.71438 GO:0042254 GO:0006412 GO:0003735 GO:0005622
GO:0005840

Cluster-22178.71440 GO:0009432 GO:0006281 GO:0051782 GO:0006310
GO:0007165 GO:0006260 GO:0051016 GO:0003723 GO:0005509
GO:0098519 GO:0009378 GO:0003724 GO:0016887 GO:0003689
GO:0005524 GO:0042575 GO:0005663 GO:0005578 GO:0009276
GO:0009379 GO:0005657 GO:0030479

Cluster-22178.71442 GO:0006886 GO:0016192 GO:0005515 GO:0005198
GO:0030117 GO:0019028

Cluster-22178.71444 GO:0005515

Cluster-22178.71445 GO:0004190 GO:0003676 GO:0016021

Cluster-22178.71446 GO:0005515

Cluster-22178.71447 GO:0007267 GO:0032259 GO:0007010 GO:0006310

GO:0006511 GO:0004175 GO:0008168 GO:0003779 GO:0003677
GO:0005515 GO:0005634 GO:0019773
Cluster-22178.71450 GO:0019497 GO:0006771 GO:0046872 GO:0005515
GO:0003993
Cluster-22178.71455 GO:0045454 GO:0000398 GO:0005681
Cluster-22178.71457 GO:0007264 GO:0007186 GO:0007165 GO:0003924
GO:0019001 GO:0031683 GO:0005524 GO:0004871 GO:0005525
GO:0003677 GO:0000166
Cluster-22178.71458 GO:0009399 GO:0008270 GO:0005515 GO:0016021
Cluster-22178.71459 GO:0006402 GO:0051252 GO:0003676 GO:0004535
GO:0046872 GO:0003723 GO:0005737 GO:0005634
Cluster-22178.71460 GO:0006525 GO:0006560 GO:0018112
Cluster-22178.71462 GO:0006397 GO:0007264 GO:0005525 GO:0003924
GO:0046872 GO:0031419 GO:0016788 GO:0016787
Cluster-22178.71463 GO:0006855 GO:0055085 GO:0006810 GO:0015893
GO:0015297 GO:0015238 GO:0016020
Cluster-22178.71464 GO:0046872
Cluster-22178.71465 GO:0016310 GO:0009069 GO:0046325 GO:0015991
GO:0004674 GO:0016773 GO:0016820 GO:0005515 GO:0000221
GO:0005737
Cluster-22178.71466 GO:0046325 GO:0005515 GO:0016773 GO:0005737
Cluster-22178.71467 GO:0016310 GO:0015991 GO:0046325 GO:0009069
GO:0004674 GO:0016773 GO:0005515 GO:0016820 GO:0000221
GO:0005737
Cluster-22178.71468 GO:0006810 GO:0007275 GO:0006508 GO:0004198
GO:0005515 GO:0005215 GO:0005576 GO:0005622
Cluster-22178.71472 GO:0007165 GO:0051258 GO:0030168 GO:0005102
GO:0030674 GO:0005577
Cluster-22178.71473 GO:0005985 GO:0005982 GO:0005978 GO:0005515
GO:0004373
Cluster-22178.71475 GO:0005524 GO:0003676
Cluster-22178.71476 GO:0055114 GO:0016491
Cluster-22178.71478 GO:0000103 GO:0006144 GO:0003824 GO:0004020

GO:0005524
Cluster-22178.71481 GO:0046872 GO:0003676 GO:0005198 GO:0005622
GO:0019028
Cluster-22178.71482 GO:0005198 GO:0003676 GO:0016787 GO:0046872
GO:0019028 GO:0005622
Cluster-22178.71483 GO:0005515 GO:0015631 GO:0045298
Cluster-22178.71484 GO:0006461 GO:0042254 GO:0019627 GO:0016151
GO:0003735 GO:0005515 GO:0005840
Cluster-22178.71488 GO:0008152 GO:0016042 GO:0007586 GO:0008047
GO:0016746 GO:0008236 GO:0005515 GO:0005509 GO:0005576
Cluster-22178.71489 GO:0015992 GO:0006123 GO:0004129 GO:0045277
GO:0005743
Cluster-22178.71491 GO:0008092 GO:0005737 GO:0019898
Cluster-22178.71493 GO:0006694 GO:0008092 GO:0008168 GO:0019898
GO:0005737
Cluster-22178.71494 GO:0042147
Cluster-22178.71495 GO:0006508 GO:0004181 GO:0003950 GO:0016787
GO:0008270 GO:0005634
Cluster-22178.71496 GO:0035556 GO:0009069 GO:0015994 GO:0016310
GO:0015996 GO:0055114 GO:0006468 GO:0004674 GO:0047134
GO:0005524 GO:0016491 GO:0016773 GO:0004672 GO:0047746
GO:0005515 GO:0016020
Cluster-22178.71497 GO:0006813 GO:0000184 GO:0005516 GO:0046872
GO:0005524 GO:0015269 GO:0008270 GO:0003924 GO:0016887
GO:0005525 GO:0016021
Cluster-22178.71499 GO:0006281 GO:0003824
Cluster-22178.715 GO:0006810 GO:0006811 GO:0005230 GO:0016020
Cluster-22178.7150 GO:0006446 GO:0003743 GO:0005840
Cluster-22178.71500 GO:0008283 GO:0040007 GO:0007165 GO:0006954
GO:0006955 GO:0005149 GO:0005509 GO:0005515 GO:0045323
Cluster-22178.71501 GO:0019064 GO:0010506 GO:0007165 GO:0004222
GO:0016788 GO:0005515 GO:0046789 GO:0009986 GO:0019031
GO:0005615

Cluster-22178.71503 GO:0016579 GO:0036459
Cluster-22178.71504 GO:0006120 GO:0006744 GO:0008152 GO:0016310
GO:0055114 GO:0015992 GO:0019049 GO:0005975 GO:0006814
GO:0009069 GO:0003824 GO:0004674 GO:0008137 GO:0016836
GO:0030246
Cluster-22178.71505 GO:0006814 GO:0008152 GO:0006744 GO:0006120
GO:0005975 GO:0019049 GO:0015992 GO:0055114 GO:0008137
GO:0030246 GO:0016836 GO:0003824
Cluster-22178.71506 GO:0055114 GO:0006355 GO:0019478 GO:0016491
GO:0043565 GO:0003676 GO:0003700 GO:0050662 GO:0016788
GO:0005515 GO:0005737 GO:0005667 GO:0016020
Cluster-22178.71507 GO:0006952 GO:0031492 GO:0005634 GO:0000785
Cluster-22178.71510 GO:0007268 GO:0033130 GO:0043495
Cluster-22178.71515 GO:0006631 GO:0016746 GO:0005515
Cluster-22178.71517 GO:0006552 GO:0006550 GO:0006568 GO:0015940
GO:0055114 GO:0019521 GO:0006574 GO:0006631 GO:0018874
GO:0006098 GO:0006633 GO:0006554 GO:0003857 GO:0008677
GO:0004616 GO:0051287 GO:0016491
Cluster-22178.71518 GO:0007304 GO:0046872 GO:0005213 GO:0042600
GO:0005576
Cluster-22178.71521 GO:0016787 GO:0005515
Cluster-22178.71522 GO:0008146 GO:0016021
Cluster-22178.71523 GO:0042742 GO:0051191 GO:0006352 GO:0005515
GO:0005576 GO:0005669
Cluster-22178.71524 GO:0051191 GO:0006352 GO:0042742 GO:0005515
GO:0005669 GO:0005576
Cluster-22178.71525 GO:0006352 GO:0051191 GO:0042742 GO:0005515
GO:0005669 GO:0005576
Cluster-22178.71527 GO:0042742 GO:0051191 GO:0006352 GO:0005515
GO:0005576 GO:0005669
Cluster-22178.71528 GO:0055114 GO:0007186 GO:0046872 GO:0004930
GO:0016972 GO:0016021
Cluster-22178.71529 GO:0016310 GO:0006144 GO:0006206 GO:0006351

GO:0009069 GO:0006468 GO:0004674 GO:0016773 GO:0005524
GO:0004672 GO:0003899 GO:0003677 GO:0016020 GO:0005730
Cluster-22178.71530 GO:0008270
Cluster-22178.71532 GO:0008270
Cluster-22178.71533 GO:0007165 GO:0004872 GO:0016021
Cluster-22178.71534 GO:0003676
Cluster-22178.71536 GO:0005515
Cluster-22178.71538 GO:0035556 GO:0016787
Cluster-22178.71541 GO:0006450 GO:0005515
Cluster-22178.71547 GO:0006144 GO:0006807 GO:0000256 GO:0004037
GO:0005634
Cluster-22178.71548 GO:0030001 GO:0008063 GO:0055085 GO:0006367
GO:0046873 GO:0005672 GO:0016020
Cluster-22178.71549 GO:0016567 GO:0004842 GO:0005515 GO:0008270
GO:0005680
Cluster-22178.71551 GO:0006813 GO:0006355 GO:0006542 GO:0006807
GO:0006811 GO:0009252 GO:0004356 GO:0003677 GO:0005249
GO:0016020 GO:0008076
Cluster-22178.71552 GO:0008063 GO:0030001 GO:0055085 GO:0006367
GO:0046873 GO:0005672 GO:0016020
Cluster-22178.71553 GO:0000398 GO:0008270 GO:0005515 GO:0005681
Cluster-22178.71556 GO:0006030 GO:0008061 GO:0005576 GO:0016020
Cluster-22178.71557 GO:0042981 GO:0008270 GO:0005515
Cluster-22178.71558 GO:0006508 GO:0006355 GO:0005515 GO:0003676
GO:0004252
Cluster-22178.71559 GO:0005515
Cluster-22178.71560 GO:0016746
Cluster-22178.71561 GO:0007165 GO:0035091 GO:0005515
Cluster-22178.71563 GO:0035556 GO:0008152 GO:0016746 GO:0005515

Cluster-22178.71564 GO:0017004 GO:0015886 GO:0016021
Cluster-22178.71565 GO:0006355 GO:0005515
Cluster-22178.71567 GO:0030036 GO:0003779 GO:0016021 GO:0030054

Cluster-22178.71569 GO:0006260 GO:0005515 GO:0003677 GO:0003887 GO:0016021 GO:0042575 GO:0009360

Cluster-22178.71570 GO:0006109 GO:0006355 GO:0016310 GO:0000160 GO:0000155 GO:0003724 GO:0003774 GO:0005524 GO:0016887 GO:0005515 GO:0008134 GO:0003723 GO:0005525 GO:0004672 GO:0003924 GO:0016459 GO:0005667 GO:0009365

Cluster-22178.71571 GO:0007165 GO:0006468 GO:0005515 GO:0005524 GO:0004672

Cluster-22178.71574 GO:0048306

Cluster-22178.71575 GO:0048306

Cluster-22178.71576 GO:0048306

Cluster-22178.71577 GO:0005515 GO:0016787

Cluster-22178.71578 GO:0007186 GO:0007275 GO:0005515 GO:0004252 GO:0003676 GO:0070008 GO:0004930 GO:0042600

Cluster-22178.71579 GO:0006281 GO:0000077 GO:0030896 GO:0005634

Cluster-22178.71581 GO:0035091 GO:0020037 GO:0005524

Cluster-22178.71582 GO:0005515

Cluster-22178.71583 GO:0007017 GO:0005875

Cluster-22178.71586 GO:0006468 GO:0004672 GO:0005524

Cluster-22178.71587 GO:0006118 GO:0045454 GO:0015035 GO:0009055 GO:0005515 GO:0005634

Cluster-22178.71590 GO:0006811 GO:0006888 GO:0007165 GO:0006810 GO:0007172 GO:0006886 GO:0006468 GO:0004713 GO:0004871 GO:0005524 GO:0004672 GO:0005230 GO:0030127 GO:0016020 GO:0005925

Cluster-22178.71592 GO:0006886 GO:0016192 GO:0005515 GO:0005198 GO:0030117

Cluster-22178.71594 GO:0043087 GO:0044419 GO:0035023 GO:0005089 GO:0005515 GO:0005887

Cluster-22178.71595 GO:0006659 GO:0006308 GO:0009405 GO:0008289 GO:0004222 GO:0008270 GO:0046872 GO:0008855 GO:0005198 GO:0005515 GO:0019031 GO:0009318 GO:0019028

Cluster-22178.71598 GO:0006874 GO:0006816 GO:0005219 GO:0003676

GO:0008270 GO:0016021 GO:0005622
Cluster-22178.716 GO:0005515
Cluster-22178.71600 GO:0006298 GO:0006308 GO:0010506 GO:0006260
GO:0030983 GO:0008855 GO:0005524 GO:0005634 GO:0009318
GO:0005615
Cluster-22178.71601 GO:0006486 GO:0006007 GO:0008373 GO:0003824
GO:0046537
Cluster-22178.71603 GO:0051289 GO:0007264 GO:0006355 GO:0008140
GO:0003700 GO:0043565 GO:0005096 GO:0005667
Cluster-22178.71606 GO:0007165 GO:0055114 GO:0005515 GO:0008270
GO:0051920 GO:0019013
Cluster-22178.71607 GO:0035434 GO:0006825 GO:0046983 GO:0005375
GO:0016021
Cluster-22178.71609 GO:0006468 GO:0006118 GO:0051536 GO:0005524
GO:0004672 GO:0009055
Cluster-22178.71611 GO:0007264 GO:0015684 GO:0007165 GO:0007186
GO:0015093 GO:0019001 GO:0031683 GO:0008270 GO:0003924
GO:0005525 GO:0004871 GO:0016021
Cluster-22178.71613 GO:0006355 GO:0042967 GO:0046854 GO:0016787
GO:0004402 GO:0003712 GO:0008270 GO:0005667 GO:0005634
GO:0019033 GO:0000123
Cluster-22178.71614 GO:0006351 GO:0009297 GO:0016567 GO:0006144
GO:0006464 GO:0006206 GO:0003899 GO:0005515 GO:0003723
GO:0016151 GO:0046872 GO:0061630 GO:0004842 GO:0008270
GO:0005634 GO:0019867 GO:0005730 GO:0005680
Cluster-22178.71616 GO:0031224
Cluster-22178.71619 GO:0006816 GO:0006874 GO:0005524 GO:0016887
GO:0005219 GO:0005622 GO:0016021
Cluster-22178.71620 GO:0010508 GO:0006351 GO:0006144 GO:0006914
GO:0006206 GO:0005515 GO:0003899 GO:0003677 GO:0008191
GO:0005730
Cluster-22178.71624 GO:0006886 GO:0005543
Cluster-22178.71625 GO:0006810 GO:0055085 GO:0030150 GO:0015979

GO:0005515 GO:0005215 GO:0016020 GO:0005742 GO:0009523
GO:0009539
Cluster-22178.71626 GO:0016021
Cluster-22178.71627 GO:0006357 GO:0006308 GO:0043087 GO:0007165
GO:0006259 GO:0007264 GO:0008270 GO:0016765 GO:0001104
GO:0005515 GO:0004531 GO:0005085 GO:0016592
Cluster-22178.71628 GO:0005515
Cluster-22178.71631 GO:0006183 GO:0006241 GO:0006206 GO:0006228
GO:0006165 GO:0006144 GO:0004550
Cluster-22178.71632 GO:0006183 GO:0006241 GO:0006165 GO:0006144
GO:0006206 GO:0006228 GO:0004550
Cluster-22178.71633 GO:0006241 GO:0006183 GO:0006206 GO:0006228
GO:0006144 GO:0006165 GO:0004550
Cluster-22178.71634 GO:0006352 GO:0005634
Cluster-22178.71637 GO:0006886 GO:0016192 GO:0006355 GO:0003700
GO:0030131 GO:0030117 GO:0005667
Cluster-22178.71640 GO:0006402 GO:0006810 GO:0051252 GO:0046872
GO:0004535 GO:0005215 GO:0003676 GO:0003723 GO:0005737
GO:0005576 GO:0005634
Cluster-22178.71641 GO:0006850 GO:0005743
Cluster-22178.71644 GO:0006468 GO:0004672 GO:0005524 GO:0003677
GO:0005515
Cluster-22178.71646 GO:0006468 GO:0046872 GO:0004672 GO:0005524
GO:0016817
Cluster-22178.71647 GO:0005643
Cluster-22178.71648 GO:0006468 GO:0004672 GO:0005524 GO:0003677
GO:0005515
Cluster-22178.71649 GO:0051090 GO:0042254 GO:0001522 GO:0005524
GO:0005515 GO:0030515 GO:0072588 GO:0005634
Cluster-22178.71655 GO:0006351 GO:0030036 GO:0008033 GO:0003677
GO:0003779 GO:0046872 GO:0030054 GO:0005634
Cluster-22178.71656 GO:0006468 GO:0005524 GO:0004672
Cluster-22178.71658 GO:0006351 GO:0008033 GO:0003677 GO:0046872

GO:0005634
Cluster-22178.71659 GO:0030036 GO:0008033 GO:0006351 GO:0003779
GO:0046872 GO:0003677 GO:0030054 GO:0005634
Cluster-22178.71661 GO:0000226 GO:0051537 GO:0015630 GO:0043231
Cluster-22178.71662 GO:0000226 GO:0051537 GO:0015630 GO:0043231
Cluster-22178.71663 GO:0016192 GO:0006886 GO:0005515 GO:0000775
GO:0030117
Cluster-22178.71665 GO:0006355 GO:0006281 GO:0019079 GO:0006310
GO:0002949 GO:0003723 GO:0016301 GO:0008134 GO:0043531
GO:0003678 GO:0009378 GO:0098519 GO:0005524 GO:0016887
GO:0003724 GO:0005667 GO:0009379 GO:0005657
Cluster-22178.71667 GO:0008152 GO:0055114 GO:0016032 GO:0016491
GO:0019033
Cluster-22178.71668 GO:0019722 GO:0035091 GO:0005515 GO:0003676
GO:0016021 GO:0005887
Cluster-22178.71669 GO:0005515 GO:0042302 GO:0005549
Cluster-22178.71670 GO:0042302 GO:0005515 GO:0005549
Cluster-22178.71671 GO:0042302 GO:0005515 GO:0005549
Cluster-22178.71673 GO:0009306 GO:0035556 GO:0051090 GO:0005515
GO:0046872 GO:0005634
Cluster-22178.71674 GO:0005634
Cluster-22178.71676 GO:0043039 GO:0006531 GO:0006522 GO:0006419
GO:0051301 GO:0005515 GO:0000166 GO:0003677 GO:0005524
GO:0016876 GO:0004813
Cluster-22178.71678 GO:0006468 GO:0016192 GO:0005524 GO:0004672
GO:0016021
Cluster-22178.71679 GO:0005975 GO:0004553
Cluster-22178.71680 GO:0006465 GO:0006355 GO:0003676 GO:0008233
GO:0016021 GO:0005622 GO:0005787
Cluster-22178.71681 GO:0006813 GO:0003676 GO:0003677 GO:0016787
GO:0005267 GO:0005524 GO:0016020
Cluster-22178.71683 GO:0006813 GO:0009405 GO:0008152 GO:0007157
GO:0016787 GO:0016301 GO:0003677 GO:0005524 GO:0005267

GO:0003676 GO:0016020 GO:0016021
Cluster-22178.71685 GO:0009405 GO:0006813 GO:0007157 GO:0045444
GO:0003677 GO:0016787 GO:0003676 GO:0005267 GO:0005524
GO:0016020 GO:0016021
Cluster-22178.71687 GO:0055114 GO:0009186 GO:0030246 GO:0016021
Cluster-22178.71688 GO:0034453 GO:0000042 GO:0005515 GO:0005815
GO:0016021
Cluster-22178.71689 GO:0006810 GO:0006334 GO:0003676 GO:0005634
GO:0005643
Cluster-22178.71690 GO:0035556 GO:0005515
Cluster-22178.71691 GO:0003677 GO:0046983
Cluster-22178.71697 GO:0000226 GO:0000166 GO:0046872 GO:0015630
GO:0016020
Cluster-22178.71698 GO:0006914 GO:0000226 GO:0055085 GO:0006260
GO:0006816 GO:0006874 GO:0006810 GO:0006270 GO:0007165
GO:0006308 GO:0030001 GO:0010508 GO:0007010 GO:0008855
GO:0005524 GO:0042626 GO:0005179 GO:0003677 GO:0003779
GO:0046872 GO:0005219 GO:0005515 GO:0005622 GO:0016021
GO:0005664 GO:0005576 GO:0015630 GO:0009318
Cluster-22178.71699 GO:0007016 GO:0007165 GO:0006412 GO:0042254
GO:0005158 GO:0003779 GO:0005200 GO:0000062 GO:0003735
GO:0005622 GO:0005856 GO:0005899 GO:0005840 GO:0001726
GO:0005925
Cluster-22178.71701 GO:0005515
Cluster-22178.71702 GO:0042254 GO:0006412 GO:0003735 GO:0005840
Cluster-22178.71703 GO:0006886 GO:0017119
Cluster-22178.71704 GO:0035023 GO:0016310 GO:0043087 GO:0007165
GO:0006468 GO:0009069 GO:0005089 GO:0004674 GO:0005096
Cluster-22178.71707 GO:0007010 GO:0006810 GO:0030001 GO:0006260
GO:0000226 GO:0055085 GO:0003677 GO:0005515 GO:0042626
GO:0046872 GO:0005524 GO:0003779 GO:0015630 GO:0005664
GO:0016021
Cluster-22178.71708 GO:0006471 GO:0003950

Cluster-22178.71710 GO:0008152 GO:0006886 GO:0016192 GO:0003824
GO:0005515 GO:0030117
Cluster-22178.71713 GO:0006355 GO:0003676 GO:0005515 GO:0005634
GO:0019028
Cluster-22178.71714 GO:0007165 GO:0007186 GO:0007264 GO:0006013
GO:0006000 GO:0003873 GO:0005525 GO:0004871 GO:0005125
GO:0005524 GO:0031683 GO:0019001 GO:0003924
Cluster-22178.71717 GO:0007165 GO:0008283 GO:0040007 GO:0008083
GO:0005515 GO:0005125 GO:0016021
Cluster-22178.71718 GO:0005524 GO:0016787 GO:0003677 GO:0003676
GO:0005515
Cluster-22178.71719 GO:0003677 GO:0005515 GO:0003676 GO:0005524
GO:0016787
Cluster-22178.71720 GO:0006281 GO:0006144 GO:0006464 GO:0046034
GO:0004017 GO:0003677 GO:0046872 GO:0008270 GO:0016151
Cluster-22178.71721 GO:0006355 GO:0009271
Cluster-22178.71723 GO:0007339 GO:0005576
Cluster-22178.71724 GO:0042710 GO:0007186 GO:0004930 GO:0016021
Cluster-22178.71728 GO:0048280 GO:0015991 GO:0006886 GO:0004869
GO:0005515 GO:0016820 GO:0005737 GO:0000221 GO:0000139
Cluster-22178.71729 GO:0006281 GO:0046872 GO:0008270
Cluster-22178.71732 GO:0003824 GO:0005886
Cluster-22178.71733 GO:0008063 GO:0006813 GO:0006814 GO:0007224
GO:0005515 GO:0008270 GO:0005890 GO:0016021
Cluster-22178.71736 GO:0005515
Cluster-22178.71737 GO:0005515
Cluster-22178.71738 GO:0055114 GO:0015992 GO:0006118 GO:0006119
GO:0016491 GO:0051536 GO:0051537 GO:0008121
Cluster-22178.7174 GO:0007010 GO:0003779
Cluster-22178.71740 GO:0006308 GO:0006281 GO:0005515 GO:0003677
GO:0004518 GO:0008855 GO:0009318
Cluster-22178.71744 GO:0006355 GO:0005634
Cluster-22178.71746 GO:0006915 GO:0006508 GO:0006260 GO:0006974

GO:0003677 GO:0003887 GO:0004181 GO:0008270 GO:0042575
Cluster-22178.71747 GO:0006260 GO:0006974 GO:0006508 GO:0006915
GO:0004181 GO:0008270 GO:0003677 GO:0003887 GO:0042575
Cluster-22178.71748 GO:0005515
Cluster-22178.7175 GO:0007155 GO:0005198 GO:0031012
Cluster-22178.71750 GO:0015031 GO:0016021
Cluster-22178.71751 GO:0048309 GO:0016032 GO:0006355 GO:0006914
GO:0051028 GO:0051607 GO:0015684 GO:0003924 GO:0043565
GO:0003700 GO:0005525 GO:0005515 GO:0015093 GO:0016021
GO:0019033 GO:0005667
Cluster-22178.71752 GO:0007423 GO:0005212
Cluster-22178.71753 GO:0015979 GO:0030145 GO:0009523 GO:0016021
Cluster-22178.71754 GO:0006265 GO:0008152 GO:0003916 GO:0008092
GO:0016491 GO:0003677 GO:0005737 GO:0019898
Cluster-22178.71756 GO:0007155 GO:0005198 GO:0005515 GO:0031012
Cluster-22178.71758 GO:0006468 GO:0005524 GO:0004672 GO:0016773
GO:0016020
Cluster-22178.71759 GO:0006508 GO:0006281 GO:0006351 GO:0006144
GO:0006206 GO:0003723 GO:0004252 GO:0003899 GO:0005524
GO:0003724 GO:0005730
Cluster-22178.71760 GO:0007283 GO:0071436 GO:0006525 GO:0006206
GO:0006144 GO:0006560 GO:0006351 GO:0006814 GO:0006281
GO:0006508 GO:0006090 GO:0003677 GO:0003724 GO:0015081
GO:0005524 GO:0004252 GO:0003899 GO:0008948 GO:0003723
GO:0016020 GO:0005730 GO:0005634 GO:0000786
Cluster-22178.71764 GO:0008270 GO:0005515
Cluster-22178.71765 GO:0035091 GO:0020037
Cluster-22178.71767 GO:0008152 GO:0016226 GO:0008168 GO:0051536
GO:0005737
Cluster-22178.71768 GO:0017004 GO:0015886 GO:0004867 GO:0016021
Cluster-22178.71769 GO:0015886 GO:0017004 GO:0004867 GO:0016021
Cluster-22178.71770 GO:0008033 GO:0055114 GO:0006769 GO:0046497
GO:0050661 GO:0051287 GO:0071949 GO:0016779 GO:0016491

GO:0008750 GO:0050660 GO:0016021
Cluster-22178.71771 GO:0046497 GO:0008033 GO:0055114 GO:0006769
GO:0016779 GO:0071949 GO:0051287 GO:0050661 GO:0050660
GO:0016491 GO:0008750 GO:0016021
Cluster-22178.71772 GO:0046497 GO:0055114 GO:0008033 GO:0006769
GO:0006813 GO:0050660 GO:0008556 GO:0016491 GO:0008750
GO:0016779 GO:0051287 GO:0050661 GO:0071949 GO:0016021
GO:0005886
Cluster-22178.71773 GO:0008033 GO:0055114 GO:0006769 GO:0046497
GO:0006813 GO:0016491 GO:0008556 GO:0008750 GO:0050660
GO:0051287 GO:0071949 GO:0030976 GO:0050661 GO:0000287
GO:0016779 GO:0005886 GO:0016021
Cluster-22178.71774 GO:0003924 GO:0005525
Cluster-22178.71775 GO:0019051 GO:0006357 GO:0001104 GO:0016592
GO:0042025
Cluster-22178.71779 GO:0042967 GO:0006950 GO:0006355 GO:0016573
GO:0006457 GO:0005524 GO:0004402 GO:0051082 GO:0000123
Cluster-22178.71780 GO:0008270 GO:0005525 GO:0005634
Cluster-22178.71783 GO:0055114 GO:0007165 GO:0016717
Cluster-22178.71785 GO:0006464 GO:0051082 GO:0031072 GO:0016151
GO:0005515
Cluster-22178.71786 GO:0006464 GO:0005515 GO:0031072 GO:0016151
GO:0051082
Cluster-22178.71787 GO:0006464 GO:0051082 GO:0005515 GO:0031072
GO:0016151
Cluster-22178.71789 GO:0055114 GO:0016620
Cluster-22178.71790 GO:0006771 GO:0006164 GO:0006144 GO:0019497
GO:0006807 GO:0050830 GO:0005515 GO:0046872 GO:0031423
GO:0004643 GO:0003937 GO:0003993 GO:0005576 GO:0019012
GO:0042720
Cluster-22178.71791 GO:0005515 GO:0008270 GO:0005783
Cluster-22178.71792 GO:0008270 GO:0005515 GO:0005783
Cluster-22178.71793 GO:0009253 GO:0006807 GO:0009252 GO:0016783

GO:0008745
Cluster-22178.71794 GO:0009252 GO:0009253 GO:0006807 GO:0008745

Cluster-22178.71795 GO:0006123 GO:0015992 GO:0004129 GO:0045277
Cluster-22178.71797 GO:0006123 GO:0015992 GO:0004129 GO:0045277
Cluster-22178.71798 GO:0043093 GO:0007165 GO:0070206 GO:0006260
GO:0007128 GO:0006355 GO:0000917 GO:0007130 GO:0070193
GO:0048870 GO:0005515 GO:0043565 GO:0003700 GO:0005667
GO:0031514 GO:0000795 GO:0005737
Cluster-22178.71799 GO:0008270 GO:0016020
Cluster-22178.71800 GO:0006813 GO:0007165 GO:0008544 GO:0048870
GO:0005515 GO:0015269 GO:0016020 GO:0031514
Cluster-22178.71802 GO:0005515
Cluster-22178.71803 GO:0007186 GO:0007165 GO:0007264 GO:0004871
GO:0005525 GO:0003924 GO:0031683 GO:0019001
Cluster-22178.71804 GO:0007264 GO:0007186 GO:0007165 GO:0003924
GO:0031683 GO:0019001 GO:0004871 GO:0005525 GO:0030976
Cluster-22178.71807 GO:0006412 GO:0042254 GO:0003735 GO:0005622
GO:0005840
Cluster-22178.71808 GO:0018279 GO:0004579 GO:0016021 GO:0008250
Cluster-22178.71809 GO:0006520 GO:0007291 GO:0016743 GO:0016597

Cluster-22178.71813 GO:0046872
Cluster-22178.71816 GO:0016021
Cluster-22178.71818 GO:0006355 GO:0003700 GO:0043565 GO:0005667
Cluster-22178.71820 GO:0007165 GO:0005515 GO:0005096
Cluster-22178.71822 GO:0006270 GO:0015684 GO:0007165 GO:0007186
GO:0008283 GO:0007264 GO:0019068 GO:0035556 GO:0043087
GO:0006576 GO:0040007 GO:0035023 GO:0016757 GO:0005089
GO:0005524 GO:0015093 GO:0016887 GO:0008083 GO:0005198
GO:0005515 GO:0005525 GO:0004871 GO:0031683 GO:0019001
GO:0046872 GO:0003924 GO:0016020 GO:0016021
Cluster-22178.71826 GO:0006508 GO:0006940 GO:0006260 GO:0008270

GO:0005515 GO:0004222
Cluster-22178.71827 GO:0016021
Cluster-22178.71831 GO:0006342 GO:0006476 GO:0006351 GO:0006355
GO:0006807 GO:0006144 GO:0006206 GO:0030976 GO:0003899
GO:0000287 GO:0008270 GO:0070403 GO:0016811 GO:0003677
GO:0051287 GO:0017136 GO:0000118 GO:0005730
Cluster-22178.71832 GO:0006367 GO:0042254 GO:0032968 GO:0015743
GO:0000226 GO:0006412 GO:0005975 GO:0000910 GO:0003677
GO:0003735 GO:0008017 GO:0004649 GO:0004190 GO:0005622
GO:0005634 GO:0005840 GO:0016021 GO:0045298
Cluster-22178.71833 GO:0000910 GO:0005975 GO:0006412 GO:0000226
GO:0032968 GO:0015743 GO:0042254 GO:0006367 GO:0004190
GO:0004649 GO:0008017 GO:0003735 GO:0003677 GO:0016021
GO:0045298 GO:0005840 GO:0005634 GO:0005622
Cluster-22178.71834 GO:0055114 GO:0008152 GO:0016491
Cluster-22178.71836 GO:0055114 GO:0008152 GO:0016491
Cluster-22178.71837 GO:0008270 GO:0008201 GO:0008134 GO:0016772
GO:0005509 GO:0005634 GO:0005667
Cluster-22178.71838 GO:0005515
Cluster-22178.71844 GO:0030683 GO:0008092 GO:0005524 GO:0003676
GO:0005737 GO:0016021 GO:0019898 GO:0019012
Cluster-22178.71846 GO:0009405 GO:0005515 GO:0005576
Cluster-22178.71847 GO:0051252 GO:0006817 GO:0006807 GO:0009245
GO:0008759 GO:0003723 GO:0004521 GO:0005315 GO:0016020
GO:0016021
Cluster-22178.71848 GO:0006355 GO:0003700 GO:0003677 GO:0043565
GO:0008270 GO:0005667
Cluster-22178.71851 GO:0007186 GO:0004930 GO:0016021
Cluster-22178.71854 GO:0016579 GO:0036459
Cluster-22178.71855 GO:0006281 GO:0031412 GO:0006260 GO:0006144
GO:0005515 GO:0003968 GO:0003911 GO:0046872 GO:0008270
GO:0031411 GO:0031379
Cluster-22178.71857 GO:0003333 GO:0009847 GO:0006508 GO:0004185

GO:0016021
Cluster-22178.71858 GO:0043531 GO:0016887 GO:0005524
Cluster-22178.71861 GO:0006281 GO:0009432 GO:0003677 GO:0003824
GO:0003697 GO:0005524 GO:0033588
Cluster-22178.71863 GO:0055114 GO:0005975 GO:0043085 GO:0006120
GO:0006814 GO:0000902 GO:0015992 GO:0006744 GO:0000290
GO:0042773 GO:0008047 GO:0005525 GO:0005515 GO:0008137
GO:0016773 GO:0016787 GO:0016020
Cluster-22178.71864 GO:0031072 GO:0051082
Cluster-22178.71865 GO:0055114 GO:0009308 GO:0048038 GO:0005507
GO:0031072 GO:0008131 GO:0051082
Cluster-22178.71867 GO:0016567 GO:0008270 GO:0046872 GO:0005515
GO:0061630 GO:0004842 GO:0005622
Cluster-22178.71871 GO:0009069 GO:0006468 GO:0016310 GO:0004672
GO:0005524 GO:0004674
Cluster-22178.71874 GO:0042802 GO:0005515
Cluster-22178.71876 GO:0006355 GO:0006118 GO:0006119 GO:0055114
GO:0015992 GO:0008152 GO:0050662 GO:0008121 GO:0003677
GO:0016491 GO:0003824
Cluster-22178.71877 GO:0005515 GO:0005509
Cluster-22178.71878 GO:0006468 GO:0009069 GO:0006915 GO:0016310
GO:0006919 GO:0004674 GO:0005524 GO:0004672 GO:0016020
GO:0005739
Cluster-22178.71879 GO:0016310 GO:0006919 GO:0006915 GO:0009069
GO:0006468 GO:0004674 GO:0005524 GO:0004672 GO:0005739
Cluster-22178.71881 GO:0006352 GO:0006355 GO:0007186 GO:0007165
GO:0003700 GO:0004871 GO:0016987 GO:0003677 GO:0005834
GO:0005667
Cluster-22178.71882 GO:0008152 GO:0016032 GO:0055114 GO:0016491
GO:0019033
Cluster-22178.71884 GO:0007005 GO:0007606 GO:0005515 GO:0043565
GO:0016021 GO:0031305 GO:0005634
Cluster-22178.71886 GO:0007606 GO:0007005 GO:0043565 GO:0005515

GO:0005634 GO:0031305 GO:0016021
Cluster-22178.71887 GO:0055085 GO:0016021
Cluster-22178.71888 GO:0055085 GO:0006811 GO:0003676 GO:0016021
GO:0016020
Cluster-22178.71889 GO:0007005 GO:0007606 GO:0005515 GO:0043565
GO:0016021 GO:0031305 GO:0005634
Cluster-22178.71895 GO:0007275 GO:0005578
Cluster-22178.71899 GO:0005515
Cluster-22178.71900 GO:0051259 GO:0005515 GO:0034618
Cluster-22178.71901 GO:0005515 GO:0008270
Cluster-22178.71902 GO:0002218 GO:0032481 GO:0008270 GO:0005515

Cluster-22178.71907 GO:0043087 GO:0007264 GO:0005085
Cluster-22178.71908 GO:0043087 GO:0007264 GO:0005085
Cluster-22178.71910 GO:0007131 GO:0009405 GO:0007283 GO:0007130
GO:0008270 GO:0016020
Cluster-22178.71911 GO:0007165 GO:0030168 GO:0051258 GO:0005102
GO:0030674 GO:0005577
Cluster-22178.71914 GO:0008283 GO:0040007 GO:0007165 GO:0008083
GO:0005615
Cluster-22178.71916 GO:0046872
Cluster-22178.71917 GO:0051607 GO:0070206 GO:0006606 GO:0009405
GO:0005515 GO:0019028 GO:0016020
Cluster-22178.71918 GO:0006355 GO:0007186 GO:0007165 GO:0055114
GO:0019337 GO:0006807 GO:0009399 GO:0005515 GO:0003677
GO:0004872 GO:0016163 GO:0004930 GO:0016610 GO:0016020
GO:0016021
Cluster-22178.71919 GO:0006914 GO:0006886 GO:0010508 GO:0008092
GO:0050840 GO:0004190 GO:0004252 GO:0005737 GO:0005886
GO:0016021 GO:0019898 GO:0005576 GO:0016020 GO:0005783
Cluster-22178.71923 GO:0030682 GO:0042147
Cluster-22178.71924 GO:0006265 GO:0005524 GO:0003916 GO:0003677
GO:0005694

Cluster-22178.71926 GO:0046034 GO:0006144 GO:0008033 GO:0006464 GO:0051252 GO:0016151 GO:0046872 GO:0004017 GO:0004540 GO:0016787

Cluster-22178.71928 GO:0003676

Cluster-22178.71929 GO:0005515

Cluster-22178.71930 GO:0006000 GO:0006013 GO:0016255 GO:0007264 GO:0007186 GO:0007165 GO:0003924 GO:0019001 GO:0031683 GO:0005524 GO:0004871 GO:0005525 GO:0003873 GO:0042765

Cluster-22178.71931 GO:0015979 GO:0042967 GO:0006468 GO:0006886 GO:0004672 GO:0005524 GO:0008080 GO:0009539 GO:0009523 GO:0030176 GO:0016020

Cluster-22178.71933 GO:0000917 GO:0015876 GO:0043093 GO:0008521 GO:0016021 GO:0005737

Cluster-22178.71937 GO:0016747

Cluster-22178.71939 GO:0007264 GO:0005525

Cluster-22178.71941 GO:0006511 GO:0046872 GO:0005515

Cluster-22178.71942 GO:0006367 GO:0005672

Cluster-22178.71943 GO:0006265 GO:0003677 GO:0005515 GO:0046872 GO:0008270 GO:0003916 GO:0005694

Cluster-22178.71945 GO:0008270 GO:0005515

Cluster-22178.71946 GO:0055114 GO:0019370 GO:0008270 GO:0008237 GO:0016491

Cluster-22178.71947 GO:0005515

Cluster-22178.71949 GO:0042967 GO:0008080 GO:0005515 GO:0016747

Cluster-22178.71950 GO:0006388 GO:0051252 GO:0000213 GO:0000214

Cluster-22178.71951 GO:0009401 GO:0006355 GO:0051087 GO:0003700 GO:0043565 GO:0016021 GO:0005667

Cluster-22178.71953 GO:0006355 GO:0009401 GO:0043565 GO:0003700 GO:0051087 GO:0005667 GO:0016021

Cluster-22178.71954 GO:0000902 GO:0006955 GO:0006355 GO:0051087 GO:0003700 GO:0043565 GO:0016020 GO:0005667

Cluster-22178.71955 GO:0006955 GO:0000902 GO:0006355 GO:0051087

GO:0003700 GO:0043565 GO:0016020 GO:0005667
Cluster-22178.71957 GO:0008152 GO:0006897 GO:0008272 GO:0015116
GO:0003824 GO:0008270 GO:0008484 GO:0005515 GO:0016021
GO:0016020
Cluster-22178.71958 GO:0007005 GO:0003676 GO:0008270 GO:0031305
Cluster-22178.71960 GO:0009405 GO:0005634 GO:0005576
Cluster-22178.71961 GO:0019051 GO:0042025
Cluster-22178.71962 GO:0042254 GO:0006412 GO:0046872 GO:0008270
GO:0005515 GO:0061630 GO:0003735 GO:0005840
Cluster-22178.71964 GO:0016579 GO:0007186 GO:0036459 GO:0004930
GO:0016020
Cluster-22178.71965 GO:0008270
Cluster-22178.71966 GO:0006355 GO:0006352 GO:0003700 GO:0016987
GO:0003677 GO:0005667
Cluster-22178.71968 GO:0016310 GO:0004673 GO:0009365
Cluster-22178.71972 GO:0019064 GO:0008053 GO:0055085 GO:0006415
GO:0007165 GO:0071973 GO:0016567 GO:0030155 GO:0006935
GO:0006308 GO:0045995 GO:0030334 GO:0006265 GO:0006449
GO:0030001 GO:0031386 GO:0003747 GO:0008855 GO:0003918
GO:0005102 GO:0046873 GO:0003774 GO:0003677 GO:0003924
GO:0016881 GO:0046789 GO:0005198 GO:0018444 GO:0019031
GO:0005840 GO:0016021 GO:0019028 GO:0009318 GO:0009986
GO:0005741 GO:0016020 GO:0009288
Cluster-22178.71973 GO:0006449 GO:0006415 GO:0006265 GO:0003677
GO:0003917 GO:0008270 GO:0003747 GO:0003918 GO:0005694
GO:0018444 GO:0005840
Cluster-22178.71974 GO:0007018 GO:0007017 GO:0008017 GO:0003777
GO:0005524 GO:0030430 GO:0045298 GO:0005874
Cluster-22178.71975 GO:0006810 GO:0009264 GO:0005515 GO:0008253
GO:0004386 GO:0003676 GO:0005524 GO:0046872 GO:0043531
GO:0016020
Cluster-22178.71980 GO:0055114 GO:0005515 GO:0005198 GO:0016616
GO:0051287

Cluster-22178.71984 GO:0015031 GO:0008565 GO:0005515 GO:0016020
GO:0016021
Cluster-22178.71985 GO:0000910 GO:0000226 GO:0008017 GO:0045298
Cluster-22178.71989 GO:0006457 GO:0005509
Cluster-22178.7199 GO:0008233
Cluster-22178.71990 GO:0051603 GO:0006333 GO:0004298 GO:0005515
GO:0003677 GO:0005634 GO:0005839
Cluster-22178.71993 GO:0006810 GO:0005515 GO:0016021
Cluster-22178.71994 GO:0003677 GO:0005634
Cluster-22178.71995 GO:0045454
Cluster-22178.71996 GO:0019370 GO:0008270 GO:0008237
Cluster-22178.71997 GO:0009878 GO:0046872
Cluster-22178.71998 GO:0016310 GO:0007178 GO:0009069 GO:0006468
GO:0004675 GO:0005524 GO:0016020
Cluster-22178.71999 GO:0006418 GO:0002161 GO:0005515
Cluster-22178.720 GO:0006508 GO:0008270 GO:0004222 GO:0031012
GO:0005576
Cluster-22178.72001 GO:0016462 GO:0005737
Cluster-22178.72002 GO:0006810 GO:0006508 GO:0006816 GO:0005246
GO:0004222 GO:0042030 GO:0008168 GO:0016020
Cluster-22178.72003 GO:0055114 GO:0008033 GO:0006355 GO:0050660
GO:0005507 GO:0003700 GO:0016491 GO:0003677 GO:0071949
GO:0005667 GO:0005634
Cluster-22178.72006 GO:0006468 GO:0005524 GO:0004672 GO:0005515

Cluster-22178.72009 GO:0016192 GO:0006904 GO:0046983 GO:0046914

Cluster-22178.72010 GO:0048013 GO:0005515
Cluster-22178.72012 GO:0007155 GO:0006606 GO:0051276 GO:0000166
GO:0003677 GO:0005515 GO:0003774 GO:0005524 GO:0016459
GO:0005694
Cluster-22178.72013 GO:0006260 GO:0006284 GO:0003906 GO:0016799

Cluster-22178.72015 GO:0007155 GO:0006816 GO:0006810 GO:0001503
GO:0042998 GO:0005246 GO:0071253 GO:0042030 GO:0019028
GO:0005802 GO:0005576 GO:0016020 GO:0016021
Cluster-22178.72016 GO:0019079 GO:0006260 GO:0008026 GO:0003887
GO:0016818 GO:0003677 GO:0003723 GO:0003676 GO:0005524
GO:0042575 GO:0005634
Cluster-22178.72017 GO:0046034 GO:0006144 GO:0006355 GO:0008270
GO:0046872 GO:0003677 GO:0004017 GO:0003682 GO:0000775
GO:0000785 GO:0005634
Cluster-22178.72019 GO:0033588
Cluster-22178.72022 GO:0019491 GO:0033990 GO:0016021
Cluster-22178.72024 GO:0009451 GO:0031167 GO:0008033 GO:0008236
GO:0003723 GO:0008168 GO:0004809
Cluster-22178.72025 GO:0006886 GO:0016192 GO:0005515 GO:0005198
GO:0003824 GO:0030126 GO:0016020 GO:0016021
Cluster-22178.72028 GO:0007165 GO:0005509 GO:0005578
Cluster-22178.72030 GO:0006468 GO:0016773 GO:0005524 GO:0004672
GO:0016020
Cluster-22178.72034 GO:0005515 GO:0003676 GO:0030250 GO:0005576
Cluster-22178.72036 GO:0043565 GO:0005515
Cluster-22178.72039 GO:0004252 GO:0008270 GO:0016021
Cluster-22178.72041 GO:0005783
Cluster-22178.72045 GO:0005515
Cluster-22178.72047 GO:0008152 GO:0008033 GO:0009451 GO:0006886
GO:0016192 GO:0007165 GO:0006400 GO:0008168 GO:0004809
GO:0046872 GO:0016301 GO:0005515 GO:0003723 GO:0008176
GO:0016021 GO:0030117
Cluster-22178.72049 GO:0006886 GO:0016021 GO:0005783
Cluster-22178.72050 GO:0007264 GO:0005525
Cluster-22178.72051 GO:0007165 GO:0035556 GO:0003774 GO:0005524
GO:0005515 GO:0016459
Cluster-22178.72052 GO:0006470 GO:0008152 GO:0030145 GO:0000287
GO:0005525 GO:0003824 GO:0004721 GO:0003924 GO:0016491

Cluster-22178.72053 GO:0008152 GO:0016846 GO:0016491 GO:0016021
Cluster-22178.72054 GO:0007219 GO:0007423 GO:0005739
Cluster-22178.72055 GO:0007165 GO:0015948 GO:0040007 GO:0046656
GO:0008283 GO:0043087 GO:0008083 GO:0030269 GO:0005125
GO:0016020 GO:0005576 GO:0016021
Cluster-22178.72056 GO:0006470 GO:0006270 GO:0003924 GO:0005525
GO:0004721 GO:0003824 GO:0000287 GO:0004190 GO:0030145
GO:0016021
Cluster-22178.72059 GO:0006355 GO:0005515 GO:0005737 GO:0005634
Cluster-22178.72061 GO:0022904 GO:0016651 GO:0005743
Cluster-22178.72062 GO:0006351 GO:0005515
Cluster-22178.72065 GO:0006505 GO:0006448 GO:0006508 GO:0006629
GO:0006886 GO:0015996 GO:0001514 GO:0008152 GO:0015994
GO:0016787 GO:0003746 GO:0003723 GO:0008236 GO:0016788
GO:0047746 GO:0005525 GO:0005576 GO:0005737 GO:0005840
Cluster-22178.72066 GO:0006886 GO:0009405 GO:0006508 GO:0006629
GO:0001514 GO:0015996 GO:0015994 GO:0008152 GO:0006810
GO:0006505 GO:0006448 GO:0008236 GO:0019871 GO:0047746
GO:0016788 GO:0003723 GO:0004190 GO:0005525 GO:0016787
GO:0003746 GO:0005840 GO:0005737 GO:0042151
Cluster-22178.72068 GO:0032968 GO:0006886 GO:0006605 GO:0006367
GO:0006270 GO:0003676 GO:0004064 GO:0005515 GO:0005509
GO:0003677 GO:0005634 GO:0005742 GO:0016020
Cluster-22178.72069 GO:0016117 GO:0009405 GO:0046872 GO:0003924
GO:0005525 GO:0003677 GO:0016705 GO:0005515
Cluster-22178.72074 GO:0005515
Cluster-22178.72077 GO:0008270 GO:0046332 GO:0046872 GO:0005201
GO:0005578
Cluster-22178.72078 GO:0006506 GO:0016021
Cluster-22178.72079 GO:0046872
Cluster-22178.72080 GO:0015991 GO:0015992 GO:0015078 GO:0033177
Cluster-22178.72081 GO:0006448 GO:0006414 GO:0003746 GO:0005840
GO:0005622

Cluster-22178.72082 GO:0007166 GO:0007165 GO:0004871 GO:0005634
Cluster-22178.72083 GO:0015992 GO:0055114 GO:0006118 GO:0006119
GO:0008121
Cluster-22178.72084 GO:0051607 GO:0006891 GO:0007049 GO:0043093
GO:0000390 GO:0000917 GO:0006886 GO:0005737 GO:0030173
GO:0016021 GO:0005801 GO:0005783 GO:0016020 GO:0071008
Cluster-22178.72085 GO:0006810 GO:0005794
Cluster-22178.72087 GO:0003824
Cluster-22178.72088 GO:0003824
Cluster-22178.72089 GO:0005515 GO:0016021 GO:0019013
Cluster-22178.72092 GO:0006144 GO:0007165 GO:0004114 GO:0005515

Cluster-22178.72095 GO:0005515 GO:0043565
Cluster-22178.72096 GO:0005515
Cluster-22178.72097 GO:0006355 GO:0005515 GO:0019031
Cluster-22178.72098 GO:0006914 GO:0000921 GO:0006413 GO:0006446
GO:0045502 GO:0042803 GO:0003743 GO:0008134 GO:0005940
GO:0030286 GO:0016021 GO:0005840 GO:0019028 GO:0005667
Cluster-22178.72099 GO:0006810 GO:0006415 GO:0055085 GO:0034508
GO:0006355 GO:0006449 GO:0055114 GO:0003700 GO:0005215
GO:0016149 GO:0016020 GO:0018444 GO:0005667 GO:0000776
GO:0005840 GO:0005737
Cluster-22178.7210 GO:0007268 GO:0006811 GO:0007165 GO:0005234
GO:0004970 GO:0016020
Cluster-22178.72100 GO:0006123 GO:0015992 GO:0004129 GO:0045277
GO:0005739
Cluster-22178.72101 GO:0055114 GO:0050660 GO:0016491
Cluster-22178.72104 GO:0042438 GO:0007186 GO:0007165 GO:0015684
GO:0007264 GO:0030036 GO:0015093 GO:0098519 GO:0004871
GO:0005507 GO:0005525 GO:0003924 GO:0003779 GO:0019001
GO:0031683 GO:0016021 GO:0009295 GO:0030054
Cluster-22178.72107 GO:0006820 GO:0042254 GO:0006412 GO:0003735
GO:0005622 GO:0016021 GO:0005840

Cluster-22178.72108 GO:0006820 GO:0016021

Cluster-22178.72113 GO:0046872

Cluster-22178.72114 GO:0055114 GO:0006281 GO:0008270 GO:0046872
GO:0005515 GO:0047134 GO:0005634

Cluster-22178.72116 GO:0015992 GO:0006123 GO:0003774 GO:0004129
GO:0005198 GO:0045277 GO:0016459 GO:0005882 GO:0005740

Cluster-22178.72117 GO:0008152 GO:0019521 GO:0055114 GO:0007005
GO:0006098 GO:0016491 GO:0004616 GO:0031305

Cluster-22178.72122 GO:0006468 GO:0007165 GO:0005125 GO:0004672
GO:0005524 GO:0005515

Cluster-22178.72123 GO:0006355 GO:0007165 GO:0048193 GO:0008270
GO:0003700 GO:0004871 GO:0005634 GO:0005667 GO:0016020

Cluster-22178.72125 GO:0006118 GO:0009060 GO:0006123 GO:0015992
GO:0055114 GO:0020037 GO:0005506 GO:0009055 GO:0004129
GO:0045277 GO:0016021

Cluster-22178.72126 GO:0006468 GO:0005524 GO:0004672 GO:0019028
GO:0016020

Cluster-22178.72127 GO:0016032 GO:0006810 GO:0042167 GO:0055114
GO:0015994 GO:0004074 GO:0046872 GO:0008270 GO:0042597
GO:0016021

Cluster-22178.72128 GO:0055114 GO:0015994 GO:0006810 GO:0042167
GO:0004074 GO:0008270 GO:0042597 GO:0016021

Cluster-22178.72129 GO:0006355 GO:0043401 GO:0043565 GO:0008270
GO:0003700 GO:0005667 GO:0005634

Cluster-22178.72130 GO:0043401 GO:0009405 GO:0006355 GO:0005515
GO:0005198 GO:0003700 GO:0008270 GO:0043565 GO:0005634
GO:0005667 GO:0016021

Cluster-22178.72131 GO:0009405 GO:0007165 GO:0043401 GO:0006355
GO:0005515 GO:0005198 GO:0008201 GO:0003700 GO:0050750
GO:0043565 GO:0008270 GO:0005783 GO:0005634 GO:0005667
GO:0016021

Cluster-22178.72132 GO:0015031 GO:0015992 GO:0006820 GO:0006352
GO:0015991 GO:0046872 GO:0008565 GO:0015078 GO:0008509

GO:0005515 GO:0016021 GO:0005669 GO:0033179
Cluster-22178.72133 GO:0007049 GO:0006886 GO:0006605 GO:0046914
GO:0004867 GO:0008201 GO:0016021 GO:0005742 GO:0017053
Cluster-22178.72136 GO:0035091
Cluster-22178.72138 GO:0006355 GO:0005515
Cluster-22178.72140 GO:0045895 GO:0007531 GO:0008301 GO:0005634
Cluster-22178.72141 GO:0016579 GO:0006276 GO:0005975 GO:0008657
GO:0004553 GO:0008270 GO:0036459 GO:0005096
Cluster-22178.72143 GO:0006508 GO:0007010 GO:0003779 GO:0008270
GO:0004222 GO:0031012
Cluster-22178.72156 GO:0006468 GO:0005515 GO:0004672 GO:0005524
GO:0016021 GO:0019012 GO:0042765
Cluster-22178.72158 GO:0007186 GO:0004930 GO:0005515 GO:0016020
Cluster-22178.72159 GO:0005515
Cluster-22178.72160 GO:0007186 GO:0006807 GO:0004930 GO:0008270
GO:0016814 GO:0016021
Cluster-22178.72162 GO:0007165 GO:0005096 GO:0005179 GO:0005634
GO:0005576
Cluster-22178.72165 GO:0016787
Cluster-22178.72167 GO:0006810 GO:0006811 GO:0051726 GO:0005230
GO:0017025 GO:0016020 GO:0005634
Cluster-22178.72169 GO:0005975 GO:0016758 GO:0016020
Cluster-22178.7217 GO:0006355 GO:0046983 GO:0003677
Cluster-22178.72170 GO:0051607
Cluster-22178.72172 GO:0004867 GO:0005524 GO:0005576
Cluster-22178.72175 GO:0019497 GO:0006771 GO:0003993
Cluster-22178.72176 GO:0042742 GO:0016301 GO:0005576
Cluster-22178.72177 GO:0006449 GO:0006415 GO:0006118 GO:0016192
GO:0006886 GO:0005506 GO:0020037 GO:0016149 GO:0009055
GO:0018444 GO:0030131 GO:0005622 GO:0005737 GO:0005840
Cluster-22178.72182 GO:0051603 GO:0006511 GO:0004175 GO:0004298
GO:0005839 GO:0019773
Cluster-22178.72183 GO:0016573 GO:0007049

Cluster-22178.72184 GO:0007049 GO:0016573
Cluster-22178.72186 GO:0043087 GO:0035023 GO:0007165 GO:0030170
GO:0005057 GO:0005515 GO:0005089
Cluster-22178.72187 GO:0046413 GO:0046983 GO:0005515 GO:0018836

Cluster-22178.72188 GO:0006508 GO:0006144 GO:0017111 GO:0005515
GO:0004197 GO:0003968 GO:0016020 GO:0031379
Cluster-22178.72189 GO:0005515
Cluster-22178.72190 GO:0019079 GO:0006260 GO:0003677 GO:0016787
GO:0003676 GO:0003678 GO:0005524 GO:0005657
Cluster-22178.72192 GO:0005515 GO:0016021
Cluster-22178.72193 GO:0007165 GO:0005057
Cluster-22178.72194 GO:0006355 GO:0010506 GO:0003677 GO:0005515
GO:0008270 GO:0043565 GO:0003700 GO:0005507 GO:0005667
GO:0005634 GO:0005615
Cluster-22178.72196 GO:0034220
Cluster-22178.72197 GO:0030244 GO:0006886 GO:0005525 GO:0003924
GO:0005515 GO:0005047 GO:0016866 GO:0005785 GO:0005786
Cluster-22178.72199 GO:0006813 GO:0000184 GO:0005242 GO:0005516
GO:0016286 GO:0046872 GO:0005524 GO:0015269 GO:0003924
GO:0016887 GO:0005525 GO:0008076 GO:0016021
Cluster-22178.72200 GO:0046486 GO:0006071 GO:0004371
Cluster-22178.72201 GO:0040007 GO:0015628 GO:0006810 GO:0006614
GO:0007165 GO:0008283 GO:0015031 GO:0008083 GO:0005515
GO:0003723 GO:0003676 GO:0005525 GO:0030145 GO:0016787
GO:0004386 GO:0016887 GO:0008565 GO:0005524 GO:0015627
Cluster-22178.72202 GO:0006313 GO:0004803 GO:0003677 GO:0046872

Cluster-22178.72203 GO:0006355 GO:0007059 GO:0051301 GO:0006457
GO:0007186 GO:0006869 GO:0042157 GO:0034080 GO:0035299
GO:0051082 GO:0003676 GO:0003700 GO:0008289 GO:0003677
GO:0004930 GO:0005524 GO:0005576 GO:0016021 GO:0000775
GO:0005667 GO:0000444 GO:0016272

Cluster-22178.72205 GO:0042254 GO:0006412 GO:0003676 GO:0003735
GO:0008270 GO:0005840 GO:0005622
Cluster-22178.72206 GO:0007186 GO:0005515 GO:0004930 GO:0016021
Cluster-22178.72207 GO:0048193 GO:0006914 GO:0006144 GO:0071988
GO:0006810 GO:0044419 GO:0007165 GO:0043093 GO:0000917
GO:0030474 GO:0006508 GO:0016747 GO:0004197 GO:0017111
GO:0003968 GO:0016301 GO:0005886 GO:0016020 GO:0031379
GO:0005737
Cluster-22178.72208 GO:0007264 GO:0005524 GO:0016787 GO:0005525
GO:0003677
Cluster-22178.72211 GO:0046872 GO:0000166
Cluster-22178.72213 GO:0006355 GO:0009271
Cluster-22178.72216 GO:0055085 GO:0006810 GO:0005975 GO:0006811
GO:0007017 GO:0007018 GO:0008270 GO:0005524 GO:0008017
GO:0005216 GO:0016868 GO:0046872 GO:0005215 GO:0003777
GO:0005515 GO:0019901 GO:0016020 GO:0045298 GO:0005874
Cluster-22178.72219 GO:0006810 GO:0006888 GO:0055085 GO:0005515
GO:0005215 GO:0022857 GO:0016020 GO:0005622 GO:0005801
GO:0019867 GO:0016021
Cluster-22178.72223 GO:0016559 GO:0005515 GO:0005779
Cluster-22178.72225 GO:0009405 GO:0006810 GO:0019871 GO:0005576
Cluster-22178.72227 GO:0006090 GO:0045116 GO:0006525 GO:0071436
GO:0006814 GO:0006560 GO:0016881 GO:0005524 GO:0015081
GO:0008948 GO:0005634 GO:0016020
Cluster-22178.72229 GO:0042254 GO:0019058 GO:0006289 GO:0006284
GO:0006508 GO:0006281 GO:0006412 GO:0006397 GO:0003906
GO:0003684 GO:0003735 GO:0004197 GO:0008242 GO:0008270
GO:0003723 GO:0016740 GO:0016799 GO:0005622 GO:0000445
GO:0005840
Cluster-22178.7223 GO:0006355 GO:0008270 GO:0005524 GO:0005509
GO:0005515 GO:0003677 GO:0016818 GO:0009986 GO:0016020
GO:0005634
Cluster-22178.72232 GO:0010506 GO:0005515 GO:0004222 GO:0005615

Cluster-22178.72234 GO:0008270 GO:0046872
Cluster-22178.72240 GO:0008270 GO:0005515
Cluster-22178.72241 GO:0015031 GO:0016021
Cluster-22178.72243 GO:0006221 GO:0006541 GO:0006206 GO:0003883
GO:0016787
Cluster-22178.72246 GO:0006570 GO:0006470 GO:0004527 GO:0008138
GO:0004725 GO:0003677
Cluster-22178.72247 GO:0015886 GO:0017004 GO:0007411 GO:0015232
GO:0019033 GO:0016020
Cluster-22178.72248 GO:0006334 GO:0030170 GO:0003723 GO:0005515
GO:0003677 GO:0000786 GO:0005634
Cluster-22178.72249 GO:0006364 GO:0005515 GO:0032040
Cluster-22178.7225 GO:0006468 GO:0005524 GO:0004672 GO:0016773
GO:0016020
Cluster-22178.72250 GO:0007165 GO:0005179 GO:0005576
Cluster-22178.72251 GO:0006260 GO:0046983 GO:0003887 GO:0003677
GO:0005515 GO:0042575
Cluster-22178.72252 GO:0006950 GO:0009415 GO:0006890 GO:0004252
GO:0016021 GO:0097197 GO:0001772
Cluster-22178.72253 GO:0006950 GO:0006890 GO:0009415 GO:0004252
GO:0001772 GO:0097197 GO:0016021
Cluster-22178.72255 GO:0044780 GO:0006816 GO:0007165 GO:0030155
GO:0030334 GO:0045995 GO:0005388 GO:0005102 GO:0016529
GO:0019028
Cluster-22178.72256 GO:0005515
Cluster-22178.72258 GO:0051603 GO:0006260 GO:0006888 GO:0003887
GO:0003677 GO:0004298 GO:0042575 GO:0005839 GO:0005801
GO:0005622
Cluster-22178.72260 GO:0071229 GO:0042710
Cluster-22178.72261 GO:0006364 GO:1901031 GO:0030688 GO:0005634
Cluster-22178.72262 GO:0006310 GO:0042138
Cluster-22178.72263 GO:0042138 GO:0006310
Cluster-22178.72264 GO:0042138 GO:0006310

Cluster-22178.72268 GO:0005515
Cluster-22178.72269 GO:0006606 GO:0006418 GO:0005524 GO:0043565 GO:0005515 GO:0004812 GO:0003723 GO:0005634
Cluster-22178.72270 GO:0006334 GO:0005515 GO:0003677 GO:0030170 GO:0003723 GO:0000786 GO:0005634
Cluster-22178.72276 GO:0006465 GO:0008233 GO:0005787 GO:0016021
Cluster-22178.72277 GO:0006355 GO:0055114 GO:0008033 GO:0071949 GO:0003677 GO:0016491 GO:0050660 GO:0005507 GO:0003700 GO:0005667 GO:0005634
Cluster-22178.72279 GO:0048870 GO:0031514
Cluster-22178.72281 GO:0015992 GO:0006123 GO:0004129 GO:0005746 GO:0045277
Cluster-22178.72283 GO:0003676 GO:0005515
Cluster-22178.72289 GO:0006355 GO:0006897 GO:0016020
Cluster-22178.72290 GO:0006897 GO:0016020
Cluster-22178.72291 GO:0008589 GO:0007218 GO:0007517
Cluster-22178.72294 GO:0043565 GO:0005515 GO:0005634
Cluster-22178.72297 GO:0015031 GO:0016021
Cluster-22178.72300 GO:0044564 GO:0000723 GO:0006281 GO:0003678 GO:0005657
Cluster-22178.72301 GO:0005515
Cluster-22178.72302 GO:0045454 GO:0055114 GO:0016491 GO:0016209

Cluster-22178.72303 GO:0055114 GO:0045454 GO:0016209 GO:0016491

Cluster-22178.72304 GO:0055114 GO:0045454 GO:0016209 GO:0016491

Cluster-22178.7231 GO:0004222
Cluster-22178.72310 GO:0003700 GO:0005515 GO:0005667
Cluster-22178.72311 GO:0005515
Cluster-22178.72312 GO:0016310 GO:0044237 GO:0016772
Cluster-22178.72313 GO:0003824
Cluster-22178.72315 GO:0042742

Cluster-22178.72318 GO:0051056 GO:0005096 GO:0005515 GO:0003676

Cluster-22178.72319 GO:0006355 GO:0003676 GO:0008017 GO:0005524
GO:0008270 GO:0016818 GO:0003677 GO:0005515 GO:0016787
GO:0045298 GO:0005634
Cluster-22178.7232 GO:0004222
Cluster-22178.72320 GO:0016020
Cluster-22178.72321 GO:0006355 GO:0016787 GO:0016818 GO:0003677
GO:0005515 GO:0005524 GO:0008270 GO:0003676 GO:0008017
GO:0005634 GO:0045298
Cluster-22178.72322 GO:0006506 GO:0006468 GO:0003676 GO:0017176
GO:0005524 GO:0004715 GO:0005515 GO:0005509 GO:0016021
GO:0005737
Cluster-22178.72324 GO:0006276 GO:0005515
Cluster-22178.72326 GO:0006355 GO:0005634
Cluster-22178.72327 GO:0006886 GO:0006605 GO:0005515 GO:0005742
GO:0016020
Cluster-22178.72330 GO:0007010 GO:0000413 GO:0006457 GO:0003755
GO:0008017 GO:0005887 GO:0045298
Cluster-22178.72334 GO:0042147 GO:0030682
Cluster-22178.72335 GO:0055114 GO:0006559 GO:0006570 GO:0042207
GO:0004411 GO:0005515
Cluster-22178.72336 GO:0016567 GO:0090263 GO:0043565
Cluster-22178.72338 GO:0090263 GO:0016567
Cluster-22178.72339 GO:0006144 GO:0006206 GO:0006289 GO:0006355
GO:0006351 GO:0003899 GO:0000439 GO:0005730
Cluster-22178.72341 GO:0006886 GO:0048280 GO:0048870 GO:0015031
GO:0008565 GO:0031514 GO:0016020 GO:0000139 GO:0005737
Cluster-22178.72343 GO:0010508 GO:0006355 GO:0006260 GO:0003677
GO:0003700 GO:0043565 GO:0005634 GO:0005667
Cluster-22178.72345 GO:0007018 GO:0007017 GO:0008017 GO:0003777
GO:0005524 GO:0045298 GO:0005874 GO:0005576
Cluster-22178.72346 GO:0006355 GO:0006260 GO:0043565 GO:0003700

GO:0005667

Cluster-22178.72349 GO:0006118 GO:0050776 GO:0014068 GO:0007165 GO:0007275 GO:0006259 GO:0020037 GO:0003824 GO:0009055 GO:0043548 GO:0003677 GO:0005102 GO:0005524 GO:0016021 GO:0016020 GO:0005694

Cluster-22178.72350 GO:0006259 GO:0007165 GO:0007275 GO:0006118 GO:0014068 GO:0050776 GO:0043548 GO:0003677 GO:0005524 GO:0005102 GO:0020037 GO:0003824 GO:0009055 GO:0005694 GO:0016020 GO:0016021

Cluster-22178.72351 GO:0006810 GO:0005622

Cluster-22178.72352 GO:0003677 GO:0046872

Cluster-22178.72353 GO:0008643 GO:0055085 GO:0005351 GO:0000139 GO:0016021 GO:0016020

Cluster-22178.72354 GO:0003676 GO:0016021

Cluster-22178.72355 GO:0003676 GO:0004527

Cluster-22178.72356 GO:0046912

Cluster-22178.72357 GO:0006171 GO:0006144 GO:0004016 GO:0000287

Cluster-22178.72358 GO:0009847 GO:0051252 GO:0016891 GO:0016021

Cluster-22178.72359 GO:0006508 GO:0003676 GO:0004190 GO:0003723

Cluster-22178.72360 GO:0046912

Cluster-22178.72363 GO:0006935 GO:0006355 GO:0071973 GO:0003774 GO:0046872 GO:0003677 GO:0009288 GO:0012511 GO:0016021 GO:0016020

Cluster-22178.72365 GO:0007186 GO:0050909 GO:0004177 GO:0004930 GO:0030145 GO:0016021

Cluster-22178.72367 GO:0048870 GO:0070206 GO:0005639 GO:0031514

Cluster-22178.72368 GO:0055085 GO:0016021 GO:0016020

Cluster-22178.72369 GO:0016021

Cluster-22178.72370 GO:0009772 GO:0009405 GO:0006118 GO:0019684 GO:0009306 GO:0045156 GO:0005576 GO:0016020

Cluster-22178.72372 GO:0008152

Cluster-22178.72374 GO:0006357 GO:0001104 GO:0016592
Cluster-22178.72376 GO:0009116 GO:0006506 GO:0016746 GO:0042393
GO:0005634 GO:0016021 GO:0005789
Cluster-22178.72377 GO:0006506 GO:0009116 GO:0016746 GO:0042393
GO:0005634 GO:0005789 GO:0016021
Cluster-22178.72379 GO:0005509 GO:0005544
Cluster-22178.72380 GO:0005509 GO:0005544
Cluster-22178.72381 GO:0006383 GO:0006351 GO:0006206 GO:0006144
GO:0003677 GO:0003899 GO:0005666 GO:0005730
Cluster-22178.72382 GO:0007275 GO:0034477 GO:0004518 GO:0005515
GO:0003700 GO:0046872 GO:0005667 GO:0005634
Cluster-22178.72383 GO:0003676
Cluster-22178.72384 GO:0043087
Cluster-22178.72386 GO:0005515
Cluster-22178.72388 GO:0016567 GO:0004842 GO:0005515 GO:0003676
GO:0046872 GO:0008270 GO:0005634
Cluster-22178.72389 GO:0042262 GO:0005509 GO:0003690
Cluster-22178.7239 GO:0007268 GO:0043495 GO:0005515 GO:0033130
Cluster-22178.72391 GO:0010506 GO:0005515 GO:0005615
Cluster-22178.72392 GO:0006468 GO:0046872 GO:0005524 GO:0004672

Cluster-22178.72393 GO:0006508 GO:0006364 GO:0008237 GO:0005515
GO:0016805 GO:0005730 GO:0005618
Cluster-22178.72394 GO:0006508 GO:0006364 GO:0008237 GO:0016805
GO:0005515 GO:0005730 GO:0005618
Cluster-22178.72395 GO:0006364 GO:0005515 GO:0005730
Cluster-22178.72396 GO:0006355 GO:0043565 GO:0003700 GO:0005667
Cluster-22178.72397 GO:0006355 GO:0043565 GO:0003700 GO:0005667
Cluster-22178.72398 GO:0016021
Cluster-22178.72400 GO:0003676 GO:0005515
Cluster-22178.72402 GO:0042967 GO:0006537 GO:0008285 GO:0055114
GO:0007050 GO:0006355 GO:0006915 GO:0003712 GO:0008270
GO:0015930 GO:0004402 GO:0016638 GO:0000123 GO:0005667

GO:0005634
Cluster-22178.72403 GO:0042967 GO:0006355 GO:0016573 GO:0004402
GO:0003713 GO:0000123 GO:0005667 GO:0005634
Cluster-22178.72404 GO:0048193 GO:0009059 GO:0070273 GO:0030176
Cluster-22178.72407 GO:0046872 GO:0008270 GO:0005515
Cluster-22178.72408 GO:0005975 GO:0016832 GO:0005515 GO:0035091

Cluster-22178.72409 GO:0007186 GO:0007165 GO:0055114 GO:0048193
GO:0050661 GO:0003924 GO:0016491 GO:0019001 GO:0031683
GO:0004871 GO:0005783
Cluster-22178.72411 GO:0006413 GO:0006446 GO:0003723 GO:0003743
GO:0005737 GO:0005840
Cluster-22178.72412 GO:0009069 GO:0006468 GO:0016310 GO:0004672
GO:0005524 GO:0004674
Cluster-22178.72413 GO:0006468 GO:0005524 GO:0004672 GO:0016773
GO:0016020
Cluster-22178.72415 GO:0043087 GO:0043248 GO:0032012 GO:0007160
GO:0005086 GO:0005509 GO:0005576 GO:0005634 GO:0005783
Cluster-22178.72419 GO:0008270
Cluster-22178.72420 GO:0006511 GO:0007005 GO:0045502 GO:0008134
GO:0042803 GO:0031305 GO:0005667 GO:0030286
Cluster-22178.72421 GO:0006886 GO:0005622
Cluster-22178.72423 GO:0006144 GO:0004114 GO:0005515 GO:0016020
Cluster-22178.72425 GO:0006769 GO:0055114 GO:0046497 GO:0070403
GO:0008750 GO:0030976 GO:0050661 GO:0000287 GO:0016021
Cluster-22178.72427 GO:0046872 GO:0003723 GO:0005515 GO:0042802
GO:0016021
Cluster-22178.72430 GO:0031175 GO:0005524 GO:0005516 GO:0008270
GO:0030507 GO:0016818 GO:0008091
Cluster-22178.72431 GO:0006355 GO:0009405 GO:0006397 GO:0006308
GO:0006302 GO:0006357 GO:0043087 GO:0006606 GO:0005515
GO:0001104 GO:0003700 GO:0008855 GO:0005634 GO:0009318
GO:0005667 GO:0016020 GO:0016592

Cluster-22178.72433 GO:0007049 GO:0009405 GO:0006486 GO:0006260
GO:0006270 GO:0007067 GO:0003980 GO:0042802 GO:0005515
GO:0003887 GO:0016757 GO:0046983 GO:0016021 GO:0000775
GO:0042575 GO:0005727 GO:0005634
Cluster-22178.72435 GO:0016579 GO:0008063 GO:0036459
Cluster-22178.72436 GO:0006446 GO:0006464 GO:0006144 GO:0007218
GO:0006413 GO:0006355 GO:0006506 GO:0005975 GO:0006808
GO:0046034 GO:0003677 GO:0004017 GO:0003743 GO:0008270
GO:0005524 GO:0004553 GO:0003723 GO:0030234 GO:0004519
GO:0005515 GO:0004672 GO:0046872 GO:0043565 GO:0016151
GO:0003700 GO:0003676 GO:0005667 GO:0005840 GO:0005789
Cluster-22178.72437 GO:0070140 GO:0032183
Cluster-22178.72440 GO:0006626 GO:0016746 GO:0005741
Cluster-22178.72442 GO:0006508 GO:0004198 GO:0005622
Cluster-22178.72443 GO:0006508 GO:0004198 GO:0005622
Cluster-22178.72445 GO:0007155 GO:0016021 GO:0016020
Cluster-22178.72446 GO:0006355 GO:0035023 GO:0007264 GO:0007186
GO:0007165 GO:0006614 GO:0006000 GO:0006013 GO:0043087
GO:0005515 GO:0003924 GO:0019001 GO:0031683 GO:0004871
GO:0005525 GO:0003873 GO:0005524 GO:0005089
Cluster-22178.72447 GO:0007186 GO:0006614 GO:0007165 GO:0007264
GO:0006000 GO:0006013 GO:0043087 GO:0006355 GO:0035023
GO:0003873 GO:0005089 GO:0005524 GO:0005515 GO:0004871
GO:0005525 GO:0003924 GO:0031683 GO:0019001
Cluster-22178.72448 GO:0006468 GO:0005524 GO:0004672
Cluster-22178.72449 GO:0071918 GO:0015840 GO:0015204 GO:0003676
GO:0016021
Cluster-22178.72450 GO:0071918 GO:0015840 GO:0015204 GO:0003676
GO:0016021
Cluster-22178.72451 GO:0006281 GO:0006355 GO:0090263 GO:0016567
GO:0006310 GO:0003678 GO:0043531 GO:0008134 GO:0003723
GO:0003724 GO:0016887 GO:0005524 GO:0003677 GO:0098519
GO:0016787 GO:0009378 GO:0005657 GO:0009379 GO:0005667

Cluster-22178.72452 GO:0015992 GO:0006281 GO:0003824 GO:0015078
GO:0008289 GO:0005515 GO:0033644 GO:0055036
Cluster-22178.72453 GO:0006355 GO:0009607 GO:0003677 GO:0005739
GO:0005747 GO:0016021
Cluster-22178.72455 GO:0006468 GO:0005515 GO:0004672 GO:0005524

Cluster-22178.72456 GO:0008270 GO:0046872
Cluster-22178.72457 GO:0006047
Cluster-22178.72459 GO:0006355 GO:0042157 GO:0007186 GO:0006869
GO:0034080 GO:0035299 GO:0003700 GO:0003676 GO:0008289
GO:0003677 GO:0005524 GO:0004930 GO:0005576 GO:0016021
GO:0000775 GO:0005667
Cluster-22178.72462 GO:0043093 GO:0006355 GO:0000917 GO:0043565
GO:0003700 GO:0005737 GO:0005794 GO:0005667
Cluster-22178.72463 GO:0000290 GO:0005975 GO:0003824 GO:0043169
GO:0016787
Cluster-22178.72466 GO:0007219 GO:0007275 GO:0030154 GO:0016021
Cluster-22178.72467 GO:0007264 GO:0007165 GO:0005515 GO:0005525

Cluster-22178.72469 GO:0005515
Cluster-22178.72470 GO:0033014 GO:0055114 GO:0008152 GO:0006783
GO:0015994 GO:0050662 GO:0004852 GO:0016491 GO:0003824
Cluster-22178.72472 GO:0015979 GO:0009523 GO:0009507
Cluster-22178.72473 GO:0000398 GO:0015979 GO:0003677 GO:0019013
GO:0009523 GO:0009507
Cluster-22178.72474 GO:0042176 GO:0000245 GO:0030234 GO:0000502
GO:0005634
Cluster-22178.72476 GO:0005198 GO:0005882
Cluster-22178.72477 GO:0042254 GO:0006412 GO:0005515 GO:0003735
GO:0005622 GO:0005840
Cluster-22178.72480 GO:0016021
Cluster-22178.72482 GO:0007275 GO:0030001 GO:0006812 GO:0055085
GO:0046873 GO:0008324 GO:0016020 GO:0016021 GO:0042600

Cluster-22178.72483 GO:0006298 GO:0005524 GO:0030983
Cluster-22178.72484 GO:0006206 GO:0006144 GO:0006508 GO:0006351
GO:0003968 GO:0004197 GO:0003677 GO:0017111 GO:0005515
GO:0003899 GO:0031379 GO:0005730
Cluster-22178.72486 GO:0006144 GO:0006206 GO:0015992 GO:0006351
GO:0015991 GO:0015078 GO:0003899 GO:0005515 GO:0003677
GO:0004190 GO:0070140 GO:0032183 GO:0016021 GO:0033179
GO:0005730
Cluster-22178.72487 GO:0070588 GO:0006816 GO:0006508 GO:0005515
GO:0005509 GO:0005262 GO:0016020 GO:0019033
Cluster-22178.72488 GO:0007166 GO:0007165 GO:0004871 GO:0005634
Cluster-22178.72489 GO:0006606 GO:0006914 GO:0016021 GO:0031083
Cluster-22178.72491 GO:0035556 GO:0009405 GO:0046488 GO:0005198
GO:0005524 GO:0046872 GO:0016307
Cluster-22178.72492 GO:0046488 GO:0009405 GO:0035556 GO:0005198
GO:0016307 GO:0046872 GO:0005524
Cluster-22178.72493 GO:0006457
Cluster-22178.72496 GO:0051016 GO:0006260 GO:0007165 GO:0006310
GO:0051782 GO:0009432 GO:0006281 GO:0005524 GO:0003689
GO:0016887 GO:0003724 GO:0009378 GO:0098519 GO:0005509
GO:0003723 GO:0030479 GO:0005657 GO:0009379 GO:0009276
GO:0005578 GO:0005663 GO:0042575
Cluster-22178.72497 GO:0046486 GO:0006687 GO:0006027 GO:0006012
GO:0005975 GO:0005515 GO:0004565 GO:0046872 GO:0009341
Cluster-22178.72498 GO:0006744 GO:0055114 GO:0009271 GO:0016567
GO:0006355 GO:0004842 GO:0046872 GO:0008270 GO:0005515
Cluster-22178.72499 GO:0006694 GO:0008299 GO:0000917 GO:0043093
GO:0050518 GO:0005525 GO:0046983 GO:0016887 GO:0005524
GO:0005737
Cluster-22178.72501 GO:0046872 GO:0000166
Cluster-22178.72507 GO:0001503 GO:0007155 GO:0046935 GO:0003676
GO:0005944 GO:0005576
Cluster-22178.72508 GO:0015979 GO:0005198 GO:0009522 GO:0016020

Cluster-22178.72509 GO:0055114 GO:0016117 GO:0006090 GO:0006099
GO:0006468 GO:0016491 GO:0004672 GO:0005524 GO:0008924
GO:0071949 GO:0016705
Cluster-22178.72514 GO:0055085 GO:0006811 GO:0005216 GO:0045735
GO:0016020
Cluster-22178.72516 GO:0048309 GO:0051028 GO:0030430
Cluster-22178.72519 GO:0006412 GO:0042254 GO:0003735 GO:0005840
GO:0005622
Cluster-22178.72522 GO:0048870 GO:0006355 GO:0010506 GO:0015031
GO:0051607 GO:0006457 GO:0006810 GO:0016310 GO:0006886
GO:0007094 GO:0006468 GO:0006606 GO:0009069 GO:0003700
GO:0008134 GO:0045502 GO:0004674 GO:0043565 GO:0042803
GO:0051082 GO:0008565 GO:0005524 GO:0016021 GO:0005801
GO:0031514 GO:0005886 GO:0030286 GO:0005615 GO:0005737
GO:0016272 GO:0016020 GO:0005667
Cluster-22178.72523 GO:0007165 GO:0035091 GO:0005515
Cluster-22178.72524 GO:0007031 GO:0000166 GO:0005779
Cluster-22178.72529 GO:0006464 GO:0051082 GO:0016151 GO:0031072

Cluster-22178.72531 GO:0005515
Cluster-22178.72532 GO:0006351 GO:0006269 GO:0035556 GO:0046034
GO:0006144 GO:0004017 GO:0005515 GO:0003896 GO:0004386
GO:0008270 GO:0005730 GO:0005657
Cluster-22178.72536 GO:0006276 GO:0015992 GO:0006123 GO:0004129
GO:0005743 GO:0045277
Cluster-22178.72538 GO:0035556 GO:0006355 GO:0005515 GO:0046872
GO:0008270 GO:0005634
Cluster-22178.72539 GO:0035556 GO:0006355 GO:0005515 GO:0008270
GO:0046872 GO:0005634
Cluster-22178.72541 GO:0004867 GO:0019031
Cluster-22178.72542 GO:0015016 GO:0008146 GO:0005515 GO:0016787

Cluster-22178.72544 GO:0006412 GO:0042254 GO:0016491 GO:0003735

GO:0005622 GO:0005840

Cluster-22178.72546 GO:0007275 GO:0030154

Cluster-22178.72551 GO:0006012 GO:0005982 GO:0005985 GO:0004564 GO:0004575 GO:0017177

Cluster-22178.72554 GO:0008270

Cluster-22178.72555 GO:0006448 GO:0001514 GO:0003746 GO:0005525 GO:0003723 GO:0005515 GO:0005737 GO:0005840

Cluster-22178.72558 GO:0006355 GO:0016579 GO:0036459 GO:0003700 GO:0005515 GO:0005667

Cluster-22178.72559 GO:0015991 GO:0006904 GO:0006397 GO:0000910 GO:0008608 GO:0007067 GO:0006914 GO:0015031 GO:0015992 GO:0000226 GO:0005198 GO:0015078 GO:0070009 GO:0008017 GO:0008239 GO:0005634 GO:0072686 GO:0019028 GO:0033179 GO:0042729 GO:0045298 GO:0019031 GO:0000445 GO:0000775 GO:0000145 GO:0005876

Cluster-22178.72561 GO:0005576

Cluster-22178.72563 GO:0008283 GO:0034968 GO:0007165 GO:0006364 GO:0006554 GO:0006379 GO:0006355 GO:0040007 GO:0009405 GO:0051252 GO:0006479 GO:0008033 GO:0006274 GO:0030674 GO:0016787 GO:0051015 GO:0003677 GO:0005509 GO:0018024 GO:0004540 GO:0003723 GO:0008083 GO:0005515 GO:0005198 GO:0008289 GO:0005694 GO:0030677 GO:0000172 GO:0005578 GO:0005737

Cluster-22178.72565 GO:0005975 GO:0006811 GO:0007049 GO:0055085 GO:0007268 GO:0051260 GO:0007165 GO:0016773 GO:0004970 GO:0005216 GO:0005515 GO:0016020

Cluster-22178.72566 GO:0006269 GO:0006351 GO:0019825 GO:0005515 GO:0005198 GO:0020037 GO:0003677 GO:0003896 GO:0005634 GO:0005730 GO:0005657

Cluster-22178.72567 GO:0006468 GO:0005524 GO:0004672

Cluster-22178.72568 GO:0006468 GO:0005524 GO:0004672 GO:0016773 GO:0016020

Cluster-22178.72569 GO:0042981 GO:0005515

Cluster-22178.72570 GO:0006355 GO:0006629 GO:0043401 GO:0008270
GO:0043565 GO:0003700 GO:0005634 GO:0005667
Cluster-22178.72571 GO:0005515
Cluster-22178.72572 GO:0006260 GO:0008152 GO:0006123 GO:0005975
GO:0015992 GO:0004129 GO:0005524 GO:0004649 GO:0003689
GO:0016624 GO:0045277 GO:0042575 GO:0005663
Cluster-22178.72573 GO:0042578
Cluster-22178.72576 GO:0005515
Cluster-22178.72578 GO:0003779 GO:0005515 GO:0005622 GO:0016020
Cluster-22178.72579 GO:0008270
Cluster-22178.72582 GO:0005634
Cluster-22178.72587 GO:0003676 GO:0005515 GO:0003723 GO:0008270

Cluster-22178.72591 GO:0019087 GO:0005515 GO:0016021
Cluster-22178.72592 GO:0006355 GO:0006351 GO:0055114 GO:0019521
GO:0008152 GO:0006098 GO:0006206 GO:0006144 GO:0003899
GO:0005515 GO:0046872 GO:0003700 GO:0051287 GO:0003677
GO:0004616 GO:0016616 GO:0008641 GO:0005634 GO:0005730
GO:0005667
Cluster-22178.72593 GO:0055114 GO:0006574 GO:0008152 GO:0006568
GO:0006552 GO:0046487 GO:0006550 GO:0009396 GO:0006554
GO:0006633 GO:0006631 GO:0018874 GO:0006298 GO:0003824
GO:0003857 GO:0016491 GO:0005524 GO:0050662 GO:0004488
Cluster-22178.72594 GO:0005525 GO:0003924
Cluster-22178.72595 GO:0016032 GO:0055114 GO:0008152 GO:0016491
GO:0019033
Cluster-22178.72596 GO:0003676
Cluster-22178.72601 GO:0051262 GO:0008219 GO:0005515 GO:0044212

Cluster-22178.72602 GO:0008152 GO:0046413 GO:0018836 GO:0005515
GO:0016491
Cluster-22178.72603 GO:0000917 GO:0006260 GO:0043093 GO:0045095
GO:0005737

Cluster-22178.72604 GO:0008152 GO:0046413 GO:0018836 GO:0005515
GO:0016491 GO:0016020
Cluster-22178.72605 GO:0006568 GO:0006552 GO:0006631 GO:0018874
GO:0006550 GO:0055114 GO:0006554 GO:0006574 GO:0008152
GO:0006633 GO:0003857 GO:0016491
Cluster-22178.72609 GO:0006874 GO:0006816 GO:0019069 GO:0006260
GO:0006468 GO:0006811 GO:0005244 GO:0005524 GO:0000049
GO:0005219 GO:0005262 GO:0004672 GO:0016020 GO:0005815
GO:0005622 GO:0071203 GO:0016021
Cluster-22178.72610 GO:0008544 GO:0008168 GO:0046872 GO:0000166

Cluster-22178.72614 GO:0006338 GO:0008152 GO:0016836 GO:0005515
GO:0003824 GO:0090544
Cluster-22178.72615 GO:0006355 GO:0003700 GO:0005515 GO:0019867
GO:0005667
Cluster-22178.72618 GO:0008608 GO:0050690 GO:0006418 GO:0043039
GO:0003676 GO:0005524 GO:0005537 GO:0000166 GO:0000049
GO:0004812 GO:0005737 GO:0042729 GO:0072686
Cluster-22178.72619 GO:0005515
Cluster-22178.72620 GO:0051252 GO:0007165 GO:0042254 GO:0006412
GO:0006355 GO:0003676 GO:0003735 GO:0004523 GO:0005840
GO:0005622
Cluster-22178.72621 GO:0032775 GO:0031167 GO:0007275 GO:0007165
GO:0006306 GO:0006298 GO:0016055 GO:0005102 GO:0008168
GO:0009007 GO:0005524 GO:0030983 GO:0098519 GO:0030414
GO:0005576
Cluster-22178.72623 GO:0071973 GO:0003774 GO:0009425
Cluster-22178.72624 GO:0008289
Cluster-22178.72628 GO:0051260 GO:0006468 GO:0005515 GO:0016773
GO:0005524 GO:0004672 GO:0016020 GO:0016021
Cluster-22178.72629 GO:0016884 GO:0016874
Cluster-22178.72631 GO:0006355 GO:0005515
Cluster-22178.72633 GO:0005515

Cluster-22178.72635 GO:0005515
Cluster-22178.72636 GO:0005515
Cluster-22178.7264 GO:0006118 GO:0055114 GO:0003995
Cluster-22178.72640 GO:0016567 GO:0006810 GO:0033014 GO:0006915
GO:0006783 GO:0015994 GO:0005515 GO:0004852 GO:0004842
GO:0005215 GO:0046872 GO:0008270 GO:0016020 GO:0005680
Cluster-22178.72643 GO:0006098 GO:0006749 GO:0007218 GO:0006260
GO:0018990 GO:0006355 GO:0007049 GO:0006006 GO:0055114
GO:0003677 GO:0008270 GO:0003887 GO:0004345 GO:0050661
GO:0003700 GO:0008255 GO:0005667 GO:0042575 GO:0005576
GO:0005634 GO:0016021
Cluster-22178.72645 GO:0006571 GO:0000162 GO:0006414 GO:0009094
GO:0006448 GO:0006432 GO:0004826 GO:0005524 GO:0003746
GO:0000287 GO:0003723 GO:0005840 GO:0009328 GO:0005853
Cluster-22178.72646 GO:0009094 GO:0006414 GO:0006448 GO:0006571
GO:0000162 GO:0006432 GO:0005524 GO:0003746 GO:0004826
GO:0000287 GO:0003723 GO:0009328 GO:0005840 GO:0005853
Cluster-22178.72647 GO:0055114 GO:0006694 GO:0007165 GO:0015936
GO:0008158 GO:0050662 GO:0004420 GO:0016020
Cluster-22178.72648 GO:0006508 GO:0005515 GO:0004190
Cluster-22178.72650 GO:0006144 GO:0051262 GO:0006206 GO:0006351
GO:0046872 GO:0005515 GO:0003899 GO:0003677 GO:0044212
GO:0005634 GO:0005730
Cluster-22178.72651 GO:0006357 GO:0005515 GO:0001104 GO:0043565
GO:0016592 GO:0005634
Cluster-22178.72652 GO:0006576 GO:0006118 GO:0006811 GO:0007264
GO:0007186 GO:0007165 GO:0015684 GO:0055085 GO:0003924
GO:0031683 GO:0019001 GO:0004871 GO:0005525 GO:0009055
GO:0003682 GO:0005524 GO:0015093 GO:0015075 GO:0005634
GO:0000785 GO:0016021 GO:0016020
Cluster-22178.72653 GO:0008219 GO:0051082 GO:0005515 GO:0005198

Cluster-22178.72655 GO:0006418 GO:0000166 GO:0004812 GO:0005524

Cluster-22178.72656 GO:0003723 GO:0003676
Cluster-22178.72657 GO:0006952 GO:0006810 GO:0009405 GO:0003723 GO:0033897 GO:0019855 GO:0005576
Cluster-22178.72659 GO:0008608 GO:0006816 GO:0006810 GO:0042030 GO:0005246 GO:0072686 GO:0016020 GO:0042729
Cluster-22178.72660 GO:0006310 GO:0006281
Cluster-22178.72663 GO:0055085 GO:0006012 GO:0009058 GO:0046486 GO:0042432 GO:0006027 GO:0016114 GO:0006355 GO:0006687 GO:0006468 GO:0004565 GO:0016773 GO:0016844 GO:0005524 GO:0004064 GO:0003700 GO:0004672 GO:0016020 GO:0005667 GO:0016021 GO:0009341
Cluster-22178.72664 GO:0006468 GO:0006355 GO:0016114 GO:0009058 GO:0042432 GO:0055085 GO:0005515 GO:0003700 GO:0004672 GO:0004064 GO:0005524 GO:0016844 GO:0016773 GO:0016021 GO:0016020 GO:0005667
Cluster-22178.72665 GO:0006412 GO:0007264 GO:0042254 GO:0006614 GO:0006270 GO:0003723 GO:0043531 GO:0003924 GO:0005525 GO:0003735 GO:0098519 GO:0016887 GO:0005524 GO:0003724 GO:0005840 GO:0005622
Cluster-22178.72666 GO:0006351 GO:0016787 GO:0015016 GO:0003677 GO:0005634
Cluster-22178.72667 GO:0043087 GO:0035023 GO:0044419 GO:0005089 GO:0005515
Cluster-22178.72668 GO:0010215 GO:0016049 GO:0031225
Cluster-22178.72672 GO:0006355 GO:0006260 GO:0016787 GO:0043565 GO:0003700 GO:0005667
Cluster-22178.72673 GO:0006355 GO:0016787 GO:0043565 GO:0005515 GO:0003700 GO:0005667
Cluster-22178.72674 GO:0007165 GO:0035556
Cluster-22178.72676 GO:0006807 GO:0003933 GO:0070403 GO:0005524

Cluster-22178.72678 GO:0006401 GO:0008272 GO:0006310 GO:0006355

GO:0006281 GO:0016887 GO:0005524 GO:0015116 GO:0003724
GO:0009378 GO:0003678 GO:0003723 GO:0004519 GO:0008134
GO:0005657 GO:0005667 GO:0009379 GO:0016021
Cluster-22178.72679 GO:0006355 GO:0006281 GO:0006955 GO:0006310
GO:0007165 GO:0003723 GO:0008134 GO:0003678 GO:0005126
GO:0009378 GO:0016887 GO:0005524 GO:0003724 GO:0005576
GO:0005667 GO:0009379 GO:0005657
Cluster-22178.72680 GO:0035556 GO:0016020
Cluster-22178.72681 GO:0006281 GO:0006355 GO:0006401 GO:0006310
GO:0008272 GO:0003678 GO:0008134 GO:0004519 GO:0005198
GO:0003723 GO:0015116 GO:0003724 GO:0016887 GO:0005524
GO:0009378 GO:0016021 GO:0019031 GO:0005657 GO:0009379
GO:0005667
Cluster-22178.72682 GO:0006355 GO:0006281 GO:0006401 GO:0006310
GO:0008272 GO:0003678 GO:0008134 GO:0004519 GO:0003723
GO:0003724 GO:0015116 GO:0016887 GO:0005524 GO:0009378
GO:0016021 GO:0005657 GO:0005667 GO:0009379
Cluster-22178.72685 GO:0016117 GO:0055114 GO:0016705 GO:0016491

Cluster-22178.72687 GO:0008152 GO:0003824
Cluster-22178.72688 GO:0006769 GO:0035023 GO:0046497 GO:0008152
GO:0006508 GO:0006741 GO:0046656 GO:0006814 GO:0007010
GO:0003333 GO:0043087 GO:0035556 GO:0046740 GO:0003779
GO:0046872 GO:0008236 GO:0016301 GO:0003951 GO:0030269
GO:0005089 GO:0005737 GO:0044219 GO:0005643 GO:0019028
GO:0012506
Cluster-22178.72692 GO:0022900 GO:0055114 GO:0006118 GO:0008827
GO:0009319 GO:0016021
Cluster-22178.72697 GO:0016192 GO:0006886 GO:0042981 GO:0005515
GO:0030117
Cluster-22178.72698 GO:0046872 GO:0008270 GO:0005515
Cluster-22178.72699 GO:0005515
Cluster-22178.72703 GO:0005515 GO:0016788 GO:0016021

Cluster-22178.72704 GO:0007165 GO:0006914 GO:0005198 GO:0031423 GO:0019012 GO:0019028

Cluster-22178.72706 GO:0008299 GO:0006570 GO:0009058 GO:0007165 GO:0035556 GO:0006694 GO:0006470 GO:0005044 GO:0005515 GO:0016779 GO:0050518 GO:0004725 GO:0016020 GO:0019031

Cluster-22178.72707 GO:0006355 GO:0016787 GO:0016818 GO:0003677 GO:0008270 GO:0005524 GO:0003676 GO:0005634

Cluster-22178.72710 GO:0005887

Cluster-22178.72711 GO:0015031 GO:0016021

Cluster-22178.72713 GO:0075521 GO:0007165 GO:0046718 GO:0006505 GO:0006886 GO:0006355 GO:0006352 GO:0019076 GO:0003677 GO:0005179 GO:0016787 GO:0016987 GO:0016788 GO:0098599 GO:0003700 GO:0030430 GO:0005667 GO:0044163 GO:0042025 GO:0005576 GO:0019028 GO:0019031

Cluster-22178.72715 GO:0016579 GO:0015940 GO:0008897 GO:0005515 GO:0000287 GO:0036459 GO:0005096

Cluster-22178.72716 GO:0016579 GO:0005096 GO:0036459 GO:0005515

Cluster-22178.72717 GO:0006355 GO:0006270 GO:0006506 GO:0017176 GO:0003700 GO:0046935 GO:0005667 GO:0005944 GO:0016021 GO:0019031

Cluster-22178.72718 GO:0007067 GO:0000090 GO:0007059 GO:0031110 GO:0051301 GO:0006914 GO:0008017 GO:0045298 GO:0005876 GO:0000940

Cluster-22178.72719 GO:0003677 GO:0005198 GO:0005515 GO:0008270 GO:0016021

Cluster-22178.72720 GO:0006508 GO:0008234

Cluster-22178.72721 GO:0006352 GO:0003676 GO:0003677 GO:0003723

Cluster-22178.72722 GO:0045892 GO:0003723 GO:0005634

Cluster-22178.72724 GO:0006811 GO:0055085 GO:0015075 GO:0005515 GO:0005886 GO:0016020

Cluster-22178.72725 GO:0035556 GO:0006464 GO:0006144 GO:0006811

GO:0046034 GO:0009405 GO:0006351 GO:0006955 GO:0000902
GO:0006355 GO:0008270 GO:0003677 GO:0004017 GO:0016151
GO:0003700 GO:0016790 GO:0003676 GO:0046872 GO:0043565
GO:0005515 GO:0005887 GO:0016020 GO:0005667 GO:0045202
Cluster-22178.72729 GO:0016021
Cluster-22178.72730 GO:0006338 GO:0016818 GO:0003676 GO:0046872
GO:0005524 GO:0005634
Cluster-22178.72731 GO:0006139 GO:0046034 GO:0006206 GO:0006144
GO:0007186 GO:0050909 GO:0004930 GO:0004127 GO:0005524
GO:0004017 GO:0016021
Cluster-22178.72732 GO:0006486 GO:0008378 GO:0016757 GO:0016020
Cluster-22178.72733 GO:0016310 GO:0004673 GO:0009365
Cluster-22178.72735 GO:0009072 GO:0015743 GO:0006570 GO:0042207
GO:0006810 GO:0004334 GO:0005886
Cluster-22178.72736 GO:0008152 GO:0009452 GO:0001510 GO:0031167
GO:0006306 GO:0008168 GO:0005201 GO:0008170 GO:0005515
GO:0003677 GO:0005581 GO:0016021 GO:0005578 GO:0019013
Cluster-22178.72737 GO:0005543
Cluster-22178.72740 GO:0000226 GO:0042254 GO:0006412 GO:0003735
GO:0015934 GO:0005840
Cluster-22178.72741 GO:0035556 GO:0008152 GO:0006144 GO:0006468
GO:0006182 GO:0046039 GO:0009190 GO:0006508 GO:0004672
GO:0005524 GO:0008236 GO:0004383 GO:0016787 GO:0016849
Cluster-22178.72744 GO:0006771 GO:0006000 GO:0019497 GO:0006013
GO:0016301 GO:0003873 GO:0003993 GO:0005524
Cluster-22178.72748 GO:0016998 GO:0006032 GO:0004568 GO:0016021
Cluster-22178.7275 GO:0043087 GO:0035023 GO:0005089
Cluster-22178.72752 GO:0006355 GO:0043565 GO:0003700 GO:0019905
GO:0005515 GO:0005667
Cluster-22178.72753 GO:0007165 GO:0043087 GO:0006812 GO:0006885
GO:0055085 GO:0035023 GO:0015299 GO:0005057 GO:0005089
GO:0016021
Cluster-22178.72755 GO:0005515

Cluster-22178.72758 GO:0008270
Cluster-22178.72762 GO:0050909 GO:0005515 GO:0016021
Cluster-22178.72764 GO:0050909 GO:0005515 GO:0016021
Cluster-22178.72765 GO:0007264 GO:0006355 GO:0006576 GO:0019068
GO:0005524 GO:0003924 GO:0005525 GO:0003700 GO:0005667
Cluster-22178.72769 GO:0051028 GO:0031012 GO:0005634
Cluster-22178.72770 GO:0003677 GO:0005634
Cluster-22178.72771 GO:0008201 GO:0009512
Cluster-22178.72773 GO:0007264 GO:0003924 GO:0005525
Cluster-22178.72775 GO:0006040 GO:0005975 GO:0016977 GO:0005576
Cluster-22178.72779 GO:0006412 GO:0042254 GO:0003735 GO:0019843
GO:0005840
Cluster-22178.72780 GO:0006570 GO:0006470 GO:0004725 GO:0008138

Cluster-22178.72783 GO:0045444 GO:0046872
Cluster-22178.72784 GO:0045444 GO:0046872
Cluster-22178.72787 GO:0005515
Cluster-22178.72788 GO:0005515
Cluster-22178.72789 GO:0006118 GO:0051536 GO:0009055
Cluster-22178.72791 GO:0046740 GO:0005515 GO:0016021
Cluster-22178.72792 GO:0006118 GO:0010508 GO:0007186 GO:0003954
GO:0004930 GO:0016020 GO:0005739 GO:0031966 GO:0016021
Cluster-22178.72793 GO:0006355 GO:0006118 GO:0006914 GO:0006260
GO:0007186 GO:0007165 GO:0042974 GO:0005198 GO:0004930
GO:0003954 GO:0016021 GO:0005667 GO:0031966 GO:0005739
GO:0016020 GO:0005882
Cluster-22178.72795 GO:0006355 GO:0042967 GO:0016747 GO:0008080

Cluster-22178.72796 GO:0006355 GO:0042967 GO:0016747 GO:0008080

Cluster-22178.72797 GO:0003677 GO:0009512
Cluster-22178.72798 GO:0055114 GO:0030699 GO:0050485 GO:0030700
Cluster-22178.72802 GO:0005201 GO:0046872 GO:0046332 GO:0008270

GO:0005578
Cluster-22178.72806 GO:0007155 GO:0006606 GO:0015031 GO:0000921
GO:0006904 GO:0051382 GO:0005198 GO:0042803 GO:0008134
GO:0045502 GO:0008289 GO:0000145 GO:0005882 GO:0005667
GO:0005940 GO:0030286 GO:0000776 GO:0016021
Cluster-22178.72807 GO:0051382 GO:0007049 GO:0006904 GO:0009405
GO:0015031 GO:0000921 GO:0006606 GO:0007155 GO:0008289
GO:0045502 GO:0005198 GO:0008134 GO:0042803 GO:0016021
GO:0000776 GO:0030286 GO:0005940 GO:0005667 GO:0005882
GO:0016020 GO:0000145
Cluster-22178.72809 GO:0042254 GO:0001522 GO:0030515 GO:0072588
Cluster-22178.72811 GO:0006629 GO:0006355 GO:0043401 GO:0043565
GO:0008270 GO:0003700 GO:0016021 GO:0005634 GO:0005667
Cluster-22178.72812 GO:0006629 GO:0006355 GO:0043401 GO:0008270
GO:0043565 GO:0003700 GO:0005667 GO:0005634
Cluster-22178.72813 GO:0043401 GO:0006355 GO:0006629 GO:0003700
GO:0008270 GO:0043565 GO:0005634 GO:0005667
Cluster-22178.72817 GO:0005515
Cluster-22178.72821 GO:0006338 GO:0006355 GO:0008270 GO:0005524
GO:0003676 GO:0016818 GO:0003677 GO:0005634 GO:0005739
Cluster-22178.72826 GO:0019058 GO:0006810 GO:0046872 GO:0016021
Cluster-22178.72827 GO:0006811 GO:0006810 GO:0051726 GO:0017025
GO:0005230 GO:0005634 GO:0016020
Cluster-22178.72828 GO:0006886 GO:0016192 GO:0005515 GO:0005198
GO:0030117 GO:0030130 GO:0030132
Cluster-22178.72831 GO:0006508 GO:0004252
Cluster-22178.72832 GO:0019042 GO:0005524 GO:0003677 GO:0000166
GO:0033644
Cluster-22178.72833 GO:0046872 GO:0016021
Cluster-22178.72834 GO:0050830 GO:0033588
Cluster-22178.72835 GO:0046872 GO:0016021
Cluster-22178.72836 GO:0055114 GO:0016491
Cluster-22178.72837 GO:0006820 GO:0004190 GO:0016021

Cluster-22178.72838 GO:0016020
Cluster-22178.72839 GO:0009058 GO:0055114 GO:0030170 GO:0016491

Cluster-22178.72840 GO:0016021
Cluster-22178.72841 GO:0051289 GO:0008140 GO:0005667
Cluster-22178.72842 GO:0051289 GO:0008140 GO:0005667
Cluster-22178.72843 GO:0030001 GO:0055085 GO:0046873 GO:0016020
Cluster-22178.72844 GO:0030001 GO:0055085 GO:0046873 GO:0016020
Cluster-22178.72845 GO:0006418 GO:0006355 GO:0005524 GO:0008270
GO:0002161 GO:0004812 GO:0016818 GO:0003677 GO:0000166
GO:0005634
Cluster-22178.72846 GO:0003676
Cluster-22178.72847 GO:0003824 GO:0005515
Cluster-22178.72849 GO:0035556 GO:0007165 GO:0004871
Cluster-22178.72850 GO:0042967 GO:0006355 GO:0003712 GO:0008270
GO:0005515 GO:0004402 GO:0000123 GO:0005634 GO:0005667
Cluster-22178.72852 GO:0007049 GO:0051301 GO:0005509 GO:0016021
Cluster-22178.72854 GO:0007017 GO:0007018 GO:0005524 GO:0003777
GO:0008017 GO:0003677 GO:0005198 GO:0005874 GO:0045298
Cluster-22178.72855 GO:0032259 GO:0008168
Cluster-22178.72857 GO:0006260
Cluster-22178.72859 GO:0005515 GO:0003676 GO:0046872 GO:0008270

Cluster-22178.72861 GO:0030472 GO:0035556 GO:0071973 GO:0055114
GO:0006935 GO:0006468 GO:0016773 GO:0005524 GO:0003774
GO:0047134 GO:0004672 GO:0005515 GO:0016779 GO:0016020
GO:0009288 GO:0072686 GO:0042729
Cluster-22178.72862 GO:0046907
Cluster-22178.72863 GO:0005975 GO:0035556 GO:0006954 GO:0007165
GO:0006952 GO:0005125 GO:0046872 GO:0004553 GO:0005515
GO:0003677 GO:0005576
Cluster-22178.72865 GO:0071816 GO:0031492 GO:0005634 GO:0000785
Cluster-22178.72868 GO:0006825 GO:0009252 GO:0006629 GO:0035434

GO:0008963 GO:0046983 GO:0003677 GO:0005375 GO:0016021
Cluster-22178.72869 GO:0000042 GO:0005515 GO:0008168
Cluster-22178.72870 GO:0008168
Cluster-22178.72872 GO:0006123 GO:0006812 GO:0006820 GO:0055114
GO:0006835 GO:0015992 GO:0004129 GO:0017153 GO:0016638
GO:0045277 GO:0016020
Cluster-22178.72873 GO:0050790 GO:0006508 GO:0009405 GO:0004197
GO:0005576
Cluster-22178.72874 GO:0005515
Cluster-22178.72877 GO:0019079 GO:0006810 GO:0006265 GO:0003917
GO:0003677 GO:0008026 GO:0003676 GO:0004386 GO:0016887
GO:0005524 GO:0005694
Cluster-22178.72878 GO:0019069 GO:0006816 GO:0006874 GO:0006260
GO:0000049 GO:0005219 GO:0071203 GO:0016020 GO:0005622
GO:0005815 GO:0016021
Cluster-22178.72879 GO:0005515
Cluster-22178.7288 GO:0055114 GO:0005507 GO:0016641
Cluster-22178.72880 GO:0006446 GO:0045454 GO:0006413 GO:0007411
GO:0055114 GO:0003743 GO:0003723 GO:0005840 GO:0005737
Cluster-22178.72881 GO:0005975 GO:0008270 GO:0005198 GO:0019028
GO:0005634
Cluster-22178.72884 GO:0043093 GO:0000226 GO:0009396 GO:0000917
GO:0046487 GO:0045132 GO:0008152 GO:0055114 GO:0004488
GO:0005509 GO:0016787 GO:0005515 GO:0003824 GO:0008092
GO:0019898 GO:0015630 GO:0005634 GO:0000775 GO:0016021
GO:0005737
Cluster-22178.72885 GO:0043087 GO:0015886 GO:0035556 GO:0005085
GO:0015232 GO:0008270 GO:0005515
Cluster-22178.72886 GO:0035556 GO:0015886 GO:0043087 GO:0005515
GO:0008270 GO:0015232 GO:0005085
Cluster-22178.72887 GO:0006308 GO:0006289 GO:0006281 GO:0006285
GO:0008534 GO:0003684
Cluster-22178.72888 GO:0000287 GO:0016787 GO:0046872 GO:0070403

GO:0030976
Cluster-22178.72889 GO:0031167 GO:0009451 GO:0006479 GO:0006306
GO:0001510 GO:0009452 GO:0008152 GO:0008033 GO:0046500
GO:0006400 GO:0006396 GO:0006464 GO:0015979 GO:0042549
GO:0008173 GO:0005515 GO:0003676 GO:0008168 GO:0004719
GO:0003677 GO:0008176 GO:0008170 GO:0009523 GO:0009539
Cluster-22178.72890 GO:0008033 GO:0008152 GO:0009452 GO:0001510
GO:0009451 GO:0031167 GO:0006306 GO:0006479 GO:0042549
GO:0015979 GO:0006464 GO:0006396 GO:0006400 GO:0046500
GO:0008168 GO:0004719 GO:0003676 GO:0005515 GO:0008173
GO:0008176 GO:0008170 GO:0003677 GO:0009523 GO:0009539
Cluster-22178.72891 GO:0006898 GO:0006508 GO:0007165 GO:0008235
GO:0005044 GO:0030145 GO:0004177 GO:0005737 GO:0016020
Cluster-22178.72892 GO:0006508 GO:0006629 GO:0006144 GO:0004252
GO:0017111 GO:0004197 GO:0003968 GO:0070008 GO:0016817
GO:0031379
Cluster-22178.72895 GO:0046660 GO:0006812 GO:0006397 GO:0003676
GO:0016887 GO:0016021 GO:0005634
Cluster-22178.72896 GO:0009847 GO:0007165 GO:0005102
Cluster-22178.72898 GO:0006308 GO:0007018 GO:0006355 GO:0007017
GO:0006812 GO:0007165 GO:0003777 GO:0005515 GO:0035257
GO:0008017 GO:0005524 GO:0008855 GO:0016887 GO:0003713
GO:0005874 GO:0016021 GO:0045298 GO:0009318 GO:0005634
GO:0005667
Cluster-22178.72899 GO:0007165 GO:0042254 GO:0006812 GO:0007018
GO:0006412 GO:0006355 GO:0007017 GO:0006308 GO:0003713
GO:0003735 GO:0008017 GO:0005524 GO:0008855 GO:0016887
[truncated: 398,516 more chars]
